# Global research trends in tuberculosis drug-resistance mechanisms: a bibliometric and cross-database analysis

**DOI:** 10.3389/fmicb.2026.1942089

**Published:** 2026-09-17

**Authors:** Jiali Lian, Hailiang Nan, Yongqiang Xing, Zhanli Wang, Jinqi Hao, Yanqin Yu

**Affiliations:** 1 School of Public Health, Baotou Medical College, Baotou, China; 2 The Fourth Hospital of Inner Mongolia Autonomous Region (Chest Hospital), Hohhot, China; 3 Inner Mongolia University of Science and Technology, Baotou, Inner Mongolia, China; 4 The Second Affiliated Hospital of Baotou Medical College, Baotou, Inner Mongolia, China

**Keywords:** bibliometrics, DR-TB, host-directed therapy, molecular pathways, One Health

## Abstract

**Background:**

Drug-resistant tuberculosis (DR-TB) remains a major challenge to global tuberculosis control. In 2024, approximately 390,000 individuals developed multidrug- or rifampicin-resistant tuberculosis (MDR/RR-TB) worldwide, while treatment coverage remained limited to 42%, highlighting persistent gaps in DR-TB management. The mechanisms underlying DR-TB involve resistance-associated mutations, bacterial adaptation, host–pathogen interactions, and drug-target-related processes. A comprehensive evaluation of research trends is needed to clarify the evolution and future directions of this field.

**Methods:**

This bibliometric review was conducted with reference to the Preferred Reporting Items for Systematic Reviews and Meta-Analyzes (PRISMA) 2020 statement. Publications related to DR-TB mechanisms from January 1, 2006, to May 10, 2026, were retrieved from the Web of Science Core Collection (WoSCC), yielding 1,043 eligible records. VOSviewer, CiteSpace, and bibliometric methods were applied to analyze countries/regions, institutions, authors, journals, keywords, and co-citation networks. An independent PubMed dataset (*n* = 787) was used for cross-database validation to assess the consistency and robustness of major findings.

**Results:**

Annual publications increased markedly after 2015. The dataset included 6,129 authors from 1,581 institutions across 60 countries/regions. The United States was the leading contributor, with the highest number of publications (248) and citations (2,460). Keyword and thematic analyzes revealed that major research areas included drug resistance mechanisms, combination therapy, microbiological analysis, antitubercular agents, and *Mycobacterium tuberculosis*. Research evolution demonstrated a transition from traditional studies of bacterial pathology and biosynthesis toward computer-aided target discovery, molecular docking, and host-directed therapies involving programmed cell death pathways such as autophagy. Descriptive country–institution–keyword mapping showed regionally patterned thematic associations, with natural products, nanomaterials, and quorum-sensing inhibition represented among associations involving institutions from Asia and Latin America, and clinical microbiology, genomics, and pathway regulation represented among those involving institutions from North America and Europe. PubMed validation demonstrated comparable publication trends, geographic distributions, and thematic patterns.

**Conclusion:**

The bibliometric landscape of DR-TB mechanism research has broadened from individual molecular targets to bacterial, host-related, and computational themes. Recent studies show increasing attention to host-directed approaches, molecular modeling, and multidimensional analyzes. These trends may inform future mechanistic and translational research, although their biological and clinical relevance requires independent validation.

## 1 Introduction

Drug-resistant tuberculosis (DR-TB) continues to pose a substantial challenge to tuberculosis prevention and treatment. In 2024, an estimated 390,000 people developed multidrug- or rifampicin-resistant tuberculosis (MDR/RR-TB) worldwide, while global treatment coverage remained limited, highlighting persistent gaps in the management of drug-resistant tuberculosis (Farhat et al., 2024). A recent meta-analysis of 148 studies involving 318,430 participants reported pooled prevalence estimates of 11.6% for MDR-TB and 2.5% for extensively drug-resistant tuberculosis (XDR-TB). The continued occurrence of MDR-TB and XDR-TB is associated with treatment failure, prolonged treatment duration, increased mortality, and substantial medical and socioeconomic burden. In addition, second-line treatment regimens may be associated with adverse effects, including neurotoxicity, hepatotoxicity, and irreversible ototoxicity (Morris et al., 2013; Matteelli et al., 2014).

The mechanisms underlying drug resistance in *Mycobacterium tuberculosis* are biologically complex. Earlier research mainly focused on resistance-associated gene mutations and their effects on susceptibility to first- and second-line anti-tuberculosis drugs. Beyond resistance-associated gene mutations, studies have also examined additional processes, including metabolic adaptation, cell-wall remodeling, compensatory evolution, epigenetic regulation, and host–pathogen interactions. These mechanisms may affect bacterial survival, drug tolerance, immune evasion, and treatment response. A clearer overview of how these research topics have developed may help contextualize current evidence and identify areas requiring further mechanistic investigation (Gopalaswamy and Subbian, 2026).

Research on DR-TB resistance mechanisms encompasses canonical resistance mutations and evolutionary processes as well as changes in the mycobacterial cell envelope, metabolic adaptation, host–pathogen interactions, and therapeutic approaches. Resistance-associated mutations and evolutionary dynamics remain central to understanding DR-TB (Gygli et al., 2017). Drug-resistant strains may also undergo structural and biochemical changes in the cell envelope that affect drug susceptibility and interactions with the host (Schami et al., 2023). Multi-omics studies have been used to identify potential therapeutic targets and clarify mechanisms of drug action in tuberculosis drug discovery (Goff et al., 2020). In addition, *Mycobacterium tuberculosis* can reprogramme its metabolism during infection, dormancy, and persistence, thereby facilitating adaptation to environmental stress and contributing to reduced antimicrobial susceptibility (Chang and Guan, 2021). Compensatory evolution may partially restore the fitness of rifampicin-resistant strains (Eckartt et al., 2024). Systems-based omics have also been applied to investigate host–pathogen metabolic interactions and immune responses (Borah K. et al., 2021). Current therapeutic research includes host-directed approaches (Mehta et al., 2022), inhibitors of mycobacterial cell-wall biosynthesis (Belete, 2022), and ferroptosis-related mechanisms involved in host–pathogen interactions (Qiang et al., 2023). However, the biological significance and translational potential of these mechanisms require further validation.

Previous reviews have summarized important aspects of DR-TB resistance mechanisms, including specific molecular pathways, drug-resistance mutations, diagnostic targets, and potential therapeutic strategies (Palomino and Martin, 2014; Swain et al., 2020; Borah P. et al., 2021). These reviews provide useful interpretations of selected biological mechanisms. However, a quantitative overview of publication trends, geographical and institutional contributions, citation patterns, and the temporal development of research topics can provide a complementary perspective on the structure of this field.

Bibliometric analysis is a quantitative method for describing patterns in scientific literature. It can summarize publication output, country and institutional contributions, author productivity, journal distribution, co-cited references, and keyword changes over time. Previous bibliometric studies have examined several areas of tuberculosis research, including drug development, diagnostic methods, and vaccine research. By focusing specifically on DR-TB resistance mechanisms, bibliometric analysis can help describe how this research area has evolved and which topics have received sustained or increasing attention.

The present study differs from previous tuberculosis-related bibliometric studies in several respects. First, it specifically focuses on the mechanistic research landscape of tuberculosis drug resistance rather than the broader fields of tuberculosis epidemiology, diagnosis, treatment, or drug development. Second, it integrates collaboration, co-citation, keyword-evolution, and thematic analyzes to characterize the development of this field and the transition from classical resistance-related mechanisms toward bacterial adaptation, host–pathogen interactions, computational approaches, and host-directed therapeutic strategies. Third, an independently retrieved PubMed dataset was used for cross-database validation of the principal temporal, geographical, and thematic patterns identified in WoSCC, thereby providing an additional assessment of the robustness of the main bibliometric findings.

The present study analyzed publications on DR-TB resistance mechanisms indexed in the Web of Science Core Collection from 1 January 2006 to 10 May 2026. Publication trends, country and institutional contributions, authors, journals, co-cited references, and keyword evolution were examined. A supplementary PubMed dataset was used to assess the consistency of the main temporal, geographical, and thematic patterns across databases. This study provides a structured bibliometric summary of DR-TB resistance-mechanism research and identifies topics that may warrant further experimental or translational investigation.

## 2 Materials and methods

### 2.1 Data sources and search strategy

The literature identification and selection process was reported with reference to the Preferred Reporting Items for Systematic Reviews and Meta-Analyzes (PRISMA) 2020 statement. The review question was defined using an adapted Population, Intervention, Comparator, Outcomes, and Study design (PICOS) framework suitable for bibliometric research. The unit of analysis comprised publications on DR-TB resistance mechanisms rather than individual patients or clinical populations; therefore, the Population component was not applicable in the conventional clinical sense. As this bibliometric review did not evaluate a therapeutic or diagnostic intervention or compare intervention groups, the Intervention and Comparator components were also not applicable. The outcomes included publication trends, geographical and institutional contributions, collaboration and citation patterns, and thematic evolution. Eligible study designs comprised English-language journal articles and reviews published between 1 January 2006 and 10 May 2026.

The Web of Science Core Collection (WoSCC) was searched on 10 May 2026 for records published between 1 January 2006 and 10 May 2026. WoSCC was used as the primary data source because it provides structured bibliographic metadata suitable for bibliometric analysis. The search strategy was as follows: TS = (“drug-resistant tuberculosis” OR “MDR-TB” OR “XDR-TB” OR “TDR-TB” OR “multidrug-resistant TB” OR “extensively drug-resistant TB”) AND TS = (“pathway” OR “mechanism” OR “signaling” OR “metabolic” OR “immune response” OR “efflux pump” OR “autophagy” OR “apoptosis” OR “cell wall” OR “biofilm” OR “gene regulation” OR “non-coding RNA”). The initial WoSCC search retrieved 1,080 records. After excluding 31 records for other reasons, comprising 2 online publications, 20 meeting abstracts, 5 editorial materials, and 4 records considered irrelevant according to the predefined eligibility criteria, 1,049 publications remained, including 803 articles and 246 reviews. Six non-English publications were subsequently excluded, resulting in a final WoSCC dataset of 1,043 publications, including 800 articles and 243 reviews. Bibliographic information, including titles, abstracts, keywords, and countries or regions of origin, was subsequently extracted for analysis.

To assess the robustness of the principal findings and reduce potential database-specific bias, an independent PubMed search was conducted on 22 July 2026 to obtain a supplementary validation dataset. PubMed was used as an independent validation source rather than being directly merged with the WoSCC dataset, allowing the reproducibility of the principal findings to be assessed across databases with different indexing characteristics. An equivalent search strategy was adapted to the title and abstract fields in PubMed, with the publication period restricted to 1 January 2006 to 10 May 2026 to ensure comparability with the WoSCC dataset. The PubMed search strategy was as follows: (“drug-resistant tuberculosis” [tiab] OR“MDR-TB” [tiab] OR “XDR-TB” [tiab] OR “TDR-TB” [tiab] OR “multidrug-resistant TB” [tiab] OR “extensively drug-resistant TB” [tiab]) AND (“pathway” [tiab] OR“mechanism” [tiab] OR “signaling” [tiab] OR “metabolic” [tiab] OR “immune response” [tiab] OR “efflux pump” [tiab] OR “autophagy” [tiab] OR “apoptosis” [tiab] OR “cell wall” [tiab] OR “biofilm” [tiab] OR “gene regulation” [tiab] OR “non-coding RNA” [tiab]). The PubMed search retrieved 802 records. After four records with ineligible publication types and 11 non-English publications were excluded, 787 publications remained for comparative cross-database validation. The complete bibliographic records retrieved from WoSCC and PubMed and used for the bibliometric analyses are provided in the Supplementary material.

### 2.2 Eligibility criteria

English-language publications classified as journal articles or reviews and published between 1 January 2006 and 10 May 2026 were eligible. Records with ineligible publication types and non-English publications were excluded. The detailed screening process is illustrated in Figure 1.

**FIGURE 1:**
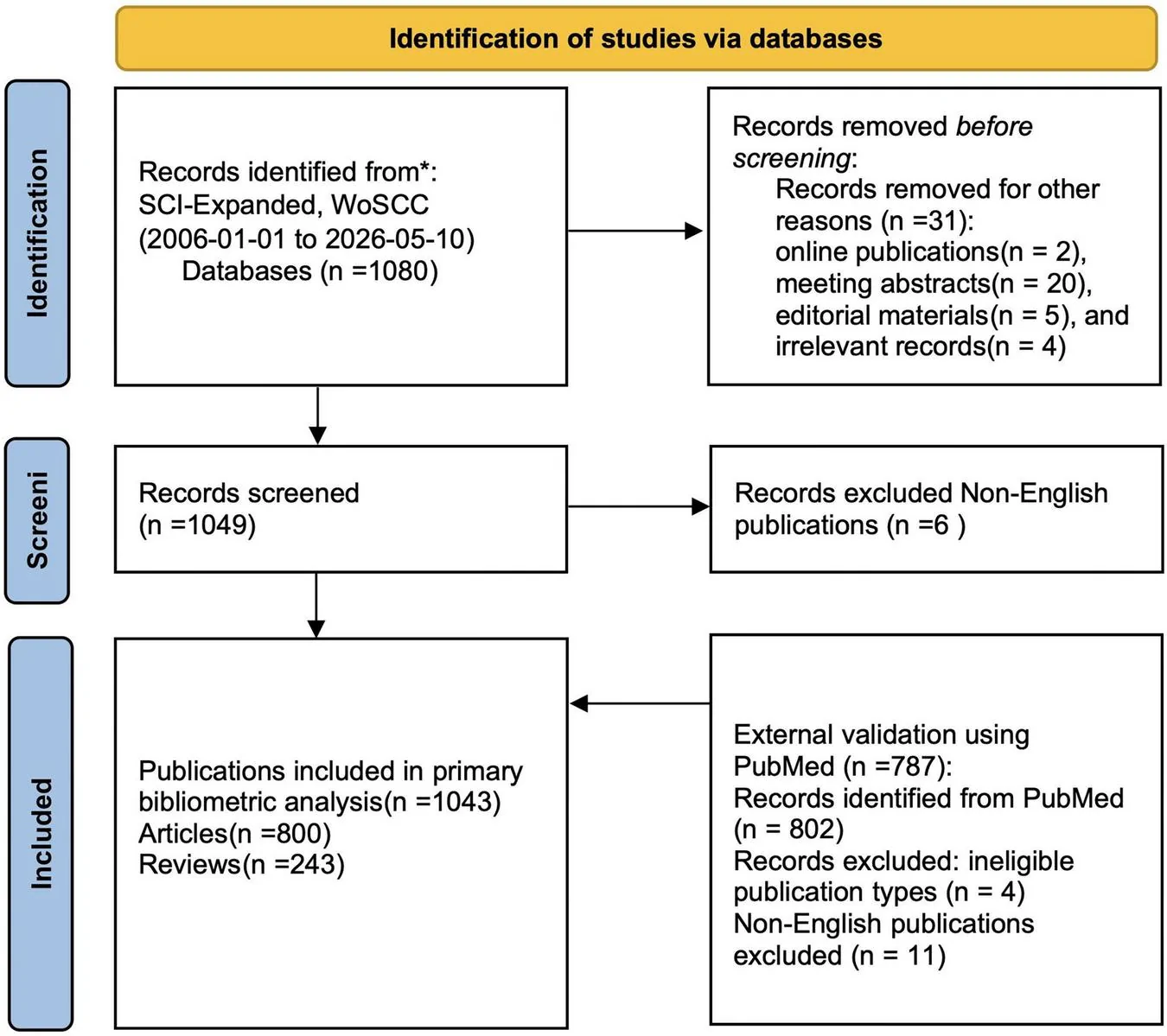
Flowchart of the publication screening process. The WoSCC search identified 1,080 records, of which 31 were excluded for other reasons and 6 non-English publications were subsequently removed, leaving 1,043 publications for the primary analysis. The PubMed search identified 802 records; after excluding 4 records with ineligible publication types and 11 non-English publications, 787 publications were included for cross-database validation.

Because this study analyzed bibliographic records rather than clinical-effectiveness evidence, conventional study-level risk-of-bias assessment was not applicable. Potential bibliometric biases, including database coverage, language restriction, indexing differences, citation time lag, and dependence on predefined search terms, are discussed in the Limitations section.

### 2.3 Data analysis

Bibliometric analyzes were performed using VOSviewer version 1.6.20, CiteSpace version 6.4.R1, R version 4.5.3 with the bibliometrix package version 4.5.3, and Microsoft Excel 2021. Bibliographic information, including titles, abstracts, keywords, authors, affiliations, citations, and references, was imported into the corresponding software for analysis.

VOSviewer was used to analyze collaboration, co-citation, and keyword co-occurrence patterns. Country- and institution-level collaboration analyzes were conducted to describe the distribution of research contributions and cooperative relationships. Co-citation analyzes were performed for cited references, authors, and journals to identify publications and sources that were frequently cited within the retrieved dataset.

CiteSpace was used to examine keyword clustering and citation-burst patterns over time. The keyword timeline was constructed from the keyword co-occurrence network; therefore, the resulting clusters represent groups of thematically related co-occurring keywords rather than document communities defined by cross-citation patterns. These analyzes were used to describe temporal changes in research topics and to identify terms or references that received increased attention during specific periods.

R, RStudio, and the bibliometrix package were used for annual publication trend analysis, three-field plots, thematic mapping, and journal co-citation analysis. For short-term forecasting of publication output, a linear regression model was fitted using complete annual publication counts from 2006 to 2025. Because the 2026 data included publications indexed only up to 10 May 2026, this incomplete year was excluded from model fitting. The fitted model was used to estimate annual publication output up to 2030, and model fit was assessed using the coefficient of determination (R^2^). In the journal co-citation analysis, journals with fewer than 20 citations were excluded to improve interpretability of the results. Because VOSviewer, CiteSpace, and bibliometrix employ different clustering, normalization, thresholding, and visualization procedures, they were used for complementary analytical purposes rather than treated as directly interchangeable methods, and their outputs were interpreted within their respective methodological frameworks. For quantitative cross-database validation, consistency between the WoSCC primary dataset (*n* = 1,043) and the PubMed validation dataset (*n* = 787) was assessed using complementary statistical measures. Spearman’s rank correlation coefficient was used to evaluate the association between annual publication counts in the two databases. The Jaccard similarity index, calculated as the size of the intersection divided by the size of the union of two sets, was used to quantify overlap in the top 10 productive countries and the top 10 predefined mechanism-related topics. Jaccard indices were also calculated for period-specific research hotspots to assess temporal thematic consistency between the two databases.

The thematic map was generated based on centrality and density. Centrality reflects the degree of association between a theme and other themes, whereas density reflects the internal development of a theme. Themes were classified into four quadrants according to their centrality and density: motor themes, niche themes, emerging or declining themes, and basic themes. These classifications were used to describe the relative development and position of major research topics within the analyzed literature.

Microsoft Excel 2021 was used to organize bibliographic data and generate descriptive charts. Lotka’s law was applied to examine the distribution of author productivity. Author productivity was assessed by fitting the observed number of publications per author to Lotka’s inverse power-law model, which is commonly used to describe the frequency distribution of scientific output within a research field. The goodness of fit was assessed using the Kolmogorov–Smirnov (KS) test, with a non-significant *P*-value indicating no statistically detectable difference between the observed author-productivity distribution and the fitted Lotka distribution.

In this study, several bibliometric indicators were used to describe publication output, citation performance, collaboration patterns, and thematic development. Publication output was assessed using the annual number of publications and the total number of publications by country, institution, author, and journal. Citation performance was evaluated using total citations, average citations per publication, Hirsch index (H-index), and local citation frequency. The H-index was used as a supplementary indicator that combines publication productivity and citation impact, particularly in the comparison of countries or regions. Total link strength was used to describe the strength of collaboration or citation relationships in VOSviewer analyzes. Co-citation analysis was used to identify references, authors, and journals that were frequently cited within the retrieved dataset. Keyword co-occurrence, keyword bursts, and thematic mapping were used to describe changes in research topics over time. In addition, Lotka’s law was applied to examine the distribution of author productivity in this field.

## 3 Results

### 3.1 Literature search results

The initial WoSCC search retrieved 1,080 records. After excluding 31 records with ineligible document types, 1,049 publications remained. Six non-English publications were subsequently excluded, resulting in 1,043 publications being included in the primary analysis, comprising 800 articles and 243 reviews. The PubMed search retrieved 802 records; after excluding four records with ineligible publication types and 11 non-English publications, 787 publications were included in the cross-database validation.

### 3.2 Analysis of publications and citations

Annual publication output showed an overall upward trend with year-to-year fluctuations, reaching a peak of 90 publications in 2025. The 2026 count (*n* = 43) represents publications indexed up to 10 May 2026 (Figure 2A). Based on the linear regression model fitted to complete annual data from 2006 to 2025, publication output was projected to reach approximately 113 publications in 2030 (*R*^2^ = 0.899; Figure 2B).

**FIGURE 2:**
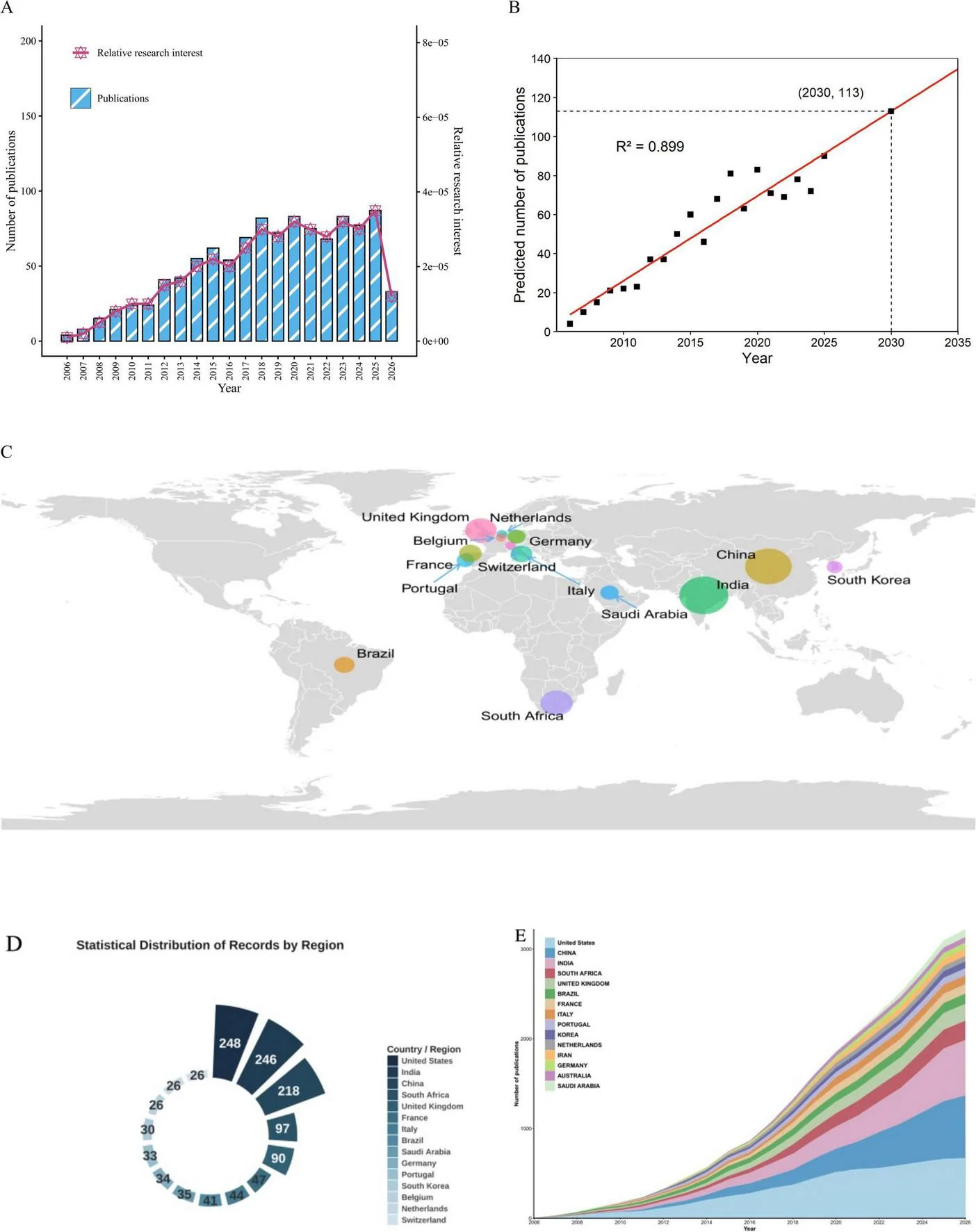
Global trends and contribution analysis of drug-resistant tuberculosis pathways (January 1, 2006–May 10, 2026). **(A)** Annual publication output and relative research interest from 2006 to 2026. **(B)** Short-term linear projection of annual publication output to 2030 based on complete annual observations from 2006 to 2025. **(C)** Global geographical distribution of publications. **(D)** Publication counts of the leading contributing countries and regions. **(E)** Cumulative publication trends of the major contributing countries and regions over time.

### 3.3 Analysis of countries/regions

The 1,043 publications in the WoSCC dataset involved authors from 60 countries or regions. Among these, 43 met the predefined threshold for inclusion in the country-level co-authorship analysis. Figure 2C shows the geographical distribution of the publications, Figures 2D,E present the countries or regions with the highest publication output, and Table 1 summarizes the top 10 countries according to publication output and citation indicators. The United States had the highest number of publications, with 248 papers, accounting for 23.77% of the total. India ranked second with 246 publications (23.59%), followed by China with 218 publications (20.90%). South Africa contributed 97 publications (9.30%), the United Kingdom 90 publications (8.62%), France 47 publications (4.51%), and Italy 44 publications (4.22%) (Figures 2D,E and Table 1). Citation indicators showed differences from the ranking based on publication output. Publications from the United States received the highest total number of citations (*n* = 2,460), followed by China (*n* = 1,204), the United Kingdom (*n* = 934), India (*n* = 802), and South Africa (*n* = 720) (Figure 3A and Table 1). For average citations per publication, the United Kingdom ranked first, with 10.38 citations per paper, followed by the United States (9.92) and Italy (7.91) (Figure 3B and Table 1). The United States also had the highest H-index (29), followed by China (20), the United Kingdom (17), India (16), and South Africa (15) (Figure 3C and Table 1). Overall, the country-level results show that publication output and citation-based indicators were not fully consistent across countries or regions. Therefore, publication counts, total citations, average citations per publication, and H-index were reported separately to describe different aspects of country-level research performance.

**TABLE 1:** Top 10 countries by publication output and their citation indicators.

Country	Publications	Total citations	Average citations per publication	H-index
United States	248	2,460	9.92	29
India	246	802	3.26	16
China	218	1,204	5.52	20
South Africa	97	720	7.42	15
United Kingdom	90	934	10.38	17
France	47	127	2.70	6
Italy	44	348	7.91	7
Brazil	41	238	5.80	6
Saudi Arabia	35	64	1.83	5
Germany	34	228	6.71	7

**FIGURE 3:**
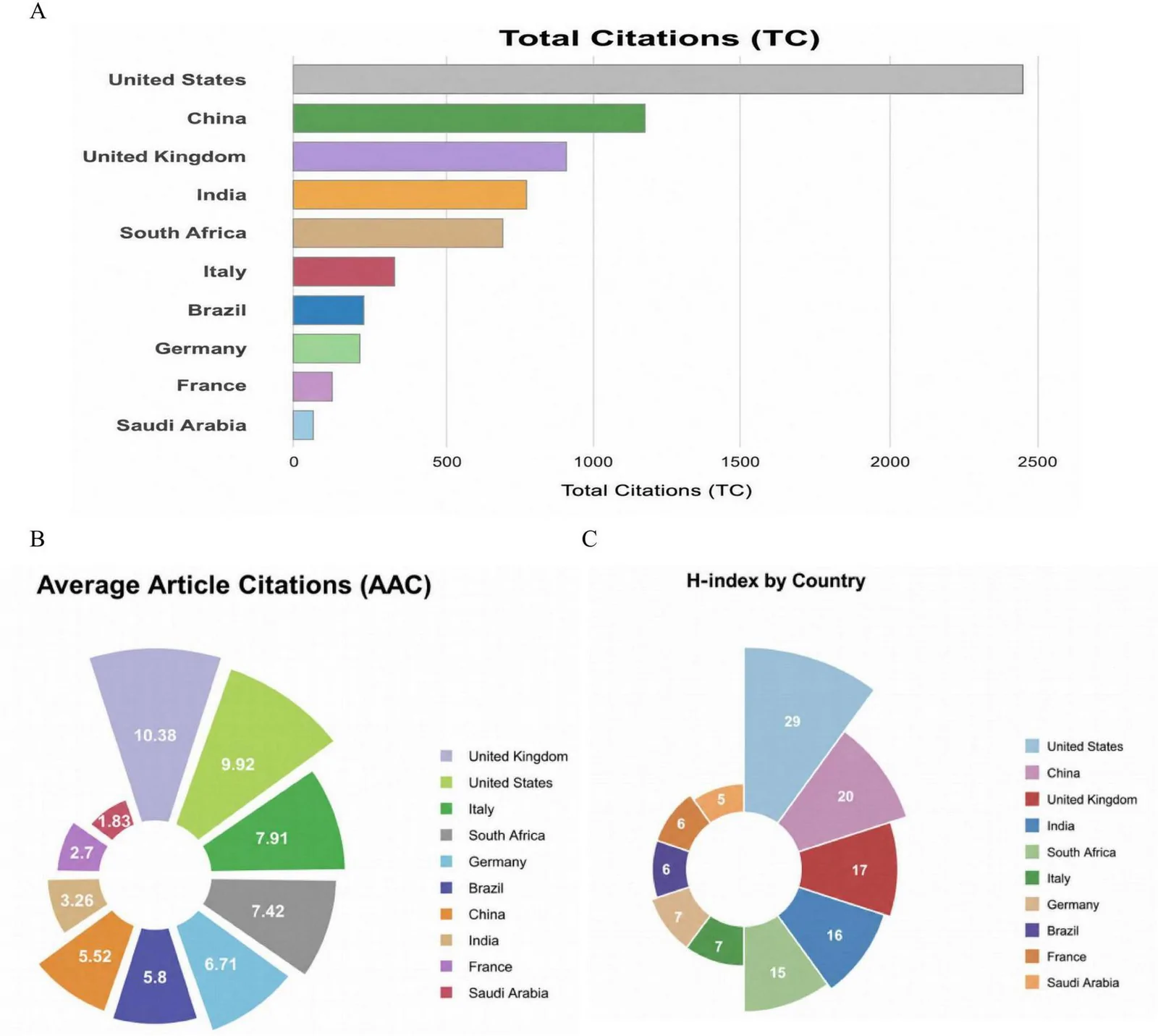
Country-level citation impact indicators. **(A)** Total citation counts for each of the top 10 countries. **(B)** Average citation counts for articles published by different countries. **(C)** List and statistics of countries with the highest h-index.

### 3.4 Analysis of collaboration among countries and institutions

This study utilized VOSviewer to conduct a visual analysis of the collaborative relationships among the countries included in the literature (Figure 4A). Within the global collaboration network, The United States, China, and India were the leading contributors in terms of publication output and total link strength, The United States had the highest total link strength (TLS = 98), followed by China (TLS = 83) and India (TLS = 79). The United Kingdom, France, and Spain ranked next, with TLS values of 71, 62, and 54, respectively.

**FIGURE 4:**
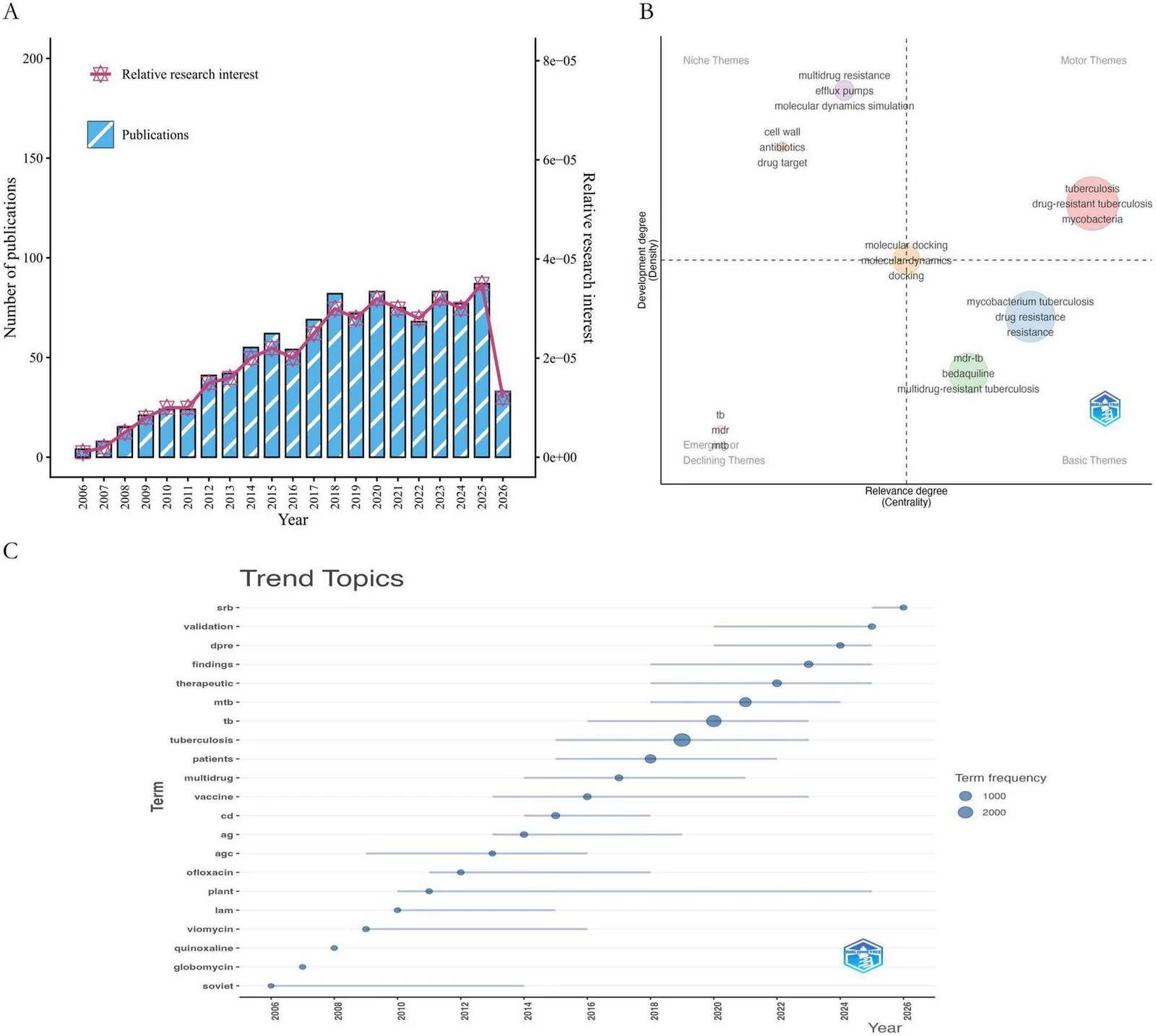
Global collaboration network and hotspot association map for DR-TB research. **(A)** Global collaboration network among countries and regions. **(B)** Collaboration network among major academic institutions. **(C)** Tri-graph association analysis of academic institutions (left), core keywords (center), and high-output countries (right).

Further analysis of institutional collaboration is shown in Figure 4B. Capital Medical University (36 publications), the University of Cape Town (29), Johns Hopkins University (27), the Chinese Academy of Sciences (22), and the University of KwaZulu-Natal (18) showed relatively high publication output and/or connectivity within the analyzed collaboration network. Network clustering also identified several transnational collaboration groups, including links involving Johns Hopkins University, the U.S. National Institute of Allergy and Infectious Diseases, and the University of Cape Town. The three-field plot in Figure 4C further illustrates associations among institutions, keywords, and high-output countries/regions. Terms including *Mycobacterium tuberculosis*, DR-TB, mechanism of action, *in vitro* studies, multidrug resistance, identification, and expression showed frequent associations with major contributing countries and institutions. These patterns describe the thematic and collaborative structure of the retrieved literature.

### 3.5 Analysis of authors, countries, and institutional collaborations

Author co-authorship analysis was performed using VOSviewer (Figure 5A). Pang Y, Viveiros M, Hernandez-Pando R, and Zhang T showed the highest total link strength values, with TLS values of 38, 33, 29, and 27, respectively (Table 2). These results indicate relatively frequent co-authorship links among these authors within the analyzed dataset.

**FIGURE 5:**
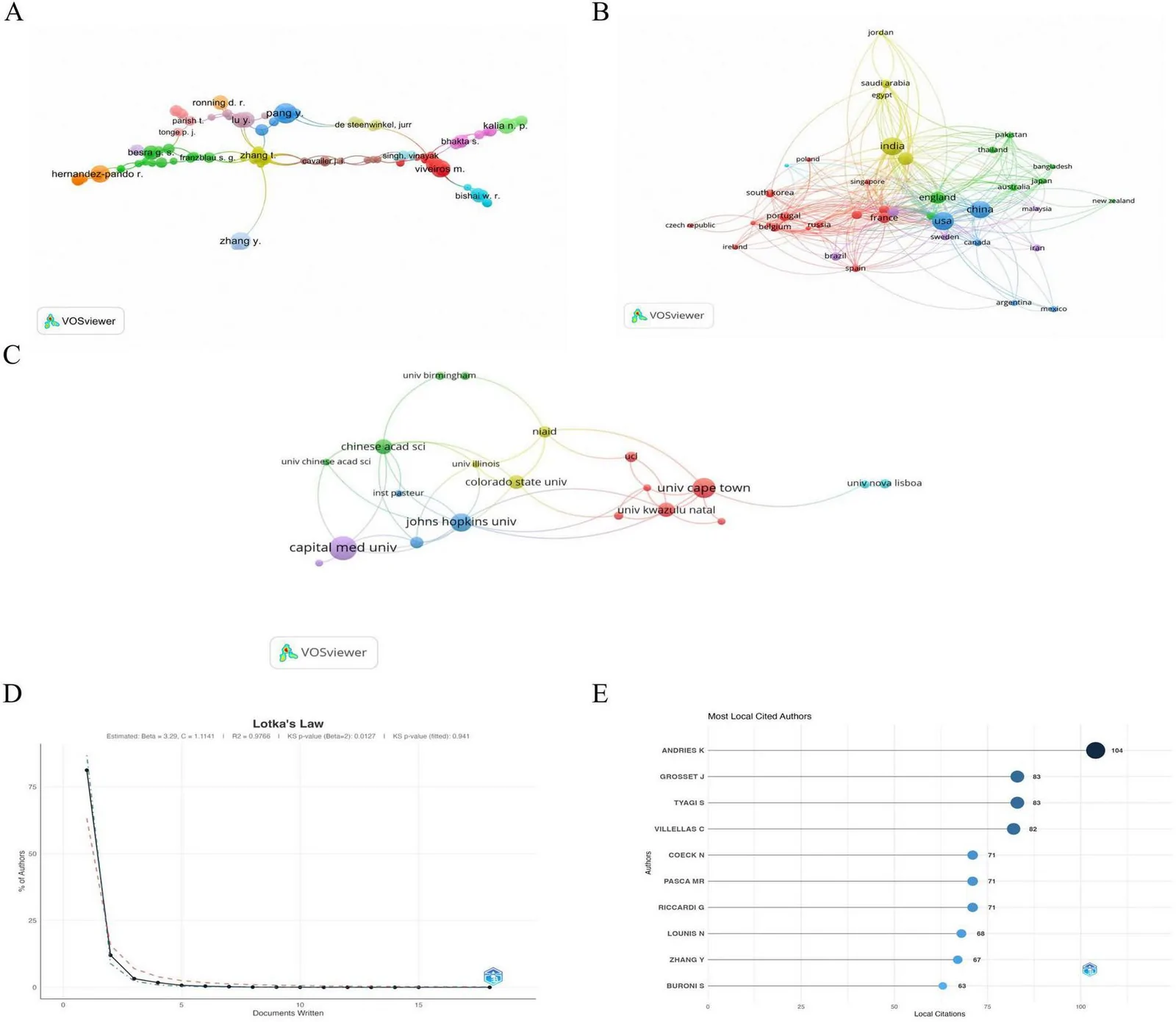
Analysis of the global co-authorship network and scholarly influence in research on the mechanisms of DR-TB. **(A)** Co-authorship co-occurrence network of core authors. **(B)** Co-authorship co-occurrence network of major countries/regions. **(C)** Co-authorship co-occurrence network of key academic institutions. **(D)** Top 10 rankings of local citation frequency (LocalCitations) for core authors. **(E)** Fitting curve of the frequency distribution of author research productivity based on Lotka’s Law.

**TABLE 2:** Analysis of authors’ publication output and collaborations (top 10).

Author	Documents	Citations	Total link strength
Pangy	14	580	38
Viveirosm	12	466	33
Hernandez-Pandor	8	373	29
Zhangt	8	351	27
Luy	7	262	26
Besrags	7	110	22
Kalianp	6	128	19
Zhangy	6	216	17
Bishaiwr	5	143	14
Ronningdr	5	127	11

At the country level, 43 of the 60 countries or regions met the predefined threshold for co-authorship analysis (Figure 5B). The United States had the highest total link strength (TLS = 98), followed by China (TLS = 83) and India (TLS = 79). The United Kingdom, France, Spain, and South Korea also showed co-authorship links with other countries or regions (Table 3).

**TABLE 3:** Top 10 countries by total link strength in the co-authorship network.

Country	Total link strength
USA	98
China	83
India	79
United Kingdom	71
France	62
Spain	54
South Korea	47
Canada	41
Saudi Arabia	36
Brazil	32

Institutional co-authorship analysis included the top 20 institutions (Figure 5C). Capital Medical University had the highest total link strength (TLS = 46), followed by the University of Cape Town (TLS = 32) and Johns Hopkins University (TLS = 27). Chinese Academy of Sciencess and the University of KwaZulu-Natal were also among the top five institutions by total link strength (Table 4).

**TABLE 4:** Analysis of publication output and collaborations by organization (top 10).

Organization	Documents	Citations	Total link strength
Capital Medical University	36	1401	46
University of Cape Town	29	1154	32
Johns Hopkins University	27	838	27
Chinese Academy of Sciences	22	716	23
University of KwaZulu-Natal	18	568	22
Colorado State University	18	591	20
National Institute of Allergy and Infectious Diseases	18	289	16
University of Birmingham	18	286	14
Institut Pasteur	16	300	11
University College London	16	240	11

Local citation analysis was used to identify authors frequently cited within the retrieved dataset (Figure 5D). Andries K had the highest number of local citations (*n* = 104), followed by Grosset J (*n* = 83), Tyagi S (*n* = 83), Villellas C (*n* = 82), Coeck N (*n* = 71), Pasca MR (*n* = 71), Riccardi G (*n* = 71), Lounis N (*n* = 68), Zhang Y (*n* = 67), and Buroni S (*n* = 63). These results reflect citation frequency within the analyzed dataset and should not be interpreted as a comprehensive measure of overall scientific contribution.

Lotka’s law was used to assess the distribution of author productivity (Figure 5E). The fitted model yielded β = 3.29, *C* = 1.11, *R*^2^ = 0.98, and a Kolmogorov–Smirnov test (KS test) *P*-value of 0.94. The non-significant KS result indicated no statistically detectable difference between the observed author-productivity distribution and the fitted Lotka distribution. Author productivity showed a skewed distribution, with 83.60% of authors contributing one publication and 1.10% contributing five or more publications. This pattern is consistent with the common distribution of scientific output, in which most authors contribute occasionally and a smaller proportion publish repeatedly within the same research field.

### 3.6 Research fields and journal analysis

A dual-map overlay of journals illustrates the citation pathways between the major disciplinary domains involved in research on DR-TB mechanisms (Figure 6A). The map indicates that publications in medicine, clinical research, and healthcare frequently draw on knowledge from molecular biology, genetics, and immunology. Citation pathways involving infectious diseases, environmental science, toxicology, and public health further demonstrate the multidisciplinary nature of this field. These patterns suggest increasing integration between basic microbiological research, drug discovery, clinical medicine, and population-health perspectives.

**FIGURE 6:**
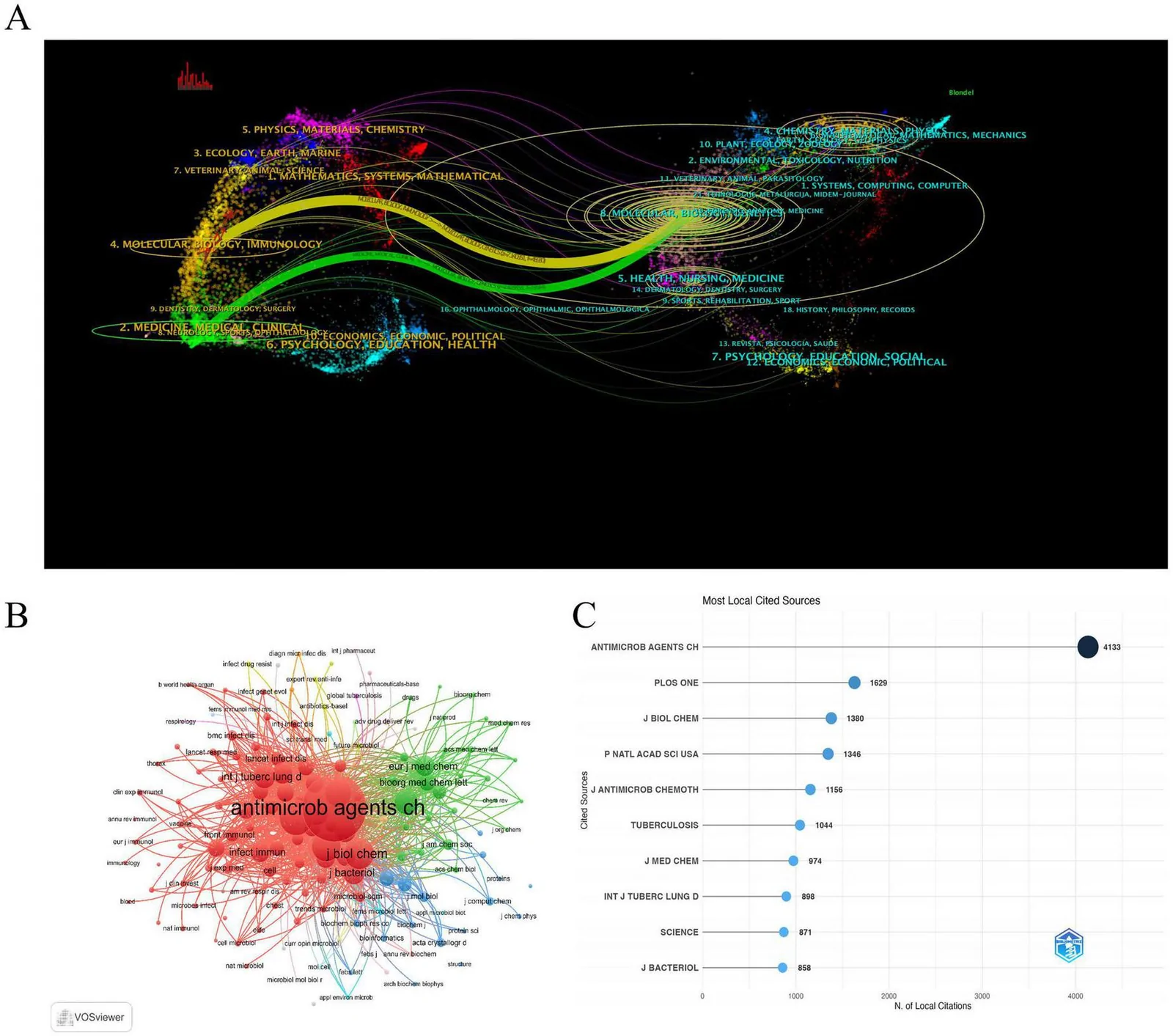
Journal-level knowledge structure and citation characteristics. **(A)** Dual-map overlay illustrating citation pathways between citing and cited disciplinary domains. **(B)** co-citation network of the principal cited journals. **(C)** Top 10 locally cited sources in the cited-journal network.

Table 5 presents the 10 most productive journals in the analyzed dataset. Scientific Reports published the largest number of articles (*n* = 11), followed by Clinical Infectious Diseases (*n* = 10) and PLOS ONE (*n* = 8). Frontiers in Microbiology and BMC Infectious Diseases each published seven articles, whereas Nature Communications published six. Among these journals, Clinical Infectious Diseases had the highest citation count and total link strength, with 459 citations and a total link strength of 479, respectively.

**TABLE 5:** Analysis of publishing journals (top 10).

Source	Documents	Citations	Total link strength
Scientific Reports	11	240	262
Clinical Infectious Diseases	10	459	479
PLOs One	8	162	178
Frontiers in Microbiology	7	98	112
BMC Infectious Diseases	7	51	65
Nature communications	6	275	287
Computational and Structural Biotechnology Journal	5	173	183
Frontiers in Pharmacology	5	165	175
Briefings in Bioinformatics	5	91	101
Diagnostics	5	27	37

Figure 6B depicts the co-citation network of cited journals, in which Antimicrobial Agents and Chemotherapy, the Journal of Biological Chemistry, and the Journal of Bacteriology occupied prominent network positions. These positions reflect their connectivity within the cited-journal network rather than publication output in the present dataset.

Figure 6C presents the 10 most locally cited sources in the cited-journal network. Antimicrobial Agents and Chemotherapy ranked first, with 4,133 local citations, followed by PLOS ONE (1,629), the Journal of Biological Chemistry (1,380), the Proceedings of the National Academy of Sciences of the United States of America (1,346), and the Journal of Antimicrobial Chemotherapy (1,156). Other prominent locally cited sources included Tuberculosis (1,044), the Journal of Medicinal Chemistry (974), the International Journal of Tuberculosis and Lung Disease (888), Science (871), and the Journal of Bacteriology (858). Their prominence in the local citation network indicates that these journals were frequently referenced by the publications included in the present dataset, but does not independently establish the quality or clinical importance of individual studies.

### 3.7 Co-citation analysis

To identify publications that were frequently cited together within the analyzed dataset, co-citation and local citation analyzes were performed using VOSviewer (Figures 7A,B). Makarov et al. (2009), Andries et al. (2014), and Almeida et al. (2016), were the three most frequently co-cited publications in the analyzed dataset. Citation-burst analysis using CiteSpace (Figure 7C) identified references that experienced marked increases in citation attention during specific periods. Andries et al. (2014) showed the strongest citation burst (strength = 8.34; 2016–2019), followed by Almeida D (6.74) and Makarov V (6.40). More recent references, including Singh et al. (2020), Allué-Guardia et al. (2021), also showed citation bursts, indicating increased citation attention to the topics represented by these studies during recent years.

**FIGURE 7:**
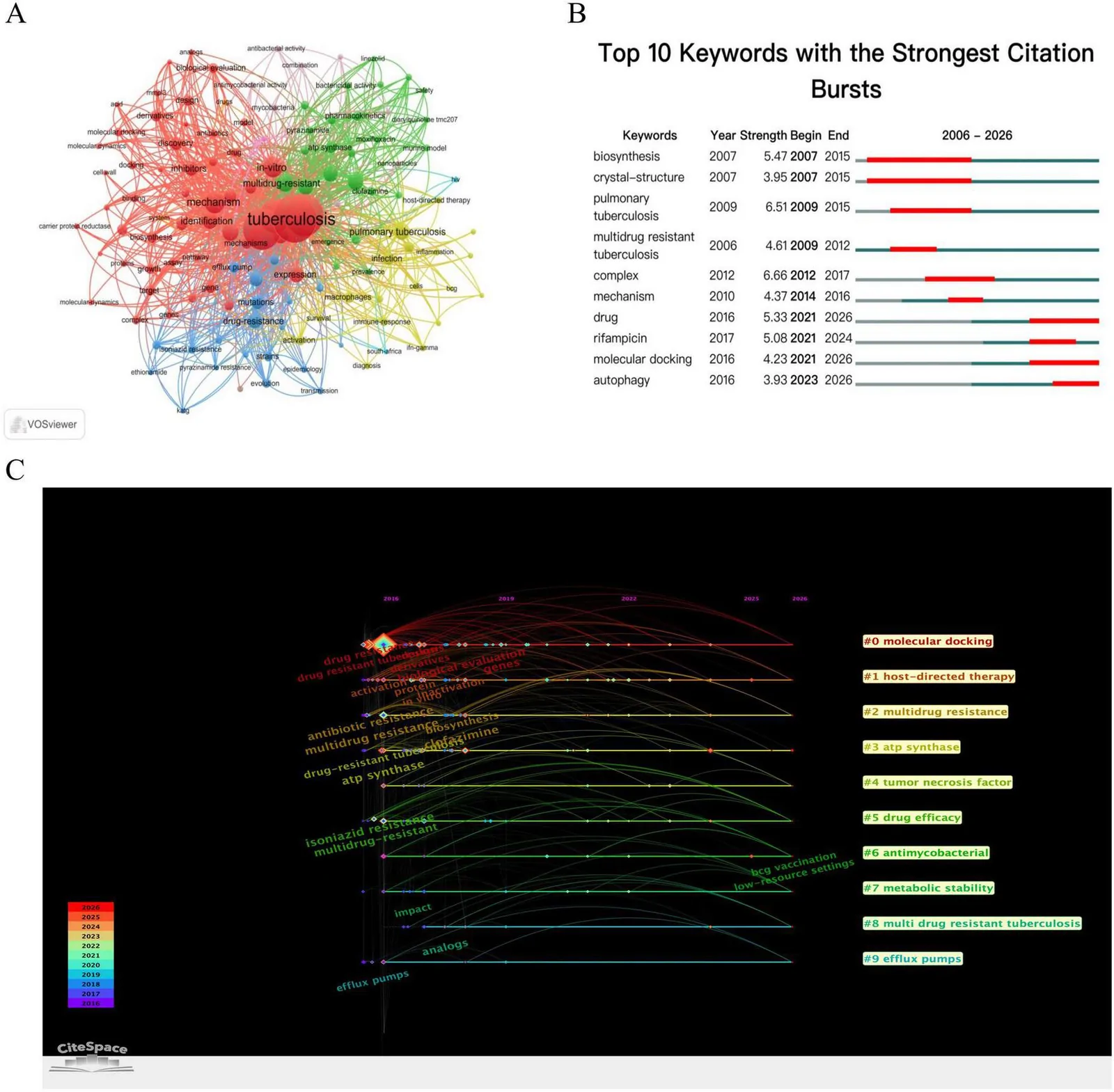
Co-citation and citation emergence analysis of core literature on the mechanisms of DR-TB**. (A)** Co-citation network of core literature. **(B)** Ranking of local citation frequencies. **(C)** Citation emergence (analysis of the top 10 references).

### 3.8 Keyword co-occurrence analysis

Figure 8A shows the keyword co-occurrence analysis generated using VOSviewer. Frequently occurring keywords included “tuberculosis,” “multidrug resistance,” “resistance mechanisms,” “molecular docking,” and “efflux pumps.” These terms suggest that the included studies mainly focused on drug-resistant *Mycobacterium tuberculosis*, mechanisms of resistance, potential drug targets, and *in vitro* evaluation of related compounds.

**FIGURE 8:**
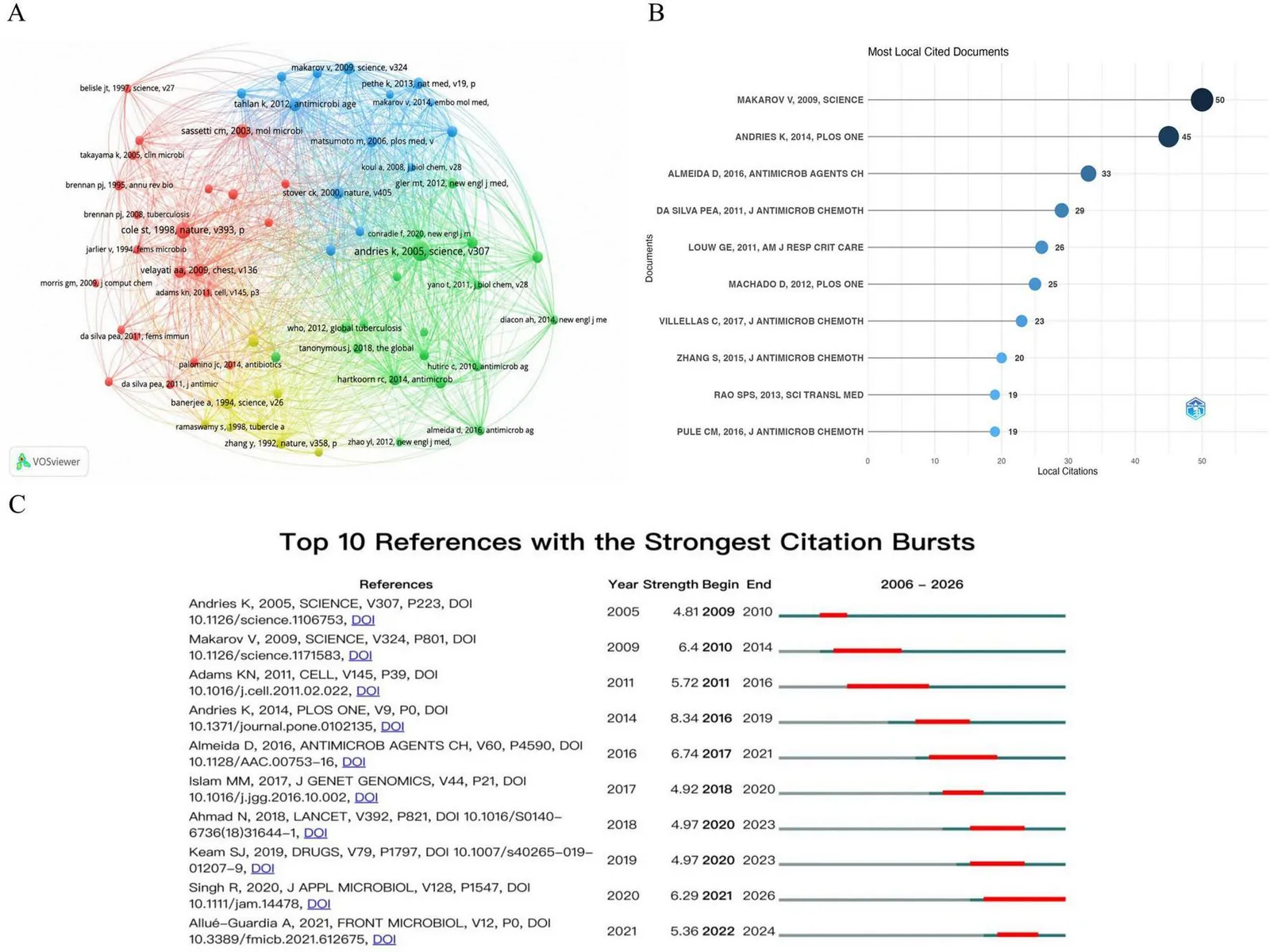
A bibliometric visualization of the mechanisms of tuberculosis drug resistance**. (A)** A co-occurrence network of keywords generated using VOSviewer. **(B)** A plot of the top 10 keywords with the strongest citation emergence. **(C)** A timeline of keyword clusters generated using CiteSpace.

Figure 8B presents the top 10 keywords with the strongest burst intensity. Earlier burst terms included “biosynthesis” and “crystal structure,” indicating attention to biological and structural features of *Mycobacterium tuberculosis*. More recent burst terms included “drug” and “molecular docking” from 2021 to 2026, “rifampicin” from 2021 to 2024, and “autophagy” from 2023 to 2026. These results suggest that recent studies have increasingly addressed computational drug-screening approaches, rifampicin-related resistance, and host-related mechanisms such as autophagy.

The timeline analysis in Figure 8C further shows the temporal distribution of keyword clusters from 2016 to 2026. Molecular docking and host-directed therapies appeared as recent co-occurrence-based keyword clusters, while Adenosine triphosphate (ATP) synthase and efflux pumps continued to appear across the analyzed period. Overall, the keyword results indicate that research attention has gradually expanded from structural and phenotypic topics toward drug-target exploration, resistance-associated pathways, and host-related mechanisms.

### 3.9 Research hotspots and trends

Figure 9A shows the annual publication trend and relative research interest in tuberculosis drug-resistance mechanism studies between 2006 and 2026. Overall, both indicators showed an upward trend during the study period, although the 2026 data only included records indexed up to 10 May 2026. These results suggest that research on DR-TB resistance mechanisms has received sustained attention in recent years. However, the clinical relevance of specific emerging topics requires further validation.

**FIGURE 9:**
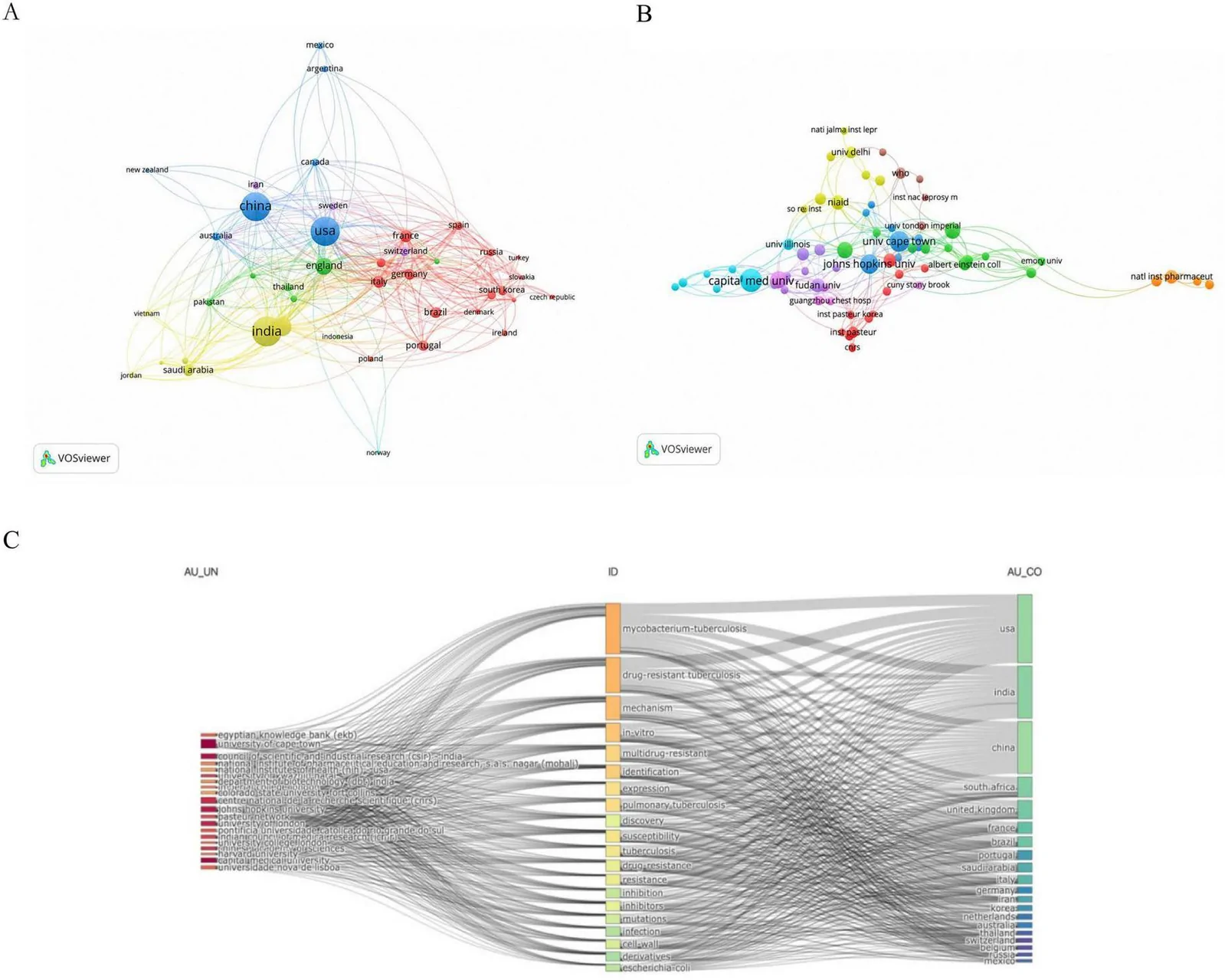
A map of bibliometric hotspots and trends in the field of tuberculosis drug resistance mechanisms. **(A)** Trend chart showing annual publication volume and relative research interest. **(B)** Thematic strategic map based on keywords. **(C)** Timeline of evolving trends.

The thematic map in Figure 9B presents the distribution of major research themes according to centrality and density. The motor-theme quadrant included “tuberculosis,” “drug-resistant tuberculosis,” and “mycobacteria,” suggesting that these topics were relatively well developed and closely associated with other themes in the analyzed literature. The basic-theme quadrant included “*Mycobacterium tuberculosis*,” “drug resistance,” “resistance,” “MDR-TB,” “bedaquiline,” and “multidrug-resistant tuberculosis.” The niche-theme quadrant included “efflux pumps,” “cell wall,” “drug targets,” and “molecular dynamics simulation,” indicating more specialized topics within the field. Molecular docking was positioned at the intersection of the median centrality and density reference lines, with a Callon centrality of 0.67 and a Callon density of 16.92. These values corresponded to the median centrality and density across the seven identified thematic clusters, quantitatively supporting its central position in the thematic map.

The trend-topic analysis in Figure 9C showed temporal changes in research terms. Earlier terms included “ofloxacin,” “viomycin,” and “quinoxaline,” whereas more recent terms included “validation,” “DprE,” “findings,” and “therapeutic.” These changes suggest that recent studies have increasingly addressed target validation, DprE-related mechanisms, and therapeutic evaluation. Overall, the thematic results indicate a gradual shift from studies of individual drugs and compounds toward mechanism-oriented and target-related research topics.

### 3.10 External validation using the PubMed database

To assess the robustness of the principal findings and reduce potential bias associated with reliance on a single bibliographic database, we conducted a cross-database validation using a separately retrieved PubMed dataset. WoSCC was retained as the primary source for bibliometric mapping, whereas PubMed served as a supplementary validation dataset. The difference in the number of included publications between PubMed (*n* = 787) and WoSCC (*n* = 1,043) may reflect differences in database coverage, indexing practices, searchable fields, and metadata structures.

Annual publication patterns were broadly comparable between the two databases (Figure 10A), with both showing sustained growth after 2015. Quantitatively, annual publication counts showed a strong positive correlation between WoSCC and PubMed (Spearman’s ρ = 0.95, *P* < 0.01). After excluding the incomplete year 2026, both databases identified 2025 as the peak publication year, with 90 publications in WoSCC and 86 in PubMed. Country-level distributions also showed substantial overlap (Figure 10B). Eight of the top 10 productive countries were shared between the two databases, corresponding to a Jaccard index of 0.67.

**FIGURE 10:**
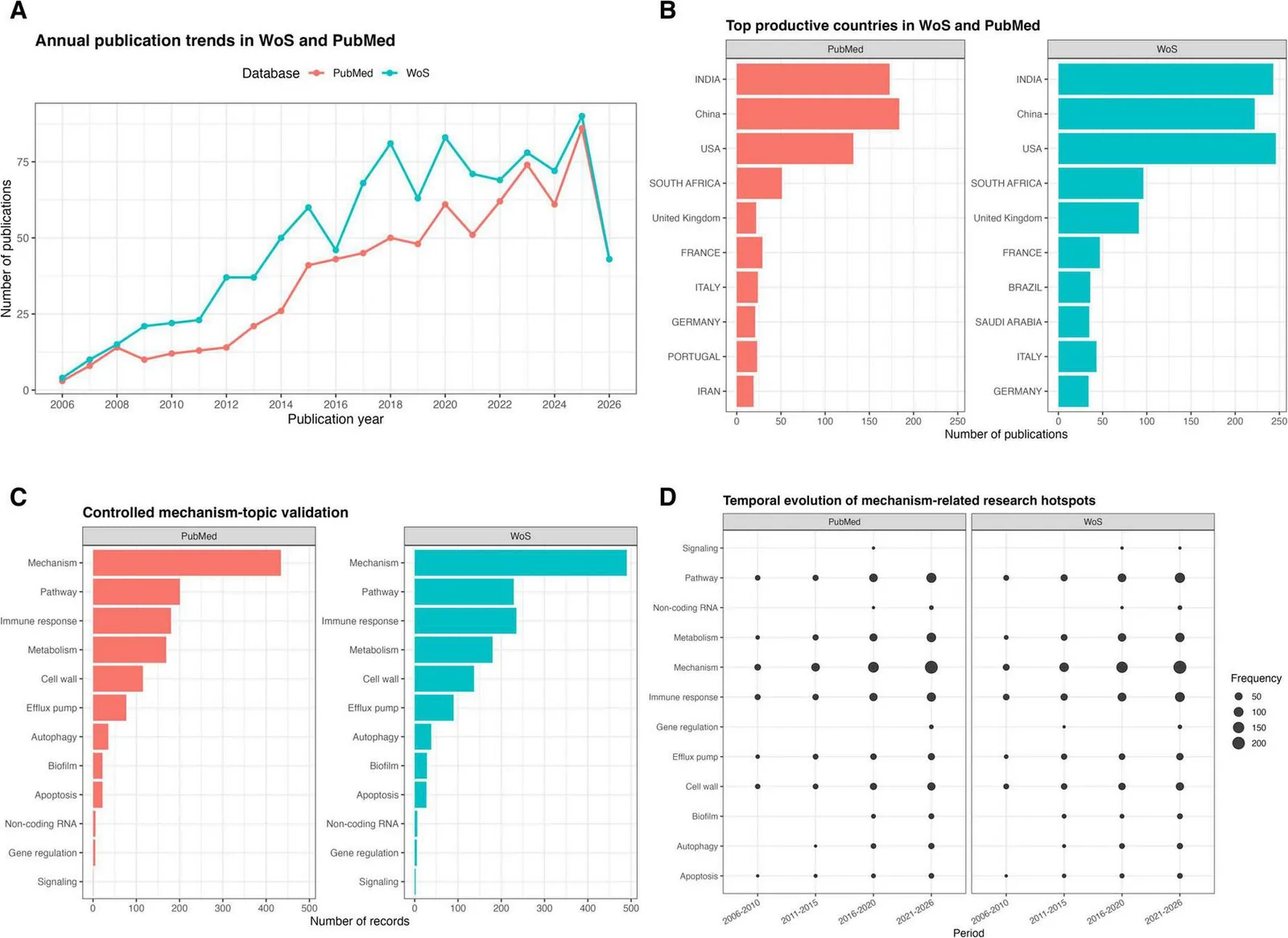
Cross-database validation of drug-resistant tuberculosis mechanism research in PubMed and WoSCC. **(A)** Annual publication trends (January 1, 2006–May 10, 2026). **(B)** Leading contributing countries. **(C)** Frequencies of predefined mechanism-related terms. **(D)** Temporal distribution of mechanism-related themes across four periods; bubble size represents term frequency. WoSCC, Web of Science Core Collection.

Thematic comparisons (Figures 10C,D) demonstrated broadly similar distributions across research on resistance mechanisms, host immune regulation, metabolic adaptation, cell-wall processes, and efflux-related mechanisms. All 10 predefined mechanism-related topics overlapped between the two datasets, corresponding to a Jaccard index of 1.00. Period-specific hotspot similarity was also high, with Jaccard indices ranging from 0.80 to 1.00 and a mean value of 0.95. Because several mechanism-related terms were predefined in the search strategy, the complete overlap of the predefined mechanism-related topics was interpreted as supportive evidence of cross-database thematic consistency rather than as independent confirmation that these topics represented research hotspots.

Overall, the PubMed-based validation showed quantitatively and qualitatively comparable temporal, geographical, and thematic patterns, reducing the likelihood that the principal findings were attributable solely to the coverage characteristics of WoSCC. Nevertheless, PubMed was used for supplementary validation rather than a fully integrated multi-database bibliometric analysis, and differences in indexing and metadata structures should be considered when interpreting the comparisons.

## 4 Discussion

### 4.1 General development of DR-TB resistance-mechanism research

The publication landscape of DR-TB resistance-mechanism research expanded steadily during the study period, with a more pronounced increase after 2015, indicating sustained scholarly attention to this field. This increase coincided with continued clinical and public-health concern regarding multidrug-resistant and extensively drug-resistant tuberculosis. More importantly, the thematic analyzes indicate that, alongside drug-susceptibility phenotypes and classical resistance-associated mutations, the literature encompasses a broader range of topics, including efflux mechanisms, cell-wall processes, metabolic adaptation, host immune responses, biofilm formation, programmed cell death, and computational approaches.

This broader thematic scope is broadly consistent with previous reviews of DR-TB resistance mechanisms, which have addressed resistance-associated mutations and canonical molecular mechanisms together with bacterial adaptation, host–pathogen interactions, and therapeutic strategies beyond conventional antibacterial targets (Palomino and Martin, 2014; Swain et al., 2020; Borah P. et al., 2021). Within this broader research landscape, the mycobacterial cell wall has continued to be investigated as a therapeutic target, including antimicrobial peptide-based approaches (Jacobo-Delgado et al., 2023). Advanced nanosystems have also been explored as drug-delivery platforms and adjunctive strategies (Sharma et al., 2025), while bacteriophage-based approaches (Yang et al., 2024; Janssen et al., 2025) and human lung organoid, animal, and genomic models have been developed for mechanistic investigation and preclinical evaluation (Bucsan et al., 2019; Kim et al., 2024). These developments illustrate the increasing methodological and translational diversity of DR-TB research.

Taken together, the bibliometric patterns indicate that DR-TB resistance-mechanism research is not confined to mutation- and phenotype-oriented questions but encompasses a broader research landscape involving bacterial adaptation, drug–target interactions, host-related mechanisms, and translational approaches. However, these findings should be interpreted within the scope of bibliometric analysis. Publication counts, citation indicators, keyword frequencies, and thematic prominence reflect patterns of research activity and scholarly attention rather than direct evidence of biological causality or clinical effectiveness. Accordingly, the prominence of a topic in the present analysis should not be interpreted as evidence that the corresponding mechanism is intrinsically more important or clinically effective.

### 4.2 Country contributions and citation performance

The geographical distribution of publications showed broad international participation but substantial variation in research output across countries and regions. The United States, India, and China were consistently among the major contributors, while South Africa and the United Kingdom also showed substantial research activity. Notably, several highly productive countries are also settings in which tuberculosis and drug-resistant tuberculosis remain important public-health concerns, indicating that the global research landscape encompasses both research-intensive systems and high-burden settings.

Publication output and citation performance, however, did not show identical geographical patterns. India provides a notable example, ranking among the leading countries in publication output while showing a comparatively lower position in total citations. Countries with high publication output did not necessarily have the highest average citation impact, suggesting that research productivity and citation-based influence represent different dimensions of bibliometric performance. Citation indicators may also be affected by publication year, journal distribution, collaboration patterns, research topic, citation practices, and the presence of highly cited studies. Differences in research priorities, publication environments, and collaboration opportunities between high-burden and research-intensive settings may also contribute to such patterns, although these factors were not formally evaluated in the present study. Therefore, country-level citation metrics should be interpreted cautiously and should not be regarded as direct measures of research quality or clinical importance.

The substantial contribution of countries with considerable tuberculosis burdens also highlights the importance of interpreting bibliometric patterns within a broader epidemiological and public-health context. Research conducted in such settings may provide access to clinical isolates, treatment-related observations, and epidemiological information relevant to mechanism-oriented studies. At the population level, One Health investigations may further complement conventional surveillance by identifying zoonotic transmission pathways and sources of exposure that may otherwise be overlooked (Martínez-Lirola et al., 2023), while long-term surveillance remains essential for characterizing the evolution and global distribution of drug-resistant tuberculosis (Dean et al., 2022). These broader considerations suggest that national research activity should be interpreted alongside disease burden, surveillance needs, and the local research environment rather than on the basis of publication volume alone.

Overall, national contributions to DR-TB resistance-mechanism research should be interpreted by considering research productivity, citation patterns, and public-health context together. The geographical patterns identified in this study describe the distribution of scholarly activity but do not, by themselves, establish the underlying reasons for differences in research output among countries.

### 4.3 International cooperation and institutional contributions

The collaboration analysis showed that international cooperation is a prominent feature of DR-TB resistance-mechanism research. The United States, China, and India were among the most highly connected countries, while several European countries also participated actively in the international collaboration network. The overlap between high publication output and strong network connectivity suggests that major contributing countries are also frequently involved in collaborative research. This pattern is consistent with the multidisciplinary nature of DR-TB mechanism studies, which often require complementary expertise in microbiology, molecular biology, pharmacology, genomics, computational analysis, and clinical research.

At the institutional level, the collaboration network included major contributors from China, South Africa, and the United States, including Capital Medical University, the University of Cape Town, Johns Hopkins University, the Chinese Academy of Sciences, and the University of KwaZulu-Natal. Their distribution across different geographical and research settings illustrates the international character of the field and the participation of institutions located in both research-intensive systems and regions with substantial tuberculosis burdens. Such geographical diversity may facilitate the integration of laboratory-based approaches with clinical, epidemiological, and population-level perspectives. However, institutional network connectivity should be interpreted as a measure of collaboration within the retrieved literature rather than as a direct indicator of research quality or institutional capacity.

The country–institution–keyword analysis further showed that major contributing countries and institutions were frequently associated with terms such as *Mycobacterium tuberculosis*, DR-TB, mechanism of action, *in vitro* studies, multidrug resistance, identification, and expression. Rather than indicating the biological priority of these topics, these associations describe the thematic structure linking major contributors within the analyzed literature. Collectively, they suggest that collaborative research in this field has centered on several interconnected areas, including resistance mechanisms, drug–target interactions, experimental evaluation, and molecular characterization.

### 4.4 Authors and cited contributors

Author-level analyzes revealed different patterns of collaboration, citation, and productivity within the DR-TB resistance-mechanism literature. Several authors, including Pang Y, Viveiros M, Hernandez-Pando R, and Zhang T, showed relatively high co-authorship connectivity, indicating frequent participation in collaborative research within the analyzed network. Such co-authorship indicators describe patterns of scientific collaboration but should not be interpreted as direct measures of research quality or individual scientific importance.

Local citation analysis identified a different group of frequently cited contributors, including Andries K, Grosset J, Tyagi S, Villellas C, Coeck N, Pasca MR, Riccardi G, Lounis N, Zhang Y, and Buroni S. Their cited work was commonly associated with anti-tuberculosis drug mechanisms, resistance-related pharmacology, experimental drug evaluation, and therapeutic target research, including topics such as bedaquiline and DprE1. This pattern suggests that drug-target and pharmacological studies constitute a prominent component of the cited knowledge base within the retrieved literature. However, local citation frequency reflects citation activity within the analyzed dataset and does not represent a comprehensive measure of an author’s overall contribution to the broader DR-TB field.

Author productivity showed a markedly skewed distribution consistent with Lotka’s law, with most authors contributing only occasionally and a much smaller proportion publishing repeatedly in the field. The non-significant KS test indicated no statistically detectable difference between the observed distribution and the fitted Lotka distribution, supporting compatibility with Lotka’s inverse power-law pattern. However, alternative productivity distributions were not formally compared in the present analysis, and the result should therefore not be interpreted as demonstrating that Lotka’s law is the uniquely optimal model. Such a pattern is commonly observed in scientific publishing and, in the context of DR-TB mechanism research, may partly reflect the specialized and multidisciplinary nature of the work. Studies in this area frequently involve microbiological experiments, drug testing, molecular assays, genomic analyzes, and computational modeling, which may favor sustained participation by specialized research groups. Nevertheless, the bibliometric data do not allow direct assessment of the underlying organizational or resource-related reasons for differences in author productivity.

### 4.5 Journal distribution and disciplinary scope

The journal distribution highlights the multidisciplinary character of DR-TB resistance-mechanism research. Publications were distributed across journals in microbiology, infectious diseases, pharmacology, molecular biology, medicinal chemistry, and general biomedical science, indicating that the field extends beyond a single disciplinary domain. This broad distribution is consistent with the complexity of tuberculosis drug resistance, which involves molecular mechanisms, bacterial physiology, antimicrobial pharmacology, drug development, clinical management, and population-level research. The comparatively high publication volume of an individual journal should not, however, be interpreted as evidence that it is the most influential outlet in the field. Journal productivity may also be affected by editorial scope, overall publication volume, publishing model, article-processing policies, and indexing coverage, factors that cannot be disentangled from the present bibliometric data.

Citation patterns further illustrate the interaction between basic and clinically oriented research. Clinically focused infectious-disease journals showed substantial citation connectivity within the analyzed dataset, while frequently cited journals in antimicrobial pharmacology, molecular biology, bacterial physiology, and medicinal chemistry formed an important part of the cited knowledge base. In particular, the prominent position of Antimicrobial Agents and Chemotherapy in the cited-journal network is consistent with the strong representation of studies addressing antimicrobial activity, mechanisms of action, resistance-associated processes, and pharmacological evaluation. However, journal-level citation and network indicators reflect citation activity and connectivity within the retrieved literature rather than the intrinsic quality or clinical importance of individual publications.

The dual-map overlay further demonstrated cross-disciplinary citation pathways. Publications in medicine, clinical research, and healthcare frequently drew on literature from molecular biology, genetics, and immunology, while additional citation links involved infectious diseases, environmental science, toxicology, and public health. These patterns suggest that DR-TB resistance-mechanism research increasingly connects laboratory-based investigation with drug discovery, clinical research, and broader population-health perspectives.

### 4.6 Co-cited references and knowledge base

Co-citation analysis identified several references that were frequently cited together by the publications included in the dataset. The most frequently co-cited studies included Makarov V et al. published in Science, Andries K et al. published in PLOS ONE, and Almeida D et al. published in Antimicrobial Agents and Chemotherapy. These publications were mainly related to novel anti-tuberculosis drug targets, bedaquiline-associated mechanisms, and preclinical or translational evaluation of anti-tuberculosis drugs.

Citation-burst analysis provided a temporal perspective on the cited knowledge base. Burst references involving studies by Andries K, Almeida D, and Makarov V indicate periods of concentrated citation attention to drug mechanisms, compound evaluation, and target identification. These patterns suggest that such topics constituted prominent components of the actively cited literature during specific periods. Such bursts may also reflect temporally clustered citation activity around particular topics or influential publications rather than sustained field-wide influence. However, citation bursts reflect rapid increases in citation frequency and should therefore be interpreted as indicators of heightened scholarly attention rather than evidence of greater biological or clinical importance.

More recent burst references, including those by Singh R and Allué-Guardia A, suggest that the cited literature has continued to evolve. The appearance of these more recent burst references alongside earlier drug-target and mechanism-of-action studies is consistent with a broadening of scholarly attention over time. However, citation bursts primarily reflect periods of rapidly increasing citation activity and should not be interpreted as direct evidence of the scientific or clinical importance of individual studies.

Overall, the co-citation and citation-burst patterns suggest a cumulative evolution of the field’s cited knowledge base. Drug-target discovery, mechanism-of-action studies, and translational drug evaluation formed prominent components of the earlier cited literature, while subsequent research has encompassed a broader range of questions related to drug resistance and its underlying mechanisms. This pattern is better interpreted as an evolution in citation structure and scholarly attention rather than as evidence that later research themes are biologically more important than earlier ones.

### 4.7 Keyword evolution and thematic development

Keyword co-occurrence analysis showed that tuberculosis, multidrug resistance, resistance mechanisms, molecular docking, and efflux pumps were among the main terms in the field. These keywords indicate that current research remains focused on resistant *Mycobacterium tuberculosis*, mechanisms of drug resistance, and the identification of potential therapeutic targets. The presence of molecular docking among the main terms suggests that computational methods have become more frequently used in recent DR-TB mechanism research. This computational shift is also reflected in model-informed strategies for the development of anti-tuberculosis drug combinations (Van Wijk et al., 2020) and network-based multi-omics integration for drug discovery (Jiang et al., 2025).

Keyword-burst analysis further revealed temporal changes in research attention. Earlier burst terms, such as biosynthesis and crystal structure, were associated mainly with biological and structural investigations of *Mycobacterium tuberculosis*, whereas more recent bursts involving molecular docking, rifampicin, and autophagy indicate a broadening of scholarly attention toward computational drug exploration, drug-specific resistance research, and host-related mechanisms. This thematic transition suggests that newer research directions have increasingly complemented, rather than replaced, classical molecular and structural approaches.

The timeline analysis showed that molecular docking and host-directed therapies were prominent recent topics, whereas ATP synthase and efflux pumps remained recurrent themes in the analyzed literature. Because the CiteSpace timeline was derived from keyword co-occurrence rather than document cross-citation, these clusters represent thematic groupings of related terms and should not be interpreted as distinct research communities with limited citation exchange. These themes are consistent with current research interests in drug-target interactions, resistance-associated transport mechanisms, and therapeutic strategies beyond conventional antibacterial approaches. However, these findings should be interpreted as indicators of research attention within the retrieved literature and do not establish the biological or clinical priority of any specific topic.

The thematic strategic map further illustrated differences in the development and connectivity of major research themes. Established motor and basic themes occupied central positions in the thematic structure, whereas topics related to efflux mechanisms, the cell wall, drug targets, and molecular dynamics simulation were more specialized. Molecular docking was positioned near the center of the map, indicating associations with multiple thematic areas and suggesting a potential interface between mechanism-oriented investigation and drug-discovery research. Overall, the coexistence of well-established core themes with more specialized and computational topics suggests increasing thematic diversification within DR-TB resistance-mechanism research rather than replacement of established research areas.

Taken together, the keyword analyzes indicate a broad thematic landscape in DR-TB resistance-mechanism research. Classical topics, including rifampicin resistance, efflux pumps, cell-wall synthesis, ATP synthase, and bedaquiline-related mechanisms, remained recurrently represented in the literature. Temporal signals were most clearly observed for molecular docking and autophagy in the keyword-burst analysis and for molecular docking and host-directed therapies in the keyword timeline. Other topics, including molecular dynamics simulation, biofilm formation, apoptosis, non-coding RNA regulation, and gene regulation, were also represented in the analyzed literature, but their occurrence—particularly for terms predefined in the search strategy—was not interpreted as independent evidence that they constituted emerging research themes. Collectively, these findings indicate that the literature encompasses broader questions involving bacterial adaptation, drug–target interactions, host immune regulation, and computational drug discovery. At the molecular level, omics-based analyzes have identified metabolism-associated determinants such as Rv3094c (Wan et al., 2023), whereas complementary combination-development frameworks increasingly seek to target phenotypic drug tolerance (Greenstein and Aldridge, 2022).

### 4.8 PubMed validation and interpretation of findings

The PubMed-based validation broadly reproduced the temporal, geographical, and thematic patterns identified in the WoSCC dataset despite differences in database coverage and indexing characteristics. These descriptive observations were further supported by the quantitative cross-database comparisons reported in the Results. Both databases showed a similar increase in publication activity after 2015 and substantial overlap among the major contributing countries. The recurrence of resistance mechanisms, host-related processes, metabolic adaptation, cell-wall processes, efflux-related mechanisms, and several complementary topics across the two datasets further supports the cross-database consistency of the principal bibliometric patterns.

The thematic concordance should nevertheless be interpreted cautiously because several mechanism-related terms were predefined in the search strategy. Their recurrence in both databases therefore provides supportive evidence of thematic consistency rather than independent confirmation that these topics represent research hotspots. In addition, differences in indexing scope, searchable fields, document coverage, and metadata structure between WoSCC and PubMed may account for variations in the number of retrieved publications and the relative representation of individual themes.

Accordingly, the PubMed analysis should be regarded as an external consistency assessment rather than a fully integrated multi-database bibliometric analysis. The broadly comparable patterns observed across the two databases reduce concern that the principal findings are unique to the WoSCC indexing environment, although database-specific coverage and indexing biases cannot be completely excluded.

### 4.9 Limitations

Although this bibliometric study provides a systematic perspective on the development, knowledge structure, and emerging trends of research into DR-TB resistance mechanisms, several considerations should be acknowledged when interpreting the findings. First, WoSCC was used as the primary database for comprehensive bibliometric and visualization analyzes. Although a supplementary PubMed dataset was included for cross-database validation and reproduced broadly comparable temporal, geographical, and thematic patterns, differences in database coverage, indexing practices, controlled-vocabulary systems, searchable fields, and metadata structures may still influence the literature retrieved from each database. In addition, the present study was restricted to English-language journal articles and reviews. Among otherwise eligible records, 6 of 1,049 WoSCC publications (0.57%) and 11 of 798 PubMed publications (1.38%) were excluded because of language. However, these proportions do not capture non-English publications not indexed in either database; therefore, the overall influence of language bias on geographical publication patterns cannot be fully quantified. In addition, although the search strategy was designed to capture the principal terminology used in DR-TB research, no bibliographic search strategy can exhaustively cover every terminology variant or evolving resistance classification, and a small number of relevant publications may therefore have been missed. Conversely, the inclusion of broad terms such as “pathway” and “mechanism” may have increased retrieval breadth at the expense of specificity, potentially including some publications in which resistance-related mechanisms were mentioned only peripherally rather than constituting the primary focus. This sensitivity–specificity trade-off may have influenced the composition of the retrieved literature and should therefore be considered when interpreting the bibliometric patterns. Second, bibliometric tools such as VOSviewer, CiteSpace, and Bibliometrix primarily analyze bibliographic metadata, including titles, abstracts, keywords, citations, and references. In addition, VOSviewer, CiteSpace, and Bibliometrix employ different analytical and visualization procedures, and differences in clustering, normalization, threshold settings, and network representation may affect the apparent structure or prominence of individual themes and nodes. The outputs of these tools should therefore be regarded as complementary representations of the bibliometric structure rather than as directly interchangeable or exactly reproducible results across software platforms. Author- and institution-level analyzes may therefore be affected by homonymous author names, spelling variants, changes in affiliations, and incomplete author disambiguation in bibliographic records. Although bibliometric software can standardize some metadata, residual misclassification of authors or institutions cannot be completely excluded. In addition, although these methods are effective for identifying large-scale research patterns and thematic relationships, they may not fully capture the deeper semantic content, methodological details, or contextual differences contained in the full texts of individual studies. Third, citation-based bibliometric indicators are inherently time dependent. Recently published studies have had less time to accumulate citations, creating a citation-lag effect that may disadvantage newer publications, while delays in database indexing may limit the timely inclusion of the latest evidence and the identification of emerging research fronts. Citation counts may also be influenced by self-citation and by differences in citation practices across authors, journals, research topics, and publication periods. Accordingly, citation frequency and network prominence should not be interpreted as direct measures of research quality, biological importance, or clinical relevance. Nevertheless, the broadly comparable patterns observed between the WoSCC and PubMed datasets support the cross-database consistency of the principal findings. Future research could combine bibliometric mapping with systematic or narrative evidence synthesis to examine the major themes identified here in greater depth and further clarify their biological and clinical translational significance.

## 5 Conclusion

This bibliometric and cross-database analysis indicates that DR-TB resistance-mechanism research encompasses a broad thematic landscape ranging from drug-susceptibility phenotypes, resistance-associated mutations, biosynthetic pathways, and structural biology to efflux systems, cell-wall processes, metabolic adaptation, host–pathogen interactions, molecular docking, molecular dynamics simulations, and host-directed approaches. Temporal analyzes identified recent scholarly attention most clearly for molecular docking and autophagy, while host-directed approaches were represented among recent keyword clusters. Other topics, including biofilm formation, programmed cell death, non-coding RNA regulation, and multi-omics approaches, were represented within the broader research landscape but should not be interpreted as emerging solely on the basis of their occurrence in the retrieved literature.

These bibliometric patterns identify areas of scholarly activity rather than establish their relative biological or clinical importance. Future experimental and translational studies may further evaluate the mechanistic and clinical relevance of these topics, including their relationships with resistance mutations, compensatory evolution, bacterial persistence, host immunity, and therapeutic responses. Integration of molecular, computational, ecological, and clinical evidence may provide a broader framework for subsequent investigation of tuberculosis drug resistance.

## Data Availability

The original contributions presented in the study are included in the article/Supplementary material, further inquiries can be directed to the corresponding authors.
