# Supplementary material for: Global research trends in tuberculosis drug-resistance mechanisms: a bibliometric and cross-database analysis
Source: Front Microbiol. 2026 Sep 17;17:1942089. doi: 10.3389/fmicb.2026.1942089 (PMC13627400; doi:10.3389/fmicb.2026.1942089)
Supplement: Supplementary file 1 [file Table_1.doc]

PMID- 41457557

OWN - NLM

STAT- MEDLINE

DCOM- 20251229

LR - 20260326

IS - 1555-8584 (Electronic)

IS - 1547-6286 (Print)

IS - 1547-6286 (Linking)

VI - 23

IP - 1

DP - 2026 Dec

TI - Different MicroRNAs expression in Mycobacterium tuberculosis and correlation with

prognosis of the disease.

PG - 1-12

LID - 10.1080/15476286.2025.2609681 [doi]

AB - Tuberculosis, caused by Mycobacterium tuberculosis, is an infectious disease

linked to high mortality and can stay in the host cell longer when inactive.

Multiple factors are linked to disease prognosis, including microRNAs. It is a

diminutive single-stranded RNA that regulates the expression of its target mRNAs.

It consists of a brief nucleotide sequence, often 19-25 nucleotides in length, of

non-coding RNA. It is also essential for early embryonic development, invasion,

cell migration, apoptosis, and cell death. The review aims to analyse the

transcriptome characteristics of various miRNAs in the tuberculosis prognosis.

However, miR-155, miR-29, circ-miRNA, and lncRNAs regulate gene expression. In TB

patients' serum exosomes, miRNA-146 expression was noticeably higher than in

healthy individuals. Drug-resistant tuberculosis was related to miR-548 m,

miR-631, miR-328-3p, and miR-let-7e-5p, as well as let-7b-5p, miR-30a-3p, IL-27,

and CXCL9/10/11 in TB patients' lesion tissue and peripheral blood. Therefore,

further miRNA research will focus on TB progression.

FAU - Sundaram, Karthikeyan

AU - Sundaram K

AUID- ORCID: 0000-0002-2674-9146

AD - Department of Herbal Pharmacology and Environmental Sustainability, Chettinad

Hospital and Research Institute, Chettinad Academy of Research and Education,

Kelambakkam, Chennai, Tamilnadu, India.

FAU - Rathinam, Sridhar

AU - Rathinam S

AD - Chettinad Hospital and Research Institute, Chettinad Academy of Research and

Education, Kelambakkam, Chennai, Tamilnadu, India.

LA - eng

PT - Journal Article

PT - Review

DEP - 20251228

PL - United States

TA - RNA Biol

JT - RNA biology

JID - 101235328

RN - 0 (MicroRNAs)

SB - IM

MH - Humans

MH - *MicroRNAs/genetics/metabolism

MH - *Mycobacterium tuberculosis/genetics/pathogenicity

MH - *Tuberculosis/genetics/microbiology/diagnosis

MH - Prognosis

MH - Gene Expression Regulation

MH - Gene Expression Profiling

MH - Host-Pathogen Interactions/genetics

MH - Transcriptome

PMC - PMC12758175

OTO - NOTNLM

OT - Tuberculosis

OT - circular-miRNA

OT - disease progression

OT - miRNA

OT - mycobacteria

COIS- No potential conflict of interest was reported by the author(s).

EDAT- 2025/12/29 07:08

MHDA- 2025/12/29 07:09

PMCR- 2025/12/28

CRDT- 2025/12/29 01:53

PHST- 2025/12/29 07:09 [medline]

PHST- 2025/12/29 07:08 [pubmed]

PHST- 2025/12/29 01:53 [entrez]

PHST- 2025/12/28 00:00 [pmc-release]

AID - 2609681 [pii]

AID - 10.1080/15476286.2025.2609681 [doi]

PST - ppublish

SO - RNA Biol. 2026 Dec;23(1):1-12. doi: 10.1080/15476286.2025.2609681. Epub 2025 Dec

28.

PMID- 42459678

OWN - NLM

STAT- MEDLINE

DCOM- 20260716

LR - 20260716

IS - 1664-3224 (Electronic)

IS - 1664-3224 (Linking)

VI - 17

DP - 2026

TI - Immunotherapy for tuberculosis: current landscape, mechanistic insights, and

translational perspectives.

PG - 1859046

LID - 10.3389/fimmu.2026.1859046 [doi]

LID - 1859046

AB - Tuberculosis (TB), caused by Mycobacterium tuberculosis (Mtb), remains the

leading cause of death from a single infectious agent worldwide, with 10.7

million new cases and 1.23 million deaths reported globally in 2024. Although

conventional chemotherapy cures most drug-susceptible TB, the rising burden of

multidrug-resistant TB (MDR-TB), prolonged treatment regimens, and suboptimal

patient adherence continue to undermine control efforts. Against this background,

immunotherapeutic approaches have gained renewed interest as rational complements

to chemotherapy, building on decades of mechanistic work that has mapped

host-pathogen interactions at unprecedented resolution. This review integrates

recent advances across the immunotherapy landscape, including next-generation

vaccines (M72/AS01E, MTBVAC, VPM1002, BNT164 mRNA candidates), host-directed

therapies (HDTs) targeting autophagy, metabolic and inflammatory pathways,

cytokine-based and antimicrobial peptide strategies, and adoptive cell therapies.

We place particular emphasis on the dual, context-dependent roles of immune

checkpoints such as PD-1/PD-L1 in TB, where checkpoint blockade can paradoxically

trigger reactivation, and on the cellular heterogeneity revealed by single-cell

and spatial transcriptomics of the granuloma. Key translational challenges-the

absence of reliable correlates of protection, the demand for precision

immunomodulation in comorbid populations (HIV, diabetes), and chronic

underfunding of TB research-are discussed alongside emerging opportunities

offered by mRNA platforms, repurposed drugs, and multi-omics-guided patient

stratification. Collectively, immunotherapy is evolving from an adjunctive

concept into a strategic pillar of TB control, with the potential to shorten

treatment, prevent relapse, and address drug-resistant disease.

CI - Copyright © 2026 He, Xie, He, Li, Zhou and Wei.

FAU - He, Yanlang

AU - He Y

AD - Department of Infectious Disease, Shaoyang Central Hospital, Shaoyang, China.

FAU - Xie, Xiangping

AU - Xie X

AD - Department of Infectious Disease, Shaoyang Central Hospital, Shaoyang, China.

FAU - He, Shuangyan

AU - He S

AD - Department of Infectious Disease, Shaoyang Central Hospital, Shaoyang, China.

FAU - Li, Sha

AU - Li S

AD - Department of General Medicine, The Second Affiliated Hospital of Wannan Medical

College, Wuhu, China.

FAU - Zhou, Haolin

AU - Zhou H

AD - Department of Clinical Medicine, Changsha Medical University, Changsha, China.

FAU - Wei, Sheng

AU - Wei S

AD - Department of General Medicine, The Second Affiliated Hospital of Wannan Medical

College, Wuhu, China.

LA - eng

PT - Journal Article

PT - Review

DEP - 20260701

PL - Switzerland

TA - Front Immunol

JT - Frontiers in immunology

JID - 101560960

RN - 0 (Tuberculosis Vaccines)

RN - 0 (Immune Checkpoint Inhibitors)

SB - IM

MH - Humans

MH - *Immunotherapy/methods

MH - *Mycobacterium tuberculosis/immunology

MH - *Tuberculosis/therapy/immunology

MH - Animals

MH - Host-Directed Therapy

MH - *Tuberculosis Vaccines/immunology/therapeutic use

MH - Host-Pathogen Interactions/immunology

MH - Translational Research, Biomedical

MH - Autophagy

MH - Immune Checkpoint Inhibitors/therapeutic use

PMC - PMC13368575

OTO - NOTNLM

OT - Mycobacterium tuberculosis

OT - adoptive cell transfer

OT - autophagy

OT - host-directed therapy

OT - immune checkpoint

OT - immunotherapy

OT - vaccines

COIS- The author(s) declared that this work was conducted in the absence of any

commercial or financial relationships that could be construed as a potential

conflict of interest.

EDAT- 2026/07/16 06:37

MHDA- 2026/07/16 13:02

PMCR- 2026/07/01

CRDT- 2026/07/16 04:38

PHST- 2026/04/18 00:00 [received]

PHST- 2026/06/12 00:00 [revised]

PHST- 2026/06/16 00:00 [accepted]

PHST- 2026/07/16 13:02 [medline]

PHST- 2026/07/16 06:37 [pubmed]

PHST- 2026/07/16 04:38 [entrez]

PHST- 2026/07/01 00:00 [pmc-release]

AID - 10.3389/fimmu.2026.1859046 [doi]

PST - epublish

SO - Front Immunol. 2026 Jul 1;17:1859046. doi: 10.3389/fimmu.2026.1859046.

eCollection 2026.

PMID- 42344733

OWN - NLM

STAT- PubMed-not-MEDLINE

DCOM- 20260625

LR - 20260625

IS - 1664-302X (Print)

IS - 1664-302X (Electronic)

IS - 1664-302X (Linking)

VI - 17

DP - 2026

TI - CRISPRi-mediated silencing of the ABC transporter Ms_5779 compromises cell

envelope integrity and impairs intracellular host lipid exploitation.

PG - 1835283

LID - 10.3389/fmicb.2026.1835283 [doi]

LID - 1835283

AB - BACKGROUND: Multidrug-resistant Mycobacterium tuberculosis (MDR-TB) poses a

severe global health threat, yet the contribution of cell envelope barriers to

intrinsic drug tolerance remains poorly understood. The ABC protein Rv0820 is

conserved across mycobacteria but functionally uncharacterized, presenting a

structural paradox: it lacks canonical transmembrane helices, making conventional

efflux pump function mechanistically implausible. METHODS: Using CRISPR

interference (CRISPRi), we silenced Ms_5779-the Mycobacterium smegmatis (M.

smegmatis) ortholog of Rv0820-to two graded depths (81% and 69% repression) and

subjected the resulting strains to systematic phenotypic interrogation spanning

drug susceptibility, cell envelope permeability, biofilm formation, multi-stress

tolerance, and macrophage infection. RESULTS: Ms_5779 silencing did not affect

growth kinetics but markedly disrupted cell envelope integrity, as evidenced by

impaired biofilm formation, dissipated membrane potential, and increased ethidium

bromide permeability. Silenced strains exhibited broad-spectrum drug

hypersusceptibility across six antibiotic classes, with the most striking effect

being a 32-fold reduction in norfloxacin MIC, alongside heightened sensitivity to

oxidative, acidic, and thermal stress. In a RAW264.7 macrophage infection model,

Ms_5779 silencing attenuated intracellular survival, impaired host cholesterol

exploitation and foamy macrophage induction, and reduced apoptosis induction.

CONCLUSION: Collectively, these findings strongly suggest that Ms_5779/Rv0820 is

a critical functional contributor indispensable for the maintenance of cell

envelope integrity, presenting a distinct mechanism from a conventional efflux

pump. Targeting this essential uncharacterized ABC protein would simultaneously

compromise the drug permeability barrier and the intracellular niche, nominating

Rv0820 as a mechanistically distinct therapeutic target for anti-tuberculosis

drug discovery.

CI - Copyright © 2026 Song, Ban, Zhang, Diao, Li, Shi, Zeng and Du.

FAU - Song, Yuhao

AU - Song Y

AD - College of Animal Science and Technology, Jilin Agricultural University,

Changchun, China.

FAU - Ban, Didi

AU - Ban D

AD - College of Animal Science and Technology, Jilin Agricultural University,

Changchun, China.

FAU - Zhang, Rui

AU - Zhang R

AD - College of Chinese Medicine Materials, Jilin Agricultural University, Changchun,

China.

FAU - Diao, Naichao

AU - Diao N

AD - College of Chinese Medicine Materials, Jilin Agricultural University, Changchun,

China.

FAU - Li, Jianming

AU - Li J

AD - College of Chinese Medicine Materials, Jilin Agricultural University, Changchun,

China.

FAU - Shi, Kun

AU - Shi K

AD - College of Chinese Medicine Materials, Jilin Agricultural University, Changchun,

China.

FAU - Zeng, Fanli

AU - Zeng F

AD - College of Chinese Medicine Materials, Jilin Agricultural University, Changchun,

China.

AD - Jilin Province Sika Deer Efficient Breeding and Product Development Technology

Engineering Research Center, Jilin Agricultural University, Changchun, China.

FAU - Du, Rui

AU - Du R

AD - College of Chinese Medicine Materials, Jilin Agricultural University, Changchun,

China.

AD - Jilin Province Sika Deer Efficient Breeding and Product Development Technology

Engineering Research Center, Jilin Agricultural University, Changchun, China.

AD - College of Agriculture, Yanbian University, Yanji, China.

LA - eng

PT - Journal Article

DEP - 20260609

PL - Switzerland

TA - Front Microbiol

JT - Frontiers in microbiology

JID - 101548977

PMC - PMC13287036

OTO - NOTNLM

OT - ABC transporter

OT - CRISPRi

OT - Mycobacterium smegmatis

OT - Rv0820/Ms_5779

OT - drug resistance

COIS- The author(s) declared that this work was conducted in the absence of any

commercial or financial relationships that could be construed as a potential

conflict of interest.

EDAT- 2026/06/25 06:35

MHDA- 2026/06/25 06:36

PMCR- 2026/06/09

CRDT- 2026/06/25 04:42

PHST- 2026/03/20 00:00 [received]

PHST- 2026/05/19 00:00 [revised]

PHST- 2026/05/28 00:00 [accepted]

PHST- 2026/06/25 06:36 [medline]

PHST- 2026/06/25 06:35 [pubmed]

PHST- 2026/06/25 04:42 [entrez]

PHST- 2026/06/09 00:00 [pmc-release]

AID - 10.3389/fmicb.2026.1835283 [doi]

PST - epublish

SO - Front Microbiol. 2026 Jun 9;17:1835283. doi: 10.3389/fmicb.2026.1835283.

eCollection 2026.

PMID- 42004739

OWN - NLM

STAT- PubMed-not-MEDLINE

DCOM- 20260421

LR - 20260421

IS - 2772-431X (Electronic)

IS - 2097-0684 (Print)

IS - 2772-431X (Linking)

VI - 5

IP - 2

DP - 2026 Jun

TI - Unequal patterns of tuberculosis in women of reproductive age: Insights from the

Global burden of disease study 2021.

PG - 100250

LID - 10.1016/j.imj.2026.100250 [doi]

LID - 100250

AB - BACKGROUND: Tuberculosis (TB) remains a major global cause of mortality and is a

leading cause of death among women of reproductive age (WRA). Untreated TB in

this population endangers both maternal and neonatal health, yet the

age-stratified burden and the shifting distribution of drug-resistant

tuberculosis remain poorly defined in global health agendas. This study aims to

employ data from the 2021 Global Burden of Disease Study to systematically

evaluate the global, regional, and age-specific burden of TB among WRA, to

delineate temporal trends in drug-resistant TB, and to assess associated risk

factors using standardized estimation methods. METHODS: Leveraging data from the

Global Burden of Disease Study 2021 covering 204 countries and territories, we

systematically assessed TB burden in WRA aged 15-49 years from 1990 to 2021. We

analyzed age-standardized incidence, prevalence, mortality, and

disability-adjusted life years across socio-demographic index (SDI) regions, TB

subtypes, and risk factors. Projections to 2030 were generated using

autoregressive integrated moving average models. RESULTS: Between 1990 and 2021,

the global prevalence and incidence of TB among WRA declined significantly.

However, this decline was uneven across age groups, disease types, and regions.

TB incidence peaked in women aged 15-24 years, while mortality and disease burden

were highest among those aged 45-49 years. Drug-resistant tuberculosis was most

prominent in the 25-29 age group. Geographically, sub-Saharan Africa remained the

core of the global TB burden, with BRICS (Brazil, Russia, India, China, South

Africa) countries carrying significantly higher burdens than G7 countries. Risk

attribution patterns revealed a shift from behavioral to metabolic drivers, with

high body mass index emerging as the leading risk factor in high-SDI regions.

Middle-SDI regions showed a combination of metabolic and behavioral risks, while

metabolic factors remained dominant in low-SDI settings. Drug-susceptible

tuberculosis was primarily linked to smoking and malnutrition, whereas

drug-resistant tuberculosis was increasingly associated with metabolic

dysfunction. CONCLUSIONS: Despite global declines in TB prevalence and incidence

among WRA, stark disparities persist. The age-stratified rise in drug-resistant

TB, particularly in resource-constrained regions, underscores the need for

targeted, gender-responsive interventions that integrate infectious disease

control with metabolic health strategies.

CI - © 2026 The Authors.

FAU - Liu, Ting

AU - Liu T

AD - Clinical Medicine Department, North Sichuan Medical College, Nanchong 637000,

China.

AD - Laboratory of Allergy and Precision Medicine, Department of Respiratory Medicine,

Chengdu Institute of Respiratory Health, The Third People's Hospital of Chengdu,

Chengdu 610041, China.

FAU - Zhang, Lei

AU - Zhang L

AD - Laboratory of Allergy and Precision Medicine, Department of Respiratory Medicine,

Chengdu Institute of Respiratory Health, The Third People's Hospital of Chengdu,

Chengdu 610041, China.

FAU - Liu, Jiao

AU - Liu J

AD - Laboratory of Allergy and Precision Medicine, Department of Respiratory Medicine,

Chengdu Institute of Respiratory Health, The Third People's Hospital of Chengdu,

Chengdu 610041, China.

AD - College of Medicine, Southwest Jiaotong University, Chengdu 611756, China.

FAU - Liu, Yao

AU - Liu Y

AD - Laboratory of Allergy and Precision Medicine, Department of Respiratory Medicine,

Chengdu Institute of Respiratory Health, The Third People's Hospital of Chengdu,

Chengdu 610041, China.

AD - State Key Laboratory of Quality Research in Chinese Medicine, Macao University of

Science and Technology, Macao Special Administrative Region 999078, China.

FAU - Xiong, Anying

AU - Xiong A

AD - Laboratory of Allergy and Precision Medicine, Department of Respiratory Medicine,

Chengdu Institute of Respiratory Health, The Third People's Hospital of Chengdu,

Chengdu 610041, China.

AD - College of Medicine, Southwest Jiaotong University, Chengdu 611756, China.

FAU - Ran, Qin

AU - Ran Q

AD - Laboratory of Allergy and Precision Medicine, Department of Respiratory Medicine,

Chengdu Institute of Respiratory Health, The Third People's Hospital of Chengdu,

Chengdu 610041, China.

FAU - He, Xiang

AU - He X

AD - Laboratory of Allergy and Precision Medicine, Department of Respiratory Medicine,

Chengdu Institute of Respiratory Health, The Third People's Hospital of Chengdu,

Chengdu 610041, China.

FAU - Jiang, Li

AU - Jiang L

AD - Clinical Medicine Department, North Sichuan Medical College, Nanchong 637000,

China.

AD - Department of Respiratory and Critical Care Medicine, The Affiliated Hospital of

North Sichuan Medical College, Nanchong 637000, China.

FAU - Li, Guoping

AU - Li G

AD - Laboratory of Allergy and Precision Medicine, Department of Respiratory Medicine,

Chengdu Institute of Respiratory Health, The Third People's Hospital of Chengdu,

Chengdu 610041, China.

AD - College of Medicine, Southwest Jiaotong University, Chengdu 611756, China.

FAU - Wang, Junyi

AU - Wang J

AD - Clinical Medicine Department, North Sichuan Medical College, Nanchong 637000,

China.

AD - Laboratory of Allergy and Precision Medicine, Department of Respiratory Medicine,

Chengdu Institute of Respiratory Health, The Third People's Hospital of Chengdu,

Chengdu 610041, China.

AD - College of Medicine, Southwest Jiaotong University, Chengdu 611756, China.

LA - eng

PT - Journal Article

DEP - 20260326

PL - Netherlands

TA - Infect Med (Beijing)

JT - Infectious medicine

JID - 9918663679306676

PMC - PMC13090512

OTO - NOTNLM

OT - Drug-resistant tuberculosis

OT - Global Burden of Disease Study 2021

OT - Time trends

OT - Tuberculosis

OT - Women of reproductive age

COIS- The authors declare that they have no known competing financial interests or

personal relationships that could have appeared to influence the work reported in

this paper.

EDAT- 2026/04/20 13:10

MHDA- 2026/04/20 13:11

PMCR- 2026/03/26

CRDT- 2026/04/20 07:32

PHST- 2025/12/22 00:00 [received]

PHST- 2026/02/02 00:00 [revised]

PHST- 2026/02/24 00:00 [accepted]

PHST- 2026/04/20 13:11 [medline]

PHST- 2026/04/20 13:10 [pubmed]

PHST- 2026/04/20 07:32 [entrez]

PHST- 2026/03/26 00:00 [pmc-release]

AID - S2772-431X(26)00016-X [pii]

AID - 100250 [pii]

AID - 10.1016/j.imj.2026.100250 [doi]

PST - epublish

SO - Infect Med (Beijing). 2026 Mar 26;5(2):100250. doi: 10.1016/j.imj.2026.100250.

eCollection 2026 Jun.

PMID- 42291305

OWN - NLM

STAT- MEDLINE

DCOM- 20260615

LR - 20260615

IS - 2235-2988 (Electronic)

IS - 2235-2988 (Linking)

VI - 16

DP - 2026

TI - Fluoroquinolones as the linchpin of tuberculosis therapy: contrasting efficacy

and resistance profiles in Mycobacterium tuberculosis versus Mycobacterium

abscessus.

PG - 1833650

LID - 10.3389/fcimb.2026.1833650 [doi]

LID - 1833650

AB - INTRODUCTION: Fluoroquinolones (FQs) are pharmacological cornerstone agents in

global tuberculosis (TB) management, critical for both accelerating

drug-sensitive regimens and defining the resistance spectrum of

multidrug-resistant TB. METHODS & RESULTS: To evaluate the therapeutic potential

of this class across the mycobacterial spectrum, we conducted a comprehensive

phenotypic screening campaign utilizing OTAVA and ECBD pilot-libraries against

reference strains of Mycobacterium tuberculosis (Mtb) and Mycobacterium abscessus

(Mab). This initial screen, followed by detailed bactericidal concentration (BC)

and cytotoxicity assessments, identified and selected potent FQ derivatives for

in-depth characterization. These FQ compounds exhibited exceptionally high

potency against Mtb, with several demonstrating robust bactericidal activity at

concentrations far below toxic levels to mammalian cells. This activity was

retained in clinically relevant, challenging states, as the compounds effectively

eliminated Mtb residing within human macrophages and persisted within protective

biofilm structures. In stark contrast, Mab displayed marked intrinsic resistance.

Most FQ derivatives highly potent against Mtb required significantly higher

concentrations for inhibition and bactericidal effect against Mab. For instance,

a leading clinical FQ showed substantially diminished activity against Mab

compared to Mtb. Analysis of acquired resistance mechanisms demonstrated that

both Mtb and Mab primarily rely on similar acquired point mutations in the

Quinolone Resistance Determining Regions (QRDRs) of the gyrA and gyrB genes.

However, molecular docking indicated a slight but significant reduction in

theoretical binding affinity to the Mab gyrase complex. DISCUSSION: We

hypothesize that the profound differential in intrinsic susceptibility is at

least partially driven by non-target factors. This work supports the continued

critical role of FQs in Mtb therapy while highlighting the importance of

structural modifications that could address hypothesized intrinsic resistance

mechanisms in Mab.

CI - Copyright © 2026 Kuzioła, Korycka-Machała, Zygała-Pytlos, Dziadek, Kawka,

Pawełczyk, Odolczyk, Zielenkiewicz, Słomka and Dziadek.

FAU - Kuzioła, Magdalena

AU - Kuzioła M

AD - Institute of Medical Biology, Polish Academy of Sciences, Łódź, Poland.

AD - The Bio-Med-Chem Doctoral School of the University of Lodz and Lodz Institutes of

the Polish Academy of Sciences, Łódź, Poland.

FAU - Korycka-Machała, Małgorzata

AU - Korycka-Machała M

AD - Institute of Medical Biology, Polish Academy of Sciences, Łódź, Poland.

FAU - Zygała-Pytlos, Daria

AU - Zygała-Pytlos D

AD - Institute of Medical Biology, Polish Academy of Sciences, Łódź, Poland.

AD - The Bio-Med-Chem Doctoral School of the University of Lodz and Lodz Institutes of

the Polish Academy of Sciences, Łódź, Poland.

FAU - Dziadek, Bożena

AU - Dziadek B

AD - Department of Molecular Microbiology, University of Łódź, Faculty of Biology and

Environmental Protection, Łódź, Poland.

FAU - Kawka, Malwina

AU - Kawka M

AD - Department of Molecular Microbiology, University of Łódź, Faculty of Biology and

Environmental Protection, Łódź, Poland.

FAU - Pawełczyk, Jakub

AU - Pawełczyk J

AD - Institute of Medical Biology, Polish Academy of Sciences, Łódź, Poland.

FAU - Odolczyk, Norbert

AU - Odolczyk N

AD - Department of Bioinformatics, Institute of Biochemistry and Biophysics, Polish

Academy of Sciences, Warsaw, Poland.

AD - Faculty of Biology, University of Warsaw, Warsaw, Poland.

FAU - Zielenkiewicz, Piotr

AU - Zielenkiewicz P

AD - Department of Bioinformatics, Institute of Biochemistry and Biophysics, Polish

Academy of Sciences, Warsaw, Poland.

AD - Faculty of Biology, University of Warsaw, Warsaw, Poland.

FAU - Słomka, Marcin

AU - Słomka M

AD - Faculty of Biology and Environmental Protection, University of Łódź, Centre for

Digital Biology and Biomedical Science - Biobank Łódź, Łódź, Poland.

FAU - Dziadek, Jarosław

AU - Dziadek J

AD - Institute of Medical Biology, Polish Academy of Sciences, Łódź, Poland.

LA - eng

PT - Journal Article

DEP - 20260528

PL - Switzerland

TA - Front Cell Infect Microbiol

JT - Frontiers in cellular and infection microbiology

JID - 101585359

RN - 0 (Fluoroquinolones)

RN - 0 (Antitubercular Agents)

RN - EC 5.99.1.3 (DNA Gyrase)

SB - IM

MH - *Fluoroquinolones/pharmacology/chemistry

MH - *Mycobacterium tuberculosis/drug effects/genetics

MH - *Mycobacterium abscessus/drug effects/genetics

MH - Humans

MH - *Antitubercular Agents/pharmacology/chemistry

MH - Microbial Sensitivity Tests

MH - Macrophages/microbiology

MH - DNA Gyrase/genetics/metabolism

MH - Drug Resistance, Multiple, Bacterial

MH - Drug Resistance, Bacterial

MH - Molecular Docking Simulation

MH - *Tuberculosis/drug therapy/microbiology

MH - Biofilms/drug effects

PMC - PMC13253442

OTO - NOTNLM

OT - DNA gyrase

OT - Mycobacterium abscessus

OT - Mycobacterium tuberculosis

OT - drug resistance

OT - fluoroquinolones

COIS- The author(s) declared that this work was conducted in the absence of any

commercial or financial relationships that could be construed as a potential

conflict of interest. The author JD declared that they were an editorial board

member of Frontiers, at the time of submission. This had no impact on the peer

review process and the final decision.

EDAT- 2026/06/15 12:53

MHDA- 2026/06/15 12:54

PMCR- 2026/05/28

CRDT- 2026/06/15 07:19

PHST- 2026/03/19 00:00 [received]

PHST- 2026/04/27 00:00 [revised]

PHST- 2026/04/30 00:00 [accepted]

PHST- 2026/06/15 12:54 [medline]

PHST- 2026/06/15 12:53 [pubmed]

PHST- 2026/06/15 07:19 [entrez]

PHST- 2026/05/28 00:00 [pmc-release]

AID - 10.3389/fcimb.2026.1833650 [doi]

PST - epublish

SO - Front Cell Infect Microbiol. 2026 May 28;16:1833650. doi:

10.3389/fcimb.2026.1833650. eCollection 2026.

PMID- 42124926

OWN - NLM

STAT- PubMed-not-MEDLINE

DCOM- 20260513

LR - 20260513

IS - 2001-0370 (Print)

IS - 2001-0370 (Electronic)

IS - 2001-0370 (Linking)

VI - 35

IP - 1

DP - 2026

TI - A Frameshift Mutation in the Methyltransferase rlmN Is Associated with Increased

Linezolid Resistance in Mycobacterium tuberculosis.

PG - 0090

LID - 10.34133/csbj.0090 [doi]

LID - 0090

AB - Background: Linezolid is a key component of treatment regimens for

multidrug-resistant and extensively drug-resistant tuberculosis, which is caused

by the pathogen Mycobacterium tuberculosis (MTB). Resistance to linezolid in MTB

has traditionally been attributed to mutations in the 23S ribosomal RNA (rrl) and

ribosomal protein L3 (rplC), but only a fraction of clinically observed linezolid

resistance is explained by mutations in these 2 genes. Results: We report that an

analysis of strains with paired whole-genome sequencing and linezolid minimum

inhibitory concentration phenotyping from the Bacterial and Viral Bioinformatics

Resource Center reveals that a relatively common frameshift mutation in MTB

methyltransferase rlmN (5.3% of assessed isolates, encompassing all isolates

known to be in the globally distributed MTB lineage 4.10) is significantly

associated with increased linezolid minimum inhibitory concentration. In addition

to statistical associations, we provide evolutionary evidence of homology to an

established linezolid resistance mechanism in Staphylococcus aureus and

structural evidence that the frameshift mutation likely ablates rlmN

methyltransferase functionality. Conclusions: We find a novel gene associated

with increased linezolid resistance in MTB, with potential implications for

resistance diagnostics and therapeutic strategies.

CI - Copyright © 2026 Bryn Marie Reimer and Anna G. Green.

FAU - Reimer, Bryn Marie

AU - Reimer BM

AUID- ORCID: 0000-0001-6133-5785

AD - Manning College of Information & Computer Sciences, University of Massachusetts

Amherst, Amherst, MA 01003, USA.

FAU - Green, Anna G

AU - Green AG

AUID- ORCID: 0000-0001-7548-3682

AD - Manning College of Information & Computer Sciences, University of Massachusetts

Amherst, Amherst, MA 01003, USA.

LA - eng

PT - Journal Article

DEP - 20260511

PL - United States

TA - Comput Struct Biotechnol J

JT - Computational and structural biotechnology journal

JID - 101585369

PMC - PMC13158455

COIS- Competing interests: The authors declare that they have no competing interests.

EDAT- 2026/05/13 06:31

MHDA- 2026/05/13 06:32

PMCR- 2026/05/11

CRDT- 2026/05/13 04:38

PHST- 2026/01/13 00:00 [received]

PHST- 2026/04/13 00:00 [revised]

PHST- 2026/04/13 00:00 [accepted]

PHST- 2026/05/13 06:32 [medline]

PHST- 2026/05/13 06:31 [pubmed]

PHST- 2026/05/13 04:38 [entrez]

PHST- 2026/05/11 00:00 [pmc-release]

AID - 0090 [pii]

AID - 10.34133/csbj.0090 [doi]

PST - epublish

SO - Comput Struct Biotechnol J. 2026 May 11;35(1):0090. doi: 10.34133/csbj.0090.

eCollection 2026.

PMID- 42094621

OWN - NLM

STAT- PubMed-not-MEDLINE

DCOM- 20260507

LR - 20260507

IS - 2398-8835 (Electronic)

IS - 2398-8835 (Linking)

VI - 9

DP - 2026 May

TI - Molecular Mechanisms of Resistance and Treatment Efficacy of Delamanid Against

Mycobacterium tuberculosis: A Systematic Review.

PG - e72481

LID - 10.1002/hsr2.72481 [doi]

LID - e72481

AB - BACKGROUND AND AIMS: Tuberculosis (TB) remains a major global health problem, and

treatment progress is increasingly threatened by rising multidrug-resistant

tuberculosis (MDR-TB). Delamanid (DLM), a nitroimidazole drug, has shown good

efficacy and safety against both drug-susceptible and drug-resistant

Mycobacterium tuberculosis (Mtb) strains. However, data on its resistance

mechanisms, drug susceptibility testing (DST), clinical effectiveness, safety,

and pharmacokinetics remain limited. This review aims to summarize the most

recent molecular, structural, and clinical evidence related to DLM. METHODS: A

comprehensive literature search was performed using WHO publications and major

scientific databases, including PubMed, Web of Science, Embase, Scopus, and the

Cochrane Library. Studies published through 2024 and early 2025 on DLM

resistance, mechanisms of action, DST, pharmacokinetics, safety, and treatment

outcomes were included. Structural analyses of key proteins involved in DLM

activation were carried out using crystal structures and AlphaFold models.

RESULTS: Recent research identified multiple mutations in the F420-dependent

activation pathway, particularly in ddn, fgd1, fbiA, fbiB, fbiC, and fbiD that

contribute to DLM resistance. Structural modeling demonstrated how these

mutations affect protein stability and cofactor binding. Clinical studies showed

that DLM-containing regimens improve culture conversion and treatment success,

especially when combined with oral agents such as bedaquiline and linezolid.

Safety data indicate that DLM is generally well tolerated, with QT prolongation

being the main but manageable adverse effect. CONCLUSION: DLM is an important and

effective component of MDR-/XDR-TB treatment. A clearer understanding of its

resistance mechanisms, pharmacological properties, and clinical outcomes can

support better regimen design and help prevent the development of further

resistance.

CI - © 2026 The Author(s). Health Science Reports published by Wiley Periodicals LLC.

FAU - Islam, Md Mahmudul

AU - Islam MM

AD - Department of Genetic Engineering and Biotechnology Daffodil International

University Dhaka Bangladesh.

AD - Department of Microbiology, Shaheed Shamsuzzoha Institute of Biosciences

Affiliated With University of Rajshahi Rajshahi Bangladesh.

AD - Department of Biochemistry and Molecular Biology Gopalganj Science and Technology

University Gopalganj Bangladesh.

FAU - Hasan, Md Zahid

AU - Hasan MZ

AD - Department of Microbiology, Rajshahi Institute of Biosciences Affiliated With

University of Rajshahi Rajshahi Bangladesh.

FAU - Tusar, Md Touki Tahamid

AU - Tusar MTT

AD - Department of Microbiology and Hygiene Bangladesh Agricultural University

Mymensingh Bangladesh.

FAU - Hossain, Md Yeamin

AU - Hossain MY

AD - Department of Fisheries University of Rajshahi Rajshahi Bangladesh.

FAU - Hossain, Md Motaher

AU - Hossain MM

AD - Department of Microbiology, Rajshahi Institute of Biosciences Affiliated With

University of Rajshahi Rajshahi Bangladesh.

FAU - Jubayed, Md Abdulla Al

AU - Jubayed MAA

AD - Department of Microbiology, Rajshahi Institute of Biosciences Affiliated With

University of Rajshahi Rajshahi Bangladesh.

FAU - Jubaer-Al-Abedin, Md

AU - Jubaer-Al-Abedin M

AD - Department of Microbiology, Rajshahi Institute of Biosciences Affiliated With

University of Rajshahi Rajshahi Bangladesh.

FAU - Soikot, Sheikh

AU - Soikot S

AD - Department of Pharmacy University of Rajshahi Rajshahi Bangladesh.

FAU - Akther, Shanzida

AU - Akther S

AD - Department of Pharmacy University of Rajshahi Rajshahi Bangladesh.

FAU - Bhuyian, Jahid

AU - Bhuyian J

AD - Department of Agriculture, Rajshahi Institute of Biosciences Affiliated With

University of Rajshahi Rajshahi Bangladesh.

FAU - Gazi, Hafizur Rahman

AU - Gazi HR

AD - Department of Biotechnology and Genetic Engineering Gopalganj Science and

Technology University Gopalganj Bangladesh.

FAU - Hasan, B M Mahmudul

AU - Hasan BMM

AD - Department of Food and Nutrition, Barishal Home Economics College Affiliated by

University of Dhaka Dhaka Bangladesh.

FAU - Azam, Md Shofiul

AU - Azam MS

AD - Department of Food Engineering Dhaka University of Engineering & Technology

Gazipur Bangladesh.

FAU - Haque, Md Enamul

AU - Haque ME

AD - Department of Biotechnology and Genetic Engineering Gopalganj Science and

Technology University Gopalganj Bangladesh.

FAU - Abdullah-Al-Jubayer

AU - Abdullah-Al-Jubayer

AD - Department of Biotechnology and Genetic Engineering Gopalganj Science and

Technology University Gopalganj Bangladesh.

FAU - Hasan, Md Faruk

AU - Hasan MF

AUID- ORCID: 0000-0003-2746-5994

AD - Department of Microbiology University of Rajshahi Bangladesh.

FAU - Haydar, F M Ali

AU - Haydar FMA

AD - Department of Botany University of Rajshahi Rajshahi Bangladesh.

FAU - Khalekuzzaman, Md

AU - Khalekuzzaman M

AD - Department of Genetic Engineering and Biotechnology University of Rajshahi

Rajshahi Bangladesh.

FAU - Islam, Md Torequl

AU - Islam MT

AD - Department of Pharmacy Gopalganj Science and Technology University Gopalganj

Bangladesh.

AD - Bioinformatics and Drug Innovation Laboratory BioLuster Research Center Ltd.

Gopalganj Bangladesh.

FAU - Hasan, Sohel

AU - Hasan S

AD - Department of Biochemistry and Molecular Biology Gopalganj Science and Technology

University Gopalganj Bangladesh.

AD - Department of Biochemistry and Molecular Biology University of Rajshahi Rajshahi

Bangladesh.

LA - eng

PT - Journal Article

PT - Review

DEP - 20260504

PL - United States

TA - Health Sci Rep

JT - Health science reports

JID - 101728855

PMC - PMC13139648

OTO - NOTNLM

OT - delamanid

OT - drug‐resistance

OT - molecular mechanism

OT - treatment efficacy

OT - tuberculosis

COIS- The authors declare no conflicts of interest.

EDAT- 2026/05/07 06:34

MHDA- 2026/05/07 06:35

PMCR- 2026/05/04

CRDT- 2026/05/07 05:15

PHST- 2025/07/02 00:00 [received]

PHST- 2026/04/17 00:00 [revised]

PHST- 2026/04/27 00:00 [accepted]

PHST- 2026/05/07 06:35 [medline]

PHST- 2026/05/07 06:34 [pubmed]

PHST- 2026/05/07 05:15 [entrez]

PHST- 2026/05/04 00:00 [pmc-release]

AID - HSR272481 [pii]

AID - 10.1002/hsr2.72481 [doi]

PST - epublish

SO - Health Sci Rep. 2026 May 4;9:e72481. doi: 10.1002/hsr2.72481. eCollection 2026

May.

PMID- 41891964

OWN - NLM

STAT- MEDLINE

DCOM- 20260713

LR - 20260713

IS - 1791-3004 (Electronic)

IS - 1791-2997 (Print)

IS - 1791-2997 (Linking)

VI - 33

IP - 5

DP - 2026 May

TI - Photodynamic therapy: A paradigm shift in overcoming drug‑resistant tuberculosis

(Review).

LID - 145 [pii]

LID - 10.3892/mmr.2026.13855 [doi]

AB - Tuberculosis (TB) poses a notable threat to global public health. Conventional

antibiotic treatments are hampered by long treatment courses, notable toxicity

and rising levels of drug resistance. By contrast, photodynamic therapy (PDT)

offers a promising alternative to antibiotics. This approach employs

photosensitizers (PSs), light and oxygen to generate reactive oxygen species,

which induce oxidative stress to eliminate Mycobacterium tuberculosis. The

present review explores the mechanisms, benefits and primary challenges of PDT in

the context of TB treatment. The present review also discusses advanced

strategies that address hurdles, such as the robust mycobacterial cell wall,

hypoxic environments and biofilm formation. These strategies include rational PS

design, innovative nanodelivery systems and synergistic combination therapies.

The present review also explores pathways for future clinical translation,

highlighting the potential of PDT as a viable supplement or alternative to

traditional TB chemotherapy through interdisciplinary innovation.

FAU - Cheng, Shuhao

AU - Cheng S

AD - Clinical Medical College, Qinghai University, Xining, Qinghai 810016, P.R. China.

FAU - Wang, Yuqing

AU - Wang Y

AD - Department of Respiratory Medicine I, The Fourth People's Hospital of Qinghai

Province, Xining, Qinghai 810000, P.R. China.

FAU - Yao, Jie

AU - Yao J

AD - Department of Respiratory Medicine I, The Fourth People's Hospital of Qinghai

Province, Xining, Qinghai 810000, P.R. China.

FAU - Cheng, Guodong

AU - Cheng G

AD - Department of Respiratory Medicine I, The Fourth People's Hospital of Qinghai

Province, Xining, Qinghai 810000, P.R. China.

FAU - Ye, Gengzhi

AU - Ye G

AD - Department of Respiratory Medicine I, The Fourth People's Hospital of Qinghai

Province, Xining, Qinghai 810000, P.R. China.

FAU - Shi, Fapeng

AU - Shi F

AD - Department of Respiratory Medicine I, The Fourth People's Hospital of Qinghai

Province, Xining, Qinghai 810000, P.R. China.

FAU - Ma, Xiaolei

AU - Ma X

AD - Department of Respiratory Medicine I, The Fourth People's Hospital of Qinghai

Province, Xining, Qinghai 810000, P.R. China.

LA - eng

PT - Journal Article

PT - Review

DEP - 20260327

PL - Greece

TA - Mol Med Rep

JT - Molecular medicine reports

JID - 101475259

RN - 0 (Photosensitizing Agents)

RN - 0 (Antitubercular Agents)

RN - 0 (Reactive Oxygen Species)

SB - IM

MH - *Photochemotherapy/methods

MH - Humans

MH - *Mycobacterium tuberculosis/drug effects

MH - *Photosensitizing Agents/therapeutic use/pharmacology

MH - *Tuberculosis, Multidrug-Resistant/drug therapy/microbiology/metabolism

MH - Antitubercular Agents/therapeutic use/pharmacology

MH - Biofilms/drug effects

MH - Animals

MH - Reactive Oxygen Species/metabolism

PMC - PMC13051530

OTO - NOTNLM

OT - MTB

OT - PDT

OT - TB

OT - biofilm

OT - drug resistance

OT - nanomedicine

OT - targeted delivery

COIS- The authors declare that they have no competing interests.

EDAT- 2026/03/27 17:37

MHDA- 2026/03/27 17:38

PMCR- 2026/03/26

CRDT- 2026/03/27 11:03

PHST- 2025/10/24 00:00 [received]

PHST- 2026/02/23 00:00 [accepted]

PHST- 2026/03/27 17:38 [medline]

PHST- 2026/03/27 17:37 [pubmed]

PHST- 2026/03/27 11:03 [entrez]

PHST- 2026/03/26 00:00 [pmc-release]

AID - 145 [pii]

AID - MMR-33-5-13855 [pii]

AID - 10.3892/mmr.2026.13855 [doi]

PST - ppublish

SO - Mol Med Rep. 2026 May;33(5):145. doi: 10.3892/mmr.2026.13855. Epub 2026 Mar 27.

PMID- 41797151

OWN - NLM

STAT- MEDLINE

DCOM- 20260710

LR - 20260710

IS - 1873-281X (Electronic)

IS - 1472-9792 (Linking)

VI - 158

DP - 2026 May

TI - Transcriptomic signatures predict severe linezolid-induced peripheral neuropathy

and reveal pre-treatment immune and antioxidant dysregulation.

PG - 102751

LID - S1472-9792(26)00021-1 [pii]

LID - 10.1016/j.tube.2026.102751 [doi]

AB - Linezolid is an essential drug for treating multidrug-resistant and extensively

drug-resistant tuberculosis, yet its long-term use is frequently limited by

peripheral neuropathy, which in severe cases may be irreversible. To investigate

biological factors underlying susceptibility, we analyzed transcriptomic profiles

of peripheral blood mononuclear cells from 51 MDR/XDR-TB patients before and

after six months of linezolid treatment, stratifying individuals into

asymptomatic, mild, and severe neuropathy groups. Patients who later developed

severe neuropathy already exhibited distinct transcriptional signatures at

baseline, including evidence of immune dysregulation and impaired antioxidant

defense, with downregulation of genes involved in immune receptor activity and

the pentose phosphate pathway. Following treatment, these patients showed limited

transcriptional responses compared with other groups, most notably a marked

downregulation of IL10, a central anti-inflammatory cytokine, suggesting

maladaptive systemic regulation. Using a machine learning-based feature selection

approach, we derived an 11-gene predictive model that achieved strong performance

with a cross-validated AUC of 0.93. Model interpretation using SHAP values

highlighted HP and HSPA1B as the most influential predictors. Overall, our

findings suggest that severe linezolid-induced peripheral neuropathy is

predisposed by baseline immune and metabolic alterations and that patients with

this complication fail to mount appropriate systemic responses during treatment.

CI - Copyright © 2026. Published by Elsevier Ltd.

FAU - Shi, Chenyan

AU - Shi C

AD - School of Public Health, Shenzhen University Medical School, Shenzhen, China;

Guangdong Provincial Key Laboratory of Infection Immunity and Inflammation,

Department of Pathogen Biology, Shenzhen University Medical School, Shenzhen,

China.

FAU - Zhang, Peize

AU - Zhang P

AD - Department of Pulmonary Medicine & Tuberculosis, Shenzhen Third People's

Hospital, National Clinical Research Center for Infectious Disease, Southern

University of Science and Technology, Shenzhen, China.

FAU - Zhen, Junfeng

AU - Zhen J

AD - Department of Pulmonary Medicine & Tuberculosis, Shenzhen Third People's

Hospital, National Clinical Research Center for Infectious Disease, Southern

University of Science and Technology, Shenzhen, China.

FAU - Zhang, Jing

AU - Zhang J

AD - School of Public Health, Shenzhen University Medical School, Shenzhen, China.

FAU - Wang, Yuxiang

AU - Wang Y

AD - Department of Pulmonary Medicine & Tuberculosis, Shenzhen Third People's

Hospital, National Clinical Research Center for Infectious Disease, Southern

University of Science and Technology, Shenzhen, China.

FAU - Xiao, Guangyang

AU - Xiao G

AD - School of Public Health, Shenzhen University Medical School, Shenzhen, China.

FAU - Yu, Xue

AU - Yu X

AD - School of Public Health, Shenzhen University Medical School, Shenzhen, China.

FAU - Wang, Tong

AU - Wang T

AD - School of Public Health, Shenzhen University Medical School, Shenzhen, China.

FAU - Wang, Wenfei

AU - Wang W

AD - National Clinical Research Center for Infectious Disease, Shenzhen Third People's

Hospital, Southern University of Science and Technology, Shenzhen, China.

FAU - Cai, Yi

AU - Cai Y

AD - Guangdong Provincial Key Laboratory of Infection Immunity and Inflammation,

Department of Pathogen Biology, Shenzhen University Medical School, Shenzhen,

China.

FAU - Chen, Xinchun

AU - Chen X

AD - Guangdong Provincial Key Laboratory of Infection Immunity and Inflammation,

Department of Pathogen Biology, Shenzhen University Medical School, Shenzhen,

China. Electronic address: chenxinchun@szu.edu.cn.

FAU - Deng, Guofang

AU - Deng G

AD - Department of Pulmonary Medicine & Tuberculosis, Shenzhen Third People's

Hospital, National Clinical Research Center for Infectious Disease, Southern

University of Science and Technology, Shenzhen, China. Electronic address:

jxxk1035@yeah.net.

LA - eng

PT - Journal Article

DEP - 20260303

PL - Scotland

TA - Tuberculosis (Edinb)

JT - Tuberculosis (Edinburgh, Scotland)

JID - 100971555

RN - ISQ9I6J12J (Linezolid)

RN - 0 (Antitubercular Agents)

RN - 0 (Antioxidants)

SB - IM

MH - Humans

MH - *Linezolid/adverse effects

MH - *Peripheral Nervous System Diseases/chemically

induced/genetics/immunology/diagnosis/metabolism

MH - *Transcriptome

MH - *Gene Expression Profiling/methods

MH - Female

MH - *Antitubercular Agents/adverse effects

MH - Male

MH - *Antioxidants/metabolism

MH - Adult

MH - Severity of Illness Index

MH - *Tuberculosis, Multidrug-Resistant/drug therapy/immunology

MH - Middle Aged

MH - Risk Factors

MH - Leukocytes, Mononuclear/metabolism/immunology

MH - Time Factors

OTO - NOTNLM

OT - Drug-resistant tuberculosis

OT - Linezolid

OT - Peripheral neuropathy

OT - Risk prediction

OT - Transcriptome

COIS- Declaration of competing interest The authors declare the following financial

interests/personal relationships which may be considered as potential competing

interests:Guofang Deng reports financial support was provided by Shenzhen Medical

Research Project. If there are other authors, they declare that they have no

known competing financial interests or personal relationships that could have

appeared to influence the work reported in this paper.

EDAT- 2026/03/09 07:41

MHDA- 2026/05/05 00:32

CRDT- 2026/03/09 01:15

PHST- 2025/12/16 00:00 [received]

PHST- 2026/02/13 00:00 [revised]

PHST- 2026/03/02 00:00 [accepted]

PHST- 2026/05/05 00:32 [medline]

PHST- 2026/03/09 07:41 [pubmed]

PHST- 2026/03/09 01:15 [entrez]

AID - S1472-9792(26)00021-1 [pii]

AID - 10.1016/j.tube.2026.102751 [doi]

PST - ppublish

SO - Tuberculosis (Edinb). 2026 May;158:102751. doi: 10.1016/j.tube.2026.102751. Epub

2026 Mar 3.

PMID- 41759600

OWN - NLM

STAT- MEDLINE

DCOM- 20260710

LR - 20260710

IS - 1872-7913 (Electronic)

IS - 0924-8579 (Linking)

VI - 67

IP - 5

DP - 2026 May

TI - L-Cycloserine, not d-Cycloserine, inhibits IscS-mediated iron-sulfur cluster

biosynthesis in Escherichia coli.

PG - 107761

LID - S0924-8579(26)00050-6 [pii]

LID - 10.1016/j.ijantimicag.2026.107761 [doi]

AB - OBJECTIVES: D-Cycloserine (DCS) is a broad-spectrum antibiotic used to treat

drug-resistant tuberculosis, primarily by inhibiting pyridoxal 5'-phosphate

(PLP)-dependent enzymes. Recent studies suggest that cycloserine may also target

cysteine desulfurases involved in iron-sulfur (Fe-S) cluster biogenesis, but the

underlying mechanism remains unclear. This study aims to elucidate the

mechanistic details of how cycloserine affects Fe-S cluster assembly and to

explore its potential against resistant strains. METHODS: The effects of DCS and

its enantiomer L-Cycloserine (LCS) on Fe-S cluster biosynthesis were investigated

using Escherichia coli (E. coli) IscS as a model cysteine desulfurase. Enzyme

activity assays were performed to assess inhibition. Liquid chromatography-mass

spectrometry (LC-MS) was employed to analyze changes in the PLP cofactor bound to

IscS. Antimicrobial efficacy was tested against the uropathogenic E. coli strain

CFT073 and experimentally evolved DCS-resistant strains in synthetic human urine

medium. RESULTS: LCS, but not DCS, inhibited the cysteine desulfurase activity of

IscS, leading to impaired Fe-S cluster biosynthesis. Specifically, LC-MS analysis

revealed that LCS converts the PLP cofactor bound to IscS to pyridoxamine

5'-phosphate (PMP). Furthermore, LCS significantly suppressed the growth of

uropathogenic E. coli CFT073 and retained effectiveness against DCS-resistant

strains in synthetic human urine medium. CONCLUSIONS: These findings demonstrate

that LCS inhibits Fe-S cluster biosynthesis by targeting the cysteine desulfurase

IscS through PLP-to-PMP conversion. The results highlight Fe-S cluster biogenesis

as a potential antimicrobial target and suggest that LCS may serve as a promising

therapeutic alternative for combating DCS-resistant bacterial infections.

CI - Copyright © 2026 Elsevier Ltd and International Society of Antimicrobial

Chemotherapy. All rights reserved.

FAU - Zhang, Lihe

AU - Zhang L

AD - Department of Rheumatology, The Second Affiliated Hospital of Wenzhou Medical

University, Wenzhou, Zhejiang, China.

FAU - Li, Yongjuan

AU - Li Y

AD - Key Laboratory of Artificial Organs and Computational Medicine in Zhejiang

Province, Shulan International Medical College, Zhejiang Shuren University,

Hangzhou, Zhejiang, China.

FAU - Bao, Yuhang

AU - Bao Y

AD - School of Laboratory Medicine and Life Sciences, Wenzhou Medical University,

Wenzhou, Zhejiang, China.

FAU - Feng, Jie

AU - Feng J

AD - School of Laboratory Medicine and Life Sciences, Wenzhou Medical University,

Wenzhou, Zhejiang, China.

FAU - Chen, Runyu

AU - Chen R

AD - School of Laboratory Medicine and Life Sciences, Wenzhou Medical University,

Wenzhou, Zhejiang, China.

FAU - Zhang, Wenjing

AU - Zhang W

AD - School of Laboratory Medicine and Life Sciences, Wenzhou Medical University,

Wenzhou, Zhejiang, China.

FAU - Zhu, Yixuan

AU - Zhu Y

AD - School of Pharmaceutical Science, Wenzhou Medical University, Wenzhou, Zhejiang,

China.

FAU - Zhang, Liwen

AU - Zhang L

AD - School of Laboratory Medicine and Life Sciences, Wenzhou Medical University,

Wenzhou, Zhejiang, China.

FAU - Zhang, Yin

AU - Zhang Y

AD - School of Laboratory Medicine and Life Sciences, Wenzhou Medical University,

Wenzhou, Zhejiang, China.

FAU - Liang, Feng

AU - Liang F

AD - Department of Clinical Laboratory, The First Affiliated Hospital of Wenzhou

Medical University, Wenzhou, Zhejiang, China.

FAU - Chen, Huimin

AU - Chen H

AD - Department of Laboratory Medicine, Taizhou Municipal Hospital (Taizhou University

Affiliated Municipal Hospital), School of Medicine, Taizhou Key Laboratory of

Infection and Tumor Immunology, Taizhou University, Taizhou, Zhejiang, China.

FAU - Luo, Xinhua

AU - Luo X

AD - Department of Laboratory Medicine, Taizhou Municipal Hospital (Taizhou University

Affiliated Municipal Hospital), School of Medicine, Taizhou Key Laboratory of

Infection and Tumor Immunology, Taizhou University, Taizhou, Zhejiang, China.

Electronic address: slyy_01541@tzc.edu.cn.

FAU - Tan, Guoqiang

AU - Tan G

AD - School of Laboratory Medicine and Life Sciences, Wenzhou Medical University,

Wenzhou, Zhejiang, China. Electronic address: tgq@wmu.edu.cn.

FAU - Wang, Wu

AU - Wang W

AD - School of Laboratory Medicine and Life Sciences, Wenzhou Medical University,

Wenzhou, Zhejiang, China. Electronic address: wangwu@wmu.edu.cn.

LA - eng

PT - Journal Article

DEP - 20260225

PL - Netherlands

TA - Int J Antimicrob Agents

JT - International journal of antimicrobial agents

JID - 9111860

RN - 95IK5KI84Z (Cycloserine)

RN - EC 4.4.1.- (cysteine desulfurase)

RN - 0 (Iron-Sulfur Proteins)

RN - EC 4.4.- (Carbon-Sulfur Lyases)

RN - 0 (Escherichia coli Proteins)

RN - 5V5IOJ8338 (Pyridoxal Phosphate)

RN - 70FD1KFU70 (Sulfur)

RN - 0 (Anti-Bacterial Agents)

SB - IM

MH - *Cycloserine/pharmacology

MH - *Escherichia coli/drug effects/metabolism

MH - *Iron-Sulfur Proteins/biosynthesis

MH - *Carbon-Sulfur Lyases/antagonists & inhibitors/metabolism

MH - *Escherichia coli Proteins/metabolism/antagonists & inhibitors

MH - Pyridoxal Phosphate/metabolism

MH - Humans

MH - Sulfur/metabolism

MH - *Anti-Bacterial Agents/pharmacology

MH - Chromatography, Liquid

MH - Mass Spectrometry

OTO - NOTNLM

OT - Cysteine desulfurases

OT - Iron-sulfur cluster assembly

OT - L-Cycloserine

OT - d-Cycloserine

EDAT- 2026/02/28 00:41

MHDA- 2026/05/05 00:32

CRDT- 2026/02/27 19:11

PHST- 2025/08/16 00:00 [received]

PHST- 2026/02/03 00:00 [revised]

PHST- 2026/02/19 00:00 [accepted]

PHST- 2026/05/05 00:32 [medline]

PHST- 2026/02/28 00:41 [pubmed]

PHST- 2026/02/27 19:11 [entrez]

AID - S0924-8579(26)00050-6 [pii]

AID - 10.1016/j.ijantimicag.2026.107761 [doi]

PST - ppublish

SO - Int J Antimicrob Agents. 2026 May;67(5):107761. doi:

10.1016/j.ijantimicag.2026.107761. Epub 2026 Feb 25.

PMID- 41197090

OWN - NLM

STAT- MEDLINE

DCOM- 20260505

LR - 20260628

IS - 1538-0254 (Electronic)

IS - 0739-1102 (Linking)

VI - 44

IP - 8

DP - 2026 May

TI - DapB is a conserved target for tackling antimicrobial resistance in Mycobacterium

tuberculosis.

PG - 4045-4056

LID - 10.1080/07391102.2025.2581074 [doi]

AB - Tuberculosis (TB) remains a major global health issue, with growing challenges

posed by drug resistance, highlighting the urgent need to identify new drug

targets. This study examines the conservation pattern of the dapB gene, which

encodes Dihydrodipicolinate reductase (DapB), a key enzyme in the lysine

biosynthesis pathway of Mycobacterium tuberculosis (Mtb), a pathway absent in

humans. The dapB gene was amplified from 72 Indian clinical isolates, sequenced

and analysed for mutations. Further, the genomic data of 310 isolates from the

Bacterial and Viral Bioinformatics Resource Center (BV-BRC) database were also

analysed. Further, atomistic simulations were performed in triplicate for the

wild-type and mutant proteins to assess the impact of mutations on protein

structure and function. Sequence analysis identified a single DapB mutation

(DapB_65), among clinical isolates. Analysis of BV-BRC isolates revealed two

synonymous mutations and one non-synonymous mutation (DapB_89). The mutations

were mapped on the surface of the protein and were found to be more than 1.0 nm

away from the active site. Simulations reveal no significant difference in the

overall structure or the binding pocket dimensions and volumes between the native

and mutant proteins. This study thus highlights the potential of DapB as a

conserved drug target for future drug development efforts aimed at TB.

FAU - Mahajan, Pratik

AU - Mahajan P

AD - ICMR - National Institute of Translational Virology and AIDS Research (formerly

NARI), Pune, India.

AD - Bioinformatics Centre, Savitribai Phule Pune University, Pune, India.

FAU - Gor, Hetarth

AU - Gor H

AD - ICMR - National Institute of Translational Virology and AIDS Research (formerly

NARI), Pune, India.

FAU - Joshi, Manali

AU - Joshi M

AD - Bioinformatics Centre, Savitribai Phule Pune University, Pune, India.

FAU - Nema, Vijay

AU - Nema V

AD - ICMR - National Institute of Translational Virology and AIDS Research (formerly

NARI), Pune, India.

AD - Academy of Scientific and Innovative Research, Ghaziabad, India.

AD - ICMR - National Institute of Research in Tribal Health, Jabalpur, India.

LA - eng

PT - Journal Article

PT - Research Support, Non-U.S. Gov't

DEP - 20251106

PL - England

TA - J Biomol Struct Dyn

JT - Journal of biomolecular structure & dynamics

JID - 8404176

RN - EC 1.17.1.8 (Dihydrodipicolinate Reductase)

RN - 0 (Bacterial Proteins)

RN - 0 (Antitubercular Agents)

SB - IM

MH - *Mycobacterium tuberculosis/drug effects/genetics/enzymology

MH - *Dihydrodipicolinate Reductase/genetics/chemistry/antagonists &

inhibitors/metabolism

MH - Mutation

MH - Humans

MH - Molecular Dynamics Simulation

MH - *Drug Resistance, Bacterial/genetics

MH - *Bacterial Proteins/genetics/chemistry/metabolism

MH - Antitubercular Agents/pharmacology/chemistry

MH - Amino Acid Sequence

MH - Tuberculosis/microbiology/drug therapy

MH - Models, Molecular

MH - Protein Conformation

OTO - NOTNLM

OT - DAP pathway

OT - MDR and XDR-TB

OT - Mycobacterium tuberculosis

OT - dapB (Rv2773c)

OT - dihydrodipicolinate reductase

EDAT- 2025/11/06 18:27

MHDA- 2026/05/05 06:33

CRDT- 2025/11/06 16:03

PHST- 2026/05/05 06:33 [medline]

PHST- 2025/11/06 18:27 [pubmed]

PHST- 2025/11/06 16:03 [entrez]

AID - 10.1080/07391102.2025.2581074 [doi]

PST - ppublish

SO - J Biomol Struct Dyn. 2026 May;44(8):4045-4056. doi:

10.1080/07391102.2025.2581074. Epub 2025 Nov 6.

PMID- 42163886

OWN - NLM

STAT- PubMed-not-MEDLINE

DCOM- 20260521

LR - 20260521

IS - 2156-6976 (Print)

IS - 2156-6976 (Electronic)

IS - 2156-6976 (Linking)

VI - 16

IP - 4

DP - 2026

TI - Diagnostic efficacy of the TSPOT assay for tuberculosis infection and drug

resistance in mycobacterium tuberculosis in patients with malignant tumors.

PG - 1608-1621

LID - 10.62347/DVJA7932 [doi]

AB - Immunosuppression in cancer patients clearly elevates the risk of acquiring

tuberculosis (TB) infection, and traditional diagnostic methods are characterized

by low sensitivity (Sen) and long turnaround times. Although the interferon-gamma

release assay (IGRA) is widely used, its ability to distinguish active

tuberculosis (ATB) from latent tuberculosis infection (LTBI) in cancer patients,

as well as its relationship with drug resistance (DR), remains unclear. This

study aimed to evaluate the diagnostic efficacy of T-SPOT for active tuberculosis

and its ability to distinguish active tuberculosis from latent tuberculosis

infection in patients with malignant tumors. A retrospective analysis was

conducted on data from 634 patients with malignant tumors, and patients were

grouped: active tuberculosis (ATB) group (AG, n = 76), LTBI group (BG, n = 184),

and non-TB group (CG, n = 374). The interferon-gamma (IFN-γ) response induced by

Early Secreted Antigenic Target 6 (ESAT-6)/Culture Filtrate Protein 10 (CFP-10)

was detected by TSPOT, and the relationship between TSPOT and DR was analyzed.

Subgroup analysis and multivariate regression were used to explore the

immune-related factors affecting the positive rate of TSPOT. The results showed

Sen for ATB was 86.8%, and specificity (Spe) was 73.3%. The Sen for screening TB

infections was 80.4%, with a Spe of 98.4% and a positive predictive value (PPV)

of 97.2%. The Spe for differentiating ATB from LTBI was only 22.3%. The overall

DR rate in the AG was 59.2% (45/76), including rifampicin (RIF) resistance

(36.8%), multidrug-resistant TB (MDR-TB) (21.1%), and pre-extensively

drug-resistant TB (Pre-XDR-TB) (7.9%). The median total spot count of TSPOT in

the drug-resistant group was significantly lower than that in the drug-sensitive

group [52 (IQR 25-85) vs. 120 (IQR 85-188), P < 0.001], and the spot count showed

a decreasing trend as the number of drug-resistant types increased (P < 0.001).

Multivariate regression showed that low lymphocyte count (< 0.8×10(9)/L) and

high-dose steroid use (> 40 mg/d) were independent factors associated with a

reduced positive rate of TSPOT. In summary, TSPOT is suitable for screening TB

infection in patients with malignant tumors but has limited capacity to

differentiate ATB from LTBI. The significant reduction in TSPOT spot counts was

closely associated with MTB drug resistance and host immunosuppressive status,

suggesting that this indicator may serve as an auxiliary early warning tool for

drug-resistant tuberculosis.

CI - AJCR Copyright © 2026.

FAU - Shi, Jianguo

AU - Shi J

AD - Department of Clinical Laboratory, First Hospital of Shanxi Medical University

Taiyuan 030001, Shanxi, China.

FAU - Shi, Zhengqin

AU - Shi Z

AD - Department of Rehabilitation, Yuci District Hospital of Traditional Chinese

Medicine Jinzhong 030600, Shanxi, China.

FAU - Li, Hao

AU - Li H

AD - Department of Clinical Laboratory, First Hospital of Shanxi Medical University

Taiyuan 030001, Shanxi, China.

FAU - Hu, Min

AU - Hu M

AD - Department of Clinical Laboratory, First Hospital of Shanxi Medical University

Taiyuan 030001, Shanxi, China.

FAU - Nan, Junbo

AU - Nan J

AD - Venous Access Center, First Hospital of Shanxi Medical University Taiyuan 030001,

Shanxi, China.

LA - eng

PT - Journal Article

DEP - 20260425

PL - United States

TA - Am J Cancer Res

JT - American journal of cancer research

JID - 101549944

PMC - PMC13184767

OTO - NOTNLM

OT - ATB

OT - LTBI

OT - TSPOT

OT - diagnostic efficacy

OT - drug-resistant TB

OT - immune response

OT - malignant tumors

COIS- None.

EDAT- 2026/05/21 06:33

MHDA- 2026/05/21 06:34

PMCR- 2026/04/25

CRDT- 2026/05/21 04:34

PHST- 2026/03/10 00:00 [received]

PHST- 2026/04/21 00:00 [accepted]

PHST- 2026/05/21 06:34 [medline]

PHST- 2026/05/21 06:33 [pubmed]

PHST- 2026/05/21 04:34 [entrez]

PHST- 2026/04/25 00:00 [pmc-release]

AID - 10.62347/DVJA7932 [doi]

PST - epublish

SO - Am J Cancer Res. 2026 Apr 25;16(4):1608-1621. doi: 10.62347/DVJA7932. eCollection

2026.

PMID- 42003198

OWN - NLM

STAT- Publisher

LR - 20260420

IS - 1875-5550 (Electronic)

IS - 1389-2037 (Linking)

DP - 2026 Apr 17

TI - Targeting Drug-Resistant Tuberculosis with Antimicrobial Peptides: Opportunities

and Challenges.

LID - 10.2174/0113892037433604260409094048 [doi]

AB - INTRODUCTION: One of the main causes of infectious disease-related deaths

globally is Mycobacterium tuberculosis (M.tb), the causative agent of

tuberculosis (TB). Novel treatment strategies are now much more urgent due to the

emergence of Extensively Drug-Resistant (XDR) and multidrug-resistant (MDR)

strains. The purpose of this study is to investigate the potential of

antimicrobial peptides (AMPs) as TB adjunctive therapeutic agents, with an

emphasis on their immunomodulatory and direct antimycobacterial effects. METHODS:

A summary of how antimicrobial peptides (AMPs) work was carried out, including

how they can damage bacterial membranes and obstruct intracellular processes.

Furthermore, the review evaluated how AMPs affect host immune responses,

including enhanced phagosome-lysosome fusion, induction of autophagy, and

cytokine production. RESULTS: Many AMPs have shown strong antimycobacterial

activity in vitro and in vivo, including defensins, cathelicidins (like LL-37),

and synthetic analogues. Even with these encouraging outcomes, issues such as

peptide stability and efficient delivery still stand in the way of clinical use.

Potential remedies for these restrictions are offered by advances in peptide

engineering, nanoparticle- based delivery methods, and structure-activity

relationship (SAR) research. DISCUSSION: Recent advances in peptide engineering,

nanoparticle-based delivery systems, and structure-activity relationship (SAR)

studies offer promising strategies to overcome the limitations associated with

AMP stability, bioavailability, and targeted delivery. These developments

significantly enhance the translational potential of AMPs for TB therapy.

CONCLUSION: Because of their distinct mechanisms of action and capacity to

overcome drug resistance, AMPs offer a novel and promising approach to the

treatment of tuberculosis. Global TB control efforts could be greatly aided by

ongoing research into maximizing their safety and effectiveness.

CI - Copyright© Bentham Science Publishers; For any queries, please email at

epub@benthamscience.net.

FAU - Ahmad, Asad

AU - Ahmad A

AD - Faculty of Pharmacy, Integral University, Lucknow, (UP), India.

FAU - Wasim, Rufaida

AU - Wasim R

AD - Faculty of Pharmacy, Integral University, Lucknow, (UP), India.

FAU - Islam, Anas

AU - Islam A

AD - Faculty of Pharmacy, Integral University, Lucknow, (UP), India.

FAU - Srivastava, Akash

AU - Srivastava A

AD - Faculty of Pharmacy, Integral University, Lucknow, (UP), India.

LA - eng

PT - Journal Article

DEP - 20260417

PL - United Arab Emirates

TA - Curr Protein Pept Sci

JT - Current protein & peptide science

JID - 100960529

SB - IM

OTO - NOTNLM

OT - Antimicrobial peptides

OT - anti-tubercular drugs

OT - antibiotic resistance

OT - immunomodulation

OT - innate immunity

OT - peptide engineering.

OT - tuberculosis

EDAT- 2026/04/20 13:10

MHDA- 2026/04/20 13:10

CRDT- 2026/04/20 02:33

PHST- 2025/09/12 00:00 [received]

PHST- 2025/12/10 00:00 [revised]

PHST- 2025/12/15 00:00 [accepted]

PHST- 2026/04/20 13:10 [medline]

PHST- 2026/04/20 13:10 [pubmed]

PHST- 2026/04/20 02:33 [entrez]

AID - CPPS-EPUB-154925 [pii]

AID - 10.2174/0113892037433604260409094048 [doi]

PST - aheadofprint

SO - Curr Protein Pept Sci. 2026 Apr 17. doi: 10.2174/0113892037433604260409094048.

PMID- 41915819

OWN - NLM

STAT- MEDLINE

DCOM- 20260714

LR - 20260714

IS - 2373-8227 (Electronic)

IS - 2373-8227 (Linking)

VI - 12

IP - 4

DP - 2026 Apr 10

TI - Simultaneous Inhibition of Cytochrome bd Oxidase and ATP Synthase for Complete

Eradication of Mycobacterium tuberculosis.

PG - 1401-1408

LID - 10.1021/acsinfecdis.5c01066 [doi]

AB - The clinical utility of bedaquiline (BDQ), a first-in-class ATP synthase

inhibitor and cornerstone of modern multidrug-resistant tuberculosis (MDR-TB)

therapy, is compromised by a delayed onset of bactericidal killing and the

emergence of resistance. This tolerance is linked to Mycobacterium tuberculosis

metabolic heterogeneity and functional redundancy within its electron transport

chain (ETC), notably the activity of the cytochrome bd oxidase (Cyt-bd). We

hypothesized that cotargeting the cytochrome bd oxidase and the F(1)F(0)-ATP

synthase would induce potent, synergistic killing by completely disrupting

bacterial energetics. Our results demonstrate that this combination drives rapid,

synergistic bactericidal activity within 09 days and complete clearance within 12

days. Mechanistic studies revealed that Cyt-bd inhibitor ND-011992 blocks the

respiratory redundancy that permits survival under BDQ pressure, leading to

catastrophic ATP depletion and a substantial arrest of oxygen consumption. This

potentiation was equally effective against nonreplicating, hypoxic bacilli and

eradicated 90% of intracellular bacteria in a macrophage model. These findings

provide critical proof-of-concept that targeting compensatory respiratory

pathways can overcome phenotypic drug tolerance. Thus, this validates Cyt-bd as a

high-value target and outlines a potent, sterilizing combination regimen capable

of countering the heterogeneity that sabotages current TB therapies.

FAU - Roy, Arnab

AU - Roy A

AD - Department of Biological Sciences (Pharmacology and Toxicology), National

Institute of Pharmaceutical Education and Research (NIPER), Hyderabad, Telangana

500037, India.

FAU - Naik, Dinesh

AU - Naik D

AD - Department of Biological Sciences (Pharmacology and Toxicology), National

Institute of Pharmaceutical Education and Research (NIPER), Hyderabad, Telangana

500037, India.

FAU - Sau, Shashikanta

AU - Sau S

AD - Department of Biological Sciences (Pharmacology and Toxicology), National

Institute of Pharmaceutical Education and Research (NIPER), Hyderabad, Telangana

500037, India.

FAU - Agnivesh, Puja Kumari

AU - Agnivesh PK

AD - Department of Biological Sciences (Pharmacology and Toxicology), National

Institute of Pharmaceutical Education and Research (NIPER), Hyderabad, Telangana

500037, India.

FAU - Parida, Kishan Kumar

AU - Parida KK

AD - Department of Biological Sciences (Pharmacology and Toxicology), National

Institute of Pharmaceutical Education and Research (NIPER), Hyderabad, Telangana

500037, India.

FAU - Kalia, Nitin Pal

AU - Kalia NP

AUID- ORCID: 0000-0003-4346-6567

AD - Department of Biological Sciences (Pharmacology and Toxicology), National

Institute of Pharmaceutical Education and Research (NIPER), Hyderabad, Telangana

500037, India.

LA - eng

PT - Journal Article

DEP - 20260331

PL - United States

TA - ACS Infect Dis

JT - ACS infectious diseases

JID - 101654580

RN - 0 (Diarylquinolines)

RN - 78846I289Y (bedaquiline)

RN - 0 (Antitubercular Agents)

RN - EC 3.6.3.- (Mitochondrial Proton-Translocating ATPases)

RN - EC 1.- (Oxidoreductases)

RN - 55G92WGH3X (telacebec)

RN - 0 (Cytochrome b Group)

RN - 8L70Q75FXE (Adenosine Triphosphate)

RN - EC 3.6.1.- (F1F0-ATP synthase)

RN - 0 (Enzyme Inhibitors)

RN - 0 (Bacterial Proteins)

RN - 0 (Imidazoles)

RN - 0 (Piperidines)

RN - 0 (Pyridines)

SB - IM

MH - *Mycobacterium tuberculosis/drug effects/enzymology

MH - Diarylquinolines/pharmacology

MH - *Antitubercular Agents/pharmacology

MH - Macrophages/microbiology

MH - Humans

MH - *Mitochondrial Proton-Translocating ATPases/antagonists & inhibitors/metabolism

MH - Animals

MH - *Oxidoreductases/antagonists & inhibitors

MH - *Cytochrome b Group/antagonists & inhibitors/metabolism

MH - Adenosine Triphosphate/metabolism

MH - Microbial Sensitivity Tests

MH - Drug Synergism

MH - *Enzyme Inhibitors/pharmacology

MH - Bacterial Proteins/antagonists & inhibitors/metabolism

MH - Imidazoles

MH - Piperidines

MH - Pyridines

OTO - NOTNLM

OT - ATP synthase

OT - Mycobacterium tuberculosis

OT - bedaquiline

OT - multidrug resistance

OT - oxidative phosphorylation

EDAT- 2026/03/31 18:49

MHDA- 2026/04/10 06:32

CRDT- 2026/03/31 15:22

PHST- 2026/04/10 06:32 [medline]

PHST- 2026/03/31 18:49 [pubmed]

PHST- 2026/03/31 15:22 [entrez]

AID - 10.1021/acsinfecdis.5c01066 [doi]

PST - ppublish

SO - ACS Infect Dis. 2026 Apr 10;12(4):1401-1408. doi: 10.1021/acsinfecdis.5c01066.

Epub 2026 Mar 31.

PMID- 41917335

OWN - NLM

STAT- MEDLINE

DCOM- 20260714

LR - 20260714

IS - 2399-3642 (Electronic)

IS - 2399-3642 (Linking)

VI - 9

IP - 1

DP - 2026 Mar 31

TI - Questiomycin A demonstrates antibacterial activity against Mycobacterium

tuberculosis by directly targeting FabD.

LID - 10.1038/s42003-026-09947-7 [doi]

LID - 709

AB - Tuberculosis (TB), caused by Mycobacterium tuberculosis (Mtb), remains a major

global health threat, particularly due to the rise of multidrug-resistant TB

(MDR-TB). Current treatment limitations highlight the urgent need for new

therapeutics to improve efficacy and shorten therapy duration. Through

high-throughput screening, we identified Questiomycin A (QM), a phenoxazinone

compound with promising anti-tuberculosis activity against drug-sensitive and

drug-resistant Mtb strains. Transcriptomic profiling indicated disruption of cell

wall-associated pathways, while thermal proteome profiling showed that QM binding

substantially increased the thermal stability of FabD (malonyl CoA-acyl carrier

protein transacylase, MCAT), a key enzyme in mycolic acid biosynthesis. Surface

plasmon resonance, enzymatic assays, and genetic manipulation confirmed QM

directly binds to and inhibits FabD. This inhibition damages cell wall integrity,

increases permeability, disturbs intracellular pH homeostasis, and collapses the

proton motive force, ultimately leading to bacterial death. Pharmacokinetic

evaluation in six-week-old male Balb/c mice following a single oral dose

demonstrates rapid absorption but limited drug exposure. Together, these findings

reveal that QM targets FabD to compromise cell wall homeostasis in Mtb, providing

a foundation for developing novel anti-tubercular agents targeting FabD.

CI - © 2026. The Author(s).

FAU - Xu, Lingling

AU - Xu L

AUID- ORCID: 0009-0002-0319-216X

AD - Department of Pharmacology, Beijing Chest Hospital, Capital Medical University,

Beijing Tuberculosis and Thoracic Tumor Research Institute, Beijing, China.

FAU - Xu, Manyi

AU - Xu M

AD - Department of Pharmacology, Beijing Chest Hospital, Capital Medical University,

Beijing Tuberculosis and Thoracic Tumor Research Institute, Beijing, China.

FAU - Wang, Bin

AU - Wang B

AD - Department of Pharmacology, Beijing Chest Hospital, Capital Medical University,

Beijing Tuberculosis and Thoracic Tumor Research Institute, Beijing, China.

FAU - Li, Xinda

AU - Li X

AD - Department of Pharmacology, Beijing Chest Hospital, Capital Medical University,

Beijing Tuberculosis and Thoracic Tumor Research Institute, Beijing, China.

FAU - Jia, Qiyue

AU - Jia Q

AD - Department of Pharmacology, Beijing Chest Hospital, Capital Medical University,

Beijing Tuberculosis and Thoracic Tumor Research Institute, Beijing, China.

FAU - Liu, Chennan

AU - Liu C

AD - Department of Pharmacology, Beijing Chest Hospital, Capital Medical University,

Beijing Tuberculosis and Thoracic Tumor Research Institute, Beijing, China.

FAU - Ye, Yangxue

AU - Ye Y

AD - Department of Pharmacology, Beijing Chest Hospital, Capital Medical University,

Beijing Tuberculosis and Thoracic Tumor Research Institute, Beijing, China.

FAU - Wang, Zimo

AU - Wang Z

AD - Department of Pharmacology, Beijing Chest Hospital, Capital Medical University,

Beijing Tuberculosis and Thoracic Tumor Research Institute, Beijing, China.

FAU - Wang, Lu

AU - Wang L

AD - Department of Pharmacology, Beijing Chest Hospital, Capital Medical University,

Beijing Tuberculosis and Thoracic Tumor Research Institute, Beijing, China.

FAU - Zhang, Weiyan

AU - Zhang W

AD - Department of Pharmacology, Beijing Chest Hospital, Capital Medical University,

Beijing Tuberculosis and Thoracic Tumor Research Institute, Beijing, China.

FAU - Lu, Yu

AU - Lu Y

AUID- ORCID: 0000-0002-9038-1884

AD - Department of Pharmacology, Beijing Chest Hospital, Capital Medical University,

Beijing Tuberculosis and Thoracic Tumor Research Institute, Beijing, China.

luyu4876@hotmail.com.

LA - eng

GR - DFL20221402/Beijing Municipal Administration of Hospitals/

PT - Journal Article

DEP - 20260331

PL - England

TA - Commun Biol

JT - Communications biology

JID - 101719179

RN - 0 (Antitubercular Agents)

RN - 0 (Bacterial Proteins)

SB - IM

MH - *Mycobacterium tuberculosis/drug effects/genetics/enzymology

MH - *Antitubercular Agents/pharmacology

MH - Animals

MH - *Bacterial Proteins/metabolism/antagonists & inhibitors

MH - Mice

MH - Microbial Sensitivity Tests

MH - Cell Wall/drug effects

MH - Tuberculosis/drug therapy/microbiology

PMC - PMC13201773

COIS- Competing interests: The authors declare no competing interests.

EDAT- 2026/04/01 00:28

MHDA- 2026/05/26 00:32

PMCR- 2026/03/31

CRDT- 2026/03/31 23:56

PHST- 2025/05/14 00:00 [received]

PHST- 2026/03/18 00:00 [accepted]

PHST- 2026/05/26 00:32 [medline]

PHST- 2026/04/01 00:28 [pubmed]

PHST- 2026/03/31 23:56 [entrez]

PHST- 2026/03/31 00:00 [pmc-release]

AID - 10.1038/s42003-026-09947-7 [pii]

AID - 9947 [pii]

AID - 10.1038/s42003-026-09947-7 [doi]

PST - epublish

SO - Commun Biol. 2026 Mar 31;9(1):709. doi: 10.1038/s42003-026-09947-7.

PMID- 41971300

OWN - NLM

STAT- MEDLINE

DCOM- 20260714

LR - 20260714

IS - 2296-2565 (Electronic)

IS - 2296-2565 (Linking)

VI - 14

DP - 2026

TI - Glucose driven bacterial persistence in extensively drug-resistant tuberculosis

with diabetes.

PG - 1746879

LID - 10.3389/fpubh.2026.1746879 [doi]

LID - 1746879

AB - BACKGROUND: Diabetes mellitus (DM) disrupts the metabolic environment of

extensively drug-resistant tuberculosis (XDR) patients, promoting mycobacterial

persistence. Key factors influencing bacterial clearance remain unclear. This

study aimed to identify critical host factors and assess the therapeutic value of

intensive glucose control. METHOD: A 48-month single-center retrospective cohort

study compared sputum smear conversion (bacterial clearance) between 57 XDR-only

(XDR) and 84 XDR with DM (XDR + DM) patients (similar treatment, consistent in

vitro MIC resistance profiles). Kaplan-Meier analysis, Cox regression,

generalized linear models, and mediation analysis were used to explore

associations between diabetic status, blood glucose (GLU) levels, and bacterial

clearance, as well as the impact of glucose control on treatment progression.

RESULTS: Compared with XDR patients, the XDR + DM group exhibited a significantly

lower bacterial clearance rate (80.7% vs. 45.2%) and longer median clearance time

(6 vs. 12 months), despite comparable baseline smear grades. Crucially, XDR + DM

patients displayed distinct immunometabolic dysregulation, characterized by

elevated inflammatory markers (CRP) and a disrupted CD4+ /CD8+ T-cell balance

(increased CD4+, decreased CD8+ counts). Multivariable analysis identified

hyperglycemia as the primary driver of delayed clearance (aHR = 1.48, 95% CI:

1.23-1.77, p < 0.001), serving as a significant mediator between DM and impaired

outcomes (mediation effect: -0.82, p = 0.006). A glucose "dose-response"

relationship was observed: severe hyperglycemia (GLU ≥ 11.1 mmol/L) markedly

increased clearance delay risk (aHR = 5.29, p < 0.001), whereas optimal control

(GLU < 7 mmol/L) mitigated these disadvantages. CONCLUSION: Dysregulated glucose

metabolism and subsequent immune imbalance in diabetes are key drivers of delayed

bacterial clearance in XDR patients, independent of the anti-tuberculosis

regimen. Precise glucose control should be regarded as a core strategy, on a par

with anti-XDR treatment.

CI - Copyright © 2026 Yao, Shi, Kong, Zhang, Chen, Wang and Liu.

FAU - Yao, Xin

AU - Yao X

AD - The Key Laboratory of Environmental Pollution Monitoring and Disease Control,

Ministry of Education, School of Public Health, Guizhou Medical University,

Guiyang, China.

AD - Shanghai Key Laboratory of Tuberculosis, Shanghai Pulmonary Hospital, Tongji

University School of Medicine, Shanghai, China.

FAU - Shi, Yarong

AU - Shi Y

AD - The Key Laboratory of Environmental Pollution Monitoring and Disease Control,

Ministry of Education, School of Public Health, Guizhou Medical University,

Guiyang, China.

AD - Shanghai Key Laboratory of Tuberculosis, Shanghai Pulmonary Hospital, Tongji

University School of Medicine, Shanghai, China.

FAU - Kong, Minghao

AU - Kong M

AD - The Key Laboratory of Environmental Pollution Monitoring and Disease Control,

Ministry of Education, School of Public Health, Guizhou Medical University,

Guiyang, China.

AD - Shanghai Key Laboratory of Tuberculosis, Shanghai Pulmonary Hospital, Tongji

University School of Medicine, Shanghai, China.

FAU - Zhang, Yujie

AU - Zhang Y

AD - The Key Laboratory of Environmental Pollution Monitoring and Disease Control,

Ministry of Education, School of Public Health, Guizhou Medical University,

Guiyang, China.

AD - Shanghai Key Laboratory of Tuberculosis, Shanghai Pulmonary Hospital, Tongji

University School of Medicine, Shanghai, China.

FAU - Chen, Jianxia

AU - Chen J

AD - Shanghai Key Laboratory of Tuberculosis, Shanghai Pulmonary Hospital, Tongji

University School of Medicine, Shanghai, China.

FAU - Wang, Wenjuan

AU - Wang W

AD - The Key Laboratory of Environmental Pollution Monitoring and Disease Control,

Ministry of Education, School of Public Health, Guizhou Medical University,

Guiyang, China.

FAU - Liu, Zhonghua

AU - Liu Z

AD - The Key Laboratory of Environmental Pollution Monitoring and Disease Control,

Ministry of Education, School of Public Health, Guizhou Medical University,

Guiyang, China.

AD - Shanghai Key Laboratory of Tuberculosis, Shanghai Pulmonary Hospital, Tongji

University School of Medicine, Shanghai, China.

LA - eng

PT - Journal Article

DEP - 20260326

PL - Switzerland

TA - Front Public Health

JT - Frontiers in public health

JID - 101616579

RN - 0 (Blood Glucose)

RN - 0 (Antitubercular Agents)

SB - IM

MH - Humans

MH - *Extensively Drug-Resistant Tuberculosis/drug therapy/microbiology

MH - Retrospective Studies

MH - Female

MH - *Blood Glucose/metabolism

MH - Male

MH - Middle Aged

MH - Antitubercular Agents/therapeutic use

MH - *Diabetes Mellitus

MH - Adult

MH - Sputum/microbiology

PMC - PMC13062200

OTO - NOTNLM

OT - bacterial clearance

OT - diabetes mellitus

OT - extensively drug-resistant tuberculosis

OT - glucose

OT - high bacterial load

COIS- The author(s) declared that this work was conducted in the absence of any

commercial or financial relationships that could be construed as a potential

conflict of interest.

EDAT- 2026/04/13 18:17

MHDA- 2026/04/13 18:18

PMCR- 2026/03/26

CRDT- 2026/04/13 06:40

PHST- 2025/12/22 00:00 [received]

PHST- 2026/03/04 00:00 [revised]

PHST- 2026/03/10 00:00 [accepted]

PHST- 2026/04/13 18:18 [medline]

PHST- 2026/04/13 18:17 [pubmed]

PHST- 2026/04/13 06:40 [entrez]

PHST- 2026/03/26 00:00 [pmc-release]

AID - 10.3389/fpubh.2026.1746879 [doi]

PST - epublish

SO - Front Public Health. 2026 Mar 26;14:1746879. doi: 10.3389/fpubh.2026.1746879.

eCollection 2026.

PMID- 41889020

OWN - NLM

STAT- MEDLINE

DCOM- 20260713

LR - 20260713

IS - 1738-8872 (Electronic)

IS - 1017-7825 (Print)

IS - 1017-7825 (Linking)

VI - 36

DP - 2026 Mar 26

TI - Philadelphus tenuifolius Leaf Extract Exhibits Anti-Tuberculosis Activity by

Enhancing Host Autophagy and Immunity: A Promising Host-Directed Therapeutic

Candidate.

PG - e2601032

LID - 10.4014/jmb.2601.01032 [doi]

LID - e2601032

AB - The global emergence of drug-resistant Mycobacterium tuberculosis (Mtb)

necessitates the urgent discovery of novel anti-tuberculosis agents. Philadelphus

tenuifolius Rupr., a Korean aromatic herb, has been historically recognized for

its traditional medicinal uses. This study aimed to scientifically investigate

the anti-tuberculosis (TB) potential of the P. tenuifolius leaf extract (PT-LE)

and elucidate its underlying mechanism of action. PT-LE was prepared by 50%

ethanolic extraction. Its anti-TB activity was evaluated against intracellular

Mtb in BMDMs. Mechanistic studies focused on the activation of the MAPK pathway

and autophagy flux. Synergistic effects with conventional anti-TB drugs were also

assessed. For the in vivo evaluation, Mtb-infected mice were orally treated daily

with PT-LE (100 mg/kg), followed by the determination of the lung bacterial

burden. PT-LE exhibits negligible host cell cytotoxicity and effectively reduced

the bacterial load of intracellular Mtb within macrophages. Mechanistically,

PT-LE was shown to significantly activate the MAPK signaling pathway, which

subsequently enhanced autophagy flux - a critical host defense mechanism against

Mtb. Furthermore, PT-LE demonstrated potent synergistic activity with existing

anti-TB drugs and modulated the host immune response by increasing the production

of the chemokine MCP-1. Critically, the in vivo experiments showed that oral

administration of PT-LE significantly reduced the Mtb bacterial burden in the

lungs of infected mice. These findings reveal a novel anti-TB function of PT-LE,

which operates by enhancing host autophagy and immunity. PT-LE represents a

promising and effective host-directed therapeutic candidate for both

drug-sensitive and drug-resistant tuberculosis.

FAU - Nguyen, Tam Doan

AU - Nguyen TD

AD - Department of Microbiology, College of Medicine, Chungnam National University,

Daejeon 35015, Republic of Korea.

AD - Department of Medical Science, College of Medicine, Chungnam National University,

Daejeon 35015, Republic of Korea.

FAU - Choi, Ji-Ae

AU - Choi JA

AD - Department of Microbiology, College of Medicine, Chungnam National University,

Daejeon 35015, Republic of Korea.

AD - Department of Medical Science, College of Medicine, Chungnam National University,

Daejeon 35015, Republic of Korea.

FAU - Son, Doyi

AU - Son D

AD - Department of Microbiology, College of Medicine, Chungnam National University,

Daejeon 35015, Republic of Korea.

AD - Department of Medical Science, College of Medicine, Chungnam National University,

Daejeon 35015, Republic of Korea.

AD - Translational Immunology Institute, Chungnam National University, Daejeon 34134,

Republic of Korea.

FAU - Yuk, Heung Joo

AU - Yuk HJ

AD - Herbal Medicine Research Division, Korea Institute of Oriental Medicine, Daejeon

34054, Republic of Korea.

FAU - Lee, Junghwan

AU - Lee J

AD - Department of Microbiology, College of Medicine, Chungnam National University,

Daejeon 35015, Republic of Korea.

AD - Department of Medical Science, College of Medicine, Chungnam National University,

Daejeon 35015, Republic of Korea.

AD - Translational Immunology Institute, Chungnam National University, Daejeon 34134,

Republic of Korea.

FAU - Son, Sanghun

AU - Son S

AD - Department of Microbiology, College of Medicine, Chungnam National University,

Daejeon 35015, Republic of Korea.

AD - Department of Medical Science, College of Medicine, Chungnam National University,

Daejeon 35015, Republic of Korea.

FAU - Kim, Jaewhan

AU - Kim J

AD - Department of Microbiology, College of Medicine, Chungnam National University,

Daejeon 35015, Republic of Korea.

AD - Department of Medical Science, College of Medicine, Chungnam National University,

Daejeon 35015, Republic of Korea.

FAU - Jo, SeoYeon

AU - Jo S

AD - Department of Microbiology, College of Medicine, Chungnam National University,

Daejeon 35015, Republic of Korea.

AD - Department of Medical Science, College of Medicine, Chungnam National University,

Daejeon 35015, Republic of Korea.

AD - Translational Immunology Institute, Chungnam National University, Daejeon 34134,

Republic of Korea.

FAU - Kim, Dong-Seon

AU - Kim DS

AD - Herbal Medicine Research Division, Korea Institute of Oriental Medicine, Daejeon

34054, Republic of Korea.

FAU - Song, Chang-Hwa

AU - Song CH

AD - Department of Microbiology, College of Medicine, Chungnam National University,

Daejeon 35015, Republic of Korea.

AD - Department of Medical Science, College of Medicine, Chungnam National University,

Daejeon 35015, Republic of Korea.

AD - Translational Immunology Institute, Chungnam National University, Daejeon 34134,

Republic of Korea.

LA - eng

PT - Journal Article

DEP - 20260326

PL - Korea (South)

TA - J Microbiol Biotechnol

JT - Journal of microbiology and biotechnology

JID - 9431852

RN - 0 (Antitubercular Agents)

RN - 0 (Plant Extracts)

SB - IM

MH - Animals

MH - *Autophagy/drug effects

MH - *Antitubercular Agents/pharmacology

MH - *Plant Extracts/pharmacology

MH - *Mycobacterium tuberculosis/drug effects

MH - Plant Leaves/chemistry

MH - Mice

MH - Host-Directed Therapy

MH - Macrophages/drug effects/microbiology/immunology

MH - Drug Synergism

MH - MAP Kinase Signaling System/drug effects

MH - Disease Models, Animal

MH - Lung/microbiology

MH - Tuberculosis/drug therapy

PMC - PMC13036502

OTO - NOTNLM

OT - Autophagy

OT - Host-directed therapy

OT - MAPK signaling

OT - Natural products

OT - Philadelphus tenuifolius

OT - Tuberculosis

COIS- Conflict of Interest The authors have no financial conflicts of interest to

declare.

EDAT- 2026/03/27 07:15

MHDA- 2026/03/27 07:16

PMCR- 2026/03/26

CRDT- 2026/03/27 01:23

PHST- 2026/01/13 00:00 [received]

PHST- 2026/03/06 00:00 [revised]

PHST- 2026/03/06 00:00 [accepted]

PHST- 2026/03/27 07:16 [medline]

PHST- 2026/03/27 07:15 [pubmed]

PHST- 2026/03/27 01:23 [entrez]

PHST- 2026/03/26 00:00 [pmc-release]

AID - jmb.2601.01032 [pii]

AID - jmb-36-e2601032 [pii]

AID - 10.4014/jmb.2601.01032 [doi]

PST - epublish

SO - J Microbiol Biotechnol. 2026 Mar 26;36:e2601032. doi: 10.4014/jmb.2601.01032.

PMID- 41498549

OWN - NLM

STAT- MEDLINE

DCOM- 20260625

LR - 20260625

IS - 1098-6618 (Electronic)

IS - 0893-8512 (Print)

IS - 0893-8512 (Linking)

VI - 39

IP - 1

DP - 2026 Mar 12

TI - The power of resistance: mechanisms of antimicrobial resistance in Mycobacterium

tuberculosis and its impact on tuberculosis management.

PG - e0019425

LID - 10.1128/cmr.00194-25 [doi]

LID - e00194-25

AB - SUMMARYThe global resurgence of drug-resistant tuberculosis (DR-TB) presents a

formidable challenge to public health, driven by a complex interplay of

mycobacterial evolution, dynamics and outcomes of host-pathogen interactions and

systemic gaps in diagnosis and treatment strategies. This comprehensive review

delineates the multifactorial basis of antimicrobial resistance (AMR) in

Mycobacterium tuberculosis (Mtb), integrating molecular, immunological, and

pharmacological perspectives to inform next-generation strategies for effective

TB control. We reconceptualize TB as a dynamic clinical spectrum-ranging from

asymptomatic infection to overt disease-shaped by granuloma biology and bacterial

adaptation. This spectrum underpins both diagnostic ambiguity and therapeutic

failure, particularly in the context of phenotypic drug tolerance/resistance to

current anti-TB drugs. We discuss Mtb's intrinsic and extrinsic resistance

mechanisms, including the lipid-rich cell envelope, efflux systems, and enzymatic

drug modification, which are compounded by acquired mutations that disrupt drug

activation, alter targets, and confer cross-resistance. These adaptations are

further potentiated by granuloma-induced pharmacokinetic heterogeneity and

host-induced metabolic quiescence. We highlight the emerging role of therapeutic

drug monitoring and pharmacokinetic/pharmacodynamic modeling in optimizing

individualized therapy, particularly for novel regimens incorporating

bedaquiline, pretomanid, and linezolid. Moreover, we underscore the diagnostic

limitations in detecting heteroresistance and early-stage disease, advocating for

expanded deployment of advanced and targeted molecular diagnostic modalities.

Finally, we propose a paradigm shift toward integrated, precision-based TB

management, leveraging host-directed therapies, biofilm-disrupting agents, and

real-time pharmacokinetics-guided dosing to preempt resistance emergence and

improve clinical outcomes. This review provides a translational framework for

addressing the biological and operational complexities of DR-TB in the era of

AMR.

FAU - Gopalaswamy, Radha

AU - Gopalaswamy R

AUID- ORCID: 0000-0001-8688-4617

AD - Madurai Kamaraj University, Madurai, India. RINGGOLD: 29944. ROR:

https://ror.org/04c8e9019

FAU - Subbian, Selvakumar

AU - Subbian S

AUID- ORCID: 0000-0003-2021-8632

AD - Public Health Research Institute, New Jersey Medical School, Rutgers, The State

University of New Jersey, Newark, New Jersey, USA. RINGGOLD: 12286. ROR:

https://ror.org/014ye1258

LA - eng

PT - Journal Article

PT - Review

DEP - 20260107

PL - United States

TA - Clin Microbiol Rev

JT - Clinical microbiology reviews

JID - 8807282

RN - 0 (Antitubercular Agents)

SB - IM

EIN - Clin Microbiol Rev. 2026 Jun 11;39(2):e0009326. doi: 10.1128/cmr.00093-26. PMID:

41925339

MH - Humans

MH - *Mycobacterium tuberculosis/drug effects/genetics

MH - *Antitubercular Agents/pharmacology/therapeutic use

MH - *Tuberculosis, Multidrug-Resistant/drug therapy/microbiology/diagnosis

MH - *Drug Resistance, Bacterial

MH - *Tuberculosis/drug therapy/microbiology

MH - Host-Pathogen Interactions

PMC - PMC12981108

OTO - NOTNLM

OT - CRISPR/Cas

OT - MALDI-ToF/MS

OT - antimicrobial resistance

OT - biofilm

OT - drug metabolism

OT - drug repurposing

OT - gene mutations

OT - granuloma

OT - therapeutic drug monitoring

COIS- The authors declare no conflict of interest.

EDAT- 2026/01/07 13:17

MHDA- 2026/03/12 13:56

PMCR- 2027/01/07

CRDT- 2026/01/07 09:03

PHST- 2026/03/12 13:56 [medline]

PHST- 2026/01/07 13:17 [pubmed]

PHST- 2026/01/07 09:03 [entrez]

PHST- 2027/01/07 00:00 [pmc-release]

AID - cmr00194-25 [pii]

AID - cmr.00194-25 [pii]

AID - 10.1128/cmr.00194-25 [doi]

PST - ppublish

SO - Clin Microbiol Rev. 2026 Mar 12;39(1):e0019425. doi: 10.1128/cmr.00194-25. Epub

2026 Jan 7.

PMID- 41868357

OWN - NLM

STAT- PubMed-not-MEDLINE

DCOM- 20260323

LR - 20260323

IS - 1664-302X (Print)

IS - 1664-302X (Electronic)

IS - 1664-302X (Linking)

VI - 17

DP - 2026

TI - Mycobacterium tuberculosis MarR family transcription factor Rv0737 regulates

bacterial growth and lipid synthesis by targeting the sigL-rslA operon.

PG - 1727573

LID - 10.3389/fmicb.2026.1727573 [doi]

LID - 1727573

AB - The MarR family of transcription factors in Mycobacterium tuberculosis plays a

critical role in bacterial adaptation to host stresses, yet the function of many

members remains unknown. Here, we characterize the novel MarR regulator Rv0737

and its homolog Ms_1492 in M. smegmatis. Overexpression of Rv0737 severely

impaired bacterial growth, cell division, and biofilm formation, while increasing

susceptibility to oxidative stress and the cell wall-targeting drug isoniazid.

Conversely, deletion of ms_1492 altered cell envelope permeability and lipid

composition, enhanced ATP synthesis, and conferred mild tolerance to H(2)O(2) and

isoniazid. Lipid profiling and transcriptomic analysis revealed significant

dysregulation of lipid metabolism genes. Crucially, electrophoretic mobility

shift assays demonstrated that both Rv0737 and Ms_1492 directly bind to the

promoter region of the sigL-rslA operon, which encodes an alternative sigma

factor and its anti-sigma factor. Our findings establish a direct regulatory

pathway wherein Rv0737/Ms_1492 modulates bacterial growth, cell envelope

integrity, and stress response by targeting the sigL-rslA operon, identifying

this system as a potential therapeutic target for combating drug-resistant

tuberculosis.

CI - Copyright © 2026 Abudukadier, Zhang, Li, Chen, Li, Gong and Xie.

FAU - Abudukadier, Abulimiti

AU - Abudukadier A

AD - School of Life Sciences, Institute of Modern Biopharmaceuticals, Southwest

University, Chongqing, China.

FAU - Zhang, Qiao

AU - Zhang Q

AD - School of Life Sciences, Institute of Modern Biopharmaceuticals, Southwest

University, Chongqing, China.

FAU - Li, Gang

AU - Li G

AD - School of Life Sciences, Institute of Modern Biopharmaceuticals, Southwest

University, Chongqing, China.

FAU - Chen, Haiqi

AU - Chen H

AD - School of Life Sciences, Institute of Modern Biopharmaceuticals, Southwest

University, Chongqing, China.

FAU - Li, Peibo

AU - Li P

AD - Chongqing Public Health Medical Center, Chongqing, China.

FAU - Gong, Zhen

AU - Gong Z

AD - Department of Clinical Laboratory, The Second Affiliated Hospital of Anhui

Medical University, Hefei, Anhui, China.

FAU - Xie, Jianping

AU - Xie J

AD - School of Life Sciences, Institute of Modern Biopharmaceuticals, Southwest

University, Chongqing, China.

AD - Chongqing Public Health Medical Center, Chongqing, China.

LA - eng

PT - Journal Article

DEP - 20260306

PL - Switzerland

TA - Front Microbiol

JT - Frontiers in microbiology

JID - 101548977

PMC - PMC13002591

OTO - NOTNLM

OT - MarR transcription factor

OT - Mycobacterium tuberculosis

OT - cell division/growth regulation

OT - lipid metabolism

OT - sigL-rslA operon

COIS- The author(s) declared that this work was conducted in the absence of any

commercial or financial relationships that could be construed as a potential

conflict of interest.

EDAT- 2026/03/23 06:31

MHDA- 2026/03/23 06:32

PMCR- 2026/03/06

CRDT- 2026/03/23 05:53

PHST- 2025/10/20 00:00 [received]

PHST- 2026/02/08 00:00 [revised]

PHST- 2026/02/13 00:00 [accepted]

PHST- 2026/03/23 06:32 [medline]

PHST- 2026/03/23 06:31 [pubmed]

PHST- 2026/03/23 05:53 [entrez]

PHST- 2026/03/06 00:00 [pmc-release]

AID - 10.3389/fmicb.2026.1727573 [doi]

PST - epublish

SO - Front Microbiol. 2026 Mar 6;17:1727573. doi: 10.3389/fmicb.2026.1727573.

eCollection 2026.

PMID- 41774181

OWN - NLM

STAT- MEDLINE

DCOM- 20260627

LR - 20260627

IS - 1617-4623 (Electronic)

IS - 1617-4623 (Linking)

VI - 301

IP - 1

DP - 2026 Mar 3

TI - Advances in functional transcriptome analysis of Mycobacterium tuberculosis: a

review.

LID - 46 [pii]

LID - 10.1007/s00438-026-02374-7 [doi]

AB - Drug-resistant tuberculosis poses a significant global challenge necessitating

the prompt advancement of novel therapeutic options. Nonetheless, disease

prognosis is contingent upon multiple factors. mRNA and other small RNAs are

essential for gene regulation and disease progression. Additionally, they are

essential for the advancement of TB mRNA therapies. The review aims to evaluate

the functions of mRNA and various small RNAs, including lncRNA, miRNA, circRNA,

and ceRNA, as interconnected components within the mRNA-miRNA-circRNA axis in

Mycobacterium tuberculosis. In this context, the analysis of various genes

expressed during transcription is essential; however, the TB group’s mRNA

expression levels of the CXCL10, CXCL9, IL1B, and PLA2G2D genes were

substantially higher compared to the control group. In addition, EspC, MetE, and

PPE15 increased IgG levels. Besides, the inadequate IgG responses to m-ESAT6 and

m-EsxI present a noteworthy research opportunity. Evidence that neutralizing

antibodies provide protection against viral infections targeted by mRNA vaccines

during the COVID-19 pandemic supports this research. mRNA-based vaccination

analogues offer potential therapeutic advantages following BCG administration.

Mycobacterium avium and Mycobacterium tuberculosis are efficiently inhibited by

the mRNA therapy, namely the repRNA-ID91/ID91 + GLA-SE vaccination, which elicits

humoral and cellular immune responses. Therefore, the therapeutic use of mRNA, as

demonstrated by numerous studies, suggests its potential as an efficacious

therapeutic vaccine subsequent to BCG treatment. Also, investigating the ceRNA

network and the relationships among miRNA, circRNA, lncRNA, and mRNA in TB study

will improve the management of this infection.

FAU - Sundaram, Karthikeyan

AU - Sundaram K

AUID- ORCID: 0000-0002-2674-9146

AD - Department of Herbal Pharmacology and Environmental Sustainability, Chettinad

Hospital and Research Institute, Chettinad Academy of Research and Education,

Kelambakkam, Chennai, Tamilnadu, India. karthikom85@gmail.com.

FAU - Rathinam, Sridhar

AU - Rathinam S

AD - Chettinad Hospital and Research Institute, Chettinad Academy of Research and

Education, Kelambakkam, Chennai, Tamilnadu, India.

LA - eng

PT - Journal Article

PT - Review

DEP - 20260303

PL - Germany

TA - Mol Genet Genomics

JT - Molecular genetics and genomics : MGG

JID - 101093320

RN - 0 (MicroRNAs)

RN - 0 (RNA, Messenger)

RN - 0 (RNA, Circular)

RN - 0 (RNA, Competitive Endogenous)

RN - 0 (RNA, Long Noncoding)

SB - IM

MH - *Mycobacterium tuberculosis/genetics/immunology/pathogenicity

MH - Humans

MH - *Gene Expression Profiling/methods

MH - MicroRNAs/genetics

MH - RNA, Messenger/genetics

MH - *Transcriptome

MH - *Tuberculosis/genetics/microbiology/immunology

MH - RNA, Circular/genetics

MH - Animals

MH - RNA, Competitive Endogenous

MH - RNA, Long Noncoding/genetics

OTO - NOTNLM

OT - Tuberculosis

OT - circRNA

OT - lncRNA

OT - mRNA

OT - miRNA

COIS- Declarations. Conflict of interest: The authors have no competing interests to

declare that are relevant to the content of this article. Ethical approval: Not

applicable. Consent for publication: Not applicable.

EDAT- 2026/03/03 12:32

MHDA- 2026/06/27 10:10

CRDT- 2026/03/03 11:16

PHST- 2025/09/28 00:00 [received]

PHST- 2026/01/24 00:00 [accepted]

PHST- 2026/06/27 10:10 [medline]

PHST- 2026/03/03 12:32 [pubmed]

PHST- 2026/03/03 11:16 [entrez]

AID - 10.1007/s00438-026-02374-7 [pii]

AID - 10.1007/s00438-026-02374-7 [doi]

PST - epublish

SO - Mol Genet Genomics. 2026 Mar 3;301(1):46. doi: 10.1007/s00438-026-02374-7.

PMID- 41704445

OWN - NLM

STAT- PubMed-not-MEDLINE

DCOM- 20260218

LR - 20260304

IS - 2046-2069 (Electronic)

IS - 2046-2069 (Linking)

VI - 16

IP - 10

DP - 2026 Feb 11

TI - Secondary metabolites from Lobaria pulmonaria (L.) Hoffm. target key metabolic

enzymes: a novel strategy against multidrug-resistant tuberculosis.

PG - 8960-8970

LID - 10.1039/d5ra05774d [doi]

AB - Numerous cultures have traditionally utilised the foliose lichen Lobaria

pulmonaria (L.) Hoffm. ("Oak Lung" or "Lungs of Oak" in English; family:

Lobariaceae) as a Tuberculosis (Tb) treatment. The present study aimed to

scientifically validate the folkloric use of L. pulmonaria in treating Tb by

investigating its antimycobacterial profile against Mycobacterium tuberculosis

H37Ra (M.tb) and six other MDR-Tb isolates. The preliminary results obtained from

XRMA revealed the notable inhibitory activity of LP and Fraction (F)-3 against

M.tb, displaying IC(50) values of 7.74 ± 0.27 and 6.26 ± 0.04 µg mL(-1),

respectively; followed by F2 (IC(50) value: 38.82 ± 0.34 µg mL(-1)) and F5

(IC(50) value: 46.69 ± 1.13 µg mL(-1)). The purification process of these

bioactive fractions resulted in the identification of four known secondary

metabolites: fukinanolide A, pinastric acid, stictic acid, and scrobiculin.

Furthermore, the MICs from REMA showed that LP, stictic acid, and fukinanolide A

have greater efficacy in controlling the growth of all six tested MDR-Tb

isolates, compared to rifampicin. Notably, LP exhibited superior

antimycobacterial activity against all six tested MDR strains as compared to all

isolated compounds and rifampicin, possibly due to the synergistic effect of its

metabolites. Furthermore, the IC(50) values of LP, stictic acid, and fukinanolide

A on THP-1 macrophages were considerably higher than MICs against the tested

mycobacterial strains, suggesting that THP-1 remained unaffected at

concentrations effective against M.tb and MDR-Tb isolates. The deliberated SI

ratio values indicated that LP, stictic acid, and fukinanolide A were more active

and less toxic to MDR-Tb strains than rifampicin. The molecular docking studies

on 1EA1, 4V1F and 3VIU revealed that fukinanolide A and stictic acid bind

effectively and selectively to 3VIU (β-ketoacyl reductase FabG4), thereby

conferring their anti-TB potential. The outcomes provide a validation for the

traditional use of L. pulmonaria in Tb treatment, with stictic acid and

fukinanolide A identified as key biomarkers. Hence, L. pulmonaria presented as a

promising source for the development of novel drugs targeting against MDR-Tb.

CI - This journal is © The Royal Society of Chemistry.

FAU - Nguyen, Ha Thi

AU - Nguyen HT

AD - Center for Pharmaceutical Biotechnology, Duy Tan University Da Nang 550000

Vietnam.

AD - School of Pharmacy and Technology Management, SVKM's Narsee Monjee Institute of

Management Studies (NMIMS), Deemed-to-be-University, Green Industrial Park, TSIIC

Jadcherla Hyderabad 509301 India.

FAU - Badavath, Vishnu Nayak

AU - Badavath VN

AUID- ORCID: 0000-0002-6433-2691

AD - School of Pharmacy & Technology Management, SVKM's Narsee Monjee Institute of

Management Studies (NMIMS) Deemed-to-University Jadcherla - 509301 Hyderabad

India.

FAU - Maji, Siddhartha

AU - Maji S

AUID- ORCID: 0000-0001-7132-9573

AD - Department of Chemistry, Oklahoma State University Stillwater 74078 Oklahoma USA.

FAU - Polimati, Haritha

AU - Polimati H

AUID- ORCID: 0000-0002-1738-1765

AD - Pharmacology Department, AU College of Pharmaceutical Sciences, Andhra University

Visakhapatnam 530003 Andhra Pradesh India.

FAU - Okello, Emmanuel

AU - Okello E

AD - Veterinary Medicine Teaching and Research Center, School of Veterinary Medicine,

University of California Davis Tulare CA USA.

FAU - Bunce, Richard A

AU - Bunce RA

AUID- ORCID: 0009-0003-7927-5655

AD - Department of Chemistry, Oklahoma State University Stillwater 74078 Oklahoma USA.

FAU - Thuan, Nguyen Huy

AU - Thuan NH

AD - Center for Pharmaceutical Biotechnology, Duy Tan University Da Nang 550000

Vietnam.

AD - School of Pharmacy and Technology Management, SVKM's Narsee Monjee Institute of

Management Studies (NMIMS), Deemed-to-be-University, Green Industrial Park, TSIIC

Jadcherla Hyderabad 509301 India.

FAU - Nuzul Hakimi Wan Salleh, Wan Mohd

AU - Nuzul Hakimi Wan Salleh WM

AUID- ORCID: 0000-0003-1408-229X

AD - Department of Chemistry, Faculty of Science and Mathematics, Universiti

Pendidikan Sultan Idris Tanjong Malim 35900 Perak Malaysia.

FAU - Tatipamula, Vinay Bharadwaj

AU - Tatipamula VB

AUID- ORCID: 0000-0002-9376-1911

AD - Center for Pharmaceutical Biotechnology, Duy Tan University Da Nang 550000

Vietnam.

AD - School of Pharmacy and Technology Management, SVKM's Narsee Monjee Institute of

Management Studies (NMIMS), Deemed-to-be-University, Green Industrial Park, TSIIC

Jadcherla Hyderabad 509301 India.

LA - eng

PT - Journal Article

DEP - 20260216

PL - England

TA - RSC Adv

JT - RSC advances

JID - 101581657

EIN - RSC Adv. 2026 Mar 2;16(13):11647. doi: 10.1039/d6ra90025a. PMID: 41777831

PMC - PMC12908696

COIS- There are no conflicts to declare.

EDAT- 2026/02/18 10:07

MHDA- 2026/02/18 10:08

PMCR- 2026/02/16

CRDT- 2026/02/18 04:25

PHST- 2025/08/07 00:00 [received]

PHST- 2026/02/09 00:00 [accepted]

PHST- 2026/02/18 10:08 [medline]

PHST- 2026/02/18 10:07 [pubmed]

PHST- 2026/02/18 04:25 [entrez]

PHST- 2026/02/16 00:00 [pmc-release]

AID - d5ra05774d [pii]

AID - 10.1039/d5ra05774d [doi]

PST - epublish

SO - RSC Adv. 2026 Feb 16;16(10):8960-8970. doi: 10.1039/d5ra05774d. eCollection 2026

Feb 11.

PMID- 41695983

OWN - NLM

STAT- PubMed-not-MEDLINE

DCOM- 20260216

LR - 20260218

IS - 1687-9686 (Print)

IS - 1687-9694 (Electronic)

IS - 1687-9686 (Linking)

VI - 2026

DP - 2026

TI - Exploring the Natural Products Atlas (NPAtlas) Database for Hunting Prospective

Irreversible Covalent DprE1 Inhibitors With Antitubercular Activity: An

Integrated In-Silico Approach.

PG - 8879019

LID - 10.1155/jotm/8879019 [doi]

LID - 8879019

AB - As the second most deadly infectious disease worldwide after COVID-19,

tuberculosis (TB) remains a pressing global health issue, further aggravated by

multidrug-resistant TB (MDR-TB) and extensively drug-resistant TB (XDR-TB)

strains. There is an urgent need to identify new anti-TB treatments and novel

therapeutics to confront drug resistance. The decaprenylphosphoryl-D-ribose

oxidase (DprE1) is an essential protein for the biosynthesis of the mycobacterial

cell wall, and its inhibition features a promising antitubercular strategy.

NPAtlas was utilized as a reference database, comprising natural products with

confirmed biological effects. The aim of the current study is to identify and

prioritize promising nitro-containing natural products from the NPAtlas as

potential covalent DprE1 inhibitors using advanced in silico approaches. Herein,

the docking scores of 133 nitro-containing NPAtlas compounds were assessed using

a covalent docking technique. Thereafter, NPAtlas compounds with docking scores

lower than PBTZ169 (calc. -7.8 kcal·mol(-1)) were subjected to molecular dynamics

simulation (MDS), accompanied by binding energy estimations utilizing the MM-GBSA

approach. Based on MM-GBSA//250 ns MDS, NPA011203, NPA013234, NPA016048,

NPA012944, NPA001712, and NPA002823 demonstrated higher binding affinities

against DprE1 with ΔG (binding) values of -75.6, -62.7, -61.6, -57.6, -54.8, and

-50.7 kcal·mol(-1), respectively, than PBTZ169 (calc. -49.4 kcal·mol(-1)). The

identified NPAtlas compounds also demonstrated structural and energetic stability

within the DprE1 active site throughout 250 ns MDS. Physicochemical and ADMET

predictions of the identified NPAtlas compounds indicated a suitable molecular

size, favorable absorption, and negligible toxicity, suggesting their potential

oral bioavailability. These in silico outcomes provide preliminary insights into

the identified NPAtlas compounds as potential DprE1 inhibitors and can guide

subsequent in vitro/in vivo experiments.

CI - Copyright © 2026 Mahmoud A. A. Ibrahim et al. Journal of Tropical Medicine

published by John Wiley & Sons Ltd.

FAU - Ibrahim, Mahmoud A A

AU - Ibrahim MAA

AUID- ORCID: 0000-0003-4819-2040

AD - Chemistry Department, Faculty of Science, Minia University, Minia, 61519, Egypt,

minia.edu.eg.

AD - School of Health Sciences, University of KwaZulu-Natal, Westville Campus, Durban,

4000, South Africa, ukzn.ac.za.

AD - Department of Supportive Requirements, University of Technology and Applied

Sciences, Nizwa, 611, Oman, hct.edu.om.

FAU - Mahmoud, Doaa G M

AU - Mahmoud DGM

AUID- ORCID: 0009-0005-3141-9458

AD - Chemistry Department, Faculty of Science, Minia University, Minia, 61519, Egypt,

minia.edu.eg.

FAU - Ebada, Sherif S

AU - Ebada SS

AUID- ORCID: 0000-0002-2753-0031

AD - Department of Pharmacognosy, Faculty of Pharmacy, Ain Shams University, Cairo,

11566, Egypt, asu.edu.eg.

AD - Department of Microbial Drugs, Helmholtz Centre for Infection Research GmbH,

Braunschweig, 38124, Germany.

FAU - Sidhom, Peter A

AU - Sidhom PA

AUID- ORCID: 0000-0003-2579-6351

AD - Department of Pharmaceutical Chemistry, Faculty of Pharmacy, Tanta University,

Tanta, 31527, Egypt, tanta.edu.eg.

FAU - Mekhemer, Gamal A H

AU - Mekhemer GAH

AUID- ORCID: 0000-0003-2474-076X

AD - Chemistry Department, Faculty of Science, Minia University, Minia, 61519, Egypt,

minia.edu.eg.

FAU - Hegazy, Mohamed-Elamir F

AU - Hegazy MF

AUID- ORCID: 0000-0002-0343-4969

AD - Chemistry of Medicinal Plants Department, National Research Centre, Giza, 12622,

Egypt, nrc.sci.eg.

FAU - Han, Yanshuo

AU - Han Y

AUID- ORCID: 0000-0002-4897-2998

AD - School of Life and Pharmaceutical Sciences, Dalian University of Technology,

Panjin, China, dlut.edu.cn.

FAU - Abalkhail, Tarad

AU - Abalkhail T

AUID- ORCID: 0009-0005-7732-9933

AD - Department of Botany and Microbiology, College of Science, King Saud University,

Riyadh, 11451, Saudi Arabia, ksu.edu.sa.

LA - eng

PT - Journal Article

DEP - 20260214

PL - United States

TA - J Trop Med

JT - Journal of tropical medicine

JID - 101524194

PMC - PMC12906241

OTO - NOTNLM

OT - DprE1

OT - NPAtlas

OT - binding energy calculation

OT - covalent docking

OT - molecular dynamics simulation

COIS- The authors declare no conflicts of interest.

EDAT- 2026/02/16 12:30

MHDA- 2026/02/16 12:31

PMCR- 2026/02/14

CRDT- 2026/02/16 06:53

PHST- 2025/07/23 00:00 [received]

PHST- 2025/12/22 00:00 [revised]

PHST- 2026/01/09 00:00 [accepted]

PHST- 2026/02/16 12:31 [medline]

PHST- 2026/02/16 12:30 [pubmed]

PHST- 2026/02/16 06:53 [entrez]

PHST- 2026/02/14 00:00 [pmc-release]

AID - JOTM8879019 [pii]

AID - 10.1155/jotm/8879019 [doi]

PST - epublish

SO - J Trop Med. 2026 Feb 14;2026:8879019. doi: 10.1155/jotm/8879019. eCollection

2026.

PMID- 41766967

OWN - NLM

STAT- MEDLINE

DCOM- 20260710

LR - 20260710

IS - 2296-2565 (Electronic)

IS - 2296-2565 (Linking)

VI - 13

DP - 2025

TI - Community-engaged clinical governance and machine learning for optimizing

tuberculosis management in rural Eastern Cape.

PG - 1713401

LID - 10.3389/fpubh.2025.1713401 [doi]

LID - 1713401

AB - Tuberculosis (TB) remains a major global health challenge, particularly in

high-burden, resource-limited settings. Community-Engaged Clinical Governance

(CE-CG) has emerged as a promising framework for strengthening accountability,

adherence, and continuity of care by integrating clinical governance and

community participation. This study examined the alignment between CE-CG

implementation and TB treatment outcomes in the rural Eastern Cape, South Africa,

using patient data from 2018 to 2020. Descriptive statistics, correlation

analysis, and explanatory machine-learning models (logistic regression, random

forest, and decision tree) were applied to address distinct research objectives,

along with scenario-based projections. CE-CG was retrospectively operationalized

as a binary programmatic indicator reflecting periods of structured governance

implementation, including community health worker tracing, digital adherence

monitoring, integrated TB-HIV care, and governance dashboard oversight.

Machine-learning models were intentionally used as explanatory tools rather than

predictive models to assess the internal coherence of the CE-CG framework. The

observed perfect classification performance reflects deterministic alignment

between governance implementation and treatment outcomes within this cohort

rather than generalizable predictive accuracy. Treatment success improved

substantially over the study period, increasing from 41.6% in 2018 to 68.3% in

2020. Scenario-based projections indicate that under a slow intervention

trajectory (3.5% annual growth), treatment success would reach only 76.6% by

2030. In contrast, a sustained governance strategy (5.34% annual growth) could

achieve the World Health Organization (WHO) target of 95%. Correlation analysis

revealed a perfect positive association between CE-CG and treatment success,

which was interpreted as an artifact of retrospective coding rather than a causal

effect. Loss to follow-up and multidrug-resistant TB demonstrated weaker

associations with outcomes, while extensively drug-resistant TB remained

negatively associated. Overall, the findings support CE-CG as a policy-relevant,

programmatic framework for strengthening adherence, retention, and accountability

in high-burden rural TB settings. Embedding CE-CG within TB programmes offers a

sustainable pathway toward achieving the WHO treatment success targets and

accelerating progress toward TB elimination.

CI - Copyright © 2026 Faye, Dlatu, Hosu, Chitha and Apalata.

FAU - Faye, Lindiwe Modest

AU - Faye LM

AD - School of Laboratory Medicine and Pathology, Faculty of Medicine and Health

Sciences, Walter Sisulu University, Mthatha, South Africa.

FAU - Dlatu, Ntandazo

AU - Dlatu N

AD - Walter Sisulu Institute for Clinical Governance, Healthcare Administration,

School of Public Health, Faculty of Medicine and Health Sciences, Walter Sisulu

University, Mthatha, South Africa.

FAU - Hosu, Mojisola Clara

AU - Hosu MC

AD - School of Laboratory Medicine and Pathology, Faculty of Medicine and Health

Sciences, Walter Sisulu University, Mthatha, South Africa.

FAU - Chitha, Wezile Wilson

AU - Chitha WW

AD - Walter Sisulu Institute for Clinical Governance, Healthcare Administration,

School of Public Health, Faculty of Medicine and Health Sciences, Walter Sisulu

University, Mthatha, South Africa.

FAU - Apalata, Teke

AU - Apalata T

AD - School of Laboratory Medicine and Pathology, Faculty of Medicine and Health

Sciences, Walter Sisulu University, Mthatha, South Africa.

LA - eng

PT - Journal Article

DEP - 20260213

PL - Switzerland

TA - Front Public Health

JT - Frontiers in public health

JID - 101616579

RN - 0 (Antitubercular Agents)

SB - IM

MH - Humans

MH - South Africa

MH - *Machine Learning

MH - *Tuberculosis/drug therapy/therapy

MH - *Clinical Governance

MH - Retrospective Studies

MH - *Rural Population

MH - Predictive Learning Models

MH - Antitubercular Agents/therapeutic use

PMC - PMC12946076

OTO - NOTNLM

OT - TB–HIV co-infection

OT - community-engaged clinical governance

OT - drug-resistant TB

OT - explanatory modeling

OT - scenario analysis

OT - tuberculosis

COIS- The author(s) declared that this work was conducted in the absence of any

commercial or financial relationships that could be construed as a potential

conflict of interest.

EDAT- 2026/03/02 13:17

MHDA- 2026/03/07 01:37

PMCR- 2026/02/13

CRDT- 2026/03/02 06:26

PHST- 2025/09/25 00:00 [received]

PHST- 2025/12/20 00:00 [revised]

PHST- 2025/12/29 00:00 [accepted]

PHST- 2026/03/07 01:37 [medline]

PHST- 2026/03/02 13:17 [pubmed]

PHST- 2026/03/02 06:26 [entrez]

PHST- 2026/02/13 00:00 [pmc-release]

AID - 10.3389/fpubh.2025.1713401 [doi]

PST - epublish

SO - Front Public Health. 2026 Feb 13;13:1713401. doi: 10.3389/fpubh.2025.1713401.

eCollection 2025.

PMID- 41680460

OWN - NLM

STAT- MEDLINE

DCOM- 20260707

LR - 20260707

IS - 2399-3642 (Electronic)

IS - 2399-3642 (Linking)

VI - 9

IP - 1

DP - 2026 Feb 12

TI - Naturally occurring dinactin targets cpsA protein and kills Mycobacterium

tuberculosis by disrupting the proton motive force.

LID - 10.1038/s42003-026-09654-3 [doi]

LID - 417

AB - Tuberculosis, especially drug-resistant tuberculosis remains a global threat, and

new drugs are desperately needed to combat the spread of multidrug-resistant

Mycobacterium tuberculosis. Here we describe a natural macrotetrolide dinactin

with anti-tuberculosis activity against susceptive and non-replicating

Mycobacterium tuberculosis. Dinactin can also synergistically enhance the

anti-tuberculosis effect of rifampicin and isoniazid against drug-resistant

strains.Furthermore, dinactin exhibited excellently antituberculosis effect in

macrophage and Galleria mellonella models. Since the ionophore properties of

dinactin, it not only enhanced cations transport and altered membrane

permeability but also caused the dissipation of proton motive force and metabolic

perturbations. Finally, through the selection of spontaneous resistant mutants

and whole genome sequencing, non-synonymous single nucleotide polymorphisms were

successfully identified in the cpsA gene of the LytR-Cps2A-Psr family. The

dinactin-resistant mutants exhibited decreased in vitro drug sensitivity to

dinactin without cross-resistance to first-line antituberculosis drugs. Genetic

studies and molecular biology assays have subsequently confirmed cpsA as one of

the potential targets for dinactin's anti-tuberculosis activity. Collectively,

these data indicate that dinactin could be a promising candidate for treating

tuberculosis.

CI - © 2026. The Author(s).

FAU - Wang, Gaoyan

AU - Wang G

AD - State Key Laboratory of Agricultural Microbiology, College of Veterinary

Medicine, Huazhong Agricultural University, Wuhan, China.

AD - Hubei Hongshan Laboratory, Wuhan, China.

AD - Key Laboratory of Preventive Veterinary Medicine in Hubei Province, Wuhan, China.

AD - International Research Center for Animal Disease, Ministry of Science and

Technology of the People's Republic of China, Wuhan, China.

FAU - Dong, Wenqi

AU - Dong W

AD - State Key Laboratory of Agricultural Microbiology, College of Veterinary

Medicine, Huazhong Agricultural University, Wuhan, China.

AD - Hubei Hongshan Laboratory, Wuhan, China.

AD - Key Laboratory of Preventive Veterinary Medicine in Hubei Province, Wuhan, China.

AD - International Research Center for Animal Disease, Ministry of Science and

Technology of the People's Republic of China, Wuhan, China.

FAU - Bai, Yajuan

AU - Bai Y

AD - State Key Laboratory of Agricultural Microbiology, College of Veterinary

Medicine, Huazhong Agricultural University, Wuhan, China.

AD - Hubei Hongshan Laboratory, Wuhan, China.

AD - Key Laboratory of Preventive Veterinary Medicine in Hubei Province, Wuhan, China.

AD - International Research Center for Animal Disease, Ministry of Science and

Technology of the People's Republic of China, Wuhan, China.

FAU - Li, Yuxin

AU - Li Y

AD - State Key Laboratory of Agricultural Microbiology, College of Veterinary

Medicine, Huazhong Agricultural University, Wuhan, China.

AD - Hubei Hongshan Laboratory, Wuhan, China.

AD - Key Laboratory of Preventive Veterinary Medicine in Hubei Province, Wuhan, China.

AD - International Research Center for Animal Disease, Ministry of Science and

Technology of the People's Republic of China, Wuhan, China.

FAU - Lu, Hao

AU - Lu H

AD - State Key Laboratory of Agricultural Microbiology, College of Veterinary

Medicine, Huazhong Agricultural University, Wuhan, China.

AD - Hubei Hongshan Laboratory, Wuhan, China.

AD - Key Laboratory of Preventive Veterinary Medicine in Hubei Province, Wuhan, China.

AD - International Research Center for Animal Disease, Ministry of Science and

Technology of the People's Republic of China, Wuhan, China.

FAU - Lu, Wenjia

AU - Lu W

AD - State Key Laboratory of Agricultural Microbiology, College of Veterinary

Medicine, Huazhong Agricultural University, Wuhan, China.

AD - Hubei Hongshan Laboratory, Wuhan, China.

AD - Key Laboratory of Preventive Veterinary Medicine in Hubei Province, Wuhan, China.

AD - International Research Center for Animal Disease, Ministry of Science and

Technology of the People's Republic of China, Wuhan, China.

FAU - Wang, Chenchen

AU - Wang C

AD - State Key Laboratory of Agricultural Microbiology, College of Veterinary

Medicine, Huazhong Agricultural University, Wuhan, China.

AD - Hubei Hongshan Laboratory, Wuhan, China.

AD - Key Laboratory of Preventive Veterinary Medicine in Hubei Province, Wuhan, China.

AD - International Research Center for Animal Disease, Ministry of Science and

Technology of the People's Republic of China, Wuhan, China.

FAU - Tang, Jia

AU - Tang J

AD - State Key Laboratory of Agricultural Microbiology, College of Veterinary

Medicine, Huazhong Agricultural University, Wuhan, China.

FAU - Li, Pei

AU - Li P

AD - National Clinical Research Center for Infectious Diseases, Guangdong Provincial

Clinical Research Center for Tuberculosis, Shenzhen Clinical Research Center for

Tuberculosis, The Third People's Hospital of Shenzhen, Shenzhen, China.

FAU - Wang, Rui

AU - Wang R

AD - Department of Experimental Animal Center, West China Hospital, Sichuan

University, Chengdu, China.

FAU - Wang, Xiangru

AU - Wang X

AUID- ORCID: 0000-0003-0191-1378

AD - State Key Laboratory of Agricultural Microbiology, College of Veterinary

Medicine, Huazhong Agricultural University, Wuhan, China.

AD - Hubei Hongshan Laboratory, Wuhan, China.

AD - Key Laboratory of Preventive Veterinary Medicine in Hubei Province, Wuhan, China.

AD - International Research Center for Animal Disease, Ministry of Science and

Technology of the People's Republic of China, Wuhan, China.

FAU - Chen, Huanchun

AU - Chen H

AD - State Key Laboratory of Agricultural Microbiology, College of Veterinary

Medicine, Huazhong Agricultural University, Wuhan, China.

AD - Hubei Hongshan Laboratory, Wuhan, China.

AD - Key Laboratory of Preventive Veterinary Medicine in Hubei Province, Wuhan, China.

AD - International Research Center for Animal Disease, Ministry of Science and

Technology of the People's Republic of China, Wuhan, China.

FAU - Tan, Chen

AU - Tan C

AUID- ORCID: 0000-0003-4622-4483

AD - State Key Laboratory of Agricultural Microbiology, College of Veterinary

Medicine, Huazhong Agricultural University, Wuhan, China.

tanchen@mail.hzau.edu.cn.

AD - Hubei Hongshan Laboratory, Wuhan, China. tanchen@mail.hzau.edu.cn.

AD - Key Laboratory of Preventive Veterinary Medicine in Hubei Province, Wuhan, China.

tanchen@mail.hzau.edu.cn.

AD - International Research Center for Animal Disease, Ministry of Science and

Technology of the People's Republic of China, Wuhan, China.

tanchen@mail.hzau.edu.cn.

LA - eng

GR - 32273008/National Natural Science Foundation of China (National Science

Foundation of China)/

PT - Journal Article

DEP - 20260212

PL - England

TA - Commun Biol

JT - Communications biology

JID - 101719179

RN - 0 (Antitubercular Agents)

RN - 0 (Bacterial Proteins)

SB - IM

MH - *Mycobacterium tuberculosis/drug effects/genetics/metabolism

MH - *Antitubercular Agents/pharmacology

MH - *Bacterial Proteins/metabolism/genetics

MH - *Proton-Motive Force/drug effects

MH - Animals

MH - Microbial Sensitivity Tests

PMC - PMC13009212

COIS- Competing interests: The authors declare no competing interests.

EDAT- 2026/02/13 01:26

MHDA- 2026/03/24 07:42

PMCR- 2026/02/12

CRDT- 2026/02/12 23:30

PHST- 2025/05/14 00:00 [received]

PHST- 2026/01/27 00:00 [accepted]

PHST- 2026/03/24 07:42 [medline]

PHST- 2026/02/13 01:26 [pubmed]

PHST- 2026/02/12 23:30 [entrez]

PHST- 2026/02/12 00:00 [pmc-release]

AID - 10.1038/s42003-026-09654-3 [pii]

AID - 9654 [pii]

AID - 10.1038/s42003-026-09654-3 [doi]

PST - epublish

SO - Commun Biol. 2026 Feb 12;9(1):417. doi: 10.1038/s42003-026-09654-3.

PMID- 41727090

OWN - NLM

STAT- PubMed-not-MEDLINE

DCOM- 20260306

LR - 20260306

IS - 2692-8205 (Electronic)

IS - 2692-8205 (Linking)

DP - 2026 Feb 10

TI - Menaquinone depletion resensitises bedaquiline-resistant tuberculosis.

LID - 2026.02.10.702511 [pii]

LID - 10.64898/2026.02.10.702511 [doi]

AB - Tuberculosis remains a leading cause of global mortality, and rising bedaquiline

resistance threatens the effectiveness of current drug-resistant treatment

regimens. Bedaquiline resistance typically arises through mutations in Rv0678

that upregulate drug efflux and confer cross-resistance to multiple drug classes.

Here, we identify and optimise a chemical series targeting MenG, a central enzyme

in the menaquinone biosynthesis pathway, yielding potent bactericidal inhibitors

with in vivo efficacy. Strikingly, MenG inhibition restored bedaquiline

susceptibility in efflux-mediated resistant strains, an effect confirmed in vivo

where combination therapy achieved a 99.8% reduction in bacterial burden compared

with bedaquiline alone. Potentiation also extended to pretomanid and other key

agents. Disruption of upstream menaquinone and shikimate pathway enzymes produced

similar resensitisation, establishing these pathways as tractable targets for

restoring drug susceptibility in Mycobacterium tuberculosis. These findings

provide a novel strategy to overcome bedaquiline resistance and strengthen future

regimens for efflux-mediated drug-resistant tuberculosis.

FAU - Wetzel, Jennefer

AU - Wetzel J

AD - Johnson & Johnson, Beerse, Belgium.

FAU - Dallow, John

AU - Dallow J

AD - Department of Infection Biology, Faculty of Infectious and Tropical Disease,

London School of Hygiene and Tropical Medicine, London, WC1E 7HT, UK.

FAU - Davis, Ellie

AU - Davis E

AD - Department of Infection Biology, Faculty of Infectious and Tropical Disease,

London School of Hygiene and Tropical Medicine, London, WC1E 7HT, UK.

FAU - Pearson, William H

AU - Pearson WH

AD - Department of Infection Biology, Faculty of Infectious and Tropical Disease,

London School of Hygiene and Tropical Medicine, London, WC1E 7HT, UK.

FAU - Daems, Stijn

AU - Daems S

AD - Johnson & Johnson, Beerse, Belgium.

FAU - Govaerts, Matthias

AU - Govaerts M

AD - Johnson & Johnson, Beerse, Belgium.

AD - Charles River Laboratories, Turnhoutseweg 30, 2340, Beerse, Belgium.

FAU - Hereijgers, Joyce

AU - Hereijgers J

AD - Charles River Laboratories, Turnhoutseweg 30, 2340, Beerse, Belgium.

FAU - Sprangers, Joke

AU - Sprangers J

AD - Charles River Laboratories, Turnhoutseweg 30, 2340, Beerse, Belgium.

FAU - Truebody, Barry

AU - Truebody B

AD - Africa Health Research Institute, University of KwaZulu Natal, Durban, South

Africa.

FAU - Maes, Valerie

AU - Maes V

AD - Johnson & Johnson, Beerse, Belgium.

AD - VIB-KU Leuven Center for Microbiology, 3001, Heverlee, Belgium.

FAU - van Hasselt, Vera

AU - van Hasselt V

AD - Charles River Laboratories, Turnhoutseweg 30, 2340, Beerse, Belgium.

FAU - Leemans, Annelies

AU - Leemans A

AD - Johnson & Johnson, Beerse, Belgium.

AD - Charles River Laboratories, Turnhoutseweg 30, 2340, Beerse, Belgium.

FAU - Pujari, Venugopal

AU - Pujari V

AD - Microbiology, Immunology, and Pathology Department, Colorado State University,

Fort Collins, CO, 80523, USA.

FAU - Vos, Ann

AU - Vos A

AD - Johnson & Johnson, Beerse, Belgium.

FAU - Viturro, Carlos M Martínez

AU - Viturro CMM

AD - Global Discovery Chemistry, Janssen-Cilag SA, a Johnson & Johnson company, C. Río

Jarama, 75A, 45007 Toledo, Spain.

FAU - Gómez, José Enrique

AU - Gómez JE

AD - Global Discovery Chemistry, Janssen-Cilag SA, a Johnson & Johnson company, C. Río

Jarama, 75A, 45007 Toledo, Spain.

FAU - Peeters, Mirte

AU - Peeters M

AD - Charles River Laboratories, Turnhoutseweg 30, 2340, Beerse, Belgium.

FAU - Gerber, Michelle

AU - Gerber M

AD - Vaccine and Infectious Disease Organization, University of Saskatchewan,

Saskatoon, Saskatchewan, S7N 5E3, Canada.

FAU - Chhabra, Nandini

AU - Chhabra N

AD - Vaccine and Infectious Disease Organization, University of Saskatchewan,

Saskatoon, Saskatchewan, S7N 5E3, Canada.

FAU - Wouters, Annelies

AU - Wouters A

AD - Johnson & Johnson, Beerse, Belgium.

FAU - Everaerts, Melissa

AU - Everaerts M

AD - Johnson & Johnson, Beerse, Belgium.

FAU - Painter, Hannah

AU - Painter H

AD - Department of Infection Biology, Faculty of Infectious and Tropical Disease,

London School of Hygiene and Tropical Medicine, London, WC1E 7HT, UK.

FAU - Fathima, Raisha

AU - Fathima R

AD - Department of Infection Biology, Faculty of Infectious and Tropical Disease,

London School of Hygiene and Tropical Medicine, London, WC1E 7HT, UK.

FAU - Willcocks, Sam J

AU - Willcocks SJ

AD - Department of Infection Biology, Faculty of Infectious and Tropical Disease,

London School of Hygiene and Tropical Medicine, London, WC1E 7HT, UK.

FAU - Davies, Cadi

AU - Davies C

AD - Department of Infection Biology, Faculty of Infectious and Tropical Disease,

London School of Hygiene and Tropical Medicine, London, WC1E 7HT, UK.

FAU - Raeymaekers, Valerie

AU - Raeymaekers V

AD - Johnson & Johnson, Beerse, Belgium.

FAU - Clark, Taane G

AU - Clark TG

AD - Department of Infection Biology, Faculty of Infectious and Tropical Disease,

London School of Hygiene and Tropical Medicine, London, WC1E 7HT, UK.

FAU - Draghia-Akli, Ruxandra

AU - Draghia-Akli R

AD - Johnson & Johnson, Spring House, Pennsylvania, USA.

FAU - Fletcher, Helen

AU - Fletcher H

AD - Department of Infection Biology, Faculty of Infectious and Tropical Disease,

London School of Hygiene and Tropical Medicine, London, WC1E 7HT, UK.

AD - Johnson & Johnson, High Wycombe, UK.

FAU - Van Loock, Marnix

AU - Van Loock M

AD - Johnson & Johnson, Beerse, Belgium.

FAU - Hibberd, Martin L

AU - Hibberd ML

AD - Department of Infection Biology, Faculty of Infectious and Tropical Disease,

London School of Hygiene and Tropical Medicine, London, WC1E 7HT, UK.

FAU - Mostowy, Serge

AU - Mostowy S

AD - Department of Infection Biology, Faculty of Infectious and Tropical Disease,

London School of Hygiene and Tropical Medicine, London, WC1E 7HT, UK.

FAU - Crick, Dean C

AU - Crick DC

AD - Microbiology, Immunology, and Pathology Department, Colorado State University,

Fort Collins, CO, 80523, USA.

FAU - Pym, Alexander S

AU - Pym AS

AD - Johnson & Johnson, High Wycombe, UK.

FAU - Samby, Kirandeep

AU - Samby K

AD - Johnson & Johnson Pvt. Ltd, Gurugram 122003, India.

FAU - Jackson, Paul

AU - Jackson P

AD - Johnson & Johnson, La Jolla, California, USA.

FAU - Trabanco, Andrés A

AU - Trabanco AA

AD - Global Discovery Chemistry, Janssen-Cilag SA, a Johnson & Johnson company, C. Río

Jarama, 75A, 45007 Toledo, Spain.

FAU - Larrouy-Maumus, Gerald

AU - Larrouy-Maumus G

AD - Centre for Bacterial Resistance Biology, Department of Life Sciences, Faculty of

Natural Sciences, Imperial College London, London, SW7 2AZ, UK.

FAU - Steyn, Adrie J C

AU - Steyn AJC

AD - Africa Health Research Institute, University of KwaZulu Natal, Durban, South

Africa.

AD - Department of Microbiology, University of Alabama at Birmingham, Birmingham, AL,

USA.

AD - Centers for AIDS Research and Free Radical Biology, University of Alabama at

Birmingham, Birmingham, AL, USA.

FAU - Stoops, Bart

AU - Stoops B

AD - Johnson & Johnson, Beerse, Belgium.

FAU - Dhar, Neeraj

AU - Dhar N

AD - Vaccine and Infectious Disease Organization, University of Saskatchewan,

Saskatoon, Saskatchewan, S7N 5E3, Canada.

FAU - Aguilar-Pérez, Clara

AU - Aguilar-Pérez C

AD - Johnson & Johnson, Beerse, Belgium.

FAU - Lamprecht, Dirk A

AU - Lamprecht DA

AD - Johnson & Johnson, Beerse, Belgium.

FAU - Wall, Richard J

AU - Wall RJ

AD - Department of Infection Biology, Faculty of Infectious and Tropical Disease,

London School of Hygiene and Tropical Medicine, London, WC1E 7HT, UK.

FAU - Koul, Anil

AU - Koul A

AD - Johnson & Johnson, Beerse, Belgium.

AD - Department of Infection Biology, Faculty of Infectious and Tropical Disease,

London School of Hygiene and Tropical Medicine, London, WC1E 7HT, UK.

LA - eng

PT - Journal Article

PT - Preprint

DEP - 20260210

PL - United States

TA - bioRxiv

JT - bioRxiv : the preprint server for biology

JID - 101680187

PMC - PMC12919086

COIS- J.W., S.D., M.G., V.M., A.L., A.V., C.M.M.V., J.E.G.P., V.R., A.W., M.E., R.D-A.,

H.F., A.S.P., M.vL., K.S., P.J., A.A.T., B.S., C.A.-P., D.A.L. and A.K. were/are

all full-time employees and potential stockholders of Johnson & Johnson

(previously Janssen Pharmaceutica). M.G. J.S., M.P., J.H., A.L. and V.vH were/are

employees of Charles River Laboratories, a contract research organisation. J.D.,

E.D., W.H.P., B.T., V.P., S.J.W, C.D., D.C., M.L.H, G.L-M., A.J.C.S., N.D.,

R.J.W. and A.K. received funding from Johnson & Johnson to perform contract

research. The other authors declare no competing interests.

EDAT- 2026/02/23 13:06

MHDA- 2026/02/23 13:07

PMCR- 2026/02/19

CRDT- 2026/02/23 06:33

PHST- 2026/02/23 13:06 [pubmed]

PHST- 2026/02/23 13:07 [medline]

PHST- 2026/02/23 06:33 [entrez]

PHST- 2026/02/19 00:00 [pmc-release]

AID - 2026.02.10.702511 [pii]

AID - 10.64898/2026.02.10.702511 [doi]

PST - epublish

SO - bioRxiv [Preprint]. 2026 Feb 10:2026.02.10.702511. doi:

10.64898/2026.02.10.702511.

PMID- 41737548

OWN - NLM

STAT- PubMed-not-MEDLINE

DCOM- 20260225

LR - 20260225

IS - 1663-9812 (Print)

IS - 1663-9812 (Electronic)

IS - 1663-9812 (Linking)

VI - 17

DP - 2026

TI - Drug resistance mechanisms in Mycobacterium tuberculosis infection and challenges

in vaccine development.

PG - 1762214

LID - 10.3389/fphar.2026.1762214 [doi]

LID - 1762214

AB - Drug-resistant Mycobacterium tuberculosis(Mtb) has become a global public health

crisis, and its diverse drug resistance jointly reduces the effectiveness of

antibacterial drugs. Mtb resistance is not merely genetic but involves a

synergistic interplay of cell wall remodeling, metabolic reprogramming, and

epigenetic regulation, all of which are closely linked to its capacity for immune

evasion. These mechanisms lead to the failure of traditional treatments,

exacerbating the prolongation of treatment duration, the increase in mortality

rate and the spread of drug-resistant bacteria. Vaccine research has gradually

become a key strategy for preventing and controlling the spread of drug-resistant

tuberculosis. This review synthesizes these multifaceted resistance pathways and

parallels them with the challenges in vaccine development, highlighting the

limited efficacy of Bacillus Calmette-Guérin and the promise of next-generation

candidates. It further explores the landscape of novel therapeutic strategies,

including new drugs like bedaquiline and host-directed therapies. In the future,

efforts should be focused on the development of multivalent vaccines, the

integration of chemoimmunotherapy, and the sharing of global monitoring data to

contribute to the ultimate goal of eliminating tuberculosis.

CI - Copyright © 2026 Zhang, Cheng and Tang.

FAU - Zhang, Shuying

AU - Zhang S

AD - Department of Laboratory Medicine, Weifang No. 2 People's Hospital, Weifang,

China.

FAU - Cheng, Juan

AU - Cheng J

AD - Department of Laboratory Medicine, Weifang No. 2 People's Hospital, Weifang,

China.

FAU - Tang, Yuanyuan

AU - Tang Y

AD - Translational Medical Center, Weifang No. 2 People's Hospital, Weifang, China.

LA - eng

PT - Journal Article

PT - Review

DEP - 20260209

PL - Switzerland

TA - Front Pharmacol

JT - Frontiers in pharmacology

JID - 101548923

PMC - PMC12926377

OTO - NOTNLM

OT - Mycobacterium tuberculosis

OT - drug resistance mechanisms

OT - immune evasion

OT - immunoinformatic

OT - vaccine development

COIS- The author(s) declared that this work was conducted in the absence of any

commercial or financial relationships that could be construed as a potential

conflict of interest.

EDAT- 2026/02/25 10:23

MHDA- 2026/02/25 10:24

PMCR- 2026/02/09

CRDT- 2026/02/25 04:51

PHST- 2025/12/06 00:00 [received]

PHST- 2026/01/15 00:00 [revised]

PHST- 2026/01/15 00:00 [accepted]

PHST- 2026/02/25 10:24 [medline]

PHST- 2026/02/25 10:23 [pubmed]

PHST- 2026/02/25 04:51 [entrez]

PHST- 2026/02/09 00:00 [pmc-release]

AID - 1762214 [pii]

AID - 10.3389/fphar.2026.1762214 [doi]

PST - epublish

SO - Front Pharmacol. 2026 Feb 9;17:1762214. doi: 10.3389/fphar.2026.1762214.

eCollection 2026.

PMID- 41736792

OWN - NLM

STAT- MEDLINE

DCOM- 20260709

LR - 20260709

IS - 2235-2988 (Electronic)

IS - 2235-2988 (Linking)

VI - 16

DP - 2026

TI - Association of vitamin D receptor genetic variants with therapeutic response in

multidrug-resistant pulmonary tuberculosis: a systematic review.

PG - 1692281

LID - 10.3389/fcimb.2026.1692281 [doi]

LID - 1692281

AB - INTRODUCTION: In high-burden nations like India, tuberculosis (TB) continues to

be a significant global public health concern. HIV infection, diabetes mellitus,

and low socioeconomic status are examples of comorbid illnesses that increase

susceptibility to tuberculosis (TB), and the introduction of multidrug-resistant

tuberculosis (MDR-TB) has made disease control even more challenging. The

time-consuming nature of conventional drug susceptibility testing (DST)

emphasizes the critical need for quick biomarkers to forecast treatment outcomes

and resistance. Because of their possible impact on host immunity and MDR-TB

risk, genetic variations particularly vitamin D receptor (VDR) polymorphisms have

drawn attention. METHODS: Studies published between 2000 and 2024 were the

subject of an extensive examination of the literature. Relevance led to the

selection of 213 articles. Keywords including vitamin D, VDR polymorphisms,

MDR-TB, pulmonary tuberculosis, and immune response were used to search databases

such as PubMed, Web of Science, and Google Scholar. To guarantee comprehensive

coverage, both original research articles and reviews were included. RESULTS: Low

serum vitamin D levels were consistently linked to an elevated risk of MDR-TB and

pulmonary tuberculosis (PTB), according to the investigation. Certain VDR

polymorphisms have often been associated with altered immunological responses and

an increased risk of disease, especially mutant forms like FokI and TaqI.

Treatment response and disease progression have also been discovered to be

influenced by immunological modulation and dietary variables. DISCUSSION: These

results imply that vitamin D levels and VDR polymorphisms could be useful

biomarkers for the diagnosis and prognosis of MDR-TB. Knowing the genetic

susceptibility of the host may help develop individualized treatment plans and

enhance the management of MDR-TB. SYSTEMATIC REVIEW REGISTRATION:

https://www.crd.york.ac.uk/PROSPERO/view/CRD420261295571, identifier

CRD420261295571.

CI - Copyright © 2026 Rathored, Budhbaware, Shende and Upadhyay.

FAU - Rathored, Jaishriram

AU - Rathored J

AD - Central Research Laboratory and Molecular Diagnostics, Datta Meghe Institute of

Higher Education and Research, Sawangi (Meghe), Wardha, Maharashtra, India.

FAU - Budhbaware, Tanushree

AU - Budhbaware T

AD - Central Research Laboratory and Molecular Diagnostics, Datta Meghe Institute of

Higher Education and Research, Sawangi (Meghe), Wardha, Maharashtra, India.

FAU - Shende, Sandesh

AU - Shende S

AD - Central Research Laboratory and Molecular Diagnostics, Datta Meghe Institute of

Higher Education and Research, Sawangi (Meghe), Wardha, Maharashtra, India.

FAU - Upadhyay, Vishwanath

AU - Upadhyay V

AD - Department of Molecular Medicine, Jamia Hamdard (Deemed to be University), New

Delhi, India.

LA - eng

PT - Journal Article

PT - Systematic Review

DEP - 20260209

PL - Switzerland

TA - Front Cell Infect Microbiol

JT - Frontiers in cellular and infection microbiology

JID - 101585359

RN - 0 (Receptors, Calcitriol)

RN - 0 (Antitubercular Agents)

RN - 1406-16-2 (Vitamin D)

RN - 0 (VDR protein, human)

SB - IM

MH - *Receptors, Calcitriol/genetics

MH - Humans

MH - *Tuberculosis, Multidrug-Resistant/genetics/drug therapy/immunology

MH - *Antitubercular Agents/therapeutic use

MH - *Tuberculosis, Pulmonary/drug therapy/genetics/immunology

MH - Vitamin D/blood

MH - Genetic Predisposition to Disease

MH - Polymorphism, Genetic

MH - Treatment Outcome

MH - Mycobacterium tuberculosis/drug effects

PMC - PMC12926482

OTO - NOTNLM

OT - genetic variants

OT - multi-drug-resistant tuberculosis (MDR-TB)

OT - polymorphism

OT - treatment response

OT - tuberculosis

OT - vitamin D

OT - vitamin D receptors (VDRs)

COIS- The author(s) declared that this work was conducted in the absence of any

commercial or financial relationships that could be construed as a potential

conflict of interest. The reviewer GK declared a shared affiliation with the

author VU to the handling editor at the time of review.

EDAT- 2026/02/25 10:20

MHDA- 2026/02/25 10:21

PMCR- 2026/02/09

CRDT- 2026/02/25 04:44

PHST- 2025/08/25 00:00 [received]

PHST- 2025/12/31 00:00 [revised]

PHST- 2026/01/13 00:00 [accepted]

PHST- 2026/02/25 10:21 [medline]

PHST- 2026/02/25 10:20 [pubmed]

PHST- 2026/02/25 04:44 [entrez]

PHST- 2026/02/09 00:00 [pmc-release]

AID - 10.3389/fcimb.2026.1692281 [doi]

PST - epublish

SO - Front Cell Infect Microbiol. 2026 Feb 9;16:1692281. doi:

10.3389/fcimb.2026.1692281. eCollection 2026.

PMID- 41652570

OWN - NLM

STAT- MEDLINE

DCOM- 20260627

LR - 20260627

IS - 1471-2334 (Electronic)

IS - 1471-2334 (Linking)

VI - 26

IP - 1

DP - 2026 Feb 6

TI - Association between overexpression of efflux pumps genes Rv0191 and Rv3008 and

pyrazinamide resistance in multidrug-resistant Mycobacterium tuberculosis: a

multi-center study.

LID - 10.1186/s12879-026-12793-x [doi]

LID - 528

AB - BACKGROUND: Pyrazinamide is a drug that helps shorten the duration of

tuberculosis treatment. Mutations in the pncA are the most common mechanism of

pyrazinamide resistance in M. tuberculosis. This study aimed to investigate the

association between overexpression of Efflux pump genes Rv0191 and Rv3008 and

pyrazinamide resistance in multidrug-resistant M. tuberculosis in a multicenter

setting. METHODS: In this study, 40 clinical isolates of pulmonary M.

tuberculosis were gathered from septum specimens; confirmation of M.tuberculosis

isolates was achieved by PCR and phenotypic methods using the IS6110 gene. The

phenotypic microdilution broth method was evaluated for drug susceptibility

testing. Frequency of pncA gene mutations associated with pyrazinamide resistance

by the PCR method in M. tuberculosis isolates. The evaluation of the Rv0191 and

Rv3008 efflux pump genes was performed on pyrazinamide-sensitive,

pyrazinamide-resistant, and multidrug-resistant M. tuberculosis isolates.

RESULTS: This study showed that, in MDR, resistance to pyrazinamide was

associated with single-resistant strains and a high percentage of mutations in

the pncA gene. The most effective method for detecting pyrazinamide resistance is

sequencing the entire pncA gene to confirm resistance, rather than the

conventional techniques that focus on mutated hotspots. CONCLUSIONS: The study

found that PZA resistance was common among MDR-TB isolates, with mutations

occurring in the pcnA gene. The findings also showed that drug resistance in

clinical isolates of M. tuberculosis is often associated with increased

expression of efflux pump genes and mutations in target genes. In cases where

there are no mutations, resistance may be related to overexpression of other

efflux pumps.

FAU - Hashemzadeh, Mohammad

AU - Hashemzadeh M

AD - Department of Microbiology, School of Medicine, Ahvaz Jundishapur University of

Medical Sciences, Ahvaz, Iran. mohammadhashemzadeh615@gmail.com.

AD - Infectious and Tropical Diseases Research Center, Health Research Institute,

Ahvaz Jundishapur University of Medical Sciences, Ahvaz, Iran.

mohammadhashemzadeh615@gmail.com.

FAU - Hamid, Sadegh

AU - Hamid S

AD - Department of Microbiology, School of Medicine, Ahvaz Jundishapur University of

Medical Sciences, Ahvaz, Iran.

FAU - Khosravi, Azar Dokht

AU - Khosravi AD

AD - Department of Microbiology, School of Medicine, Ahvaz Jundishapur University of

Medical Sciences, Ahvaz, Iran.

AD - Infectious and Tropical Diseases Research Center, Health Research Institute,

Ahvaz Jundishapur University of Medical Sciences, Ahvaz, Iran.

FAU - Soleimanpour, Saman

AU - Soleimanpour S

AD - Antimicrobial Resistance Research Centre, Bu-Ali Research Institute, Mashhad

University of Medical Sciences, Mashhad, Iran.

FAU - Savari, Mohammad

AU - Savari M

AD - Department of Microbiology, School of Medicine, Ahvaz Jundishapur University of

Medical Sciences, Ahvaz, Iran.

AD - Infectious and Tropical Diseases Research Center, Health Research Institute,

Ahvaz Jundishapur University of Medical Sciences, Ahvaz, Iran.

LA - eng

PT - Journal Article

PT - Multicenter Study

DEP - 20260206

PL - England

TA - BMC Infect Dis

JT - BMC infectious diseases

JID - 100968551

RN - 2KNI5N06TI (Pyrazinamide)

RN - 0 (Antitubercular Agents)

RN - 0 (Bacterial Proteins)

RN - 0 (Membrane Transport Proteins)

RN - EC 3.5.- (Amidohydrolases)

RN - EC 3.5.- (PncA protein, Mycobacterium tuberculosis)

SB - IM

MH - *Pyrazinamide/pharmacology

MH - *Mycobacterium tuberculosis/drug effects/genetics/isolation & purification

MH - *Antitubercular Agents/pharmacology

MH - Humans

MH - Microbial Sensitivity Tests

MH - *Tuberculosis, Multidrug-Resistant/microbiology

MH - *Drug Resistance, Multiple, Bacterial/genetics

MH - *Bacterial Proteins/genetics/metabolism

MH - *Membrane Transport Proteins/genetics/metabolism

MH - Amidohydrolases/genetics

MH - Mutation

PMC - PMC12977539

OTO - NOTNLM

OT - Mycobacterium tuberculosis

OT - Efflux pumps

OT - Multidrug resistance

OT - Pyrazinamide

OT - Real-time PCR

COIS- Declarations. Ethics approval and consent to participate: This research was

conducted in accordance with the Helsinki Declaration. Following the submission

of the preliminary proposal, the study received approval from the Institutional

Ethics and Review Board of Ahvaz Jundishapur University of Medical Sciences,

Ahvaz, Iran, and the necessary permission for sample collection was granted

(IR.AJUMS.MEDICINE.REC.1400.001). Written informed consent was obtained from all

participants. After the preliminary proposal was submitted, permission to collect

the sample was granted. The Tuberculosis Center Data Protection Authority also

approved the study. All respondents were provided with an information letter

about the study and asked to give written consent to participate. We made it

clear that participation was voluntary and that individuals could withdraw from

the study at any time. Furthermore, all procedures involving human participants

confirmed that written consent had been obtained for the use of their

information. In this study, personal information such as names, surnames,

ethnicity, and personal identification numbers, as well as any information

participants requested remain unpublished, was excluded. Consent for publication:

Not applicable. Competing interests: The authors declare no competing interests.

EDAT- 2026/02/07 06:33

MHDA- 2026/06/27 06:45

PMCR- 2026/02/06

CRDT- 2026/02/07 00:09

PHST- 2025/08/30 00:00 [received]

PHST- 2026/02/02 00:00 [accepted]

PHST- 2026/06/27 06:45 [medline]

PHST- 2026/02/07 06:33 [pubmed]

PHST- 2026/02/07 00:09 [entrez]

PHST- 2026/02/06 00:00 [pmc-release]

AID - 10.1186/s12879-026-12793-x [pii]

AID - 12793 [pii]

AID - 10.1186/s12879-026-12793-x [doi]

PST - epublish

SO - BMC Infect Dis. 2026 Feb 6;26(1):528. doi: 10.1186/s12879-026-12793-x.

PMID- 41478405

OWN - NLM

STAT- MEDLINE

DCOM- 20260219

LR - 20260228

IS - 1520-6017 (Electronic)

IS - 0022-3549 (Linking)

VI - 115

IP - 2

DP - 2026 Feb

TI - Interaction of isoniazid derivatives active against drug-resistant tuberculosis

with models of the lung surfactant and of the Mycobacterium tuberculosis cell

wall.

PG - 104151

LID - S0022-3549(25)00605-7 [pii]

LID - 10.1016/j.xphs.2025.104151 [doi]

AB - Tuberculosis is one of the most important causes of death in the world. The

emergence and increased prominence of multidrug-resistant strains of

Mycobacterium tuberculosis (Mtb), non-susceptible to currently available

therapies, has toughened the fight to eradicate this disease. This study focuses

on further investigating the therapeutic potential of promising antitubercular

compounds, namely, isoniazid (INH), and three INH derivatives,

N'-decanoylisonicotinohydrazide (INH-C10),

N'-(E)-(4-phenoxybenzylidene)isonicotinohydrazide (N34) and

N'-(4-phenoxybenzyl)isonicotinohydrazide (N34red). INH-C10 and N34 have been

selected due to their high selectivity index against the Mtb mutant bearing the

primary mutation responsible for INH drug resistance. In opposition, N34red,

which differs from N34 only in the saturation of the N' = C bond, exhibits a poor

selectivity index. Moreover, INH-C10 and N34 interact with human serum albumin

and model lipid membranes mimicking the plasma membrane of human cells, showing

their promising potential. In the current study, the interaction of these

compounds with models of the lung surfactant (LS) and of the mycolic acid

(MA)-enriched Mtb cell wall was assessed, in order to further explore their

ability to interact with and cross the various biological barriers to be

encountered on their way to the molecular target inside Mtb. We show that all the

INH derivatives were able to interact with both the LS and the mycolic

acid-enriched cell wall models. INH-C10 and N34 had a smaller impact than N34red

on the pulmonary surfactant model. On the other hand, INH-C10 promoted the most

extensive perturbation of the MA-enriched cell wall model, which correlates well

with the previously shown ability of this compound to incorporate into and

disturb gel-phase lipid bilayers. This indicates that INH-C10 may penetrate a MA

rich barrier more easily, reaching higher intracellular levels, and increase its

permeability. These traits contribute to explain the high antimicrobial activity

of this derivative against the most common drug-resistant Mtb mutant.

CI - Copyright © 2025 American Pharmacists Association. Published by Elsevier Inc. All

rights reserved.

FAU - Śliżewska, Weronika

AU - Śliżewska W

AD - Institute of Molecular and Industrial Biotechnology, Faculty of Biotechnology and

Food Sciences, Lodz University of Technology, Lodz, Poland; Centro de Química

Estrutural, Institute of Molecular Sciences, Departamento de Química e

Bioquímica, Faculdade de Ciências, Universidade de Lisboa, 1749-016 Campo Grande,

Portugal.

FAU - Martins, Filomena

AU - Martins F

AD - Centro de Química Estrutural, Institute of Molecular Sciences, Departamento de

Química e Bioquímica, Faculdade de Ciências, Universidade de Lisboa, 1749-016

Campo Grande, Portugal.

FAU - de Almeida, Rodrigo F M

AU - de Almeida RFM

AD - Centro de Química Estrutural, Institute of Molecular Sciences, Departamento de

Química e Bioquímica, Faculdade de Ciências, Universidade de Lisboa, 1749-016

Campo Grande, Portugal.

FAU - Marquês, Joaquim T

AU - Marquês JT

AD - Centro de Química Estrutural, Institute of Molecular Sciences, Departamento de

Química e Bioquímica, Faculdade de Ciências, Universidade de Lisboa, 1749-016

Campo Grande, Portugal. Electronic address: jmtmarques@ciencias.ulisboa.pt.

LA - eng

PT - Journal Article

DEP - 20251230

PL - United States

TA - J Pharm Sci

JT - Journal of pharmaceutical sciences

JID - 2985195R

RN - V83O1VOZ8L (Isoniazid)

RN - 0 (Antitubercular Agents)

RN - 0 (Pulmonary Surfactants)

SB - IM

MH - *Mycobacterium tuberculosis/drug effects/metabolism

MH - *Isoniazid/pharmacology/analogs & derivatives/metabolism

MH - *Antitubercular Agents/pharmacology/chemistry

MH - *Cell Wall/drug effects/metabolism

MH - Humans

MH - *Tuberculosis, Multidrug-Resistant/drug therapy/metabolism/microbiology

MH - *Pulmonary Surfactants/metabolism

MH - Microbial Sensitivity Tests

MH - Cell Membrane/metabolism

OTO - NOTNLM

OT - Antitubercular compounds

OT - Langmuir monolayers

OT - Multidrug-resistant tuberculosis

OT - Mycolic acids

OT - Pulmonary surfactant model

COIS- Declaration of competing interest The authors declare that they have no known

competing financial interests or personal relationships that could have appeared

to influence the work reported in this paper.

EDAT- 2026/01/02 00:33

MHDA- 2026/02/20 00:32

CRDT- 2026/01/01 19:34

PHST- 2025/10/20 00:00 [received]

PHST- 2025/12/22 00:00 [revised]

PHST- 2025/12/26 00:00 [accepted]

PHST- 2026/02/20 00:32 [medline]

PHST- 2026/01/02 00:33 [pubmed]

PHST- 2026/01/01 19:34 [entrez]

AID - S0022-3549(25)00605-7 [pii]

AID - 10.1016/j.xphs.2025.104151 [doi]

PST - ppublish

SO - J Pharm Sci. 2026 Feb;115(2):104151. doi: 10.1016/j.xphs.2025.104151. Epub 2025

Dec 30.

PMID- 41443014

OWN - NLM

STAT- MEDLINE

DCOM- 20260119

LR - 20260417

IS - 1090-2120 (Electronic)

IS - 0045-2068 (Linking)

VI - 169

DP - 2026 Feb

TI - Synthesis, antitubercular activity and computational studies of click-tethered

phosphonium salts.

PG - 109390

LID - S0045-2068(25)01270-2 [pii]

LID - 10.1016/j.bioorg.2025.109390 [doi]

AB - This study reports the synthesis of a library comprising three series of 21

phosphonium salt hybrids (1a-1 g, 2a-2 g, 3a-3 g) generated via a click reaction

yielding 1, 2, 3-triazoles with potential antitubercular activity. The

phosphonium salts were synthesized through a 1, 3-dipolar cycloaddition (click)

reaction. The structures of all synthesized compounds were elucidated using (1)H

NMR, (13)C NMR, AT-FTIR, and HR-LCMS spectroscopy. The compounds were screened

for antitubercular activity against Mycobacterium tuberculosis H37Rv and

multidrug-resistant TB (MDR-TB) with Rifampicin and Isoniazid, Notably,

derivatives 1d and 1e demonstrated the most potent activity, achieving 100 %

inhibition of MDR-TB at a minimum inhibitory concentration (MIC) of 6.25 μg/mL.

At 100 μg/mL, these compounds reduced the colony-forming units (CFU) of MDR-TB by

96 % and 64 %, respectively. Compounds 2e, 3e, and 3f also displayed inhibitory

activity against H37Rv, with 2e showing moderate efficacy (68 % inhibition,

6.25 μg/mL) towards MDR-TB and the highest selectivity index against murine

macrophages. Molecular docking studies against seventeen (17) M. tuberculosis

target proteins revealed that compounds 1d, 1e, and 2e displayed the strongest

binding affinity for InhA (PDB ID: 5MTP), a key enzyme in mycolic acid

biosynthesis, interacting critically with residues TYR158, PHE149, MET199,

ILE194, and SER94. MM/GBSA calculations further confirmed the favorable binding

free energies of these complexes. All compounds exhibited favorable predicted

oral bioavailability and drug-like properties. These findings highlight the

click-tethered phosphonium salts, particularly 1d, as promising lead candidates

for the development of novel antitubercular agents targeting InhA through a

mechanism of dynamic stabilization.

CI - Copyright © 2024. Published by Elsevier Inc.

FAU - Amengor, Cedric Dzidzor Kodjo

AU - Amengor CDK

AD - Department of Pharmaceutical Chemistry, Drug Discovery Unit, School of Pharmacy,

University of Health and Allied Sciences, Ho, Ghana. Electronic address:

camengor@uhas.edu.gh.

FAU - Biniyam, Prince Danan

AU - Biniyam PD

AD - Department of Pharmaceutical Chemistry, Computational Medicinal Chemistry Unit,

School of Pharmacy, University of Health and Allied Sciences, Ho, Ghana.

FAU - Ohene-Adu, Victoria

AU - Ohene-Adu V

AD - Department of Pharmaceutical Chemistry, Computational Medicinal Chemistry Unit,

School of Pharmacy, University of Health and Allied Sciences, Ho, Ghana.

FAU - Adu-Adjei, Kwabena

AU - Adu-Adjei K

AD - Department of Pharmaceutical Chemistry, Computational Medicinal Chemistry Unit,

School of Pharmacy, University of Health and Allied Sciences, Ho, Ghana.

FAU - Osei, Michael

AU - Osei M

AD - Department of Pharmaceutical Chemistry, Computational Medicinal Chemistry Unit,

School of Pharmacy, University of Health and Allied Sciences, Ho, Ghana.

FAU - Gyan, Patrick

AU - Gyan P

AD - Department of Pharmaceutical Chemistry, Drug Discovery Unit, School of Pharmacy,

University of Health and Allied Sciences, Ho, Ghana.

FAU - Quansah, Paul

AU - Quansah P

AD - Department of Pharmaceutical Chemistry, Drug Discovery Unit, School of Pharmacy,

University of Health and Allied Sciences, Ho, Ghana.

FAU - Odame, Felix

AU - Odame F

AD - Department of Basic Sciences, School of Basic and Biomedical Sciences, University

of Health and Allied Sciences, Ho, Ghana.

FAU - Oyeh, Ernest

AU - Oyeh E

AD - Department of Nursing, Faculty of Science, Methodist University, Ghana.

FAU - Amengor, Cyril Makafui

AU - Amengor CM

AD - Department of Mathematics, College of Science, University of Ghana, Legon, Ghana.

FAU - Borquaye, Lawrence Sheringham

AU - Borquaye LS

AD - Department of Chemistry, Kwame Nkrumah University of Science and Technology,

Ghana.

LA - eng

PT - Journal Article

PT - Research Support, Non-U.S. Gov't

DEP - 20251219

PL - United States

TA - Bioorg Chem

JT - Bioorganic chemistry

JID - 1303703

RN - 0 (Antitubercular Agents)

RN - 0 (Organophosphorus Compounds)

RN - 0 (Salts)

SB - IM

MH - *Antitubercular Agents/pharmacology/chemical synthesis/chemistry

MH - *Mycobacterium tuberculosis/drug effects

MH - Microbial Sensitivity Tests

MH - Structure-Activity Relationship

MH - Molecular Structure

MH - *Organophosphorus Compounds/pharmacology/chemistry/chemical synthesis

MH - Molecular Docking Simulation

MH - Mice

MH - Animals

MH - Salts/pharmacology/chemistry/chemical synthesis

MH - Click Chemistry

MH - Dose-Response Relationship, Drug

OTO - NOTNLM

OT - Antitubercular

OT - H37Rv

OT - InhA inhibition

OT - MDR-TB

OT - Molecular dynamics

OT - Phosphonium salts

OT - Triazoles

COIS- Declaration of competing interest The authors declare that they have no known

competing financial interests or personal relationships that could have appeared

to influence the work reported in this paper.

EDAT- 2025/12/25 00:29

MHDA- 2026/01/20 00:37

CRDT- 2025/12/24 18:08

PHST- 2025/10/11 00:00 [received]

PHST- 2025/12/07 00:00 [revised]

PHST- 2025/12/14 00:00 [accepted]

PHST- 2026/01/20 00:37 [medline]

PHST- 2025/12/25 00:29 [pubmed]

PHST- 2025/12/24 18:08 [entrez]

AID - S0045-2068(25)01270-2 [pii]

AID - 10.1016/j.bioorg.2025.109390 [doi]

PST - ppublish

SO - Bioorg Chem. 2026 Feb;169:109390. doi: 10.1016/j.bioorg.2025.109390. Epub 2025

Dec 19.

PMID- 41371315

OWN - NLM

STAT- MEDLINE

DCOM- 20260110

LR - 20260110

IS - 1096-1208 (Electronic)

IS - 0882-4010 (Linking)

VI - 211

DP - 2026 Feb

TI - Host-directed insights into mycobacterial infections: Toward targeted

immunomodulation.

PG - 108233

LID - S0882-4010(25)00958-1 [pii]

LID - 10.1016/j.micpath.2025.108233 [doi]

AB - Mycobacterium tuberculosis, the etiological agent of tuberculosis, is a

significant worldwide health threat, especially in resource-limited environments.

This review emphasizes that manipulating host metabolic and epigenetic pathways

can bolster immune defences and potentially shorten the duration of tuberculosis

treatment. The growing prevalence of multidrug-resistant TB and the protracted

nature of standard treatments underscore the urgent need for alternative

therapeutic approaches. Host-directed treatment has arisen as a promising

approach that aims to enhance or redirect the host's innate and adaptive

responses rather than targeting the pathogen directly. This strategy focuses on

counteracting M. tuberculosis-induced subversion of immune and cellular

processes. Approaches under investigation include modulation of host metabolic

pathways, stimulation of autophagy, epigenetic reprogramming, and strengthening

of immune defense mechanisms to control or eliminate infection. These

interventions hold potential for not only overcoming traditional drug resistance

but also for accelerating recovery and reducing immunopathology. In this review,

we explore recent advances in host-directed therapy research, with particular

emphasis on mechanisms involving immunometabolic regulation, epigenetic

remodeling, and enhancement of intercellular antimicrobial responses. The

advancement of these methodologies may facilitate the creation of more accurate

and efficacious tuberculosis treatments. We highlight recent work on three linked

areas-immunometabolic regulation, epigenetic control, and autophagy driven

antimicrobial activity drawing out the main advances and remaining debates that

guide future host-directed approaches. Viewing host-directed therapy through this

integrated lens outline a path toward more targeted, durable, and

resistance-resilient interventions for tuberculosis.

CI - Copyright © 2025 Elsevier Ltd. All rights reserved.

FAU - Menon, Arjun M

AU - Menon AM

AD - School of Biotechnology, Amrita Vishwa Vidyapeetham, Amritapuri, Kollam, 690525,

Kerala, India.

FAU - Kuniyil, Abhinand

AU - Kuniyil A

AD - School of Biotechnology, Amrita Vishwa Vidyapeetham, Amritapuri, Kollam, 690525,

Kerala, India.

FAU - Thomas, Shwetha Susan

AU - Thomas SS

AD - School of Biotechnology, Amrita Vishwa Vidyapeetham, Amritapuri, Kollam, 690525,

Kerala, India.

FAU - Salini, S

AU - Salini S

AD - National Centre for Aquatic Animal Health, CUSAT, Kochi, Kerala, 682016, India.

FAU - Edison, Lekshmi K

AU - Edison LK

AD - Department of Comparative, Diagnostic, and Population Medicine, College of

Veterinary Medicine, University of Florida, Gainesville, FL, 32610, USA.

FAU - Parvathi Mohanan, P C

AU - Parvathi Mohanan PC

AD - School of Biotechnology, Amrita Vishwa Vidyapeetham, Amritapuri, Kollam, 690525,

Kerala, India.

FAU - Arun, K B

AU - Arun KB

AD - Department of Life Sciences, CHRIST (Deemed to be University), Bangalore, 560029,

Karnataka, India.

FAU - Babu, Pradeesh

AU - Babu P

AD - School of Biotechnology, Amrita Vishwa Vidyapeetham, Amritapuri, Kollam, 690525,

Kerala, India.

FAU - Kumar, Geetha B

AU - Kumar GB

AD - School of Biotechnology, Amrita Vishwa Vidyapeetham, Amritapuri, Kollam, 690525,

Kerala, India.

FAU - Nair, Bipin G

AU - Nair BG

AD - School of Biotechnology, Amrita Vishwa Vidyapeetham, Amritapuri, Kollam, 690525,

Kerala, India.

FAU - Madhavan, Aravind

AU - Madhavan A

AD - School of Biotechnology, Amrita Vishwa Vidyapeetham, Amritapuri, Kollam, 690525,

Kerala, India. Electronic address: aravind7933@gmail.com.

LA - eng

PT - Journal Article

PT - Review

DEP - 20251208

PL - England

TA - Microb Pathog

JT - Microbial pathogenesis

JID - 8606191

RN - 0 (Antitubercular Agents)

SB - IM

MH - Humans

MH - *Mycobacterium tuberculosis/immunology/drug effects

MH - Epigenesis, Genetic

MH - *Tuberculosis/immunology/drug therapy/microbiology/therapy

MH - *Immunomodulation

MH - Autophagy

MH - *Host-Pathogen Interactions/immunology

MH - Antitubercular Agents/therapeutic use/pharmacology

MH - Animals

COIS- Declaration of competing interest The authors declare that they have no known

competing financial interests or personal relationships that could have appeared

to influence the work reported in this paper.

EDAT- 2025/12/11 01:22

MHDA- 2026/01/11 00:34

CRDT- 2025/12/10 19:30

PHST- 2025/07/26 00:00 [received]

PHST- 2025/10/15 00:00 [revised]

PHST- 2025/12/07 00:00 [accepted]

PHST- 2026/01/11 00:34 [medline]

PHST- 2025/12/11 01:22 [pubmed]

PHST- 2025/12/10 19:30 [entrez]

AID - S0882-4010(25)00958-1 [pii]

AID - 10.1016/j.micpath.2025.108233 [doi]

PST - ppublish

SO - Microb Pathog. 2026 Feb;211:108233. doi: 10.1016/j.micpath.2025.108233. Epub 2025

Dec 8.

PMID- 41075756

OWN - NLM

STAT- MEDLINE

DCOM- 20251211

LR - 20260610

IS - 1476-928X (Electronic)

IS - 1476-9271 (Linking)

VI - 120

IP - Pt 2

DP - 2026 Feb

TI - An integrated computational bioprospection of flavonoids as modulators of

Mycobacterium tuberculosis decaprenylphosphoryl-β-d-ribose-2'-epimerase 1.

PG - 108719

LID - S1476-9271(25)00380-9 [pii]

LID - 10.1016/j.compbiolchem.2025.108719 [doi]

AB - Tuberculosis (TB) remains a significant global health threat, claiming millions

of lives annually despite being preventable. The emergence of drug-resistant

strains, including extensively drug-resistant TB (XDR-TB) and multidrug-resistant

TB (MDR-TB), severely limits conventional treatment options. Furthermore,

commonly used TB medications like isoniazid (INH) and rifampicin (RIF) are

associated with adverse side effects. Consequently, researchers increasingly

explore natural products as potential sources for novel anti-TB therapeutics.

This study investigated the inhibitory potential of 103 flavonoid compounds with

documented antimycobacterial activity against TB. Focusing on

decaprenylphosphoryl-β-D-ribose 2'-epimerase 1 (DprE1) as a druggable target, we

employed molecular docking, pharmacokinetic evaluation, and 200-ns molecular

dynamics simulations to assess stability and energy refinement. Our results

showed that the top five compounds exhibited more favourable binding free energy

values against DprE1 than the standard, PBTZ169. Notably, cycloartobiloxanthone

demonstrated a binding free energy of -63.67 kcal/mol, surpassing PBTZ169

(-37.78 kcal/mol). Structural analysis revealed that cycloartobiloxanthone

stabilised the protein and formed additional interactions without compromising

its integrity. These findings suggest a potential structural mechanism for the

inhibitory action of cycloartobiloxanthone against Mycobacterium tuberculosis

DprE1. While this study highlights the potential of cycloartobiloxanthone as a

lead compound, further validation through in vivo and in vitro studies is

recommended.

CI - Copyright © 2025 The Authors. Published by Elsevier Ltd.. All rights reserved.

FAU - Kisimba, Kakudji

AU - Kisimba K

AD - Department of Pharmaceutics, Discipline of Pharmaceutical Sciences, College of

Health Sciences, University of KwaZulu-Natal, Private Bag X54001, Durban, South

Africa.

FAU - Abdulsalam, Rukayat Abiola

AU - Abdulsalam RA

AD - Department of Biotechnology and Food Science, Faculty of Applied Sciences, Durban

University of Technology, Durban 4000, South Africa.

FAU - Salifu, Elliasu Y

AU - Salifu EY

AD - Department of Pharmaceutical Sciences, Tshwane University of Technology, Private

Bag X680, Pretoria 0001, South Africa.

FAU - Sabiu, Saheed

AU - Sabiu S

AD - Department of Biotechnology and Food Science, Faculty of Applied Sciences, Durban

University of Technology, Durban 4000, South Africa.

FAU - Faya, Mbuso

AU - Faya M

AD - Department of Pharmaceutics, Discipline of Pharmaceutical Sciences, College of

Health Sciences, University of KwaZulu-Natal, Private Bag X54001, Durban, South

Africa. Electronic address: FayaA@ukzn.ac.za.

LA - eng

PT - Journal Article

PT - Research Support, Non-U.S. Gov't

DEP - 20251008

PL - England

TA - Comput Biol Chem

JT - Computational biology and chemistry

JID - 101157394

RN - 0 (Flavonoids)

RN - 0 (Antitubercular Agents)

RN - EC 1.1.- (DprE1 protein, Mycobacterium tuberculosis)

RN - EC 5.1.3.- (Carbohydrate Epimerases)

RN - 0 (Bacterial Proteins)

RN - 0 (Enzyme Inhibitors)

RN - EC 1.1.- (Alcohol Oxidoreductases)

SB - IM

MH - *Mycobacterium tuberculosis/enzymology/drug effects

MH - *Flavonoids/pharmacology/chemistry

MH - Molecular Docking Simulation

MH - *Antitubercular Agents/pharmacology/chemistry

MH - Molecular Dynamics Simulation

MH - *Carbohydrate Epimerases/metabolism/antagonists & inhibitors

MH - *Bacterial Proteins/antagonists & inhibitors/metabolism

MH - Microbial Sensitivity Tests

MH - *Enzyme Inhibitors/pharmacology/chemistry

MH - Molecular Structure

MH - Humans

MH - Alcohol Oxidoreductases

OTO - NOTNLM

OT - DprE1

OT - Flavonoid

OT - Molecular dynamics simulation

OT - Molecular fingerprinting

OT - Multidrug-resistant TB

OT - Mycobacterium tuberculosis

COIS- Declaration of Competing Interest The authors declare that they have no known

competing financial interests or personal relationships that could have appeared

to influence the work reported in this paper.

EDAT- 2025/10/12 00:33

MHDA- 2025/12/12 03:29

CRDT- 2025/10/11 18:12

PHST- 2025/07/22 00:00 [received]

PHST- 2025/09/29 00:00 [revised]

PHST- 2025/10/07 00:00 [accepted]

PHST- 2025/12/12 03:29 [medline]

PHST- 2025/10/12 00:33 [pubmed]

PHST- 2025/10/11 18:12 [entrez]

AID - S1476-9271(25)00380-9 [pii]

AID - 10.1016/j.compbiolchem.2025.108719 [doi]

PST - ppublish

SO - Comput Biol Chem. 2026 Feb;120(Pt 2):108719. doi:

10.1016/j.compbiolchem.2025.108719. Epub 2025 Oct 8.

PMID- 40730708

OWN - NLM

STAT- MEDLINE

DCOM- 20260222

LR - 20260222

IS - 1573-501X (Electronic)

IS - 1381-1991 (Linking)

VI - 30

IP - 1

DP - 2026 Feb

TI - Identifying dormancy-associated enzymes in Mycobacterium tuberculosis through a

computational pipeline integrating flux balance analysis and metabolic modeling.

PG - 1599-1621

LID - 10.1007/s11030-025-11300-9 [doi]

AB - Tuberculosis, caused by Mycobacterium tuberculosis (Mtb), remains a critical

global health challenge due to rising drug resistance and the pathogen's ability

to persist in hostile host environments. Identifying novel molecular targets that

underlie Mtb's unique survival mechanisms is essential for developing more

effective therapies. In this study, we developed an integrative computational

pipeline combining genome-scale metabolic modeling, flux balance analysis (FBA),

comparative genomics, and network-based prioritization to uncover metabolic

vulnerabilities specific to Mtb. Comparative analysis with the reductively

evolved Mycobacterium leprae revealed significant differences in pathways

involved in pantothenate biosynthesis (PanB), peptidoglycan synthesis (GlmU), and

branched-chain amino acid metabolism (IlvN). These targets were prioritized based

on gene essentiality, dormancy-associated expression, druggability, and absence

of human homologs to maximize therapeutic selectivity. Molecular docking,

followed by MM-GBSA binding free energy calculations, identified high-affinity

ligands from LifeChemicals and ChEMBL libraries interacting strongly with

active-site residues. Molecular dynamics simulations were performed to further

validate target engagement and ligand retention, revealing stable conformational

behavior and persistent protein-ligand interactions across 300 ns. Similarly,

metabolite flux analysis and pathway enrichment highlighted adaptive rewiring in

glycine, serine, pyruvate, and nitrogen metabolism, reflecting Mtb's persistence

strategies under host-imposed stress. This study provides a robust, generalizable

pipeline for pathogen-specific drug target and ligand discovery and supports the

rational development of new therapies against drug-resistant tuberculosis.

CI - © 2025. The Author(s), under exclusive licence to Springer Nature Switzerland AG.

FAU - Imran, Mohd

AU - Imran M

AD - Department of Pharmaceutical Chemistry, College of Pharmacy, Northern Border

University, 91911, Rafha, Saudi Arabia. mohammad.baks@nbu.edu.sa.

AD - King Salman Center for Disability Research, 11614, Riyadh, Saudi Arabia.

mohammad.baks@nbu.edu.sa.

AD - Center For Health Research, Northern Border University, 73213, Arar, Saudi

Arabia. mohammad.baks@nbu.edu.sa.

FAU - Alshrari, Ahmed S

AU - Alshrari AS

AD - Department of Medical Laboratory Technology, College of Applied Medical Sciences,

Northern Border University, 91431, Arar, Saudi Arabia.

FAU - Khan, Abida

AU - Khan A

AD - Department of Pharmaceutical Chemistry, College of Pharmacy, Northern Border

University, 91911, Rafha, Saudi Arabia.

AD - Center For Health Research, Northern Border University, 73213, Arar, Saudi

Arabia.

LA - eng

GR - KSRG-2024-088/King Salman center For Disability Research/

PT - Journal Article

DEP - 20250729

PL - Netherlands

TA - Mol Divers

JT - Molecular diversity

JID - 9516534

RN - 0 (Bacterial Proteins)

RN - 0 (Ligands)

SB - IM

MH - *Mycobacterium tuberculosis/enzymology/genetics/metabolism

MH - Molecular Docking Simulation

MH - Molecular Dynamics Simulation

MH - *Metabolic Flux Analysis/methods

MH - Metabolic Networks and Pathways

MH - *Computational Biology/methods

MH - *Bacterial Proteins/metabolism/chemistry

MH - Ligands

OTO - NOTNLM

OT - Mycobacterium leprae

OT - Mycobacterium tuberculosis

OT - Comparative metabolic modeling

OT - Flux balance analysis

OT - Molecular modeling

OT - Systems biology

COIS- Declarations. Competing interests: The authors declare no competing interests.

EDAT- 2025/07/30 00:31

MHDA- 2026/02/22 15:51

CRDT- 2025/07/29 23:23

PHST- 2025/05/23 00:00 [received]

PHST- 2025/07/15 00:00 [accepted]

PHST- 2026/02/22 15:51 [medline]

PHST- 2025/07/30 00:31 [pubmed]

PHST- 2025/07/29 23:23 [entrez]

AID - 10.1007/s11030-025-11300-9 [pii]

AID - 10.1007/s11030-025-11300-9 [doi]

PST - ppublish

SO - Mol Divers. 2026 Feb;30(1):1599-1621. doi: 10.1007/s11030-025-11300-9. Epub 2025

Jul 29.

PMID- 39714098

OWN - NLM

STAT- MEDLINE

DCOM- 20260222

LR - 20260629

IS - 1538-0254 (Electronic)

IS - 0739-1102 (Linking)

VI - 44

IP - 3

DP - 2026 Feb

TI - Integrating structure-guided and fragment-based inhibitor design to combat

bedaquiline resistant Mycobacterium tuberculosis: a molecular dynamics study.

PG - 1475-1513

LID - 10.1080/07391102.2024.2441426 [doi]

AB - The first FDA approved, MDR-TB inhibitory drug bedaquiline (BDQ), entraps the

c-ring of the proton-translocating F(0) region of enzyme ATP synthase of

Mycobacterium tuberculosis, thus obstructing successive ATP production.

Present-day BDQ-resistance has been associated with cardiotoxicity and

mutation(s) in the atpE gene encoding the c subunit of ATP synthase (ATPc)

generating five distinct ATPc mutants: Ala63→Pro, Ile66→Met, Asp28→Gly, Asp28→Val

and Glu61→Asp. We created three discrete libraries, first by repurposing

bedaquiline via scaffold hopping approach, second one having natural plant

compounds and the third being experimentally derived analogues of BDQ to identify

one drug candidate that can inhibit ATPc activity more efficiently with less

toxic properties. For this purpose, we adopted techniques like molecular dynamics

simulation, virtual screening, PCA, DCCM, binding affinity analysis to gauge

structure-function relationship of the L136-ATPc complexes. L136 was found to

induce a distinguishable conformational change in the bound ATPc which captivated

the c(9) rotor ring. L136 displays a binding free energy of -57.294, -59.027,

-57.273, -58.726, -55.889 and -58.651 kcal/mol for ATPc_WT and the five

respective mutants. The pIC(50) value for the L136 ligand for the same proteins

was unveiled to be 6.760, 7.285, 6.898, 7.222, 6.987 and 7.687. Moreover, L136

exhibited a strong ADMET profile. Furthermore, we discovered that the change in

the hydrophobic platform in ATPc mutants hinders BDQ binding, which is overcome

by L136, ensuring efficient binding and providing an assessment of L136's

mechanism of ATPc inhibition. L136 provides a scope for in vivo test for future

clinical drug trials.

FAU - Roy, Alankar

AU - Roy A

AD - Amity Institute of Biotechnology, Amity University, Kolkata, India.

FAU - Banerjee, Prantik

AU - Banerjee P

AD - Amity Institute of Biotechnology, Amity University, Kolkata, India.

FAU - Paul, Ishani

AU - Paul I

AD - Amity Institute of Biotechnology, Amity University, Kolkata, India.

FAU - Ghosh, Ritam

AU - Ghosh R

AD - Amity Institute of Biotechnology, Amity University, Kolkata, India.

FAU - Ray, Sujay

AU - Ray S

AD - Amity Institute of Biotechnology, Amity University, Kolkata, India.

LA - eng

PT - Journal Article

PT - Research Support, Non-U.S. Gov't

DEP - 20241223

PL - England

TA - J Biomol Struct Dyn

JT - Journal of biomolecular structure & dynamics

JID - 8404176

RN - 78846I289Y (bedaquiline)

RN - 0 (Diarylquinolines)

RN - 0 (Antitubercular Agents)

RN - 0 (Bacterial Proteins)

SB - IM

MH - *Mycobacterium tuberculosis/drug effects/enzymology/genetics

MH - *Molecular Dynamics Simulation

MH - *Diarylquinolines/chemistry/pharmacology

MH - *Antitubercular Agents/pharmacology/chemistry

MH - *Drug Design

MH - *Drug Resistance, Bacterial/drug effects

MH - Structure-Activity Relationship

MH - Protein Binding

MH - Molecular Docking Simulation

MH - Mutation

MH - Bacterial Proteins/chemistry/antagonists & inhibitors

MH - Binding Sites

OTO - NOTNLM

OT - ATP synthase

OT - Multi drug resistant tuberculosis

OT - bedaquiline analogues

OT - c-ring

OT - fragment replacement

OT - natural plant products

EDAT- 2024/12/23 12:22

MHDA- 2026/02/23 01:34

CRDT- 2024/12/23 08:32

PHST- 2026/02/23 01:34 [medline]

PHST- 2024/12/23 12:22 [pubmed]

PHST- 2024/12/23 08:32 [entrez]

AID - 10.1080/07391102.2024.2441426 [doi]

PST - ppublish

SO - J Biomol Struct Dyn. 2026 Feb;44(3):1475-1513. doi:

10.1080/07391102.2024.2441426. Epub 2024 Dec 23.

PMID- 41745461

OWN - NLM

STAT- MEDLINE

DCOM- 20260709

LR - 20260709

IS - 1660-3397 (Electronic)

IS - 1660-3397 (Linking)

VI - 24

IP - 2

DP - 2026 Jan 31

TI - Deep-Sea Marine Metabolites as Promising Anti-Tubercular Agents: CADD-Guided

Targeting of the F(420)-Dependent Oxidoreductase.

LID - 10.3390/md24020058 [doi]

LID - 58

AB - Tuberculosis, caused by Mycobacterium tuberculosis (M. tb), remains a leading

global threat, escalated now by the rise of multidrug-resistant (MDR-TB) and

extensively drug-resistant (XDR-TB) strains. In search of a novel anti-tubercular

agent with a distinct mechanism of action, this study explores deep-sea marine

metabolites as potential inhibitors of the F(420)-dependent oxidoreductase

Rv1155, a redox enzyme essential for M. tb survival. A total of 2773

marine-derived compounds curated from the CMNPD, Reaxys, and MarinLit databases

were screened using an integrated CADD workflow combining molecular docking,

in-silico ADMET profiling, and molecular dynamics (MD) simulations. Docking

identified 68 metabolites with strong affinity (-10.98 to -15.95 kcal/mol) for

the Rv1155 binding pocket, and from which three compounds, Upenamide

(CMNPD_22964), Aspyronol (Compound_1749), and Fiscpropionate F (Compound_1796),

were shortlisted as hit candidates. Among these, Upenamide displayed the

strongest binding (ΔG = -28.56 kcal/mol) with stable RMSD and hydrogen bond

persistence during 100 ns MD simulation, while Aspyronol demonstrated a promising

ADMET profile comparable to the native cofactor F(4202). MM-GBSA analysis further

confirmed the strong binding strength (ΔG _bind = -24.77 to -34.07 kcal/mol) for

all three hit candidates. These findings confirm the strong and stable

interaction of selected deep-sea marine metabolites with Rv1155. This validated

screening pipeline established here provides a cost-effective framework for

future experimental validation and expansion to additional F(420)-related drug

targets in M. tb.

FAU - Desai, Ria

AU - Desai R

AUID- ORCID: 0009-0000-3997-7570

AD - Marine Biodiscovery Centre, Department of Chemistry, University of Aberdeen,

Aberdeen AB24 3UE, UK.

FAU - Alaroud, Amane A

AU - Alaroud AA

AUID- ORCID: 0009-0003-5974-5605

AD - Honorary Research Fellow, Rowett Institute, University of Aberdeen, Aberdeen AB25

2ZD, UK.

AD - Department of Pharmaceutics and Pharmaceutical Biotechnology, Faculty of

Pharmacy, Mutah University, Karak 61710, Jordan.

FAU - Preet, Gagan

AU - Preet G

AUID- ORCID: 0000-0002-7978-7306

AD - Marine Biodiscovery Centre, Department of Chemistry, University of Aberdeen,

Aberdeen AB24 3UE, UK.

FAU - Astakala, Rishi Vachaspathy

AU - Astakala RV

AUID- ORCID: 0000-0003-4034-1873

AD - Marine Biodiscovery Centre, Department of Chemistry, University of Aberdeen,

Aberdeen AB24 3UE, UK.

FAU - Ebel, Rainer

AU - Ebel R

AUID- ORCID: 0000-0002-9702-2235

AD - Marine Biodiscovery Centre, Department of Chemistry, University of Aberdeen,

Aberdeen AB24 3UE, UK.

FAU - Jaspars, Marcel

AU - Jaspars M

AUID- ORCID: 0000-0002-2426-6028

AD - Marine Biodiscovery Centre, Department of Chemistry, University of Aberdeen,

Aberdeen AB24 3UE, UK.

LA - eng

GR - 101072475/UK Research and Innovation (HOTBIO)/

GR - DEA15275/Department for Environment Food and Rural Affairs (DEEPEND)/

PT - Journal Article

DEP - 20260131

PL - Switzerland

TA - Mar Drugs

JT - Marine drugs

JID - 101213729

RN - 0 (Antitubercular Agents)

RN - EC 1.- (Oxidoreductases)

RN - 0 (Bacterial Proteins)

SB - IM

MH - *Antitubercular Agents/pharmacology/chemistry

MH - *Mycobacterium tuberculosis/drug effects

MH - Molecular Docking Simulation

MH - *Oxidoreductases/antagonists & inhibitors/metabolism

MH - *Aquatic Organisms/metabolism

MH - Molecular Dynamics Simulation

MH - Humans

MH - Bacterial Proteins/antagonists & inhibitors/metabolism

PMC - PMC12942459

OTO - NOTNLM

OT - BBNJ

OT - F420-dependent oxidoreductase (Rv1155)

OT - Mycobacterium tuberculosis

OT - deep-sea metabolites

OT - drug discovery

COIS- The authors declare no conflicts of interest.

EDAT- 2026/02/26 13:40

MHDA- 2026/03/06 09:03

PMCR- 2026/01/31

CRDT- 2026/02/26 09:14

PHST- 2025/11/25 00:00 [received]

PHST- 2026/01/22 00:00 [revised]

PHST- 2026/01/24 00:00 [accepted]

PHST- 2026/03/06 09:03 [medline]

PHST- 2026/02/26 13:40 [pubmed]

PHST- 2026/02/26 09:14 [entrez]

PHST- 2026/01/31 00:00 [pmc-release]

AID - md24020058 [pii]

AID - marinedrugs-24-00058 [pii]

AID - 10.3390/md24020058 [doi]

PST - epublish

SO - Mar Drugs. 2026 Jan 31;24(2):58. doi: 10.3390/md24020058.

PMID- 41618188

OWN - NLM

STAT- MEDLINE

DCOM- 20260627

LR - 20260627

IS - 1471-2334 (Electronic)

IS - 1471-2334 (Linking)

VI - 26

IP - 1

DP - 2026 Jan 30

TI - Mutations in sugar metabolism-related genes driving global drug-resistant

Mycobacterium tuberculosis transmission revealed by whole-genome sequencing.

LID - 10.1186/s12879-026-12559-5 [doi]

LID - 456

AB - BACKGROUND: Tuberculosis (TB) continues to represent a significant global health

challenge, particularly due to the emergence of drug-resistant strains that

hinder TB control initiatives. Elucidating the mechanisms underlying the

proliferation of drug-resistant strains is essential for developing effective

strategies to address this public health threat. METHODS: Whole-genome sequencing

was conducted on 13,525 Mycobacterium tuberculosis isolates. Genes associated

with sugar metabolism were obtained from the National Center for Biotechnology

Information (NCBI) Gene database. Analytical approaches, including Random

Forests, Gradient Boosting Decision Trees, and Generalized Linear Mixed Models

were used to identify mutation sites in sugar metabolism genes that contribute to

transmission of Multidrug-Resistant Tuberculosis (MDR-TB). RESULTS: Significant

associations were identified between specific gene mutations and transmission

clusters in MDR-TB. Notable mutations include Rv0650 (C113T, C316T), sugB C734A,

epiA C43T, Rv151c C8G, Rv1520 (C138T, T618C, A649C), Rv2038c G282A, Rv2039c

(G391T, T283G), Rv2040c G835C, Rv2316 C161G, and sugI C1268T. Additionally,

mutations associated with MDR-TB transmission clusters, include Rv0650 C316T,

Rv151c T809C, and Rv2039c C794T. Certain mutations, such as Rv0539 G588T and uspA

G379C, were found to increase the risk of cross-regional transmission in MDR

clades. The presence of Rv1512 (epiA, C462T) and Rv2038c T161G was associated

with an increased therisk of developing MDR isolates compared to single drug

resistant (SDR) isolates. CONCLUSION: Mutations in sugar metabolism genes

significantly contribute to the global transmission of MDR-TB. Identifing these

genetic determinants canguide targeted interventions to control drug-resistant

strains and improve TB management. Further research is required to elucidate

mechanism underlying mechanisms of transmission and resistance development.

CLINICAL TRIAL NUMBER: Not applicable.

FAU - Li, Yameng

AU - Li Y

AD - Department of Pulmonary and Critical Care Medicine, The Third Affiliated Hospital

of Shandong First Medical University (Affiliated Hospital of Shandong Academy of

Medical Sciences), Jinan, Shandong, 250031, China.

FAU - Li, Yifan

AU - Li Y

AD - Department of Pulmonary and Critical Care Medicine, The Third Affiliated Hospital

of Shandong First Medical University (Affiliated Hospital of Shandong Academy of

Medical Sciences), Jinan, Shandong, 250031, China.

FAU - Kong, Xianglong

AU - Kong X

AD - Artificial Intelligence Institute, Qilu University of Technology (Shandong

Academy of Sciences), Jinan, Shandong, 250011, China.

FAU - Tao, Ningning

AU - Tao N

AD - Department of Pulmonary and Critical Care Medicine, Shandong Provincial Hospital

Affiliated to Shandong First Medical University, Jinan, Shandong, 250021, China.

FAU - Hou, Yawei

AU - Hou Y

AD - Qilu Second Hospital of Shandong University, Jinan, Shandong, 250033, China.

FAU - Han, Qilin

AU - Han Q

AD - Shandong First Medical University & Shandong Academy of Medical Sciences, Jinan,

Shandong, 250117, China.

FAU - Zhang, Yuzhen

AU - Zhang Y

AD - Shandong First Medical University & Shandong Academy of Medical Sciences, Jinan,

Shandong, 250117, China.

FAU - Long, Fei

AU - Long F

AD - Department of Pulmonary and Critical Care Medicine, The Third Affiliated Hospital

of Shandong First Medical University (Affiliated Hospital of Shandong Academy of

Medical Sciences), Jinan, Shandong, 250031, China. earlf792002@163.com.

FAU - Liu, Yao

AU - Liu Y

AD - Department of Pulmonary and Critical Care Medicine, Shandong Provincial Hospital

Affiliated to Shandong First Medical University, Jinan, Shandong, 250021, China.

doctorliu@126.com.

FAU - Li, Huaichen

AU - Li H

AD - Department of Pulmonary and Critical Care Medicine, Shandong Provincial Hospital

Affiliated to Shandong First Medical University, Jinan, Shandong, 250021, China.

lihuaichen@163.com.

LA - eng

GR - No. ZR2020KH013; No. ZR2021MH006/Natural Science Foundation of Shandong Province/

GR - No. 2007GG30002033; No. 2017GSF218052/Department of Science & Technology of

Shandong Province/

GR - No. 201704100/Jinan Science and Technology Bureau/

PT - Journal Article

DEP - 20260130

PL - England

TA - BMC Infect Dis

JT - BMC infectious diseases

JID - 100968551

RN - 0 (Antitubercular Agents)

RN - 0 (Bacterial Proteins)

SB - IM

MH - *Mycobacterium tuberculosis/genetics/drug effects/metabolism/isolation &

purification

MH - *Whole Genome Sequencing

MH - *Tuberculosis, Multidrug-Resistant/transmission/microbiology

MH - *Mutation

MH - Humans

MH - *Carbohydrate Metabolism/genetics

MH - Genome, Bacterial

MH - Drug Resistance, Multiple, Bacterial/genetics

MH - Antitubercular Agents/pharmacology

MH - Bacterial Proteins/genetics

PMC - PMC12930972

OTO - NOTNLM

OT - Mycobacterium tuberculosis

OT - Gene mutation

OT - Multidrug-resistant tuberculosis

OT - Sugar metabolism

OT - Whole-genome sequencing

COIS- Declarations. Ethics approval and consent to participate: This study complied

with the Declaration of Helsinki, and was approved by the Ethics Committee of

Shandong Provincial Hospital, affiliated with Shandong University (SPH), the

Ethics Weifang Respiratory Disease Hospital (WRDH) and the Ethics Committee of

Shandong Provincial Chest Hospital (SPCH), which waived informed patient consent

because all patient records and information were anonymized and deidentified

before the analysis. Consent for publication: Not applicable. Competing

interests: The authors declare no competing interests.

EDAT- 2026/01/31 00:29

MHDA- 2026/06/27 10:10

PMCR- 2026/01/30

CRDT- 2026/01/30 23:56

PHST- 2024/09/05 00:00 [received]

PHST- 2026/01/08 00:00 [accepted]

PHST- 2026/06/27 10:10 [medline]

PHST- 2026/01/31 00:29 [pubmed]

PHST- 2026/01/30 23:56 [entrez]

PHST- 2026/01/30 00:00 [pmc-release]

AID - 10.1186/s12879-026-12559-5 [pii]

AID - 12559 [pii]

AID - 10.1186/s12879-026-12559-5 [doi]

PST - epublish

SO - BMC Infect Dis. 2026 Jan 30;26(1):456. doi: 10.1186/s12879-026-12559-5.

PMID- 41693862

OWN - NLM

STAT- MEDLINE

DCOM- 20260708

LR - 20260708

IS - 2235-2988 (Electronic)

IS - 2235-2988 (Linking)

VI - 15

DP - 2025

TI - Metagenomic and metatranscriptomic profiling of bronchoalveolar lavage fluid

identifies microbial and host biomarkers of drug-resistant tuberculosis.

PG - 1726935

LID - 10.3389/fcimb.2025.1726935 [doi]

LID - 1726935

AB - BACKGROUND: Drug-resistant tuberculosis (DR-TB) undermines global TB control, yet

how resistant Mycobacterium tuberculosis strains interact with the lung

microbiome, phage communities, and local host immunity remains poorly defined.

METHODS: In a prospective cohort of 130 pulmonary TB patients (49 DR-TB, 81

drug-susceptible TB [DS-TB] patients), bronchoalveolar lavage fluid (BALF) was

subjected to paired metagenomic and transcriptomic profiling. Microbial and

bacteriophage community structures were assessed by diversity metrics and

differential abundance testing, whereas host responses were characterized by gene

expression, pathway enrichment, and immune cell deconvolution. A Random Forest

model was trained to evaluate the diagnostic potential of host transcriptional

signatures. RESULTS: DR-TB airways presented distinct microbial beta diversity,

with enrichment of Streptococcus spp. and streptococcal-targeting phages (e.g.,

Javan variants, phi-Ssu5SJ28rum). Transcriptomic analysis revealed 494

differentially expressed genes, which were associated with increased oxidative

phosphorylation, suppressed ion channel and transporter activity, and enrichment

of extracellular matrix remodeling pathways. Immune profiling demonstrated a

significant reduction in γδ T cells in DR-TB patients (P = 0.0059). An 8-gene

host-derived signature (ARHGEF5, PTGES3L, GAL3ST1, RANBP17, ACTA2_AS1, CBY3,

MAMSTR, and LOC102031319) discriminated DR-TB from DS-TB with high accuracy (AUC

= 0.837). CONCLUSION: This dual-omics study defines the airway niche of DR-TB as

a convergence of microbial dysbiosis, phage imbalance, and host immune-metabolic

dysfunction. By uncovering DR-TB-specific microbial and transcriptional

signatures, and deriving a predictive host-based classifier, our findings provide

mechanistic insights and highlight novel opportunities for microbiome- and

host-directed interventions in drug-resistant tuberculosis.

CI - Copyright © 2026 Zhang, Zhang, Yang, Gao, Liu, Zhang and Chen.

FAU - Zhang, Haiqing

AU - Zhang H

AD - Department of Tuberculosis, Xuzhou Hospital for Infectious Diseases, Xuzhou,

Jiangsu, China.

FAU - Zhang, Limao

AU - Zhang L

AD - Department of Tuberculosis, Xuzhou Hospital for Infectious Diseases, Xuzhou,

Jiangsu, China.

FAU - Yang, Bin

AU - Yang B

AD - Center for Infectious Diseases, Vision Medicals Co., Ltd, Guangzhou,

Guangdong, China.

FAU - Gao, Chunjing

AU - Gao C

AD - Department of Tuberculosis, Xuzhou Hospital for Infectious Diseases, Xuzhou,

Jiangsu, China.

FAU - Liu, Huimei

AU - Liu H

AD - Department of Tuberculosis, Xuzhou Hospital for Infectious Diseases, Xuzhou,

Jiangsu, China.

FAU - Zhang, Yunshi

AU - Zhang Y

AD - Department of Science and Education, Xuzhou Hospital for Infectious Diseases,

Xuzhou, Jiangsu, China.

FAU - Chen, Xiaoyou

AU - Chen X

AD - Infectious Diseases Department, Beijing Ditan Hospital, Capital Medical

University, Beijing, China.

LA - eng

PT - Journal Article

DEP - 20260129

PL - Switzerland

TA - Front Cell Infect Microbiol

JT - Frontiers in cellular and infection microbiology

JID - 101585359

RN - 0 (Biomarkers)

SB - IM

EIN - Front Cell Infect Microbiol. 2026 Mar 26;16:1826950. doi:

10.3389/fcimb.2026.1826950. PMID: 41969652

MH - Humans

MH - *Bronchoalveolar Lavage Fluid/microbiology

MH - *Mycobacterium tuberculosis/genetics/drug effects

MH - *Metagenomics

MH - *Gene Expression Profiling

MH - *Tuberculosis, Multidrug-Resistant/microbiology/diagnosis/immunology

MH - Biomarkers/analysis

MH - Prospective Studies

MH - Microbiota

MH - Transcriptome

MH - Lung/microbiology

MH - Multiomics

PMC - PMC12894345

OTO - NOTNLM

OT - Mycobacterium tuberculosis

OT - drug resistance

OT - host response

OT - microbiome

OT - transcriptomics

COIS- Author BY was employed by the company Vision Medicals Co., Ltd. The remaining

author(s) declared that this work was conducted in the absence of any commercial

or financial relationships that could be construed as a potential conflict of

interest.

EDAT- 2026/02/16 12:31

MHDA- 2026/02/16 12:32

PMCR- 2026/01/29

CRDT- 2026/02/16 06:35

PHST- 2025/10/17 00:00 [received]

PHST- 2025/12/11 00:00 [revised]

PHST- 2025/12/12 00:00 [accepted]

PHST- 2026/02/16 12:32 [medline]

PHST- 2026/02/16 12:31 [pubmed]

PHST- 2026/02/16 06:35 [entrez]

PHST- 2026/01/29 00:00 [pmc-release]

AID - 10.3389/fcimb.2025.1726935 [doi]

PST - epublish

SO - Front Cell Infect Microbiol. 2026 Jan 29;15:1726935. doi:

10.3389/fcimb.2025.1726935. eCollection 2025.

PMID- 41746042

OWN - NLM

STAT- PubMed-not-MEDLINE

DCOM- 20260226

LR - 20260308

IS - 2076-393X (Print)

IS - 2076-393X (Electronic)

IS - 2076-393X (Linking)

VI - 14

IP - 2

DP - 2026 Jan 27

TI - Pediatric Tuberculosis: Unraveling Immunity, Clinical Complexities, and

Resource-Driven Disparities in the Pursuit of Prevention.

LID - 10.3390/vaccines14020119 [doi]

LID - 119

AB - Pediatric tuberculosis (TB) remains a critically underrecognized contributor to

global childhood morbidity and mortality, with the highest burden concentrated in

low-resource settings. Although children comprise a minority of overall TB cases,

mortality is disproportionately high, particularly among those under five years

of age, driven largely by delayed diagnosis, inadequate linkage to care, and

limited access to effective treatment. The continued rise of pediatric

multidrug-resistant TB (MDR-TB), especially in regions with low sociodemographic

development, further highlights persistent gaps in current control strategies.

This review synthesizes key aspects of pediatric TB pathogenesis and host immune

responses that predispose young children to rapid disease progression and severe

outcomes, including immune immaturity and paucibacillary infection. We summarize

pulmonary and extrapulmonary disease manifestations and identify populations at

heightened risk, including children with HIV, malnutrition, type 1 diabetes

mellitus, and congenital or treatment-related immunosuppression. Ongoing

challenges in diagnosis and treatment are discussed, including limitations of

existing microbiologic and immunologic tests, specimen collection constraints,

regimen toxicity, and barriers to adherence. Prevention remains central to

reducing pediatric TB mortality. We highlight the sustained importance of bacille

Calmette-Guérin (BCG) vaccination in preventing severe disease and death, the

context-dependent variability in vaccine effectiveness, and the structural and

socioeconomic determinants of vaccine coverage. We conclude that integrating

equitable vaccine delivery, scalable preventive therapy, and child-adapted

diagnostic strategies is essential to meaningfully reduce the global pediatric TB

burden.

FAU - Mashiach, Daniel

AU - Mashiach D

AUID- ORCID: 0000-0002-4025-7412

AD - College of Osteopathic Medicine of the Pacific, Western University of Health

Sciences, Pomona, CA 91766, USA.

FAU - Shon, Justin

AU - Shon J

AD - College of Osteopathic Medicine of the Pacific, Western University of Health

Sciences, Pomona, CA 91766, USA.

FAU - Mashiach, Raquel

AU - Mashiach R

AD - College of Osteopathic Medicine of the Pacific, Western University of Health

Sciences, Pomona, CA 91766, USA.

FAU - Ayzenberg, Gregory

AU - Ayzenberg G

AD - College of Osteopathic Medicine of the Pacific, Western University of Health

Sciences, Pomona, CA 91766, USA.

FAU - Barazani, Osnat

AU - Barazani O

AD - College of Osteopathic Medicine of the Pacific, Western University of Health

Sciences, Pomona, CA 91766, USA.

FAU - Aabedi, Andre

AU - Aabedi A

AD - College of Osteopathic Medicine of the Pacific, Western University of Health

Sciences, Pomona, CA 91766, USA.

FAU - Venketaraman, Vishwanath

AU - Venketaraman V

AUID- ORCID: 0000-0002-2586-1160

AD - College of Osteopathic Medicine of the Pacific, Western University of Health

Sciences, Pomona, CA 91766, USA.

LA - eng

GR - R15 HL143545/HL/NHLBI NIH HHS/United States

PT - Journal Article

PT - Review

DEP - 20260127

PL - Switzerland

TA - Vaccines (Basel)

JT - Vaccines

JID - 101629355

PMC - PMC12945030

OTO - NOTNLM

OT - bacille Calmette–Guérin (BCG)

OT - child health disparities

OT - extrapulmonary tuberculosis

OT - latent tuberculosis infection

OT - low-resource settings

OT - multidrug-resistant tuberculosis

OT - pediatric immune response

OT - pediatric tuberculosis

OT - tuberculosis prevention

OT - vaccine equity

COIS- The authors declare no conflicts of interest.

EDAT- 2026/02/26 13:40

MHDA- 2026/02/26 13:41

PMCR- 2026/01/27

CRDT- 2026/02/26 09:24

PHST- 2025/12/24 00:00 [received]

PHST- 2026/01/21 00:00 [revised]

PHST- 2026/01/24 00:00 [accepted]

PHST- 2026/02/26 13:41 [medline]

PHST- 2026/02/26 13:40 [pubmed]

PHST- 2026/02/26 09:24 [entrez]

PHST- 2026/01/27 00:00 [pmc-release]

AID - vaccines14020119 [pii]

AID - vaccines-14-00119 [pii]

AID - 10.3390/vaccines14020119 [doi]

PST - epublish

SO - Vaccines (Basel). 2026 Jan 27;14(2):119. doi: 10.3390/vaccines14020119.

PMID- 41601639

OWN - NLM

STAT- MEDLINE

DCOM- 20260701

LR - 20260701

IS - 1664-3224 (Electronic)

IS - 1664-3224 (Linking)

VI - 16

DP - 2025

TI - Research progress on protein tyrosine phosphatase A from Mycobacterium

tuberculosis.

PG - 1754992

LID - 10.3389/fimmu.2025.1754992 [doi]

LID - 1754992

AB - Mycobacterium tuberculosis (Mtb) protein tyrosine phosphatase A (PtpA) is a

crucial tyrosine phosphatase involved in the pathogenesis of tuberculosis.

Structural analyses reveal that the W-loop and conserved cysteine residues are

essential for the catalytic activity of PtpA, with modifications induced by

reactive oxygen species playing a significant role in its function. PtpA

suppresses key cellular processes, including phagosome-lysosome fusion and host

cell apoptosis, while promoting ferroptosis and disrupting cytokine production to

evade host immune responses. Its activity is enhanced by various

post-translational modifications, including ubiquitination and phosphorylation,

which facilitate its interactions with key cellular pathways. Recent research has

identified several selective inhibitors that present promising therapeutic

avenues against drug-resistant tuberculosis. This review synthesizes current

knowledge on the characteristics, functions, and potential inhibitors of PtpA,

underscoring its significance as a therapeutic target in the ongoing battle

against tuberculosis and its associated challenges.

CI - Copyright © 2026 Cai and Zhang.

FAU - Cai, Yifei

AU - Cai Y

AD - Department of Clinical Laboratory Medicine, The First Affiliated Hospital of

Shandong First Medical University and Shandong Provincial Qianfoshan Hospital,

Jinan, Shandong, China.

AD - Department of Pathogen Biology, School of Clinical and Basic Medical Sciences,

Shandong First Medical University and Shandong Academy of Medical Sciences,

Jinan, Shandong, China.

FAU - Zhang, Leiliang

AU - Zhang L

AD - Department of Clinical Laboratory Medicine, The First Affiliated Hospital of

Shandong First Medical University and Shandong Provincial Qianfoshan Hospital,

Jinan, Shandong, China.

AD - Department of Pathogen Biology, School of Clinical and Basic Medical Sciences,

Shandong First Medical University and Shandong Academy of Medical Sciences,

Jinan, Shandong, China.

LA - eng

PT - Journal Article

PT - Review

DEP - 20260112

PL - Switzerland

TA - Front Immunol

JT - Frontiers in immunology

JID - 101560960

RN - EC 3.1.3.48 (Protein Tyrosine Phosphatases)

RN - 0 (Bacterial Proteins)

RN - 0 (MptpA protein, Mycobacterium tuberculosis)

RN - 0 (Reactive Oxygen Species)

RN - 0 (Antitubercular Agents)

SB - IM

MH - *Mycobacterium tuberculosis/enzymology/immunology

MH - Humans

MH - *Tuberculosis/drug therapy/microbiology/immunology

MH - Animals

MH - *Protein Tyrosine Phosphatases/metabolism/antagonists & inhibitors/chemistry

MH - Apoptosis

MH - *Bacterial Proteins/metabolism/antagonists & inhibitors/chemistry

MH - Protein Processing, Post-Translational

MH - Reactive Oxygen Species/metabolism

MH - Host-Pathogen Interactions

MH - Ferroptosis

MH - Antitubercular Agents/pharmacology/therapeutic use

PMC - PMC12832872

OTO - NOTNLM

OT - PtpA

OT - apoptosis

OT - ferroptosis

OT - inhibitor

OT - mycobacterium tuberculosis

COIS- The author(s) declared that this work was conducted in the absence of any

commercial or financial relationships that could be construed as a potential

conflict of interest. The author LZ declared that they were an editorial board

member of Frontiers, at the time of submission. This had no impact on the peer

review process and the final decision.

EDAT- 2026/01/28 07:47

MHDA- 2026/01/28 19:20

PMCR- 2026/01/12

CRDT- 2026/01/28 04:55

PHST- 2025/11/26 00:00 [received]

PHST- 2025/12/17 00:00 [revised]

PHST- 2025/12/22 00:00 [accepted]

PHST- 2026/01/28 19:20 [medline]

PHST- 2026/01/28 07:47 [pubmed]

PHST- 2026/01/28 04:55 [entrez]

PHST- 2026/01/12 00:00 [pmc-release]

AID - 10.3389/fimmu.2025.1754992 [doi]

PST - epublish

SO - Front Immunol. 2026 Jan 12;16:1754992. doi: 10.3389/fimmu.2025.1754992.

eCollection 2025.

PMID- 41457858

OWN - NLM

STAT- MEDLINE

DCOM- 20260108

LR - 20260108

IS - 1520-4804 (Electronic)

IS - 0022-2623 (Linking)

VI - 69

IP - 1

DP - 2026 Jan 8

TI - Discovery and Optimization of Phenoxazinone Derivatives as Potent Antitubercular

Agents Targeting FabD Protein.

PG - 163-182

LID - 10.1021/acs.jmedchem.5c02148 [doi]

AB - Tuberculosis (TB) therapy demands novel agents with distinct structures, targets,

and mechanisms. Herein, through phenotypic screening, we identified a

phenoxazinone scaffold questiomycin A (QA) with potent activity against

Mycobacterium tuberculosis (MTB) H(37)Rv strain (MIC: 0.41 μg/mL) and

broad-spectrum activity against drug-resistant clinical isolates (MIC: 0.16-0.31

μg/mL). Structural optimization of QA yielded lead B10 with enhanced activity

against drug-resistance MTB (MIC: 0.063-0.25 μg/mL) and good intracellular

antimycobacterial activity. In addition, B10 showed improved in vitro and in vivo

safety, optimized pharmacokinetic profiles (t(1/2), 5.34 h; C(max), 229.97 ng/mL;

AUC(0-24 h), 738.12 ng·h/mL). Preliminary mechanistic investigations revealed

that B10 disrupts mycobacterial envelope integrity by inhibiting Malonyl CoA-acyl

carrier protein transacylase (encoded by FabD gene), an unexploited target in the

essential mycolic acid biosynthesis pathway. This work establishes QA derivatives

as first-in-class fresh scaffolds against FabD as a potential target for MDR-TB

therapy, offering an effectively strategy to discover novel anti-TB lead

compounds.

FAU - Xu, Manyi

AU - Xu M

AUID- ORCID: 0009-0006-0211-2753

AD - Beijing Key Laboratory of Drug Resistance Tuberculosis Research, Department of

Pharmacology, Beijing Chest Hospital, Capital Medical University, Beijing

Tuberculosis and Thoracic Tumor Research Institute, Beijing 101149, China.

FAU - Xu, Lingling

AU - Xu L

AD - Beijing Key Laboratory of Drug Resistance Tuberculosis Research, Department of

Pharmacology, Beijing Chest Hospital, Capital Medical University, Beijing

Tuberculosis and Thoracic Tumor Research Institute, Beijing 101149, China.

FAU - Wang, Bin

AU - Wang B

AD - Beijing Key Laboratory of Drug Resistance Tuberculosis Research, Department of

Pharmacology, Beijing Chest Hospital, Capital Medical University, Beijing

Tuberculosis and Thoracic Tumor Research Institute, Beijing 101149, China.

FAU - Zhang, Wei-Yan

AU - Zhang WY

AD - Beijing Key Laboratory of Drug Resistance Tuberculosis Research, Department of

Pharmacology, Beijing Chest Hospital, Capital Medical University, Beijing

Tuberculosis and Thoracic Tumor Research Institute, Beijing 101149, China.

FAU - Wang, Lu

AU - Wang L

AD - Beijing Key Laboratory of Drug Resistance Tuberculosis Research, Department of

Pharmacology, Beijing Chest Hospital, Capital Medical University, Beijing

Tuberculosis and Thoracic Tumor Research Institute, Beijing 101149, China.

FAU - Wang, Apeng

AU - Wang A

AUID- ORCID: 0009-0004-8648-5164

AD - Institute of Medicinal Biotechnology, Chinese Academy of Medical Sciences and

Peking Union Medical College, Beijing 100050, China.

FAU - Lu, Yu

AU - Lu Y

AD - Beijing Key Laboratory of Drug Resistance Tuberculosis Research, Department of

Pharmacology, Beijing Chest Hospital, Capital Medical University, Beijing

Tuberculosis and Thoracic Tumor Research Institute, Beijing 101149, China.

LA - eng

PT - Journal Article

DEP - 20251229

PL - United States

TA - J Med Chem

JT - Journal of medicinal chemistry

JID - 9716531

RN - 0 (Antitubercular Agents)

RN - 0 (Oxazines)

RN - 0 (Bacterial Proteins)

SB - IM

MH - *Antitubercular Agents/pharmacology/chemistry/pharmacokinetics/chemical synthesis

MH - *Mycobacterium tuberculosis/drug effects

MH - Microbial Sensitivity Tests

MH - Structure-Activity Relationship

MH - Animals

MH - Humans

MH - *Drug Discovery

MH - *Oxazines/pharmacology/chemistry/chemical synthesis/pharmacokinetics

MH - *Bacterial Proteins/antagonists & inhibitors/metabolism

MH - Mice

EDAT- 2025/12/29 07:08

MHDA- 2026/01/08 07:13

CRDT- 2025/12/29 04:12

PHST- 2026/01/08 07:13 [medline]

PHST- 2025/12/29 07:08 [pubmed]

PHST- 2025/12/29 04:12 [entrez]

AID - 10.1021/acs.jmedchem.5c02148 [doi]

PST - ppublish

SO - J Med Chem. 2026 Jan 8;69(1):163-182. doi: 10.1021/acs.jmedchem.5c02148. Epub

2025 Dec 29.

PMID- 41647442

OWN - NLM

STAT- Publisher

LR - 20260422

IS - 2632-8682 (Electronic)

IS - 2632-8682 (Linking)

VI - 17

IP - 3

DP - 2026 Jan 5

TI - Inhibitors of type II NADH-dehydrogenase, cytochrome bd oxidase, and ATP synthase

for anti-tubercular response.

PG - 1271-88

LID - 10.1039/d5md00950b [doi]

AB - The identification of novel anti-tubercular agents capable of eliciting lethal

responses against drug-resistant tuberculosis is critically needed to address the

escalating mortality from tuberculosis. Mycobacterium tuberculosis, the

etiological agent of this disease, employs a highly efficient energy-producing

machinery, the oxidative phosphorylation pathway. Mycobacterium can withstand

extreme environmental conditions due to the robust functionality of this

multicomponent pathway, which satisfies its energy requirements, during both the

persistent phase under stress and the active growth phase. Considering the

significance of this biological pathway, in this review we described the dynamics

of oxidative phosphorylation and the rationale for targeting its essential

components. Furthermore, we provide a comprehensive overview of

literature-reported inhibitors, targeting key elements of this pathway, namely,

type II NADH dehydrogenase, cytochrome-bd oxidase, and ATP synthase.

CI - This journal is © The Royal Society of Chemistry.

FAU - Saha, Pallavi

AU - Saha P

AUID- ORCID: 0000-0003-1652-8275

AD - Department of Pharmaceutical Engg. and Tech, IIT-Banaras Hindu University

Varanasi UP India-221005 deepak.phe@itbhu.ac.in.

FAU - Kumar, Mohit

AU - Kumar M

AD - Department of Pharmaceutical Engg. and Tech, IIT-Banaras Hindu University

Varanasi UP India-221005 deepak.phe@itbhu.ac.in.

FAU - Sharma, Deepak K

AU - Sharma DK

AUID- ORCID: 0000-0002-1579-5008

AD - Department of Pharmaceutical Engg. and Tech, IIT-Banaras Hindu University

Varanasi UP India-221005 deepak.phe@itbhu.ac.in.

LA - eng

PT - Journal Article

PT - Review

DEP - 20260105

PL - England

TA - RSC Med Chem

JT - RSC medicinal chemistry

JID - 101759460

PMC - PMC12869301

COIS- There are no conflicts to declare.

EDAT- 2026/02/06 13:17

MHDA- 2026/02/06 13:17

PMCR- 2027/01/05

CRDT- 2026/02/06 06:13

PHST- 2025/10/22 00:00 [received]

PHST- 2026/01/02 00:00 [accepted]

PHST- 2026/02/06 13:17 [medline]

PHST- 2026/02/06 13:17 [pubmed]

PHST- 2026/02/06 06:13 [entrez]

PHST- 2027/01/05 00:00 [pmc-release]

AID - d5md00950b [pii]

AID - 10.1039/d5md00950b [doi]

PST - aheadofprint

SO - RSC Med Chem. 2026 Jan 5;17(3):1271-88. doi: 10.1039/d5md00950b.

PMID- 41599169

OWN - NLM

STAT- PubMed-not-MEDLINE

DCOM- 20260128

LR - 20260131

IS - 1999-4923 (Print)

IS - 1999-4923 (Electronic)

IS - 1999-4923 (Linking)

VI - 18

IP - 1

DP - 2026 Jan 1

TI - Advancing Tuberculosis Treatment with Next-Generation Drugs and Smart Delivery

Systems.

LID - 10.3390/pharmaceutics18010060 [doi]

LID - 60

AB - Tuberculosis (TB) remains a leading infectious killer, increasingly complicated

by multidrug-resistant (MDR) and extensively drug-resistant (XDR) disease;

current regimens, although effective, are prolonged, toxic, and often fail to

reach intracellular bacilli in heterogeneous lung lesions. This narrative review

synthesizes how next-generation antimycobacterial strategies can be translated

"from molecule to patient" by coupling potent therapeutics with delivery

platforms tailored to the lesion microenvironment. We survey emerging

small-molecule classes, including decaprenylphosphoryl-β-D-ribose 2'-epimerase

(DprE1) inhibitors, mycobacterial membrane protein large 3 (MmpL3) inhibitors,

and respiratory chain blockers, alongside optimized uses of established agents

and host-directed therapies (HDTs). These are mapped to inhalable and nanocarrier

systems that improve intralesional exposure, macrophage uptake, and targeted

release while reducing systemic toxicity. Particular emphasis is placed on

pulmonary dry powder inhalers (DPIs) and aerosols for direct lung targeting,

stimuli-responsive carriers that trigger release through pH, redox, or enzymatic

cues, and long-acting depots or implants that shift daily dosing to monthly or

quarterly schedules to enhance adherence, safety, and access. We also outline

translational enablers, including model-informed pharmacokinetic/pharmacodynamic

(PK/PD) integration, device formulation co-design, manufacturability, regulatory

quality frameworks, and patient-centered implementation. Overall, aligning

stronger drugs with smart delivery platforms offers a practical pathway to

shorter, safer, and more easily completed TB therapy, improving both individual

outcomes and public health impact.

FAU - Elbehiry, Ayman

AU - Elbehiry A

AUID- ORCID: 0000-0001-7726-9532

AD - Department of Public Health, College of Applied Medical Sciences, Qassim

University, P.O. Box 6666, Buraydah 51452, Saudi Arabia.

FAU - Marzouk, Eman

AU - Marzouk E

AUID- ORCID: 0000-0003-1699-6263

AD - Department of Public Health, College of Applied Medical Sciences, Qassim

University, P.O. Box 6666, Buraydah 51452, Saudi Arabia.

FAU - Abalkhail, Adil

AU - Abalkhail A

AUID- ORCID: 0000-0002-2294-9837

AD - Department of Public Health, College of Applied Medical Sciences, Qassim

University, P.O. Box 6666, Buraydah 51452, Saudi Arabia.

LA - eng

PT - Journal Article

PT - Review

DEP - 20260101

PL - Switzerland

TA - Pharmaceutics

JT - Pharmaceutics

JID - 101534003

PMC - PMC12845121

OTO - NOTNLM

OT - drug-resistant tuberculosis

OT - host-directed therapy

OT - inhaled drug delivery

OT - long-acting formulations

OT - nanocarriers

OT - precision pharmaceutics

OT - public health

OT - tuberculosis

COIS- The authors declare that they have no conflicts of interest.

EDAT- 2026/01/28 07:47

MHDA- 2026/01/28 07:48

PMCR- 2026/01/01

CRDT- 2026/01/28 01:20

PHST- 2025/11/27 00:00 [received]

PHST- 2025/12/21 00:00 [revised]

PHST- 2025/12/31 00:00 [accepted]

PHST- 2026/01/28 07:48 [medline]

PHST- 2026/01/28 07:47 [pubmed]

PHST- 2026/01/28 01:20 [entrez]

PHST- 2026/01/01 00:00 [pmc-release]

AID - pharmaceutics18010060 [pii]

AID - pharmaceutics-18-00060 [pii]

AID - 10.3390/pharmaceutics18010060 [doi]

PST - epublish

SO - Pharmaceutics. 2026 Jan 1;18(1):60. doi: 10.3390/pharmaceutics18010060.

PMID- 41329266

OWN - NLM

STAT- PubMed-not-MEDLINE

DCOM- 20260108

LR - 20260111

IS - 2193-8229 (Print)

IS - 2193-6382 (Electronic)

IS - 2193-6382 (Linking)

VI - 15

IP - 1

DP - 2026 Jan

TI - The Structure, Properties, and Clinical Utility of Contezolid for

Antituberculosis: A Narrative Review.

PG - 43-56

LID - 10.1007/s40121-025-01256-6 [doi]

AB - Tuberculosis (TB) continues to represent a significant global public health

concern, with China being a country that bears the burden of a high incidence of

TB cases on a global scale. Although linezolid (LZD) has been recommended for

treating drug-resistant tuberculosis (DR-TB), its intolerability and adverse

events, such as myelosuppression, neurotoxicity, etc., have limited its long-term

usage in anti-TB treatment. Contezolid (CZD), a new generation of oxazolidinone

drug, shows comparable or superior antibacterial activity to LZD, with lower

risks of myelosuppressive toxicity, neurotoxicity, and lactic acidosis. Its

unique metabolic pathway and favorable pharmacokinetic profile render it a

promising alternative to LZD for TB treatment. Recent years have seen mounting

evidence of the potential of CZD in treating TB. In this paper, the development

history, the mode of action, resistance mechanisms, and research progress on CZD

for TB treatment are reviewed, aiming to enhance understanding of its role in

anti-TB therapy and to provide valuable references for clinical use and future

research.

CI - © 2025. The Author(s).

FAU - Chen, Tao

AU - Chen T

AD - Shanghai Institute of Infectious Disease and Biosecurity, School of Public

Health, Fudan University, Shanghai, China.

AD - Shanghai Micurx Pharmaceutical Co., Ltd., Shanghai, China.

FAU - Li, Weili

AU - Li W

AD - Shanghai Micurx Pharmaceutical Co., Ltd., Shanghai, China.

FAU - Shen, Fangxue

AU - Shen F

AD - Shanghai Micurx Pharmaceutical Co., Ltd., Shanghai, China.

FAU - Nie, Wenjuan

AU - Nie W

AD - Tuberculosis Department, Beijing Chest Hospital affiliated to Capital Medical

University, Beijing, China. 94642975@qq.com.

AD - Tuberculosis and Thoracic Tumor Research Institute, Beijing, China.

94642975@qq.com.

FAU - Wu, Fan

AU - Wu F

AUID- ORCID: 0000-0003-1039-3752

AD - Shanghai Institute of Infectious Disease and Biosecurity, School of Public

Health, Fudan University, Shanghai, China. wufan@shmu.edu.cn.

LA - eng

GR - BJRID225-013/Beijing Major Respiratory Infectious Disease Research Center

Project/

GR - BRWEP2024W042160109/Excellent Clinical Research Program for Research Wards of

Beijing Municipal Health Commission/

GR - ZLRK202331/Beijing Medical Management Center Yangfan 3.0 Project/

PT - Journal Article

PT - Review

DEP - 20251202

PL - New Zealand

TA - Infect Dis Ther

JT - Infectious diseases and therapy

JID - 101634499

PMC - PMC12783479

OTO - NOTNLM

OT - Adverse events

OT - Contezolid

OT - Linezolid

OT - Tuberculosis

COIS- Declarations. Conflict of Interest: Weili Li, Tao Chen, and Fangxue Shen are

employed by Shanghai MicuRx Pharmaceutical Co., Ltd. Wenjuan Nie and Fan Wu have

nothing to disclose. Ethical Approval: This article is based on previously

conducted studies and does not contain any new studies with human participants or

animals performed by any of the authors.

EDAT- 2025/12/02 12:56

MHDA- 2025/12/02 12:57

PMCR- 2025/12/02

CRDT- 2025/12/02 11:05

PHST- 2025/08/25 00:00 [received]

PHST- 2025/10/20 00:00 [accepted]

PHST- 2025/12/02 12:57 [medline]

PHST- 2025/12/02 12:56 [pubmed]

PHST- 2025/12/02 11:05 [entrez]

PHST- 2025/12/02 00:00 [pmc-release]

AID - 10.1007/s40121-025-01256-6 [pii]

AID - 1256 [pii]

AID - 10.1007/s40121-025-01256-6 [doi]

PST - ppublish

SO - Infect Dis Ther. 2026 Jan;15(1):43-56. doi: 10.1007/s40121-025-01256-6. Epub 2025

Dec 2.

PMID- 41207942

OWN - NLM

STAT- MEDLINE

DCOM- 20251112

LR - 20260522

IS - 0065-2598 (Print)

IS - 0065-2598 (Linking)

VI - 1484

DP - 2026

TI - Structural and Functional Characterization of Protein Adhesins and Pili in

Mycobacterium tuberculosis.

PG - 249-296

LID - 10.1007/978-3-031-96883-9_8 [doi]

AB - Mycobacterium tuberculosis (Mtb) is a highly adaptable pathogen whose ability to

establish infection and persist within the host is primarily driven by a diverse

array of adhesins, surface proteins, and structures that mediate attachment to

host cells and tissues. These adhesins play critical roles in bacterial

colonization, invasion, immune evasion, and long-term persistence, making them

central to Mtb pathogenesis. Mtb adhesins can be broadly categorized based on

their structural features, functional roles, and binding specificities. Protein

adhesins such as LpqH (the 19-kDa lipoprotein), PstS-1 (phosphate-binding

protein), Cpn60.2 (chaperonin GroEL2), Apa (alanine- and proline-rich antigen),

members of the PE-PGRS protein family, the antigen 85 complex (Ag85A, Ag85B, and

Ag85C), glutamine synthetase A1, malate synthase, N-acetylmuramoyl-L-alanine

amidase, the histone-like HU protein, protein kinase D, ESAT-6, Mce1, and HBHA

(heparin-binding hemagglutinin adhesin) exhibit specific affinities for host cell

receptors and extracellular matrix components such as fibronectin, laminin, and

epithelial surfaces. Many of these proteins are multifunctional "moonlighting"

enzymes, performing both metabolic and adhesive roles, thereby enhancing

bacterial survival and virulence. In addition to protein adhesins, Mtb expresses

a variety of pili and fimbrial structures that further augment its capacity to

interact with the host. Recent atomic force microscopy studies have identified

seven distinct pili types on the mycobacterial surface: Type III (secretion

needle pili), Type IV secretion pili, Type IV-like pili, curli-like pili (MTP),

and the newly described Types V (relief funnel pili), VI (adhesion tapering

pili), and VII (adhesion flap pili). These pili exhibit remarkable structural

diversity, with Types VI and VII found exclusively in extensively drug-resistant

(XDR) and totally drug-resistant (TDR) Mtb strains, suggesting a role in

adaptation to antibiotic pressure and enhanced survival. Functionally, curli-like

and Type IV pili contribute to biofilm formation, bacterial aggregation, and

robust adhesion to macrophages and epithelial cells, while the novel pili types

(V, VI, and VII) may mediate specialized adhesion and intercellular interactions,

particularly in drug-resistant isolates. The adhesins and pili target diverse

host molecules: some facilitate macrophage binding (e.g., LpqH, PstS-1, Cpn60.2,

Apa), others bind fibronectin and laminin (e.g., PE-PGRS proteins, antigen 85

complex, malate synthase, curli-like pili), and yet others adhere to epithelial

cells (e.g., Mce1, HBHA, Type IV pili). This multifaceted adhesion strategy

enables Mtb to colonize a wide range of tissue environments, evade immune

defenses, and establish latent or persistent infections. The redundancy and

multifunctionality of these adhesins and pili underscore their evolutionary

significance in Mtb's lifecycle. Collectively, the surface adhesins and pili

constitute a complex and versatile toolkit that underpins Mtb pathogenicity. The

discovery of multiple distinct pili types, especially those associated with drug

resistance, opens new avenues for research into bacterial adaptation,

persistence, and immune evasion mechanisms. A comprehensive understanding of

these structures and their interactions with host tissues holds promise for the

development of innovative diagnostics, vaccines, and antiadhesin therapies aimed

at combating both drug-sensitive and drug-resistant tuberculosis.

CI - © 2026. The Author(s), under exclusive license to Springer Nature Switzerland AG.

FAU - Farnia, Parissa

AU - Farnia P

AD - Shahid Beheshti University of Medical Sciences, Mycobacteriology Research Centre

(MRC), National Research Institute of Tuberculosis and Lung Diseases (NRITLD),

Tehran, Iran. farnia@theaasm.org.

FAU - Velayati, Ali Akbar

AU - Velayati AA

AD - Shahid Beheshti University of Medical Sciences, Mycobacteriology Research Centre

(MRC), National Research Institute of Tuberculosis and Lung Diseases (NRITLD),

Tehran, Iran.

FAU - Ghanavi, Jalaledin

AU - Ghanavi J

AD - Shahid Beheshti University of Medical Sciences, Mycobacteriology Research Centre

(MRC), National Research Institute of Tuberculosis and Lung Diseases (NRITLD),

Tehran, Iran.

FAU - Farnia, Poopak

AU - Farnia P

AD - Shahid Beheshti University of Medical Sciences, Mycobacteriology Research Centre

(MRC), National Research Institute of Tuberculosis and Lung Diseases (NRITLD),

Tehran, Iran.

LA - eng

PT - Journal Article

PL - United States

TA - Adv Exp Med Biol

JT - Advances in experimental medicine and biology

JID - 0121103

RN - 0 (Adhesins, Bacterial)

RN - 0 (Bacterial Proteins)

SB - IM

MH - Animals

MH - Humans

MH - *Adhesins, Bacterial/chemistry/metabolism/genetics

MH - Bacterial Adhesion

MH - Bacterial Proteins/chemistry/metabolism

MH - *Fimbriae, Bacterial/metabolism/chemistry/genetics/ultrastructure

MH - *Mycobacterium tuberculosis/pathogenicity/metabolism/genetics/physiology

MH - Tuberculosis/microbiology

OTO - NOTNLM

OT - Curli-like pili (MTP)

OT - Mycobacterium tuberculosis

OT - Protein adhesins

OT - Secretion needle pili

OT - Types V (relief funnel pili)

OT - Types VI (adhesion tapering pili)

OT - Types VII (adhesion flap pili)

EDAT- 2025/11/10 00:53

MHDA- 2025/11/12 23:29

CRDT- 2025/11/09 23:14

PHST- 2025/11/12 23:29 [medline]

PHST- 2025/11/10 00:53 [pubmed]

PHST- 2025/11/09 23:14 [entrez]

AID - 10.1007/978-3-031-96883-9_8 [doi]

PST - ppublish

SO - Adv Exp Med Biol. 2026;1484:249-296. doi: 10.1007/978-3-031-96883-9_8.

PMID- 41187886

OWN - NLM

STAT- MEDLINE

DCOM- 20251206

LR - 20260603

IS - 1873-2968 (Electronic)

IS - 0006-2952 (Linking)

VI - 243

IP - Pt 1

DP - 2026 Jan

TI - Mitoxantrone elicits ROS-dependent elimination of dysfunctional mitochondria

through mitophagy to control viability of intracellular mycobacteria in human

macrophages.

PG - 117512

LID - S0006-2952(25)00777-4 [pii]

LID - 10.1016/j.bcp.2025.117512 [doi]

AB - Autophagy plays a critical role in clearance of Mycobacterium tuberculosis. It

has emerged as a promising target for host-directed therapies against

drug-resistant tuberculosis (TB). This insight opens up promising therapeutic

avenues, suggesting that pharmacological activation of autophagy could

effectively combat this highly persistent and harmful bacterium. The current

study investigates the anti-mycobacterial properties of the anthracene-dione

compound Mitoxantrone (MTX) through the activation of autophagy in differentiated

THP-1 cells. The non-cytotoxic dose of MTX reduced the intracellular viability of

mycobacteria compared to the control cells, and inhibition of autophagy reversed

the effect of MTX on intracellular bacterial burden. Through multiparametric

approaches, our investigation established the effect of MTX on mitochondria, the

principal source of endogenous reactive oxygen species (ROS), acting as essential

signal transducers that promote autophagy. Further, we have demonstrated that MTX

decreased ATP production, which caused disruption of mitochondrial membrane

proteins and increased mitochondrial ROS generation, resulting in mitochondrial

fission and accelerating the initiation of mitophagy, leading to the elimination

of intracellular mycobacteria. Our findings collectively demonstrated that

MTX-induced mitochondrial dysfunction triggered interplay between two selective

autophagic responses, diminishing mycobacterial infection and promoting its

clearance. This study highlights MTX as a potential host-directed therapeutic

candidate against TB through modulation of mitochondrial signaling pathways and

autophagic responses.

CI - Copyright © 2025 Elsevier Inc. All rights reserved.

FAU - Das, Mousumi

AU - Das M

AD - Laboratory of Mycobacterial Immunology, Department of Life Science, National

Institute of Technology Rourkela, 769008, India.

FAU - Nayak, Dev Kiran

AU - Nayak DK

AD - Laboratory of Mycobacterial Immunology, Department of Life Science, National

Institute of Technology Rourkela, 769008, India.

FAU - Patel, Salina

AU - Patel S

AD - Laboratory of Mycobacterial Immunology, Department of Life Science, National

Institute of Technology Rourkela, 769008, India.

FAU - Kumar, Ashish

AU - Kumar A

AD - Laboratory of Mycobacterial Immunology, Department of Life Science, National

Institute of Technology Rourkela, 769008, India.

FAU - Quaderi, Mustafeez Ali

AU - Quaderi MA

AD - Laboratory of Mycobacterial Immunology, Department of Life Science, National

Institute of Technology Rourkela, 769008, India.

FAU - Dandsena, Pramathesh Kumar

AU - Dandsena PK

AD - Laboratory of Mycobacterial Immunology, Department of Life Science, National

Institute of Technology Rourkela, 769008, India.

FAU - Naik, Lincoln

AU - Naik L

AD - Laboratory of Mycobacterial Immunology, Department of Life Science, National

Institute of Technology Rourkela, 769008, India.

FAU - Mishra, Abtar

AU - Mishra A

AD - Laboratory of Mycobacterial Immunology, Department of Life Science, National

Institute of Technology Rourkela, 769008, India.

FAU - Mishra, Amit

AU - Mishra A

AD - Cellular and Molecular Neurobiology Unit, Indian Institute of Technology Jodhpur,

Rajasthan 342011, India.

FAU - Singh, Ramandeep

AU - Singh R

AD - Tuberculosis Research Laboratory, Translational Health Science and Technology

Institute, NCR, Biotech Science Cluster, 3rd Milestone, Faridabad-Gurugram

Expressway, PO Box # 4, Faridabad, Haryana 121001, India.

FAU - Bhutia, Sujit Kumar

AU - Bhutia SK

AD - Cancer and Cell Death Laboratory, Department of Life Science, National Institute

of Technology Rourkela, 769008, India.

FAU - Behura, Assirbad

AU - Behura A

AD - Laboratory of Mycobacterial Immunology, Department of Life Science, National

Institute of Technology Rourkela, 769008, India. Electronic address:

ashu.hura@gmail.com.

FAU - Dhiman, Rohan

AU - Dhiman R

AD - Laboratory of Mycobacterial Immunology, Department of Life Science, National

Institute of Technology Rourkela, 769008, India. Electronic address:

dhimanr@nitrkl.ac.in.

LA - eng

PT - Journal Article

PT - Research Support, Non-U.S. Gov't

DEP - 20251102

PL - England

TA - Biochem Pharmacol

JT - Biochemical pharmacology

JID - 0101032

RN - 0 (Reactive Oxygen Species)

RN - BZ114NVM5P (Mitoxantrone)

SB - IM

MH - Humans

MH - *Reactive Oxygen Species/metabolism

MH - *Mitoxantrone/pharmacology

MH - *Mitophagy/drug effects/physiology

MH - *Mitochondria/drug effects/metabolism

MH - *Macrophages/drug effects/microbiology/metabolism

MH - THP-1 Cells

MH - *Mycobacterium tuberculosis/drug effects

MH - Cell Survival/drug effects/physiology

MH - Autophagy/drug effects

MH - *Microbial Viability/drug effects

MH - Dose-Response Relationship, Drug

OTO - NOTNLM

OT - Autophagy

OT - Mitochondria

OT - Mitophagy

OT - Mitoxantrone

OT - Mycobacteria

COIS- Declaration of competing interest The authors declare that they have no known

competing financial interests or personal relationships that could have appeared

to influence the work reported in this paper.

EDAT- 2025/11/05 00:28

MHDA- 2025/12/07 00:41

CRDT- 2025/11/04 19:11

PHST- 2025/07/09 00:00 [received]

PHST- 2025/10/28 00:00 [revised]

PHST- 2025/10/31 00:00 [accepted]

PHST- 2025/12/07 00:41 [medline]

PHST- 2025/11/05 00:28 [pubmed]

PHST- 2025/11/04 19:11 [entrez]

AID - S0006-2952(25)00777-4 [pii]

AID - 10.1016/j.bcp.2025.117512 [doi]

PST - ppublish

SO - Biochem Pharmacol. 2026 Jan;243(Pt 1):117512. doi: 10.1016/j.bcp.2025.117512.

Epub 2025 Nov 2.

PMID- 40947707

OWN - NLM

STAT- MEDLINE

DCOM- 20260422

LR - 20260630

IS - 1875-5550 (Electronic)

IS - 1389-2037 (Linking)

VI - 27

IP - 2

DP - 2026

TI - MmpS5-MmpL5 Transporters Deliver M. tuberculosis Resistance to Bedaquiline (BDQ)

and Delamanid (DLM).

PG - 182-188

LID - 10.2174/0113892037383330250807101618 [doi]

AB - INTRODUCTION: One of the earliest illnesses that has been identified is

tuberculosis (TB). The largest challenge in managing tuberculosis today is the

growing number of individuals infected with TB bacilli, particularly those that

are Extensively drug resistant-tuberculosis (XDR-TB) and Multidrug-resistant

tuberculosis (MDR-TB). However, by figuring out the resistance's molecular

mechanism, Advanced molecular methods may be used to rapidly determine therapy

plans. Combining Delamanid (DLM) with Bedaquiline (BDQ), one of the recently

authorized medications, indicates that the therapy is effective. METHODS: We aim

to investigate efflux-mediated resistance mechanisms in M. Tuberculosis by using

quantitative real-time PCR to assess the expression level of mmpS5 and mmpL5.

RESULTS: The median (M) and interquartile range (Iqr) of mmpL5 and mmpS5

expression varied from 5.65 to 9.01 and 7.95 to 10.74, respectively, when

resistant strains were compared with sensitive ones. M and Iqr of mmpL5 and mmpS5

expression, however, ranged from 0.08-3.04 and 0.05- 1.61 for sensitive strains,

correspondingly. DISCUSSION: Our findings have implications for the development

of fast genotypic drug susceptibility testing (DST). Quantitative real-time PCR

to measure the expression level of mmpS5 and mmpL5 of baseline and post-baseline

isolates is important to track the development of BDQ and DLM resistance.

CONCLUSION: Thus, when developing anti-tuberculosis drugs, mycobacterial

MmpS5-MmpL5 transporters should be taken into consideration early on, as they are

an MDR-efflux system.

CI - Copyright© Bentham Science Publishers; For any queries, please email at

epub@benthamscience.net.

FAU - Balgouthi, Khaoula

AU - Balgouthi K

AD - Department of Biotechnology, Institute of Natural and Applied Sciences, Çukurova

University, 01330, Balcalı, Adana, Türkiye.

FAU - Eker, Emel

AU - Eker E

AD - Department of Pharmaceutical Microbiology, Faculty of Pharmacy, Harran

University, Sanlıurfa, 63300, Turkiye.

FAU - AlMatar, Manaf

AU - AlMatar M

AD - Department of Biology, Faculty of Education and Arts, Sohar University, Sohar,

311, Sultanate of Oman.

LA - eng

PT - Journal Article

PL - United Arab Emirates

TA - Curr Protein Pept Sci

JT - Current protein & peptide science

JID - 100960529

RN - 78846I289Y (bedaquiline)

RN - 0 (Diarylquinolines)

RN - 0 (Antitubercular Agents)

RN - 0 (OPC-67683)

RN - 0 (Oxazoles)

RN - 0 (Nitroimidazoles)

RN - 0 (Bacterial Proteins)

RN - 0 (Membrane Transport Proteins)

SB - IM

MH - *Diarylquinolines/pharmacology

MH - *Mycobacterium tuberculosis/drug effects/genetics/metabolism

MH - *Antitubercular Agents/pharmacology

MH - *Oxazoles/pharmacology

MH - *Nitroimidazoles/pharmacology

MH - Humans

MH - *Bacterial Proteins/genetics/metabolism

MH - Microbial Sensitivity Tests

MH - Tuberculosis, Multidrug-Resistant/drug therapy/microbiology

MH - *Membrane Transport Proteins/genetics/metabolism

MH - Gene Expression Regulation, Bacterial/drug effects

OTO - NOTNLM

OT - BDQ

OT - DLM

OT - M. Tuberculosis

OT - MmpS5-MmpL5

OT - drug resistance

OT - efflux

EDAT- 2025/09/15 06:32

MHDA- 2026/04/22 06:32

CRDT- 2025/09/15 02:14

PHST- 2025/01/04 00:00 [received]

PHST- 2025/04/10 00:00 [revised]

PHST- 2025/04/30 00:00 [accepted]

PHST- 2026/04/22 06:32 [medline]

PHST- 2025/09/15 06:32 [pubmed]

PHST- 2025/09/15 02:14 [entrez]

AID - CPPS-EPUB-150485 [pii]

AID - 10.2174/0113892037383330250807101618 [doi]

PST - ppublish

SO - Curr Protein Pept Sci. 2026;27(2):182-188. doi:

10.2174/0113892037383330250807101618.

PMID- 41454966

OWN - NLM

STAT- MEDLINE

DCOM- 20251227

LR - 20260210

IS - 1432-0991 (Electronic)

IS - 0343-8651 (Linking)

VI - 83

IP - 2

DP - 2025 Dec 27

TI - Whole Genome Sequencing and Metabolomic Profiling of Six Clinical Mycobacterium

Tuberculosis Isolates Reveal Drug Resistance-Associated Features.

PG - 102

LID - 10.1007/s00284-025-04677-1 [doi]

AB - Drug-resistant tuberculosis (DR-TB), resulting from newly emerging strains of

Mycobacterium tuberculosis (Mtb), and the World Health Organisation (WHO)

included it as a top-priority antimicrobial-resistant pathogen. Whole genome

sequence analysis (WGS) of clinical Mtb isolates could correlate to their drug

resistance phenotype and may also reflect their metabolome. In this report,

clinical Mtb isolates (S1, S4, S5, S6, S7, S10) harvested from the sputum of

tuberculosis patients were characterized using drug sensitive test (DST),

electron microscope, WGS and untargeted Gas chromatography and mass spectrometry

(GC-MS) based metabolomics analysis. The majority of these Mtb isolates showed

similar size (length: 1.0-3.2 μm; width: 0.32-0.52 μm) to the H37Rv Mtb strain,

whereas significant variations were observed in their growth kinetics, WGS and

metabolome profiles. In-silico drug resistance prediction, from the WGS data

(single-nucleotide polymorphisms (SNP) pattern) of these Mtb isolates, showed

resistance to tuberculosis drugs and matched with DST results. Differences in the

genes involved in stress response, pathogenicity, and drug efflux pumps were

observed between isolates, but genes of the central carbon metabolic pathways and

amino acid metabolism were conserved. GC-MS-based metabolite profiling of these

clinical isolates identified 291 metabolites involved in various metabolic

pathways, and a subset of these metabolites (glutamic acid, aspartic acid and

serine) contributed to the drug resistance patterns. These clinical Mtb isolates

could be useful as an alternate reagent for understanding host-pathogen

interaction. The pipeline used for WGS analysis could be used to predict the drug

resistance pattern of new Mtb isolates.

CI - © 2025. The Author(s), under exclusive licence to Springer Science+Business

Media, LLC, part of Springer Nature.

FAU - Sahoo, Ashutosh

AU - Sahoo A

AUID- ORCID: 0000-0003-0879-6683

AD - School of Biosciences and Bioengineering, Indian Institute of Technology Mandi,

Kamand, 175005, Himachal Pradesh, India.

FAU - Mohapatra, Amit Kumar

AU - Mohapatra AK

AD - Translational Health Group, International Centre for Genetic Engineering and

Biotechnology, New Delhi, 110067, India.

AD - School of Life Sciences, Sambalpur University, Sambalpur, 768019, Odisha, India.

FAU - Priyadarsini, Haripriya

AU - Priyadarsini H

AD - Translational Health Group, International Centre for Genetic Engineering and

Biotechnology, New Delhi, 110067, India.

FAU - Pemmadi, Raghuveer Varma

AU - Pemmadi RV

AD - Translational Health Group, International Centre for Genetic Engineering and

Biotechnology, New Delhi, 110067, India.

FAU - Das, Anjan

AU - Das A

AD - Department of Respiratory Medicine, Agartala Government Medical College,

Kunjaban, Agartala, Tripura, 799006, India. anjan8870@gmail.com.

FAU - Choedon, Tenzin

AU - Choedon T

AD - Structural Biology Group, International Centre for Genetic Engineering and

Biotechnology, New Delhi, 110067, India.

FAU - Nikam, Chaitali

AU - Nikam C

AD - Thyrocare Technologies, Mumbai, 110067, Maharashtra, India.

FAU - Behera, Rajendra Kumar

AU - Behera RK

AD - School of Life Sciences, Sambalpur University, Sambalpur, 768019, Odisha, India.

FAU - Masakapalli, Shyam Kumar

AU - Masakapalli SK

AUID- ORCID: 0000-0003-1988-5569

AD - School of Biosciences and Bioengineering, Indian Institute of Technology Mandi,

Kamand, 175005, Himachal Pradesh, India. shyam@iitmandi.ac.in.

FAU - Nanda, Ranjan Kumar

AU - Nanda RK

AD - Translational Health Group, International Centre for Genetic Engineering and

Biotechnology, New Delhi, 110067, India. ranjan@icgeb.res.in.

LA - eng

GR - BT/PR23238/NER/95/636/2017/Department of Biotechnology, Ministry of Science and

Technology, India/

PT - Journal Article

DEP - 20251227

PL - United States

TA - Curr Microbiol

JT - Current microbiology

JID - 7808448

RN - 0 (Antitubercular Agents)

SB - IM

MH - *Mycobacterium tuberculosis/genetics/drug effects/metabolism/isolation &

purification

MH - Humans

MH - Whole Genome Sequencing

MH - Antitubercular Agents/pharmacology

MH - Metabolomics

MH - Genome, Bacterial

MH - Microbial Sensitivity Tests

MH - *Metabolome

MH - Polymorphism, Single Nucleotide

MH - *Tuberculosis, Multidrug-Resistant/microbiology

MH - *Drug Resistance, Bacterial

MH - Gas Chromatography-Mass Spectrometry

OTO - NOTNLM

OT - Drug resistance

OT - Metabolome

OT - Mycobacterium tuberculosis

OT - Tuberculosis

OT - Whole genome sequencing

COIS- Declarations. Competing Interests: The authors have no relevant financial or

non-financial interests to disclose. Consent for Publication: Not applicable.

Ethics Approval and Consent to Participate: This study was approved by the human

ethics committees of the Agartala Government Medical College, Agartala (F.4

[6–9]/AGMC/Academic/IEC Committee/2015/8965, dated 25 April 2018) and

International Centre for Genetic Engineering and Biotechnology, New Delhi

(ICGEB/IEC/2017/07). Subjects or patients with symptoms of tuberculosis disease

like cough for 2 weeks or more, weight loss, fever, and night sweats reporting to

the outpatient department of the hospital were recruited after receiving their

signed informed consent forms by the individual or their concerned guardian.

Sputum samples (day 0 and day 1) of these subjects were taken for sputum

microscopy at the hospital and culture test for drug sensitivity test using

Mycobacteria Growth Indicator Tube (MGIT) in BACTEC MGIT 960 System at a National

Accreditation Board for Testing and Calibration Laboratories (NABL) accredited

laboratory following WHO guidelines. All the methods and experimental procedure

related with clinical samples was carried out in accordance with ethical

guidelines and regulations.

EDAT- 2025/12/27 17:51

MHDA- 2025/12/27 17:52

CRDT- 2025/12/27 11:05

PHST- 2025/05/10 00:00 [received]

PHST- 2025/12/06 00:00 [accepted]

PHST- 2025/12/27 17:52 [medline]

PHST- 2025/12/27 17:51 [pubmed]

PHST- 2025/12/27 11:05 [entrez]

AID - 10.1007/s00284-025-04677-1 [pii]

AID - 10.1007/s00284-025-04677-1 [doi]

PST - epublish

SO - Curr Microbiol. 2025 Dec 27;83(2):102. doi: 10.1007/s00284-025-04677-1.

PMID- 41313016

OWN - NLM

STAT- MEDLINE

DCOM- 20251223

LR - 20251225

IS - 2379-5042 (Electronic)

IS - 2379-5042 (Linking)

VI - 10

IP - 12

DP - 2025 Dec 23

TI - Analysis of the drug target of the anti-tuberculosis compound OCT313:

phosphotransacetylase is a potential drug target for anti-mycobacterial agents.

PG - e0046325

LID - 10.1128/msphere.00463-25 [doi]

LID - e00463-25

AB - Tuberculosis (TB) is one of the most common infectious diseases caused by

bacteria worldwide. The increasing prevalence of multidrug-resistant TB (MDR-TB)

and latent TB infection (LTBI) has intensified the global TB burden. Therefore,

the development of new drugs for MDR-TB and LTBI is urgently required. We have

reported that the derivative of dithiocarbamate sugar derivative,

2-acetamido-2-deoxy-β-D-glucopyranosyl N,N-dimethyldithiocarbamate (OCT313),

exhibits anti-mycobacterial activity against MDR-MTB. Here, we identified the

target of OCT313. In experimentally generated OCT313-resistant bacteria, adenine

at position 1,092 in the metabolic enzyme phosphotransacetylase (PTA) gene was

replaced with cytosine. This mutation is a nonsynonymous mutation that converts

methionine to leucine at position 365 in the PTA protein. OCT313 inhibited the

enzymatic activity of recombinant wild-type PTA, but not of the mutant PTA

(M365L). PTA is an enzyme that produces acetyl-coenzyme A (acetyl-CoA) from

acetyl phosphate and CoA and is involved in metabolic pathways; therefore, it was

expected to also be active against dormant Mycobacterium tuberculosis bacilli.

OCT313 exhibits antibacterial activity in the Wayne model of dormancy using

Mycobacterium bovis BCG, and overexpression of PTA in OCT313-resistant bacilli

restored sensitivity to OCT313. Collectively, the target of OCT313 is PTA, and

OCT313 is a promising antimicrobial candidate for MDR-TB and

LTBI.IMPORTANCEThrough this study, we propose a new target for the development of

medicines to treat multidrug-resistant tuberculosis and latent tuberculosis

infection. The target enzyme phosphotransacetylase (PTA) is a key enzyme that

functions in major metabolic pathways, and the homologous structures of PTA

enzymes vary greatly among bacterial species. Since the treatment of

mycobacterial disease is long term, the development of antibiotics targeting PTA

is useful for species-specific therapy.

FAU - Takii, Takemasa

AU - Takii T

AUID- ORCID: 0000-0002-5005-0941

AD - Department of Mycobacterium Reference and Research, the Research Institute of

Tuberculosis, Japan Anti-Tuberculosis Association, Kiyose, Tokyo, Japan.

RINGGOLD: 46635

AD - Department of Hygienic Chemistry, Graduate School of Pharmaceutical Sciences,

Nagoya City University, Nagoya, Aichi, Japan. RINGGOLD: 12963. ROR:

https://ror.org/04wn7wc95

AD - Ōmura Satoshi Memorial Institute, Kitasato University, Minato-ku, Tokyo, Japan.

RINGGOLD: 12877. ROR: https://ror.org/00f2txz25

FAU - Hasegawa, Tomohiro

AU - Hasegawa T

AD - Department of Hygienic Chemistry, Graduate School of Pharmaceutical Sciences,

Nagoya City University, Nagoya, Aichi, Japan. RINGGOLD: 12963. ROR:

https://ror.org/04wn7wc95

FAU - Itoh, Saotomo

AU - Itoh S

AD - Department of Hygienic Chemistry, Graduate School of Pharmaceutical Sciences,

Nagoya City University, Nagoya, Aichi, Japan. RINGGOLD: 12963. ROR:

https://ror.org/04wn7wc95

AD - Laboratory of Hygienic Chemistry, School of Pharmacy, Aichi Gakuin University,

Nagoya, Aichi, Japan. RINGGOLD: 57898. ROR: https://ror.org/01rwx7470

FAU - Maeda, Shinji

AU - Maeda S

AD - Graduate School of Pharmaceutical Sciences, Hokkaido University of Sciences,

Sapporo, Hokkaido, Japan. ROR: https://ror.org/02e16g702

FAU - Wada, Takayuki

AU - Wada T

AD - Department of Microbiology, Graduate School of Human Life and Ecology, Osaka

Metropolitan University, Osaka, Japan. RINGGOLD: 12936. ROR:

https://ror.org/01hvx5h04

AD - Osaka International Research Center for Infectious Diseases, Osaka Metropolitan

University, Osaka, Osaka, Japan.

FAU - Horita, Yasuhiro

AU - Horita Y

AD - Department of Clinical Pharmaceutics, Graduate School of Medical Sciences, Nagoya

City University, Nagoya, Aichi, Japan. RINGGOLD: 12963. ROR:

https://ror.org/04wn7wc95

FAU - Nishiyama, Akihito

AU - Nishiyama A

AD - Department of Bacteriology, Niigata University School of Medicine, Niigata,

Japan. RINGGOLD: 12978. ROR: https://ror.org/04ww21r56

FAU - Matsumoto, Sohkichi

AU - Matsumoto S

AUID- ORCID: 0000-0002-6106-7538

AD - Department of Bacteriology, Niigata University School of Medicine, Niigata,

Japan. RINGGOLD: 12978. ROR: https://ror.org/04ww21r56

AD - Department of Bacteriology, Osaka Metropolitan University Graduate School of

Medicine, Osaka, Japan. RINGGOLD: 12936. ROR: https://ror.org/01hvx5h04

AD - Division of Research Aids, Hokkaido University Institute for Vaccine Research &

Development, Sapporo, Hokkaido, Japan. RINGGOLD: 12810. ROR:

https://ror.org/02e16g702

AD - Laboratory of Tuberculosis, Institute of Tropical Disease, Universitas Airlangga,

Surabaya, Indonesia. RINGGOLD: 148005. ROR: https://ror.org/04ctejd88

FAU - Ohara, Naoya

AU - Ohara N

AD - Department of Oral Microbiology, Graduate School of Medicine, Dentistry and

Pharmaceutical Sciences, Okayama University, Okayama, Japan. RINGGOLD: 12997.

ROR: https://ror.org/02pc6pc55

FAU - Kimishima, Aoi

AU - Kimishima A

AD - Laboratory of Applied Microbial Chemistry, Ōmura Satoshi Memorial Institute,

Kitasato University, Minato-ku, Tokyo, Japan. RINGGOLD: 12877. ROR:

https://ror.org/00f2txz25

FAU - Asami, Yukihiro

AU - Asami Y

AD - Laboratory of Applied Microbial Chemistry, Ōmura Satoshi Memorial Institute,

Kitasato University, Minato-ku, Tokyo, Japan. RINGGOLD: 12877. ROR:

https://ror.org/00f2txz25

FAU - Hida, Shigeaki

AU - Hida S

AD - Department of Hygienic Chemistry, Graduate School of Pharmaceutical Sciences,

Nagoya City University, Nagoya, Aichi, Japan. RINGGOLD: 12963. ROR:

https://ror.org/04wn7wc95

FAU - Onozaki, Kikuo

AU - Onozaki K

AD - Department of Hygienic Chemistry, Graduate School of Pharmaceutical Sciences,

Nagoya City University, Nagoya, Aichi, Japan. RINGGOLD: 12963. ROR:

https://ror.org/04wn7wc95

LA - eng

GR - 20K07125, 24K09872/Japan Society for the Promotion of Science/

GR - JP21fk0108590, JP23fk0108591, JP223fa627005/Japan Agency for Medical Research and

Development/

PT - Journal Article

DEP - 20251128

PL - United States

TA - mSphere

JT - mSphere

JID - 101674533

RN - 0 (Antitubercular Agents)

RN - EC 2.3.1.8 (Phosphate Acetyltransferase)

RN - 0 (Bacterial Proteins)

SB - IM

MH - *Antitubercular Agents/pharmacology

MH - *Mycobacterium tuberculosis/drug effects/enzymology/genetics

MH - *Phosphate Acetyltransferase/genetics/antagonists & inhibitors

MH - Microbial Sensitivity Tests

MH - Tuberculosis, Multidrug-Resistant/drug therapy/microbiology

MH - Humans

MH - Mutation

MH - Animals

MH - Bacterial Proteins/genetics/antagonists & inhibitors

PMC - PMC12724200

OTO - NOTNLM

OT - N-acetyl glucosamine

OT - acetyl coenzyme A

OT - anti-mycobacterial agents

OT - dithiocarbamate

OT - latent tuberculosis infection

OT - phosphotransacetylase

COIS- The authors declare no conflict of interest.

EDAT- 2025/11/28 13:30

MHDA- 2025/12/23 12:48

PMCR- 2025/11/28

CRDT- 2025/11/28 08:03

PHST- 2025/12/23 12:48 [medline]

PHST- 2025/11/28 13:30 [pubmed]

PHST- 2025/11/28 08:03 [entrez]

PHST- 2025/11/28 00:00 [pmc-release]

AID - msphere00463-25 [pii]

AID - msphere.00463-25 [pii]

AID - 10.1128/msphere.00463-25 [doi]

PST - ppublish

SO - mSphere. 2025 Dec 23;10(12):e0046325. doi: 10.1128/msphere.00463-25. Epub 2025

Nov 28.

PMID- 41598998

OWN - NLM

STAT- MEDLINE

DCOM- 20260701

LR - 20260701

IS - 2076-0817 (Electronic)

IS - 2076-0817 (Linking)

VI - 15

IP - 1

DP - 2025 Dec 22

TI - Alterations in the Immune Response in Individuals with Latent Tuberculosis

Infection.

LID - 10.3390/pathogens15010014 [doi]

LID - 14

AB - Latent tuberculosis infection (LTBI) represents a biologically active yet

clinically asymptomatic stage of Mycobacterium tuberculosis (Mtb) persistence.

This condition is characterized by subtle immunometabolic alterations reflecting

the host-pathogen equilibrium. Understanding the mechanisms and biomarkers

associated with the preclinical phase of LTBI is crucial for preventing

progression to active tuberculosis (ATB). Recent advances have identified

multiple immunological, transcriptomic, metabolic, and imaging-based approaches

that enable stratification of individuals at increased risk of LTBI reactivation.

Quantitative assays such as IGRA, multiplex and T-cell activation marker (TAM)

tests, as well as interferon-related transcriptional signatures, demonstrate

predictive potential when combined with functional assays (MGIA) and metabolic

imaging (PET/CT). Experimental primate models faithfully reproduce the spectrum

from latency to reactivation, allowing for validation of biomarkers and vaccine

or immunomodulatory strategies. The review also highlights the particular

challenges of multidrug-resistant LTBI (MDR-LTBI), where standard

chemoprophylaxis is less effective and immune control plays a decisive role. The

preclinical phase of LTBI constitutes a key point in the TB control cascade.

Integrating immunological, transcriptomic, and radiological data into risk-based

screening algorithms could substantially improve early detection and targeted

prevention. Translating research-derived signatures into clinically applicable,

standardized, and cost-effective diagnostic tools requires coordinated

international efforts, technological transfer, and policy-level support to reduce

TB reactivation and transmission, including MDR-TB.

FAU - Starshinova, Anna

AU - Starshinova A

AUID- ORCID: 0000-0002-9023-6986

AD - Department of Mathematics and Computer Science, Saint Petersburg State

University, 199034 Saint Petersburg, Russia.

AD - Almazov National Medical Research Center of the Ministry of Health of the Russian

Federation, 2, Akkuratov Str., 197341 Saint Petersburg, Russia.

FAU - Sabirova, Adilya

AU - Sabirova A

AD - Department of Mathematics and Computer Science, Saint Petersburg State

University, 199034 Saint Petersburg, Russia.

AD - Medicine Department, Bashkir State Medical University, 450008 Ufa, Russia.

FAU - Kudryavtsev, Igor

AU - Kudryavtsev I

AD - Institute of Experimental Medicine, 197376 Saint Petersburg, Russia.

FAU - Rubinstein, Artem

AU - Rubinstein A

AUID- ORCID: 0000-0002-8493-5211

AD - Institute of Experimental Medicine, 197376 Saint Petersburg, Russia.

FAU - Aquino, Arthur

AU - Aquino A

AUID- ORCID: 0000-0001-6516-7184

AD - Almazov National Medical Research Center of the Ministry of Health of the Russian

Federation, 2, Akkuratov Str., 197341 Saint Petersburg, Russia.

FAU - Churilov, Leonid P

AU - Churilov LP

AUID- ORCID: 0000-0001-6359-0026

AD - Department of Medicine, Saint Petersburg State University, 199034 Saint

Petersburg, Russia.

FAU - Belyaeva, Ekaterina

AU - Belyaeva E

AD - Department of Mathematics and Computer Science, Saint Petersburg State

University, 199034 Saint Petersburg, Russia.

FAU - Kulpina, Anastasia

AU - Kulpina A

AUID- ORCID: 0000-0001-7059-3436

AD - Department of Mathematics and Computer Science, Saint Petersburg State

University, 199034 Saint Petersburg, Russia.

AD - Almazov National Medical Research Center of the Ministry of Health of the Russian

Federation, 2, Akkuratov Str., 197341 Saint Petersburg, Russia.

FAU - Sharipov, Raul A

AU - Sharipov RA

AUID- ORCID: 0000-0002-7720-4832

AD - Medicine Department, Bashkir State Medical University, 450008 Ufa, Russia.

FAU - Tukfatullin, Ravil K

AU - Tukfatullin RK

AUID- ORCID: 0009-0003-4100-6839

AD - Medicine Department, Bashkir State Medical University, 450008 Ufa, Russia.

FAU - Nikolenko, Nikolay

AU - Nikolenko N

AUID- ORCID: 0000-0002-1071-2680

AD - The Moscow Research and Clinical Center for Tuberculosis Control of the Moscow

Government Department of Health, 127006 Moscow, Russia.

FAU - Kudlay, Dmitry

AU - Kudlay D

AUID- ORCID: 0000-0003-1878-4467

AD - Department of Pharmacology, Institute of Pharmacy, Sechenov University, 119991

Moscow, Russia.

AD - NRC Institute of Immunology FMBA of Russia, 115522 Moscow, Russia.

AD - Department of Bioengineering and Bioinformatics, Lomonosov Moscow State

University, 119991 Moscow, Russia.

LA - eng

GR - 075-15-2025-013/the Ministry of Science and Higher Education of the Russian

Federation/

PT - Journal Article

PT - Review

DEP - 20251222

PL - Switzerland

TA - Pathogens

JT - Pathogens (Basel, Switzerland)

JID - 101596317

RN - 0 (Biomarkers)

SB - IM

MH - *Latent Tuberculosis/immunology/diagnosis/microbiology/metabolism

MH - Humans

MH - *Mycobacterium tuberculosis/immunology

MH - Animals

MH - Biomarkers

MH - *Host-Pathogen Interactions/immunology

PMC - PMC12844760

OTO - NOTNLM

OT - Mycobacterium tuberculosis

OT - PET/CT imaging

OT - extracellular vesicles

OT - immune biomarkers

OT - immunodiagnostics

OT - interferon signature

OT - latent tuberculosis infection

OT - multidrug-resistant tuberculosis

OT - preclinical stage

OT - transcriptomics

COIS- The authors declare no conflicts of interest.

EDAT- 2026/01/28 07:47

MHDA- 2026/01/28 19:20

PMCR- 2025/12/22

CRDT- 2026/01/28 01:20

PHST- 2025/10/26 00:00 [received]

PHST- 2025/12/15 00:00 [revised]

PHST- 2025/12/17 00:00 [accepted]

PHST- 2026/01/28 19:20 [medline]

PHST- 2026/01/28 07:47 [pubmed]

PHST- 2026/01/28 01:20 [entrez]

PHST- 2025/12/22 00:00 [pmc-release]

AID - pathogens15010014 [pii]

AID - pathogens-15-00014 [pii]

AID - 10.3390/pathogens15010014 [doi]

PST - epublish

SO - Pathogens. 2025 Dec 22;15(1):14. doi: 10.3390/pathogens15010014.

PMID- 41425329

OWN - NLM

STAT- PubMed-not-MEDLINE

DCOM- 20251222

LR - 20251224

IS - 2694-250X (Electronic)

IS - 2694-250X (Linking)

VI - 5

IP - 6

DP - 2025 Dec 17

TI - Integrating Docking, Dynamics, and Assays to Predict Antimicrobial Peptide

Interactions with Mycolic Acid Membranes in Mycobacterium tuberculosis.

PG - 981-1000

LID - 10.1021/acsmeasuresciau.5c00126 [doi]

AB - The global burden of multidrug-resistant tuberculosis (MDR-TB) underscores the

urgent need for novel therapeutics with distinct mechanisms of action. Here, we

report a comparative evaluation of four antimicrobial peptides (AMPs) derived

from the amphibian peptide B1CTcu5, integrating experimental validation with

molecular modeling to elucidate structure-activity relationships. Among them,

W-B1CTcu5, featuring a single N-terminal tryptophan substitution, exhibited the

most potent antimycobacterial activity (MIC = 3.2 μg/mL) against Mycobacterium

tuberculosis (MTB) combined with high structural stability, persistent membrane

interaction, and multitarget affinity against key MTB proteins, including the

porin MspA, the transporter CpnT, and the cell wall enzyme Ag85B. In contrast,

analogs with reduced hydrophobic anchoring or dynamic instability demonstrated

diminished efficacy despite partial membrane insertion or surface affinity.

Molecular dynamics simulations revealed that peptides with low root-mean-square

deviation and minimal residue fluctuation retained compact, α-helical

conformations and maintained productive bilayer engagement, which are traits

correlated with antimicrobial performance. However, the hemolytic properties of

W-B1CTcu5 highlight a therapeutic trade-off between potency and host toxicity.

Together, these findings emphasize the predictive power of dynamic structural

descriptors in AMP design, and identify W-B1CTcu5 as a promising, yet

optimization-requiring, scaffold for future design of anti-TB AMPs.

CI - © 2025 The Authors. Published by American Chemical Society.

FAU - Roque-Borda, Cesar Augusto

AU - Roque-Borda CA

AUID- ORCID: 0000-0002-9262-0383

AD - Department of Biological Sciences, School of Pharmaceutical Sciences,

Universidade Estadual Paulista (UNESP), 14800901 Araraquara, Brazil. RINGGOLD:

28108

AD - iMed.ULisboa-Institute for Medicines Research, Faculty of Pharmacy, University of

Lisbon, 1649004 Lisbon, Portugal. RINGGOLD: 37809

FAU - Ramirez Delgado, Oswaldo Julio

AU - Ramirez Delgado OJ

AD - Department of Biological Sciences, School of Pharmaceutical Sciences,

Universidade Estadual Paulista (UNESP), 14800901 Araraquara, Brazil. RINGGOLD:

28108

FAU - Duran Gleriani Primo, Laura Maria

AU - Duran Gleriani Primo LM

AD - Department of Biological Sciences, School of Pharmaceutical Sciences,

Universidade Estadual Paulista (UNESP), 14800901 Araraquara, Brazil. RINGGOLD:

28108

FAU - Dyhr, Emma

AU - Dyhr E

AUID- ORCID: 0009-0008-1806-0761

AD - Faculty of Health and Medical Sciences, Department of Drug Design and

Pharmacology, University of Copenhagen, 2100 Copenhagen, Denmark. RINGGOLD: 4321

FAU - Sæbø, Ingvill Pedersen

AU - Sæbø IP

AD - Department of Microbiology, Oslo University Hospital and the University of Oslo,

Rikshospitalet, 0373 Oslo, Norway. RINGGOLD: 6305

FAU - Helgesen, Emily

AU - Helgesen E

AD - Department of Microbiology, Oslo University Hospital and the University of Oslo,

Rikshospitalet, 0373 Oslo, Norway. RINGGOLD: 6305

AD - Department of Clinical and Molecular Medicine, Norwegian University of Science

and Technology, and Clinic of Laboratory Medicine, St. Olavs Hospital, 7491

Trondheim, Norway.

FAU - Booth, James

AU - Booth J

AD - Department of Microbiology, Oslo University Hospital and the University of Oslo,

Rikshospitalet, 0373 Oslo, Norway. RINGGOLD: 6305

AD - Department of Clinical and Molecular Medicine, Norwegian University of Science

and Technology, and Clinic of Laboratory Medicine, St. Olavs Hospital, 7491

Trondheim, Norway.

FAU - Franzyk, Henrik

AU - Franzyk H

AUID- ORCID: 0000-0002-2822-1927

AD - Faculty of Health and Medical Sciences, Department of Drug Design and

Pharmacology, University of Copenhagen, 2100 Copenhagen, Denmark. RINGGOLD: 4321

FAU - Hansen, Paul R

AU - Hansen PR

AD - Faculty of Health and Medical Sciences, Department of Drug Design and

Pharmacology, University of Copenhagen, 2100 Copenhagen, Denmark. RINGGOLD: 4321

FAU - Morales-Navarrete, Hernan

AU - Morales-Navarrete H

AUID- ORCID: 0000-0002-9578-2556

AD - Bio-Cheminformatics Research Group, Universidad de Las Américas, 170504 Quito,

Ecuador.

FAU - de la Torre, Beatriz G

AU - de la Torre BG

AUID- ORCID: 0000-0001-8521-9172

AD - School of Laboratory Medicine and Medical Sciences, College of Health Sciences,

University of KwaZulu-Natal, 4041 Durban, South Africa. RINGGOLD: 56394

AD - Peptide Science Laboratory, School of Chemistry and Physics, University of

KwaZulu-Natal, 4001 Durban, South Africa.

FAU - Albericio, Fernando

AU - Albericio F

AUID- ORCID: 0000-0002-8946-0462

AD - Peptide Science Laboratory, School of Chemistry and Physics, University of

KwaZulu-Natal, 4001 Durban, South Africa.

AD - Department of Organic Chemistry, University of Barcelona, 08028 Barcelona, Spain.

FAU - Perdigão, João

AU - Perdigão J

AD - iMed.ULisboa-Institute for Medicines Research, Faculty of Pharmacy, University of

Lisbon, 1649004 Lisbon, Portugal. RINGGOLD: 37809

FAU - Pavan, Fernando Rogério

AU - Pavan FR

AUID- ORCID: 0000-0002-6969-3963

AD - Department of Biological Sciences, School of Pharmaceutical Sciences,

Universidade Estadual Paulista (UNESP), 14800901 Araraquara, Brazil. RINGGOLD:

28108

LA - eng

PT - Journal Article

DEP - 20251014

PL - United States

TA - ACS Meas Sci Au

JT - ACS measurement science au

JID - 9918232604306676

PMC - PMC12715740

OTO - NOTNLM

OT - Mycobacterium tuberculosis

OT - antimicrobial peptides

OT - membrane-targeting agents

OT - molecular dynamics simulations

OT - multidrug resistance

OT - structure–activity relationship

EDAT- 2025/12/22 18:09

MHDA- 2025/12/22 18:10

PMCR- 2025/10/14

CRDT- 2025/12/22 06:43

PHST- 2025/08/14 00:00 [received]

PHST- 2025/10/07 00:00 [revised]

PHST- 2025/10/07 00:00 [accepted]

PHST- 2025/12/22 18:10 [medline]

PHST- 2025/12/22 18:09 [pubmed]

PHST- 2025/12/22 06:43 [entrez]

PHST- 2025/10/14 00:00 [pmc-release]

AID - 10.1021/acsmeasuresciau.5c00126 [doi]

PST - epublish

SO - ACS Meas Sci Au. 2025 Oct 14;5(6):981-1000. doi: 10.1021/acsmeasuresciau.5c00126.

eCollection 2025 Dec 17.

PMID- 41243230

OWN - NLM

STAT- MEDLINE

DCOM- 20251212

LR - 20251212

IS - 2373-8227 (Electronic)

IS - 2373-8227 (Linking)

VI - 11

IP - 12

DP - 2025 Dec 12

TI - Succinylation of GyrB Is a Mechanism Conferring Fluoroquinolone Resistance.

PG - 3607-3619

LID - 10.1021/acsinfecdis.5c00781 [doi]

AB - Being a persistent and deadly infection, tuberculosis (TB) caused by

Mycobacterium tuberculosis remains a global health challenge. Despite having a

well-established 4-drug combination therapy for drug-sensitive TB, the emergence

of drug-resistant TB has rendered the treatment less effective. Although

fluoroquinolones (FQs) are among the prominent drugs in the second-line treatment

for multidrug-resistant tuberculosis (MDR-TB), FQ resistance has readily emerged

in cases of extensively drug-resistant tuberculosis (XDR-TB). Other than the

mutations in DNA gyrase, a universally conserved bacterial enzyme targeted by

FQs, several mechanisms contribute to the emergence of FQ resistance. Recently,

post-translational modification of DNA gyrase is implicated as one of the

mechanisms for FQ resistance. Here, we describe succinylation of GyrB by a

promiscuous acetyltransferase, Eis of M. tuberculosis, as a new mechanism

contributing to FQ resistance in mycobacteria. Lysine succinylation of GyrB

results in a reduced interaction of FQs with the enzyme, thereby decreasing the

DNA cleavage by DNA gyrase in the presence of FQs. Accordingly, Eis

overexpressing mycobacterial strains exhibit increased minimum inhibitory

concentration (MIC) to FQs. Thus, succinylation of DNA gyrase is yet another

resistance mechanism against the FQs.

FAU - Jha, Abodh Kumar

AU - Jha AK

AD - Department of Microbiology and Cell Biology, Indian Institute of Science (IISc),

Bangalore 560012, India.

FAU - Faheem, Iqball

AU - Faheem I

AD - Department of Microbiology and Cell Biology, Indian Institute of Science (IISc),

Bangalore 560012, India.

FAU - Nagaraja, Valakunja

AU - Nagaraja V

AUID- ORCID: 0000-0002-9768-1505

AD - Department of Microbiology and Cell Biology, Indian Institute of Science (IISc),

Bangalore 560012, India.

AD - Indian Institute of Science Education and Research (IISER), Bhopal 462066, India.

LA - eng

PT - Journal Article

DEP - 20251117

PL - United States

TA - ACS Infect Dis

JT - ACS infectious diseases

JID - 101654580

RN - EC 5.99.1.3 (DNA Gyrase)

RN - 0 (Fluoroquinolones)

RN - 0 (Antitubercular Agents)

RN - EC 2.3.1.- (Acetyltransferases)

RN - 0 (Bacterial Proteins)

SB - IM

MH - *DNA Gyrase/metabolism/genetics

MH - *Fluoroquinolones/pharmacology

MH - *Mycobacterium tuberculosis/drug effects/genetics/enzymology/metabolism

MH - *Drug Resistance, Bacterial

MH - *Antitubercular Agents/pharmacology

MH - Microbial Sensitivity Tests

MH - Acetyltransferases/metabolism/genetics

MH - Bacterial Proteins/metabolism/genetics

MH - Protein Processing, Post-Translational

OTO - NOTNLM

OT - DNA gyrase

OT - Mycobacterium tuberculosis

OT - fluoroquinolone resistance

OT - gyrase succinylation

OT - post-translational modification

EDAT- 2025/11/17 09:08

MHDA- 2025/12/12 07:20

CRDT- 2025/11/17 01:13

PHST- 2025/12/12 07:20 [medline]

PHST- 2025/11/17 09:08 [pubmed]

PHST- 2025/11/17 01:13 [entrez]

AID - 10.1021/acsinfecdis.5c00781 [doi]

PST - ppublish

SO - ACS Infect Dis. 2025 Dec 12;11(12):3607-3619. doi: 10.1021/acsinfecdis.5c00781.

Epub 2025 Nov 17.

PMID- 41118320

OWN - NLM

STAT- MEDLINE

DCOM- 20251210

LR - 20260513

IS - 1098-6596 (Electronic)

IS - 0066-4804 (Print)

IS - 0066-4804 (Linking)

VI - 69

IP - 12

DP - 2025 Dec 10

TI - Variability in intrinsic drug tolerance in Mycobacterium tuberculosis corresponds

with phylogenetic lineage.

PG - e0099625

LID - 10.1128/aac.00996-25 [doi]

LID - e00996-25

AB - Drug tolerance allows bacteria to survive extended exposure to bactericidal drugs

and is thought to play a role in drug resistance evolution. In Mycobacterium

tuberculosis (Mtb), the causative agent of tuberculosis (TB), multidrug-resistant

TB outbreaks are frequently caused by strains belonging to two phylogenetic

lineages of the human-adapted strains of the Mtb complex, namely, lineages (L) 2

and L4. We hypothesized that members of L2 and L4 are more intrinsically drug

tolerant and, as such, more readily evolve drug resistance. To explore this, we

devised a high-throughput in vitro assay to measure drug tolerance in Mtb. We

selected a cohort of strains representative of the globally most frequent

lineages, L1-L4. We measured tolerance to rifampicin and bedaquiline and found L3

and L4 strains to have higher tolerance compared to L1 and L2 strains. In

addition, phylogenetically closely related strains exhibited similar levels of

tolerance, suggesting that tolerance is heritable. Finally, we explored genes

previously reported to be associated with tolerance in Mtb and found significant

enrichment in mutations in genes involved in cell wall and cell processes,

intermediary metabolism and respiration, as well as lipid metabolism in

high-tolerance strains.

FAU - March, Valerie F A

AU - March VFA

AD - Swiss Tropical and Public Health Institute, Allschwil, Switzerland.

AD - University of Basel, Basel, Switzerland.

FAU - Zwyer, Michaela

AU - Zwyer M

AD - Swiss Tropical and Public Health Institute, Allschwil, Switzerland.

AD - University of Basel, Basel, Switzerland.

FAU - Loiseau, Chloé

AU - Loiseau C

AD - Swiss Tropical and Public Health Institute, Allschwil, Switzerland.

AD - University of Basel, Basel, Switzerland.

FAU - Brites, Daniela

AU - Brites D

AD - Swiss Tropical and Public Health Institute, Allschwil, Switzerland.

AD - University of Basel, Basel, Switzerland.

FAU - Goig, Galo A

AU - Goig GA

AD - Swiss Tropical and Public Health Institute, Allschwil, Switzerland.

AD - University of Basel, Basel, Switzerland.

FAU - Bouaouina, Selim

AU - Bouaouina S

AD - Swiss Tropical and Public Health Institute, Allschwil, Switzerland.

AD - University of Basel, Basel, Switzerland.

FAU - Doetsch, Anna

AU - Doetsch A

AD - Swiss Tropical and Public Health Institute, Allschwil, Switzerland.

AD - University of Basel, Basel, Switzerland.

FAU - Reinhard, Miriam

AU - Reinhard M

AD - Swiss Tropical and Public Health Institute, Allschwil, Switzerland.

AD - University of Basel, Basel, Switzerland.

FAU - Kalkan, Sevda

AU - Kalkan S

AD - Swiss Tropical and Public Health Institute, Allschwil, Switzerland.

AD - University of Basel, Basel, Switzerland.

FAU - Gagneux, Sebastien

AU - Gagneux S

AUID- ORCID: 0000-0001-7783-9048

AD - Swiss Tropical and Public Health Institute, Allschwil, Switzerland.

AD - University of Basel, Basel, Switzerland.

FAU - Borrell, Sonia

AU - Borrell S

AUID- ORCID: 0000-0001-6661-6957

AD - Swiss Tropical and Public Health Institute, Allschwil, Switzerland.

AD - University of Basel, Basel, Switzerland.

LA - eng

GR - 883582-ECOEVODRTB/ERC_/European Research Council/International

GR - 320030-227432/SNSF_/Swiss National Science Foundation/Switzerland

GR - 320030L-231163/SNSF_/Swiss National Science Foundation/Switzerland

GR - CRSII5_213514/SNSF_/Swiss National Science Foundation/Switzerland

PT - Journal Article

DEP - 20251021

PL - United States

TA - Antimicrob Agents Chemother

JT - Antimicrobial agents and chemotherapy

JID - 0315061

RN - 0 (Antitubercular Agents)

RN - 0 (Diarylquinolines)

RN - 78846I289Y (bedaquiline)

RN - VJT6J7R4TR (Rifampin)

SB - IM

MH - *Mycobacterium tuberculosis/drug effects/genetics/classification

MH - *Antitubercular Agents/pharmacology

MH - Phylogeny

MH - Diarylquinolines/pharmacology

MH - Rifampin/pharmacology

MH - Microbial Sensitivity Tests

MH - Humans

MH - Drug Resistance, Multiple, Bacterial/genetics

MH - Tuberculosis, Multidrug-Resistant/microbiology/drug therapy

MH - Mutation

MH - Cell Wall/genetics/drug effects

PMC - PMC12691649

OTO - NOTNLM

OT - Mycobacterium tuberculosis

OT - bedaquiline

OT - drug tolerance

OT - genetic diversity

OT - rifampicin

COIS- The authors declare no conflict of interest.

EDAT- 2025/10/21 18:30

MHDA- 2025/12/10 13:52

PMCR- 2025/10/21

CRDT- 2025/10/21 13:13

PHST- 2025/12/10 13:52 [medline]

PHST- 2025/10/21 18:30 [pubmed]

PHST- 2025/10/21 13:13 [entrez]

PHST- 2025/10/21 00:00 [pmc-release]

AID - aac00996-25 [pii]

AID - aac.00996-25 [pii]

AID - 10.1128/aac.00996-25 [doi]

PST - ppublish

SO - Antimicrob Agents Chemother. 2025 Dec 10;69(12):e0099625. doi:

10.1128/aac.00996-25. Epub 2025 Oct 21.

PMID- 41364252

OWN - NLM

STAT- MEDLINE

DCOM- 20251209

LR - 20260206

IS - 1432-0991 (Electronic)

IS - 0343-8651 (Linking)

VI - 83

IP - 1

DP - 2025 Dec 9

TI - Bedaquiline Resistance: A Looming Global Threat in Tuberculosis Management.

PG - 57

LID - 10.1007/s00284-025-04655-7 [doi]

AB - The development of bedaquiline holds promise for treating multidrug-resistant

tuberculosis (MDR-TB). Still, the inflation in bedaquiline resistance has alarmed

the global public health crisis, indicating the need for a new treatment

strategy. Several scientific studies have reported a high incidence of resistance

among MDR-TB patients who had prior exposure to bedaquiline for treating

Mycobacterium tuberculosis infections. Disparate research findings indicate that

individuals previously administered this drug exhibit a remarkable prevalence of

resistance. Studies have further demonstrated that prior treatment often

correlates with the emergence of resistant Mycobacterium tuberculosis strains.

Overall, evidence from multiple investigations highlights a concerning trend of

increased resistance in MDR-TB cases with a history of bedaquiline therapy.

Moreover, bedaquiline resistance in MDR-TB strains has been linked to mutations

in several chromosomal genes, including atpE, Rv0677c, Rv0678, and pepQ.

Consequently, it is imperative to mitigate the burden of MDR-TB and bedaquiline

resistance. Herein, this article emphasizes structural features, mechanism of

action, emergence of underlying resistance mechanisms, pharmacokinetic &

pharmacodynamic properties, clinical toxicity, and strategies to combat

resistance associated with bedaquiline.

CI - © 2025. The Author(s), under exclusive licence to Springer Science+Business

Media, LLC, part of Springer Nature.

FAU - Singh, Indu

AU - Singh I

AD - School of Pharmacy, Graphic Era Hill University, Dehradun, 248002, India.

AD - Department of Biotechnology, Graphic Era (Deemed to be) University, Dehradun,

248002, India.

FAU - Sharma, Juhi

AU - Sharma J

AD - School of Life Science, Jaipur National University, Jaipur, India.

FAU - Sharma, Divakar

AU - Sharma D

AUID- ORCID: 0000-0002-8735-5562

AD - Department of Biotechnology, Graphic Era (Deemed to be) University, Dehradun,

248002, India. divakarsharma88@gmail.com.

FAU - Ratn, Arun

AU - Ratn A

AD - Department of Zoology, Sanatan Dharm College, Muzaffarnagar, 251001, India.

FAU - Bisht, Deepa

AU - Bisht D

AD - Department of Biochemistry, ICMR-National JALMA Institute for Leprosy and other

Mycobacterial Diseases, Tajganj, Agra, India.

FAU - Sharma, Sandeep

AU - Sharma S

AD - Department of Medical Laboratory Sciences, Lovely Professional University,

Phagwara, India.

FAU - Dhakad, Megh Singh

AU - Dhakad MS

AD - Department of Microbiology, Maulana Azad Medical College and Associated Hospital,

New Delhi, 110002, India.

FAU - Dalal, Jasbir

AU - Dalal J

AD - Department of Pharmacology, Faculty of Medicine and Health Sciences, SGT

University, Gurugram, Haryana, India.

FAU - Beg, Mirza Masroor Ali

AU - Beg MMA

AD - Faculty of Medicine, Alatoo International University, Bishkek, Kyrgyzstan.

LA - eng

PT - Journal Article

PT - Review

DEP - 20251209

PL - United States

TA - Curr Microbiol

JT - Current microbiology

JID - 7808448

RN - 78846I289Y (bedaquiline)

RN - 0 (Diarylquinolines)

RN - 0 (Antitubercular Agents)

SB - IM

MH - *Diarylquinolines/pharmacology/therapeutic use

MH - Humans

MH - *Antitubercular Agents/pharmacology/therapeutic use

MH - *Mycobacterium tuberculosis/drug effects/genetics

MH - *Tuberculosis, Multidrug-Resistant/drug therapy/microbiology/epidemiology

MH - Global Health

MH - *Drug Resistance, Multiple, Bacterial

COIS- Declarations. Conflict of interest: The authors declare that they have no

conflicts of interest. Ethical Approval: Not applicable. Consent to Participate:

Not applicable. Consent for Publication: Not applicable.

EDAT- 2025/12/09 13:09

MHDA- 2025/12/09 13:10

CRDT- 2025/12/09 11:14

PHST- 2025/01/23 00:00 [received]

PHST- 2025/11/28 00:00 [accepted]

PHST- 2025/12/09 13:10 [medline]

PHST- 2025/12/09 13:09 [pubmed]

PHST- 2025/12/09 11:14 [entrez]

AID - 10.1007/s00284-025-04655-7 [pii]

AID - 10.1007/s00284-025-04655-7 [doi]

PST - epublish

SO - Curr Microbiol. 2025 Dec 9;83(1):57. doi: 10.1007/s00284-025-04655-7.

PMID- 41353661

OWN - NLM

STAT- Publisher

LR - 20260226

IS - 1471-8847 (Electronic)

IS - 1471-8731 (Linking)

DP - 2025 Dec 7

TI - Contributions of Community Advisory Board and Experience of Study Participants in

TB/MDR-TB Clinical Trial Management in Addis Ababa, Ethiopia: A Qualitative

Study.

LID - 10.1111/dewb.70014 [doi]

AB - Community Advisory Boards (CABs), as a form of community engagement, provide an

important mechanism to ensure that research aligns with community needs and

priorities by representing broader community interests and guiding research

accordingly. Tuberculosis (TB) and multidrug-resistant tuberculosis (MDR-TB)

remain significant public health concerns, particularly due to resistance to key

first-line anti-TB medications, and individuals affected by these conditions

often face stigma and discrimination that hinder timely diagnosis and treatment

adherence. Addressing these challenges requires community-driven strategies and

improved access to health services, with CABs serving a central role in bridging

gaps between researchers, healthcare providers, and affected communities. This

study explored the contributions of CAB members in supporting TB/MDR-TB clinical

trials, the challenges experienced by trial participants, and the strategies

employed to address these challenges. Using an exploratory qualitative design,

in-depth interviews were conducted with 17 stakeholders involved in TB/MDR-TB

clinical trials, and data were analyzed using deductive thematic analysis. The

findings highlight persistent stigma and discrimination, limited understanding of

the role and functions of CABs, inconsistent budget allocation, reduced

participation, and diminished CAB influence throughout the research process. The

study underscores the essential role of CABs in enhancing community engagement in

TB/MDR-TB clinical trials and emphasizes the need for greater awareness,

advocacy, and institutional support to strengthen their contributions. Adequate

and sustained funding, along with systematic planning and implementation, is

critical for reinforcing CAB roles and promoting more ethical, inclusive, and

responsive public health research practices in Ethiopia and other low- and

middle-income countries.

CI - © 2025 John Wiley & Sons Ltd.

FAU - Seralegne, Yemisrach

AU - Seralegne Y

AUID- ORCID: 0000-0001-7092-1006

AD - Armauer Hansen Research Institute (AHRI), Addis Ababa, Ethiopia.

AD - University of Oxford, Oxford, UK.

AD - University of Oslo, Oslo, Norway.

FAU - G/Medihin, Abaraham

AU - G/Medihin A

AD - Armauer Hansen Research Institute (AHRI), Addis Ababa, Ethiopia.

FAU - Hundito, Ermiyas

AU - Hundito E

AD - Armauer Hansen Research Institute (AHRI), Addis Ababa, Ethiopia.

FAU - Adane, Hawult

AU - Adane H

AD - Armauer Hansen Research Institute (AHRI), Addis Ababa, Ethiopia.

FAU - Sintayehu, Bizunesh

AU - Sintayehu B

AD - Armauer Hansen Research Institute (AHRI), Addis Ababa, Ethiopia.

LA - eng

PT - Journal Article

DEP - 20251207

PL - England

TA - Dev World Bioeth

JT - Developing world bioethics

JID - 101120122

SB - IM

OTO - NOTNLM

OT - Community Advisory Board

OT - Community Engagement and TB/MDRTB

OT - clinical trial study

EDAT- 2025/12/07 20:36

MHDA- 2025/12/07 20:36

CRDT- 2025/12/07 10:43

PHST- 2025/10/30 00:00 [revised]

PHST- 2025/10/30 00:00 [received]

PHST- 2025/11/20 00:00 [accepted]

PHST- 2025/12/07 20:36 [pubmed]

PHST- 2025/12/07 20:36 [medline]

PHST- 2025/12/07 10:43 [entrez]

AID - 10.1111/dewb.70014 [doi]

PST - aheadofprint

SO - Dev World Bioeth. 2025 Dec 7. doi: 10.1111/dewb.70014.

PMID- 41338905

OWN - NLM

STAT- MEDLINE

DCOM- 20251203

LR - 20251203

IS - 1757-790X (Electronic)

IS - 1757-790X (Linking)

VI - 18

IP - 12

DP - 2025 Dec 3

TI - Levofloxacin-induced focal seizure in a patient with multidrug-resistant

tuberculosis and chronic kidney disease.

LID - e269834 [pii]

LID - 10.1136/bcr-2025-269834 [doi]

AB - Levofloxacin, a fluoroquinolone antibiotic, is essential in the treatment

protocols for multidrug-resistant tuberculosis (MDR-TB). Generally well

tolerated, it infrequently induces central nervous system adverse effects,

especially in patients with renal impairment. We present a woman in her early 50s

with chronic renal disease and hypothyroidism who experienced focal seizures

after 48 hours of commencing levofloxacin as part of her multidrug-resistant

tuberculosis treatment regimen. The seizures exhibited unilateral aberrant

movements with initially intact consciousness, thereafter followed by brief

episodes of altered awareness. Thorough investigations ruled out metabolic,

structural and other pharmacological reasons. Seizure activity fully resolved

following 3 days of discontinuing levofloxacin, with no recurrence documented

during follow-up. This case highlights the importance of recognising

levofloxacin-induced neurotoxicity in patients with chronic kidney disease. Dose

adjustment, close monitoring and early detection of neurological symptoms are

crucial to ensure safe MDR-TB management.

CI - © BMJ Publishing Group Limited 2025. No commercial re-use. See rights and

permissions. Published by BMJ Group.

FAU - Ramanuj, Priyanka

AU - Ramanuj P

AD - Department of Pharmacology, Pt. Jawahar Lal Nehru Memorial Medical College,

Raipur, Chhattisgarh, India.

FAU - Khasbage, Sameer Uttamrao

AU - Khasbage SU

AUID- ORCID: 0000-0003-3235-7197

AD - Department of Pharmacology, All India Institute Of Medical Sciences (AIIMS),

Raipur, Chhattisgarh, India samkhasbage@gmail.com.

LA - eng

PT - Case Reports

PT - Journal Article

DEP - 20251203

PL - England

TA - BMJ Case Rep

JT - BMJ case reports

JID - 101526291

RN - 6GNT3Y5LMF (Levofloxacin)

RN - 0 (Antitubercular Agents)

RN - 0 (Anti-Bacterial Agents)

SB - IM

MH - Humans

MH - *Levofloxacin/adverse effects

MH - Female

MH - *Tuberculosis, Multidrug-Resistant/drug therapy/complications

MH - *Seizures/chemically induced

MH - Middle Aged

MH - *Renal Insufficiency, Chronic/complications

MH - Antitubercular Agents/adverse effects/therapeutic use

MH - *Anti-Bacterial Agents/adverse effects

OTO - NOTNLM

OT - Chronic renal failure

OT - Respiratory medicine

OT - Tuberculosis

COIS- Competing interests: None declared.

EDAT- 2025/12/04 02:11

MHDA- 2025/12/04 02:12

CRDT- 2025/12/03 21:13

PHST- 2025/12/04 02:12 [medline]

PHST- 2025/12/04 02:11 [pubmed]

PHST- 2025/12/03 21:13 [entrez]

AID - 18/12/e269834 [pii]

AID - 10.1136/bcr-2025-269834 [doi]

PST - epublish

SO - BMJ Case Rep. 2025 Dec 3;18(12):e269834. doi: 10.1136/bcr-2025-269834.

PMID- 41235782

OWN - NLM

STAT- MEDLINE

DCOM- 20251114

LR - 20260306

IS - 1099-0801 (Electronic)

IS - 0269-3879 (Linking)

VI - 39

IP - 12

DP - 2025 Dec

TI - Quantitation of Plasma Bedaquiline Concentrations in Indian MDR-TB Patients Using

Liquid Chromatography-Tandem Mass Spectrometry.

PG - e70243

LID - 10.1002/bmc.70243 [doi]

AB - Bedaquiline (BDQ) is a diarylquinoline used for the treatment of

multidrug-resistant tuberculosis (MDR-TB) with a mechanism of action that

inhibits the mycobacterial adenosine triphosphate synthase. There is a limited

literature on the pharmacokinetics (PK) of BDQ in MDR-TB patients globally and

even less from India. We aimed to develop a rapid, selective, sensitive, and

robust high-throughput liquid chromatography-tandem mass spectrometry (LC-MS/MS)

method to evaluate the PK of BDQ from the plasma of people with MDR-TB. The

analyte was extracted from plasma by protein precipitation. The separation was

achieved on a reverse phase C18 column with a mixture of aqueous and organic

mobile phase in gradient mode at a flow rate of 0.3 mL/min and detected on a

triple quadrupole mass spectrometer. Linearity was obtained between 0.0313

to 4.0 mg/L, and the method was validated following bioanalytical method

validation guidelines. BDQ concentrations were determined from plasma samples,

and intensive PK testing was performed in samples from 23 patients. The median

Cmax attained was 1.59 mg/L with a Tmax around 6 h for most of our patients. This

method was successfully developed and validated for a pilot PK study in Indian

MDR-TB patients.

CI - © 2025 John Wiley & Sons Ltd.

FAU - Lokhande, Rohan V

AU - Lokhande RV

AUID- ORCID: 0000-0002-3737-698X

AD - Research Laboratories, P.D. Hinduja Hospital and Medical Research Centre, Mumbai,

India.

AD - Department of Chemistry, Satish Pradhan Dnyanasadhana College, Thane, India.

FAU - Arora, Prerna R

AU - Arora PR

AUID- ORCID: 0000-0002-7015-0376

AD - Research Laboratories, P.D. Hinduja Hospital and Medical Research Centre, Mumbai,

India.

FAU - Joubert, Anton

AU - Joubert A

AD - Division of Clinical Pharmacology, Department of Medicine, University of Cape

Town, Cape Town, South Africa.

FAU - Bhagure, Ganesh R

AU - Bhagure GR

AD - Department of Chemistry, Satish Pradhan Dnyanasadhana College, Thane, India.

FAU - Udwadia, Zarir F

AU - Udwadia ZF

AD - Research Laboratories, P.D. Hinduja Hospital and Medical Research Centre, Mumbai,

India.

FAU - Gupta, Amita

AU - Gupta A

AD - Center for Infectious Diseases in India, Division of Infectious Diseases, Johns

Hopkins University School of Medicine, Baltimore, Maryland, USA.

AD - Center for Tuberculosis Research, Division of Infectious Diseases, Johns Hopkins

University School of Medicine, Baltimore, Maryland, USA.

AD - Bloomberg School of Public Health, Johns Hopkins University School of Medicine,

Baltimore, Maryland, USA.

FAU - Rodrigues, Camilla

AU - Rodrigues C

AD - Research Laboratories, P.D. Hinduja Hospital and Medical Research Centre, Mumbai,

India.

FAU - Tornheim, Jeffrey A

AU - Tornheim JA

AD - Center for Infectious Diseases in India, Division of Infectious Diseases, Johns

Hopkins University School of Medicine, Baltimore, Maryland, USA.

AD - Center for Tuberculosis Research, Division of Infectious Diseases, Johns Hopkins

University School of Medicine, Baltimore, Maryland, USA.

AD - Bloomberg School of Public Health, Johns Hopkins University School of Medicine,

Baltimore, Maryland, USA.

FAU - Wiesner, Lubbe

AU - Wiesner L

AD - Division of Clinical Pharmacology, Department of Medicine, University of Cape

Town, Cape Town, South Africa.

FAU - Ashavaid, Tester F

AU - Ashavaid TF

AUID- ORCID: 0000-0002-2740-0819

AD - Research Laboratories, P.D. Hinduja Hospital and Medical Research Centre, Mumbai,

India.

CN - MDR‐TB MUKT and RePORT India Study Team

CN - Indo‐South Africa Study Team

LA - eng

GR - DAA9-19-65351-1/RePORT International Coordinating Center/

GR - R01AI16837101/NH/NIH HHS/United States

GR - K23AI135102/NH/NIH HHS/United States

GR - P30AI16843602/NH/NIH HHS/United States

GR - R01AI134430/NH/NIH HHS/United States

GR - DST/INT/SOUTH AFRICA/P-24/2017/Department of Science and Technology, Ministry of

Science and Technology, India/

GR - R01 AI168371/AI/NIAID NIH HHS/United States

GR - R01AI17561801/NH/NIH HHS/United States

GR - BT/PR24492/MED/29/1219/2017/Department of Biotechnology, Ministry of Science and

Technology, India/

GR - National Health and Education Society/

PT - Journal Article

PL - England

TA - Biomed Chromatogr

JT - Biomedical chromatography : BMC

JID - 8610241

RN - 78846I289Y (bedaquiline)

RN - 0 (Diarylquinolines)

RN - 0 (Antitubercular Agents)

SB - IM

MH - Humans

MH - *Tandem Mass Spectrometry/methods

MH - *Diarylquinolines/blood/pharmacokinetics/therapeutic use/chemistry

MH - *Tuberculosis, Multidrug-Resistant/drug therapy/blood

MH - India

MH - Reproducibility of Results

MH - *Antitubercular Agents/blood/pharmacokinetics/therapeutic use

MH - Male

MH - Linear Models

MH - Chromatography, Liquid/methods

MH - Adult

MH - Female

MH - Limit of Detection

MH - Middle Aged

MH - Sensitivity and Specificity

OTO - NOTNLM

OT - bedaquiline

OT - liquid chromatography–tandem mass spectrometry

OT - multidrug‐resistant tuberculosis

OT - pharmacokinetics

OT - plasma

EDAT- 2025/11/14 12:32

MHDA- 2025/11/14 12:33

CRDT- 2025/11/14 07:52

PHST- 2025/10/04 00:00 [revised]

PHST- 2025/07/03 00:00 [received]

PHST- 2025/10/07 00:00 [accepted]

PHST- 2025/11/14 12:33 [medline]

PHST- 2025/11/14 12:32 [pubmed]

PHST- 2025/11/14 07:52 [entrez]

AID - 10.1002/bmc.70243 [doi]

PST - ppublish

SO - Biomed Chromatogr. 2025 Dec;39(12):e70243. doi: 10.1002/bmc.70243.

PMID- 40259579

OWN - NLM

STAT- MEDLINE

DCOM- 20250422

LR - 20250425

IS - 1365-2060 (Electronic)

IS - 0785-3890 (Print)

IS - 0785-3890 (Linking)

VI - 57

IP - 1

DP - 2025 Dec

TI - Care-seeking and treatment pathways of multidrug-resistant tuberculosis patients:

an analysis of real-world data from regional health information system in Ningbo

City in Eastern China.

PG - 2496405

LID - 10.1080/07853890.2025.2496405 [doi]

LID - 2496405

AB - OBJECTIVES: The patient pathway of multidrug-resistant tuberculosis (MDR-TB) in

China is an essential but not well-studied area. This study aimed to understand

the alignment between patient-initiated care-seeking demands for MDR-TB and the

availability of diagnostic and treatment services in Ningbo, a city in eastern

China, using patient pathway analysis (PPA). METHODS: We collected the diagnostic

and treatment data of 240 patients with MDR-TB in Ningbo from 2015 to 2019. Using

patient pathway analysis, we matched the medical data of patients from different

medical institutions and mapped their care pathways to illustrate their access to

medical services. RESULT: Our study indicated that the proportion of patients

with MDR-TB who chose non-TB-designated medical institutions (55%) was higher

than those who chose TB-designated medical institutions (45%) at their initial

visit. An estimated 69% of patients with MDR-TB patients received initial TB

screening services during their first visit. In this study, 47% of patients

needed to visit 4-7 medical institutions to be diagnosed with MDR-TB. Overall,

80% (n = 192) of patients were diagnosed with MDR-TB within four visits, while

13% (n = 30), 4% (n = 10), and 3% (n = 8) of patients were not diagnosed at the

fourth visit and remained at level 2, 1, and 0 medical institutions,

respectively. CONCLUSION: The care-seeking pathway of patients with MDR-TB in

Ningbo is complex. This indicates room for improvement in local diagnosis and

referral services. There is a need to promote the deployment of MDR-TB screening,

diagnosis, and treatment services at lower-level institutions.

FAU - Chen, Bin

AU - Chen B

AD - Department of Tuberculosis Control and Prevention, Zhejiang Provincial Center for

Disease Control and Prevention, Hangzhou, People's Republic of China.

FAU - Li, Yang

AU - Li Y

AD - School of Public Health, Hangzhou Normal University, Hangzhou, People's Republic

of China.

FAU - Peng, Ying

AU - Peng Y

AD - Department of Tuberculosis Control and Prevention, Zhejiang Provincial Center for

Disease Control and Prevention, Hangzhou, People's Republic of China.

FAU - Zhou, Lin

AU - Zhou L

AD - Department of Tuberculosis Control and Prevention, Zhejiang Provincial Center for

Disease Control and Prevention, Hangzhou, People's Republic of China.

FAU - Chen, Xin-Yi

AU - Chen XY

AD - Department of Tuberculosis Control and Prevention, Zhejiang Provincial Center for

Disease Control and Prevention, Hangzhou, People's Republic of China.

FAU - Ren, Yan-Li

AU - Ren YL

AD - Songzi Center for Disease Control and Prevention, Jingzhou, People's Republic of

China.

FAU - Luo, Dan

AU - Luo D

AD - School of Public Health, Hangzhou Medical College, Hangzhou, People's Republic of

China.

FAU - Ling, Yu-Xiao

AU - Ling YX

AD - School of Public Health, Health Science Center, Ningbo University, Ningbo,

People's Republic of China.

FAU - Zhou, Yi-Qing

AU - Zhou YQ

AD - School of Public Health, Hangzhou Medical College, Hangzhou, People's Republic of

China.

FAU - Jiang, Jian-Min

AU - Jiang JM

AD - Department of Tuberculosis Control and Prevention, Zhejiang Provincial Center for

Disease Control and Prevention, Hangzhou, People's Republic of China.

AD - Key Laboratory of Vaccine, Prevention and Control of Infectious Disease of

Zhejiang Province, Zhejiang Provincial Center for Disease Control and Prevention,

Hangzhou, People's Republic of China.

FAU - Xu, Biao

AU - Xu B

AD - Department of Epidemiology, School of Public Health, Fudan University, Shanghai,

People's Republic of China.

AD - Key Laboratory of Health Technology Assessment, National Health Commission of the

People's Republic of China (Fudan University), Shanghai, People's Republic of

China.

LA - eng

PT - Journal Article

DEP - 20250421

PL - England

TA - Ann Med

JT - Annals of medicine

JID - 8906388

RN - 0 (Antitubercular Agents)

SB - IM

MH - Humans

MH - *Tuberculosis, Multidrug-Resistant/diagnosis/drug therapy/epidemiology/therapy

MH - China/epidemiology

MH - Male

MH - Female

MH - *Patient Acceptance of Health Care/statistics & numerical data

MH - Adult

MH - Middle Aged

MH - Antitubercular Agents/therapeutic use

MH - Health Information Systems/statistics & numerical data

MH - Health Services Accessibility/statistics & numerical data

MH - Aged

MH - Young Adult

PMC - PMC12016273

OTO - NOTNLM

OT - Multidrug-resistant tuberculosis

OT - care seeking

OT - ningbo

OT - patient pathway analysis

OT - real-world

COIS- No potential conflict of interest was reported by the author(s).

EDAT- 2025/04/22 06:26

MHDA- 2025/04/22 06:27

PMCR- 2025/04/21

CRDT- 2025/04/22 01:33

PHST- 2025/04/22 06:27 [medline]

PHST- 2025/04/22 06:26 [pubmed]

PHST- 2025/04/22 01:33 [entrez]

PHST- 2025/04/21 00:00 [pmc-release]

AID - 2496405 [pii]

AID - 10.1080/07853890.2025.2496405 [doi]

PST - ppublish

SO - Ann Med. 2025 Dec;57(1):2496405. doi: 10.1080/07853890.2025.2496405. Epub 2025

Apr 21.

PMID- 40085403

OWN - NLM

STAT- MEDLINE

DCOM- 20251121

LR - 20251121

IS - 1573-501X (Electronic)

IS - 1381-1991 (Linking)

VI - 29

IP - 6

DP - 2025 Dec

TI - New pyrazole-based derivatives targeting MmpL3 transporter in Mycobacterium

tuberculosis: design, synthesis, biological evaluation and molecular docking

studies.

PG - 6437-6458

LID - 10.1007/s11030-025-11152-3 [doi]

AB - This study addresses the urgent need for new drugs to combat multi-drug-resistant

tuberculosis (MDR-TB). Focusing on MmpL3, a protein essential for mycobacterial

cell wall synthesis, we designed and synthesised 50 new pyrazole-based amide

derivatives. These compounds were then tested for their ability to inhibit the

growth of various Mycobacterium tuberculosis (Mtb) strains, including both

drug-susceptible and drug-resistant strains (resistant to isoniazid, rifampicin,

or both). Two compounds, 15 and 35, emerged as potent inhibitors. They showed

strong activity against both drug-susceptible and drug-resistant Mtb strains,

with low minimum inhibitory concentration (MIC) values of 2 µg/mL and 2-4 µg/mL,

respectively. Importantly, these compounds also demonstrated a high selectivity

index, meaning they were significantly more toxic to Mtb cells than to human

liver cells (HepG2). Compound 15 further proved to be bactericidal, effectively

killing Mtb within six days. Interestingly, compounds 15 and 35 were inactive

against lab-generated Mtb strains resistant to SQ109, a known MmpL3 inhibitor.

This finding, supported by molecular docking, molecular dynamics simulations, and

genetic analysis of the mmpl3 gene in the SQ109-resistant strains, strongly

suggests that these novel compounds also target MmpL3. This research highlights

the potential of pyrazole-based amides as a promising new class of anti-TB drugs.

By targeting MmpL3, these compounds offer a novel mechanism of action to combat

drug-resistant TB, potentially leading to improved treatment outcomes.

CI - © 2025. The Author(s), under exclusive licence to Springer Nature Switzerland AG.

FAU - Maddipatla, Sarvan

AU - Maddipatla S

AD - Department of Chemical Sciences, National Institute of Pharmaceutical Education

and Research (NIPER), Balanagar, Hyderabad, Telangana, 500037, India.

FAU - Agnivesh, Puja Kumari

AU - Agnivesh PK

AD - Department of Biological Sciences, National Institute of Pharmaceutical Education

and Research (NIPER), Balanagar, Hyderabad, Telangana, 500037, India.

FAU - Bakchi, Bulti

AU - Bakchi B

AD - Department of Chemical Sciences, National Institute of Pharmaceutical Education

and Research (NIPER), Balanagar, Hyderabad, Telangana, 500037, India.

FAU - Nanduri, Srinivas

AU - Nanduri S

AD - Department of Chemical Sciences, National Institute of Pharmaceutical Education

and Research (NIPER), Balanagar, Hyderabad, Telangana, 500037, India.

FAU - Kalia, Nitin Pal

AU - Kalia NP

AD - Department of Biological Sciences, National Institute of Pharmaceutical Education

and Research (NIPER), Balanagar, Hyderabad, Telangana, 500037, India.

nitin.kalia@niperhyd.ac.in.

FAU - Yaddanapudi, Venkata Madhavi

AU - Yaddanapudi VM

AD - Department of Chemical Sciences, National Institute of Pharmaceutical Education

and Research (NIPER), Balanagar, Hyderabad, Telangana, 500037, India.

yssmadhavi@gmail.com.

LA - eng

PT - Journal Article

DEP - 20250314

PL - Netherlands

TA - Mol Divers

JT - Molecular diversity

JID - 9516534

RN - 0 (Pyrazoles)

RN - 0 (Antitubercular Agents)

RN - 0 (Bacterial Proteins)

RN - 0 (MmpL3 protein, Mycobacterium tuberculosis)

RN - 0 (Membrane Transport Proteins)

RN - 3QD5KJZ7ZJ (pyrazole)

SB - IM

MH - *Mycobacterium tuberculosis/drug effects/metabolism

MH - *Pyrazoles/chemistry/pharmacology/chemical synthesis

MH - *Molecular Docking Simulation

MH - *Antitubercular Agents/pharmacology/chemistry/chemical synthesis

MH - *Bacterial Proteins/antagonists & inhibitors/metabolism/chemistry

MH - *Drug Design

MH - Humans

MH - Microbial Sensitivity Tests

MH - *Membrane Transport Proteins/metabolism/chemistry

MH - Structure-Activity Relationship

OTO - NOTNLM

OT - Antitubercular

OT - Drug resistance

OT - MmpL3

OT - Molecular dynamics

OT - Pyrazole

COIS- Declarations. Conflict of interest: The authors declare no competing interests.

EDAT- 2025/03/14 18:28

MHDA- 2025/11/21 18:25

CRDT- 2025/03/14 12:23

PHST- 2024/09/19 00:00 [received]

PHST- 2025/02/27 00:00 [accepted]

PHST- 2025/11/21 18:25 [medline]

PHST- 2025/03/14 18:28 [pubmed]

PHST- 2025/03/14 12:23 [entrez]

AID - 10.1007/s11030-025-11152-3 [pii]

AID - 10.1007/s11030-025-11152-3 [doi]

PST - ppublish

SO - Mol Divers. 2025 Dec;29(6):6437-6458. doi: 10.1007/s11030-025-11152-3. Epub 2025

Mar 14.

PMID- 40021588

OWN - NLM

STAT- MEDLINE

DCOM- 20251121

LR - 20251121

IS - 1573-501X (Electronic)

IS - 1381-1991 (Linking)

VI - 29

IP - 6

DP - 2025 Dec

TI - Synthesis, DFT study, in silico ADMET evaluation, molecular docking, and QSAR

analysis of new anti-tuberculosis drugs derived from 2-hydroxybenzohydrazide

derivatives.

PG - 6343-6362

LID - 10.1007/s11030-025-11130-9 [doi]

AB - This study investigates the potential of novel thiazole and hydroxybenzohydrazide

derivatives as antitubercular agents. Using molecular docking and density

functional theory (DFT) calculations, the binding affinities of these derivatives

to the enoyl-acyl carrier protein reductase (InhA) enzyme of M. tb were assessed.

InhA is crucial for the mycobacterial fatty acid synthase II (FAS-II) pathway,

making it a prime target for drug development. QSAR analysis was employed to

relate molecular descriptors to biological activity, and ADMET descriptors

evaluated the pharmacokinetics and toxicity of the compounds. Experimental

synthesis of the compounds and their characterization via IR and NMR spectroscopy

confirmed their structures. DFT calculations revealed multiple conformers for

each compound, with specific isomers showing enhanced stability and favorable

binding interactions with InhA. These findings suggest that the synthesized

derivatives have potential as new antitubercular agents, offering a basis for

future drug development strategies against multidrug-resistant TB.

CI - © 2025. The Author(s), under exclusive licence to Springer Nature Switzerland AG.

FAU - Hassanien, Alaa E

AU - Hassanien AE

AD - Basic Science Department, Future Higher Institute of Engineering and Technology

in Mansoura, Mansoura, Egypt. 3alaa.hassanien@gmail.com.

AD - Department of Chemistry, Faculty of Science, Mansoura University, El-Gomhoria

Street, Mansoura, ET- 35516, Egypt. 3alaa.hassanien@gmail.com.

FAU - Elsherbiny, Ghada

AU - Elsherbiny G

AD - Botany Department, Faculty of Science, Mansoura University, Mansoura, Egypt.

FAU - Abdelfattah, Gamal M

AU - Abdelfattah GM

AD - Botany Department, Faculty of Science, Mansoura University, Mansoura, Egypt.

FAU - Abdel-Aziz, Marwa M

AU - Abdel-Aziz MM

AD - Medical Microbiology Department, Regional Center for Mycology and Biotechnology,

Al-Azhar University, Cairo, Egypt.

FAU - El-Hagrassey, Eman A

AU - El-Hagrassey EA

AD - Botany Department, Faculty of Science, Mansoura University, Mansoura, Egypt.

LA - eng

PT - Journal Article

DEP - 20250301

PL - Netherlands

TA - Mol Divers

JT - Molecular diversity

JID - 9516534

RN - 0 (Antitubercular Agents)

RN - 0 (Hydrazines)

RN - 0 (Bacterial Proteins)

RN - EC 1.3.1.9 (InhA protein, Mycobacterium)

RN - EC 1.- (Oxidoreductases)

SB - IM

MH - *Antitubercular Agents/chemistry/chemical synthesis/pharmacology/pharmacokinetics

MH - *Quantitative Structure-Activity Relationship

MH - *Molecular Docking Simulation

MH - *Hydrazines/chemistry/pharmacology/chemical synthesis

MH - Mycobacterium tuberculosis/drug effects/enzymology

MH - Density Functional Theory

MH - Bacterial Proteins/metabolism/chemistry/antagonists & inhibitors

MH - Oxidoreductases/metabolism/chemistry

OTO - NOTNLM

OT - Antituberculotic

OT - Benzohydrazide

OT - DFT

OT - Molecular docking

OT - Thiazole

COIS- Declarations. Competing interests: The authors declare no competing interests.

EDAT- 2025/03/01 14:40

MHDA- 2025/11/21 12:50

CRDT- 2025/02/28 23:17

PHST- 2024/11/10 00:00 [received]

PHST- 2025/02/07 00:00 [accepted]

PHST- 2025/11/21 12:50 [medline]

PHST- 2025/03/01 14:40 [pubmed]

PHST- 2025/02/28 23:17 [entrez]

AID - 10.1007/s11030-025-11130-9 [pii]

AID - 10.1007/s11030-025-11130-9 [doi]

PST - ppublish

SO - Mol Divers. 2025 Dec;29(6):6343-6362. doi: 10.1007/s11030-025-11130-9. Epub 2025

Mar 1.

PMID- 41328075

OWN - NLM

STAT- PubMed-not-MEDLINE

DCOM- 20251202

LR - 20251204

IS - 2633-4690 (Electronic)

IS - 2633-4682 (Print)

IS - 2633-4690 (Linking)

VI - 2025

DP - 2025

TI - Novel Pharmacological Approaches for Multidrug-Resistant Tuberculosis: Review.

PG - 8849786

LID - 10.1155/adpp/8849786 [doi]

LID - 8849786

AB - BACKGROUND: Due to its resistance to common anti-TB drugs, multidrug-resistant

tuberculosis (MDR-TB) presents substantial treatment problems. Optimizing

therapeutic outcomes requires individualized treatment plans that take into

account patient comorbidities, medication susceptibility profiles, and past

treatment history. The significance of individualized medication in the treatment

of MDR-TB is emphasized in this study. METHODS OF REVIEW: Current research on

tailored treatment plans for MDR-TB is summarized in this review. It highlights

how pharmacogenomics, medication sensitivity testing, and patient-centered care

can be used to customize treatment plans. The utilization of combination

therapies, monitoring and adaptation techniques, and novel treatment options-such

as adjuvant therapy and newer agents-are also covered in the review. FINDINGS:

Important findings show that thorough medication susceptibility testing is

essential for directing wise treatment decisions. Dosage modifications based on

individual metabolic responses can be informed by pharmacogenomic data. Treatment

regimen adherence is improved when patients participate in decision-making.

Combination therapy involving new drugs has demonstrated potential for increasing

therapeutic effectiveness while reducing the emergence of resistance. Frequent

monitoring makes it possible to promptly modify therapy in response to the

patient response. CONCLUSIONS: Treatment for MDR-TB must be individualized and

comprehensive due to its complexity. For individuals with MDR-TB, therapy

outcomes can be greatly enhanced while lowering the risk of further resistance by

combining host-directed therapies, pharmacological breakthroughs, and continuous

patient monitoring. Enhancing customized care solutions in this difficult field

of infectious illness management requires ongoing research and innovation.

CI - Copyright © 2025 Kassahun Dires Ayenew et al. Advances in Pharmacological and

Pharmaceutical Sciences published by John Wiley & Sons Ltd.

FAU - Ayenew, Kassahun Dires

AU - Ayenew KD

AUID- ORCID: 0000-0001-7262-8663

AD - Department of Pharmacy, Asrat Woldeyes Health Science Campus, Debre Berhan

University, Debre Berhan, Ethiopia, dbu.edu.et.

FAU - Habteweld, Habtemariam Alekaw

AU - Habteweld HA

AD - Department of Pharmacy, Asrat Woldeyes Health Science Campus, Debre Berhan

University, Debre Berhan, Ethiopia, dbu.edu.et.

FAU - Tsige, Abate Wondesen

AU - Tsige AW

AD - Department of Pharmacy, Asrat Woldeyes Health Science Campus, Debre Berhan

University, Debre Berhan, Ethiopia, dbu.edu.et.

FAU - Bizu, Yihenew Sewale

AU - Bizu YS

AUID- ORCID: 0000-0003-3606-9803

AD - Department of Adult Health Nursing, Medicine and Health Science Campus, Debre

Markos University, Debre Markos, Ethiopia, dmu.edu.et.

LA - eng

PT - Journal Article

PT - Review

DEP - 20251129

PL - United States

TA - Adv Pharmacol Pharm Sci

JT - Advances in pharmacological and pharmaceutical sciences

JID - 101762941

PMC - PMC12664762

OTO - NOTNLM

OT - MDR-TB

OT - combination therapies

OT - novel approaches

COIS- The authors declare no conflicts of interest.

EDAT- 2025/12/02 07:36

MHDA- 2025/12/02 07:37

PMCR- 2025/11/29

CRDT- 2025/12/02 04:03

PHST- 2025/04/17 00:00 [received]

PHST- 2025/11/13 00:00 [revised]

PHST- 2025/11/15 00:00 [accepted]

PHST- 2025/12/02 07:37 [medline]

PHST- 2025/12/02 07:36 [pubmed]

PHST- 2025/12/02 04:03 [entrez]

PHST- 2025/11/29 00:00 [pmc-release]

AID - ADPP8849786 [pii]

AID - 10.1155/adpp/8849786 [doi]

PST - epublish

SO - Adv Pharmacol Pharm Sci. 2025 Nov 29;2025:8849786. doi: 10.1155/adpp/8849786.

eCollection 2025.

PMID- 41083112

OWN - NLM

STAT- MEDLINE

DCOM- 20251029

LR - 20260127

IS - 1879-0038 (Electronic)

IS - 0378-1119 (Linking)

VI - 972

DP - 2025 Nov 20

TI - Associations between host efflux pump gene polymorphisms (ABCB1, ABCC1, ABCG2)

and drug-resistant tuberculosis in a Chinese cohort.

PG - 149826

LID - S0378-1119(25)00615-8 [pii]

LID - 10.1016/j.gene.2025.149826 [doi]

AB - BACKGROUND: Drug-resistant tuberculosis (DR-TB) is associated with alterations in

drug pharmacokinetics (PK), aberrant expression of efflux pump transporters, and

genetic mutations. Polymorphisms in host genes encoding efflux pumps may affect

drug concentrations, potentially contributing to the emergence of drug

resistance. This study aims to investigate the association between single

nucleotide polymorphisms (SNPs) in host efflux pump transporter genes and the

development of drug resistance in tuberculosis. METHODS: This study enrolled 117

DR-TB patients, 193 patients with drug-sensitive tuberculosis (DS-TB) and 398

healthy control subjects (HCS). SNPs associated with drug resistance were

selected based a comprehensive literature review and publicly available

databases. A total of 12 SNPs within four ABC transporter genes were selected and

genotyped: ABCB1 (rs1128503, rs2032582, rs3842, rs1045642); ABCC1 (rs212091,

rs3743527); ABCC2 (rs717620, rs2273697, rs3740066, rs8187710); and ABCG2

(rs2231142, rs2231137). The relationship between SNPs and phenotype was evaluated

using logistic regression analysis, with adjustments for potential confounding

factors including sex, age and ethnicity. RESULTS: All 12 SNPs were in

Hardy-Weinberg genetic equilibrium (P > 0.5). Genetic analysis revealed

statistically significant associations between efflux pump gene polymorphisms and

distinct drug-resistant tuberculosis profiles. Specifically, the ABCC1 rs212091

CC genotype was significantly associated with an increased risk of both overall

drug-resistant TB (OR = 4.84, 95 % CI: 1.24-18.92) and multidrug-resistant TB (OR

= 8.45, 95 % CI: 1.50-47.65). In contrast, the ABCB1 rs3842 CC genotype

demonstrated a protective effect against MDR-TB (OR = 0.37, 95 % CI: 0.14-0.99),

whereas the ABCG2 rs2231142 TT genotype was linked to a decreased risk of

pre-extensively drug-resistant TB (OR = 0.41, 95 % CI: 0.17-0.97). CONCLUSION:

Our findings suggest that polymorphisms in host efflux pump genes may affect

susceptibility to different DR-TB phenotypes. The identified risk (ABCC1

rs212091) and protective variants (ABCB1 rs3842, ABCG2 rs2231142) reveal genetic

mechanisms underlying drug resistance. Further validation in larger, more diverse

populations is needed.

CI - Copyright © 2025 Elsevier B.V. All rights reserved.

FAU - Tang, Daoyan

AU - Tang D

AD - Department of Respiratory and Critical Care Medicine, West China Hospital of

Sichuan University, China.

FAU - La, Ba

AU - La B

AD - Department of Tuberculosis, Third People's Hospital of Tibet Autonomous Region,

Nongke Road, Chengguan District, Lhasa, Tibet Autonomous Region, China.

FAU - He, Jian-Qing

AU - He JQ

AD - Department of Respiratory and Critical Care Medicine, West China Hospital of

Sichuan University, China. Electronic address: jianqing_he@scu.edu.cn.

LA - eng

PT - Journal Article

DEP - 20251011

PL - Netherlands

TA - Gene

JT - Gene

JID - 7706761

RN - 0 (Antitubercular Agents)

RN - 0 (ATP Binding Cassette Transporter, Subfamily B)

RN - 0 (ATP Binding Cassette Transporter, Subfamily G, Member 2)

RN - 0 (Multidrug Resistance-Associated Protein 2)

RN - 0 (ATP-Binding Cassette, Sub-Family C Proteins)

RN - 0 (Neoplasm Proteins)

RN - 0 (ABCB1 protein, human)

RN - 0 (ABCC2 protein, human)

RN - 0 (ABCG2 protein, human)

RN - Y49M64GZ4Q (multidrug resistance-associated protein 1)

RN - Chinese people

SB - IM

MH - Adult

MH - Female

MH - Humans

MH - Male

MH - Middle Aged

MH - Antitubercular Agents/therapeutic use

MH - ATP Binding Cassette Transporter, Subfamily B/genetics

MH - *ATP Binding Cassette Transporter, Subfamily G, Member 2/genetics

MH - Case-Control Studies

MH - China

MH - Cohort Studies

MH - Genetic Predisposition to Disease

MH - Genotype

MH - Multidrug Resistance-Associated Protein 2

MH - *ATP-Binding Cassette, Sub-Family C Proteins/genetics

MH - *Neoplasm Proteins/genetics

MH - *Polymorphism, Single Nucleotide

MH - *Tuberculosis, Multidrug-Resistant/genetics

MH - East Asian People/genetics

OTO - NOTNLM

OT - Drug transporter

OT - Drug-resistant tuberculosis

OT - Efflux pump

OT - Pharmacokinetics

OT - Single nucleotide polymorphisms

COIS- Declaration of competing interest The authors declare that they have no known

competing financial interests or personal relationships that could have appeared

to influence the work reported in this paper.

EDAT- 2025/10/14 00:30

MHDA- 2025/10/30 00:27

CRDT- 2025/10/13 19:29

PHST- 2025/07/01 00:00 [received]

PHST- 2025/08/26 00:00 [revised]

PHST- 2025/10/09 00:00 [accepted]

PHST- 2025/10/30 00:27 [medline]

PHST- 2025/10/14 00:30 [pubmed]

PHST- 2025/10/13 19:29 [entrez]

AID - S0378-1119(25)00615-8 [pii]

AID - 10.1016/j.gene.2025.149826 [doi]

PST - ppublish

SO - Gene. 2025 Nov 20;972:149826. doi: 10.1016/j.gene.2025.149826. Epub 2025 Oct 11.

PMID- 41305415

OWN - NLM

STAT- MEDLINE

DCOM- 20251127

LR - 20260116

IS - 2076-0817 (Electronic)

IS - 2076-0817 (Linking)

VI - 14

IP - 11

DP - 2025 Nov 18

TI - Proposing Bromo-Epi-Androsterone for Host-Directed Therapy Against Tuberculosis.

LID - 10.3390/pathogens14111179 [doi]

LID - 1179

AB - Bromoepiandrosterone (BEA), a synthetic analog of the adrenal steroid DHEA, holds

promise as a host-directed therapy for both active and latent tuberculosis (TB).

Unlike DHEA, BEA lacks hormonal side effects yet retains potent immunomodulatory

activity. It promotes a Th1-skewed immune response by enhancing interferon-γ

(IFN-γ) and tumor necrosis factor-α (TNF-α), critical cytokines for macrophage

activation and intracellular control of Mycobacterium tuberculosis (Mtb), while

suppressing Th2 cytokines such as IL-4. BEA also inhibits 11β-hydroxysteroid

dehydrogenase-1, lowering intracellular cortisol levels and reversing the local

immunosuppression commonly seen in TB. These features enable BEA to restore

immune competency in TB-infected tissues. In murine TB models, BEA halted

bacterial growth, reduced pulmonary inflammation, and synergized with standard

anti-TB drugs to enhance bacterial clearance. Additionally, DHEA and its

analogues have demonstrated direct antimycobacterial activity, likely by

interfering with Mtb mycolic acid synthesis, a property BEA is believed to share.

For latent TB, BEA's ability to sustain Th1-mediated immunity and counteract

immune suppression could help maintain latency and prevent reactivation,

especially in immunocompromised individuals. By boosting immune surveillance and

potentially contributing to bacillary clearance, BEA offers a unique adjunctive

approach that complements existing TB treatments without contributing to drug

resistance. Its dual function, an immune modulator and antimicrobial agent,

supports its use across the TB disease spectrum. These properties position BEA as

a novel candidate for host-directed therapy aimed at improving outcomes in both

drug-sensitive and drug-resistant TB, as well as therapies aimed at enhancing

long-term containment of latent infection.

FAU - Dow, Coad Thomas

AU - Dow CT

AUID- ORCID: 0000-0003-4301-6101

AD - McPherson Eye Research Institute, University of Wisconsin-Madison, Madison, WI

53705, USA.

FAU - Obaid, Liam

AU - Obaid L

AD - Keck School of Medicine, University of Southern California, Los Angeles, CA

90089, USA.

LA - eng

PT - Journal Article

PT - Review

DEP - 20251118

PL - Switzerland

TA - Pathogens

JT - Pathogens (Basel, Switzerland)

JID - 101596317

RN - 0 (Antitubercular Agents)

RN - 459AG36T1B (Dehydroepiandrosterone)

SB - IM

MH - Animals

MH - Humans

MH - Mice

MH - *Antitubercular Agents/therapeutic use/pharmacology

MH - *Dehydroepiandrosterone/analogs & derivatives/therapeutic use/pharmacology

MH - *Mycobacterium tuberculosis/drug effects/immunology

MH - *Tuberculosis/drug therapy/immunology

PMC - PMC12655360

OTO - NOTNLM

OT - BEA

OT - DHEA

OT - MDR-TB

OT - Mtb

OT - Mycobacterium tuberculosis

OT - bromoepiandrosterone

OT - host-directed immuno-therapy

OT - immunosenescence

OT - murine model

COIS- C.T.D. serves as the chief medical officer of Protibea Therapeutics, Inc., the

company that holds patents on BEA.

EDAT- 2025/11/27 06:27

MHDA- 2025/11/27 06:28

PMCR- 2025/11/18

CRDT- 2025/11/27 01:22

PHST- 2025/10/08 00:00 [received]

PHST- 2025/11/02 00:00 [revised]

PHST- 2025/11/12 00:00 [accepted]

PHST- 2025/11/27 06:28 [medline]

PHST- 2025/11/27 06:27 [pubmed]

PHST- 2025/11/27 01:22 [entrez]

PHST- 2025/11/18 00:00 [pmc-release]

AID - pathogens14111179 [pii]

AID - pathogens-14-01179 [pii]

AID - 10.3390/pathogens14111179 [doi]

PST - epublish

SO - Pathogens. 2025 Nov 18;14(11):1179. doi: 10.3390/pathogens14111179.

PMID- 41334161

OWN - NLM

STAT- PubMed-not-MEDLINE

DCOM- 20251203

LR - 20251205

IS - 1664-302X (Print)

IS - 1664-302X (Electronic)

IS - 1664-302X (Linking)

VI - 16

DP - 2025

TI - The burden of tuberculosis and drug resistance in 22 Sub-Saharan African

countries, 1990-2021: a GBD 2021 analysis and progress towards WHO 2035 targets

with projections to 2050.

PG - 1695592

LID - 10.3389/fmicb.2025.1695592 [doi]

LID - 1695592

AB - BACKGROUND: Tuberculosis (TB) remains a major public health challenge in

Sub-Saharan Africa (SSA), compounded by rising multidrug-resistant (MDR-TB), and

extensively drug-resistant tuberculosis (XDR-TB) strains. This study aimed to

quantify the burden, temporal trends, and subregional heterogeneity of TB across

22 selected SSA countries; project future trends to 2050; and evaluate the

alignment of national TB policies with WHO End TB 2035 targets. METHODS: We

conducted a mixed-methods analysis using Global Burden of Disease (GBD) 2021

data. Age- and sex-specific TB incidence, prevalence, mortality, and

disability-adjusted life years (DALYs) were analyzed across 22 SSA countries from

1990 to 2021. BAPC model projected disease burden to 2050. National TB policy

alignment with WHO targets was assessed qualitatively. Drug-susceptible (DS-TB),

MDR-TB, and XDR-TB forms were evaluated alongside key attributable risk factors.

RESULTS: Between 1990 and 2021, absolute TB incidence in SSA increased by 25.6%

and prevalence by 44.2%, while mortality and DALYs declined by 14.0 and 24.8%,

respectively. Age-standardized rates declined significantly across all metrics;

incidence (-46.2%), prevalence (-35.2%), mortality (-56.4%), and DALYs (-60.9%).

Progress varied substantially by region: Western SSA showed the greatest

improvement, while Southern and Central SSA continued to face high burdens, with

rising mortality in some areas. MDR-TB incidence surged by 743.2%, with XDR-TB

also increasing markedly, particularly in Eastern and Central SSA. HIV

co-infection amplified MDR/XDR-TB mortality, with Southern SSA most affected.

Age- and sex-specific analyses revealed early-adulthood incidence peaks, male

predominance in mortality and DALYs, and the highest MDR-TB burden among older

adults. Leading risk factors for TB mortality included high alcohol use, elevated

fasting plasma glucose, tobacco use, and high body mass index. Projections

indicate SSA is unlikely to meet the WHO 2035 mortality reduction target, though

Ghana, Guinea, and Tanzania are projected to achieve the incidence target.

CONCLUSION: Despite overall declines in TB mortality, the growing

DR-TB/MDR-TB/XDR-TB epidemic, significant subregional disparities, and systemic

health system challenges threaten progress toward WHO End TB goals. Strengthening

diagnostics, expanding treatment access, integrating care services, and

addressing key metabolic and behavioral risk factors are essential to accelerate

TB control efforts and align SSA with the 2035 targets.

CI - Copyright © 2025 Li, Mensah, Liu, Pan, Lu, Zhou, Zhang, Cheng, Zhao, Wei and Zha.

FAU - Li, Shirong

AU - Li S

AD - Department of Respiratory and Critical Care Medicine, The Second Affiliated

Hospital of Anhui Medical University, Hefei, Anhui, China.

AD - Department of Pulmonary and Critical Care, Wuhu Hospital of East China Normal

University (The Second People's Hospital of Wuhu), Wuhu, Anhui, China.

FAU - Mensah, Emmanuel

AU - Mensah E

AD - Department of Pulmonary and Critical Care Medicine, The First Affiliated Hospital

of Wannan Medical College (Yijishan Hospital of Wannan Medical College), Wuhu,

Anhui, China.

AD - Takoradi Hospital, Takoradi, Ghana.

FAU - Liu, Min

AU - Liu M

AD - Graduate School of Bengbu Medical University, Bengbu Medical University, Bengbu,

Anhui, China.

FAU - Pan, Lingling

AU - Pan L

AD - Department of Cardiology, The First Affiliated Hospital of Wannan Medical College

(Yijishan Hospital of Wannan Medical College), Wuhu, Anhui, China.

FAU - Lu, Wei

AU - Lu W

AD - Department of Pulmonary and Critical Care Medicine, The First Affiliated Hospital

of Wannan Medical College (Yijishan Hospital of Wannan Medical College), Wuhu,

Anhui, China.

FAU - Zhou, Susheng

AU - Zhou S

AD - Department of Pulmonary and Critical Care Medicine, The First Affiliated Hospital

of Wannan Medical College (Yijishan Hospital of Wannan Medical College), Wuhu,

Anhui, China.

FAU - Zhang, Liqin

AU - Zhang L

AD - Department of Pulmonary and Critical Care Medicine, The First Affiliated Hospital

of Wannan Medical College (Yijishan Hospital of Wannan Medical College), Wuhu,

Anhui, China.

FAU - Cheng, Yusheng

AU - Cheng Y

AD - Department of Pulmonary and Critical Care Medicine, The First Affiliated Hospital

of Wannan Medical College (Yijishan Hospital of Wannan Medical College), Wuhu,

Anhui, China.

FAU - Zhao, Hui

AU - Zhao H

AD - Department of Respiratory and Critical Care Medicine, The Second Affiliated

Hospital of Anhui Medical University, Hefei, Anhui, China.

FAU - Wei, Shuoshuo

AU - Wei S

AD - Department of Pulmonary and Critical Care Medicine, The First Affiliated Hospital

of Wannan Medical College (Yijishan Hospital of Wannan Medical College), Wuhu,

Anhui, China.

FAU - Zha, Lei

AU - Zha L

AD - Department of Pulmonary and Critical Care Medicine, The First Affiliated Hospital

of Wannan Medical College (Yijishan Hospital of Wannan Medical College), Wuhu,

Anhui, China.

LA - eng

PT - Journal Article

DEP - 20251117

PL - Switzerland

TA - Front Microbiol

JT - Frontiers in microbiology

JID - 101548977

PMC - PMC12665714

OTO - NOTNLM

OT - Sub-Saharan Africa

OT - WHO End TB targets

OT - antibiotic resistance

OT - drug-resistant tuberculosis

OT - epidemiology

OT - extensively drug-resistant tuberculosis

OT - multidrug-resistant tuberculosis

OT - tuberculosis

COIS- The authors declare that the research was conducted in the absence of any

commercial or financial relationships that could be construed as a potential

conflict of interest.

EDAT- 2025/12/03 07:41

MHDA- 2025/12/03 07:42

PMCR- 2025/11/17

CRDT- 2025/12/03 05:47

PHST- 2025/09/01 00:00 [received]

PHST- 2025/10/23 00:00 [accepted]

PHST- 2025/12/03 07:42 [medline]

PHST- 2025/12/03 07:41 [pubmed]

PHST- 2025/12/03 05:47 [entrez]

PHST- 2025/11/17 00:00 [pmc-release]

AID - 10.3389/fmicb.2025.1695592 [doi]

PST - epublish

SO - Front Microbiol. 2025 Nov 17;16:1695592. doi: 10.3389/fmicb.2025.1695592.

eCollection 2025.

PMID- 41134923

OWN - NLM

STAT- MEDLINE

DCOM- 20251114

LR - 20251114

IS - 2373-8227 (Electronic)

IS - 2373-8227 (Linking)

VI - 11

IP - 11

DP - 2025 Nov 14

TI - Deciphering the Role of pafBC in Mycobacteriophage Resistance and Biofilm

Formation.

PG - 3251-3262

LID - 10.1021/acsinfecdis.5c00627 [doi]

AB - Tuberculosis (TB) remains the world's deadliest bacterial infection, with 8.2

million newly notified cases and an estimated 1.25 million deaths in 2023.

Alarmingly, ∼19% of multidrug- or rifampicin-resistant (MDR/RR) strains already

meet the World Health Organization (WHO) definition of pre-XDR-TB because they

are resistant to at least one fluoroquinolone (FQ). Although gyrA/gyrB

target-site mutations dominate clinical FQ resistance, Mycobacteria also rely on

transcriptional networks that help them withstand the oxidative and DNA

strand-breaking stress caused by these drugs. Central to this response is the

heterodimeric transcription factor pafBC, whose WYL domain binds to

single-stranded DNA and redirects RNA polymerase to a dedicated promoter set,

thereby orchestrating a LexA-independent DNA-damage response (DDR). Up-regulation

of pafBC has been linked to enhanced intracellular survival of M. tuberculosis

and nontuberculous mycobacteria after FQ exposure, yet the downstream phenotypes

and their connection to drug or phage resistance have remained unclear. Here, we

demonstrate that deletion of pafBC in Mycobacterium smegmatis profoundly remodels

the cell envelope, as evidenced by altered colony rugosity, reduced sliding

motility, enhanced aggregation, and a three- to 5-fold decline in quantitative

biofilm biomass. Untargeted lipid profiling revealed the selective depletion of

long-chain trehalose polyphosphates and other apolar glycolipids that normally

decorate the outer membrane─lipid classes that have recently been shown in other

studies to serve as essential receptors for therapeutic mycobacteriophages such

as BPs and Muddy. Consistent with this lipid deficit, the pafBC mutant exhibited

markedly reduced phage adsorption and plaque formation; ectopic expression of

RecA restored adsorption efficiency, implicating DDR envelope crosstalk in

antiphage defense. Complementation with wild-type pafBC rescued lipid

composition, biofilm mass, and phage resistance, whereas a WYL-domain mutant that

cannot bind single-stranded DNA failed to do so, underscoring the necessity of

canonical pafBC activation for envelope homeostasis. Immunoprofiling in THP-1

macrophages further showed that pafBC-proficient bacilli induce significantly

higher secretion of IL-1β, TNF-α, and IL-6 compared to their isogenic mutant.

This effect correlated with the presence of intact surface glycolipids, molecules

known to interact with scavenger and Toll-like receptors on phagocytes and to

enhance opsonizing antibody deposition at the host-pathogen interface. Overall,

our findings connect the molecular mechanisms of the pafBC DDR with observable

phenotypes such as fluoroquinolone tolerance, biofilm structure, phage

resistance, and host immune recognition, by highlighting cell-envelope remodeling

as the central factor.

FAU - Rafique, Hafiza Amina

AU - Rafique HA

AUID- ORCID: 0009-0002-5190-7609

AD - Institute of Modern Biopharmaceuticals, State Key Laboratory Breeding Base of

Eco-Environment and Bio-Resource of the Three Gorges Area, Key Laboratory of

Eco-environments in Three Gorges Reservoir Region, Ministry of Education, School

of Life Sciences, Southwest University, Beibei, Chongqing 400715, China.

FAU - Yu, Huang

AU - Yu H

AD - Institute of Modern Biopharmaceuticals, State Key Laboratory Breeding Base of

Eco-Environment and Bio-Resource of the Three Gorges Area, Key Laboratory of

Eco-environments in Three Gorges Reservoir Region, Ministry of Education, School

of Life Sciences, Southwest University, Beibei, Chongqing 400715, China.

FAU - Abudukadier, Abulimiti

AU - Abudukadier A

AD - Institute of Modern Biopharmaceuticals, State Key Laboratory Breeding Base of

Eco-Environment and Bio-Resource of the Three Gorges Area, Key Laboratory of

Eco-environments in Three Gorges Reservoir Region, Ministry of Education, School

of Life Sciences, Southwest University, Beibei, Chongqing 400715, China.

FAU - Suleiman, Ismail Mohamed

AU - Suleiman IM

AUID- ORCID: 0000-0003-3063-6627

AD - Department of Science and Laboratory Technology, Dar-es-Salaam Institute of

Technology, Bibititi and Morogoro Rd Junction, P.O. Box 2958, Dar-es-salaam 255,

Tanzania.

FAU - Zhang, Tianyu

AU - Zhang T

AD - Institute of Modern Biopharmaceuticals, State Key Laboratory Breeding Base of

Eco-Environment and Bio-Resource of the Three Gorges Area, Key Laboratory of

Eco-environments in Three Gorges Reservoir Region, Ministry of Education, School

of Life Sciences, Southwest University, Beibei, Chongqing 400715, China.

FAU - Le, Thi Thu Thuy

AU - Le TTT

AD - Institute of Modern Biopharmaceuticals, State Key Laboratory Breeding Base of

Eco-Environment and Bio-Resource of the Three Gorges Area, Key Laboratory of

Eco-environments in Three Gorges Reservoir Region, Ministry of Education, School

of Life Sciences, Southwest University, Beibei, Chongqing 400715, China.

FAU - Chen, Haiqi

AU - Chen H

AD - Institute of Modern Biopharmaceuticals, State Key Laboratory Breeding Base of

Eco-Environment and Bio-Resource of the Three Gorges Area, Key Laboratory of

Eco-environments in Three Gorges Reservoir Region, Ministry of Education, School

of Life Sciences, Southwest University, Beibei, Chongqing 400715, China.

FAU - Xie, Jianping

AU - Xie J

AD - Institute of Modern Biopharmaceuticals, State Key Laboratory Breeding Base of

Eco-Environment and Bio-Resource of the Three Gorges Area, Key Laboratory of

Eco-environments in Three Gorges Reservoir Region, Ministry of Education, School

of Life Sciences, Southwest University, Beibei, Chongqing 400715, China.

LA - eng

PT - Journal Article

DEP - 20251024

PL - United States

TA - ACS Infect Dis

JT - ACS infectious diseases

JID - 101654580

RN - 0 (Bacterial Proteins)

SB - IM

MH - *Biofilms/growth & development

MH - *Mycobacteriophages/physiology/genetics/drug effects

MH - *Bacterial Proteins/genetics/metabolism

MH - Humans

MH - *Mycobacterium smegmatis/virology/genetics/drug effects

MH - Mycobacterium tuberculosis/genetics/virology/drug effects

MH - Gene Expression Regulation, Bacterial

OTO - NOTNLM

OT - DNA-damage response

OT - biofilm architecture

OT - cell-envelope remodeling

OT - fluoroquinolone resistance

OT - mycobacteriophage adsorption

OT - pafBC

EDAT- 2025/10/24 18:27

MHDA- 2025/11/14 06:28

CRDT- 2025/10/24 14:13

PHST- 2025/11/14 06:28 [medline]

PHST- 2025/10/24 18:27 [pubmed]

PHST- 2025/10/24 14:13 [entrez]

AID - 10.1021/acsinfecdis.5c00627 [doi]

PST - ppublish

SO - ACS Infect Dis. 2025 Nov 14;11(11):3251-3262. doi: 10.1021/acsinfecdis.5c00627.

Epub 2025 Oct 24.

PMID- 41362315

OWN - NLM

STAT- PubMed-not-MEDLINE

DCOM- 20251209

LR - 20251211

IS - 2666-5174 (Electronic)

IS - 2666-5174 (Linking)

VI - 9

DP - 2025

TI - Microevolutionary models and multi-omics analysis uncover the cross-drug

resistance characteristics of capreomycin-selected Mycobacterium tuberculosis.

PG - 100507

LID - 10.1016/j.crmicr.2025.100507 [doi]

LID - 100507

AB - The drug-resistant tuberculosis (TB) is a major public health problem worldwide.

Capreomycin (Cm) has been used as an effective drug to treat multidrug-resistant

tuberculosis (MDR-TB) since 1960s. Although Cm and amikacin (Am) are historically

grouped as second-line injectable drugs (SLIDs) with shared resistance mechanisms

(e.g., rrs mutations), recent clinical and genetic data suggests divergent

evolutionary pathways. It remains unclear how Mtb evolves from sensitive to

resistant phenotypes under Cm pressure. In this study, we generate in vitro

drug-resistant Mtb models to systematically study the evolutionary mechanisms of

Cm and Am cross-resistance. By integrating transcriptome/proteome/metabolome

analyses, we identify both genetic mutations and non-genetic factors contributing

to Cm resistance. We show that Cm- and Am-selective pressures trigger independent

cross-resistance mechanisms revealed by distinct genetic mutations.

(Gly232Asp)tlyA and (Trp120fs)tlyA mutations are directly associated with low

level resistance to Cm, whereas (Ala48Val)mmaA2, (Gln19Arg)rpmA and variant at

the promoter of eis (which encodes an N-acetyltransferase; c.-14c>t) mediate

cross-resistance pathways specific to Cm selection. In addition, dysregulations

of non-genetic factors including metal ions transportations and lipids metabolism

also lead to drug-resistance. In summary, our findings systematically

characterize the molecular mechanisms of Cm resistance, demonstrating that the

"genetic factors" (DNA mutations) are often the drivers that induce the stable

"non-genetic factors" (dysregulated pathways, such as eis overexpression, metal

ion transport, and metabolic reprogramming), which ultimately mediate the

resistance phenotype. These insights uncover the evolutionary trajectory of Cm

and Am cross-resistance and provide potential strategies for optimizing Cm use to

treat MDR-TB. IMPORTANCE: Tuberculosis, caused by Mycobacterium tuberculosis, is

responsible for the highest mortality rate worldwide among single pathogen

infections. The diagnosis and treatment of drug-resistant tuberculosis pose a

global challenge. Capreomycin (Cm) is one of the drugs utilized in the management

of multidrug-resistant tuberculosis (MDR-TB) and extensively drug-resistant

tuberculosis (XDR-TB). Currently, the clinical detection of mutations in rrs gene

is employed to identify Cm resistance. However, the sensitivity and specificity

of this method are suboptimal. To further investigate the mechanisms underlying

Cm resistance, this study established a microevolution model in vitro, thereby

simulating the progression of MTB from susceptible strains to drug-resistant

strains. The genomes of each generation were sequenced throughout the

evolutionary process. In addition, the transcriptome-proteome-lipid metabolome

analysis of representative strains were performed. Our findings reveal that Cm

resistance is a combinatory effect of both genetic and non-genetic changes,

providing potential optimization strategies to the molecular drug sensitivity

testing used in clinical practice.

CI - © 2025 The Authors. Published by Elsevier B.V.

FAU - Zhao, Yuchuan

AU - Zhao Y

AD - Center for Tuberculosis Control of Guangdong Province, Guangzhou 510630, China.

FAU - Zhang, Chenchen

AU - Zhang C

AD - Center for Tuberculosis Control of Guangdong Province, Guangzhou 510630, China.

FAU - Wang, Xuezhi

AU - Wang X

AD - Foshan Fourth People's Hospital, Foshan 528000, China.

FAU - Feng, Huiying

AU - Feng H

AD - Center for Tuberculosis Control of Guangdong Province, Guangzhou 510630, China.

FAU - Yu, Meiling

AU - Yu M

AD - Center for Tuberculosis Control of Guangdong Province, Guangzhou 510630, China.

FAU - Chen, Xunxun

AU - Chen X

AD - Center for Tuberculosis Control of Guangdong Province, Guangzhou 510630, China.

FAU - Liang, Jingjing

AU - Liang J

AD - Dongguan Key Laboratory of tuberculosis Control, Dongguan Sixth People's

Hospital, Dongguan 523008, China.

FAU - Hong, Jianming

AU - Hong J

AD - Dongguan Key Laboratory of tuberculosis Control, Dongguan Sixth People's

Hospital, Dongguan 523008, China.

FAU - Huang, Shanshan

AU - Huang S

AD - Center for Tuberculosis Control of Guangdong Province, Guangzhou 510630, China.

FAU - Lin, Yuqi

AU - Lin Y

AD - Center for Tuberculosis Control of Guangdong Province, Guangzhou 510630, China.

FAU - Chen, Yuhui

AU - Chen Y

AD - Center for Tuberculosis Control of Guangdong Province, Guangzhou 510630, China.

FAU - Wei, Wenjing

AU - Wei W

AD - Center for Tuberculosis Control of Guangdong Province, Guangzhou 510630, China.

LA - eng

PT - Journal Article

DEP - 20251113

PL - Netherlands

TA - Curr Res Microb Sci

JT - Current research in microbial sciences

JID - 101773003

PMC - PMC12681867

OTO - NOTNLM

OT - Capreomycin

OT - Cross-resistance

OT - Microevolution

OT - Multi-omics

OT - Mycobacterium tuberculosis

COIS- The authors declare that they have no known competing financial interests or

personal relationships that could have appeared to influence the work reported in

this paper.

EDAT- 2025/12/09 07:38

MHDA- 2025/12/09 07:39

PMCR- 2025/11/13

CRDT- 2025/12/09 04:13

PHST- 2025/12/09 07:39 [medline]

PHST- 2025/12/09 07:38 [pubmed]

PHST- 2025/12/09 04:13 [entrez]

PHST- 2025/11/13 00:00 [pmc-release]

AID - S2666-5174(25)00167-1 [pii]

AID - 100507 [pii]

AID - 10.1016/j.crmicr.2025.100507 [doi]

PST - epublish

SO - Curr Res Microb Sci. 2025 Nov 13;9:100507. doi: 10.1016/j.crmicr.2025.100507.

eCollection 2025.

PMID- 41209293

OWN - NLM

STAT- Publisher

LR - 20260422

IS - 2632-8682 (Electronic)

IS - 2632-8682 (Linking)

VI - 16

IP - 12

DP - 2025 Nov 7

TI - Design and synthesis of isoxazole-functionalized benzene sulphonamides as novel

inhibitors of Mycobacterium tuberculosis β-carbonic anhydrases.

PG - 6368-79

LID - 10.1039/d5md00744e [doi]

AB - The escalating prevalence of multidrug-resistant tuberculosis (MDR-TB)

underscores the urgent need for new classes of antitubercular agents targeting

novel pathways. Carbonic anhydrase, a ubiquitous metalloenzyme, catalyses the

reversible hydration of carbon dioxide in the CO(2) + H(2)O ⇋ HCO(3) (-) + H(+)

reaction. Suppressing this enzymatic activity has recently been identified as a

new pathway for the treatment of Mycobacterium tuberculosis. To address this, a

series of isoxazole-sulphonamides was rationally designed, incorporating an

isoxazole pharmacophore as the aromatic tail, amide as a linker, and sulphonamide

as the zinc-binding group. These compounds were evaluated against Mycobacterium

tuberculosis carbonic anhydrases (MtCA 1 and 3) and two human carbonic anhydrases

(hCA I and II) to identify selective inhibitors of the bacterial enzymes. The

findings indicated that molecules containing an isoxazole pharmacophore with

amide-linked benzene-3-sulphonamide were significantly more selective for MtCA 3

than hCA I and II. Among these compounds, 12c, 12e, and 19b had the highest

inhibition against the MtCA 3 with K (i) values between 0.08-0.09 μM compared to

the standard acetazolamide with a K (i) value of 0.10 μM. Some of the best

compounds exhibited potent and selective inhibition of MtCA 3 over hCA I and II,

with the meta- and para-substituted derivatives demonstrating higher selectivity

and stronger inhibition. Specifically, compound 19b proved to be 199 and 38 times

more selective for MtCA 3 than hCA I and hCA II respectively, compared to the

standard drug acetazolamide, which is a non-selective CA inhibitor. The potential

of compound 19b as a promising antitubercular agent with a MIC value of 8 μg

mL(-1) against mc(2) 6230 was further strengthened by in silico ligand-target

interaction studies. Thus, compound 19b is emphasised as a promising lead in the

pursuit of new, selective agents targeting MtCA 3.

CI - This journal is © The Royal Society of Chemistry.

FAU - Bandela, Rani

AU - Bandela R

AD - Department of Chemical Sciences, National Institute of Pharmaceutical Education

and Research (NIPER) Balanagar Hyderabad 500037 Telangana India

yssmadhavi@gmail.com.

FAU - Singampalli, Anuradha

AU - Singampalli A

AD - Department of Chemical Sciences, National Institute of Pharmaceutical Education

and Research (NIPER) Balanagar Hyderabad 500037 Telangana India

yssmadhavi@gmail.com.

FAU - Maddipatla, Sarvan

AU - Maddipatla S

AUID- ORCID: 0000-0002-9828-1124

AD - Department of Chemical Sciences, National Institute of Pharmaceutical Education

and Research (NIPER) Balanagar Hyderabad 500037 Telangana India

yssmadhavi@gmail.com.

FAU - Kumar, Pardeep

AU - Kumar P

AD - Department of Chemical Sciences, National Institute of Pharmaceutical Education

and Research (NIPER) Balanagar Hyderabad 500037 Telangana India

yssmadhavi@gmail.com.

FAU - Bellapukonda, Sri Mounika

AU - Bellapukonda SM

AD - Department of Chemical Sciences, National Institute of Pharmaceutical Education

and Research (NIPER) Balanagar Hyderabad 500037 Telangana India

yssmadhavi@gmail.com.

FAU - Ramavath, Rajendhar

AU - Ramavath R

AD - Department of Chemical Sciences, National Institute of Pharmaceutical Education

and Research (NIPER) Balanagar Hyderabad 500037 Telangana India

yssmadhavi@gmail.com.

FAU - Mahajan, Lina S

AU - Mahajan LS

AD - Department of Chemical Sciences, National Institute of Pharmaceutical Education

and Research (NIPER) Balanagar Hyderabad 500037 Telangana India

yssmadhavi@gmail.com.

FAU - Nanduri, Srinivas

AU - Nanduri S

AUID- ORCID: 0000-0002-6671-2022

AD - Department of Chemical Sciences, National Institute of Pharmaceutical Education

and Research (NIPER) Balanagar Hyderabad 500037 Telangana India

yssmadhavi@gmail.com.

FAU - Vemula, Divya

AU - Vemula D

AD - Department of Biological Sciences, National Institute of Pharmaceutical Education

and Research (NIPER) Balanagar Hyderabad 500037 Telangana India.

FAU - Dalal, Aman

AU - Dalal A

AD - Department of Biological Sciences, National Institute of Pharmaceutical Education

and Research (NIPER) Balanagar Hyderabad 500037 Telangana India.

FAU - Kalia, Nitin Pal

AU - Kalia NP

AD - Department of Biological Sciences, National Institute of Pharmaceutical Education

and Research (NIPER) Balanagar Hyderabad 500037 Telangana India.

FAU - Bhandari, Vasundhra

AU - Bhandari V

AD - Department of Biological Sciences, National Institute of Pharmaceutical Education

and Research (NIPER) Balanagar Hyderabad 500037 Telangana India.

FAU - Gratteri, Paola

AU - Gratteri P

AUID- ORCID: 0000-0002-9137-2509

AD - Department NEUROFARBA - Pharmaceutical and Nutraceutical Section, Laboratory of

Molecular Modeling Cheminformatics & QSAR, University of Florence Via Ugo Schiff

6 50019 Sesto Fiorentino (Florence) Italy.

FAU - Paoletti, Niccolò

AU - Paoletti N

AD - Department of NEUROFARBA, Pharmaceutical and Nutraceutical Section, University of

Florence Sesto Fiorentino (Florence) 50019 Italy claudiu.supuran@unifi.it.

AD - Department NEUROFARBA - Pharmaceutical and Nutraceutical Section, Laboratory of

Molecular Modeling Cheminformatics & QSAR, University of Florence Via Ugo Schiff

6 50019 Sesto Fiorentino (Florence) Italy.

FAU - Bonardi, Alessandro

AU - Bonardi A

AD - Department of NEUROFARBA, Pharmaceutical and Nutraceutical Section, University of

Florence Sesto Fiorentino (Florence) 50019 Italy claudiu.supuran@unifi.it.

AD - Department NEUROFARBA - Pharmaceutical and Nutraceutical Section, Laboratory of

Molecular Modeling Cheminformatics & QSAR, University of Florence Via Ugo Schiff

6 50019 Sesto Fiorentino (Florence) Italy.

FAU - Supuran, Claudiu T

AU - Supuran CT

AUID- ORCID: 0000-0003-4262-0323

AD - Department of NEUROFARBA, Pharmaceutical and Nutraceutical Section, University of

Florence Sesto Fiorentino (Florence) 50019 Italy claudiu.supuran@unifi.it.

FAU - Yaddanapudi, Venkata Madhavi

AU - Yaddanapudi VM

AUID- ORCID: 0000-0002-9813-8694

AD - Department of Chemical Sciences, National Institute of Pharmaceutical Education

and Research (NIPER) Balanagar Hyderabad 500037 Telangana India

yssmadhavi@gmail.com.

LA - eng

PT - Journal Article

DEP - 20251107

PL - England

TA - RSC Med Chem

JT - RSC medicinal chemistry

JID - 101759460

PMC - PMC12593002

COIS- The authors declare no conflict of interest.

EDAT- 2025/11/10 06:29

MHDA- 2025/11/10 06:29

PMCR- 2026/11/07

CRDT- 2025/11/10 05:58

PHST- 2025/08/25 00:00 [received]

PHST- 2025/10/13 00:00 [accepted]

PHST- 2025/11/10 06:29 [medline]

PHST- 2025/11/10 06:29 [pubmed]

PHST- 2025/11/10 05:58 [entrez]

PHST- 2026/11/07 00:00 [pmc-release]

AID - d5md00744e [pii]

AID - 10.1039/d5md00744e [doi]

PST - aheadofprint

SO - RSC Med Chem. 2025 Nov 7;16(12):6368-79. doi: 10.1039/d5md00744e.

PMID- 40592183

OWN - NLM

STAT- MEDLINE

DCOM- 20250804

LR - 20250812

IS - 1768-3254 (Electronic)

IS - 0223-5234 (Linking)

VI - 297

DP - 2025 Nov 5

TI - Discovery of novel fluorescent amino-pyrazolines that detect and kill

Mycobacterium tuberculosis.

PG - 117889

LID - S0223-5234(25)00654-3 [pii]

LID - 10.1016/j.ejmech.2025.117889 [doi]

AB - The emergence of multidrug-resistant Mycobacterium tuberculosis (MDR-TB)

necessitates novel therapeutics with distinct mechanisms. Here, we report

amino-pyrazoline derivatives as a new class of dual-functional antimycobacterial

agents, integrating potent bactericidal activity with fluorescence-based

bacterial imaging. Initial screening identified AP-07 as a promising hit compound

(MIC(99): 40 μM against Mycobacterium smegmatis, 49 μM against Mycobacterium

bovis BCG). Structure-based optimization led to the discovery of AP-02 and AP-05

as lead compounds, with enhanced activity (MIC(99): 13-16 μM against M.

smegmatis; 20-25 μM against M. bovis BCG). Additionally, spontaneous resistance

assays detected no resistant colonies, suggesting a low risk of resistance

development. Mechanistic studies confirmed Ag85C as the primary molecular target,

disrupting late-stage mycolic acid biosynthesis and impairing cell wall

integrity. Notably, pyrazoline derivatives exhibit intrinsic fluorescence,

selectively labeling intracellular mycobacteria while remaining non-toxic to host

macrophages, enabling real-time bacterial imaging. This work establishes

fluorescent amino-pyrazolines as a promising foundation for next-generation

antitubercular agents, bridging diagnostics and therapy in tuberculosis drug

discovery.

CI - Copyright © 2025 The Authors. Published by Elsevier Masson SAS.. All rights

reserved.

FAU - Cui, Yixin

AU - Cui Y

AD - School of Chemistry, University of Birmingham, Edgbaston, Birmingham, West

Midlands, B15 2TT, UK.

FAU - Lanne, Alice

AU - Lanne A

AD - Institute of Microbiology and Infection, School of Biosciences, University of

Birmingham, Edgbaston, Birmingham, West Midlands, B15 2TT, UK.

FAU - Avula, Sreenivas

AU - Avula S

AD - Centre for Regenerative, Medicine and Health, Hong Kong Institute of Science

Innovation, Chinese Academy of Sciences, 5/F, 15W Science Park West Avenue, the

Hong Kong Special Administrative Region of China.

FAU - Hama Salih, Mariwan A

AU - Hama Salih MA

AD - School of Chemistry, University of Birmingham, Edgbaston, Birmingham, West

Midlands, B15 2TT, UK; College of Health and Medical Technology, Sulaimani

Polytechnic University, Sulaimani, Iraq.

FAU - Peng, Xudan

AU - Peng X

AD - Guangzhou National Laboratory, Guangzhou International Bio Island, Guangzhou

510005, China.

FAU - Milne, Gavin

AU - Milne G

AD - Sygnature Discovery, The Discovery Building, BioCity, Pennyfoot Street,

Nottingham, NG1 1GR, UK.

FAU - Jones, Geraint

AU - Jones G

AD - Sygnature Discovery, The Discovery Building, BioCity, Pennyfoot Street,

Nottingham, NG1 1GR, UK.

FAU - Ritchie, John

AU - Ritchie J

AD - Sygnature Discovery, The Discovery Building, BioCity, Pennyfoot Street,

Nottingham, NG1 1GR, UK.

FAU - Zhao, Yiming

AU - Zhao Y

AD - School of Chemistry, University of Birmingham, Edgbaston, Birmingham, West

Midlands, B15 2TT, UK; X-Ray Crystallography Facility, School of Chemistry,

University of Birmingham, Edgbaston, Birmingham, West Midlands, B15 2TT, UK.

FAU - Frampton, Jon

AU - Frampton J

AD - College of Medical and Dental Sciences, University of Birmingham, Edgbaston,

Birmingham, West Midlands, B15 2TT, UK.

FAU - Tortorella, Micky

AU - Tortorella M

AD - Centre for Regenerative, Medicine and Health, Hong Kong Institute of Science

Innovation, Chinese Academy of Sciences, 5/F, 15W Science Park West Avenue, the

Hong Kong Special Administrative Region of China.

FAU - Fossey, John S

AU - Fossey JS

AD - School of Chemistry, University of Birmingham, Edgbaston, Birmingham, West

Midlands, B15 2TT, UK.

FAU - Alderwick, Luke J

AU - Alderwick LJ

AD - Institute of Microbiology and Infection, School of Biosciences, University of

Birmingham, Edgbaston, Birmingham, West Midlands, B15 2TT, UK; Discovery

Sciences, Charles River Laboratories, Chesterford Research Park, CB10 1XL, UK.

Electronic address: luke.alderwick@crl.com.

FAU - Neagoie, Cleopatra

AU - Neagoie C

AD - Centre for Regenerative, Medicine and Health, Hong Kong Institute of Science

Innovation, Chinese Academy of Sciences, 5/F, 15W Science Park West Avenue, the

Hong Kong Special Administrative Region of China. Electronic address:

cleopatra.neagoie@crmh-cas.org.hk.

LA - eng

PT - Journal Article

DEP - 20250624

PL - France

TA - Eur J Med Chem

JT - European journal of medicinal chemistry

JID - 0420510

RN - 0 (Pyrazoles)

RN - 0 (Antitubercular Agents)

RN - 0 (Fluorescent Dyes)

SB - IM

MH - *Mycobacterium tuberculosis/drug effects

MH - *Pyrazoles/pharmacology/chemistry/chemical synthesis

MH - *Antitubercular Agents/pharmacology/chemistry/chemical synthesis

MH - Microbial Sensitivity Tests

MH - Structure-Activity Relationship

MH - *Fluorescent Dyes/chemistry/pharmacology/chemical synthesis

MH - Molecular Structure

MH - *Drug Discovery

MH - Humans

MH - Dose-Response Relationship, Drug

OTO - NOTNLM

OT - Pyrazoline

OT - TB detection

OT - Tuberculosis

COIS- Declaration of competing interest The authors declare that they have no known

competing financial interests or personal relationships that could have appeared

to influence the work reported in this paper.

EDAT- 2025/07/02 00:27

MHDA- 2025/08/05 00:29

CRDT- 2025/07/01 18:09

PHST- 2024/11/21 00:00 [received]

PHST- 2025/06/16 00:00 [revised]

PHST- 2025/06/17 00:00 [accepted]

PHST- 2025/08/05 00:29 [medline]

PHST- 2025/07/02 00:27 [pubmed]

PHST- 2025/07/01 18:09 [entrez]

AID - S0223-5234(25)00654-3 [pii]

AID - 10.1016/j.ejmech.2025.117889 [doi]

PST - ppublish

SO - Eur J Med Chem. 2025 Nov 5;297:117889. doi: 10.1016/j.ejmech.2025.117889. Epub

2025 Jun 24.

PMID- 41126479

OWN - NLM

STAT- MEDLINE

DCOM- 20251119

LR - 20260127

IS - 1938-3673 (Electronic)

IS - 0741-5400 (Linking)

VI - 117

IP - 11

DP - 2025 Nov 4

TI - Dectin-1 (CLEC-7A) and Mincle (CLEC-4E) suppress protective responses of

macrophages during mycobacterial infection.

LID - qiaf146 [pii]

LID - 10.1093/jleuko/qiaf146 [doi]

AB - Mycobacterium tuberculosis (M. tb), the causative agent of tuberculosis (TB), is

responsible for extreme mortality and morbidity across the globe. The bacteria

have evolved multiple strategies for their successful prevalence. The emergence

of multidrug-resistant TB (MDR-TB) has established the importance of eliciting

host-pathogen interactions at cellular and molecular levels. Various pattern

recognition receptors play determinant roles when encountering M. tb infection.

Here, we investigated the regulation of key defense responses from Dectin-1 and

Mincle during mycobacterial infection in THP-1-derived macrophages, the long-term

hosts for mycobacteria. Our data indicate that infection of THP-1 macrophages

with either M. bovis Bacillus Calmette-Guérin (BCG) or M. tb H37Rv increases the

surface expression of Dectin-1 and Mincle. This increase translated directly to

increased intracellular bacterial survival within macrophages. Likewise, M. bovis

BCG infection of human peripheral blood mononuclear cell-derived macrophages also

led to an increased expression of Dectin-1 and Mincle. Stimulation of Dectin-1 or

Mincle along with BCG infection induces suppressor responses such as an

attenuated oxidative burst and mitochondrial membrane potential intactness. In

addition, decreased apoptosis and autophagy induction was also observed following

stimulation of Dectin-1 and Mincle. Conversely, RNA interference-mediated

knockdown of Dectin-1 or Mincle reversed the previous responses, resulting in

higher oxidative burst, mitochondrial membrane potential disruption, increased

mitochondrial reactive oxygen species production, and increased apoptosis. This

results in a significant decrease in intracellular mycobacterial survival. These

results point toward a well-orchestrated strategy of fine-tuning the host's

defense machinery of Dectin-1 and Mincle adopted by mycobacteria to suppress

protective responses mounted against it and prepare the macrophages for prolonged

persistent infection.

CI - © The Author(s) 2025. Published by Oxford University Press on behalf of Society

for Leukocyte Biology. All rights reserved. For commercial re-use, please contact

reprints@oup.com for reprints and translation rights for reprints. All other

permissions can be obtained through our RightsLink service via the Permissions

link on the article page on our site—for further information please contact

journals.permissions@oup.com.

FAU - Singh, Akshita

AU - Singh A

AUID- ORCID: 0009-0009-0239-1854

AD - Infectious Disease Immunology Laboratory, Dr. B.R. Ambedkar Center for Biomedical

Research, University of Delhi, North Campus, University Enclave, Delhi 110007,

India.

FAU - Singh, Aarti

AU - Singh A

AD - Infectious Disease Immunology Laboratory, Dr. B.R. Ambedkar Center for Biomedical

Research, University of Delhi, North Campus, University Enclave, Delhi 110007,

India.

FAU - Sinha, Vishal

AU - Sinha V

AD - Infectious Disease Immunology Laboratory, Dr. B.R. Ambedkar Center for Biomedical

Research, University of Delhi, North Campus, University Enclave, Delhi 110007,

India.

FAU - Rana, Ankush K

AU - Rana AK

AD - Infectious Disease Immunology Laboratory, Dr. B.R. Ambedkar Center for Biomedical

Research, University of Delhi, North Campus, University Enclave, Delhi 110007,

India.

FAU - Saraswati, Shakuntala S K

AU - Saraswati SSK

AD - Cellular Immunology Group, International Centre for Genetic Engineering and

Biotechnology, Aruna Asaf Ali Marg, New Delhi 110067, India.

FAU - Singh, Aayushi

AU - Singh A

AD - Infectious Disease Immunology Laboratory, Dr. B.R. Ambedkar Center for Biomedical

Research, University of Delhi, North Campus, University Enclave, Delhi 110007,

India.

FAU - Kalra, Kanika

AU - Kalra K

AD - Infectious Disease Immunology Laboratory, Dr. B.R. Ambedkar Center for Biomedical

Research, University of Delhi, North Campus, University Enclave, Delhi 110007,

India.

FAU - Thakur, Sunil

AU - Thakur S

AD - W.U.S. Health Centre, University of Delhi, Chhatra Marg, Art Faculty, University

Enclave, New Delhi 110007, India.

FAU - Kumar, Dhiraj

AU - Kumar D

AD - Cellular Immunology Group, International Centre for Genetic Engineering and

Biotechnology, Aruna Asaf Ali Marg, New Delhi 110067, India.

FAU - Natarajan, Krishnamurthy

AU - Natarajan K

AD - Infectious Disease Immunology Laboratory, Dr. B.R. Ambedkar Center for Biomedical

Research, University of Delhi, North Campus, University Enclave, Delhi 110007,

India.

LA - eng

GR - University of Delhi/

GR - BT/PR41505/Department of Biotechnology, Ministry of Science and Technology,

Government of India/

GR - MED/29/1551/2020/Department of Biotechnology, Ministry of Science and Technology,

Government of India/

GR - Department of Biotechnology/

PT - Journal Article

PL - England

TA - J Leukoc Biol

JT - Journal of leukocyte biology

JID - 8405628

RN - 0 (Lectins, C-Type)

RN - 0 (Membrane Proteins)

RN - 0 (Reactive Oxygen Species)

RN - 0 (Dectin-1)

RN - 0 (CLEC7A protein, human)

SB - IM

MH - *Lectins, C-Type/immunology/genetics/metabolism

MH - Humans

MH - *Macrophages/immunology/microbiology/metabolism/pathology

MH - *Membrane Proteins/immunology/genetics/metabolism

MH - *Mycobacterium bovis/immunology

MH - *Mycobacterium tuberculosis/immunology

MH - Reactive Oxygen Species/metabolism

MH - *Tuberculosis/immunology

MH - THP-1 Cells

MH - Dectin-1

OTO - NOTNLM

OT - C-Type Lectin receptor

OT - Tuberculosis

OT - apoptosis

OT - autophagy

OT - oxidative burst

COIS- Conflict of interest: None.

EDAT- 2025/10/23 06:27

MHDA- 2025/11/19 18:27

CRDT- 2025/10/23 00:52

PHST- 2025/01/07 00:00 [received]

PHST- 2025/05/21 00:00 [revised]

PHST- 2025/10/06 00:00 [accepted]

PHST- 2025/11/19 18:27 [medline]

PHST- 2025/10/23 06:27 [pubmed]

PHST- 2025/10/23 00:52 [entrez]

AID - 8299827 [pii]

AID - 10.1093/jleuko/qiaf146 [doi]

PST - ppublish

SO - J Leukoc Biol. 2025 Nov 4;117(11):qiaf146. doi: 10.1093/jleuko/qiaf146.

PMID- 40784165

OWN - NLM

STAT- MEDLINE

DCOM- 20250821

LR - 20250825

IS - 1464-3391 (Electronic)

IS - 0968-0896 (Linking)

VI - 129

DP - 2025 Nov 1

TI - Synthesis of 1,3-diaryl substituted pyrazole-based imidazo[1,2-a]pyridine

carboxamides and evaluation of their antitubercular activity.

PG - 118341

LID - S0968-0896(25)00282-2 [pii]

LID - 10.1016/j.bmc.2025.118341 [doi]

AB - The rise of drug-resistant tuberculosis (TB) has created an urgent need to

discover and develop new anti-mycobacterial agents. Herein, we report the

synthesis and evaluation of a library of 1,3-diaryl substituted pyrazole-based

imidazo[1,2-a]pyridine carboxamides as promising anti-TB agents. In preliminary

screening, 10 out of 26 compounds displayed potent in vitro inhibition against

Mtb H37Rv with a MIC value of 0.03 μg/mL, which is 17-fold more potent than the

first-line TB drug streptomycin, 33-fold more potent than ethambutol, and

equipotent with isoniazid and rifampicin. Encouragingly, most of these compounds

exhibited a selectivity index (SI) >3333.3 and CC₅₀ values >100 μg/mL against

Vero cells, indicating they are over 3000 times more toxic to M. tuberculosis

than to mammalian cells and demonstrate absence of cytotoxicity at concentrations

effective against TB (MIC = 0.03 μg/mL). Among them, 12a, 14a, and 14d

demonstrated remarkable activity against drug-resistant strains of Mtb with an

MIC of 0.03 μM. Time-kill kinetic studies revealed that 12a, 14a, and 14d

exhibited bacteriostatic properties. Furthermore, 12a, 14a, and 14d demonstrated

synergistic effects with the FDA-approved anti-TB drugs rifampicin (ƩFIC 0.093),

ethambutol (ƩFIC 0.061), and moxifloxacin (ƩFIC 0.154-0.281), exhibiting

bactericidal time-kill properties in combination with these drugs. Additionally,

12a, 14a, and 14d exhibited acceptable metabolic stability (CL(int)

11.49-14.62 μL/min/mg microsomal protein), indicating effective drug levels and

bioavailability. Also, 12a, 14a, and 14d showed stable interactions with QcrB in

docking studies. These findings highlight 12a, 14a, and 14d as potential

candidates for in vivo evaluation and further development as novel

anti-tubercular drugs.

CI - Copyright © 2025 Elsevier Ltd. All rights reserved.

FAU - Ommi, Ojaswitha

AU - Ommi O

AD - Department of Chemical Sciences, National Institute of Pharmaceutical Education

and Research (NIPER), Balanagar, Hyderabad 500037, Telangana, India.

FAU - Bhalerao, Harshada Anil

AU - Bhalerao HA

AD - Department of Pharmaceutical Analysis, National Institute of Pharmaceutical

Education and Research (NIPER), Balanagar, Hyderabad 500037, Telangana, India.

FAU - Malik, Pradip

AU - Malik P

AD - Division of Molecular Microbiology and Immunology, CSIR-Central Drug Research

Institute, Sector 10, Janakipuram Extension, Sitapur Road, Lucknow 226031, UP,

India; AcSIR: Academy of Scientific and Innovative Research (AcSIR), Ghaziabad

201002, India.

FAU - Ali, Juned

AU - Ali J

AD - Division of Molecular Microbiology and Immunology, CSIR-Central Drug Research

Institute, Sector 10, Janakipuram Extension, Sitapur Road, Lucknow 226031, UP,

India; AcSIR: Academy of Scientific and Innovative Research (AcSIR), Ghaziabad

201002, India.

FAU - Saxena, Deepanshi

AU - Saxena D

AD - Division of Molecular Microbiology and Immunology, CSIR-Central Drug Research

Institute, Sector 10, Janakipuram Extension, Sitapur Road, Lucknow 226031, UP,

India; AcSIR: Academy of Scientific and Innovative Research (AcSIR), Ghaziabad

201002, India.

FAU - Nanduri, Srinivas

AU - Nanduri S

AD - Department of Chemical Sciences, National Institute of Pharmaceutical Education

and Research (NIPER), Balanagar, Hyderabad 500037, Telangana, India.

FAU - Sonti, Rajesh

AU - Sonti R

AD - Department of Pharmaceutical Analysis, National Institute of Pharmaceutical

Education and Research (NIPER), Balanagar, Hyderabad 500037, Telangana, India.

Electronic address: rajesh.sonti@niperhyd.ac.in.

FAU - Dasgupta, Arunava

AU - Dasgupta A

AD - Division of Molecular Microbiology and Immunology, CSIR-Central Drug Research

Institute, Sector 10, Janakipuram Extension, Sitapur Road, Lucknow 226031, UP,

India; AcSIR: Academy of Scientific and Innovative Research (AcSIR), Ghaziabad

201002, India. Electronic address: a.dasgupta@cdri.res.in.

FAU - Chopra, Sidharth

AU - Chopra S

AD - Division of Molecular Microbiology and Immunology, CSIR-Central Drug Research

Institute, Sector 10, Janakipuram Extension, Sitapur Road, Lucknow 226031, UP,

India; AcSIR: Academy of Scientific and Innovative Research (AcSIR), Ghaziabad

201002, India. Electronic address: skchopra007@gmail.com.

FAU - Yaddanapudi, Venkata Madhavi

AU - Yaddanapudi VM

AD - Department of Chemical Sciences, National Institute of Pharmaceutical Education

and Research (NIPER), Balanagar, Hyderabad 500037, Telangana, India. Electronic

address: yssmadhavi@gmail.com.

LA - eng

PT - Journal Article

DEP - 20250806

PL - England

TA - Bioorg Med Chem

JT - Bioorganic & medicinal chemistry

JID - 9413298

RN - 0 (Antitubercular Agents)

RN - 0 (Imidazoles)

RN - 0 (Pyrazoles)

RN - 0 (Pyridines)

RN - VJT6J7R4TR (Rifampin)

RN - U188XYD42P (Moxifloxacin)

SB - IM

MH - Animals

MH - Humans

MH - *Antitubercular Agents/pharmacology/chemical synthesis/chemistry

MH - Chlorocebus aethiops

MH - Dose-Response Relationship, Drug

MH - *Imidazoles/chemistry/pharmacology/chemical synthesis

MH - Microbial Sensitivity Tests

MH - Molecular Docking Simulation

MH - Molecular Structure

MH - *Mycobacterium tuberculosis/drug effects

MH - *Pyrazoles/chemistry/pharmacology/chemical synthesis

MH - *Pyridines/chemistry/pharmacology/chemical synthesis

MH - Structure-Activity Relationship

MH - Vero Cells

MH - Rifampin/chemistry/pharmacology

MH - Moxifloxacin/chemistry/pharmacology

OTO - NOTNLM

OT - 1,3-Diaryl substituted pyrazole

OT - Antitubercular

OT - Drug resistance

OT - Imidazo[1,2-a]pyridine

OT - Microsomal stability

OT - Synergism

COIS- Declaration of competing interest The authors declare that they have no known

competing financial interests or personal relationships that could have appeared

to influence the work reported in this paper. Acknowledgements Authors thank

NIPER Hyderabad for facilities. OO, HAR are thankful to the department of

Pharmaceuticals, Ministry of Chemicals and Fertilizers, Govt. of India, New Delhi

for the award of NIPER fellowship. PM, JA, and DS thank UGC for their

fellowships.

EDAT- 2025/08/11 00:26

MHDA- 2025/08/31 20:07

CRDT- 2025/08/10 18:05

PHST- 2025/05/29 00:00 [received]

PHST- 2025/08/04 00:00 [revised]

PHST- 2025/08/04 00:00 [accepted]

PHST- 2025/08/31 20:07 [medline]

PHST- 2025/08/11 00:26 [pubmed]

PHST- 2025/08/10 18:05 [entrez]

AID - S0968-0896(25)00282-2 [pii]

AID - 10.1016/j.bmc.2025.118341 [doi]

PST - ppublish

SO - Bioorg Med Chem. 2025 Nov 1;129:118341. doi: 10.1016/j.bmc.2025.118341. Epub 2025

Aug 6.

PMID- 40326994

OWN - NLM

STAT- MEDLINE

DCOM- 20260129

LR - 20260129

IS - 1538-0254 (Electronic)

IS - 0739-1102 (Linking)

VI - 43

IP - 18

DP - 2025 Nov

TI - Computational structure-guided approach to simulate delamanid and pretomanid

binding to mycobacterial F420 redox cycling proteins: identification of key

determinants of resistance.

PG - 11177-11197

LID - 10.1080/07391102.2025.2498621 [doi]

AB - The recently approved delamanid (DLM) and pretomanid (PTM) improved the existing

options to treat multidrug-resistant tuberculosis (MDR-TB). However, the high

spontaneous mutation rates in mycobacterial F420 genes ddn, fgd1, fbiA, fbiB,

fbiC, and fbiD create a bottleneck to successful anti-TB treatments. Of known

mutations, identifying the therapeutically relevant ones is a prerequisite for

understanding the drug resistance mechanism. Here, we applied a multistep

computational pipeline to rank the mutations in F420 genes associated with

DLM/PTM resistance. The DLM-/PTM-resistant protein mutants were built and

simulated their innate sensitivity towards the drugs. The molecular dynamics (MD)

and molecular mechanics Poisson-Boltzmann surface area (MM-PBSA) calculations

quantified the effect of key mutations on drug union. The dynamic

cross-correlated map (DCCM) and principal component analysis (PCA) showed a

substantial link between the drug binding region and other sections in the

mutants, hints to their potential role as an allosteric site. Also, the

alterations induced conformationally unstable proteins with decreased DLM/PTM

affinity. These investigations highlighted the DLM-tolerant G53D and Y65S and

PTM-resilient Y133M (Ddn), L308P (FbiA), and C562W (FbiC) as candidate

loss-of-function mutants of progressive research. The present results and

interpretations could supply vital clues for protein engineering and drug

development.

FAU - Chakraborty, Gourav

AU - Chakraborty G

AD - Indian Institute of Technology (Indian School of Mines), Dhanbad, Jharkhand,

India.

FAU - Kolpe, Mahima Sudhir

AU - Kolpe MS

AD - AarogyaAI Innovations Private Limited, Bengaluru, Karnataka, India.

FAU - Nath, I V Ambily

AU - Nath IVA

AD - AarogyaAI Innovations Private Limited, Bengaluru, Karnataka, India.

FAU - Tiwari, Avlokita

AU - Tiwari A

AD - AarogyaAI Innovations Private Limited, Bengaluru, Karnataka, India.

FAU - Jayaswal, Praapti

AU - Jayaswal P

AD - AarogyaAI Innovations Private Limited, Bengaluru, Karnataka, India.

FAU - Patra, Niladri

AU - Patra N

AD - Indian Institute of Technology (Indian School of Mines), Dhanbad, Jharkhand,

India.

LA - eng

PT - Journal Article

DEP - 20250506

PL - England

TA - J Biomol Struct Dyn

JT - Journal of biomolecular structure & dynamics

JID - 8404176

RN - 0 (Nitroimidazoles)

RN - 0 (Antitubercular Agents)

RN - 0 (OPC-67683)

RN - 0 (Oxazoles)

RN - 0 (Bacterial Proteins)

SB - IM

MH - Molecular Dynamics Simulation

MH - *Nitroimidazoles/chemistry/pharmacology/metabolism

MH - *Antitubercular Agents/chemistry/pharmacology

MH - *Oxazoles/chemistry/pharmacology/metabolism

MH - *Bacterial Proteins/chemistry/genetics/metabolism

MH - Protein Binding

MH - Mutation

MH - *Mycobacterium tuberculosis/drug effects/genetics

MH - Oxidation-Reduction

MH - Binding Sites

MH - *Drug Resistance, Bacterial

MH - Protein Conformation

OTO - NOTNLM

OT - F420 proteins

OT - Mycobacterium tuberculosis

OT - molecular dynamics

OT - mutation

OT - nitroimidazole resistance

OT - principal component analysis

EDAT- 2025/05/06 12:42

MHDA- 2026/01/29 14:01

CRDT- 2025/05/06 10:24

PHST- 2026/01/29 14:01 [medline]

PHST- 2025/05/06 12:42 [pubmed]

PHST- 2025/05/06 10:24 [entrez]

AID - 10.1080/07391102.2025.2498621 [doi]

PST - ppublish

SO - J Biomol Struct Dyn. 2025 Nov;43(18):11177-11197. doi:

10.1080/07391102.2025.2498621. Epub 2025 May 6.

PMID- 41116166

OWN - NLM

STAT- MEDLINE

DCOM- 20251020

LR - 20260710

IS - 2054-9369 (Electronic)

IS - 2095-7467 (Print)

IS - 2054-9369 (Linking)

VI - 12

IP - 1

DP - 2025 Oct 20

TI - Immunotherapy for tuberculosis: current strategies and future directions.

PG - 68

LID - 10.1186/s40779-025-00655-7 [doi]

LID - 68

AB - The worldwide dissemination of drug-resistant tuberculosis (TB) presents

significant obstacles to conventional anti-TB treatment and prevention methods

based on bactericidal antimicrobial drugs, greatly impeding advancements in

combating this most lethal disease. With growing insights into the

immunopathogenesis of TB, we are increasingly recognizing the potential of

immunotherapeutic strategies aimed at targeting the host. After invading the

host, Mycobacterium tuberculosis (M. tuberculosis) induces host cell exhaustion

through its own molecules, such as early secretory antigen target-6 (ESAT-6) and

di-O-acyl-trehalose, manifested as suppressed proliferative capacity, cytokine

production, and cytotoxicity, thereby triggering the onset of TB. In response to

this pathogenic mechanism, immunotherapeutic strategies, including cell therapy

and immune checkpoint inhibitors, have been developed to promote cytokine

production, activate immune cells to exhibit anti-TB activities such as

autophagy, and restore immune homeostasis, including the balance between T helper

1 (Th1) and Th2 responses. These approaches have shown promise in restoring host

immunity and demonstrating therapeutic effects against TB. However, a

comprehensive evaluation of factors such as drug safety, optimal treatment

duration, and others, is essential before these strategies can be integrated into

routine clinical TB management. The advancement of immunotherapy has the

potential to revolutionize current TB management and provide further benefits to

patients. This review aims to comprehensively explore the advancements in diverse

TB immunotherapeutic strategies, including efficacy, safety, and administration

methods, and to explore the challenges and prospects of TB immunotherapy.

CI - © 2025. The Author(s).

FAU - Lyu, Meng-Yuan

AU - Lyu MY

AD - Department of Laboratory Medicine/Clinical Laboratory Medicine Research Center,

West China Hospital, Sichuan University, Chengdu, 610041, China.

AD - Sichuan Clinical Research Center for Laboratory Medicine, Chengdu, 610041, China.

FAU - Lai, Hong-Li

AU - Lai HL

AD - Department of Laboratory Medicine/Clinical Laboratory Medicine Research Center,

West China Hospital, Sichuan University, Chengdu, 610041, China.

FAU - Peng, Hao-Ran

AU - Peng HR

AD - West China School of Medicine, Sichuan University, Chengdu, 610041, China.

FAU - Luo, Han

AU - Luo H

AD - Department of Division of Thyroid and Parathyroid SurgeryWest China Hospital,

Sichuan University, Chengdu, 610041, China.

FAU - Zhou, Jian

AU - Zhou J

AD - Department of Thoracic Surgery, West China Hospital, Sichuan University, Chengdu,

610041, China.

FAU - Ma, Wan-An-Qi

AU - Ma WA

AD - West China School of Medicine, Sichuan University, Chengdu, 610041, China.

FAU - Zhang, Chun-Ying

AU - Zhang CY

AD - Department of Laboratory Medicine/Clinical Laboratory Medicine Research Center,

West China Hospital, Sichuan University, Chengdu, 610041, China.

AD - Sichuan Clinical Research Center for Laboratory Medicine, Chengdu, 610041, China.

FAU - Ruan, Hong-Xia

AU - Ruan HX

AD - Department of Laboratory Medicine/Clinical Laboratory Medicine Research Center,

West China Hospital, Sichuan University, Chengdu, 610041, China.

AD - Sichuan Clinical Research Center for Laboratory Medicine, Chengdu, 610041, China.

FAU - Liu, Yang

AU - Liu Y

AD - Department of Laboratory Medicine/Clinical Laboratory Medicine Research Center,

West China Hospital, Sichuan University, Chengdu, 610041, China.

FAU - Chen, Jie

AU - Chen J

AD - Department of Laboratory Medicine/Clinical Laboratory Medicine Research Center,

West China Hospital, Sichuan University, Chengdu, 610041, China.

chenjiewch@wchscu.cn.

FAU - Ying, Bin-Wu

AU - Ying BW

AD - Department of Laboratory Medicine/Clinical Laboratory Medicine Research Center,

West China Hospital, Sichuan University, Chengdu, 610041, China.

yingbinwu@wchscu.cn.

AD - Sichuan Clinical Research Center for Laboratory Medicine, Chengdu, 610041, China.

yingbinwu@wchscu.cn.

LA - eng

PT - Journal Article

PT - Research Support, Non-U.S. Gov't

PT - Review

DEP - 20251020

PL - Netherlands

TA - Mil Med Res

JT - Military Medical Research

JID - 101643181

SB - IM

MH - Humans

MH - *Immunotherapy/methods/trends/standards

MH - *Tuberculosis/therapy/drug therapy

MH - Mycobacterium tuberculosis/pathogenicity/drug effects

PMC - PMC12536540

OTO - NOTNLM

OT - Cell exhaustion

OT - Clinical trials

OT - Immunotherapy

OT - Molecular mechanisms

OT - Tuberculosis (TB)

COIS- Declarations. Ethics approval and consent to participate: Not applicable. Consent

for publication: Not applicable. Competing interests: All the authors declare

that they have no competing interests.

EDAT- 2025/10/21 00:28

MHDA- 2025/10/21 00:29

PMCR- 2025/10/20

CRDT- 2025/10/20 23:28

PHST- 2024/10/31 00:00 [received]

PHST- 2025/09/28 00:00 [accepted]

PHST- 2025/10/21 00:29 [medline]

PHST- 2025/10/21 00:28 [pubmed]

PHST- 2025/10/20 23:28 [entrez]

PHST- 2025/10/20 00:00 [pmc-release]

AID - 10.1186/s40779-025-00655-7 [pii]

AID - 655 [pii]

AID - 10.1186/s40779-025-00655-7 [doi]

PST - epublish

SO - Mil Med Res. 2025 Oct 20;12(1):68. doi: 10.1186/s40779-025-00655-7.

PMID- 41102315

OWN - NLM

STAT- MEDLINE

DCOM- 20251016

LR - 20251019

IS - 2045-2322 (Electronic)

IS - 2045-2322 (Linking)

VI - 15

IP - 1

DP - 2025 Oct 16

TI - Machine learning identifies MiRNA biomarkers and immune mechanisms in active

tuberculosis.

PG - 36246

LID - 10.1038/s41598-025-20112-8 [doi]

LID - 36246

AB - Tuberculosis (TB), caused by Mycobacterium tuberculosis (Mtb), remains a major

global public health threat. The rising prevalence of HIV/TB co-infection and

multidrug-resistant tuberculosis (MDR-TB) has further intensified this challenge.

This study aims to explore the role of microRNAs (miRNAs) in the immune response

to Mtb infection and to identify potential miRNA biomarkers for active TB

diagnosis using machine learning techniques. miRNA expression profiles were

retrieved from the Gene Expression Omnibus (GEO) database (accession number:

GSE70425). Differential expression analysis between active TB and latent TB

infection (LTBI) patients was conducted using the "limma" package, with a

screening threshold of |logFC| > 0.25 and p-value < 0.05. Key differentially

expressed miRNAs (DE-miRNAs) were further refined using machine learning

algorithms, including least absolute shrinkage and selection operator (LASSO)

regression, support vector machine-recursive feature elimination (SVM-RFE), and

Boruta. TargetScan was employed to predict miRNA target genes, and a regulatory

network was visualized using Cytoscape. Subsequently, Gene Ontology (GO) and

Kyoto Encyclopedia of Genes and Genomes (KEGG) pathway enrichment analyses were

conducted to elucidate the functional roles of target mRNAs. Nine machine

learning models were developed based on the selected miRNAs, and their predictive

performance was assessed using metrics including AUROC, accuracy, sensitivity,

specificity, positive predictive value (PPV), and negative predictive value

(NPV). In vitro experiments were performed using THP-1 macrophages to establish

an Mtb infection model. Cells were transfected with miR-3607-3p mimics and

negative controls, followed by flow cytometry for apoptosis detection, Western

blot for protein expression analysis, and Quantitative Real-Time PCR (qRT-PCR)

for validation of apoptosis-related gene expression. A total of 72 differentially

expressed miRNAs were identified. Key miRNAs, including hsa-miR-3607-3p,

hsa-miR-148b, and hsa-miR-519e, were identified through multiple machine learning

methods, with hsa-miR-3607-3p emerging as the primary candidate miRNA. The nine

machine learning models exhibited robust predictive performance in both the

training and test sets. PPI network and functional enrichment analyses indicated

that the target genes of hsa-miR-3607-3p are primarily associated with cell

growth and apoptosis-related pathways. In vitro experiments further suggested

that hsa-miR-3607-3p may modulate the apoptotic response of THP-1 cells to Mtb

infection via a caspase-dependent mechanism. miR-3607-3p was upregulated in

active TB patients and may modulate the apoptosis of THP-1 cells during Mtb

infection via a caspase-dependent pathway. The mechanism of this miRNA offers

preliminary insights into the immune regulation of tuberculosis. miR-3607-3p may

serve as a potential biomarker for the early diagnosis and intervention of active

TB; however, its clinical applicability necessitates further validation through

larger sample sizes and multicenter studies.

CI - © 2025. The Author(s).

FAU - Cai, Zihan

AU - Cai Z

AD - Department of Medical Laboratory, Siyang Hospital, Siyang, 223700, China.

AD - Oncology and Laboratory Immunology Research Innovation Center, Siyang Hospital,

Siyang, 223700, China.

FAU - Huang, Chunxiao

AU - Huang C

AD - Department of Laboratory Medicine, Gutian County Hospital, Gutian, 352200, China.

FAU - Zhou, Yuyang

AU - Zhou Y

AD - Department of Medical Laboratory, Siyang Hospital, Siyang, 223700, China.

AD - Oncology and Laboratory Immunology Research Innovation Center, Siyang Hospital,

Siyang, 223700, China.

FAU - Wu, Lixian

AU - Wu L

AD - Department of Microbiology and Immunology, School of Basic Medical Sciences, Dali

University, Dali, 671000, China.

FAU - Ding, Shoupeng

AU - Ding S

AD - Department of Laboratory Medicine, Gutian County Hospital, Gutian, 352200, China.

zihancai001@163.com.

LA - eng

GR - 2024SY005/Siyang Hospital and the Affiliated Hospital of Jiangsu University in

the first session of the hospital science and technology programmed projects to

set up the topic/

GR - 2024SY005/Siyang Hospital and the Affiliated Hospital of Jiangsu University in

the first session of the hospital science and technology programmed projects to

set up the topic/

PT - Journal Article

DEP - 20251016

PL - England

TA - Sci Rep

JT - Scientific reports

JID - 101563288

RN - 0 (MicroRNAs)

RN - 0 (Biomarkers)

SB - IM

MH - *MicroRNAs/genetics

MH - Humans

MH - *Machine Learning

MH - Biomarkers/metabolism

MH - *Tuberculosis/immunology/genetics/diagnosis

MH - Gene Regulatory Networks

MH - Mycobacterium tuberculosis/immunology

MH - Gene Expression Profiling

PMC - PMC12533189

OTO - NOTNLM

OT - Biomarker

OT - Functional enrichment analysis

OT - Machine learning

OT - MicroRNA

OT - Tuberculosis

COIS- Declarations. Competing interests: The authors declare no competing interests.

Ethics approval and consent to participate: This study has been approved by the

Ethics Committee of Siyang Hospital with the approval number HY2024005.

EDAT- 2025/10/17 00:31

MHDA- 2025/10/17 00:32

PMCR- 2025/10/16

CRDT- 2025/10/16 23:31

PHST- 2025/03/31 00:00 [received]

PHST- 2025/09/12 00:00 [accepted]

PHST- 2025/10/17 00:32 [medline]

PHST- 2025/10/17 00:31 [pubmed]

PHST- 2025/10/16 23:31 [entrez]

PHST- 2025/10/16 00:00 [pmc-release]

AID - 10.1038/s41598-025-20112-8 [pii]

AID - 20112 [pii]

AID - 10.1038/s41598-025-20112-8 [doi]

PST - epublish

SO - Sci Rep. 2025 Oct 16;15(1):36246. doi: 10.1038/s41598-025-20112-8.

PMID- 41170421

OWN - NLM

STAT- PubMed-not-MEDLINE

DCOM- 20251031

LR - 20251102

IS - 1664-302X (Print)

IS - 1664-302X (Electronic)

IS - 1664-302X (Linking)

VI - 16

DP - 2025

TI - Mycobacterium tuberculosis genomic surveillance in Mexico. Characterization of

variants in drug resistance and efflux pump genes.

PG - 1666838

LID - 10.3389/fmicb.2025.1666838 [doi]

LID - 1666838

AB - Tuberculosis (TB) remains a persistent global public health challenge, with the

rise of drug-resistant tuberculosis (DR-TB) complicating all the disease control

efforts. The World Health Organization (WHO) has advocated for molecular

diagnostic techniques, including whole-genome sequencing (WGS), to enhance TB

diagnosis and treatment strategies. In this study, we performed WGS analysis on

49 pulmonary tuberculosis isolates from Mexican patients to identify mutations

conferring resistance to 11 key antimicrobial agents: four first-line drugs

(isoniazid, rifampicin, ethambutol, and pyrazinamide) and 7 second line drugs

(fluoroquinolones, ethionamide/prothionamide, amikacin, kanamycin, capreomycin,

streptomycin, and bedaquiline). We identified 89 novel variants: 48 in genes

previously associated with drug resistance and 41 in genes not previously linked

to resistance mechanisms, including potential novel mutations associated with

delamanid resistance. Additionally, we detected 31 mutations across three efflux

pump superfamilies (ABC, RND, and MFS); all of these variants warrant further

investigation regarding their contribution to antibiotic resistance. This

analysis represents approximately 10% of Mexico's national variant registry,

providing substantial insight into the molecular epidemiology of drug-resistant

tuberculosis within the country. The identification of new resistance-associated

variants (RAV) from clinical isolates underrepresented in global databases,

contributes to develop improved diagnostic tools, optimize treatment regimens,

and probably to elucidate antibiotic resistance mechanisms. Specifically, the

identification of RAVs for new drugs like bedaquiline, pretomanid, delamanid, and

linezolid, which are central to the most recent schemes of treatment (BPaLM,

BPaL, BDLLfxC, BLMZ), is key to the improvement of patient outcomes and

preventing the emergence of resistance to these critical therapeutic options.

CI - Copyright © 2025 Alvarado-Peña, Muñoz Torrico, Narváez-Díaz, Mejía-Ponce,

Becerril Vargas, Zúñiga, Muñiz-Salazar, Laniado-Laborín, Licona-Cassani, Soberón

and Silva-Herzog.

FAU - Alvarado-Peña, Néstor

AU - Alvarado-Peña N

AD - Clínica de Tuberculosis, Instituto Nacional de Enfermedades Respiratorias "Ismael

Cosío Villegas", Mexico City, Mexico.

AD - Laboratorio de Vinculación Científica, Facultad de Medicina-Universidad Nacional

Autonoma de Mexico-Instituto Nacional de Medicina Genómica (UNAM-INMEGEN), Mexico

City, Mexico.

FAU - Muñoz Torrico, Marcela

AU - Muñoz Torrico M

AD - Clínica de Tuberculosis, Instituto Nacional de Enfermedades Respiratorias "Ismael

Cosío Villegas", Mexico City, Mexico.

FAU - Narváez-Díaz, Luis

AU - Narváez-Díaz L

AD - Laboratorio de Microbiología, Instituto Nacional de Enfermedades Respiratorias

(INER), Mexico City, Mexico.

FAU - Mejía-Ponce, Paulina M

AU - Mejía-Ponce PM

AD - Escuela de Ingeniería y Ciencias, Tecnológico de Monterrey, Monterrey, Mexico.

FAU - Becerril Vargas, Eduardo

AU - Becerril Vargas E

AD - Laboratorio de Microbiología, Instituto Nacional de Enfermedades Respiratorias

(INER), Mexico City, Mexico.

FAU - Zúñiga, Joaquín

AU - Zúñiga J

AD - Laboratorio de Inmunobiología y Genética, Instituto Nacional de Enfermedades

Respiratorias "Ismael Cosío Villegas" and Tecnológico de Monterrey, Escuela de

Medicina y Ciencias de la Salud, Mexico City, Mexico.

FAU - Muñiz-Salazar, Raquel

AU - Muñiz-Salazar R

AD - Escuela de Ciencias de la Salud, Universidad Autónoma de Baja California,

Ensenada, Mexico.

FAU - Laniado-Laborín, Rafael

AU - Laniado-Laborín R

AD - Facultad de Medicina, Universidad Autónoma de Baja California, Tijuana, Mexico.

FAU - Licona-Cassani, Cuauhtémoc

AU - Licona-Cassani C

AD - Escuela de Ingeniería y Ciencias, Tecnológico de Monterrey, Monterrey, Mexico.

AD - Tecnológico de Monterrey, The Institute for Obesity Research, Monterrey, Mexico.

FAU - Soberón, Xavier

AU - Soberón X

AD - Departamento de Ingeniería Celular y Biocatálisis, Instituto de Biotecnología,

Universidad Nacional Autónoma de México, Cuernavaca, Mexico.

FAU - Silva-Herzog, Eugenia

AU - Silva-Herzog E

AD - Laboratorio de Vinculación Científica, Facultad de Medicina-Universidad Nacional

Autonoma de Mexico-Instituto Nacional de Medicina Genómica (UNAM-INMEGEN), Mexico

City, Mexico.

LA - eng

PT - Journal Article

DEP - 20251015

PL - Switzerland

TA - Front Microbiol

JT - Frontiers in microbiology

JID - 101548977

PMC - PMC12568502

OTO - NOTNLM

OT - Mycobacterium tuberculosis complex

OT - drug-resistant tuberculosis

OT - efflux pumps

OT - resistance-associated variants

OT - tuberculosis

OT - whole genome sequencing

COIS- The authors declare that the research was conducted in the absence of any

commercial or financial relationships that could be construed as a potential

conflict of interest.

EDAT- 2025/10/31 06:26

MHDA- 2025/10/31 06:27

PMCR- 2025/10/15

CRDT- 2025/10/31 04:57

PHST- 2025/07/15 00:00 [received]

PHST- 2025/09/19 00:00 [accepted]

PHST- 2025/10/31 06:27 [medline]

PHST- 2025/10/31 06:26 [pubmed]

PHST- 2025/10/31 04:57 [entrez]

PHST- 2025/10/15 00:00 [pmc-release]

AID - 10.3389/fmicb.2025.1666838 [doi]

PST - epublish

SO - Front Microbiol. 2025 Oct 15;16:1666838. doi: 10.3389/fmicb.2025.1666838.

eCollection 2025.

PMID- 41094671

OWN - NLM

STAT- MEDLINE

DCOM- 20251016

LR - 20251019

IS - 1471-2334 (Electronic)

IS - 1471-2334 (Linking)

VI - 25

IP - 1

DP - 2025 Oct 15

TI - Association of vitamin D receptor mRNA expression, vitamin D deficiency and

genetic variant in patients with multi-drug resistant pulmonary tuberculosis.

PG - 1334

LID - 10.1186/s12879-025-11707-7 [doi]

LID - 1334

AB - BACKGROUND: Multi-drug resistant pulmonary tuberculosis (MDR-TB), is a serious

threat to world health. Serum levels of vitamin D, a ligand for the VDR that

controls VDR mRNA expression, are still poorly understood in MDR-TB. OBJECTIVE:

To study the association of mRNA expression with low vitamin D levels and VDR

polymorphisms in patients with MDR-TB compared to normal controls. METHODS: Study

groups consisted of sputum smears and culture-positive MDR-TB at two hospitals in

New Delhi, and normal controls were enrolled from a North Indian population. A

total 100 (50 MDR-TB subjects and 50 controls) were consecutively enrolled. VDR

mRNA expression in peripheral blood mononuclear cells (PBMC) was analysed by

Real-time PCR. Serum 25-hydroxyvitamin D, intact parathyroid hormone (iPTH), and

calcium (ionized and total) levels were measured, and the correlation between

variables was determined. The association between VDR genotype and VDR mRNA

expression was studied between MDR-TB and normal controls together with the

genotypic and allelic frequencies of the FokI, BsmI, and TaqI VDR polymorphisms

were also assessed in between the two groups. RESULTS: To investigate the role of

VDR gene expression and FokI polymorphism in MDR-TB, a total of 100 patients were

split into two groups. VDR mRNA expression significantly decreased in MDR-TB

patients, being 0.6 times lower than in healthy controls. Notably, the ff

genotype was associated with reduced VDR expression, indicating a functional

impact on gene regulation. However, there was no appreciable variation in the

groups' distribution of FokI alleles and genotypes. These findings highlight the

importance of merging genetic and expression data, showing that while the ff

variation influences individual expression, it does not distinguish MDR-TB

patients from controls. CONCLUSION: In present study, the VDR gene's FokI

polymorphism affects the levels of VDR mRNA expression, with the ff genotype

linked to lower expression in both MDR-TB patients and healthy individuals.

Nonetheless, there were no appreciable differences in the genotypic and allelic

frequencies of FokI between the groups. These findings suggest that the location

of the FokI variation in the population may not be as important to MDR-TB

susceptibility as its functional impact on gene expression.

CI - © 2025. The Author(s).

FAU - Rathored, Jaishriram

AU - Rathored J

AD - Department of Medicine, All India Institute of Medical Sciences, New Delhi,

India. jaishriz@gmail.com.

AD - In-charge Central Research laboratory (CRL) and Molecular Diagnostics, School of

Allied Health Sciences, Datta Meghe Institute of Higher Education and Research

(DU), Sawangi (Meghe), Wardha, 442107, Maharashtra, India. jaishriz@gmail.com.

FAU - Sharma, Surendra Kumar

AU - Sharma SK

AD - Department of Medicine, All India Institute of Medical Sciences, New Delhi,

India.

FAU - Sreenivas, V

AU - Sreenivas V

AD - Department of Biostatistics, All India Institute of Medical Sciences, New Delhi,

India.

FAU - Srivastava, Abhay Krishna

AU - Srivastava AK

AD - Department of Laboratory Medicine, All India Institute of Medical Sciences, New

Delhi, India.

LA - eng

PT - Journal Article

DEP - 20251015

PL - England

TA - BMC Infect Dis

JT - BMC infectious diseases

JID - 100968551

RN - 0 (Receptors, Calcitriol)

RN - 0 (RNA, Messenger)

RN - 0 (VDR protein, human)

RN - 1406-16-2 (Vitamin D)

SB - IM

MH - Humans

MH - *Receptors, Calcitriol/genetics

MH - Male

MH - Female

MH - Adult

MH - *Tuberculosis, Multidrug-Resistant/genetics/complications

MH - *RNA, Messenger/genetics

MH - Middle Aged

MH - *Vitamin D Deficiency/complications/genetics

MH - *Tuberculosis, Pulmonary/genetics/complications

MH - Vitamin D/blood/analogs & derivatives

MH - Genotype

MH - Gene Frequency

MH - India

MH - Young Adult

MH - Leukocytes, Mononuclear/metabolism

MH - Polymorphism, Genetic

MH - Case-Control Studies

MH - Genetic Predisposition to Disease

PMC - PMC12522335

OTO - NOTNLM

OT - Calcium levels

OT - Intact parathyroid hormone

OT - Multi-drug-resistant pulmonary tuberculosis

OT - Vitamin d mRNA expression

OT - Vitamin d receptor

COIS- Declarations. Ethics approval and consent to participate: The study was approved

by the Institutional Ethical committee of All India Institute of Medical Sciences

(IEC-AIIMS) Reference Number: A-08/5.5, New Delhi, India, and written informed

consent was obtained from all subjects, including patients and normal controls.

Ethics committee (AIIMS-IEC) approved the procedure involved in the present

research. Consent for publication: All authors agreed and give their consent for

publication. Competing interests: The authors declare no competing interests.

EDAT- 2025/10/16 06:29

MHDA- 2025/10/16 06:30

PMCR- 2025/10/15

CRDT- 2025/10/16 00:03

PHST- 2024/05/09 00:00 [received]

PHST- 2025/09/16 00:00 [accepted]

PHST- 2025/10/16 06:30 [medline]

PHST- 2025/10/16 06:29 [pubmed]

PHST- 2025/10/16 00:03 [entrez]

PHST- 2025/10/15 00:00 [pmc-release]

AID - 10.1186/s12879-025-11707-7 [pii]

AID - 11707 [pii]

AID - 10.1186/s12879-025-11707-7 [doi]

PST - epublish

SO - BMC Infect Dis. 2025 Oct 15;25(1):1334. doi: 10.1186/s12879-025-11707-7.

PMID- 40885237

OWN - NLM

STAT- MEDLINE

DCOM- 20250916

LR - 20250916

IS - 1879-0712 (Electronic)

IS - 0014-2999 (Linking)

VI - 1005

DP - 2025 Oct 15

TI - Tioconazole exerts anti-TB activity by destroying cell integrity.

PG - 178102

LID - S0014-2999(25)00856-8 [pii]

LID - 10.1016/j.ejphar.2025.178102 [doi]

AB - The increasing prevalence of drug-resistant tuberculosis (TB), coupled with the

lengthy and toxic nature of conventional treatments and the complicating factor

of Human Immunodeficiency Virus (HIV) co-infection, underscores the urgent need

for the development of effective anti-TB drugs and robust stockpiles to combat

this global health crisis. In this study, the anti-tubercular activity and

potential antibacterial mechanisms of tioconazole were investigated. Tioconazole

exhibited remarkable antibacterial activity against Mtb H37Ra and H37Rv, with

minimum inhibitory concentrations (MICs) of 4 μg/mL and 1 μg/mL respectively.

While cholesterol or palmitic acid was utilized as a solo carbon source, its MIC

against Mtb H37Ra decreased to 2 μg/mL. Mtb H37Ra CYP121 conditional mutant was

more sensitive to tioconazole in comparison with Mtb H37Ra in the presence of the

pristinamycin (0.1 μg/mL and 0.001 μg/mL). Further experiments demonstrated that

treatment with 0.5 × MIC of tioconazole (2 μg/mL) for five days resulted in the

formation of invagination and bulge on the surfaces of Mtb cells. Concurrently,

intracellular ATP levels decreased by nearly 50 %, likely due to the inhibition

of lipid metabolism by tioconazole, which compromises the integrity of the cell

wall and cell membrane. Taken together, these findings suggest that tioconazole

may serve as a novel anti-TB agent, exerting anti-TB activities by disrupting the

structural integrity of the pathogen's cells.

CI - Copyright © 2025 Elsevier B.V. All rights reserved.

FAU - Chen, Zhanpeng

AU - Chen Z

AD - National Clinical Research Center for Infectious Diseases, Shenzhen Third

People's Hospital, Shenzhen, Guangdong, China.

FAU - Wang, Xiaoming

AU - Wang X

AD - National Clinical Research Center for Infectious Diseases, Shenzhen Third

People's Hospital, Shenzhen, Guangdong, China.

FAU - Zhang, He

AU - Zhang H

AD - National Clinical Research Center for Infectious Diseases, Shenzhen Third

People's Hospital, Shenzhen, Guangdong, China.

FAU - Kong, Xingxing

AU - Kong X

AD - National Clinical Research Center for Infectious Diseases, Shenzhen Third

People's Hospital, Shenzhen, Guangdong, China.

FAU - Chen, Jinyun

AU - Chen J

AD - National Clinical Research Center for Infectious Diseases, Shenzhen Third

People's Hospital, Shenzhen, Guangdong, China.

FAU - Wang, Qiong

AU - Wang Q

AD - Department of Pharmacy, Shenzhen University General Hospital, Shenzhen, China.

FAU - Meng, Jianzhou

AU - Meng J

AD - National Clinical Research Center for Infectious Diseases, Shenzhen Third

People's Hospital, Shenzhen, Guangdong, China. Electronic address:

mengjianzhou@126.com.

FAU - Lu, Shuihua

AU - Lu S

AD - National Clinical Research Center for Infectious Diseases, Shenzhen Third

People's Hospital, Shenzhen, Guangdong, China. Electronic address:

lushuihua66@126.com.

LA - eng

PT - Journal Article

DEP - 20250828

PL - Netherlands

TA - Eur J Pharmacol

JT - European journal of pharmacology

JID - 1254354

RN - 0 (Antitubercular Agents)

RN - S57Y5X1117 (tioconazole)

RN - 0 (Imidazoles)

RN - 8L70Q75FXE (Adenosine Triphosphate)

SB - IM

MH - Microbial Sensitivity Tests

MH - *Antitubercular Agents/pharmacology

MH - *Mycobacterium tuberculosis/drug effects/cytology/metabolism

MH - Humans

MH - *Imidazoles/pharmacology

MH - Adenosine Triphosphate/metabolism

MH - Lipid Metabolism/drug effects

MH - Cell Wall/drug effects/metabolism

OTO - NOTNLM

OT - CYP121

OT - Cell integrity

OT - Integrity

OT - Mtb

OT - TB

OT - Tioconazole

COIS- Declaration of competing interest The author(s) declared no potential conflicts

of interest with respect to the research, authorship, and/or publication of this

article.

EDAT- 2025/08/31 04:44

MHDA- 2025/09/16 18:31

CRDT- 2025/08/30 19:12

PHST- 2025/03/03 00:00 [received]

PHST- 2025/08/13 00:00 [revised]

PHST- 2025/08/28 00:00 [accepted]

PHST- 2025/09/16 18:31 [medline]

PHST- 2025/08/31 04:44 [pubmed]

PHST- 2025/08/30 19:12 [entrez]

AID - S0014-2999(25)00856-8 [pii]

AID - 10.1016/j.ejphar.2025.178102 [doi]

PST - ppublish

SO - Eur J Pharmacol. 2025 Oct 15;1005:178102. doi: 10.1016/j.ejphar.2025.178102. Epub

2025 Aug 28.

PMID- 41060999

OWN - NLM

STAT- PubMed-not-MEDLINE

DCOM- 20251008

LR - 20251011

IS - 2767-3375 (Electronic)

IS - 2767-3375 (Linking)

VI - 5

IP - 10

DP - 2025

TI - Children, caregivers and health workers' perceptions and experiences of the

XTEMP-R tool to improve tuberculosis treatment.

PG - e0005269

LID - 10.1371/journal.pgph.0005269 [doi]

LID - e0005269

AB - Treating drug-resistant tuberculosis (DR-TB) in children remains a significant

challenge for patients, caregivers, and health systems, despite advances in

child-friendly drug formulations. While new formulations offer benefits, their

widespread availability is limited, and many exhibit poor palatability. A key

strategy to improve administration and mask the taste of paediatric TB

medications involves creating extemporaneous suspensions. However, this often

requires pharmaceutical services not readily available in high-burden settings.

To address this, the Global Alliance for TB Drug Development (TB Alliance)

developed XTEMP-R, an inexpensive prototype tool designed to facilitate

home-based preparation of liquid TB medication suspensions. This study explored

the experiences and perceptions of children, their caregivers, and health workers

regarding the XTEMP-R tool for preparing extemporaneous DR-TB treatment

suspensions. We collected qualitative data from two sites in South Africa. The

first component involved interviews with 17 caregivers and 12 health workers,

followed by focus group discussions, with participants directly interacting with

the XTEMP-R tool. The second component comprised 31 interviews with 11 caregivers

of 13 children who used the XTEMP-R tool for home administration. Case

descriptions were iteratively refined and analyzed using deductive thematic

analysis. Findings indicate that children, caregivers, and health workers found

the XTEMP-R tool easy to use, clean, and store, appreciating its appealing color

and durability. Home users reported that the tool simplified treatment

preparation and administration, reducing time and relational burdens associated

with DR-TB treatment. While XTEMP-R effectively addressed usability challenges

related to drug preparation, fundamental obstacles concerning medication

palatability, nausea, and side effects remain significant barriers. Importantly,

the tool appeared to foster increased treatment responsibility among some

children, suggesting a potential pathway to improve therapeutic engagement and

agency. This research underscores the XTEMP-R tool's potential to ease paediatric

DR-TB treatment and highlights crucial areas for design refinement, ultimately

aiming to enhance adherence and overall outcomes.

CI - Copyright: © 2025 Wademan et al. This is an open access article distributed under

the terms of the Creative Commons Attribution License, which permits unrestricted

use, distribution, and reproduction in any medium, provided the original author

and source are credited.

FAU - Wademan, Dillon T

AU - Wademan DT

AUID- ORCID: 0000-0003-2222-7401

AD - Desmond Tutu TB Centre, Department of Paediatrics and Child Health, Faculty of

Medicine and Health Sciences, Stellenbosch University, Cape Town, South Africa.

FAU - Filander, Willdon J

AU - Filander WJ

AD - Desmond Tutu TB Centre, Department of Paediatrics and Child Health, Faculty of

Medicine and Health Sciences, Stellenbosch University, Cape Town, South Africa.

FAU - Mlomzale, Mfundo

AU - Mlomzale M

AUID- ORCID: 0000-0002-0549-0630

AD - Desmond Tutu TB Centre, Department of Paediatrics and Child Health, Faculty of

Medicine and Health Sciences, Stellenbosch University, Cape Town, South Africa.

FAU - Sibisi, Ntokozo

AU - Sibisi N

AD - Tuberculosis and HIV Investigative Network (THINK), Durban, South Africa.

FAU - Thwala, Cyril

AU - Thwala C

AD - Tuberculosis and HIV Investigative Network (THINK), Durban, South Africa.

FAU - Memela, Phumlani

AU - Memela P

AD - Tuberculosis and HIV Investigative Network (THINK), Durban, South Africa.

FAU - Vanqa, Nosivuyile

AU - Vanqa N

AUID- ORCID: 0000-0002-2774-5622

AD - Desmond Tutu TB Centre, Department of Paediatrics and Child Health, Faculty of

Medicine and Health Sciences, Stellenbosch University, Cape Town, South Africa.

FAU - Palmer, Megan

AU - Palmer M

AD - Desmond Tutu TB Centre, Department of Paediatrics and Child Health, Faculty of

Medicine and Health Sciences, Stellenbosch University, Cape Town, South Africa.

FAU - Sachs, Tina

AU - Sachs T

AD - Desmond Tutu TB Centre, Department of Paediatrics and Child Health, Faculty of

Medicine and Health Sciences, Stellenbosch University, Cape Town, South Africa.

FAU - Khan, Munira

AU - Khan M

AUID- ORCID: 0000-0003-2083-2106

AD - Tuberculosis and HIV Investigative Network (THINK), Durban, South Africa.

FAU - Taneja, Rajneesh

AU - Taneja R

AUID- ORCID: 0000-0003-2418-9680

AD - Global Alliance for TB Drug Development, New York, New York, united States of

America.

FAU - Pande, Poonam

AU - Pande P

AUID- ORCID: 0000-0001-6830-0083

AD - Global Alliance for TB Drug Development, New York, New York, united States of

America.

FAU - Inabathina, Koteswara Rao

AU - Inabathina KR

AUID- ORCID: 0009-0005-4832-1414

AD - Global Alliance for TB Drug Development, New York, New York, united States of

America.

FAU - Hesseling, Anneke C

AU - Hesseling AC

AD - Desmond Tutu TB Centre, Department of Paediatrics and Child Health, Faculty of

Medicine and Health Sciences, Stellenbosch University, Cape Town, South Africa.

FAU - Garcia-Prats, Anthony J

AU - Garcia-Prats AJ

AD - Desmond Tutu TB Centre, Department of Paediatrics and Child Health, Faculty of

Medicine and Health Sciences, Stellenbosch University, Cape Town, South Africa.

AD - Department of Paediatrics, University of Wisconsin School of Medicine and Public

Health, Madison, Wisconsin, United States of America.

FAU - Hoddinott, Graeme

AU - Hoddinott G

AUID- ORCID: 0000-0001-5915-8126

AD - Desmond Tutu TB Centre, Department of Paediatrics and Child Health, Faculty of

Medicine and Health Sciences, Stellenbosch University, Cape Town, South Africa.

AD - Sydney School of Public Health, Faculty of Medicine and Health, University of

Sydney, Camperdown, Australia.

LA - eng

PT - Journal Article

DEP - 20251008

PL - United States

TA - PLOS Glob Public Health

JT - PLOS global public health

JID - 9918283779606676

PMC - PMC12507288

COIS- The authors have declared that no competing interests exist.

EDAT- 2025/10/08 18:33

MHDA- 2025/10/08 18:34

PMCR- 2025/10/08

CRDT- 2025/10/08 13:44

PHST- 2025/06/12 00:00 [received]

PHST- 2025/09/15 00:00 [accepted]

PHST- 2025/10/08 18:34 [medline]

PHST- 2025/10/08 18:33 [pubmed]

PHST- 2025/10/08 13:44 [entrez]

PHST- 2025/10/08 00:00 [pmc-release]

AID - PGPH-D-25-01547 [pii]

AID - 10.1371/journal.pgph.0005269 [doi]

PST - epublish

SO - PLOS Glob Public Health. 2025 Oct 8;5(10):e0005269. doi:

10.1371/journal.pgph.0005269. eCollection 2025.

PMID- 40827962

OWN - NLM

STAT- MEDLINE

DCOM- 20251001

LR - 20260518

IS - 1098-6596 (Electronic)

IS - 0066-4804 (Print)

IS - 0066-4804 (Linking)

VI - 69

IP - 10

DP - 2025 Oct

TI - Microbiological evidence for the trisubstituted benzimidazoles targeting MmpL3 in

Mycobacterium tuberculosis.

PG - e0036825

LID - 10.1128/aac.00368-25 [doi]

LID - e00368-25

AB - New anti-tuberculosis (TB) drugs with novel modes of action are in great demand

due to the complex treatment regimens as well as the rising number of

multidrug-resistant TB cases. We recently re-evaluated a few 2,5,6-trisubstituted

benzimidazole derivatives (SBZ) previously demonstrated to have potent

antitubercular activity. These compounds displayed favorable MICs and

significantly reduced bacterial counts in an acute mouse infection model.

Although this antitubercular lead series was initially reported to inhibit

mycobacterial cell division, our findings suggest that its primary activity

likely involves other cellular targets. By using bacterial cytological profiling,

we observed that SBZ-treated Mycobacterium tuberculosis cells exhibit cell

wall-damaging phenotypes resembling those caused by known cell wall biosynthesis

inhibitors, such as AU1235 and SQ109, that mostly target the membrane protein

large 3 (MmpL3). Whole-cell assays further supported the findings by showing

activation of the iniBAC operon and accumulation of intracellular ATP. The

antitubercular activity of SBZs was tested against engineered mycobacterial

strains that have the transcriptionally regulated mmpL3 gene expression,

confirming that SBZs engage the MmpL3 target in the cell. Strains with mutations

in mmpL3 exhibited either low- or high-level resistance to the SBZs. A molecule

docking model is proposed, based on a high-resolution crystal structure of MmpL3,

which could be useful in reconciling the inhibition mechanism and suggesting a

further development of MmpL3 inhibitor starting with the SBZ scaffold.

FAU - Zhang, Mengyun

AU - Zhang M

AUID- ORCID: 0009-0004-9911-3212

AD - Global Health Drug Discovery Institute, Beijing, China.

FAU - Allen, Renee

AU - Allen R

AD - Center for Global Infectious Disease Research, Seattle Children's Research

Institute, Seattle, Washington, USA.

FAU - Ames, Lauren

AU - Ames L

AD - Center for Global Infectious Disease Research, Seattle Children's Research

Institute, Seattle, Washington, USA.

FAU - Engelhart, Curtis A

AU - Engelhart CA

AD - Department of Microbiology and Immunology, Weill Cornell Medicine, New York, New

York, USA.

FAU - Quach, Diana

AU - Quach D

AD - Linnaeus Bioscience Inc., San Diego, California, USA.

FAU - Lv, Xiaoying

AU - Lv X

AD - Global Health Drug Discovery Institute, Beijing, China.

FAU - Xiao, Genhui

AU - Xiao G

AD - Global Health Drug Discovery Institute, Beijing, China.

FAU - Wang, Heng

AU - Wang H

AD - Global Health Drug Discovery Institute, Beijing, China.

FAU - Wang, Jinglan

AU - Wang J

AD - Global Health Drug Discovery Institute, Beijing, China.

FAU - Zhou, Liangliang

AU - Zhou L

AD - Global Health Drug Discovery Institute, Beijing, China.

FAU - Pan, Miaomiao

AU - Pan M

AD - Global Health Drug Discovery Institute, Beijing, China.

FAU - Sugie, Joseph

AU - Sugie J

AD - Linnaeus Bioscience Inc., San Diego, California, USA.

FAU - Pogliano, Joe

AU - Pogliano J

AD - Linnaeus Bioscience Inc., San Diego, California, USA.

FAU - Schnappinger, Dirk

AU - Schnappinger D

AUID- ORCID: 0000-0002-4445-7219

AD - Department of Microbiology and Immunology, Weill Cornell Medicine, New York, New

York, USA.

FAU - Parish, Tanya

AU - Parish T

AUID- ORCID: 0000-0001-7507-0423

AD - Center for Global Infectious Disease Research, Seattle Children's Research

Institute, Seattle, Washington, USA.

AD - Department of Pediatrics, University of Washington School of Medicine, Seattle,

Washington, USA.

FAU - Chen, Shawn

AU - Chen S

AUID- ORCID: 0000-0002-6678-3293

AD - Global Health Drug Discovery Institute, Beijing, China.

LA - eng

GR - INV-008249/GATES/Gates Foundation/United States

GR - INV-008249/GATES/Gates Foundation/United States

GR - INV-056399/Bill and Melinda Gates Foundation/

PT - Journal Article

DEP - 20250819

PL - United States

TA - Antimicrob Agents Chemother

JT - Antimicrobial agents and chemotherapy

JID - 0315061

RN - 0 (Benzimidazoles)

RN - 0 (Antitubercular Agents)

RN - 0 (Bacterial Proteins)

RN - 0 (MmpL3 protein, Mycobacterium tuberculosis)

RN - 0 (Membrane Transport Proteins)

SB - IM

MH - *Mycobacterium tuberculosis/drug effects/genetics/metabolism

MH - *Benzimidazoles/pharmacology/chemistry

MH - *Antitubercular Agents/pharmacology/chemistry

MH - *Bacterial Proteins/metabolism/genetics/antagonists & inhibitors

MH - Microbial Sensitivity Tests

MH - Cell Wall/drug effects/metabolism

MH - Mice

MH - Molecular Docking Simulation

MH - Animals

MH - Membrane Transport Proteins

PMC - PMC12486814

OTO - NOTNLM

OT - MmpL3

OT - Mycobacterium tuberculosis

OT - mechanisms of action

COIS- The authors declare no conflict of interest.

EDAT- 2025/08/19 12:32

MHDA- 2025/10/01 12:31

PMCR- 2025/08/19

CRDT- 2025/08/19 09:42

PHST- 2025/10/01 12:31 [medline]

PHST- 2025/08/19 12:32 [pubmed]

PHST- 2025/08/19 09:42 [entrez]

PHST- 2025/08/19 00:00 [pmc-release]

AID - aac00368-25 [pii]

AID - aac.00368-25 [pii]

AID - 10.1128/aac.00368-25 [doi]

PST - ppublish

SO - Antimicrob Agents Chemother. 2025 Oct;69(10):e0036825. doi: 10.1128/aac.00368-25.

Epub 2025 Aug 19.

PMID- 40681004

OWN - NLM

STAT- MEDLINE

DCOM- 20250810

LR - 20250810

IS - 1096-1208 (Electronic)

IS - 0882-4010 (Linking)

VI - 207

DP - 2025 Oct

TI - Immune defense and immune evasion in Mycobacterium tuberculosis Infection:

Inspirations and challenges for host directed therapy.

PG - 107914

LID - S0882-4010(25)00639-4 [pii]

LID - 10.1016/j.micpath.2025.107914 [doi]

AB - Tuberculosis (TB), caused by Mycobacterium tuberculosis (Mtb), remains a major

global public health issue, despite improvements in socioeconomic conditions and

widespread use of antibiotics. Host immune defense against Mtb infection involve

various cells like macrophages, dendritic cells, natural killer cells and T cell

subsets, which play distinct roles. By inhibiting phagosome maturation,

modulating reactive oxygen and nitrogen species production, regulating host cell

death pathway, as well as suppressing antigen presentation and T cell immune

responses, the immune escape help Mtb to survive and replicate in macrophages,

which ultimately contributes to the development of latent or active TB. While

traditional TB treatment strategy suffers challenges like low efficacy, long

treatment durations and side effects, the emergence of drug-resistant TB (DR-TB)

and multidrug-resistant TB (MDR-TB), which further highlight the therapeutic

challenges due to the low cure rate. Host Directed Therapy (HDT) is an emerging

supplementary approach to TB treatment, which leverages insights into how host

immune cells defend Mtb infection, as well as how pathogens manipulate host

immune defense mechanisms. HDT is an approach for treating TB that appropriately

modulates host immune responses, which aims to enhance the antimicrobial activity

of the host. In this review, we summarized the host immune defense mechanisms, as

well as analyzed how Mtb evades host immunological killings, thus potentially

providing new insights into the host-pathogen interactions during Mtb infection

and TB development. Furthermore, we reviewed recent advances in exploring HDT

strategies for effective anti-TB interventions, which may highlight more

effective therapeutics to fight against TB.

CI - Copyright © 2025 Elsevier Ltd. All rights reserved.

FAU - Li, Jiaxiang

AU - Li J

AD - Dongguan Key Laboratory for Pathogenesis and Experimental Diagnosis of Infectious

Diseases, The First Dongguan Affiliated Hospital, School of Medical Technology,

Guangdong Medical University, Dongguan, 523808, China; Research Center of Nano

Technology and Application Engineering, Dongguan Innovation Institute, School of

Medical Technology, Guangdong Medical University, Dongguan, 523808, China.

FAU - Liu, Yilin

AU - Liu Y

AD - Dongguan Key Laboratory for Pathogenesis and Experimental Diagnosis of Infectious

Diseases, The First Dongguan Affiliated Hospital, School of Medical Technology,

Guangdong Medical University, Dongguan, 523808, China; Research Center of Nano

Technology and Application Engineering, Dongguan Innovation Institute, School of

Medical Technology, Guangdong Medical University, Dongguan, 523808, China.

FAU - Ruan, Yongdui

AU - Ruan Y

AD - Dongguan Key Laboratory for Pathogenesis and Experimental Diagnosis of Infectious

Diseases, The First Dongguan Affiliated Hospital, School of Medical Technology,

Guangdong Medical University, Dongguan, 523808, China.

FAU - Kong, Xinen

AU - Kong X

AD - Dongguan Key Laboratory for Pathogenesis and Experimental Diagnosis of Infectious

Diseases, The First Dongguan Affiliated Hospital, School of Medical Technology,

Guangdong Medical University, Dongguan, 523808, China; Research Center of Nano

Technology and Application Engineering, Dongguan Innovation Institute, School of

Medical Technology, Guangdong Medical University, Dongguan, 523808, China.

FAU - Jin, Xiaoying

AU - Jin X

AD - Dongguan Key Laboratory for Pathogenesis and Experimental Diagnosis of Infectious

Diseases, The First Dongguan Affiliated Hospital, School of Medical Technology,

Guangdong Medical University, Dongguan, 523808, China; Research Center of Nano

Technology and Application Engineering, Dongguan Innovation Institute, School of

Medical Technology, Guangdong Medical University, Dongguan, 523808, China.

FAU - Wang, Jiajun

AU - Wang J

AD - Dongguan Key Laboratory for Pathogenesis and Experimental Diagnosis of Infectious

Diseases, The First Dongguan Affiliated Hospital, School of Medical Technology,

Guangdong Medical University, Dongguan, 523808, China; Research Center of Nano

Technology and Application Engineering, Dongguan Innovation Institute, School of

Medical Technology, Guangdong Medical University, Dongguan, 523808, China.

FAU - Liao, Kangsheng

AU - Liao K

AD - Dongguan Key Laboratory for Pathogenesis and Experimental Diagnosis of Infectious

Diseases, The First Dongguan Affiliated Hospital, School of Medical Technology,

Guangdong Medical University, Dongguan, 523808, China; Research Center of Nano

Technology and Application Engineering, Dongguan Innovation Institute, School of

Medical Technology, Guangdong Medical University, Dongguan, 523808, China.

Electronic address: liaoks@yeah.net.

FAU - Shen, Ling

AU - Shen L

AD - Department of Microbiology and Immunology, University of Illinois at Chicago,

Chicago, IL, 60607, USA. Electronic address: lshen@uic.edu.

FAU - Pi, Jiang

AU - Pi J

AD - Dongguan Key Laboratory for Pathogenesis and Experimental Diagnosis of Infectious

Diseases, The First Dongguan Affiliated Hospital, School of Medical Technology,

Guangdong Medical University, Dongguan, 523808, China; Research Center of Nano

Technology and Application Engineering, Dongguan Innovation Institute, School of

Medical Technology, Guangdong Medical University, Dongguan, 523808, China.

Electronic address: jiangpi@gdmu.edu.cn.

LA - eng

PT - Journal Article

PT - Review

DEP - 20250716

PL - England

TA - Microb Pathog

JT - Microbial pathogenesis

JID - 8606191

RN - 0 (Antitubercular Agents)

SB - IM

MH - *Mycobacterium tuberculosis/immunology/pathogenicity/drug effects

MH - Humans

MH - *Immune Evasion

MH - *Tuberculosis/immunology/microbiology/drug therapy/therapy

MH - *Host-Pathogen Interactions/immunology

MH - Macrophages/immunology/microbiology

MH - Animals

MH - Antitubercular Agents/therapeutic use

MH - Dendritic Cells/immunology

MH - Tuberculosis, Multidrug-Resistant/immunology

OTO - NOTNLM

OT - Host directed therapy (HDT)

OT - Host immunity

OT - Immune evasion

OT - Mycobacterium tuberculosis (Mtb)

OT - Tuberculosis (TB)

COIS- Declaration of competing interest The authors declare that they have no competing

interests.

EDAT- 2025/07/19 16:46

MHDA- 2025/08/11 00:26

CRDT- 2025/07/18 19:36

PHST- 2025/05/22 00:00 [received]

PHST- 2025/07/13 00:00 [revised]

PHST- 2025/07/14 00:00 [accepted]

PHST- 2025/08/11 00:26 [medline]

PHST- 2025/07/19 16:46 [pubmed]

PHST- 2025/07/18 19:36 [entrez]

AID - S0882-4010(25)00639-4 [pii]

AID - 10.1016/j.micpath.2025.107914 [doi]

PST - ppublish

SO - Microb Pathog. 2025 Oct;207:107914. doi: 10.1016/j.micpath.2025.107914. Epub 2025

Jul 16.

PMID- 40650441

OWN - NLM

STAT- MEDLINE

DCOM- 20250924

LR - 20250924

IS - 1520-5762 (Electronic)

IS - 0363-9045 (Linking)

VI - 51

IP - 10

DP - 2025 Oct

TI - Antimycobacterial and autophagic activity of nebulized delamanid microemulsion

targeting alveolar macrophage.

PG - 1347-1362

LID - 10.1080/03639045.2025.2532762 [doi]

AB - OBJECTIVE: The aerosol delivery of the novel anti-tubercular drug delamanid for

pulmonary targeting holds promise for the effective treatment of

multi-drug-resistant tuberculosis. Here, we developed a microemulsion formulation

of delamanid for nebulization, evaluating its potential bioactivity. METHODS:

Herein, we attempted to develop microemulsion formulations loaded delamanid for

nebulization and their physicochemical characteristics were assessed. The

formulation was checked for aerosol characteristics, cytotoxic potential, in

vitro antimycobacterial activity, and autophagy. RESULTS: The optimized

formulations, nebulized into a next-generation impactor, exhibited a fine

particle fraction of 63.12%, ensuring efficient in vitro deposition. In vitro

evaluations revealed that formulation did not demonstrate any cytotoxic potential

on respiratory cell lines up to 2.5 µg/mL and lack of inflammatory cytokines

production and nitric oxide from macrophage NR8383 cells, signified its safety.

The flow cytometry analysis revealed that the elimination of Mycobacterium bovis

by formulation after day 4, with considerably low minimum bactericidal

concentration. The formulation demonstrated adequate macrophage autophagic

activity when evaluated using confocal laser scanning microscopy and Western

blot. CONCLUSION: Consequently, the aerosolized microemulsion of delamanid for

pulmonary delivery may serve as an effective therapy for MDR-TB, necessitating

further in vivo studies.

FAU - Paliwal, Himanshu

AU - Paliwal H

AD - Department of Pharmacy, Marwadi University Research Center, Marwadi University,

Rajkot, India.

FAU - Tongkanarak, Krittawan

AU - Tongkanarak K

AD - Drug Delivery System Excellence Center, Department of Pharmaceutical Technology,

Faculty of Pharmaceutical Sciences, Prince of Songkla University, Hat Yai,

Songkhla, Thailand.

FAU - Aekwattanaphol, Nattanit

AU - Aekwattanaphol N

AD - Drug Delivery System Excellence Center, Department of Pharmaceutical Technology,

Faculty of Pharmaceutical Sciences, Prince of Songkla University, Hat Yai,

Songkhla, Thailand.

FAU - Srichana, Teerapol

AU - Srichana T

AD - Drug Delivery System Excellence Center, Department of Pharmaceutical Technology,

Faculty of Pharmaceutical Sciences, Prince of Songkla University, Hat Yai,

Songkhla, Thailand.

LA - eng

PT - Journal Article

DEP - 20250724

PL - England

TA - Drug Dev Ind Pharm

JT - Drug development and industrial pharmacy

JID - 7802620

RN - 0 (Emulsions)

RN - 0 (Nitroimidazoles)

RN - 0 (Oxazoles)

RN - 0 (OPC-67683)

RN - 0 (Antitubercular Agents)

RN - 0 (Aerosols)

SB - IM

MH - Emulsions/chemistry

MH - *Autophagy/drug effects

MH - *Macrophages, Alveolar/drug effects/metabolism

MH - Animals

MH - Cell Line

MH - *Nitroimidazoles/pharmacology/administration & dosage/chemistry

MH - *Oxazoles/pharmacology/administration & dosage/chemistry

MH - Administration, Inhalation

MH - *Antitubercular Agents/administration & dosage/pharmacology/chemistry

MH - Nebulizers and Vaporizers

MH - Mycobacterium bovis/drug effects

MH - Aerosols

MH - Particle Size

MH - Microbial Sensitivity Tests

MH - Drug Delivery Systems/methods

OTO - NOTNLM

OT - Delamanid

OT - autophagy

OT - microemulsion

OT - nebulizer

OT - pulmonary tuberculosis

EDAT- 2025/07/13 03:38

MHDA- 2025/09/24 13:00

CRDT- 2025/07/12 04:03

PHST- 2025/09/24 13:00 [medline]

PHST- 2025/07/13 03:38 [pubmed]

PHST- 2025/07/12 04:03 [entrez]

AID - 10.1080/03639045.2025.2532762 [doi]

PST - ppublish

SO - Drug Dev Ind Pharm. 2025 Oct;51(10):1347-1362. doi:

10.1080/03639045.2025.2532762. Epub 2025 Jul 24.

PMID- 40175253

OWN - NLM

STAT- MEDLINE

DCOM- 20250904

LR - 20250904

IS - 1995-9133 (Electronic)

IS - 1684-1182 (Linking)

VI - 58

IP - 5

DP - 2025 Oct

TI - Recent advances in research on Mycobacterium tuberculosis virulence factors and

their role in pathogenesis.

PG - 497-507

LID - S1684-1182(25)00079-9 [pii]

LID - 10.1016/j.jmii.2025.03.017 [doi]

AB - Mycobacterium tuberculosis (Mtb) is the causative agent of tuberculosis (TB) in

humans and animals. Mtb invades the host's lungs via airborne transmission,

infecting macrophages and causing TB. In some cases, the infection can spread to

other tissues and organs. Despite the availability of several drugs for TB

treatment, the emergence of multidrug-resistant TB has led to high morbidity and

mortality rates worldwide. Therefore, it is urgent to discover new

anti-tuberculosis drugs for more effective treatment. Recent studies have shown

that Mtb virulence factors play a crucial role in its pathogenicity. By evading

the host's immune surveillance through mechanisms such as anti-oxidative stress,

nutrient synthesis and metabolism, and apoptosis in host cells, Mtb can achieve

long-term survival in the host. Understanding the pathogenicity mechanisms of Mtb

will aid the development of new vaccines and anti-tuberculosis drugs. In this

review, we summarize the latest research progress on Mtb virulence factors to

provide a reference for targeted TB treatment.

CI - Copyright © 2025. Published by Elsevier B.V.

FAU - Sun, Ming-Rui

AU - Sun MR

AD - Key Laboratory of Respiratory Tract Pathogens and Drug Therapy, School of Life

Science and Technology, Shandong Second Medical University, Weifang, 261053,

China.

FAU - Xing, Jia-Yin

AU - Xing JY

AD - Key Laboratory of Respiratory Tract Pathogens and Drug Therapy, School of Life

Science and Technology, Shandong Second Medical University, Weifang, 261053,

China.

FAU - Li, Xiao-Tian

AU - Li XT

AD - Key Laboratory of Respiratory Tract Pathogens and Drug Therapy, School of Life

Science and Technology, Shandong Second Medical University, Weifang, 261053,

China.

FAU - Fang, Ren

AU - Fang R

AD - Key Laboratory of Respiratory Tract Pathogens and Drug Therapy, School of Life

Science and Technology, Shandong Second Medical University, Weifang, 261053,

China.

FAU - Zhang, Yang

AU - Zhang Y

AD - Key Laboratory of Respiratory Tract Pathogens and Drug Therapy, School of Life

Science and Technology, Shandong Second Medical University, Weifang, 261053,

China.

FAU - Li, Zhao-Li

AU - Li ZL

AD - SAFE Pharmaceutical Technology Co., Ltd., Beijing, 100000, China. Electronic

address: lizhaoli@safeglp.com.

FAU - Song, Ning-Ning

AU - Song NN

AD - Key Laboratory of Respiratory Tract Pathogens and Drug Therapy, School of Life

Science and Technology, Shandong Second Medical University, Weifang, 261053,

China. Electronic address: songningning@sdsmu.edu.cn.

LA - eng

PT - Journal Article

PT - Review

DEP - 20250327

PL - England

TA - J Microbiol Immunol Infect

JT - Journal of microbiology, immunology, and infection = Wei mian yu gan ran za zhi

JID - 100956211

RN - 0 (Virulence Factors)

RN - 0 (Antitubercular Agents)

SB - IM

MH - *Virulence Factors/metabolism/genetics

MH - *Mycobacterium tuberculosis/pathogenicity/genetics

MH - Humans

MH - Animals

MH - *Tuberculosis/microbiology/drug therapy

MH - Antitubercular Agents/pharmacology/therapeutic use

MH - Virulence

MH - Host-Pathogen Interactions

OTO - NOTNLM

OT - Drug targets

OT - Mycobacterium tuberculosis

OT - TB treatment

OT - Virulence factors

COIS- Declaration of competing interest The authors declare no potential conflicts of

interest with regards to the research, authorship, or publication of this

article.

EDAT- 2025/04/03 00:25

MHDA- 2025/09/05 00:35

CRDT- 2025/04/02 21:59

PHST- 2024/10/22 00:00 [received]

PHST- 2025/02/28 00:00 [revised]

PHST- 2025/03/25 00:00 [accepted]

PHST- 2025/09/05 00:35 [medline]

PHST- 2025/04/03 00:25 [pubmed]

PHST- 2025/04/02 21:59 [entrez]

AID - S1684-1182(25)00079-9 [pii]

AID - 10.1016/j.jmii.2025.03.017 [doi]

PST - ppublish

SO - J Microbiol Immunol Infect. 2025 Oct;58(5):497-507. doi:

10.1016/j.jmii.2025.03.017. Epub 2025 Mar 27.

PMID- 39446300

OWN - NLM

STAT- MEDLINE

DCOM- 20250930

LR - 20250930

IS - 1559-0305 (Electronic)

IS - 1073-6085 (Linking)

VI - 67

IP - 10

DP - 2025 Oct

TI - Molecular Indicator for Distinguishing Multi-drug-Resistant Tuberculosis from

Drug Sensitivity Tuberculosis and Potential Medications for Treatment.

PG - 3946-3959

LID - 10.1007/s12033-024-01299-z [doi]

AB - The issue of multi-drug-resistant tuberculosis (MDR-TB) presents a substantial

challenge to global public health. Regrettably, the diagnosis of drug-resistant

tuberculosis (DR-TB) frequently necessitates an extended period or more extensive

laboratory resources. The swift identification of MDR-TB poses a particularly

challenging endeavor. To identify the biomarkers indicative of multi-drug

resistance, we conducted a screening of the GSE147689 dataset for differentially

expressed genes (DEGs) and subsequently conducted a gene enrichment analysis. Our

analysis identified a total of 117 DEGs, concentrated in pathways related to the

immune response. Three machine learning methods, namely random forest, decision

tree, and support vector machine recursive feature elimination (SVM-RFE), were

implemented to identify the top 10 genes according to their feature importance

scores. A4GALT and S1PR1, which were identified as common genes among the three

methods, were selected as potential molecular markers for distinguishing between

MDR-TB and drug-susceptible tuberculosis (DS-TB). These markers were subsequently

validated using the GSE147690 dataset. The findings suggested that A4GALT

exhibited area under the curve (AUC) values of 0.8571 and 0.7121 in the training

and test datasets, respectively, for distinguishing between MDR-TB and DS-TB.

S1PR1 demonstrated AUC values of 0.8163 and 0.5404 in the training and test

datasets, respectively. When A4GALT and S1PR1 were combined, the AUC values in

the training and test datasets were 0.881 and 0.7551, respectively. The

relationship between hub genes and 28 immune cells infiltrating MDR-TB was

investigated using single sample gene enrichment analysis (ssGSEA). The findings

indicated that MDR-TB samples exhibited a higher proportion of type 1 T helper

cells and a lower proportion of activated dendritic cells in contrast to DS-TB

samples. A negative correlation was observed between A4GALT and type 1 T helper

cells, whereas a positive correlation was found with activated dendritic cells.

S1PR1 exhibited a positive correlation with type 1 T helper cells and a negative

correlation with activated dendritic cells. Furthermore, our study utilized

connectivity map analysis to identify nine potential medications, including

verapamil, for treating MDR-TB. In conclusion, our research identified two

molecular indicators for the differentiation between MDR-TB and DS-TB and

identified a total of nine potential medications for MDR-TB.

CI - © 2024. The Author(s), under exclusive licence to Springer Science+Business

Media, LLC, part of Springer Nature.

FAU - Song, Shulin

AU - Song S

AD - Department of Radiology, Guangxi Medical University Cancer Hospital, Nanning,

530023, Guangxi, China.

AD - Department of Radiology, The Fourth People's Hospital of Nanning, Nanning,

530023, Guangxi, China.

FAU - Gan, Donghui

AU - Gan D

AD - Department of Radiology, The Fourth People's Hospital of Nanning, Nanning,

530023, Guangxi, China.

FAU - Wu, Di

AU - Wu D

AD - Department of Radiology, The Fourth People's Hospital of Nanning, Nanning,

530023, Guangxi, China.

FAU - Li, Ting

AU - Li T

AD - Department of Radiology, Guangxi Medical University Cancer Hospital, Nanning,

530023, Guangxi, China.

FAU - Zhang, Shiqian

AU - Zhang S

AD - Department of Radiology, The Fourth People's Hospital of Nanning, Nanning,

530023, Guangxi, China.

FAU - Lu, Yibo

AU - Lu Y

AD - Department of Radiology, The Fourth People's Hospital of Nanning, Nanning,

530023, Guangxi, China. bobosunny@163.com.

FAU - Jin, Guanqiao

AU - Jin G

AUID- ORCID: 0000-0003-1183-6228

AD - Department of Radiology, Guangxi Medical University Cancer Hospital, Nanning,

530023, Guangxi, China. jinguanqiao77@gxmu.edu.cn.

LA - eng

GR - 20233069/Key Research and Development Program of Nanning/

PT - Journal Article

DEP - 20241024

PL - Switzerland

TA - Mol Biotechnol

JT - Molecular biotechnology

JID - 9423533

RN - 0 (Antitubercular Agents)

RN - 0 (Biomarkers)

SB - IM

MH - *Tuberculosis, Multidrug-Resistant/diagnosis/genetics/drug therapy/microbiology

MH - Humans

MH - *Antitubercular Agents/therapeutic use/pharmacology

MH - Machine Learning

MH - *Mycobacterium tuberculosis/drug effects/genetics

MH - Gene Expression Profiling/methods

MH - Support Vector Machine

MH - Biomarkers

MH - Computational Biology/methods

MH - Databases, Genetic

MH - Drug Resistance, Multiple, Bacterial/genetics

OTO - NOTNLM

OT - Biomarker

OT - Drug

OT - Immune infiltrating

OT - Machine learning

OT - Multi-drug-resistant tuberculosis

COIS- Declarations. Conflict of interest: The authors declare that they have no

competing interests. Ethical Approval: Not applicable. Consent to participate:

Not applicable. Consent for publication: Not applicable.

EDAT- 2024/10/24 12:32

MHDA- 2025/09/30 14:17

CRDT- 2024/10/24 11:18

PHST- 2023/11/26 00:00 [received]

PHST- 2024/09/30 00:00 [accepted]

PHST- 2025/09/30 14:17 [medline]

PHST- 2024/10/24 12:32 [pubmed]

PHST- 2024/10/24 11:18 [entrez]

AID - 10.1007/s12033-024-01299-z [pii]

AID - 10.1007/s12033-024-01299-z [doi]

PST - ppublish

SO - Mol Biotechnol. 2025 Oct;67(10):3946-3959. doi: 10.1007/s12033-024-01299-z. Epub

2024 Oct 24.

PMID- 41063792

OWN - NLM

STAT- Publisher

LR - 20260422

IS - 2632-8682 (Electronic)

IS - 2632-8682 (Linking)

VI - 16

IP - 12

DP - 2025 Sep 25

TI - Tanshinones target drug-resistant tuberculosis: efficacy, selectivity, and

potential mechanism of action.

PG - 6020-30

LID - 10.1039/d5md00637f [doi]

AB - This study evaluates the antimycobacterial potential of tanshinone I (TI),

tanshinone IIA (TIIA), and cryptotanshinone (CPT), natural compounds isolated

from Salvia miltiorrhiza, against Mycobacterium tuberculosis, the primary

etiological agent of tuberculosis. Given the global challenge posed by

antimicrobial resistance and the complexity of current treatment regimens, we

aimed to identify effective and safe alternative therapies. The compounds' in

vitro activity was initially assessed via minimum inhibitory concentration

(MIC(90)) and cytotoxicity index (CI(50)) determinations, yielding MIC(90) values

of 1.03, 0.38, and 1.21 μg mL(-1) for TI, TIIA, and CPT, respectively, with low

toxicity and high selectivity indices. A narrow antimicrobial spectrum was

observed upon testing against representative bacteria, fungi, and non-tuberculous

mycobacteria (NTM). Combination assays with rifampicin revealed synergism for TI

and indifference for TIIA and CPT, as determined by the fractional inhibitory

concentration index (FICI). Scanning electron microscopy (SEM) revealed

morphological alterations in the bacilli's cell wall, suggesting it as a possible

target of the compounds' mechanism of action. Whole genome sequencing (WGS) of

resistant strains identified mutations predominantly in PE_PGRS family genes,

supporting the hypothesis that tanshinones modulate cell wall structure. Finally,

efficacy was confirmed against multidrug-resistant clinical isolates, with

MIC(90) values near 1 μg mL(-1). These findings position TI, TIIA, and CPT as

promising candidates for developing new therapies against drug-resistant

tuberculosis.

CI - This journal is © The Royal Society of Chemistry.

FAU - Polinário, Giulia

AU - Polinário G

AD - Tuberculosis Research Laboratory, School of Pharmaceutical Sciences, São Paulo

State University - UNESP Araraquara São Paulo Brazil fernando.pavan@unesp.br.

FAU - Rosa, Maiara A B C

AU - Rosa MABC

AD - Tuberculosis Research Laboratory, School of Pharmaceutical Sciences, São Paulo

State University - UNESP Araraquara São Paulo Brazil fernando.pavan@unesp.br.

FAU - Campos, Débora L

AU - Campos DL

AD - Tuberculosis Research Laboratory, School of Pharmaceutical Sciences, São Paulo

State University - UNESP Araraquara São Paulo Brazil fernando.pavan@unesp.br.

FAU - Moraes, Liliana L S

AU - Moraes LLS

AD - Department of Clinical and Toxicological Analysis, Federal University of Santa

Maria (UFSM) Santa Maria RS Brazil.

FAU - de Campos, Marli M A

AU - de Campos MMA

AD - Department of Clinical and Toxicological Analysis, Federal University of Santa

Maria (UFSM) Santa Maria RS Brazil.

FAU - Silva, Ingrid Gracielle M

AU - Silva IGM

AD - Microscopy and Microanalysis Laboratory, Department of Cell Biology, Institute of

Biological Sciences, University of Brasília Brasília DF Brazil.

FAU - Barros-Cordeiro, Karine B

AU - Barros-Cordeiro KB

AD - Microscopy and Microanalysis Laboratory, Department of Cell Biology, Institute of

Biological Sciences, University of Brasília Brasília DF Brazil.

FAU - Báo, Sônia N

AU - Báo SN

AD - Microscopy and Microanalysis Laboratory, Department of Cell Biology, Institute of

Biological Sciences, University of Brasília Brasília DF Brazil.

FAU - Pavan, Fernando R

AU - Pavan FR

AUID- ORCID: 0000-0002-6969-3963

AD - Tuberculosis Research Laboratory, School of Pharmaceutical Sciences, São Paulo

State University - UNESP Araraquara São Paulo Brazil fernando.pavan@unesp.br.

LA - eng

PT - Journal Article

DEP - 20250925

PL - England

TA - RSC Med Chem

JT - RSC medicinal chemistry

JID - 101759460

PMC - PMC12503125

COIS- There is no conflict of interest to declare.

EDAT- 2025/10/09 06:28

MHDA- 2025/10/09 06:28

PMCR- 2026/09/25

CRDT- 2025/10/09 04:55

PHST- 2025/07/21 00:00 [received]

PHST- 2025/09/23 00:00 [accepted]

PHST- 2025/10/09 06:28 [medline]

PHST- 2025/10/09 06:28 [pubmed]

PHST- 2025/10/09 04:55 [entrez]

PHST- 2026/09/25 00:00 [pmc-release]

AID - d5md00637f [pii]

AID - 10.1039/d5md00637f [doi]

PST - aheadofprint

SO - RSC Med Chem. 2025 Sep 25;16(12):6020-30. doi: 10.1039/d5md00637f.

PMID- 40976821

OWN - NLM

STAT- Publisher

LR - 20250921

IS - 1874-9356 (Electronic)

IS - 0015-5632 (Linking)

DP - 2025 Sep 22

TI - Flurbiprofen restores rifampicin and isoniazid sensitivity in multidrug-resistant

Mycobacterium tuberculosis putatively by inhibiting efflux pumps Rv0194 and

Rv0933.

LID - 10.1007/s12223-025-01319-8 [doi]

AB - Multidrug-resistant tuberculosis remains a global health challenge, necessitating

novel therapeutic approaches. Efflux pumps, including Rv0194 and Rv0933,

contribute to Mycobacterium tuberculosis resistance by actively extruding

first-line drugs such as rifampicin and isoniazid. This study aimed to identify

small-molecule inhibitors that target these pumps to restore drug susceptibility.

Through in silico screening, six lead compounds were selected and evaluated for

antimicrobial activity against ten MDR-TB clinical isolates. Among them,

flurbiprofen and trichlorocarbinalide exhibited significant inhibitory effects,

enhancing rifampicin and isoniazid activity in checkerboard synergy assays. These

combinations reduced the minimum inhibitory concentrations of both drugs,

confirming their potential to reverse resistance. Cytotoxicity assessments of

peripheral blood mononuclear and THP-1 cells demonstrated favourable safety

profiles. Mechanistic studies revealed increased expression of Rv0194 and Rv0933

upon rifampicin and isoniazid exposure, underscoring their role in drug

resistance. Flurbiprofen and trichlorocarbinalide may enhance intracellular drug

retention by inhibiting these efflux pumps, improving therapeutic efficacy.

However, trichlorocarbinalide did not restore rifampicin or isoniazid sensitivity

as efficiently as flurbiprofen did. These findings highlight flurbiprofen as a

promising efflux pump inhibitor that could potentiate standard TB treatments and

counteract resistance. Further studies using diverse clinical isolates and in

vivo models are needed to validate its therapeutic potential.

CI - © 2025. Institute of Microbiology, Academy of Sciences of the Czech Republic,

v.v.i.

FAU - Elango, Padmasini

AU - Elango P

AD - Department of Bacteriology, ICMR-National Institute for Research in Tuberculosis,

No.1, Mayor Sathyamoorthy Salai, Chetpet, Chennai, 600031, India.

AD - Department of Molecular Analytics, SIMATS Engineering, Saveetha University,

Chennai, India.

FAU - Nirmal, Christy Rosaline

AU - Nirmal CR

AD - Department of Bacteriology, ICMR-National Institute for Research in Tuberculosis,

No.1, Mayor Sathyamoorthy Salai, Chetpet, Chennai, 600031, India.

AD - Achira Labs Pvt. Ltd., No. 66B, 13th Cross, Dollar Layout, JP Nagar III Phase,

Bangalore, Karnataka, India.

FAU - Rajadas, Sam Ebenezer

AU - Rajadas SE

AD - Department of Bacteriology, ICMR-National Institute for Research in Tuberculosis,

No.1, Mayor Sathyamoorthy Salai, Chetpet, Chennai, 600031, India.

AD - Centre for Drug Discovery and Development, Sathyabama Institute of Science and

Technology, Chennai, 600119, India.

FAU - Ravi, Rajkumar

AU - Ravi R

AD - Department of Bacteriology, ICMR-National Institute for Research in Tuberculosis,

No.1, Mayor Sathyamoorthy Salai, Chetpet, Chennai, 600031, India.

FAU - Chilamakuru, Naresh Babu

AU - Chilamakuru NB

AD - Department of Pharmaceutical Chemistry, Raghavendra Institute of Pharmaceutical

Education and Research (RIPER)-Autonomous, K.R.Palli Cross, Anantapur, 515721,

India.

FAU - V N, Azger Dusthackeer

AU - V N AD

AD - Department of Bacteriology, ICMR-National Institute for Research in Tuberculosis,

No.1, Mayor Sathyamoorthy Salai, Chetpet, Chennai, 600031, India.

azgerdusthackeer.vn@icmr.gov.in.

LA - eng

GR - Fellowship/Bact/RA/Padmasini/31.10.2022/National Institute for Research in

Tuberculosis/

GR - F.No. 3/1/3/PDF(21)/2019-HRD dt 18.03.2020/National Institute for Research in

Tuberculosis/

GR - No. Fellowship/TB/40/2020-7030/National Institute for Research in Tuberculosis/

PT - Journal Article

DEP - 20250922

PL - United States

TA - Folia Microbiol (Praha)

JT - Folia microbiologica

JID - 0376757

SB - IM

OTO - NOTNLM

OT - Drug synergy

OT - Efflux pump inhibitors

OT - Flurbiprofen

OT - Multidrug-resistant tuberculosis

OT - Rv0194

OT - Rv0933

COIS- Declarations. Ethical approval: This study was approved by the Institutional

Ethics Committee of the ICMR-National Institute for Research in Tuberculosis

(NIRT, Chennai) before its implementation (No: 021/NIRT-IEC/2021; dtd 25/2/2021).

This study was also submitted and issued approval from the institutional

bioethics committee for Category III as per the guidelines of DBT held at ICMR

NIRT, Chennai, on 06/10/2021. Conflict of interest: The authors declare no

competing interests.

EDAT- 2025/09/22 00:30

MHDA- 2025/09/22 00:30

CRDT- 2025/09/21 23:14

PHST- 2025/03/28 00:00 [received]

PHST- 2025/08/07 00:00 [accepted]

PHST- 2025/09/22 00:30 [medline]

PHST- 2025/09/22 00:30 [pubmed]

PHST- 2025/09/21 23:14 [entrez]

AID - 10.1007/s12223-025-01319-8 [pii]

AID - 10.1007/s12223-025-01319-8 [doi]

PST - aheadofprint

SO - Folia Microbiol (Praha). 2025 Sep 22. doi: 10.1007/s12223-025-01319-8.

PMID- 40970967

OWN - NLM

STAT- MEDLINE

DCOM- 20260626

LR - 20260626

IS - 1432-1831 (Electronic)

IS - 0300-8584 (Print)

IS - 0300-8584 (Linking)

VI - 214

IP - 1

DP - 2025 Sep 19

TI - Therapeutic potential of Bacillus sonorensis PMC204 membrane vesicles against

drug-resistant Mycobacterium tuberculosis.

PG - 43

LID - 10.1007/s00430-025-00851-1 [doi]

LID - 43

AB - Tuberculosis remains a severe global health threat, exacerbated by the rising

prevalence of multidrug-resistant (MDR) and extensively drug-resistant (XDR)

Mycobacterium tuberculosis. Despite the urgent need for effective interventions,

the development of anti-tuberculosis drugs has been slow, and the emergence of

pan-drug-resistant strains underscores the critical need for innovative

therapeutic strategies. This study introduces Bacillus sonorensis PMC204, a novel

probiotic strain with potent anti-tuberculosis properties identified through

extensive screening. PMC204 significantly reduced M. tuberculosis H37Rv and XDR

strains within Raw 264.7 macrophage cells. Moreover, membrane vesicles (MVs)

derived from this strain exhibited superior inhibitory effects against both

standard and XDR strains of M. tuberculosis. Proteomic analysis of the isolated

MVs revealed a high abundance of flagellin proteins, which are hypothesized to

play a pivotal role in the observed anti-tuberculosis effects. These findings

also suggest a close link between the therapeutic efficacy of PMC204 and

autophagy activation. Safety assessments further demonstrated the feasibility of

PMC204 as a potential anti-tuberculosis therapeutic. The anti-tuberculosis

activity of bacterial MVs represents an innovative approach in microbiome

therapeutics, positioning PMC204 as a next-generation probiotic distinct from

conventional strains. This study contributes to advancing the field of

microbiome-based therapeutics and presents promising avenues for managing

drug-resistant tuberculosis.

CI - © 2025. The Author(s).

FAU - Lee, Youngkyoung

AU - Lee Y

AUID- ORCID: 0000-0001-5426-4050

AD - Department of Microbiology and Immunology, School of Medicine, Soonchunhyang

University, Cheonan, Chungnam, 31151, Korea.

AD - Human Microbiome Medical Research Center (HM·MRC), School of Medicine,

Soonchunhyang University, Asan, Chungnam, 31538, Korea.

FAU - Seo, Hoonhee

AU - Seo H

AUID- ORCID: 0000-0002-5932-6633

AD - Human Microbiome Medical Research Center (HM·MRC), School of Medicine,

Soonchunhyang University, Asan, Chungnam, 31538, Korea.

FAU - Lee, Soyeon

AU - Lee S

AUID- ORCID: 0000-0001-6284-9999

AD - Department of Microbiology and Immunology, School of Medicine, Soonchunhyang

University, Cheonan, Chungnam, 31151, Korea.

FAU - Choi, Dongsic

AU - Choi D

AUID- ORCID: 0000-0002-2516-5616

AD - Department of Biochemistry, School of Medicine, Soonchunhyang University,

Cheonan, Chungnam, 31151, Korea.

FAU - Kim, Sukyung

AU - Kim S

AD - Human Microbiome Medical Research Center (HM·MRC), School of Medicine,

Soonchunhyang University, Asan, Chungnam, 31538, Korea.

FAU - Rahim, Md Abdur

AU - Rahim MA

AUID- ORCID: 0000-0003-2000-5602

AD - Human Microbiome Medical Research Center (HM·MRC), School of Medicine,

Soonchunhyang University, Asan, Chungnam, 31538, Korea.

FAU - Song, Ho-Yeon

AU - Song HY

AUID- ORCID: 0000-0003-4429-1383

AD - Department of Microbiology and Immunology, School of Medicine, Soonchunhyang

University, Cheonan, Chungnam, 31151, Korea. songmic@sch.ac.kr.

AD - Human Microbiome Medical Research Center (HM·MRC), School of Medicine,

Soonchunhyang University, Asan, Chungnam, 31538, Korea. songmic@sch.ac.kr.

LA - eng

GR - RS-2023-00219563/Ministry of Science and ICT, South Korea/

PT - Journal Article

DEP - 20250919

PL - Germany

TA - Med Microbiol Immunol

JT - Medical microbiology and immunology

JID - 0314524

RN - 0 (Antitubercular Agents)

SB - IM

MH - *Mycobacterium tuberculosis/drug effects

MH - Animals

MH - Mice

MH - RAW 264.7 Cells

MH - *Antitubercular Agents/pharmacology

MH - *Probiotics/pharmacology

MH - Macrophages/microbiology

MH - *Bacillus

MH - Autophagy

MH - *Tuberculosis, Multidrug-Resistant/therapy

PMC - PMC12449405

OTO - NOTNLM

OT - Bacillus sonorensis PMC204

OT - Autophagy

OT - Drug-resistant Mycobacterium tuberculosis

OT - Membrane vesicles (MVs)

OT - Next-generation probiotics

COIS- Declarations. Conflict of interest: The authors declare no competing interests.

Ethical approval: This study was conducted in Animal Biosafety Level 3 (ABL-3)

and Level 2 (ABL-2) laboratories at Soonchunhyang University. (SCH ABL-3 facility

approval number: KDCA-20-3-04; ABL-2 facility approval number: LML20-591). All

protocols and procedures were approved by the Institutional Animal Care and Use

Committee (IACUC) and the Institutional Biosafety Committee (IBC) of

Soonchunhyang University (SCH) and the HMMRC Center (IACUC approval numbers:

SCH22-0042, SCH21-0049; IBC approval number: SCH22-002).

EDAT- 2025/09/19 12:51

MHDA- 2025/09/19 12:52

PMCR- 2025/09/19

CRDT- 2025/09/19 11:15

PHST- 2025/01/16 00:00 [received]

PHST- 2025/08/11 00:00 [accepted]

PHST- 2025/09/19 12:52 [medline]

PHST- 2025/09/19 12:51 [pubmed]

PHST- 2025/09/19 11:15 [entrez]

PHST- 2025/09/19 00:00 [pmc-release]

AID - 10.1007/s00430-025-00851-1 [pii]

AID - 851 [pii]

AID - 10.1007/s00430-025-00851-1 [doi]

PST - epublish

SO - Med Microbiol Immunol. 2025 Sep 19;214(1):43. doi: 10.1007/s00430-025-00851-1.

PMID- 40980328

OWN - NLM

STAT- PubMed-not-MEDLINE

DCOM- 20250922

LR - 20250924

IS - 1664-302X (Print)

IS - 1664-302X (Electronic)

IS - 1664-302X (Linking)

VI - 16

DP - 2025

TI - Progress of anti-tuberculosis drug targets and novel therapeutic strategies.

PG - 1637254

LID - 10.3389/fmicb.2025.1637254 [doi]

LID - 1637254

AB - Tuberculosis, a chronic infectious disease caused by Mycobacterium tuberculosis

complex, has re-emerged as the leading cause of death worldwide as a single

infectious agent. The increasing prevalence of multidrug-resistant tuberculosis

and extensively drug-resistant tuberculosis poses a severe and growing threat to

global health. Therefore, it is urgent to find new drug targets. Recently,

significant advancements have been made in the research of drug targets and novel

therapeutic strategies for tuberculosis. This review summarizes recent processes

on anti-tuberculosis drug targets, such as cell wall synthesis, nucleic acid

replication and transcription, energy metabolism, and ferroptosis. Furthermore,

this review summarizes the research progress of three innovative tuberculosis

treatment strategies, including antimicrobial peptides, host-directed therapies,

and nanoparticle-based drug delivery systems, aiming to provide a theoretical

foundation and new research perspectives for the clinical development of new

drugs.

CI - Copyright © 2025 Zhang, Wu, Sun, Li, Fang, Xing, Li, Wen and Song.

FAU - Zhang, Yang

AU - Zhang Y

AD - Weifang Key Laboratory of Respiratory Tract Pathogens and Drug Therapy, School of

Life Science and Technology, Shandong Second Medical University, Weifang, China.

FAU - Wu, Ruiying

AU - Wu R

AD - Weifang Key Laboratory of Respiratory Tract Pathogens and Drug Therapy, School of

Life Science and Technology, Shandong Second Medical University, Weifang, China.

FAU - Sun, Mingrui

AU - Sun M

AD - Weifang Key Laboratory of Respiratory Tract Pathogens and Drug Therapy, School of

Life Science and Technology, Shandong Second Medical University, Weifang, China.

FAU - Li, Xiaotian

AU - Li X

AD - Weifang Key Laboratory of Respiratory Tract Pathogens and Drug Therapy, School of

Life Science and Technology, Shandong Second Medical University, Weifang, China.

FAU - Fang, Ren

AU - Fang R

AD - Weifang Key Laboratory of Respiratory Tract Pathogens and Drug Therapy, School of

Life Science and Technology, Shandong Second Medical University, Weifang, China.

FAU - Xing, Jiayin

AU - Xing J

AD - Weifang Key Laboratory of Respiratory Tract Pathogens and Drug Therapy, School of

Life Science and Technology, Shandong Second Medical University, Weifang, China.

FAU - Li, Zhaoli

AU - Li Z

AD - SAFE Pharmaceutical Technology Co., Ltd., Beijing, China.

FAU - Wen, Yurong

AU - Wen Y

AD - The First Affiliated Hospital of Xi'an Jiaotong University, Xi'an, China.

FAU - Song, Ningning

AU - Song N

AD - Weifang Key Laboratory of Respiratory Tract Pathogens and Drug Therapy, School of

Life Science and Technology, Shandong Second Medical University, Weifang, China.

LA - eng

PT - Journal Article

PT - Review

DEP - 20250905

PL - Switzerland

TA - Front Microbiol

JT - Frontiers in microbiology

JID - 101548977

PMC - PMC12446298

OTO - NOTNLM

OT - Mycobacterium tuberculosis

OT - anti-tuberculosis drugs

OT - drug target

OT - novel therapeutics

OT - tuberculosis treatment

COIS- ZL was employed by the SAFE Pharmaceutical Technology Co., Ltd. The remaining

authors declare that the research was conducted in the absence of any commercial

or financial relationships that could be construed as a potential conflict of

interest.

EDAT- 2025/09/22 12:46

MHDA- 2025/09/22 12:47

PMCR- 2025/09/05

CRDT- 2025/09/22 08:22

PHST- 2025/05/29 00:00 [received]

PHST- 2025/08/21 00:00 [accepted]

PHST- 2025/09/22 12:47 [medline]

PHST- 2025/09/22 12:46 [pubmed]

PHST- 2025/09/22 08:22 [entrez]

PHST- 2025/09/05 00:00 [pmc-release]

AID - 10.3389/fmicb.2025.1637254 [doi]

PST - epublish

SO - Front Microbiol. 2025 Sep 5;16:1637254. doi: 10.3389/fmicb.2025.1637254.

eCollection 2025.

PMID- 40901459

OWN - NLM

STAT- PubMed-not-MEDLINE

DCOM- 20250915

LR - 20250915

IS - 2046-2069 (Electronic)

IS - 2046-2069 (Linking)

VI - 15

IP - 38

DP - 2025 Aug 29

TI - Advancements in the design and development of pyrazoline-based antimycobacterial

agents: an update and future perspectives.

PG - 31360-31401

LID - 10.1039/d5ra03759j [doi]

AB - Pyrazoline scaffolds have attracted significant interest in medicinal chemistry

due to their broad spectrum of pharmacological activities. Pyrazole-based drugs

are either already approved or are currently undergoing clinical trials across a

range of therapeutic areas. Pyrazolines (Δ(2)-pyrazolines or 2-pyrazoline or

4,5-dihydropyrazoles) evolved as cyclic analogues of thioacetazone and were

explored for enhanced antitubercular activity over the past five decades. The

scope of this review focused on how extensively the chemical space around

pyrazolines has been explored in relation to their antitubercular activity,

rather than presenting a general structure-activity relationship (SAR) account.

In this exercise, we covered key molecular modifications, including rationale

substitutions and conjugations, aimed at enhancing the potency in general.

Additionally, information pertaining to in vitro/in silico target interaction and

ADMET studies are also covered. A dedicated section is included to showcase

target-oriented strategies (InhA, cytochrome P450 14α-sterol demethylase, and

enzymes involved in the mycobactin biosynthesis pathway), recent patents,

suggested schemes for reported pyrazolines, and an overview of research

methodologies and evaluation models. We believe that this review will enable

medicinal chemists to map unexplored chemical space in identifying critical

research gaps. This is essential for the rational design and development of

potent antitubercular agents against tuberculosis (TB), drug-resistant

tuberculosis (DR-TB), and other non-tubercular mycobacterial diseases (NTMD).

CI - This journal is © The Royal Society of Chemistry.

FAU - Rakshit, Gourav

AU - Rakshit G

AUID- ORCID: 0000-0003-1341-7289

AD - Department of Pharmaceutical Sciences and Technology, Birla Institute of

Technology Mesra Ranchi 835215 India venkatesanj@bitmesra.ac.in.

AD - Mycobacteria Research Laboratory, Institute of Structural and Molecular Biology,

School of Natural Sciences, Birkbeck, University of London London WCIE 7HX UK

s.bhakta@bbk.ac.uk.

FAU - Chakraborty, Soumi

AU - Chakraborty S

AUID- ORCID: 0009-0005-7764-7472

AD - Department of Pharmaceutical Sciences and Technology, Birla Institute of

Technology Mesra Ranchi 835215 India venkatesanj@bitmesra.ac.in.

FAU - Bhakta, Sanjib

AU - Bhakta S

AUID- ORCID: 0000-0002-1165-9295

AD - Mycobacteria Research Laboratory, Institute of Structural and Molecular Biology,

School of Natural Sciences, Birkbeck, University of London London WCIE 7HX UK

s.bhakta@bbk.ac.uk.

FAU - Jayaprakash, Venkatesan

AU - Jayaprakash V

AUID- ORCID: 0000-0002-9724-4153

AD - Department of Pharmaceutical Sciences and Technology, Birla Institute of

Technology Mesra Ranchi 835215 India venkatesanj@bitmesra.ac.in.

LA - eng

PT - Journal Article

PT - Review

DEP - 20250901

PL - England

TA - RSC Adv

JT - RSC advances

JID - 101581657

PMC - PMC12401172

COIS- There are no conflicts of interest to declare.

EDAT- 2025/09/03 18:35

MHDA- 2025/09/03 18:36

PMCR- 2025/09/01

CRDT- 2025/09/03 12:14

PHST- 2025/05/28 00:00 [received]

PHST- 2025/08/14 00:00 [accepted]

PHST- 2025/09/03 18:36 [medline]

PHST- 2025/09/03 18:35 [pubmed]

PHST- 2025/09/03 12:14 [entrez]

PHST- 2025/09/01 00:00 [pmc-release]

AID - d5ra03759j [pii]

AID - 10.1039/d5ra03759j [doi]

PST - epublish

SO - RSC Adv. 2025 Sep 1;15(38):31360-31401. doi: 10.1039/d5ra03759j. eCollection 2025

Aug 29.

PMID- 40782406

OWN - NLM

STAT- MEDLINE

DCOM- 20250916

LR - 20260420

IS - 1090-2120 (Electronic)

IS - 0045-2068 (Linking)

VI - 164

DP - 2025 Sep

TI - Anti-Mycobacterium tuberculosis activity of 17-hydroxy-jolkinolide B by

interacting with RNA polymerase.

PG - 108851

LID - S0045-2068(25)00731-X [pii]

LID - 10.1016/j.bioorg.2025.108851 [doi]

AB - Euphorbia fischeriana has been traditionally used in Chinese medicine for

tuberculosis (TB) treatment since ancient times. In this study, we first report

the identification of an abietane-type diterpenoid, 17-hydroxy-jolkinolide B

(HJKB), from E. fischeriana, which exhibits potent antimycobacterial activity

against both Mycobacterium tuberculosis H37Ra strain and clinical isolates. The

minimum inhibitory concentrations (MICs) of HJKB against diverse M. tuberculosis

strains range from 1 to 12 μg/mL. Notably, HJKB demonstrates significant

bactericidal activity against intracellular M. tuberculosis H37Ra in macrophage

models, accompanied by anti-inflammatory effects at concentrations of 2-5 μg/mL.

Using a combination of chemoproteomic analysis and pull-down assays, we explored

the preliminary antimycobacterial mechanism of HJKB. Results indicate that HJKB

interacts with the target proteins RpoB and RpoC in M. tuberculosis H37Ra, a

finding further corroborated by molecular docking studies. RpoB and RpoC are

essential subunits of the DNA-directed RNA polymerase holoenzyme, which is

critical for bacterial ribosomal transcription regulation. In summary, HJKB

represents a bioactive constituent of E. fischeriana with anti-TB efficacy,

acting as a transcription inhibitor against M. tuberculosis. This study not only

elucidates its antimycobacterial mechanism but also provides a preclinical

foundation for the development of natural product-based TB therapeutics.

CI - Copyright © 2025 Elsevier Inc. All rights reserved.

FAU - Han, Xiuyan

AU - Han X

AD - Pharmaceutical Research Center, Second Affiliated Hospital of Dalian Medical

University, Dalian 116044, People's Republic of China.

FAU - Chen, Changming

AU - Chen C

AD - Department of Rheumatology and Immunology, The Second Affiliated Hospital of

Guizhou University of Traditional Chinese Medicine, Guiyang 550001, People's

Republic of China.

FAU - Zhao, Renxiang

AU - Zhao R

AD - College of Pharmacy, Hangzhou Normal University, Hangzhou 311121, People's

Republic of China.

FAU - Wu, Jing

AU - Wu J

AD - Beijing Key Laboratory of Drug Resistance Tuberculosis Research, Beijing

Tuberculosis and Thoracic Tumor Research Institute, Beijing Chest Hospital,

Capital Medical University, Beijing 101149, People's Republic of China.

FAU - Zhang, Shujing

AU - Zhang S

AD - Pharmaceutical Research Center, Second Affiliated Hospital of Dalian Medical

University, Dalian 116044, People's Republic of China.

FAU - Wu, Yuzhuo

AU - Wu Y

AD - Pharmaceutical Research Center, Second Affiliated Hospital of Dalian Medical

University, Dalian 116044, People's Republic of China.

FAU - Ma, Yufang

AU - Ma Y

AD - College of Basic Medical Sciences, Dalian Medical University, Dalian 116044,

People's Republic of China. Electronic address: yufangma@dmu.edu.cn.

FAU - Wang, Chao

AU - Wang C

AD - College of Pharmacy, Dalian Medical University, Dalian 116044, People's Republic

of China. Electronic address: wach_edu@sina.com.

FAU - Ma, Xiaochi

AU - Ma X

AD - Pharmaceutical Research Center, Second Affiliated Hospital of Dalian Medical

University, Dalian 116044, People's Republic of China. Electronic address:

maxc1978@163.com.

LA - eng

PT - Journal Article

PT - Research Support, Non-U.S. Gov't

DEP - 20250805

PL - United States

TA - Bioorg Chem

JT - Bioorganic chemistry

JID - 1303703

RN - 0 (Diterpenes)

RN - 0 (Antitubercular Agents)

RN - EC 2.7.7.6 (DNA-Directed RNA Polymerases)

RN - 0 (Bacterial Proteins)

RN - 0 (17-hydroxy-jolkinolide B)

RN - 0 (Terpenes)

SB - IM

MH - *Mycobacterium tuberculosis/drug effects/enzymology

MH - *Diterpenes/pharmacology/chemistry/isolation & purification

MH - *Antitubercular Agents/pharmacology/chemistry/isolation & purification

MH - *DNA-Directed RNA Polymerases/metabolism/antagonists & inhibitors

MH - Microbial Sensitivity Tests

MH - Molecular Docking Simulation

MH - Humans

MH - Structure-Activity Relationship

MH - Molecular Structure

MH - Dose-Response Relationship, Drug

MH - Animals

MH - Mice

MH - Bacterial Proteins/metabolism/antagonists & inhibitors

MH - Terpenes

OTO - NOTNLM

OT - 17-hydroxy-Jolkinolide B

OT - Euphorbia fischeriana

OT - MDR/XDR-TB

OT - Mycobacterium tuberculosis

OT - RNA polymerase

COIS- Declaration of competing interest The authors declare that they have no known

competing financial interests or personal relationships that could have appeared

to influence the work reported in this paper.

EDAT- 2025/08/10 06:13

MHDA- 2025/09/16 13:02

CRDT- 2025/08/09 18:02

PHST- 2025/04/02 00:00 [received]

PHST- 2025/07/20 00:00 [revised]

PHST- 2025/08/03 00:00 [accepted]

PHST- 2025/09/16 13:02 [medline]

PHST- 2025/08/10 06:13 [pubmed]

PHST- 2025/08/09 18:02 [entrez]

AID - S0045-2068(25)00731-X [pii]

AID - 10.1016/j.bioorg.2025.108851 [doi]

PST - ppublish

SO - Bioorg Chem. 2025 Sep;164:108851. doi: 10.1016/j.bioorg.2025.108851. Epub 2025

Aug 5.

PMID- 40650508

OWN - NLM

STAT- MEDLINE

DCOM- 20250901

LR - 20250901

IS - 1746-045X (Electronic)

IS - 1746-0441 (Linking)

VI - 20

IP - 9

DP - 2025 Sep

TI - Understanding the key challenges in tuberculosis drug discovery: what does the

future hold?

PG - 1115-1130

LID - 10.1080/17460441.2025.2531229 [doi]

AB - INTRODUCTION: Tuberculosis (TB), caused by Mycobacterium tuberculosis (Mtb),

remains a major global health concern. It spreads through airborne droplets and

has a high mortality rate, particularly without treatment. Drug resistance is

rising, with treatments against multidrug-resistant TB (MDR-TB) showing poor

treatment success rates. The thick, lipid-rich wall of Mtb and its slow growth

reduce antibiotic effectiveness, requiring long treatment courses of 4-6 months.

Current therapies often fail against drug-resistant strains, highlighting the

urgent need for new, short-course treatment, affordable, and combination-friendly

drugs. AREAS COVERED: Within this perspective, the authors review and comment on

the following topics regarding Mtb resistance emergence and treatment strategies:

i) Existing treatment ii) Resistance evolution in Mtb; iii) Key challenges in

drug discovery targeting Mtb; iv) emerging strategies and recent advances in Mtb

drug discovery, and v) Next-generation approaches. Literature was identified

through a search of PubMed, google scholar, and web of science, from January 2010

to March 2025. EXPERT OPINION: AI is accelerating the discovery of bioavailable

and safe preclinical drug candidates for TB, though data limitations and

biological complexity remain challenging. Future progress requires multi-modal

models, open-access datasets, and interdisciplinary collaboration.

FAU - Zein-Eddine, Rima

AU - Zein-Eddine R

AUID- ORCID: 0000-0001-9965-0027

AD - Laboratoire d'Optique et Biosciences (LOB), Ecole Polytechnique, Inserm U1182,

CNRS UMR7645, Institut Polytechnique de Paris, Palaiseau, France.

FAU - Ramuz, Masoud

AU - Ramuz M

AD - Laboratoire d'Informatique (LIX), Ecole Polytechnique, CNRS, UMR7161, Institut

Polytechnique de Paris, Palaiseau, France.

FAU - Refrégier, Guislaine

AU - Refrégier G

AD - Laboratoire Ecologie, Société et Evolution (ESE), Université Paris-Saclay, CNRS,

AgroParisTech, Gif-sur-Yvette, France.

FAU - Lutzeyer, Johannes F

AU - Lutzeyer JF

AUID- ORCID: 0000-0001-9512-7917

AD - Laboratoire d'Informatique (LIX), Ecole Polytechnique, CNRS, UMR7161, Institut

Polytechnique de Paris, Palaiseau, France.

FAU - Aleksandrov, Alexey

AU - Aleksandrov A

AUID- ORCID: 0000-0001-7868-165X

AD - Laboratoire d'Optique et Biosciences (LOB), Ecole Polytechnique, Inserm U1182,

CNRS UMR7645, Institut Polytechnique de Paris, Palaiseau, France.

FAU - Myllykallio, Hannu

AU - Myllykallio H

AUID- ORCID: 0000-0002-0541-1197

AD - Laboratoire d'Optique et Biosciences (LOB), Ecole Polytechnique, Inserm U1182,

CNRS UMR7645, Institut Polytechnique de Paris, Palaiseau, France.

LA - eng

PT - Journal Article

PT - Review

DEP - 20250712

PL - England

TA - Expert Opin Drug Discov

JT - Expert opinion on drug discovery

JID - 101295755

RN - 0 (Antitubercular Agents)

SB - IM

MH - Humans

MH - *Antitubercular Agents/pharmacology/administration & dosage

MH - *Drug Discovery/methods

MH - Mycobacterium tuberculosis/drug effects/isolation & purification

MH - *Tuberculosis, Multidrug-Resistant/drug therapy/microbiology

MH - *Tuberculosis/drug therapy/microbiology

MH - Animals

MH - Drug Resistance, Multiple, Bacterial

MH - Drug Development/methods

OTO - NOTNLM

OT - AI-driven drug discovery

OT - Anti-microbial resistance (AMR)

OT - anti-evolution drugs

OT - drug discovery

OT - drug-resistance mechanism

OT - mycobacterium tuberculosis (mtb)

EDAT- 2025/07/13 03:32

MHDA- 2025/09/01 08:44

CRDT- 2025/07/12 06:33

PHST- 2025/09/01 08:44 [medline]

PHST- 2025/07/13 03:32 [pubmed]

PHST- 2025/07/12 06:33 [entrez]

AID - 10.1080/17460441.2025.2531229 [doi]

PST - ppublish

SO - Expert Opin Drug Discov. 2025 Sep;20(9):1115-1130. doi:

10.1080/17460441.2025.2531229. Epub 2025 Jul 12.

PMID- 38726567

OWN - NLM

STAT- MEDLINE

DCOM- 20251104

LR - 20251104

IS - 1538-0254 (Electronic)

IS - 0739-1102 (Linking)

VI - 43

IP - 14

DP - 2025 Sep

TI - In silico analysis and characterization of potential inhibitors of MmaA3, a

methoxy mycolic acid synthase from Mycobacterium tuberculosis.

PG - 7771-7796

LID - 10.1080/07391102.2024.2349545 [doi]

AB - The emergence of the multi-and extensively drug-resistant (MDR and XDR) strains

of Mycobacterium tuberculosis (M.tb), necessitates paradigm-shifting therapeutic

approaches. The impermeable waxy lipid layer, primarily composed of mycolic

acids, is a key factor in conferring resistance to conventional drugs. This study

introduces a novel strategy to combat drug resistance by targeting Methoxy

mycolic acid synthase 3 (MmaA3), a critical enzyme in the mycolic acid

biosynthesis pathway. MmaA3 is responsible for the O-methylation of

hydroxymycolate precursors and emerges as a promising therapeutic target. Through

homology-based modeling, we generated a three-dimensional structure of MmaA3,

providing crucial insights into its structural characteristics. High throughput

virtual screening was performed against the MmaA3 model, using diverse sources:

knowledge-based, FDA-approved Drugbank, and Asinex-Elite libraries. Through

rigorous computational analyses, including binding affinity assessments,

molecular interactions analysis, and binding free energy calculations, potential

inhibitors of MmaA3 have been identified. Subsequent validation studies evaluated

the stability of top protein-ligand complexes, and free energy calculations using

molecular dynamics simulations. The stability of complexes within the catalytic

site was confirmed through RMSD and RMSF profile analyses. Furthermore, binding

free energy calculations using the MM-GBSA approach revealed significant binding

affinity of identified ligands for MmaA3 target protein, comparable to its

substrate/cofactors. These findings underscore the potential of the proposed

molecules as candidates for further experimental exploration, offering promising

avenues for the development of effective inhibitors against M.tb. Overall, our

research contributes to significantly advancing the formulation of progressive

therapeutic strategies in combating drug-resistant tuberculosis.

FAU - Chaudhary, Bhawna

AU - Chaudhary B

AD - Structural and Molecular Biology Laboratory (SMBL), Department of Biotechnology,

TERI School of Advanced Studies (TERI SAS), New Delhi, India.

FAU - Sisodia, Rinki

AU - Sisodia R

AD - Structural and Molecular Biology Laboratory (SMBL), Department of Biotechnology,

TERI School of Advanced Studies (TERI SAS), New Delhi, India.

FAU - Sarmadhikari, Debapriyo

AU - Sarmadhikari D

AD - Translational Health Science and Technology Institute (THSTI), NCR Biotech

Science Cluster, Faridabad, Haryana, India.

FAU - Mazumdar, Pooja Anjali

AU - Mazumdar PA

AD - Independent Researcher, New Delhi, India.

FAU - Asthana, Shailendra

AU - Asthana S

AD - Translational Health Science and Technology Institute (THSTI), NCR Biotech

Science Cluster, Faridabad, Haryana, India.

FAU - Madhurantakam, Chaithanya

AU - Madhurantakam C

AD - Structural and Molecular Biology Laboratory (SMBL), Department of Biotechnology,

TERI School of Advanced Studies (TERI SAS), New Delhi, India.

LA - eng

PT - Journal Article

DEP - 20240510

PL - England

TA - J Biomol Struct Dyn

JT - Journal of biomolecular structure & dynamics

JID - 8404176

RN - 0 (Mycolic Acids)

RN - 0 (Enzyme Inhibitors)

RN - 0 (Bacterial Proteins)

RN - 0 (Antitubercular Agents)

RN - 0 (Ligands)

SB - IM

MH - *Mycobacterium tuberculosis/enzymology/drug effects

MH - Molecular Dynamics Simulation

MH - *Mycolic Acids/metabolism/chemistry

MH - *Enzyme Inhibitors/chemistry/pharmacology

MH - Molecular Docking Simulation

MH - Protein Binding

MH - *Bacterial Proteins/chemistry/antagonists & inhibitors

MH - *Antitubercular Agents/pharmacology/chemistry

MH - Ligands

OTO - NOTNLM

OT - MD simulations

OT - MMGBSA

OT - Methyl transferases

OT - MmaA3

OT - Mycobacterium tuberculosis

OT - virtual screening

EDAT- 2024/05/10 06:42

MHDA- 2025/11/04 12:27

CRDT- 2024/05/10 04:12

PHST- 2025/11/04 12:27 [medline]

PHST- 2024/05/10 06:42 [pubmed]

PHST- 2024/05/10 04:12 [entrez]

AID - 10.1080/07391102.2024.2349545 [doi]

PST - ppublish

SO - J Biomol Struct Dyn. 2025 Sep;43(14):7771-7796. doi:

10.1080/07391102.2024.2349545. Epub 2024 May 10.

PMID- 40855526

OWN - NLM

STAT- MEDLINE

DCOM- 20250826

LR - 20250829

IS - 1471-2334 (Electronic)

IS - 1471-2334 (Linking)

VI - 25

IP - 1

DP - 2025 Aug 25

TI - Plasma exosomal miR-122-5p_R-1, miR-23b-3p_R + 1, and miR-15a-5p_R-1 are

associated with multidrug-resistant tuberculosis.

PG - 1063

LID - 10.1186/s12879-025-11487-0 [doi]

LID - 1063

AB - BACKGROUND: Patients with multidrug-resistant tuberculosis (MDR-TB) who are

resistant to at least both rifampicin and isoniazid, lack effective treatment

options in clinic. The gold standard for the diagnosis of MDR-TB is drug

sensitivity test, which is time-consuming and has a relatively low positive

detection rate. Screening early diagnostic biomarker for MDR-TB is urgent need in

clinical practice. METHODS: A total of 33 patients with MDR-TB, healthy controls

and drug-sensitive tuberculosis (DS-TB) were included in this study. Total plasma

exosomal RNA was extracted from the subjects, and the MDR-TB plasma-specific

exosomal miRNAs were obtained by Illumina sequencing. RESULTS: There were 644 and

647 differentially expressed miRNAs in the plasma exosomes of MDR-TB patients

obtained by sequencing and biogenic analysis compared with DS-TB patients and

healthy controls, respectively. Differential miRNAs are mainly involved in the

biological function of regulation of transcription and protein binding, and

enriched in the pathways in cancer and MAPK signaling pathway. Moreover, seven

plasma exosomal miRNAs in MDR-TB patients were significantly different from those

in DS-TB patients and healthy controls. Among them, three of the miRNAs

(hsa-miR-122-5p_R-1, hsa-miR-23b-3p_R + 1, and hsa-miR-15a-5p_R-1) were found to

be in target relationship with MDR-TB related genes (NTRK2, KIDINS220, NCKAP1,

MAPK9, NFAT5, ATF6 and SLC11A2) by target gene prediction analysis. Further the

bioinformatic analysis showed that hsa-miR-122-5p_R-1 targets the protein

PGLYRP2, a diagnostic biomarker identified in our previous study. CONCLUSIONS: We

suggest that hsa-miR-122-5p_R-1, hsa-miR-23b-3p_R + 1, and hsa-miR-15a-5p_R-1 are

closely related to MDR-TB.

CI - © 2025. The Author(s).

FAU - Zhang, Yang

AU - Zhang Y

AD - Center for Pancreatic Cancer Research, The South China University of Technology

School of Medicine, 382 Waihuan Road, Guangzhou, Guangdong, 510006, China.

FAU - Zhang, Linyu

AU - Zhang L

AD - Center for Pancreatic Cancer Research, The South China University of Technology

School of Medicine, 382 Waihuan Road, Guangzhou, Guangdong, 510006, China.

FAU - Shi, Liying

AU - Shi L

AD - Department of Clinical Laboratory, Zhejiang Hospital, 310030, Hangzhou, China.

FAU - Wei, Liliang

AU - Wei L

AD - Department of Pneumology, Affiliated Hospital of Shaoxing University, 312000,

Shaoxing, China.

FAU - Gan, Lin

AU - Gan L

AD - Center for Pancreatic Cancer Research, The South China University of Technology

School of Medicine, 382 Waihuan Road, Guangzhou, Guangdong, 510006, China.

FAU - Hu, Yuting

AU - Hu Y

AD - Center for Pancreatic Cancer Research, The South China University of Technology

School of Medicine, 382 Waihuan Road, Guangzhou, Guangdong, 510006, China.

FAU - Huang, Huai

AU - Huang H

AD - Center for Pancreatic Cancer Research, The South China University of Technology

School of Medicine, 382 Waihuan Road, Guangzhou, Guangdong, 510006, China.

FAU - Xie, Keping

AU - Xie K

AD - Center for Pancreatic Cancer Research, The South China University of Technology

School of Medicine, 382 Waihuan Road, Guangzhou, Guangdong, 510006, China.

mcxiekeping@scut.edu.cn.

FAU - Jiang, Tingting

AU - Jiang T

AD - Center for Pancreatic Cancer Research, The South China University of Technology

School of Medicine, 382 Waihuan Road, Guangzhou, Guangdong, 510006, China.

ttjiang@scut.edu.cn.

FAU - Li, Ji-Cheng

AU - Li JC

AD - Institute of Cell Biology, Zhejiang University, Hangzhou, 310031, China.

Zjulijicheng@163.com.

LA - eng

GR - 2025072985/The Zhejiang Province Traditional Chinese Medicine Science and

Technology Plan Project/

GR - 20230330/Guangzhou Ruiqian Biological Technology Co., LTD/

PT - Journal Article

DEP - 20250825

PL - England

TA - BMC Infect Dis

JT - BMC infectious diseases

JID - 100968551

RN - 0 (MicroRNAs)

RN - 0 (Biomarkers)

RN - 0 (MIRN122 microRNA, human)

RN - 0 (MIRN15 microRNA, human)

SB - IM

MH - Humans

MH - *MicroRNAs/blood/genetics

MH - *Tuberculosis, Multidrug-Resistant/blood/genetics/diagnosis

MH - Male

MH - *Exosomes/genetics

MH - Female

MH - Middle Aged

MH - Adult

MH - Biomarkers/blood

MH - Case-Control Studies

MH - Aged

PMC - PMC12376478

OTO - NOTNLM

OT - Biomarker.

OT - Exosome

OT - MDR-TB

OT - MiRNA

OT - Plasma

COIS- Declarations. Ethics approval and consent to participate: The research was

approved by the Ethics Committee of South China University of Technology School

of Medicine (2018-No.5), and the Ethics Committee of Zhejiang University School

of Medicine (2017-No.023), and written informed consent was obtained from all

subjects before blood sampling. All procedures were in accordance with the

ethical standards of the Committee responsible for human experimentation

(institutional and national) and followed the Declaration of Helsinki. Consent

for publication: Written informed consent for publication was acquired from the

patients for the publication of the identity revealing information. Competing

interests: The authors declare no competing interests.

EDAT- 2025/08/28 04:46

MHDA- 2025/08/28 04:47

PMCR- 2025/08/25

CRDT- 2025/08/26 00:36

PHST- 2025/01/11 00:00 [received]

PHST- 2025/08/01 00:00 [accepted]

PHST- 2025/08/28 04:47 [medline]

PHST- 2025/08/28 04:46 [pubmed]

PHST- 2025/08/26 00:36 [entrez]

PHST- 2025/08/25 00:00 [pmc-release]

AID - 10.1186/s12879-025-11487-0 [pii]

AID - 11487 [pii]

AID - 10.1186/s12879-025-11487-0 [doi]

PST - epublish

SO - BMC Infect Dis. 2025 Aug 25;25(1):1063. doi: 10.1186/s12879-025-11487-0.

PMID- 40558048

OWN - NLM

STAT- MEDLINE

DCOM- 20250827

LR - 20260708

IS - 2150-7511 (Electronic)

VI - 16

IP - 8

DP - 2025 Aug 13

TI - Vitamin C potentiates the killing of Mycobacterium tuberculosis by bedaquiline

through metabolic disruption.

PG - e0148425

LID - 10.1128/mbio.01484-25 [doi]

LID - e01484-25

AB - Tuberculosis (TB), a disease caused by the bacterium Mycobacterium tuberculosis

(Mtb), continues to pose a major global health threat, exacerbated by the

emergence of drug-resistant strains and the lengthy treatment regimens required

for effective management. Bedaquiline (BDQ), a key component in novel regimens

for multidrug-resistant (MDR) TB, has demonstrated significant efficacy but is

threatened by rising resistance. Our study investigates the potential of vitamin

C to enhance BDQ's activity and prevent resistance. We found that combining BDQ

with vitamin C sterilized drug-susceptible and MDR Mtb cultures in vitro within

21 days, achieving a 6-log reduction in colony-forming units. This combination

also enhanced Mtb killing in infected human macrophages and peripheral blood

mononuclear cells. Transcriptomic analysis revealed that the BDQ/vitamin C

combination induces widespread metabolic disruption in Mtb, characterized by

upregulation of stress response and metal ion homeostasis genes and

downregulation of energy metabolism and cell wall biosynthesis genes. Mechanistic

studies implicated reactive oxygen species and disrupted copper homeostasis as

contributing factors to the sterilization effect. These findings highlight the

potential of using vitamin C as an adjunct therapy with BDQ, offering a promising

strategy to enhance drug efficacy and mitigate emerging drug resistance during

MDR-TB treatment. IMPORTANCE: Tuberculosis (TB) remains a major global health

problem, especially as drug-resistant forms become more common and harder to

treat. Bedaquiline is one of the most important new drugs for treating these

resistant infections, but resistance to bedaquiline is also starting to appear.

This study found that the combination of vitamin C and bedaquiline sterilizes

Mycobacterium tuberculosis cultures in vitro while potentiating bedaquiline

activity in infected human macrophage cells. The combination appears to overwhelm

the bacteria by creating stress and disrupting essential functions, like energy

production and metal balance. These results suggest that vitamin C, a safe and

inexpensive supplement, could be used alongside existing drugs to make treatment

faster and more effective while also helping to prevent resistance.

FAU - Vilchèze, Catherine

AU - Vilchèze C

AUID- ORCID: 0000-0001-5960-6670

AD - Department of Microbiology and Immunology, Albert Einstein College of Medicine,

Bronx, New York, USA.

FAU - Rajagopalan, Saranathan

AU - Rajagopalan S

AD - Department of Microbiology and Immunology, Albert Einstein College of Medicine,

Bronx, New York, USA.

FAU - Kalluru, Raja Sab

AU - Kalluru RS

AD - Department of Pathology, Stanford University School of Medicine, Stanford,

California, USA.

FAU - Banaei, Niaz

AU - Banaei N

AD - Department of Pathology, Stanford University School of Medicine, Stanford,

California, USA.

FAU - Jacobs, William R Jr

AU - Jacobs WR Jr

AUID- ORCID: 0000-0003-3321-3080

AD - Department of Microbiology and Immunology, Albert Einstein College of Medicine,

Bronx, New York, USA.

LA - eng

SI - BioProject/PRJNA1206187

SI - SRA/SRR31886240

SI - SRA/SRR31886241

SI - SRA/SRR31886242

SI - SRA/SRR31886243

SI - SRA/SRR31886244

SI - SRA/SRR31886245

SI - SRA/SRR31886246

SI - SRA/SRR31886247.

GR - R01 AI026170/AI/NIAID NIH HHS/United States

GR - R37 AI026170/AI/NIAID NIH HHS/United States

GR - AI026170/National Institute of Allergy and Infectious Diseases/

PT - Journal Article

PT - Research Support, N.I.H., Extramural

DEP - 20250625

PL - United States

TA - mBio

JT - mBio

JID - 101519231

RN - PQ6CK8PD0R (Ascorbic Acid)

RN - 78846I289Y (bedaquiline)

RN - 0 (Antitubercular Agents)

RN - 0 (Diarylquinolines)

RN - 789U1901C5 (Copper)

RN - 0 (Reactive Oxygen Species)

SB - IM

MH - *Ascorbic Acid/pharmacology/therapeutic use

MH - *Mycobacterium tuberculosis/drug effects/genetics/metabolism

MH - *Antitubercular Agents/pharmacology/therapeutic use

MH - Drug Synergism

MH - *Diarylquinolines/pharmacology/therapeutic use

MH - *Tuberculosis, Multidrug-Resistant/drug therapy/microbiology

MH - Drug Resistance, Multiple, Bacterial/drug effects/genetics

MH - Drug Therapy, Combination

MH - Gene Expression Regulation, Bacterial/drug effects

MH - Metabolic Networks and Pathways/drug effects/genetics

MH - Homeostasis/drug effects

MH - Copper/metabolism

MH - Reactive Oxygen Species/metabolism

MH - Microbial Sensitivity Tests

MH - Humans

MH - THP-1 Cells

MH - Primary Cell Culture

MH - Microbial Viability/drug effects/genetics

PMC - PMC12345178

OTO - NOTNLM

OT - antibiotics

OT - bedaquiline

OT - gene expression

OT - metabolism

OT - persistence

OT - sterilization

OT - tuberculosis

COIS- The authors declare no conflict of interest.

EDAT- 2025/06/25 12:27

MHDA- 2025/08/31 20:02

PMCR- 2025/06/25

CRDT- 2025/06/25 09:03

PHST- 2025/08/31 20:02 [medline]

PHST- 2025/06/25 12:27 [pubmed]

PHST- 2025/06/25 09:03 [entrez]

PHST- 2025/06/25 00:00 [pmc-release]

AID - mbio01484-25 [pii]

AID - mbio.01484-25 [pii]

AID - 10.1128/mbio.01484-25 [doi]

PST - ppublish

SO - mBio. 2025 Aug 13;16(8):e0148425. doi: 10.1128/mbio.01484-25. Epub 2025 Jun 25.

PMID- 40770126

OWN - NLM

STAT- MEDLINE

DCOM- 20260626

LR - 20260626

IS - 1573-0972 (Electronic)

IS - 0959-3993 (Linking)

VI - 41

IP - 8

DP - 2025 Aug 7

TI - S292L mutation in Rv1258c efflux pump drives pyrazinamide efflux and a novel

inhibitor designed for co-therapy to improve MDR-TB treatment outcomes.

PG - 300

LID - 10.1007/s11274-025-04510-8 [doi]

AB - Tuberculosis (TB) remains a major global health challenge, exacerbated by the

rise of drug-resistant Mycobacterium tuberculosis (Mtb) strains that undermine

first-line therapies. Resistance to pyrazinamide (PZA), an essential anti-TB

drug, is often linked to mutations in the pncA gene and efflux pump genes such as

Rv1258c. The Rv1258c efflux pump not only expels antibiotics, reducing

intracellular drug concentrations, but also enhances bacterial survival under

stress, making it a promising therapeutic target. This study investigated the

effects of two critical Rv1258c mutations, V219A and S292L, on drug efflux

function and inhibitor binding. Molecular dynamics simulations revealed that both

mutations significantly altered the thermodynamic stability and binding

energetics of the efflux pump. Comparative MM/GBSA analysis showed that the

binding free energy (ΔG) of PZA with wild-type Rv1258c (-17.08 ± 1.66 kcal/mol)

was moderately weakened in mutants S292L (-16.01 ± 0.82 kcal/mol) and V219A

(-11.68 ± 3.31 kcal/mol), suggesting reduced drug affinity. Virtual screening

further identified three potent inhibitors that met all ADMET criteria. MM/GBSA

calculations indicated these inhibitors had substantially stronger binding

affinities than PZA, with ΔG values of -46.49 ± 3.97 kcal/mol,

-19.61 ± 4.58 kcal/mol, and -28.14 ± 3.61 kcal/mol for ZINC000000035064,

ZINC000000040452, and ZINC000000040453, respectively. These findings suggest that

targeting Rv1258c could effectively restore intracellular drug concentrations

even in resistant strains. Overall, this study provides a strong foundation for

the development and experimental validation of efflux pump inhibitors as a novel

therapeutic strategy against multidrug-resistant TB.

FAU - Singh, Garima

AU - Singh G

AD - Department of Biotechnology, Babasaheb Bhimrao Ambedkar University, Lucknow,

226025, Uttar Pradesh, India.

FAU - Shreya, Shriti

AU - Shreya S

AD - Department of Biotechnology, Babasaheb Bhimrao Ambedkar University, Lucknow,

226025, Uttar Pradesh, India.

FAU - Yadav, Saurabh

AU - Yadav S

AD - Department of Biotechnology, Babasaheb Bhimrao Ambedkar University, Lucknow,

226025, Uttar Pradesh, India.

FAU - Akhter, Yusuf

AU - Akhter Y

AD - Department of Biotechnology, Babasaheb Bhimrao Ambedkar University, Lucknow,

226025, Uttar Pradesh, India. yusuf@daad-alumni.de.

LA - eng

PT - Journal Article

DEP - 20250807

PL - Germany

TA - World J Microbiol Biotechnol

JT - World journal of microbiology & biotechnology

JID - 9012472

RN - 2KNI5N06TI (Pyrazinamide)

RN - 0 (Antitubercular Agents)

RN - 0 (Membrane Transport Proteins)

RN - 0 (Bacterial Proteins)

RN - 0 (Rv1258c protein, Mycobacterium tuberculosis)

RN - 0 (ATP-Binding Cassette Transporters)

SB - IM

MH - *Pyrazinamide/pharmacology/metabolism

MH - *Mycobacterium tuberculosis/genetics/drug effects/metabolism

MH - *Antitubercular Agents/pharmacology

MH - Molecular Dynamics Simulation

MH - *Membrane Transport Proteins/genetics/metabolism/chemistry

MH - *Bacterial Proteins/genetics/metabolism/chemistry

MH - Mutation

MH - *Tuberculosis, Multidrug-Resistant/drug therapy/microbiology

MH - Microbial Sensitivity Tests

MH - Drug Resistance, Multiple, Bacterial/genetics

MH - Thermodynamics

MH - Humans

MH - ATP-Binding Cassette Transporters

OTO - NOTNLM

OT - Mycobacterium tuberculosis

OT - ADMET filtering

OT - MM/GBSA

OT - Molecular dynamics simulation

OT - Multidrug resistance

OT - Pyrazinamide

OT - Rv1258c efflux pump

COIS- Declarations. Competing interests: The authors declare no competing interests.

EDAT- 2025/08/07 00:27

MHDA- 2026/06/27 00:49

CRDT- 2025/08/06 23:28

PHST- 2025/06/20 00:00 [received]

PHST- 2025/07/23 00:00 [accepted]

PHST- 2026/06/27 00:49 [medline]

PHST- 2025/08/07 00:27 [pubmed]

PHST- 2025/08/06 23:28 [entrez]

AID - 10.1007/s11274-025-04510-8 [pii]

AID - 10.1007/s11274-025-04510-8 [doi]

PST - epublish

SO - World J Microbiol Biotechnol. 2025 Aug 7;41(8):300. doi:

10.1007/s11274-025-04510-8.

PMID- 40799345

OWN - NLM

STAT- PubMed-not-MEDLINE

LR - 20260208

IS - 2752-6542 (Electronic)

IS - 2752-6542 (Linking)

VI - 4

IP - 8

DP - 2025 Aug

TI - Mechanism of the dual action self-potentiating antitubercular drug

morphazinamide.

PG - pgaf242

LID - 10.1093/pnasnexus/pgaf242 [doi]

LID - pgaf242

AB - Pyrazinamide (PZA) is a cornerstone of first-line antitubercular drug therapy and

is unique in its ability to kill nongrowing populations of Mycobacterium

tuberculosis through disruption of coenzyme A (CoA) metabolism. Unlike other

drugs, PZA action is conditional and requires potentiation by host-relevant

environmental stressors, such as low pH and nutrient limitation. Despite its

pivotal role in tuberculosis therapy, the durability of this crucial drug is

challenged by the emergent spread of drug resistance. To advance drug discovery

efforts, we characterized the activity of a more potent PZA analog,

morphazinamide (MZA). Here, we demonstrate that like PZA, MZA acts in part

through impairment of CoA metabolism. Unexpectedly, we find that, in contrast to

PZA, MZA does not require potentiation and maintains bactericidal activity

against PZA-resistant strains due to an additional mechanism involving aldehyde

release. Further, we find that the principal mechanism for resistance to the

aldehyde component is through promoter mutations that increase expression of the

mycothiol oxidoreductase MscR. Our findings reveal a dual-action synergistic

mechanism of MZA that results in a faster kill rate and a higher barrier to

resistance. These observations provide new insights for the discovery of improved

therapeutic approaches for addressing the growing problem of drug-resistant

tuberculosis.

CI - © The Author(s) 2025. Published by Oxford University Press on behalf of National

Academy of Sciences.

FAU - Ostrer, Lev

AU - Ostrer L

AD - Department of Microbiology and Immunology, University of Minnesota Medical

School, Minneapolis, MN 55455, USA.

FAU - Crooks, Taylor A

AU - Crooks TA

AUID- ORCID: 0000-0001-7608-1992

AD - Department of Microbiology and Immunology, University of Minnesota Medical

School, Minneapolis, MN 55455, USA.

FAU - Howe, Michael D

AU - Howe MD

AD - Department of Microbiology and Immunology, University of Minnesota Medical

School, Minneapolis, MN 55455, USA.

FAU - Vo, Sang

AU - Vo S

AUID- ORCID: 0000-0002-2005-2372

AD - Department of Microbiology and Immunology, University of Minnesota Medical

School, Minneapolis, MN 55455, USA.

AD - Department of Medicinal Chemistry, College of Pharmacy, University of Minnesota

Medical School, Minneapolis, MN 55455, USA.

FAU - Jia, Ziyi

AU - Jia Z

AUID- ORCID: 0000-0001-8279-5561

AD - Department of Microbiology and Immunology, University of Minnesota Medical

School, Minneapolis, MN 55455, USA.

FAU - Hegde, Pooja

AU - Hegde P

AD - Department of Medicinal Chemistry, College of Pharmacy, University of Minnesota

Medical School, Minneapolis, MN 55455, USA.

FAU - Schacht, Nathan

AU - Schacht N

AUID- ORCID: 0009-0007-7081-7648

AD - Department of Microbiology and Immunology, University of Minnesota Medical

School, Minneapolis, MN 55455, USA.

FAU - Aldrich, Courtney C

AU - Aldrich CC

AD - Department of Medicinal Chemistry, College of Pharmacy, University of Minnesota

Medical School, Minneapolis, MN 55455, USA.

FAU - Baughn, Anthony D

AU - Baughn AD

AUID- ORCID: 0000-0003-1188-4238

AD - Department of Microbiology and Immunology, University of Minnesota Medical

School, Minneapolis, MN 55455, USA.

LA - eng

GR - R01 AI123146/AI/NIAID NIH HHS/United States

GR - T32 GM008244/GM/NIGMS NIH HHS/United States

GR - T32 HL007741/HL/NHLBI NIH HHS/United States

PT - Journal Article

DEP - 20250729

PL - England

TA - PNAS Nexus

JT - PNAS nexus

JID - 9918367777906676

UOF - bioRxiv. 2025 Jan 31:2024.10.08.617272. doi: 10.1101/2024.10.08.617272. PMID:

39416067

PMC - PMC12342587

OTO - NOTNLM

OT - coenzyme A

OT - drug discovery

OT - pyrazinamide

OT - thiol stress

OT - tuberculosis

EDAT- 2025/08/13 06:30

MHDA- 2025/08/13 06:31

PMCR- 2025/07/29

CRDT- 2025/08/13 05:48

PHST- 2024/10/08 00:00 [received]

PHST- 2025/07/16 00:00 [accepted]

PHST- 2025/08/13 06:31 [medline]

PHST- 2025/08/13 06:30 [pubmed]

PHST- 2025/08/13 05:48 [entrez]

PHST- 2025/07/29 00:00 [pmc-release]

AID - pgaf242 [pii]

AID - 10.1093/pnasnexus/pgaf242 [doi]

PST - epublish

SO - PNAS Nexus. 2025 Jul 29;4(8):pgaf242. doi: 10.1093/pnasnexus/pgaf242. eCollection

2025 Aug.

PMID- 40627906

OWN - NLM

STAT- MEDLINE

DCOM- 20250809

LR - 20260422

IS - 1090-2120 (Electronic)

IS - 0045-2068 (Linking)

VI - 163

DP - 2025 Aug

TI - Targeting drug-resistant tuberculosis: Thioacetamide-substituted benzothiazoles

as bactericidal agents inhibiting type II NADH dehydrogenase.

PG - 108718

LID - S0045-2068(25)00598-X [pii]

LID - 10.1016/j.bioorg.2025.108718 [doi]

AB - The identification of novel anti-tubercular agents that inhibit the function of

less-explored yet essential components of Mycobacterium tuberculosis is

critically needed to address the increasing prevalence of drug-resistant

tuberculosis. The type II NADH dehydrogenase, an indispensable component of the

oxidative phosphorylation pathway, is the entry point of electrons into the

electron transport chain, facilitating ATP production. This enzyme is a promising

drug target because its inhibition leads to a bactericidal response through

energy starvation. In our study, we synthesized a series of 6-isopropyl

benzothiazole derivatives, with various thioacetamide side chains; and assessed

their anti-tubercular efficacy, and specificity toward NADH dehydrogenase through

in vitro studies. The most potent compounds, C4-1 (MIC = 4 μg/mL, 13.042 μM), and

C4-11 (MIC = 8 μg/mL, 24.80 μM), exhibited inhibitory effects against

mycobacterial strains, exhibiting resistance to FDA approved anti-tubercular

drugs; and demonstrated bactericidal activity with a safety index exceeding 10.

The primary target for these derivatives is proposed to be type II NADH

dehydrogenase, indicated by increased NADH/NAD(+) ratios in the peredox mCherry

assay, and from detection of single-nucleotide polymorphisms at multiple points

in the type II NADH dehydrogenase gene coding sequence of the strains, exhibiting

resistance to the compounds C4-1, and C4-11. Additionally, molecular docking,

molecular dynamics, and in silico ADME profiling were performed on the active

compounds.

CI - Copyright © 2025 Elsevier Inc. All rights reserved.

FAU - Saha, Pallavi

AU - Saha P

AD - Department of Pharmaceutical Engg. and Tech, IIT-Banaras Hindu University,

Varanasi, UP 221005, India.

FAU - Sau, Shashikanta

AU - Sau S

AD - Department of Pharmacology and Toxicology, NIPER-Hyderabad, Hyderabad, 500037,

India.

FAU - Kalia, Nitin Pal

AU - Kalia NP

AD - Department of Pharmacology and Toxicology, NIPER-Hyderabad, Hyderabad, 500037,

India. Electronic address: nitin.kalia@niperhyd.ac.in.

FAU - Sharma, Deepak K

AU - Sharma DK

AD - Department of Pharmaceutical Engg. and Tech, IIT-Banaras Hindu University,

Varanasi, UP 221005, India. Electronic address: deepak.phe@itbhu.ac.in.

LA - eng

PT - Journal Article

PT - Research Support, Non-U.S. Gov't

DEP - 20250703

PL - United States

TA - Bioorg Chem

JT - Bioorganic chemistry

JID - 1303703

RN - 0 (Antitubercular Agents)

RN - EC 1.6.99.3 (NADH Dehydrogenase)

RN - 0 (Benzothiazoles)

RN - EC 1.6.99.- (NADH dehydrogenase II)

RN - 075T165X8M (Thioacetamide)

RN - 0 (Enzyme Inhibitors)

SB - IM

MH - *Mycobacterium tuberculosis/drug effects/enzymology

MH - *Antitubercular Agents/pharmacology/chemistry/chemical synthesis

MH - *NADH Dehydrogenase/antagonists & inhibitors/metabolism

MH - *Benzothiazoles/pharmacology/chemistry/chemical synthesis

MH - Microbial Sensitivity Tests

MH - Structure-Activity Relationship

MH - *Thioacetamide/chemistry/pharmacology

MH - Molecular Structure

MH - Humans

MH - Dose-Response Relationship, Drug

MH - *Enzyme Inhibitors/pharmacology/chemistry/chemical synthesis

MH - Molecular Docking Simulation

MH - Tuberculosis, Multidrug-Resistant/drug therapy

OTO - NOTNLM

OT - Benzothiazole

OT - Drug-resistant tuberculosis

OT - Mycobacterium tuberculosis

OT - Single-nucleotide polymorphisms

OT - Type II NADH dehydrogenase enzyme

OT - electron transport chain

COIS- Declaration of competing interest The authors declare that they have no known

competing financial interests or personal relationships that could have appeared

to influence the work reported in this paper.

EDAT- 2025/07/09 00:27

MHDA- 2025/08/09 13:48

CRDT- 2025/07/08 18:01

PHST- 2025/05/16 00:00 [received]

PHST- 2025/06/28 00:00 [revised]

PHST- 2025/06/30 00:00 [accepted]

PHST- 2025/08/09 13:48 [medline]

PHST- 2025/07/09 00:27 [pubmed]

PHST- 2025/07/08 18:01 [entrez]

AID - S0045-2068(25)00598-X [pii]

AID - 10.1016/j.bioorg.2025.108718 [doi]

PST - ppublish

SO - Bioorg Chem. 2025 Aug;163:108718. doi: 10.1016/j.bioorg.2025.108718. Epub 2025

Jul 3.

PMID- 40533558

OWN - NLM

STAT- MEDLINE

DCOM- 20250807

LR - 20260405

IS - 1476-4687 (Electronic)

IS - 0028-0836 (Print)

IS - 0028-0836 (Linking)

VI - 644

IP - 8075

DP - 2025 Aug

TI - Targeting de novo purine biosynthesis for tuberculosis treatment.

PG - 214-220

LID - 10.1038/s41586-025-09177-7 [doi]

AB - Tuberculosis remains the leading cause of death from an infectious disease(1,2).

Here we report the discovery of a first-in-class small-molecule inhibitor

targeting PurF, the first enzyme in the mycobacterial de novo purine biosynthesis

pathway. The lead candidate, JNJ-6640, exhibited nanomolar bactericidal activity

in vitro. Comprehensive genetic and biochemical approaches confirmed that

JNJ-6640 was highly selective for mycobacterial PurF. Single-cell-level

microscopy demonstrated a downstream effect on DNA replication. We determined the

physiologically relevant concentrations of nucleobases in human and mouse lung

tissue, showing that these levels were insufficient to salvage PurF inhibition.

Indeed, proof-of-concept studies using a long-acting injectable formulation

demonstrated the in vivo efficacy of the compound. Finally, we show that

inclusion of JNJ-6640 could have a crucial role in improving current treatment

regimens for drug-resistant tuberculosis. Together, we demonstrate that JNJ-6640

is a promising chemical lead and that targeting de novo purine biosynthesis

represents a novel strategy for tuberculosis drug development.

CI - © 2025. The Author(s).

FAU - Lamprecht, Dirk A

AU - Lamprecht DA

AUID- ORCID: 0000-0003-1066-9026

AD - Janssen Global Public Health, LLC, Janssen Pharmaceutica NV, Antwerp, Belgium.

dirk.lamprecht@uct.ac.za.

AD - Holistic Drug Discovery and Development (H3D) Centre, University of Cape Town,

Rondebosch, South Africa. dirk.lamprecht@uct.ac.za.

FAU - Wall, Richard J

AU - Wall RJ

AUID- ORCID: 0000-0003-3487-3187

AD - Department of Infection Biology, Faculty of Infectious and Tropical Diseases,

London School of Hygiene and Tropical Medicine, London, UK.

FAU - Leemans, Annelies

AU - Leemans A

AD - Janssen Global Public Health, LLC, Janssen Pharmaceutica NV, Antwerp, Belgium.

FAU - Truebody, Barry

AU - Truebody B

AD - Africa Health Research Institute, University of KwaZulu Natal, Durban, South

Africa.

FAU - Sprangers, Joke

AU - Sprangers J

AD - Janssen Global Public Health, LLC, Janssen Pharmaceutica NV, Antwerp, Belgium.

AD - Charles River Laboratories, Antwerp, Belgium.

FAU - Fiogbe, Patricia

AU - Fiogbe P

AD - Janssen Global Public Health, LLC, Janssen Pharmaceutica NV, Antwerp, Belgium.

AD - Charles River Laboratories, Antwerp, Belgium.

FAU - Davies, Cadi

AU - Davies C

AD - Department of Infection Biology, Faculty of Infectious and Tropical Diseases,

London School of Hygiene and Tropical Medicine, London, UK.

FAU - Wetzel, Jennefer

AU - Wetzel J

AD - Janssen Global Public Health, LLC, Janssen Pharmaceutica NV, Antwerp, Belgium.

FAU - Daems, Stijn

AU - Daems S

AUID- ORCID: 0009-0005-6778-203X

AD - Janssen Global Public Health, LLC, Janssen Pharmaceutica NV, Antwerp, Belgium.

FAU - Pearson, William

AU - Pearson W

AUID- ORCID: 0000-0003-4146-9422

AD - Department of Infection Biology, Faculty of Infectious and Tropical Diseases,

London School of Hygiene and Tropical Medicine, London, UK.

FAU - Pillay, Vanessa

AU - Pillay V

AD - Africa Health Research Institute, University of KwaZulu Natal, Durban, South

Africa.

FAU - Saylock, Samantha

AU - Saylock S

AD - Janssen Research and Development, LLC, Janssen Pharmaceutica, Spring House, PA,

USA.

FAU - Ricketts, M Daniel

AU - Ricketts MD

AD - Janssen Research and Development, LLC, Janssen Pharmaceutica, Spring House, PA,

USA.

FAU - Davis, Ellie

AU - Davis E

AD - Department of Infection Biology, Faculty of Infectious and Tropical Diseases,

London School of Hygiene and Tropical Medicine, London, UK.

FAU - Huff, Adam

AU - Huff A

AD - Janssen Research and Development, LLC, Janssen Pharmaceutica, Spring House, PA,

USA.

FAU - Grell, Tsehai

AU - Grell T

AD - Janssen Research and Development, LLC, Janssen Pharmaceutica, Spring House, PA,

USA.

FAU - Lin, Shiming

AU - Lin S

AD - Vaccine and Infectious Disease Organization, University of Saskatchewan,

Saskatoon, Saskatchewan, Canada.

FAU - Gerber, Michelle

AU - Gerber M

AD - Vaccine and Infectious Disease Organization, University of Saskatchewan,

Saskatoon, Saskatchewan, Canada.

FAU - Vos, Ann

AU - Vos A

AUID- ORCID: 0000-0001-9018-5878

AD - Janssen Research and Development, LLC, Janssen Pharmaceutica NV, Antwerp,

Belgium.

FAU - Dallow, John

AU - Dallow J

AD - Department of Infection Biology, Faculty of Infectious and Tropical Diseases,

London School of Hygiene and Tropical Medicine, London, UK.

FAU - Willcocks, Sam J

AU - Willcocks SJ

AD - Department of Infection Biology, Faculty of Infectious and Tropical Diseases,

London School of Hygiene and Tropical Medicine, London, UK.

AD - Department of Life Sciences, College of Health, Medicine and Life Sciences,

Brunel University of London, Uxbridge, UK.

FAU - Roubert, Christine

AU - Roubert C

AD - Evotec ID (LYON) SAS, Lyon, France.

FAU - Sans, Stéphanie

AU - Sans S

AD - Evotec ID (LYON) SAS, Lyon, France.

FAU - Desorme, Amandine

AU - Desorme A

AD - Evotec ID (LYON) SAS, Lyon, France.

FAU - Chappat, Nicolas

AU - Chappat N

AD - Evotec ID (LYON) SAS, Lyon, France.

FAU - Ray, Aurélie

AU - Ray A

AD - Evotec ID (LYON) SAS, Lyon, France.

FAU - Pereira Moraes, Mariana

AU - Pereira Moraes M

AUID- ORCID: 0009-0004-0962-2620

AD - Department of Molecular Biology and Microbiology, Tufts University School of

Medicine and Stuart B. Levy Center for Integrated Management of Antimicrobial

Resistance, Boston, MA, USA.

FAU - Washington, Tracy

AU - Washington T

AD - Department of Molecular Biology and Microbiology, Tufts University School of

Medicine and Stuart B. Levy Center for Integrated Management of Antimicrobial

Resistance, Boston, MA, USA.

FAU - D'Erasmo, Hope

AU - D'Erasmo H

AUID- ORCID: 0009-0003-5714-7724

AD - Department of Molecular Biology and Microbiology, Tufts University School of

Medicine and Stuart B. Levy Center for Integrated Management of Antimicrobial

Resistance, Boston, MA, USA.

FAU - Sancheti, Pavankumar

AU - Sancheti P

AD - Janssen Research and Development, LLC, Janssen Pharmaceutica NV, Antwerp,

Belgium.

FAU - Everaerts, Melissa

AU - Everaerts M

AD - Janssen Research and Development, LLC, Janssen Pharmaceutica NV, Antwerp,

Belgium.

FAU - Monshouwer, Mario

AU - Monshouwer M

AD - Janssen Global Public Health, LLC, Janssen Pharmaceutica NV, Antwerp, Belgium.

FAU - Esquivias, Jorge

AU - Esquivias J

AUID- ORCID: 0000-0001-6050-5092

AD - Janssen Research and Development, LLC, Janssen Pharmaceutica, Toledo, Spain.

FAU - Larrouy-Maumus, Gerald

AU - Larrouy-Maumus G

AUID- ORCID: 0000-0001-6614-8698

AD - Centre for Bacterial Resistance Biology, Department of Life Sciences, Faculty of

Natural Sciences, Imperial College London, London, UK.

FAU - Draghia Akli, Ruxandra

AU - Draghia Akli R

AUID- ORCID: 0000-0001-6372-4039

AD - Janssen Research and Development, LLC, Janssen Pharmaceutica, Spring House, PA,

USA.

AD - Research and Development, Novavax, Inc., Gaithersburg, MD, USA.

FAU - Fletcher, Helen

AU - Fletcher H

AD - Department of Infection Biology, Faculty of Infectious and Tropical Diseases,

London School of Hygiene and Tropical Medicine, London, UK.

AD - Janssen Global Public Health, LLC, Janssen Pharmaceutica, High Wycombe, UK.

FAU - Pym, Alexander S

AU - Pym AS

AD - Janssen Global Public Health, LLC, Janssen Pharmaceutica, High Wycombe, UK.

FAU - Aldridge, Bree B

AU - Aldridge BB

AUID- ORCID: 0000-0003-2236-1424

AD - Department of Molecular Biology and Microbiology, Tufts University School of

Medicine and Stuart B. Levy Center for Integrated Management of Antimicrobial

Resistance, Boston, MA, USA.

AD - Department of Biomedical Engineering, Tufts University School of Engineering,

Medford, MA, USA.

FAU - Sarathy, Jansy P

AU - Sarathy JP

AUID- ORCID: 0000-0002-1532-146X

AD - Center for Discovery and Innovation, Hackensack Meridian Health, Nutley, NJ, USA.

FAU - Clancy, Kathleen W

AU - Clancy KW

AD - Janssen Research and Development, LLC, Janssen Pharmaceutica, Spring House, PA,

USA.

FAU - Stoops, Bart

AU - Stoops B

AD - Janssen Research and Development, LLC, Janssen Pharmaceutica NV, Antwerp,

Belgium.

FAU - Dhar, Neeraj

AU - Dhar N

AUID- ORCID: 0000-0002-5887-8137

AD - Vaccine and Infectious Disease Organization, University of Saskatchewan,

Saskatoon, Saskatchewan, Canada.

FAU - Steyn, Adrie J C

AU - Steyn AJC

AUID- ORCID: 0000-0001-9177-8827

AD - Africa Health Research Institute, University of KwaZulu Natal, Durban, South

Africa.

AD - Department of Microbiology, University of Alabama at Birmingham, Birmingham, AL,

USA.

AD - Centers for AIDS Research and Free Radical Biology, University of Alabama at

Birmingham, Birmingham, AL, USA.

FAU - Jackson, Paul

AU - Jackson P

AD - Janssen Global Public Health, LLC, Janssen Pharmaceutica, La Jolla, CA, USA.

FAU - Aguilar-Pérez, Clara

AU - Aguilar-Pérez C

AUID- ORCID: 0000-0001-7438-9141

AD - Janssen Global Public Health, LLC, Janssen Pharmaceutica NV, Antwerp, Belgium.

FAU - Koul, Anil

AU - Koul A

AUID- ORCID: 0009-0009-5542-4568

AD - Janssen Global Public Health, LLC, Janssen Pharmaceutica NV, Antwerp, Belgium.

anil.koul@lshtm.ac.uk.

AD - Department of Infection Biology, Faculty of Infectious and Tropical Diseases,

London School of Hygiene and Tropical Medicine, London, UK.

anil.koul@lshtm.ac.uk.

LA - eng

GR - WT_/Wellcome Trust/United Kingdom

GR - INV-027276/GATES/Gates Foundation/United States

GR - R24 AI186591/AI/NIAID NIH HHS/United States

GR - R33 AI138280/AI/NIAID NIH HHS/United States

PT - Journal Article

PT - Research Support, N.I.H., Extramural

PT - Research Support, Non-U.S. Gov't

DEP - 20250618

PL - England

TA - Nature

JT - Nature

JID - 0410462

RN - 0 (Purines)

RN - 0 (Antitubercular Agents)

RN - W60KTZ3IZY (purine)

SB - IM

MH - *Purines/biosynthesis

MH - Mice

MH - Animals

MH - Humans

MH - *Mycobacterium tuberculosis/drug effects/enzymology/genetics/metabolism

MH - *Antitubercular Agents/pharmacology/chemistry/therapeutic use/administration &

dosage

MH - Female

MH - *Tuberculosis/drug therapy/microbiology

MH - Lung/metabolism/drug effects/microbiology

MH - Male

MH - DNA Replication/drug effects

MH - Tuberculosis, Multidrug-Resistant/drug therapy/microbiology

MH - Mice, Inbred C57BL

MH - Proof of Concept Study

PMC - PMC12328218

COIS- Competing interests: D.A.L., A.L., J.S., P.F., J.W., S.D., S. Saylock, M.D.R.,

A.H., T.G., A.V., K.W.C., P.S., M.E., M.M., J.E., R.D.A., H.F., A.S.P., B.S.,

C.A.-P., P.J. and A.K. were or are all full-time employees of Janssen

Pharmaceutica, a Johnson & Johnson company, and/or potential stockholders of

Johnson & Johnson. J.S. and P.F. were or are employees of Charles River

Laboratories, a contract research organization. A.R., C.R., N.C. and S. Sans were

or are all full-time employees of Evotec. R.J.W., C.D., W.P., E.D., S.L., M.G.,

J.D., S.J.W., G.L.-M., N.D. and A.K. received funding from Janssen Pharmaceutica

to perform contract research. The other authors declare no competing interests.

EDAT- 2025/06/19 00:25

MHDA- 2025/08/07 06:26

PMCR- 2025/06/18

CRDT- 2025/06/18 23:18

PHST- 2024/08/14 00:00 [received]

PHST- 2025/05/20 00:00 [accepted]

PHST- 2025/08/07 06:26 [medline]

PHST- 2025/06/19 00:25 [pubmed]

PHST- 2025/06/18 23:18 [entrez]

PHST- 2025/06/18 00:00 [pmc-release]

AID - 10.1038/s41586-025-09177-7 [pii]

AID - 9177 [pii]

AID - 10.1038/s41586-025-09177-7 [doi]

PST - ppublish

SO - Nature. 2025 Aug;644(8075):214-220. doi: 10.1038/s41586-025-09177-7. Epub 2025

Jun 18.

PMID- 38895953

OWN - NLM

STAT- MEDLINE

DCOM- 20250918

LR - 20250918

IS - 1538-0254 (Electronic)

IS - 0739-1102 (Linking)

VI - 43

IP - 12

DP - 2025 Aug

TI - Screening of bioactive compounds from selected mushroom species against putative

drug targets in Mycobacterium tuberculosis: a multi-target approach.

PG - 6269-6284

LID - 10.1080/07391102.2024.2335292 [doi]

AB - Mycobacterium tuberculosis (Mtb) is a notorious pathogen that causes one of the

highest mortalities globally. Due to a pressing demand to identify novel

therapeutic alternatives, the present study aims to focus on screening the

putative drug targets and prioritizing their role in antibacterial drug

development. The most vital proteins involved in the Biotin biosynthesis pathway

and the Lipoarabinomannan (LAM) pathway such as biotin synthase (bioB) and

alpha-(1->6)-mannopyranosyltransferase A (mptA) respectively, along with other

essential virulence proteins of Mtb were selected as drug targets. Among these,

the ones without native structures were modelled and validated using standard

bioinformatics tools. Further, the interactions were performed with naturally

available lead molecules present in selected mushroom species such as Agaricus

bisporus, Pleurotus djamor, Hypsizygus ulmarius. Through Gas Chromatography-Mass

Spectrometry (GC-MS), 15 bioactive compounds from the methanolic extract of

mushrooms were identified. Further, 4 were selected based on drug-likeness and

pharmacokinetic screening for molecular docking analysis against our prioritized

targets wherein Benz[e]azulene from Pleurotus djamor illustrated a good binding

affinity with a LF rank score of -9.036 kcal mol (-1) against nuoM (NADH quinone

oxidoreductase subunit M) and could be used as a prospective candidate in order

to combat Tuberculosis (TB). Furthermore, the stability of the complex are

validated using MD Simulations and subsequently, the binding free energy was

calculated using MM-GBSA analysis. Thus, the current in silico analysis suggests

a promising role of compounds extracted from mushrooms in tackling the TB burden.

FAU - Prasanna, Akshatha

AU - Prasanna A

AD - Department of Biotechnology, Dayananda Sagar College of Engineering (Affiliated

to Visvesvaraya Technological University, Belagavi), Bengaluru, Karnataka, India.

FAU - Karunakar, Prashantha

AU - Karunakar P

AD - Department of Biotechnology, Dayananda Sagar College of Engineering (Affiliated

to Visvesvaraya Technological University, Belagavi), Bengaluru, Karnataka, India.

FAU - Pillai, Anushka

AU - Pillai A

AD - Department of Biotechnology, Dayananda Sagar College of Engineering (Affiliated

to Visvesvaraya Technological University, Belagavi), Bengaluru, Karnataka, India.

FAU - Mukundan, Shreyashree

AU - Mukundan S

AD - Department of Biotechnology, Dayananda Sagar College of Engineering (Affiliated

to Visvesvaraya Technological University, Belagavi), Bengaluru, Karnataka, India.

FAU - Y V, Mansi

AU - Y V M

AD - Department of Biotechnology, Dayananda Sagar College of Engineering (Affiliated

to Visvesvaraya Technological University, Belagavi), Bengaluru, Karnataka, India.

FAU - Balaji, Renu

AU - Balaji R

AD - Department of Biotechnology, Dayananda Sagar College of Engineering (Affiliated

to Visvesvaraya Technological University, Belagavi), Bengaluru, Karnataka, India.

FAU - Niranjan, Vidya

AU - Niranjan V

AUID- ORCID: 0000-0001-9187-7753

AD - Department of Biotechnology, RV College of Engineering, Bengaluru, Karnataka,

India.

FAU - Skariyachan, Sinosh

AU - Skariyachan S

AD - Department of Microbiology, St. Pius X College Rajapuram, Kasaragod, Kerala,

India.

FAU - Narayanappa, Rajeswari

AU - Narayanappa R

AD - Department of Biotechnology, Dayananda Sagar College of Engineering (Affiliated

to Visvesvaraya Technological University, Belagavi), Bengaluru, Karnataka, India.

LA - eng

PT - Journal Article

DEP - 20240619

PL - England

TA - J Biomol Struct Dyn

JT - Journal of biomolecular structure & dynamics

JID - 8404176

RN - 0 (Antitubercular Agents)

RN - 0 (Bacterial Proteins)

SB - IM

MH - Molecular Docking Simulation

MH - *Mycobacterium tuberculosis/drug effects

MH - *Antitubercular Agents/pharmacology/chemistry

MH - *Agaricales/chemistry

MH - Molecular Dynamics Simulation

MH - Bacterial Proteins/chemistry/antagonists & inhibitors/metabolism

MH - Gas Chromatography-Mass Spectrometry

OTO - NOTNLM

OT - Biotin

OT - Lipoarabinomannan (LAM)

OT - Mycobacterium tuberculosis

OT - Tuberculosis (TB)

OT - multidrug resistant TB

EDAT- 2024/06/19 12:43

MHDA- 2025/09/18 18:31

CRDT- 2024/06/19 08:03

PHST- 2025/09/18 18:31 [medline]

PHST- 2024/06/19 12:43 [pubmed]

PHST- 2024/06/19 08:03 [entrez]

AID - 10.1080/07391102.2024.2335292 [doi]

PST - ppublish

SO - J Biomol Struct Dyn. 2025 Aug;43(12):6269-6284. doi:

10.1080/07391102.2024.2335292. Epub 2024 Jun 19.

PMID- 40761881

OWN - NLM

STAT- PubMed-not-MEDLINE

LR - 20250807

IS - 1178-6973 (Print)

IS - 1178-6973 (Electronic)

IS - 1178-6973 (Linking)

VI - 18

DP - 2025

TI - Progress on the Global Research Agenda for Antimicrobial Resistance in Human

Health in Pakistan: Findings and Implications.

PG - 3795-3828

LID - 10.2147/IDR.S531874 [doi]

AB - BACKGROUND AND OBJECTIVE: Antimicrobial resistance (AMR) poses a formidable

challenge to global public health, with low- and middle-income countries (LMICs)

including Pakistan being particularly vulnerable. This study assesses the

progress made in Pakistan following the Global Research Agenda for AMR, which

builds on the key activities and goals of its national action plan to reduce AMR.

The intention is to identify key gaps, achievements, and future areas of focus to

help reduce rising AMR rates in Pakistan. METHODS: Utilizing a

systematic-narrative hybrid literature review methodology approach, recent

research publication and policy initiatives related to AMR, including those

published on the internet, were examined and documented. FINDINGS: The findings

from 349 published studies were divided into the 40 research priority areas. This

included 23 papers (9.95%) specifically related to prevention and 55 (22.9%) to

diagnosis, 64 (26.7%) for treatment and care of patients with infectious

diseases, 59 (24.5%) for cross-cutting, and 44 (18.33%) for drug-resistant

tuberculosis (TB). Currently, research on AMR in Pakistan is primarily

concentrated in major urban centers across a limited number of cities. This needs

addressing going forward. To effectively combat AMR in Pakistan, prioritizing

prevention is crucial to curb disease spread and reduce reliance on prophylactic

treatments, especially inappropriate prescribing and dispensing of

antimicrobials. Enhancing diagnostic facilities, strengthening antimicrobial

surveillance systems and promoting appropriate management of patients with

infectious diseases, supported by robust antimicrobial stewardship programs, can

also help enhance judicious antibiotic use in Pakistan and reduce AMR going

forward. CONCLUSION AND INTERPRETATION: There are ongoing concerns regarding

current research activities in Pakistan to reduce AMR. The pathway forward in

Pakistan includes leveraging global partnerships to share knowledge, resources,

and strategies to enhance the use of Access antibiotics as well as reduce AMR to

reach agreed United Nations' goals.

CI - © 2025 Maryam et al.

FAU - Maryam, Sharmeen

AU - Maryam S

AD - Department of Pharmaceutical Practices, College of Pharmacy, Umm Al-Qura

University, Makkah, Saudi Arabia.

FAU - Saleem, Zikria

AU - Saleem Z

AUID- ORCID: 0000-0003-3202-6347

AD - Department of Pharmacy Practice, College of Pharmacy, Qassim University, Qassim,

Saudi Arabia.

FAU - Haseeb, Abdul

AU - Haseeb A

AD - Clinical Pharmacy Department, Al Rayan National College of Health Sciences and

Nursing, Al Madinah Al Munawarrah, Saudi Arabia.

FAU - Qamar, Muhammad Usman

AU - Qamar MU

AUID- ORCID: 0000-0002-2636-238X

AD - Institute of Microbiology, Faculty of Life Sciences, Government College

University Faisalabad, Faisalabad, 38000, Pakistan.

AD - Division of Infectious Diseases, Department of Medicine, Geneva University

Hospitals and Medical School, Geneva, Switzerland.

FAU - Amir, Afreenish

AU - Amir A

AUID- ORCID: 0000-0002-1802-1737

AD - Department of Microbiology, National University of Medical Sciences, Rawalpindi,

46000, Pakistan.

AD - Department of Microbiology, National Institute of Health, Islamabad, 45501,

Pakistan.

FAU - Almarzoky Abuhussain, Safa S

AU - Almarzoky Abuhussain SS

AD - Department of Pharmaceutical Practices, College of Pharmacy, Umm Al-Qura

University, Makkah, 21955, Saudi Arabia.

FAU - Imam, Mohammad Tarique

AU - Imam MT

AD - Department of Clinical Pharmacy, College of Pharmacy, Prince Sattam Bin Abdulaziz

University, Al Kharj, 11942, Saudi Arabia.

FAU - Afzal, Shairyar

AU - Afzal S

AUID- ORCID: 0009-0008-8733-1748

AD - Department of Pharmacy, DHQ Hospital Jhelum, Jhelum, Pakistan.

FAU - Meyer, Johanna C

AU - Meyer JC

AUID- ORCID: 0000-0003-0462-5713

AD - Department of Public Health Pharmacy and Management, School of Pharmacy, Sefako

Makgatho Health Sciences University, Pretoria, 0208, South Africa.

AD - South African Vaccination and Immunisation Centre, Sefako Makgatho Health

Sciences University, Ga-Rankuwa, 0208, South Africa.

FAU - Mudenda, Steward

AU - Mudenda S

AUID- ORCID: 0000-0003-1692-8981

AD - Department of Pharmacy, School of Health Sciences, University of Zambia, Lusaka,

50110, Zambia.

FAU - Godman, Brian

AU - Godman B

AUID- ORCID: 0000-0001-6539-6972

AD - Department of Public Health Pharmacy and Management, School of Pharmacy, Sefako

Makgatho Health Sciences University, Pretoria, 0208, South Africa.

AD - Strathclyde Institute of Pharmacy and Biomedical Science (SIPBS), University of

Strathclyde, Glasgow, G4 0RE, UK.

AD - Antibiotic Policy Group, Institute for Infection and Immunity, City St. George's,

University of London, London, SW17 0RE, UK.

LA - eng

PT - Journal Article

PT - Review

DEP - 20250729

PL - New Zealand

TA - Infect Drug Resist

JT - Infection and drug resistance

JID - 101550216

PMC - PMC12318840

OAB - Antimicrobial resistance (AMR) poses a substantial threat to public health, as it

reduces the number of effective antibiotics to combat infections, as well as

increasing costs and the number of deaths. Pakistan is a critical country, with

high and growing rates of AMR. Consequently, this issue must be addressed. The

Global Research Agenda for AMR provided guidance on approaches that key

stakeholders in Pakistan should undertake to reduce AMR. Our findings uncovered

20 published studies covering multiple aspects to reduce AMR. These included

studies related to ways to reduce infections and the associated use of

antibiotics and improve the care of patients with infectious diseases, including

tuberculosis (TB), resistant to the current antimicrobials. Going forward, health

authorities and others in Pakistan need to prioritize activities to reduce

infections, including better hygiene, as well as reduce unnecessary prescribing

and dispensing of antibiotics. This includes activities called antimicrobial

stewardship programs to improve antibiotic use. The pathway forward also included

leveraging global partnerships to share knowledge, resources, activities and

strategies to improve the future use of antibiotics. We believe that this is the

first study among low- and middle-income countries to fully explore current

activities as part of the Global Research Agenda for AMR to provide guidance to

other researchers and countries operating in this field.

OABL- eng

OTO - NOTNLM

OT - AWaRe

OT - Pakistan

OT - antibiotics

OT - antimicrobial resistance

OT - antimicrobial surveillance

OT - global research

OT - health policy

COIS- The authors declare no relevant conflicts of interest.

EDAT- 2025/08/05 06:28

MHDA- 2025/08/05 06:29

PMCR- 2025/07/29

CRDT- 2025/08/05 04:56

PHST- 2025/04/11 00:00 [received]

PHST- 2025/06/20 00:00 [accepted]

PHST- 2025/08/05 06:29 [medline]

PHST- 2025/08/05 06:28 [pubmed]

PHST- 2025/08/05 04:56 [entrez]

PHST- 2025/07/29 00:00 [pmc-release]

AID - 531874 [pii]

AID - 10.2147/IDR.S531874 [doi]

PST - epublish

SO - Infect Drug Resist. 2025 Jul 29;18:3795-3828. doi: 10.2147/IDR.S531874.

eCollection 2025.

PMID- 40703475

OWN - NLM

STAT- MEDLINE

DCOM- 20250724

LR - 20250728

IS - 1178-2013 (Electronic)

IS - 1176-9114 (Print)

IS - 1176-9114 (Linking)

VI - 20

DP - 2025

TI - Nitric Oxide Therapeutics: New Hopes for More Effective Tuberculosis Treatment

Combine with Targeted and Controlled Nanotechnology.

PG - 9195-9218

LID - 10.2147/IJN.S531255 [doi]

AB - Tuberculosis (TB) caused by Mycobacterium tuberculosis (Mtb) is one of the most

prevalent infectious diseases worldwide. Nitric oxide (NO) is produced by the

reaction of arginine and oxygen catalyzed by nitric oxide synthase (NOS) in

mammals. Several studies have highlighted the potential therapeutic use of NO for

the treatment of various diseases, including infectious diseases. NO plays a

direct bactericidal role by damaging the bacterial DNA, proteins, and enzymes.

Additionally, it plays a role in modulating immune cell function, contributing to

their anti-tuberculosis (anti-TB) effects by regulating macrophage activity. NO

has also been shown to eliminate bacterial biofilms, thereby increasing drug

sensitivity of drug-resistant bacteria. Therefore, combining NO with antibiotics

may be a strategy for treating drug-resistant tuberculosis (DR-TB). However,

owing to the limitations of NO, including their short half-life, instability, and

cytotoxicity, exogenous supplementation with NO donors has emerged as a promising

alternative therapy. Rapid advancements in nanotechnology have led to the

development of nanoparticles (NPs) as drug delivery platforms, at the same time,

using strategies such as introducing selective organ targeting (SORT) molecules

into nanocarrier systems or preparing nanodrugs in inhalable or dry powder

inhalation forms can increase the accumulation of nanodrugs in the lungs.

Combined with host-directed therapy strategies, this can improve the therapeutic

effect on tuberculosis and shorten the treatment time. This review summarizes the

biological activities of NO and introduces their applications in the treatment of

several major infectious diseases, followed by a systemic analysis of the role

and mechanism of action of NO in TB treatment. Moreover, nanotechnology-assisted

NO therapeutics are also summarized to explore the potential for more effective

Mtb killing based on the advantages of targeted NO release at the infected site

and host cells, thus benefiting the development of more effective therapeutics

against TB and drug-resistant TB.

CI - © 2025 Jin et al.

FAU - Jin, Xiaoying

AU - Jin X

AD - Research Center of Nanotechnology and Application Engineering, School of Medical

Technology, The First Dongguan Affiliated Hospital, Guangdong Medical University,

Dongguan, People's Republic of China.

AD - Dongguan Key Laboratory for Pathogenesis and Experimental Diagnosis of Infectious

Diseases, The First Dongguan Affiliated Hospital, School of Medical Technology,

Guangdong Medical University, Dongguan, People's Republic of China.

FAU - Wang, Jiajun

AU - Wang J

AUID- ORCID: 0000-0003-1824-6837

AD - Research Center of Nanotechnology and Application Engineering, School of Medical

Technology, The First Dongguan Affiliated Hospital, Guangdong Medical University,

Dongguan, People's Republic of China.

AD - Dongguan Key Laboratory for Pathogenesis and Experimental Diagnosis of Infectious

Diseases, The First Dongguan Affiliated Hospital, School of Medical Technology,

Guangdong Medical University, Dongguan, People's Republic of China.

FAU - Ruan, Yongdui

AU - Ruan Y

AD - Research Center of Nanotechnology and Application Engineering, School of Medical

Technology, The First Dongguan Affiliated Hospital, Guangdong Medical University,

Dongguan, People's Republic of China.

FAU - Li, Jiaxiang

AU - Li J

AD - Research Center of Nanotechnology and Application Engineering, School of Medical

Technology, The First Dongguan Affiliated Hospital, Guangdong Medical University,

Dongguan, People's Republic of China.

AD - Dongguan Key Laboratory for Pathogenesis and Experimental Diagnosis of Infectious

Diseases, The First Dongguan Affiliated Hospital, School of Medical Technology,

Guangdong Medical University, Dongguan, People's Republic of China.

FAU - Kong, Xinen

AU - Kong X

AD - Research Center of Nanotechnology and Application Engineering, School of Medical

Technology, The First Dongguan Affiliated Hospital, Guangdong Medical University,

Dongguan, People's Republic of China.

AD - Dongguan Key Laboratory for Pathogenesis and Experimental Diagnosis of Infectious

Diseases, The First Dongguan Affiliated Hospital, School of Medical Technology,

Guangdong Medical University, Dongguan, People's Republic of China.

FAU - Xia, Jiaojiao

AU - Xia J

AD - Department of Biochemistry and Molecular Biology, School of Basic Medical

Sciences, Kunming Medical University, Kunming, Yunnan, 650500, People's Republic

of China.

FAU - Yang, Jiayi

AU - Yang J

AD - Research Center of Nanotechnology and Application Engineering, School of Medical

Technology, The First Dongguan Affiliated Hospital, Guangdong Medical University,

Dongguan, People's Republic of China.

AD - Dongguan Key Laboratory for Pathogenesis and Experimental Diagnosis of Infectious

Diseases, The First Dongguan Affiliated Hospital, School of Medical Technology,

Guangdong Medical University, Dongguan, People's Republic of China.

FAU - Zhang, Qiao

AU - Zhang Q

AD - Department of Biochemistry and Molecular Biology, School of Basic Medical

Sciences, Kunming Medical University, Kunming, Yunnan, 650500, People's Republic

of China.

FAU - Liu, Juan

AU - Liu J

AD - Research Center of Nanotechnology and Application Engineering, School of Medical

Technology, The First Dongguan Affiliated Hospital, Guangdong Medical University,

Dongguan, People's Republic of China.

AD - Dongguan Key Laboratory for Pathogenesis and Experimental Diagnosis of Infectious

Diseases, The First Dongguan Affiliated Hospital, School of Medical Technology,

Guangdong Medical University, Dongguan, People's Republic of China.

FAU - Pi, Jiang

AU - Pi J

AUID- ORCID: 0000-0003-4418-1248

AD - Research Center of Nanotechnology and Application Engineering, School of Medical

Technology, The First Dongguan Affiliated Hospital, Guangdong Medical University,

Dongguan, People's Republic of China.

AD - Dongguan Key Laboratory for Pathogenesis and Experimental Diagnosis of Infectious

Diseases, The First Dongguan Affiliated Hospital, School of Medical Technology,

Guangdong Medical University, Dongguan, People's Republic of China.

LA - eng

PT - Journal Article

PT - Review

DEP - 20250719

PL - New Zealand

TA - Int J Nanomedicine

JT - International journal of nanomedicine

JID - 101263847

RN - 31C4KY9ESH (Nitric Oxide)

RN - 0 (Antitubercular Agents)

RN - 0 (Nanoparticle Drug Delivery System)

SB - IM

MH - Humans

MH - *Nitric Oxide/therapeutic use/pharmacology/administration & dosage/metabolism

MH - Mycobacterium tuberculosis/drug effects

MH - *Antitubercular Agents/administration & dosage/pharmacology/therapeutic use

MH - *Tuberculosis/drug therapy

MH - Animals

MH - Drug Delivery Systems/methods

MH - Nanoparticles/chemistry

MH - Nanotechnology

MH - Tuberculosis, Multidrug-Resistant/drug therapy

MH - Nanomedicine

MH - Nanoparticle Drug Delivery System/chemistry

PMC - PMC12285888

OTO - NOTNLM

OT - nanotechnology

OT - nitric oxide

OT - nitric oxide donors

OT - tuberculosis

COIS- The authors declare no competing interests.

EDAT- 2025/07/24 06:31

MHDA- 2025/07/24 06:32

PMCR- 2025/07/19

CRDT- 2025/07/24 05:07

PHST- 2025/03/31 00:00 [received]

PHST- 2025/07/09 00:00 [accepted]

PHST- 2025/07/24 06:32 [medline]

PHST- 2025/07/24 06:31 [pubmed]

PHST- 2025/07/24 05:07 [entrez]

PHST- 2025/07/19 00:00 [pmc-release]

AID - 531255 [pii]

AID - 10.2147/IJN.S531255 [doi]

PST - epublish

SO - Int J Nanomedicine. 2025 Jul 19;20:9195-9218. doi: 10.2147/IJN.S531255.

eCollection 2025.

PMID- 40707100

OWN - NLM

STAT- MEDLINE

DCOM- 20250724

LR - 20250724

IS - 0019-5707 (Print)

IS - 0019-5707 (Linking)

VI - 72

IP - 3

DP - 2025 Jul

TI - Structure and mechanism basis of β-lactam activity against Mycobacterium

tuberculosis: A review of literature.

PG - 424-437

LID - S0019-5707(25)00001-0 [pii]

LID - 10.1016/j.ijtb.2025.01.001 [doi]

AB - Tuberculosis (TB), caused by the infectious agent Mycobacterium tuberculosis

(Mtb), has resulted in the highest mortality rates, even surpassing HIV/AIDS. The

rise of Drug-resistant TB has worsened the health crisis and urgently requires

new treatment approaches. The WHO has approved the repurposing of β-lactam in

combination with β-lactamase (BlaC) inhibitor for treating MDR/XDR-TB. Numerous

targets of β lactams present in the Mtb's cell wall are involved in its

structural cytoskeleton, peptidoglycan (PG) biosynthesis. Delving into the

mechanistic basis of β-lactam activity against Mtb has become a holistic approach

towards developing new kinds of β-lactams and ß-lactamase-inhibitors against Mtb.

This work comprehensively reviews the literature-landscape of the structure and

mechanism of β-lactams binding to different PG enzymes and the β-lactamase

inhibitors that can inhibit BlaC in Mtb.

CI - Copyright © 2025 Tuberculosis Association of India. Published by Elsevier B.V.

All rights reserved.

FAU - Ahmad, Nazia

AU - Ahmad N

AD - Department of Biochemistry, School of Chemical and Life Sciences, Jamia Hamdard,

New Delhi, 110062, India. Electronic address: 14nadia.ahmad@gmail.com.

FAU - Islam, Zeyaul

AU - Islam Z

AD - Qatar Biomedical Research Institute (QBRI), Hamad Bin Khalifa University, Qatar

(HBKU), Qatar Foundation, Doha, Qatar. Electronic address: zislam@hbku.edu.qa.

FAU - Dhar, Sohan

AU - Dhar S

AD - University of California, Davis, CA, 95616, USA. Electronic address:

sandhar@ucdavis.edu.

FAU - Kumar, Pankaj

AU - Kumar P

AD - Department of Biochemistry, School of Chemical and Life Sciences, Jamia Hamdard,

New Delhi, 110062, India. Electronic address: pankajkumar@jamiahamdard.ac.in.

FAU - Zaidi, Rana

AU - Zaidi R

AD - Department of Biochemistry, Jamia Hamdard, New Delhi, 110062, India. Electronic

address: rzaidi@jamiahamdard.ac.in.

LA - eng

PT - Journal Article

PT - Review

DEP - 20250110

PL - India

TA - Indian J Tuberc

JT - The Indian journal of tuberculosis

JID - 0373027

RN - 0 (beta-Lactams)

RN - 0 (beta-Lactamase Inhibitors)

RN - 0 (Antitubercular Agents)

RN - EC 3.5.2.6 (beta-Lactamases)

RN - 0 (Peptidoglycan)

SB - IM

MH - *Mycobacterium tuberculosis/drug effects

MH - *beta-Lactams/pharmacology/chemistry

MH - Humans

MH - *beta-Lactamase Inhibitors/pharmacology/chemistry

MH - *Antitubercular Agents/pharmacology/chemistry

MH - Tuberculosis, Multidrug-Resistant/drug therapy

MH - beta-Lactamases/metabolism

MH - Peptidoglycan

OTO - NOTNLM

OT - BlaC

OT - DDT

OT - LDT

OT - Mycobacterium tuberculosis (Mtb)

OT - Peptidoglycan

OT - β-lactams

COIS- Declaration of competing interest The authors declare there is no competing

interest.

EDAT- 2025/07/25 00:28

MHDA- 2025/07/25 00:29

CRDT- 2025/07/24 20:54

PHST- 2024/09/12 00:00 [received]

PHST- 2024/09/24 00:00 [revised]

PHST- 2025/01/10 00:00 [accepted]

PHST- 2025/07/25 00:29 [medline]

PHST- 2025/07/25 00:28 [pubmed]

PHST- 2025/07/24 20:54 [entrez]

AID - S0019-5707(25)00001-0 [pii]

AID - 10.1016/j.ijtb.2025.01.001 [doi]

PST - ppublish

SO - Indian J Tuberc. 2025 Jul;72(3):424-437. doi: 10.1016/j.ijtb.2025.01.001. Epub

2025 Jan 10.

PMID- 40396746

OWN - NLM

STAT- MEDLINE

DCOM- 20250625

LR - 20260127

IS - 2379-5042 (Electronic)

IS - 2379-5042 (Linking)

VI - 10

IP - 6

DP - 2025 Jun 25

TI - Sudapyridine (WX-081) inhibits Mycobacterium tuberculosis by targeting ATP

synthase and upregulating host innate immunity.

PG - e0014925

LID - 10.1128/msphere.00149-25 [doi]

LID - e00149-25

AB - Drug-resistant tuberculosis (DR-TB) urgently requires safer, more accessible

alternatives to bedaquiline (BDQ), which faces critical flaws like

cardiotoxicity, high costs, and emerging resistance. WX-081, a promising BDQ

alternative, has demonstrated superior anti-TB activity and improved safety in

clinical studies. However, its mechanism of action remains unexplored,

underscoring the need for further research to optimize its potential in advancing

global TB elimination efforts. This study reveals WX-081's dual mechanisms:

targeting atpE to disrupt ATP synthase and proton motive force via resistance

screening, gene sequencing, and functional assays while enhancing host immunity

through macrophage transcriptomics. Molecular docking confirmed atpE binding

sites, and immune activation pathways (NF-κB/MAPK) were identified, positioning

WX-081 as a potent, safe anti-DR-TB candidate despite unresolved mechanistic

details.IMPORTANCEBedaquiline, a key drug for drug-resistant tuberculosis, is

restricted by safety issues impacting its clinical utility. Its next-generation

alternative, WX-081, has advanced to Phase III trials but lacks in-depth studies

on its mechanism and host immune-modulatory effects, necessitating further

research before broad clinical adoption.

FAU - Li, Xinda

AU - Li X

AUID- ORCID: 0009-0006-8278-9413

AD - Department of Pharmacology, Beijing Chest Hospital, Capital Medical University,

Beijing, China. RINGGOLD: 12517. ROR: https://ror.org/013xs5b60

AD - Beijing Key Laboratory of Drug Resistance Tuberculosis Research, Beijing

Tuberculosis and Thoracic Tumor Research Institute, Beijing, China. RINGGOLD:

117550

FAU - Luo, Xiaoyi

AU - Luo X

AD - Department of Pharmacology, Beijing Chest Hospital, Capital Medical University,

Beijing, China. RINGGOLD: 12517. ROR: https://ror.org/013xs5b60

AD - Beijing Key Laboratory of Drug Resistance Tuberculosis Research, Beijing

Tuberculosis and Thoracic Tumor Research Institute, Beijing, China. RINGGOLD:

117550

FAU - Wang, Bin

AU - Wang B

AD - Department of Pharmacology, Beijing Chest Hospital, Capital Medical University,

Beijing, China. RINGGOLD: 12517. ROR: https://ror.org/013xs5b60

AD - Beijing Key Laboratory of Drug Resistance Tuberculosis Research, Beijing

Tuberculosis and Thoracic Tumor Research Institute, Beijing, China. RINGGOLD:

117550

FAU - Fu, Lei

AU - Fu L

AD - Department of Pharmacology, Beijing Chest Hospital, Capital Medical University,

Beijing, China. RINGGOLD: 12517. ROR: https://ror.org/013xs5b60

AD - Beijing Key Laboratory of Drug Resistance Tuberculosis Research, Beijing

Tuberculosis and Thoracic Tumor Research Institute, Beijing, China. RINGGOLD:

117550

FAU - Chen, Xi

AU - Chen X

AD - Department of Pharmacology, Beijing Chest Hospital, Capital Medical University,

Beijing, China. RINGGOLD: 12517. ROR: https://ror.org/013xs5b60

AD - Beijing Key Laboratory of Drug Resistance Tuberculosis Research, Beijing

Tuberculosis and Thoracic Tumor Research Institute, Beijing, China. RINGGOLD:

117550

FAU - Lu, Yu

AU - Lu Y

AUID- ORCID: 0000-0002-9038-1884

AD - Department of Pharmacology, Beijing Chest Hospital, Capital Medical University,

Beijing, China. RINGGOLD: 12517. ROR: https://ror.org/013xs5b60

AD - Beijing Key Laboratory of Drug Resistance Tuberculosis Research, Beijing

Tuberculosis and Thoracic Tumor Research Institute, Beijing, China. RINGGOLD:

117550

LA - eng

GR - 82304570/National Natural Science Foundation of China/

GR - DFL20221402/Beijing Municipal Administration of Hospitals/

PT - Journal Article

DEP - 20250521

PL - United States

TA - mSphere

JT - mSphere

JID - 101674533

RN - 0 (Antitubercular Agents)

RN - 0 (Diarylquinolines)

RN - 0 (sudapyridine)

RN - 0 (Pyridines)

SB - IM

MH - *Mycobacterium tuberculosis/drug effects/enzymology

MH - *Immunity, Innate/drug effects

MH - *Antitubercular Agents/pharmacology

MH - Humans

MH - Macrophages/drug effects/immunology

MH - Molecular Docking Simulation

MH - Mice

MH - Animals

MH - Up-Regulation

MH - Tuberculosis, Multidrug-Resistant/drug therapy

MH - Diarylquinolines/pharmacology

MH - Pyridines

PMC - PMC12188713

OTO - NOTNLM

OT - ATP synthase

OT - WX-081

OT - drug-resistant tuberculosis

OT - immunomodulation

COIS- The authors declare no conflict of interest.

EDAT- 2025/05/21 12:31

MHDA- 2025/06/25 12:28

PMCR- 2025/05/21

CRDT- 2025/05/21 09:04

PHST- 2025/06/25 12:28 [medline]

PHST- 2025/05/21 12:31 [pubmed]

PHST- 2025/05/21 09:04 [entrez]

PHST- 2025/05/21 00:00 [pmc-release]

AID - msphere00149-25 [pii]

AID - msphere.00149-25 [pii]

AID - 10.1128/msphere.00149-25 [doi]

PST - ppublish

SO - mSphere. 2025 Jun 25;10(6):e0014925. doi: 10.1128/msphere.00149-25. Epub 2025 May

21.

PMID- 40667120

OWN - NLM

STAT- PubMed-not-MEDLINE

DCOM- 20250930

LR - 20250930

IS - 2692-8205 (Electronic)

IS - 2692-8205 (Linking)

DP - 2025 Jun 24

TI - Structural and functional analysis of the Mycobacterium tuberculosis MmpS5L5

efflux pump presages a pathway to increased bedaquiline resistance.

LID - 2025.06.24.661325 [pii]

LID - 10.1101/2025.06.24.661325 [doi]

AB - Bedaquiline, an antitubercular drug that targets ATP-synthase, is a key component

of a new oral drug regimen that has revolutionized the treatment of multi drug

resistant tuberculosis. Clinical bedaquiline resistance in Mycobacterium

tuberculosis has rapidly emerged, primarily due to mutations in the

transcriptional repressor, Rv0678 that result in upregulation of the

Resistance-Nodulation-Division (RND) efflux pump MmpS5/MmpL5 (MmpS5L5). Here, to

understand how MmpS5L5 effluxes bedaquiline, we determined the structure of the

MmpS5L5 complex using cryo-electron microscopy, revealing a novel trimeric

architecture distinct from the canonical tripartite RND efflux pumps of

Gram-negative bacteria. Structure prediction modelling in conjunction with

functional genetic analysis indicates that it uses a periplasmic coiled-coil tube

to transport molecules across the cell wall. Structure-guided genetic approaches

identify MmpL5 mutations that alter bedaquiline transport; these mutations

converge on a region in MmpL5 located in the lower portion of the periplasmic

cavity, proximal to the outer leaflet of the inner membrane, suggesting a route

for bedaquiline entry into the pump. While currently known clinical resistance to

bedaquiline is due to pump upregulation, our findings that several MmpL5 variants

increase bedaquiline efflux may presage the emergence of additional modes of

clinical resistance. SIGNIFICANCE STATEMENT: Resistance to bedaquiline, a

cornerstone drug for treating multidrug-resistant tuberculosis, is rapidly

emerging due to mutations that upregulate expression of the MmpS5L5 efflux pump.

Here, we reveal the cryo-EM structure of this pump, showing a novel trimeric

architecture and a unique α-helical coiled-coil tube for drug transport.

Structure-guided genetic analysis identifies MmpL5 variants that further increase

bedaquiline efflux, suggesting potential resistance mechanisms beyond pump

upregulation.

FAU - Fountain, Adam J

AU - Fountain AJ

AUID- ORCID: 0000-0003-4232-3791

FAU - Böhning, Jan

AU - Böhning J

AUID- ORCID: 0000-0002-6242-1041

FAU - McLaughlin, Stephen H

AU - McLaughlin SH

AUID- ORCID: 0000-0001-9135-6253

FAU - Morgan, Tomos E

AU - Morgan TE

AUID- ORCID: 0000-0002-3366-8889

FAU - Edelstein, Paul H

AU - Edelstein PH

AUID- ORCID: 0000-0002-4069-5279

FAU - Troll, Mark

AU - Troll M

AUID- ORCID: 0000-0002-0137-7307

FAU - Lamers, Meindert H

AU - Lamers MH

AUID- ORCID: 0000-0002-4205-1338

FAU - Bharat, Tanmay A M

AU - Bharat TAM

AUID- ORCID: 0000-0002-0168-0277

FAU - Luisi, Ben F

AU - Luisi BF

AUID- ORCID: 0000-0003-1144-9877

FAU - Ramakrishnan, Lalita

AU - Ramakrishnan L

AUID- ORCID: 0000-0003-0692-5533

LA - eng

GR - WT_/Wellcome Trust/United Kingdom

GR - R01 AI054503/AI/NIAID NIH HHS/United States

PT - Journal Article

PT - Preprint

DEP - 20250624

PL - United States

TA - bioRxiv

JT - bioRxiv : the preprint server for biology

JID - 101680187

UIN - Proc Natl Acad Sci U S A. 2025 Sep 30;122(39):e2516660122. doi:

10.1073/pnas.2516660122. PMID: 40986343

PMC - PMC12262577

EDAT- 2025/07/16 06:25

MHDA- 2025/07/16 06:26

PMCR- 2025/07/15

CRDT- 2025/07/16 05:39

PHST- 2025/07/16 06:25 [pubmed]

PHST- 2025/07/16 06:26 [medline]

PHST- 2025/07/16 05:39 [entrez]

PHST- 2025/07/15 00:00 [pmc-release]

AID - 2025.06.24.661325 [pii]

AID - 10.1101/2025.06.24.661325 [doi]

PST - epublish

SO - bioRxiv [Preprint]. 2025 Jun 24:2025.06.24.661325. doi:

10.1101/2025.06.24.661325.

PMID- 40666898

OWN - NLM

STAT- PubMed-not-MEDLINE

DCOM- 20251223

LR - 20260109

IS - 2692-8205 (Electronic)

IS - 2692-8205 (Linking)

DP - 2025 Jun 23

TI - Tre-DST: A new drug susceptibility test for Mycobacterium tuberculosis using

solvatochromic trehalose probes.

LID - 2025.06.23.661142 [pii]

LID - 10.1101/2025.06.23.661142 [doi]

AB - Tuberculosis (TB) is the most lethal cause of death from a single infectious

agent. In 2024, an estimated 10 million people developed TB, nearly half a

million of which were infected with drug-resistant tuberculosis (DR-TB). Early

detection of infection and drug resistance is critical to controlling DR-TB as

this enables rapid engagement into effective care. Currently, bacterial culture

and nucleic acid testing remain the primary methods for diagnosing infection,

with smear microscopy being phased out. However, these methods present

significant limitations for diagnosing drug resistance such as lengthy

time-to-result for phenotypic tests, as well as the need for prior knowledge of

resistance mutations and prohibitive cost for molecular tests. To address this,

we developed a rapid phenotypic TB drug susceptibility test, termed Tre-DST,

based on novel trehalose probes, which upon metabolic conversion emit enhanced

fluorescence signal, giving them their unique ability to specifically detect live

mycobacteria. We used the nonpathogenic Mycobacterium smegmatis and the

virulence-attenuated Mycobacterium tuberculosis (Mtb) H37Ra or auxotrophic Mtb to

demonstrate a strong correlation between cost-effective plate reader results and

flow cytometry data, suggesting the fluorescence plate reader is a suitable

fluorescence detector for Tre-DST. We determined that adding a one-week

incubation step for Mtb allowed samples originally seeded at 10(4) CFU/mL to

become detectable, over two weeks earlier than colony forming unit analysis.

Importantly, we found that Tre-DST reports on drug susceptibility in a

drug-agnostic manner, demonstrating loss of fluorescence with frontline TB drugs

rifampicin (RIF), isoniazid (INH), and ethambutol, as well as the newer drug

bedaquiline. Finally, Tre-DST distinguished RIF- and INH-resistant auxotrophs

from susceptible controls and accurately reported resistance activity.

Ultimately, because Tre-DST is agnostic to mechanisms of drug resistance, this

assay is likely compatible with all WHO-recommended DR-TB drugs as well as any

future TB drugs as a diagnostic in reference laboratories.

FAU - Schwartz, Lilith A

AU - Schwartz LA

AUID- ORCID: 0009-0002-3439-790X

AD - UCLA Department of Chemistry and Biochemistry, Los Angeles, CA, USA.

FAU - Brodeth, Adriann L

AU - Brodeth AL

AUID- ORCID: 0009-0004-3084-0024

AD - UCLA Department of Bioengineering, Los Angeles, CA, USA.

FAU - Susilo, Cara T

AU - Susilo CT

AUID- ORCID: 0009-0000-5059-4001

AD - UCLA Department of Bioengineering, Los Angeles, CA, USA.

FAU - Rodolf, Amelia A

AU - Rodolf AA

AUID- ORCID: 0009-0003-3296-5392

AD - UCLA Department of Bioengineering, Los Angeles, CA, USA.

FAU - Ivanov, Tanya

AU - Ivanov T

AUID- ORCID: 0009-0004-9692-7624

AD - UCLA Molecular Biology Institute, Los Angeles, CA, USA.

FAU - Corrales, Esmeralda Mendoza

AU - Corrales EM

AUID- ORCID: 0000-0001-9182-0829

AD - UCLA Molecular Biology Institute, Los Angeles, CA, USA.

FAU - Kumar, Shivani S

AU - Kumar SS

AUID- ORCID: 0009-0007-3561-7923

AD - UCLA Department of Bioengineering, Los Angeles, CA, USA.

FAU - Kamariza, Mireille

AU - Kamariza M

AUID- ORCID: 0000-0003-1255-0803

AD - UCLA Department of Bioengineering, Los Angeles, CA, USA.

AD - UCLA Molecular Biology Institute, Los Angeles, CA, USA.

LA - eng

GR - R01 AI179891/AI/NIAID NIH HHS/United States

GR - S10 OD025017/OD/NIH HHS/United States

GR - T32 GM136614/GM/NIGMS NIH HHS/United States

PT - Journal Article

PT - Preprint

DEP - 20250623

PL - United States

TA - bioRxiv

JT - bioRxiv : the preprint server for biology

JID - 101680187

UIN - ACS Infect Dis. 2026 Jan 9;12(1):460-470. doi: 10.1021/acsinfecdis.5c01008. PMID:

41416726

PMC - PMC12262397

EDAT- 2025/07/16 06:25

MHDA- 2025/07/16 06:26

PMCR- 2025/07/15

CRDT- 2025/07/16 05:36

PHST- 2025/07/16 06:25 [pubmed]

PHST- 2025/07/16 06:26 [medline]

PHST- 2025/07/16 05:36 [entrez]

PHST- 2025/07/15 00:00 [pmc-release]

AID - 2025.06.23.661142 [pii]

AID - 10.1101/2025.06.23.661142 [doi]

PST - epublish

SO - bioRxiv [Preprint]. 2025 Jun 23:2025.06.23.661142. doi:

10.1101/2025.06.23.661142.

PMID- 40396760

OWN - NLM

STAT- MEDLINE

DCOM- 20250617

LR - 20260324

IS - 1098-5514 (Electronic)

IS - 0022-538X (Print)

IS - 0022-538X (Linking)

VI - 99

IP - 6

DP - 2025 Jun 17

TI - Comparative study on the virulence of mycobacteriophages.

PG - e0192024

LID - 10.1128/jvi.01920-24 [doi]

LID - e01920-24

AB - The global tuberculosis (TB) epidemic affected 10 million people and caused 1.3

million deaths in 2022 alone. Multidrug-resistant TB is successfully treated in

less than 60% of cases by long, expensive, and aggressive treatments.

Mycobacteriophages, viruses that can infect bacteria such as Mycobacterium

tuberculosis-the species responsible for TB-have the potential to redefine TB

prevention and treatments. However, the development of phage-based products

necessitates the assessment of numerous parameters, including virulence and

adsorption, to ensure their performance and quality. In this work, we

characterized the virulence of three different mycobacteriophages (Fionnbharth,

Muddy, and D29), alone and as cocktails, against a TB model host (Mycobacterium

smegmatis) under planktonic and early-stage biofilm growth conditions. Phage D29

and cocktails containing D29 had the highest virulence under all conditions.

Interestingly, phages Fionnbharth and Muddy and their combination showed higher

virulence against early-stage biofilm than against the planktonic phenotype.

Adsorption assays indicated that all three phages had lower adsorption

efficiencies on the early-stage biofilm phenotype than on the planktonic one,

suggesting a reduced availability of receptors in the former. Given that, despite

these lower adsorption efficiencies, the virulence of the phages and phage

cocktails was either unchanged or higher against the early-stage biofilm, this

phenotype must display properties that are favorable to other steps of the

infection process. These results inform us on the dynamics of mycobacteriophage

infections, both alone and in cocktail formulations, under different host growth

conditions, serving as a basis for the development of phage products targeting

mycobacteria biofilms. IMPORTANCE: This study provides a systematic investigation

of the virulence of three mycobacteriophages, Fionnbharth, Muddy, and D29, and

their combinations as cocktails against Mycobacterium smegmatis. We also included

considerations on the hydrodynamic conditions (shaking and not shaking) and host

phenotype (planktonic and early-onset biofilm cultures) during the infection

process and adsorption of the phage to the host. We showed that virulence was

strongly affected by phenotype and that higher virulence shown against the

early-onset biofilm phenotype was not linked to faster adsorption to the host. We

also showed that phage D29 and cocktails containing this phage had the highest

virulence. These results are important as they provide a framework for a better

evaluation and development of phage-based treatment against mycobacterial

infections.

FAU - Rubino, Ilaria

AU - Rubino I

AUID- ORCID: 0000-0003-1589-1566

AD - Department of Chemical and Materials Engineering, University of Alberta,

Edmonton, Alberta, Canada.

AD - Department of Chemical and Biotechnological Engineering, Université de

Sherbrooke, Sherbrooke, Québec, Canada.

FAU - Guerrero-Bustamante, Carlos A

AU - Guerrero-Bustamante CA

AUID- ORCID: 0000-0002-7256-5188

AD - Department of Biological Sciences, University of Pittsburgh, Pittsburgh,

Pennsylvania, USA.

FAU - Harrison, Melissa

AU - Harrison M

AD - Department of Chemical and Materials Engineering, University of Alberta,

Edmonton, Alberta, Canada.

FAU - Co, Sheila

AU - Co S

AD - Department of Chemical and Materials Engineering, University of Alberta,

Edmonton, Alberta, Canada.

FAU - Tetreau, Isobel

AU - Tetreau I

AD - Department of Mechanical Engineering, University of Alberta, Edmonton, Alberta,

Canada.

FAU - Ordoubadi, Mani

AU - Ordoubadi M

AD - Department of Mechanical Engineering, University of Alberta, Edmonton, Alberta,

Canada.

FAU - Larsen, Sasha E

AU - Larsen SE

AUID- ORCID: 0000-0001-5034-9727

AD - Seattle Children's Research Institute, Center for Global Infectious Disease

Research, Seattle, Washington, USA.

FAU - Coler, Rhea N

AU - Coler RN

AD - Seattle Children's Research Institute, Center for Global Infectious Disease

Research, Seattle, Washington, USA.

AD - Department of Pediatrics, University of Washington School of Medicine, Seattle,

Washington, USA.

AD - Department of Global Health, University of Washington, Seattle, Washington, USA.

FAU - Vehring, Reinhard

AU - Vehring R

AD - Department of Mechanical Engineering, University of Alberta, Edmonton, Alberta,

Canada.

FAU - Hatfull, Graham F

AU - Hatfull GF

AUID- ORCID: 0000-0002-6705-6821

AD - Department of Biological Sciences, University of Pittsburgh, Pittsburgh,

Pennsylvania, USA.

FAU - Sauvageau, Dominic

AU - Sauvageau D

AUID- ORCID: 0000-0002-1995-5523

AD - Department of Chemical and Materials Engineering, University of Alberta,

Edmonton, Alberta, Canada.

LA - eng

GR - R35 GM131729/GM/NIGMS NIH HHS/United States

GR - R21 AI156807/AI/NIAID NIH HHS/United States

PT - Comparative Study

PT - Journal Article

PT - Research Support, N.I.H., Extramural

DEP - 20250521

PL - United States

TA - J Virol

JT - Journal of virology

JID - 0113724

SB - IM

UOF - bioRxiv. 2024 Oct 29:2024.10.23.619922. doi: 10.1101/2024.10.23.619922. PMID:

39554140

MH - *Mycobacteriophages/pathogenicity/physiology

MH - Virulence

MH - Biofilms/growth & development

MH - *Mycobacterium smegmatis/virology

MH - *Mycobacterium tuberculosis/virology

PMC - PMC12172466

OTO - NOTNLM

OT - D29

OT - Fionnbharth

OT - Muddy

OT - Mycobacterium smegmatis

OT - bacteriophages

OT - infection dynamics

OT - mycobacteriophages

OT - phage adsorption

OT - phages

OT - virulence

OT - virulence index

COIS- The authors declare no conflict of interest.

EDAT- 2025/05/21 12:31

MHDA- 2025/06/17 14:35

PMCR- 2025/05/21

CRDT- 2025/05/21 09:04

PHST- 2025/06/17 14:35 [medline]

PHST- 2025/05/21 12:31 [pubmed]

PHST- 2025/05/21 09:04 [entrez]

PHST- 2025/05/21 00:00 [pmc-release]

AID - jvi01920-24 [pii]

AID - jvi.01920-24 [pii]

AID - 10.1128/jvi.01920-24 [doi]

PST - ppublish

SO - J Virol. 2025 Jun 17;99(6):e0192024. doi: 10.1128/jvi.01920-24. Epub 2025 May 21.

PMID- 40279721

OWN - NLM

STAT- MEDLINE

DCOM- 20250516

LR - 20250516

IS - 1873-2542 (Electronic)

IS - 0378-1135 (Linking)

VI - 305

DP - 2025 Jun

TI - Heat-inactivated Mycobacterium bovis and P22PI protein immunocomplex: Two

candidates for use as immunostimulants of innate immune response.

PG - 110527

LID - S0378-1135(25)00162-2 [pii]

LID - 10.1016/j.vetmic.2025.110527 [doi]

AB - Tuberculosis (TB), caused by members of the Mycobacterium tuberculosis complex,

remains a critical global health challenge, affecting humans and a wide range of

domestic and wild animals. Despite the availability of anti-TB drugs, cure rates

remain suboptimal, exacerbated by the rise of multidrug-resistant TB strains. The

Bacille Calmette-Guérin (BCG) vaccine, the only licensed vaccine against TB, has

demonstrated efficacy in reducing lesion severity and bacterial burden in

animals, as well as lowering TB-related and all-cause mortality in infants.

However, BCG presents several safety concerns inherent to live vaccines. To

overcome these limitations, exploring alternative vaccine candidates that do not

incorporate live mycobacteria is crucial. This study aimed to evaluate and

compare the immunostimulatory potential of two candidates based in mycobacteria

inactivated or their derivatives, heat-inactivated Mycobacterium bovis (HIMB) and

P22PI protein immunocomplex (P22PI), in bovine foetal lung cells. To assess the

expression of innate immune components, including Toll-like receptors (TLRs),

cathelicidins, and cytokines, bovine foetal lung were exposed to different

concentrations of HIMB and P22PI immunostimulants, starting at 7.8 × 10⁶ CFU/ml

and 10 µg/ml, respectively. These initial concentrations were subsequently

diluted to 1/2 and 1/10 to evaluate dose-dependent effects. Our findings reveal

that both HIMB and P22PI significantly stimulate innate immune mechanisms, as

evidenced by the upregulation of TLR2 and TLR4, alongside the induction of BMAP28

cathelicidin, tumour necrosis factor alpha (TNFA) and interferons (IFNs). These

results suggest their potential to orchestrate a robust innate immune response

providing valuable insights into the immunological mechanisms underlying the

protective effects of these immunostimulants. This underscores their potential

role in in vivo studies as vaccine candidates. Furthermore, their ability to

enhance antigen recognition via TLR and induce pro-inflammatory cytokines also

indicates broader applications in immune modulation, potentially extending

protection against heterologous pathogens through trained immunity.

CI - Copyright © 2025 The Authors. Published by Elsevier B.V. All rights reserved.

FAU - Agulló-Ros, Irene

AU - Agulló-Ros I

AD - Departamento de Anatomía y Anatomía Patológica Comparadas y Toxicología, Grupo de

Investigación en Sanidad Animal y Zoonosis (GISAZ), UIC Zoonosis y Enfermedades

Emergentes ENZOEM, Universidad de Córdoba, Córdoba, Spain.

FAU - Burucúa, Mercedes M

AU - Burucúa MM

AD - Departamento de Biología, Facultad de Ciencias Exactas y Naturales, Universidad

Nacional de Mar del Plata, Mar del Plata, Buenos Aires, Argentina.

FAU - Cheuquepán, Felipe A

AU - Cheuquepán FA

AD - Departamento de Biología, Facultad de Ciencias Exactas y Naturales, Universidad

Nacional de Mar del Plata, Mar del Plata, Buenos Aires, Argentina; Consejo

Nacional de Investigaciones Científicas y Técnicas (CONICET), Buenos Aires,

Argentina.

FAU - Domínguez, Mercedes

AU - Domínguez M

AD - Servicio de Inmunología Microbiana, Centro Nacional de Microbiología, Instituto

de Salud Carlos III, Majadahonda, Madrid, Spain.

FAU - Sevilla, Iker A

AU - Sevilla IA

AD - Animal Health Department, NEIKER-Instituto Vasco de Investigación y Desarrollo

Agrario, Derio, Bizkaia, Spain.

FAU - Martínez, Remigio

AU - Martínez R

AD - Departamento de Sanidad Animal, Grupo de Investigación en Sanidad Animal y

Zoonosis (GISAZ), UIC Zoonosis y Enfermedades Emergentes ENZOEM, Universidad de

Córdoba, Córdoba, Spain.

FAU - Plá, Natalia

AU - Plá N

AD - Facultad de Ciencias Agrarias, Universidad Nacional de Mar del Plata, Mar del

Plata, Buenos Aires, Argentina.

FAU - Risalde, María A

AU - Risalde MA

AD - Departamento de Anatomía y Anatomía Patológica Comparadas y Toxicología, Grupo de

Investigación en Sanidad Animal y Zoonosis (GISAZ), UIC Zoonosis y Enfermedades

Emergentes ENZOEM, Universidad de Córdoba, Córdoba, Spain; CIBERINFEC, ISCIII -

CIBER de Enfermedades Infecciosas, Instituto de Salud Carlos III, Madrid, Spain.

Electronic address: v12rimom@uco.es.

FAU - Marin, Maia S

AU - Marin MS

AD - Departamento de Biología, Facultad de Ciencias Exactas y Naturales, Universidad

Nacional de Mar del Plata, Mar del Plata, Buenos Aires, Argentina; Consejo

Nacional de Investigaciones Científicas y Técnicas (CONICET), Buenos Aires,

Argentina.

LA - eng

PT - Journal Article

DEP - 20250421

PL - Netherlands

TA - Vet Microbiol

JT - Veterinary microbiology

JID - 7705469

RN - 0 (Adjuvants, Immunologic)

RN - 0 (Cytokines)

RN - 0 (Bacterial Proteins)

RN - 0 (Vaccines, Inactivated)

RN - 0 (BCG Vaccine)

RN - 0 (Toll-Like Receptors)

SB - IM

MH - Animals

MH - *Immunity, Innate

MH - *Mycobacterium bovis/immunology

MH - Cattle

MH - *Adjuvants, Immunologic/pharmacology

MH - Cytokines/genetics/immunology

MH - Lung/immunology/cytology/embryology

MH - Hot Temperature

MH - *Bacterial Proteins/immunology

MH - Vaccines, Inactivated/immunology

MH - BCG Vaccine/immunology

MH - Toll-Like Receptors/genetics

OTO - NOTNLM

OT - Cathelicidins

OT - Cytokines

OT - HIMB

OT - Innate immunity

OT - P22PI

OT - Toll-like receptors

COIS- Declaration of Competing Interest None of the authors of this study has a

financial or personal relationship with other people or organizations that could

inappropriately influence or bias the content of the paper.

EDAT- 2025/04/26 16:16

MHDA- 2025/05/16 11:42

CRDT- 2025/04/25 18:03

PHST- 2024/09/20 00:00 [received]

PHST- 2025/03/22 00:00 [revised]

PHST- 2025/04/18 00:00 [accepted]

PHST- 2025/05/16 11:42 [medline]

PHST- 2025/04/26 16:16 [pubmed]

PHST- 2025/04/25 18:03 [entrez]

AID - S0378-1135(25)00162-2 [pii]

AID - 10.1016/j.vetmic.2025.110527 [doi]

PST - ppublish

SO - Vet Microbiol. 2025 Jun;305:110527. doi: 10.1016/j.vetmic.2025.110527. Epub 2025

Apr 21.

PMID- 40074018

OWN - NLM

STAT- MEDLINE

DCOM- 20250517

LR - 20260127

IS - 1879-0720 (Electronic)

IS - 0928-0987 (Linking)

VI - 209

DP - 2025 Jun 1

TI - DprE1 inhibitors: An insight into the recent developments and synthetic

approaches.

PG - 107062

LID - S0928-0987(25)00061-2 [pii]

LID - 10.1016/j.ejps.2025.107062 [doi]

AB - In the current medical era, the proliferation and dissemination of drug-resistant

strains of Mycobacterium tuberculosis (Mtb) continue to pose a significant

worldwide health hazard, necessitating the development of new and innovative

medications to combat tuberculosis. Decaprenylphosphoryl-β-D-ribose 2'-epimerase

(DprE1) is a crucial enzyme for cell wall synthesis in Mtb. Its importance is due

to its eminent contribution in forming lipoarabinomannan and arabinogalactan, key

components of the mycobacterial cell wall. The emergence of the DprE1 enzyme as a

druggable target was based on inhibitors discovered in high-throughput screening.

Since then, inhibitors with different types of chemical scaffolds have been

reported for their activity against it. DprE1 inhibitors can be categorized

according to the formation of a covalent or non-covalent bond in the enzyme's

active site, causing a loss of its catalytic activity, leading to Mtb's demise.

Herein, we describe diverse DprE1 inhibitors that have had anti-tubercular

activity reported over the past fifteen years and till the present time.

CI - Copyright © 2025. Published by Elsevier B.V.

FAU - Shahin, Mai I

AU - Shahin MI

AD - Department of Pharmaceutical Chemistry, Faculty of Pharmacy, Ain Shams

University, Abassia, Cairo 11566, Egypt.

FAU - Elyamani, Mai A

AU - Elyamani MA

AD - Scientific Research and Innovation Support Unit, Faculty of Pharmacy,

Kafrelsheikh University, Kafrelsheikh, Egypt.

FAU - Elsawi, Ahmed E

AU - Elsawi AE

AD - Department of Pharmaceutical Chemistry, Faculty of Pharmacy, Kafrelsheikh

University, Kafrelsheikh 33516, Egypt.

FAU - Negmeldin, Ahmed T

AU - Negmeldin AT

AD - Department of Pharmaceutical Sciences, College of Pharmacy and Thumbay Research

Institute for Precision Medicine, Gulf Medical University, Ajman, United Arab

Emirates; Department of Pharmaceutical Organic Chemistry, Faculty of Pharmacy,

Cairo University, Cairo, Egypt. Electronic address: ahmed.n@pharma.cu.edu.eg.

FAU - Naguib, Shahenda

AU - Naguib S

AD - Scientific Research and Innovation Support Unit, Faculty of Pharmacy,

Kafrelsheikh University, Kafrelsheikh, Egypt.

FAU - Safwat, Heba

AU - Safwat H

AD - Scientific Research and Innovation Support Unit, Faculty of Pharmacy,

Kafrelsheikh University, Kafrelsheikh, Egypt.

FAU - Abdel-Aziz, Hatem A

AU - Abdel-Aziz HA

AD - Department of Applied Organic Chemistry, National Research Center, Dokki, Cairo

12622, Egypt.

FAU - Eldehna, Wagdy M

AU - Eldehna WM

AD - Department of Pharmaceutical Chemistry, Faculty of Pharmacy, Kafrelsheikh

University, Kafrelsheikh 33516, Egypt; Department of Pharmaceutical Chemistry,

Faculty of Pharmacy, Pharos University in Alexandria; Canal El Mahmoudia St.,

Alexandria 21648, Egypt. Electronic address: wagdy2000@gmail.com.

LA - eng

PT - Journal Article

PT - Review

DEP - 20250310

PL - Netherlands

TA - Eur J Pharm Sci

JT - European journal of pharmaceutical sciences : official journal of the European

Federation for Pharmaceutical Sciences

JID - 9317982

RN - 0 (Antitubercular Agents)

RN - EC 1.1.- (DprE1 protein, Mycobacterium tuberculosis)

RN - 0 (Enzyme Inhibitors)

RN - 0 (Bacterial Proteins)

RN - EC 5.1.3.- (Carbohydrate Epimerases)

RN - EC 1.1.- (Alcohol Oxidoreductases)

SB - IM

MH - *Antitubercular Agents/pharmacology/chemistry

MH - *Mycobacterium tuberculosis/drug effects/enzymology

MH - Humans

MH - *Enzyme Inhibitors/pharmacology/chemistry

MH - *Bacterial Proteins/antagonists & inhibitors/metabolism

MH - *Carbohydrate Epimerases/antagonists & inhibitors/metabolism

MH - Animals

MH - Tuberculosis/drug therapy

MH - Alcohol Oxidoreductases

OTO - NOTNLM

OT - Anti-tubercular agents

OT - Benzothiazinones

OT - Covalent inhibitors

OT - PBTZ169

OT - XDR-TB

COIS- Declaration of competing interest The authors declare no conflicts of interest.

EDAT- 2025/03/13 00:33

MHDA- 2025/05/18 17:01

CRDT- 2025/03/12 20:14

PHST- 2024/12/24 00:00 [received]

PHST- 2025/03/04 00:00 [revised]

PHST- 2025/03/06 00:00 [accepted]

PHST- 2025/05/18 17:01 [medline]

PHST- 2025/03/13 00:33 [pubmed]

PHST- 2025/03/12 20:14 [entrez]

AID - S0928-0987(25)00061-2 [pii]

AID - 10.1016/j.ejps.2025.107062 [doi]

PST - ppublish

SO - Eur J Pharm Sci. 2025 Jun 1;209:107062. doi: 10.1016/j.ejps.2025.107062. Epub

2025 Mar 10.

PMID- 39292406

OWN - NLM

STAT- MEDLINE

DCOM- 20250515

LR - 20250515

IS - 1573-501X (Electronic)

IS - 1381-1991 (Linking)

VI - 29

IP - 3

DP - 2025 Jun

TI - A network pharmacology-based approach to understand the mechanism of action of

anti-mycobacterial activity of Acacia nilotica: a modelling and experimental

study.

PG - 2227-2242

LID - 10.1007/s11030-024-10985-8 [doi]

AB - The rapid rise in drug-resistant tuberculosis poses a serious threat to public

health and demands the discovery of new anti-mycobacterial agents. Medicinal

plants are a proven potential source of bioactive compounds; however, identifying

those responsible for the putative anti-mycobacterial action still remains a

challenging task. In this study, we undertook a systematic network pharmacology

approach to identify and evaluate anti-mycobacterial compounds from a traditional

plant, Acacia nilotica, as a model system. The protein-protein interaction

network revealed 17 key pathways in M. tuberculosis encompassing 40 unique

druggable targets that are necessary for its growth and survival. The

phytochemicals of A. nilotica were preferentially found to interfere with the

cell division and cell wall biogenesis proteins, especially FtsZ and Mur.

Notably, the compounds epigallocatechin, ellagic acid, chlorogenic acid, and

D-pinitol were found to exhibit a potential polypharmacological effect against

multiple proteins. Further, in vitro studies confirmed that the selected

candidates, chlorogenic acid, and ellagic acid exhibited potent

anti-mycobacterial activity (against M. smegmatis) with specific inhibition of

purified M.tb FtsZ enzyme. Taken together, the present study demonstrates that

network pharmacology combined with molecular docking can be utilized as an

efficient approach to identify potential bioactive phytochemicals from natural

products along with their mechanism of action. Hence, the compounds identified in

this study can be potential lead candidates for developing novel

anti-mycobacterial drugs, while the key proteins identified here can be potential

drug targets.

CI - © 2024. The Author(s), under exclusive licence to Springer Nature Switzerland AG.

FAU - Suresh, Madhumitha

AU - Suresh M

AD - Bioengineering and Drug Design Lab, Department of Biotechnology, Indian Institute

of Technology Madras, Chennai, 600036, India.

FAU - Sai, Kadambari Vijay

AU - Sai KV

AD - Bioengineering and Drug Design Lab, Department of Biotechnology, Indian Institute

of Technology Madras, Chennai, 600036, India.

FAU - Mitra, Kartik

AU - Mitra K

AUID- ORCID: 0000-0003-4891-1463

AD - Bioengineering and Drug Design Lab, Department of Biotechnology, Indian Institute

of Technology Madras, Chennai, 600036, India. bt17d200@smail.iitm.ac.in.

FAU - Ravindran, Radhika

AU - Ravindran R

AD - Bioengineering and Drug Design Lab, Department of Biotechnology, Indian Institute

of Technology Madras, Chennai, 600036, India.

FAU - Doble, Mukesh

AU - Doble M

AD - Department of Biotechnology, Theevanam Additives Nutraceuts Pvt Ltd, IITM

Bioincubator, IIT Madras, Chennai, 600036, India.

AD - Saveetha Dental College and Hospitals, SIMATS, Chennai, 600077, India.

LA - eng

PT - Journal Article

DEP - 20240918

PL - Netherlands

TA - Mol Divers

JT - Molecular diversity

JID - 9516534

RN - 0 (Bacterial Proteins)

RN - 0 (Antitubercular Agents)

RN - 0 (Plant Extracts)

RN - 0 (Anti-Bacterial Agents)

RN - 0 (Phytochemicals)

SB - IM

MH - *Acacia/chemistry

MH - *Network Pharmacology/methods

MH - *Mycobacterium tuberculosis/drug effects/metabolism

MH - Molecular Docking Simulation

MH - Bacterial Proteins/metabolism/antagonists & inhibitors

MH - *Antitubercular Agents/pharmacology/chemistry

MH - *Plant Extracts/pharmacology/chemistry

MH - *Anti-Bacterial Agents/pharmacology/chemistry

MH - Phytochemicals/pharmacology/chemistry

MH - Protein Interaction Maps/drug effects

MH - Microbial Sensitivity Tests

OTO - NOTNLM

OT - Acacia nilotica

OT - Mycobacterium tuberculosis

OT - Cell division

OT - Molecular docking

OT - Network pharmacology

COIS- Declarations. Conflict of interest: The authors declare no competing interests.

EDAT- 2024/09/18 12:51

MHDA- 2025/05/15 12:37

CRDT- 2024/09/18 11:14

PHST- 2024/05/28 00:00 [received]

PHST- 2024/08/29 00:00 [accepted]

PHST- 2025/05/15 12:37 [medline]

PHST- 2024/09/18 12:51 [pubmed]

PHST- 2024/09/18 11:14 [entrez]

AID - 10.1007/s11030-024-10985-8 [pii]

AID - 10.1007/s11030-024-10985-8 [doi]

PST - ppublish

SO - Mol Divers. 2025 Jun;29(3):2227-2242. doi: 10.1007/s11030-024-10985-8. Epub 2024

Sep 18.

PMID- 40507146

OWN - NLM

STAT- MEDLINE

DCOM- 20250613

LR - 20250615

IS - 2072-6643 (Electronic)

IS - 2072-6643 (Linking)

VI - 17

IP - 11

DP - 2025 May 30

TI - Nutritional Deficiencies and Management in Tuberculosis: Pharmacotherapeutic and

Clinical Implications.

LID - 10.3390/nu17111878 [doi]

LID - 1878

AB - Tuberculosis is an infectious condition caused by Mycobacterium tuberculosis,

primarily targeting the pulmonary system, with the potential to disseminate to

various other organs via the haematogenous pathway, ranking among the top ten

causes of global mortality. Tuberculosis remains a serious public health problem

worldwide. This narrative review aims to emphasise the clinical importance of the

inter-relationships between nutrition, pharmacotherapy, and the most common

drug-nutrient interactions in the context of tuberculosis and

multi-drug-resistant tuberculosis management. Nowadays, pharmacologic approaches

utilise polytherapeutic regimens that, although showing increased efficacy,

prominently affect the nutritional status of patients and modify multiple

metabolic pathways, thus influencing both the effectiveness of therapy and the

patient outcomes. There is much evidence that antituberculosis drugs are

associated with deficiencies in essential vitamins and various micronutrients,

leading to serious adverse consequences. Moreover, poor nutrition exacerbates TB

outcomes, and TB further exacerbates nutritional status, a vicious cycle that is

particularly prevalent in low-resource environments. Nutritional support is

necessary, and clinicians ought to evaluate it on a patient-by-patient basis, as

empirical evidence has shown that it can improve immune recovery, decrease

tuberculosis-associated morbidity, and increase adherence to therapy. However,

drug-food interactions are increasingly prevalent, and patients with tuberculosis

require personalised dietary and pharmacological regimens. In this context,

antituberculosis treatment requires a holistic approach, based on the

collaboration of the prescribing physician, pharmacist, and nutritionist, to

assess the patient's needs from a nutritional and pharmacological perspective,

with the ultimate goal of decreasing mortality and improving the prognosis of

patients through personalised therapies.

FAU - Fâcă, Anca Ionela

AU - Fâcă AI

AD - Faculty of Pharmacy, Carol Davila University of Medicine and Pharmacy, 6, Traian

Vuia Street, 020956 Bucharest, Romania.

AD - Marius Nasta Institute of Pneumology, 90, Viilor Street, 050159 Bucharest,

Romania.

FAU - Udeanu, Denisa Ioana

AU - Udeanu DI

AUID- ORCID: 0000-0001-5266-2530

AD - Faculty of Pharmacy, Carol Davila University of Medicine and Pharmacy, 6, Traian

Vuia Street, 020956 Bucharest, Romania.

AD - Marius Nasta Institute of Pneumology, 90, Viilor Street, 050159 Bucharest,

Romania.

FAU - Arsene, Andreea Letiția

AU - Arsene AL

AUID- ORCID: 0000-0002-9478-6176

AD - Faculty of Pharmacy, Carol Davila University of Medicine and Pharmacy, 6, Traian

Vuia Street, 020956 Bucharest, Romania.

AD - Marius Nasta Institute of Pneumology, 90, Viilor Street, 050159 Bucharest,

Romania.

FAU - Mahler, Beatrice

AU - Mahler B

AUID- ORCID: 0000-0002-9240-922X

AD - Marius Nasta Institute of Pneumology, 90, Viilor Street, 050159 Bucharest,

Romania.

AD - Faculty of Medicine, Carol Davila University of Medicine and Pharmacy, 8, Eroii

Sanitari Street, 050474 Bucharest, Romania.

FAU - Drăgănescu, Doina

AU - Drăgănescu D

AD - Faculty of Pharmacy, Carol Davila University of Medicine and Pharmacy, 6, Traian

Vuia Street, 020956 Bucharest, Romania.

FAU - Apetroaei, Miruna-Maria

AU - Apetroaei MM

AD - Faculty of Pharmacy, Carol Davila University of Medicine and Pharmacy, 6, Traian

Vuia Street, 020956 Bucharest, Romania.

LA - eng

PT - Journal Article

PT - Review

DEP - 20250530

PL - Switzerland

TA - Nutrients

JT - Nutrients

JID - 101521595

RN - 0 (Antitubercular Agents)

RN - 0 (Micronutrients)

SB - IM

MH - Humans

MH - *Antitubercular Agents/therapeutic use/adverse effects

MH - Nutritional Status

MH - *Tuberculosis/drug therapy/complications

MH - *Malnutrition/therapy

MH - Tuberculosis, Multidrug-Resistant/drug therapy

MH - Food-Drug Interactions

MH - Nutritional Support

MH - Micronutrients/deficiency

PMC - PMC12157128

OTO - NOTNLM

OT - antituberculosis therapy

OT - clinical nutrition

OT - drug–food interactions

OT - nutritional support

OT - tuberculosis deficiencies

OT - tuberculosis management

COIS- The authors declare no conflicts of interest.

EDAT- 2025/06/13 06:34

MHDA- 2025/06/13 06:35

PMCR- 2025/05/30

CRDT- 2025/06/13 01:01

PHST- 2025/05/15 00:00 [received]

PHST- 2025/05/27 00:00 [revised]

PHST- 2025/05/29 00:00 [accepted]

PHST- 2025/06/13 06:35 [medline]

PHST- 2025/06/13 06:34 [pubmed]

PHST- 2025/06/13 01:01 [entrez]

PHST- 2025/05/30 00:00 [pmc-release]

AID - nu17111878 [pii]

AID - nutrients-17-01878 [pii]

AID - 10.3390/nu17111878 [doi]

PST - epublish

SO - Nutrients. 2025 May 30;17(11):1878. doi: 10.3390/nu17111878.

PMID- 40214257

OWN - NLM

STAT- MEDLINE

DCOM- 20250509

LR - 20250509

IS - 2373-8227 (Electronic)

IS - 2373-8227 (Linking)

VI - 11

IP - 5

DP - 2025 May 9

TI - Florasulam Is a Potent Inhibitor of Mycobacterium tuberculosis Acetohydroxyacid

Synthase and Possesses In Vivo Antituberculosis Activity.

PG - 1180-1189

LID - 10.1021/acsinfecdis.4c01028 [doi]

AB - Tuberculosis (TB) remains as a leading cause of morbidity and mortality,

accounting for ∼1.3 million fatalities worldwide per year. There are two major

concerns: (i) the rise in the number of multi- and extensively drug-resistant

strains of TB and (ii) the significant side-effects related to the use of many of

the current therapies to treat drug-resistant and drug-sensitive TB alike. Thus,

there is an ongoing need to discover new drugs and drug targets to combat this

disease. Here, acetohydroxyacid synthase (AHAS), the first enzyme in the

branched-chain amino acids (BCAAs) biosynthesis pathway, is comprehensively

investigated as such a drug target. All five chemical classes of plant AHAS

inhibitors, established as commercial herbicides, were assessed as leads. Members

of the triazolopyrimidine family (e.g., metosulam, penoxsulam, and florasulam)

are the most potent inhibitors of Mycobacterium tuberculosis AHAS (MtbAHAS) with

K(i) values as low as 20 nM. These compounds also exhibit the property of

accumulative time-dependent inhibition, a feature that appears to be crucial for

herbicidal activity and more generally for biocidal activity. Of these, the

anti-TB activity of florasulam was the most effective, with an MIC of 500 nM

against virulent Mtb grown in culture. This compound is also effective in killing

intramacrophage Mtb and reduces bacterial load, as compared to vehicle-only by

13-fold in the lungs of mice infected with Mtb. Thus, triazolopyrimidines as AHAS

inhibitors, and in-particular florasulam, represents a promising new class of

leads for anti-TB drug development.

FAU - Wun, Shun Jie

AU - Wun SJ

AD - School of Chemistry and Molecular Biosciences, Australian Infectious Diseases

Research Centre, The University of Queensland, St Lucia, Queensland 4072,

Australia.

FAU - Tan, Lendl

AU - Tan L

AUID- ORCID: 0000-0002-9955-2440

AD - School of Chemistry and Molecular Biosciences, Australian Infectious Diseases

Research Centre, The University of Queensland, St Lucia, Queensland 4072,

Australia.

FAU - Lonhienne, Thierry G

AU - Lonhienne TG

AD - School of Chemistry and Molecular Biosciences, Australian Infectious Diseases

Research Centre, The University of Queensland, St Lucia, Queensland 4072,

Australia.

FAU - Low, Yu Shang

AU - Low YS

AD - School of Chemistry and Molecular Biosciences, Australian Infectious Diseases

Research Centre, The University of Queensland, St Lucia, Queensland 4072,

Australia.

FAU - Josh, Peter

AU - Josh P

AD - School of Chemistry and Molecular Biosciences, Australian Infectious Diseases

Research Centre, The University of Queensland, St Lucia, Queensland 4072,

Australia.

FAU - Kuo, Andy

AU - Kuo A

AUID- ORCID: 0000-0001-6395-3114

AD - School of Biomedical Sciences, The University of Queensland, St Lucia, Queensland

4072, Australia.

FAU - Smith, Maree T

AU - Smith MT

AUID- ORCID: 0000-0003-2281-3734

AD - School of Biomedical Sciences, The University of Queensland, St Lucia, Queensland

4072, Australia.

FAU - Gao, Yanhua

AU - Gao Y

AUID- ORCID: 0000-0002-1211-2291

AD - School of Chemistry and Molecular Biosciences, Australian Infectious Diseases

Research Centre, The University of Queensland, St Lucia, Queensland 4072,

Australia.

FAU - Pierens, Gregory K

AU - Pierens GK

AD - UQ Centre for Advanced Imaging, St Lucia, Queensland 4072, Australia.

FAU - Guddat, Luke W

AU - Guddat LW

AUID- ORCID: 0000-0002-8204-8408

AD - School of Chemistry and Molecular Biosciences, Australian Infectious Diseases

Research Centre, The University of Queensland, St Lucia, Queensland 4072,

Australia.

FAU - West, Nicholas P

AU - West NP

AUID- ORCID: 0000-0003-0955-9890

AD - School of Chemistry and Molecular Biosciences, Australian Infectious Diseases

Research Centre, The University of Queensland, St Lucia, Queensland 4072,

Australia.

LA - eng

PT - Journal Article

DEP - 20250411

PL - United States

TA - ACS Infect Dis

JT - ACS infectious diseases

JID - 101654580

RN - EC 2.2.1.6 (Acetolactate Synthase)

RN - 0 (Antitubercular Agents)

RN - 0 (Enzyme Inhibitors)

SB - IM

MH - *Mycobacterium tuberculosis/drug effects/enzymology

MH - *Acetolactate Synthase/antagonists & inhibitors

MH - Animals

MH - *Antitubercular Agents/pharmacology/chemistry/therapeutic use

MH - Mice

MH - *Enzyme Inhibitors/pharmacology/chemistry

MH - Tuberculosis/drug therapy/microbiology

MH - Microbial Sensitivity Tests

MH - Female

MH - Disease Models, Animal

OTO - NOTNLM

OT - MDR-TB

OT - Mycobacterium tuberculosis

OT - acetohydroxyacid synthase

OT - herbicide

OT - tuberculosis

EDAT- 2025/04/11 12:28

MHDA- 2025/05/09 12:36

CRDT- 2025/04/11 09:03

PHST- 2025/05/09 12:36 [medline]

PHST- 2025/04/11 12:28 [pubmed]

PHST- 2025/04/11 09:03 [entrez]

AID - 10.1021/acsinfecdis.4c01028 [doi]

PST - ppublish

SO - ACS Infect Dis. 2025 May 9;11(5):1180-1189. doi: 10.1021/acsinfecdis.4c01028.

Epub 2025 Apr 11.

PMID- 40168905

OWN - NLM

STAT- MEDLINE

DCOM- 20250503

LR - 20250503

IS - 1873-281X (Electronic)

IS - 1472-9792 (Linking)

VI - 152

DP - 2025 May

TI - Construction and expression of multi-stage antigen fusion protein RPC4 vaccine

for Mycobacterium tuberculosis and its immunogenicity analysis in combination

with adjuvant DIMQ.

PG - 102635

LID - S1472-9792(25)00030-7 [pii]

LID - 10.1016/j.tube.2025.102635 [doi]

AB - Mycobacterium tuberculosis (M. tb) serves as the main pathogen responsible for

Tuberculosis (TB). It predominantly targets the lungs and leads to a persistent

infectious disease. The spread of drug-resistant tuberculosis and the

exacerbation of economic burdens due to co-infections with Human Immunodeficiency

Virus (HIV)/M. tb pose significant challenges in prevention and treatment. The

BCG vaccine is currently the only approved (TB) vaccine, but its protective

effect is limited for adults. In this research, we engineered the fusion protein

gene RPC4, incorporating four crucial antigens from M. tb. The study revealed

that the IFN-γ levels in the peripheral blood of infected patients significantly

surpassed those in healthy individuals. To assess the immune response of RPC4 as

a BCG-enhanced vaccine following initial immunity, researchers administered it

alongside the novel adjuvant DIMQ to immunize mice. Experiments revealed that the

BCG + RPC4/DIMQ vaccine induces a substantial immunogenic response in the mice.

CI - Copyright © 2025 Elsevier Ltd. All rights reserved.

FAU - Wang, Xiaochun

AU - Wang X

AD - First Affiliated Hospital, Anhui University of Science and Technology, Huainan,

232001, China; Department of Pathogen Biology, School of Medicine, Anhui

University of Science and Technology, Huainan, 232001, China. Electronic address:

wxcvieri@126.com.

FAU - Xu, Yun

AU - Xu Y

AD - Department of Pathogen Biology, School of Medicine, Anhui University of Science

and Technology, Huainan, 232001, China.

FAU - Zhong, Qiangsen

AU - Zhong Q

AD - Department of Pathogen Biology, School of Medicine, Anhui University of Science

and Technology, Huainan, 232001, China.

FAU - Zhang, Zian

AU - Zhang Z

AD - Department of Immunology, School of Medicine, Anhui University of Science and

Technology, Huainan, 232001, China.

FAU - Kong, LingYun

AU - Kong L

AD - Department of Pathogen Biology, School of Medicine, Anhui University of Science

and Technology, Huainan, 232001, China.

FAU - Zhou, Mingming

AU - Zhou M

AD - Department of Immunology, School of Medicine, Anhui University of Science and

Technology, Huainan, 232001, China.

FAU - Wang, Runlin

AU - Wang R

AD - Department of Pathogen Biology, School of Medicine, Anhui University of Science

and Technology, Huainan, 232001, China.

FAU - Pi, Xinxin

AU - Pi X

AD - Department of Pathogen Biology, School of Medicine, Anhui University of Science

and Technology, Huainan, 232001, China.

FAU - Qiao, Suwen

AU - Qiao S

AD - Department of Pathogen Biology, School of Medicine, Anhui University of Science

and Technology, Huainan, 232001, China.

LA - eng

PT - Journal Article

DEP - 20250326

PL - Scotland

TA - Tuberculosis (Edinb)

JT - Tuberculosis (Edinburgh, Scotland)

JID - 100971555

RN - 0 (Antigens, Bacterial)

RN - 0 (Recombinant Fusion Proteins)

RN - 0 (Tuberculosis Vaccines)

RN - 82115-62-6 (Interferon-gamma)

RN - 0 (Adjuvants, Immunologic)

RN - 0 (BCG Vaccine)

RN - 0 (Bacterial Proteins)

RN - 0 (IFNG protein, human)

SB - IM

MH - Animals

MH - *Mycobacterium tuberculosis/immunology/genetics

MH - *Antigens, Bacterial/immunology/genetics

MH - Humans

MH - *Recombinant Fusion Proteins/immunology/genetics

MH - Female

MH - *Tuberculosis Vaccines/immunology/genetics/administration & dosage

MH - *Immunogenicity, Vaccine

MH - Interferon-gamma/blood

MH - *Tuberculosis/immunology/prevention & control/microbiology

MH - *Adjuvants, Immunologic/administration & dosage

MH - BCG Vaccine/immunology/administration & dosage

MH - *Bacterial Proteins/immunology/genetics

MH - Adult

MH - Mice

MH - Mice, Inbred C57BL

MH - Male

OTO - NOTNLM

OT - Fusion proteins

OT - Immunogenicity

OT - Mycobacterium tuberculosis

OT - Subunit vaccines

COIS- Declaration of competing interest The authors declare that they have no known

competing financial interests or personal relationships that could have appeared

to influence the work reported in this paper.

EDAT- 2025/04/02 00:25

MHDA- 2025/05/03 22:25

CRDT- 2025/04/01 18:09

PHST- 2024/09/22 00:00 [received]

PHST- 2025/03/01 00:00 [revised]

PHST- 2025/03/17 00:00 [accepted]

PHST- 2025/05/03 22:25 [medline]

PHST- 2025/04/02 00:25 [pubmed]

PHST- 2025/04/01 18:09 [entrez]

AID - S1472-9792(25)00030-7 [pii]

AID - 10.1016/j.tube.2025.102635 [doi]

PST - ppublish

SO - Tuberculosis (Edinb). 2025 May;152:102635. doi: 10.1016/j.tube.2025.102635. Epub

2025 Mar 26.

PMID- 40244664

OWN - NLM

STAT- MEDLINE

DCOM- 20250417

LR - 20250608

IS - 1091-6490 (Electronic)

IS - 0027-8424 (Print)

IS - 0027-8424 (Linking)

VI - 122

IP - 16

DP - 2025 Apr 22

TI - Verapamil and its metabolite norverapamil inhibit the Mycobacterium tuberculosis

MmpS5L5 efflux pump to increase bedaquiline activity.

PG - e2426827122

LID - 10.1073/pnas.2426827122 [doi]

LID - e2426827122

AB - Bedaquiline is the cornerstone of a new regimen for the treatment of

drug-resistant tuberculosis. However, its clinical use is threatened by the

emergence of bedaquiline-resistant strains of Mycobacterium tuberculosis.

Bedaquiline targets mycobacterial ATP synthase but the predominant route to

clinical bedaquiline resistance is via upregulation of the MmpS5L5 efflux pump

due to mutations that inactivate the transcriptional repressor Rv0678. Here, we

show that the MmpS5L5 efflux pump reduces susceptibility to bedaquiline as well

as its new, more potent derivative TBAJ-876 and other antimicrobial substrates,

including clofazimine and the DprE1 inhibitors PBTZ-169 and OPC-167832.

Furthermore, the increased resistance of Rv0678 mutants stems entirely from

increased MmpS5L5 expression. These results highlight the potential of a

pharmacological MmpS5L5 inhibitor to increase drug efficacy. Verapamil, primarily

used as a calcium channel inhibitor, is known to inhibit diverse efflux pumps and

to potentiate bedaquiline and clofazimine activity in M. tuberculosis. Here, we

show that verapamil potentiates the activity of multiple diverse MmpS5L5

substrates. Using biochemical approaches, we demonstrate that verapamil does not

exert this effect by acting as a disruptor of the protonmotive force used to

power MmpS5L5, as previously proposed, suggesting that verapamil inhibits the

function of the MmpS5L5 pump. Finally, norverapamil, the major verapamil

metabolite, which has greatly reduced calcium channel activity, has equal potency

in reducing resistance to MmpS5L5 substrates. Our findings highlight verapamil's

potential for enhancing bedaquiline TB treatment, for preventing acquired

resistance to bedaquiline and other MmpS5L5 substrates, while also providing the

impetus to identify additional MmpS5L5 inhibitors.

FAU - Fountain, Adam J

AU - Fountain AJ

AD - Medical Research Council Laboratory of Molecular Biology, Cambridge CB2 0QH,

United Kingdom.

AD - Molecular Immunity Unit, Cambridge Institute of Therapeutic Immunology and

Infectious Diseases, Department of Medicine, University of Cambridge, Cambridge

CB2 0AW, United Kingdom.

FAU - Waller, Natalie J E

AU - Waller NJE

AD - Department of Microbiology and Immunology, University of Otago, Dunedin 9053, New

Zealand.

FAU - Cheung, Chen-Yi

AU - Cheung CY

AD - Department of Microbiology and Immunology, University of Otago, Dunedin 9053, New

Zealand.

FAU - Jowsey, William

AU - Jowsey W

AD - Department of Microbiology and Immunology, University of Otago, Dunedin 9053, New

Zealand.

FAU - Chrisp, Michael T

AU - Chrisp MT

AD - Department of Microbiology and Immunology, University of Otago, Dunedin 9053, New

Zealand.

FAU - Troll, Mark

AU - Troll M

AD - Medical Research Council Laboratory of Molecular Biology, Cambridge CB2 0QH,

United Kingdom.

AD - Molecular Immunity Unit, Cambridge Institute of Therapeutic Immunology and

Infectious Diseases, Department of Medicine, University of Cambridge, Cambridge

CB2 0AW, United Kingdom.

FAU - Edelstein, Paul H

AU - Edelstein PH

AUID- ORCID: 0000-0002-4069-5279

AD - Medical Research Council Laboratory of Molecular Biology, Cambridge CB2 0QH,

United Kingdom.

AD - Molecular Immunity Unit, Cambridge Institute of Therapeutic Immunology and

Infectious Diseases, Department of Medicine, University of Cambridge, Cambridge

CB2 0AW, United Kingdom.

FAU - Cook, Gregory M

AU - Cook GM

AUID- ORCID: 0000-0001-8349-1500

AD - Department of Microbiology and Immunology, University of Otago, Dunedin 9053, New

Zealand.

FAU - McNeil, Matthew B

AU - McNeil MB

AUID- ORCID: 0000-0001-7747-7745

AD - Department of Microbiology and Immunology, University of Otago, Dunedin 9053, New

Zealand.

FAU - Ramakrishnan, Lalita

AU - Ramakrishnan L

AUID- ORCID: 0000-0003-0692-5533

AD - Medical Research Council Laboratory of Molecular Biology, Cambridge CB2 0QH,

United Kingdom.

AD - Molecular Immunity Unit, Cambridge Institute of Therapeutic Immunology and

Infectious Diseases, Department of Medicine, University of Cambridge, Cambridge

CB2 0AW, United Kingdom.

LA - eng

GR - 223103/Z/21/Z/Wellcome Trust (WT)/

GR - UOO1807/Maurice Wilkins Centre for Molecular Biodiscovery and the Marsden Fund

(Royal Society of New Zealand)/

GR - 223103/WT_/Wellcome Trust/United Kingdom

GR - R37 AI054503/AI/NIAID NIH HHS/United States

GR - R01 AI054503/AI/NIAID NIH HHS/United States

PT - Journal Article

DEP - 20250417

PL - United States

TA - Proc Natl Acad Sci U S A

JT - Proceedings of the National Academy of Sciences of the United States of America

JID - 7505876

RN - 78846I289Y (bedaquiline)

RN - 0 (Diarylquinolines)

RN - CJ0O37KU29 (Verapamil)

RN - 0 (Antitubercular Agents)

RN - 0 (Bacterial Proteins)

RN - 0 (Membrane Transport Proteins)

SB - IM

MH - *Mycobacterium tuberculosis/drug effects/metabolism/genetics

MH - *Diarylquinolines/pharmacology

MH - *Verapamil/pharmacology/analogs & derivatives

MH - *Antitubercular Agents/pharmacology

MH - *Bacterial Proteins/metabolism/antagonists & inhibitors/genetics

MH - Microbial Sensitivity Tests

MH - *Membrane Transport Proteins/metabolism/genetics

MH - Drug Resistance, Bacterial/drug effects

PMC - PMC12036985

OTO - NOTNLM

OT - MmpS5L5 drug efflux pump

OT - Mycobacterium tuberculosis

OT - TBAJ-587 and TBAJ-876

OT - bedaquiline

OT - verapamil and norverapamil

COIS- Competing interests statement:The authors declare no competing interest.

EDAT- 2025/04/17 12:26

MHDA- 2025/04/17 18:24

PMCR- 2025/04/17

CRDT- 2025/04/17 11:53

PHST- 2025/04/17 18:24 [medline]

PHST- 2025/04/17 12:26 [pubmed]

PHST- 2025/04/17 11:53 [entrez]

PHST- 2025/04/17 00:00 [pmc-release]

AID - 202426827 [pii]

AID - 10.1073/pnas.2426827122 [doi]

PST - ppublish

SO - Proc Natl Acad Sci U S A. 2025 Apr 22;122(16):e2426827122. doi:

10.1073/pnas.2426827122. Epub 2025 Apr 17.

PMID- 40214493

OWN - NLM

STAT- MEDLINE

DCOM- 20250411

LR - 20250417

IS - 2073-4409 (Electronic)

IS - 2073-4409 (Linking)

VI - 14

IP - 7

DP - 2025 Apr 3

TI - Insights into Autophagy in Microbiome Therapeutic Approaches for Drug-Resistant

Tuberculosis.

LID - 10.3390/cells14070540 [doi]

LID - 540

AB - Tuberculosis, primarily caused by Mycobacterium tuberculosis, is an airborne lung

disease and continues to pose a significant global health threat, resulting in

millions of deaths annually. The current treatment for tuberculosis involves a

prolonged regimen of antibiotics, which leads to complications such as

recurrence, drug resistance, reinfection, and a range of side effects. This

scenario underscores the urgent need for novel therapeutic strategies to combat

this lethal pathogen. Over the last two decades, microbiome therapeutics have

emerged as promising next-generation drug candidates, offering advantages over

traditional medications. In 2022, the Food and Drug Administration approved the

first microbiome therapeutic for recurrent Clostridium infections, and extensive

research is underway on microbiome treatments for various challenging diseases,

including metabolic disorders and cancer. Research on microbiomes concerning

tuberculosis commenced roughly a decade ago, and the scope of this research has

broadened considerably over the last five years, with microbiome therapeutics now

viewed as viable options for managing drug-resistant tuberculosis. Nevertheless,

the understanding of their mechanisms is still in its infancy. Although autophagy

has been extensively studied in other diseases, research into its role in

tuberculosis is just beginning, with preliminary developments in progress.

Against this backdrop, this comprehensive review begins by succinctly outlining

tuberculosis' characteristics and assessing existing treatments' strengths and

weaknesses, followed by a detailed examination of microbiome-based therapeutic

approaches for drug-resistant tuberculosis. Additionally, this review focuses on

establishing a basic understanding of microbiome treatments for tuberculosis,

mainly through the lens of autophagy as a mechanism of action. Ultimately, this

review aims to contribute to the foundational comprehension of microbiome-based

therapies for tuberculosis, thereby setting the stage for the further advancement

of microbiome therapeutics for drug-resistant tuberculosis.

FAU - Rahim, Md Abdur

AU - Rahim MA

AUID- ORCID: 0000-0003-2000-5602

AD - Department of Microbiology and Immunology, School of Medicine, Soonchunhyang

University, 31, Suncheonhyang 6-gil, Dongnam-gu, Cheonan-si 31151, Republic of

Korea.

AD - Human Microbiome Medical Research Center (HM·MRC), School of Medicine,

Soonchunhyang University, 22, Soonchunhyang-ro, Sinchang-myeon, Asan-si 31538,

Republic of Korea.

FAU - Seo, Hoonhee

AU - Seo H

AD - Human Microbiome Medical Research Center (HM·MRC), School of Medicine,

Soonchunhyang University, 22, Soonchunhyang-ro, Sinchang-myeon, Asan-si 31538,

Republic of Korea.

AD - Probiotics Microbiome Commercialization Research Center (PMC), Soonchunhyang

University, 22, Soonchunhyang-ro, Sinchang-myeon, Asan-si 31538, Republic of

Korea.

FAU - Barman, Indrajeet

AU - Barman I

AUID- ORCID: 0000-0003-1180-0380

AD - Department of Microbiology and Immunology, School of Medicine, Soonchunhyang

University, 31, Suncheonhyang 6-gil, Dongnam-gu, Cheonan-si 31151, Republic of

Korea.

AD - Human Microbiome Medical Research Center (HM·MRC), School of Medicine,

Soonchunhyang University, 22, Soonchunhyang-ro, Sinchang-myeon, Asan-si 31538,

Republic of Korea.

FAU - Hossain, Mohammed Solayman

AU - Hossain MS

AD - Department of Microbiology and Immunology, School of Medicine, Soonchunhyang

University, 31, Suncheonhyang 6-gil, Dongnam-gu, Cheonan-si 31151, Republic of

Korea.

AD - Human Microbiome Medical Research Center (HM·MRC), School of Medicine,

Soonchunhyang University, 22, Soonchunhyang-ro, Sinchang-myeon, Asan-si 31538,

Republic of Korea.

FAU - Shuvo, Md Sarower Hossen

AU - Shuvo MSH

AUID- ORCID: 0009-0006-1785-1639

AD - Department of Microbiology and Immunology, School of Medicine, Soonchunhyang

University, 31, Suncheonhyang 6-gil, Dongnam-gu, Cheonan-si 31151, Republic of

Korea.

AD - Human Microbiome Medical Research Center (HM·MRC), School of Medicine,

Soonchunhyang University, 22, Soonchunhyang-ro, Sinchang-myeon, Asan-si 31538,

Republic of Korea.

FAU - Song, Ho-Yeon

AU - Song HY

AD - Department of Microbiology and Immunology, School of Medicine, Soonchunhyang

University, 31, Suncheonhyang 6-gil, Dongnam-gu, Cheonan-si 31151, Republic of

Korea.

AD - Human Microbiome Medical Research Center (HM·MRC), School of Medicine,

Soonchunhyang University, 22, Soonchunhyang-ro, Sinchang-myeon, Asan-si 31538,

Republic of Korea.

AD - Probiotics Microbiome Commercialization Research Center (PMC), Soonchunhyang

University, 22, Soonchunhyang-ro, Sinchang-myeon, Asan-si 31538, Republic of

Korea.

LA - eng

GR - RS-2023-00219563/National Research Foundation of Korea/

GR - P248400003/Korea Institute for Advancement of Technology (KIAT)/

GR - Soonchunhyang University Research Fund/Soonchunhyang University/

PT - Journal Article

PT - Research Support, Non-U.S. Gov't

PT - Review

DEP - 20250403

PL - Switzerland

TA - Cells

JT - Cells

JID - 101600052

RN - 0 (Antitubercular Agents)

SB - IM

MH - Humans

MH - *Autophagy

MH - *Tuberculosis, Multidrug-Resistant/therapy/microbiology

MH - *Microbiota

MH - Mycobacterium tuberculosis/drug effects

MH - Animals

MH - Antitubercular Agents/therapeutic use/pharmacology

PMC - PMC11989032

OTO - NOTNLM

OT - autophagy

OT - drug-resistant tuberculosis

OT - microbiome therapeutics

COIS- The authors declare no conflicts of interest.

EDAT- 2025/04/11 12:28

MHDA- 2025/04/11 12:29

PMCR- 2025/04/03

CRDT- 2025/04/11 09:53

PHST- 2025/02/21 00:00 [received]

PHST- 2025/03/23 00:00 [revised]

PHST- 2025/04/02 00:00 [accepted]

PHST- 2025/04/11 12:29 [medline]

PHST- 2025/04/11 12:28 [pubmed]

PHST- 2025/04/11 09:53 [entrez]

PHST- 2025/04/03 00:00 [pmc-release]

AID - cells14070540 [pii]

AID - cells-14-00540 [pii]

AID - 10.3390/cells14070540 [doi]

PST - epublish

SO - Cells. 2025 Apr 3;14(7):540. doi: 10.3390/cells14070540.

PMID- 40196755

OWN - NLM

STAT- MEDLINE

DCOM- 20250409

LR - 20260127

IS - 1177-8881 (Electronic)

IS - 1177-8881 (Linking)

VI - 19

DP - 2025

TI - Bibliometric and Visualization Analysis of DprE1 Inhibitors to Combat

Tuberculosis.

PG - 2577-2596

LID - 10.2147/DDDT.S515049 [doi]

AB - BACKGROUND: Tuberculosis (TB) poses a serious threat to public health,

particularly owing to the increase in multidrug-resistant tuberculosis (MDR-TB)

and extremely drug-resistant tuberculosis (XDR-TB); thus, there is an imperative

need for novel treatments to tackle this issue. Decaprenylphosphoryl-β-D-ribose

2'-epimerase (DprE1) is essential for mycobacterial cell wall integrity and

viability. As no relevant bibliometric study has been reported, we performed

bibliometric and visual analyses to depict the knowledge framework of research

related to the involvement of DprE1 in TB. METHODS: Relevant studies were sourced

from the Web of Science Core Collection database. VOSviewer, CiteSpace, and

bibliometrics (http://bibliometric.com/) were used to construct networks based on

an analysis of journals, countries, funding, institutions, authors, references,

and keywords. RESULTS: A total of 184 publications were retrieved; the total

citations were 3405 times and the mean citation was 17.28 per article. The annual

number of publications on DprE1 in TB has shown a significantly increasing trend.

The European Journal of Medicinal Chemistry is the most published journal, with

19 articles. Lu Yu and Bin Wang contributed the most prolific authors with 18

articles. Stratified by the number of publications, India was the most prolific

country that cooperated closely with the USA, UK, Japan, and United Arab

Emirates. Burstness analysis of references and keywords showed that the

developing research trends in this field mainly woven around "Mtb", "DprE1" and

"inhibitors" during the past years. CONCLUSION: A systematic bibliometric study

indicates that DprE1 remains a focal point in the anti-TB domain. These results

can serve as a data-driven reference for future research and offer precise

insights into the development of anti-TB agents associated with DprE1. To the

best of our knowledge, this study is the first to comprehensively investigate

DprE1 in TB by means of bibliometric analysis.

CI - © 2025 Wang et al.

FAU - Wang, Qingqing

AU - Wang Q

AUID- ORCID: 0009-0007-9195-7690

AD - College of Pharmacy, Guizhou University of Traditional Chinese Medicine/National

Engineering Technology Research Center for Miao Medicine/Guizhou Engineering

Technology Research Center for Processing and Preparation of Traditional Chinese

Medicine and Ethnic Medicine, Guiyang, People's Republic of China.

FAU - Fu, Huixiao

AU - Fu H

AD - Department of Science and Education, The First People's Hospital of Guiyang,

Guiyang, People's Republic of China.

FAU - Zhang, Yining

AU - Zhang Y

AUID- ORCID: 0009-0005-4236-8182

AD - College of Traditional Chinese Medicine, Tianjin University of Traditional

Chinese Medicine, Tianjin, People's Republic of China.

FAU - Zhang, Man

AU - Zhang M

AUID- ORCID: 0009-0009-0012-5819

AD - College of Pharmacy, Guizhou University of Traditional Chinese Medicine/National

Engineering Technology Research Center for Miao Medicine/Guizhou Engineering

Technology Research Center for Processing and Preparation of Traditional Chinese

Medicine and Ethnic Medicine, Guiyang, People's Republic of China.

FAU - Xu, Jian

AU - Xu J

AD - College of Pharmacy, Guizhou University of Traditional Chinese Medicine/National

Engineering Technology Research Center for Miao Medicine/Guizhou Engineering

Technology Research Center for Processing and Preparation of Traditional Chinese

Medicine and Ethnic Medicine, Guiyang, People's Republic of China.

FAU - Fu, Jian

AU - Fu J

AD - College of Pharmacy, Guizhou University of Traditional Chinese Medicine/National

Engineering Technology Research Center for Miao Medicine/Guizhou Engineering

Technology Research Center for Processing and Preparation of Traditional Chinese

Medicine and Ethnic Medicine, Guiyang, People's Republic of China.

AD - State Key Laboratory of Functions and Applications of Medicinal Plants, Guizhou

Medical University, Guiyang, People's Republic of China.

LA - eng

PT - Journal Article

DEP - 20250403

PL - New Zealand

TA - Drug Des Devel Ther

JT - Drug design, development and therapy

JID - 101475745

RN - 0 (Antitubercular Agents)

RN - EC 1.1.- (DprE1 protein, Mycobacterium tuberculosis)

RN - 0 (Bacterial Proteins)

RN - 0 (Enzyme Inhibitors)

RN - EC 1.1.- (Alcohol Oxidoreductases)

SB - IM

MH - *Bibliometrics

MH - *Antitubercular Agents/pharmacology/chemistry

MH - Humans

MH - *Mycobacterium tuberculosis/drug effects/enzymology

MH - *Tuberculosis/drug therapy

MH - *Bacterial Proteins/antagonists & inhibitors/metabolism

MH - *Enzyme Inhibitors/pharmacology/chemistry

MH - Tuberculosis, Multidrug-Resistant/drug therapy

MH - Alcohol Oxidoreductases

PMC - PMC11974558

OTO - NOTNLM

OT - CiteSpace

OT - DprE1 inhibitors

OT - R-bibliometrix

OT - VOSviewer

OT - drug target

OT - tuberculosis

COIS- The authors declare no conflicts of interest in this work.

EDAT- 2025/04/08 06:24

MHDA- 2025/04/08 06:25

PMCR- 2025/04/03

CRDT- 2025/04/08 04:35

PHST- 2025/01/07 00:00 [received]

PHST- 2025/03/18 00:00 [accepted]

PHST- 2025/04/08 06:25 [medline]

PHST- 2025/04/08 06:24 [pubmed]

PHST- 2025/04/08 04:35 [entrez]

PHST- 2025/04/03 00:00 [pmc-release]

AID - 515049 [pii]

AID - 10.2147/DDDT.S515049 [doi]

PST - epublish

SO - Drug Des Devel Ther. 2025 Apr 3;19:2577-2596. doi: 10.2147/DDDT.S515049.

eCollection 2025.

PMID- 40164948

OWN - NLM

STAT- MEDLINE

DCOM- 20250515

LR - 20251231

IS - 1420-908X (Electronic)

IS - 1023-3830 (Print)

IS - 1023-3830 (Linking)

VI - 74

IP - 1

DP - 2025 Apr 1

TI - Identification of inflammatory protein biomarkers for predicting the different

subtype of adult with tuberculosis: an Olink proteomic study.

PG - 60

LID - 10.1007/s00011-025-02020-9 [doi]

LID - 60

AB - OBJECTIVE: This study aimed to identify the potential inflammatory molecular

biomarkers that could be utilized for the early prediction of different subtypes

of tuberculosis (TB) in adults. METHODS: Plasma samples were obtained from a

cohort of adults diagnosed with 48 cases of active TB, including drug-susceptible

TB (S-TB, n = 28), multidrug-resistant TB (R-TB, n = 20), latent TB infection

(LTBI, n = 20), as well as a control group of healthy individuals without any

infection (HC, n = 20). The expression level of 92 inflammatory-related proteins

was detected by using the high-throughput Olink proteomics platform. RESULTS:

There were 47 inflammatory proteins showing a significant difference (p < 0.05)

among TB, LTBI, and HC groups, and 7 of them differed significantly between HC

and LTBI groups, 43 proteins differed considerably between LTBI and TB groups,

and overall, CXCL10 and TGF-alpha proteins differed substantially among the three

groups which could be used as potential diagnostic biomarkers. Furthermore, SCF

demonstrates remarkable discriminatory power in distinguishing TB from LTBI, with

an area under the curve (AUC) score of 0.920. SLAMF1 has emerged as the top

predictor for distinguishing Sputum Culture-Negative from positive tuberculosis

cases, with an AUC of 0.779. The Correlation analyses showed various

relationships among co-differentiated proteins. In LTBI versus HC, TGF-alpha and

CXCL10 had a strong positive correlation. In non-severe versus severe TB, CXCL10

and CXCL9, as well as TNF and CCL3, were strongly positively correlated, while

IL-6 and SCF had a negative correlation. These co-differentiated proteins were

found to be enriched in various biological processes and molecular functions

related to immune regulation and signaling pathways, such as the p53 signaling

pathway, the TNF signaling pathway, and the NF-kappa B signaling pathway,

highlighting the complex interplay of these proteins in the immune response to TB

infection. CONCLUSION: Inflammation-related proteins exhibited distinct

expression profiles in various conditions of TB. These proteins are

intercorrelated and involve the pathogenesis of tuberculosis by activating

diverse immune cells and promoting the secretion of pro-inflammatory cytokines.

Their functions influence cellular phenotypes, which play a crucial regulatory

role in the interaction between the host and Mycobacterium tuberculosis. These

findings suggest that these proteins are potential disease prevention and

treatment targets.

CI - © 2025. The Author(s).

FAU - Song, Yunlin

AU - Song Y

AD - Department of Intensive Care Unit, The First Affiliated Hospital of Xinjiang

Medical University, Urumqi, 830054, China.

AD - Department of Intensive Care Unit, State Key Laboratory of Pathogenesis,

Prevention and Treatment of High Incidence Diseases in Central Asia, The First

Affiliated Hospital of Xinjiang Medical University, 393 South Li Yu Shan Road,

Urumqi, 830054, Xinjiang, China.

FAU - Abuduaini, Buzukela

AU - Abuduaini B

AD - Department of Intensive Care Unit, The First Affiliated Hospital of Xinjiang

Medical University, Urumqi, 830054, China.

FAU - Yang, Xinting

AU - Yang X

AD - Tuberculosis Department, Beijing Chest Hospital, Capital Medical University,

Beijing, 101149, China.

FAU - Zhang, Jiyuan

AU - Zhang J

AD - First Clinical Institute of Xinjiang Medical University, Urumqi, 830054,

Xinjiang, China.

FAU - Wang, Guirong

AU - Wang G

AD - Department of Clinical Laboratory, Beijing Chest Hospital, Capital Medical

University, Beijing, 101149, China. wangguirong1230@ccmu.edu.cn.

FAU - Lu, Xiaobo

AU - Lu X

AD - Center of Infection, The First Affiliated Hospital of Xinjiang Medical

University, 393 South Li Yu Shan Road, Urumqi, 830054, Xinjiang, China.

xjykdluxiaobo@126.com.

LA - eng

GR - SKL-HIDCA-2021-JH13/Treatment of Central Asian High Incidence Diseases Fund of

China/

GR - TSYC202301B013/Tianshan Outstanding Medical and Health High-level Talent Training

Project/

GR - 82360381/the National Natural Science Foundation of China/

PT - Journal Article

DEP - 20250401

PL - Switzerland

TA - Inflamm Res

JT - Inflammation research : official journal of the European Histamine Research

Society ... [et al.]

JID - 9508160

RN - 0 (Biomarkers)

RN - 0 (Chemokine CXCL10)

RN - 0 (Transforming Growth Factor alpha)

SB - IM

MH - Humans

MH - Adult

MH - Biomarkers/blood

MH - Male

MH - Proteomics

MH - Female

MH - Middle Aged

MH - *Tuberculosis/blood/diagnosis

MH - Young Adult

MH - Latent Tuberculosis/blood/diagnosis

MH - Chemokine CXCL10/blood

MH - Transforming Growth Factor alpha/blood

PMC - PMC11958430

OTO - NOTNLM

OT - Tuberculosis

OT - Biomarker

OT - Inflammation

OT - Olink proteomics

COIS- Declarations. Conflict of interests: The authors declare no competing interests.

EDAT- 2025/04/01 00:27

MHDA- 2025/04/01 00:28

PMCR- 2025/04/01

CRDT- 2025/03/31 23:33

PHST- 2024/08/07 00:00 [received]

PHST- 2025/03/02 00:00 [accepted]

PHST- 2025/02/20 00:00 [revised]

PHST- 2025/04/01 00:28 [medline]

PHST- 2025/04/01 00:27 [pubmed]

PHST- 2025/03/31 23:33 [entrez]

PHST- 2025/04/01 00:00 [pmc-release]

AID - 10.1007/s00011-025-02020-9 [pii]

AID - 2020 [pii]

AID - 10.1007/s00011-025-02020-9 [doi]

PST - epublish

SO - Inflamm Res. 2025 Apr 1;74(1):60. doi: 10.1007/s00011-025-02020-9.

PMID- 38111165

OWN - NLM

STAT- MEDLINE

DCOM- 20250421

LR - 20250513

IS - 1538-0254 (Electronic)

IS - 0739-1102 (Linking)

VI - 43

IP - 6

DP - 2025 Apr

TI - Deciphering insights into the binding mechanism and plasticity of Telacebec with

M. tuberculosis cytochrome bcc-aa3 supercomplex through an unbiased molecular

dynamics simulation, free-energy analysis, and DFT study.

PG - 2968-2981

LID - 10.1080/07391102.2023.2294833 [doi]

AB - The cytochrome bcc-aa3 supercomplex, a key component in the electron transport

chain pathway involved in bacterial energy production and homeostasis, is a

clinically validated target for tuberculosis (TB), leading to Telacebec (Q203).

Telacebec is a potent candidate drug under Phase II clinical development for the

treatment of drug-sensitive and drug-resistant TB. Recently, the cryo-electron

microscopy structure of this supercomplex from Mycobacterium tuberculosis (Mtb)

complexed with Q203 was resolved at 6.9 Å resolution (PDB ID: 7E1W). To

understand the binding site (Q(P) site) flexibility and Q203's stability at the

Q(P) site of the Mtb cytochrome bcc complex, we conducted molecular dynamics (MD)

simulation and free energy analysis on this complex in an explicit hydrated lipid

bilayer environment for 500 ns. Through this study, the persistence of a range of

direct and indirect interactions was observed over the course of the simulation.

The significance of the interactions with His375, Tyr161, Ala178, Ala179, Ile183,

His355, Leu356, and Thr313 is underlined. Electrostatic energy was the primary

source of the net binding free energy, regardless of the important interacting

residues. The overall binding free energy for Q203 was -112.84 ± 7.73 kcal/mol,

of which the electrostatic and lipophilic energy contributions were

-116.31 ± 1.14 and -21.32 ± 2.35 kcal/mol, respectively. Meanwhile, DFT

calculations were utilized to elucidate Q203's molecular properties. Overall,

this study deciphers key insights into the cytochrome bcc-aa3 supercomplex with

Q203 on the ground of molecular mechanics and quantum mechanics that may

facilitate structure-based drug design and optimization for the discovery of the

next-generation antitubercular drug(s).

FAU - Ray, Bedabrata

AU - Ray B

AD - Department of Pharmaceutical Sciences, School of Health Sciences and Technology,

UPES, Dehradun, Uttarakhand, India.

FAU - Roy, Kuldeep K

AU - Roy KK

AUID- ORCID: 0000-0001-9623-0617

AD - Department of Pharmaceutical Sciences, School of Health Sciences and Technology,

UPES, Dehradun, Uttarakhand, India.

LA - eng

PT - Journal Article

DEP - 20231218

PL - England

TA - J Biomol Struct Dyn

JT - Journal of biomolecular structure & dynamics

JID - 8404176

RN - 0 (Bacterial Proteins)

RN - 0 (Antitubercular Agents)

SB - IM

MH - *Molecular Dynamics Simulation

MH - *Mycobacterium tuberculosis/metabolism/enzymology

MH - Binding Sites

MH - Protein Binding

MH - Thermodynamics

MH - Density Functional Theory

MH - *Bacterial Proteins/chemistry/metabolism

MH - Antitubercular Agents/chemistry/pharmacology

OTO - NOTNLM

OT - DFT

OT - MDR-TB

OT - Telacebec

OT - Tuberculosis

OT - cytochrome bcc complex

OT - molecular dynamics

EDAT- 2023/12/19 06:42

MHDA- 2025/02/18 12:25

CRDT- 2023/12/19 01:03

PHST- 2025/02/18 12:25 [medline]

PHST- 2023/12/19 06:42 [pubmed]

PHST- 2023/12/19 01:03 [entrez]

AID - 10.1080/07391102.2023.2294833 [doi]

PST - ppublish

SO - J Biomol Struct Dyn. 2025 Apr;43(6):2968-2981. doi:

10.1080/07391102.2023.2294833. Epub 2023 Dec 18.

PMID- 40284559

OWN - NLM

STAT- PubMed-not-MEDLINE

LR - 20250428

IS - 2076-2607 (Print)

IS - 2076-2607 (Electronic)

IS - 2076-2607 (Linking)

VI - 13

IP - 4

DP - 2025 Mar 24

TI - Innovative Strategies for Combating Multidrug-Resistant Tuberculosis: Advances in

Drug Delivery Systems and Treatment.

LID - 10.3390/microorganisms13040722 [doi]

LID - 722

AB - Multidrug-resistant tuberculosis (MDR-TB) is a significant public health

challenge globally, exacerbated by the limited efficacy of existing therapeutic

approaches, prolonged treatment duration, and severe side effects. As drug

resistance continues to emerge, innovative drug delivery systems and treatment

strategies are critical to combating this crisis. This review highlights the

molecular mechanisms underlying resistance to drugs in Mycobacterium

tuberculosis, such as genetic mutation, efflux pump activity, and biofilm

formation, contributing to the persistence and difficulty in eradicating MDR-TB.

Current treatment options, including second-line drugs, offer limited

effectiveness, prompting the need for innovation of advanced therapies and drug

delivery systems. The progression in drug discovery has resulted in the approval

of innovative therapeutics, including bedaquiline and delamanid, amongst other

promising candidates under investigation. However, overcoming the limitations of

traditional drug delivery remains a significant challenge. Nanotechnology has

emerged as a promising solution, with nanoparticle-based drug delivery systems

offering improved bioavailability and targeted and controlled release delivery,

particularly for pulmonary targeting and intracellular delivery to macrophages.

Furthermore, the development of inhalable formulations and the potential of

nanomedicines to bypass drug resistance mechanisms presents a novel approach to

enhancing drug efficacy. Moreover, adjunctive therapies, including immune

modulation and host-directed therapies, are being explored to improve treatment

outcomes. Immunotherapies, such as cytokine modulation and novel TB vaccines,

offer complementary strategies to the use of antibiotics in combating MDR-TB.

Personalized medicine approaches, leveraging genomic profiling of both the

pathogen and the host, offer promise in optimizing treatment regimens and

minimizing drug resistance. This review underscores the importance of

multidisciplinary approaches, combining drug discovery, advanced delivery system

development, and immune modulation to address the complexities of treating

MDR-TB. Continued innovation, global collaboration, and improved diagnostics are

essential to developing practical, accessible, and affordable treatments for

MDR-TB.

FAU - Omoteso, Omobolanle A

AU - Omoteso OA

AUID- ORCID: 0000-0003-3059-7381

AD - Division of Pharmaceutics, Faculty of Pharmacy, Rhodes University, Makhanda 6139,

South Africa.

FAU - Fadaka, Adewale O

AU - Fadaka AO

AD - Department of Anesthesia, Division of Pain Management, Cincinnati Children's

Hospital Medical Center, Cincinnati, OH 45229, USA.

FAU - Walker, Roderick B

AU - Walker RB

AUID- ORCID: 0000-0003-2781-4154

AD - Division of Pharmaceutics, Faculty of Pharmacy, Rhodes University, Makhanda 6139,

South Africa.

FAU - Khamanga, Sandile M

AU - Khamanga SM

AUID- ORCID: 0000-0003-2374-8460

AD - Division of Pharmaceutics, Faculty of Pharmacy, Rhodes University, Makhanda 6139,

South Africa.

LA - eng

GR - N/A/Rhodes University/

PT - Journal Article

PT - Review

DEP - 20250324

PL - Switzerland

TA - Microorganisms

JT - Microorganisms

JID - 101625893

PMC - PMC12029526

OTO - NOTNLM

OT - drug delivery systems

OT - drug resistance mechanisms

OT - host-directed therapies

OT - multidrug-resistant tuberculosis

OT - nanotechnology

COIS- The authors declare no conflicts of interest.

EDAT- 2025/04/26 16:07

MHDA- 2025/04/26 16:08

PMCR- 2025/03/24

CRDT- 2025/04/26 01:18

PHST- 2025/02/28 00:00 [received]

PHST- 2025/03/18 00:00 [revised]

PHST- 2025/03/21 00:00 [accepted]

PHST- 2025/04/26 16:08 [medline]

PHST- 2025/04/26 16:07 [pubmed]

PHST- 2025/04/26 01:18 [entrez]

PHST- 2025/03/24 00:00 [pmc-release]

AID - microorganisms13040722 [pii]

AID - microorganisms-13-00722 [pii]

AID - 10.3390/microorganisms13040722 [doi]

PST - epublish

SO - Microorganisms. 2025 Mar 24;13(4):722. doi: 10.3390/microorganisms13040722.

PMID- 40161687

OWN - NLM

STAT- PubMed-not-MEDLINE

LR - 20250721

IS - 2692-8205 (Electronic)

IS - 2692-8205 (Linking)

DP - 2025 Mar 15

TI - Multiomic analysis identifies suppressive myeloid cell populations in human TB

granulomas.

LID - 2025.03.10.642376 [pii]

LID - 10.1101/2025.03.10.642376 [doi]

AB - Tuberculosis (TB) remains a major global health challenge, particularly in the

context of multidrug-resistant (MDR) Mycobacterium tuberculosis (Mtb).

Host-directed therapies (HDTs) have been proposed as adjunctive therapy to

enhance immune control of infection. Recently, one such HDT, pharmacologic

modulation of myeloid-derived suppressor cells (MDSCs), has been proposed to

treat MDR-TB. While MDSCs have been well characterized in cancer, their role in

TB pathogenesis remains unclear. To investigate whether MDSCs or other myeloid

suppressor populations contribute to TB granuloma microenvironments (GME), we

performed spatial transcriptional profiling and single-cell immunophenotyping on

eighty-four granulomas in lung specimens from three individuals with active

disease. Granulomas were histologically classified based on H&E staining, and

transcriptional signatures were compared across regions of interest (ROIs) at

different states of granuloma maturation. Our analysis revealed that immune

suppression within granuloma was not primarily driven by classical MDSCs but

rather by multiple myeloid cell subsets, including dendritic cells expressing

indoleamine 2,3 dioxygenase-1 expressing (IDO1+ DCs). IDO1+ DCs were the most

frequently observed suppressive myeloid cells, particularly in cellular regions,

and their spatial proximity to activated T cells suggested localized

immunosuppression. Importantly, granulomas at different stages contained distinct

proportions of suppressor myeloid cells, with necrotic and cellular regions

showing different myeloid phenotypes that may influence granuloma progression.

Gene set enrichment analysis (GSEA) further indicated that elevated IDO1

expression was associated with a complex immune response that balanced

suppressive signaling, immune activation, and cellular metabolism. These findings

suggest that classical MDSCs, as defined in tumor microenvironments, likely play

a minor role in TB, whereas IDO1+ DCs may be key regulators of immune suppression

in granulomas influencing local Mtb control in infected lung. A deeper

understanding of the role of IDO1+ suppressive myeloid cells in TB granulomas is

essential to assessing their potential as therapeutic targets in TB treatment.

FAU - Jain, Neharika

AU - Jain N

FAU - Ogbonna, Emmanuel C

AU - Ogbonna EC

FAU - Maliga, Zoltan

AU - Maliga Z

FAU - Jacobson, Connor

AU - Jacobson C

FAU - Zhang, Liang

AU - Zhang L

FAU - Shih, Angela

AU - Shih A

FAU - Rosenberg, Jacob

AU - Rosenberg J

FAU - Kalam, Haroon

AU - Kalam H

FAU - Gagné, Andréanne

AU - Gagné A

FAU - Solomon, Isaac H

AU - Solomon IH

FAU - Santagata, Sandro

AU - Santagata S

FAU - Sorger, Peter K

AU - Sorger PK

AUID- ORCID: 0000-0002-3364-1838

FAU - Aldridge, Bree B

AU - Aldridge BB

AUID- ORCID: 0000-0003-2236-1424

FAU - Martinot, Amanda J

AU - Martinot AJ

AUID- ORCID: 0000-0001-6237-6191

LA - eng

GR - R21 AI155003/AI/NIAID NIH HHS/United States

GR - UC7 AI180310/AI/NIAID NIH HHS/United States

PT - Journal Article

PT - Preprint

DEP - 20250315

PL - United States

TA - bioRxiv

JT - bioRxiv : the preprint server for biology

JID - 101680187

PMC - PMC11952478

EDAT- 2025/03/31 21:17

MHDA- 2025/03/31 21:18

PMCR- 2025/03/28

CRDT- 2025/03/31 06:23

PHST- 2025/03/31 21:18 [medline]

PHST- 2025/03/31 21:17 [pubmed]

PHST- 2025/03/31 06:23 [entrez]

PHST- 2025/03/28 00:00 [pmc-release]

AID - 2025.03.10.642376 [pii]

AID - 10.1101/2025.03.10.642376 [doi]

PST - epublish

SO - bioRxiv [Preprint]. 2025 Mar 15:2025.03.10.642376. doi:

10.1101/2025.03.10.642376.

PMID- 40225858

OWN - NLM

STAT- MEDLINE

DCOM- 20250414

LR - 20250415

IS - 1449-1907 (Electronic)

IS - 1449-1907 (Linking)

VI - 22

IP - 8

DP - 2025

TI - Assessing the Role of Polyamine Metabolites in Blood and the DNA Methylation of

Mycobacterium Tuberculosis in Patients with Multidrug-Resistant Tuberculosis.

PG - 1762-1772

LID - 10.7150/ijms.102568 [doi]

AB - Background: Tuberculosis (TB) is the second largest infectious disease killer in

China, and the increasing prevalence of drug-resistant TB patients complicates

treatment efforts and raises associated costs. Research on the mechanisms and

characteristics of drug-resistant TB contributes to the discovery of new drug

targets and the development of new anti-tuberculosis drugs. Methods: In this

study, high-performance liquid chromatography (HPLC) was used to detect the

content of polyamine metabolites, while western blotting, qPCR and ELISA were

used to detect the expression of polyamine metabolism-related enzymes. The Oxford

Nanopore Technologies (ONT) sequencing was applied to profile DNA methylation in

multidrug-resistant Mycobacterium tuberculosis (Mtb). Gene ontology (GO) analysis

and Kyoto Encyclopedia of Genes and Genomes (KEGG) pathway enrichment analysis

were performed on the screened differentially hypermethylated genes. Furthermore,

STRING and Cytoscape software were used to construct a protein-protein

interaction (PPI) network to identify the key genes. Results: The findings

indicated the spermidine (SPD) and polyamine metabolism-related enzymes were

elevated in the peripheral blood of TB patients. In addition, the production of

polyamines and polyamine metabolism-related enzymes was increased in the

peripheral blood of multidrug-resistant tuberculosis (MDR-TB) patients. GO and

KEGG analyses showed that the differentially hypermethylated genes were mainly

enriched in arginine metabolism. The PPI network analysis identified the top five

key genes with the highest degrees: moaX, vapC49, vapB49, highA3 and nuoC.

Conclusions: Polyamine metabolites were increased in the peripheral blood of

MDR-TB patients. The differentially hypermethylated genes in multidrug-resistant

Mtb are involved in the arginine biosynthetic process, the differentially

methylated genes may play an important biological role in the multidrug

resistance of Mtb.

CI - © The author(s).

FAU - Yan, Li

AU - Yan L

AD - Dongguan Key Laboratory of Tuberculosis Prevention and Control, Dongguan Sixth

People's Hospital, Dongguan 523008, Guangdong, China.

FAU - Niu, Xinxin

AU - Niu X

AD - Dongguan Key Laboratory of Medical Bioactive Molecular Developmental and

Translational Research, Guangdong Provincial Key Laboratory of Medical Immunology

and Molecular Diagnostics, Guangdong Medical University, Dongguan 523808,

Guangdong, China.

FAU - Liang, Kuidi

AU - Liang K

AD - Dongguan Key Laboratory of Tuberculosis Prevention and Control, Dongguan Sixth

People's Hospital, Dongguan 523008, Guangdong, China.

FAU - Guan, Feifeng

AU - Guan F

AD - Dongguan Key Laboratory of Tuberculosis Prevention and Control, Dongguan Sixth

People's Hospital, Dongguan 523008, Guangdong, China.

FAU - Yu, Xiaolin

AU - Yu X

AD - Dongguan Key Laboratory of Tuberculosis Prevention and Control, Dongguan Sixth

People's Hospital, Dongguan 523008, Guangdong, China.

FAU - Ye, Ziyu

AU - Ye Z

AD - Xinghai Institute of Cell, Guangdong Xianhua Institute for Medical Research,

Dongguan 523808, Guangdong, China.

FAU - Huang, Mingyuan

AU - Huang M

AD - Dongguan Key Laboratory of Medical Bioactive Molecular Developmental and

Translational Research, Guangdong Provincial Key Laboratory of Medical Immunology

and Molecular Diagnostics, Guangdong Medical University, Dongguan 523808,

Guangdong, China.

FAU - Liang, Hancheng

AU - Liang H

AD - Dongguan Key Laboratory of Tuberculosis Prevention and Control, Dongguan Sixth

People's Hospital, Dongguan 523008, Guangdong, China.

FAU - Zhong, Xinguang

AU - Zhong X

AD - Dongguan Key Laboratory of Tuberculosis Prevention and Control, Dongguan Sixth

People's Hospital, Dongguan 523008, Guangdong, China.

FAU - Zeng, Jincheng

AU - Zeng J

AD - Dongguan Key Laboratory of Medical Bioactive Molecular Developmental and

Translational Research, Guangdong Provincial Key Laboratory of Medical Immunology

and Molecular Diagnostics, Guangdong Medical University, Dongguan 523808,

Guangdong, China.

AD - Xinghai Institute of Cell, Guangdong Xianhua Institute for Medical Research,

Dongguan 523808, Guangdong, China.

LA - eng

PT - Journal Article

DEP - 20250310

PL - Australia

TA - Int J Med Sci

JT - International journal of medical sciences

JID - 101213954

RN - 0 (Polyamines)

RN - U87FK77H25 (Spermidine)

SB - IM

MH - Humans

MH - *Tuberculosis, Multidrug-Resistant/microbiology/blood/genetics/drug therapy

MH - *DNA Methylation/genetics

MH - *Mycobacterium tuberculosis/genetics/metabolism

MH - *Polyamines/blood/metabolism

MH - Female

MH - Male

MH - Adult

MH - Protein Interaction Maps/genetics

MH - Spermidine/blood

MH - Middle Aged

MH - Chromatography, High Pressure Liquid

PMC - PMC11983307

OTO - NOTNLM

OT - DNA methylation.

OT - Drug-resistant tuberculosis

OT - Polyamine

OT - Tuberculosis

COIS- Competing Interests: The authors have declared that no competing interest exists.

EDAT- 2025/04/14 17:28

MHDA- 2025/04/14 17:29

PMCR- 2025/01/01

CRDT- 2025/04/14 07:14

PHST- 2024/08/20 00:00 [received]

PHST- 2025/01/23 00:00 [accepted]

PHST- 2025/04/14 17:29 [medline]

PHST- 2025/04/14 17:28 [pubmed]

PHST- 2025/04/14 07:14 [entrez]

PHST- 2025/01/01 00:00 [pmc-release]

AID - ijmsv22p1762 [pii]

AID - 10.7150/ijms.102568 [doi]

PST - epublish

SO - Int J Med Sci. 2025 Mar 10;22(8):1762-1772. doi: 10.7150/ijms.102568. eCollection

2025.

PMID- 39996595

OWN - NLM

STAT- MEDLINE

DCOM- 20250509

LR - 20250509

IS - 1549-960X (Electronic)

IS - 1549-9596 (Linking)

VI - 65

IP - 5

DP - 2025 Mar 10

TI - Mechanistic Insights into the Reversible Inhibition of d-Cycloserine in Alanine

Racemase from Mycobacterium tuberculosis.

PG - 2610-2622

LID - 10.1021/acs.jcim.4c01763 [doi]

AB - d-Cycloserine (DCS), an antibiotic used in the treatment of drug-resistant

tuberculosis, was traditionally believed to irreversibly inhibit the

pyridoxal-5'-phosphate (PLP)-dependent alanine racemase from Mycobacterium

tuberculosis (MtAlr). However, recent research suggests that the inhibition is

reversible, as MtAlr can be reactivated by destructing DCS. This study employs

the hybrid quantum mechanics/molecular mechanics (QM/MM) method to investigate

the mechanisms of MtAlr inhibition and DCS destruction. Computational results

indicate that the inhibition reaction via an "isoxazole-forming" pathway is

kinetically favorable, while the DCS destruction reaction via an "oxime-forming"

pathway is thermodynamically favorable, explaining the irreversible inhibition of

DCS. For the inhibition reaction, the isoxazole product was found to prefer the

keto form, contrary to the previously proposed enol form. Moreover, K44 and D322'

were identified as key residues. K44 transfers the proton from Cα and Cβ of DCS,

while D322' stabilizes the carbanion intermediate and isoxazole product via

electrostatic interaction with the protonated K44. Such electrostatic interaction

was eliminated in the DCS-resistance variant, D322'N, making the inhibition

reaction unfavorable. For DCS destruction, an "up-to-down" conformational change

is required to place the isoxazolidinone ring in an appropriate position for

hydrolysis. The deprotonated Y273' facilitates the hydrolysis reaction by

enhancing the nucleophilicity of the water molecule. Throughout the whole

reaction of MtAlr, PLP plays multiple roles, including stabilizing the carbanion

intermediate and acting as a proton shuttle. Overall, this study provides deeper

insight into the catalytic mechanism of MtAlr and offers valuable insights for

drug development.

FAU - Zhuang, Jingyuan

AU - Zhuang J

AD - Warshel Institute for Computational Biology, School of Medicine, The Chinese

University of Hong Kong, Shenzhen 518172, China.

FAU - Yang, Wei

AU - Yang W

AUID- ORCID: 0000-0002-9396-329X

AD - Warshel Institute for Computational Biology, School of Medicine, The Chinese

University of Hong Kong, Shenzhen 518172, China.

AD - National Clinical Research Center for Infectious Diseases, Shenzhen Third

People's Hospital, Shenzhen 518112, China.

FAU - Cheng, Gui-Juan

AU - Cheng GJ

AUID- ORCID: 0000-0002-2818-2235

AD - Warshel Institute for Computational Biology, School of Medicine, The Chinese

University of Hong Kong, Shenzhen 518172, China.

LA - eng

PT - Journal Article

DEP - 20250225

PL - United States

TA - J Chem Inf Model

JT - Journal of chemical information and modeling

JID - 101230060

RN - 95IK5KI84Z (Cycloserine)

RN - EC 5.1.1.1 (Alanine Racemase)

RN - 0 (Enzyme Inhibitors)

SB - IM

MH - *Mycobacterium tuberculosis/enzymology

MH - *Cycloserine/pharmacology/chemistry/metabolism

MH - *Alanine Racemase/antagonists & inhibitors/metabolism/chemistry

MH - Quantum Theory

MH - *Enzyme Inhibitors/pharmacology/chemistry

MH - Molecular Dynamics Simulation

EDAT- 2025/02/25 12:24

MHDA- 2025/03/10 08:00

CRDT- 2025/02/25 07:53

PHST- 2025/03/10 08:00 [medline]

PHST- 2025/02/25 12:24 [pubmed]

PHST- 2025/02/25 07:53 [entrez]

AID - 10.1021/acs.jcim.4c01763 [doi]

PST - ppublish

SO - J Chem Inf Model. 2025 Mar 10;65(5):2610-2622. doi: 10.1021/acs.jcim.4c01763.

Epub 2025 Feb 25.

PMID- 38088766

OWN - NLM

STAT- MEDLINE

DCOM- 20250421

LR - 20260630

IS - 1538-0254 (Electronic)

IS - 0739-1102 (Linking)

VI - 43

IP - 4

DP - 2025 Mar

TI - Virtual screening and identification of promising therapeutic compounds against

drug-resistant Mycobacterium tuberculosis β-ketoacyl-acyl carrier protein

synthase I (KasA).

PG - 2029-2041

LID - 10.1080/07391102.2023.2293276 [doi]

AB - The emergence of new Mycobacterium tuberculosis (Mtb) strains resistant to the

key drugs currently used in the clinic for tuberculosis treatment can

substantially reduce the probability of therapy success, causing the relevance

and importance of studies on the development of novel potent antibacterial agents

targeting different vulnerable spots of Mtb. In this study, 28,860 compounds from

the library of bioactive molecules were screened to identify novel potential

inhibitors of β-ketoacyl-acyl carrier protein synthase I (KasA), one of the key

enzymes involved in the biosynthesis of mycolic acids of the Mtb cell wall. In

doing so, we used a structure-based virtual screening approach to drug

repurposing that included high-throughput docking of the C171Q KasA enzyme with

compounds from the library of bioactive molecules including the FDA-approved

drugs and investigational drug candidates, assessment of the binding affinity for

the docked ligand/C171Q KasA complexes, and molecular dynamics simulations

followed by binding free energy calculations. As a result, post-modeling analysis

revealed 6 top-ranking compounds exhibiting a strong attachment to the malonyl

binding site of the enzyme, as evidenced by the values of binding free energy

which are significantly lower than those predicted for the KasA inhibitor TLM5

used in the calculations as a positive control. In light of the data obtained,

the identified compounds are suggested to form a good basis for the development

of new antitubercular molecules of clinical significance with activity against

the KasA enzyme of Mtb.Communicated by Ramaswamy H. Sarma.

FAU - Andrianov, Alexander M

AU - Andrianov AM

AD - Institute of Bioorganic Chemistry, National Academy of Sciences of Belarus,

Minsk, Republic of Belarus.

FAU - Furs, Konstantin V

AU - Furs KV

AD - United Institute of Informatics Problems, National Academy of Sciences of

Belarus, Minsk, Republic of Belarus.

FAU - Gonchar, Anna V

AU - Gonchar AV

AD - United Institute of Informatics Problems, National Academy of Sciences of

Belarus, Minsk, Republic of Belarus.

FAU - Skrahina, Alena M

AU - Skrahina AM

AD - Republican Scientific and Practical Center of Pulmonology and Tuberculosis,

Minsk, Republic of Belarus.

FAU - Wang, Yixin

AU - Wang Y

AD - Key Laboratory of Medical Molecular Virology of the Ministry of

Education/Ministry of Health Commission, School of Basic Medical Sciences, Fudan

University, Shanghai, People's Republic of China.

FAU - Lyu, Liang-Dong

AU - Lyu LD

AD - Key Laboratory of Medical Molecular Virology of the Ministry of

Education/Ministry of Health Commission, School of Basic Medical Sciences, Fudan

University, Shanghai, People's Republic of China.

AD - Shanghai Clinical Research Center for Infectious Disease (Tuberculosis), Shanghai

Key Laboratory of Tuberculosis, Shanghai Pulmonary Hospital, Shanghai, People's

Republic of China.

FAU - Tuzikov, Alexander V

AU - Tuzikov AV

AD - United Institute of Informatics Problems, National Academy of Sciences of

Belarus, Minsk, Republic of Belarus.

LA - eng

PT - Journal Article

PT - Research Support, Non-U.S. Gov't

DEP - 20231213

PL - England

TA - J Biomol Struct Dyn

JT - Journal of biomolecular structure & dynamics

JID - 8404176

RN - EC 2.3.1.41 (3-Oxoacyl-(Acyl-Carrier-Protein) Synthase)

RN - 0 (Antitubercular Agents)

RN - 0 (Enzyme Inhibitors)

RN - 0 (Ligands)

RN - 0 (Bacterial Proteins)

SB - IM

MH - *Mycobacterium tuberculosis/drug effects/enzymology

MH - Molecular Docking Simulation

MH - *3-Oxoacyl-(Acyl-Carrier-Protein) Synthase/antagonists &

inhibitors/chemistry/metabolism

MH - Molecular Dynamics Simulation

MH - *Antitubercular Agents/pharmacology/chemistry

MH - *Enzyme Inhibitors/chemistry/pharmacology

MH - Protein Binding

MH - *Drug Resistance, Bacterial/drug effects

MH - Binding Sites

MH - Drug Repositioning

MH - Ligands

MH - Drug Evaluation, Preclinical

MH - Bacterial Proteins/chemistry/antagonists & inhibitors

OTO - NOTNLM

OT - Mycobacterium tuberculosis

OT - antitubercular agents

OT - binding free energy calculations

OT - drug repurposing

OT - drug-resistant tuberculosis

OT - molecular docking

OT - molecular dynamics

OT - β-ketoacyl-acyl carrier protein synthase I (KasA)

EDAT- 2023/12/13 12:43

MHDA- 2025/01/31 06:16

CRDT- 2023/12/13 09:33

PHST- 2025/01/31 06:16 [medline]

PHST- 2023/12/13 12:43 [pubmed]

PHST- 2023/12/13 09:33 [entrez]

AID - 10.1080/07391102.2023.2293276 [doi]

PST - ppublish

SO - J Biomol Struct Dyn. 2025 Mar;43(4):2029-2041. doi:

10.1080/07391102.2023.2293276. Epub 2023 Dec 13.

PMID- 39825760

OWN - NLM

STAT- MEDLINE

DCOM- 20250502

LR - 20260602

IS - 2373-8227 (Electronic)

IS - 2373-8227 (Linking)

VI - 11

IP - 2

DP - 2025 Feb 14

TI - Multifunctional Mycobacterial Topoisomerases with Distinctive Features.

PG - 366-385

LID - 10.1021/acsinfecdis.4c00880 [doi]

AB - Tuberculosis (TB) continues to be a major cause of death worldwide despite having

an effective combinatorial therapeutic regimen and vaccine. Being one of the most

successful human pathogens, Mycobacterium tuberculosis retains the ability to

adapt to diverse intracellular and extracellular environments encountered by it

during infection, persistence, and transmission. Designing and developing new

therapeutic strategies to counter the emergence of multidrug-resistant and

extensively drug-resistant TB remains a major task. DNA topoisomerases make up a

unique class of ubiquitous enzymes that ensure steady-state level supercoiling

and solve topological problems occurring during DNA transactions in cells. They

continue to be attractive targets for the discovery of novel classes of

antibacterials and to develop better molecules from existing drugs by virtue of

their reaction mechanism. The limited repertoire of topoisomerases in M.

tuberculosis, key differences in their properties compared to topoisomerases from

other bacteria, their essentiality for the pathogen's survival, and validation as

candidates for drug discovery provide an opportunity to exploit them in drug

discovery efforts. The present review provides insights into their organization,

structure, function, and regulation to further efforts in targeting them for new

inhibitor discovery. First, the structure and biochemical properties of DNA

gyrase and Topoisomerase I (TopoI) of mycobacteria are described compared to the

well-studied counterparts from other bacteria. Next, we provide an overview of

known inhibitors of DNA gyrase and emerging novel bacterial topoisomerase

inhibitors (NBTIs). We also provide an update on TopoI-specific compounds,

highlighting mycobacteria-specific inhibitors.

FAU - Faheem, Iqball

AU - Faheem I

AD - Department of Microbiology and Cell Biology, Indian Institute of Science, C.V.

Raman Avenue, Bangalore 560012, India.

FAU - Nagaraja, Valakunja

AU - Nagaraja V

AUID- ORCID: 0000-0002-9768-1505

AD - Department of Microbiology and Cell Biology, Indian Institute of Science, C.V.

Raman Avenue, Bangalore 560012, India.

AD - Jawaharlal Nehru Centre for Advanced Scientific Research, Bangalore 560064,

India.

AD - Indian Institute of Science Education and Research, Bhopal 462066, India.

LA - eng

PT - Journal Article

PT - Research Support, Non-U.S. Gov't

PT - Review

DEP - 20250118

PL - United States

TA - ACS Infect Dis

JT - ACS infectious diseases

JID - 101654580

RN - EC 5.99.1.- (DNA Topoisomerases)

RN - 0 (Topoisomerase Inhibitors)

RN - 0 (Antitubercular Agents)

RN - 0 (Bacterial Proteins)

SB - IM

MH - *Mycobacterium tuberculosis/enzymology/drug effects/genetics

MH - Humans

MH - *DNA Topoisomerases/metabolism/chemistry/genetics

MH - Topoisomerase Inhibitors/pharmacology

MH - Antitubercular Agents/pharmacology

MH - Drug Discovery

MH - Bacterial Proteins/metabolism/chemistry

MH - Tuberculosis/microbiology/drug therapy

OTO - NOTNLM

OT - DNA Topology

OT - DNA gyrase

OT - Mycobacterium tuberculosis

OT - Topoisomerase I

OT - Topoisomerase Inhibitors

EDAT- 2025/01/18 15:22

MHDA- 2025/02/14 06:20

CRDT- 2025/01/18 08:03

PHST- 2025/02/14 06:20 [medline]

PHST- 2025/01/18 15:22 [pubmed]

PHST- 2025/01/18 08:03 [entrez]

AID - 10.1021/acsinfecdis.4c00880 [doi]

PST - ppublish

SO - ACS Infect Dis. 2025 Feb 14;11(2):366-385. doi: 10.1021/acsinfecdis.4c00880. Epub

2025 Jan 18.

PMID- 39674553

OWN - NLM

STAT- MEDLINE

DCOM- 20250427

LR - 20250518

IS - 1879-0720 (Electronic)

IS - 0928-0987 (Linking)

VI - 205

DP - 2025 Feb 1

TI - Membrane interaction studies of isoniazid derivatives active against

drug-resistant tuberculosis.

PG - 106986

LID - S0928-0987(24)00299-9 [pii]

LID - 10.1016/j.ejps.2024.106986 [doi]

AB - Tuberculosis is one of the leading causes of mortality worldwide due to the

growth of multi-drug resistant strains unsusceptible to currently available

therapies. Four compounds, isoniazid (INH) and three derivatives,

N'-decanoylisonicotinohydrazide (INHC10),

N'-(E)-(4-phenoxybenzylidene)isonicotinohydrazide (N34) and

N'-(4-phenoxybenzyl)isonicotinohydrazide (N34red), were studied. Owing to their

advantageous in vitro selectivity index against the primary mutation responsible

for drug resistance in Mycobacterium tuberculosis (Mtb), as well as their

suitable lipophilicity and interaction with human serum albumin, INHC10 and N34

were deemed promising antitubercular compounds. N34red, despite differing from

N34 only in the saturation of the N' = C bond, presents a poor selectivity index.

To delve deeper into the therapeutic potential of these compounds, their

interaction with biomembrane models, mimicking biological barriers on the way to

the target inside Mtb cells, was herein evaluated. All compounds, except N34red,

weakened the packing of the acyl chains in the rigid lipid gel phase, especially

INHC10, which was the only compound disturbing liquid disordered membranes.

Notably, all compounds except INH decreased membrane dipole potential, across all

types of bilayers studied, but only N34red had a drastic effect. The insertion in

gel phase bilayers suggests that the compounds may be able to penetrate the rigid

cell wall of Mtb. Förster's resonance energy transfer (FRET) assays in ternary

bilayers with liquid ordered/liquid disordered lateral heterogeneity mimicking

human cell membranes, showed that the compounds affected neither the size nor the

organization of lipid domains. These results provide molecular insights into the

low toxicity against human cell lines and improved activity against

drug-resistant Mtb of INHC10 and N34.

CI - Copyright © 2024 The Author(s). Published by Elsevier B.V. All rights reserved.

FAU - Terreiro, Joana F P R

AU - Terreiro JFPR

AD - Departamento de Química e Bioquímica, Faculdade de Ciências, Centro de Química

Estrutural, Institute of Molecular Sciences, Universidade de Lisboa, Campo Grande

1749-016, Portugal.

FAU - Marquês, Joaquim T

AU - Marquês JT

AD - Departamento de Química e Bioquímica, Faculdade de Ciências, Centro de Química

Estrutural, Institute of Molecular Sciences, Universidade de Lisboa, Campo Grande

1749-016, Portugal. Electronic address: jmtmarques@ciencias.ulisboa.pt.

FAU - Antunes, Inês

AU - Antunes I

AD - Departamento de Química e Bioquímica, Faculdade de Ciências, Centro de Química

Estrutural, Institute of Molecular Sciences, Universidade de Lisboa, Campo Grande

1749-016, Portugal.

FAU - de Faria, Catarina Frazão

AU - de Faria CF

AD - Departamento de Química e Bioquímica, Faculdade de Ciências, Centro de Química

Estrutural, Institute of Molecular Sciences, Universidade de Lisboa, Campo Grande

1749-016, Portugal.

FAU - Santos, Susana

AU - Santos S

AD - Departamento de Química e Bioquímica, Faculdade de Ciências, Centro de Química

Estrutural, Institute of Molecular Sciences, Universidade de Lisboa, Campo Grande

1749-016, Portugal.

FAU - Martins, Filomena

AU - Martins F

AD - Departamento de Química e Bioquímica, Faculdade de Ciências, Centro de Química

Estrutural, Institute of Molecular Sciences, Universidade de Lisboa, Campo Grande

1749-016, Portugal. Electronic address: filomena.martins@ciencias.ulisboa.pt.

FAU - de Almeida, Rodrigo F M

AU - de Almeida RFM

AD - Departamento de Química e Bioquímica, Faculdade de Ciências, Centro de Química

Estrutural, Institute of Molecular Sciences, Universidade de Lisboa, Campo Grande

1749-016, Portugal. Electronic address: rfalmeida@fc.ul.pt.

LA - eng

PT - Journal Article

DEP - 20241212

PL - Netherlands

TA - Eur J Pharm Sci

JT - European journal of pharmaceutical sciences : official journal of the European

Federation for Pharmaceutical Sciences

JID - 9317982

RN - V83O1VOZ8L (Isoniazid)

RN - 0 (Antitubercular Agents)

RN - 0 (Lipid Bilayers)

SB - IM

MH - *Isoniazid/analogs & derivatives/pharmacology/chemistry

MH - *Antitubercular Agents/pharmacology/chemistry

MH - *Mycobacterium tuberculosis/drug effects

MH - Humans

MH - *Tuberculosis, Multidrug-Resistant/drug therapy

MH - *Cell Membrane/metabolism/drug effects

MH - Lipid Bilayers/metabolism/chemistry

MH - Microbial Sensitivity Tests

OTO - NOTNLM

OT - Antitubercular compounds

OT - Drug-membrane interactions

OT - FRET

OT - Fluorescence spectroscopy

OT - Lipid rafts

OT - Multidrug-resistant tuberculosis

COIS- Declaration of competing interest None.

EDAT- 2024/12/15 00:41

MHDA- 2025/01/14 00:20

CRDT- 2024/12/14 19:18

PHST- 2024/09/19 00:00 [received]

PHST- 2024/11/22 00:00 [revised]

PHST- 2024/12/09 00:00 [accepted]

PHST- 2025/01/14 00:20 [medline]

PHST- 2024/12/15 00:41 [pubmed]

PHST- 2024/12/14 19:18 [entrez]

AID - S0928-0987(24)00299-9 [pii]

AID - 10.1016/j.ejps.2024.106986 [doi]

PST - ppublish

SO - Eur J Pharm Sci. 2025 Feb 1;205:106986. doi: 10.1016/j.ejps.2024.106986. Epub

2024 Dec 12.

PMID- 39631241

OWN - NLM

STAT- MEDLINE

DCOM- 20250115

LR - 20250319

IS - 1464-3391 (Electronic)

IS - 0968-0896 (Linking)

VI - 118

DP - 2025 Feb 1

TI - Structural toxicity relationship (STR) of linezolid to mitigate myelosuppression

and serotonergic toxicity.

PG - 118025

LID - S0968-0896(24)00439-5 [pii]

LID - 10.1016/j.bmc.2024.118025 [doi]

AB - Tuberculosis (TB) remains a significant global health challenge, with

multidrug-resistant (MDR-TB) and extensively drug-resistant (XDR-TB) strains

posing severe threats to treatment efficacy. Linezolid, a key component of the

BPaL (Bedaquiline, Pretomanid and Linezolid) regimen, has demonstrated

substantial efficacy against MDR-TB and XDR-TB. However, its clinical utility is

often limited by side effects such as myelosuppression and monoamine oxidase

(MAO) inhibition, linked to its mechanism of action. This perspective centres on

the structural toxicity relationship (STR) of Linezolid and its analogues,

exploring modifications to the C-ring and C-5 position that aim to reduce these

toxicities while maintaining or enhancing antibacterial activity. Several

promising analogues have been identified that exhibit reduced myelosuppression

and MAO inhibition, highlighting the potential for developing safer Linezolid

derivatives. The findings underscore the importance of continued research into

the structure toxicity relationships of oxazolidinones to improve the therapeutic

profiles of these essential drugs in combating drug-resistant TB.

CI - Copyright © 2024 Elsevier Ltd. All rights reserved.

FAU - Shaikh, Matin

AU - Shaikh M

AD - Division of Computer Aided Drug Design, Department of Pharmaceutical Chemistry,

R. C. Patel Institute of Pharmaceutical Education and Research, Shirpur, Dhule

425405, Maharashtra, India; Department of Pharmaceutical Chemistry, H. R. Patel

Institute of Pharmaceutical Education and Research, Shirpur, Dhule 425405,

Maharashtra, India. Electronic address: skm00707@gmail.com.

FAU - Patel, Harun

AU - Patel H

AD - Division of Computer Aided Drug Design, Department of Pharmaceutical Chemistry,

R. C. Patel Institute of Pharmaceutical Education and Research, Shirpur, Dhule

425405, Maharashtra, India. Electronic address: hpatel_38@yahoo.com.

LA - eng

PT - Journal Article

PT - Review

DEP - 20241129

PL - England

TA - Bioorg Med Chem

JT - Bioorganic & medicinal chemistry

JID - 9413298

RN - ISQ9I6J12J (Linezolid)

RN - 0 (Antitubercular Agents)

RN - 0 (Monoamine Oxidase Inhibitors)

RN - EC 1.4.3.4 (Monoamine Oxidase)

SB - IM

MH - *Linezolid/pharmacology/chemistry

MH - Structure-Activity Relationship

MH - Humans

MH - Antitubercular Agents/pharmacology/chemistry

MH - Monoamine Oxidase Inhibitors/pharmacology/chemistry/chemical synthesis

MH - Molecular Structure

MH - Microbial Sensitivity Tests

MH - Animals

MH - Monoamine Oxidase/metabolism

MH - Mycobacterium tuberculosis/drug effects

MH - Dose-Response Relationship, Drug

OTO - NOTNLM

OT - Linezolid

OT - Myelosuppression

OT - Serotonergic toxicity

OT - Structural toxicity relationship

COIS- Declaration of competing interest The authors declare that they have no known

competing financial interests or personal relationships that could have appeared

to influence the work reported in this paper.

EDAT- 2024/12/05 05:32

MHDA- 2025/01/16 00:20

CRDT- 2024/12/04 18:06

PHST- 2024/10/15 00:00 [received]

PHST- 2024/11/21 00:00 [revised]

PHST- 2024/11/25 00:00 [accepted]

PHST- 2025/01/16 00:20 [medline]

PHST- 2024/12/05 05:32 [pubmed]

PHST- 2024/12/04 18:06 [entrez]

AID - S0968-0896(24)00439-5 [pii]

AID - 10.1016/j.bmc.2024.118025 [doi]

PST - ppublish

SO - Bioorg Med Chem. 2025 Feb 1;118:118025. doi: 10.1016/j.bmc.2024.118025. Epub 2024

Nov 29.

PMID- 39416067

OWN - NLM

STAT- PubMed-not-MEDLINE

DCOM- 20250911

LR - 20250911

IS - 2692-8205 (Electronic)

IS - 2692-8205 (Linking)

DP - 2025 Jan 31

TI - Mechanism of the Dual Action Self-Potentiating Antitubercular Drug

Morphazinamide.

LID - 2024.10.08.617272 [pii]

LID - 10.1101/2024.10.08.617272 [doi]

AB - Pyrazinamide (PZA) is a cornerstone of first-line antitubercular drug therapy and

is unique in its ability to kill nongrowing populations of Mycobacterium

tuberculosis through disruption of coenzyme A synthesis. Unlike other drugs, PZA

action is conditional and requires potentiation by host-relevant environmental

stressors, such as low pH and nutrient limitation. Despite its pivotal role in

tuberculosis therapy, the durability of this crucial drug is challenged by the

emergent spread of drug-resistance. To advance drug discovery efforts, we

characterized the activity of a more potent PZA analog, morphazinamide (MZA).

Here, we demonstrate that like PZA, MZA acts in part through impairment of

coenzyme A synthesis. Unexpectedly, we find that, in contrast to PZA, MZA does

not require potentiation and maintains bactericidal activity against

PZA-resistant strains due to an additional mechanism involving aldehyde release.

Further, we find that the principal mechanism for resistance to the aldehyde

component is through promoter mutations that increase expression of the mycothiol

oxidoreductase MscR. Our findings reveal a dual action synergistic mechanism of

MZA that results in a faster kill rate and a higher barrier to resistance. These

observations provide new insights for discovery of improved therapeutic

approaches for addressing the growing problem of drug-resistant tuberculosis.

FAU - Ostrer, Lev

AU - Ostrer L

AUID- ORCID: 0000-0001-8047-393X

AD - Department of Microbiology and Immunology, University of Minnesota Medical

School, Minneapolis, Minnesota USA.

FAU - Crooks, Taylor A

AU - Crooks TA

AUID- ORCID: 0000-0001-7608-1992

AD - Department of Microbiology and Immunology, University of Minnesota Medical

School, Minneapolis, Minnesota USA.

FAU - Howe, Michael D

AU - Howe MD

AD - Department of Microbiology and Immunology, University of Minnesota Medical

School, Minneapolis, Minnesota USA.

FAU - Vo, Sang

AU - Vo S

AD - Department of Microbiology and Immunology, University of Minnesota Medical

School, Minneapolis, Minnesota USA.

AD - Department of Medicinal Chemistry, College of Pharmacy, University of Minnesota

Medical School, Minneapolis, Minnesota USA.

FAU - Jia, Ziyi

AU - Jia Z

AUID- ORCID: 0000-0001-8279-5561

AD - Department of Microbiology and Immunology, University of Minnesota Medical

School, Minneapolis, Minnesota USA.

FAU - Hegde, Pooja

AU - Hegde P

AD - Department of Medicinal Chemistry, College of Pharmacy, University of Minnesota

Medical School, Minneapolis, Minnesota USA.

FAU - Schacht, Nathan

AU - Schacht N

AD - Department of Microbiology and Immunology, University of Minnesota Medical

School, Minneapolis, Minnesota USA.

FAU - Aldrich, Courtney C

AU - Aldrich CC

AD - Department of Medicinal Chemistry, College of Pharmacy, University of Minnesota

Medical School, Minneapolis, Minnesota USA.

FAU - Baughn, Anthony D

AU - Baughn AD

AUID- ORCID: 0000-0003-1188-4238

AD - Department of Microbiology and Immunology, University of Minnesota Medical

School, Minneapolis, Minnesota USA.

LA - eng

GR - R01 AI123146/AI/NIAID NIH HHS/United States

GR - T32 GM008244/GM/NIGMS NIH HHS/United States

GR - T32 HL007741/HL/NHLBI NIH HHS/United States

PT - Journal Article

PT - Preprint

DEP - 20250131

PL - United States

TA - bioRxiv

JT - bioRxiv : the preprint server for biology

JID - 101680187

UIN - PNAS Nexus. 2025 Jul 29;4(8):pgaf242. doi: 10.1093/pnasnexus/pgaf242. PMID:

40799345

PMC - PMC11482805

EDAT- 2024/10/17 10:05

MHDA- 2024/10/17 10:06

PMCR- 2025/02/27

CRDT- 2024/10/17 04:49

PHST- 2024/10/17 10:05 [pubmed]

PHST- 2024/10/17 10:06 [medline]

PHST- 2024/10/17 04:49 [entrez]

PHST- 2025/02/27 00:00 [pmc-release]

AID - 2024.10.08.617272 [pii]

AID - 10.1101/2024.10.08.617272 [doi]

PST - epublish

SO - bioRxiv [Preprint]. 2025 Jan 31:2024.10.08.617272. doi:

10.1101/2024.10.08.617272.

PMID- 39812792

OWN - NLM

STAT- MEDLINE

DCOM- 20250501

LR - 20250521

IS - 1432-072X (Electronic)

IS - 0302-8933 (Linking)

VI - 207

IP - 2

DP - 2025 Jan 15

TI - Targeting InhA in drug-resistant Mycobacterium tuberculosis: potent

antimycobacterial activity of diaryl ether dehydrozingerone derivatives.

PG - 34

LID - 10.1007/s00203-025-04238-x [doi]

AB - Tuberculosis (TB) remains a major global threat, with 10 million new cases and

1.5 million deaths each year. In multidrug-resistant tuberculosis (MDR-TB),

resistance is most commonly observed against isoniazid (INH) and rifampicin

(RIF), the two frontline drugs. Isoniazid resistance is predominantly linked to

mutations in the InhA gene, which encodes an enzyme involved in mycolic acid

synthesis, a vital component of the mycobacterial cell wall. Mutations in InhA

reduce drug binding, rendering INH ineffective. These morbidity and mortality

figures, along with the fact that the rise and global spread of drug-resistant

TB, underscores the need for the discovery of novel therapeutics. In this

direction, we have previously synthesized, characterized, and screened a library

of diaryl ether dehydrozingerone derivatives against mycobacteria and identified

two best hits, 7 and 14, based on bacteriostatic activities. The present study

aimed to thoroughly investigate the antituberculosis potential of these

compounds, particularly regarding drug-resistant TB. Our findings revealed that

both compounds exhibited tuberculocidal activity against the standard laboratory

strain Mycobacterium tuberculosis (M. tb) H37Rv, with minimal bactericidal

concentrations (MBC) of 4μg/ml for compound 7 and 8 μg/ml for compound 14. Next,

concentration vs time-kill kinetics of both these compounds showed

concentration-dependent bactericidal activities against M. tb and complete

pathogen eradication from culture at just 16× MIC. Both compounds were found to

be suitable for combination regimens as their interactions with isoniazid and

rifampicin against M. tb were observed to be synergistic. Additionally, 7 and 14

exhibited minimal hemolysis against human RBCs and less cytotoxicity was observed

against three human cell lines up to 1000 μM. Molecular docking revealed that

these compounds bind more effectively to M. tb InhA, including its mutant forms

where isoniazid binding is impaired, outperforming both isoniazid and triclosan

in binding affinity. Importantly 7 and 14 showed potent activity against

drug-susceptible clinical isolates and two isoniazid-resistant M. tb clinical

isolates equivalent to that against M. tb H37Rv. The most interesting observation

was that both compounds were found to be effective against three multi-drug

resistant (MDR) strains of M. tb, thereby depicting their potential against

drug-resistant TB. An ex vivo assay on RAW 264 cells infected with M. tb

demonstrated a significant reduction in bacterial load at 8× MIC, revealing the

fact that these compounds are highly effective against intracellular M. tb H37Rv.

To the best of our knowledge, this is the first study that reports promising

antimycobacterial potential of 7 and 14 against drug-susceptible,

isoniazid-resistant, and MDR tuberculosis which warrants further exploration

considering the need for new anti-TB medicine.

CI - © 2025. The Author(s), under exclusive licence to Springer-Verlag GmbH Germany,

part of Springer Nature.

FAU - Mubarak, Mohamad Mosa

AU - Mubarak MM

AD - Clinical Microbiology and PK-PD Division, CSIR-Indian Institute of Integrative

Medicine, Sanatnagar, Srinagar, J&K, 190005, India.

AD - Academy of Scientific and Innovative Research (AcSIR), Ghaziabad, 201002, India.

FAU - Kantroo, Hadiya Amin

AU - Kantroo HA

AD - Clinical Microbiology and PK-PD Division, CSIR-Indian Institute of Integrative

Medicine, Sanatnagar, Srinagar, J&K, 190005, India.

AD - Academy of Scientific and Innovative Research (AcSIR), Ghaziabad, 201002, India.

FAU - Mir, Firdoous Ahmad

AU - Mir FA

AD - Clinical Microbiology and PK-PD Division, CSIR-Indian Institute of Integrative

Medicine, Sanatnagar, Srinagar, J&K, 190005, India.

AD - Genetics Resources and Agrotechnology Division, CSIR-Indian Institute of

Integrative Medicine, Sanatnagar, Srinagar, J&K, 190005, India.

FAU - Kumar, Chetan

AU - Kumar C

AD - School of Pharmaceutical and Populations Health Informatics, DIT University,

Dehradun, 248009, India. chetan.kumar@dituniversity.edu.in.

FAU - Ahmad, Zahoor

AU - Ahmad Z

AD - Clinical Microbiology and PK-PD Division, CSIR-Indian Institute of Integrative

Medicine, Sanatnagar, Srinagar, J&K, 190005, India. zahoorap@iiim.res.in.

AD - Academy of Scientific and Innovative Research (AcSIR), Ghaziabad, 201002, India.

zahoorap@iiim.res.in.

LA - eng

GR - GAP-2176/Department of Biotechnology, Ministry of Science and Technology, India/

GR - GAP-2192/Indian Council of Medical Research/

PT - Journal Article

DEP - 20250115

PL - Germany

TA - Arch Microbiol

JT - Archives of microbiology

JID - 0410427

RN - 0 (Antitubercular Agents)

RN - EC 1.3.1.9 (InhA protein, Mycobacterium)

RN - 0 (Bacterial Proteins)

RN - EC 1.- (Oxidoreductases)

RN - V83O1VOZ8L (Isoniazid)

RN - VJT6J7R4TR (Rifampin)

SB - IM

MH - *Mycobacterium tuberculosis/drug effects/genetics

MH - *Antitubercular Agents/pharmacology/chemistry

MH - *Bacterial Proteins/genetics/metabolism/antagonists & inhibitors

MH - Microbial Sensitivity Tests

MH - Humans

MH - *Oxidoreductases/genetics/metabolism/antagonists & inhibitors

MH - Tuberculosis, Multidrug-Resistant/microbiology/drug therapy

MH - Isoniazid/pharmacology

MH - Molecular Docking Simulation

MH - Rifampin/pharmacology

MH - Drug Resistance, Multiple, Bacterial

OTO - NOTNLM

OT - Antituberculosis potential

OT - Diaryl ether dehydrozingerone derivatives

OT - Isoniazid resistance

OT - Multidrug-resistant

OT - Mycobacterium tuberculosis

OT - Tuberculosis

COIS- Declarations. Conflict of interest: The authors declare that they have no

competing interests.

EDAT- 2025/01/15 12:24

MHDA- 2025/01/15 12:25

CRDT- 2025/01/15 11:13

PHST- 2024/11/12 00:00 [received]

PHST- 2025/01/06 00:00 [accepted]

PHST- 2024/12/23 00:00 [revised]

PHST- 2025/01/15 12:25 [medline]

PHST- 2025/01/15 12:24 [pubmed]

PHST- 2025/01/15 11:13 [entrez]

AID - 10.1007/s00203-025-04238-x [pii]

AID - 10.1007/s00203-025-04238-x [doi]

PST - epublish

SO - Arch Microbiol. 2025 Jan 15;207(2):34. doi: 10.1007/s00203-025-04238-x.

PMID- 39400949

OWN - NLM

STAT- MEDLINE

DCOM- 20250106

LR - 20260624

IS - 1521-3773 (Electronic)

IS - 1433-7851 (Print)

IS - 1433-7851 (Linking)

VI - 64

IP - 1

DP - 2025 Jan 2

TI - Arenicolide Family Macrolides Provide a New Therapeutic Lead Combating

Multidrug-Resistant Tuberculosis.

PG - e202412994

LID - 10.1002/anie.202412994 [doi]

LID - e202412994

AB - The emergence of multidrug-resistant (MDR) and extensively drug-resistant (XDR)

strains of Mycobacterium tuberculosis (Mtb) poses a significant threat to health

globally. During searching for new chemical entities regulating MDR- and XDR-Mtb,

chemical investigation of the black oil beetle gut bacterium Micromonospora sp.

GR10 led to the discovery of eight new members of arenicolides along with the

identification of arenicolide A (Ar-A, 1), which was a previously reported

macrolide with incomplete configuration. Genomic analysis of the bacterial strain

GR10 revealed their putative biosynthetic pathway. Quantum mechanics-based

computation, chemical derivatizations, and bioinformatic analysis established the

absolute stereochemistry of Ar-A and arenicolides D-K (Ar-D-K, 2-9) completely

for the first time. Biological studies of 1-9 revealed their antimicrobial

activity against MDR and XDR strains of Mtb. Ar-A had the most potent in vitro

antimicrobial efficacy against MDR- and XDR-Mtb. Mechanistically, Ar-A induced

ATP depletion and destabilized Mtb cell wall, thereby inhibiting growth. Notably,

Ar-A exerted a significant antimicrobial effect against Mtb in macrophages, was

effective in the treatment of Mtb infections, and showed a synergistic effect

with amikacin (AMK) in a mouse model of MDR-Mtb lung infection. Collectively, our

findings indicate Ar-A to be a promising drug lead for drug-resistant

tuberculosis.

CI - © 2024 The Author(s). Angewandte Chemie International Edition published by

Wiley-VCH GmbH.

FAU - Hwang, Sunghoon

AU - Hwang S

AD - Natural Products Research Institute, College of Pharmacy, Seoul National

University, 1 Gwanak-ro, Gwanak-gu, 08826, Seoul, Republic of Korea.

FAU - Heo, Bo Eun

AU - Heo BE

AD - Division of Life Science, Department of Bio & Medical Big Data (BK21 Four

Program), Research Institute of Life Science, Gyeongsang National University,

52828, Jinju, Republic of Korea.

FAU - Nguyen, Thanh Quang

AU - Nguyen TQ

AD - Division of Life Science, Department of Bio & Medical Big Data (BK21 Four

Program), Research Institute of Life Science, Gyeongsang National University,

52828, Jinju, Republic of Korea.

FAU - Kim, Young Jae

AU - Kim YJ

AD - Department of Microbiology, College of Medicine, Chungnam National University,

35015, Daejeon, Republic of Korea.

AD - Department of Medical Science, College of Medicine, Chungnam National University,

35015, Daejeon, Republic of Korea.

FAU - Lee, Sung-Gwon

AU - Lee SG

AD - Section of Genetics and Physiology, Laboratory of Molecular and Cellular Biology,

National Institute of Diabetes and Digestive and Kidney Diseases (NIDDK),

National Institutes of Health (NIH), Bethesda, 20892, MD, USA.

FAU - Huynh, Thanh-Hau

AU - Huynh TH

AD - Natural Products Research Institute, College of Pharmacy, Seoul National

University, 1 Gwanak-ro, Gwanak-gu, 08826, Seoul, Republic of Korea.

FAU - Kim, Eunji

AU - Kim E

AD - Natural Products Research Institute, College of Pharmacy, Seoul National

University, 1 Gwanak-ro, Gwanak-gu, 08826, Seoul, Republic of Korea.

FAU - Jo, Shin-Il

AU - Jo SI

AD - Institute of Korean Insect Life, 23 Banchuk-ro 83 beon-gil, Dong-gu, 22530,

Incheon, Republic of Korea.

FAU - Baek, Min-Jun

AU - Baek MJ

AD - College of Pharmacy and Research Institute of Pharmaceutical Sciences, 1

Gwanak-ro, Gwanak-gu, 08826, Seoul, Republic of Korea.

FAU - Shin, Eun-Kyung

AU - Shin EK

AD - College of Pharmacy and Research Institute of Pharmaceutical Sciences, 1

Gwanak-ro, Gwanak-gu, 08826, Seoul, Republic of Korea.

FAU - Oh, Joonseok

AU - Oh J

AD - Institute of Biomolecular Design & Discovery and Department of Chemistry, Yale

University, West Haven, 06516, CT, USA.

FAU - Park, Chungoo

AU - Park C

AD - School of Biological Sciences and Technology, Chonnam National University, 61186,

Gwangju, Republic of Korea.

FAU - Yoon, Yeo Joon

AU - Yoon YJ

AD - Natural Products Research Institute, College of Pharmacy, Seoul National

University, 1 Gwanak-ro, Gwanak-gu, 08826, Seoul, Republic of Korea.

AD - MolGenBio Co., Ltd., 08826, Seoul, Republic of Korea.

FAU - Park, Eun-Jin

AU - Park EJ

AD - Department of Microbiology, College of Medicine, Chungnam National University,

35015, Daejeon, Republic of Korea.

AD - Department of Medical Science, College of Medicine, Chungnam National University,

35015, Daejeon, Republic of Korea.

FAU - Kim, Kyung Tae

AU - Kim KT

AD - Department of Microbiology, College of Medicine, Chungnam National University,

35015, Daejeon, Republic of Korea.

AD - Department of Medical Science, College of Medicine, Chungnam National University,

35015, Daejeon, Republic of Korea.

FAU - Ryoo, Sungweon

AU - Ryoo S

AD - Clinical Research Centre, Masan National Tuberculosis Hospital, 51755,

Changwon-si, Gyeongsangnam-do, Republic of Korea.

FAU - Lee, Da-Gyum

AU - Lee DG

AD - Clinical Research Centre, Masan National Tuberculosis Hospital, 51755,

Changwon-si, Gyeongsangnam-do, Republic of Korea.

FAU - Wood, Connor

AU - Wood C

AD - Tuberculosis Research Laboratory, Institute Pasteur Korea, 13488, Seongnam-si,

Gyeonggi-do, Republic of Korea.

FAU - Woo, Minjeong

AU - Woo M

AD - Tuberculosis Research Laboratory, Institute Pasteur Korea, 13488, Seongnam-si,

Gyeonggi-do, Republic of Korea.

FAU - Kim, Dae-Duk

AU - Kim DD

AD - Natural Products Research Institute, College of Pharmacy, Seoul National

University, 1 Gwanak-ro, Gwanak-gu, 08826, Seoul, Republic of Korea.

AD - College of Pharmacy and Research Institute of Pharmaceutical Sciences, 1

Gwanak-ro, Gwanak-gu, 08826, Seoul, Republic of Korea.

FAU - Paik, Seungwha

AU - Paik S

AD - Department of Microbiology, College of Medicine, Chungnam National University,

35015, Daejeon, Republic of Korea.

AD - Department of Medical Science, College of Medicine, Chungnam National University,

35015, Daejeon, Republic of Korea.

FAU - Jo, Eun-Kyeong

AU - Jo EK

AD - Department of Microbiology, College of Medicine, Chungnam National University,

35015, Daejeon, Republic of Korea.

AD - Department of Medical Science, College of Medicine, Chungnam National University,

35015, Daejeon, Republic of Korea.

FAU - Jang, Jichan

AU - Jang J

AD - Division of Life Science, Department of Bio & Medical Big Data (BK21 Four

Program), Research Institute of Life Science, Gyeongsang National University,

52828, Jinju, Republic of Korea.

FAU - Oh, Dong-Chan

AU - Oh DC

AUID- ORCID: 0000-0001-6405-5535

AD - Natural Products Research Institute, College of Pharmacy, Seoul National

University, 1 Gwanak-ro, Gwanak-gu, 08826, Seoul, Republic of Korea.

LA - eng

GR - HI22C1361/Korea Health Industry Development Institute/

GR - RS-2023-00255021/National Research Foundation of Korea/

GR - RS-2024-00352229/National Research Foundation of Korea/

GR - RS-2023-00301974/Learning &amp; Academic research institution for Master's·PhD

students, and Postdocs (LAMP) Program of the National Research Foundation of

Korea/

PT - Journal Article

PT - Research Support, Non-U.S. Gov't

DEP - 20241113

PL - Germany

TA - Angew Chem Int Ed Engl

JT - Angewandte Chemie (International ed. in English)

JID - 0370543

RN - 0 (Antitubercular Agents)

RN - 0 (Macrolides)

SB - IM

MH - *Mycobacterium tuberculosis/drug effects

MH - *Antitubercular Agents/pharmacology/chemistry

MH - *Macrolides/pharmacology/chemistry

MH - Animals

MH - Mice

MH - *Tuberculosis, Multidrug-Resistant/drug therapy

MH - *Microbial Sensitivity Tests

MH - Humans

MH - Molecular Structure

PMC - PMC11701349

OTO - NOTNLM

OT - anti-tubercular mechanism

OT - multidrug resistance

OT - natural product

OT - structure determination

OT - tuberculosis

COIS- The authors submitted patent applications for arenicolides and their biological

activities.

EDAT- 2024/10/14 12:23

MHDA- 2025/01/07 04:51

PMCR- 2025/01/06

CRDT- 2024/10/14 11:25

PHST- 2024/07/10 00:00 [received]

PHST- 2025/01/07 04:51 [medline]

PHST- 2024/10/14 12:23 [pubmed]

PHST- 2024/10/14 11:25 [entrez]

PHST- 2025/01/06 00:00 [pmc-release]

AID - ANIE202412994 [pii]

AID - 10.1002/anie.202412994 [doi]

PST - ppublish

SO - Angew Chem Int Ed Engl. 2025 Jan 2;64(1):e202412994. doi: 10.1002/anie.202412994.

Epub 2024 Nov 13.

PMID- 40417764

OWN - NLM

STAT- MEDLINE

DCOM- 20260218

LR - 20260218

IS - 2212-3989 (Electronic)

IS - 1871-5265 (Linking)

VI - 25

IP - 7

DP - 2025

TI - Proteomic Analysis and Sequential Events During the in-vivo Acquisition of Drug

Resistance in Clinical Isolates of Mycobacterium tuberculosis.

PG - e18715265356091

LID - 10.2174/0118715265356091250519032548 [doi]

AB - AIM: This study was undertaken to compare the proteomic profile of sequential

isolates of Beijing lineage Mycobacterium tuberculosis (M. tuberculosis) from a

patient who developed drugresistant tuberculosis (TB) in vivo during

anti-tuberculosis therapy (ATT). INTRODUCTION: Various studies have found the

Beijing lineage of M. tuberculosis strongly associated with multidrug resistance

(MDR) development. OBJECTIVES: To identify and characterize the differentially

expressed proteins during the in-vivo drug resistance conversion in M.

tuberculosis Beijing lineage clinical isolates. METHODS: Drug-susceptible and

drug-resistant M. tuberculosis isolates were confirmed as Beijing lineage. The

isolates were grown in Middlebrook 7H9 medium for two weeks, and whole-cell

lysate was prepared. Two-dimensional gel electrophoresis (2DGE) was used for

proteomic analysis, and differentially expressed proteins were identified using

MALDI-TOF-MS. Bioinformatics tools were used for molecular docking,

phosphorylation, and pupylation site prediction. RESULTS: Seventeen proteins were

found overexpressed in drug-resistant isolates as compared to drugsusceptible

isolates, including the six proteins with unknown functions. Molecular docking

showed that Isoniazid (INH) and Rifampicin (RIF) interacted with their conserved

domains/active sites of these proteins. DISCUSSION: We characterized two paired

clinical isolates from a patient, one being INH and RIF susceptible and other

resistant. The comparative analysis of over expressed proteins showed that 5 of

17 proteins belonged to the cell wall and cell processes functional group, 3 to

virulence, detoxification, adaptation functional group, and 3 to information

pathways functional group, 2 proteins belonged to insertion sequences and phage

functional group, and 1 each (Rv0242c, Rv2970c and Rv3208A) to lipid metabolism,

intermediary metabolism & respiration and regulatory functional group. We found

that the Rv1827, Rv2626c, Rv2714, Rv2970c, Rv3208A, and Rv3881c proteins showed

significant interaction in-silico with INH and RIF. CONCLUSIONS: These

over-expressed proteins probably play an important role in drug resistance

development, and further studies on drug resistance mechanisms could provide more

details. We also believe that these over-expressed proteins could be used as

biomarkers for early prediction of in-vivo drug-resistance development.

CI - Copyright© Bentham Science Publishers; For any queries, please email at

epub@benthamscience.net.

FAU - Singh, Amit

AU - Singh A

AD - Department of Laboratory Medicine, Division of Clinical Microbiology and

Molecular Medicine, All India Institute of Medical Sciences, New Delhi, India.

AD - Department of Microbiology, Central University of Punjab, Bathinda, India.

FAU - Sharma, Divakar

AU - Sharma D

AD - Department of Biochemistry, National JALMA Institute for Leprosy and Other

Mycobacterial Diseases, Tajganj, Agra, 282004, India.

AD - Department of Biotechnology, Graphic Era Deemed to be University, Dehradun,

248002, India.

FAU - Gopinath, Krishnamoorthy

AU - Gopinath K

AD - Department of Laboratory Medicine, Division of Clinical Microbiology and

Molecular Medicine, All India Institute of Medical Sciences, New Delhi, India.

AD - Division of Pulmonary Inflammation, Charité-Universitätsmedizin Berlin, 10115

Berlin, Germany.

FAU - Gupta, Anil Kumar

AU - Gupta AK

AD - Department of Laboratory Medicine, Division of Clinical Microbiology and

Molecular Medicine, All India Institute of Medical Sciences, New Delhi, India.

AD - Department of Biotechnology, Graphic Era Deemed to be University, Dehradun,

248002, India.

FAU - Sharma, Prashant

AU - Sharma P

AD - Department of Microbiology, Central University of Punjab, Bathinda, India.

FAU - Bisht, Deepa

AU - Bisht D

AD - Department of Microbiology, Central University of Punjab, Bathinda, India.

FAU - Singh, Sarman

AU - Singh S

AUID- ORCID: 0000-0002-0749-9647

AD - Department of Laboratory Medicine, Division of Clinical Microbiology and

Molecular Medicine, All India Institute of Medical Sciences, New Delhi, India.

AD - Aarupadai Veedu Medical College & Hospital, Vinayaka Mission's Research

Foundation (Deemed University), Pondicherry, India.

LA - eng

PT - Journal Article

PL - United Arab Emirates

TA - Infect Disord Drug Targets

JT - Infectious disorders drug targets

JID - 101269158

RN - 0 (Antitubercular Agents)

RN - 0 (Bacterial Proteins)

RN - V83O1VOZ8L (Isoniazid)

RN - VJT6J7R4TR (Rifampin)

SB - IM

MH - *Mycobacterium tuberculosis/drug effects/isolation &

purification/metabolism/genetics

MH - Humans

MH - Proteomics

MH - *Antitubercular Agents/pharmacology/therapeutic use

MH - *Bacterial Proteins/metabolism/genetics/chemistry

MH - *Tuberculosis, Multidrug-Resistant/microbiology/drug therapy

MH - Isoniazid/pharmacology

MH - Rifampin/pharmacology

MH - Molecular Docking Simulation

MH - *Drug Resistance, Multiple, Bacterial

MH - Microbial Sensitivity Tests

MH - Electrophoresis, Gel, Two-Dimensional

MH - Spectrometry, Mass, Matrix-Assisted Laser Desorption-Ionization

OTO - NOTNLM

OT - 2D-gel electrophoresis

OT - Beijing genotype

OT - MDR-TB

OT - Proteomics

OT - docking.

OT - mass spectrometry

EDAT- 2025/05/26 12:37

MHDA- 2026/02/18 10:07

CRDT- 2025/05/26 06:18

PHST- 2024/10/09 00:00 [received]

PHST- 2025/03/23 00:00 [revised]

PHST- 2025/03/25 00:00 [accepted]

PHST- 2026/02/18 10:07 [medline]

PHST- 2025/05/26 12:37 [pubmed]

PHST- 2025/05/26 06:18 [entrez]

AID - IDDT-EPUB-148560 [pii]

AID - 10.2174/0118715265356091250519032548 [doi]

PST - ppublish

SO - Infect Disord Drug Targets. 2025;25(7):e18715265356091. doi:

10.2174/0118715265356091250519032548.

PMID- 39718911

OWN - NLM

STAT- PubMed-not-MEDLINE

LR - 20250205

IS - 0970-2113 (Print)

IS - 0974-598X (Electronic)

IS - 0970-2113 (Linking)

VI - 42

IP - 1

DP - 2025 Jan 1

TI - Evaluation of the Drug-Resistant Tuberculosis (DR-TB) management component under

the National Tuberculosis Elimination Program (NTEP) in Kerala, India, 2021-22.

PG - 16-24

LID - 10.4103/lungindia.lungindia_355_24 [doi]

AB - BACKGROUND AND OBJECTIVE: We evaluated the DR-TB component of the National

Tuberculosis Elimination Program (NTEP) in a high-burden district in Kerala to

identify the programmatic gaps, if any, in screening, diagnosis, treatment, and

follow-up of notified DR-TB patients. METHODS: A mixed-methods design was used,

and the evaluation was performed in two steps. In the first step, we reviewed the

program documents and conducted stakeholder interviews to develop a detailed

description of the program design and developed a logical framework to evaluate

program performance. Consequently, in the next step, we conducted programmatic

data reviews, facility surveys, and in-depth interviews with key stakeholders to

identify the programmatic gaps in implementation, guided by the logic framework.

RESULTS: Of the 494 microbiologically confirmed TB patients during 2021-22, 342

(69%) were tested for drug sensitivity, and 30 DR-TB patients were identified.

There was no separate district DR-TB treatment center with airborne infection

control facilities, and only 16% (66/422) of the various categories of staff were

trained in recent guidelines. Only 30% (9/30) of DR-TB patients were provided

with any psychological assessment. The favorable treatment outcome was 80%

Interviews revealed poor readiness and motivation from the private sector for

screening, contextual barriers in human resource availability, transportation,

and financial barriers to the beneficiary despite providing financial benefits.

CONCLUSION: Prioritizing the establishment of a district DR-TB treatment center

and sputum transport mechanism, posting a clinical psychologist dedicated to

counseling patients on therapy, and training all categories of staff on DR-TB

management guidelines will significantly contribute to improving program

outcomes.

CI - Copyright © 2024 Indian Chest Society.

FAU - Vaman, Raman Swathy

AU - Vaman RS

AD - Assistant Director of Health Services, District Hospital, Kanhangad, Kasaragod,

Kerala, India.

FAU - Kalyanasundaram, Madhanraj

AU - Kalyanasundaram M

AD - Scientist E, ICMR-National Institute of Epidemiology, Chennai, Tamil Nadu, India.

FAU - Mohan, Malu

AU - Mohan M

AD - Public Health Researcher, Women's Institute for Social and Health Science Studies

(WISHS), Kerala, India.

FAU - Pradeepa, Narayana

AU - Pradeepa N

AD - Junior Consultant-Respiratory Medicine, District TB Centre, Kasaragod, Kerala,

India.

FAU - Murhekar, Manoj V

AU - Murhekar MV

AD - Scientist G and Director, ICMR-National Institute of Epidemiology, Chennai, Tamil

Nadu, India.

LA - eng

PT - Journal Article

DEP - 20241224

PL - India

TA - Lung India

JT - Lung India : official organ of Indian Chest Society

JID - 8405380

PMC - PMC11789963

COIS- There are no conflicts of interest.

EDAT- 2024/12/24 18:20

MHDA- 2024/12/24 18:21

PMCR- 2025/01/01

CRDT- 2024/12/24 12:23

PHST- 2024/07/24 00:00 [received]

PHST- 2024/11/05 00:00 [accepted]

PHST- 2024/12/24 18:21 [medline]

PHST- 2024/12/24 18:20 [pubmed]

PHST- 2024/12/24 12:23 [entrez]

PHST- 2025/01/01 00:00 [pmc-release]

AID - 01408641-202501000-00004 [pii]

AID - LI-42-16 [pii]

AID - 10.4103/lungindia.lungindia_355_24 [doi]

PST - ppublish

SO - Lung India. 2025 Jan 1;42(1):16-24. doi: 10.4103/lungindia.lungindia_355_24. Epub

2024 Dec 24.

PMID- 39626090

OWN - NLM

STAT- MEDLINE

DCOM- 20250109

LR - 20260102

IS - 1365-2958 (Electronic)

IS - 0950-382X (Print)

IS - 0950-382X (Linking)

VI - 123

IP - 1

DP - 2025 Jan

TI - ClpS Directs Degradation of N-Degron Substrates With Primary Destabilizing

Residues in Mycolicibacterium smegmatis.

PG - 16-30

LID - 10.1111/mmi.15334 [doi]

AB - Drug-resistant tuberculosis infections are a major threat to global public

health. The essential mycobacterial ClpC1P1P2 protease has received attention as

a prospective target for novel antibacterial therapeutics. However, efforts to

probe its function in cells are constrained by our limited knowledge of its

physiological proteolytic repertoire. Here, we interrogate the role of

mycobacterial ClpS in directing N-degron pathway proteolysis by ClpC1P1P2 in

Mycolicibacterium smegmatis. Binding assays demonstrate that mycobacterial ClpS

binds canonical primary destabilizing residues (Leu, Phe, Tyr, Trp) with moderate

affinity. N-degron binding restricts the conformational flexibility of a loop

adjacent to the ClpS N-degron binding pocket and strengthens ClpS•ClpC1 binding

affinity ~30-fold, providing a mechanism for cells to prioritize N-degron

proteolysis when substrates are abundant. Proteolytic reporter assays in M.

smegmatis confirm degradation of substrates bearing primary N-degrons, but

suggest that secondary N-degrons are absent in mycobacteria. This work expands

our understanding of the mycobacterial N-degron pathway and identifies ClpS as a

critical component for substrate specificity, providing insights that may support

the development of improved Clp protease inhibitors.

CI - © 2024 John Wiley & Sons Ltd.

FAU - Presloid, Christopher J

AU - Presloid CJ

AD - Department of Biological Sciences, University of Delaware, Newark, Delaware, USA.

FAU - Jiang, Jialiu

AU - Jiang J

AD - Department of Chemistry & Biochemistry, University of Delaware, Newark, Delaware,

USA.

FAU - Kandel, Pratistha

AU - Kandel P

AD - Department of Biological Sciences, University of Delaware, Newark, Delaware, USA.

FAU - Anderson, Henry R

AU - Anderson HR

AD - Department of Chemistry & Biochemistry, University of Delaware, Newark, Delaware,

USA.

FAU - Beardslee, Patrick C

AU - Beardslee PC

AD - Department of Chemistry & Biochemistry, University of Delaware, Newark, Delaware,

USA.

FAU - Swayne, Thomas M

AU - Swayne TM

AD - Department of Chemistry & Biochemistry, University of Delaware, Newark, Delaware,

USA.

FAU - Schmitz, Karl R

AU - Schmitz KR

AUID- ORCID: 0000-0003-1411-7624

AD - Department of Biological Sciences, University of Delaware, Newark, Delaware, USA.

AD - Department of Chemistry & Biochemistry, University of Delaware, Newark, Delaware,

USA.

LA - eng

GR - T32GM133395/GM/NIGMS NIH HHS/United States

GR - T32 GM133395/GM/NIGMS NIH HHS/United States

GR - P20GM103446/GM/NIGMS NIH HHS/United States

GR - P20 GM103446/GM/NIGMS NIH HHS/United States

GR - 19A00938/University of Delaware Research Foundation/

GR - R01 AI171196/AI/NIAID NIH HHS/United States

GR - P20 GM104316/GM/NIGMS NIH HHS/United States

GR - R01AI171196/National Institute of Allergy and Infectious Diseases/

GR - S10 OD026896/OD/NIH HHS/United States

GR - P20GM104316/GM/NIGMS NIH HHS/United States

GR - S10OD26896/GM/NIGMS NIH HHS/United States

PT - Journal Article

PT - Research Support, N.I.H., Extramural

DEP - 20241203

PL - England

TA - Mol Microbiol

JT - Molecular microbiology

JID - 8712028

RN - 0 (Bacterial Proteins)

RN - EC 3.4.21.92 (Endopeptidase Clp)

SB - IM

MH - *Mycobacterium smegmatis/metabolism/genetics

MH - *Bacterial Proteins/metabolism/genetics

MH - *Proteolysis

MH - Endopeptidase Clp/metabolism

MH - Substrate Specificity

MH - Protein Binding

MH - Degrons

PMC - PMC11717620

MID - NIHMS2037769

OTO - NOTNLM

OT - N‐degron

OT - mycobacteria

OT - proteolysis

EDAT- 2024/12/03 18:22

MHDA- 2025/01/09 06:21

PMCR- 2026/01/01

CRDT- 2024/12/03 15:03

PHST- 2024/11/06 00:00 [revised]

PHST- 2024/08/01 00:00 [received]

PHST- 2024/11/13 00:00 [accepted]

PHST- 2025/01/09 06:21 [medline]

PHST- 2024/12/03 18:22 [pubmed]

PHST- 2024/12/03 15:03 [entrez]

PHST- 2026/01/01 00:00 [pmc-release]

AID - 10.1111/mmi.15334 [doi]

PST - ppublish

SO - Mol Microbiol. 2025 Jan;123(1):16-30. doi: 10.1111/mmi.15334. Epub 2024 Dec 3.

PMID- 39579511

OWN - NLM

STAT- MEDLINE

DCOM- 20250106

LR - 20260127

IS - 1873-281X (Electronic)

IS - 1472-9792 (Linking)

VI - 150

DP - 2025 Jan

TI - Identification of BMVC-8C3O as a novel Pks13 inhibitor with anti-tuberculosis

activity.

PG - 102579

LID - S1472-9792(24)00105-7 [pii]

LID - 10.1016/j.tube.2024.102579 [doi]

AB - Given the increasing prevalence of drug-resistant tuberculosis (TB), there is an

urgent demand in developing novel anti-TB medications with highly effective,

safe, and utilize innovative mechanisms of action. Blocking the mycolic acid

synthesis pathway is well-established to be a significant strategy in developing

anti-TB drugs, and Pks13 was identified as a crucial enzyme in this process.

Importantly, the modes of action of recognized Pks13 inhibitors differ from

traditional anti-TB medications, highlighting Pks13 as a potential and promising

target in drug development within TB treatment. In this study, we discovered a

compound named BMVC-8C3O that effectively inhibited the activity of Pks13 with a

6.94 μM IC(50) value. The binding between BMVC-8C3O and Pks13 was validated

through surface plasmon resonance (SPR) assay as well as molecular docking

analysis. Moreover, the SPR assay showed that the mutation of Asn1640 and Ser1533

resulted in decreased affinity of BMVC-8C3O to Pks13. Additionally, BMVC-8C3O not

only exhibited activity against Mycobacterium tuberculosis (MTB), but also

displayed potential intracellular anti-TB activity in macrophages. In summary,

our findings indicate that BMVC-8C3O holds great potential as a lead compound

against TB.

CI - Copyright © 2024 Elsevier Ltd. All rights reserved.

FAU - Liu, Tianjun

AU - Liu T

AD - State Key Laboratory of Bioactive Substances and Functions of Natural Medicines,

NHC Key Laboratory of Biotechnology for Microbial Drugs, National Center for New

Microbial Drug Screening, Institute of Medicinal Biotechnology, Chinese Academy

of Medical Sciences and Peking Union Medical College (CAMS & PUMC), Tiantan Xili

#1, Beijing, 100050, China.

FAU - Meng, Jianzhou

AU - Meng J

AD - National Clinical Research Center for Infectious Disease, Shenzhen Third People's

Hospital, The Second Affiliated Hospital, School of Medicine, Southern University

of Science and Technology, Bougibland Road #29, Shenzhen, 518112, China.

FAU - Wang, Bin

AU - Wang B

AD - Department of Pharmacology, Beijing Key Laboratory of Drug Resistance

Tuberculosis Research, Beijing Chest Hospital, Capital Medical University,

Beijing Tuberculosis and Thoracic Tumor Research Institute, 97 Ma Chang Street,

Beijing, 101149, China.

FAU - Li, Xiaohui

AU - Li X

AD - State Key Laboratory of Bioactive Substances and Functions of Natural Medicines,

NHC Key Laboratory of Biotechnology for Microbial Drugs, National Center for New

Microbial Drug Screening, Institute of Medicinal Biotechnology, Chinese Academy

of Medical Sciences and Peking Union Medical College (CAMS & PUMC), Tiantan Xili

#1, Beijing, 100050, China.

FAU - Wang, Qian

AU - Wang Q

AD - State Key Laboratory of Bioactive Substances and Functions of Natural Medicines,

NHC Key Laboratory of Biotechnology for Microbial Drugs, National Center for New

Microbial Drug Screening, Institute of Medicinal Biotechnology, Chinese Academy

of Medical Sciences and Peking Union Medical College (CAMS & PUMC), Tiantan Xili

#1, Beijing, 100050, China.

FAU - Liu, Sihan

AU - Liu S

AD - State Key Laboratory of Bioactive Substances and Functions of Natural Medicines,

NHC Key Laboratory of Biotechnology for Microbial Drugs, National Center for New

Microbial Drug Screening, Institute of Medicinal Biotechnology, Chinese Academy

of Medical Sciences and Peking Union Medical College (CAMS & PUMC), Tiantan Xili

#1, Beijing, 100050, China.

FAU - Guan, Yan

AU - Guan Y

AD - State Key Laboratory of Bioactive Substances and Functions of Natural Medicines,

NHC Key Laboratory of Biotechnology for Microbial Drugs, National Center for New

Microbial Drug Screening, Institute of Medicinal Biotechnology, Chinese Academy

of Medical Sciences and Peking Union Medical College (CAMS & PUMC), Tiantan Xili

#1, Beijing, 100050, China.

FAU - Wang, Xiao

AU - Wang X

AD - State Key Laboratory of Bioactive Substances and Functions of Natural Medicines,

NHC Key Laboratory of Biotechnology for Microbial Drugs, National Center for New

Microbial Drug Screening, Institute of Medicinal Biotechnology, Chinese Academy

of Medical Sciences and Peking Union Medical College (CAMS & PUMC), Tiantan Xili

#1, Beijing, 100050, China. Electronic address: wangxiao_1107@163.com.

FAU - Liu, Yishuang

AU - Liu Y

AD - State Key Laboratory of Bioactive Substances and Functions of Natural Medicines,

NHC Key Laboratory of Biotechnology for Microbial Drugs, National Center for New

Microbial Drug Screening, Institute of Medicinal Biotechnology, Chinese Academy

of Medical Sciences and Peking Union Medical College (CAMS & PUMC), Tiantan Xili

#1, Beijing, 100050, China. Electronic address: liuys@imb.pumc.edu.cn.

LA - eng

PT - Journal Article

DEP - 20241119

PL - Scotland

TA - Tuberculosis (Edinb)

JT - Tuberculosis (Edinburgh, Scotland)

JID - 100971555

RN - 0 (Antitubercular Agents)

RN - 0 (polyketide synthase Pks13, Mycobacterium tuberculosis)

RN - 0 (Bacterial Proteins)

RN - EC 2.3.- (Acyltransferases)

RN - 79956-01-7 (Polyketide Synthases)

SB - IM

MH - *Antitubercular Agents/pharmacology/chemistry

MH - *Mycobacterium tuberculosis/drug effects/enzymology/genetics

MH - *Molecular Docking Simulation

MH - Humans

MH - Bacterial Proteins/antagonists & inhibitors/metabolism/genetics

MH - Animals

MH - Microbial Sensitivity Tests

MH - Acyltransferases/antagonists & inhibitors/metabolism/genetics

MH - Macrophages/drug effects/metabolism/microbiology

MH - Mice

MH - Polyketide Synthases

OTO - NOTNLM

OT - Anti-TB drugs

OT - BMVC-8C3O

OT - Pks13 inhibitor

OT - Tuberculosis

COIS- Declaration of competing interest The authors declare that the research was

conducted in the absence of any commercial or financial relationships that could

be construed as a potential conflict of interest.

EDAT- 2024/11/24 00:44

MHDA- 2025/01/07 00:21

CRDT- 2024/11/23 18:04

PHST- 2024/07/18 00:00 [received]

PHST- 2024/11/13 00:00 [revised]

PHST- 2024/11/17 00:00 [accepted]

PHST- 2025/01/07 00:21 [medline]

PHST- 2024/11/24 00:44 [pubmed]

PHST- 2024/11/23 18:04 [entrez]

AID - S1472-9792(24)00105-7 [pii]

AID - 10.1016/j.tube.2024.102579 [doi]

PST - ppublish

SO - Tuberculosis (Edinb). 2025 Jan;150:102579. doi: 10.1016/j.tube.2024.102579. Epub

2024 Nov 19.

PMID- 39702827

OWN - NLM

STAT- MEDLINE

DCOM- 20241220

LR - 20260624

IS - 2399-3642 (Electronic)

IS - 2399-3642 (Linking)

VI - 7

IP - 1

DP - 2024 Dec 19

TI - Structural insights into the enzymatic breakdown of azomycin-derived antibiotics

by 2-nitroimdazole hydrolase (NnhA).

PG - 1676

LID - 10.1038/s42003-024-07336-6 [doi]

LID - 1676

AB - The antibiotic 2-nitroimidazole (2NI) or azomycin, used for treating

drug-resistant tuberculosis and imaging tumor hypoxia, requires activation by

bacterial nitroreductases for its antibiotic and cytotoxic effect. Mycobacterium

sp. JS330 produces 2-nitroimidazole nitrohydrolase (NnhA) that circumvents 2NI

activation, conferring 2NI resistance by hydrolysing it to nitrite and

imidazol-2-one (IM2O) instead. This study elucidates NnhA's structure, catalytic

mechanism, and evolutionary background within the guanidino-group modifying

enzyme (GME) superfamily, aided by a more soluble protein variant engineered

through directed evolution. Despite low sequence similarity and limited

occurrence in a few soil-dwelling mycobacteria and Actinomycetota, NnhA maintains

the α/β propeller fold characteristic of GME superfamily enzymes and forms an

unusual hexameric ring structure formed by a trimer of domain-swapped dimers. The

similarity of its active site to arginine deiminases (ADIs) and human

dimethylarginine dimethylaminohydrolases (DDAHs), along with molecular dynamics

simulations, suggests NnhA's catalytic mechanism resembles the hydrolysis

reactions of these related enzymes.

CI - © 2024. Crown.

FAU - Ahmed, F Hafna

AU - Ahmed FH

AUID- ORCID: 0000-0002-9153-2699

AD - Environment, CSIRO, Canberra, ACT, 2601, Australia. hafna.ahmed@csiro.au.

AD - Advanced Engineering Biology Future Science Platform, CSIRO, Canberra, ACT, 2601,

Australia. hafna.ahmed@csiro.au.

FAU - Liu, Jian-Wei

AU - Liu JW

AD - Environment, CSIRO, Canberra, ACT, 2601, Australia.

FAU - Royan, Santana

AU - Royan S

AD - Manufacturing, CSIRO, 343 Royal Parade, Parkville, VIC, 3052, Australia.

FAU - Warden, Andrew C

AU - Warden AC

AUID- ORCID: 0000-0003-2683-9798

AD - Environment, CSIRO, Canberra, ACT, 2601, Australia.

AD - Advanced Engineering Biology Future Science Platform, CSIRO, Canberra, ACT, 2601,

Australia.

FAU - Esquirol, Lygie

AU - Esquirol L

AUID- ORCID: 0000-0002-4887-3260

AD - Environment, CSIRO, Canberra, ACT, 2601, Australia.

AD - Advanced Engineering Biology Future Science Platform, CSIRO, Canberra, ACT, 2601,

Australia.

FAU - Pandey, Gunjan

AU - Pandey G

AUID- ORCID: 0000-0002-3514-4624

AD - Environment, CSIRO, Canberra, ACT, 2601, Australia.

FAU - Newman, Janet

AU - Newman J

AD - Manufacturing, CSIRO, 343 Royal Parade, Parkville, VIC, 3052, Australia.

AD - BABS, UNSW, Kensington, NSW, 2052, Australia.

FAU - Scott, Colin

AU - Scott C

AUID- ORCID: 0000-0001-6110-6982

AD - Environment, CSIRO, Canberra, ACT, 2601, Australia.

AD - Advanced Engineering Biology Future Science Platform, CSIRO, Canberra, ACT, 2601,

Australia.

AD - ARC Centre of Excellence in Synthetic Biology, CSIRO, Canberra, ACT, 2601,

Australia.

FAU - Peat, Thomas S

AU - Peat TS

AUID- ORCID: 0000-0002-6488-0831

AD - Manufacturing, CSIRO, 343 Royal Parade, Parkville, VIC, 3052, Australia.

t.peat@unsw.edu.au.

AD - BABS, UNSW, Kensington, NSW, 2052, Australia. t.peat@unsw.edu.au.

LA - eng

PT - Journal Article

DEP - 20241219

PL - England

TA - Commun Biol

JT - Communications biology

JID - 101719179

RN - EC 3.- (Hydrolases)

RN - 0 (Anti-Bacterial Agents)

RN - 0 (Nitroimidazoles)

RN - 0 (Bacterial Proteins)

SB - IM

MH - *Hydrolases/metabolism/chemistry

MH - *Anti-Bacterial Agents/metabolism/chemistry

MH - Nitroimidazoles/metabolism/chemistry

MH - Mycobacterium/enzymology

MH - Bacterial Proteins/metabolism/chemistry/genetics

MH - Humans

MH - Catalytic Domain

PMC - PMC11659421

COIS- Competing interests: The authors declare no competing interests.

EDAT- 2024/12/20 06:24

MHDA- 2024/12/20 06:25

PMCR- 2024/12/19

CRDT- 2024/12/20 00:56

PHST- 2024/04/17 00:00 [received]

PHST- 2024/11/29 00:00 [accepted]

PHST- 2024/12/20 06:25 [medline]

PHST- 2024/12/20 06:24 [pubmed]

PHST- 2024/12/20 00:56 [entrez]

PHST- 2024/12/19 00:00 [pmc-release]

AID - 10.1038/s42003-024-07336-6 [pii]

AID - 7336 [pii]

AID - 10.1038/s42003-024-07336-6 [doi]

PST - epublish

SO - Commun Biol. 2024 Dec 19;7(1):1676. doi: 10.1038/s42003-024-07336-6.

PMID- 39772057

OWN - NLM

STAT- PubMed-not-MEDLINE

LR - 20250115

IS - 2076-393X (Print)

IS - 2076-393X (Electronic)

IS - 2076-393X (Linking)

VI - 12

IP - 12

DP - 2024 Dec 12

TI - Challenges of Multidrug-Resistant Tuberculosis Meningitis: Current Treatments and

the Role of Glutathione as an Adjunct Therapy.

LID - 10.3390/vaccines12121397 [doi]

LID - 1397

AB - Multidrug-resistant tuberculosis (MDR-TB) poses a significant global health

threat, especially when it involves the central nervous system (CNS). Tuberculous

meningitis (TBM), a severe manifestation of TB, is linked to high mortality rates

and long-term neurological complications, further exacerbated by drug resistance

and immune evasion mechanisms employed by Mycobacterium tuberculosis (Mtb).

Although pulmonary TB remains the primary focus of research, MDR-TBM introduces

unique challenges in diagnosis, treatment, and patient outcomes. The

effectiveness of current treatments is frequently compromised by poor CNS

penetration of anti-TB drugs and the necessity for prolonged therapy, which often

involves considerable toxicity. This review explores the potential of

cytokine-based adjunct immunotherapies for MDR-TBM, addressing the challenges of

balancing pro-inflammatory and anti-inflammatory signals within the CNS. A

central focus is the prospective role of glutathione, not only in reducing

oxidative stress but also in enhancing host immune defenses against Mtb's immune

evasion strategies. Furthermore, the development of vaccines aimed at

upregulating glutathione synthesis in macrophages represents a promising strategy

to bolster the immune response and improve treatment outcomes. By integrating

glutathione and innovative vaccine approaches into MDR-TBM management, this

review proposes a comprehensive strategy that targets Mtb directly while

supporting immune modulation, with the potential to enhance patient outcomes and

reduce treatment related adverse effects. We underscore the urgent need for

further research into adjunctive therapies and immunomodulatory strategies to

more effectively combat MDR-TBM.

FAU - Nasiri, Mohammad J

AU - Nasiri MJ

AUID- ORCID: 0000-0002-3279-0671

AD - Department of Microbiology, School of Medicine, Shahid Beheshti University of

Medical Sciences, Tehran 19839-69411, Iran.

FAU - Lutfy, Kabir

AU - Lutfy K

AUID- ORCID: 0000-0003-0350-3693

AD - College of Pharmacy, Western University of Health Sciences, Pomona, CA

91766-1854, USA.

FAU - Venketaraman, Vishwanath

AU - Venketaraman V

AUID- ORCID: 0000-0002-2586-1160

AD - College of Osteopathic Medicine of the Pacific, Western University of Health

Sciences, Pomona, CA 91766-1854, USA.

LA - eng

GR - R15 HL143545/HL/NHLBI NIH HHS/United States

GR - 2R15HL143545-02/NIH-NHLBI/

PT - Journal Article

PT - Review

DEP - 20241212

PL - Switzerland

TA - Vaccines (Basel)

JT - Vaccines

JID - 101629355

PMC - PMC11728556

OTO - NOTNLM

OT - adjunct therapy

OT - antioxidants

OT - central nervous system

OT - cytokines

OT - glutathione

OT - immune modulation

OT - immune response

OT - multidrug-resistant tuberculosis

OT - mycobacterium tuberculosis

OT - oxidative stress

OT - therapeutic strategies

OT - tuberculosis

OT - tuberculosis meningitis

OT - vaccines

COIS- The authors declare no conflicts of interest. The funders had no role in the

design of the study; in the collection, analyses, or interpretation of the data;

in the writing of the manuscript; or in the decision to publish the results.

EDAT- 2025/01/08 06:20

MHDA- 2025/01/08 06:21

PMCR- 2024/12/12

CRDT- 2025/01/08 01:41

PHST- 2024/11/07 00:00 [received]

PHST- 2024/12/02 00:00 [revised]

PHST- 2024/12/10 00:00 [accepted]

PHST- 2025/01/08 06:21 [medline]

PHST- 2025/01/08 06:20 [pubmed]

PHST- 2025/01/08 01:41 [entrez]

PHST- 2024/12/12 00:00 [pmc-release]

AID - vaccines12121397 [pii]

AID - vaccines-12-01397 [pii]

AID - 10.3390/vaccines12121397 [doi]

PST - epublish

SO - Vaccines (Basel). 2024 Dec 12;12(12):1397. doi: 10.3390/vaccines12121397.

PMID- 39593272

OWN - NLM

STAT- MEDLINE

DCOM- 20241211

LR - 20241213

IS - 1098-2825 (Electronic)

IS - 0887-8013 (Print)

IS - 0887-8013 (Linking)

VI - 38

IP - 23

DP - 2024 Dec

TI - Mycobacterium tuberculosis: The Mechanism of Pathogenicity, Immune Responses, and

Diagnostic Challenges.

PG - e25122

LID - 10.1002/jcla.25122 [doi]

LID - e25122

AB - BACKGROUND: The infection caused by Mycobacterium tuberculosis arises from a

complex interplay between the host immune system and the bacteria. Early and

effective treatment of this disease is of great importance in order to prevent

the emergence of drug-resistant strains. This necessitates the availability of

fast and reliable diagnostic methods for managing affected cases. One reason why

this study is significant is the lack of a comprehensive review in this field

that thoroughly examines the importance, pathogenesis, and diagnosis of M.

tuberculosis. Therefore, the aim of this review is to provide updated information

on M. tuberculosis. METHODS: We investigate the virulence factors, pathogenicity,

and diagnostic methods of this bacterium, alongside the clinical symptoms and

interpretation of different types of tuberculosis, including cerebral, miliary,

nerve, and tubercular tuberculosis. RESULTS: Mycobacterium tuberculosis acts as

the causative agent of human tuberculosis and is regarded as one of the most

adaptable human pathogens. M. tuberculosis possesses several virulence factors

that help the bacterium evade mucous barriers. The rise of multidrug-resistant

tuberculosis (MDR-TB) in both developing and industrialized countries emphasizes

the need for rapid diagnostic methods. CONCLUSIONS: Non-protein virulence factors

play a crucial role in the pathogenicity of Mycobacterium tuberculosis (M.

tuberculosis). The bacterial cell membrane contains proteins that modulate the

host immune response. For instance, ESAT-6, either alone or in combination with

CFP-10, reduces immune activity. While molecular techniques-such as DNA

microarray, luciferase reporter assay, polymerase chain reaction (PCR), DNA and

RNA probes, next-generation sequencing, and whole-genome sequencing-offer rapid,

sensitive, and specific detection of M. tuberculosis, these methods are expensive

and require technical expertise.

CI - © 2024 The Author(s). Journal of Clinical Laboratory Analysis published by Wiley

Periodicals LLC.

FAU - Mohammadnabi, Noura

AU - Mohammadnabi N

AD - Student Research Committee, Khomein University of Medical Sciences, Khomein,

Iran.

FAU - Shamseddin, Jebreil

AU - Shamseddin J

AD - Infectious and Tropical Diseases Research Center, Hormozgan Health Institute,

Hormozgan University of Medical Sciences, Bandar Abbas, Iran.

FAU - Emadi, Mobina

AU - Emadi M

AD - Student Research Committee, Khomein University of Medical Sciences, Khomein,

Iran.

FAU - Bodaghi, Ali Bayat

AU - Bodaghi AB

AD - Student Research Committee, Khomein University of Medical Sciences, Khomein,

Iran.

FAU - Varseh, Mahdieh

AU - Varseh M

AD - Student Research Committee, Khomein University of Medical Sciences, Khomein,

Iran.

FAU - Shariati, Aref

AU - Shariati A

AD - Infectious Diseases Research Center (IDRC), Arak University of Medical Sciences,

Arak, Iran.

FAU - Rezaei, Mina

AU - Rezaei M

AD - School of Environment, College of Engineering, University of Tehran, Tehran,

Iran.

FAU - Dastranj, Mahsa

AU - Dastranj M

AD - Microbiology Department, Kurdistan Science and Research Branch, Islamic Azad

University, Sanandaj, Iran.

FAU - Farahani, Abbas

AU - Farahani A

AUID- ORCID: 0000-0002-0245-2612

AD - Molecular Medicine Research Center, Khomein University of Medical Sciences,

Khomein, Iran.

AD - Department of Basic Sciences, Khomein University of Medical Sciences, Khomein,

Iran.

LA - eng

PT - Journal Article

PT - Review

DEP - 20241126

PL - United States

TA - J Clin Lab Anal

JT - Journal of clinical laboratory analysis

JID - 8801384

RN - 0 (Virulence Factors)

SB - IM

MH - Humans

MH - *Mycobacterium tuberculosis/pathogenicity/immunology

MH - *Tuberculosis/immunology/microbiology/diagnosis

MH - Virulence Factors/genetics

PMC - PMC11632860

OTO - NOTNLM

OT - Mycobacterium tuberculosis

OT - diagnostic method

OT - immune response

OT - pathogenicity

COIS- The authors declare no conflicts of interest.

EDAT- 2024/11/27 06:20

MHDA- 2024/12/11 12:35

PMCR- 2024/11/26

CRDT- 2024/11/27 00:53

PHST- 2024/09/27 00:00 [revised]

PHST- 2024/03/20 00:00 [received]

PHST- 2024/10/30 00:00 [accepted]

PHST- 2024/12/11 12:35 [medline]

PHST- 2024/11/27 06:20 [pubmed]

PHST- 2024/11/27 00:53 [entrez]

PHST- 2024/11/26 00:00 [pmc-release]

AID - JCLA25122 [pii]

AID - 10.1002/jcla.25122 [doi]

PST - ppublish

SO - J Clin Lab Anal. 2024 Dec;38(23):e25122. doi: 10.1002/jcla.25122. Epub 2024 Nov

26.

PMID- 39528171

OWN - NLM

STAT- MEDLINE

DCOM- 20241217

LR - 20241217

IS - 1879-0003 (Electronic)

IS - 0141-8130 (Linking)

VI - 283

IP - Pt 2

DP - 2024 Dec

TI - Treatment of Mycobacterium tuberculosis infected macrophages with Rifabutin

loaded β-glucan microparticles induces macroautophagy mediated bacillary killing.

PG - 137256

LID - S0141-8130(24)08065-6 [pii]

LID - 10.1016/j.ijbiomac.2024.137256 [doi]

AB - Tuberculosis (TB), attributable to Mycobacterium tuberculosis (M.tb.),

constitutes a formidable global health challenge, particularly with the

proliferation of multidrug-resistant (MDR-TB) strains. The efficacious clearance

of M.tb. from host cells is imperative for mitigating infection and averting

disease progression. Autophagy, an intricate cellular mechanism for degrading and

recycling biomolecules, plays a pivotal role in the immune response to M.tb. by

facilitating the degradation of intracellular pathogen through the formation of

autophagosomes and their subsequent fusion with lysosomes. The present study

elucidates the therapeutic efficacy of Rifabutin loaded YDGP (DYDGP)

microparticles within M.tb.infected macrophage. Our results show that the

administration of DYDGP improve the membrane integrity of macrophage infected

with H37Rv as well as MDR strains, as compared to that of untreated controls at

30 min, 6 h and 24 h post-exposure time points. DCFHDA staining elucidated that

DYDGP treatment significantly enhances intracellular reactive oxygen species

(ROS) production compared to blank YDGP, even in the presence of NOX-2

inhibitors. Furthermore, DYDGP promotes the biogenesis of acidic vesicular

organelles and phago-lysosomal maturation, as corroborated by acridine orange and

Lysotracker Red staining. Immunofluorescence and dansylcadaverine dual staining

data evidenced that DYDGP treatment enhances autophagosome formation, autophagy

induction and LC3 puncta formation within M.tb. infected macrophage at both

30 min and 24 h post-exposure time points. Further, protein expression analyses

demonstrated that DYDGP treatment enhances the expression levels of NOX-2 and

LC3, thereby confirming autophagy induction within M.tb. infected macrophage.

Antimycobacterial efficacy assessments revealed that DYDGP treatment engendered

significant reductions in colony-forming units (CFUs) of H37Rv (64, 40, 19),

MDR32420 (44, 35, 18), MDR32422 (44, 39, 21), and MDR32521 (38, 22, 18) after

30 min, 24 h, and 48 h, exposure respectively. These findings accentuate DYDGP's

potential to substantially attenuate M.tb. burden, including the addressal of MDR

strains, thereby positioning it as a promising adjunctive therapy for augmenting

TB treatment.

CI - Copyright © 2024 The Authors. Published by Elsevier B.V. All rights reserved.

FAU - Ahmad, Firoz

AU - Ahmad F

AD - Department of Biosciences, Integral University, Lucknow 226026, U.P., India;

Department of Clinical Immunology & Rheumatology, Sanjay Gandhi Post Graduate

Institute of Medical Sciences, Lucknow 226014, U.P., India; Department of

Physiological Sciences, Oklahoma Centre for Respiratory and Infectious Diseases,

Oklahoma State University, Stillwater, OK 74078, United States of America.

FAU - Fatima, Nida

AU - Fatima N

AD - Department of Biosciences, Integral University, Lucknow 226026, U.P., India.

FAU - Ahmad, Shad

AU - Ahmad S

AD - Department of Biochemistry, Dr. Ram Manohar Lohia Avadh University, Faizabad

224001, U.P., India.

FAU - Upadhyay, Tarun Kumar

AU - Upadhyay TK

AD - Department of Biotechnology, Parul Institute of Applied Sciences and Research and

Development Cell, Parul University, Vadodara 391760, Gujarat, India.

FAU - Jain, Parul

AU - Jain P

AD - Department of Microbiology, King George Medical University, Lucknow 226003, U.P.,

India.

FAU - Saeed, Mohd

AU - Saeed M

AD - Department of Biology, College of Sciences, University of Hail, Hail 34464, Saudi

Arabia.

FAU - Ahmad, Irfan

AU - Ahmad I

AD - Department of Clinical Laboratory Sciences, College of Applied Medical Science,

King Khalid University, Abha, Saudi Arabia.

FAU - Al-Keridis, Lamya Ahmed

AU - Al-Keridis LA

AD - Department of Biology, Faculty of Science, Princess Nourah Bint Abdulrahman

University, P.O.Box 84428, Riyadh 11671, Saudi Arabia.

FAU - Khubaib, Mohd

AU - Khubaib M

AD - Department of Biosciences, Integral University, Lucknow 226026, U.P., India.

FAU - Sharma, Rolee

AU - Sharma R

AD - Department of Life Sciences & Biotechnology, CSJM University, Kanpur 228024,

U.P., India. Electronic address: roleesh@gmail.com.

LA - eng

PT - Journal Article

DEP - 20241109

PL - Netherlands

TA - Int J Biol Macromol

JT - International journal of biological macromolecules

JID - 7909578

RN - 1W306TDA6S (Rifabutin)

RN - 0 (Reactive Oxygen Species)

SB - IM

MH - *Mycobacterium tuberculosis/drug effects

MH - *Macrophages/drug effects/metabolism/microbiology

MH - *Rifabutin/pharmacology

MH - *Reactive Oxygen Species/metabolism

MH - Macroautophagy/drug effects

MH - Animals

MH - Mice

MH - Humans

MH - Tuberculosis/drug therapy/microbiology

MH - Autophagy/drug effects

MH - Lysosomes/metabolism/drug effects

MH - RAW 264.7 Cells

OTO - NOTNLM

OT - Autophagy

OT - Macrophage cells

OT - Mycobacterium tuberculosis

OT - β-Glucan

COIS- Declaration of competing interest The authors declare no conflict of interest.

EDAT- 2024/11/13 13:53

MHDA- 2024/12/17 11:48

CRDT- 2024/11/11 19:23

PHST- 2024/08/12 00:00 [received]

PHST- 2024/10/29 00:00 [revised]

PHST- 2024/11/02 00:00 [accepted]

PHST- 2024/12/17 11:48 [medline]

PHST- 2024/11/13 13:53 [pubmed]

PHST- 2024/11/11 19:23 [entrez]

AID - S0141-8130(24)08065-6 [pii]

AID - 10.1016/j.ijbiomac.2024.137256 [doi]

PST - ppublish

SO - Int J Biol Macromol. 2024 Dec;283(Pt 2):137256. doi:

10.1016/j.ijbiomac.2024.137256. Epub 2024 Nov 9.

PMID- 39373613

OWN - NLM

STAT- MEDLINE

DCOM- 20250109

LR - 20251008

IS - 1757-6199 (Electronic)

IS - 1757-6180 (Print)

IS - 1757-6180 (Linking)

VI - 16

IP - 23-24

DP - 2024 Dec-Dec

TI - Quantification of MDR-TB drug JBD0131 and its metabolite in plasma via

UPLC-MS/MS: application in first-in-human study.

PG - 1229-1240

LID - 10.1080/17576180.2024.2404311 [doi]

AB - Aim: JBD0131, a novel anti-multidrug-resistant tuberculosis (MDR-TB) drug, can

target and inhibit the synthesis of mycolic acids, which are crucial components

of the cell wall of the Mycobacterium tuberculosis complex. To support the

results of this clinical trial in healthy subjects, development of a specific and

accurate quantification method for detecting JBD0131 and its metabolite DM131 in

human plasma is needed.Materials & methods: Samples with prior added stabilizer

were pretreated by protein precipitation method and the extracts were subjected

to ultra-performance liquid chromatography-tandem mass spectrometry (UPLC-MS/MS).

The m/z transitions for the precursor/product ion pairs were 402.1/273 for

JBD0131, 333.1/273 for DM131 and 386.1/257 for the internal standard

(IS).Results: This method showed good linearity from 1 to 2000 ng/ml for JBD0131

and 0.25 to 500 ng/ml for DM131 and was validated in terms of selectivity,

linearity, accuracy, precision, matrix effect, recovery of pretreament and

stability.Conclusion: This method was sensitive and specific for measuring the

plasma concentrations of JBD0131 and its metabolites. And it was applied for the

investigation of the pharmacokinetics of JBD0131 and DM131 in a clinical trial.

FAU - Gao, Tiantao

AU - Gao T

AD - Department of Pharmacy, West China Hospital, Sichuan University, Chengdu, 610041,

China.

AD - NMPA Key Laboratory for Clinical Research and Evaluation of Innovative Drug,

Clinical Trial Center, West China Hospital, Sichuan University, Chengdu, 610041,

China.

FAU - Ou, Xiaoxue

AU - Ou X

AD - Department of Pharmacy, West China Hospital, Sichuan University, Chengdu, 610041,

China.

AD - NMPA Key Laboratory for Clinical Research and Evaluation of Innovative Drug,

Clinical Trial Center, West China Hospital, Sichuan University, Chengdu, 610041,

China.

FAU - Miao, Jia

AU - Miao J

AD - NMPA Key Laboratory for Clinical Research and Evaluation of Innovative Drug,

Clinical Trial Center, West China Hospital, Sichuan University, Chengdu, 610041,

China.

FAU - Qin, Yongping

AU - Qin Y

AD - Department of Pharmacy, West China Hospital, Sichuan University, Chengdu, 610041,

China.

AD - NMPA Key Laboratory for Clinical Research and Evaluation of Innovative Drug,

Clinical Trial Center, West China Hospital, Sichuan University, Chengdu, 610041,

China.

LA - eng

PT - Journal Article

DEP - 20241007

PL - England

TA - Bioanalysis

JT - Bioanalysis

JID - 101512484

RN - 0 (Antitubercular Agents)

SB - IM

MH - Humans

MH - *Tandem Mass Spectrometry/methods

MH - Chromatography, High Pressure Liquid/methods

MH - *Antitubercular Agents/blood

MH - *Tuberculosis, Multidrug-Resistant/drug therapy/blood

MH - Liquid Chromatography-Mass Spectrometry

PMC - PMC11702988

OAB - [Box: see text].

OABL- eng

OTO - NOTNLM

OT - JBD0131 and metabolite

OT - UPLC-MS/MS

OT - multidrug-resistant tuberculosis

OT - pharmacokinetics

OT - stabilizer

COIS- The authors have no relevant affiliations or financial involvement with any

organization or entity with a financial interest in or financial conflict with

the subject matter or materials discussed in the manuscript. This includes

employment, consultancies, honoraria, stock ownership or options, expert

testimony, grants or patents received or pending, or royalties.

EDAT- 2024/10/07 12:58

MHDA- 2025/01/09 06:22

PMCR- 2025/10/07

CRDT- 2024/10/07 09:34

PHST- 2025/01/09 06:22 [medline]

PHST- 2024/10/07 12:58 [pubmed]

PHST- 2024/10/07 09:34 [entrez]

PHST- 2025/10/07 00:00 [pmc-release]

AID - 2404311 [pii]

AID - 10.1080/17576180.2024.2404311 [doi]

PST - ppublish

SO - Bioanalysis. 2024 Dec-Dec;16(23-24):1229-1240. doi:

10.1080/17576180.2024.2404311. Epub 2024 Oct 7.

PMID- 39667968

OWN - NLM

STAT- MEDLINE

DCOM- 20241212

LR - 20241218

IS - 2214-0190 (Electronic)

IS - 0895-3988 (Linking)

VI - 37

IP - 11

DP - 2024 Nov 20

TI - Therapeutic Strategies for Tuberculosis: Progress and Lessons Learned.

PG - 1310-1323

LID - 10.3967/bes2024.168 [doi]

AB - Tuberculosis (TB) remains a significant global health challenge, ranking second

only to COVID-19 as the leading cause of death from a single infectious agent,

with 1.3 million TB-related deaths reported in 2022. Treatment efficacy has been

compromised by the emergence of drug-resistant strains, including

rifampin-resistant TB (RR-TB), multidrug-resistant TB (MDR-TB), and extensively

drug-resistant TB (XDR-TB). Although first-line drugs like isoniazid, rifampicin,

pyrazinamide, and ethambutol form the cornerstone of TB therapy, the rise of

resistant strains necessitates the use of second-line drugs, which often come

with increased toxicity and limited accessibility. Recent advances have focused

on repurposing existing compounds and developing new drugs with novel mechanisms

of action. Promising agents such as second-generation bedaquiline analogs

(TBAJ-587, TBAJ-876), sudapyridine (WX-081), delamanid, pretomanid, and TBI-166

(pyrifazimine) have shown efficacy against resistant Mtb strains. Innovative

treatment regimens like the BPaLM protocol-combining bedaquiline, pretomanid,

linezolid, and moxifloxacin-offer shorter, all-oral therapies with higher cure

rates. Personalized treatment durations and dose optimizations are becoming

feasible through risk stratification algorithms and

pharmacokinetic/pharmacodynamic studies. Immunotherapy is emerging as a

complementary strategy to enhance the host's immune response against Mtb. Agents

such as vitamin D, corticosteroids, non-steroidal anti-inflammatory drugs

(NSAIDs), statins, metformin, and biological agents like interleukins and

granulocyte-macrophage colony-stimulating factor are under exploration.

Additionally, cell therapies involving mesenchymal stem cells and immune effector

cells present new therapeutic avenues. Despite these advancements, significant

challenges remain in achieving the World Health Organization's "End TB Strategy"

goals, particularly as the COVID-19 pandemic has diverted resources and

attention. Ongoing research and global collaboration are crucial to develop novel

therapeutic strategies, optimize treatment regimens, and ultimately reduce the

global burden of TB.

CI - Copyright © 2024 The Editorial Board of Biomedical and Environmental Sciences.

Published by China CDC. All rights reserved.

FAU - Sun, Qingfeng

AU - Sun Q

AD - Department of Bacteriology and Immunology, Beijing Chest Hospital, Capital

Medical University/Beijing Tuberculosis and Thoracic Tumor Research Institute,

Beijing 101149, China;The Second Department of Tuberculosis, Beijing Chest

Hospital, Capital Medical University/Beijing Tuberculosis and Thoracic Tumor

Research Institute, Beijing 101149, China.

FAU - Li, Shanshan

AU - Li S

AD - Department of Bacteriology and Immunology, Beijing Chest Hospital, Capital

Medical University/Beijing Tuberculosis and Thoracic Tumor Research Institute,

Beijing 101149, China.

FAU - Gao, Mengqiu

AU - Gao M

AD - The Second Department of Tuberculosis, Beijing Chest Hospital, Capital Medical

University/Beijing Tuberculosis and Thoracic Tumor Research Institute, Beijing

101149, China.

FAU - Pang, Yu

AU - Pang Y

AD - Department of Bacteriology and Immunology, Beijing Chest Hospital, Capital

Medical University/Beijing Tuberculosis and Thoracic Tumor Research Institute,

Beijing 101149, China.

LA - eng

PT - Journal Article

PT - Review

PL - China

TA - Biomed Environ Sci

JT - Biomedical and environmental sciences : BES

JID - 8909524

RN - 0 (Antitubercular Agents)

SB - IM

MH - Humans

MH - *Antitubercular Agents/pharmacology/therapeutic use

MH - COVID-19/epidemiology

MH - *Tuberculosis/drug therapy/epidemiology

EDAT- 2024/12/13 00:23

MHDA- 2024/12/13 00:24

CRDT- 2024/12/12 22:13

PHST- 2024/06/03 00:00 [received]

PHST- 2024/10/12 00:00 [accepted]

PHST- 2024/12/13 00:24 [medline]

PHST- 2024/12/13 00:23 [pubmed]

PHST- 2024/12/12 22:13 [entrez]

AID - 10.3967/bes2024.168 [doi]

PST - ppublish

SO - Biomed Environ Sci. 2024 Nov 20;37(11):1310-1323. doi: 10.3967/bes2024.168.

PMID- 39537610

OWN - NLM

STAT- MEDLINE

DCOM- 20250311

LR - 20250709

IS - 2041-1723 (Electronic)

IS - 2041-1723 (Linking)

VI - 15

IP - 1

DP - 2024 Nov 13

TI - Catalase activity deficiency sensitizes multidrug-resistant Mycobacterium

tuberculosis to the ATP synthase inhibitor bedaquiline.

PG - 9792

LID - 10.1038/s41467-024-53933-8 [doi]

LID - 9792

AB - Multidrug-resistant tuberculosis (MDR-TB), defined as resistance to the

first-line drugs isoniazid and rifampin, is a growing source of global mortality

and threatens global control of tuberculosis disease. The diarylquinoline

bedaquiline has recently emerged as a highly efficacious drug against MDR-TB and

kills Mycobacterium tuberculosis by inhibiting mycobacterial ATP synthase.

However, the mechanisms underlying bedaquiline's efficacy against MDR-TB remain

unknown. Here we investigate bedaquiline hyper-susceptibility in drug-resistant

Mycobacterium tuberculosis using systems biology approaches. We discovered that

MDR clinical isolates are commonly sensitized to bedaquiline. This

hypersensitization is caused by several physiological changes induced by

deficient catalase activity. These include enhanced accumulation of reactive

oxygen species, increased susceptibility to DNA damage, induction of sensitizing

transcriptional programs, and metabolic repression of several biosynthetic

pathways. In this work we demonstrate how resistance-associated changes in

bacterial physiology can mechanistically induce collateral antimicrobial drug

sensitivity and reveal druggable vulnerabilities in antimicrobial resistant

pathogens.

CI - © 2024. The Author(s).

FAU - Ofori-Anyinam, Boatema

AU - Ofori-Anyinam B

AD - Ruy V. Lourenço Center for Emerging and Re-Emerging Pathogens, Rutgers New Jersey

Medical School, Newark, NJ, 07103, USA.

AD - Department of Microbiology, Biochemistry, and Molecular Genetics, Rutgers New

Jersey Medical School, Newark, NJ, 07103, USA.

FAU - Hamblin, Meagan

AU - Hamblin M

AD - Infectious Disease and Microbiome Program, Broad Institute of MIT and Harvard,

Cambridge, MA, 02142, USA.

AD - Eversana Consulting, Boston, MA, 02120, USA.

FAU - Coldren, Miranda L

AU - Coldren ML

AD - Center for Global Infectious Disease Research, Seattle Children's Research

Institute, Seattle, WA, 98105, USA.

FAU - Li, Barry

AU - Li B

AUID- ORCID: 0000-0003-1102-561X

AD - Ruy V. Lourenço Center for Emerging and Re-Emerging Pathogens, Rutgers New Jersey

Medical School, Newark, NJ, 07103, USA.

AD - Department of Microbiology, Biochemistry, and Molecular Genetics, Rutgers New

Jersey Medical School, Newark, NJ, 07103, USA.

FAU - Mereddy, Gautam

AU - Mereddy G

AD - Ruy V. Lourenço Center for Emerging and Re-Emerging Pathogens, Rutgers New Jersey

Medical School, Newark, NJ, 07103, USA.

AD - Department of Microbiology, Biochemistry, and Molecular Genetics, Rutgers New

Jersey Medical School, Newark, NJ, 07103, USA.

FAU - Shaikh, Mustafa

AU - Shaikh M

AD - Ruy V. Lourenço Center for Emerging and Re-Emerging Pathogens, Rutgers New Jersey

Medical School, Newark, NJ, 07103, USA.

AD - Department of Microbiology, Biochemistry, and Molecular Genetics, Rutgers New

Jersey Medical School, Newark, NJ, 07103, USA.

FAU - Shah, Avi

AU - Shah A

AD - Ruy V. Lourenço Center for Emerging and Re-Emerging Pathogens, Rutgers New Jersey

Medical School, Newark, NJ, 07103, USA.

AD - Department of Microbiology, Biochemistry, and Molecular Genetics, Rutgers New

Jersey Medical School, Newark, NJ, 07103, USA.

FAU - Grady, Courtney

AU - Grady C

AD - Ruy V. Lourenço Center for Emerging and Re-Emerging Pathogens, Rutgers New Jersey

Medical School, Newark, NJ, 07103, USA.

AD - Public Health Research Institute, Rutgers New Jersey Medical School, Newark, NJ,

07103, USA.

FAU - Ranu, Navpreet

AU - Ranu N

AD - Infectious Disease and Microbiome Program, Broad Institute of MIT and Harvard,

Cambridge, MA, 02142, USA.

AD - Department of Biological Engineering, Massachusetts Institute of Technology,

Cambridge, MA, 02139, USA.

AD - insitro, South San Francisco, CA, 94080, USA.

FAU - Lu, Sean

AU - Lu S

AD - Ruy V. Lourenço Center for Emerging and Re-Emerging Pathogens, Rutgers New Jersey

Medical School, Newark, NJ, 07103, USA.

AD - Department of Microbiology, Biochemistry, and Molecular Genetics, Rutgers New

Jersey Medical School, Newark, NJ, 07103, USA.

FAU - Blainey, Paul C

AU - Blainey PC

AUID- ORCID: 0000-0002-4889-8783

AD - Infectious Disease and Microbiome Program, Broad Institute of MIT and Harvard,

Cambridge, MA, 02142, USA.

AD - Department of Biological Engineering, Massachusetts Institute of Technology,

Cambridge, MA, 02139, USA.

AD - Koch Institute of Integrative Cancer Research at MIT, Cambridge, MA, 02139, USA.

FAU - Ma, Shuyi

AU - Ma S

AUID- ORCID: 0000-0003-0783-1328

AD - Center for Global Infectious Disease Research, Seattle Children's Research

Institute, Seattle, WA, 98105, USA.

AD - Department of Pediatrics, University of Washington, Seattle, WA, 98195, USA.

AD - Department of Chemical Engineering, University of Washington, Seattle, WA, 98195,

USA.

AD - Pathobiology Graduate Program, Department of Global Health, University of

Washington, Seattle, WA, 98195, USA.

FAU - Collins, James J

AU - Collins JJ

AUID- ORCID: 0000-0002-5560-8246

AD - Infectious Disease and Microbiome Program, Broad Institute of MIT and Harvard,

Cambridge, MA, 02142, USA.

AD - Department of Biological Engineering, Massachusetts Institute of Technology,

Cambridge, MA, 02139, USA.

AD - Institute for Medical Engineering and Science, Massachusetts Institute of

Technology, Cambridge, MA, 02139, USA.

FAU - Yang, Jason H

AU - Yang JH

AUID- ORCID: 0000-0003-0921-4657

AD - Ruy V. Lourenço Center for Emerging and Re-Emerging Pathogens, Rutgers New Jersey

Medical School, Newark, NJ, 07103, USA. jason.y@rutgers.edu.

AD - Department of Microbiology, Biochemistry, and Molecular Genetics, Rutgers New

Jersey Medical School, Newark, NJ, 07103, USA. jason.y@rutgers.edu.

LA - eng

SI - BioProject/PRJNA1139169

GR - U19 AI162598/AI/NIAID NIH HHS/United States

GR - R00 GM118907/GM/NIGMS NIH HHS/United States

GR - HDTRA12210032/United States Department of Defense | Defense Threat Reduction

Agency (DTRA)/

GR - RM1-HG006193/U.S. Department of Health &amp; Human Services | National Institutes

of Health (NIH)/

GR - U19-AI62598/U.S. Department of Health &amp; Human Services | National Institutes

of Health (NIH)/

GR - U19-AI11276/U.S. Department of Health &amp; Human Services | National Institutes

of Health (NIH)/

GR - U19-AI62589/U.S. Department of Health &amp; Human Services | National Institutes

of Health (NIH)/

GR - R01-AI146194/U.S. Department of Health &amp; Human Services | National Institutes

of Health (NIH)/

GR - U24 AI118668/AI/NIAID NIH HHS/United States

GR - R21 AI121932/AI/NIAID NIH HHS/United States

GR - R01 AI146194/AI/NIAID NIH HHS/United States

GR - DP2 AI164249/AI/NIAID NIH HHS/United States

GR - R00-GM118907/U.S. Department of Health &amp; Human Services | National Institutes

of Health (NIH)/

GR - RM1 HG006193/HG/NHGRI NIH HHS/United States

GR - R21-AI121932/U.S. Department of Health &amp; Human Services | National Institutes

of Health (NIH)/

PT - Journal Article

PT - Research Support, N.I.H., Extramural

PT - Research Support, Non-U.S. Gov't

PT - Research Support, U.S. Gov't, Non-P.H.S.

DEP - 20241113

PL - England

TA - Nat Commun

JT - Nature communications

JID - 101528555

RN - 78846I289Y (bedaquiline)

RN - 0 (Antitubercular Agents)

RN - 0 (Diarylquinolines)

RN - EC 3.6.3.- (Mitochondrial Proton-Translocating ATPases)

RN - 0 (Reactive Oxygen Species)

RN - EC 1.11.1.6 (katG protein, Mycobacterium tuberculosis)

RN - EC 1.11.1.6 (Catalase)

RN - 0 (Bacterial Proteins)

RN - EC 3.6.1.- (Bacterial Proton-Translocating ATPases)

SB - IM

UOF - bioRxiv. 2023 Oct 17:2023.10.17.562707. doi: 10.1101/2023.10.17.562707. PMID:

37905073

MH - *Tuberculosis, Multidrug-Resistant/drug therapy/microbiology

MH - Antitubercular Agents/pharmacology/therapeutic use

MH - *Diarylquinolines/pharmacology/therapeutic use

MH - *Mycobacterium tuberculosis/drug effects/enzymology/genetics/metabolism

MH - Mitochondrial Proton-Translocating ATPases

MH - Systems Biology

MH - Reactive Oxygen Species/metabolism

MH - DNA Damage/drug effects/genetics

MH - Drug Resistance, Multiple, Bacterial/genetics

MH - Microbial Sensitivity Tests

MH - *Catalase/genetics/metabolism

MH - *Bacterial Proteins/genetics/metabolism

MH - *Bacterial Proton-Translocating ATPases/antagonists & inhibitors/metabolism

MH - Biosynthetic Pathways/drug effects/genetics

MH - Humans

PMC - PMC11561320

COIS- Competing interests J.J.C. is a co-founder and board member of Phare Bio, a

nonprofit venture focused on antibiotic drug development. P.C.B. is a consultant

to or holds equity in 10X Genomics, General Automation Lab Technologies/Isolation

Bio, Celsius Therapeutics, Next Gen Diagnostics, Cache DNA, Concerto Biosciences,

Stately Bio, Ramona Optics, Bifrost Biosystems, and Amber Bio. His laboratory has

received research funding from Calico Life Sciences, Merck, and Genentech for

unrelated work. None of these interests are connected to this study. The

remaining authors declare no competing interests.

EDAT- 2024/11/14 03:56

MHDA- 2024/11/14 03:57

PMCR- 2024/11/13

CRDT- 2024/11/13 23:15

PHST- 2024/01/01 00:00 [received]

PHST- 2024/10/28 00:00 [accepted]

PHST- 2024/11/14 03:57 [medline]

PHST- 2024/11/14 03:56 [pubmed]

PHST- 2024/11/13 23:15 [entrez]

PHST- 2024/11/13 00:00 [pmc-release]

AID - 10.1038/s41467-024-53933-8 [pii]

AID - 53933 [pii]

AID - 10.1038/s41467-024-53933-8 [doi]

PST - epublish

SO - Nat Commun. 2024 Nov 13;15(1):9792. doi: 10.1038/s41467-024-53933-8.

PMID- 39361873

OWN - NLM

STAT- MEDLINE

DCOM- 20241107

LR - 20260709

IS - 2165-0497 (Electronic)

IS - 2165-0497 (Linking)

VI - 12

IP - 11

DP - 2024 Nov 5

TI - Discovery of benzo[c]phenanthridine derivatives with potent activity against

multidrug-resistant Mycobacterium tuberculosis.

PG - e0124624

LID - 10.1128/spectrum.01246-24 [doi]

LID - e01246-24

AB - Mycobacterium tuberculosis (Mtb), the pathogen responsible for tuberculosis (TB),

is the leading cause of bacterial disease-related death worldwide. Current

antibiotic regimens for the treatment of TB remain dated and suffer from long

treatment times as well as the development of drug resistance. As such, the

search for novel chemical modalities that have selective or potent anti-Mtb

properties remains an urgent priority, particularly against multidrug-resistant

(MDR) Mtb strains. Herein, we design and synthesize 35 novel

benzo[c]phenanthridine derivatives (BPDs). The two most potent compounds, BPD-6

and BPD-9, accumulated within the bacterial cell and exhibited strong inhibitory

activity (MIC(90) ~2 to 10 µM) against multiple Mycobacterium strains while

remaining inactive against a range of other Gram-negative and Gram-positive

bacteria. BPD-6 and BPD-9 were also effective in reducing Mtb survival within

infected macrophages, and BPD-9 reduced the burden of Mycobacterium bovis BCG in

the lungs of infected mice. The two BPD compounds displayed comparable efficacy

to rifampicin (RIF) against non-replicating Mtb (NR-Mtb). Importantly, BPD-6 and

BPD-9 inhibited the growth of multiple MDR Mtb clinical isolates. Generation of

BPD-9-resistant mutants identified the involvement of the Mmr efflux pump as an

indirect resistance mechanism. The unique specificity of BPDs to Mycobacterium

spp. and their efficacy against MDR Mtb isolates suggest a potential novel

mechanism of action. The discovery of BPDs provides novel chemical scaffolds for

anti-TB drug discovery.IMPORTANCEThe emergence of drug-resistant tuberculosis

(TB) is a serious global health threat. There remains an urgent need to discover

new antibiotics with unique mechanisms of action that are effective against

drug-resistant Mycobacterium tuberculosis (Mtb). This study shows that novel

semi-synthetic compounds can be derived from natural compounds to produce potent

activity against Mtb. Importantly, the identified compounds have narrow spectrum

activity against Mycobacterium species, including clinical multidrug-resistant

(MDR) strains, are effective in infected macrophages and against non-replicating

Mtb (NR-Mtb), and show anti-mycobacterial activity in mice. These new compounds

provide promising chemical scaffolds to develop potent anti-Mtb drugs of the

future.

FAU - Liang, Yi Chu

AU - Liang YC

AUID- ORCID: 0000-0002-0797-126X

AD - Department of Microbiology and Immunology, University of British Columbia,

Vancouver, Canada.

AD - Department of Biochemistry, Microbiology and Immunology, University of Ottawa,

Ottawa, Canada.

FAU - Sun, Zhiqi

AU - Sun Z

AD - Department of Biochemistry, Microbiology and Immunology, University of Ottawa,

Ottawa, Canada.

FAU - Lu, Chen

AU - Lu C

AD - Chinese Academy of Sciences Key Laboratory of Receptor Research, Shanghai

Institute of Materia Medica (SIMM), Chinese Academy of Sciences, Shanghai, China.

AD - University of Chinese Academy of Sciences, Beijing, China.

FAU - Lupien, Andréanne

AU - Lupien A

AD - Infectious Diseases and Immunity in Global Health Program, Research Institute of

the McGill University Health Centre, Montréal, Canada.

AD - McGill International TB Centre, Montréal, Canada.

AD - Department of Microbiology and Immunology, McGill University, Montréal, Canada.

AD - Department of Medicine, McGill University Health Centre, Montréal, Canada.

FAU - Xu, Zhongliang

AU - Xu Z

AD - Chinese Academy of Sciences Key Laboratory of Receptor Research, Shanghai

Institute of Materia Medica (SIMM), Chinese Academy of Sciences, Shanghai, China.

AD - University of Chinese Academy of Sciences, Beijing, China.

FAU - Berton, Stefania

AU - Berton S

AUID- ORCID: 0000-0003-2961-6100

AD - Department of Biochemistry, Microbiology and Immunology, University of Ottawa,

Ottawa, Canada.

FAU - Xu, Peng

AU - Xu P

AD - Department of Microbiology and Immunology, University of British Columbia,

Vancouver, Canada.

FAU - Behr, Marcel A

AU - Behr MA

AUID- ORCID: 0000-0003-0402-4467

AD - Infectious Diseases and Immunity in Global Health Program, Research Institute of

the McGill University Health Centre, Montréal, Canada.

AD - McGill International TB Centre, Montréal, Canada.

AD - Department of Microbiology and Immunology, McGill University, Montréal, Canada.

AD - Department of Medicine, McGill University Health Centre, Montréal, Canada.

FAU - Yang, Weibo

AU - Yang W

AUID- ORCID: 0000-0003-1633-7655

AD - Chinese Academy of Sciences Key Laboratory of Receptor Research, Shanghai

Institute of Materia Medica (SIMM), Chinese Academy of Sciences, Shanghai, China.

AD - University of Chinese Academy of Sciences, Beijing, China.

AD - School of Pharmaceutical Science and Technology, Hangzhou Institute for Advanced

Study, University of Chinese Academy of Sciences, Hangzhou, China.

FAU - Sun, Jim

AU - Sun J

AUID- ORCID: 0000-0002-6873-6052

AD - Department of Microbiology and Immunology, University of British Columbia,

Vancouver, Canada.

AD - Department of Biochemistry, Microbiology and Immunology, University of Ottawa,

Ottawa, Canada.

LA - eng

SI - BioProject/PRJNA1107169.

GR - PJT-162424, PPE-185827/Canadian Government | Canadian Institutes of Health

Research (CIHR)/

GR - Scholars Program/National Sanitarium Association (NSA)/

GR - FDN-148362/Canadian Government | Canadian Institutes of Health Research (CIHR)/

GR - 23XD1424400/STCSM | Program of Shanghai Academic Research Leader (Shanghai

Academic Research Leader)/

GR - 82373708, 22171275/MOST | National Natural Science Foundation of China (NSFC)/

PT - Journal Article

PT - Research Support, Non-U.S. Gov't

DEP - 20241003

PL - United States

TA - Microbiol Spectr

JT - Microbiology spectrum

JID - 101634614

RN - 0 (Antitubercular Agents)

RN - 0 (Phenanthridines)

SB - IM

MH - *Mycobacterium tuberculosis/drug effects/genetics

MH - Animals

MH - Mice

MH - *Antitubercular Agents/pharmacology/chemistry

MH - *Microbial Sensitivity Tests

MH - *Tuberculosis, Multidrug-Resistant/drug therapy/microbiology

MH - *Phenanthridines/pharmacology/chemistry

MH - *Drug Resistance, Multiple, Bacterial/drug effects

MH - Humans

MH - Female

MH - Macrophages/microbiology/drug effects

PMC - PMC11537118

OTO - NOTNLM

OT - Mycobacterium tuberculosis

OT - anti-mycobacterial compounds

OT - benzophenanthridine

OT - multidrug-resistant TB

OT - natural compounds

OT - non-replicating bacteria

OT - tuberculosis

COIS- The authors declare no conflict of interest.

EDAT- 2024/10/04 07:19

MHDA- 2024/11/12 04:44

PMCR- 2024/10/03

CRDT- 2024/10/03 15:23

PHST- 2024/11/12 04:44 [medline]

PHST- 2024/10/04 07:19 [pubmed]

PHST- 2024/10/03 15:23 [entrez]

PHST- 2024/10/03 00:00 [pmc-release]

AID - spectrum01246-24 [pii]

AID - spectrum.01246-24 [pii]

AID - 10.1128/spectrum.01246-24 [doi]

PST - ppublish

SO - Microbiol Spectr. 2024 Nov 5;12(11):e0124624. doi: 10.1128/spectrum.01246-24.

Epub 2024 Oct 3.

PMID- 39563136

OWN - NLM

STAT- MEDLINE

DCOM- 20241120

LR - 20241120

IS - 0004-5772 (Print)

IS - 0004-5772 (Linking)

VI - 72

IP - 11

DP - 2024 Nov

TI - A Case Involving Linezolid-induced Fatal Lactic Acidosis and Pancytopenia.

PG - e45-e46

LID - 10.59556/japi.72.0735 [doi]

AB - We present a case of a 77-year-old female diagnosed with multidrug-resistant

(MDR) pulmonary tuberculosis (PTB), on all oral longer regimen [according to

Indian Programmatic Management of Drug Resistant Tuberculosis (PMDT) guidelines]

for a month. She presented with nausea, vomiting, worsening of breathlessness for

a week, pancytopenia, and gradually increasing lactate levels. Various causes of

lactic acidosis including metabolic, septic, and hypoxic were ruled out. Despite

our dedicated efforts, she tragically passed away due to severe lactic acidosis,

a complication associated with linezolid treatment.

CI - © Journal of the Association of Physicians of India 2024.

FAU - Mehta, Kundan

AU - Mehta K

AD - Associate Professor, Department of Respiratory Medicine, Dr D Y Patil Medical

College, Hospital and Research Centre, Dr D Y Patil Vidyapeeth University, Pune,

Maharashtra, India.

FAU - Gandhi, Rhea

AU - Gandhi R

AD - Resident, Department of Respiratory Medicine, Dr D Y Patil Medical College,

Hospital and Research Centre, Dr D Y Patil Vidyapeeth University, Pune,

Maharashtra, India, Corresponding Author.

FAU - Ansari, Islahuzzama Salahuddin

AU - Ansari IS

AD - Resident, Department of Respiratory Medicine, Dr D Y Patil Medical College,

Hospital and Research Centre, Dr D Y Patil Vidyapeeth University, Pune,

Maharashtra, India.

FAU - Barthwal, M S

AU - Barthwal MS

AD - Professor and HOD, Department of Respiratory Medicine, Dr D Y Patil Medical

College, Hospital and Research Centre, Dr D Y Patil Vidyapeeth University, Pune,

Maharashtra, India.

LA - eng

PT - Case Reports

PT - Journal Article

PL - India

TA - J Assoc Physicians India

JT - The Journal of the Association of Physicians of India

JID - 7505585

RN - ISQ9I6J12J (Linezolid)

RN - 0 (Anti-Infective Agents)

SB - IM

MH - Humans

MH - *Linezolid/adverse effects

MH - Female

MH - *Acidosis, Lactic/chemically induced

MH - *Pancytopenia/chemically induced

MH - Aged

MH - Fatal Outcome

MH - Tuberculosis, Multidrug-Resistant/drug therapy

MH - Tuberculosis, Pulmonary/drug therapy

MH - Anti-Infective Agents/adverse effects

EDAT- 2024/11/20 11:04

MHDA- 2024/11/20 11:05

CRDT- 2024/11/20 02:18

PHST- 2024/11/20 11:05 [medline]

PHST- 2024/11/20 11:04 [pubmed]

PHST- 2024/11/20 02:18 [entrez]

AID - 10.59556/japi.72.0735 [doi]

PST - ppublish

SO - J Assoc Physicians India. 2024 Nov;72(11):e45-e46. doi: 10.59556/japi.72.0735.

PMID- 39393282

OWN - NLM

STAT- MEDLINE

DCOM- 20241110

LR - 20260523

IS - 1532-2084 (Electronic)

IS - 1368-7646 (Print)

IS - 1368-7646 (Linking)

VI - 77

DP - 2024 Nov

TI - Widespread loss-of-function mutations implicating preexisting resistance to new

or repurposed anti-tuberculosis drugs.

PG - 101156

LID - S1368-7646(24)00114-6 [pii]

LID - 10.1016/j.drup.2024.101156 [doi]

AB - BACKGROUND: Five New or Repurposed Drugs (NRDs) were approved in the last decade

for treatment of multi-drug resistant tuberculosis: bedaquiline, clofazimine,

linezolid, delamanid, and pretomanid. Unfortunately, resistance to these drugs

emerged faster than anticipated, potentially due to preexisting resistance in

naïve strains. Previous investigations into the rapid emergence have mostly

included short variants. For the first time, we utilize de novo-assembled

genomes, and systematically include Structural Variations (SV) and heterogeneity

to comprehensively study this rapid emergence. We show high prevalence of

preexisting resistance, identify novel markers of resistance, and lay the

foundation for preventing preexisting resistance in future drug development.

METHODS: First, a systematic literature review revealed 313 NRD resistance

variants in 13 genes. Next, 409 globally diverse clinical isolates collected

prior to the drugs' programmatic use (308 were multidrug resistant, 106 had de

novo assembled genomes) were utilized to study the 13 genes comprehensively for

conventional, structural, and heterogeneous variants. FINDINGS: We identified 5

previously reported and 67 novel putative NRD resistance variants. These variants

were 2 promoter mutations (in 8/409 isolates), 13 frameshifts (21/409), 6 SVs

(9/409), 35 heterogeneous frameshifts (32/409) and 11 heterogeneous SVs (12/106).

Delamanid and pretomanid resistance mutations were most prevalent (48/409), while

linezolid resistance mutations were least prevalent (8/409). INTERPRETATION:

Preexisting mutations implicated in resistance to at least one NRD was highly

prevalent (85/409, 21 %). This was mostly caused by loss-of-function mutations in

genes responsible for prodrug activation and efflux pump regulation. These

preexisting mutations may have emerged through a bet-hedging strategy, or through

cross-resistance with non-tuberculosis drugs such as metronidazole. Future drugs

that could be resisted through loss-of-function in non-essential genes may suffer

from preexisting resistance. The methods used here for comprehensive preexisting

resistance assessment (especially SVs and heterogeneity) may mitigate this risk

during early-stage drug development.

CI - Copyright © 2024. Published by Elsevier Ltd.

FAU - Conkle-Gutierrez, Derek

AU - Conkle-Gutierrez D

AD - Laboratory for Pathogenesis of Clinical Drug Resistance and Persistence, San

Diego State University, San Diego, CA, USA.

FAU - Gorman, Bria M

AU - Gorman BM

AD - Laboratory for Pathogenesis of Clinical Drug Resistance and Persistence, San

Diego State University, San Diego, CA, USA.

FAU - Thosar, Nachiket

AU - Thosar N

AD - Laboratory for Pathogenesis of Clinical Drug Resistance and Persistence, San

Diego State University, San Diego, CA, USA.

FAU - Elghraoui, Afif

AU - Elghraoui A

AD - Laboratory for Pathogenesis of Clinical Drug Resistance and Persistence, San

Diego State University, San Diego, CA, USA.

FAU - Modlin, Samuel J

AU - Modlin SJ

AD - Laboratory for Pathogenesis of Clinical Drug Resistance and Persistence, San

Diego State University, San Diego, CA, USA.

FAU - Valafar, Faramarz

AU - Valafar F

AD - Laboratory for Pathogenesis of Clinical Drug Resistance and Persistence, San

Diego State University, San Diego, CA, USA. Electronic address:

faramarz@sdsu.edu.

LA - eng

SI - SRA/PRJNA353873

SI - SRA/PRJNA555636

SI - SRA/PRJEB8783

GR - R01 AI105185/AI/NIAID NIH HHS/United States

GR - R01 AI163202/AI/NIAID NIH HHS/United States

PT - Journal Article

PT - Research Support, N.I.H., Extramural

DEP - 20241005

PL - Scotland

TA - Drug Resist Updat

JT - Drug resistance updates : reviews and commentaries in antimicrobial and

anticancer chemotherapy

JID - 9815369

RN - 0 (Antitubercular Agents)

RN - 78846I289Y (bedaquiline)

RN - 0 (Diarylquinolines)

SB - IM

MH - *Antitubercular Agents/pharmacology

MH - Humans

MH - *Mycobacterium tuberculosis/drug effects/genetics

MH - *Tuberculosis, Multidrug-Resistant/drug therapy/genetics/microbiology

MH - *Drug Repositioning/methods

MH - *Drug Resistance, Multiple, Bacterial/genetics

MH - Loss of Function Mutation

MH - Microbial Sensitivity Tests

MH - Diarylquinolines

PMC - PMC12981229

MID - NIHMS2151417

OTO - NOTNLM

OT - Drug resistance

OT - Heterogeneity

OT - Preexisting resistance

OT - Structural variation

OT - Tuberculosis

COIS- Declaration of Competing Interest The authors declare the following financial

interests/personal relationships which may be considered as potential competing

interests: Faramarz Valafar reports financial support was provided by National

Institute of Allergy and Infectious Diseases. If there are other authors, they

declare that they have no known competing financial interests or personal

relationships that could have appeared to influence the work reported in this

paper.

EDAT- 2024/10/12 13:18

MHDA- 2024/11/13 13:53

PMCR- 2026/03/12

CRDT- 2024/10/11 18:06

PHST- 2023/11/11 00:00 [received]

PHST- 2024/09/05 00:00 [revised]

PHST- 2024/09/28 00:00 [accepted]

PHST- 2024/11/13 13:53 [medline]

PHST- 2024/10/12 13:18 [pubmed]

PHST- 2024/10/11 18:06 [entrez]

PHST- 2026/03/12 00:00 [pmc-release]

AID - S1368-7646(24)00114-6 [pii]

AID - 10.1016/j.drup.2024.101156 [doi]

PST - ppublish

SO - Drug Resist Updat. 2024 Nov;77:101156. doi: 10.1016/j.drup.2024.101156. Epub 2024

Oct 5.

PMID- 39353459

OWN - NLM

STAT- MEDLINE

DCOM- 20241110

LR - 20260319

IS - 2666-5247 (Electronic)

IS - 2666-5247 (Linking)

VI - 5

IP - 11

DP - 2024 Nov

TI - The catalogue of Mycobacterium tuberculosis mutations associated with drug

resistance to 12 drugs in China from a nationwide survey: a genomic analysis.

PG - 100899

LID - S2666-5247(24)00131-9 [pii]

LID - 10.1016/S2666-5247(24)00131-9 [doi]

AB - BACKGROUND: WHO issued the first edition catalogue of Mycobacterium tuberculosis

complex (MTBC) mutations associated with drug resistance in 2021. However,

country-specific issues might lead to arising complex and additional

drug-resistant mutations. We aimed to fully reflect the characteristics of drug

resistance mutations in China. METHODS: We analysed MTBC isolates from the

nationwide drug-resistant tuberculosis surveillance with 70 counties in

31 provinces, municipalities, and autonomous regions in China. Three types of

MYCOTB plates were used to perform drug susceptibility testing for 12 antibiotics

(rifampicin, isoniazid, ethambutol, levofloxacin, moxifloxacin, amikacin,

kanamycin, ethionamide, clofazimine, linezolid, delamanid, and bedaquiline).

Mutations were divided into five groups according to their odds ratios, positive

predictive values, false discovery rate-corrected p values, and 95% CIs: (1)

associated with resistance; (2) associated with resistance-interim; (3) uncertain

significance; (4) not associated with resistance-interim; and (5) not associated

with resistance. The Wilcoxon rank-sum and Kruskal-Wallis tests were used to

quantify the association between mutations and minimum inhibitory concentrations

(MICs). Our dataset was compared with the first edition of the WHO catalogue.

FINDINGS: We analysed 10 146 MTBC isolates, of which 9071 (89·4%) isolates were

included in the final analysis. 744 (8·2%) isolates were resistant to rifampicin

and 1339 (14·8%) to isoniazid. 208 (1·9%) of 11 065 mutations were classified as

associated with resistance or associated with resistance-interim. 33 (97·1%) of

34 mutations in group 1 and 92 (52·9%) of 174 in group 2 also appeared in groups

1 or 2 of the WHO catalogue. Of 81 indel mutations in group 2, 15 (18·5%) were in

the WHO catalogue. The newly discovered mutation gyrA_Ala288Asp was associated

with levofloxacin resistance. MIC values for rifampicin, isoniazid, moxifloxacin,

and levofloxacin corresponding to resistance mutations in group 1 were

significantly different (p<0·0001), and 12 high-level resistance mutations were

detected. 61 mutations in group 3 occurred as solo in at least five

phenotypically susceptible isolates, but with MIC values moderately higher than

other susceptible isolates. Among 945 phenotypically resistant but genotypically

susceptible isolates, 433 (45·8%) were mutated for at least one efflux pump gene.

INTERPRETATION: Our analysis reflects the complexity of drug resistance mutations

in China and suggests that indel mutations, efflux pump genes, protein structure,

and MICs should be fully considered in the WHO catalogue, especially in countries

with a high tuberculosis burden. FUNDING: National Key Research and Development

Program of China and the Science and Technology Major Project of Tibetan

Autonomous Region of China.

CI - Copyright © 2024 The Author(s). Published by Elsevier Ltd.. All rights reserved.

FAU - Pei, Shaojun

AU - Pei S

AD - Department of Global Health, School of Public Health, Peking University, Beijing,

China; National Center for Tuberculosis Control and Prevention, Chinese Center

for Disease Control and Prevention, Beijing, China; National Key Laboratory of

Intelligent Tracking and Forecasting for Infectious Diseases, Chinese Center for

Disease Control and Prevention, Beijing, China.

FAU - Song, Zexuan

AU - Song Z

AD - National Center for Tuberculosis Control and Prevention, Chinese Center for

Disease Control and Prevention, Beijing, China.

FAU - Yang, Wei

AU - Yang W

AD - National Clinical Research Center for Infectious Diseases, Shenzhen Third

People's Hospital, Shenzhen, China.

FAU - He, Wencong

AU - He W

AD - National Center for Tuberculosis Control and Prevention, Chinese Center for

Disease Control and Prevention, Beijing, China.

FAU - Ou, Xichao

AU - Ou X

AD - National Center for Tuberculosis Control and Prevention, Chinese Center for

Disease Control and Prevention, Beijing, China; National Key Laboratory of

Intelligent Tracking and Forecasting for Infectious Diseases, Chinese Center for

Disease Control and Prevention, Beijing, China.

FAU - Zhao, Bing

AU - Zhao B

AD - National Center for Tuberculosis Control and Prevention, Chinese Center for

Disease Control and Prevention, Beijing, China; National Key Laboratory of

Intelligent Tracking and Forecasting for Infectious Diseases, Chinese Center for

Disease Control and Prevention, Beijing, China.

FAU - He, Ping

AU - He P

AD - National Center for Tuberculosis Control and Prevention, Chinese Center for

Disease Control and Prevention, Beijing, China.

FAU - Zhou, Yang

AU - Zhou Y

AD - National Center for Tuberculosis Control and Prevention, Chinese Center for

Disease Control and Prevention, Beijing, China; National Key Laboratory of

Intelligent Tracking and Forecasting for Infectious Diseases, Chinese Center for

Disease Control and Prevention, Beijing, China.

FAU - Xia, Hui

AU - Xia H

AD - National Center for Tuberculosis Control and Prevention, Chinese Center for

Disease Control and Prevention, Beijing, China; National Key Laboratory of

Intelligent Tracking and Forecasting for Infectious Diseases, Chinese Center for

Disease Control and Prevention, Beijing, China.

FAU - Wang, Shengfen

AU - Wang S

AD - National Center for Tuberculosis Control and Prevention, Chinese Center for

Disease Control and Prevention, Beijing, China; National Key Laboratory of

Intelligent Tracking and Forecasting for Infectious Diseases, Chinese Center for

Disease Control and Prevention, Beijing, China.

FAU - Jia, Zhongwei

AU - Jia Z

AD - Department of Global Health, School of Public Health, Peking University, Beijing,

China. Electronic address: urchinjj@163.com.

FAU - Walker, Timothy M

AU - Walker TM

AD - Oxford University Clinical Research Unit, Ho Chi Minh City, Viet Nam; Nuffield

Department of Medicine, Centre for Tropical Medicine and Global Health,

University of Oxford, Oxford, UK. Electronic address:

timothy.walker@ndm.ox.ac.uk.

FAU - Zhao, Yanlin

AU - Zhao Y

AD - National Center for Tuberculosis Control and Prevention, Chinese Center for

Disease Control and Prevention, Beijing, China; National Key Laboratory of

Intelligent Tracking and Forecasting for Infectious Diseases, Chinese Center for

Disease Control and Prevention, Beijing, China; National Clinical Research Center

for Infectious Diseases, Shenzhen Third People's Hospital, Shenzhen, China.

Electronic address: zhaoyl@chinacdc.cn.

LA - eng

GR - WT_/Wellcome Trust/United Kingdom

PT - Journal Article

PT - Research Support, Non-U.S. Gov't

DEP - 20240928

PL - England

TA - Lancet Microbe

JT - The Lancet. Microbe

JID - 101769019

RN - 0 (Antitubercular Agents)

SB - IM

MH - *Mycobacterium tuberculosis/drug effects/genetics

MH - China/epidemiology

MH - *Antitubercular Agents/pharmacology

MH - *Mutation

MH - Humans

MH - *Microbial Sensitivity Tests

MH - *Tuberculosis, Multidrug-Resistant/microbiology/epidemiology/drug

therapy/genetics

MH - Drug Resistance, Multiple, Bacterial/genetics

MH - Genomics

PMC - PMC11543636

COIS- Declaration of interests We declare no competing interests.

EDAT- 2024/10/03 03:38

MHDA- 2024/11/13 13:48

PMCR- 2024/11/01

CRDT- 2024/10/01 18:52

PHST- 2023/03/21 00:00 [received]

PHST- 2024/05/05 00:00 [revised]

PHST- 2024/05/10 00:00 [accepted]

PHST- 2024/11/13 13:48 [medline]

PHST- 2024/10/03 03:38 [pubmed]

PHST- 2024/10/01 18:52 [entrez]

PHST- 2024/11/01 00:00 [pmc-release]

AID - S2666-5247(24)00131-9 [pii]

AID - 100899 [pii]

AID - 10.1016/S2666-5247(24)00131-9 [doi]

PST - ppublish

SO - Lancet Microbe. 2024 Nov;5(11):100899. doi: 10.1016/S2666-5247(24)00131-9. Epub

2024 Sep 28.

PMID- 39478003

OWN - NLM

STAT- MEDLINE

DCOM- 20241031

LR - 20241106

IS - 2045-2322 (Electronic)

IS - 2045-2322 (Linking)

VI - 14

IP - 1

DP - 2024 Oct 30

TI - The CRISPR-dCas9 interference system suppresses inhA gene expression in

Mycobacterium smegmatis.

PG - 26116

LID - 10.1038/s41598-024-77442-2 [doi]

LID - 26116

AB - CRISPR-dead Cas9 interference (CRISPRi) has become a valuable tool for precise

gene regulation. In this study, CRISPRi was designed to target the inhA gene of

Mycobacterium smegmatis (Msm), a gene necessary for mycolic acid synthesis. Our

findings revealed that sgRNA2 induced with 100 ng/ml aTc achieved over 90%

downregulation of inhA gene expression and inhibited bacterial viability by

approximately 1,000-fold. Furthermore, CRISPRi enhanced the susceptibility of M.

smegmatis to isoniazid and rifampicin, which are both 50% and 90% lower than

those of the wild-type strain or other strains, respectively. This study

highlights the ability of CRISPRi to silence the inhA gene, which impacts

bacterial viability and drug susceptibility. The findings provide valuable

insights into the utility of CRISPRi as an alternative tool for gene regulation.

CRISPRi might be further assessed for its synergistic effect with current

anti-tuberculosis drugs and its possible implications for combating mycobacterial

infections, especially drug-resistant tuberculosis.

CI - © 2024. The Author(s).

FAU - Singpanomchai, Nuntita

AU - Singpanomchai N

AD - Inter-Department Program of Biomedical Sciences, Faculty of Graduate School,

Chulalongkorn University, Bangkok, 10330, Thailand.

FAU - Ratthawongjirakul, Panan

AU - Ratthawongjirakul P

AD - Center of Excellence for Innovative Diagnosis of Antimicrobial Resistance,

Faculty of Allied Health Sciences, Chulalongkorn University, Bangkok, 10330,

Thailand. panan_etc@yahoo.com.

LA - eng

GR - CU_FRB65_hea (81)_176_37_06/Thailand Science Research and Innovation Fund of

Chulalongkorn University/

PT - Journal Article

DEP - 20241030

PL - England

TA - Sci Rep

JT - Scientific reports

JID - 101563288

RN - 0 (Bacterial Proteins)

RN - EC 1.3.1.9 (InhA protein, Mycobacterium)

RN - EC 1.- (Oxidoreductases)

RN - V83O1VOZ8L (Isoniazid)

RN - 0 (Antitubercular Agents)

RN - VJT6J7R4TR (Rifampin)

SB - IM

MH - *Mycobacterium smegmatis/genetics/drug effects

MH - *Bacterial Proteins/genetics/metabolism

MH - *CRISPR-Cas Systems

MH - *Gene Expression Regulation, Bacterial/drug effects

MH - *Oxidoreductases/genetics/metabolism

MH - *Isoniazid/pharmacology

MH - *Antitubercular Agents/pharmacology

MH - Rifampin/pharmacology

MH - Microbial Sensitivity Tests

MH - Clustered Regularly Interspaced Short Palindromic Repeats/genetics

PMC - PMC11525817

OTO - NOTNLM

OT - inhA

OT - CRISPR interference

OT - DCas9

OT - Mycobacteria

COIS- The authors declare no competing interests.

EDAT- 2024/10/31 10:18

MHDA- 2024/10/31 10:19

PMCR- 2024/10/30

CRDT- 2024/10/31 00:50

PHST- 2024/06/28 00:00 [received]

PHST- 2024/10/22 00:00 [accepted]

PHST- 2024/10/31 10:19 [medline]

PHST- 2024/10/31 10:18 [pubmed]

PHST- 2024/10/31 00:50 [entrez]

PHST- 2024/10/30 00:00 [pmc-release]

AID - 10.1038/s41598-024-77442-2 [pii]

AID - 77442 [pii]

AID - 10.1038/s41598-024-77442-2 [doi]

PST - epublish

SO - Sci Rep. 2024 Oct 30;14(1):26116. doi: 10.1038/s41598-024-77442-2.

PMID- 39554140

OWN - NLM

STAT- PubMed-not-MEDLINE

LR - 20250617

IS - 2692-8205 (Electronic)

IS - 2692-8205 (Linking)

DP - 2024 Oct 29

TI - Comparative study on the virulence of mycobacteriophages.

LID - 2024.10.23.619922 [pii]

LID - 10.1101/2024.10.23.619922 [doi]

AB - The global tuberculosis (TB) epidemic affected 10 million people and caused 1.3

million deaths in 2022 alone. Multidrug-resistant TB is successfully treated in

less than 60% of cases by long, expensive and aggressive treatments.

Mycobacteriophages, viruses that can infect bacteria such as Mycobacterium

tuberculosis-the species responsible for TB, have the potential to redefine TB

prevention and treatments. However, the development of phage-based products

necessitates the assessment of numerous parameters, including virulence and

adsorption, to ensure their performance and quality. In this work, we

characterized the virulence of three different mycobacteriophages (Fionnbharth,

Muddy and D29), alone and as cocktails, against a TB model host (Mycobacterium

smegmatis) under planktonic and early-stage biofilm growth conditions. Phage D29

and cocktails containing D29 had the highest virulence under all conditions.

Interestingly, phages Fionnbharth and Muddy and their combination showed higher

virulence against early-stage biofilm than against the planktonic phenotype.

Adsorption assays indicated that all three phages had lower adsorption

efficiencies on the early-stage biofilm phenotype than on the planktonic one,

suggesting a reduced availability of receptors in the former. Given that, despite

these lower adsorption efficiencies, the virulence of the phages and phage

cocktails was either unchanged or higher against the early-stage biofilm, this

phenotype must display properties that are favorable to other steps of the

infection process. These results inform us on the dynamics of mycobacteriophage

infections, alone and in cocktail formulations, under different host growth

conditions, and serve as a basis for the development of phage products targeting

mycobacteria biofilms.

FAU - Rubino, Ilaria

AU - Rubino I

AUID- ORCID: 0000-0003-1589-1566

AD - Department of Chemical and Materials Engineering, University of Alberta,

Edmonton, Alberta, Canada.

AD - Département de Génie Chimique et de Génie Biotechnologique, Université de

Sherbrooke, Sherbrooke, Québec, Canada.

FAU - Guerrero-Bustamante, Carlos A

AU - Guerrero-Bustamante CA

AD - Department of Biological Sciences, University of Pittsburgh, Pennsylvania, USA.

FAU - Harrison, Melissa

AU - Harrison M

AD - Department of Chemical and Materials Engineering, University of Alberta,

Edmonton, Alberta, Canada.

FAU - Co, Sheila

AU - Co S

AD - Department of Chemical and Materials Engineering, University of Alberta,

Edmonton, Alberta, Canada.

FAU - Tetreau, Isobel

AU - Tetreau I

AD - Department of Mechanical Engineering, University of Alberta, Edmonton, Alberta,

Canada.

FAU - Ordoubadi, Mani

AU - Ordoubadi M

AUID- ORCID: 0000-0002-2000-1604

AD - Department of Mechanical Engineering, University of Alberta, Edmonton, Alberta,

Canada.

FAU - Larsen, Sasha E

AU - Larsen SE

AUID- ORCID: 0000-0001-5034-9727

AD - Center for Global Infectious Disease Research, Seattle Children's Research

Institute, Seattle, Washington, USA.

FAU - Coler, Rhea N

AU - Coler RN

AD - Center for Global Infectious Disease Research, Seattle Children's Research

Institute, Seattle, Washington, USA.

AD - University of Washington School of Medicine, Department of Pediatrics, Seattle,

WA, USA.

AD - University of Washington, Department of Global Health, Seattle, WA, USA.

FAU - Vehring, Reinhard

AU - Vehring R

AUID- ORCID: 0000-0002-4446-9826

AD - Department of Mechanical Engineering, University of Alberta, Edmonton, Alberta,

Canada.

FAU - Hatfull, Graham F

AU - Hatfull GF

AUID- ORCID: 0000-0002-6705-6821

AD - Department of Biological Sciences, University of Pittsburgh, Pennsylvania, USA.

FAU - Sauvageau, Dominic

AU - Sauvageau D

AUID- ORCID: 0000-0002-1995-5523

AD - Department of Chemical and Materials Engineering, University of Alberta,

Edmonton, Alberta, Canada.

LA - eng

GR - R21 AI156807/AI/NIAID NIH HHS/United States

GR - R35 GM131729/GM/NIGMS NIH HHS/United States

PT - Journal Article

PT - Preprint

DEP - 20241029

PL - United States

TA - bioRxiv

JT - bioRxiv : the preprint server for biology

JID - 101680187

UIN - J Virol. 2025 Jun 17;99(6):e0192024. doi: 10.1128/jvi.01920-24. PMID: 40396760

PMC - PMC11565895

OTO - NOTNLM

OT - Bacteriophages

OT - D29

OT - Fionnbharth

OT - Muddy

OT - Mycobacterium smegmatis

OT - infection dynamics

OT - mycobacteriophages

OT - phage adsorption

OT - phages

OT - virulence

OT - virulence index

COIS- Declaration of Competing Interest The authors declare no competing interest.

EDAT- 2024/11/18 13:19

MHDA- 2024/11/18 13:20

PMCR- 2024/11/15

CRDT- 2024/11/18 06:19

PHST- 2024/11/18 13:19 [pubmed]

PHST- 2024/11/18 13:20 [medline]

PHST- 2024/11/18 06:19 [entrez]

PHST- 2024/11/15 00:00 [pmc-release]

AID - 2024.10.23.619922 [pii]

AID - 10.1101/2024.10.23.619922 [doi]

PST - epublish

SO - bioRxiv [Preprint]. 2024 Oct 29:2024.10.23.619922. doi:

10.1101/2024.10.23.619922.

PMID- 39305944

OWN - NLM

STAT- MEDLINE

DCOM- 20241017

LR - 20250610

IS - 1878-7568 (Electronic)

IS - 1742-7061 (Linking)

VI - 188

DP - 2024 Oct 15

TI - Nanoparticles target M2 macrophages to silence kallikrein-related peptidase 12

for the treatment of tuberculosis and drug-resistant tuberculosis.

PG - 358-373

LID - S1742-7061(24)00542-7 [pii]

LID - 10.1016/j.actbio.2024.09.026 [doi]

AB - Matrix metalloproteinases (MMPs) are involved in the breakdown of lung

extracellular matrix and the consequent release of Mycobacterium tuberculosis

into the airways. Recent studies indicate that kallikrein-related peptidase 12

(KLK12) regulate MMP-1 and MMP-9, suggesting that targeting the KLK12 gene could

be a promising tuberculosis (TB) treatment. To maximise therapeutic potential,

this strategy of silencing KLK12 needs to be delivered to the pathogenic cell

population while preserving the immunoprotective and tissue homeostatic functions

of other lung macrophages. Our research found that KLK12 is highly expressed in

M2 macrophages, leading us to design mannose-based bovine serum albumin

nanoparticles (MBNPs) for delivering siRNA to silence KLK12 in these cells. The

results of in vitro experiments showed that MBNPs could accurately enter M2

macrophages and sustainably release KLK12-siRNA with the help of mannose and

mannose receptor targeting. The results of the in vivo experiments showed that

MBNPs could reach the lungs within 1 h after intraperitoneal injection and peaked

at 6 h. MBNPs increased collagen fibre content in the lungs by decreasing the

levels of KLK12/MMPs thereby limiting the progression of TB. Importantly, MBNPs

provided greater alleviation of pulmonary TB symptoms and reduced bacterial load

in both TB and drug-resistant TB models. These findings provide an alternative

and effective option for the treatment of TB, especially when drug resistance

occurs. STATEMENT OF SIGNIFICANCE: RNA interference using small interfering RNA

(siRNA) can target various genes and has potential for treating diseases such as

tuberculosis (TB). However, siRNAs are unstable in the blood and within cells.

This study presents bovine serum albumin nanoparticles encapsulating KLK12-siRNA

(BNPs) synthesized via desolvation. A mannose layer was added (MBNPs) to target

mannose receptors on M2 macrophages, facilitating endocytosis. The low

pH-responsive MBNPs enhance lysosomal escape for siRNA delivery, downregulating

the KLK12 pathway. Tests confirmed that MBNPs effectively inhibited Mycobacterium

bovis proliferation, reduced granulomas, and decreased inflammation in a mouse

model. This research aims to reduce antibiotic use, shorten treatment duration,

and provide a novel TB treatment option.

CI - Copyright © 2024 Acta Materialia Inc. Published by Elsevier Ltd. All rights

reserved.

FAU - Wang, Yuanzhi

AU - Wang Y

AD - National Key Laboratory of Veterinary Public Health and Safety, College of

Veterinary Medicine, China Agricultural University, Beijing 100193, China.

FAU - Liu, Yiduo

AU - Liu Y

AD - National Key Laboratory of Veterinary Public Health and Safety, College of

Veterinary Medicine, China Agricultural University, Beijing 100193, China.

FAU - Long, Meizhen

AU - Long M

AD - National Key Laboratory of Veterinary Public Health and Safety, College of

Veterinary Medicine, China Agricultural University, Beijing 100193, China.

FAU - Dong, Yuhui

AU - Dong Y

AD - National Key Laboratory of Veterinary Public Health and Safety, College of

Veterinary Medicine, China Agricultural University, Beijing 100193, China.

FAU - Li, Lin

AU - Li L

AD - National Key Laboratory of Veterinary Public Health and Safety, College of

Veterinary Medicine, China Agricultural University, Beijing 100193, China.

FAU - Zhou, Xiangmei

AU - Zhou X

AD - National Key Laboratory of Veterinary Public Health and Safety, College of

Veterinary Medicine, China Agricultural University, Beijing 100193, China.

Electronic address: zhouxm@cau.edu.cn.

LA - eng

PT - Journal Article

PT - Research Support, Non-U.S. Gov't

DEP - 20240919

PL - England

TA - Acta Biomater

JT - Acta biomaterialia

JID - 101233144

RN - 0 (RNA, Small Interfering)

RN - EC 3.4.21.- (Kallikreins)

RN - 27432CM55Q (Serum Albumin, Bovine)

SB - IM

MH - Animals

MH - *Nanoparticles/chemistry

MH - Mice

MH - *Macrophages/metabolism/drug effects

MH - RNA, Small Interfering/metabolism

MH - Tuberculosis, Multidrug-Resistant/drug therapy/pathology

MH - Gene Silencing/drug effects

MH - Kallikreins/metabolism

MH - Mycobacterium tuberculosis/drug effects

MH - Tuberculosis/drug therapy/pathology

MH - Serum Albumin, Bovine/chemistry

MH - Humans

MH - Mice, Inbred BALB C

MH - Lung/pathology

OTO - NOTNLM

OT - KLK12

OT - M2 macrophages

OT - ManLAM

OT - Nanoparticles

OT - Tuberculosis

COIS- Declaration of competing interest The authors declare that they have no known

competing financial interests or personal relationships that could have appeared

to influence the work reported in this paper.

EDAT- 2024/09/22 00:42

MHDA- 2024/10/17 10:05

CRDT- 2024/09/21 19:24

PHST- 2024/05/14 00:00 [received]

PHST- 2024/09/08 00:00 [revised]

PHST- 2024/09/16 00:00 [accepted]

PHST- 2024/10/17 10:05 [medline]

PHST- 2024/09/22 00:42 [pubmed]

PHST- 2024/09/21 19:24 [entrez]

AID - S1742-7061(24)00542-7 [pii]

AID - 10.1016/j.actbio.2024.09.026 [doi]

PST - ppublish

SO - Acta Biomater. 2024 Oct 15;188:358-373. doi: 10.1016/j.actbio.2024.09.026. Epub

2024 Sep 19.

PMID- 39377599

OWN - NLM

STAT- Publisher

LR - 20241008

IS - 1538-4683 (Electronic)

IS - 1061-5377 (Linking)

DP - 2024 Oct 8

TI - QT Prolongation Associated with Administration of Bedaquiline, a Novel

Anti-Tuberculosis Drug.

LID - 10.1097/CRD.0000000000000790 [doi]

AB - Bedaquiline is a diarylquinoline compound that has recently been introduced and

approved for use in the treatment of multidrug-resistant tuberculosis (MDR-TB).

Its mechanism of action is inhibition of adenosine triphosphate-synthase. In

combination with other antibiotics, bedaquiline-containing regimens administered

for 6 months achieve cure rates of roughly 90%, in contrast to the previously

used, 24-month-long WHO-recommended regimens for the treatment of MDR-TB.

However, since its introduction, concerns have been raised about its effects on

QT prolongation and its safety in routine clinical use. We reviewed the published

experience regarding bedaquiline use, QT prolongation, and adverse cardiac events

when the drug was used alone or in combination. Overall, data are reassuring that

bedaquiline use in clinical practice is not associated with an excess of cardiac

deaths or other clinically meaningful cardiac events. This review provides

reassurance and support for the continued use of bedaquiline in the treatment of

MDR-TB.

CI - Copyright © 2024 The Author(s). Published by Wolters Kluwer Health, Inc.

FAU - Gavras, Nicholas

AU - Gavras N

AD - From the Department of Medicine, New York Medical College, Valhalla, NY.

FAU - Schluger, Neil W

AU - Schluger NW

LA - eng

PT - Journal Article

DEP - 20241008

PL - United States

TA - Cardiol Rev

JT - Cardiology in review

JID - 9304686

SB - IM

COIS- Disclosure: The authors have no conflicts of interest to report.

EDAT- 2024/10/08 12:24

MHDA- 2024/10/08 12:24

CRDT- 2024/10/08 09:03

PHST- 2024/10/08 12:24 [medline]

PHST- 2024/10/08 12:24 [pubmed]

PHST- 2024/10/08 09:03 [entrez]

AID - 00045415-990000000-00342 [pii]

AID - 10.1097/CRD.0000000000000790 [doi]

PST - aheadofprint

SO - Cardiol Rev. 2024 Oct 8. doi: 10.1097/CRD.0000000000000790.

PMID- 39278679

OWN - NLM

STAT- MEDLINE

DCOM- 20240915

LR - 20240915

IS - 0019-5707 (Print)

IS - 0019-5707 (Linking)

VI - 71

IP - 4

DP - 2024 Oct

TI - Tuberculosis vaccine - A timely analysis of the drawbacks for the development of

novel vaccines.

PG - 453-459

LID - S0019-5707(23)00219-6 [pii]

LID - 10.1016/j.ijtb.2023.12.002 [doi]

AB - The BCG vaccine, Bacille Calmette Guerin, holds the distinction of being the most

widely administered vaccine. Remarkably, a century has passed since its

discovery; however, puzzlingly, questions persist regarding the effectiveness of

the immune response it triggers. After years of diligent observation, it has been

deduced that BCG imparts immunity primarily to a specific age group, namely

children. This prompts a significant query: the rationale behind BCG's limited

efficacy against TB in particular age groups and populations remains elusive.

Beyond vaccinations, drug therapy has emerged as an alternative route for TB

prevention. Nonetheless, this approach faces challenges in the contemporary

landscape, marked by the emergence of new instances of MDR-TB and XDR-TB,

compounded by the financial burden of treatment. It's noteworthy that BCG remains

the sole WHO-approved vaccine for TB. This comprehensive review delves into

several aspects, encompassing the immune response during infection, the

shortcomings of BCG in conferring immunity, and the various factors contributing

to its limitations. Within this discourse, we explore potential explanations for

the observed deficiencies of the BCG vaccine and consider how these insights

could catalyze the development of future vaccines. The current landscape of novel

vaccine development for TB is illuminated, including a spotlight on the latest

vaccine candidates.

CI - Copyright © 2023. Published by Elsevier B.V.

FAU - Jeyachandran, Dr Sivakamavalli

AU - Jeyachandran DS

AD - Lab in Biotechnology and Biosignal Transduction, Department of Orthodontics,

Saveetha Dental College & Hospitals, Saveetha Institute of Medical and Technical

Sciences (SIMATS), Saveetha University, Chennai, 77, Tamil Nadu, India.

Electronic address: sivakamavalli.sdc@saveetha.com.

FAU - Pusam, Yashika

AU - Pusam Y

AD - PG & Research Department of Biotechnology & Microbiology, National College

Autonomous, Tiruchirappalli, Tamil Nadu, India. Electronic address:

yashikapusam09@gmail.com.

LA - eng

PT - Journal Article

PT - Review

DEP - 20231223

PL - India

TA - Indian J Tuberc

JT - The Indian journal of tuberculosis

JID - 0373027

RN - 0 (BCG Vaccine)

RN - 0 (Tuberculosis Vaccines)

SB - IM

MH - Humans

MH - *BCG Vaccine/immunology

MH - *Tuberculosis Vaccines/immunology

MH - Tuberculosis/prevention & control

MH - Vaccine Development

MH - Tuberculosis, Multidrug-Resistant/prevention & control

OTO - NOTNLM

OT - BCG vaccine

OT - Epidemiology

OT - Immune response

OT - Tuberculosis

EDAT- 2024/09/16 00:42

MHDA- 2024/09/16 00:43

CRDT- 2024/09/15 20:57

PHST- 2022/07/01 00:00 [received]

PHST- 2023/08/10 00:00 [revised]

PHST- 2023/12/21 00:00 [accepted]

PHST- 2024/09/16 00:43 [medline]

PHST- 2024/09/16 00:42 [pubmed]

PHST- 2024/09/15 20:57 [entrez]

AID - S0019-5707(23)00219-6 [pii]

AID - 10.1016/j.ijtb.2023.12.002 [doi]

PST - ppublish

SO - Indian J Tuberc. 2024 Oct;71(4):453-459. doi: 10.1016/j.ijtb.2023.12.002. Epub

2023 Dec 23.

PMID- 39159075

OWN - NLM

STAT- MEDLINE

DCOM- 20241023

LR - 20260309

IS - 2198-3844 (Electronic)

IS - 2198-3844 (Linking)

VI - 11

IP - 39

DP - 2024 Oct

TI - Automated Diagnosis and Phenotyping of Tuberculosis Using Serum Metabolic

Fingerprints.

PG - e2406233

LID - 10.1002/advs.202406233 [doi]

LID - 2406233

AB - Tuberculosis (TB) stands as the second most fatal infectious disease after

COVID-19, the effective treatment of which depends on accurate diagnosis and

phenotyping. Metabolomics provides valuable insights into the identification of

differential metabolites for disease diagnosis and phenotyping. However, TB

diagnosis and phenotyping remain great challenges due to the lack of a

satisfactory metabolic approach. Here, a metabolomics-based diagnostic method for

rapid TB detection is reported. Serum metabolic fingerprints are examined via an

automated nanoparticle-enhanced laser desorption/ionization mass spectrometry

platform outstanding by its rapid detection speed (measured in seconds), minimal

sample consumption (in nanoliters), and cost-effectiveness (approximately $3). A

panel of 14 m z(-1) features is identified as biomarkers for TB diagnosis and a

panel of 4 m z(-1) features for TB phenotyping. Based on the acquired biomarkers,

TB metabolic models are constructed through advanced machine learning algorithms.

The robust metabolic model yields a 97.8% (95% confidence interval (CI),

0.964-0.986) area under the curve (AUC) in TB diagnosis and an 85.7% (95% CI,

0.806-0.891) AUC in phenotyping. In this study, serum metabolic biomarker panels

are revealed and develop an accurate metabolic tool with desirable diagnostic

performance for TB diagnosis and phenotyping, which may expedite the effective

implementation of the end-TB strategy.

CI - © 2024 The Author(s). Advanced Science published by Wiley‐VCH GmbH.

FAU - Liu, Yajing

AU - Liu Y

AD - Department of Ultrasound in Medicine, The Second Affiliated Hospital of Zhejiang

University School of Medicine, Zhejiang University, Hangzhou, 310009, P. R.

China.

FAU - Wang, Ruimin

AU - Wang R

AD - State Key Laboratory for Oncogenes and Related Genes School of Biomedical

Engineering Institute of Medical Robotics and Med-X Research Institute, Shanghai

Jiao Tong University, Shanghai, 200030, P. R. China.

FAU - Zhang, Chao

AU - Zhang C

AD - Department of Ultrasound in Medicine, The Second Affiliated Hospital of Zhejiang

University School of Medicine, Zhejiang University, Hangzhou, 310009, P. R.

China.

FAU - Huang, Lin

AU - Huang L

AD - State Key Laboratory for Oncogenes and Related Genes School of Biomedical

Engineering Institute of Medical Robotics and Med-X Research Institute, Shanghai

Jiao Tong University, Shanghai, 200030, P. R. China.

FAU - Chen, Jifan

AU - Chen J

AD - Department of Ultrasound in Medicine, The Second Affiliated Hospital of Zhejiang

University School of Medicine, Zhejiang University, Hangzhou, 310009, P. R.

China.

FAU - Zeng, Yiqing

AU - Zeng Y

AD - Department of Ultrasound in Medicine, The Second Affiliated Hospital of Zhejiang

University School of Medicine, Zhejiang University, Hangzhou, 310009, P. R.

China.

FAU - Chen, Hongjian

AU - Chen H

AD - Post-Doctoral Research Center, Zhejiang SUKEAN Pharmaceutical Co., Ltd, Hangzhou,

311225, P. R. China.

FAU - Wang, Guowei

AU - Wang G

AD - Department of Ultrasound in Medicine, The Second Affiliated Hospital of Zhejiang

University School of Medicine, Zhejiang University, Hangzhou, 310009, P. R.

China.

FAU - Qian, Kun

AU - Qian K

AD - State Key Laboratory for Oncogenes and Related Genes School of Biomedical

Engineering Institute of Medical Robotics and Med-X Research Institute, Shanghai

Jiao Tong University, Shanghai, 200030, P. R. China.

FAU - Huang, Pintong

AU - Huang P

AUID- ORCID: 0000-0003-0747-5765

AD - Department of Ultrasound in Medicine, The Second Affiliated Hospital of Zhejiang

University School of Medicine, Zhejiang University, Hangzhou, 310009, P. R.

China.

AD - Research Center for Life Science and Human Health, Binjiang Institute of Zhejiang

University, Hangzhou, 310053, P. R. China.

LA - eng

GR - 82001818/National Natural Science Foundation of China/

GR - 82230069/National Natural Science Foundation of China/

GR - 82030048/National Natural Science Foundation of China/

GR - 82102191/National Natural Science Foundation of China/

GR - LQ21H180007/Zhejiang Science and Technology Project/

GR - 2024YFHZ0176/Sichuan Provincial Department of Science and Technology/

GR - 2019C03077/Key Research and Development Program of Zhejiang Province/

GR - 20DZ2220400/Science and Technology Commission of Shanghai Municipality/

GR - 2021SHZDZX/Science and Technology Commission of Shanghai Municipality/

GR - ZXWF082101/Shanghai Municipal Education Commission/

GR - 2019CXJQ03/Shanghai Municipal Health Commission/

GR - SHSMU-ZDCX20210700/Innovative Research Team of High-level Local University in

Shanghai/

GR - 2021-01-07-00-02-E00083/Program for Professor of Special Appointment (Eastern

Scholar) at Shanghai Institutions of Higher Learning/

PT - Journal Article

PT - Research Support, Non-U.S. Gov't

DEP - 20240819

PL - Germany

TA - Adv Sci (Weinh)

JT - Advanced science (Weinheim, Baden-Wurttemberg, Germany)

JID - 101664569

RN - 0 (Biomarkers)

SB - IM

MH - Humans

MH - *Tuberculosis/diagnosis/blood

MH - *Biomarkers/blood

MH - *Metabolomics/methods

MH - *Phenotype

MH - Spectrometry, Mass, Matrix-Assisted Laser Desorption-Ionization/methods

MH - COVID-19/diagnosis/blood

MH - Male

MH - Female

MH - Adult

MH - Middle Aged

MH - Machine Learning

PMC - PMC11497029

OTO - NOTNLM

OT - diagnosis and phenotyping

OT - drug resistant tuberculosis

OT - nanoparticle enhanced laser desorption/ionization mass spectrometry

OT - serum metabolic fingerprints

OT - tuberculosis

COIS- The authors declare no conflict of interest.

EDAT- 2024/08/19 18:42

MHDA- 2024/10/23 12:40

PMCR- 2024/08/19

CRDT- 2024/08/19 13:03

PHST- 2024/07/23 00:00 [revised]

PHST- 2024/06/05 00:00 [received]

PHST- 2024/10/23 12:40 [medline]

PHST- 2024/08/19 18:42 [pubmed]

PHST- 2024/08/19 13:03 [entrez]

PHST- 2024/08/19 00:00 [pmc-release]

AID - ADVS9293 [pii]

AID - 10.1002/advs.202406233 [doi]

PST - ppublish

SO - Adv Sci (Weinh). 2024 Oct;11(39):e2406233. doi: 10.1002/advs.202406233. Epub 2024

Aug 19.

PMID- 39142196

OWN - NLM

STAT- MEDLINE

DCOM- 20240828

LR - 20250304

IS - 1090-2120 (Electronic)

IS - 0045-2068 (Linking)

VI - 151

DP - 2024 Oct

TI - N-Acyl phenothiazines as mycobacterial ATP synthase inhibitors: Rational design,

synthesis and in vitro evaluation against drug sensitive, RR and MDR-TB.

PG - 107702

LID - S0045-2068(24)00607-2 [pii]

LID - 10.1016/j.bioorg.2024.107702 [doi]

AB - The mycobacterial F-ATP synthase is responsible for the optimal growth,

metabolism and viability of Mycobacteria, establishing it as a validated target

for the development of anti-TB therapeutics. Herein, we report the discovery of

an N-acyl phenothiazine derivative, termed PT6, targeting the mycobacterial F-ATP

synthase. PT6 is bactericidal and active against the drug sensitive,

Rifampicin-resistant as well as Multidrug-resistant tuberculosis strains.

Compound PT6 showed noteworthy inhibition of F-ATP synthesis, exhibiting an

IC(50) of 0.788 µM in M. smegmatis IMVs and was observed that it could deplete

intracellular ATP levels, exhibiting an IC(50) of 30 µM. PT6 displayed a high

selectivity towards mycobacterial ATP synthase compared to mitochondrial ATP

synthase. Compound PT6 showed a minor synergistic effect in combination with

Rifampicin and Isoniazid. PT6 demonstrated null cytotoxicity as confirmed by

assessing its toxicity against VERO cell lines. Further, the binding mechanism

and the activity profile of PT6 were validated by employing in silico techniques

such as molecular docking, Prime MM/GBSA, DFT and ADMET analysis. These results

suggest that PT6 presents an attractive lead for the discovery of a novel class

of mycobacterial F-ATP synthase inhibitors.

CI - Copyright © 2024 Elsevier Inc. All rights reserved.

FAU - Reddyrajula, Rajkumar

AU - Reddyrajula R

AD - Central Research Facility, National Institute of Technology Karnataka, Surathkal,

Mangalore 575 025, India; Department of Chemical Sciences, National Institute of

Pharmaceutical Education and Research (NIPER), Hyderabad 500 037, India.

FAU - Perveen, Summaya

AU - Perveen S

AD - Infectious Diseases Division, CSIR- Indian Institute of Integrative Medicine,

Jammu 180 001, India; Academy of Scientific and Innovative Research (AcSIR),

Ghaziabad 201 002, India.

FAU - Negi, Anjali

AU - Negi A

AD - Infectious Diseases Division, CSIR- Indian Institute of Integrative Medicine,

Jammu 180 001, India; Academy of Scientific and Innovative Research (AcSIR),

Ghaziabad 201 002, India.

FAU - Etikyala, Umadevi

AU - Etikyala U

AD - Medicinal Chemistry Laboratory, Department of Chemistry, Osmania University,

Hyderabad 500 076, India.

FAU - Manga, Vijjulatha

AU - Manga V

AD - Medicinal Chemistry Laboratory, Department of Chemistry, Osmania University,

Hyderabad 500 076, India.

FAU - Sharma, Rashmi

AU - Sharma R

AD - Infectious Diseases Division, CSIR- Indian Institute of Integrative Medicine,

Jammu 180 001, India; Academy of Scientific and Innovative Research (AcSIR),

Ghaziabad 201 002, India. Electronic address: rashmi.sharma.09@iiim.res.in.

FAU - Dalimba, Udaya Kumar

AU - Dalimba UK

AD - Organic and Materials Chemistry Laboratory, Department of Chemistry, National

Institute of Technology Karnataka, Surathkal, Mangalore 575 025, India.

Electronic address: udayakumar@nitk.edu.in.

LA - eng

PT - Journal Article

PT - Research Support, Non-U.S. Gov't

DEP - 20240807

PL - United States

TA - Bioorg Chem

JT - Bioorganic chemistry

JID - 1303703

RN - 0 (Phenothiazines)

RN - 0 (Antitubercular Agents)

RN - 0 (Enzyme Inhibitors)

SB - IM

MH - *Phenothiazines/pharmacology/chemistry/chemical synthesis

MH - *Antitubercular Agents/pharmacology/chemical synthesis/chemistry

MH - *Drug Design

MH - *Mycobacterium tuberculosis/drug effects/enzymology

MH - Structure-Activity Relationship

MH - *Microbial Sensitivity Tests

MH - Molecular Structure

MH - *Enzyme Inhibitors/pharmacology/chemical synthesis/chemistry

MH - Dose-Response Relationship, Drug

MH - Animals

MH - Chlorocebus aethiops

MH - Vero Cells

MH - Molecular Docking Simulation

MH - Tuberculosis, Multidrug-Resistant/drug therapy

OTO - NOTNLM

OT - Molecular docking and DFT analysis

OT - Mycobacterial F-ATP synthase

OT - Mycobacterium tuberculosis

OT - RR/MDR-TB

COIS- Declaration of competing interest The authors declare that they have no known

competing financial interests or personal relationships that could have appeared

to influence the work reported in this paper.

EDAT- 2024/08/15 00:42

MHDA- 2024/08/31 09:44

CRDT- 2024/08/14 18:08

PHST- 2024/05/22 00:00 [received]

PHST- 2024/07/31 00:00 [revised]

PHST- 2024/08/05 00:00 [accepted]

PHST- 2024/08/31 09:44 [medline]

PHST- 2024/08/15 00:42 [pubmed]

PHST- 2024/08/14 18:08 [entrez]

AID - S0045-2068(24)00607-2 [pii]

AID - 10.1016/j.bioorg.2024.107702 [doi]

PST - ppublish

SO - Bioorg Chem. 2024 Oct;151:107702. doi: 10.1016/j.bioorg.2024.107702. Epub 2024

Aug 7.

PMID- 39309911

OWN - NLM

STAT- PubMed-not-MEDLINE

LR - 20240924

IS - 2405-8440 (Print)

IS - 2405-8440 (Electronic)

IS - 2405-8440 (Linking)

VI - 10

IP - 18

DP - 2024 Sep 30

TI - Association of bacteriomes with drug susceptibility in lesions of pulmonary

tuberculosis patients.

PG - e37583

LID - 10.1016/j.heliyon.2024.e37583 [doi]

LID - e37583

AB - Understanding how the bacteriomes in tuberculous lesions can be influenced by the

susceptibility of Mycobacterium tuberculosis (MTB) can provide valuable

information for preventing and treating drug resistant tuberculosis (DR-TB).

High-throughput 16S rRNA sequencing was employed to analyze the bacteriome in

pulmonary TB lesions from 14 patients with DR-TB and 47 patients with drug

sensitive tuberculosis (DS-TB), along with 18 normal lung tissues (NT) from 18

lung cancer patients serving as the bacterial baseline. The phylogenetic

investigation of communities by reconstruction of unobserved states2 (PICRUSt2)

algorithm was utilized to predict bacterial metabolic functions. The major phyla

of pulmonary bacteriomes included Proteobacteria, Actinobacteria, Bacteroidetes,

Firmicutes and Fusobacteria. Alpha diversity indices, including ACE, Chao1,

Shannon and OTU observed, all demonstrated different bacterial communities of

DS-TB samples from that of NT samples; while only Shannon indicated difference

between DR-TB and NT samples. The analysis of similarity (ANOSIM) showed

significantly different bacterial communities within TB lesions compared to NT

samples (R = 0.418, p = 0.001). However, difference was not observed between

DR-TB and DS-TB samples (ANOSIM, R = 0.069, p = 0.173). The bacterial profiles

within each DR-TB individual appeared unique, with no obvious clusters

corresponding to drug-resistant phenotypes. Nevertheless, indicator genera

identified in DR-TB and DS-TB lesions demonstrated distinctive micro-ecological

environments. Most COG functions were enriched in TB lesions, and the most

significant one was [J] translation, ribosomal structure and biogenesis. The

distinct enrichment patterns of bacterial enzymes in DR-TB and DS-TB lesions

suggest that pulmonary bacterial activities can be modulated by the

susceptibility of MTB bacilli. This study provides fresh perspectives and

strategies for the precise diagnosis and assessment of drug resistance

tuberculosis.

CI - © 2024 The Authors. Published by Elsevier Ltd.

FAU - Du, Weili

AU - Du W

AD - Department of Pathology, Beijing Key Laboratory for Drug Resistant Tuberculosis

Research, Beijing Chest Hospital, Capital Medical University, Beijing

Tuberculosis and Thoracic Tumor Research Institute, Beiguandajie 9#, Tongzhou

Dist, Beijing, 101149, China.

FAU - Zhao, Yingli

AU - Zhao Y

AD - Department of Pathology, Beijing Key Laboratory for Drug Resistant Tuberculosis

Research, Beijing Chest Hospital, Capital Medical University, Beijing

Tuberculosis and Thoracic Tumor Research Institute, Beiguandajie 9#, Tongzhou

Dist, Beijing, 101149, China.

FAU - Zhang, Chen

AU - Zhang C

AD - Department of Pathology, Beijing Key Laboratory for Drug Resistant Tuberculosis

Research, Beijing Chest Hospital, Capital Medical University, Beijing

Tuberculosis and Thoracic Tumor Research Institute, Beiguandajie 9#, Tongzhou

Dist, Beijing, 101149, China.

FAU - Zhang, Li

AU - Zhang L

AD - Department of Pathology, Beijing Key Laboratory for Drug Resistant Tuberculosis

Research, Beijing Chest Hospital, Capital Medical University, Beijing

Tuberculosis and Thoracic Tumor Research Institute, Beiguandajie 9#, Tongzhou

Dist, Beijing, 101149, China.

FAU - Zhou, Lijuan

AU - Zhou L

AD - Department of Pathology, Beijing Key Laboratory for Drug Resistant Tuberculosis

Research, Beijing Chest Hospital, Capital Medical University, Beijing

Tuberculosis and Thoracic Tumor Research Institute, Beiguandajie 9#, Tongzhou

Dist, Beijing, 101149, China.

FAU - Sun, Zuyu

AU - Sun Z

AD - Department of Pathology, Beijing Key Laboratory for Drug Resistant Tuberculosis

Research, Beijing Chest Hospital, Capital Medical University, Beijing

Tuberculosis and Thoracic Tumor Research Institute, Beiguandajie 9#, Tongzhou

Dist, Beijing, 101149, China.

FAU - Huang, Xiaojie

AU - Huang X

AD - Department of Pathology, Beijing Key Laboratory for Drug Resistant Tuberculosis

Research, Beijing Chest Hospital, Capital Medical University, Beijing

Tuberculosis and Thoracic Tumor Research Institute, Beiguandajie 9#, Tongzhou

Dist, Beijing, 101149, China.

FAU - Zhang, Nana

AU - Zhang N

AD - Department of Pathology, Beijing Key Laboratory for Drug Resistant Tuberculosis

Research, Beijing Chest Hospital, Capital Medical University, Beijing

Tuberculosis and Thoracic Tumor Research Institute, Beiguandajie 9#, Tongzhou

Dist, Beijing, 101149, China.

FAU - Liu, Zichen

AU - Liu Z

AD - Department of Pathology, Beijing Key Laboratory for Drug Resistant Tuberculosis

Research, Beijing Chest Hospital, Capital Medical University, Beijing

Tuberculosis and Thoracic Tumor Research Institute, Beiguandajie 9#, Tongzhou

Dist, Beijing, 101149, China.

FAU - Li, Kun

AU - Li K

AD - Department of Pathology, Beijing Key Laboratory for Drug Resistant Tuberculosis

Research, Beijing Chest Hospital, Capital Medical University, Beijing

Tuberculosis and Thoracic Tumor Research Institute, Beiguandajie 9#, Tongzhou

Dist, Beijing, 101149, China.

FAU - Che, Nanying

AU - Che N

AD - Department of Pathology, Beijing Key Laboratory for Drug Resistant Tuberculosis

Research, Beijing Chest Hospital, Capital Medical University, Beijing

Tuberculosis and Thoracic Tumor Research Institute, Beiguandajie 9#, Tongzhou

Dist, Beijing, 101149, China.

LA - eng

PT - Journal Article

DEP - 20240906

PL - England

TA - Heliyon

JT - Heliyon

JID - 101672560

PMC - PMC11414563

OTO - NOTNLM

OT - Bacterial metabolic functions

OT - Drug susceptibility

OT - Pulmonary bacteriomes

OT - TB lesions

COIS- All authors declare that they have no conflicts of interests.

EDAT- 2024/09/23 11:43

MHDA- 2024/09/23 11:44

PMCR- 2024/09/06

CRDT- 2024/09/23 05:56

PHST- 2024/04/24 00:00 [received]

PHST- 2024/09/03 00:00 [revised]

PHST- 2024/09/05 00:00 [accepted]

PHST- 2024/09/23 11:44 [medline]

PHST- 2024/09/23 11:43 [pubmed]

PHST- 2024/09/23 05:56 [entrez]

PHST- 2024/09/06 00:00 [pmc-release]

AID - S2405-8440(24)13614-6 [pii]

AID - e37583 [pii]

AID - 10.1016/j.heliyon.2024.e37583 [doi]

PST - epublish

SO - Heliyon. 2024 Sep 6;10(18):e37583. doi: 10.1016/j.heliyon.2024.e37583.

eCollection 2024 Sep 30.

PMID- 39342331

OWN - NLM

STAT- MEDLINE

DCOM- 20240929

LR - 20260127

IS - 1476-0711 (Electronic)

IS - 1476-0711 (Linking)

VI - 23

IP - 1

DP - 2024 Sep 28

TI - Deciphering the possible role of MmpL7 efflux pump in SQ109 resistance in

Mycobacterium tuberculosis.

PG - 87

LID - 10.1186/s12941-024-00746-8 [doi]

LID - 87

AB - BACKGROUND: SQ109 is a promising candidate drug for the treatment of patients

with drug-resistant tuberculosis (DR-TB). The purpose of this study was to

investigate the activity of SQ109 against clinical isolates of Mycobacterium

tuberculosis (MTB) from patients with multidrug-resistant TB (MDR-TB) and

pre-extensively drug-resistant TB (pre-XDR-TB), and to explore new drug-resistant

mechanisms of SQ109. METHODS: We evaluated the in vitro activity of SQ109 against

clinical isolates from patients with MDR-TB and pre-XDR-TB using minimal

inhibitory concentration (MIC) assay. The drug-resistant gene, mmpL3 of

SQ109-resistant strains was sequenced, and a quantitative real-time PCR assay was

used to analyze 28 efflux pump genes in SQ109-resistant strains without mmpL3

mutations. The role of candidate efflux pumps mmpL5 and mmpL7 on the MIC of SQ109

was evaluated using recombinantly cloned MmpL5 and MmpL7 expressed in

Mycobacterium smegmatis. RESULTS: The MIC(90), MIC(95) and MIC(99) values of

SQ109 for 225 clinical isolates of MTB were 0.25 mg/L, 0.5 mg/L and 1.0 mg/L,

respectively. Among the pre-XDR strains, six showed resistance to SQ109 despite

the absence of gene mutations in mmpL3. In six resistant pre-XDR strains, the MIC

of SQ109 decreased with the use of an efflux pump inhibitor, and there was

significant upregulation of mmpL5 and mmpL7 in two strains after exposure to

SQ109. The presence of MmpL7 in Mycobacterium smegmatis resulted in decreased

susceptibility to SQ109, with the MIC increasing from 16 mg/L to 32 mg/L.

CONCLUSIONS: Our data demonstrated that SQ109 exhibited excellent levels of in

vitro activity against MTB. MmpL7 may be a potential gene for MTB resistance to

SQ109, providing a useful target for detecting SQ109 resistance in MTB.

CI - © 2024. The Author(s).

FAU - Jing, Wei

AU - Jing W

AD - Department of Tuberculosis, Beijing Chest Hospital, Capital Medical University,

Beijing Thoracic Tumor Research Institute, No. 9, Beiguan Street, Tongzhou

District, Beijing, 101149, PR China.

FAU - Zhang, Fuzhen

AU - Zhang F

AD - Department of Bacteriology and Immunology, Beijing Chest Hospital, Capital

Medical University, Beijing Tuberculosis & Thoracic Tumor Research Institute, No.

9, Beiguan Street, Tongzhou District, Beijing, 101149, PR China.

AD - Department of Epidemiology, School of Public Health, Cheeloo College of Medicine,

Shandong University, Jinan, 250012, PR China.

FAU - Shang, Yuanyuan

AU - Shang Y

AD - Department of Bacteriology and Immunology, Beijing Chest Hospital, Capital

Medical University, Beijing Tuberculosis & Thoracic Tumor Research Institute, No.

9, Beiguan Street, Tongzhou District, Beijing, 101149, PR China.

FAU - Shi, Wenhui

AU - Shi W

AD - Department of Tuberculosis, Beijing Chest Hospital, Capital Medical University,

Beijing Thoracic Tumor Research Institute, No. 9, Beiguan Street, Tongzhou

District, Beijing, 101149, PR China.

FAU - Yao, Cong

AU - Yao C

AD - Department of Bacteriology and Immunology, Beijing Chest Hospital, Capital

Medical University, Beijing Tuberculosis & Thoracic Tumor Research Institute, No.

9, Beiguan Street, Tongzhou District, Beijing, 101149, PR China.

FAU - Zhang, Xuxia

AU - Zhang X

AD - Department of Bacteriology and Immunology, Beijing Chest Hospital, Capital

Medical University, Beijing Tuberculosis & Thoracic Tumor Research Institute, No.

9, Beiguan Street, Tongzhou District, Beijing, 101149, PR China.

FAU - Chu, Naihui

AU - Chu N

AD - Department of Tuberculosis, Beijing Chest Hospital, Capital Medical University,

Beijing Thoracic Tumor Research Institute, No. 9, Beiguan Street, Tongzhou

District, Beijing, 101149, PR China. chunaihui1994@sina.com.

FAU - Lu, Jie

AU - Lu J

AD - Beijing Key Laboratory for Pediatric Diseases of Otolaryngology, Head and Neck

Surgery, Beijing Pediatric Research Institute, Beijing Children's Hospital,

Capital Medical University, National Center for Children's Health, Beijing,

101149, PR China. lujiebch@163.com.

FAU - Yuan, Jinfeng

AU - Yuan J

AD - Department of Bacteriology and Immunology, Beijing Chest Hospital, Capital

Medical University, Beijing Tuberculosis & Thoracic Tumor Research Institute, No.

9, Beiguan Street, Tongzhou District, Beijing, 101149, PR China.

yuanjinfeng0920@163.com.

LA - eng

GR - No.7224324/Beijing Natural Science Foundation (Youth Program)/

GR - QML20231606/"Young talents" program of Beijing hospitals Authority/

PT - Journal Article

DEP - 20240928

PL - England

TA - Ann Clin Microbiol Antimicrob

JT - Annals of clinical microbiology and antimicrobials

JID - 101152152

RN - 0 (Antitubercular Agents)

RN - 0 (Membrane Transport Proteins)

RN - 0 (Bacterial Proteins)

RN - 0 (N-geranyl-N'-(2-adamantyl)ethane-1,2-diamine)

RN - PJY633525U (Adamantane)

RN - 0 (Ethylenediamines)

SB - IM

MH - *Mycobacterium tuberculosis/drug effects/genetics/metabolism

MH - *Microbial Sensitivity Tests

MH - Humans

MH - *Antitubercular Agents/pharmacology

MH - *Membrane Transport Proteins/genetics/metabolism

MH - *Bacterial Proteins/genetics/metabolism

MH - *Tuberculosis, Multidrug-Resistant/microbiology

MH - Drug Resistance, Multiple, Bacterial/genetics

MH - Adamantane/pharmacology/analogs & derivatives

MH - Mutation

MH - Mycobacterium smegmatis/genetics/drug effects/metabolism

MH - Ethylenediamines

PMC - PMC11439249

OTO - NOTNLM

OT - Mycobacterium tuberculosis

OT - MmpL7

OT - SQ109

OT - Susceptibility

COIS- The authors declare no competing interests.

EDAT- 2024/09/29 00:43

MHDA- 2024/09/29 10:18

PMCR- 2024/09/28

CRDT- 2024/09/28 23:44

PHST- 2024/03/27 00:00 [received]

PHST- 2024/09/20 00:00 [accepted]

PHST- 2024/09/29 10:18 [medline]

PHST- 2024/09/29 00:43 [pubmed]

PHST- 2024/09/28 23:44 [entrez]

PHST- 2024/09/28 00:00 [pmc-release]

AID - 10.1186/s12941-024-00746-8 [pii]

AID - 746 [pii]

AID - 10.1186/s12941-024-00746-8 [doi]

PST - epublish

SO - Ann Clin Microbiol Antimicrob. 2024 Sep 28;23(1):87. doi:

10.1186/s12941-024-00746-8.

PMID- 39315249

OWN - NLM

STAT- PubMed-not-MEDLINE

LR - 20250715

IS - 2693-5015 (Electronic)

IS - 2693-5015 (Linking)

DP - 2024 Sep 13

TI - Trehalose catalytic shift is an intrinsic factor in Mycobacterium tuberculosis

that enhances phenotypic heterogeneity and multidrug resistance.

LID - rs.3.rs-4999164 [pii]

LID - 10.21203/rs.3.rs-4999164/v1 [doi]

AB - Drug-resistance (DR) in many bacterial pathogens often arises from the repetitive

formation of drug-tolerant bacilli, known as persisters. However, it is unclear

whether Mycobacterium tuberculosis (Mtb), the bacterium that causes tuberculosis

(TB), undergoes a similar phenotypic transition. Recent metabolomics studies have

identified that a change in trehalose metabolism is necessary for Mtb to develop

persisters and plays a crucial role in metabolic networks of DR-TB strains. The

present study used Mtb mutants lacking the trehalose catalytic shift and showed

that the mutants exhibited a significantly lower frequency of the emergence of DR

mutants compared to wildtype, due to reduced persister formation. The trehalose

catalytic shift enables Mtb persisters to survive under bactericidal antibiotics

by increasing metabolic heterogeneity and drug tolerance, ultimately leading to

development of DR. Intriguingly, rifampicin (RIF)-resistant bacilli exhibit

cross-resistance to a second antibiotic, due to a high trehalose catalytic shift

activity. This phenomenon explains how the development of multidrug resistance

(MDR) is facilitated by the acquisition of RIF resistance. In this context, the

heightened risk of MDR-TB in the lineage 4 HN878 W-Beijing strain can be

attributed to its greater trehalose catalytic shift. Genetic and pharmacological

inactivation of the trehalose catalytic shift significantly reduced persister

formation, subsequently decreasing the incidence of MDR-TB in HN878 W-Beijing

strain. Collectively, the trehalose catalytic shift serves as an intrinsic factor

of Mtb responsible for persister formation, cross-resistance to multiple

antibiotics, and the emergence of MDR-TB. This study aids in the discovery of new

TB therapeutics by targeting the trehalose catalytic shift of Mtb.

FAU - Eoh, Hyungjin

AU - Eoh H

AUID- ORCID: 0000-0001-8774-6400

AD - University of Southern California.

FAU - Lee, Jae Jin

AU - Lee JJ

AUID- ORCID: 0000-0001-8317-437X

AD - University of Southern California.

FAU - Swanson, Daniel

AU - Swanson D

AD - Central Michigan University.

FAU - Lee, Sun-Kyung

AU - Lee SK

AD - Internal Tuberculosis Research Center.

FAU - Dihardjo, Stephanie

AU - Dihardjo S

AD - University of Southern California.

FAU - Lee, Gi Yong

AU - Lee GY

AD - University of Southern California.

FAU - Sree, Gelle

AU - Sree G

AD - University of Southern California.

FAU - Maskill, Emily

AU - Maskill E

AD - Central Michigan University.

FAU - Taylor, Zachary

AU - Taylor Z

AD - Indiana University Bloomington.

FAU - Van Nieuwenhze, Michael

AU - Van Nieuwenhze M

AD - Baylor University.

FAU - Singh, Abhyudai

AU - Singh A

AUID- ORCID: 0000-0002-1451-2838

AD - University of Delaware.

FAU - Lee, Jong-Seok

AU - Lee JS

AD - International Tuberculosis Research Center.

FAU - Eum, Seok-Yong

AU - Eum SY

AD - Internal Tuberculosis Research Center.

FAU - Cho, Sang-Nae

AU - Cho SN

AD - Yonsei University College of Medicine.

FAU - Swarts, Benjamin

AU - Swarts B

AD - Central Michigan University.

LA - eng

GR - R01 AI168088/AI/NIAID NIH HHS/United States

GR - R21 AI139386/AI/NIAID NIH HHS/United States

GR - R35 GM148351/GM/NIGMS NIH HHS/United States

PT - Journal Article

PT - Preprint

DEP - 20240913

PL - United States

TA - Res Sq

JT - Research square

JID - 101768035

UIN - Nat Commun. 2025 Jul 11;16(1):6442. doi: 10.1038/s41467-025-61703-3. PMID:

40645928

PMC - PMC11419184

EDAT- 2024/09/24 10:19

MHDA- 2024/09/24 10:20

PMCR- 2024/09/23

CRDT- 2024/09/24 04:50

PHST- 2024/09/24 10:19 [pubmed]

PHST- 2024/09/24 10:20 [medline]

PHST- 2024/09/24 04:50 [entrez]

PHST- 2024/09/23 00:00 [pmc-release]

AID - rs.3.rs-4999164 [pii]

AID - 10.21203/rs.3.rs-4999164/v1 [doi]

PST - epublish

SO - Res Sq [Preprint]. 2024 Sep 13:rs.3.rs-4999164. doi: 10.21203/rs.3.rs-4999164/v1.

PMID- 39150887

OWN - NLM

STAT- MEDLINE

DCOM- 20240913

LR - 20260127

IS - 2373-8227 (Electronic)

IS - 2373-8227 (Linking)

VI - 10

IP - 9

DP - 2024 Sep 13

TI - Development and Evaluation of Bis-benzothiazoles as a New Class of Benzothiazoles

Targeting DprE1 as Antitubercular Agents.

PG - 3320-3331

LID - 10.1021/acsinfecdis.4c00415 [doi]

AB - Benzothiazole-bearing compounds have emerged as potential noncovalent DprE1

(decaprenylphosphoryl-β-d-ribose-2'-epimerase) inhibitors active against

Mycobacterium tuberculosis. Based on structure-based virtual screening (PDB ID:

4KW5), a focused library of thirty-one skeletally diverse benzothiazole amides

was prepared, and the compounds were assessed for their antitubercular activity

against M.tb H37Ra. Most potent compounds 3b and 3n were further evaluated

against the M.tb H37Rv strain by the microdilution assay method. Among the

compounds evaluated, bis-benzothiazole amide 3n emerged as a hit molecule and

demonstrated promising antitubercular activity with minimum inhibitory

concentration (MIC) values of 0.45 μg/mL and 8.0 μg/mL against H(37)Ra and

H(37)Rv, respectively. Based on the preliminary hit molecule (3n), a focused

library of 12 more bis-benzothiazole amide derivatives was further prepared by

varying the substituents on either side to obtain new leads and generate a

structure-activity relationship (SAR). Among these compounds, 6a, 6c, and 6d

demonstrated remarkable antitubercular activity with MIC values of 0.5 μg/mL

against H37Ra and 1.0, 2.0, and 8.0 μg/mL against H(37)Rv, respectively. The most

active compound, 6a, also displayed significant efficacy against four

drug-resistant tuberculosis strains. Compound 6a was assessed for in vitro

cytotoxicity against the HepG2 cell line, and it displayed insignificant

cytotoxicity. Furthermore, time-kill kinetic studies demonstrated time- and

dose-dependent bactericidal activity of this compound. The GFP release assay

revealed that compound 6a targets the inhibition of a cell wall component. SNPs

in dprE-1 gene assessment revealed that compound 6a binds to tyrosine at position

314 of DprE1 and replaces it with histidine, causing resistance similar to that

of standard TCA1. In silico docking studies further suggest that the strong

noncovalent interactions of these compounds may lead to the development of potent

noncovalent DprE1 inhibitors.

FAU - Samoon, Rabiya

AU - Samoon R

AD - Department of Chemistry, School of Chemical and Life science, Jamia Hamdard, New

Delhi 110062, India.

FAU - Sau, Shashikanta

AU - Sau S

AD - Department of Biological Sciences (Pharmacology and Toxicology), National

Institute of Pharmaceutical Education and Research (NIPER), Hyderabad, Telangana

500037, India.

FAU - Roy, Arnab

AU - Roy A

AD - Department of Biological Sciences (Pharmacology and Toxicology), National

Institute of Pharmaceutical Education and Research (NIPER), Hyderabad, Telangana

500037, India.

FAU - Parida, Kishan Kumar

AU - Parida KK

AD - Department of Biological Sciences (Pharmacology and Toxicology), National

Institute of Pharmaceutical Education and Research (NIPER), Hyderabad, Telangana

500037, India.

FAU - Sharma, Kalicharan

AU - Sharma K

AUID- ORCID: 0000-0003-0333-2521

AD - Department of Pharmaceutical Chemistry, ISF College of Pharmacy, Moga, Punjab

142001, India.

FAU - Yakkala, Prasanna Anjaneyulu

AU - Yakkala PA

AD - Department of Pharmaceutical Chemistry, School of Pharmaceutical Education and

Research, Jamia Hamdard, New Delhi 110062, India.

FAU - Dewangan, Rikeshwer Prasad

AU - Dewangan RP

AD - Department of Pharmaceutical Chemistry, School of Pharmaceutical Education and

Research, Jamia Hamdard, New Delhi 110062, India.

FAU - Abdin, Malik Zainul

AU - Abdin MZ

AD - Centre for Transgenic Plants, Department of Biotechnology, School of Chemical and

Life Sciences, Jamia Hamdard India, New Delhi 110062, India.

FAU - Kalia, Nitin Pal

AU - Kalia NP

AD - Department of Biological Sciences (Pharmacology and Toxicology), National

Institute of Pharmaceutical Education and Research (NIPER), Hyderabad, Telangana

500037, India.

FAU - Shafi, Syed

AU - Shafi S

AUID- ORCID: 0000-0001-7657-0630

AD - Department of Chemistry, School of Chemical and Life science, Jamia Hamdard, New

Delhi 110062, India.

LA - eng

PT - Journal Article

DEP - 20240816

PL - United States

TA - ACS Infect Dis

JT - ACS infectious diseases

JID - 101654580

RN - 0 (Antitubercular Agents)

RN - 0 (Benzothiazoles)

RN - EC 1.1.- (DprE1 protein, Mycobacterium tuberculosis)

RN - 0 (Bacterial Proteins)

RN - EC 1.1.- (Alcohol Oxidoreductases)

SB - IM

MH - *Antitubercular Agents/pharmacology/chemistry/chemical synthesis

MH - *Mycobacterium tuberculosis/drug effects

MH - *Benzothiazoles/pharmacology/chemistry

MH - *Microbial Sensitivity Tests

MH - Humans

MH - *Bacterial Proteins/antagonists & inhibitors/metabolism

MH - Structure-Activity Relationship

MH - Molecular Docking Simulation

MH - Alcohol Oxidoreductases

OTO - NOTNLM

OT - DprE1

OT - SAR

OT - TCA1

OT - antitubercular

OT - benzothiazole

OT - in silico

OT - noncovalent inhibitors

EDAT- 2024/08/16 18:42

MHDA- 2024/09/15 21:13

CRDT- 2024/08/16 13:12

PHST- 2024/09/15 21:13 [medline]

PHST- 2024/08/16 18:42 [pubmed]

PHST- 2024/08/16 13:12 [entrez]

AID - 10.1021/acsinfecdis.4c00415 [doi]

PST - ppublish

SO - ACS Infect Dis. 2024 Sep 13;10(9):3320-3331. doi: 10.1021/acsinfecdis.4c00415.

Epub 2024 Aug 16.

PMID- 39282290

OWN - NLM

STAT- PubMed-not-MEDLINE

LR - 20250509

IS - 2692-8205 (Electronic)

IS - 2692-8205 (Linking)

DP - 2024 Sep 4

TI - Chemical genetic interactions elucidate pathways controlling tuberculosis

antibiotic efficacy during infection.

LID - 2024.09.04.609063 [pii]

LID - 10.1101/2024.09.04.609063 [doi]

AB - Successful tuberculosis therapy requires treatment with an unwieldy multidrug

combination for several months. Thus, there is a growing need to identify novel

genetic vulnerabilities that can be leveraged to develop new, more effective

antitubercular drugs. Consequently, recent efforts to optimize TB therapy have

exploited Mtb chemical genetics to identify pathways influencing antibiotic

efficacy, novel mechanisms of antibiotic action, and new targets for TB drug

discovery. However, the influence of the complex host environment on these

interactions remains largely unknown, leaving the therapeutic potential of the

identified targets unclear. In this study, we leveraged a library of conditional

mutants targeting 467 essential Mtb genes to characterize the chemical-genetic

interactions (CGIs) with TB drugs directly in the mouse infection model. We found

that these in vivo CGIs differ significantly from those identified in vitro .

Both drug-specific and drug-agnostic effects were identified, and many were

preserved during treatment with a multidrug combination, suggesting numerous

strategies for enhancing therapy. This work also elucidated the complex effects

of pyrazinamide (PZA), a drug that relies on aspects of the infection environment

for efficacy. Specifically, our work supports the importance of coenzyme A

synthesis inhibition during infection, as well as the antagonistic effect of iron

limitation on PZA activity. In addition, we found that inhibition of thiamine and

purine synthesis increases PZA efficacy, suggesting novel therapeutically

exploitable metabolic dependencies. Our findings present a map of the unique in

vivo CGIs, characterizing the mechanism of PZA activity in vivo and identifying

novel targets for TB drug development. SIGNIFICANCE: The inevitable rise of

multi-drug-resistant tuberculosis underscores the urgent need for new TB drugs

and novel drug targets while prioritizing synergistic drug combinations.

Chemical-genetic interaction (CGI) studies have delineated bacterial pathways

influencing antibiotic efficacy and uncovered druggable pathways that synergize

with TB drugs. However, most studies are conducted in vitro , limiting our

understanding of how the host environment influences drug-mutant interactions.

Using an inducible mutant library targeting essential Mtb genes to characterize

CGIs during infection, this study reveals that CGIs are both drug-specific and

drug-agnostic and differ significantly from those observed in vitro . Synergistic

CGIs comprised distinct metabolic pathways mediating antibiotic efficacy,

revealing novel drug mechanisms of action, and defining potential drug targets

that would synergize with frontline antitubercular drugs.

FAU - Oluoch, Peter O

AU - Oluoch PO

AUID- ORCID: 0000-0001-7451-4993

FAU - Koh, Eun-Ik

AU - Koh EI

FAU - Proulx, Megan K

AU - Proulx MK

AUID- ORCID: 0000-0002-9524-8302

FAU - Reames, Charlotte J

AU - Reames CJ

AUID- ORCID: 0000-0001-5579-5881

FAU - Papavinasasundaram, Kadamba G

AU - Papavinasasundaram KG

AUID- ORCID: 0000-0001-7837-1344

FAU - Murphy, Kenan C

AU - Murphy KC

AUID- ORCID: 0000-0002-3677-2876

FAU - Zimmerman, Matthew D

AU - Zimmerman MD

FAU - Dartois, Véronique

AU - Dartois V

AUID- ORCID: 0000-0001-9470-5009

FAU - Sassetti, Christopher M

AU - Sassetti CM

AUID- ORCID: 0000-0001-6178-4329

LA - eng

PT - Journal Article

PT - Preprint

DEP - 20240904

PL - United States

TA - bioRxiv

JT - bioRxiv : the preprint server for biology

JID - 101680187

UIN - Proc Natl Acad Sci U S A. 2025 Mar 04;122(9):e2417525122. doi:

10.1073/pnas.2417525122. PMID: 39993187

PMC - PMC11398305

EDAT- 2024/09/17 10:42

MHDA- 2024/09/17 10:43

PMCR- 2024/09/13

CRDT- 2024/09/16 06:25

PHST- 2024/09/17 10:43 [medline]

PHST- 2024/09/17 10:42 [pubmed]

PHST- 2024/09/16 06:25 [entrez]

PHST- 2024/09/13 00:00 [pmc-release]

AID - 2024.09.04.609063 [pii]

AID - 10.1101/2024.09.04.609063 [doi]

PST - epublish

SO - bioRxiv [Preprint]. 2024 Sep 4:2024.09.04.609063. doi: 10.1101/2024.09.04.609063.

PMID- 39227770

OWN - NLM

STAT- MEDLINE

DCOM- 20240904

LR - 20240906

IS - 1471-2180 (Electronic)

IS - 1471-2180 (Linking)

VI - 24

IP - 1

DP - 2024 Sep 4

TI - Antibacterial efficacy of mycobacteriophages against virulent Mycobacterium

tuberculosis.

PG - 320

LID - 10.1186/s12866-024-03474-3 [doi]

LID - 320

AB - Tuberculosis (TB) remains a major global health concern, with drug-resistant

strains posing a significant challenge to effective treatment. Bacteriophage

(phage) therapy has emerged as a potential alternative to combat antibiotic

resistance. In this study, we investigated the efficacy of widely used

mycobacteriophages (D29, TM4, DS6A) against Mycobacterium tuberculosis (M.

tuberculosis) under pathophysiological conditions associated with TB, such as low

pH and hypoxia. We found that even at low multiplicity of infection (MOI),

mycobacteriophages effectively infected M. tuberculosis, got rapidly amplified,

and lysed M. tuberculosis, demonstrating their potential as therapeutic agents.

Furthermore, we observed a novel phage tolerance mechanism with bacteria forming

aggregates after several days of phage treatment. These aggregates were enriched

with biofilm components and metabolically active bacteria. However, no phage

tolerance was observed upon treatment with the three-phage mixture, highlighting

the dynamic interplay between phages and bacteria and emphasizing the importance

of phage cocktails. We also observed that phages were effective in lysing

bacteria even under low pH and low oxygen concentrations as well as

antibiotic-resistant bacteria. Our results provide key insights into phage

infection of slow-growing bacteria and suggest that mycobacteriophages can

effectively eliminate M. tuberculosis in complex pathophysiological environments

like hypoxia and acidic pH. These results can aid in developing targeted

phage-based therapies to combat antibiotic-resistant mycobacterial infections.

CI - © 2024. The Author(s).

FAU - Jeyasankar, Sharumathi

AU - Jeyasankar S

AD - Department of Bioengineering, Indian Institute of Science, Bengaluru, India.

FAU - Kalapala, Yeswanth Chakravarthy

AU - Kalapala YC

AD - Department of Bioengineering, Indian Institute of Science, Bengaluru, India.

FAU - Sharma, Pallavi Raj

AU - Sharma PR

AD - Department of Bioengineering, Indian Institute of Science, Bengaluru, India.

FAU - Agarwal, Rachit

AU - Agarwal R

AD - Department of Bioengineering, Indian Institute of Science, Bengaluru, India.

rachit@iisc.ac.in.

LA - eng

GR - OPP1210498/Bill and Melinda Gates Foundation/

PT - Journal Article

DEP - 20240904

PL - England

TA - BMC Microbiol

JT - BMC microbiology

JID - 100966981

SB - IM

MH - *Mycobacterium tuberculosis/virology/drug effects/pathogenicity

MH - *Mycobacteriophages/physiology

MH - Hydrogen-Ion Concentration

MH - *Phage Therapy

MH - Tuberculosis/microbiology/therapy

MH - Biofilms/drug effects/growth & development

MH - Humans

PMC - PMC11373169

OTO - NOTNLM

OT - Drug-resistant tuberculosis

OT - Hypoxia

OT - Low pH

OT - Multiplicity of infection (MOI)

OT - Pathophysiological conditions

OT - Phage cocktails

OT - Phage resistance

OT - Phage therapy

COIS- The authors declare no competing interests.

EDAT- 2024/09/04 01:01

MHDA- 2024/09/04 06:42

PMCR- 2024/09/04

CRDT- 2024/09/03 23:45

PHST- 2024/07/15 00:00 [received]

PHST- 2024/08/23 00:00 [accepted]

PHST- 2024/09/04 06:42 [medline]

PHST- 2024/09/04 01:01 [pubmed]

PHST- 2024/09/03 23:45 [entrez]

PHST- 2024/09/04 00:00 [pmc-release]

AID - 10.1186/s12866-024-03474-3 [pii]

AID - 3474 [pii]

AID - 10.1186/s12866-024-03474-3 [doi]

PST - epublish

SO - BMC Microbiol. 2024 Sep 4;24(1):320. doi: 10.1186/s12866-024-03474-3.

PMID- 39032811

OWN - NLM

STAT- MEDLINE

DCOM- 20240902

LR - 20240905

IS - 1878-5832 (Electronic)

IS - 1359-6446 (Linking)

VI - 29

IP - 9

DP - 2024 Sep

TI - P-glycoprotein inhibitors as an adjunct therapy for TB.

PG - 104108

LID - S1359-6446(24)00233-2 [pii]

LID - 10.1016/j.drudis.2024.104108 [doi]

AB - The primary challenge in TB treatment is the emergence of multidrug-resistant TB

(MDR-TB). One of the major factors responsible for MDR is the upregulation of

efflux pumps. Permeation-glycoprotein (P-gp), an efflux pump, hinders the

bioavailability of the administered drugs inside the infected cells.

Simultaneously, angiogenesis, the formation of new blood vessels, contributes to

drug delivery complexities. TB infection triggers a cascade of events that

upregulates the expression of angiogenic factors and P-gp. The combined action of

P-gp and angiogenesis foster the emergence of MDR-TB. Understanding these

mechanisms is pivotal for developing targeted interventions to overcome MDR in

TB. P-gp inhibitors, such as verapamil, and anti-angiogenic drugs, including

bevacizumab, have shown improvement in TB drug delivery to granuloma. In this

review, we discuss the potential of P-gp inhibitors as an adjunct therapy to

shorten TB treatment.

CI - Copyright © 2024. Published by Elsevier Ltd.

FAU - Parida, Kishan Kumar

AU - Parida KK

AD - Department of Biological Sciences (Pharmacology and Toxicology), National

Institute of Pharmaceutical Education and Research, Hyderabad, Telangana, India.

FAU - Lahiri, Monali

AU - Lahiri M

AD - Department of Biological Sciences (Pharmacology and Toxicology), National

Institute of Pharmaceutical Education and Research, Hyderabad, Telangana, India.

FAU - Ghosh, Mainak

AU - Ghosh M

AD - Department of Biological Sciences (Pharmacology and Toxicology), National

Institute of Pharmaceutical Education and Research, Hyderabad, Telangana, India.

FAU - Dalal, Aman

AU - Dalal A

AD - Department of Biological Sciences (Pharmacology and Toxicology), National

Institute of Pharmaceutical Education and Research, Hyderabad, Telangana, India.

FAU - Kalia, Nitin Pal

AU - Kalia NP

AD - Department of Biological Sciences (Pharmacology and Toxicology), National

Institute of Pharmaceutical Education and Research, Hyderabad, Telangana, India.

Electronic address: kalianpk@gmail.com.

LA - eng

PT - Journal Article

PT - Review

DEP - 20240719

PL - England

TA - Drug Discov Today

JT - Drug discovery today

JID - 9604391

RN - 0 (Antitubercular Agents)

RN - 0 (ATP Binding Cassette Transporter, Subfamily B, Member 1)

SB - IM

MH - Humans

MH - *Antitubercular Agents/pharmacology/therapeutic use

MH - Animals

MH - *ATP Binding Cassette Transporter, Subfamily B, Member 1/antagonists &

inhibitors/metabolism

MH - *Tuberculosis, Multidrug-Resistant/drug therapy

OTO - NOTNLM

OT - P-glycoprotein

OT - TB

OT - angiogenesis

OT - drug efflux

OT - granuloma

OT - macrophages

OT - multidrug resistance

EDAT- 2024/07/21 15:43

MHDA- 2024/09/02 08:45

CRDT- 2024/07/20 19:30

PHST- 2024/03/21 00:00 [received]

PHST- 2024/07/08 00:00 [revised]

PHST- 2024/07/15 00:00 [accepted]

PHST- 2024/09/02 08:45 [medline]

PHST- 2024/07/21 15:43 [pubmed]

PHST- 2024/07/20 19:30 [entrez]

AID - S1359-6446(24)00233-2 [pii]

AID - 10.1016/j.drudis.2024.104108 [doi]

PST - ppublish

SO - Drug Discov Today. 2024 Sep;29(9):104108. doi: 10.1016/j.drudis.2024.104108. Epub

2024 Jul 19.

PMID- 39002701

OWN - NLM

STAT- MEDLINE

DCOM- 20240902

LR - 20240902

IS - 1872-7913 (Electronic)

IS - 0924-8579 (Linking)

VI - 64

IP - 3

DP - 2024 Sep

TI - Anti-tuberculosis effect of microbiome therapeutic PMC205 in extensively

drug-resistant pulmonary tuberculosis in vivo.

PG - 107274

LID - S0924-8579(24)00192-4 [pii]

LID - 10.1016/j.ijantimicag.2024.107274 [doi]

AB - BACKGROUND: Tuberculosis is a highly contagious disease caused by Mycobacterium

tuberculosis, and the increase in antibiotic resistance threatens humankind.

Therefore, there is an urgent need to develop new anti-tuberculosis drugs that

can overcome the limitations of existing drugs. Here, we report the

anti-tuberculosis effect of microbiome therapeutic PMC205, a strain of Bacillus

subtilis. METHODS: The anti-tuberculosis activity of probiotics was evaluated in

mouse models of lethal and latent pulmonary tuberculosis induced by high or

low-dose infection of the extensively drug-resistant strain. Probiotics were

administered by inhalation, and the burden of M. tuberculosis in the lungs, along

with mortality and clinical observations, were monitored for 12 weeks and 8

months, respectively. For an in-depth understanding, analysis of the microbiome

and inflammatory profile of the lung microenvironment and induction of autophagy

in vitro were explored. RESULTS: After inhalation administration of PMC205 for 3

months, the survival rate was 100%, unlike all deaths in the saline-treated

group, and the burden of M. tuberculosis in the lungs was reduced by log 1.3 in

the 8-month latent tuberculosis model. Moreover, PMC205 induced recovery of

disrupted lung microflora, increased butyric acid, and suppressed excessive

inflammation. It also promoted autophagy. CONCLUSIONS: These results confirm

PMC205's anti-tuberculosis effect, suggesting that it can be developed as an

adjuvant to current antibiotic therapy to solve the drug-resistant tuberculosis

problem.

CI - Copyright © 2024. Published by Elsevier Ltd.

FAU - Seo, Hoonhee

AU - Seo H

AD - Probiotics Microbiome Convergence Center, Soonchunhyang University, Asan-si,

Chungnam, Republic of Korea.

FAU - Yoon, Youjin

AU - Yoon Y

AD - Department of Microbiology and Immunology, School of Medicine, Soonchunhyang

University, Cheonan-si, Chungnam, Republic of Korea.

FAU - Kim, Sukyung

AU - Kim S

AD - Probiotics Microbiome Convergence Center, Soonchunhyang University, Asan-si,

Chungnam, Republic of Korea.

FAU - Ghorbanian, Fatemeh

AU - Ghorbanian F

AD - Department of Microbiology and Immunology, School of Medicine, Soonchunhyang

University, Cheonan-si, Chungnam, Republic of Korea.

FAU - Tajdozian, Hanieh

AU - Tajdozian H

AD - Department of Microbiology and Immunology, School of Medicine, Soonchunhyang

University, Cheonan-si, Chungnam, Republic of Korea.

FAU - Jo, Sujin

AU - Jo S

AD - Department of Microbiology and Immunology, School of Medicine, Soonchunhyang

University, Cheonan-si, Chungnam, Republic of Korea.

FAU - Barman, Indrajeet

AU - Barman I

AD - Department of Microbiology and Immunology, School of Medicine, Soonchunhyang

University, Cheonan-si, Chungnam, Republic of Korea.

FAU - Lee, Soyeon

AU - Lee S

AD - Department of Microbiology and Immunology, School of Medicine, Soonchunhyang

University, Cheonan-si, Chungnam, Republic of Korea.

FAU - Lee, Youngkyoung

AU - Lee Y

AD - Department of Microbiology and Immunology, School of Medicine, Soonchunhyang

University, Cheonan-si, Chungnam, Republic of Korea.

FAU - Rahim, Md Abdur

AU - Rahim MA

AD - Department of Microbiology and Immunology, School of Medicine, Soonchunhyang

University, Cheonan-si, Chungnam, Republic of Korea.

FAU - Hossain, Mohammed Solayman

AU - Hossain MS

AD - Department of Microbiology and Immunology, School of Medicine, Soonchunhyang

University, Cheonan-si, Chungnam, Republic of Korea.

FAU - Lee, Saebim

AU - Lee S

AD - Department of Microbiology and Immunology, School of Medicine, Soonchunhyang

University, Cheonan-si, Chungnam, Republic of Korea; Probiotics Microbiome

Convergence Center, Soonchunhyang University, Asan-si, Chungnam, Republic of

Korea.

FAU - Song, Ho-Yeon

AU - Song HY

AD - Department of Microbiology and Immunology, School of Medicine, Soonchunhyang

University, Cheonan-si, Chungnam, Republic of Korea; Probiotics Microbiome

Convergence Center, Soonchunhyang University, Asan-si, Chungnam, Republic of

Korea. Electronic address: songmic@sch.ac.kr.

LA - eng

PT - Journal Article

DEP - 20240711

PL - Netherlands

TA - Int J Antimicrob Agents

JT - International journal of antimicrobial agents

JID - 9111860

RN - 0 (Antitubercular Agents)

SB - IM

MH - Animals

MH - Mice

MH - *Mycobacterium tuberculosis/drug effects

MH - *Probiotics/therapeutic use/administration & dosage

MH - *Disease Models, Animal

MH - *Extensively Drug-Resistant Tuberculosis/drug therapy/microbiology

MH - *Antitubercular Agents/therapeutic use/pharmacology

MH - *Lung/microbiology/pathology

MH - *Microbiota/drug effects

MH - *Tuberculosis, Pulmonary/drug therapy/microbiology

MH - Bacillus subtilis/drug effects

MH - Female

MH - Autophagy/drug effects

MH - Mice, Inbred C57BL

MH - Humans

OTO - NOTNLM

OT - Adjuvant microbiome therapeutic

OT - Autophagy

OT - Mycobacterium tuberculosis

OT - Tuberculosis

OT - XDR (extensively drug-resistant)

EDAT- 2024/07/14 02:43

MHDA- 2024/09/03 01:42

CRDT- 2024/07/13 19:26

PHST- 2023/06/15 00:00 [received]

PHST- 2024/06/05 00:00 [revised]

PHST- 2024/07/06 00:00 [accepted]

PHST- 2024/09/03 01:42 [medline]

PHST- 2024/07/14 02:43 [pubmed]

PHST- 2024/07/13 19:26 [entrez]

AID - S0924-8579(24)00192-4 [pii]

AID - 10.1016/j.ijantimicag.2024.107274 [doi]

PST - ppublish

SO - Int J Antimicrob Agents. 2024 Sep;64(3):107274. doi:

10.1016/j.ijantimicag.2024.107274. Epub 2024 Jul 11.

PMID- 38925196

OWN - NLM

STAT- MEDLINE

DCOM- 20240730

LR - 20240730

IS - 1879-0003 (Electronic)

IS - 0141-8130 (Linking)

VI - 274

IP - Pt 2

DP - 2024 Aug

TI - Identification of potent indolizine derivatives against Mycobacterial

tuberculosis: In vitro anti-TB properties, in silico target validation, molecular

docking and dynamics studies.

PG - 133285

LID - S0141-8130(24)04090-X [pii]

LID - 10.1016/j.ijbiomac.2024.133285 [doi]

AB - In the current study, two sets of compounds:

(E)-1-(2-(4-substitutedphenyl)-2-oxoethyl)-4-((hydroxyimino)methyl)pyridinium

derivatives (3a-3e); and

(E)-3-(substitutedbenzoyl)-7-((hydroxyimino)methyl)-2-substitutedindolizine-1-carboxylate

derivatives (5a-5j), were synthesized and biologically evaluated against two

strains of Mycobacterial tuberculosis (ATCC 25177) and multi-drug resistant (MDR)

strains. Further, they were also tested in vitro against the mycobacterial InhA

enzyme. The in vitro results showed excellent inhibitory activities against both

MTB strains and compounds 5a-5j were found to be more potent, and their MIC

values ranged from 5 to 16 μg/mL and 16-64 μg/mL against the M. tuberculosis

(ATCC 25177) and MDR-TB strains, respectively. Compound 5h with phenyl and

4-fluorobenzoyl groups attached to the 2- and 3-position of the indolizine core

was found to be the most active against both strains with MIC values of 5 μg/mL

and 16 μg/mL, respectively. On the other hand, the two sets of compounds showed

weak to moderate inhibition of InhA enzyme activity that ranged from 5 to 17 %

and 10-52 %, respectively, with compound 5f containing 4-fluoro benzoyl group

attached to the 3-position of the indolizine core being the most active (52 %

inhibition of InhA). Unfortunately, there was no clear correlation between the

InhA inhibitory activity and MIC values of the tested compounds, indicating the

probability that they might have different modes of action other than InhA

inhibition. Therefore, a computational investigation was conducted by employing

molecular docking to identify their putative drug target(s) and, consequently,

understand their mechanism of action. A panel of 20 essential mycobacterial

enzymes was investigated, of which β-ketoacyl acyl carrier protein synthase I

(KasA) and pyridoxal-5'-phosphate (PLP)-dependent aminotransferase (BioA) enzymes

were revealed as putative targets for compounds 3a-3e and 5a-5j, respectively.

Moreover, in silico ADMET predictions showed adequate properties for these

compounds, making them promising leads worthy of further optimization.

CI - Copyright © 2024 Elsevier B.V. All rights reserved.

FAU - Venugopala, Katharigatta N

AU - Venugopala KN

AD - Department of Pharmaceutical Sciences, College of Clinical Pharmacy, King Faisal

University, Al-Ahsa 31982, Saudi Arabia; Department of Biotechnology and Food

Science, Faculty of Applied Sciences, Durban University of Technology, Durban

4000, South Africa. Electronic address: kvenugopala@kfu.edu.sa.

FAU - Chandrashekharappa, Sandeep

AU - Chandrashekharappa S

AD - Department of Medicinal Chemistry, National Institute of Pharmaceutical Education

and Research (NIPER-R), Raebareli, Lucknow, UP 226002, India. Electronic address:

c.sandeep@niperraebareli.edu.in.

FAU - Deb, Pran Kishore

AU - Deb PK

AD - Department of Pharmaceutical Sciences and Technology, Birla Institute of

Technology (BIT), Mesra, Ranchi 835215, Jharkhand, India. Electronic address:

prankishoredeb@bitmesra.ac.in.

FAU - Al-Shar'i, Nizar A

AU - Al-Shar'i NA

AD - Department of Medicinal Chemistry and Pharmacognosy, Faculty of Pharmacy, Jordan

University of Science and Technology, P.O. Box 3030, Irbid 22110, Jordan;

Department of Pharmaceutical Sciences, College of Pharmacy, Qatar University,

P.O. Box: 2713, Doha, Qatar.

FAU - Pillay, Melendhran

AU - Pillay M

AD - Department of Microbiology, National Health Laboratory Services, KZN Academic

Complex, Inkosi Albert Luthuli Central Hospital, Durban 4001, South Africa.

FAU - Tiwari, Priya

AU - Tiwari P

AD - Department of Medicinal Chemistry, National Institute of Pharmaceutical Education

and Research (NIPER-R), Raebareli, Lucknow, UP 226002, India.

FAU - Chopra, Deepak

AU - Chopra D

AD - Department of Chemistry, Indian Institute of Science Education and Research

Bhopal, Bhopal By-pass Road, Bhauri, Bhopal 462066, Madhya Pradesh, India.

FAU - Borah, Pobitra

AU - Borah P

AD - Department of Biological Sciences and Bioengineering, Indian Institute of

Technology (IIT), Kanpur, 208016, Uttar Pradesh, India.

FAU - Tamhaev, Rasoul

AU - Tamhaev R

AD - Synthèse et Physico-Chimie de Molécules d'Intérêt Biologique (LSPCMIB), UMR 5068,

CNRS, Université Toulouse III - Paul Sabatier (UT3), Toulouse, France; Institut

de Pharmacologie et de Biologie Structurale (IPBS), Université de Toulouse, CNRS,

Université Toulouse III - Paul Sabatier (UPS), Toulouse, France.

FAU - Mourey, Lionel

AU - Mourey L

AD - Institut de Pharmacologie et de Biologie Structurale (IPBS), Université de

Toulouse, CNRS, Université Toulouse III - Paul Sabatier (UPS), Toulouse, France.

FAU - Lherbet, Christian

AU - Lherbet C

AD - Synthèse et Physico-Chimie de Molécules d'Intérêt Biologique (LSPCMIB), UMR 5068,

CNRS, Université Toulouse III - Paul Sabatier (UT3), Toulouse, France.

FAU - Aldhubiab, Bandar E

AU - Aldhubiab BE

AD - Department of Pharmaceutical Sciences, College of Clinical Pharmacy, King Faisal

University, Al-Ahsa 31982, Saudi Arabia.

FAU - Tratrat, Christophe

AU - Tratrat C

AD - Department of Pharmaceutical Sciences, College of Clinical Pharmacy, King Faisal

University, Al-Ahsa 31982, Saudi Arabia.

FAU - Attimarad, Mahesh

AU - Attimarad M

AD - Department of Pharmaceutical Sciences, College of Clinical Pharmacy, King Faisal

University, Al-Ahsa 31982, Saudi Arabia.

FAU - Nair, Anroop B

AU - Nair AB

AD - Department of Pharmaceutical Sciences, College of Clinical Pharmacy, King Faisal

University, Al-Ahsa 31982, Saudi Arabia.

FAU - Sreeharsha, Nagaraja

AU - Sreeharsha N

AD - Department of Pharmaceutical Sciences, College of Clinical Pharmacy, King Faisal

University, Al-Ahsa 31982, Saudi Arabia; Department of Pharmaceutics, Vidya Siri

College of Pharmacy, Off Sarjapura Road, Bangalore 560035, India.

FAU - Mailavaram, Raghu Prasad

AU - Mailavaram RP

AD - Department of Pharmaceutical Chemistry, Shri Vile Parle Kelavani Mandal's

Institute of Pharmacy, Samtanagar, Dhule 424 001, Maharashtra, India.

FAU - Venugopala, Rashmi

AU - Venugopala R

AD - Department of Public Health Medicine, Howard College Campus, University of

KwaZulu-Natal, Durban 4001, South Africa.

FAU - Mohanlall, Viresh

AU - Mohanlall V

AD - Department of Biotechnology and Food Science, Faculty of Applied Sciences, Durban

University of Technology, Durban 4000, South Africa.

FAU - Morsy, Mohamed A

AU - Morsy MA

AD - Department of Pharmaceutical Sciences, College of Clinical Pharmacy, King Faisal

University, Al-Ahsa 31982, Saudi Arabia; Department of Pharmacology, Faculty of

Medicine, Minia University, El-Minia 61511, Egypt.

LA - eng

PT - Journal Article

DEP - 20240624

PL - Netherlands

TA - Int J Biol Macromol

JT - International journal of biological macromolecules

JID - 7909578

RN - 0 (Antitubercular Agents)

RN - 0 (Indolizines)

RN - 0 (Bacterial Proteins)

RN - EC 1.3.1.9 (InhA protein, Mycobacterium)

RN - EC 1.- (Oxidoreductases)

RN - 274-40-8 (indolizine)

SB - IM

MH - *Mycobacterium tuberculosis/drug effects/enzymology

MH - *Antitubercular Agents/pharmacology/chemistry

MH - *Molecular Docking Simulation

MH - *Indolizines/chemistry/pharmacology

MH - *Microbial Sensitivity Tests

MH - Molecular Dynamics Simulation

MH - Bacterial Proteins/antagonists & inhibitors/metabolism/chemistry

MH - Oxidoreductases/antagonists & inhibitors/metabolism

MH - Structure-Activity Relationship

OTO - NOTNLM

OT - 2-Substituted indolizine

OT - MTB target validation

OT - Molecular docking

OT - Multidrug-resistant Mycobacterial tuberculosis

OT - Mycobacterial tuberculosis

COIS- Declaration of competing interest Authors declare that they have no competing

interest.

EDAT- 2024/06/27 00:42

MHDA- 2024/07/30 06:42

CRDT- 2024/06/26 19:24

PHST- 2023/11/20 00:00 [received]

PHST- 2024/06/17 00:00 [revised]

PHST- 2024/06/18 00:00 [accepted]

PHST- 2024/07/30 06:42 [medline]

PHST- 2024/06/27 00:42 [pubmed]

PHST- 2024/06/26 19:24 [entrez]

AID - S0141-8130(24)04090-X [pii]

AID - 10.1016/j.ijbiomac.2024.133285 [doi]

PST - ppublish

SO - Int J Biol Macromol. 2024 Aug;274(Pt 2):133285. doi:

10.1016/j.ijbiomac.2024.133285. Epub 2024 Jun 24.

PMID- 38761495

OWN - NLM

STAT- MEDLINE

DCOM- 20240606

LR - 20240606

IS - 1873-4367 (Electronic)

IS - 0927-7765 (Linking)

VI - 239

DP - 2024 Jul

TI - Evaluating the biomolecular interaction between delamanid/formulations and human

serum albumin by fluorescence, CD spectroscopy and SPR: Effects on protein

conformation, kinetic and thermodynamic parameters.

PG - 113964

LID - S0927-7765(24)00223-6 [pii]

LID - 10.1016/j.colsurfb.2024.113964 [doi]

AB - Delamanid is an anti-tuberculosis drug used for the treatment of drug-resistant

tuberculosis. Since delamanid has a high protein bound potential, even patients

with low albumin levels should experience high and rapid delamanid clearance.

However, the interaction between delamanid and albumin should be better

controlled to optimize drug efficacy. This study was designed to evaluate the

binding characteristics of delamanid to human serum albumin (HSA) using various

methods: fluorescence spectroscopy, circular dichroism (CD), surface plasmon

resonance (SPR), and molecular docking simulation. The fluorescence emission band

without any shift indicated the interaction was not affected by the polarity of

the fluorophore microenvironment. The reduction of fluorescence intensity at

344 nm was proportional to the increment of delamanid concentration as a

fluorescence quencher. UV-absorbance measurement at the maximum wavelength

(λ(max), 280 nm) was evaluated using inner filter effect correction. The HSA

conformation change was explained by the intermolecular energy transfer between

delamanid and HSA during complex formation. The study, which was conducted at

temperatures of 298 K, 303 K, and 310 K, revealed a static quenching mechanism

that correlated with a decreased of bimolecular quenching rate constant (kq) and

binding constant (Ka) at increased temperatures. The Ka was 1.75-3.16 × 10(4)

M(-1) with a specific binding site with stoichiometry 1:1. The negative enthalpy

change, negative entropy change, and negative Gibbs free energy change

demonstrated an exothermic-spontaneous reaction while van der Waals forces and

hydrogen bonds played a vital role in the binding. The molecular displacement

approach and molecular docking confirmed that the binding occurred mainly in

subdomain IIA, which is a hydrophobic pocket of HSA, with a theoretical binding

free energy of -9.33 kcal/mol. SPR exhibited a real time negative sensorgram that

resulted from deviation of the reflex angle due to ligand delamanid-HSA complex

forming. The binding occurred spontaneously after delamanid was presented to the

HSA surface. The SPR mathematical fitting model revealed that the association

rate constant (k(on)) was 2.62 × 10(8) s(-1)M(-1) and the dissociation rate

constant (k(off)) was 5.65 × 10(-3) s(-1). The complexes were performed with an

association constant (K(A)) of 4.64 × 10(10) M(-1) and the dissociation constant

(K(D)) of 2.15 × 10(-11) M. The binding constant indicated high binding affinity

and high stability of the complex in an equilibrium. Modified CD spectra revealed

that conformation of the HSA structure was altered by the presence of delamanid

during preparation of the proliposomes that led to the reduction of secondary

structure stabilization. This was indicated by the percentage decrease of

α-helix. These findings are beneficial to understanding delamanid-HSA binding

characteristics as well as the drug administration regimen.

CI - Copyright © 2024 Elsevier B.V. All rights reserved.

FAU - Tongkanarak, Krittawan

AU - Tongkanarak K

AD - Drug Delivery System Excellence Center, Department of Pharmaceutical Technology,

Faculty of Pharmaceutical Sciences, Prince of Songkla University, Hat

Yai, Songkhla 90110, Thailand.

FAU - Loupiac, Camille

AU - Loupiac C

AD - Univ. Bourgogne Franche - Comté, L'Institut Agro, Université de Bourgogne, INRAE,

UMR PAM 1517, Joint Unit Food Processing and Microbiology, Food and Wine

Physico-Chemistry Unit, 1 esplanade Erasme, Dijon 21000, France.

FAU - Neiers, Fabrice

AU - Neiers F

AD - Flavour Perception: Molecular Mechanisms (Flavours), Université de Bourgogne, 7

bd Jeanne d'Arc, Dijon 21000, France.

FAU - Chambin, Odile

AU - Chambin O

AD - Univ. Bourgogne Franche - Comté, L'Institut Agro, Université de Bourgogne, INRAE,

UMR PAM 1517, Joint Unit Food Processing and Microbiology, Food and Wine

Physico-Chemistry Unit, 1 esplanade Erasme, Dijon 21000, France; Department of

Pharmaceutical Technology, Faculty of Health Sciences, Université de Bourgogne, 7

bd Jeanne d'Arc, Dijon Cedex 21079, France.

FAU - Srichana, Teerapol

AU - Srichana T

AD - Drug Delivery System Excellence Center, Department of Pharmaceutical Technology,

Faculty of Pharmaceutical Sciences, Prince of Songkla University, Hat

Yai, Songkhla 90110, Thailand. Electronic address: teerapol.s@psu.ac.th.

LA - eng

PT - Journal Article

DEP - 20240514

PL - Netherlands

TA - Colloids Surf B Biointerfaces

JT - Colloids and surfaces. B, Biointerfaces

JID - 9315133

RN - ZIF514RVZR (Serum Albumin, Human)

RN - 0 (Oxazoles)

SB - IM

MH - Humans

MH - *Thermodynamics

MH - *Serum Albumin, Human/chemistry/metabolism

MH - Kinetics

MH - *Molecular Docking Simulation

MH - *Spectrometry, Fluorescence

MH - *Circular Dichroism

MH - *Surface Plasmon Resonance

MH - Protein Conformation

MH - Protein Binding

MH - Oxazoles/chemistry/metabolism

OTO - NOTNLM

OT - Delamanid

OT - HSA conformation

OT - Human serum albumin

OT - Interaction

OT - Proliposomes

COIS- Declaration of Competing Interest There are no conflicts of interest to declare.

EDAT- 2024/05/19 00:42

MHDA- 2024/06/07 00:43

CRDT- 2024/05/18 18:03

PHST- 2024/03/22 00:00 [received]

PHST- 2024/04/27 00:00 [revised]

PHST- 2024/05/10 00:00 [accepted]

PHST- 2024/06/07 00:43 [medline]

PHST- 2024/05/19 00:42 [pubmed]

PHST- 2024/05/18 18:03 [entrez]

AID - S0927-7765(24)00223-6 [pii]

AID - 10.1016/j.colsurfb.2024.113964 [doi]

PST - ppublish

SO - Colloids Surf B Biointerfaces. 2024 Jul;239:113964. doi:

10.1016/j.colsurfb.2024.113964. Epub 2024 May 14.

PMID- 38697608

OWN - NLM

STAT- MEDLINE

DCOM- 20240530

LR - 20240530

IS - 1878-3511 (Electronic)

IS - 1201-9712 (Linking)

VI - 144

DP - 2024 Jul

TI - Delineating the evolutionary pathway to multidrug-resistant outbreaks of a

Mycobacterium tuberculosis L4.1.2.1/Haarlem sublineage.

PG - 107077

LID - S1201-9712(24)00148-6 [pii]

LID - 10.1016/j.ijid.2024.107077 [doi]

AB - OBJECTIVES: We sought to capture the evolutionary itinerary of the Mycobacterium

tuberculosis L4.1.2.1/Haarlem sublineage in northern Tunisia, where it caused a

major multidrug-resistant (MDR) tuberculosis outbreak in a context strictly

negative for HIV infection. METHODS: We combined whole genome sequencing and

Bayesian approaches using a representative collection of drug-susceptible and

drug-resistant L4.1.2.1/Haarlem clinical strains (n = 121) recovered from the

outbreak region over 16 years. RESULTS: In the absence of drug resistance, the

L4.1.2.1/Haarlem sublineage showed a propensity for rapid transmission as

witnessed by the high clustering (44.6%) and recent transmission rates (25%), as

well as the reduced mean distance between genome pairs. The entire pool of

L4.1.2.1/Haarlem MDR strains was found to be linked to either the aforementioned

major outbreak (68 individuals, 2001-2016) or to a minor, newly uncovered

outbreak (six cases, 2001-2011). Strikingly, the two outbreaks descended

independently from a common ancestor that can be dated back to 1886. CONCLUSIONS:

Our data point to the intrinsic propensity for rapid transmission of the M.

tuberculosis L4.1.2.1/Haarlem sublineage in northern Tunisia, linking the overall

MDR tuberculosis epidemic to a single ancestor. These findings bring out the

important role of the bacillus' genetic background in the emergence of successful

MDR M. tuberculosis clones.

CI - Copyright © 2024 Institut Pasteur of Tunis. Published by Elsevier Ltd.. All

rights reserved.

FAU - Dekhil, Naira

AU - Dekhil N

AD - Unit of Typing & Genetics of Mycobacteria, Laboratory of Molecular Microbiology,

Vaccinology, and Biotechnology Development, Institut Pasteur de Tunis, Université

de Tunis El Manar, Tunis, Tunisia.

FAU - Mardassi, Helmi

AU - Mardassi H

AD - Unit of Typing & Genetics of Mycobacteria, Laboratory of Molecular Microbiology,

Vaccinology, and Biotechnology Development, Institut Pasteur de Tunis, Université

de Tunis El Manar, Tunis, Tunisia. Electronic address: helmi.mardassi@mesrs.tn.

LA - eng

PT - Journal Article

DEP - 20240430

PL - Canada

TA - Int J Infect Dis

JT - International journal of infectious diseases : IJID : official publication of the

International Society for Infectious Diseases

JID - 9610933

RN - 0 (Antitubercular Agents)

SB - IM

MH - *Mycobacterium tuberculosis/genetics/drug effects

MH - *Tuberculosis, Multidrug-Resistant/epidemiology/microbiology/transmission

MH - Humans

MH - *Disease Outbreaks

MH - Tunisia/epidemiology

MH - *Whole Genome Sequencing

MH - Antitubercular Agents/pharmacology/therapeutic use

MH - Bayes Theorem

MH - Phylogeny

MH - Drug Resistance, Multiple, Bacterial/genetics

MH - Evolution, Molecular

MH - Male

MH - Genome, Bacterial

MH - Female

MH - Adult

MH - Microbial Sensitivity Tests

MH - Genotype

OTO - NOTNLM

OT - Bayesian analysis

OT - L4.1.2.1/Haarlem sublineage

OT - MDR/XDR-TB

OT - Mycobacterium tuberculosis

OT - Whole genome sequencing

OT - outbreaks

COIS- Declarations of competing interest The authors have no competing interests to

declare.

EDAT- 2024/05/03 00:50

MHDA- 2024/05/31 00:42

CRDT- 2024/05/02 19:31

PHST- 2024/02/20 00:00 [received]

PHST- 2024/04/23 00:00 [revised]

PHST- 2024/04/28 00:00 [accepted]

PHST- 2024/05/31 00:42 [medline]

PHST- 2024/05/03 00:50 [pubmed]

PHST- 2024/05/02 19:31 [entrez]

AID - S1201-9712(24)00148-6 [pii]

AID - 10.1016/j.ijid.2024.107077 [doi]

PST - ppublish

SO - Int J Infect Dis. 2024 Jul;144:107077. doi: 10.1016/j.ijid.2024.107077. Epub 2024

Apr 30.

PMID- 39065554

OWN - NLM

STAT- PubMed-not-MEDLINE

LR - 20240729

IS - 1999-4923 (Print)

IS - 1999-4923 (Electronic)

IS - 1999-4923 (Linking)

VI - 16

IP - 7

DP - 2024 Jun 25

TI - TB/FLU-06E Influenza Vector-Based Vaccine in the Complex Therapy of

Drug-Susceptible and Drug-Resistant Experimental Tuberculosis.

LID - 10.3390/pharmaceutics16070857 [doi]

LID - 857

AB - The steady rise of drug-resistant tuberculosis (TB), which renders standard

therapy regimens ineffective, necessitates the development of innovative

treatment approaches. Immunotherapeutic vaccines have the potential to

effectively regulate the anti-TB immune response and enhance the efficacy of

anti-TB treatment. In the present study, we aimed to evaluate the potency of the

mucosal vector vaccine TB/FLU-06E as part of a complex treatment regimen for

drug-susceptible (DS) or drug-resistant (DR) tuberculosis in C57BL/6 mice.

Incorporating TB/FLU-06E into the treatment protocol significantly increased the

effectiveness of therapy for both forms of tuberculosis. It was evidenced by

higher survival rates and reduced pulmonary bacterial load (1.83 lg CFU for DS

tuberculosis and 0.93 lg CFU for DR tuberculosis). Furthermore, the treatment

reduced pathomorphological lesions in the lungs and stimulated the local and

systemic T-helper 1 (Th1) and cytotoxic T-lymphocyte (CTL) anti-TB immune

responses. Thus, therapeutic immunization with the TB/FLU-06E vaccine

significantly enhances the efficacy of tuberculosis treatment, which is

particularly important in DR tuberculosis.

FAU - Shurygina, Anna-Polina S

AU - Shurygina AS

AUID- ORCID: 0000-0003-3685-7068

AD - Smorodintsev Research Institute of Influenza, The Ministry of Health of the

Russian Federation, 197022 Saint-Petersburg, Russia.

FAU - Zabolotnykh, Natalia V

AU - Zabolotnykh NV

AD - Saint-Petersburg State Research Institute of Phthisiopulmonology, The Ministry of

Health of the Russian Federation, 194064 Saint-Petersburg, Russia.

FAU - Vinogradova, Tatiana I

AU - Vinogradova TI

AD - Saint-Petersburg State Research Institute of Phthisiopulmonology, The Ministry of

Health of the Russian Federation, 194064 Saint-Petersburg, Russia.

FAU - Vitovskaya, Maria L

AU - Vitovskaya ML

AD - Saint-Petersburg State Research Institute of Phthisiopulmonology, The Ministry of

Health of the Russian Federation, 194064 Saint-Petersburg, Russia.

FAU - Dogonadze, Marine Z

AU - Dogonadze MZ

AD - Saint-Petersburg State Research Institute of Phthisiopulmonology, The Ministry of

Health of the Russian Federation, 194064 Saint-Petersburg, Russia.

FAU - Vasilyev, Kirill A

AU - Vasilyev KA

AD - Smorodintsev Research Institute of Influenza, The Ministry of Health of the

Russian Federation, 197022 Saint-Petersburg, Russia.

FAU - Buzitskaya, Zhanna V

AU - Buzitskaya ZV

AD - Smorodintsev Research Institute of Influenza, The Ministry of Health of the

Russian Federation, 197022 Saint-Petersburg, Russia.

FAU - Yablonskiy, Petr K

AU - Yablonskiy PK

AD - Saint-Petersburg State Research Institute of Phthisiopulmonology, The Ministry of

Health of the Russian Federation, 194064 Saint-Petersburg, Russia.

FAU - Lioznov, Dmitriy A

AU - Lioznov DA

AUID- ORCID: 0000-0003-3643-7354

AD - Smorodintsev Research Institute of Influenza, The Ministry of Health of the

Russian Federation, 197022 Saint-Petersburg, Russia.

FAU - Stukova, Marina A

AU - Stukova MA

AD - Smorodintsev Research Institute of Influenza, The Ministry of Health of the

Russian Federation, 197022 Saint-Petersburg, Russia.

LA - eng

GR - 121112600145-2/Ministry of Health of the Russian Federation/

PT - Journal Article

DEP - 20240625

PL - Switzerland

TA - Pharmaceutics

JT - Pharmaceutics

JID - 101534003

PMC - PMC11279844

OTO - NOTNLM

OT - ESAT6

OT - TB-specific immunotherapy

OT - drug-resistant tuberculosis

OT - influenza vector

OT - mucosal vaccine

COIS- The authors declare no conflicts of interest. The funders had no role in the

design of the study; in the collection, analyses, or interpretation of data; in

the writing of the manuscript; or in the decision to publish the results.

EDAT- 2024/07/27 10:46

MHDA- 2024/07/27 10:47

PMCR- 2024/06/25

CRDT- 2024/07/27 01:27

PHST- 2024/04/30 00:00 [received]

PHST- 2024/06/14 00:00 [revised]

PHST- 2024/06/21 00:00 [accepted]

PHST- 2024/07/27 10:47 [medline]

PHST- 2024/07/27 10:46 [pubmed]

PHST- 2024/07/27 01:27 [entrez]

PHST- 2024/06/25 00:00 [pmc-release]

AID - pharmaceutics16070857 [pii]

AID - pharmaceutics-16-00857 [pii]

AID - 10.3390/pharmaceutics16070857 [doi]

PST - epublish

SO - Pharmaceutics. 2024 Jun 25;16(7):857. doi: 10.3390/pharmaceutics16070857.

PMID- 38927114

OWN - NLM

STAT- MEDLINE

DCOM- 20240627

LR - 20260413

IS - 2218-273X (Electronic)

IS - 2218-273X (Linking)

VI - 14

IP - 6

DP - 2024 Jun 16

TI - Insights into the Effects of Ligand Binding on Leucyl-tRNA Synthetase Inhibitors

for Tuberculosis: In Silico Analysis and Isothermal Titration Calorimetry

Validation.

LID - 10.3390/biom14060711 [doi]

LID - 711

AB - Incidences of drug-resistant tuberculosis have become common and are rising at an

alarming rate. Aminoacyl t-RNA synthetase has been validated as a newer target

against Mycobacterium tuberculosis. Leucyl t-RNA synthetase (LeuRS) is

ubiquitously found in all organisms and regulates transcription, protein

synthesis, mitochondrial RNA cleavage, and proofreading of matured t-RNA. Leucyl

t-RNA synthetase promotes growth and development and is the key enzyme needed for

biofilm formation in Mycobacterium. Inhibition of this enzyme could restrict the

growth and development of the mycobacterial population. A database consisting of

2734 drug-like molecules was screened against leucyl t-RNA synthetase enzymes

through virtual screening. Based on the docking scores and MMGBSA energy values,

the top three compounds were selected for molecular dynamics simulation. The

druggable nature of the top three hits was confirmed by predicting their

pharmacokinetic parameters. The top three hits-compounds 1035 (ZINC000001543916),

1054 (ZINC000001554197), and 2077 (ZINC000008214483)-were evaluated for their

binding affinity toward leucyl t-RNA synthetase by an isothermal titration

calorimetry study. The inhibitory activity of these compounds was tested against

antimycobacterial activity, biofilm formation, and LeuRS gene expression

potential. Compound 1054 (Macimorelin) was found to be the most potent molecule,

with better antimycobacterial activity, enzyme binding affinity, and significant

inhibition of biofilm formation, as well as inhibition of the LeuRS gene

expression. Compound 1054, the top hit compound, has the potential to be used as

a lead to develop successful leucyl t-RNA synthetase inhibitors.

FAU - Rehman, Zia Ur

AU - Rehman ZU

AUID- ORCID: 0000-0002-6540-7966

AD - Department of Pharmaceutical Chemistry, Faculty of Pharmacy, Jazan University,

P.O. Box 114, Jazan 45142, Saudi Arabia.

FAU - Najmi, Asim

AU - Najmi A

AUID- ORCID: 0000-0002-7529-5523

AD - Department of Pharmaceutical Chemistry, Faculty of Pharmacy, Jazan University,

P.O. Box 114, Jazan 45142, Saudi Arabia.

FAU - Zoghebi, Khalid

AU - Zoghebi K

AD - Department of Pharmaceutical Chemistry, Faculty of Pharmacy, Jazan University,

P.O. Box 114, Jazan 45142, Saudi Arabia.

LA - eng

GR - ISP22-16/Deanship for Research and Innovation, Ministry of Education Saudi

Arabia/

PT - Journal Article

PT - Research Support, Non-U.S. Gov't

DEP - 20240616

PL - Switzerland

TA - Biomolecules

JT - Biomolecules

JID - 101596414

RN - 0 (Ligands)

RN - 0 (Antitubercular Agents)

RN - EC 6.1.1.4 (Leucine-tRNA Ligase)

RN - 0 (Enzyme Inhibitors)

SB - IM

MH - *Mycobacterium tuberculosis/enzymology/drug effects

MH - Ligands

MH - *Antitubercular Agents/pharmacology/chemistry

MH - *Leucine-tRNA Ligase/antagonists & inhibitors/metabolism

MH - *Molecular Docking Simulation

MH - *Enzyme Inhibitors/pharmacology/chemistry

MH - Calorimetry

MH - Molecular Dynamics Simulation

MH - Tuberculosis/drug therapy/microbiology

MH - Computer Simulation

MH - Protein Binding

MH - Humans

PMC - PMC11201714

OTO - NOTNLM

OT - computer-aided drug design

OT - leucyl t-RNA synthetase inhibition

OT - molecular dynamics simulation

OT - virtual screening

COIS- The authors declare no conflicts of interest.

EDAT- 2024/06/27 06:42

MHDA- 2024/06/27 06:43

PMCR- 2024/06/16

CRDT- 2024/06/27 01:00

PHST- 2024/04/27 00:00 [received]

PHST- 2024/06/09 00:00 [revised]

PHST- 2024/06/12 00:00 [accepted]

PHST- 2024/06/27 06:43 [medline]

PHST- 2024/06/27 06:42 [pubmed]

PHST- 2024/06/27 01:00 [entrez]

PHST- 2024/06/16 00:00 [pmc-release]

AID - biom14060711 [pii]

AID - biomolecules-14-00711 [pii]

AID - 10.3390/biom14060711 [doi]

PST - epublish

SO - Biomolecules. 2024 Jun 16;14(6):711. doi: 10.3390/biom14060711.

PMID- 38733886

OWN - NLM

STAT- MEDLINE

DCOM- 20240525

LR - 20240531

IS - 1768-3254 (Electronic)

IS - 0223-5234 (Linking)

VI - 272

DP - 2024 Jun 5

TI - New synergistic benzoquinone scaffolds as inhibitors of mycobacterial cytochrome

bc1 complex to treat multi-drug resistant tuberculosis.

PG - 116479

LID - S0223-5234(24)00359-3 [pii]

LID - 10.1016/j.ejmech.2024.116479 [doi]

AB - Through a comprehensive molecular docking study, a unique series of

naphthoquinones clubbed azetidinone scaffolds was arrived with promising binding

affinity to Mycobacterial Cytbc1 complex, a drug target chosen to kill multi-drug

resistant Mycobacterium tuberculosis (MDR-Mtb). Five compounds from series-2, 2a,

2c, 2g, 2h, and 2j, showcased significant in vitro anti-tubercular activities

against Mtb H(37)Rv and MDR clinical isolates. Further, synergistic studies of

these compounds in combination with INH and RIF revealed a potent bactericidal

effect of compound 2a at concentration of 0.39 μg/mL, and remaining (2c, 2g, 2h,

and 2j) at 0.78 μg/mL. Exploration into the mechanism study through chemo-stress

assay and proteome profiling uncovered the down-regulation of key proteins of

electron-transport chain and Cytbc1 inhibition pathway. Metabolomics corroborated

these proteome findings, and heightened further understanding of the underlying

mechanism. Notably, in vitro and in vivo animal toxicity studies demonstrated

minimal toxicity, thus underscoring the potential of these compounds as promising

anti-TB agents in combination with RIF and INH. These active compounds adhered to

Lipinski's Rule of Five, indicating the suitability of these compounds for drug

development. Particular significance of molecules NQ02, 2a, and 2h, which have

been patented (Published 202141033473).

CI - Copyright © 2024 Elsevier Masson SAS. All rights reserved.

FAU - Chilamakuru, Naresh Babu

AU - Chilamakuru NB

AD - Research Scholar, Manipal Academy of Higher Education, Manipal, 576104,

Karnataka, India; RERDS-CPR, Raghavendra Institute of Pharmaceutical Education

and Research Campus, Ananthapuramu, 515721, Andhra Pradesh, India.

FAU - Vn, Azger Dusthackeer

AU - Vn AD

AD - ICMR-National Institute for Research in Tuberculosis (NIRT), Chennai, 600031,

Tamil Nadu, India.

FAU - G, Varadaraj Bhat

AU - G VB

AD - Department of Pharmaceutical Chemistry, Manipal College of Pharmaceutical

Sciences, Manipal Academy of Higher Education, Manipal 576104, Karnataka, India.

FAU - Pallaprolu, Nikhil

AU - Pallaprolu N

AD - Department of Pharmaceutical Analysis, National Institute of Pharmaceutical

Education and Research (NIPER), Hajipur 844102, Bihar, India.

FAU - Dande, Aishwarya

AU - Dande A

AD - Department of Pharmaceutical Analysis, National Institute of Pharmaceutical

Education and Research (NIPER), Hajipur 844102, Bihar, India.

FAU - Nair, Dina

AU - Nair D

AD - ICMR-National Institute for Research in Tuberculosis (NIRT), Chennai, 600031,

Tamil Nadu, India.

FAU - Pemmadi, Raghuveer Varma

AU - Pemmadi RV

AD - RERDS-CPR, Raghavendra Institute of Pharmaceutical Education and Research Campus,

Ananthapuramu, 515721, Andhra Pradesh, India; Department of Pharmaceutical

Chemistry, A.K.R.G College of Pharmacy, Nallajerla, Andhra Pradesh 534112.

Electronic address: drraghuveervarma@gmail.com.

FAU - Reddy Y, Padmanabha

AU - Reddy Y P

AD - RERDS-CPR, Raghavendra Institute of Pharmaceutical Education and Research Campus,

Ananthapuramu, 515721, Andhra Pradesh, India.

FAU - Peraman, Ramalingam

AU - Peraman R

AD - RERDS-CPR, Raghavendra Institute of Pharmaceutical Education and Research Campus,

Ananthapuramu, 515721, Andhra Pradesh, India; Department of Pharmaceutical

Analysis, National Institute of Pharmaceutical Education and Research (NIPER),

Hajipur 844102, Bihar, India. Electronic address: drram@niperhajipur.ac.in.

LA - eng

PT - Journal Article

DEP - 20240507

PL - France

TA - Eur J Med Chem

JT - European journal of medicinal chemistry

JID - 0420510

RN - 0 (Antitubercular Agents)

RN - EC 7.1.1.8 (Electron Transport Complex III)

RN - 0 (Benzoquinones)

RN - 0 (Enzyme Inhibitors)

SB - IM

MH - *Mycobacterium tuberculosis/drug effects

MH - *Antitubercular Agents/pharmacology/chemistry/chemical synthesis

MH - *Tuberculosis, Multidrug-Resistant/drug therapy

MH - *Electron Transport Complex III/antagonists & inhibitors/metabolism

MH - *Microbial Sensitivity Tests

MH - Structure-Activity Relationship

MH - Molecular Structure

MH - Molecular Docking Simulation

MH - Benzoquinones/chemistry/pharmacology

MH - Animals

MH - Humans

MH - Dose-Response Relationship, Drug

MH - Enzyme Inhibitors/pharmacology/chemistry/chemical synthesis

MH - Drug Synergism

OTO - NOTNLM

OT - Chemo-proteomics

OT - Cytochrome bc1 complex inhibitors

OT - MDR-TB

OT - Mycobacterium tuberculosis

OT - Naphthoquinone

COIS- Declaration of competing interest The authors declare the following financial

interests/personal relationships which may be considered as potential competing

interests: Dr. Ramalingam Peraman reports financial support was provided by DST -

Science and Engineering Research Board, Government of India. Ramalingam Peraman

has patent #202141033473 pending to Ramalingam Peraman. If there are other

authors, they declare that they have no known competing financial interests or

personal relationships that could have appeared to influence the work reported in

this paper.

EDAT- 2024/05/12 00:44

MHDA- 2024/05/26 18:10

CRDT- 2024/05/11 18:11

PHST- 2024/03/18 00:00 [received]

PHST- 2024/04/25 00:00 [revised]

PHST- 2024/05/04 00:00 [accepted]

PHST- 2024/05/26 18:10 [medline]

PHST- 2024/05/12 00:44 [pubmed]

PHST- 2024/05/11 18:11 [entrez]

AID - S0223-5234(24)00359-3 [pii]

AID - 10.1016/j.ejmech.2024.116479 [doi]

PST - ppublish

SO - Eur J Med Chem. 2024 Jun 5;272:116479. doi: 10.1016/j.ejmech.2024.116479. Epub

2024 May 7.

PMID- 38738892

OWN - NLM

STAT- MEDLINE

DCOM- 20240605

LR - 20260627

IS - 2165-0497 (Electronic)

IS - 2165-0497 (Linking)

VI - 12

IP - 6

DP - 2024 Jun 4

TI - Discordant results between Xpert MTB/RIF assay and Bactec MGIT 960 culture system

regarding the detection of rifampin-resistant Mycobacterium tuberculosis isolates

in Wenzhou, China.

PG - e0385923

LID - 10.1128/spectrum.03859-23 [doi]

LID - e03859-23

AB - This study aimed to assess the possible causes of discordant results between

Xpert MTB/RIF (Xpert) and Bactec MGIT 960 Culture System (MGIT960) regarding

rifampicin (RIF) susceptibility in Mycobacterium tuberculosis. Patients with

previous RIF-resistant tuberculosis who were admitted to Wenzhou Central Hospital

from January 2020 to December 2022 were enrolled. The isolates obtained from

these patients were subjected to RIF susceptibility tests using Xpert and

MGIT960, and the minimum inhibitory concentration (MIC) of RIF was determined by

the MYCOTB MIC plate test. Additionally, molecular docking and molecular dynamics

(MD) simulations were performed to evaluate the binding efficacy of rpoB and RIF

based on rpoB mutations detected in the isolates with discordant RIF

susceptibility results. A total of 28 isolates with discordant RIF susceptibility

test results were detected, 15 of them were RIF susceptible with MICs ≤ 0.5

µg/mL. Twelve out of 15 isolates contained borderline RIF resistance-associated

mutations [L430P (n = 6), H445N (n = 6)], 1 isolate had D435Y and Q429H double

mutation, and the remaining 2 isolates had a silent (Q432Q) mutation. Compared

with the affinity of RIF toward the wild type (WT) (-45.83 kcal/mol) by MD, its

affinity toward L452P (-55.52 kcal/mol), D435Y (-47.39 kcal/mol), L430P

(approximately -69.72 kcal/mol), H445N (-49.53 kcal/mol), and Q429H (-55.67

kcal/mol) increased. Borderline RIF resistance-associated mutations were the main

cause for the discordant RIF susceptibility results between Xpert and MGIT960,

and the mechanisms of the resistance need further investigated.IMPORTANCEThis

study is aimed at assessing discordant results between Xpert MTB/RIF (Xpert)

assay and Bactec MGIT 960 Culture System (MGIT960) regarding the detection of

rifampicin (RIF)-resistant Mycobacterium tuberculosis isolates in Wenzhou, China.

The discordant results of RIF between these two assays were mainly caused by

borderline RIF resistance-associated mutations, subsequently by silent mutations

of rpoB. Borderline RIF resistance- associated mutations detected in our study

were demonstrated to not be affected by the affinity of rpoB and RIF by molecular

dynamics, and the mechanism of resistance was needed to be clarified. For the

discordant results of RIF by Xpert and MGIT960 that occurred, rpoB DNA sequencing

was recommended to investigate its association with resistance to RIF.

FAU - He, Guiqing

AU - He G

AUID- ORCID: 0000-0002-6700-6920

AD - Department of Infectious Diseases, Wenzhou Central Hospital, The Dingli Clinical

College of Wenzhou Medical University, The Second Affiliated Hospital of Shanghai

University, Wenzhou, China.

AD - Laboratory of Infectious Diseases, Wenzhou Central Hospital, The Dingli Clinical

College of Wenzhou Medical University, The Second Affiliated Hospital of Shanghai

University, Wenzhou, China.

FAU - Zheng, Qingyong

AU - Zheng Q

AD - Laboratory of Infectious Diseases, Wenzhou Central Hospital, The Dingli Clinical

College of Wenzhou Medical University, The Second Affiliated Hospital of Shanghai

University, Wenzhou, China.

FAU - Wu, Jing

AU - Wu J

AD - National Clinical Laboratory on Tuberculosis, Beijing Key Laboratory on

Drug-Resistant Tuberculosis, Beijing Chest Hospital, Capital Medical University,

Beijing, China.

FAU - Wu, Lianpeng

AU - Wu L

AD - Department of Clinical Laboratory Medicine, Wenzhou Central Hospital, The Dingli

Clinical College of Wenzhou Medical University, The Second Affiliated Hospital of

Shanghai University, Wenzhou, China.

FAU - Geng, Zhi

AU - Geng Z

AD - Beijing Synchrotron Radiation Facility, Institute of High Energy Physics, Chinese

Academy of Sciences, Beijing, China.

FAU - Jiang, Guanglu

AU - Jiang G

AD - National Clinical Laboratory on Tuberculosis, Beijing Key Laboratory on

Drug-Resistant Tuberculosis, Beijing Chest Hospital, Capital Medical University,

Beijing, China.

FAU - Huang, Hairong

AU - Huang H

AUID- ORCID: 0000-0002-4295-4262

AD - National Clinical Laboratory on Tuberculosis, Beijing Key Laboratory on

Drug-Resistant Tuberculosis, Beijing Chest Hospital, Capital Medical University,

Beijing, China.

FAU - Jiang, Xiangao

AU - Jiang X

AUID- ORCID: 0000-0002-8527-1015

AD - Department of Infectious Diseases, Wenzhou Central Hospital, The Dingli Clinical

College of Wenzhou Medical University, The Second Affiliated Hospital of Shanghai

University, Wenzhou, China.

FAU - Yu, Xia

AU - Yu X

AUID- ORCID: 0000-0002-1400-7321

AD - National Clinical Laboratory on Tuberculosis, Beijing Key Laboratory on

Drug-Resistant Tuberculosis, Beijing Chest Hospital, Capital Medical University,

Beijing, China.

LA - eng

PT - Journal Article

PT - Research Support, Non-U.S. Gov't

DEP - 20240513

PL - United States

TA - Microbiol Spectr

JT - Microbiology spectrum

JID - 101634614

RN - VJT6J7R4TR (Rifampin)

RN - 0 (Bacterial Proteins)

RN - 0 (Antitubercular Agents)

RN - EC 2.7.7.6 (DNA-Directed RNA Polymerases)

RN - 0 (rpoB protein, Mycobacterium tuberculosis)

SB - IM

MH - *Mycobacterium tuberculosis/drug effects/genetics/isolation & purification

MH - *Rifampin/pharmacology

MH - *Microbial Sensitivity Tests

MH - Humans

MH - China

MH - *Bacterial Proteins/genetics/metabolism

MH - *Mutation

MH - *Tuberculosis, Multidrug-Resistant/microbiology

MH - Antitubercular Agents/pharmacology

MH - DNA-Directed RNA Polymerases/genetics

MH - Drug Resistance, Bacterial/genetics

MH - Molecular Docking Simulation

PMC - PMC11237732

OTO - NOTNLM

OT - Xpert MTB/RIF assay, Bactec MGIT 960

OT - borderline RIF resistance

OT - drug-resistant tuberculosis

OT - rpoB mutations

COIS- The authors declare no conflict of interest.

EDAT- 2024/05/13 12:53

MHDA- 2024/06/05 06:42

PMCR- 2024/05/13

CRDT- 2024/05/13 09:04

PHST- 2024/06/05 06:42 [medline]

PHST- 2024/05/13 12:53 [pubmed]

PHST- 2024/05/13 09:04 [entrez]

PHST- 2024/05/13 00:00 [pmc-release]

AID - 03859-23 [pii]

AID - spectrum.03859-23 [pii]

AID - 10.1128/spectrum.03859-23 [doi]

PST - ppublish

SO - Microbiol Spectr. 2024 Jun 4;12(6):e0385923. doi: 10.1128/spectrum.03859-23. Epub

2024 May 13.

PMID- 38615748

OWN - NLM

STAT- MEDLINE

DCOM- 20240427

LR - 20240624

IS - 1879-0631 (Electronic)

IS - 0024-3205 (Linking)

VI - 346

DP - 2024 Jun 1

TI - Synchronization of Mycobacterium life cycle: A possible novel mechanism of

antimycobacterial drug resistance evolution and its manipulation.

PG - 122632

LID - S0024-3205(24)00222-4 [pii]

LID - 10.1016/j.lfs.2024.122632 [doi]

AB - Mycobacterium Tuberculosis (Mtb) causing Tuberculosis (TB) is a widespread

disease infecting millions of people worldwide. Additionally, emergence of drug

resistant tuberculosis is a major challenge and concern in high TB burden

countries. Most of the drug resistance in mycobacteria is attributed to

developing acquired resistance due to spontaneous mutations or intrinsic

resistance mechanisms. In this review, we emphasize on the role of bacterial cell

cycle synchronization as one of the intrinsic mechanisms used by the bacteria to

cope with stress response and perhaps involved in evolution of its drug

resistance. The importance of cell cycle synchronization and its function in drug

resistance in cancer cells, malarial and viral pathogens is well understood, but

its role in bacterial pathogens has yet to be established. From the extensive

literature survey, we could collect information regarding how mycobacteria use

synchronization to overcome the stress response. Additionally, it has been

observed that most of the microbial pathogens including mycobacteria are

responsive to drugs predominantly in their logarithmic phase, while they show

resistance to antibiotics when they are in the lag or stationary phase.

Therefore, we speculate that Mtb might use this novel strategy wherein they

regulate their cell cycle upon antibiotic pressure such that they either enter in

their low metabolic phase i.e., either the lag or stationary phase to overcome

the antibiotic pressure and function as persister cells. Thus, we propose that

manipulating the mycobacterial drug resistance could be possible by fine-tuning

its cell cycle.

CI - Copyright © 2024 Elsevier Inc. All rights reserved.

FAU - Verma, Hritika

AU - Verma H

AD - Amity Institute of Molecular Medicine and Stem Cell Research, Amity University

Uttar Pradesh, Noida 201313, India.

FAU - Chauhan, Aditi

AU - Chauhan A

AD - Amity Institute of Molecular Medicine and Stem Cell Research, Amity University

Uttar Pradesh, Noida 201313, India.

FAU - Kumar, Awanish

AU - Kumar A

AD - Department of Bio Technology, National Institute of Technology, Raipur, India.

FAU - Kumar, Manoj

AU - Kumar M

AD - Amity Institute of Genome Engineering, Amity University Uttar Pradesh, Noida

201313, India.

FAU - Kanchan, Kajal

AU - Kanchan K

AD - Amity Institute of Molecular Medicine and Stem Cell Research, Amity University

Uttar Pradesh, Noida 201313, India. Electronic address: kkanchan@amity.edu.

LA - eng

PT - Journal Article

PT - Review

DEP - 20240413

PL - Netherlands

TA - Life Sci

JT - Life sciences

JID - 0375521

RN - 0 (Antitubercular Agents)

SB - IM

MH - *Mycobacterium tuberculosis/drug effects/genetics

MH - Humans

MH - *Antitubercular Agents/pharmacology

MH - Cell Cycle/drug effects

MH - Drug Resistance, Bacterial

MH - Mycobacterium/drug effects/genetics

MH - Tuberculosis, Multidrug-Resistant/drug therapy/microbiology

MH - Tuberculosis/microbiology/drug therapy

OTO - NOTNLM

OT - Antimycobacterial drugs

OT - Cell cycle synchronization

OT - Evolution

OT - Mycobacterium tuberculosis

COIS- Declaration of competing interest There is no conflict of interest among authors.

EDAT- 2024/04/15 00:42

MHDA- 2024/04/28 07:24

CRDT- 2024/04/14 19:23

PHST- 2023/10/14 00:00 [received]

PHST- 2024/03/26 00:00 [revised]

PHST- 2024/04/10 00:00 [accepted]

PHST- 2024/04/28 07:24 [medline]

PHST- 2024/04/15 00:42 [pubmed]

PHST- 2024/04/14 19:23 [entrez]

AID - S0024-3205(24)00222-4 [pii]

AID - 10.1016/j.lfs.2024.122632 [doi]

PST - ppublish

SO - Life Sci. 2024 Jun 1;346:122632. doi: 10.1016/j.lfs.2024.122632. Epub 2024 Apr

13.

PMID- 38873419

OWN - NLM

STAT- PubMed-not-MEDLINE

LR - 20240615

IS - 1663-9812 (Print)

IS - 1663-9812 (Electronic)

IS - 1663-9812 (Linking)

VI - 15

DP - 2024

TI - Identification of urine biomarkers predictive of prolonged QTc interval in

multidrug-resistant tuberculosis patients treated with bedaquiline.

PG - 1362544

LID - 10.3389/fphar.2024.1362544 [doi]

LID - 1362544

AB - The most frequent adverse event associated with bedaquiline (BDQ) is the QTc

interval prolongation; however, there was no biomarkers that could be used to

predict the occurrence of QTc prolongation in BDQ-treated patients. In this

study, we employed the ultra-high performance liquid chromatography-MS/MS

(UHPLC-MS/MS) to generate metabolic profiling for the discovery of potential

predictive urine biomarkers of QTc prolongation in these patients. Untargeted

metabolomic technique was used to concentrate the differential metabolic pathway,

and targeted metabolomic technique was subsequently performed to identify

predictive biomarkers for QTc prolongation. A total of 45 rifampicin-resistant TB

(RR-TB) and multidrug-resistant TB (MDR-TB) patients were enrolled in our study,

including 15 RR/MDR-TB patients with QTc interval prolongation (QIP) and 30

RR/MDR-TB patients with QTc interval un-prolongations (QIU). Untargeted technique

revealed that the lipid metabolism was the most differential metabolic pathway

between two groups. Further targeted technique identified four differential

metabolites, including betaine, LPE (18:2), LPE (20:3), and LPE (20:4). The

combined analysis of metabolisms revealed that the combined use of LPE (20:3) and

LPE (20:4) had the best performance for predicting the occurrence of QTc

prolongation in TB patients, yielding a sensitivity of 87.4% and a specificity of

78.5%. In addition, with the progression of BDQ treatment, the LPEs exhibited

persistent difference in the BDQ-treated TB patients experiencing QTc interval

prolongation. In conclusion, our data demonstrate that the combined use of LPE

(20:3) and LPE (20:4) yields promising performance for predicting the occurrence

of QTc interval prolongation in BDQ-treated patients.

CI - Copyright © 2024 Yu, Ren, Yuan, Liu, Ma, Tang and Pang.

FAU - Yu, Jiajia

AU - Yu J

AD - Department of Bacteriology and Immunology, Beijing Chest Hospital, Capital

Medical University/Beijing Tuberculosis and Thoracic Tumor Research Institute,

Beijing, China.

FAU - Ren, Weicong

AU - Ren W

AD - Department of Bacteriology and Immunology, Beijing Chest Hospital, Capital

Medical University/Beijing Tuberculosis and Thoracic Tumor Research Institute,

Beijing, China.

FAU - Yuan, Jinfeng

AU - Yuan J

AD - Department of Bacteriology and Immunology, Beijing Chest Hospital, Capital

Medical University/Beijing Tuberculosis and Thoracic Tumor Research Institute,

Beijing, China.

FAU - Liu, Rongmei

AU - Liu R

AD - Department of Tuberculosis, Beijing Chest Hospital, Capital Medical

University/Beijing Tuberculosis and Thoracic Tumor Research Institute, Beijing,

China.

FAU - Ma, Liping

AU - Ma L

AD - Department of Tuberculosis, Beijing Chest Hospital, Capital Medical

University/Beijing Tuberculosis and Thoracic Tumor Research Institute, Beijing,

China.

FAU - Tang, Shenjie

AU - Tang S

AD - Clinical Center on Tuberculosis, Beijing Chest Hospital, Capital Medical

University, Beijing Tuberculosis and Thoracic Tumor Research Institute, Beijing,

China.

FAU - Pang, Yu

AU - Pang Y

AD - Department of Bacteriology and Immunology, Beijing Chest Hospital, Capital

Medical University/Beijing Tuberculosis and Thoracic Tumor Research Institute,

Beijing, China.

LA - eng

PT - Journal Article

DEP - 20240530

PL - Switzerland

TA - Front Pharmacol

JT - Frontiers in pharmacology

JID - 101548923

PMC - PMC11169739

OTO - NOTNLM

OT - bedaquiline

OT - biomarkers

OT - metabolomics

OT - tuberculosis

OT - urine

COIS- The authors declare that the research was conducted in the absence of any

commercial or financial relationships that could be construed as a potential

conflict of interest.

EDAT- 2024/06/14 06:42

MHDA- 2024/06/14 06:43

PMCR- 2024/05/30

CRDT- 2024/06/14 04:27

PHST- 2023/12/28 00:00 [received]

PHST- 2024/05/15 00:00 [accepted]

PHST- 2024/06/14 06:43 [medline]

PHST- 2024/06/14 06:42 [pubmed]

PHST- 2024/06/14 04:27 [entrez]

PHST- 2024/05/30 00:00 [pmc-release]

AID - 1362544 [pii]

AID - 10.3389/fphar.2024.1362544 [doi]

PST - epublish

SO - Front Pharmacol. 2024 May 30;15:1362544. doi: 10.3389/fphar.2024.1362544.

eCollection 2024.

PMID- 38745294

OWN - NLM

STAT- MEDLINE

DCOM- 20240515

LR - 20260203

IS - 1471-2164 (Electronic)

IS - 1471-2164 (Linking)

VI - 25

IP - 1

DP - 2024 May 14

TI - Identification of novel single nucleotide variants in the drug resistance

mechanism of Mycobacterium tuberculosis isolates by whole-genome analysis.

PG - 478

LID - 10.1186/s12864-024-10390-3 [doi]

LID - 478

AB - BACKGROUND: Tuberculosis (TB) represents a major global health challenge. Drug

resistance in Mycobacterium tuberculosis (MTB) poses a substantial obstacle to

effective TB treatment. Identifying genomic mutations in MTB isolates holds

promise for unraveling the underlying mechanisms of drug resistance in this

bacterium. METHODS: In this study, we investigated the roles of single nucleotide

variants (SNVs) in MTB isolates resistant to four antibiotics (moxifloxacin,

ofloxacin, amikacin, and capreomycin) through whole-genome analysis. We

identified the drug-resistance-associated SNVs by comparing the genomes of MTB

isolates with reference genomes using the MuMmer4 tool. RESULTS: We observed a

strikingly high proportion (94.2%) of MTB isolates resistant to ofloxacin,

underscoring the current prevalence of drug resistance in MTB. An average of 3529

SNVs were detected in a single ofloxacin-resistant isolate, indicating a mutation

rate of approximately 0.08% under the selective pressure of ofloxacin exposure.

We identified a set of 60 SNVs associated with extensively drug-resistant

tuberculosis (XDR-TB), among which 42 SNVs were non-synonymous mutations located

in the coding regions of nine key genes (ctpI, desA3, mce1R, moeB1, ndhA,

PE_PGRS4, PPE18, rpsA, secF). Protein structure modeling revealed that SNVs of

three genes (PE_PGRS4, desA3, secF) are close to the critical catalytic active

sites in the three-dimensional structure of the coding proteins. CONCLUSION: This

comprehensive study elucidates novel resistance mechanisms in MTB against

antibiotics, paving the way for future design and development of

anti-tuberculosis drugs.

CI - © 2024. The Author(s).

FAU - Qian, Weiye

AU - Qian W

AD - School of Artificial Intelligence, Hangzhou Dianzi University, Hangzhou, 310018,

China.

FAU - Ma, Nan

AU - Ma N

AD - School of Artificial Intelligence, Hangzhou Dianzi University, Hangzhou, 310018,

China.

FAU - Zeng, Xi

AU - Zeng X

AD - Agricultural Bioinformatics Key Laboratory of Hubei Province and 3D Genomics

Research Centre, College of Informatics, Huazhong Agricultural University, Wuhan,

430070, China.

FAU - Shi, Mai

AU - Shi M

AD - School of Biomedical Sciences, The Chinese University of Hong Kong, Hong Kong

SAR, China.

FAU - Wang, Mingqiang

AU - Wang M

AD - Stanford Cardiovascular Institute, Stanford University School of Medicine,

Stanford, CA, 94305, USA.

FAU - Yang, Zhiyuan

AU - Yang Z

AD - School of Artificial Intelligence, Hangzhou Dianzi University, Hangzhou, 310018,

China. yangzhiyuan@link.cuhk.edu.hk.

AD - School of Biomedical Sciences, The Chinese University of Hong Kong, Hong Kong

SAR, China. yangzhiyuan@link.cuhk.edu.hk.

FAU - Tsui, Stephen Kwok-Wing

AU - Tsui SK

AD - School of Biomedical Sciences, The Chinese University of Hong Kong, Hong Kong

SAR, China. kwtsui@cuhk.edu.hk.

AD - Hong Kong Bioinformatics Centre, The Chinese University of Hong Kong, Hong Kong

SAR, China. kwtsui@cuhk.edu.hk.

LA - eng

GR - 61903107/National Natural Science Foundation of China/

PT - Journal Article

DEP - 20240514

PL - England

TA - BMC Genomics

JT - BMC genomics

JID - 100965258

RN - 0 (Antitubercular Agents)

RN - 0 (Bacterial Proteins)

SB - IM

MH - *Mycobacterium tuberculosis/genetics/drug effects

MH - *Polymorphism, Single Nucleotide

MH - *Whole Genome Sequencing

MH - Genome, Bacterial

MH - Humans

MH - Drug Resistance, Bacterial/genetics

MH - Microbial Sensitivity Tests

MH - Mutation

MH - Antitubercular Agents/pharmacology

MH - Bacterial Proteins/genetics

PMC - PMC11094924

OTO - NOTNLM

OT - Mycobacterium tuberculosis

OT - Single nucleotide variant

OT - Whole-genome sequencing

COIS- The authors declare no competing interests.

EDAT- 2024/05/15 05:48

MHDA- 2024/05/15 06:43

PMCR- 2024/05/14

CRDT- 2024/05/14 23:57

PHST- 2024/01/11 00:00 [received]

PHST- 2024/05/08 00:00 [accepted]

PHST- 2024/05/15 06:43 [medline]

PHST- 2024/05/15 05:48 [pubmed]

PHST- 2024/05/14 23:57 [entrez]

PHST- 2024/05/14 00:00 [pmc-release]

AID - 10.1186/s12864-024-10390-3 [pii]

AID - 10390 [pii]

AID - 10.1186/s12864-024-10390-3 [doi]

PST - epublish

SO - BMC Genomics. 2024 May 14;25(1):478. doi: 10.1186/s12864-024-10390-3.

PMID- 38783951

OWN - NLM

STAT- PubMed-not-MEDLINE

LR - 20240525

IS - 1663-9812 (Print)

IS - 1663-9812 (Electronic)

IS - 1663-9812 (Linking)

VI - 15

DP - 2024

TI - Assessing the safety of bedaquiline: insight from adverse event reporting system

analysis.

PG - 1382441

LID - 10.3389/fphar.2024.1382441 [doi]

LID - 1382441

AB - BACKGROUND: The development and marketing of Bedaquiline (BDQ) represent

significant advancements in treating tuberculosis, particularly

multidrug-resistant forms. However, comprehensive research into BDQ's real-world

safety remains limited. PURPOSE: We obtained BDQ related adverse event (AE)

information from the US Food and Drug Administration's Adverse Event Reporting

System (FAERS) to assess its safety and inform drug usage. METHODS: The AE data

for BDQ from 2012 Q4 to 2023 Q3 was collected and standardized.

Disproportionality analysis, including Reporting Odds Ratio (ROR), Proportional

Reporting Ratio (PRR), Multi-item Gamma Poisson Shrinker (MGPS), and Bayesian

Confidence Propagation Neural Network (BCPNN) was used to quantify signals of

BDQ-related AEs. Logistic regression was used to analyze the individual data of

hepatotoxicity and drug-induced liver injury, and multiple linear regression

models were established. Additionally, network pharmacology was employed to

identify the potential biological mechanisms of BDQ-induced liver injury.

RESULTS: We identified 2017 case reports directly related to BDQ. Our analysis

identified 341 Preferred Terms (PTs) characterizing these AEs across 27 System

Organ Classes (SOC). An important discovery was the identification of AEs

associated with ear and labyrinth disorders, which had not been documented in the

drug's official leaflet before. Subgroup analysis revealed a negative correlation

between BDQ-related liver injury and females (OR: 0.4, 95%CI: 0.3-0.6). In

addition, via network pharmacology approach, a total of 76 potential targets for

BDQ related liver injury were predicted, and 11 core target genes were selected

based on the characterization of protein-protein interactions. The pathway linked

to BDQ-induced liver injury was identified, and it was determined that the

PI3K-Akt signaling pathway contained the highest number of associated genes.

CONCLUSION: The analysis of the FAERS database revealed adverse events linked to

BDQ, prompting the use of a network pharmacology approach to study the potential

molecular mechanism of BDQ-induced liver injury. These findings emphasized the

significance of drug safety and offered understanding into the mechanisms behind

BDQ-induced liver injury. BDQ demonstrated distinct advantages, including reduced

incidence of certain adverse events compared to traditional treatments such as

injectable agents and second-line drugs. However, it is important to acknowledge

the limitations of this analysis, including potential underreporting and

confounding factors. This study provides valuable insights into the safety of BDQ

and its role in the management of MDR-TB, emphasizing the need for continued

surveillance and monitoring to ensure its safe and effective use.

CI - Copyright © 2024 Wu, Pan, Shen and Zhao.

FAU - Wu, Jiaqiang

AU - Wu J

AD - School of Life Sciences and Biopharmaceutical Science, Shenyang Pharmaceutical

University, Shenyang, China.

FAU - Pan, Hong

AU - Pan H

AD - Department of Pharmacy, Wuxi No.5 People's Hospital, Wuxi, Jiangsu, China.

FAU - Shen, Li

AU - Shen L

AD - Department of Pharmacy, Suzhou Hospital, Affiliated Hospital of Medical School,

Nanjing University, Suzhou, Jiangsu, China.

FAU - Zhao, Mingyi

AU - Zhao M

AD - School of Life Sciences and Biopharmaceutical Science, Shenyang Pharmaceutical

University, Shenyang, China.

LA - eng

PT - Journal Article

DEP - 20240509

PL - Switzerland

TA - Front Pharmacol

JT - Frontiers in pharmacology

JID - 101548923

PMC - PMC11111899

OTO - NOTNLM

OT - FAERS database

OT - adverse event

OT - bedaquiline

OT - drug induced liver injury

OT - network pharmacology

COIS- The authors declare that the research was conducted in the absence of any

commercial or financial relationships that could be construed as a potential

conflict of interest.

EDAT- 2024/05/24 06:42

MHDA- 2024/05/24 06:43

PMCR- 2024/05/09

CRDT- 2024/05/24 03:45

PHST- 2024/02/06 00:00 [received]

PHST- 2024/04/29 00:00 [accepted]

PHST- 2024/05/24 06:43 [medline]

PHST- 2024/05/24 06:42 [pubmed]

PHST- 2024/05/24 03:45 [entrez]

PHST- 2024/05/09 00:00 [pmc-release]

AID - 1382441 [pii]

AID - 10.3389/fphar.2024.1382441 [doi]

PST - epublish

SO - Front Pharmacol. 2024 May 9;15:1382441. doi: 10.3389/fphar.2024.1382441.

eCollection 2024.

PMID- 38460337

OWN - NLM

STAT- MEDLINE

DCOM- 20240415

LR - 20250730

IS - 1090-2120 (Electronic)

IS - 0045-2068 (Linking)

VI - 146

DP - 2024 May

TI - Unlocking InhA: Novel approaches to inhibit Mycobacterium tuberculosis.

PG - 107250

LID - S0045-2068(24)00155-X [pii]

LID - 10.1016/j.bioorg.2024.107250 [doi]

AB - Multidrug-resistant tuberculosis continues to pose a health security risk and

remains a public health emergency. Antimicrobial resistance result from treatment

regimens that are both insufficient and incomplete leading to the emergence of

multidrug-resistant tuberculosis, extensively drug-resistant tuberculosis and

totally drug-resistant tuberculosis. The impact of tuberculosis on the people

suffering from HIV (Human immunodeficiency virus infection) have resulted in the

increased research efforts in designing and discovery of novel antitubercular

drugs that may result in decreasing treatment duration, minimising the need for

multiple drug intake, minimising cytotoxicity and enhancing the mechanism of

action of drug. While many drugs are available to treat tuberculosis, a precise

and timely cure is still absent. Consequently, further investigation is needed to

identify more recent molecular equivalents that have the potential to swiftly

remove this disease. Isoniazid (INH), a treatment for tuberculosis (TB), targets

the enzyme InhA (mycobacterium enoyl acyl carrier protein reductase), the

Mycobacterium tuberculosis enoyl-acyl carrier protein (ACP) reductase, most

common INH resistance is circumvented by InhA inhibitors that do not require KatG

(catalase-peroxidase) activation, as a result, researchers are trying to work in

the area of development of InhA inhibitors which could help in eradicating the

era of tuberculosis from the world.

CI - Copyright © 2024 Elsevier Inc. All rights reserved.

FAU - Wahan, Simranpreet K

AU - Wahan SK

AD - Department of Chemical Sciences, I.K. Gujral Punjab Technical University,

Kapurthala, India.

FAU - Bhargava, Gaurav

AU - Bhargava G

AD - Department of Chemical Sciences, I.K. Gujral Punjab Technical University,

Kapurthala, India.

FAU - Chawla, Viney

AU - Chawla V

AD - University Institute of Pharmaceutical Sciences and Research, Baba Farid

University of Health Sciences, Faridkot, Punjab 151203, India.

FAU - Chawla, Pooja A

AU - Chawla PA

AD - University Institute of Pharmaceutical Sciences and Research, Baba Farid

University of Health Sciences, Faridkot, Punjab 151203, India. Electronic

address: pvchawla@gmail.com.

LA - eng

PT - Journal Article

PT - Review

DEP - 20240305

PL - United States

TA - Bioorg Chem

JT - Bioorganic chemistry

JID - 1303703

RN - 0 (Acyl Carrier Protein)

RN - 0 (Antitubercular Agents)

RN - V83O1VOZ8L (Isoniazid)

RN - 0 (Bacterial Proteins)

SB - IM

MH - Humans

MH - *Mycobacterium tuberculosis

MH - Acyl Carrier Protein

MH - Antitubercular Agents/pharmacology/therapeutic use

MH - Isoniazid/pharmacology

MH - *Tuberculosis/drug therapy/microbiology

MH - *Tuberculosis, Multidrug-Resistant/drug therapy

MH - Bacterial Proteins/metabolism

MH - Mutation

MH - Microbial Sensitivity Tests

OTO - NOTNLM

OT - Inhibitory activity

OT - KatG activation

OT - Multidrug-resistant tuberculosis

OT - Mycobacterium tuberculosis enoyl-ACP reductase

OT - Structural activity relationship studies

COIS- Declaration of competing interest The authors declare that they have no known

competing financial interests or personal relationships that could have appeared

to influence the work reported in this paper.

EDAT- 2024/03/10 00:42

MHDA- 2024/04/15 06:42

CRDT- 2024/03/09 18:08

PHST- 2023/12/21 00:00 [received]

PHST- 2024/01/29 00:00 [revised]

PHST- 2024/02/28 00:00 [accepted]

PHST- 2024/04/15 06:42 [medline]

PHST- 2024/03/10 00:42 [pubmed]

PHST- 2024/03/09 18:08 [entrez]

AID - S0045-2068(24)00155-X [pii]

AID - 10.1016/j.bioorg.2024.107250 [doi]

PST - ppublish

SO - Bioorg Chem. 2024 May;146:107250. doi: 10.1016/j.bioorg.2024.107250. Epub 2024

Mar 5.

PMID- 38422860

OWN - NLM

STAT- MEDLINE

DCOM- 20240318

LR - 20240318

IS - 1618-0623 (Electronic)

IS - 0944-5013 (Linking)

VI - 282

DP - 2024 May

TI - 4-(Benzyloxy)phenol-induced p53 exhibits antimycobacterial response triggering

phagosome-lysosome fusion through ROS-dependent intracellular Ca(2+) pathway in

THP-1 cells.

PG - 127664

LID - S0944-5013(24)00065-X [pii]

LID - 10.1016/j.micres.2024.127664 [doi]

AB - Drug-resistant tuberculosis (TB) outbreak has emerged as a global public health

crisis. Therefore, new and innovative therapeutic options like host-directed

therapies (HDTs) through novel modulators are urgently required to overcome the

challenges associated with TB. In the present study, we have investigated the

anti-mycobacterial effect of 4-(Benzyloxy)phenol. Cell-viability assay asserted

that 50 μM of 4-(Benzyloxy)phenol was not cytotoxic to phorbol 12-myristate

13-acetate (PMA) differentiated THP-1 (dTHP-1) cells. It was observed that

4-(Benzyloxy)phenol activates p53 expression by hindering its association with

KDM1A. Increased ROS, intracellular Ca(2+) and phagosome-lysosome fusion, were

also observed upon 4-(Benzyloxy)phenol treatment. 4-(Benzyloxy)phenol mediated

killing of intracellular mycobacteria was abrogated in the presence of specific

inhibitors of ROS, Ca(2+) and phagosome-lysosome fusion like NAC, BAPTA-AM, and

W7, respectively. We further demonstrate that 4-(Benzyloxy)phenol mediated

enhanced ROS production is mediated by acetylation of p53. Blocking of p53

acetylation by Pifithrin-α (PFT- α) enhanced intracellular mycobacterial growth

by blocking the mycobactericidal effect of 4-(Benzyloxy)phenol. Altogether, the

results showed that 4-(Benzyloxy)phenol executed its anti-mycobacterial effect by

modulating p53-mediated ROS production to regulate phagosome-lysosome fusion

through Ca(2+) production.

CI - Copyright © 2024 Elsevier GmbH. All rights reserved.

FAU - Naik, Lincoln

AU - Naik L

AD - Laboratory of Mycobacterial Immunology, Department of Life Science, National

Institute of Technology, Rourkela, Odisha 769008, India.

FAU - Patel, Salina

AU - Patel S

AD - Laboratory of Mycobacterial Immunology, Department of Life Science, National

Institute of Technology, Rourkela, Odisha 769008, India.

FAU - Kumar, Ashish

AU - Kumar A

AD - Laboratory of Mycobacterial Immunology, Department of Life Science, National

Institute of Technology, Rourkela, Odisha 769008, India.

FAU - Ghosh, Abhirupa

AU - Ghosh A

AD - Divison of Bioinformatics, Bose Institute Kolkata, West Bengal 700054, India.

FAU - Mishra, Abtar

AU - Mishra A

AD - Laboratory of Mycobacterial Immunology, Department of Life Science, National

Institute of Technology, Rourkela, Odisha 769008, India.

FAU - Das, Mousumi

AU - Das M

AD - Laboratory of Mycobacterial Immunology, Department of Life Science, National

Institute of Technology, Rourkela, Odisha 769008, India.

FAU - Nayak, Dev Kiran

AU - Nayak DK

AD - Laboratory of Mycobacterial Immunology, Department of Life Science, National

Institute of Technology, Rourkela, Odisha 769008, India.

FAU - Saha, Sudipto

AU - Saha S

AD - Divison of Bioinformatics, Bose Institute Kolkata, West Bengal 700054, India.

FAU - Mishra, Amit

AU - Mishra A

AD - Cellular and Molecular Neurobiology Unit, Indian Institute of Technology

Jodhpur, Rajasthan 342011, India.

FAU - Singh, Ramandeep

AU - Singh R

AD - Tuberculosis Research Laboratory, Translational Health Science and Technology

Institute, NCR Biotech Science Cluster, Faridabad-Gurugram Expressway, 3rd

Milestone, PO Box # 4, Faridabad, Haryana 121001, India.

FAU - Behura, Assirbad

AU - Behura A

AD - Laboratory of Mycobacterial Immunology, Department of Life Science, National

Institute of Technology, Rourkela, Odisha 769008, India. Electronic address:

ashu.hura@gmail.com.

FAU - Dhiman, Rohan

AU - Dhiman R

AD - Laboratory of Mycobacterial Immunology, Department of Life Science, National

Institute of Technology, Rourkela, Odisha 769008, India. Electronic address:

dhimanr@nitrkl.ac.in.

LA - eng

PT - Journal Article

DEP - 20240224

PL - Germany

TA - Microbiol Res

JT - Microbiological research

JID - 9437794

RN - 0 (Reactive Oxygen Species)

RN - 0 (Tumor Suppressor Protein p53)

RN - 339NCG44TV (Phenol)

RN - 0 (Phenols)

SB - IM

MH - Humans

MH - Reactive Oxygen Species/metabolism

MH - *Tumor Suppressor Protein p53/metabolism/pharmacology

MH - Macrophages

MH - Phenol

MH - THP-1 Cells

MH - Phagosomes/metabolism/microbiology

MH - Lysosomes/metabolism

MH - *Mycobacterium/metabolism

MH - Phenols/pharmacology/metabolism

OTO - NOTNLM

OT - 4-(Benzyloxy)phenol

OT - Ca(2+)

OT - Mycobacteria

OT - Phagosome-lysosome fusion

OT - ROS

OT - p53

COIS- Declaration of Competing Interest The authors state that they do not have any

identifiable financial conflicts of interest or personal relationships that might

be perceived as exerting an influence on the research presented in this paper.

EDAT- 2024/03/01 01:22

MHDA- 2024/03/18 06:44

CRDT- 2024/02/29 18:13

PHST- 2023/11/09 00:00 [received]

PHST- 2024/02/19 00:00 [revised]

PHST- 2024/02/20 00:00 [accepted]

PHST- 2024/03/18 06:44 [medline]

PHST- 2024/03/01 01:22 [pubmed]

PHST- 2024/02/29 18:13 [entrez]

AID - S0944-5013(24)00065-X [pii]

AID - 10.1016/j.micres.2024.127664 [doi]

PST - ppublish

SO - Microbiol Res. 2024 May;282:127664. doi: 10.1016/j.micres.2024.127664. Epub 2024

Feb 24.

PMID- 38665829

OWN - NLM

STAT- PubMed-not-MEDLINE

LR - 20250320

IS - 2632-8682 (Electronic)

IS - 2632-8682 (Linking)

VI - 15

IP - 4

DP - 2024 Apr 24

TI - Synthesis and biological evaluation of new naphthalimide-thiourea derivatives as

potent antimicrobial agents active against multidrug-resistant Staphylococcus

aureus and Mycobacterium tuberculosis.

PG - 1381-1391

LID - 10.1039/d4md00062e [doi]

AB - The emergence of antibiotic resistance to S. aureus and M. tuberculosis,

particularly MRSA, VRSA, and drug-resistant tuberculosis, poses a serious threat

to human health. Towards discovering new antibacterial agents, we designed and

synthesized a series of new naphthalimide-thiourea derivatives and evaluated them

against a panel of bacterial strains consisting of E. coli, S. aureus, K.

pneumoniae, P. aeruginosa, A. baumannii and various mycobacterial pathogens.

Compounds 4a, 4l, 4m, 4n, 4q, 9f, 9l, 13a, 13d, 13e, 17a, 17b, 17c, 17d, and 17e

demonstrated potent antibacterial activity against S. aureus with MIC 0.03-8 μg

mL(-1). In addition, these compounds have also exhibited potent inhibition

against MDR strains of S. aureus, including VRSA with MICs 0.06-4 μg mL(-1).

Compounds 4h, 4j, 4l, 4m, 4q, 4r, 9a, 9b, 9c, 9d, 9e, 9g, 9h, 9j, 13f and 17e

also exhibited good antimycobacterial activity against M. tuberculosis with MIC

2-64 μg mL(-1). The cytotoxicity assay using Vero cells revealed that all the

compounds were non-toxic and exhibited a favorable selectivity index (SI >40).

Time kill kinetics data indicated that compounds exhibited

concentration-dependent killing. Furthermore, in silico studies were performed to

decipher the possible mechanism of action. Comprehensively, these results

highlight the potential of naphthalimide-thiourea derivatives as promising

antibacterial agents.

CI - This journal is © The Royal Society of Chemistry.

FAU - Rana, Preeti

AU - Rana P

AD - Department of Chemical Sciences, National Institute of Pharmaceutical Education

and Research (NIPER) Hyderabad Telangana-500037 India nandurisrini92@gmail.com.

FAU - Parupalli, Ramulu

AU - Parupalli R

AD - Department of Chemical Sciences, National Institute of Pharmaceutical Education

and Research (NIPER) Hyderabad Telangana-500037 India nandurisrini92@gmail.com.

FAU - Akhir, Abdul

AU - Akhir A

AD - Division of Molecular Microbiology and Immunology, CSIR-Central Drug Research

Institute Sitapur Road, Sector 10, Janakipuram Extension Lucknow-226031 Uttar

Pradesh India skchopra007@gmail.com.

FAU - Saxena, Deepanshi

AU - Saxena D

AD - Division of Molecular Microbiology and Immunology, CSIR-Central Drug Research

Institute Sitapur Road, Sector 10, Janakipuram Extension Lucknow-226031 Uttar

Pradesh India skchopra007@gmail.com.

FAU - Maitra, Rahul

AU - Maitra R

AD - Division of Molecular Microbiology and Immunology, CSIR-Central Drug Research

Institute Sitapur Road, Sector 10, Janakipuram Extension Lucknow-226031 Uttar

Pradesh India skchopra007@gmail.com.

FAU - Imran, Mohmmad

AU - Imran M

AD - Division of Molecular Microbiology and Immunology, CSIR-Central Drug Research

Institute Sitapur Road, Sector 10, Janakipuram Extension Lucknow-226031 Uttar

Pradesh India skchopra007@gmail.com.

FAU - Malik, Pradip

AU - Malik P

AD - Division of Molecular Microbiology and Immunology, CSIR-Central Drug Research

Institute Sitapur Road, Sector 10, Janakipuram Extension Lucknow-226031 Uttar

Pradesh India skchopra007@gmail.com.

FAU - Mahammad Ghouse, Shaik

AU - Mahammad Ghouse S

AD - Department of Chemical Sciences, National Institute of Pharmaceutical Education

and Research (NIPER) Hyderabad Telangana-500037 India nandurisrini92@gmail.com.

FAU - Joshi, Swanand Vinayak

AU - Joshi SV

AD - Department of Chemical Sciences, National Institute of Pharmaceutical Education

and Research (NIPER) Hyderabad Telangana-500037 India nandurisrini92@gmail.com.

FAU - Srikanth, Danaboina

AU - Srikanth D

AD - Department of Chemical Sciences, National Institute of Pharmaceutical Education

and Research (NIPER) Hyderabad Telangana-500037 India nandurisrini92@gmail.com.

FAU - Madhavi, Y V

AU - Madhavi YV

AUID- ORCID: 0000-0002-9813-8694

AD - Department of Chemical Sciences, National Institute of Pharmaceutical Education

and Research (NIPER) Hyderabad Telangana-500037 India nandurisrini92@gmail.com.

FAU - Dasgupta, Arunava

AU - Dasgupta A

AD - Division of Molecular Microbiology and Immunology, CSIR-Central Drug Research

Institute Sitapur Road, Sector 10, Janakipuram Extension Lucknow-226031 Uttar

Pradesh India skchopra007@gmail.com.

AD - AcSIR: Academy of Scientific and Innovative Research (AcSIR) Ghaziabad 201002

India.

FAU - Chopra, Sidharth

AU - Chopra S

AUID- ORCID: 0000-0001-8823-6074

AD - Division of Molecular Microbiology and Immunology, CSIR-Central Drug Research

Institute Sitapur Road, Sector 10, Janakipuram Extension Lucknow-226031 Uttar

Pradesh India skchopra007@gmail.com.

AD - AcSIR: Academy of Scientific and Innovative Research (AcSIR) Ghaziabad 201002

India.

FAU - Nanduri, Srinivas

AU - Nanduri S

AUID- ORCID: 0000-0002-6671-2022

AD - Department of Chemical Sciences, National Institute of Pharmaceutical Education

and Research (NIPER) Hyderabad Telangana-500037 India nandurisrini92@gmail.com.

LA - eng

PT - Journal Article

DEP - 20240319

PL - England

TA - RSC Med Chem

JT - RSC medicinal chemistry

JID - 101759460

PMC - PMC11042119

COIS- There are no conflicts to declare.

EDAT- 2024/04/26 06:56

MHDA- 2024/04/26 06:57

PMCR- 2025/03/19

CRDT- 2024/04/26 03:56

PHST- 2024/01/23 00:00 [received]

PHST- 2024/02/21 00:00 [accepted]

PHST- 2024/04/26 06:57 [medline]

PHST- 2024/04/26 06:56 [pubmed]

PHST- 2024/04/26 03:56 [entrez]

PHST- 2025/03/19 00:00 [pmc-release]

AID - d4md00062e [pii]

AID - 10.1039/d4md00062e [doi]

PST - epublish

SO - RSC Med Chem. 2024 Mar 19;15(4):1381-1391. doi: 10.1039/d4md00062e. eCollection

2024 Apr 24.

PMID- 38628245

OWN - NLM

STAT- PubMed-not-MEDLINE

LR - 20240425

IS - 1178-6973 (Print)

IS - 1178-6973 (Electronic)

IS - 1178-6973 (Linking)

VI - 17

DP - 2024

TI - Identification of Mycobacterium tuberculosis Resistance to Common Antibiotics: An

Overview of Current Methods and Techniques.

PG - 1491-1506

LID - 10.2147/IDR.S457308 [doi]

AB - Multidrug-resistant tuberculosis (MDR-TB) is an essential cause of tuberculosis

treatment failure and death of tuberculosis patients. The rapid and reliable

profiling of Mycobacterium tuberculosis (MTB) drug resistance in the early stage

is a critical research area for public health. Then, most traditional approaches

for detecting MTB are time-consuming and costly, leading to the inappropriate

therapeutic schedule resting on the ambiguous information of MTB drug resistance,

increasing patient economic burden, morbidity, and mortality. Therefore, novel

diagnosis methods are frequently required to meet the emerging challenges of MTB

drug resistance distinguish. Considering the difficulty in treating MDR-TB, it is

urgently required for the development of rapid and accurate methods in the

identification of drug resistance profiles of MTB in clinical diagnosis. This

review discussed recent advances in MTB drug resistance detection, focusing on

developing emerging approaches and their applications in tangled clinical

situations. In particular, a brief overview of antibiotic resistance to MTB was

present, referred to as intrinsic bacterial resistance, consisting of cell wall

barriers and efflux pumping action and acquired resistance caused by genetic

mutations. Then, different drug susceptibility test (DST) methods were described,

including phenotype DST, genotype DST and novel DST methods. The phenotype DST

includes nitrate reductase assay, Roche(TM) solid ratio method, and liquid

culture method and genotype DST includes fluorescent PCR, GeneXpert, PCR reverse

dot hybridization, ddPCR, next-generation sequencing and gene chips. Then, novel

DST methods were described, including metabolism testing, cell-free DNA probe,

CRISPR assay, and spectral analysis technique. The limitations, challenges, and

perspectives of different techniques for drug resistance are also discussed.

These methods significantly improve the detection sensitivity and accuracy of

multidrug-resistant tuberculosis (MRT) and can effectively curb the incidence of

drug-resistant tuberculosis and accelerate the process of tuberculosis

eradication.

CI - © 2024 Xiong et al.

FAU - Xiong, Xue-Song

AU - Xiong XS

AUID- ORCID: 0009-0001-3637-3760

AD - Department of Laboratory Medicine, The Affiliated Huai'an Hospital of Yangzhou

University, Huai'an, Jiangsu Province, People's Republic of China.

AD - Department of Laboratory Medicine, The Fifth People's Hospital of Huai'an,

Huai'an, Jiangsu Province, People's Republic of China.

FAU - Zhang, Xue-Di

AU - Zhang XD

AD - Department of Laboratory Medicine, Xuzhou Infectious Diseases Hospital, Xuzhou,

Jiangsu Province, People's Republic of China.

FAU - Yan, Jia-Wei

AU - Yan JW

AD - Department of Laboratory Medicine, Xuzhou Infectious Diseases Hospital, Xuzhou,

Jiangsu Province, People's Republic of China.

FAU - Huang, Ting-Ting

AU - Huang TT

AUID- ORCID: 0009-0001-5465-8612

AD - Department of Laboratory Medicine, The Affiliated Huai'an Hospital of Yangzhou

University, Huai'an, Jiangsu Province, People's Republic of China.

AD - Department of Laboratory Medicine, The Fifth People's Hospital of Huai'an,

Huai'an, Jiangsu Province, People's Republic of China.

FAU - Liu, Zhan-Zhong

AU - Liu ZZ

AD - Department of Pharmacy, Xuzhou Infectious Diseases Hospital, Xuzhou, Jiangsu

Province, People's Republic of China.

FAU - Li, Zheng-Kang

AU - Li ZK

AD - Department of Laboratory Medicine, Guangdong Provincial People's Hospital

(Guangdong Academy of Medical Sciences), Southern Medical University, Guangzhou,

Guangdong Province, People's Republic of China.

FAU - Wang, Liang

AU - Wang L

AUID- ORCID: 0000-0001-5339-7484

AD - Department of Laboratory Medicine, Guangdong Provincial People's Hospital

(Guangdong Academy of Medical Sciences), Southern Medical University, Guangzhou,

Guangdong Province, People's Republic of China.

FAU - Li, Fen

AU - Li F

AD - Department of Laboratory Medicine, The Affiliated Huai'an Hospital of Yangzhou

University, Huai'an, Jiangsu Province, People's Republic of China.

AD - Department of Laboratory Medicine, The Fifth People's Hospital of Huai'an,

Huai'an, Jiangsu Province, People's Republic of China.

LA - eng

PT - Journal Article

PT - Review

DEP - 20240412

PL - New Zealand

TA - Infect Drug Resist

JT - Infection and drug resistance

JID - 101550216

PMC - PMC11020249

OTO - NOTNLM

OT - MTB

OT - Raman spectroscopy

OT - antibiotic resistance

OT - rapid detection

COIS- The authors declare that the research was conducted without any commercial or

financial relationships that could be construed as a potential conflict of

interest.

EDAT- 2024/04/17 06:43

MHDA- 2024/04/17 06:44

PMCR- 2024/04/12

CRDT- 2024/04/17 03:35

PHST- 2024/01/14 00:00 [received]

PHST- 2024/03/26 00:00 [accepted]

PHST- 2024/04/17 06:44 [medline]

PHST- 2024/04/17 06:43 [pubmed]

PHST- 2024/04/17 03:35 [entrez]

PHST- 2024/04/12 00:00 [pmc-release]

AID - 457308 [pii]

AID - 10.2147/IDR.S457308 [doi]

PST - epublish

SO - Infect Drug Resist. 2024 Apr 12;17:1491-1506. doi: 10.2147/IDR.S457308.

eCollection 2024.

PMID- 38411952

OWN - NLM

STAT- MEDLINE

DCOM- 20240404

LR - 20250306

IS - 1098-6596 (Electronic)

IS - 0066-4804 (Print)

IS - 0066-4804 (Linking)

VI - 68

IP - 4

DP - 2024 Apr 3

TI - Ethambutol and meropenem/clavulanate synergy promotes enhanced extracellular and

intracellular killing of Mycobacterium tuberculosis.

PG - e0158623

LID - 10.1128/aac.01586-23 [doi]

LID - e01586-23

AB - Increasing evidence supports the repositioning of beta-lactams for tuberculosis

(TB) therapy, but further research on their interaction with conventional anti-TB

agents is still warranted. Moreover, the complex cell envelope of Mycobacterium

tuberculosis (Mtb) may pose an additional obstacle to beta-lactam diffusion. In

this context, we aimed to identify synergies between beta-lactams and anti-TB

drugs ethambutol (EMB) and isoniazid (INH) by assessing antimicrobial effects,

intracellular activity, and immune responses. Checkerboard assays with H37Rv and

eight clinical isolates, including four drug-resistant strains, exposed that only

treatments containing EMB and beta-lactams achieved synergistic effects.

Meanwhile, the standard EMB and INH association failed to produce any synergy. In

Mtb-infected THP-1 macrophages, combinations of EMB with increasing meropenem

(MEM) concentrations consistently displayed superior killing activities over the

individual antibiotics. Flow cytometry with BODIPY FL vancomycin, which binds

directly to the peptidoglycan (PG), confirmed an increased exposure of this layer

after co-treatment. This was reinforced by the high IL-1β secretion levels found

in infected macrophages after incubation with MEM concentrations above 5 mg/L,

indicating an exposure of the host innate response sensors to pathogen-associated

molecular patterns in the PG. Our findings show that the proposed impaired access

of beta-lactams to periplasmic transpeptidases is counteracted by concomitant

administration with EMB. The efficiency of this combination may be attributed to

the synchronized inhibition of arabinogalactan and PG synthesis, two key cell

wall components. Given that beta-lactams exhibit a time-dependent bactericidal

activity, a more effective pathogen recognition and killing prompted by this

association may be highly beneficial to optimize TB regimens containing

carbapenems.IMPORTANCEAddressing drug-resistant tuberculosis with existing

therapies is challenging and the treatment success rate is lower when compared to

drug-susceptible infection. This study demonstrates that pairing beta-lactams

with ethambutol (EMB) significantly improves their efficacy against Mycobacterium

tuberculosis (Mtb). The presence of EMB enhances beta-lactam access through the

cell wall, which may translate into a prolonged contact between the drug and its

targets at a concentration that effectively kills the pathogen. Importantly, we

showed that the effects of the EMB and meropenem (MEM)/clavulanate combination

were maintained intracellularly. These results are of high significance

considering that the time above the minimum inhibitory concentration is the main

determinant of beta-lactam efficacy. Moreover, a correlation was established

between incubation with higher MEM concentrations during macrophage infection and

increased IL-1β secretion. This finding unveils a previously overlooked aspect of

carbapenem repurposing against tuberculosis, as certain Mtb strains suppress the

secretion of this key pro-inflammatory cytokine to evade host surveillance.

FAU - Olivença, Francisco

AU - Olivença F

AUID- ORCID: 0000-0001-7973-2631

AD - Research Institute for Medicines (iMed.ULisboa), Faculty of Pharmacy,

Universidade de Lisboa, Lisbon, Portugal.

FAU - Pires, David

AU - Pires D

AUID- ORCID: 0000-0001-9602-1516

AD - Research Institute for Medicines (iMed.ULisboa), Faculty of Pharmacy,

Universidade de Lisboa, Lisbon, Portugal.

AD - Universidade Católica Portuguesa, Católica Medical School, Centre for

Interdisciplinary Research in Health, Lisbon, Portugal.

FAU - Silveiro, Cátia

AU - Silveiro C

AD - Research Institute for Medicines (iMed.ULisboa), Faculty of Pharmacy,

Universidade de Lisboa, Lisbon, Portugal.

FAU - Gama, Bianca

AU - Gama B

AD - Research Institute for Medicines (iMed.ULisboa), Faculty of Pharmacy,

Universidade de Lisboa, Lisbon, Portugal.

FAU - Holtreman, Frederico

AU - Holtreman F

AD - Research Institute for Medicines (iMed.ULisboa), Faculty of Pharmacy,

Universidade de Lisboa, Lisbon, Portugal.

FAU - Anes, Elsa

AU - Anes E

AUID- ORCID: 0000-0001-5934-0198

AD - Research Institute for Medicines (iMed.ULisboa), Faculty of Pharmacy,

Universidade de Lisboa, Lisbon, Portugal.

FAU - Catalão, Maria João

AU - Catalão MJ

AUID- ORCID: 0000-0002-3794-457X

AD - Research Institute for Medicines (iMed.ULisboa), Faculty of Pharmacy,

Universidade de Lisboa, Lisbon, Portugal.

LA - eng

GR - PTDC/BIA-MIC/31233/2017/MEC | Fundação para a Ciência e a Tecnologia (FCT)/

GR - ESCMID Research Grant 2018/European Society of Clinical Microbiology and

Infectious Diseases (ESCMID)/

GR - SFRH/BD/136853/2018/MEC | Fundação para a Ciência e a Tecnologia (FCT)/

GR - 2021.05446.BD/MEC | Fundação para a Ciência e a Tecnologia (FCT)/

PT - Journal Article

PT - Research Support, Non-U.S. Gov't

DEP - 20240227

PL - United States

TA - Antimicrob Agents Chemother

JT - Antimicrobial agents and chemotherapy

JID - 0315061

RN - 8G167061QZ (Ethambutol)

RN - FV9J3JU8B1 (Meropenem)

RN - 23521W1S24 (Clavulanic Acid)

RN - 0 (Antitubercular Agents)

RN - 0 (Carbapenems)

RN - 0 (beta-Lactams)

SB - IM

MH - Humans

MH - Ethambutol/pharmacology/therapeutic use

MH - *Mycobacterium tuberculosis

MH - Meropenem/pharmacology/therapeutic use

MH - Clavulanic Acid/pharmacology

MH - Antitubercular Agents/pharmacology/therapeutic use

MH - *Tuberculosis, Multidrug-Resistant/drug therapy

MH - *Tuberculosis/microbiology

MH - Carbapenems/pharmacology

MH - beta-Lactams/pharmacology/therapeutic use

MH - Microbial Sensitivity Tests

PMC - PMC10989012

OTO - NOTNLM

OT - Mycobacterium tuberculosis

OT - antibiotic synergy

OT - antimicrobial resistance

OT - carbapenems

COIS- The authors declare no conflict of interest.

EDAT- 2024/02/27 12:47

MHDA- 2024/04/04 06:44

PMCR- 2024/02/27

CRDT- 2024/02/27 11:23

PHST- 2024/04/04 06:44 [medline]

PHST- 2024/02/27 12:47 [pubmed]

PHST- 2024/02/27 11:23 [entrez]

PHST- 2024/02/27 00:00 [pmc-release]

AID - 01586-23 [pii]

AID - aac.01586-23 [pii]

AID - 10.1128/aac.01586-23 [doi]

PST - ppublish

SO - Antimicrob Agents Chemother. 2024 Apr 3;68(4):e0158623. doi:

10.1128/aac.01586-23. Epub 2024 Feb 27.

PMID- 38612573

OWN - NLM

STAT- MEDLINE

DCOM- 20240415

LR - 20240430

IS - 1422-0067 (Electronic)

IS - 1422-0067 (Linking)

VI - 25

IP - 7

DP - 2024 Mar 28

TI - Revealing the Interaction Mechanism between Mycobacterium tuberculosis GyrB and

Novobiocin, SPR719 through Binding Thermodynamics and Dissociation Kinetics

Analysis.

LID - 10.3390/ijms25073764 [doi]

LID - 3764

AB - With the rapid emergence of drug-resistant strains of Mycobacterium tuberculosis

(Mtb), various levels of resistance against existing anti-tuberculosis (TB) drugs

have developed. Consequently, the identification of new anti-TB targets and drugs

is critically urgent. DNA gyrase subunit B (GyrB) has been identified as a

potential anti-TB target, with novobiocin and SPR719 proposed as inhibitors

targeting GyrB. Therefore, elucidating the molecular interactions between GyrB

and its inhibitors is crucial for the discovery and design of efficient GyrB

inhibitors for combating multidrug-resistant TB. In this study, we revealed the

detailed binding mechanisms and dissociation processes of the representative

inhibitors, novobiocin and SPR719, with GyrB using classical molecular dynamics

(MD) simulations, tau-random acceleration molecular dynamics (τ-RAMD)

simulations, and steered molecular dynamics (SMD) simulations. Our simulation

results demonstrate that both electrostatic and van der Waals interactions

contribute favorably to the inhibitors' binding to GyrB, with Asn52, Asp79,

Arg82, Lys108, Tyr114, and Arg141 being key residues for the inhibitors'

attachment to GyrB. The τ-RAMD simulations indicate that the inhibitors primarily

dissociate from the ATP channel. The SMD simulation results reveal that both

inhibitors follow a similar dissociation mechanism, requiring the overcoming of

hydrophobic interactions and hydrogen bonding interactions formed with the ATP

active site. The binding and dissociation mechanisms of GyrB with inhibitors

novobiocin and SPR719 obtained in our work will provide new insights for the

development of promising GyrB inhibitors.

FAU - Qiu, Xiaofei

AU - Qiu X

AD - School of Pharmacy, Lanzhou University, Lanzhou 730000, China.

FAU - Zhang, Qianqian

AU - Zhang Q

AUID- ORCID: 0000-0002-4422-4968

AD - Faculty of Applied Science, Macao Polytechnic University, Macao SAR, China.

FAU - Li, Zhaoguo

AU - Li Z

AUID- ORCID: 0009-0000-1702-9182

AD - School of Pharmacy, Lanzhou University, Lanzhou 730000, China.

FAU - Zhang, Juan

AU - Zhang J

AD - School of Pharmacy, Lanzhou University, Lanzhou 730000, China.

FAU - Liu, Huanxiang

AU - Liu H

AUID- ORCID: 0000-0002-9284-3667

AD - Faculty of Applied Science, Macao Polytechnic University, Macao SAR, China.

LA - eng

GR - 0091/2022/A2/The Science and Technology Development Fund, Macau, SAR/

GR - RP/ FCA-02/2023/Macao Polytechnic University/

PT - Journal Article

DEP - 20240328

PL - Switzerland

TA - Int J Mol Sci

JT - International journal of molecular sciences

JID - 101092791

RN - 17EC19951N (Novobiocin)

RN - 0 (Antitubercular Agents)

RN - 8L70Q75FXE (Adenosine Triphosphate)

SB - IM

MH - *Mycobacterium tuberculosis

MH - Novobiocin/pharmacology

MH - Thermodynamics

MH - Antitubercular Agents/pharmacology

MH - Molecular Dynamics Simulation

MH - Adenosine Triphosphate

PMC - PMC11011931

OTO - NOTNLM

OT - GyrB

OT - SPR719

OT - binding mechanism

OT - dissociation mechanism

OT - molecular dynamics (MD) simulations

OT - novobiocin

OT - tuberculosis

COIS- The authors declare no conflict of interest.

EDAT- 2024/04/13 10:43

MHDA- 2024/04/15 06:42

PMCR- 2024/03/28

CRDT- 2024/04/13 01:14

PHST- 2024/02/22 00:00 [received]

PHST- 2024/03/22 00:00 [revised]

PHST- 2024/03/24 00:00 [accepted]

PHST- 2024/04/15 06:42 [medline]

PHST- 2024/04/13 10:43 [pubmed]

PHST- 2024/04/13 01:14 [entrez]

PHST- 2024/03/28 00:00 [pmc-release]

AID - ijms25073764 [pii]

AID - ijms-25-03764 [pii]

AID - 10.3390/ijms25073764 [doi]

PST - epublish

SO - Int J Mol Sci. 2024 Mar 28;25(7):3764. doi: 10.3390/ijms25073764.

PMID- 38966407

OWN - NLM

STAT- PubMed-not-MEDLINE

LR - 20250530

IS - 3005-7590 (Electronic)

IS - 3005-7590 (Linking)

VI - 1

IP - 3

DP - 2024 Mar

TI - Operational research as a mechanism to improve treatment outcomes for

drug-resistant TB in the WHO European Region.

PG - 103-110

LID - 10.5588/ijtldopen.24.0035 [doi]

AB - In 2022, the WHO European Region accounted for 15.1% of all incident

rifampicin-resistant/multidrug-resistant TB (RR/MDR-TB) cases. Most occurred in

18 high-priority countries of eastern Europe and central Asia, many of which

joined an initiative led by the WHO Regional Office for Europe. The aim was to

introduce three, fully oral, 9-month modified shorter treatment regimens (mSTR)

to treat RR/MDR-TB under operational research conditions. The three regimens

were: 1) bedaquiline + linezolid + levofloxacin + clofazimine + cycloserine

(BdqLzdLfxCfzCs); 2) BdqLzdLfxCfz + delamanid (Dlm) for children over 6 years of

age and adults; and 3) DlmLzdLfxCfz for children under 6 years of age. The

project aimed to enhance treatment success, facilitate mSTR implementation,

promote quality of care and build research capacity, while also contributing to

global knowledge on all-oral mSTR use. Between April 2020 and June 2022, >2,800

patients underwent mSTR treatment in the WHO European Region. This unique

experience promoted further collaboration with national tuberculosis programmes,

health authorities, experts and donors within and outside Europe, with a focus on

implementing operational research and improving the quality of care in high TB

burden countries of the region. In the hope of encouraging others to adopt this

model, we have described the principles of the initiative, its strengths and

weaknesses and next steps.

CI - © 2024 The Authors.

FAU - Migliori, G B

AU - Migliori GB

AD - Servizio di Epidemiologia Clinica delle Malattie Respiratorie, Istituti Clinici

Scientifici Maugeri, Istituto di Ricovero e Cura a Carattere Scientifico,

Tradate, Italy.

FAU - Korotych, O

AU - Korotych O

AD - Joint Infectious Diseases Unit, World Health Organization Regional Office for

Europe, Copenhagen, Denmark.

FAU - Achar, J

AU - Achar J

AD - Department of Global Public Health, Karolinska Institutet, Stockholm, Sweden.

AD - Department of Science and Innovation-National Research Foundation Centre of

Excellence for Biomedical Tuberculosis Research, South Africa Medical Research

Council Centre for Tuberculosis Research, Division of Molecular Biology and Human

Genetics, Faculty of Medicine and Health Sciences, Stellenbosch University, Cape

Town, South Africa.

FAU - Ciobanu, A

AU - Ciobanu A

AD - Joint Infectious Diseases Unit, World Health Organization Regional Office for

Europe, Copenhagen, Denmark.

FAU - Dravniece, G

AU - Dravniece G

AD - PATH, Kyiv, Ukraine.

FAU - Germanovych, M

AU - Germanovych M

AD - Joint Infectious Diseases Unit, World Health Organization Regional Office for

Europe, Copenhagen, Denmark.

FAU - Gurbanova, E

AU - Gurbanova E

AD - Lung Clinic, University of Tartu, Tartu, Estonia.

FAU - Hovhannesyan, A

AU - Hovhannesyan A

AD - Joint Infectious Diseases Unit, World Health Organization Regional Office for

Europe, Copenhagen, Denmark.

FAU - Khachatryan, N

AU - Khachatryan N

AD - National Center of Pulmonology, Ministry of Health, Abovyan, Armenia.

FAU - Kuksa, L

AU - Kuksa L

AD - Tuberculosis and Lung Disease Clinic, Riga East Univesity Hospital, Riga, Latvia.

FAU - Lomtadze, N

AU - Lomtadze N

AD - National Center for Tuberculosis and Lung Diseases, Tbilisi, Georgia.

AD - The University of Georgia, Tbilisi, Georgia.

FAU - Rich, M L

AU - Rich ML

AD - Division of Global Health Equity, Brigham and Women's Hospital, Boston, MA, USA.

FAU - Skrahina, A

AU - Skrahina A

AD - Republican Scientific and Practical Centre for Pulmonology and Tuberculosis,

Minsk, Belarus.

FAU - Yedilbayev, A

AU - Yedilbayev A

AD - Joint Infectious Diseases Unit, World Health Organization Regional Office for

Europe, Copenhagen, Denmark.

LA - eng

GR - 001/WHO_/World Health Organization/International

PT - Journal Article

DEP - 20240301

PL - France

TA - IJTLD Open

JT - IJTLD open

JID - 9918803487706676

PMC - PMC11221584

OTO - NOTNLM

OT - MDR-TB

OT - mSTR

OT - rifampicin resistant

OT - shorter regimens

OT - tuberculosis

COIS- Conflicts of interest: none declared.

EDAT- 2024/07/05 06:41

MHDA- 2024/07/05 06:42

PMCR- 2024/03/01

CRDT- 2024/07/05 04:09

PHST- 2024/01/23 00:00 [received]

PHST- 2024/02/07 00:00 [accepted]

PHST- 2024/07/05 06:42 [medline]

PHST- 2024/07/05 06:41 [pubmed]

PHST- 2024/07/05 04:09 [entrez]

PHST- 2024/03/01 00:00 [pmc-release]

AID - 0035 [pii]

AID - 10.5588/ijtldopen.24.0035 [doi]

PST - epublish

SO - IJTLD Open. 2024 Mar 1;1(3):103-110. doi: 10.5588/ijtldopen.24.0035. eCollection

2024 Mar.

PMID- 38159468

OWN - NLM

STAT- MEDLINE

DCOM- 20240129

LR - 20240408

IS - 1096-0023 (Electronic)

IS - 1043-4666 (Linking)

VI - 175

DP - 2024 Mar

TI - NK-derived exosome miR-1249-3p inhibits Mycobacterium tuberculosis survival in

macrophages by targeting SKOR1.

PG - 156481

LID - S1043-4666(23)00359-9 [pii]

LID - 10.1016/j.cyto.2023.156481 [doi]

AB - Murine Natural Killer cells were cultivated in vitro to isolate NK-derived

exosomes. Subsequent quantification via qPCR confirmed enrichment of miR-1249-3p.

Ana-1 murine macrophages were cultured in vitro and subsequently inoculated with

Mycobacterium tuberculosis (MTB) strain H37Rv. NK-exo and NK-exo miR-1249-3p were

separately applied to the infection model, followed by immunological assays

conducted post-48-hour co-culture. Western blot analyses corroborated that NK-exo

exhibited exosomal marker proteins Granzyme A (GzmA), Granzyme B (GzmB), and

Perforin (PFN), alongside a notable enrichment of miR-1249-3p. Functionally,

NK-exo augmented the expression levels of Caspase-9,-8, and -3, as well as PARP,

while attenuating the expression of NLRP3, ASC, and Cleaved-Caspase-1.

Furthermore, qPCR demonstrated an up-regulation of Caspase-9, -8, and -3, along

with pro-apoptotic factors Bax and Bid, and a concomitant down-regulation of the

anti-apoptotic factor Bcl-2. The expression levels of inflammatory markers ASC,

NLRP3, Cleaved-Caspase-1, and IL-1β were concomitantly decreased. ELISA findings

indicated diminished levels of TNF-α and ROS secretion. NK-exo miR-1249-3p

specifically targeted and attenuated the expression of SKOR-1, engendering

up-regulation of apoptosis-associated proteins and down-regulation of

inflammation-related proteins, consequently affecting cellular fate.Our empirical

evidence substantiates that NK-exo induces macrophage apoptosis, thereby

mitigating MTB survival. Furthermore, NK-exo miR-1249-3p directly targets and

inhibits SKOR-1 expression, leading to macrophage apoptosis and consequently

hampering the proliferation of MTB. The data implicate the potential therapeutic

relevance of NK-exo and miR-1249-3p in managing drug-resistant tuberculosis.

CI - Copyright © 2023 Elsevier Ltd. All rights reserved.

FAU - Ma, Fengqian

AU - Ma F

AD - Department of Microbiology and Immunology, School of Basic Medical Sciences, Dali

University, Dali 671000, China.

FAU - Wang, Xuan

AU - Wang X

AD - Nanchang University Queen Mary School, Nan Chang 330031, China.

FAU - Qiu, Zhanghua

AU - Qiu Z

AD - School of Medicine, Kunming University of Science and Technology, Kunming 650093,

China.

FAU - Ding, Shoupeng

AU - Ding S

AD - Department of Laboratory Medicine, Gutian General Hospital, Fujian Province,

Gutian 352200, China.

FAU - Du, Wenya

AU - Du W

AD - Department of Microbiology and Immunology, School of Basic Medical Sciences, Dali

University, Dali 671000, China.

FAU - Dai, Yumei

AU - Dai Y

AD - Department of Microbiology and Immunology, School of Basic Medical Sciences, Dali

University, Dali 671000, China.

FAU - Ma, Tao

AU - Ma T

AD - Department of Microbiology and Immunology, School of Basic Medical Sciences, Dali

University, Dali 671000, China.

FAU - Yue, Linzhi

AU - Yue L

AD - Department of Microbiology and Immunology, School of Basic Medical Sciences, Dali

University, Dali 671000, China.

FAU - Wang, Guofu

AU - Wang G

AD - Department of Microbiology and Immunology, School of Basic Medical Sciences, Dali

University, Dali 671000, China.

FAU - Wang, Tao

AU - Wang T

AD - Department of Microbiology and Immunology, School of Basic Medical Sciences, Dali

University, Dali 671000, China.

FAU - Geng, Ling

AU - Geng L

AD - Department of Microbiology and Immunology, School of Basic Medical Sciences, Dali

University, Dali 671000, China. Electronic address: w_lixian@163.com.

FAU - Wu, Lixian

AU - Wu L

AD - Department of Microbiology and Immunology, School of Basic Medical Sciences, Dali

University, Dali 671000, China. Electronic address: wulixianwgf@163.com.

LA - eng

PT - Journal Article

PT - Research Support, Non-U.S. Gov't

DEP - 20231229

PL - England

TA - Cytokine

JT - Cytokine

JID - 9005353

RN - 0 (NLR Family, Pyrin Domain-Containing 3 Protein)

RN - EC 3.4.22.- (Caspase 9)

RN - 0 (MicroRNAs)

SB - IM

MH - Animals

MH - Mice

MH - NLR Family, Pyrin Domain-Containing 3 Protein/genetics/metabolism

MH - Caspase 9/metabolism

MH - *Mycobacterium tuberculosis/metabolism

MH - *Exosomes/metabolism

MH - Macrophages/metabolism

MH - *MicroRNAs/metabolism

OTO - NOTNLM

OT - Apoptosis

OT - Macrophage

OT - Mycobacterium tuberculosis

OT - NK-exo

OT - SKOR-1

OT - miR-1249-3P

COIS- Declaration of competing interest The authors declare that they have no known

competing financial interests or personal relationships that could have appeared

to influence the work reported in this paper.

EDAT- 2024/01/02 11:46

MHDA- 2024/01/29 06:42

CRDT- 2023/12/30 18:08

PHST- 2023/10/23 00:00 [received]

PHST- 2023/12/16 00:00 [revised]

PHST- 2023/12/19 00:00 [accepted]

PHST- 2024/01/29 06:42 [medline]

PHST- 2024/01/02 11:46 [pubmed]

PHST- 2023/12/30 18:08 [entrez]

AID - S1043-4666(23)00359-9 [pii]

AID - 10.1016/j.cyto.2023.156481 [doi]

PST - ppublish

SO - Cytokine. 2024 Mar;175:156481. doi: 10.1016/j.cyto.2023.156481. Epub 2023 Dec 29.

PMID- 38145750

OWN - NLM

STAT- MEDLINE

DCOM- 20240320

LR - 20240813

IS - 1769-714X (Electronic)

IS - 1286-4579 (Linking)

VI - 26

IP - 3

DP - 2024 Mar-Apr

TI - Unravelling the potential of Triflusal as an anti-TB repurposed drug by targeting

replication protein DciA.

PG - 105284

LID - S1286-4579(23)00197-1 [pii]

LID - 10.1016/j.micinf.2023.105284 [doi]

AB - The increasing prevalence of drug-resistant Tuberculosis (TB) is imposing extreme

difficulties in controlling the TB infection rate globally, making treatment

critically challenging. To combat the prevailing situation, it is crucial to

explore new anti-TB drugs with a novel mechanism of action and high efficacy. The

Mycobacterium tuberculosis (M.tb)DciA is an essential protein involved in

bacterial replication and regulates its growth. DciA interacts with DNA and

provides critical help in binding other replication machinery proteins to the

DNA. Moreover, the lack of any structural homology of M.tb DciA with human

proteins makes it an appropriate target for drug development. In this study,

FDA-approved drugs were virtually screened against M.tb DciA to identify

potential inhibitors. Four drugs namely Lanreotide, Risedronate, Triflusal, and

Zoledronic acid showed higher molecular docking scores. Further, molecular

dynamics simulations analysis of DciA-drugs complexes reported stable

interaction, more compactness, and reduced atomic motion. The anti-TB activity of

drugs was further evaluated under in vitro and ex vivo conditions where Triflusal

was observed to have the best possible activity with the MIC of 25 μg/ml. Our

findings present novel DciA inhibitors and anti-TB activity of Triflusal. Further

investigations on the use of Triflusal may lead to the discovery of a new anti-TB

drug.

CI - Copyright © 2023 Institut Pasteur. Published by Elsevier Masson SAS. All rights

reserved.

FAU - Ali, Waseem

AU - Ali W

AD - Jamia Hamdard, Department of Molecular Medicine, New Delhi 110062, India.

Electronic address: waseemiamr@gmail.com.

FAU - Jamal, Salma

AU - Jamal S

AD - Jamia Hamdard, Department of Molecular Medicine, New Delhi 110062, India.

Electronic address: jamalsalma1@gmail.com.

FAU - Gangwar, Rishabh

AU - Gangwar R

AD - Jamia Hamdard, Department of Molecular Medicine, New Delhi 110062, India.

Electronic address: rishabhgangwar@hotmail.com.

FAU - Ahmed, Faraz

AU - Ahmed F

AD - Jamia Hamdard, Department of Molecular Medicine, New Delhi 110062, India.

Electronic address: ahmedfaraz9419@gmail.com.

FAU - Pahuja, Isha

AU - Pahuja I

AD - Immunobiology Group, International Centre for Genetic Engineering and

Biotechnology (ICGEB), New Delhi, India. Electronic address:

ishapahuja7@gmail.com.

FAU - Sharma, Rahul

AU - Sharma R

AD - Department of Molecular Medicine Jamia Hamdard, New Delhi 110062, India.

Electronic address: rahulsharma_sch@jamiahamdard.ac.in.

FAU - Prakash Dwivedi, Ved

AU - Prakash Dwivedi V

AD - Immunobiology Group, International Centre for Genetic Engineering and

Biotechnology (ICGEB), New Delhi, India. Electronic address:

vedprakashbt@gmail.com.

FAU - Agarwal, Meetu

AU - Agarwal M

AD - Jamia Hamdard, Department of Molecular Medicine, New Delhi 110062, India.

Electronic address: meetuagarwal2388@gmail.com.

FAU - Grover, Sonam

AU - Grover S

AD - Jamia Hamdard, Department of Molecular Medicine, New Delhi 110062, India.

Electronic address: sonamgrover@jamiahamdard.ac.in.

LA - eng

PT - Journal Article

DEP - 20231223

PL - France

TA - Microbes Infect

JT - Microbes and infection

JID - 100883508

RN - 0 (Antitubercular Agents)

RN - 1Z0YFI05OO (triflusal)

RN - 9007-49-2 (DNA)

RN - 0 (Salicylates)

SB - IM

MH - Humans

MH - Antitubercular Agents/pharmacology/therapeutic use

MH - Molecular Docking Simulation

MH - *Mycobacterium tuberculosis

MH - *Tuberculosis/microbiology

MH - DNA/therapeutic use

MH - *Salicylates

OTO - NOTNLM

OT - CFU

OT - Drug repurposing

OT - M.tb DciA

OT - Molecular dynamics simulations

OT - Replisome

OT - Tuberculosis

COIS- Declaration of competing interest The authors declare that they have no competing

interests.

EDAT- 2023/12/26 00:42

MHDA- 2024/03/20 06:45

CRDT- 2023/12/25 19:24

PHST- 2023/06/02 00:00 [received]

PHST- 2023/11/25 00:00 [revised]

PHST- 2023/12/20 00:00 [accepted]

PHST- 2024/03/20 06:45 [medline]

PHST- 2023/12/26 00:42 [pubmed]

PHST- 2023/12/25 19:24 [entrez]

AID - S1286-4579(23)00197-1 [pii]

AID - 10.1016/j.micinf.2023.105284 [doi]

PST - ppublish

SO - Microbes Infect. 2024 Mar-Apr;26(3):105284. doi: 10.1016/j.micinf.2023.105284.

Epub 2023 Dec 23.

PMID- 38128751

OWN - NLM

STAT- MEDLINE

DCOM- 20240320

LR - 20240813

IS - 1769-714X (Electronic)

IS - 1286-4579 (Linking)

VI - 26

IP - 3

DP - 2024 Mar-Apr

TI - Bioactives from medicinal herb against bedaquiline resistant tuberculosis:

removing the dark clouds from the horizon.

PG - 105279

LID - S1286-4579(23)00192-2 [pii]

LID - 10.1016/j.micinf.2023.105279 [doi]

AB - Tuberculosis is a contagious bacterial ailment that primarily affects the lungs

and is brought on by the bacterium Mycobacterium tuberculosis (MTB). An

antimycobacterial medication called bedaquiline (BQ) is specified to treat

multidrug-resistant tuberculosis (MDR-TB). Despite its contemporary use in

clinical practice, the mutations (D32 A/G/N/V/P) constrain the potential of BQ by

causing transitions in the structural conformation of the atpE subunit-c after

binding. In this study, we have taken the benzylisoquinoline alkaloids from

thalictrum foliolosum due to its antimicrobial activity reported in prior

literature. We used an efficient and optimized structure-based strategy to

examine the wild type (WT) and mutated protein upon molecule binding. Our results

emphasize the drastic decline in BQ binding affinity of mutant and WT atpE

subunit-c complexes compared to thalirugidine (top hit) from thalictrum

foliolosum. The decrease in BQ binding free energy is due to electrostatic energy

because nearly every atom in a macromolecule harbors a partial charge, and

molecules taking part in molecular recognition will interact electrostatically.

Similarly, the high potential mean force of thalirugidine than BQ in WT and

mutant complexes demonstrated the remarkable ability to eradicate mycobacteria

efficiently. Furthermore, the Alamar blue cell viability and ATP determination

assay were performed to validate the computational outcomes in search of novel

antimycobacterial. Upon closer examination of the ATP determination assay, it

became apparent that both BQ and thalirugidine showed similar reductions in ATP

levels at their respective MICs, presenting a potential common mechanism of

action.

CI - Copyright © 2023 Institut Pasteur. Published by Elsevier Masson SAS. All rights

reserved.

FAU - Singh, Rahul

AU - Singh R

AD - Structural Bioinformatics Lab, Biotechnology Division, CSIR-Institute of

Himalayan Bioresource Technology, Palampur, 176061, Himachal Pradesh, India;

Academy of Scientific and Innovative Research (AcSIR), Ghaziabad, 201002, India.

FAU - Manna, Souvik

AU - Manna S

AD - CSIR-Institute of Microbial Technology, Sector-39, Chandigarh, 160036, India;

Academy of Scientific and Innovative Research (AcSIR), Ghaziabad, 201002, India.

FAU - Nandanwar, Hemraj

AU - Nandanwar H

AD - CSIR-Institute of Microbial Technology, Sector-39, Chandigarh, 160036, India;

Academy of Scientific and Innovative Research (AcSIR), Ghaziabad, 201002, India.

FAU - Purohit, Rituraj

AU - Purohit R

AD - Structural Bioinformatics Lab, Biotechnology Division, CSIR-Institute of

Himalayan Bioresource Technology, Palampur, 176061, Himachal Pradesh, India;

Academy of Scientific and Innovative Research (AcSIR), Ghaziabad, 201002, India.

Electronic address: rituraj@ihbt.res.in.

LA - eng

PT - Journal Article

PT - Research Support, Non-U.S. Gov't

DEP - 20231219

PL - France

TA - Microbes Infect

JT - Microbes and infection

JID - 100883508

RN - 78846I289Y (bedaquiline)

RN - 0 (Antitubercular Agents)

RN - 8L70Q75FXE (Adenosine Triphosphate)

RN - 0 (Diarylquinolines)

SB - IM

MH - Humans

MH - Antitubercular Agents/pharmacology/therapeutic use

MH - *Plants, Medicinal

MH - *Tuberculosis/microbiology

MH - *Mycobacterium tuberculosis/genetics

MH - Microbial Sensitivity Tests

MH - Adenosine Triphosphate

MH - *Diarylquinolines

OTO - NOTNLM

OT - Bedaquiline

OT - MDR-TB

OT - Thalictrum foliolosum

OT - Thalirugidine

OT - atpE

COIS- Declaration of competing interest There are no competing interests declared by

the authors.

EDAT- 2023/12/22 00:42

MHDA- 2024/03/20 06:45

CRDT- 2023/12/21 19:27

PHST- 2023/04/17 00:00 [received]

PHST- 2023/12/15 00:00 [revised]

PHST- 2023/12/15 00:00 [accepted]

PHST- 2024/03/20 06:45 [medline]

PHST- 2023/12/22 00:42 [pubmed]

PHST- 2023/12/21 19:27 [entrez]

AID - S1286-4579(23)00192-2 [pii]

AID - 10.1016/j.micinf.2023.105279 [doi]

PST - ppublish

SO - Microbes Infect. 2024 Mar-Apr;26(3):105279. doi: 10.1016/j.micinf.2023.105279.

Epub 2023 Dec 19.

PMID- 38289177

OWN - NLM

STAT- MEDLINE

DCOM- 20240226

LR - 20240311

IS - 1520-6025 (Electronic)

IS - 0163-3864 (Linking)

VI - 87

IP - 2

DP - 2024 Feb 23

TI - Amycolatopsis mediterranei: A Sixty-Year Journey from Strain Isolation to

Unlocking Its Potential of Rifamycin Analogue Production by Combinatorial

Biosynthesis.

PG - 424-438

LID - 10.1021/acs.jnatprod.3c00686 [doi]

AB - Ever since the isolation of Amycolatopsis mediterranei in 1957, this strain has

been the focus of research worldwide. In the last 60 years or more, our

understanding of the taxonomy, development of cloning vectors and conjugation

system, physiology, genetics, genomics, and biosynthetic pathway of rifamycin B

production in A. mediterranei has substantially increased. In particular, the

development of cloning vectors, transformation system, characterization of the

rifamycin biosynthetic gene cluster, and the regulation of rifamycin B production

by the pioneering work of Heinz Floss have made the rifamycin polyketide

biosynthetic gene cluster (PKS) an attractive target for extensive genetic

manipulations to produce rifamycin B analogues which could be effective against

multi-drug-resistant tuberculosis. Additionally, a better understanding of the

regulation of rifamycin B production and the application of newer genomics tools,

including CRISPR-assisted genome editing systems, might prove useful to overcome

the limitations associated with low production of rifamycin analogues.

FAU - Sood, Utkarsh

AU - Sood U

AD - Department of Zoology, Kirori Mal College, University of Delhi, Delhi-110007,

India.

FAU - Müller, Moritz

AU - Müller M

AD - Institute of Pharmaceutical Biology and Biotechnology,

Albert-Ludwigs-Universität, Stefan-Meier-Straße 19, 79104, Freiburg, Germany.

FAU - Lan, Tian

AU - Lan T

AUID- ORCID: 0000-0002-4099-5386

AD - Department of Medicinal Chemistry, College of Pharmacy, University of Minnesota,

Minneapolis, Minnesota 55455, United States.

FAU - Garg, Gauri

AU - Garg G

AD - Department of Zoology, Kirori Mal College, University of Delhi, Delhi-110007,

India.

FAU - Singhvi, Nirjara

AU - Singhvi N

AD - School of Allied Sciences, Dev Bhoomi Uttarakhand University, Dehradun,

Uttarakhand 248007, India.

FAU - Hira, Princy

AU - Hira P

AD - Department of Zoology, Maitreyi College, University of Delhi, Delhi-110003,

India.

FAU - Singh, Priya

AU - Singh P

AD - Department of Zoology, Maitreyi College, University of Delhi, Delhi-110003,

India.

FAU - Nigam, Aeshna

AU - Nigam A

AD - Department of Zoology, Shivaji College, University of Delhi, Delhi-110027, India.

FAU - Verma, Mansi

AU - Verma M

AD - Department of Zoology, Hansraj College, University of Delhi, Delhi-110007, India.

FAU - Lata, Pushp

AU - Lata P

AD - Department of Zoology, University of Delhi, Delhi-110007, India.

FAU - Kaur, Hardeep

AU - Kaur H

AD - Department of Zoology, Ramjas College, University of Delhi, Delhi-110007, India.

FAU - Kumar, Abhilash

AU - Kumar A

AD - Department of Zoology, Ramjas College, University of Delhi, Delhi-110007, India.

FAU - Rawat, Charu Dogra

AU - Rawat CD

AD - Department of Zoology, Ramjas College, University of Delhi, Delhi-110007, India.

FAU - Lal, Sukanya

AU - Lal S

AD - PhiXGen Private Limited, Gurugram, Haryana-122001, India.

FAU - Aldrich, Courtney

AU - Aldrich C

AUID- ORCID: 0000-0001-9261-594X

AD - Department of Medicinal Chemistry, College of Pharmacy, University of Minnesota,

Minneapolis, Minnesota 55455, United States.

FAU - Bechthold, Andreas

AU - Bechthold A

AUID- ORCID: 0000-0003-3594-6851

AD - Institute of Pharmaceutical Biology and Biotechnology,

Albert-Ludwigs-Universität, Stefan-Meier-Straße 19, 79104, Freiburg, Germany.

FAU - Lal, Rup

AU - Lal R

AUID- ORCID: 0000-0001-8364-8547

AD - PhiXGen Private Limited, Gurugram, Haryana-122001, India.

AD - Acharya Narendra Dev College, University of Delhi, Delhi-110019, India.

LA - eng

PT - Journal Article

PT - Research Support, Non-U.S. Gov't

PT - Review

DEP - 20240130

PL - United States

TA - J Nat Prod

JT - Journal of natural products

JID - 7906882

RN - 0 (Rifamycins)

RN - 8DDB535DJI (rifamycin B)

RN - Amycolatopsis mediterranei

SB - IM

MH - *Actinomycetales

MH - Amycolatopsis

MH - Biosynthetic Pathways/genetics

MH - *Rifamycins/metabolism

EDAT- 2024/01/30 12:42

MHDA- 2024/02/26 06:43

CRDT- 2024/01/30 09:32

PHST- 2024/02/26 06:43 [medline]

PHST- 2024/01/30 12:42 [pubmed]

PHST- 2024/01/30 09:32 [entrez]

AID - 10.1021/acs.jnatprod.3c00686 [doi]

PST - ppublish

SO - J Nat Prod. 2024 Feb 23;87(2):424-438. doi: 10.1021/acs.jnatprod.3c00686. Epub

2024 Jan 30.

PMID- 38331432

OWN - NLM

STAT- MEDLINE

DCOM- 20240223

LR - 20241002

IS - 1520-4804 (Electronic)

IS - 0022-2623 (Print)

IS - 0022-2623 (Linking)

VI - 67

IP - 4

DP - 2024 Feb 22

TI - Azetidines Kill Multidrug-Resistant Mycobacterium tuberculosis without Detectable

Resistance by Blocking Mycolate Assembly.

PG - 2529-2548

LID - 10.1021/acs.jmedchem.3c01643 [doi]

AB - Tuberculosis (TB) is the leading cause of global morbidity and mortality

resulting from infectious disease, with over 10.6 million new cases and 1.4

million deaths in 2021. This global emergency is exacerbated by the emergence of

multidrug-resistant MDR-TB and extensively drug-resistant XDR-TB; therefore, new

drugs and new drug targets are urgently required. From a whole cell phenotypic

screen, a series of azetidines derivatives termed BGAz, which elicit potent

bactericidal activity with MIC(99) values <10 μM against drug-sensitive

Mycobacterium tuberculosis and MDR-TB, were identified. These compounds

demonstrate no detectable drug resistance. The mode of action and target

deconvolution studies suggest that these compounds inhibit mycobacterial growth

by interfering with cell envelope biogenesis, specifically late-stage mycolic

acid biosynthesis. Transcriptomic analysis demonstrates that the BGAz compounds

tested display a mode of action distinct from the existing mycobacterial cell

wall inhibitors. In addition, the compounds tested exhibit toxicological and

PK/PD profiles that pave the way for their development as antitubercular

chemotherapies.

FAU - Cui, Yixin

AU - Cui Y

AD - School of Chemistry, University of Birmingham, Edgbaston, Birmingham, West

Midlands B15 2TT, U.K.

FAU - Lanne, Alice

AU - Lanne A

AD - Institute of Microbiology and Infection, School of Biosciences, University of

Birmingham, Edgbaston, Birmingham, West Midlands B15 2TT, U.K.

FAU - Peng, Xudan

AU - Peng X

AD - State Key Laboratory of Respiratory Disease, China-New Zealand Joint Laboratory

on Biomedicine and Health, Guangzhou Institutes of Biomedicine and Health,

Chinese Academy of Science, 190 Kai Yuan Avenue, Science Park, Guangzhou 510530,

China.

FAU - Browne, Edward

AU - Browne E

AD - Sygnature Discovery, The Discovery Building, BioCity, Pennyfoot Street,

Nottingham NG1 1GR, U.K.

FAU - Bhatt, Apoorva

AU - Bhatt A

AD - Institute of Microbiology and Infection, School of Biosciences, University of

Birmingham, Edgbaston, Birmingham, West Midlands B15 2TT, U.K.

FAU - Coltman, Nicholas J

AU - Coltman NJ

AUID- ORCID: 0000-0002-8210-9178

AD - School of Biosciences, University of Birmingham, Edgbaston, Birmingham, West

Midlands B15 2TT, U.K.

FAU - Craven, Philip

AU - Craven P

AUID- ORCID: 0000-0002-7617-132X

AD - School of Chemistry, University of Birmingham, Edgbaston, Birmingham, West

Midlands B15 2TT, U.K.

FAU - Cox, Liam R

AU - Cox LR

AUID- ORCID: 0000-0001-7018-3904

AD - School of Chemistry, University of Birmingham, Edgbaston, Birmingham, West

Midlands B15 2TT, U.K.

FAU - Cundy, Nicholas J

AU - Cundy NJ

AUID- ORCID: 0000-0001-6783-5422

AD - School of Chemistry, University of Birmingham, Edgbaston, Birmingham, West

Midlands B15 2TT, U.K.

FAU - Dale, Katie

AU - Dale K

AD - Institute of Microbiology and Infection, School of Biosciences, University of

Birmingham, Edgbaston, Birmingham, West Midlands B15 2TT, U.K.

FAU - Feula, Antonio

AU - Feula A

AD - School of Chemistry, University of Birmingham, Edgbaston, Birmingham, West

Midlands B15 2TT, U.K.

FAU - Frampton, Jon

AU - Frampton J

AD - College of Medical and Dental Sciences, University of Birmingham, Edgbaston,

Birmingham, West Midlands B15 2TT, U.K.

FAU - Fung, Martin

AU - Fung M

AD - Centre for Regenerative Medicine and Health, Hong Kong Institute of Science &

Innovation, Chinese Academy of Sciences, 15 Science Park West Avenue NT, Hong

Kong SAR.

FAU - Morton, Michael

AU - Morton M

AD - ApconiX Ltd, BIOHUB at Alderly Park, Nether Alderly, Cheshire SK10 4TG, U.K.

FAU - Goff, Aaron

AU - Goff A

AD - Department of Global Health and Infection, Brighton and Sussex Medical School,

University of Sussex, Falmer BN1 9PX, U.K.

FAU - Salih, Mariwan

AU - Salih M

AD - School of Chemistry, University of Birmingham, Edgbaston, Birmingham, West

Midlands B15 2TT, U.K.

FAU - Lang, Xingfen

AU - Lang X

AD - State Key Laboratory of Respiratory Disease, China-New Zealand Joint Laboratory

on Biomedicine and Health, Guangzhou Institutes of Biomedicine and Health,

Chinese Academy of Science, 190 Kai Yuan Avenue, Science Park, Guangzhou 510530,

China.

FAU - Li, Xingjian

AU - Li X

AD - School of Chemistry, University of Birmingham, Edgbaston, Birmingham, West

Midlands B15 2TT, U.K.

AD - State Key Laboratory of Respiratory Disease, China-New Zealand Joint Laboratory

on Biomedicine and Health, Guangzhou Institutes of Biomedicine and Health,

Chinese Academy of Science, 190 Kai Yuan Avenue, Science Park, Guangzhou 510530,

China.

FAU - Moon, Chris

AU - Moon C

AD - TB Research Group, National Infection Service, Public Health England (UKHSA),

Manor Farm Road, Porton, Salisbury SP4 0JG, U.K.

FAU - Pascoe, Jordan

AU - Pascoe J

AD - TB Research Group, National Infection Service, Public Health England (UKHSA),

Manor Farm Road, Porton, Salisbury SP4 0JG, U.K.

FAU - Portman, Vanessa

AU - Portman V

AD - Sygnature Discovery, The Discovery Building, BioCity, Pennyfoot Street,

Nottingham NG1 1GR, U.K.

FAU - Press, Cara

AU - Press C

AD - Institute of Microbiology and Infection, School of Biosciences, University of

Birmingham, Edgbaston, Birmingham, West Midlands B15 2TT, U.K.

FAU - Schulz-Utermoehl, Timothy

AU - Schulz-Utermoehl T

AD - Sygnature Discovery, The Discovery Building, BioCity, Pennyfoot Street,

Nottingham NG1 1GR, U.K.

FAU - Lee, Suki

AU - Lee S

AD - Centre for Regenerative Medicine and Health, Hong Kong Institute of Science &

Innovation, Chinese Academy of Sciences, 15 Science Park West Avenue NT, Hong

Kong SAR.

FAU - Tortorella, Micky D

AU - Tortorella MD

AD - State Key Laboratory of Respiratory Disease, China-New Zealand Joint Laboratory

on Biomedicine and Health, Guangzhou Institutes of Biomedicine and Health,

Chinese Academy of Science, 190 Kai Yuan Avenue, Science Park, Guangzhou 510530,

China.

AD - Centre for Regenerative Medicine and Health, Hong Kong Institute of Science &

Innovation, Chinese Academy of Sciences, 15 Science Park West Avenue NT, Hong

Kong SAR.

FAU - Tu, Zhengchao

AU - Tu Z

AD - State Key Laboratory of Respiratory Disease, China-New Zealand Joint Laboratory

on Biomedicine and Health, Guangzhou Institutes of Biomedicine and Health,

Chinese Academy of Science, 190 Kai Yuan Avenue, Science Park, Guangzhou 510530,

China.

FAU - Underwood, Zoe E

AU - Underwood ZE

AUID- ORCID: 0000-0002-4777-5757

AD - TB Research Group, National Infection Service, Public Health England (UKHSA),

Manor Farm Road, Porton, Salisbury SP4 0JG, U.K.

FAU - Wang, Changwei

AU - Wang C

AD - State Key Laboratory of Respiratory Disease, China-New Zealand Joint Laboratory

on Biomedicine and Health, Guangzhou Institutes of Biomedicine and Health,

Chinese Academy of Science, 190 Kai Yuan Avenue, Science Park, Guangzhou 510530,

China.

FAU - Yoshizawa, Akina

AU - Yoshizawa A

AD - School of Chemistry, University of Birmingham, Edgbaston, Birmingham, West

Midlands B15 2TT, U.K.

FAU - Zhang, Tianyu

AU - Zhang T

AUID- ORCID: 0000-0001-5647-6014

AD - State Key Laboratory of Respiratory Disease, China-New Zealand Joint Laboratory

on Biomedicine and Health, Guangzhou Institutes of Biomedicine and Health,

Chinese Academy of Science, 190 Kai Yuan Avenue, Science Park, Guangzhou 510530,

China.

FAU - Waddell, Simon J

AU - Waddell SJ

AUID- ORCID: 0000-0002-3684-9116

AD - Department of Global Health and Infection, Brighton and Sussex Medical School,

University of Sussex, Falmer BN1 9PX, U.K.

FAU - Bacon, Joanna

AU - Bacon J

AD - TB Research Group, National Infection Service, Public Health England (UKHSA),

Manor Farm Road, Porton, Salisbury SP4 0JG, U.K.

FAU - Alderwick, Luke

AU - Alderwick L

AUID- ORCID: 0000-0002-1257-6053

AD - Institute of Microbiology and Infection, School of Biosciences, University of

Birmingham, Edgbaston, Birmingham, West Midlands B15 2TT, U.K.

AD - Discovery Sciences, Charles River Laboratories, Chesterford Research Park,

Saffron Walden CB10 1XL, U.K.

FAU - Fossey, John S

AU - Fossey JS

AUID- ORCID: 0000-0002-2626-5117

AD - School of Chemistry, University of Birmingham, Edgbaston, Birmingham, West

Midlands B15 2TT, U.K.

FAU - Neagoie, Cleopatra

AU - Neagoie C

AUID- ORCID: 0000-0003-2289-3949

AD - State Key Laboratory of Respiratory Disease, China-New Zealand Joint Laboratory

on Biomedicine and Health, Guangzhou Institutes of Biomedicine and Health,

Chinese Academy of Science, 190 Kai Yuan Avenue, Science Park, Guangzhou 510530,

China.

AD - Centre for Regenerative Medicine and Health, Hong Kong Institute of Science &

Innovation, Chinese Academy of Sciences, 15 Science Park West Avenue NT, Hong

Kong SAR.

AD - Visiting Scientist, School of Chemistry, University of Birmingham, Edgbaston,

Birmingham, West Midlands B15 2TT, U.K.

LA - eng

PT - Journal Article

PT - Research Support, Non-U.S. Gov't

DEP - 20240208

PL - United States

TA - J Med Chem

JT - Journal of medicinal chemistry

JID - 9716531

RN - 0 (Azetidines)

RN - 0 (Antitubercular Agents)

SB - IM

MH - Humans

MH - *Mycobacterium tuberculosis

MH - *Azetidines/pharmacology/therapeutic use

MH - Antitubercular Agents/pharmacology/therapeutic use

MH - *Tuberculosis, Multidrug-Resistant/drug therapy

MH - *Extensively Drug-Resistant Tuberculosis/drug therapy

MH - Microbial Sensitivity Tests

PMC - PMC10895678

COIS- The authors declare the following competing financial interest(s): A patent

application disclosing aspects of this study has been filed by the University of

Birmingham. The views expressed in this publication are those of the authors and

not necessarily those of Public Health England (UKHSA), or the Department of

Health. The authors declare no other competing interests.

EDAT- 2024/02/09 00:42

MHDA- 2024/02/23 06:43

PMCR- 2024/02/26

CRDT- 2024/02/08 20:13

PHST- 2024/02/23 06:43 [medline]

PHST- 2024/02/09 00:42 [pubmed]

PHST- 2024/02/08 20:13 [entrez]

PHST- 2024/02/26 00:00 [pmc-release]

AID - 10.1021/acs.jmedchem.3c01643 [doi]

PST - ppublish

SO - J Med Chem. 2024 Feb 22;67(4):2529-2548. doi: 10.1021/acs.jmedchem.3c01643. Epub

2024 Feb 8.

PMID- 38295025

OWN - NLM

STAT- MEDLINE

DCOM- 20240214

LR - 20240613

IS - 2373-8227 (Electronic)

IS - 2373-8227 (Linking)

VI - 10

IP - 2

DP - 2024 Feb 9

TI - Unlocking Opportunities for Mycobacterium leprae and Mycobacterium ulcerans.

PG - 251-269

LID - 10.1021/acsinfecdis.3c00371 [doi]

AB - In the recent decade, scientific communities have toiled to tackle the emerging

burden of drug-resistant tuberculosis (DR-TB) and rapidly growing opportunistic

nontuberculous mycobacteria (NTM). Among these, two neglected mycobacteria

species of the Acinetobacter family, Mycobacterium leprae and Mycobacterium

ulcerans, are the etiological agents of leprosy and Buruli ulcer infections,

respectively, and fall under the broad umbrella of neglected tropical diseases

(NTDs). Unfortunately, lackluster drug discovery efforts have been made against

these pathogenic bacteria in the recent decade, resulting in the discovery of

only a few countable hits and majorly repurposing anti-TB drug candidates such as

telacebec (Q203), P218, and TB47 for current therapeutic interventions. Major

ignorance in drug candidate identification might aggravate the dramatic

consequences of rapidly spreading mycobacterial NTDs in the coming days.

Therefore, this Review focuses on an up-to-date account of drug discovery efforts

targeting selected druggable targets from both bacilli, including the

accompanying challenges that have been identified and are responsible for the

slow drug discovery. Furthermore, a succinct discussion of the all-new

possibilities that could be alternative solutions to mitigate the neglected

mycobacterial NTD burden and subsequently accelerate the drug discovery effort is

also included. We anticipate that the state-of-the-art strategies discussed here

may attract major attention from the scientific community to navigate and expand

the roadmap for the discovery of next-generation therapeutics against these NTDs.

FAU - Shyam, Mousumi

AU - Shyam M

AUID- ORCID: 0000-0002-0791-3772

AD - Department of Pharmaceutical Sciences & Technology, Birla Institute of

Technology, Mersa, Ranchi, Jharkhand 835215, India.

FAU - Kumar, Sumit

AU - Kumar S

AUID- ORCID: 0000-0002-4588-7237

AD - Holistic Drug Discovery and Development (H3D) Centre, University of Cape Town,

Rondebosch 7701, South Africa.

FAU - Singh, Vinayak

AU - Singh V

AUID- ORCID: 0000-0001-9002-2489

AD - Holistic Drug Discovery and Development (H3D) Centre, University of Cape Town,

Rondebosch 7701, South Africa.

AD - South African Medical Research Council Drug Discovery and Development Research

Unit, University of Cape Town, Rondebosch 7701, South Africa.

AD - Institute of Infectious Disease and Molecular Medicine (IDM), University of Cape

Town, Observatory 7925, Cape Town, South Africa.

LA - eng

PT - Journal Article

PT - Research Support, Non-U.S. Gov't

PT - Review

DEP - 20240131

PL - United States

TA - ACS Infect Dis

JT - ACS infectious diseases

JID - 101654580

SB - IM

MH - Humans

MH - *Mycobacterium ulcerans

MH - Mycobacterium leprae

MH - *Buruli Ulcer/drug therapy/microbiology/pathology

MH - *Mycobacterium

PMC - PMC10862552

OTO - NOTNLM

OT - 3M mechanisms

OT - Buruli ulcer

OT - Mycobacterium leprae

OT - Mycobacterium ulcerans

OT - PKS inhibitors

OT - antituberculars

OT - drug repurposing

OT - efflux pump inhibitors

OT - leprosy

OT - mycolactone analogues

OT - mycolactone biosynthesis

COIS- The authors declare no competing financial interest.

EDAT- 2024/01/31 18:42

MHDA- 2024/02/10 14:47

PMCR- 2024/02/13

CRDT- 2024/01/31 13:33

PHST- 2024/02/10 14:47 [medline]

PHST- 2024/01/31 18:42 [pubmed]

PHST- 2024/01/31 13:33 [entrez]

PHST- 2024/02/13 00:00 [pmc-release]

AID - 10.1021/acsinfecdis.3c00371 [doi]

PST - ppublish

SO - ACS Infect Dis. 2024 Feb 9;10(2):251-269. doi: 10.1021/acsinfecdis.3c00371. Epub

2024 Jan 31.

PMID- 38193667

OWN - NLM

STAT- MEDLINE

DCOM- 20240208

LR - 20250306

IS - 1098-6596 (Electronic)

IS - 0066-4804 (Print)

IS - 0066-4804 (Linking)

VI - 68

IP - 2

DP - 2024 Feb 7

TI - Identification and optimization of pyridine carboxamide-based scaffold as a drug

lead for Mycobacterium tuberculosis.

PG - e0076623

LID - 10.1128/aac.00766-23 [doi]

LID - e00766-23

AB - New drugs with novel mechanisms of action are urgently needed to tackle the issue

of drug-resistant tuberculosis. Here, we have performed phenotypic screening

using the Pathogen Box library obtained from the Medicines for Malaria Venture

against Mycobacterium tuberculosis in vitro. We have identified a pyridine

carboxamide derivative, MMV687254, as a promising hit. This molecule is

specifically active against M. tuberculosis and Mycobacterium bovis Bacillus

Calmette-Guérin (M. bovis BCG) but inactive against Enterococcus faecalis,

Staphylococcus aureus, Klebsiella pneumoniae, Acinetobacter baumanii, Pseudomonas

aeruginosa, and Escherichia coli pathogens. We demonstrate that MMV687254

inhibits M. tuberculosis growth in liquid cultures in a bacteriostatic manner.

Surprisingly, MMV687254 was as active as isoniazid in macrophages and inhibited

M. tuberculosis growth in a bactericidal manner. Mechanistic studies revealed

that MMV687254 is a prodrug and that its anti-mycobacterial activity requires

AmiC-dependent hydrolysis. We further demonstrate that MMV687254 inhibits M.

tuberculosis growth in macrophages by inducing autophagy. In the present study,

we have also carried out a detailed structure-activity relationship study and

identified a promising novel lead candidate. The identified novel series of

compounds also showed activity against drug-resistant M. bovis BCG and M.

tuberculosis clinical strains. Finally, we demonstrate that in contrast to

MMV687254, the lead molecule was able to inhibit M. tuberculosis growth in a

chronic mouse model of infection. Taken together, we have identified a novel lead

molecule with a dual mechanism of action that can be further optimized to design

more potent anti-tubercular agents.

FAU - Singh, Padam

AU - Singh P

AUID- ORCID: 0000-0002-8844-9157

AD - Translational Health Sciences and Technology Institute, NCR Biotech Science

Cluster, Faridabad, Haryana, India.

FAU - Kumar, Arun

AU - Kumar A

AD - Translational Health Sciences and Technology Institute, NCR Biotech Science

Cluster, Faridabad, Haryana, India.

FAU - Sharma, Pankaj

AU - Sharma P

AD - Translational Health Sciences and Technology Institute, NCR Biotech Science

Cluster, Faridabad, Haryana, India.

FAU - Chugh, Saurabh

AU - Chugh S

AD - Translational Health Sciences and Technology Institute, NCR Biotech Science

Cluster, Faridabad, Haryana, India.

FAU - Kumar, Ashish

AU - Kumar A

AD - Department of Life Science, Laboratory of Mycobacterial Immunology, National

Institute of Technology, Rourkela, India.

FAU - Sharma, Nidhi

AU - Sharma N

AD - Translational Health Sciences and Technology Institute, NCR Biotech Science

Cluster, Faridabad, Haryana, India.

FAU - Gupta, Sonu

AU - Gupta S

AD - Translational Health Sciences and Technology Institute, NCR Biotech Science

Cluster, Faridabad, Haryana, India.

FAU - Singh, Manisha

AU - Singh M

AD - Translational Health Sciences and Technology Institute, NCR Biotech Science

Cluster, Faridabad, Haryana, India.

FAU - Kidwai, Saqib

AU - Kidwai S

AD - Translational Health Sciences and Technology Institute, NCR Biotech Science

Cluster, Faridabad, Haryana, India.

FAU - Sankar, Jishnu

AU - Sankar J

AD - Translational Health Sciences and Technology Institute, NCR Biotech Science

Cluster, Faridabad, Haryana, India.

FAU - Taneja, Neha

AU - Taneja N

AD - Translational Health Sciences and Technology Institute, NCR Biotech Science

Cluster, Faridabad, Haryana, India.

FAU - Kumar, Yashwant

AU - Kumar Y

AD - Translational Health Sciences and Technology Institute, NCR Biotech Science

Cluster, Faridabad, Haryana, India.

FAU - Dhiman, Rohan

AU - Dhiman R

AD - Department of Life Science, Laboratory of Mycobacterial Immunology, National

Institute of Technology, Rourkela, India.

FAU - Mahajan, Dinesh

AU - Mahajan D

AUID- ORCID: 0000-0002-7939-9506

AD - Translational Health Sciences and Technology Institute, NCR Biotech Science

Cluster, Faridabad, Haryana, India.

FAU - Singh, Ramandeep

AU - Singh R

AUID- ORCID: 0000-0003-2705-3826

AD - Translational Health Sciences and Technology Institute, NCR Biotech Science

Cluster, Faridabad, Haryana, India.

LA - eng

GR - BT/PR30159/MED/15/188/2018/Department of Biotechnology, Ministry of Science and

Technology, India (DBT)/

GR - BT/PR30159/MED/15/188/2018/Department of Biotechnology, Ministry of Science and

Technology, India (DBT)/

PT - Journal Article

PT - Research Support, Non-U.S. Gov't

DEP - 20240109

PL - United States

TA - Antimicrob Agents Chemother

JT - Antimicrobial agents and chemotherapy

JID - 0315061

RN - 0 (Antitubercular Agents)

RN - V83O1VOZ8L (Isoniazid)

SB - IM

MH - Mice

MH - Animals

MH - *Mycobacterium tuberculosis

MH - Antitubercular Agents/pharmacology

MH - *Mycobacterium bovis

MH - Isoniazid

MH - *Tuberculosis/prevention & control

PMC - PMC10848774

OTO - NOTNLM

OT - 1-aminoisoquinoline

OT - 5-butyl-2-pyridinecarboxylic acid

OT - AmiC

OT - Mycobacterium tuberculosis

OT - autophagy

OT - pyridine carboxamide

COIS- The authors declare no conflict of interest.

EDAT- 2024/01/09 13:42

MHDA- 2024/02/08 06:42

PMCR- 2024/07/09

CRDT- 2024/01/09 09:04

PHST- 2024/02/08 06:42 [medline]

PHST- 2024/01/09 13:42 [pubmed]

PHST- 2024/01/09 09:04 [entrez]

PHST- 2024/07/09 00:00 [pmc-release]

AID - 00766-23 [pii]

AID - aac.00766-23 [pii]

AID - 10.1128/aac.00766-23 [doi]

PST - ppublish

SO - Antimicrob Agents Chemother. 2024 Feb 7;68(2):e0076623. doi:

10.1128/aac.00766-23. Epub 2024 Jan 9.

PMID- 38070474

OWN - NLM

STAT- MEDLINE

DCOM- 20240124

LR - 20250221

IS - 1090-2120 (Electronic)

IS - 0045-2068 (Linking)

VI - 143

DP - 2024 Feb

TI - Novel indolinone-tethered benzothiophenes as anti-tubercular agents against

MDR/XDR M. tuberculosis: Design, synthesis, biological evaluation and in vivo

pharmacokinetic study.

PG - 107009

LID - S0045-2068(23)00670-3 [pii]

LID - 10.1016/j.bioorg.2023.107009 [doi]

AB - Joining the global effort to eradicate tuberculosis, one of the deadliest

infectious killers in the world, we disclose in this paper the design and

synthesis of new indolinone-tethered benzothiophene hybrids 6a-i and 7a-i as

potential anti-tubercular agents. The MICs were determined in vitro for the

synthesized compounds against the sensitive M. tuberculosis strain ATCC 25177.

Potent compounds 6b, 6d, 6f, 6h, 7a, 7b, 7d, 7f, 7h and 7i were furtherly

assessed versus resistant MDR-TB and XDR-TB. Structure activity relationship

investigation of the synthesized compounds was illustrated, accordingly.

Superlative potency was unveiled for compound 6h (MIC = 0.48, 1.95 and 7.81 µg/mL

for ATCC 25177 sensitive TB strain, resistant MDR-TB and XDR-TB, respectively).

Moreover, validated in vivo pharmacokinetic study was performed for the most

potent derivative 6h revealing superior pharmacokinetic profile over the

reference drug. For further exploration of the anti-tubercular mechanism of

action, molecular docking was carried out for the former compound in DprE1 active

site as one of the important biological targets of TB. The binding mode and the

docking score uncovered exceptional binding when compared to the co-crystallized

ligand suggesting that it maybe the underlying target for its outstanding

anti-tubercular potency.

CI - Copyright © 2023 Elsevier Inc. All rights reserved.

FAU - Eldehna, Wagdy M

AU - Eldehna WM

AD - Department of Pharmaceutical Chemistry, Faculty of Pharmacy, Kafrelsheikh

University, Kafrelsheikh 33516, Egypt; Scientific Research and Innovation Support

Unit, Faculty of Pharmacy, Kafrelsheikh University, Kafrelsheikh, Egypt.

Electronic address: wagdy2000@gmail.com.

FAU - Mahmoud, Sally Tarek

AU - Mahmoud ST

AD - Pharmaceutical Chemistry Department, Faculty of Pharmacy, Cairo University, Kasr

Elini St., Cairo, 11562, Egypt.

FAU - Elshnawey, Esraa R

AU - Elshnawey ER

AD - Scientific Research and Innovation Support Unit, Faculty of Pharmacy,

Kafrelsheikh University, Kafrelsheikh, Egypt.

FAU - Elsayed, Zainab M

AU - Elsayed ZM

AD - Scientific Research and Innovation Support Unit, Faculty of Pharmacy,

Kafrelsheikh University, Kafrelsheikh, Egypt.

FAU - Majrashi, Taghreed A

AU - Majrashi TA

AD - Department of Pharmacognosy, College of Pharmacy, King Khalid University, Asir

61421, Saudi Arabia.

FAU - El-Ashrey, Mohamed K

AU - El-Ashrey MK

AD - Pharmaceutical Chemistry Department, Faculty of Pharmacy, Cairo University, Kasr

Elini St., Cairo, 11562, Egypt; Medicinal Chemistry Department, Faculty of

Pharmacy, King Salman International University, Ras-Sedr, South Sinai, 46612,

Egypt.

FAU - Rashed, Mahmoud

AU - Rashed M

AD - Pharmaceutical Medicinal Chemistry & Drug Design Department, Faculty of Pharmacy,

Al-Azhar University, Cairo, Egypt.

FAU - Hemeda, Loah R

AU - Hemeda LR

AD - Department of Medicinal Chemistry, Faculty of Pharmacy, Beni-Suef University,

Beni-Suef 62514, Egypt.

FAU - Shoun, Aly A

AU - Shoun AA

AD - Microbiology and Immunology Department, Faculty of Pharmacy, El Saleheya El

Gadida University, El Saleheya El Gadida, 44813, Egypt.

FAU - Elkaeed, Eslam B

AU - Elkaeed EB

AD - Department of Pharmaceutical Sciences, College of Pharmacy, AlMaarefa University,

Riyadh 13713, Saudi Arabia.

FAU - El Hassab, Mahmoud A

AU - El Hassab MA

AD - Medicinal Chemistry Department, Faculty of Pharmacy, King Salman International

University, Ras-Sedr, South Sinai, 46612, Egypt.

FAU - Abdel-Aziz, Marwa M

AU - Abdel-Aziz MM

AD - The Regional Center for Mycology and Biotechnology, Al-Azhar University, Cairo

11651, Egypt.

FAU - Shahin, Mai I

AU - Shahin MI

AD - Department of Pharmaceutical Chemistry, Faculty of Pharmacy, Ain Shams

University, Abassia, Cairo 11566, Egypt.

LA - eng

PT - Journal Article

PT - Research Support, Non-U.S. Gov't

DEP - 20231129

PL - United States

TA - Bioorg Chem

JT - Bioorganic chemistry

JID - 1303703

RN - 0 (Antitubercular Agents)

RN - 073790YQ2G (benzothiophene)

RN - 0 (Thiophenes)

SB - IM

MH - Humans

MH - Antitubercular Agents/chemistry

MH - *Extensively Drug-Resistant Tuberculosis

MH - Molecular Docking Simulation

MH - *Mycobacterium tuberculosis

MH - *Tuberculosis, Multidrug-Resistant/drug therapy

MH - Structure-Activity Relationship

MH - Microbial Sensitivity Tests

MH - *Thiophenes

OTO - NOTNLM

OT - Anti-mycobacterial activity

OT - DprE1

OT - Molecular docking

OT - Pharmacokinetics

OT - Synthesis

COIS- Declaration of competing interest The authors declare that they have no known

competing financial interests or personal relationships that could have appeared

to influence the work reported in this paper.

EDAT- 2023/12/10 06:42

MHDA- 2024/01/24 06:43

CRDT- 2023/12/09 18:14

PHST- 2023/09/14 00:00 [received]

PHST- 2023/11/04 00:00 [revised]

PHST- 2023/11/27 00:00 [accepted]

PHST- 2024/01/24 06:43 [medline]

PHST- 2023/12/10 06:42 [pubmed]

PHST- 2023/12/09 18:14 [entrez]

AID - S0045-2068(23)00670-3 [pii]

AID - 10.1016/j.bioorg.2023.107009 [doi]

PST - ppublish

SO - Bioorg Chem. 2024 Feb;143:107009. doi: 10.1016/j.bioorg.2023.107009. Epub 2023

Nov 29.

PMID- 37097976

OWN - NLM

STAT- MEDLINE

DCOM- 20240221

LR - 20240221

IS - 1538-0254 (Electronic)

IS - 0739-1102 (Linking)

VI - 42

IP - 4

DP - 2024 Feb-Mar

TI - Bioisosteric modification of Linezolid identified the potential M. tuberculosis

protein synthesis inhibitors to overcome the myelosuppression and serotonergic

toxicity associated with Linezolid in the treatment of the multi-drug resistance

tuberculosis (MDR-TB).

PG - 2111-2126

LID - 10.1080/07391102.2023.2203254 [doi]

AB - Linezolid is the first and only oxazolidinone antibacterial drug was approved in

the last 35 years. It exhibits bacteriostatic efficacy against M. tuberculosis

and is a crucial constituent of the BPaL regimen (Bedaquiline, Pretomanid, and

Linezolid), which was authorized by the FDA in 2019 for the treatment of XDR-TB

or MDR-TB. Despite its unique mechanism of action, Linezolid carries a

considerable risk of toxicity, including myelosuppression and serotonin syndrome

(SS), which is caused by inhibition of mitochondrial protein synthesis (MPS) and

monoamine oxidase (MAO), respectively. Based on the structure toxicity

relationship (STR) of Linezolid, in this work, we used a bioisosteric replacement

approach to optimize the structure of Linezolid at the C-ring and/or C-5 position

for myelosuppression and serotogenic toxicity. Extensive hierarchical multistep

docking, drug likeness prediction, molecular binding interactions analyses, and

toxicity assessment identified three promising compounds (3071, 7549 and 9660) as

less toxic potential modulators of Mtb EthR protein. Compounds 3071, 7549 and

9660 were having the significant docking score of -12.696 Kcal/mol,

-12.681 Kcal/mol and -15.293 Kcal/mol towards the Mtb EthR protein with less

MAO-A and B affinity [compound 3071: MAO A (-4.799 Kcal/mol) and MAO B

(-6.552 Kcal/mol); compound 7549: MAO A (> -2.00 Kcal/mol) and MAO B (>

-2.00 Kcal/mol) and compound 9660: MAO A (> -5.678 Kcal/mol) and MAO B (>

-6.537Kcal/mol) and none of them shown the Leukopenia as a side effect due to the

Myelosuppression. The MD simulation results and binding free energy estimations

correspond well with docking analyses, indicating that the proposed compounds

bind and inhibit the EthR protein more effectively than Linezolid. The quantum

mechanical and electrical characteristics were evaluated using density functional

theory (DFT), which also demonstrated that the proposed compounds are more

reactive than Linezolid.Communicated by Ramaswamy H. Sarma.

FAU - Girase, Rukaiyya

AU - Girase R

AD - Division of Computer Aided Drug Design, Department of Pharmaceutical Chemistry,

R. C. Patel Institute of Pharmaceutical Education and Research, Shirpur, India.

FAU - Ahmad, Iqrar

AU - Ahmad I

AUID- ORCID: 0000-0002-7697-9572

AD - Division of Computer Aided Drug Design, Department of Pharmaceutical Chemistry,

R. C. Patel Institute of Pharmaceutical Education and Research, Shirpur, India.

FAU - Patel, Harun

AU - Patel H

AD - Division of Computer Aided Drug Design, Department of Pharmaceutical Chemistry,

R. C. Patel Institute of Pharmaceutical Education and Research, Shirpur, India.

LA - eng

PT - Journal Article

DEP - 20230425

PL - England

TA - J Biomol Struct Dyn

JT - Journal of biomolecular structure & dynamics

JID - 8404176

RN - ISQ9I6J12J (Linezolid)

RN - 0 (Protein Synthesis Inhibitors)

RN - EC 1.4.3.4 (Monoamine Oxidase)

SB - IM

MH - Humans

MH - Linezolid/adverse effects

MH - Protein Synthesis Inhibitors/pharmacology/therapeutic use

MH - *Tuberculosis, Multidrug-Resistant/drug therapy/microbiology

MH - *Mycobacterium tuberculosis

MH - Monoamine Oxidase

MH - Drug Resistance, Multiple

MH - Molecular Docking Simulation

OTO - NOTNLM

OT - DFT

OT - Linezolid

OT - M. tuberculosis

OT - MM-GBSA

OT - molecular docking

OT - molecular dynamics

EDAT- 2023/04/25 18:42

MHDA- 2024/02/21 11:15

CRDT- 2023/04/25 13:22

PHST- 2024/02/21 11:15 [medline]

PHST- 2023/04/25 18:42 [pubmed]

PHST- 2023/04/25 13:22 [entrez]

AID - 10.1080/07391102.2023.2203254 [doi]

PST - ppublish

SO - J Biomol Struct Dyn. 2024 Feb-Mar;42(4):2111-2126. doi:

10.1080/07391102.2023.2203254. Epub 2023 Apr 25.

PMID- 38118315

OWN - NLM

STAT- MEDLINE

DCOM- 20240118

LR - 20240118

IS - 1878-1705 (Electronic)

IS - 1567-5769 (Linking)

VI - 127

DP - 2024 Jan 25

TI - Defensins: A novel weapon against Mycobacterium tuberculosis?

PG - 111383

LID - S1567-5769(23)01710-1 [pii]

LID - 10.1016/j.intimp.2023.111383 [doi]

AB - Tuberculosis (TB) is a serious airborne communicable disease caused by organisms

of the Mycobacterium tuberculosis (Mtb) complex. Although the standard treatment

antimicrobials, including isoniazid, rifampicin, pyrazinamide, and ethambutol,

have made great progress in the treatment of TB, problems including the rising

incidence of multidrug-resistant tuberculosis (MDR-TB) and extensively

drug-resistant tuberculosis (XDR-TB), the severe toxicity and side effects of

antimicrobials, and the low immunity of TB patients have become the bottlenecks

of the current TB treatments. Therefore, both safe and effective new strategies

to prevent and treat TB have become a top priority. As a subfamily of cationic

antimicrobial peptides, defensins are rich in cysteine and play a vital role in

resisting the invasion of microorganisms and regulating the immune response.

Inspired by studies on the roles of defensins in host defence, we describe their

research history and then review their structural features and antimicrobial

mechanisms, specifically for fighting Mtb in detail. Finally, we discuss the

clinical relevance, therapeutic potential, and potential challenges of defensins

in anti-TB therapy. We further debate the possible solutions of the current

application of defensins to provide new insights for eliminating Mtb.

CI - Copyright © 2023 Elsevier B.V. All rights reserved.

FAU - Gao, Xuehan

AU - Gao X

AD - Department of Immunology, Center of Immunomolecular Engineering, Innovation &

Practice Base for Graduate Students Education, Special Key Laboratory of Gene

Detection & Therapy, Zunyi Medical University, Zunyi, Guizhou 563000, China.

FAU - Feng, Jihong

AU - Feng J

AD - Department of Oncology, The Sixth Affiliated Hospital of Wenzhou Medical

University, Lishui People's Hospital, Lishui 323000, China.

FAU - Wei, Linna

AU - Wei L

AD - Department of Immunology, Center of Immunomolecular Engineering, Innovation &

Practice Base for Graduate Students Education, Special Key Laboratory of Gene

Detection & Therapy, Zunyi Medical University, Zunyi, Guizhou 563000, China.

FAU - Dong, Pinzhi

AU - Dong P

AD - Department of Immunology, Center of Immunomolecular Engineering, Innovation &

Practice Base for Graduate Students Education, Special Key Laboratory of Gene

Detection & Therapy, Zunyi Medical University, Zunyi, Guizhou 563000, China.

FAU - Chen, Jin

AU - Chen J

AD - Department of Immunology, Center of Immunomolecular Engineering, Innovation &

Practice Base for Graduate Students Education, Special Key Laboratory of Gene

Detection & Therapy, Zunyi Medical University, Zunyi, Guizhou 563000, China.

FAU - Zhang, Langlang

AU - Zhang L

AD - Department of Immunology, Center of Immunomolecular Engineering, Innovation &

Practice Base for Graduate Students Education, Special Key Laboratory of Gene

Detection & Therapy, Zunyi Medical University, Zunyi, Guizhou 563000, China.

FAU - Yang, Yuhan

AU - Yang Y

AD - Department of Immunology, Center of Immunomolecular Engineering, Innovation &

Practice Base for Graduate Students Education, Special Key Laboratory of Gene

Detection & Therapy, Zunyi Medical University, Zunyi, Guizhou 563000, China.

FAU - Xu, Lin

AU - Xu L

AD - Department of Immunology, Center of Immunomolecular Engineering, Innovation &

Practice Base for Graduate Students Education, Special Key Laboratory of Gene

Detection & Therapy, Zunyi Medical University, Zunyi, Guizhou 563000, China.

FAU - Wang, Haiyan

AU - Wang H

AD - Department of Epidemiology and Health Statistics, Zunyi Medical University,

Zunyi, Guizhou 563000, China.

FAU - Luo, Junmin

AU - Luo J

AD - Department of Immunology, Center of Immunomolecular Engineering, Innovation &

Practice Base for Graduate Students Education, Special Key Laboratory of Gene

Detection & Therapy, Zunyi Medical University, Zunyi, Guizhou 563000, China.

Electronic address: luojm16128@163.com.

FAU - Qin, Ming

AU - Qin M

AD - Department of Immunology, Center of Immunomolecular Engineering, Innovation &

Practice Base for Graduate Students Education, Special Key Laboratory of Gene

Detection & Therapy, Zunyi Medical University, Zunyi, Guizhou 563000, China; Key

Laboratory of Basic Pharmacology of Ministry of Education and Joint International

Research Laboratory of Ethnomedicine of Ministry of Education, Zunyi Medical

University, Zunyi, Guizhou 563000, China. Electronic address: 158277563@qq.com.

LA - eng

PT - Journal Article

PT - Review

DEP - 20231220

PL - Netherlands

TA - Int Immunopharmacol

JT - International immunopharmacology

JID - 100965259

RN - 0 (Antitubercular Agents)

RN - 0 (Defensins)

SB - IM

MH - Humans

MH - *Mycobacterium tuberculosis

MH - Antitubercular Agents/pharmacology/therapeutic use

MH - *Tuberculosis, Multidrug-Resistant/drug therapy/microbiology

MH - *Extensively Drug-Resistant Tuberculosis/drug therapy/epidemiology/microbiology

MH - Defensins/therapeutic use/pharmacology

OTO - NOTNLM

OT - Defensins

OT - Dendritic cells

OT - Macrophages

OT - Mycobacterium tuberculosis

OT - Neutrophils

OT - Tuberculosis

EDAT- 2023/12/21 00:41

MHDA- 2024/01/18 06:42

CRDT- 2023/12/20 18:08

PHST- 2023/10/10 00:00 [received]

PHST- 2023/12/04 00:00 [revised]

PHST- 2023/12/12 00:00 [accepted]

PHST- 2024/01/18 06:42 [medline]

PHST- 2023/12/21 00:41 [pubmed]

PHST- 2023/12/20 18:08 [entrez]

AID - S1567-5769(23)01710-1 [pii]

AID - 10.1016/j.intimp.2023.111383 [doi]

PST - ppublish

SO - Int Immunopharmacol. 2024 Jan 25;127:111383. doi: 10.1016/j.intimp.2023.111383.

Epub 2023 Dec 20.

PMID- 38243296

OWN - NLM

STAT- MEDLINE

DCOM- 20240131

LR - 20240131

IS - 1745-6215 (Electronic)

IS - 1745-6215 (Linking)

VI - 25

IP - 1

DP - 2024 Jan 19

TI - Quabodepistat in combination with delamanid and bedaquiline in participants with

drug-susceptible pulmonary tuberculosis: protocol for a multicenter, phase 2b/c,

open-label, randomized, dose-finding trial to evaluate safety and efficacy.

PG - 70

LID - 10.1186/s13063-024-07912-5 [doi]

LID - 70

AB - BACKGROUND: Delamanid and bedaquiline are two of the most recently developed

antituberculosis (TB) drugs that have been extensively studied in patients with

multidrug-resistant TB. There is currently a need for more potent, less-toxic

drugs with novel mechanisms of action that can be used in combination with these

newer agents to shorten the duration of treatment as well as prevent the

development of drug resistance. Quabodepistat (QBS) is a newly discovered

inhibitor of decaprenylphosphoryl-β-D-ribose-2'-oxidase, an essential enzyme for

Mycobacterium tuberculosis to synthesize key components of its cell wall. The

objective of this study is to evaluate the safety, efficacy, and appropriate

dosing of a 4-month regimen of QBS in combination with delamanid and bedaquiline

in participants with drug-susceptible pulmonary TB in comparison with the 6-month

standard treatment (i.e., rifampicin, isoniazid, ethambutol, and pyrazinamide).

METHODS: This phase 2b/c, open-label, randomized, parallel group, dose-finding

trial will enroll approximately 120 participants (including no more than 15% with

human immunodeficiency virus [HIV] coinfection) aged ≥ 18 to ≤ 65 years at

screening with newly diagnosed pulmonary drug-sensitive TB from ~8 sites in South

Africa. Following a screening period of up to 14 days, eligible participants will

be randomized in a ratio of 1:2:2:1 to one of four arms. Randomization will be

stratified by HIV status and the presence of bilateral cavitation on a screening

chest x-ray. After the end of the treatment period, participants will be followed

until 12 months post randomization. The primary efficacy endpoint is the

proportion of participants achieving sputum culture conversion in Mycobacteria

Growth Indicator Tube by the end of the treatment period. The safety endpoints

consist of adverse events, clinical laboratory tests, vital signs, physical

examination findings, and electrocardiographic changes. DISCUSSION: QBS's potent

bactericidal activity and distinct mechanism of action (compared with other TB

drugs currently available for human use) may make it an ideal candidate for

inclusion in a novel treatment regimen to improve efficacy and potentially

prevent resistance to concomitant TB drugs. This trial will assess the

effectiveness, safety, and dosing of a new, shorter, QBS-based, combination

anti-TB treatment regimen. TRIAL STATUS: ClinicalTrials.gov NCT05221502.

Registered on February 3, 2022.

CI - © 2024. The Author(s).

FAU - Dawson, Rodney

AU - Dawson R

AD - Division of Pulmonology, Department of Medicine, University of Cape Town and

University of Cape Town Lung Institute, Cape Town, South Africa.

FAU - Diacon, Andreas H

AU - Diacon AH

AD - Department of Medicine, Stellenbosch University, Cape Town, South Africa.

AD - TASK Applied Science, Cape Town, South Africa.

FAU - Takuva, Simbarashe

AU - Takuva S

AD - Otsuka Novel Products GmbH, Munich, Germany.

AD - Faculty of Health Sciences, School of Health Systems and Public Health,

University of Pretoria, Pretoria, South Africa.

FAU - Liu, Yongge

AU - Liu Y

AD - Otsuka Pharmaceutical Development & Commercialization, Inc., Rockville, MD, USA.

FAU - Zheng, Bo

AU - Zheng B

AD - Otsuka Pharmaceutical Development & Commercialization, Inc., Rockville, MD, USA.

FAU - Karwe, Vatsala

AU - Karwe V

AD - Otsuka Pharmaceutical Development & Commercialization, Inc., Rockville, MD, USA.

FAU - Hafkin, Jeffrey

AU - Hafkin J

AD - Otsuka Pharmaceutical Development & Commercialization, Inc., Rockville, MD, USA.

jeffrey.hafkin@otsuka-us.com.

LA - eng

SI - ClinicalTrials.gov/NCT05221502

PT - Clinical Trial Protocol

PT - Journal Article

DEP - 20240119

PL - England

TA - Trials

JT - Trials

JID - 101263253

RN - 0 (Antitubercular Agents)

RN - 78846I289Y (bedaquiline)

RN - 0 (Diarylquinolines)

RN - 0 (Nitroimidazoles)

RN - 0 (OPC-67683)

RN - 0 (Oxazoles)

SB - IM

MH - Humans

MH - Antitubercular Agents

MH - Clinical Trials, Phase II as Topic

MH - *Diarylquinolines

MH - Drug Therapy, Combination

MH - *HIV Infections/drug therapy

MH - Multicenter Studies as Topic

MH - *Nitroimidazoles

MH - *Oxazoles

MH - Randomized Controlled Trials as Topic

MH - Treatment Outcome

MH - *Tuberculosis, Multidrug-Resistant/diagnosis

MH - *Tuberculosis, Pulmonary/diagnosis/drug therapy/microbiology

MH - Adolescent

MH - Young Adult

MH - Adult

MH - Middle Aged

MH - Aged

PMC - PMC10799444

OTO - NOTNLM

OT - Antituberculosis agent

OT - Bedaquiline

OT - Delamanid

OT - DprE1 inhibitor

OT - Drug-sensitive tuberculosis

OT - Quabodepistat

OT - Tuberculosis

COIS- R. D. is an investigator for the study described in this paper, and they and/or

their institutions will receive funding support from Otsuka Pharmaceutical

Development & Commercialization, Inc. to conduct this study. A. H. D. is an

investigator for the study described in this paper, and their institution will

receive funding support from Otsuka Pharmaceutical Development &

Commercialization, Inc. to conduct this study. S. T., Y. L., B. Z., V. K., and J.

H. are employees of Otsuka Pharmaceutical Development & Commercialization, Inc.

EDAT- 2024/01/20 05:42

MHDA- 2024/01/22 06:43

PMCR- 2024/01/19

CRDT- 2024/01/19 23:42

PHST- 2023/10/19 00:00 [received]

PHST- 2024/01/03 00:00 [accepted]

PHST- 2024/01/22 06:43 [medline]

PHST- 2024/01/20 05:42 [pubmed]

PHST- 2024/01/19 23:42 [entrez]

PHST- 2024/01/19 00:00 [pmc-release]

AID - 10.1186/s13063-024-07912-5 [pii]

AID - 7912 [pii]

AID - 10.1186/s13063-024-07912-5 [doi]

PST - epublish

SO - Trials. 2024 Jan 19;25(1):70. doi: 10.1186/s13063-024-07912-5.

PMID- 38256079

OWN - NLM

STAT- MEDLINE

DCOM- 20240124

LR - 20241023

IS - 1422-0067 (Electronic)

IS - 1422-0067 (Linking)

VI - 25

IP - 2

DP - 2024 Jan 13

TI - Free Energy Barriers for Passive Drug Transport through the Mycobacterium

tuberculosis Outer Membrane: A Molecular Dynamics Study.

LID - 10.3390/ijms25021006 [doi]

LID - 1006

AB - The emergence of multi-drug-resistant tuberculosis strains poses a significant

challenge to modern medicine. The development of new antituberculosis drugs is

hindered by the low permeability of many active compounds through the extremely

strong bacterial cell wall of mycobacteria. In order to estimate the ability of

potential antimycobacterial agents to diffuse through the outer mycolate

membrane, the free energy profiles, the corresponding activation barriers, and

possible permeability modes of passive transport for a series of known

antibiotics, modern antituberculosis drugs, and prospective active drug-like

molecules were determined using molecular dynamics simulations with the all-atom

force field and potential of mean-force calculations. The membranes of different

chemical and conformational compositions, density, thickness, and ionization

states were examined. The typical activation barriers for the low-mass molecules

penetrating through the most realistic membrane model were 6-13 kcal/mol for

isoniazid, pyrazinamide, and etambutol, and 19 and 25 kcal/mol for bedaquilin and

rifampicin. The barriers for the ionized molecules are usually in the range of

37-63 kcal/mol. The linear regression models were derived from the obtained data,

allowing one to estimate the permeability barriers from simple physicochemical

parameters of the diffusing molecules, notably lipophilicity and molecular

polarizability.

FAU - Steshin, Ilya S

AU - Steshin IS

AUID- ORCID: 0000-0002-3330-716X

AD - Department of Chemistry, Lobachevsky State University of Nizhny Novgorod, Nizhny

Novgorod 603022, Russia.

FAU - Vasyankin, Alexander V

AU - Vasyankin AV

AD - Department of Chemistry, Lobachevsky State University of Nizhny Novgorod, Nizhny

Novgorod 603022, Russia.

FAU - Shirokova, Ekaterina A

AU - Shirokova EA

AUID- ORCID: 0000-0002-6252-5158

AD - Department of Chemistry, Lobachevsky State University of Nizhny Novgorod, Nizhny

Novgorod 603022, Russia.

FAU - Rozhkov, Alexey V

AU - Rozhkov AV

AD - Department of Chemistry, Lobachevsky State University of Nizhny Novgorod, Nizhny

Novgorod 603022, Russia.

FAU - Livshits, Grigory D

AU - Livshits GD

AUID- ORCID: 0000-0002-0856-5140

AD - Department of Chemistry, Lobachevsky State University of Nizhny Novgorod, Nizhny

Novgorod 603022, Russia.

FAU - Panteleev, Sergey V

AU - Panteleev SV

AD - Department of Chemistry, Lobachevsky State University of Nizhny Novgorod, Nizhny

Novgorod 603022, Russia.

FAU - Radchenko, Eugene V

AU - Radchenko EV

AUID- ORCID: 0000-0002-2091-7022

AD - Department of Chemistry, Lobachevsky State University of Nizhny Novgorod, Nizhny

Novgorod 603022, Russia.

AD - Department of Chemistry, Lomonosov Moscow State University, Leninskie Gory 1/3,

Moscow 119991, Russia.

FAU - Ignatov, Stanislav K

AU - Ignatov SK

AUID- ORCID: 0000-0002-6039-3905

AD - Department of Chemistry, Lobachevsky State University of Nizhny Novgorod, Nizhny

Novgorod 603022, Russia.

FAU - Palyulin, Vladimir A

AU - Palyulin VA

AUID- ORCID: 0000-0001-8792-8386

AD - Department of Chemistry, Lobachevsky State University of Nizhny Novgorod, Nizhny

Novgorod 603022, Russia.

AD - Department of Chemistry, Lomonosov Moscow State University, Leninskie Gory 1/3,

Moscow 119991, Russia.

LA - eng

GR - N-473-99/N. I. Lobachevsky State University of Nizhny Novgorod/

PT - Journal Article

DEP - 20240113

PL - Switzerland

TA - Int J Mol Sci

JT - International journal of molecular sciences

JID - 101092791

RN - 0 (Antitubercular Agents)

SB - IM

MH - Humans

MH - Molecular Dynamics Simulation

MH - *Mycobacterium tuberculosis

MH - Prospective Studies

MH - Cell Wall

MH - *Tuberculosis

MH - Antitubercular Agents/pharmacology

PMC - PMC10815926

OTO - NOTNLM

OT - activation barrier

OT - cell wall

OT - drug permeability

OT - free energy profile

OT - molecular dynamics

OT - mycobacterium

OT - outer membrane

OT - passive transport

OT - potential of mean force

OT - tuberculosis

COIS- The authors declare no conflict of interest. The funders had no role in the

design of this study, in the collection, analysis, or interpretation of data, in

the writing of this manuscript, or in the decision to publish these results.

EDAT- 2024/01/23 06:43

MHDA- 2024/01/24 06:43

PMCR- 2024/01/13

CRDT- 2024/01/23 01:12

PHST- 2023/12/05 00:00 [received]

PHST- 2024/01/04 00:00 [revised]

PHST- 2024/01/09 00:00 [accepted]

PHST- 2024/01/24 06:43 [medline]

PHST- 2024/01/23 06:43 [pubmed]

PHST- 2024/01/23 01:12 [entrez]

PHST- 2024/01/13 00:00 [pmc-release]

AID - ijms25021006 [pii]

AID - ijms-25-01006 [pii]

AID - 10.3390/ijms25021006 [doi]

PST - epublish

SO - Int J Mol Sci. 2024 Jan 13;25(2):1006. doi: 10.3390/ijms25021006.

PMID- 38274444

OWN - NLM

STAT- PubMed-not-MEDLINE

LR - 20241023

IS - 2296-858X (Print)

IS - 2296-858X (Electronic)

IS - 2296-858X (Linking)

VI - 10

DP - 2023

TI - Molecular mechanisms of resistance and treatment efficacy of clofazimine and

bedaquiline against Mycobacterium tuberculosis.

PG - 1304857

LID - 10.3389/fmed.2023.1304857 [doi]

LID - 1304857

AB - Clofazimine (CFZ) and bedaquiline (BDQ) are currently used for the treatment of

multidrug-resistant (MDR) Mycobacterium tuberculosis (Mtb) strains. In recent

years, adding CFZ and BDQ to tuberculosis (TB) drug regimens against MDR Mtb

strains has significantly improved treatment results, but these improvements are

threatened by the emergence of MDR and extensively drug-resistant (XDR) Mtb

strains. Recently, CFZ and BDQ have attracted much attention for their strong

clinical efficacy, although very little is known about the mechanisms of action,

drug susceptibility test (DST), resistance mechanisms, cross-resistance, and

pharmacokinetics of these two drugs. In this current review, we provide recent

updates on the mechanisms of action, DST, associated mutations with individual

resistance and cross-resistance, clinical efficacy, and pharmacokinetics of CFZ

and BDQ against Mtb strains. Presently, known mechanisms of resistance for CFZ

and/or BDQ include mutations within the Rv0678, pepQ, Rv1979c, and atpE genes.

The cross-resistance between CFZ and BDQ may reduce available MDR-/XDR-TB

treatment options. The use of CFZ and BDQ for treatment in the setting of limited

DST could allow further spread of drug resistance. The DST and resistance

knowledge are urgently needed where CFZ and BDQ resistance do emerge. Therefore,

an in-depth understanding of clinical efficacy, DST, cross-resistance, and

pharmacokinetics for CFZ and BDQ against Mtb can provide new ideas for improving

treatment outcomes, reducing mortality, preventing drug resistance, and TB

transmission. Along with this, it will also help to develop rapid molecular

diagnostic tools as well as novel therapeutic drugs for TB.

CI - Copyright © 2024 Islam, Alam, Liu, Khatun, Yusuf, Hameed, Tian, Chhotaray,

Basnet, Abraha, Zhang, Khan, Fang, Li, Hasan, Tan, Zhong, Hu and Zhang.

FAU - Islam, Md Mahmudul

AU - Islam MM

AD - State Key Laboratory of Respiratory Disease, Guangzhou Institutes of Biomedicine

and Health, Chinese Academy of Sciences, Guangzhou, China.

AD - Guangdong-Hong Kong-Macao Joint Laboratory of Respiratory Infectious Diseases,

Guangzhou Institutes of Biomedicine and Health, Chinese Academy of Sciences,

Guangzhou, China.

AD - University of Chinese Academy of Sciences, Beijing, China.

AD - China-New Zealand Joint Laboratory on Biomedicine and Health, Guangzhou

Institutes of Biomedicine and Health, Chinese Academy of Sciences, Guangzhou,

China.

AD - Department of Microbiology, Shaheed Shamsuzzoha Institute of Biosciences,

Affiliated with University of Rajshahi, Rajshahi, Bangladesh.

FAU - Alam, Md Shah

AU - Alam MS

AD - State Key Laboratory of Respiratory Disease, Guangzhou Institutes of Biomedicine

and Health, Chinese Academy of Sciences, Guangzhou, China.

AD - Guangdong-Hong Kong-Macao Joint Laboratory of Respiratory Infectious Diseases,

Guangzhou Institutes of Biomedicine and Health, Chinese Academy of Sciences,

Guangzhou, China.

AD - University of Chinese Academy of Sciences, Beijing, China.

AD - China-New Zealand Joint Laboratory on Biomedicine and Health, Guangzhou

Institutes of Biomedicine and Health, Chinese Academy of Sciences, Guangzhou,

China.

FAU - Liu, Zhiyong

AU - Liu Z

AD - State Key Laboratory of Respiratory Disease, Guangzhou Institutes of Biomedicine

and Health, Chinese Academy of Sciences, Guangzhou, China.

AD - Guangdong-Hong Kong-Macao Joint Laboratory of Respiratory Infectious Diseases,

Guangzhou Institutes of Biomedicine and Health, Chinese Academy of Sciences,

Guangzhou, China.

AD - China-New Zealand Joint Laboratory on Biomedicine and Health, Guangzhou

Institutes of Biomedicine and Health, Chinese Academy of Sciences, Guangzhou,

China.

AD - Guangzhou Medical University, Guangzhou, China.

AD - Guangzhou National Laboratory, Guangzhou, China.

FAU - Khatun, Mst Sumaia

AU - Khatun MS

AD - State Key Laboratory of Respiratory Disease, Guangzhou Institutes of Biomedicine

and Health, Chinese Academy of Sciences, Guangzhou, China.

AD - Guangdong-Hong Kong-Macao Joint Laboratory of Respiratory Infectious Diseases,

Guangzhou Institutes of Biomedicine and Health, Chinese Academy of Sciences,

Guangzhou, China.

AD - University of Chinese Academy of Sciences, Beijing, China.

AD - China-New Zealand Joint Laboratory on Biomedicine and Health, Guangzhou

Institutes of Biomedicine and Health, Chinese Academy of Sciences, Guangzhou,

China.

FAU - Yusuf, Buhari

AU - Yusuf B

AD - State Key Laboratory of Respiratory Disease, Guangzhou Institutes of Biomedicine

and Health, Chinese Academy of Sciences, Guangzhou, China.

AD - Guangdong-Hong Kong-Macao Joint Laboratory of Respiratory Infectious Diseases,

Guangzhou Institutes of Biomedicine and Health, Chinese Academy of Sciences,

Guangzhou, China.

AD - University of Chinese Academy of Sciences, Beijing, China.

AD - China-New Zealand Joint Laboratory on Biomedicine and Health, Guangzhou

Institutes of Biomedicine and Health, Chinese Academy of Sciences, Guangzhou,

China.

FAU - Hameed, H M Adnan

AU - Hameed HMA

AD - State Key Laboratory of Respiratory Disease, Guangzhou Institutes of Biomedicine

and Health, Chinese Academy of Sciences, Guangzhou, China.

AD - Guangdong-Hong Kong-Macao Joint Laboratory of Respiratory Infectious Diseases,

Guangzhou Institutes of Biomedicine and Health, Chinese Academy of Sciences,

Guangzhou, China.

AD - University of Chinese Academy of Sciences, Beijing, China.

AD - China-New Zealand Joint Laboratory on Biomedicine and Health, Guangzhou

Institutes of Biomedicine and Health, Chinese Academy of Sciences, Guangzhou,

China.

FAU - Tian, Xirong

AU - Tian X

AD - State Key Laboratory of Respiratory Disease, Guangzhou Institutes of Biomedicine

and Health, Chinese Academy of Sciences, Guangzhou, China.

AD - Guangdong-Hong Kong-Macao Joint Laboratory of Respiratory Infectious Diseases,

Guangzhou Institutes of Biomedicine and Health, Chinese Academy of Sciences,

Guangzhou, China.

AD - University of Chinese Academy of Sciences, Beijing, China.

AD - China-New Zealand Joint Laboratory on Biomedicine and Health, Guangzhou

Institutes of Biomedicine and Health, Chinese Academy of Sciences, Guangzhou,

China.

FAU - Chhotaray, Chiranjibi

AU - Chhotaray C

AD - Department of Medicine, Center for Emerging Pathogens, Rutgers-New Jersey Medical

School, Newark, NJ, United States.

FAU - Basnet, Rajesh

AU - Basnet R

AD - State Key Laboratory of Respiratory Disease, Guangzhou Institutes of Biomedicine

and Health, Chinese Academy of Sciences, Guangzhou, China.

AD - Guangdong-Hong Kong-Macao Joint Laboratory of Respiratory Infectious Diseases,

Guangzhou Institutes of Biomedicine and Health, Chinese Academy of Sciences,

Guangzhou, China.

AD - University of Chinese Academy of Sciences, Beijing, China.

AD - China-New Zealand Joint Laboratory on Biomedicine and Health, Guangzhou

Institutes of Biomedicine and Health, Chinese Academy of Sciences, Guangzhou,

China.

FAU - Abraha, Haftay

AU - Abraha H

AD - State Key Laboratory of Respiratory Disease, Guangzhou Institutes of Biomedicine

and Health, Chinese Academy of Sciences, Guangzhou, China.

AD - Guangdong-Hong Kong-Macao Joint Laboratory of Respiratory Infectious Diseases,

Guangzhou Institutes of Biomedicine and Health, Chinese Academy of Sciences,

Guangzhou, China.

AD - University of Chinese Academy of Sciences, Beijing, China.

AD - China-New Zealand Joint Laboratory on Biomedicine and Health, Guangzhou

Institutes of Biomedicine and Health, Chinese Academy of Sciences, Guangzhou,

China.

FAU - Zhang, Xiaofan

AU - Zhang X

AD - State Key Laboratory of Respiratory Disease, Guangzhou Institutes of Biomedicine

and Health, Chinese Academy of Sciences, Guangzhou, China.

AD - Guangdong-Hong Kong-Macao Joint Laboratory of Respiratory Infectious Diseases,

Guangzhou Institutes of Biomedicine and Health, Chinese Academy of Sciences,

Guangzhou, China.

AD - University of Chinese Academy of Sciences, Beijing, China.

AD - China-New Zealand Joint Laboratory on Biomedicine and Health, Guangzhou

Institutes of Biomedicine and Health, Chinese Academy of Sciences, Guangzhou,

China.

FAU - Khan, Shahzad Akbar

AU - Khan SA

AD - State Key Laboratory of Respiratory Disease, Guangzhou Institutes of Biomedicine

and Health, Chinese Academy of Sciences, Guangzhou, China.

AD - Guangdong-Hong Kong-Macao Joint Laboratory of Respiratory Infectious Diseases,

Guangzhou Institutes of Biomedicine and Health, Chinese Academy of Sciences,

Guangzhou, China.

AD - University of Chinese Academy of Sciences, Beijing, China.

AD - China-New Zealand Joint Laboratory on Biomedicine and Health, Guangzhou

Institutes of Biomedicine and Health, Chinese Academy of Sciences, Guangzhou,

China.

AD - Laboratory of Pathology, Department of Pathobiology, University of Poonch

Rawalakot, Azad Kashmir, Pakistan.

FAU - Fang, Cuiting

AU - Fang C

AD - State Key Laboratory of Respiratory Disease, Guangzhou Institutes of Biomedicine

and Health, Chinese Academy of Sciences, Guangzhou, China.

AD - Guangdong-Hong Kong-Macao Joint Laboratory of Respiratory Infectious Diseases,

Guangzhou Institutes of Biomedicine and Health, Chinese Academy of Sciences,

Guangzhou, China.

AD - University of Chinese Academy of Sciences, Beijing, China.

AD - China-New Zealand Joint Laboratory on Biomedicine and Health, Guangzhou

Institutes of Biomedicine and Health, Chinese Academy of Sciences, Guangzhou,

China.

FAU - Li, Chunyu

AU - Li C

AD - State Key Laboratory of Respiratory Disease, Guangzhou Institutes of Biomedicine

and Health, Chinese Academy of Sciences, Guangzhou, China.

AD - Guangdong-Hong Kong-Macao Joint Laboratory of Respiratory Infectious Diseases,

Guangzhou Institutes of Biomedicine and Health, Chinese Academy of Sciences,

Guangzhou, China.

AD - University of Chinese Academy of Sciences, Beijing, China.

AD - China-New Zealand Joint Laboratory on Biomedicine and Health, Guangzhou

Institutes of Biomedicine and Health, Chinese Academy of Sciences, Guangzhou,

China.

FAU - Hasan, Sohel

AU - Hasan S

AD - Department of Biochemistry and Molecular Biology, University of Rajshahi,

Rajshahi, Bangladesh.

FAU - Tan, Shouyong

AU - Tan S

AD - Guangzhou National Laboratory, Guangzhou, China.

AD - State Key Laboratory of Respiratory Disease, Guangzhou Chest Hospital, Guangzhou,

China.

FAU - Zhong, Nanshan

AU - Zhong N

AD - Guangdong-Hong Kong-Macao Joint Laboratory of Respiratory Infectious Diseases,

Guangzhou Institutes of Biomedicine and Health, Chinese Academy of Sciences,

Guangzhou, China.

AD - Guangzhou National Laboratory, Guangzhou, China.

AD - State Key Laboratory of Respiratory Disease, National Clinical Research Center

for Respiratory Disease, The National Center for Respiratory Medicine, The First

Affiliated Hospital of Guangzhou Medical University, Guangzhou, China.

FAU - Hu, Jinxing

AU - Hu J

AD - Guangzhou National Laboratory, Guangzhou, China.

AD - State Key Laboratory of Respiratory Disease, Guangzhou Chest Hospital, Guangzhou,

China.

FAU - Zhang, Tianyu

AU - Zhang T

AD - State Key Laboratory of Respiratory Disease, Guangzhou Institutes of Biomedicine

and Health, Chinese Academy of Sciences, Guangzhou, China.

AD - Guangdong-Hong Kong-Macao Joint Laboratory of Respiratory Infectious Diseases,

Guangzhou Institutes of Biomedicine and Health, Chinese Academy of Sciences,

Guangzhou, China.

AD - University of Chinese Academy of Sciences, Beijing, China.

AD - China-New Zealand Joint Laboratory on Biomedicine and Health, Guangzhou

Institutes of Biomedicine and Health, Chinese Academy of Sciences, Guangzhou,

China.

LA - eng

PT - Journal Article

PT - Review

DEP - 20240110

PL - Switzerland

TA - Front Med (Lausanne)

JT - Frontiers in medicine

JID - 101648047

PMC - PMC10809401

OTO - NOTNLM

OT - cross-resistance

OT - drug resistance

OT - mechanism of action

OT - molecular diagnostic

OT - regimens

OT - treatment

COIS- The authors declare that the research was conducted in the absence of any

commercial or financial relationships that could be construed as a potential

conflict of interest. The author(s) declared that they were an editorial board

member of Frontiers, at the time of submission. This had no impact on the peer

review process and the final decision.

EDAT- 2024/01/26 06:44

MHDA- 2024/01/26 06:45

PMCR- 2024/01/10

CRDT- 2024/01/26 03:54

PHST- 2023/09/30 00:00 [received]

PHST- 2023/11/21 00:00 [accepted]

PHST- 2024/01/26 06:45 [medline]

PHST- 2024/01/26 06:44 [pubmed]

PHST- 2024/01/26 03:54 [entrez]

PHST- 2024/01/10 00:00 [pmc-release]

AID - 10.3389/fmed.2023.1304857 [doi]

PST - epublish

SO - Front Med (Lausanne). 2024 Jan 10;10:1304857. doi: 10.3389/fmed.2023.1304857.

eCollection 2023.

PMID- 39317997

OWN - NLM

STAT- MEDLINE

DCOM- 20241129

LR - 20241129

IS - 1874-4702 (Electronic)

IS - 1874-4672 (Linking)

VI - 17

DP - 2024

TI - Mutations in Rv0678, Rv2535c, and Rv1979c Confer Resistance to Bedaquiline in

Clinical Isolates of Mycobacterium Tuberculosis.

PG - e18761429314641

LID - 10.2174/0118761429314641240815080447 [doi]

AB - INTRODUCTION: Reduced bedaquiline (BDQ) sensitivity to antimycobacterial drugs

has been linked to mutations in the Rv0678, pepQ, and Rv1979c genes of

Mycobacterium tuberculosis (MTB). Resistance-causing mutations in MTB strains

under treatment may have an impact on novel BDQ-based medication regimens

intended to reduce treatment time. Due to this, we investigated the genetic basis

of BDQ resistance in Turkish TB patients with MTB clinical isolates. Furthermore,

mutations in the genes linked to efflux pumps were examined as a backup

resistance mechanism. METHODS: We scrutinized 100 MTB clinical isolates from TB

patients using convenience sampling. Eighty MDR and twenty pan-drug susceptible

MTB strains were among these isolates. Sequencing was performed on all strains,

and genomic analyses were performed to find mutations in BDQ

resistance-associated genes, including Rv0678 and pepQ(Rv2535c), which correspond

to a putative Xaa-Pro aminopeptidase, and Rv1979c. Of the 74 isolates with PepQ

(Rv2535c) mutations, four isolates (2.96%) exhibited MGIT-BDQ susceptibility.

RESULTS: Twenty-one (19.11%) of the ninety-one isolates carrying mutations,

including Rv1979c, were MGIT-BDQ-sensitive. Nonetheless, out of the 39 isolates

with Rv0678 mutations, four (2.96%) were sensitive to MGIT-BDQ. It was found that

resistance-associated variants (RAVs) in Rv0678, pepQ, and Rv1979c are often

linked to BDQ resistance. CONCLUSION: In order to include variations in efflux

pump genes in genome-based diagnostics for drug-resistant MTB, further evidence

about their involvement in resistance is needed.

FAU - Balgouthi, Khaoula

AU - Balgouthi K

AD - Department of Biotechnology, Institute of Natural and Applied Sciences, Çukurova

University, 01330, Balcalı, Adana, Türkiye.

FAU - AlMatar, Manaf

AU - AlMatar M

AD - Biology Department, Faculty of Education and Arts, Sohar University, Sohar, 311,

Sultanate of Oman.

FAU - Saghrouchni, Hamza

AU - Saghrouchni H

AD - Department of Biotechnology, Institute of Natural and Applied Sciences, Çukurova

University, 01330, Balcalı, Adana, Türkiye.

FAU - Albarri, Osman

AU - Albarri O

AD - Department of Biotechnology, Institute of Natural and Applied Sciences, Çukurova

University, 01330, Balcalı, Adana, Türkiye.

FAU - Var, Işıl

AU - Var I

AD - Department of Food Engineering, Agricultural Faculty, Çukurova University, Adana,

Turkey.

LA - eng

PT - Journal Article

PL - Netherlands

TA - Curr Mol Pharmacol

JT - Current molecular pharmacology

JID - 101467997

RN - 78846I289Y (bedaquiline)

RN - 0 (Diarylquinolines)

RN - 0 (Antitubercular Agents)

RN - 0 (Bacterial Proteins)

SB - IM

MH - *Mycobacterium tuberculosis/genetics/drug effects/isolation & purification

MH - *Diarylquinolines/pharmacology

MH - Humans

MH - *Mutation

MH - *Antitubercular Agents/pharmacology

MH - *Microbial Sensitivity Tests

MH - *Bacterial Proteins/genetics/metabolism

MH - Tuberculosis, Multidrug-Resistant/microbiology/drug therapy

MH - Drug Resistance, Bacterial/genetics

MH - Male

MH - Female

OTO - NOTNLM

OT - Antimycobacterial drugs

OT - Bedaquiline resistance

OT - Drug-resistant tuberculosis

OT - PepQ

OT - Rv0678

OT - Rv1979c

EDAT- 2024/09/25 10:18

MHDA- 2024/11/29 12:22

CRDT- 2024/09/25 00:23

PHST- 2024/04/08 00:00 [received]

PHST- 2024/06/05 00:00 [revised]

PHST- 2024/06/20 00:00 [accepted]

PHST- 2024/11/29 12:22 [medline]

PHST- 2024/09/25 10:18 [pubmed]

PHST- 2024/09/25 00:23 [entrez]

AID - CMP-EPUB-143243 [pii]

AID - 10.2174/0118761429314641240815080447 [doi]

PST - ppublish

SO - Curr Mol Pharmacol. 2024;17:e18761429314641. doi:

10.2174/0118761429314641240815080447.

PMID- 39222997

OWN - NLM

STAT- MEDLINE

DCOM- 20240902

LR - 20240902

IS - 0423-2607 (Print)

IS - 1874-6047 (Linking)

VI - 55

DP - 2024

TI - Mycobacterial β-carbonic anhydrases: Molecular biology, role in the pathogenesis

of tuberculosis and inhibition studies.

PG - 343-381

LID - S1874-6047(24)00012-X [pii]

LID - 10.1016/bs.enz.2024.05.012 [doi]

AB - Mycobacterium tuberculosis (Mtb), which causes tuberculosis (TB), is still a

major global health problem. According to the World Health Organization (WHO), TB

still causes more deaths worldwide than any other infectious agent.

Drug-sensitive TB is treatable using first-line drugs; treatment of

multidrug-resistant (MDR) and extensively drug-resistant (XDR) TB requires

second- and third-line drugs. However, due to the long duration of treatment, the

noncompliance of patients with different levels of resistance of Mtb to these

drugs has worsened the situation. Previously developed anti-TB drugs targeted the

replication machinery, protein synthesis, and cell wall biosynthesis pathways of

Mtb. Therefore, novel drugs targeting alternate pathways crucial for the survival

and pathogenesis of Mtb in the human host are needed. The genome of Mtb encodes

three β-carbonic anhydrases (CAs) that are fundamental for pH homeostasis,

hypoxia, survival, and pathogenesis. Recently, several studies have shown that

the β-CAs of Mtb could be inhibited both in vitro and in vivo using small

chemical molecules, suggesting that these enzymes could be novel targets for

developing anti-TB compounds that are devoid of resistance by Mtb. In addition,

homologs of β-CAs are absent in humans; therefore, drugs developed to target

these enzymes might have minimal off-target effects. In this work, we describe

the roles of β-CAs in Mtb and discuss bioinformatics and cheminformatics tools

used in development and discovery of novel inhibitors of these enzymes. In

addition, we summarize the in vitro and in vivo studies demonstrating that the

β-CAs of Mtb are indeed druggable targets.

CI - Copyright © 2024. Published by Elsevier Inc.

FAU - Parkkinen, Jenny

AU - Parkkinen J

AD - Faculty of Medicine and Health Technology, Tampere University, Tampere, Finland.

FAU - Bhowmik, Ratul

AU - Bhowmik R

AD - Faculty of Medicine and Health Technology, Tampere University, Tampere, Finland.

FAU - Tolvanen, Martti

AU - Tolvanen M

AD - Department of Computing, University of Turku, Finland.

FAU - Carta, Fabrizio

AU - Carta F

AD - Neurofarba Department, Sezione di Chimica Farmaceutica e Nutraceutica, Università

degli Studi di Firenze, Firenze, Italy.

FAU - Supuran, Claudiu T

AU - Supuran CT

AD - Neurofarba Department, Sezione di Chimica Farmaceutica e Nutraceutica, Università

degli Studi di Firenze, Firenze, Italy.

FAU - Parkkila, Seppo

AU - Parkkila S

AD - Faculty of Medicine and Health Technology, Tampere University, Tampere, Finland;

Fimlab Ltd. and Tampere University Hospital, Tampere, Finland.

FAU - Aspatwar, Ashok

AU - Aspatwar A

AD - Faculty of Medicine and Health Technology, Tampere University, Tampere, Finland.

Electronic address: ashok.aspatwar@tuni.fi.

LA - eng

PT - Journal Article

PT - Review

DEP - 20240806

PL - United States

TA - Enzymes

JT - The Enzymes

JID - 101637215

RN - EC 4.2.1.1 (Carbonic Anhydrases)

RN - 0 (Antitubercular Agents)

RN - 0 (Carbonic Anhydrase Inhibitors)

RN - 0 (Bacterial Proteins)

SB - IM

MH - *Mycobacterium tuberculosis/drug effects/enzymology

MH - Humans

MH - *Carbonic Anhydrases/metabolism

MH - *Antitubercular Agents/pharmacology

MH - *Carbonic Anhydrase Inhibitors/pharmacology/therapeutic use

MH - *Tuberculosis/drug therapy/microbiology

MH - Animals

MH - Bacterial Proteins/metabolism/antagonists & inhibitors/genetics

OTO - NOTNLM

OT - Drug development

OT - Inhibitors

OT - Mycobacterium tuberculosis

OT - Tuberculosis

OT - β-Carbonic anhydrases

EDAT- 2024/09/03 01:42

MHDA- 2024/09/03 01:43

CRDT- 2024/09/02 20:59

PHST- 2024/09/03 01:43 [medline]

PHST- 2024/09/03 01:42 [pubmed]

PHST- 2024/09/02 20:59 [entrez]

AID - S1874-6047(24)00012-X [pii]

AID - 10.1016/bs.enz.2024.05.012 [doi]

PST - ppublish

SO - Enzymes. 2024;55:343-381. doi: 10.1016/bs.enz.2024.05.012. Epub 2024 Aug 6.

PMID- 38919089

OWN - NLM

STAT- MEDLINE

DCOM- 20241002

LR - 20241002

IS - 1873-4294 (Electronic)

IS - 1568-0266 (Linking)

VI - 24

IP - 21

DP - 2024

TI - Recent Biochemical Advances in Antitubercular Drugs: Challenges and Future.

PG - 1829-1855

LID - 10.2174/0115680266286294240610102911 [doi]

AB - Tuberculosis (TB) is one of the leading causes of death world-wide after AIDS. It

infects around one-third of global population and approximately two million

people die annually from this disease because it is a very contagious disease

spread by Mycobacterium tuberculosis. The increasing number of drug-resistant

strains and the failure of conventional treatments against this strain are the

challenges of the coming decades. New therapeutic techniques aim to confirm cure

without deterioration, to reduce deaths, contagions and the formation of

drug-resistant strains. A plethora of new diagnostic tests are available to

diagnose the active tuberculosis, screen latent M. tuberculosis infection, and to

identify drug-resistant strains of M. tuberculosis. When effective prevention

strategies do not prevail, high rates of early case detection and successive

cures to control TB emergence would not be possible. In this review, we discussed

the structural features of M. tuberculosis, Multi drug resistance tuberculosis

(MDR-TB), extremely drug-resistant tuberculosis (XDR-TB), the mechanism of M.

tuberculosis infection, the mode of action of first and second-line

antitubercular drugs, the mechanism of resistance to the existing drugs,

compounds in preclinical and clinical trial and drugs presently available for the

treatment of tuberculosis. Moreover, the new diagnostic techniques to detect M.

tuberculosis are also discussed in this review.

CI - Copyright© Bentham Science Publishers; For any queries, please email at

epub@benthamscience.net.

FAU - Jain, Akanksha

AU - Jain A

AD - Department of Food and Nutrition, Bhagini Nivedita College, University of Delhi,

Kair Near Najafgarh, Delhi, 110043, India.

FAU - Kumar, Rajesh

AU - Kumar R

AD - P.G. Department of Chemistry, R.D.S. College, B.R.A. Bihar University,

Muzaffarpur, 842002, India.

FAU - Mothsra, Poonam

AU - Mothsra P

AD - Department of Chemistry, Bhagini Nivedita College, University of Delhi, Kair Near

Najafgarh, Delhi, 110043, India.

FAU - Sharma, Atul Kumar

AU - Sharma AK

AD - Department of Chemistry, Deshbandhu College, University of Delhi, 110019, India.

FAU - Singh, Anil Kumar

AU - Singh AK

AD - Department of Chemistry, School of Physical Sciences, Mahatma Gandhi Central

University, Motihari, Bihar, 845401, India.

FAU - Kumar, Yogesh

AU - Kumar Y

AUID- ORCID: 0000-0002-8200-5838

AD - Department of Chemistry, Bhagini Nivedita College, University of Delhi, Kair Near

Najafgarh, Delhi, 110043, India.

LA - eng

PT - Journal Article

PT - Review

PL - United Arab Emirates

TA - Curr Top Med Chem

JT - Current topics in medicinal chemistry

JID - 101119673

RN - 0 (Antitubercular Agents)

SB - IM

MH - *Antitubercular Agents/pharmacology/chemistry

MH - Humans

MH - *Mycobacterium tuberculosis/drug effects

MH - Microbial Sensitivity Tests

MH - Tuberculosis, Multidrug-Resistant/drug therapy/diagnosis

MH - Tuberculosis/drug therapy/diagnosis

OTO - NOTNLM

OT - Anti-tuberculosis drugs

OT - First line anti-tubercular drugs

OT - Mechanism of drug resistance.

OT - Mode of action of anti-tubercular drugs

OT - Multidrug resistant tuberculosis

OT - Second line anti-tubercular drugs

EDAT- 2024/06/26 06:42

MHDA- 2024/10/02 07:02

CRDT- 2024/06/26 03:23

PHST- 2023/12/31 00:00 [received]

PHST- 2024/03/29 00:00 [revised]

PHST- 2024/04/29 00:00 [accepted]

PHST- 2024/10/02 07:02 [medline]

PHST- 2024/06/26 06:42 [pubmed]

PHST- 2024/06/26 03:23 [entrez]

AID - CTMC-EPUB-141265 [pii]

AID - 10.2174/0115680266286294240610102911 [doi]

PST - ppublish

SO - Curr Top Med Chem. 2024;24(21):1829-1855. doi:

10.2174/0115680266286294240610102911.

PMID- 37888812

OWN - NLM

STAT- MEDLINE

DCOM- 20240606

LR - 20240606

IS - 1873-4316 (Electronic)

IS - 1389-2010 (Linking)

VI - 25

IP - 6

DP - 2024

TI - How do Mutations of Mycobacterium Genes Cause Drug Resistance in Tuberculosis?

PG - 724-736

LID - 10.2174/0113892010257816230920053547 [doi]

AB - A steady increase in the prevalence of drug-resistant tuberculosis (DR-TB) has

already been reported in Pakistan. In addition, DR-TB is gradually changing from

one-drug resistance to multi-drug resistance, which is a serious challenge for

tuberculosis treatment. This review provides an overview of the anti-tuberculosis

drugs and focuses on the molecular mechanisms of drug resistance in Mycobacterium

tuberculosis, with the hope that it will contribute to the study of drug

resistance in response to the emergence of multidrug-resistant tuberculosis.

CI - Copyright© Bentham Science Publishers; For any queries, please email at

epub@benthamscience.net.

FAU - Hou, Kaiying

AU - Hou K

AD - School of Life Sciences, Henan University, Kaifeng, 475004, China.

FAU - Jabeen, Riffat

AU - Jabeen R

AD - School of Life Sciences, Henan University, Kaifeng, 475004, China.

FAU - Sun, Lin

AU - Sun L

AD - College of Chemistry and Chemical Engineering, Henan University, Kaifeng, 475004,

China.

FAU - Wei, Jianshe

AU - Wei J

AD - School of Life Sciences, Henan University, Kaifeng, 475004, China.

LA - eng

GR - 81271410/National Natural Science Foundation of China/

GR - 182300410313/Natural Science Foundation of Henan Province/

PT - Journal Article

PT - Review

PL - Netherlands

TA - Curr Pharm Biotechnol

JT - Current pharmaceutical biotechnology

JID - 100960530

RN - 0 (Antitubercular Agents)

RN - 0 (Bacterial Proteins)

SB - IM

MH - *Antitubercular Agents/pharmacology/therapeutic use

MH - *Mycobacterium tuberculosis/genetics/drug effects

MH - Humans

MH - *Mutation

MH - *Tuberculosis, Multidrug-Resistant/drug therapy/genetics/microbiology

MH - Drug Resistance, Multiple, Bacterial/genetics

MH - Bacterial Proteins/genetics

OTO - NOTNLM

OT - Mycobacterium tuberculosis

OT - Tuberculosis

OT - anti-tuberculosis drugs

OT - drug-resistant tuberculosis

OT - gene mutation.

OT - molecular mechanism

EDAT- 2023/10/27 12:44

MHDA- 2024/06/06 12:45

CRDT- 2023/10/27 07:34

PHST- 2023/05/05 00:00 [received]

PHST- 2023/07/28 00:00 [revised]

PHST- 2023/08/07 00:00 [accepted]

PHST- 2024/06/06 12:45 [medline]

PHST- 2023/10/27 12:44 [pubmed]

PHST- 2023/10/27 07:34 [entrez]

AID - CPB-EPUB-134929 [pii]

AID - 10.2174/0113892010257816230920053547 [doi]

PST - ppublish

SO - Curr Pharm Biotechnol. 2024;25(6):724-736. doi:

10.2174/0113892010257816230920053547.

PMID- 37715690

OWN - NLM

STAT- MEDLINE

DCOM- 20231206

LR - 20250206

IS - 1365-2567 (Electronic)

IS - 0019-2805 (Linking)

VI - 171

IP - 1

DP - 2024 Jan

TI - Transforming growth factor-β, Interleukin-23 and interleukin-1β modulate TH22

response during active multidrug-resistant tuberculosis.

PG - 45-59

LID - 10.1111/imm.13698 [doi]

AB - We previously reported that patients with multidrug-resistant tuberculosis

(MDR-TB) showed low systemic and Mtb-induced Th22 responses associated to high

sputum bacillary load and severe lung lesions suggesting that Th22 response could

influence the ability of these patients to control bacillary growth and tissue

damage. In MDR-TB patients, the percentage of IL-22(+) cells inversely correlates

with the proportion of senescent PD-1(+) T cells. Herein, we aimed to evaluate

the pathways involved on the regulation of systemic and Mtb-induced Th22 response

in MDR-TB and fully drug-susceptible TB patients (S-TB) and healthy donors. Our

results show that while IL-1β and IL-23 promote Mtb-induced IL-22 secretion and

expansion of IL-22(+) cells, TGF-β inhibits this response. Systemic and in vitro

Mtb-induced Th22 response inversely correlates with TGF-β amounts in plasma and

in PBMC cultures respectively. The number of circulating PD-1(+) T cells directly

correlates with plasmatic TGF-β levels and blockade of PD-1/PD-L1 signalling

enhances in vitro Mtb-induced expansion of IL-22(+) cells. Thus, TGF-β could also

inhibit Th22 response through upregulation of PD-1 expression in T cells. Higher

percentage of IL-23(+) monocytes was observed in TB patients. In contrast, the

proportion of IL-1β(+) monocytes was lower in TB patients with bilateral lung

cavities (BCC) compared to those patients with unilateral cavities (UCC).

Interestingly, TB patients with BCC showed higher plasmatic and Mtb-induced TGF-β

secretion than patients with UCC. Thus, high TGF-β secretion and subtle

differences in IL-23 and IL-1β expression could diminish systemic and in vitro

Mtb-induced Th22 response along disease progression in TB patients.

CI - © 2023 John Wiley & Sons Ltd.

FAU - Imperiale, Belén R

AU - Imperiale BR

AUID- ORCID: 0000-0003-0198-8746

AD - Institute of Experimental Medicine (IMEX)-CONICET, National Academy of Medicine,

Buenos Aires City, Argentina.

FAU - Gamberale, Ana

AU - Gamberale A

AD - Dr. Francisco Javier Muñiz Hospital, Buenos Aires City, Argentina.

FAU - Yokobori, Noemí

AU - Yokobori N

AD - National Institute of Infectious Diseases, ANLIS Carlos G. Malbrán, Buenos Aires

City, Argentina.

FAU - García, Ana

AU - García A

AD - Dr. Francisco Javier Muñiz Hospital, Buenos Aires City, Argentina.

FAU - Bartoletti, Bruno

AU - Bartoletti B

AD - Dr. Francisco Javier Muñiz Hospital, Buenos Aires City, Argentina.

FAU - Aidar, Omar

AU - Aidar O

AD - Dr. Francisco Javier Muñiz Hospital, Buenos Aires City, Argentina.

FAU - López, Beatriz

AU - López B

AD - National Institute of Infectious Diseases, ANLIS Carlos G. Malbrán, Buenos Aires

City, Argentina.

FAU - Cruz, Victor

AU - Cruz V

AD - Dr. Francisco Javier Muñiz Hospital, Buenos Aires City, Argentina.

FAU - González Montaner, Pablo

AU - González Montaner P

AD - Dr. Francisco Javier Muñiz Hospital, Buenos Aires City, Argentina.

AD - Vaccareza Institute, Buenos Aires City, Argentina.

FAU - Palmero, Domingo J

AU - Palmero DJ

AD - Dr. Francisco Javier Muñiz Hospital, Buenos Aires City, Argentina.

AD - Vaccareza Institute, Buenos Aires City, Argentina.

FAU - de la Barrera, Silvia

AU - de la Barrera S

AD - Institute of Experimental Medicine (IMEX)-CONICET, National Academy of Medicine,

Buenos Aires City, Argentina.

LA - eng

GR - PIP 112-2013-0100202)/CONICET/

GR - PICT 2015-0491/National Agency of Scientific and Technological Promotion/

GR - PICT 2020-1134/National Agency of Scientific and Technological Promotion/

PT - Journal Article

PT - Research Support, Non-U.S. Gov't

DEP - 20230916

PL - England

TA - Immunology

JT - Immunology

JID - 0374672

RN - 0 (Transforming Growth Factor beta)

RN - 0 (Interleukin-23)

RN - 0 (Interleukin-1beta)

RN - 0 (Programmed Cell Death 1 Receptor)

RN - 76057-06-2 (Transforming Growth Factors)

SB - IM

MH - Humans

MH - Transforming Growth Factor beta/metabolism

MH - Interleukin-23

MH - Leukocytes, Mononuclear/metabolism

MH - Interleukin-1beta

MH - Programmed Cell Death 1 Receptor

MH - *Tuberculosis, Multidrug-Resistant/drug therapy

MH - Transforming Growth Factors

MH - *Mycobacterium tuberculosis

OTO - NOTNLM

OT - IL-1β

OT - IL-22

OT - IL-23

OT - M. tuberculosis strains

OT - TGF-β

OT - TH22 regulation

OT - multidrug-resistant tuberculosis

EDAT- 2023/09/16 20:42

MHDA- 2023/12/06 06:42

CRDT- 2023/09/16 08:23

PHST- 2023/01/23 00:00 [received]

PHST- 2023/09/08 00:00 [accepted]

PHST- 2023/12/06 06:42 [medline]

PHST- 2023/09/16 20:42 [pubmed]

PHST- 2023/09/16 08:23 [entrez]

AID - 10.1111/imm.13698 [doi]

PST - ppublish

SO - Immunology. 2024 Jan;171(1):45-59. doi: 10.1111/imm.13698. Epub 2023 Sep 16.

PMID- 38138601

OWN - NLM

STAT- MEDLINE

DCOM- 20231225

LR - 20231225

IS - 1420-3049 (Electronic)

IS - 1420-3049 (Linking)

VI - 28

IP - 24

DP - 2023 Dec 15

TI - Rationally Designed Novel Phenyloxazoline Synthase Inhibitors: Chemical Synthesis

and Biological Evaluation to Accelerate the Discovery of New Antimycobacterial

Antibiotics.

LID - 10.3390/molecules28248115 [doi]

LID - 8115

AB - The uncontrolled spread of drug-resistant tuberculosis (DR-TB) clinical cases

necessitates the urgent discovery of newer chemotypes with novel mechanisms of

action. Here, we report the chemical synthesis of rationally designed novel

transition-state analogues (TSAs) by targeting the cyclization (Cy) domain of

phenyloxazoline synthase (MbtB), a key enzyme of the conditionally essential

siderophore biosynthesis pathway. Following bio-assay-guided evaluation of TSA

analogues preferentially in iron-deprived and iron-rich media to understand

target preferentiality against a panel of pathogenic and non-pathogenic

mycobacteria strains, we identified a hit, i.e., TSA-5. Molecular docking,

dynamics, and MMPBSA calculations enabled us to comprehend TSA-5's stable binding

at the active site pocket of MbtB_Cy and the results imply that the MbtB_Cy

binding pocket has a strong affinity for electron-withdrawing functional groups

and contributes to stable polar interactions between enzyme and ligand.

Furthermore, enhanced intracellular killing efficacy (8 μg/mL) of TSA-5 against

Mycobacterium aurum in infected macrophages is noted in comparison to moderate in

vitro antimycobacterial efficacy (64 μg/mL) against M. aurum. TSA-5 also

demonstrates whole-cell efflux pump inhibitory activity against Mycobacterium

smegmatis. Identification of TSA-5 by focusing on the modular MbtB_Cy domain

paves the way for accelerating novel anti-TB antibiotic discoveries.

FAU - Shyam, Mousumi

AU - Shyam M

AD - Department of Pharmaceutical Sciences & Technology, Birla Institute of

Technology, Mesra, Ranchi 835215, India.

AD - Mycobacteria Research Laboratory, School of Natural Sciences, Institute of

Structural and Molecular Biology, Birkbeck, University of London, Malet Street,

London WC1E 7HX, UK.

FAU - Bhattacharje, Gourab

AU - Bhattacharje G

AUID- ORCID: 0000-0001-7823-8033

AD - Department of Biotechnology, Indian Institute of Technology Kharagpur, Kharagpur

721302, India.

FAU - Daniel, Chris

AU - Daniel C

AUID- ORCID: 0000-0002-8650-516X

AD - Mycobacteria Research Laboratory, School of Natural Sciences, Institute of

Structural and Molecular Biology, Birkbeck, University of London, Malet Street,

London WC1E 7HX, UK.

FAU - Kumar, Amrendra

AU - Kumar A

AD - Division of Medicinal & Process Chemistry, CSIR-Central Drug Research Institute,

Sector 10 Janakipuram Extension, Sitapur Road, Lucknow 226031, India.

FAU - Yadav, Pragya

AU - Yadav P

AD - Division of Medicinal & Process Chemistry, CSIR-Central Drug Research Institute,

Sector 10 Janakipuram Extension, Sitapur Road, Lucknow 226031, India.

FAU - Mukherjee, Piyali

AU - Mukherjee P

AD - Department of Microbiology and Cell Biology, Centre for Infectious Disease

Research, Indian Institute of Science, CV Raman Avenue, Bengaluru 560012, India.

FAU - Singh, Samsher

AU - Singh S

AD - Department of Microbiology and Cell Biology, Centre for Infectious Disease

Research, Indian Institute of Science, CV Raman Avenue, Bengaluru 560012, India.

FAU - Das, Amit Kumar

AU - Das AK

AUID- ORCID: 0000-0001-9703-1898

AD - Department of Biotechnology, Indian Institute of Technology Kharagpur, Kharagpur

721302, India.

FAU - Narender, Tadigoppula

AU - Narender T

AD - Division of Medicinal & Process Chemistry, CSIR-Central Drug Research Institute,

Sector 10 Janakipuram Extension, Sitapur Road, Lucknow 226031, India.

FAU - Singh, Amit

AU - Singh A

AUID- ORCID: 0000-0001-6761-1664

AD - Department of Microbiology and Cell Biology, Centre for Infectious Disease

Research, Indian Institute of Science, CV Raman Avenue, Bengaluru 560012, India.

FAU - Jayaprakash, Venkatesan

AU - Jayaprakash V

AD - Department of Pharmaceutical Sciences & Technology, Birla Institute of

Technology, Mesra, Ranchi 835215, India.

FAU - Bhakta, Sanjib

AU - Bhakta S

AUID- ORCID: 0000-0002-1165-9295

AD - Mycobacteria Research Laboratory, School of Natural Sciences, Institute of

Structural and Molecular Biology, Birkbeck, University of London, Malet Street,

London WC1E 7HX, UK.

LA - eng

PT - Journal Article

DEP - 20231215

PL - Switzerland

TA - Molecules

JT - Molecules (Basel, Switzerland)

JID - 100964009

RN - 0 (Anti-Bacterial Agents)

RN - E1UOL152H7 (Iron)

RN - 0 (Antitubercular Agents)

SB - IM

MH - *Anti-Bacterial Agents/pharmacology/metabolism

MH - *Mycobacterium tuberculosis

MH - Molecular Docking Simulation

MH - Iron/metabolism

MH - Mycobacterium smegmatis

MH - Antitubercular Agents/chemistry

PMC - PMC10745776

OTO - NOTNLM

OT - MD simulations

OT - MMPBSA studies

OT - cyclization domain

OT - efflux pump

OT - intracellular killing

OT - mycobactin metabolism

OT - phenyloxazoline synthase

OT - transition-state analogues

COIS- The authors declare no conflict of interest.

EDAT- 2023/12/23 12:43

MHDA- 2023/12/25 06:42

PMCR- 2023/12/15

CRDT- 2023/12/23 01:17

PHST- 2023/10/30 00:00 [received]

PHST- 2023/12/05 00:00 [revised]

PHST- 2023/12/07 00:00 [accepted]

PHST- 2023/12/25 06:42 [medline]

PHST- 2023/12/23 12:43 [pubmed]

PHST- 2023/12/23 01:17 [entrez]

PHST- 2023/12/15 00:00 [pmc-release]

AID - molecules28248115 [pii]

AID - molecules-28-08115 [pii]

AID - 10.3390/molecules28248115 [doi]

PST - epublish

SO - Molecules. 2023 Dec 15;28(24):8115. doi: 10.3390/molecules28248115.

PMID- 37344177

OWN - NLM

STAT- MEDLINE

DCOM- 20231218

LR - 20241023

IS - 1468-3296 (Electronic)

IS - 0040-6376 (Print)

IS - 0040-6376 (Linking)

VI - 79

IP - 1

DP - 2023 Dec 15

TI - Outcomes from a national screening program for Ukrainian refugees at risk of drug

resistant tuberculosis in Wales.

PG - 86-89

LID - 10.1136/thorax-2023-220161 [doi]

AB - High rates of drug-resistant tuberculosis in Ukraine suggest screening is

necessary to mitigate public health hazards for host populations. A pathway was

implemented in Wales and data prospectively collected Between 8 April and 21

December 2022. Of 5425 Ukrainian arrivals, notifications were received by TB

teams on 2395 (44%) of whom 1955 (82%) were screened. The refugees were young

(median age 30, IQR 14-41), and predominantly female (66.1%). Interferon- gamma

release assay (IGRA) tests were positive in 112 (6.5%). One Case of active

tuberculosis was identified (0.05%). Our data supports European guidelines that

routine screening of this population is not recommended, but we remain uncertain

as to the risks of this population going forwards.

CI - © Author(s) (or their employer(s)) 2024. Re-use permitted under CC BY-NC. No

commercial re-use. See rights and permissions. Published by BMJ.

FAU - Barry, Simon M

AU - Barry SM

AD - Respiratory Medicine, Cardiff and Vale UHB, Cardiff, UK simon.barry@wales.nhs.uk.

AD - Respiratory Health Implementation Group, Cardiff, UK.

FAU - Davies, Gareth

AU - Davies G

AD - Institute for Clinical Science and Technology, Cardiff, UK.

FAU - Barry, Tamas D

AU - Barry TD

AD - Division of infection and immunity, Cardiff University, Cardiff, UK.

FAU - Evans, Jennifer

AU - Evans J

AD - Child Health, Cardiff and Vale University Health Board, Cardiff, UK.

FAU - Backx, Matthijs

AU - Backx M

AD - Infectious Disease, Cardiff and Vale University Health Board, Cardiff, UK.

FAU - Brouns, Mattheus

AU - Brouns M

AD - Department of Respiratory Medicine, Aneurin Bevan University Health Board,

Abergavenny, UK.

FAU - Mughal, Ahsan

AU - Mughal A

AD - Respiratory Medicine, Swansea Bay University Health Board, Swansea, UK.

FAU - Kelly, Stephen

AU - Kelly S

AD - Respiratory Medicine, Betsi Cadwaladr University Health Board, Wrexham, UK.

FAU - Collier, Gareth

AU - Collier G

AD - Hywel Dda University Health Board, Carmarthen, UK.

FAU - Ambalavanan, Sakkarai

AU - Ambalavanan S

AD - Betsi Cadwaladr University Health Board, Rhyl, UK.

FAU - Davies, Chris

AU - Davies C

AD - Institute for Clinical Science and Technology, Cardiff, UK.

FAU - Sharp, Hannah

AU - Sharp H

AD - Institute for Clinical Science and Technology, Cardiff, UK.

FAU - Lloyd, Pam

AU - Lloyd P

AD - Respiratory Medicine, Betsi Cadwaladr University Health Board, Wrexham, UK.

FAU - Hester, Yvonne

AU - Hester Y

AD - Respiratory Medicine, Cardiff and Vale University Health Board, Cardiff, UK.

FAU - Murray, Natalie

AU - Murray N

AD - Respiratory Medicine, Cwm Taf Morgannwg University Health Board, Llantrisant, UK.

FAU - Goddard, Kelly

AU - Goddard K

AD - Respiratory Medicine, Hywel Dda University Health Board, Carmarthen, UK.

FAU - Johnstone, Linzi

AU - Johnstone L

AD - Respiratory Medicine, Swansea Bay University Health Board, Swansea, UK.

FAU - Parry, Jane

AU - Parry J

AD - Respiratory Medicine, Betsi Cadwaladr University Health Board, Bangor, UK.

FAU - Davies, Olwen

AU - Davies O

AD - Respiratory Medicine, Betsi Cadwaladr University Health Board, Bangor, UK.

FAU - Williams, Rhian

AU - Williams R

AD - Respiratory Medicine, Cwm Taf Morgannwg University Health Board, Llantrisant, UK.

FAU - Ahern, George

AU - Ahern G

AD - Public Health Wales, Cardiff, UK.

FAU - Smith, Josie

AU - Smith J

AD - Public Health Wales, Cardiff, UK.

LA - eng

PT - Journal Article

DEP - 20231215

PL - England

TA - Thorax

JT - Thorax

JID - 0417353

SB - IM

CIN - doi: 10.1136/thorax-2023-220502

MH - Humans

MH - Female

MH - Adult

MH - Male

MH - *Latent Tuberculosis/diagnosis/epidemiology

MH - Tuberculin Test

MH - Wales/epidemiology

MH - *Refugees

MH - Interferon-gamma Release Tests

MH - *Tuberculosis, Multidrug-Resistant/diagnosis/epidemiology

MH - Mass Screening

PMC - PMC10804008

OTO - NOTNLM

OT - Infection Control

OT - Tuberculosis

COIS- Competing interests: None declared.

EDAT- 2023/06/22 01:07

MHDA- 2023/12/18 06:42

PMCR- 2024/01/23

CRDT- 2023/06/21 21:37

PHST- 2023/12/18 06:42 [medline]

PHST- 2023/06/22 01:07 [pubmed]

PHST- 2023/06/21 21:37 [entrez]

PHST- 2024/01/23 00:00 [pmc-release]

AID - thorax-2023-220161 [pii]

AID - 10.1136/thorax-2023-220161 [doi]

PST - epublish

SO - Thorax. 2023 Dec 15;79(1):86-89. doi: 10.1136/thorax-2023-220161.

PMID- 37845182

OWN - NLM

STAT- MEDLINE

DCOM- 20231216

LR - 20260127

IS - 1860-7187 (Electronic)

IS - 1860-7179 (Linking)

VI - 18

IP - 24

DP - 2023 Dec 14

TI - Structural Optimization of Antimycobacterial Azaaurones Towards Improved

Solubility and Metabolic Stability.

PG - e202300410

LID - 10.1002/cmdc.202300410 [doi]

AB - While N-acetyl azaaurones have already been disclosed for their potential against

tuberculosis (TB), their low metabolic stability remains an unaddressed

liability. We now report a study designed to improve the metabolic stability and

solubility of the azaaurone scaffold and to identify the structural requirements

for antimycobacterial activity. Replacing the N-acetyl moiety for a N-carbamoyl

group led to analogues with sub- and nanomolar potencies against M. tuberculosis

H37Rv, as well as equipotent against drug-susceptible and drug-resistant M.

tuberculosis isolates. The new N-carbamoyl azaaurones exhibited improved

microsomal stability, compared to their N-acetylated counterparts, with several

compounds displaying moderate to high kinetic solubility. The frequency of

spontaneous resistance to azaaurones was observed to be in the range of 10(-8) ,

a value that is comparable to current TB drugs in the market. Overall, these

results reveal that azaaurones are amenable to structural modifications to

improve metabolic and solubility liabilities, and highlight their potential as

antimycobacterial agents.

CI - © 2023 Wiley-VCH GmbH.

FAU - Campaniço, André

AU - Campaniço A

AUID- ORCID: 0000-0002-0560-4597

AD - Instituto de Investigação do Medicamento (iMed.ULisboa), Faculdade de Farmácia,

Universidade de Lisboa, Av. Prof. Gama Pinto, 1649-003, Lisboa, Portugal.

FAU - Harjivan, Shrika G

AU - Harjivan SG

AUID- ORCID: 0000-0002-1975-1571

AD - Instituto de Investigação do Medicamento (iMed.ULisboa), Faculdade de Farmácia,

Universidade de Lisboa, Av. Prof. Gama Pinto, 1649-003, Lisboa, Portugal.

FAU - Freitas, Elisabete

AU - Freitas E

AD - Instituto de Investigação do Medicamento (iMed.ULisboa), Faculdade de Farmácia,

Universidade de Lisboa, Av. Prof. Gama Pinto, 1649-003, Lisboa, Portugal.

FAU - Serafini, Marco

AU - Serafini M

AUID- ORCID: 0000-0003-4687-2640

AD - Instituto de Investigação do Medicamento (iMed.ULisboa), Faculdade de Farmácia,

Universidade de Lisboa, Av. Prof. Gama Pinto, 1649-003, Lisboa, Portugal.

FAU - Gaspar, M Manuela

AU - Gaspar MM

AUID- ORCID: 0000-0001-6814-7226

AD - Instituto de Investigação do Medicamento (iMed.ULisboa), Faculdade de Farmácia,

Universidade de Lisboa, Av. Prof. Gama Pinto, 1649-003, Lisboa, Portugal.

FAU - Capela, Rita

AU - Capela R

AUID- ORCID: 0000-0001-9637-3714

AD - Instituto de Investigação do Medicamento (iMed.ULisboa), Faculdade de Farmácia,

Universidade de Lisboa, Av. Prof. Gama Pinto, 1649-003, Lisboa, Portugal.

FAU - Gomes, Pedro

AU - Gomes P

AD - Instituto de Investigação do Medicamento (iMed.ULisboa), Faculdade de Farmácia,

Universidade de Lisboa, Av. Prof. Gama Pinto, 1649-003, Lisboa, Portugal.

FAU - Jordaan, Audrey

AU - Jordaan A

AD - Molecular Mycobacteriology Research Unit, Department of Pathology and Institute

of Infectious Disease and Molecular Medicine, Faculty of Health Sciences,

University of Cape Town, Cape Town, Rondebosch, 7701, South Africa.

FAU - Madureira, Ana M

AU - Madureira AM

AUID- ORCID: 0000-0001-5973-2183

AD - Instituto de Investigação do Medicamento (iMed.ULisboa), Faculdade de Farmácia,

Universidade de Lisboa, Av. Prof. Gama Pinto, 1649-003, Lisboa, Portugal.

FAU - André, Vânia

AU - André V

AUID- ORCID: 0000-0001-5599-8355

AD - Centro de Química Estrutural, Institute of Molecular Sciences, Instituto Superior

Técnico, Universidade de Lisboa, Av. Rovisco Pais 1, 1049-001, Lisboa, Portugal.

AD - Associação do Instituto Superior Técnico para a Investigação e Desenvolvimento

(IST-ID), Avenida António José de Almeida, n.° 12, 1000-043, Lisboa, Portugal.

FAU - Silva, Andreia B

AU - Silva AB

AUID- ORCID: 0000-0002-3047-0586

AD - Faculdade de Farmácia, Universidade de Lisboa, Av. Prof. Gama Pinto, 1649-003,

Lisboa, Portugal.

FAU - Duarte, M Teresa

AU - Duarte MT

AUID- ORCID: 0000-0003-0994-1352

AD - Centro de Química Estrutural, Institute of Molecular Sciences, Instituto Superior

Técnico, Universidade de Lisboa, Av. Rovisco Pais 1, 1049-001, Lisboa, Portugal.

FAU - Portugal, Isabel

AU - Portugal I

AUID- ORCID: 0000-0002-7843-0006

AD - Instituto de Investigação do Medicamento (iMed.ULisboa), Faculdade de Farmácia,

Universidade de Lisboa, Av. Prof. Gama Pinto, 1649-003, Lisboa, Portugal.

FAU - Perdigão, João

AU - Perdigão J

AUID- ORCID: 0000-0002-0339-1305

AD - Instituto de Investigação do Medicamento (iMed.ULisboa), Faculdade de Farmácia,

Universidade de Lisboa, Av. Prof. Gama Pinto, 1649-003, Lisboa, Portugal.

FAU - Moreira, Rui

AU - Moreira R

AUID- ORCID: 0000-0003-0727-9852

AD - Instituto de Investigação do Medicamento (iMed.ULisboa), Faculdade de Farmácia,

Universidade de Lisboa, Av. Prof. Gama Pinto, 1649-003, Lisboa, Portugal.

FAU - Warner, Digby F

AU - Warner DF

AUID- ORCID: 0000-0002-4146-0930

AD - Molecular Mycobacteriology Research Unit, Department of Pathology and Institute

of Infectious Disease and Molecular Medicine, Faculty of Health Sciences,

University of Cape Town, Cape Town, Rondebosch, 7701, South Africa.

AD - Wellcome Centre for Infectious Diseases Research in Africa, University of Cape

Town, Cape Town, Rondebosch, 7701, South Africa.

FAU - Lopes, Francisca

AU - Lopes F

AUID- ORCID: 0000-0002-5350-147X

AD - Instituto de Investigação do Medicamento (iMed.ULisboa), Faculdade de Farmácia,

Universidade de Lisboa, Av. Prof. Gama Pinto, 1649-003, Lisboa, Portugal.

LA - eng

GR - UIDB/04138/2020/Fundação para a Ciência e Tecnologia/

GR - UIDP/04138/2020/Fundação para a Ciência e Tecnologia/

GR - PTDC/MED-FAR/30266/2017/FCT and FEDER/

GR - LISBOA-01-0145-FEDER-030266/FCT and FEDER/

GR - SFRH/BD/131896/2017/FCT/

GR - Strategic Health Innovation 532 Partnerships/

GR - South African Medical Research Council/

GR - UIDB/00100/2020/National 533 Treasury under its Economic Competitiveness and

Support Package/

GR - UIDP/00100/2020/National 533 Treasury under its Economic Competitiveness and

Support Package/

GR - LA/P/0056/2020/National 533 Treasury under its Economic Competitiveness and

Support Package/

GR - CEECIND/00283/2018/National 533 Treasury under its Economic Competitiveness and

Support Package/

GR - PTDC/MED-FAR/30266/2017/FCT/

GR - LISBOA-01-0145-FEDER-030266/FCT/

GR - SFRH/BD/131896/2017/FEDER/

PT - Journal Article

PT - Research Support, Non-U.S. Gov't

DEP - 20231106

PL - Germany

TA - ChemMedChem

JT - ChemMedChem

JID - 101259013

RN - 0 (Antitubercular Agents)

RN - 0 (azaaurone)

RN - 0 (Benzofurans)

SB - IM

MH - Humans

MH - Antitubercular Agents/pharmacology/chemistry

MH - Solubility

MH - *Mycobacterium tuberculosis

MH - *Tuberculosis

MH - Microbial Sensitivity Tests

MH - Benzofurans

OTO - NOTNLM

OT - M. tuberculosis

OT - azaaurones

OT - drug discovery

OT - drug-resistant tuberculosis

OT - structure-activity relationships

EDAT- 2023/10/17 00:42

MHDA- 2023/12/17 09:43

CRDT- 2023/10/16 22:23

PHST- 2023/10/09 00:00 [revised]

PHST- 2023/08/02 00:00 [received]

PHST- 2023/12/17 09:43 [medline]

PHST- 2023/10/17 00:42 [pubmed]

PHST- 2023/10/16 22:23 [entrez]

AID - 10.1002/cmdc.202300410 [doi]

PST - ppublish

SO - ChemMedChem. 2023 Dec 14;18(24):e202300410. doi: 10.1002/cmdc.202300410. Epub

2023 Nov 6.

PMID- 37922420

OWN - NLM

STAT- MEDLINE

DCOM- 20231216

LR - 20240613

IS - 2373-8227 (Electronic)

IS - 2373-8227 (Linking)

VI - 9

IP - 12

DP - 2023 Dec 8

TI - Quinoline Compounds Targeting the c-Ring of ATP Synthase Inhibit Drug-Resistant

Pseudomonas aeruginosa.

PG - 2448-2456

LID - 10.1021/acsinfecdis.3c00317 [doi]

AB - Pseudomonas aeruginosa (PA) is a Gram-negative, biofilm-forming bacterium and an

opportunistic pathogen. The growing drug resistance of PA is a serious threat

that necessitates the discovery of novel antibiotics, ideally with previously

underexplored mechanisms of action. Due to their central role in cell metabolism,

bacterial bioenergetic processes are of increasing interest as drug targets,

especially with the success of the ATP synthase inhibitor bedaquiline to treat

drug-resistant tuberculosis. Like Mycobacterium tuberculosis, PA requires

F(1)F(o) ATP synthase for growth, even under anaerobic conditions, making the PA

ATP synthase an ideal drug target for the treatment of drug-resistant infection.

In previous work, we conducted an initial screen for quinoline compounds that

inhibit ATP synthesis activity in PA. In the present study, we report additional

quinoline derivatives, including one with increased potency against PA ATP

synthase in vitro and antibacterial activity against drug-resistant PA. Moreover,

by expressing the PA ATP synthase in Escherichia coli, we show that mutations in

the H(+) binding site on the membrane-embedded rotor ring alter inhibition by the

reported quinoline compounds. Identification of a potent inhibitor and its

probable binding site on ATP synthase enables further development of promising

quinoline derivatives into a viable treatment for drug-resistant PA infection.

FAU - Fraunfelter, Vesper M

AU - Fraunfelter VM

AD - Department of Chemistry and Biochemistry, University of North Carolina Asheville,

One University Heights, Asheville, North Carolina 28804, United States.

FAU - Pugh, Bryce A

AU - Pugh BA

AD - Department of Chemistry and Biochemistry, University of North Carolina Asheville,

One University Heights, Asheville, North Carolina 28804, United States.

FAU - Williams, Alexander P L

AU - Williams APL

AD - Department of Chemistry and Biochemistry, University of North Carolina Asheville,

One University Heights, Asheville, North Carolina 28804, United States.

FAU - Ward, Katie T

AU - Ward KT

AD - Department of Chemistry and Biochemistry, University of North Carolina Asheville,

One University Heights, Asheville, North Carolina 28804, United States.

FAU - Jackson, Dietrich O

AU - Jackson DO

AD - Department of Chemistry and Biochemistry, University of North Carolina Asheville,

One University Heights, Asheville, North Carolina 28804, United States.

FAU - Austin, Molly

AU - Austin M

AD - Department of Chemistry and Biochemistry, University of North Carolina Asheville,

One University Heights, Asheville, North Carolina 28804, United States.

FAU - Ciprich, John F

AU - Ciprich JF

AD - Department of Chemistry and Biochemistry, University of North Carolina Asheville,

One University Heights, Asheville, North Carolina 28804, United States.

FAU - Dippy, Lorelei

AU - Dippy L

AD - Department of Chemistry and Biochemistry, University of North Carolina Asheville,

One University Heights, Asheville, North Carolina 28804, United States.

FAU - Dunford, Jason

AU - Dunford J

AD - Department of Chemistry and Biochemistry, University of North Carolina Asheville,

One University Heights, Asheville, North Carolina 28804, United States.

FAU - Edwards, G Nathaniel

AU - Edwards GN

AD - Department of Chemistry and Biochemistry, University of North Carolina Asheville,

One University Heights, Asheville, North Carolina 28804, United States.

FAU - Glass, Evan

AU - Glass E

AD - Department of Chemistry and Biochemistry, University of North Carolina Asheville,

One University Heights, Asheville, North Carolina 28804, United States.

FAU - Handy, Kyle M

AU - Handy KM

AD - Department of Chemistry and Biochemistry, University of North Carolina Asheville,

One University Heights, Asheville, North Carolina 28804, United States.

FAU - Kellogg, Casey N

AU - Kellogg CN

AD - Department of Chemistry and Biochemistry, University of North Carolina Asheville,

One University Heights, Asheville, North Carolina 28804, United States.

FAU - Llewellyn, Kaitlyn

AU - Llewellyn K

AD - Department of Chemistry and Biochemistry, University of North Carolina Asheville,

One University Heights, Asheville, North Carolina 28804, United States.

FAU - Nyberg, K Quinn

AU - Nyberg KQ

AD - Department of Chemistry and Biochemistry, University of North Carolina Asheville,

One University Heights, Asheville, North Carolina 28804, United States.

FAU - Shepard, Sam J

AU - Shepard SJ

AD - Department of Chemistry and Biochemistry, University of North Carolina Asheville,

One University Heights, Asheville, North Carolina 28804, United States.

FAU - Thomas, Casey

AU - Thomas C

AD - Department of Chemistry and Biochemistry, University of North Carolina Asheville,

One University Heights, Asheville, North Carolina 28804, United States.

FAU - Wolfe, Amanda L

AU - Wolfe AL

AUID- ORCID: 0000-0003-1867-6861

AD - Department of Chemistry and Biochemistry, University of North Carolina Asheville,

One University Heights, Asheville, North Carolina 28804, United States.

FAU - Steed, P Ryan

AU - Steed PR

AUID- ORCID: 0000-0001-5445-0080

AD - Department of Chemistry and Biochemistry, University of North Carolina Asheville,

One University Heights, Asheville, North Carolina 28804, United States.

LA - eng

GR - R15 AI163474/AI/NIAID NIH HHS/United States

GR - R15 GM134453/GM/NIGMS NIH HHS/United States

PT - Journal Article

PT - Research Support, N.I.H., Extramural

PT - Research Support, Non-U.S. Gov't

DEP - 20231103

PL - United States

TA - ACS Infect Dis

JT - ACS infectious diseases

JID - 101654580

RN - 0 (Anti-Bacterial Agents)

RN - 0 (Anti-Infective Agents)

RN - 8L70Q75FXE (Adenosine Triphosphate)

SB - IM

MH - Pseudomonas aeruginosa/genetics/metabolism

MH - Anti-Bacterial Agents/pharmacology

MH - *Mycobacterium tuberculosis/genetics/metabolism

MH - *Anti-Infective Agents

MH - Adenosine Triphosphate

PMC - PMC10714390

OTO - NOTNLM

OT - ATP synthase

OT - Pseudomonas aeruginosa

OT - antibiotic resistance

OT - antibiotics

OT - bioenergetics

OT - quinolines

COIS- The authors declare no competing financial interest.

EDAT- 2023/11/03 18:43

MHDA- 2023/12/17 13:19

PMCR- 2023/12/12

CRDT- 2023/11/03 15:43

PHST- 2023/12/17 13:19 [medline]

PHST- 2023/11/03 18:43 [pubmed]

PHST- 2023/11/03 15:43 [entrez]

PHST- 2023/12/12 00:00 [pmc-release]

AID - 10.1021/acsinfecdis.3c00317 [doi]

PST - ppublish

SO - ACS Infect Dis. 2023 Dec 8;9(12):2448-2456. doi: 10.1021/acsinfecdis.3c00317.

Epub 2023 Nov 3.

PMID- 38138078

OWN - NLM

STAT- PubMed-not-MEDLINE

LR - 20231225

IS - 2076-2607 (Print)

IS - 2076-2607 (Electronic)

IS - 2076-2607 (Linking)

VI - 11

IP - 12

DP - 2023 Dec 7

TI - Adenoviral Vector Codifying for TNF as a Co-Adjuvant Therapy against

Multi-Drug-Resistant Tuberculosis.

LID - 10.3390/microorganisms11122934 [doi]

LID - 2934

AB - Mycobacterium tuberculosis is the main causal agent of pulmonary tuberculosis

(TB); the treatment of this disease is long and involves a mix of at least four

different antibiotics that frequently lead to abandonment, favoring the surge of

drug-resistant mycobacteria (MDR-TB), whose treatment becomes more aggressive,

being longer and more toxic. Thus, the search for novel strategies for treatment

that improves time or efficiency is of relevance. In this work, we used a murine

model of pulmonary TB produced by the MDR-TB strain to test the efficiency of

gene therapy with adenoviral vectors codifying TNF (AdTNF), a pro-inflammatory

cytokine that has protective functions in TB by inducing apoptosis, granuloma

formation and expression of other Th1-like cytokines. When compared to the

control group that received an adenoviral vector that codifies for the green

fluorescent protein (AdGFP), a single dose of AdTNF at the chronic active stage

of the disease produced total survival, decreasing bacterial load and tissue

damage (pneumonia), which correlated with an increase in cells expressing IFN-γ,

iNOS and TNF in pneumonic areas and larger granulomas that efficiently contain

and eliminate mycobacteria. Second-line antibiotic treatment against MDR-TB plus

AdTNF gene therapy reduced bacterial load faster within a week of treatment

compared to empty vector plus antibiotics or antibiotics alone, suggesting that

AdTNF is a new potential type of treatment against MDR-TB that can shorten

second-line chemotherapy but which requires further experimentation in other

animal models (non-human primates) that develop a more similar disease to human

pulmonary TB.

FAU - Hernández-Bazán, Sujhey

AU - Hernández-Bazán S

AUID- ORCID: 0000-0002-3251-2479

AD - Sección de Patología Experimental, Departamento de Patología, Instituto Nacional

de Ciencias Médicas y Nutrición Salvador Zubirán, Mexico City 14080, Mexico.

FAU - Mata-Espinosa, Dulce

AU - Mata-Espinosa D

AD - Sección de Patología Experimental, Departamento de Patología, Instituto Nacional

de Ciencias Médicas y Nutrición Salvador Zubirán, Mexico City 14080, Mexico.

FAU - Ramos-Espinosa, Octavio

AU - Ramos-Espinosa O

AUID- ORCID: 0000-0002-9010-4625

AD - Sección de Patología Experimental, Departamento de Patología, Instituto Nacional

de Ciencias Médicas y Nutrición Salvador Zubirán, Mexico City 14080, Mexico.

FAU - Lozano-Ordaz, Vasti

AU - Lozano-Ordaz V

AD - Sección de Patología Experimental, Departamento de Patología, Instituto Nacional

de Ciencias Médicas y Nutrición Salvador Zubirán, Mexico City 14080, Mexico.

FAU - Barrios-Payán, Jorge

AU - Barrios-Payán J

AUID- ORCID: 0000-0002-5134-5573

AD - Sección de Patología Experimental, Departamento de Patología, Instituto Nacional

de Ciencias Médicas y Nutrición Salvador Zubirán, Mexico City 14080, Mexico.

FAU - López-Casillas, Fernando

AU - López-Casillas F

AD - Departamento de Biología Celular, Instituto de Fisiología Celular, Universidad

Nacional Autónoma de México, Mexico City 14080, Mexico.

FAU - Hernández-Pando, Rogelio

AU - Hernández-Pando R

AD - Sección de Patología Experimental, Departamento de Patología, Instituto Nacional

de Ciencias Médicas y Nutrición Salvador Zubirán, Mexico City 14080, Mexico.

LA - eng

GR - Fon Inst 58/2016/Consejo Nacional de Humanidades, Ciencias y Tecnologías/

PT - Journal Article

DEP - 20231207

PL - Switzerland

TA - Microorganisms

JT - Microorganisms

JID - 101625893

PMC - PMC10745769

OTO - NOTNLM

OT - Tuberculosis

OT - Tumor Necrosis Factor

OT - adenoviral vectors

OT - gene therapy

OT - multi-drug resistance

COIS- The authors declare no conflict of interest.

EDAT- 2023/12/23 12:44

MHDA- 2023/12/23 12:45

PMCR- 2023/12/07

CRDT- 2023/12/23 01:13

PHST- 2023/10/30 00:00 [received]

PHST- 2023/11/29 00:00 [revised]

PHST- 2023/12/02 00:00 [accepted]

PHST- 2023/12/23 12:45 [medline]

PHST- 2023/12/23 12:44 [pubmed]

PHST- 2023/12/23 01:13 [entrez]

PHST- 2023/12/07 00:00 [pmc-release]

AID - microorganisms11122934 [pii]

AID - microorganisms-11-02934 [pii]

AID - 10.3390/microorganisms11122934 [doi]

PST - epublish

SO - Microorganisms. 2023 Dec 7;11(12):2934. doi: 10.3390/microorganisms11122934.

PMID- 38144646

OWN - NLM

STAT- PubMed-not-MEDLINE

LR - 20241202

IS - 0377-1237 (Print)

IS - 2213-4743 (Electronic)

IS - 0377-1237 (Linking)

VI - 79

IP - Suppl 1

DP - 2023 Dec

TI - An unusual complication secondary to kanamycin use in a patient of

multidrug-resistant pulmonary tuberculosis.

PG - S280-S282

LID - 10.1016/j.mjafi.2021.09.008 [doi]

AB - Gitelman syndrome (GS) is a rare genetic renal disease characterized by

hypomagnesemia, hypokalemia, hypocalciuria, and metabolic alkalosis. It usually

presents in late childhood or early adulthood. A 30-year-old female diagnosed

case of multidrug-resistant (MDR-TB) pulmonary tuberculosis 2 months ago

presented to our outpatient department with intermittent painful spasms in all

four limb muscles. Her treatment regimen consisted of kanamycin, levofloxacin,

cycloserine, and ethionamide. On further evaluation, her investigations revealed

hypokalemia, hypocalcemia, hypomagnesemia, metabolic alkalosis with normal serum

creatinine level. She was initially treated with intravenous calcium and

potassium. However, the electrolyte abnormalities and metabolic alkalosis

persisted. All her lab parameters became normal after discontinuing kanamycin and

electrolyte replacement for 4 weeks. She was discharged and advised to continue

her antituberculosis treatment. There was no recurrence of symptoms on further

follow up.

CI - © 2021 Director General, Armed Forces Medical Services. Published by Elsevier, a

division of RELX India Pvt. Ltd.

FAU - Mathews, Sajin

AU - Mathews S

AD - Junior Resident (Respiratory Medicine), Dr DY Patil Medical College, Hospital &

Research Centre, Dr DY Patil Vidyapeeth, Pimpri, Pune, India.

FAU - Dole, Sachinkumar

AU - Dole S

AD - Associate Professor (Respiratory Medicine), Dr DY Patil Medical College, Hospital

& Research Centre, Dr DY Patil Vidyapeeth, Pimpri, Pune, India.

FAU - Barthwal, Madhusudan

AU - Barthwal M

AD - Professor & Head (Respiratory Medicine), Dr DY Patil Medical College, Hospital &

Research Centre, Dr DY Patil Vidyapeeth, Pimpri, Pune, India.

FAU - Sahasrabudhe, Tushar

AU - Sahasrabudhe T

AD - Professor (Respiratory Medicine), Dr DY Patil Medical College, Hospital &

Research Centre, Dr DY Patil Vidyapeeth, Pimpri, Pune, India.

LA - eng

PT - Case Reports

PT - Journal Article

DEP - 20211128

PL - India

TA - Med J Armed Forces India

JT - Medical journal, Armed Forces India

JID - 7602492

PMC - PMC10746731

OTO - NOTNLM

OT - Gitelman

OT - Hypokalemia

OT - Hypomagnesemia

OT - Kanamycin

OT - Tuberculosis

COIS- The authors have none to declare.

EDAT- 2023/12/25 06:42

MHDA- 2023/12/25 06:43

PMCR- 2024/12/01

CRDT- 2023/12/25 04:39

PHST- 2021/01/18 00:00 [received]

PHST- 2021/09/21 00:00 [accepted]

PHST- 2023/12/25 06:43 [medline]

PHST- 2023/12/25 06:42 [pubmed]

PHST- 2023/12/25 04:39 [entrez]

PHST- 2024/12/01 00:00 [pmc-release]

AID - S0377-1237(21)00264-1 [pii]

AID - 10.1016/j.mjafi.2021.09.008 [doi]

PST - ppublish

SO - Med J Armed Forces India. 2023 Dec;79(Suppl 1):S280-S282. doi:

10.1016/j.mjafi.2021.09.008. Epub 2021 Nov 28.

PMID- 37864894

OWN - NLM

STAT- MEDLINE

DCOM- 20231130

LR - 20240206

IS - 1950-6007 (Electronic)

IS - 0753-3322 (Print)

IS - 0753-3322 (Linking)

VI - 168

DP - 2023 Dec

TI - Combination of MCL-1 and BCL-2 inhibitors is a promising approach for a

host-directed therapy for tuberculosis.

PG - 115738

LID - S0753-3322(23)01536-6 [pii]

LID - 10.1016/j.biopha.2023.115738 [doi]

AB - Tuberculosis (TB) accounts for 1.6 million deaths annually and over 25% of deaths

due to antimicrobial resistance. Mycobacterium tuberculosis (M.tb) drives MCL-1

expression (family member of anti-apoptotic BCL-2 proteins) to limit apoptosis

and grow intracellularly in human macrophages. The feasibility of re-purposing

specific MCL-1 and BCL-2 inhibitors to limit M.tb growth, using inhibitors that

are in clinical trials and FDA-approved for cancer treatment has not be tested

previously. We show that specifically inhibiting MCL-1 and BCL-2 induces

apoptosis of M.tb-infected macrophages, and markedly reduces M.tb growth in human

and murine macrophages, and in a pre-clinical model of human granulomas. MCL-1

and BCL-2 inhibitors limit growth of drug resistant and susceptible M.tb in

macrophages and act in additive fashion with the antibiotics isoniazid and

rifampicin. This exciting work uncovers targeting the intrinsic apoptosis pathway

as a promising approach for TB host-directed therapy. Since safety and activity

studies are underway in cancer clinics for MCL-1 and BCL-2 inhibitors, we expect

that re-purposing them for TB treatment should translate more readily and rapidly

to the clinic. Thus, the work supports further development of this host-directed

therapy approach to augment current TB treatment.

CI - Copyright © 2023 The Authors. Published by Elsevier Masson SAS.. All rights

reserved.

FAU - Arnett, Eusondia

AU - Arnett E

AD - Host Pathogen Interactions Program, Texas Biomedical Research Institute, San

Antonio, TX 78227, USA. Electronic address: earnett@txbiomed.org.

FAU - Pahari, Susanta

AU - Pahari S

AD - Host Pathogen Interactions Program, Texas Biomedical Research Institute, San

Antonio, TX 78227, USA.

FAU - Leopold Wager, Chrissy M

AU - Leopold Wager CM

AD - Host Pathogen Interactions Program, Texas Biomedical Research Institute, San

Antonio, TX 78227, USA.

FAU - Hernandez, Elizabeth

AU - Hernandez E

AD - Host Pathogen Interactions Program, Texas Biomedical Research Institute, San

Antonio, TX 78227, USA.

FAU - Bonifacio, Jordan R

AU - Bonifacio JR

AD - Host Pathogen Interactions Program, Texas Biomedical Research Institute, San

Antonio, TX 78227, USA.

FAU - Lumbreras, Miranda

AU - Lumbreras M

AD - Host Pathogen Interactions Program, Texas Biomedical Research Institute, San

Antonio, TX 78227, USA.

FAU - Renshaw, Charles

AU - Renshaw C

AD - Host Pathogen Interactions Program, Texas Biomedical Research Institute, San

Antonio, TX 78227, USA.

FAU - Montoya, Maria J

AU - Montoya MJ

AD - Host Pathogen Interactions Program, Texas Biomedical Research Institute, San

Antonio, TX 78227, USA.

FAU - Opferman, Joseph T

AU - Opferman JT

AD - St. Jude Children's Research Hospital, Memphis, TN 38105, USA.

FAU - Schlesinger, Larry S

AU - Schlesinger LS

AD - Host Pathogen Interactions Program, Texas Biomedical Research Institute, San

Antonio, TX 78227, USA. Electronic address: lschlesinger@txbiomed.org.

LA - eng

GR - P30 AI168439/AI/NIAID NIH HHS/United States

GR - R01 AI136831/AI/NIAID NIH HHS/United States

GR - R21 AI145539/AI/NIAID NIH HHS/United States

PT - Journal Article

DEP - 20231019

PL - France

TA - Biomed Pharmacother

JT - Biomedicine & pharmacotherapy = Biomedecine & pharmacotherapie

JID - 8213295

RN - 0 (Antineoplastic Agents)

RN - 0 (Antitubercular Agents)

RN - 0 (Myeloid Cell Leukemia Sequence 1 Protein)

RN - 0 (Proto-Oncogene Proteins c-bcl-2)

SB - IM

MH - Animals

MH - Humans

MH - Mice

MH - *Antineoplastic Agents/pharmacology/therapeutic use

MH - *Antitubercular Agents/pharmacology/therapeutic use/metabolism

MH - Macrophages/drug effects

MH - *Mycobacterium tuberculosis/drug effects

MH - *Myeloid Cell Leukemia Sequence 1 Protein/antagonists & inhibitors/metabolism

MH - *Proto-Oncogene Proteins c-bcl-2/antagonists & inhibitors/metabolism

MH - *Tuberculosis/drug therapy/microbiology

MH - *Drug Repositioning

PMC - PMC10841846

MID - NIHMS1944570

OTO - NOTNLM

OT - Apoptosis

OT - Drug resistant tuberculosis

OT - Granuloma

OT - Host-directed therapy

OT - Macrophage

OT - Tuberculosis

COIS- Declaration of Competing Interest The following patents have been submitted:

Provisional patent number: 63/069,086, international patent number:

PCT/US2021/047074.

EDAT- 2023/10/22 00:41

MHDA- 2023/11/15 06:43

PMCR- 2024/02/05

CRDT- 2023/10/21 18:01

PHST- 2023/06/02 00:00 [received]

PHST- 2023/10/04 00:00 [revised]

PHST- 2023/10/14 00:00 [accepted]

PHST- 2023/11/15 06:43 [medline]

PHST- 2023/10/22 00:41 [pubmed]

PHST- 2023/10/21 18:01 [entrez]

PHST- 2024/02/05 00:00 [pmc-release]

AID - S0753-3322(23)01536-6 [pii]

AID - 10.1016/j.biopha.2023.115738 [doi]

PST - ppublish

SO - Biomed Pharmacother. 2023 Dec;168:115738. doi: 10.1016/j.biopha.2023.115738. Epub

2023 Oct 19.

PMID- 37642861

OWN - NLM

STAT- MEDLINE

DCOM- 20231102

LR - 20231201

IS - 1573-4951 (Electronic)

IS - 0920-654X (Linking)

VI - 37

IP - 12

DP - 2023 Dec

TI - Discovery of novel and potent InhA direct inhibitors by ensemble docking-based

virtual screening and biological assays.

PG - 695-706

LID - 10.1007/s10822-023-00530-4 [doi]

AB - Multidrug-resistant tuberculosis (MDR-TB) continues to spread worldwide and

remains one of the leading causes of death among infectious diseases. The

enoyl-acyl carrier protein reductase (InhA) belongs to FAS-II family and is

essential for the formation of the Mycobacterium tuberculosis cell wall. Recent

years, InhA direct inhibitors have been extensively studied to overcome MDR-TB.

However, there are still no inhibitors that have entered clinical research. Here,

the ensemble docking-based virtual screening along with biological assay were

used to identify potent InhA direct inhibitors from Chembridge, Chemdiv, and

Specs. Ultimately, 34 compounds were purchased and first assayed for the binding

affinity, of which four compounds can bind InhA well with K(D) values ranging

from 48.4 to 56.2 µM. Among them, compound 9,222,034 has the best inhibitory

activity against InhA enzyme with an IC(50) value of 18.05 µM. In addition, the

molecular dynamic simulation and binding free energy calculation indicate that

the identified compounds bind to InhA with "extended" conformation. Residue

energy decomposition shows that residues such as Tyr158, Met161, and Met191 have

higher energy contributions in the binding of compounds. By analyzing the binding

modes, we found that these compounds can bind to a hydrophobic sub-pocket formed

by residues Tyr158, Phe149, Ile215, Leu218, etc., resulting in extensive van der

Waals interactions. In summary, this study proposed an efficient strategy for

discovering InhA direct inhibitors through ensemble docking-based virtual

screening, and finally identified four active compounds with new skeletons, which

can provide valuable information for the discovery and optimization of InhA

direct inhibitors.

CI - © 2023. The Author(s), under exclusive licence to Springer Nature Switzerland AG.

FAU - Zhang, Qianqian

AU - Zhang Q

AD - Faculty of Applied Sciences, Macao Polytechnic University, Macao, SAR, China.

AD - School of Pharmacy, Lanzhou University, Lanzhou, 730000, China.

FAU - Han, Jianting

AU - Han J

AD - College of Chemistry and Chemical Engineering, Lanzhou University, Lanzhou,

China.

FAU - Zhu, Yongchang

AU - Zhu Y

AD - College of Chemistry and Chemical Engineering, Lanzhou University, Lanzhou,

China.

FAU - Yu, Fansen

AU - Yu F

AD - School of Pharmacy, Lanzhou University, Lanzhou, 730000, China.

FAU - Hu, Xiaopeng

AU - Hu X

AD - Faculty of Applied Sciences, Macao Polytechnic University, Macao, SAR, China.

FAU - Tong, Henry H Y

AU - Tong HHY

AD - Faculty of Applied Sciences, Macao Polytechnic University, Macao, SAR, China.

FAU - Liu, Huanxiang

AU - Liu H

AD - Faculty of Applied Sciences, Macao Polytechnic University, Macao, SAR, China.

hxliu@mpu.edu.mo.

LA - eng

PT - Journal Article

PT - Research Support, Non-U.S. Gov't

DEP - 20230829

PL - Netherlands

TA - J Comput Aided Mol Des

JT - Journal of computer-aided molecular design

JID - 8710425

RN - 0 (Antitubercular Agents)

RN - 0 (Bacterial Proteins)

RN - 0 (Enzyme Inhibitors)

SB - IM

MH - Humans

MH - Antitubercular Agents/pharmacology/chemistry

MH - *Mycobacterium tuberculosis

MH - Molecular Dynamics Simulation

MH - Molecular Conformation

MH - *Tuberculosis, Multidrug-Resistant

MH - Bacterial Proteins/chemistry

MH - Molecular Docking Simulation

MH - Enzyme Inhibitors/pharmacology/chemistry

OTO - NOTNLM

OT - Binding mode analysis

OT - Biological assays

OT - Ensemble docking-based virtual screening

OT - InhA direct inhibitors

OT - MD simulation

OT - MDR-TB

OT - MM-GBSA calculation

EDAT- 2023/08/29 12:42

MHDA- 2023/11/02 12:46

CRDT- 2023/08/29 11:15

PHST- 2023/05/09 00:00 [received]

PHST- 2023/08/15 00:00 [accepted]

PHST- 2023/11/02 12:46 [medline]

PHST- 2023/08/29 12:42 [pubmed]

PHST- 2023/08/29 11:15 [entrez]

AID - 10.1007/s10822-023-00530-4 [pii]

AID - 10.1007/s10822-023-00530-4 [doi]

PST - ppublish

SO - J Comput Aided Mol Des. 2023 Dec;37(12):695-706. doi: 10.1007/s10822-023-00530-4.

Epub 2023 Aug 29.

PMID- 37871508

OWN - NLM

STAT- MEDLINE

DCOM- 20231115

LR - 20260127

IS - 1464-3391 (Electronic)

IS - 0968-0896 (Linking)

VI - 95

DP - 2023 Nov 15

TI - Expanding the squaramide library as mycobacterial ATP synthase inhibitors:

Innovative synthetic pathway and biological evaluation.

PG - 117504

LID - S0968-0896(23)00352-8 [pii]

LID - 10.1016/j.bmc.2023.117504 [doi]

AB - Mycobacterial ATP synthase is a validated therapeutic target for combating

drug-resistant tuberculosis. Inhibition of this enzyme has been featured as an

efficient strategy for the development of new antimycobacterial agents against

drug-resistant pathogens. In this study, we synthesised and explored two distinct

series of squaric acid analogues designed to inhibit mycobacterial ATP synthase.

Among the extensive array of compounds investigated, members of the

phenyl-substituted sub-library emerged as primary hits. To gain deeper insights

into their mechanisms of action, we conducted advanced biological studies,

focusing on the compounds displaying a direct binding of a nitrogen heteroatom to

the phenyl ring, resulting in the highest potency. Our investigations into

spontaneous mutants led to the validation of a single point mutation within the

atpB gene (Rv1304), responsible for encoding the ATP synthase subunit a. This

genetic alteration sheds light on the molecular basis of resistance to

squaramides. Furthermore, we explored the possibility of synergy between

squaramides and the reference drug clofazimine using a checkerboard assay,

highlighting the promising avenue for enhancing the effectiveness of existing

treatments through combined therapeutic approaches. This study contributes to the

expansion of investigating squaramides as promising drug candidates in the

ongoing battle against drug-resistant tuberculosis.

CI - Copyright © 2023 Elsevier Ltd. All rights reserved.

FAU - Chasák, Jan

AU - Chasák J

AD - Department of Organic Chemistry, Faculty of Science, Palacký University, 17.

listopadu 12, 77146, Olomouc, Czech Republic.

FAU - Oorts, Lauren

AU - Oorts L

AD - Laboratory of Microbiology, Parasitology and Hygiene (LMPH), S7, Faculty of

Pharmaceutical, Biomedical and Veterinary Sciences, University of Antwerp,

Wilrijk, Belgium.

FAU - Dak, Milan

AU - Dak M

AD - Department of Organic Chemistry, Faculty of Science, Palacký University, 17.

listopadu 12, 77146, Olomouc, Czech Republic.

FAU - Šlachtová, Veronika

AU - Šlachtová V

AD - Department of Organic Chemistry, Faculty of Science, Palacký University, 17.

listopadu 12, 77146, Olomouc, Czech Republic.

FAU - Bazgier, Václav

AU - Bazgier V

AD - Department of Physical Chemistry, Faculty of Science, Palacký University, 17.

listopadu 12, 771 46, Olomouc, Czech Republic.

FAU - Berka, Karel

AU - Berka K

AD - Department of Physical Chemistry, Faculty of Science, Palacký University, 17.

listopadu 12, 771 46, Olomouc, Czech Republic.

FAU - De Vooght, Linda

AU - De Vooght L

AD - Laboratory of Microbiology, Parasitology and Hygiene (LMPH), S7, Faculty of

Pharmaceutical, Biomedical and Veterinary Sciences, University of Antwerp,

Wilrijk, Belgium.

FAU - Smiejkowska, Natalia

AU - Smiejkowska N

AD - Laboratory of Microbiology, Parasitology and Hygiene (LMPH), S7, Faculty of

Pharmaceutical, Biomedical and Veterinary Sciences, University of Antwerp,

Wilrijk, Belgium.

FAU - Calster, Kevin Van

AU - Calster KV

AD - Laboratory of Microbiology, Parasitology and Hygiene (LMPH), S7, Faculty of

Pharmaceutical, Biomedical and Veterinary Sciences, University of Antwerp,

Wilrijk, Belgium.

FAU - Van Moll, Laurence

AU - Van Moll L

AD - Laboratory of Microbiology, Parasitology and Hygiene (LMPH), S7, Faculty of

Pharmaceutical, Biomedical and Veterinary Sciences, University of Antwerp,

Wilrijk, Belgium.

FAU - Cappoen, Davie

AU - Cappoen D

AD - Laboratory of Microbiology, Parasitology and Hygiene (LMPH), S7, Faculty of

Pharmaceutical, Biomedical and Veterinary Sciences, University of Antwerp,

Wilrijk, Belgium.

FAU - Cos, Paul

AU - Cos P

AD - Laboratory of Microbiology, Parasitology and Hygiene (LMPH), S7, Faculty of

Pharmaceutical, Biomedical and Veterinary Sciences, University of Antwerp,

Wilrijk, Belgium.

FAU - Brulíková, Lucie

AU - Brulíková L

AD - Department of Organic Chemistry, Faculty of Science, Palacký University, 17.

listopadu 12, 77146, Olomouc, Czech Republic. Electronic address:

lucie.brulikova@upol.cz.

LA - eng

PT - Journal Article

PT - Research Support, Non-U.S. Gov't

DEP - 20231018

PL - England

TA - Bioorg Med Chem

JT - Bioorganic & medicinal chemistry

JID - 9413298

RN - 8L70Q75FXE (Adenosine Triphosphate)

RN - 0 (Antitubercular Agents)

RN - EC 3.6.3.- (Mitochondrial Proton-Translocating ATPases)

RN - 0 (squaramide)

RN - A7V27PHC7A (Quinine)

SB - IM

MH - Humans

MH - Adenosine Triphosphate/metabolism

MH - Antitubercular Agents/chemistry

MH - *Mycobacterium tuberculosis

MH - *Tuberculosis, Multidrug-Resistant

MH - Mitochondrial Proton-Translocating ATPases/chemistry/metabolism

MH - Quinine/analogs & derivatives

OTO - NOTNLM

OT - ATP synthase

OT - Antimycobacterial agents

OT - MDR-TB

OT - Mycobacterium tuberculosis

OT - Squaramides

OT - Tuberculosis

COIS- Declaration of Competing Interest The authors declare that they have no known

competing financial interests or personal relationships that could have appeared

to influence the work reported in this paper.

EDAT- 2023/10/24 00:41

MHDA- 2023/11/14 06:42

CRDT- 2023/10/23 18:08

PHST- 2023/07/28 00:00 [received]

PHST- 2023/10/11 00:00 [revised]

PHST- 2023/10/16 00:00 [accepted]

PHST- 2023/11/14 06:42 [medline]

PHST- 2023/10/24 00:41 [pubmed]

PHST- 2023/10/23 18:08 [entrez]

AID - S0968-0896(23)00352-8 [pii]

AID - 10.1016/j.bmc.2023.117504 [doi]

PST - ppublish

SO - Bioorg Med Chem. 2023 Nov 15;95:117504. doi: 10.1016/j.bmc.2023.117504. Epub 2023

Oct 18.

PMID- 38020140

OWN - NLM

STAT- PubMed-not-MEDLINE

LR - 20231201

IS - 2296-858X (Print)

IS - 2296-858X (Electronic)

IS - 2296-858X (Linking)

VI - 10

DP - 2023

TI - Prolonged survival of a patient with active MDR-TB HIV co-morbidity: insights

from a Mycobacterium tuberculosis strain with a unique genomic deletion.

PG - 1292665

LID - 10.3389/fmed.2023.1292665 [doi]

LID - 1292665

AB - Coinfection of HIV and multidrug-resistant tuberculosis (MDR-TB) presents

significant challenges in terms of the treatment and prognosis of tuberculosis,

leading to complexities in managing the disease and impacting the overall outcome

for TB patients. This study presents a remarkable case of a patient with MDR-TB

and HIV coinfection who survived for over 8 years, despite poor treatment

adherence and comorbidities. Whole genome sequencing (WGS) of the infecting

Mycobacterium tuberculosis (Mtb) strain revealed a unique genomic deletion,

spanning 18 genes, including key genes involved in hypoxia response,

intracellular survival, immunodominant antigens, and dormancy. This deletion,

that we have called "Del-X," potentially exerts a profound influence on the

bacterial physiology and its virulence. Only few similar deletions were detected

in other non-related Mtb genomes worldwide. In vivo evolution analysis identified

drug resistance and metabolic adaptation mutations and their temporal dynamics

during the patient's treatment course.

CI - Copyright © 2023 Rubinstein, Makhon, Losev, Valenci, Gatt, Margalit, Fass,

Kutikov, Murik, Zeevi, Savyon, Tau, Kaidar Shwartz, Dveyrin, Rorman and Nissan.

FAU - Rubinstein, Mor

AU - Rubinstein M

AD - National Public Health Laboratory, Public Health Directorate, Ministry of Health,

Tel Aviv, Israel.

FAU - Makhon, Andrei

AU - Makhon A

AD - National Public Health Laboratory, Public Health Directorate, Ministry of Health,

Tel Aviv, Israel.

FAU - Losev, Yelena

AU - Losev Y

AD - National Public Health Laboratory, Public Health Directorate, Ministry of Health,

Tel Aviv, Israel.

FAU - Valenci, Gal Zizelski

AU - Valenci GZ

AD - National Public Health Laboratory, Public Health Directorate, Ministry of Health,

Tel Aviv, Israel.

FAU - Gatt, Yair E

AU - Gatt YE

AD - Department of Microbiology and Molecular Genetics, Institute for Medical Research

Israel-Canada, Faculty of Medicine, The Hebrew University of Jerusalem,

Jerusalem, Israel.

FAU - Margalit, Hanah

AU - Margalit H

AD - Department of Microbiology and Molecular Genetics, Institute for Medical Research

Israel-Canada, Faculty of Medicine, The Hebrew University of Jerusalem,

Jerusalem, Israel.

FAU - Fass, Ephraim

AU - Fass E

AD - National Public Health Laboratory, Public Health Directorate, Ministry of Health,

Tel Aviv, Israel.

FAU - Kutikov, Ina

AU - Kutikov I

AD - National Public Health Laboratory, Public Health Directorate, Ministry of Health,

Tel Aviv, Israel.

FAU - Murik, Omer

AU - Murik O

AD - Translational Genomics Laboratory, Medical Genetics Institute, Shaare Zedek

Medical Center, Jerusalem, Israel.

FAU - Zeevi, David A

AU - Zeevi DA

AD - Translational Genomics Laboratory, Medical Genetics Institute, Shaare Zedek

Medical Center, Jerusalem, Israel.

FAU - Savyon, Michal

AU - Savyon M

AD - Tel Aviv District Health Office, Ministry of Health, Tel Aviv, Israel.

FAU - Tau, Luba

AU - Tau L

AD - Tel Aviv Sourasky Medical Center, Tel Aviv, Israel.

AD - Faculty of Medicine, Tel Aviv University, Tel Aviv, Israel.

FAU - Kaidar Shwartz, Hasia

AU - Kaidar Shwartz H

AD - National Public Health Laboratory, Public Health Directorate, Ministry of Health,

Tel Aviv, Israel.

FAU - Dveyrin, Zeev

AU - Dveyrin Z

AD - National Public Health Laboratory, Public Health Directorate, Ministry of Health,

Tel Aviv, Israel.

FAU - Rorman, Efrat

AU - Rorman E

AD - National Public Health Laboratory, Public Health Directorate, Ministry of Health,

Tel Aviv, Israel.

FAU - Nissan, Israel

AU - Nissan I

AD - National Public Health Laboratory, Public Health Directorate, Ministry of Health,

Tel Aviv, Israel.

LA - eng

PT - Journal Article

DEP - 20231106

PL - Switzerland

TA - Front Med (Lausanne)

JT - Frontiers in medicine

JID - 101648047

PMC - PMC10657812

OTO - NOTNLM

OT - HspX

OT - Mycobacterium tuberculosis (MTB)

OT - acquired immunodeficiency syndrome (AIDS)

OT - dormancy

OT - human immunodeficiency virus (HIV)

OT - in vivo evolution

OT - multidrug-resistant tuberculosis (MDR-TB)

OT - whole genome sequencing (WGS)

COIS- The authors declare that the research was conducted in the absence of any

commercial or financial relationships that could be construed as a potential

conflict of interest.

EDAT- 2023/11/29 18:42

MHDA- 2023/11/29 18:43

PMCR- 2023/11/06

CRDT- 2023/11/29 14:39

PHST- 2023/09/11 00:00 [received]

PHST- 2023/10/16 00:00 [accepted]

PHST- 2023/11/29 18:43 [medline]

PHST- 2023/11/29 18:42 [pubmed]

PHST- 2023/11/29 14:39 [entrez]

PHST- 2023/11/06 00:00 [pmc-release]

AID - 10.3389/fmed.2023.1292665 [doi]

PST - epublish

SO - Front Med (Lausanne). 2023 Nov 6;10:1292665. doi: 10.3389/fmed.2023.1292665.

eCollection 2023.

PMID- 37290049

OWN - NLM

STAT- MEDLINE

DCOM- 20231109

LR - 20231114

IS - 1537-6613 (Electronic)

IS - 0022-1899 (Linking)

VI - 228

IP - 9

DP - 2023 Nov 2

TI - Cotreatment With Clofazimine and Rapamycin Eliminates Drug-Resistant Tuberculosis

by Inducing Polyfunctional Central Memory T-Cell Responses.

PG - 1166-1178

LID - 10.1093/infdis/jiad214 [doi]

AB - Mycobacterium tuberculosis, the causative agent of tuberculosis, is acquiring

drug resistance at a faster rate than the discovery of new antibiotics.

Therefore, alternate therapies that can limit the drug resistance and disease

recurrence are urgently needed. Emerging evidence indicates that combined

treatment with antibiotics and an immunomodulator provides superior treatment

efficacy. Clofazimine (CFZ) enhances the generation of T central memory (TCM)

cells by blocking the Kv1.3+ potassium channels. Rapamycin (RAPA) facilitates M.

tuberculosis clearance by inducing autophagy. In this study, we observed that

cotreatment with CFZ and RAPA potently eliminates both multiple and extensively

drug-resistant (MDR and XDR) clinical isolates of M. tuberculosis in a mouse

model by inducing robust T-cell memory and polyfunctional TCM responses.

Furthermore, cotreatment reduces the expression of latency-associated genes of M.

tuberculosis in human macrophages. Therefore, CFZ and RAPA cotherapy holds

promise for treating patients infected with MDR and XDR strains of M.

tuberculosis.

CI - © The Author(s) 2023. Published by Oxford University Press on behalf of

Infectious Diseases Society of America. All rights reserved. For permissions,

please e-mail: journals.permissions@oup.com.

FAU - Singh, Dhiraj Kumar

AU - Singh DK

AD - Immunobiology Group, International Centre for Genetic Engineering and

Biotechnology, New Delhi, India.

AD - Special Center for Molecular Medicine, Jawaharlal Nehru University, New Delhi,

India.

FAU - Bhaskar, Ashima

AU - Bhaskar A

AD - Immunobiology Group, International Centre for Genetic Engineering and

Biotechnology, New Delhi, India.

FAU - Pahuja, Isha

AU - Pahuja I

AD - Immunobiology Group, International Centre for Genetic Engineering and

Biotechnology, New Delhi, India.

FAU - Shaji, Aishwarya

AU - Shaji A

AD - Immunobiology Group, International Centre for Genetic Engineering and

Biotechnology, New Delhi, India.

FAU - Moitra, Barnani

AU - Moitra B

AD - Special Center for Molecular Medicine, Jawaharlal Nehru University, New Delhi,

India.

FAU - Shi, Yufang

AU - Shi Y

AD - State Key Laboratory of Radiation Medicine and Protection, Suzhou Medical College

of Soochow University, Suzhou, Jiangsu, People's Republic of China.

FAU - Dwivedi, Ved Prakash

AU - Dwivedi VP

AUID- ORCID: 0000-0003-4321-2567

AD - Immunobiology Group, International Centre for Genetic Engineering and

Biotechnology, New Delhi, India.

FAU - Das, Gobardhan

AU - Das G

AD - Special Center for Molecular Medicine, Jawaharlal Nehru University, New Delhi,

India.

LA - eng

PT - Journal Article

PT - Research Support, Non-U.S. Gov't

PL - United States

TA - J Infect Dis

JT - The Journal of infectious diseases

JID - 0413675

RN - D959AE5USF (Clofazimine)

RN - 0 (Antitubercular Agents)

RN - W36ZG6FT64 (Sirolimus)

SB - IM

MH - Animals

MH - Mice

MH - Humans

MH - Clofazimine/adverse effects

MH - Antitubercular Agents/pharmacology/therapeutic use

MH - Sirolimus/pharmacology/therapeutic use

MH - Memory T Cells

MH - *Tuberculosis, Multidrug-Resistant/drug therapy/microbiology

MH - *Mycobacterium tuberculosis

MH - Microbial Sensitivity Tests

MH - Drug Resistance, Multiple, Bacterial

OTO - NOTNLM

OT - central memory T cells

OT - clofazimine

OT - extensively drug-resistant tuberculosis

OT - helper T cells

OT - immunotherapy

OT - multidrug-resistance

OT - rapamycin

OT - tuberculosis

EDAT- 2023/06/08 19:14

MHDA- 2023/11/09 06:42

CRDT- 2023/06/08 16:13

PHST- 2023/03/14 00:00 [received]

PHST- 2023/06/07 00:00 [accepted]

PHST- 2023/11/09 06:42 [medline]

PHST- 2023/06/08 19:14 [pubmed]

PHST- 2023/06/08 16:13 [entrez]

AID - 7192348 [pii]

AID - 10.1093/infdis/jiad214 [doi]

PST - ppublish

SO - J Infect Dis. 2023 Nov 2;228(9):1166-1178. doi: 10.1093/infdis/jiad214.

PMID- 37740791

OWN - NLM

STAT- MEDLINE

DCOM- 20260311

LR - 20260311

IS - 1435-4373 (Electronic)

IS - 0934-9723 (Linking)

VI - 42

IP - 11

DP - 2023 Nov

TI - Exploring and exploiting the host cell autophagy during Mycobacterium

tuberculosis infection.

PG - 1297-1315

LID - 10.1007/s10096-023-04663-0 [doi]

AB - Tuberculosis, caused by Mycobacterium tuberculosis, is a fatal infectious disease

that prevails to be the second leading cause of death from a single infectious

agent despite the availability of multiple drugs for treatment. The current

treatment regimen involves the combination of several drugs for 6 months that

remain ineffective in completely eradicating the infection because of several

drawbacks, such as the long duration of treatment and the side effects of drugs

causing non-adherence of patients to the treatment regimen. Autophagy is an

intracellular degradative process that eliminates pathogens at the early stages

of infection. Mycobacterium tuberculosis's unique autophagy-blocking capability

makes it challenging to eliminate compared to usual pathogens. The present review

discusses recent advances in autophagy-inhibiting factors and mechanisms that

could be exploited to identify autophagy-inducing chemotherapeutics that could be

used as adjunctive therapy with the existing first-line anti-TB agent to shorten

the duration of therapy and enhance cure rates from multidrug-resistant

tuberculosis (MDR-TB) and extreme drug-resistant tuberculosis (XDR-TB).

CI - © 2023. The Author(s), under exclusive licence to Springer-Verlag GmbH Germany,

part of Springer Nature.

FAU - Nagdev, Pavan Kumar

AU - Nagdev PK

AD - Department of Biological Sciences (Pharmacology and Toxicology), National

Institute of Pharmaceutical Education and Research (NIPER), Hyderabad, Telangana,

500037, India.

FAU - Agnivesh, Puja Kumari

AU - Agnivesh PK

AD - Department of Biological Sciences (Pharmacology and Toxicology), National

Institute of Pharmaceutical Education and Research (NIPER), Hyderabad, Telangana,

500037, India.

FAU - Roy, Arnab

AU - Roy A

AD - Department of Biological Sciences (Pharmacology and Toxicology), National

Institute of Pharmaceutical Education and Research (NIPER), Hyderabad, Telangana,

500037, India.

FAU - Sau, Shashikanta

AU - Sau S

AD - Department of Biological Sciences (Pharmacology and Toxicology), National

Institute of Pharmaceutical Education and Research (NIPER), Hyderabad, Telangana,

500037, India.

FAU - Kalia, Nitin Pal

AU - Kalia NP

AUID- ORCID: 0000-0003-4346-6567

AD - Department of Biological Sciences (Pharmacology and Toxicology), National

Institute of Pharmaceutical Education and Research (NIPER), Hyderabad, Telangana,

500037, India. nitin.kalia@niperhyd.ac.in.

LA - eng

GR - D.O.NO.BT/HRD/35/02/2006; NO. BT/RLF/Re-entry 66/2017; GAP-39/Department of

Biotechnology, Ministry of Science and Technology, India/

PT - Journal Article

PT - Review

DEP - 20230923

PL - Germany

TA - Eur J Clin Microbiol Infect Dis

JT - European journal of clinical microbiology & infectious diseases : official

publication of the European Society of Clinical Microbiology

JID - 8804297

RN - 0 (Antitubercular Agents)

SB - IM

MH - *Autophagy/drug effects

MH - Humans

MH - *Mycobacterium tuberculosis/drug effects/physiology

MH - Antitubercular Agents/therapeutic use/pharmacology

MH - *Tuberculosis/drug therapy/microbiology/immunology

MH - *Host-Pathogen Interactions

MH - Tuberculosis, Multidrug-Resistant/drug therapy/microbiology

MH - Animals

OTO - NOTNLM

OT - Autophagy

OT - Host cell inhibitors

OT - Host defense

OT - Tuberculosis

EDAT- 2023/09/23 20:41

MHDA- 2026/03/12 01:09

CRDT- 2023/09/23 11:05

PHST- 2023/04/10 00:00 [received]

PHST- 2023/09/06 00:00 [accepted]

PHST- 2026/03/12 01:09 [medline]

PHST- 2023/09/23 20:41 [pubmed]

PHST- 2023/09/23 11:05 [entrez]

AID - 10.1007/s10096-023-04663-0 [pii]

AID - 10.1007/s10096-023-04663-0 [doi]

PST - ppublish

SO - Eur J Clin Microbiol Infect Dis. 2023 Nov;42(11):1297-1315. doi:

10.1007/s10096-023-04663-0. Epub 2023 Sep 23.

PMID- 37211822

OWN - NLM

STAT- MEDLINE

DCOM- 20231030

LR - 20250626

IS - 1973-9478 (Electronic)

IS - 1120-009X (Linking)

VI - 35

IP - 7

DP - 2023 Nov

TI - Global status of phenotypic pyrazinamide resistance in Mycobacterium tuberculosis

clinical isolates: an updated systematic review and meta-analysis.

PG - 583-595

LID - 10.1080/1120009X.2023.2214473 [doi]

AB - Pyrazinamide (PZA) is an essential first-line tuberculosis drug for its unique

mechanism of action active against multidrug-resistant-TB (MDR-TB). Thus, the aim

of updated meta-analysis was to estimate the PZA weighted pooled resistance (WPR)

rate in M. tuberculosis isolates based on publication date and WHO regions. We

systematically searched the related reports in PubMed, Scopus, and Embase (from

January 2015 to July 2022). Statistical analyses were performed using STATA

software. The 115 final reports in the analysis investigated phenotypic PZA

resistance data. The WPR of PZA was 57% (95% CI 48-65%) in MDR-TB cases.

According to the WHO regions, the higher WPRs of PZA were reported in the Western

Pacific (32%; 95% CI 18-46%), South East Asian region (37%; 95% CI 31-43%), and

the Eastern Mediterranean (78%; 95% CI 54-95%) among any-TB patients, high risk

of MDR-TB patients, and MDR-TB patients, respectively. A negligible increase in

the rate of PZA resistance were showed in MDR-TB cases (55% to 58%). The rate of

PZA resistance has been rising in recent years among MDR-TB cases, underlines the

essential for both standard and novel drug regimens development.

FAU - Wang, Zheming

AU - Wang Z

AD - Department of Pharmacy, Shaoxing People's Hospital, Shaoxing, China.

FAU - Tang, Zhihua

AU - Tang Z

AD - Department of Pharmacy, Shaoxing People's Hospital, Shaoxing, China.

FAU - Heidari, Hamid

AU - Heidari H

AD - Department of Microbiology, Faculty of Medicine, Shahid Sadoughi University of

Medical Sciences, Yazd, Iran.

FAU - Molaeipour, Leila

AU - Molaeipour L

AD - Department of Epidemiology, School of Public Health, University of Medical

Sciences, Tehran, Iran.

FAU - Ghanavati, Roya

AU - Ghanavati R

AD - Behbahan Faculty of Medical Sciences, Behbahan, Iran.

FAU - Kazemian, Hossein

AU - Kazemian H

AD - Clinical Microbiology Research Center, Ilam University of Medical Sciences, Ilam,

Iran.

FAU - Koohsar, Faramarz

AU - Koohsar F

AD - Laboratory Sciences Research Center, Golestan University of Medical Sciences,

Gorgan, Iran.

FAU - Kouhsari, Ebrahim

AU - Kouhsari E

AD - Laboratory Sciences Research Center, Golestan University of Medical Sciences,

Gorgan, Iran.

AD - Department of Laboratory Sciences, Faculty of Paramedicine, Golestan University

of Medical Sciences, Gorgan, Iran.

LA - eng

PT - Journal Article

PT - Meta-Analysis

PT - Systematic Review

DEP - 20230521

PL - England

TA - J Chemother

JT - Journal of chemotherapy (Florence, Italy)

JID - 8907348

RN - 2KNI5N06TI (Pyrazinamide)

RN - 0 (Antitubercular Agents)

RN - EC 3.5.- (Amidohydrolases)

SB - IM

MH - Humans

MH - Pyrazinamide/pharmacology/therapeutic use

MH - *Mycobacterium tuberculosis

MH - Antitubercular Agents/pharmacology/therapeutic use

MH - *Tuberculosis, Multidrug-Resistant/drug therapy/microbiology

MH - Drug Resistance, Multiple, Bacterial

MH - Amidohydrolases/genetics/pharmacology

MH - Mutation

MH - Microbial Sensitivity Tests

MH - *Tuberculosis/drug therapy/microbiology

OTO - NOTNLM

OT - MDR

OT - Tuberculosis

OT - pyrazinamide

OT - resistance

OT - systematic review and meta-analysis

EDAT- 2023/05/22 06:42

MHDA- 2023/10/30 06:46

CRDT- 2023/05/22 03:46

PHST- 2023/10/30 06:46 [medline]

PHST- 2023/05/22 06:42 [pubmed]

PHST- 2023/05/22 03:46 [entrez]

AID - 10.1080/1120009X.2023.2214473 [doi]

PST - ppublish

SO - J Chemother. 2023 Nov;35(7):583-595. doi: 10.1080/1120009X.2023.2214473. Epub

2023 May 21.

PMID- 37859726

OWN - NLM

STAT- PubMed-not-MEDLINE

LR - 20240907

IS - 2632-8682 (Electronic)

IS - 2632-8682 (Linking)

VI - 14

IP - 10

DP - 2023 Oct 18

TI - Targeting Mycobacterium tuberculosis iron-scavenging tools: a recent update on

siderophores inhibitors.

PG - 1885-1913

LID - 10.1039/d3md00201b [doi]

AB - Among the various bacterial infections, tuberculosis (TB) remains a

life-threatening infectious disease responsible as the most significant cause of

mortality and morbidity worldwide. The co-infection of human immunodeficiency

virus (HIV) in association with TB burdens the healthcare system substantially.

Notably, M.tb possesses defence against most antitubercular antibiotic drugs, and

the efficacy of existing frontline anti-TB drugs is waning. Also, new and

recurring cases of TB from resistant bacteria such as multidrug-resistant TB

(MDR), extensively drug-resistant TB (XDR), and totally drug-resistant TB (TDR)

strains are increasing. Hence, TB begs the scientific community to explore the

new therapeutic class of compounds with their novel mechanism. M.tb requires iron

from host cells to sustain, grow, and carry out several biological processes.

M.tb has developed strategic methods of acquiring iron from the surrounding

environment. In this communication, we discuss an overview of M.tb

iron-scavenging tools. Also, we have summarized recently identified MbtA and MbtI

inhibitors, which prevent M.tb from scavenging iron. These iron-scavenging tool

inhibitors have the potential to be developed as anti-TB agents/drugs.

CI - This journal is © The Royal Society of Chemistry.

FAU - Kumar, Gautam

AU - Kumar G

AUID- ORCID: 0000-0003-4047-1416

AD - Department of Natural Products, Chemical Sciences, National Institute of

Pharmaceutical Education and Research-Hyderabad (NIPER-Hyderabad) Balanagar

Hyderabad 500037 India gautam.kumar2@niperhyd.ac.in.

FAU - Adhikrao, Patil Amruta

AU - Adhikrao PA

AD - Department of Natural Products, Chemical Sciences, National Institute of

Pharmaceutical Education and Research-Hyderabad (NIPER-Hyderabad) Balanagar

Hyderabad 500037 India gautam.kumar2@niperhyd.ac.in.

LA - eng

PT - Journal Article

PT - Review

DEP - 20230906

PL - England

TA - RSC Med Chem

JT - RSC medicinal chemistry

JID - 101759460

PMC - PMC10583813

COIS- There is no conflict of interest to declare.

EDAT- 2023/10/20 06:42

MHDA- 2023/10/20 06:43

PMCR- 2024/09/06

CRDT- 2023/10/20 04:18

PHST- 2023/04/28 00:00 [received]

PHST- 2023/08/22 00:00 [accepted]

PHST- 2023/10/20 06:43 [medline]

PHST- 2023/10/20 06:42 [pubmed]

PHST- 2023/10/20 04:18 [entrez]

PHST- 2024/09/06 00:00 [pmc-release]

AID - d3md00201b [pii]

AID - 10.1039/d3md00201b [doi]

PST - epublish

SO - RSC Med Chem. 2023 Sep 6;14(10):1885-1913. doi: 10.1039/d3md00201b. eCollection

2023 Oct 18.

PMID- 37905073

OWN - NLM

STAT- PubMed-not-MEDLINE

LR - 20250708

IS - 2692-8205 (Electronic)

IS - 2692-8205 (Linking)

DP - 2023 Oct 17

TI - KatG catalase deficiency confers bedaquiline hyper-susceptibility to isoniazid

resistant Mycobacterium tuberculosis.

LID - 2023.10.17.562707 [pii]

LID - 10.1101/2023.10.17.562707 [doi]

AB - Multidrug-resistant tuberculosis (MDR-TB) is a growing source of global mortality

and threatens global control of tuberculosis (TB) disease. The diarylquinoline

bedaquiline (BDQ) recently emerged as a highly efficacious drug against MDR-TB,

defined as resistance to the first-line drugs isoniazid (INH) and rifampin. INH

resistance is primarily caused by loss-of-function mutations in the catalase

KatG, but mechanisms underlying BDQ's efficacy against MDR-TB remain unknown.

Here we employ a systems biology approach to investigate BDQ hyper-susceptibility

in INH-resistant Mycobacterium tuberculosis . We found hyper-susceptibility to

BDQ in INH-resistant cells is due to several physiological changes induced by

KatG deficiency, including increased susceptibility to reactive oxygen species

and DNA damage, remodeling of transcriptional programs, and metabolic repression

of folate biosynthesis. We demonstrate BDQ hyper-susceptibility is common in

INH-resistant clinical isolates. Collectively, these results highlight how

altered bacterial physiology can impact drug efficacy in drug-resistant bacteria.

FAU - Ofori-Anyinam, N

AU - Ofori-Anyinam N

FAU - Hamblin, M

AU - Hamblin M

FAU - Coldren, M L

AU - Coldren ML

FAU - Li, B

AU - Li B

FAU - Mereddy, G

AU - Mereddy G

FAU - Shaikh, M

AU - Shaikh M

FAU - Shah, A

AU - Shah A

FAU - Ranu, N

AU - Ranu N

FAU - Lu, S

AU - Lu S

FAU - Blainey, P C

AU - Blainey PC

FAU - Ma, S

AU - Ma S

FAU - Collins, J J

AU - Collins JJ

FAU - Yang, J H

AU - Yang JH

LA - eng

PT - Journal Article

PT - Preprint

DEP - 20231017

PL - United States

TA - bioRxiv

JT - bioRxiv : the preprint server for biology

JID - 101680187

UIN - Nat Commun. 2024 Nov 13;15(1):9792. doi: 10.1038/s41467-024-53933-8. PMID:

39537610

PMC - PMC10614911

EDAT- 2023/10/31 06:42

MHDA- 2023/10/31 06:43

PMCR- 2023/10/30

CRDT- 2023/10/31 04:01

PHST- 2023/10/31 06:42 [pubmed]

PHST- 2023/10/31 06:43 [medline]

PHST- 2023/10/31 04:01 [entrez]

PHST- 2023/10/30 00:00 [pmc-release]

AID - 2023.10.17.562707 [pii]

AID - 10.1101/2023.10.17.562707 [doi]

PST - epublish

SO - bioRxiv [Preprint]. 2023 Oct 17:2023.10.17.562707. doi:

10.1101/2023.10.17.562707.

PMID- 37615440

OWN - NLM

STAT- PubMed-not-MEDLINE

DCOM- 20260326

LR - 20260326

IS - 2165-0497 (Electronic)

IS - 2165-0497 (Linking)

VI - 11

IP - 5

DP - 2023 Oct 17

TI - Rv1453 is associated with clofazimine resistance in Mycobacterium tuberculosis.

PG - e0000223

LID - 10.1128/spectrum.00002-23 [doi]

LID - e00002-23

AB - Tuberculosis (TB) is an infectious disease caused by the bacterium Mycobacterium

tuberculosis, especially the emergence of multidrug-resistant tuberculosis

(MDR-TB) brings great distress to humans. Clofazimine (CFZ) plays an important

role in the treatment of MDR-TB. To understand the underlying mechanism of

clofazimine resistance better, in this study, we review and detail the findings

of the mutation of intergenic region of Rv1453 and find additional evidence that

this mutation is related to clofazimine resistance in 30 additional isolates. The

significance of our research is to contribute to a comprehensive understanding of

clofazimine-resistant mechanisms, which is critical for reducing the emergence of

resistance and for anti-TB drug discovery.

FAU - Zhang, Lei

AU - Zhang L

AD - Department of Pharmacology, Beijing Key Laboratory of Drug Resistance

Tuberculosis Research, Beijing Chest Hospital, Capital Medical University,

Beijing Tuberculosis and Thoracic Tumor Research Institute , Beijing, China.

FAU - Zhang, Ye

AU - Zhang Y

AD - Department of Pharmacology, Beijing Key Laboratory of Drug Resistance

Tuberculosis Research, Beijing Chest Hospital, Capital Medical University,

Beijing Tuberculosis and Thoracic Tumor Research Institute , Beijing, China.

FAU - Li, Yuanyuan

AU - Li Y

AD - Department of Pharmacology, Beijing Key Laboratory of Drug Resistance

Tuberculosis Research, Beijing Chest Hospital, Capital Medical University,

Beijing Tuberculosis and Thoracic Tumor Research Institute , Beijing, China.

FAU - Huo, Fengmin

AU - Huo F

AD - National Clinical Laboratory on Tuberculosis, Beijing Key Laboratory for

Drug-Resistant Tuberculosis Research, Beijing Chest Hospital, Capital Medical

University, Beijing Tuberculosis and Thoracic Tumor Institute , Beijing, China.

FAU - Chen, Xi

AU - Chen X

AD - Department of Pharmacology, Beijing Key Laboratory of Drug Resistance

Tuberculosis Research, Beijing Chest Hospital, Capital Medical University,

Beijing Tuberculosis and Thoracic Tumor Research Institute , Beijing, China.

FAU - Zhu, Hui

AU - Zhu H

AD - Department of Pharmacology, Beijing Key Laboratory of Drug Resistance

Tuberculosis Research, Beijing Chest Hospital, Capital Medical University,

Beijing Tuberculosis and Thoracic Tumor Research Institute , Beijing, China.

FAU - Guo, Shaochen

AU - Guo S

AD - Department of Pharmacology, Beijing Key Laboratory of Drug Resistance

Tuberculosis Research, Beijing Chest Hospital, Capital Medical University,

Beijing Tuberculosis and Thoracic Tumor Research Institute , Beijing, China.

FAU - Fu, Lei

AU - Fu L

AD - Department of Pharmacology, Beijing Key Laboratory of Drug Resistance

Tuberculosis Research, Beijing Chest Hospital, Capital Medical University,

Beijing Tuberculosis and Thoracic Tumor Research Institute , Beijing, China.

FAU - Wang, Bin

AU - Wang B

AD - Department of Pharmacology, Beijing Key Laboratory of Drug Resistance

Tuberculosis Research, Beijing Chest Hospital, Capital Medical University,

Beijing Tuberculosis and Thoracic Tumor Research Institute , Beijing, China.

FAU - Lu, Yu

AU - Lu Y

AUID- ORCID: 0000-0002-9038-1884

AD - Department of Pharmacology, Beijing Key Laboratory of Drug Resistance

Tuberculosis Research, Beijing Chest Hospital, Capital Medical University,

Beijing Tuberculosis and Thoracic Tumor Research Institute , Beijing, China.

LA - eng

GR - 81973367/MOST | National Natural Science Foundation of China (NSFC)/

GR - ZYLX202123/Beijing Hospitals Authority Clinical Medicine Development of Special

Funding Support/

GR - DFL20221402/Beijing Municipal Administration of Hospitals' Ascent Plan/

PT - Journal Article

DEP - 20230824

PL - United States

TA - Microbiol Spectr

JT - Microbiology spectrum

JID - 101634614

SB - IM

PMC - PMC10580819

OTO - NOTNLM

OT - Mycobacterium tuberculosis

OT - Rv1453

OT - clofazimine

OT - drug resistant

COIS- The authors declare no conflict of interest.

EDAT- 2023/08/24 13:43

MHDA- 2023/08/24 13:44

PMCR- 2023/08/24

CRDT- 2023/08/24 09:03

PHST- 2023/08/24 13:44 [medline]

PHST- 2023/08/24 13:43 [pubmed]

PHST- 2023/08/24 09:03 [entrez]

PHST- 2023/08/24 00:00 [pmc-release]

AID - 00002-23 [pii]

AID - spectrum.00002-23 [pii]

AID - 10.1128/spectrum.00002-23 [doi]

PST - ppublish

SO - Microbiol Spectr. 2023 Oct 17;11(5):e0000223. doi: 10.1128/spectrum.00002-23.

Epub 2023 Aug 24.

PMID- 37895946

OWN - NLM

STAT- PubMed-not-MEDLINE

LR - 20250622

IS - 1424-8247 (Print)

IS - 1424-8247 (Electronic)

IS - 1424-8247 (Linking)

VI - 16

IP - 10

DP - 2023 Oct 16

TI - The Use of Viral Vectors for Gene Therapy and Vaccination in Tuberculosis.

LID - 10.3390/ph16101475 [doi]

LID - 1475

AB - Tuberculosis (TB), an infection caused by Mycobacterium tuberculosis (Mtb), is

one of the primary causes of death globally. The treatment of TB is long and

based on several drugs, producing problems in compliance and toxicity, increasing

Mtb resistance to first-line antibiotics that result in multidrug-resistant TB

and extensively drug-resistant TB. Thus, the need for new anti-TB treatments has

increased. Here, we review some model strategies to study gene therapy based on

the administration of a recombinant adenovirus that encodes diverse cytokines,

such as IFNγ, IL12, GM/CSF, OPN, TNFα, and antimicrobial peptides to enhance the

protective immune response against Mtb. These models include a model of

progressive pulmonary TB, a model of chronic infection similar to latent TB, and

a murine model of pulmonary Mtb transmission to close contacts. We also review

new vaccines that deliver Mtb antigens via particle- or virus-based vectors and

trigger protective immune responses. The results obtained in this type of

research suggest that this is an alternative therapy that has the potential to

treat active TB as an adjuvant to conventional antibiotics and a promising

preventive treatment for latent TB reactivation and Mtb transmission. Moreover,

Ad vector vaccines are adequate for preventing infectious diseases, including TB.

FAU - Mata-Espinosa, Dulce

AU - Mata-Espinosa D

AD - Sección de Patología Experimental, Instituto Nacional de Ciencias Médicas y

Nutrición Salvador Zubirán, Vasco de Quiroga 15, Belisario Domínguez Sección 16,

Tlalpan, Mexico City 14080, Mexico.

FAU - Lara-Espinosa, Jacqueline V

AU - Lara-Espinosa JV

AUID- ORCID: 0000-0001-6635-2939

AD - Sección de Patología Experimental, Instituto Nacional de Ciencias Médicas y

Nutrición Salvador Zubirán, Vasco de Quiroga 15, Belisario Domínguez Sección 16,

Tlalpan, Mexico City 14080, Mexico.

FAU - Barrios-Payán, Jorge

AU - Barrios-Payán J

AUID- ORCID: 0000-0002-5134-5573

AD - Sección de Patología Experimental, Instituto Nacional de Ciencias Médicas y

Nutrición Salvador Zubirán, Vasco de Quiroga 15, Belisario Domínguez Sección 16,

Tlalpan, Mexico City 14080, Mexico.

FAU - Hernández-Pando, Rogelio

AU - Hernández-Pando R

AUID- ORCID: 0000-0003-1292-3833

AD - Sección de Patología Experimental, Instituto Nacional de Ciencias Médicas y

Nutrición Salvador Zubirán, Vasco de Quiroga 15, Belisario Domínguez Sección 16,

Tlalpan, Mexico City 14080, Mexico.

LA - eng

PT - Journal Article

PT - Review

DEP - 20231016

PL - Switzerland

TA - Pharmaceuticals (Basel)

JT - Pharmaceuticals (Basel, Switzerland)

JID - 101238453

PMC - PMC10610538

OTO - NOTNLM

OT - adenovirus

OT - gene therapy

OT - tuberculosis

OT - vaccines

COIS- The authors declare no conflict of interest.

EDAT- 2023/10/28 11:42

MHDA- 2023/10/28 11:43

PMCR- 2023/10/16

CRDT- 2023/10/28 01:24

PHST- 2023/08/19 00:00 [received]

PHST- 2023/10/10 00:00 [revised]

PHST- 2023/10/11 00:00 [accepted]

PHST- 2023/10/28 11:43 [medline]

PHST- 2023/10/28 11:42 [pubmed]

PHST- 2023/10/28 01:24 [entrez]

PHST- 2023/10/16 00:00 [pmc-release]

AID - ph16101475 [pii]

AID - pharmaceuticals-16-01475 [pii]

AID - 10.3390/ph16101475 [doi]

PST - epublish

SO - Pharmaceuticals (Basel). 2023 Oct 16;16(10):1475. doi: 10.3390/ph16101475.

PMID- 37810827

OWN - NLM

STAT- PubMed-not-MEDLINE

LR - 20231010

IS - 2405-8440 (Print)

IS - 2405-8440 (Electronic)

IS - 2405-8440 (Linking)

VI - 9

IP - 10

DP - 2023 Oct

TI - Prevalence, risk factors and treatment outcomes of fluoroquinolones-associated

tendinopathy in tuberculosis patients at university hospital, Thailand.

PG - e20331

LID - 10.1016/j.heliyon.2023.e20331 [doi]

LID - e20331

AB - BACKGROUND: Tuberculosis (TB) is an epidemic disease in Thailand.

Fluoroquinolones are used to treat TB and have a lengthy treatment course.

Therefore, many patients may have adverse effects from these medications.

Tendinopathy has been reported as a significant adverse effect of

fluoroquinolones. Although the mechanism of tendinopathy from fluoroquinolones is

not fully understood, it can progress to a more serious consequence such as a

ruptured tendon which can result in morbidity if not treated effectively.

METHODS: This study was a single-centered, retrospective descriptive study

conducted in patients at a tertiary-level university hospital in Thailand. TB

patients who received fluoroquinolones for the treatment of TB from January 2017

to December 2019 were enrolled. This study assessed the prevalence, clinical

characteristics, treatment, treatment outcomes for fluoroquinolones-associated

tendinopathy, and treatment outcomes of TB. RESULTS: During the study period, 184

participants that were diagnosed with TB and used fluoroquinolones were enrolled

in the study. 34 (18.5%) participants developed tendinopathy. The risk factors

that were associated with fluoroquinolones-associated tendinopathy were younger

age (<60 years) (Odd ratio (OR) 3.61; 95% CI 1.16-11.23), female (OR 3.54; 95% CI

1.58-7.90), and prolonged usage of levofloxacin (>180 days) (OR 2.61; 95%CI

1.12-6.08). All participants who developed tendinopathy received conservative

treatment; the dose of fluoroquinolones was reduced in 9 (26.4%) participants,

fluoroquinolones were discontinued in 7 (20.6%) participants and the rest of the

participants (n = 18; 52.9%) had conservative treatment. After conservative

treatment, 25 (73.5%) participants recovered from tendinopathy. For the TB

treatment, 27 (79.4%) participants in the tendinopathy group completed TB

treatment and none of them experienced treatment failure. On the other hand, 89

(59.3%) participants in the no tendinopathy group had completed their TB

treatment and 3 (2%) of them experienced treatment failure. CONCLUSIONS: The

prevalence of fluoroquinolones-associated tendinopathy was not uncommon, and the

risk of fluoroquinolones-associated tendinopathy was high in young and female

patients. Levofloxacin use was related to an elevated risk of developing

tendinopathy, which was dose- and duration-dependent. Conservative treatment,

reducing the dose or discontinuation of fluoroquinolones successfully improved

the symptoms of tendinopathy. Fluoroquinolones-associated tendinopathy did not

affect the treatment of TB.

CI - © 2023 The Authors. Published by Elsevier Ltd.

FAU - Chongboonwatana, Jirarat

AU - Chongboonwatana J

AD - Department of Medicine, Faculty of Medicine, Chulalongkorn University, and King

Chulalongkorn Memorial Hospital, Thai Red Cross Society, Bangkok, Thailand.

FAU - Terbsiri, Varalee

AU - Terbsiri V

AD - Division of Infectious Diseases, Faculty of Medicine, Chulalongkorn University,

and King Chulalongkorn Memorial Hospital, Thai Red Cross Society, Bangkok,

Thailand.

FAU - Suwanpimolkul, Gompol

AU - Suwanpimolkul G

AD - Division of Infectious Diseases, Faculty of Medicine, Chulalongkorn University,

and King Chulalongkorn Memorial Hospital, Thai Red Cross Society, Bangkok,

Thailand.

AD - Center of Excellence in Tuberculosis, Faculty of Medicine, Chulalongkorn

University, Bangkok, Thailand.

AD - Emerging Infectious Diseases Clinical Center, Thai Red Cross Society, Bangkok,

Thailand.

LA - eng

PT - Journal Article

DEP - 20230920

PL - England

TA - Heliyon

JT - Heliyon

JID - 101672560

PMC - PMC10550594

OTO - NOTNLM

OT - Drug resistant tuberculosis

OT - Fluoroquinolones

OT - Tendinopathy

OT - Tuberculosis

COIS- The authors declare that they have no known competing financial interests or

personal relationships that could have appeared to influence the work reported in

this paper.

EDAT- 2023/10/09 12:42

MHDA- 2023/10/09 12:43

PMCR- 2023/09/20

CRDT- 2023/10/09 06:09

PHST- 2022/12/10 00:00 [received]

PHST- 2023/09/10 00:00 [revised]

PHST- 2023/09/19 00:00 [accepted]

PHST- 2023/10/09 12:43 [medline]

PHST- 2023/10/09 12:42 [pubmed]

PHST- 2023/10/09 06:09 [entrez]

PHST- 2023/09/20 00:00 [pmc-release]

AID - S2405-8440(23)07539-4 [pii]

AID - e20331 [pii]

AID - 10.1016/j.heliyon.2023.e20331 [doi]

PST - epublish

SO - Heliyon. 2023 Sep 20;9(10):e20331. doi: 10.1016/j.heliyon.2023.e20331.

eCollection 2023 Oct.

PMID- 37736177

OWN - NLM

STAT- PubMed-not-MEDLINE

LR - 20230923

IS - 1948-5875 (Print)

IS - 1948-5875 (Electronic)

IS - 1948-5875 (Linking)

VI - 14

IP - 9

DP - 2023 Sep 14

TI - Synthesis and Structure-Activity Relationships of a New Class of Oxadiazoles

Targeting DprE1 as Antitubercular Agents.

PG - 1275-1283

LID - 10.1021/acsmedchemlett.3c00295 [doi]

AB - The continuing prevalence of drug-resistant tuberculosis threatens global TB

control programs, highlighting the need to discover new drug candidates to feed

the drug development pipeline. In this study, we describe a high-throughput

screening hit

(4-benzylpiperidin-1-yl)(1-(5-phenyl-1,3,4-oxadiazol-2-yl)piperidin-4-yl)methanone

(P1) as a potent antitubercular agent. Structure-activity guided synthesis led to

the discovery of several analogs with high in vitro potency. P1 was found to have

promising potency against many drug-resistant strains, as well as

drug-susceptible clinical isolates. It also showed cidality against Mtb growing

in host macrophages. Whole genome sequencing of genomic DNA from resistant

mutants raised to P1 revealed mutations in decaprenylphosphoryl-β-d-ribose

2'-oxidase (DprE1). This novel oxadiazole scaffold expands the set of chemical

tools for targeting a well-validated pathway to treat tuberculosis.

CI - © 2023 The Authors. Published by American Chemical Society.

FAU - Yadav, Veena D

AU - Yadav VD

AD - Tuberculosis Research Section, Laboratory of Clinical Immunology and

Microbiology, National Institute of Allergy and Infectious Diseases (NIAID),

National Institutes of Health (NIH), Bethesda, Maryland 20892, United States.

FAU - Boshoff, Helena I

AU - Boshoff HI

AUID- ORCID: 0000-0002-4333-206X

AD - Tuberculosis Research Section, Laboratory of Clinical Immunology and

Microbiology, National Institute of Allergy and Infectious Diseases (NIAID),

National Institutes of Health (NIH), Bethesda, Maryland 20892, United States.

FAU - Trifonov, Lena

AU - Trifonov L

AD - Tuberculosis Research Section, Laboratory of Clinical Immunology and

Microbiology, National Institute of Allergy and Infectious Diseases (NIAID),

National Institutes of Health (NIH), Bethesda, Maryland 20892, United States.

FAU - Roma, Jose Santinni O

AU - Roma JSO

AD - Tuberculosis Research Section, Laboratory of Clinical Immunology and

Microbiology, National Institute of Allergy and Infectious Diseases (NIAID),

National Institutes of Health (NIH), Bethesda, Maryland 20892, United States.

FAU - Ioerger, Thomas R

AU - Ioerger TR

AD - Department of Computer Science and Engineering, Texas A&M University, College

Station, Texas 77843, United States.

FAU - Barry, Clifton E 3rd

AU - Barry CE 3rd

AD - Tuberculosis Research Section, Laboratory of Clinical Immunology and

Microbiology, National Institute of Allergy and Infectious Diseases (NIAID),

National Institutes of Health (NIH), Bethesda, Maryland 20892, United States.

FAU - Oh, Sangmi

AU - Oh S

AUID- ORCID: 0000-0003-0895-1110

AD - Tuberculosis Research Section, Laboratory of Clinical Immunology and

Microbiology, National Institute of Allergy and Infectious Diseases (NIAID),

National Institutes of Health (NIH), Bethesda, Maryland 20892, United States.

LA - eng

PT - Journal Article

DEP - 20230815

PL - United States

TA - ACS Med Chem Lett

JT - ACS medicinal chemistry letters

JID - 101521073

PMC - PMC10510505

COIS- The authors declare no competing financial interest.

EDAT- 2023/09/22 06:42

MHDA- 2023/09/22 06:43

PMCR- 2023/08/15

CRDT- 2023/09/22 03:40

PHST- 2023/07/05 00:00 [received]

PHST- 2023/08/09 00:00 [accepted]

PHST- 2023/09/22 06:43 [medline]

PHST- 2023/09/22 06:42 [pubmed]

PHST- 2023/09/22 03:40 [entrez]

PHST- 2023/08/15 00:00 [pmc-release]

AID - 10.1021/acsmedchemlett.3c00295 [doi]

PST - epublish

SO - ACS Med Chem Lett. 2023 Aug 15;14(9):1275-1283. doi:

10.1021/acsmedchemlett.3c00295. eCollection 2023 Sep 14.

PMID- 37523915

OWN - NLM

STAT- MEDLINE

DCOM- 20230811

LR - 20230811

IS - 1876-035X (Electronic)

IS - 1876-0341 (Linking)

VI - 16

IP - 9

DP - 2023 Sep

TI - Deciphering the emerging role of phytocompounds: Implications in the management

of drug-resistant tuberculosis and ATDs-induced hepatic damage.

PG - 1443-1459

LID - S1876-0341(23)00250-2 [pii]

LID - 10.1016/j.jiph.2023.07.016 [doi]

AB - Tuberculosis is a disease of poverty, discrimination, and socioeconomic burden.

Epidemiological studies suggest that the mortality and incidence of tuberculosis

are unacceptably higher worldwide. Genomic mutations in embCAB, embR, katG, inhA,

ahpC, rpoB, pncA, rrs, rpsL, gyrA, gyrB, and ethR contribute to drug resistance

reducing the susceptibility of Mycobacterium tuberculosis to many antibiotics.

Additionally, treating tuberculosis with antibiotics also poses a serious risk of

hepatotoxicity in the patient's body. Emerging data on drug-induced liver injury

showed that anti-tuberculosis drugs remarkably altered levels of hepatotoxicity

biomarkers. The review is an attempt to explore the anti-mycobacterial potential

of selected, commonly available, and well-known phytocompounds and extracts of

medicinal plants against strains of Mycobacterium tuberculosis. Many studies have

demonstrated that phytocompounds such as flavonoids, alkaloids, terpenoids, and

phenolic compounds have antibacterial action against Mycobacterium species,

inhibiting the bacteria's growth and replication, and sometimes, causing cell

death. Phytocompounds act by disrupting bacterial cell walls and membranes,

reducing enzyme activity, and interfering with essential metabolic processes. The

combination of these processes reduces the overall survivability of the bacteria.

Moreover, several phytochemicals have synergistic effects with antibiotics

routinely used to treat TB, improving their efficacy and decreasing the risk of

resistance development. Interestingly, phytocompounds have been presented to

reduce isoniazid- and ethambutol-induced hepatotoxicity by reversing serum levels

of AST, ALP, ALT, bilirubin, MDA, urea, creatinine, and albumin to their normal

range, leading to attenuation of inflammation and hepatic necrosis. As a result,

phytochemicals represent a promising field of research for the development of new

TB medicines.

CI - Copyright © 2023 The Authors. Published by Elsevier Ltd.. All rights reserved.

FAU - Ansari, Mohammad Azam

AU - Ansari MA

AD - Department of Epidemic Disease Research, Institute for Research and Medical

Consultations (IRMC), Imam Abdulrahman Bin Faisal University, 31441 Dammam, Saudi

Arabia. Electronic address: maansari@iau.edu.sa.

FAU - Shoaib, Shoaib

AU - Shoaib S

AD - Department Biochemistry, Faculty of Medicine, Aligarh Muslim University, Aligarh,

Uttar Pradesh 202002, India.

FAU - Alomary, Mohammad N

AU - Alomary MN

AD - Advanced Diagnostic and Therapeutic Institute, King Abdulaziz City for Science

and Technology (KACST), Riyadh 11442, Saudi Arabia.

FAU - Ather, Hissana

AU - Ather H

AD - Department of Pharmaceutical Chemistry, College of Pharmacy, King Khalid

University, Abha 62529, Saudi Arabia.

FAU - Ansari, Shaik Mohammad Asif

AU - Ansari SMA

AD - Department of Clinical Pharmacy, College of Pharmacy, King Khalid University,

Abha 62529, Saudi Arabia.

FAU - Hani, Umme

AU - Hani U

AD - Department of Pharmaceutics, College of Pharmacy, King Khalid University, Abha

62529, Saudi Arabia.

FAU - Jamous, Yahya F

AU - Jamous YF

AD - Vaccine and Bioprocessing Center, King Abdulaziz City for Science and Technology

(KACST), Riyadh 11442, Saudi Arabia.

FAU - Alyahya, Sami A

AU - Alyahya SA

AD - Wellness and Preventive Medicine Institute, King Abdulaziz City for Science and

Technology (KACST), Riyadh 11442, Saudi Arabia.

FAU - Alharbi, Jameela Naif

AU - Alharbi JN

AD - Department of Epidemic Disease Research, Institute for Research and Medical

Consultations (IRMC), Imam Abdulrahman Bin Faisal University, 31441 Dammam, Saudi

Arabia.

FAU - Imran, Mohammad Azhar

AU - Imran MA

AD - Department of Internal Medicine, Gangnam Severance Hospital, Yonsei University

College of Medicine, Seoul 120752, Republic of Korea.

FAU - Wahab, Shadma

AU - Wahab S

AD - Department of Pharmacognosy, College of Pharmacy, King Khalid University, Abha

61421, Saudi Arabia.

FAU - Ahmad, Wasim

AU - Ahmad W

AD - Department of Pharmacy, Mohammed Al-Mana College for Medical Sciences, Dammam

34222, Saudi Arabia.

FAU - Islam, Najmul

AU - Islam N

AD - Department Biochemistry, Faculty of Medicine, Aligarh Muslim University, Aligarh,

Uttar Pradesh 202002, India. Electronic address: nxi7@hotmail.com.

LA - eng

PT - Journal Article

PT - Review

DEP - 20230726

PL - England

TA - J Infect Public Health

JT - Journal of infection and public health

JID - 101487384

RN - 0 (Bacterial Proteins)

RN - 0 (Antitubercular Agents)

RN - V83O1VOZ8L (Isoniazid)

SB - IM

MH - Humans

MH - Bacterial Proteins/genetics

MH - *Tuberculosis, Multidrug-Resistant/microbiology

MH - Antitubercular Agents/adverse effects

MH - *Mycobacterium tuberculosis/genetics

MH - *Tuberculosis/drug therapy/microbiology

MH - Isoniazid/pharmacology

MH - Mutation

MH - *Liver Diseases

MH - *Chemical and Drug Induced Liver Injury/drug therapy

MH - Microbial Sensitivity Tests

MH - Drug Resistance, Multiple, Bacterial/genetics

OTO - NOTNLM

OT - Hepatoprotection

OT - Multi drug resistance

OT - Mycobacterium tuberculosis

OT - Phytocompounds

OT - Prevention

OT - Synergism

COIS- Declaration of Competing Interest The authors declare no competing interests.

EDAT- 2023/08/01 01:08

MHDA- 2023/08/11 06:43

CRDT- 2023/07/31 18:06

PHST- 2022/12/12 00:00 [received]

PHST- 2023/05/05 00:00 [revised]

PHST- 2023/07/22 00:00 [accepted]

PHST- 2023/08/11 06:43 [medline]

PHST- 2023/08/01 01:08 [pubmed]

PHST- 2023/07/31 18:06 [entrez]

AID - S1876-0341(23)00250-2 [pii]

AID - 10.1016/j.jiph.2023.07.016 [doi]

PST - ppublish

SO - J Infect Public Health. 2023 Sep;16(9):1443-1459. doi:

10.1016/j.jiph.2023.07.016. Epub 2023 Jul 26.

PMID- 37382506

OWN - NLM

STAT- MEDLINE

DCOM- 20230904

LR - 20230906

IS - 2150-7511 (Electronic)

VI - 14

IP - 4

DP - 2023 Aug 31

TI - Berbamine promotes macrophage autophagy to clear Mycobacterium tuberculosis by

regulating the ROS/Ca(2+) axis.

PG - e0027223

LID - 10.1128/mbio.00272-23 [doi]

LID - e00272-23

AB - Drug-resistant tuberculosis (TB) poses a major threat to global TB control;

consequently, there is an urgent need to develop novel anti-TB drugs or

strategies. Host-directed therapy (HDT) is emerging as an effective treatment

strategy, especially for drug-resistant TB. This study evaluated the effects of

berbamine (BBM), a bisbenzylisoquinoline alkaloid, on mycobacterial growth in

macrophages. BBM inhibited intracellular Mycobacterium tuberculosis (Mtb) growth

by promoting autophagy and silencing ATG5, partially abolishing the inhibitory

effect. In addition, BBM increased intracellular reactive oxygen species (ROS),

while the antioxidant N-acetyl-L-cysteine (NAC) abolished BBM-induced autophagy

and the ability to inhibit Mtb survival. Furthermore, the increased intracellular

Ca(2+) concentration induced by BBM was regulated by ROS, and BAPTA-AM, an

intracellular Ca(2+)-chelating agent, could block ROS-mediated autophagy and Mtb

clearance. Finally, BBM could inhibit the survival of drug-resistant Mtb.

Collectively, these findings provide evidence that BBM, a Food and Drug

Administration (FDA)-approved drug, could effectively clear drug-sensitive and

-resistant Mtb through regulating ROS/Ca(2+) axis-mediated autophagy and has

potential as an HDT candidate for TB therapy. IMPORTANCE It is urgent to develop

novel treatment strategies against drug-resistant TB, and HDT provides a

promising approach to fight drug-resistant TB by repurposing old drugs. Our

studies demonstrate, for the first time, that BBM, an FDA-approved drug, not only

potently inhibits intracellular drug-sensitive Mtb growth but also restricts

drug-resistant Mtb by promoting macrophage autophagy. Mechanistically, BBM

activates macrophage autophagy by regulating the ROS/Ca(2+) axis. In conclusion,

BBM could be considered as an HDT candidate and may contribute to improving the

outcomes or shortening the treatment course of drug-resistant TB.

FAU - Zhang, Su

AU - Zhang S

AD - National Clinical Research Center for Infectious Diseases, Guangdong Provincial

Clinical Research Center for Tuberculosis, Shenzhen Third People's Hospital,

Southern University of Science and Technology , Shenzhen, China.

FAU - Zhou, Xuefeng

AU - Zhou X

AD - Guangzhou Medical University , Guangzhou, China.

FAU - Ou, Min

AU - Ou M

AD - National Clinical Research Center for Infectious Diseases, Guangdong Provincial

Clinical Research Center for Tuberculosis, Shenzhen Third People's Hospital,

Southern University of Science and Technology , Shenzhen, China.

FAU - Fu, Xiangdong

AU - Fu X

AD - National Clinical Research Center for Infectious Diseases, Guangdong Provincial

Clinical Research Center for Tuberculosis, Shenzhen Third People's Hospital,

Southern University of Science and Technology , Shenzhen, China.

FAU - Lin, Qiao

AU - Lin Q

AD - Department of Clinical Laboratory, The Baoan People's Hospital of Shenzhen, The

Second Affiliated Hospital of Shenzhen University , Shenzhen, China.

FAU - Tao, Xiaoyu

AU - Tao X

AD - National Clinical Research Center for Infectious Diseases, Guangdong Provincial

Clinical Research Center for Tuberculosis, Shenzhen Third People's Hospital,

Southern University of Science and Technology , Shenzhen, China.

FAU - Wang, Zhaoqin

AU - Wang Z

AD - National Clinical Research Center for Infectious Diseases, Guangdong Provincial

Clinical Research Center for Tuberculosis, Shenzhen Third People's Hospital,

Southern University of Science and Technology , Shenzhen, China.

FAU - Liu, Aimei

AU - Liu A

AD - Department of Tuberculosis, Guangxi Chest Hospital , Liuzhou, China.

FAU - Li, Guobao

AU - Li G

AD - National Clinical Research Center for Infectious Diseases, Guangdong Provincial

Clinical Research Center for Tuberculosis, Shenzhen Third People's Hospital,

Southern University of Science and Technology , Shenzhen, China.

FAU - Xu, Yuzhong

AU - Xu Y

AD - Department of Clinical Laboratory, The Baoan People's Hospital of Shenzhen, The

Second Affiliated Hospital of Shenzhen University , Shenzhen, China.

FAU - Zhang, Guoliang

AU - Zhang G

AUID- ORCID: 0000-0002-3617-5449

AD - National Clinical Research Center for Infectious Diseases, Guangdong Provincial

Clinical Research Center for Tuberculosis, Shenzhen Third People's Hospital,

Southern University of Science and Technology , Shenzhen, China.

LA - eng

PT - Journal Article

PT - Research Support, Non-U.S. Gov't

DEP - 20230629

PL - United States

TA - mBio

JT - mBio

JID - 101519231

RN - V5KM4XJ0WM (berbamine)

RN - 0 (Reactive Oxygen Species)

RN - 0 (Benzylisoquinolines)

SB - IM

MH - Humans

MH - *Mycobacterium tuberculosis

MH - Reactive Oxygen Species

MH - Macrophages/microbiology

MH - *Benzylisoquinolines/pharmacology

MH - Autophagy

MH - *Tuberculosis, Multidrug-Resistant

PMC - PMC10470588

OTO - NOTNLM

OT - Mycobacterium tuberculosis

OT - ROS

OT - autophagy

OT - berbamine

OT - calcium

COIS- The authors declare no conflict of interest.

EDAT- 2023/06/29 13:42

MHDA- 2023/09/04 06:42

PMCR- 2023/06/29

CRDT- 2023/06/29 09:53

PHST- 2023/09/04 06:42 [medline]

PHST- 2023/06/29 13:42 [pubmed]

PHST- 2023/06/29 09:53 [entrez]

PHST- 2023/06/29 00:00 [pmc-release]

AID - 00272-23 [pii]

AID - mbio.00272-23 [pii]

AID - 10.1128/mbio.00272-23 [doi]

PST - ppublish

SO - mBio. 2023 Aug 31;14(4):e0027223. doi: 10.1128/mbio.00272-23. Epub 2023 Jun 29.

PMID- 37455004

OWN - NLM

STAT- MEDLINE

DCOM- 20230831

LR - 20230831

IS - 1879-0003 (Electronic)

IS - 0141-8130 (Linking)

VI - 247

DP - 2023 Aug 30

TI - Cell wall and immune modulation by Rv1800 (PPE28) helps M. smegmatis to evade

intracellular killing.

PG - 125837

LID - S0141-8130(23)02732-0 [pii]

LID - 10.1016/j.ijbiomac.2023.125837 [doi]

AB - Rv1800 is predicted as PPE family protein found in pathogenic mycobacteria only.

Under acidic stress, the rv1800 gene was expressed in M. tuberculosis H37Ra.

In-silico study showed lipase/esterase activity in C-terminus PE-PPE domain

having pentapeptide motif with catalytic Ser-Asp-His residue. Full-length Rv1800

and C-terminus PE-PPE domain proteins showed esterase activity with pNP-C4 at the

optimum temperature of 40 °C and pH 8.0. However, the N-terminus PPE domain

showed no esterase activity, but involved in thermostability of Rv1800

full-length protein. M. smegmatis expressing rv1800 (MS_Rv1800) showed altered

colony morphology and a significant resistance to numerous environmental

stresses, antibiotics and higher lipid content. In extracellular and membrane

fraction, Rv1800 protein was detected, while C terminus PE-PPE was present in

cytoplasm, suggesting the role of N-terminus PPE domain in transportation of

protein. MS_Rv1800 infected macrophage showed higher intracellular survival and

low production of ROS, NO and expression levels of iNOS and pro-inflammatory

cytokines, while induced expression of the anti-inflammatory cytokines. The

Rv1800, PPE and PE-PPE showed antibody-mediated immunity in MDR-TB and PTB

patients. Overall, these results confirmed the esterase activity in the

C-terminus and function of N-terminus in thermostabilization and transportation;

predicting the role of Rv1800 in immune/lipid modulation to support intracellular

mycobacterium survival.

CI - Copyright © 2023. Published by Elsevier B.V.

FAU - Anand, Pradeep Kumar

AU - Anand PK

AD - Department of Biotechnology, BMS Block-1, South Campus, Panjab University,

Chandigarh, 160014, India.

FAU - Saini, Varinder

AU - Saini V

AD - Department of Pulmonary Medicine, Government Medical College and Hospital,

Chandigarh, India.

FAU - Kaur, Jasbinder

AU - Kaur J

AD - Department of Biochemistry, Government Medical College and Hospital, Chandigarh,

India.

FAU - Kumar, Arbind

AU - Kumar A

AD - Department of Biotechnology, BMS Block-1, South Campus, Panjab University,

Chandigarh, 160014, India.

FAU - Kaur, Jagdeep

AU - Kaur J

AD - Department of Biotechnology, BMS Block-1, South Campus, Panjab University,

Chandigarh, 160014, India. Electronic address: jagsekhon@pu.ac.in.

LA - eng

PT - Journal Article

DEP - 20230716

PL - Netherlands

TA - Int J Biol Macromol

JT - International journal of biological macromolecules

JID - 7909578

RN - EC 3.1.- (Esterases)

RN - EC 3.1.1.3 (Lipase)

RN - 0 (Cytokines)

RN - 0 (Lipids)

SB - IM

MH - Humans

MH - *Esterases/chemistry

MH - Lipase/chemistry

MH - *Mycobacterium tuberculosis/genetics/metabolism

MH - Cytokines/metabolism

MH - Cell Wall/metabolism

MH - Lipids

OTO - NOTNLM

OT - Cell wall modulation

OT - Immune modulation

OT - Infection

OT - Intracellular survival

OT - Mycobacterium tuberculosis

COIS- Declaration of competing interest The authors declare no conflict of interest

regarding the publication of this article.

EDAT- 2023/07/17 00:42

MHDA- 2023/08/31 06:41

CRDT- 2023/07/16 19:29

PHST- 2023/04/03 00:00 [received]

PHST- 2023/06/27 00:00 [revised]

PHST- 2023/07/13 00:00 [accepted]

PHST- 2023/08/31 06:41 [medline]

PHST- 2023/07/17 00:42 [pubmed]

PHST- 2023/07/16 19:29 [entrez]

AID - S0141-8130(23)02732-0 [pii]

AID - 10.1016/j.ijbiomac.2023.125837 [doi]

PST - ppublish

SO - Int J Biol Macromol. 2023 Aug 30;247:125837. doi: 10.1016/j.ijbiomac.2023.125837.

Epub 2023 Jul 16.

PMID- 37590225

OWN - NLM

STAT- MEDLINE

DCOM- 20230821

LR - 20230823

IS - 1932-6203 (Electronic)

IS - 1932-6203 (Linking)

VI - 18

IP - 8

DP - 2023

TI - Vulnerability and tuberculosis treatment outcomes in urban settings in England: A

mixed-methods study.

PG - e0281918

LID - 10.1371/journal.pone.0281918 [doi]

LID - e0281918

AB - BACKGROUND: Evidence on factors contributing to poor treatment outcome and

healthcare priorities in vulnerable populations affected by tuberculosis (TB) in

urban areas of England other than London is needed to inform setting-specific

prevention and care policies. We addressed this knowledge gap in a cohort of TB

patients and healthcare providers in Birmingham and Leicester, UK. METHODS: A

mixed-methods study was performed. Logistic regression was used to identify TB

patients more likely to have poor treatment outcomes according to clinical and

demographic characteristics and social risk factors (SRFs) in a 2013-18 cohort.

25 semi-structured interviews were undertaken in purposely selected individuals

(9 patients and 16 healthcare professionals) to glean insights on their

healthcare priorities and the factors that contribute to poor treatment outcome.

RESULTS: The quantitative cohort comprised 2252 patients. Those who were ≥ 55

years of age, foreign-born from Central Europe, East Asia and Sub Saharan Africa

and with MDR-TB were more likely to have poor treatment outcomes. According to

patients and healthcare professionals, the factors that contribute to

vulnerability to develop TB and poor treatment outcomes include poor working and

living conditions, inadequate or absent welfare protection, poor primary

healthcare responsiveness, treatment duration and side effects. These factors

could be addressed by increased networking, partnership and integration between

healthcare and social services and better integration between primary and

secondary healthcare. CONCLUSIONS: In both cities, being ≥ 55 years of age,

having MDR-TB and being of foreign-birth are predictors of unfavourable treatment

outcome. Risk of poor treatment outcome and vulnerability seem to be

multidimensional. A better understanding of specific vulnerabilities and how they

affect patient care pathway is needed to design adequate support programmes.

CI - Copyright: © 2023 Berrocal-Almanza et al. This is an open access article

distributed under the terms of the Creative Commons Attribution License, which

permits unrestricted use, distribution, and reproduction in any medium, provided

the original author and source are credited.

FAU - Berrocal-Almanza, Luis C

AU - Berrocal-Almanza LC

AUID- ORCID: 0000-0003-1634-9971

AD - National Heart and Lung Institute, NIHR Health Protection Research Unit in

Respiratory Infections, Imperial College London, London, United Kingdom.

FAU - Lima, Marcela

AU - Lima M

AD - National Heart and Lung Institute, NIHR Health Protection Research Unit in

Respiratory Infections, Imperial College London, London, United Kingdom.

FAU - Piotrowski, Helen

AU - Piotrowski H

AD - National Heart and Lung Institute, NIHR Health Protection Research Unit in

Respiratory Infections, Imperial College London, London, United Kingdom.

FAU - Botticello, Julie

AU - Botticello J

AD - Department of Allied and Public Health, School of Health, Sport and Bioscience,

University of East London, London, United Kingdom.

FAU - Badhan, Amarjit

AU - Badhan A

AD - National Heart and Lung Institute, NIHR Health Protection Research Unit in

Respiratory Infections, Imperial College London, London, United Kingdom.

FAU - Karnani, Nisha

AU - Karnani N

AD - National Heart and Lung Institute, NIHR Health Protection Research Unit in

Respiratory Infections, Imperial College London, London, United Kingdom.

FAU - Kaur, Hanna

AU - Kaur H

AD - Birmingham & Solihull TB Service, University Hospitals Birmingham NHS Foundation

Trust, Birmingham, United Kingdom.

FAU - Pareek, Manish

AU - Pareek M

AD - Department of Respiratory Sciences, University of Leicester, Leicester, United

Kingdom.

FAU - Haldar, Pranabashis

AU - Haldar P

AUID- ORCID: 0000-0002-1572-5421

AD - Department of Respiratory Sciences, Institute for Lung Health, Respiratory

Biomedical Research Centre, University of Leicester, Leicester, United Kingdom.

FAU - Dedicoat, Martin

AU - Dedicoat M

AD - Birmingham & Solihull TB Service, University Hospitals Birmingham NHS Foundation

Trust, Birmingham, United Kingdom.

FAU - Kon, Onn Min

AU - Kon OM

AD - National Heart and Lung Institute, NIHR Health Protection Research Unit in

Respiratory Infections, Imperial College London, London, United Kingdom.

AD - St Mary's Hospital, Imperial Healthcare NHS Trust, London, United Kingdom.

FAU - Zenner, Dominik

AU - Zenner D

AD - National Heart and Lung Institute, NIHR Health Protection Research Unit in

Respiratory Infections, Imperial College London, London, United Kingdom.

AD - Wolfson Institute for Population Health, Queen Mary University, London, United

Kingdom.

FAU - Lalvani, Ajit

AU - Lalvani A

AD - National Heart and Lung Institute, NIHR Health Protection Research Unit in

Respiratory Infections, Imperial College London, London, United Kingdom.

AD - National Heart and Lung Institute, Tuberculosis Research Centre, Respiratory

Medicine, Imperial College London, London, United Kingdom.

LA - eng

GR - MR/V027549/1/MRC_/Medical Research Council/United Kingdom

PT - Journal Article

PT - Research Support, Non-U.S. Gov't

DEP - 20230817

PL - United States

TA - PLoS One

JT - PloS one

JID - 101285081

SB - IM

MH - Humans

MH - *Tuberculosis/drug therapy

MH - Treatment Outcome

MH - England

MH - Duration of Therapy

MH - *Tuberculosis, Multidrug-Resistant

PMC - PMC10434856

COIS- The authors have declared that no competing interests exist.

EDAT- 2023/08/17 18:42

MHDA- 2023/08/21 06:42

PMCR- 2023/08/17

CRDT- 2023/08/17 13:35

PHST- 2022/10/17 00:00 [received]

PHST- 2023/02/05 00:00 [accepted]

PHST- 2023/08/21 06:42 [medline]

PHST- 2023/08/17 18:42 [pubmed]

PHST- 2023/08/17 13:35 [entrez]

PHST- 2023/08/17 00:00 [pmc-release]

AID - PONE-D-22-28286 [pii]

AID - 10.1371/journal.pone.0281918 [doi]

PST - epublish

SO - PLoS One. 2023 Aug 17;18(8):e0281918. doi: 10.1371/journal.pone.0281918.

eCollection 2023.

PMID- 37310268

OWN - NLM

STAT- MEDLINE

DCOM- 20230821

LR - 20260127

IS - 2165-0497 (Electronic)

IS - 2165-0497 (Linking)

VI - 11

IP - 4

DP - 2023 Aug 17

TI - Effects of Bedaquiline Combined with Fluoroquinolone and/or Clofazimine on QT

Interval in Patients with Multidrug-Resistant Tuberculosis: a Retrospective

Study.

PG - e0104823

LID - 10.1128/spectrum.01048-23 [doi]

LID - e01048-23

AB - With the application of bedaquiline (Bdq), the success rate of

multidrug-resistant tuberculosis (MDR-TB) treatment has been significantly

improved; however, the cardiac safety of the patients during treatment cannot be

ignored. Hence, this study compared the effects of bedaquiline alone and

bedaquiline combined with fluoroquinolones (FQs) and/or clofazimine (CFZ) on the

QT interval. This single-center retrospective cohort study analyzed the clinical

data of MDR-TB patients treated with bedaquiline for 24 weeks from January 2020

to May 2021 in Xi'an Chest Hospital and compared the changes in QTcF between the

two groups. Eighty-five patients were included in the study and grouped by types

of anti-TB drugs affecting the QT interval they used. Group A included

bedaquiline (n = 33), and group B included bedaquiline in combination with

fluoroquinolones and/or clofazimine (n = 52). Out of patients with available

corrected QT interval by Fridericia's formula (QTcF) data, 2.4% (2/85)

experienced a postbaseline QTcF of ≥500 ms, and 24.7% (21/85) had at least one

change of QTcF of ≥60 ms from baseline. In group A, 9.1% (3/33) had at least one

ΔQTcF of >60 ms, as did 34.6% (18/52) of group B. Multivariate Cox regression

analysis showed that the adjusted risk of QT prolongation was 4.82 times higher

in group B (95% confidence interval [CI], 1.406 to 16.488). Bedaquiline combined

with other anti-TB drugs affecting QT interval significantly increased the

incidence of grade 3 or 4 QT prolongation; however, no serious ventricular

arrhythmia and permanent drug withdrawal occurred. The use of bedaquiline

combined with fluoroquinolone and/or clofazimine is an independent risk factor

affecting QT interval. IMPORTANCE Tuberculosis (TB) is a chronic infectious

disease caused by Mycobacterium tuberculosis. The emergence of MDR-TB is caused

by an organism that is resistant to at least isoniazid and rifampin and is

currently considered the major challenge for the global control of TB.

Bedaquiline is the first new TB drug in 50 years with a unique mechanism of

action, strong anti-M. tuberculosis activity. Yet unexplained excess deaths in

the bedaquiline arms have been found in some phase II clinical trials; thus, the

FDA has issued a "boxed warning." However, the cardiac safety of the patients

during treatment cannot be ignored. Accordingly, further investigations are

needed to establish whether bedaquiline combined with clofazimine,

fluoroquinolones, or anti-TB drugs affecting the QT interval in a long-course or

short-course treatment increases the risk of QT prolongation.

FAU - Li, Rong

AU - Li R

AD - Department of Drug-resistance tuberculosis, Xi'an Chest Hospital, Xi'an, China.

FAU - Ma, Jin-Bao

AU - Ma JB

AD - Department of Drug-resistance tuberculosis, Xi'an Chest Hospital, Xi'an, China.

FAU - Yang, Hong

AU - Yang H

AD - Department of Drug-resistance tuberculosis, Xi'an Chest Hospital, Xi'an, China.

FAU - Yang, Han

AU - Yang H

AD - Medical Transformation Center of Xi'an Chest Hospital, Xi'an, China.

FAU - Yang, Xin-Jun

AU - Yang XJ

AD - Department of Drug-resistance tuberculosis, Xi'an Chest Hospital, Xi'an, China.

FAU - Wu, Yan-Qin

AU - Wu YQ

AD - Department of Drug-resistance tuberculosis, Xi'an Chest Hospital, Xi'an, China.

FAU - Ren, Fei

AU - Ren F

AUID- ORCID: 0000-0001-7308-686X

AD - Department of Drug-resistance tuberculosis, Xi'an Chest Hospital, Xi'an, China.

LA - eng

PT - Journal Article

PT - Research Support, Non-U.S. Gov't

DEP - 20230613

PL - United States

TA - Microbiol Spectr

JT - Microbiology spectrum

JID - 101634614

RN - D959AE5USF (Clofazimine)

RN - 0 (Antitubercular Agents)

RN - 0 (Fluoroquinolones)

RN - 78846I289Y (bedaquiline)

RN - 0 (Diarylquinolines)

SB - IM

MH - Humans

MH - Clofazimine/adverse effects

MH - Antitubercular Agents/adverse effects

MH - Retrospective Studies

MH - Fluoroquinolones/adverse effects

MH - *Tuberculosis, Multidrug-Resistant/drug therapy

MH - *Long QT Syndrome/chemically induced/drug therapy

MH - Diarylquinolines

PMC - PMC10434111

OTO - NOTNLM

OT - bedaquiline

OT - drug toxicity

OT - electrocardiography

OT - multidrug resistance

OT - pulmonary

OT - tuberculosis

COIS- The authors declare no conflict of interest.

EDAT- 2023/06/13 13:12

MHDA- 2023/08/21 06:43

PMCR- 2023/06/13

CRDT- 2023/06/13 10:13

PHST- 2023/08/21 06:43 [medline]

PHST- 2023/06/13 13:12 [pubmed]

PHST- 2023/06/13 10:13 [entrez]

PHST- 2023/06/13 00:00 [pmc-release]

AID - 01048-23 [pii]

AID - spectrum.01048-23 [pii]

AID - 10.1128/spectrum.01048-23 [doi]

PST - ppublish

SO - Microbiol Spectr. 2023 Aug 17;11(4):e0104823. doi: 10.1128/spectrum.01048-23.

Epub 2023 Jun 13.

PMID- 37306591

OWN - NLM

STAT- MEDLINE

DCOM- 20230821

LR - 20230830

IS - 2165-0497 (Electronic)

IS - 2165-0497 (Linking)

VI - 11

IP - 4

DP - 2023 Aug 17

TI - Effect of Stenotrophomonas maltophilia on Tuberculosis.

PG - e0094423

LID - 10.1128/spectrum.00944-23 [doi]

LID - e00944-23

AB - Tuberculosis (TB) is an important infectious disease suffered by many countries,

including China. In this stage, accurate diagnosis and treatment are key measures

for the prevention and control of TB. Stenotrophomonas maltophilia is a global

emerging Gram-negative, multidrug-resistant (MDR) organism characterized by its

high contribution to the increase in crude mortality rates. By single cell

preparation and strain identification, we isolated S. maltophilia from stored

cultures of Mycobacterium tuberculosis (Mtb). We found that S. maltophilia could

not be removed from sputum by alkali treatment or inhibited by antibiotic mixture

added to MGIT 960 indicator tubes. When co-cultured with Mtb on a

Löwenstein-Jensen (L-J) slant, it could inhibit the growth of Mtb and liquefy the

medium. More seriously, it was resistant to 10 of the 12 anti-TB drugs, including

isoniazid and rifampin, and made the mixed samples display multidrug-resistant

Mtb (MDR-TB) results in the drug sensitivity test, which might change a treatment

regimen and increase disease burden. Following, we conducted a small-scale

surveillance which showed that the isolation rate of S. maltophilia in TB

patients was 6.74%, but these patients had no special characteristics and the

presence of S. maltophilia was hidden. The effect of S. maltophilus on TB and its

mechanism are unclear and require more attention. IMPORTANCE China is a

high-burden country for tuberculosis (TB),

multidrug-resistant/rifampicin-resistant tuberculosis (MDR/RR-TB), and

HIV-associated TB. Increasing the positive rate of culture and the accuracy of

antibiotic susceptibility testing (AST) are important for diagnosis, treatment,

and control of TB. In our study, we found that the isolation rate of

Stenotrophomonas maltophilia in TB patients was not neglectable and that this

bacterium affects the isolation and AST results of TB. Due to a lack of relevant

research, the impact of S. maltophilia on the course and outcome of TB is

unclear. However, the characteristics of S. maltophilia that increase disease

mortality require attention. Therefore, in the clinical testing of TB, in

addition to mycobacteria, it is recommended to increase the detection of

co-infected bacteria and improve the awareness of TB clinicians of these

bacteria.

FAU - Li, Yue

AU - Li Y

AD - State Key Laboratory for Infectious Disease Prevention and Control, National

Institute for Communicable Disease Control and Prevention, Chinese Centre for

Disease Control and Prevention, Beijing, China.

FAU - Zhao, Ailan

AU - Zhao A

AD - Tuberculosis Clinic, Chaoyang District Centre for Disease Control and Prevention,

Beijing, China.

FAU - Yu, Qin

AU - Yu Q

AD - Tuberculosis Clinic, Chaoyang District Centre for Disease Control and Prevention,

Beijing, China.

FAU - Yu, Nan

AU - Yu N

AD - Tuberculosis Clinic, Chaoyang District Centre for Disease Control and Prevention,

Beijing, China.

FAU - Cui, Yao

AU - Cui Y

AD - State Key Laboratory for Infectious Disease Prevention and Control, National

Institute for Communicable Disease Control and Prevention, Chinese Centre for

Disease Control and Prevention, Beijing, China.

FAU - Ma, Xiaohan

AU - Ma X

AD - State Key Laboratory for Infectious Disease Prevention and Control, National

Institute for Communicable Disease Control and Prevention, Chinese Centre for

Disease Control and Prevention, Beijing, China.

FAU - Liu, Haican

AU - Liu H

AD - State Key Laboratory for Infectious Disease Prevention and Control, National

Institute for Communicable Disease Control and Prevention, Chinese Centre for

Disease Control and Prevention, Beijing, China.

FAU - Wang, Ruibai

AU - Wang R

AUID- ORCID: 0000-0002-2258-3599

AD - State Key Laboratory for Infectious Disease Prevention and Control, National

Institute for Communicable Disease Control and Prevention, Chinese Centre for

Disease Control and Prevention, Beijing, China.

LA - eng

PT - Journal Article

DEP - 20230612

PL - United States

TA - Microbiol Spectr

JT - Microbiology spectrum

JID - 101634614

RN - 0 (Antitubercular Agents)

RN - VJT6J7R4TR (Rifampin)

SB - IM

MH - Humans

MH - *Stenotrophomonas maltophilia

MH - Antitubercular Agents/pharmacology/therapeutic use

MH - *Tuberculosis/drug therapy

MH - *Mycobacterium tuberculosis

MH - Rifampin/pharmacology

MH - *Tuberculosis, Multidrug-Resistant/diagnosis/drug therapy/epidemiology

MH - Microbial Sensitivity Tests

PMC - PMC10433947

OTO - NOTNLM

OT - Stenotrophomonas maltophilia

OT - co-infection

OT - decay

OT - multidrug-resistant

OT - tuberculosis

COIS- The authors declare no conflict of interest.

EDAT- 2023/06/12 13:06

MHDA- 2023/08/21 06:42

PMCR- 2023/06/12

CRDT- 2023/06/12 10:07

PHST- 2023/08/21 06:42 [medline]

PHST- 2023/06/12 13:06 [pubmed]

PHST- 2023/06/12 10:07 [entrez]

PHST- 2023/06/12 00:00 [pmc-release]

AID - 00944-23 [pii]

AID - spectrum.00944-23 [pii]

AID - 10.1128/spectrum.00944-23 [doi]

PST - ppublish

SO - Microbiol Spectr. 2023 Aug 17;11(4):e0094423. doi: 10.1128/spectrum.00944-23.

Epub 2023 Jun 12.

PMID- 37377118

OWN - NLM

STAT- MEDLINE

DCOM- 20230803

LR - 20260127

IS - 1460-2075 (Electronic)

IS - 0261-4189 (Print)

IS - 0261-4189 (Linking)

VI - 42

IP - 15

DP - 2023 Aug 1

TI - Mechanism of mycobacterial ATP synthase inhibition by squaramides and second

generation diarylquinolines.

PG - e113687

LID - 10.15252/embj.2023113687 [doi]

LID - e113687

AB - Mycobacteria, such as Mycobacterium tuberculosis, depend on the activity of

adenosine triphosphate (ATP) synthase for growth. The diarylquinoline bedaquiline

(BDQ), a mycobacterial ATP synthase inhibitor, is an important medication for

treatment of drug-resistant tuberculosis but suffers from off-target effects and

is susceptible to resistance mutations. Consequently, both new and improved

mycobacterial ATP synthase inhibitors are needed. We used electron cryomicroscopy

and biochemical assays to study the interaction of Mycobacterium smegmatis ATP

synthase with the second generation diarylquinoline TBAJ-876 and the squaramide

inhibitor SQ31f. The aryl groups of TBAJ-876 improve binding compared with BDQ,

while SQ31f, which blocks ATP synthesis ~10 times more potently than ATP

hydrolysis, binds a previously unknown site in the enzyme's proton-conducting

channel. Remarkably, BDQ, TBAJ-876, and SQ31f all induce similar conformational

changes in ATP synthase, suggesting that the resulting conformation is

particularly suited for drug binding. Further, high concentrations of the

diarylquinolines uncouple the transmembrane proton motive force while for SQ31f

they do not, which may explain why high concentrations of diarylquinolines, but

not SQ31f, have been reported to kill mycobacteria.

CI - © 2023 The Authors.

FAU - Courbon, Gautier M

AU - Courbon GM

AD - Molecular Medicine Program, The Hospital for Sick Children, Toronto, ON, Canada.

AD - Department of Medical Biophysics, The University of Toronto, Toronto, ON, Canada.

FAU - Palme, Paul R

AU - Palme PR

AD - Institut für Pharmazie, Martin-Luther-Universität Halle-Wittenberg, Halle, Saale,

Germany.

FAU - Mann, Lea

AU - Mann L

AD - Institut für Pharmazie, Martin-Luther-Universität Halle-Wittenberg, Halle, Saale,

Germany.

FAU - Richter, Adrian

AU - Richter A

AD - Institut für Pharmazie, Martin-Luther-Universität Halle-Wittenberg, Halle, Saale,

Germany.

FAU - Imming, Peter

AU - Imming P

AUID- ORCID: 0000-0003-2178-3887

AD - Institut für Pharmazie, Martin-Luther-Universität Halle-Wittenberg, Halle, Saale,

Germany.

FAU - Rubinstein, John L

AU - Rubinstein JL

AUID- ORCID: 0000-0003-0566-2209

AD - Molecular Medicine Program, The Hospital for Sick Children, Toronto, ON, Canada.

AD - Department of Medical Biophysics, The University of Toronto, Toronto, ON, Canada.

AD - Department of Biochemistry, The University of Toronto, Toronto, ON, Canada.

LA - eng

SI - PDB/8G07

SI - PDB/8G08

SI - PDB/8G09

SI - PDB/8G0A

SI - PDB/8G0B

SI - PDB/8G0C

SI - PDB/8G0D

SI - PDB/8G0E

GR - PJT162186/CIHR/Canada

PT - Journal Article

PT - Research Support, Non-U.S. Gov't

DEP - 20230628

PL - England

TA - EMBO J

JT - The EMBO journal

JID - 8208664

RN - 0 (Diarylquinolines)

RN - 0 (Antitubercular Agents)

RN - 8L70Q75FXE (Adenosine Triphosphate)

RN - 0 (squaramide)

RN - A7V27PHC7A (Quinine)

SB - IM

CIN - EMBO J. 2023 Aug 1;42(15):e114912. doi: 10.15252/embj.2023114912. PMID: 37435707

MH - *Diarylquinolines/pharmacology

MH - Antitubercular Agents/pharmacology/chemistry/metabolism

MH - Adenosine Triphosphate/metabolism

MH - *Mycobacterium tuberculosis/genetics

MH - Quinine/analogs & derivatives

PMC - PMC10390873

OTO - NOTNLM

OT - ATP synthase

OT - diarylquinoline

OT - inhibitor

OT - mycobacterial

OT - squaramide

COIS- The authors declare that they have no conflict of interest.

EDAT- 2023/06/28 13:08

MHDA- 2023/08/03 06:43

PMCR- 2024/06/28

CRDT- 2023/06/28 06:34

PHST- 2023/06/03 00:00 [revised]

PHST- 2023/02/06 00:00 [received]

PHST- 2023/06/05 00:00 [accepted]

PHST- 2023/08/03 06:43 [medline]

PHST- 2023/06/28 13:08 [pubmed]

PHST- 2023/06/28 06:34 [entrez]

PHST- 2024/06/28 00:00 [pmc-release]

AID - EMBJ2023113687 [pii]

AID - 10.15252/embj.2023113687 [doi]

PST - ppublish

SO - EMBO J. 2023 Aug 1;42(15):e113687. doi: 10.15252/embj.2023113687. Epub 2023 Jun

28.

PMID- 37254945

OWN - NLM

STAT- MEDLINE

DCOM- 20230811

LR - 20230811

IS - 1099-0461 (Electronic)

IS - 1095-6670 (Linking)

VI - 37

IP - 8

DP - 2023 Aug

TI - Molecular and cellular remodeling of HepG2 cells upon treatment with

antitubercular drugs.

PG - e23386

LID - 10.1002/jbt.23386 [doi]

AB - Drug-induced liver injury (DILI) is an adverse outcome of the currently used

tuberculosis treatment regimen, which results in patient noncompliance, poor

treatment outcomes, and the emergence of drug-resistant tuberculosis. DILI is

primarily caused by the toxicity of the drugs and their metabolites, which affect

liver cells, biliary epithelial cells, and liver vasculature. However, the

precise mechanism behind the cellular damage attributable to first-line

antitubercular drugs (ATDs), as well as the effect of toxicity on the cell

survival strategies, is yet to be elucidated. In the current study, HepG2 cells

upon treatment with a high concentration of ATDs showed increased perforation

within the cell, cuboidal shape, and membrane blebbing as compared with

control/untreated cells. It was observed that ATD-induced toxicity in HepG2 cells

leads to altered mitochondrial membrane permeability, which was depicted by the

decreased fluorescence intensity of the MitoRed tracker dye at higher drug

concentrations. In addition, high doses of ATDs caused cell damage through an

increase in reactive oxygen species production in HepG2 cells and a simultaneous

reduction in glutathione levels. Further, high dose of isoniazid (50-200 mM),

pyrazinamide (50-200 mM), and rifampicin (20-100 µM) causes cell apoptosis and

affects cell survival during toxic conditions by decreasing the expression of

potent autophagy markers Atg5, Atg7, and LC3B. Thus, ATD-mediated toxicity

contributes to the reduced ability of hepatocytes to tolerate cellular damage

caused by altered mitochondrial membrane permeability, increased apoptosis, and

decreased autophagy. These findings further emphasize the need to develop

adjuvant therapies that can mitigate ATD-induced toxicity for the effective

treatment of tuberculosis.

CI - © 2023 Wiley Periodicals LLC.

FAU - Bakshi, Shikha

AU - Bakshi S

AD - Department of Biochemistry, Post Graduate Institute of Medical Education and

Research, Chandigarh, India.

FAU - Kaur, Maninder

AU - Kaur M

AD - Department of Biochemistry, Post Graduate Institute of Medical Education and

Research, Chandigarh, India.

FAU - Verma, Arpana

AU - Verma A

AD - Department of Biochemistry, Post Graduate Institute of Medical Education and

Research, Chandigarh, India.

FAU - Sharma, Sadhna

AU - Sharma S

AUID- ORCID: 0000-0002-6631-1704

AD - Department of Biochemistry, Post Graduate Institute of Medical Education and

Research, Chandigarh, India.

LA - eng

GR - Indian Council of Medical Research/

PT - Journal Article

DEP - 20230531

PL - United States

TA - J Biochem Mol Toxicol

JT - Journal of biochemical and molecular toxicology

JID - 9717231

RN - 0 (Antitubercular Agents)

RN - V83O1VOZ8L (Isoniazid)

RN - 2KNI5N06TI (Pyrazinamide)

SB - IM

MH - Humans

MH - Antitubercular Agents/pharmacology

MH - Hep G2 Cells

MH - Isoniazid/pharmacology

MH - Pyrazinamide/adverse effects

MH - *Tuberculosis/chemically induced/drug therapy

MH - *Chemical and Drug Induced Liver Injury/drug therapy

OTO - NOTNLM

OT - antitubercular drug-induced liver injury

OT - antitubercular drugs

OT - toxicity

OT - tuberculosis

EDAT- 2023/05/31 13:11

MHDA- 2023/08/11 06:42

CRDT- 2023/05/31 06:53

PHST- 2023/02/20 00:00 [revised]

PHST- 2022/06/13 00:00 [received]

PHST- 2023/05/17 00:00 [accepted]

PHST- 2023/08/11 06:42 [medline]

PHST- 2023/05/31 13:11 [pubmed]

PHST- 2023/05/31 06:53 [entrez]

AID - 10.1002/jbt.23386 [doi]

PST - ppublish

SO - J Biochem Mol Toxicol. 2023 Aug;37(8):e23386. doi: 10.1002/jbt.23386. Epub 2023

May 31.

PMID- 35969333

OWN - NLM

STAT- MEDLINE

DCOM- 20230814

LR - 20230814

IS - 1573-501X (Electronic)

IS - 1381-1991 (Linking)

VI - 27

IP - 4

DP - 2023 Aug

TI - Discovery of newer pyrazole derivatives with potential anti-tubercular activity

via 3D-QSAR based pharmacophore modelling, virtual screening, molecular docking

and molecular dynamics simulation studies.

PG - 1547-1566

LID - 10.1007/s11030-022-10511-8 [doi]

AB - Tuberculosis is one of the leading causes of death of at least one million people

annually. The deadliest infectious disease has caused more than 120 million

deaths in humans since 1882. The cell wall structure of Mycobacterium

tuberculosis is important for survival in the host environment. InhA is the

foremost target for the development of novel anti-tubercular agents. Therefore,

we report pharmacophore-based virtual screening (ZINC and ASINEX databases) and

molecular docking study (PDB Code: 4TZK) to identify and design potent inhibitors

targeting to InhA. A five-point pharmacophore model AADHR_1 (with R(2) = 0.97 and

Q(2) = 0.77) was developed by using 47 compounds with its reported MIC values.

Further, to identify and design potent hit molecules based on lead identification

and modification, generated hypothesis employed for virtual screening using ZINC

and ASINEX databases. Predicted pyrazole derivatives further gauged for drug

likeliness and docked against enoyl acyl carrier protein reductase to categorize

the essential amino acid interactions to the active site of the enzyme. Structure

elucidation of these synthesized compounds was carried out using IR, MS, 1H-NMR

and 13C-NMR spectroscopy. Amongst all the synthesized compounds, some of the

compounds 5a, 5c, 5d and 5e were found to be potent with their MIC ranging from

2.23 to 4.61 µM. Based on preliminary anti-tubercular activity synthesized potent

molecules were further assessed for MDR-TB, XDR-TB and cytotoxic study.

CI - © 2022. The Author(s), under exclusive licence to Springer Nature Switzerland AG.

FAU - Modi, Palmi

AU - Modi P

AD - Department of Pharmaceutical Chemistry, L. M. College of Pharmacy, Ahmedabad,

Gujarat, 380009, India.

AD - L. J. Institute of Pharmacy, L J University, Ahmedabad, Gujarat, 382 210, India.

FAU - Patel, Shivani

AU - Patel S

AD - Department of Pharmaceutical Chemistry, L. M. College of Pharmacy, Ahmedabad,

Gujarat, 380009, India.

AD - Division of Biological and Life Sciences, Ahmedabad University, Ahmedabad,

Gujarat, 380009, India.

FAU - Chhabria, Mahesh

AU - Chhabria M

AUID- ORCID: 0000-0002-0303-5503

AD - Department of Pharmaceutical Chemistry, L. M. College of Pharmacy, Ahmedabad,

Gujarat, 380009, India. mahesh.chhabria@rediffmail.com.

LA - eng

PT - Journal Article

DEP - 20220815

PL - Netherlands

TA - Mol Divers

JT - Molecular diversity

JID - 9516534

RN - 0 (Pyrazoles)

RN - J41CSQ7QDS (Zinc)

RN - 0 (Antitubercular Agents)

SB - IM

MH - Humans

MH - Molecular Docking Simulation

MH - *Molecular Dynamics Simulation

MH - *Quantitative Structure-Activity Relationship

MH - Pharmacophore

MH - Pyrazoles/pharmacology

MH - Zinc

MH - Antitubercular Agents/pharmacology/chemistry

OTO - NOTNLM

OT - 3D-QSAR

OT - Anti-tubercular activity

OT - Molecular docking

OT - Pharmacophore modelling

OT - Pyrazole

OT - Virtual screening

EDAT- 2022/08/16 06:00

MHDA- 2023/08/14 06:42

CRDT- 2022/08/15 11:17

PHST- 2022/05/10 00:00 [received]

PHST- 2022/07/30 00:00 [accepted]

PHST- 2023/08/14 06:42 [medline]

PHST- 2022/08/16 06:00 [pubmed]

PHST- 2022/08/15 11:17 [entrez]

AID - 10.1007/s11030-022-10511-8 [pii]

AID - 10.1007/s11030-022-10511-8 [doi]

PST - ppublish

SO - Mol Divers. 2023 Aug;27(4):1547-1566. doi: 10.1007/s11030-022-10511-8. Epub 2022

Aug 15.

PMID- 37255473

OWN - NLM

STAT- MEDLINE

DCOM- 20230717

LR - 20231120

IS - 1098-6596 (Electronic)

IS - 0066-4804 (Print)

IS - 0066-4804 (Linking)

VI - 67

IP - 7

DP - 2023 Jul 18

TI - Bedquiline Resistance Mutations: Correlations with Drug Exposures and Impact on

the Proteome in M. tuberculosis.

PG - e0153222

LID - 10.1128/aac.01532-22 [doi]

LID - e01532-22

AB - Bedaquiline (BDQ) is an effective drug for the treatment of drug-resistant

tuberculosis. Mutations in atpE, which encodes the target of BDQ, are associated

with large increases in MICs. Mutations in Rv0678 that derepress the

transcription of the MmpL5-MmpS5 efflux transporter are associated with smaller

increases in MICs. However, Rv0678 mutations are the most common mutations that

are associated with BDQ resistance in clinical isolates, and they also confer

cross-resistance to clofazimine (CFZ). To investigate the mechanism of BDQ

resistance and the correlation between Rv0678 mutations and target-based atpE

mutations, M. tuberculosis strains were exposed to different concentrations of

BDQ or CFZ to select Rv0678 mutations and atpE mutations. Gene overexpression

strains were constructed to illustrate the roles of MmpL5 and MmpS5. A

quantitative proteome analysis was performed to compare the BDQ-resistant mutants

to the isogenic strain H37Rv. Here, we report that the Rv0678 mutations were more

readily selected than were the atpE mutations at low concentrations of BDQ or

CFZ. The atpE mutations were selected by high concentrations of BDQ exposure. The

overexpression of both mmpL5 and mmpS5 reduced the susceptibility of

Mycobacterium tuberculosis to BDQ and CFZ. Secreted immunogenic proteins and

proteins involved in the biosynthesis and transport of phthiocerol

dimycocerosates were associated with Rv0678 mutations conferring BDQ resistance

in the proteome analysis. In conclusion, exposure to different bedaquiline

concentrations resulted in the selection of different mutations. The coexpression

of MmpL5 and MmpS5 contributed to drug resistance and upregulated pathogenic

proteins in M. tuberculosis, suggesting MmpL5-MmpS5 as a new potential target for

antituberculosis drug development. These results warrant further surveillance for

the evolution of BDQ resistance during clinical usage.

FAU - Xu, Jian

AU - Xu J

AUID- ORCID: 0000-0001-6100-0547

AD - Beijing Key Laboratory of Drug Resistance Tuberculosis Research, Beijing

Tuberculosis and Thoracic Tumor Research Institute, and Beijing Chest Hospital,

Capital Medical University, Beijing, China.

FAU - Li, Dongshuo

AU - Li D

AD - Beijing Key Laboratory of Drug Resistance Tuberculosis Research, Beijing

Tuberculosis and Thoracic Tumor Research Institute, and Beijing Chest Hospital,

Capital Medical University, Beijing, China.

FAU - Shi, Jinghua

AU - Shi J

AD - Beijing Key Laboratory of Drug Resistance Tuberculosis Research, Beijing

Tuberculosis and Thoracic Tumor Research Institute, and Beijing Chest Hospital,

Capital Medical University, Beijing, China.

FAU - Wang, Bin

AU - Wang B

AD - Beijing Key Laboratory of Drug Resistance Tuberculosis Research, Beijing

Tuberculosis and Thoracic Tumor Research Institute, and Beijing Chest Hospital,

Capital Medical University, Beijing, China.

FAU - Ge, Fei

AU - Ge F

AD - Beijing Key Laboratory of Drug Resistance Tuberculosis Research, Beijing

Tuberculosis and Thoracic Tumor Research Institute, and Beijing Chest Hospital,

Capital Medical University, Beijing, China.

FAU - Guo, Zhenyong

AU - Guo Z

AD - Beijing Key Laboratory of Drug Resistance Tuberculosis Research, Beijing

Tuberculosis and Thoracic Tumor Research Institute, and Beijing Chest Hospital,

Capital Medical University, Beijing, China.

FAU - Mu, Xiaopan

AU - Mu X

AD - Beijing Key Laboratory of Drug Resistance Tuberculosis Research, Beijing

Tuberculosis and Thoracic Tumor Research Institute, and Beijing Chest Hospital,

Capital Medical University, Beijing, China.

FAU - Nuermberger, Eric

AU - Nuermberger E

AUID- ORCID: 0000-0003-1440-9889

AD - Center for Tuberculosis Research, Department of Medicine, Johns Hopkins

University School of Medicine, Baltimore, Maryland, USA.

AD - Department of International Health, Johns Hopkins Bloomberg School of Public

Health, Baltimore, Maryland, USA.

FAU - Lu, Yu

AU - Lu Y

AUID- ORCID: 0000-0002-9038-1884

AD - Beijing Key Laboratory of Drug Resistance Tuberculosis Research, Beijing

Tuberculosis and Thoracic Tumor Research Institute, and Beijing Chest Hospital,

Capital Medical University, Beijing, China.

LA - eng

PT - Journal Article

PT - Research Support, Non-U.S. Gov't

DEP - 20230531

PL - United States

TA - Antimicrob Agents Chemother

JT - Antimicrobial agents and chemotherapy

JID - 0315061

RN - 0 (Proteome)

RN - 0 (Antitubercular Agents)

RN - 0 (Diarylquinolines)

RN - D959AE5USF (Clofazimine)

SB - IM

MH - Humans

MH - *Mycobacterium tuberculosis

MH - Proteome/genetics

MH - Antitubercular Agents/pharmacology/therapeutic use

MH - Diarylquinolines/pharmacology/therapeutic use

MH - *Tuberculosis, Multidrug-Resistant/drug therapy/genetics

MH - Clofazimine/pharmacology/therapeutic use

MH - Mutation/genetics

MH - Microbial Sensitivity Tests

PMC - PMC10353445

OTO - NOTNLM

OT - Mycobacterium tuberculosis

OT - Rv0678

OT - atpE

OT - bedaquiline

OT - exposure

OT - proteome

OT - resistance

COIS- The authors declare no conflict of interest.

EDAT- 2023/05/31 13:11

MHDA- 2023/07/17 06:42

PMCR- 2023/05/31

CRDT- 2023/05/31 10:12

PHST- 2023/07/17 06:42 [medline]

PHST- 2023/05/31 13:11 [pubmed]

PHST- 2023/05/31 10:12 [entrez]

PHST- 2023/05/31 00:00 [pmc-release]

AID - 01532-22 [pii]

AID - aac.01532-22 [pii]

AID - 10.1128/aac.01532-22 [doi]

PST - ppublish

SO - Antimicrob Agents Chemother. 2023 Jul 18;67(7):e0153222. doi:

10.1128/aac.01532-22. Epub 2023 May 31.

PMID- 37492259

OWN - NLM

STAT- PubMed-not-MEDLINE

LR - 20230727

IS - 1664-302X (Print)

IS - 1664-302X (Electronic)

IS - 1664-302X (Linking)

VI - 14

DP - 2023

TI - In vitro modeling of isoniazid resistance mechanisms in Mycobacterium

tuberculosis H37Rv.

PG - 1171861

LID - 10.3389/fmicb.2023.1171861 [doi]

LID - 1171861

AB - INTRODUCTION: Mycobacterium tuberculosis (MTB), the causative agent of

tuberculosis, has been a global threat to human beings for several decades.

Treating tuberculosis has become more difficult as the prevalence of

drug-resistant tuberculosis has increased globally. Evidence suggests that the

comprehensive landscape of resistance mechanisms in MTB is ambiguous. More

importantly, little is known regarding the series of events connected to

resistance mechanisms in MTB before exposure to anti-TB drugs, during exposure to

the drugs, and finally, when the MTB becomes resistant after exposure, upon

analyses of its genome. METHODS: We used the wild-type strain of MTB (H37Rv) in

an in vitro model for generating induced resistance using a sub-inhibitory

concentration of isoniazid, and the generated resistance-associated variants

(RAVs) were identified using the whole genome sequencing method. RESULTS: The

detection of an inhA promoter mutation (fabG1-15C>T), which results in increased

production of InhA protein, was found to be a major mechanism for developing

resistance to isoniazid in the first place. We observed adaptation of MTB

resistance mechanisms in high isoniazid stress by alteration and abolishment of

KatG due to the detection of katG S315N, the common region of mutation that

confers isoniazid resistance, along with katG K414N, katG N138S, and katG A162E.

Furthermore, we detected the ahpC-72C>T and ahpC 21C>A mutations, but further

investigation is needed to determine their role in compensating for the loss of

KatG activity. DISCUSSION: This suggests that increased InhA production is the

main mechanism where there are low levels of isoniazid, whereas the alteration of

KatG was found to be utilized in mycobacterium with a high concentration of

isoniazid. Our work demonstrates that this in vitro approach of generating

induced resistance could provide clinically relevant information after the

fabG1-15C>T mutation, which is the common mutation found in clinical isolates.

Moreover, other mutations detected in this work can also be found in clinical

isolates. These findings may shed light on the impact of isoniazid in generating

RAV and the resistance mechanism scenario that mycobacterium used under various

isoniazid-pressuring conditions. More research is needed to understand better the

role of RAV and mechanical resistance events within the mycobacterium genome in

promoting a promising drug prediction platform that could lead to the right

treatment for patients with MDR-TB and XDR-TB.

CI - Copyright © 2023 Dokrungkoon, Tulyaprawat, Suwannakarn and Ngamskulrungroj.

FAU - Dokrungkoon, Thanadon

AU - Dokrungkoon T

AD - Department of Microbiology, Faculty of Medicine Siriraj Hospital, Mahidol

University, Bangkoknoi, Bangkok, Thailand.

FAU - Tulyaprawat, Orawan

AU - Tulyaprawat O

AD - Department of Microbiology, Faculty of Medicine Siriraj Hospital, Mahidol

University, Bangkoknoi, Bangkok, Thailand.

FAU - Suwannakarn, Kamol

AU - Suwannakarn K

AD - Department of Microbiology, Faculty of Medicine Siriraj Hospital, Mahidol

University, Bangkoknoi, Bangkok, Thailand.

FAU - Ngamskulrungroj, Popchai

AU - Ngamskulrungroj P

AD - Department of Microbiology, Faculty of Medicine Siriraj Hospital, Mahidol

University, Bangkoknoi, Bangkok, Thailand.

LA - eng

PT - Journal Article

DEP - 20230710

PL - Switzerland

TA - Front Microbiol

JT - Frontiers in microbiology

JID - 101548977

PMC - PMC10364472

OTO - NOTNLM

OT - Mycobacterium tuberculosis

OT - in vitro model

OT - inhA

OT - isoniazid (INH)

OT - katG

OT - resistance-associated variant (RAV)

COIS- The authors declare that the research was conducted in the absence of any

commercial or financial relationships that could be construed as a potential

conflict of interest.

EDAT- 2023/07/26 06:42

MHDA- 2023/07/26 06:43

PMCR- 2023/07/10

CRDT- 2023/07/26 03:48

PHST- 2023/02/22 00:00 [received]

PHST- 2023/06/06 00:00 [accepted]

PHST- 2023/07/26 06:43 [medline]

PHST- 2023/07/26 06:42 [pubmed]

PHST- 2023/07/26 03:48 [entrez]

PHST- 2023/07/10 00:00 [pmc-release]

AID - 10.3389/fmicb.2023.1171861 [doi]

PST - epublish

SO - Front Microbiol. 2023 Jul 10;14:1171861. doi: 10.3389/fmicb.2023.1171861.

eCollection 2023.

PMID- 37120997

OWN - NLM

STAT- MEDLINE

DCOM- 20230522

LR - 20230522

IS - 1768-3254 (Electronic)

IS - 0223-5234 (Linking)

VI - 255

DP - 2023 Jul 5

TI - Comprehensive coverage on anti-mycobacterial endeavour reported during 2022.

PG - 115409

LID - S0223-5234(23)00375-6 [pii]

LID - 10.1016/j.ejmech.2023.115409 [doi]

AB - TB being one of the deadliest diseases and second most common infectious cause of

deaths, poses the severe threat to global health. The extended duration of

therapy owing to resistance and its upsurge in immune-compromised patients have

been the driving force for the development of novel of anti-TB scaffolds.

Recently, we have compiled the account of anti-mycobacterial scaffolds published

during 2015-2020 and updated them in 2021. The present work involves the insights

on the anti-mycobacterial scaffolds reported in 2022 with their mechanism of

action, structure activity relationships, along with the key perceptions for the

design of newer anti-TB agents for the broader interests of medicinal chemists.

CI - Copyright © 2023 Elsevier Masson SAS. All rights reserved.

FAU - Dhameliya, Tejas M

AU - Dhameliya TM

AD - L. M. College of Pharmacy, Navrangpura, Ahmedabad, 380 009, Gujarat, India.

Electronic address: tejasm.dhameliya@nirmauni.ac.in.

FAU - Vekariya, Drashtiben D

AU - Vekariya DD

AD - L. M. College of Pharmacy, Navrangpura, Ahmedabad, 380 009, Gujarat, India.

FAU - Patel, Heta Y

AU - Patel HY

AD - L. M. College of Pharmacy, Navrangpura, Ahmedabad, 380 009, Gujarat, India.

FAU - Patel, Janvi T

AU - Patel JT

AD - L. M. College of Pharmacy, Navrangpura, Ahmedabad, 380 009, Gujarat, India.

LA - eng

PT - Journal Article

PT - Review

DEP - 20230421

PL - France

TA - Eur J Med Chem

JT - European journal of medicinal chemistry

JID - 0420510

RN - 0 (Antitubercular Agents)

SB - IM

MH - Humans

MH - *Antitubercular Agents/chemistry

MH - *Mycobacterium tuberculosis

OTO - NOTNLM

OT - Anti-TB activity

OT - Heterocyclic scaffolds

OT - M. tuberculosis

OT - MDR-TB

OT - Mycobacterium

COIS- Declaration of competing interest The authors declare that they have no known

competing financial interests or personal relationships that could have appeared

to influence the work reported in this paper.

EDAT- 2023/05/01 00:42

MHDA- 2023/05/22 06:42

CRDT- 2023/04/30 18:05

PHST- 2023/02/25 00:00 [received]

PHST- 2023/04/07 00:00 [revised]

PHST- 2023/04/20 00:00 [accepted]

PHST- 2023/05/22 06:42 [medline]

PHST- 2023/05/01 00:42 [pubmed]

PHST- 2023/04/30 18:05 [entrez]

AID - S0223-5234(23)00375-6 [pii]

AID - 10.1016/j.ejmech.2023.115409 [doi]

PST - ppublish

SO - Eur J Med Chem. 2023 Jul 5;255:115409. doi: 10.1016/j.ejmech.2023.115409. Epub

2023 Apr 21.

PMID- 37182451

OWN - NLM

STAT- MEDLINE

DCOM- 20230616

LR - 20260127

IS - 1878-1705 (Electronic)

IS - 1567-5769 (Linking)

VI - 120

DP - 2023 Jul

TI - Cisatracurium besylate rescues Mycobacterium Tuberculosis-infected macrophages

from necroptosis and enhances the bactericidal effect of isoniazid.

PG - 110291

LID - S1567-5769(23)00614-8 [pii]

LID - 10.1016/j.intimp.2023.110291 [doi]

AB - OBJECTIVE: Tuberculosis is the leading killer among the chronic single-source

infectious diseases. Mycobacterium tuberculosis can induce necrotic-dominant

multiple modes of cell death in macrophages, which accelerates bacterium

dissemination and expands tissue injury in host lungs. Mining drugs to counteract

Mycobacterium tuberculosis-induced cell death would be beneficial to tuberculosis

patients. METHODS: In this study, the protective drug was screened out from the

FDA-approved drug library in Mycobacterium tuberculosis-infected macrophages with

CCK-8 assay. The death mode regulated by the drug was identified using

transcriptomic sequencing, cytomorphological observation, and in the experimental

mouse Mycobacterium tuberculosis-infection model. The functional mechanism was

explored using western blot, co-immunoprecipitation, and DARTS assay. The

intracellular bacterial survival was detected using colony forming unit assays.

RESULTS: Cisatracurium besylate was identified to be highly protective for the

viability of macrophages during Mycobacterium tuberculosis infection via

inhibiting necroptosis. Cisatracurium besylate prevented RIPK3 to be associated

with the executive molecule MLKL for forming the necroptotic complex, resulting

in the inhibition of MLKL phosphorylation and pore formation on cell membrane.

However, Cisatracurium besylate did not interfere with the association between

RIPK3 with its upstream kinase RIPK1 or ZBP1 but regulated RIPK3

autophosphorylation. Moreover, Cisatracurium besylate significantly inhibited the

expansion of intracellular Mycobacterium tuberculosis both in vitro and in vivo,

which also displayed a strong auxiliary bacteriostatic effect to support the

therapeutic efficacy of isoniazid and rifampicin, the first-line anti-tubercular

drugs. CONCLUSION: Cisatracurium besylate performs anti-Mycobacterium

tuberculosis and anti-necroptotic roles, which potentiates its application to be

an adjuvant drug for antituberculosis therapy to assist the battle against

drug-resistant tuberculosis.

CI - Copyright © 2023 Elsevier B.V. All rights reserved.

FAU - Wen, Qian

AU - Wen Q

AD - Institute of Molecular Immunology, School of Laboratory Medicine and

Biotechnology, Southern Medical University, Guangzhou, Guangdong, China.

FAU - Zhang, Jing

AU - Zhang J

AD - Institute of Molecular Immunology, School of Laboratory Medicine and

Biotechnology, Southern Medical University, Guangzhou, Guangdong, China.

FAU - Zhang, Zhanqing

AU - Zhang Z

AD - Institute of Molecular Immunology, School of Laboratory Medicine and

Biotechnology, Southern Medical University, Guangzhou, Guangdong, China.

FAU - Chen, Liru

AU - Chen L

AD - Institute of Molecular Immunology, School of Laboratory Medicine and

Biotechnology, Southern Medical University, Guangzhou, Guangdong, China.

FAU - Liu, Honglin

AU - Liu H

AD - Institute of Molecular Immunology, School of Laboratory Medicine and

Biotechnology, Southern Medical University, Guangzhou, Guangdong, China.

FAU - Han, Zhenyu

AU - Han Z

AD - Institute of Molecular Immunology, School of Laboratory Medicine and

Biotechnology, Southern Medical University, Guangzhou, Guangdong, China.

FAU - Chen, Yaoxin

AU - Chen Y

AD - Institute of Molecular Immunology, School of Laboratory Medicine and

Biotechnology, Southern Medical University, Guangzhou, Guangdong, China.

FAU - Wang, Ke

AU - Wang K

AD - Institute of Molecular Immunology, School of Laboratory Medicine and

Biotechnology, Southern Medical University, Guangzhou, Guangdong, China.

FAU - Liu, Jieyu

AU - Liu J

AD - Institute of Molecular Immunology, School of Laboratory Medicine and

Biotechnology, Southern Medical University, Guangzhou, Guangdong, China.

FAU - Sai, Na

AU - Sai N

AD - Institute of Molecular Immunology, School of Laboratory Medicine and

Biotechnology, Southern Medical University, Guangzhou, Guangdong, China.

FAU - Zhou, Xinying

AU - Zhou X

AD - Institute of Molecular Immunology, School of Laboratory Medicine and

Biotechnology, Southern Medical University, Guangzhou, Guangdong, China.

FAU - Zhou, Chaoying

AU - Zhou C

AD - Institute of Molecular Immunology, School of Laboratory Medicine and

Biotechnology, Southern Medical University, Guangzhou, Guangdong, China.

FAU - Hu, Shengfeng

AU - Hu S

AD - Institute of Molecular Immunology, School of Laboratory Medicine and

Biotechnology, Southern Medical University, Guangzhou, Guangdong, China.

FAU - Ma, Li

AU - Ma L

AD - Institute of Molecular Immunology, School of Laboratory Medicine and

Biotechnology, Southern Medical University, Guangzhou, Guangdong, China.

Electronic address: mali_61648322@smu.edu.cn.

LA - eng

PT - Journal Article

DEP - 20230512

PL - Netherlands

TA - Int Immunopharmacol

JT - International immunopharmacology

JID - 100965259

RN - V83O1VOZ8L (Isoniazid)

RN - EC 2.7.- (Protein Kinases)

RN - 0 (Anti-Bacterial Agents)

RN - EC 2.7.11.1 (Receptor-Interacting Protein Serine-Threonine Kinases)

RN - QX62KLI41N (cisatracurium)

RN - 2GQ1IRY63P (Atracurium)

SB - IM

MH - Mice

MH - Animals

MH - Apoptosis

MH - *Mycobacterium tuberculosis/metabolism

MH - Isoniazid/pharmacology/therapeutic use

MH - Necroptosis

MH - Protein Kinases/metabolism

MH - *Tuberculosis/drug therapy/metabolism

MH - Anti-Bacterial Agents/pharmacology

MH - Receptor-Interacting Protein Serine-Threonine Kinases/metabolism

MH - Macrophages/metabolism

MH - Atracurium/analogs & derivatives

OTO - NOTNLM

OT - Cisatracurium besylate

OT - MLKL

OT - Mycobacterium tuberculosis

OT - Necroptosis

OT - RIPK3

COIS- Declaration of Competing Interest The authors declare that they have no known

competing financial interests or personal relationships that could have appeared

to influence the work reported in this paper.

EDAT- 2023/05/15 00:42

MHDA- 2023/06/16 06:42

CRDT- 2023/05/14 18:09

PHST- 2023/01/15 00:00 [received]

PHST- 2023/05/02 00:00 [revised]

PHST- 2023/05/03 00:00 [accepted]

PHST- 2023/06/16 06:42 [medline]

PHST- 2023/05/15 00:42 [pubmed]

PHST- 2023/05/14 18:09 [entrez]

AID - S1567-5769(23)00614-8 [pii]

AID - 10.1016/j.intimp.2023.110291 [doi]

PST - ppublish

SO - Int Immunopharmacol. 2023 Jul;120:110291. doi: 10.1016/j.intimp.2023.110291. Epub

2023 May 12.

PMID- 35574601

OWN - NLM

STAT- MEDLINE

DCOM- 20230612

LR - 20230612

IS - 1538-0254 (Electronic)

IS - 0739-1102 (Linking)

VI - 41

IP - 10

DP - 2023 Jul

TI - Molecular dynamic assisted investigation on impact of mutations in deazaflavin

dependent nitroreductase against pretomanid: a computational study.

PG - 4421-4443

LID - 10.1080/07391102.2022.2069156 [doi]

AB - In the past decade, TB drugs belonging to the nitroimidazole class, pretomanid

and delamanid, have been authorised to treat MDR-TB and XDR-TB. With a novel

inhibition mechanism and a reduction in the span of treatment, it is now being

administered in various combinations. This approach is not the ultimate remedy

since the target protein Deazaflavin dependent nitroreductase (Ddn) has a high

mutation frequency, and already pretomanid resistant clinical isolates are

reported in various studies. Ddn is essential for M.tuberculosis to emerge from

hypoxia, and point mutations in critical residues confer resistance to

Nitro-imidazoles. Among the pool of available mutants, we have selected seven

mutants viz Ddn(L49P), Ddn(Y65S), Ddn(S78Y), Ddn(K79Q), Ddn(W88R), Ddn(Y133C),

and Ddn(Y136S), all of which exhibited resistance to pretomanid. To address this

issue, through computational study primarily by MD simulation, we attempted to

elucidate these point mutations' impact and investigate the resistance mechanism.

Hence, the Ddn(WT) and mutant (MT) complexes were subjected to all-atom molecular

dynamics (MD) simulations for 100 ns. Interestingly, we observed the escalation

of the distance between cofactor and ligand in some mutants, along with a

significant change in ligand conformation relative to the Ddn(WT). Moreover, we

confirmed that mutations rendered ligand instability and were ejected from the

binding pocket as a result. In conclusion, the results obtained provide a new

structural insight and vital clues for designing novel inhibitors to combat

nitroimidazole resistanceCommunicated by Ramaswamy H. Sarma.

FAU - Singh, Ravi

AU - Singh R

AD - Department of Pharmacoinformatics, National Institute of Pharmaceutical Education

and Research (NIPER), Punjab, India.

FAU - Shaheer, Muhammed

AU - Shaheer M

AD - Department of Pharmacoinformatics, National Institute of Pharmaceutical Education

and Research (NIPER), Punjab, India.

FAU - Sobhia, M Elizabeth

AU - Sobhia ME

AUID- ORCID: 0000-0002-9763-8814

AD - Department of Pharmacoinformatics, National Institute of Pharmaceutical Education

and Research (NIPER), Punjab, India.

LA - eng

PT - Journal Article

DEP - 20220514

PL - England

TA - J Biomol Struct Dyn

JT - Journal of biomolecular structure & dynamics

JID - 8404176

RN - 0 (pretomanid)

RN - 0 (Ligands)

RN - 0 (Nitroimidazoles)

RN - EC 1.7.- (Nitroreductases)

RN - 0 (Antitubercular Agents)

SB - IM

MH - Molecular Dynamics Simulation

MH - Ligands

MH - *Nitroimidazoles/pharmacology/chemistry/metabolism

MH - *Mycobacterium tuberculosis/genetics

MH - Mutation

MH - Nitroreductases/genetics/chemistry/metabolism

MH - Antitubercular Agents/pharmacology

OTO - NOTNLM

OT - Ddn

OT - F420H2

OT - FDOR

OT - MD simulation

OT - delamanid

OT - docking

OT - flexibility

OT - hydrophobic shielding

OT - mutation

OT - pretomanid

EDAT- 2022/05/17 06:00

MHDA- 2023/06/12 06:42

CRDT- 2022/05/16 04:52

PHST- 2023/06/12 06:42 [medline]

PHST- 2022/05/17 06:00 [pubmed]

PHST- 2022/05/16 04:52 [entrez]

AID - 10.1080/07391102.2022.2069156 [doi]

PST - ppublish

SO - J Biomol Struct Dyn. 2023 Jul;41(10):4421-4443. doi:

10.1080/07391102.2022.2069156. Epub 2022 May 14.

PMID- 37457712

OWN - NLM

STAT- MEDLINE

DCOM- 20230718

LR - 20230718

IS - 1664-3224 (Electronic)

IS - 1664-3224 (Linking)

VI - 14

DP - 2023

TI - Downregulation of monocyte miRNAs: implications for immune dysfunction and

disease severity in drug-resistant tuberculosis.

PG - 1197805

LID - 10.3389/fimmu.2023.1197805 [doi]

LID - 1197805

AB - BACKGROUND: Monocyte miRNAs govern both protective and pathological responses

during tuberculosis (TB) through their differential expression and emerged as

potent targets for biomarker discovery and host-directed therapeutics. Thus, this

study examined the miRNA profile of sorted monocytes across the TB disease

spectrum [drug-resistant TB (DR-TB), drug-sensitive TB (DS-TB), and latent TB]

and in healthy individuals (HC) to understand the underlying pathophysiology and

their regulatory mechanism. METHODS: We sorted total monocytes including three

subsets (HLA-DR(+)CD14(+), HLA-DR(+)CD14(+)CD16(+), and HLA-DR(+)CD16(+)cells)

from peripheral blood mononuclear cells (PBMCs) of healthy and TB-infected

individuals through flow cytometry and subjected them to NanoString-based miRNA

profiling. RESULTS: The outcome was the differential expression of 107 miRNAs

particularly the downregulation of miRNAs in the active TB groups (both

drug-resistant and drug-sensitive). The miRNA profile revealed differential

expression signatures: i) decline of miR-548m in DR-TB alone, ii) decline of

miR-486-3p in active TB but significant elevation only in LTB iii) elevation of

miR-132-3p only in active TB (DR-TB and DS-TB) and iv) elevation of miR-150-5p in

DR-TB alone. The directionality of functions mediated by monocyte miRNAs from

Gene Set Enrichment Analysis (GSEA) facilitated two phenomenal findings: i) a

bidirectional response between active disease (activation profile in DR-TB and

DS-TB compared to LTB and HC) and latent infection (suppression profile in LTB vs

HC) and ii) hyper immune activation in the DR-TB group compared to DS-TB.

CONCLUSION: Thus, monocyte miRNA signatures provide pathological clues for

altered monocyte function, drug resistance, and disease severity. Further studies

on monocyte miRNAs may shed light on the immune regulatory mechanism for

tuberculosis.

CI - Copyright © 2023 Sampath, Moorthy, Menon, Madhav, Janaki, Dhanapal, Natarajan,

Hissar, Ranganathan, Ramaswamy and Bethunaickan.

FAU - Sampath, Pavithra

AU - Sampath P

AD - Department of Immunology, Indian Council of Medical Research (ICMR)-National

Institute for Research in Tuberculosis (NIRT), Chennai, India.

FAU - Moorthy, Manju

AU - Moorthy M

AD - TheraCUES Innovations Pvt. Ltd, Bangalore, India.

FAU - Menon, Athul

AU - Menon A

AD - TheraCUES Innovations Pvt. Ltd, Bangalore, India.

FAU - Madhav, Lekshmi

AU - Madhav L

AD - TheraCUES Innovations Pvt. Ltd, Bangalore, India.

FAU - Janaki, Aishwarya

AU - Janaki A

AD - Department of Immunology, Indian Council of Medical Research (ICMR)-National

Institute for Research in Tuberculosis (NIRT), Chennai, India.

FAU - Dhanapal, Madhavan

AU - Dhanapal M

AD - Department of Immunology, Indian Council of Medical Research (ICMR)-National

Institute for Research in Tuberculosis (NIRT), Chennai, India.

FAU - Natarajan, Alangudi Palaniappan

AU - Natarajan AP

AD - Department of Clinical Research, ICMR-National Institute of Research in

Tuberculosis (NIRT), Chennai, India.

FAU - Hissar, Syed

AU - Hissar S

AD - Department of Clinical Research, ICMR-National Institute of Research in

Tuberculosis (NIRT), Chennai, India.

FAU - Ranganathan, Uma Devi

AU - Ranganathan UD

AD - Department of Immunology, Indian Council of Medical Research (ICMR)-National

Institute for Research in Tuberculosis (NIRT), Chennai, India.

FAU - Ramaswamy, Gopalakrishna

AU - Ramaswamy G

AD - TheraCUES Innovations Pvt. Ltd, Bangalore, India.

FAU - Bethunaickan, Ramalingam

AU - Bethunaickan R

AD - Department of Immunology, Indian Council of Medical Research (ICMR)-National

Institute for Research in Tuberculosis (NIRT), Chennai, India.

LA - eng

PT - Journal Article

PT - Research Support, Non-U.S. Gov't

DEP - 20230629

PL - Switzerland

TA - Front Immunol

JT - Frontiers in immunology

JID - 101560960

RN - 0 (MicroRNAs)

RN - 0 (HLA-DR Antigens)

SB - IM

MH - Humans

MH - Monocytes

MH - *MicroRNAs/genetics/metabolism

MH - Leukocytes, Mononuclear

MH - Down-Regulation

MH - *Tuberculosis

MH - HLA-DR Antigens

MH - *Tuberculosis, Multidrug-Resistant/drug therapy/metabolism

MH - Patient Acuity

PMC - PMC10345223

OTO - NOTNLM

OT - Mycobacterium tuberculosis

OT - NanoString

OT - drug-resistant -TB

OT - immune dysfunction

OT - monocyte miRNA

OT - monocyte sorting

COIS- Authors MM, AM, LM, and GR have been employed by TheraCUES Innovations PVT. Ltd.

The remaining authors declare that the research was conducted in the absence of

any commercial or financial relationships that could be construed as a potential

conflict of interest.

EDAT- 2023/07/17 06:42

MHDA- 2023/07/18 13:08

PMCR- 2023/01/01

CRDT- 2023/07/17 04:43

PHST- 2023/03/31 00:00 [received]

PHST- 2023/06/02 00:00 [accepted]

PHST- 2023/07/18 13:08 [medline]

PHST- 2023/07/17 06:42 [pubmed]

PHST- 2023/07/17 04:43 [entrez]

PHST- 2023/01/01 00:00 [pmc-release]

AID - 10.3389/fimmu.2023.1197805 [doi]

PST - epublish

SO - Front Immunol. 2023 Jun 29;14:1197805. doi: 10.3389/fimmu.2023.1197805.

eCollection 2023.

PMID- 37369740

OWN - NLM

STAT- MEDLINE

DCOM- 20230629

LR - 20230701

IS - 2045-2322 (Electronic)

IS - 2045-2322 (Linking)

VI - 13

IP - 1

DP - 2023 Jun 27

TI - Bedaquiline- and clofazimine- selected Mycobacterium tuberculosis mutants:

further insights on resistance driven largely by Rv0678.

PG - 10444

LID - 10.1038/s41598-023-36955-y [doi]

LID - 10444

AB - Drug-resistant tuberculosis is a serious global health threat. Bedaquiline (BDQ)

is a relatively new core drug, targeting the respiratory chain in Mycobacterium

tuberculosis (Mtb). While mutations in the BDQ target gene, atpE, are rare in

clinical isolates, mutations in the Rv0678 gene, a transcriptional repressor

regulating the efflux pump MmpS5-MmpL5, are increasingly observed, and have been

linked to worse treatment outcomes. Nevertheless, underlying mechanisms of

(cross)-resistance remain incompletely resolved. Our study aims to distinguish

resistance associated variants from other polymorphisms, by assessing the in

vitro onset of mutations under drug pressure, combined with their impact on

minimum inhibitory concentrations (MICs) and on protein stability. For this

purpose, isolates were exposed in vitro to sub-lethal concentrations of BDQ or

clofazimine (CFZ). Selected colonies had BDQ- and CFZ-MICs determined on 7H10 and

7H11 agar. Sanger sequencing and additional Deeplex Myc-TB and whole genome

sequencing (WGS) for a subset of isolates were used to search for mutations in

Rv0678, atpE and pepQ. In silico characterization of relevant mutations was

performed using computational tools. We found that colonies that grew on BDQ

medium had mutations in Rv0678, atpE or pepQ, while CFZ-exposed isolates

presented mutations in Rv0678 and pepQ, but none in atpE. Twenty-eight Rv0678

mutations had previously been described among in vitro selected mutants or in

patients' isolates, while 85 were new. Mutations were scattered across the Rv0678

gene without apparent hotspot. While most Rv0678 mutations led to an increased

BDQ- and/or CFZ-MIC, only a part of them surpassed the critical concentration

(69.1% for BDQ and 87.9% for CFZ). Among the mutations leading to elevated MICs

for BDQ and CFZ, we report a synonymous Val1Val mutation in the Rv0678 start

codon. Finally, in silico characterization of Rv0678 mutations suggests that

especially the C46R mutant may render Rv0678 less stable.

CI - © 2023. The Author(s).

FAU - Snobre, J

AU - Snobre J

AD - Mycobacteriology Unit, Biomedical Sciences, Institute of Tropical Medicine,

Antwerp, Belgium.

AD - Internal Medicine Department, UZ Brussel, Brussels, Belgium.

AD - Doctoral School of Life Sciences & Medicine, Vrije Universiteit Brussel,

Brussels, Belgium.

FAU - Villellas, M C

AU - Villellas MC

AD - Department of Infectious Diseases, Janssen Pharmaceutica, Beerse, Belgium.

FAU - Coeck, N

AU - Coeck N

AD - Mycobacteriology Unit, Biomedical Sciences, Institute of Tropical Medicine,

Antwerp, Belgium.

FAU - Mulders, W

AU - Mulders W

AD - Mycobacteriology Unit, Biomedical Sciences, Institute of Tropical Medicine,

Antwerp, Belgium.

FAU - Tzfadia, O

AU - Tzfadia O

AD - Mycobacteriology Unit, Biomedical Sciences, Institute of Tropical Medicine,

Antwerp, Belgium.

FAU - de Jong, B C

AU - de Jong BC

AD - Mycobacteriology Unit, Biomedical Sciences, Institute of Tropical Medicine,

Antwerp, Belgium.

FAU - Andries, K

AU - Andries K

AD - Department of Infectious Diseases, Janssen Pharmaceutica, Beerse, Belgium.

FAU - Rigouts, L

AU - Rigouts L

AD - Mycobacteriology Unit, Biomedical Sciences, Institute of Tropical Medicine,

Antwerp, Belgium. lrigouts@itg.be.

LA - eng

PT - Journal Article

DEP - 20230627

PL - England

TA - Sci Rep

JT - Scientific reports

JID - 101563288

RN - 78846I289Y (bedaquiline)

RN - D959AE5USF (Clofazimine)

RN - 0 (Antitubercular Agents)

RN - 0 (Diarylquinolines)

SB - IM

MH - Humans

MH - *Mycobacterium tuberculosis

MH - Clofazimine/pharmacology/therapeutic use

MH - Antitubercular Agents/pharmacology/therapeutic use

MH - Diarylquinolines/pharmacology

MH - *Tuberculosis, Multidrug-Resistant/drug therapy

MH - Microbial Sensitivity Tests

PMC - PMC10300004

COIS- J. S. is supported by an FWO PhD fellowship for fundamental research. C. V. and

K. A. are employees of Janssen Pharmaceutica. All other authors: none to declare.

EDAT- 2023/06/28 01:06

MHDA- 2023/06/29 06:43

PMCR- 2023/06/27

CRDT- 2023/06/27 23:21

PHST- 2023/04/05 00:00 [received]

PHST- 2023/06/13 00:00 [accepted]

PHST- 2023/06/29 06:43 [medline]

PHST- 2023/06/28 01:06 [pubmed]

PHST- 2023/06/27 23:21 [entrez]

PHST- 2023/06/27 00:00 [pmc-release]

AID - 10.1038/s41598-023-36955-y [pii]

AID - 36955 [pii]

AID - 10.1038/s41598-023-36955-y [doi]

PST - epublish

SO - Sci Rep. 2023 Jun 27;13(1):10444. doi: 10.1038/s41598-023-36955-y.

PMID- 37363078

OWN - NLM

STAT- PubMed-not-MEDLINE

LR - 20250530

IS - 2694-2437 (Electronic)

IS - 2694-2437 (Linking)

VI - 3

IP - 3

DP - 2023 Jun 21

TI - Structure of the d-Cycloserine-Resistant Variant D322N of Alanine Racemase from

Mycobacterium tuberculosis.

PG - 233-239

LID - 10.1021/acsbiomedchemau.2c00074 [doi]

AB - Alanine racemase (Alr) is a pyridoxal 5'-phosphate-dependent enzyme that

catalyzes the racemization of l-alanine to d-alanine. Alr is one of the two

targets of the broad-spectrum antibiotic d-cycloserine (DCS), a structural

analogue of d-alanine. Despite being an essential component of regimens used to

treat multi- and extensively drug-resistant tuberculosis for almost seven

decades, resistance to DCS has not been observed in patients. We previously

demonstrated that DCS evades resistance due to an ultralow rate of emergence of

mutations. Yet, we identified a single polymorphism (converting Asp322 to Asn) in

the alr gene, which arose in 8 out of 11 independent variants identified and that

confers resistance. Here, we present the crystal structure of the Alr variant

D322N in both the free and DCS-inactivated forms and the characterization of its

DCS inactivation mechanism by UV-visible and fluorescence spectroscopy.

Comparison of these results with those obtained with wild-type Alr reveals the

structural basis of the 240-fold reduced inhibition observed in Alr D322N.

CI - © 2023 The Authors. Published by American Chemical Society.

FAU - de Chiara, Cesira

AU - de Chiara C

AD - Mycobacterial Metabolism and Antibiotic Research Laboratory, The Francis Crick

Institute, London, NW1 1AT, United Kingdom.

FAU - Prosser, Gareth A

AU - Prosser GA

AD - Mycobacterial Metabolism and Antibiotic Research Laboratory, The Francis Crick

Institute, London, NW1 1AT, United Kingdom.

FAU - Ogrodowicz, Roksana

AU - Ogrodowicz R

AD - Structural Biology Science Technology Platform, The Francis Crick Institute,

London, NW1 1AT, United Kingdom.

FAU - de Carvalho, Luiz P S

AU - de Carvalho LPS

AUID- ORCID: 0000-0003-2875-4552

AD - Mycobacterial Metabolism and Antibiotic Research Laboratory, The Francis Crick

Institute, London, NW1 1AT, United Kingdom.

AD - Department of Chemistry, The Herbert Wertheim UF Scripps Institute for Biomedical

Innovation & Technology, Jupiter, Florida 33458, United States.

LA - eng

GR - CC2000/WT_/Wellcome Trust/United Kingdom

PT - Journal Article

DEP - 20230327

PL - United States

TA - ACS Bio Med Chem Au

JT - ACS bio & med chem Au

JID - 9918232604406676

PMC - PMC10288493

COIS- The authors declare no competing financial interest.

EDAT- 2023/06/26 19:07

MHDA- 2023/06/26 19:08

PMCR- 2023/03/27

CRDT- 2023/06/26 13:13

PHST- 2022/11/10 00:00 [received]

PHST- 2023/03/17 00:00 [revised]

PHST- 2023/03/21 00:00 [accepted]

PHST- 2023/06/26 19:08 [medline]

PHST- 2023/06/26 19:07 [pubmed]

PHST- 2023/06/26 13:13 [entrez]

PHST- 2023/03/27 00:00 [pmc-release]

AID - 10.1021/acsbiomedchemau.2c00074 [doi]

PST - epublish

SO - ACS Bio Med Chem Au. 2023 Mar 27;3(3):233-239. doi:

10.1021/acsbiomedchemau.2c00074. eCollection 2023 Jun 21.

PMID- 37125940

OWN - NLM

STAT- MEDLINE

DCOM- 20230619

LR - 20260127

IS - 2165-0497 (Electronic)

IS - 2165-0497 (Linking)

VI - 11

IP - 3

DP - 2023 Jun 15

TI - Baicalein Suppresses NLRP3 and AIM2 Inflammasome-Mediated Pyroptosis in

Macrophages Infected by Mycobacterium tuberculosis via Induced Autophagy.

PG - e0471122

LID - 10.1128/spectrum.04711-22 [doi]

LID - e04711-22

AB - Mycobacterium tuberculosis (Mtb) continues to pose a significant threat to global

health because it causes granulomas and systemic inflammatory responses during

active tuberculosis (TB). Mtb can induce macrophage pyroptosis, which results in

the release of IL-1β and causes tissue damage, thereby promoting its spread. In

the absence of anti-TB drugs, host-directed therapy (HDT) has been demonstrated

to be an effective strategy against TB. In this study, we used an in vitro

Mtb-infected macrophage model to assess the effect of baicalein, derived from

Scutellariae radix, on pyroptosis induced in Mtb-infected macrophages. Further,

we investigated the molecular mechanisms underlying the actions of baicalein. The

results of the study suggest that baicalein inhibits pyroptosis in Mtb-infected

macrophages by downregulating the assembly of AIM2 and NLRP3 inflammasome and

promoting autophagy. Further research has also shown that the mechanism by which

baicalein promotes autophagy may involve the inhibition of the activation of the

Akt/mTOR pathway and the inhibition of the AIM2 protein, which affects the levels

of CHMP2A protein required to promote autophagy. Thus, our data show that

baicalein can inhibit Mtb infection-induced macrophage pyroptosis and has the

potential to be a new adjunctive HDT drug. IMPORTANCE Current strategies for

treating drug-resistant tuberculosis have limited efficacy and undesirable side

effects; hence, research on new treatments, including innovative medications, is

required. Host-directed therapy (HDT) has emerged as a viable strategy for

modulating host cell responses in order to enhance protective immunity against

infections. Baicalein, extracted from Scutellariae radix, was shown to inhibit

pyroptosis caused by Mycobacterium tuberculosis-infected macrophages and was

associated with autophagy. Our findings reveal that baicalein can be used as an

adjunctive treatment for tuberculosis or other inflammatory diseases by

regulating immune function and enhancing the antibacterial ability of the host.

It also provides a new idea for exploring the anti-inflammatory mechanism of

baicalein.

FAU - Ning, Bangzuo

AU - Ning B

AD - Center for Traditional Chinese Medicine and Immunology Research, School of Basic

Medical Sciences, Shanghai University of Traditional Chinese Medicine, Shanghai,

China.

FAU - Shen, Jingjing

AU - Shen J

AD - Center for Traditional Chinese Medicine and Immunology Research, School of Basic

Medical Sciences, Shanghai University of Traditional Chinese Medicine, Shanghai,

China.

FAU - Liu, Fanglin

AU - Liu F

AD - Center for Traditional Chinese Medicine and Immunology Research, School of Basic

Medical Sciences, Shanghai University of Traditional Chinese Medicine, Shanghai,

China.

FAU - Zhang, Hemin

AU - Zhang H

AD - Center for Traditional Chinese Medicine and Immunology Research, School of Basic

Medical Sciences, Shanghai University of Traditional Chinese Medicine, Shanghai,

China.

FAU - Jiang, Xin

AU - Jiang X

AUID- ORCID: 0000-0003-0447-0168

AD - Center for Traditional Chinese Medicine and Immunology Research, School of Basic

Medical Sciences, Shanghai University of Traditional Chinese Medicine, Shanghai,

China.

LA - eng

PT - Journal Article

PT - Research Support, Non-U.S. Gov't

DEP - 20230501

PL - United States

TA - Microbiol Spectr

JT - Microbiology spectrum

JID - 101634614

RN - 0 (Inflammasomes)

RN - 0 (NLR Family, Pyrin Domain-Containing 3 Protein)

RN - 0 (DNA-Binding Proteins)

RN - 49QAH60606 (baicalein)

RN - 0 (Flavanones)

RN - 0 (AIM2 protein, human)

SB - IM

MH - Humans

MH - *Mycobacterium tuberculosis

MH - Inflammasomes/metabolism

MH - NLR Family, Pyrin Domain-Containing 3 Protein/metabolism

MH - Pyroptosis

MH - Macrophages

MH - *Tuberculosis

MH - Autophagy

MH - DNA-Binding Proteins/metabolism

MH - Flavanones

PMC - PMC10269511

OTO - NOTNLM

OT - AIM2 inflammasome

OT - Mycobacterium tuberculosis

OT - NLRP3 inflammasome

OT - autophagy

OT - baicalein

OT - pyroptosis

COIS- The authors declare no conflict of interest.

EDAT- 2023/05/01 12:43

MHDA- 2023/06/19 13:08

PMCR- 2023/05/01

CRDT- 2023/05/01 10:08

PHST- 2023/06/19 13:08 [medline]

PHST- 2023/05/01 12:43 [pubmed]

PHST- 2023/05/01 10:08 [entrez]

PHST- 2023/05/01 00:00 [pmc-release]

AID - 04711-22 [pii]

AID - spectrum.04711-22 [pii]

AID - 10.1128/spectrum.04711-22 [doi]

PST - ppublish

SO - Microbiol Spectr. 2023 Jun 15;11(3):e0471122. doi: 10.1128/spectrum.04711-22.

Epub 2023 May 1.

PMID- 37384221

OWN - NLM

STAT- MEDLINE

DCOM- 20230703

LR - 20260127

IS - 2235-2988 (Electronic)

IS - 2235-2988 (Linking)

VI - 13

DP - 2023

TI - Advances of new drugs bedaquiline and delamanid in the treatment of multi-drug

resistant tuberculosis in children.

PG - 1183597

LID - 10.3389/fcimb.2023.1183597 [doi]

LID - 1183597

AB - Tuberculosis (TB) is a major public health problem, with nearly 10 million new

cases and millions of deaths each year. Around 10% of these cases are in

children, but only a fraction receive proper diagnosis and treatment. The spread

of drug-resistant (DR) strain of TB has made it difficult to control, with only

60% of patients responding to treatment. Multi-drug resistant TB (MDR-TB) is

often undiagnosed in children due to lack of awareness or under-diagnosis, and

the target for children's DR-TB treatment has only been met in 15% of goals. New

medications such as bedaquiline and delamanid have been approved for treating

DR-TB. However, due to age and weight differences, adults and children require

different dosages. The availability of child-friendly formulations is limited by

a lack of clinical data in children. This paper reviews the development history

of these drugs, their mechanism of action, efficacy, safety potential problems

and current use in treating DR-TB in children.

CI - Copyright © 2023 Zhu, Zhou, Zhuang, Li, Bi and Mi.

FAU - Zhu, Hanzhao

AU - Zhu H

AD - Chinese Academy of Science (CAS) Key Laboratory of Pathogen Microbiology and

Immunology, Institute of Microbiology, Chinese Academy of Sciences, Beijing,

China.

AD - Savaid Medical School, University of Chinese Academy of Sciences, Beijing, China.

FAU - Zhou, Xintong

AU - Zhou X

AD - Chinese Academy of Science (CAS) Key Laboratory of Pathogen Microbiology and

Immunology, Institute of Microbiology, Chinese Academy of Sciences, Beijing,

China.

FAU - Zhuang, Zengfang

AU - Zhuang Z

AD - Chinese Academy of Science (CAS) Key Laboratory of Pathogen Microbiology and

Immunology, Institute of Microbiology, Chinese Academy of Sciences, Beijing,

China.

AD - Savaid Medical School, University of Chinese Academy of Sciences, Beijing, China.

FAU - Li, Lianju

AU - Li L

AD - Chinese Academy of Science (CAS) Key Laboratory of Pathogen Microbiology and

Immunology, Institute of Microbiology, Chinese Academy of Sciences, Beijing,

China.

AD - School of Basic Medicine, Shandong First Medical University and Shandong Academy

of Medical Sciences, Jinan, China.

FAU - Bi, Jing

AU - Bi J

AD - Baoding Hospital of Beijing Children's Hospital, Capital Medical University,

Baoding Key Laboratory for Precision Diagnosis and Treatment of Infectious

Diseases in Children, Baoding, China.

FAU - Mi, Kaixia

AU - Mi K

AD - Chinese Academy of Science (CAS) Key Laboratory of Pathogen Microbiology and

Immunology, Institute of Microbiology, Chinese Academy of Sciences, Beijing,

China.

AD - Savaid Medical School, University of Chinese Academy of Sciences, Beijing, China.

LA - eng

PT - Journal Article

PT - Research Support, Non-U.S. Gov't

PT - Review

DEP - 20230613

PL - Switzerland

TA - Front Cell Infect Microbiol

JT - Frontiers in cellular and infection microbiology

JID - 101585359

RN - 0 (Diarylquinolines)

RN - 0 (Nitroimidazoles)

RN - 78846I289Y (bedaquiline)

RN - 0 (OPC-67683)

RN - 0 (Oxazoles)

SB - IM

MH - Adult

MH - Humans

MH - *Tuberculosis, Multidrug-Resistant/drug therapy

MH - Diarylquinolines/therapeutic use

MH - *Nitroimidazoles/therapeutic use

MH - Oxazoles

PMC - PMC10293792

OTO - NOTNLM

OT - bedaquiline

OT - children

OT - delamanid

OT - multi-drug resistant TB

OT - tuberculosis (TB)

COIS- The authors declare that the research was conducted in the absence of any

commercial or financial relationships that could be construed as a potential

conflict of interest.

EDAT- 2023/06/29 13:42

MHDA- 2023/07/03 06:41

PMCR- 2023/01/01

CRDT- 2023/06/29 12:03

PHST- 2023/03/10 00:00 [received]

PHST- 2023/05/30 00:00 [accepted]

PHST- 2023/07/03 06:41 [medline]

PHST- 2023/06/29 13:42 [pubmed]

PHST- 2023/06/29 12:03 [entrez]

PHST- 2023/01/01 00:00 [pmc-release]

AID - 10.3389/fcimb.2023.1183597 [doi]

PST - epublish

SO - Front Cell Infect Microbiol. 2023 Jun 13;13:1183597. doi:

10.3389/fcimb.2023.1183597. eCollection 2023.

PMID- 37197901

OWN - NLM

STAT- MEDLINE

DCOM- 20230615

LR - 20230615

IS - 1365-2672 (Electronic)

IS - 1364-5072 (Linking)

VI - 134

IP - 6

DP - 2023 Jun 1

TI - Role of non-coding RNAs in tuberculosis and their potential for clinical

applications.

LID - lxad104 [pii]

LID - 10.1093/jambio/lxad104 [doi]

AB - Tuberculosis (TB), caused by Mycobacterium tuberculosis (Mtb), remains the

leading cause of mortality due to infectious diseases, only surpassed in 2020 by

COVID-19. Despite the development in diagnostics, therapeutics, and evaluation of

new vaccines for TB, this infectious disease remains uncontrollable due to the

emergence of multidrug-resistant (MDR) and extremely drug-resistant (XDR) TB,

among other factors. The development in transcriptomics (RNomics) has enabled the

study of gene expression in TB. It is considered that non-coding RNAs (ncRNAs)

from host [microRNAs (miRNAs)] and Mtb [small RNAs (sRNAs)] are important

elements in TB pathogenesis, immune resistance, and susceptibility. Many studies

have shown the importance of host miRNAs in regulating immune response against

Mtb via in vitro and in vivo mice models. The bacterial sRNAs play a major role

in survival, adaptation, and virulence. Here, we review the characterization and

function of host and bacteria ncRNAs in TB and their potential use in clinical

applications as diagnostic, prognostic, and therapeutic biomarkers.

CI - © The Author(s) 2023. Published by Oxford University Press on behalf of Applied

Microbiology International.

FAU - Jumat, Mohd Iskandar

AU - Jumat MI

AD - Faculty of Medicine and Health Sciences, Universiti Malaysia Sabah, 88400 Kota

Kinabalu, Sabah, Malaysia.

FAU - Sarmiento, Maria E

AU - Sarmiento ME

AD - School of Health Sciences, Universiti Sains Malaysia, 16150 Kubang Kerian,

Kelantan, Malaysia.

FAU - Acosta, Armando

AU - Acosta A

AD - School of Health Sciences, Universiti Sains Malaysia, 16150 Kubang Kerian,

Kelantan, Malaysia.

FAU - Chin, Kai Ling

AU - Chin KL

AUID- ORCID: 0000-0003-4362-1731

AD - Faculty of Medicine and Health Sciences, Universiti Malaysia Sabah, 88400 Kota

Kinabalu, Sabah, Malaysia.

LA - eng

GR - FRGS/1/2022/SKK12/UMS/01/2/Malaysia Ministry of Education/

GR - GUG0502-2/2020/Universiti Malaysia Sabah/

PT - Journal Article

PT - Review

PL - England

TA - J Appl Microbiol

JT - Journal of applied microbiology

JID - 9706280

RN - 0 (Antitubercular Agents)

RN - 0 (MicroRNAs)

SB - IM

MH - Animals

MH - Mice

MH - Antitubercular Agents/therapeutic use

MH - *COVID-19/genetics

MH - *Tuberculosis/genetics/drug therapy

MH - *Mycobacterium tuberculosis/genetics

MH - *MicroRNAs/therapeutic use

MH - *Tuberculosis, Multidrug-Resistant/microbiology

OTO - NOTNLM

OT - biomarkers

OT - diagnosis

OT - non-coding RNA

OT - prognostic

OT - therapeutic

OT - tuberculosis

EDAT- 2023/05/18 01:07

MHDA- 2023/06/15 06:42

CRDT- 2023/05/17 21:12

PHST- 2022/10/28 00:00 [received]

PHST- 2023/04/27 00:00 [revised]

PHST- 2023/05/16 00:00 [accepted]

PHST- 2023/06/15 06:42 [medline]

PHST- 2023/05/18 01:07 [pubmed]

PHST- 2023/05/17 21:12 [entrez]

AID - 7170045 [pii]

AID - 10.1093/jambio/lxad104 [doi]

PST - ppublish

SO - J Appl Microbiol. 2023 Jun 1;134(6):lxad104. doi: 10.1093/jambio/lxad104.

PMID- 35109718

OWN - NLM

STAT- MEDLINE

DCOM- 20230517

LR - 20230517

IS - 1531-1937 (Electronic)

IS - 0897-1900 (Linking)

VI - 36

IP - 3

DP - 2023 Jun

TI - D-Cycloserine-Induced Seizure Activity in the Emergency Department: A Case

Report.

PG - 716-718

LID - 10.1177/08971900221074955 [doi]

AB - D-cycloserine (DCS) is an anti-tuberculosis medication that has been utilized for

years for drug-resistant tuberculosis. DCS works via a centrally acting mechanism

which can cause neurotoxic adverse effects which has limited its use. This

centrally acting mechanism also allows for DCS to be utilized for various

neuropsychiatric purposes. Our patient was on high-dose DCS for autism spectrum

disorder and presented to the emergency department (ED) with a seizure. The

seizure episode was managed with both anti-epileptics and pyridoxine. With

increasing novel use of this older medication, it is imperative for ED clinicians

to be aware of the different management strategies that may be required when a

patient presents with a neurotoxic effect, specifically seizures, secondary to

DCS.

FAU - Kuhrau, Shannon

AU - Kuhrau S

AUID- ORCID: 0000-0002-5066-293X

AD - Department of Pharmacy, Loyola University Medical Center, Maywood, IL, USA.

RINGGOLD: 25815

FAU - Boykin, Tracy

AU - Boykin T

AD - Department of Emergency Medicine, Stritch School of Medicine, Loyola University

Chicago, Maywood, IL, USA. RINGGOLD: 25815

FAU - Rech, Megan A

AU - Rech MA

AUID- ORCID: 0000-0001-6542-518X

AD - Department of Pharmacy, Loyola University Medical Center, Maywood, IL, USA.

RINGGOLD: 25815

AD - Department of Emergency Medicine, Stritch School of Medicine, Loyola University

Chicago, Maywood, IL, USA. RINGGOLD: 25815

LA - eng

PT - Case Reports

PT - Journal Article

DEP - 20220203

PL - United States

TA - J Pharm Pract

JT - Journal of pharmacy practice

JID - 8900945

RN - 95IK5KI84Z (Cycloserine)

RN - 0 (Anticonvulsants)

SB - IM

MH - Humans

MH - *Cycloserine/adverse effects

MH - *Autism Spectrum Disorder/drug therapy

MH - Anticonvulsants/therapeutic use

MH - Seizures/chemically induced

MH - Emergency Service, Hospital

OTO - NOTNLM

OT - cycloserine

OT - emergency medicine

OT - seizures

EDAT- 2022/02/04 06:00

MHDA- 2023/05/17 06:42

CRDT- 2022/02/03 05:27

PHST- 2023/05/17 06:42 [medline]

PHST- 2022/02/04 06:00 [pubmed]

PHST- 2022/02/03 05:27 [entrez]

AID - 10.1177/08971900221074955 [doi]

PST - ppublish

SO - J Pharm Pract. 2023 Jun;36(3):716-718. doi: 10.1177/08971900221074955. Epub 2022

Feb 3.

PMID- 37244948

OWN - NLM

STAT- MEDLINE

DCOM- 20230529

LR - 20230602

IS - 2045-2322 (Electronic)

IS - 2045-2322 (Linking)

VI - 13

IP - 1

DP - 2023 May 27

TI - Metabolomic analysis of Mycobacterium tuberculosis reveals metabolic profiles for

identification of drug-resistant tuberculosis.

PG - 8655

LID - 10.1038/s41598-023-35882-2 [doi]

LID - 8655

AB - The detection of pre-extensively (pre-XDR) and extensively drug-resistant

tuberculosis (XDR-TB) is challenging. Drug-susceptibility tests for some anti-TB

drugs, especially ethambutol (ETH) and ethionamide (ETO), are problematic due to

overlapping thresholds to differentiate between susceptible and resistant

phenotypes. We aimed to identify possible metabolomic markers to detect

Mycobacterium tuberculosis (Mtb) strains causing pre-XDR and XDR-TB. The

metabolic patterns of ETH- and ETO-resistant Mtb isolates were also investigated.

Metabolomics of 150 Mtb isolates (54 pre-XDR, 63 XDR-TB and 33 pan-susceptible;

pan-S) were investigated. Metabolomics of ETH and ETO phenotypically resistant

subgroups were analyzed using UHPLC-ESI-QTOF-MS/MS. Orthogonal partial

least-squares discriminant analysis revealed distinct separation in all pairwise

comparisons among groups. Two metabolites (meso-hydroxyheme and itaconic

anhydride) were able to differentiate the pre-XDR and XDR-TB groups from the

pan-S group with 100% sensitivity and 100% specificity. In comparisons of the ETH

and ETO phenotypically resistant subsets, sets of increased (ETH = 15, ETO = 7)

and decreased (ETH = 1, ETO = 6) metabolites specific for the resistance

phenotype of each drug were found. We demonstrated the potential for metabolomics

of Mtb to differentiate among types of DR-TB as well as between isolates that

were phenotypically resistant to ETO and ETH. Thus, metabolomics might be further

applied for DR-TB diagnosis and patient management.

CI - © 2023. The Author(s).

FAU - Chaiyachat, Pratchakan

AU - Chaiyachat P

AD - Department of Microbiology, Faculty of Medicine, Khon Kaen University, Khon Kaen,

Thailand.

AD - Research and Diagnostic Center for Emerging Infectious Diseases (RCEID), Khon

Kaen University, Khon Kaen, Thailand.

FAU - Kaewseekhao, Benjawan

AU - Kaewseekhao B

AD - Department of Microbiology, Faculty of Medicine, Khon Kaen University, Khon Kaen,

Thailand.

AD - Research and Diagnostic Center for Emerging Infectious Diseases (RCEID), Khon

Kaen University, Khon Kaen, Thailand.

FAU - Chaiprasert, Angkana

AU - Chaiprasert A

AD - Office for Research and Development, Faculty of Medicine Siriraj Hospital,

Mahidol University, Bangkok, Thailand.

FAU - Kamolwat, Phalin

AU - Kamolwat P

AD - Bureau of Tuberculosis, Department of Disease Control, Ministry of Public Health,

Nonthaburi, Thailand.

FAU - Nonghanphithak, Ditthawat

AU - Nonghanphithak D

AD - Department of Microbiology, Faculty of Medicine, Khon Kaen University, Khon Kaen,

Thailand.

AD - Research and Diagnostic Center for Emerging Infectious Diseases (RCEID), Khon

Kaen University, Khon Kaen, Thailand.

FAU - Phetcharaburanin, Jutarop

AU - Phetcharaburanin J

AD - Department of Systems Biosciences and Computational Medicine, Faculty of

Medicine, Khon Kaen University, Khon Kaen, Thailand.

FAU - Sirichoat, Auttawit

AU - Sirichoat A

AD - Department of Microbiology, Faculty of Medicine, Khon Kaen University, Khon Kaen,

Thailand.

AD - Research and Diagnostic Center for Emerging Infectious Diseases (RCEID), Khon

Kaen University, Khon Kaen, Thailand.

FAU - Ong, Rick Twee-Hee

AU - Ong RT

AD - Saw Swee Hock School of Public Health, National University of Singapore and

National University Health System, Singapore, Singapore.

FAU - Faksri, Kiatichai

AU - Faksri K

AD - Department of Microbiology, Faculty of Medicine, Khon Kaen University, Khon Kaen,

Thailand. kiatichai@kku.ac.th.

AD - Research and Diagnostic Center for Emerging Infectious Diseases (RCEID), Khon

Kaen University, Khon Kaen, Thailand. kiatichai@kku.ac.th.

LA - eng

PT - Journal Article

PT - Research Support, Non-U.S. Gov't

DEP - 20230527

PL - England

TA - Sci Rep

JT - Scientific reports

JID - 101563288

RN - 0 (Antitubercular Agents)

RN - OAY8ORS3CQ (Ethionamide)

RN - 8G167061QZ (Ethambutol)

SB - IM

MH - Humans

MH - *Mycobacterium tuberculosis/genetics

MH - Antitubercular Agents/therapeutic use

MH - *Extensively Drug-Resistant Tuberculosis/microbiology

MH - Tandem Mass Spectrometry

MH - Drug Resistance, Multiple, Bacterial/genetics

MH - *Tuberculosis, Multidrug-Resistant/drug therapy

MH - Ethionamide

MH - Ethambutol/pharmacology

MH - Metabolome

MH - Microbial Sensitivity Tests

PMC - PMC10224971

COIS- The authors declare no competing interests.

EDAT- 2023/05/28 01:07

MHDA- 2023/05/29 06:41

PMCR- 2023/05/27

CRDT- 2023/05/27 23:18

PHST- 2022/08/22 00:00 [received]

PHST- 2023/05/25 00:00 [accepted]

PHST- 2023/05/29 06:41 [medline]

PHST- 2023/05/28 01:07 [pubmed]

PHST- 2023/05/27 23:18 [entrez]

PHST- 2023/05/27 00:00 [pmc-release]

AID - 10.1038/s41598-023-35882-2 [pii]

AID - 35882 [pii]

AID - 10.1038/s41598-023-35882-2 [doi]

PST - epublish

SO - Sci Rep. 2023 May 27;13(1):8655. doi: 10.1038/s41598-023-35882-2.

PMID- 37187890

OWN - NLM

STAT- PubMed-not-MEDLINE

LR - 20230918

IS - 1177-9322 (Print)

IS - 1177-9322 (Electronic)

IS - 1177-9322 (Linking)

VI - 17

DP - 2023

TI - Bioinformatics Analysis to Uncover the Potential Drug Targets Responsible for

Mycobacterium tuberculosis Peptidoglycan and Lysine Biosynthesis.

PG - 11779322231171774

LID - 10.1177/11779322231171774 [doi]

LID - 11779322231171774

AB - Drug-resistant tuberculosis (TB), which results mainly from the selection of

naturally resistant strains of Mycobacterium tuberculosis (MTB) due to mismanaged

treatment, poses a severe challenge to the global control of TB. Therefore,

screening novel and unique drug targets against this pathogen is urgently needed.

The metabolic pathways of Homo sapiens and MTB were compared using the Kyoto

Encyclopedia of Genes and Genomes tool, and further, the proteins that are

involved in the metabolic pathways of MTB were subtracted and proceeded to

protein-protein interaction network analysis, subcellular localization, drug

ability testing, and gene ontology. The study aims to identify enzymes for the

unique pathways for further screening to determine the feasibility of the

therapeutic targets. The qualitative characteristics of 28 proteins identified as

drug target candidates were studied. The results showed that 12 were cytoplasmic,

2 were extracellular, 12 were transmembrane, and 3 were unknown. Furthermore,

druggability analysis revealed 14 druggable proteins, of which 12 were novel and

responsible for MTB peptidoglycan and lysine biosynthesis. The novel targets

obtained in this study are used to develop antimicrobial treatments against

pathogenic bacteria. Future studies should further shed light on the clinical

implementation to identify antimicrobial therapies against MTB.

CI - © The Author(s) 2023.

FAU - Ayu Eka Pitaloka, Dian

AU - Ayu Eka Pitaloka D

AD - Department of Pharmacology and Clinical Pharmacy, Faculty of Pharmacy,

Universitas Padjadjaran, Sumedang, Indonesia.

AD - Center for Translational Biomarker Research, Universitas Padjadjaran, Sumedang,

Indonesia.

FAU - Izzati, Afifah

AU - Izzati A

AD - Department of Pharmacology and Clinical Pharmacy, Faculty of Pharmacy,

Universitas Padjadjaran, Sumedang, Indonesia.

FAU - Rafa Amirah, Siti

AU - Rafa Amirah S

AD - Department of Pharmacology and Clinical Pharmacy, Faculty of Pharmacy,

Universitas Padjadjaran, Sumedang, Indonesia.

FAU - Abdan Syakuran, Luqman

AU - Abdan Syakuran L

AD - Genetics and Molecular Laboratory, Faculty of Biology, Jenderal Soedirman

University, Purwokerto, Indonesia.

FAU - Muhammad Irham, Lalu

AU - Muhammad Irham L

AD - Faculty of Pharmacy, Universitas Ahmad Dahlan, Yogyakarta, Indonesia.

AD - Research Center for Pharmaceutical Ingrediensts and Traditional Medicine,

National Research and Inovation Agency (BRIN), South Tangerang, Indonesia.

FAU - Darumas Putri, Athika

AU - Darumas Putri A

AD - Semarang College of Pharmaceutical Sciences (STIFAR), Semarang, Indonesia.

FAU - Adikusuma, Wirawan

AU - Adikusuma W

AD - Department of Pharmacy, Faculty of Health Science, Universitas Muhammadiyah

Mataram, Mataram, Indonesia.

AD - Research Center for Vaccine and Drugs, National Research and Inovation Agency

(BRIN), South Tangerang, Indonesia.

LA - eng

PT - Journal Article

DEP - 20230510

PL - United States

TA - Bioinform Biol Insights

JT - Bioinformatics and biology insights

JID - 101467187

PMC - PMC10176782

OTO - NOTNLM

OT - In silico drug targets

OT - Mycobacterium tuberculosis

OT - metabolic pathways

OT - protein-protein interaction

COIS- The author(s) declared no potential conflicts of interest with respect to the

research, authorship, and/or publication of this article.

EDAT- 2023/05/16 01:09

MHDA- 2023/05/16 01:10

PMCR- 2023/05/10

CRDT- 2023/05/15 19:33

PHST- 2022/11/29 00:00 [received]

PHST- 2023/04/07 00:00 [accepted]

PHST- 2023/05/16 01:09 [pubmed]

PHST- 2023/05/16 01:10 [medline]

PHST- 2023/05/15 19:33 [entrez]

PHST- 2023/05/10 00:00 [pmc-release]

AID - 10.1177_11779322231171774 [pii]

AID - 10.1177/11779322231171774 [doi]

PST - epublish

SO - Bioinform Biol Insights. 2023 May 10;17:11779322231171774. doi:

10.1177/11779322231171774. eCollection 2023.

PMID- 36963294

OWN - NLM

STAT- MEDLINE

DCOM- 20230602

LR - 20230627

IS - 1873-281X (Electronic)

IS - 1472-9792 (Linking)

VI - 140

DP - 2023 May

TI - In vitro activity of tetracycline analogs against multidrug-resistant and

extensive drug resistance clinical isolates of Mycobacterium tuberculosis.

PG - 102336

LID - S1472-9792(23)00034-3 [pii]

LID - 10.1016/j.tube.2023.102336 [doi]

AB - BACKGROUND: Multidrug-resistant tuberculosis (MDR-TB) has become a big threaten

to global health. The current strategy for treatment of MDR-TB and extensive drug

resistant tuberculosis (XDR-TB) is with low efficacy and high side effect. While

new drug is fundamental for cure MDR-TB, repurposing the Food and Drug

Administration (FDA)-approved drugs represents an alternative soluation with less

cost. METHODS: The activity of 8 tetracycline-class antibiotics against

mycobacterium tuberculosis (M.tb) were determined by Minimum Inhibitory

Concentration (MIC) in vitro. A transposon M.smeg libraries was generated by

using the Harm phage and then used to isolate the conditional growth mutants in

doxycycline containing plate. Eleven mutants were isolated and genomic DNAs were

extracted using the cetyltrimethyl ammonium bromide (CTAB) method and analyzed by

whole genome sequencing. RESULTS: We found that three of eight drugs efficiently

inhibited mycobacteria growth under the peak plasma concentration in the human

body. Further tests showed these three tetracycline analogs (demeclocycline,

doxycycline and methacycline) had antimicrobial activity against seven clinical

isolates, including MDR and XDR strains. Among them, Doxycycline had the lowest

MICs in all mycobacteria strains tested in this study. By using a transposon

library, we identify the insertion of transposon in two genes, porin and MshA,

associatewith the resistant to doxycycline. CONCLUSIONS: Our findings show that

tetracycline analogs such as doxycycline, has bactericidal activity against not

only drug sensitive M.tb, but also clinical MDR and XDR strains, provided proof

of concept to repurpose doxycycline to fight MDR-TB and XDR-TB. Further

investigations are warranted to clarify the underlying mechanism and optimize the

strategy in combination with other anti-TB drugs.

CI - Copyright © 2023 The Authors. Published by Elsevier Ltd.. All rights reserved.

FAU - Zhang, Chi

AU - Zhang C

AD - Department of Laboratory Medicine, Shenzhen University General Hospital, Shenzhen

University, Shenzhen, China.

FAU - Ouyang, Qi

AU - Ouyang Q

AD - Guangdong Key Lab of Regional Immunity and Diseases, Department of Pathogen

Biology, Shenzhen University School of Medicine, Shenzhen University, Shenzhen,

China.

FAU - Zhou, Xianyuan

AU - Zhou X

AD - Department of Laboratory Medicine, Shenzhen University General Hospital, Shenzhen

University, Shenzhen, China.

FAU - Huang, Yingfeng

AU - Huang Y

AD - Department of Laboratory Medicine, Shenzhen University General Hospital, Shenzhen

University, Shenzhen, China.

FAU - Zeng, Yu

AU - Zeng Y

AD - Department of Laboratory Medicine, Shenzhen University General Hospital, Shenzhen

University, Shenzhen, China.

FAU - Deng, Li

AU - Deng L

AD - Department of Laboratory Medicine, Shenzhen University General Hospital, Shenzhen

University, Shenzhen, China.

FAU - Lin, Dachuan

AU - Lin D

AD - Guangdong Key Lab of Regional Immunity and Diseases, Department of Pathogen

Biology, Shenzhen University School of Medicine, Shenzhen University, Shenzhen,

China. Electronic address: lindachuan@szu.edu.cn.

FAU - Zheng, Weidong

AU - Zheng W

AD - Department of Laboratory Medicine, Shenzhen University General Hospital, Shenzhen

University, Shenzhen, China. Electronic address: zwdin@szu.edu.cn.

LA - eng

PT - Journal Article

PT - Research Support, Non-U.S. Gov't

DEP - 20230311

PL - Scotland

TA - Tuberculosis (Edinb)

JT - Tuberculosis (Edinburgh, Scotland)

JID - 100971555

RN - 0 (Antitubercular Agents)

RN - N12000U13O (Doxycycline)

RN - F8VB5M810T (Tetracycline)

SB - IM

MH - Humans

MH - *Mycobacterium tuberculosis

MH - Antitubercular Agents/pharmacology/therapeutic use

MH - *Extensively Drug-Resistant Tuberculosis/microbiology

MH - Doxycycline/pharmacology/therapeutic use

MH - *Tuberculosis, Multidrug-Resistant/drug therapy/microbiology

MH - Tetracycline/pharmacology/therapeutic use

MH - Drug Resistance

MH - Microbial Sensitivity Tests

MH - Drug Resistance, Multiple, Bacterial/genetics

OTO - NOTNLM

OT - Doxycycline

OT - Multiple-drug resistance

OT - Mycobacterium tuberculosis

OT - Tetracycline

COIS- Declaration of competing interest The authors declare that they have no competing

interests.

EDAT- 2023/03/25 06:00

MHDA- 2023/06/02 06:43

CRDT- 2023/03/24 19:07

PHST- 2022/12/08 00:00 [received]

PHST- 2023/02/27 00:00 [revised]

PHST- 2023/03/10 00:00 [accepted]

PHST- 2023/06/02 06:43 [medline]

PHST- 2023/03/25 06:00 [pubmed]

PHST- 2023/03/24 19:07 [entrez]

AID - S1472-9792(23)00034-3 [pii]

AID - 10.1016/j.tube.2023.102336 [doi]

PST - ppublish

SO - Tuberculosis (Edinb). 2023 May;140:102336. doi: 10.1016/j.tube.2023.102336. Epub

2023 Mar 11.

PMID- 37117557

OWN - NLM

STAT- MEDLINE

DCOM- 20230501

LR - 20230510

IS - 2045-2322 (Electronic)

IS - 2045-2322 (Linking)

VI - 13

IP - 1

DP - 2023 Apr 28

TI - An integrated computational approach towards novel drugs discovery against

polyketide synthase 13 thioesterase domain of Mycobacterium tuberculosis.

PG - 7014

LID - 10.1038/s41598-023-34222-8 [doi]

LID - 7014

AB - The acquired drug resistance by Mycobacterium tuberculosis (M. tuberculosis) to

antibiotics urges the need for developing novel anti-M. tuberculosis drugs that

possess novel mechanism of action. Since traditional drug discovery is a

labor-intensive and costly process, computer aided drug design is highly

appreciated tool as it speeds up and lower the cost of drug development process.

Herein, Asinex antibacterial compounds were virtually screened against

thioesterase domain of Polyketide synthase 13, a unique enzyme that forms α-alkyl

β-ketoesters as a direct precursor of mycolic acids which are essential

components of the lipid-rich cell wall of M. tuberculosis. The study identified

three drug-like compounds as the most promising leads; BBB_26582140, BBD_30878599

and BBC_29956160 with binding energy value of - 11.25 kcal/mol, - 9.87 kcal/mol

and - 9.33 kcal/mol, respectively. The control molecule binding energy score is

-9.25 kcal/mol. Also, the docked complexes were dynamically stable with maximum

root mean square deviation (RMSD) value of 3 Å. Similarly, the MM-GB\PBSA method

revealed highly stable complexes with mean energy values < - 75 kcal/mol for all

three systems. The net binding energy scores are validated by WaterSwap and

entropy energy analysis. Furthermore, The in silico druglike and pharmacokinetic

investigation revealed that the compounds could be suitable candidates for

additional experimentations. In summary, the study findings are significant, and

the compounds may be used in experimental validation pipeline to develop

potential drugs against drug-resistant tuberculosis.

CI - © 2023. The Author(s).

FAU - Altharawi, Ali

AU - Altharawi A

AD - Department of Pharmaceutical Chemistry, College of Pharmacy, Prince Sattam Bin

Abdulaziz University, Al-Kharj, 11942, Saudi Arabia.

FAU - Alossaimi, Manal A

AU - Alossaimi MA

AD - Department of Pharmaceutical Chemistry, College of Pharmacy, Prince Sattam Bin

Abdulaziz University, Al-Kharj, 11942, Saudi Arabia.

FAU - Alanazi, Mohammed M

AU - Alanazi MM

AD - Department of Pharmaceutical Chemistry, College of Pharmacy, King Saud

University, Riyadh, 11451, Saudi Arabia.

FAU - Alqahatani, Safar M

AU - Alqahatani SM

AD - Department of Pharmaceutical Chemistry, College of Pharmacy, Prince Sattam Bin

Abdulaziz University, Al-Kharj, 11942, Saudi Arabia.

FAU - Tahir Ul Qamar, Muhammad

AU - Tahir Ul Qamar M

AD - Department of Bioinformatics and Biotechnology, Government College University

Faisalabad (GCUF), Faisalabad, 38000, Pakistan. tahirulqamar@gcuf.edu.pk.

LA - eng

PT - Journal Article

PT - Research Support, Non-U.S. Gov't

DEP - 20230428

PL - England

TA - Sci Rep

JT - Scientific reports

JID - 101563288

RN - 79956-01-7 (Polyketide Synthases)

RN - 0 (Antitubercular Agents)

SB - IM

MH - Humans

MH - *Mycobacterium tuberculosis

MH - Polyketide Synthases

MH - Molecular Dynamics Simulation

MH - Antitubercular Agents/pharmacology/chemistry

MH - Drug Discovery

MH - *Tuberculosis/drug therapy/microbiology

MH - Molecular Docking Simulation

PMC - PMC10147368

COIS- The authors declare no competing interests.

EDAT- 2023/04/29 06:04

MHDA- 2023/05/01 06:42

PMCR- 2023/04/28

CRDT- 2023/04/28 23:27

PHST- 2023/02/22 00:00 [received]

PHST- 2023/04/26 00:00 [accepted]

PHST- 2023/05/01 06:42 [medline]

PHST- 2023/04/29 06:04 [pubmed]

PHST- 2023/04/28 23:27 [entrez]

PHST- 2023/04/28 00:00 [pmc-release]

AID - 10.1038/s41598-023-34222-8 [pii]

AID - 34222 [pii]

AID - 10.1038/s41598-023-34222-8 [doi]

PST - epublish

SO - Sci Rep. 2023 Apr 28;13(1):7014. doi: 10.1038/s41598-023-34222-8.

PMID- 37239338

OWN - NLM

STAT- MEDLINE

DCOM- 20230529

LR - 20240919

IS - 2073-4425 (Electronic)

IS - 2073-4425 (Linking)

VI - 14

IP - 5

DP - 2023 Apr 26

TI - MOSR and NDHA Genes Comprising G-Quadruplex as Promising Therapeutic Targets

against Mycobacterium tuberculosis: Molecular Recognition by Mitoxantrone

Suppresses Replication and Gene Regulation.

LID - 10.3390/genes14050978 [doi]

LID - 978

AB - Occurrence of non-canonical G-quadruplex (G4) DNA structures in the genome have

been recognized as key factors in gene regulation and several other cellular

processes. The mosR and ndhA genes involved in pathways of oxidation sensing

regulation and ATP generation, respectively, make Mycobacterium tuberculosis

(Mtb) bacteria responsible for oxidative stress inside host macrophage cells.

Circular Dichroism spectra demonstrate stable hybrid G4 DNA conformations of

mosR/ndhA DNA sequences. Real-time binding of mitoxantrone to G4 DNA with an

affinity constant ~10(5)-10(7) M(-1), leads to hypochromism with a red shift of

~18 nm, followed by hyperchromism in the absorption spectra. The corresponding

fluorescence is quenched with a red shift ~15 nm followed by an increase in

intensity. A change in conformation of the G4 DNA accompanies the formation of

multiple stoichiometric complexes with a dual binding mode. The external binding

of mitoxantrone with a partial stacking with G-quartets and/or groove binding

induces significant thermal stabilization, ~20-29 °C in ndhA/mosR G4 DNA. The

interaction leads to a two/four-fold downregulation of transcriptomes of

mosR/ndhA genes apart from the suppression of DNA replication by Taq polymerase

enzyme, establishing the role of mitoxantrone in targeting G4 DNA, as an

alternate strategy for effective anti-tuberculosis action in view of deadly

multi-drug resistant tuberculosis disease causing bacterial strains t that arise

from existing therapeutic treatments.

FAU - Dey, Arpita

AU - Dey A

AD - Department of Biosciences and Bioengineering, Indian Institute of Technology

Roorkee, Roorkee 247667, Uttarakhand, India.

FAU - Anand, Kushi

AU - Anand K

AUID- ORCID: 0000-0001-5929-3176

AD - Centre for Infectious Disease Research, Indian Institute of Science, Bengaluru

560012, Karnataka, India.

FAU - Singh, Amit

AU - Singh A

AD - Centre for Infectious Disease Research, Indian Institute of Science, Bengaluru

560012, Karnataka, India.

FAU - Prasad, Ramasare

AU - Prasad R

AUID- ORCID: 0000-0001-7341-2346

AD - Department of Biosciences and Bioengineering, Indian Institute of Technology

Roorkee, Roorkee 247667, Uttarakhand, India.

FAU - Barthwal, Ritu

AU - Barthwal R

AUID- ORCID: 0000-0002-4038-2256

AD - Department of Biosciences and Bioengineering, Indian Institute of Technology

Roorkee, Roorkee 247667, Uttarakhand, India.

LA - eng

GR - WT_/Wellcome Trust/United Kingdom

PT - Journal Article

PT - Research Support, Non-U.S. Gov't

DEP - 20230426

PL - Switzerland

TA - Genes (Basel)

JT - Genes

JID - 101551097

RN - BZ114NVM5P (Mitoxantrone)

RN - 9007-49-2 (DNA)

SB - IM

MH - Mitoxantrone/pharmacology/chemistry

MH - *Mycobacterium tuberculosis/genetics/metabolism

MH - DNA/genetics

MH - *G-Quadruplexes

MH - Base Sequence

PMC - PMC10217741

OTO - NOTNLM

OT - DNA replication

OT - DNA stabilization

OT - DNA targeting by mitoxantrone

OT - G-quadruplex

OT - Mtb

OT - downregulation

OT - mosR/ndhA genes

OT - potent G-quadruplex ligand

OT - spectroscopy and calorimetry

COIS- The authors declare no competing interest.

EDAT- 2023/05/27 09:42

MHDA- 2023/05/29 06:42

PMCR- 2023/04/26

CRDT- 2023/05/27 01:10

PHST- 2022/11/15 00:00 [received]

PHST- 2022/12/19 00:00 [revised]

PHST- 2023/04/18 00:00 [accepted]

PHST- 2023/05/29 06:42 [medline]

PHST- 2023/05/27 09:42 [pubmed]

PHST- 2023/05/27 01:10 [entrez]

PHST- 2023/04/26 00:00 [pmc-release]

AID - genes14050978 [pii]

AID - genes-14-00978 [pii]

AID - 10.3390/genes14050978 [doi]

PST - epublish

SO - Genes (Basel). 2023 Apr 26;14(5):978. doi: 10.3390/genes14050978.

PMID- 37017524

OWN - NLM

STAT- MEDLINE

DCOM- 20230427

LR - 20230509

IS - 2150-7511 (Electronic)

VI - 14

IP - 2

DP - 2023 Apr 25

TI - A Novel Tool to Identify Bactericidal Compounds against Vulnerable Targets in

Drug-Tolerant M. tuberculosis found in Caseum.

PG - e0059823

LID - 10.1128/mbio.00598-23 [doi]

LID - e00598-23

AB - Caseous necrosis is a hallmark of tuberculosis (TB) pathology and creates a niche

for drug-tolerant persisters within the host. Cavitary TB and high bacterial

burden in caseum require longer treatment duration. An in vitro model that

recapitulates the major features of Mycobacterium tuberculosis (Mtb) in caseum

would accelerate the identification of compounds with treatment-shortening

potential. We have developed a caseum surrogate model consisting of lysed and

denatured foamy macrophages. Upon inoculation of Mtb from replicating cultures,

the pathogen adapts to the lipid-rich matrix and gradually adopts a

nonreplicating state. We determined that the lipid composition of ex vivo caseum

and the surrogate matrix are similar. We also observed that Mtb in caseum

surrogate accumulates intracellular lipophilic inclusions (ILI), a distinctive

characteristic of quiescent and drug-tolerant Mtb. Expression profiling of a

representative gene subset revealed common signatures between the models.

Comparison of Mtb drug susceptibility in caseum and caseum surrogate revealed

that both populations are similarly tolerant to a panel of TB drugs. By screening

drug candidates in the surrogate model, we determined that the bedaquiline

analogs TBAJ876 and TBAJ587, currently in clinical development, exhibit superior

bactericidal against caseum-resident Mtb, both alone and as substitutions for

bedaquiline in the bedaquiline-pretomanid-linezolid regimen approved for the

treatment of multidrug-resistant TB. In summary, we have developed a

physiologically relevant nonreplicating persistence model that reflects the

distinct metabolic and drug-tolerant state of Mtb in caseum. IMPORTANCE M.

tuberculosis (Mtb) within the caseous core of necrotic granulomas and cavities is

extremely drug tolerant and presents a significant hurdle to treatment success

and relapse prevention. Many in vitro models of nonreplicating persistence have

been developed to characterize the physiologic and metabolic adaptations of Mtb

and identify compounds active against this treatment-recalcitrant population.

However, there is little consensus on their relevance to in vivo infection. Using

lipid-laden macrophage lysates, we have designed and validated a surrogate matrix

that closely mimics caseum and in which Mtb develops a phenotype similar to that

of nonreplicating bacilli in vivo. The assay is well suited to screen for

bactericidal compounds against caseum-resident Mtb in a medium-throughput format,

allowing for reduced reliance on resource intensive animal models that present

large necrotic lesions and cavities. Importantly, this approach will aid the

identification of vulnerable targets in caseum Mtb and can accelerate the

development of novel TB drugs with treatment-shortening potential.

FAU - Sarathy, Jansy P

AU - Sarathy JP

AD - Center for Discovery and Innovation, Hackensack Meridian Health, Nutley, New

Jersey, USA.

FAU - Xie, Min

AU - Xie M

AD - Center for Discovery and Innovation, Hackensack Meridian Health, Nutley, New

Jersey, USA.

FAU - Jones, Richard M

AU - Jones RM

AD - Department of Microbiology, University of Washington, Seattle, Washington, USA.

FAU - Chang, Adrienne

AU - Chang A

AD - Nancy E. and Peter C. Meinig School of Biomedical Engineering, Cornell

University, Ithaca, New York, USA.

FAU - Osiecki, Paulina

AU - Osiecki P

AD - Center for Discovery and Innovation, Hackensack Meridian Health, Nutley, New

Jersey, USA.

FAU - Weiner, Danielle

AU - Weiner D

AD - Tuberculosis Research Section, Laboratory of Clinical Infectious Diseases, NIAID,

NIH, Bethesda, Maryland, USA.

AD - Tuberculosis Imaging Program, Division of Intramural Research, NIAID, NIH,

Bethesda, Maryland, USA.

FAU - Tsao, Wen-Shan

AU - Tsao WS

AD - Center for Discovery and Innovation, Hackensack Meridian Health, Nutley, New

Jersey, USA.

FAU - Dougher, Maureen

AU - Dougher M

AD - Center for Discovery and Innovation, Hackensack Meridian Health, Nutley, New

Jersey, USA.

FAU - Blanc, Landry

AU - Blanc L

AD - University of Bordeaux, CNRS, CBMN, UMR 5248, Pessac, France.

FAU - Fotouhi, Nader

AU - Fotouhi N

AD - Global Alliance for TB Drug Development, New York, New York, USA.

FAU - Via, Laura E

AU - Via LE

AD - Tuberculosis Research Section, Laboratory of Clinical Infectious Diseases, NIAID,

NIH, Bethesda, Maryland, USA.

AD - Tuberculosis Imaging Program, Division of Intramural Research, NIAID, NIH,

Bethesda, Maryland, USA.

FAU - Barry, Clifton E 3rd

AU - Barry CE 3rd

AD - Tuberculosis Research Section, Laboratory of Clinical Infectious Diseases, NIAID,

NIH, Bethesda, Maryland, USA.

AD - Institute of Infectious Disease and Molecular Medicine, Faculty of Health

Sciences, University of Cape Town, Cape Town, South Africa.

FAU - De Vlaminck, Iwijn

AU - De Vlaminck I

AD - Nancy E. and Peter C. Meinig School of Biomedical Engineering, Cornell

University, Ithaca, New York, USA.

FAU - Sherman, David R

AU - Sherman DR

AD - Department of Microbiology, University of Washington, Seattle, Washington, USA.

FAU - Dartois, Véronique A

AU - Dartois VA

AUID- ORCID: 0000-0001-9470-5009

AD - Center for Discovery and Innovation, Hackensack Meridian Health, Nutley, New

Jersey, USA.

AD - Hackensack Meridian School of Medicine, Department of Medical Sciences, Nutley,

New Jersey, USA.

LA - eng

PT - Journal Article

PT - Research Support, N.I.H., Intramural

PT - Research Support, Non-U.S. Gov't

DEP - 20230405

PL - United States

TA - mBio

JT - mBio

JID - 101519231

RN - 0 (Antitubercular Agents)

RN - 0 (Lipids)

SB - IM

MH - Animals

MH - *Mycobacterium tuberculosis/genetics

MH - Antitubercular Agents/pharmacology/therapeutic use

MH - *Tuberculosis/drug therapy/microbiology

MH - *Tuberculosis, Multidrug-Resistant

MH - Lipids

PMC - PMC10127596

OTO - NOTNLM

OT - caseum

OT - nonreplicating Mycobacterium tuberculosis

OT - persister

OT - tuberculosis

OT - vulnerable target

COIS- The authors declare no conflict of interest.

EDAT- 2023/04/06 06:00

MHDA- 2023/04/27 06:42

PMCR- 2023/04/05

CRDT- 2023/04/05 10:05

PHST- 2023/04/27 06:42 [medline]

PHST- 2023/04/06 06:00 [pubmed]

PHST- 2023/04/05 10:05 [entrez]

PHST- 2023/04/05 00:00 [pmc-release]

AID - 00598-23 [pii]

AID - mbio.00598-23 [pii]

AID - 10.1128/mbio.00598-23 [doi]

PST - ppublish

SO - mBio. 2023 Apr 25;14(2):e0059823. doi: 10.1128/mbio.00598-23. Epub 2023 Apr 5.

PMID- 37153159

OWN - NLM

STAT- MEDLINE

DCOM- 20230509

LR - 20240917

IS - 2235-2988 (Electronic)

IS - 2235-2988 (Linking)

VI - 13

DP - 2023

TI - A pro-oxidant property of vitamin C to overcome the burden of latent

Mycobacterium tuberculosis infection: A cross-talk review with Fenton reaction.

PG - 1152269

LID - 10.3389/fcimb.2023.1152269 [doi]

LID - 1152269

AB - Tuberculosis (TB), caused by the bacillus M. tuberculosis, is one of the

deadliest infectious illnesses of our day, along with HIV and

malaria.Chemotherapy, the cornerstone of TB control efforts, is jeopardized by

the advent of M. tuberculosis strains resistant to many, if not all, of the

existing medications.Isoniazid (INH), rifampicin (RIF), pyrazinamide, and

ethambutol are used to treat drug-susceptible TB for two months, followed by four

months of INH and RIF, but chemotherapy with potentially harmful side effects is

sometimes needed to treat multidrug-resistant (MDR) TB for up to two years.

Chemotherapy might be greatly shortened by drugs that kill M. tuberculosis more

quickly while simultaneously limiting the emergence of drug resistance.Regardless

of their intended target, bactericidal medicines commonly kill pathogenic

bacteria (gram-negative and gram-positive) by producing hydroxyl radicals via the

Fenton reaction.Researchers have concentrated on vitamins with bactericidal

properties to address the rising cases globally and have discovered that these

vitamins are effective when given along with first-line drugs. The presence of

elevated iron content, reactive oxygen species (ROS) generation, and DNA damage

all contributed to VC's sterilizing action on M. tb in vitro. Moreover, it has a

pleiotropic effect on a variety of biological processes such as detoxification,

protein folding - chaperons, cell wall processes, information pathways,

regulatory, virulence, metabolism etc.In this review report, the authors

extensively discussed the effects of VC on M. tb., such as the generation of free

radicals and bactericidal mechanisms with existing treatments, and their further

drug development based on ROS production.

CI - Copyright © 2023 Gaglani, Dwivedi, Upadhyay, Kaushal, Ahmad and Saeed.

FAU - Gaglani, Pratikkumar

AU - Gaglani P

AD - Department of Life Sciences, Parul Institute of Applied Sciences and Biophysics

and Structural Biology Laboratory, Center of Research for Development, Parul

University, Vadodara, Gujarat, India.

FAU - Dwivedi, Manish

AU - Dwivedi M

AD - Amity Institute of Biotechnology, Amity University, Lucknow, Uttar Pradesh,

India.

FAU - Upadhyay, Tarun Kumar

AU - Upadhyay TK

AD - Department of Life Sciences, Parul Institute of Applied Sciences and Animal Cell

Culture and Immunobiochemistry Lab, Center of Research for Development, Parul

University, Vadodara, Gujarat, India.

FAU - Kaushal, Radhey Shyam

AU - Kaushal RS

AD - Department of Life Sciences, Parul Institute of Applied Sciences and Biophysics

and Structural Biology Laboratory, Center of Research for Development, Parul

University, Vadodara, Gujarat, India.

FAU - Ahmad, Irfan

AU - Ahmad I

AD - Department of Clinical Laboratory Sciences, College of Applied Medical Sciences,

King Khalid University, Abha, Saudi Arabia.

FAU - Saeed, Mohd

AU - Saeed M

AD - Department of Biology, College of Sciences, University of Hail, Hail, Saudi

Arabia.

LA - eng

PT - Journal Article

PT - Research Support, Non-U.S. Gov't

PT - Review

DEP - 20230419

PL - Switzerland

TA - Front Cell Infect Microbiol

JT - Frontiers in cellular and infection microbiology

JID - 101585359

RN - 0 (Antitubercular Agents)

RN - 0 (Reactive Oxygen Species)

RN - PQ6CK8PD0R (Ascorbic Acid)

RN - VJT6J7R4TR (Rifampin)

RN - 0 (Vitamins)

SB - IM

MH - Humans

MH - Antitubercular Agents/pharmacology/therapeutic use

MH - Reactive Oxygen Species

MH - Ascorbic Acid/pharmacology

MH - *Tuberculosis/drug therapy

MH - *Mycobacterium tuberculosis/genetics

MH - *Tuberculosis, Multidrug-Resistant/drug therapy

MH - Rifampin/pharmacology

MH - *Latent Tuberculosis

MH - Vitamins

MH - Microbial Sensitivity Tests

PMC - PMC10155705

OTO - NOTNLM

OT - Fenton reaction

OT - Mycobacterium tuberculosis

OT - drug resistance

OT - oxidative burst

OT - pro-oxidant property

OT - reactive oxygen species (ROS)

OT - vitamin C

COIS- The authors declare that the research was conducted in the absence of any

commercial or financial relationships that could be construed as a potential

conflict of interest.

EDAT- 2023/05/08 06:41

MHDA- 2023/05/09 10:16

PMCR- 2023/01/01

CRDT- 2023/05/08 04:12

PHST- 2023/01/27 00:00 [received]

PHST- 2023/03/17 00:00 [accepted]

PHST- 2023/05/09 10:16 [medline]

PHST- 2023/05/08 06:41 [pubmed]

PHST- 2023/05/08 04:12 [entrez]

PHST- 2023/01/01 00:00 [pmc-release]

AID - 10.3389/fcimb.2023.1152269 [doi]

PST - epublish

SO - Front Cell Infect Microbiol. 2023 Apr 19;13:1152269. doi:

10.3389/fcimb.2023.1152269. eCollection 2023.

PMID- 36975792

OWN - NLM

STAT- MEDLINE

DCOM- 20230420

LR - 20230929

IS - 1098-6596 (Electronic)

IS - 0066-4804 (Print)

IS - 0066-4804 (Linking)

VI - 67

IP - 4

DP - 2023 Apr 18

TI - Pharmacokinetics and Efficacy of the Benzothiazinone BTZ-043 against Tuberculous

Mycobacteria inside Granulomas in the Guinea Pig Model.

PG - e0143822

LID - 10.1128/aac.01438-22 [doi]

LID - e01438-22

AB - Tuberculosis (TB), caused by Mycobacterium tuberculosis, is the world's leading

cause of mortality from a single bacterial pathogen. With increasing frequency,

emergence of drug-resistant mycobacteria leads to failures of standard TB

treatment regimens. Therefore, new anti-TB drugs are urgently required. BTZ-043

belongs to a novel class of nitrobenzothiazinones, which inhibit mycobacterial

cell wall formation by covalent binding of an essential cysteine in the catalytic

pocket of decaprenylphosphoryl-β-d-ribose oxidase (DprE1). Thus, the compound

blocks the formation of decaprenylphosphoryl-β-d-arabinose, a precursor for the

synthesis of arabinans. An excellent in vitro efficacy against M. tuberculosis

has been demonstrated. Guinea pigs are an important small-animal model to study

anti-TB drugs, as they are naturally susceptible to M. tuberculosis and develop

human-like granulomas after infection. In the current study, dose-finding

experiments were conducted to establish the appropriate oral dose of BTZ-043 for

the guinea pig. Subsequently, it could be shown that the active compound was

present at high concentrations in Mycobacterium bovis BCG-induced granulomas. To

evaluate its therapeutic effect, guinea pigs were subcutaneously infected with

virulent M. tuberculosis and treated with BTZ-043 for 4 weeks. BTZ-043-treated

guinea pigs had reduced and less necrotic granulomas than vehicle-treated

controls. In comparison to the vehicle controls a highly significant reduction of

the bacterial burden was observed after BTZ-043 treatment at the site of

infection and in the draining lymph node and spleen. Together, these findings

indicate that BTZ-043 holds great promise as a new antimycobacterial drug.

FAU - Eckhardt, Emmelie

AU - Eckhardt E

AD - Friedrich-Loeffler-Institut, Federal Research Institute for Animal Health,

Greifswald, Germany.

FAU - Li, Yan

AU - Li Y

AD - Transfer Group Anti-infectives, Leibniz Institute for Natural Product Research

and Infection Biology, Leibniz-HKI, Jena, Germany.

FAU - Mamerow, Svenja

AU - Mamerow S

AD - Friedrich-Loeffler-Institut, Federal Research Institute for Animal Health, Jena,

Germany.

FAU - Schinköthe, Jan

AU - Schinköthe J

AD - Institute of Veterinary Pathology, Faculty of Veterinary Medicine, Leipzig

University, Leipzig, Germany.

FAU - Sehl-Ewert, Julia

AU - Sehl-Ewert J

AD - Friedrich-Loeffler-Institut, Federal Research Institute for Animal Health,

Greifswald, Germany.

FAU - Dreisbach, Julia

AU - Dreisbach J

AD - Division of Infectious Diseases and Tropical Medicine, University Hospital of the

University of Munich (LMU), Munich, Germany.

AD - German Center for Infection Research (DZIF), Partner Site Munich, Munich,

Germany.

FAU - Corleis, Björn

AU - Corleis B

AD - Friedrich-Loeffler-Institut, Federal Research Institute for Animal Health,

Greifswald, Germany.

FAU - Dorhoi, Anca

AU - Dorhoi A

AD - Friedrich-Loeffler-Institut, Federal Research Institute for Animal Health,

Greifswald, Germany.

FAU - Teifke, Jens

AU - Teifke J

AD - Friedrich-Loeffler-Institut, Federal Research Institute for Animal Health,

Greifswald, Germany.

FAU - Menge, Christian

AU - Menge C

AD - Friedrich-Loeffler-Institut, Federal Research Institute for Animal Health, Jena,

Germany.

FAU - Kloss, Florian

AU - Kloss F

AD - Transfer Group Anti-infectives, Leibniz Institute for Natural Product Research

and Infection Biology, Leibniz-HKI, Jena, Germany.

FAU - Bastian, Max

AU - Bastian M

AUID- ORCID: 0000-0001-7926-8568

AD - Friedrich-Loeffler-Institut, Federal Research Institute for Animal Health,

Greifswald, Germany.

LA - eng

PT - Journal Article

PT - Research Support, Non-U.S. Gov't

DEP - 20230328

PL - United States

TA - Antimicrob Agents Chemother

JT - Antimicrobial agents and chemotherapy

JID - 0315061

RN - 0 (Antitubercular Agents)

RN - EC 1.- (Oxidoreductases)

SB - IM

MH - Guinea Pigs

MH - Animals

MH - Humans

MH - *Tuberculosis/drug therapy/microbiology

MH - *Mycobacterium tuberculosis

MH - Antitubercular Agents/pharmacology/therapeutic use/chemistry

MH - *Mycobacterium bovis

MH - Oxidoreductases

PMC - PMC10112198

OTO - NOTNLM

OT - BTZ-043

OT - MDR-TB

OT - Mycobacterium bovis BCG

OT - Mycobacterium tuberculosis

OT - guinea pig

OT - new antibiotics

OT - treatment

COIS- The authors declare no conflict of interest.

EDAT- 2023/03/29 06:00

MHDA- 2023/04/20 06:42

PMCR- 2023/09/28

CRDT- 2023/03/28 10:06

PHST- 2023/04/20 06:42 [medline]

PHST- 2023/03/29 06:00 [pubmed]

PHST- 2023/03/28 10:06 [entrez]

PHST- 2023/09/28 00:00 [pmc-release]

AID - 01438-22 [pii]

AID - aac.01438-22 [pii]

AID - 10.1128/aac.01438-22 [doi]

PST - ppublish

SO - Antimicrob Agents Chemother. 2023 Apr 18;67(4):e0143822. doi:

10.1128/aac.01438-22. Epub 2023 Mar 28.

PMID- 36943050

OWN - NLM

STAT- PubMed-not-MEDLINE

DCOM- 20260326

LR - 20260326

IS - 2165-0497 (Electronic)

IS - 2165-0497 (Linking)

VI - 11

IP - 2

DP - 2023 Apr 13

TI - Identification and Quantification of S-Sulfenylation Proteome of Mycobacterium

tuberculosis under Oxidative Stress.

PG - e0338622

LID - 10.1128/spectrum.03386-22 [doi]

LID - e03386-22

AB - The ability to maintain redox homeostasis is critical for Mycobacterium

tuberculosis (Mtb) to survive the redox stress of the host. There are many

antioxidant systems in Mtb to ensure its normal replication and survival in the

host, and cysteine thiols are one of them. S-sulfenylation is one of the

reversible modifications of cysteine thiols to resist oxidative stress. In the

study, we investigated the total cysteine thiols modification and S-sulfenylation

modification of Mtb proteome under the oxidative stress provided by hydrogen

peroxide. To determine and quantify the S-sulfenylation modified proteins, high

specific IodoTMT6plex reagents and high resolution mass spectrometry were used to

label and quantify the peptides and proteins modified. There are significant

differences for the total cysteine modification levels of 279 proteins and

S-sulfenylation modification levels of 297 proteins under hydrogen peroxide

stress. Functional enrichment analysis indicated that these cysteine-modified

proteins were involved in the oxidation-reduction process, fatty acid

biosynthetic process, stress response, protein repair, cell wall, etc. In

conclusion, our study provides a view of cysteine modifications of the Mtb

proteome under oxidative stress, revealing a series of proteins that may play a

role in maintaining redox homeostasis. IMPORTANCE With the continuous spread of

drug-resistant tuberculosis, there is an urgent need for new antituberculosis

drugs with new mechanisms. The ability of Mtb to resist oxidative stress is

extremely important for maintaining redox homeostasis and survival in the host.

The reversible modifications of cysteine residues have a dual role of protection

from irreversible damage to protein functions and regulation, which plays an

important role in the redox homeostasis system. Thus, to discover cysteine

modification changes in the proteome level under oxidative stress is

quintessential to elucidate its antioxidant mechanism. Our results provided a

list of proteins involved in the antioxidant process that potentially could be

considered targets for drug discovery and vaccine development. Furthermore, it is

the first study to determine and quantify the S-sulfenylation-modified proteins

in Mtb, which provided better insight into the Mtb response to the host oxidative

defense and enable a deeper understanding of Mtb survival strategies.

FAU - Lu, Yun

AU - Lu Y

AD - Beijing Key Laboratory of Antimicrobial Agents, Institute of Medicinal

Biotechnology, Chinese Academy of Medical Sciences and Peking Union Medical

College, Beijing, China.

FAU - Chen, Hongtong

AU - Chen H

AD - Beijing Key Laboratory of Antimicrobial Agents, Institute of Medicinal

Biotechnology, Chinese Academy of Medical Sciences and Peking Union Medical

College, Beijing, China.

FAU - Wang, Penghe

AU - Wang P

AD - Beijing Key Laboratory of Antimicrobial Agents, Institute of Medicinal

Biotechnology, Chinese Academy of Medical Sciences and Peking Union Medical

College, Beijing, China.

FAU - Pang, Jing

AU - Pang J

AUID- ORCID: 0000-0002-0538-0327

AD - Beijing Key Laboratory of Antimicrobial Agents, Institute of Medicinal

Biotechnology, Chinese Academy of Medical Sciences and Peking Union Medical

College, Beijing, China.

FAU - Lu, Xi

AU - Lu X

AD - Beijing Key Laboratory of Antimicrobial Agents, Institute of Medicinal

Biotechnology, Chinese Academy of Medical Sciences and Peking Union Medical

College, Beijing, China.

FAU - Li, Guoqing

AU - Li G

AD - Beijing Key Laboratory of Antimicrobial Agents, Institute of Medicinal

Biotechnology, Chinese Academy of Medical Sciences and Peking Union Medical

College, Beijing, China.

FAU - Hu, Xinxin

AU - Hu X

AD - Beijing Key Laboratory of Antimicrobial Agents, Institute of Medicinal

Biotechnology, Chinese Academy of Medical Sciences and Peking Union Medical

College, Beijing, China.

FAU - Wang, Xiukun

AU - Wang X

AD - Beijing Key Laboratory of Antimicrobial Agents, Institute of Medicinal

Biotechnology, Chinese Academy of Medical Sciences and Peking Union Medical

College, Beijing, China.

FAU - Yang, Xinyi

AU - Yang X

AD - Beijing Key Laboratory of Antimicrobial Agents, Institute of Medicinal

Biotechnology, Chinese Academy of Medical Sciences and Peking Union Medical

College, Beijing, China.

FAU - Li, Congran

AU - Li C

AD - Beijing Key Laboratory of Antimicrobial Agents, Institute of Medicinal

Biotechnology, Chinese Academy of Medical Sciences and Peking Union Medical

College, Beijing, China.

FAU - Lu, Yu

AU - Lu Y

AUID- ORCID: 0000-0002-9038-1884

AD - Department of Pharmacology, Beijing Chest Hospital, Capital Medical University;

Beijing Key Laboratory of Drug Resistance Tuberculosis Research, Beijing

Tuberculosis and Thoracic Tumor Research Institute, Beijing, China.

FAU - You, Xuefu

AU - You X

AUID- ORCID: 0000-0001-5024-9697

AD - Beijing Key Laboratory of Antimicrobial Agents, Institute of Medicinal

Biotechnology, Chinese Academy of Medical Sciences and Peking Union Medical

College, Beijing, China.

LA - eng

GR - 81803593/National Natural Science Foundation of China (NSFC)/

GR - 81273427/National Natural Science Foundation of China (NSFC)/

GR - 2021-I2M-1-026/CAMS Innovation Fund for Medical Sciences(CIFMS)/

GR - 2021-PT350-001/Fundamental Research Funds for the Central Universities/

GR - 3332018094/Fundamental Research Funds for the Central Universities/

GR - NPRC-32/National Science and Technology Infrastructure of China/

PT - Journal Article

DEP - 20230321

PL - United States

TA - Microbiol Spectr

JT - Microbiology spectrum

JID - 101634614

SB - IM

PMC - PMC10101118

OTO - NOTNLM

OT - Mycobacterium tuberculosis

OT - S-sulfenylation

OT - cysteine residue

OT - hydrogen peroxide

OT - redox homeostasis

COIS- The authors declare no conflict of interest.

EDAT- 2023/03/22 06:00

MHDA- 2023/03/22 06:01

PMCR- 2023/03/21

CRDT- 2023/03/21 10:05

PHST- 2023/03/22 06:01 [medline]

PHST- 2023/03/22 06:00 [pubmed]

PHST- 2023/03/21 10:05 [entrez]

PHST- 2023/03/21 00:00 [pmc-release]

AID - 03386-22 [pii]

AID - spectrum.03386-22 [pii]

AID - 10.1128/spectrum.03386-22 [doi]

PST - ppublish

SO - Microbiol Spectr. 2023 Apr 13;11(2):e0338622. doi: 10.1128/spectrum.03386-22.

Epub 2023 Mar 21.

PMID- 37031225

OWN - NLM

STAT- MEDLINE

DCOM- 20230411

LR - 20231130

IS - 2041-1723 (Electronic)

IS - 2041-1723 (Linking)

VI - 14

IP - 1

DP - 2023 Apr 8

TI - The relative transmission fitness of multidrug-resistant Mycobacterium

tuberculosis in a drug resistance hotspot.

PG - 1988

LID - 10.1038/s41467-023-37719-y [doi]

LID - 1988

AB - Multidrug-resistant tuberculosis (MDR-TB) is among the most frequent causes of

death due to antimicrobial resistance. Although only 3% of global TB cases are

MDR, geographical hotspots with up to 40% of MDR-TB have been observed in

countries of the former Soviet Union. While the quality of TB control and

patient-related factors are known contributors to such hotspots, the role of the

pathogen remains unclear. Here we show that in the country of Georgia, a known

hotspot of MDR-TB, MDR Mycobacterium tuberculosis strains of lineage 4 (L4)

transmit less than their drug-susceptible counterparts, whereas most MDR strains

of L2 suffer no such defect. Our findings further indicate that the high

transmission fitness of these L2 strains results from epistatic interactions

between the rifampicin resistance-conferring mutation RpoB S450L, compensatory

mutations in the RNA polymerase, and other pre-existing genetic features of

L2/Beijing clones that circulate in Georgia. We conclude that the transmission

fitness of MDR M. tuberculosis strains is heterogeneous, but can be as high as

drug-susceptible forms, and that such highly drug-resistant and transmissible

strains contribute to the emergence and maintenance of hotspots of MDR-TB. As

these strains successfully overcome the metabolic burden of drug resistance, and

given the ongoing rollout of new treatment regimens against MDR-TB, proper

surveillance should be implemented to prevent these strains from acquiring

resistance to the additional drugs.

CI - © 2023. The Author(s).

FAU - Loiseau, Chloé

AU - Loiseau C

AD - Swiss Tropical and Public Health Institute, Allschwil, Switzerland.

AD - University of Basel, Basel, Switzerland.

FAU - Windels, Etthel M

AU - Windels EM

AUID- ORCID: 0000-0003-2106-6765

AD - Department of Biosystems Science and Engineering, ETH Zürich, Basel, Switzerland.

etthel.windels@bsse.ethz.ch.

AD - Swiss Institute of Bioinformatics, Lausanne, Switzerland.

etthel.windels@bsse.ethz.ch.

FAU - Gygli, Sebastian M

AU - Gygli SM

AD - Swiss Tropical and Public Health Institute, Allschwil, Switzerland.

AD - University of Basel, Basel, Switzerland.

FAU - Jugheli, Levan

AU - Jugheli L

AD - Swiss Tropical and Public Health Institute, Allschwil, Switzerland.

AD - University of Basel, Basel, Switzerland.

AD - National Center for Tuberculosis and Lung Diseases (NCTLD), Tbilisi, Georgia.

FAU - Maghradze, Nino

AU - Maghradze N

AD - Swiss Tropical and Public Health Institute, Allschwil, Switzerland.

AD - University of Basel, Basel, Switzerland.

AD - National Center for Tuberculosis and Lung Diseases (NCTLD), Tbilisi, Georgia.

FAU - Brites, Daniela

AU - Brites D

AUID- ORCID: 0000-0002-8090-2287

AD - Swiss Tropical and Public Health Institute, Allschwil, Switzerland.

AD - University of Basel, Basel, Switzerland.

FAU - Ross, Amanda

AU - Ross A

AD - Swiss Tropical and Public Health Institute, Allschwil, Switzerland.

AD - University of Basel, Basel, Switzerland.

FAU - Goig, Galo

AU - Goig G

AD - Swiss Tropical and Public Health Institute, Allschwil, Switzerland.

AD - University of Basel, Basel, Switzerland.

FAU - Reinhard, Miriam

AU - Reinhard M

AD - Swiss Tropical and Public Health Institute, Allschwil, Switzerland.

AD - University of Basel, Basel, Switzerland.

FAU - Borrell, Sonia

AU - Borrell S

AD - Swiss Tropical and Public Health Institute, Allschwil, Switzerland.

AD - University of Basel, Basel, Switzerland.

FAU - Trauner, Andrej

AU - Trauner A

AD - Swiss Tropical and Public Health Institute, Allschwil, Switzerland.

AD - University of Basel, Basel, Switzerland.

FAU - Dötsch, Anna

AU - Dötsch A

AD - Swiss Tropical and Public Health Institute, Allschwil, Switzerland.

AD - University of Basel, Basel, Switzerland.

FAU - Aspindzelashvili, Rusudan

AU - Aspindzelashvili R

AD - National Center for Tuberculosis and Lung Diseases (NCTLD), Tbilisi, Georgia.

FAU - Denes, Rebecca

AU - Denes R

AD - Department of Biosystems Science and Engineering, ETH Zürich, Basel, Switzerland.

FAU - Reither, Klaus

AU - Reither K

AD - Swiss Tropical and Public Health Institute, Allschwil, Switzerland.

AD - University of Basel, Basel, Switzerland.

FAU - Beisel, Christian

AU - Beisel C

AUID- ORCID: 0000-0001-5360-2193

AD - Department of Biosystems Science and Engineering, ETH Zürich, Basel, Switzerland.

FAU - Tukvadze, Nestani

AU - Tukvadze N

AD - Swiss Tropical and Public Health Institute, Allschwil, Switzerland.

AD - University of Basel, Basel, Switzerland.

AD - National Center for Tuberculosis and Lung Diseases (NCTLD), Tbilisi, Georgia.

FAU - Avaliani, Zaza

AU - Avaliani Z

AD - National Center for Tuberculosis and Lung Diseases (NCTLD), Tbilisi, Georgia.

FAU - Stadler, Tanja

AU - Stadler T

AUID- ORCID: 0000-0001-6431-535X

AD - Department of Biosystems Science and Engineering, ETH Zürich, Basel, Switzerland.

AD - Swiss Institute of Bioinformatics, Lausanne, Switzerland.

FAU - Gagneux, Sebastien

AU - Gagneux S

AUID- ORCID: 0000-0001-7783-9048

AD - Swiss Tropical and Public Health Institute, Allschwil, Switzerland.

sebastien.gagneux@swisstph.ch.

AD - University of Basel, Basel, Switzerland. sebastien.gagneux@swisstph.ch.

LA - eng

GR - D43 TW007124/TW/FIC NIH HHS/United States

PT - Journal Article

PT - Research Support, Non-U.S. Gov't

DEP - 20230408

PL - England

TA - Nat Commun

JT - Nature communications

JID - 101528555

RN - 0 (Antitubercular Agents)

RN - VJT6J7R4TR (Rifampin)

SB - IM

MH - Humans

MH - *Mycobacterium tuberculosis/genetics

MH - Antitubercular Agents/pharmacology/therapeutic use

MH - *Tuberculosis, Multidrug-Resistant/drug therapy/epidemiology/microbiology

MH - Mutation

MH - Rifampin/pharmacology/therapeutic use

MH - Drug Resistance, Multiple, Bacterial/genetics

MH - Microbial Sensitivity Tests

PMC - PMC10082831

COIS- The authors declare no competing interests.

EDAT- 2023/04/09 06:00

MHDA- 2023/04/11 06:42

PMCR- 2023/04/08

CRDT- 2023/04/08 23:17

PHST- 2022/11/09 00:00 [received]

PHST- 2023/03/28 00:00 [accepted]

PHST- 2023/04/11 06:42 [medline]

PHST- 2023/04/08 23:17 [entrez]

PHST- 2023/04/09 06:00 [pubmed]

PHST- 2023/04/08 00:00 [pmc-release]

AID - 10.1038/s41467-023-37719-y [pii]

AID - 37719 [pii]

AID - 10.1038/s41467-023-37719-y [doi]

PST - epublish

SO - Nat Commun. 2023 Apr 8;14(1):1988. doi: 10.1038/s41467-023-37719-y.

PMID- 37456186

OWN - NLM

STAT- PubMed-not-MEDLINE

LR - 20231031

IS - 0972-3919 (Print)

IS - 0974-0244 (Electronic)

IS - 0974-0244 (Linking)

VI - 38

IP - 2

DP - 2023 Apr-Jun

TI - A Rare Presentation of Tuberculosis in Transplanted Kidney on Fluorodeoxyglucose

Positron Emission Tomography with Computed Tomography Scan.

PG - 177-179

LID - 10.4103/ijnm.ijnm_129_22 [doi]

AB - Tuberculosis (TB) is currently the world's leading cause of infectious mortality.

Infective complications are common after renal transplantation. TB is one of the

leading infections following renal transplantation; however, TB affecting the

transplanted kidney is a rare presentation. Reactivation of the Mycobacterium

tuberculosis is the most common mode of infection. The use of immunosuppressive

agents such as cyclosporin, azathioprine, and steroids advance the onset of TB to

an earlier date which most often presents as a fever or pyrexia of unknown origin

(PUO). The use of 18F-fluorodeoxyglucose positron emission tomography with

computed tomography (18F-FDG-PET/CT) scan images of the whole body and provides

the metabolic map of the infection as well as also helps in its radiological

localization and characterization and selecting the most appropriate site of the

biopsy. Currently, the combined FDG-PET/CT scan modality is the investigation of

the choice of physicians for the diagnosis of PUO. Not only the diagnosis but

18F-FDG-PET/CT is also very valuable in assessing early disease response to

therapy, and plays an important role in cases where conventional microbiological

methods are unavailable for monitoring response to the therapy in cases of

pulmonary, extrapulmonary, or multidrug resistant TB.

CI - Copyright: © 2023 Indian Journal of Nuclear Medicine.

FAU - Rathore, Hemant

AU - Rathore H

AD - Department of Nuclear Medicine and PET CT, Bombay Hospital and Medical Research

Centre, Mumbai, Maharashtra, India.

FAU - Thaker, Nirav

AU - Thaker N

AD - Department of Radiodiagnosis, Bombay Hospital and Medical Research Centre,

Mumbai, Maharashtra, India.

LA - eng

PT - Journal Article

DEP - 20230608

PL - United States

TA - Indian J Nucl Med

JT - Indian journal of nuclear medicine : IJNM : the official journal of the Society

of Nuclear Medicine, India

JID - 8901274

PMC - PMC10348506

OTO - NOTNLM

OT - Fluorodeoxyglucose positron emission tomography

OT - pyrexia of unknown origin

OT - transplant kidney

OT - tuberculosis

COIS- There are no conflicts of interest.

EDAT- 2023/07/17 06:42

MHDA- 2023/07/17 06:43

PMCR- 2023/04/01

CRDT- 2023/07/17 04:21

PHST- 2022/07/27 00:00 [received]

PHST- 2022/11/07 00:00 [revised]

PHST- 2022/12/24 00:00 [accepted]

PHST- 2023/07/17 06:43 [medline]

PHST- 2023/07/17 06:42 [pubmed]

PHST- 2023/07/17 04:21 [entrez]

PHST- 2023/04/01 00:00 [pmc-release]

AID - IJNM-38-177 [pii]

AID - 10.4103/ijnm.ijnm_129_22 [doi]

PST - ppublish

SO - Indian J Nucl Med. 2023 Apr-Jun;38(2):177-179. doi: 10.4103/ijnm.ijnm_129_22.

Epub 2023 Jun 8.

PMID- 36749527

OWN - NLM

STAT- MEDLINE

DCOM- 20230329

LR - 20230329

IS - 1573-4978 (Electronic)

IS - 0301-4851 (Linking)

VI - 50

IP - 4

DP - 2023 Apr

TI - A host blood transcriptional signature differentiates

multi-drug/rifampin-resistant tuberculosis (MDR/RR-TB) from drug susceptible

tuberculosis: a pilot study.

PG - 3935-3943

LID - 10.1007/s11033-023-08307-6 [doi]

AB - BACKGROUND: Tuberculosis (TB), caused by the bacterium Mycobacterium tuberculosis

is one of the top thirteen causes of death worldwide. The major challenge to

control TB is the emergence of drug-resistant tuberculosis (DR-TB); specifically,

multi-drug resistant TB which are resistant to the most potent drugs; rifampin

and isoniazid. Owing to the inconsistencies of the current diagnostic methods, a

single test cannot identify the whole spectrum of DR-TB associated mutations.

Recently, host blood transcriptomics has gained attention as a promising

technique that develops disease-specific RNA signatures/biomarkers. However,

studies on host transcriptomics infected with DR-TB is limited. Herein, we

intended to identify genes/pathways that are differentially expressed in

multi-drug/rifampin resistant TB (MDR/RR-TB) in contrast to drug susceptible TB.

METHOD AND RESULTS: We conducted blood RNA sequencing of 10 pulmonary TB patients

(4; drug susceptible and 6; DR-TB) and 55 genes that were differentially

expressed in MDR/RR-TB from drug-susceptible/mono-resistant TB were identified.

CD300LD, MYL9, VAMP5, CARD17, CLEC2B, GBP6, BATF2, ETV7, IFI27 and FCGR1CP were

found to be upregulated in MDR/RR-TB in all comparisons, among which CLEC2B and

CD300LD were not previously linked to TB. In comparison pathway analysis,

interferon alpha/gamma response was upregulated while Wnt/beta catenin signaling,

lysosome, microtubule nucleation and notch signaling were downregulated.

CONCLUSION: Up/down-regulation of immunity related genes/pathways speculate the

collective effect of hosts' attempt to fight against continuously multiplying

DR-TB bacteria and the bacterial factors to fight against the host defense. The

identified genes/pathways could act as MDR/RR-TB biomarkers, hence, further

research on their clinical use should be encouraged.

CI - © 2023. The Author(s), under exclusive licence to Springer Nature B.V.

FAU - Madamarandawala, Pavithra

AU - Madamarandawala P

AUID- ORCID: 0000-0003-4608-8143

AD - Molecular Microbiology & Human Diseases Project, National Institute of

Fundamental Studies, Hantana Road, Kandy, 20000, Sri Lanka.

FAU - Rajapakse, Sanath

AU - Rajapakse S

AUID- ORCID: 0000-0002-0014-5811

AD - Department of Molecular Biology and Biotechnology, Faculty of Science, University

of Peradeniya, Peradeniya, 20400, Sri Lanka.

FAU - Gunasena, Bandu

AU - Gunasena B

AD - National Hospital for Respiratory Diseases, Welisara, 11010, Sri Lanka.

FAU - Madegedara, Dushantha

AU - Madegedara D

AUID- ORCID: 0000-0003-3978-4722

AD - Respiratory Diseases Treatment Unit, General Teaching Hospital, Kandy, 20000, Sri

Lanka.

FAU - Magana-Arachchi, Dhammika

AU - Magana-Arachchi D

AUID- ORCID: 0000-0001-5825-4626

AD - Molecular Microbiology & Human Diseases Project, National Institute of

Fundamental Studies, Hantana Road, Kandy, 20000, Sri Lanka.

dhammika.ma@nifs.ac.lk.

LA - eng

GR - NSF PSF ICRP 2017 HS 01/National Science Foundation of Sri Lanka/

PT - Journal Article

DEP - 20230207

PL - Netherlands

TA - Mol Biol Rep

JT - Molecular biology reports

JID - 0403234

RN - VJT6J7R4TR (Rifampin)

RN - 0 (Antitubercular Agents)

RN - V83O1VOZ8L (Isoniazid)

SB - IM

MH - Humans

MH - Rifampin/pharmacology

MH - Pilot Projects

MH - Antitubercular Agents/pharmacology

MH - *Tuberculosis/microbiology

MH - *Tuberculosis, Multidrug-Resistant/drug therapy/genetics

MH - *Mycobacterium tuberculosis/genetics

MH - Isoniazid/pharmacology

MH - Microbial Sensitivity Tests

OTO - NOTNLM

OT - Biomarkers

OT - Drug resistance

OT - Gene signature

OT - Transcriptome

OT - Tuberculosis

EDAT- 2023/02/08 06:00

MHDA- 2023/03/29 06:05

CRDT- 2023/02/07 11:22

PHST- 2022/09/28 00:00 [received]

PHST- 2023/01/27 00:00 [accepted]

PHST- 2023/03/29 06:05 [medline]

PHST- 2023/02/08 06:00 [pubmed]

PHST- 2023/02/07 11:22 [entrez]

AID - 10.1007/s11033-023-08307-6 [pii]

AID - 10.1007/s11033-023-08307-6 [doi]

PST - ppublish

SO - Mol Biol Rep. 2023 Apr;50(4):3935-3943. doi: 10.1007/s11033-023-08307-6. Epub

2023 Feb 7.

PMID- 35613080

OWN - NLM

STAT- MEDLINE

DCOM- 20230426

LR - 20230426

IS - 2046-5556 (Electronic)

IS - 0264-8725 (Linking)

VI - 39

IP - 1

DP - 2023 Apr

TI - The chamber of secretome in Mycobacterium tuberculosis as a potential therapeutic

target.

PG - 1-44

LID - 10.1080/02648725.2022.2076031 [doi]

AB - Mycobacterium tuberculosis (MTB) causes one of the ancient diseases,

Tuberculosis, affects people around the globe and its severity can be understood

by its classification as a second infectious disease after COVID-19 and the 13th

leading cause of death according to a WHO report. Despite having advanced

diagnostic approaches and therapeutic strategies, unfortunately, TB is still

spreading across the population due to the emergence of drug-resistance MTB and

Latent TB infection (LTBI). We are seeking for effective approaches to overcome

these hindrances and efficient treatment for this perilous disease. Therefore,

there is an urgent need to develop drugs based on operative targeting of the

bacterial system that could result in both efficient treatment and lesser

emergence of MDR-TB. One such promising target could be the secretory systems and

especially the Type 7 secretory system (T7SS-ESX) of Mycobacterium tuberculosis,

which is crucial for the secretion of effector proteins as well as in

establishing host-pathogen interactions of the tubercle bacilli. The five

paralogous ESX systems (ESX-1 to EXS-5) have been observed by in silico genome

analysis of MTB, among which ESX-1 and ESX-5 are substantial for virulence and

mediating host cellular inflammasome. The bacterium growth and virulence can be

modulated by targeting the T7SS. In the present review, we demonstrate the

current status of therapeutics against MTB and focus on the function and

cruciality of T7SS along with other secretory systems as a promising therapeutic

target against Tuberculosis.

FAU - Dwivedi, Manish

AU - Dwivedi M

AUID- ORCID: 0000-0001-5217-9039

AD - Amity Institute of Biotechnology, Amity University Uttar Pradesh, Lucknow, India.

FAU - Bajpai, Kriti

AU - Bajpai K

AD - Department of Biotechnology, Himachal Pradesh University, Shimla, India.

LA - eng

PT - Journal Article

PT - Review

DEP - 20220525

PL - England

TA - Biotechnol Genet Eng Rev

JT - Biotechnology & genetic engineering reviews

JID - 8510274

RN - 0 (Bacterial Proteins)

SB - IM

MH - Humans

MH - *Mycobacterium tuberculosis/genetics/metabolism

MH - Bacterial Proteins/metabolism

MH - Secretome

MH - *COVID-19

MH - *Tuberculosis/microbiology

OTO - NOTNLM

OT - ESX

OT - Sec pathway

OT - Tat pathway

OT - Tuberculosis

OT - drug resistant

OT - drug target

OT - secretory system

EDAT- 2022/05/26 06:00

MHDA- 2023/04/26 06:42

CRDT- 2022/05/25 13:33

PHST- 2023/04/26 06:42 [medline]

PHST- 2022/05/26 06:00 [pubmed]

PHST- 2022/05/25 13:33 [entrez]

AID - 10.1080/02648725.2022.2076031 [doi]

PST - ppublish

SO - Biotechnol Genet Eng Rev. 2023 Apr;39(1):1-44. doi:

10.1080/02648725.2022.2076031. Epub 2022 May 25.

PMID- 37032734

OWN - NLM

STAT- PubMed-not-MEDLINE

LR - 20240604

IS - 2090-150X (Print)

IS - 2090-1518 (Electronic)

IS - 2090-150X (Linking)

VI - 2023

DP - 2023

TI - Drug Resistance in Tuberculous Lymphadenitis: Molecular Characterization.

PG - 3291538

LID - 10.1155/2023/3291538 [doi]

LID - 3291538

AB - BACKGROUND: Drug-resistant tuberculosis (TB) epidemic in high-TB-incidence

countries, particularly Ethiopia, remains a significant challenge. As a result,

we investigated the drug resistance, common gene mutation, and molecular

characterization of mycobacterial isolates from patients with suspected

tuberculous lymphadenitis (TBLN). Methodology. A cross-sectional study of 218 FNA

samples from TBLN patients inoculated on Lowenstein-Jensen media was carried out.

The culture isolates were identified as MTB by polymerase chain reaction (PCR)

and the difference-9 (RD9) test region. In addition, the GenoType MTBDRplus assay

tested the first and second-line MTB drugs, and the spoligotyping

strain-dependent polymorphism test was determined. RESULTS: Among the 50

culture-positive isolates, 14% (7/50) had drug resistance caused by a gene

mutation. Out of these, 4 (8%) isolates were mono-resistant to isoniazid drug,

which is caused by a gene mutation in katG in the region of interrogated at codon

315 in the amino acid sequence of S315T1, and 3 (6%) isolates were resistant to

both rifampicin and isoniazid drugs. The mutation was observed for katG (at codon

315 with a change in the sequence of amino acid S315T) and rpoB (at codon 530-533

with a change in the sequence of amino acid S531L (S450L)) genes. The most

prevalent spoligotypes were orphan and SIT53 strains. CONCLUSION: The

predominance of INH mono-resistance poses a critical risk for the potential

development of MDR-TB, as INH mono-resistance is a typical pathway to the

occurrence of MDR-TB. The orphan and SIT53 (T) strains were the most common in

the study area, and a drug-resistant strain caused by a common gene mutation

could indicate the transmission of clonal-resistant strains in the community.

CI - Copyright © 2023 Gebeyehu Assefa et al.

FAU - Assefa, Gebeyehu

AU - Assefa G

AUID- ORCID: 0000-0003-1308-7566

AD - Armauer Hansen Research Institute, AHRI, Addis Ababa, Ethiopia.

AD - Department of Medical Laboratory Sciences, College of Health Sciences, Addis

Ababa University, Addis Ababa, Ethiopia.

FAU - Desta, Kassu

AU - Desta K

AD - Department of Medical Laboratory Sciences, College of Health Sciences, Addis

Ababa University, Addis Ababa, Ethiopia.

FAU - Araya, Shambel

AU - Araya S

AUID- ORCID: 0000-0002-0109-6062

AD - Department of Medical Laboratory Sciences, College of Health Sciences, Addis

Ababa University, Addis Ababa, Ethiopia.

FAU - Girma, Selfu

AU - Girma S

AUID- ORCID: 0000-0002-1866-0950

AD - Armauer Hansen Research Institute, AHRI, Addis Ababa, Ethiopia.

FAU - Hailu, Elena

AU - Hailu E

AUID- ORCID: 0000-0002-9358-0838

AD - Armauer Hansen Research Institute, AHRI, Addis Ababa, Ethiopia.

FAU - Mihret, Adane

AU - Mihret A

AUID- ORCID: 0000-0003-3183-4772

AD - Armauer Hansen Research Institute, AHRI, Addis Ababa, Ethiopia.

FAU - Hailu, Tsegaye

AU - Hailu T

AD - Armauer Hansen Research Institute, AHRI, Addis Ababa, Ethiopia.

FAU - Tilahun, Melaku

AU - Tilahun M

AUID- ORCID: 0000-0002-3101-3926

AD - Armauer Hansen Research Institute, AHRI, Addis Ababa, Ethiopia.

FAU - Diriba, Getu

AU - Diriba G

AUID- ORCID: 0000-0002-0166-682X

AD - Ethiopian Public Health Institute, EPHI, Addis Ababa, Ethiopia.

FAU - Dagne, Biniyam

AU - Dagne B

AUID- ORCID: 0000-0002-5930-2690

AD - Ethiopian Public Health Institute, EPHI, Addis Ababa, Ethiopia.

FAU - Atnafu, Abay

AU - Atnafu A

AUID- ORCID: 0000-0001-7594-4004

AD - Armauer Hansen Research Institute, AHRI, Addis Ababa, Ethiopia.

FAU - Endalafer, Nigatu

AU - Endalafer N

AD - Armauer Hansen Research Institute, AHRI, Addis Ababa, Ethiopia.

FAU - Abera, Adugna

AU - Abera A

AD - Ethiopian Public Health Institute, EPHI, Addis Ababa, Ethiopia.

FAU - Bekele, Shiferaw

AU - Bekele S

AD - Armauer Hansen Research Institute, AHRI, Addis Ababa, Ethiopia.

FAU - Mengistu, Yordanos

AU - Mengistu Y

AD - Haramaya University, Harar, Ethiopia.

FAU - Bobosha, Kidist

AU - Bobosha K

AUID- ORCID: 0000-0001-8091-7182

AD - Armauer Hansen Research Institute, AHRI, Addis Ababa, Ethiopia.

FAU - Aseffa, Abraham

AU - Aseffa A

AUID- ORCID: 0000-0002-8028-1150

AD - Armauer Hansen Research Institute, AHRI, Addis Ababa, Ethiopia.

LA - eng

GR - D43 TW009127/TW/FIC NIH HHS/United States

PT - Journal Article

DEP - 20230329

PL - United States

TA - Tuberc Res Treat

JT - Tuberculosis research and treatment

JID - 101576351

PMC - PMC10076118

COIS- The authors declare that they have no competing interests.

EDAT- 2023/04/11 06:00

MHDA- 2023/04/11 06:01

PMCR- 2023/03/29

CRDT- 2023/04/10 03:45

PHST- 2022/05/27 00:00 [received]

PHST- 2023/02/27 00:00 [revised]

PHST- 2023/03/11 00:00 [accepted]

PHST- 2023/04/11 06:01 [medline]

PHST- 2023/04/10 03:45 [entrez]

PHST- 2023/04/11 06:00 [pubmed]

PHST- 2023/03/29 00:00 [pmc-release]

AID - 10.1155/2023/3291538 [doi]

PST - epublish

SO - Tuberc Res Treat. 2023 Mar 29;2023:3291538. doi: 10.1155/2023/3291538.

eCollection 2023.

PMID- 37123717

OWN - NLM

STAT- PubMed-not-MEDLINE

LR - 20250929

IS - 2168-8184 (Print)

IS - 2168-8184 (Electronic)

IS - 2168-8184 (Linking)

VI - 15

IP - 3

DP - 2023 Mar

TI - Unraveling the Secrets Behind the Multidrug-Resistant Tuberculosis Treatment

Outcome in Chronic Renal Failure Patients Requiring Hemodialysis: A Systematic

Review.

PG - e36833

LID - 10.7759/cureus.36833 [doi]

LID - e36833

AB - Multidrug-resistant/rifampicin-resistant tuberculosis (MDR/RR TB) is a global

concern, with 450,000 new cases and 191,000 deaths in 2021. TB and chronic kidney

disease (CKD) have been associated since 1974, with suggested explanations such

as oxidative stress, malnutrition, dysfunction in vitamin D metabolism, and a

compromised cell-mediated immune response. End-stage renal failure patients are

more likely to acquire drug resistance due to poor adherence, adverse drug

reactions, and inappropriate dose adjustment. We then aim to evaluate the

therapeutic outcome of multidrug-resistant TB of the lungs in patients who

require hemodialysis in terms of successful treatment (cured and treatment

completed) and the associated factors for a favorable outcome. Our secondary goal

is to identify unfavorable treatment outcomes (treatment failed, patient died, or

patient lost to follow-up) and the underlying associated factors. We conformed to

the Preferred Reporting Items for Systematic Reviews and Meta-Analyses (PRISMA)

2020 Guidelines for this systematic review. We included adults (>19 years old)

with chronic kidney disease who needed hemodialysis and had microbiologically

confirmed multidrug-resistant pulmonary TB, excluding patients who had a renal

allograft transplant, were on peritoneal dialysis, had extrapulmonary TB, were

children and pregnant patients. We searched PubMed, MEDLINE, PubMed Central,

ScienceDirect, Public Library of Science (PLOS), and Google Scholar. Keywords

were combined with the Boolean "AND" operator to gather results as well as the

medical subject heading (MeSH) search strategy. After screening study articles by

reading their titles and abstracts, the following tools were used to assess the

risk of bias: the Newcastle-Ottawa scale for observational studies, the

Assessment of Multiple Systematic Reviews (AMSTAR) checklist for systematic

reviews, and the Joanna Briggs Institute (JBI) assessment tool for case

reports. Primary and secondary outcomes were assessed, and a conclusion was made.

We gathered 21,570 studies from the databases between 2013 and 2023, with 30,062

total participants. There were eight eligible studies for review. Patients with

CKD, particularly those on dialysis, are at increased risk of TB due to a

combination of factors that contribute to immunosuppression. TB reactivation is

common in chronic renal failure patients. Diagnostic samples such as sputum and

pleural fluid had lower sensitivity rates compared to tissue samples, which led

to delays in diagnosis and treatment and, most importantly, contributed to drug

resistance. All new dialysis patients should undergo interferon-gamma release

assay testing. TB-infected patients with severe renal disease (eGFR 30 ml/min)

had increased morbidity and mortality; however, the use of directly observed

treatment, short-course (DOTS), and renal-dose adjustment of anti-TB medications

significantly reduced these risks. Drug-induced hepatitis and cutaneous reactions

were common adverse effects of anti-TB medications. A therapeutic drug monitoring

guideline is required to reduce these adverse events and even mortality.

Additional research is required to assess the safety and efficacy of therapeutic

regimens, as well as their outcomes, in this population with multidrug-resistant

TB.

CI - Copyright © 2023, Hernandez et al.

FAU - Hernandez, Grethel N

AU - Hernandez GN

AD - Internal Medicine, California Institute of Behavioral Neurosciences & Psychology,

Fairfield, USA.

FAU - Seffah, Kofi

AU - Seffah K

AD - Internal Medicine, California Institute of Behavioral Neurosciences & Psychology,

Fairfield, USA.

AD - Internal Medicine, Piedmont Athens Regional, Athens, USA.

FAU - Zaman, Mustafa Abrar

AU - Zaman MA

AD - Internal Medicine, St. George's University School of Medicine, Newcastle upon

Tyne, GBR.

AD - Internal Medicine, California Institute of Behavioral Neurosciences & Psychology,

Fairfield, USA.

FAU - Awais, Nimra

AU - Awais N

AD - Research, California Institute of Behavioral Neurosciences & Psychology,

Fairfield, USA.

FAU - Satnarine, Travis

AU - Satnarine T

AD - Pediatrics, California Institute of Behavioral Neurosciences & Psychology,

Fairfield, USA.

FAU - Haq, Ayesha

AU - Haq A

AD - Internal Medicine, California Institute of Behavioral Neurosciences & Psychology,

Fairfield, USA.

FAU - Patel, Deepkumar

AU - Patel D

AD - Neurology, California Institute of Behavioral Neurosciences & Psychology,

Fairfield, USA.

FAU - Gutlapalli, Sai Dheeraj

AU - Gutlapalli SD

AD - Internal Medicine, Richmond University Medical Center, Staten Island, USA.

AD - Internal Medicine Clinical Research, California Institute of Behavioral

Neurosciences & Psychology, Fairfield, USA.

FAU - Ahmed, Areeg

AU - Ahmed A

AD - Internal Medicine, California Institute of Behavioral Neurosciences & Psychology,

Fairfield, USA.

FAU - Khan, Safeera

AU - Khan S

AD - Internal Medicine, California Institute of Behavioral Neurosciences & Psychology,

Fairfield, USA.

LA - eng

PT - Journal Article

PT - Review

DEP - 20230328

PL - United States

TA - Cureus

JT - Cureus

JID - 101596737

EIN - Cureus. 2025 Sep 25;17(9):c320. doi: 10.7759/cureus.c320. PMID: 41020011

PMC - PMC10147484

OTO - NOTNLM

OT - chronic kidney disease

OT - multidrug-resistant pulmonary tb

OT - mycobacterium tuberculosis

OT - pulmonary tuberculosis

OT - renal failure

OT - treatment outcome

COIS- The authors have declared that no competing interests exist.

EDAT- 2023/05/01 06:42

MHDA- 2023/05/01 06:43

PMCR- 2023/03/28

CRDT- 2023/05/01 03:34

PHST- 2023/03/01 00:00 [received]

PHST- 2023/03/27 00:00 [accepted]

PHST- 2023/05/01 06:43 [medline]

PHST- 2023/05/01 06:42 [pubmed]

PHST- 2023/05/01 03:34 [entrez]

PHST- 2023/03/28 00:00 [pmc-release]

AID - 10.7759/cureus.36833 [doi]

PST - epublish

SO - Cureus. 2023 Mar 28;15(3):e36833. doi: 10.7759/cureus.36833. eCollection 2023

Mar.

PMID- 37013167

OWN - NLM

STAT- PubMed-not-MEDLINE

LR - 20230405

IS - 1178-6973 (Print)

IS - 1178-6973 (Electronic)

IS - 1178-6973 (Linking)

VI - 16

DP - 2023

TI - ApoE Mimetic Peptide COG1410 Exhibits Strong Additive Interaction with

Antibiotics Against Mycobacterium smegmatis.

PG - 1801-1812

LID - 10.2147/IDR.S403232 [doi]

AB - BACKGROUND: Drug-resistant tuberculosis (TB) is an emerging threat to public

health worldwide. Antimicrobial peptide (AMP) is a promising solution to solve

the antimicrobial resistance crisis. The apolipoprotein E mimetic peptide COG1410

has been confirmed to simultaneously have neuroprotective, anti-inflammatory, and

antibacterial activity. However, whether it is effective to inhibit growth of

mycobacteria has not been investigated yet. METHODS: The peptide COG1410 was

synthesized with conventional solid-phase peptide synthesis and qualified by HPLC

and mass spectrometry. Micro-dilution method was used to determine the minimal

inhibitory concentration. A time-kill assay was used to determine the

bactericidal dynamics of antimicrobial peptide and relative antibiotics. Static

biofilm formation was conducted in 24-well plate and the biofilm was separated

from planktonic cells and collected. The mechanism of action of COG1410 was

explored by TEM observation and ATP leak assay. The localization of COG1410 was

observed by confocal laser scan microscopy. The drug-drug interaction was

determined by a checkerboard assay. RESULTS: COG1410 was a potent bactericidal

agent against M. smegmatis in vitro and within the macrophages with MIC 16 μg/mL,

but invalid against M. abscess and M. tuberculosis. A time-kill assay showed that

COG1410 killed M. smegmatis as potent as clarithromycin, but faster than LL-37,

another short synthetic cationic peptide. 1× MIC COG1410 almost reduced 90%

biofilm formation of M. smegmatis. Additionally, COG1410 was able to penetrate

the cell membrane of macrophage and inhibit intracellular M. smegmatis growth.

TEM observation and ATP leak assay found that COG1410 disrupted cell membrane and

caused release of cell contents. Confocal fluorescence microscopy showed that

FITC-COG1410 aggregated around cell membrane instead of entering the cytoplasm.

Although COG1410 had relative high cytotoxicity, it exhibited strong additive

interaction with regular anti-TB antibiotics, which reduced the working

concentration of COG1410 and expanding safety window. After 30 passages, there

was no induced drug resistance for COG1410. CONCLUSION: COG1410 was a novel and

potent AMP against M. smegmatis by disrupting the integrity of cell membrane.

CI - © 2023 Zhao et al.

FAU - Zhao, Yan-Yan

AU - Zhao YY

AD - Department of Tuberculosis, the Second Hospital of Nanjing, Nanjing University of

Chinese Medicine, Nanjing, 210003, People's Republic of China.

FAU - Wang, Chun

AU - Wang C

AD - Department of Tuberculosis, the Second Hospital of Nanjing, Nanjing University of

Chinese Medicine, Nanjing, 210003, People's Republic of China.

FAU - Wang, Wei-Xiao

AU - Wang WX

AD - Clinical Research Center, the Second Hospital of Nanjing, Nanjing University of

Chinese Medicine, Nanjing, 210003, People's Republic of China.

FAU - Han, Li-Mei

AU - Han LM

AD - Department of Tuberculosis, the Second Hospital of Nanjing, Nanjing University of

Chinese Medicine, Nanjing, 210003, People's Republic of China.

FAU - Zhang, Caiyun

AU - Zhang C

AD - Clinical Research Center, the Second Hospital of Nanjing, Nanjing University of

Chinese Medicine, Nanjing, 210003, People's Republic of China.

FAU - Yu, Jiao-Yang

AU - Yu JY

AD - Key Laboratory of Resources Biology and Biotechnology in Western China, Ministry

of Education, College of Life Sciences, Northwest University, Xi'an, 710069,

People's Republic of China.

FAU - Chen, Wei

AU - Chen W

AUID- ORCID: 0000-0003-1526-4677

AD - Clinical Research Center, the Second Hospital of Nanjing, Nanjing University of

Chinese Medicine, Nanjing, 210003, People's Republic of China.

FAU - Hu, Chun-Mei

AU - Hu CM

AUID- ORCID: 0000-0002-3788-4191

AD - Department of Tuberculosis, the Second Hospital of Nanjing, Nanjing University of

Chinese Medicine, Nanjing, 210003, People's Republic of China.

AD - The Clinical Infectious Disease Center of Nanjing, Nanjing, 210003, People's

Republic of China.

LA - eng

PT - Journal Article

DEP - 20230328

PL - New Zealand

TA - Infect Drug Resist

JT - Infection and drug resistance

JID - 101550216

PMC - PMC10066718

OTO - NOTNLM

OT - COG1410

OT - Mycobacterium smegmatis

OT - additive interaction

OT - antimicrobial peptide

COIS- The authors report no conflicts of interest in this work.

EDAT- 2023/04/05 06:00

MHDA- 2023/04/05 06:01

PMCR- 2023/03/28

CRDT- 2023/04/04 02:01

PHST- 2023/01/17 00:00 [received]

PHST- 2023/03/22 00:00 [accepted]

PHST- 2023/04/05 06:01 [medline]

PHST- 2023/04/04 02:01 [entrez]

PHST- 2023/04/05 06:00 [pubmed]

PHST- 2023/03/28 00:00 [pmc-release]

AID - 403232 [pii]

AID - 10.2147/IDR.S403232 [doi]

PST - epublish

SO - Infect Drug Resist. 2023 Mar 28;16:1801-1812. doi: 10.2147/IDR.S403232.

eCollection 2023.

PMID- 37009497

OWN - NLM

STAT- MEDLINE

DCOM- 20230404

LR - 20230407

IS - 2235-2988 (Electronic)

IS - 2235-2988 (Linking)

VI - 13

DP - 2023

TI - CRISPRi-mediated characterization of novel anti-tuberculosis targets:

Mycobacterial peptidoglycan modifications promote beta-lactam resistance and

intracellular survival.

PG - 1089911

LID - 10.3389/fcimb.2023.1089911 [doi]

LID - 1089911

AB - The lack of effective therapeutics against emerging multi-drug resistant strains

of Mycobacterium tuberculosis (Mtb) prompts the identification of novel

anti-tuberculosis targets. The essential nature of the peptidoglycan (PG) layer

of the mycobacterial cell wall, which features several distinctive modifications,

such as the N-glycolylation of muramic acid and the amidation of D-iso-glutamate,

makes it a target of particular interest. To understand their role in

susceptibility to beta-lactams and in the modulation of host-pathogen

interactions, the genes encoding the enzymes responsible for these PG

modifications (namH and murT/gatD, respectively) were silenced in the model

organism Mycobacterium smegmatis using CRISPR interference (CRISPRi). Although

beta-lactams are not included in TB-therapy, their combination with

beta-lactamase inhibitors is a prospective strategy to treat MDR-TB. To uncover

synergistic effects between the action of beta-lactams and the depletion of these

PG modifications, knockdown mutants were also constructed in strains lacking the

major beta-lactamase of M. smegmatis BlaS, PM965 (M. smegmatis ΔblaS1) and PM979

(M. smegmatis ΔblaS1 ΔnamH). The phenotyping assays affirmed the essentiality of

the amidation of D-iso-glutamate to the survival of mycobacteria, as opposed to

the N-glycolylation of muramic acid. The qRT-PCR assays confirmed the successful

repression of the target genes, along with few polar effects and differential

knockdown level depending on PAM strength and target site. Both PG modifications

were found to contribute to beta-lactam resistance. While the amidation of

D-iso-glutamate impacted cefotaxime and isoniazid resistance, the N-glycolylation

of muramic acid substantially promoted resistance to the tested beta-lactams.

Their simultaneous depletion provoked synergistic reductions in beta-lactam MICs.

Moreover, the depletion of these PG modifications promoted a significantly faster

bacilli killing by J774 macrophages. Whole-genome sequencing revealed that these

PG modifications are highly conserved in a set of 172 clinical strains of Mtb,

demonstrating their potential as therapeutic targets against TB. Our results

support the development of new therapeutic agents targeting these distinctive

mycobacterial PG modifications.

CI - Copyright © 2023 Silveiro, Marques, Olivença, Pires, Mortinho, Nunes, Pimentel,

Anes and Catalão.

FAU - Silveiro, Cátia

AU - Silveiro C

AD - Research Institute for Medicines (iMed.ULisboa), Faculty of Pharmacy,

Universidade de Lisboa, Lisboa, Portugal.

FAU - Marques, Mariana

AU - Marques M

AD - Research Institute for Medicines (iMed.ULisboa), Faculty of Pharmacy,

Universidade de Lisboa, Lisboa, Portugal.

FAU - Olivença, Francisco

AU - Olivença F

AD - Research Institute for Medicines (iMed.ULisboa), Faculty of Pharmacy,

Universidade de Lisboa, Lisboa, Portugal.

FAU - Pires, David

AU - Pires D

AD - Research Institute for Medicines (iMed.ULisboa), Faculty of Pharmacy,

Universidade de Lisboa, Lisboa, Portugal.

AD - Universidade Católica Portuguesa, Católica Medical School, Centre for

Interdisciplinary Research in Health, Lisbon, Portugal.

FAU - Mortinho, Diana

AU - Mortinho D

AD - Research Institute for Medicines (iMed.ULisboa), Faculty of Pharmacy,

Universidade de Lisboa, Lisboa, Portugal.

FAU - Nunes, Alexandra

AU - Nunes A

AD - Genomics and Bioinformatics Unit, Department of Infectious Diseases, National

Institute of Health, Lisbon, Portugal.

AD - Faculty of Veterinary Medicine, Universidade Lusófona, Lisbon, Portugal.

FAU - Pimentel, Madalena

AU - Pimentel M

AD - Research Institute for Medicines (iMed.ULisboa), Faculty of Pharmacy,

Universidade de Lisboa, Lisboa, Portugal.

FAU - Anes, Elsa

AU - Anes E

AD - Research Institute for Medicines (iMed.ULisboa), Faculty of Pharmacy,

Universidade de Lisboa, Lisboa, Portugal.

FAU - Catalão, Maria João

AU - Catalão MJ

AD - Research Institute for Medicines (iMed.ULisboa), Faculty of Pharmacy,

Universidade de Lisboa, Lisboa, Portugal.

LA - eng

PT - Journal Article

PT - Research Support, Non-U.S. Gov't

DEP - 20230315

PL - Switzerland

TA - Front Cell Infect Microbiol

JT - Frontiers in cellular and infection microbiology

JID - 101585359

RN - 0 (Peptidoglycan)

RN - 0 (Muramic Acids)

RN - 0 (beta-Lactams)

RN - 0 (Glutamates)

RN - 0 (Anti-Bacterial Agents)

SB - IM

MH - Humans

MH - Peptidoglycan/genetics

MH - Muramic Acids/pharmacology

MH - Clustered Regularly Interspaced Short Palindromic Repeats

MH - *Tuberculosis/microbiology

MH - *Mycobacterium tuberculosis

MH - beta-Lactam Resistance

MH - Cell Wall

MH - beta-Lactams/pharmacology

MH - Glutamates/genetics/pharmacology

MH - Anti-Bacterial Agents/pharmacology

PMC - PMC10050696

OTO - NOTNLM

OT - CRISPR interference

OT - anti-TB targets

OT - antibiotic resistance

OT - beta-lactams

OT - host-pathogen interactions

OT - intracellular survival

OT - peptidoglycan modifications

OT - tuberculosis

COIS- The authors declare that the research was conducted in the absence of any

commercial or financial relationships that could be construed as a potential

conflict of interest.

EDAT- 2023/04/04 06:00

MHDA- 2023/04/04 06:42

PMCR- 2023/01/01

CRDT- 2023/04/03 04:28

PHST- 2022/11/04 00:00 [received]

PHST- 2023/03/03 00:00 [accepted]

PHST- 2023/04/04 06:42 [medline]

PHST- 2023/04/03 04:28 [entrez]

PHST- 2023/04/04 06:00 [pubmed]

PHST- 2023/01/01 00:00 [pmc-release]

AID - 10.3389/fcimb.2023.1089911 [doi]

PST - epublish

SO - Front Cell Infect Microbiol. 2023 Mar 15;13:1089911. doi:

10.3389/fcimb.2023.1089911. eCollection 2023.

PMID- 36731361

OWN - NLM

STAT- MEDLINE

DCOM- 20230223

LR - 20230320

IS - 1879-0534 (Electronic)

IS - 0010-4825 (Linking)

VI - 154

DP - 2023 Mar

TI - Deciphering the mechanism of resistance by novel double mutations in pncA in

Mycobacterium tuberculosis using protein structural graphs (PSG) and structural

bioinformatic approaches.

PG - 106599

LID - S0010-4825(23)00064-1 [pii]

LID - 10.1016/j.compbiomed.2023.106599 [doi]

AB - The evolution of MDR and XDR-TB is a growing concern and public health safety

threat around the world. Gene mutations are the prime cause of drug resistance in

tuberculosis, however the reports of double mutations further aggravated the

situation. Despite the large-scale genomic sequencing and identification of novel

mutations, structure investigation of the protein is still required to

structurally and functionally characterize these novel mutations to design novel

drugs for improved clinical outcome. Hence, we used structural bioinformatics

approaches i.e. molecular modeling, residues communication and molecular

simulation to understand the impact of novel double S59Y-L85P, D86G-V180F and

S104G-V130 M mutation on the structure, function of pncA encoded Pyrazinamidase

(PZase) and resistance of Pyrazinamide (PZA). Our results revealed that these

mutations alter the binding paradigm and destabilize the protein to release the

drug. Protein commination network (PCN) revealed variations in the hub residues

and sub-networks which consequently alter the internal communication and

signaling. The region 1-75 demonstrated higher flexibility in the mutant

structures and minimal by the wild type which destabilize of the internally

arranged beta-sheets which consequently reduce the binding of PZA and potentially

Fe ion in the mutants. Hydrogen bonding analysis further validated the findings.

The total binding free energy (ΔG) for each complex i.e. wild type

-7.46 kcal/mol, S59Y-L85P -5.21 kcal/mol, S104G-V130 M -5.33 kcal/mol while for

the D86G-V180F mutant the TBE was calculated to be -6.26 kcal/mol. This further

confirms that these mutations reduce the binding energy of PZA for PZase and

causes resistance in the effective therapy for TB. The trajectories motion was

also observed to be affected by these mutations. In conclusion, these mutations

use destabilizing approach to reduce the binding of PZA and causes resistance.

These features can be used to design novel structure-based drugs against

Tuberculosis.

CI - Copyright © 2023 Elsevier Ltd. All rights reserved.

FAU - Alshabrmi, Fahad M

AU - Alshabrmi FM

AD - Department of Medical Laboratories, College of Applied Medical Sciences, Qassim

University, Buraydah, 51452, Saudi Arabia. Electronic address: Fshbrmy@qu.edu.sa.

FAU - Alatawi, Eid A

AU - Alatawi EA

AD - Department of Medical Laboratory Technology, Faculty of Applied Medical Sciences,

University of Tabuk, Tabuk, 71491, Saudi Arabia. Electronic address:

eid.alatawi@ut.edu.sa.

LA - eng

PT - Journal Article

PT - Research Support, Non-U.S. Gov't

DEP - 20230128

PL - United States

TA - Comput Biol Med

JT - Computers in biology and medicine

JID - 1250250

RN - 0 (Antitubercular Agents)

RN - 2KNI5N06TI (Pyrazinamide)

SB - IM

MH - Humans

MH - *Mycobacterium tuberculosis/genetics

MH - Antitubercular Agents/pharmacology

MH - Pyrazinamide/pharmacology

MH - *Tuberculosis/drug therapy/genetics

MH - Mutation

MH - Computational Biology

MH - Microbial Sensitivity Tests

MH - Drug Resistance, Bacterial/genetics

OTO - NOTNLM

OT - Mutations

OT - PZA

OT - Resistance

OT - Simulation

OT - Tuberculosis

OT - pncA

COIS- Declaration of competing interest Authors declare there is no declaration of

interest.

EDAT- 2023/02/03 06:00

MHDA- 2023/02/25 06:00

CRDT- 2023/02/02 18:15

PHST- 2022/11/03 00:00 [received]

PHST- 2022/12/17 00:00 [revised]

PHST- 2023/01/22 00:00 [accepted]

PHST- 2023/02/03 06:00 [pubmed]

PHST- 2023/02/25 06:00 [medline]

PHST- 2023/02/02 18:15 [entrez]

AID - S0010-4825(23)00064-1 [pii]

AID - 10.1016/j.compbiomed.2023.106599 [doi]

PST - ppublish

SO - Comput Biol Med. 2023 Mar;154:106599. doi: 10.1016/j.compbiomed.2023.106599. Epub

2023 Jan 28.

PMID- 36455142

OWN - NLM

STAT- MEDLINE

DCOM- 20230214

LR - 20230214

IS - 1078-6791 (Print)

IS - 1078-6791 (Linking)

VI - 29

IP - 2

DP - 2023 Mar

TI - Analysis of the Mechanism of Action of Kushen in the Treatment of Tuberculosis

Based on Network Pharmacology.

PG - 155-161

LID - AT7586 [pii]

AB - CONTEXT: Drug-resistant tuberculosis (TB), especially multidrug-resistant TB, has

continued to increase and pan-drug-resistant TB and even fully drug-resistant TB

have emerged, bringing great challenges to the treatment of TB. Development of

new, safe, and effective antituberculosis drugs is an urgent need. OBJECTIVE: The

study intended to evaluate the use of the network pharmacology method to

comprehensively and systematically analyze the network relationship of Kushen's

main components, targets, and signaling pathways, aiming to provide new ideas and

clues for an in-depth study of the mechanism of Kushen's main components in the

treatment of pulmonary TB. DESIGN: The research team performed a Network

pharmacology analysis. SETTING: The study took place in the Department of

Respiratory and Critical Care Medicine at the Third People's Hospital of Yichang

City in Yichang, Hubei, China. OUTCOME MEASURES: The research team: (1) screened

Kushen's active ingredients and related targets using the Traditional Chinese

Medicine System Pharmacology (TCMSP) database and analysis platform; (2) used the

GeneCards database and the Online Mendelian Inheritance in Man (OMIM) database to

search for disease targets, (3) connected the active ingredient's targets to the

disease targets to obtain predictive targets for Kushen to act against TB, (4)

used the STRING database to construct a protein-protein interaction (PPI) network

map, (5) used the Database for Annotation, Visualization and Integrated Discovery

(DAVID) to subject the intersecting genes to gene ontology (GO) and Kyoto

Encyclopedia of Genes and Genomes (KEGG) enrichment analyses, and (6) used the

TCMSP and Protein Data Bank (PDB) databases to dock the active ingredients with

target-protein molecules. RESULTS: The research team found 45 active ingredients

for Kushen and 177 target-protein genes related to active ingredients. The PPI

network map of the Kushen-TB targets and found that the top 10 targets of Kushen

were: (1) mitogen-activated protein kinase 8 (MAPK8); (2) protein kinase B

(AKT1); (3) MAPK1, (4) estrogen receptor 1 (ESR1), (5) rel avian

reticuloendotheliosis viral oncogene homolog A (RELA), (6) interleukin-6 (IL6),

(7) MYC proto-oncogene, basic helix-loop-helix (bHLH) transcription factor MYC),

(8) retinoid X receptor alpha (RXRA), (9) FOS proto-oncogene activator protein 1

(AP-1) transcription factor subunit (FOS), and (10) JUN proto-oncogene AP-1

transcription factor subunit (JUN). The KEGG analysis suggested that Kushen can

intervene in TB through the hypoxia-inducible factor 1 (HIF-1) signaling pathway.

CONCLUSIONS: The network pharmacology analysis showed that Kushen's active

ingredients can play a role in the treatment of TB through the HIF-1 signaling

pathway.

FAU - Lin, Min

AU - Lin M

FAU - Zhou, Huan

AU - Zhou H

FAU - Li, Rong

AU - Li R

FAU - Quan, Li-Li

AU - Quan LL

FAU - Jin, Zhu

AU - Jin Z

FAU - Tong, Xiao-Wei

AU - Tong XW

LA - eng

PT - Journal Article

PL - United States

TA - Altern Ther Health Med

JT - Alternative therapies in health and medicine

JID - 9502013

RN - IYR6K8KQ5K (kushen)

RN - 0 (Transcription Factor AP-1)

RN - 0 (Drugs, Chinese Herbal)

SB - IM

MH - Humans

MH - Network Pharmacology

MH - Transcription Factor AP-1

MH - *Tuberculosis

MH - *Tuberculosis, Multidrug-Resistant

MH - Medicine, Chinese Traditional

MH - *Drugs, Chinese Herbal/pharmacology/therapeutic use

EDAT- 2022/12/02 06:00

MHDA- 2023/02/15 06:00

CRDT- 2022/12/01 15:23

PHST- 2022/12/02 06:00 [pubmed]

PHST- 2023/02/15 06:00 [medline]

PHST- 2022/12/01 15:23 [entrez]

AID - AT7586 [pii]

PST - ppublish

SO - Altern Ther Health Med. 2023 Mar;29(2):155-161.

PMID- 36855179

OWN - NLM

STAT- MEDLINE

DCOM- 20230302

LR - 20260127

IS - 1476-0711 (Electronic)

IS - 1476-0711 (Linking)

VI - 22

IP - 1

DP - 2023 Feb 28

TI - Investigation of bedaquiline resistance and genetic mutations in multi-drug

resistant Mycobacterium tuberculosis clinical isolates in Chongqing, China.

PG - 19

LID - 10.1186/s12941-023-00568-0 [doi]

LID - 19

AB - BACKGROUND: To investigate the prevalence and molecular characterization of

bedaquiline resistance among MDR-TB isolates collected from Chongqing, China.

METHODS: A total of 205 MDR-TB isolates were collected from Chongqing

Tuberculosis Control Institute between March 2019 and June 2020. The MICs of BDQ

were determined by microplate alamarblue assay. All strains were genotyped by

melting curve spoligotyping, and were subjected to WGS. RESULTS: Among the 205

MDR isolates, the resistance rate of BDQ was 4.4% (9/205). The 55 (26.8%) were

from male patients and 50 (24.4%) were new cases. Furthermore, 81 (39.5%) of

these patients exhibited lung cavitation, 13 (6.3%) patients afflicted with

diabetes mellitus, and 170 (82.9%) isolates belonged to Beijing family. However,

the distribution of BDQ resistant isolates showed no significant difference among

these characteristics. Of the 86 OFX resistant isolates, 8 isolates were XDR

(9.3%, 8/86). Six BDQ resistant isolates (66.7%, 6/9) and two BDQ susceptible

isolates (1.0%, 2/196) carried mutations in Rv0678. A total of 4 mutations types

were identified in BDQ resistant isolates, including mutation in A152G (50%,

3/6), T56C (16.7%, 1/6), GA492 insertion (16.7%, 1/6), and A274 insertion (16.7%,

1/6). BDQ showed excellent activity against MDR-TB in Chongqing. CONCLUSIONS: BDQ

showed excellent activity against MDR-TB in Chongqing. The resistance rate of BDQ

was not related to demographic and clinical characteristics. Mutations in Rv0678

gene were the major mechanism to BDQ resistance, with A152G as the most common

mutation type. WGS has a good popularize value and application prospect in the

rapid detection of BDQ resistance.

CI - © 2023. The Author(s).

FAU - Hu, Yan

AU - Hu Y

AD - Tuberculosis Reference Laboratory, Chongqing Tuberculosis Control Institute,

Chongqing, China.

FAU - Fan, Jun

AU - Fan J

AD - Tuberculosis Reference Laboratory, Chongqing Tuberculosis Control Institute,

Chongqing, China.

FAU - Zhu, Damin

AU - Zhu D

AD - Tuberculosis Reference Laboratory, Chongqing Tuberculosis Control Institute,

Chongqing, China.

FAU - Liu, Wenguo

AU - Liu W

AD - Tuberculosis Reference Laboratory, Chongqing Tuberculosis Control Institute,

Chongqing, China.

FAU - Li, Feina

AU - Li F

AD - Laboratory of Respiratory Diseases, Beijing Key Laboratory of Pediatric

Respiratory Infection Diseases, Beijing Pediatric Research Institute, Key

Laboratory of Major Diseases in Children, Ministry of Education, National

Clinical Research Center for Respiratory Diseases, Beijing Children's Hospital,

Capital Medical University, National Center for Children's Health, Beijing,

100045, China.

FAU - Li, Tongxin

AU - Li T

AD - Central Laboratory, Chongqing Public Health Medical Center, Chongqing, 400036,

China. cqltxin@163.com.

FAU - Zheng, Huiwen

AU - Zheng H

AD - Laboratory of Respiratory Diseases, Beijing Key Laboratory of Pediatric

Respiratory Infection Diseases, Beijing Pediatric Research Institute, Key

Laboratory of Major Diseases in Children, Ministry of Education, National

Clinical Research Center for Respiratory Diseases, Beijing Children's Hospital,

Capital Medical University, National Center for Children's Health, Beijing,

100045, China. hanhui820@126.com.

LA - eng

GR - 2020MSXM089/Chongqing medical scientific research project/

GR - 2021MSXM300/Chongqing medical scientific research project/

GR - 2022MSXM061/Chongqing medical scientific research project/

GR - 7224328/Beijing Natural Science Foundation/

PT - Journal Article

DEP - 20230228

PL - England

TA - Ann Clin Microbiol Antimicrob

JT - Annals of clinical microbiology and antimicrobials

JID - 101152152

RN - 78846I289Y (bedaquiline)

RN - 0 (Diarylquinolines)

SB - IM

MH - Humans

MH - Male

MH - *Mycobacterium tuberculosis/genetics

MH - China

MH - Beijing

MH - *Tuberculosis, Multidrug-Resistant

MH - Diarylquinolines

PMC - PMC9976417

OTO - NOTNLM

OT - Bedaquiline

OT - MDR

OT - Mutation

OT - Resistance

COIS- The authors declare that there is no competing interests regarding the

publication of this paper.

EDAT- 2023/03/02 06:00

MHDA- 2023/03/03 06:00

PMCR- 2023/02/28

CRDT- 2023/03/01 00:47

PHST- 2022/12/05 00:00 [received]

PHST- 2023/02/15 00:00 [accepted]

PHST- 2023/03/01 00:47 [entrez]

PHST- 2023/03/02 06:00 [pubmed]

PHST- 2023/03/03 06:00 [medline]

PHST- 2023/02/28 00:00 [pmc-release]

AID - 10.1186/s12941-023-00568-0 [pii]

AID - 568 [pii]

AID - 10.1186/s12941-023-00568-0 [doi]

PST - epublish

SO - Ann Clin Microbiol Antimicrob. 2023 Feb 28;22(1):19. doi:

10.1186/s12941-023-00568-0.

PMID- 36915381

OWN - NLM

STAT- PubMed-not-MEDLINE

LR - 20240914

IS - 2001-0370 (Print)

IS - 2001-0370 (Electronic)

IS - 2001-0370 (Linking)

VI - 21

DP - 2023

TI - In silico analyses of isoniazid and streptomycin resistance-associated mutations

in Mycobacterium tuberculosis.

PG - 1874-1884

LID - 10.1016/j.csbj.2023.02.035 [doi]

AB - Multi-drug resistant tuberculosis is categorised by the World Health Organisation

(WHO) as a public health crisis. In silico techniques were used to probe the

structural basis of Mycobacterium tuberculosis resistance to isoniazid and

streptomycin. Isoniazid resistance-associated mutations in InhA were predicted to

reduce the binding affinity of NADH to InhA, without affecting INH-NAD

(competitive-inhibitor) binding. Perturbation of the mutated residues was

predicted (with the AlloSigMA server) to modulate the free energy of allosteric

modulation of key binding site residues F41, F149, Y158 and W222. These results

suggest that allosteric modulation of the protein structure may be key to the

mechanism by which isoniazid resistance-associated mutations act. Mutations in

the methyltransferase glucose-inhibited division gene B (GidB) are associated

with streptomycin resistance. Molecular docking was carried out to predict the

structure of the GidB bound to its substrate (s-adenosyl methionine). The effects

of streptomycin resistance-associated mutations in GidB on protein stability and

substrate binding were predicted (using SDM and mCSM-lig). All GidB mutants were

predicted to disfavour SAM binding.

CI - © 2023 The Authors.

FAU - Dasoondi, Rushikesh Singh

AU - Dasoondi RS

AD - Department of Biochemistry, University of Cambridge, United Kingdom.

FAU - Blundell, Tom L

AU - Blundell TL

AD - Department of Biochemistry, University of Cambridge, United Kingdom.

FAU - Pandurangan, Arun Prasad

AU - Pandurangan AP

AD - Department of Biochemistry, University of Cambridge, United Kingdom.

LA - eng

GR - WT_/Wellcome Trust/United Kingdom

PT - Journal Article

DEP - 20230221

PL - United States

TA - Comput Struct Biotechnol J

JT - Computational and structural biotechnology journal

JID - 101585369

PMC - PMC10006719

COIS- The authors declare the following financial interests/personal relationships

which may be considered as potential competing interests: Arun Prasad Pandurangan

is an Editorial Board member of the Computational and Structural Biotechnology

Journal.

EDAT- 2023/03/15 06:00

MHDA- 2023/03/15 06:01

PMCR- 2023/02/21

CRDT- 2023/03/14 02:05

PHST- 2022/09/09 00:00 [received]

PHST- 2023/02/18 00:00 [revised]

PHST- 2023/02/18 00:00 [accepted]

PHST- 2023/03/14 02:05 [entrez]

PHST- 2023/03/15 06:00 [pubmed]

PHST- 2023/03/15 06:01 [medline]

PHST- 2023/02/21 00:00 [pmc-release]

AID - S2001-0370(23)00081-8 [pii]

AID - 10.1016/j.csbj.2023.02.035 [doi]

PST - epublish

SO - Comput Struct Biotechnol J. 2023 Feb 21;21:1874-1884. doi:

10.1016/j.csbj.2023.02.035. eCollection 2023.

PMID- 36891238

OWN - NLM

STAT- PubMed-not-MEDLINE

LR - 20230310

IS - 2296-889X (Print)

IS - 2296-889X (Electronic)

IS - 2296-889X (Linking)

VI - 10

DP - 2023

TI - Biomarker discovery for tuberculosis using metabolomics.

PG - 1099654

LID - 10.3389/fmolb.2023.1099654 [doi]

LID - 1099654

AB - Tuberculosis (TB) is the leading cause of death among infectious diseases, and

the ratio of cases in which its pathogen Mycobacterium tuberculosis (Mtb) is drug

resistant has been increasing worldwide, whereas latent tuberculosis infection

(LTBI) may develop into active TB. Thus it is important to understand the

mechanism of drug resistance, find new drugs, and find biomarkers for TB

diagnosis. The rapid progress of metabolomics has enabled quantitative metabolite

profiling of both the host and the pathogen. In this context, we provide recent

progress in the application of metabolomics toward biomarker discovery for

tuberculosis. In particular, we first focus on biomarkers based on blood or other

body fluids for diagnosing active TB, identifying LTBI and predicting the risk of

developing active TB, as well as monitoring the effectiveness of anti-TB drugs.

Then we discuss the pathogen-based biomarker research for identifying drug

resistant TB. While there have been many reports of potential candidate

biomarkers, validations and clinical testing as well as improved bioinformatics

analysis are needed to further substantiate and select key biomarkers before they

can be made clinically applicable.

CI - Copyright © 2023 Yu, Jiang and Li.

FAU - Yu, Yi

AU - Yu Y

AD - Center for Analyses and Measurements, College of Chemical Engineering, Zhejiang

University of Technology, Hangzhou, China.

FAU - Jiang, Xin-Xin

AU - Jiang XX

AD - Clinical Research Laboratory, Shaoxing Seventh People's Hospital, Shaoxing,

China.

FAU - Li, Ji-Cheng

AU - Li JC

AD - Clinical Research Laboratory, Shaoxing Seventh People's Hospital, Shaoxing,

China.

AD - Institute of Cell Biology, Zhejiang University Medical School, Hangzhou, China.

LA - eng

PT - Journal Article

PT - Review

DEP - 20230220

PL - Switzerland

TA - Front Mol Biosci

JT - Frontiers in molecular biosciences

JID - 101653173

PMC - PMC9986447

OTO - NOTNLM

OT - Mycobacterium tuberculosis

OT - biomarker

OT - drug resistant tuberculosis

OT - metabolomics

OT - pulmonary tuberculosis

OT - tuberculosis

COIS- The authors declare that the research was conducted in the absence of any

commercial or financial relationships that could be construed as a potential

conflict of interest.

EDAT- 2023/03/10 06:00

MHDA- 2023/03/10 06:01

PMCR- 2023/01/01

CRDT- 2023/03/09 02:11

PHST- 2022/11/16 00:00 [received]

PHST- 2023/02/06 00:00 [accepted]

PHST- 2023/03/09 02:11 [entrez]

PHST- 2023/03/10 06:00 [pubmed]

PHST- 2023/03/10 06:01 [medline]

PHST- 2023/01/01 00:00 [pmc-release]

AID - 1099654 [pii]

AID - 10.3389/fmolb.2023.1099654 [doi]

PST - epublish

SO - Front Mol Biosci. 2023 Feb 20;10:1099654. doi: 10.3389/fmolb.2023.1099654.

eCollection 2023.

PMID- 36819973

OWN - NLM

STAT- PubMed-not-MEDLINE

LR - 20240912

IS - 2168-8184 (Print)

IS - 2168-8184 (Electronic)

IS - 2168-8184 (Linking)

VI - 15

IP - 2

DP - 2023 Feb

TI - Multidrug-Resistant TB (MDR-TB) and Extensively Drug-Resistant TB (XDR-TB) Among

Children: Where We Stand Now.

PG - e35154

LID - 10.7759/cureus.35154 [doi]

LID - e35154

AB - Drug-resistant tuberculosis (DR-TB) has continued to be a global health

cataclysm. It is an arduous condition to tackle but is curable with the proper

choice of drug and adherence to the drug therapy. WHO has introduced newer drugs

with all-oral shorter regimens, but the COVID-19 pandemic has disrupted the

achievements and raised the severity. The COVID-19 controlling mechanism is based

on social distancing, using face masks, personal protective equipment, medical

glove, head shoe cover, face shield, goggles, hand hygiene, and many more. Around

the globe, national and international health authorities impose lockdown and

movement control orders to ensure social distancing and prevent transmission of

COVID-19 infection. Therefore, WHO proposed a TB control program impaired during

a pandemic. Children, the most vulnerable group, suffer more from the

drug-resistant form and act as the storehouse of future fatal cases. It has dire

effects on physical health and hampers their mental health and academic career.

Treatment of drug-resistant cases has more success stories in children than

adults, but enrollment for treatment has been persistently low in this age group.

Despite that, drug-resistant childhood tuberculosis has been neglected, and

proper surveillance has not yet been achieved. Insufficient reporting, lack of

appropriate screening tools for children, less accessibility to the treatment

facility, inadequate awareness, and reduced funding for TB have worsened the

situation. All these have resulted in jeopardizing our dream to terminate this

deadly condition. So, it is high time to focus on this issue to achieve our

Sustainable Development Goals (SDGs), the goal of ending TB by 2030, as planned

by WHO. This review explores childhood TB's current position and areas to

improve. This review utilized electronic-based data searched through PubMed,

Google Scholar, Google Search Engine, Science Direct, and Embase.

CI - Copyright © 2023, Chowdhury et al.

FAU - Chowdhury, Kona

AU - Chowdhury K

AD - Pediatrics, Gonoshasthaya Samaj Vittik Medical College, Dhaka, BGD.

FAU - Ahmad, Rahnuma

AU - Ahmad R

AD - Physiology, Medical College for Women and Hospital, Dhaka, BGD.

FAU - Sinha, Susmita

AU - Sinha S

AD - Physiology, Khulna City Medical College, Khulna, BGD.

FAU - Dutta, Siddhartha

AU - Dutta S

AD - Pharmacology, All India Institute of Medical Sciences, Rajkot, IND.

FAU - Haque, Mainul

AU - Haque M

AD - Pharmacology and Therapeutics, National Defence University of Malaysia, Kuala

Lumpur, MYS.

LA - eng

PT - Journal Article

PT - Review

DEP - 20230218

PL - United States

TA - Cureus

JT - Cureus

JID - 101596737

PMC - PMC9938784

OTO - NOTNLM

OT - extensively drug-resistant tb (xdr tb)

OT - multidrug-resistant tb (mdr tb)

OT - mycobacterium tuberculosis

OT - new financial investment.

OT - pediatric population

OT - sustainable development goals (sdgs)

OT - target regarding tb management

OT - treatment success

OT - tuberculosis

OT - who- world health organization

COIS- The authors have declared that no competing interests exist.

EDAT- 2023/02/24 06:00

MHDA- 2023/02/24 06:01

PMCR- 2023/02/18

CRDT- 2023/02/23 10:10

PHST- 2023/02/18 00:00 [accepted]

PHST- 2023/02/23 10:10 [entrez]

PHST- 2023/02/24 06:00 [pubmed]

PHST- 2023/02/24 06:01 [medline]

PHST- 2023/02/18 00:00 [pmc-release]

AID - 10.7759/cureus.35154 [doi]

PST - epublish

SO - Cureus. 2023 Feb 18;15(2):e35154. doi: 10.7759/cureus.35154. eCollection 2023

Feb.

PMID- 36876077

OWN - NLM

STAT- PubMed-not-MEDLINE

LR - 20230307

IS - 1664-302X (Print)

IS - 1664-302X (Electronic)

IS - 1664-302X (Linking)

VI - 14

DP - 2023

TI - Endogenous relapse and exogenous reinfection in recurrent pulmonary tuberculosis:

A retrospective study revealed by whole genome sequencing.

PG - 1115295

LID - 10.3389/fmicb.2023.1115295 [doi]

LID - 1115295

AB - BACKGROUND: Tuberculosis may reoccur due to reinfection or relapse after

initially successful treatment. Distinguishing the cause of TB recurrence is

crucial to guide TB control and treatment. This study aimed to investigate the

source of TB recurrence and risk factors related to relapse in Hunan province, a

high TB burden region in southern China. METHODS: A population-based

retrospective study was conducted on all culture-positive TB cases in Hunan

province, China from 2013 to 2020. Phenotypic drug susceptibility testing and

whole-genome sequencing were used to detect drug resistance and distinguish

between relapse and reinfection. Pearson chi-square test and Fisher exact test

were applied to compare differences in categorical variables between relapse and

reinfection. The Kaplan-Meier curve was generated in R studio (4.0.4) to describe

and compare the time to recurrence between different groups. p < 0.05 was

considered statistically significant. RESULTS: Of 36 recurrent events, 27 (75.0%,

27/36) paired isolates were caused by relapse, and reinfection accounted for

25.0% (9/36) of recurrent cases. No significant difference in characteristics was

observed between relapse and reinfection (all p > 0.05). In addition, TB relapse

occurs earlier in patients of Tu ethnicity compared to patients of Han ethnicity

(p < 0.0001), whereas no significant differences in the time interval to relapse

were noted in other groups. Moreover, 83.3% (30/36) of TB recurrence occurred

within 3 years. Overall, these recurrent TB isolates were predominantly

pan-susceptible strains (71.0%, 49/69), followed by DR-TB (17.4%, 12/69) and

MDR-TB (11.6%, 8/69), with mutations mainly in codon 450 of the rpoB gene and

codon 315 of the katG gene. 11.1% (3/27) of relapse cases had acquired new

resistance during treatment, with fluoroquinolone resistance occurring most

frequently (7.4%, 2/27), both with mutations in codon 94 of gyrA. CONCLUSION:

Endogenous relapse is the main mechanism leading to TB recurrences in Hunan

province. Given that TB recurrences can occur more than 4 years after treatment

completion, it is necessary to extend the post-treatment follow-up period to

achieve better management of TB patients. Moreover, the relatively high frequency

of fluoroquinolone resistance in the second episode of relapse suggests that

fluoroquinolones should be used with caution when treating TB cases with relapse,

preferably guided by DST results.

CI - Copyright © 2023 He, Tan, Song, Liu, Wang, He, Xia, Huang, Liu, Zheng, Pei, Liu,

Ma, Cao, Zhao, Ou, Wang and Zhao.

FAU - He, Wencong

AU - He W

AD - National Institute for Communicable Disease Control and Prevention, Chinese

Center for Disease Control and Prevention, Beijing, China.

FAU - Tan, Yunhong

AU - Tan Y

AD - Hunan Provincial Chest Hospital, Tuberculosis Control Institution of Hunan

Province, Changsha, Hunan, China.

FAU - Song, Zexuan

AU - Song Z

AD - National Institute for Communicable Disease Control and Prevention, Chinese

Center for Disease Control and Prevention, Beijing, China.

FAU - Liu, Binbin

AU - Liu B

AD - Hunan Provincial Chest Hospital, Tuberculosis Control Institution of Hunan

Province, Changsha, Hunan, China.

FAU - Wang, Yiting

AU - Wang Y

AD - National Institute for Communicable Disease Control and Prevention, Chinese

Center for Disease Control and Prevention, Beijing, China.

FAU - He, Ping

AU - He P

AD - National Institute for Communicable Disease Control and Prevention, Chinese

Center for Disease Control and Prevention, Beijing, China.

FAU - Xia, Hui

AU - Xia H

AD - National Tuberculosis Reference Laboratory, Chinese Center for Disease Control

and Prevention, Beijing, China.

FAU - Huang, Fei

AU - Huang F

AD - National Tuberculosis Reference Laboratory, Chinese Center for Disease Control

and Prevention, Beijing, China.

FAU - Liu, Chunfa

AU - Liu C

AD - National Tuberculosis Reference Laboratory, Chinese Center for Disease Control

and Prevention, Beijing, China.

FAU - Zheng, Huiwen

AU - Zheng H

AD - Laboratory of Respiratory Diseases, Beijing Key Laboratory of Pediatric

Respiratory Infection Diseases, Beijing Pediatric Research Institute, Beijing

Children's Hospital, Capital Medical University, Beijing, China.

FAU - Pei, Shaojun

AU - Pei S

AD - School of Public Health, Peking University, Beijing, China.

FAU - Liu, Dongxin

AU - Liu D

AD - Shenzhen Third People's Hospital, Shenzhen, China.

FAU - Ma, Aijing

AU - Ma A

AD - National Tuberculosis Reference Laboratory, Chinese Center for Disease Control

and Prevention, Beijing, China.

FAU - Cao, Xiaolong

AU - Cao X

AD - National Tuberculosis Reference Laboratory, Chinese Center for Disease Control

and Prevention, Beijing, China.

FAU - Zhao, Bing

AU - Zhao B

AD - National Tuberculosis Reference Laboratory, Chinese Center for Disease Control

and Prevention, Beijing, China.

FAU - Ou, Xichao

AU - Ou X

AD - National Tuberculosis Reference Laboratory, Chinese Center for Disease Control

and Prevention, Beijing, China.

FAU - Wang, Shengfen

AU - Wang S

AD - National Tuberculosis Reference Laboratory, Chinese Center for Disease Control

and Prevention, Beijing, China.

FAU - Zhao, Yanlin

AU - Zhao Y

AD - National Tuberculosis Reference Laboratory, Chinese Center for Disease Control

and Prevention, Beijing, China.

LA - eng

PT - Journal Article

DEP - 20230217

PL - Switzerland

TA - Front Microbiol

JT - Frontiers in microbiology

JID - 101548977

PMC - PMC9981662

OTO - NOTNLM

OT - recurrence

OT - reinfection

OT - relapse

OT - tuberculosis

OT - whole genome sequencing

COIS- The authors declare that the research was conducted in the absence of any

commercial or financial relationships that could be construed as a potential

conflict of interest.

EDAT- 2023/03/07 06:00

MHDA- 2023/03/07 06:01

PMCR- 2023/02/17

CRDT- 2023/03/06 04:19

PHST- 2022/12/06 00:00 [received]

PHST- 2023/02/02 00:00 [accepted]

PHST- 2023/03/06 04:19 [entrez]

PHST- 2023/03/07 06:00 [pubmed]

PHST- 2023/03/07 06:01 [medline]

PHST- 2023/02/17 00:00 [pmc-release]

AID - 10.3389/fmicb.2023.1115295 [doi]

PST - epublish

SO - Front Microbiol. 2023 Feb 17;14:1115295. doi: 10.3389/fmicb.2023.1115295.

eCollection 2023.

PMID- 36621541

OWN - NLM

STAT- MEDLINE

DCOM- 20230131

LR - 20260127

IS - 1879-0631 (Electronic)

IS - 0024-3205 (Linking)

VI - 315

DP - 2023 Feb 15

TI - Molecular mechanism for the involvement of CYP2E1/NF-κB axis in

bedaquiline-induced hepatotoxicity.

PG - 121375

LID - S0024-3205(23)00009-7 [pii]

LID - 10.1016/j.lfs.2023.121375 [doi]

AB - Bedaquiline (BDQ) is a new class of anti-tubercular (anti-TB) drugs and is

currently reserved for multiple drug resistance (MDR-TB). However, after

receiving fast-track approval, its clinical studies demonstrate that its

treatment is associated with hepatotoxicity and labeled as 'boxed warning' by the

USFDA. No data is available on BDQ to understand the mechanism for drug-induced

liver injury (DILI), a severe concern for therapeutic failure/unbearable

tolerated toxicities leading to drug resistance. Therefore, we performed

mechanistic studies to decipher the potential of BDQ at three dose levels (80 to

320 mg/kg) upon the repeated dose administration orally using a widely used mice

model for TB. Results of BDQ treatment at the highest dose level showed that

substantial increase of hepatic marker enzymes (SGPT and SGOT) in serum,

oxidative stress marker levels (MDA and GSH) in hepatic tissue, and

pro-inflammatory cytokine levels (TNF-α, IL-6, and IL-1β) in serum compared to

control animals. Induction of liver injury situation was further evaluated by

Western blotting for various protein expressions linked to oxidative stress (SOD,

Nrf2, and Keap1), inflammation (NF-ĸB and IKKβ), apoptosis (BAX, Bcl-2, and

Caspase-3) and drug metabolism enzymes (CYP3A4 and CYP2E1). The elevated plasma

level of BDQ and its metabolite (N-desmethyl BDQ) were observed, corresponding to

BDQ doses. Histopathological examination and SEM analysis of the liver tissue

corroborate the above-mentioned findings. Overall results suggest that BDQ

treatment-associated generation of its cytotoxic metabolite could act on

CYP2E1/NF-kB pathway to aggravate the condition of oxidative stress,

inflammation, and apoptosis in the liver and precipitating hepatotoxicity.

CI - Copyright © 2023 Elsevier Inc. All rights reserved.

FAU - Kotwal, Pankul

AU - Kotwal P

AD - Pharmacology Division, CSIR-Indian Institute of Integrative Medicine, Jammu

180001, India; Academy of Scientific and Innovative Research (AcSIR), Ghaziabad

201002, India.

FAU - Khajuria, Parul

AU - Khajuria P

AD - Pharmacology Division, CSIR-Indian Institute of Integrative Medicine, Jammu

180001, India; Academy of Scientific and Innovative Research (AcSIR), Ghaziabad

201002, India.

FAU - Dhiman, Sumit

AU - Dhiman S

AD - Pharmacology Division, CSIR-Indian Institute of Integrative Medicine, Jammu

180001, India.

FAU - Kour, Dilpreet

AU - Kour D

AD - Pharmacology Division, CSIR-Indian Institute of Integrative Medicine, Jammu

180001, India; Academy of Scientific and Innovative Research (AcSIR), Ghaziabad

201002, India.

FAU - Dhiman, Shakti Kumar

AU - Dhiman SK

AD - Quality Management and Instrumentation Division, CSIR-Indian Institute of

Integrative Medicine, Jammu 180001, India.

FAU - Kumar, Ajay

AU - Kumar A

AD - Pharmacology Division, CSIR-Indian Institute of Integrative Medicine, Jammu

180001, India; Academy of Scientific and Innovative Research (AcSIR), Ghaziabad

201002, India.

FAU - Nandi, Utpal

AU - Nandi U

AD - Pharmacology Division, CSIR-Indian Institute of Integrative Medicine, Jammu

180001, India; Academy of Scientific and Innovative Research (AcSIR), Ghaziabad

201002, India. Electronic address: utpal.nandi@iiim.res.in.

LA - eng

PT - Journal Article

DEP - 20230106

PL - Netherlands

TA - Life Sci

JT - Life sciences

JID - 0375521

RN - 0 (NF-kappa B)

RN - EC 1.14.13.- (Cytochrome P-450 CYP2E1)

RN - 0 (Kelch-Like ECH-Associated Protein 1)

RN - 0 (NF-E2-Related Factor 2)

RN - 78846I289Y (bedaquiline)

RN - 0 (Diarylquinolines)

SB - IM

MH - Mice

MH - Animals

MH - *NF-kappa B/metabolism

MH - Cytochrome P-450 CYP2E1/metabolism

MH - Kelch-Like ECH-Associated Protein 1/metabolism

MH - NF-E2-Related Factor 2/metabolism

MH - Oxidative Stress

MH - Liver/metabolism

MH - Inflammation/pathology

MH - *Chemical and Drug Induced Liver Injury/metabolism

MH - Diarylquinolines

OTO - NOTNLM

OT - Bedaquiline

OT - CYP2E1

OT - Drug-induced liver injury

OT - Hepatotoxicity

OT - N-desmethyl bedaquiline

OT - Oxidative stress

COIS- Declaration of competing interest The authors have no potential conflict of

interest to declare.

EDAT- 2023/01/10 06:00

MHDA- 2023/02/01 06:00

CRDT- 2023/01/09 04:55

PHST- 2022/10/20 00:00 [received]

PHST- 2022/12/26 00:00 [revised]

PHST- 2023/01/03 00:00 [accepted]

PHST- 2023/01/10 06:00 [pubmed]

PHST- 2023/02/01 06:00 [medline]

PHST- 2023/01/09 04:55 [entrez]

AID - S0024-3205(23)00009-7 [pii]

AID - 10.1016/j.lfs.2023.121375 [doi]

PST - ppublish

SO - Life Sci. 2023 Feb 15;315:121375. doi: 10.1016/j.lfs.2023.121375. Epub 2023 Jan

6.

PMID- 36817137

OWN - NLM

STAT- PubMed-not-MEDLINE

LR - 20240426

IS - 1663-9812 (Print)

IS - 1663-9812 (Electronic)

IS - 1663-9812 (Linking)

VI - 14

DP - 2023

TI - Clofazimine for the treatment of tuberculosis.

PG - 1100488

LID - 10.3389/fphar.2023.1100488 [doi]

LID - 1100488

AB - Shorter (6-9 months), fully oral regimens containing new and repurposed drugs are

now the first-choice option for the treatment of drug-resistant tuberculosis

(DR-TB). Clofazimine, long used in the treatment of leprosy, is one such

repurposed drug that has become a cornerstone of DR-TB treatment and ongoing

trials are exploring novel, shorter clofazimine-containing regimens for

drug-resistant as well as drug-susceptible tuberculosis. Clofazimine's

repurposing was informed by evidence of potent activity against DR-TB strains in

vitro and in mice and a treatment-shortening effect in DR-TB patients as part of

a multidrug regimen. Clofazimine entered clinical use in the 1950s without the

rigorous safety and pharmacokinetic evaluation which is part of modern drug

development and current dosing is not evidence-based. Recent studies have begun

to characterize clofazimine's exposure-response relationship for safety and

efficacy in populations with TB. Despite being better tolerated than some other

second-line TB drugs, the extent and impact of adverse effects including skin

discolouration and cardiotoxicity are not well understood and together with

emergent resistance, may undermine clofazimine use in DR-TB programmes.

Furthermore, clofazimine's precise mechanism of action is not well established,

as is the genetic basis of clofazimine resistance. In this narrative review, we

present an overview of the evidence base underpinning the use and limitations of

clofazimine as an antituberculosis drug and discuss advances in the understanding

of clofazimine pharmacokinetics, toxicity, and resistance. The unusual

pharmacokinetic properties of clofazimine and how these relate to its putative

mechanism of action, antituberculosis activity, dosing considerations and adverse

effects are highlighted. Finally, we discuss the development of novel

riminophenazine analogues as antituberculosis drugs.

CI - Copyright © 2023 Stadler, Maartens, Meintjes and Wasserman.

FAU - Stadler, Jacob A M

AU - Stadler JAM

AD - Department of Medicine, University of Cape Town, Cape Town, South Africa.

AD - Wellcome Centre for Infectious Diseases Research in Africa, Institute of

Infectious Disease and Molecular Medicine, University of Cape Town, Cape Town,

South Africa.

FAU - Maartens, Gary

AU - Maartens G

AD - Wellcome Centre for Infectious Diseases Research in Africa, Institute of

Infectious Disease and Molecular Medicine, University of Cape Town, Cape Town,

South Africa.

AD - Department of Medicine, Division of Clinical Pharmacology, University of Cape

Town, Cape Town, South Africa.

FAU - Meintjes, Graeme

AU - Meintjes G

AD - Department of Medicine, University of Cape Town, Cape Town, South Africa.

AD - Wellcome Centre for Infectious Diseases Research in Africa, Institute of

Infectious Disease and Molecular Medicine, University of Cape Town, Cape Town,

South Africa.

FAU - Wasserman, Sean

AU - Wasserman S

AD - Wellcome Centre for Infectious Diseases Research in Africa, Institute of

Infectious Disease and Molecular Medicine, University of Cape Town, Cape Town,

South Africa.

AD - Division of Infectious Diseases and HIV Medicine, Department of Medicine,

University of Cape Town, Cape Town, South Africa.

LA - eng

GR - WT_/Wellcome Trust/United Kingdom

GR - K43 TW011421/TW/FIC NIH HHS/United States

GR - U01 AI170426/AI/NIAID NIH HHS/United States

PT - Journal Article

PT - Review

DEP - 20230202

PL - Switzerland

TA - Front Pharmacol

JT - Frontiers in pharmacology

JID - 101548923

PMC - PMC9932205

OTO - NOTNLM

OT - B663

OT - clofazimine

OT - drug-resistant tuberculosis (DR-TB)

OT - multidrug-resistant tuberculosis (MDR-TB)

OT - riminophenazines

OT - tuberculosis

COIS- The authors declare that the research was conducted in the absence of any

commercial or financial relationships that could be construed as a potential

conflict of interest.

EDAT- 2023/02/24 06:00

MHDA- 2023/02/24 06:01

PMCR- 2023/02/02

CRDT- 2023/02/23 09:31

PHST- 2022/11/16 00:00 [received]

PHST- 2023/01/19 00:00 [accepted]

PHST- 2023/02/23 09:31 [entrez]

PHST- 2023/02/24 06:00 [pubmed]

PHST- 2023/02/24 06:01 [medline]

PHST- 2023/02/02 00:00 [pmc-release]

AID - 1100488 [pii]

AID - 10.3389/fphar.2023.1100488 [doi]

PST - epublish

SO - Front Pharmacol. 2023 Feb 2;14:1100488. doi: 10.3389/fphar.2023.1100488.

eCollection 2023.

PMID- 37202938

OWN - NLM

STAT- MEDLINE

DCOM- 20230522

LR - 20260127

IS - 0971-5916 (Print)

IS - 0975-9174 (Electronic)

IS - 0971-5916 (Linking)

VI - 157

IP - 2&3

DP - 2023 Feb-Mar

TI - Dodecanoic acid & palmitic acid disarms rifampicin resistance by putatively

targeting mycobacterial efflux pump Rv1218c.

PG - 192-203

LID - 10.4103/ijmr.ijmr_1610_22 [doi]

AB - BACKGROUND & OBJECTIVES: Drug-resistant tuberculosis (TB) jeopardizes the

treatment process with poor outcomes. Efflux pumps (EPs) belonging to the ABC

transporter family in Mycobacterium tuberculosis confer resistance to rifampicin

(RMP) besides genetic mutations thus serving as a target for a potential adjunct

therapeutic inhibitory molecule. Rv1218c is one such pump that was previously

reported to be active in multidrug-resistant TB clinical isolates. METHODS: In

this study, the inhibition potential of Rv1218c-EP was tested on 8 molecules that

were shortlisted by in silico methods. These molecules were subjected to the

minimum inhibitory concentration (MIC) determination, checkerboard drug

combination assay, ethidium bromide-DNA binding assay, and in vitro and ex vivo

cytotoxicity assay. RESULTS: Based on the outcome of the study, two molecules

dodecanoic acid (DA) and palmitic acid (PA) were found to be potential enough to

decrease the MIC of RMP by 8 to 1000 folds against multidrug-resistant clinical

isolates and Rv1218c expressing recombinant Mycobacterium smegmatis.

INTERPRETATION & CONCLUSIONS: These molecules were also found to reduce the time

taken by RMP to kill these drug-resistant Mycobacteria to 48 h, unlike control

isolates that survived more than 240 h of RMP exposure. The functional

concentration of both molecules was non-toxic to the epithelial and blood

mononuclear cells. With further comprehensive scientific validation, PA and DA

could be recommended as adjunct therapeutic molecules with first-line anti-TB

drugs to treat drug-resistant TB.

FAU - Nirmal, Christy Rosaline

AU - Nirmal CR

AD - Department of Bacteriology, ICMR-National Institute for Research in Tuberculosis,

Chennai, Tamil Nadu; Achira Labs Pvt. Ltd., Bengaluru, Karnataka, India.

FAU - Rajadas, Sam Ebenezer

AU - Rajadas SE

AD - Department of Bacteriology, ICMR-National Institute for Research in Tuberculosis,

Chennai; Department of Biotechnology, VSB Engineering College, Karur, Tamil Nadu,

India.

FAU - Balasubramanian, Mahizhaveni

AU - Balasubramanian M

AD - Department of Bacteriology, ICMR-National Institute for Research in Tuberculosis,

Chennai, Tamil Nadu, India.

FAU - Magdaline, Divya

AU - Magdaline D

AD - Department of Bacteriology, ICMR-National Institute for Research in Tuberculosis,

Chennai, Tamil Nadu, India.

FAU - Chilamakuru, Naresh Babu

AU - Chilamakuru NB

AD - Department of Pharmaceutical Chemistry, Raghavendra Institute of Pharmaceutical

Education & Research, Chiyyedu, Andhra Pradesh, India.

FAU - Dinesh, Rajkumar

AU - Dinesh R

AD - Department of Bacteriology, ICMR-National Institute for Research in Tuberculosis,

Chennai, Tamil Nadu, India.

FAU - Radhakrishnan, A

AU - Radhakrishnan A

AD - Department of Bacteriology, ICMR-National Institute for Research in Tuberculosis,

Chennai, Tamil Nadu, India.

FAU - Paraman, Ramalingam

AU - Paraman R

AD - Department of Pharmaceutical Analysis, National Institute of Pharmaceutical

Education & Research, Hajipur, Bihar, India.

FAU - Mondal, Rajesh

AU - Mondal R

AD - Department of Biochemistry, ICMR-National Institute for Research in Environmental

Health, Bhopal, Madhya Pradesh, India.

FAU - Azger Dusthackeer, V N

AU - Azger Dusthackeer VN

AD - Department of Bacteriology, ICMR-National Institute for Research in Tuberculosis,

Chennai, Tamil Nadu, India.

LA - eng

PT - Journal Article

PL - United States

TA - Indian J Med Res

JT - The Indian journal of medical research

JID - 0374701

RN - VJT6J7R4TR (Rifampin)

RN - 2V16EO95H1 (Palmitic Acid)

RN - 0 (Antitubercular Agents)

RN - 0 (Bacterial Proteins)

RN - 1160N9NU9U (lauric acid)

RN - 0 (Lauric Acids)

SB - IM

MH - Humans

MH - Rifampin/pharmacology/therapeutic use

MH - Palmitic Acid/pharmacology/metabolism/therapeutic use

MH - Antitubercular Agents/pharmacology/therapeutic use

MH - *Mycobacterium tuberculosis/genetics

MH - *Tuberculosis, Multidrug-Resistant/drug therapy/genetics

MH - Microbial Sensitivity Tests

MH - Bacterial Proteins/genetics

MH - Lauric Acids

PMC - PMC10319381

OTO - NOTNLM

OT - Adjunct therapy

OT - Rv1218c

OT - efflux inhibitors

OT - efflux pump

OT - rifampicin-resistance

OT - tuberculosis

COIS- Conflicts of Interest: None.

EDAT- 2023/05/19 06:42

MHDA- 2023/05/22 06:42

PMCR- 2023/02/01

CRDT- 2023/05/19 01:49

PHST- 2023/05/22 06:42 [medline]

PHST- 2023/05/19 06:42 [pubmed]

PHST- 2023/05/19 01:49 [entrez]

PHST- 2023/02/01 00:00 [pmc-release]

AID - IndianJMedRes_2023_157_2_192_375568 [pii]

AID - IJMR-157-192 [pii]

AID - 10.4103/ijmr.ijmr_1610_22 [doi]

PST - ppublish

SO - Indian J Med Res. 2023 Feb-Mar;157(2&3):192-203. doi: 10.4103/ijmr.ijmr_1610_22.

PMID- 36662223

OWN - NLM

STAT- MEDLINE

DCOM- 20230124

LR - 20230202

IS - 1660-3397 (Electronic)

IS - 1660-3397 (Linking)

VI - 21

IP - 1

DP - 2023 Jan 12

TI - Secondary Metabolites from Marine-Derived Bacteria with Antibiotic and

Antibiofilm Activities against Drug-Resistant Pathogens.

LID - 10.3390/md21010050 [doi]

LID - 50

AB - The search for new antibiotics against drug-resistant microbes has been expanded

to marine bacteria. Marine bacteria have been proven to be a prolific source of a

myriad of novel compounds with potential biological activities. Therefore, this

review highlights novel and bioactive compounds from marine bacteria reported

during the period of January 2016 to December 2021. Published articles containing

novel marine bacterial secondary metabolites that are active against

drug-resistant pathogens were collected. Previously described compounds (prior to

January 2016) are not included in this review. Unreported compounds during this

period that exhibited activity against pathogenic microbes were discussed and

compared in order to find the cue of the structure-bioactivity relationship. The

results showed that Streptomyces are the most studied bacteria with undescribed

bioactive compounds, followed by other genera in the Actinobacteria. We have

categorized the structures of the compounds in the present review into four

groups, based on their biosynthetic origins, as polyketide derivatives, amino

acid derivatives, terpenoids, as well as compounds with mixed origin. These

compounds were active against one or more drug-resistant pathogens, such as

methicillin-resistant Staphylococcus aureus (MRSA), methicillin-resistant

Staphylococcus epidermidis (MRSE), vancomycin-resistant Enterococci (VRE),

multidrug-resistant Mycobacterium tuberculosis (MDR-TB), and amphotericin

B-resistant Candida albicans. In addition, some of the compounds also showed

activity against biofilm formation of the test bacteria. Some previously

undescribed compounds, isolated from marine-derived bacteria during this period,

could have a good potential as lead compounds for the development of drug

candidates to overcome multidrug-resistant pathogens.

FAU - Wibowo, Joko Tri

AU - Wibowo JT

AUID- ORCID: 0000-0003-3467-6263

AD - Research Center for Vaccine and Drug, Research Organization for Health, National

Research and Innovation Agency (BRIN), KST Soekarno Jl. Raya Bogor Km. 46,

Cibinong 16911, Indonesia.

FAU - Bayu, Asep

AU - Bayu A

AD - Research Center for Vaccine and Drug, Research Organization for Health, National

Research and Innovation Agency (BRIN), KST Soekarno Jl. Raya Bogor Km. 46,

Cibinong 16911, Indonesia.

FAU - Aryati, Widya Dwi

AU - Aryati WD

AD - Faculty of Pharmacy, Universitas Indonesia, Depok 16424, Indonesia.

FAU - Fernandes, Carla

AU - Fernandes C

AUID- ORCID: 0000-0003-0940-9163

AD - Laboratório de Química Orgânica e Farmacêutica, Departamento de Ciências

Químicas, Faculdade de Farmácia, Universidade do Porto and CIIMAR, Rua de Jorge

Viterbo Ferreira 228, 4050-313 Porto, Portugal.

FAU - Yanuar, Arry

AU - Yanuar A

AD - Faculty of Pharmacy, Universitas Indonesia, Depok 16424, Indonesia.

AD - National Metabolomics Collaborative Research Center, Faculty of Pharmacy,

Universitas Indonesia, Kampus UI Depok, Depok 16424, Indonesia.

FAU - Kijjoa, Anake

AU - Kijjoa A

AUID- ORCID: 0000-0002-3321-1061

AD - ICBAS-Instituto de Ciências Biomédicas Abel Salazar and CIIMAR, Universidade do

Porto, Rua de Jorge Viterbo Ferreira 228, 4050-313 Porto, Portugal.

FAU - Putra, Masteria Yunovilsa

AU - Putra MY

AUID- ORCID: 0000-0002-9879-3293

AD - Research Center for Vaccine and Drug, Research Organization for Health, National

Research and Innovation Agency (BRIN), KST Soekarno Jl. Raya Bogor Km. 46,

Cibinong 16911, Indonesia.

AD - National Metabolomics Collaborative Research Center, Faculty of Pharmacy,

Universitas Indonesia, Kampus UI Depok, Depok 16424, Indonesia.

LA - eng

GR - NKB-741/UN2.RST/HKP.05.00/2022, UIDB/04423/2020, UIDP/04423/2020/Hibah Publikasi

Terindeks Internasional (PUTI) Review Article Q1 Tahun Anggaran 2022 No-mor:

NKB-741/UN2.RST/HKP.05.00/2022 and by the national funds through the

FCT-Foundation for Science and Technology of Portugal with the scope of

UIDB/04423/2020 and U/

GR - NKB-741/UN2.RST/HKP.05.00/2022, UIDB/04423/2020 and UIDP/04423/2020/Hibah

Publikasi Terindeks Internasional (PUTI) and FCT-Foundation for Science and

Technology of Portugal/

PT - Journal Article

PT - Review

DEP - 20230112

PL - Switzerland

TA - Mar Drugs

JT - Marine drugs

JID - 101213729

RN - 0 (Anti-Bacterial Agents)

SB - IM

MH - Anti-Bacterial Agents/pharmacology

MH - *Methicillin-Resistant Staphylococcus aureus

MH - Bacteria

MH - *Actinobacteria

MH - Biofilms

MH - Microbial Sensitivity Tests

PMC - PMC9861457

OTO - NOTNLM

OT - drug-resistant pathogens

OT - marine-derived bacteria

OT - novel antibiotics

OT - secondary metabolites

EDAT- 2023/01/21 06:00

MHDA- 2023/01/25 06:00

PMCR- 2023/01/12

CRDT- 2023/01/20 11:03

PHST- 2022/12/07 00:00 [received]

PHST- 2023/01/09 00:00 [revised]

PHST- 2023/01/10 00:00 [accepted]

PHST- 2023/01/20 11:03 [entrez]

PHST- 2023/01/21 06:00 [pubmed]

PHST- 2023/01/25 06:00 [medline]

PHST- 2023/01/12 00:00 [pmc-release]

AID - md21010050 [pii]

AID - marinedrugs-21-00050 [pii]

AID - 10.3390/md21010050 [doi]

PST - epublish

SO - Mar Drugs. 2023 Jan 12;21(1):50. doi: 10.3390/md21010050.

PMID- 36974959

OWN - NLM

STAT- MEDLINE

DCOM- 20231216

LR - 20260127

IS - 1538-0254 (Electronic)

IS - 0739-1102 (Linking)

VI - 41

IP - 23

DP - 2023

TI - An overview of mechanism and chemical inhibitors of shikimate kinase.

PG - 14582-14598

LID - 10.1080/07391102.2023.2193985 [doi]

AB - Tuberculosis is a highly infectious disease other than HIV/AIDS and it is one of

the top ten causes of death worldwide. Resistance development in the bacteria

occurs because of genetic alterations, and the molecular insights suggest that

the accumulation of mutation in the individual drug target genes is the primary

mechanism of multi-drug resistant tuberculosis. Chorismate is an essential

structural fragment for the synthesis of aromatic amino acids and synthesized

biochemically by a number of bacteria, including Mycobacterium tuberculosis,

utilizing the shikimate pathway. This shikimate kinase is the newer possible

target for the generation of novel antitubercular drug because this pathway is

expressed only in mycobacterium and not in Mammals. The discovery and development

of shikimate kinase inhibitors provide an opportunity for the development of

novel selective medications. Multiple shikimate kinase inhibitors have been

identified via insilico virtual screening and related protein-ligand interactions

along with their in-vitro studies. These inhibitors bind to the active site in a

similar fashion to shikimate. In the current review, we present an overview of

the biology and chemistry of the shikimate kinase protein and its inhibitors,

with special emphasis on the various active scaffold against the enzyme. A

variety of chemically diversified synthetic scaffolds including Benzothiazoles,

Oxadiazoles, Thiobarbiturates, Naphthoquinones, Thiazoleacetonitriles, Hybridized

Pyrazolone derivatives, Orthologous biological macromolecule derivatives,

Manzamine Alkaloids derivatives, Dipeptide inhibitor, and Chalcones are discussed

in detail. These derivatives bind to the specific target appropriately proving

their potential ability through different binding interactions and effectively

explored as an effective and selective Sk inhibitor.Communicated by Ramaswamy H.

Sarma.

FAU - Chagaleti, Bharath Kumar

AU - Chagaleti BK

AD - Department of Pharmaceutical Chemistry, SRM College of Pharmacy, SRM IST

Kattankulathur, Kancheepuram, Tamil Nadu, India.

FAU - Reddy, M B Rahul

AU - Reddy MBR

AD - Department of Pharmaceutical Chemistry, SRM College of Pharmacy, SRM IST

Kattankulathur, Kancheepuram, Tamil Nadu, India.

FAU - Saravanan, Venkatesan

AU - Saravanan V

AD - Department of Pharmaceutical Chemistry, SRM College of Pharmacy, SRM IST

Kattankulathur, Kancheepuram, Tamil Nadu, India.

FAU - B, Shanthakumar

AU - B S

AD - Department of Pharmaceutical Chemistry, SRM College of Pharmacy, SRM IST

Kattankulathur, Kancheepuram, Tamil Nadu, India.

FAU - D, Priya

AU - D P

AD - Department of Pharmaceutical Chemistry, SRM College of Pharmacy, SRM IST

Kattankulathur, Kancheepuram, Tamil Nadu, India.

FAU - Senthil Kumar, P

AU - Senthil Kumar P

AD - Faculty of Pharmacy, Karpagam Academy of Higher Education, Coimbatore, Tamil

Nadu, India.

FAU - Kathiravan, M K

AU - Kathiravan MK

AD - 209, Dr. APJ Abdul Kalam Research Lab, Dept of Pharmaceutical Chemistry, SRM

College of Pharmacy, SRM IST Kattankulathur, Kancheepuram, Tamil Nadu, India.

LA - eng

PT - Journal Article

PT - Research Support, Non-U.S. Gov't

PT - Review

DEP - 20230328

PL - England

TA - J Biomol Struct Dyn

JT - Journal of biomolecular structure & dynamics

JID - 8404176

RN - EC 2.7.1.71 (shikimate kinase)

RN - 0 (shikimate)

RN - 29MS2WI2NU (Shikimic Acid)

RN - 0 (Antitubercular Agents)

RN - EC 2.7.1.- (Phosphotransferases (Alcohol Group Acceptor))

RN - 0 (Enzyme Inhibitors)

SB - IM

MH - Animals

MH - *Shikimic Acid/metabolism/pharmacology/analogs & derivatives

MH - Antitubercular Agents/chemistry

MH - *Mycobacterium tuberculosis

MH - Phosphotransferases (Alcohol Group Acceptor)/chemistry/metabolism

MH - Enzyme Inhibitors/chemistry

MH - Mammals/metabolism

OTO - NOTNLM

OT - Antitubercular

OT - molecular docking

OT - shikimate kinase (Sk) inhibitors

OT - structural activity relationship

OT - synthesis

EDAT- 2023/03/29 06:00

MHDA- 2023/12/17 09:44

CRDT- 2023/03/28 06:43

PHST- 2023/12/17 09:44 [medline]

PHST- 2023/03/29 06:00 [pubmed]

PHST- 2023/03/28 06:43 [entrez]

AID - 10.1080/07391102.2023.2193985 [doi]

PST - ppublish

SO - J Biomol Struct Dyn. 2023;41(23):14582-14598. doi: 10.1080/07391102.2023.2193985.

Epub 2023 Mar 28.

PMID- 36924097

OWN - NLM

STAT- MEDLINE

DCOM- 20230601

LR - 20230601

IS - 1875-5305 (Electronic)

IS - 0929-8665 (Linking)

VI - 30

IP - 4

DP - 2023

TI - Antimicrobial Peptides: A Promising Strategy for Anti-tuberculosis Therapeutics.

PG - 280-294

LID - 10.2174/0929866530666230315113624 [doi]

AB - The high global burden of tuberculosis (TB) and the increasing emergence of the

drugresistant (DR) strain of Mycobacterium tuberculosis (Mtb) emphasize the

urgent need for novel antimycobacterial agents. Antimicrobial peptides (AMPs) are

small peptides widely existing in a variety of organisms and usually have

amphiphilic cationic structures, which have a selective affinity to the

negatively charged bacterial cell wall. Besides direct bactericidal mechanisms,

including interacting with the bacterial cell membrane and interfering with the

biosynthesis of the cell wall, DNA, or protein, some AMPs are involved in the

host's innate immunity. AMPs are promising alternative or complementary agents

for the treatment of DR-TB, given their various antibacterial mechanisms and low

cytotoxicity. A large number of AMPs, synthetic or natural, from human to

bacteriophage sources, have displayed potent anti-mycobacterial activity in vitro

and in vivo. In this review, we summarized the features, antimycobacterial

activity, and mechanisms of action of the AMPs according to their sources.

Although AMPs have not yet met the expectations for clinical application due to

their low bioavailabilities, high cost, and difficulties in large-scale

production, their potent antimycobacterial activity and action mechanisms, which

are different from conventional antibiotics, make them promising antibacterial

agents against DR-Mtb in the future.

CI - Copyright© Bentham Science Publishers; For any queries, please email at

epub@benthamscience.net.

FAU - Ning, Yu

AU - Ning Y

AD - Department of Medical Laboratory, Weifang Medical University, Weifang, Shandong,

261053, P.R. China.

FAU - Wang, Lujuan

AU - Wang L

AD - Department of Medical Laboratory, Weifang Medical University, Weifang, Shandong,

261053, P.R. China.

AD - Institutional Key Laboratory of Clinical Laboratory Diagnostics, 12th 5-Year

Project of Shandong Province, Weifang Medical University, Weifang, Shandong,

261053, P.R. China.

FAU - Wang, Menglu

AU - Wang M

AD - Department of Medical Laboratory, Weifang Medical University, Weifang, Shandong,

261053, P.R. China.

FAU - Meng, Xiangying

AU - Meng X

AD - Department of Medical Laboratory, Weifang Medical University, Weifang, Shandong,

261053, P.R. China.

AD - Institutional Key Laboratory of Clinical Laboratory Diagnostics, 12th 5-Year

Project of Shandong Province, Weifang Medical University, Weifang, Shandong,

261053, P.R. China.

FAU - Qiao, Jinjuan

AU - Qiao J

AD - Department of Medical Laboratory, Weifang Medical University, Weifang, Shandong,

261053, P.R. China.

AD - Institutional Key Laboratory of Clinical Laboratory Diagnostics, 12th 5-Year

Project of Shandong Province, Weifang Medical University, Weifang, Shandong,

261053, P.R. China.

LA - eng

GR - No. 2019WS593,No. ZR2020MH305,ZR2020MH305,ZR2020MH305,No. ZR2020MH305/Natural

Science Foundation of Shandong Province/

PT - Journal Article

PT - Review

PL - Netherlands

TA - Protein Pept Lett

JT - Protein and peptide letters

JID - 9441434

RN - 0 (Antitubercular Agents)

RN - 0 (Antimicrobial Peptides)

RN - 0 (Anti-Bacterial Agents)

RN - 0 (Antimicrobial Cationic Peptides)

SB - IM

MH - Humans

MH - Antitubercular Agents/pharmacology/therapeutic use

MH - Antimicrobial Peptides

MH - *Tuberculosis/drug therapy

MH - *Mycobacterium tuberculosis/genetics

MH - Anti-Bacterial Agents

MH - Antimicrobial Cationic Peptides/pharmacology/therapeutic use

OTO - NOTNLM

OT - <i>Mycobacterium tuberculosis</i>

OT - antibacterial activity

OT - antimicrobial peptides

OT - antimycobacterial peptides

OT - drug-resistant tuberculosis

OT - mechanisms of action

EDAT- 2023/03/17 06:00

MHDA- 2023/06/01 06:42

CRDT- 2023/03/16 03:44

PHST- 2022/12/14 00:00 [received]

PHST- 2023/01/25 00:00 [revised]

PHST- 2023/01/31 00:00 [accepted]

PHST- 2023/06/01 06:42 [medline]

PHST- 2023/03/17 06:00 [pubmed]

PHST- 2023/03/16 03:44 [entrez]

AID - PPL-EPUB-130160 [pii]

AID - 10.2174/0929866530666230315113624 [doi]

PST - ppublish

SO - Protein Pept Lett. 2023;30(4):280-294. doi: 10.2174/0929866530666230315113624.

PMID- 36892024

OWN - NLM

STAT- MEDLINE

DCOM- 20230517

LR - 20230517

IS - 1873-4286 (Electronic)

IS - 1381-6128 (Linking)

VI - 29

IP - 8

DP - 2023

TI - An Insight into MptpB Inhibitors as a Key Strategy to Treat MDR and

XDRTuberculosis.

PG - 562-575

LID - 10.2174/1381612829666230308112634 [doi]

AB - Tuberculosis (TB) is a chronic, air-borne infectious disease caused by

Mycobacterium tuberculosis (Mtb), which prominently affects the lungs and usually

manifests in other organs. TB is preventable and curable but what makes it

challenging is the emergence of resistance to the available treatment options.

MDR-continued TB's expansion is one of the world's most pressing and difficult

problems. Mtb revives via the reciprocity between Mycobacterium and host

signalling pathways. Mtb secretes a virulence component called Mycobacterium

tuberculosis protein tyrosine phosphatase (MptpB), which helps to survive against

host macrophages. It indicates that targeting secreted virulence factors offers

more benefits to circumvent the emergence of resistance. Many effective

inhibitors of MptpA and MptpB have been discovered, providing a solid foundation

for future research and development. Aside from possessing a structurally unique

binding site in the Mtb enzyme, MptpB's minimal resemblance to other human

phosphatases provides a broad platform for improving selectivity over host PTPs.

We believe that addressing several parts of infection processes in the host and

bacteria with combination therapy is the greatest way to reduce treatment burden

and medication resistance. We have discussed the recent potent, selective, and

efficacious MptpB inhibitors, such as natural and marine-based, isoxazole- linked

carboxylic acid-based, oxamic acid-based, and lactone-based inhibitors, as

potential strategies for treating TB.

CI - Copyright© Bentham Science Publishers; For any queries, please email at

epub@benthamscience.net.

FAU - Jain, Madhur

AU - Jain M

AD - Department of Medicinal Chemistry, National Institute of Pharmaceutical Education

and Research, Raebareli, New Transit Campus, Bijnor-Sisendi Road, Sarojini Nagar,

Near CRPF Base Camp, Lucknow, Uttar Pradesh, 226002, India.

FAU - Gollapudi, Sirisha

AU - Gollapudi S

AD - Department of Medicinal Chemistry, National Institute of Pharmaceutical Education

and Research, Raebareli, New Transit Campus, Bijnor-Sisendi Road, Sarojini Nagar,

Near CRPF Base Camp, Lucknow, Uttar Pradesh, 226002, India.

FAU - Khatik, Gopal L

AU - Khatik GL

AD - Department of Medicinal Chemistry, National Institute of Pharmaceutical Education

and Research, Raebareli, New Transit Campus, Bijnor-Sisendi Road, Sarojini Nagar,

Near CRPF Base Camp, Lucknow, Uttar Pradesh, 226002, India.

LA - eng

PT - Journal Article

PT - Research Support, Non-U.S. Gov't

PL - United Arab Emirates

TA - Curr Pharm Des

JT - Current pharmaceutical design

JID - 9602487

RN - 0 (Antitubercular Agents)

RN - EC 3.1.3.48 (Protein Tyrosine Phosphatases)

SB - IM

MH - Humans

MH - Antitubercular Agents/pharmacology/therapeutic use/chemistry

MH - *Mycobacterium tuberculosis

MH - *Tuberculosis/drug therapy

MH - Signal Transduction

MH - Protein Tyrosine Phosphatases/metabolism/pharmacology

OTO - NOTNLM

OT - MDR-TB

OT - MptpB inhibitors

OT - Mycobacterium tuberculosis

OT - XDR-tuberculosis

OT - drug resistance

OT - protein tyrosine phosphatases

EDAT- 2023/03/10 06:00

MHDA- 2023/05/17 06:42

CRDT- 2023/03/09 06:23

PHST- 2022/08/21 00:00 [received]

PHST- 2023/01/11 00:00 [accepted]

PHST- 2023/05/17 06:42 [medline]

PHST- 2023/03/10 06:00 [pubmed]

PHST- 2023/03/09 06:23 [entrez]

AID - CPD-EPUB-130052 [pii]

AID - 10.2174/1381612829666230308112634 [doi]

PST - ppublish

SO - Curr Pharm Des. 2023;29(8):562-575. doi: 10.2174/1381612829666230308112634.

PMID- 36740319

OWN - NLM

STAT- MEDLINE

DCOM- 20230211

LR - 20230211

IS - 0019-5707 (Print)

IS - 0019-5707 (Linking)

VI - 70

IP - 1

DP - 2023 Jan

TI - Correlation expression Toll-like receptor 4 with multidrugs resistant

tuberculosis in diabetes mellitus condition.

PG - 59-64

LID - S0019-5707(22)00041-5 [pii]

LID - 10.1016/j.ijtb.2022.03.012 [doi]

AB - BACKGROUND: Toll-like receptor (TLR) are ligand homologous protein in the APC

cell membrane that has functions as a receptor to triger leukocytes and innate

immune responses. When there is a Microbacterium tuberculosis (MTB) infection

enters from droplets to the lungs, the alveolar macrophages perform a phagocytic

function. The interaction between M. tuberculosis and the TLR macrophage

receptors produces chemokines which induce migration of monocytes and dendrite

cells for destruction. Diabetes militus (DM) has become risk factor for

developing tuberculosis. DM condition will reduce immunity and the ability of

immune cell phagocytes bactery and triger severe infections. The consequences of

more severe infection and metabolic disorders that occur make a person more

likely to experience Multidrugs resistant MTB. Not much data that reports on the

expression of TLR4 as a ligand that triggers an immune response in conditions of

MDR and DM. We try to find out correlation between TLR-4 in MDR MTB, diabetes and

level of MTB bacteria in experimental animals. METHODS: We conducted an

experimental study on 30 experimental mice weighing 25 grams consisting of

negative control grub, infected with MTB, infected with MDR MTB, negative control

diabetes, MTB DM, MDR MTB DM. DM animals were induced by streptozosin to

experience DM, then in the treatment of infection, intraperitoneal MTB and MDR

MTB bacterial injections were given. Termination was carried out on day 14. We

count number of bacteria level in the lungs and perform evaluation TLR4 from

blood sampel. RESULTS: The negative control group had mean TLR value of 1.47 (±

0.46) while the MTB group showed an increase in TLR 9.22 (± 0.39) followed by MDR

MTB 9.50 (± 0.29), DM negative control 9, 21 (± 0.24) and more increasing in

conditions of DM MTB 13.36 (± 0.32) and DM MDR MTB 13.35 (± 0.34). ANOVA analysis

showed a significant difference (P = 0.00). pearson correlation analysis find

strong correlation TLR4 in MTB and MDR MTB with diabetes. CONCLUSION: there were

a significant difference level TLR4 between MTB and MDR TB infection with

diabetes. higher TLR4 level higher in DM MTB, DM MDR MTB. TLR 4 strong correlates

with an increase in the number of MTB bacteria.

CI - Copyright © 2022. Published by Elsevier B.V.

FAU - Agustin, Heidy

AU - Agustin H

AD - Faculty of Medicine, Hasanuddin University, Indonesia. Electronic address:

heidy_agst@yahoo.com.

FAU - Massi, Muhammad Nasrum

AU - Massi MN

AD - Faculty of Medicine, Hasanuddin University, Indonesia.

FAU - Djaharuddin, Irawaty

AU - Djaharuddin I

AD - Faculty of Medicine, Hasanuddin University, Indonesia.

FAU - Patellongi, Ilhamjaya

AU - Patellongi I

AD - Faculty of Medicine, Hasanuddin University, Indonesia. Electronic address:

ilham_pt@yahoo.com.

FAU - Susanto, Agus Dwi

AU - Susanto AD

AD - Department of Pulmonology and Respiratory Medicine, Faculty of Medicine,

Indonesia University, Indonesia. Electronic address: Agus_ds2000@yahoo.com.

FAU - Islam, Andi Asadul

AU - Islam AA

AD - Faculty of Medicine, Hasanuddin University, Indonesia.

FAU - Hatta, Mochammad

AU - Hatta M

AD - Faculty of Medicine, Hasanuddin University, Indonesia.

FAU - Bukhari, Agussalim

AU - Bukhari A

AD - Faculty of Medicine, Hasanuddin University, Indonesia.

FAU - Tabri, Nur Ahmad

AU - Tabri NA

AD - Faculty of Medicine, Hasanuddin University, Indonesia.

FAU - Santoso, Arif

AU - Santoso A

AD - Faculty of Medicine, Hasanuddin University, Indonesia.

FAU - Burhan, Erlina

AU - Burhan E

AD - Department of Pulmonology and Respiratory Medicine, Faculty of Medicine,

Indonesia University, Indonesia. Electronic address: Erlina_burhan@yahoo.com.

FAU - Isbaniyah, Fathiyah

AU - Isbaniyah F

AD - Department of Pulmonology and Respiratory Medicine, Faculty of Medicine,

Indonesia University, Indonesia.

FAU - Farsida

AU - Farsida

AD - Faculty of Medicine and Health, University of Muhammadiyah Jakarta, Indonesia.

FAU - Effendy, Zulham

AU - Effendy Z

AD - Faculty of Medicine, Syiah Kuala University, Indonesia.

LA - eng

PT - Journal Article

DEP - 20220317

PL - India

TA - Indian J Tuberc

JT - The Indian journal of tuberculosis

JID - 0373027

RN - 0 (Ligands)

RN - 0 (Toll-Like Receptor 4)

RN - 0 (Toll-Like Receptors)

RN - 0 (Tlr4 protein, mouse)

SB - IM

MH - Animals

MH - Mice

MH - *Diabetes Mellitus

MH - Ligands

MH - *Mycobacterium tuberculosis

MH - Toll-Like Receptor 4

MH - Toll-Like Receptors

MH - *Tuberculosis

OTO - NOTNLM

OT - Diabetes militus

OT - Microbacterium tuberculosis

OT - Multidrugs resistance

OT - TLR4

COIS- Conflicts of interest The authors have none to declare.

EDAT- 2023/02/06 06:00

MHDA- 2023/02/08 06:00

CRDT- 2023/02/05 20:56

PHST- 2021/03/18 00:00 [received]

PHST- 2022/03/09 00:00 [accepted]

PHST- 2023/02/05 20:56 [entrez]

PHST- 2023/02/06 06:00 [pubmed]

PHST- 2023/02/08 06:00 [medline]

AID - S0019-5707(22)00041-5 [pii]

AID - 10.1016/j.ijtb.2022.03.012 [doi]

PST - ppublish

SO - Indian J Tuberc. 2023 Jan;70(1):59-64. doi: 10.1016/j.ijtb.2022.03.012. Epub 2022

Mar 17.

PMID- 35546770

OWN - NLM

STAT- MEDLINE

DCOM- 20230208

LR - 20260127

IS - 1875-5704 (Electronic)

IS - 1567-2018 (Linking)

VI - 20

IP - 3

DP - 2023

TI - Intracellular Activity of Poly (DL-Lactide-co-Glycolide) Nanoparticles

Encapsulated with Prothionamide, Pyrazinamide, Levofloxacin, Linezolid, or

Ethambutol on Multidrug-Resistant Mycobacterium tuberculosis.

PG - 306-316

LID - 10.2174/1567201819666220511120215 [doi]

AB - BACKGROUND: Multidrug-resistant Mycobacterium tuberculosis (MDR-TB) is a major

cause of death amongst tuberculosis patients. Nanomedicine avoids some

limitations of conventional drug treatment and increases therapeutic efficacy

against bacterial infections. However, the effect of anti-TB drug nanoparticle

(NP) compounds in anti-TB regimens against MDR-TB remains unclear. OBJECTIVE: The

objective of this article is to prepare levofloxacin, linezolid, ethambutol,

prothionamide, and pyrazinamide encapsulated NPs and to evaluate their

therapeutic efficacy against MDR-TB in macrophages. METHODS: Drug-loaded PLGA NPs

were prepared by the multiple emulsion method. The colocalization, intracellular

release, and anti-TB activity of these NPs were investigated on cultured

macrophages. The immune phenotype of the macrophages, including their

mitochondrial membrane potential, reactive oxygen species (ROS), and nitric oxide

(NO) production, was evaluated following treatment with NPs or free drug

compounds. RESULTS: All drug-loaded PLGA NPs were spherical in shape, 150 to 210

nm in size, and showed 14.22% to 43.51% encapsulation efficiencies and

long-duration release. Drug-loaded PLGA NPs were mainly distributed in the

cytoplasm of macrophages, showed high cellular compatibility, and maintained

their concentration for at least 13 days. Compared with the free drug compounds,

the number of colonies after exposure to PLGA NP compounds was significantly

less. The enhanced antibacterial activity of the NP compounds may be due to the

enhanced levels of ROS and NO and the increased early apoptosis stress within M.

tuberculosis-infected macrophages additionally. CONCLUSION: The application of

PLGA NP compounds not only enhances drug efficacy but also induces innate

bactericidal events in macrophages, confirming this as a promising approach for

MDR-TB therapy.

CI - Copyright© Bentham Science Publishers; For any queries, please email at

epub@benthamscience.net.

FAU - Jiang, Huixian

AU - Jiang H

AD - School of Pharmaceutical Sciences, Southern Medical University, Guangzhou, China.

FAU - Li, Xiang

AU - Li X

AD - Department of Pharmacy, Guangzhou Chest Hospital, Guangzhou, China.

FAU - Xing, Zhenjian

AU - Xing Z

AD - Department of Pharmacy, Guangzhou Chest Hospital, Guangzhou, China.

FAU - Niu, Qun

AU - Niu Q

AD - Pulmonary Disease Institute, Guangzhou Chest Hospital, Guangzhou, China.

FAU - Xu, Jiangping

AU - Xu J

AD - School of Pharmaceutical Sciences, Southern Medical University, Guangzhou, China.

LA - eng

PT - Journal Article

PL - United Arab Emirates

TA - Curr Drug Deliv

JT - Current drug delivery

JID - 101208455

RN - 2KNI5N06TI (Pyrazinamide)

RN - 76YOO33643 (Prothionamide)

RN - 8G167061QZ (Ethambutol)

RN - 6GNT3Y5LMF (Levofloxacin)

RN - ISQ9I6J12J (Linezolid)

RN - 0 (Reactive Oxygen Species)

RN - 0 (Anti-Bacterial Agents)

RN - 1SIA8062RS (Polylactic Acid-Polyglycolic Acid Copolymer)

RN - 0 (Antitubercular Agents)

RN - 95-96-5 (dilactide)

RN - 0 (Dioxanes)

SB - IM

MH - Humans

MH - *Mycobacterium tuberculosis

MH - Pyrazinamide/pharmacology/therapeutic use

MH - Prothionamide/pharmacology/therapeutic use

MH - Ethambutol/pharmacology/therapeutic use

MH - Levofloxacin/pharmacology

MH - Linezolid/pharmacology/therapeutic use

MH - Reactive Oxygen Species

MH - *Tuberculosis

MH - Anti-Bacterial Agents/pharmacology/therapeutic use

MH - Polylactic Acid-Polyglycolic Acid Copolymer/therapeutic use

MH - *Tuberculosis, Multidrug-Resistant/drug therapy/microbiology

MH - *Nanoparticles

MH - Antitubercular Agents

MH - Dioxanes

OTO - NOTNLM

OT - Multidrug-resistant Mycobacterium tuberculosis

OT - anti-TB regimens

OT - bactericidal activity

OT - macrophage

OT - nanoparticle

OT - poly (DL-lactide-co-glycolide)

EDAT- 2022/05/14 06:00

MHDA- 2023/02/09 06:00

CRDT- 2022/05/13 11:04

PHST- 2022/01/17 00:00 [received]

PHST- 2022/03/27 00:00 [revised]

PHST- 2022/03/30 00:00 [accepted]

PHST- 2022/05/14 06:00 [pubmed]

PHST- 2023/02/09 06:00 [medline]

PHST- 2022/05/13 11:04 [entrez]

AID - CDD-EPUB-123363 [pii]

AID - 10.2174/1567201819666220511120215 [doi]

PST - ppublish

SO - Curr Drug Deliv. 2023;20(3):306-316. doi: 10.2174/1567201819666220511120215.

PMID- 36915970

OWN - NLM

STAT- MEDLINE

DCOM- 20230315

LR - 20231028

IS - 1008-9292 (Print)

IS - 1008-9292 (Linking)

VI - 51

IP - 6

DP - 2022 Dec 25

TI - The past, present and future of tuberculosis treatment.

PG - 657-668

LID - 10.3724/zdxbyxb-2022-0454 [doi]

AB - Tuberculosis (TB) is an ancient infectious disease. Before the availability of

effective drug therapy, it had high morbidity and mortality. In the past 100

years, the discovery of revolutionary anti-TB drugs such as streptomycin,

isoniazid, pyrazinamide, ethambutol and rifampicin, along with drug combination

treatment, has greatly improved TB control globally. As anti-TB drugs were widely

used, multidrug-resistant (MDR) and extensively drug-resistant (XDR) strains of

Mycobacterium tuberculosis emerged due to acquired genetic mutations, and this

now presents a major problem for effective treatment. Genes associated with drug

resistance have been identified, including katG mutations in isoniazid

resistance, rpoB mutations in rifampin resistance, pncA mutations in pyrazinamide

resistance, and gyrA mutations in quinolone resistance. The major mechanisms of

drug resistance include loss of enzyme activity in prodrug activation, drug

target alteration, overexpression of drug target, and overexpression of the

efflux pump. During the disease process, Mycobacterium tuberculosis may reside in

different microenvironments where it is expose to acidic pH, low oxygen, reactive

oxygen species and anti-TB drugs, which can facilitate the development of

non-replicating persisters and promote bacterial survival. The mechanisms of

persister formation may include toxin-antitoxin (TA) modules, DNA protection and

repair, protein degradation such as trans-translation, efflux, and altered

metabolism. In recent years, the use of new anti-TB drugs, repurposed drugs, and

their drug combinations has greatly improved treatment outcomes in patients with

both drug-susceptible TB and MDR/XDR-TB. The importance of developing more

effective drugs targeting persisters of Mycobacterium tuberculosis is emphasized.

In addition, host-directed therapeutics using both conventional drugs and herbal

medicines for more effective TB treatment should also be explored. In this

article, we review historical aspects of the research on anti-TB drugs and

discuss the current understanding and treatments of drug resistant and persistent

tuberculosis to inform future therapeutic development.

FAU - Bi, Kefan

AU - Bi K

AD - 1. The First Affiliated Hospital, Zhejiang University School of Medicine, State

Key Laboratory for Diagnosis and Treatment of Infectious Diseases, National

Clinical Research Center for Infectious Diseases, National Medical Center for

Infectious Diseases, Collaborative Innovation Center for Diagnosis and Treatment

of Infectious Diseases, Hangzhou 310003,China.

AD - 2. Jinan Microecological Biomedicine Shandong Laboratory, Jinan 250117, China.

FAU - Cao, Dan

AU - Cao D

AD - 1. The First Affiliated Hospital, Zhejiang University School of Medicine, State

Key Laboratory for Diagnosis and Treatment of Infectious Diseases, National

Clinical Research Center for Infectious Diseases, National Medical Center for

Infectious Diseases, Collaborative Innovation Center for Diagnosis and Treatment

of Infectious Diseases, Hangzhou 310003,China.

AD - 2. Jinan Microecological Biomedicine Shandong Laboratory, Jinan 250117, China.

FAU - Ding, Cheng

AU - Ding C

AD - 1. The First Affiliated Hospital, Zhejiang University School of Medicine, State

Key Laboratory for Diagnosis and Treatment of Infectious Diseases, National

Clinical Research Center for Infectious Diseases, National Medical Center for

Infectious Diseases, Collaborative Innovation Center for Diagnosis and Treatment

of Infectious Diseases, Hangzhou 310003,China.

FAU - Lu, Shuihua

AU - Lu S

AD - 3. Department for Infectious Diseases, Shenzhen Third People's Hospital, National

Clinical Research Center for Infectious Diseases, Shenzhen 518000, Guangdong

Province, China.

FAU - Lu, Hongzhou

AU - Lu H

AD - 3. Department for Infectious Diseases, Shenzhen Third People's Hospital, National

Clinical Research Center for Infectious Diseases, Shenzhen 518000, Guangdong

Province, China.

FAU - Zhang, Guangyu

AU - Zhang G

AD - 4. Shulan (Hangzhou) Hospital Affiliated to Shulan International Medical College,

Zhejiang Shuren University, Hangzhou 310015, China.

FAU - Zhang, Wenhong

AU - Zhang W

AD - 5. Department of Infectious Diseases, Huashan Hospital, Fudan University,

National Medical Center for Infectious Diseases, Shanghai 200040, China.

FAU - Li, Liang

AU - Li L

AD - 6. Beijing Chest Hospital, Capital Medical University, Beijing 101199, China.

FAU - Xu, Kaijin

AU - Xu K

AD - 1. The First Affiliated Hospital, Zhejiang University School of Medicine, State

Key Laboratory for Diagnosis and Treatment of Infectious Diseases, National

Clinical Research Center for Infectious Diseases, National Medical Center for

Infectious Diseases, Collaborative Innovation Center for Diagnosis and Treatment

of Infectious Diseases, Hangzhou 310003,China.

FAU - Li, Lanjuan

AU - Li L

AD - 1. The First Affiliated Hospital, Zhejiang University School of Medicine, State

Key Laboratory for Diagnosis and Treatment of Infectious Diseases, National

Clinical Research Center for Infectious Diseases, National Medical Center for

Infectious Diseases, Collaborative Innovation Center for Diagnosis and Treatment

of Infectious Diseases, Hangzhou 310003,China.

AD - 2. Jinan Microecological Biomedicine Shandong Laboratory, Jinan 250117, China.

FAU - Zhang, Ying

AU - Zhang Y

AD - 1. The First Affiliated Hospital, Zhejiang University School of Medicine, State

Key Laboratory for Diagnosis and Treatment of Infectious Diseases, National

Clinical Research Center for Infectious Diseases, National Medical Center for

Infectious Diseases, Collaborative Innovation Center for Diagnosis and Treatment

of Infectious Diseases, Hangzhou 310003,China.

AD - 2. Jinan Microecological Biomedicine Shandong Laboratory, Jinan 250117, China.

LA - eng

PT - Journal Article

PT - Review

PL - China

TA - Zhejiang Da Xue Xue Bao Yi Xue Ban

JT - Zhejiang da xue xue bao. Yi xue ban = Journal of Zhejiang University. Medical

sciences

JID - 100927946

RN - 2KNI5N06TI (Pyrazinamide)

RN - V83O1VOZ8L (Isoniazid)

RN - 0 (Antitubercular Agents)

RN - VJT6J7R4TR (Rifampin)

SB - IM

MH - Humans

MH - Pyrazinamide/pharmacology/therapeutic use

MH - Isoniazid/pharmacology/therapeutic use

MH - Antitubercular Agents/pharmacology/therapeutic use

MH - *Tuberculosis, Multidrug-Resistant/drug therapy/microbiology

MH - *Mycobacterium tuberculosis/genetics

MH - *Tuberculosis/drug therapy

MH - Rifampin/pharmacology/therapeutic use

MH - Mutation

MH - Drug Resistance, Multiple, Bacterial/genetics

PMC - PMC10262004

OAB - Tuberculosis (TB) is an ancient infectious disease. Before the availability of

effective drug therapy, it had high morbidity and mortality. In the past 100

years, the discovery of revolutionary anti-TB drugs such as streptomycin,

isoniazid, pyrazinamide, ethambutol and rifampicin, along with drug combination

treatment, has greatly improved TB control globally. As anti-TB drugs were widely

used, multidrug-resistant (MDR) and extensively drug-resistant (XDR) strains of

Mycobacterium tuberculosis emerged due to acquired genetic mutations, and this

now presents a major problem for effective treatment. Genes associated with drug

resistance have been identified, including katG mutations in isoniazid

resistance, rpoB mutations in rifampin resistance, pncA mutations in pyrazinamide

resistance, and gyrA mutations in quinolone resistance. The major mechanisms of

drug resistance include loss of enzyme activity in prodrug activation, drug

target alteration, overexpression of drug target, and overexpression of the

efflux pump. During the disease process, Mycobacterium tuberculosis may reside in

different microenvironments where it is expose to acidic pH, low oxygen, reactive

oxygen species and anti-TB drugs, which can facilitate the development of

non-replicating persisters and promote bacterial survival. The mechanisms of

persister formation may include toxin-antitoxin (TA) modules, DNA protection and

repair, protein degradation such as trans-translation, efflux, and altered

metabolism. In recent years, the use of new anti-TB drugs, repurposed drugs, and

their drug combinations has greatly improved treatment outcomes in patients with

both drug-susceptible TB and MDR/XDR-TB. The importance of developing more

effective drugs targeting persisters of Mycobacterium tuberculosis is emphasized.

In addition, host-directed therapeutics using both conventional drugs and herbal

medicines for more effective TB treatment should also be explored. In this

article, we review historical aspects of the research on anti-TB drugs and

discuss the current understanding and treatments of drug resistant and persistent

tuberculosis to inform future therapeutic development.

OABL- eng

OTO - NOTNLM

OT - Diagnosis

OT - Resistant tuberculosis

OT - Review

OT - Treatment

OT - Tuberculosis

COIS- 所有作者均声明不存在利益冲突

EDAT- 2023/03/15 06:00

MHDA- 2023/03/16 06:00

PMCR- 2022/12/01

CRDT- 2023/03/14 02:23

PHST- 2023/03/14 02:23 [entrez]

PHST- 2023/03/15 06:00 [pubmed]

PHST- 2023/03/16 06:00 [medline]

PHST- 2022/12/01 00:00 [pmc-release]

AID - 10.3724/zdxbyxb-2022-0454 [doi]

PST - ppublish

SO - Zhejiang Da Xue Xue Bao Yi Xue Ban. 2022 Dec 25;51(6):657-668. doi:

10.3724/zdxbyxb-2022-0454.

PMID- 36550444

OWN - NLM

STAT- MEDLINE

DCOM- 20221226

LR - 20221226

IS - 1471-2334 (Electronic)

IS - 1471-2334 (Linking)

VI - 22

IP - 1

DP - 2022 Dec 22

TI - Bacteriophages of Mycobacterium tuberculosis, their diversity, and potential

therapeutic uses: a review.

PG - 957

LID - 10.1186/s12879-022-07944-9 [doi]

LID - 957

AB - Tuberculosis (TB) caused by Mycobacterium tuberculosis (M. tuberculosis) is a

highly infectious disease and worldwide health problem. Based on the WHO TB

report, 9 million active TB cases are emerging, leading to 2 million deaths each

year. The recent emergence of multidrug-resistant tuberculosis (MDR-TB) and

extensively drug-resistant tuberculosis (XDR-TB) strains emphasizes the necessity

to improve novel therapeutic plans. Among the various developing antibacterial

approaches, phage therapy is thought to be a precise hopeful resolution.

Mycobacteriophages are viruses that infect bacteria such as Mycobacterium spp.,

containing the M. tuberculosis complex. Phages and phage-derived proteins can act

as promising antimicrobial agents. Also, phage cocktails can broaden the spectrum

of lysis activity against bacteria. Recent researches have also shown the

effective combination of antibiotics and phages to defeat the infective bacteria.

There are limitations and concerns about phage therapy. For example, human immune

response to phage therapy, transferring antibiotic resistance genes, emerging

resistance to phages, and safety issues. So, in the present study, we introduced

mycobacteriophages, their use as therapeutic agents, and their advantages and

limitations as therapeutic applications.

CI - © 2022. The Author(s).

FAU - Zeynali Kelishomi, Fatemeh

AU - Zeynali Kelishomi F

AD - Medical Microbiology Research Center, Qazvin University of Medical Sciences,

Qazvin, Iran.

FAU - Khanjani, Susan

AU - Khanjani S

AD - Medical Microbiology Research Center, Qazvin University of Medical Sciences,

Qazvin, Iran.

FAU - Fardsanei, Fatemeh

AU - Fardsanei F

AD - Medical Microbiology Research Center, Qazvin University of Medical Sciences,

Qazvin, Iran.

FAU - Saghi Sarabi, Hediyeh

AU - Saghi Sarabi H

AD - Medical Microbiology Research Center, Qazvin University of Medical Sciences,

Qazvin, Iran.

FAU - Nikkhahi, Farhad

AU - Nikkhahi F

AD - Medical Microbiology Research Center, Qazvin University of Medical Sciences,

Qazvin, Iran. farhadnikkhahi@gmail.com.

FAU - Dehghani, Behzad

AU - Dehghani B

AD - Department of Bacteriology-Virology, Shiraz University of Medical Sciences,

Shiraz, Iran.

LA - eng

PT - Journal Article

PT - Review

DEP - 20221222

PL - England

TA - BMC Infect Dis

JT - BMC infectious diseases

JID - 100968551

RN - 0 (Antitubercular Agents)

SB - IM

MH - Humans

MH - *Mycobacterium tuberculosis/genetics

MH - Antitubercular Agents/therapeutic use/pharmacology

MH - *Bacteriophages

MH - *Tuberculosis, Multidrug-Resistant/drug therapy

MH - *Extensively Drug-Resistant Tuberculosis/drug therapy

PMC - PMC9773572

OTO - NOTNLM

OT - Drug resistance

OT - Mycobacteriophages

OT - Mycobacterium tuberculosis

OT - Phage therapy

COIS- The authors declare no conflict of interest.

EDAT- 2022/12/23 06:00

MHDA- 2022/12/27 06:00

PMCR- 2022/12/22

CRDT- 2022/12/22 23:47

PHST- 2022/08/04 00:00 [received]

PHST- 2022/12/12 00:00 [accepted]

PHST- 2022/12/22 23:47 [entrez]

PHST- 2022/12/23 06:00 [pubmed]

PHST- 2022/12/27 06:00 [medline]

PHST- 2022/12/22 00:00 [pmc-release]

AID - 10.1186/s12879-022-07944-9 [pii]

AID - 7944 [pii]

AID - 10.1186/s12879-022-07944-9 [doi]

PST - epublish

SO - BMC Infect Dis. 2022 Dec 22;22(1):957. doi: 10.1186/s12879-022-07944-9.

PMID- 36620019

OWN - NLM

STAT- PubMed-not-MEDLINE

LR - 20230111

IS - 1664-302X (Print)

IS - 1664-302X (Electronic)

IS - 1664-302X (Linking)

VI - 13

DP - 2022

TI - Anti-tuberculosis drug development via targeting the cell envelope of

Mycobacterium tuberculosis.

PG - 1056608

LID - 10.3389/fmicb.2022.1056608 [doi]

LID - 1056608

AB - Mycobacterium tuberculosis possesses a dynamic cell envelope, which consists of a

peptidoglycan layer, a mycolic acid layer, and an arabinogalactan polysaccharide.

This envelope possesses a highly complex and unique structure representing a

barrier that protects and assists the growth of M. tuberculosis and allows its

adaptation to the host. It regulates the immune response of the host cells,

causing their damage. Therefore, the cell envelope of M. tuberculosis is an

attractive target for vaccine and drug development. The emergence of

multidrug-resistant as well as extensively drug resistant tuberculosis and

co-infection with HIV prevented an effective control of this disease. Thus, the

discovery and development of new drugs is a major keystone for TB treatment and

control. This review mainly summarizes the development of drug enzymes involved

in the biosynthesis of the cell wall in M. tuberculosis, and other potential drug

targets in this pathway, to provide more effective strategies for the development

of new drugs.

CI - Copyright © 2022 Xu, Dong, Peng, Gao, He, Wang and Zeng.

FAU - Xu, Xinyue

AU - Xu X

AD - West China-PUMC CC Chen Institute of Health, West China School of Public Health

and West China Fourth Hospital, Sichuan University, Chengdu, China.

FAU - Dong, Baoyu

AU - Dong B

AD - West China-PUMC CC Chen Institute of Health, West China School of Public Health

and West China Fourth Hospital, Sichuan University, Chengdu, China.

FAU - Peng, Lijun

AU - Peng L

AD - West China-PUMC CC Chen Institute of Health, West China School of Public Health

and West China Fourth Hospital, Sichuan University, Chengdu, China.

FAU - Gao, Chao

AU - Gao C

AD - State Key Laboratory of Biotherapy, Sichuan University, Chengdu, China.

AD - Laboratory of Human Diseases and Immunotherapies, West China Hospital, Sichuan

University, Chengdu, China.

FAU - He, Zhiqun

AU - He Z

AD - West China-PUMC CC Chen Institute of Health, West China School of Public Health

and West China Fourth Hospital, Sichuan University, Chengdu, China.

FAU - Wang, Chuan

AU - Wang C

AD - West China-PUMC CC Chen Institute of Health, West China School of Public Health

and West China Fourth Hospital, Sichuan University, Chengdu, China.

FAU - Zeng, Jumei

AU - Zeng J

AD - West China-PUMC CC Chen Institute of Health, West China School of Public Health

and West China Fourth Hospital, Sichuan University, Chengdu, China.

LA - eng

PT - Journal Article

PT - Review

DEP - 20221221

PL - Switzerland

TA - Front Microbiol

JT - Frontiers in microbiology

JID - 101548977

PMC - PMC9810820

OTO - NOTNLM

OT - Mycobacterium tuberculosis

OT - anti-tuberculosis drug

OT - cell envelope

OT - drug target

OT - lead compounds

COIS- The authors declare that the research was conducted in the absence of any

commercial or financial relationships that could be construed as a potential

conflict of interest.

EDAT- 2023/01/10 06:00

MHDA- 2023/01/10 06:01

PMCR- 2022/12/21

CRDT- 2023/01/09 04:04

PHST- 2022/09/29 00:00 [received]

PHST- 2022/11/25 00:00 [accepted]

PHST- 2023/01/09 04:04 [entrez]

PHST- 2023/01/10 06:00 [pubmed]

PHST- 2023/01/10 06:01 [medline]

PHST- 2022/12/21 00:00 [pmc-release]

AID - 10.3389/fmicb.2022.1056608 [doi]

PST - epublish

SO - Front Microbiol. 2022 Dec 21;13:1056608. doi: 10.3389/fmicb.2022.1056608.

eCollection 2022.

PMID- 36453945

OWN - NLM

STAT- MEDLINE

DCOM- 20230106

LR - 20231020

IS - 2165-0497 (Electronic)

IS - 2165-0497 (Linking)

VI - 10

IP - 6

DP - 2022 Dec 21

TI - Mycobacterium Fluoroquinolone Resistance Protein D (MfpD), a GTPase-Activating

Protein of GTPase MfpB, Is Involved in Fluoroquinolones Potency.

PG - e0209822

LID - 10.1128/spectrum.02098-22 [doi]

LID - e02098-22

AB - Tuberculosis (TB) caused by Mycobacterium tuberculosis infection remains one of

the most serious global health problems. Fluoroquinolones (FQs) are an important

component of drug regimens against multidrug-resistant tuberculosis, but

challenged by the emergence of FQ-resistant strains. Mycobacterium

fluoroquinolone resistance protein A (MfpA) is a pentapeptide protein that

confers resistance to FQs. MfpA is the fifth gene in the mfp operon among most

Mycobacterium, implying other mfp genes might regulate the activity of MfpA. To

elucidate the function of this operon, we constructed deletion mutants and

rescued strains and found that MfpD is a GTPase-activating protein (GAP) involved

in FQs activity. We showed that the recombinant strains overexpressing mfpD

became more sensitive to FQs, whereas an mfpD deletion mutant was more resistant

to FQs. By using site-directed mutagenesis and mycobacterial protein fragment

complementation, we genetically demonstrated that mfpD participated in FQs

susceptibility via directly acting on mfpB. We further biochemically demonstrated

that MfpD was a GAP capable of stimulating the GTPase activity of MfpB. Our

studies suggest that MfpD, a GAP of MfpB, is involved in MfpA-mediated FQs

resistance. The function of MfpD adds new insights into the role of the mfp

operon in Mycobacterium fluoroquinolone resistance. IMPORTANCE Tuberculosis is

one of the leading causes of morbidity and mortality worldwide largely due to

increasingly prevalent drug-resistant strains. Fluoroquinolones are important

antibiotics used for treating multidrug-resistant tuberculosis (MDR-TB). The

resistance mechanism mediated by the Mycobacterium fluoroquinolone resistance

protein (MfpA) is unique in Mycobacterium. However, the regulatory mechanism of

MfpA remains largely unclear. In this study, we first report that MfpD acts as a

GAP for MfpB and characterize a novel pathway that controls Mycobacterium small G

proteins. Our findings provide new insights into the regulation of MfpA and

inspiration for new candidate targets for the discovery and development of

anti-TB drugs.

FAU - Huang, Yu

AU - Huang Y

AUID- ORCID: 0000-0003-4053-3648

AD - Institute of Modern Biopharmaceuticals, State Key Laboratory Breeding Base of

Eco-Environment and Bio-Resource of the Three Gorges Area, Key Laboratory of

Eco-Environments in Three Gorges Reservoir Region, Ministry of Education, School

of Life Sciences, Southwest University, Chongqing, China.

FAU - Yan, Shuangquan

AU - Yan S

AD - Institute of Modern Biopharmaceuticals, State Key Laboratory Breeding Base of

Eco-Environment and Bio-Resource of the Three Gorges Area, Key Laboratory of

Eco-Environments in Three Gorges Reservoir Region, Ministry of Education, School

of Life Sciences, Southwest University, Chongqing, China.

FAU - Li, Yuzhu

AU - Li Y

AD - Institute of Modern Biopharmaceuticals, State Key Laboratory Breeding Base of

Eco-Environment and Bio-Resource of the Three Gorges Area, Key Laboratory of

Eco-Environments in Three Gorges Reservoir Region, Ministry of Education, School

of Life Sciences, Southwest University, Chongqing, China.

FAU - Ai, Xuefeng

AU - Ai X

AD - Institute of Modern Biopharmaceuticals, State Key Laboratory Breeding Base of

Eco-Environment and Bio-Resource of the Three Gorges Area, Key Laboratory of

Eco-Environments in Three Gorges Reservoir Region, Ministry of Education, School

of Life Sciences, Southwest University, Chongqing, China.

FAU - Yu, Xi

AU - Yu X

AD - Institute of Modern Biopharmaceuticals, State Key Laboratory Breeding Base of

Eco-Environment and Bio-Resource of the Three Gorges Area, Key Laboratory of

Eco-Environments in Three Gorges Reservoir Region, Ministry of Education, School

of Life Sciences, Southwest University, Chongqing, China.

FAU - Ge, Yan

AU - Ge Y

AD - Institute of Modern Biopharmaceuticals, State Key Laboratory Breeding Base of

Eco-Environment and Bio-Resource of the Three Gorges Area, Key Laboratory of

Eco-Environments in Three Gorges Reservoir Region, Ministry of Education, School

of Life Sciences, Southwest University, Chongqing, China.

FAU - Lv, Xi

AU - Lv X

AD - Institute of Modern Biopharmaceuticals, State Key Laboratory Breeding Base of

Eco-Environment and Bio-Resource of the Three Gorges Area, Key Laboratory of

Eco-Environments in Three Gorges Reservoir Region, Ministry of Education, School

of Life Sciences, Southwest University, Chongqing, China.

FAU - Fan, Lin

AU - Fan L

AD - Shanghai Clinic and Research Center of Tuberculosis, Shanghai Pulmonary Hospital,

Tongji University School of Medicine, Shanghai Key Laboratory of Tuberculosis,

Shanghai, China.

FAU - Xie, Jianping

AU - Xie J

AUID- ORCID: 0000-0002-8959-8146

AD - Institute of Modern Biopharmaceuticals, State Key Laboratory Breeding Base of

Eco-Environment and Bio-Resource of the Three Gorges Area, Key Laboratory of

Eco-Environments in Three Gorges Reservoir Region, Ministry of Education, School

of Life Sciences, Southwest University, Chongqing, China.

LA - eng

PT - Journal Article

PT - Research Support, Non-U.S. Gov't

DEP - 20221201

PL - United States

TA - Microbiol Spectr

JT - Microbiology spectrum

JID - 101634614

RN - 0 (Antitubercular Agents)

RN - 0 (Fluoroquinolones)

RN - EC 3.6.1.- (GTP Phosphohydrolases)

RN - 0 (GTPase-Activating Proteins)

SB - IM

MH - Humans

MH - *Antitubercular Agents/pharmacology

MH - Fluoroquinolones/pharmacology

MH - GTP Phosphohydrolases/genetics

MH - GTPase-Activating Proteins/genetics

MH - Microbial Sensitivity Tests

MH - *Mycobacterium tuberculosis/drug effects/genetics

MH - *Tuberculosis, Multidrug-Resistant/microbiology

PMC - PMC9769811

OTO - NOTNLM

OT - GTPase activating protein

OT - MfpB

OT - MfpD

OT - Mycobacterium fluoroquinolone resistance protein

OT - mycobacterial protein fragment complementation

OT - small GTPase

COIS- The authors declare no conflict of interest.

EDAT- 2022/12/02 06:00

MHDA- 2022/12/28 06:00

PMCR- 2022/12/01

CRDT- 2022/12/01 09:14

PHST- 2022/12/02 06:00 [pubmed]

PHST- 2022/12/28 06:00 [medline]

PHST- 2022/12/01 09:14 [entrez]

PHST- 2022/12/01 00:00 [pmc-release]

AID - 02098-22 [pii]

AID - spectrum.02098-22 [pii]

AID - 10.1128/spectrum.02098-22 [doi]

PST - ppublish

SO - Microbiol Spectr. 2022 Dec 21;10(6):e0209822. doi: 10.1128/spectrum.02098-22.

Epub 2022 Dec 1.

PMID- 36314972

OWN - NLM

STAT- MEDLINE

DCOM- 20221227

LR - 20231020

IS - 2165-0497 (Electronic)

IS - 2165-0497 (Linking)

VI - 10

IP - 6

DP - 2022 Dec 21

TI - NSC19723, a Thiacetazone-Like Benzaldehyde Thiosemicarbazone Improves the

Efficacy of TB Drugs In Vitro and In Vivo.

PG - e0259222

LID - 10.1128/spectrum.02592-22 [doi]

LID - e02592-22

AB - The complexity and duration of tuberculosis (TB) treatment contributes to the

emergence of drug resistant tuberculosis (DR-TB) and drug-associated side

effects. Alternate chemotherapeutic agents are needed to shorten the time and

improve efficacy of current treatment. In this study, we have assessed the

antitubercular activity of NSC19723, a benzaldehyde thiosemicarbazone molecule.

NSC19723 is structurally similar to thiacetazone (TAC), a second-line anti-TB

drug used to treat individuals with DR-TB. NSC19723 displayed better MIC values

than TAC against Mycobacterium tuberculosis and Mycobacterium bovis BCG. In our

checkerboard experiments, NSC19723 displayed better profiles than TAC in

combination with known first-line and recently approved drugs. Mechanistic

studies revealed that NSC19723 inhibits mycolic acid biosynthesis by targeting

the HadABC complex. Computational studies revealed that the binding pocket of

HadAB is similarly occupied by NSC19723 and TAC. NSC19723 also improved the

efficacy of isoniazid in macrophages and mouse models of infection. Cumulatively,

we have identified a benzaldehyde thiosemicarbazone scaffold that improved the

activity of TB drugs in liquid cultures, macrophages, and mice. IMPORTANCE

Mycobacterium tuberculosis, the causative agent of TB is among the leading causes

of death among infectious diseases in humans. This situation has worsened due to

the failure of BCG vaccines and the increased number of cases with HIV-TB

coinfections and drug-resistant strains. Another challenge in the field is the

lengthy duration of therapy for drug-sensitive and -resistant TB. Here, we have

deciphered the mechanism of action of NSC19723, benzaldehyde thiosemicarbazone.

We show that NSC19723 targets HadABC complex and inhibits mycolic acid

biosynthesis. We also show that NSC19723 enhances the activity of known drugs in

liquid cultures, macrophages, and mice. We have also performed molecular docking

studies to identify the interacting residues of HadAB with NSC19723. Taken

together, we demonstrate that NSC19723, a benzaldehyde thiosemicarbazone, has

better antitubercular activity than thiacetazone.

FAU - Singh, Padam

AU - Singh P

AD - Translational Health Science and Technology Institute, NCR Biotech Science

Cluster, Faridabad, Haryana, India.

FAU - Rawat, Srishti

AU - Rawat S

AD - Department of Chemistry, University of Delhi, Delhi, India.

FAU - Agrahari, Ashish Kumar

AU - Agrahari AK

AD - Translational Health Science and Technology Institute, NCR Biotech Science

Cluster, Faridabad, Haryana, India.

FAU - Singh, Manisha

AU - Singh M

AD - Translational Health Science and Technology Institute, NCR Biotech Science

Cluster, Faridabad, Haryana, India.

FAU - Chugh, Saurabh

AU - Chugh S

AD - Translational Health Science and Technology Institute, NCR Biotech Science

Cluster, Faridabad, Haryana, India.

FAU - Gurcha, Sudagar

AU - Gurcha S

AD - Institute of Microbiology and Infection, School of Biosciences, University of

Birminghamgrid.6572.6, Birmingham, United Kingdom.

FAU - Singh, Albel

AU - Singh A

AD - Institute of Microbiology and Infection, School of Biosciences, University of

Birminghamgrid.6572.6, Birmingham, United Kingdom.

FAU - Abrahams, Katherine

AU - Abrahams K

AD - Institute of Microbiology and Infection, School of Biosciences, University of

Birminghamgrid.6572.6, Birmingham, United Kingdom.

FAU - Besra, Gurdyal S

AU - Besra GS

AD - Institute of Microbiology and Infection, School of Biosciences, University of

Birminghamgrid.6572.6, Birmingham, United Kingdom.

FAU - Asthana, Shailendra

AU - Asthana S

AD - Translational Health Science and Technology Institute, NCR Biotech Science

Cluster, Faridabad, Haryana, India.

FAU - Rawat, Diwan S

AU - Rawat DS

AD - Department of Chemistry, University of Delhi, Delhi, India.

FAU - Singh, Ramandeep

AU - Singh R

AUID- ORCID: 0000-0003-2705-3826

AD - Translational Health Science and Technology Institute, NCR Biotech Science

Cluster, Faridabad, Haryana, India.

LA - eng

PT - Journal Article

PT - Research Support, Non-U.S. Gov't

DEP - 20221031

PL - United States

TA - Microbiol Spectr

JT - Microbiology spectrum

JID - 101634614

RN - MMG78X7SSR (Thioacetazone)

RN - 0 (Thiosemicarbazones)

RN - 0 (BCG Vaccine)

RN - 0 (Mycolic Acids)

RN - TA269SD04T (benzaldehyde)

RN - 0 (Benzaldehydes)

RN - 0 (Antitubercular Agents)

SB - IM

MH - Humans

MH - Animals

MH - Mice

MH - *Thioacetazone/pharmacology

MH - *Thiosemicarbazones/pharmacology

MH - BCG Vaccine

MH - Mycolic Acids/pharmacology

MH - Benzaldehydes/pharmacology

MH - Molecular Docking Simulation

MH - Antitubercular Agents/pharmacology/therapeutic use

MH - *Mycobacterium tuberculosis

PMC - PMC9769743

OTO - NOTNLM

OT - HadABC

OT - Mycobacterium tuberculosis

OT - NSC19723

OT - synergy

OT - thiacetazone

COIS- The authors declare no conflict of interest.

EDAT- 2022/11/01 06:00

MHDA- 2022/12/28 06:00

PMCR- 2022/10/31

CRDT- 2022/10/31 10:11

PHST- 2022/11/01 06:00 [pubmed]

PHST- 2022/12/28 06:00 [medline]

PHST- 2022/10/31 10:11 [entrez]

PHST- 2022/10/31 00:00 [pmc-release]

AID - 02592-22 [pii]

AID - spectrum.02592-22 [pii]

AID - 10.1128/spectrum.02592-22 [doi]

PST - ppublish

SO - Microbiol Spectr. 2022 Dec 21;10(6):e0259222. doi: 10.1128/spectrum.02592-22.

Epub 2022 Oct 31.

PMID- 36448833

OWN - NLM

STAT- MEDLINE

DCOM- 20221230

LR - 20260127

IS - 1098-6596 (Electronic)

IS - 0066-4804 (Print)

IS - 0066-4804 (Linking)

VI - 66

IP - 12

DP - 2022 Dec 20

TI - Spontaneous Mutational Patterns and Novel Mutations for Delamanid Resistance in

Mycobacterium tuberculosis.

PG - e0053122

LID - 10.1128/aac.00531-22 [doi]

LID - e00531-22

AB - Delamanid (DLM) and pretomanid (PTM) are recent additions to the

anti-tuberculosis (TB) drug armamentarium, and they offer more effective options

for drug-resistant TB treatment. In particular, DLM is included in Group C, which

is recommended for use in longer multidrug-resistant (MDR)-TB regimens. Previous

studies have shown that resistance to DLM/PTM is caused by mutations in the ddn,

fgd1, fbiA, fbiB, fbiC, and fbiD genes, which are related to the F(420)-dependent

bioactivation pathway. Herein, we conduct in vitro selection of DLM-resistant

strains using clinical Mycobacterium tuberculosis (MTB) isolates with various

drug resistance profiles. The spontaneous resistance frequency of

drug-susceptible (DS) MTB (1.14 × 10(-6) to 1.04 × 10(-4)) to DLM was similar to

that of H37Rv (8.88 × 10(-6) to 9.96 × 10(-6)) but higher than those of

multidrug-resistant MTB (2.03 × 10(-7) to 3.18 × 10(-6)) and extensively

drug-resistant (XDR) MTB (4.67 × 10(-8) to 3.60 × 10(-6)). Of the 100

independently selected DLM-resistant MTB mutants, 65% harbored mutations in genes

associated with either DLM prodrug activation (ddn, 39.73%; fgd1, 16.44%) or the

F(420) biosynthetic pathway (fbiA, 16.44%; fbiB, 5.48%; fbiC, 21.92%). Of the 45

mutations we identified, 38 were not previously reported. A structure analysis

revealed that several point mutations affected the ligand binding or structural

stability of enzymes related to DLM resistance, which would block the enzyme

activity required for prodrug activation. Our results elucidate the in vitro

spontaneous DLM-resistance patterns of different clinical strains, which could

improve the understanding of the causes of DLM resistance in clinical strains and

of the effects on drug resistance of different mutations in genes that are

related to the DLM activation pathway.

FAU - Liu, Yuanyuan

AU - Liu Y

AD - Beijing Key Laboratory for Pediatric Diseases of Otolaryngology, Head and Neck

Surgery, Beijing Pediatric Research Institute, Beijing Children's Hospital,

Capital Medical University, National Center for Children's Health, Beijing,

People's Republic of China.

FAU - Shi, Jin

AU - Shi J

AD - Department of Tuberculosis, Beijing Chest Hospital, Capital Medical

University/Beijing Tuberculosis & Thoracic Tumor Research Institute, Beijing,

People's Republic of China.

FAU - Li, Lu

AU - Li L

AD - Department of Bacteriology and Immunology, Beijing Chest Hospital, Capital

Medical University/Beijing Tuberculosis & Thoracic Tumor Research Institute,

Beijing, People's Republic of China.

FAU - Wu, Tuoya

AU - Wu T

AD - Department of Tuberculosis Diseases, Tongliao Infectious Disease Hospital,

Tongliao, Inner Mongolia, People's Republic of China.

FAU - Chu, Ping

AU - Chu P

AD - Beijing Key Laboratory for Pediatric Diseases of Otolaryngology, Head and Neck

Surgery, Beijing Pediatric Research Institute, Beijing Children's Hospital,

Capital Medical University, National Center for Children's Health, Beijing,

People's Republic of China.

FAU - Pang, Yu

AU - Pang Y

AUID- ORCID: 0000-0001-6803-9807

AD - Department of Bacteriology and Immunology, Beijing Chest Hospital, Capital

Medical University/Beijing Tuberculosis & Thoracic Tumor Research Institute,

Beijing, People's Republic of China.

FAU - Guo, Yongli

AU - Guo Y

AD - Beijing Key Laboratory for Pediatric Diseases of Otolaryngology, Head and Neck

Surgery, Beijing Pediatric Research Institute, Beijing Children's Hospital,

Capital Medical University, National Center for Children's Health, Beijing,

People's Republic of China.

FAU - Gao, Mengqiu

AU - Gao M

AD - Department of Tuberculosis, Beijing Chest Hospital, Capital Medical

University/Beijing Tuberculosis & Thoracic Tumor Research Institute, Beijing,

People's Republic of China.

FAU - Lu, Jie

AU - Lu J

AUID- ORCID: 0000-0003-1097-4450

AD - Beijing Key Laboratory for Pediatric Diseases of Otolaryngology, Head and Neck

Surgery, Beijing Pediatric Research Institute, Beijing Children's Hospital,

Capital Medical University, National Center for Children's Health, Beijing,

People's Republic of China.

LA - eng

PT - Journal Article

PT - Research Support, Non-U.S. Gov't

DEP - 20221130

PL - United States

TA - Antimicrob Agents Chemother

JT - Antimicrobial agents and chemotherapy

JID - 0315061

RN - 0 (Antitubercular Agents)

RN - 0 (Prodrugs)

RN - 0 (OPC-67683)

RN - 0 (Nitroimidazoles)

RN - 0 (Oxazoles)

SB - IM

MH - *Antitubercular Agents/pharmacology

MH - Drug Resistance, Multiple, Bacterial/genetics

MH - Microbial Sensitivity Tests

MH - Mutation

MH - *Mycobacterium tuberculosis/drug effects/genetics

MH - *Prodrugs/pharmacology

MH - Nitroimidazoles

MH - Oxazoles

PMC - PMC9765178

OTO - NOTNLM

OT - F420

OT - MTB

OT - delamanid

OT - pretomanid

OT - resistance

COIS- The authors declare no conflict of interest.

EDAT- 2022/12/01 06:00

MHDA- 2022/12/23 06:00

PMCR- 2023/05/30

CRDT- 2022/11/30 09:19

PHST- 2022/12/01 06:00 [pubmed]

PHST- 2022/12/23 06:00 [medline]

PHST- 2022/11/30 09:19 [entrez]

PHST- 2023/05/30 00:00 [pmc-release]

AID - 00531-22 [pii]

AID - aac.00531-22 [pii]

AID - 10.1128/aac.00531-22 [doi]

PST - ppublish

SO - Antimicrob Agents Chemother. 2022 Dec 20;66(12):e0053122. doi:

10.1128/aac.00531-22. Epub 2022 Nov 30.

PMID- 36545199

OWN - NLM

STAT- PubMed-not-MEDLINE

LR - 20230724

IS - 1664-302X (Print)

IS - 1664-302X (Electronic)

IS - 1664-302X (Linking)

VI - 13

DP - 2022

TI - Localization of Mycobacterium tuberculosis topoisomerase I C-terminal sequence

motif required for inhibition by endogenous toxin MazF4.

PG - 1032320

LID - 10.3389/fmicb.2022.1032320 [doi]

LID - 1032320

AB - Only about half the multi-drug resistant tuberculosis (MDR-TB) cases are

successfully cured. Thus, there is an urgent need of new TB treatment against a

novel target. Mycobacterium tuberculosis (Mtb) topoisomerase I (TopA) is the only

type IA topoisomerase in this organism and has been validated as an essential

target for TB drug discovery. Toxin-antitoxin (TA) systems participate as gene

regulators within bacteria. The TA systems contribute to the long-term dormancy

of Mtb within the host-cell environment. Mtb's toxin MazF4 (Rv1495) that is part

of the MazEF4 TA system has been shown to have dual activities as

endoribonuclease and topoisomerase I inhibitor. We have developed a complementary

assay using an Escherichia coli strain with temperature-sensitive topA mutation

to provide new insights into the MazF4 action. The assay showed that E. coli is

not sensitive to the endoribonuclease activity of Mtb MazF4 but became vulnerable

to MazF4 growth inhibition when recombinant Mtb TopA relaxation activity is

required for growth. Results from the complementation by Mtb TopA mutants with

C-terminal deletions showed that the lysine-rich C-terminal tail is required for

interaction with MazF4. Site-directed mutagenesis is utilized to identify two

lysine residues within a conserved motif in this C-terminal tail that are

critical for MazF4 inhibition. We performed molecular dynamics simulations to

predict the Mtb TopA-MazF4 complex. Our simulation results show that the complex

is stabilized by hydrogen bonds and electrostatic interactions established by

residues in the TopA C-terminal tail including the two conserved lysines. The

mechanism of Mtb TopA inhibition by MazF4 could be useful for the discovery of

novel inhibitors against a new antibacterial target in pathogenic mycobacteria

for treatment of both TB and diseases caused by the non-tuberculosis mycobacteria

(NTM).

CI - Copyright © 2022 Garcia, Martinez Borrero, Annamalai, Diaz, Balarezo, Tiwari and

Tse-Dinh.

FAU - Garcia, Pamela K

AU - Garcia PK

AD - Department of Chemistry and Biochemistry, Florida International University,

Miami, FL, United States.

FAU - Martinez Borrero, Rosemarie

AU - Martinez Borrero R

AD - Department of Chemistry and Biochemistry, Florida International University,

Miami, FL, United States.

FAU - Annamalai, Thirunavukkarasu

AU - Annamalai T

AD - Department of Chemistry and Biochemistry, Florida International University,

Miami, FL, United States.

AD - Biomolecular Sciences Institute, Florida International University, Miami, FL,

United States.

FAU - Diaz, Esnel

AU - Diaz E

AD - Department of Chemistry and Biochemistry, Florida International University,

Miami, FL, United States.

FAU - Balarezo, Steve

AU - Balarezo S

AD - Department of Chemistry and Biochemistry, Florida International University,

Miami, FL, United States.

FAU - Tiwari, Purushottam B

AU - Tiwari PB

AD - Department of Oncology, Georgetown University, Washington, DC, United States.

FAU - Tse-Dinh, Yuk-Ching

AU - Tse-Dinh YC

AD - Department of Chemistry and Biochemistry, Florida International University,

Miami, FL, United States.

AD - Biomolecular Sciences Institute, Florida International University, Miami, FL,

United States.

LA - eng

GR - R01 GM054226/GM/NIGMS NIH HHS/United States

GR - R35 GM139817/GM/NIGMS NIH HHS/United States

PT - Journal Article

DEP - 20221205

PL - Switzerland

TA - Front Microbiol

JT - Frontiers in microbiology

JID - 101548977

PMC - PMC9760754

OTO - NOTNLM

OT - MazF toxin

OT - TopA

OT - mycobacteria

OT - topoisomerase

OT - tuberculosis

COIS- The authors declare that the research was conducted in the absence of any

commercial or financial relationships that could be construed as a potential

conflict of interest.

EDAT- 2022/12/23 06:00

MHDA- 2022/12/23 06:01

PMCR- 2022/12/05

CRDT- 2022/12/22 02:31

PHST- 2022/08/30 00:00 [received]

PHST- 2022/11/17 00:00 [accepted]

PHST- 2022/12/22 02:31 [entrez]

PHST- 2022/12/23 06:00 [pubmed]

PHST- 2022/12/23 06:01 [medline]

PHST- 2022/12/05 00:00 [pmc-release]

AID - 10.3389/fmicb.2022.1032320 [doi]

PST - epublish

SO - Front Microbiol. 2022 Dec 5;13:1032320. doi: 10.3389/fmicb.2022.1032320.

eCollection 2022.

PMID- 36126606

OWN - NLM

STAT- MEDLINE

DCOM- 20221108

LR - 20221222

IS - 1090-2120 (Electronic)

IS - 0045-2068 (Linking)

VI - 129

DP - 2022 Dec

TI - Novel diaryl ether derivatives as InhA inhibitors: Design, synthesis and

antimycobacterial activity.

PG - 106125

LID - S0045-2068(22)00531-4 [pii]

LID - 10.1016/j.bioorg.2022.106125 [doi]

AB - A new series of triclosan (TCL)-mimicking diaryl ether derivatives 7-25 were

synthesized and evaluated as inhibitors of enoyl acyl carrier protein reductase

InhA enzyme. In addition, these derivatives were screened as inhibitors of

drug-susceptible (DS), multidrug-resistant (MDR), and extensive drug-resistant

(XDR) Mycobacterium tuberculosis (MTB) strains. Most compounds exihibted superior

anti-TB activities and improved ClogP compared to TCL as a standard drug. The

present work has led to the identification of compounds 14, 19 and 24 which

possess remarkable activities against DS, MDR and XDR MTB strains with MIC values

of 1.95, 3.9 and 15.63 µg/ml, respectively for compound 14, 1.95, 3.9 and

7.81 µg/ml, respectively for compound 19 and 0.98, 1.95 and 3.9 µg/ml,

respectively for compound 24. Most compounds did not exhibit toxicity to HePG2

normal cell line. Compounds 14, 19 and 24, presenting the best MIC values, were

further evaluated as inhibitors of InhA enzyme. They showed high binding

affinities in the micromolar range with IC(50) values of 1.33, 0.6, and 0.29 µM

for compounds 14, 19, and 24, respectively. Furthermore, molecular docking

approach was utilized to understand the difference in bioactivities between the

new compounds. In particular, the results revealed strong binding interactions

and high docking scores of compounds 14, 19 and 24, which could correlate with

their high activities. Mainly, the molecular modelling study of compound 24

provides an excellent platform for understanding the molecular mechanism

regarding InhA inhibition. Thus, compound 24 could be a lead compound for future

development of new antitubercular drugs.

CI - Copyright © 2022 Elsevier Inc. All rights reserved.

FAU - Abdelaziz, Ola A

AU - Abdelaziz OA

AD - Department of Pharmaceutical Organic Chemistry, Faculty of Pharmacy, Mansoura

University, Mansoura 35516, Egypt.

FAU - Othman, Dina I A

AU - Othman DIA

AD - Department of Pharmaceutical Organic Chemistry, Faculty of Pharmacy, Mansoura

University, Mansoura 35516, Egypt. Electronic address: dr_dinaali@mans.edu.eg.

FAU - Abdel-Aziz, Marwa M

AU - Abdel-Aziz MM

AD - The Regional Center for Mycology and Biotechnology, Al-Azhar University, Cairo,

Egypt.

FAU - Badr, Sahar M I

AU - Badr SMI

AD - Department of Pharmaceutical Organic Chemistry, Faculty of Pharmacy, Mansoura

University, Mansoura 35516, Egypt.

FAU - Eisa, Hassan M

AU - Eisa HM

AD - Department of Pharmaceutical Organic Chemistry, Faculty of Pharmacy, Mansoura

University, Mansoura 35516, Egypt.

LA - eng

PT - Journal Article

DEP - 20220906

PL - United States

TA - Bioorg Chem

JT - Bioorganic chemistry

JID - 1303703

RN - 0F5N573A2Y (Ether)

RN - 0 (Antitubercular Agents)

RN - 4NM5039Y5X (Triclosan)

RN - 0 (Bacterial Proteins)

RN - 0 (Enzyme Inhibitors)

SB - IM

MH - Molecular Docking Simulation

MH - Microbial Sensitivity Tests

MH - Ether

MH - Antitubercular Agents/chemistry

MH - *Mycobacterium tuberculosis

MH - *Triclosan/pharmacology

MH - Bacterial Proteins/metabolism

MH - Structure-Activity Relationship

MH - Enzyme Inhibitors/pharmacology/chemistry

OTO - NOTNLM

OT - Anti-TB

OT - DS

OT - InhA

OT - MDR-TB

OT - MTB

OT - Triclosan

OT - XDR

COIS- Declaration of Competing Interest The authors declare that they have no known

competing financial interests or personal relationships that could have appeared

to influence the work reported in this paper.

EDAT- 2022/09/21 06:00

MHDA- 2022/11/09 06:00

CRDT- 2022/09/20 18:28

PHST- 2022/07/07 00:00 [received]

PHST- 2022/08/16 00:00 [revised]

PHST- 2022/08/31 00:00 [accepted]

PHST- 2022/09/21 06:00 [pubmed]

PHST- 2022/11/09 06:00 [medline]

PHST- 2022/09/20 18:28 [entrez]

AID - S0045-2068(22)00531-4 [pii]

AID - 10.1016/j.bioorg.2022.106125 [doi]

PST - ppublish

SO - Bioorg Chem. 2022 Dec;129:106125. doi: 10.1016/j.bioorg.2022.106125. Epub 2022

Sep 6.

PMID- 34935599

OWN - NLM

STAT- MEDLINE

DCOM- 20220214

LR - 20220214

IS - 2222-1751 (Electronic)

IS - 2222-1751 (Linking)

VI - 11

IP - 1

DP - 2022 Dec

TI - Cryo-EM structure of Mycobacterium tuberculosis 50S ribosomal subunit bound with

clarithromycin reveals dynamic and specific interactions with macrolides.

PG - 293-305

LID - 10.1080/22221751.2021.2022439 [doi]

AB - Tuberculosis (TB) is the leading infectious disease caused by Mycobacterium

tuberculosis (Mtb). Clarithromycin (CTY), an analog of erythromycin (ERY), is

more potent against multidrug-resistance (MDR) TB. ERY and CTY were previously

reported to bind to the nascent polypeptide exit tunnel (NPET) near peptidyl

transferase center (PTC), but the only available CTY structure in complex with D.

radiodurans (Dra) ribosome could be misinterpreted due to resolution limitation.

To date, the mechanism of specificity and efficacy of CTY for Mtb remains elusive

since the Mtb ribosome-CTY complex structure is still unknown. Here, we employed

new sample preparation methods and solved the Mtb ribosome-CTY complex structure

at 3.3Å with cryo-EM technique, where the crucial gate site A2062 (E. coli

numbering) is located at the CTY binding site within NPET. Two alternative

conformations of A2062, a novel syn-conformation as well as a swayed conformation

bound with water molecule at interface, may play a role in coordinating the

binding of specific drug molecules. The previously overlooked C-H hydrogen bond

(H-bond) and π interaction may collectively contribute to the enhanced binding

affinity. Together, our structure data provide a structural basis for the dynamic

binding as well as the specificity of CTY and explain of how a single methyl

group in CTY improves its potency, which provides new evidence to reveal

previously unclear mechanism of translational modulation for future drug design

and anti-TB therapy. Furthermore, our sample preparation method may facilitate

drug discovery based on the complexes with low water solubility drugs by cryo-EM

technique.

FAU - Zhang, Wen

AU - Zhang W

AUID- ORCID: 0000-0002-4973-1168

AD - Institutes of Biomedical Sciences, Fudan University, Shanghai, People's Republic

of China.

AD - Fudan University Pudong Medical Center, Department of Systems Biology for

Medicine, School of Basic Medical Sciences, Fudan University, Shanghai, China.

FAU - Li, ZhiFei

AU - Li Z

AD - State Key Laboratory of Membrane Biology, National Biomedical Imaging Center,

Peking-Tsinghua Center for Life Sciences, School of Life Sciences, Peking

University, Beijing, People's Republic of China.

AD - China National Center for Biotechnology Development, Beijing, People's Republic

of China.

FAU - Sun, Yufan

AU - Sun Y

AD - Department of Medical Microbiology, Key Laboratory of Medical Molecular Virology

of Ministries of Education and Health, School of Basic Medical Sciences, Fudan

University, Shanghai, People's Republic of China.

FAU - Cui, Peng

AU - Cui P

AD - Department of Infectious Diseases, National Medical Center for Infectious

Diseases, Shanghai Key Laboratory of Infectious Diseases and Biosafety Emergency

Response, Huashan Hospital, Shanghai Medical College, Fudan University, Shanghai,

People's Republic of China.

FAU - Liang, Jianhua

AU - Liang J

AD - School of Chemistry and Chemical Engineering, Beijing Institute of Technology,

Beijing, People's Republic of China.

FAU - Xing, Qinghe

AU - Xing Q

AD - Institutes of Biomedical Sciences, Fudan University, Shanghai, People's Republic

of China.

FAU - Wu, Jing

AU - Wu J

AD - Department of Infectious Diseases, National Medical Center for Infectious

Diseases, Shanghai Key Laboratory of Infectious Diseases and Biosafety Emergency

Response, Huashan Hospital, Shanghai Medical College, Fudan University, Shanghai,

People's Republic of China.

FAU - Xu, Yanhui

AU - Xu Y

AD - Institutes of Biomedical Sciences, Fudan University, Shanghai, People's Republic

of China.

FAU - Zhang, Wenhong

AU - Zhang W

AD - Department of Infectious Diseases, National Medical Center for Infectious

Diseases, Shanghai Key Laboratory of Infectious Diseases and Biosafety Emergency

Response, Huashan Hospital, Shanghai Medical College, Fudan University, Shanghai,

People's Republic of China.

FAU - Zhang, Ying

AU - Zhang Y

AD - Department of Infectious Diseases, National Medical Center for Infectious

Diseases, Shanghai Key Laboratory of Infectious Diseases and Biosafety Emergency

Response, Huashan Hospital, Shanghai Medical College, Fudan University, Shanghai,

People's Republic of China.

AD - State Key Laboratory for the Diagnosis and Treatment of Infectious Diseases, The

First Affiliated Hospital, Zhejiang University School of Medicine, Hangzhou,

People's Republic of China.

FAU - He, Lin

AU - He L

AD - Institutes of Biomedical Sciences, Fudan University, Shanghai, People's Republic

of China.

AD - Bio-X Institute, Key Laboratory for the Genetics of Developmental and

Neuropsychiatric Disorders (Ministry of Education), Shanghai Jiao Tong

University, Shanghai, People's Republic of China.

FAU - Gao, Ning

AU - Gao N

AUID- ORCID: 0000-0003-3067-9993

AD - State Key Laboratory of Membrane Biology, National Biomedical Imaging Center,

Peking-Tsinghua Center for Life Sciences, School of Life Sciences, Peking

University, Beijing, People's Republic of China.

LA - eng

PT - Journal Article

PL - United States

TA - Emerg Microbes Infect

JT - Emerging microbes & infections

JID - 101594885

RN - 0 (Macrolides)

RN - H1250JIK0A (Clarithromycin)

SB - IM

MH - Clarithromycin/pharmacology

MH - Cryoelectron Microscopy

MH - Escherichia coli

MH - Macrolides

MH - *Mycobacterium tuberculosis/genetics

MH - Ribosome Subunits

PMC - PMC8786254

OTO - NOTNLM

OT - 50S ribosomal subunit

OT - Cryo-EM

OT - Mycobacterium tuberculosis

OT - clarithromycin

OT - dynamic interaction

OT - gate site a2062

COIS- No potential conflict of interest was reported by the author(s).

EDAT- 2021/12/23 06:00

MHDA- 2022/02/15 06:00

PMCR- 2022/01/21

CRDT- 2021/12/22 12:32

PHST- 2021/12/23 06:00 [pubmed]

PHST- 2022/02/15 06:00 [medline]

PHST- 2021/12/22 12:32 [entrez]

PHST- 2022/01/21 00:00 [pmc-release]

AID - 2022439 [pii]

AID - 10.1080/22221751.2021.2022439 [doi]

PST - ppublish

SO - Emerg Microbes Infect. 2022 Dec;11(1):293-305. doi:

10.1080/22221751.2021.2022439.

PMID- 35973312

OWN - NLM

STAT- MEDLINE

DCOM- 20220920

LR - 20220920

IS - 1768-3254 (Electronic)

IS - 0223-5234 (Linking)

VI - 242

DP - 2022 Nov 15

TI - Design, synthesis and biological evaluation of (Quinazoline 4-yloxy)acetamide and

(4-oxoquinazoline-3(4H)-yl)acetamide derivatives as inhibitors of Mycobacterium

tuberculosis bd oxidase.

PG - 114639

LID - S0223-5234(22)00541-4 [pii]

LID - 10.1016/j.ejmech.2022.114639 [doi]

AB - New chemical scaffolds with novel mechanism of action are urgently needed for the

treatment of drug resistant tuberculosis. The oxidative phosphorylation pathway

of Mycobacterium tuberculosis consists of multiple clinically validated drug

targets. This pathway can function through any one of the two terminal

oxidases-the proton pumping cytochrome bc(1)-aa(3) supercomplex, or the less

energy efficient but high affinity cytochrome bd oxidase. Inhibiting the bc(1)

complex alone has been found bacteriostatic and not bactericidal. On the other

hand, inhibition of both these oxidases turns lethal to the pathogen. In the

present study, we used a bc(1) complex mutant of M. tuberculosis to screen

(Quinazoline 4-yloxy)acetamide and (4-oxoquinazoline-3(4H)-yl)acetamide

derivatives against the alternate oxidase, i.e., cytochrome bd oxidase. Two

molecules, S-021-0601 and S-021-0607 were found to inhibit the mutant with MICs 8

and 16 μM respectively, compared to MICs of 128 and 256 μM against the wild type

M. tuberculosis. In the wild type, one of the compounds showed synergism with

Q203, an inhibitor of bc(1) complex, in inhibiting growth under aerobic

conditions. Both compounds showed synergism with Q203 in depleting bacterial ATP

and inhibiting oxygen consumption. Both the compounds at 32 μM (one-fourth or

one-eighth of their MICs for wild type) were bactericidal to wild type bacteria

under hypoxic condition, causing ∼1.9 log(10) reduction in viable counts which

increased to ∼4-log(10) when combined with Q203.

CI - Copyright © 2022 Elsevier Masson SAS. All rights reserved.

FAU - Kumar, Amit

AU - Kumar A

AD - Medicinal & Process Chemistry Division, CSIR-Central Drug Research Institute,

Sector 10, Jankipuram Extension, Sitapur Road, Lucknow, 226031, UP, India.

FAU - Kumari, Neetu

AU - Kumari N

AD - Molecular Microbiology and Immunology Division, CSIR-Central Drug Research

Institute, Sector 10, Jankipuram Extension, Sitapur Road, Lucknow, 226031, UP,

India.

FAU - Bhattacherjee, Sandeep

AU - Bhattacherjee S

AD - Medicinal & Process Chemistry Division, CSIR-Central Drug Research Institute,

Sector 10, Jankipuram Extension, Sitapur Road, Lucknow, 226031, UP, India.

FAU - Venugopal, Umamageswaran

AU - Venugopal U

AD - Molecular Microbiology and Immunology Division, CSIR-Central Drug Research

Institute, Sector 10, Jankipuram Extension, Sitapur Road, Lucknow, 226031, UP,

India.

FAU - Parwez, Shahid

AU - Parwez S

AD - Biochemistry and Structural Biology, CSIR-Central Drug Research Institute, Sector

10, Jankipuram Extension, Sitapur Road, Lucknow, 226031, UP, India; Academy of

Scientific and Innovative Research (AcSIR), Ghaziabad, 201002, India.

FAU - Siddiqi, Mohammad Imran

AU - Siddiqi MI

AD - Biochemistry and Structural Biology, CSIR-Central Drug Research Institute, Sector

10, Jankipuram Extension, Sitapur Road, Lucknow, 226031, UP, India; Academy of

Scientific and Innovative Research (AcSIR), Ghaziabad, 201002, India.

FAU - Krishnan, Manju Y

AU - Krishnan MY

AD - Molecular Microbiology and Immunology Division, CSIR-Central Drug Research

Institute, Sector 10, Jankipuram Extension, Sitapur Road, Lucknow, 226031, UP,

India; Academy of Scientific and Innovative Research (AcSIR), Ghaziabad, 201002,

India. Electronic address: manju@cdri.res.in.

FAU - Panda, Gautam

AU - Panda G

AD - Medicinal & Process Chemistry Division, CSIR-Central Drug Research Institute,

Sector 10, Jankipuram Extension, Sitapur Road, Lucknow, 226031, UP, India;

Academy of Scientific and Innovative Research (AcSIR), Ghaziabad, 201002, India.

Electronic address: gautam.panda@gmail.com.

LA - eng

PT - Journal Article

DEP - 20220806

PL - France

TA - Eur J Med Chem

JT - European journal of medicinal chemistry

JID - 0420510

RN - 0 (Acetamides)

RN - 0 (Amides)

RN - 0 (Cytochromes)

RN - 0 (Protons)

RN - 0 (Quinazolines)

RN - 8L70Q75FXE (Adenosine Triphosphate)

RN - EC 1.- (Oxidoreductases)

RN - EC 1.9.3.1 (Electron Transport Complex IV)

SB - IM

MH - Acetamides/metabolism/pharmacology

MH - Adenosine Triphosphate/metabolism

MH - Amides/metabolism

MH - Cytochromes/metabolism

MH - Electron Transport Complex IV/metabolism

MH - Humans

MH - *Mycobacterium tuberculosis/metabolism

MH - Oxidoreductases/metabolism

MH - Protons

MH - Quinazolines/metabolism/pharmacology

MH - *Tuberculosis, Multidrug-Resistant

OTO - NOTNLM

OT - (4-oxoquinazoline-3(4H)-yl)acetamide

OT - (Quinazoline 4-yloxy)acetamide

OT - Mycobacterium tuberculosis bd-oxidase

COIS- Declaration of competing interest The authors declare that they have no known

competing financial interests or personal relationships that could have appeared

to influence the work reported in this paper.

EDAT- 2022/08/17 06:00

MHDA- 2022/09/21 06:00

CRDT- 2022/08/16 18:19

PHST- 2022/05/25 00:00 [received]

PHST- 2022/07/25 00:00 [revised]

PHST- 2022/07/25 00:00 [accepted]

PHST- 2022/08/17 06:00 [pubmed]

PHST- 2022/09/21 06:00 [medline]

PHST- 2022/08/16 18:19 [entrez]

AID - S0223-5234(22)00541-4 [pii]

AID - 10.1016/j.ejmech.2022.114639 [doi]

PST - ppublish

SO - Eur J Med Chem. 2022 Nov 15;242:114639. doi: 10.1016/j.ejmech.2022.114639. Epub

2022 Aug 6.

PMID- 36426362

OWN - NLM

STAT- MEDLINE

DCOM- 20221128

LR - 20260127

IS - 1664-3224 (Electronic)

IS - 1664-3224 (Linking)

VI - 13

DP - 2022

TI - Delamanid suppresses CXCL10 expression via regulation of JAK/STAT1 signaling and

correlates with reduced inflammation in tuberculosis patients.

PG - 923492

LID - 10.3389/fimmu.2022.923492 [doi]

LID - 923492

AB - BACKGROUND: Apart from bactericidal effects, anti-tuberculosis drugs can

interfere with the host's immune system. In this study, we analyzed the role of

delamanid (DLM), an inhibitor of mycolic acid synthesis of mycobacterial cell

wall, on human macrophages. METHODS: Based on a cohort of multidrug-resistant

tuberculosis (MDR-TB) patients treated with DLM, the levels of C-reaction protein

(CRP) and cytokines in the plasma were monitored using immunoturbidimetric assay

and flow cytometry, respectively. We investigated the role of DLM on CXCL10

expression in U937 cell model using the following methods: cell viability assay,

reverse transcription-quantitative polymerase chain reaction, enzyme linked

immunosorbent assay, immunoblot, and transwell co-culture assay. RESULTS: A total

of 23 MDR-TB patients were included, comprising of 13 patients treated with

optimized background therapeutic regimen (OBR) plus DLM regimen (OBR+DLM) and 10

patients treated with OBR plus placebo. DLM administration was associated with a

significant reduce in circulating CRP level. Correspondingly, after treatment,

the level of CXCL10 in patients treated with OBR+DLM was significantly lower than

that with control. Using cell model, DLM dramatically suppressed CXCL10

expression, which majorly depended on inhibiting the JAK/STAT pathway, and

impaired the migration of PBMCs. CONCLUSION: Our data firstly demonstrate that

DLM suppresses CXCL10 expression via regulation of JAK2/STAT1 signaling and

correlates with reduced inflammation in MDR-TB patients. DLM could be used as a

potential drug for immunotherapy of patients with overactive immune response due

to CXCL10.

CI - Copyright © 2022 Qiao, Li, Yuan, Ren, Shang, Wang, Liu, Zhang, Li, Wu, Lu, Gao

and Pang.

FAU - Qiao, Min

AU - Qiao M

AD - Department of Bacteriology and Immunology, Beijing Chest Hospital, Capital

Medical University/Beijing Tuberculosis & Thoracic Tumor Research Institute,

Beijing, China.

AD - Department of Gastroenterology, Shanxi Provincial People's Hospital, Affiliate of

Shanxi Medical University, Taiyuan, Shanxi, China.

AD - Department of Tuberculosis, Beijing Chest Hospital, Capital Medical

University/Beijing Tuberculosis & Thoracic Tumor Research Institute, Beijing,

China.

FAU - Li, Shanshan

AU - Li S

AD - Department of Bacteriology and Immunology, Beijing Chest Hospital, Capital

Medical University/Beijing Tuberculosis & Thoracic Tumor Research Institute,

Beijing, China.

FAU - Yuan, Jinfeng

AU - Yuan J

AD - Department of Bacteriology and Immunology, Beijing Chest Hospital, Capital

Medical University/Beijing Tuberculosis & Thoracic Tumor Research Institute,

Beijing, China.

FAU - Ren, Weicong

AU - Ren W

AD - Department of Bacteriology and Immunology, Beijing Chest Hospital, Capital

Medical University/Beijing Tuberculosis & Thoracic Tumor Research Institute,

Beijing, China.

FAU - Shang, Yuanyuan

AU - Shang Y

AD - Department of Bacteriology and Immunology, Beijing Chest Hospital, Capital

Medical University/Beijing Tuberculosis & Thoracic Tumor Research Institute,

Beijing, China.

FAU - Wang, Wei

AU - Wang W

AD - Department of Bacteriology and Immunology, Beijing Chest Hospital, Capital

Medical University/Beijing Tuberculosis & Thoracic Tumor Research Institute,

Beijing, China.

FAU - Liu, Rongmei

AU - Liu R

AD - Department of Tuberculosis, Beijing Chest Hospital, Capital Medical

University/Beijing Tuberculosis & Thoracic Tumor Research Institute, Beijing,

China.

FAU - Zhang, Fuzhen

AU - Zhang F

AD - Department of Bacteriology and Immunology, Beijing Chest Hospital, Capital

Medical University/Beijing Tuberculosis & Thoracic Tumor Research Institute,

Beijing, China.

FAU - Li, Qing

AU - Li Q

AD - Department of Bacteriology and Immunology, Beijing Chest Hospital, Capital

Medical University/Beijing Tuberculosis & Thoracic Tumor Research Institute,

Beijing, China.

FAU - Wu, Xiao

AU - Wu X

AD - Department of Bacteriology and Immunology, Beijing Chest Hospital, Capital

Medical University/Beijing Tuberculosis & Thoracic Tumor Research Institute,

Beijing, China.

FAU - Lu, Jie

AU - Lu J

AD - Beijing Key Laboratory for Pediatric Diseases of Otolaryngology, Head and Neck

Surgery, Beijing Pediatric Research Institute, Beijing Children's Hospital,

Capital Medical University, National Center for Children's Health, Beijing,

China.

FAU - Gao, Mengqiu

AU - Gao M

AD - Department of Tuberculosis, Beijing Chest Hospital, Capital Medical

University/Beijing Tuberculosis & Thoracic Tumor Research Institute, Beijing,

China.

FAU - Pang, Yu

AU - Pang Y

AD - Department of Bacteriology and Immunology, Beijing Chest Hospital, Capital

Medical University/Beijing Tuberculosis & Thoracic Tumor Research Institute,

Beijing, China.

LA - eng

PT - Journal Article

PT - Research Support, Non-U.S. Gov't

DEP - 20221108

PL - Switzerland

TA - Front Immunol

JT - Frontiers in immunology

JID - 101560960

RN - EC 2.7.10.2 (Janus Kinases)

RN - 0 (STAT Transcription Factors)

RN - 0 (Chemokine CXCL10)

RN - 0 (STAT1 Transcription Factor)

RN - 0 (OPC-67683)

RN - 0 (Nitroimidazoles)

RN - 0 (Oxazoles)

RN - 0 (CXCL10 protein, human)

RN - 0 (STAT1 protein, human)

SB - IM

MH - Humans

MH - Signal Transduction

MH - U937 Cells

MH - Janus Kinases

MH - STAT Transcription Factors

MH - Inflammation

MH - *Tuberculosis

MH - *Tuberculosis, Multidrug-Resistant

MH - Chemokine CXCL10

MH - STAT1 Transcription Factor

MH - Nitroimidazoles

MH - Oxazoles

PMC - PMC9679411

OTO - NOTNLM

OT - CXCL10

OT - JAK/STAT1

OT - delamanid

OT - inflammation

OT - tuberculosis

COIS- The authors declare that the research was conducted in the absence of any

commercial or financial relationships that could be construed as a potential

conflict of interest.

EDAT- 2022/11/26 06:00

MHDA- 2022/11/29 06:00

PMCR- 2022/01/01

CRDT- 2022/11/25 03:04

PHST- 2022/06/07 00:00 [received]

PHST- 2022/10/24 00:00 [accepted]

PHST- 2022/11/25 03:04 [entrez]

PHST- 2022/11/26 06:00 [pubmed]

PHST- 2022/11/29 06:00 [medline]

PHST- 2022/01/01 00:00 [pmc-release]

AID - 10.3389/fimmu.2022.923492 [doi]

PST - epublish

SO - Front Immunol. 2022 Nov 8;13:923492. doi: 10.3389/fimmu.2022.923492. eCollection

2022.

PMID- 36335391

OWN - NLM

STAT- MEDLINE

DCOM- 20221108

LR - 20221109

IS - 1476-0711 (Electronic)

IS - 1476-0711 (Linking)

VI - 21

IP - 1

DP - 2022 Nov 5

TI - Determination of critical concentration for drug susceptibility testing of

Mycobacterium tuberculosis against para-aminosalicylic acid with clinical

isolates with thyA, folC and dfrA mutations.

PG - 48

LID - 10.1186/s12941-022-00537-z [doi]

LID - 48

AB - BACKGROUND & OBJECTIVES: Accurate determination of antimicrobial resistance

profiles is of great importance to formulate optimal regimens against

multidrug-resistant tuberculosis (MDR-TB). Although para-aminosalicylic acid

(PAS) has been widely used clinically, the reliable testing methods for PAS

susceptibility were not established. Herein, we aimed to establish critical test

concentration for PAS on the Mycobacterial Growth Indicator Tube (MGIT) 960 in

our laboratory settings. METHODS: A total of 102 clinical isolates were included

in this study, including 82 wild-type and 20 resistotype isolates. Minimum

inhibitory concentration (MIC) was determined by MGIT 960. Whole-genome

sequencing was used to identify the mutation patterns potentially conferring PAS

resistance. Sequence alignment and structure modelling were carried out to

analyze potential drug-resistant mechanism of folC mutant. RESULTS: Overall, the

Minimum inhibitory concentration (MIC) distribution demonstrated excellent

separation between wild-type and resistotype isolates. The wild-type population

were all at least 1 dilution below 4 μg/ml, and the resistotype population were

no lower than 4 μg/ml, indicating that 4 μg/ml was appropriate critical

concentration to separate these two populations. Of 20 mutant isolates, 12

(60.0%) harbored thyA mutations, 2 (10%) had a mutation on upstream of dfrA, and

the remaining isolates had folC mutations. Overall, thyA and folC mutations were

scattered throughout the whole gene without any one mutation predominating. All

mutations within thyA resulted in high-level resistance to PAS (MIC > 32 μg/ml);

whereas the MICs of isolates with folC mutations exhibited great diversity,

ranged from 4 to > 32 μg/ml, and sequence and structure analysis partially

provided the possible reasons for this diversity. CONCLUSIONS: We propose 4 μg/ml

as tentative critical concentration for MGIT 960. The major mechanism of PAS

resistance is mutations within thyA and folC in MTB isolations. The whole-gene

deletion of thyA locus confers high-level resistance to PAS. The diversity of

many distinct mutations scattered throughout the full-length folC gene challenges

the PCR-based mutation analysis for PAS susceptibility.

CI - © 2022. The Author(s).

FAU - Wang, Wei

AU - Wang W

AD - Department of Bacteriology and Immunology, Beijing Key Laboratory On

Drug-Resistant Tuberculosis Research, Beijing Tuberculosis and Thoracic Tumor

Research Institute/Beijing Chest Hospital, Capital Medical University, Beijing,

101149, People's Republic of China.

FAU - Li, Shanshan

AU - Li S

AD - Department of Bacteriology and Immunology, Beijing Key Laboratory On

Drug-Resistant Tuberculosis Research, Beijing Tuberculosis and Thoracic Tumor

Research Institute/Beijing Chest Hospital, Capital Medical University, Beijing,

101149, People's Republic of China.

FAU - Ge, Qiping

AU - Ge Q

AD - Department of Tuberculosis, Beijing Tuberculosis and Thoracic Tumor Research

Institute/Beijing Chest Hospital, Capital Medical University, Beijing, 101149,

People's Republic of China.

FAU - Guo, Haiping

AU - Guo H

AD - Department of Bacteriology and Immunology, Beijing Key Laboratory On

Drug-Resistant Tuberculosis Research, Beijing Tuberculosis and Thoracic Tumor

Research Institute/Beijing Chest Hospital, Capital Medical University, Beijing,

101149, People's Republic of China.

FAU - Shang, Yuanyuan

AU - Shang Y

AD - Department of Bacteriology and Immunology, Beijing Key Laboratory On

Drug-Resistant Tuberculosis Research, Beijing Tuberculosis and Thoracic Tumor

Research Institute/Beijing Chest Hospital, Capital Medical University, Beijing,

101149, People's Republic of China.

FAU - Ren, Weicong

AU - Ren W

AD - Department of Bacteriology and Immunology, Beijing Key Laboratory On

Drug-Resistant Tuberculosis Research, Beijing Tuberculosis and Thoracic Tumor

Research Institute/Beijing Chest Hospital, Capital Medical University, Beijing,

101149, People's Republic of China.

FAU - Wang, Yufeng

AU - Wang Y

AD - Innovation Alliance On Tuberculosis Diagnosis and Treatment, Beijing, 101149,

People's Republic of China.

FAU - Xue, Zhongtan

AU - Xue Z

AD - Innovation Alliance On Tuberculosis Diagnosis and Treatment, Beijing, 101149,

People's Republic of China.

FAU - Lu, Jie

AU - Lu J

AD - Beijing Key Laboratory for Pediatric Diseases of Otolaryngology, Head and Neck

Surgery, Beijing Pediatric Research Institute, National Center for Children's

Health, Beijing Children's Hospital, Capital Medical University, Beijing, 100045,

People's Republic of China. lujiebch@163.com.

FAU - Pang, Yu

AU - Pang Y

AD - Department of Bacteriology and Immunology, Beijing Key Laboratory On

Drug-Resistant Tuberculosis Research, Beijing Tuberculosis and Thoracic Tumor

Research Institute/Beijing Chest Hospital, Capital Medical University, Beijing,

101149, People's Republic of China. pangyupound@163.com.

LA - eng

GR - 2020-1-1041/the Capital's Funds for Health Improvement and Research/

GR - DFL20191601/Beijing Hospitals Authority Ascent Plan/

GR - ZYLX202122/Beijing Hospitals Authority Clinical Medicine Development of Special

Funding/

PT - Journal Article

DEP - 20221105

PL - England

TA - Ann Clin Microbiol Antimicrob

JT - Annals of clinical microbiology and antimicrobials

JID - 101152152

RN - 5B2658E0N2 (Aminosalicylic Acid)

RN - 0 (Antitubercular Agents)

SB - IM

MH - Humans

MH - *Mycobacterium tuberculosis/genetics

MH - *Aminosalicylic Acid/pharmacology

MH - Microbial Sensitivity Tests

MH - Antitubercular Agents/pharmacology

MH - Drug Resistance, Bacterial/genetics

MH - *Tuberculosis, Multidrug-Resistant/microbiology

MH - Mutation

PMC - PMC9637297

OTO - NOTNLM

OT - Critical Concentration

OT - Drug Susceptibility Testing

OT - Mycobacterium tuberculosis

OT - Para-Aminosalicylic Acid

COIS- The authors declare no competing interests.

EDAT- 2022/11/06 06:00

MHDA- 2022/11/09 06:00

PMCR- 2022/11/05

CRDT- 2022/11/06 00:57

PHST- 2022/07/28 00:00 [received]

PHST- 2022/10/18 00:00 [accepted]

PHST- 2022/11/06 00:57 [entrez]

PHST- 2022/11/06 06:00 [pubmed]

PHST- 2022/11/09 06:00 [medline]

PHST- 2022/11/05 00:00 [pmc-release]

AID - 10.1186/s12941-022-00537-z [pii]

AID - 537 [pii]

AID - 10.1186/s12941-022-00537-z [doi]

PST - epublish

SO - Ann Clin Microbiol Antimicrob. 2022 Nov 5;21(1):48. doi:

10.1186/s12941-022-00537-z.

PMID- 36359313

OWN - NLM

STAT- PubMed-not-MEDLINE

LR - 20221126

IS - 2227-9059 (Print)

IS - 2227-9059 (Electronic)

IS - 2227-9059 (Linking)

VI - 10

IP - 11

DP - 2022 Nov 3

TI - MmpL3 Inhibition as a Promising Approach to Develop Novel Therapies against

Tuberculosis: A Spotlight on SQ109, Clinical Studies, and Patents Literature.

LID - 10.3390/biomedicines10112793 [doi]

LID - 2793

AB - Tuberculosis (TB) is accountable for considerable global morbidity and mortality.

Effective TB therapy with multiple drugs completes in about six months. The

longer duration of TB therapy challenges patient compliance and contributes to

treatment collapse and drug resistance (DR) progress. Therefore, new medications

with an innovative mechanism of action are desperately required to shorten the TB

therapy's duration and effective TB control. The mycobacterial membrane protein

Large 3 (MmpL3) is a novel, mycobacteria-conserved and recognized promiscuous

drug target used in the development of better treatments for multi-drug

resistance TB (MDR-TB) and extensively drug-resistant TB (XDR-TB). This article

spotlights MmpL3, the clinical studies of its inhibitor (SQ109), and the patent

literature. The literature on MmpL3 inhibitors was searched on PubMed and freely

available patent databases (Espacenet, USPTO, and PatentScope). SQ109, an analog

of ethambutol (EMB), is an established MmpL3 inhibitor and has completed Phase

2b-3 clinical trials. Infectex and Sequella are developing orally active SQ109 in

partnership to treat MDR pulmonary TB. SQ109 has demonstrated activity against

drug-sensitive (DS) and drug-resistant (DR) Mycobacterium tuberculosis (Mtb) and

a synergistic effect with isoniazid (INH), rifampicin (RIF), clofazimine (CFZ),

and bedaquiline (BNQ). The combination of SQ109, clofazimine, bedaquiline, and

pyrazinamide (PZA) has been patented due to its excellent anti-TB activity

against MDR-TB, XDR-TB, and latent-TB. The combinations of SQ109 with other

anti-TB drugs (chloroquine, hydroxychloroquine, and sutezolid) have also been

claimed in the patent literature. SQ109 is more potent than EMB and could

substitute EMB in the intensive stage of TB treatment with the three- or

four-drug combination. Developing MmpL3 inhibitors is a promising approach to

fighting the challenges associated with DS-TB and DR-TB. The authors foresee

MmpL3 inhibitors such as SQ109 as future drugs for TB treatment.

FAU - Imran, Mohd

AU - Imran M

AUID- ORCID: 0000-0002-6064-1040

AD - Department of Pharmaceutical Chemistry, Faculty of Pharmacy, Northern Border

University, Rafha 91911, Saudi Arabia.

FAU - Arora, Mandeep Kumar

AU - Arora MK

AD - School of Pharmaceutical and Population Health Informatics, DIT University,

Dehradun 248009, India.

FAU - Chaudhary, Anurag

AU - Chaudhary A

AUID- ORCID: 0000-0002-1657-811X

AD - Department of Pharmaceutical Technology, Meerut Institute of Engineering and

Technology, Meerut 250005, India.

FAU - Khan, Shah Alam

AU - Khan SA

AUID- ORCID: 0000-0002-0729-3403

AD - Department of Pharmaceutical Chemistry, College of Pharmacy, National University

of Science and Technology, Muscat 130, Oman.

FAU - Kamal, Mehnaz

AU - Kamal M

AUID- ORCID: 0000-0002-3585-0828

AD - Department of Pharmaceutical Chemistry, College of Pharmacy, Prince Sattam Bin

Abdulaziz University, Al-Kharj 11942, Saudi Arabia.

FAU - Alshammari, Manal Mutlaq

AU - Alshammari MM

AD - Pharmacy Department, Hotat Bani Tamim General Hospital, Hotat Bani Tamim 16631,

Saudi Arabia.

FAU - Alharbi, Raghad Mohammad

AU - Alharbi RM

AD - Department of Pharmacy, Umm-Alqura University, Makkah 24225, Saudi Arabia.

FAU - Althomali, Nuha Abdullah

AU - Althomali NA

AD - Department of Community Pharmacy, Kol Alosra, Taif 26512, Saudi Arabia.

FAU - Alzimam, Ibrahim Mohammed

AU - Alzimam IM

AD - Faculty of Pharmacy, Northern Border University, Rafha 91911, Saudi Arabia.

FAU - Alshammari, Abdullah Ayed

AU - Alshammari AA

AD - Nahdi Medical Company, Riyadh 12211, Saudi Arabia.

FAU - Alharbi, Bashair Hamed

AU - Alharbi BH

AD - Department of Clinical Pharmacy, Umm-Alqura University, Jeddah 21921, Saudi

Arabia.

FAU - Alshengeti, Amer

AU - Alshengeti A

AUID- ORCID: 0000-0001-6861-6934

AD - Department of Pediatrics, College of Medicine, Taibah University, Al-Madinah

41491, Saudi Arabia.

AD - Department of Infection Prevention and Control, Prince Mohammad Bin Abdulaziz

Hospital, National Guard Health Affairs, Al-Madinah 41491, Saudi Arabia.

FAU - Alsaleh, Abdulmonem A

AU - Alsaleh AA

AUID- ORCID: 0000-0002-1188-1451

AD - Clinical Laboratory Science Department, Mohammed Al-Mana College for Medical

Sciences, Dammam 34222, Saudi Arabia.

FAU - Alqahtani, Shayea A

AU - Alqahtani SA

AD - Medical Laboratory Department, Erhadh Hospital, Dammam 32434, Saudi Arabia.

FAU - Rabaan, Ali A

AU - Rabaan AA

AUID- ORCID: 0000-0002-6774-9847

AD - Molecular Diagnostic Laboratory, Johns Hopkins Aramco Healthcare, Dhahran 31311,

Saudi Arabia.

AD - College of Medicine, Alfaisal University, Riyadh 11533, Saudi Arabia.

AD - Department of Public Health and Nutrition, The University of Haripur, Haripur

22610, Pakistan.

LA - eng

PT - Journal Article

PT - Review

DEP - 20221103

PL - Switzerland

TA - Biomedicines

JT - Biomedicines

JID - 101691304

PMC - PMC9687596

OTO - NOTNLM

OT - Mmpl3

OT - SQ109

OT - clinical studies

OT - drug-resistance

OT - patent

OT - tuberculosis

COIS- The authors declare no conflict of interest.

EDAT- 2022/11/12 06:00

MHDA- 2022/11/12 06:01

PMCR- 2022/11/03

CRDT- 2022/11/11 01:10

PHST- 2022/10/11 00:00 [received]

PHST- 2022/10/24 00:00 [revised]

PHST- 2022/10/26 00:00 [accepted]

PHST- 2022/11/11 01:10 [entrez]

PHST- 2022/11/12 06:00 [pubmed]

PHST- 2022/11/12 06:01 [medline]

PHST- 2022/11/03 00:00 [pmc-release]

AID - biomedicines10112793 [pii]

AID - biomedicines-10-02793 [pii]

AID - 10.3390/biomedicines10112793 [doi]

PST - epublish

SO - Biomedicines. 2022 Nov 3;10(11):2793. doi: 10.3390/biomedicines10112793.

PMID- 35753313

OWN - NLM

STAT- MEDLINE

DCOM- 20221111

LR - 20221123

IS - 2692-9368 (Electronic)

IS - 2692-9368 (Linking)

VI - 14

IP - 6

DP - 2022 Nov

TI - Resistance and tolerance of Mycobacterium tuberculosis to antimicrobial

agents-How M. tuberculosis can escape antibiotics.

PG - e1573

LID - 10.1002/wsbm.1573 [doi]

AB - Tuberculosis (TB) poses a serious threat to public health worldwide since it was

discovered. Until now, TB has been one of the top 10 causes of death from a

single infectious disease globally. The treatment of active TB cases majorly

relies on various anti-tuberculosis drugs. However, under the selection pressure

by drugs, the continuous evolution of Mycobacterium tuberculosis (Mtb)

facilitates the emergence of drug-resistant strains, further resulting in the

accumulation of tubercle bacilli with multiple drug resistance, especially deadly

multidrug-resistant TB and extensively drug-resistant TB. Researches on the

mechanism of drug action and drug resistance of Mtb provide a new scheme for

clinical management of TB patients, and prevention of drug resistance. In this

review, we summarized the molecular mechanisms of drug resistance of existing

anti-TB drugs to better understand the evolution of drug resistance of Mtb, which

will provide more effective strategies against drug-resistant TB, and accelerate

the achievement of the EndTB Strategy by 2035. This article is categorized under:

Infectious Diseases > Molecular and Cellular Physiology.

CI - © 2022 Wiley Periodicals LLC.

FAU - Li, Haoran

AU - Li H

AD - Department of Bacteriology and Immunology, Beijing Chest Hospital, Capital

Medical University/Beijing Tuberculosis and Thoracic Tumor Research Institute,

Beijing, China.

FAU - Yuan, Jinfeng

AU - Yuan J

AD - Department of Bacteriology and Immunology, Beijing Chest Hospital, Capital

Medical University/Beijing Tuberculosis and Thoracic Tumor Research Institute,

Beijing, China.

FAU - Duan, Shujuan

AU - Duan S

AD - School of Medical Technology, Guangdong Medical University, Dongguan, China.

FAU - Pang, Yu

AU - Pang Y

AUID- ORCID: 0000-0001-6803-9807

AD - Department of Bacteriology and Immunology, Beijing Chest Hospital, Capital

Medical University/Beijing Tuberculosis and Thoracic Tumor Research Institute,

Beijing, China.

LA - eng

PT - Journal Article

PT - Research Support, Non-U.S. Gov't

PT - Review

DEP - 20220626

PL - United States

TA - WIREs Mech Dis

JT - WIREs mechanisms of disease

JID - 9918227353306676

RN - 0 (Antitubercular Agents)

SB - IM

MH - Humans

MH - *Mycobacterium tuberculosis

MH - Antitubercular Agents/pharmacology

MH - *Tuberculosis, Multidrug-Resistant/drug therapy

MH - *Extensively Drug-Resistant Tuberculosis/drug therapy

MH - *Tuberculosis, Lymph Node/drug therapy

OTO - NOTNLM

OT - Mycobacterium tuberculosis

OT - drug

OT - resistance

OT - tolerance

EDAT- 2022/06/27 06:00

MHDA- 2022/11/15 06:00

CRDT- 2022/06/26 18:52

PHST- 2022/05/22 00:00 [revised]

PHST- 2022/03/15 00:00 [received]

PHST- 2022/05/30 00:00 [accepted]

PHST- 2022/06/27 06:00 [pubmed]

PHST- 2022/11/15 06:00 [medline]

PHST- 2022/06/26 18:52 [entrez]

AID - 10.1002/wsbm.1573 [doi]

PST - ppublish

SO - WIREs Mech Dis. 2022 Nov;14(6):e1573. doi: 10.1002/wsbm.1573. Epub 2022 Jun 26.

PMID- 35701239

OWN - NLM

STAT- MEDLINE

DCOM- 20220913

LR - 20250728

IS - 1090-2120 (Electronic)

IS - 0045-2068 (Linking)

VI - 128

DP - 2022 Nov

TI - Discovery of new riminophenazine analogues as antimycobacterial agents against

drug-resistant Mycobacterium tuberculosis.

PG - 105929

LID - S0045-2068(22)00334-0 [pii]

LID - 10.1016/j.bioorg.2022.105929 [doi]

AB - Twenty-three new riminophenazine and pyrido[3,2-b]quinoxaline derivatives were

prepared and examined for their antimycobacterial activities against

Mycobacterium marinum and Mycobacterium tuberculosis H37Rv, taking clofazimine

(1) as the lead. Structure-activity relationship (SAR) analysis revealed that the

introduction of a heterocycle or diethylamine substituted benzene moiety on the

N-5 atom might be beneficial for activity. The most potent compound 7m also

displayed enhanced activity against wild-type as well as multidrug-resistant

(MDR) and extensively drug-resistant (XDR) TB clinical isolates, with the MICs

ranging from 0.08 to 1.25 μg/mL, especially effective toward strain M20A507,

resistant to 1. Further mechanism study indicated that its anti-TB activity was

independent of cell membrane disruption, but related to NDH-2 reduction and the

resulting high ROS production. Our study provides instructive guidance for the

further development of clofazimine derivatives into promising antimicrobial

agents against MDR and XDR TB.

CI - Copyright © 2022 Elsevier Inc. All rights reserved.

FAU - Zhao, Xiaoqiang

AU - Zhao X

AD - Beijing Key Laboratory of Antimicrobial Agents, Institute of Medicinal

Biotechnology, Chinese Academy of Medical Sciences & Peking Union Medical

College, Beijing 100050, China.

FAU - Mei, Yuheng

AU - Mei Y

AD - Beijing Key Laboratory of Antimicrobial Agents, Institute of Medicinal

Biotechnology, Chinese Academy of Medical Sciences & Peking Union Medical

College, Beijing 100050, China.

FAU - Guo, Zhihao

AU - Guo Z

AD - Beijing Key Laboratory of Antimicrobial Agents, Institute of Medicinal

Biotechnology, Chinese Academy of Medical Sciences & Peking Union Medical

College, Beijing 100050, China.

FAU - Si, Shuyi

AU - Si S

AD - Beijing Key Laboratory of Antimicrobial Agents, Institute of Medicinal

Biotechnology, Chinese Academy of Medical Sciences & Peking Union Medical

College, Beijing 100050, China.

FAU - Ma, Xican

AU - Ma X

AD - Beijing Key Laboratory of Antimicrobial Agents, Institute of Medicinal

Biotechnology, Chinese Academy of Medical Sciences & Peking Union Medical

College, Beijing 100050, China.

FAU - Li, Yinghong

AU - Li Y

AD - Beijing Key Laboratory of Antimicrobial Agents, Institute of Medicinal

Biotechnology, Chinese Academy of Medical Sciences & Peking Union Medical

College, Beijing 100050, China. Electronic address: liyinghong@imb.pumc.edu.cn.

FAU - Li, Yan

AU - Li Y

AD - Beijing Key Laboratory of Antimicrobial Agents, Institute of Medicinal

Biotechnology, Chinese Academy of Medical Sciences & Peking Union Medical

College, Beijing 100050, China. Electronic address: liyan@imb.pumc.edu.cn.

FAU - Song, Danqing

AU - Song D

AD - Beijing Key Laboratory of Antimicrobial Agents, Institute of Medicinal

Biotechnology, Chinese Academy of Medical Sciences & Peking Union Medical

College, Beijing 100050, China.

LA - eng

PT - Journal Article

PT - Research Support, Non-U.S. Gov't

DEP - 20220607

PL - United States

TA - Bioorg Chem

JT - Bioorganic chemistry

JID - 1303703

RN - 0 (Antitubercular Agents)

RN - D959AE5USF (Clofazimine)

SB - IM

MH - Antitubercular Agents/pharmacology

MH - Clofazimine/pharmacology

MH - Humans

MH - Microbial Sensitivity Tests

MH - *Mycobacterium tuberculosis

MH - *Tuberculosis, Multidrug-Resistant/drug therapy/microbiology

OTO - NOTNLM

OT - Antitubercular

OT - Drug−resistance

OT - Pyrido[3,2-b]quinoxaline derivatives

OT - Riminophenazine analogues

OT - Structure−activity relationship

COIS- Declaration of Competing Interest The authors declare that they have no known

competing financial interests or personal relationships that could have appeared

to influence the work reported in this paper.

EDAT- 2022/06/15 06:00

MHDA- 2022/09/14 06:00

CRDT- 2022/06/14 22:02

PHST- 2022/04/22 00:00 [received]

PHST- 2022/05/30 00:00 [revised]

PHST- 2022/06/02 00:00 [accepted]

PHST- 2022/06/15 06:00 [pubmed]

PHST- 2022/09/14 06:00 [medline]

PHST- 2022/06/14 22:02 [entrez]

AID - S0045-2068(22)00334-0 [pii]

AID - 10.1016/j.bioorg.2022.105929 [doi]

PST - ppublish

SO - Bioorg Chem. 2022 Nov;128:105929. doi: 10.1016/j.bioorg.2022.105929. Epub 2022

Jun 7.

PMID- 36316637

OWN - NLM

STAT- MEDLINE

DCOM- 20221102

LR - 20221104

IS - 1471-2334 (Electronic)

IS - 1471-2334 (Linking)

VI - 22

IP - 1

DP - 2022 Oct 31

TI - Regional distribution of Mycobacterium tuberculosis infection and resistance to

rifampicin and isoniazid as determined by high-resolution melt analysis.

PG - 812

LID - 10.1186/s12879-022-07792-7 [doi]

LID - 812

AB - BACKGROUND: Identifying the transmission mode and resistance mechanism of

Mycobacterium tuberculosis (MTB) is key to prevent disease transmission. However,

there is a lack of regional data. Therefore, the aim of this study was to

identify risk factors associated with the transmission of MTB and regional

patterns of resistance to isoniazid (INH) and rifampicin (RFP), as well as the

prevalence of multidrug-resistant tuberculosis (MDR-TB). METHODS: High-resolution

melt (HRM) analysis was conducted using sputum, alveolar lavage fluid, and

pleural fluid samples collected from 17,515 patients with suspected or confirmed

MTB infection in the downtown area and nine counties of Luoyang City from 2019 to

2021. RESULTS: Of the 17,515 patients, 82.6% resided in rural areas, and 96.0%

appeared for an initial screening. The HRM positivity rate was 16.8%, with a

higher rate in males than females (18.0% vs. 14.1%, p < 0.001). As expected, a

positive sputum smear was correlated with a positive result for HRM analysis. By

age, the highest rates of MTB infection occurred in males (22.9%) aged 26-30

years and females (28.1%) aged 21-25. The rates of resistance to RFP and INH and

the incidence of MDR were higher in males than females (20.5% vs. 16.1%,

p < 0.001, 15.9% vs. 12.0%, p < 0.001 and 12.9% vs. 10.2%, p < 0.001,

respectively). The HRM positivity rate was much higher in previously treated

patients than those newly diagnosed for MTB infection. Notably, males at the

initial screening had significantly higher rates of HRM positive, INH resistance,

RFP resistance, and MDR-TB than females (all, p < 0.05), but not those previously

treated for MTB infection. The HRM positivity and drug resistance rates were much

higher in the urban vs. rural population. By multivariate analyses, previous

treatment, age < 51 years, residing in an urban area, and male sex were

significantly and positively associated with drug resistance after adjusting for

smear results and year of testing. CONCLUSION: Males were at higher risks for MTB

infection and drug resistance, while a younger age was associated with MTB

infection, resistance to INH and RFP, and MDR-TB. Further comprehensive

monitoring of resistance patterns is needed to control the spread of MTB

infection and manage drug resistance locally.

CI - © 2022. The Author(s).

FAU - Wang, Zhenzhen

AU - Wang Z

AD - The First Affiliated Hospital and Clinical Medical College, Henan University of

Science and Technology, 471000, Luo Yang, China.

AD - School of Medical Technology and Engineering, Henan University of Science and

Technology, Luo Yang, 471000, China.

FAU - Guo, Tengfei

AU - Guo T

AD - The First Affiliated Hospital and Clinical Medical College, Henan University of

Science and Technology, 471000, Luo Yang, China.

FAU - Jiang, Tao

AU - Jiang T

AD - The First Affiliated Hospital and Clinical Medical College, Henan University of

Science and Technology, 471000, Luo Yang, China.

FAU - Zhao, Zhanqin

AU - Zhao Z

AD - Animal Science and Technology, Henan University of Science and Technology, Luo

Yang, 471000, China. zhaozhanqin@126.com.

FAU - Zu, Xiangyang

AU - Zu X

AD - School of Medical Technology and Engineering, Henan University of Science and

Technology, Luo Yang, 471000, China.

FAU - Li, Long

AU - Li L

AD - The First Affiliated Hospital and Clinical Medical College, Henan University of

Science and Technology, 471000, Luo Yang, China.

FAU - Zhang, Qing

AU - Zhang Q

AD - The First Affiliated Hospital and Clinical Medical College, Henan University of

Science and Technology, 471000, Luo Yang, China.

FAU - Hou, Yi

AU - Hou Y

AD - Luoyang City CDC, Luo Yang, 471000, China.

FAU - Song, Kena

AU - Song K

AD - School of Medical Technology and Engineering, Henan University of Science and

Technology, Luo Yang, 471000, China.

FAU - Xue, Yun

AU - Xue Y

AD - School of Medical Technology and Engineering, Henan University of Science and

Technology, Luo Yang, 471000, China. xueyun6688@163.com.

LA - eng

GR - LHGJ20200589/Joint construction project of Henan Province/

GR - 32072899/National Nature Science Foundation of China/

GR - 212102310741/Henan Science and Technology Development Plan/

PT - Journal Article

DEP - 20221031

PL - England

TA - BMC Infect Dis

JT - BMC infectious diseases

JID - 100968551

RN - VJT6J7R4TR (Rifampin)

RN - V83O1VOZ8L (Isoniazid)

RN - 0 (Antitubercular Agents)

SB - IM

MH - Female

MH - Humans

MH - Male

MH - Adult

MH - Rifampin/pharmacology/therapeutic use

MH - Isoniazid/pharmacology/therapeutic use

MH - Antitubercular Agents/adverse effects

MH - *Tuberculosis, Multidrug-Resistant/drug therapy/epidemiology/microbiology

MH - *Mycobacterium tuberculosis/genetics

MH - Microbial Sensitivity Tests

PMC - PMC9620668

OTO - NOTNLM

OT - Molecular epidemiology

OT - Multidrug-resistant tuberculosis

OT - Resistance model

OT - Transmission

COIS- The authors declare that they have no competing interests.

EDAT- 2022/11/02 06:00

MHDA- 2022/11/03 06:00

PMCR- 2022/10/31

CRDT- 2022/11/01 00:43

PHST- 2022/06/27 00:00 [received]

PHST- 2022/10/19 00:00 [accepted]

PHST- 2022/09/29 00:00 [revised]

PHST- 2022/11/01 00:43 [entrez]

PHST- 2022/11/02 06:00 [pubmed]

PHST- 2022/11/03 06:00 [medline]

PHST- 2022/10/31 00:00 [pmc-release]

AID - 10.1186/s12879-022-07792-7 [pii]

AID - 7792 [pii]

AID - 10.1186/s12879-022-07792-7 [doi]

PST - epublish

SO - BMC Infect Dis. 2022 Oct 31;22(1):812. doi: 10.1186/s12879-022-07792-7.

PMID- 36358911

OWN - NLM

STAT- MEDLINE

DCOM- 20221114

LR - 20221201

IS - 2218-273X (Electronic)

IS - 2218-273X (Linking)

VI - 12

IP - 11

DP - 2022 Oct 26

TI - Adenosine-Mimicking Derivatives of 3-Aminopyrazine-2-Carboxamide: Towards

Inhibitors of Prolyl-tRNA Synthetase with Antimycobacterial Activity.

LID - 10.3390/biom12111561 [doi]

LID - 1561

AB - Multidrug-resistant tuberculosis (MDR-TB) poses a significant threat to mankind

and as such earned its place on the WHO list of priority pathogens. New

antimycobacterials with a mechanism of action different to currently used agents

are highly required. This study presents the design, synthesis, and biological

evaluation of 3-acylaminopyrazine-2-carboxamides derived from a previously

reported inhibitor of human prolyl-tRNA synthetase. Compounds were evaluated in

vitro against various strains of mycobacteria, pathogenic bacteria, and fungi of

clinical significance. In general, high activity against mycobacteria was noted,

while the antibacterial and antifungal activity was minimal. The most active

compounds were 4'-substituted 3-(benzamido)pyrazine-2-carboxamides, exerting MIC

(Minimum Inhibitory Concentration) from 1.95 to 31.25 µg/mL. Detailed

structure-activity relationships were established and rationalized in silico with

regard to mycobacterial ProRS as a probable target. The active compounds

preserved their activity even against multidrug-resistant strains of

Mycobacterium tuberculosis. At the same time, they were non-cytotoxic against

HepG2 human hepatocellular carcinoma cells. This project is the first step in the

successful repurposing of inhibitors of human ProRS to inhibitors of

mycobacterial ProRS with antimycobacterial activity.

FAU - Pallabothula, Vinod Sukanth Kumar

AU - Pallabothula VSK

AUID- ORCID: 0000-0002-3327-3078

AD - Faculty of Pharmacy in Hradec Králové, Charles University, Ak. Heyrovského

1203/8, 500 05 Hradec Králové, Czech Republic.

FAU - Kerda, Marek

AU - Kerda M

AUID- ORCID: 0000-0002-5850-5381

AD - Faculty of Pharmacy in Hradec Králové, Charles University, Ak. Heyrovského

1203/8, 500 05 Hradec Králové, Czech Republic.

FAU - Juhás, Martin

AU - Juhás M

AUID- ORCID: 0000-0002-1890-9082

AD - Faculty of Pharmacy in Hradec Králové, Charles University, Ak. Heyrovského

1203/8, 500 05 Hradec Králové, Czech Republic.

FAU - Janďourek, Ondřej

AU - Janďourek O

AUID- ORCID: 0000-0003-4633-2062

AD - Faculty of Pharmacy in Hradec Králové, Charles University, Ak. Heyrovského

1203/8, 500 05 Hradec Králové, Czech Republic.

FAU - Konečná, Klára

AU - Konečná K

AUID- ORCID: 0000-0001-5670-7767

AD - Faculty of Pharmacy in Hradec Králové, Charles University, Ak. Heyrovského

1203/8, 500 05 Hradec Králové, Czech Republic.

FAU - Bárta, Pavel

AU - Bárta P

AUID- ORCID: 0000-0001-5445-5658

AD - Faculty of Pharmacy in Hradec Králové, Charles University, Ak. Heyrovského

1203/8, 500 05 Hradec Králové, Czech Republic.

FAU - Paterová, Pavla

AU - Paterová P

AUID- ORCID: 0000-0003-0192-7345

AD - Department of Clinical Microbiology, University Hospital Hradec Králové, Sokolská

581, 500 05 Hradec Králové, Czech Republic.

FAU - Zitko, Jan

AU - Zitko J

AUID- ORCID: 0000-0003-0104-9925

AD - Faculty of Pharmacy in Hradec Králové, Charles University, Ak. Heyrovského

1203/8, 500 05 Hradec Králové, Czech Republic.

LA - eng

PT - Journal Article

PT - Research Support, Non-U.S. Gov't

DEP - 20221026

PL - Switzerland

TA - Biomolecules

JT - Biomolecules

JID - 101596414

RN - 0 (Antitubercular Agents)

RN - K72T3FS567 (Adenosine)

RN - EC 6.1.1.- (Amino Acyl-tRNA Synthetases)

SB - IM

MH - Humans

MH - Antitubercular Agents/pharmacology

MH - Adenosine/pharmacology

MH - Microbial Sensitivity Tests

MH - *Mycobacterium tuberculosis

MH - *Amino Acyl-tRNA Synthetases

PMC - PMC9687610

OTO - NOTNLM

OT - 3-aminopyrazinamide

OT - antibacterial

OT - antimycobacterial

OT - homology model

OT - molecular dynamics

OT - prolyl-tRNA synthetase

OT - repurposing

OT - tuberculosis

COIS- Authors declare no conflict of interest.

EDAT- 2022/11/12 06:00

MHDA- 2022/11/15 06:00

PMCR- 2022/10/26

CRDT- 2022/11/11 01:08

PHST- 2022/09/25 00:00 [received]

PHST- 2022/10/11 00:00 [revised]

PHST- 2022/10/13 00:00 [accepted]

PHST- 2022/11/11 01:08 [entrez]

PHST- 2022/11/12 06:00 [pubmed]

PHST- 2022/11/15 06:00 [medline]

PHST- 2022/10/26 00:00 [pmc-release]

AID - biom12111561 [pii]

AID - biomolecules-12-01561 [pii]

AID - 10.3390/biom12111561 [doi]

PST - epublish

SO - Biomolecules. 2022 Oct 26;12(11):1561. doi: 10.3390/biom12111561.

PMID- 36129262

OWN - NLM

STAT- MEDLINE

DCOM- 20221028

LR - 20240205

IS - 2165-0497 (Electronic)

IS - 2165-0497 (Linking)

VI - 10

IP - 5

DP - 2022 Oct 26

TI - FDA-Approved Amoxapine Effectively Promotes Macrophage Control of Mycobacteria by

Inducing Autophagy.

PG - e0250922

LID - 10.1128/spectrum.02509-22 [doi]

LID - e02509-22

AB - Antibiotic resistance poses a significant hurdle in combating global public

health crises, prompting the development of novel therapeutics. Strategies to

enhance the intracellular killing of mycobacteria by targeting host defense

mechanisms offer numerous beneficial effects, which include reducing cytotoxicity

caused by current lengthy anti-tubercular treatment regimens and slowing or

circumventing the development of multidrug-resistant strains. The intracellular

pathogen Mycobacterium tuberculosis infects macrophages and exploits host

machinery to survive and multiply. Using a cell-based screen of FDA-approved

drugs, we identified an antidepressant, Amoxapine, capable of inhibiting

macrophage cytotoxicity during mycobacterial infection. Notably, this reduced

cytotoxicity was related to the enhanced intracellular killing of Mycobacterium

bovis BCG and M. tuberculosis within human and murine macrophages. Interestingly,

we discovered that postinfection treatment with Amoxapine inhibited mTOR

(mammalian target of rapamycin) activation, resulting in the induction of

autophagy without affecting autophagic flux in macrophages. Also, inhibition of

autophagy by chemical inhibitor 3-MA or knockdown of an essential component of

the autophagic pathway, ATG16L1, significantly diminished Amoxapine's

intracellular killing effects against mycobacteria in the host cells. Finally, we

demonstrated that Amoxapine treatment enhanced host defense against M.

tuberculosis in mice. In conclusion, our study identified Amoxapine as a novel

host-directed drug that enhances the intracellular killing of mycobacteria by

induction of autophagy, with concomitant protection of macrophages against death.

IMPORTANCE The emergence and spread of multidrug-resistant (MDR) and extensive

drug-resistant (XDR) TB urges the development of new therapeutics. One promising

approach to combat drug resistance is targeting host factors necessary for the

bacteria to survive or replicate while simultaneously minimizing the dosage of

traditional agents. Moreover, repurposing FDA-approved drugs presents an

attractive avenue for reducing the cost and time associated with new drug

development. Using a cell-based screen of FDA-approved host-directed therapies

(HDTs), we showed that Amoxapine inhibits macrophage cytotoxicity during

mycobacterial infection and enhances the intracellular killing of mycobacteria

within macrophages by activating the autophagy pathway, both in vitro and in

vivo. These findings confirm targeted autophagy as an effective strategy for

developing new HDT against mycobacteria.

FAU - Wang, Jia

AU - Wang J

AD - Department of Microbiology and Immunology, University of Texas Medical Branch,

Galveston, Texas, USA.

FAU - Sha, Jian

AU - Sha J

AD - Department of Microbiology and Immunology, University of Texas Medical Branch,

Galveston, Texas, USA.

FAU - Strong, Emily

AU - Strong E

AD - Department of Microbiology and Immunology, University of Texas Medical Branch,

Galveston, Texas, USA.

FAU - Chopra, Ashok K

AU - Chopra AK

AD - Department of Microbiology and Immunology, University of Texas Medical Branch,

Galveston, Texas, USA.

FAU - Lee, Sunhee

AU - Lee S

AUID- ORCID: 0000-0002-2599-6938

AD - Department of Microbiology and Immunology, University of Texas Medical Branch,

Galveston, Texas, USA.

LA - eng

GR - R01 AI127711/AI/NIAID NIH HHS/United States

GR - R56 AI132682/AI/NIAID NIH HHS/United States

PT - Journal Article

PT - Research Support, N.I.H., Extramural

PT - Research Support, Non-U.S. Gov't

DEP - 20220921

PL - United States

TA - Microbiol Spectr

JT - Microbiology spectrum

JID - 101634614

RN - R63VQ857OT (Amoxapine)

RN - 0 (BCG Vaccine)

RN - EC 2.7.11.1 (TOR Serine-Threonine Kinases)

SB - IM

MH - Mice

MH - Humans

MH - Animals

MH - *Amoxapine/metabolism/pharmacology

MH - BCG Vaccine

MH - *Mycobacterium tuberculosis/metabolism

MH - Macrophages

MH - Autophagy

MH - TOR Serine-Threonine Kinases/metabolism

MH - *Tuberculosis/drug therapy/metabolism

MH - Mammals/metabolism

PMC - PMC9602717

OTO - NOTNLM

OT - Mycobacterium tuberculosis

OT - antidepressant

OT - autophagy

OT - host-directed therapeutics

OT - mTOR

OT - macrophages

COIS- The authors declare no conflict of interest.

EDAT- 2022/09/22 06:00

MHDA- 2022/10/29 06:00

PMCR- 2022/09/21

CRDT- 2022/09/21 09:03

PHST- 2022/09/22 06:00 [pubmed]

PHST- 2022/10/29 06:00 [medline]

PHST- 2022/09/21 09:03 [entrez]

PHST- 2022/09/21 00:00 [pmc-release]

AID - 02509-22 [pii]

AID - spectrum.02509-22 [pii]

AID - 10.1128/spectrum.02509-22 [doi]

PST - ppublish

SO - Microbiol Spectr. 2022 Oct 26;10(5):e0250922. doi: 10.1128/spectrum.02509-22.

Epub 2022 Sep 21.

PMID- 36000734

OWN - NLM

STAT- MEDLINE

DCOM- 20221028

LR - 20221129

IS - 2150-7511 (Electronic)

VI - 13

IP - 5

DP - 2022 Oct 26

TI - Mycobacterium tuberculosis Utilizes Host Histamine Receptor H1 to Modulate

Reactive Oxygen Species Production and Phagosome Maturation via the p38MAPK-NOX2

Axis.

PG - e0200422

LID - 10.1128/mbio.02004-22 [doi]

LID - e02004-22

AB - Tuberculosis (TB), which is caused by the single pathogenic bacterium,

Mycobacterium tuberculosis, is among the top 10 lethal diseases worldwide. This

situation has been exacerbated by the increasing number of cases of

multidrug-resistant TB (MDR-TB) and extensively drug-resistant TB (XDR-TB).

Histamine is an organic nitrogenous compound that mediates a plethora of cell

processes via different receptors. The expression of histamine receptor H1

(HRH1), one of the four histamine receptors identified to date was previously

reported to be augmented by M. tuberculosis infection, although the underlying

mechanism is unclear. In the present study, we applied confocal microscopy, flow

cytometry, and Western blotting to show that HRH1 expression was enhanced in

macrophages following mycobacterial infection. Furthermore, by combining

techniques of gene knockdown, immunoprecipitation, intracellular bacterial burden

analysis, fluorescence labeling, and imaging, we found that M. tuberculosis

targeted the host HRH1 to suppress NOX2-mediated cROS production and inhibit

phagosome maturation and acidification via the GRK2-p38MAPK signaling pathway.

Our findings clarified the underlying mechanism of the M. tuberculosis and host

HRH1 interaction and may provide useful information for the development of novel

antituberculosis treatments. IMPORTANCE Once engulfed in macrophage phagosomes,

M. tuberculosis adopts various strategies to take advantage of the host

environment for its intracellular survival. Histamine is an organic

nitrogen-containing compound that mediates a plethora of cellular processes via

different receptors, but the crosstalk mechanism between M. tuberculosis and HRH1

in macrophages is not clear. Our results revealed that M. tuberculosis infection

enhanced HRH1 expression, which in turn restrained macrophage bactericidal

activity by modulating the GRK2-p38MAPK signaling pathway, inhibiting

NOX2-mediated cROS production and phagosome maturation. Clarification of the

underlying mechanism by which M. tuberculosis utilizes host HRH1 to favor its

intracellular survival may provide useful information for the development of

novel antituberculosis treatments.

FAU - Mo, Siwei

AU - Mo S

AD - Guangdong Provincial Key Laboratory of Regional Immunity and Diseases, Department

of Pathogen Biology, School of Medicine, Shenzhen Universitygrid.263488.3,

Shenzhen, China.

AD - Department of Respiratory Medicine, Children's Hospital of Chongqing Medical

University, Chongqing Key Laboratory of Pediatrics, Chongqing, China.

AD - National Clinical Research Center for Child Health and Disorders, Ministry of

Education Key Laboratory of Child Development and Disorders, China International

Science and Technology Cooperation Base of Child Development and Critical

Disorders, Chongqing Key Laboratory of Pediatrics, Children's Hospital of

Chongqing Medical University, Chongqing, China.

FAU - Guo, Jiubiao

AU - Guo J

AD - College of Pharmacy, Shenzhen Technology University, Shenzhen, China.

FAU - Ye, Taosheng

AU - Ye T

AD - Pulmonary Diseases Department Two, National Clinical Research Center for

Infectious Disease (Shenzhen), Guangdong Provincial Clinical Research Center for

Infectious Diseases (Tuberculosis), Shenzhen Third People's Hospital, Southern

University of Science and Technology, Shenzhen, China.

FAU - Zhang, Ximeng

AU - Zhang X

AD - Guangdong Provincial Key Laboratory of Regional Immunity and Diseases, Department

of Pathogen Biology, School of Medicine, Shenzhen Universitygrid.263488.3,

Shenzhen, China.

FAU - Zeng, Jiang

AU - Zeng J

AD - The Fifth People's Hospital of Ganzhou, Ganzhou, Jiangxi Province, China.

FAU - Xu, Yuzhong

AU - Xu Y

AD - Department of Clinical Laboratory, Shenzhen Baoan Hospital, Shenzhen

Universitygrid.263488.3, Shenzhen, China.

FAU - Peng, Bin

AU - Peng B

AD - Guangdong Provincial Key Laboratory of Regional Immunity and Diseases, Department

of Pathogen Biology, School of Medicine, Shenzhen Universitygrid.263488.3,

Shenzhen, China.

FAU - Dai, Youchao

AU - Dai Y

AD - Guangdong Provincial Key Laboratory of Regional Immunity and Diseases, Department

of Pathogen Biology, School of Medicine, Shenzhen Universitygrid.263488.3,

Shenzhen, China.

FAU - Xiao, Wei

AU - Xiao W

AD - Guangdong Provincial Key Laboratory of Regional Immunity and Diseases, Department

of Pathogen Biology, School of Medicine, Shenzhen Universitygrid.263488.3,

Shenzhen, China.

FAU - Zhang, Peize

AU - Zhang P

AD - Pulmonary Diseases Department Two, National Clinical Research Center for

Infectious Disease (Shenzhen), Guangdong Provincial Clinical Research Center for

Infectious Diseases (Tuberculosis), Shenzhen Third People's Hospital, Southern

University of Science and Technology, Shenzhen, China.

FAU - Deng, Guofang

AU - Deng G

AD - Pulmonary Diseases Department Two, National Clinical Research Center for

Infectious Disease (Shenzhen), Guangdong Provincial Clinical Research Center for

Infectious Diseases (Tuberculosis), Shenzhen Third People's Hospital, Southern

University of Science and Technology, Shenzhen, China.

FAU - Xu, Dechang

AU - Xu D

AD - The Fifth People's Hospital of Ganzhou, Ganzhou, Jiangxi Province, China.

FAU - Long, Xiaoru

AU - Long X

AD - Department of Respiratory Medicine, Children's Hospital of Chongqing Medical

University, Chongqing Key Laboratory of Pediatrics, Chongqing, China.

AD - National Clinical Research Center for Child Health and Disorders, Ministry of

Education Key Laboratory of Child Development and Disorders, China International

Science and Technology Cooperation Base of Child Development and Critical

Disorders, Chongqing Key Laboratory of Pediatrics, Children's Hospital of

Chongqing Medical University, Chongqing, China.

FAU - Cai, Yi

AU - Cai Y

AUID- ORCID: 0000-0002-1363-2328

AD - Guangdong Provincial Key Laboratory of Regional Immunity and Diseases, Department

of Pathogen Biology, School of Medicine, Shenzhen Universitygrid.263488.3,

Shenzhen, China.

FAU - Chen, Xinchun

AU - Chen X

AUID- ORCID: 0000-0002-2101-9013

AD - Guangdong Provincial Key Laboratory of Regional Immunity and Diseases, Department

of Pathogen Biology, School of Medicine, Shenzhen Universitygrid.263488.3,

Shenzhen, China.

LA - eng

PT - Journal Article

PT - Research Support, Non-U.S. Gov't

DEP - 20220824

PL - United States

TA - mBio

JT - mBio

JID - 101519231

RN - 0 (Reactive Oxygen Species)

RN - EC 2.7.11.24 (p38 Mitogen-Activated Protein Kinases)

RN - 820484N8I3 (Histamine)

RN - 0 (Antitubercular Agents)

RN - N762921K75 (Nitrogen)

SB - IM

MH - Humans

MH - *Mycobacterium tuberculosis/genetics

MH - Reactive Oxygen Species/metabolism

MH - p38 Mitogen-Activated Protein Kinases/metabolism

MH - Histamine

MH - *Tuberculosis/microbiology

MH - Antitubercular Agents

MH - Phagosomes/microbiology

MH - Nitrogen/metabolism

PMC - PMC9600773

OTO - NOTNLM

OT - HRH1

OT - Mycobacterium tuberculosis

OT - NOX2

OT - ROS

OT - p38MAPK

COIS- The authors declare no conflict of interest.

EDAT- 2022/08/25 06:00

MHDA- 2022/10/29 06:00

PMCR- 2022/08/24

CRDT- 2022/08/24 08:23

PHST- 2022/08/25 06:00 [pubmed]

PHST- 2022/10/29 06:00 [medline]

PHST- 2022/08/24 08:23 [entrez]

PHST- 2022/08/24 00:00 [pmc-release]

AID - 02004-22 [pii]

AID - mbio.02004-22 [pii]

AID - 10.1128/mbio.02004-22 [doi]

PST - ppublish

SO - mBio. 2022 Oct 26;13(5):e0200422. doi: 10.1128/mbio.02004-22. Epub 2022 Aug 24.

PMID- 36275035

OWN - NLM

STAT- MEDLINE

DCOM- 20221025

LR - 20221222

IS - 2235-2988 (Electronic)

IS - 2235-2988 (Linking)

VI - 12

DP - 2022

TI - Meta-analysis of active tuberculosis gene expression ascertains host directed

drug targets.

PG - 1010771

LID - 10.3389/fcimb.2022.1010771 [doi]

LID - 1010771

AB - Multi-drug resistant tuberculosis still remains a major public health crisis

globally. With the emergence of newer active tuberculosis disease, the

requirement of prolonged treatment time and adherence to therapy till its

completion necessitates the search of newer therapeutics, targeting human host

factors. The current work utilized statistical meta-analysis of human gene

transcriptomes of active pulmonary tuberculosis disease obtained from six public

datasets. The meta-analysis resulted in the identification of 2038 significantly

differentially expressed genes (DEGs) in the active tuberculosis disease. The

gene ontology (GO) analysis revealed that these genes were major contributors in

immune responses. The pathway enrichment analyses identified from various human

canonical pathways are related to other infectious diseases. In addition, the

comparison of the DEGs with the tuberculosis genome wide association study (GWAS)

datasets revealed the presence of few genetic variants in their proximity. The

analysis of protein interaction networks (human and Mycobacterium tuberculosis)

and host directed drug-target interaction network led to new candidate drug

targets for drug repurposing studies. The current work sheds light on host genes

and pathways enriched in active tuberculosis disease and suggest potential drug

repurposing targets for host-directed therapies.

CI - Copyright © 2022 Ponnusamy and Arumugam.

FAU - Ponnusamy, Nirmaladevi

AU - Ponnusamy N

AD - Department of Biotechnology, School of Biosciences and Technology, Vellore

Institute of Technology, Vellore, Tamil Nadu, India.

FAU - Arumugam, Mohanapriya

AU - Arumugam M

AD - Department of Biotechnology, School of Biosciences and Technology, Vellore

Institute of Technology, Vellore, Tamil Nadu, India.

LA - eng

PT - Journal Article

PT - Meta-Analysis

DEP - 20221005

PL - Switzerland

TA - Front Cell Infect Microbiol

JT - Frontiers in cellular and infection microbiology

JID - 101585359

SB - IM

MH - Humans

MH - Genome-Wide Association Study

MH - *Tuberculosis/drug therapy/genetics

MH - *Mycobacterium tuberculosis/genetics

MH - *Tuberculosis, Pulmonary/drug therapy/genetics

MH - Transcriptome

PMC - PMC9581169

OTO - NOTNLM

OT - drug repurposing

OT - gene ontology

OT - genetic variants

OT - meta-analysis

OT - pathway enrichment

OT - tuberculosis

COIS- The authors declare that the research was conducted in the absence of any

commercial or financial relationships that could be construed as a potential

conflict of interest.

EDAT- 2022/10/25 06:00

MHDA- 2022/10/26 06:00

PMCR- 2022/01/01

CRDT- 2022/10/24 03:51

PHST- 2022/08/03 00:00 [received]

PHST- 2022/09/21 00:00 [accepted]

PHST- 2022/10/24 03:51 [entrez]

PHST- 2022/10/25 06:00 [pubmed]

PHST- 2022/10/26 06:00 [medline]

PHST- 2022/01/01 00:00 [pmc-release]

AID - 10.3389/fcimb.2022.1010771 [doi]

PST - epublish

SO - Front Cell Infect Microbiol. 2022 Oct 5;12:1010771. doi:

10.3389/fcimb.2022.1010771. eCollection 2022.

PMID- 36297325

OWN - NLM

STAT- PubMed-not-MEDLINE

LR - 20221030

IS - 1424-8247 (Print)

IS - 1424-8247 (Electronic)

IS - 1424-8247 (Linking)

VI - 15

IP - 10

DP - 2022 Sep 30

TI - Fluoroquinolone Derivatives in the Treatment of Mycobacterium tuberculosis

Infection.

LID - 10.3390/ph15101213 [doi]

LID - 1213

AB - Tuberculosis (TB) is currently one of the leading causes of death due to

infective agents, and the growing rate of multidrug-resistant tuberculosis (MDR

TB) cases poses an emergent public health threat. Fluoroquinolones are commonly

used in the treatment of both MDR TB and drug-sensitive tuberculosis patients who

are intolerant to first-line antitubercular agents. Unfortunately, these drugs

have mild side effects, relevant to the prolonged treatment regimens and

diminished bioavailability due to binding of metal ions. Moreover, the resistance

to fluoroquinolones is also on the rise, a characteristic of extensively

drug-resistant TB (XDR TB). Here, we developed esters as prodrugs of the

fluoroquinolones levofloxacin and ciprofloxacin, with long-chain fatty alcohols.

Both the alcohols and the quinolone have previously shown antimycobacterial

activity and the aim was to develop esters with improved lipophilicity and

capable of delivering the free acid inside mycobacterial cells. The carboxylic

acid group of fluoroquinolones is essential to the mode of action but is also

responsible for many of its side effects and metal-chelating properties. The

synthesis, stability in biological media, and antibacterial activity were

evaluated, the latter not only against Mycobacterium tuberculosis but also

against other clinically relevant bacterial species, since the parent compounds

display a broad spectrum of activity. The biological results show a reduction in

the antitubercular activity of the synthesized derivatives, probably due to

deficient activation of the ester prodrug. Despite this, it was found that the

derivatives exhibit bioactivity against other fluoroquinolone-resistant bacteria,

indicating a different mode of action and suggesting that it may be worthwhile to

research further modifications to the carboxylic acid group. This might lead to

new compounds that are efficient against resistant strains. This idea that the

compounds may act by a different mechanism of action was further supported by a

brief computer investigation that demonstrated the potential lack of selectivity

of the esters to the fluoroquinolone target.

FAU - Pais, João Pedro

AU - Pais JP

AUID- ORCID: 0000-0002-3865-5913

AD - Research Institute for Medicines and Pharmaceutical Sciences (iMed.UL), Av. Prof.

Gama Pinto, 1649-003 Lisboa, Portugal.

FAU - Policarpo, Margarida

AU - Policarpo M

AD - Research Institute for Medicines and Pharmaceutical Sciences (iMed.UL), Av. Prof.

Gama Pinto, 1649-003 Lisboa, Portugal.

FAU - Pires, David

AU - Pires D

AUID- ORCID: 0000-0001-9602-1516

AD - Research Institute for Medicines and Pharmaceutical Sciences (iMed.UL), Av. Prof.

Gama Pinto, 1649-003 Lisboa, Portugal.

FAU - Francisco, Ana Paula

AU - Francisco AP

AUID- ORCID: 0000-0001-6602-2075

AD - Research Institute for Medicines and Pharmaceutical Sciences (iMed.UL), Av. Prof.

Gama Pinto, 1649-003 Lisboa, Portugal.

AD - Faculty of Pharmacy, University of Lisbon, Av. Prof. Gama Pinto, 1649-003 Lisboa,

Portugal.

FAU - Madureira, Ana Margarida

AU - Madureira AM

AD - Research Institute for Medicines and Pharmaceutical Sciences (iMed.UL), Av. Prof.

Gama Pinto, 1649-003 Lisboa, Portugal.

AD - Faculty of Pharmacy, University of Lisbon, Av. Prof. Gama Pinto, 1649-003 Lisboa,

Portugal.

FAU - Testa, Bernard

AU - Testa B

AD - University of Lausanne, 1015 Lausanne, Switzerland.

FAU - Anes, Elsa

AU - Anes E

AUID- ORCID: 0000-0001-5934-0198

AD - Research Institute for Medicines and Pharmaceutical Sciences (iMed.UL), Av. Prof.

Gama Pinto, 1649-003 Lisboa, Portugal.

AD - Faculty of Pharmacy, University of Lisbon, Av. Prof. Gama Pinto, 1649-003 Lisboa,

Portugal.

FAU - Constantino, Luís

AU - Constantino L

AUID- ORCID: 0000-0002-6847-4830

AD - Research Institute for Medicines and Pharmaceutical Sciences (iMed.UL), Av. Prof.

Gama Pinto, 1649-003 Lisboa, Portugal.

AD - Faculty of Pharmacy, University of Lisbon, Av. Prof. Gama Pinto, 1649-003 Lisboa,

Portugal.

LA - eng

GR - PTDC/SAU-INF/28080/2017/Fundação para a Ciência e Tecnologia/

GR - EXPL/SAU-INF/1097/2021/Fundação para a Ciência e Tecnologia/

GR - UIDB/04138/2020/Fundação para a Ciência e Tecnologia/

GR - UIDP/04138/2020/Fundação para a Ciência e Tecnologia/

PT - Journal Article

DEP - 20220930

PL - Switzerland

TA - Pharmaceuticals (Basel)

JT - Pharmaceuticals (Basel, Switzerland)

JID - 101238453

PMC - PMC9609866

OTO - NOTNLM

OT - esters

OT - fluoroquinolones

OT - mycobacteria

OT - prodrugs

OT - tuberculosis

COIS- The authors declare no conflict of interest.

EDAT- 2022/10/28 06:00

MHDA- 2022/10/28 06:01

PMCR- 2022/09/30

CRDT- 2022/10/27 01:49

PHST- 2022/08/31 00:00 [received]

PHST- 2022/09/23 00:00 [revised]

PHST- 2022/09/26 00:00 [accepted]

PHST- 2022/10/27 01:49 [entrez]

PHST- 2022/10/28 06:00 [pubmed]

PHST- 2022/10/28 06:01 [medline]

PHST- 2022/09/30 00:00 [pmc-release]

AID - ph15101213 [pii]

AID - pharmaceuticals-15-01213 [pii]

AID - 10.3390/ph15101213 [doi]

PST - epublish

SO - Pharmaceuticals (Basel). 2022 Sep 30;15(10):1213. doi: 10.3390/ph15101213.

PMID- 36250047

OWN - NLM

STAT- MEDLINE

DCOM- 20221018

LR - 20221019

IS - 2235-2988 (Electronic)

IS - 2235-2988 (Linking)

VI - 12

DP - 2022

TI - Fluoroquinolone heteroresistance, antimicrobial tolerance, and lethality

enhancement.

PG - 938032

LID - 10.3389/fcimb.2022.938032 [doi]

LID - 938032

AB - With tuberculosis, the emergence of fluoroquinolone resistance erodes the ability

of treatment to interrupt the progression of MDR-TB to XDR-TB. One way to reduce

the emergence of resistance is to identify heteroresistant infections in which

subpopulations of resistant mutants are likely to expand and make the infections

fully resistant: treatment modification can be instituted to suppress mutant

enrichment. Rapid DNA-based detection methods exploit the finding that

fluoroquinolone-resistant substitutions occur largely in a few codons of DNA

gyrase. A second approach for restricting the emergence of resistance involves

understanding fluoroquinolone lethality through studies of antimicrobial

tolerance, a condition in which bacteria fail to be killed even though their

growth is blocked by lethal agents. Studies with Escherichia coli guide work with

Mycobacterium tuberculosis. Lethal action, which is mechanistically distinct from

blocking growth, is associated with a surge in respiration and reactive oxygen

species (ROS). Mutations in carbohydrate metabolism that attenuate ROS

accumulation create pan-tolerance to antimicrobials, disinfectants, and

environmental stressors. These observations indicate the existence of a general

death pathway with respect to stressors. M. tuberculosis displays a variation on

the death pathway idea, as stress-induced ROS is generated by NADH-mediated

reductive stress rather than by respiration. A third approach, which emerges from

lethality studies, uses a small molecule, N-acetyl cysteine, to artificially

increase respiration and additional ROS accumulation. That enhances moxifloxacin

lethality with M. tuberculosis in culture, during infection of cultured

macrophages, and with infection of mice. Addition of ROS stimulators to

fluoroquinolone treatment of tuberculosis constitutes a new direction for

suppressing the transition of MDR-TB to XDR-TB.

CI - Copyright © 2022 Singh, Zhao and Drlica.

FAU - Singh, Amit

AU - Singh A

AD - Department of Microbiology and Cell Biology, Indian Institute of Science,

Bangalore, India.

AD - Centre for Infectious Disease Research, Indian Institute of Science, Bangalore,

India.

FAU - Zhao, Xilin

AU - Zhao X

AD - Public Health Research Institute and Department of Microbiology, Biochemistry and

Molecular Genetics, New Jersey Medical School, Rutgers Biomedical and Health

Sciences, Rutgers University, Newark, NJ, United States.

AD - State Key Laboratory of Molecular Vaccinology and Molecular Diagnostics, School

of Public Health, Xiamen University, Xiamen, China.

FAU - Drlica, Karl

AU - Drlica K

AD - Public Health Research Institute and Department of Microbiology, Biochemistry and

Molecular Genetics, New Jersey Medical School, Rutgers Biomedical and Health

Sciences, Rutgers University, Newark, NJ, United States.

LA - eng

PT - Journal Article

PT - Research Support, Non-U.S. Gov't

PT - Review

DEP - 20220929

PL - Switzerland

TA - Front Cell Infect Microbiol

JT - Frontiers in cellular and infection microbiology

JID - 101585359

RN - 0 (Anti-Bacterial Agents)

RN - 0 (Antitubercular Agents)

RN - 0 (Disinfectants)

RN - 0 (Fluoroquinolones)

RN - 0 (Reactive Oxygen Species)

RN - 0U46U6E8UK (NAD)

RN - EC 5.99.1.3 (DNA Gyrase)

RN - K848JZ4886 (Cysteine)

RN - U188XYD42P (Moxifloxacin)

SB - IM

MH - Animals

MH - Anti-Bacterial Agents/pharmacology

MH - Antitubercular Agents/pharmacology/therapeutic use

MH - Cysteine

MH - DNA Gyrase/genetics

MH - *Disinfectants

MH - *Extensively Drug-Resistant Tuberculosis/drug therapy

MH - Fluoroquinolones/pharmacology

MH - Mice

MH - Microbial Sensitivity Tests

MH - Moxifloxacin

MH - *Mycobacterium tuberculosis/genetics/metabolism

MH - NAD

MH - Reactive Oxygen Species

MH - *Tuberculosis, Multidrug-Resistant/drug therapy

PMC - PMC9559723

OTO - NOTNLM

OT - N-acetyl cysteine

OT - antimycobacterial

OT - fluoroquinolone

OT - oxidative stress

OT - redox biosensor

OT - reductive stress

OT - resistance

OT - respiration

COIS- The authors declare that the research was conducted in the absence of any

commercial or financial relationship that could be construed as a potential

conflict of interest.

EDAT- 2022/10/18 06:00

MHDA- 2022/10/19 06:00

PMCR- 2022/01/01

CRDT- 2022/10/17 05:33

PHST- 2022/05/06 00:00 [received]

PHST- 2022/07/21 00:00 [accepted]

PHST- 2022/10/17 05:33 [entrez]

PHST- 2022/10/18 06:00 [pubmed]

PHST- 2022/10/19 06:00 [medline]

PHST- 2022/01/01 00:00 [pmc-release]

AID - 10.3389/fcimb.2022.938032 [doi]

PST - epublish

SO - Front Cell Infect Microbiol. 2022 Sep 29;12:938032. doi:

10.3389/fcimb.2022.938032. eCollection 2022.

PMID- 36211964

OWN - NLM

STAT- MEDLINE

DCOM- 20221011

LR - 20221012

IS - 2235-2988 (Electronic)

IS - 2235-2988 (Linking)

VI - 12

DP - 2022

TI - Drug resistant tuberculosis: Implications for transmission, diagnosis, and

disease management.

PG - 943545

LID - 10.3389/fcimb.2022.943545 [doi]

LID - 943545

AB - Drug resistant tuberculosis contributes significantly to the global burden of

antimicrobial resistance, often consuming a large proportion of the healthcare

budget and associated resources in many endemic countries. The rapid emergence of

resistance to newer tuberculosis therapies signals the need to ensure appropriate

antibiotic stewardship, together with a concerted drive to develop new regimens

that are active against currently circulating drug resistant strains. Herein, we

highlight that the current burden of drug resistant tuberculosis is driven by a

combination of ongoing transmission and the intra-patient evolution of resistance

through several mechanisms. Global control of tuberculosis will require

interventions that effectively address these and related aspects. Interrupting

tuberculosis transmission is dependent on the availability of novel rapid

diagnostics which provide accurate results, as near-patient as is possible,

together with appropriate linkage to care. Contact tracing, longitudinal

follow-up for symptoms and active mapping of social contacts are essential

elements to curb further community-wide spread of drug resistant strains.

Appropriate prophylaxis for contacts of drug resistant index cases is imperative

to limit disease progression and subsequent transmission. Preventing the

evolution of drug resistant strains will require the development of shorter

regimens that rapidly eliminate all populations of mycobacteria, whilst

concurrently limiting bacterial metabolic processes that drive drug tolerance,

mutagenesis and the ultimate emergence of resistance. Drug discovery programs

that specifically target bacterial genetic determinants associated with these

processes will be paramount to tuberculosis eradication. In addition, the

development of appropriate clinical endpoints that quantify drug tolerant

organisms in sputum, such as differentially culturable/detectable tubercle

bacteria is necessary to accurately assess the potential of new therapies to

effectively shorten treatment duration. When combined, this holistic approach to

addressing the critical problems associated with drug resistance will support

delivery of quality care to patients suffering from tuberculosis and bolster

efforts to eradicate this disease.

CI - Copyright © 2022 Liebenberg, Gordhan and Kana.

FAU - Liebenberg, Dale

AU - Liebenberg D

AD - Department of Science and Innovation/National Research Foundation (DSI/NRF)

Centre of Excellence for Biomedical Tuberculosis Research (TB), Faculty of Health

Sciences, University of the Witwatersrand, National Health Laboratory Service,

Johannesburg, South Africa.

FAU - Gordhan, Bhavna Gowan

AU - Gordhan BG

AD - Department of Science and Innovation/National Research Foundation (DSI/NRF)

Centre of Excellence for Biomedical Tuberculosis Research (TB), Faculty of Health

Sciences, University of the Witwatersrand, National Health Laboratory Service,

Johannesburg, South Africa.

FAU - Kana, Bavesh Davandra

AU - Kana BD

AD - Department of Science and Innovation/National Research Foundation (DSI/NRF)

Centre of Excellence for Biomedical Tuberculosis Research (TB), Faculty of Health

Sciences, University of the Witwatersrand, National Health Laboratory Service,

Johannesburg, South Africa.

LA - eng

PT - Journal Article

PT - Research Support, Non-U.S. Gov't

PT - Review

DEP - 20220923

PL - Switzerland

TA - Front Cell Infect Microbiol

JT - Frontiers in cellular and infection microbiology

JID - 101585359

RN - 0 (Antitubercular Agents)

SB - IM

MH - Antitubercular Agents/pharmacology/therapeutic use

MH - Disease Management

MH - Humans

MH - *Mycobacterium tuberculosis/genetics

MH - Sputum

MH - *Tuberculosis/diagnosis/drug therapy/epidemiology

MH - *Tuberculosis, Multidrug-Resistant/diagnosis/drug therapy/epidemiology

PMC - PMC9538507

OTO - NOTNLM

OT - acquired drug resistance

OT - health systems strengthening

OT - persisters

OT - tolerance

OT - transmitted drug resistance

COIS- The authors declare that the research was conducted in the absence of any

commercial or financial relationships that could be construed as a potential

conflict of interest.

EDAT- 2022/10/11 06:00

MHDA- 2022/10/12 06:00

PMCR- 2022/01/01

CRDT- 2022/10/10 04:03

PHST- 2022/05/13 00:00 [received]

PHST- 2022/09/06 00:00 [accepted]

PHST- 2022/10/10 04:03 [entrez]

PHST- 2022/10/11 06:00 [pubmed]

PHST- 2022/10/12 06:00 [medline]

PHST- 2022/01/01 00:00 [pmc-release]

AID - 10.3389/fcimb.2022.943545 [doi]

PST - epublish

SO - Front Cell Infect Microbiol. 2022 Sep 23;12:943545. doi:

10.3389/fcimb.2022.943545. eCollection 2022.

PMID- 36131811

OWN - NLM

STAT- PubMed-not-MEDLINE

LR - 20220924

IS - 1178-6973 (Print)

IS - 1178-6973 (Electronic)

IS - 1178-6973 (Linking)

VI - 15

DP - 2022

TI - High Pretreatment Level of Neutrophil to Lymphocyte Ratio, Monocyte to Lymphocyte

Ratio and Other Factors Associated with Delayed Sputum Conversion in Patients

with Pulmonary Tuberculosis.

PG - 5455-5462

LID - 10.2147/IDR.S380166 [doi]

AB - INTRODUCTION: Patients with delayed intensive phase sputum conversion have a

higher risk of multidrug resistant-tuberculosis (MDR-TB) and poorer treatment

outcomes. Both, host (immune response and comorbidity) and pathogen factors play

important roles in determining sputum conversion after treatment initiation.

Impaired host immune response, especially the cellular components, as defined by

the increased pre-treatment level of neutrophil-to-lymphocyte ratio (NLR),

monocyte-to-lymphocyte ratio (MLR) and other additional factors, were associated

with severe active TB. PURPOSE: To evaluate whether impaired immune responses

(high pre-treatment level of NLR and MLR) and other factors associate with

delayed sputum conversion at the end of the intensive phase treatment. PATIENTS

AND METHODS: This was a case-control study from 2016 to 2020, which

retrospectively analyzed the pre-treatment level of NLR, MLR and other factors

among patients with new cases of pulmonary tuberculosis (PTB). RESULTS: A total

of 62 patients (31 cases and 31 control). The cut-off value of high pretreatment

level of NLR and MLR was 5.065 and 0.585, respectively. Bivariate analysis showed

that pretreatment NLR ≥5.065 (OR 8.23, CI 95% 2.48-27.32, p < 0.001), MLR ≥0.585

(OR 10.18, 95% CI 3.13-33.18, p < 0.001) and BMI <18.5 (OR 2.91, 95% CI

1.03-8.20, p = 0.041) were associated with an increased risk of delayed sputum

conversion. Multivariate analysis, however, showed that pretreatment NLR ≥5.065

was not significantly associated with delayed sputum conversion (AOR 3.370, 95%

CI 0.71-15.91, p value 0.125). A high pretreatment of MLR (AOR 30.802, 95% CI

3.22-287.55, p value 0.003) and lower BMI (AOR 10.942, 95% CI 1.121-98.563, p

value 0.033) were significantly associated with an increased risk of delayed

intensive phase sputum conversion. CONCLUSION: High MLR pretreatment and a low

BMI were significantly associated with an increased risk of delayed sputum

conversion at the end of the PTB intensive phase treatment. High NLR

pretreatment, smoking, diabetes, and HIV were not associated with sputum

conversion.

CI - © 2022 Suryana et al.

FAU - Suryana, Ketut

AU - Suryana K

AUID- ORCID: 0000-0003-4465-1705

AD - Division of Clinical Allergy-Immunology, Department of Internal Medicine, Faculty

of Medicine, Udayana University, Denpasar, Bali, Indonesia.

AD - Department of Internal Medicine, Merpati Clinic, Wangaya Hospital, Denpasar,

Bali, Indonesia.

FAU - Dharmesti, Ni Wayan Wina

AU - Dharmesti NWW

AD - Department of Internal Medicine, Faculty of Medicine, Udayana University,

Denpasar, Bali, Indonesia.

FAU - Rai, I B Ngurah

AU - Rai IBN

AD - Division of Pulmonology, Department of Internal Medicine, Faculty of Medicine,

Udayana University, Sanglah Hospital, Denpasar, Bali, Indonesia.

LA - eng

PT - Journal Article

DEP - 20220915

PL - New Zealand

TA - Infect Drug Resist

JT - Infection and drug resistance

JID - 101550216

PMC - PMC9484577

OTO - NOTNLM

OT - delayed sputum conversion

OT - intensive phase treatment

OT - monocyte to lymphocyte ratio

OT - neutrophil to lymphocyte ratio

OT - pulmonary tuberculosis

COIS- The authors report no conflicts of interest in this work.

EDAT- 2022/09/23 06:00

MHDA- 2022/09/23 06:01

PMCR- 2022/09/15

CRDT- 2022/09/22 03:26

PHST- 2022/07/05 00:00 [received]

PHST- 2022/08/29 00:00 [accepted]

PHST- 2022/09/22 03:26 [entrez]

PHST- 2022/09/23 06:00 [pubmed]

PHST- 2022/09/23 06:01 [medline]

PHST- 2022/09/15 00:00 [pmc-release]

AID - 380166 [pii]

AID - 10.2147/IDR.S380166 [doi]

PST - epublish

SO - Infect Drug Resist. 2022 Sep 15;15:5455-5462. doi: 10.2147/IDR.S380166.

eCollection 2022.

PMID- 36160387

OWN - NLM

STAT- PubMed-not-MEDLINE

LR - 20220928

IS - 1663-9812 (Print)

IS - 1663-9812 (Electronic)

IS - 1663-9812 (Linking)

VI - 13

DP - 2022

TI - Mechanism underlying linezolid-induced peripheral neuropathy in

multidrug-resistant tuberculosis.

PG - 946058

LID - 10.3389/fphar.2022.946058 [doi]

LID - 946058

AB - Multidrug-resistant tuberculosis (MDR-TB) remains a main global health concern as

there is no comprehensive therapeutic intervention yet and numerous adverse

effects follow the therapeutic process. In recent years, linezolid has been

frequently used for treating MDR-TB. However, peripheral neuropathy associated

with linezolid has reduced patient compliance. The current study explored the

mechanism underlying linezolid-induced peripheral neuropathy in MDR-TB. Autophagy

plays a neuroprotective role against peripheral nerve injury. We hypothesized

that autophagy might also play a neuroprotective role against linezolid-induced

peripheral neuropathy. In this study, we collected 12 questionnaires from MDR-TB

patients in our hospital, and 10 of them developed linezolid-induced pain. The

pain is mainly concentrated in the feet and accompanied by numbness.

Subsequently, we used Sprague-Dawley (SD) rats and Schwann cells (SCs) to explore

the mechanism. We found that linezolid causes a sparse arrangement of sciatic

nerve tissue with associated loss of neurons, myelin sheaths, and down-regulation

of LC3B expression. These results were also confirmed by in vitro experiments,

showing that linezolid inhibited the proliferation of SCs. And the expression of

P-AKT and P62 was elevated, and the expression of LC3B declined compared with the

control group. Moreover, chloroquine (CQ), an autophagy inhibitor, also exhibited

experimental results similar to linezolid. In summary, we conclude that

linezolid-induced peripheral neuropathy is associated with the inhibition of

autophagy flux.

CI - Copyright © 2022 Yuan, Li, Chen, Cai, Xu, Lin, Lang, Guo, Zhang and Cai.

FAU - Yuan, Yuan

AU - Yuan Y

AD - Zhejiang University School of Medicine, Affiliated Hangzhou Chest Hospital,

Hangzhou, Zhejiang, China.

FAU - Li, Jinmeng

AU - Li J

AD - Zhejiang University School of Medicine, Affiliated Hangzhou Chest Hospital,

Hangzhou, Zhejiang, China.

FAU - Chen, Yanhong

AU - Chen Y

AD - Laboratory Animal Center of Zhejiang University, Hangzhou, Zhejiang, China.

FAU - Cai, Qingshan

AU - Cai Q

AD - Zhejiang University School of Medicine, Affiliated Hangzhou Chest Hospital,

Hangzhou, Zhejiang, China.

FAU - Xu, Yingying

AU - Xu Y

AD - Zhejiang University School of Medicine, Affiliated Hangzhou Chest Hospital,

Hangzhou, Zhejiang, China.

FAU - Lin, Luting

AU - Lin L

AD - College of Pharmacy, Zhejiang Chinese Medical University, Hangzhou, Zhejiang,

China.

FAU - Lang, Yazhen

AU - Lang Y

AD - Zhejiang University School of Medicine, Affiliated Hangzhou Chest Hospital,

Hangzhou, Zhejiang, China.

FAU - Guo, Suhang

AU - Guo S

AD - Zhejiang University School of Medicine, Affiliated Hangzhou Chest Hospital,

Hangzhou, Zhejiang, China.

FAU - Zhang, Ruoying

AU - Zhang R

AD - Zhejiang University School of Medicine, Affiliated Hangzhou Chest Hospital,

Hangzhou, Zhejiang, China.

FAU - Cai, Xinjun

AU - Cai X

AD - Zhejiang University School of Medicine, Affiliated Hangzhou Chest Hospital,

Hangzhou, Zhejiang, China.

LA - eng

PT - Journal Article

DEP - 20220909

PL - Switzerland

TA - Front Pharmacol

JT - Frontiers in pharmacology

JID - 101548923

PMC - PMC9500448

OTO - NOTNLM

OT - autophagy

OT - linezolid

OT - multidrug-resistant tuberculosis

OT - peripheral neuropathy

OT - schwann cells

COIS- The authors declare that the research was conducted in the absence of any

commercial or financial relationships that could be construed as a potential

conflict of interest.

EDAT- 2022/09/27 06:00

MHDA- 2022/09/27 06:01

PMCR- 2022/09/09

CRDT- 2022/09/26 17:50

PHST- 2022/05/27 00:00 [received]

PHST- 2022/08/12 00:00 [accepted]

PHST- 2022/09/26 17:50 [entrez]

PHST- 2022/09/27 06:00 [pubmed]

PHST- 2022/09/27 06:01 [medline]

PHST- 2022/09/09 00:00 [pmc-release]

AID - 946058 [pii]

AID - 10.3389/fphar.2022.946058 [doi]

PST - epublish

SO - Front Pharmacol. 2022 Sep 9;13:946058. doi: 10.3389/fphar.2022.946058.

eCollection 2022.

PMID- 36077494

OWN - NLM

STAT- MEDLINE

DCOM- 20220912

LR - 20250728

IS - 1422-0067 (Electronic)

IS - 1422-0067 (Linking)

VI - 23

IP - 17

DP - 2022 Sep 3

TI - Binding Thermodynamics and Dissociation Kinetics Analysis Uncover the Key

Structural Motifs of Phenoxyphenol Derivatives as the Direct InhA Inhibitors and

the Hotspot Residues of InhA.

LID - 10.3390/ijms231710102 [doi]

LID - 10102

AB - Given the current epidemic of multidrug-resistant tuberculosis, there is an

urgent need to develop new drugs to combat drug-resistant tuberculosis. Direct

inhibitors of the InhA target do not require activation and thus can overcome

drug resistance caused by mutations in drug-activating enzymes. In this work, the

binding thermodynamic and kinetic information of InhA to its direct inhibitors,

phenoxyphenol derivatives, were explored through multiple computer-aided drug

design (CADD) strategies. The results show that the van der Waals interactions

were the main driving force for protein-ligand binding, among which hydrophobic

residues such as Tyr158, Phe149, Met199 and Ile202 have high energy contribution.

The AHRR pharmacophore model generated by multiple ligands demonstrated that

phenoxyphenol derivatives inhibitors can form pi-pi stacking and hydrophobic

interactions with InhA target. In addition, the order of residence time predicted

by random acceleration molecular dynamics was consistent with the experimental

values. The intermediate states of these inhibitors could form hydrogen bonds and

van der Waals interactions with surrounding residues during dissociation.

Overall, the binding and dissociation mechanisms at the atomic level obtained in

this work can provide important theoretical guidance for the development of InhA

direct inhibitors with higher activity and proper residence time.

FAU - Zhang, Qianqian

AU - Zhang Q

AD - Faculty of Applied Science, Macao Polytechnic University, Macao, China.

AD - School of Pharmacy, Lanzhou University, Lanzhou 730000, China.

FAU - Han, Jianting

AU - Han J

AD - College of Chemistry and Chemical Engineering, Lanzhou University, Lanzhou

730000, China.

FAU - Zhu, Yongchang

AU - Zhu Y

AD - College of Chemistry and Chemical Engineering, Lanzhou University, Lanzhou

730000, China.

FAU - Tan, Shuoyan

AU - Tan S

AD - College of Chemistry and Chemical Engineering, Lanzhou University, Lanzhou

730000, China.

FAU - Liu, Huanxiang

AU - Liu H

AUID- ORCID: 0000-0002-9284-3667

AD - Faculty of Applied Science, Macao Polytechnic University, Macao, China.

LA - eng

GR - RP/FCA-01/2022/Macao Polytechnic University/

PT - Journal Article

DEP - 20220903

PL - Switzerland

TA - Int J Mol Sci

JT - International journal of molecular sciences

JID - 101092791

RN - 0 (Antitubercular Agents)

RN - 0 (Bacterial Proteins)

RN - 0 (Ligands)

RN - EC 1.- (Oxidoreductases)

SB - IM

MH - *Antitubercular Agents/chemistry/pharmacology

MH - Bacterial Proteins/metabolism

MH - Ligands

MH - *Mycobacterium tuberculosis/metabolism

MH - Oxidoreductases/metabolism

MH - Thermodynamics

PMC - PMC9456180

OTO - NOTNLM

OT - InhA direct inhibitors

OT - dissociation pathway

OT - hotspot residues

OT - molecular dynamics simulation

OT - pharmacophore model

OT - phenoxyphenol derivatives

OT - residence time

COIS- The researcher claims no conflicts of interests.

EDAT- 2022/09/10 06:00

MHDA- 2022/09/14 06:00

PMCR- 2022/09/03

CRDT- 2022/09/09 01:09

PHST- 2022/07/28 00:00 [received]

PHST- 2022/08/19 00:00 [revised]

PHST- 2022/08/23 00:00 [accepted]

PHST- 2022/09/09 01:09 [entrez]

PHST- 2022/09/10 06:00 [pubmed]

PHST- 2022/09/14 06:00 [medline]

PHST- 2022/09/03 00:00 [pmc-release]

AID - ijms231710102 [pii]

AID - ijms-23-10102 [pii]

AID - 10.3390/ijms231710102 [doi]

PST - epublish

SO - Int J Mol Sci. 2022 Sep 3;23(17):10102. doi: 10.3390/ijms231710102.

PMID- 36120339

OWN - NLM

STAT- PubMed-not-MEDLINE

LR - 20220920

IS - 1663-9812 (Print)

IS - 1663-9812 (Electronic)

IS - 1663-9812 (Linking)

VI - 13

DP - 2022

TI - Tibetan medicine salidroside improves host anti-mycobacterial response by

boosting inflammatory cytokine production in zebrafish.

PG - 936295

LID - 10.3389/fphar.2022.936295 [doi]

LID - 936295

AB - The treatment for tuberculosis (TB), especially multidrug-resistant TB (MDR-TB),

has a prolonged cycle which can last up to a year. This is partially due to the

lack of effective therapies. The development of novel anti-TB drugs from the

perspective of host immune regulation can provide an important supplement for

conventional treatment strategies. Salidroside (SAL), a bioactive component from

the Tibetan medicine Rhodiola rosea, has been used in the treatment of TB,

although its mechanism remains unclear. Here, the bacteriostatic effect of SAL in

vivo was first demonstrated using a zebrafish-M. marinum infection model. To

further investigate the underlying mechanism, we then examined the impact of SAL

on immune cell recruitment during wound and infection. Increased macrophage and

neutrophil infiltrations were found both in the vicinity of the wound and

infection sites after SAL treatment compared with control, which might be due to

the elevated chemokine expression levels after SAL treatment. SAL treatment alone

was also demonstrated to improve the survival of infected zebrafish larvae, an

effect that was amplified when combining SAL treatment with isoniazid or

rifampicin. Interestingly, the reduced bacterial burden and improved survival

rate under SAL treatment were compromised in tnfα-deficient embryos which

suggests a requirement of Tnfα signaling on the anti-mycobacterial effects of

SAL. In summary, this study provides not only the cellular and molecular

mechanisms for the host anti-mycobacterial effects of the Tibetan medicine SAL

but also proof of concept that combined application of SAL with traditional

first-line anti-TB drugs could be a novel strategy to improve treatment efficacy.

CI - Copyright © 2022 He, Fan, Sun, Yang, Liu, Yang, Liu, Luo, Chen, Zhou, Xia, Zhang

and Yan.

FAU - He, Shumei

AU - He S

AD - Key Laboratory of Molecular Mechanistic and Interventional Research of Plateau

Diseases in Tibet Autonomous Region, Key Laboratory of High Altitude Hypoxia

Environment and Life Health, Joint Central Laboratory for Active Components and

Pharmacological Mechanism of Tibetan Medicine, School of Medicine, Xizang Minzu

University, Xianyang, China.

FAU - Fan, Hongyan

AU - Fan H

AD - Key Laboratory of Molecular Mechanistic and Interventional Research of Plateau

Diseases in Tibet Autonomous Region, Key Laboratory of High Altitude Hypoxia

Environment and Life Health, Joint Central Laboratory for Active Components and

Pharmacological Mechanism of Tibetan Medicine, School of Medicine, Xizang Minzu

University, Xianyang, China.

AD - Shanghai Public Health Clinical Center, Fudan University, Shanghai, China.

FAU - Sun, Bin

AU - Sun B

AD - Department of Stomatology, The First Affiliated Hospital of Shihezi University

Medical College, Shihezi, China.

FAU - Yang, Meipan

AU - Yang M

AD - Key Laboratory of Molecular Mechanistic and Interventional Research of Plateau

Diseases in Tibet Autonomous Region, Key Laboratory of High Altitude Hypoxia

Environment and Life Health, Joint Central Laboratory for Active Components and

Pharmacological Mechanism of Tibetan Medicine, School of Medicine, Xizang Minzu

University, Xianyang, China.

FAU - Liu, Hongxu

AU - Liu H

AD - Key Laboratory of Molecular Mechanistic and Interventional Research of Plateau

Diseases in Tibet Autonomous Region, Key Laboratory of High Altitude Hypoxia

Environment and Life Health, Joint Central Laboratory for Active Components and

Pharmacological Mechanism of Tibetan Medicine, School of Medicine, Xizang Minzu

University, Xianyang, China.

AD - Shanghai Public Health Clinical Center, Fudan University, Shanghai, China.

FAU - Yang, Jianwei

AU - Yang J

AD - Key Laboratory of Molecular Mechanistic and Interventional Research of Plateau

Diseases in Tibet Autonomous Region, Key Laboratory of High Altitude Hypoxia

Environment and Life Health, Joint Central Laboratory for Active Components and

Pharmacological Mechanism of Tibetan Medicine, School of Medicine, Xizang Minzu

University, Xianyang, China.

AD - Shanghai Public Health Clinical Center, Fudan University, Shanghai, China.

FAU - Liu, Jianxin

AU - Liu J

AD - Key Laboratory of Molecular Mechanistic and Interventional Research of Plateau

Diseases in Tibet Autonomous Region, Key Laboratory of High Altitude Hypoxia

Environment and Life Health, Joint Central Laboratory for Active Components and

Pharmacological Mechanism of Tibetan Medicine, School of Medicine, Xizang Minzu

University, Xianyang, China.

AD - Shanghai Public Health Clinical Center, Fudan University, Shanghai, China.

FAU - Luo, Sizhu

AU - Luo S

AD - Key Laboratory of Molecular Mechanistic and Interventional Research of Plateau

Diseases in Tibet Autonomous Region, Key Laboratory of High Altitude Hypoxia

Environment and Life Health, Joint Central Laboratory for Active Components and

Pharmacological Mechanism of Tibetan Medicine, School of Medicine, Xizang Minzu

University, Xianyang, China.

AD - Shanghai Public Health Clinical Center, Fudan University, Shanghai, China.

FAU - Chen, Zihan

AU - Chen Z

AD - Shanghai Public Health Clinical Center, Fudan University, Shanghai, China.

AD - Medical College, China Three Gorges University, Yichang, China.

FAU - Zhou, Jing

AU - Zhou J

AD - Shanghai Public Health Clinical Center, Fudan University, Shanghai, China.

AD - Medical College, China Three Gorges University, Yichang, China.

FAU - Xia, Lu

AU - Xia L

AD - Shanghai Public Health Clinical Center, Fudan University, Shanghai, China.

FAU - Zhang, Shulin

AU - Zhang S

AD - Shanghai Public Health Clinical Center, Fudan University, Shanghai, China.

AD - Department of Immunology and Microbiology, Shanghai Jiao Tong University School

of Medicine, Shanghai, China.

FAU - Yan, Bo

AU - Yan B

AD - Shanghai Public Health Clinical Center, Fudan University, Shanghai, China.

LA - eng

PT - Journal Article

DEP - 20220831

PL - Switzerland

TA - Front Pharmacol

JT - Frontiers in pharmacology

JID - 101548923

PMC - PMC9470765

OTO - NOTNLM

OT - Tnfα

OT - innate immunity

OT - macrophage

OT - mycobacterium

OT - neutrophil

OT - salidroside

OT - zebrafish

COIS- The authors declare that the research was conducted in the absence of any

commercial or financial relationships that could be construed as a potential

conflict of interest.

EDAT- 2022/09/20 06:00

MHDA- 2022/09/20 06:01

PMCR- 2022/08/31

CRDT- 2022/09/19 04:39

PHST- 2022/05/05 00:00 [received]

PHST- 2022/07/29 00:00 [accepted]

PHST- 2022/09/19 04:39 [entrez]

PHST- 2022/09/20 06:00 [pubmed]

PHST- 2022/09/20 06:01 [medline]

PHST- 2022/08/31 00:00 [pmc-release]

AID - 936295 [pii]

AID - 10.3389/fphar.2022.936295 [doi]

PST - epublish

SO - Front Pharmacol. 2022 Aug 31;13:936295. doi: 10.3389/fphar.2022.936295.

eCollection 2022.

PMID- 35862980

OWN - NLM

STAT- MEDLINE

DCOM- 20220908

LR - 20250728

IS - 2165-0497 (Electronic)

IS - 2165-0497 (Linking)

VI - 10

IP - 4

DP - 2022 Aug 31

TI - Evaluation of 3-Deoxy-D-Arabino-Heptulosonate 7-Phosphate Synthase (DAHPS) as a

Vulnerable Target in Mycobacterium tuberculosis.

PG - e0072822

LID - 10.1128/spectrum.00728-22 [doi]

LID - e00728-22

AB - Tuberculosis (TB) remains one of the leading causes of death due to a single

pathogen. The emergence and proliferation of multidrug-resistant (MDR-TB) and

extensively drug-resistant strains (XDR-TB) represent compelling reasons to

invest in the pursuit of new anti-TB agents. The shikimate pathway, responsible

for chorismate biosynthesis, which is a precursor of important aromatic

compounds, is required for Mycobacterium tuberculosis growth. The enzyme

3-deoxy-d-arabino-heptulosonate 7-phosphate synthase (MtbDAHPS) catalyzes the

first step in the shikimate pathway and it is an attractive target for

anti-tubercular agents. Here, we used a CRISPRi system to evaluate the DAHPS as a

vulnerable target in M. tuberculosis. The silencing of aroG significantly reduces

the M. tuberculosis growth in both rich medium and, especially, in infected

murine macrophages. The supplementation with amino acids was only able to

partially rescue the growth of bacilli, whereas the Aro supplement (aromix) was

enough to sustain the bacterial growth at lower rates. This study shows that

MtbDAHPS protein is vulnerable and, therefore, an attractive target to develop

new anti-TB agents. In addition, the study contributes to a better understanding

of the biosynthesis of aromatic compounds and the bacillus physiology. IMPORTANCE

Determining the vulnerability of a potential target allows us to assess whether

its partial inhibition will impact bacterial growth. Here, we evaluated the

vulnerability of the enzyme 3-deoxy-d-arabino-heptulosonate 7-phosphate synthase

(DAHPS) from M. tuberculosis by silencing the DAHPS-coding aroG gene in different

contexts. These results could lead to the development of novel and potent

anti-tubercular agents in the near future.

FAU - Galina, Luiza

AU - Galina L

AUID- ORCID: 0000-0003-4394-221X

AD - Centro de Pesquisas em Biologia Molecular e Funcional (CPBMF) and Instituto

Nacional de Ciência e Tecnologia em Tuberculose (INCT-TB), Pontifícia

Universidade Católica do Rio Grande do Sul (PUCRS), Porto Alegre, Rio Grande do

Sul, Brazil.

AD - Programa de Pós-Graduação em Medicina e Ciências da Saúde, Pontifícia

Universidade Católica do Rio Grande do Sul, Porto Alegre, Rio Grande do Sul,

Brazil.

FAU - Hopf, Fernanda S M

AU - Hopf FSM

AD - Centro de Pesquisas em Biologia Molecular e Funcional (CPBMF) and Instituto

Nacional de Ciência e Tecnologia em Tuberculose (INCT-TB), Pontifícia

Universidade Católica do Rio Grande do Sul (PUCRS), Porto Alegre, Rio Grande do

Sul, Brazil.

AD - Programa de Pós-Graduação em Biologia Celular e Molecular, Pontifícia

Universidade Católica do Rio Grande do Sul, Porto Alegre, Rio Grande do Sul,

Brazil.

FAU - Abbadi, Bruno Lopes

AU - Abbadi BL

AUID- ORCID: 0000-0002-4336-1282

AD - Centro de Pesquisas em Biologia Molecular e Funcional (CPBMF) and Instituto

Nacional de Ciência e Tecnologia em Tuberculose (INCT-TB), Pontifícia

Universidade Católica do Rio Grande do Sul (PUCRS), Porto Alegre, Rio Grande do

Sul, Brazil.

FAU - Sperotto, Nathalia D de Moura

AU - Sperotto NDM

AD - Centro de Pesquisas em Biologia Molecular e Funcional (CPBMF) and Instituto

Nacional de Ciência e Tecnologia em Tuberculose (INCT-TB), Pontifícia

Universidade Católica do Rio Grande do Sul (PUCRS), Porto Alegre, Rio Grande do

Sul, Brazil.

FAU - Czeczot, Alexia M

AU - Czeczot AM

AD - Centro de Pesquisas em Biologia Molecular e Funcional (CPBMF) and Instituto

Nacional de Ciência e Tecnologia em Tuberculose (INCT-TB), Pontifícia

Universidade Católica do Rio Grande do Sul (PUCRS), Porto Alegre, Rio Grande do

Sul, Brazil.

AD - Programa de Pós-Graduação em Medicina e Ciências da Saúde, Pontifícia

Universidade Católica do Rio Grande do Sul, Porto Alegre, Rio Grande do Sul,

Brazil.

FAU - Duque-Villegas, Mario A

AU - Duque-Villegas MA

AUID- ORCID: 0000-0002-1800-0450

AD - Centro de Pesquisas em Biologia Molecular e Funcional (CPBMF) and Instituto

Nacional de Ciência e Tecnologia em Tuberculose (INCT-TB), Pontifícia

Universidade Católica do Rio Grande do Sul (PUCRS), Porto Alegre, Rio Grande do

Sul, Brazil.

FAU - Perello, Marcia Alberton

AU - Perello MA

AD - Centro de Pesquisas em Biologia Molecular e Funcional (CPBMF) and Instituto

Nacional de Ciência e Tecnologia em Tuberculose (INCT-TB), Pontifícia

Universidade Católica do Rio Grande do Sul (PUCRS), Porto Alegre, Rio Grande do

Sul, Brazil.

FAU - Matter, Letícia Beatriz

AU - Matter LB

AD - Centro de Pesquisas em Biologia Molecular e Funcional (CPBMF) and Instituto

Nacional de Ciência e Tecnologia em Tuberculose (INCT-TB), Pontifícia

Universidade Católica do Rio Grande do Sul (PUCRS), Porto Alegre, Rio Grande do

Sul, Brazil.

AD - Programa de Pós-Graduação em Biologia Celular e Molecular, Pontifícia

Universidade Católica do Rio Grande do Sul, Porto Alegre, Rio Grande do Sul,

Brazil.

FAU - de Souza, Eduardo Vieira

AU - de Souza EV

AD - Centro de Pesquisas em Biologia Molecular e Funcional (CPBMF) and Instituto

Nacional de Ciência e Tecnologia em Tuberculose (INCT-TB), Pontifícia

Universidade Católica do Rio Grande do Sul (PUCRS), Porto Alegre, Rio Grande do

Sul, Brazil.

FAU - Parish, Tanya

AU - Parish T

AUID- ORCID: 0000-0001-7507-0423

AD - Center for Global Infectious Disease Research, Seattle Children's Research

Institute, Seattle, Washington, USA.

FAU - Machado, Pablo

AU - Machado P

AD - Centro de Pesquisas em Biologia Molecular e Funcional (CPBMF) and Instituto

Nacional de Ciência e Tecnologia em Tuberculose (INCT-TB), Pontifícia

Universidade Católica do Rio Grande do Sul (PUCRS), Porto Alegre, Rio Grande do

Sul, Brazil.

AD - Programa de Pós-Graduação em Biologia Celular e Molecular, Pontifícia

Universidade Católica do Rio Grande do Sul, Porto Alegre, Rio Grande do Sul,

Brazil.

FAU - Basso, Luiz A

AU - Basso LA

AD - Centro de Pesquisas em Biologia Molecular e Funcional (CPBMF) and Instituto

Nacional de Ciência e Tecnologia em Tuberculose (INCT-TB), Pontifícia

Universidade Católica do Rio Grande do Sul (PUCRS), Porto Alegre, Rio Grande do

Sul, Brazil.

AD - Programa de Pós-Graduação em Medicina e Ciências da Saúde, Pontifícia

Universidade Católica do Rio Grande do Sul, Porto Alegre, Rio Grande do Sul,

Brazil.

AD - Programa de Pós-Graduação em Biologia Celular e Molecular, Pontifícia

Universidade Católica do Rio Grande do Sul, Porto Alegre, Rio Grande do Sul,

Brazil.

FAU - Bizarro, Cristiano V

AU - Bizarro CV

AUID- ORCID: 0000-0002-2609-8996

AD - Centro de Pesquisas em Biologia Molecular e Funcional (CPBMF) and Instituto

Nacional de Ciência e Tecnologia em Tuberculose (INCT-TB), Pontifícia

Universidade Católica do Rio Grande do Sul (PUCRS), Porto Alegre, Rio Grande do

Sul, Brazil.

AD - Programa de Pós-Graduação em Biologia Celular e Molecular, Pontifícia

Universidade Católica do Rio Grande do Sul, Porto Alegre, Rio Grande do Sul,

Brazil.

LA - eng

PT - Journal Article

PT - Research Support, Non-U.S. Gov't

DEP - 20220714

PL - United States

TA - Microbiol Spectr

JT - Microbiology spectrum

JID - 101634614

RN - 0 (Antitubercular Agents)

RN - 0 (Phosphates)

RN - EC 2.5.1.54 (3-Deoxy-7-Phosphoheptulonate Synthase)

SB - IM

MH - *3-Deoxy-7-Phosphoheptulonate Synthase/chemistry/genetics/metabolism

MH - Animals

MH - Antitubercular Agents/pharmacology

MH - Mice

MH - *Mycobacterium tuberculosis/metabolism

MH - Phosphates

PMC - PMC9430761

OTO - NOTNLM

OT - CRISPRi

OT - aroG

OT - macrophages

OT - shikimate pathway

OT - vulnerability

COIS- The authors declare no conflict of interest.

EDAT- 2022/07/22 06:00

MHDA- 2022/09/09 06:00

PMCR- 2022/07/14

CRDT- 2022/07/21 16:47

PHST- 2022/07/22 06:00 [pubmed]

PHST- 2022/09/09 06:00 [medline]

PHST- 2022/07/21 16:47 [entrez]

PHST- 2022/07/14 00:00 [pmc-release]

AID - 00728-22 [pii]

AID - spectrum.00728-22 [pii]

AID - 10.1128/spectrum.00728-22 [doi]

PST - ppublish

SO - Microbiol Spectr. 2022 Aug 31;10(4):e0072822. doi: 10.1128/spectrum.00728-22.

Epub 2022 Jul 14.

PMID- 36185922

OWN - NLM

STAT- PubMed-not-MEDLINE

LR - 20221004

IS - 2168-8184 (Print)

IS - 2168-8184 (Electronic)

IS - 2168-8184 (Linking)

VI - 14

IP - 8

DP - 2022 Aug

TI - Bedaquiline: A Novel Diarylquinoline for Multidrug-Resistant Pulmonary

Tuberculosis.

PG - e28519

LID - 10.7759/cureus.28519 [doi]

LID - e28519

AB - A new drug Bedaquiline, a diarylquinoline agent has been approved by the Food and

Drug Administration for the treatment of pulmonary multidrug-resistant

tuberculosis. It has been given approval for use along with the basic regimen

with only conditional access through the National Program for tuberculosis in

India. The major problem with existing antitubercular drugs used for the

treatment of multi-drug resistant tuberculosis is antimicrobial resistance, less

efficacy, and poor side effect profile. Bedaquiline might be a solution to these

issues. Bedaquiline is a first of its class drug with a unique and specific

mechanism of action. It inhibits mycobacterial adenosine triphosphate (ATP)

synthase's proton pump. There are many randomized clinical trials and cohort

studies that reported a higher culture conversion rate with bedaquiline

treatment as compared to the control group. Many meta-analyses and systematic

reviews have reported higher culture conversion rate, higher cure rate, and lower

mortality rate in patients with drug-resistant tuberculosis treated with a

bedaquiline-containing regimen. Here is a detailed drug profile of bedaquiline to

help health care workers treat tuberculosis patients. Keywords.

CI - Copyright © 2022, Deshkar et al.

FAU - Deshkar, Anuradha T

AU - Deshkar AT

AD - Department of Pharmacology, Datta Meghe Institute of Medical Sciences, Nagpur,

IND.

FAU - Shirure, Prashant A

AU - Shirure PA

AD - Department of Pharmacology, Dr. Vaishampayan Memorial Government Medical College,

Solapur, IND.

LA - eng

PT - Journal Article

PT - Review

DEP - 20220829

PL - United States

TA - Cureus

JT - Cureus

JID - 101596737

PMC - PMC9516320

OTO - NOTNLM

OT - bedaquiline

OT - culture conversion rate

OT - diarylquinoline

OT - drug resistance

OT - heteroresistance

OT - multidrug-resistant tuberculosis

OT - pulmonary tuberculosis

COIS- The authors have declared that no competing interests exist.

EDAT- 2022/10/04 06:00

MHDA- 2022/10/04 06:01

PMCR- 2022/08/29

CRDT- 2022/10/03 04:27

PHST- 2022/06/25 00:00 [received]

PHST- 2022/08/26 00:00 [accepted]

PHST- 2022/10/03 04:27 [entrez]

PHST- 2022/10/04 06:00 [pubmed]

PHST- 2022/10/04 06:01 [medline]

PHST- 2022/08/29 00:00 [pmc-release]

AID - 10.7759/cureus.28519 [doi]

PST - epublish

SO - Cureus. 2022 Aug 29;14(8):e28519. doi: 10.7759/cureus.28519. eCollection 2022

Aug.

PMID- 36015371

OWN - NLM

STAT- PubMed-not-MEDLINE

LR - 20220830

IS - 1999-4923 (Print)

IS - 1999-4923 (Electronic)

IS - 1999-4923 (Linking)

VI - 14

IP - 8

DP - 2022 Aug 21

TI - Development of Inhalable ATRA-Loaded PLGA Nanoparticles as Host-Directed

Immunotherapy against Tuberculosis.

LID - 10.3390/pharmaceutics14081745 [doi]

LID - 1745

AB - Developing new effective treatment strategies to overcome the rise in multi-drug

resistant tuberculosis cases (MDR-TB) represents a global challenge. A

host-directed therapy (HDT), acting on the host immune response rather than Mtb

directly, could address these resistance issues. We developed an HDT for targeted

TB treatment, using All Trans Retinoic Acid (ATRA)-loaded nanoparticles (NPs)

that are suitable for nebulization. Efficacy studies conducted on THP-1

differentiated cells infected with the H37Ra avirulent Mycobacterium tuberculosis

(Mtb) strain, have shown a dose-dependent reduction in H37Ra growth as determined

by the BACT/ALERT(®) system. Confocal microscopy images showed efficient and

extensive cellular delivery of ATRA-PLGA NPs into THP-1-derived macrophages. A

commercially available vibrating mesh nebulizer was used to generate

nanoparticle-loaded droplets with a mass median aerodynamic diameter of 2.13 μm

as measured by cascade impaction, and a volumetric median diameter of 4.09 μm as

measured by laser diffraction. In an adult breathing simulation experiment, 65.1%

of the ATRA PLGA-NP dose was inhaled. This targeted inhaled HDT could offer a new

adjunctive TB treatment option that could enhance current dosage regimens leading

to better patient prognosis and a decreasing incidence of MDR-TB.

FAU - Bahlool, Ahmad Z

AU - Bahlool AZ

AUID- ORCID: 0000-0002-9992-7476

AD - School of Pharmacy and Biomolecular Sciences, Royal College of Surgeons in

Ireland (RCSI), 123 St. Stephens Green, D02 YN77 Dublin, Ireland.

AD - Tissue Engineering Research Group, Royal College of Surgeons in Ireland (RCSI),

123 St. Stephens Green, D02 YN77 Dublin, Ireland.

AD - Department of Clinical Medicine, Trinity Translational Medicine Institute, St.

James's Hospital, Trinity College Dublin, The University of Dublin, D08 9WRT

Dublin, Ireland.

FAU - Fattah, Sarinj

AU - Fattah S

AD - School of Pharmacy and Biomolecular Sciences, Royal College of Surgeons in

Ireland (RCSI), 123 St. Stephens Green, D02 YN77 Dublin, Ireland.

AD - Tissue Engineering Research Group, Royal College of Surgeons in Ireland (RCSI),

123 St. Stephens Green, D02 YN77 Dublin, Ireland.

FAU - O'Sullivan, Andrew

AU - O'Sullivan A

AUID- ORCID: 0000-0002-5434-1260

AD - School of Pharmacy and Biomolecular Sciences, Royal College of Surgeons in

Ireland (RCSI), 123 St. Stephens Green, D02 YN77 Dublin, Ireland.

AD - Research and Development, Science and Emerging Technologies, Aerogen Ltd., Galway

Business Park, Dangan, H91 HE94 Galway, Ireland.

FAU - Cavanagh, Brenton

AU - Cavanagh B

AD - Cellular and Molecular Imaging Core, Royal College of Surgeons in Ireland RCSI,

D02 YN77 Dublin, Ireland.

FAU - MacLoughlin, Ronan

AU - MacLoughlin R

AUID- ORCID: 0000-0002-3164-1607

AD - School of Pharmacy and Biomolecular Sciences, Royal College of Surgeons in

Ireland (RCSI), 123 St. Stephens Green, D02 YN77 Dublin, Ireland.

AD - Research and Development, Science and Emerging Technologies, Aerogen Ltd., Galway

Business Park, Dangan, H91 HE94 Galway, Ireland.

AD - School of Pharmacy and Pharmaceutical Sciences, Trinity College, D02 PN40 Dublin,

Ireland.

FAU - Keane, Joseph

AU - Keane J

AD - Department of Clinical Medicine, Trinity Translational Medicine Institute, St.

James's Hospital, Trinity College Dublin, The University of Dublin, D08 9WRT

Dublin, Ireland.

FAU - O'Sullivan, Mary P

AU - O'Sullivan MP

AUID- ORCID: 0000-0002-6104-8943

AD - Department of Clinical Medicine, Trinity Translational Medicine Institute, St.

James's Hospital, Trinity College Dublin, The University of Dublin, D08 9WRT

Dublin, Ireland.

FAU - Cryan, Sally-Ann

AU - Cryan SA

AD - School of Pharmacy and Biomolecular Sciences, Royal College of Surgeons in

Ireland (RCSI), 123 St. Stephens Green, D02 YN77 Dublin, Ireland.

AD - Tissue Engineering Research Group, Royal College of Surgeons in Ireland (RCSI),

123 St. Stephens Green, D02 YN77 Dublin, Ireland.

AD - SFI Advanced Materials and Bioengineering Research (AMBER) Centre, RCSI and

Trinity College Dublin, D02 PN40 Dublin, Ireland.

AD - SFI Centre for Research in Medical Devices (CÚRAM), NUIG & RCSI, H91 W2TY Galway,

Ireland.

LA - eng

GR - 19179A06/Royal College of Surgeons in Ireland/

PT - Journal Article

DEP - 20220821

PL - Switzerland

TA - Pharmaceutics

JT - Pharmaceutics

JID - 101534003

PMC - PMC9415714

OTO - NOTNLM

OT - ATRA

OT - PLGA

OT - all trans retinoic acid

OT - host-directed therapy

OT - immunotherapy

OT - inhalation

OT - nanoparticles

OT - nebulization

OT - pulmonary drug delivery

OT - tuberculosis

COIS- Ronan MacLoughlin and Andrew O’Sullivan reports an employment relationship with

Aerogen Ltd. Other Authors declare no conflict of interest.

EDAT- 2022/08/27 06:00

MHDA- 2022/08/27 06:01

PMCR- 2022/08/21

CRDT- 2022/08/26 01:42

PHST- 2022/07/26 00:00 [received]

PHST- 2022/08/11 00:00 [revised]

PHST- 2022/08/20 00:00 [accepted]

PHST- 2022/08/26 01:42 [entrez]

PHST- 2022/08/27 06:00 [pubmed]

PHST- 2022/08/27 06:01 [medline]

PHST- 2022/08/21 00:00 [pmc-release]

AID - pharmaceutics14081745 [pii]

AID - pharmaceutics-14-01745 [pii]

AID - 10.3390/pharmaceutics14081745 [doi]

PST - epublish

SO - Pharmaceutics. 2022 Aug 21;14(8):1745. doi: 10.3390/pharmaceutics14081745.

PMID- 35915958

OWN - NLM

STAT- MEDLINE

DCOM- 20220812

LR - 20250728

IS - 1520-4804 (Electronic)

IS - 0022-2623 (Print)

IS - 0022-2623 (Linking)

VI - 65

IP - 15

DP - 2022 Aug 11

TI - Design, Synthesis, and Biological Evaluation of Pyrrole-2-carboxamide Derivatives

as Mycobacterial Membrane Protein Large 3 Inhibitors for Treating Drug-Resistant

Tuberculosis.

PG - 10534-10553

LID - 10.1021/acs.jmedchem.2c00718 [doi]

AB - In this work, pyrrole-2-carboxamides were designed with a structure-guided

strategy based on the crystal structure of MmpL3 and a pharmacophore model. The

structure-activity relationship studies revealed that attaching phenyl and

pyridyl groups with electron-withdrawing substituents to the pyrrole ring and

attaching bulky substituents to the carboxamide greatly improved anti-TB

activity. Most compounds showed potent anti-TB activity (MIC < 0.016 μg/mL) and

low cytotoxicity (IC(50) > 64 μg/mL). Compound 32 displayed excellent activity

against drug-resistant tuberculosis, good microsomal stability, almost no

inhibition of the hERG K(+) channel, and good in vivo efficacy. Furthermore, the

target of the pyrrole-2-carboxamides was identified by measuring their potency

against M. smegmatis expressing wild-type and mutated variants of the mmpL3 gene

from M. tuberculosis (mmpL3tb) and determining their effect on mycolic acid

biosynthesis using a [(14)C] acetate metabolic labeling assay. The present study

provides new MmpL3 inhibitors that are promising anti-TB agents.

FAU - Zhao, Hongyi

AU - Zhao H

AD - Beijing Key Laboratory of Active Substance Discovery and Druggability Evaluation,

Chinese Academy of Medical Sciences Key Laboratory of Anti-DR TB Innovative Drug

Research, Institute of Materia Medica, Peking Union Medical College and Chinese

Academy of Medical Sciences, 1 Xian Nong Tan Street, Beijing 100050, P. R. China.

FAU - Gao, Yongxin

AU - Gao Y

AD - Beijing Key Laboratory of Active Substance Discovery and Druggability Evaluation,

Chinese Academy of Medical Sciences Key Laboratory of Anti-DR TB Innovative Drug

Research, Institute of Materia Medica, Peking Union Medical College and Chinese

Academy of Medical Sciences, 1 Xian Nong Tan Street, Beijing 100050, P. R. China.

FAU - Li, Wei

AU - Li W

AD - Mycobacteria Research Laboratories, Department of Microbiology, Immunology and

Pathology, Colorado State University, Fort Collins, Colorado 80523, United

States.

FAU - Sheng, Li

AU - Sheng L

AD - Beijing Key Laboratory of Non-Clinical Drug Metabolism and PK/PD Study, Chinese

Academy of Medical Sciences Key Laboratory of Anti-DR TB Innovative Drug

Research, Institute of Materia Medica, Peking Union Medical College and Chinese

Academy of Medical Sciences, 1 Xian Nong Tan Street, Beijing 100050, P. R. China.

FAU - Cui, Keli

AU - Cui K

AD - College of Life Science and Bio-engineering, Beijing University of Technology,

100 Ping Le Yuan, Beijing 100124, P. R. China.

FAU - Wang, Bin

AU - Wang B

AD - Beijing Key Laboratory of Drug Resistance Tuberculosis Research, Department of

Pharmacology, Beijing Tuberculosis and Thoracic Tumor Research Institute, Beijing

Chest Hospital, Capital Medical University, 97 Ma Chang Street, Beijing 101149,

P. R. China.

FAU - Fu, Lei

AU - Fu L

AD - Beijing Key Laboratory of Drug Resistance Tuberculosis Research, Department of

Pharmacology, Beijing Tuberculosis and Thoracic Tumor Research Institute, Beijing

Chest Hospital, Capital Medical University, 97 Ma Chang Street, Beijing 101149,

P. R. China.

FAU - Gao, Meng

AU - Gao M

AD - Beijing Key Laboratory of Active Substance Discovery and Druggability Evaluation,

Chinese Academy of Medical Sciences Key Laboratory of Anti-DR TB Innovative Drug

Research, Institute of Materia Medica, Peking Union Medical College and Chinese

Academy of Medical Sciences, 1 Xian Nong Tan Street, Beijing 100050, P. R. China.

FAU - Lin, Ziyun

AU - Lin Z

AD - Beijing Key Laboratory of Active Substance Discovery and Druggability Evaluation,

Chinese Academy of Medical Sciences Key Laboratory of Anti-DR TB Innovative Drug

Research, Institute of Materia Medica, Peking Union Medical College and Chinese

Academy of Medical Sciences, 1 Xian Nong Tan Street, Beijing 100050, P. R. China.

FAU - Zou, Xiaowen

AU - Zou X

AD - Beijing Key Laboratory of Non-Clinical Drug Metabolism and PK/PD Study, Chinese

Academy of Medical Sciences Key Laboratory of Anti-DR TB Innovative Drug

Research, Institute of Materia Medica, Peking Union Medical College and Chinese

Academy of Medical Sciences, 1 Xian Nong Tan Street, Beijing 100050, P. R. China.

FAU - Jackson, Mary

AU - Jackson M

AUID- ORCID: 0000-0002-9212-0258

AD - Mycobacteria Research Laboratories, Department of Microbiology, Immunology and

Pathology, Colorado State University, Fort Collins, Colorado 80523, United

States.

FAU - Huang, Haihong

AU - Huang H

AD - Beijing Key Laboratory of Active Substance Discovery and Druggability Evaluation,

Chinese Academy of Medical Sciences Key Laboratory of Anti-DR TB Innovative Drug

Research, Institute of Materia Medica, Peking Union Medical College and Chinese

Academy of Medical Sciences, 1 Xian Nong Tan Street, Beijing 100050, P. R. China.

FAU - Lu, Yu

AU - Lu Y

AD - Beijing Key Laboratory of Drug Resistance Tuberculosis Research, Department of

Pharmacology, Beijing Tuberculosis and Thoracic Tumor Research Institute, Beijing

Chest Hospital, Capital Medical University, 97 Ma Chang Street, Beijing 101149,

P. R. China.

FAU - Zhang, Dongfeng

AU - Zhang D

AUID- ORCID: 0000-0003-0870-3782

AD - Beijing Key Laboratory of Active Substance Discovery and Druggability Evaluation,

Chinese Academy of Medical Sciences Key Laboratory of Anti-DR TB Innovative Drug

Research, Institute of Materia Medica, Peking Union Medical College and Chinese

Academy of Medical Sciences, 1 Xian Nong Tan Street, Beijing 100050, P. R. China.

LA - eng

GR - R01 AI116525/AI/NIAID NIH HHS/United States

PT - Journal Article

PT - Research Support, N.I.H., Extramural

PT - Research Support, Non-U.S. Gov't

DEP - 20220801

PL - United States

TA - J Med Chem

JT - Journal of medicinal chemistry

JID - 9716531

RN - 0 (Antitubercular Agents)

RN - 0 (Bacterial Proteins)

RN - 0 (Membrane Proteins)

RN - 0 (Pyrroles)

SB - IM

MH - Antitubercular Agents/pharmacology/therapeutic use

MH - Bacterial Proteins/metabolism

MH - Humans

MH - Membrane Proteins/metabolism

MH - Microbial Sensitivity Tests

MH - *Mycobacterium tuberculosis

MH - Pyrroles/chemistry

MH - Structure-Activity Relationship

MH - *Tuberculosis, Multidrug-Resistant

PMC - PMC9379527

MID - NIHMS1828531

COIS- The authors declare no competing financial interest.

EDAT- 2022/08/03 06:00

MHDA- 2022/08/13 06:00

PMCR- 2023/08/11

CRDT- 2022/08/02 02:52

PHST- 2022/08/03 06:00 [pubmed]

PHST- 2022/08/13 06:00 [medline]

PHST- 2022/08/02 02:52 [entrez]

PHST- 2023/08/11 00:00 [pmc-release]

AID - 10.1021/acs.jmedchem.2c00718 [doi]

PST - ppublish

SO - J Med Chem. 2022 Aug 11;65(15):10534-10553. doi: 10.1021/acs.jmedchem.2c00718.

Epub 2022 Aug 1.

PMID- 35597009

OWN - NLM

STAT- MEDLINE

DCOM- 20220623

LR - 20220623

IS - 1768-3254 (Electronic)

IS - 0223-5234 (Linking)

VI - 238

DP - 2022 Aug 5

TI - Emerging impact of triazoles as anti-tubercular agent.

PG - 114454

LID - S0223-5234(22)00356-7 [pii]

LID - 10.1016/j.ejmech.2022.114454 [doi]

AB - Tuberculosis, a disease of poverty is a communicable infection with a reasonably

high mortality rate worldwide. 10 Million new cases of TB were reported with

approx 1.4 million deaths in the year 2019. Due to the growing number of

drug-sensitive and drug-resistant tuberculosis cases, there is a vital need to

develop new and effective candidates useful to combat this deadly disease.

Despite tremendous efforts to identify a mechanism-based novel antitubercular

agent, only a few have entered into clinical trials in the last six decades. In

recent years, triazoles have been well explored as the most valuable scaffolds in

drug discovery and development. Triazole framework possesses favorable properties

like hydrogen bonding, moderate dipole moment, enhanced water solubility, and

also the ability to bind effectively with biomolecular targets of M. tuberculosis

and therefore this scaffold displayed excellent potency against TB. This review

is an endeavor to summarize an up-to-date innovation of triazole-appended hybrids

during the last 10 years having potential in vitro and in vivo antitubercular

activity with structure activity relationship analysis. This review may help

medicinal chemists to explore the triazole scaffolds for the rational design of

potent drug candidates having better efficacy, improved selectivity and minimal

toxicity so that these hybrid NCEs can effectively be explored as potential lead

to fight against M. tuberculosis.

CI - Copyright © 2022 Elsevier Masson SAS. All rights reserved.

FAU - Sharma, Anindra

AU - Sharma A

AD - Department of Chemistry, A.P.S.M. College, Barauni (A Constituent Unit of Lalit

Narayan Mithila University, Darbhanga), Begusarai, 851112, Bihar, India.

Electronic address: anindrasharma2007@gmail.com.

FAU - Agrahari, Anand K

AU - Agrahari AK

AD - Department of Chemistry, Institute of Science, Banaras Hindu University,

Varanasi, 221005, India.

FAU - Rajkhowa, Sanchayita

AU - Rajkhowa S

AD - Department of Chemistry, The Assam Royal Global University, Guwahati, 781035,

Assam, India.

FAU - Tiwari, Vinod K

AU - Tiwari VK

AD - Department of Chemistry, Institute of Science, Banaras Hindu University,

Varanasi, 221005, India. Electronic address: Vinod.Tiwari@bhu.ac.in.

LA - eng

PT - Journal Article

PT - Review

DEP - 20220513

PL - France

TA - Eur J Med Chem

JT - European journal of medicinal chemistry

JID - 0420510

RN - 0 (Antitubercular Agents)

RN - 0 (Triazoles)

SB - IM

MH - Antitubercular Agents/chemistry

MH - Humans

MH - Microbial Sensitivity Tests

MH - *Mycobacterium tuberculosis

MH - Structure-Activity Relationship

MH - Triazoles/chemistry

MH - *Tuberculosis/drug therapy

OTO - NOTNLM

OT - Click chemistry

OT - Drug development

OT - Molecular hybridization

OT - Triazole

OT - Tuberculosis

EDAT- 2022/05/22 06:00

MHDA- 2022/06/24 06:00

CRDT- 2022/05/21 18:16

PHST- 2021/07/05 00:00 [received]

PHST- 2022/05/04 00:00 [revised]

PHST- 2022/05/08 00:00 [accepted]

PHST- 2022/05/22 06:00 [pubmed]

PHST- 2022/06/24 06:00 [medline]

PHST- 2022/05/21 18:16 [entrez]

AID - S0223-5234(22)00356-7 [pii]

AID - 10.1016/j.ejmech.2022.114454 [doi]

PST - ppublish

SO - Eur J Med Chem. 2022 Aug 5;238:114454. doi: 10.1016/j.ejmech.2022.114454. Epub

2022 May 13.

PMID- 35866418

OWN - NLM

STAT- MEDLINE

DCOM- 20220815

LR - 20250728

IS - 1756-8927 (Electronic)

IS - 1756-8919 (Linking)

VI - 14

IP - 15

DP - 2022 Aug

TI - Recent advances in oxazolidinones as antituberculosis agents.

PG - 1149-1165

LID - 10.4155/fmc-2022-0079 [doi]

AB - Tuberculosis (TB) is an infectious and fatal disease caused by Mycobacterium

tuberculosis (Mtb) and remains a serious public health threat; therefore, the

development of new antitubercular agents is a priority for the World Health

Organization's End TB strategy and the United Nations' Sustainable Development

Goals to eradicate TB. Oxazolidinones are a class of synthetic antibacterial

agents with a distinct mode of action developed for the treatment of

Gram-positive bacterial infections. Many oxazolidinones exhibit good activity

against Mtb, and some are currently in clinical trials for multidrug-resistant

TB and extensively drug-resistant TB therapy. In this review, the mechanism of

action, activity and toxicity of oxazolidinones and recent progress in the

research and development of oxazolidinones as anti-TB agents are summarized.

FAU - Lu, Haijia

AU - Lu H

AD - Beijing Key Laboratory of Active Substance Discovery & Druggability Evaluation,

Chinese Academy of Medical Sciences Key Laboratory of Anti-DR TB Innovative Drug

Research, Institute of Materia Medica, Peking Union Medical College & Chinese

Academy of Medical Sciences, 1 Xian Nong Tan Street, Beijing, 100050, China.

FAU - Wang, Huahao

AU - Wang H

AD - Beijing Key Laboratory of Active Substance Discovery & Druggability Evaluation,

Chinese Academy of Medical Sciences Key Laboratory of Anti-DR TB Innovative Drug

Research, Institute of Materia Medica, Peking Union Medical College & Chinese

Academy of Medical Sciences, 1 Xian Nong Tan Street, Beijing, 100050, China.

FAU - Zhao, Hongyi

AU - Zhao H

AD - Beijing Key Laboratory of Active Substance Discovery & Druggability Evaluation,

Chinese Academy of Medical Sciences Key Laboratory of Anti-DR TB Innovative Drug

Research, Institute of Materia Medica, Peking Union Medical College & Chinese

Academy of Medical Sciences, 1 Xian Nong Tan Street, Beijing, 100050, China.

FAU - Zhang, Dongfeng

AU - Zhang D

AUID- ORCID: 0000-0003-0870-3782

AD - Beijing Key Laboratory of Active Substance Discovery & Druggability Evaluation,

Chinese Academy of Medical Sciences Key Laboratory of Anti-DR TB Innovative Drug

Research, Institute of Materia Medica, Peking Union Medical College & Chinese

Academy of Medical Sciences, 1 Xian Nong Tan Street, Beijing, 100050, China.

LA - eng

PT - Journal Article

PT - Research Support, Non-U.S. Gov't

PT - Review

DEP - 20220722

PL - England

TA - Future Med Chem

JT - Future medicinal chemistry

JID - 101511162

RN - 0 (Antitubercular Agents)

RN - 0 (Oxazolidinones)

SB - IM

MH - Antitubercular Agents/pharmacology/therapeutic use

MH - Humans

MH - *Mycobacterium tuberculosis

MH - *Oxazolidinones/pharmacology/therapeutic use

MH - *Tuberculosis/drug therapy/microbiology

MH - *Tuberculosis, Multidrug-Resistant/drug therapy

OTO - NOTNLM

OT - Mycobacterium tuberculosis

OT - antituberculosis agents

OT - mechanism of action

OT - oxazolidinones

OT - toxicity

EDAT- 2022/07/23 06:00

MHDA- 2022/08/16 06:00

CRDT- 2022/07/22 05:03

PHST- 2022/07/23 06:00 [pubmed]

PHST- 2022/08/16 06:00 [medline]

PHST- 2022/07/22 05:03 [entrez]

AID - 10.4155/fmc-2022-0079 [doi]

PST - ppublish

SO - Future Med Chem. 2022 Aug;14(15):1149-1165. doi: 10.4155/fmc-2022-0079. Epub 2022

Jul 22.

PMID- 35577251

OWN - NLM

STAT- MEDLINE

DCOM- 20220614

LR - 20220614

IS - 1878-3511 (Electronic)

IS - 1201-9712 (Linking)

VI - 121

DP - 2022 Aug

TI - Minimum inhibitory concentration of cycloserine against Mycobacterium

tuberculosis using the MGIT 960 system and a proposed critical concentration.

PG - 148-151

LID - S1201-9712(22)00293-4 [pii]

LID - 10.1016/j.ijid.2022.05.030 [doi]

AB - OBJECTIVES: We aimed to determine the breakpoint of cycloserine (CS)

susceptibility in MGIT and to describe the molecular characteristics of

CS-resistant Mycobacterium tuberculosis (MTB) isolates. METHODS: A total of 124

MTB isolates were recruited in our analysis. Minimum inhibitory concentration

(MIC) was determined using the MGIT system. The mutations of MTB isolates within

alr, ddl, ald, and cycA, potentially conferring CS resistance were analyzed by

the whole-genome sequencing. RESULTS: In vitro drug susceptibility testing of

isolates with doubling concentrations of CS revealed that the modal MIC values

was 4 mg/L for MGIT, accounting for 35.5% (44/124) of isolates tested. Seven

isolates harbored mutations conferring CS resistance, consisting of five with alr

mutations and two with ald mutations. On the basis of the MIC distributions of

wild-type and resistotype populations, we proposed a tentative epidemiologic

cut-off value of 16 mg/l. The proportion of CS resistance in extensively

drug-resistant TB was significantly higher than that of multidrug-resistant TB.

CONCLUSION: In conclusion, we propose critical concentration for MGIT 960 to

properly diagnose CS-resistant MTB and demonstrate that mutations in alr and ald

genes are the major mechanism conferring CS resistance in clinical isolates.

CI - Copyright © 2022 The Authors. Published by Elsevier Ltd.. All rights reserved.

FAU - Wu, Xiao

AU - Wu X

AD - Department of Bacteriology and Immunology, Beijing Key Laboratory on

Drug-Resistant Tuberculosis Research, Beijing Tuberculosis and Thoracic Tumor

Research Institute/Beijing Chest Hospital, Capital Medical University, Beijing,

People's Republic of China.

FAU - Shang, Yuanyuan

AU - Shang Y

AD - Department of Bacteriology and Immunology, Beijing Key Laboratory on

Drug-Resistant Tuberculosis Research, Beijing Tuberculosis and Thoracic Tumor

Research Institute/Beijing Chest Hospital, Capital Medical University, Beijing,

People's Republic of China.

FAU - Ren, Weicong

AU - Ren W

AD - Department of Bacteriology and Immunology, Beijing Key Laboratory on

Drug-Resistant Tuberculosis Research, Beijing Tuberculosis and Thoracic Tumor

Research Institute/Beijing Chest Hospital, Capital Medical University, Beijing,

People's Republic of China.

FAU - Wang, Wei

AU - Wang W

AD - Department of Bacteriology and Immunology, Beijing Key Laboratory on

Drug-Resistant Tuberculosis Research, Beijing Tuberculosis and Thoracic Tumor

Research Institute/Beijing Chest Hospital, Capital Medical University, Beijing,

People's Republic of China.

FAU - Wang, Yufeng

AU - Wang Y

AD - Innovation Alliance on Tuberculosis Diagnosis and Treatment, Beijing, People's

Republic of China.

FAU - Xue, Zhongtan

AU - Xue Z

AD - Department of Bacteriology and Immunology, Beijing Key Laboratory on

Drug-Resistant Tuberculosis Research, Beijing Tuberculosis and Thoracic Tumor

Research Institute/Beijing Chest Hospital, Capital Medical University, Beijing,

People's Republic of China.

FAU - Li, Shanshan

AU - Li S

AD - Department of Bacteriology and Immunology, Beijing Key Laboratory on

Drug-Resistant Tuberculosis Research, Beijing Tuberculosis and Thoracic Tumor

Research Institute/Beijing Chest Hospital, Capital Medical University, Beijing,

People's Republic of China. Electronic address: lss9011@126.com.

FAU - Pang, Yu

AU - Pang Y

AD - Department of Bacteriology and Immunology, Beijing Key Laboratory on

Drug-Resistant Tuberculosis Research, Beijing Tuberculosis and Thoracic Tumor

Research Institute/Beijing Chest Hospital, Capital Medical University, Beijing,

People's Republic of China. Electronic address: pangyupound@163.com.

LA - eng

PT - Journal Article

DEP - 20220513

PL - Canada

TA - Int J Infect Dis

JT - International journal of infectious diseases : IJID : official publication of the

International Society for Infectious Diseases

JID - 9610933

RN - 0 (Antitubercular Agents)

RN - 95IK5KI84Z (Cycloserine)

SB - IM

MH - Antitubercular Agents/pharmacology/therapeutic use

MH - Cycloserine/pharmacology

MH - Humans

MH - Microbial Sensitivity Tests

MH - Mutation

MH - *Mycobacterium tuberculosis

MH - *Tuberculosis, Lymph Node/drug therapy

MH - *Tuberculosis, Multidrug-Resistant/microbiology

OTO - NOTNLM

OT - Cycloserine

OT - Drug susceptibility testing

OT - Tuberculosis

COIS- Conflict of interest The authors have no conflict of interests to declare.

EDAT- 2022/05/17 06:00

MHDA- 2022/06/15 06:00

CRDT- 2022/05/16 19:35

PHST- 2022/04/06 00:00 [received]

PHST- 2022/05/10 00:00 [revised]

PHST- 2022/05/10 00:00 [accepted]

PHST- 2022/05/17 06:00 [pubmed]

PHST- 2022/06/15 06:00 [medline]

PHST- 2022/05/16 19:35 [entrez]

AID - S1201-9712(22)00293-4 [pii]

AID - 10.1016/j.ijid.2022.05.030 [doi]

PST - ppublish

SO - Int J Infect Dis. 2022 Aug;121:148-151. doi: 10.1016/j.ijid.2022.05.030. Epub

2022 May 13.

PMID- 35957807

OWN - NLM

STAT- PubMed-not-MEDLINE

LR - 20220813

IS - 2214-2509 (Print)

IS - 2214-2509 (Electronic)

IS - 2214-2509 (Linking)

VI - 29

DP - 2022

TI - Anemia with elevation of growth differentiation factor-15 level in linezolid

treated multidrug-resistant tuberculosis: Case series of three patients.

PG - e01591

LID - 10.1016/j.idcr.2022.e01591 [doi]

LID - e01591

AB - Linezolid is now recommended as a first line drug for Multidrug Resistant

Tuberculosis (MDR-TB). Previous studies reported hematologic toxicity as one of

the main side effects. The mechanism of this toxicity is mitochondrial

dysfunction, for which a biomarker is Growth differentiation factor-15 (GDF-15).

There is no previous report about GDF-15 and its association with hematologic

toxicity from Linezolid in the treatment of MDR-TB. We present three cases of

MDR-TB involving severe anemia associated with linezolid who had GDF-15

elevation. These cases highlight the need for more research into the relationship

between GDF-15 and hematologic toxicity in MDR-TB patients treated with

linezolid.

CI - © 2022 The Authors.

FAU - Oehadian, Amaylia

AU - Oehadian A

AD - Division of Hematology and Oncology Medic, Department of Internal Medicine

Faculty of Medicine, Universitas Padjadjaran/ Dr. Hasan Sadikin General Hospital,

Bandung, Indonesia.

AD - Doctoral Study Program, Faculty of Medicine, Universitas Padjadjaran, Bandung,

West Java, Indonesia.

FAU - Santoso, Prayudi

AU - Santoso P

AD - Division of Respirology and Critical Care, Department of Internal Medicine

Faculty of Medicine, Universitas Padjadjaran/ Dr. Hasan Sadikin General Hospital,

Bandung, Indonesia.

FAU - Menzies, Dick

AU - Menzies D

AD - McGill International TB Centre Respiratory Epidemiology and Clinical Research

Unit, Montreal, Canada.

FAU - Ruslami, Rovina

AU - Ruslami R

AD - Department of Biomedical Science, Division of Pharmacology, Universitas

Padjadjaran/ Dr. Hasan Sadikin General Hospital, Bandung, Indonesia.

LA - eng

PT - Case Reports

PT - Journal Article

DEP - 20220729

PL - Netherlands

TA - IDCases

JT - IDCases

JID - 101634540

PMC - PMC9358446

OTO - NOTNLM

OT - Anemia

OT - Case Series

OT - Growth Differentiation Factor-15

OT - Linezolid

OT - Multi-Drug Resistant Tuberculosis

COIS- The authors report no conflicts of interest.

EDAT- 2022/08/13 06:00

MHDA- 2022/08/13 06:01

PMCR- 2022/07/29

CRDT- 2022/08/12 02:46

PHST- 2022/06/09 00:00 [received]

PHST- 2022/07/28 00:00 [accepted]

PHST- 2022/08/12 02:46 [entrez]

PHST- 2022/08/13 06:00 [pubmed]

PHST- 2022/08/13 06:01 [medline]

PHST- 2022/07/29 00:00 [pmc-release]

AID - S2214-2509(22)00219-0 [pii]

AID - e01591 [pii]

AID - 10.1016/j.idcr.2022.e01591 [doi]

PST - epublish

SO - IDCases. 2022 Jul 29;29:e01591. doi: 10.1016/j.idcr.2022.e01591. eCollection

2022.

PMID- 35992375

OWN - NLM

STAT- PubMed-not-MEDLINE

LR - 20240902

IS - 2590-2571 (Electronic)

IS - 2590-2571 (Linking)

VI - 3

DP - 2022

TI - Indole: A promising scaffold for the discovery and development of potential

anti-tubercular agents.

PG - 100119

LID - 10.1016/j.crphar.2022.100119 [doi]

LID - 100119

AB - Indole-containing small molecules have been reported to have diverse

pharmacological activities. The aromatic heterocyclic scaffold, which resembles

various protein structures, has received attention from organic and medicinal

chemists. Exploration of indole derivatives in drug discovery has rapidly yielded

a vast array of biologically active compounds with broad therapeutic potential.

Nature is the major source of indole scaffolds, but various classical and

advanced synthesis methods for indoles have also been reported. One-pot synthesis

is widely considered an efficient approach in synthetic organic chemistry and has

been used to synthesize some indole compounds. The rapid emergence of

drug-resistant tuberculosis is a major challenge to be addressed. Identifying

novel targets and drug candidates for tuberculosis is therefore crucial.

Researchers have extensively explored indole derivatives as potential

anti-tubercular agents or drugs. Indole scaffolds containing the novel

non-covalent (decaprenylphosphoryl-β-D-ribose2'-epimerase) DprE1 inhibitor

1,4-azaindole is currently in clinical trials to treat Mycobacterium

tuberculosis. In addition, DG167 indazole sulfonamide with potent anti-tubercular

activity is undergoing early-stage development in preclinical studies. Indole

bearing cationic amphiphiles with high chemical diversity have been reported to

depolarize and disrupt the mycobacterial membrane. Some indole-based compounds

have potential inhibitory activities against distinct anti-tubercular targets,

including the inhibition of cell wall synthesis, replication, transcription, and

translation, as summarized in the graphical abstract. The success of

computer-aided drug design in the fields of cancer and anti-viral drugs has

accelerated in silico studies in antibacterial drug development. This review

describes the sources of indole scaffolds, the potential for novel indole

derivatives to serve as anti-tubercular agents, in silico findings, and proposed

actions to facilitate the design of novel compounds with anti-tubercular

activity.

CI - © 2022 The Authors.

FAU - Bajad, Nilesh Gajanan

AU - Bajad NG

AD - Department of Pharmaceutical Engineering & Technology, Indian Institute of

Technology (Banaras Hindu University), Varanasi, 221005, India.

FAU - Singh, Sudhir Kumar

AU - Singh SK

AD - Department of Microbiology, Institute of Medical Sciences, Banaras Hindu

University, Varanasi, 221005, India.

FAU - Singh, Sushil Kumar

AU - Singh SK

AD - Department of Pharmaceutical Engineering & Technology, Indian Institute of

Technology (Banaras Hindu University), Varanasi, 221005, India.

FAU - Singh, Tryambak Deo

AU - Singh TD

AD - Department of Medicinal Chemistry, Faculty of Ayurveda, Institute of Medical

Sciences, Banaras Hindu University, Varanasi, 221005, India.

FAU - Singh, Meenakshi

AU - Singh M

AD - Department of Medicinal Chemistry, Faculty of Ayurveda, Institute of Medical

Sciences, Banaras Hindu University, Varanasi, 221005, India.

LA - eng

PT - Journal Article

PT - Review

DEP - 20220716

PL - Netherlands

TA - Curr Res Pharmacol Drug Discov

JT - Current research in pharmacology and drug discovery

JID - 9918300982506676

PMC - PMC9389259

OTO - NOTNLM

OT - CM, chorismate mutase

OT - Clinical trials

OT - Computer-aided drug design

OT - DHFR, Dihydrofolate reductase

OT - DprE1, decaprenylphosphoryl-β-D-ribose2′-epimerase

OT - Drug development

OT - Drug discovery

OT - In silico

OT - In vitro

OT - Indole

OT - Indole alkaloids

OT - KasA, β-ketoacyl ACP synthase I

OT - MmpL3, mycobacterial membrane protein large 3

OT - SBDD, Structure-based drug design

OT - Synthesis

OT - Tuberculosis

COIS- The authors declare that they have no known competing financial interests or

personal relationships that could have appeared to influence the work reported in

this paper.

EDAT- 2022/08/23 06:00

MHDA- 2022/08/23 06:01

PMCR- 2022/07/16

CRDT- 2022/08/22 04:26

PHST- 2021/12/27 00:00 [received]

PHST- 2022/04/13 00:00 [revised]

PHST- 2022/07/05 00:00 [accepted]

PHST- 2022/08/22 04:26 [entrez]

PHST- 2022/08/23 06:00 [pubmed]

PHST- 2022/08/23 06:01 [medline]

PHST- 2022/07/16 00:00 [pmc-release]

AID - S2590-2571(22)00039-6 [pii]

AID - 100119 [pii]

AID - 10.1016/j.crphar.2022.100119 [doi]

PST - epublish

SO - Curr Res Pharmacol Drug Discov. 2022 Jul 16;3:100119. doi:

10.1016/j.crphar.2022.100119. eCollection 2022.

PMID- 35898907

OWN - NLM

STAT- PubMed-not-MEDLINE

LR - 20220729

IS - 1664-302X (Print)

IS - 1664-302X (Electronic)

IS - 1664-302X (Linking)

VI - 13

DP - 2022

TI - Deficiency of GntR Family Regulator MSMEG_5174 Promotes Mycobacterium smegmatis

Resistance to Aminoglycosides via Manipulating Purine Metabolism.

PG - 919538

LID - 10.3389/fmicb.2022.919538 [doi]

LID - 919538

AB - The increasing incidence of drug-resistant tuberculosis is still an emergency for

global public health and a major obstacle to tuberculosis treatment. Therefore,

deciphering the novel mechanisms of mycobacterial antibiotic resistance is

crucial for combatting the rapid emergence of drug-resistant strains. In this

study, we identified an unexpected role of Mycobacterium smegmatis GntR family

transcriptional regulator MSMEG_5174 and its homologous gene Mycobacterium

tuberculosis Rv1152 in aminoglycoside antibiotic resistance. Deficiency of

MSMEG_5174 rendered Mycobacterium smegmatis highly resistant to aminoglycoside

antibiotic treatment, and ectopic expression of Rv1152 in MSMEG_5174 mutants

restored antibiotic-induced bacterial killing. We further demonstrated that

MSMEG_5174 negatively regulates the expression of purine metabolism-related genes

and the accumulation of purine metabolites. Moreover, overexpression of xanthine

dehydrogenase MSMEG_0871 or xanthine treatment elicited a significant decrease in

aminoglycoside antibiotic lethality for Mycobacterium smegmatis. Together, our

findings revealed MSMEG_5174 as a metabolic regulator and hint toward unexplored

crosstalk between purine metabolism and antibiotic resistance.

CI - Copyright © 2022 Deng, Zheng, Chen, Yang, Yan, Li, Zeng, Xie, Gong and Zeng.

FAU - Deng, Wanyan

AU - Deng W

AD - The Joint Center for Infection and Immunity, Guangzhou Institute of Pediatrics,

Guangzhou Women and Children's Medical Center, Guangzhou, China.

AD - Institut Pasteur of Shanghai, Chinese Academy of Sciences, Shanghai, China.

AD - Key Laboratory of Molecular Biology for Infectious Diseases (Ministry of

Education), Department of Infectious Diseases, Institute for Viral Hepatitis, The

Second Affiliated Hospital of Chongqing Medical University, Chongqing, China.

FAU - Zheng, Zengzhang

AU - Zheng Z

AD - The Joint Center for Infection and Immunity, Guangzhou Institute of Pediatrics,

Guangzhou Women and Children's Medical Center, Guangzhou, China.

AD - Institut Pasteur of Shanghai, Chinese Academy of Sciences, Shanghai, China.

FAU - Chen, Yi

AU - Chen Y

AD - Key Laboratory of Molecular Biology for Infectious Diseases (Ministry of

Education), Department of Infectious Diseases, Institute for Viral Hepatitis, The

Second Affiliated Hospital of Chongqing Medical University, Chongqing, China.

FAU - Yang, Maoyi

AU - Yang M

AD - Key Laboratory of Molecular Biology for Infectious Diseases (Ministry of

Education), Department of Infectious Diseases, Institute for Viral Hepatitis, The

Second Affiliated Hospital of Chongqing Medical University, Chongqing, China.

FAU - Yan, Jun

AU - Yan J

AD - Key Laboratory of Molecular Biology for Infectious Diseases (Ministry of

Education), Department of Infectious Diseases, Institute for Viral Hepatitis, The

Second Affiliated Hospital of Chongqing Medical University, Chongqing, China.

FAU - Li, Wu

AU - Li W

AD - The Joint Center for Infection and Immunity, Guangzhou Institute of Pediatrics,

Guangzhou Women and Children's Medical Center, Guangzhou, China.

AD - Institut Pasteur of Shanghai, Chinese Academy of Sciences, Shanghai, China.

FAU - Zeng, Jie

AU - Zeng J

AD - Department of Respiratory Medicine, The First People's Hospital of Yunnan

Province, Kunming, China.

AD - Affiliated Hospital of Kunming University of Science and Technology, Kunming,

China.

FAU - Xie, Jianping

AU - Xie J

AD - State Key Laboratory Breeding Base of Eco-Environment and Bio-Resource of the

Three Gorges Area, Key Laboratory of Eco-Environments in Three Gorges Reservoir

Region, Ministry of Education, School of Life Sciences, Institute of Modern

Biopharmaceuticals, Southwest University, Chongqing, China.

FAU - Gong, Sitang

AU - Gong S

AD - The Joint Center for Infection and Immunity, Guangzhou Institute of Pediatrics,

Guangzhou Women and Children's Medical Center, Guangzhou, China.

AD - Institut Pasteur of Shanghai, Chinese Academy of Sciences, Shanghai, China.

FAU - Zeng, Huasong

AU - Zeng H

AD - The Joint Center for Infection and Immunity, Guangzhou Institute of Pediatrics,

Guangzhou Women and Children's Medical Center, Guangzhou, China.

AD - Institut Pasteur of Shanghai, Chinese Academy of Sciences, Shanghai, China.

LA - eng

PT - Journal Article

DEP - 20220711

PL - Switzerland

TA - Front Microbiol

JT - Frontiers in microbiology

JID - 101548977

PMC - PMC9309504

OTO - NOTNLM

OT - GntR

OT - MSMEG_5174

OT - Mycobacterium smegmatis

OT - aminoglycoside antibiotics resistance

OT - purine metabolism

COIS- The authors declare that the research was conducted in the absence of any

commercial or financial relationships that could be construed as a potential

conflict of interest.

EDAT- 2022/07/29 06:00

MHDA- 2022/07/29 06:01

PMCR- 2022/07/11

CRDT- 2022/07/28 02:17

PHST- 2022/04/13 00:00 [received]

PHST- 2022/06/20 00:00 [accepted]

PHST- 2022/07/28 02:17 [entrez]

PHST- 2022/07/29 06:00 [pubmed]

PHST- 2022/07/29 06:01 [medline]

PHST- 2022/07/11 00:00 [pmc-release]

AID - 10.3389/fmicb.2022.919538 [doi]

PST - epublish

SO - Front Microbiol. 2022 Jul 11;13:919538. doi: 10.3389/fmicb.2022.919538.

eCollection 2022.

PMID- 35617724

OWN - NLM

STAT- MEDLINE

DCOM- 20220624

LR - 20220729

IS - 1879-0534 (Electronic)

IS - 0010-4825 (Linking)

VI - 146

DP - 2022 Jul

TI - A multidrug efflux protein in Mycobacterium tuberculosis; tap as a potential drug

target for drug repurposing.

PG - 105607

LID - S0010-4825(22)00399-7 [pii]

LID - 10.1016/j.compbiomed.2022.105607 [doi]

AB - Tuberculosis (TB) is a serious communicative disease caused by Mycobacterium

tuberculosis. Although there are vaccines and drugs available to treat the

disease, they are not efficient, moreover, multidrug-resistant TB (MDR-TB) become

a major hurdle in its therapy. These MDR strains utilize the multidrug efflux

pump as a decisive weapon to fight against antitubercular drugs. Tap membrane

protein was observed as a crucial multidrug efflux pump in M. tuberculosis and

its critical implication in MDR-MTB development makes it an effective drug

target. In the present study, we have utilized various in silico approaches to

predict the applicability of FDA-approved ion channel inhibitors and blockers as

therapeutic leads against Tuberculosis by targeting multidrug efflux protein; Tap

in MTB. Tap protein structure is predicted by Phyre2 server followed by model

refinement, validation, physio-chemical catheterization and target prediction.

Further, the interaction between Tap protein and ligands were analysed by

molecular docking and MD simulation run of 100 ns. Based on implication and

compatibility, 18 FDA-approved ion channel inhibitors and blockers are selected

as a ligand against the Tap protein and eventually observed five ligands;

Glimepiride, Flecainide, Flupiritine, Nimodipine and Amlodipine as promising

compounds which have exhibited the significant stable interaction with Tap

protein and are proposed to modulate or interfere with its activity. These

compounds illustrated the substantial docking score and total binding enthalpy

more than -7 kcal/mol and -42 kcal/mol respectively which implies that the

selected FDA-approved compounds can spontaneously interact with the Tap protein

to modulate its function. This study proposed Tap protein as a prominent drug

target in MTB and investigated compounds that show considerable interaction with

the Tap protein as potential therapeutic molecules. These interactions may lead

to modulating or inhibit the activity of drug efflux protein thereby making MTB

susceptible to antitubercular drugs.

CI - Copyright © 2022 Elsevier Ltd. All rights reserved.

FAU - Dwivedi, Manish

AU - Dwivedi M

AD - Amity Institute of Biotechnology, Amity University Uttar Pradesh, Lucknow,

226028, India. Electronic address: mdwivedi@lko.amity.edu.

FAU - Mukhopadhyay, Sutanu

AU - Mukhopadhyay S

AD - Department of Chemistry, Ramakrishna Mission Vivekananda Centenary College, West

Bengal, India.

FAU - Yadav, Shalini

AU - Yadav S

AD - Department of Chemistry, Shiv Nadar University, Greater Noida, 201314, India.

FAU - Dubey, Kshatresh Dutta

AU - Dubey KD

AD - Department of Chemistry, Shiv Nadar University, Greater Noida, 201314, India.

LA - eng

PT - Journal Article

PT - Research Support, Non-U.S. Gov't

DEP - 20220516

PL - United States

TA - Comput Biol Med

JT - Computers in biology and medicine

JID - 1250250

RN - 0 (Antitubercular Agents)

RN - 0 (Bacterial Proteins)

RN - 0 (Ligands)

SB - IM

MH - Antitubercular Agents/chemistry/pharmacology/therapeutic use

MH - Bacterial Proteins/chemistry

MH - Drug Repositioning

MH - Humans

MH - Ligands

MH - Molecular Docking Simulation

MH - *Mycobacterium tuberculosis/metabolism

MH - *Tuberculosis/drug therapy/microbiology

OTO - NOTNLM

OT - Drug repurposing

OT - FDA-Approved

OT - Homology modelling

OT - In silico

OT - MD simulation

OT - MD simulations

OT - Molecular docking

OT - Mycobacterium tuberculosis

OT - Tap

EDAT- 2022/05/27 06:00

MHDA- 2022/06/25 06:00

CRDT- 2022/05/26 18:13

PHST- 2022/02/26 00:00 [received]

PHST- 2022/05/02 00:00 [revised]

PHST- 2022/05/09 00:00 [accepted]

PHST- 2022/05/27 06:00 [pubmed]

PHST- 2022/06/25 06:00 [medline]

PHST- 2022/05/26 18:13 [entrez]

AID - S0010-4825(22)00399-7 [pii]

AID - 10.1016/j.compbiomed.2022.105607 [doi]

PST - ppublish

SO - Comput Biol Med. 2022 Jul;146:105607. doi: 10.1016/j.compbiomed.2022.105607. Epub

2022 May 16.

PMID- 34453865

OWN - NLM

STAT- MEDLINE

DCOM- 20220720

LR - 20221025

IS - 1742-4658 (Electronic)

IS - 1742-464X (Linking)

VI - 289

IP - 14

DP - 2022 Jul

TI - Epigenetic code during mycobacterial infections: therapeutic implications for

tuberculosis.

PG - 4172-4191

LID - 10.1111/febs.16170 [doi]

AB - Epigenetics involves changing the gene function without any change in the

sequence of the genes. In the case of tuberculosis (TB) infections, the bacilli,

Mycobacterium tuberculosis (M.tb), uses epigenetics as a tool to protect itself

from the host immune system. TB is a deadly disease-causing maximum death per

year due to a single infectious agent. In the case of TB, there is an urgent need

for novel host-directed therapies which can effectively target the survival and

long-term persistence of the bacteria without developing drug resistance in the

bacterial strains while also reducing the duration and toxicity associated with

the mainstream anti-TB drugs. Recent studies have suggested that TB infection has

a significant effect on the host epigenome thereby manipulating the host immune

response in the favor of the pathogen. M.tb alters the activation status of key

genes involved in the immune response against TB to promote its survival and

subvert the antibacterial strategies of the host. These changes are reversible

and can be exploited to design very efficient host-directed therapies to fight

against TB. This review has been written with the purpose of discussing the role

of epigenetic changes in TB pathogenesis and the therapeutic approaches involving

epigenetics, which can be utilized for targeting the pathogen.

CI - © 2021 Federation of European Biochemical Societies.

FAU - Fatima, Samreen

AU - Fatima S

AD - Immunobiology Group, International Centre for Genetic Engineering and

Biotechnology, New Delhi, India.

FAU - Kumari, Anjna

AU - Kumari A

AD - Immunobiology Group, International Centre for Genetic Engineering and

Biotechnology, New Delhi, India.

FAU - Agarwal, Meetu

AU - Agarwal M

AD - Department of Biosciences, Jamia Hamdard University, New Delhi, India.

FAU - Pahuja, Isha

AU - Pahuja I

AD - Immunobiology Group, International Centre for Genetic Engineering and

Biotechnology, New Delhi, India.

FAU - Yadav, Vinod

AU - Yadav V

AD - Department of Microbiology, Central University of Haryana, Mahendragarh, India.

FAU - Dwivedi, Ved Prakash

AU - Dwivedi VP

AD - Immunobiology Group, International Centre for Genetic Engineering and

Biotechnology, New Delhi, India.

FAU - Bhaskar, Ashima

AU - Bhaskar A

AUID- ORCID: 0000-0002-7367-874X

AD - Immunobiology Group, International Centre for Genetic Engineering and

Biotechnology, New Delhi, India.

LA - eng

PT - Journal Article

PT - Research Support, Non-U.S. Gov't

PT - Review

DEP - 20210917

PL - England

TA - FEBS J

JT - The FEBS journal

JID - 101229646

RN - 0 (Antitubercular Agents)

SB - IM

MH - Antitubercular Agents/pharmacology/therapeutic use

MH - Epigenesis, Genetic

MH - Epigenomics

MH - Humans

MH - *Mycobacterium tuberculosis/genetics

MH - *Tuberculosis/drug therapy/genetics/microbiology

OTO - NOTNLM

OT - Mycobacterium tuberculosis

OT - drug-resistant tuberculosis

OT - epigenetics

OT - immunotherapy

OT - vaccine

EDAT- 2021/08/29 06:00

MHDA- 2022/07/22 06:00

CRDT- 2021/08/28 17:06

PHST- 2021/07/23 00:00 [revised]

PHST- 2021/06/11 00:00 [received]

PHST- 2021/08/26 00:00 [accepted]

PHST- 2021/08/29 06:00 [pubmed]

PHST- 2022/07/22 06:00 [medline]

PHST- 2021/08/28 17:06 [entrez]

AID - 10.1111/febs.16170 [doi]

PST - ppublish

SO - FEBS J. 2022 Jul;289(14):4172-4191. doi: 10.1111/febs.16170. Epub 2021 Sep 17.

PMID- 35612308

OWN - NLM

STAT- MEDLINE

DCOM- 20220701

LR - 20220801

IS - 2165-0497 (Electronic)

IS - 2165-0497 (Linking)

VI - 10

IP - 3

DP - 2022 Jun 29

TI - Chemical Exploration of a Highly Selective Scaffold with Activity against

Intracellular Mycobacterium tuberculosis.

PG - e0116122

LID - 10.1128/spectrum.01161-22 [doi]

LID - e01161-22

AB - We previously identified a phenylthiourea series with activity against

intracellular Mycobacterium tuberculosis using a high-throughput, high-content

assay. We conducted a catalog structure-activity relationship study with a

collection of 35 analogs. We identified several thiourea derivatives with

excellent potency against intracellular bacteria and good selectivity over

eukaryotic cells. Compounds had much lower activity against extracellular

bacteria, which was not increased by using cholesterol as the sole carbon source.

Compounds were equally active against strains with mutations in QcrB or MmpL3,

thereby excluding common, promiscuous targets as the mode of action. The

phenylthiourea series represents a good starting point for further exploration to

develop novel antitubercular agents. IMPORTANCE Mycobacterium tuberculosis is

responsible for the highest number of deaths from a bacterial pathogen, with >1.5

million in 2020. M. tuberculosis is a sophisticated pathogen that can replicate

inside immune cells. There is an urgent need for new drugs to combat M.

tuberculosis and to shorten therapy from 6 to 24 months. We have identified a

series of molecules that inhibit the growth of M. tuberculosis inside

macrophages; we tested a number of derivatives to link structural features to

biological activity. The compounds are likely to have novel mechanism of action

and so could be developed as new agents for drug-resistant tuberculosis.

FAU - Njikan, Samuel

AU - Njikan S

AD - Infectious Disease Research Institute, Seattle, Washington, USA.

AD - Center for Global Infectious Disease Research Seattle, Seattle Children's

Research Institute, Seattle, Washington, USA.

FAU - Ahmed, Sara

AU - Ahmed S

AD - Infectious Disease Research Institute, Seattle, Washington, USA.

AD - Center for Global Infectious Disease Research Seattle, Seattle Children's

Research Institute, Seattle, Washington, USA.

FAU - Manning, Alyssa

AU - Manning A

AD - Infectious Disease Research Institute, Seattle, Washington, USA.

FAU - Awasthi, Divya

AU - Awasthi D

AD - Infectious Disease Research Institute, Seattle, Washington, USA.

FAU - Ovechkina, Yulia

AU - Ovechkina Y

AD - Infectious Disease Research Institute, Seattle, Washington, USA.

AD - Center for Global Infectious Disease Research Seattle, Seattle Children's

Research Institute, Seattle, Washington, USA.

FAU - Chowdhury, Sultan

AU - Chowdhury S

AD - Infectious Disease Research Institute, Seattle, Washington, USA.

AD - Center for Global Infectious Disease Research Seattle, Seattle Children's

Research Institute, Seattle, Washington, USA.

FAU - Butts, Arielle

AU - Butts A

AD - Infectious Disease Research Institute, Seattle, Washington, USA.

AD - Center for Global Infectious Disease Research Seattle, Seattle Children's

Research Institute, Seattle, Washington, USA.

FAU - Parish, Tanya

AU - Parish T

AUID- ORCID: 0000-0001-7507-0423

AD - Infectious Disease Research Institute, Seattle, Washington, USA.

AD - Center for Global Infectious Disease Research Seattle, Seattle Children's

Research Institute, Seattle, Washington, USA.

LA - eng

GR - R01 AI132634/AI/NIAID NIH HHS/United States

PT - Journal Article

PT - Research Support, N.I.H., Extramural

DEP - 20220525

PL - United States

TA - Microbiol Spectr

JT - Microbiology spectrum

JID - 101634614

RN - 0 (Antitubercular Agents)

RN - 6F82C6Q54C (Phenylthiourea)

SB - IM

MH - Antitubercular Agents/chemistry/pharmacology

MH - Humans

MH - Microbial Sensitivity Tests

MH - *Mycobacterium tuberculosis

MH - Phenylthiourea

MH - *Tuberculosis, Lymph Node

MH - *Tuberculosis, Multidrug-Resistant

PMC - PMC9241686

OTO - NOTNLM

OT - Mycobacterium tuberculosis

OT - antibacterial drug discovery

OT - high-content analysis

OT - phenotypic screening

COIS- The authors declare no conflict of interest.

EDAT- 2022/05/26 06:00

MHDA- 2022/07/02 06:00

PMCR- 2022/05/25

CRDT- 2022/05/25 09:03

PHST- 2022/05/26 06:00 [pubmed]

PHST- 2022/07/02 06:00 [medline]

PHST- 2022/05/25 09:03 [entrez]

PHST- 2022/05/25 00:00 [pmc-release]

AID - 01161-22 [pii]

AID - spectrum.01161-22 [pii]

AID - 10.1128/spectrum.01161-22 [doi]

PST - ppublish

SO - Microbiol Spectr. 2022 Jun 29;10(3):e0116122. doi: 10.1128/spectrum.01161-22.

Epub 2022 May 25.

PMID- 35978978

OWN - NLM

STAT- PubMed-not-MEDLINE

LR - 20220819

IS - 1949-8470 (Print)

IS - 1949-8470 (Electronic)

IS - 1949-8470 (Linking)

VI - 14

IP - 6

DP - 2022 Jun 28

TI - Tuberculosis conundrum - current and future scenarios: A proposed comprehensive

approach combining laboratory, imaging, and computing advances.

PG - 114-136

LID - 10.4329/wjr.v14.i6.114 [doi]

AB - Tuberculosis (TB) remains a global threat, with the rise of multiple and

extensively drug resistant TB posing additional challenges. The International

health community has set various 5-yearly targets for TB elimination:

mathematical modelling suggests that a 2050 target is feasible with a strategy

combining better diagnostics, drugs, and vaccines to detect and treat both latent

and active infection. The availability of rapid and highly sensitive diagnostic

tools (Gene-Xpert, TB-Quick) will vastly facilitate population-level

identification of TB (including rifampicin resistance and through it,

multi-drug-resistant TB). Basic-research advances have illuminated molecular

mechanisms in TB, including the protective role of Vitamin D. Also, Mycobacterium

tuberculosis impairs the host immune response through epigenetic mechanisms

(histone-binding modulation). Imaging will continue to be key, both for initial

diagnosis and follow-up. We discuss advances in multiple imaging modalities to

evaluate TB tissue changes, such as molecular imaging techniques (including

pathogen-specific positron emission tomography imaging agents), non-invasive

temporal monitoring, and computing enhancements to improve data acquisition and

reduce scan times. Big data analysis and Artificial Intelligence (AI) algorithms,

notably in the AI sub-field called "Deep Learning", can potentially increase the

speed and accuracy of diagnosis. Additionally, Federated learning makes

multi-institutional/multi-city AI-based collaborations possible without sharing

identifiable patient data. More powerful hardware designs - e.g., Edge and

Quantum Computing- will facilitate the role of computing applications in TB.

However, "Artificial Intelligence needs real Intelligence to guide it!" To have

maximal impact, AI must use a holistic approach that incorporates time tested

human wisdom gained over decades from the full gamut of TB, i.e., key imaging and

clinical parameters, including prognostic indicators, plus bacterial and

epidemiologic data. We propose a similar holistic approach at the level of

national/international policy formulation and implementation, to enable effective

culmination of TB's endgame, summarizing it with the acronym "TB - REVISITED".

CI - ©The Author(s) 2022. Published by Baishideng Publishing Group Inc. All rights

reserved.

FAU - Merchant, Suleman Adam

AU - Merchant SA

AD - Lokmanya Tilak Municipal Medical College and General Hospital, Mumbai 400022,

Maharashtra, India. suleman.a.merchant@gmail.com.

FAU - Shaikh, Mohd Javed Saifullah

AU - Shaikh MJS

AD - Department of Radiology, North Bengal Neuro Centre, Jupiter magnetic resonance

imaging, Diagnostic Centre, Siliguri 734003, West Bengal, India.

FAU - Nadkarni, Prakash

AU - Nadkarni P

AD - College of Nursing, University of Iowa, Iowa 52242, IA, United States.

LA - eng

PT - Journal Article

PT - Review

PL - United States

TA - World J Radiol

JT - World journal of radiology

JID - 101538184

PMC - PMC9258306

OTO - NOTNLM

OT - Artificial intelligence

OT - GenXpert

OT - Molecular imaging

OT - Quantum computing

OT - Radiology

OT - Tuberculosis

COIS- Conflict-of-interest statement: All authors declare no conflict of interests for

this article.

EDAT- 2022/08/19 06:00

MHDA- 2022/08/19 06:01

PMCR- 2022/06/28

CRDT- 2022/08/18 02:09

PHST- 2022/01/17 00:00 [received]

PHST- 2022/04/17 00:00 [revised]

PHST- 2022/05/27 00:00 [accepted]

PHST- 2022/08/18 02:09 [entrez]

PHST- 2022/08/19 06:00 [pubmed]

PHST- 2022/08/19 06:01 [medline]

PHST- 2022/06/28 00:00 [pmc-release]

AID - 10.4329/wjr.v14.i6.114 [doi]

PST - ppublish

SO - World J Radiol. 2022 Jun 28;14(6):114-136. doi: 10.4329/wjr.v14.i6.114.

PMID- 35615956

OWN - NLM

STAT- MEDLINE

DCOM- 20221222

LR - 20221222

IS - 2379-5077 (Print)

IS - 2379-5077 (Electronic)

IS - 2379-5077 (Linking)

VI - 7

IP - 3

DP - 2022 Jun 28

TI - Single-Fluorescence ATP Sensor Based on Fluorescence Resonance Energy Transfer

Reveals Role of Antibiotic-Induced ATP Perturbation in Mycobacterial Killing.

PG - e0020922

LID - 10.1128/msystems.00209-22 [doi]

LID - e00209-22

AB - The rapid emergence of multidrug-resistant/extensively drug-resistant

tuberculosis (TB) is responsible for treatment failure in patients with TB and

significantly endangers global public health. Recently, bioenergetics has become

a new paradigm for anti-TB drug discovery and is based on the link between

bacterial ATP levels and drug efficacy. A better understanding of the role of ATP

fluctuations during antibiotic treatment may provide insight into

antibiotic-mediated killing of mycobacteria. Here, we employed an advanced

single-fluorescence FRET (fluorescence resonance energy transfer)-based ATP

biosensor, ATPser, for the stable and convenient detection of intracellular ATP

fluctuations in mycobacteria. This strategy correlated closely with the results

obtained from conventional luminescence ATP assays, indicating the reliability of

the system for bioenergetics analysis in mycobacteria. Moreover, the reporter

strains expressing ATPser displayed obvious ATP changes when subjected to

different stresses, such as starvation and ATP depletion. Interestingly, we

observed that different antibiotics induced fluctuations in cellular ATP levels

in individual cells of various magnitudes, revealing a strong connection between

ATP fluctuations and drug efficacy. Furthermore, drug combinations accelerated

ATP perturbation, resulting in increased cell death. We concluded that ATPser

enabled real-time measurement of ATP at the single-cell level in mycobacteria,

and monitoring ATP dynamics in drug-treated bacteria may shed light on novel

treatment strategies. IMPORTANCE Bioenergetics has emerged as a new paradigm for

antituberculosis (anti-TB) drug discovery, and the cellular ATP level is the core

indicator reflecting bacterial metabolic homeostasis. Although several bulk

assays have been designed for the measurement of cellular ATP content, a more

convenient strategy is required for real-time ATP measurement of single viable

cells. In this study, by combining the ε-subunit of Bacillus subtilis

F(o)F(1)-ATP synthase with a circularly permuted green fluorescent protein

[(cp)GFP], we constructed a FRET-based single-fluorescence ATP sensor, ATPser,

for real-time single-cell ATP detection among a mycobacterial population. Using

the ATPser, we designed different drug combinations containing components that

have similar/opposite effects on ATP alternation. Our results demonstrated that

increased cellular ATP fluctuations were associated with depletion of

mycobacterial viability, while counteracting ATP fluctuations weakened the

killing effect of the drug regime. Thus, potentially efficient drug combinations

can be considered based on their similar effects on mycobacterial ATP levels, and

ATPser may be a useful tool to study mycobacterial bioenergetics and to guide

drug regime design.

FAU - Liang, Lujie

AU - Liang L

AD - Department of Microbiology, Zhongshan School of Medicine, Sun Yat-sen

Universitygrid.12981.33, Guangzhou, China.

AD - Key Laboratory of Tropical Diseases Control (Sun Yat-sen University), Ministry of

Education, Guangzhou, China.

FAU - Lin, Daixi

AU - Lin D

AD - Department of Microbiology, Zhongshan School of Medicine, Sun Yat-sen

Universitygrid.12981.33, Guangzhou, China.

AD - Key Laboratory of Tropical Diseases Control (Sun Yat-sen University), Ministry of

Education, Guangzhou, China.

FAU - Chen, Yishen

AU - Chen Y

AD - Department of Pharmacy, The Fifth Affiliated Hospital of Sun Yat-sen

Universitygrid.12981.33, Zhuhai, China.

FAU - Li, Jiachen

AU - Li J

AD - Department of Microbiology, Zhongshan School of Medicine, Sun Yat-sen

Universitygrid.12981.33, Guangzhou, China.

AD - Key Laboratory of Tropical Diseases Control (Sun Yat-sen University), Ministry of

Education, Guangzhou, China.

FAU - Liang, Wanfei

AU - Liang W

AD - Department of Microbiology, Zhongshan School of Medicine, Sun Yat-sen

Universitygrid.12981.33, Guangzhou, China.

AD - Key Laboratory of Tropical Diseases Control (Sun Yat-sen University), Ministry of

Education, Guangzhou, China.

FAU - Zhao, Hui

AU - Zhao H

AD - Guangdong Provincial Key Laboratory of Biotechnology for Plant Development,

School of Life Sciences, South China Normal University, Guangzhou, China.

FAU - Luo, Wenji

AU - Luo W

AD - Department of Pharmacy, The Fifth Affiliated Hospital of Sun Yat-sen

Universitygrid.12981.33, Zhuhai, China.

FAU - Tian, Guo-Bao

AU - Tian GB

AUID- ORCID: 0000-0001-6750-5482

AD - Department of Microbiology, Zhongshan School of Medicine, Sun Yat-sen

Universitygrid.12981.33, Guangzhou, China.

AD - Key Laboratory of Tropical Diseases Control (Sun Yat-sen University), Ministry of

Education, Guangzhou, China.

FAU - Feng, Siyuan

AU - Feng S

AUID- ORCID: 0000-0003-2416-0693

AD - Department of Microbiology, Zhongshan School of Medicine, Sun Yat-sen

Universitygrid.12981.33, Guangzhou, China.

AD - Key Laboratory of Tropical Diseases Control (Sun Yat-sen University), Ministry of

Education, Guangzhou, China.

LA - eng

PT - Journal Article

PT - Research Support, Non-U.S. Gov't

DEP - 20220526

PL - United States

TA - mSystems

JT - mSystems

JID - 101680636

RN - 0 (Antitubercular Agents)

RN - 8L70Q75FXE (Adenosine Triphosphate)

MH - Humans

MH - *Fluorescence Resonance Energy Transfer

MH - Reproducibility of Results

MH - *Mycobacterium/metabolism

MH - Antitubercular Agents/pharmacology

MH - Adenosine Triphosphate/metabolism

PMC - PMC9238375

OTO - NOTNLM

OT - ATP

OT - FRET

OT - Mycobacterium

OT - drug combination

COIS- The authors declare no conflict of interest.

EDAT- 2022/05/27 06:00

MHDA- 2022/05/27 06:01

PMCR- 2022/05/26

CRDT- 2022/05/26 04:32

PHST- 2022/05/27 06:00 [pubmed]

PHST- 2022/05/27 06:01 [medline]

PHST- 2022/05/26 04:32 [entrez]

PHST- 2022/05/26 00:00 [pmc-release]

AID - 00209-22 [pii]

AID - msystems.00209-22 [pii]

AID - 10.1128/msystems.00209-22 [doi]

PST - ppublish

SO - mSystems. 2022 Jun 28;7(3):e0020922. doi: 10.1128/msystems.00209-22. Epub 2022

May 26.

PMID- 35680110

OWN - NLM

STAT- MEDLINE

DCOM- 20220621

LR - 20221221

IS - 1873-3476 (Electronic)

IS - 0378-5173 (Print)

IS - 0378-5173 (Linking)

VI - 622

DP - 2022 Jun 25

TI - Insights into innovative therapeutics for drug-resistant tuberculosis:

Host-directed therapy and autophagy inducing modified nanoparticles.

PG - 121893

LID - S0378-5173(22)00448-3 [pii]

LID - 10.1016/j.ijpharm.2022.121893 [doi]

FAU - Khoza, Leon J

AU - Khoza LJ

AD - Wits Advanced Drug Delivery Platform Research Unit, Department of Pharmacy and

Pharmacology, School of Therapeutic Science, Faculty of Health Sciences,

University of Witwatersrand, Johannesburg, Parktown 2193, South Africa.

FAU - Kumar, Pradeep

AU - Kumar P

AD - Wits Advanced Drug Delivery Platform Research Unit, Department of Pharmacy and

Pharmacology, School of Therapeutic Science, Faculty of Health Sciences,

University of Witwatersrand, Johannesburg, Parktown 2193, South Africa.

FAU - Dube, Admire

AU - Dube A

AD - School of Pharmacy, Faculty of Natural Sciences, University of the Western Cape,

Cape Town, South Africa.

FAU - Demana, Patrick H

AU - Demana PH

AD - Division of Pharmaceutical Sciences, School of Pharmacy, Sefako Makgatho Health

Sciences University, Pretoria 0208, South Africa.

FAU - Choonara, Yahya E

AU - Choonara YE

AD - Wits Advanced Drug Delivery Platform Research Unit, Department of Pharmacy and

Pharmacology, School of Therapeutic Science, Faculty of Health Sciences,

University of Witwatersrand, Johannesburg, Parktown 2193, South Africa.

Electronic address: yahya.choonara@wits.ac.za.

LA - eng

PT - Journal Article

PT - Research Support, Non-U.S. Gov't

DEP - 20220606

PL - Netherlands

TA - Int J Pharm

JT - International journal of pharmaceutics

JID - 7804127

SB - IM

MH - Autophagy

MH - Humans

MH - Immunity, Innate

MH - *Mycobacterium tuberculosis

MH - *Nanoparticles

MH - *Tuberculosis

MH - *Tuberculosis, Multidrug-Resistant

PMC - PMC9169426

OAB - [Figure: see text]

OABL- eng

OTO - NOTNLM

OT - Alveolar macrophages

OT - Autophagy induction

OT - Host-directed therapy

OT - Innate immunity

OT - Mycobacterium tuberculosis

OT - Nanoparticles

COIS- The authors declare that they have no known competing financial interests or

personal relationships that could have appeared to influence the work reported in

this paper.

EDAT- 2022/06/10 06:00

MHDA- 2022/06/22 06:00

PMCR- 2022/06/06

CRDT- 2022/06/09 19:27

PHST- 2022/03/24 00:00 [received]

PHST- 2022/05/31 00:00 [revised]

PHST- 2022/06/02 00:00 [accepted]

PHST- 2022/06/10 06:00 [pubmed]

PHST- 2022/06/22 06:00 [medline]

PHST- 2022/06/09 19:27 [entrez]

PHST- 2022/06/06 00:00 [pmc-release]

AID - S0378-5173(22)00448-3 [pii]

AID - 121893 [pii]

AID - 10.1016/j.ijpharm.2022.121893 [doi]

PST - ppublish

SO - Int J Pharm. 2022 Jun 25;622:121893. doi: 10.1016/j.ijpharm.2022.121893. Epub

2022 Jun 6.

PMID- 35694543

OWN - NLM

STAT- MEDLINE

DCOM- 20220614

LR - 20220716

IS - 2235-2988 (Electronic)

IS - 2235-2988 (Linking)

VI - 12

DP - 2022

TI - Whole Genome Sequencing Identifies Novel Mutations Associated With Bedaquiline

Resistance in Mycobacterium tuberculosis.

PG - 807095

LID - 10.3389/fcimb.2022.807095 [doi]

LID - 807095

AB - Bedaquiline (BDQ), a new antitubercular agent, has been used to treat

drug-resistant tuberculosis (TB). Although mutations in atpE, rv0678, and pepQ

confer major resistance to BDQ, the mechanisms of resistance to BDQ in vitro and

in clinical settings have not been fully elucidated. We selected BDQ-resistant

mutants from 7H10 agar plates containing 0.5 mg/L BDQ (the critical

concentration) and identified mutations associated with BDQ resistance through

whole genome sequencing and Sanger sequencing. A total of 1,025 mutants were

resistant to BDQ. We randomly selected 168 mutants for further analysis and

discovered that 157/168 BDQ-resistant mutants harbored mutations in rv0678, which

encodes a transcriptional regulator that represses the expression of the efflux

pump, MmpS5-MmpL5. Moreover, we found two mutations with high frequency in rv0678

at nucleotide positions 286-287 (CG286-287 insertion; accounting for 26.8%

[45/168]) and 198-199 (G198, G199 insertion, and G198 deletion; accounting for

14.3% [24/168]). The other mutations were dispersed covering the entire rv0678

gene. Moreover, we found that one new gene, glpK, harbors a G572 insertion; this

mutation has a high prevalence (85.7%; 144/168) in the isolated mutants, and the

minimum inhibitory concentration (MIC) assay demonstrated that it is closely

associated with BDQ resistance. In summary, we characterized 168/1,025 mutants

resistant to BDQ and found that mutations in rv0678 confer the primary mechanism

of BDQ resistance. Moreover, we identified a new gene (glpK) involved in BDQ

resistance. Our study offers new insights and valuable information that will

contribute to rapid identification of BDQ-resistant isolates in clinical

settings.

CI - Copyright © 2022 Guo, Bi, Lin, Ye, Wang, Wang, Liu and Zhang.

FAU - Guo, Qinglong

AU - Guo Q

AD - National Clinical Research Center for Infectious Diseases, Guangdong Provincial

Clinical Research Center for Tuberculosis, Shenzhen Third People's Hospital,

Southern University of Science and Technology, Shenzhen, China.

FAU - Bi, Jing

AU - Bi J

AD - National Clinical Research Center for Infectious Diseases, Guangdong Provincial

Clinical Research Center for Tuberculosis, Shenzhen Third People's Hospital,

Southern University of Science and Technology, Shenzhen, China.

FAU - Lin, Qiao

AU - Lin Q

AD - Department of Traditional Chinese Medicine, The Baoan People's Hospital of

Shenzhen, Shenzhen University, Shenzhen, China.

FAU - Ye, Taosheng

AU - Ye T

AD - National Clinical Research Center for Infectious Diseases, Guangdong Provincial

Clinical Research Center for Tuberculosis, Shenzhen Third People's Hospital,

Southern University of Science and Technology, Shenzhen, China.

FAU - Wang, Zhongyuan

AU - Wang Z

AD - National Clinical Research Center for Infectious Diseases, Guangdong Provincial

Clinical Research Center for Tuberculosis, Shenzhen Third People's Hospital,

Southern University of Science and Technology, Shenzhen, China.

FAU - Wang, Zhaoqin

AU - Wang Z

AD - National Clinical Research Center for Infectious Diseases, Guangdong Provincial

Clinical Research Center for Tuberculosis, Shenzhen Third People's Hospital,

Southern University of Science and Technology, Shenzhen, China.

FAU - Liu, Lei

AU - Liu L

AD - National Clinical Research Center for Infectious Diseases, Guangdong Provincial

Clinical Research Center for Tuberculosis, Shenzhen Third People's Hospital,

Southern University of Science and Technology, Shenzhen, China.

FAU - Zhang, Guoliang

AU - Zhang G

AD - National Clinical Research Center for Infectious Diseases, Guangdong Provincial

Clinical Research Center for Tuberculosis, Shenzhen Third People's Hospital,

Southern University of Science and Technology, Shenzhen, China.

LA - eng

PT - Journal Article

PT - Research Support, Non-U.S. Gov't

DEP - 20220527

PL - Switzerland

TA - Front Cell Infect Microbiol

JT - Frontiers in cellular and infection microbiology

JID - 101585359

RN - 0 (Antitubercular Agents)

RN - 0 (Diarylquinolines)

RN - 78846I289Y (bedaquiline)

SB - IM

MH - Antitubercular Agents/pharmacology/therapeutic use

MH - Diarylquinolines/pharmacology

MH - Humans

MH - Microbial Sensitivity Tests

MH - Mutation

MH - *Mycobacterium tuberculosis

MH - *Tuberculosis, Lymph Node

MH - Whole Genome Sequencing

PMC - PMC9184757

OTO - NOTNLM

OT - Mycobacterium tuberculosis

OT - bedaquiline

OT - drug resistance

OT - glpK

OT - rv0678

COIS- The authors declare that the research was conducted in the absence of any

commercial or financial relationships that could be construed as a potential

conflict of interest.

EDAT- 2022/06/14 06:00

MHDA- 2022/06/15 06:00

PMCR- 2022/01/01

CRDT- 2022/06/13 03:33

PHST- 2021/11/01 00:00 [received]

PHST- 2022/04/26 00:00 [accepted]

PHST- 2022/06/13 03:33 [entrez]

PHST- 2022/06/14 06:00 [pubmed]

PHST- 2022/06/15 06:00 [medline]

PHST- 2022/01/01 00:00 [pmc-release]

AID - 10.3389/fcimb.2022.807095 [doi]

PST - epublish

SO - Front Cell Infect Microbiol. 2022 May 27;12:807095. doi:

10.3389/fcimb.2022.807095. eCollection 2022.

PMID- 35645569

OWN - NLM

STAT- PubMed-not-MEDLINE

LR - 20220716

IS - 1178-6361 (Print)

IS - 1178-6361 (Electronic)

IS - 1178-6361 (Linking)

VI - 15

DP - 2022

TI - Recent Progress in the Development of Novel Mycobacterium Cell Wall Inhibitor to

Combat Drug-Resistant Tuberculosis.

PG - 11786361221099878

LID - 10.1177/11786361221099878 [doi]

LID - 11786361221099878

AB - Despite decades of research in drug development against TB, it is still the

leading cause of death due to infectious diseases. The long treatment duration,

patient noncompliance coupled with the ability of the tuberculosis bacilli to

resist the current drugs increases multidrug-resistant tuberculosis that

exacerbates the situation. Identification of novel drug targets is important for

the advancement of drug development against Mycobacterium tuberculosis. The

development of an effective treatment course that could help us eradicates TB.

Hence, we require drugs that could eliminate the bacteria and shorten the

treatment duration. This review briefly describes the available data on the

peptidoglycan component structural characterization, identification of the

metabolic pathway, and the key enzymes involved in the peptidoglycan synthesis,

like N-Acetylglucosamine-1-phosphate uridyltransferase, mur enzyme, alanine

racemase as well as their inhibition. Besides, this paper also provides studies

on mycolic acid and arabinogalactan synthesis and the transport mechanisms that

show considerable promise as new targets to develop a new product with their

inhibiter.

CI - © The Author(s) 2022.

FAU - Belete, Tafere Mulaw

AU - Belete TM

AUID- ORCID: 0000-0002-6634-5971

AD - Department of Pharmacology, College of Medicine and Health Sciences, University

of Gondar, Gondar, Ethiopia.

LA - eng

PT - Journal Article

PT - Review

DEP - 20220523

PL - United States

TA - Microbiol Insights

JT - Microbiology insights

JID - 101622216

PMC - PMC9131376

OTO - NOTNLM

OT - Mycobacterium tuberculosis

OT - Tuberculosis drugs

OT - drug discovery

OT - drug regimens

COIS- Declaration of Conflicting Interests: The author declared no potential conflicts

of interest with respect to the research, authorship, and/or publication of this

article.

EDAT- 2022/06/02 06:00

MHDA- 2022/06/02 06:01

PMCR- 2022/05/23

CRDT- 2022/06/01 11:24

PHST- 2021/11/26 00:00 [received]

PHST- 2022/04/21 00:00 [accepted]

PHST- 2022/06/01 11:24 [entrez]

PHST- 2022/06/02 06:00 [pubmed]

PHST- 2022/06/02 06:01 [medline]

PHST- 2022/05/23 00:00 [pmc-release]

AID - 10.1177_11786361221099878 [pii]

AID - 10.1177/11786361221099878 [doi]

PST - epublish

SO - Microbiol Insights. 2022 May 23;15:11786361221099878. doi:

10.1177/11786361221099878. eCollection 2022.

PMID- 35646807

OWN - NLM

STAT- MEDLINE

DCOM- 20220603

LR - 20220721

IS - 2296-2565 (Electronic)

IS - 2296-2565 (Linking)

VI - 10

DP - 2022

TI - "DOST" Model to Link and Support Drug Resistant TB Patients From Private Sector:

An Experience From Delhi, India.

PG - 835055

LID - 10.3389/fpubh.2022.835055 [doi]

LID - 835055

AB - BACKGROUND: The National TB Elimination Programme (NTEP) has quite successfully

involved private sector for referral of presumptive drug resistant TB (DR-TB)

patients for molecular testing and referral for DR-TB management. There was a

challenge as all the referred patients were not reaching to the facilities. A

"DOST" intervention model was implemented to strengthen the patient care pathway.

We conducted this study to describe the patient care cascade, the

clinico-demographic characteristics of patients linked to the treatment and to

estimate the mean turn-around time for drug resistant TB care services. METHODS:

It is a cross-sectional study conducted at New Delhi during the period July

2019-December 2020 under programmatic settings. RESULTS: A total of 9,331

patients were subjected to CB-NAAT test and 382 (4%) were found to be resistant

for rifampicin and 231 (76%) were initiated on treatment in the public sector

under NTEP. CONCLUSION: The DOST intervention model developed to link the DR-TB

patients from private sector to the public sector DR-TB centers is found to be

efficient and effective.

CI - Copyright © 2022 Vatsyayan, Pattery, Sayyad, Williams, Pal, Panibatla and Khanna.

FAU - Vatsyayan, Vindhya

AU - Vatsyayan V

AD - Clinton Health Access Initiative (CHAI), New Delhi, India.

FAU - Pattery, Theresa

AU - Pattery T

AD - Johnson & Johnson Global Public Health R&D, Beerse, Belgium.

FAU - Sayyad, Khasim

AU - Sayyad K

AD - TB Alert India, Hyderabad, India.

FAU - Williams, Jason

AU - Williams J

AD - Johnson & Johnson Global Public Health R&D, Beerse, Belgium.

FAU - Pal, Arnab

AU - Pal A

AD - Clinton Health Access Initiative (CHAI), New Delhi, India.

FAU - Panibatla, Vikas

AU - Panibatla V

AD - TB Alert India, Hyderabad, India.

FAU - Khanna, Ashwani

AU - Khanna A

AD - State TB Office, New Delhi, India.

LA - eng

PT - Journal Article

PT - Research Support, Non-U.S. Gov't

DEP - 20220511

PL - Switzerland

TA - Front Public Health

JT - Frontiers in public health

JID - 101616579

SB - IM

MH - Cross-Sectional Studies

MH - Humans

MH - India

MH - *Private Sector

MH - Public Sector

MH - *Tuberculosis, Multidrug-Resistant/drug therapy

PMC - PMC9131023

OTO - NOTNLM

OT - DOST model

OT - Delhi

OT - India

OT - drug resistant tuberculosis

OT - linkages

OT - private sector

COIS- VV and AP were employed by Clinton Health Access Initiative (CHAI). TP and JW

were employed by Johnson & Johnson Global Public Health R&D. KS and VP were

employed by TB Alert India. The remaining author declares that the research was

conducted in the absence of any commercial or financial relationships that could

be construed as a potential conflict of interest.

EDAT- 2022/06/02 06:00

MHDA- 2022/06/07 06:00

PMCR- 2022/05/11

CRDT- 2022/06/01 11:41

PHST- 2021/12/14 00:00 [received]

PHST- 2022/04/07 00:00 [accepted]

PHST- 2022/06/01 11:41 [entrez]

PHST- 2022/06/02 06:00 [pubmed]

PHST- 2022/06/07 06:00 [medline]

PHST- 2022/05/11 00:00 [pmc-release]

AID - 10.3389/fpubh.2022.835055 [doi]

PST - epublish

SO - Front Public Health. 2022 May 11;10:835055. doi: 10.3389/fpubh.2022.835055.

eCollection 2022.

PMID- 35364247

OWN - NLM

STAT- MEDLINE

DCOM- 20220524

LR - 20220607

IS - 1096-1186 (Electronic)

IS - 1043-6618 (Linking)

VI - 179

DP - 2022 May

TI - Epigenetic changes in Mycobacterium tuberculosis and its host provide potential

targets or biomarkers for drug discovery and clinical diagnosis.

PG - 106195

LID - S1043-6618(22)00140-2 [pii]

LID - 10.1016/j.phrs.2022.106195 [doi]

AB - Tuberculosis infection caused by the contagious pathogen Mycobacterium

tuberculosis (MTB) is one of the ancient diseases in the world. The problem of

drug resistance is a difficulty in tuberculosis treatment. MTB engendered

epigenetic changes play vital parts in escaping the host immune response and

bring about the persistence as well as bacterial expansion. This article

describes the epigenetic changes that occur in the pathogen MTB and its host

during infection, including DNA methylation, histone modification and microRNA,

and summarizes their research progress in drug discovery and tuberculosis

diagnosis, providing new ideas and strategies to combat against drug-resistant

tuberculosis.

CI - Copyright © 2022 Elsevier Ltd. All rights reserved.

FAU - Sui, Jing

AU - Sui J

AD - State Key Laboratory of Biotherapy and Cancer Center, West China Hospital,

Sichuan University, Chengdu 610041, China.

FAU - Qiao, Wenliang

AU - Qiao W

AD - Lung Cancer Center, Laboratory of Lung Cancer, Western China Hospital of Sichuan

University, China.

FAU - Xiang, Xinrong

AU - Xiang X

AD - State Key Laboratory of Biotherapy and Cancer Center, West China Hospital,

Sichuan University, Chengdu 610041, China.

FAU - Luo, Youfu

AU - Luo Y

AD - State Key Laboratory of Biotherapy and Cancer Center, West China Hospital,

Sichuan University, Chengdu 610041, China. Electronic address:

luo_youfu@scu.edu.cn.

LA - eng

PT - Journal Article

PT - Research Support, Non-U.S. Gov't

DEP - 20220329

PL - Netherlands

TA - Pharmacol Res

JT - Pharmacological research

JID - 8907422

RN - 0 (Biomarkers)

SB - IM

MH - Biomarkers

MH - Drug Discovery

MH - Epigenesis, Genetic

MH - Host-Pathogen Interactions

MH - Humans

MH - *Mycobacterium tuberculosis/genetics

MH - *Tuberculosis/diagnosis/drug therapy/genetics

OTO - NOTNLM

OT - DNA methylation

OT - Epigenetics

OT - Histone modification

OT - MicroRNA

OT - Mycobacterium tuberculosis

EDAT- 2022/04/02 06:00

MHDA- 2022/05/25 06:00

CRDT- 2022/04/01 20:11

PHST- 2022/01/20 00:00 [received]

PHST- 2022/03/14 00:00 [revised]

PHST- 2022/03/25 00:00 [accepted]

PHST- 2022/04/02 06:00 [pubmed]

PHST- 2022/05/25 06:00 [medline]

PHST- 2022/04/01 20:11 [entrez]

AID - S1043-6618(22)00140-2 [pii]

AID - 10.1016/j.phrs.2022.106195 [doi]

PST - ppublish

SO - Pharmacol Res. 2022 May;179:106195. doi: 10.1016/j.phrs.2022.106195. Epub 2022

Mar 29.

PMID- 34533236

OWN - NLM

STAT- MEDLINE

DCOM- 20220425

LR - 20220516

IS - 1938-3673 (Electronic)

IS - 0741-5400 (Linking)

VI - 111

IP - 5

DP - 2022 May

TI - Flunarizine suppresses Mycobacterium tuberculosis growth via calmodulin-dependent

phagosome maturation.

PG - 1021-1029

LID - 10.1002/JLB.4A0221-119RR [doi]

AB - Tuberculosis (TB), an infectious bacterial disease caused by Mycobacterium

tuberculosis (Mtb), is a major cause of death worldwide. Multidrug-resistant TB

remains a public health crisis and thus novel effective treatments, such as

host-directed therapies (HDTs), are urgently required to overcome the challenges

of TB infection. In this study, we evaluated 4 calcium modulators for their

effects on Mtb growth in macrophages. Only flunarizine enhanced the bactericidal

ability of macrophages against Mtb, which was induced by an increase in

phosphorylated calcium/calmodulin (CaM)-dependent protein kinase II (pCaMKII)

levels. We further discovered that the expression of CaM was decreased in

Mtb-infected macrophages and restored following flunarizine treatment; this was

associated with phagolysosome maturation and acidification. Consistent with these

findings, the anti-TB ability of macrophages was reduced following the silencing

of CaM or inhibition of CAMKII activity. In conclusion, our results demonstrated

that flunarizine enhanced the bactericidal ability of macrophages and clarified

its CaM-pCAMKII-dependent mechanism. Therefore, our findings strongly support

further studies of this currently approved drug as an HDT candidate for TB

therapy.

CI - ©2021 Society for Leukocyte Biology.

FAU - Mo, Siwei

AU - Mo S

AD - Guangdong Provincial Key Laboratory of Regional Immunity and Diseases, Department

of Pathogen Biology, School of Medicine, Shenzhen University, Shenzhen, China.

FAU - Liu, Xiaoqian

AU - Liu X

AD - Department of Infectious Disease, Shenzhen People's Hospital, Second Clinical

Medical College of Jinan University, Shenzhen, Guangdong Province, China.

AD - Integrated Chinese and Western Medicine Postdoctoral Research Station, Jinan

University, Guangzhou, Guangdong Province, China.

FAU - Zhang, Kehong

AU - Zhang K

AD - Guangdong Provincial Key Laboratory of Regional Immunity and Diseases, Department

of Pathogen Biology, School of Medicine, Shenzhen University, Shenzhen, China.

AD - Department of Pharmaceutical/Medicinal Chemistry, Institute of Pharmacy,

Friedrich-Schiller-University, Jena, Germany.

FAU - Wang, Wenfei

AU - Wang W

AD - Guangdong Provincial Key Laboratory of Regional Immunity and Diseases, Department

of Pathogen Biology, School of Medicine, Shenzhen University, Shenzhen, China.

AD - Department of Pharmaceutical/Medicinal Chemistry, Institute of Pharmacy,

Friedrich-Schiller-University, Jena, Germany.

FAU - Cai, Yi

AU - Cai Y

AD - Guangdong Provincial Key Laboratory of Regional Immunity and Diseases, Department

of Pathogen Biology, School of Medicine, Shenzhen University, Shenzhen, China.

FAU - Ouyang, Qi

AU - Ouyang Q

AD - Guangdong Provincial Key Laboratory of Regional Immunity and Diseases, Department

of Pathogen Biology, School of Medicine, Shenzhen University, Shenzhen, China.

FAU - Zhu, Chuanzhi

AU - Zhu C

AD - Laboratory of Molecular Biology, Beijing Key Laboratory for Drug Resistance

Tuberculosis Research, Beijing Chest Hospital, Capital Medical University,

Beijing Tuberculosis and Thoracic Tumor Research Institute, Beijing, China.

FAU - Lin, Dachuan

AU - Lin D

AD - Guangdong Provincial Key Laboratory of Regional Immunity and Diseases, Department

of Pathogen Biology, School of Medicine, Shenzhen University, Shenzhen, China.

FAU - Wan, Haoqiang

AU - Wan H

AD - Department of Infectious Disease, Shenzhen People's Hospital, Second Clinical

Medical College of Jinan University, Shenzhen, Guangdong Province, China.

FAU - Li, Dechang

AU - Li D

AD - Yuebei Second People's Hospital, Shaoguan, Guangdong, China.

FAU - Wen, Zhihua

AU - Wen Z

AD - Yuebei Second People's Hospital, Shaoguan, Guangdong, China.

FAU - Chen, Xinchun

AU - Chen X

AUID- ORCID: 0000-0002-2101-9013

AD - Guangdong Provincial Key Laboratory of Regional Immunity and Diseases, Department

of Pathogen Biology, School of Medicine, Shenzhen University, Shenzhen, China.

LA - eng

PT - Journal Article

PT - Research Support, Non-U.S. Gov't

DEP - 20210917

PL - England

TA - J Leukoc Biol

JT - Journal of leukocyte biology

JID - 8405628

RN - 0 (Anti-Bacterial Agents)

RN - 0 (Calmodulin)

RN - R7PLA2DM0J (Flunarizine)

RN - SY7Q814VUP (Calcium)

SB - IM

MH - Anti-Bacterial Agents/pharmacology

MH - Calcium/metabolism

MH - Calmodulin/metabolism

MH - Flunarizine/pharmacology

MH - Humans

MH - *Mycobacterium tuberculosis

MH - Phagosomes/metabolism

MH - *Tuberculosis/microbiology

OTO - NOTNLM

OT - HDT

OT - calcium modulators

OT - pCAMKII

EDAT- 2021/09/18 06:00

MHDA- 2022/04/26 06:00

CRDT- 2021/09/17 08:47

PHST- 2021/09/18 06:00 [pubmed]

PHST- 2022/04/26 06:00 [medline]

PHST- 2021/09/17 08:47 [entrez]

AID - 10.1002/JLB.4A0221-119RR [doi]

PST - ppublish

SO - J Leukoc Biol. 2022 May;111(5):1021-1029. doi: 10.1002/JLB.4A0221-119RR. Epub

2021 Sep 17.

PMID- 35470432

OWN - NLM

STAT- MEDLINE

DCOM- 20220427

LR - 20250623

IS - 1469-493X (Electronic)

IS - 1361-6137 (Linking)

VI - 4

IP - 4

DP - 2022 Apr 26

TI - Rapid molecular tests for tuberculosis and tuberculosis drug resistance: a

qualitative evidence synthesis of recipient and provider views.

PG - CD014877

LID - 10.1002/14651858.CD014877.pub2 [doi]

LID - CD014877

AB - BACKGROUND: Programmes that introduce rapid molecular tests for tuberculosis and

tuberculosis drug resistance aim to bring tests closer to the community, and

thereby cut delay in diagnosis, ensure early treatment, and improve health

outcomes, as well as overcome problems with poor laboratory infrastructure and

inadequately trained personnel. Yet, diagnostic technologies only have an impact

if they are put to use in a correct and timely manner. Views of the intended

beneficiaries are important in uptake of diagnostics, and their effective use

also depends on those implementing testing programmes, including providers,

laboratory professionals, and staff in health ministries. Otherwise, there is a

risk these technologies will not fit their intended use and setting, cannot be

made to work and scale up, and are not used by, or not accessible to, those in

need. OBJECTIVES: To synthesize end-user and professional user perspectives and

experiences with low-complexity nucleic acid amplification tests (NAATs) for

detection of tuberculosis and tuberculosis drug resistance; and to identify

implications for effective implementation and health equity. SEARCH METHODS: We

searched MEDLINE, Embase, CINAHL, PsycInfo and Science Citation Index Expanded

databases for eligible studies from 1 January 2007 up to 20 October 2021. We

limited all searches to 2007 onward because the development of Xpert MTB/RIF, the

first rapid molecular test in this review, was completed in 2009. SELECTION

CRITERIA: We included studies that used qualitative methods for data collection

and analysis, and were focused on perspectives and experiences of users and

potential users of low-complexity NAATs to diagnose tuberculosis and

drug-resistant tuberculosis. NAATs included Xpert MTB/RIF, Xpert MTB/RIF Ultra,

Xpert MTB/XDR, and the Truenat assays. Users were people with presumptive or

confirmed tuberculosis and drug-resistant tuberculosis (including

multidrug-resistant (MDR-TB)) and their caregivers, healthcare providers,

laboratory technicians and managers, and programme officers and staff; and were

from any type of health facility and setting globally. MDR-TB is tuberculosis

caused by resistance to at least rifampicin and isoniazid, the two most effective

first-line drugs used to treat tuberculosis. DATA COLLECTION AND ANALYSIS: We

used a thematic analysis approach for data extraction and synthesis, and assessed

confidence in the findings using GRADE CERQual approach. We developed a

conceptual framework to illustrate how the findings relate. MAIN RESULTS: We

found 32 studies. All studies were conducted in low- and middle-income countries.

Twenty-seven studies were conducted in high-tuberculosis burden countries and 21

studies in high-MDR-TB burden countries. Only one study was from an Eastern

European country. While the studies covered a diverse use of low-complexity

NAATs, in only a minority of studies was it used as the initial diagnostic test

for all people with presumptive tuberculosis. We identified 18 review findings

and grouped them into three overarching categories. Critical aspects users value

People with tuberculosis valued reaching diagnostic closure with an accurate

diagnosis, avoiding diagnostic delays, and keeping diagnostic-associated cost

low. Similarly, healthcare providers valued aspects of accuracy and the resulting

confidence in low-complexity NAAT results, rapid turnaround times, and keeping

cost to people seeking a diagnosis low. In addition, providers valued diversity

of sample types (for example, gastric aspirate specimens and stool in children)

and drug resistance information. Laboratory professionals appreciated the

improved ease of use, ergonomics, and biosafety of low-complexity NAATs compared

to sputum microscopy, and increased staff satisfaction. Challenges reported to

realizing those values People with tuberculosis and healthcare workers were

reluctant to test for tuberculosis (including MDR-TB) due to fears, stigma, or

cost concerns. Thus, low-complexity NAAT testing is not implemented with

sufficient support or discretion to overcome barriers that are common to other

approaches to testing for tuberculosis. Delays were reported at many steps of the

diagnostic pathway owing to poor sample quality; difficulties with transporting

specimens; lack of sufficient resources; maintenance of low-complexity NAATs;

increased workload; inefficient work and patient flows; over-reliance on

low-complexity NAAT results in lieu of clinical judgement; and lack of

data-driven and inclusive implementation processes. These challenges were

reported to lead to underutilization.  Concerns for access and equity The

reported concerns included sustainable funding and maintenance and equitable use

of resources to access low-complexity NAATs, as well as conflicts of interest

between donors and people implementing the tests. Also, lengthy diagnostic

delays, underutilization of low-complexity NAATs, lack of tuberculosis diagnostic

facilities in the community, and too many eligibility restrictions hampered

access to prompt and accurate testing and treatment. This was particularly the

case for vulnerable groups, such as children, people with MDR-TB, or people with

limited ability to pay. We had high confidence in most of our findings. AUTHORS'

CONCLUSIONS: Low-complexity diagnostics have been presented as a solution to

overcome deficiencies in laboratory infrastructure and lack of skilled

professionals. This review indicates this is misleading. The lack of

infrastructure and human resources undermine the added value new diagnostics of

low complexity have for recipients and providers. We had high confidence in the

evidence contributing to these review findings. Implementation of new diagnostic

technologies, like those considered in this review, will need to tackle the

challenges identified in this review including weak infrastructure and systems,

and insufficient data on ground level realities prior and during implementation,

as well as problems of conflicts of interest in order to ensure equitable use of

resources.

CI - Copyright © 2022 The Authors. Cochrane Database of Systematic Reviews published

by John Wiley & Sons, Ltd. on behalf of The Cochrane Collaboration.

FAU - Engel, Nora

AU - Engel N

AD - Department of Health, Ethics & Society, School of Public Health and Primary Care

(CAPHRI), Maastricht University, Maastricht, Netherlands.

FAU - Ochodo, Eleanor A

AU - Ochodo EA

AD - Centre for Evidence-based Health Care, Department of Global Health, Faculty of

Medicine and Health Sciences, Stellenbosch University, Cape Town, South Africa.

AD - Centre for Global Health Research, Kenya Medical Research Institute, Kisumu,

Kenya.

FAU - Karanja, Perpetua Wanjiku

AU - Karanja PW

AD - Department of Health, Kirinyaga County, Kerugoya, Kenya.

FAU - Schmidt, Bey-Marrié

AU - Schmidt BM

AD - School of Public Health, University of the Western Cape, Cape Town, South Africa.

FAU - Janssen, Ricky

AU - Janssen R

AD - Department of Health, Ethics & Society, School of Public Health and Primary Care

(CAPHRI), Maastricht University, Maastricht, Netherlands.

FAU - Steingart, Karen R

AU - Steingart KR

AD - Honorary Research Fellow, Department of Clinical Sciences, Liverpool School of

Tropical Medicine, Liverpool, UK.

FAU - Oliver, Sandy

AU - Oliver S

AD - EPPI-Centre, Social Science Research Unit, UCL Institute of Education, University

College London, London, UK.

AD - Africa Centre for Evidence, Faculty of Humanities, University of Johannesburg,

Johannesburg, South Africa.

LA - eng

GR - MR/T008768/1/MRC_/Medical Research Council/United Kingdom

PT - Journal Article

PT - Research Support, Non-U.S. Gov't

PT - Systematic Review

DEP - 20220426

PL - England

TA - Cochrane Database Syst Rev

JT - The Cochrane database of systematic reviews

JID - 100909747

RN - VJT6J7R4TR (Rifampin)

SB - IM

UOF - doi: 10.1002/14651858.CD014877

MH - Child

MH - Drug Resistance

MH - Humans

MH - Nucleic Acid Amplification Techniques

MH - Rifampin/therapeutic use

MH - *Tuberculosis/diagnosis/drug therapy

MH - *Tuberculosis, Multidrug-Resistant/diagnosis/drug therapy

PMC - PMC9038447

COIS- NE received funding from the World Health Organization (WHO) Global Tuberculosis

Programme, Switzerland. She was first author on one study included in this review

(Engel 2015a, funded by the Bill and Melinda Gates Foundation) and senior author

on a second included study (Mwaura 2020, funded by Foundation for Innovative New

Diagnostics (FIND)) For both studies the funders had no role in study design or

interpretation of results. Assessment of study eligibility and data extraction

were checked independently by other review authors. NE did not assess the

methodological limitations of these studies.  EAO received funding from the World

Health Organization (WHO) Global Tuberculosis Programme, Switzerland. PWK has no

known conflicts of interest. BS has no known conflicts of interest. RJ has no

known conflicts of interest. KRS received funding from the World Health

Organization (WHO) Global Tuberculosis Programme, Switzerland and Maastricht

University, Maastricht. She has received additional financial support from

Cochrane Infectious Diseases, UK; McGill University, Canada; University of

Washington, Seattle; Baylor College of Medicine, Houston; and the World Health

Organization Global Tuberculosis Programme, Switzerland, for the preparation of

related systematic reviews and educational materials; consultancy fees from

Foundation for Innovative New Diagnostics (FIND), Switzerland (for the

preparation of systematic reviews and GRADE tables); consultancy fees from

Stellenbosch University, Stellenbosch (for guidance on evidence syntheses); and

honoraria and travel support to attend WHO guideline meetings. SO has no known

conflicts of interest.

EDAT- 2022/04/27 06:00

MHDA- 2022/04/28 06:00

PMCR- 2022/04/26

CRDT- 2022/04/26 05:59

PHST- 2022/04/26 05:59 [entrez]

PHST- 2022/04/27 06:00 [pubmed]

PHST- 2022/04/28 06:00 [medline]

PHST- 2022/04/26 00:00 [pmc-release]

AID - CD014877.pub2 [pii]

AID - 10.1002/14651858.CD014877.pub2 [doi]

PST - epublish

SO - Cochrane Database Syst Rev. 2022 Apr 26;4(4):CD014877. doi:

10.1002/14651858.CD014877.pub2.

PMID- 35379406

OWN - NLM

STAT- MEDLINE

DCOM- 20220406

LR - 20220406

IS - 0019-5707 (Print)

IS - 0019-5707 (Linking)

VI - 69

IP - 2

DP - 2022 Apr

TI - Drug resistant tuberculosis: Current scenario and impending challenges.

PG - 227-233

LID - S0019-5707(21)00080-9 [pii]

LID - 10.1016/j.ijtb.2021.04.008 [doi]

AB - Tuberculosis is still one of the ten leading causes for death worldwide. In spite

of the latest medical and health advance gained over a period of time,

tuberculosis effectively evades the successful targeting by drugs. The

persistence abilities demonstrated by the mycobacteria had surprised the global

community, since its discovery and pathogenesis in humans. Emergence and

detection of drug resistant mycobacteria (MDR-TB, XDR-TB) had further complicated

the treatment regime. Under the aegis of WHO, there is a concerted understanding

and effort by the global community to eradicate TB. Towards this goal, novel drug

molecules, new vaccine and treatment regime are being developed. Here, our

current understanding pertaining to mode of action, molecular mechanisms of novel

as well as traditional drug molecules and possible drug resistance mechanism in

M. Tuberculosis is reviewed. Recent advances on new vaccination regime are also

reviewed as it demonstrated huge potential in containing TB. This knowledge is

essential for the development of more effective drug molecules, vaccines and may

help in devising new strategy for containing and eradicating TB.

CI - Copyright © 2021 Tuberculosis Association of India. Published by Elsevier B.V.

All rights reserved.

FAU - Singh Dewhare, Shivendra

AU - Singh Dewhare S

AD - School of Studies in Life Science, Pt. RavishankarShukla University, Raipur,

492010, Chhattisgarh, India. Electronic address: shivendraprsu@gmail.com.

LA - eng

PT - Journal Article

PT - Review

DEP - 20210420

PL - India

TA - Indian J Tuberc

JT - The Indian journal of tuberculosis

JID - 0373027

RN - 0 (Antitubercular Agents)

SB - IM

MH - Antitubercular Agents/pharmacology/therapeutic use

MH - *Extensively Drug-Resistant Tuberculosis/diagnosis/drug therapy/epidemiology

MH - Humans

MH - *Mycobacterium tuberculosis

MH - *Tuberculosis, Multidrug-Resistant/diagnosis/drug therapy/epidemiology

OTO - NOTNLM

OT - Drug

OT - MDR-TB

OT - Mycobacteria

OT - Tuberculosis

OT - Vaccine

COIS- Conflicts of interest None.

EDAT- 2022/04/06 06:00

MHDA- 2022/04/07 06:00

CRDT- 2022/04/05 05:28

PHST- 2021/03/05 00:00 [received]

PHST- 2021/04/05 00:00 [accepted]

PHST- 2022/04/05 05:28 [entrez]

PHST- 2022/04/06 06:00 [pubmed]

PHST- 2022/04/07 06:00 [medline]

AID - S0019-5707(21)00080-9 [pii]

AID - 10.1016/j.ijtb.2021.04.008 [doi]

PST - ppublish

SO - Indian J Tuberc. 2022 Apr;69(2):227-233. doi: 10.1016/j.ijtb.2021.04.008. Epub

2021 Apr 20.

PMID- 35136191

OWN - NLM

STAT- MEDLINE

DCOM- 20220506

LR - 20220506

IS - 1881-1469 (Electronic)

IS - 0021-8820 (Linking)

VI - 75

IP - 4

DP - 2022 Apr

TI - Chrysomycin A inhibits the topoisomerase I of Mycobacterium tuberculosis.

PG - 226-235

LID - 10.1038/s41429-022-00503-z [doi]

AB - Novel anti-tuberculosis drugs are essential to manage drug-resistant

tuberculosis, caused by Mycobacterium tuberculosis. We recently reported the

antimycobacterial activity of chrysomycin A in vitro and in infected macrophages.

In this study, we report that it inhibits the growth of drug-resistant clinical

strains of M. tuberculosis and acts in synergy with anti-TB drugs such as

ethambutol, ciprofloxacin, and novobiocin. In pursuit of its mechanism of action,

it was found that chrysomycin A is bactericidal and exerts this activity by

interacting with DNA at specific sequences and by inhibiting the topoisomerase I

activity of M. tuberculosis. It also exhibits weak inhibition of the DNA gyrase

enzyme of the pathogen.

CI - © 2022. The Author(s), under exclusive licence to the Japan Antibiotics Research

Association.

FAU - Muralikrishnan, Balaji

AU - Muralikrishnan B

AD - Mycobacterium Research Laboratory, Rajiv Gandhi Centre for Biotechnology,

Thiruvananthapuram, Kerala, India.

FAU - Edison, Lekshmi K

AU - Edison LK

AD - Mycobacterium Research Laboratory, Rajiv Gandhi Centre for Biotechnology,

Thiruvananthapuram, Kerala, India.

FAU - Dusthackeer, Azger

AU - Dusthackeer A

AD - Department of Bacteriology, National Institute for Research in Tuberculosis,

Chennai, Tamil Nadu, India.

FAU - Jijimole, G R

AU - Jijimole GR

AD - Mycobacterium Research Laboratory, Rajiv Gandhi Centre for Biotechnology,

Thiruvananthapuram, Kerala, India.

FAU - Ramachandran, Ranjit

AU - Ramachandran R

AD - Mycobacterium Research Laboratory, Rajiv Gandhi Centre for Biotechnology,

Thiruvananthapuram, Kerala, India.

FAU - Madhavan, Aravind

AU - Madhavan A

AD - Mycobacterium Research Laboratory, Rajiv Gandhi Centre for Biotechnology,

Thiruvananthapuram, Kerala, India.

FAU - Kumar, Ramakrishnan Ajay

AU - Kumar RA

AD - Mycobacterium Research Laboratory, Rajiv Gandhi Centre for Biotechnology,

Thiruvananthapuram, Kerala, India. rakumar@rgcb.res.in.

LA - eng

PT - Journal Article

PT - Research Support, Non-U.S. Gov't

DEP - 20220208

PL - England

TA - J Antibiot (Tokyo)

JT - The Journal of antibiotics

JID - 0151115

RN - 0 (Aminoglycosides)

RN - 0 (Antitubercular Agents)

RN - 82196-88-1 (chrysomycin A)

RN - EC 5.99.1.2 (DNA Topoisomerases, Type I)

SB - IM

MH - Aminoglycosides

MH - Antitubercular Agents/pharmacology

MH - DNA Topoisomerases, Type I

MH - Humans

MH - Microbial Sensitivity Tests

MH - *Mycobacterium tuberculosis

MH - *Tuberculosis

EDAT- 2022/02/10 06:00

MHDA- 2022/05/07 06:00

CRDT- 2022/02/09 05:43

PHST- 2021/08/17 00:00 [received]

PHST- 2021/10/15 00:00 [accepted]

PHST- 2021/10/03 00:00 [revised]

PHST- 2022/02/10 06:00 [pubmed]

PHST- 2022/05/07 06:00 [medline]

PHST- 2022/02/09 05:43 [entrez]

AID - 10.1038/s41429-022-00503-z [pii]

AID - 10.1038/s41429-022-00503-z [doi]

PST - ppublish

SO - J Antibiot (Tokyo). 2022 Apr;75(4):226-235. doi: 10.1038/s41429-022-00503-z. Epub

2022 Feb 8.

PMID- 35045688

OWN - NLM

STAT- PubMed-not-MEDLINE

LR - 20220419

IS - 1738-3536 (Print)

IS - 2005-6184 (Electronic)

IS - 1738-3536 (Linking)

VI - 85

IP - 2

DP - 2022 Apr

TI - Concise Clinical Review of Hematologic Toxicity of Linezolid in

Multidrug-Resistant and Extensively Drug-Resistant Tuberculosis: Role of

Mitochondria.

PG - 111-121

LID - 10.4046/trd.2021.0122 [doi]

AB - Multidrug-resistant tuberculosis (MDR-TB) is caused by an organism that is

resistant to both rifampicin and isoniazid. Extensively drug-resistant TB, a rare

type of MDR-TB, is caused by an organism that is resistant to quinolone and one

of group A TB drugs (i.e., linezolid and bedaquiline). In 2018, the World Health

Organization revised the groupings of TB medicines and reclassified linezolid as

a group A drug for the treatment of MDR-TB. Linezolid is a synthetic

antimicrobial agent in the oxazolidinone class. Although linezolid has a good

efficacy, it can cause substantial adverse events, especially hematologic

toxicity. In both TB infection and linezolid mechanism of action, mitochondrial

dysfunction plays an important role. In this concise review, characteristics of

linezolid as an anti-TB drug are summarized, including its efficacy, pathogenesis

of hematologic toxicity highlighting mitochondrial dysfunction, and the

monitoring and management of hematologic toxicity.

FAU - Oehadian, Amaylia

AU - Oehadian A

AD - Division of Hematology and Oncology Medic, Department of Internal Medicine

Faculty of Medicine, Padjadjaran University/Dr. Hasan Sadikin General Hospital,

Bandung, Indonesia.

FAU - Santoso, Prayudi

AU - Santoso P

AD - Division of Hematology and Oncology Medic, Department of Internal Medicine

Faculty of Medicine, Padjadjaran University/Dr. Hasan Sadikin General Hospital,

Bandung, Indonesia.

FAU - Menzies, Dick

AU - Menzies D

AD - McGill International TB Centre Respiratory Epidemiology and Clinical Research

Unit, Montreal, Canada.

FAU - Ruslami, Rovina

AU - Ruslami R

AD - Division of Pharmacology, Department of Biomedical Science, Padjadjaran

University/Dr. Hasan Sadikin General Hospital, Bandung, Indonesia.

AD - Jl. Prof. Eyckman No. 38, Bandung, Indonesia.

LA - eng

GR - 1595/UN6.3.1/PT.00/2021/Universitas Padjadjaran/

GR - 3855/UN.C/LT/2019/Universitas Padjadjaran/

PT - Journal Article

PT - Review

DEP - 20220120

PL - Korea (South)

TA - Tuberc Respir Dis (Seoul)

JT - Tuberculosis and respiratory diseases

JID - 101479418

PMC - PMC8987663

OTO - NOTNLM

OT - Hematologic Toxicity

OT - Linezolid

OT - MDR-TB

OT - Mitochondria

OT - XDR-TB

COIS- Conflicts of Interest No potential conflict of interest relevant to this article

was reported.

EDAT- 2022/01/21 06:00

MHDA- 2022/01/21 06:01

PMCR- 2022/04/01

CRDT- 2022/01/20 03:00

PHST- 2021/08/07 00:00 [received]

PHST- 2022/01/16 00:00 [accepted]

PHST- 2022/01/21 06:00 [pubmed]

PHST- 2022/01/21 06:01 [medline]

PHST- 2022/01/20 03:00 [entrez]

PHST- 2022/04/01 00:00 [pmc-release]

AID - trd.2021.0122 [pii]

AID - trd-2021-0122 [pii]

AID - 10.4046/trd.2021.0122 [doi]

PST - ppublish

SO - Tuberc Respir Dis (Seoul). 2022 Apr;85(2):111-121. doi: 10.4046/trd.2021.0122.

Epub 2022 Jan 20.

PMID- 34414591

OWN - NLM

STAT- MEDLINE

DCOM- 20220502

LR - 20220524

IS - 1098-2299 (Electronic)

IS - 0272-4391 (Linking)

VI - 83

IP - 2

DP - 2022 Apr

TI - Conventional and microwave-assisted organic synthesis of novel antimycobacterial

agents bearing furan and pyridine hybrids.

PG - 416-431

LID - 10.1002/ddr.21872 [doi]

AB - Drug resistance in tuberculosis poses a serious threat to humanity because

currently available antitubercular drugs are ineffective against Mycobacterium

tuberculosis (M. tuberculosis). As a result, the approval of Bedaquiline and

Delamanid for the treatment of drug-resistant tuberculosis was accelerated.

Still, there is an urgent need to search for new antitubercular drugs with novel

mechanisms of action (MoA). Due to this, we have designed a synthetic strategy by

utilizing microwave-assisted organic synthesis. We have compared our method with

the conventional procedure, and the data show that our procedure is more

effective in the preparation of title compounds. A unique series of

1-(2-(furan-2-yl)-5-(pyridin-4-yl)-1,3,4-oxadiazol-3(2H)-yl)-3-(aryl)-prop-2-en-1-ones

(5a-o) was synthesized utilizing conventional and microwave-assisted techniques.

Synthetic compounds were investigated for antitubercular activity against

Mycobacterium TB H(37) Ra and Mycobacterium bovis (M. bovis). Compound 5b was

reported to be the most effective against M. tuberculosis H(37) Ra (97.69 percent

inhibition at 30 μg/ml) and M. bovis (97.09 percent inhibition at 30 μg/ml). An

in silico binding affinity study of mycobacterial enoyl-acyl carrier protein

reductase (InhA) reveals the binding mechanism and thermodynamic interactions

that determine these molecule's binding affinity. Compound 5b had a high glide

score of -8.991 and low glide energy of -49.893 kcal/mol.

CI - © 2021 Wiley Periodicals LLC.

FAU - Desai, Nisheeth C

AU - Desai NC

AUID- ORCID: 0000-0001-6864-8661

AD - Division of Medicinal Chemistry, Department of Chemistry, Mahatma Gandhi Campus,

Maharaja Krishnakumarsinhji Bhavnagar University, Bhavnagar, India.

FAU - Bhatt, Kandarp

AU - Bhatt K

AD - Division of Medicinal Chemistry, Department of Chemistry, Mahatma Gandhi Campus,

Maharaja Krishnakumarsinhji Bhavnagar University, Bhavnagar, India.

FAU - Jadeja, Dharmpalsinh J

AU - Jadeja DJ

AD - Division of Medicinal Chemistry, Department of Chemistry, Mahatma Gandhi Campus,

Maharaja Krishnakumarsinhji Bhavnagar University, Bhavnagar, India.

FAU - Mehta, Harsh K

AU - Mehta HK

AD - Division of Medicinal Chemistry, Department of Chemistry, Mahatma Gandhi Campus,

Maharaja Krishnakumarsinhji Bhavnagar University, Bhavnagar, India.

FAU - Khedkar, Vijay M

AU - Khedkar VM

AD - School of Pharmacy, Vishwakarma University, Pune, India.

FAU - Sarkar, Dhiman

AU - Sarkar D

AD - Combichem Bio-resource Centre, CSIR-National Chemical Laboratory, Pune, India.

LA - eng

PT - Journal Article

DEP - 20210820

PL - United States

TA - Drug Dev Res

JT - Drug development research

JID - 8204468

RN - 0 (Antitubercular Agents)

RN - 0 (Furans)

RN - 0 (Pyridines)

SB - IM

MH - Antitubercular Agents/chemistry

MH - Chemistry Techniques, Synthetic

MH - Furans/pharmacology/therapeutic use

MH - Humans

MH - Microbial Sensitivity Tests

MH - Microwaves

MH - Molecular Docking Simulation

MH - *Mycobacterium tuberculosis

MH - Pyridines/pharmacology

MH - Structure-Activity Relationship

MH - *Tuberculosis/drug therapy

OTO - NOTNLM

OT - antitubercular activity

OT - microwave-assisted organic synthesis

OT - molecular docking

EDAT- 2021/08/21 06:00

MHDA- 2022/05/03 06:00

CRDT- 2021/08/20 07:22

PHST- 2021/07/21 00:00 [revised]

PHST- 2021/06/12 00:00 [received]

PHST- 2021/07/23 00:00 [accepted]

PHST- 2021/08/21 06:00 [pubmed]

PHST- 2022/05/03 06:00 [medline]

PHST- 2021/08/20 07:22 [entrez]

AID - 10.1002/ddr.21872 [doi]

PST - ppublish

SO - Drug Dev Res. 2022 Apr;83(2):416-431. doi: 10.1002/ddr.21872. Epub 2021 Aug 20.

PMID- 35089314

OWN - NLM

STAT- MEDLINE

DCOM- 20220401

LR - 20220602

IS - 1460-2091 (Electronic)

IS - 0305-7453 (Print)

IS - 0305-7453 (Linking)

VI - 77

IP - 4

DP - 2022 Mar 31

TI - Delamanid or pretomanid? A Solomonic judgement!

PG - 880-902

LID - 10.1093/jac/dkab505 [doi]

AB - Given the low treatment success rates of drug-resistant tuberculosis (TB), novel

TB drugs are urgently needed. The landscape of TB treatment has changed

considerably over the last decade with the approval of three new compounds:

bedaquiline, delamanid and pretomanid. Of these, delamanid and pretomanid belong

to the same class of drugs, the nitroimidazoles. In order to close the knowledge

gap on how delamanid and pretomanid compare with each other, we summarize the

main findings from preclinical research on these two compounds. We discuss the

compound identification, mechanism of action, drug resistance, in vitro activity,

in vivo pharmacokinetic profiles, and preclinical in vivo activity and efficacy.

Although delamanid and pretomanid share many similarities, several differences

could be identified. One finding of particular interest is that certain

Mycobacterium tuberculosis isolates have been described that are resistant to

either delamanid or pretomanid, but with preserved susceptibility to the other

compound. This might imply that delamanid and pretomanid could replace one

another in certain regimens. Regarding bactericidal activity, based on in vitro

and preclinical in vivo activity, delamanid has lower MICs and higher

mycobacterial load reductions at lower drug concentrations and doses compared

with pretomanid. However, when comparing in vivo preclinical bactericidal

activity at dose levels equivalent to currently approved clinical doses based on

drug exposure, this difference in activity between the two compounds fades.

However, it is important to interpret these comparative results with caution

knowing the variability inherent in preclinical in vitro and in vivo models.

CI - © The Author(s) 2022. Published by Oxford University Press on behalf of British

Society for Antimicrobial Chemotherapy.

FAU - Mudde, Saskia E

AU - Mudde SE

AD - Department of Medical Microbiology and Infectious Diseases, Erasmus University

Medical Center, Rotterdam, The Netherlands.

FAU - Upton, Anna M

AU - Upton AM

AD - Evotec, Princeton, New Jersey, USA.

FAU - Lenaerts, Anne

AU - Lenaerts A

AD - Mycobacteria Research Laboratories, Department of Microbiology, Immunology, and

Pathology, Colorado State University, Fort Collins, CO, USA.

FAU - Bax, Hannelore I

AU - Bax HI

AD - Department of Medical Microbiology and Infectious Diseases, Erasmus University

Medical Center, Rotterdam, The Netherlands.

AD - Department of Internal Medicine, Section of Infectious Diseases, Erasmus

University Medical Center, Rotterdam, The Netherlands.

FAU - De Steenwinkel, Jurriaan E M

AU - De Steenwinkel JEM

AUID- ORCID: 0000-0002-9910-156X

AD - Department of Medical Microbiology and Infectious Diseases, Erasmus University

Medical Center, Rotterdam, The Netherlands.

LA - eng

PT - Journal Article

PT - Research Support, Non-U.S. Gov't

PL - England

TA - J Antimicrob Chemother

JT - The Journal of antimicrobial chemotherapy

JID - 7513617

RN - 0 (Antitubercular Agents)

RN - 0 (Diarylquinolines)

RN - 0 (Nitroimidazoles)

RN - 0 (OPC-67683)

RN - 0 (Oxazoles)

RN - 0 (pretomanid)

SB - IM

MH - Antitubercular Agents/pharmacology/therapeutic use

MH - Diarylquinolines/therapeutic use

MH - Humans

MH - *Mycobacterium tuberculosis

MH - *Nitroimidazoles/pharmacology/therapeutic use

MH - Oxazoles/pharmacology/therapeutic use

MH - *Tuberculosis, Multidrug-Resistant/drug therapy

PMC - PMC8969540

EDAT- 2022/01/29 06:00

MHDA- 2022/04/02 06:00

PMCR- 2022/01/28

CRDT- 2022/01/28 12:12

PHST- 2022/01/29 06:00 [pubmed]

PHST- 2022/04/02 06:00 [medline]

PHST- 2022/01/28 12:12 [entrez]

PHST- 2022/01/28 00:00 [pmc-release]

AID - 6516247 [pii]

AID - dkab505 [pii]

AID - 10.1093/jac/dkab505 [doi]

PST - ppublish

SO - J Antimicrob Chemother. 2022 Mar 31;77(4):880-902. doi: 10.1093/jac/dkab505.

PMID- 35496824

OWN - NLM

STAT- PubMed-not-MEDLINE

LR - 20240827

IS - 2590-2555 (Electronic)

IS - 2590-2555 (Linking)

VI - 3

DP - 2022

TI - All trans retinoic acid as a host-directed immunotherapy for tuberculosis.

PG - 54-72

LID - 10.1016/j.crimmu.2022.03.003 [doi]

AB - Tuberculosis (TB) is the top bacterial infectious disease killer and one of the

top ten causes of death worldwide. The emergence of strains of multiple

drug-resistant tuberculosis (MDR-TB) has pushed our available stock of anti-TB

agents to the limit of effectiveness. This has increased the urgent need to

develop novel treatment strategies using currently available resources. An

adjunctive, host-directed therapy (HDT) designed to act on the host, instead of

the bacteria, by boosting the host immune response through activation of

intracellular pathways could be the answer. The integration of multidisciplinary

approaches of repurposing currently FDA-approved drugs, with a targeted

drug-delivery platform is a very promising option to reduce the long timeline

associated with the approval of new drugs - time that cannot be afforded given

the current levels of morbidity and mortality associated with TB infection. The

deficiency of vitamin A has been reported to be highly associated with the

increased susceptibility of TB. All trans retinoic acid (ATRA), the active

metabolite of vitamin A, has proven to be very efficacious against TB both in

vitro and in vivo. In this review, we discuss and summarise the importance of

vitamin A metabolites in the fight against TB and what is known regarding the

molecular mechanisms of ATRA as a host-directed therapy for TB including its

effect on macrophages cytokine profile and cellular pathways. Furthermore, we

focus on the issues behind why previous clinical trials with vitamin A

supplementation have failed, and how these issues might be overcome.

CI - © 2022 The Authors.

FAU - Bahlool, Ahmad Z

AU - Bahlool AZ

AD - School of Pharmacy and Biomolecular Sciences (PBS), Royal College of Surgeons in

Ireland (RCSI), 123 St Stephens Green, Dublin 2, Ireland.

AD - Tissue Engineering Research Group (TERG), Royal College of Surgeons in Ireland

(RCSI), 123 St Stephens Green, Dublin 2, Ireland.

AD - Department of Clinical Medicine, Trinity Translational Medicine Institute, St.

James's Hospital, Trinity College Dublin, The University of Dublin, Dublin 8,

Ireland.

FAU - Grant, Conor

AU - Grant C

AD - Department of Clinical Medicine, Trinity Translational Medicine Institute, St.

James's Hospital, Trinity College Dublin, The University of Dublin, Dublin 8,

Ireland.

FAU - Cryan, Sally-Ann

AU - Cryan SA

AD - School of Pharmacy and Biomolecular Sciences (PBS), Royal College of Surgeons in

Ireland (RCSI), 123 St Stephens Green, Dublin 2, Ireland.

AD - Tissue Engineering Research Group (TERG), Royal College of Surgeons in Ireland

(RCSI), 123 St Stephens Green, Dublin 2, Ireland.

AD - SFI Advanced Materials and Bioengineering Research (AMBER) Centre, RCSI & TCD,

Dublin, Ireland.

AD - SFI Centre for Research in Medical Devices (CURAM), RCSI, Dublin and National

University of Ireland, Galway, Ireland.

FAU - Keane, Joseph

AU - Keane J

AD - Department of Clinical Medicine, Trinity Translational Medicine Institute, St.

James's Hospital, Trinity College Dublin, The University of Dublin, Dublin 8,

Ireland.

FAU - O'Sullivan, Mary P

AU - O'Sullivan MP

AD - Department of Clinical Medicine, Trinity Translational Medicine Institute, St.

James's Hospital, Trinity College Dublin, The University of Dublin, Dublin 8,

Ireland.

LA - eng

GR - WT_/Wellcome Trust/United Kingdom

PT - Journal Article

PT - Review

DEP - 20220330

PL - Netherlands

TA - Curr Res Immunol

JT - Current research in immunology

JID - 101776898

PMC - PMC9040133

OTO - NOTNLM

OT - Host-directed therapy (HDT)

OT - Macrophage

OT - Retinoic acid (ATRA)

OT - Tuberculosis (TB)

COIS- The authors declare that they have no known competing financial interests or

personal relationships that could have appeared to influence the work reported in

this paper.

EDAT- 2022/05/03 06:00

MHDA- 2022/05/03 06:01

PMCR- 2022/03/30

CRDT- 2022/05/02 06:55

PHST- 2021/12/07 00:00 [received]

PHST- 2022/03/11 00:00 [revised]

PHST- 2022/03/22 00:00 [accepted]

PHST- 2022/05/02 06:55 [entrez]

PHST- 2022/05/03 06:00 [pubmed]

PHST- 2022/05/03 06:01 [medline]

PHST- 2022/03/30 00:00 [pmc-release]

AID - S2590-2555(22)00004-X [pii]

AID - 10.1016/j.crimmu.2022.03.003 [doi]

PST - epublish

SO - Curr Res Immunol. 2022 Mar 30;3:54-72. doi: 10.1016/j.crimmu.2022.03.003.

eCollection 2022.

PMID- 35240539

OWN - NLM

STAT- MEDLINE

DCOM- 20220504

LR - 20220531

IS - 1873-281X (Electronic)

IS - 1472-9792 (Linking)

VI - 133

DP - 2022 Mar

TI - Characterization of embB mutations involved in ethambutol resistance in

multi-drug resistant Mycobacterium tuberculosis isolates in Zambia.

PG - 102184

LID - S1472-9792(22)00021-X [pii]

LID - 10.1016/j.tube.2022.102184 [doi]

AB - BACKGROUND: Ethambutol (EMB) is an important anti-tuberculosis drug used in the

management of multi-drug resistant tuberculosis (MDR-TB). Mutations in embB are

the major mechanism of resistance. This study investigated embB mutations among

MDR-TB isolates and analyzed their correlations with phenotypic drug

susceptibility testing (DST) in Zambia. METHOD: A total of 132 MDR-TB isolates

were collected from January 2014 to April 2017 and characterized using MGIT 960

systems, embB sequencing, and spoligotyping. RESULTS: Out of 61 phenotypically

EMB resistant isolates, 53 had mutations in embB. Among the 71 EMB susceptible

isolates, 47 had embB mutations. Sensitivity of embB mutations was 86.9% while

specificity was 33.8%. CAS1_Kili (SIT21) had high odds of having embB mutations,

particularly, G918A (Met306eIl) (Odds ratio 16.7, p < 0.0001). CONCLUSION:

Molecular EMB resistance testing by DNA sequencing can improve detection of EMB

resistance among MDR-TB patients in Zambia. Additionally, CAS1_Kili was

associated with embB amino acid substitution Met306Ile suggesting transmission. A

detailed investigation to track and determine transmission hotspot area for

MDR-TB could help optimize control strategies.

CI - Copyright © 2022 Elsevier Ltd. All rights reserved.

FAU - Bwalya, Precious

AU - Bwalya P

AD - Division of Bioresources, Hokkaido University International Institute for

Zoonosis Control, Sapporo, 001-0020, Japan; Department of Pathology and

Microbiology, University Teaching Hospital, Ministry of Health, Lusaka, 10101,

Zambia.

FAU - Solo, Eddie S

AU - Solo ES

AD - Department of Pathology and Microbiology, University Teaching Hospital, Ministry

of Health, Lusaka, 10101, Zambia.

FAU - Chizimu, Joseph Y

AU - Chizimu JY

AD - Division of Bioresources, Hokkaido University International Institute for

Zoonosis Control, Sapporo, 001-0020, Japan; Zambia National Public Health

Institute, Ministry Health, Lusaka, 10101, Zambia.

FAU - Shrestha, Dipti

AU - Shrestha D

AD - Division of Bioresources, Hokkaido University International Institute for

Zoonosis Control, Sapporo, 001-0020, Japan; Department of Microbiology, Kathmandu

College of Science and Technology, Tribhuvan University, Kathmandu, Nepal.

FAU - Mbulo, Grace

AU - Mbulo G

AD - Department of Pathology and Microbiology, University Teaching Hospital, Ministry

of Health, Lusaka, 10101, Zambia.

FAU - Thapa, Jeewan

AU - Thapa J

AD - Division of Bioresources, Hokkaido University International Institute for

Zoonosis Control, Sapporo, 001-0020, Japan.

FAU - Nakajima, Chie

AU - Nakajima C

AD - Division of Bioresources, Hokkaido University International Institute for

Zoonosis Control, Sapporo, 001-0020, Japan; International Collaboration Unit,

Hokkaido University International Institute for Zoonosis Control, Sapporo,

001-0020, Japan.

FAU - Suzuki, Yasuhiko

AU - Suzuki Y

AD - Division of Bioresources, Hokkaido University International Institute for

Zoonosis Control, Sapporo, 001-0020, Japan; International Collaboration Unit,

Hokkaido University International Institute for Zoonosis Control, Sapporo,

001-0020, Japan. Electronic address: suzuki@czc.hokudai.ac.jp.

LA - eng

PT - Journal Article

PT - Research Support, Non-U.S. Gov't

DEP - 20220224

PL - Scotland

TA - Tuberculosis (Edinb)

JT - Tuberculosis (Edinburgh, Scotland)

JID - 100971555

RN - 0 (Antitubercular Agents)

RN - 8G167061QZ (Ethambutol)

RN - EC 2.4.2.- (Pentosyltransferases)

SB - IM

MH - Antitubercular Agents/metabolism/pharmacology/therapeutic use

MH - Ethambutol/pharmacology/therapeutic use

MH - Humans

MH - Microbial Sensitivity Tests

MH - Mutation

MH - *Mycobacterium tuberculosis

MH - Pentosyltransferases/genetics

MH - *Tuberculosis, Multidrug-Resistant/diagnosis/drug therapy/genetics

MH - Zambia/epidemiology

OTO - NOTNLM

OT - Ethambutol

OT - Multi-drug resistance

OT - Mycobacterium tuberculosis

OT - Zambia

OT - embB mutations

EDAT- 2022/03/04 06:00

MHDA- 2022/05/06 06:00

CRDT- 2022/03/03 20:19

PHST- 2021/10/30 00:00 [received]

PHST- 2022/02/14 00:00 [revised]

PHST- 2022/02/20 00:00 [accepted]

PHST- 2022/03/04 06:00 [pubmed]

PHST- 2022/05/06 06:00 [medline]

PHST- 2022/03/03 20:19 [entrez]

AID - S1472-9792(22)00021-X [pii]

AID - 10.1016/j.tube.2022.102184 [doi]

PST - ppublish

SO - Tuberculosis (Edinb). 2022 Mar;133:102184. doi: 10.1016/j.tube.2022.102184. Epub

2022 Feb 24.

PMID- 33074049

OWN - NLM

STAT- MEDLINE

DCOM- 20220315

LR - 20220315

IS - 1538-0254 (Electronic)

IS - 0739-1102 (Linking)

VI - 40

IP - 5

DP - 2022 Mar

TI - Development of potential proteasome inhibitors against Mycobacterium

tuberculosis.

PG - 2189-2203

LID - 10.1080/07391102.2020.1835722 [doi]

AB - Tuberculosis (TB) has been recently declared as a health emergency because of

sporadic increase in Multidrug-resistant Tuberculosis (MDR-TB) problem throughout

the world. TB causing bacteria, Mycobacterium tuberculosis has become resistant

to the first line of treatment along with second line of treatment and drugs,

which are accessible to us. Thus, there is an urgent need of identification of

key targets and development of potential therapeutic approach(s), which can

overcome the Mycobacterium tuberculosis complications. In the present study,

Mycobacterium tuberculosis proteasome has been taken as a potential target as it

is one of the key regulatory proteins in Mycobacterium tuberculosis propagation.

Further, a library of 400 compounds (small molecule) from Medicines for Malaria

Venture (MMV) were screened against the target (proteasome) using molecular

docking and simulation approach, and selected lead compounds were validated in in

vitro model. In this study, we have identified two potent small molecules from

the MMV Pathogen Box library, MMV019838 and MMV687146 with -9.8 kcal/mol and

-8.7 kcal/mol binding energy respectively, which actively interact with the

catalytic domain/active domain of Mycobacterium tuberculosis proteasome and

inhibit the Mycobacterium tuberculosis growth in in vitro culture. Furthermore,

the molecular docking and simulation study of MMV019838 and MMV687146 with

proteasome show strong and stable interaction with Mycobacterium tuberculosis

compared to human proteasome and show no cytotoxicity effect. A better

understanding of proteasome inhibition in Mycobacterium tuberculosis in in vitro

and in vivo model would eventually allow us to understand the molecular

mechanism(s) and discover a novel and potent therapeutic agent against

Tuberculosis. Active efflux of drugs mediated by efflux pumps that confer drug

resistance is one of the mechanisms developed by bacteria to counter the adverse

effects of antibiotics and chemicals. Efflux pump activity was tested for a

specific compound MMV019838 which was showing good in silico results than MIC

values.Communicated by Ramaswamy H. Sarma.

FAU - Tyagi, Rashmi

AU - Tyagi R

AD - Centre for Drug Design Discovery and Development (C4D), SRM University, Delhi,

Haryana, India.

FAU - Srivastava, Mitul

AU - Srivastava M

AD - Translational Health Science and Technology Institute, NCR Biotech Science

Cluster, Faridabad, India.

FAU - Jain, Preeti

AU - Jain P

AD - Special Centre for Molecular Medicine, Jawaharlal Nehru University, New Delhi,

India.

FAU - Pandey, Ramendra Pati

AU - Pandey RP

AD - Centre for Drug Design Discovery and Development (C4D), SRM University, Delhi,

Haryana, India.

FAU - Asthana, Shailendra

AU - Asthana S

AD - Translational Health Science and Technology Institute, NCR Biotech Science

Cluster, Faridabad, India.

FAU - Kumar, Dhruv

AU - Kumar D

AD - Amity Institute of Molecular Medicine and Stem Cell Research, Amity University

Uttar Pradesh, Noida, India.

FAU - Raj, V Samuel

AU - Raj VS

AD - Centre for Drug Design Discovery and Development (C4D), SRM University, Delhi,

Haryana, India.

LA - eng

PT - Journal Article

DEP - 20201019

PL - England

TA - J Biomol Struct Dyn

JT - Journal of biomolecular structure & dynamics

JID - 8404176

RN - 0 (Antitubercular Agents)

RN - 0 (Proteasome Inhibitors)

SB - IM

MH - Antitubercular Agents/chemistry

MH - Humans

MH - Molecular Docking Simulation

MH - *Mycobacterium tuberculosis

MH - Proteasome Inhibitors/metabolism/pharmacology/therapeutic use

MH - *Tuberculosis, Multidrug-Resistant/drug therapy

OTO - NOTNLM

OT - Autodock

OT - MTT assay

OT - Mycobacterium tuberculosis

OT - molecular docking

OT - molecular dynamics

OT - proteasome

EDAT- 2020/10/20 06:00

MHDA- 2022/03/16 06:00

CRDT- 2020/10/19 12:12

PHST- 2020/10/20 06:00 [pubmed]

PHST- 2022/03/16 06:00 [medline]

PHST- 2020/10/19 12:12 [entrez]

AID - 10.1080/07391102.2020.1835722 [doi]

PST - ppublish

SO - J Biomol Struct Dyn. 2022 Mar;40(5):2189-2203. doi:

10.1080/07391102.2020.1835722. Epub 2020 Oct 19.

PMID- 35336089

OWN - NLM

STAT- PubMed-not-MEDLINE

LR - 20220329

IS - 2076-2607 (Print)

IS - 2076-2607 (Electronic)

IS - 2076-2607 (Linking)

VI - 10

IP - 3

DP - 2022 Feb 26

TI - Pre-Clinical Tools for Predicting Drug Efficacy in Treatment of Tuberculosis.

LID - 10.3390/microorganisms10030514 [doi]

LID - 514

AB - Combination therapy has, to some extent, been successful in limiting the

emergence of drug-resistant tuberculosis. Drug combinations achieve this

advantage by simultaneously acting on different targets and metabolic pathways.

Additionally, drug combination therapies are shown to shorten the duration of

therapy for tuberculosis. As new drugs are being developed, to overcome the

challenge of finding new and effective drug combinations, systems biology

commonly uses approaches that analyse mycobacterial cellular processes. These

approaches identify the regulatory networks, metabolic pathways, and signaling

programs associated with M. tuberculosis infection and survival. Different

preclinical models that assess anti-tuberculosis drug activity are available, but

the combination of models that is most predictive of clinical treatment efficacy

remains unclear. In this structured literature review, we appraise the options to

accelerate the TB drug development pipeline through the evaluation of preclinical

testing assays of drug combinations.

FAU - Margaryan, Hasmik

AU - Margaryan H

AD - UCL Centre for Clinical Microbiology, Division of Infection & Immunity, UCL,

Royal Free Campus, London NW3 2PF, UK.

FAU - Evangelopoulos, Dimitrios D

AU - Evangelopoulos DD

AUID- ORCID: 0000-0003-0472-7576

AD - Department of Microbial Diseases, Eastman Dental Institute, UCL, Royal Free

Campus, Rowland Hill Street, London NW3 2PF, UK.

FAU - Muraro Wildner, Leticia

AU - Muraro Wildner L

AD - UCL Centre for Clinical Microbiology, Division of Infection & Immunity, UCL,

Royal Free Campus, London NW3 2PF, UK.

FAU - McHugh, Timothy D

AU - McHugh TD

AUID- ORCID: 0000-0003-4658-8594

AD - UCL Centre for Clinical Microbiology, Division of Infection & Immunity, UCL,

Royal Free Campus, London NW3 2PF, UK.

LA - eng

PT - Journal Article

PT - Review

DEP - 20220226

PL - Switzerland

TA - Microorganisms

JT - Microorganisms

JID - 101625893

PMC - PMC8956012

OTO - NOTNLM

OT - MDR-TB

OT - drug activity

OT - drug combinations

OT - drug efficacy

OT - high order combinations

OT - in vitro preclinical modelling

OT - synergism

OT - transcriptomics

OT - tuberculosis

COIS- The authors declare no conflict of interest.

EDAT- 2022/03/27 06:00

MHDA- 2022/03/27 06:01

PMCR- 2022/02/26

CRDT- 2022/03/26 01:05

PHST- 2022/01/28 00:00 [received]

PHST- 2022/02/22 00:00 [accepted]

PHST- 2022/03/26 01:05 [entrez]

PHST- 2022/03/27 06:00 [pubmed]

PHST- 2022/03/27 06:01 [medline]

PHST- 2022/02/26 00:00 [pmc-release]

AID - microorganisms10030514 [pii]

AID - microorganisms-10-00514 [pii]

AID - 10.3390/microorganisms10030514 [doi]

PST - epublish

SO - Microorganisms. 2022 Feb 26;10(3):514. doi: 10.3390/microorganisms10030514.

PMID- 34896768

OWN - NLM

STAT- MEDLINE

DCOM- 20220203

LR - 20220203

IS - 1873-3344 (Electronic)

IS - 0162-0134 (Linking)

VI - 227

DP - 2022 Feb

TI - New silver(I) phosphino complexes: Evaluation of their potential as prospective

agents against Mycobacterium tuberculosis.

PG - 111683

LID - S0162-0134(21)00330-5 [pii]

LID - 10.1016/j.jinorgbio.2021.111683 [doi]

AB - Despite being a preventable and curable disease, Tuberculosis (TB) is the world's

top infectious killer. Development of new drugs is urgently needed. In this work,

the synthesis and characterization of new silver(I) complexes, that include

N'-[(E)-(pyridine-2-ylmethylene)pyrazine-2-carbohydrazide, HPCPH, as main ligand

and substituted aryl-phosphines as auxiliary ligands, is reported. HPCPH was

synthesized from pyrazinoic acid, the active metabolite of the first-line

antimycobacterial drug pyrazinamide. Complexes [Ag(HPCPH)(PPh(3))(2)]OTf (1),

[Ag(HPCPH)((P(p-tolyl)(3))(2)]OTf (2) and [Ag(HPCPH)(P(p-anisyl)(3))(2)]OTf (3)

were characterized in solid state and in solution by elemental analysis and FTIR

and NMR spectroscopies (OTftriflate). Crystal structures of (1,2) were determined

by XRD. The Ag atom is coordinated to azomethine and pyridine nitrogen atoms of

HPCPH ligand and to the phosphorous atom of each aryl-phosphine co-ligand.

Although HPCPH did not show activity, the Ag(I) compounds demonstrated activity

against Mycobacterium tuberculosis (MTB), H(37)Rv strain, and multi-drug

resistant clinical isolates (MDR-TB). Globally, results showed that the compounds

are not only effective against the sensitive strain, but are more potent against

MDR-TB than antimycobacterial drugs used in therapy. The compounds showed low to

moderate selectivity index values (SI) towards the bacteria, using MRC-5 cells

(ATCC CCL-171) as mammalian cell model. Interaction with DNA was explored to get

insight into the potential mechanism of action against the pathogen. No

significant interaction was detected, allowing to discard this biomolecule as a

potential molecular target. Compound 1 was identified as a hit compound (MIC(90)

2.23 μM; SI 4.4) to develop further chemical modifications in the search for new

drugs.

CI - Copyright © 2021 Elsevier Inc. All rights reserved.

FAU - Maldonado, Yndira Dolores

AU - Maldonado YD

AD - Escuela de Ingeniería Química, Facultad de Ingeniería Química y Textil,

Universidad Nacional de Ingeniería, Lima, Peru; Laboratorio de Biopolímeros y

Metalofármacos, LIBIPMET, Facultad de Ciencias, Universidad Nacional de

Ingeniería, Lima, Peru.

FAU - Scalese, Gonzalo

AU - Scalese G

AD - Área Química Inorgánica, Facultad de Química, Universidad de la República,

Montevideo, Uruguay.

FAU - Manieri, Karyn Fernanda

AU - Manieri KF

AD - Faculdade de Ciências Farmacêuticas, UNESP, Araraquara, Brazil.

FAU - Pavan, Fernando R

AU - Pavan FR

AD - Faculdade de Ciências Farmacêuticas, UNESP, Araraquara, Brazil.

FAU - Aguirre Méndez, Larry D

AU - Aguirre Méndez LD

AD - Laboratorio de Biopolímeros y Metalofármacos, LIBIPMET, Facultad de Ciencias,

Universidad Nacional de Ingeniería, Lima, Peru; Facultad de Ingeniería Eléctrica

y Electrónica, Universidad Nacional de Ingeniería, Lima, Peru.

FAU - Gambino, Dinorah

AU - Gambino D

AD - Área Química Inorgánica, Facultad de Química, Universidad de la República,

Montevideo, Uruguay. Electronic address: dgambino@fq.edu.uy.

LA - eng

PT - Journal Article

PT - Research Support, Non-U.S. Gov't

DEP - 20211203

PL - United States

TA - J Inorg Biochem

JT - Journal of inorganic biochemistry

JID - 7905788

RN - 0 (Antitubercular Agents)

RN - 0 (Coordination Complexes)

RN - 0 (Hydrazines)

RN - 3M4G523W1G (Silver)

SB - IM

MH - *Antitubercular Agents/chemical synthesis/chemistry/pharmacology

MH - Cell Line

MH - *Coordination Complexes/chemical synthesis/chemistry/pharmacology

MH - Drug Evaluation, Preclinical

MH - Humans

MH - *Hydrazines/chemistry/pharmacology

MH - Mycobacterium tuberculosis/*growth & development

MH - *Silver/chemistry/pharmacology

OTO - NOTNLM

OT - Arylphosphines

OT - DNA interaction

OT - Lipophilicity

OT - Mycobacterium tuberculosis

OT - Pyrazinoic acid derivative

OT - Silver(I)

EDAT- 2021/12/14 06:00

MHDA- 2022/02/04 06:00

CRDT- 2021/12/13 12:55

PHST- 2021/08/17 00:00 [received]

PHST- 2021/11/19 00:00 [revised]

PHST- 2021/11/28 00:00 [accepted]

PHST- 2021/12/14 06:00 [pubmed]

PHST- 2022/02/04 06:00 [medline]

PHST- 2021/12/13 12:55 [entrez]

AID - S0162-0134(21)00330-5 [pii]

AID - 10.1016/j.jinorgbio.2021.111683 [doi]

PST - ppublish

SO - J Inorg Biochem. 2022 Feb;227:111683. doi: 10.1016/j.jinorgbio.2021.111683. Epub

2021 Dec 3.

PMID- 34601761

OWN - NLM

STAT- MEDLINE

DCOM- 20220314

LR - 20220314

IS - 1097-0134 (Electronic)

IS - 0887-3585 (Linking)

VI - 90

IP - 2

DP - 2022 Feb

TI - Molecular insights into the differential efflux mechanism of Rv1634 protein, a

multidrug transporter of major facilitator superfamily in Mycobacterium

tuberculosis.

PG - 566-578

LID - 10.1002/prot.26253 [doi]

AB - Currently, multidrug-resistant tuberculosis (MDR-TB) is a public health crisis

and a major health security threat globally. In Mycobacterium tuberculosis (Mtb),

major facilitator superfamily (MFS) is the largest group of secondary active

transporters. Along with the transport of their natural substrates, MFS proteins

were involved in a drug efflux mechanism that ultimately lead to resistance

against available anti-TB drugs in Mtb. In the present study, the

three-dimensional structure model of an MFS protein, Rv1634, a probable multidrug

transporter from Mtb, was generated using homology modeling. The protein

structure model was found in inward-open conformation having 14 transmembrane

helices. In addition, a central transport channel was deduced across the protein,

and a single binding pocket was identified halfway through the central cavity by

structural alignment with the homologous protein structures. Further, Rv1634

protein was studied based on the differential structural behavior of apo and

ligand-bound forms. All the protein systems were inserted into a phospholipid

bilayer to characterize the conformational dynamics of the protein using

molecular dynamics (MD) simulations. Detailed analysis of the MD trajectories

showed the diverse substrate specificity of the binding pocket for the

antibiotics that caused differential movement in the ciprofloxacin and

norfloxacin, to which Mtb strains have now become resistant. The expulsion of the

drugs outside the bacterial cell occurs through the alternating-access mechanism

of N and C-terminal domains, which is intriguing and essential to the

understanding the drug resistance mechanism in pathogenic bacteria.

CI - © 2021 Wiley Periodicals LLC.

FAU - Singh, Garima

AU - Singh G

AD - Department of Biotechnology, Babasaheb Bhimrao Ambedkar University, Lucknow,

Uttar Pradesh, India.

FAU - Akhter, Yusuf

AU - Akhter Y

AUID- ORCID: 0000-0001-9564-9790

AD - Department of Biotechnology, Babasaheb Bhimrao Ambedkar University, Lucknow,

Uttar Pradesh, India.

LA - eng

PT - Journal Article

PT - Research Support, Non-U.S. Gov't

DEP - 20211019

PL - United States

TA - Proteins

JT - Proteins

JID - 8700181

RN - 0 (Bacterial Proteins)

SB - IM

MH - Bacterial Proteins/*metabolism

MH - *Drug Resistance, Multiple, Bacterial

MH - Humans

MH - Mycobacterium tuberculosis/*metabolism

MH - Protein Structure, Secondary

MH - Tuberculosis/*virology

OTO - NOTNLM

OT - Mycobacterium tuberculosis

OT - Rv1634 protein

OT - major facilitator superfamily

OT - molecular dynamics simulations

OT - multidrug-resistance

OT - tuberculosis

EDAT- 2021/10/04 06:00

MHDA- 2022/03/15 06:00

CRDT- 2021/10/03 21:13

PHST- 2021/09/20 00:00 [revised]

PHST- 2021/03/03 00:00 [received]

PHST- 2021/09/27 00:00 [accepted]

PHST- 2021/10/04 06:00 [pubmed]

PHST- 2022/03/15 06:00 [medline]

PHST- 2021/10/03 21:13 [entrez]

AID - 10.1002/prot.26253 [doi]

PST - ppublish

SO - Proteins. 2022 Feb;90(2):566-578. doi: 10.1002/prot.26253. Epub 2021 Oct 19.

PMID- 35111403

OWN - NLM

STAT- MEDLINE

DCOM- 20230109

LR - 20230111

IS - 2167-8359 (Print)

IS - 2167-8359 (Electronic)

IS - 2167-8359 (Linking)

VI - 10

DP - 2022

TI - Screening and identification of differentially expressed long non-coding RNAs in

multidrug-resistant tuberculosis.

PG - e12776

LID - 10.7717/peerj.12776 [doi]

LID - e12776

AB - BACKGROUND: Efforts to eradicate tuberculosis are largely threatened by

drug-resistant tuberculosis, particularly, multidrug-resistant tuberculosis

(MDR-TB). Screening and identification potential biomarkers for MDR-TB is crucial

to diagnose early and reduce the incidence of MDR-TB. METHODS: To screen the

differentially expressed long non-coding RNAs in MDR-TB, the lncRNA and mRNA

expression profiles in serum derived from healthy controls (HCs), individuals

with MDR-TB and drug-sensitive tuberculosis (DS-TB) were analyzed by microarray

assay and 10 lncRNAs were randomly selected for further validation by reverse

transcription-quantitative real-time PCR(RT-qPCR). The biological functions of

differentially expressed mRNAs as well as relationships between genes and

signaling pathways were investigated using Gene Ontology (GO) and the Kyoto

Encyclopedia of Genes and Genomes (KEGG), respectively. RESULTS: A total of 353

differentially expressed lncRNAs (312 upregulated) and 202 mRNAs (99 upregulated)

were found in the MDR-TB group compared to HCs. And compared with the DS-TB

group, 442 differentially expressed lncRNAs (115 upregulated) and 190 mRNAs (87

upregulated) were found in the MDR-TB group. The expression levels of lncRNA

n335659 were found to differ significantly between each group by RT-qPCR.

Compared with DS-TB group, the GO analysis showed that the differential mRNAs

were mainly enriched in the processes associated with the detection of the

chemical stimulus, the regulation of mRNA metabolic process and neutrophil

activation in the MDR-TB group; the KEGG analysis indicated that the differential

mRNAs between DS-TB and MDR-TB were mainly enriched in proteasome and Notch

signaling pathway, which might reveal a fraction of the mechanism of MDR-TB. The

discovery of the serum lncRNA n335659 might serve as a potential biomarker for

MDR-TB and Notch signaling pathway provided a new clue for the investigation of

the pathological mechanism of MDR-TB.

CI - ©2022 Zhao et al.

FAU - Zhao, Junwei

AU - Zhao J

AD - Department of Clinical Laboratory, Key Clinical Laboratory of Henan Province, The

First Affiliated Hospital of Zhengzhou University, Zhengzhou, China.

FAU - Gao, ShuHui

AU - Gao S

AD - Department of Clinical Laboratory, Key Clinical Laboratory of Henan Province, The

First Affiliated Hospital of Zhengzhou University, Zhengzhou, China.

FAU - Chen, Chunguang

AU - Chen C

AD - Department of Clinical Laboratory, Henan Provincial Infectious Disease Hospital,

Zhengzhou, China.

FAU - Li, Hui

AU - Li H

AD - Tuberculosis Reference Laboratory, Centers for Disease Control and Prevention of

Henan Province, Zhengzhou, China.

FAU - Wang, Shaohua

AU - Wang S

AD - Tuberculosis Reference Laboratory, Centers for Disease Control and Prevention of

Henan Province, Zhengzhou, China.

FAU - Yu, Yongmin

AU - Yu Y

AD - Department of Clinical Laboratory, Henan Provincial Infectious Disease Hospital,

Zhengzhou, China.

FAU - Ming, Liang

AU - Ming L

AD - Department of Clinical Laboratory, Key Clinical Laboratory of Henan Province, The

First Affiliated Hospital of Zhengzhou University, Zhengzhou, China.

LA - eng

PT - Journal Article

PT - Research Support, Non-U.S. Gov't

DEP - 20220117

PL - United States

TA - PeerJ

JT - PeerJ

JID - 101603425

RN - 0 (RNA, Long Noncoding)

RN - 0 (RNA, Messenger)

RN - 0 (Biomarkers)

MH - Humans

MH - *RNA, Long Noncoding/genetics

MH - *Tuberculosis

MH - *Tuberculosis, Multidrug-Resistant/diagnosis

MH - Microarray Analysis

MH - RNA, Messenger/genetics

MH - Biomarkers

PMC - PMC8772445

OTO - NOTNLM

OT - Biomarkers

OT - Long non-coding RNAs

OT - Multidrug-resistant tuberculosis

OT - Serum

OT - n335659

COIS- The authors declare there are no competing interests.

EDAT- 2022/02/04 06:00

MHDA- 2022/02/04 06:01

PMCR- 2022/01/17

CRDT- 2022/02/03 05:34

PHST- 2021/09/22 00:00 [received]

PHST- 2021/12/21 00:00 [accepted]

PHST- 2022/02/03 05:34 [entrez]

PHST- 2022/02/04 06:00 [pubmed]

PHST- 2022/02/04 06:01 [medline]

PHST- 2022/01/17 00:00 [pmc-release]

AID - 12776 [pii]

AID - 10.7717/peerj.12776 [doi]

PST - epublish

SO - PeerJ. 2022 Jan 17;10:e12776. doi: 10.7717/peerj.12776. eCollection 2022.

PMID- 34951359

OWN - NLM

STAT- MEDLINE

DCOM- 20221024

LR - 20221024

IS - 1873-4316 (Electronic)

IS - 1389-2010 (Linking)

VI - 23

IP - 14

DP - 2022

TI - Strategies Employed to Evade the Host Immune Response and the Mechanism of Drug

Resistance in Mycobacterium tuberculosis: In Search of Finding New Targets.

PG - 1704-1720

LID - 10.2174/1389201023666211222164938 [doi]

AB - The partial effectiveness of the host immune response to M. tuberculosis drives

bacteria into a latent state, but it is difficult to eliminate the bacteria

completely. Usually, this latent condition of M. tuberculosis is reversible, and

reactivation of tuberculosis is the leading cause of the majority of

transmission. A number of studies performed on animal models and humans have not

yet provided a detailed understanding of the mechanisms or correlates of immunity

of M. tuberculosis infection or why there is a significant immunity failure to

remove the pathogen. Moreover, the mechanism of resistance involved in

drug-resistant M. tuberculosis leads to the emergence of strains of bacteria that

show significant resistance to the majority of anti-tuberculosis drugs. We have

also provided the recent findings and trends regarding the development of new

drug molecules to treat drug and multidrug-resistant tuberculosis and the

advancements in immunotherapy in the treatment of drug-resistant tuberculosis.

This article provides an in-depth and critical analysis of various strategies

employed by the drug-resistant M. tuberculosis to escape the host immune

response. This bacterium persists in the host for a longer period of time and

leads to the development of tuberculosis infection. Furthermore, we also

discussed the new targets for the effective treatment of drug-resistant

tuberculosis.

CI - Copyright© Bentham Science Publishers; For any queries, please email at

epub@benthamscience.net.

FAU - Sheikh, Bashir A

AU - Sheikh BA

AD - Department of Bioresources, School of Biological Sciences, University of Kashmir,

Srinagar, 190006, J&K, India.

FAU - Bhat, Basharat A

AU - Bhat BA

AD - Department of Bioresources, School of Biological Sciences, University of Kashmir,

Srinagar, 190006, J&K, India.

FAU - Ahmad, Zahoor

AU - Ahmad Z

AD - Clinical Microbiology PK/PD/ Laboratory, IIIM, Sanatnagar, Srinagar, 190005,

India.

FAU - Mir, Manzoor A

AU - Mir MA

AD - Department of Bioresources, School of Biological Sciences, University of Kashmir,

Srinagar, 190006, J&K, India.

LA - eng

GR - TAR/001213/2018/Science and Engineering Research Board, Department of Science and

Technology (SERB-DST) Govt. of India/

PT - Journal Article

PL - Netherlands

TA - Curr Pharm Biotechnol

JT - Current pharmaceutical biotechnology

JID - 100960530

RN - 0 (Antitubercular Agents)

SB - IM

MH - Humans

MH - Animals

MH - *Mycobacterium tuberculosis

MH - Antitubercular Agents/pharmacology/therapeutic use

MH - *Tuberculosis, Multidrug-Resistant/drug therapy

MH - *Tuberculosis, Lymph Node

MH - Drug Resistance

MH - Immunity

OTO - NOTNLM

OT - Tuberculosis

OT - V5 immunitor

OT - anti-tuberculosis drugs

OT - cytokines

OT - drug delivery

OT - drug resistance

OT - immunotherapy

EDAT- 2021/12/25 06:00

MHDA- 2022/10/25 06:00

CRDT- 2021/12/24 08:42

PHST- 2021/07/11 00:00 [received]

PHST- 2021/09/25 00:00 [revised]

PHST- 2021/10/13 00:00 [accepted]

PHST- 2021/12/25 06:00 [pubmed]

PHST- 2022/10/25 06:00 [medline]

PHST- 2021/12/24 08:42 [entrez]

AID - CPB-EPUB-119632 [pii]

AID - 10.2174/1389201023666211222164938 [doi]

PST - ppublish

SO - Curr Pharm Biotechnol. 2022;23(14):1704-1720. doi:

10.2174/1389201023666211222164938.

PMID- 35140414

OWN - NLM

STAT- MEDLINE

DCOM- 20220211

LR - 20220211

IS - 1999-6217 (Electronic)

IS - 1727-5482 (Linking)

VI - 19

IP - 3

DP - 2021 Dec 10

TI - Barriers to Treatment Compliance of Directly Observed Treatment Shortcourse among

Pulmunary Tuberculosis Patients.

PG - 450-459

LID - 10.33314/jnhrc.v19i3.3478 [doi]

AB - BACKGROUND: Treatment compliance is an important aspect for tuberculosis

prevention and control. Poor compliance to treatment can lead to the development

of drug-resistant tuberculosis. The aim of this study was to explore the factors

affecting treatment compliance for tuberculosis patients. METHODS: Facility based

unmatched case control study was done among the forty non-compliance and eighty

compliance pulmonary tuberculosis patients registered at selected directly

observed treatment short-course centers of six districts. Data were collected

using in-depth interview guideline with the tuberculosis focal person and Focus

Group Discussion with tuberculosis patients. RESULTS: A total of 120 respondents,

40 cases and 80 controls were enrolled in the study. About 72.5% of the cases and

56.2% of the controls were male. Five significant independent risk factors for

non-compliance to TB treatment were identified. The qualitative session confirmed

geographical barriers, inaccessibility to health facility, economic barriers,

difficulty in convincing people, knowledge about Directly observed treatment

shortcourse program, longer medication period, migration and stigma as a major

barrier for treatment compliance. CONCLUSIONS: Wider ranges of barriers are

prevalent in context of tuberculosis treatment pathway and outcome. Knowledge of

the tuberculosis patients and attitude of the family plays a vital role in

treatment compliance. Directly observed treatment shortcourse playing tremendous

role to ensure treatment adherence has been identified as major barrier to

adherence as well. Enablers of adherence need to be emphasized to address the

barriers.

FAU - Marahatta, Sujan Babu

AU - Marahatta SB

AD - Manmohan Memorial Institute of Health Sciences, Kathmandu, Nepal.

FAU - Yadav, Rajesh Kumar

AU - Yadav RK

AD - Health Research Together Initiative (HeaRT-Initiaitve), Kathmandu, Nepal.

FAU - Baral, Sushila

AU - Baral S

AD - Health Research Together Initiative (HeaRT-Initiaitve), Kathmandu, Nepal.

FAU - Aryal, Neeta

AU - Aryal N

AD - Health Research Together Initiative (HeaRT-Initiaitve), Kathmandu, Nepal.

FAU - Paudel, Srijana

AU - Paudel S

AD - National Academy of Medical Sciences, Kathmandu, Nepal.

FAU - Shah, Naveen Prakash

AU - Shah NP

AD - National Tuberculosis Centre, Ministry of Health and Population, Bhaktapur,

Nepal.

FAU - Yadav, Punita

AU - Yadav P

AD - Krishan Medial and Technical Research Centre, Janakpurdham, Nepal.

FAU - Gurung, Suman Chandra

AU - Gurung SC

AD - Liverpool School of Tropical Medicine, Department of Clinical Sciences,

Liverpool, UK.

FAU - Khatri, Elina

AU - Khatri E

AD - Nepal Health Research Council, Kathmandu, Nepal.

LA - eng

PT - Journal Article

DEP - 20211210

PL - Nepal

TA - J Nepal Health Res Counc

JT - Journal of Nepal Health Research Council

JID - 101292936

RN - 0 (Antitubercular Agents)

SB - IM

MH - Antitubercular Agents/therapeutic use

MH - Case-Control Studies

MH - Humans

MH - Male

MH - Nepal

MH - Patient Compliance

MH - *Tuberculosis/drug therapy

MH - *Tuberculosis, Pulmonary/drug therapy

OTO - NOTNLM

OT - Barriers; DOTS; mixed method; Nepal; treatment compliance; tuberculosis.

EDAT- 2022/02/11 06:00

MHDA- 2022/02/12 06:00

CRDT- 2022/02/10 05:32

PHST- 2021/03/15 00:00 [received]

PHST- 2021/12/10 00:00 [accepted]

PHST- 2022/02/10 05:32 [entrez]

PHST- 2022/02/11 06:00 [pubmed]

PHST- 2022/02/12 06:00 [medline]

AID - 10.33314/jnhrc.v19i3.3478 [doi]

PST - epublish

SO - J Nepal Health Res Counc. 2021 Dec 10;19(3):450-459. doi:

10.33314/jnhrc.v19i3.3478.

PMID- 34872558

OWN - NLM

STAT- MEDLINE

DCOM- 20220309

LR - 20220309

IS - 1471-2466 (Electronic)

IS - 1471-2466 (Linking)

VI - 21

IP - 1

DP - 2021 Dec 6

TI - Association between body mass index and newly diagnosed drug-resistant pulmonary

tuberculosis in Shandong, China from 2004 to 2019.

PG - 399

LID - 10.1186/s12890-021-01774-2 [doi]

LID - 399

AB - BACKGROUND: Drug-resistant tuberculosis (DR-TB), obesity, and malnutrition are

growing public health problems in the world. However, little has discussed the

impact of different BMI status on the emergence of TB drug resistance. We aimed

to explore the drug-resistant profiles of DR-TB and its clinical predictors among

underweight, overweight or obesity population. METHODS: 8957 newly diagnosed TB

cases with drug susceptibility results and BMI data in Shandong China, from 2004

to 2019 were enrolled. Multivariable and univariable logistic regression models

were applied to investigate the impact of BMI on different drug-resistance.

Clinical predicators and drug-resistant profiles of DR-TB among obesity,

underweight, normal TB group were also described. RESULTS: Among 8957 TB cases,

6417 (71.64%) were normal weight, 2121 (23.68%) were underweight, 373 (4.16%)

were overweight, and 46 (0.51%) were obese. The proportion of drug resistance and

co-morbidity among normal weight, underweight, overweight, obese TB groups were

18.86%/18.25%/20.38%/23.91% (DR-TB), 11.19%/11.74%/9.65%/17.39% (mono-resistant

tuberculosis, MR-TB), 3.41%/3.06%/5.36%/0.00% (multidrug resistant tuberculosis,

MDR-TB), 4.21%/3.39%/5.36%/6.52% (polydrug resistant tuberculosis, PDR-TB),

10.57%/8.44%/19.57%/23.91% (co-morbidity), respectively. Compared with normal

weight group, underweight were associated with lower risk of streptomycin-related

resistance (OR 0.844, 95% CI 0.726-0.982), but contributed to a higher risk of

MR-TB (isoniazid) (odds ratio (OR) 1.347, 95% CI 1.049-1.730; adjusted OR (aOR)

1.31, 95% CI 1.017-1.686), P < 0.05. In addition, overweight were positively

associated with MDR-TB (OR 1.603, 95% CI 1.002-2.566; aOR 1.639, 95% CI

1.02-2.633), isoniazid + rifampicin + streptomycin resistance (OR 1.948, 95%

confidence interval (CI): 1.061-3.577; aOR 2.113, 95% CI 1.141-3.912), Any

isoniazid + streptomycin resistance (OR 1.472, 95% CI 1.013-2.14; aOR 1.483, 95%

CI 1.017-2.164), P < 0.05. CONCLUSIONS: The higher risk of MDR-TB,

isoniazid + rifampicin + streptomycin resistance, Any isoniazid + streptomycin

resistance, and co-morbidity among overweight population implies that routine

screening for drug sensitivity and more attention on co-morbidity among

overweight TB cases may be necessary. In addition, underweight TB cases have a

higher risk of isoniazid resistance. Our study suggests that an in-depth study of

the interaction between host metabolic activity and infection of DR-TB may

contribute more to novel treatment options or preventive measures, and accelerate

the implementation of the STOP TB strategy.

CI - © 2021. The Author(s).

FAU - Song, Wan-Mei

AU - Song WM

AD - Department of Respiratory and Critical Care Medicine, Shandong Provincial

Hospital Affiliated to Shandong University, Shandong Provincial Hospital

Affiliated to Shandong First Medical University, Jinan, 250021, Shandong,

People's Republic of China.

AD - Cheeloo College of Medicine, Shandong University, Jinan, 250012, Shandong,

People's Republic of China.

FAU - Guo, Jing

AU - Guo J

AD - Department of Respiratory and Critical Care Medicine, Shandong Provincial

Hospital Affiliated to Shandong University, Shandong Provincial Hospital

Affiliated to Shandong First Medical University, Jinan, 250021, Shandong,

People's Republic of China.

AD - School of Medicine and Life Sciences, Shandong First Medical University and

Shandong Academy of Medical Sciences, Taian, 271016, Shandong, People's Republic

of China.

AD - Department of Emergency, The Fifth People's Hospital of Jinan, Jinan, 250031,

Shandong, People's Republic of China.

FAU - Xu, Ting-Ting

AU - Xu TT

AD - Department of Respiratory and Critical Care Medicine, Shandong Provincial

Hospital Affiliated to Shandong University, Shandong Provincial Hospital

Affiliated to Shandong First Medical University, Jinan, 250021, Shandong,

People's Republic of China.

AD - School of Medicine and Life Sciences, Shandong First Medical University and

Shandong Academy of Medical Sciences, Taian, 271016, Shandong, People's Republic

of China.

FAU - Li, Shi-Jin

AU - Li SJ

AD - Department of Respiratory and Critical Care Medicine, Shandong Provincial

Hospital Affiliated to Shandong University, Shandong Provincial Hospital

Affiliated to Shandong First Medical University, Jinan, 250021, Shandong,

People's Republic of China.

AD - Cheeloo College of Medicine, Shandong University, Jinan, 250012, Shandong,

People's Republic of China.

FAU - Liu, Jin-Yue

AU - Liu JY

AD - Department of Intensive Care Unit, Shandong Provincial Third Hospital, Jinan,

100191, Shandong, People's Republic of China.

FAU - Tao, Ning-Ning

AU - Tao NN

AD - Department of Respiratory and Critical Care Medicine, Beijing Hospital, Beijing,

100730, People's Republic of China.

AD - Graduate School of Peking Union Medical College, Chinese Academy of Medical

Sciences and Peking Union Medical College, Beijing, 100730, People's Republic of

China.

FAU - Liu, Yao

AU - Liu Y

AD - Department of Respiratory and Critical Care Medicine, Shandong Provincial

Hospital Affiliated to Shandong University, Shandong Provincial Hospital

Affiliated to Shandong First Medical University, Jinan, 250021, Shandong,

People's Republic of China.

FAU - Zhang, Qian-Yun

AU - Zhang QY

AD - Department of Respiratory and Critical Care Medicine, Shandong Provincial

Hospital Affiliated to Shandong University, Shandong Provincial Hospital

Affiliated to Shandong First Medical University, Jinan, 250021, Shandong,

People's Republic of China.

AD - Cheeloo College of Medicine, Shandong University, Jinan, 250012, Shandong,

People's Republic of China.

FAU - Liu, Si-Qi

AU - Liu SQ

AD - Department of Respiratory and Critical Care Medicine, Shandong Provincial

Hospital Affiliated to Shandong University, Shandong Provincial Hospital

Affiliated to Shandong First Medical University, Jinan, 250021, Shandong,

People's Republic of China.

AD - Cheeloo College of Medicine, Shandong University, Jinan, 250012, Shandong,

People's Republic of China.

FAU - An, Qi-Qi

AU - An QQ

AD - Department of Respiratory and Critical Care Medicine, Shandong Provincial

Hospital Affiliated to Shandong University, Shandong Provincial Hospital

Affiliated to Shandong First Medical University, Jinan, 250021, Shandong,

People's Republic of China.

AD - Cheeloo College of Medicine, Shandong University, Jinan, 250012, Shandong,

People's Republic of China.

FAU - Li, Yi-Fan

AU - Li YF

AD - Department of Respiratory and Critical Care Medicine, Shandong Provincial

Hospital Affiliated to Shandong University, Shandong Provincial Hospital

Affiliated to Shandong First Medical University, Jinan, 250021, Shandong,

People's Republic of China.

FAU - Yu, Chun-Bao

AU - Yu CB

AD - Katharine Hsu International Research Center of Human Infectious Diseases,

Shandong Provincial Chest Hospital, Jinan, 250013, Shandong, People's Republic of

China.

FAU - Dong, Ji-Hua

AU - Dong JH

AD - Department of Respiratory Medicine, Heze Mudan People's Hospital, Heze, 274000,

Shandong, People's Republic of China. dongjihua321@sohu.com.

FAU - Li, Huai-Chen

AU - Li HC

AD - Department of Respiratory and Critical Care Medicine, Shandong Provincial

Hospital Affiliated to Shandong University, Shandong Provincial Hospital

Affiliated to Shandong First Medical University, Jinan, 250021, Shandong,

People's Republic of China. lihuaichen@163.com.

AD - College of Traditional Chinese Medicine, Shandong University of Traditional

Chinese Medicine, Jinan, 250355, Shandong, People's Republic of China.

lihuaichen@163.com.

LA - eng

GR - ZR2020KH013/Natural Science Foundation of Shandong Province/

GR - No.2007GG30002033/Department of Science & Technology of Shandong Province/

GR - No.2017GSF218052/Department of Science & Technology of Shandong Province/

GR - 201704100/Jinan Science and Technology Bureau/

PT - Comparative Study

PT - Journal Article

DEP - 20211206

PL - England

TA - BMC Pulm Med

JT - BMC pulmonary medicine

JID - 100968563

RN - 0 (Antitubercular Agents)

SB - IM

MH - Adolescent

MH - Adult

MH - Aged

MH - Antitubercular Agents/pharmacology

MH - Body Mass Index

MH - Child

MH - Child, Preschool

MH - China/epidemiology

MH - Drug Resistance, Multiple, Bacterial

MH - Female

MH - Humans

MH - Infant

MH - Male

MH - Middle Aged

MH - Overweight/*complications/*epidemiology

MH - Risk Factors

MH - Tuberculosis, Multidrug-Resistant/*complications/drug therapy/*epidemiology

MH - Young Adult

PMC - PMC8647447

OTO - NOTNLM

OT - Body mass index

OT - Drug resistance

OT - Overweight

OT - Tuberculosis

OT - Underweight

COIS- The authors declare that they have no competing interests.

EDAT- 2021/12/08 06:00

MHDA- 2022/03/11 06:00

PMCR- 2021/12/06

CRDT- 2021/12/07 05:40

PHST- 2021/03/30 00:00 [received]

PHST- 2021/12/01 00:00 [accepted]

PHST- 2021/12/07 05:40 [entrez]

PHST- 2021/12/08 06:00 [pubmed]

PHST- 2022/03/11 06:00 [medline]

PHST- 2021/12/06 00:00 [pmc-release]

AID - 10.1186/s12890-021-01774-2 [pii]

AID - 1774 [pii]

AID - 10.1186/s12890-021-01774-2 [doi]

PST - epublish

SO - BMC Pulm Med. 2021 Dec 6;21(1):399. doi: 10.1186/s12890-021-01774-2.

PMID- 34974855

OWN - NLM

STAT- MEDLINE

DCOM- 20220104

LR - 20260127

IS - 1550-7033 (Print)

IS - 1550-7033 (Linking)

VI - 17

IP - 12

DP - 2021 Dec 1

TI - Nano-Drug Delivery Systems: Possible End to the Rising Threats of Tuberculosis.

PG - 2298-2318

LID - 10.1166/jbn.2021.3201 [doi]

AB - Tuberculosis (TB) is still one of the deadliest disease across the globe caused

by Mycobacterium tuberculosis (Mtb). Mtb invades host macrophages and other

immune cells, modifies their lysosome trafficking proteins, prevents

phagolysosomes formation, and inhibits the TNF receptor-dependent apoptosis in

macrophages and monocytes. Tuberculosis (TB) killed 1.4 million people worldwide

in the year 2019. Despite the advancements in tuberculosis (TB) treatments,

multidrugresistant tuberculosis (MDR-TB) remains a severe threat to human health.

The complications are further compounded by the emergence of MDR/XDR strains and

the failure of conventional drug regimens to eradicate the resistant bacterial

strains. Thus, new therapeutic approaches aim to ensure cure without relapse, to

prevent the occurrence of deaths and emergence of drug-resistant strains. In this

context, this review article summarises the essential nanotechnology-related

research outcomes in the treatment of tuberculosis (TB), including

drug-susceptible and drug-resistant strains of Mtb. The novel anti-tuberculosis

drug delivery systems are also being detailed. This article highlights recent

advances in tuberculosis (TB) treatments, including the use of novel drug

delivery technologies such as solid lipid nanoparticles, liposomes, polymeric

micelles, nano-suspensions, nano-emulsion, niosomes, liposomes, polymeric

nanoparticles and microparticles for the delivery of anti-TB drugs and hence

eradication and control of both drug-susceptible as well as drug-resistant

strains of Mtb.

FAU - Sheikh, Bashir A

AU - Sheikh BA

AD - Department of Bio-Resources, School of Biological Sciences, University of

Kashmir, Srinagar 190006, J&K, India.

FAU - Bhat, Basharat A

AU - Bhat BA

AD - Department of Bio-Resources, School of Biological Sciences, University of

Kashmir, Srinagar 190006, J&K, India.

FAU - Alshehri, Bader

AU - Alshehri B

AD - Department of Medical Laboratory Sciences, College of Applied Medical Sciences,

Majmaah University KSA, Almajmaah, 11952, Saudi Arabia.

FAU - Mir, Rakeeb A

AU - Mir RA

AD - Department of Biotechnology, School of Biosciences and Biotechnology, BGSB

University, Rajouri 185234, J&K, India.

FAU - Mir, Wajahat R

AU - Mir WR

AD - Department of Bio-Resources, School of Biological Sciences, University of

Kashmir, Srinagar 190006, J&K, India.

FAU - Parry, Zahoor A

AU - Parry ZA

AD - Clinical Microbiology PK/PD/Laboratory, Indian Institute of Integrated Medicine

(IIIM)-Srinagar 190005, J&K, India.

FAU - Mir, Manzoor A

AU - Mir MA

AD - Department of Bio-Resources, School of Biological Sciences, University of

Kashmir, Srinagar 190006, J&K, India.

LA - eng

PT - Journal Article

PT - Review

PL - United States

TA - J Biomed Nanotechnol

JT - Journal of biomedical nanotechnology

JID - 101230869

RN - 0 (Antitubercular Agents)

RN - 0 (Lipid Nanoparticles)

RN - 0 (Liposomes)

RN - 0 (Nanoparticle Drug Delivery System)

SB - IM

MH - *Antitubercular Agents/administration & dosage/therapeutic use

MH - Humans

MH - Liposomes

MH - *Nanoparticle Drug Delivery System

MH - *Tuberculosis/drug therapy

MH - Nanoparticles

EDAT- 2022/01/04 06:00

MHDA- 2022/01/05 06:00

CRDT- 2022/01/03 05:23

PHST- 2022/01/03 05:23 [entrez]

PHST- 2022/01/04 06:00 [pubmed]

PHST- 2022/01/05 06:00 [medline]

AID - 10.1166/jbn.2021.3201 [doi]

PST - ppublish

SO - J Biomed Nanotechnol. 2021 Dec 1;17(12):2298-2318. doi: 10.1166/jbn.2021.3201.

PMID- 34886926

OWN - NLM

STAT- MEDLINE

DCOM- 20220323

LR - 20220323

IS - 1815-7920 (Electronic)

IS - 1027-3719 (Linking)

VI - 25

IP - 12

DP - 2021 Dec 1

TI - Efflux pump gene expression study using RNA-seq in multidrug-resistant TB.

PG - 974-981

LID - 10.5588/ijtld.21.0117 [doi]

AB - BACKGROUND: The mechanism underlying kanamycin (KM) resistance in Mycobacterium

tuberculosis is not well understood, although efflux pump proteins are thought to

play a role. This study used RNA-seq data to investigate changes in the

expression levels of efflux pump genes following exposure to KM.METHODS: RNA

expression of efflux pump and regulatory genes following exposure to different

concentrations of KM (minimum inhibitory concentration MIC 25 and MIC50) in rrs

wild-type strain and rrs A1401G mutated strain were compared with the control

group.RESULTS: The selected strains had differential RNA expression patterns.

Among the 71 putative efflux pump and regulatory genes, 46 had significant fold

changes, and 12 genes (Rv0842, Rv1146, Rv1258c, Rv1473, Rv1686c, Rv1687c, Rv1877,

Rv2038c, Rv3065, Rv3197a, Rv3728 and Rv3789) that were overexpressed following

exposure to KM were thought to contribute to drug resistance. Rv3197A (whiB7)

showed a distinct fold change based on the concentration of KM.CONCLUSION: The

significant changes in the expression of the efflux pump and regulatory genes

following exposure to KM may provide insights into the identification of a new

resistance mechanism.

FAU - Lee, J J

AU - Lee JJ

AD - Department of Laboratory Medicine, Graduate School, Kyung Hee University, Seoul,

Korea, Department of Laboratory Medicine, Kyung Hee University Hospital, Kyung

Hee University School of Medicine, Seoul, Korea.

FAU - Kang, H Y

AU - Kang HY

AD - Department of Laboratory Medicine, Kyung Hee University Hospital, Kyung Hee

University School of Medicine, Seoul, Korea.

FAU - Lee, W-I

AU - Lee WI

AD - Department of Laboratory Medicine, Kyung Hee University Hospital at Gangdong,

Kyung Hee University School of Medicine, Seoul, Korea.

FAU - Cho, S Y

AU - Cho SY

AD - Department of Laboratory Medicine, Kyung Hee University Hospital, Kyung Hee

University School of Medicine, Seoul, Korea.

FAU - Kim, Y J

AU - Kim YJ

AD - Department of Laboratory Medicine, Kyung Hee University Hospital, Kyung Hee

University School of Medicine, Seoul, Korea.

FAU - Lee, H J

AU - Lee HJ

AD - Korean National Tuberculosis Association, Seoul, Korea.

LA - eng

PT - Journal Article

PL - France

TA - Int J Tuberc Lung Dis

JT - The international journal of tuberculosis and lung disease : the official journal

of the International Union against Tuberculosis and Lung Disease

JID - 9706389

RN - 0 (Antitubercular Agents)

RN - 0 (Bacterial Proteins)

SB - IM

MH - Antitubercular Agents/pharmacology/therapeutic use

MH - Bacterial Proteins/genetics

MH - Drug Resistance, Multiple, Bacterial/genetics

MH - Gene Expression

MH - Humans

MH - Microbial Sensitivity Tests

MH - *Mycobacterium tuberculosis/genetics

MH - RNA-Seq

MH - *Tuberculosis, Multidrug-Resistant/drug therapy/genetics/microbiology

EDAT- 2021/12/11 06:00

MHDA- 2022/03/24 06:00

CRDT- 2021/12/10 05:44

PHST- 2021/12/10 05:44 [entrez]

PHST- 2021/12/11 06:00 [pubmed]

PHST- 2022/03/24 06:00 [medline]

AID - 10.5588/ijtld.21.0117 [doi]

PST - ppublish

SO - Int J Tuberc Lung Dis. 2021 Dec 1;25(12):974-981. doi: 10.5588/ijtld.21.0117.

PMID- 34841463

OWN - NLM

STAT- MEDLINE

DCOM- 20211208

LR - 20211214

IS - 1432-0614 (Electronic)

IS - 0175-7598 (Linking)

VI - 105

IP - 24

DP - 2021 Dec

TI - Antimycobacterial compound of chitosan and ethambutol: ultrastructural biological

evaluation in vitro against Mycobacterium tuberculosis.

PG - 9167-9179

LID - 10.1007/s00253-021-11690-4 [doi]

AB - Chitosan (CS) is a promising biopolymer and has been tested as a complement to

the action and compensation of toxicity presented by anti-tuberculosis drugs. The

present work studied the adjuvant effect of CS with the drug ethambutol (EMB) as

a compound (CS-EMB), to explore its antimicrobial and cytotoxic activity, using

transmission electron microscopy (TEM), to examine ultracellular changes that

represent possible antimycobacterial action of CS on Mycobacterium tuberculosis

(Mtb). Antimycobacterial activities were tested against reference strains Mtb

ATCC® H37Rv and multidrug resistant (MDR). In vitro cytotoxicity tests were

performed on Raw 264.7. For the studied compounds, morphological,

ultrastructural, and physical-chemical analyses were performed. Drug-polymer

interactions that occur through the H bridges were confirmed by physical-chemical

analyses. The CS-EMB compound is stable at pHs of 6.5-7.5, allowing its release

at physiological pH. The antibacterial activity (minimum inhibitory

concentration) of the CS-EMB compound was 50% greater than that of the EMB in the

H37Rv and MDR strains and the ultrastructural changes in the bacilli observed by

TEM proved that the CS-EMB compound has a bactericidal action, allowing it to

break down the Mtb cell wall. The cytotoxicity of CS-EMB was higher than that of

isolated EMB, IC50 279, and 176 μg/mL, respectively. It is concluded that CS-EMB

forms a promising composite against strains Mtb H37Rv and multidrug resistant

(MDR-TB).Key points• Our study will be the first to observe ultrastructurally the

effects of the CS-EMB compound on Mtb cells.• CS-EMB antimicrobial activity in a

multidrug-resistant clinical strain.• The CS-EMB compound has promising potential

for the development of a new drug to fight tuberculosis.

CI - © 2021. The Author(s), under exclusive licence to Springer-Verlag GmbH Germany,

part of Springer Nature.

FAU - Oliveira, M E F A G

AU - Oliveira MEFAG

AD - Programa de Pós-Graduação Em Morfotecnologia, Universidade Federal de Pernambuco

(UFPE), Recife, PE, 50670-420, Brazil. m.dudafago@gmail.com.

FAU - Silva, Y J A

AU - Silva YJA

AD - Programa de Pós-Graduação Em Ciência de Materiais, Universidade Federal de

Pernambuco (UFPE), Recife, PE, 50740-560, Brazil.

FAU - Azevedo, L A

AU - Azevedo LA

AD - Programa de Pós-Graduação Em Ciência de Materiais, Universidade Federal de

Pernambuco (UFPE), Recife, PE, 50740-560, Brazil.

FAU - Linhares, L A

AU - Linhares LA

AD - Instituto Aggeu Magalhães/Fundação Oswaldo Cruz (IAM/FIOCRUZ), 50740-465,

Recife-PE, Brazil.

FAU - Montenegro, L M L

AU - Montenegro LML

AD - Instituto Aggeu Magalhães/Fundação Oswaldo Cruz (IAM/FIOCRUZ), 50740-465,

Recife-PE, Brazil.

FAU - Alves, S Jr

AU - Alves S Jr

AD - Departamento de Química Fundamental (dQF), Universidade Federal de Pernambuco

(UFPE), Recife, PE, 50740-560, Brazil.

FAU - Amorim, R V S

AU - Amorim RVS

AD - Departamento de Histologia E Embriologia (DHE-CB), Universidade Federal de

Pernambuco (UFPE), Recife, PE, 50670-420, Brazil.

LA - eng

PT - Journal Article

DEP - 20211129

PL - Germany

TA - Appl Microbiol Biotechnol

JT - Applied microbiology and biotechnology

JID - 8406612

RN - 0 (Antitubercular Agents)

RN - 8G167061QZ (Ethambutol)

RN - 9012-76-4 (Chitosan)

SB - IM

MH - Antitubercular Agents/toxicity

MH - *Chitosan/pharmacology

MH - Ethambutol/pharmacology

MH - Humans

MH - Microbial Sensitivity Tests

MH - *Mycobacterium tuberculosis

MH - *Tuberculosis, Multidrug-Resistant

OTO - NOTNLM

OT - Chitosan

OT - Cytotoxicity

OT - Ethambutol

OT - Multidrug-resistant

OT - Mycobacterium tuberculosis

OT - Ultrastructural

EDAT- 2021/11/30 06:00

MHDA- 2021/12/15 06:00

CRDT- 2021/11/29 06:40

PHST- 2021/04/23 00:00 [received]

PHST- 2021/11/08 00:00 [accepted]

PHST- 2021/10/14 00:00 [revised]

PHST- 2021/11/30 06:00 [pubmed]

PHST- 2021/12/15 06:00 [medline]

PHST- 2021/11/29 06:40 [entrez]

AID - 10.1007/s00253-021-11690-4 [pii]

AID - 10.1007/s00253-021-11690-4 [doi]

PST - ppublish

SO - Appl Microbiol Biotechnol. 2021 Dec;105(24):9167-9179. doi:

10.1007/s00253-021-11690-4. Epub 2021 Nov 29.

PMID- 34816020

OWN - NLM

STAT- PubMed-not-MEDLINE

LR - 20240407

IS - 2405-5794 (Electronic)

IS - 2405-5794 (Linking)

VI - 25

DP - 2021 Dec

TI - The pipeline of new molecules and regimens against drug-resistant tuberculosis.

PG - 100285

LID - 10.1016/j.jctube.2021.100285 [doi]

LID - 100285

AB - The clinical development and regulatory approval of bedaquiline, delamanid and

pretomanid over the last decade brought about significant progress in the

management of drug-resistant tuberculosis, providing all-oral regimens with

favorable safety profiles. The Nix-TB and ZeNix trials of a bedaquiline -

pretomanid - linezolid regimen demonstrated for the first time that certain forms

of drug-resistant tuberculosis can be cured in the majority of patients within

6 months. Ongoing Phase 3 studies containing these drugs may further advance

optimized regimen compositions. Investigational drugs in clinical development

that target clinically validated mechanisms, such as second generation

oxazolidinones (sutezolid, delpazolid, TBI-223) and diarylquinolines (TBAJ-876

and TBAJ-587) promise improved potency and/or safety compared to the

first-in-class drugs. Compounds with novel targets involved in diverse bacterial

functions such as cell wall synthesis (DrpE1, MmpL3), electron transport, DNA

synthesis (GyrB), cholesterol metabolism and transcriptional regulation of

ethionamide bioactivation pathways have advanced to early clinical studies with

the potential to enhance antibacterial activity when added to new or established

anti-TB drug regimens. Clinical validation of preclinical in vitro and animal

model predictions of new anti-TB regimens may further improve the translational

value of these models to identify optimal anti-TB therapies.

CI - © 2021 The Authors.

FAU - Black, Todd A

AU - Black TA

AD - Global Alliance for TB Drug Development, 40 Wall Street, 24th Floor, New York, NY

10005, USA.

FAU - Buchwald, Ulrike K

AU - Buchwald UK

AD - Global Alliance for TB Drug Development, 40 Wall Street, 24th Floor, New York, NY

10005, USA.

LA - eng

PT - Journal Article

PT - Review

DEP - 20211105

PL - England

TA - J Clin Tuberc Other Mycobact Dis

JT - Journal of clinical tuberculosis and other mycobacterial diseases

JID - 101682877

PMC - PMC8593651

OTO - NOTNLM

OT - Animal models

OT - Bedaquiline

OT - Delamanid

OT - Drug-resistant tuberculosis

OT - Pretomanid

OT - Regimen development

COIS- The authors declare the following financial interests/personal relationships

which may be considered as potential competing interests: Todd A. Black reports a

relationship with Merck & Co Inc that includes: employment and equity or stocks.

Ulrike K. Buchwald reports a relationship with Merck & Co Inc that includes:

prior employment.

EDAT- 2021/11/25 06:00

MHDA- 2021/11/25 06:01

PMCR- 2021/11/05

CRDT- 2021/11/24 06:35

PHST- 2021/11/24 06:35 [entrez]

PHST- 2021/11/25 06:00 [pubmed]

PHST- 2021/11/25 06:01 [medline]

PHST- 2021/11/05 00:00 [pmc-release]

AID - S2405-5794(21)00074-7 [pii]

AID - 100285 [pii]

AID - 10.1016/j.jctube.2021.100285 [doi]

PST - epublish

SO - J Clin Tuberc Other Mycobact Dis. 2021 Nov 5;25:100285. doi:

10.1016/j.jctube.2021.100285. eCollection 2021 Dec.

PMID- 34779708

OWN - NLM

STAT- MEDLINE

DCOM- 20220113

LR - 20220113

IS - 2222-1751 (Electronic)

IS - 2222-1751 (Linking)

VI - 10

IP - 1

DP - 2021 Dec

TI - IMB-XMA0038, a new inhibitor targeting aspartate-semialdehyde dehydrogenase of

Mycobacterium tuberculosis.

PG - 2291-2299

LID - 10.1080/22221751.2021.2006578 [doi]

AB - The emergence of drug-resistant tuberculosis (TB) constitutes a major challenge

to TB control programmes. There is an urgent need to develop effective anti-TB

drugs with novel mechanisms of action. Aspartate-semialdehyde dehydrogenase

(ASADH) is the second enzyme in the aspartate metabolic pathway. The absence of

the pathway in humans and the absolute requirement of aspartate in bacteria make

ASADH a highly attractive drug target. In this study, we used ASADH coupled with

Escherichia coli type III aspartate kinase (LysC) to establish a high-throughput

screening method to find new anti-TB inhibitors. IMB-XMA0038 was identified as an

inhibitor of MtASADH with an IC(50) value of 0.59 μg/mL through screening. The

interaction between IMB-XMA0038 and MtASADH was confirmed by surface plasmon

resonance (SPR) assay and molecular docking analysis. Furthermore, IMB-XMA0038

was found to inhibit various drug-resistant MTB strains potently with minimal

inhibitory concentrations (MICs) of 0.25-0.5 μg/mL. The conditional mutant strain

MTB::asadh cultured with different concentrations of inducer (10(-5) or 10(-1)

μg/mL pristinamycin) resulted in a maximal 16 times difference in MICs. At the

same time, IMB-XMA0038 showed low cytotoxicity in vitro and vivo. In mouse model,

it encouragingly declined the MTB colony forming units (CFU) in lung by 1.67

log10 dosed at 25 mg/kg for 15 days. In conclusion, our data demonstrate that

IMB-XMA0038 is a promising lead compound against drug-resistant tuberculosis.

FAU - Wang, Xiao

AU - Wang X

AD - National Laboratory for Screening New Microbial Drugs, Institute of Medicinal

Biotechnology, Peking Union Medical College and Chinese Academy of Medical

Sciences, Beijing, People's Republic of China.

FAU - Yang, Ruifang

AU - Yang R

AD - Department of Bacteriology and Immunology, Beijing Key Laboratory on

Drug-Resistant Tuberculosis Research, Beijing Tuberculosis and Thoracic Tumor

Research Institute/Beijing Chest Hospital, Capital Medical University, Beijing,

People's Republic of China.

FAU - Liu, Sihan

AU - Liu S

AD - National Laboratory for Screening New Microbial Drugs, Institute of Medicinal

Biotechnology, Peking Union Medical College and Chinese Academy of Medical

Sciences, Beijing, People's Republic of China.

FAU - Guan, Yan

AU - Guan Y

AD - National Laboratory for Screening New Microbial Drugs, Institute of Medicinal

Biotechnology, Peking Union Medical College and Chinese Academy of Medical

Sciences, Beijing, People's Republic of China.

FAU - Xiao, Chunling

AU - Xiao C

AD - National Laboratory for Screening New Microbial Drugs, Institute of Medicinal

Biotechnology, Peking Union Medical College and Chinese Academy of Medical

Sciences, Beijing, People's Republic of China.

FAU - Li, Chuanyou

AU - Li C

AD - Department of Bacteriology and Immunology, Beijing Key Laboratory on

Drug-Resistant Tuberculosis Research, Beijing Tuberculosis and Thoracic Tumor

Research Institute/Beijing Chest Hospital, Capital Medical University, Beijing,

People's Republic of China.

FAU - Meng, Jianzhou

AU - Meng J

AD - National Laboratory for Screening New Microbial Drugs, Institute of Medicinal

Biotechnology, Peking Union Medical College and Chinese Academy of Medical

Sciences, Beijing, People's Republic of China.

FAU - Pang, Yu

AU - Pang Y

AD - Department of Bacteriology and Immunology, Beijing Key Laboratory on

Drug-Resistant Tuberculosis Research, Beijing Tuberculosis and Thoracic Tumor

Research Institute/Beijing Chest Hospital, Capital Medical University, Beijing,

People's Republic of China.

FAU - Liu, Yishuang

AU - Liu Y

AD - National Laboratory for Screening New Microbial Drugs, Institute of Medicinal

Biotechnology, Peking Union Medical College and Chinese Academy of Medical

Sciences, Beijing, People's Republic of China.

LA - eng

PT - Journal Article

PL - United States

TA - Emerg Microbes Infect

JT - Emerging microbes & infections

JID - 101594885

RN - 0 (Antitubercular Agents)

RN - 0 (Bacterial Proteins)

RN - 0 (Enzyme Inhibitors)

RN - EC 1.2.1.11 (Aspartate-Semialdehyde Dehydrogenase)

SB - IM

MH - Animals

MH - Antitubercular Agents/administration & dosage/*chemistry

MH - Aspartate-Semialdehyde Dehydrogenase/*antagonists &

inhibitors/chemistry/metabolism

MH - Bacterial Proteins/*antagonists & inhibitors/chemistry/genetics/metabolism

MH - Enzyme Inhibitors/administration & dosage/*chemistry

MH - Humans

MH - Male

MH - Mice

MH - Molecular Docking Simulation

MH - Mycobacterium tuberculosis/*drug effects/*enzymology/genetics

MH - Tuberculosis/drug therapy/*microbiology

PMC - PMC8648042

OTO - NOTNLM

OT - Mycobacteria tuberculosis

OT - aspartate-semialdehyde dehydrogenase

OT - drug-resistance

OT - high-throughput screening

OT - inhibitor

COIS- According to the journal rules, there is a conflict of interest that should be

stated. One patent applied for by our laboratory about the anti-mycobacterial

effect of IMB-XMA0038 was authorized by the Chinese patent office (Patent number:

ZL201710859051.3).

EDAT- 2021/11/16 06:00

MHDA- 2022/01/14 06:00

PMCR- 2021/12/02

CRDT- 2021/11/15 12:17

PHST- 2021/11/16 06:00 [pubmed]

PHST- 2022/01/14 06:00 [medline]

PHST- 2021/11/15 12:17 [entrez]

PHST- 2021/12/02 00:00 [pmc-release]

AID - 2006578 [pii]

AID - 10.1080/22221751.2021.2006578 [doi]

PST - ppublish

SO - Emerg Microbes Infect. 2021 Dec;10(1):2291-2299. doi:

10.1080/22221751.2021.2006578.

PMID- 34867965

OWN - NLM

STAT- MEDLINE

DCOM- 20220119

LR - 20220119

IS - 1664-3224 (Electronic)

IS - 1664-3224 (Linking)

VI - 12

DP - 2021

TI - Modulation of Cystatin C in Human Macrophages Improves Anti-Mycobacterial Immune

Responses to Mycobacterium tuberculosis Infection and Coinfection With HIV.

PG - 742822

LID - 10.3389/fimmu.2021.742822 [doi]

LID - 742822

AB - Tuberculosis owes its resurgence as a major global health threat mostly to the

emergence of drug resistance and coinfection with HIV. The synergy between HIV

and Mycobacterium tuberculosis (Mtb) modifies the host immune environment to

enhance both viral and bacterial replication and spread. In the lung immune

context, both pathogens infect macrophages, establishing favorable intracellular

niches. Both manipulate the endocytic pathway in order to avoid destruction.

Relevant players of the endocytic pathway to control pathogens include

endolysosomal proteases, cathepsins, and their natural inhibitors, cystatins.

Here, a mapping of the human macrophage transcriptome for type I and II cystatins

during Mtb, HIV, or Mtb-HIV infection displayed different profiles of gene

expression, revealing cystatin C as a potential target to control mycobacterial

infection as well as HIV coinfection. We found that cystatin C silencing in

macrophages significantly improves the intracellular killing of Mtb, which was

concomitant with an increased general proteolytic activity of cathepsins. In

addition, downmodulation of cystatin C led to an improved expression of the human

leukocyte antigen (HLA) class II in macrophages and an increased CD4(+)

T-lymphocyte proliferation along with enhanced IFN-γ secretion. Overall, our

results suggest that the targeting of cystatin C in human macrophages represents

a promising approach to improve the control of mycobacterial infections including

multidrug-resistant (MDR) TB.

CI - Copyright © 2021 Pires, Calado, Velez, Mandal, Catalão, Neyrolles,

Lugo-Villarino, Vérollet, Azevedo-Pereira and Anes.

FAU - Pires, David

AU - Pires D

AD - Host-Pathogen Interactions, Research Institute for Medicines, iMed-ULisboa,

Faculty of Pharmacy, Universidade de Lisboa, Lisbon, Portugal.

FAU - Calado, Marta

AU - Calado M

AD - Host-Pathogen Interactions, Research Institute for Medicines, iMed-ULisboa,

Faculty of Pharmacy, Universidade de Lisboa, Lisbon, Portugal.

FAU - Velez, Tomás

AU - Velez T

AD - Host-Pathogen Interactions, Research Institute for Medicines, iMed-ULisboa,

Faculty of Pharmacy, Universidade de Lisboa, Lisbon, Portugal.

FAU - Mandal, Manoj

AU - Mandal M

AD - Host-Pathogen Interactions, Research Institute for Medicines, iMed-ULisboa,

Faculty of Pharmacy, Universidade de Lisboa, Lisbon, Portugal.

FAU - Catalão, Maria João

AU - Catalão MJ

AD - Host-Pathogen Interactions, Research Institute for Medicines, iMed-ULisboa,

Faculty of Pharmacy, Universidade de Lisboa, Lisbon, Portugal.

FAU - Neyrolles, Olivier

AU - Neyrolles O

AD - Institut de Pharmacologie et Biologie Structurale, IPBS, Université de Toulouse,

Centre National de la Recherche Scientifique (CNRS), Toulouse, France.

FAU - Lugo-Villarino, Geanncarlo

AU - Lugo-Villarino G

AD - Institut de Pharmacologie et Biologie Structurale, IPBS, Université de Toulouse,

Centre National de la Recherche Scientifique (CNRS), Toulouse, France.

FAU - Vérollet, Christel

AU - Vérollet C

AD - Institut de Pharmacologie et Biologie Structurale, IPBS, Université de Toulouse,

Centre National de la Recherche Scientifique (CNRS), Toulouse, France.

FAU - Azevedo-Pereira, José Miguel

AU - Azevedo-Pereira JM

AD - Host-Pathogen Interactions, Research Institute for Medicines, iMed-ULisboa,

Faculty of Pharmacy, Universidade de Lisboa, Lisbon, Portugal.

FAU - Anes, Elsa

AU - Anes E

AD - Host-Pathogen Interactions, Research Institute for Medicines, iMed-ULisboa,

Faculty of Pharmacy, Universidade de Lisboa, Lisbon, Portugal.

LA - eng

PT - Journal Article

PT - Research Support, Non-U.S. Gov't

DEP - 20211118

PL - Switzerland

TA - Front Immunol

JT - Frontiers in immunology

JID - 101560960

RN - 0 (CST3 protein, human)

RN - 0 (Cystatin C)

RN - 82115-62-6 (Interferon-gamma)

SB - IM

MH - CD4-Positive T-Lymphocytes/immunology

MH - Cells, Cultured

MH - Coinfection/*immunology

MH - Cystatin C/genetics/*immunology

MH - HIV Infections/*immunology

MH - HIV-1

MH - Humans

MH - Interferon-gamma/immunology

MH - Macrophages/*immunology/microbiology

MH - Mycobacterium tuberculosis

MH - Tuberculosis/*immunology

PMC - PMC8637326

OTO - NOTNLM

OT - HIV/Mtb coinfection

OT - cathepsins

OT - cystatins

OT - host-directed therapies

OT - tuberculosis

COIS- The authors declare that the research was conducted in the absence of any

commercial or financial relationships that could be construed as a potential

conflict of interest.

EDAT- 2021/12/07 06:00

MHDA- 2022/01/20 06:00

PMCR- 2021/01/01

CRDT- 2021/12/06 09:07

PHST- 2021/07/16 00:00 [received]

PHST- 2021/10/21 00:00 [accepted]

PHST- 2021/12/06 09:07 [entrez]

PHST- 2021/12/07 06:00 [pubmed]

PHST- 2022/01/20 06:00 [medline]

PHST- 2021/01/01 00:00 [pmc-release]

AID - 10.3389/fimmu.2021.742822 [doi]

PST - epublish

SO - Front Immunol. 2021 Nov 18;12:742822. doi: 10.3389/fimmu.2021.742822. eCollection

2021.

PMID- 34899300

OWN - NLM

STAT- PubMed-not-MEDLINE

LR - 20211215

IS - 1663-9812 (Print)

IS - 1663-9812 (Electronic)

IS - 1663-9812 (Linking)

VI - 12

DP - 2021

TI - Alginate-Capped Silver Nanoparticles as a Potent Anti-mycobacterial Agent Against

Mycobacterium tuberculosis.

PG - 746496

LID - 10.3389/fphar.2021.746496 [doi]

LID - 746496

AB - Tuberculosis (TB) is a leading cause of death from a single infectious agent,

Mycobacterium tuberculosis (Mtb). Although progress has been made in TB control,

still about 10 million people worldwide develop TB annually and 1.5 million die

of the disease. The rapid emergence of aggressive, drug-resistant strains and

latent infections have caused TB to remain a global health challenge. TB

treatments are lengthy and their side effects lead to poor patient compliance,

which in turn has contributed to the drug resistance and exacerbated the TB

epidemic. The relatively low output of newly approved antibiotics has spurred

research interest toward alternative antibacterial molecules such as silver

nanoparticles (AgNPs). In the present study, we use the natural biopolymer

alginate to serve as a stabilizer and/or reductant to green synthesize AgNPs,

which improves their biocompatibility and avoids the use of toxic chemicals. The

average size of the alginate-capped AgNPs (ALG-AgNPs) was characterized as

nanoscale, and the particles were round in shape. Drug susceptibility tests

showed that these ALG-AgNPs are effective against both drug-resistant Mtb strains

and dormant Mtb. A bacterial cell-wall permeability assay showed that the

anti-mycobacterial action of ALG-AgNPs is mediated through an increase in

cell-wall permeability. Notably, the anti-mycobacterial potential of ALG-AgNPs

was effective in both zebrafish and mouse TB animal models in vivo. These results

suggest that ALG-AgNPs could provide a new therapeutic option to overcome the

difficulties of current TB treatments.

CI - Copyright © 2021 Chen, Chen, Yeh, Hsu, Yu, Hsu, Wei, Ho, Tsai, Song, Yen, Chen,

Young, Chuang and Dou.

FAU - Chen, Cheng-Cheung

AU - Chen CC

AD - Institute of Preventive Medicine, National Defense Medical Center, Taipei,

Taiwan.

AD - Graduate Institute of Medical Science, National Defense Medical Center, Taipei,

Taiwan.

FAU - Chen, Yih-Yuan

AU - Chen YY

AD - Department of Biochemical Science and Technology, National Chiayi University,

Chia-Yi, Taiwan.

FAU - Yeh, Chang-Ching

AU - Yeh CC

AD - National Institute of Infectious Diseases and Vaccinology, National Health

Research Institutes, Zhunan, Taiwan.

FAU - Hsu, Chia-Wei

AU - Hsu CW

AD - National Institute of Infectious Diseases and Vaccinology, National Health

Research Institutes, Zhunan, Taiwan.

FAU - Yu, Shang-Jie

AU - Yu SJ

AD - National Institute of Infectious Diseases and Vaccinology, National Health

Research Institutes, Zhunan, Taiwan.

FAU - Hsu, Chih-Hao

AU - Hsu CH

AD - National Institute of Infectious Diseases and Vaccinology, National Health

Research Institutes, Zhunan, Taiwan.

FAU - Wei, Ting-Chun

AU - Wei TC

AD - National Institute of Infectious Diseases and Vaccinology, National Health

Research Institutes, Zhunan, Taiwan.

FAU - Ho, Sin-Ni

AU - Ho SN

AD - National Institute of Infectious Diseases and Vaccinology, National Health

Research Institutes, Zhunan, Taiwan.

FAU - Tsai, Pei-Chu

AU - Tsai PC

AD - National Institute of Infectious Diseases and Vaccinology, National Health

Research Institutes, Zhunan, Taiwan.

FAU - Song, Yung-Deng

AU - Song YD

AD - National Institute of Infectious Diseases and Vaccinology, National Health

Research Institutes, Zhunan, Taiwan.

FAU - Yen, Hui-Ju

AU - Yen HJ

AD - Institute of Preventive Medicine, National Defense Medical Center, Taipei,

Taiwan.

AD - School of Pharmacy, National Defense Medical Center, Taipei, Taiwan.

FAU - Chen, Xin-An

AU - Chen XA

AD - Institute of Preventive Medicine, National Defense Medical Center, Taipei,

Taiwan.

FAU - Young, Jenn-Jong

AU - Young JJ

AD - Institute of Preventive Medicine, National Defense Medical Center, Taipei,

Taiwan.

FAU - Chuang, Chuan-Chung

AU - Chuang CC

AD - School of Dentistry and Graduate Institute of Dental Science, National Defense

Medical Center, Taipei, Taiwan.

AD - Department of Dentistry, Tri-Service General Hospital, Taipei, Taiwan.

FAU - Dou, Horng-Yunn

AU - Dou HY

AD - National Institute of Infectious Diseases and Vaccinology, National Health

Research Institutes, Zhunan, Taiwan.

AD - Department of Biological Science and Technology, National Yang Ming Chiao Tung

University, Hsinchu, Taiwan.

LA - eng

PT - Journal Article

DEP - 20211117

PL - Switzerland

TA - Front Pharmacol

JT - Frontiers in pharmacology

JID - 101548923

PMC - PMC8660078

OTO - NOTNLM

OT - MDR TB

OT - antimycobacterial agent

OT - dormant TB

OT - mycobacterium tubeculosis

OT - silver nanopaiticles

COIS- The authors declare that the research was conducted in the absence of any

commercial or financial relationships that could be construed as a potential

conflict of interest.

EDAT- 2021/12/14 06:00

MHDA- 2021/12/14 06:01

PMCR- 2021/11/17

CRDT- 2021/12/13 17:53

PHST- 2021/07/23 00:00 [received]

PHST- 2021/10/27 00:00 [accepted]

PHST- 2021/12/13 17:53 [entrez]

PHST- 2021/12/14 06:00 [pubmed]

PHST- 2021/12/14 06:01 [medline]

PHST- 2021/11/17 00:00 [pmc-release]

AID - 746496 [pii]

AID - 10.3389/fphar.2021.746496 [doi]

PST - epublish

SO - Front Pharmacol. 2021 Nov 17;12:746496. doi: 10.3389/fphar.2021.746496.

eCollection 2021.

PMID- 34830070

OWN - NLM

STAT- MEDLINE

DCOM- 20220119

LR - 20220119

IS - 1422-0067 (Electronic)

IS - 1422-0067 (Linking)

VI - 22

IP - 22

DP - 2021 Nov 10

TI - The Effect of Tuberculosis Antimicrobials on the Immunometabolic Profiles of

Primary Human Macrophages Stimulated with Mycobacterium tuberculosis.

LID - 10.3390/ijms222212189 [doi]

LID - 12189

AB - Tuberculosis (TB) remains a global health challenge. Patients with drug-sensitive

and drug-resistant TB undergo long, arduous, and complex treatment regimens,

often involving multiple antimicrobials. While these drugs were initially

implemented based on their bactericidal effects, some studies show that TB

antimicrobials can also directly affect cells of the immune system, altering

their immune function. As use of these antimicrobials has been the mainstay of TB

therapy for over fifty years now, it is more important than ever to understand

how these antimicrobials affect key pathways of the immune system. One such

central pathway, which underpins the immune response to a variety of infections,

is immunometabolism, namely glycolysis and oxidative phosphorylation (OXPHOS). We

hypothesise that in addition to their direct bactericidal effect on Mycobacterium

tuberculosis (Mtb), current TB antimicrobials can modulate immunometabolic

profiles and alter mitochondrial function in primary human macrophages. Human

monocyte-derived macrophages (hMDMs) were differentiated from PBMCs isolated from

healthy blood donors, and treated with four first-line and six second-line TB

antimicrobials three hours post stimulation with either iH37Rv-Mtb or

lipopolysaccharide (LPS). 24 h post stimulation, baseline metabolism and

mitochondrial function were determined using the Seahorse Extracellular Flux

Analyser. The effect of these antimicrobials on cytokine and chemokine production

was also assayed using Meso Scale Discovery Multi-Array technology. We show that

some of the TB antimicrobials tested can significantly alter OXPHOS and

glycolysis in uninfected, iH37Rv-Mtb, and LPS-stimulated hMDMs. We also

demonstrate how these antimicrobial-induced immunometabolic effects are linked

with alterations in mitochondrial function. Our results show that TB

antimicrobials, specifically clofazimine, can modify host immunometabolism and

mitochondrial function. Moreover, clofazimine significantly increased the

production of IL-6 in human macrophages that were stimulated with iH37Rv-Mtb.

This provides further insight into the use of some of these TB antimicrobials as

potential host-directed therapies in patients with early and active disease,

which could help to inform TB treatment strategies in the future.

FAU - Cahill, Christina

AU - Cahill C

AUID- ORCID: 0000-0002-2472-9627

AD - TB Immunology Group, Department of Clinical Medicine, Trinity Translational

Medicine Institute, Trinity College, Dublin, Ireland.

FAU - Cox, Dónal J

AU - Cox DJ

AUID- ORCID: 0000-0002-8523-3836

AD - TB Immunology Group, Department of Clinical Medicine, Trinity Translational

Medicine Institute, Trinity College, Dublin, Ireland.

FAU - O'Connell, Fiona

AU - O'Connell F

AUID- ORCID: 0000-0002-3641-4374

AD - Department of Surgery, Trinity Translational Medicine Institute, Trinity College

Dublin, St James's Hospital, Dublin 8, Ireland.

FAU - Basdeo, Sharee A

AU - Basdeo SA

AUID- ORCID: 0000-0001-5616-6665

AD - TB Immunology Group, Department of Clinical Medicine, Trinity Translational

Medicine Institute, Trinity College, Dublin, Ireland.

FAU - Gogan, Karl M

AU - Gogan KM

AD - TB Immunology Group, Department of Clinical Medicine, Trinity Translational

Medicine Institute, Trinity College, Dublin, Ireland.

FAU - Ó'Maoldomhnaigh, Cilian

AU - Ó'Maoldomhnaigh C

AD - TB Immunology Group, Department of Clinical Medicine, Trinity Translational

Medicine Institute, Trinity College, Dublin, Ireland.

FAU - O'Sullivan, Jacintha

AU - O'Sullivan J

AD - Department of Surgery, Trinity Translational Medicine Institute, Trinity College

Dublin, St James's Hospital, Dublin 8, Ireland.

FAU - Keane, Joseph

AU - Keane J

AD - TB Immunology Group, Department of Clinical Medicine, Trinity Translational

Medicine Institute, Trinity College, Dublin, Ireland.

FAU - Phelan, James J

AU - Phelan JJ

AUID- ORCID: 0000-0001-9431-2002

AD - TB Immunology Group, Department of Clinical Medicine, Trinity Translational

Medicine Institute, Trinity College, Dublin, Ireland.

LA - eng

GR - 18/TIDA/6026/SFI_/Science Foundation Ireland/Ireland

GR - BaggotStreetHospital/Dublin 4/Ireland/The Royal City of Dublin Hospital Trust/

GR - GOIPD/2020/16/Irish Research Council/

PT - Journal Article

DEP - 20211110

PL - Switzerland

TA - Int J Mol Sci

JT - International journal of molecular sciences

JID - 101092791

RN - 0 (Antitubercular Agents)

RN - 0 (Cytokines)

RN - 0 (Lipopolysaccharides)

RN - D959AE5USF (Clofazimine)

SB - IM

MH - Antitubercular Agents/*immunology/*pharmacology

MH - Clofazimine/pharmacology

MH - Cytokines/metabolism

MH - Glycolysis/drug effects

MH - Humans

MH - Lipopolysaccharides/toxicity

MH - Macrophages/drug effects/*immunology/*metabolism/microbiology

MH - Mitochondria/drug effects

MH - Mycobacterium tuberculosis/*drug effects

MH - Oxidative Phosphorylation/drug effects

MH - Primary Cell Culture

MH - Tuberculosis/*drug therapy

PMC - PMC8624646

OTO - NOTNLM

OT - antimicrobials

OT - bioenergetics

OT - drug-resistant tuberculosis

OT - glycolysis

OT - host-directed therapy

OT - lipopolysaccharide

OT - mitochondrial function

OT - oxidative phosphorylation

OT - tuberculosis

COIS- The authors declare no conflict of interest.

EDAT- 2021/11/28 06:00

MHDA- 2022/01/20 06:00

PMCR- 2021/11/10

CRDT- 2021/11/27 01:10

PHST- 2021/07/30 00:00 [received]

PHST- 2021/11/07 00:00 [revised]

PHST- 2021/11/08 00:00 [accepted]

PHST- 2021/11/27 01:10 [entrez]

PHST- 2021/11/28 06:00 [pubmed]

PHST- 2022/01/20 06:00 [medline]

PHST- 2021/11/10 00:00 [pmc-release]

AID - ijms222212189 [pii]

AID - ijms-22-12189 [pii]

AID - 10.3390/ijms222212189 [doi]

PST - epublish

SO - Int J Mol Sci. 2021 Nov 10;22(22):12189. doi: 10.3390/ijms222212189.

PMID- 34198149

OWN - NLM

STAT- MEDLINE

DCOM- 20211012

LR - 20211012

IS - 1768-3254 (Electronic)

IS - 0223-5234 (Linking)

VI - 223

DP - 2021 Nov 5

TI - Design and synthesis of 2-(2-isonicotinoylhydrazineylidene)propanamides as InhA

inhibitors with high antitubercular activity.

PG - 113668

LID - S0223-5234(21)00517-1 [pii]

LID - 10.1016/j.ejmech.2021.113668 [doi]

AB - Based on successful antitubercular isoniazid scaffold we have designed its

"mee-too" analogues by a combination of this drug linked with substituted

anilines through pyruvic acid as a bridge. Lipophilicity important for passive

diffusion through impenetrable mycobacterial cell wall was increased by halogen

substitution on the aniline. We prepared twenty new

2-(2-isonicotinoylhydrazineylidene)propanamides that were assayed against

susceptible Mycobacterium tuberculosis H(37)Rv, nontuberculous mycobacteria, and

also multidrug-resistant tuberculous strains (MDR-TB). All the compounds showed

excellent activity not only against Mtb. (minimum inhibitory concentrations, MIC,

from ≤0.03 μM), but also against M. kansasii (MIC ≥2 μM). The most active

molecules have CF(3) and OCF(3) substituent in the position 4 on the aniline

ring. MIC against MDR-TB were from 8 μM. The most effective derivatives were used

for the mechanism of action investigation. The treatment of Mtb. H(37)Ra with

tested compounds led to decreased production of mycolic acids and the strains

overproducing InhA were more resistant to them. These results confirm that

studied compounds inhibit the enoyl-acyl carrier protein reductase (InhA) in

mycobacteria. The compounds did not show any cytotoxic and cytostatic activity

for HepG2 cells. The amides can be considered as a promising scaffold for

antitubercular drug discovery having better antimicrobial properties than

original isoniazid together with a significantly improved pharmaco-toxicological

profile.

CI - Copyright © 2021 Elsevier Masson SAS. All rights reserved.

FAU - Pflégr, Václav

AU - Pflégr V

AD - Department of Organic and Bioorganic Chemistry, Faculty of Pharmacy in Hradec

Králové, Charles University, Akademika Heyrovského 1203, 500 05, Hradec Králové,

Czech Republic.

FAU - Horváth, Lilla

AU - Horváth L

AD - MTA-ELTE Research Group of Peptide Chemistry, Eötvös Loránd Research Network

(ELKH), Institute of Chemistry, Eötvös Loránd University, Pázmány Péter Sétány

1/A, Budapest, H-1117, P.O. Box 32, 1518, Budapest 112, Hungary.

FAU - Stolaříková, Jiřina

AU - Stolaříková J

AD - Laboratory for Mycobacterial Diagnostics and Tuberculosis, Regional Institute of

Public Health in Ostrava, Partyzánské náměstí 7, 702 00, Ostrava, Czech Republic.

FAU - Pál, Adrián

AU - Pál A

AD - Department of Biochemistry, Faculty of Natural Sciences, Comenius University in

Bratislava, Mlynská dolina CH-1, Ilkovičova 6, 842 15, Bratislava, Slovakia.

FAU - Korduláková, Jana

AU - Korduláková J

AD - Department of Biochemistry, Faculty of Natural Sciences, Comenius University in

Bratislava, Mlynská dolina CH-1, Ilkovičova 6, 842 15, Bratislava, Slovakia.

FAU - Bősze, Szilvia

AU - Bősze S

AD - MTA-ELTE Research Group of Peptide Chemistry, Eötvös Loránd Research Network

(ELKH), Institute of Chemistry, Eötvös Loránd University, Pázmány Péter Sétány

1/A, Budapest, H-1117, P.O. Box 32, 1518, Budapest 112, Hungary.

FAU - Vinšová, Jarmila

AU - Vinšová J

AD - Department of Organic and Bioorganic Chemistry, Faculty of Pharmacy in Hradec

Králové, Charles University, Akademika Heyrovského 1203, 500 05, Hradec Králové,

Czech Republic.

FAU - Krátký, Martin

AU - Krátký M

AD - Department of Organic and Bioorganic Chemistry, Faculty of Pharmacy in Hradec

Králové, Charles University, Akademika Heyrovského 1203, 500 05, Hradec Králové,

Czech Republic. Electronic address: martin.kratky@faf.cuni.cz.

LA - eng

PT - Journal Article

DEP - 20210623

PL - France

TA - Eur J Med Chem

JT - European journal of medicinal chemistry

JID - 0420510

RN - 0 (Amides)

RN - 0 (Aniline Compounds)

RN - 0 (Antitubercular Agents)

RN - 0 (Bacterial Proteins)

RN - 8558G7RUTR (Pyruvic Acid)

RN - EC 1.- (Oxidoreductases)

RN - EC 1.3.1.9 (InhA protein, Mycobacterium)

SB - IM

MH - Amides/*chemistry/metabolism/pharmacology/therapeutic use

MH - Aniline Compounds/chemistry

MH - Antitubercular Agents/*chemical synthesis/metabolism/pharmacology/therapeutic use

MH - Bacterial Proteins/*antagonists & inhibitors/metabolism

MH - Cell Survival/drug effects

MH - *Drug Design

MH - Drug Resistance, Bacterial/drug effects

MH - Hep G2 Cells

MH - Humans

MH - Microbial Sensitivity Tests

MH - Mycobacterium tuberculosis/drug effects/metabolism

MH - Oxidoreductases/*antagonists & inhibitors/metabolism

MH - Pyruvic Acid/chemistry

MH - Structure-Activity Relationship

MH - Tuberculosis/drug therapy

OTO - NOTNLM

OT - Antimycobacterial activity

OT - InhA

OT - Isoniazid

OT - Mechanism of action

OT - Multidrug resistance

OT - Pyruvic acid

OT - Tuberculosis

COIS- Declaration of competing interest The authors declare that they have no known

competing financial interests or personal relationships that could have appeared

to influence the work reported in this paper.

EDAT- 2021/07/02 06:00

MHDA- 2021/10/13 06:00

CRDT- 2021/07/01 20:23

PHST- 2021/06/02 00:00 [received]

PHST- 2021/06/18 00:00 [revised]

PHST- 2021/06/21 00:00 [accepted]

PHST- 2021/07/02 06:00 [pubmed]

PHST- 2021/10/13 06:00 [medline]

PHST- 2021/07/01 20:23 [entrez]

AID - S0223-5234(21)00517-1 [pii]

AID - 10.1016/j.ejmech.2021.113668 [doi]

PST - ppublish

SO - Eur J Med Chem. 2021 Nov 5;223:113668. doi: 10.1016/j.ejmech.2021.113668. Epub

2021 Jun 23.

PMID- 34795672

OWN - NLM

STAT- MEDLINE

DCOM- 20220217

LR - 20240404

IS - 1664-3224 (Electronic)

IS - 1664-3224 (Linking)

VI - 12

DP - 2021

TI - Dissecting Host-Pathogen Interactions in TB Using Systems-Based Omic Approaches.

PG - 762315

LID - 10.3389/fimmu.2021.762315 [doi]

LID - 762315

AB - Tuberculosis (TB) is a devastating infectious disease that kills over a million

people every year. There is an increasing burden of multi drug resistance (MDR)

and extensively drug resistance (XDR) TB. New and improved therapies are urgently

needed to overcome the limitations of current treatment. The causative agent,

Mycobacterium tuberculosis (Mtb) is one of the most successful pathogens that can

manipulate host cell environment for adaptation, evading immune defences,

virulence, and pathogenesis of TB infection. Host-pathogen interaction is

important to establish infection and it involves a complex set of processes.

Metabolic cross talk between the host and pathogen is a facet of TB infection and

has been an important topic of research where there is growing interest in

developing therapies and drugs that target these interactions and metabolism of

the pathogen in the host. Mtb scavenges multiple nutrient sources from the host

and has adapted its metabolism to survive in the intracellular niche.

Advancements in systems-based omic technologies have been successful to unravel

host-pathogen interactions in TB. In this review we discuss the application and

usefulness of omics in TB research that provides promising interventions for

developing anti-TB therapies.

CI - Copyright © 2021 Borah, Xu and McFadden.

FAU - Borah, Khushboo

AU - Borah K

AD - School of Biosciences and Medicine, Faculty of Health and Medical Sciences,

University of Surrey, Guildford, United Kingdom.

FAU - Xu, Ye

AU - Xu Y

AD - School of Biosciences and Medicine, Faculty of Health and Medical Sciences,

University of Surrey, Guildford, United Kingdom.

FAU - McFadden, Johnjoe

AU - McFadden J

AD - School of Biosciences and Medicine, Faculty of Health and Medical Sciences,

University of Surrey, Guildford, United Kingdom.

LA - eng

GR - BB/V010611/1/BB_/Biotechnology and Biological Sciences Research Council/United

Kingdom

PT - Journal Article

PT - Research Support, Non-U.S. Gov't

PT - Review

DEP - 20211102

PL - Switzerland

TA - Front Immunol

JT - Frontiers in immunology

JID - 101560960

SB - IM

MH - Animals

MH - Genomics

MH - *Host-Pathogen Interactions

MH - Humans

MH - Metabolomics

MH - Models, Biological

MH - Mycobacterium tuberculosis/*physiology

MH - Systems Biology

MH - Tuberculosis/*genetics/*metabolism

PMC - PMC8593131

OTO - NOTNLM

OT - Mycobacterium tuberculosis

OT - macrophage

OT - omic technology

OT - systems biology

OT - tuberculosis

COIS- The authors declare that the research was conducted in the absence of any

commercial or financial relationships that could be construed as a potential

conflict of interest.

EDAT- 2021/11/20 06:00

MHDA- 2022/02/19 06:00

PMCR- 2021/01/01

CRDT- 2021/11/19 06:54

PHST- 2021/08/21 00:00 [received]

PHST- 2021/10/18 00:00 [accepted]

PHST- 2021/11/19 06:54 [entrez]

PHST- 2021/11/20 06:00 [pubmed]

PHST- 2022/02/19 06:00 [medline]

PHST- 2021/01/01 00:00 [pmc-release]

AID - 10.3389/fimmu.2021.762315 [doi]

PST - epublish

SO - Front Immunol. 2021 Nov 2;12:762315. doi: 10.3389/fimmu.2021.762315. eCollection

2021.

PMID- 34244055

OWN - NLM

STAT- MEDLINE

DCOM- 20210914

LR - 20210914

IS - 1437-7780 (Electronic)

IS - 1341-321X (Linking)

VI - 27

IP - 11

DP - 2021 Nov

TI - Profiling and identification of novel rpoB mutations in rifampicin-resistant

Mycobacterium tuberculosis clinical isolates from Pakistan.

PG - 1578-1583

LID - S1341-321X(21)00181-1 [pii]

LID - 10.1016/j.jiac.2021.06.020 [doi]

AB - INTRODUCTION: Rifampicin (RIF) is one of the most effective anti-tuberculosis

first-line drugs prescribed along with isoniazid. However, the emergence of RIF

resistance Mycobacterium tuberculosis (MTB) isolates is a major issue towards

tuberculosis (TB) control program in high MDR TB-burdened countries including

Pakistan. Molecular data behind phenotypic resistance is essential for better

management of RIF resistance which has been linked with mutations in rpoB gene.

Since molecular studies on RIF resistance is limited in Pakistan, the current

study was aimed to investigate the molecular data of mutations in rpoB gene

behind phenotypic RIF resistance isolates in Pakistan. METHOD: A total of 322

phenotypically RIF-resistant isolates were randomly selected from National TB

Reference Laboratory, Pakistan for sequencing while 380 RIF resistance

whole-genome sequencing (WGS) of Pakistani isolates (BioProject PRJEB25972), were

also analyzed for rpoB mutations. RESULT: Among the 702 RIF resistance samples,

675 (96.1%) isolates harbored mutations in rpoB in which 663 (94.4%) were

detected within the Rifampicin Resistance Determining Region (RRDR) also known as

a mutation hot spot region, including three novel. Among these mutations, 657

(97.3%) were substitutions including 603 (89.3%) single nucleotide polymorphism,

49 (7.25%) double and five (0.8%) triple. About 94.4% of Phenotypic RIF

resistance strains, exhibited mutations in RRDR, which were also detectable by

GeneXpert. CONCLUSION: Mutations in the RRDR region of rpoB is a major mechanism

of RIF resistance in MTB circulating isolates in Pakistan. Molecular detection of

drug resistance is a faster and better approach than phenotypic drug

susceptibility testing to reduce the time for transmission of RIF resistance

strains in population. Such insights will inform the deployment of anti-TB drug

regimens and disease control tools and strategies in high burden settings, such

as Pakistan.

CI - Copyright © 2021. Published by Elsevier Ltd.

FAU - Qadir, Mehmood

AU - Qadir M

AD - Department of Microbiology, Quaid-i-Azam University, Islamabad, Pakistan.

FAU - Tahseen, Sabira

AU - Tahseen S

AD - National TB Reference Laboratory, National TB Control Program, Islamabad,

Pakistan.

FAU - McHugh, Timothy D

AU - McHugh TD

AD - Centre for Clinical Microbiology, Division of Infection and Immunity, University

College London, London, UK.

FAU - Hussain, Alamdar

AU - Hussain A

AD - National TB Reference Laboratory, National TB Control Program, Islamabad,

Pakistan.

FAU - Masood, Faisal

AU - Masood F

AD - National TB Reference Laboratory, National TB Control Program, Islamabad,

Pakistan.

FAU - Ahmed, Niaz

AU - Ahmed N

AD - National TB Reference Laboratory, National TB Control Program, Islamabad,

Pakistan.

FAU - Faryal, Rani

AU - Faryal R

AD - Department of Microbiology, Quaid-i-Azam University, Islamabad, Pakistan.

Electronic address: ranifaryal@qau.edu.pk.

LA - eng

PT - Journal Article

DEP - 20210707

PL - Netherlands

TA - J Infect Chemother

JT - Journal of infection and chemotherapy : official journal of the Japan Society of

Chemotherapy

JID - 9608375

RN - 0 (Antitubercular Agents)

RN - 0 (Bacterial Proteins)

RN - EC 2.7.7.6 (DNA-Directed RNA Polymerases)

RN - VJT6J7R4TR (Rifampin)

SB - IM

MH - Antitubercular Agents/pharmacology

MH - Bacterial Proteins/genetics

MH - DNA-Directed RNA Polymerases/genetics

MH - Humans

MH - Microbial Sensitivity Tests

MH - Mutation

MH - *Mycobacterium tuberculosis/genetics

MH - Pakistan

MH - Rifampin/pharmacology

MH - *Tuberculosis, Multidrug-Resistant/drug therapy

OTO - NOTNLM

OT - Anti-TB drug

OT - Mutations

OT - Mycobacterium tuberculosis

OT - Rifampicin-resistant

OT - rpoB

EDAT- 2021/07/11 06:00

MHDA- 2021/09/15 06:00

CRDT- 2021/07/10 05:36

PHST- 2021/03/06 00:00 [received]

PHST- 2021/06/07 00:00 [revised]

PHST- 2021/06/27 00:00 [accepted]

PHST- 2021/07/11 06:00 [pubmed]

PHST- 2021/09/15 06:00 [medline]

PHST- 2021/07/10 05:36 [entrez]

AID - S1341-321X(21)00181-1 [pii]

AID - 10.1016/j.jiac.2021.06.020 [doi]

PST - ppublish

SO - J Infect Chemother. 2021 Nov;27(11):1578-1583. doi: 10.1016/j.jiac.2021.06.020.

Epub 2021 Jul 7.

PMID- 34126456

OWN - NLM

STAT- MEDLINE

DCOM- 20210901

LR - 20210901

IS - 1768-3254 (Electronic)

IS - 0223-5234 (Linking)

VI - 222

DP - 2021 Oct 15

TI - Identification of novel benzothiopyranones with ester and amide motifs derived

from active metabolite as promising leads against Mycobacterium tuberculosis.

PG - 113603

LID - S0223-5234(21)00452-9 [pii]

LID - 10.1016/j.ejmech.2021.113603 [doi]

AB - We reported three distinct series of novel benzothiopyranones, derived from an

active metabolite (M-1) of anti-TB agent 6b. These small molecules were evaluated

for their biological activities against a range of Mycobacterium tuberculosis

(M. tuberculosis) strains. Preliminary druggability evaluation demonstrated that

M-1 showed good aqueous solubility and hepatocyte stability. Benzothiopyranones

with acyl, sulfonyl and phosphoryl groups exhibited potent in vitro inhibitory

activity against M. tuberculosis H(37)Rv and low cytotoxicity. In particular,

compound 3d, containing a benzoate fragment, displayed marked metabolic stability

and potent in vitro activity against drug-resistant tuberculosis clinical

strains. Further druggability evaluation based on the identified compounds 3d, 4e

and 5b is ongoing for the discovery of promising anti-TB agents.

CI - Copyright © 2021 Elsevier Masson SAS. All rights reserved.

FAU - Li, Peng

AU - Li P

AD - Beijing Key Laboratory of Active Substance Discovery and Druggability Evaluation,

Institute of Materia Medica, Peking Union Medical College and Chinese Academy of

Medical Sciences, 1 Xian Nong Tan Street, Beijing, 100050, PR China; Chinese

Academy of Medical Sciences Key Laboratory of Anti-DR TB Innovative Drug

Research, Institute of Materia Medica, Peking Union Medical College and Chinese

Academy of Medical Sciences, 1 Xian Nong Tan Street, Beijing, 100050, PR China.

FAU - Wang, Bin

AU - Wang B

AD - Beijing Key Laboratory of Drug Resistance Tuberculosis Research, Department of

Pharmacology, Beijing Tuberculosis and Thoracic Tumor Research Institute, Beijing

Chest Hospital, Capital Medical University, 97 Ma Chang Street, Beijing, 101149,

PR China.

FAU - Fu, Lei

AU - Fu L

AD - Beijing Key Laboratory of Drug Resistance Tuberculosis Research, Department of

Pharmacology, Beijing Tuberculosis and Thoracic Tumor Research Institute, Beijing

Chest Hospital, Capital Medical University, 97 Ma Chang Street, Beijing, 101149,

PR China.

FAU - Guo, Kaijing

AU - Guo K

AD - Chinese Academy of Medical Sciences Key Laboratory of Anti-DR TB Innovative Drug

Research, Institute of Materia Medica, Peking Union Medical College and Chinese

Academy of Medical Sciences, 1 Xian Nong Tan Street, Beijing, 100050, PR China.

FAU - Ma, Chen

AU - Ma C

AD - Chinese Academy of Medical Sciences Key Laboratory of Anti-DR TB Innovative Drug

Research, Institute of Materia Medica, Peking Union Medical College and Chinese

Academy of Medical Sciences, 1 Xian Nong Tan Street, Beijing, 100050, PR China.

FAU - Wang, Baolian

AU - Wang B

AD - Chinese Academy of Medical Sciences Key Laboratory of Anti-DR TB Innovative Drug

Research, Institute of Materia Medica, Peking Union Medical College and Chinese

Academy of Medical Sciences, 1 Xian Nong Tan Street, Beijing, 100050, PR China.

FAU - Lin, Ziyun

AU - Lin Z

AD - Beijing Key Laboratory of Active Substance Discovery and Druggability Evaluation,

Institute of Materia Medica, Peking Union Medical College and Chinese Academy of

Medical Sciences, 1 Xian Nong Tan Street, Beijing, 100050, PR China; Chinese

Academy of Medical Sciences Key Laboratory of Anti-DR TB Innovative Drug

Research, Institute of Materia Medica, Peking Union Medical College and Chinese

Academy of Medical Sciences, 1 Xian Nong Tan Street, Beijing, 100050, PR China.

FAU - Li, Gang

AU - Li G

AD - Beijing Key Laboratory of Active Substance Discovery and Druggability Evaluation,

Institute of Materia Medica, Peking Union Medical College and Chinese Academy of

Medical Sciences, 1 Xian Nong Tan Street, Beijing, 100050, PR China; Chinese

Academy of Medical Sciences Key Laboratory of Anti-DR TB Innovative Drug

Research, Institute of Materia Medica, Peking Union Medical College and Chinese

Academy of Medical Sciences, 1 Xian Nong Tan Street, Beijing, 100050, PR China.

Electronic address: ligang@imm.ac.cn.

FAU - Huang, Haihong

AU - Huang H

AD - Beijing Key Laboratory of Active Substance Discovery and Druggability Evaluation,

Institute of Materia Medica, Peking Union Medical College and Chinese Academy of

Medical Sciences, 1 Xian Nong Tan Street, Beijing, 100050, PR China; Chinese

Academy of Medical Sciences Key Laboratory of Anti-DR TB Innovative Drug

Research, Institute of Materia Medica, Peking Union Medical College and Chinese

Academy of Medical Sciences, 1 Xian Nong Tan Street, Beijing, 100050, PR China.

Electronic address: joyce@imm.ac.cn.

FAU - Lu, Yu

AU - Lu Y

AD - Beijing Key Laboratory of Drug Resistance Tuberculosis Research, Department of

Pharmacology, Beijing Tuberculosis and Thoracic Tumor Research Institute, Beijing

Chest Hospital, Capital Medical University, 97 Ma Chang Street, Beijing, 101149,

PR China. Electronic address: luyu4876@hotmail.com.

LA - eng

PT - Journal Article

DEP - 20210605

PL - France

TA - Eur J Med Chem

JT - European journal of medicinal chemistry

JID - 0420510

RN - 0 (Amides)

RN - 0 (Antitubercular Agents)

RN - 0 (Benzopyrans)

RN - 0 (Esters)

SB - IM

MH - Amides/chemistry/metabolism/*pharmacology

MH - Antitubercular Agents/chemistry/metabolism/*pharmacology

MH - Benzopyrans/chemistry/metabolism/*pharmacology

MH - Dose-Response Relationship, Drug

MH - Esters/chemistry/metabolism/*pharmacology

MH - Microbial Sensitivity Tests

MH - Molecular Structure

MH - Mycobacterium tuberculosis/*drug effects

MH - Structure-Activity Relationship

OTO - NOTNLM

OT - Active metabolite

OT - Benzothiopyranones

OT - Drug-resistant tuberculosis

OT - Metabolic stability

COIS- Declaration of competing interest The authors declare that they have no known

competing financial interests or personal relationships that could have appeared

to influence the work reported in this paper.

EDAT- 2021/06/15 06:00

MHDA- 2021/09/02 06:00

CRDT- 2021/06/14 20:25

PHST- 2021/01/20 00:00 [received]

PHST- 2021/05/19 00:00 [revised]

PHST- 2021/05/30 00:00 [accepted]

PHST- 2021/06/15 06:00 [pubmed]

PHST- 2021/09/02 06:00 [medline]

PHST- 2021/06/14 20:25 [entrez]

AID - S0223-5234(21)00452-9 [pii]

AID - 10.1016/j.ejmech.2021.113603 [doi]

PST - ppublish

SO - Eur J Med Chem. 2021 Oct 15;222:113603. doi: 10.1016/j.ejmech.2021.113603. Epub

2021 Jun 5.

PMID- 34118719

OWN - NLM

STAT- MEDLINE

DCOM- 20210831

LR - 20210831

IS - 1768-3254 (Electronic)

IS - 0223-5234 (Linking)

VI - 222

DP - 2021 Oct 15

TI - The Mur Enzymes Chink in the Armour of Mycobacterium tuberculosis cell wall.

PG - 113568

LID - S0223-5234(21)00417-7 [pii]

LID - 10.1016/j.ejmech.2021.113568 [doi]

AB - TUBERCULOSIS: (TB) transmitted by Mycobacterium tuberculosis (Mtb) is one of the

top 10 causes of death globally. Currently, the widespread occurrence of

resistance toward Mtb strains is becoming a significant concern to public health.

This scenario exaggerated the need for the discovery of novel targets and their

inhibitors. Targeting the "Mtb cell wall peptidoglycan synthesis" is an

attractive strategy to overcome drug resistance. Mur enzymes (MurA-MurF) play

essential roles in the peptidoglycan synthesis by catalyzing the ligation of key

amino acid residues to the stem peptide. These enzymes are unique and confined to

the eubacteria and are absent in humans, representing potential targets for

anti-tubercular drug discovery. Mtb Mur ligases with the same catalytic mechanism

share conserved amino acid regions and structural features that can conceivably

exploit for the designing of the inhibitors, which can simultaneously target more

than one isoforms (MurC-MurF) of the enzyme. In light of these findings in the

current review, we have discussed the recent advances in medicinal chemistry of

Mtb Mur enzymes (MurA-MurF) and their inhibitors, offering attractive

multi-targeted strategies to combat the problem of drug-resistant in

M. tuberculosis.

CI - Copyright © 2021 Elsevier Masson SAS. All rights reserved.

FAU - Shinde, Yashodeep

AU - Shinde Y

AD - Department of Pharmaceutical Chemistry, R. C. Patel Institute of Pharmaceutical

Education and Research, Shirpur, District Dhule, 425405, Maharashtra, India.

FAU - Ahmad, Iqrar

AU - Ahmad I

AD - Department of Pharmaceutical Chemistry, R. C. Patel Institute of Pharmaceutical

Education and Research, Shirpur, District Dhule, 425405, Maharashtra, India.

FAU - Surana, Sanjay

AU - Surana S

AD - Department of Pharmaceutical Chemistry, R. C. Patel Institute of Pharmaceutical

Education and Research, Shirpur, District Dhule, 425405, Maharashtra, India.

FAU - Patel, Harun

AU - Patel H

AD - Department of Pharmaceutical Chemistry, R. C. Patel Institute of Pharmaceutical

Education and Research, Shirpur, District Dhule, 425405, Maharashtra, India.

Electronic address: hpatel_38@yahoo.com.

LA - eng

PT - Journal Article

PT - Review

DEP - 20210602

PL - France

TA - Eur J Med Chem

JT - European journal of medicinal chemistry

JID - 0420510

RN - 0 (Antitubercular Agents)

RN - 0 (Enzyme Inhibitors)

RN - 0 (Peptidoglycan)

RN - EC 6.3.2.- (Peptide Synthases)

SB - IM

MH - Antitubercular Agents/chemical synthesis/chemistry/*pharmacology

MH - Cell Wall/drug effects/metabolism

MH - Enzyme Inhibitors/chemical synthesis/chemistry/*pharmacology

MH - Molecular Structure

MH - Mycobacterium tuberculosis/cytology/*enzymology

MH - Peptide Synthases/*antagonists & inhibitors/metabolism

MH - Peptidoglycan/chemistry/*pharmacology

OTO - NOTNLM

OT - MDR-TB

OT - Mur ligase

OT - Tuberculosis

OT - XDR-TB

COIS- Declaration of competing interest The authors declare that they have no known

competing financial interests or personal relationships that could have appeared

to influence the work reported in this paper.

EDAT- 2021/06/13 06:00

MHDA- 2021/09/01 06:00

CRDT- 2021/06/12 20:21

PHST- 2021/02/22 00:00 [received]

PHST- 2021/05/11 00:00 [revised]

PHST- 2021/05/13 00:00 [accepted]

PHST- 2021/06/13 06:00 [pubmed]

PHST- 2021/09/01 06:00 [medline]

PHST- 2021/06/12 20:21 [entrez]

AID - S0223-5234(21)00417-7 [pii]

AID - 10.1016/j.ejmech.2021.113568 [doi]

PST - ppublish

SO - Eur J Med Chem. 2021 Oct 15;222:113568. doi: 10.1016/j.ejmech.2021.113568. Epub

2021 Jun 2.

PMID- 34690963

OWN - NLM

STAT- PubMed-not-MEDLINE

LR - 20231107

IS - 1664-302X (Print)

IS - 1664-302X (Electronic)

IS - 1664-302X (Linking)

VI - 12

DP - 2021

TI - Mechanism of Action, Resistance, Synergism, and Clinical Implications of

Delamanid Against Multidrug-Resistant Mycobacterium tuberculosis.

PG - 717045

LID - 10.3389/fmicb.2021.717045 [doi]

LID - 717045

AB - Multidrug-resistant (MDR) isolates of Mycobacterium tuberculosis (MTB) remain a

primary global threat to the end of tuberculosis (TB) era. Delamanid (DLM) is a

nitro-dihydro-imidazooxazole derivative utilized to treat MDR-TB. DLM has

distinct mechanism of action, inhibiting methoxy- and keto-mycolic acid (MA)

synthesis through the F420 coenzyme mycobacteria system and generating nitrous

oxide. While DLM resistance among MTB strains is uncommon, there are increasing

reports in Asia and Europe, and such resistance will prolong the treatment

courses of patients infected with MDR-TB. In this review, we address the

antimycobacterial properties of DLM, report the global prevalence of DLM

resistance, discuss the synergism of DLM with other anti-TB drugs, and evaluate

the documented clinical trials to provide new insights into the clinical use of

this antibiotic.

CI - Copyright © 2021 Khoshnood, Taki, Sadeghifard, Kaviar, Haddadi, Farshadzadeh,

Kouhsari, Goudarzi and Heidary.

FAU - Khoshnood, Saeed

AU - Khoshnood S

AD - Clinical Microbiology Research Center, Ilam University of Medical Sciences, Ilam,

Iran.

FAU - Taki, Elahe

AU - Taki E

AD - Department of Microbiology, School of Medicine, Tehran University of Medical

Sciences, Tehran, Iran.

FAU - Sadeghifard, Nourkhoda

AU - Sadeghifard N

AD - Clinical Microbiology Research Center, Ilam University of Medical Sciences, Ilam,

Iran.

FAU - Kaviar, Vahab Hassan

AU - Kaviar VH

AD - Clinical Microbiology Research Center, Ilam University of Medical Sciences, Ilam,

Iran.

FAU - Haddadi, Mohammad Hossein

AU - Haddadi MH

AD - Clinical Microbiology Research Center, Ilam University of Medical Sciences, Ilam,

Iran.

FAU - Farshadzadeh, Zahra

AU - Farshadzadeh Z

AD - Infectious and Tropical Diseases Research Center, Health Research Institute,

Ahvaz Jundishapur University of Medical Sciences, Ahvaz, Iran.

AD - Department of Microbiology, School of Medicine, Ahvaz Jundishapur University of

Medical Sciences, Ahvaz, Iran.

FAU - Kouhsari, Ebrahim

AU - Kouhsari E

AD - Laboratory Sciences Research Center, Golestan University of Medical Sciences,

Gorgan, Iran.

FAU - Goudarzi, Mehdi

AU - Goudarzi M

AD - Department of Microbiology, School of Medicine, Shahid Beheshti University of

Medical Sciences, Tehran, Iran.

FAU - Heidary, Mohsen

AU - Heidary M

AD - Department of Laboratory Sciences, School of Paramedical Sciences, Sabzevar

University of Medical Sciences, Sabzevar, Iran.

AD - Cellular and Molecular Research Center, Sabzevar University of Medical Sciences,

Sabzevar, Iran.

LA - eng

PT - Journal Article

PT - Review

DEP - 20211007

PL - Switzerland

TA - Front Microbiol

JT - Frontiers in microbiology

JID - 101548977

PMC - PMC8529252

OTO - NOTNLM

OT - Mycobacterium tuberculosis

OT - TB

OT - delamanid

OT - review

OT - tuberculosis

COIS- The authors declare that the research was conducted in the absence of any

commercial or financial relationships that could be construed as a potential

conflict of interest.

EDAT- 2021/10/26 06:00

MHDA- 2021/10/26 06:01

PMCR- 2021/10/07

CRDT- 2021/10/25 06:25

PHST- 2021/06/02 00:00 [received]

PHST- 2021/09/02 00:00 [accepted]

PHST- 2021/10/25 06:25 [entrez]

PHST- 2021/10/26 06:00 [pubmed]

PHST- 2021/10/26 06:01 [medline]

PHST- 2021/10/07 00:00 [pmc-release]

AID - 10.3389/fmicb.2021.717045 [doi]

PST - epublish

SO - Front Microbiol. 2021 Oct 7;12:717045. doi: 10.3389/fmicb.2021.717045.

eCollection 2021.

PMID- 34875838

OWN - NLM

STAT- MEDLINE

DCOM- 20211209

LR - 20220210

IS - 1210-7816 (Print)

IS - 1210-7816 (Linking)

VI - 70

IP - 5

DP - 2021 Fall

TI - Telacebec (Q203): Is there a novel effective and safe anti-tuberculosis drug on

the horizon?

PG - 164–171

AB - High prevalence and stronger emergency of various forms of drug-resistant

tuberculosis (DR-TB), including the multidrug-resistant (MDR-TB) as well as

extensively drug-resistant (XDR-TB) ones, caused by variously resistant

Mycobacterium tuberculosis pathogens, make first-line anti-tuberculosis (anti-TB)

agents therapeutically more and more ineffective. Therefore, there is an

imperative to develop novel highly efficient (synthetic) agents against both

drug-sensitive-TB and DR-TB. The exploration of various heterocycles as

prospective core scaffolds for the discovery, development and optimization of

anti-TB drugs remains an intriguing scientific endeavour. Telacebec (Q203; TCB),

a molecule containing an imidazo[1,2-a]pyridine-3-carboxamide (IPA) structural

motif, is considered a novel very promising anti-TB agent showing a unique

mechanism of action. The compound blocks oxidative phosphorylation by inhibiting

a mycobacterial respiratory chain due to interference with a specific cytochrome

b subunit (QcrB) of transmembrane bc1 menaquinol-cytochrome c oxidoreductase as

an essential component for transporting electrons across the membrane from

menaquinol to other specific subunit, cytochrome c (QcrC). Thus, the ability of

mycobacteria to synthesize adenosine-5´-triphosphate is limited and energy

generating machinery is disabled. The TCB molecule effectively fights

drug-susceptible, MDR as well as XDR M. tuberculosis strains. The article briefly

explains a mechanism of an anti-TB action related to the compounds containing a

variously substituted IPA scaffold and is focused on their fundamental

structure-anti-TB activity relationships as well. Special consideration is paid

to TCB indicating the importance of particular structural fragments for

maintaining (or even improving) favourable pharmacodynamic, pharmacokinetic

and/or toxicological properties. High lipophilicity of TCB might be regarded as

one of the key physicochemical properties with positive impact on anti-TB effect

of the drug. In January 2021, the TCB molecule was also involved in phase-II

clinical trials focused on the treatment of Coronavirus Disease-19 caused by

Severe Acute Respiratory Syndrome Coronavirus 2.

FAU - Malík, Ivan

AU - Malík I

FAU - Čižmárik, Jozef

AU - Čižmárik J

FAU - Kováč, Gustáv

AU - Kováč G

FAU - Pecháčová, Mária

AU - Pecháčová M

FAU - Hudecova, Lucia

AU - Hudecova L

LA - eng

PT - Journal Article

TT - Telacebek (Q203): Vynára sa na obzore nové efektívne a bezpe&#269;né

antituberkulotikum?

PL - Czech Republic

TA - Ceska Slov Farm

JT - Ceska a Slovenska farmacie : casopis Ceske farmaceuticke spolecnosti a Slovenske

farmaceuticke spolecnosti

JID - 9433765

RN - 0 (Antitubercular Agents)

RN - 0 (Imidazoles)

RN - 0 (Piperidines)

RN - 0 (Pyridines)

RN - 55G92WGH3X (telacebec)

SB - IM

MH - Antitubercular Agents

MH - *COVID-19

MH - Humans

MH - Imidazoles

MH - *Mycobacterium tuberculosis

MH - Piperidines

MH - Prospective Studies

MH - Pyridines

MH - SARS-CoV-2

MH - *Tuberculosis, Multidrug-Resistant

OTO - NOTNLM

OT - Mycobacterium tuberculosis

OT - drug-resistant tuberculosis

OT - imidazo[1,2-a]pyridine-3-carboxamides

OT - respiratory chain

OT - telacebec (Q203)

EDAT- 2021/12/09 06:00

MHDA- 2021/12/15 06:00

CRDT- 2021/12/08 05:25

PHST- 2021/12/08 05:25 [entrez]

PHST- 2021/12/09 06:00 [pubmed]

PHST- 2021/12/15 06:00 [medline]

AID - 128806 [pii]

AID - 10.5817/CSF2021-5-164 [doi]

PST - ppublish

SO - Ceska Slov Farm. 2021 Fall;70(5):164–171. doi: 10.5817/CSF2021-5-164.

PMID- 34594117

OWN - NLM

STAT- PubMed-not-MEDLINE

LR - 20220427

IS - 1178-6973 (Print)

IS - 1178-6973 (Electronic)

IS - 1178-6973 (Linking)

VI - 14

DP - 2021

TI - The Transcription Factor Rv1453 Regulates the Expression of qor and Confers

Resistant to Clofazimine in Mycobacterium tuberculosis.

PG - 3937-3948

LID - 10.2147/IDR.S324043 [doi]

AB - OBJECTIVE: Clofazimine plays an important role in the treatment of drug-resistant

tuberculosis. However, the mechanism of clofazimine resistance remains unclear.

In order to slow down the occurrence of clofazimine resistance, it is necessary

to study its resistance mechanism. METHODS: In this study, we constructed Rv1453

knockout, complementary and overexpressed strain. The minimum inhibitory

concentration (MIC) of clofazimine against Mycobacterium tuberculosis was

detected by microplate alamar blue assay (MABA). The transcription levels of

Rv1453 and its adjacent genes were detected by quantitative reverse transcriptase

PCR. The purified Rv1453 protein was used for electrophoretic mobility shift

assay (EMSA) to identify the binding site of Rv1453 protein. RESULTS: The minimum

inhibitory concentration (MIC) of clofazimine increased about 4-fold for the

Rv1453 knockout strain and decreased about 4-fold for the Rv1453 overexpressed

strain compared with Mycobacterium tuberculosis H37Rv. Further analysis showed

that Rv1453 protein, as a regulatory protein, binds to the RNA polymerase binding

site of qor and blocks the transcription process. CONCLUSION: This study

preliminarily revealed that Rv1453 protein of Mycobacterium tuberculosis affects

its susceptibility to clofazimine by regulating the transcription level of qor,

which is shedding a new light on the mechanism of clofazimine resistance.

CI - © 2021 Li et al.

FAU - Li, Yuanyuan

AU - Li Y

AD - Beijing Key Laboratory of Drug Resistance Tuberculosis Research, Department of

Pharmacology, Beijing Tuberculosis and Thoracic Tumor Research Institute, Beijing

Chest Hospital, Capital Medical University, Beijing, 101149, People's Republic of

China.

FAU - Fu, Lei

AU - Fu L

AD - Beijing Key Laboratory of Drug Resistance Tuberculosis Research, Department of

Pharmacology, Beijing Tuberculosis and Thoracic Tumor Research Institute, Beijing

Chest Hospital, Capital Medical University, Beijing, 101149, People's Republic of

China.

FAU - Zhang, Weiyan

AU - Zhang W

AD - Beijing Key Laboratory of Drug Resistance Tuberculosis Research, Department of

Pharmacology, Beijing Tuberculosis and Thoracic Tumor Research Institute, Beijing

Chest Hospital, Capital Medical University, Beijing, 101149, People's Republic of

China.

FAU - Chen, Xi

AU - Chen X

AD - Beijing Key Laboratory of Drug Resistance Tuberculosis Research, Department of

Pharmacology, Beijing Tuberculosis and Thoracic Tumor Research Institute, Beijing

Chest Hospital, Capital Medical University, Beijing, 101149, People's Republic of

China.

FAU - Lu, Yu

AU - Lu Y

AD - Beijing Key Laboratory of Drug Resistance Tuberculosis Research, Department of

Pharmacology, Beijing Tuberculosis and Thoracic Tumor Research Institute, Beijing

Chest Hospital, Capital Medical University, Beijing, 101149, People's Republic of

China.

LA - eng

PT - Journal Article

DEP - 20210924

PL - New Zealand

TA - Infect Drug Resist

JT - Infection and drug resistance

JID - 101550216

PMC - PMC8478341

OTO - NOTNLM

OT - electrophoretic mobility shift assay

OT - redox

OT - resistance mechanisms

OT - transcriptional regulation

COIS- Miss Yuanyuan Li reports grants from National Natural Science Foundation of

China, grants from Beijing Hospitals Authority Clinical Medicine Development of

Special Funding Support, during the conduct of the study Mr Lei Fu reports grants

from National Natural Science Foundation of China, grants from Beijing Hospitals

Authority Clinical Medicine Development of Special Funding Support, during the

conduct of the study. Mrs Weiyan Zhang reports grants from the National Natural

Science Foundation of China, grants from Beijing Hospitals Authority Clinical

Medicine Development of Special Funding Support, during the conduct of the study.

Mrs Xi Chen reports grants from the National Natural Science Foundation of China,

grants from Beijing Hospitals Authority Clinical Medicine Development of Special

Funding Support, during the conduct of the study. Professor Yu Lu reports grants

from the National Natural Science Foundation of China, grants from Beijing

Hospitals Authority Clinical Medicine Development of Special Funding Support,

outside the submitted work. The authors report no conflicts of interest in this

work.

EDAT- 2021/10/02 06:00

MHDA- 2021/10/02 06:01

PMCR- 2021/09/24

CRDT- 2021/10/01 07:23

PHST- 2021/06/11 00:00 [received]

PHST- 2021/08/27 00:00 [accepted]

PHST- 2021/10/01 07:23 [entrez]

PHST- 2021/10/02 06:00 [pubmed]

PHST- 2021/10/02 06:01 [medline]

PHST- 2021/09/24 00:00 [pmc-release]

AID - 324043 [pii]

AID - 10.2147/IDR.S324043 [doi]

PST - epublish

SO - Infect Drug Resist. 2021 Sep 24;14:3937-3948. doi: 10.2147/IDR.S324043.

eCollection 2021.

PMID- 34559866

OWN - NLM

STAT- MEDLINE

DCOM- 20211124

LR - 20221202

IS - 1553-7374 (Electronic)

IS - 1553-7366 (Print)

IS - 1553-7366 (Linking)

VI - 17

IP - 9

DP - 2021 Sep

TI - TCA cycle remodeling drives proinflammatory signaling in humans with pulmonary

tuberculosis.

PG - e1009941

LID - 10.1371/journal.ppat.1009941 [doi]

LID - e1009941

AB - The metabolic signaling pathways that drive pathologic tissue inflammation and

damage in humans with pulmonary tuberculosis (TB) are not well understood. Using

combined methods in plasma high-resolution metabolomics, lipidomics and cytokine

profiling from a multicohort study of humans with pulmonary TB disease, we

discovered that IL-1β-mediated inflammatory signaling was closely associated with

TCA cycle remodeling, characterized by accumulation of the proinflammatory

metabolite succinate and decreased concentrations of the anti-inflammatory

metabolite itaconate. This inflammatory metabolic response was particularly

active in persons with multidrug-resistant (MDR)-TB that received at least 2

months of ineffective treatment and was only reversed after 1 year of appropriate

anti-TB chemotherapy. Both succinate and IL-1β were significantly associated with

proinflammatory lipid signaling, including increases in the products of

phospholipase A2, increased arachidonic acid formation, and metabolism of

arachidonic acid to proinflammatory eicosanoids. Together, these results indicate

that decreased itaconate and accumulation of succinate and other TCA cycle

intermediates is associated with IL-1β-mediated proinflammatory eicosanoid

signaling in pulmonary TB disease. These findings support host metabolic

remodeling as a key driver of pathologic inflammation in human TB disease.

FAU - Collins, Jeffrey M

AU - Collins JM

AUID- ORCID: 0000-0002-6574-0873

AD - Department of Medicine, Emory University School of Medicine, Atlanta, Georgia,

United States of America.

FAU - Jones, Dean P

AU - Jones DP

AUID- ORCID: 0000-0002-2090-0677

AD - Department of Medicine, Emory University School of Medicine, Atlanta, Georgia,

United States of America.

FAU - Sharma, Ashish

AU - Sharma A

AD - Department of Pathology, Emory University School of Medicine, Atlanta, Georgia,

United States of America.

FAU - Khadka, Manoj

AU - Khadka M

AUID- ORCID: 0000-0002-4003-7049

AD - Department of Biochemistry, Emory University, Atlanta, Georgia, United States of

America.

FAU - Liu, Ken H

AU - Liu KH

AUID- ORCID: 0000-0002-9736-5828

AD - Department of Medicine, Emory University School of Medicine, Atlanta, Georgia,

United States of America.

FAU - Kempker, Russell R

AU - Kempker RR

AUID- ORCID: 0000-0002-7188-4996

AD - Department of Medicine, Emory University School of Medicine, Atlanta, Georgia,

United States of America.

FAU - Prideaux, Brendan

AU - Prideaux B

AD - Department of Neuroscience, Cell Biology, & Anatomy, University of Texas Medical

Branch, Galveston, Texas, United States of America.

FAU - Maner-Smith, Kristal

AU - Maner-Smith K

AUID- ORCID: 0000-0002-2274-4284

AD - Department of Biochemistry, Emory University, Atlanta, Georgia, United States of

America.

FAU - Tukvadze, Nestani

AU - Tukvadze N

AD - National Center for Tuberculosis and Lung Diseases, Tbilisi, Georgia.

FAU - Shah, N Sarita

AU - Shah NS

AD - Department of Medicine, Emory University School of Medicine, Atlanta, Georgia,

United States of America.

AD - Department of Global Health, Emory University Rollins School of Public Health,

Atlanta, Georgia, United States of America.

AD - Department of Epidemiology, Emory University Rollins School of Public Health,

Atlanta, Georgia, United States of America.

FAU - Brust, James C M

AU - Brust JCM

AUID- ORCID: 0000-0003-3362-1818

AD - Department of Medicine, Albert Einstein College of Medicine and Montefiore

Medical Center, Bronx, New York, United States of America.

FAU - Sékaly, Rafick-Pierre

AU - Sékaly RP

AD - Department of Pathology, Emory University School of Medicine, Atlanta, Georgia,

United States of America.

FAU - Gandhi, Neel R

AU - Gandhi NR

AD - Department of Medicine, Emory University School of Medicine, Atlanta, Georgia,

United States of America.

AD - Department of Global Health, Emory University Rollins School of Public Health,

Atlanta, Georgia, United States of America.

AD - Department of Epidemiology, Emory University Rollins School of Public Health,

Atlanta, Georgia, United States of America.

FAU - Blumberg, Henry M

AU - Blumberg HM

AUID- ORCID: 0000-0002-3110-2443

AD - Department of Medicine, Emory University School of Medicine, Atlanta, Georgia,

United States of America.

AD - Department of Global Health, Emory University Rollins School of Public Health,

Atlanta, Georgia, United States of America.

AD - Department of Epidemiology, Emory University Rollins School of Public Health,

Atlanta, Georgia, United States of America.

FAU - Ortlund, Eric A

AU - Ortlund EA

AUID- ORCID: 0000-0001-8855-3029

AD - Department of Biochemistry, Emory University, Atlanta, Georgia, United States of

America.

FAU - Ziegler, Thomas R

AU - Ziegler TR

AD - Department of Medicine, Emory University School of Medicine, Atlanta, Georgia,

United States of America.

AD - Section of Endocrinology, Atlanta Veterans Affairs Medical Center, Atlanta,

Georgia, United States of America.

LA - eng

GR - K23 AI144040/AI/NIAID NIH HHS/United States

GR - D43 TW007124/TW/FIC NIH HHS/United States

GR - R01 AI145679/AI/NIAID NIH HHS/United States

GR - UL1 TR001073/TR/NCATS NIH HHS/United States

GR - UL1 TR002378/TR/NCATS NIH HHS/United States

GR - P30 ES019776/ES/NIEHS NIH HHS/United States

GR - K24 AI155045/AI/NIAID NIH HHS/United States

GR - U19 AI111211/AI/NIAID NIH HHS/United States

GR - P30 AI050409/AI/NIAID NIH HHS/United States

GR - R21 AI130918/AI/NIAID NIH HHS/United States

GR - T32 AI074492/AI/NIAID NIH HHS/United States

GR - R01 AI087465/AI/NIAID NIH HHS/United States

GR - K24 AI114444/AI/NIAID NIH HHS/United States

GR - R01 AI114304/AI/NIAID NIH HHS/United States

GR - P30 AI124414/AI/NIAID NIH HHS/United States

GR - UL1 TR002556/TR/NCATS NIH HHS/United States

PT - Journal Article

PT - Research Support, N.I.H., Extramural

PT - Research Support, Non-U.S. Gov't

DEP - 20210924

PL - United States

TA - PLoS Pathog

JT - PLoS pathogens

JID - 101238921

SB - IM

MH - Citric Acid Cycle/*physiology

MH - Humans

MH - Inflammation/*metabolism

MH - Signal Transduction/*physiology

MH - Tuberculosis, Pulmonary/*metabolism

PMC - PMC8494353

COIS- The authors have declared that no competing interests exist.

EDAT- 2021/09/25 06:00

MHDA- 2021/11/25 06:00

PMCR- 2021/09/24

CRDT- 2021/09/24 17:20

PHST- 2021/04/27 00:00 [received]

PHST- 2021/09/03 00:00 [accepted]

PHST- 2021/10/06 00:00 [revised]

PHST- 2021/09/25 06:00 [pubmed]

PHST- 2021/11/25 06:00 [medline]

PHST- 2021/09/24 17:20 [entrez]

PHST- 2021/09/24 00:00 [pmc-release]

AID - PPATHOGENS-D-21-00892 [pii]

AID - 10.1371/journal.ppat.1009941 [doi]

PST - epublish

SO - PLoS Pathog. 2021 Sep 24;17(9):e1009941. doi: 10.1371/journal.ppat.1009941.

eCollection 2021 Sep.

PMID- 34174037

OWN - NLM

STAT- PubMed-not-MEDLINE

DCOM- 20251003

LR - 20251003

IS - 1588-2640 (Electronic)

IS - 1217-8950 (Linking)

VI - 68

IP - 3

DP - 2021 Sep 8

TI - Linezolid resistance among multidrug-resistant Mycobacterium tuberculosis

clinical isolates in Iran.

PG - 203-207

LID - 10.1556/030.2021.01490 [doi]

AB - The management of multidrug-resistant (MDR) and extensively drug-resistant

tuberculosis (XDR-TB) presents a main challenge and the drug options for treating

these infections are very limited. Linezolid (LNZ) has recently been approved for

the treatment of MDR and XDR-TB. But, there are narrow data on genotypic and

phenotypic LNZ resistance in clinical isolates. So, we aimed to determine the

prevalence of LNZ resistance and to identify the mutations associated with LNZ

resistance among clinical MDR-TB isolates. The minimum inhibitory concentration

(MIC) values of LNZ for 22 MDR-TB isolates were determined by broth microdilution

method. All MDR-TB isolates were sequenced in the rrl and rplC genes conferring

LNZ resistance. LNZ resistance was found in 3 (13.6%) of 22 MDR-TB isolates. The

MICs of LNZ were 8 μg/mL for two isolates and 16 μg/mL for one isolate. The 421

(A/G) and 449 (T/A) mutations in rplC gene were detected in one of the

LNZ-resistant isolates. There was no mutation in rrl gene. The results reveal

that the prevalence of LNZ-resistant isolates is 13.6% among MDR-TB isolates and

drug susceptibility testing (DST) against LNZ is useful in the management of

complicated and drug-resistant cases. However, further studies could identify

other possible genetic mechanism of resistance in TB.

FAU - Khosravi, Azar Dokht

AU - Khosravi AD

AD - 1 Infectious and Tropical Diseases Research Center, Health Research Institute,

Ahvaz Jundishapur University of Medical Sciences, Ahvaz, Iran.

AD - 2 Department of Microbiology, School of Medicine, Ahvaz Jundishapur University of

Medical Sciences, Ahvaz, Iran.

AD - 3 Iranian Study Group on Microbial Drug Resistance, Iran.

FAU - Tabandeh, Mohammad Reza

AU - Tabandeh MR

AD - 4 Department of Basic Sciences, Division of Biochemistry and Molecular Biology,

Faculty of Veterinary Medicine, Shahid Chamran University of Ahvaz, Ahvaz, Iran.

FAU - Shahi, Fatemeh

AU - Shahi F

AUID- ORCID: 0000-0003-4069-7423

AD - 1 Infectious and Tropical Diseases Research Center, Health Research Institute,

Ahvaz Jundishapur University of Medical Sciences, Ahvaz, Iran.

AD - 2 Department of Microbiology, School of Medicine, Ahvaz Jundishapur University of

Medical Sciences, Ahvaz, Iran.

FAU - Salmanzadeh, Shokrollah

AU - Salmanzadeh S

AD - 1 Infectious and Tropical Diseases Research Center, Health Research Institute,

Ahvaz Jundishapur University of Medical Sciences, Ahvaz, Iran.

AD - 5 Tropical Medicine Ward, Razi Teaching Hospital, Ahvaz Jundishapur University of

Medical Sciences, Ahvaz, Iran.

LA - eng

PT - Journal Article

DEP - 20210624

PL - Hungary

TA - Acta Microbiol Immunol Hung

JT - Acta microbiologica et immunologica Hungarica

JID - 9434021

SB - IM

OTO - NOTNLM

OT - Mycobacterium tuberculosis

OT - linezolid

OT - minimum inhibitory concentration

OT - multidrug-resistance

OT - mutation

EDAT- 2021/06/27 06:00

MHDA- 2021/06/27 06:01

CRDT- 2021/06/26 17:04

PHST- 2021/05/04 00:00 [received]

PHST- 2021/06/09 00:00 [accepted]

PHST- 2021/06/27 06:01 [medline]

PHST- 2021/06/27 06:00 [pubmed]

PHST- 2021/06/26 17:04 [entrez]

AID - 10.1556/030.2021.01490 [doi]

PST - epublish

SO - Acta Microbiol Immunol Hung. 2021 Jun 24;68(3):203-207. doi:

10.1556/030.2021.01490. Print 2021 Sep 8.

PMID- 34287057

OWN - NLM

STAT- MEDLINE

DCOM- 20220106

LR - 20220106

IS - 2165-0497 (Electronic)

IS - 2165-0497 (Linking)

VI - 9

IP - 1

DP - 2021 Sep 3

TI - Intragenic Distribution of IS6110 in Clinical Mycobacterium tuberculosis Strains:

Bioinformatic Evidence for Gene Disruption Leading to Underdiagnosed Antibiotic

Resistance.

PG - e0001921

LID - 10.1128/Spectrum.00019-21 [doi]

LID - 10.1128/spectrum.00019-21

AB - Antibiotic resistance is a global challenge for tuberculosis control, and

accelerating its diagnosis is critical for therapy decisions and controlling

transmission. Genotype-based molecular diagnostics now play an increasing role in

accelerating the detection of such antibiotic resistance, but their accuracy

depends on the instructed detection of genetic variations. Genetic mobile

elements such as IS6110 are established sources of genetic variation in

Mycobacterium tuberculosis, but their implication in clinical antibiotic

resistance has thus far been unclear. Here, we describe the discovery of an

intragenic IS6110 insertion into Rv0678 that caused antibiotic resistance in an

in vitro-selected M. tuberculosis isolate. The subsequent development of

bioinformatics scripts allowed genome-wide analysis of intragenic IS6110

insertions causing gene disruptions in 6,426 clinical M. tuberculosis strains.

This analysis identified 10,070 intragenic IS6110 insertions distributed among

333 different genes. Focusing on genes whose disruption leads to antibiotic

resistance, 12 clinical isolates were identified with high confidence to be

resistant to bedaquiline, clofazimine, pyrazinamide, ethionamide, and

para-aminosalicylic acid because of an IS6110-mediated gene disruption event. A

number of these IS6110-mediated resistant strains had identical genomic

distributions of IS6110 elements and likely represent transmission events of a

single resistant isolate. These data provide strong evidence that IS6110-mediated

gene disruption is a clinically relevant mechanism of antibiotic resistance in M.

tuberculosis that should be considered for molecular diagnostics. Concomitantly,

this analysis provides a list of 333 IS6110-disrupted genes in clinical

tuberculosis isolates that can be deemed nonessential for human infection.

IMPORTANCE To help control the spread of drug-resistant tuberculosis and to guide

treatment choices, it is important that rapid and accurate molecular diagnostic

tools are used. Current molecular diagnostic tools detect the most common

antibiotic-resistance-conferring mutations in the form of single nucleotide

changes, small deletions, or insertions. Mobile genetic elements, named IS6110,

are also known to move within the M. tuberculosis genome and cause significant

genetic variations, although the role of this variation in clinical drug

resistance remains unclear. In this work, we show that both in vitro and in data

analyzed from 6,426 clinical M. tuberculosis strains, IS6110 elements are found

that disrupt specific genes essential for the function of a number of pivotal

antituberculosis drugs. By providing ample evidence of clinically relevant

IS6110-mediated drug resistance, we believe that this shows that this form of

genetic variation must not be overlooked in molecular diagnostics of drug

resistance.

FAU - Antoine, Rudy

AU - Antoine R

AD - Univ. Lille, CNRS, Inserm, CHU Lille, Institut Pasteur de Lille, U1019-UMR 9017 -

CIIL- Center for Infection and Immunity of Lille, Lille, France.

FAU - Gaudin, Cyril

AU - Gaudin C

AD - Univ. Lille, CNRS, Inserm, CHU Lille, Institut Pasteur de Lille, U1019-UMR 9017 -

CIIL- Center for Infection and Immunity of Lille, Lille, France.

FAU - Hartkoorn, Ruben C

AU - Hartkoorn RC

AUID- ORCID: 0000-0001-7315-1553

AD - Univ. Lille, CNRS, Inserm, CHU Lille, Institut Pasteur de Lille, U1019-UMR 9017 -

CIIL- Center for Infection and Immunity of Lille, Lille, France.

LA - eng

PT - Journal Article

PT - Research Support, Non-U.S. Gov't

DEP - 20210721

PL - United States

TA - Microbiol Spectr

JT - Microbiology spectrum

JID - 101634614

RN - 0 (Antitubercular Agents)

RN - 0 (DNA Transposable Elements)

RN - D959AE5USF (Clofazimine)

RN - OAY8ORS3CQ (Ethionamide)

SB - IM

MH - Antitubercular Agents/*pharmacology

MH - Clofazimine/pharmacology

MH - Computational Biology

MH - *DNA Transposable Elements

MH - *Drug Resistance, Bacterial

MH - Ethionamide/pharmacology

MH - Humans

MH - Mutation

MH - Mycobacterium tuberculosis/*drug effects/*genetics/isolation & purification

MH - Tuberculosis/*microbiology

PMC - PMC8552512

OTO - NOTNLM

OT - IS6110

OT - Mycobacterium tuberculosis

OT - antibiotic resistance

OT - diagnostics

OT - genetic polymorphisms

EDAT- 2021/07/22 06:00

MHDA- 2022/01/07 06:00

PMCR- 2021/07/21

CRDT- 2021/07/21 12:35

PHST- 2021/07/22 06:00 [pubmed]

PHST- 2022/01/07 06:00 [medline]

PHST- 2021/07/21 12:35 [entrez]

PHST- 2021/07/21 00:00 [pmc-release]

AID - 00019-21 [pii]

AID - 10.1128/Spectrum.00019-21 [doi]

PST - ppublish

SO - Microbiol Spectr. 2021 Sep 3;9(1):e0001921. doi: 10.1128/Spectrum.00019-21. Epub

2021 Jul 21.

PMID- 34466004

OWN - NLM

STAT- PubMed-not-MEDLINE

LR - 20220426

IS - 1178-6973 (Print)

IS - 1178-6973 (Electronic)

IS - 1178-6973 (Linking)

VI - 14

DP - 2021

TI - Prevalence and Molecular Characteristics Based on Whole Genome Sequencing of

Mycobacterium tuberculosis Resistant to Four Anti-Tuberculosis Drugs from

Southern Xinjiang, China.

PG - 3379-3391

LID - 10.2147/IDR.S320024 [doi]

AB - OBJECTIVE: Drug-resistant tuberculosis is a major public health problem,

especially in the southern region of Xinjiang, China; however, there is little

information regarding drug resistance profiles and mechanism of Mycobacterium

tuberculosis in this area. The aim of this study was to determine the prevalence

and molecular characteristics of M. tuberculosis resistant to four

anti-tuberculosis drugs from this area. METHODS: Three hundred and forty-six

isolates from the southern region of Xinjiang, China were included and used to

perform phenotypic drug susceptibility testing and whole genome sequencing (WGS).

Mutations in seven loci associated with drug resistance, including rpoB for

rifampicin (RMP), katG, inhA promoter and oxyR-ahpC for isoniazid (INH), rrs 530

and 912 loops and rpsL for streptomycin (STR), and embB for ethambutol (EMB),

were characterized. RESULTS: Among 346 isolates, 106, 60, 70 and 29 were

resistant to INH, RMP, STR and EMB, respectively; 132 were resistant to at least

one of the four anti-tuberculosis drugs and 51 were multi-drug resistant (MDR).

Beijing genotype and retreated patients showed a significantly increased risk for

developing MDR tuberculosis. Compared with the phenotypic data, the sensitivity

and specificity for WGS to predict resistance were 96.7% and 98.6% for RMP, 75.5%

and 97.1% for INH, 68.6% and 99.6% for STR, 93.1% and 93.7% for EMB,

respectively. The most common mutations conferring RMP, INH, STR and EMB

resistance were Ser450Leu (51.7%) in rpoB, Ser315Thr (44.3%) in katG, Lys43Arg

(35.7%) in rpsL and Met306Val (24.1%) in embB. CONCLUSION: This study provides

the first information on the prevalence and molecular characters of drug

resistant M. tuberculosis in the southern region of Xinjiang, China, which will

be helpful for choosing early detection methods for drug resistance (ig,

molecular methods) and subsequently initiation of proper therapy of tuberculosis

in this area.

CI - © 2021 Anwaierjiang et al.

FAU - Anwaierjiang, Aiketaguli

AU - Anwaierjiang A

AD - College of Public Health, Xinjiang Medical University, Wulumuqi, 830011, People's

Republic of China.

FAU - Wang, Quan

AU - Wang Q

AD - The Eighth Affiliated Hospital of Xinjiang Medical University, Wulumuqi, 830001,

People's Republic of China.

FAU - Liu, Haican

AU - Liu H

AUID- ORCID: 0000-0002-9420-1211

AD - State Key Laboratory for Infectious Disease Prevention and Control, Collaborative

Innovation Center for Diagnosis and Treatment of Infectious Diseases, National

Institute for Communicable Disease Control and Prevention, Chinese Center for

Disease Control and Prevention, Beijing, 102206, People's Republic of China.

FAU - Yin, Chunjie

AU - Yin C

AD - College of Public Health, Xinjiang Medical University, Wulumuqi, 830011, People's

Republic of China.

FAU - Xu, Miao

AU - Xu M

AD - The Eighth Affiliated Hospital of Xinjiang Medical University, Wulumuqi, 830001,

People's Republic of China.

FAU - Li, Machao

AU - Li M

AD - State Key Laboratory for Infectious Disease Prevention and Control, Collaborative

Innovation Center for Diagnosis and Treatment of Infectious Diseases, National

Institute for Communicable Disease Control and Prevention, Chinese Center for

Disease Control and Prevention, Beijing, 102206, People's Republic of China.

FAU - Liu, Mengwen

AU - Liu M

AD - College of Public Health, Xinjiang Medical University, Wulumuqi, 830011, People's

Republic of China.

FAU - Liu, Yan

AU - Liu Y

AD - The Eighth Affiliated Hospital of Xinjiang Medical University, Wulumuqi, 830001,

People's Republic of China.

FAU - Zhao, Xiuqin

AU - Zhao X

AD - State Key Laboratory for Infectious Disease Prevention and Control, Collaborative

Innovation Center for Diagnosis and Treatment of Infectious Diseases, National

Institute for Communicable Disease Control and Prevention, Chinese Center for

Disease Control and Prevention, Beijing, 102206, People's Republic of China.

FAU - Liu, Jinbao

AU - Liu J

AD - College of Public Health, Xinjiang Medical University, Wulumuqi, 830011, People's

Republic of China.

FAU - Li, Guilian

AU - Li G

AD - State Key Laboratory for Infectious Disease Prevention and Control, Collaborative

Innovation Center for Diagnosis and Treatment of Infectious Diseases, National

Institute for Communicable Disease Control and Prevention, Chinese Center for

Disease Control and Prevention, Beijing, 102206, People's Republic of China.

FAU - Mijiti, Xiaokaiti

AU - Mijiti X

AD - The Eighth Affiliated Hospital of Xinjiang Medical University, Wulumuqi, 830001,

People's Republic of China.

FAU - Wan, Kanglin

AU - Wan K

AD - State Key Laboratory for Infectious Disease Prevention and Control, Collaborative

Innovation Center for Diagnosis and Treatment of Infectious Diseases, National

Institute for Communicable Disease Control and Prevention, Chinese Center for

Disease Control and Prevention, Beijing, 102206, People's Republic of China.

LA - eng

PT - Journal Article

DEP - 20210824

PL - New Zealand

TA - Infect Drug Resist

JT - Infection and drug resistance

JID - 101550216

PMC - PMC8402983

OTO - NOTNLM

OT - Mycobacterium tuberculosis

OT - ethambutol

OT - isoniazid

OT - mutation

OT - prevalence

OT - resistance

OT - rifampicin

OT - streptomycin

OT - whole-genome sequencing

COIS- The authors declare that they have no competing interests.

EDAT- 2021/09/02 06:00

MHDA- 2021/09/02 06:01

PMCR- 2021/08/24

CRDT- 2021/09/01 07:21

PHST- 2021/05/13 00:00 [received]

PHST- 2021/08/07 00:00 [accepted]

PHST- 2021/09/01 07:21 [entrez]

PHST- 2021/09/02 06:00 [pubmed]

PHST- 2021/09/02 06:01 [medline]

PHST- 2021/08/24 00:00 [pmc-release]

AID - 320024 [pii]

AID - 10.2147/IDR.S320024 [doi]

PST - epublish

SO - Infect Drug Resist. 2021 Aug 24;14:3379-3391. doi: 10.2147/IDR.S320024.

eCollection 2021.

PMID- 34255743

OWN - NLM

STAT- MEDLINE

DCOM- 20211108

LR - 20211117

IS - 1558-8238 (Electronic)

IS - 0021-9738 (Print)

IS - 0021-9738 (Linking)

VI - 131

IP - 16

DP - 2021 Aug 16

TI - WNT6/ACC2-induced storage of triacylglycerols in macrophages is exploited by

Mycobacterium tuberculosis.

LID - 10.1172/JCI141833 [doi]

LID - e141833

AB - In view of emerging drug-resistant tuberculosis (TB), host-directed adjunct

therapies are urgently needed to improve treatment outcomes with currently

available anti-TB therapies. One approach is to interfere with the formation of

lipid-laden "foamy" macrophages in the host, as they provide a nutrient-rich host

cell environment for Mycobacterium tuberculosis (Mtb). Here, we provide evidence

that Wnt family member 6 (WNT6), a ligand of the evolutionarily conserved

Wingless/Integrase 1 (WNT) signaling pathway, promotes foam cell formation by

regulating key lipid metabolic genes including acetyl-CoA carboxylase 2 (ACC2)

during pulmonary TB. Using genetic and pharmacological approaches, we

demonstrated that lack of functional WNT6 or ACC2 significantly reduced

intracellular triacylglycerol (TAG) levels and Mtb survival in macrophages.

Moreover, treatment of Mtb-infected mice with a combination of a pharmacological

ACC2 inhibitor and the anti-TB drug isoniazid (INH) reduced lung TAG and cytokine

levels, as well as lung weights, compared with treatment with INH alone. This

combination also reduced Mtb bacterial numbers and the size of mononuclear cell

infiltrates in livers of infected mice. In summary, our findings demonstrate that

Mtb exploits WNT6/ACC2-induced storage of TAGs in macrophages to facilitate its

intracellular survival, a finding that opens new perspectives for host-directed

adjunctive treatment of pulmonary TB.

FAU - Brandenburg, Julius

AU - Brandenburg J

AD - Microbial Interface Biology, Research Center Borstel, Leibniz Lung Center,

Borstel, Germany.

AD - German Center for Infection Research (DZIF), Site Hamburg-Lübeck-Borstel-Riems,

Hamburg, Germany.

FAU - Marwitz, Sebastian

AU - Marwitz S

AD - Pathology, Research Center Borstel, Borstel, Germany.

AD - Airway Research Center North (ARCN), Member of the German Center for Lung

Research (DZL), Grosshansdorf, Germany.

FAU - Tazoll, Simone C

AU - Tazoll SC

AD - Microbial Interface Biology, Research Center Borstel, Leibniz Lung Center,

Borstel, Germany.

FAU - Waldow, Franziska

AU - Waldow F

AD - German Center for Infection Research (DZIF), Site Hamburg-Lübeck-Borstel-Riems,

Hamburg, Germany.

AD - Bioanalytical Chemistry.

FAU - Kalsdorf, Barbara

AU - Kalsdorf B

AD - German Center for Infection Research (DZIF), Site Hamburg-Lübeck-Borstel-Riems,

Hamburg, Germany.

AD - Clinical Infectious Diseases.

FAU - Vierbuchen, Tim

AU - Vierbuchen T

AD - Innate Immunity.

FAU - Scholzen, Thomas

AU - Scholzen T

AD - Fluorescence Cytometry Core Unit.

FAU - Gross, Annette

AU - Gross A

AD - Microbial Interface Biology, Research Center Borstel, Leibniz Lung Center,

Borstel, Germany.

FAU - Goldenbaum, Svenja

AU - Goldenbaum S

AD - Microbial Interface Biology, Research Center Borstel, Leibniz Lung Center,

Borstel, Germany.

FAU - Hölscher, Alexandra

AU - Hölscher A

AD - Infection Immunology, and.

FAU - Hein, Martina

AU - Hein M

AD - Fluorescence Cytometry Core Unit.

FAU - Linnemann, Lara

AU - Linnemann L

AD - Cellular Microbiology, Research Center Borstel, Borstel, Germany.

FAU - Reimann, Maja

AU - Reimann M

AD - Clinical Infectious Diseases.

FAU - Kispert, Andreas

AU - Kispert A

AD - Institute for Molecular Biology, Hannover Medical School, Hannover, Germany.

FAU - Leitges, Michael

AU - Leitges M

AD - Division of BioMedical Sciences/Faculty of Medicine, Memorial University of

Newfoundland, St. John's, Newfoundland, Canada.

FAU - Rupp, Jan

AU - Rupp J

AD - German Center for Infection Research (DZIF), Site Hamburg-Lübeck-Borstel-Riems,

Hamburg, Germany.

AD - Department of Infectious Diseases and Microbiology and.

FAU - Lange, Christoph

AU - Lange C

AD - German Center for Infection Research (DZIF), Site Hamburg-Lübeck-Borstel-Riems,

Hamburg, Germany.

AD - Clinical Infectious Diseases.

AD - Respiratory Medicine & International Health, University of Lübeck, Lübeck,

Germany.

AD - Baylor College of Medicine and Texas Children's Hospital, Houston, Texas, USA.

FAU - Niemann, Stefan

AU - Niemann S

AD - German Center for Infection Research (DZIF), Site Hamburg-Lübeck-Borstel-Riems,

Hamburg, Germany.

AD - Molecular and Experimental Mycobacteriology, Research Center Borstel, Borstel,

Germany.

FAU - Behrends, Jochen

AU - Behrends J

AD - Fluorescence Cytometry Core Unit.

FAU - Goldmann, Torsten

AU - Goldmann T

AD - Pathology, Research Center Borstel, Borstel, Germany.

AD - Airway Research Center North (ARCN), Member of the German Center for Lung

Research (DZL), Grosshansdorf, Germany.

FAU - Heine, Holger

AU - Heine H

AD - Innate Immunity.

FAU - Schaible, Ulrich E

AU - Schaible UE

AD - German Center for Infection Research (DZIF), Site Hamburg-Lübeck-Borstel-Riems,

Hamburg, Germany.

AD - Cellular Microbiology, Research Center Borstel, Borstel, Germany.

FAU - Hölscher, Christoph

AU - Hölscher C

AD - German Center for Infection Research (DZIF), Site Hamburg-Lübeck-Borstel-Riems,

Hamburg, Germany.

AD - Infection Immunology, and.

FAU - Schwudke, Dominik

AU - Schwudke D

AD - German Center for Infection Research (DZIF), Site Hamburg-Lübeck-Borstel-Riems,

Hamburg, Germany.

AD - Airway Research Center North (ARCN), Member of the German Center for Lung

Research (DZL), Grosshansdorf, Germany.

AD - Bioanalytical Chemistry.

FAU - Reiling, Norbert

AU - Reiling N

AD - Microbial Interface Biology, Research Center Borstel, Leibniz Lung Center,

Borstel, Germany.

AD - German Center for Infection Research (DZIF), Site Hamburg-Lübeck-Borstel-Riems,

Hamburg, Germany.

LA - eng

PT - Journal Article

PT - Research Support, Non-U.S. Gov't

PL - United States

TA - J Clin Invest

JT - The Journal of clinical investigation

JID - 7802877

RN - 0 (Antitubercular Agents)

RN - 0 (Enzyme Inhibitors)

RN - 0 (Proto-Oncogene Proteins)

RN - 0 (Triglycerides)

RN - 0 (WNT6 protein, human)

RN - 0 (Wnt Proteins)

RN - 0 (Wnt6 protein, mouse)

RN - EC 6.4.1.2 (ACACB protein, human)

RN - EC 6.4.1.2 (Acacb protein, mouse)

RN - EC 6.4.1.2 (Acetyl-CoA Carboxylase)

RN - V83O1VOZ8L (Isoniazid)

SB - IM

MH - Acetyl-CoA Carboxylase/antagonists & inhibitors/*metabolism

MH - Animals

MH - Antitubercular Agents/administration & dosage

MH - Enzyme Inhibitors/administration & dosage

MH - Foam Cells/metabolism

MH - Host Microbial Interactions/drug effects/physiology

MH - Humans

MH - Isoniazid/administration & dosage

MH - Lung/drug effects/metabolism/microbiology

MH - Macrophages/*metabolism/*microbiology

MH - Mice

MH - Mice, Inbred C57BL

MH - Mice, Knockout

MH - Mycobacterium tuberculosis/drug effects/*metabolism/*pathogenicity

MH - Proto-Oncogene Proteins/deficiency/genetics/*metabolism

MH - Signal Transduction/drug effects

MH - Triglycerides/*metabolism

MH - Tuberculosis, Pulmonary/drug therapy/metabolism/microbiology

MH - Wnt Proteins/deficiency/genetics/*metabolism

PMC - PMC8363280

OTO - NOTNLM

OT - Infectious disease

OT - Macrophages

OT - Metabolism

OT - Tuberculosis

COIS- Conflict of interest: N. Reiling and J. Brandenburg have filed a patent

application titled “ACC inhibitors as means and methods for treating

mycobacterial diseases” (WO2018007430A1).

EDAT- 2021/07/14 06:00

MHDA- 2021/11/09 06:00

PMCR- 2021/11/16

CRDT- 2021/07/13 17:23

PHST- 2020/07/06 00:00 [received]

PHST- 2021/07/06 00:00 [accepted]

PHST- 2021/07/14 06:00 [pubmed]

PHST- 2021/11/09 06:00 [medline]

PHST- 2021/07/13 17:23 [entrez]

PHST- 2021/11/16 00:00 [pmc-release]

AID - e141833 [pii]

AID - 141833 [pii]

AID - 10.1172/JCI141833 [doi]

PST - ppublish

SO - J Clin Invest. 2021 Aug 16;131(16):e141833. doi: 10.1172/JCI141833.

PMID- 34401121

OWN - NLM

STAT- PubMed-not-MEDLINE

LR - 20220425

IS - 2049-0801 (Print)

IS - 2049-0801 (Electronic)

IS - 2049-0801 (Linking)

VI - 68

DP - 2021 Aug

TI - Analysis of CD4 and CD8 expression in multidrug-resistant tuberculosis infection

with diabetes mellitus: An experimental study in mice.

PG - 102596

LID - 10.1016/j.amsu.2021.102596 [doi]

LID - 102596

AB - BACKGROUND: Tuberculosis (TB) remains a major global health problem, in the top

10 causes of death. As a regulator of the immune response, T-helper (Th) cells

activate other lymphocytes from the immune system, such as B cells, to destroy

the TB pathogen by releasing CD4 and CD8 Th cells. Diabetes mellitus (DM) is a

known cause of developing active pulmonary TB. Few studies have examined the

biomolecular expression affecting Mycobacterium tuberculosis (MTB) and

multidrug-resistant (MDR) MTB, which are associated with low immunity represented

by TB in diabetes and CD4 and CD8 levels. MATERIALS AND METHODS: This animal

study used a post-test control group design. We performed an experimental study

using 30 BALB/c mice, each weighing 25 g. It included six experimental animal

groups, of which three had a diabetes condition induced using intraperitoneal

streptozotocin, and all were infected with MTB or MDR TB. We evaluated the CD4

and CD8 levels in each group and analyzed the differences. RESULTS: We found a

significant difference in CD4 and CD8 levels in MTB and MDR TB conditions.

CONCLUSION: This study shows that acute infection in experimental mice with MTB

and MDR TB with or without diabetes had the highest levels of both CD4 and CD8

cells, which can be a sign of increased cellular immunity in a mice model.

CI - © 2021 The Authors.

FAU - Agustin, Heidy

AU - Agustin H

AD - Doctoral Program of Medical Sciences, Faculty of Medicine, Hasanuddin University,

Makassar, Indonesia.

AD - Departement of Pulmonology and Respiratory Medicine, Faculty of Medicine,

Indonesia University, Jakarta, Indonesia.

AD - Departement of Pulmonology and Respiratory Medicine, Persahabatan Hospital,

Jakarta, Indonesia.

FAU - Massi, Muhammad Nasrum

AU - Massi MN

AD - Departement of Microbiology, Faculty of Medicine, Hasanuddin University,

Makassar, Indonesia.

FAU - Djaharuddin, Irawati

AU - Djaharuddin I

AD - Departement of Pulmonology and Respiratory Medicine, Faculty of Medicine,

Hasanuddin University, Makassar, Indonesia.

FAU - Susanto, Agus Dwi

AU - Susanto AD

AD - Departement of Pulmonology and Respiratory Medicine, Faculty of Medicine,

Indonesia University, Jakarta, Indonesia.

AD - Departement of Pulmonology and Respiratory Medicine, Persahabatan Hospital,

Jakarta, Indonesia.

FAU - Islam, Andi Asadul

AU - Islam AA

AD - Department of Neurosurgery, Faculty of Medicine, Hasanuddin University, Makassar,

Indonesia.

FAU - Hatta, Mochammad

AU - Hatta M

AD - Clinical Microbiologist Program, Faculty of Medicine, Hasanuddin University,

Makassar, Indonesia.

FAU - Bukhari, Agussalim

AU - Bukhari A

AD - Department of Clinical Nutrition, Faculty of Medicine, Hasanuddin University,

Makassar, Indonesia.

FAU - Tabri, Nur Ahmad

AU - Tabri NA

AD - Departement of Pulmonology and Respiratory Medicine, Faculty of Medicine,

Hasanuddin University, Makassar, Indonesia.

FAU - Santoso, Arif

AU - Santoso A

AD - Departement of Pulmonology and Respiratory Medicine, Faculty of Medicine,

Hasanuddin University, Makassar, Indonesia.

FAU - Patellongi, Ilhamjaya

AU - Patellongi I

AD - Department of Biostatistics, Faculty of Public Health, Hasanuddin University,

Sulawesi Selatan, Indonesia.

LA - eng

PT - Journal Article

DEP - 20210727

PL - England

TA - Ann Med Surg (Lond)

JT - Annals of medicine and surgery (2012)

JID - 101616869

PMC - PMC8350178

OTO - NOTNLM

OT - CD4

OT - CD8

OT - Diabetes mellitus

OT - Multidrug resistance

OT - Mycobacterium tuberculosis

EDAT- 2021/08/18 06:00

MHDA- 2021/08/18 06:01

PMCR- 2021/07/27

CRDT- 2021/08/17 07:29

PHST- 2021/06/23 00:00 [received]

PHST- 2021/07/19 00:00 [revised]

PHST- 2021/07/25 00:00 [accepted]

PHST- 2021/08/17 07:29 [entrez]

PHST- 2021/08/18 06:00 [pubmed]

PHST- 2021/08/18 06:01 [medline]

PHST- 2021/07/27 00:00 [pmc-release]

AID - S2049-0801(21)00546-X [pii]

AID - 102596 [pii]

AID - 10.1016/j.amsu.2021.102596 [doi]

PST - epublish

SO - Ann Med Surg (Lond). 2021 Jul 27;68:102596. doi: 10.1016/j.amsu.2021.102596.

eCollection 2021 Aug.

PMID- 33709862

OWN - NLM

STAT- MEDLINE

DCOM- 20210805

LR - 20220425

IS - 1744-7674 (Electronic)

IS - 1354-3776 (Linking)

VI - 31

IP - 8

DP - 2021 Aug

TI - Synthetic molecules as DprE1 inhibitors: A patent review.

PG - 759-772

LID - 10.1080/13543776.2021.1902990 [doi]

AB - INTRODUCTION: In recent years, the advent of multidrug-resistant tuberculosis

(MDR-TB), the extensively-resistant TB (XDR-TB), and the total drug-resistant-TB

(TDR-TB) have led the community to develop new antitubercular molecules. The

decaprenylphosphoryl-β-D-ribose-2'-epimerase-1 (DprE1) is an established target

to developed new anti-TB drugs. This enzyme is required to synthesize the cell

wall of Mycobacterium tuberculosis (Mtb). AREA COVERED: This patent review

focuses on the granted patents and patent applications related to the chemical

entities developed as DprE1 inhibitors for TB treatment from the publication year

of the BTZ-043 compound patent application (2007) till 30 September 2020. EXPERT

OPINION: The DprE1 has many advantages in the development of new antitubercular

molecules, for example, its location in the periplasm of the Mtb cell wall and

its absence in the human body. This indicates that the DprE1 inhibitors are

selective for Mtb, and their toxic and side effects on the human body may be

negligible or small. Accordingly, the use of DprE1 inhibitors may be benefic for

patients with drug-resistant bacteria that require long-term medication. Four

molecules are in clinical trials, which could become the drugs of the future for

TB-therapy.

FAU - Imran, Mohd

AU - Imran M

AUID- ORCID: 0000-0002-6064-1040

AD - Department of Pharmaceutical Chemistry, Faculty of Pharmacy, Northern Border

University, Rafha, Saudi Arabia.

FAU - A S, Alshrari

AU - A S A

AUID- ORCID: 0000-0002-3666-3848

AD - Department of Biological Sciences, Faculty of Science, Northern Border

University, Arar, Saudi Arabia.

FAU - Thabet, Hamdy Kh

AU - Thabet HK

AD - cDepartment of Chemistry, Faculty of Arts and Science, Northern Border

University, Rafha, Saudi Arabia.

FAU - Abida

AU - Abida

AUID- ORCID: 0000-0001-6186-461X

AD - Department of Pharmaceutical Chemistry, Faculty of Pharmacy, Northern Border

University, Rafha, Saudi Arabia.

FAU - Afroz Bakht, Md

AU - Afroz Bakht M

AUID- ORCID: 0000-0002-3382-0457

AD - dDepartment of Chemistry, College of Science and Humanities, Prince Sattam Bin

Abdulaziz University, Al-Kharj, Saudi Arabia.

LA - eng

PT - Journal Article

PT - Review

DEP - 20210413

PL - England

TA - Expert Opin Ther Pat

JT - Expert opinion on therapeutic patents

JID - 9516419

RN - 0 (Antitubercular Agents)

RN - 0 (Bacterial Proteins)

RN - EC 1.1.- (Alcohol Oxidoreductases)

RN - EC 1.1.- (DprE1 protein, Mycobacterium tuberculosis)

SB - IM

MH - Alcohol Oxidoreductases/*antagonists & inhibitors

MH - Animals

MH - Antitubercular Agents/*pharmacology

MH - Bacterial Proteins/*antagonists & inhibitors

MH - Drug Design

MH - Drug Development

MH - Drug Resistance, Multiple, Bacterial

MH - Humans

MH - Mycobacterium tuberculosis/drug effects

MH - Patents as Topic

MH - Tuberculosis/*drug therapy/microbiology

OTO - NOTNLM

OT - Azaindole

OT - benzothiazinone

OT - dpre1 inhibitors

OT - patent review

OT - synthetic molecules

OT - tuberculosis

EDAT- 2021/03/13 06:00

MHDA- 2021/08/06 06:00

CRDT- 2021/03/12 12:25

PHST- 2021/03/13 06:00 [pubmed]

PHST- 2021/08/06 06:00 [medline]

PHST- 2021/03/12 12:25 [entrez]

AID - 10.1080/13543776.2021.1902990 [doi]

PST - ppublish

SO - Expert Opin Ther Pat. 2021 Aug;31(8):759-772. doi: 10.1080/13543776.2021.1902990.

Epub 2021 Apr 13.

PMID- 34429848

OWN - NLM

STAT- PubMed-not-MEDLINE

LR - 20240506

IS - 2001-0370 (Print)

IS - 2001-0370 (Electronic)

IS - 2001-0370 (Linking)

VI - 19

DP - 2021

TI - Deciphering the mechanism of action of antitubercular compounds with

metabolomics.

PG - 4284-4299

LID - 10.1016/j.csbj.2021.07.034 [doi]

AB - Tuberculosis (TB), one of the oldest and deadliest bacterial diseases, continues

to cause serious global economic, health, and social problems. Current TB

treatments are lengthy, expensive, and routinely ineffective against emerging

drug resistant strains. Thus, there is an urgent need for the identification and

development of novel TB drugs possessing comprehensive and specific mechanisms of

action (MoAs). Metabolomics is a valuable approach to elucidating the MoA,

toxicity, and potency of promising chemical leads, which is a critical step of

the drug discovery process. Recent advances in metabolomics methodologies for

deciphering MoAs include high-throughput screening techniques, the integration of

multiple omics methods, mass spectrometry imaging, and software for automated

analysis. This review describes recently introduced metabolomics methodologies

and techniques for drug discovery, highlighting specific applications to the

discovery of new antitubercular drugs and the elucidation of their MoAs.

CI - © 2021 The Author(s).

FAU - Sakallioglu, Isin T

AU - Sakallioglu IT

AD - Department of Chemistry, University of Nebraska-Lincoln, Lincoln, NE 68588-0304,

USA.

FAU - Barletta, Raúl G

AU - Barletta RG

AD - School of Veterinary Medicine and Biomedical Sciences, University of Nebraska

Lincoln, Lincoln, NE 68583-0905, USA.

FAU - Dussault, Patrick H

AU - Dussault PH

AD - Department of Chemistry, University of Nebraska-Lincoln, Lincoln, NE 68588-0304,

USA.

FAU - Powers, Robert

AU - Powers R

AD - Department of Chemistry, University of Nebraska-Lincoln, Lincoln, NE 68588-0304,

USA.

AD - Nebraska Center for Integrated Biomolecular Communication, University of

Nebraska-Lincoln, Lincoln, NE 68588-0304, USA.

LA - eng

GR - P20 GM113126/GM/NIGMS NIH HHS/United States

GR - P30 GM103335/GM/NIGMS NIH HHS/United States

PT - Journal Article

PT - Review

DEP - 20210730

PL - United States

TA - Comput Struct Biotechnol J

JT - Computational and structural biotechnology journal

JID - 101585369

PMC - PMC8358470

OTO - NOTNLM

OT - Antitubercular drugs

OT - Drug discovery

OT - Mechanisms of action

OT - Metabolomics

OT - Multi-drug resistant tuberculosis

OT - Pharmacometabolomics

OT - Tuberculosis

COIS- The authors declare that they have no known competing financial interests or

personal relationships that could have appeared to influence the work reported in

this paper.

EDAT- 2021/08/26 06:00

MHDA- 2021/08/26 06:01

PMCR- 2021/07/30

CRDT- 2021/08/25 06:23

PHST- 2021/03/23 00:00 [received]

PHST- 2021/07/26 00:00 [revised]

PHST- 2021/07/28 00:00 [accepted]

PHST- 2021/08/25 06:23 [entrez]

PHST- 2021/08/26 06:00 [pubmed]

PHST- 2021/08/26 06:01 [medline]

PHST- 2021/07/30 00:00 [pmc-release]

AID - S2001-0370(21)00324-X [pii]

AID - 10.1016/j.csbj.2021.07.034 [doi]

PST - epublish

SO - Comput Struct Biotechnol J. 2021 Jul 30;19:4284-4299. doi:

10.1016/j.csbj.2021.07.034. eCollection 2021.

PMID- 34261449

OWN - NLM

STAT- MEDLINE

DCOM- 20220113

LR - 20220113

IS - 1528-3658 (Electronic)

IS - 1076-1551 (Print)

IS - 1076-1551 (Linking)

VI - 27

IP - 1

DP - 2021 Jul 14

TI - Anti-tuberculosis chemotherapy alters TNFR2 expression on CD4+ lymphocytes in

both drug-sensitive and -resistant tuberculosis: however, only drug-resistant

tuberculosis maintains a pro-inflammatory profile after a long time.

PG - 76

LID - 10.1186/s10020-021-00320-4 [doi]

LID - 76

AB - BACKGROUND: Tuberculosis (TB) is an infectious disease. During TB, regulatory T

cells (Treg) are related to poor prognosis. However, information about

conventional and unconventional Treg (cTreg and uTreg, respectively) is limited.

The tumour necrosis factor (TNF) and its receptors (TNFR1 and TNFR2) are

necessary for mycobacterial infection, and TNFR2 signalling is required to

maintain Treg. METHODS: A blood sample of drug-susceptible (DS-TB) and

drug-resistant tuberculosis (DR-TB) patients was obtained before (basal) and

after 2 and 6 months of anti-TB therapy. Expression of TNF, TNFR1, and TNFR2

(transmembrane form, tm) on cTreg, uTreg, activated CD4+ (actCD4+), and CD4+

CD25- (CD4+) T cell subpopulations were evaluated. The main objective was to

identify immunological changes associated with sensitive/resistant Mtb strains

and with the use of anti-TB therapy. RESULTS: We found that after 6 months of

anti-TB therapy, both DS- and DR-TB patients have decreased the frequency of

cTreg tmTNF+, CD4+ tmTNFR1+ and CD4+ tmTNFR2+. Nevertheless, after 6 months of

therapy, only DR-TB patients decreased the frequency of actCD4+ tmTNF+ and

actCD4+ tmTNFR2+, exhibited a systemic inflammatory status (high levels of TNF,

IFN-γ and IL-12), and their purified CD4+ T cells showed that TNF and TNFR2 are

up-regulated at the transcriptional level. Moreover, DS- and DR-TB down-regulated

TNFR1 and other proteins associated with Treg (FOXP3 and TGFβ1) in response to

the anti-TB therapy. CONCLUSION: These results partially explain the differences

in the immune response of DS-TB vs DR-TB. The frequency of actCD4+ tmTNFR2+ cells

and inflammatory status should be considered in the follow-up of therapy in DR-TB

patients.

CI - © 2021. The Author(s).

FAU - Téllez-Navarrete, Norma A

AU - Téllez-Navarrete NA

AD - Laboratory of Integrative Immunology, Instituto Nacional de Enfermedades

Respiratorias "Ismael Cosío Villegas", Calzada de Tlalpan No. 4510, CP. 14080,

Mexico City, Mexico.

FAU - Ramon-Luing, Lucero A

AU - Ramon-Luing LA

AD - Laboratory of Integrative Immunology, Instituto Nacional de Enfermedades

Respiratorias "Ismael Cosío Villegas", Calzada de Tlalpan No. 4510, CP. 14080,

Mexico City, Mexico.

FAU - Muñoz-Torrico, Marcela

AU - Muñoz-Torrico M

AD - Clinic of Tuberculosis, Instituto Nacional de Enfermedades Respiratorias "Ismael

Cosío Villegas", Mexico City, Mexico.

FAU - Preciado-García, Mario

AU - Preciado-García M

AD - Laboratory of Integrative Immunology, Instituto Nacional de Enfermedades

Respiratorias "Ismael Cosío Villegas", Calzada de Tlalpan No. 4510, CP. 14080,

Mexico City, Mexico.

FAU - Medina-Quero, Karen

AU - Medina-Quero K

AD - Laboratory of Immunology, Escuela Militar de Graduados en Sanidad, Mexico City,

Mexico.

FAU - Hernandez-Pando, Rogelio

AU - Hernandez-Pando R

AD - Experimental Pathology Section, Department of Pathology, Instituto Nacional de

Ciencias Médicas y Nutrición Salvador Zubirán, Mexico City, Mexico.

FAU - Chavez-Galan, Leslie

AU - Chavez-Galan L

AUID- ORCID: 0000-0002-2334-0361

AD - Laboratory of Integrative Immunology, Instituto Nacional de Enfermedades

Respiratorias "Ismael Cosío Villegas", Calzada de Tlalpan No. 4510, CP. 14080,

Mexico City, Mexico. lchavez_galan@iner.gob.mx.

LA - eng

PT - Journal Article

DEP - 20210714

PL - England

TA - Mol Med

JT - Molecular medicine (Cambridge, Mass.)

JID - 9501023

RN - 0 (Antitubercular Agents)

RN - 0 (Biomarkers)

RN - 0 (Cytokines)

RN - 0 (Inflammation Mediators)

RN - 0 (Receptors, Tumor Necrosis Factor, Type II)

SB - IM

MH - Adult

MH - Aged

MH - Antitubercular Agents/pharmacology/therapeutic use

MH - Biomarkers

MH - CD4 Lymphocyte Count

MH - CD4-Positive T-Lymphocytes/*immunology/*metabolism

MH - Cytokines/metabolism

MH - Disease Susceptibility/immunology

MH - Female

MH - Gene Expression Regulation/*drug effects

MH - Host-Pathogen Interactions/genetics/immunology

MH - Humans

MH - Immunophenotyping

MH - Inflammation Mediators/metabolism

MH - Male

MH - Middle Aged

MH - Mycobacterium tuberculosis/*immunology

MH - Receptors, Tumor Necrosis Factor, Type II/*genetics/metabolism

MH - Time Factors

MH - Treatment Outcome

MH - Tuberculosis/diagnosis/drug therapy/*etiology/*metabolism

MH - Tuberculosis, Multidrug-Resistant/diagnosis/drug therapy/etiology/metabolism

PMC - PMC8278684

OTO - NOTNLM

OT - Drug-resistance

OT - Receptors

OT - TNF

OT - Tregs

OT - Tuberculosis

COIS- The authors declare no potential conflicts of interest.

EDAT- 2021/07/16 06:00

MHDA- 2022/01/14 06:00

PMCR- 2021/07/14

CRDT- 2021/07/15 05:29

PHST- 2020/12/23 00:00 [received]

PHST- 2021/05/27 00:00 [accepted]

PHST- 2021/07/15 05:29 [entrez]

PHST- 2021/07/16 06:00 [pubmed]

PHST- 2022/01/14 06:00 [medline]

PHST- 2021/07/14 00:00 [pmc-release]

AID - 10.1186/s10020-021-00320-4 [pii]

AID - 320 [pii]

AID - 10.1186/s10020-021-00320-4 [doi]

PST - epublish

SO - Mol Med. 2021 Jul 14;27(1):76. doi: 10.1186/s10020-021-00320-4.

PMID- 34337183

OWN - NLM

STAT- PubMed-not-MEDLINE

LR - 20220425

IS - 2405-8440 (Print)

IS - 2405-8440 (Electronic)

IS - 2405-8440 (Linking)

VI - 7

IP - 7

DP - 2021 Jul

TI - Investigation of the Rv3065, Rv2942, Rv1258c, Rv1410c, and Rv2459 efflux pump

genes expression among multidrug-resistant Mycobacterium tuberculosis clinical

isolates.

PG - e07566

LID - 10.1016/j.heliyon.2021.e07566 [doi]

LID - e07566

AB - BACKGROUND: Different resistance mechanisms for multidrug-resistant tuberculosis

(MDR-TB) and extensively drug-resistant tuberculosis (XDR-TB) have been reported.

Although mutations in target genes are the main cause of drug resistance, efflux

pumps (Eps) also play an important role in this process. Here, we investigated

the overexpression of five putative EP genes plus gene mutations in MDR-TB

clinical isolates. METHODS: A total of 27 M. tuberculosis (Mtb) clinical isolates

including, 22 MDR and 5 sensitive isolates were analyzed. Minimum inhibitory

concentrations (MIC) were determined in the absence and presence of efflux

inhibitor. The expression level of 5 EP genes (Rv3065, Rv2942, Rv1258c, Rv1410c,

Rv2459) was investigated by quantitative real time PCR (RT-qPCR). DNA sequencing

of rpoB, katG, and inhA promoter was done. RESULTS: Among the 22 MDR-TB isolates,

13 (59.1%) showed significant overexpression (>4-fold) for at least one EP gene.

The expression levels of 5 genes were significantly higher (P < 0.05) in MDR-TB

isolates than sensitive isolates. The Rv3065 (22.7%), and Rv1410c (18.2%) were

found to be the most commonly overexpressed EPs. The observed MICs were as

follows: RIF (2 to >128 μg/ml) and INH (2-32 μg/ml). After efflux pump inhibitor

exposure, 10/22 (45.45%) isolates showed a decrease in MIC of INH, and 17/22

(77.27%) isolates showed a decrease in MIC of RIF. Of the isolates that

overexpressed, 4 isolates lacked mutation in inhA, rpoB, and katG genes and 10

ones lacked mutation in inhA and katG. CONCLUSION: The results showed that

overexpression of EP genes in Mtb isolates, besides target gene mutations can

contribute to the development of MDR phenotype.

CI - © 2021 The Authors.

FAU - Shahi, Fatemeh

AU - Shahi F

AD - Infectious and Tropical Diseases Research Center, Health Research Institute,

Ahvaz Jundishapur University of Medical Sciences, Ahvaz, Iran.

AD - Department of Microbiology, School of Medicine, Ahvaz Jundishapur University of

Medical Sciences, Ahvaz, Iran.

FAU - Khosravi, Azar Dokht

AU - Khosravi AD

AD - Infectious and Tropical Diseases Research Center, Health Research Institute,

Ahvaz Jundishapur University of Medical Sciences, Ahvaz, Iran.

AD - Department of Microbiology, School of Medicine, Ahvaz Jundishapur University of

Medical Sciences, Ahvaz, Iran.

AD - Iranian Study Group on Microbial Drug Resistance, Iran.

FAU - Tabandeh, Mohammad Reza

AU - Tabandeh MR

AD - Department of Basic Sciences, Division of Biochemistry and Molecular Biology,

Faculty of Veterinary Medicine, Shahid Chamran University of Ahvaz, Ahvaz, Iran.

FAU - Salmanzadeh, Shokrollah

AU - Salmanzadeh S

AD - Infectious and Tropical Diseases Research Center, Health Research Institute,

Ahvaz Jundishapur University of Medical Sciences, Ahvaz, Iran.

AD - Tropical Medicine Ward, Razi Teaching Hospital, Ahvaz Jundishapur University of

Medical Sciences, Ahvaz, Iran.

LA - eng

PT - Journal Article

DEP - 20210712

PL - England

TA - Heliyon

JT - Heliyon

JID - 101672560

PMC - PMC8318855

OTO - NOTNLM

OT - Efflux pumps

OT - MDR-TB

OT - Mutation

OT - Tuberculosis

EDAT- 2021/08/03 06:00

MHDA- 2021/08/03 06:01

PMCR- 2021/07/12

CRDT- 2021/08/02 06:11

PHST- 2021/01/10 00:00 [received]

PHST- 2021/02/23 00:00 [revised]

PHST- 2021/07/09 00:00 [accepted]

PHST- 2021/08/02 06:11 [entrez]

PHST- 2021/08/03 06:00 [pubmed]

PHST- 2021/08/03 06:01 [medline]

PHST- 2021/07/12 00:00 [pmc-release]

AID - S2405-8440(21)01669-8 [pii]

AID - e07566 [pii]

AID - 10.1016/j.heliyon.2021.e07566 [doi]

PST - epublish

SO - Heliyon. 2021 Jul 12;7(7):e07566. doi: 10.1016/j.heliyon.2021.e07566. eCollection

2021 Jul.

PMID- 34361913

OWN - NLM

STAT- PubMed-not-MEDLINE

LR - 20210809

IS - 2076-2607 (Print)

IS - 2076-2607 (Electronic)

IS - 2076-2607 (Linking)

VI - 9

IP - 7

DP - 2021 Jul 9

TI - Common and Rare Hematological Manifestations and Adverse Drug Events during

Treatment of Active TB: A State of Art.

LID - 10.3390/microorganisms9071477 [doi]

LID - 1477

AB - BACKGROUND: Tuberculosis (TB) can seriously affect the hematopoietic system, with

involvement of both myeloid and lymphoid cell lines as well as plasma components.

These hematological changes act as a marker for the diagnosis, prognosis and

response to therapy. METHODS: We searched PubMed, Scopus, Google Scholar, EMBASE,

Cochrane Library and WHO websites from 1950 to May 2021 for papers on the

interaction between TB and common and rare hematological manifestation. RESULTS:

Hematological reactions in patients with TB are possible in both young and old

women and men but seem more frequent in the elderly, and they can be predictors

of both diagnosis and worse outcome for TB, regardless of whether it is

pulmonary, extra pulmonary or miliary. Even anti-TB therapies can cause

hematological adverse events, among which some are serious and rare and can

compromise the patient's recovery pathway to completing treatment. CONCLUSION:

Hematological screening and follow-up, including complete blood count and

coagulation, are always necessary both at the diagnosis of TB and during

antitubercular treatment in order to monitor hematological parameters. Short

therapy regimens for multidrug-resistant TB (MDR-TB) may also be useful for

reducing hematological toxicity, especially in contexts where this cannot be

monitored. Close monitoring of drug interactions and hematological adverse events

is always recommended.

FAU - Minardi, Maria Letizia

AU - Minardi ML

AD - National Institute for Infectious Diseases "L. Spallanzani" IRCCS, 00149 Rome,

Italy.

AD - Clinic of Infectious Diseases, Policlinico Tor Vergata, V. Montpelier 1, 00133

Rome, Italy.

FAU - Fato, Ilenia

AU - Fato I

AD - National Institute for Infectious Diseases "L. Spallanzani" IRCCS, 00149 Rome,

Italy.

AD - Clinic of Infectious Diseases, Policlinico Tor Vergata, V. Montpelier 1, 00133

Rome, Italy.

FAU - Di Gennaro, Francesco

AU - Di Gennaro F

AUID- ORCID: 0000-0003-3453-5647

AD - National Institute for Infectious Diseases "L. Spallanzani" IRCCS, 00149 Rome,

Italy.

AD - Department of Biomedical Sciences and Human Oncology, Clinic of Infectious

Diseases, University of Bari "Aldo Moro", 70124 Bari, Italy.

FAU - Mosti, Silvia

AU - Mosti S

AD - National Institute for Infectious Diseases "L. Spallanzani" IRCCS, 00149 Rome,

Italy.

FAU - Mastrobattista, Annelisa

AU - Mastrobattista A

AD - National Institute for Infectious Diseases "L. Spallanzani" IRCCS, 00149 Rome,

Italy.

FAU - Cerva, Carlotta

AU - Cerva C

AD - National Institute for Infectious Diseases "L. Spallanzani" IRCCS, 00149 Rome,

Italy.

FAU - Libertone, Raffaella

AU - Libertone R

AD - National Institute for Infectious Diseases "L. Spallanzani" IRCCS, 00149 Rome,

Italy.

FAU - Saracino, Annalisa

AU - Saracino A

AD - Department of Biomedical Sciences and Human Oncology, Clinic of Infectious

Diseases, University of Bari "Aldo Moro", 70124 Bari, Italy.

FAU - Goletti, Delia

AU - Goletti D

AUID- ORCID: 0000-0001-8360-4376

AD - National Institute for Infectious Diseases "L. Spallanzani" IRCCS, 00149 Rome,

Italy.

FAU - Girardi, Enrico

AU - Girardi E

AUID- ORCID: 0000-0001-8182-5896

AD - National Institute for Infectious Diseases "L. Spallanzani" IRCCS, 00149 Rome,

Italy.

FAU - Andreoni, Massimo

AU - Andreoni M

AD - Clinic of Infectious Diseases, Policlinico Tor Vergata, V. Montpelier 1, 00133

Rome, Italy.

FAU - Palmieri, Fabrizio

AU - Palmieri F

AUID- ORCID: 0000-0001-8184-6291

AD - National Institute for Infectious Diseases "L. Spallanzani" IRCCS, 00149 Rome,

Italy.

FAU - Gualano, Gina

AU - Gualano G

AD - National Institute for Infectious Diseases "L. Spallanzani" IRCCS, 00149 Rome,

Italy.

LA - eng

PT - Journal Article

PT - Review

DEP - 20210709

PL - Switzerland

TA - Microorganisms

JT - Microorganisms

JID - 101625893

PMC - PMC8304680

OTO - NOTNLM

OT - adverse events

OT - anemia

OT - hematopoietic system

OT - rare hematologic disorders

OT - tuberculosis

COIS- The authors declare no conflict of interest.

EDAT- 2021/08/08 06:00

MHDA- 2021/08/08 06:01

PMCR- 2021/07/09

CRDT- 2021/08/07 01:11

PHST- 2021/06/07 00:00 [received]

PHST- 2021/07/02 00:00 [revised]

PHST- 2021/07/08 00:00 [accepted]

PHST- 2021/08/07 01:11 [entrez]

PHST- 2021/08/08 06:00 [pubmed]

PHST- 2021/08/08 06:01 [medline]

PHST- 2021/07/09 00:00 [pmc-release]

AID - microorganisms9071477 [pii]

AID - microorganisms-09-01477 [pii]

AID - 10.3390/microorganisms9071477 [doi]

PST - epublish

SO - Microorganisms. 2021 Jul 9;9(7):1477. doi: 10.3390/microorganisms9071477.

PMID- 34214859

OWN - NLM

STAT- MEDLINE

DCOM- 20220107

LR - 20220107

IS - 1873-281X (Electronic)

IS - 1472-9792 (Linking)

VI - 129

DP - 2021 Jul

TI - A multi-targeting pre-clinical candidate against drug-resistant tuberculosis.

PG - 102104

LID - S1472-9792(21)00054-8 [pii]

LID - 10.1016/j.tube.2021.102104 [doi]

AB - FNDR-20081 [4-{4-[5-(4-Isopropyl-phenyl)-

[1,2,4]oxadiazol-3-ylmethyl]-piperazin-1-yl}-7-pyridin-3-yl-quinoline] is a

novel, first in class anti-tubercular pre-clinical candidate against sensitive

and drug-resistant Mycobacterium tuberculosis (Mtb). In-vitro combination studies

of FNDR-20081 with first- and second-line drugs exhibited no antagonism,

suggesting its compatibility for developing new combination-regimens. FNDR-20081,

which is non-toxic with no CYP3A4 liability, demonstrated exposure-dependent

killing of replicating-Mtb, as well as the non-replicating-Mtb, and efficacy in a

mouse model of infection. Whole genome sequencing (WGS) of FNDR-20081 resistant

mutants revealed the identification of pleotropic targets: marR (Rv0678), a

regulator of MmpL5, a transporter/efflux pump mechanism for drug resistance; and

Rv3683, a putative metalloprotease potentially involved in peptidoglycan

biosynthesis. In summary, FNDR-20081 is a promising first in class compound with

the potential to form a new combination regimen for MDR-TB treatment.

CI - Copyright © 2021 Elsevier Ltd. All rights reserved.

FAU - Kaur, Parvinder

AU - Kaur P

AD - Foundation for Neglected Disease Research, Bangalore, India. Electronic address:

parvinder.kaur@fndr.in.

FAU - Potluri, Vijay

AU - Potluri V

AD - Foundation for Neglected Disease Research, Bangalore, India.

FAU - Ahuja, Vijay Kamal

AU - Ahuja VK

AD - Foundation for Neglected Disease Research, Bangalore, India.

FAU - Naveenkumar, C N

AU - Naveenkumar CN

AD - Foundation for Neglected Disease Research, Bangalore, India.

FAU - Krishnamurthy, Ramya Vadageri

AU - Krishnamurthy RV

AD - Foundation for Neglected Disease Research, Bangalore, India.

FAU - Gangadharaiah, Shruthi Thimmalapura

AU - Gangadharaiah ST

AD - Anthem BioSciences. Pvt. Ltd., No 49, Canara Bank Road, Hosur Rd, Electronics

City Phase 1, Bommasandra Industrial Area, Bengaluru, Karnataka, 560099, India.

FAU - Shivarudraiah, Prasad

AU - Shivarudraiah P

AD - Anthem BioSciences. Pvt. Ltd., No 49, Canara Bank Road, Hosur Rd, Electronics

City Phase 1, Bommasandra Industrial Area, Bengaluru, Karnataka, 560099, India.

FAU - Eswaran, Sumesh

AU - Eswaran S

AD - Anthem BioSciences. Pvt. Ltd., No 49, Canara Bank Road, Hosur Rd, Electronics

City Phase 1, Bommasandra Industrial Area, Bengaluru, Karnataka, 560099, India.

FAU - Nirmal, Christy Rosaline

AU - Nirmal CR

AD - National Institute for Research in Tuberculosis, No.1, Mayor Sathiyamoorthy St,

Chetpet, Chennai, Tamil Nadu, 600031, India.

FAU - Mahizhaveni, Balasubramanian

AU - Mahizhaveni B

AD - National Institute for Research in Tuberculosis, No.1, Mayor Sathiyamoorthy St,

Chetpet, Chennai, Tamil Nadu, 600031, India.

FAU - Dusthackeer, Azger

AU - Dusthackeer A

AD - National Institute for Research in Tuberculosis, No.1, Mayor Sathiyamoorthy St,

Chetpet, Chennai, Tamil Nadu, 600031, India.

FAU - Mondal, Rajesh

AU - Mondal R

AD - National Institute for Research in Tuberculosis, No.1, Mayor Sathiyamoorthy St,

Chetpet, Chennai, Tamil Nadu, 600031, India.

FAU - Batt, Sarah M

AU - Batt SM

AD - Insitiute of Microbiology & Infection, School of Biosciences, University of

Birmingham, Edgbaston, Birmingham, B15 2TT, UK.

FAU - Richardson, Emily J

AU - Richardson EJ

AD - Insitiute of Microbiology & Infection, School of Biosciences, University of

Birmingham, Edgbaston, Birmingham, B15 2TT, UK.

FAU - Loman, Nicholas J

AU - Loman NJ

AD - Insitiute of Microbiology & Infection, School of Biosciences, University of

Birmingham, Edgbaston, Birmingham, B15 2TT, UK.

FAU - Besra, Gurdyal Singh

AU - Besra GS

AD - Insitiute of Microbiology & Infection, School of Biosciences, University of

Birmingham, Edgbaston, Birmingham, B15 2TT, UK.

FAU - Shandil, Radha Krishan

AU - Shandil RK

AD - Foundation for Neglected Disease Research, Bangalore, India.

FAU - Narayanan, Shridhar

AU - Narayanan S

AD - Foundation for Neglected Disease Research, Bangalore, India.

LA - eng

PT - Journal Article

PT - Research Support, Non-U.S. Gov't

DEP - 20210618

PL - Scotland

TA - Tuberculosis (Edinb)

JT - Tuberculosis (Edinburgh, Scotland)

JID - 100971555

RN - 0 (Antitubercular Agents)

RN - 0 (Quinolines)

SB - IM

MH - Animals

MH - Antitubercular Agents/*pharmacology

MH - Drug Evaluation, Preclinical

MH - Drug Resistance, Multiple, Bacterial

MH - Hep G2 Cells

MH - Humans

MH - Male

MH - Mice

MH - Mice, Inbred BALB C

MH - Microbial Sensitivity Tests

MH - Molecular Structure

MH - Mycobacterium tuberculosis

MH - Quinolines/*pharmacology

MH - THP-1 Cells

MH - Tuberculosis, Multidrug-Resistant/*drug therapy

OTO - NOTNLM

OT - Drug resistance

OT - First-in-class

OT - Multi-target

OT - Mycobacterium tuberculosis

OT - Pre-clinical candidate

EDAT- 2021/07/03 06:00

MHDA- 2022/01/08 06:00

CRDT- 2021/07/02 20:22

PHST- 2021/03/31 00:00 [received]

PHST- 2021/06/11 00:00 [revised]

PHST- 2021/06/13 00:00 [accepted]

PHST- 2021/07/03 06:00 [pubmed]

PHST- 2022/01/08 06:00 [medline]

PHST- 2021/07/02 20:22 [entrez]

AID - S1472-9792(21)00054-8 [pii]

AID - 10.1016/j.tube.2021.102104 [doi]

PST - ppublish

SO - Tuberculosis (Edinb). 2021 Jul;129:102104. doi: 10.1016/j.tube.2021.102104. Epub

2021 Jun 18.

PMID- 34234413

OWN - NLM

STAT- MEDLINE

DCOM- 20211208

LR - 20250530

IS - 1177-8881 (Electronic)

IS - 1177-8881 (Linking)

VI - 15

DP - 2021

TI - Profiling Pretomanid as a Therapeutic Option for TB Infection: Evidence to Date.

PG - 2815-2830

LID - 10.2147/DDDT.S281639 [doi]

AB - Tuberculosis (TB) is the most deadly infectious disease globally. Although most

individuals achieve a cure, a substantial portion develop multi-drug resistant TB

which is exceedingly difficult to treat, and the number of effective agents is

dwindling. Development of new anti-tubercular medications is imperative to combat

existing drug resistance and accelerate global eradication of TB. Pretomanid

(PA-824) represents one of the newest drug classes (ie, nitroimidazooxazines)

approved in 2019 by the United States Food and Drug Administration as part of a

multi-drug regimen (with bedaquiline and linezolid, BPaL) and recommended by the

World Health Organization (WHO) to treat extensively-resistant (XR-TB) and

multi-drug resistant tuberculosis (MDR-TB). Approval was granted through the

FDA's Limited Population Pathway for Antibacterial and Antifungal Drugs, which

accelerates approval for antimicrobial drugs used to treat life-threatening or

serious infections in a limited population with unmet need. This review details

the pharmacology, efficacy, and safety of this new agent and describes evidence

to date for its role in the treatment of drug resistant TB including published,

ongoing, and planned studies.

CI - © 2021 Stancil et al.

FAU - Stancil, Stephani L

AU - Stancil SL

AD - Division of Adolescent Medicine, Children's Mercy Kansas City, Kansas City, MO,

64108, USA.

AD - Division of Clinical Pharmacology, Toxicology and Therapeutic Innovation,

Children's Mercy Kansas City, Kansas City, MO, 64108, USA.

AD - Department of Pediatrics, University of Missouri-Kansas City School of Medicine,

Kansas City, MO, 64108, USA.

FAU - Mirzayev, Fuad

AU - Mirzayev F

AD - World Health Organization, Geneva, Switzerland.

FAU - Abdel-Rahman, Susan M

AU - Abdel-Rahman SM

AD - Division of Clinical Pharmacology, Toxicology and Therapeutic Innovation,

Children's Mercy Kansas City, Kansas City, MO, 64108, USA.

AD - Department of Pediatrics, University of Missouri-Kansas City School of Medicine,

Kansas City, MO, 64108, USA.

LA - eng

GR - 001/WHO_/World Health Organization/International

PT - Journal Article

PT - Review

DEP - 20210628

PL - New Zealand

TA - Drug Des Devel Ther

JT - Drug design, development and therapy

JID - 101475745

RN - 0 (Antitubercular Agents)

RN - 0 (Nitroimidazoles)

RN - 0 (pretomanid)

SB - IM

MH - Animals

MH - Antitubercular Agents/*administration & dosage/adverse effects/pharmacology

MH - Drug Therapy, Combination

MH - Extensively Drug-Resistant Tuberculosis/drug therapy/microbiology

MH - Humans

MH - Nitroimidazoles/*administration & dosage/adverse effects/pharmacology

MH - Tuberculosis, Multidrug-Resistant/*drug therapy/microbiology

PMC - PMC8253981

OTO - NOTNLM

OT - antimicrobial

OT - multi-drug resistant

OT - pretomanid

OT - tuberculosis

COIS- The authors report no conflicts of interest in this work.

EDAT- 2021/07/09 06:00

MHDA- 2021/12/15 06:00

PMCR- 2021/06/28

CRDT- 2021/07/08 06:36

PHST- 2021/04/01 00:00 [received]

PHST- 2021/05/28 00:00 [accepted]

PHST- 2021/07/08 06:36 [entrez]

PHST- 2021/07/09 06:00 [pubmed]

PHST- 2021/12/15 06:00 [medline]

PHST- 2021/06/28 00:00 [pmc-release]

AID - 281639 [pii]

AID - 10.2147/DDDT.S281639 [doi]

PST - epublish

SO - Drug Des Devel Ther. 2021 Jun 28;15:2815-2830. doi: 10.2147/DDDT.S281639.

eCollection 2021.

PMID- 34171989

OWN - NLM

STAT- MEDLINE

DCOM- 20210706

LR - 20210706

IS - 1471-2334 (Electronic)

IS - 1471-2334 (Linking)

VI - 21

IP - 1

DP - 2021 Jun 25

TI - Phenotypic and molecular characterization of pyrazinamide resistance among

multidrug-resistant Mycobacterium tuberculosis isolates in Ningbo, China.

PG - 605

LID - 10.1186/s12879-021-06306-1 [doi]

LID - 605

AB - BACKGROUND: Detection of pyrazinamide (PZA) resistance in Mycobacterium

tuberculosis (TB) patients is critical, especially in dealing with

multidrug-resistant Mycobacterium tuberculosis (MDR-TB) case. Up to date, PZA

drug susceptibility testing (DST) has not been regularly performed in China. The

prevalence and molecular characteristics of PZA resistance in M.tuberculosis

isolates, especially MDR-TB have not been studied in Ningbo, China. This study

aimed to analyze the phenotypic and molecular characterization of PZA resistance

among MDR-TB isolates in Ningbo. METHODS: A total of 110 MDR-TB isolates were

collected from the TB patients who were recorded at local TB dispensaries in

Ningbo. All clinical isolates were examined by drug susceptibility testing and

genotyping. DNA sequencing was used to detect mutations in the pncA gene

associated with PZA resistance. RESULTS: The prevalence of PZA resistance among

MDR-TB strains in Ningbo was 59.1%. With regard to the history and the outcome of

treatments among MDR-TB cases, the percentages of re-treated MDR-TB patients in

the PZA-resistant group and of successful patients in PZA-susceptible group were

significantly higher than the ones in the PZA-susceptible group and in the

PZA-resistant group, respectively (P = 0.027, P = 0.020). The results showed that

the resistance of streptomycin (67.7% vs 46.7%, P = 0.027), ethambutol (56.9% vs

33.3%, P = 0.015), ofloxacin (43.1% vs 11.1%, P = 0.000), levofloxacin (43.1% vs

11.1%, P = 0.000), pre-XDR (pre-Xtensively Drug Resistance) (38.5% vs 15.6%,

P = 0.009), were more frequently adverted among PZA-resistant isolates compared

with PZA-susceptible isolates. In addition, 110 MDR-TB was composed of 87 (PZA

resistant, 78.5%) Beijing strains and 23 (PZA resistant, 21.5%) non-Beijing

strains. Fifty-four out of 65 (83.1%) PZA-resistant MDR strains harbored a

mutation located in the pncA gene and the majority (90.7%) were point mutations.

Compared with the phenotypic characterization, DNA sequencing of pncA has

sensitivity and specificity of 83.1 and 95.6%. CONCLUSION: The mutations within

pncA gene was the primary mechanism of PZA resistance among MDR-TB and DNA

sequencing of pncA gene could provide a rapid detection evidence in PZA drug

resistance of MDR-TB in Ningbo.

FAU - Che, Yang

AU - Che Y

AD - Institute of Tuberculosis Prevention and Control, Ningbo Municipal Center for

Disease Control and Prevention, Ningbo, 315010, Zhejiang, China.

FAU - Bo, Dingyi

AU - Bo D

AD - Institute of Tuberculosis Prevention and Control, Haishu Municipal Center for

Disease Control and Prevention, Ningbo, Zhejiang, China.

FAU - Lin, Xiang

AU - Lin X

AD - Institute of Tuberculosis Prevention and Control, Ningbo Municipal Center for

Disease Control and Prevention, Ningbo, 315010, Zhejiang, China.

FAU - Chen, Tong

AU - Chen T

AD - Institute of Tuberculosis Prevention and Control, Ningbo Municipal Center for

Disease Control and Prevention, Ningbo, 315010, Zhejiang, China.

FAU - He, Tianfeng

AU - He T

AD - Institute of Tuberculosis Prevention and Control, Ningbo Municipal Center for

Disease Control and Prevention, Ningbo, 315010, Zhejiang, China.

hetf@nbcdc.org.cn.

FAU - Lin, Yi

AU - Lin Y

AD - Center for Health Economics, Faculty of Humanities and Social Sciences,

University of Nottingham, Ningbo, Zhejiang, China. Lily.Lin@nottingham.edu.cn.

LA - eng

PT - Journal Article

DEP - 20210625

PL - England

TA - BMC Infect Dis

JT - BMC infectious diseases

JID - 100968551

RN - 0 (Antitubercular Agents)

RN - 0 (DNA, Bacterial)

RN - 2KNI5N06TI (Pyrazinamide)

RN - EC 3.5.- (Amidohydrolases)

RN - EC 3.5.- (PncA protein, Mycobacterium tuberculosis)

SB - IM

MH - Adult

MH - Aged

MH - Aged, 80 and over

MH - Amidohydrolases/genetics

MH - Antitubercular Agents/*pharmacology

MH - Beijing

MH - China

MH - DNA, Bacterial

MH - Humans

MH - Male

MH - Microbial Sensitivity Tests

MH - Middle Aged

MH - Mutation

MH - Mycobacterium tuberculosis/*drug effects/isolation & purification

MH - Phenotype

MH - Pyrazinamide/*pharmacology

MH - Sequence Analysis, DNA

MH - Tuberculosis, Multidrug-Resistant/*microbiology

MH - Young Adult

PMC - PMC8228925

OTO - NOTNLM

OT - Multidrug-resistant

OT - Mycobacterium tuberculosis

OT - Resistance

OT - pncA gene

COIS- The authors declare that they have no competing interests.

EDAT- 2021/06/27 06:00

MHDA- 2021/07/07 06:00

PMCR- 2021/06/25

CRDT- 2021/06/26 05:24

PHST- 2021/03/25 00:00 [received]

PHST- 2021/06/07 00:00 [accepted]

PHST- 2021/06/26 05:24 [entrez]

PHST- 2021/06/27 06:00 [pubmed]

PHST- 2021/07/07 06:00 [medline]

PHST- 2021/06/25 00:00 [pmc-release]

AID - 10.1186/s12879-021-06306-1 [pii]

AID - 6306 [pii]

AID - 10.1186/s12879-021-06306-1 [doi]

PST - epublish

SO - BMC Infect Dis. 2021 Jun 25;21(1):605. doi: 10.1186/s12879-021-06306-1.

PMID- 33915409

OWN - NLM

STAT- MEDLINE

DCOM- 20210527

LR - 20210527

IS - 1878-5905 (Electronic)

IS - 0142-9612 (Linking)

VI - 273

DP - 2021 Jun

TI - Extracellular matrix-inspired inhalable aerogels for rapid clearance of pulmonary

tuberculosis.

PG - 120848

LID - S0142-9612(21)00204-0 [pii]

LID - 10.1016/j.biomaterials.2021.120848 [doi]

AB - Tuberculosis (TB) remains a leading cause of death from a single infectious

agent, and limiting the spread of multidrug-resistant TB (MDR-TB) is now an

urgent global health priority. Essential to the persistence of this disease is

the ability of Mycobacterium tuberculosis (Mtb) to circumvent host defenses by

infecting lung macrophages to create a cellular niche for its survival and

proliferation. This has urged the development of new therapeutic strategies that

act through mechanisms distinct from conventional antibiotics, and thus are

effective against MDR bacteria, while being able to efficiently kill persister

Mtb cells in infected host macrophages. Here, we report a new class of gel-like

microparticle aerosols, or 'aerogels', designed to exploit metabolic

vulnerabilities of Mtb pathogens and TB-infected macrophages to enable

preferential delivery of synergistic peptide-antibiotic combinations for potent

and rapid antitubercular therapy. This is achieved by formulating aerogels

through the supramolecular assembly of a de novo designed anti-TB peptide and the

extracellular matrix (ECM)-derived polysaccharide, hyaluronic acid (HA).

Importantly, HA serves as a nutrient source for Mtb cells during tissue invasion

and proliferation, and is recognized by CD44 receptors highly expressed on lung

macrophages during TB infection. By exploiting this metabolic substrate for

pathogen targeting, HA aerogels are shown to avidly bind and kill both

drug-sensitive and drug-resistant mycobacteria, while being efficiently

internalized into macrophage host cells in vitro and in vivo to clear Mtb

persisters. This multifaceted bioactivity suggests aerogels may serve as a

versatile inhalable platform upon which novel biomaterials-enabled therapeutics

can be developed to rapidly clear pulmonary MDR-TB.

CI - Copyright © 2021 Elsevier Ltd. All rights reserved.

FAU - Simonson, Andrew W

AU - Simonson AW

AD - Department of Biomedical Engineering, Pennsylvania State University, University

Park, PA, 16802, USA.

FAU - Umstead, Todd M

AU - Umstead TM

AD - Department of Pediatrics, Penn State College of Medicine, Hershey, PA, 17033,

USA; Pulmonary Immunology and Physiology Laboratory, Penn State College of

Medicine, Hershey, PA, 17033, USA.

FAU - Lawanprasert, Atip

AU - Lawanprasert A

AD - Department of Biomedical Engineering, Pennsylvania State University, University

Park, PA, 16802, USA.

FAU - Klein, Bailey

AU - Klein B

AD - Department of Biomedical Engineering, Pennsylvania State University, University

Park, PA, 16802, USA.

FAU - Almarzooqi, Sarah

AU - Almarzooqi S

AD - Department of Biomedical Engineering, Pennsylvania State University, University

Park, PA, 16802, USA.

FAU - Halstead, E Scott

AU - Halstead ES

AD - Department of Pediatrics, Penn State College of Medicine, Hershey, PA, 17033,

USA; Pulmonary Immunology and Physiology Laboratory, Penn State College of

Medicine, Hershey, PA, 17033, USA.

FAU - Medina, Scott H

AU - Medina SH

AD - Department of Biomedical Engineering, Pennsylvania State University, University

Park, PA, 16802, USA; Huck Institutes of the Life Sciences, Penn State

University, University Park, PA, 16802, USA. Electronic address: shm126@psu.edu.

LA - eng

PT - Journal Article

PT - Research Support, Non-U.S. Gov't

DEP - 20210422

PL - Netherlands

TA - Biomaterials

JT - Biomaterials

JID - 8100316

RN - 0 (Antitubercular Agents)

SB - IM

MH - Antitubercular Agents

MH - Extracellular Matrix

MH - Humans

MH - *Mycobacterium tuberculosis

MH - *Tuberculosis

MH - *Tuberculosis, Pulmonary/drug therapy

OTO - NOTNLM

OT - Aerosol

OT - Drug delivery

OT - Hyaluronic acid

OT - Macrophage

OT - Peptide

OT - Tuberculosis

EDAT- 2021/04/30 06:00

MHDA- 2021/05/28 06:00

CRDT- 2021/04/29 20:22

PHST- 2020/11/30 00:00 [received]

PHST- 2021/04/16 00:00 [revised]

PHST- 2021/04/17 00:00 [accepted]

PHST- 2021/04/30 06:00 [pubmed]

PHST- 2021/05/28 06:00 [medline]

PHST- 2021/04/29 20:22 [entrez]

AID - S0142-9612(21)00204-0 [pii]

AID - 10.1016/j.biomaterials.2021.120848 [doi]

PST - ppublish

SO - Biomaterials. 2021 Jun;273:120848. doi: 10.1016/j.biomaterials.2021.120848. Epub

2021 Apr 22.

PMID- 33826064

OWN - NLM

STAT- MEDLINE

DCOM- 20210817

LR - 20211213

IS - 1559-0291 (Electronic)

IS - 0273-2289 (Print)

IS - 0273-2289 (Linking)

VI - 193

IP - 6

DP - 2021 Jun

TI - In Silico Approach for Phytocompound-Based Drug Designing to Fight Efflux

Pump-Mediated Multidrug-Resistant Mycobacterium tuberculosis.

PG - 1757-1779

LID - 10.1007/s12010-021-03557-1 [doi]

AB - Tuberculosis (TB), caused by the bacteria Mycobacterium tuberculosis, is one of

the principal causes of death in the world despite the existence of a significant

number of antibiotics aimed against it. This is mainly due to the drug resistance

mechanisms present in the bacterium, which leads to multidrug-resistant

tuberculosis (MDR-TB). Additionally, the development of new antibiotics has

become limited over the years. Although there are various drug resistance

mechanisms present, efflux pumps are of utmost importance because they extrude

out several dissimilar antitubercular drugs out of the cell. There are many

efflux pump proteins present in Mycobacterium tuberculosis. Therefore, blocking

these efflux pumps by inhibitors can raise the efficacy of the existing

antibiotics and may also pave the path for the discovery and synthesis of new

drugs. Plant compounds can act as a resource for the development of efflux pump

inhibitors (EPIs), which may eventually replace or augment the current

therapeutic options. This is mainly because plants have been traditionally used

for ages for food or treatment and are considered safe with little or no side

effects. Various computational tools are available which are used for the virtual

screening of a large number of phytocompounds within a short span of time. This

review aims to highlight the mechanism and appearance of drug resistance in

Mycobacterium tuberculosis with emphasis on efflux pumps along with the

significance of phytochemicals as inhibitors of these pumps and their screening

strategy by computational approaches.

FAU - Biswas, Santasree Sarma

AU - Biswas SS

AD - Department of Biosciences, Assam Don Bosco University, Tapesia Gardens, Sonapur,

Assam, 782102, India. sarma.santasree@gmail.com.

FAU - Browne, Rene Barbie

AU - Browne RB

AD - Department of Biosciences, Assam Don Bosco University, Tapesia Gardens, Sonapur,

Assam, 782102, India.

FAU - Borah, Vedant Vikrom

AU - Borah VV

AD - Department of Biosciences, Assam Don Bosco University, Tapesia Gardens, Sonapur,

Assam, 782102, India.

FAU - Roy, Jayanti Datta

AU - Roy JD

AD - Department of Biosciences, Assam Don Bosco University, Tapesia Gardens, Sonapur,

Assam, 782102, India.

LA - eng

PT - Journal Article

PT - Review

DEP - 20210407

PL - United States

TA - Appl Biochem Biotechnol

JT - Applied biochemistry and biotechnology

JID - 8208561

RN - 0 (Antitubercular Agents)

RN - 0 (Bacterial Proteins)

RN - 0 (Membrane Transport Proteins)

RN - 0 (Phytochemicals)

SB - IM

MH - Animals

MH - *Antitubercular Agents/chemistry/therapeutic use

MH - *Bacterial Proteins/antagonists & inhibitors/metabolism

MH - *Computer Simulation

MH - Drug Resistance, Multiple, Bacterial/*drug effects

MH - Humans

MH - Membrane Transport Proteins/*metabolism

MH - Mycobacterium tuberculosis/*metabolism

MH - *Phytochemicals/chemistry/therapeutic use

MH - *Tuberculosis, Multidrug-Resistant/drug therapy/metabolism

PMC - PMC8024441

OTO - NOTNLM

OT - Efflux pump inhibitors

OT - Efflux pumps

OT - Multidrug-resistant tuberculosis

OT - Phytocompounds

COIS- The authors declare no competing interests.

EDAT- 2021/04/08 06:00

MHDA- 2021/08/18 06:00

PMCR- 2021/04/07

CRDT- 2021/04/07 12:33

PHST- 2020/11/03 00:00 [received]

PHST- 2021/03/22 00:00 [accepted]

PHST- 2021/04/08 06:00 [pubmed]

PHST- 2021/08/18 06:00 [medline]

PHST- 2021/04/07 12:33 [entrez]

PHST- 2021/04/07 00:00 [pmc-release]

AID - 10.1007/s12010-021-03557-1 [pii]

AID - 3557 [pii]

AID - 10.1007/s12010-021-03557-1 [doi]

PST - ppublish

SO - Appl Biochem Biotechnol. 2021 Jun;193(6):1757-1779. doi:

10.1007/s12010-021-03557-1. Epub 2021 Apr 7.

PMID- 33751730

OWN - NLM

STAT- MEDLINE

DCOM- 20210727

LR - 20210727

IS - 1099-0461 (Electronic)

IS - 1095-6670 (Linking)

VI - 35

IP - 6

DP - 2021 Jun

TI - Monocarbonyl curcuminoids as antituberculosis agents with their moderate in-vitro

metabolic stability on human liver microsomes.

PG - 1-10

LID - 10.1002/jbt.22754 [doi]

AB - Tuberculosis, an airborne infectious disease, results in a high morbidity and

mortality rate. The continuous emergence of TB resistance strains including MDR

(multidrug-resistant tuberculosis), XDR (extensive drug-resistant tuberculosis),

and especially TDR (totally drug-resistant tuberculosis) is a major public health

threat and has intensified the need to develop new antitubercular agents. A

natural product, curcumin, possesses diverse biological activities but suffers

due to a lack of water solubility and bioavailability. To overcome these

limitations, a series of 17 water-soluble monocarbonyl curcuminoids was

synthesized and evaluated for antimycobacterial activity. All compounds exhibited

good to moderate anti-TB activity with MIC(99) in the range of 3.12-25.0 µM, out

of which 7c and 7p were found the most potent compounds with MIC(99) in the range

of 3.12-6.25 µM. Furthermore, these compounds were observed to be nonhaemolytic,

nontoxic, and stable under both physiological as well as reducing conditions.

In-vitro metabolic stability data of the representative compound 7p with the

human liver microsome revealed that these compounds possess a moderate metabolism

with a half-life of 1.2 h and an intrinsic clearance of 1.12 ml/h/mg.

CI - © 2021 Wiley Periodicals LLC.

FAU - Gagandeep

AU - Gagandeep

AD - Department of Chemistry, University of Delhi, New Delhi, India.

FAU - Singh, Manisha

AU - Singh M

AD - Tuberculosis Research Laboratory, Translational Health Science and Technology

Institute, Faridabad, India.

FAU - Kidawi, Saqib

AU - Kidawi S

AD - Tuberculosis Research Laboratory, Translational Health Science and Technology

Institute, Faridabad, India.

FAU - Das, Ujjalkumar S

AU - Das US

AD - Department of Ocular Pharmacology, Dr. R. P. Centre for Ophthalmic Sciences, All

India Institute of Medical Sciences, New Delhi, India.

FAU - Velpandian, Thirumurthy

AU - Velpandian T

AD - Department of Ocular Pharmacology, Dr. R. P. Centre for Ophthalmic Sciences, All

India Institute of Medical Sciences, New Delhi, India.

FAU - Singh, Ramandeep

AU - Singh R

AD - Tuberculosis Research Laboratory, Translational Health Science and Technology

Institute, Faridabad, India.

FAU - Rawat, Diwan S

AU - Rawat DS

AUID- ORCID: 0000-0002-5473-7476

AD - Department of Chemistry, University of Delhi, New Delhi, India.

LA - eng

GR - 02(0318)/17/EMR-II/Council of Scientific and Industrial Research, India/

PT - Journal Article

DEP - 20210310

PL - United States

TA - J Biochem Mol Toxicol

JT - Journal of biochemical and molecular toxicology

JID - 9717231

RN - 0 (Antitubercular Agents)

RN - 0 (Diarylheptanoids)

SB - IM

MH - *Antitubercular Agents/pharmacokinetics/pharmacology

MH - *Diarylheptanoids/pharmacokinetics/pharmacology

MH - Humans

MH - Microbial Sensitivity Tests

MH - Microsomes, Liver/*metabolism

MH - Mycobacterium tuberculosis/*growth & development

OTO - NOTNLM

OT - Mycobacterium tuberculosis

OT - curcuminoids

OT - human liver microsomes

OT - metabolic stability

EDAT- 2021/03/23 06:00

MHDA- 2021/07/28 06:00

CRDT- 2021/03/22 19:34

PHST- 2021/01/19 00:00 [revised]

PHST- 2020/12/02 00:00 [received]

PHST- 2021/02/24 00:00 [accepted]

PHST- 2021/03/23 06:00 [pubmed]

PHST- 2021/07/28 06:00 [medline]

PHST- 2021/03/22 19:34 [entrez]

AID - 10.1002/jbt.22754 [doi]

PST - ppublish

SO - J Biochem Mol Toxicol. 2021 Jun;35(6):1-10. doi: 10.1002/jbt.22754. Epub 2021 Mar

10.

PMID- 33886255

OWN - NLM

STAT- MEDLINE

DCOM- 20210820

LR - 20240924

IS - 1520-4898 (Electronic)

IS - 0001-4842 (Print)

IS - 0001-4842 (Linking)

VI - 54

IP - 10

DP - 2021 May 18

TI - Strategies to Combat Multi-Drug Resistance in Tuberculosis.

PG - 2361-2376

LID - 10.1021/acs.accounts.0c00878 [doi]

AB - "Drug resistance is an unavoidable consequence of the use of drugs; however, the

emergence of multi-drug resistance can be managed by accurate diagnosis and

tailor-made regimens."Antimicrobial resistance (AMR), is one of the most

paramount health perils that has emerged in the 21st century. The global increase

in drug-resistant strains of various bacterial pathogens prompted the World

Health Organization (WHO) to develop a priority list of AMR pathogens.

Mycobacterium tuberculosis (Mtb), an acid-fast bacillus that causes tuberculosis

(TB), merits being one of the highest priority pathogens on this list since

drug-resistant TB (DR-TB) accounts for ∼29% of deaths attributable to AMR. In

recent years, funded collaborative efforts of researchers from academia,

not-for-profit virtual R&D organizations and industry have resulted in the

continuous growth of the TB drug discovery and development pipeline. This has so

far led to the accelerated regulatory approval of bedaquiline and delamanid for

the treatment of DR-TB. However, despite the availability of drug regimes, the

current cure rate for multi-drug-resistant TB (MDR-TB) and extensively

drug-resistant TB (XDR-TB) treatment regimens is 50% and 30%, respectively. It is

to be noted that these regimens are administered over a long duration and have a

serious side effect profile. Coupled with poor patient adherence, this has led to

further acquisition of drug resistance and treatment failure. There is therefore

an urgent need to develop new TB drugs with novel mechanism of actions (MoAs) and

associated regimens.This Account recapitulates drug resistance in TB, existing

challenges in addressing DR-TB, new drugs and regimens in development, and

potential ways to treat DR-TB. We highlight our research aimed at identifying

novel small molecule leads and associated targets against TB toward contributing

to the global TB drug discovery and development pipeline. Our work mainly

involves screening of various small molecule chemical libraries in phenotypic

whole-cell based assays to identify hits for medicinal chemistry optimization,

with attendant deconvolution of the MoA. We discuss the identification of small

molecule chemotypes active against Mtb and subsequent structure-activity

relationships (SAR) and MoA deconvolution studies. This is followed by a

discussion on a chemical series identified by whole-cell cross-screening against

Mtb, for which MoA deconvolution studies revealed a pathway that explained the

lack of in vivo efficacy in a mouse model of TB and reiterated the importance of

selecting an appropriate growth medium during phenotypic screening. We also

discuss our efforts on drug repositioning toward addressing DR-TB. In the

concluding section, we preview some promising future directions and the

challenges inherent in advancing the drug pipeline to address DR-TB.

FAU - Singh, Vinayak

AU - Singh V

AUID- ORCID: 0000-0001-9002-2489

AD - Drug Discovery and Development Centre (H3D), University of Cape Town, Rondebosch

7701, South Africa.

AD - South African Medical Research Council Drug Discovery and Development Research

Unit, Department of Chemistry and Institute of Infectious Disease and Molecular

Medicine, University of Cape Town, Rondebosch 7701, South Africa.

FAU - Chibale, Kelly

AU - Chibale K

AUID- ORCID: 0000-0002-1327-4727

AD - Drug Discovery and Development Centre (H3D), University of Cape Town, Rondebosch

7701, South Africa.

AD - South African Medical Research Council Drug Discovery and Development Research

Unit, Department of Chemistry and Institute of Infectious Disease and Molecular

Medicine, University of Cape Town, Rondebosch 7701, South Africa.

LA - eng

PT - Journal Article

PT - Research Support, Non-U.S. Gov't

PT - Review

DEP - 20210422

PL - United States

TA - Acc Chem Res

JT - Accounts of chemical research

JID - 0157313

RN - 0 (Antitubercular Agents)

SB - IM

MH - Animals

MH - Antitubercular Agents/chemistry/*pharmacology

MH - Drug Resistance, Multiple/*drug effects

MH - Humans

MH - Mycobacterium tuberculosis/*drug effects

MH - Structure-Activity Relationship

MH - Tuberculosis/*drug therapy

PMC - PMC8154215

COIS- The authors declare no competing financial interest.

EDAT- 2021/04/23 06:00

MHDA- 2021/08/21 06:00

PMCR- 2021/05/26

CRDT- 2021/04/22 17:12

PHST- 2021/04/23 06:00 [pubmed]

PHST- 2021/08/21 06:00 [medline]

PHST- 2021/04/22 17:12 [entrez]

PHST- 2021/05/26 00:00 [pmc-release]

AID - 10.1021/acs.accounts.0c00878 [doi]

PST - ppublish

SO - Acc Chem Res. 2021 May 18;54(10):2361-2376. doi: 10.1021/acs.accounts.0c00878.

Epub 2021 Apr 22.

PMID- 34063426

OWN - NLM

STAT- PubMed-not-MEDLINE

LR - 20210615

IS - 2076-0817 (Print)

IS - 2076-0817 (Electronic)

IS - 2076-0817 (Linking)

VI - 10

IP - 5

DP - 2021 May 15

TI - Label-Free Comparative Proteomics of Differentially Expressed Mycobacterium

tuberculosis Protein in Rifampicin-Related Drug-Resistant Strains.

LID - 10.3390/pathogens10050607 [doi]

LID - 607

AB - Rifampicin (RIF) is one of the most important first-line anti-tuberculosis (TB)

drugs, and more than 90% of RIF-resistant (RR) Mycobacterium tuberculosis

clinical isolates belong to multidrug-resistant (MDR) and extensively

drug-resistant (XDR) TB. In order to identify specific candidate target proteins

as diagnostic markers or drug targets, differential protein expression between

drug-sensitive (DS) and drug-resistant (DR) strains remains to be investigated.

In the present study, a label-free, quantitative proteomics technique was

performed to compare the proteome of DS, RR, MDR, and XDR clinical strains. We

found iniC, Rv2141c, folB, and Rv2561 were up-regulated in both RR and MDR

strains, while fadE9, espB, espL, esxK, and Rv3175 were down-regulated in the

three DR strains when compared to the DS strain. In addition, lprF, mce2R, mce2B,

and Rv2627c were specifically expressed in the three DR strains, and 41 proteins

were not detected in the DS strain. Functional category showed that these

differentially expressed proteins were mainly involved in the cell wall and cell

processes. When compared to the RR strain, Rv2272, smtB, lpqB, icd1, and folK

were up-regulated, while esxK, PPE19, Rv1534, rpmI, ureA, tpx, mpt64, frr,

Rv3678c, esxB, esxA, and espL were down-regulated in both MDR and XDR strains.

Additionally, nrp, PPE3, mntH, Rv1188, Rv1473, nadB, PPE36, and sseA were

specifically expressed in both MDR and XDR strains, whereas 292 proteins were not

identified when compared to the RR strain. When compared between MDR and XDR

strains, 52 proteins were up-regulated, while 45 proteins were down-regulated in

the XDR strain. 316 proteins were especially expressed in the XDR strain, while

92 proteins were especially detected in the MDR strain. Protein interaction

networks further revealed the mechanism of their involvement in virulence and

drug resistance. Therefore, these differentially expressed proteins are of great

significance for exploring effective control strategies of DR-TB.

FAU - Ullah, Nadeem

AU - Ullah N

AUID- ORCID: 0000-0001-6089-8906

AD - Department of Pathogen Biology, School of Basic Medicine, Tongji Medical College,

Huazhong University of Science and Technology, Wuhan 430030, China.

FAU - Hao, Ling

AU - Hao L

AD - Department of Pathogen Biology, School of Basic Medicine, Tongji Medical College,

Huazhong University of Science and Technology, Wuhan 430030, China.

FAU - Banga Ndzouboukou, Jo-Lewis

AU - Banga Ndzouboukou JL

AUID- ORCID: 0000-0003-1501-8361

AD - Department of Pathogen Biology, School of Basic Medicine, Tongji Medical College,

Huazhong University of Science and Technology, Wuhan 430030, China.

FAU - Chen, Shiyun

AU - Chen S

AD - CAS Key Laboratory of Special Pathogens and Biosafety, Institute of Virology,

Chinese Academy of Sciences, Wuhan 430071, China.

FAU - Wu, Yaqi

AU - Wu Y

AD - Department of Pathogen Biology, School of Basic Medicine, Tongji Medical College,

Huazhong University of Science and Technology, Wuhan 430030, China.

FAU - Li, Longmeng

AU - Li L

AD - Department of Pathogen Biology, School of Basic Medicine, Tongji Medical College,

Huazhong University of Science and Technology, Wuhan 430030, China.

FAU - Borham Mohamed, Eman

AU - Borham Mohamed E

AD - Department of Pathogen Biology, School of Basic Medicine, Tongji Medical College,

Huazhong University of Science and Technology, Wuhan 430030, China.

FAU - Hu, Yangbo

AU - Hu Y

AD - CAS Key Laboratory of Special Pathogens and Biosafety, Institute of Virology,

Chinese Academy of Sciences, Wuhan 430071, China.

FAU - Fan, Xionglin

AU - Fan X

AUID- ORCID: 0000-0001-9754-372X

AD - Department of Pathogen Biology, School of Basic Medicine, Tongji Medical College,

Huazhong University of Science and Technology, Wuhan 430030, China.

LA - eng

GR - 2018ZX10302302002-001/National Mega-Projects of Science Research for the 13th

Five-year Plan of China/

GR - 81971909/Open Research Fund Program of CAS Key Laboratory of Special Pathogens

and Biosafety, Chinese Academy of Sciences, the Natural Science Foundation of

China/

GR - 2020kfyXGYJ040/HUST COVID-19 Rapid Response Call/

PT - Journal Article

DEP - 20210515

PL - Switzerland

TA - Pathogens

JT - Pathogens (Basel, Switzerland)

JID - 101596317

PMC - PMC8157059

OTO - NOTNLM

OT - Mycobacterium tuberculosis

OT - drug resistance

OT - label-free

OT - proteomics

OT - rifampicin

COIS- The authors declare no conflict of interest.

EDAT- 2021/06/03 06:00

MHDA- 2021/06/03 06:01

PMCR- 2021/05/15

CRDT- 2021/06/02 01:06

PHST- 2021/03/19 00:00 [received]

PHST- 2021/04/28 00:00 [revised]

PHST- 2021/05/10 00:00 [accepted]

PHST- 2021/06/02 01:06 [entrez]

PHST- 2021/06/03 06:00 [pubmed]

PHST- 2021/06/03 06:01 [medline]

PHST- 2021/05/15 00:00 [pmc-release]

AID - pathogens10050607 [pii]

AID - pathogens-10-00607 [pii]

AID - 10.3390/pathogens10050607 [doi]

PST - epublish

SO - Pathogens. 2021 May 15;10(5):607. doi: 10.3390/pathogens10050607.

PMID- 33410233

OWN - NLM

STAT- MEDLINE

DCOM- 20220111

LR - 20220111

IS - 1860-7187 (Electronic)

IS - 1860-7179 (Linking)

VI - 16

IP - 8

DP - 2021 Apr 20

TI - Benzofuroxan Derivatives as Potent Agents against Multidrug-Resistant

Mycobacterium tuberculosis.

PG - 1268-1282

LID - 10.1002/cmdc.202000899 [doi]

AB - Tuberculosis (TB) is currently the leading cause of death related to infectious

diseases worldwide, as reported by the World Health Organization. Moreover, the

increasing number of multidrug-resistant tuberculosis (MDR-TB) cases has alarmed

health agencies, warranting extensive efforts to discover novel drugs that are

effective and also safe. In this study, 23 new compounds were synthesized and

evaluated in vitro against the drug-resistant strains of M. tuberculosis. The

compound 6-((3-fluoro-4-thiomorpholinophenyl)carbamoyl)benzo[c][1,2,5]oxadiazole

1-N-oxide (5 b) was particularly remarkable in this regard as it demonstrated

MIC(90) values below 0.28 μM against all the MDR strains evaluated, thus

suggesting that this compound might have a different mechanism of action.

Benzofuroxans are an attractive new class of anti-TB agents, exemplified by

compound 5 b, with excellent potency against the replicating and drug-resistant

strains of M. tuberculosis.

CI - © 2021 Wiley-VCH GmbH.

FAU - Fernandes, Guilherme F S

AU - Fernandes GFS

AUID- ORCID: 0000-0002-7644-5466

AD - School of Pharmaceutical Sciences, São Paulo State University (UNESP), Araraquara

Jaú Highway KM 01, 14800903, Araraquara, Brazil.

AD - Institute of Chemistry, São Paulo State University (UNESP), Francisco Degni

Street 55, 14800060, Araraquara, Brazil.

FAU - Campos, Débora L

AU - Campos DL

AD - School of Pharmaceutical Sciences, São Paulo State University (UNESP), Araraquara

Jaú Highway KM 01, 14800903, Araraquara, Brazil.

FAU - Da Silva, Isabel C

AU - Da Silva IC

AD - School of Pharmaceutical Sciences, São Paulo State University (UNESP), Araraquara

Jaú Highway KM 01, 14800903, Araraquara, Brazil.

FAU - Prates, João L B

AU - Prates JLB

AD - School of Pharmaceutical Sciences, São Paulo State University (UNESP), Araraquara

Jaú Highway KM 01, 14800903, Araraquara, Brazil.

AD - Institute of Chemistry, São Paulo State University (UNESP), Francisco Degni

Street 55, 14800060, Araraquara, Brazil.

FAU - Pavan, Aline R

AU - Pavan AR

AD - School of Pharmaceutical Sciences, São Paulo State University (UNESP), Araraquara

Jaú Highway KM 01, 14800903, Araraquara, Brazil.

AD - Institute of Chemistry, São Paulo State University (UNESP), Francisco Degni

Street 55, 14800060, Araraquara, Brazil.

FAU - Pavan, Fernando R

AU - Pavan FR

AD - School of Pharmaceutical Sciences, São Paulo State University (UNESP), Araraquara

Jaú Highway KM 01, 14800903, Araraquara, Brazil.

FAU - Dos Santos, Jean L

AU - Dos Santos JL

AD - School of Pharmaceutical Sciences, São Paulo State University (UNESP), Araraquara

Jaú Highway KM 01, 14800903, Araraquara, Brazil.

AD - Institute of Chemistry, São Paulo State University (UNESP), Francisco Degni

Street 55, 14800060, Araraquara, Brazil.

LA - eng

GR - 2016/09502-7/Fundação de Amparo à Pesquisa do Estado de São Paulo/

GR - 2018/17739-2/Fundação de Amparo à Pesquisa do Estado de São Paulo/

GR - 2018/11079-0/Fundação de Amparo à Pesquisa do Estado de São Paulo/

GR - 2018/00163-0/Fundação de Amparo à Pesquisa do Estado de São Paulo/

GR - 2018/21778-3/Fundação de Amparo à Pesquisa do Estado de São Paulo/

GR - 2018/24783-8/Fundação de Amparo à Pesquisa do Estado de São Paulo/

GR - 001/Coordenação de Aperfeiçoamento de Pessoal de Nível Superior-Brasil (CAPES)/

GR - 404181/2019-8/Conselho Nacional de Desenvolvimento Científico e Tecnológico/

GR - 429139/2018-7/Conselho Nacional de Desenvolvimento Científico e Tecnológico/

PT - Journal Article

PT - Research Support, Non-U.S. Gov't

DEP - 20210209

PL - Germany

TA - ChemMedChem

JT - ChemMedChem

JID - 101259013

RN - 0 (Antitubercular Agents)

RN - 0 (Benzoxazoles)

RN - 0 (Oxadiazoles)

RN - 480-96-6 (benzofuroxan)

SB - IM

MH - Antitubercular Agents/chemical synthesis/*pharmacology

MH - Benzoxazoles/chemical synthesis/*pharmacology

MH - Drug Design

MH - Drug Resistance, Multiple/drug effects

MH - Microbial Sensitivity Tests

MH - Molecular Structure

MH - Mycobacterium tuberculosis/*drug effects

MH - Oxadiazoles/chemical synthesis/*pharmacology

MH - Structure-Activity Relationship

OTO - NOTNLM

OT - N-oxide

OT - antitubercular agents

OT - benzofuroxan

OT - multidrug-resistant TB

OT - tuberculosis

EDAT- 2021/01/08 06:00

MHDA- 2022/01/12 06:00

CRDT- 2021/01/07 06:16

PHST- 2021/01/06 00:00 [revised]

PHST- 2020/11/18 00:00 [received]

PHST- 2021/01/08 06:00 [pubmed]

PHST- 2022/01/12 06:00 [medline]

PHST- 2021/01/07 06:16 [entrez]

AID - 10.1002/cmdc.202000899 [doi]

PST - ppublish

SO - ChemMedChem. 2021 Apr 20;16(8):1268-1282. doi: 10.1002/cmdc.202000899. Epub 2021

Feb 9.

PMID- 33945243

STAT- Publisher

CTDT- 20210418

PB - MDText.com, Inc.

DP - 2000

TI - Diabetes Mellitus and Tuberculosis.

BTI - Endotext

AB - The converging epidemics of non-communicable disease like DM (DM) and an

infectious disease like tuberculosis (TB) is a double burden. DM is increasing in

the same population that is at high risk for developing TB. There is a

two-to-four-fold higher risk of active TB in individuals with DM and up to 30% of

individuals with TB are likely to have DM. Immune deficiency either in absolute

or relative quantities are sufficient for re-activation of latent TB. From a 10%

risk of reactivation over the whole lifetime of an immunocompetent individual,

the risk of reactivation increases to 10% every year in immune-deficient

individuals. DM impairs cell mediated immunity and poor glycemic control affects

cytokine response and alters the defenses in the alveolar macrophages. Fever,

hemoptysis, extensive parenchymal lesions, and lung cavities are more common in

those with DM particularly heavier and older males. DM increases the risk of

treatment failure, death, and relapse. Evidence collected from meta-analysis

conclude that DM can increase the odds of developing Multi Drug resistant TB

(MDR-TB). The synergism between DM and TB necessitates bi-directional screening.

Sputum examination for Ziehl-Neelsen staining is both a sensitive and specific

screening test. Rapid molecular diagnostic tests like cartridge based nucleic

acid amplification tests (CB-NAAT) are useful in cases where there is a

high-index of suspicion and difficulty in arriving at a definitive diagnosis

exists. Random plasma glucose and HbA1c (glycosylated Hemoglobin) measurements

are convenient tests for DM screening that can be done in non-fasting

individuals. Screening for DM more than once during the course of illness is

sensible so that transient DM and new-onset DM can be identified. Anti-TB drugs

affect glycemic control as they interact with anti-diabetic drugs by either

stimulating or inhibiting the metabolizing enzymes. They may also aggravate

metabolic, ocular, and neuropathic complications of DM. Insulin is the preferred

drug in most instances. The presence of renal and hepatic dysfunction affects TB

and hyperglycemic management. For complete coverage of all related areas of

Endocrinology, please visit our on-line FREE web-text, WWW.ENDOTEXT.ORG.

CI - Copyright © 2000-2026, MDText.com, Inc.

FED - Feingold, Kenneth R

ED - Feingold KR

AD - Professor of Medicine Emeritus, University of California, San Francisco, CA

FED - Adler, Robert A

ED - Adler RA

AD - Chief of Endocrinology and Metabolism at the Richmond Veterans Affairs Medical

Center and Professor of Internal Medicine at Virginia Commonwealth University.

FED - Ahmed, S Faisal

ED - Ahmed SF

AD - Developmental Endocrinology Research Group, University of Glasgow, Glasgow, UK

FED - Anawalt, Bradley

ED - Anawalt B

AD - Chief of Medicine at the University of Washington Medical Center and Professor

and Vice Chair of the Department of Medicine, University of Washington

FED - Blackman, Marc R

ED - Blackman MR

AD - Sr. Physician Scientist, Washington DC VA Medical Center; Professor of Medicine &

Rehabilitation Medicine, Georgetown University; Clinical Professor of Medicine,

Biochemistry and Molecular Medicine, George Washington University; and Professor

of Medicine (Part-time), Johns Hopkins University

FED - Chrousos, George

ED - Chrousos G

AD - Professor of Pediatrics and Endocrinology, Division of Endocrinology, Metabolism

and Diabetes, First Department of Pediatrics, National and Kapodistrian

University of Athens Medical School, "Aghia Sophia" Children's Hospital, Athens,

Greece

FED - Corpas, Emiliano

ED - Corpas E

AD - M.D. Ph.D in Gerontology. Honorary Professor of Medicine, Universidad de Alcalá,

Madrid. Consultant in Endocrinology, Hospital HLA Guadalajara (Spain).

FED - de Herder, Wouter W

ED - de Herder WW

AD - Professor of Endocrine Oncology, Erasmus MC and Erasmus MC Cancer Center,

Rotterdam, the Netherlands

FED - Dhatariya, Ketan

ED - Dhatariya K

AD - Consultant in Diabetes, Endocrinology and General Medicine, Norfolk and Norwich

University Hospitals NHS Foundation Trust and University of East Anglia, Norwich,

UK.

FED - Dungan, Kathleen

ED - Dungan K

AD - Professor of Medicine, Division of Endocrinology, Diabetes, and Metabolism, Ohio

State University

FED - Hamilton, Emma

ED - Hamilton E

AD - Consultant Endocrinologist at Fiona Stanley (FSH) and Fremantle Hospitals (FH) in

Perth, Western Australia

FED - Hofland, Johannes

ED - Hofland J

AD - Consultant Endocrinologist, Erasmus MC and Erasmus MC Cancer Center, Rotterdam,

the Netherlands

FED - Jan de Beur, Suzanne

ED - Jan de Beur S

AD - Professor of Endocrinology and Chief of the Division of Endocrinology and

Metabolism at the University of Virginia School of Medicine.

FED - Kalra, Sanjay

ED - Kalra S

AD - Consultant Endocrinologist, Department of Endocrinology, Bharti Hospital, Karnal,

India

FED - Kaltsas, Gregory

ED - Kaltsas G

AD - Professor of General Medicine-Endocrinology, 1st Department of Propaedeutic

Medicine, National and Kapodistrian University of Athens, Athens, Greece

FED - Kapoor, Nitin

ED - Kapoor N

AD - Professor of Endocrinology, Department of Endocrinology, Diabetes and Metabolism,

Christian Medical College & Hospital, Vellore, Tamil Nadu, India, Melbourne

School of Population and Global Health, Faculty of Medicine, Dentistry and Health

Science, The University of Melbourne, Australia.

FED - Kim, Matthew

ED - Kim M

AD - Clinical Director of the Division of Endocrinology, Diabetes and Hypertension at

Brigham and Women’s Hospital and an Assistant Professor of Medicine at Harvard

Medical School. 18. Professor, Division of Endocrinology, Diabetes, Metabolism,

Department of Medicine, The University of Tennessee Health Science Center,

Memphis, TN, and University of Florida, Jacksonville, FL.

FED - Koch, Christian

ED - Koch C

AD - Professor, The University of Tennessee Health Science Center, Memphis, Tennessee

FED - Kopp, Peter

ED - Kopp P

AD - Professor of Medicine and Chief of the Division of Endocrinology, Diabetology and

Metabolism, University of Lausanne, Switzerland

FED - Korbonits, Márta

ED - Korbonits M

AD - Professor of Endocrinology and Metabolism, Centre Lead for Endocrinology and

Deputy Institute Director, William Harvey Research Institute, Barts and the

London School of Medicine and Dentistry, Queen Mary University of London, London,

England

FED - Kovacs, Christopher S

ED - Kovacs CS

AD - University Research Professor and Professor of Medicine (Endocrinology and

Metabolism), Obstetrics & Gynecology, and BioMedical Sciences, at Memorial

University of Newfoundland in St. John’s, Newfoundland, Canada.

FED - Kuohung, Wendy

ED - Kuohung W

AD - Director of the Division of Reproductive Endocrinology at Boston Medical Center

and an Associate Professor of Obstetrics and Gynecology at the Boston University

School of Medicine

FED - Laferrère, Blandine

ED - Laferrère B

AD - Professor of Medicine, New York Nutrition Obesity Research Center, Division of

Endocrinology, Department of Medicine, Columbia University Irving Medical Center,

New York, NY, USA.

FED - Levy, Miles

ED - Levy M

AD - Consultant endocrinologist at University Hospitals of Leicester and Honorary

Associate Professor at Leicester University

FED - McGee, Elizabeth A

ED - McGee EA

AD - Professor of Obstetrics and Gynecology at the University of Vermont and Director

of the Division of Reproductive Endocrinology and Infertility. Burlington,

Vermont

FED - McLachlan, Robert

ED - McLachlan R

AD - Director of Clinical Research, Hudson Institute of Medical Research; Consultant

Endocrinologist, Monash Medical Centre, Melbourne, Australia

FED - Muzumdar, Radhika

ED - Muzumdar R

AD - Allan L. Drash Endowed Chair, Chief, Division of Endocrinology and Diabetes, UPMC

Children’s Hospital of Pittsburgh, Professor of Pediatrics and Cell Biology,

University of Pittsburgh School of Medicine, Pittsburgh, PA

FED - Purnell, Jonathan

ED - Purnell J

AD - Professor of Medicine, Knight Cardiovascular Institute and the Division of

Endocrinology, and Associate Director, Bob and Charlee Moore Institute for

Nutrition and Wellness, Oregon Health and Science University, Portland, OR

FED - Rey, Rodolfo

ED - Rey R

AD - Centro de Investigaciones Endocrinológicas “Dr. César Bergadá” (CEDIE),

CONICET-FEI-División de Endocrinología, Hospital de Niños Ricardo Gutiérrez,

Gallo 1330, C1425EFD Buenos Aires; and Departamento de Histología, Biología

Celular, Embriología y Genética, Facultad de Medicina, Universidad de Buenos

Aires, C1121ABG Buenos Aires, Argentina

FED - Sahay, Rakesh

ED - Sahay R

AD - Professor and Head of Department of Endocrinology, Osmania Medical College and

Osmania General Hospital, Hyderabad, India.

FED - Shah, Amy S

ED - Shah AS

AD - Professor of Pediatrics, The University of Cincinnati, Department of Pediatrics

and Cincinnati Children’s Hospital Medical Center, Division of Endocrinology,

Cincinnati, OH, USA

FED - Sperling, Mark A

ED - Sperling MA

AD - Professorial Lecturer, Division of Pediatric Endocrinology and Diabetes, Icahn

School of Medicine at Mount Sinai, New York, NY. Emeritus Professor and Chair,

Department of Pediatrics, University of Pittsburgh.

FED - Stratakis, Constantine A

ED - Stratakis CA

AD - CSO, ELPEN, Inc. & Director, Research Institute, Athens, Greece & Senior

Investigator, Human Genetics & Precision Medicine, FORTH (ITE), Heraklion,

Greece. Emeritus Scientific Director & Senior Investigator, NICHD, NIH, Bethesda,

MD, USA

FED - Trence, Dace L

ED - Trence DL

AD - Professor of Medicine, Emeritus, University of Washington, Seattle, WA

FED - Wilson, Don P

ED - Wilson DP

AD - Endowed Chair, Cardiovascular Health and Risk Prevention, Pediatric Endocrinology

and Diabetes, Cook Children's Medical Center, Fort Worth, TX

FAU - Krishna, Sudeep

AU - Krishna S

AD - Associate Professor and Head, Department of Endocrinology, DM and Metabolism,

Father Muller Medical College Hospital, Mangalore, 575002. Karnataka, India

FAU - Jacob, Jubbin Jagan

AU - Jacob JJ

AD - Professor and Head, Department of Endocrinology, Christian Medical College and

Hospital, Ludhiana 141008, Punjab, India

LA - eng

PT - Review

PT - Book Chapter

PL - South Dartmouth (MA)

EDAT- 2021/04/18 00:00

CRDT- 2021/04/18 00:00

AID - NBK570126 [bookaccession]

PMID- 33161886

OWN - NLM

STAT- PubMed-not-MEDLINE

LR - 20240827

IS - 1937-190X (Electronic)

IS - 1937-190X (Linking)

VI - 36

IP - 3

DP - 2021 Apr 3

TI - Statement of Removal: Role and Impact of Engaging Private Pharmacies in TB Care

and Control in Selected Districts of Pakistan.

PG - 330-343

LID - 10.1080/19371918.2020.1826382 [doi]

AB - Tuberculosis (TB) constitutes a major public health challenge and is one of the

top 10 causes of death worldwide. In 2015, over 10.4 million people fell ill with

TB and 1.8 million died from the disease; an estimated 480,000 people developed

multidrug-resistant TB (MDR-TB); and an estimated 1 million children became ill

with TB, and 170,000 children died due to TB. According to the WHO, the incidence

of TB cases in Pakistan for all types are 270/100,000. A cross-sectional study

was designed, and a potential 2,000 registered pharmacies from different rural

and urban/peri-urban areas of 12 districts across Pakistan were selected.

Supervisors, pharmacy focal persons, students and faculty coordinators from seven

universities were trained on TB case identification, a referral mechanism, and

collaboration with the program, followed by signing MOU. In phase 1 an entire

(100%) pharmacy staff was trained; followed by the second phase, in which 96% of

focal persons were trained from private pharmacies nation-wide. A total of 4,085

referrals were sent to the trained doctors, out of which 622 cases were

identified. The percentage of case registration was 14.5% of total referrals. The

highest number of TB presumptives (527) were referred from private pharmacies of

district Muzafargarh, and 136 TB cases were registered out of these referrals.

For a signiﬁcant impact on case detection, it is important to acknowledge a

number of concerns expressed by pharmacists and attempt to change both client's

and provider's expectations with regard to the pharmacist's role.

FAU - Kakar, Maria

AU - Kakar M

AD - Department of Pharmacy, Abasyn University, Peshawar, Pakistan.

FAU - Afandi, Noorulain

AU - Afandi N

AD - Department of Pharmacy, Abasyn University, Peshawar, Pakistan.

LA - eng

PT - Journal Article

PT - Retracted Publication

DEP - 20201109

PL - United States

TA - Soc Work Public Health

JT - Social work in public health

JID - 101308228

RIN - 10.1080/19371918.2020.1826382

OTO - NOTNLM

OT - Case detection

OT - new referral system

OT - private pharmacies

OT - public-private mix

OT - tuberculosis

EDAT- 2020/11/10 06:00

MHDA- 2020/11/10 06:01

CRDT- 2020/11/09 05:22

PHST- 2020/11/10 06:00 [pubmed]

PHST- 2020/11/10 06:01 [medline]

PHST- 2020/11/09 05:22 [entrez]

AID - 10.1080/19371918.2020.1826382 [doi]

PST - ppublish

SO - Soc Work Public Health. 2021 Apr 3;36(3):330-343. doi:

10.1080/19371918.2020.1826382. Epub 2020 Nov 9.

PMID- 33845969

OWN - NLM

STAT- MEDLINE

DCOM- 20211012

LR - 20211012

IS - 0019-5707 (Print)

IS - 0019-5707 (Linking)

VI - 68

IP - 2

DP - 2021 Apr

TI - Pretomanid: The latest USFDA-approved anti-tuberculosis drug.

PG - 287-291

LID - S0019-5707(20)30151-7 [pii]

LID - 10.1016/j.ijtb.2020.09.003 [doi]

AB - Pretomanid is a nitroimidazooxazine drug which inhibits synthesis of mycolic

acid. This leads to defective cell wall formation, ultimately causing bacterial

cell death. It is active against both replicating and non-replicating M.

tuberculosis. Following promising result in a phase III trial, pretomanid was

approved by United States Food and Drug Administration in August 2019. This

orally active drug has been approved as part of a combination regimen of

bedaquiline, pretomanid and linezolid (BPaL regimen) to treat adults with

pulmonary extensive drug resistant tuberculosis (TB) or treatment-intolerant or

non-responsive multidrug resistant TB. Peripheral neuropathy and increased liver

enzymes are some of the reported adverse events associated with pretomanid.

However, more studies are required to confirm the role of pretomanid in

paediatric, geriatric and HIV co-infection cases.

CI - Copyright © 2020 Tuberculosis Association of India. Published by Elsevier B.V.

All rights reserved.

FAU - Deb, Uttiya

AU - Deb U

AD - Department of Pharmacology, Burdwan Medical College, Burdwan. Electronic address:

dr.uttiyadeb@gmail.com.

FAU - Biswas, Supreeti

AU - Biswas S

AD - Department of Pharmacology, Burdwan Medical College, Burdwan.

LA - eng

PT - Journal Article

PT - Review

DEP - 20200906

PL - India

TA - Indian J Tuberc

JT - The Indian journal of tuberculosis

JID - 0373027

RN - 0 (Antitubercular Agents)

RN - 0 (Nitroimidazoles)

RN - 0 (pretomanid)

SB - IM

MH - Antitubercular Agents/administration & dosage/*therapeutic use

MH - Drug Approval

MH - Drug Therapy, Combination

MH - Extensively Drug-Resistant Tuberculosis/*drug therapy

MH - Humans

MH - Nitroimidazoles/administration & dosage/*therapeutic use

MH - United States

MH - United States Food and Drug Administration

OTO - NOTNLM

OT - Extensive drug resistance

OT - Multidrug resistance

OT - Pretomanid

OT - Tuberculosis

EDAT- 2021/04/14 06:00

MHDA- 2021/10/13 06:00

CRDT- 2021/04/13 05:42

PHST- 2020/08/13 00:00 [received]

PHST- 2020/09/03 00:00 [accepted]

PHST- 2021/04/13 05:42 [entrez]

PHST- 2021/04/14 06:00 [pubmed]

PHST- 2021/10/13 06:00 [medline]

AID - S0019-5707(20)30151-7 [pii]

AID - 10.1016/j.ijtb.2020.09.003 [doi]

PST - ppublish

SO - Indian J Tuberc. 2021 Apr;68(2):287-291. doi: 10.1016/j.ijtb.2020.09.003. Epub

2020 Sep 6.

PMID- 33679685

OWN - NLM

STAT- MEDLINE

DCOM- 20210622

LR - 20230919

IS - 1664-3224 (Electronic)

IS - 1664-3224 (Linking)

VI - 11

DP - 2020

TI - The Cholinergic System Contributes to the Immunopathological Progression of

Experimental Pulmonary Tuberculosis.

PG - 581911

LID - 10.3389/fimmu.2020.581911 [doi]

LID - 581911

AB - The cholinergic system is present in both bacteria and mammals and regulates

inflammation during bacterial respiratory infections through neuronal and

non-neuronal production of acetylcholine (ACh) and its receptors. However, the

presence of this system during the immunopathogenesis of pulmonary tuberculosis

(TB) in vivo and in its causative agent Mycobacterium tuberculosis (Mtb) has not

been studied. Therefore, we used an experimental model of progressive pulmonary

TB in BALB/c mice to quantify pulmonary ACh using high-performance liquid

chromatography during the course of the disease. In addition, we performed

immunohistochemistry in lung tissue to determine the cellular expression of

cholinergic system components, and then administered nicotinic receptor (nAChR)

antagonists to validate their effect on lung bacterial burden, inflammation, and

pro-inflammatory cytokines. Finally, we subjected Mtb cultures to colorimetric

analysis to reveal the production of ACh and the effect of ACh and nAChR

antagonists on Mtb growth. Our results show high concentrations of ACh and

expression of its synthesizing enzyme choline acetyltransferase (ChAT) during

early infection in lung epithelial cells and macrophages. During late progressive

TB, lung ACh upregulation was even higher and coincided with ChAT and α7 nAChR

subunit expression in immune cells. Moreover, the administration of nAChR

antagonists increased pro-inflammatory cytokines, reduced bacillary loads and

synergized with antibiotic therapy in multidrug resistant TB. Finally, in vitro

studies revealed that the bacteria is capable of producing nanomolar

concentrations of ACh in liquid culture. In addition, the administration of ACh

and nicotinic antagonists to Mtb cultures induced or inhibited bacterial

proliferation, respectively. These results suggest that Mtb possesses a

cholinergic system and upregulates the lung non-neuronal cholinergic system,

particularly during late progressive TB. The upregulation of the cholinergic

system during infection could aid both bacterial growth and immunomodulation

within the lung to favor disease progression. Furthermore, the therapeutic

efficacy of modulating this system suggests that it could be a target for

treating the disease.

CI - Copyright © 2021 Islas-Weinstein, Marquina-Castillo, Mata-Espinosa,

Paredes-González, Chávez, Balboa, Marín Franco, Guerrero-Romero, Barrios-Payan

and Hernandez-Pando.

FAU - Islas-Weinstein, Leon

AU - Islas-Weinstein L

AD - Division of Experimental Pathology, Department of Pathology, National Institute

of Medical Sciences and Nutrition Salvador Zubirán, México City, Mexico.

FAU - Marquina-Castillo, Brenda

AU - Marquina-Castillo B

AD - Division of Experimental Pathology, Department of Pathology, National Institute

of Medical Sciences and Nutrition Salvador Zubirán, México City, Mexico.

FAU - Mata-Espinosa, Dulce

AU - Mata-Espinosa D

AD - Division of Experimental Pathology, Department of Pathology, National Institute

of Medical Sciences and Nutrition Salvador Zubirán, México City, Mexico.

FAU - Paredes-González, Iris S

AU - Paredes-González IS

AD - Division of Experimental Pathology, Department of Pathology, National Institute

of Medical Sciences and Nutrition Salvador Zubirán, México City, Mexico.

FAU - Chávez, Jaime

AU - Chávez J

AD - Department of Bronchial Hyperreactivity, National Institute of Respiratory

Diseases (Mexico), Mexico City, Mexico.

FAU - Balboa, Luciana

AU - Balboa L

AD - Laboratorio de Inmunología de Enfermedades Respiratorias, Instituto de Medicina

Experimental del National Scientific and Technical Research Council (CONICET),

Academia Nacional de Medicina, Buenos Aires, Argentina.

FAU - Marín Franco, José Luis

AU - Marín Franco JL

AD - Laboratorio de Inmunología de Enfermedades Respiratorias, Instituto de Medicina

Experimental del National Scientific and Technical Research Council (CONICET),

Academia Nacional de Medicina, Buenos Aires, Argentina.

FAU - Guerrero-Romero, Daniel

AU - Guerrero-Romero D

AD - Departamento de Matemáticas, Escuela Superior de Física y Matemáticas, Instituto

Politécnico Nacional, Mexico City, Mexico.

FAU - Barrios-Payan, Jorge Alberto

AU - Barrios-Payan JA

AD - Division of Experimental Pathology, Department of Pathology, National Institute

of Medical Sciences and Nutrition Salvador Zubirán, México City, Mexico.

FAU - Hernandez-Pando, Rogelio

AU - Hernandez-Pando R

AD - Division of Experimental Pathology, Department of Pathology, National Institute

of Medical Sciences and Nutrition Salvador Zubirán, México City, Mexico.

LA - eng

PT - Journal Article

PT - Research Support, Non-U.S. Gov't

DEP - 20210218

PL - Switzerland

TA - Front Immunol

JT - Frontiers in immunology

JID - 101560960

RN - 0 (Cytokines)

RN - 0 (Nicotinic Antagonists)

RN - 0 (Receptors, Nicotinic)

RN - 0 (alpha7 Nicotinic Acetylcholine Receptor)

RN - EC 2.3.1.6 (Choline O-Acetyltransferase)

RN - N9YNS0M02X (Acetylcholine)

SB - IM

MH - Acetylcholine/metabolism

MH - Animals

MH - Choline O-Acetyltransferase/metabolism

MH - Cytokines/metabolism

MH - Disease Models, Animal

MH - Disease Progression

MH - Epithelial Cells/metabolism/pathology

MH - Inflammation/metabolism/pathology

MH - Lung/metabolism/pathology

MH - Macrophages/metabolism/pathology

MH - Male

MH - Mice

MH - Mice, Inbred BALB C

MH - Mycobacterium tuberculosis/drug effects

MH - Neurons/metabolism/pathology

MH - Nicotinic Antagonists/pharmacology

MH - Non-Neuronal Cholinergic System/drug effects/*physiology

MH - Receptors, Nicotinic/metabolism

MH - Tuberculosis, Pulmonary/*metabolism/*pathology

MH - Up-Regulation/physiology

MH - alpha7 Nicotinic Acetylcholine Receptor/metabolism

PMC - PMC7930380

OTO - NOTNLM

OT - acetylcholine

OT - choline acetyltransferase

OT - cholinergic

OT - immune response

OT - mycobacterium tuberculosis

OT - nAChR antagonism

OT - pulmonary inflammation

OT - tuberculosis

COIS- The authors declare that the research was conducted in the absence of any

commercial or financial relationships that could be construed as a potential

conflict of interest.

EDAT- 2021/03/09 06:00

MHDA- 2021/06/23 06:00

PMCR- 2020/01/01

CRDT- 2021/03/08 05:50

PHST- 2020/07/10 00:00 [received]

PHST- 2020/12/22 00:00 [accepted]

PHST- 2021/03/08 05:50 [entrez]

PHST- 2021/03/09 06:00 [pubmed]

PHST- 2021/06/23 06:00 [medline]

PHST- 2020/01/01 00:00 [pmc-release]

AID - 10.3389/fimmu.2020.581911 [doi]

PST - epublish

SO - Front Immunol. 2021 Feb 18;11:581911. doi: 10.3389/fimmu.2020.581911. eCollection

2020.

PMID- 33361298

OWN - NLM

STAT- MEDLINE

DCOM- 20210617

LR - 20210818

IS - 1098-6596 (Electronic)

IS - 0066-4804 (Print)

IS - 0066-4804 (Linking)

VI - 65

IP - 3

DP - 2021 Feb 17

TI - Systematic Review of Mutations Associated with Isoniazid Resistance Points to

Continuing Evolution and Subsequent Evasion of Molecular Detection, and Potential

for Emergence of Multidrug Resistance in Clinical Strains of Mycobacterium

tuberculosis.

LID - 10.1128/AAC.02091-20 [doi]

LID - e02091-20

AB - Molecular testing is rapidly becoming an integral component of global

tuberculosis (TB) control. Uncommon mechanisms of resistance escape detection by

these platforms and undermine our ability to contain outbreaks. This article is a

systematic review of published articles that reported isoniazid (INH)

resistance-conferring mutations between September 2013 and December 2019. The

genes katG, inhA, and fabG1, and the intergenic region oxyR'-ahpC were considered

in this review. Fifty-two articles were included that described 9,306 clinical

isolates (5,804 INH resistant [INH(r)] and 3,502 INH susceptible [INH(s)]) from

31 countries. The three most frequently mutated loci continue to be locus 315 of

katG (katG315; n = 4,271), locus -15 of inhA (inhA-15; n = 787), and locus -8 of

inhA (inhA-8; 106). However, the diagnostic value of inhA-8 is far lower than

previously thought, as it only appears in 25 (0.4%) of the INH(r) isolates

lacking the first two mutations. I catalogued 45 new loci (29 katG, nine inhA,

and seven ahpC) associated with INH resistance and identified 59 loci (common to

this and previous reviews) as a reliable basis for molecular diagnostics.

Including all observed mutations provides a cumulative sensitivity of 85.6%. In

14.4% of resistant isolates, no mechanism of resistance was detected, making them

likely to escape molecular detection, and in the case of INH monoresistance,

likely to convert to multidrug-resistant TB (MDR-TB). Integrating the information

cataloged in this study into current diagnostic tools is essential for combating

the emergence of MDR-TB, and its exclusion can lead to an unintended selection

against common mechanisms and to diversifying evolution. Observation of many

low-frequency resistance-conferring mutations points to an advantage of

whole-genome sequencing (WGS) for diagnostics. Finally, I provide five

recommendations for future diagnostic platforms.

CI - Copyright © 2021 American Society for Microbiology.

FAU - Valafar, Siavash J

AU - Valafar SJ

AUID- ORCID: 0000-0001-5421-6059

AD - Department of Biology, University of California, Irvine, Irvine, California, USA

svalafar@uci.edu.

LA - eng

PT - Journal Article

PT - Systematic Review

DEP - 20210217

PL - United States

TA - Antimicrob Agents Chemother

JT - Antimicrobial agents and chemotherapy

JID - 0315061

RN - 0 (Antitubercular Agents)

RN - 0 (Bacterial Proteins)

RN - 0 (DNA, Bacterial)

RN - V83O1VOZ8L (Isoniazid)

SB - IM

MH - Antitubercular Agents/pharmacology/therapeutic use

MH - Bacterial Proteins/genetics

MH - DNA, Bacterial

MH - Drug Resistance, Multiple

MH - Humans

MH - Isoniazid/pharmacology

MH - Microbial Sensitivity Tests

MH - Mutation

MH - *Mycobacterium tuberculosis/genetics

MH - *Tuberculosis, Multidrug-Resistant/diagnosis/drug therapy

PMC - PMC8092511

OTO - NOTNLM

OT - drug resistance evolution

OT - drug resistance mechanisms

OT - genotypic identification

OT - isoniazid

OT - phenotypic identification

OT - tuberculosis

EDAT- 2020/12/29 06:00

MHDA- 2021/06/22 06:00

PMCR- 2021/08/17

CRDT- 2020/12/28 11:41

PHST- 2020/10/06 00:00 [received]

PHST- 2020/12/13 00:00 [accepted]

PHST- 2020/12/29 06:00 [pubmed]

PHST- 2021/06/22 06:00 [medline]

PHST- 2020/12/28 11:41 [entrez]

PHST- 2021/08/17 00:00 [pmc-release]

AID - AAC.02091-20 [pii]

AID - 02091-20 [pii]

AID - 10.1128/AAC.02091-20 [doi]

PST - epublish

SO - Antimicrob Agents Chemother. 2021 Feb 17;65(3):e02091-20. doi:

10.1128/AAC.02091-20. Print 2021 Feb 17.

PMID- 33215746

OWN - NLM

STAT- MEDLINE

DCOM- 20210303

LR - 20210303

IS - 1521-3765 (Electronic)

IS - 0947-6539 (Linking)

VI - 27

IP - 9

DP - 2021 Feb 10

TI - Discovery of a Pyrimidinedione Derivative with Potent Inhibitory Activity against

Mycobacterium tuberculosis Ketol-Acid Reductoisomerase.

PG - 3130-3141

LID - 10.1002/chem.202004665 [doi]

AB - New drugs aimed at novel targets are urgently needed to combat the increasing

rate of drug-resistant tuberculosis (TB). Herein, the National Cancer Institute

Developmental Therapeutic Program (NCI-DTP) chemical library was screened against

a promising new target, ketol-acid reductoisomerase (KARI), the second enzyme in

the branched-chain amino acid (BCAA) biosynthesis pathway. From this library,

6-hydroxy-2-methylthiazolo[4,5-d]pyrimidine-5,7(4H,6H)-dione (NSC116565) was

identified as a potent time-dependent inhibitor of Mycobacterium tuberculosis

(Mt) KARI with a K(i) of 95.4 nm. Isothermal titration calorimetry studies showed

that this inhibitor bound to MtKARI in the presence and absence of the cofactor,

nicotinamide adenine dinucleotide phosphate (NADPH), which was confirmed by

crystal structures of the compound in complex with closely related

Staphylococcus aureus KARI. It is also shown that NSC116565 inhibits the growth

of H37Ra and H37Rv strains of Mt with MIC(50) values of 2.93 and 6.06 μm,

respectively. These results further validate KARI as a TB drug target and show

that NSC116565 is a promising lead for anti-TB drug development.

CI - © 2020 Wiley-VCH GmbH.

FAU - Lin, Xin

AU - Lin X

AD - School of Chemistry and Molecular Biosciences, The University of Queensland,

Brisbane, 4072, Australia.

FAU - Kurz, Julia L

AU - Kurz JL

AD - School of Chemistry and Molecular Biosciences, The University of Queensland,

Brisbane, 4072, Australia.

FAU - Patel, Khushboo M

AU - Patel KM

AD - School of Chemistry and Molecular Biosciences, The University of Queensland,

Brisbane, 4072, Australia.

FAU - Wun, Shun Jie

AU - Wun SJ

AD - School of Chemistry and Molecular Biosciences, The University of Queensland,

Brisbane, 4072, Australia.

FAU - Hussein, Waleed M

AU - Hussein WM

AD - School of Chemistry and Molecular Biosciences, The University of Queensland,

Brisbane, 4072, Australia.

AD - Pharmaceutical Organic Chemistry Department, Faculty of Pharmacy, Ein Helwan,

Helwan University, Helwan, Egypt.

FAU - Lonhienne, Thierry

AU - Lonhienne T

AD - School of Chemistry and Molecular Biosciences, The University of Queensland,

Brisbane, 4072, Australia.

FAU - West, Nicholas P

AU - West NP

AD - School of Chemistry and Molecular Biosciences, The University of Queensland,

Brisbane, 4072, Australia.

FAU - McGeary, Ross P

AU - McGeary RP

AD - School of Chemistry and Molecular Biosciences, The University of Queensland,

Brisbane, 4072, Australia.

FAU - Schenk, Gerhard

AU - Schenk G

AD - School of Chemistry and Molecular Biosciences, The University of Queensland,

Brisbane, 4072, Australia.

FAU - Guddat, Luke W

AU - Guddat LW

AUID- ORCID: 0000-0002-8204-8408

AD - School of Chemistry and Molecular Biosciences, The University of Queensland,

Brisbane, 4072, Australia.

LA - eng

GR - 114729/National Health and Medical Research Council/

PT - Journal Article

DEP - 20210112

PL - Germany

TA - Chemistry

JT - Chemistry (Weinheim an der Bergstrasse, Germany)

JID - 9513783

RN - 0 (Antitubercular Agents)

RN - 0 (Pyrimidinones)

RN - 53-59-8 (NADP)

RN - EC 1.1.1.86 (Ketol-Acid Reductoisomerase)

SB - IM

MH - Antitubercular Agents/*pharmacology

MH - Cell Line

MH - Humans

MH - Ketol-Acid Reductoisomerase/*antagonists & inhibitors/metabolism

MH - Mycobacterium tuberculosis/drug effects/*enzymology

MH - NADP/metabolism

MH - Pyrimidinones/*pharmacology

MH - Staphylococcus aureus/enzymology

MH - Tuberculosis/drug therapy/microbiology

OTO - NOTNLM

OT - crystal growth

OT - drug design

OT - ketol-acid reductoisomerase

OT - library screening

OT - tuberculosis

EDAT- 2020/11/21 06:00

MHDA- 2021/03/04 06:00

CRDT- 2020/11/20 08:40

PHST- 2020/10/21 00:00 [received]

PHST- 2020/11/21 06:00 [pubmed]

PHST- 2021/03/04 06:00 [medline]

PHST- 2020/11/20 08:40 [entrez]

AID - 10.1002/chem.202004665 [doi]

PST - ppublish

SO - Chemistry. 2021 Feb 10;27(9):3130-3141. doi: 10.1002/chem.202004665. Epub 2021

Jan 12.

PMID- 33249329

OWN - NLM

STAT- MEDLINE

DCOM- 20210512

LR - 20210512

IS - 1878-1667 (Electronic)

IS - 0147-9571 (Linking)

VI - 74

DP - 2021 Feb

TI - Drug resistant Tuberculosis: A review.

PG - 101574

LID - S0147-9571(20)30163-6 [pii]

LID - 10.1016/j.cimid.2020.101574 [doi]

AB - Tuberculosis (TB) was announced as a global emergency in 1993. There was an

alarming counter attack of TB worldwide. However, when it was known that TB can

be cured completely, the general public became ignorant towards the infection.

The pathogenic organism Mycobacterium tuberculosis continuously evolved to resist

the antagonist drugs. This has led to the outbreak of resistant strain that gave

rise to "Multi Drug Resistant-Tuberculosis" and "Extensively Drug Resistant

Tuberculosis" that can still be cured with a lower success rate. While the

mechanism of resistance proceeds further, it ultimately causes unmanageable

totally drug resistant TB (TDR-TB). Studying the molecular mechanisms underlying

the resistance to drugs would help us grasp the genetics and pathophysiology of

the disease. In this review, we present the molecular mechanisms behind

Mycobacterium tolerance to drugs and their approach towards the development of

multi-drug resistant, extremely drug resistant and totally drug resistant TB.

CI - Copyright © 2020 Elsevier Ltd. All rights reserved.

FAU - Khawbung, Jessy Lallungawi

AU - Khawbung JL

AD - Department of Biotechnology, Assam University, Silchar, 788011, Assam, India.

FAU - Nath, Durbba

AU - Nath D

AD - Department of Biotechnology, Assam University, Silchar, 788011, Assam, India.

FAU - Chakraborty, Supriyo

AU - Chakraborty S

AD - Department of Biotechnology, Assam University, Silchar, 788011, Assam, India.

Electronic address: supriyoch_2008@rediffmail.com.

LA - eng

PT - Journal Article

PT - Review

DEP - 20201116

PL - England

TA - Comp Immunol Microbiol Infect Dis

JT - Comparative immunology, microbiology and infectious diseases

JID - 7808924

RN - 0 (Antitubercular Agents)

SB - IM

MH - Animals

MH - Antitubercular Agents/pharmacology/therapeutic use

MH - Disease Outbreaks

MH - Drug Resistance, Multiple, Bacterial

MH - *Extensively Drug-Resistant Tuberculosis/drug therapy/epidemiology/veterinary

MH - *Mycobacterium tuberculosis/genetics

OTO - NOTNLM

OT - Drug resistance

OT - MDR-TB

OT - Mycobacterium tuberculosis

OT - TDR-TB

OT - Tuberculosis

OT - XDR-TB

EDAT- 2020/11/30 06:00

MHDA- 2021/05/13 06:00

CRDT- 2020/11/29 20:31

PHST- 2020/09/19 00:00 [received]

PHST- 2020/11/05 00:00 [accepted]

PHST- 2020/11/30 06:00 [pubmed]

PHST- 2021/05/13 06:00 [medline]

PHST- 2020/11/29 20:31 [entrez]

AID - S0147-9571(20)30163-6 [pii]

AID - 10.1016/j.cimid.2020.101574 [doi]

PST - ppublish

SO - Comp Immunol Microbiol Infect Dis. 2021 Feb;74:101574. doi:

10.1016/j.cimid.2020.101574. Epub 2020 Nov 16.

PMID- 33584600

OWN - NLM

STAT- PubMed-not-MEDLINE

LR - 20230919

IS - 1664-302X (Print)

IS - 1664-302X (Electronic)

IS - 1664-302X (Linking)

VI - 11

DP - 2020

TI - The Prospective Synergy of Antitubercular Drugs With NAD Biosynthesis Inhibitors.

PG - 634640

LID - 10.3389/fmicb.2020.634640 [doi]

LID - 634640

AB - Given the upsurge of drug-resistant tuberculosis worldwide, there is much focus

on developing novel drug combinations allowing shorter treatment duration and a

lower toxicity profile. Nicotinamide adenine dinucleotide (NAD) biosynthesis

targeting is acknowledged as a promising strategy to combat drug-susceptible,

drug-resistant, and latent tuberculosis (TB) infections. In this review, we

describe the potential synergy of NAD biosynthesis inhibitors with several

TB-drugs in prospective novel combination therapy. Despite not directly targeting

the essential NAD cofactor's biosynthesis, several TB prodrugs either require a

NAD biosynthesis enzyme to be activated or form a toxic chemical adduct with

NAD(H) itself. For example, pyrazinamide requires the action of nicotinamidase

(PncA), often referred to as pyrazinamidase, to be converted into its active

form. PncA is an essential player in NAD salvage and recycling. Since most

pyrazinamide-resistant strains are PncA-defective, a combination with downstream

NAD-blocking molecules may enhance pyrazinamide activity and possibly overcome

the resistance mechanism. Isoniazid, ethionamide, and delamanid form NAD adducts

in their active form, partly perturbing the redox cofactor metabolism. Indeed,

NAD depletion has been observed in Mycobacterium tuberculosis (Mtb) during

isoniazid treatment, and activation of the intracellular NAD phosphorylase MbcT

toxin potentiates its effect. Due to the NAD cofactor's crucial role in cellular

energy production, additional synergistic correlations of NAD biosynthesis

blockade can be envisioned with bedaquiline and other drugs targeting

energy-metabolism in mycobacteria. In conclusion, future strategies targeting NAD

metabolism in Mtb should consider its potential synergy with current and other

forthcoming TB-drugs.

CI - Copyright © 2021 Rohde and Sorci.

FAU - Rohde, Kyle H

AU - Rohde KH

AD - Burnett School of Biomedical Sciences, College of Medicine, University of Central

Florida, Orlando, FL, United States.

FAU - Sorci, Leonardo

AU - Sorci L

AD - Division of Bioinformatics and Biochemistry, Department of Materials,

Environmental Sciences and Urban Planning, Polytechnic University of Marche,

Ancona, Italy.

LA - eng

PT - Journal Article

PT - Review

DEP - 20210126

PL - Switzerland

TA - Front Microbiol

JT - Frontiers in microbiology

JID - 101548977

PMC - PMC7873932

OTO - NOTNLM

OT - NAD biosynthesis inhibition

OT - delamanid

OT - ethionamide

OT - isoniazid

OT - nicotinamide

OT - pyrazinamide

OT - toxin-antitoxin system

OT - tuberculosis

COIS- The authors declare that the research was conducted in the absence of any

commercial or financial relationships that could be construed as a potential

conflict of interest.

EDAT- 2021/02/16 06:00

MHDA- 2021/02/16 06:01

PMCR- 2021/01/26

CRDT- 2021/02/15 06:08

PHST- 2020/11/28 00:00 [received]

PHST- 2020/12/23 00:00 [accepted]

PHST- 2021/02/15 06:08 [entrez]

PHST- 2021/02/16 06:00 [pubmed]

PHST- 2021/02/16 06:01 [medline]

PHST- 2021/01/26 00:00 [pmc-release]

AID - 10.3389/fmicb.2020.634640 [doi]

PST - epublish

SO - Front Microbiol. 2021 Jan 26;11:634640. doi: 10.3389/fmicb.2020.634640.

eCollection 2020.

PMID- 35114793

OWN - NLM

STAT- MEDLINE

DCOM- 20220207

LR - 20221207

IS - 1210-7816 (Print)

IS - 1210-7816 (Linking)

VI - 70

IP - 5

DP - 2021 Winter

TI - Telacebec (Q203): Is there a novel effective and safe anti-tuberculosis drug on

the horizon?

PG - 164-171

LID - 10.5817/CSF2021-5-164 [doi]

AB - High prevalence and stronger emergency of various forms of drug-resistant

tuberculosis (DR-TB), including the multidrug-resistant (MDR-TB) as well as

extensively drug-resistant (XDR-TB) ones, caused by variously resistant

Mycobacterium tuberculosis pathogens, make first-line anti-tuberculosis (anti-TB)

agents therapeutically more and more ineffective. Therefore, there is an

imperative to develop novel highly efficient (synthetic) agents against both

drug-sensitive-TB and DR-TB. The exploration of various heterocycles as

prospective core scaffolds for the discovery, development and optimization of

anti-TB drugs remains an intriguing scientific endeavour. Telacebec (Q203; TCB),

a molecule containing an imidazo[1,2-a]pyridine-3-carboxamide (IPA) structural

motif, is considered a novel very promising anti-TB agent showing a unique

mechanism of action. The compound blocks oxidative phosphorylation by inhibiting

a mycobacterial respiratory chain due to interference with a specific cytochrome

b subunit (QcrB) of transmembrane bc1 menaquinol-cytochrome c oxidoreductase as

an essential component for transporting electrons across the membrane from

menaquinol to other specific subunit, cytochrome c (QcrC). Thus, the ability of

mycobacteria to synthesize adenosine-5´-triphosphate is limited and energy

generating machinery is disabled. The TCB molecule effectively fights

drug-susceptible, MDR as well as XDR M. tuberculosis strains. The article briefly

explains a mechanism of an anti-TB action related to the compounds containing a

variously substituted IPA scaffold and is focused on their fundamental

structure-anti-TB activity relationships as well. Special consideration is paid

to TCB indicating the importance of particular structural fragments for

maintaining (or even improving) favourable pharmacodynamic, pharmacokinetic

and/or toxicological properties. High lipophilicity of TCB might be regarded as

one of the key physicochemical properties with positive impact on anti-TB effect

of the drug. In January 2021, the TCB molecule was also involved in phase-II

clinical trials focused on the treatment of Coronavirus Disease-19 caused by

Severe Acute Respiratory Syndrome Coronavirus 2.

FAU - Malík, Ivan

AU - Malík I

FAU - Čižmárik, Jozef

AU - Čižmárik J

FAU - Kováč, Gustáv

AU - Kováč G

FAU - Pecháčová, Mária

AU - Pecháčová M

FAU - Hudecova, Lucia

AU - Hudecova L

LA - eng

PT - Journal Article

TT - Telacebek (Q203): Vynára sa na obzore nové efektívne a bezpe&#269;né

antituberkulotikum?

PL - Czech Republic

TA - Ceska Slov Farm

JT - Ceska a Slovenska farmacie : casopis Ceske farmaceuticke spolecnosti a Slovenske

farmaceuticke spolecnosti

JID - 9433765

RN - 0 (Antitubercular Agents)

RN - 0 (Imidazoles)

RN - 0 (Piperidines)

RN - 0 (Pyridines)

RN - 55G92WGH3X (telacebec)

SB - IM

MH - *Antitubercular Agents/adverse effects/pharmacology

MH - Clinical Trials, Phase II as Topic

MH - Humans

MH - *Imidazoles/adverse effects/pharmacology

MH - Mycobacterium tuberculosis/drug effects

MH - *Piperidines/adverse effects/pharmacology

MH - *Pyridines/adverse effects/pharmacology

MH - SARS-CoV-2

MH - *Tuberculosis, Multidrug-Resistant/drug therapy

MH - COVID-19 Drug Treatment

OTO - NOTNLM

OT - Mycobacterium tuberculosis

OT - drug-resistant tuberculosis

OT - imidazo[1,2-a]pyridine-3-carboxamides

OT - respiratory chain

OT - telacebec (Q203)

EDAT- 2022/02/05 06:00

MHDA- 2022/02/08 06:00

CRDT- 2022/02/04 05:34

PHST- 2022/02/04 05:34 [entrez]

PHST- 2022/02/05 06:00 [pubmed]

PHST- 2022/02/08 06:00 [medline]

AID - 128806 [pii]

AID - 10.5817/CSF2021-5-164 [doi]

PST - ppublish

SO - Ceska Slov Farm. 2021 Winter;70(5):164-171. doi: 10.5817/CSF2021-5-164.

PMID- 34340773

OWN - NLM

STAT- MEDLINE

DCOM- 20210902

LR - 20210902

IS - 1876-1631 (Electronic)

IS - 1876-1623 (Linking)

VI - 127

DP - 2021

TI - A systemic approach to explore the mechanisms of drug resistance and altered

signaling cascades in extensively drug-resistant tuberculosis.

PG - 343-364

LID - S1876-1623(21)00020-1 [pii]

LID - 10.1016/bs.apcsb.2021.02.002 [doi]

AB - BACKGROUND AND AIM: The persistence of extensively drug-resistant (XDR) strains

of Mycobacterium tuberculosis (MTB) continue to pose a significant challenge to

the treatment and control of tuberculosis infections worldwide. XDR-MTB strains

exhibit resistance against first-line anti-TB drugs, fluoroquinolones, and

second-line injectable drugs. The mechanisms of drug resistance of MTB remains

poorly understood. Our study aims at identifying the differentially expressed

genes (DEGs), associated gene networks, and signaling cascades involved in

rendering this pathogen resistant to multiple drugs, namely, isoniazid,

rifampicin, and capreomycin. METHODS: We used the microarray dataset GSE53843.

The GEO2R tool was used to prioritize the most significant DEGs (top 250) of each

drug exposure sample between XDR strains and non-resistant strains. The

validation of the 250 DEGs was performed using volcano plots. Protein-protein

interaction networks of the DEGs were created using STRING and Cytoscape tools,

which helped decipher the relationship between these genes. The significant DEGs

were functionally annotated using DAVID and ClueGO. The concomitant biological

processes (BP) and molecular functions (MF) were represented as dot plots.

RESULTS AND CONCLUSION: We identified relevant molecular pathways and biological

processes, such as cell wall biogenesis, lipid metabolic process, ion transport,

phosphopantetheine binding, and triglyceride lipase activity. These processes

indicated the involvement of multiple interconnected mechanisms in drug

resistance. Our study highlighted the impact of cell wall permeability, with the

dysregulation of the mur family of proteins, as essential factors in the

inference of resistance. Additionally, upregulation of genes responsible for ion

transport such as ctpF, arsC, and nark3, emphasizes the importance of transport

channels and efflux pumps in potentially driving out stress-inducing compounds.

This study investigated the upregulation of the Lip family of proteins, which

play a crucial role in triglyceride lipase activity. Thereby illuminating the

potential role of drug-induced dormancy and subsequent resistance in the

mycobacterial strains. Multiple mechanisms such as carboxylic acid metabolic

process, NAD biosynthetic process, triglyceride lipase activity,

phosphopantetheine binding, organic acid biosynthetic process, and growth of

symbiont in host cell were observed to partake in resistance of XDR-MTB. This

study ultimately provides a platform for important mapping targets for potential

therapeutics against XDR-MTB.

CI - Copyright © 2021 Elsevier Inc. All rights reserved.

FAU - Udhaya Kumar, S

AU - Udhaya Kumar S

AD - School of Biosciences and Technology, Vellore Institute of Technology, Vellore,

Tamil Nadu, India.

FAU - Saleem, Aisha

AU - Saleem A

AD - School of Biosciences and Technology, Vellore Institute of Technology, Vellore,

Tamil Nadu, India.

FAU - Thirumal Kumar, D

AU - Thirumal Kumar D

AD - Department of Bioinformatics, Saveetha School of Engineering, Saveetha Institute

of Medical and Technical Sciences, Chennai, Tamil Nadu, India.

FAU - Anu Preethi, V

AU - Anu Preethi V

AD - School of Computer Science and Engineering, Vellore Institute of Technology,

Vellore, Tamil Nadu, India.

FAU - Younes, Salma

AU - Younes S

AD - Translational Research Institute, Women's Wellness and Research Center, Hamad

Medical Corporation, Doha, Qatar.

FAU - Zayed, Hatem

AU - Zayed H

AD - Department of Biomedical Sciences, College of Health and Sciences, QU Health,

Qatar University, Doha, Qatar.

FAU - Tayubi, Iftikhar Aslam

AU - Tayubi IA

AD - Faculty of Computing and Information Technology, King Abdul-Aziz University,

Rabigh, Saudi Arabia.

FAU - George Priya Doss, C

AU - George Priya Doss C

AD - School of Biosciences and Technology, Vellore Institute of Technology, Vellore,

Tamil Nadu, India. Electronic address: georgepriyadoss@vit.ac.in.

LA - eng

PT - Journal Article

PT - Research Support, Non-U.S. Gov't

DEP - 20210324

PL - Netherlands

TA - Adv Protein Chem Struct Biol

JT - Advances in protein chemistry and structural biology

JID - 101497281

RN - 0 (Bacterial Proteins)

SB - IM

MH - *Bacterial Proteins/genetics/metabolism

MH - Drug Resistance, Bacterial/*genetics

MH - *Extensively Drug-Resistant Tuberculosis/genetics/metabolism

MH - *Gene Expression Regulation, Bacterial

MH - Humans

MH - *Mycobacterium tuberculosis

MH - *Systems Biology

OTO - NOTNLM

OT - Capreomycin

OT - Cluego

OT - Cluepedia

OT - Extensively drug-resistant

OT - Functional enrichment analysis

OT - Gene ontology

OT - Isoniazid

OT - Mechanisms

OT - Mycobacterium tuberculosis

OT - Regulatory networks

OT - Rifampicin

COIS- Conflict of interest The authors have declared that no conflicts of interest

exist.

EDAT- 2021/08/04 06:00

MHDA- 2021/09/03 06:00

CRDT- 2021/08/03 05:38

PHST- 2021/08/03 05:38 [entrez]

PHST- 2021/08/04 06:00 [pubmed]

PHST- 2021/09/03 06:00 [medline]

AID - S1876-1623(21)00020-1 [pii]

AID - 10.1016/bs.apcsb.2021.02.002 [doi]

PST - ppublish

SO - Adv Protein Chem Struct Biol. 2021;127:343-364. doi:

10.1016/bs.apcsb.2021.02.002. Epub 2021 Mar 24.

PMID- 33837735

OWN - NLM

STAT- MEDLINE

DCOM- 20210824

LR - 20260127

IS - 1083-351X (Electronic)

IS - 0021-9258 (Print)

IS - 0021-9258 (Linking)

VI - 296

DP - 2021 Jan-Jun

TI - Rediscovery of PF-3845 as a new chemical scaffold inhibiting phenylalanyl-tRNA

synthetase in Mycobacterium tuberculosis.

PG - 100257

LID - S0021-9258(21)00024-7 [pii]

LID - 10.1016/j.jbc.2021.100257 [doi]

LID - 100257

AB - Mycobacterium tuberculosis (Mtb) remains the deadliest pathogenic bacteria

worldwide. The search for new antibiotics to treat drug-sensitive as well as

drug-resistant tuberculosis has become a priority. The essential enzyme

phenylalanyl-tRNA synthetase (PheRS) is an antibacterial drug target because of

the large differences between bacterial and human PheRS counterparts. In a

high-throughput screening of 2148 bioactive compounds, PF-3845, which is a known

inhibitor of human fatty acid amide hydrolase, was identified inhibiting Mtb

PheRS at K(i) ∼ 0.73 ± 0.06 μM. The inhibition mechanism was studied with enzyme

kinetics, protein structural modeling, and crystallography, in comparison to a

PheRS inhibitor of the noted phenyl-thiazolylurea-sulfonamide class. The 2.3-Å

crystal structure of Mtb PheRS in complex with PF-3845 revealed its novel binding

mode, in which a trifluoromethyl-pyridinylphenyl group occupies the phenylalanine

pocket, whereas a piperidine-piperazine urea group binds into the ATP pocket

through an interaction network enforced by a sulfate ion. It represents the first

non-nucleoside bisubstrate competitive inhibitor of bacterial PheRS. PF-3845

inhibits the in vitro growth of Mtb H37Rv at ∼24 μM, and the potency of PF-3845

increased against an engineered strain Mtb pheS-FDAS, suggesting on target

activity in mycobacterial whole cells. PF-3845 does not inhibit human cytoplasmic

or mitochondrial PheRS in biochemical assay, which can be explained from the

crystal structures. Further medicinal chemistry efforts focused on the

piperidine-piperazine urea moiety may result in the identification of a selective

antibacterial lead compound.

CI - Copyright © 2021 The Authors. Published by Elsevier Inc. All rights reserved.

FAU - Wang, Heng

AU - Wang H

AD - Global Health Drug Discovery Institute, Haidian, Beijing, China.

FAU - Xu, Min

AU - Xu M

AD - Global Health Drug Discovery Institute, Haidian, Beijing, China.

FAU - Engelhart, Curtis A

AU - Engelhart CA

AD - Department of Microbiology and Immunology, Weill Cornell Medical College, New

York, New York, USA.

FAU - Zhang, Xi

AU - Zhang X

AD - Global Health Drug Discovery Institute, Haidian, Beijing, China.

FAU - Yan, Baohua

AU - Yan B

AD - Center of Protein Science Facility, Tsinghua University, Beijing, China.

FAU - Pan, Miaomiao

AU - Pan M

AD - Global Health Drug Discovery Institute, Haidian, Beijing, China.

FAU - Xu, Yuanyuan

AU - Xu Y

AD - Global Health Drug Discovery Institute, Haidian, Beijing, China.

FAU - Fan, Shilong

AU - Fan S

AD - Center of Protein Science Facility, Tsinghua University, Beijing, China.

FAU - Liu, Renhe

AU - Liu R

AD - Global Health Drug Discovery Institute, Haidian, Beijing, China.

FAU - Xu, Lan

AU - Xu L

AD - Global Health Drug Discovery Institute, Haidian, Beijing, China.

FAU - Hua, Lan

AU - Hua L

AD - Global Health Drug Discovery Institute, Haidian, Beijing, China.

FAU - Schnappinger, Dirk

AU - Schnappinger D

AD - Department of Microbiology and Immunology, Weill Cornell Medical College, New

York, New York, USA.

FAU - Chen, Shawn

AU - Chen S

AD - Global Health Drug Discovery Institute, Haidian, Beijing, China. Electronic

address: schen169@hotmail.com.

LA - eng

PT - Journal Article

PT - Research Support, Non-U.S. Gov't

DEP - 20210108

PL - United States

TA - J Biol Chem

JT - The Journal of biological chemistry

JID - 2985121R

RN - EC 3.5.- (Amidohydrolases)

RN - 0 (Anti-Bacterial Agents)

RN - EC 6.1.1.20 (Phenylalanine-tRNA Ligase)

RN - 0 (Piperidines)

RN - 0 (Pyridines)

RN - EC 3.5.1.- (Fatty Acid Amide Hydrolases)

RN - 0 (PF 3845)

SB - IM

MH - Amidohydrolases/antagonists & inhibitors/chemistry

MH - Amino Acid Sequence/genetics

MH - Anti-Bacterial Agents/chemistry

MH - Binding Sites/drug effects

MH - Crystallography, X-Ray

MH - Humans

MH - Kinetics

MH - Mycobacterium tuberculosis/drug effects/*enzymology/pathogenicity

MH - Phenylalanine-tRNA Ligase/antagonists & inhibitors/chemistry/*ultrastructure

MH - Piperidines/chemistry/pharmacology

MH - *Protein Conformation

MH - Pyridines/chemistry/pharmacology

MH - Tuberculosis, Multidrug-Resistant/*drug therapy/enzymology/genetics

MH - Fatty Acid Amide Hydrolases

PMC - PMC7948948

OTO - NOTNLM

OT - Mycobacterium tuberculosis

OT - crystallography

OT - high-throughput screening

OT - inhibition mechanism

OT - phenylalanyl-tRNA synthetase (PheRS)

COIS- Conflict of interest The authors declare that they have no conflicts of interest

with the contents of this article.

EDAT- 2021/04/11 06:00

MHDA- 2021/08/25 06:00

PMCR- 2021/01/08

CRDT- 2021/04/10 12:06

PHST- 2020/10/27 00:00 [received]

PHST- 2020/12/24 00:00 [revised]

PHST- 2021/01/04 00:00 [accepted]

PHST- 2021/04/11 06:00 [pubmed]

PHST- 2021/08/25 06:00 [medline]

PHST- 2021/04/10 12:06 [entrez]

PHST- 2021/01/08 00:00 [pmc-release]

AID - S0021-9258(21)00024-7 [pii]

AID - 100257 [pii]

AID - 10.1016/j.jbc.2021.100257 [doi]

PST - ppublish

SO - J Biol Chem. 2021 Jan-Jun;296:100257. doi: 10.1016/j.jbc.2021.100257. Epub 2021

Jan 8.

PMID- 33276981

OWN - NLM

STAT- MEDLINE

DCOM- 20210908

LR - 20220102

IS - 1090-2120 (Electronic)

IS - 0045-2068 (Print)

IS - 0045-2068 (Linking)

VI - 106

DP - 2021 Jan

TI - Design, synthesis and antimycobacterial evaluation of novel adamantane and

adamantanol analogues effective against drug-resistant tuberculosis.

PG - 104486

LID - S0045-2068(20)31784-3 [pii]

LID - 10.1016/j.bioorg.2020.104486 [doi]

AB - The treacherous nature of tuberculosis (TB) combined with the ubiquitous presence

of the drug-resistant (DR) forms pose this disease as a growing public health

menace. Therefore, it is imperative to develop new chemotherapeutic agents with a

novel mechanism of action to circumvent the cross-resistance problems. The unique

architecture of the Mycobacterium tuberculosis (M. tb) outer envelope plays a

predominant role in its pathogenesis, contributing to its intrinsic resistance

against available therapeutic agents. The mycobacterial membrane protein large 3

(MmpL3), which is a key player in forging the M. tb rigid cell wall, represents

an emerging target for TB drug development. Several indole-2-carboxamides were

previously identified in our group as potent anti-TB agents that act as inhibitor

of MmpL3 transporter protein. Despite their highly potent in vitro activities,

the lingering Achilles heel of these indoleamides can be ascribed to their high

lipophilicity as well as low water solubility. In this study, we report our

attempt to improve the aqueous solubility of these indole-2-carboxamides while

maintaining an adequate lipophilicity to allow effective M. tb cell wall

penetration. A more polar adamantanol moiety was incorporated into the framework

of several indole-2-carboxamides, whereupon the corresponding analogues were

tested for their anti-TB activity against drug-sensitive (DS) M. tb H37Rv strain.

Three adamantanol derivatives 8i, 8j and 8l showed nearly 2- and 4-fold higher

activity (MIC = 1.32 - 2.89 µM) than ethambutol (MIC = 4.89 µM). Remarkably, the

most potent adamantanol analogue 8j demonstrated high selectivity towards DS and

DR M. tb strains over mammalian cells [IC(50) (Vero cells) ≥ 169 µM], evincing

its lack of cytotoxicity. The top eight active compounds 8b, 8d, 8f, 8i, 8j, 8k,

8l and 10a retained their in vitro potency against DR M. tb strains and were

docked into the MmpL3 active site. The most potent adamantanol/adamantane-based

indoleamides 8j/8k displayed a two-fold surge in potency against extensively DR

(XDR) M. tb strains with MIC values of 0.66 and 0.012 µM, respectively. The

adamantanol-containing indole-2-carboxamides exhibited improved water solubility

both in silico and experimentally, relative to the adamantane counterparts.

Overall, the observed antimycobacterial and physicochemical profiles support the

notion that adamantanol moiety is a suitable replacement to the adamantane

scaffold within the series of indole-2-carboxamide-based MmpL3 inhibitors.

CI - Copyright © 2020 Elsevier Inc. All rights reserved.

FAU - Alsayed, Shahinda S R

AU - Alsayed SSR

AD - School of Pharmacy and Biomedical Sciences, Faculty of Health Sciences, Curtin

University, Bentley, Perth, WA 6102, Australia.

FAU - Lun, Shichun

AU - Lun S

AD - Center for Tuberculosis Research, Department of Medicine, Division of Infectious

Disease, Johns Hopkins School of Medicine, 1550, Orleans Street, Baltimore, MD

21231-1044, United States.

FAU - Payne, Alan

AU - Payne A

AD - School of Molecular and Life Sciences, Curtin University, Perth, WA 6102,

Australia.

FAU - Bishai, William R

AU - Bishai WR

AD - Center for Tuberculosis Research, Department of Medicine, Division of Infectious

Disease, Johns Hopkins School of Medicine, 1550, Orleans Street, Baltimore, MD

21231-1044, United States; Howard Hughes Medical Institute, 4000 Jones Bridge

Road, Chevy Chase, Maryland 20815-6789, United States. Electronic address:

wbishai1@jhmi.edu.

FAU - Gunosewoyo, Hendra

AU - Gunosewoyo H

AD - School of Pharmacy and Biomedical Sciences, Faculty of Health Sciences, Curtin

University, Bentley, Perth, WA 6102, Australia. Electronic address:

Hendra.Gunosewoyo@curtin.edu.au.

LA - eng

GR - R01 AI037856/AI/NIAID NIH HHS/United States

GR - R01 HL133190/HL/NHLBI NIH HHS/United States

PT - Journal Article

PT - Research Support, N.I.H., Extramural

PT - Research Support, Non-U.S. Gov't

DEP - 20201119

PL - United States

TA - Bioorg Chem

JT - Bioorganic chemistry

JID - 1303703

RN - 0 (Antitubercular Agents)

RN - PJY633525U (Adamantane)

SB - IM

MH - Adamantane/analogs & derivatives/chemistry/*pharmacology

MH - Antitubercular Agents/chemical synthesis/chemistry/*pharmacology

MH - Dose-Response Relationship, Drug

MH - *Drug Design

MH - Microbial Sensitivity Tests

MH - Models, Molecular

MH - Molecular Structure

MH - Mycobacterium tuberculosis/*drug effects

MH - Structure-Activity Relationship

MH - Tuberculosis, Multidrug-Resistant/*drug therapy

PMC - PMC7775894

MID - NIHMS1651220

OTO - NOTNLM

OT - Adamantane

OT - Adamantanol

OT - Cytotoxicity

OT - Indoleamides

OT - MDR-TB

OT - MmpL3

OT - Tuberculosis

OT - Water solubility

OT - XDR-TB

EDAT- 2020/12/06 06:00

MHDA- 2021/09/09 06:00

PMCR- 2022/01/01

CRDT- 2020/12/05 05:24

PHST- 2020/10/03 00:00 [received]

PHST- 2020/11/11 00:00 [revised]

PHST- 2020/11/16 00:00 [accepted]

PHST- 2020/12/06 06:00 [pubmed]

PHST- 2021/09/09 06:00 [medline]

PHST- 2020/12/05 05:24 [entrez]

PHST- 2022/01/01 00:00 [pmc-release]

AID - S0045-2068(20)31784-3 [pii]

AID - 10.1016/j.bioorg.2020.104486 [doi]

PST - ppublish

SO - Bioorg Chem. 2021 Jan;106:104486. doi: 10.1016/j.bioorg.2020.104486. Epub 2020

Nov 19.

PMID- 33130209

OWN - NLM

STAT- MEDLINE

DCOM- 20210315

LR - 20210315

IS - 1878-3511 (Electronic)

IS - 1201-9712 (Linking)

VI - 102

DP - 2021 Jan

TI - Acquisition of clofazimine resistance following bedaquiline treatment for

multidrug-resistant tuberculosis.

PG - 392-396

LID - S1201-9712(20)32285-2 [pii]

LID - 10.1016/j.ijid.2020.10.081 [doi]

AB - OBJECTIVES: We described the prevalence of clofazimine (CFZ) resistance in a

multidrug-resistant tuberculosis (MDR-TB) cohort in China. We also aimed to

identify dynamic changes in CFZ susceptibility and its molecular mechanism after

exposure to bedaquiline (BDQ) and/or CFZ. METHODS: The experimental settings were

conducted based on our MDR-TB cohort receiving BDQ-containing regimens.

Sequential isolates were obtained from patients. CFZ and BDQ susceptibility of

isolates were determined using the minimum inhibitory concentration (MIC) method.

The fragments of Rv0678 and pepQ were sequenced. RESULTS: A total of 277 patients

infected with MDR-TB were included in our study. CFZ resistance was noted in 23

(23/277, 8.3%) isolates. The rate of acquired CFZ resistance (12/189, 6.3%) was

significantly greater than that of primary resistance (11/88, 12.5%, P = 0.028).

Out of 23 CFZ-resistant isolates, five (5/23) were BDQ-resistant, and the other

18 (18/23) were susceptible to BDQ. Of note, nine 9/23) out of 23 CFZ-resistant

isolates had mutations within either target genes. Kaplan-Meier analysis

demonstrated that the baseline CFZ resistance had no influence on time to culture

conversion in our cohort (P = 0.828). Acquired CFZ resistance emerged in eight

(8/94, 8.5%) patients during treatment for MDR-TB, including three patients

receiving regimens without CFZ. CONCLUSIONS: Our results demonstrate the high

rate of CFZ resistance among MDR-TB patients in China. Patients treated with

BDQ-containing regimens achieve comparative culture conversion rate regardless of

baseline CFZ susceptibility. The presence of acquired CFZ-resistance following

BDQ treatment without known mutation indicates that other mechanisms conferring

cross resistance to these two compounds may exist.

CI - Copyright © 2020 The Authors. Published by Elsevier Ltd.. All rights reserved.

FAU - Liu, Yuhong

AU - Liu Y

AD - Clinical Center on TB, Beijing Chest Hospital, Capital Medical University/

Beijing Tuberculosis & Thoracic Tumor Research Institute, Beijing, 101149, P.R.

China.

FAU - Gao, Jingtao

AU - Gao J

AD - Clinical Center on TB, Beijing Chest Hospital, Capital Medical University/

Beijing Tuberculosis & Thoracic Tumor Research Institute, Beijing, 101149, P.R.

China.

FAU - Du, Jian

AU - Du J

AD - Clinical Center on TB, Beijing Chest Hospital, Capital Medical University/

Beijing Tuberculosis & Thoracic Tumor Research Institute, Beijing, 101149, P.R.

China.

FAU - Shu, Wei

AU - Shu W

AD - Clinical Center on TB, Beijing Chest Hospital, Capital Medical University/

Beijing Tuberculosis & Thoracic Tumor Research Institute, Beijing, 101149, P.R.

China.

FAU - Wang, Lu

AU - Wang L

AD - National Clinical Laboratory on Tuberculosis, Beijing Chest Hospital, Capital

Medical University/Beijing Tuberculosis & Thoracic Tumor Research Institute,

Beijing, 101149, P.R. China.

FAU - Wang, Yufeng

AU - Wang Y

AD - Department of Laboratory Quality Control, Innovation Alliance on Tuberculosis

Diagnosis and Treatment (Beijing), Beijing, 101149, P. R. China.

FAU - Xue, Zhongtan

AU - Xue Z

AD - Department of Laboratory Quality Control, Innovation Alliance on Tuberculosis

Diagnosis and Treatment (Beijing), Beijing, 101149, P. R. China.

FAU - Li, Liang

AU - Li L

AD - Clinical Center on TB, Beijing Chest Hospital, Capital Medical University/

Beijing Tuberculosis & Thoracic Tumor Research Institute, Beijing, 101149, P.R.

China. Electronic address: liliang69@tb123.org.

FAU - Xu, Shaofa

AU - Xu S

AD - Clinical Center on TB, Beijing Chest Hospital, Capital Medical University/

Beijing Tuberculosis & Thoracic Tumor Research Institute, Beijing, 101149, P.R.

China. Electronic address: xushaofa@263.net.

FAU - Pang, Yu

AU - Pang Y

AD - National Clinical Laboratory on Tuberculosis, Beijing Chest Hospital, Capital

Medical University/Beijing Tuberculosis & Thoracic Tumor Research Institute,

Beijing, 101149, P.R. China. Electronic address: pangyupound@163.com.

LA - eng

PT - Journal Article

DEP - 20201029

PL - Canada

TA - Int J Infect Dis

JT - International journal of infectious diseases : IJID : official publication of the

International Society for Infectious Diseases

JID - 9610933

RN - 0 (Antitubercular Agents)

RN - 0 (Diarylquinolines)

RN - 78846I289Y (bedaquiline)

RN - D959AE5USF (Clofazimine)

SB - IM

MH - Antitubercular Agents/*therapeutic use

MH - China

MH - Clofazimine/*pharmacology

MH - Cohort Studies

MH - Diarylquinolines/*therapeutic use

MH - Drug Resistance, Multiple, Bacterial/*drug effects

MH - Female

MH - Genotype

MH - Humans

MH - Male

MH - Microbial Sensitivity Tests

MH - Middle Aged

MH - Mutation

MH - Mycobacterium tuberculosis/*drug effects/genetics

MH - Tuberculosis, Multidrug-Resistant/*drug therapy/microbiology

OTO - NOTNLM

OT - acquisition resistance

OT - bedaquiline

OT - clofazimine

OT - multidrug-resistant

OT - tuberculosis

EDAT- 2020/11/02 06:00

MHDA- 2021/03/16 06:00

CRDT- 2020/11/01 20:31

PHST- 2020/09/08 00:00 [received]

PHST- 2020/10/20 00:00 [revised]

PHST- 2020/10/23 00:00 [accepted]

PHST- 2020/11/02 06:00 [pubmed]

PHST- 2021/03/16 06:00 [medline]

PHST- 2020/11/01 20:31 [entrez]

AID - S1201-9712(20)32285-2 [pii]

AID - 10.1016/j.ijid.2020.10.081 [doi]

PST - ppublish

SO - Int J Infect Dis. 2021 Jan;102:392-396. doi: 10.1016/j.ijid.2020.10.081. Epub

2020 Oct 29.

PMID- 32745362

OWN - NLM

STAT- MEDLINE

DCOM- 20210923

LR - 20210923

IS - 1747-0285 (Electronic)

IS - 1747-0277 (Linking)

VI - 97

IP - 1

DP - 2021 Jan

TI - Novel peptidomimetic peptide deformylase (PDF) inhibitors of Mycobacterium

tuberculosis.

PG - 148-156

LID - 10.1111/cbdd.13769 [doi]

AB - Emergence of MDR-TB and XDR-TB led to the failure of available anti-tubercular

drugs. In order to explore, identify and develop new anti-tubercular drugs, novel

peptidomimetic series of Mtb-peptide deformylase (PDF) inhibitors was designed

and synthesized. In vitro antimycobacterial potential of compounds was

established by screening of compounds against Mycobacterium tuberculosis H37Rv

strain using MABA. Among them, ester series of compounds 4a, 4b, 4c, 4d, and 4e

were found most active, with compound 4c being highly active and exhibiting

minimum inhibitory concentration of 6.25 µg/ml against M. tb H37Rv strain.

Additionally, the compounds were docked to determine the probable binding

interactions and understand the mechanism of action of most active molecules on

Mtb-peptide deformylase (PDF), which is involved in the mycobacterium protein

synthesis.

CI - © 2020 John Wiley & Sons A/S.

FAU - Gokhale, Kunal M

AU - Gokhale KM

AUID- ORCID: 0000-0002-0629-9686

AD - Department of Pharmaceutical Sciences and Technology, Institute of Chemical

Technology, Mumbai, India.

FAU - Telvekar, Vikas N

AU - Telvekar VN

AD - Department of Pharmaceutical Sciences and Technology, Institute of Chemical

Technology, Mumbai, India.

LA - eng

PT - Journal Article

DEP - 20200824

PL - England

TA - Chem Biol Drug Des

JT - Chemical biology & drug design

JID - 101262549

RN - 0 (Antitubercular Agents)

RN - 0 (Bacterial Proteins)

RN - 0 (Enzyme Inhibitors)

RN - 0 (Peptidomimetics)

RN - EC 3.5.- (Amidohydrolases)

RN - EC 3.5.1.88 (peptide deformylase)

SB - IM

MH - Amidohydrolases/*antagonists & inhibitors/metabolism

MH - Antitubercular Agents/chemistry/metabolism/pharmacology

MH - Bacterial Proteins/*antagonists & inhibitors/metabolism

MH - Binding Sites

MH - Cell Line

MH - Cell Survival/drug effects

MH - Enzyme Inhibitors/*chemistry/metabolism/pharmacology

MH - Humans

MH - Microbial Sensitivity Tests

MH - Molecular Docking Simulation

MH - Mycobacterium tuberculosis/*enzymology

MH - Peptidomimetics/*chemistry/metabolism/pharmacology

MH - Structure-Activity Relationship

OTO - NOTNLM

OT - Mycobacterium tuberculosis

OT - MABA

OT - Mtb

OT - docking

OT - peptide deformylase (PDF) inhibitor

OT - peptidomimetic

EDAT- 2020/08/04 06:00

MHDA- 2021/09/24 06:00

CRDT- 2020/08/04 06:00

PHST- 2020/05/31 00:00 [received]

PHST- 2020/07/15 00:00 [revised]

PHST- 2020/07/25 00:00 [accepted]

PHST- 2020/08/04 06:00 [pubmed]

PHST- 2021/09/24 06:00 [medline]

PHST- 2020/08/04 06:00 [entrez]

AID - 10.1111/cbdd.13769 [doi]

PST - ppublish

SO - Chem Biol Drug Des. 2021 Jan;97(1):148-156. doi: 10.1111/cbdd.13769. Epub 2020

Aug 24.

PMID- 32691718

OWN - NLM

STAT- MEDLINE

DCOM- 20211015

LR - 20211015

IS - 2212-3989 (Electronic)

IS - 1871-5265 (Linking)

VI - 21

IP - 4

DP - 2021

TI - An Experience with Delamanid in an XDR TB Case - Case Report.

PG - 637-639

LID - 10.2174/1871526520666200720113711 [doi]

AB - INTRODUCTION: Tuberculosis is an endemic infection and a serious public health

problem in India. India constitutes one-fourth of the global TB population. The

emerging drug resistance is a major threat to global tuberculosis care and

control. CASE REPORT: We present a case of newly diagnosed microbiologically

confirmed, extremely drugresistant primary pulmonary tuberculosis which was

treated with Delamanid and was found to be cured of tuberculosis. DISCUSSION:

Delamanid is a new anti-tubercular drug, which is thought to primarily inhibit

the synthesis of methoxy-mycolic, and keto-mycolic acid, which are components of

the mycobacterial cell wall. In our patient who was a newly diagnosed case of

MDR-TB converted to XDR-TB in little course of time and we were successful in

treating him with Delamanid therapy. His sputum culture conversion was achieved

in 20 days. Even though the patient did not tolerate well because of the side

effects of the drug, still he became microbiologically negative for tuberculosis.

CONCLUSION: Delamanid fulfills many target criteria for new TB drugs and may be

particularly useful for the treatment of MDR-TB. It can be administered orally

and its bactericidal properties make it suitable in regimens designed to shorten

treatment duration. Clinical efficacy data, while limited, are reassuring.

CI - Copyright© Bentham Science Publishers; For any queries, please email at

epub@benthamscience.net.

FAU - Mohan, Nimitha

AU - Mohan N

AD - Department of Microbiology, Government Medical College Hospital-32, Sector 32,

Chandigarh, India.

FAU - Kaur, Manharpreet

AU - Kaur M

AD - Department of Microbiology, Government Medical College Hospital-32, Sector 32,

Chandigarh, India.

FAU - Singhal, Lipika

AU - Singhal L

AD - Department of Pulmonary Medicine, Government Medical College Hospital-32, Sector

32, Chandigarh, India.

FAU - Saini, Varinder

AU - Saini V

AD - Department of Microbiology, Government Medical College Hospital-32, Sector 32,

Chandigarh, India.

FAU - Chander, Jagdish

AU - Chander J

AD - Department of Microbiology, Government Medical College Hospital-32, Sector 32,

Chandigarh, India.

LA - eng

PT - Case Reports

PT - Journal Article

PL - United Arab Emirates

TA - Infect Disord Drug Targets

JT - Infectious disorders drug targets

JID - 101269158

RN - 0 (Antitubercular Agents)

RN - 0 (Nitroimidazoles)

RN - 0 (OPC-67683)

RN - 0 (Oxazoles)

SB - IM

MH - Antitubercular Agents/therapeutic use

MH - *Extensively Drug-Resistant Tuberculosis/drug therapy

MH - Humans

MH - Male

MH - *Mycobacterium tuberculosis

MH - *Nitroimidazoles/therapeutic use

MH - Oxazoles/therapeutic use

OTO - NOTNLM

OT - MDR-TB.

OT - XDR-TB

OT - cure of TB

OT - delamanid

OT - drug defaulter

OT - methoxy-mycolic

EDAT- 2020/07/22 06:00

MHDA- 2021/10/16 06:00

CRDT- 2020/07/22 06:00

PHST- 2020/03/12 00:00 [received]

PHST- 2020/05/22 00:00 [revised]

PHST- 2020/05/31 00:00 [accepted]

PHST- 2020/07/22 06:00 [pubmed]

PHST- 2021/10/16 06:00 [medline]

PHST- 2020/07/22 06:00 [entrez]

AID - IDDT-EPUB-108315 [pii]

AID - 10.2174/1871526520666200720113711 [doi]

PST - ppublish

SO - Infect Disord Drug Targets. 2021;21(4):637-639. doi:

10.2174/1871526520666200720113711.

PMID- 32188396

OWN - NLM

STAT- MEDLINE

DCOM- 20210921

LR - 20210921

IS - 2212-3989 (Electronic)

IS - 1871-5265 (Linking)

VI - 21

IP - 1

DP - 2021

TI - Simultaneous Detection of Drug-resistant Mutations in Mycobacterium tuberculosis

and Determining their Role through In Silico Docking.

PG - 134-141

LID - 10.2174/1871526520666200318111140 [doi]

AB - A molecular method for diagnosis of drug-resistant Tuberculosis is Multiplex

allele-specific PCR (MAS-PCR), which is more time-efficient, and its accuracy is

studied using DNA sequencing. Also, understanding the role of mutations, when

translated to protein, in causing resistance helps in better drug designing. AIM:

To study MAS-PCR in the detection of drug resistance in comparison to DNA

sequencing in Mycobacterium tuberculosis, and understand the mechanism of

interaction of drugs with mutant proteins. METHODS: MAS-PCR was used for the

detection of drug-resistant mutations and validation was done through DNA

sequencing. MAS-PCR targeted four genes, iniA for the drug Ethambutol, rpsL and

rrs for Streptomycin, and gyrA for Fluoroquinolone resistance, respectively.

Further, the sequence data was analysed and modeled for in silico docking to

study the effect on the interaction of the anti-TB drug molecule with the target

protein. RESULTS: We identified drug-resistant mutations in four out of 95

isolates with one of them carrying a mutation at codon iniA501, two at gyrA94,

and one for both iniA501 and gyrA94 using MASPCR. DNA sequencing confirmed

drug-resistant mutations in only two isolates, whereas two others had mutation

adjacent to the target allele. Drug-protein docking showed Estimated Free Energy

of Binding to be higher for Fluoroquinolone binding with GyrA D94V mutant. Both

wild and mutant IniA interact with EMB but had no significant effect on binding

energy. CONCLUSION: DNA sequencing-based drug resistance detection of TB is more

accurate than MASPCR. Understanding the role of mutations in influencing the

drug-protein interaction will help in designing effective drug alternatives.

CI - Copyright© Bentham Science Publishers; For any queries, please email at

epub@benthamscience.net.

FAU - Nachappa, Somanna Ajjamada

AU - Nachappa SA

AD - Department of Studies in Genetics and Genomics, Genetics and Genomics Lab,

University of Mysore, Mysuru, Karnataka, India.

FAU - Neelambike, Sumana M

AU - Neelambike SM

AD - Department of Microbiology, JSS Medical College and Hospital, Mysuru, Karnataka,

India.

FAU - Sarikhani, Ahmad

AU - Sarikhani A

AD - Department of Studies in Genetics and Genomics, Genetics and Genomics Lab,

University of Mysore, Mysuru, Karnataka, India.

FAU - Ramachandra, Nallur B

AU - Ramachandra NB

AD - Department of Studies in Genetics and Genomics, Genetics and Genomics Lab,

University of Mysore, Mysuru, Karnataka, India.

LA - eng

PT - Journal Article

PL - United Arab Emirates

TA - Infect Disord Drug Targets

JT - Infectious disorders drug targets

JID - 101269158

RN - 0 (Antitubercular Agents)

RN - 0 (Pharmaceutical Preparations)

SB - IM

MH - Antitubercular Agents

MH - Computer Simulation

MH - Humans

MH - Microbial Sensitivity Tests

MH - Mutation

MH - *Mycobacterium tuberculosis

MH - *Pharmaceutical Preparations

OTO - NOTNLM

OT - Tuberculosis

OT - docking

OT - drug-resistant

OT - ethambutol

OT - fluoroquinolone

OT - mutations

OT - resistance

EDAT- 2020/03/20 06:00

MHDA- 2021/09/22 06:00

CRDT- 2020/03/20 06:00

PHST- 2019/10/04 00:00 [received]

PHST- 2020/02/12 00:00 [revised]

PHST- 2020/02/15 00:00 [accepted]

PHST- 2020/03/20 06:00 [pubmed]

PHST- 2021/09/22 06:00 [medline]

PHST- 2020/03/20 06:00 [entrez]

AID - IDDT-EPUB-105341 [pii]

AID - 10.2174/1871526520666200318111140 [doi]

PST - ppublish

SO - Infect Disord Drug Targets. 2021;21(1):134-141. doi:

10.2174/1871526520666200318111140.

PMID- 33374765

OWN - NLM

STAT- PubMed-not-MEDLINE

LR - 20210218

IS - 2079-6382 (Print)

IS - 2079-6382 (Electronic)

IS - 2079-6382 (Linking)

VI - 10

IP - 1

DP - 2020 Dec 23

TI - Identification of Mutations Conferring Tryptanthrin Resistance to Mycobacterium

smegmatis.

LID - 10.3390/antibiotics10010006 [doi]

LID - 6

AB - Tuberculosis (TB), caused by Mycobacterium tuberculosis, is a global burden,

responsible for over 1 million deaths annually. The emergence and spread of

drug-resistant M. tuberculosis strains (MDR-, XDR- and TDR-TB) is the main

challenge in global TB-control, requiring the development of novel drugs acting

on new biotargets, thus able to overcome the drug-resistance. Tryptanthrin is a

natural alkaloid, with great therapeutic potential due to its simple way of

synthesis and wide spectrum of biological activities including high bactericidal

activity on both drug-susceptible and MDR M. tuberculosis strains. InhA was

suggested as the target of tryptanthrins by in silico modeling, making it a

promising alternative to isoniazid, able to overcome drug resistance provided by

katG mutations. However, neither the mechanism of action of tryptanthrin nor the

mechanism of resistance to tryptanthrins was ever confirmed in vitro. We show

that the MmpS5-MmpL5 efflux system is able to provide resistance to tryptanthrins

using an in-house test-system. Comparative genomic analysis of spontaneous

tryptanthrin-resistant M. smegmatis mutants showed that mutations in MSMEG_1963

(EmbR transcriptional regulator) lead to a high-level resistance, while those in

MSMEG_5597 (TetR transcriptional regulator) to a low-level one. Mutations in an

MFS transporter gene (MSMEG_4427) were also observed, which might be involved in

providing a basal level of tryptanthrins-resistance.

FAU - Frolova, Svetlana G

AU - Frolova SG

AD - Laboratory of Bacterial Genetics, Vavilov Institute of General Genetics, Russian

Academy of Sciences, Moscow 119333, Russia.

AD - Moscow Institute of Physics and Technology (State University), Dolgoprudny

141701, Russia.

FAU - Klimina, Ksenia M

AU - Klimina KM

AD - Laboratory of Bacterial Genetics, Vavilov Institute of General Genetics, Russian

Academy of Sciences, Moscow 119333, Russia.

AD - Department of Molecular Biology and Genetics, Federal Research and Clinical

Center of Physical-Chemical Medicine of Federal Medical Biological Agency, Moscow

119435, Russia.

FAU - Kumar, Ravinder

AU - Kumar R

AD - Department of Chemistry and Centre of Advanced Studies in Chemistry, Panjab

University, Chandigarh 160014, India.

FAU - Vatlin, Aleksey A

AU - Vatlin AA

AD - Laboratory of Bacterial Genetics, Vavilov Institute of General Genetics, Russian

Academy of Sciences, Moscow 119333, Russia.

AD - Department of Design of Functional Foods and Nutritionology, Moscow State

University of Food Production, Moscow 109316, Russia.

FAU - Salunke, Deepak B

AU - Salunke DB

AD - Department of Chemistry and Centre of Advanced Studies in Chemistry, Panjab

University, Chandigarh 160014, India.

AD - National Interdisciplinary Centre of Vaccine, Immunotherapeutics and

Antimicrobials, Panjab University, Chandigarh 160014, India.

FAU - Kendrekar, Pravin

AU - Kendrekar P

AUID- ORCID: 0000-0002-1308-4210

AD - Unit for Drug Discovery Research (UDDR), Department of Health Sciences, Central

University of Technology, Bloemfontein 9300, South Africa.

FAU - Danilenko, Valery N

AU - Danilenko VN

AD - Laboratory of Bacterial Genetics, Vavilov Institute of General Genetics, Russian

Academy of Sciences, Moscow 119333, Russia.

FAU - Maslov, Dmitry A

AU - Maslov DA

AUID- ORCID: 0000-0003-4296-5815

AD - Laboratory of Bacterial Genetics, Vavilov Institute of General Genetics, Russian

Academy of Sciences, Moscow 119333, Russia.

LA - eng

GR - 17-75-20060-P/Russian Science Foundation/

GR - MK-797.2020.4/Grant of the President of Russian Federation for young scientists -

candidates of science/

PT - Journal Article

DEP - 20201223

PL - Switzerland

TA - Antibiotics (Basel)

JT - Antibiotics (Basel, Switzerland)

JID - 101637404

PMC - PMC7823563

OTO - NOTNLM

OT - drug resistance

OT - efflux

OT - mycobacterium smegmatis

OT - tryptanthrin

OT - tuberculosis

COIS- The authors declare no conflict of interest.

EDAT- 2020/12/31 06:00

MHDA- 2020/12/31 06:01

PMCR- 2020/12/23

CRDT- 2020/12/30 01:02

PHST- 2020/12/03 00:00 [received]

PHST- 2020/12/21 00:00 [revised]

PHST- 2020/12/22 00:00 [accepted]

PHST- 2020/12/30 01:02 [entrez]

PHST- 2020/12/31 06:00 [pubmed]

PHST- 2020/12/31 06:01 [medline]

PHST- 2020/12/23 00:00 [pmc-release]

AID - antibiotics10010006 [pii]

AID - antibiotics-10-00006 [pii]

AID - 10.3390/antibiotics10010006 [doi]

PST - epublish

SO - Antibiotics (Basel). 2020 Dec 23;10(1):6. doi: 10.3390/antibiotics10010006.

PMID- 33308139

OWN - NLM

STAT- MEDLINE

DCOM- 20210104

LR - 20231110

IS - 1471-2105 (Electronic)

IS - 1471-2105 (Linking)

VI - 21

IP - Suppl 17

DP - 2020 Dec 14

TI - Moving forward through the in silico modeling of tuberculosis: a further step

with UISS-TB.

PG - 458

LID - 10.1186/s12859-020-03762-5 [doi]

LID - 458

AB - BACKGROUND: In 2018, about 10 million people were found infected by tuberculosis,

with approximately 1.2 million deaths worldwide. Despite these numbers have been

relatively stable in recent years, tuberculosis is still considered one of the

top 10 deadliest diseases worldwide. Over the years, Mycobacterium tuberculosis

has developed a form of resistance to first-line tuberculosis treatments,

specifically to isoniazid, leading to multi-drug-resistant tuberculosis. In this

context, the EU and Indian DBT funded project STriTuVaD-In Silico Trial for

Tuberculosis Vaccine Development-is supporting the identification of new

interventional strategies against tuberculosis thanks to the use of Universal

Immune System Simulator (UISS), a computational framework capable of predicting

the immunity induced by specific drugs such as therapeutic vaccines and

antibiotics. RESULTS: Here, we present how UISS accurately simulates tuberculosis

dynamics and its interaction within the immune system, and how it predicts the

efficacy of the combined action of isoniazid and RUTI vaccine in a specific

digital population cohort. Specifically, we simulated two groups of 100 digital

patients. The first group was treated with isoniazid only, while the second one

was treated with the combination of RUTI vaccine and isoniazid, according to the

dosage strategy described in the clinical trial design. UISS-TB shows to be in

good agreement with clinical trial results suggesting that RUTI vaccine may favor

a partial recover of infected lung tissue. CONCLUSIONS: In silico trials

innovations represent a powerful pipeline for the prediction of the effects of

specific therapeutic strategies and related clinical outcomes. Here, we present a

further step in UISS framework implementation. Specifically, we found that the

simulated mechanism of action of RUTI and INH are in good alignment with the

results coming from past clinical phase IIa trials.

FAU - Russo, Giulia

AU - Russo G

AD - Department of Drug Sciences, University of Catania, 95125, Catania, Italy.

FAU - Sgroi, Giuseppe

AU - Sgroi G

AD - Department of Mathematics and Computer Science, University of Catania, 95125,

Catania, Italy.

FAU - Parasiliti Palumbo, Giuseppe Alessandro

AU - Parasiliti Palumbo GA

AD - Department of Mathematics and Computer Science, University of Catania, 95125,

Catania, Italy.

FAU - Pennisi, Marzio

AU - Pennisi M

AD - Computer Science Institute, DiSIT, University of Eastern Piedmont, 15121,

Alessandria, Italy.

FAU - Juarez, Miguel A

AU - Juarez MA

AD - School of Mathematics and Statistics, University of Sheffield, Sheffield, S3 7RH,

UK.

FAU - Cardona, Pere-Joan

AU - Cardona PJ

AD - Archivel Farma, S.L., 08916, Badalona, Spain.

AD - Experimental Tuberculosis Unit (UTE), Fundació Institut Germans Trias I Pujol

(IGTP), Universitat Autònoma de Barcelona (UAB), Badalona, Spain.

AD - Centro de Investigación Biomédica en Red (CIBER) de Enfermedades Respiratorias,

Madrid, Spain.

FAU - Motta, Santo

AU - Motta S

AD - National Research Council of Italy, 00185, Rome, Italy.

FAU - Walker, Kenneth B

AU - Walker KB

AD - TuBerculosis Vaccine Initiative (TBVI), Lelystad, 8219, The Netherlands.

FAU - Fichera, Epifanio

AU - Fichera E

AD - Etna Biotech S.r.l., 95121, Catania, Italy.

FAU - Viceconti, Marco

AU - Viceconti M

AD - Department of Industrial Engineering, University of Bologna, 40136, Bologna,

Italy.

FAU - Pappalardo, Francesco

AU - Pappalardo F

AD - Department of Drug Sciences, University of Catania, 95125, Catania, Italy.

francesco.pappalardo@unict.it.

LA - eng

GR - 777123/Horizon 2020 Framework Programme/

PT - Journal Article

DEP - 20201214

PL - England

TA - BMC Bioinformatics

JT - BMC bioinformatics

JID - 100965194

RN - 0 (Antitubercular Agents)

RN - 0 (Tuberculosis Vaccines)

RN - V83O1VOZ8L (Isoniazid)

SB - IM

MH - Antitubercular Agents/therapeutic use

MH - Computational Biology/*methods

MH - Immune System/immunology

MH - Isoniazid/therapeutic use

MH - Treatment Outcome

MH - Tuberculosis/drug therapy/*immunology/metabolism/prevention & control

MH - Tuberculosis Vaccines/immunology

MH - *User-Computer Interface

PMC - PMC7733696

OTO - NOTNLM

OT - Computational modeling

OT - Immunity

OT - In silico trials

OT - Isoniazid

OT - RUTI

OT - Therapeutic strategies

OT - Tuberculosis

COIS- The authors declare that they have no competing interests.

EDAT- 2020/12/15 06:00

MHDA- 2021/01/05 06:00

PMCR- 2020/12/14

CRDT- 2020/12/14 09:51

PHST- 2020/09/14 00:00 [received]

PHST- 2020/09/17 00:00 [accepted]

PHST- 2020/12/14 09:51 [entrez]

PHST- 2020/12/15 06:00 [pubmed]

PHST- 2021/01/05 06:00 [medline]

PHST- 2020/12/14 00:00 [pmc-release]

AID - 10.1186/s12859-020-03762-5 [pii]

AID - 3762 [pii]

AID - 10.1186/s12859-020-03762-5 [doi]

PST - epublish

SO - BMC Bioinformatics. 2020 Dec 14;21(Suppl 17):458. doi:

10.1186/s12859-020-03762-5.

PMID- 33277558

OWN - NLM

STAT- MEDLINE

DCOM- 20210329

LR - 20240501

IS - 2045-2322 (Electronic)

IS - 2045-2322 (Linking)

VI - 10

IP - 1

DP - 2020 Dec 4

TI - High-throughput screening to discover inhibitors of the CarD·RNA polymerase

protein-protein interaction in Mycobacterium tuberculosis.

PG - 21309

LID - 10.1038/s41598-020-78269-3 [doi]

LID - 21309

AB - Multidrug-resistant Mycobacterium tuberculosis (MDR-TB) accounts for 3.7% of new

cases of TB annually worldwide and is a major threat to global public health. Due

to the prevalence of the MDR-TB and extensively drug resistant tuberculosis

(XDR-TB) cases, there is an urgent need for new drugs with novel mechanisms of

action. CarD, a global transcription regulator in MTB, binds RNAP and activates

transcription by stabilizing the transcription initiation open-promoter complex

(RPo). CarD is required for MTB viability and it has highly conserved homologues

in many eubacteria. A fluorescence polarization (FP) assay which monitors the

association of MTB RNAP, native rRNA promoter DNA and CarD has been developed.

Overall, our objective is to identify and characterize small molecule inhibitors

which block the CarD/RNAP interaction and to understand the mechanisms by which

CarD interacts with the molecules. We expect that the development of a new and

improved anti-TB compound with a novel mechanism of action will relieve the

burden of resistance. This CarD FP assay is amenable to HTS and is an enabling

tool for future novel therapeutic discovery.

FAU - Stefan, Maxwell A

AU - Stefan MA

AD - Department of Medicinal Chemistry, University of Michigan, Ann Arbor, MI, USA.

FAU - Velazquez, Glory M

AU - Velazquez GM

AD - Department of Medicinal Chemistry, University of Michigan, Ann Arbor, MI, USA.

FAU - Garcia, George A

AU - Garcia GA

AD - Department of Medicinal Chemistry, University of Michigan, Ann Arbor, MI, USA.

gagarcia@umich.edu.

LA - eng

GR - R01 AI110780/AI/NIAID NIH HHS/United States

GR - R01-AI110780/NH/NIH HHS/United States

PT - Journal Article

PT - Research Support, N.I.H., Extramural

PT - Research Support, Non-U.S. Gov't

PT - Validation Study

DEP - 20201204

PL - England

TA - Sci Rep

JT - Scientific reports

JID - 101563288

RN - EC 2.7.7.6 (DNA-Directed RNA Polymerases)

SB - IM

MH - DNA-Directed RNA Polymerases/*antagonists & inhibitors/metabolism

MH - *Fluorescence Polarization

MH - High-Throughput Screening Assays

MH - Mycobacterium tuberculosis/*enzymology

PMC - PMC7718890

COIS- The authors declare no competing interests.

EDAT- 2020/12/06 06:00

MHDA- 2021/03/30 06:00

PMCR- 2020/12/04

CRDT- 2020/12/05 05:30

PHST- 2020/10/07 00:00 [received]

PHST- 2020/11/23 00:00 [accepted]

PHST- 2020/12/05 05:30 [entrez]

PHST- 2020/12/06 06:00 [pubmed]

PHST- 2021/03/30 06:00 [medline]

PHST- 2020/12/04 00:00 [pmc-release]

AID - 10.1038/s41598-020-78269-3 [pii]

AID - 78269 [pii]

AID - 10.1038/s41598-020-78269-3 [doi]

PST - epublish

SO - Sci Rep. 2020 Dec 4;10(1):21309. doi: 10.1038/s41598-020-78269-3.

PMID- 33343518

OWN - NLM

STAT- PubMed-not-MEDLINE

LR - 20210110

IS - 1664-302X (Print)

IS - 1664-302X (Electronic)

IS - 1664-302X (Linking)

VI - 11

DP - 2020

TI - Characterization of the Mycobacterial MSMEG-3762/63 Efflux Pump in Mycobacterium

smegmatis Drug Efflux.

PG - 575828

LID - 10.3389/fmicb.2020.575828 [doi]

LID - 575828

AB - Multi-drug resistant tuberculosis (MDR-TB) represents a major health problem

worldwide. Drug efflux and the activity of efflux transporters likely play

important roles in the development of drug-tolerant and drug-resistant

mycobacterial phenotypes. This study is focused on the action of a mycobacterial

efflux pump as a mechanism of drug resistance. Previous studies demonstrated

up-regulation of the TetR-like transcriptional regulator MSMEG_3765 in

Mycobacterium smegmatis and its ortholog Rv1685c in Mycobacterium tuberculosis

(Mtb) in acid-nitrosative stress conditions. MSMEG-3765 regulates the expression

of the MSMEG_3762/63/65 operon, and of the orthologous region in Mtb

(Rv1687c/86c/85c). MSMEG-3762 and Rv1687c are annotated as ATP-binding proteins,

while MSMEG-3763 and Rv1686c are annotated as trans-membrane polypeptides,

defining an ABC efflux pump in both M. smegmatis and Mtb. The two putative efflux

systems share a high percentage of identity. To examine the role of the putative

efflux system MSMEG-3762/63, we constructed and characterized a MSMEG-3763

deletion mutant in M. smegmatis (∆MSMEG_3763). By comparative analysis of wild

type, knockout, and complemented strains, together with structural modeling and

molecular docking bioinformatics analyses of the MSMEG-3763 trans-membrane

protein, we define the protein complex MSMEG-3762/63 as an efflux pump. Moreover,

we demonstrate involvement of this pump in biofilm development and in the

extrusion of rifampicin and ciprofloxacin (CIP), antimicrobial drugs used in

first- and second-line anti-TB therapies.

CI - Copyright © 2020 De Siena, Campolattano, D’Abrosca, Russo, Cantillon, Marasco,

Muscariello, Waddell and Sacco.

FAU - De Siena, Barbara

AU - De Siena B

AD - Dipartimento di Scienze e Tecnologie Ambientali Biologiche e Farmaceutiche,

Università degli Studi della Campania Luigi Vanvitelli, Caserta, Italy.

FAU - Campolattano, Nicoletta

AU - Campolattano N

AD - Dipartimento di Scienze e Tecnologie Ambientali Biologiche e Farmaceutiche,

Università degli Studi della Campania Luigi Vanvitelli, Caserta, Italy.

FAU - D'Abrosca, Gianluca

AU - D'Abrosca G

AD - Dipartimento di Scienze e Tecnologie Ambientali Biologiche e Farmaceutiche,

Università degli Studi della Campania Luigi Vanvitelli, Caserta, Italy.

FAU - Russo, Luigi

AU - Russo L

AD - Dipartimento di Scienze e Tecnologie Ambientali Biologiche e Farmaceutiche,

Università degli Studi della Campania Luigi Vanvitelli, Caserta, Italy.

FAU - Cantillon, Daire

AU - Cantillon D

AD - Department of Global Health and Infection, Brighton and Sussex Medical School,

University of Sussex, Brighton, United Kingdom.

FAU - Marasco, Rosangela

AU - Marasco R

AD - Dipartimento di Scienze e Tecnologie Ambientali Biologiche e Farmaceutiche,

Università degli Studi della Campania Luigi Vanvitelli, Caserta, Italy.

FAU - Muscariello, Lidia

AU - Muscariello L

AD - Dipartimento di Scienze e Tecnologie Ambientali Biologiche e Farmaceutiche,

Università degli Studi della Campania Luigi Vanvitelli, Caserta, Italy.

FAU - Waddell, Simon J

AU - Waddell SJ

AD - Department of Global Health and Infection, Brighton and Sussex Medical School,

University of Sussex, Brighton, United Kingdom.

FAU - Sacco, Margherita

AU - Sacco M

AD - Dipartimento di Scienze e Tecnologie Ambientali Biologiche e Farmaceutiche,

Università degli Studi della Campania Luigi Vanvitelli, Caserta, Italy.

LA - eng

GR - WT_/Wellcome Trust/United Kingdom

PT - Journal Article

DEP - 20201203

PL - Switzerland

TA - Front Microbiol

JT - Frontiers in microbiology

JID - 101548977

PMC - PMC7744416

OTO - NOTNLM

OT - 3D structure modeling

OT - antimicrobial drugs

OT - biofilm

OT - efflux pump

OT - multi drug resistance

OT - mycobacteria

OT - transmembrane protein

COIS- The authors declare that the research was conducted in the absence of any

commercial or financial relationships that could be construed as a potential

conflict of interest.

EDAT- 2020/12/22 06:00

MHDA- 2020/12/22 06:01

PMCR- 2020/12/03

CRDT- 2020/12/21 06:04

PHST- 2020/06/24 00:00 [received]

PHST- 2020/11/10 00:00 [accepted]

PHST- 2020/12/21 06:04 [entrez]

PHST- 2020/12/22 06:00 [pubmed]

PHST- 2020/12/22 06:01 [medline]

PHST- 2020/12/03 00:00 [pmc-release]

AID - 10.3389/fmicb.2020.575828 [doi]

PST - epublish

SO - Front Microbiol. 2020 Dec 3;11:575828. doi: 10.3389/fmicb.2020.575828.

eCollection 2020.

PMID- 33032201

OWN - NLM

STAT- MEDLINE

DCOM- 20210621

LR - 20210621

IS - 1873-4243 (Electronic)

IS - 1093-3263 (Linking)

VI - 101

DP - 2020 Dec

TI - An integrated computational investigation to unveil the structural impacts of

mutation on the InhA structural gene of Mycobacterium tuberculosis.

PG - 107768

LID - S1093-3263(20)30557-X [pii]

LID - 10.1016/j.jmgm.2020.107768 [doi]

AB - Growing concern about the difficulty in diagnosis and treatments of

drug-resistant tuberculosis falls under the major global health issues. There is

an urgent need for finding novel strategies to develop drugs or bioactive

molecules against the global threat of Mycobacterium tuberculosis (MTB).

Isoniazid (INH) is a front line drug against tuberculosis; it primarily targets

the enoyl-acyl carrier protein reductase (InhA), a potent drug target in the

mycolic acid pathway of MTB. To gain deeper insight into the impact of INH

resistant mutation and its influence on the structural dynamics of InhA, combined

conformational dynamics and residue interaction network (RIN) studies were

performed. The molecular dynamics investigation provided a hint about the

structural changes altering protein activity. The principal component analysis

(PCA) based free energy landscape plot highlighted the highest stable part of

wild-type (WT) and mutant structures. Intriguingly, the mutation at the 78(th)

position of InhA from its native residue valine to alanine increases the

structural stability with higher NADH binding affinity. The MM-PBSA based binding

energy calculations confirm that electrostatic interactions played a critical

role in the binding of NADH at the binding site of InhA. The calculated binding

energy score, as well as potential hydrogen bonds and salt bridge networks,

proved the strong binding of mutant InhA as compared to WT. Further, the mutation

potentially altered the protein network topology, thereby subsequently affected

the landscape of NADH binding. The present study is an attempt to understand the

structural and functional impact associated with a drug-resistant mutation (V78A)

thus it will be helpful in designing potent inhibitors against drug-resistant

tuberculosis.

CI - Copyright © 2020 Elsevier Inc. All rights reserved.

FAU - Jayaraman, Manikandan

AU - Jayaraman M

AD - Centre for Bioinformatics, School of Life Sciences, Pondicherry University,

Puducherry, 605014, India.

FAU - Ramadas, Krishna

AU - Ramadas K

AD - Centre for Bioinformatics, School of Life Sciences, Pondicherry University,

Puducherry, 605014, India. Electronic address: ramadaskr@gmail.com.

LA - eng

PT - Journal Article

PT - Research Support, Non-U.S. Gov't

DEP - 20200930

PL - United States

TA - J Mol Graph Model

JT - Journal of molecular graphics & modelling

JID - 9716237

RN - 0 (Antitubercular Agents)

RN - 0 (Bacterial Proteins)

RN - EC 1.- (Oxidoreductases)

RN - V83O1VOZ8L (Isoniazid)

SB - IM

MH - Antitubercular Agents/pharmacology

MH - Bacterial Proteins/genetics

MH - Isoniazid/pharmacology

MH - Mutation

MH - *Mycobacterium tuberculosis/genetics

MH - Oxidoreductases/genetics

OTO - NOTNLM

OT - Binding free energy

OT - Free energy landscape

OT - InhA

OT - Molecular dynamics simulation

OT - Principal component analysis

OT - Residue interaction network

COIS- Declaration of competing interest The authors declare that they have no known

competing financial interests or personal relationships that could have appeared

to influence the work reported in this paper.

EDAT- 2020/10/09 06:00

MHDA- 2021/06/22 06:00

CRDT- 2020/10/08 20:19

PHST- 2020/07/28 00:00 [received]

PHST- 2020/09/26 00:00 [revised]

PHST- 2020/09/27 00:00 [accepted]

PHST- 2020/10/09 06:00 [pubmed]

PHST- 2021/06/22 06:00 [medline]

PHST- 2020/10/08 20:19 [entrez]

AID - S1093-3263(20)30557-X [pii]

AID - 10.1016/j.jmgm.2020.107768 [doi]

PST - ppublish

SO - J Mol Graph Model. 2020 Dec;101:107768. doi: 10.1016/j.jmgm.2020.107768. Epub

2020 Sep 30.

PMID- 32848049

OWN - NLM

STAT- MEDLINE

DCOM- 20240724

LR - 20240726

IS - 1539-7262 (Electronic)

IS - 0022-2275 (Print)

IS - 0022-2275 (Linking)

VI - 61

IP - 12

DP - 2020 Dec

TI - The anti-tubercular activity of simvastatin is mediated by cholesterol-driven

autophagy via the AMPK-mTORC1-TFEB axis.

PG - 1617-1628

LID - S0022-2275(20)60023-2 [pii]

LID - 10.1194/jlr.RA120000895 [doi]

AB - The rise of drug-resistant tuberculosis poses a major risk to public health.

Statins, which inhibit both cholesterol biosynthesis and protein prenylation

branches of the mevalonate pathway, increase anti-tubercular antibiotic efficacy

in animal models. However, the underlying molecular mechanisms are unknown. In

this study, we used an in vitro macrophage infection model to investigate

simvastatin's anti-tubercular activity by systematically inhibiting each branch

of the mevalonate pathway and evaluating the effects of the branch-specific

inhibitors on mycobacterial growth. The anti-tubercular activity of simvastatin

used at clinically relevant doses specifically targeted the cholesterol

biosynthetic branch rather than the prenylation branches of the mevalonate

pathway. Using Western blot analysis and AMP/ATP measurements, we found that

simvastatin treatment blocked activation of mechanistic target of rapamycin

complex 1 (mTORC1), activated AMP-activated protein kinase (AMPK) through

increased intracellular AMP:ATP ratios, and favored nuclear translocation of

transcription factor EB (TFEB). These mechanisms all induce autophagy, which is

anti-mycobacterial. The biological effects of simvastatin on the

AMPK-mTORC1-TFEB-autophagy axis were reversed by adding exogenous cholesterol to

the cells. Our data demonstrate that the anti-tubercular activity of simvastatin

requires inhibiting cholesterol biosynthesis, reveal novel links between

cholesterol homeostasis, the AMPK-mTORC1-TFEB axis, and Mycobacterium

tuberculosis infection control, and uncover new anti-tubercular therapy targets.

CI - Copyright © 2020 Bruiners et al. Published by The American Society for

Biochemistry and Molecular Biology, Inc.

FAU - Bruiners, Natalie

AU - Bruiners N

AD - Public Health Research Institute, New Jersey Medical School, Rutgers, The State

University of New Jersey, Newark, NJ, USA.

FAU - Dutta, Noton K

AU - Dutta NK

AD - Center for Tuberculosis Research, Department of Medicine, Johns Hopkins

University School of Medicine, Baltimore, MD, USA.

FAU - Guerrini, Valentina

AU - Guerrini V

AD - Public Health Research Institute, New Jersey Medical School, Rutgers, The State

University of New Jersey, Newark, NJ, USA.

FAU - Salamon, Hugh

AU - Salamon H

AD - Knowledge Synthesis Inc., Berkeley, CA, USA.

FAU - Yamaguchi, Ken D

AU - Yamaguchi KD

AD - Knowledge Synthesis Inc., Berkeley, CA, USA.

FAU - Karakousis, Petros C

AU - Karakousis PC

AD - Center for Tuberculosis Research, Department of Medicine, Johns Hopkins

University School of Medicine, Baltimore, MD, USA; Department of International

Health, Johns Hopkins Bloomberg School of Public Health, Baltimore, MD, USA.

FAU - Gennaro, Maria L

AU - Gennaro ML

AD - Public Health Research Institute, New Jersey Medical School, Rutgers, The State

University of New Jersey, Newark, NJ, USA. Electronic address:

marila.gennaro@rutgers.edu.

LA - eng

GR - R01 AI104615/AI/NIAID NIH HHS/United States

GR - UH2 AI122309/AI/NIAID NIH HHS/United States

GR - UH3 AI122309/AI/NIAID NIH HHS/United States

GR - UL1 TR003017/TR/NCATS NIH HHS/United States

GR - R01 HL149450/HL/NHLBI NIH HHS/United States

PT - Journal Article

PT - Research Support, N.I.H., Extramural

DEP - 20200826

PL - United States

TA - J Lipid Res

JT - Journal of lipid research

JID - 0376606

RN - AGG2FN16EV (Simvastatin)

RN - EC 2.7.11.1 (Mechanistic Target of Rapamycin Complex 1)

RN - EC 2.7.11.31 (AMP-Activated Protein Kinases)

RN - 97C5T2UQ7J (Cholesterol)

RN - 0 (Antitubercular Agents)

RN - 0 (Basic Helix-Loop-Helix Leucine Zipper Transcription Factors)

RN - 0 (TFEB protein, human)

MH - *Simvastatin/pharmacology

MH - *Mechanistic Target of Rapamycin Complex 1/metabolism

MH - *Autophagy/drug effects

MH - *AMP-Activated Protein Kinases/metabolism

MH - *Cholesterol/metabolism/biosynthesis

MH - *Antitubercular Agents/pharmacology

MH - *Basic Helix-Loop-Helix Leucine Zipper Transcription Factors/metabolism

MH - Humans

MH - Mycobacterium tuberculosis/drug effects

MH - Animals

MH - Mice

MH - Macrophages/metabolism/drug effects/microbiology

PMC - PMC7707180

OTO - NOTNLM

OT - Mycobacterium tuberculosis

OT - adenosine 5′-monophosphate-activated protein kinase-mechanistic target of

rapamycin complex 1-transcription factor EB axis

OT - immunology

OT - lipids

OT - macrophages/monocytes

OT - mechanistic target of rapamycin complex 1 regulation

OT - statins

COIS- Conflict of interest—The authors declare that they have no conflicts of interest

with the contents of this article.

EDAT- 2020/08/28 06:00

MHDA- 2021/10/26 06:00

PMCR- 2020/08/26

CRDT- 2020/08/28 06:00

PHST- 2020/08/28 06:00 [pubmed]

PHST- 2021/10/26 06:00 [medline]

PHST- 2020/08/28 06:00 [entrez]

PHST- 2020/08/26 00:00 [pmc-release]

AID - S0022-2275(20)60023-2 [pii]

AID - rA120000895 [pii]

AID - 10.1194/jlr.RA120000895 [doi]

PST - ppublish

SO - J Lipid Res. 2020 Dec;61(12):1617-1628. doi: 10.1194/jlr.RA120000895. Epub 2020

Aug 26.

PMID- 32829153

OWN - NLM

STAT- MEDLINE

DCOM- 20211101

LR - 20211101

IS - 1873-281X (Electronic)

IS - 1472-9792 (Linking)

VI - 125

DP - 2020 Dec

TI - Molecular analysis of streptomycin-resistance associating genes in Mycobacterium

tuberculosis isolates from Nepal.

PG - 101985

LID - S1472-9792(20)30152-9 [pii]

LID - 10.1016/j.tube.2020.101985 [doi]

AB - Mutation in rpsL (encoding ribosomal protein S12), rrs (encoding 16S ribosomal

RNA) and gidB (encoding 7-methylguanosine methyltransferase) are associated with

resistance to streptomycin (STR), which is used for the treatment of multi-drug

resistant tuberculosis (MDR-TB) in Nepal. The aim of our study is to analyze the

correlation between mutations in the target genes and STR-resistance in 197

Mycobacterium tuberculosis (MTB) isolates from Nepal. Mutations in rpsL was

harbored by 65.9% of isolates, in which the most common mutation in rpsL is

caused by K43R (58.8%) and were significantly associated with Beijing genotype

(P < 0.001). About 13.2% of isolates harbored mutations in two highly mutable

regions of rrs, the 530 loop and the 912 region. About 13.2% of gidB mutants do

not show any mutation in rpsL and rrs, which might suggest the role of gidB

mutations in STR-resistance in MTB. In addition, 5.6% of isolates do not show any

mutations in three genes examined, suggesting the involvement of other mechanism

in STR-resistance in MTB. Our findings can be implemented for the establishment

of molecular STR-susceptibility testing, in which tuberculosis can be treated

with appropriate drugs and can improve control strategies for DR-TB.

CI - Copyright © 2020 Elsevier Ltd. All rights reserved.

FAU - Shrestha, Dipti

AU - Shrestha D

AD - Division of Bioresources, Hokkaido University Research Center for Zoonosis

Control, Kita 20, Nishi 10, Kita-ku, Sapporo, 001-0020, Japan.

FAU - Maharjan, Bhagwan

AU - Maharjan B

AD - German Nepal Tuberculosis Project c/o Nepal Anti-Tuberculosis Association,

Kalimati, Kathmandu, Post Box 1494, Nepal.

FAU - Thida Oo, Nan Aye

AU - Thida Oo NA

AD - Division of Bioresources, Hokkaido University Research Center for Zoonosis

Control, Kita 20, Nishi 10, Kita-ku, Sapporo, 001-0020, Japan; Bacteriology

Research Division, Department of Medical Research, Ministry of Health and Sports,

Myanmar.

FAU - Isoda, Norikazu

AU - Isoda N

AD - The Global Station for Zoonosis Control, Global Institution for Collaborative

Research and Education (GI-Core), Hokkaido University, Kita 20, Nishi 10,

Kita-ku, Sapporo, 001-0020, Japan; Unit of Risk Analysis and Management, Hokkaido

University Research Center for Zoonosis Control, Kita 20, Nishi 10, Kita-ku,

Sapporo, 001-0020, Japan.

FAU - Nakajima, Chie

AU - Nakajima C

AD - Division of Bioresources, Hokkaido University Research Center for Zoonosis

Control, Kita 20, Nishi 10, Kita-ku, Sapporo, 001-0020, Japan; The Global Station

for Zoonosis Control, Global Institution for Collaborative Research and Education

(GI-Core), Hokkaido University, Kita 20, Nishi 10, Kita-ku, Sapporo, 001-0020,

Japan.

FAU - Suzuki, Yasuhiko

AU - Suzuki Y

AD - Division of Bioresources, Hokkaido University Research Center for Zoonosis

Control, Kita 20, Nishi 10, Kita-ku, Sapporo, 001-0020, Japan; The Global Station

for Zoonosis Control, Global Institution for Collaborative Research and Education

(GI-Core), Hokkaido University, Kita 20, Nishi 10, Kita-ku, Sapporo, 001-0020,

Japan. Electronic address: suzuki@czc.hokudai.ac.jp.

LA - eng

PT - Journal Article

PT - Research Support, Non-U.S. Gov't

DEP - 20200808

PL - Scotland

TA - Tuberculosis (Edinb)

JT - Tuberculosis (Edinburgh, Scotland)

JID - 100971555

RN - 0 (Anti-Bacterial Agents)

RN - 0 (DNA, Bacterial)

RN - 0 (RNA, Ribosomal, 16S)

RN - 0 (Ribosomal Proteins)

RN - 0 (ribosomal protein S12)

RN - Y45QSO73OB (Streptomycin)

SB - IM

MH - Anti-Bacterial Agents/pharmacology

MH - DNA Mutational Analysis

MH - DNA, Bacterial/genetics

MH - Drug Resistance, Bacterial/*genetics

MH - Genotype

MH - Humans

MH - Incidence

MH - Microbial Sensitivity Tests

MH - *Mutation

MH - Mycobacterium tuberculosis/*genetics

MH - Nepal/epidemiology

MH - RNA, Ribosomal, 16S/*genetics/metabolism

MH - Retrospective Studies

MH - Ribosomal Proteins/*genetics/metabolism

MH - Streptomycin/*pharmacology

MH - Tuberculosis, Multidrug-Resistant/epidemiology/*genetics/microbiology

OTO - NOTNLM

OT - Drug-resistance

OT - Mycobacterium tuberculosis

OT - Streptomycin

OT - gidB

OT - rpsL

OT - rrs

EDAT- 2020/08/24 06:00

MHDA- 2021/11/03 06:00

CRDT- 2020/08/24 06:00

PHST- 2020/05/27 00:00 [received]

PHST- 2020/07/30 00:00 [revised]

PHST- 2020/08/03 00:00 [accepted]

PHST- 2020/08/24 06:00 [pubmed]

PHST- 2021/11/03 06:00 [medline]

PHST- 2020/08/24 06:00 [entrez]

AID - S1472-9792(20)30152-9 [pii]

AID - 10.1016/j.tube.2020.101985 [doi]

PST - ppublish

SO - Tuberculosis (Edinb). 2020 Dec;125:101985. doi: 10.1016/j.tube.2020.101985. Epub

2020 Aug 8.

PMID- 32483653

OWN - NLM

STAT- MEDLINE

DCOM- 20210514

LR - 20210514

IS - 1619-7089 (Electronic)

IS - 1619-7070 (Linking)

VI - 47

IP - 13

DP - 2020 Dec

TI - Sequential (18)F-fluorodeoxyglucose positron emission tomography ((18)F-FDG PET)

scan findings in patients with extrapulmonary tuberculosis during the course of

treatment-a prospective observational study.

PG - 3118-3129

LID - 10.1007/s00259-020-04888-7 [doi]

AB - BACKGROUND: Initial studies of tuberculosis (TB) in macaques and humans using

(18)F-FDG positron emission tomography (PET) imaging as a research tool suggest

its usefulness in localising disease sites and as a clinical biomarker.

Sequential serial scans in patients with extrapulmonary TB (EPTB) could inform on

the value of PET-CT for monitoring response to treatment and defining cure.

PATIENTS AND METHODS: HIV-negative adults with EPTB from eight sites across six

countries had three (18)F-FDG PET/CT scans: (i) within 2 weeks of enrolment, (ii)

at 2 months into TB treatment and (iii) at end of ATT treatment. Scanning was

performed according to the EANM guidelines. (18)F-FDG PET/CT scans were performed

60 ± 10 min after intravenous injection of 2.5-5.0 MBq/kg of (18)F-FDG. FINDINGS:

One hundred and forty-seven patients with EPTB underwent 3 sequential scans. A

progressive reduction over time of both the number of active sites and the uptake

level (SUVmax) at these sites was seen. At the end of WHO recommended treatment,

53/147 (36.0%) patients had negative PET/CT scans, and 94/147 (63.9%) patients

remained PET/CT positive, of which 12 patients had developed MDR TB. One died of

brain tuberculoma. INTERPRETATION: Current (18)F-FDG PET/CT imaging technology

cannot be used clinically as a biomarker of treatment response, cure or for

decision-making on when to stop EPTB treatment. PET/CT remains a research tool

for TB and further development of PET/CT is required using new Mycobacterium

tuberculosis-specific radiopharmaceuticals targeting high-density surface

epitopes, gene targets or metabolic pathways.

FAU - Bomanji, Jamshed

AU - Bomanji J

AD - Institute of Nuclear Medicine, 5th Floor, UCLH NHS Foundation Trust, 235 Euston

Road, London, NW1 2BU, UK. jamshed.bomanji@nhs.net.

FAU - Sharma, Rajnish

AU - Sharma R

AD - Division of Nuclear Medicine & PET Imaging, Specialist in Nuclear Medicine

&Thyroid diseases, Molecular Imaging & Research Center (MIRC), INMAS, Delhi,

India.

FAU - Mittal, Bhagwant R

AU - Mittal BR

AD - Department of Nuclear Medicine & PET, Post Graduate Institute of Medical

Education and Research, Sector 12, Chandigarh, 160012, India.

FAU - Gambhir, Sanjay

AU - Gambhir S

AD - Department of Nuclear Medicine, SGPGIMS, Rae Bareli Road, Lucknow, 226014, India.

FAU - Qureshy, Ahmad

AU - Qureshy A

AD - Institute of Nuclear Medicine and Oncology (INMOL) Hospital, New Campus Road,

Lahore, 54600, Pakistan.

FAU - Begum, Shamim M F

AU - Begum SMF

AD - National Institute of Nuclear Medicine & Allied Sciences (NINMAS), BSM Medical

University Campus, 7th-10th Floor, Block-D, Shahbag, Dhaka, 1000, Bangladesh.

FAU - Paez, Diana

AU - Paez D

AD - Nuclear Medicine and Diagnostic Imaging Section, Division of Human Health, IAEA,

Vienna, Austria.

FAU - Sathekge, Mike

AU - Sathekge M

AD - Department of Nuclear Medicine, Steve Biko Academic Hospital, University of

Pretoria, Pretoria, South Africa.

FAU - Vorster, Mariza

AU - Vorster M

AD - Department of Nuclear Medicine, Steve Biko Academic Hospital, University of

Pretoria, Pretoria, South Africa.

FAU - Sobic Saranovic, Dragana

AU - Sobic Saranovic D

AD - Faculty of Medicine University of Belgrade and Center of Nuclear Medicine

Clinical Center of Serbia, Belgrade, Serbia.

FAU - Pusuwan, Pawana

AU - Pusuwan P

AD - Division of Nuclear Medicine, Faculty of Medicine, Siriraj Hospital, Mahidol

University, Bangkok-noi, Bangkok, 10700, Thailand.

FAU - Mann, Vera

AU - Mann V

AD - Institute of Nuclear Medicine, 5th Floor, UCLH NHS Foundation Trust, 235 Euston

Road, London, NW1 2BU, UK.

FAU - Vinjamuri, Sobhan

AU - Vinjamuri S

AD - Royal Liverpool University Hospital, Liverpool, L7 8XP, UK.

FAU - Zumla, Alimuddin

AU - Zumla A

AD - Center for Clinical Microbiology, Division of Infection and Immunity, University

College London, and the National Institute of Health Research Biomedical Research

Centre at UCL Hospitals, London, UK.

FAU - Pascual, Thomas N B

AU - Pascual TNB

AD - Section of Nuclear Medicine and Diagnostic Imaging, Division of Human Health,

Department of Nuclear Sciences and Applications, International Atomic Energy

Agency, Vienna International Centre, PO Box 100, 1400, Vienna, Austria.

LA - eng

GR - CRPE15021/International Atomic Energy Agency/

PT - Journal Article

PT - Observational Study

PT - Research Support, Non-U.S. Gov't

DEP - 20200602

PL - Germany

TA - Eur J Nucl Med Mol Imaging

JT - European journal of nuclear medicine and molecular imaging

JID - 101140988

RN - 0 (Radiopharmaceuticals)

RN - 0Z5B2CJX4D (Fluorodeoxyglucose F18)

SB - IM

MH - Adult

MH - *Fluorodeoxyglucose F18

MH - Humans

MH - Positron Emission Tomography Computed Tomography

MH - Positron-Emission Tomography

MH - Radiopharmaceuticals

MH - *Tuberculosis/diagnostic imaging

OTO - NOTNLM

OT - 18F-FDG PET/CT scan

OT - Biomarker

OT - Cure

OT - Disease activity

OT - Extrapulmonary tuberculosis

OT - Imaging

OT - Monitoring treatment

OT - Relapse

OT - Tuberculosis

EDAT- 2020/06/03 06:00

MHDA- 2021/05/15 06:00

CRDT- 2020/06/03 06:00

PHST- 2020/05/05 00:00 [received]

PHST- 2020/05/21 00:00 [accepted]

PHST- 2020/06/03 06:00 [pubmed]

PHST- 2021/05/15 06:00 [medline]

PHST- 2020/06/03 06:00 [entrez]

AID - 10.1007/s00259-020-04888-7 [pii]

AID - 10.1007/s00259-020-04888-7 [doi]

PST - ppublish

SO - Eur J Nucl Med Mol Imaging. 2020 Dec;47(13):3118-3129. doi:

10.1007/s00259-020-04888-7. Epub 2020 Jun 2.

PMID- 33007556

OWN - NLM

STAT- MEDLINE

DCOM- 20210721

LR - 20210721

IS - 1464-3391 (Electronic)

IS - 0968-0896 (Linking)

VI - 28

IP - 22

DP - 2020 Nov 15

TI - Inhibition of Mycobacterium tuberculosis InhA: Design, synthesis and evaluation

of new di-triclosan derivatives.

PG - 115744

LID - S0968-0896(20)30574-5 [pii]

LID - 10.1016/j.bmc.2020.115744 [doi]

AB - Multi-drug resistant tuberculosis (MDR-TB) represents a growing problem for

global healthcare systems. In addition to 1.3 million deaths in 2018, the World

Health Organisation reported 484,000 new cases of MDR-TB. Isoniazid is a key

anti-TB drug that inhibits InhA, a crucial enzyme in the cell wall biosynthesis

pathway and identical in Mycobacterium tuberculosis and M. bovis. Isoniazid is a

pro-drug which requires activation by the enzyme KatG, mutations in KatG prevent

activation and confer INH-resistance. 'Direct inhibitors' of InhA are attractive

as they would circumvent the main clinically observed resistance mechanisms. A

library of new 1,5-triazoles, designed to mimic the structures of both triclosan

molecules uniquely bound to InhA have been synthesised. The inhibitory activity

of these compounds was evaluated using isolated enzyme assays with 2

(5-chloro-2-(4-(5-(((4-(4-chloro-2-hydroxyphenoxy)benzyl)oxy)methyl)-1H-1,2,3-triazol-1-yl)phenoxy)phenol)

exhibiting an IC(50) of 5.6 µM. Whole-cell evaluation was also performed, with 11

(5-chloro-2-(4-(5-(((4-(cyclopropylmethoxy)benzyl)oxy)methyl)-1H-1,2,3-triazol-1-yl)phenoxy)phenol)

showing the greatest potency, with an MIC(99) of 12.9 µM against M. bovis.

CI - Copyright © 2020. Published by Elsevier Ltd.

FAU - Armstrong, Tom

AU - Armstrong T

AD - Biodiscovery Institute, School of Chemistry, University of Nottingham, University

Park, Nottingham NG7 2RD, United Kingdom.

FAU - Lamont, Malcolm

AU - Lamont M

AD - Biodiscovery Institute, School of Chemistry, University of Nottingham, University

Park, Nottingham NG7 2RD, United Kingdom.

FAU - Lanne, Alice

AU - Lanne A

AD - Institute of Microbiology and Infection, School of Bioscience, University of

Birmingham, Birmingham B15 2TT, United Kingdom.

FAU - Alderwick, Luke J

AU - Alderwick LJ

AD - Institute of Microbiology and Infection, School of Bioscience, University of

Birmingham, Birmingham B15 2TT, United Kingdom.

FAU - Thomas, Neil R

AU - Thomas NR

AD - Biodiscovery Institute, School of Chemistry, University of Nottingham, University

Park, Nottingham NG7 2RD, United Kingdom. Electronic address:

neil.thomas@nottingham.ac.uk.

LA - eng

GR - 203974/Z/17/A/WT_/Wellcome Trust/United Kingdom

GR - BB/M008770/1/BB_/Biotechnology and Biological Sciences Research Council/United

Kingdom

GR - 215154/Z/18/Z/WT_/Wellcome Trust/United Kingdom

PT - Journal Article

PT - Research Support, Non-U.S. Gov't

DEP - 20200908

PL - England

TA - Bioorg Med Chem

JT - Bioorganic & medicinal chemistry

JID - 9413298

RN - 0 (Antitubercular Agents)

RN - 0 (Bacterial Proteins)

RN - 0 (Enzyme Inhibitors)

RN - 4NM5039Y5X (Triclosan)

RN - EC 1.- (Oxidoreductases)

RN - EC 1.3.1.9 (InhA protein, Mycobacterium)

SB - IM

MH - Antitubercular Agents/chemical synthesis/chemistry/*pharmacology

MH - Bacterial Proteins/*antagonists & inhibitors/metabolism

MH - Dose-Response Relationship, Drug

MH - *Drug Design

MH - Enzyme Inhibitors/chemical synthesis/chemistry/*pharmacology

MH - Microbial Sensitivity Tests

MH - Molecular Structure

MH - Mycobacterium tuberculosis/*drug effects/metabolism

MH - Oxidoreductases/*antagonists & inhibitors/metabolism

MH - Structure-Activity Relationship

MH - Triclosan/chemical synthesis/chemistry/*pharmacology

MH - Tuberculosis, Multidrug-Resistant/drug therapy/metabolism

OTO - NOTNLM

OT - InhA

OT - Isoniazid

OT - Mycobacterium tuberculosis

OT - TB

OT - Triazole

OT - Triclosan

EDAT- 2020/10/03 06:00

MHDA- 2021/07/22 06:00

CRDT- 2020/10/02 20:11

PHST- 2020/07/10 00:00 [received]

PHST- 2020/08/24 00:00 [revised]

PHST- 2020/08/27 00:00 [accepted]

PHST- 2020/10/03 06:00 [pubmed]

PHST- 2021/07/22 06:00 [medline]

PHST- 2020/10/02 20:11 [entrez]

AID - S0968-0896(20)30574-5 [pii]

AID - 10.1016/j.bmc.2020.115744 [doi]

PST - ppublish

SO - Bioorg Med Chem. 2020 Nov 15;28(22):115744. doi: 10.1016/j.bmc.2020.115744. Epub

2020 Sep 8.

PMID- 32927218

OWN - NLM

STAT- MEDLINE

DCOM- 20210419

LR - 20210419

IS - 1768-3254 (Electronic)

IS - 0223-5234 (Linking)

VI - 206

DP - 2020 Nov 15

TI - Design, synthesis and biological evaluation of diamino substituted

cyclobut-3-ene-1,2-dione derivatives for the treatment of drug-resistant

tuberculosis.

PG - 112538

LID - S0223-5234(20)30510-9 [pii]

LID - 10.1016/j.ejmech.2020.112538 [doi]

AB - Mycobacterium tuberculosis (Mtb) ATP synthase is an important target for treating

drug-resistant infections and sterilizing the bacteria, spurring intensive

efforts to develop new TB therapeutics based on this target. In this work, four

novel series including furan-2(5H)-ketone (3, 4), maleimide (5) and squaramide

(6) derivatives were designed, respectively, through the strategy of scaffold

morphing and hydrogen-bond introduction, using the selective Mtb ATP synthase

inhibitor compound 2 as the lead compound. The result demonstrated that diamino

substituted cyclobut-3-ene-1,2-dione compounds 6ab and 6ah displayed good to

excellent in vitro anti-TB activities (MIC 0.452-0.963 μg/mL) with low

cytotoxicity (IC(50) > 64 μg/mL). In addition, not only did compound 6ab show

effective activity against clinically isolated resistant strains, it also

revealed good druggability profiles including improved metabolic stability, no

hERG channel inhibition potential, and acceptable oral bioavailability. The

preliminary result of docking study and in vitro anti-bedaquiline-resistant

strain test compared to compound 2 suggested that Mtb ATP synthase is most likely

the target of compound 6ab. The structure-activity relationship laid a good

foundation for the identification of novel squaramides as a potential treatment

of drug-resistant tuberculosis.

CI - Copyright © 2020 Elsevier Masson SAS. All rights reserved.

FAU - Li, Peng

AU - Li P

AD - Beijing Key Laboratory of Active Substance Discovery and Druggability Evaluation,

Institute of Materia Medica, Peking Union Medical College and Chinese Academy of

Medical Sciences, 1 Xian Nong Tan Street, Beijing, 100050, PR China; Chinese

Academy of Medical Sciences Key Laboratory of Anti-DR TB Innovative Drug

Research, Institute of Materia Medica, Peking Union Medical College and Chinese

Academy of Medical Sciences, 1 Xian Nong Tan Street, Beijing, 100050, PR China.

FAU - Wang, Bin

AU - Wang B

AD - Beijing Key Laboratory of Drug Resistance Tuberculosis Research, Department of

Pharmacology, Beijing Tuberculosis and Thoracic Tumor Research Institute, Beijing

Chest Hospital, Capital Medical University, 97 Ma Chang Street, Beijing, 101149,

PR China.

FAU - Li, Gang

AU - Li G

AD - Beijing Key Laboratory of Active Substance Discovery and Druggability Evaluation,

Institute of Materia Medica, Peking Union Medical College and Chinese Academy of

Medical Sciences, 1 Xian Nong Tan Street, Beijing, 100050, PR China; Chinese

Academy of Medical Sciences Key Laboratory of Anti-DR TB Innovative Drug

Research, Institute of Materia Medica, Peking Union Medical College and Chinese

Academy of Medical Sciences, 1 Xian Nong Tan Street, Beijing, 100050, PR China.

FAU - Fu, Lei

AU - Fu L

AD - Beijing Key Laboratory of Drug Resistance Tuberculosis Research, Department of

Pharmacology, Beijing Tuberculosis and Thoracic Tumor Research Institute, Beijing

Chest Hospital, Capital Medical University, 97 Ma Chang Street, Beijing, 101149,

PR China.

FAU - Zhang, Dongfeng

AU - Zhang D

AD - Beijing Key Laboratory of Active Substance Discovery and Druggability Evaluation,

Institute of Materia Medica, Peking Union Medical College and Chinese Academy of

Medical Sciences, 1 Xian Nong Tan Street, Beijing, 100050, PR China; Chinese

Academy of Medical Sciences Key Laboratory of Anti-DR TB Innovative Drug

Research, Institute of Materia Medica, Peking Union Medical College and Chinese

Academy of Medical Sciences, 1 Xian Nong Tan Street, Beijing, 100050, PR China.

FAU - Lin, Ziyun

AU - Lin Z

AD - Beijing Key Laboratory of Active Substance Discovery and Druggability Evaluation,

Institute of Materia Medica, Peking Union Medical College and Chinese Academy of

Medical Sciences, 1 Xian Nong Tan Street, Beijing, 100050, PR China; Chinese

Academy of Medical Sciences Key Laboratory of Anti-DR TB Innovative Drug

Research, Institute of Materia Medica, Peking Union Medical College and Chinese

Academy of Medical Sciences, 1 Xian Nong Tan Street, Beijing, 100050, PR China.

FAU - Huang, Haihong

AU - Huang H

AD - Beijing Key Laboratory of Active Substance Discovery and Druggability Evaluation,

Institute of Materia Medica, Peking Union Medical College and Chinese Academy of

Medical Sciences, 1 Xian Nong Tan Street, Beijing, 100050, PR China; Chinese

Academy of Medical Sciences Key Laboratory of Anti-DR TB Innovative Drug

Research, Institute of Materia Medica, Peking Union Medical College and Chinese

Academy of Medical Sciences, 1 Xian Nong Tan Street, Beijing, 100050, PR China.

Electronic address: joyce@imm.ac.cn.

FAU - Lu, Yu

AU - Lu Y

AD - Beijing Key Laboratory of Drug Resistance Tuberculosis Research, Department of

Pharmacology, Beijing Tuberculosis and Thoracic Tumor Research Institute, Beijing

Chest Hospital, Capital Medical University, 97 Ma Chang Street, Beijing, 101149,

PR China. Electronic address: luyu4876@hotmail.com.

LA - eng

PT - Journal Article

DEP - 20200719

PL - France

TA - Eur J Med Chem

JT - European journal of medicinal chemistry

JID - 0420510

RN - 0 (Anti-Bacterial Agents)

RN - 0 (Cycloparaffins)

RN - EC 3.6.3.- (Mitochondrial Proton-Translocating ATPases)

SB - IM

MH - Animals

MH - Anti-Bacterial Agents/*chemical

synthesis/chemistry/pharmacokinetics/*pharmacology

MH - Chemistry Techniques, Synthetic

MH - Cycloparaffins/*chemical synthesis/chemistry/pharmacokinetics/*pharmacology

MH - *Drug Design

MH - Drug Resistance, Bacterial/*drug effects

MH - Drug Stability

MH - Humans

MH - Inhibitory Concentration 50

MH - Male

MH - Mice

MH - Microbial Sensitivity Tests

MH - Mitochondrial Proton-Translocating ATPases/chemistry/metabolism

MH - Molecular Docking Simulation

MH - Mycobacterium tuberculosis/*drug effects/enzymology/physiology

MH - Protein Conformation

OTO - NOTNLM

OT - ATP synthase inhibitor

OT - Drug-resistant tuberculosis

OT - Squaramide

OT - Structure-activity relationship

COIS- Declaration of competing interest The authors declare that they have no known

competing financial interests or personal relationships that could have appeared

to influence the work reported in this paper.

EDAT- 2020/09/15 06:00

MHDA- 2021/04/20 06:00

CRDT- 2020/09/14 20:14

PHST- 2020/03/30 00:00 [received]

PHST- 2020/05/24 00:00 [revised]

PHST- 2020/06/03 00:00 [accepted]

PHST- 2020/09/15 06:00 [pubmed]

PHST- 2021/04/20 06:00 [medline]

PHST- 2020/09/14 20:14 [entrez]

AID - S0223-5234(20)30510-9 [pii]

AID - 10.1016/j.ejmech.2020.112538 [doi]

PST - ppublish

SO - Eur J Med Chem. 2020 Nov 15;206:112538. doi: 10.1016/j.ejmech.2020.112538. Epub

2020 Jul 19.

PMID- 33182483

OWN - NLM

STAT- PubMed-not-MEDLINE

LR - 20201201

IS - 1999-4923 (Print)

IS - 1999-4923 (Electronic)

IS - 1999-4923 (Linking)

VI - 12

IP - 11

DP - 2020 Nov 9

TI - Antimicrobial Peptide against Mycobacterium Tuberculosis That Activates Autophagy

Is an Effective Treatment for Tuberculosis.

LID - 10.3390/pharmaceutics12111071 [doi]

LID - 1071

AB - Mycobacterium tuberculosis (MTB) is the principal cause of human tuberculosis

(TB), which is a serious health problem worldwide. The development of innovative

therapeutic modalities to treat TB is mainly due to the emergence of multi drug

resistant (MDR) TB. Autophagy is a cell-host defense process. Previous studies

have reported that autophagy-activating agents eliminate intracellular MDR MTB.

Thus, combining a direct antibiotic activity against circulating bacteria with

autophagy activation to eliminate bacteria residing inside cells could treat MDR

TB. We show that the synthetic peptide, IP-1 (KFLNRFWHWLQLKPGQPMY), induced

autophagy in HEK293T cells and macrophages at a low dose (10 μM), while

increasing the dose (50 μM) induced cell death; IP-1 induced the secretion of

TNFα in macrophages and killed Mtb at a dose where macrophages are not killed by

IP-1. Moreover, IP-1 showed significant therapeutic activity in a mice model of

progressive pulmonary TB. In terms of the mechanism of action, IP-1 sequesters

ATP in vitro and inside living cells. Thus, IP-1 is the first antimicrobial

peptide that eliminates MDR MTB infection by combining four activities: reducing

ATP levels, bactericidal activity, autophagy activation, and TNFα secretion.

FAU - Peláez Coyotl, Erika A

AU - Peláez Coyotl EA

AD - Department of Biochemistry and Structural Biology, Institute of Cellular

Physiology, National Autonomous University of Mexico (UNAM), 04510 Mexico City,

Mexico.

FAU - Barrios Palacios, Jacqueline

AU - Barrios Palacios J

AD - Experimental Pathology Section, National Institute of Medical Sciences and

Nutrition Salvador Zubirán, 14080 Mexico City, Mexico.

FAU - Muciño, Gabriel

AU - Muciño G

AD - Department of Neurodevelopment and Physiology, Instituto de Fisiologia Celular,

National Autonomous University of Mexico, 04510 Mexico City, Mexico.

FAU - Moreno-Blas, Daniel

AU - Moreno-Blas D

AUID- ORCID: 0000-0002-8394-8205

AD - Department of Neurodevelopment and Physiology, Instituto de Fisiologia Celular,

National Autonomous University of Mexico, 04510 Mexico City, Mexico.

FAU - Costas, Miguel

AU - Costas M

AUID- ORCID: 0000-0001-8604-6381

AD - Laboratorio de Biofisicoquímica, Departamento de Fisicoquímica, Facultad de

Química, Universidad Nacional Autonoma de Mexico, 04510 Mexico City, Mexico.

FAU - Montiel Montes, Teresa

AU - Montiel Montes T

AD - Department of Molecular Neuropathology, Institute of Cellular Physiology,

National Autonomous University of Mexico (UNAM), 04510 Mexico City, Mexico.

FAU - Diener, Christian

AU - Diener C

AD - Department of Biochemistry and Structural Biology, Institute of Cellular

Physiology, National Autonomous University of Mexico (UNAM), 04510 Mexico City,

Mexico.

FAU - Uribe-Carvajal, Salvador

AU - Uribe-Carvajal S

AD - Department of Molecular Genetics, Institute of Cellular Physiology, National

Autonomous University of Mexico, 04510 Mexico City, Mexico.

FAU - Massieu, Lourdes

AU - Massieu L

AD - Department of Molecular Neuropathology, Institute of Cellular Physiology,

National Autonomous University of Mexico (UNAM), 04510 Mexico City, Mexico.

FAU - Castro-Obregón, Susana

AU - Castro-Obregón S

AUID- ORCID: 0000-0003-0549-5690

AD - Department of Neurodevelopment and Physiology, Instituto de Fisiologia Celular,

National Autonomous University of Mexico, 04510 Mexico City, Mexico.

FAU - Espinosa, Octavio Ramos

AU - Espinosa OR

AUID- ORCID: 0000-0002-9010-4625

AD - Experimental Pathology Section, National Institute of Medical Sciences and

Nutrition Salvador Zubirán, 14080 Mexico City, Mexico.

FAU - Mata Espinosa, Dulce

AU - Mata Espinosa D

AD - Experimental Pathology Section, National Institute of Medical Sciences and

Nutrition Salvador Zubirán, 14080 Mexico City, Mexico.

FAU - Barrios-Payan, Jorge

AU - Barrios-Payan J

AD - Experimental Pathology Section, National Institute of Medical Sciences and

Nutrition Salvador Zubirán, 14080 Mexico City, Mexico.

FAU - León Contreras, Juan Carlos

AU - León Contreras JC

AUID- ORCID: 0000-0001-8827-1670

AD - Experimental Pathology Section, National Institute of Medical Sciences and

Nutrition Salvador Zubirán, 14080 Mexico City, Mexico.

FAU - Corzo, Gerardo

AU - Corzo G

AUID- ORCID: 0000-0002-9513-1622

AD - Department of Molecular Medicine and Bioprocesses, Institute of Biotechnology,

National Autonomous University of Mexico (UNAM), 62210 Cuernavaca Morelos,

Mexico.

FAU - Hernández-Pando, Rogelio

AU - Hernández-Pando R

AD - Experimental Pathology Section, National Institute of Medical Sciences and

Nutrition Salvador Zubirán, 14080 Mexico City, Mexico.

FAU - Del Rio, Gabriel

AU - Del Rio G

AUID- ORCID: 0000-0002-8118-4985

AD - Department of Biochemistry and Structural Biology, Institute of Cellular

Physiology, National Autonomous University of Mexico (UNAM), 04510 Mexico City,

Mexico.

LA - eng

GR - IG100513/Dirección General de Asuntos del Personal Académico, Universidad

Nacional Autónoma de México/

GR - IG100416/Dirección General de Asuntos del Personal Académico, Universidad

Nacional Autónoma de México/

GR - IN208014/Dirección General de Asuntos del Personal Académico, Universidad

Nacional Autónoma de México/

GR - IN208817/Dirección General de Asuntos del Personal Académico, Universidad

Nacional Autónoma de México/

GR - IN206518/Dirección General de Asuntos del Personal Académico, Universidad

Nacional Autónoma de México/

GR - FOIN-219/Consejo Nacional de Ciencia y Tecnología/

GR - CB252316/Consejo Nacional de Ciencia y Tecnología/

GR - 2015-2 FC-921/Consejo Nacional de Ciencia y Tecnología/

GR - FC2015-1/115/Consejo Nacional de Ciencia y Tecnología/

PT - Journal Article

DEP - 20201109

PL - Switzerland

TA - Pharmaceutics

JT - Pharmaceutics

JID - 101534003

PMC - PMC7697726

OTO - NOTNLM

OT - antimicrobial peptide

OT - autophagy

OT - iztli peptide

OT - multidrug resistant

OT - tuberculosis

COIS- The authors declare no conflict of interest. The funders had no role in the

design of the study; in the collection, analyses, or interpretation of data; in

the writing of the manuscript, or in the decision to publish the results.

EDAT- 2020/11/14 06:00

MHDA- 2020/11/14 06:01

PMCR- 2020/11/01

CRDT- 2020/11/13 01:03

PHST- 2020/09/07 00:00 [received]

PHST- 2020/10/29 00:00 [revised]

PHST- 2020/11/03 00:00 [accepted]

PHST- 2020/11/13 01:03 [entrez]

PHST- 2020/11/14 06:00 [pubmed]

PHST- 2020/11/14 06:01 [medline]

PHST- 2020/11/01 00:00 [pmc-release]

AID - pharmaceutics12111071 [pii]

AID - pharmaceutics-12-01071 [pii]

AID - 10.3390/pharmaceutics12111071 [doi]

PST - epublish

SO - Pharmaceutics. 2020 Nov 9;12(11):1071. doi: 10.3390/pharmaceutics12111071.

PMID- 32777127

OWN - NLM

STAT- MEDLINE

DCOM- 20210423

LR - 20240330

IS - 1365-313X (Electronic)

IS - 0960-7412 (Print)

IS - 0960-7412 (Linking)

VI - 104

IP - 3

DP - 2020 Nov

TI - The biosynthesis of the anti-microbial diterpenoid leubethanol in Leucophyllum

frutescens proceeds via an all-cis prenyl intermediate.

PG - 693-705

LID - 10.1111/tpj.14957 [doi]

AB - Serrulatane diterpenoids are natural products found in plants from a subset of

genera within the figwort family (Scrophulariaceae). Many of these compounds have

been characterized as having anti-microbial properties and share a common

diterpene backbone. One example, leubethanol from Texas sage (Leucophyllum

frutescens) has demonstrated activity against multi-drug-resistant tuberculosis.

Leubethanol is the only serrulatane diterpenoid identified from this genus;

however, a range of such compounds have been found throughout the closely related

Eremophila genus. Despite their potential therapeutic relevance, the biosynthesis

of serrulatane diterpenoids has not been previously reported. Here we leverage

the simple product profile and high accumulation of leubethanol in the roots of

L. frutescens and compare tissue-specific transcriptomes with existing data from

Eremophila serrulata to decipher the biosynthesis of leubethanol. A short-chain

cis-prenyl transferase (LfCPT1) first produces the rare diterpene precursor

nerylneryl diphosphate, which is cyclized by an unusual plastidial terpene

synthase (LfTPS1) into the characteristic serrulatane diterpene backbone. Final

conversion to leubethanol is catalyzed by a cytochrome P450 (CYP71D616) of the

CYP71 clan. This pathway documents the presence of a short-chain cis-prenyl

diphosphate synthase, previously only found in Solanaceae, which is likely

involved in the biosynthesis of other known diterpene backbones in Eremophila.

LfTPS1 represents neofunctionalization of a compartment-switching terpene

synthase accepting a novel substrate in the plastid. Biosynthetic access to

leubethanol will enable pathway discovery to more complex serrulatane

diterpenoids which share this common starting structure and provide a platform

for the production and diversification of this class of promising anti-microbial

therapeutics in heterologous systems.

CI - © 2020 The Authors. The Plant Journal published by Society for Experimental

Biology and John Wiley & Sons Ltd.

FAU - Miller, Garret P

AU - Miller GP

AUID- ORCID: 0000-0002-0222-0184

AD - Biochemistry and Molecular Biology, Michigan State University, East Lansing, MI,

USA.

FAU - Bhat, Wajid Waheed

AU - Bhat WW

AUID- ORCID: 0000-0002-7049-8671

AD - Biochemistry and Molecular Biology, Michigan State University, East Lansing, MI,

USA.

FAU - Lanier, Emily R

AU - Lanier ER

AUID- ORCID: 0000-0002-1878-4566

AD - Biochemistry and Molecular Biology, Michigan State University, East Lansing, MI,

USA.

FAU - Johnson, Sean R

AU - Johnson SR

AUID- ORCID: 0000-0001-8261-9015

AD - Biochemistry and Molecular Biology, Michigan State University, East Lansing, MI,

USA.

FAU - Mathieu, Davis T

AU - Mathieu DT

AD - Biochemistry and Molecular Biology, Michigan State University, East Lansing, MI,

USA.

FAU - Hamberger, Björn

AU - Hamberger B

AUID- ORCID: 0000-0003-1249-1807

AD - Biochemistry and Molecular Biology, Michigan State University, East Lansing, MI,

USA.

LA - eng

GR - T32 GM110523/GM/NIGMS NIH HHS/United States

GR - T32-GM110523/NH/NIH HHS/United States

PT - Journal Article

PT - Research Support, N.I.H., Extramural

PT - Research Support, Non-U.S. Gov't

PT - Research Support, U.S. Gov't, Non-P.H.S.

DEP - 20200828

PL - England

TA - Plant J

JT - The Plant journal : for cell and molecular biology

JID - 9207397

RN - 0 (Diterpenes)

RN - 0 (Plant Proteins)

RN - 0 (Polyisoprenyl Phosphates)

RN - 0 (leubethanol)

RN - 0 (prenyl)

RN - 9010-98-4 (Neoprene)

RN - 9035-51-2 (Cytochrome P-450 Enzyme System)

RN - EC 2.- (Transferases)

RN - EC 2.5.- (Alkyl and Aryl Transferases)

RN - EC 2.5.1.- (cis-prenyl transferase)

RN - EC 2.5.1.- (terpene synthase)

RN - N21T0D88LX (geranylgeranyl pyrophosphate)

SB - IM

MH - Alkyl and Aryl Transferases/metabolism

MH - Cytochrome P-450 Enzyme System/genetics/metabolism

MH - Diterpenes/*metabolism

MH - Eremophila Plant/genetics

MH - Escherichia coli/genetics

MH - Neoprene/metabolism

MH - Plant Proteins/genetics/metabolism

MH - Plant Roots/metabolism

MH - Plants, Genetically Modified

MH - Polyisoprenyl Phosphates/metabolism

MH - Scrophulariaceae/genetics/*metabolism

MH - Nicotiana/genetics/metabolism

MH - Transferases/genetics/metabolism

PMC - PMC7649979

MID - NIHMS1640112

OTO - NOTNLM

OT - all-cis prenyl diphosphate

OT - anti-microbial

OT - cytochrome P450

OT - leubethanol

OT - terpene biosynthesis

COIS- BH, SJ, WWB, GM, EL, and DM have filed a provisional patent application (US

Patent Application Serial No.: 62/986,286) describing the method to produce

leubethanol and related diterpenes.

EDAT- 2020/08/11 06:00

MHDA- 2021/04/24 06:00

PMCR- 2020/11/27

CRDT- 2020/08/11 06:00

PHST- 2020/03/15 00:00 [received]

PHST- 2020/06/17 00:00 [revised]

PHST- 2020/07/16 00:00 [accepted]

PHST- 2020/08/11 06:00 [pubmed]

PHST- 2021/04/24 06:00 [medline]

PHST- 2020/08/11 06:00 [entrez]

PHST- 2020/11/27 00:00 [pmc-release]

AID - TPJ14957 [pii]

AID - 10.1111/tpj.14957 [doi]

PST - ppublish

SO - Plant J. 2020 Nov;104(3):693-705. doi: 10.1111/tpj.14957. Epub 2020 Aug 28.

PMID- 32900827

OWN - NLM

STAT- MEDLINE

DCOM- 20210407

LR - 20230726

IS - 1098-5530 (Electronic)

IS - 0021-9193 (Print)

IS - 0021-9193 (Linking)

VI - 202

IP - 22

DP - 2020 Oct 22

TI - Nanoluciferase Reporter Mycobacteriophage for Sensitive and Rapid Detection of

Mycobacterium tuberculosis Drug Susceptibility.

LID - 10.1128/JB.00411-20 [doi]

LID - e00411-20

AB - Phenotypic testing for drug susceptibility of Mycobacterium tuberculosis is

critical to basic research and managing the evolving problem of antimicrobial

resistance in tuberculosis management, but it remains a specialized technique to

which access is severely limited. Here, we report on the development and

validation of an improved phage-mediated detection system for M. tuberculosis We

incorporated a nanoluciferase (Nluc) reporter gene cassette into the TM4

mycobacteriophage genome to create phage TM4-nluc. We assessed the performance of

this reporter phage in the context of cellular limit of detection and drug

susceptibility testing using multiple biosafety level 2 drug-sensitive and

-resistant auxotrophs as well as virulent M. tuberculosis strains. For both limit

of detection and drug susceptibility testing, we developed a standardized method

consisting of a 96-hour cell preculture followed by a 72-hour experimental window

for M. tuberculosis detection with or without antibiotic exposure. The cellular

limit of detection of M. tuberculosis in a 96-well plate batch culture was ≤10(2)

CFU. Consistent with other phenotypic methods for drug susceptibility testing, we

found TM4-nluc to be compatible with antibiotics representing multiple classes

and mechanisms of action, including inhibition of core central dogma functions,

cell wall homeostasis, metabolic inhibitors, compounds currently in clinical

trials (SQ109 and Q203), and susceptibility testing for bedaquiline, pretomanid,

and linezolid (components of the BPaL regimen for the treatment of multi- and

extensively drug-resistant tuberculosis). Using the same method, we accurately

identified rifampin-resistant and multidrug-resistant M. tuberculosis

strains.IMPORTANCEMycobacterium tuberculosis, the causative agent of tuberculosis

disease, remains a public health crisis on a global scale, and development of new

interventions and identification of drug resistance are pillars in the World

Health Organization End TB Strategy. Leveraging the tractability of the TM4

mycobacteriophage and the sensitivity of the nanoluciferase reporter enzyme, the

present work describes an evolution of phage-mediated detection and drug

susceptibility testing of M. tuberculosis, adding a valuable tool in drug

discovery and basic biology research. With additional validation, this system may

play a role as a quantitative phenotypic reference method and complement to

genotypic methods for diagnosis and antibiotic susceptibility testing.

CI - Copyright © 2020 Jain et al.

FAU - Jain, Paras

AU - Jain P

AD - Intellectual Ventures Laboratory, Bellevue, Washington, USA parasjain29@gmail.com

kyle.minch@ghlabs.org.

AD - Department of Microbiology and Immunology, Albert Einstein College of Medicine,

Bronx, New York, USA.

AD - Trudeau Institute, Saranac Lake, New York, USA.

FAU - Garing, Spencer

AU - Garing S

AD - Intellectual Ventures Laboratory, Bellevue, Washington, USA.

FAU - Verma, Deepshikha

AU - Verma D

AD - Mycobacteria Research Laboratories, Department of Microbiology, Immunology and

Pathology, Colorado State University, Fort Collins, Colorado, USA.

FAU - Saranathan, Rajagopalan

AU - Saranathan R

AD - Department of Microbiology and Immunology, Albert Einstein College of Medicine,

Bronx, New York, USA.

FAU - Clute-Reinig, Nicholas

AU - Clute-Reinig N

AD - Intellectual Ventures Laboratory, Bellevue, Washington, USA.

FAU - Gadwa, Jacob

AU - Gadwa J

AD - Mycobacteria Research Laboratories, Department of Microbiology, Immunology and

Pathology, Colorado State University, Fort Collins, Colorado, USA.

FAU - Peterson, Chelsea

AU - Peterson C

AD - Mycobacteria Research Laboratories, Department of Microbiology, Immunology and

Pathology, Colorado State University, Fort Collins, Colorado, USA.

FAU - Hermansky, Gleda

AU - Hermansky G

AD - Intellectual Ventures Laboratory, Bellevue, Washington, USA.

FAU - Astashkina Fernandez, Anna

AU - Astashkina Fernandez A

AD - Intellectual Ventures Laboratory, Bellevue, Washington, USA.

FAU - Asare, Emmanuel

AU - Asare E

AD - Department of Microbiology and Immunology, Albert Einstein College of Medicine,

Bronx, New York, USA.

AD - Department of Pathology, Albert Einstein College of Medicine and Montefiore

Medical Center, Bronx, New York, USA.

FAU - Weisbrod, Torin R

AU - Weisbrod TR

AD - Department of Microbiology and Immunology, Albert Einstein College of Medicine,

Bronx, New York, USA.

FAU - Spencer, Ethan

AU - Spencer E

AD - Intellectual Ventures Laboratory, Bellevue, Washington, USA.

FAU - Mulholland, Claire V

AU - Mulholland CV

AD - Department of Microbiology and Immunology, Albert Einstein College of Medicine,

Bronx, New York, USA.

FAU - Berney, Michael

AU - Berney M

AD - Department of Microbiology and Immunology, Albert Einstein College of Medicine,

Bronx, New York, USA.

FAU - Bell, David

AU - Bell D

AD - Intellectual Ventures' Global Good Fund, Bellevue, Washington, USA.

FAU - Nichols, Kevin P

AU - Nichols KP

AD - Intellectual Ventures Laboratory, Bellevue, Washington, USA.

FAU - Le Ny, Anne-Laure M

AU - Le Ny AM

AD - Intellectual Ventures Laboratory, Bellevue, Washington, USA.

FAU - Ordway, Diane

AU - Ordway D

AD - Mycobacteria Research Laboratories, Department of Microbiology, Immunology and

Pathology, Colorado State University, Fort Collins, Colorado, USA.

FAU - Jacobs, William R Jr

AU - Jacobs WR Jr

AUID- ORCID: 0000-0003-3321-3080

AD - Department of Microbiology and Immunology, Albert Einstein College of Medicine,

Bronx, New York, USA.

AD - Department of Molecular Genetics, Albert Einstein College of Medicine, Bronx, New

York, USA.

FAU - Somoskovi, Akos

AU - Somoskovi A

AD - Intellectual Ventures' Global Good Fund, Bellevue, Washington, USA.

FAU - Minch, Kyle J

AU - Minch KJ

AUID- ORCID: 0000-0003-2447-5759

AD - Intellectual Ventures Laboratory, Bellevue, Washington, USA parasjain29@gmail.com

kyle.minch@ghlabs.org.

LA - eng

GR - R37 AI026170/AI/NIAID NIH HHS/United States

PT - Journal Article

PT - Research Support, Non-U.S. Gov't

DEP - 20201022

PL - United States

TA - J Bacteriol

JT - Journal of bacteriology

JID - 2985120R

RN - 0 (Antitubercular Agents)

RN - EC 1.13.12.- (Luciferases)

RN - VJT6J7R4TR (Rifampin)

SB - IM

MH - Antitubercular Agents/*pharmacology

MH - *Drug Resistance, Bacterial

MH - Humans

MH - Luciferases/genetics/metabolism

MH - Luminescent Measurements

MH - Microbial Sensitivity Tests/*methods

MH - Mycobacteriophages/*genetics

MH - Mycobacterium tuberculosis/*drug effects/genetics/virology

MH - Rifampin/*pharmacology

MH - Tuberculosis, Multidrug-Resistant/microbiology

MH - Tuberculosis, Pulmonary/microbiology

PMC - PMC7585058

OTO - NOTNLM

OT - Mycobacterium tuberculosis

OT - bacteriophages

OT - drug screening

OT - drug susceptibility testing

OT - nanoluciferase

OT - phage

EDAT- 2020/09/10 06:00

MHDA- 2021/04/10 06:00

PMCR- 2020/10/22

CRDT- 2020/09/09 05:25

PHST- 2020/07/21 00:00 [received]

PHST- 2020/09/01 00:00 [accepted]

PHST- 2020/09/10 06:00 [pubmed]

PHST- 2021/04/10 06:00 [medline]

PHST- 2020/09/09 05:25 [entrez]

PHST- 2020/10/22 00:00 [pmc-release]

AID - JB.00411-20 [pii]

AID - 00411-20 [pii]

AID - 10.1128/JB.00411-20 [doi]

PST - epublish

SO - J Bacteriol. 2020 Oct 22;202(22):e00411-20. doi: 10.1128/JB.00411-20. Print 2020

Oct 22.

PMID- 32781051

OWN - NLM

STAT- MEDLINE

DCOM- 20201119

LR - 20201119

IS - 1096-0384 (Electronic)

IS - 0003-9861 (Linking)

VI - 692

DP - 2020 Oct 15

TI - Orphan response regulator Rv3143 increases antibiotic sensitivity by regulating

cell wall permeability in Mycobacterium smegmatis.

PG - 108522

LID - S0003-9861(20)30531-2 [pii]

LID - 10.1016/j.abb.2020.108522 [doi]

AB - About one quarter of people worldwide are infected with tuberculosis, and

multi-drug resistant tuberculosis (MDR-TB) remains a health threat. It is known

that two-Component Signal Transduction Systems (TCSs) of Mycobacterium

tuberculosis are closely related to tuberculosis resistance, but the mechanism by

which orphan response protein Rv3143 regulates strain sensitivity to drug is

still unclear. This study found that Rv3143 overexpression resulted in

approximately two-fold increase in Mycobacterium smegmatis antibiotic

sensitivity. Transcriptome sequencing indicated that 198 potential genes were

regulated by Rv3143, affecting the sensitivity of the strain to rifampicin (RIF).

MSMEG_4740 promoter binding with Rv3143, was screened out by surface plasmon

resonance (SPR). Rv1524, the homologous gene of MSMEG_4740, belonging to the

glycosyltransferase (Gtf) family, was related to cell wall modification. By

measuring ethidium bromide (EB) accumulation, we found when Rv3143 or MSMEG_4740,

or Rv1524 was overexpressed, the cell wall permeability of Mycobacterium

smegmatis was increased. In addition, a combination of Rv3143 and RIF was

observed. Our findings provide a new strategy for treating drug-resistant

tuberculosis by increasing the expression of Rv3143 to enhance the strain

sensitivity to antibiotics.

CI - Copyright © 2020 Elsevier Inc. All rights reserved.

FAU - Dong, Wenqi

AU - Dong W

AD - State Key Laboratory of Agricultural Microbiology, College of Veterinary

Medicine, Huazhong Agricultural University, Wuhan, Hubei, 430070, China.

FAU - Wang, Rui

AU - Wang R

AD - State Key Laboratory of Agricultural Microbiology, College of Veterinary

Medicine, Huazhong Agricultural University, Wuhan, Hubei, 430070, China.

FAU - Li, Pei

AU - Li P

AD - State Key Laboratory of Agricultural Microbiology, College of Veterinary

Medicine, Huazhong Agricultural University, Wuhan, Hubei, 430070, China;

Department of Gastrointestinal Surgery, The Second Clinical Medical College of

Jinan University, Shenzhen, Guangdong, 518020, China.

FAU - Wang, Gaoyan

AU - Wang G

AD - State Key Laboratory of Agricultural Microbiology, College of Veterinary

Medicine, Huazhong Agricultural University, Wuhan, Hubei, 430070, China.

FAU - Ren, Xuanxiu

AU - Ren X

AD - State Key Laboratory of Agricultural Microbiology, College of Veterinary

Medicine, Huazhong Agricultural University, Wuhan, Hubei, 430070, China.

FAU - Feng, Jiajia

AU - Feng J

AD - State Key Laboratory of Agricultural Microbiology, College of Veterinary

Medicine, Huazhong Agricultural University, Wuhan, Hubei, 430070, China.

FAU - Lu, Hao

AU - Lu H

AD - State Key Laboratory of Agricultural Microbiology, College of Veterinary

Medicine, Huazhong Agricultural University, Wuhan, Hubei, 430070, China.

FAU - Lu, Wenjia

AU - Lu W

AD - State Key Laboratory of Agricultural Microbiology, College of Veterinary

Medicine, Huazhong Agricultural University, Wuhan, Hubei, 430070, China.

FAU - Wang, Xiangru

AU - Wang X

AD - State Key Laboratory of Agricultural Microbiology, College of Veterinary

Medicine, Huazhong Agricultural University, Wuhan, Hubei, 430070, China; Key

Laboratory of Preventive Veterinary Medicine in Hubei Province, Wuhan, Hubei,

430070, China; Key Laboratory of Development of Veterinary Diagnostic Products,

Ministry of Agriculture of the People's Republic of China, Wuhan, Hubei, 430070,

China.

FAU - Chen, Huanchun

AU - Chen H

AD - State Key Laboratory of Agricultural Microbiology, College of Veterinary

Medicine, Huazhong Agricultural University, Wuhan, Hubei, 430070, China; Key

Laboratory of Preventive Veterinary Medicine in Hubei Province, Wuhan, Hubei,

430070, China; Key Laboratory of Development of Veterinary Diagnostic Products,

Ministry of Agriculture of the People's Republic of China, Wuhan, Hubei, 430070,

China.

FAU - Tan, Chen

AU - Tan C

AD - State Key Laboratory of Agricultural Microbiology, College of Veterinary

Medicine, Huazhong Agricultural University, Wuhan, Hubei, 430070, China; Key

Laboratory of Preventive Veterinary Medicine in Hubei Province, Wuhan, Hubei,

430070, China; Key Laboratory of Development of Veterinary Diagnostic Products,

Ministry of Agriculture of the People's Republic of China, Wuhan, Hubei, 430070,

China. Electronic address: tanchen@mail.hzau.edu.cn.

LA - eng

PT - Journal Article

PT - Research Support, Non-U.S. Gov't

DEP - 20200808

PL - United States

TA - Arch Biochem Biophys

JT - Archives of biochemistry and biophysics

JID - 0372430

RN - 0 (Bacterial Proteins)

RN - VJT6J7R4TR (Rifampin)

SB - IM

MH - Bacterial Proteins/genetics/*metabolism

MH - Cell Wall/genetics/*metabolism

MH - *Drug Resistance, Bacterial

MH - Gene Expression Regulation, Bacterial/drug effects

MH - Mycobacterium smegmatis/genetics/*metabolism

MH - Permeability/drug effects

MH - Rifampin/pharmacology

MH - Transcriptome/drug effects

OTO - NOTNLM

OT - Antibiotic sensitivity

OT - Cell wall permeability

OT - Rv3143

OT - Two-component signal transduction systems

EDAT- 2020/08/12 06:00

MHDA- 2020/11/20 06:00

CRDT- 2020/08/12 06:00

PHST- 2020/05/04 00:00 [received]

PHST- 2020/07/23 00:00 [revised]

PHST- 2020/07/28 00:00 [accepted]

PHST- 2020/08/12 06:00 [pubmed]

PHST- 2020/11/20 06:00 [medline]

PHST- 2020/08/12 06:00 [entrez]

AID - S0003-9861(20)30531-2 [pii]

AID - 10.1016/j.abb.2020.108522 [doi]

PST - ppublish

SO - Arch Biochem Biophys. 2020 Oct 15;692:108522. doi: 10.1016/j.abb.2020.108522.

Epub 2020 Aug 8.

PMID- 34485463

OWN - NLM

STAT- PubMed-not-MEDLINE

LR - 20220426

IS - 2313-1810 (Electronic)

IS - 2312-0053 (Print)

IS - 2312-0053 (Linking)

VI - 35

IP - 1

DP - 2020

TI - Adverse effects of bedaquiline in patients with extensively drug-resistant

tuberculosis.

PG - 23

LID - 10.4102/sajid.v35i1.23 [doi]

LID - 23

AB - BACKGROUND: The World Health Organisation (WHO) guidelines recommend that,

because of the resistance patterns of extensively drug-resistant (XDR)

tuberculosis (TB) and its unique mechanism of action, bedaquiline be included in

the regimen. Although the results of clinical trials have shown bedaquiline to be

beneficial, it also carries the risk of adverse effects, some potentially

life-threatening. The aim of the study was to determine the incidence of adverse

effects caused by bedaquiline in patients diagnosed with XDR-TB. The subsequent

management of these adverse effects was also analysed. METHODS: The medical

records of patients aged 18 years or older living with XDR-TB who were prescribed

bedaquiline in combination with a background regimen at a public-sector

drug-resistant TB hospital in the Eastern Cape were reviewed. RESULTS: Thirty

records were reviewed in September 2016. Female patients constituted 66.67% (n =

20) of the sample. Nearly half (46.67%; n = 14) of the patients were living with

human immunodeficiency virus, and six (42.86%) of them were female. Adverse

effects were recorded for 26 patients (86.67%) including corrected QT

prolongation (40%; n = 12), skin rash (33.33%; n = 10) and hyperlactataemia

(33.33%; n = 10) as the most common. There were no treatment discontinuations or

deaths. The management of adverse effects varied from omitting doses of

bedaquiline to pharmacological intervention. CONCLUSION: All patients completed

bedaquiline treatment, indicating that the adverse effects did not require

discontinuation of the drug. However, when pharmacological intervention is

required for the management of adverse effects, care should be taken to ensure

that there is minimal interaction with other TB drugs and a low risk of further

adverse effects.

CI - © 2020. The Authors.

FAU - Gaida, Razia

AU - Gaida R

AUID- ORCID: 0000-0003-2895-4669

AD - Drug Utilization Research Unit (DURU), Department of Pharmacy, Faculty of Health

Sciences, Nelson Mandela University, Port Elizabeth, South Africa.

AD - Social Aspects of Public Health Unit, Human Sciences Research Council, Port

Elizabeth, South Africa.

FAU - Truter, Ilse

AU - Truter I

AUID- ORCID: 0000-0002-6527-0306

AD - Drug Utilization Research Unit (DURU), Department of Pharmacy, Faculty of Health

Sciences, Nelson Mandela University, Port Elizabeth, South Africa.

FAU - Peters, Charles A

AU - Peters CA

AUID- ORCID: 0000-0002-6739-0686

AD - Jose Pearson TB Hospital, Eastern Cape Department of Health, Port Elizabeth,

South Africa.

LA - eng

PT - Journal Article

DEP - 20201014

PL - South Africa

TA - S Afr J Infect Dis

JT - Southern African journal of infectious diseases

JID - 101646666

PMC - PMC8378113

OTO - NOTNLM

OT - Eastern Cape

OT - World Health Organisation

OT - adverse effects

OT - bedaquiline

OT - extensively drug resistant tuberculosis (XDR-TB)

COIS- The authors declare that they have no financial or personal relationships that

may have inappropriately influenced them in writing this article.

EDAT- 2021/09/07 06:00

MHDA- 2021/09/07 06:01

PMCR- 2020/10/14

CRDT- 2021/09/06 06:08

PHST- 2019/05/10 00:00 [received]

PHST- 2020/07/27 00:00 [accepted]

PHST- 2021/09/06 06:08 [entrez]

PHST- 2021/09/07 06:00 [pubmed]

PHST- 2021/09/07 06:01 [medline]

PHST- 2020/10/14 00:00 [pmc-release]

AID - SAJID-35-23 [pii]

AID - 10.4102/sajid.v35i1.23 [doi]

PST - epublish

SO - S Afr J Infect Dis. 2020 Oct 14;35(1):23. doi: 10.4102/sajid.v35i1.23.

eCollection 2020.

PMID- 33100904

OWN - NLM

STAT- MEDLINE

DCOM- 20211004

LR - 20211004

IS - 1466-1861 (Electronic)

IS - 0962-9351 (Print)

IS - 0962-9351 (Linking)

VI - 2020

DP - 2020

TI - Guttiferone K Exerts the Anti-inflammatory Effect on Mycobacterium Tuberculosis-

(H37Ra-) Infected Macrophages by Targeting the TLR/IRAK-1 Mediated Akt and NF-κB

Pathway.

PG - 8528901

LID - 10.1155/2020/8528901 [doi]

LID - 8528901

AB - Mycobacterium tuberculosis (Mtb) remains a great threat to global health, killing

more people than any other single infectious agent and causing uncontrollable

inflammation in the host. Poorly controlled inflammatory processes can be

deleterious and result in immune exhaustion. The current tuberculosis (TB)

control is facing the challenge of drugs deficiency, especially in the context of

increasingly multidrug resistant (MDR) TB. Under this circumstance, alternative

host-directed therapy (HDT) emerges timely which can be exploited to improve the

efficacy of TB treatment and disease prognosis by targeting the host. Here, we

established the in vitro infection model of Mtb macrophages with H37Ra strain to

seek effective anti-TB active agent. The present study showed that Guttiferone K,

isolated from Garcinia yunnanensis, could significantly inhibit Mtb-induced

inflammation in RAW264.7 and primary peritoneal macrophages. It was evidenced by

the decreased production of inflammatory mediators, including interleukin-1β

(IL-1β), tumor necrosis factor-α (TNF-α), interleukin-6 (IL-6), inducible nitric

oxide synthase (iNOS), and cyclooxygenase-2 (COX-2). Further studies with

immunoblotting and immunofluorescence revealed that Guttiferone K obviously

inhibits the nuclear factor-kappa B (NF-κB) both in RAW264.7 and primary

peritoneal macrophages relying on the TLR/IRAK-1 pathway. Guttiferone K could

also suppress the NLRP3 inflammasome activity and induce autophagy by inhibiting

the protein kinase B (p-Akt) and mammalian target of rapamycin (mTOR)

phosphorylation at Ser473 and Ser2448 in both cell lines. Thus, Guttiferone K

possesses significant anti-inflammatory effect, alleviating Mtb-induced

inflammation with an underlying mechanism that targeting on the TLR/IRAK-1

pathway and inhibiting the downstream NF-κB and Akt/mTOR signaling pathways.

Together, Guttiferone K can be an anti-inflammatory agent candidate for the

design of new adjunct HDT drugs fighting against tuberculosis.

CI - Copyright © 2020 Qingwen Zhang et al.

FAU - Zhang, Qingwen

AU - Zhang Q

AD - Center for Traditional Chinese Medicine and Immunology Research, School of Basic

Medical Sciences, Shanghai University of Traditional Chinese Medicine, 201203

Shanghai, China.

AD - Shanghai Key Laboratory of Molecular Imaging, Shanghai University of Medicine &

Health Sciences, 201318 Shanghai, China.

FAU - Sun, Jinxia

AU - Sun J

AD - Center for Traditional Chinese Medicine and Immunology Research, School of Basic

Medical Sciences, Shanghai University of Traditional Chinese Medicine, 201203

Shanghai, China.

FAU - Fu, Yan

AU - Fu Y

AD - Center for Traditional Chinese Medicine and Immunology Research, School of Basic

Medical Sciences, Shanghai University of Traditional Chinese Medicine, 201203

Shanghai, China.

FAU - He, Weigang

AU - He W

AD - Center for Traditional Chinese Medicine and Immunology Research, School of Basic

Medical Sciences, Shanghai University of Traditional Chinese Medicine, 201203

Shanghai, China.

FAU - Li, Yinhong

AU - Li Y

AD - Center for Traditional Chinese Medicine and Immunology Research, School of Basic

Medical Sciences, Shanghai University of Traditional Chinese Medicine, 201203

Shanghai, China.

FAU - Tan, Hongsheng

AU - Tan H

AD - Clinical Research Center, Shanghai Jiao Tong University School of Medicine,

200240 Shanghai, China.

FAU - Xu, Hongxi

AU - Xu H

AUID- ORCID: 0000-0001-6238-4511

AD - Shuguang Hospital, Shanghai University of Traditional Chinese Medicine, Shanghai

201203, China.

AD - School of Pharmacy, Shanghai University of Traditional Chinese Medicine, 201203

Shanghai, China.

FAU - Jiang, Xin

AU - Jiang X

AUID- ORCID: 0000-0001-7007-0483

AD - Center for Traditional Chinese Medicine and Immunology Research, School of Basic

Medical Sciences, Shanghai University of Traditional Chinese Medicine, 201203

Shanghai, China.

LA - eng

PT - Journal Article

DEP - 20201010

PL - United States

TA - Mediators Inflamm

JT - Mediators of inflammation

JID - 9209001

RN - 0 (Anti-Inflammatory Agents)

RN - 0 (Benzophenones)

RN - 0 (NF-kappa B)

RN - 0 (guttiferone K)

RN - EC 2.7.11.1 (Interleukin-1 Receptor-Associated Kinases)

RN - EC 2.7.11.1 (Irak1 protein, mouse)

RN - EC 2.7.11.1 (Proto-Oncogene Proteins c-akt)

SB - IM

MH - Animals

MH - Anti-Inflammatory Agents/*therapeutic use

MH - Benzophenones/*therapeutic use

MH - Blotting, Western

MH - Cell Survival/drug effects

MH - Female

MH - Immunoprecipitation

MH - Interleukin-1 Receptor-Associated Kinases/*metabolism

MH - Macrophages/*drug effects/*metabolism

MH - Mice

MH - Mycobacterium tuberculosis/*drug effects/*pathogenicity

MH - NF-kappa B/*metabolism

MH - Proto-Oncogene Proteins c-akt/*metabolism

MH - RAW 264.7 Cells

PMC - PMC7569438

COIS- The authors declare no conflict of interest.

EDAT- 2020/10/27 06:00

MHDA- 2021/10/05 06:00

PMCR- 2020/10/10

CRDT- 2020/10/26 05:20

PHST- 2020/06/05 00:00 [received]

PHST- 2020/08/26 00:00 [revised]

PHST- 2020/09/16 00:00 [accepted]

PHST- 2020/10/26 05:20 [entrez]

PHST- 2020/10/27 06:00 [pubmed]

PHST- 2021/10/05 06:00 [medline]

PHST- 2020/10/10 00:00 [pmc-release]

AID - 10.1155/2020/8528901 [doi]

PST - epublish

SO - Mediators Inflamm. 2020 Oct 10;2020:8528901. doi: 10.1155/2020/8528901.

eCollection 2020.

PMID- 33323653

OWN - NLM

STAT- MEDLINE

DCOM- 20210924

LR - 20210924

IS - 2212-554X (Electronic)

IS - 2212-5531 (Linking)

VI - 9

IP - 4

DP - 2020 Oct-Dec

TI - Association of disease severity with toll-like receptor polymorphisms in

multidrug-resistant tuberculosis patients.

PG - 380-390

LID - 10.4103/ijmy.ijmy_175_20 [doi]

AB - BACKGROUND: The disease severity in pulmonary Multidrug-resistant tuberculosis

(MDR-TB) varies from mild to severe, which is determined by host and pathogen

virulence factors. The difference of symptoms felt by TB patients were
[truncated: 1,698,784 more chars]
