# Supplementary material for: Global research trends in tuberculosis drug-resistance mechanisms: a bibliometric and cross-database analysis
Source: Front Microbiol. 2026 Sep 17;17:1942089. doi: 10.3389/fmicb.2026.1942089 (PMC13627400; doi:10.3389/fmicb.2026.1942089)
Supplement: Supplementary file 2 [file Table_2.doc]

FN Clarivate Analytics Web of Science

VR 1.0

PT J

AU Jing, W

Zhang, FZ

Shang, YY

Shi, WH

Yao, C

Zhang, XX

Chu, NH

Lu, J

Yuan, JF

AF Jing, Wei

Zhang, Fuzhen

Shang, Yuanyuan

Shi, Wenhui

Yao, Cong

Zhang, Xuxia

Chu, Naihui

Lu, Jie

Yuan, Jinfeng

TI Deciphering the possible role of MmpL7 efflux pump in SQ109 resistance

in Mycobacterium tuberculosis

SO ANNALS OF CLINICAL MICROBIOLOGY AND ANTIMICROBIALS

LA English

DT Article

DE Mycobacterium tuberculosis; SQ109; Susceptibility; MmpL7

ID ANTITUBERCULAR DRUG; SUSCEPTIBILITY; DIAMINE; GENE

AB Background SQ109 is a promising candidate drug for the treatment of patients with drug-resistant tuberculosis (DR-TB). The purpose of this study was to investigate the activity of SQ109 against clinical isolates of Mycobacterium tuberculosis (MTB) from patients with multidrug-resistant TB (MDR-TB) and pre-extensively drug-resistant TB (pre-XDR-TB), and to explore new drug-resistant mechanisms of SQ109. Methods We evaluated the in vitro activity of SQ109 against clinical isolates from patients with MDR-TB and pre-XDR-TB using minimal inhibitory concentration (MIC) assay. The drug-resistant gene, mmpL3 of SQ109-resistant strains was sequenced, and a quantitative real-time PCR assay was used to analyze 28 efflux pump genes in SQ109-resistant strains without mmpL3 mutations. The role of candidate efflux pumps mmpL5 and mmpL7 on the MIC of SQ109 was evaluated using recombinantly cloned MmpL5 and MmpL7 expressed in Mycobacterium smegmatis. Results The MIC90, MIC95 and MIC99 values of SQ109 for 225 clinical isolates of MTB were 0.25 mg/L, 0.5 mg/L and 1.0 mg/L, respectively. Among the pre-XDR strains, six showed resistance to SQ109 despite the absence of gene mutations in mmpL3. In six resistant pre-XDR strains, the MIC of SQ109 decreased with the use of an efflux pump inhibitor, and there was significant upregulation of mmpL5 and mmpL7 in two strains after exposure to SQ109. The presence of MmpL7 in Mycobacterium smegmatis resulted in decreased susceptibility to SQ109, with the MIC increasing from 16 mg/L to 32 mg/L.<br /> Conclusions Our data demonstrated that SQ109 exhibited excellent levels of in vitro activity against MTB. MmpL7 may be a potential gene for MTB resistance to SQ109, providing a useful target for detecting SQ109 resistance in MTB.

C1 [Jing, Wei; Shi, Wenhui; Chu, Naihui] Capital Med Univ, Beijing Chest Hosp, Beijing Thorac Tumor Res Inst, Dept TB, 9 Beiguan St, Beijing 101149, Peoples R China.

[Zhang, Fuzhen; Shang, Yuanyuan; Yao, Cong; Zhang, Xuxia; Yuan, Jinfeng] Capital Med Univ, Beijing Chest Hosp, Beijing TB & Thorac Tumor Res Inst, Dept Bacteriol & Immunol, 9 Beiguan St, Beijing 101149, Peoples R China.

[Lu, Jie] Capital Med Univ, Beijing Childrens Hosp, Beijing Pediat Res Inst, Natl Ctr Childrens Hlth,Beijing Key Lab Pediat Dis, Beijing 101149, Peoples R China.

[Zhang, Fuzhen] Shandong Univ, Cheeloo Coll Med, Sch Publ Hlth, Dept Epidemiol, Jinan 250012, Peoples R China.

C3 Capital Medical University; Capital Medical University; Capital Medical

University; Shandong University

RP Chu, NH (corresponding author), Capital Med Univ, Beijing Chest Hosp, Beijing Thorac Tumor Res Inst, Dept TB, 9 Beiguan St, Beijing 101149, Peoples R China.; Yuan, JF (corresponding author), Capital Med Univ, Beijing Chest Hosp, Beijing TB & Thorac Tumor Res Inst, Dept Bacteriol & Immunol, 9 Beiguan St, Beijing 101149, Peoples R China.; Lu, J (corresponding author), Capital Med Univ, Beijing Childrens Hosp, Beijing Pediat Res Inst, Natl Ctr Childrens Hlth,Beijing Key Lab Pediat Dis, Beijing 101149, Peoples R China.

EM chunaihui1994@sina.com; lujiebch@163.com; yuanjinfeng0920@163.com

RI 袁, 金锋/LZE-8341-2025; Zhang, Fuzhen/LZE-8185-2025

FU Beijing Natural Science Foundation (Youth Program)

FX Not applicable.

CR [Anonymous], 2018, The Global Tuberculosis Report

Black TA, 2021, J CLIN TUBER OTH MYC, V25, DOI 10.1016/j.jctube.2021.100285

Bolla JR, 2020, BIOCHEM SOC T, V48, P1463, DOI 10.1042/BST20190950

Camacho LR, 2001, J BIOL CHEM, V276, P19845, DOI 10.1074/jbc.M100662200

Chen P, 2006, J ANTIMICROB CHEMOTH, V58, P332, DOI 10.1093/jac/dkl227

Franzen O, 2011, Plos Negl Trop D, V5

Imran M, 2022, BIOMEDICINES, V10, DOI 10.3390/biomedicines10112793

Jia J, 2005, BRIT J PHARMACOL, V144, P80, DOI 10.1038/sj.bjp.0705984

Lee RE, 2003, J COMB CHEM, V5, P172, DOI 10.1021/cc020071p

Livak KJ, 2001, METHODS, V25, P402, DOI 10.1006/meth.2001.1262

Moscow Municipal Scientific Practical Center of Tuberculosis Control Health Department of Moscow Russian Federation, 2018, Tuberculosis and lung diseases, V96, P6, DOI [10.21292/2075-1230-2018-96-3-6-18, 10.21292/2075-1230-2018-96-3-6-18, DOI 10.21292/2075-1230-2018-96-3-6-18]

Pang Y, 2011, J MICROBIOL METH, V86, P291, DOI 10.1016/j.mimet.2011.05.016

Pasca MR, 2005, ANTIMICROB AGENTS CH, V49, P4775, DOI 10.1128/AAC.49.11.4775-4777.2005

Protopopova M, 2005, J ANTIMICROB CHEMOTH, V56, P968, DOI 10.1093/jac/dki319

Reddy VM, 2010, ANTIMICROB AGENTS CH, V54, P2840, DOI 10.1128/AAC.01601-09

Sacksteder KA, 2012, FUTURE MICROBIOL, V7, P823, DOI [10.2217/fmb.12.56, 10.2217/FMB.12.56]

Tahlan K, 2012, ANTIMICROB AGENTS CH, V56, P1797, DOI 10.1128/AAC.05708-11

van Klingeren B, 2007, J CLIN MICROBIOL, V45, P2662, DOI 10.1128/JCM.00244-07

Vanino E, 2023, INT J INFECT DIS, V130, pS12, DOI 10.1016/j.ijid.2023.03.013

WHO, 2021, World Health Organization WHO Announces Updated Definitions of Extensively Drug-Resistant Tuberculosis

Xu J, 2023, ANTIMICROB AGENTS CH, V67, DOI 10.1128/aac.01532-22

Zhang FZ, 2020, INFECT DRUG RESIST, V13, P815, DOI 10.2147/IDR.S240716

Zhang MW, 2021, EUR J MED RES, V26, DOI 10.1186/s40001-021-00502-0

Zhang ZJ, 2015, INT J ANTIMICROB AG, V45, P491, DOI 10.1016/j.ijantimicag.2015.01.012

NR 24

TC 0

Z9 2

U1 2

U2 4

PU BMC

PI LONDON

PA CAMPUS, 4 CRINAN ST, LONDON N1 9XW, ENGLAND

EI 1476-0711

J9 ANN CLIN MICROB ANTI

JI Ann. Clin. Microbiol. Antimicrob.

PD SEP 28

PY 2024

VL 23

IS 1

AR 87

DI 10.1186/s12941-024-00746-8

PG 8

WC Microbiology

WE Science Citation Index Expanded (SCI-EXPANDED)

SC Microbiology

GA H6U5O

UT WOS:001324772900002

OA Green Submitted, gold

DA 2026-05-07

ER

PT J

AU Khawbung, JL

Nath, D

Chakraborty, S

AF Khawbung, Jessy Lallungawi

Nath, Durbba

Chakraborty, Supriyo

TI Drug resistant Tuberculosis: A review

SO COMPARATIVE IMMUNOLOGY MICROBIOLOGY AND INFECTIOUS DISEASES

LA English

DT Review

DE Tuberculosis; Mycobacterium tuberculosis; Drug resistance; MDR-TB;

XDR-TB; TDR-TB

AB Tuberculosis (TB) was announced as a global emergency in 1993. There was an alarming counter attack of TB worldwide. However, when it was known that TB can be cured completely, the general public became ignorant towards the infection. The pathogenic organism Mycobacterium tuberculosis continuously evolved to resist the antagonist drugs. This has led to the outbreak of resistant strain that gave rise to "Multi Drug Resistant -Tuberculosis" and "Extensively Drug Resistant Tuberculosis" that can still be cured with a lower success rate. While the mechanism of resistance proceeds further, it ultimately causes unmanageable totally drug resistant TB (TDR-TB). Studying the molecular mechanisms underlying the resistance to drugs would help us grasp the genetics and pathophysiology of the disease. In this review, we present the molecular mechanisms behind Mycobacterium tolerance to drugs and their approach towards the development of multi-drug resistant, extremely drug resistant and totally drug resistant TB.

C1 [Khawbung, Jessy Lallungawi; Nath, Durbba; Chakraborty, Supriyo] Assam Univ, Dept Biotechnol, Silchar 788011, Assam, India.

C3 Assam University

RP Chakraborty, S (corresponding author), Assam Univ, Dept Biotechnol, Silchar 788011, Assam, India.

EM supriyoch_2008@rediffmail.com

CR Agyeman AA, 2017, J PUBLIC HLTH EMERG, V1, P7, DOI [10.21037/jphe.2016.12.08, DOI 10.21037/JPHE.2016.12.08, 10.21037/JPHE.2016.12.08, 10.21037/jphe.2016.12.08]

Ahmad S, 2007, TUBERCULOSIS, V87, P123, DOI 10.1016/j.tube.2006.05.004

Alangaden GJ, 1998, ANTIMICROB AGENTS CH, V42, P1295, DOI 10.1128/AAC.42.5.1295

Da Silva PEA, 2011, J ANTIMICROB CHEMOTH, V66, P1417, DOI 10.1093/jac/dkr173

Amaral L, 2012, ACTA MEDICA PORT, V25, P118

Andini N, 2006, ANTIMICROB AGENTS CH, V50, P2560, DOI 10.1128/AAC.00264-06

Andries K, 2005, SCIENCE, V307, P223, DOI 10.1126/science.1106753

[Anonymous], 2017, Review of available evidence on the use of bedaquiline for the treatment of multidrug-resistant tuberculosis: data analysis report March 8, Version 2017

[Anonymous], 2014, ANT RES GLOB REP SUR

ARMSTRONG JA, 1975, J EXP MED, V142, P1, DOI 10.1084/jem.142.1.1

Aubry A, 2004, ANTIMICROB AGENTS CH, V48, P1281, DOI 10.1128/AAC.48.4.1281-1288.2004

Bloemberg GV, 2015, NEW ENGL J MED, V373, P1986, DOI 10.1056/NEJMc1505196

Blower SM, 2004, NAT MED, V10, P1111, DOI 10.1038/nm1102

Bosne-David S, 2000, J ANTIMICROB CHEMOTH, V46, P391, DOI 10.1093/jac/46.3.391

C. Consortium, 2018, NEW ENGL J MED, V379, P1403

Caceres NE, 1997, J BACTERIOL, V179, P5046, DOI 10.1128/jb.179.16.5046-5055.1997

Caminero JA, 2010, LANCET INFECT DIS, V10, P621, DOI 10.1016/S1473-3099(10)70139-0

Campbell IA, 2006, BRIT MED J, V332, P1194, DOI 10.1136/bmj.332.7551.1194

Cf.D. Control, 1993, MMWR MORB MORTAL WKL, V42, P1

Chahine EB, 2014, ANN PHARMACOTHER, V48, P107, DOI 10.1177/1060028013504087

Cheng AFB, 2004, ANTIMICROB AGENTS CH, V48, P596, DOI 10.1128/AAC.48.2.596-601.2004

Crofton J, 1970, J Ir Med Assoc, V63, P75

CROWLE AJ, 1984, AM REV RESPIR DIS, V130, P839

Cui P., 2017, CLOFAZIMINE TARGETS

Dannenberg Arthur M. Jr., 1994, P459

Desjardins CA, 2016, NAT GENET, V48, P544, DOI 10.1038/ng.3548

Dey T, 2013, J ANTIMICROB CHEMOTH, V68, P284, DOI 10.1093/jac/dks389

Eker B, 2008, EMERG INFECT DIS, V14, P1700, DOI 10.3201/eid1411.080729

Everett MJ, 1996, ANTIMICROB AGENTS CH, V40, P2380, DOI 10.1128/AAC.40.10.2380

Falzon D., 2011, EUR RESP SOC

Falzon D, 2017, EUR RESPIR J, V49, DOI 10.1183/13993003.02308-2016

Fenner L, 2012, ANTIMICROB AGENTS CH, V56, P3047, DOI 10.1128/AAC.06460-11

FINKEN M, 1993, MOL MICROBIOL, V9, P1239, DOI 10.1111/j.1365-2958.1993.tb01253.x

Flores AR, 2005, MICROBIOL-SGM, V151, P521, DOI 10.1099/mic.0.27629-0

Franke MF, 2008, CLIN INFECT DIS, V46, P1844, DOI 10.1086/588292

Furin J, 2007, CURR OPIN PULM MED, V13, P212, DOI 10.1097/MCP.0b013e3280f3c0b2

Furin J, 2019, LANCET, V393, P1642, DOI 10.1016/S0140-6736(19)30308-3

Gandhi NR, 2006, LANCET, V368, P1575, DOI 10.1016/S0140-6736(06)69573-1

Gandhi NR, 2010, LANCET, V375, P1830, DOI 10.1016/S0140-6736(10)60410-2

Gao R., 2020, INT J INFECT DIS

Gopalan N, 2018, JAMA INTERN MED, V178, P485, DOI 10.1001/jamainternmed.2018.0141

GOSS WA, 1965, J BACTERIOL, V89, P1068, DOI 10.1128/JB.89.4.1068-1074.1965

Grosset J, 2003, ANTIMICROB AGENTS CH, V47, P833, DOI 10.1128/AAC.47.3.833-836.2003

Gupta-Wright A, 2018, LANCET, V392, P292, DOI [10.1016/s0140-6736(18)31267-4, 10.1016/S0140-6736(18)31267-4]

Gurumurthy P, 2004, CLIN INFECT DIS, V38, P280, DOI 10.1086/380795

Harding E, 2020, LANCET RESP MED, V8, P19, DOI 10.1016/S2213-2600(19)30418-7

Hartkoorn RC, 2014, ANTIMICROB AGENTS CH, V58, P2979, DOI 10.1128/AAC.00037-14

Hazbón MH, 2006, ANTIMICROB AGENTS CH, V50, P2640, DOI 10.1128/AAC.00112-06

He L, 2015, TUBERCULOSIS, V95, P149, DOI 10.1016/j.tube.2014.12.002

Hillemann D, 2008, ANTIMICROB AGENTS CH, V52, P800, DOI 10.1128/AAC.01189-07

Jassal M, 2009, LANCET INFECT DIS, V9, P19, DOI 10.1016/S1473-3099(08)70260-3

Jin DJ, 1996, METHOD ENZYMOL, V273, P300

Johansen SK, 2006, MOL CELL, V23, P173, DOI 10.1016/j.molcel.2006.05.044

Joshi R, 2006, PLOS MED, V3, P2376, DOI 10.1371/journal.pmed.0030494

Kambli P, 2016, INT J MYCOBACT, V5, P1, DOI 10.1016/j.ijmyco.2015.09.001

Klopper M, 2013, EMERG INFECT DIS, V19, P449, DOI [10.3201/EID1903.120246, 10.3201//EID1903.120246]

Koch A, 2018, CURR OPIN PHARMACOL, V42, P7, DOI 10.1016/j.coph.2018.05.013

KOCHI A, 1993, RES MICROBIOL, V144, P104, DOI 10.1016/0923-2508(93)90023-U

KONNO K, 1967, AM REV RESPIR DIS, V95, P461

KOSEKI Y, 1963, JPN J MED SCI BIOL, V16, P31, DOI 10.7883/yoken1952.16.31

Koul A, 2008, J BIOL CHEM, V283, P25273, DOI 10.1074/jbc.M803899200

Kucers A, 1979, HEINEMANN MED BOOKS

Laurenzo D, 2011, ACTA TROP, V119, P5, DOI 10.1016/j.actatropica.2011.04.008

Leung ETY, 2006, ANTIMICROB AGENTS CH, V50, P1075, DOI 10.1128/AAC.50.3.1075-1078.2006

Nguyen L, 2016, ARCH TOXICOL, V90, P1585, DOI 10.1007/s00204-016-1727-6

Lipsitch M, 1998, INT J TUBERC LUNG D, V2, P187

Lu J, 2014, FEMS MICROBIOL LETT, V353, P40, DOI 10.1111/1574-6968.12401

Machado D, 2013, J ANTIMICROB CHEMOTH, V68, P1728, DOI 10.1093/jac/dkt090

Malik ZA, 2000, J EXP MED, V191, P287, DOI 10.1084/jem.191.2.287

Mangtani P, 2014, CLIN INFECT DIS, V58, P470, DOI 10.1093/cid/cit790

Matteelli A, 2014, CLIN EPIDEMIOL, V6, P111, DOI 10.2147/CLEP.S35839

Maus CE, 2005, ANTIMICROB AGENTS CH, V49, P571, DOI 10.1128/AAC.49.2.571-577.2005

Meier A., 1996, ANTIMICROB AGENTS CH, V40

Migliori GB, 2013, EUR RESPIR J, V42, P169, DOI 10.1183/09031936.00136312

Miotto P, 2018, RESPIROLOGY, V23, P1098, DOI 10.1111/resp.13393

Miotto P, 2015, CHEST, V147, P1135, DOI 10.1378/chest.14-1286

Nguyen L, 2006, TRENDS MICROBIOL, V14, P304, DOI 10.1016/j.tim.2006.05.005

Nguyen VAT, 2015, CLIN MICROBIOL INFEC, V21, P1084, DOI 10.1016/j.cmi.2015.08.024

Niki M, 2012, J BIOL CHEM, V287, P27743, DOI 10.1074/jbc.M111.333385

Ofori-Asenso R., 2017, J Public Health Emerg, V1

OVCHINNIKOV YA, 1981, MOL GEN GENET, V184, P536, DOI 10.1007/BF00352535

Palomino JC, 2014, ANTIBIOTICS-BASEL, V3, P317, DOI 10.3390/antibiotics3030317

Pande T, 2016, INT J TUBERC LUNG D, V20, P1226, DOI 10.5588/ijtld.15.0926

Parida SK, 2015, J INTERN MED, V277, P388, DOI 10.1111/joim.12264

Pasipanodya JG, 2011, CURR OPIN PHARMACOL, V11, P457, DOI 10.1016/j.coph.2011.07.001

PEARSON ML, 1992, ANN INTERN MED, V117, P191, DOI 10.7326/0003-4819-117-3-191

Rattan A, 1998, EMERG INFECT DIS, V4, P195, DOI 10.3201/eid0402.980207

REITZ RH, 1967, BIOCHEMISTRY-US, V6, P2561, DOI 10.1021/bi00860a038

Rengarajan J, 2004, MOL MICROBIOL, V53, P275, DOI 10.1111/j.1365-2958.2004.04120.x

Richter E, 2007, ANTIMICROB AGENTS CH, V51, P1534, DOI 10.1128/AAC.01113-06

Safi H, 2008, ANTIMICROB AGENTS CH, V52, P2027, DOI 10.1128/AAC.01486-07

Saliu OY, 2007, J ANTIMICROB CHEMOTH, V60, P994, DOI 10.1093/jac/dkm291

Satta G, 2018, CLIN MICROBIOL INFEC, V24, P604, DOI 10.1016/j.cmi.2017.10.030

Schena E., 2016, J ANTIMICROB CHEMOTH, V71

Schluger NW, 2005, AM J RESP CELL MOL, V32, P251, DOI 10.1165/rcmb.F293

Scorpio A, 1996, NAT MED, V2, P662, DOI 10.1038/nm0696-662

Scorpio A, 1997, ANTIMICROB AGENTS CH, V41, P540, DOI 10.1128/AAC.41.3.540

Seki M, 2018, EUR J CLIN MICROBIOL, V37, P1405, DOI 10.1007/s10096-018-3258-y

Sharma SK, 2004, INDIAN J MED RES, V120, P354

Sharma SK, 2006, CHEST, V130, P261, DOI [10.1378/chest.130.1.261, 10.1016/S0012-3692(15)50981-1]

Singh R, 2020, J APPL MICROBIOL, V128, P1547, DOI 10.1111/jam.14478

Song H, 2008, TUBERCULOSIS, V88, P526, DOI 10.1016/j.tube.2008.02.004

Sotgiu G, 2012, EUR RESPIR J, V40, P1430, DOI 10.1183/09031936.00022912

SPRATT BG, 1994, SCIENCE, V264, P388, DOI 10.1126/science.8153626

Stover CK, 2000, NATURE, V405, P962, DOI 10.1038/35016103

Suchindran S, 2009, PLOS ONE, V4, DOI 10.1371/journal.pone.0005561

Tahlan K, 2012, ANTIMICROB AGENTS CH, V56, P1797, DOI 10.1128/AAC.05708-11

TAKAYAMA K, 1989, ANTIMICROB AGENTS CH, V33, P1493, DOI 10.1128/AAC.33.9.1493

Takiff HE, 2000, RESURG EMERGING INFE, V1, P77

TAKIFF HE, 1994, ANTIMICROB AGENTS CH, V38, P773, DOI 10.1128/AAC.38.4.773

Telenti A, 1997, NAT MED, V3, P567, DOI 10.1038/nm0597-567

Thiam S, 2007, JAMA-J AM MED ASSOC, V297, P380, DOI 10.1001/jama.297.4.380

Tonge P.J, 2003, P NATL ACAD SCI, V100

Van der Meeren O, 2018, NEW ENGL J MED, V379, P1621, DOI [10.1056/NEJMoa1803484, 10.1056/nejmoa1803484]

van der Werf MJ, 2012, INT J TUBERC LUNG D, V16, P426, DOI 10.5588/ijtld.11.0605

Velayati A., 2010, EUR RESPIR J, V36

Velayati AA, 2013, INT J CLIN EXP MED, V6, P307

Velayati AA, 2009, CHEST, V136, P420, DOI 10.1378/chest.08-2427

W.H. Organisation, 2017, GLOBAL TUBERCULOSIS

W.H. Organization, 2009, WHO POLIC TB INF CON

W.H. Organization, 2010, MULT EXT DRUG RES T

W.H. Organization, 2010, Treatment of Tuberculosis Guidelines

W.H. Organization, 2010, GUID PAP WHO SUPP CO

W.H. Organization, 2008, GUID PROGR MAN DRUG

W.H. Organization, 2010, WHO MULT EXT DRUGR T

Whittle CA, 2016, GENOME BIOL EVOL, V8, P2722, DOI 10.1093/gbe/evw068

Wrande M, 2008, P NATL ACAD SCI USA, V105, P11863, DOI 10.1073/pnas.0804739105

Xu J, 2017, ANTIMICROB AGENTS CH, V61, DOI [10.1128/AAC.00239-17, 10.1128/aac.00239-17]

Ying Z, 2005, ANNU REV PHARMACOL, V45, P529, DOI 10.1146/annurev.pharmtox.45.120403.100120

Zhang Y, 2015, INT J TUBERC LUNG D, V19, P1276, DOI 10.5588/ijtld.15.0389

ZHANG Y, 1992, NATURE, V358, P591, DOI 10.1038/358591a0

Zhang Y, 2014, EMERG MICROBES INFEC, V3, DOI 10.1038/emi.2014.3

Zhang ZJ, 2014, ANTIMICROB AGENTS CH, V58, P364, DOI 10.1128/AAC.01228-13

Zhao F, 2014, ANTIMICROB AGENTS CH, V58, P1479, DOI 10.1128/AAC.01775-13

Zheng J, 2013, J BIOL CHEM, V288, P23447, DOI 10.1074/jbc.M113.475798

Zumla AI, 2014, LANCET INFECT DIS, V14, P327, DOI 10.1016/S1473-3099(13)70328-1

NR 136

TC 165

Z9 208

U1 9

U2 172

PU ELSEVIER SCI LTD

PI London

PA 125 London Wall, London, ENGLAND

SN 0147-9571

EI 1878-1667

J9 COMP IMMUNOL MICROB

JI Comp. Immunol. Microbiol. Infect. Dis.

PD FEB

PY 2021

VL 74

AR 101574

DI 10.1016/j.cimid.2020.101574

PG 9

WC Immunology; Microbiology; Veterinary Sciences

WE Science Citation Index Expanded (SCI-EXPANDED)

SC Immunology; Microbiology; Veterinary Sciences

GA QK4OR

UT WOS:000620367000001

DA 2026-05-07

ER

PT J

AU Porwal, C

Kaushik, A

Makkar, N

Banavaliker, JN

Hanif, M

Singla, R

Bhatnagar, AK

Behera, D

Pande, JN

Singh, UB

AF Porwal, Chhavi

Kaushik, Amit

Makkar, Nayani

Banavaliker, Jayant N.

Hanif, Mahmud

Singla, Rupak

Bhatnagar, Anuj K.

Behera, Digambar

Pande, Jitendra Nath

Singh, Urvashi B.

TI Incidence and Risk Factors for Extensively Drug-Resistant Tuberculosis

in Delhi Region

SO PLOS ONE

LA English

DT Article

ID MYCOBACTERIUM-TUBERCULOSIS; MULTIDRUG-RESISTANT; 2ND-LINE DRUGS; XDR-TB;

SOUTH-AFRICA; INDIA; PREVALENCE; SYSTEM

AB Background: India with a major burden of multidrug-resistant tuberculosis (MDR-TB) does not have national level data on this hazardous disease. Since 2006, emergence of extensively drug-resistant TB (XDR-TB) is considered a serious threat to global TB control. This study highlights the demographic and clinical risk factors associated with XDR-TB in Delhi.

Methods: The study was conducted during April 2007 to May 2010. Six hundred eleven MDR-TB suspects were enrolled from four tertiary care hospitals, treating TB patients in Delhi and the demographic details recorded. Sputum samples were cultured using rapid, automated liquid culture system (MGIT 960). Drug susceptibility testing (DST) for Rifampicin (RIF) and Isoniazid (INH) was performed for all positive M. tuberculosis (M.tb) cultures. All MDR-TB isolates were tested for sensitivity to second-line drugs [Amikacin (AMK), Capreomycin (CAP), Ofloxacin (OFX), Ethionamide (ETA)].

Results/Findings: Of 611, 483 patients were infected with MDR M. tuberculosis (M. tb) strains. Eighteen MDR-TB isolates (3.7%) were XDR M.tb strains. Family history of TB (p 0.045), socioeconomic status (p 0.013), concomitant illness (p 0.001) and previous intake of 2nd line injectable drugs (p 0.001) were significantly associated with occurrence of XDR-TB. Only two of the patients enrolled were HIV seropositive, but had a negative culture for M. tuberculosis. 56/483 isolates were pre-XDR M. tuberculosis, though the occurrence of pre-XDR-TB did not show any significant demographical associations.

Conclusions: The actual incidence and prevalence rate of XDR-TB in India is not available, although some scattered data is available. This study raises a concern about existence of XDR-TB in India, though small, signaling a need to strengthen the TB control program for early diagnosis of both tuberculosis and drug resistance in order to break the chains of transmission.

C1 [Porwal, Chhavi; Kaushik, Amit; Makkar, Nayani; Singh, Urvashi B.] All India Inst Med Sci, Dept Microbiol, New Delhi 110029, India.

[Banavaliker, Jayant N.; Bhatnagar, Anuj K.] Rajan Babu Inst Pulm Med & TB, Delhi, India.

[Hanif, Mahmud] New Delhi TB Ctr, New Delhi, India.

[Singla, Rupak; Behera, Digambar] Lala Ram Sarup Inst TB & Resp Dis, New Delhi, India.

[Pande, Jitendra Nath] Sitaram Bhartia Inst Sci & Res, New Delhi, India.

C3 All India Institute of Medical Sciences (AIIMS) New Delhi; Sitaram

Bhartia Institute of Science & Research

RP Singh, UB (corresponding author), All India Inst Med Sci, Dept Microbiol, New Delhi 110029, India.

EM drurvashi@gmail.com

RI , Amit/J-8836-2019; Kaushik, Amit/J-8836-2019

OI , Amit/0000-0002-5622-1721;

FU Indian Council of Medical Research (ICMR), New Delhi; Council of

Scientific and Industrial Research (CSIR), New Delhi

FX Chhavi Porwal and Amit Kaushik gratefully acknowledge the research

fellowships from the Indian Council of Medical Research (ICMR) and

Council of Scientific and Industrial Research (CSIR), New Delhi

respectively.

CR Ajbani K, 2011, J CLIN MICROBIOL, V49, P1588, DOI 10.1128/JCM.00113-11

[Anonymous], 1985, Public health mycobacteriology: a guide for the level III laboratory

[Anonymous], 2006, JAMA: the Journal of the American Medical Association (American Medical Association), V296, P2792

[Anonymous], 2006, Weekly Epidemiol Rec, V81, P430

[Anonymous], 2010, WHO/HTM/TB/2010.3

Balaji V, 2010, PLOS ONE, V5, DOI 10.1371/journal.pone.0009527

Banerjee R, 2008, CLIN INFECT DIS, V47, P450, DOI 10.1086/590009

Chan ED, 2009, CLIN INFECT DIS, V48, pE50, DOI 10.1086/597010

Dalton T, 2012, LANCET, V380, P1406, DOI 10.1016/S0140-6736(12)60734-X

Jain A, 2008, J BIOSCIENCES, V33, P605, DOI 10.1007/s12038-008-0078-8

Jassal M, 2009, LANCET INFECT DIS, V9, P19, DOI 10.1016/S1473-3099(08)70260-3

Jeon CY, 2008, CLIN INFECT DIS, V46, P42, DOI 10.1086/524017

Khanna A, 2010, ANTIMICROB AGENTS CH, V54, P4789, DOI 10.1128/AAC.00661-10

Lin SYG, 2009, J CLIN MICROBIOL, V47, P3630, DOI 10.1128/JCM.00803-09

Martin A, 2008, J CLIN MICROBIOL, V46, P3952, DOI 10.1128/JCM.01171-08

Maus CE, 2005, ANTIMICROB AGENTS CH, V49, P3192, DOI 10.1128/AAC.49.8.3192-3197.2005

Migliori GB, 2007, EUR RESPIR J, V29, P423, DOI 10.1183/09031936.00001307

Mlambo CK, 2008, INT J TUBERC LUNG D, V12, P99

Mondal R, 2007, EMERG INFECT DIS, V13, P1429, DOI 10.3201/eid1309.070443

Paramasivan CN, 2010, INT J TUBERC LUNG D, V14, P243

Raviglione MC, 2007, NEW ENGL J MED, V356, P656, DOI 10.1056/NEJMp068273

Rodrigues C, 2008, INT J TUBERC LUNG D, V12, P1449

Rüsch-Gerdes S, 2006, J CLIN MICROBIOL, V44, P688, DOI 10.1128/JCM.44.3.688-692.2006

Sharma SK, 2009, INDIAN J MED RES, V130, P392

Siddiqi S., 2006, MGIT Procedure Manual: For BACTEC MGIT 960 TB System

Siddiqi S, 2006, WHOHTMTB2008392

Singh JA, 2007, PLOS MED, V4, P19, DOI 10.1371/journal.pmed.0040050

Singh S, 2007, AIDS, V21, P2345, DOI 10.1097/QAD.0b013e3282f125c9

World Health Organization, 2012, WHOHTMTB20126

NR 29

TC 29

Z9 43

U1 0

U2 14

PU PUBLIC LIBRARY SCIENCE

PI SAN FRANCISCO

PA 1160 BATTERY STREET, STE 100, SAN FRANCISCO, CA 94111 USA

SN 1932-6203

J9 PLOS ONE

JI PLoS One

PD FEB 4

PY 2013

VL 8

IS 2

AR e55299

DI 10.1371/journal.pone.0055299

PG 7

WC Multidisciplinary Sciences

WE Science Citation Index Expanded (SCI-EXPANDED)

SC Science & Technology - Other Topics

GA 086NH

UT WOS:000314691100040

OA Green Submitted, gold

DA 2026-05-07

ER

PT J

AU Sheikh, BA

Bhat, BA

Mehraj, U

Mir, W

Hamadani, S

Mir, MA

AF Sheikh, Bashir A.

Bhat, Basharat A.

Mehraj, Umar

Mir, Wajahat

Hamadani, Suhail

Mir, Manzoor A.

TI Development of New Therapeutics to Meet the Current Challenge of Drug

Resistant Tuberculosis

SO CURRENT PHARMACEUTICAL BIOTECHNOLOGY

LA English

DT Review

DE Tuberculosis; multidrug resistant TB; gene mutations; drug targets;

extensively drug resistant TB (XDR-TB); totally drug resistant TB

(TDR-TB)

ID PARA-AMINOSALICYLIC ACID; MYCOBACTERIUM-TUBERCULOSIS; ISONIAZID

RESISTANCE; STREPTOMYCIN RESISTANCE; RIFAMPICIN-RESISTANCE; PYRAZINAMIDE

RESISTANCE; MOLECULAR-BASIS; CELL-WALL; IN-VITRO;

DIHYDROFOLATE-REDUCTASE

AB Tuberculosis (TB) is a prominent infective disease and a major reason of mortality/morbidity globally. Mycobacterium tuberculosis causes a long-lasting latent infection in a significant proportion of human population. The increasing burden of tuberculosis is mainly caused due to multi drug-resistance. The failure of conventional treatment has been observed in large number of cases. Drugs that are used to treat extensively drug-resistant tuberculosis are expensive, have limited efficacy, and have more side effects for a longer duration of time and are often associated with poor prognosis. To regulate the emergence of multidrug resistant tuberculosis, extensively drug-resistant tuberculosis and totally drug resistant tuberculosis, efforts are being made to understand the genetic/molecular basis of target drug delivery and mechanisms of drug resistance. Understanding the molecular approaches and pathology of Mycobacterium tuberculosis through whole genome sequencing may further help in the improvement of new therapeutics to meet the current challenge of global health. Understanding cellular mechanisms that trigger resistance to Mycobacterium tuberculosis infection may expose immune associates of protection, which could be an important way for vaccine development, diagnostics, and novel host-directed therapeutic strategies. The recent development of new drugs and combinational therapies for drug-resistant tuberculosis through major collaboration between industry, donors, and academia gives an improved hope to overcome the challenges in tuberculosis treatment. In this review article, an attempt was made to highlight the new developments of drug resistance to the conventional drugs and the recent progress in the development of new therapeutics for the treatment of drug-resistant and non-resistant cases.

C1 [Sheikh, Bashir A.; Bhat, Basharat A.; Mehraj, Umar; Mir, Wajahat; Hamadani, Suhail; Mir, Manzoor A.] Univ Kashmir, Sch Biol Sci, Dept Bioresources, Srinagar 190006, J&k, India.

C3 University of Kashmir

RP Mir, MA (corresponding author), Univ Kashmir, Sch Biol Sci, Dept Bioresources, Srinagar 190006, J&k, India.

EM drmanzoor@kashmiruniversity.ac.in

RI ; Mir, Manzoor/W-8905-2018; Mehraj, Umar/MXJ-7443-2025

OI Haani, Syed suhail/0000-0002-5325-7163; sheikh, Bashir

ahmad/0000-0002-1087-564X; Mir, Manzoor/0000-0003-3297-1402;

FU Science and Engineering Research Board, Department of Science and

Technology (SERB-DST) [TAR/2018/00l213]

FX This work was supported by the Science and Engineering Research Board,

Department of Science and Technology (SERB-DST), Govt. of India via

Grant (FILENO.TAR/2018/00l213).

CR Ahmad S, 2007, TUBERCULOSIS, V87, P123, DOI 10.1016/j.tube.2006.05.004

Alangaden GJ, 1998, ANTIMICROB AGENTS CH, V42, P1295, DOI 10.1128/AAC.42.5.1295

Alexander DC, 2012, J CLIN MICROBIOL, V50, P3726, DOI 10.1128/JCM.00620-12

Da Silva PEA, 2011, J ANTIMICROB CHEMOTH, V66, P1417, DOI 10.1093/jac/dkr173

Andini N, 2006, ANTIMICROB AGENTS CH, V50, P2560, DOI 10.1128/AAC.00264-06

Ando H, 2014, MOL MICROBIOL, V91, P538, DOI 10.1111/mmi.12476

Ando H, 2011, MOL MICROBIOL, V79, P1615, DOI 10.1111/j.1365-2958.2011.07547.x

Andries K, 2005, SCIENCE, V307, P223, DOI 10.1126/science.1106753

[Anonymous], 2013, The use of Bedaquiline in the treatment of m ultidrug-resistant tuberculosis. Interim policy guidance

[Anonymous], 1999, WORLD HLTH REPORT 19

[Anonymous], 2013, TECHNOLOGY RAPID SIM

Argyrou A, 2006, NAT STRUCT MOL BIOL, V13, P408, DOI 10.1038/nsmb1089

Argyrou A, 2006, BIOCHEMISTRY-US, V45, P13947, DOI 10.1021/bi061874m

Arjanova OV, 2011, IMMUNOTHERAPY-UK, V3, P181, DOI [10.2217/imt.10.96, 10.2217/IMT.10.96]

Aubry A, 2004, ANTIMICROB AGENTS CH, V48, P1281, DOI 10.1128/AAC.48.4.1281-1288.2004

Aubry A, 2006, ANTIMICROB AGENTS CH, V50, P104, DOI 10.1128/AAC.50.1.104-112.2006

BANERJEE A, 1994, SCIENCE, V263, P227, DOI 10.1126/science.8284673

Banu S, 2014, J CLIN MICROBIOL, V52, P156, DOI 10.1128/JCM.02378-13

Barnard FM, 2001, ANTIMICROB AGENTS CH, V45, P1994, DOI 10.1128/AAC.45.7.1994-2000.2001

BARRY VC, 1957, NATURE, V179, P1013, DOI 10.1038/1791013a0

Batdelger D, 2008, VACCINE, V26, P2733, DOI 10.1016/j.vaccine.2008.03.021

Beckert P, 2012, ANTIMICROB AGENTS CH, V56, P2743, DOI 10.1128/AAC.06227-11

Blanchard JS, 1996, ANNU REV BIOCHEM, V65, P215, DOI 10.1146/annurev.bi.65.070196.001243

Borrell S, 2011, CLIN MICROBIOL INFEC, V17, P815, DOI 10.1111/j.1469-0691.2011.03556.x

Bosne-David S, 2000, J ANTIMICROB CHEMOTH, V46, P391, DOI 10.1093/jac/46.3.391

Böttger EC, 1998, NAT MED, V4, P1343, DOI 10.1038/3906

Boyce John M., 2002, Infection Control and Hospital Epidemiology, V23, pS3, DOI 10.1086/502007

Brandis G, 2013, J ANTIMICROB CHEMOTH, V68, P2493, DOI 10.1093/jac/dkt224

Brossier F, 2011, ANTIMICROB AGENTS CH, V55, P355, DOI 10.1128/AAC.01030-10

BROWNE S. G., 1962, LEPROSY REV, V33, P6

Burman WJ, 2001, AM J RESP CRIT CARE, V164, P7, DOI 10.1164/ajrccm.164.1.2101133

Butov D.A., 2011, VACCINES, V9, P3

Butov DA, 2013, IMMUNOTHERAPY-UK, V5, P1047, DOI 10.2217/imt.13.110

Caceres NE, 1997, J BACTERIOL, V179, P5046, DOI 10.1128/jb.179.16.5046-5055.1997

Campbell EA, 2001, CELL, V104, P901, DOI 10.1016/S0092-8674(01)00286-0

Campbell PJ, 2011, ANTIMICROB AGENTS CH, V55, P2032, DOI 10.1128/AAC.01550-10

Cardosa RF, 2007, MEM I OSWALDO CRUZ, V102, P59, DOI 10.1590/S0074-02762007000100009

Carette X, 2012, NUCLEIC ACIDS RES, V40, P3018, DOI 10.1093/nar/gkr1113

Cavusoglu C, 2004, CLIN MICROBIOL INFEC, V10, P662, DOI 10.1111/j.1469-0691.2004.00917.x

Caws M, 2008, PLOS PATHOG, V4, DOI 10.1371/journal.ppat.1000034

Chen JM, 2012, PLOS ONE, V7, DOI 10.1371/journal.pone.0043467

Chen P, 2006, J ANTIMICROB CHEMOTH, V58, P332, DOI 10.1093/jac/dkl227

Cheng AFB, 2004, ANTIMICROB AGENTS CH, V48, P596, DOI 10.1128/AAC.48.2.596-601.2004

Cheng SJ, 2000, ANTIMICROB AGENTS CH, V44, P528, DOI 10.1128/AAC.44.3.528-532.2000

Cholo MC, 2012, J ANTIMICROB CHEMOTH, V67, P290, DOI 10.1093/jac/dkr444

Cohen T, 2012, CLIN MICROBIOL REV, V25, P708, DOI 10.1128/CMR.00021-12

Comas I, 2013, NAT GENET, V45, P1176, DOI 10.1038/ng.2744

Comas I, 2012, NAT GENET, V44, P106, DOI 10.1038/ng.1038

CROFTON J, 1948, BMJ-BRIT MED J, V2, P1009, DOI 10.1136/bmj.2.4588.1009

Das S, 2017, International Journal of Medical and Dental Sciences, V6, P1449, DOI [10.19056/ijmdsjssmes/2017/v6i1/125574, 10.19056/ijmdsjssmes/2017/v6i1/125574, DOI 10.19056/IJMDSJSSMES/2017/V6I1/125574]

Dawson R., 2017, C RETR OPP INF CROI

Dawson R, 2009, PLOS ONE, V4, DOI 10.1371/journal.pone.0006984

de Vos M, 2013, ANTIMICROB AGENTS CH, V57, P827, DOI 10.1128/AAC.01541-12

DeBarber AE, 2000, P NATL ACAD SCI USA, V97, P9677, DOI 10.1073/pnas.97.17.9677

Dorjee K., 2019, INDIAN J TUBERC, V67, P59

Drobniewski F, 2005, JAMA-J AM MED ASSOC, V293, P2726, DOI 10.1001/jama.293.22.2726

Dye C, 1999, JAMA-J AM MED ASSOC, V282, P677, DOI 10.1001/jama.282.7.677

EDLIN BR, 1992, NEW ENGL J MED, V326, P1514, DOI 10.1056/NEJM199206043262302

Elliott AM, 2004, J INFECT DIS, V190, P869, DOI 10.1086/422257

ELLIOTT AM, 1992, Q J MED, V85, P855

Escribano I, 2007, CHEMOTHERAPY, V53, P397, DOI 10.1159/000109769

Espinal MA, 2001, NEW ENGL J MED, V344, P1294, DOI 10.1056/NEJM200104263441706

Fàbrega A, 2009, MICROB BIOTECHNOL, V2, P40, DOI 10.1111/j.1751-7915.2008.00063.x

Falzon D, 2017, EUR RESPIR J, V49, DOI 10.1183/13993003.02308-2016

Farhat MR, 2013, NAT GENET, V45, P1183, DOI 10.1038/ng.2747

Fenner L, 2012, ANTIMICROB AGENTS CH, V56, P3047, DOI 10.1128/AAC.06460-11

Field S, 2013, DERM-ENDOCRINOL, V5, DOI 10.4161/derm.25244

FINKEN M, 1993, MOL MICROBIOL, V9, P1239, DOI 10.1111/j.1365-2958.1993.tb01253.x

FISCHL MA, 1992, ANN INTERN MED, V117, P177, DOI 10.7326/0003-4819-117-3-177

FRIEDEN TR, 1993, NEW ENGL J MED, V328, P521, DOI 10.1056/NEJM199302253280801

Gagneux S, 2007, LANCET INFECT DIS, V7, P328, DOI 10.1016/S1473-3099(07)70108-1

Gandhi NR, 2006, LANCET, V368, P1575, DOI 10.1016/S0140-6736(06)69573-1

Gandhi NR, 2010, LANCET, V375, P1830, DOI 10.1016/S0140-6736(10)60410-2

García-García MD, 2000, ARCH INTERN MED, V160, P630, DOI 10.1001/archinte.160.5.630

Georghiou SB, 2012, PLOS ONE, V7, DOI 10.1371/journal.pone.0033275

Gillespie SH, 2002, ANTIMICROB AGENTS CH, V46, P267, DOI 10.1128/AAC.46.2.267-274.2002

Gopaul KK, 2006, J CLIN MICROBIOL, V44, P2492, DOI 10.1128/JCM.01428-05

GOSS WA, 1965, J BACTERIOL, V89, P1068, DOI 10.1128/JB.89.4.1068-1074.1965

Gov C, 2000, SAF EFF VAR DOS TREA

Grzegorzewicz AE, 2012, J BIOL CHEM, V287, P38434, DOI 10.1074/jbc.M112.400994

Grzegorzewicz AE, 2012, NAT CHEM BIOL, V8, P334, DOI [10.1038/NCHEMBIO.794, 10.1038/nchembio.794]

Gupta A, 2012, PLOS ONE, V7, DOI 10.1371/journal.pone.0039215

Hameed HMA, 2018, FRONT CELL INFECT MI, V8, DOI 10.3389/fcimb.2018.00114

Hartkoorn RC, 2014, ANTIMICROB AGENTS CH, V58, P2979, DOI 10.1128/AAC.00037-14

Hazbón MH, 2008, ANTIMICROB AGENTS CH, V52, P3369, DOI 10.1128/AAC.00309-08

Hazbón MH, 2006, ANTIMICROB AGENTS CH, V50, P2640, DOI 10.1128/AAC.00112-06

Heep M, 2000, ANTIMICROB AGENTS CH, V44, P1075, DOI 10.1128/AAC.44.4.1075-1077.2000

Helb D, 2010, J CLIN MICROBIOL, V48, P229, DOI 10.1128/JCM.01463-09

Heym B, 1997, INFECT IMMUN, V65, P1395, DOI 10.1128/IAI.65.4.1395-1401.1997

Hillemann D, 2008, ANTIMICROB AGENTS CH, V52, P800, DOI 10.1128/AAC.01189-07

Himasree Y., 2017, MYCOBACT DIS, V7, P250

Ho YM, 2009, ANTIMICROB AGENTS CH, V53, P4010, DOI 10.1128/AAC.00433-09

Hofman S, 2016, EXPERT OPIN EMERG DR, V21, P103, DOI 10.1517/14728214.2016.1151000

Holden C, 1999, SCIENCE, V286, P1670, DOI 10.1126/science.286.5445.1670

Huang YZ, 2010, BIOMATERIALS, V31, P9086, DOI 10.1016/j.biomaterials.2010.08.046

Huh AJ, 2011, J CONTROL RELEASE, V156, P128, DOI 10.1016/j.jconrel.2011.07.002

India T., 2017, RNTCP STATUS REPORT

Ioerger TR, 2010, BMC GENOMICS, V11, DOI 10.1186/1471-2164-11-670

Jia J, 2005, BRIT J PHARMACOL, V144, P80, DOI 10.1038/sj.bjp.0705984

Jiao XN, 2002, J IMMUNOL, V168, P1294, DOI 10.4049/jimmunol.168.3.1294

Johansen SK, 2006, MOL CELL, V23, P173, DOI 10.1016/j.molcel.2006.05.044

Kim SJ, 2005, EUR RESPIR J, V25, P564, DOI 10.1183/09031936.05.00111304

Kimerling ME, 1999, INT J TUBERC LUNG D, V3, P451

KONNO K, 1967, AM REV RESPIR DIS, V95, P461

Koul A, 2007, NAT CHEM BIOL, V3, P323, DOI 10.1038/nchembio884

Krüüner A, 2003, ANTIMICROB AGENTS CH, V47, P2971, DOI 10.1128/AAC.47.9.2971-2973.2003

Lanzas F, 2013, J CLIN MICROBIOL

Larsen MH, 2002, MOL MICROBIOL, V46, P453, DOI 10.1046/j.1365-2958.2002.03162.x

Laurenzo D, 2011, ACTA TROP, V119, P5, DOI 10.1016/j.actatropica.2011.04.008

Law S, 2017, LANCET PUBLIC HEALTH, V2, pE47, DOI 10.1016/S2468-2667(16)30035-4

Lawn SD, 2013, LANCET INFECT DIS, V13, P349, DOI 10.1016/S1473-3099(13)70008-2

Leach KL, 2011, ANN NY ACAD SCI, V1222, P49, DOI 10.1111/j.1749-6632.2011.05962.x

Lew W, 2008, ANN INTERN MED, V149, P123, DOI 10.7326/0003-4819-149-2-200807150-00008

Li J, 2014, EMERG MICROBES INFEC, V3, DOI 10.1038/emi.2014.21

Li XZ, 2009, DRUGS, V69, P1555, DOI 10.2165/11317030-000000000-00000

Liu CH, 2017, CELL MOL IMMUNOL, V14, P963, DOI 10.1038/cmi.2017.88

Machado D, 2013, J ANTIMICROB CHEMOTH, V68, P1728, DOI 10.1093/jac/dkt090

Machado D, 2012, PLOS ONE, V7, DOI 10.1371/journal.pone.0034538

Maruri F, 2012, J ANTIMICROB CHEMOTH, V67, P819, DOI 10.1093/jac/dkr566

Mathema B, 2006, CLIN MICROBIOL REV, V19, P658, DOI 10.1128/CMR.00061-05

Mathur ML, 2006, CURR RESPIR MED REV, V2, P183, DOI 10.2174/157339806776843120

Mathuria JP, 2009, DIG J NANOMATER BIOS, V4, P309

Mathys V, 2009, ANTIMICROB AGENTS CH, V53, P2100, DOI 10.1128/AAC.01197-08

Mayosi BM, 2014, NEW ENGL J MED, V371, P1121, DOI 10.1056/NEJMoa1407380

MCCLATCHY JK, 1977, TUBERCLE, V58, P29, DOI 10.1016/S0041-3879(77)80007-X

Miesel L, 1998, J BACTERIOL, V180, P2459, DOI 10.1128/JB.180.9.2459-2467.1998

Migliori GB, 2012, CLIN INFECT DIS, V54, P1379, DOI 10.1093/cid/cis128

MIKUSOVA K, 1995, ANTIMICROB AGENTS CH, V39, P2484, DOI 10.1128/AAC.39.11.2484

Millet JP, 2013, EUR SPINE J, V22, P539, DOI 10.1007/s00586-012-2334-8

MITCHISON DA, 1979, CHEST, V76, P771, DOI 10.1378/chest.76.6.771

MITCHISON DA, 1985, TUBERCLE, V66, P219, DOI 10.1016/0041-3879(85)90040-6

MOAZED D, 1987, NATURE, V327, P389, DOI 10.1038/327389a0

Müller B, 2013, PLOS ONE, V8, DOI 10.1371/journal.pone.0070919

Müller B, 2013, TRENDS GENET, V29, P160, DOI 10.1016/j.tig.2012.11.005

MUSSER JM, 1995, CLIN MICROBIOL REV, V8, P496, DOI 10.1128/CMR.8.4.496

O'Garra A, 2013, ANNU REV IMMUNOL, V31, P475, DOI 10.1146/annurev-immunol-032712-095939

Okamoto S, 2007, MOL MICROBIOL, V63, P1096, DOI 10.1111/j.1365-2958.2006.05585.x

Organization W.H., 2017, Declaration on Good Off-Label Use Practice

Organization WH, 1997, WHOTB97229

Organization WH, 2009, Pathways to better diagnostics for tuberculosis: a blueprint for the development of TB diagnostics by the new diagnostics working group of the Stop TB Partnership

Palomino JC, 2013, CURR MED CHEM, V20, P3785, DOI 10.2174/09298673113209990166

Pietersen E, 2014, LANCET, V383, P1230, DOI 10.1016/S0140-6736(13)62675-6

Portaels F, 1999, INT J TUBERC LUNG D, V3, P582

Pym AS, 2016, EUR RESPIR J, V47, P564, DOI 10.1183/13993003.00724-2015

Ramaswamy S., 1998, Tubercle and Lung Disease, V79, P3, DOI 10.1054/tuld.1998.0002

Ramaswamy SV, 2003, ANTIMICROB AGENTS CH, V47, P1241, DOI 10.1128/AAC.47.4.1241-1250.2003

Raviglione M C, 1997, AIDS, V11 Suppl B, pS115

RAVIGLIONE MC, 1995, JAMA-J AM MED ASSOC, V273, P220, DOI 10.1001/jama.273.3.220

Rawat R, 2003, P NATL ACAD SCI USA, V100, P13881, DOI 10.1073/pnas.2235848100

Reddy VM, 2010, ANTIMICROB AGENTS CH, V54, P2840, DOI 10.1128/AAC.01601-09

Rengarajan J, 2004, MOL MICROBIOL, V53, P275, DOI 10.1111/j.1365-2958.2004.04120.x

Richter E, 2007, ANTIMICROB AGENTS CH, V51, P1534, DOI 10.1128/AAC.01113-06

Rinder H, 1998, EUR J CLIN MICROBIOL, V17, P508

Rouse DA, 1996, MOL MICROBIOL, V22, P583, DOI 10.1046/j.1365-2958.1996.00133.x

Rozwarski DA, 1998, SCIENCE, V279, P98, DOI 10.1126/science.279.5347.98

Rustomjee R, 2008, INT J TUBERC LUNG D, V12, P128

Rustomjee R, 2015, INFECT DRUG RESIST, V8, P359, DOI 10.2147/IDR.S62119

Sacksteder KA, 2012, FUTURE MICROBIOL, V7, P823, DOI [10.2217/fmb.12.56, 10.2217/FMB.12.56]

Salk JJ, 2018, NAT REV GENET, V19, P269, DOI 10.1038/nrg.2017.117

Schieffer A, 2014, TRANSFORM INNOV SER, P1

Schmalstieg AM, 2012, ANTIMICROB AGENTS CH, V56, P4806, DOI 10.1128/AAC.05546-11

Scorpio A, 1996, NAT MED, V2, P662, DOI 10.1038/nm0696-662

Scorpio A, 1997, ANTIMICROB AGENTS CH, V41, P540, DOI 10.1128/AAC.41.3.540

Sherman DR, 1996, SCIENCE, V272, P1641, DOI 10.1126/science.272.5268.1641

Silva DR, 2018, J BRAS PNEUMOL, V44, P153, DOI [10.1590/s1806-37562017000000436, 10.1590/S1806-37562017000000436]

Simons SO, 2013, J CLIN MICROBIOL, V51, P382, DOI 10.1128/JCM.02739-12

Siu GKH, 2011, J ANTIMICROB CHEMOTH, V66, P730, DOI 10.1093/jac/dkq519

Sloan DJ, 2016, T ROY SOC TROP MED H, V110, P163, DOI 10.1093/trstmh/trv107

Somoskovi A, 2001, RESP RES, V2, P164, DOI 10.1186/rr54

Spies FS, 2008, ANTIMICROB AGENTS CH, V52, P2947, DOI 10.1128/AAC.01570-07

Srajal P, REV NOVEL APROACHES, V3, P4

Sreevatsan S, 1997, ANTIMICROB AGENTS CH, V41, P1677, DOI 10.1128/AAC.41.8.1677

Stanley RE, 2010, NAT STRUCT MOL BIOL, V17, P289, DOI 10.1038/nsmb.1755

Stover CK, 2000, NATURE, V405, P962, DOI 10.1038/35016103

Sun ZG, 2008, INT J ANTIMICROB AG, V31, P115, DOI 10.1016/j.ijantimicag.2007.10.014

Suzuki Y, 1998, J CLIN MICROBIOL, V36, P1220, DOI 10.1128/JCM.36.5.1220-1225.1998

Tahlan K, 2012, ANTIMICROB AGENTS CH, V56, P1797, DOI 10.1128/AAC.05708-11

TAKAYAMA K, 1989, ANTIMICROB AGENTS CH, V33, P1493, DOI 10.1128/AAC.33.9.1493

TAKIFF HE, 1994, ANTIMICROB AGENTS CH, V38, P773, DOI 10.1128/AAC.38.4.773

Tan YJ, 2014, J CLIN MICROBIOL, V52, P291, DOI 10.1128/JCM.01903-13

TELENTI A, 1993, LANCET, V341, P647, DOI 10.1016/0140-6736(93)90417-F

Telenti A, 1997, NAT MED, V3, P567, DOI 10.1038/nm0597-567

TRIVEDI SS, 1988, TUBERCLE, V69, P37, DOI 10.1016/0041-3879(88)90038-4

Udwadia ZF, 2012, CLIN INFECT DIS, V54, P579, DOI 10.1093/cid/cir889

Upton AM, 2015, ANTIMICROB AGENTS CH, V59, P136, DOI 10.1128/AAC.03823-14

Van Deun A, 2011, INT J TUBERC LUNG D, V15, P116

Varela C, 2012, CHEM BIOL, V19, P498, DOI 10.1016/j.chembiol.2012.03.006

Velayati AA, 2009, CHEST, V136, P420, DOI 10.1378/chest.08-2427

Vilchèze C, 2005, ANTIMICROB AGENTS CH, V49, P708, DOI 10.1128/AAC.49.2.708-720.2005

Vilchèze C, 2008, MOL MICROBIOL, V69, P1316, DOI 10.1111/j.1365-2958.2008.06365.x

Vilchéze C, 2007, ANNU REV MICROBIOL, V61, P35, DOI 10.1146/annurev.micro.61.111606.122346

Wang F, 2010, ANTIMICROB AGENTS CH, V54, P3776, DOI 10.1128/AAC.00453-10

Wang LL, 2017, INT J NANOMED, V12, P1227, DOI 10.2147/IJN.S121956

Willcox P A, 2001, Curr Opin Pulm Med, V7, P148, DOI 10.1097/00063198-200105000-00006

Winston CA, 2012, EMERG INFECT DIS, V18, P1201, DOI 10.3201/eid1807.120261

World Health Organization, SuRF report; 2)

Wright A., 2006, Morbidity and Mortality Weekly Report, V55, P301

Xavier AS, 2014, J PHARMACOL PHARMACO, V5, P222, DOI 10.4103/0976-500X.136121

Xu P, 2015, SARCOIDOSIS VASC DIF, V32, P83

Yang B, 1998, J ANTIMICROB CHEMOTH, V42, P621, DOI 10.1093/jac/42.5.621

Yano T, 2011, J BIOL CHEM, V286, P10276, DOI 10.1074/jbc.M110.200501

Ying Z, 2005, ANNU REV PHARMACOL, V45, P529, DOI 10.1146/annurev.pharmtox.45.120403.100120

Zaunbrecher MA, 2009, P NATL ACAD SCI USA, V106, P20004, DOI 10.1073/pnas.0907925106

Zhang Y, 2003, INT J TUBERC LUNG D, V7, P6

Zhang Y, 2009, INT J TUBERC LUNG D, V13, P1320

ZHANG Y, 1992, NATURE, V358, P591, DOI 10.1038/358591a0

Zhang Y, 2012, ANTIMICROB AGENTS CH, V56, P2223, DOI 10.1128/AAC.06288-11

Zhao F, 2014, ANTIMICROB AGENTS CH, V58, P1479, DOI 10.1128/AAC.01775-13

Zimhony O, 2000, NAT MED, V6, P1043, DOI 10.1038/79558

Zimhony O, 2007, ANTIMICROB AGENTS CH, V51, P752, DOI 10.1128/AAC.01369-06

Zumla A, 2015, LANCET GLOB HEALTH, V3, pE10, DOI 10.1016/S2214-109X(14)70361-4

NR 211

TC 25

Z9 29

U1 0

U2 23

PU BENTHAM SCIENCE PUBL LTD

PI SHARJAH

PA EXECUTIVE STE Y-2, PO BOX 7917, SAIF ZONE, 1200 BR SHARJAH, U ARAB

EMIRATES

SN 1389-2010

EI 1873-4316

J9 CURR PHARM BIOTECHNO

JI Curr. Pharm. Biotechnol.

PY 2021

VL 22

IS 4

BP 480

EP 500

DI 10.2174/1389201021666200628021702

PG 21

WC Biochemistry & Molecular Biology; Pharmacology & Pharmacy

WE Science Citation Index Expanded (SCI-EXPANDED)

SC Biochemistry & Molecular Biology; Pharmacology & Pharmacy

GA QU2WW

UT WOS:000627145200004

DA 2026-05-07

ER

PT J

AU Tang, DY

La, B

He, JQ

AF Tang, Daoyan

La, Ba

He, Jian-Qing

TI Associations between host efflux pump gene polymorphisms (ABCB1, ABCC1,

ABCG2) and drug-resistant tuberculosis in a Chinese cohort

SO GENE

LA English

DT Article

DE Drug-resistant tuberculosis; Pharmacokinetics; Drug transporter; Efflux

pump; Single nucleotide polymorphisms

ID P-GLYCOPROTEIN; COMMON VARIANTS; CHEMOTHERAPY; TRANSPORTER;

SUSCEPTIBILITY; INHIBITION; EXPRESSION

AB Background Drug-resistant tuberculosis (DR-TB) is associated with alterations in drug pharmacokinetics (PK), aberrant expression of efflux pump transporters, and genetic mutations. Polymorphisms in host genes encoding efflux pumps may affect drug concentrations, potentially contributing to the emergence of drug resistance. This study aims to investigate the association between single nucleotide polymorphisms (SNPs) in host efflux pump transporter genes and the development of drug resistance in tuberculosis. Methods This study enrolled 117 DR-TB patients, 193 patients with drug-sensitive tuberculosis (DS-TB) and 398 healthy control subjects (HCS). SNPs associated with drug resistance were selected based a comprehensive literature review and publicly available databases. A total of 12 SNPs within four ABC transporter genes were selected and genotyped: ABCB1 (rs1128503, rs2032582, rs3842, rs1045642); ABCC1 (rs212091, rs3743527); ABCC2 (rs717620, rs2273697, rs3740066, rs8187710); and ABCG2 (rs2231142, rs2231137). The relationship between SNPs and phenotype was evaluated using logistic regression analysis, with adjustments for potential confounding factors including sex, age and ethnicity. Results All 12 SNPs were in Hardy-Weinberg genetic equilibrium (P > 0.5). Genetic analysis revealed statistically significant associations between efflux pump gene polymorphisms and distinct drug-resistant tuberculosis profiles. Specifically, the ABCC1 rs212091 CC genotype was significantly associated with an increased risk of both overall drug-resistant TB (OR = 4.84, 95 % CI: 1.24-18.92) and multidrug-resistant TB (OR = 8.45, 95 % CI: 1.50-47.65). In contrast, the ABCB1 rs3842 CC genotype demonstrated a protective effect against MDR-TB (OR = 0.37, 95 % CI: 0.14-0.99), whereas the ABCG2 rs2231142 TT genotype was linked to a decreased risk of pre-extensively drug-resistant TB (OR = 0.41, 95 % CI: 0.17-0.97). Conclusion Our findings suggest that polymorphisms in host efflux pump genes may affect susceptibility to different DR-TB phenotypes. The identified risk (ABCC1 rs212091) and protective variants (ABCB1 rs3842, ABCG2 rs2231142) reveal genetic mechanisms underlying drug resistance. Further validation in larger, more diverse populations is needed.

C1 [Tang, Daoyan; He, Jian-Qing] Sichuan Univ, West China Hosp, Dept Resp & Crit Care Med, 37 Guo Xue Alley, Chengdu 610041, Peoples R China.

[La, Ba] Third Peoples Hosp Tibet Autonomous Reg, Dept TB, Nongke Rd, Lhasa, Tibet, Peoples R China.

C3 Sichuan University

RP He, JQ (corresponding author), Sichuan Univ, West China Hosp, Dept Resp & Crit Care Med, 37 Guo Xue Alley, Chengdu 610041, Peoples R China.

EM jianqing_he@scu.edu.cn

CR Adams KN, 2011, CELL, V145, P39, DOI 10.1016/j.cell.2011.02.022

Rodríguez-Castillo JA, 2015, TUBERCULOSIS, V95, P532, DOI 10.1016/j.tube.2015.04.004

[Anonymous], 2019, Global tuberculosis report 2019

[Anonymous], 2021, Meeting report of the WHO expert consultation on the definition of extensively drug-resistant tuberculosis, 27-29 October 2020

Avramovic MZ, 2017, J RHEUMATOL, V44, P1216, DOI 10.3899/jrheum.160950

Beceiro A, 2013, CLIN MICROBIOL REV, V26, P185, DOI 10.1128/CMR.00059-12

Campa D, 2012, INT J CANCER, V131, P2920, DOI 10.1002/ijc.27567

Chen KG, 2012, CLIN CANCER RES, V18, P1863, DOI 10.1158/1078-0432.CCR-11-1590

Coelho AVC, 2013, J CLIN PHARMACOL, V53, P1286, DOI 10.1002/jcph.165

De Marchi P, 2019, ORAL ONCOL, V98, P48, DOI 10.1016/j.oraloncology.2019.09.013

Dheda K, 2017, LANCET RESP MED, V5, P291, DOI [10.1016/s2213-2600(17)30079-6, 10.1016/S2213-2600(17)30079-6]

Freedman JA, 2018, CARCINOGENESIS, V39, P879, DOI 10.1093/carcin/bgy062

GOLLAPUDI S, 1994, BIOCHEM BIOPH RES CO, V199, P1181, DOI 10.1006/bbrc.1994.1355

Gumbo T, 2010, ANTIMICROB AGENTS CH, V54, P1484, DOI 10.1128/AAC.01474-09

Jain N, 2020, NAT COMMUN, V11, DOI 10.1038/s41467-020-16877-3

Katayama Kazuhiro., 2014, New J. Sci, V2014, P1, DOI DOI 10.1155/2014/476974

Kaur S, 2021, INFECT GENET EVOL, V87, DOI 10.1016/j.meegid.2020.104662

Kunadt D, 2020, ANN HEMATOL, V99, P2173, DOI 10.1007/s00277-020-04163-7

Lange C, 2019, LANCET, V394, P953, DOI 10.1016/S0140-6736(19)31882-3

Lian Zhan, 2011, Zhonghua Jie He He Hu Xi Za Zhi, V34, P520

Liu J, 2017, INT J MOL SCI, V18, DOI 10.3390/ijms18030603

Maliepaard M, 2001, CANCER RES, V61, P3458

Matt U, 2017, INT J ANTIMICROB AG, V50, P55, DOI 10.1016/j.ijantimicag.2017.02.022

Pasipanodya JG, 2013, J INFECT DIS, V208, P1464, DOI 10.1093/infdis/jit352

Pedersen JM, 2008, J MED CHEM, V51, P3275, DOI 10.1021/jm7015683

Pontual Y, 2017, CLIN SCI, V131, P1831, DOI 10.1042/CS20170277

Qian CY, 2016, CHIN J CANCER, V35, DOI 10.1186/s40880-016-0145-8

Qu J, 2012, CNS NEUROSCI THER, V18, P647, DOI 10.1111/j.1755-5949.2012.00336.x

Rajman I, 2020, CTS-CLIN TRANSL SCI, V13, P848, DOI 10.1111/cts.12769

Robey RW, 2009, ADV DRUG DELIVER REV, V61, P3, DOI 10.1016/j.addr.2008.11.003

Roy U, 2015, FRONT MICROBIOL, V6, DOI 10.3389/fmicb.2015.00948

Schuetz EG, 1996, P NATL ACAD SCI USA, V93, P4001, DOI 10.1073/pnas.93.9.4001

Sosnik A, 2013, ADV DRUG DELIVER REV, V65, P1828, DOI 10.1016/j.addr.2013.09.002

Thomas L, 2020, ANTIBIOTICS-BASEL, V9, DOI 10.3390/antibiotics9060307

Tian CQ, 2012, GYNECOL ONCOL, V124, P575, DOI 10.1016/j.ygyno.2011.11.022

Wang HJ, 2009, CANCER-AM CANCER SOC, V115, P595, DOI 10.1002/cncr.24042

Wolking S, 2015, CLIN PHARMACOKINET, V54, P709, DOI 10.1007/s40262-015-0267-1

NR 37

TC 0

Z9 0

U1 1

U2 1

PU ELSEVIER

PI AMSTERDAM

PA RADARWEG 29, 1043 NX AMSTERDAM, NETHERLANDS

SN 0378-1119

EI 1879-0038

J9 GENE

JI Gene

PD NOV 20

PY 2025

VL 972

AR 149826

DI 10.1016/j.gene.2025.149826

EA OCT 2025

PG 8

WC Genetics & Heredity

WE Science Citation Index Expanded (SCI-EXPANDED)

SC Genetics & Heredity

GA 8VB0H

UT WOS:001598017600001

DA 2026-05-07

ER

PT J

AU Mlambo, CK

Warren, RM

Poswa, X

Victor, TC

Duse, AG

Marais, E

AF Mlambo, C. K.

Warren, R. M.

Poswa, X.

Victor, T. C.

Duse, A. G.

Marais, E.

TI Genotypic diversity of extensively drug-resistant tuberculosis (XDR-TB)

in South Africa

SO INTERNATIONAL JOURNAL OF TUBERCULOSIS AND LUNG DISEASE

LA English

DT Article

DE mycobacterium tuberculosis; MDR-TB; XDR-TB

ID MYCOBACTERIUM-TUBERCULOSIS; MOLECULAR EPIDEMIOLOGY; WESTERN CAPE

AB SETTING: The epidemiology of extensively drug-resistant tuberculosis (XDR-TB), an emerging threat to TB control, is not well understood.

OBJECTIVE: To gain insight into the genotypic population structure of XDR Mycobacterium tuberculosis strains in South Africa using a molecular approach and thereby determine whether XDR-TB is mainly acquired or transmitted.

DESIGN: Sputum isolates from patients with multidrug-resistant tuberculosis (MDR-TB) were submitted to the National Referral Laboratory for second-line drug susceptibility testing. The XDR-TB isolates were spoligotyped and these data were compared to the geographic origin of the isolate.

RESULTS: Of the 699 MDR-TB isolates submitted for testing between June 2005 and December 2006, 101 (17%) patients had a culture that was resistant to either ofloxacin or kanamycin, and 41 (6%) were resistant to both drugs (XDR-TB). Spoligotyping of the XDR-TB isolates identified 17 genotypes. As a result of the high genotypic diversity and geographical distribution, we estimate that between 63% and 75% of cases developed XDR-TB through acquisition.

CONCLUSION: Acquisition of extensive drug resistance appears to be the primary mechanism driving the XDR-TB epidemic in South Africa. This urgent TB control issue has to be addressed to prevent the spread of this potentially incurable disease.

C1 [Mlambo, C. K.; Duse, A. G.; Marais, E.] Natl Hlth Lab Serv, Johannesburg, Gauteng, South Africa.

[Mlambo, C. K.; Duse, A. G.; Marais, E.] Univ Witwatersrand, Johannesburg, Gauteng, South Africa.

[Warren, R. M.; Victor, T. C.] Univ Stellenbosch, Dept Sci & Technol, Natl Res Fdn, Ctr Excellence Biomed TB Res,MRC,Ctr Mol & Cellul, Western Cape, South Africa.

[Poswa, X.] Natl Hlth Lab Serv, TB Referral Lab, Johannesburg, Gauteng, South Africa.

C3 National Health Laboratory Service; University of Witwatersrand;

Stellenbosch University; National Research Foundation - South Africa;

National Health Laboratory Service

RP Marais, E (corresponding author), POB 2115, ZA-2041 Johannesburg, South Africa.

EM else.marais@wits.ac.za

OI Warren, Robin/0000-0001-5741-7358

CR Bifani PJ, 1999, JAMA-J AM MED ASSOC, V282, P2321, DOI 10.1001/jama.282.24.2321

Brudey K, 2006, BMC MICROBIOL, V6, DOI 10.1186/1471-2180-6-23

*CDCP, 2006, EMERG INFECT DIS, V2, P736

Gandhi NR, 2006, LANCET, V368, P1575, DOI 10.1016/S0140-6736(06)69573-1

Grimaldo ER, 2001, INT J TUBERC LUNG D, V5, P546

Holtz TH, 2006, INT J TUBERC LUNG D, V10, P649

Kamerbeek J, 1997, J CLIN MICROBIOL, V35, P907, DOI 10.1128/JCM.35.4.907-914.1997

Marais BJ, 2006, J CLIN MICROBIOL, V44, P3539, DOI 10.1128/JCM.01291-06

Masjedi MR, 2006, CLIN INFECT DIS, V43, P841, DOI 10.1086/507542

Mathema B, 2006, CLIN MICROBIOL REV, V19, P658, DOI 10.1128/CMR.00061-05

Richardson M, 2002, INT J TUBERC LUNG D, V6, P1001

SHISANA O, 2005, HIV INC BEH COMM SUR

SMALL PM, 1994, NEW ENGL J MED, V330, P1703, DOI 10.1056/NEJM199406163302402

Toungoussova OS, 2003, CLIN INFECT DIS, V37, P665, DOI 10.1086/377205

van Rie A, 1999, J INFECT DIS, V180, P1608, DOI 10.1086/315054

Van Rie A, 2006, LANCET, V368, P1554, DOI 10.1016/S0140-6736(06)69575-5

WEYER K, 1995, S AFR MED J, V85, P499

WHO, 2006, WHO MED REP WHO GLOB

*WHO INT UN AG TUB, 2004, ANT TUB DRUG RES WOR

Wright A., 2006, Morbidity and Mortality Weekly Report, V55, P301

NR 20

TC 95

Z9 107

U1 0

U2 8

PU INT UNION AGAINST TUBERCULOSIS LUNG DISEASE (I U A T L D)

PI PARIS

PA 68 BOULEVARD SAINT-MICHEL,, 75006 PARIS, FRANCE

SN 1027-3719

J9 INT J TUBERC LUNG D

JI Int. J. Tuberc. Lung Dis.

PD JAN

PY 2008

VL 12

IS 1

BP 99

EP 104

PG 6

WC Infectious Diseases; Respiratory System

WE Science Citation Index Expanded (SCI-EXPANDED)

SC Infectious Diseases; Respiratory System

GA 244LY

UT WOS:000251868600019

DA 2026-05-07

ER

PT J

AU Sun, ZG

Xu, YH

Sun, Y

Liu, Y

Zhang, XX

Huang, HR

Li, CY

AF Sun, Zhaogang

Xu, Yuhui

Sun, Yong

Liu, Yi

Zhang, Xuxia

Huang, Hairong

Li, Chuanyou

TI Ofloxacin Resistance in Mycobacterium tuberculosis Is Associated

with Efflux Pump Activity Independent of Resistance Pattern and Genotype

SO MICROBIAL DRUG RESISTANCE

LA English

DT Article

ID FLUOROQUINOLONE RESISTANCE; DRUG-RESISTANCE; ANTIBIOTIC-RESISTANCE;

MUTATIONS; STRAINS; SUSCEPTIBILITY; TRANSPORTERS; EVOLUTION; GYRASE;

LEVEL

AB Drug-resistance to ofloxacin (OFX) in Mycobacterium tuberculosis is due to missense mutations in gyrA and other factors, such as alterations in the activity of drug efflux pumps. In this study, we identified 8 extensively drug resistant tuberculosis (XDR-TB), 40 multidrug resistant TB (MDR-TB), 38 polydrug resistant TB (PDR-TB), and 16 single OFX-resistant TB from 102 clinical isolates. We tested the effect of three efflux inhibitors, reserpine, verapamil, and carbonyl cyanide m-chlorophenyl hydrazone (CCCP), on changes in the OFX minimum inhibitory concentration (MIC) using Resazurin microtitre assay. These three inhibitors changed the MICs from 2- to 32-fold, with CCCP having the strongest effect. A total of 55%, 74%, and 83% of the tested isolates had changes in MIC of more than two-fold by reserpine, verapamil, and CCCP, respectively. The inhibitors led to similar fold-changes of OFX MICs in the XDR, MDR, PDR, and single OFX-resistant isolates. For each inhibitor, a higher resistance to OFX was associated with the greater efflux pump activity. There were no significant differences in the effect of efflux pump inhibitors upon Beijing and non-Beijing M. tuberculosis genotypes. Taken together, these results indicate that the efflux pump activity was greater in the isolates higher resistant to OFX and had similar effects on isolates with different drug resistant pattern, and had similar effects on Beijing and non-Beijing genotypes.

C1 [Sun, Zhaogang; Xu, Yuhui; Sun, Yong; Liu, Yi; Zhang, Xuxia; Huang, Hairong; Li, Chuanyou] Capital Med Univ, Beijing Key Lab Drug Resistant TB Res, Beijing TB & Thorac Tumor Res Inst, Beijing Chest Hosp, Beijing 101149, Peoples R China.

C3 Capital Medical University

RP Li, CY (corresponding author), Capital Med Univ, Beijing Key Lab Drug Resistant TB Res, Beijing TB & Thorac Tumor Res Inst, Beijing Chest Hosp, 97 Machang Rd, Beijing 101149, Peoples R China.

EM lichuanyou6688@hotmail.com

RI Li, Chuanyou/ABI-4553-2020; Sun, Zhaogang/OZD-6869-2025

FU National Special Key Project of China [2013ZX 10003009-001]; National

Natural Science Foundation of China [30901283]; High-Level Technical

Personnel Training Project in Health of Beijing [2011-3-069]

FX This work was supported by the National Special Key Project of China on

Major Infectious Disease (2013ZX 10003009-001), National Natural Science

Foundation of China (30901283) and High-Level Technical Personnel

Training Project in Health of Beijing (2011-3-069).

CR Amaral L, 2007, J ANTIMICROB CHEMOTH, V59, P1237, DOI 10.1093/jac/dkl500

Banerjee SK, 1996, BIOCHEM BIOPH RES CO, V226, P362, DOI 10.1006/bbrc.1996.1362

Chatterjee A, 2013, PLOS ONE, V8, DOI 10.1371/journal.pone.0054717

Colangeli R, 2005, MOL MICROBIOL, V55, P1829, DOI 10.1111/j.1365-2958.2005.04510.x

Committee of Chinese anti-Tuberculosis Association, 2006, CHIN ANT TUB ASS PRO

De Rossi E, 2006, FEMS MICROBIOL REV, V30, P36, DOI 10.1111/j.1574-6976.2005.00000.x

De Rossi Edda, 2002, Molecular Medicine (Baltimore), V8, P714

Escribano I, 2007, CHEMOTHERAPY, V53, P397, DOI 10.1159/000109769

Fattorini L, 1999, MICROB DRUG RESIST, V5, P265, DOI 10.1089/mdr.1999.5.265

Gupta S, 2013, AM J RESP CRIT CARE, V188, P600, DOI 10.1164/rccm.201304-0650OC

Hatfull G. F., 2000, MOL GENETICS MYCOBAC, P235

Helal ZH, 2009, J CLIN MICROBIOL, V47, P2794, DOI 10.1128/JCM.00360-09

Kamerbeek J, 1997, J CLIN MICROBIOL, V35, P907, DOI 10.1128/JCM.35.4.907-914.1997

Li XZ, 2004, ANTIMICROB AGENTS CH, V48, P2415, DOI 10.1128/AAC.48.7.2415-2423.2004

Long QX, 2012, INT J ANTIMICROB AG, V39, P486, DOI 10.1016/j.ijantimicag.2012.02.015

Louw GE, 2011, AM J RESP CRIT CARE, V184, P269, DOI 10.1164/rccm.201011-1924OC

Machado D, 2012, PLOS ONE, V7, DOI 10.1371/journal.pone.0034538

Malik SZ, 2012, PLOS ONE, V7, DOI [10.1371/journal.pone.0036016, 10.1371/journal.pone.0039754]

Martin A, 2003, ANTIMICROB AGENTS CH, V47, P3616, DOI 10.1128/AAC.47.11.3616-3619.2003

Mukherjee JS, 2004, LANCET, V363, P474, DOI 10.1016/S0140-6736(04)15496-2

Nebenzahl-Guimaraes H, 2014, J ANTIMICROB CHEMOTH, V69, P331, DOI 10.1093/jac/dkt358

Pasca MR, 2004, ANTIMICROB AGENTS CH, V48, P3175, DOI 10.1128/AAC.48.8.3175-3178.2004

Pillay M, 2007, CLIN INFECT DIS, V45, P1409, DOI 10.1086/522987

Rabori Eshagh Dortaj, 2006, Journal of Communicable Diseases, V37, P249

Ryan BM, 2001, EXPERT OPIN INV DRUG, V10, P1409, DOI 10.1517/13543784.10.8.1409

Schmalstieg AM, 2012, ANTIMICROB AGENTS CH, V56, P4806, DOI 10.1128/AAC.05546-11

Siddiqi S, 2006, WHOHTMTB2008392

Singh M, 2011, INDIAN J MED RES, V133, P535

Singh R, 2012, ANTIMICROB AGENTS CH, V56, P1680, DOI 10.1128/AAC.05693-11

Sulochana S, 2007, J CHEMOTHER, V19, P89

Sun Z, 2010, INT J TUBERC LUNG D, V14, P1436

Sun ZG, 2008, INT J ANTIMICROB AG, V31, P115, DOI 10.1016/j.ijantimicag.2007.10.014

Tao J, 2013, NUCLEIC ACIDS RES, V41, P2370, DOI 10.1093/nar/gks1351

World Health Organization, 2013, GLOB TUB REP 2012

Xu C, 1996, J INFECT DIS, V174, P1127, DOI 10.1093/infdis/174.5.1127

NR 35

TC 20

Z9 25

U1 0

U2 27

PU MARY ANN LIEBERT, INC

PI NEW ROCHELLE

PA 140 HUGUENOT STREET, 3RD FL, NEW ROCHELLE, NY 10801 USA

SN 1076-6294

EI 1931-8448

J9 MICROB DRUG RESIST

JI Microb. Drug Resist.

PD DEC 1

PY 2014

VL 20

IS 6

BP 525

EP 532

DI 10.1089/mdr.2013.0171

PG 8

WC Infectious Diseases; Microbiology; Pharmacology & Pharmacy

WE Science Citation Index Expanded (SCI-EXPANDED)

SC Infectious Diseases; Microbiology; Pharmacology & Pharmacy

GA AU4ZG

UT WOS:000345616200003

DA 2026-05-07

ER

PT J

AU Hameed, HMA

Islam, MM

Chhotaray, C

Wang, CW

Liu, Y

Tan, YJ

Li, XJ

Tan, SY

Delorme, V

Yew, WW

Liu, JX

Zhang, TY

AF Hameed, H. M. Adnan

Islam, Md Mahmudul

Chhotaray, Chiranjibi

Wang, Changwei

Liu, Yang

Tan, Yaoju

Li, Xinjie

Tan, Shouyong

Delorme, Vincent

Yew, Wing W.

Liu, Jianxiong

Zhang, Tianyu

TI Molecular Targets Related Drug Resistance Mechanisms in MDR-, XDR-, and

TDR-Mycobacterium tuberculosis Strains

SO FRONTIERS IN CELLULAR AND INFECTION MICROBIOLOGY

LA English

DT Review

DE Mycobacterium tuberculosis; drug resistance; molecular; comorbidities;

therapeutic; drug targets

ID PARA-AMINOSALICYLIC ACID; HIGH-LEVEL RESISTANCE; PYRAZINAMIDE

RESISTANCE; RIFAMPICIN-RESISTANCE; STREPTOMYCIN RESISTANCE; ETHAMBUTOL

RESISTANCE; CROSS-RESISTANCE; ATP SYNTHASE; CELL-WALL; FLUOROQUINOLONE

RESISTANCE

AB Tuberculosis (TB) is a formidable infectious disease that remains a major cause of death worldwide today. Escalating application of genomic techniques has expedited the identification of increasing number of mutations associated with drug resistance in Mycobacterium tuberculosis. Unfortunately the prevalence of bacillary resistance becomes alarming in many parts of the world, with the daunting scenarios of multidrug-resistant tuberculosis (MDR-TB), extensively drug-resistant tuberculosis (XDR-TB) and total drug-resistant tuberculosis (TDR-TB), due to number of resistance pathways, alongside some apparently obscure ones. Recent advances in the understanding of the molecular/genetic basis of drug targets and drug resistance mechanisms have been steadily made. Intriguing findings through whole genome sequencing and other molecular approaches facilitate the further understanding of biology and pathology of M. tuberculosis for the development of new therapeutics to meet the immense challenge of global health.

C1 [Hameed, H. M. Adnan; Islam, Md Mahmudul; Chhotaray, Chiranjibi; Wang, Changwei; Liu, Yang; Zhang, Tianyu] Chinese Acad Sci, Guangzhou Inst Biomed & Hlth, Guangzhou Regenerat Med & Hlth Guangdong Lab, State Key Lab Resp Dis, Guangzhou, Guangdong, Peoples R China.

[Hameed, H. M. Adnan; Islam, Md Mahmudul; Chhotaray, Chiranjibi; Zhang, Tianyu] Univ Chinese Acad Sci, Beijing, Peoples R China.

[Liu, Yang] Anhui Univ, Inst Hlth Sci, Hefei, Anhui, Peoples R China.

[Tan, Yaoju; Li, Xinjie; Tan, Shouyong; Liu, Jianxiong] Guangzhou Chest Hosp, State Key Lab Resp Dis, Guangzhou, Guangdong, Peoples R China.

[Delorme, Vincent] Inst Pasteur Korea, TB Res Lab, Seongnam Si, South Korea.

[Yew, Wing W.] Chinese Univ Hong Kong, Stanley Ho Ctr Emerging Infect Dis, Hong Kong, Hong Kong, Peoples R China.

C3 State Key Laboratory of Respiratory Disease; Bioland Lab; Chinese

Academy of Sciences; Guangzhou Institute of Biomedicine & Health, CAS;

Chinese Academy of Sciences; University of Chinese Academy of Sciences,

CAS; Anhui University; State Key Laboratory of Respiratory Disease;

Institut Pasteur Korea; Chinese University of Hong Kong

RP Zhang, TY (corresponding author), Chinese Acad Sci, Guangzhou Inst Biomed & Hlth, Guangzhou Regenerat Med & Hlth Guangdong Lab, State Key Lab Resp Dis, Guangzhou, Guangdong, Peoples R China.; Zhang, TY (corresponding author), Univ Chinese Acad Sci, Beijing, Peoples R China.; Liu, JX (corresponding author), Guangzhou Chest Hosp, State Key Lab Resp Dis, Guangzhou, Guangdong, Peoples R China.

EM ljxer64@qq.com; zhang_tianyu@gibh.ac.cn

RI li, xinjie/LJK-4266-2024; Zhang, Tianyu/K-4801-2015

OI Zhang, Tianyu/0000-0001-5647-6014; Chhotaray,

Chiranjibi/0000-0002-5310-8758; 刘, 洋/0000-0001-9971-8191; Islam,

Mahmudul/0000-0001-7264-5180; Delorme, Vincent/0000-0001-5235-7069; Ali,

Sagor/0009-0000-8890-7675

FU National Natural Science Foundation of China [81572037]; Chinese Academy

of Sciences [154144KYSB20150045, KFZD-SW-207]; National Mega-project of

China for Innovative Drugs [2018ZX09721001-003-003]; National

Mega-project of China for Main Infectious Diseases

[2017ZX10302301-003-002]; State Key Lab of Respiratory Disease,

Guangzhou Institute of Respiratory Disease, First Affiliated Hospital of

Guangzhou Medical University [SKLRD2016ZJ003, 2014SKLRD-O06]; UCAS Ph.D.

Fellowship Program; CAS-TWAS President's Ph.D. Fellowship Program;

Guangzhou Municipal Industry and Research Collaborative Innovation

Program [201508020248, 201604020019]; Guangzhou Municipal Clinical

Medical Center Program [155700012]; National Research Foundation in

Korea (NRF) - Ministry of Science, ICT and future Planning (MSIP)

[NRF-2017M3A9G6068246, 2015R1C1A1A01053355]; French ministry of Foreign

Affairs; Valery N. Danilenko, Vavilov Institute of General Genetics,

Russian Academy of Sciences

FX This work was supported by the National Natural Science Foundation of

China (81572037), the Chinese Academy of Sciences (154144KYSB20150045,

KFZD-SW-207), the National Mega-project of China for Innovative Drugs

(2018ZX09721001-003-003), the National Mega-project of China for Main

Infectious Diseases (2017ZX10302301-003-002), the Key Project Grant

(SKLRD2016ZJ003) and the Open Project Grant (2014SKLRD-O06) from the

State Key Lab of Respiratory Disease, Guangzhou Institute of Respiratory

Disease, First Affiliated Hospital of Guangzhou Medical University, the

UCAS Ph.D. Fellowship Program (to HH) and CAS-TWAS President's Ph.D.

Fellowship Program (to MI and CC) for International Students, and

partially supported by Guangzhou Municipal Industry and Research

Collaborative Innovation Program (201508020248, 201604020019) and

Guangzhou Municipal Clinical Medical Center Program (155700012). TZ

received Science and Technology Innovation Leader of Guangdong Province

(2016TX03R095). VD was supported by the National Research Foundation in

Korea (NRF), funded by the Ministry of Science, ICT and future Planning

(MSIP, grant numbers NRF-2017M3A9G6068246 and 2015R1C1A1A01053355), as

well as the French ministry of Foreign Affairs. We acknowledge the

support of Valery N. Danilenko, Vavilov Institute of General Genetics,

Russian Academy of Sciences.

CR Ajbani K, 2011, J CLIN MICROBIOL, V49, P1588, DOI 10.1128/JCM.00113-11

Alexander DC, 2012, J CLIN MICROBIOL, V50, P3726, DOI 10.1128/JCM.00620-12

Almeida D, 2016, ANTIMICROB AGENTS CH, V60, P4590, DOI 10.1128/AAC.00753-16

Alvarez N, 2014, BIOMED RES INT-UK, V2014, DOI 10.1155/2014/367268

Andini N, 2006, ANTIMICROB AGENTS CH, V50, P2560, DOI 10.1128/AAC.00264-06

Andries K, 2005, SCIENCE, V307, P223, DOI 10.1126/science.1106753

Andries K, 2014, PLOS ONE, V9, DOI 10.1371/journal.pone.0102135

[Anonymous], 2016, GLOB TUB REP

[Anonymous], EMERG INFECT DIS

[Anonymous], 2017, GLOB TUB REP

Arnold C, 2007, CLIN MICROBIOL INFEC, V13, P120, DOI 10.1111/j.1469-0691.2006.01637.x

Aubry A, 2004, ANTIMICROB AGENTS CH, V48, P1281, DOI 10.1128/AAC.48.4.1281-1288.2004

Aubry A, 2006, ANTIMICROB AGENTS CH, V50, P104, DOI 10.1128/AAC.50.1.104-112.2006

Aye KS, 2016, J INFECT CHEMOTHER, V22, P174, DOI 10.1016/j.jiac.2015.12.009

BANERJEE A, 1994, SCIENCE, V263, P227, DOI 10.1126/science.8284673

Bantubani N, 2014, PLOS ONE, V9, DOI 10.1371/journal.pone.0090868

Barnard M, 2012, AM J RESP CRIT CARE, V186, P1298, DOI 10.1164/rccm.201205-0960OC

Beceiro A, 2013, CLIN MICROBIOL REV, V26, P185, DOI 10.1128/CMR.00059-12

Beckert P, 2012, ANTIMICROB AGENTS CH, V56, P2743, DOI 10.1128/AAC.06227-11

Bhuju S, 2013, INFECT GENET EVOL, V19, P1, DOI 10.1016/j.meegid.2013.06.008

Bloemberg GV, 2015, NEW ENGL J MED, V373, P1986, DOI 10.1056/NEJMc1505196

Böttger EC, 2011, CLIN MICROBIOL INFEC, V17, P1128, DOI 10.1111/j.1469-0691.2011.03551.x

Borrell S, 2011, CLIN MICROBIOL INFEC, V17, P815, DOI 10.1111/j.1469-0691.2011.03556.x

Boshoff HIM, 2004, J BIOL CHEM, V279, P40174, DOI 10.1074/jbc.M406796200

Brandis G, 2013, J ANTIMICROB CHEMOTH, V68, P2493, DOI 10.1093/jac/dkt224

Brigden G, 2014, B WORLD HEALTH ORGAN, V92, P68, DOI 10.2471/BLT.13.122028

Brossier F, 2015, ANTIMICROB AGENTS CH, V59, P4800, DOI 10.1128/AAC.00150-15

Caceres NE, 1997, J BACTERIOL, V179, P5046, DOI 10.1128/jb.179.16.5046-5055.1997

Carette X, 2012, NUCLEIC ACIDS RES, V40, P3018, DOI 10.1093/nar/gkr1113

Carreira S, 2012, REV PORT PNEUMOL, V18, P239, DOI 10.1016/j.rppneu.2012.04.001

Cavusoglu C, 2004, CLIN MICROBIOL INFEC, V10, P662, DOI 10.1111/j.1469-0691.2004.00917.x

Chakraborty S, 2013, SCIENCE, V339, P88, DOI 10.1126/science.1228980

Chen JM, 2012, PLOS ONE, V7, DOI 10.1371/journal.pone.0043467

Coban AY, 2016, SCI REP-UK, V6, DOI 10.1038/srep39050

Cohen T, 2003, LANCET INFECT DIS, V3, P13, DOI 10.1016/S1473-3099(03)00483-3

Comas I, 2012, NAT GENET, V44, P106, DOI 10.1038/ng.1038

CROFTON J, 1948, BMJ-BRIT MED J, V2, P1009, DOI 10.1136/bmj.2.4588.1009

Cui ZL, 2011, BMC INFECT DIS, V11, DOI 10.1186/1471-2334-11-78

Dalton T, 2012, LANCET, V380, P1406, DOI 10.1016/S0140-6736(12)60734-X

Daniel TM, 2006, RESP MED, V100, P1862, DOI 10.1016/j.rmed.2006.08.006

Das P, 2010, LANCET, V375, P1755, DOI 10.1016/S0140-6736(10)60600-9

Datta G, 2016, TUBERCULOSIS, V98, P50, DOI 10.1016/j.tube.2016.02.004

Day WA Jr, 2001, INFECT IMMUN, V69, P7471, DOI 10.1128/IAI.69.12.7471-7480.2001

de Vos M, 2013, ANTIMICROB AGENTS CH, V57, P827, DOI 10.1128/AAC.01541-12

Deggim-Messmer V, 2016, EBIOMEDICINE, V9, P228, DOI 10.1016/j.ebiom.2016.06.016

Desjardins CA, 2016, NAT GENET, V48, P544, DOI 10.1038/ng.3548

Dharmadhikari AS, 2013, ANTIMICROB AGENTS CH, V57, P2613, DOI 10.1128/AAC.02346-12

Dheda K, 2014, LANCET RESP MED, V2, P321, DOI 10.1016/S2213-2600(14)70031-1

dos Santos GF, 2015, Biochemistry & Pharmacology Open Access, V04, DOI 10.4172/2167-0501.1000e178

Du QL, 2013, DIAGN MICR INFEC DIS, V77, P138, DOI 10.1016/j.diagmicrobio.2013.06.031

Dye C, 2002, SCIENCE, V295, P2042, DOI 10.1126/science.1063814

Eniyan K, 2016, SCI REP-UK, V6, DOI 10.1038/srep35134

Escribano I, 2007, CHEMOTHERAPY, V53, P397, DOI 10.1159/000109769

Fenner L, 2012, ANTIMICROB AGENTS CH, V56, P3047, DOI 10.1128/AAC.06460-11

FINKEN M, 1993, MOL MICROBIOL, V9, P1239, DOI 10.1111/j.1365-2958.1993.tb01253.x

Fogel N, 2015, TUBERCULOSIS, V95, P527, DOI 10.1016/j.tube.2015.05.017

Forrellad MA, 2013, VIRULENCE, V4, P3, DOI 10.4161/viru.22329

Freihofer P, 2016, RNA, V22, P1836, DOI 10.1261/rna.057257.116

FRIEDEN TR, 1993, NEW ENGL J MED, V328, P521, DOI 10.1056/NEJM199302253280801

Gandhi NR, 2006, LANCET, V368, P1575, DOI 10.1016/S0140-6736(06)69573-1

Gao P, 2012, EUR J PHARMACOL, V694, P45, DOI 10.1016/j.ejphar.2012.08.012

Gao W, 2015, ANTIMICROB AGENTS CH, V59, P880, DOI 10.1128/AAC.04054-14

Gavalda S, 2009, J BIOL CHEM, V284, P19255, DOI 10.1074/jbc.M109.006940

Gavrish E, 2014, CHEM BIOL, V21, P509, DOI 10.1016/j.chembiol.2014.01.014

Geldmacher C, 2012, CURR OPIN HIV AIDS, V7, P268, DOI 10.1097/COH.0b013e3283524e32

Georghiou SB, 2017, J CLIN MICROBIOL, V55, P1928, DOI 10.1128/JCM.00152-17

Gilchrist CA, 2015, CLIN MICROBIOL REV, V28, P541, DOI 10.1128/CMR.00075-13

Gillespie SH, 2002, ANTIMICROB AGENTS CH, V46, P267, DOI 10.1128/AAC.46.2.267-274.2002

Gler MT, 2012, NEW ENGL J MED, V366, P2151, DOI 10.1056/NEJMoa1112433

Glickman MS, 2016, CELL HOST MICROBE, V19, P432, DOI 10.1016/j.chom.2016.04.001

Grant SS, 2016, CELL CHEM BIOL, V23, P666, DOI 10.1016/j.chembiol.2016.05.011

Grossman RF, 2014, INT J INFECT DIS, V18, P14, DOI 10.1016/j.ijid.2013.09.013

Grzegorzewicz AE, 2012, J BIOL CHEM, V287, P38434, DOI 10.1074/jbc.M112.400994

Haagsma AC, 2009, ANTIMICROB AGENTS CH, V53, P1290, DOI 10.1128/AAC.01393-08

Hartkoorn RC, 2014, ANTIMICROB AGENTS CH, V58, P2979, DOI 10.1128/AAC.00037-14

Haver HL, 2015, ANTIMICROB AGENTS CH, V59, P5316, DOI 10.1128/AAC.00308-15

Hillemann D, 2008, ANTIMICROB AGENTS CH, V52, P800, DOI 10.1128/AAC.01189-07

Huitric E, 2010, ANTIMICROB AGENTS CH, V54, P1022, DOI 10.1128/AAC.01611-09

Islam MM, 2017, J GENET GENOMICS, V44, P21, DOI 10.1016/j.jgg.2016.10.002

Jacobson KR, 2013, CLIN INFECT DIS, V56, P503, DOI 10.1093/cid/cis920

Jagielski T, 2014, PLOS ONE, V9, DOI 10.1371/journal.pone.0100078

Jenkins C, 2009, J ANTIMICROB CHEMOTH, V63, P1118, DOI 10.1093/jac/dkp125

Jeon CY, 2008, PLOS MED, V5, P1091, DOI 10.1371/journal.pmed.0050152

Jia L, 2005, J PHARMACOL EXP THER, V315, P905, DOI 10.1124/jpet.105.087817

Jnawali HN, 2013, DIAGN MICR INFEC DIS, V76, P187, DOI 10.1016/j.diagmicrobio.2013.02.035

Juréen P, 2008, ANTIMICROB AGENTS CH, V52, P1852, DOI 10.1128/AAC.00110-08

Keshavjee S, 2012, NEW ENGL J MED, V367, P931, DOI 10.1056/NEJMra1205429

Kisacik B, 2016, J RHEUMATOL, V43, P524, DOI 10.3899/jrheum.150177

Klopper M, 2013, EMERG INFECT DIS, V19, P449, DOI [10.3201/EID1903.120246, 10.3201//EID1903.120246]

Kolly GS, 2014, MOL MICROBIOL, V92, P194, DOI 10.1111/mmi.12546

Koul A, 2007, NAT CHEM BIOL, V3, P323, DOI 10.1038/nchembio884

Krieger IV, 2012, CHEM BIOL, V19, P1556, DOI 10.1016/j.chembiol.2012.09.018

Lange C, 2014, EUR RESPIR J, V44, P23, DOI 10.1183/09031936.00188313

Lata M, 2015, J PROTEOMICS, V127, P114, DOI 10.1016/j.jprot.2015.07.031

Lata M, 2015, PROTEIN PEPTIDE LETT, V22, P362, DOI 10.2174/0929866522666150209113708

Lawn SD, 2013, LANCET INFECT DIS, V13, P349, DOI 10.1016/S1473-3099(13)70008-2

Li J, 2014, EMERG MICROBES INFEC, V3, DOI 10.1038/emi.2014.21

Lillebaek T, 2016, INT J MED MICROBIOL, V306, P580, DOI 10.1016/j.ijmm.2016.05.017

Long QX, 2012, INT J ANTIMICROB AG, V39, P486, DOI 10.1016/j.ijantimicag.2012.02.015

Machado D, 2013, J ANTIMICROB CHEMOTH, V68, P1728, DOI 10.1093/jac/dkt090

Machado D, 2012, PLOS ONE, V7, DOI 10.1371/journal.pone.0034538

Maeurer M, 2014, LANCET RESP MED, V2, P256, DOI 10.1016/S2213-2600(14)70020-7

Makafe GG, 2016, ANTIMICROB AGENTS CH, V60, P3202, DOI 10.1128/AAC.00152-16

Makarov V, 2014, EMBO MOL MED, V6, P372, DOI 10.1002/emmm.201303575

Manina G, 2010, MOL MICROBIOL, V77, P1172, DOI 10.1111/j.1365-2958.2010.07277.x

Manjunatha UH, 2006, P NATL ACAD SCI USA, V103, P431, DOI 10.1073/pnas.0508392103

Manson AL, 2017, NAT GENET, V49, P395, DOI 10.1038/ng.3767

Maslov DA, 2015, TUBERCULOSIS, V95, P608, DOI 10.1016/j.tube.2015.05.013

Mathys V, 2009, ANTIMICROB AGENTS CH, V53, P2100, DOI 10.1128/AAC.01197-08

Matsumoto M, 2006, PLOS MED, V3, P2131, DOI 10.1371/journal.pmed.0030466

Mboowa G, 2014, BMC INFECT DIS, V14, DOI 10.1186/1471-2334-14-481

McGrath M, 2014, J ANTIMICROB CHEMOTH, V69, P292, DOI 10.1093/jac/dkt364

Meumann EM, 2015, MICROB GENOMICS, V1, DOI 10.1099/mgen.0.000037

Migliori GB, 2007, EMERG INFECT DIS, V13, P780, DOI 10.3201/eid1305.070200

MIKUSOVA K, 1995, ANTIMICROB AGENTS CH, V39, P2484, DOI 10.1128/AAC.39.11.2484

Minion J, 2010, INT J TUBERC LUNG D, V14, P941

Mori G, 2017, DRUG DISCOV TODAY, V22, P519, DOI 10.1016/j.drudis.2016.09.006

Mori G, 2015, CHEM BIOL, V22, P917, DOI 10.1016/j.chembiol.2015.05.016

Moure R, 2014, J ANTIMICROB CHEMOTH, V69, P947, DOI 10.1093/jac/dkt448

Müller B, 2011, INT J TUBERC LUNG D, V15, P344

Negin J, 2015, INT J INFECT DIS, V32, P135, DOI 10.1016/j.ijid.2014.11.018

Njire M, 2017, ANTIMICROB AGENTS CH, V61, DOI [10.1128/AAC.00070-17, 10.1128/aac.00070-17]

Njire M, 2016, ADV MED SCI-POLAND, V61, P63, DOI 10.1016/j.advms.2015.09.007

Nosova EY, 2013, J MED MICROBIOL, V62, P108, DOI 10.1099/jmm.0.046821-0

Ocheretina O, 2014, PLOS ONE, V9, DOI 10.1371/journal.pone.0090569

Palomino JC, 2014, ANTIBIOTICS-BASEL, V3, P317, DOI 10.3390/antibiotics3030317

Palomino JC, 2013, CURR MED CHEM, V20, P3785, DOI 10.2174/09298673113209990166

Pandey B, 2016, GENE, V581, P31, DOI 10.1016/j.gene.2016.01.024

Pantel A, 2012, ANTIMICROB AGENTS CH, V56, P1990, DOI 10.1128/AAC.06272-11

Park Y, 2017, ACS INFECT DIS, V3, P18, DOI 10.1021/acsinfecdis.6b00103

Perdigao J, 2014, CLIN MICROBIOL INFEC, V20, pO278, DOI 10.1111/1469-0691.12392

Perdigao J, 2009, INT J ANTIMICROB AG, V33, P334, DOI 10.1016/j.ijantimicag.2008.09.021

Peter S, 2016, EUR RESPIR J, V48, DOI 10.1183/13993003.congress-2016.PA1908

Piccaro G, 2014, ANTIMICROB AGENTS CH, V58, P7527, DOI 10.1128/AAC.03169-14

Puckett S, 2017, P NATL ACAD SCI USA, V114, pE2225, DOI 10.1073/pnas.1617655114

Ramaswamy S., 1998, Tubercle and Lung Disease, V79, P3, DOI 10.1054/tuld.1998.0002

Ramaswamy SV, 2003, ANTIMICROB AGENTS CH, V47, P1241, DOI 10.1128/AAC.47.4.1241-1250.2003

Reeves AZ, 2013, ANTIMICROB AGENTS CH, V57, P1857, DOI 10.1128/AAC.02191-12

Ren JX, 2015, COMPUT BIOL MED, V58, P110, DOI 10.1016/j.compbiomed.2014.12.020

Rengarajan J, 2004, MOL MICROBIOL, V53, P275, DOI 10.1111/j.1365-2958.2004.04120.x

Roca I., 2015, New Microbes and New Infections, V6, P22, DOI [10.1016/j.nmni.2015.02.007, 10.1016/j.nmni.2015.02.007]

Rozwarski DA, 1998, SCIENCE, V279, P98, DOI 10.1126/science.279.5347.98

Safi H, 2013, NAT GENET, V45, P1190, DOI 10.1038/ng.2743

Schmitt EK, 2011, ANGEW CHEM INT EDIT, V50, P5889, DOI 10.1002/anie.201101740

Schroeder M, 2017, GENES-BASEL, V8, DOI 10.3390/genes8010039

Seifert M, 2015, PLOS ONE, V10, DOI 10.1371/journal.pone.0119628

Sharma D, 2016, BIOCHEM BIOPH RES CO, V478, P908, DOI 10.1016/j.bbrc.2016.08.049

Sharma D, 2015, CURR PROTEOMICS, V12, P96, DOI 10.2174/157016461202150903113053

Sharma D, 2007, J MOL BIOL, V374, P1065, DOI 10.1016/j.jmb.2007.10.003

Shen X, 2007, ANTIMICROB AGENTS CH, V51, P2618, DOI 10.1128/AAC.01516-06

Shi DW, 2011, J ANTIMICROB CHEMOTH, V66, P2240, DOI 10.1093/jac/dkr284

Shi WL, 2014, EMERG MICROBES INFEC, V3, DOI 10.1038/emi.2014.61

Shi WL, 2011, SCIENCE, V333, P1630, DOI 10.1126/science.1208813

Shimokawa Y, 2014, BIOL PHARM BULL, V37, P1727, DOI 10.1248/bpb.b14-00311

Singh V, 2017, ACS INFECT DIS, V3, P5, DOI 10.1021/acsinfecdis.6b00102

Skrahina A, 2012, EUR RESPIR J, V39, P1425, DOI 10.1183/09031936.00145411

Smith T, 2014, CURR TOP MICROBIOL, V374, P53, DOI 10.1007/82_2012_279

Smittipat N, 2016, J GLOB ANTIMICROB RE, V4, P5, DOI 10.1016/j.jgar.2015.11.009

Sowajassatakul A, 2014, BMC MICROBIOL, V14, DOI 10.1186/1471-2180-14-165

Srivastava S, 2011, J INFECT DIS, V204, P1951, DOI 10.1093/infdis/jir658

Stanley RE, 2010, NAT STRUCT MOL BIOL, V17, P289, DOI 10.1038/nsmb.1755

Stanley SA, 2013, P NATL ACAD SCI USA, V110, P11565, DOI 10.1073/pnas.1302114110

Stoffels K, 2013, PLOS ONE, V8, DOI 10.1371/journal.pone.0063128

Stover CK, 2000, NATURE, V405, P962, DOI 10.1038/35016103

Tahlan K, 2012, ANTIMICROB AGENTS CH, V56, P1797, DOI 10.1128/AAC.05708-11

Tan YJ, 2014, J CLIN MICROBIOL, V52, P291, DOI 10.1128/JCM.01903-13

Tan YJ, 2012, J CLIN MICROBIOL, V50, P81, DOI 10.1128/JCM.05092-11

TELENTI A, 1993, LANCET, V341, P647, DOI 10.1016/0140-6736(93)90417-F

Thee S, 2016, TUBERCULOSIS, V97, P126, DOI 10.1016/j.tube.2015.09.007

Thirumurugan R, 2015, J INFECT PUBLIC HEAL, V8, P619, DOI 10.1016/j.jiph.2015.05.003

Toms C, 2015, COMMUN DIS INTELL, V39, pE217, DOI [10.33321/cdi.2015.39.21, 10.33321/cdi.2015.39.21]

Trefzer C, 2010, J AM CHEM SOC, V132, P13663, DOI 10.1021/ja106357w

Tye GJ, 2015, J IMMUNOL RES, V2015, DOI 10.1155/2015/916780

Udwadia ZF, 2012, CLIN INFECT DIS, V54, P579, DOI 10.1093/cid/cir889

Vadwai V, 2011, PLOS ONE, V6, DOI 10.1371/journal.pone.0024770

Vaubourgeix J, 2009, J BIOL CHEM, V284, P19321, DOI 10.1074/jbc.M809599200

Velayati AA, 2009, CHEST, V136, P420, DOI 10.1378/chest.08-2427

Vento S, 2011, LANCET ONCOL, V12, P520, DOI 10.1016/S1470-2045(11)70105-X

Vilchèze C, 2008, MOL MICROBIOL, V69, P1316, DOI 10.1111/j.1365-2958.2008.06365.x

Vilchéze C, 2007, ANNU REV MICROBIOL, V61, P35, DOI 10.1146/annurev.micro.61.111606.122346

Vilchèze C, 2006, NAT MED, V12, P1027, DOI 10.1038/nm1466

Vincent V, 2012, INT J TUBERC LUNG D, V16, P24, DOI 10.5588/ijtld.11.0223

Von Groll A, 2009, ANTIMICROB AGENTS CH, V53, P4498, DOI 10.1128/AAC.00287-09

Wang F, 2010, ANTIMICROB AGENTS CH, V54, P3776, DOI 10.1128/AAC.00453-10

Watanabe M, 2002, MICROBIOL-SGM, V148, P1881, DOI 10.1099/00221287-148-6-1881

Williams KN, 2009, ANTIMICROB AGENTS CH, V53, P1314, DOI 10.1128/AAC.01182-08

Wilson R, 2013, NAT CHEM BIOL, V9, P499, DOI 10.1038/nchembio.1277

Workneh MH, 2016, INFECT DIS POVERTY, V5, DOI 10.1186/s40249-016-0115-z

Wu FL, 2017, MOL CELL PROTEOMICS, V16, P1491, DOI 10.1074/mcp.M116.065771

Wu VC, 2013, PLOS ONE, V8, DOI 10.1371/journal.pone.0069556

Xia Q, 2015, ANTIMICROB AGENTS CH, V59, P1695, DOI 10.1128/AAC.04541-14

Xu P, 2016, TUBERCULOSIS, V98, P56, DOI 10.1016/j.tube.2016.02.008

Xu YH, 2015, BIOMED RES INT, V2015, DOI 10.1155/2015/951706

Xue WF, 2016, J MICROBIOL BIOTECHN, V26, P180, DOI 10.4014/jmb.1501.01015

Yano T, 2011, J BIOL CHEM, V286, P10276, DOI 10.1074/jbc.M110.200501

Yoon JH, 2013, J MICROBIOL METH, V92, P301, DOI 10.1016/j.mimet.2012.12.014

Yuan XL, 2012, J CLIN MICROBIOL, V50, P2404, DOI 10.1128/JCM.06860-11

Zhang HT, 2013, NAT GENET, V45, P1255, DOI 10.1038/ng.2735

Zhang S, 2016, ANTIMICROB AGENTS CH, V60, P2542, DOI 10.1128/AAC.02941-15

Zhang SW, 2017, J ADV CERAM, V6, P1, DOI 10.1007/s40145-016-0209-x

Zhang Y, 2015, INT J TUBERC LUNG D, V19, P1276, DOI 10.5588/ijtld.15.0389

ZHANG Y, 1992, NATURE, V358, P591, DOI 10.1038/358591a0

Zhang ZJ, 2014, J CLIN MICROBIOL, V52, P638, DOI 10.1128/JCM.02713-13

Zhao F, 2014, ANTIMICROB AGENTS CH, V58, P1479, DOI 10.1128/AAC.01775-13

Zimhony O, 2007, ANTIMICROB AGENTS CH, V51, P752, DOI 10.1128/AAC.01369-06

NR 205

TC 130

Z9 158

U1 2

U2 47

PU FRONTIERS MEDIA SA

PI LAUSANNE

PA AVENUE DU TRIBUNAL FEDERAL 34, LAUSANNE, CH-1015, SWITZERLAND

SN 2235-2988

J9 FRONT CELL INFECT MI

JI Front. Cell. Infect. Microbiol.

PD APR 10

PY 2018

VL 8

AR 114

DI 10.3389/fcimb.2018.00114

PG 21

WC Immunology; Microbiology

WE Science Citation Index Expanded (SCI-EXPANDED)

SC Immunology; Microbiology

GA GC2DH

UT WOS:000429591900001

OA Green Submitted, gold

DA 2026-05-07

ER

PT J

AU Raqib, R

Sarker, P

AF Raqib, Rubhana

Sarker, Protim

TI Repurposed Drugs and Plant-Derived Natural Products as Potential

Host-Directed Therapeutic Candidates for Tuberculosis

SO BIOMOLECULES

LA English

DT Review

DE phytochemicals; <italic>Mycobacterium tuberculosis</italic>; MDR; XDR;

anti-TB drugs

ID MULTIDRUG-RESISTANT TUBERCULOSIS; RANDOMIZED DOUBLE-BLIND;

MYCOBACTERIUM-TUBERCULOSIS; INDUCED HEPATOTOXICITY; BACTERIAL-GROWTH;

PHASE-I; EFFICACY; CURCUMIN; SAFETY; AUTOPHAGY

AB Tuberculosis (TB) is one of the leading causes of death due to infectious disease. It is a treatable disease; however, conventional treatment requires a lengthy treatment regimen with severe side effects, resulting in poor compliance among TB patients. Intermittent drug use, the non-compliance of patients, and prescription errors, among other factors, have led to the emergence of multidrug-resistant TB, while the mismanagement of multidrug-resistant TB (MDR-TB) has eventually led to the development of extensively drug-resistant tuberculosis (XDR-TB). Thus, there is an urgent need for new drug development, but due to the enormous expenses and time required (up to 20 years) for new drug research and development, new therapeutic approaches to TB are required. Host-directed therapies (HDT) could be a most attractive strategy, as they target the host defense processes instead of the microbe and thereby may prevent the alarming rise of MDR- and XDR-TB. This paper reviews the progress in HDT for the treatment of TB using repurposed drugs which have been investigated in clinical trials (completed or ongoing) and plant-derived natural products that are in clinical or preclinical trial stages. Additionally, this review describes the existing challenges to the development and future research directions in the implementation of HDT.

C1 [Raqib, Rubhana; Sarker, Protim] Bangladesh Icddr B, Int Ctr Diarrhoeal Dis Res, Nutr Res Div, Immunobiol Nutr & Toxicol Unit, Dhaka 1212, Bangladesh.

C3 International Centre for Diarrhoeal Disease Research (ICDDR)

RP Raqib, R (corresponding author), Bangladesh Icddr B, Int Ctr Diarrhoeal Dis Res, Nutr Res Div, Immunobiol Nutr & Toxicol Unit, Dhaka 1212, Bangladesh.

EM rubhana@icddrb.org; protim@icddrb.org

OI Raqib, Rubhana/0000-0002-8116-4511

FU Governments of Bangladesh and Canada

FX This research received no external funding. icddr,b is grateful to the

Governments of Bangladesh and Canada for providing core/unrestricted

support for its operations and research.

CR Adewole OO, 2023, INT J TUBERC LUNG D, V27, P912, DOI 10.5588/ijtld.23.0190

Adikesavalu H, 2021, MEDICINA-LITHUANIA, V57, DOI 10.3390/medicina57060522

Aerts L, 2019, J IMMUNOL, V202, P421, DOI 10.4049/jimmunol.1800840

Agarwal A, 2010, PHYTOMEDICINE, V17, P23, DOI 10.1016/j.phymed.2009.10.019

Aghababaei F, 2023, PHARMACEUTICALS-BASE, V16, DOI 10.3390/ph16071020

Ahmad S, 2019, INFECT IMMUN, V87, DOI 10.1128/IAI.00291-19

Ahmed S, 2020, ANTIBIOTICS-BASEL, V9, DOI 10.3390/antibiotics9010021

Alonzi T, 2024, BIOMED PHARMACOTHER, V178, DOI 10.1016/j.biopha.2024.117153

Anand PK, 2006, INT J BIOCHEM CELL B, V38, P600, DOI 10.1016/j.biocel.2005.10.021

Andres S, 2018, MOL NUTR FOOD RES, V62, DOI 10.1002/mnfr.201700447

[Anonymous], 2023, Global Tuberculosis Report

Arias L, 2023, TRIALS, V24, DOI 10.1186/s13063-023-07448-0

Aryan H, 2022, PHYTOTHER RES, V36, P3924, DOI 10.1002/ptr.7537

Augenstreich J, 2017, CELL MICROBIOL, V19, DOI 10.1111/cmi.12726

Babunovic GH, 2022, MBIO, V13, DOI 10.1128/mbio.03683-21

Bai XY, 2016, RESPIROLOGY, V21, P951, DOI 10.1111/resp.12762

Baldwin PR, 2015, EUR J MED CHEM, V92, P693, DOI 10.1016/j.ejmech.2015.01.020

Bandala C, 2024, NUTRIENTS, V16, DOI 10.3390/nu16142284

Bekele A, 2018, J INTERN MED, V284, P292, DOI 10.1111/joim.12767

Bellmann-Strobl J, 2021, NEUROL-NEUROIMMUNOL, V8, DOI 10.1212/NXI.0000000000000981

Bergman P, 2020, FRONT IMMUNOL, V11, DOI 10.3389/fimmu.2020.01209

Bhaskar A, 2020, INT IMMUNOPHARMACOL, V87, DOI 10.1016/j.intimp.2020.106809

Bodke H, 2023, CUREUS J MED SCIENCE, V15, DOI 10.7759/cureus.49374

Böhme J, 2020, NAT COMMUN, V11, DOI 10.1038/s41467-020-19095-z

Boland R, 2023, MBIO, V14, DOI 10.1128/mbio.03024-22

Braian C, 2013, J INNATE IMMUN, V5, P591, DOI 10.1159/000348676

Brandenburg J, 2021, J CLIN INVEST, V131, DOI 10.1172/JCI141833

Bussi C, 2019, FEMS MICROBIOL REV, V43, P341, DOI 10.1093/femsre/fuz006

Butov D, 2016, INT J MYCOBACT, V5, P446, DOI 10.1016/j.ijmyco.2016.06.012

Butova Tatyana, 2016, Int J Mycobacteriol, V5 Suppl 1, pS103, DOI [10.1016/j.ijmyco.2016.09.037, 10.1016/j.ijmyco.2016.09.037]

Carrillo-Martinez EJ, 2024, MOLECULES, V29, DOI 10.3390/molecules29051000

Chatree S, 2021, EXP BIOL MED, V246, P163, DOI 10.1177/1535370220962708

Chen MS, 2015, BMC PUBLIC HEALTH, V15, DOI 10.1186/s12889-015-1855-6

Chen Q, 2022, FRONT PHARMACOL, V13, DOI 10.3389/fphar.2022.813682

Cheng AL, 2001, ANTICANCER RES, V21, P2895

Cho JH, 2017, ONCOTARGET, V8, P24932, DOI 10.18632/oncotarget.15326

Cleverley TL, 2023, PLOS PATHOG, V19, DOI 10.1371/journal.ppat.1011387

Crichton M, 2024, J ACAD NUTR DIET, V124, DOI 10.1016/j.jand.2023.09.003

Crichton M, 2023, J NUTR, V153, P3193, DOI 10.1016/j.tjnut.2023.09.002

Cross GB, 2023, LANCET INFECT DIS, V23, P847, DOI 10.1016/S1473-3099(23)00067-1

Cubillos-Angulo JM, 2022, FRONT MED-LAUSANNE, V9, DOI 10.3389/fmed.2022.970408

Cui YL, 2024, N-S ARCH PHARMACOL, V397, P8485, DOI 10.1007/s00210-024-03199-0

Dallenga T, 2017, CELL HOST MICROBE, V22, P519, DOI 10.1016/j.chom.2017.09.003

de Almeida PE, 2023, FRONT CELL INFECT MI, V13, DOI 10.3389/fcimb.2023.1102643

Deng YP, 2023, J AGR FOOD CHEM, V71, P19207, DOI 10.1021/acs.jafc.3c05602

Di Pierro F, 2021, INT J GEN MED, V14, P2807, DOI 10.2147/IJGM.S318949

Di Stadio A, 2022, CURR NEUROPHARMACOL, V20, P2001, DOI 10.2174/1570159X20666220420113513

Diriba G, 2023, INT J INFECT DIS, V132, P50, DOI 10.1016/j.ijid.2023.04.392

Dludla PV, 2023, MOLECULES, V28, DOI 10.3390/molecules28186569

Donovan J, 2023, NEW ENGL J MED, V389, P1357, DOI 10.1056/NEJMoa2216218

Doostkam A, 2024, SCI REP-UK, V14, DOI 10.1038/s41598-024-68572-8

Dwivedi VP, 2017, FRONT CELL INFECT MI, V7, DOI 10.3389/fcimb.2017.00149

Dwivedi VP, 2019, J ETHNOPHARMACOL, V243, DOI 10.1016/j.jep.2018.12.008

Flaig TW, 2007, INVEST NEW DRUG, V25, P139, DOI 10.1007/s10637-006-9019-2

Fu L, 2024, INFECT DRUG RESIST, V17, P595, DOI 10.2147/IDR.S443897

Gabor KA, 2017, CURR MOL PHARMACOL, V10, P27, DOI 10.2174/1874467209666160112123603

Giver CR, 2019, BLOOD, V134, DOI 10.1182/blood-2019-130275

Guler R, 2015, NAT CHEM BIOL, V11, P748, DOI 10.1038/nchembio.1917

Gupta PK, 2023, FRONT IMMUNOL, V14, DOI 10.3389/fimmu.2023.1233630

Gupta PK, 2016, PLOS ONE, V11, DOI 10.1371/journal.pone.0154725

Han MK, 2020, BMJ OPEN RESPIR RES, V7, DOI 10.1136/bmjresp-2018-000392

Harrison SA, 2021, NAT COMMUN, V12, DOI 10.1038/s41467-021-25701-5

Hasan N, 2006, FEBS LETT, V580, P2517, DOI 10.1016/j.febslet.2006.03.071

Hawn TR, 2015, IMMUNOL REV, V264, P344, DOI 10.1111/imr.12255

Heo E, 2017, TUBERC RESPIR DIS, V80, P265, DOI 10.4046/trd.2017.80.3.265

Hsu CY, 2024, PHYTOTHER RES, V38, P4336, DOI 10.1002/ptr.8255

Hu J, 2018, REGUL TOXICOL PHARM, V95, P412, DOI 10.1016/j.yrtph.2018.03.019

Huang XJ, 2024, BIOMED PHARMACOTHER, V171, DOI 10.1016/j.biopha.2023.116087

Inbaraj LR, 2024, TRIALS, V25, DOI 10.1186/s13063-024-08133-6

García-Martínez BI, 2023, INT J MOL SCI, V24, DOI 10.3390/ijms24087422

Ivashkin VT, 2022, PLOS ONE, V17, DOI 10.1371/journal.pone.0263880

Jahagirdar PS, 2020, J PHARM SCI-US, V109, P2262, DOI 10.1016/j.xphs.2020.03.018

Jena P, 2012, PLOS ONE, V7, DOI 10.1371/journal.pone.0050345

Jenum S, 2021, NAT COMMUN, V12, DOI 10.1038/s41467-021-27029-6

Jeong EK, 2022, PATHOGENS, V11, DOI 10.3390/pathogens11111291

Kaufmann SHE, 2018, NAT REV DRUG DISCOV, V17, P35, DOI 10.1038/nrd.2017.162

Kim JJ, 2012, CELL HOST MICROBE, V11, P457, DOI 10.1016/j.chom.2012.03.008

Kozhukhov S, 2024, HELL J CARDIOL, V76, P68, DOI 10.1016/j.hjc.2023.08.004

Krismawati Hana, 2023, Wellcome Open Res, V8, P289, DOI [10.12688/wellcomeopenres.19455.1, 10.12688/wellcomeopenres.19455.2]

Krug S, 2023, NAT COMMUN, V14, DOI 10.1038/s41467-023-43937-1

Kumar NP, 2024, TUBERCULOSIS, V148, DOI 10.1016/j.tube.2024.102523

Kumar S, 2019, J BIOL CHEM, V294, P8555, DOI 10.1074/jbc.RA119.008005

Lachmandas E, 2019, J INFECT DIS, V220, P139, DOI 10.1093/infdis/jiz064

Lara-Espinosa JV, 2022, INT J MOL SCI, V23, DOI 10.3390/ijms23041964

Larsen SE, 2022, FRONT IMMUNOL, V13, DOI 10.3389/fimmu.2022.840225

Lee HJ, 2020, FRONT MICROBIOL, V11, DOI 10.3389/fmicb.2020.01684

Lee YY, 2023, DRUGS CONTEXT, V12, DOI 10.7573/dic.2023-2-11

Lewinsohn DM, 2022, FRONT IMMUNOL, V13, DOI 10.3389/fimmu.2022.869057

Li JL, 2024, PLOS ONE, V19, DOI 10.1371/journal.pone.0309144

Li MY, 2014, MOL MED REP, V10, P3261, DOI 10.3892/mmr.2014.2615

Li Y, 2022, J APPL TOXICOL, V42, P1192, DOI 10.1002/jat.4288

Lim YJ, 2011, PLOS ONE, V6, DOI 10.1371/journal.pone.0028531

Lin PL, 2015, SEMIN IMMUNOPATHOL, V37, P239, DOI 10.1007/s00281-015-0490-8

Lu CC, 2014, J LEUKOCYTE BIOL, V96, P1119, DOI 10.1189/jlb.4A0713-363RR

Luangchosiri C, 2015, BMC COMPLEM ALTERN M, V15, DOI 10.1186/s12906-015-0861-7

Madaan R, 2022, CURR TOP MED CHEM, V22, P132, DOI 10.2174/1568026621666211015092654

Maglione PJ, 2007, J IMMUNOL, V178, P7222, DOI 10.4049/jimmunol.178.11.7222

Mahjabeen W, 2022, COMPLEMENT THER MED, V66, DOI 10.1016/j.ctim.2022.102819

Mahmoud AM, 2014, INT J PHARMACOL, V10, P451, DOI 10.3923/ijp.2014.451.460

Mai NTH, 2018, ELIFE, V7, DOI [10.7554/elife.33478, 10.7554/eLife.33478]

Maitre T, 2022, TRIALS, V23, DOI 10.1186/s13063-022-06772-1

Mankowski RT, 2020, EXP GERONTOL, V131, DOI 10.1016/j.exger.2019.110821

Mannick JB, 2014, SCI TRANSL MED, V6, DOI 10.1126/scitranslmed.3009892

Manzanillo PS, 2013, NATURE, V501, P512, DOI 10.1038/nature12566

Marjani Majid, 2019, Gastroenterol Hepatol Bed Bench, V12, P138

Marjani M, 2016, IRAN J PHARM RES, V15, P247

Mehmood S, 2022, J FOOD BIOCHEM, V46, DOI 10.1111/jfbc.14189

Mi J, 2024, FRONT MICROBIOL, V15, DOI 10.3389/fmicb.2024.1380848

Mihret A, 2012, VIRULENCE, V3, P654, DOI 10.4161/viru.22586

Mily A, 2015, PLOS ONE, V10, DOI 10.1371/journal.pone.0138340

Mily A, 2013, BMC PULM MED, V13, DOI 10.1186/1471-2466-13-23

Ming J, 2021, GENOME MED, V13, DOI 10.1186/s13073-021-00942-7

Miow QH, 2021, J CLIN INVEST, V131, DOI 10.1172/JCI141895

Miranda MS, 2012, CLIN DEV IMMUNOL, DOI 10.1155/2012/139127

Misra UK, 2018, NEUROL INDIA, V66, P1672, DOI 10.4103/0028-3886.246278

Mukhopadhyay S, 2024, TUBERCULOSIS, V147, DOI 10.1016/j.tube.2024.102517

Muvva JR, 2021, J INFECT DIS, V224, P332, DOI 10.1093/infdis/jiab100

Nasim N, 2022, NUCLEUS CALCUTTA, V65, P399, DOI 10.1007/s13237-022-00405-3

Noppakun K, 2024, CARDIORENAL MED, V14, P160, DOI 10.1159/000537710

Ntalouka F, 2023, FRONT PAIN RES, V4, DOI 10.3389/fpain.2023.1114428

Nyasulu PS, 2024, CURR OPIN PULM MED, V30, P217, DOI 10.1097/MCP.0000000000001070

Oh JE, 2014, FRONT IMMUNOL, V5, DOI 10.3389/fimmu.2014.00300

Ouyang J, 2020, OXID MED CELL LONGEV, V2020, DOI 10.1155/2020/9723686

Ozturk M, 2021, FRONT IMMUNOL, V12, DOI 10.3389/fimmu.2021.656419

Padmapriydarsini C, 2022, CLIN INFECT DIS, V75, P425, DOI 10.1093/cid/ciab964

Pahuja I, 2023, PLOS PATHOG, V19, DOI 10.1371/journal.ppat.1011165

Panigrahi A, 2023, BMC ENDOCR DISORD, V23, DOI 10.1186/s12902-023-01442-y

Parihar SP, 2019, NAT REV IMMUNOL, V19, P104, DOI 10.1038/s41577-018-0094-3

Patel Naresh, 2017, J Assoc Physicians India, V65, P48

Pennini ME, 2007, J IMMUNOL, V179, P6910, DOI 10.4049/jimmunol.179.10.6910

Periyasamy KM, 2020, MOL IMMUNOL, V127, P238, DOI 10.1016/j.molimm.2020.08.007

Popat R, 2013, BRIT J HAEMATOL, V160, P714, DOI 10.1111/bjh.12154

PREISLER HD, 1986, CANCER CHEMOTH PHARM, V18, P257

Prendergast KA, 2013, TUBERCULOSIS, V93, P115, DOI 10.1016/j.tube.2012.10.008

Qiao M, 2023, PHARM BIOL, V61, P1474, DOI 10.1080/13880209.2023.2248167

Qin S, 2021, INT IMMUNOPHARMACOL, V99, DOI 10.1016/j.intimp.2021.107898

Raien A, 2023, CELLS-BASEL, V12, DOI 10.3390/cells12222653

Rana SV, 2011, NUTR RES REV, V24, P60, DOI 10.1017/S0954422410000338

Ravesloot-Chávez MM, 2021, ANNU REV IMMUNOL, V39, P611, DOI 10.1146/annurev-immunol-093019-010426

Rekha RS, 2018, BMC INFECT DIS, V18, DOI 10.1186/s12879-018-3203-9

Rekha RS, 2015, AUTOPHAGY, V11, P1688, DOI 10.1080/15548627.2015.1075110

Rodríguez-Carlos A, 2023, MEM I OSWALDO CRUZ, V118, DOI 10.1590/0074-02760230143

Rodríguez-Flores EM, 2019, PLOS ONE, V14, DOI 10.1371/journal.pone.0217457

Rondanelli M, 2023, EUR REV MED PHARMACO, V27, P6718, DOI 10.26355/eurrev_202307_33142

Rust R, 2021, NEUROL-NEUROIMMUNOL, V8, DOI 10.1212/NXI.0000000000000964

Safe IP, 2021, FRONT IMMUNOL, V11, DOI 10.3389/fimmu.2020.602589

Safe IP, 2020, PLOS ONE, V15, DOI 10.1371/journal.pone.0235381

Saini Neha, 2023, Curr Drug Res Rev, V15, P105, DOI [10.2174/2589977515666221101153730, 10.2174/2589977515666221101153730]

Salimo ZM, 2023, BIOMOLECULES, V13, DOI 10.3390/biom13030403

Sanjay S, 2021, CAN J PHYSIOL PHARM, V99, P952, DOI 10.1139/cjpp-2021-0008

Schiebler M, 2015, EMBO MOL MED, V7, P127, DOI 10.15252/emmm.201404137

Schoeman JF, 2011, J CHILD NEUROL, V26, P956, DOI 10.1177/0883073811398132

Shaito A, 2020, INT J MOL SCI, V21, DOI 10.3390/ijms21062084

Sharifi-Rad J, 2020, FRONT PHARMACOL, V11, DOI 10.3389/fphar.2020.01021

Sharma A, 2020, ACS BIOMATER SCI ENG, V6, P4126, DOI 10.1021/acsbiomaterials.0c00823

Sharma S, 2014, TUBERCULOSIS, V94, P389, DOI 10.1016/j.tube.2014.04.007

Shen L, 2016, SCI REP-UK, V6, DOI 10.1038/srep20872

Singh DK, 2021, PLOS PATHOG, V17, DOI 10.1371/journal.ppat.1009805

Singh R, 2020, J APPL MICROBIOL, V128, P1547, DOI 10.1111/jam.14478

Singhal A, 2014, SCI TRANSL MED, V6, DOI 10.1126/scitranslmed.3009885

Soh AZ, 2017, NUTRIENTS, V9, DOI 10.3390/nu9060544

Soltani M, 2024, TRIALS, V25, DOI 10.1186/s13063-024-08354-9

Stan D, 2021, FRONT PHARMACOL, V12, DOI 10.3389/fphar.2021.723233

Steiger J, 2016, J IMMUNOL, V197, P222, DOI 10.4049/jimmunol.1502407

Talebi A, 2023, INT J PREVENTIVE MED, V14, DOI 10.4103/ijpvm.ijpvm_81_22

Tamara L, 2022, J GLOB HEALTH, V12, DOI 10.7189/jogh.12.04015

Terzo S, 2023, NUTRIENTS, V15, DOI 10.3390/nu15020462

Tiberi S, 2018, LANCET INFECT DIS, V18, pE183, DOI 10.1016/S1473-3099(18)30110-5

Tousif S, 2017, FRONT IMMUNOL, V8, DOI 10.3389/fimmu.2017.00739

Vora Agam, 2016, J Assoc Physicians India, V64, P20

Wallis RS, 2024, NEJM EVID, V3, DOI [10.1056/evidoa2300332, 10.1056/EVIDoa2300332]

Wallis RS, 2023, NAT REV IMMUNOL, V23, P121, DOI 10.1038/s41577-022-00734-z

Wallis RS, 2022, PLOS ONE, V17, DOI 10.1371/journal.pone.0252097

Wallis RS, 2021, LANCET RESP MED, V9, P897, DOI 10.1016/S2213-2600(20)30448-3

Watson RO, 2012, CELL, V150, P803, DOI 10.1016/j.cell.2012.06.040

Wik JA, 2022, FRONT IMMUNOL, V13, DOI 10.3389/fimmu.2022.840610

World Health Organization, 2024, Consolidated guidelines on differentiated HIV testing services: web annex C: GRADE table and systematic review: should HIV self-testing be used to support PrEP delivery

Xu P, 2022, FRONT CELL INFECT MI, V12, DOI 10.3389/fcimb.2022.877265

Yaikwawong M, 2024, NUTR J, V23, DOI 10.1186/s12937-024-01022-3

Yaikwawong M, 2024, NUTRIENTS, V16, DOI 10.3390/nu16152441

Yang CT, 2012, CELL HOST MICROBE, V12, P301, DOI 10.1016/j.chom.2012.07.009

Yang H, 2021, INT IMMUNOPHARMACOL, V91, DOI 10.1016/j.intimp.2020.107283

Yang H, 2020, ARCH BIOCHEM BIOPHYS, V694, DOI 10.1016/j.abb.2020.108612

Yang H, 2019, ARCH BIOCHEM BIOPHYS, V667, P49, DOI 10.1016/j.abb.2019.04.006

Yang JX, 2023, MEDCOMM, V4, DOI 10.1002/mco2.353

Yang KL, 2022, FRONT IMMUNOL, V13, DOI 10.3389/fimmu.2022.949746

Yin XY, 2024, BMC CANCER, V24, DOI 10.1186/s12885-024-12228-3

Younes M, 2018, EFSA J, V16, DOI 10.2903/j.efsa.2018.5239

Yücel Ç, 2022, FRONT PHARMACOL, V13, DOI 10.3389/fphar.2022.902551

Zhang SM, 2016, J GASTROEN HEPATOL, V31, P409, DOI 10.1111/jgh.13070

Zhang WY, 2021, FOOD FUNCT, V12, P8867, DOI [10.1039/D1FO01971F, 10.1039/d1fo01971f]

Zhang YM, 2020, INT IMMUNOPHARMACOL, V85, DOI 10.1016/j.intimp.2020.106634

Zhang ZH, 2022, FOOD RES INT, V156, DOI 10.1016/j.foodres.2022.111314

Zhao JV, 2021, NUTRIENTS, V13, DOI 10.3390/nu13082550

Zhou YQ, 2023, NUTRIENTS, V15, DOI 10.3390/nu15030492

Ziegenhagen R, 2021, FOODS, V10, DOI 10.3390/foods10092121

NR 196

TC 4

Z9 4

U1 1

U2 6

PU MDPI

PI BASEL

PA ST ALBAN-ANLAGE 66, CH-4052 BASEL, SWITZERLAND

EI 2218-273X

J9 BIOMOLECULES

JI Biomolecules

PD DEC

PY 2024

VL 14

IS 12

AR 1497

DI 10.3390/biom14121497

PG 32

WC Biochemistry & Molecular Biology

WE Science Citation Index Expanded (SCI-EXPANDED)

SC Biochemistry & Molecular Biology

GA Q8M2S

UT WOS:001387143200001

OA Green Submitted, gold

DA 2026-05-07

ER

PT J

AU Kumar, A

Karkara, BB

Panda, G

AF Kumar, Amit

Karkara, Bidhu Bhusan

Panda, Gautam

TI Novel candidates in the clinical development pipeline for TB drug

development and their synthetic approaches

SO CHEMICAL BIOLOGY & DRUG DESIGN

LA English

DT Review

DE bedaquiline; delamanid; MDR-Tb; Mycobacterium tuberculosis; pretomanid;

XDR-Tb

ID IN-VIVO ACTIVITIES; NONREPLICATING MYCOBACTERIUM-TUBERCULOSIS; CELL-WALL

CORE; ATP SYNTHASE; BIOLOGICAL EVALUATION; ANTITUBERCULOSIS AGENTS; LEAD

OPTIMIZATION; DNA GYRASE; ANTIMYCOBACTERIAL ACTIVITIES; ANTIBACTERIAL

ACTIVITY

AB Tuberculosis (TB) is an infection caused by Mycobacterium tuberculosis (Mtb) and one of the deadliest infectious diseases in the world. Mtb has the ability to become dormant within the host and to develop resistance. Hence, new antitubercular agents are required to overcome problems in the treatment of multi-drug-resistant Tb (MDR-Tb) and extensively drug-resistant Tb (XDR-Tb) along with shortening the treatment time. Several efforts are being made to develop very effective new drugs for Tb, within the pharmaceutical industry, the academia and through public-private partnerships. This review will address the antitubercular activities, biological target, mode of action, synthetic approaches and thoughtful concept for the development of several new drugs currently in the clinical trial pipeline (up to October 2019) for tuberculosis. The aim of this review may be very useful in scheming new chemical entities (NCEs) for Mtb.

C1 [Kumar, Amit; Panda, Gautam] CSIR, Cent Drug Res Inst, Med & Proc Chem Div, Sect 10,Jankipuram Extens,Sitapur Rd, Lucknow 226031, UP, India.

[Karkara, Bidhu Bhusan] Vignans Fdn Sci Technol & Res Univ, Dept Pharmaceut Sci, Guntur, Andhra Pradesh, India.

C3 Council of Scientific & Industrial Research (CSIR) - India; CSIR -

Central Drug Research Institute (CDRI); Vignan's Foundation for Science,

Technology & Research (VFSTR)

RP Panda, G (corresponding author), CSIR, Cent Drug Res Inst, Med & Proc Chem Div, Sect 10,Jankipuram Extens,Sitapur Rd, Lucknow 226031, UP, India.

EM gautam.panda@gmail.com

RI ; Karkara, BIDHU/AAW-4286-2020

OI Karkara, BIDHU/0000-0003-3445-9728;

FU Science and Engineering Research Board

FX Science and Engineering Research Board

CR Abrahams KA, 2017, SCI REP-UK, V7, DOI 10.1038/s41598-017-09642-y

Abrahams KA, 2016, NAT COMMUN, V7, DOI 10.1038/ncomms12581

Ali MM, 2001, SYNLETT, P251

Ananthan S, 2009, TUBERCULOSIS, V89, P334, DOI 10.1016/j.tube.2009.05.008

Andreotti D, 1996, BIOORG MED CHEM LETT, V6, P491, DOI 10.1016/0960-894X(96)00056-X

Andries K, 2005, SCIENCE, V307, P223, DOI 10.1126/science.1106753

Andries K, 2014, PLOS ONE, V9, DOI 10.1371/journal.pone.0102135

Arora S. K., 2004, Patent No. [WO2004/026828, 2004026828]

ASHTEKAR DR, 1993, ANTIMICROB AGENTS CH, V37, P183, DOI 10.1128/AAC.37.2.183

Baker W.R., 1997, PCT publication, Patent No. [WO9701562A1, 9701562]

Balasubramanian V, 2014, ANTIMICROB AGENTS CH, V58, P495, DOI 10.1128/AAC.01903-13

Ballell L, 2013, CHEMMEDCHEM, V8, P313, DOI 10.1002/cmdc.201200428

Ballell Pages L., 2017, Antituberculosis Agent, Patent No. [2017162591, WO2017162591A1]

Barbachyn MR, 1996, J MED CHEM, V39, P680, DOI 10.1021/jm950956y

Barry CE III, 1998, PROG LIPID RES, V37, P143, DOI 10.1016/S0163-7827(98)00008-3

Barthod L, 2018, J CHEMOTHERAPY, V30, P1, DOI 10.1080/1120009X.2017.1338845

Bellale E, 2014, J MED CHEM, V57, P6572, DOI 10.1021/jm500833f

Bogatcheva E, 2011, BIOORG MED CHEM LETT, V21, P5353, DOI 10.1016/j.bmcl.2011.07.015

Brecik M, 2015, ACS CHEM BIOL, V10, P1631, DOI 10.1021/acschembio.5b00237

Brickner SJ, 1996, J MED CHEM, V39, P673, DOI 10.1021/jm9509556

Brooks JV, 1999, ANTIMICROB AGENTS CH, V43, P1285, DOI 10.1128/AAC.43.5.1285

Brown DS, 1998, TETRAHEDRON LETT, V39, P8533, DOI 10.1016/S0040-4039(98)01892-9

Brown-Elliott BA, 2018, ANTIMICROB AGENTS CH, V62, DOI [10.1128/AAC.01503-18, 10.1128/aac.01503-18]

Bugg Timothy D. H., 2006, Infectious Disorders - Drug Targets, V6, P85, DOI 10.2174/187152606784112128

Carlos, 2019, International Publication Number, Patent No. [WO2019034729A1, 2019034729]

Chandran M, 2015, INT J MYCOBACT, V4, P104, DOI 10.1016/j.ijmyco.2015.02.002

Chandrasekhar S, 2011, EUR J ORG CHEM, V2011, P2057, DOI 10.1002/ejoc.201001720

Cho YL, 2020, APPL SCI-BASEL, V10, DOI 10.3390/app10072211

Choi PJ, 2017, BIOORG MED CHEM LETT, V27, P5190, DOI 10.1016/j.bmcl.2017.10.042

Christophe T, 2009, PLOS PATHOG, V5, DOI 10.1371/journal.ppat.1000645

Cooper CB, 2017, US patent, Patent No. [WO/2017/015106, 2017015106]

Cunningham F, 2020, ACS INFECT DIS, V6, P1098, DOI 10.1021/acsinfecdis.9b00493

Das SK, 2007, BIOORG MED CHEM LETT, V17, P5586, DOI 10.1016/j.bmcl.2007.07.089

Dey R, 2020, CURR TOP MED CHEM, V20, P2662, DOI 10.2174/1568026620666200903163921

Diacon AH, 2012, ANTIMICROB AGENTS CH, V56, P3271, DOI 10.1128/AAC.06126-11

Dubuisson T, 2010, J ANTIMICROB CHEMOTH, V65, P2590, DOI 10.1093/jac/dkq372

EDWARDS DI, 1993, J ANTIMICROB CHEMOTH, V31, P9, DOI 10.1093/jac/31.1.9

Falzari K, 2005, ANTIMICROB AGENTS CH, V49, P1447, DOI 10.1128/AAC.49.4.1447-1454.2005

Freundlich, Patent No. [WO2019046467A1, 2019046467]

GAO Y, 1987, J AM CHEM SOC, V109, P5765, DOI 10.1021/ja00253a032

Garrido-Cardenas JA, 2020, TUBERCULOSIS, V121, DOI 10.1016/j.tube.2020.101917

Gestel, 2005, PCT publication, Patent No. [US20050148581A1, 20050148581]

Gestel J. F. E. V., 2004, [No title captured], Patent No. [2004011436, WO 2004011436]

Goulooze SC, 2015, BRIT J CLIN PHARMACO, V80, P182, DOI 10.1111/bcp.12613

Grant SS, 2013, ACS CHEM BIOL, V8, P2224, DOI 10.1021/cb4004817

Grzegorzewicz AE, 2012, NAT CHEM BIOL, V8, P334, DOI [10.1038/NCHEMBIO.794, 10.1038/nchembio.794]

Guillemont J., 2009, CURR BIOACT COMPD, V5, P137, DOI [DOI 10.2174/157340709788451975, 10.2174/157340709788451975]

Guillemont J, 2011, FUTURE MED CHEM, V3, P1345, DOI [10.4155/FMC.11.79, 10.4155/fmc.11.79]

Haagsma AC, 2011, PLOS ONE, V6, DOI 10.1371/journal.pone.0023575

Haagsma AC, 2009, ANTIMICROB AGENTS CH, V53, P1290, DOI 10.1128/AAC.01393-08

Hariguchi N, 2020, ANTIMICROB AGENTS CH, V64, DOI 10.1128/AAC.02020-19

Hartkoorn RC, 2014, ANTIMICROB AGENTS CH, V58, P2979, DOI 10.1128/AAC.00037-14

Hoagland DT, 2016, ADV DRUG DELIVER REV, V102, P55, DOI 10.1016/j.addr.2016.04.026

Ioerger TR, 2013, PLOS ONE, V8, DOI 10.1371/journal.pone.0075245

Ishizaki Y, 2013, J BIOL CHEM, V288, P30309, DOI 10.1074/jbc.M113.492173

Jain PP, 2013, BIOORG MED CHEM LETT, V23, P6097, DOI 10.1016/j.bmcl.2013.09.027

Jansen RS, 2017, TRENDS PHARMACOL SCI, V38, P393, DOI 10.1016/j.tips.2017.01.005

Jeankumar VU, 2015, BIOORGAN MED CHEM, V23, P588, DOI 10.1016/j.bmc.2014.12.001

Jeong JW, 2010, ANTIMICROB AGENTS CH, V54, P5359, DOI 10.1128/AAC.00723-10

Jia J, 2005, BRIT J PHARMACOL, V144, P80, DOI 10.1038/sj.bjp.0705984

Rebollo-Lopez MJ, 2015, PLOS ONE, V10, DOI 10.1371/journal.pone.0142293

Kakkar AK, 2014, TUBERCULOSIS, V94, P357, DOI 10.1016/j.tube.2014.04.001

Kalia D, 2015, MEDCHEMCOMM, V6, P1554, DOI 10.1039/c5md00131e

Kang S, 2014, J MED CHEM, V57, P5293, DOI 10.1021/jm5003606

Karkara BB, 2020, BIOORG CHEM, V99, DOI 10.1016/j.bioorg.2020.103775

Keam SJ, 2019, DRUGS, V79, P1797, DOI 10.1007/s40265-019-01207-9

Khan SR, 2013, INT J ANTIMICROB AG, V41, P41, DOI 10.1016/j.ijantimicag.2012.09.012

Kimura K, 2003, NAT PROD REP, V20, P252, DOI 10.1039/b202149h

Koga T, 2004, J ANTIMICROB CHEMOTH, V54, P755, DOI 10.1093/jac/dkh417

Koul A, 2008, J BIOL CHEM, V283, P25273, DOI 10.1074/jbc.M803899200

Koul A, 2007, NAT CHEM BIOL, V3, P323, DOI 10.1038/nchembio884

Kumar P, 2018, MBIO, V9, DOI 10.1128/mBio.02101-17

Kumar S, 2013, BIOORG MED CHEM LETT, V23, P2404, DOI 10.1016/j.bmcl.2013.02.030

Lakshmanan M, 2013, J YOUNG PHARM, V5, P112, DOI 10.1016/j.jyp.2013.12.002

Lee RE, 2003, J COMB CHEM, V5, P172, DOI 10.1021/cc020071p

Lee RE, 2014, NAT MED, V20, P152, DOI 10.1038/nm.3458

Li W, 2016, ANTIMICROB AGENTS CH, V60, P5198, DOI 10.1128/AAC.00826-16

Li XF, 2017, J MED CHEM, V60, P8011, DOI 10.1021/acs.jmedchem.7b00631

Liu JY, 2017, ACS INFECT DIS, V3, P72, DOI 10.1021/acsinfecdis.6b00158

Locher CP, 2015, ANTIMICROB AGENTS CH, V59, P1455, DOI 10.1128/AAC.04347-14

Lu P, 2014, BBA-BIOENERGETICS, V1837, P1208, DOI 10.1016/j.bbabio.2014.01.022

Lu XY, 2019, ACS INFECT DIS, V5, P239, DOI 10.1021/acsinfecdis.8b00225

Macikenas, 2014, PCT publication, Patent No. [WO, 2014/015105A1, 2014015105]

Maitra A, 2015, INT J INFECT DIS, V32, P50, DOI 10.1016/j.ijid.2014.12.031

Makarov V.A., 2011, PCT publication, Patent No. [WO2007134625A1, 2007134625]

Makarov V, 2014, EMBO MOL MED, V6, P372, DOI 10.1002/emmm.201303575

Makarov V, 2009, SCIENCE, V324, P801, DOI 10.1126/science.1171583

Manjunatha U., 2009, Communicative and Integrative Biology, V2, P215, DOI [10.4161/cib.2.3.7926, DOI 10.4161/CIB.2.3.7926]

Manjunatha UH, 2006, P NATL ACAD SCI USA, V103, P431, DOI 10.1073/pnas.0508392103

Matsoso LG, 2005, J BACTERIOL, V187, P6300, DOI 10.1128/JB.187.18.6300-6308.2005

Matsumoto M, 2007, CURR TOP MED CHEM, V7, P499, DOI 10.2174/156802607780059727

McKee EE, 2006, ANTIMICROB AGENTS CH, V50, P2042, DOI 10.1128/AAC.01411-05

Mdluli K., 2017, P ASM MICR 2017 NEW

Medapi B, 2016, BIOORGAN MED CHEM, V24, P877, DOI 10.1016/j.bmc.2016.01.011

Moscow Municipal Scientific Practical Center of Tuberculosis Control Health Department of Moscow Russian Federation, 2018, Tuberculosis and lung diseases, V96, P6, DOI [10.21292/2075-1230-2018-96-3-6-18, 10.21292/2075-1230-2018-96-3-6-18, DOI 10.21292/2075-1230-2018-96-3-6-18]

Muramatsu Y, 2003, J ANTIBIOT, V56, P253, DOI 10.7164/antibiotics.56.253

NAGARAJAN K, 1989, EUR J MED CHEM, V24, P631, DOI 10.1016/0223-5234(89)90034-2

Nagiec EE, 2005, ANTIMICROB AGENTS CH, V49, P3896, DOI 10.1128/AAC.49.9.3896-3902.2005

Nakamura H, 2016, ORG LETT, V18, P2300, DOI 10.1021/acs.orglett.6b00943

Nikonenko BV, 2007, ANTIMICROB AGENTS CH, V51, P1563, DOI 10.1128/AAC.01326-06

No, 2011, PCT publication, Patent No. [WO 2011085990A1, 2011085990]

No Z, 2011, Patent No. [2011113606, WO 2011113606A1]

O'Dowd H, 2015, ACS MED CHEM LETT, V6, P822, DOI 10.1021/acsmedchemlett.5b00196

Orita A, 2007, ADV SYNTH CATAL, V349, P2136, DOI 10.1002/adsc.200700119

Panda G, 2005, BIOORG MED CHEM LETT, V15, P5222, DOI 10.1016/j.bmcl.2005.08.045

Panda G, 2007, EUR J MED CHEM, V42, P410, DOI 10.1016/j.ejmech.2006.09.020

Panunzio M, 1996, TETRAHEDRON-ASYMMETR, V7, P2929, DOI 10.1016/0957-4166(96)00384-9

Parai MK, 2008, BIOORG MED CHEM LETT, V18, P289, DOI 10.1016/j.bmcl.2007.10.083

Patil TS, 2018, EXPERT OPIN THER PAT, V28, P615, DOI 10.1080/13543776.2018.1508454

Pethe K, 2013, NAT MED, V19, P1157, DOI 10.1038/nm.3262

Pethe K, 2010, NAT COMMUN, V1, DOI 10.1038/ncomms1060

Phetsuksiri B, 1999, ANTIMICROB AGENTS CH, V43, P1042, DOI 10.1128/AAC.43.5.1042

Porstmann F. R., 2006, [No title captured], Patent No. [2006125769, WO2006125769A1]

Protopopova M, 2005, J ANTIMICROB CHEMOTH, V56, P968, DOI 10.1093/jac/dki319

Rao SPS, 2008, P NATL ACAD SCI USA, V105, P11945, DOI 10.1073/pnas.0711697105

Reddy VM, 2008, ANTIMICROB AGENTS CH, V52, P719, DOI 10.1128/AAC.01469-07

Roy KK, 2011, BIOORG MED CHEM LETT, V21, P5589, DOI 10.1016/j.bmcl.2011.06.076

Ryan NJ, 2014, DRUGS, V74, P1041, DOI 10.1007/s40265-014-0241-5

Saga Y, 2010, J AM CHEM SOC, V132, P7905, DOI 10.1021/ja103183r

Saha T, 2017, RSC ADV, V7, P6966, DOI 10.1039/c6ra25429b

Sasaki H, 2006, J MED CHEM, V49, P7854, DOI 10.1021/jm060957y

Sassetti CM, 2003, MOL MICROBIOL, V48, P77, DOI 10.1046/j.1365-2958.2003.03425.x

Saxena AK, 2013, INT J ANTIMICROB AG, V42, P27, DOI 10.1016/j.ijantimicag.2013.03.007

Saxena AK, 2019, CURR TOP MED CHEM, V19, P337, DOI 10.2174/1568026619666190219105722

Shirude PS, 2013, J MED CHEM, V56, P9701, DOI 10.1021/jm401382v

Singh, 2013, PCT publication, Patent No. [WO2013102936A1, 2013102936]

Singh P, 2015, EUR J MED CHEM, V95, P357, DOI 10.1016/j.ejmech.2015.03.036

Singh R, 2008, SCIENCE, V322, P1392, DOI 10.1126/science.1164571

Singh S., 2012, Patent No. [WO2012164572A1, 2012164572]

Singh S, 2015, BIOORGAN MED CHEM, V23, P742, DOI 10.1016/j.bmc.2014.12.060

Singh V, 2017, DRUG DISCOV TODAY, V22, P503, DOI 10.1016/j.drudis.2016.09.010

Singh VK, 2014, TUBERCULOSIS, V94, P252, DOI 10.1016/j.tube.2014.02.001

Spigelman M., 2008, 1 INT WORKSH CLIN PH

Stover CK, 2000, NATURE, V405, P962, DOI 10.1038/35016103

Sutherland HS, 2019, BIOORGAN MED CHEM, V27, P1292, DOI 10.1016/j.bmc.2019.02.026

Swaminathan S, 2016, TUBERCULOSIS, V101, P31, DOI 10.1016/j.tube.2016.08.002

Tahlan K, 2012, ANTIMICROB AGENTS CH, V56, P1797, DOI 10.1128/AAC.05708-11

Takahashi Y, 2013, J ANTIBIOT, V66, P171, DOI 10.1038/ja.2013.9

Tang J, 2015, ACS MED CHEM LETT, V6, P814, DOI 10.1021/acsmedchemlett.5b00176

Tantry SJ, 2017, J MED CHEM, V60, P1379, DOI 10.1021/acs.jmedchem.6b01358

Tantry SJ, 2015, BIOORG MED CHEM LETT, V25, P3234, DOI 10.1016/j.bmcl.2015.05.087

Thompson AM, 2009, J MED CHEM, V52, P637, DOI 10.1021/jm801087e

Warrier T, 2015, ACS INFECT DIS, V1, P580, DOI 10.1021/acsinfecdis.5b00025

Wilson R, 2013, NAT CHEM BIOL, V9, P499, DOI 10.1038/nchembio.1277

Worley MV, 2014, PHARMACOTHERAPY, V34, P1187, DOI 10.1002/phar.1482

Xu ZJ, 2017, P NATL ACAD SCI USA, V114, P7993, DOI 10.1073/pnas.1700062114

Zhang DF, 2012, J MED CHEM, V55, P8409, DOI 10.1021/jm300828h

Zhang YM, 2009, EUR J ORG CHEM, V2009, P2983, DOI 10.1002/ejoc.200900059

Zhu T, 2014, ANTIMICROB AGENTS CH, V58, P3306, DOI 10.1128/AAC.01920-13

NR 149

TC 11

Z9 15

U1 0

U2 14

PU WILEY

PI HOBOKEN

PA 111 RIVER ST, HOBOKEN 07030-5774, NJ USA

SN 1747-0277

EI 1747-0285

J9 CHEM BIOL DRUG DES

JI Chem. Biol. Drug Des.

PD NOV

PY 2021

VL 98

IS 5

BP 787

EP 827

DI 10.1111/cbdd.13934

EA SEP 2021

PG 41

WC Biochemistry & Molecular Biology; Chemistry, Medicinal

WE Science Citation Index Expanded (SCI-EXPANDED)

SC Biochemistry & Molecular Biology; Pharmacology & Pharmacy

GA WO4HY

UT WOS:000696408400001

DA 2026-05-07

ER

PT J

AU Ma, Z

Lienhardt, C

AF Ma, Zhenkun

Lienhardt, Christian

TI Toward an Optimized Therapy for Tuberculosis? Drugs in Clinical Trials

and in Preclinical Development

SO CLINICS IN CHEST MEDICINE

LA English

DT Article

DE Tuberculosis; Chemotherapy; Novel mechanism of action; Drug combination;

Drug resistance; Drug persistence; MDR-TB; XDR-TB

ID EARLY BACTERICIDAL ACTIVITY; MULTIDRUG-RESISTANT TUBERCULOSIS; IN-VITRO

ACTIVITY; MYCOBACTERIUM-TUBERCULOSIS; PULMONARY TUBERCULOSIS;

DIARYLQUINOLINE TMC207; STERILIZING ACTIVITIES; PHASE-II; MOXIFLOXACIN;

GATIFLOXACIN

AB Tuberculosis (TB) continues to be one of the greatest challenges in the global public health arena. Current therapeutic agents against TB are old and inadequate, particularly in the face of many new challenges. Multidrug-resistant TB (MDR-TB) has become prevalent in many parts of the world and extensively drug-resistant TB (XDR-TB) is rapidly emerging. There are few or essentially no effective drugs available to treat these drug-resistant forms of TB. TB and human immunodeficiency virus (HIV) coinfection has become another major problem in areas with high prevalence of HIV infection. Simultaneous treatment of TB and HIV is difficult due to the severe drug-drug interactions between the first-line rifamycin-containing TB therapy and antiretroviral agents. However, there have been some encouraging developments in TB drug research and development within the past decade. At present there are 6 compounds, including 3 novel agents, in late stages of clinical development. There are even larger numbers of compounds and projects in the TB drug pipeline at the discovery stage and in early stages of clinical development, mainly targeting treatment shortening and drug resistance. Despite these encouraging developments, the current TB drug pipeline is not sufficient to address the multitude of challenges inherent in the current standard of TB therapy. A stronger TB drug pipeline and a new paradigm for the development of novel TB drug combinations are needed.

C1 [Ma, Zhenkun] Global Alliance TB Drug Dev, New York, NY 10005 USA.

[Lienhardt, Christian] WHO, Stop TB Partnership & Stop TB Dept, CH-1211 Geneva 27, Switzerland.

C3 World Health Organization

RP Ma, Z (corresponding author), Global Alliance TB Drug Dev, New York, NY 10005 USA.

EM zhenkun.ma@tballiance.org

RI Lienhardt, Christian/AAU-4969-2021

OI Lienhardt, Christian/0000-0003-0233-893X

CR Alvirez-Freites EJ, 2002, ANTIMICROB AGENTS CH, V46, P1022, DOI 10.1128/AAC.46.4.1022-1025.2002

Andries K, 2005, SCIENCE, V307, P223, DOI 10.1126/science.1106753

[Anonymous], 2000, AM J RESP CRIT CARE, V161, pS221, DOI [DOI 10.1164/AJRCCM.161.SUPPLEMENT_, 10.1164/ajrccm.161.supplement_3.ats600, DOI 10.1164/AJRCCM.161.SUPPLEMENT_3.ATS600]

[Anonymous], CLIN INFECT DIS

[Anonymous], 2003, Treatment of Tuberculosis: Guidelines for National Programmes

[Anonymous], 2025, National guidelines for management of drug-resistant tuberculosis

[Anonymous], 2009, GLOBAL TUBERCULOSIS

Benator D, 2002, LANCET, V360, P528, DOI 10.1016/S0140-6736(02)09742-8

Brown D, 2003, DRUG DISCOV TODAY, V8, P1067, DOI 10.1016/S1359-6446(03)02902-7

Burman WJ, 2006, AM J RESP CRIT CARE, V174, P331, DOI 10.1164/rccm.200603-360OC

Cobelens FGJ, 2008, PLOS MED, V5, P1037, DOI 10.1371/journal.pmed.0050150

COMSTOCK GW, 1979, AM REV RESPIR DIS, V119, P827

Conde MB, 2009, LANCET, V373, P1183, DOI 10.1016/S0140-6736(09)60333-0

Corbett EL, 2003, ARCH INTERN MED, V163, P1009, DOI 10.1001/archinte.163.9.1009

Cynamon MH, 1999, ANTIMICROB AGENTS CH, V43, P1189, DOI 10.1128/AAC.43.5.1189

Cynamon MH, 2003, ANTIMICROB AGENTS CH, V47, P2442, DOI 10.1128/AAC.47.8.2442-2444.2003

Davies G, 2007, COCHRANE DB SYST REV, DOI 10.1002/14651858.CD005159.pub2

Diacon AH, 2009, NEW ENGL J MED, V360, P2397, DOI 10.1056/NEJMoa0808427

Diekema DJ, 2001, LANCET, V358, P1975, DOI 10.1016/S0140-6736(01)06964-1

Dietze R, 2008, AM J RESP CRIT CARE, V178, P1180, DOI 10.1164/rccm.200806-892OC

Espinal M, 2009, INT J TUBERC LUNG D, V13, P1

FOX W, 1981, BRIT J DIS CHEST, V75, P331, DOI 10.1016/0007-0971(81)90022-X

Fox W., 1999, Int J Tuberc Lung Dis, V3, pS231

GINSBERG AM, 2008, 39 UN WORLD C LUNG H

Ginsberg AM, 2007, NAT MED, V13, P290, DOI 10.1038/nm0307-290

Gosling RD, 2003, AM J RESP CRIT CARE, V168, P1342, DOI 10.1164/rccm.200305-682OC

Gray DM, 2009, COCHRANE DB SYST REV, DOI 10.1002/14651858.CD006418.pub2

Hu YM, 2003, ANTIMICROB AGENTS CH, V47, P653, DOI 10.1128/AAC.47.2.653-657.2003

*INT UN TUB LUNG D, 1994, TUBERCLE LUNG DIS, V75, P180

Jereb J, 2003, INT J TUBERC LUNG D, V7, pS384

Jia L, 2006, BRIT J PHARMACOL, V147, P476, DOI 10.1038/sj.bjp.0706650

Jindani A, 2004, LANCET, V364, P1244, DOI 10.1016/S0140-6736(04)17141-9

Johnson JL, 2006, INT J TUBERC LUNG D, V10, P605

Jones D, 1944, SCIENCE, V100, P103, DOI 10.1126/science.100.2588.103

Lawn SD, 2005, LANCET INFECT DIS, V5, P361, DOI 10.1016/S1473-3099(05)70140-7

Lawn SD, 2005, AM J RESP CRIT CARE, V172, P1348, DOI 10.1164/ajrccm.172.10.1348

Lenaerts AJ, 2007, ANTIMICROB AGENTS CH, V51, P3338, DOI 10.1128/AAC.00276-07

Lounis N, 2006, ANTIMICROB AGENTS CH, V50, P3543, DOI 10.1128/AAC.00766-06

Ma Z., 2007, Compreh. Med. Chem. II, V7, P699, DOI [DOI 10.1016/B0-08-045044-X/00224-8, 10.1016/B0-08-045044-X/00224-8]

MADDIX DS, 1994, ANN PHARMACOTHER, V28, P1250, DOI 10.1177/106002809402801108

Matsumoto M, 2006, PLOS MED, V3, P2131, DOI 10.1371/journal.pmed.0030466

MCCUNE RM, 1956, J EXP MED, V104, P737, DOI 10.1084/jem.104.5.737

MITCHISON DA, 1979, CHEST, V76, P771, DOI 10.1378/chest.76.6.771

MITCHISON DA, 1980, J ROY COLL PHYS LOND, V14, P91

Miyazaki E, 1999, ANTIMICROB AGENTS CH, V43, P85, DOI 10.1128/AAC.43.1.85

Mukherjee JS, 2004, LANCET, V363, P474, DOI 10.1016/S0140-6736(04)15496-2

Munsiff SS, 2006, CLIN INFECT DIS, V43, P1468, DOI 10.1086/508278

Nathanson E, 2004, INT J TUBERC LUNG D, V8, P1382

Niemi M, 2003, CLIN PHARMACOKINET, V42, P819, DOI 10.2165/00003088-200342090-00003

Nikonenko BV, 2007, ANTIMICROB AGENTS CH, V51, P1563, DOI 10.1128/AAC.01326-06

Ntziora F, 2007, INT J TUBERC LUNG D, V11, P606

Nuermberger EL, 2004, AM J RESP CRIT CARE, V169, P421, DOI 10.1164/rccm.200310-1380OC

Ormerod P, 2000, THORAX, V55, P887

Paramasivan CN, 2005, ANTIMICROB AGENTS CH, V49, P627, DOI 10.1128/AAC.49.2.627-631.2005

Pletz MWR, 2004, ANTIMICROB AGENTS CH, V48, P780, DOI 10.1128/AAC.48.3.780-782.2004

Protopopova M, 2005, J ANTIMICROB CHEMOTH, V56, P968, DOI 10.1093/jac/dki319

Rodríguez JC, 2001, INT J ANTIMICROB AG, V17, P229, DOI 10.1016/S0924-8579(00)00337-X

Rosenthal IM, 2008, AM J RESP CRIT CARE, V178, P989, DOI 10.1164/rccm.200807-1029OC

Rosenthal IM, 2007, PLOS MED, V4, P1931, DOI 10.1371/journal.pmed.0040344

Rustomjee R, 2008, ANTIMICROB AGENTS CH, V52, P2831, DOI 10.1128/AAC.01204-07

Rustomjee R, 2008, INT J TUBERC LUNG D, V12, P128

Schluger N, 2007, PLOS MED, V4, P1726, DOI 10.1371/journal.pmed.0040302

Singh R, 2008, SCIENCE, V322, P1392, DOI 10.1126/science.1164571

Smieja M J, 2000, Cochrane Database Syst Rev, pCD001363, DOI 10.1002/14651858.CD001363

Sood R., 2006, Infectious Disorders - Drug Targets, V6, P343, DOI 10.2174/187152606779025860

*STOP TB PARTN WOR, WORK GROUP TB DRUG R

Stover CK, 2000, NATURE, V405, P962, DOI 10.1038/35016103

Sulochana S, 2005, J CHEMOTHERAPY, V17, P169, DOI 10.1179/joc.2005.17.2.169

Temple ME, 1999, ANN PHARMACOTHER, V33, P1203, DOI 10.1345/aph.18450

THOMPSON NJ, 1982, B WORLD HEALTH ORGAN, V60, P555

Tyagi S, 2005, ANTIMICROB AGENTS CH, V49, P2289, DOI 10.1128/AAC.49.6.2289-2293.2005

Veziris N, 2009, AM J RESP CRIT CARE, V179, P75, DOI 10.1164/rccm.200711-1736OC

Williams KN, 2009, ANTIMICROB AGENTS CH, V53, P1314, DOI 10.1128/AAC.01182-08

WOLDEHANNA S, 2006, COCHRANE DB SYST REV, P171

1999, INT J TUBERC LUNG DI, V3, pS281

NR 75

TC 15

Z9 20

U1 0

U2 8

PU W B SAUNDERS CO-ELSEVIER INC

PI PHILADELPHIA

PA 1600 JOHN F KENNEDY BOULEVARD, STE 1800, PHILADELPHIA, PA 19103-2899 USA

SN 0272-5231

EI 1557-8216

J9 CLIN CHEST MED

JI Clin. Chest Med.

PD DEC

PY 2009

VL 30

IS 4

BP 755

EP +

DI 10.1016/j.ccm.2009.08.011

PG 15

WC Respiratory System

WE Science Citation Index Expanded (SCI-EXPANDED)

SC Respiratory System

GA 617BQ

UT WOS:000279256200009

DA 2026-05-07

ER

PT J

AU Oh, TS

Kim, YJ

Kang, HY

Kim, CK

Cho, SY

Lee, HJ

AF Oh, Tae Sang

Kim, Young Jin

Kang, Hee Yoon

Kim, Chang-Ki

Cho, Sun Young

Lee, Hee Joo

TI RNA expression analysis of efflux pump genes in clinical isolates of

multidrug-resistant and extensively drug-resistant Mycobacterium

tuberculosis in South Korea

SO INFECTION GENETICS AND EVOLUTION

LA English

DT Article

DE Aminoglycosides; Efflux pump; Fluoroquinolones; MDR-TB; Tuberculosis;

XDR-TB

ID TETRACYCLINE RESISTANCE; MEDIATED RESISTANCE; ACTIVE EFFLUX; MECHANISMS;

FLUOROQUINOLONES; TRANSPORTER; BACTERIA; TAP; PCR

AB Tuberculosis (TB), caused by infection with Mycobacterium tuberculosis, is an important communicable disease. Various mechanisms of resistance to antituberculosis drugs have been reported; these are principally mutations in target genes. However, not all M. tuberculosis resistance can be explained by mutations in such genes. Other resistance mechanisms associated with drug transport, such as efflux pumps, have also been reported. In this study, we investigated the expression levels of three putative efflux pumps and mutations in target genes associatedwith injectable agents and fluoroquinoloneswith clinicalMDR and XDR-TB isolates. Thirty clinical isolates of M. tuberculosis that had been phenotypically characterized were obtained from the Korean Institute of Tuberculosis. Of these, 14 were MDR-TB isolates resistant to at least one injectable aminoglycoside (amikacin; AMK, kanamycin; KAN, and/or capreomycin; CPM) and 16 were XDR-TB isolates. M. tuberculosis H37Rv (ATCC 27249) was used as a reference strain. Five putative genes (Rv1258c, Rv2686c, Rv2687c, Rv2688c and pstB) were selected for analysis in this study. Sequencing was performed to detect mutations in rrs and eis genes. qRT-PCR was performed to investigate expression levels of five efflux pump genes. Of the 30 isolates, 25 strains hadmutations in rrs associated with resistance to KAN, CPMand AMK and two strains had eis mutations, as well as mutations in rrs. pstB (Rv0933) exhibited increased expression and Rv2687c and Rv2688c exhibited decreased expression compared to the reference strain. Increased expression of pstB in clinical drug-resistant tuberculosis isolates may contribute to drug resistance in M. tuberculosis. In our case, overexpression of Rv1258c may have been associated with resistance to kanamycin. No correlation was evident between Rv2686c, Rv2687c or Rv2688c expression and fluoroquinolone resistance. To explore the details of efflux pump drug-resistancemechanisms, further studies on efflux pump inhibitors, transcriptional regulators, such as whiB7, and additional efflux pump genes are needed. (C) 2017 Elsevier B. V. All rights reserved.

C1 [Oh, Tae Sang] Kyung Hee Univ Hosp, Dept Lab Med, Seoul, South Korea.

[Kim, Young Jin; Kang, Hee Yoon; Cho, Sun Young; Lee, Hee Joo] Kyung Hee Univ, Sch Med, Dept Lab Med, Seoul, South Korea.

[Kim, Chang-Ki] Hanyang Univ, Guri Hosp, Dept Lab Med, Guri, South Korea.

C3 Kyung Hee University; Kyung Hee University Hospital; Kyung Hee

University; Hanyang University

RP Lee, HJ (corresponding author), Kyung Hee Univ, Sch Med Hosp, 23 Kyung Hee Dae, Seoul 02447, South Korea.

EM leehejo@gmail.com

RI Kim, Young-jin/GSD-3168-2022; Cho, Sun Young/H-8599-2013

FU Basic Science Research Program of the National Research Foundation of

Korea (NRF) - Ministry of Science, ICT & Future Planning

[NRF-2014R1A1A2004931]

FX This research was supported by the Basic Science Research Program of the

National Research Foundation of Korea (NRF) funded by the Ministry of

Science, ICT & Future Planning (NRF-2014R1A1A2004931).

CR Aínsa JA, 1998, J BACTERIOL, V180, P5836

BALL PR, 1980, BIOCHEM BIOPH RES CO, V93, P74, DOI 10.1016/S0006-291X(80)80247-6

Banerjee SK, 1998, FEBS LETT, V425, P151, DOI 10.1016/S0014-5793(98)00214-2

Bhatt K, 2000, EUR J BIOCHEM, V267, P4028, DOI 10.1046/j.1432-1327.2000.01437.x

Bustin SA, 2010, BMC MOL BIOL, V11, DOI 10.1186/1471-2199-11-74

De Rossi E, 2006, FEMS MICROBIOL REV, V30, P36, DOI 10.1111/j.1574-6976.2005.00000.x

DeMarco CE, 2007, ANTIMICROB AGENTS CH, V51, P3235, DOI 10.1128/AAC.00430-07

Joo Lee Hee, 2014, Annals of Clinical Microbiology, V17, P65

김창기, 2014, Annals of Clinical Microbiology, V17, P80

오태상, 2016, Annals of Clinical Microbiology, V19, P20

Korea Centers for Disease Control and Prevention (KCDC), 2016, ANN REP NOT TUB KOR

Levy SB, 2002, J APPL MICROBIOL, V92, p65S, DOI 10.1046/j.1365-2672.92.5s1.4.x

Li GL, 2015, J ANTIBIOT, V68, P431, DOI 10.1038/ja.2015.9

Livak KJ, 2001, METHODS, V25, P402, DOI 10.1006/meth.2001.1262

Löffler FE, 2000, APPL ENVIRON MICROB, V66, P1369, DOI 10.1128/AEM.66.4.1369-1374.2000

Lu J, 2014, FEMS MICROBIOL LETT, V353, P40, DOI 10.1111/1574-6968.12401

Mangan JA, 1997, NUCLEIC ACIDS RES, V25, P675, DOI 10.1093/nar/25.3.675

MCMURRY L, 1980, P NATL ACAD SCI-BIOL, V77, P3974, DOI 10.1073/pnas.77.7.3974

Morris RP, 2005, P NATL ACAD SCI USA, V102, P12200, DOI 10.1073/pnas.0505446102

NIKAIDO H, 1994, SCIENCE, V264, P382, DOI 10.1126/science.8153625

Pasca MR, 2004, ANTIMICROB AGENTS CH, V48, P3175, DOI 10.1128/AAC.48.8.3175-3178.2004

Piddock LJV, 2006, NAT REV MICROBIOL, V4, P629, DOI 10.1038/nrmicro1464

Poole K, 2000, ANTIMICROB AGENTS CH, V44, P2595, DOI 10.1128/AAC.44.10.2595-2599.2000

Poole K, 2000, ANTIMICROB AGENTS CH, V44, P2233, DOI 10.1128/AAC.44.9.2233-2241.2000

Reeves AZ, 2013, ANTIMICROB AGENTS CH, V57, P1857, DOI 10.1128/AAC.02191-12

Sarathy JP, 2012, PHARMACEUTICALS-BASE, V5, P1210, DOI 10.3390/ph5111210

Sharma S, 2010, J ANTIMICROB CHEMOTH, V65, P1694, DOI 10.1093/jac/dkq186

Siddiqi N, 2004, INFECTION, V32, P109, DOI 10.1007/s15010-004-3097-x

Stavri M, 2007, J ANTIMICROB CHEMOTH, V59, P1247, DOI 10.1093/jac/dkl460

WHO, 2015, GLOB TUB REP 2015

Zhang Y, 2009, INT J TUBERC LUNG D, V13, P1320

NR 31

TC 18

Z9 25

U1 0

U2 30

PU ELSEVIER SCIENCE BV

PI AMSTERDAM

PA PO BOX 211, 1000 AE AMSTERDAM, NETHERLANDS

SN 1567-1348

EI 1567-7257

J9 INFECT GENET EVOL

JI Infect. Genet. Evol.

PD APR

PY 2017

VL 49

BP 111

EP 115

DI 10.1016/j.meegid.2017.01.002

PG 5

WC Infectious Diseases

WE Science Citation Index Expanded (SCI-EXPANDED)

SC Infectious Diseases

GA EM7AN

UT WOS:000395463900016

DA 2026-05-07

ER

PT J

AU Varshney, D

Singh, SV

Mohanty, KK

Kumar, S

Varshney, N

Sinha, E

Barik, SK

AF Varshney, Deepika

Singh, Shoor Vir

Mohanty, Keshar Kunja

Kumar, Santosh

Varshney, Nitin

Sinha, Ekata

Barik, Sushanta Kumar

TI Toll-like receptor 2 (-196 to-174) del and TLR1 743 A &gt;

G gene polymorphism-a possible association with drug-resistant

tuberculosis in the north Indian population

SO FRONTIERS IN MICROBIOLOGY

LA English

DT Article

DE Toll-like receptors; drug-resistant tuberculosis; genotyping; TNF-alpha;

IL-6; IFN-gamma

ID TOLL-LIKE RECEPTORS; TUMOR-NECROSIS-FACTOR; MYCOBACTERIUM-TUBERCULOSIS;

PULMONARY TUBERCULOSIS; IMMUNE-RESPONSE; IFN-GAMMA; TNF-ALPHA;

SUSCEPTIBILITY; MACROPHAGES; DISEASE

AB Objectives: The objective of this study is to analyze the association between TLR2 deletion (-196 to -174) and TLR1 743 A > G gene polymorphism with drug resistant tuberculosis (PTB, MDR-TB, and XDR-TB) in a population from Agra, Uttar Pradesh. Methods: The present case-control study included 101 pulmonary TB patients, 104 multidrug-resistant TB patients, 48 extremely drug-resistant TB patients, and 130 healthy and unrelated controls residing in the same locality. The genotyping method for TLR2 deletion (-196 to -174) was carried out by allele-specific polymerase chain reaction (PCR), and TLR1 743 A > G gene polymorphism was performed by hybridization probe chemistry in Roche Real-Time PCR. Genotype and allele frequencies were analyzed by the chi-square test. Cytokine levels were measured by ELISA and compared using Mann-Whitney and Kruskal-Wallis tests. Results: The frequency of heterozygous (Ins/del) genotypes for TLR2 (-196 to -174) polymorphism was predominant in XDR-TB patients (0.57), whereas heterozygous A/G genotype for TLR1 743 A > G single nucleotide polymorphism (SNP) was predominant in healthy controls (0.57) for TLR1 743 A > G gene polymorphism. The heterozygous genotype of TLR2 deletion polymorphism was found to be significantly higher in XDR-TB (p = 0.0001). TLR1 743 A > G SNP, AG genotypes were found to be significantly associated with healthy controls than PTB (p = 0.047). The level of serum cytokines (IL-6, TNF-alpha, and IFN-gamma) was also found to be significantly different among TB patients and healthy controls. Conclusion: The findings suggested that in the present population, the heterozygous (Ins/Del) genotype and deletion allele of TLR2 deletion (-196 to -174) polymorphism are associated with the risk for the development of drug-resistant TB. Furthermore, for TLR1 743 A > G gene polymorphism, A/G genotype, and G allele are found associated with healthy controls, suggesting the protective role against TB.

C1 [Varshney, Deepika; Mohanty, Keshar Kunja; Sinha, Ekata; Barik, Sushanta Kumar] ICMR Natl JALMA Inst Leprosy & Other Mycobacterial, Agra, Uttar Pradesh, India.

[Singh, Shoor Vir] GLA Univ, Inst Appl Sci & Humanities, Dept Biotechnol, Mathura, Uttar Pradesh, India.

[Kumar, Santosh] S N Med Coll & Hosp, Dept Chest & TB, Agra, Uttar Pradesh, India.

[Varshney, Nitin] Navsari Agr Univ, N M Coll Agr, Dept Agr Stat, Navsari, Gujarat, India.

[Varshney, Nitin] Navsari Agr Univ, N M Coll Agr, Comp Ctr, Navsari, Gujarat, India.

C3 Indian Council of Medical Research (ICMR); ICMR - National JALMA

Institute for Leprosy & Other Mycobacterial Diseases, Agra; GLA

University

RP Mohanty, KK (corresponding author), ICMR Natl JALMA Inst Leprosy & Other Mycobacterial, Agra, Uttar Pradesh, India.

EM keshar63@yahoo.com

RI MOHANTY, KESHAR/AAN-4814-2021; Varshney, Nitin/LRB-4577-2024

OI Varshney, Nitin/0000-0001-9144-5475

FU Department of Science and Technology (DST), New Delhi [IF180309]

FX The author(s) declare financial support was received for the research,

authorship, and/or publication of this article. This study is funded by

intramural research funding of ICMR-National JALMA Institute for Leprosy

and Other Mycobacterial Diseases, Agra. Raj Pal Suman is acknowledged

for technical help. DV was financially supported as PhD scholar by the

Department of Science and Technology (DST), New Delhi (Award letter

code. IF180309).

CR Akira S, 2006, CELL, V124, P783, DOI 10.1016/j.cell.2006.02.015

[Anonymous], 2009, J. Adv. Med. Biomed. Res

[Anonymous], 2020, World Health Organization: Global Tuberculosis Report 2019

BARNES PF, 1993, INFECT IMMUN, V61, P3482, DOI 10.1128/IAI.61.8.3482-3489.1993

Basingnaa A, 2019, DISEASES-BASEL, V7, DOI 10.3390/diseases7010002

Basu J, 2012, FRONT CELL INFECT MI, V2, DOI 10.3389/fcimb.2012.00145

Ben-Kahla I, 2016, INT J ANTIMICROB AG, V48, P353, DOI 10.1016/j.ijantimicag.2016.07.010

Berrington WR, 2007, IMMUNOL REV, V219, P167, DOI 10.1111/j.1600-065X.2007.00545.x

Brightbill HD, 1999, SCIENCE, V285, P732, DOI 10.1126/science.285.5428.732

Buwitt-Beckmann U, 2005, EUR J IMMUNOL, V35, P282, DOI 10.1002/eji.200424955

Central TB Division Ministry of Health and Family Welfare, 2017, TB India 2017 Revised National Tubercuosis Control Programme Annual Status Report

Chen YC, 2010, BMC MED GENET, V11, DOI 10.1186/1471-2350-11-17

Cicchese JM, 2018, IMMUNOL REV, V285, P147, DOI 10.1111/imr.12671

Cooper AM, 2008, IMMUNOL REV, V226, P191, DOI 10.1111/j.1600-065X.2008.00702.x

Cooper AM, 2009, ANNU REV IMMUNOL, V27, P393, DOI 10.1146/annurev.immunol.021908.132703

Dittrich N, 2015, TUBERCULOSIS, V95, P328, DOI 10.1016/j.tube.2015.02.045

DONNELLY RP, 1995, J IMMUNOL, V155, P1420

Ghasemian R., 2009, Iranian Journal of Clinical Infectious Diseases, V4, P135

Grassin-Delyle Stanislas, 2020, J Innate Immun, V12, P63, DOI [10.1159/000494463, 10.1159/000494463]

Guan Y, 2010, J IMMUNOL, V184, P5094, DOI 10.4049/jimmunol.0901888

Guidelines on Programmatic Management of Drug-Resistant Tuberculosis in India, 2017, Revised National Tuberculosis Control Programme

Haehnel V, 2002, J IMMUNOL, V168, P5629, DOI 10.4049/jimmunol.168.11.5629

Harling K, 2019, CELL MOL IMMUNOL, V16, P275, DOI 10.1038/cmi.2018.5

HernandezPando R, 1997, IMMUNOLOGY, V90, P607, DOI 10.1046/j.1365-2567.1997.00193.x

Hook JS, 2020, J IMMUNOL, V204, P671, DOI 10.4049/jimmunol.1900919

Indian Council of Medical Research, 2017, Ethical Guidelines for Biomedical Research

Jahantigh D, 2013, SCI WORLD J, DOI 10.1155/2013/534053

Juffermans NP, 1998, AM J RESP CRIT CARE, V157, P1328, DOI 10.1164/ajrccm.157.4.9709126

Keane J, 2000, J IMMUNOL, V164, P2016, DOI 10.4049/jimmunol.164.4.2016

Khan AUH, 2014, INT J IMMUNOGENET, V41, P105, DOI 10.1111/iji.12086

Kumar NP, 2019, OPEN FORUM INFECT DI, V6, DOI 10.1093/ofid/ofz257

Ma X, 2007, PLOS ONE, V2, DOI 10.1371/journal.pone.0001318

Mattos AMM, 2010, INT IMMUNOL, V22, P775, DOI 10.1093/intimm/dxq429

MILLER SA, 1988, NUCLEIC ACIDS RES, V16, P1215, DOI 10.1093/nar/16.3.1215

Mittal M, 2018, MOL BIOL REP, V45, P469, DOI 10.1007/s11033-018-4182-z

Nakaya M, 1995, Kekkaku, V70, P461

Nemeth J, 2011, CLIN IMMUNOL, V138, P50, DOI 10.1016/j.clim.2010.09.005

Noguchi E, 2004, CLIN EXP ALLERGY, V34, P177, DOI 10.1111/j.1365-2222.2004.01839.x

Ogus AC, 2004, EUR RESPIR J, V23, P219, DOI 10.1183/09031936.03.00061703

Omueti KO, 2005, J BIOL CHEM, V280, P36616, DOI 10.1074/jbc.M504320200

PANKOW W, 1991, LUNG, V169, P255, DOI 10.1007/BF02714161

Salie M, 2015, INFECT GENET EVOL, V34, P221, DOI 10.1016/j.meegid.2015.07.004

Schuring RP, 2009, J INFECT DIS, V199, P1816, DOI 10.1086/599121

Schurz H, 2015, PLOS ONE, V10, DOI 10.1371/journal.pone.0139711

Sia JK, 2019, MICROBIOL SPECTR, V7, DOI 10.1128/microbiolspec.GPP3-0022-2018

Sinha E, 2014, HUM IMMUNOL, V75, P880, DOI 10.1016/j.humimm.2014.06.014

Tahara T, 2008, DIGEST DIS SCI, V53, P919, DOI 10.1007/s10620-007-9950-x

Takeuchi O, 2002, J IMMUNOL, V169, P10, DOI 10.4049/jimmunol.169.1.10

Thada S, 2013, SCAND J IMMUNOL, V78, P221, DOI 10.1111/sji.12066

Tötemeyer S, 2006, J IMMUNOL, V176, P4804, DOI 10.4049/jimmunol.176.8.4804

Uciechowski P, 2011, J LEUKOCYTE BIOL, V90, P377, DOI 10.1189/jlb.0409233

Varshney D, 2022, CYTOKINE, V152, DOI 10.1016/j.cyto.2021.155791

Velez DR, 2010, HUM GENET, V127, P65, DOI 10.1007/s00439-009-0741-7

Vidya MK, 2018, INT REV IMMUNOL, V37, P20, DOI 10.1080/08830185.2017.1380200

Vijay K, 2018, INT IMMUNOPHARMACOL, V59, P391, DOI 10.1016/j.intimp.2018.03.002

Wu LL, 2015, BMC MED GENET, V16, DOI 10.1186/s12881-015-0166-1

Yao XY, 2017, TUBERCULOSIS, V107, P88, DOI 10.1016/j.tube.2017.07.013

YU GP, 1988, TUBERCLE, V69, P105, DOI 10.1016/0041-3879(88)90072-4

Zarember KA, 2002, J IMMUNOL, V168, P554, DOI 10.4049/jimmunol.168.2.554

Zhou Y, 2020, INNATE IMMUN-LONDON, V26, P75, DOI 10.1177/1753425919862354

NR 60

TC 0

Z9 0

U1 0

U2 2

PU FRONTIERS MEDIA SA

PI LAUSANNE

PA AVENUE DU TRIBUNAL FEDERAL 34, LAUSANNE, CH-1015, SWITZERLAND

EI 1664-302X

J9 FRONT MICROBIOL

JI Front. Microbiol.

PD JAN 24

PY 2024

VL 14

AR 1305974

DI 10.3389/fmicb.2023.1305974

PG 11

WC Microbiology

WE Science Citation Index Expanded (SCI-EXPANDED)

SC Microbiology

GA LA5U2

UT WOS:001184076100001

OA Green Submitted, gold

DA 2026-05-07

ER

PT J

AU Lilienkampf, A

Mao, JL

Wan, BJ

Wang, YH

Franzblau, SG

Kozikowski, AP

AF Lilienkampf, Annamaria

Mao, Jialin

Wan, Baojie

Wang, Yuehong

Franzblau, Scott G.

Kozikowski, Alan P.

TI Structure-Activity Relationships for a Series of Quinoline-Based

Compounds Active against Replicating and Nonreplicating Mycobacterium

tuberculosis

SO JOURNAL OF MEDICINAL CHEMISTRY

LA English

DT Article

ID MEFLOQUINE-BASED LIGANDS; METABOLISM; DESIGN; FLUOROQUINOLONES;

RESISTANCE; INHIBITORS; MECHANISM; TOXICITY; TARGET

AB Tuberculosis (TB) remains as a global pandemic that is aggravated by a lack of health care, the spread of HIV, and the emergence of multidrug-resistant TB (MDR-TB) and extensively drug-resistant TB (XDR-TB) strains. New anti-TB drugs are urgently required to shorten the long 6-12 month treatment regimen and to battle drug-resistant Mtb strains. We have identified several potent quinoline-based anti-TB compounds, bearing an isoxazole containing side-chain. The most potent compounds, 7g and 13, exhibited submicromolar activity against the replicating bacteria (R-TB), with minimum inhibitory concentrations (MICs) of 0.77 and 0.95 mu M, respectively. In general, these compounds also had micromolar activity against the nonreplicating persistent bacteria (NRP-TB) and did not show toxicity on Vero cells up to 128 mu M concentration. Compounds 7g and 13 were shown to retain their anti-TB activity against rifampin, isoniazid, and streptomycin resistant Mtb strains. The results suggest that quinoline-isoxazole-based anti-TB compounds are promising leads for new TB drug development.

C1 [Mao, Jialin; Wan, Baojie; Wang, Yuehong; Franzblau, Scott G.] Univ Illinois, Coll Pharm, Inst TB Res, Chicago, IL 60612 USA.

[Lilienkampf, Annamaria; Mao, Jialin; Kozikowski, Alan P.] Univ Illinois, Coll Pharm, Dept Med Chem & Pharmacognosy, Drug Discovery Program, Chicago, IL 60612 USA.

C3 University of Illinois System; University of Illinois Chicago;

University of Illinois Chicago Hospital; University of Illinois System;

University of Illinois Chicago; University of Illinois Chicago Hospital

RP Franzblau, SG (corresponding author), Univ Illinois, Coll Pharm, Inst TB Res, 833 S Wood St, Chicago, IL 60612 USA.

EM sgf@uic.edu; kozikowa@uic.edu

RI ; Mao, Jialin/L-4639-2013

OI Franzblau, Scott/0000-0002-8698-0243;

FU Academy of Finland [120441]; Academy of Finland (AKA) [120441] Funding

Source: Academy of Finland (AKA)

FX A.L. thanks The Academy of Finland for a fellowship (grant 120441). TB

Alliance is acknowledged for financial support.

CR Andries K, 2005, SCIENCE, V307, P223, DOI 10.1126/science.1106753

[Anonymous], 2008, Global Tuberculosis Control: Surveillance, Planning, and Financing; WHO Report 2008

Boshoff HIM, 2005, NAT REV MICROBIOL, V3, P70, DOI 10.1038/nrmicro1065

Charbonnière LJ, 2007, CHEM-EUR J, V13, P346, DOI 10.1002/chem.200600915

Cho SH, 2007, ANTIMICROB AGENTS CH, V51, P1380, DOI 10.1128/AAC.00055-06

Dan A, 2005, BIOORG MED CHEM LETT, V15, P4085, DOI 10.1016/j.bmcl.2005.06.016

De Angelis I, 1999, XENOBIOTICA, V29, P1157, DOI 10.1080/004982599238029

Drlica K, 1999, CURR OPIN MICROBIOL, V2, P504, DOI 10.1016/S1369-5274(99)00008-9

Escribano I, 2007, CHEMOTHERAPY, V53, P397, DOI 10.1159/000109769

Franzblau SG, 1998, J CLIN MICROBIOL, V36, P362, DOI 10.1128/JCM.36.2.362-366.1998

Jain R, 2003, BIOORG MED CHEM LETT, V13, P1051, DOI 10.1016/S0960-894X(03)00074-X

Janin YL, 2007, BIOORGAN MED CHEM, V15, P2479, DOI 10.1016/j.bmc.2007.01.030

Jayaprakash S, 2006, CHEMMEDCHEM, V1, P593, DOI 10.1002/cmdc.200600010

Koul A, 2007, NAT CHEM BIOL, V3, P323, DOI 10.1038/nchembio884

Lee CB, 2001, J AM CHEM SOC, V123, P5249, DOI 10.1021/ja010039j

LIU W, 2007, Patent No. 2007146072

Mao J, 2007, CHEMMEDCHEM, V2, P1624, DOI 10.1002/cmdc.200700112

Mao JL, 2007, CHEMMEDCHEM, V2, P811, DOI 10.1002/cmdc.200700048

Martín-Galiano AJ, 2002, ANTIMICROB AGENTS CH, V46, P1680, DOI 10.1128/AAC.46.6.1680-1687.2002

MICETICH RG, 1985, HETEROCYCLES, V23, P571

Moadebi S, 2007, DRUGS, V67, P2077, DOI 10.2165/00003495-200767140-00007

Monga V, 2004, BIOORGAN MED CHEM, V12, P6465, DOI 10.1016/j.bmc.2004.09.017

MORENO SNJ, 1985, ENVIRON HEALTH PERSP, V64, P199, DOI 10.2307/3430010

Nayyar A, 2006, BIOORGAN MED CHEM, V14, P847, DOI 10.1016/j.bmc.2005.09.018

Rae JM, 2001, J PHARMACOL EXP THER, V299, P849

Singh R, 2003, MOL MICROBIOL, V50, P751, DOI 10.1046/j.1365-2958.2003.03712.x

Soellner MB, 2007, J AM CHEM SOC, V129, P9613, DOI 10.1021/ja0727520

Spigelman MK, 2007, J INFECT DIS, V196, pS28, DOI 10.1086/518663

Tangallapally RP, 2005, J MED CHEM, V48, P8261, DOI 10.1021/jm050765n

Wang JY, 2007, J ANTIMICROB CHEMOTH, V59, P860, DOI 10.1093/jac/dkm061

Wayne LG, 2001, ANNU REV MICROBIOL, V55, P139, DOI 10.1146/annurev.micro.55.1.139

NR 31

TC 318

Z9 335

U1 1

U2 48

PU AMER CHEMICAL SOC

PI WASHINGTON

PA 1155 16TH ST, NW, WASHINGTON, DC 20036 USA

SN 0022-2623

EI 1520-4804

J9 J MED CHEM

JI J. Med. Chem.

PD APR 9

PY 2009

VL 52

IS 7

BP 2109

EP 2118

DI 10.1021/jm900003c

PG 10

WC Chemistry, Medicinal

WE Science Citation Index Expanded (SCI-EXPANDED); Index Chemicus (IC)

SC Pharmacology & Pharmacy

GA 428GK

UT WOS:000264835800032

DA 2026-05-07

ER

PT J

AU Parrish, N

Carroll, K

AF Parrish, Nicole

Carroll, Karen

TI Importance of improved TB diagnostics in addressing the extensively

drug-resistant TB crisis

SO FUTURE MICROBIOLOGY

LA English

DT Article

DE diagnostics; drug resistance; MDR-TB; susceptibility testing;

tuberculosis; XDR-TB

ID MULTIDRUG-RESISTANT; MYCOBACTERIUM-TUBERCULOSIS; SMEAR MICROSCOPY;

FLUOROQUINOLONE RESISTANCE; SPUTUM; SUSCEPTIBILITY; SENSITIVITY

AB There is an urgent need for new, rapid diagnostics to stem the tide of multidrug-resistant (MDR) and extensively drug-resistant (XDR) TB. Current identification and detection of resistance for the majority of Mycobacteria, including Mycobacterium tuberculosis, require weeks and multiple methodologies to complete. The sheer number and variety of tests required often exceeds the capacity of many laboratories. Current strategies center on improvement of existing tests and development of novel ones. Ultimately, what is needed is a single, rapid, diagnostic for identification and determination of drug resistance in M. tuberculosis. Such a method should be capable of detecting antibiotic resistance for all first- and second-line agents irrespective of the genetic mechanism of resistance. Detection should be achievable directly from clinical specimens. Yet, improved diagnostics are only part of a larger solution. Such a solution must include a comprehensive approach in which careful consideration is given to each particular setting relative to local resources and overall burden of disease. Such an approach is essential for the future of TB control and stemming the tide of MDR-TB and XDR-TB.

C1 [Parrish, Nicole; Carroll, Karen] Johns Hopkins Univ, Johns Hopkins Med Inst, Baltimore, MD 21287 USA.

C3 Johns Hopkins University; Johns Hopkins Medicine

RP Parrish, N (corresponding author), Johns Hopkins Univ, Johns Hopkins Med Inst, 600 N Wolfe St,Meyer B1-193, Baltimore, MD 21287 USA.

EM nparrish@jhmi.edu; kcarrol7@jhmi.edu

CR Andrews JR, 2007, J INFECT DIS, V196, pS482, DOI 10.1086/521121

[Anonymous], GLOB TUB CONTR SURV

Apers L, 2003, INT J TUBERC LUNG D, V7, P376

Bateman C, 2007, SAMJ S AFR MED J, V97, P1233

*CDC, 2002, P 4 NAT C LAB ASP TU

Centers for Disease Control and Prevention (CDC), 2006, MMWR-MORBID MORTAL W, V55, P301

Cohen T, 2004, NAT MED, V10, P1117, DOI 10.1038/nm1110

Crampin AC, 2001, INT J TUBERC LUNG D, V5, P994

DOOLEY SW, 1992, ANN INTERN MED, V117, P257, DOI 10.7326/0003-4819-117-3-257

Dye C, 1999, JAMA-J AM MED ASSOC, V282, P677, DOI 10.1001/jama.282.7.677

ELLIOTT AM, 1993, TUBERCLE LUNG DIS, V74, P191, DOI 10.1016/0962-8479(93)90010-U

Farnia P, 2002, J CLIN MICROBIOL, V40, P508, DOI 10.1128/JCM.40.2.508-511.2002

Fluit AC, 2001, CLIN MICROBIOL REV, V14, P836, DOI 10.1128/CMR.14.4.836-871.2001

FRIEDEN TR, 1993, NEW ENGL J MED, V328, P521, DOI 10.1056/NEJM199302253280801

Gandhi NR, 2006, LANCET, V368, P1575, DOI 10.1016/S0140-6736(06)69573-1

Ginsburg AS, 2003, CLIN INFECT DIS, V37, P1448, DOI 10.1086/379328

Ginsburg AS, 2003, NEW ENGL J MED, V349, P1977, DOI 10.1056/NEJM200311133492023

Goldman Robert C., 2007, Infectious Disorders - Drug Targets, V7, P73

Institute of Medicine, 2000, END NEGL EL TUB US

Jenkins C, 2005, EMERG INFECT DIS, V11, P931

Johnson JL, 1998, INT J TUBERC LUNG D, V2, P397

Jones KDJ, 2008, T ROY SOC TROP MED H, V102, P219, DOI 10.1016/j.trstmh.2007.11.014

Maurice E, 2006, JAMA-J AM MED ASSOC, V296, P2792

Palomino JC, 2005, EUR RESPIR J, V26, P339, DOI 10.1183/09031936.05.00050305

Palomino JC, 2006, CURR OPIN PULM MED, V12, P172, DOI 10.1097/01.mcp.0000219265.50310.9b

Park WG, 2002, J CLIN MICROBIOL, V40, P4750, DOI 10.1128/JCM.40.12.4750-4752.2002

Parrish N, 2007, J CLIN MICROBIOL, V45, P3915, DOI 10.1128/JCM.02528-06

Perkins MD, 2007, J INFECT DIS, V196, pS15, DOI 10.1086/518656

Piatek AS, 2000, ANTIMICROB AGENTS CH, V44, P103, DOI 10.1128/AAC.44.1.103-110.2000

Piersimoni C, 2001, J CLIN MICROBIOL, V39, P651, DOI 10.1128/JCM.39.2.651-657.2001

Steingart K, 2006, LANCET INFECT DIS, V6, P570, DOI 10.1016/S1473-3099(06)70578-3

SCARPARO C, 2002, DIAGN MICROBIOL INFE, V44, P17

Selvakumar N, 2002, J CLIN MICROBIOL, V40, P3017, DOI 10.1128/JCM.40.8.3017-3020.2002

Shah N. S., 2007, Morbidity and Mortality Weekly Report, V56, P250

Shamputa IC, 2004, APMIS, V112, P728, DOI 10.1111/j.1600-0463.2004.apm11211-1203.x

Steingart KR, 2006, LANCET INFECT DIS, V6, P664, DOI 10.1016/S1473-3099(06)70602-8

NR 36

TC 15

Z9 18

U1 0

U2 3

PU FUTURE MEDICINE LTD

PI LONDON

PA UNITEC HOUSE, 3RD FLOOR, 2 ALBERT PLACE, FINCHLEY CENTRAL, LONDON, N3

1QB, ENGLAND

SN 1746-0913

EI 1746-0921

J9 FUTURE MICROBIOL

JI Future Microbiol.

PD AUG

PY 2008

VL 3

IS 4

BP 405

EP 413

DI 10.2217/17460913.3.4.405

PG 9

WC Microbiology

WE Science Citation Index Expanded (SCI-EXPANDED)

SC Microbiology

GA 364SM

UT WOS:000260355600011

DA 2026-05-07

ER

PT J

AU Kisimba, K

Abdulsalam, RA

Salifu, EY

Sabiu, S

Faya, M

AF Kisimba, Kakudji

Abdulsalam, Rukayat Abiola

Salifu, Elliasu Y.

Sabiu, Saheed

Faya, Mbuso

TI An integrated computational bioprospection of flavonoids as modulators

of Mycobacterium tuberculosis

decaprenylphosphoryl-β-d-ribose-2′-epimerase 1

SO COMPUTATIONAL BIOLOGY AND CHEMISTRY

LA English

DT Article

DE Mycobacterium tuberculosis; Multidrug-resistant TB; DprE1; Flavonoid;

Molecular dynamics simulation; Molecular fingerprinting

ID ANTITUBERCULOSIS ACTIVITY; ANTIMYCOBACTERIAL; IDENTIFICATION; DOCKING

AB Tuberculosis (TB) remains a significant global health threat, claiming millions of lives annually despite being preventable. The emergence of drug-resistant strains, including extensively drug-resistant TB (XDR-TB) and multidrug-resistant TB (MDR-TB), severely limits conventional treatment options. Furthermore, commonly used TB medications like isoniazid (INH) and rifampicin (RIF) are associated with adverse side effects. Consequently, researchers increasingly explore natural products as potential sources for novel anti-TB therapeutics. This study investigated the inhibitory potential of 103 flavonoid compounds with documented antimycobacterial activity against TB. Focusing on decaprenylphosphoryl-beta-D-ribose 2 '-epimerase 1 (DprE1) as a druggable target, we employed molecular docking, pharmacokinetic evaluation, and 200-ns molecular dynamics simulations to assess stability and energy refinement. Our results showed that the top five compounds exhibited more favourable binding free energy values against DprE1 than the standard, PBTZ169. Notably, cycloartobiloxanthone demonstrated a binding free energy of-63.67 kcal/mol, surpassing PBTZ169 (-37.78 kcal/mol). Structural analysis revealed that cycloartobiloxanthone stabilised the protein and formed additional interactions without compromising its integrity. These findings suggest a potential structural mechanism for the inhibitory action of cycloartobiloxanthone against Mycobacterium tuberculosis DprE1. While this study highlights the potential of cycloartobiloxanthone as a lead compound, further validation through in vivo and in vitro studies is recommended.

C1 [Kisimba, Kakudji; Faya, Mbuso] Univ KwaZulu Natal, Coll Hlth Sci, Dept Pharmaceut, Discipline Pharmaceut Sci, Private Bag X54001, Durban, South Africa.

[Abdulsalam, Rukayat Abiola; Sabiu, Saheed] Durban Univ Technol, Fac Appl Sci, Dept Biotechnol & Food Sci, ZA-4000 Durban, South Africa.

[Salifu, Elliasu Y.] Tshwane Univ Technol, Dept Pharmaceut Sci, Private Bag X680, ZA-0001 Pretoria, South Africa.

C3 University of Kwazulu Natal; Durban University of Technology; Tshwane

University of Technology

RP Faya, M (corresponding author), Univ KwaZulu Natal, Coll Hlth Sci, Dept Pharmaceut, Discipline Pharmaceut Sci, Private Bag X54001, Durban, South Africa.

EM FayaA@ukzn.ac.za

RI Abdulsalam, Rukayat/JGM-7251-2023; Abdulsalam, Rukayat/JGM-7251-2023;

Sabiu, Saheed/AAA-2497-2021; Faya, Mbuso/V-3039-2019

OI Abdulsalam, Rukayat/0000-0003-4996-3441; Faya,

Mbuso/0000-0003-0315-2619; Salifu, Elliasu Yakubu/0000-0001-8713-5001

CR Abdulsalam R.A., 2023, Cheminformatics Validation of Plazomicin and Bioprospection of Putative Modulators of Aminoglycoside Modifying Enzymes from Gnaphalium affine towards Interventive Antibacterial Therapy

Al-Karmalawy AA, 2021, FRONT CHEM, V9, DOI 10.3389/fchem.2021.661230

Alam R, 2021, RSC ADV, V11, P40120, DOI 10.1039/d1ra06842c

Alonso H, 2006, MED RES REV, V26, P531, DOI 10.1002/med.20067

Altharawi A, 2023, SCI REP-UK, V13, DOI 10.1038/s41598-023-34222-8

Appetecchia F., 2022, Flavonoids Phenolics, V1, P145

Aribisala JO, 2024, SCI REP-UK, V14, DOI 10.1038/s41598-024-59489-3

Aribisala JO, 2022, COMPUT BIOL MED, V145, DOI 10.1016/j.compbiomed.2022.105432

Askun T, 2013, BMC COMPLEM ALTERN M, V13, DOI 10.1186/1472-6882-13-365

Bajorath J, 2001, J CHEM INF COMP SCI, V41, P233, DOI 10.1021/ci0001482

Banerjee P, 2018, NUCLEIC ACIDS RES, V46, pW257, DOI 10.1093/nar/gky318

Begum S, 2008, NAT PROD RES, V22, P467, DOI 10.1080/14786410600898714

Biharee A, 2020, FITOTERAPIA, V146, DOI 10.1016/j.fitote.2020.104720

BIOVIA D.S., 2021, Discovery Studio, P627

Boniface P.K., 2020, Stud. Nat. Prod. Chem., P85, DOI DOI 10.1016/B978-0-12-817905-5.00003-2

Campanico A., 2018, N. Drug Targets antimycobacterial Agents, V150, P525

Cao RQ, 2019, MOLECULES, V24, DOI 10.3390/molecules24050851

Arévalo JMC, 2022, SCI REP-UK, V12, DOI 10.1038/s41598-022-08359-x

Chokchaisiri R, 2009, CHEM PHARM BULL, V57, P428, DOI 10.1248/cpb.57.428

Dallakyan S, 2015, METHODS MOL BIOL, V1263, P243, DOI 10.1007/978-1-4939-2269-7_19

Dash S, 2024, SCI REP-UK, V14, DOI 10.1038/s41598-024-61901-x

Dash S, 2024, J BIOMOL STRUCT DYN, V42, P6472, DOI 10.1080/07391102.2023.2230312

Davis Charles Kozhikkadan, 2018, In Silico Pharmacology, V6, P8, DOI [10.1007/s40203-018-0045-5, 10.1007/s40203-018-0045-5]

Deschenes LA, 2000, J AM CHEM SOC, V122, P9567, DOI 10.1021/ja004761d

Durham E, 2009, J MOL MODEL, V15, P1093, DOI 10.1007/s00894-009-0454-9

Elsaman T, 2025, FRONT CHEM, V13, DOI 10.3389/fchem.2025.1524607

Favela-Hernandez J., 2012, Antibacterial and antimycobacterial lignans and flavonoids from Larrea tridentata, V26, P1957

Gao F, 2019, EUR J MED CHEM, V165, P323, DOI 10.1016/j.ejmech.2019.01.042

Gordon SV, 2018, MICROBIOL-SGM, V164, P437, DOI 10.1099/mic.0.000601

Halder SK, 2021, J CLIN TUBER OTH MYC, V24, DOI 10.1016/j.jctube.2021.100246

Hosseinzadeh S, 2015, International Journal of Clinical Medicine, V06, P635, DOI [10.4236/ijcm.2015.69084, 10.4236/ijcm.2015.69084, DOI 10.4236/IJCM.2015.69084]

Hu XP, 2022, ACTA PHARMACOL SIN, V43, P1605, DOI 10.1038/s41401-021-00779-1

Ismail EA, 2024, INT J PHARMACEUT, V662, DOI 10.1016/j.ijpharm.2024.124493

Jnawali HN, 2016, J NAT PROD, V79, P961, DOI 10.1021/acs.jnatprod.5b01033

Kharwar S., 2018, Mechanistic Insight Flavonoids against Enoyl-ACP-Reductase for Effective Management of Mycobacterium Tuberculosis: In-Silico Validation

Khezri A, 2018, INT J BIOL MACROMOL, V114, P972, DOI 10.1016/j.ijbiomac.2018.03.100

Khumbulani M, 2022, EVID-BASED COMPL ALT, V2022, DOI 10.1155/2022/3809232

Kuete V, 2008, J ETHNOPHARMACOL, V120, P17, DOI 10.1016/j.jep.2008.07.026

Lanrewaju AA, 2024, SCI AFR, V23, DOI 10.1016/j.sciaf.2024.e02109

Lipinski CA, 1997, ADV DRUG DELIVER REV, V23, P3, DOI 10.1016/S0169-409X(96)00423-1

Ma N, 2019, ACS CHEM BIOL, V14, P2546, DOI 10.1021/acschembio.9b00784

Mazlun MH, 2019, MOLECULES, V24, DOI 10.3390/molecules24132449

Michaud-Agrawal N, 2011, J COMPUT CHEM, V32, P2319, DOI 10.1002/jcc.21787

Mtetwa LM, 2023, COMPUT BIOL CHEM, V105, DOI 10.1016/j.compbiolchem.2023.107909

Murillo JI, 2003, FITOTERAPIA, V74, P226, DOI 10.1016/S0367-326X(03)00033-9

Nayeem N., 2018, J. North Basic Appl. Sci., V3, P44

Opo FADM, 2021, SCI REP-UK, V11, DOI 10.1038/s41598-021-83626-x

Pawar A, 2020, SCI REP-UK, V10, DOI 10.1038/s41598-020-57658-8

Pecorini G, 2023, PHARMACEUTICS, V15, DOI 10.3390/pharmaceutics15020628

Pettersen EF, 2004, J COMPUT CHEM, V25, P1605, DOI 10.1002/jcc.20084

Rabaan A. A., 2023, Mol. Divers., P1

Rabaan AA, 2024, MOL DIVERS, V28, P1057, DOI 10.1007/s11030-023-10632-8

Ramírez D, 2018, MOLECULES, V23, DOI 10.3390/molecules23051038

Rathod S, 2023, J MOL MODEL, V29, DOI 10.1007/s00894-023-05521-8

Remko M, 2011, STRUCT CHEM, V22, P635, DOI 10.1007/s11224-011-9741-z

Richter A, 2018, SCI REP-UK, V8, DOI 10.1038/s41598-018-31316-6

Saiz-Poseu J, 2019, ANGEW CHEM INT EDIT, V58, P696, DOI 10.1002/anie.201801063

Sasikumar K., 2018, Rv. 3 Biotech., V8, P1

Shamsudin NF, 2022, MOLECULES, V27, DOI 10.3390/molecules27041149

Shode FO, 2022, J BIOMOL STRUCT DYN, V40, P6587, DOI 10.1080/07391102.2021.1886993

Shode FO, 2022, METABOLITES, V12, DOI 10.3390/metabo12111155

Suksamrarn A, 2003, ARCH PHARM RES, V26, P816, DOI 10.1007/BF02980026

Tewari AK, 2008, BIOORGAN MED CHEM, V16, P126, DOI 10.1016/j.bmc.2007.09.023

Uhomoibhi JOO, 2022, J MOL GRAPH MODEL, V114, DOI 10.1016/j.jmgm.2022.108185

Ullah A, 2020, MOLECULES, V25, DOI 10.3390/molecules25225243

Verma K, 2021, BRAZ ARCH BIOL TECHN, V64, DOI 10.1590/1678-4324-2021200402

Yildirim K, 2024, INT J TUBERC LUNG D, V28, P37, DOI 10.5588/ijtld.23.0224

Zheng YJ, 2014, BMC COMPLEM ALTERN M, V14, DOI 10.1186/1472-6882-14-400

NR 68

TC 0

Z9 0

U1 0

U2 0

PU ELSEVIER SCI LTD

PI London

PA 125 London Wall, London, ENGLAND

SN 1476-9271

EI 1476-928X

J9 COMPUT BIOL CHEM

JI Comput. Biol. Chem.

PD FEB

PY 2026

VL 120

AR 108719

DI 10.1016/j.compbiolchem.2025.108719

EA OCT 2025

PN 2

PG 10

WC Biology; Computer Science, Interdisciplinary Applications

WE Science Citation Index Expanded (SCI-EXPANDED)

SC Life Sciences & Biomedicine - Other Topics; Computer Science

GA 8TJ1S

UT WOS:001596869100001

OA hybrid

DA 2026-05-07

ER

PT J

AU Li, SR

Mensah, E

Liu, M

Pan, LL

Lu, W

Zhou, SS

Zhang, LQ

Cheng, YS

Zhao, H

Wei, SS

Zha, L

AF Li, Shirong

Mensah, Emmanuel

Liu, Min

Pan, Lingling

Lu, Wei

Zhou, Susheng

Zhang, Liqin

Cheng, Yusheng

Zhao, Hui

Wei, Shuoshuo

Zha, Lei

TI The burden of tuberculosis and drug resistance in 22 Sub-Saharan African

countries, 1990-2021: a GBD 2021 analysis and progress towards WHO 2035

targets with projections to 2050

SO FRONTIERS IN MICROBIOLOGY

LA English

DT Article

DE tuberculosis; Sub-Saharan Africa; drug-resistant tuberculosis;

multidrug-resistant tuberculosis; extensively drug-resistant

tuberculosis; antibiotic resistance; WHO End TB targets; epidemiology

ID GLOBAL BURDEN; HIV; DETERMINANTS; MTBDRPLUS

AB Background Tuberculosis (TB) remains a major public health challenge in Sub-Saharan Africa (SSA), compounded by rising multidrug-resistant (MDR-TB), and extensively drug-resistant tuberculosis (XDR-TB) strains. This study aimed to quantify the burden, temporal trends, and subregional heterogeneity of TB across 22 selected SSA countries; project future trends to 2050; and evaluate the alignment of national TB policies with WHO End TB 2035 targets.Methods We conducted a mixed-methods analysis using Global Burden of Disease (GBD) 2021 data. Age- and sex-specific TB incidence, prevalence, mortality, and disability-adjusted life years (DALYs) were analyzed across 22 SSA countries from 1990 to 2021. BAPC model projected disease burden to 2050. National TB policy alignment with WHO targets was assessed qualitatively. Drug-susceptible (DS-TB), MDR-TB, and XDR-TB forms were evaluated alongside key attributable risk factors.Results Between 1990 and 2021, absolute TB incidence in SSA increased by 25.6% and prevalence by 44.2%, while mortality and DALYs declined by 14.0 and 24.8%, respectively. Age-standardized rates declined significantly across all metrics; incidence (-46.2%), prevalence (-35.2%), mortality (-56.4%), and DALYs (-60.9%). Progress varied substantially by region: Western SSA showed the greatest improvement, while Southern and Central SSA continued to face high burdens, with rising mortality in some areas. MDR-TB incidence surged by 743.2%, with XDR-TB also increasing markedly, particularly in Eastern and Central SSA. HIV co-infection amplified MDR/XDR-TB mortality, with Southern SSA most affected. Age- and sex-specific analyses revealed early-adulthood incidence peaks, male predominance in mortality and DALYs, and the highest MDR-TB burden among older adults. Leading risk factors for TB mortality included high alcohol use, elevated fasting plasma glucose, tobacco use, and high body mass index. Projections indicate SSA is unlikely to meet the WHO 2035 mortality reduction target, though Ghana, Guinea, and Tanzania are projected to achieve the incidence target.Conclusion Despite overall declines in TB mortality, the growing DR-TB/MDR-TB/XDR-TB epidemic, significant subregional disparities, and systemic health system challenges threaten progress toward WHO End TB goals. Strengthening diagnostics, expanding treatment access, integrating care services, and addressing key metabolic and behavioral risk factors are essential to accelerate TB control efforts and align SSA with the 2035 targets.

C1 [Li, Shirong; Zhao, Hui] Anhui Med Univ, Dept Resp & Crit Care Med, Affiliated Hosp 2, Hefei, Peoples R China.

[Li, Shirong] East China Normal Univ, Peoples Hosp Wuhu 2, Dept Pulm & Crit Care, Wuhu Hosp, Wuhu, Anhui, Peoples R China.

[Mensah, Emmanuel; Lu, Wei; Zhou, Susheng; Zhang, Liqin; Cheng, Yusheng; Wei, Shuoshuo; Zha, Lei] Wannan Med Coll, Yijishan Hosp, Dept Pulm & Crit Care Med, Affiliated Hosp 1, Wuhu, Anhui, Peoples R China.

[Mensah, Emmanuel] Takoradi Hosp, Takoradi, Ghana.

[Liu, Min] Bengbu Med Univ, Grad Sch, Bengbu, Anhui, Peoples R China.

[Pan, Lingling] Wannan Med Coll, Yijishan Hosp, Dept Cardiol, Affiliated Hosp 1, Wuhu, Anhui, Peoples R China.

C3 Anhui Medical University; East China Normal University; Wannan Medical

College; Bengbu Medical University; Wannan Medical College

RP Zhao, H (corresponding author), Anhui Med Univ, Dept Resp & Crit Care Med, Affiliated Hosp 2, Hefei, Peoples R China.; Wei, SS; Zha, L (corresponding author), Wannan Med Coll, Yijishan Hosp, Dept Pulm & Crit Care Med, Affiliated Hosp 1, Wuhu, Anhui, Peoples R China.

EM zhaohuichenxi@126.com; weishuoshuo@yjsyy.com; zhalei@yjsyy.com

RI Li, Shirong/PCT-9035-2025

FU Special Research Start-up Fund for Recruited Talent of the First

Affiliated Hospital of Wannan Medical College [YR202408]; Special

Research Start-Up Fund for Talent Introduction [YR202450]; Wannan

Medical College High-Level Talent Introduction Program [GCCRCJB202320]

FX The author(s) declare that financial support was received for the

research and/or publication of this article. The study was supported by

the Special Research Start-up Fund for Recruited Talent of the First

Affiliated Hospital of Wannan Medical College (YR202408); Wannan Medical

College High-Level Talent Introduction Program (No. GCCRCJB202320); and

Special Research Start-Up Fund for Talent Introduction (YR202450).

CR Adewole A., 2024, Antimicrobial resistance and tuberculosis prevalence in Africa: A public health concern, V8

Affolabi D, 2017, CAN J INFECT DIS MED, V2017, DOI 10.1155/2017/3276240

Ahmed S.K., 2024, J MED SURG PUBLIC HL, V2, DOI [10.1016/j.glmedi.2024.100081, 10.1016/j.glmedi.2024.100081, DOI 10.1016/J.GLMEDI.2024.100081]

Ambia J, 2017, BMC HEALTH SERV RES, V17, DOI 10.1186/s12913-017-2678-1

Amendah A. I. D. D., 2019, Domestic financial contributions to HIV, TB, and malaria

Amu H, 2023, ARCH PUBLIC HEALTH, V81, DOI 10.1186/s13690-023-01181-5

Antimicrobial Resistance Collaborators, 2022, Lancet, V399, P629, DOI [10.1016/S0140-6736(21)02724-0, DOI 10.1016/S0140-6736(21)02724-0]

Asante-Poku A, 2015, INT J TUBERC LUNG D, V19, P954, DOI 10.5588/ijtld.14.0864

Atake EH, 2023, COST EFFECT RESOUR A, V21, DOI 10.1186/s12962-023-00485-1

Bayigga J, 2025, SUBST ABUSE TREAT PR, V20, DOI 10.1186/s13011-024-00629-3

Cattamanchi A, 2015, BMC HEALTH SERV RES, V15, DOI 10.1186/s12913-014-0668-0

Cazabon Danielle, 2018, Gates Open Res, V2, P35, DOI 10.12688/gatesopenres.12842.2

Chala B., 2020, Prevalence of multidrug resistant Mycobacterium tuberculosis among tuberculosis patients admitted to Adama hospital medical college, Adama, Ethiopia: a retrospective study open access, DOI [10.4236/jtr.2020.83013, DOI 10.4236/JTR.2020.83013]

Chanda-Kapata P, 2022, INT J INFECT DIS, V124, pS41, DOI 10.1016/j.ijid.2022.03.033

Chapman HJ, 2021, GLOB HEALTH-SCI PRAC, V9, P964, DOI 10.9745/GHSP-D-21-00368

Chen YM, 2024, SCI ONE HEALTH, V3, DOI [10.1016/j.soh.2024.100072, 10.1016/j.soh.2024.100072]

Dean AS, 2022, LANCET INFECT DIS, V22, P191, DOI 10.1016/S1473-3099(21)00808-2

Dheda K, 2022, LANCET RESP MED, V10, P603, DOI 10.1016/S2213-2600(22)00092-3

Dheda K, 2017, LANCET RESP MED, V5, P291, DOI [10.1016/s2213-2600(17)30079-6, 10.1016/S2213-2600(17)30079-6]

Dolan K, 2016, LANCET, V388, P1089, DOI 10.1016/S0140-6736(16)30466-4

Emerson C, 2016, INT J TUBERC LUNG D, V20, P941, DOI 10.5588/ijtld.15.0892

Engelbrecht MC, 2018, AFR J PRIM HEALTH CA, V10, DOI [10.4102/phcfm.v10i1.1628, 10.4102/phcfm.vl10il.1628]

Eze P, 2022, B WORLD HEALTH ORGAN, V100, P337, DOI 10.2471/BLT.21.287673

Fadare JO, 2019, J GLOB ANTIMICROB RE, V17, P132, DOI 10.1016/j.jgar.2018.11.025

Gele Abdi A, 2010, Confl Health, V4, P1, DOI 10.1186/1752-1505-4-1

Gouda HN, 2019, LANCET GLOB HEALTH, V7, pE1375, DOI 10.1016/S2214-109X(19)30374-2

Gulumbe BH, 2019, MEDITERR J INFECT M, V8, DOI 10.4274/mjima.galenos.2019.2019.25

Gyimah FT, 2019, GLOBALIZATION HEALTH, V15, DOI 10.1186/s12992-019-0459-9

Hajissa K, 2021, ANTIBIOTICS-BASEL, V10, DOI 10.3390/antibiotics10080932

Hargreaves JR, 2011, AM J PUBLIC HEALTH, V101, P654, DOI 10.2105/AJPH.2010.199505

Hayward SE, 2024, BMC GLOB PUBLIC HEAL, V2, DOI [10.1186/s44263-024-00084-z, 10.1186/s44263-024-00084-z]

Hogan AB, 2020, LANCET GLOB HEALTH, V8, pE1132, DOI 10.1016/S2214-109X(20)30288-6

Izudi J, 2022, IJID REG, V2, P107, DOI 10.1016/j.ijregi.2022.01.001

Jagger P, 2024, BMJ PUBLIC HEALTH, V2, DOI [10.1136/bmjph-2023-000176, 10.1136/bmjph-2023-000176]

John MA, 2022, International Journal of Scientific Reports, V8, P221, DOI [10.18203/issn.2454-2156.intjscirep20221842, 10.18203/issn.2454-2156.IntJSciRep20221842, DOI 10.18203/ISSN.2454-2156.INTJSCIREP20221842]

Kadia BM, 2021, AIDS RES THER, V18, DOI 10.1186/s12981-021-00395-3

Kallon II, 2022, BMC HEALTH SERV RES, V22, DOI 10.1186/s12913-022-07540-2

Kapata N, 2025, IJID REG, V14, DOI 10.1016/j.ijregi.2025.100585

Kaumba PC, 2023, Social Sciences & Humanities Open, V7, P100399, DOI [10.1016/j.ssaho.2023.100399, 10.1016/j.ssaho.2023.100399, DOI 10.1016/J.SSAHO.2023.100399]

Kerrigan D, 2017, HEALTH RES POLICY SY, V15, DOI 10.1186/s12961-017-0206-8

Kuupiel D, 2020, BMC INFECT DIS, V20, DOI 10.1186/s12879-020-05283-1

Kwabla MP, 2025, BMC INFECT DIS, V25, DOI 10.1186/s12879-025-10622-1

Lamuka P., 2018, Prevalence of antibiotic resistance among Mycobacterium tuberculosis complex species from camel milk in Isiolo County, Kenya

Laokri S, 2014, TROP MED INT HEALTH, V19, P1249, DOI 10.1111/tmi.12365

Lasebikan VO, 2020, PAN AFR MED J, V36, DOI 10.11604/pamj.2020.36.321.17118

Lawn SD, 2009, CURR OPIN HIV AIDS, V4, P325, DOI 10.1097/COH.0b013e32832c7d61

Ledesma JR, 2024, LANCET INFECT DIS, V24, P698, DOI 10.1016/S1473-3099(24)00007-0

Ledesma JR, 2022, LANCET INFECT DIS, V22, P222, DOI 10.1016/S1473-3099(21)00449-7

Liebenberg D, 2022, FRONT CELL INFECT MI, V12, DOI 10.3389/fcimb.2022.943545

Liyew AM, 2025, INT J INFECT DIS, V153, DOI 10.1016/j.ijid.2025.107777

Long Q, 2021, LANCET PUBLIC HEALTH, V6, pE948, DOI 10.1016/S2468-2667(21)00203-6

Lorent N, 2015, PLOS ONE, V10, DOI 10.1371/journal.pone.0130179

Lukoye D, 2015, BMC PUBLIC HEALTH, V15, DOI 10.1186/s12889-015-1614-8

MacPherson P, 2019, BMC INFECT DIS, V19, DOI 10.1186/s12879-019-4502-5

Mahmud O, 2011, INT J ENV RES PUB HE, V8, P2524, DOI 10.3390/ijerph8062524

Marou V, 2024, CONFL HEALTH, V18, DOI 10.1186/s13031-023-00568-z

Mbunka MA, 2024, BMJ OPEN RESPIR RES, V11, DOI 10.1136/bmjresp-2023-001727

Mbuthia GW, 2020, PLOS ONE, V15, DOI 10.1371/journal.pone.0240457

Megerso A, 2020, BMC HEALTH SERV RES, V20, DOI 10.1186/s12913-020-05787-1

Molla KA, 2022, PLOS ONE, V17, DOI 10.1371/journal.pone.0270272

Monde N, Front Trop Dis, V2, DOI [10.3389/fitd.2021.735028, 10.3389/fitd.2021.735028, DOI 10.3389/FITD.2021.735028]

Msoka EF, 2021, BMJ OPEN, V11, DOI 10.1136/bmjopen-2021-050911

Mtetwa HN, 2023, HELIYON, V9, DOI 10.1016/j.heliyon.2023.e18302

Mugauri H, 2022, IJID REG, V3, P37, DOI 10.1016/j.ijregi.2022.03.004

Mukora R, 2023, BMJ OPEN, V13, DOI 10.1136/bmjopen-2022-065202

Muluneh MA, 2021, CAN J INFECT DIS MED, V2021, DOI 10.1155/2021/6696199

Naghavi M, 2024, LANCET, V404, P1199, DOI 10.1016/S0140-6736(24)01867-1

Naidoo P, 2013, BMC PUBLIC HEALTH, V13, DOI 10.1186/1471-2458-13-396

Nidoi J, 2021, BMC PUBLIC HEALTH, V21, DOI 10.1186/s12889-021-12056-1

Njagi P, 2018, SYST REV-LONDON, V7, DOI 10.1186/s13643-018-0799-1

Ntinginya NE, 2021, BMJ GLOB HEALTH, V6, DOI 10.1136/bmjgh-2021-005357

Ntshanka NGM, 2023, CAN J INFECT DIS MED, V2023, DOI 10.1155/2023/6659212

Nyasulu P, 2018, J MULTIDISCIP HEALTH, V11, P375, DOI 10.2147/JMDH.S156949

Nyirenda T, 2021, INT J INFECT DIS, V110, P54, DOI 10.1016/j.ijid.2021.06.061

Obels I, 2022, TROP MED INT HEALTH, V27, P369, DOI 10.1111/tmi.13733

Oga-Omenka Charity, 2020, BMJ Glob Health, V5, DOI [10.1136/bmjgh-2019-002280, 10.1136/bmjgh-2019-002280]

Ogbuabor DC, 2019, INFECT DIS POVERTY, V8, DOI 10.1186/s40249-019-0556-2

Okello G, 2018, ENVIRON INT, V119, P429, DOI 10.1016/j.envint.2018.07.002

Ombura IP, 2016, PLOS ONE, V11, DOI 10.1371/journal.pone.0163994

Omer ZB, 2016, INT J MYCOBACT, V5, P475, DOI 10.1016/j.ijmyco.2016.06.005

Osman M, 2021, BMC HEALTH SERV RES, V21, DOI 10.1186/s12913-021-06398-0

Otieno J, 2024, PLOS ONE, V19, DOI 10.1371/journal.pone.0298268

Otim ME, 2020, FRONT PUBLIC HEALTH, V8, DOI 10.3389/fpubh.2020.584547

Pedrazzoli D, 2021, SOC SCI MED, V277, DOI 10.1016/j.socscimed.2021.113875

Peltzer K, 2014, AFR HEALTH SCI, V14, P157, DOI 10.4314/ahs.v14i1.24

Peresu E, 2020, HUM RESOUR HEALTH, V18, DOI 10.1186/s12960-020-00541-4

Perumal R, 2018, BMC PUBLIC HEALTH, V18, DOI 10.1186/s12889-018-5362-4

Pradipta IS, 2021, BMC PUBLIC HEALTH, V21, DOI 10.1186/s12889-021-12005-y

Robbiati C, 2022, FRONT PUBLIC HEALTH, V10, DOI 10.3389/fpubh.2022.745928

Sahal MR, 2023, PLOS NEGLECT TROP D, V17, DOI 10.1371/journal.pntd.0011619

Salam MA, 2023, HEALTHCARE-BASEL, V11, DOI 10.3390/healthcare11131946

Salomon A, 2022, BMJ GLOB HEALTH, V7, DOI 10.1136/bmjgh-2021-007456

Sekyere JO, 2019, J INFECTION, V79, P550, DOI 10.1016/j.jinf.2019.10.006

Shegaze M, 2022, J CLIN TUBER OTH MYC, V29, DOI 10.1016/j.jctube.2022.100338

Telisinghe L, 2016, LANCET, V388, P1215, DOI 10.1016/S0140-6736(16)30578-5

The Global Fund to Fight AIDS Tuberculosis and Malaria, 2024, Tuberculosis

Timire C, 2021, TROP MED INT HEALTH, V26, P1248, DOI 10.1111/tmi.13647

Tiruneh MG, 2023, BMJ OPEN, V13, DOI 10.1136/bmjopen-2023-073634

Tukamuhebwa PM, 2024, BMC INFECT DIS, V24, DOI 10.1186/s12879-024-09798-9

Useni S., 2016, J. Public Health, V4, P352, DOI [10.11648/j.sjph.20160404.23, DOI 10.11648/J.SJPH.20160404.23]

Vellekoop H, 2022, COST EFFECT RESOUR A, V20, DOI 10.1186/s12962-022-00365-0

Wang XY, 2025, DRUG RESIST UPDATE, V82, DOI 10.1016/j.drup.2025.101265

Williams H, 2025, SCI REP-UK, V15, DOI 10.1038/s41598-024-81413-y

Williams V, 2022, AFR J LAB MED, V11, DOI 10.4102/ajlm.v11i1.1811

World Health Organization, 2024, Global tuberculosis report 2024

World Health Organization, 2023, Costs faced by people with TB and their households, social protection and human rights

World Health Organization, 2025, WHO tuberculosis facts-sheets

World Health Organization, 2021, WHO global air quality guidelines: Particulate matter (PM. and PM), ozone, nitrogen dioxide

World Health Organization African Region, 2025, Tuberculosis (TB) WHO African region-fact-sheets

World Health Organization Regional Office for Africa, 2024, Rate of TB diagnosis, treatment in Africa increasing

Xue Y, 2022, FRONT MED-LAUSANNE, V9, DOI 10.3389/fmed.2022.905245

Yang HF, 2024, BMC PUBLIC HEALTH, V24, DOI 10.1186/s12889-024-20664-w

Zegeye B, 2023, REPROD HEALTH, V20, DOI 10.1186/s12978-023-01675-z

Zhang T, 2023, J INFECT PUBLIC HEAL, V16, P368, DOI 10.1016/j.jiph.2023.01.014

Zhong YY, 2025, PEDIATR RES, V98, P901, DOI 10.1038/s41390-025-03917-1

NR 115

TC 3

Z9 5

U1 1

U2 1

PU FRONTIERS MEDIA SA

PI LAUSANNE

PA AVENUE DU TRIBUNAL FEDERAL 34, LAUSANNE, CH-1015, SWITZERLAND

EI 1664-302X

J9 FRONT MICROBIOL

JI Front. Microbiol.

PD NOV 17

PY 2025

VL 16

AR 1695592

DI 10.3389/fmicb.2025.1695592

PG 25

WC Microbiology

WE Science Citation Index Expanded (SCI-EXPANDED)

SC Microbiology

GA J1015

UT WOS:001627211600001

OA Green Submitted, gold

DA 2026-05-07

ER

PT J

AU Zhang, ZJ

Pang, Y

Wang, YF

Liu, CT

Zhao, YL

AF Zhang, Zhijian

Pang, Yu

Wang, Yufeng

Liu, Changting

Zhao, Yanlin

TI Beijing genotype of Mycobacterium tuberculosis is significantly

associated with linezolid resistance in multidrug-resistant and

extensively drug-resistant tuberculosis in China

SO INTERNATIONAL JOURNAL OF ANTIMICROBIAL AGENTS

LA English

DT Article

DE Tuberculosis; Linezolid; Multidrug resistance

ID STRAINS; OXAZOLIDINONES; MECHANISM

AB Linezolid (LNZ) is a promising antimicrobial agent for the treatment of multidrug-resistant (MDR) and extensively drug-resistant (XDR) tuberculosis (TB). To investigate the efficacy of LNZ among MDR-TB and XDR-TB in China, the LNZ susceptibility of 158 MDR-TB isolates from the national drug resistance survey was determined by the minimum inhibitory concentration method. The 158 MDR-TB isolates were also sequenced in the 23S rRNA, rplC and rplD genes conferring LNZ resistance and were typed using spoligotyping to identify the Beijing genotype of Mycobacterium tuberculosis. Overall, the prevalence of LNZ-resistant isolates was 10.8% (171158) among MDR-TB isolates circulating in China. Beijing genotype was significantly associated with LNZ resistance in MDR-TB and XDR-TB (odds ratio=4.66, 95% confidence interval 1.03-21.16; P=0.033). In addition, a higher frequency of LNZ-resistant isolates was observed among XDR-TB strains (60%) compared with the MDR (5.6%; P<0.001) and pre-XDR groups (12.2%; P=0.004). Mutations in 23S rRNA and rplC were responsible for only 29.4% of LNZ-resistant M. tuberculosis among MDR-TB isolates, and a novel non-synonymous substitution His155Asp in rplC was first identified to be contributing to low-level LNZ resistance (2 mu g/mL) in M. tuberculosis. The unsatisfactory correlation between mutant genotypes highlights the urgent need to investigate another mechanism for LNZ resistance that has not yet been described. (C) 2013 Elsevier B.V. and the International Society of Chemotherapy. All rights reserved.

C1 [Zhang, Zhijian; Pang, Yu; Liu, Changting] Chinese Peoples Liberat Army Gen Hosp, Resp Dis Dept Nanlou, Beijing 100853, Peoples R China.

[Pang, Yu; Wang, Yufeng; Zhao, Yanlin] Chinese Ctr Dis Control & Prevent, Natl Ctr TB Control & Prevent, Beijing 102206, Peoples R China.

C3 Chinese People's Liberation Army General Hospital; Chinese Center for

Disease Control & Prevention

RP Pang, Y (corresponding author), Chinese Ctr Dis Control & Prevent, Natl Ctr TB Control & Prevent, 155 Chang Bai Rd, Beijing 102206, Peoples R China.

EM pangyu@chinatb.org; liuchangting301@163.com; zhaoyanlin@chinatb.org

FU National Key Project [2013ZX10003003]

FX This study was supported by the National Key Project [2013ZX10003003].

CR Alcala L, ANTIMICROB AGENTS CH

Amaral L, 2012, INT J ANTIMICROB AG, V39, P376, DOI 10.1016/j.ijantimicag.2012.01.012

An DD, 2009, ANTIMICROB AGENTS CH, V53, P4835, DOI 10.1128/AAC.00541-09

Anger HA, 2010, J ANTIMICROB CHEMOTH, V65, P775, DOI 10.1093/jac/dkq017

Anh DD, 2000, EMERG INFECT DIS, V6, P302

[Anonymous], 2010, Multidrug and extensively drug-resistant TB(M/XDR-TB): 2010 Global Report on Surveillance and Response

Beckert P, 2012, ANTIMICROB AGENTS CH, V56, P2743, DOI 10.1128/AAC.06227-11

Bozdogan B, 2004, INT J ANTIMICROB AG, V23, P113, DOI 10.1016/j.ijantimicag.2003.11.003

Buu TN, 2009, INT J TUBERC LUNG D, V13, P900

Cynamon MH, 1999, ANTIMICROB AGENTS CH, V43, P1189, DOI 10.1128/AAC.43.5.1189

Dietze R, 2008, AM J RESP CRIT CARE, V178, P1180, DOI 10.1164/rccm.200806-892OC

Escribano I, 2007, CHEMOTHERAPY, V53, P397, DOI 10.1159/000109769

Hillemann D, 2005, ANTIMICROB AGENTS CH, V49, P1229, DOI 10.1128/AAC.49.3.1229-1231.2005

Hillemann D, 2008, ANTIMICROB AGENTS CH, V52, P800, DOI 10.1128/AAC.01189-07

JARLIER V, 1994, FEMS MICROBIOL LETT, V123, P11, DOI 10.1111/j.1574-6968.1994.tb07194.x

Lee MM, N ENGL J MED

Long KS, 2012, ANTIMICROB AGENTS CH, V56, P603, DOI 10.1128/AAC.05702-11

Mokrousov I, 2002, ANTIMICROB AGENTS CH, V46, P1417, DOI 10.1128/AAC.46.5.1417-1424.2002

Pang Y, 2013, ANTIMICROB AGENTS CH, V57, P893, DOI 10.1128/AAC.01024-12

Pang Y, 2012, PLOS ONE, V7, DOI 10.1371/journal.pone.0032976

Pang Y, 2011, J MICROBIOL METH, V86, P291, DOI 10.1016/j.mimet.2011.05.016

Parwati I, 2010, LANCET INFECT DIS, V10, P103, DOI 10.1016/S1473-3099(09)70330-5

Prodinger WM, 2001, EMERG INFECT DIS, V7, P483, DOI 10.3201/eid0703.017330

Schecter GF, 2010, CLIN INFECT DIS, V50, P49, DOI 10.1086/648675

Toungoussova OS, 2002, J CLIN MICROBIOL, V40, P1930, DOI 10.1128/JCM.40.6.1930-1937.2002

Varghese B, 2012, INFECT GENET EVOL, V12, P549, DOI 10.1016/j.meegid.2012.01.024

Velayati AA, 2009, CHEMOTHERAPY, V55, P303, DOI 10.1159/000226425

Wang LD, 2007, LANCET, V369, P691, DOI 10.1016/S0140-6736(07)60316-X

WHO, 2012, GLOBAL TUBERCULOSIS REPORT 2012, P1

Zhao YL, 2012, NEW ENGL J MED, V366, P2161, DOI 10.1056/NEJMoa1108789

NR 30

TC 74

Z9 86

U1 0

U2 15

PU ELSEVIER SCIENCE BV

PI AMSTERDAM

PA PO BOX 211, 1000 AE AMSTERDAM, NETHERLANDS

SN 0924-8579

EI 1872-7913

J9 INT J ANTIMICROB AG

JI Int. J. Antimicrob. Agents

PD MAR

PY 2014

VL 43

IS 3

BP 231

EP 235

DI 10.1016/j.ijantimicag.2013.12.007

PG 5

WC Infectious Diseases; Microbiology; Pharmacology & Pharmacy

WE Science Citation Index Expanded (SCI-EXPANDED)

SC Infectious Diseases; Microbiology; Pharmacology & Pharmacy

GA AD5AH

UT WOS:000333263200005

DA 2026-05-07

ER

PT J

AU Park, SK

Hong, S

Eum, SY

Lee, IH

Shin, DO

Cho, JE

Cho, S

Cho, SN

AF Park, Seung-Kyu

Hong, Sunghee

Eum, Seok-Yong

Lee, In Hee

Shin, Donk Ok

Cho, Jang Eun

Cho, Sungae

Cho, Sang-Nae

TI Changes in cell-mediated immune response after lung resection surgery

for MDR-TB patients

SO TUBERCULOSIS

LA English

DT Article

DE Mycobacterium tuberculosis (Mtb); MDR-TB (Multidrug-resistant

tuberculosis); IFN-gamma; IL-10; Lung resection surgery

ID MULTIDRUG-RESISTANT TUBERCULOSIS; NECROSIS-FACTOR-ALPHA;

INTERFERON-GAMMA; PULMONARY TUBERCULOSIS; SURGICAL-MANAGEMENT; DRUG

SENSITIVITY; IFN-GAMMA; MYCOBACTERIAL; THERAPY; DISEASE

AB The immune responses of multidrug-resistant tuberculosis (MDR-TB) patients undergoing lung resection surgery were investigated in order to understand the mechanism of strong immune suppression in MDR-TB. We examined changes in cell-mediated immune response (CMI) of a total of sixteen MDR-TB patients, three of them extensively drug-resistant tuberculosis (XDR-TB) patients, after the removal of the heavily diseased lung section. The IFN-gamma response to Mycobacterium tuberculosis culture filtrate proteins (Mtb-CFP), one of the most important CMI to defend TB, showed a statistically significant elevation in 2-4 months after operation when compared to the preoperative CMI in patients who were converted into AFB negative and cured in two years' follow-up, suggesting that the recovery of CMI may be one of the key factors in the successful treatment of MDR-TB. Interestingly, IL-10 response to Mtb-CFP was also elevated in 2-4 months after surgery in cured patients although both proliferative response and PBMC composition were not significantly changed. Infection with first- or second-line drugs resistant Mtb reduces the efficiency of chemotherapeutic treatment of MDR-TB to about 50%. Thus, this study suggests that chemotherapeutic treatment of MDR-TB may be more effective when combined with accompanying therapy that increases CMI, includes lung resection surgery. (C) 2011 Elsevier Ltd. All rights reserved.

C1 [Hong, Sunghee; Cho, Jang Eun; Cho, Sungae; Cho, Sang-Nae] Yonsei Univ, Dept Microbiol, Inst Immunol & Immunol Dis, Coll Med, Seoul 120752, South Korea.

[Park, Seung-Kyu; Lee, In Hee; Shin, Donk Ok] Natl Masan TB Hosp, Masan 631710, South Korea.

[Eum, Seok-Yong] Int TB Res Ctr, Masan 631710, South Korea.

C3 Yonsei University; Yonsei University Health System

RP Cho, S (corresponding author), Yonsei Univ, Dept Microbiol, Inst Immunol & Immunol Dis, Coll Med, 134 Shinchon Dong, Seoul 120752, South Korea.

EM pulmo116@korea.kr; h22h22h22@naver.com; syeumkr@gmail.com;

Lihkor@unitel.co.kr; doshin55@hanmail.net; jangyang@hanmail.net;

scho@yuhs.ac; raycho@yuhs.ac

RI CHO, SANG NAE/JVM-8918-2024

FU Ministry of Health and Welfare, Republic of Korea [A010381]; Yonsei

University

FX This work was supported by a grant from the Korea Health 21 R & D

Project, Ministry of Health and Welfare, Republic of Korea (A010381),

and by a grant from the Brain Korea Project for Medical Sciences at

Yonsei University.

CR Abebe F, 2009, CLIN EXP IMMUNOL, V157, P235, DOI 10.1111/j.1365-2249.2009.03967.x

Aguilar D, 2004, CIRCULATION, V110, P1572, DOI 10.1161/01.CIR.0000142047.28024.F2

Alexander J, 1937, COLLAPSE THERAPY TUB

BLOOM BR, 1992, SCIENCE, V257, P1055, DOI 10.1126/science.257.5073.1055

Boussiotis VA, 2000, J CLIN INVEST, V105, P1317, DOI 10.1172/JCI9918

CANETTI G, 1963, B WORLD HEALTH ORGAN, V29, P565

CANETTI G, 1969, B WORLD HEALTH ORGAN, V41, P21

Chen XC, 2007, CLIN IMMUNOL, V123, P50, DOI 10.1016/j.clim.2006.11.009

COOPER AM, 1993, J EXP MED, V178, P2243, DOI 10.1084/jem.178.6.2243

Cooper AM, 1997, J EXP MED, V186, P39, DOI 10.1084/jem.186.1.39

Corbett EL, 2003, ARCH INTERN MED, V163, P1009, DOI 10.1001/archinte.163.9.1009

de Jong R, 1998, SCIENCE, V280, P1435

Dorman SE, 2000, CYTOKINE GROWTH F R, V11, P321, DOI 10.1016/S1359-6101(00)00010-1

Dye C, 1999, JAMA-J AM MED ASSOC, V282, P677, DOI 10.1001/jama.282.7.677

Erb KJ, 1998, IMMUNOL CELL BIOL, V76, P41, DOI 10.1046/j.1440-1711.1998.00719.x

Fortes A, 2005, CLIN EXP IMMUNOL, V141, P541, DOI 10.1111/j.1365-2249.2005.02872.x

Gandhi NR, 2006, LANCET, V368, P1575, DOI 10.1016/S0140-6736(06)69573-1

Gandhi NR, 2010, LANCET, V375, P1830, DOI 10.1016/S0140-6736(10)60410-2

Geffner L, 2009, INFECT IMMUN, V77, P5025, DOI 10.1128/IAI.00224-09

Hernandez-Pando R, 2009, ARCH IMMUNOL THER EX, V57, P355, DOI 10.1007/s00005-009-0042-9

Hirsch CS, 1999, J INFECT DIS, V180, P2069, DOI 10.1086/315114

I-Shehadat S, 2004, PROGR TUBERCULOSIS R

ISEMAN MD, 1994, P NATL ACAD SCI USA, V91, P2428, DOI 10.1073/pnas.91.7.2428

ISEMAN MD, 1990, AM REV RESPIR DIS, V141, P623, DOI 10.1164/ajrccm/141.3.623

ISEMAN MD, 1993, NEW ENGL J MED, V329, P784, DOI 10.1056/NEJM199309093291108

Johnson BJ, 1997, TUBERCLE LUNG DIS, V78, P195, DOI 10.1016/S0962-8479(97)90026-5

JOHNSON BJ, 1995, CYTOKINES MOL THER, V1, P185

Jouanguy E, 1997, J CLIN INVEST, V100, P2658, DOI 10.1172/JCI119810

Kaplan G, 2003, INFECT IMMUN, V71, P7099, DOI 10.1128/IAI.71.12.7099-7108.2003

Kim HJ, 2008, INT J TUBERC LUNG D, V12, P1071

Kim JY, 2010, B WORLD HEALTH ORGAN, P5

LANGSTON HT, 1966, ANN SURG, V164, P567

Lee JS, 2002, CLIN EXP IMMUNOL, V128, P516, DOI 10.1046/j.1365-2249.2002.01858.x

Mann DL, 2004, CIRCULATION, V109, P1594, DOI 10.1161/01.CIR.0000124490.27666.B2

McDyer JF, 1997, J IMMUNOL, V158, P492

O'Brien R J, 1994, Semin Respir Infect, V9, P104

Orlovic D, 2001, INT J TUBERC LUNG D, V5, P370

Ottenhoff THM, 2002, NAT GENET, V32, P97, DOI 10.1038/ng0902-97

Park SK, 2009, INT J INFECT DIS, V13, P170, DOI 10.1016/j.ijid.2008.06.001

POMERANTZ M, 1991, ANN THORAC SURG, V52, P1108, DOI 10.1016/0003-4975(91)91289-8

RIZZI A, 1995, ANN THORAC SURG, V59, P896, DOI 10.1016/0003-4975(95)00011-9

SANCHEZ FO, 1994, INFECT IMMUN, V62, P5673

Seah GT, 2000, J INFECT DIS, V181, P385, DOI 10.1086/315200

Shahemabadi AS, 2007, CLIN EXP IMMUNOL, V149, P285, DOI 10.1111/j.1365-2249.2007.03406.x

Sharma S, 2004, IMMUNOL CELL BIOL, V82, P377, DOI 10.1111/j.0818-9641.2004.01245.x

Silva CL, 2005, GENE THER, V12, P281, DOI 10.1038/sj.gt.3302418

SURCEL HM, 1994, IMMUNOLOGY, V81, P171

TREASURE RL, 1995, ANN THORAC SURG, V59, P1405, DOI 10.1016/0003-4975(95)00145-B

Tufariello JM, 2003, LANCET INFECT DIS, V3, P578, DOI 10.1016/S1473-3099(03)00741-2

van de Vosse E, 2004, LANCET INFECT DIS, V4, P739, DOI 10.1016/S1473-3099(04)01203-4

Veenstra H, 2007, CLIN IMMUNOL, V124, P336, DOI 10.1016/j.clim.2007.05.014

NR 51

TC 5

Z9 5

U1 0

U2 5

PU CHURCHILL LIVINGSTONE

PI EDINBURGH

PA JOURNAL PRODUCTION DEPT, ROBERT STEVENSON HOUSE, 1-3 BAXTERS PLACE,

LEITH WALK, EDINBURGH EH1 3AF, MIDLOTHIAN, SCOTLAND

SN 1472-9792

J9 TUBERCULOSIS

JI Tuberculosis

PD JUL

PY 2011

VL 91

IS 4

BP 300

EP 307

DI 10.1016/j.tube.2011.02.003

PG 8

WC Immunology; Microbiology; Respiratory System

WE Science Citation Index Expanded (SCI-EXPANDED)

SC Immunology; Microbiology; Respiratory System

GA 791AJ

UT WOS:000292631900004

OA Green Submitted

DA 2026-05-07

ER

PT J

AU Kashyap, A

Singh, PK

Silakari, O

AF Kashyap, Aanchal

Singh, Pankaj Kumar

Silakari, Om

TI Mechanistic investigation of resistance via drug-inactivating enzymes in

Mycobacterium tuberculosis

SO DRUG METABOLISM REVIEWS

LA English

DT Review

DE Resistant tuberculosis; drug inactivation; Eis; BlaC; NAT

ID ARYLAMINE N-ACETYLTRANSFERASE; BETA-LACTAM ANTIBIOTICS; MULTIDRUG EFFLUX

PUMP; AMINOGLYCOSIDE 2'-N-ACETYLTRANSFERASE; KANAMYCIN RESISTANCE; EIS

PROTEIN; INHIBITORS; SMEGMATIS; DISCOVERY; GENE

AB Tuberculosis (TB) is a serious major health concern that has existed from millennia. According to annual WHO report 2016, it is considered as world's ninth highest killer disease by single infectious agent, ranking above HIV/AIDS. To worsen the scenario the development of multi-drug resistant tuberculosis (MDR-TB) and extremely drug-resistant tuberculosis (XDR-TB) have significantly reduced the success rate of TB treatment. Several efforts are being made to handle pharmacodynamic resistance (MDR and XDR-TB) involving designing of new inhibitors, targeting mutated target or by multi-targeting agents. However, the issue of pharmacokinetic resistance in TB is not being addressed appropriately till date. Pharmacokinetic mode of resistance involves an intrinsic mechanism of bacterial drug resistance via expression of various enzymes and efflux pumps that are responsible for the loss of activity of the therapeutic agents. Mycobacterium tuberculosis is also intrinsically resistant to various approved agents via pharmacokinetic mechanism of resistance. Several bacterial enzymes are encoded that either degrade or modifies the drugs and renders them ineffective. Targeting such inactivating bacterial enzymes provides a novel approach to make the current therapy effective and combat the problem of resistance. This review provides an insight into different bacterial enzymes which are responsible for pharmacokinetic drug resistance in TB. The structure attributes and mechanism of catalysis employed by these enzymes to inactivate drug have also been discussed which may provide basis for developing novel therapeutic agents for resistant TB.

C1 [Kashyap, Aanchal; Singh, Pankaj Kumar; Silakari, Om] Punjabi Univ, Dept Pharmaceut Sci & Drug Res, MML, Patiala 147002, Punjab, India.

C3 Punjabi University

RP Silakari, O (corresponding author), Punjabi Univ, Dept Pharmaceut Sci & Drug Res, MML, Patiala 147002, Punjab, India.

EM omsilakari@gmail.com

RI Singh, Pankaj Kumar/AAG-1935-2019

OI Singh, Pankaj Kumar/0000-0002-4774-6358

CR Aad G, 2012, PHYS LETT B, V716, P1, DOI 10.1016/j.physletb.2012.08.020

Abuhammad A, 2014, MOLECULES, V19, P16274, DOI 10.3390/molecules191016274

Abuhammad A, 2013, ACTA CRYSTALLOGR D, V69, P1433, DOI 10.1107/S0907444913015126

Abuhammad A, 2012, PLOS ONE, V7, DOI 10.1371/journal.pone.0052790

Aínsa JA, 1998, J BACTERIOL, V180, P5836

Ainsa JA, 1997, MOL MICROBIOL, V24, P431, DOI 10.1046/j.1365-2958.1997.3471717.x

[Anonymous], 2018, WHO | Global tuberculosis report

Brooke EW, 2003, BIOORGAN MED CHEM, V11, P1227, DOI 10.1016/S0968-0896(02)00642-9

CHAMBERS HF, 1995, ANTIMICROB AGENTS CH, V39, P2620, DOI 10.1128/AAC.39.12.2620

Chen WJ, 2011, P NATL ACAD SCI USA, V108, P9804, DOI 10.1073/pnas.1105379108

Chopra S, 2012, J ANTIMICROB CHEMOTH, V67, P415, DOI 10.1093/jac/dkr449

Danilchanka O, 2008, ANTIMICROB AGENTS CH, V52, P2503, DOI 10.1128/AAC.00298-08

De Rossi Edda, 2002, Molecular Medicine (Baltimore), V8, P714

Dubée V, 2015, ANTIMICROB AGENTS CH, V59, P2938, DOI 10.1128/AAC.05080-14

Egesborg P, 2015, PROTEIN SCI, V24, P534, DOI 10.1002/pro.2617

Flores AR, 2005, J BACTERIOL, V187, P1892, DOI 10.1128/JB.187.6.1892-1900.2005

Flores AR, 2005, MICROBIOL-SGM, V151, P521, DOI 10.1099/mic.0.27629-0

Garzan A, 2017, ACS INFECT DIS, V3, P302, DOI 10.1021/acsinfecdis.6b00193

Garzan A, 2016, ACS MED CHEM LETT, V7, P1219, DOI 10.1021/acsmedchemlett.6b00261

Garzan A, 2016, J MED CHEM, V59, P10619, DOI 10.1021/acs.jmedchem.6b01161

Gler MT, 2012, NEW ENGL J MED, V366, P2151, DOI 10.1056/NEJMoa1112433

Green KD, 2012, CHEMMEDCHEM, V7, P73, DOI 10.1002/cmdc.201100332

Hegde SS, 2001, J BIOL CHEM, V276, P45876, DOI 10.1074/jbc.M108810200

Hoagland DT, 2016, ADV DRUG DELIVER REV, V102, P55, DOI 10.1016/j.addr.2016.04.026

Houghton JL, 2013, CHEMBIOCHEM, V14, P2127, DOI 10.1002/cbic.201300359

Houghton JL, 2013, J ANTIMICROB CHEMOTH, V68, P800, DOI 10.1093/jac/dks497

Hugonnet JE, 2007, BIOCHEMISTRY-US, V46, P11998, DOI 10.1021/bi701506h

JARLIER V, 1991, ANTIMICROB AGENTS CH, V35, P1937, DOI 10.1128/AAC.35.9.1937

Johansen SK, 2006, MOL CELL, V23, P173, DOI 10.1016/j.molcel.2006.05.044

Kashyap A, 2018, TUBERCULOSIS, V113, P43, DOI 10.1016/j.tube.2018.09.001

Kashyap A, 2018, TUBERCULOSIS, V112, P83, DOI 10.1016/j.tube.2018.08.005

Kashyap A, 2019, J CELL BIOCHEM, V120, P3149, DOI 10.1002/jcb.27579

Kim KH, 2012, P NATL ACAD SCI USA, V109, P7729, DOI 10.1073/pnas.1120251109

Kumar B, 2013, J PROTEOMICS, V94, P68, DOI 10.1016/j.jprot.2013.08.025

Mishra MN, 2013, J BACTERIOL, V195, P1610, DOI 10.1128/JB.01724-12

Molodtsov V, 2017, MOL MICROBIOL, V103, P1034, DOI 10.1111/mmi.13606

Payton M, 1999, J BACTERIOL, V181, P1343, DOI 10.1128/JB.181.4.1343-1347.1999

Payton M, 2001, MICROBIOL-SGM, V147, P3295, DOI 10.1099/00221287-147-12-3295

Rodrigues L, 2013, ANTIMICROB AGENTS CH, V57, P751, DOI 10.1128/AAC.01482-12

Samuel LP, 2007, MICROBIOL-SGM, V153, P529, DOI 10.1099/mic.0.2006/002642-0

Sandy J, 2002, J MOL BIOL, V318, P1071, DOI 10.1016/S0022-2836(02)00141-9

Sharma D, 2018, PROTEOMES, V6, DOI 10.3390/proteomes6020026

Sharma D, 2017, FRONT CELL INFECT MI, V7, DOI 10.3389/fcimb.2017.00240

Sharma D, 2017, FRONT MICROBIOL, V8, DOI 10.3389/fmicb.2017.00465

Sharma D, 2015, PLOS ONE, V10, DOI 10.1371/journal.pone.0139414

Sherman DR, 2001, P NATL ACAD SCI USA, V98, P7534, DOI 10.1073/pnas.121172498

Sikora AL, 2008, BIOCHEMISTRY-US, V47, P10781, DOI 10.1021/bi800398c

Sinclair JC, 2000, NAT STRUCT BIOL, V7, P560

Singh PK, 2017, LIFE SCI, V186, P25, DOI 10.1016/j.lfs.2017.08.001

Singh PK, 2017, FUTURE MED CHEM, V9, P469, DOI 10.4155/fmc-2016-0234

Speck-Planche A, 2012, COMB CHEM HIGH T SCR, V15, P666, DOI 10.2174/138620712802650487

Suarez J, 2009, J BIOL CHEM, V284, P7017, DOI 10.1074/jbc.M808106200

Tremblay LV, 2010, BIOCHEMISTRY-US, V49, P9685, DOI 10.1021/bi1015088

Udwadia ZF, 2012, CLIN INFECT DIS, V54, P579, DOI 10.1093/cid/cir889

Upton AM, 2001, MOL MICROBIOL, V42, P309, DOI 10.1046/j.1365-2958.2001.02648.x

Velayati AA, 2009, CHEST, V136, P420, DOI 10.1378/chest.08-2427

Vetting MW, 2002, NAT STRUCT BIOL, V9, P653, DOI 10.1038/nsb830

Wang F, 2006, ANTIMICROB AGENTS CH, V50, P2762, DOI 10.1128/AAC.00320-06

Warrier T, 2016, P NAT ACAD SCI, V113

Westwood IM, 2011, COMB CHEM HIGH T SCR, V14, P117

Whitfield MG, 2015, PLOS ONE, V10, DOI 10.1371/journal.pone.0133869

Willby MJ, 2016, ACS CHEM BIOL, V11, P1639, DOI 10.1021/acschembio.6b00110

Yu SW, 2003, J BIOL CHEM, V278, P14769, DOI 10.1074/jbc.M300326200

Zaunbrecher MA, 2009, P NATL ACAD SCI USA, V106, P20004, DOI 10.1073/pnas.0907925106

ZHANG YS, 1992, AM REV RESPIR DIS, V145, P657, DOI 10.1164/ajrccm/145.3.657

NR 65

TC 12

Z9 15

U1 0

U2 13

PU TAYLOR & FRANCIS LTD

PI ABINGDON

PA 2-4 PARK SQUARE, MILTON PARK, ABINGDON OR14 4RN, OXON, ENGLAND

SN 0360-2532

EI 1097-9883

J9 DRUG METAB REV

JI Drug Metab. Rev.

PY 2018

VL 50

IS 4

BP 448

EP 465

DI 10.1080/03602532.2018.1533966

PG 18

WC Pharmacology & Pharmacy

WE Science Citation Index Expanded (SCI-EXPANDED)

SC Pharmacology & Pharmacy

GA HI4LZ

UT WOS:000456423500004

DA 2026-05-07

ER

PT J

AU Ahmad, S

Mokaddas, E

AF Ahmad, Suhail

Mokaddas, Eiman

TI New Approaches in the Diagnosis and Treatment of Susceptible,

Multidrug-Resistant and Extensively Drug Resistant Tuberculosis

SO KUWAIT MEDICAL JOURNAL

LA English

DT Review

DE diagnosis; MDR-TB; treatment; tuberculosis; XDR-TB

ID EARLY BACTERICIDAL ACTIVITY; NONREPLICATING MYCOBACTERIUM-TUBERCULOSIS;

SHORT-COURSE CHEMOTHERAPY; CATALASE-PEROXIDASE GENE; HIV-INFECTED

PATIENTS; LINE PROBE ASSAY; THIN-LAYER AGAR; EMBB CODON 306; PULMONARY

TUBERCULOSIS; RAPID DETECTION

AB Tuberculosis (TB) is killing nearly two million people worldwide every year. The current global burden of TB is mainly due to the expanding human immunodeficiency-virus infection and its association with active TB disease and increasing resistance of Mycobacterium tuberculosis strains to most-effective (first-line) anti-TBdrugs. Incomplete/improper treatment of TB patients leads to evolution of drug-resistant M. tuberculosis strains as a result of chromosomal mutations in genes encoding drug targets. Sequential accumulation of mutations in target genes generate multidrug-resistant (resistant atleast to rifampin and isoniazid) M. tuberculosis (MDR-TB) and extensively drug-resistant (additionally resistant to fluoroquinolones and an injectable anti-TB agent) M. tuberculosis (XDR-TB) strains. While proper treatment of susceptible TB has > 95% cure rate, effective treatment of MDR-TB is difficult in developing countries as it is heavily dependent on rapid diagnosis, supervised aggressive therapy with several (5 - 6) expensive, toxic and less efficacious drugs for 18-24 months and regular monitoring for bacteriological and clinical improvement. Treatment of XDR-TB is far more difficult even in developed countries. Several anti-TB drugs with novel mechanism of action are under clinical development, which may shorten treatment duration of susceptible TB to around three months and also help in effective treatment of MDR-TB / XDR-TB.

C1 [Ahmad, Suhail; Mokaddas, Eiman] Kuwait Univ, Dept Microbiol, Fac Med, Safat 13110, Kuwait.

C3 Kuwait University

RP Ahmad, S (corresponding author), Kuwait Univ, Dept Microbiol, Fac Med, POB 24923, Safat 13110, Kuwait.

EM suhail_ah@hsc.edu.kw

RI Ahmad, Suhail/AAJ-5456-2021

FU Kuwait University Research Administration [MI 06/02]

FX This study was supported by Kuwait University Research Administration

grant MI 06/02.

CR Abal AT, 2002, MICROB DRUG RESIST, V8, P99, DOI 10.1089/107662902760190644

Affolabi D, 2007, J CLIN MICROBIOL, V45, P2123, DOI 10.1128/JCM.00691-07

Ahmad S, 2008, J CHEMOTHERAPY, V20, P285, DOI 10.1179/joc.2008.20.2.285

Ahmad S, 2005, INT J ANTIMICROB AG, V26, P205, DOI 10.1016/j.ijantimicag.2005.06.009

Ahmad S, 2007, TUBERCULOSIS, V87, P123, DOI 10.1016/j.tube.2006.05.004

Ahmad S, 2004, MOL CELL PROBE, V18, P299, DOI 10.1016/j.mcp.2004.04.001

Ahmad S, 2004, INT J ANTIMICROB AG, V23, P473, DOI 10.1016/j.ijantimicag.2003.10.004

Ahmad S, 2002, DIAGN MICR INFEC DIS, V44, P245, DOI 10.1016/S0732-8893(02)00457-1

Ahmad S, 2002, INT J TUBERC LUNG D, V6, P920

Ahmad S, 2000, DIAGN MICR INFEC DIS, V38, P227, DOI 10.1016/S0732-8893(00)00200-5

Ahmad S, 2011, CLIN DEV IMMUNOL, DOI 10.1155/2011/814943

Ahmad S, 2009, RESP MED, V103, P1777, DOI 10.1016/j.rmed.2009.07.010

Ahmad S, 2009, INDIAN J EXP BIOL, V47, P454

Al-Mutairi NM, 2011, INT J TUBERC LUNG D, V15, P110

Alcaide F, 1997, ANTIMICROB AGENTS CH, V41, P2270, DOI 10.1128/AAC.41.10.2270

Alffenaar JWC, 2010, CLIN PHARMACOKINET, V49, P559, DOI 10.2165/11532080-000000000-00000

Andries K, 2005, SCIENCE, V307, P223, DOI 10.1126/science.1106753

[Anonymous], WHOHTMTB2008402

[Anonymous], 2003, MORBIDITY MORTALITY, V52

[Anonymous], 2009, Treatment of tuberculosis guidelines

[Anonymous], 2009, WHOHTMTB2009411

[Anonymous], 2008, WHO/HTM/TB/2008.394

[Anonymous], 2010, WHO/HTM/TB/2010.3

Aziz MA, 2006, LANCET, V368, P2142, DOI 10.1016/S0140-6736(06)69863-2

Banaiee N, 2008, TUBERCULOSIS, V88, P64, DOI 10.1016/j.tube.2007.08.006

Behbehani N, 2002, INT J TUBERC LUNG D, V6, P465

Bergal IL, 2008, J CLIN MICROBIOL, V46, P689, DOI 10.1128/JCM.01821-07

Blanc FX, 2007, J INFECT DIS, V196, pS46, DOI 10.1086/518658

Boulanger C, 2009, CLIN INFECT DIS, V49, P1305, DOI 10.1086/606056

Boulle A, 2008, JAMA-J AM MED ASSOC, V300, P530, DOI 10.1001/jama.300.5.530

Brodie D, 2005, CLIN CHEST MED, V26, P247, DOI 10.1016/j.ccm.2005.02.012

Burman WJ, 2010, CLIN INFECT DIS, V50, pS165, DOI 10.1086/651487

Burman WJ, 2005, CLIN CHEST MED, V26, P283, DOI 10.1016/j.ccm.2005.02.002

Bwanga F, 2010, INT J TUBERC LUNG D, V14, P890

Cailhol J, 2005, J CLIN EPIDEMIOL, V58, P1066, DOI 10.1016/j.jclinepi.2005.02.023

Causse M, 2008, INT J TUBERC LUNG D, V12, P1456

Centers for Disease Control and Prevention (CDC), 2006, MMWR Morb Mortal Wkly Rep, V55, P301

Chan ED, 2004, AM J RESP CRIT CARE, V169, P1103, DOI 10.1164/rccm.200308-1159OC

Chan ED, 2008, CURR OPIN INFECT DIS, V21, P587, DOI 10.1097/QCO.0b013e328319bce6

Chen P, 2006, J ANTIMICROB CHEMOTH, V58, P332, DOI 10.1093/jac/dkl227

Cheng SJ, 2000, ANTIMICROB AGENTS CH, V44, P528, DOI 10.1128/AAC.44.3.528-532.2000

Cheng VCC, 2005, EUR J CLIN MICROBIOL, V24, P711, DOI 10.1007/s10096-005-0039-1

COCKERILL FR, 1995, J INFECT DIS, V171, P240, DOI 10.1093/infdis/171.1.240

Conde MB, 2009, LANCET, V373, P1183, DOI 10.1016/S0140-6736(09)60333-0

Connolly LE, 2007, PLOS MED, V4, P435, DOI 10.1371/journal.pmed.0040120

Crampin AC, 2001, INT J TUBERC LUNG D, V5, P994

DAVID HL, 1970, APPL MICROBIOL, V20, P810, DOI 10.1128/AEM.20.5.810-814.1970

Dheda K, 2010, INFECT DIS CLIN N AM, V24, P705, DOI 10.1016/j.idc.2010.05.001

Dhillon J, 2010, TUBERCULOSIS, V90, P301, DOI 10.1016/j.tube.2010.07.004

Diacon AH, 2007, ANTIMICROB AGENTS CH, V51, P2994, DOI 10.1128/AAC.01474-06

Diacon AH, 2010, ANTIMICROB AGENTS CH, V54, P3402, DOI 10.1128/AAC.01354-09

Diacon AH, 2009, NEW ENGL J MED, V360, P2397, DOI 10.1056/NEJMoa0808427

Dietze R, 2008, AM J RESP CRIT CARE, V178, P1180, DOI 10.1164/rccm.200806-892OC

Donald PR, 2008, TUBERCULOSIS, V88, pS75, DOI 10.1016/S1472-9792(08)70038-6

Donald PR, 2010, TUBERCULOSIS, V90, P279, DOI 10.1016/j.tube.2010.07.002

Dooley KE, 2009, LANCET INFECT DIS, V9, P737, DOI 10.1016/S1473-3099(09)70282-8

Dorman SE, 2009, AM J RESP CRIT CARE, V180, P273, DOI 10.1164/rccm.200901-0078OC

Dravniece G, 2009, EUR RESPIR J, V34, P180, DOI 10.1183/09031936.00047208

Dye C, 1999, JAMA-J AM MED ASSOC, V282, P677, DOI 10.1001/jama.282.7.677

Ejigu GS, 2008, INT J TUBERC LUNG D, V12, P332

Espinal MA, 2000, JAMA-J AM MED ASSOC, V283, P2537, DOI 10.1001/jama.283.19.2537

Evans J, 2009, J ANTIMICROB CHEMOTH, V63, P11, DOI 10.1093/jac/dkn433

Férriz JM, 2010, BIOORGAN MED CHEM, V18, P1054, DOI 10.1016/j.bmc.2009.12.055

Furin J, 2007, CURR OPIN PULM MED, V13, P212, DOI 10.1097/MCP.0b013e3280f3c0b2

Gandhi NR, 2006, LANCET, V368, P1575, DOI 10.1016/S0140-6736(06)69573-1

Garrigó M, 2007, J CLIN MICROBIOL, V45, P1766, DOI 10.1128/JCM.02162-06

Ginsberg AM, 2010, TUBERCULOSIS, V90, P162, DOI 10.1016/j.tube.2010.03.003

Ginsberg AM, 2009, ANTIMICROB AGENTS CH, V53, P3720, DOI 10.1128/AAC.00106-09

Golden MP, 2005, AM FAM PHYSICIAN, V72, P1761

Guo SP, 2010, J MED CHEM, V53, P649, DOI 10.1021/jm901235p

Harries AD, 2006, LANCET, V367, P944, DOI 10.1016/S0140-6736(06)68387-6

Heep M, 2001, J CLIN MICROBIOL, V39, P107, DOI 10.1128/JCM.39.1.107-110.2001

Hillemann D, 2007, J CLIN MICROBIOL, V45, P2635, DOI 10.1128/JCM.00521-07

Hillemann D, 2009, J CLIN MICROBIOL, V47, P1767, DOI 10.1128/JCM.00081-09

Holtz TH, 2006, ANN INTERN MED, V144, P650, DOI 10.7326/0003-4819-144-9-200605020-00008

Huang QQ, 2009, J MED CHEM, V52, P6757, DOI 10.1021/jm901112f

Huang WL, 2009, J CLIN MICROBIOL, V47, P2520, DOI 10.1128/JCM.02499-08

Hugonnet JE, 2009, SCIENCE, V323, P1215, DOI 10.1126/science.1167498

Huitric E, 2010, ANTIMICROB AGENTS CH, V54, P1022, DOI 10.1128/AAC.01611-09

Ibrahim M, 2009, AM J RESP CRIT CARE, V180, P553, DOI 10.1164/rccm.200807-1152OC

Jacobson KR, 2010, CLIN INFECT DIS, V51, P6, DOI 10.1086/653115

Johnson JL, 2006, INT J TUBERC LUNG D, V10, P605

Johnston JC, 2009, PLOS ONE, V4, DOI 10.1371/journal.pone.0006914

Kalscheuer R, 2010, NAT CHEM BIOL, V6, P376, DOI 10.1038/nchembio.340

Kana BD, 2008, MOL MICROBIOL, V67, P672, DOI 10.1111/j.1365-2958.2007.06078.x

Kang MW, 2010, ANN THORAC SURG, V89, P1597, DOI 10.1016/j.athoracsur.2010.02.020

KAPUR V, 1994, J CLIN MICROBIOL, V32, P1095, DOI 10.1128/JCM.32.4.1095-1098.1994

Kliiman K, 2009, EUR RESPIR J, V33, P1085, DOI 10.1183/09031936.00155708

Koul A, 2008, J BIOL CHEM, V283, P25273, DOI 10.1074/jbc.M803899200

Kritski AL, 1997, CHEST, V111, P1162, DOI 10.1378/chest.111.5.1162

Lawn SD, 2005, LANCET INFECT DIS, V5, P361, DOI 10.1016/S1473-3099(05)70140-7

Lew W, 2008, ANN INTERN MED, V149, P123, DOI 10.7326/0003-4819-149-2-200807150-00008

Lienhardt C, 2010, CURR OPIN PULM MED, V16, P186, DOI 10.1097/MCP.0b013e328337580c

Lilienkampf A, 2010, J MED CHEM, V53, P678, DOI 10.1021/jm901273n

Lilienkampf A, 2009, J MED CHEM, V52, P2109, DOI 10.1021/jm900003c

Lillebaek T, 2002, J INFECT DIS, V185, P401, DOI 10.1086/338342

LoBue P, 2009, CURR OPIN INFECT DIS, V22, P167, DOI 10.1097/QCO.0b013e3283229fab

Lounis N, 2006, ANTIMICROB AGENTS CH, V50, P3543, DOI 10.1128/AAC.00766-06

Ma ZK, 2010, LANCET, V375, P2100, DOI 10.1016/S0140-6736(10)60359-9

Makarov V, 2009, SCIENCE, V324, P801, DOI 10.1126/science.1171583

Malhotra S, 2010, TUBERCULOSIS, V90, P152, DOI 10.1016/j.tube.2010.01.001

Manjunatha UH, 2006, P NATL ACAD SCI USA, V103, P431, DOI 10.1073/pnas.0508392103

Manosuthi W, 2009, CLIN INFECT DIS, V48, P1752, DOI 10.1086/599114

Marais BJ, 2008, CLIN INFECT DIS, V47, P203, DOI 10.1086/589248

Martin A, 2009, INT J TUBERC LUNG D, V13, P1301

Martin A, 2007, J ANTIMICROB CHEMOTH, V59, P175, DOI 10.1093/jac/dkl477

Marttila HJ, 1998, ANTIMICROB AGENTS CH, V42, P2443, DOI 10.1128/AAC.42.9.2443

Mathema B, 2008, SEMIN RESP CRIT CARE, V29, P467, DOI 10.1055/s-0028-1085699

Matsumoto M, 2006, PLOS MED, V3, P2131, DOI 10.1371/journal.pmed.0030466

Maus CE, 2005, ANTIMICROB AGENTS CH, V49, P3192, DOI 10.1128/AAC.49.8.3192-3197.2005

Meintjes G, 2008, LANCET INFECT DIS, V8, P516, DOI 10.1016/S1473-3099(08)70184-1

Menzies D, 2009, PLOS MED, V6, DOI [10.1371/journal.pmed.1000150, 10.1371/journal.pmed.1000146]

Migliori GB, 2008, EUR RESPIR J, V31, P1155, DOI 10.1183/09031936.00028708

Migliori GB, 2009, EUR RESPIR J, V34, P387, DOI 10.1183/09031936.00009509

Minion J, 2010, INT J TUBERC LUNG D, V14, P941

Minion J, 2010, LANCET INFECT DIS, V10, P688, DOI 10.1016/S1473-3099(10)70165-1

Mitchison DA, 2000, INT J TUBERC LUNG D, V4, P796

Mitchison DA, 2004, SEMIN RESP CRIT CARE, V25, P307, DOI 10.1055/s-2004-829503

MITCHISON DA, 1985, TUBERCLE, V66, P219, DOI 10.1016/0041-3879(85)90040-6

Mitnick CD, 2008, SEMIN RESP CRIT CARE, V29, P499, DOI 10.1055/s-0028-1085702

Mitnick CD, 2008, NEW ENGL J MED, V359, P563, DOI 10.1056/NEJMoa0800106

Mitnick CD, 2007, PLOS MED, V4, P1730, DOI 10.1371/journal.pmed.0040292

Mokaddas E, 2008, INT J TUBERC LUNG D, V12, P319

Mokaddas E, 2005, ANN SAUDI MED, V25, P150, DOI 10.5144/0256-4947.2005.150

Mokaddas E, 2008, CURR MICROBIOL, V56, P413, DOI 10.1007/s00284-008-9102-3

Mokaddas E, 2007, JPN J INFECT DIS, V60, P140

Mokrousov I, 2004, J MICROBIOL METH, V57, P323, DOI 10.1016/j.mimet.2004.02.006

Mokrousov I, 2002, ANTIMICROB AGENTS CH, V46, P1417, DOI 10.1128/AAC.46.5.1417-1424.2002

Moore DAJ, 2006, NEW ENGL J MED, V355, P1539, DOI 10.1056/NEJMoa055524

Mukherjee JS, 2004, LANCET, V363, P474, DOI 10.1016/S0140-6736(04)15496-2

Musser JM, 1996, J INFECT DIS, V173, P196, DOI 10.1093/infdis/173.1.196

Nam HS, 2009, INT J ANTIMICROB AG, V33, P92, DOI 10.1016/j.ijantimicag.2008.06.014

Nikonenko BV, 2007, ANTIMICROB AGENTS CH, V51, P1563, DOI 10.1128/AAC.01326-06

Nuermberger E, 2008, ANTIMICROB AGENTS CH, V52, P1522, DOI 10.1128/AAC.00074-08

Nuermberger EL, 2010, RESPIROLOGY, V15, P764, DOI 10.1111/j.1440-1843.2010.01775.x

Nyendak MR, 2009, CURR OPIN INFECT DIS, V22, P174, DOI 10.1097/QCO.0b013e3283262fe9

O'Brien RJ, 2003, AM J RESP CRIT CARE, V168, P1266, DOI 10.1164/rccm.2309011

Onajole OK, 2010, EUR J MED CHEM, V45, P2075, DOI 10.1016/j.ejmech.2010.01.046

Orenstein EW, 2009, LANCET INFECT DIS, V9, P153, DOI 10.1016/S1473-3099(09)70041-6

Palaci M, 2007, J CLIN MICROBIOL, V45, P4064, DOI 10.1128/JCM.01780-07

Palomino JC, 2007, CLIN MICROBIOL INFEC, V13, P754, DOI 10.1111/j.1469-0691.2007.01698.x

Palomino JC, 2005, EUR RESPIR J, V26, P339, DOI 10.1183/09031936.05.00050305

Palomino JC, 2008, J MICROBIOL METH, V75, P161, DOI 10.1016/j.mimet.2008.06.015

Parsons LA, 2005, ANTIMICROB AGENTS CH, V49, P2218, DOI 10.1128/AAC.49.6.2218-2225.2005

Perkins MD, 2007, J INFECT DIS, V196, pS15, DOI 10.1086/518656

Piersimoni C, 2006, J CLIN MICROBIOL, V44, P20, DOI 10.1128/JCM.44.1.20-28.2006

Protopopova M, 2005, J ANTIMICROB CHEMOTH, V56, P968, DOI 10.1093/jac/dki319

Quezada CM, 2007, J CLIN MICROBIOL, V45, P3111, DOI 10.1128/JCM.00590-07

Ramaswamy SV, 2003, ANTIMICROB AGENTS CH, V47, P1241, DOI 10.1128/AAC.47.4.1241-1250.2003

Ramaswamy SV, 2000, ANTIMICROB AGENTS CH, V44, P326, DOI 10.1128/AAC.44.2.326-336.2000

Rich ML, 2006, INT J TUBERC LUNG D, V10, P290

Riekstina V, 2007, INT J TUBERC LUNG D, V11, P585

Robledo J, 2008, INT J TUBERC LUNG D, V12, P1482

Rosenthal IM, 2008, AM J RESP CRIT CARE, V178, P989, DOI 10.1164/rccm.200807-1029OC

Rosenthal IM, 2007, PLOS MED, V4, P1931, DOI 10.1371/journal.pmed.0040344

Rustomjee R, 2008, ANTIMICROB AGENTS CH, V52, P2831, DOI 10.1128/AAC.01204-07

Rustomjee R, 2008, INT J TUBERC LUNG D, V12, P128

Sandman L, 1999, AM J RESP CRIT CARE, V159, P468, DOI 10.1164/ajrccm.159.2.9805097

Schecter GF, 2010, CLIN INFECT DIS, V50, P49, DOI 10.1086/648675

Scorpio A, 1996, NAT MED, V2, P662, DOI 10.1038/nm0696-662

Sekiguchi JI, 2007, J CLIN MICROBIOL, V45, P2802, DOI 10.1128/JCM.00352-07

Shiloh MU, 2010, CURR OPIN MICROBIOL, V13, P86, DOI 10.1016/j.mib.2009.11.006

Singh R, 2008, SCIENCE, V322, P1392, DOI 10.1126/science.1164571

Sonnenberg P, 2005, J INFECT DIS, V191, P150, DOI 10.1086/426827

Sreevatsan S, 1996, ANTIMICROB AGENTS CH, V40, P1024, DOI 10.1128/AAC.40.4.1024

Starks AM, 2009, ANTIMICROB AGENTS CH, V53, P1061, DOI 10.1128/AAC.01357-08

Surekha V, 1997, NATL MED J INDIA, V10, P11

Suzuki Y, 1998, J CLIN MICROBIOL, V36, P1220, DOI 10.1128/JCM.36.5.1220-1225.1998

Tasneen R, 2008, ANTIMICROB AGENTS CH, V52, P3664, DOI 10.1128/AAC.00686-08

TELENTI A, 1993, LANCET, V341, P647, DOI 10.1016/0140-6736(93)90417-F

Van Deun A, 2009, J CLIN MICROBIOL, V47, P3501, DOI 10.1128/JCM.01209-09

Van Rie A, 2001, J CLIN MICROBIOL, V39, P636, DOI 10.1128/JCM.39.2.636-641.2001

Veziris N, 2009, AM J RESP CRIT CARE, V179, P75, DOI 10.1164/rccm.200711-1736OC

Vijdea R, 2008, EUR J CLIN MICROBIOL, V27, P1079, DOI 10.1007/s10096-008-0548-9

Wallis RS, 2010, J INFECT DIS, V202, P745, DOI 10.1086/655471

WHO, 2009, WHOHTMTB2009426

Williams KN, 2009, ANTIMICROB AGENTS CH, V53, P1314, DOI 10.1128/AAC.01182-08

Williams KN, 2009, AM J RESP CRIT CARE, V180, P371, DOI 10.1164/rccm.200904-0611OC

World Health Organization, 2008, WHOCDSTB2008108

Wright A, 2009, LANCET, V373, P1861, DOI 10.1016/S0140-6736(09)60331-7

Yee D, 2003, AM J RESP CRIT CARE, V167, P1472, DOI 10.1164/rccm.200206-626OC

Yew WW, 2009, EUR RESPIR J, V34, P1492, DOI 10.1183/09031936.00111009

Yew WW, 2008, RESPIROLOGY, V13, P21, DOI 10.1111/j.1440-1843.2007.01180.x

Zhang Y, 2009, INT J TUBERC LUNG D, V13, P1320

ZHANG Y, 1992, NATURE, V358, P591, DOI 10.1038/358591a0

NR 185

TC 2

Z9 3

U1 0

U2 11

PU KUWAIT MEDICAL ASSOC

PI SAFAT

PA PO BOX 1202, SAFAT, 13013, KUWAIT

SN 0023-5776

J9 KUWAIT MED J

JI Kuwait Med. J.

PD MAR

PY 2012

VL 44

IS 1

BP 3

EP 19

PG 17

WC Medicine, General & Internal

WE Science Citation Index Expanded (SCI-EXPANDED)

SC General & Internal Medicine

GA 916XJ

UT WOS:000302136200002

DA 2026-05-07

ER

PT J

AU Sotgiu, G

Pontali, E

Centis, R

D'Ambrosio, L

Migliori, GB

AF Sotgiu, Giovanni

Pontali, Emanuele

Centis, Rosella

D'Ambrosio, Lia

Migliori, Giovanni Battista

TI Delamanid (OPC-67683) for treatment of multi-drug-resistant tuberculosis

SO EXPERT REVIEW OF ANTI-INFECTIVE THERAPY

LA English

DT Article

DE delamanid; MDR-TB prevention; mycolic acid inhibition;

nitro-dihydro-imidazooxazole; tuberculosis

ID PULMONARY TUBERCULOSIS; BACTERICIDAL ACTIVITY; TB; ELIMINATION;

TOLERABILITY; EFFICACY; SAFETY

AB The research and development of delamanid was carried out by Otsuka Pharmaceutical Development and Commercialization (Osaka, Tokyo, Japan). It belongs to the group of nitroimidazoles. It inhibits the synthesis of mycolic acids, crucial component of the cell wall of the Mycobacterium tuberculosis complex. It is insoluble in water and its activity was proven in several in vitro and in vivo studies. Its market approval was obtained in April 2014 in Europe. Its bactericidal activity was demonstrated in individuals with drug-susceptible and drug-resistant tuberculosis (MDR- and XDR-TB). The safety and tolerability profile was good; the notified increased QT interval was not clinically relevant. It was approved for adults but ongoing clinical trials and clinical experiences have been proving its efficacy in the pediatric population.

C1 [Sotgiu, Giovanni] Univ Sassari, Dept Biomed Sci, Clin Epidemiol & Med Stat Unit, I-07100 Sassari, Italy.

[Sotgiu, Giovanni] AOU Sassari, Res Med Educ & Profess Dev Unit, Sassari, Italy.

[Pontali, Emanuele] Galliera Hosp, Dept Infect Dis, Genoa, Italy.

[Centis, Rosella; D'Ambrosio, Lia; Migliori, Giovanni Battista] WHO, Collaborating Ctr TB & Lung Dis, Fdn S Maugeri, Care & Res Inst, Tradate, Italy.

C3 University of Sassari; University of Genoa; Ente Ospedaliero Ospedali

Galliera; Istituti Clinici Scientifici Maugeri IRCCS; World Health

Organization

RP Sotgiu, G (corresponding author), Univ Sassari, Dept Biomed Sci, Clin Epidemiol & Med Stat Unit, I-07100 Sassari, Italy.

EM giovannibattista.migliori@fsm.it

RI Centis, Rosella/E-8882-2018; Migliori, Giovanni Battista/AAB-8589-2020;

Sotgiu, Giovanni/N-1032-2017

OI Centis, Rosella/0000-0002-8551-3598; Migliori, Giovanni

Battista/0000-0002-2597-574X; Pontali, Emanuele/0000-0002-1085-0442;

Sotgiu, Giovanni/0000-0002-1600-4474

CR Abubakar I, 2013, LANCET INFECT DIS, V13, P529, DOI 10.1016/S1473-3099(13)70030-6

Acosta CD, 2014, PUBLIC HEALTH ACTION, V4, pS3, DOI 10.5588/pha.14.0087

Acosta CD, 2014, LANCET GLOB HEALTH, V2, pE15, DOI 10.1016/S2214-109X(13)70153-0

Adada H, 2014, INT J TUBERC LUNG D, V18, P1516, DOI 10.5588/ijtld.14.0319

Ahuja SD, 2012, PLOS MED, V9, DOI 10.1371/journal.pmed.1001300

[Anonymous], 2011, WHO/HTM/TB/2011.3

[Anonymous], 2014, USE DELAMANID TREATM

[Anonymous], 2007, EURO SURVEILL

[Anonymous], 2015, WHO-HTM-TB

[Anonymous], 2014, Guidance for national tuberculosis programmes on the management of tuberculosis in children

[Anonymous], WHO/HTM/TB/2014.13

Blasi F, 2013, EUR RESPIR J, V41, P491, DOI 10.1183/09031936.00196712

Codecasa LR, 2015, EUR RESPIR J, V45, P292, DOI 10.1183/09031936.00134114

Cole ST, 1998, NATURE, V393, P537, DOI 10.1038/31159

Cox H, 2012, INT J TUBERC LUNG D, V16, P447, DOI 10.5588/ijtld.11.0451

D'Ambrosio L, 2015, INT J INFEC IN PRESS

D'Ambrosio L, 2014, EUR RESPIR J, V43, P1410, DOI 10.1183/09031936.00198813

D'Ambrosio L, 2014, EUR RESPIR J, V43, P286, DOI 10.1183/09031936.00125813

Daskapan A, 2014, EUR RESP J IN PRESS

Diacon AH, 2011, INT J TUBERC LUNG D, V15, P949, DOI 10.5588/ijtld.10.0616

Diel R, 2013, EUR RESPIR J, V42, P785, DOI 10.1183/09031936.00205512

Diel R, 2012, EUR RESPIR J, V40, P143, DOI 10.1183/09031936.00204611

Engohang-Ndong J, 2012, EXPERT OPIN INV DRUG, V21, P1789, DOI 10.1517/13543784.2012.724397

Esposito S, 2014, EUR RESPIR J, V44, P811, DOI 10.1183/09031936.00060414

Falzon D, EUR RESP J

Falzon D, 2013, EUR RESPIR J, V42, P156, DOI 10.1183/09031936.00134712

Gler MT, 2012, NEW ENGL J MED, V366, P2151, DOI 10.1056/NEJMoa1112433

Lee M, 2012, NEW ENGL J MED, V367, P1508, DOI 10.1056/NEJMoa1201964

Lonnroth K, 2015, EUR RESP J IN PRESS

Maher D, 2006, INT J TUBERC LUNG D, V10, P1205

Matsumoto M, 2006, PLOS MED, V3, P2131, DOI 10.1371/journal.pmed.0030466

Migliori GB, 2012, CLIN INFECT DIS, V54, P1379, DOI 10.1093/cid/cis128

Migliori GB, 2009, EUR RESPIR J, V34, P387, DOI 10.1183/09031936.00009509

Migliori GB, 2014, EUR RESPIR J, V44, P1412, DOI 10.1183/09031936.00132114

Migliori GB, 2013, EUR RESPIR J, V42, P169, DOI 10.1183/09031936.00136312

Paccaly A, 19 INT AIDS C

Petersen C, 2012, ICAAC C 2012

Pontali E, 2013, CURR OPIN PULM MED, V19, P266, DOI 10.1097/MCP.0b013e32835f1bf3

Saliu OY, 2007, J ANTIMICROB CHEMOTH, V60, P994, DOI 10.1093/jac/dkm291

Sandgren A, 2013, EUROSURVEILLANCE, V18, P12

Shimokawa Y, 2014, BIOL PHARM BULL, V37, P1727, DOI 10.1248/bpb.b14-00311

Skrahina A, 2012, EUR RESPIR J, V39, P1425, DOI 10.1183/09031936.00145411

Skripconoka V, 2013, EUR RESPIR J, V41, P1393, DOI 10.1183/09031936.00125812

Sotgiu G, 2015, EUR RESPIR J, V45, P25, DOI 10.1183/09031936.00145014

Sotgiu G, 2014, INT J TUBERC LUNG D, V18, P1389, DOI 10.5588/ijtld.14.0569

Sotgiu G, 2014, EUR RESPIR J, V43, P1563, DOI 10.1183/09031936.00064314

Sotgiu G, 2014, LANCET INFECT DIS, V14, P364, DOI 10.1016/S1473-3099(14)70030-1

Sotgiu G, 2013, EUR RESPIR J, V42, P288, DOI 10.1183/09031936.00191712

Sotgiu G, 2012, EUR RESPIR J, V40, P1430, DOI 10.1183/09031936.00022912

Tang SJ, 2015, EUR RESPIR J, V45, P161, DOI 10.1183/09031936.00035114

Udwadia Z, 2013, J EPIDEMIOL COMMUN H, V67, P471, DOI 10.1136/jech-2012-201640

Ulmasova DJ, 2013, EUROSURVEILLANCE, V18, P42

Upton AM, 2015, ANTIMICROB AGENTS CH, V59, P136, DOI 10.1128/AAC.03823-14

Voniatis C, 2014, EUR RESPIR J, V44, P543, DOI 10.1183/09031936.00044314

Wells CD, 2015, EUR RESP J IN PRESS

Weyer K, 2013, EUR RESPIR J, V42, P252, DOI 10.1183/09031936.00157212

WHO, 2013, USE BEDAQUILINE TREA, P57

World Health Organization, 2014, WHO POL IMPL PACK NE

World Health Organization, 2014, WHOHTMTB201408

Zignol M, 2013, DRUG RESIST UPDATE, V16, P108, DOI 10.1016/j.drup.2014.02.003

Zumla A, 2012, J INFECT DIS, V205, pS228, DOI 10.1093/infdis/jir858

NR 61

TC 45

Z9 51

U1 0

U2 32

PU TAYLOR & FRANCIS LTD

PI ABINGDON

PA 2-4 PARK SQUARE, MILTON PARK, ABINGDON OR14 4RN, OXON, ENGLAND

SN 1478-7210

EI 1744-8336

J9 EXPERT REV ANTI-INFE

JI Expert Rev. Anti-Infect. Ther.

PD MAR

PY 2015

VL 13

IS 3

BP 305

EP 315

DI 10.1586/14787210.2015.1011127

PG 11

WC Infectious Diseases; Microbiology; Pharmacology & Pharmacy

WE Science Citation Index Expanded (SCI-EXPANDED)

SC Infectious Diseases; Microbiology; Pharmacology & Pharmacy

GA CB4RT

UT WOS:000349616300003

DA 2026-05-07

ER

PT J

AU Lu, HJ

Wang, HH

Zhao, HY

Zhang, DF

AF Lu, Haijia

Wang, Huahao

Zhao, Hongyi

Zhang, Dongfeng

TI Recent advances in oxazolidinones as antituberculosis agents

SO FUTURE MEDICINAL CHEMISTRY

LA English

DT Review

DE antituberculosis agents; mechanism of action; Mycobacterium

tuberculosis; oxazolidinones; toxicity

ID IN-VITRO ACTIVITIES; DRUG-RESISTANT TUBERCULOSIS;

MYCOBACTERIUM-TUBERCULOSIS; ANTIBACTERIAL ACTIVITY; MULTIDRUG-RESISTANT;

INVIVO ACTIVITIES; VIVO ACTIVITIES; HIGHLY POTENT; PNU-100480; DISCOVERY

AB Tuberculosis (TB) is an infectious and fatal disease caused by Mycobacterium tuberculosis (Mtb) and remains a serious public health threat; therefore, the development of new antitubercular agents is a priority for the World Health Organization's End TB strategy and the United Nations' Sustainable Development Goals to eradicate TB. Oxazolidinones are a class of synthetic antibacterial agents with a distinct mode of action developed for the treatment of Gram-positive bacterial infections. Many oxazolidinones exhibit good activity against Mtb, and some are currently in clinical trials for multidrug-resistant TB and extensively drug-resistant TB therapy. In this review, the mechanism of action, activity and toxicity of oxazolidinones and recent progress in the research and development of oxazolidinones as anti-TB agents are summarized.

C1 [Lu, Haijia; Wang, Huahao; Zhao, Hongyi; Zhang, Dongfeng] Peking Union Med Coll & Chinese Acad Med Sci, Beijing Key Lab Active Substance Discovery & Drug, Chinese Acad Med Sci Key Lab AntiDR TB Innovat Dr, Inst Mat Med, 1 Xian Nong Tan St, Beijing 100050, Peoples R China.

C3 Chinese Academy of Medical Sciences - Peking Union Medical College;

Peking Union Medical College

RP Zhang, DF (corresponding author), Peking Union Med Coll & Chinese Acad Med Sci, Beijing Key Lab Active Substance Discovery & Drug, Chinese Acad Med Sci Key Lab AntiDR TB Innovat Dr, Inst Mat Med, 1 Xian Nong Tan St, Beijing 100050, Peoples R China.

EM zdf@imm.ac.cn

OI Zhang, Dongfeng/0000-0003-0870-3782; Wang, Huahao/0009-0004-2016-1829

FU National Natural Science Foundation of China [81502917]

FX We gratefully acknowledged the financial support from the National

Natural Science Foundation of China (no. 81502917). The authors have no

other relevant affiliations or financial involvement with any

organization or entity with a financial interest in or financial

conflict with the subject matter or materials discussed in the

manuscript apart from those disclosed.

CR Ahmad N, 2018, LANCET, V392, P821, DOI 10.1016/S0140-6736(18)31644-1

Al-Tannak NF, 2017, SCI PHARM, V85, DOI 10.3390/scipharm85040034

Amunts A, 2015, SCIENCE, V348, P95, DOI 10.1126/science.aaa1193

Andrews J, 2016, LANCET GLOB HEALTH, V4, pE89, DOI 10.1016/S2214-109X(15)00285-5

Ang W, 2014, BIOORG MED CHEM LETT, V24, P1496, DOI 10.1016/j.bmcl.2014.02.025

[Anonymous], TBI 223

[Anonymous], 2020, GLOBAL TUBERCULOSIS

[Anonymous], URGENT THREAT TB DRU

[Anonymous], CLIN FIELD ADV VALUE

[Anonymous], DELPAZOLID LEGOCHEM

[Anonymous], PHASE 1 CLIN TRIAL T

[Anonymous], MPP MEDIA POSTTHE ME

[Anonymous], PHASE 2 CLIN STUDY L

[Anonymous], 2014, REV SUTEZOLID PNU 10

[Anonymous], 2014, The End TB Strategy

[Anonymous], 2012, ZYVOX LINEZOLID INJE

[Anonymous], 2017, MOSCOW DECLARATION E

[Anonymous], 2020, COVID-19: Considerations for tuberculosis (TB) care: World Health Organization (WHO) Information Note

[Anonymous], 2021, WORKING GROUP NEW TB

[Anonymous], SIVEXTRO TEDIZOLID P

Antal EJ, 2001, J CLIN PHARMACOL, V41, P552, DOI 10.1177/00912700122010294

Aoki H, 2002, ANTIMICROB AGENTS CH, V46, P1080, DOI 10.1128/AAC.46.4.1080-1085.2002

ASHTEKAR DR, 1991, DIAGN MICR INFEC DIS, V14, P465, DOI 10.1016/0732-8893(91)90002-W

Barbachyn M. R., 2005, Patent WO, Patent No. [2005113520A1, 2005113520]

Barbachyn MR, 2003, ANGEW CHEM INT EDIT, V42, P2010, DOI 10.1002/anie.200200528

Barbachyn MR, 1996, J MED CHEM, V39, P680, DOI 10.1021/jm950956y

Beckert P, 2012, ANTIMICROB AGENTS CH, V56, P2743, DOI 10.1128/AAC.06227-11

BERNARD S, 1995, EUR J MED CHEM, V30, P471, DOI 10.1016/0223-5234(96)88258-4

Bhattarai D, 2014, CHEM PHARM BULL, V62, P1214, DOI 10.1248/cpb.c14-00510

Bhattarai D, 2012, CHEM BIOL DRUG DES, V80, P388, DOI 10.1111/j.1747-0285.2012.01404.x

Billett EE, 2004, NEUROTOXICOLOGY, V25, P139, DOI 10.1016/S0161-813X(03)00094-9

BROWN C, 1989, J CLIN PHARMACOL, V29, P529, DOI 10.1002/j.1552-4604.1989.tb03376.x

Cantarini MV, 2004, BRIT J CLIN PHARMACO, V58, P470, DOI 10.1111/j.1365-2125.2004.02186.x

Cho YS, 2018, ANTIMICROB AGENTS CH, V62, DOI [10.1128/AAC.00451-18, 10.1128/aac.00451-18]

Cho YL, 2020, APPL SCI-BASEL, V10, DOI 10.3390/app10072211

Choi Y, 2018, J ANTIMICROB CHEMOTH, V73, P183, DOI 10.1093/jac/dkx367

Cole ST, 2017, DRUG DISCOV TODAY, V22, P477, DOI 10.1016/j.drudis.2016.09.026

Cooper, 2017, Patent WO, Patent No. 2017015106

Desai R., 2020, Patent WO, Patent No. 2020021468

DING CZ, 1993, J MED CHEM, V36, P3606, DOI 10.1021/jm00075a015

Ebner DC, 2008, BIOORGAN MED CHEM, V16, P2651, DOI 10.1016/j.bmc.2007.11.040

Gordeev MF, 2014, J MED CHEM, V57, P4487, DOI 10.1021/jm401931e

Gravestock MB, 2003, BIOORG MED CHEM LETT, V13, P4179, DOI 10.1016/j.bmcl.2003.07.033

GREGORY WA, 1989, J MED CHEM, V32, P1673, DOI 10.1021/jm00128a003

Guo SC, 2021, ANTIMICROB AGENTS CH, V65, DOI 10.1128/AAC.00974-21

Gutierrez-Lugo MT, 2008, J MED CHEM, V51, P2606, DOI 10.1021/jm070719i

Harris CR., 2006, 46 INTERSCIENCE C AN

Hendershot PE, 2001, J CLIN PHARMACOL, V41, P563, DOI 10.1177/00912700122010302

Ippoliti JT., 2018, Patent WO, Patent No. 2018157043

Ippolito JA, 2008, J MED CHEM, V51, P3353, DOI 10.1021/jm800379d

Jadhavar PS, 2015, CURR MED CHEM, V22, P4379, DOI 10.2174/0929867323666151106125759

Jeong JW, 2010, ANTIMICROB AGENTS CH, V54, P5359, DOI 10.1128/AAC.00723-10

Ji C, 2012, BIOORGAN MED CHEM, V20, P3422, DOI 10.1016/j.bmc.2012.04.026

Jiang JW, 2021, J CHROMATOGR B, V1167, DOI 10.1016/j.jchromb.2021.122552

Kamal A, 2013, EUR J MED CHEM, V64, P239, DOI 10.1016/j.ejmech.2013.03.027

Kamal A, 2011, EUR J MED CHEM, V46, P893, DOI 10.1016/j.ejmech.2010.12.028

Kanafani ZA, 2012, EXPERT OPIN INV DRUG, V21, P515, DOI 10.1517/13543784.2012.660250

Kaushik A, 2016, BIOORG MED CHEM LETT, V26, P3572, DOI 10.1016/j.bmcl.2016.06.019

Leach KL, 2007, MOL CELL, V26, P393, DOI 10.1016/j.molcel.2007.04.005

Leach KL, 2011, ANN NY ACAD SCI, V1222, P49, DOI 10.1111/j.1749-6632.2011.05962.x

Lee J., 2013, United States Patent, Patent No. [US8507540, 8507540]

Liang JC, 2012, CURR MICROBIOL, V64, P530, DOI 10.1007/s00284-012-0104-9

Livingston MG, 1996, DRUG SAFETY, V14, P219, DOI 10.2165/00002018-199614040-00002

Mai A, 2002, J MED CHEM, V45, P1180, DOI 10.1021/jm015578d

Makafe GG, 2016, ANTIMICROB AGENTS CH, V60, P3202, DOI 10.1128/AAC.00152-16

Mandal MB., 2017, Patent WO, Patent No. 2017070024

McKee EE, 2006, ANTIMICROB AGENTS CH, V50, P2042, DOI 10.1128/AAC.01411-05

McNeil MB, 2017, ANTIMICROB AGENTS CH, V61, DOI [10.1128/AAC.01296-17, 10.1128/aac.01296-17]

Vendrell MM, 2020, ANTIMICROB AGENTS CH, V64, DOI 10.1128/AAC.00356-20

Mikusová K, 2017, DRUG DISCOV TODAY, V22, P534, DOI 10.1016/j.drudis.2016.09.025

Mushtaq A, 2019, LANCET RESP MED, V7, P1011, DOI 10.1016/S2213-2600(19)30376-5

Phillips Oludotun Adebayo, 2012, Tuberc Res Treat, V2012, P289136, DOI 10.1155/2012/289136

Ramsay RR, 2003, MINI-REV MED CHEM, V3, P129, DOI 10.2174/1389557033405287

Richter E, 2007, ANTIMICROB AGENTS CH, V51, P1534, DOI 10.1128/AAC.01113-06

Ruiz P, 2019, ANTIMICROB AGENTS CH, V63, DOI 10.1128/AAC.01939-18

Shoen C, 2018, ANTIMICROB AGENTS CH, V62, DOI [10.1128/aac.00493-18, 10.1128/ACC00493-18;e00493-18, 10.1128/AAC.00493-18, 10.1128/aac.00493-18]

SLEE AM, 1987, ANTIMICROB AGENTS CH, V31, P1791, DOI 10.1128/AAC.31.11.1791

Stevens Dennis L, 2004, Expert Rev Anti Infect Ther, V2, P51, DOI 10.1586/14787210.2.1.51

Su J., 2018, Patent WO, Patent No. 2018175185

Sunwoo J, 2018, DRUG DES DEV THER, V12, P1707, DOI 10.2147/DDDT.S155657

Thomas KD, 2011, EUR J MED CHEM, V46, P4834, DOI 10.1016/j.ejmech.2011.07.049

Tiberi S, 2018, LANCET INFECT DIS, V18, pE183, DOI 10.1016/S1473-3099(18)30110-5

Vera-Cabrera L, 2006, ANTIMICROB AGENTS CH, V50, P3170, DOI 10.1128/AAC.00571-06

Vinh DC, 2009, J INFECTION, V59, pS59, DOI 10.1016/S0163-4453(09)60009-8

Wallis RS, 2014, PLOS ONE, V9, DOI 10.1371/journal.pone.0094462

Wallis RS, 2011, ANTIMICROB AGENTS CH, V55, P567, DOI 10.1128/AAC.01179-10

Wallis RS, 2010, J INFECT DIS, V202, P745, DOI 10.1086/655471

Wang D, 2014, J ANTIMICROB CHEMOTH, V69, P1711, DOI 10.1093/jac/dkt539

Wasserman S, 2016, EXPERT REV ANTI-INFE, V14, P901, DOI 10.1080/14787210.2016.1225498

WHO, 2010, TREATMENT OF TUBERCULOSIS: GUIDELINES, FOURTH EDITION, P1

Williams KN, 2009, ANTIMICROB AGENTS CH, V53, P1314, DOI 10.1128/AAC.01182-08

Williams KN, 2009, AM J RESP CRIT CARE, V180, P371, DOI 10.1164/rccm.200904-0611OC

Wilson DN, 2008, P NATL ACAD SCI USA, V105, P13339, DOI 10.1073/pnas.0804276105

Wu YC, 2019, BIOORG MED CHEM LETT, V29, P496, DOI 10.1016/j.bmcl.2018.12.012

Wu YC, 2018, EUR J MED CHEM, V158, P247, DOI 10.1016/j.ejmech.2018.09.004

Wu YQ, 2021, J MED CHEM, V64, P3234, DOI 10.1021/acs.jmedchem.0c02153

Xin QS, 2011, J MED CHEM, V54, P7493, DOI 10.1021/jm200614t

Yip PCW, 2013, INT J ANTIMICROB AG, V42, P96, DOI 10.1016/j.ijantimicag.2013.03.002

Zhang M, 2014, ANTIMICROB AGENTS CH, V58, P3217, DOI 10.1128/AAC.02410-14

Zhang S, 2016, ANTIMICROB AGENTS CH, V60, P2542, DOI 10.1128/AAC.02941-15

Zhang ZJ, 2014, INT J ANTIMICROB AG, V43, P231, DOI 10.1016/j.ijantimicag.2013.12.007

Zhao HY, 2020, J MED CHEM, V63, P9316, DOI 10.1021/acs.jmedchem.0c00500

Zhao HY, 2017, ACS MED CHEM LETT, V8, P533, DOI 10.1021/acsmedchemlett.7b00068

Zhao Y., 2018, China Patent, Patent No. [CN108976222, 108976222]

Zhao Y., 2018, China Patent, Patent No. [CN107721943, 107721943]

Zhu T, 2014, ANTIMICROB AGENTS CH, V58, P3306, DOI 10.1128/AAC.01920-13

Zong Zhaojing, 2018, Antimicrob Agents Chemother, V62, DOI 10.1128/AAC.00165-18

Zumla A, 2013, NEW ENGL J MED, V368, P745, DOI 10.1056/NEJMra1200894

Zurenko GE, 1996, ANTIMICROB AGENTS CH, V40, P839, DOI 10.1128/AAC.40.4.839

NR 109

TC 14

Z9 15

U1 3

U2 31

PU Newlands Press Ltd

PI London

PA 2 Albert Pl, London, UNITED KINGDOM

SN 1756-8919

EI 1756-8927

J9 FUTURE MED CHEM

JI Future Med. Chem.

PD AUG

PY 2022

VL 14

IS 15

BP 1149

EP 1165

DI 10.4155/fmc-2022-0079

EA JUL 2022

PG 17

WC Chemistry, Medicinal

WE Science Citation Index Expanded (SCI-EXPANDED)

SC Pharmacology & Pharmacy

GA 3S3RB

UT WOS:000829850000001

DA 2026-05-07

ER

PT J

AU Shahi, F

Khosravi, AD

Tabandeh, MR

Salmanzadeh, S

AF Shahi, Fatemeh

Khosravi, Azar Dokht

Tabandeh, Mohammad Reza

Salmanzadeh, Shokrollah

TI Investigation of the Rv3065, Rv2942, Rv1258c, Rv1410c, and

Rv2459 efflux pump genes expression among multidrug-resistant

Mycobacterium tuberculosis clinical isolates

SO HELIYON

LA English

DT Article

DE Efflux pumps; MDR-TB; Mutation; Tuberculosis

ID DRUG-RESISTANCE; SUSCEPTIBILITY; MECHANISMS; STRAINS; ASSAY

AB Background: Different resistance mechanisms for multidrug-resistant tuberculosis (MDR-TB) and extensively drug-resistant tuberculosis (XDR-TB) have been reported. Although mutations in target genes are the main cause of drug resistance, efflux pumps (Eps) also play an important role in this process. Here, we investigated the over-expression of five putative EP genes plus gene mutations in MDR-TB clinical isolates.

Methods: A total of 27 M. tuberculosis (Mtb) clinical isolates including, 22 MDR and 5 sensitive isolates were analyzed. Minimum inhibitory concentrations (MIC) were determined in the absence and presence of efflux in-hibitor. The expression level of 5 EP genes (Rv3065, Rv2942, Rv1258c, Rv1410c, Rv2459) was investigated by quantitative real time PCR (RT-qPCR). DNA sequencing of rpoB, katG, and inhA promoter was done.

Results: Among the 22 MDR-TB isolates, 13 (59.1%) showed significant overexpression (>4-fold) for at least one EP gene. The expression levels of 5 genes were significantly higher (P < 0.05) in MDR-TB isolates than sensitive isolates. The Rv3065 (22.7%), and Rv1410c (18.2%) were found to be the most commonly overexpressed EPs. The observed MICs were as follows: RIF (2 to >128 mu g/ml) and INH (2-32 mu g/ml). After efflux pump inhibitor exposure, 10/22 (45.45%) isolates showed a decrease in MIC of INH, and 17/22 (77.27%) isolates showed a decrease in MIC of RIF. Of the isolates that overexpressed, 4 isolates lacked mutation in inhA, rpoB, and katG genes and 10 ones lacked mutation in inhA and katG.

Conclusion: The results showed that overexpression of EP genes in Mtb isolates, besides target gene mutations can contribute to the development of MDR phenotype.

C1 [Shahi, Fatemeh; Khosravi, Azar Dokht; Salmanzadeh, Shokrollah] Ahvaz Jundishapur Univ Med Sci, Infect & Trop Dis Res Ctr, Hlth Res Inst, Ahvaz, Iran.

[Shahi, Fatemeh; Khosravi, Azar Dokht] Ahvaz Jundishapur Univ Med Sci, Sch Med, Dept Microbiol, Ahvaz, Iran.

[Khosravi, Azar Dokht] Iranian Study Grp Microbial Drug Resistance, Ahvaz, Iran.

[Tabandeh, Mohammad Reza] Shahid Chamran Univ Ahvaz, Fac Vet Med, Dept Basic Sci, Div Biochem & Mol Biol, Ahvaz, Iran.

[Salmanzadeh, Shokrollah] Ahvaz Jundishapur Univ Med Sci, Razi Teaching Hosp, Trop Med Ward, Ahvaz, Iran.

C3 Ahvaz Jundishapur University of Medical Sciences (AJUMS); Ahvaz

Jundishapur University of Medical Sciences (AJUMS); Shahid Chamran

University of Ahvaz; Ahvaz Jundishapur University of Medical Sciences

(AJUMS)

RP Khosravi, AD (corresponding author), Ahvaz Jundishapur Univ Med Sci, Infect & Trop Dis Res Ctr, Hlth Res Inst, Ahvaz, Iran.; Khosravi, AD (corresponding author), Ahvaz Jundishapur Univ Med Sci, Sch Med, Dept Microbiol, Ahvaz, Iran.; Khosravi, AD (corresponding author), Iranian Study Grp Microbial Drug Resistance, Ahvaz, Iran.

EM azarkhosravi69@gmail.com

RI Khosravi, Azar/M-9244-2017; tabandeh, mohammad/AAA-4855-2019; shahi,

fatemeh/F-9947-2019; Salmanzadeh, Shokrollah/R-7800-2019

FU Ahvaz Jundishapur University of Medical Sciences, Ahvaz, Iran [OG-9736]

FX This study is a part of Ph.D. thesis by Fatemeh Shahi, which was

approved in Infectious and Tropical Diseases Research Center, and was

financially supported by a grant (OG-9736) from Research Affairs, Ahvaz

Jundishapur University of Medical Sciences, Ahvaz, Iran.

CR [Anonymous], 2019, WHO GUIDELINE RECOMM

Balganesh M, 2012, ANTIMICROB AGENTS CH, V56, P2643, DOI 10.1128/AAC.06003-11

Böttger EC, 2011, CLIN MICROBIOL INFEC, V17, P1128, DOI 10.1111/j.1469-0691.2011.03551.x

Calgin MK, 2013, DIAGN MICR INFEC DIS, V76, P291, DOI 10.1016/j.diagmicrobio.2013.02.033

Fonseca JD, 2015, INT J INFECT DIS, V32, P94, DOI 10.1016/j.ijid.2015.01.014

Ghajavand H, 2019, ANTIMICROB RESIST IN, V8, DOI 10.1186/s13756-019-0516-4

Iacobino A, 2020, APPL SCI-BASEL, V10, DOI 10.3390/app10062153

Jaiswal I, 2017, LUNG INDIA, V34, P499, DOI 10.4103/0970-2113.217567

Jiang X, 2008, MICROB DRUG RESIST, V14, P7, DOI 10.1089/mdr.2008.0772

Kardan-Yamchi J, 2019, J GLOB ANTIMICROB RE, V17, P201, DOI 10.1016/j.jgar.2019.01.003

Li GL, 2015, PLOS ONE, V10, DOI 10.1371/journal.pone.0119013

Livak KJ, 2001, METHODS, V25, P402, DOI 10.1006/meth.2001.1262

Machado D, 2017, FRONT MICROBIOL, V8, DOI 10.3389/fmicb.2017.00711

Machado D, 2016, PLOS ONE, V11, DOI 10.1371/journal.pone.0149326

Machado D, 2012, PLOS ONE, V7, DOI 10.1371/journal.pone.0034538

Matteelli A, 2014, CLIN EPIDEMIOL, V6, P111, DOI 10.2147/CLEP.S35839

Narang A, 2019, PLOS ONE, V14, DOI 10.1371/journal.pone.0223163

Nateche F, 2006, J MED MICROBIOL, V55, P857, DOI 10.1099/jmm.0.46513-0

Oh TS, 2017, INFECT GENET EVOL, V49, P111, DOI 10.1016/j.meegid.2017.01.002

Palomino JC, 2002, ANTIMICROB AGENTS CH, V46, P2720, DOI 10.1128/AAC.46.8.2720-2722.2002

Palomino JC, 2014, ANTIBIOTICS-BASEL, V3, P317, DOI 10.3390/antibiotics3030317

Raviglione M, 2016, INFECT DIS REP, V8, P33, DOI 10.4081/idr.2016.6570

Sarathy JP, 2012, PHARMACEUTICALS-BASE, V5, P1210, DOI 10.3390/ph5111210

Seifert M, 2015, PLOS ONE, V10, DOI 10.1371/journal.pone.0119628

Sirous M, 2018, INFECT DRUG RESIST, V11, P1819, DOI 10.2147/IDR.S178831

Taneja NK, 2007, J ANTIMICROB CHEMOTH, V60, P288, DOI 10.1093/jac/dkm207

Velayati AA, 2009, CHEST, V136, P420, DOI 10.1378/chest.08-2427

WHO, 2019, Joint Special Administrators' Second Progress Report (Report Period 5 February 2020 to 4 August 2020)

Yamchi JK, 2015, INFECT GENET EVOL, V36, P23, DOI 10.1016/j.meegid.2015.08.036

NR 31

TC 14

Z9 17

U1 2

U2 9

PU CELL PRESS

PI CAMBRIDGE

PA 50 HAMPSHIRE ST, FLOOR 5, CAMBRIDGE, MA 02139 USA

EI 2405-8440

J9 HELIYON

JI Heliyon

PD JUL

PY 2021

VL 7

IS 7

AR e07566

DI 10.1016/j.heliyon.2021.e07566

EA JUL 2021

PG 6

WC Multidisciplinary Sciences

WE Science Citation Index Expanded (SCI-EXPANDED)

SC Science & Technology - Other Topics

GA UD5UJ

UT WOS:000687271200038

OA Green Submitted, gold

DA 2026-05-07

ER

PT J

AU Khosravi, AD

Tabandeh, MR

Shahi, F

Salmanzadeh, S

AF Khosravi, Azar Dokht

Tabandeh, Mohammad Reza

Shahi, Fatemeh

Salmanzadeh, Shokrollah

TI Linezolid resistance among multidrug-resistant Mycobacterium

tuberculosis clinical isolates in Iran

SO ACTA MICROBIOLOGICA ET IMMUNOLOGICA HUNGARICA

LA English

DT Article

DE Mycobacterium tuberculosis; multidrug resistance; linezolid; mutation;

minimum inhibitory concentration

ID EXTENSIVELY DRUG-RESISTANT; MUTATIONS; STRAINS

AB The management of multidrug-resistant (MDR) and extensively drug-resistant tuberculosis (XDR-TB) presents a main challenge and the drug options for treating these infections are very limited. Linezolid (LNZ) has recently been approved for the treatment of MDR and XDR-TB. But, there are narrow data on genotypic and phenotypic LNZ resistance in clinical isolates. So, we aimed to determine the prevalence of LNZ resistance and to identify the mutations associated with LNZ resistance among clinical MDR-TB isolates. The minimum inhibitory concentration (MIC) values of LNZ, for 22 MDR-TB isolates were determined by broth microdilution method. All MDR-TB isolates were sequenced in the rrl and rp1C genes conferring LNZ, resistance. LNZ, resistance was found in 3 (13.6%) of 22 MDR-TB isolates. The MICs of LNZ were 8 mu g/mL for two isolates and 16 mu g/mL for one isolate. The 421 (A/G) and 449 (T/A) mutations in rplC gene were detected in one of the LNZ-resistant isolates. There was no mutation in rrl gene. The results reveal that the prevalence of LNZ-resistant isolates is 13.6% among MDR-TB isolates and drug susceptibility testing (DST) against LNZ is useful in the management of complicated and drug-resistant cases. However, further studies could identify other possible genetic mechanism of resistance in TB.

C1 [Khosravi, Azar Dokht; Shahi, Fatemeh; Salmanzadeh, Shokrollah] Ahvaz Jundishapur Univ Med Sci, Infect & Trop Dis Res Ctr, Hlth Res Inst, Ahvaz, Iran.

[Khosravi, Azar Dokht; Shahi, Fatemeh] Ahvaz Jundishapur Univ Med Sci, Sch Med, Dept Microbiol, Ahvaz, Iran.

[Khosravi, Azar Dokht] Iranian Study Grp Microbial Drug Resistance, Ahvaz, Iran.

[Tabandeh, Mohammad Reza] Shahid Chamran Univ Ahvaz, Fac Vet Med, Dept Basic Sci, Div Biochem & Mol Biol, Ahvaz, Iran.

[Salmanzadeh, Shokrollah] Ahvaz Jundishapur Univ Med Sci, Razi Teaching Hosp, Trop Med Ward, Ahvaz, Iran.

C3 Ahvaz Jundishapur University of Medical Sciences (AJUMS); Ahvaz

Jundishapur University of Medical Sciences (AJUMS); Shahid Chamran

University of Ahvaz; Ahvaz Jundishapur University of Medical Sciences

(AJUMS)

RP Shahi, F (corresponding author), Ahvaz Jundishapur Univ Med Sci, Infect & Trop Dis Res Ctr, Hlth Res Inst, Ahvaz, Iran.; Shahi, F (corresponding author), Ahvaz Jundishapur Univ Med Sci, Sch Med, Dept Microbiol, Ahvaz, Iran.

EM fatemehshahi87@gmail.com

RI Shahi, Fatemeh/F-9947-2019; Salmanzadeh, Shokrollah/R-7800-2019;

Khosravi, Azar/M-9244-2017; tabandeh, mohammad/AAA-4855-2019

OI Shahi, Fatemeh/0000-0003-4069-7423;

FU Research Affairs, Ahvaz Jundishapur University of Medical Sciences,

Ahvaz, Iran [OG-9736]

FX This study is a part of Ph.D. thesis by Fatemeh Shahi, which was

approved in Infectious and Tropical Diseases Research Center, and was

financially supported by a grant (OG-9736) from Research Affairs, Ahvaz

Jundishapur University of Medical Sciences, Ahvaz, Iran. We are grateful

to the Research Affairs of the university for the financial support of

the study. We are also greatly thankful to the staff of Regional TB

Reference Laboratory especially Nazanin Ahmad Khosravi for their

collaboration.

CR Agyeman AA, 2016, ANN CLIN MICROB ANTI, V15, DOI 10.1186/s12941-016-0156-y

Ahmed I, 2013, ANTIMICROB AGENTS CH, V57, P2522, DOI 10.1128/AAC.02020-12

Da Silva PEA, 2011, J ANTIMICROB CHEMOTH, V66, P1417, DOI 10.1093/jac/dkr173

Beckert P, 2012, ANTIMICROB AGENTS CH, V56, P2743, DOI 10.1128/AAC.06227-11

Escribano I, 2007, CHEMOTHERAPY, V53, P397, DOI 10.1159/000109769

Fortún J, 2005, J ANTIMICROB CHEMOTH, V56, P180, DOI 10.1093/jac/dki148

Hillemann D, 2008, ANTIMICROB AGENTS CH, V52, P800, DOI 10.1128/AAC.01189-07

Hu MH, 2020, INFECT DRUG RESIST, V13, P1751, DOI 10.2147/IDR.S257145

Kazemian H, 2015, INT J ANTIMICROB AG, V45, P668, DOI 10.1016/j.ijantimicag.2015.02.004

Klitgaard RN, 2015, ANTIMICROB AGENTS CH, V59, P3518, DOI 10.1128/AAC.00179-15

Organization WHO, 2020, WHO CONS GUID TUB MO

Richter E, 2007, ANTIMICROB AGENTS CH, V51, P1534, DOI 10.1128/AAC.01113-06

Sander P, 2002, MOL MICROBIOL, V46, P1295, DOI 10.1046/j.1365-2958.2002.03242.x

Singh B, 2019, COCHRANE DB SYST REV, DOI 10.1002/14651858.CD012836.pub2

Singla R, 2012, EUR RESPIR J, V39, P956, DOI 10.1183/09031936.00076811

Sotgiu G, 2012, EUR RESPIR J, V40, P1430, DOI 10.1183/09031936.00022912

Tornheim JA, 2020, J CLIN TUBER OTH MYC, V20, DOI 10.1016/j.jctube.2020.100175

Wasserman S, 2019, J ANTIMICROB CHEMOTH, V74, P2377, DOI 10.1093/jac/dkz206

WHO, 2019, GLOBAL TUBERCULOSIS

World Health Organization, 2018, WHO/HTM/TB/2018.392

Yang JS, 2018, ANN LAB MED, V38, P563, DOI 10.3343/alm.2018.38.6.563

Zhang S, 2016, ANTIMICROB AGENTS CH, V60, P2542, DOI 10.1128/AAC.02941-15

Zhang ZJ, 2014, INT J ANTIMICROB AG, V43, P231, DOI 10.1016/j.ijantimicag.2013.12.007

NR 23

TC 5

Z9 5

U1 0

U2 3

PU AKADEMIAI KIADO ZRT

PI BUDAPEST

PA BUDAFOKI UT 187-189-A-3, H-1117 BUDAPEST, HUNGARY

SN 1217-8950

EI 1588-2640

J9 ACTA MICROBIOL IMM H

JI Acta Microbiol. Immunol. Hung.

PD SEP

PY 2021

VL 68

IS 3

BP 203

EP 207

DI 10.1556/030.2021.01490

PG 5

WC Immunology; Microbiology

WE Science Citation Index Expanded (SCI-EXPANDED)

SC Immunology; Microbiology

GA UN4WX

UT WOS:000694018400010

OA hybrid

DA 2026-05-07

ER

PT J

AU Singh, R

Kumar, S

Bhardwaj, VK

Purohit, R

AF Singh, Rahul

Kumar, Sachin

Bhardwaj, Vijay Kumar

Purohit, Rituraj

TI Screening and reckoning of potential therapeutic agents against DprE1

protein of Mycobacterium tuberculosis

SO JOURNAL OF MOLECULAR LIQUIDS

LA English

DT Article

DE Mycobacterium tuberculosis; DprE1; Molecular dynamic simulation;

MM-PBSA; MDR-TB; XDR-TB

ID INHIBITION; TOOL

AB Tuberculosis is caused by Mycobacterium tuberculosis, a resilient microbe that can survive within the long courses of medications as well as several years of dormancy in the host system. Although tuberculosis is a preventable and curable disease, however, the rise of multidrug-resistant tuberculosis (MDR-TB) and extensively drug-resistant tuberculosis (XDR-TB) have made remedy more difficult and a major concern to public health. In Mycobacterium tuberculosis, DprE1 is a key isomerase enzyme that has a vital function in cell wall synthesis. Moreover, DprE1 has also been recognized as a crucial target for drug development against tuberculosis. In the present study, molecular docking, dynamics simulations, and thermodynamic free energy calculations were used to analyze the ability of aminoarylbenzosuberene (AAB) molecules to inhibit DprE1 in comparison to six co-crystallized DprE1 inhibitors. This study identified a promising molecule (AAB4) with better stability and binding affinity than the co-crystallized inhibitors. Moreover, AAB4 molecule could also inhibit (Y314C)-DprE1 protein (a TCA1 inhibitor resistance mutant). Thus, the molecule AAB4 could be used as potential lead against tuberculosis and would be an easy pick for in-vitro and in-vivo studies.(c) 2022 Elsevier B.V. All rights reserved.

C1 [Singh, Rahul; Kumar, Sachin; Bhardwaj, Vijay Kumar; Purohit, Rituraj] Inst Himalayan Bioresource Technol CSIR IHBT, Struct Bioinformat Lab, CSIR, Palampur 176061, Himachal Prades, India.

[Singh, Rahul; Kumar, Sachin; Bhardwaj, Vijay Kumar; Purohit, Rituraj] CSIR IHBT, Biotechnol Div, Palampur 176061, Himachal Prades, India.

[Singh, Rahul; Kumar, Sachin; Bhardwaj, Vijay Kumar; Purohit, Rituraj] Acad Sci & Innovat Res AcSIR, Ghaziabad, Uttar Pradesh, India.

C3 Council of Scientific & Industrial Research (CSIR) - India; CSIR -

Institute of Himalayan Bioresource Technology (IHBT); Council of

Scientific & Industrial Research (CSIR) - India; CSIR - Institute of

Himalayan Bioresource Technology (IHBT); Academy of Scientific &

Innovative Research (AcSIR)

RP Purohit, R (corresponding author), Inst Himalayan Bioresource Technol CSIR IHBT, Struct Bioinformat Lab, CSIR, Palampur 176061, Himachal Prades, India.

EM rituraj@ihbt.res.in

RI ; Singh, Rahul/U-1327-2018; Bhardwaj, Vijay/LMO-7994-2024; Purohit,

Rituraj/AAF-6257-2021

OI Kumar, Sachin/0000-0002-6675-3553; Kumar, Sachin/0000-0003-3862-5287;

Singh, Rahul/0000-0003-1213-2708; Bhardwaj, Vijay/0000-0001-9511-6206;

FU Department of Science and Technology, New Delhi [ECR/2016/000031]; Board

of Research in Nuclear Sciences, Department of Atomic Energy, Mumbai,

India [37 (1) /14/26/2015/BRNS]; Indian Council of Medical Research, New

Delhi, India; Council of Scientific and Industrial Research, New Delhi,

India; CSIR

FX RP gratefully acknowledges the Department of Science and Technology, New

Delhi (SERB File No: ECR/2016/000031) and Board of Research in Nuclear

Sciences, Department of Atomic Energy, Mumbai, India for financial

support (Letter No: 37 (1) /14/26/2015/BRNS) . R. S. expresses gratitude

to the Indian Council of Medical Research, New Delhi, India for

providing Senior Research Fellowship. S. K. expresses gratitude to the

Council of Scientific and Industrial Research, New Delhi, India for

providing the Junior and Senior Research Fellowship and V. K. B.

expresses grat-itude to the Council of Scientific and Industrial

Research of India for GATE Fellowship New Delhi, India. The CSIR support

in the form of projects MLP:0201 for bioinformatics studies is highly

acknowl-edged. We also acknowledge the CSIR-Institute of Himalayan

Bioresource Technology, Palampur for providing the facilities to carry

out this work. This manuscript represents CSIR-IHBT commu-nication no.

5040.

CR Abraham Mark James, 2015, SoftwareX, V1-2, P19, DOI [10.1016/j.softx.2015.06.001, 10.1016/j.softx.2015.06.001]

Aljindan RY, 2021, COMPUT BIOL MED, V135, DOI 10.1016/j.compbiomed.2021.104654

Barry CE, 1996, TRENDS MICROBIOL, V4, P275, DOI 10.1016/0966-842X(96)10031-7

Batt SM, 2012, P NATL ACAD SCI USA, V109, P11354, DOI 10.1073/pnas.1205735109

BERENDSEN HJC, 1984, J CHEM PHYS, V81, P3684, DOI 10.1063/1.448118

Bhardwaj VK, 2022, INT J BIOL MACROMOL, V208, P11, DOI 10.1016/j.ijbiomac.2022.03.021

Bhardwaj VK, 2021, J MOL LIQ, V328, DOI 10.1016/j.molliq.2021.115419

Bhardwaj VK, 2021, FOOD CHEM, V347, DOI 10.1016/j.foodchem.2020.128932

Bharti R, 2017, APPL ORGANOMET CHEM, V31, DOI 10.1002/aoc.3749

Brecik M, 2015, ACS CHEM BIOL, V10, P1631, DOI 10.1021/acschembio.5b00237

BRENNAN PJ, 1995, ANNU REV BIOCHEM, V64, P29, DOI 10.1146/annurev.bi.64.070195.000333

Dupont C, 2019, J BIOL CHEM, V294, P17512, DOI 10.1074/jbc.RA119.010135

Gao Y, 2019, BIOORG CHEM, V85, P168, DOI 10.1016/j.bioorg.2018.12.018

Gopalakrishnan C, 2019, J CELL BIOCHEM, V120, P18496, DOI 10.1002/jcb.29172

Kamaraj B, 2021, J BIOMOL STRUCT DYN, V39, P5235, DOI 10.1080/07391102.2020.1784790

Kaufmann SHE, 2017, INT J INFECT DIS, V56, P263, DOI 10.1016/j.ijid.2016.10.018

Kim S, 2016, NUCLEIC ACIDS RES, V44, pD1202, DOI 10.1093/nar/gkv951

Kumar S, 2022, J CELL BIOCHEM, V123, P935, DOI 10.1002/jcb.30237

Kumar S, 2021, J MOL LIQ, V339, DOI 10.1016/j.molliq.2021.116798

Kumar SU, 2020, MOLECULES, V25, DOI 10.3390/molecules25235543

Kumari R, 2014, J CHEM INF MODEL, V54, P1951, DOI 10.1021/ci500020m

Kurz SG, 2016, INFECT DIS CLIN N AM, V30, P509, DOI 10.1016/j.idc.2016.02.010

Liu RH, 2017, ANGEW CHEM INT EDIT, V56, P13011, DOI 10.1002/anie.201707324

Naik M, 2014, J MED CHEM, V57, P5419, DOI 10.1021/jm5005978

Neres J, 2015, ACS CHEM BIOL, V10, P705, DOI 10.1021/cb5007163

Organization W.H., 2020, World Health Organization Global Tuberculosis Report 2020, P232

Richter A, 2018, SCI REP-UK, V8, DOI 10.1038/s41598-018-31316-6

San Diego: Accelrys Software Inc, 2012, Discovery Studio Modeling Environment

Schüttelkopf AW, 2004, ACTA CRYSTALLOGR D, V60, P1355, DOI 10.1107/S0907444904011679

Singh R, 2022, CHEM COMMUN, V58, P5005, DOI 10.1039/d1cc06894f

Turner P., 2005, Center for Coastal and Land-Margin Research

Umare MD, 2021, CHEMMEDCHEM, V16, P3136, DOI 10.1002/cmdc.202100359

Warrier T, 2016, P NATL ACAD SCI USA, V113, pE4523, DOI 10.1073/pnas.1606590113

Wu GS, 2003, J COMPUT CHEM, V24, P1549, DOI 10.1002/jcc.10306

Zheng JJ, 2017, J CHEM THEORY COMPUT, V13, P6424, DOI 10.1021/acs.jctc.7b00719

NR 35

TC 21

Z9 21

U1 0

U2 6

PU ELSEVIER

PI AMSTERDAM

PA RADARWEG 29, 1043 NX AMSTERDAM, NETHERLANDS

SN 0167-7322

EI 1873-3166

J9 J MOL LIQ

JI J. Mol. Liq.

PD JUL 15

PY 2022

VL 358

AR 119101

DI 10.1016/j.molliq.2022.119101

EA APR 2022

PG 8

WC Chemistry, Physical; Physics, Atomic, Molecular & Chemical

WE Science Citation Index Expanded (SCI-EXPANDED)

SC Chemistry; Physics

GA 1L4PF

UT WOS:000799271500003

DA 2026-05-07

ER

PT J

AU Sowajassatakul, A

Prammananan, T

Chaiprasert, A

Phunpruch, S

AF Sowajassatakul, Angkanang

Prammananan, Therdsak

Chaiprasert, Angkana

Phunpruch, Saranya

TI Molecular characterization of amikacin, kanamycin and capreomycin

resistance in M/XDR-TB strains isolated in Thailand

SO BMC MICROBIOLOGY

LA English

DT Article

DE Tuberculosis; Second-line drug; Resistance; Aminoglycoside

ID MYCOBACTERIUM-TUBERCULOSIS STRAINS; GENOTYPE MTBDRPLUS ASSAY;

CROSS-RESISTANCE; ACETYLTRANSFERASE EIS; DRUG-RESISTANCE;

GENETIC-MARKER; MUTATIONS; MULTIDRUG; RIFAMPIN; IDENTIFICATION

AB Background: The emergence of multidrug-resistant tuberculosis (MDR-TB) and extensively drug-resistant tuberculosis (XDR-TB) makes the treatment and control of tuberculosis difficult. Rapid detection of drug-resistant strains is important for the successful treatment of drug-resistant tuberculosis; however, not all resistance mechanisms to the injectable second-line drugs such as amikacin (AK), kanamycin (KM), and capreomycin (CAP) are well understood. This study aims to validate the mechanisms associated with AK, KM, and CAP resistance in M. tuberculosis clinical strains isolated in Thailand.

Results: A total of 15,124 M. tuberculosis clinical strains were isolated from 23,693 smear-positive sputum samples sent from 288 hospitals in 46 of 77 provinces of Thailand. Phenotypic analysis identified 1,294 strains as MDR-TB and second-line drugs susceptibility was performed in all MDR-TB strains and revealed 58 XDR-TB strains. Twenty-nine KM-resistant strains (26 XDR-TB and 3 MDR-TB) could be retrieved and their genes associated with AK, KM, and CAP resistance were investigated compared with 27 KM-susceptible strains. Mutation of the rrs (A1401G) was found in 21 out of 29 KM-resistant strains whereas mutations of eis either at C-14 T or at G-37 T were found in 5 strains. Three remaining KM-resistant strains did not contain any known mutations. Capreomycin resistance was determined in 28 of 29 KM-resistant strains. Analysis of tlyA revealed that the A33G mutation was found in all CAP-resistant strains and also in susceptible strains. In contrast, the recently identified tlyA mutation T539G and the novel Ins49GC were found in two and one CAP-resistant strains, respectively. In addition, our finding demonstrated the insertion of cytosine at position 581 of the tap, a putative drug efflux encoding gene, in both KM-resistant and KM-susceptible strains.

Conclusions: Our finding demonstrated that the majority of KM resistance mechanism in Thai M. tuberculosis clinical strains was rrs mutation at A1401G. Mutations of the eis promoter region either at C-14 T or G-37 T was found in 5 of 29 strains whereas three strains did not contain any known mutations. For CAP resistance, 3 of 28 CAP-resistant strains contained either T539G or Ins49GC mutations at tlyA that might be associated with the resistant phenotype.

C1 [Sowajassatakul, Angkanang; Phunpruch, Saranya] King Mongkuts Inst Technol Ladkrabang, Fac Sci, Dept Biol, Bangkok 10520, Thailand.

[Prammananan, Therdsak] Natl Sci & Technol Dev Agcy, Natl Ctr Genet Engn & Biotechnol, TB Res Lab, Pathum Thani 12120, Thailand.

[Chaiprasert, Angkana] Mahidol Univ, Siriraj Hosp, Fac Med, Dept Microbiol, Bangkok 10700, Thailand.

[Prammananan, Therdsak; Chaiprasert, Angkana] Siriraj Fdn, Drug Resistance TB Res Fund, Bangkok 10700, Thailand.

C3 King Mongkuts Institute of Technology Ladkrabang; National Science &

Technology Development Agency - Thailand; National Center Genetic

Engineering & Biotechnology (BIOTEC); Mahidol University

RP Phunpruch, S (corresponding author), King Mongkuts Inst Technol Ladkrabang, Fac Sci, Dept Biol, Bangkok 10520, Thailand.

EM kpsarany@kmitl.ac.th

OI Phunpruch, Saranya/0000-0003-0755-0833

FU Faculty of Science, King Mongkut's Institute of Technology Ladkrabang

(KMITL); Drug-Resistant Tuberculosis Research Fund, Siriraj Foundation,

Faculty of Medicine Siriraj Hospital, Mahidol University; Thailand

Graduate Institute of Science and Technology (TGIST), National Science

and Technology Development Agency (NSTDA)

FX This study was financially supported by the Faculty of Science, King

Mongkut's Institute of Technology Ladkrabang (KMITL) and the

Drug-Resistant Tuberculosis Research Fund, Siriraj Foundation, Faculty

of Medicine Siriraj Hospital, Mahidol University. A. Sowajassatakul is

also thankful for a scholarship for the Ph.D. Program that was provided

by the Thailand Graduate Institute of Science and Technology (TGIST),

National Science and Technology Development Agency (NSTDA).

CR Aínsa JA, 1998, J BACTERIOL, V180, P5836

Ajbani K, 2011, J CLIN MICROBIOL, V49, P1588, DOI 10.1128/JCM.00113-11

Akbergenov R, 2011, ANTIMICROB AGENTS CH, V55, P4712, DOI 10.1128/AAC.00628-11

[Anonymous], 2003, M24A NAT COMM CLIN L

[Anonymous], 2015, GLOBAL TUBERCULOSIS

Blakemore R, 2010, J CLIN MICROBIOL, V48, P2495, DOI 10.1128/JCM.00128-10

Boehme CC, 2010, NEW ENGL J MED, V363, P1005, DOI 10.1056/NEJMoa0907847

Brossier F, 2010, J CLIN MICROBIOL, V48, P1683, DOI 10.1128/JCM.01947-09

Campbell PJ, 2011, ANTIMICROB AGENTS CH, V55, P2032, DOI 10.1128/AAC.01550-10

Chaiprasert Angkana, 2006, Southeast Asian Journal of Tropical Medicine and Public Health, V37, P494

Chen WJ, 2011, P NATL ACAD SCI USA, V108, P9804, DOI 10.1073/pnas.1105379108

Daum LT, 2012, J CLIN MICROBIOL, V50, P3831, DOI 10.1128/JCM.01893-12

De Rossi Edda, 2002, Molecular Medicine (Baltimore), V8, P714

Engström A, 2011, J ANTIMICROB CHEMOTH, V66, P1247, DOI 10.1093/jac/dkr109

Evans J, 2010, J ANTIMICROB CHEMOTH, V65, P897, DOI 10.1093/jac/dkq047

Feuerriegel S, 2009, ANTIMICROB AGENTS CH, V53, P3353, DOI 10.1128/AAC.00050-09

Georghiou SB, 2012, PLOS ONE, V7, DOI 10.1371/journal.pone.0033275

Gikalo MB, 2012, J ANTIMICROB CHEMOTH, V67, P2107, DOI 10.1093/jac/dks178

Helb D, 2010, J CLIN MICROBIOL, V48, P229, DOI 10.1128/JCM.01463-09

Hillemann D, 2007, J CLIN MICROBIOL, V45, P2635, DOI 10.1128/JCM.00521-07

Jin J, 2012, INT J TUBERC LUNG D, V16, P521, DOI 10.5588/ijtld.11.0269

Jnawali HN, 2013, WORLD J MICROB BIOT, V29, P975, DOI 10.1007/s11274-013-1256-x

Johansen SK, 2006, MOL CELL, V23, P173, DOI 10.1016/j.molcel.2006.05.044

Jugheli L, 2009, ANTIMICROB AGENTS CH, V53, P5064, DOI 10.1128/AAC.00851-09

Köser CU, 2013, ANTIMICROB AGENTS CH, V57, P3461, DOI 10.1128/AAC.00626-13

Krüüner A, 2003, ANTIMICROB AGENTS CH, V47, P2971, DOI 10.1128/AAC.47.9.2971-2973.2003

Lacoma A, 2008, J CLIN MICROBIOL, V46, P3660, DOI 10.1128/JCM.00618-08

Laszlo A, 2002, INT J TUBERC LUNG D, V6, P748

Maus CE, 2005, ANTIMICROB AGENTS CH, V49, P3192, DOI 10.1128/AAC.49.8.3192-3197.2005

Maus CE, 2005, ANTIMICROB AGENTS CH, V49, P571, DOI 10.1128/AAC.49.2.571-577.2005

Morris RP, 2005, P NATL ACAD SCI USA, V102, P12200, DOI 10.1073/pnas.0505446102

Perdigao J, 2010, J ANTIMICROB CHEMOTH, V65, P224, DOI 10.1093/jac/dkp452

Recht MI, 1999, EMBO J, V18, P3133, DOI 10.1093/emboj/18.11.3133

Reeves AZ, 2013, ANTIMICROB AGENTS CH, V57, P1857, DOI 10.1128/AAC.02191-12

Siddiqi N, 2004, INFECTION, V32, P109, DOI 10.1007/s15010-004-3097-x

Sirgel FA, 2012, MICROB DRUG RESIST, V18, P193, DOI 10.1089/mdr.2011.0063

Suzuki Y, 1998, J CLIN MICROBIOL, V36, P1220, DOI 10.1128/JCM.36.5.1220-1225.1998

THOMPSON JD, 1994, NUCLEIC ACIDS RES, V22, P4673, DOI 10.1093/nar/22.22.4673

Via LE, 2010, J CLIN MICROBIOL, V48, P402, DOI 10.1128/JCM.01476-09

Villellas C, 2013, J CLIN MICROBIOL, V51, P2124, DOI 10.1128/JCM.01944-12

Vo SK, 2010, J CLIN MICROBIOL, V48, P2934, DOI 10.1128/JCM.00201-10

Yuan XL, 2012, J CLIN MICROBIOL, V50, P2404, DOI 10.1128/JCM.06860-11

Zaunbrecher MA, 2009, P NATL ACAD SCI USA, V106, P20004, DOI 10.1073/pnas.0907925106

NR 43

TC 58

Z9 69

U1 0

U2 17

PU BMC

PI LONDON

PA CAMPUS, 4 CRINAN ST, LONDON N1 9XW, ENGLAND

SN 1471-2180

J9 BMC MICROBIOL

JI BMC Microbiol.

PD JUN 22

PY 2014

VL 14

AR 165

DI 10.1186/1471-2180-14-165

PG 7

WC Microbiology

WE Science Citation Index Expanded (SCI-EXPANDED)

SC Microbiology

GA AK1GY

UT WOS:000338164700001

OA Green Submitted, gold

DA 2026-05-07

ER

PT J

AU Ouyang, Q

Zhang, KH

Lin, DC

Feng, CG

Cai, Y

Chen, XC

AF Ouyang, Qi

Zhang, Kehong

Lin, Dachuan

Feng, Carl G.

Cai, Yi

Chen, Xinchun

TI Bazedoxifene Suppresses Intracellular Mycobacterium tuberculosis

Growth by Enhancing Autophagy

SO MSPHERE

LA English

DT Article

DE Mycobacterium tuberculosis; autophagy; bazedoxifene; host-directed

therapy

ID ESTROGEN-RECEPTOR MODULATOR; BREAST-CANCER; MITOCHONDRIA; RESISTANT;

TAMOXIFEN; RELAPSE; INNATE; DOTS

AB Tuberculosis (TB) is still the leading killer caused by Mycobacterium tuberculosis infection. There is a clear need for new treatment strategy against TB. It has been reported that tamoxifen, known as a selective estrogen receptor modulator (SERM), exhibits antimycobacterial activity and inhibits M. tuberculosis growth in macrophages. However, it remains unknown whether such antimicrobial activity is a general property of all SERMs and how it works. In this study, we identified that bazedoxifene (BZA), a newer SERM, inhibits intracellular M. tuberculosis growth in macrophages. BZA treatment increases autophagosome formation and LC3B-II protein expression in M. tuberculosis-infected macrophages. We further demonstrated that the enhancement of autophagy by BZA is dependent on increased reactive oxygen species (ROS) production and associated with phosphorylation of Akt/mTOR signaling. In summary, our data reveal a previously unappreciated antimicrobial function of BZA and suggest that future investigation focusing on the mechanism of action of SERMs in macrophages may lead to new host-directed therapies against TB.

IMPORTANCE Since current strategies for the treatment of multidrug-resistant tuberculosis (MDR-TB) and extensively drug-resistant tuberculosis (XDR-TB) have low efficacy and highly negative side effects, research on new treatments including novel drugs is essential for curing drug-resistant tuberculosis. Host-directed therapy (HDT) has become a promising idea to modulate host cell responses to enhance protective immunity against pathogens. Bazedoxifene (BZA), which belongs to a new generation of SERMs, shows the ability to inhibit the growth of M. tuberculosis in macrophages and is associated with autophagy. Our findings reveal a previously unrecognized antibacterial function of BZA. We propose that the mechanism of SERMs action in macrophages may provide a new potential measure for host-directed therapies against TB.

C1 [Ouyang, Qi; Zhang, Kehong; Lin, Dachuan; Feng, Carl G.; Cai, Yi; Chen, Xinchun] Shenzhen Univ, Sch Med, Dept Pathogen Biol, Guangdong Prov Key Lab Reg Immun & Dis, Shenzhen, Peoples R China.

[Zhang, Kehong] Friedrich Schiller Univ Jena, Inst Pharm, Dept Pharmaceut Med Chem, Jena, Germany.

[Feng, Carl G.] Univ Sydney, Fac Med & Hlth, Dept Infect Dis & Immunol, Immunol & Host Def Grp, Sydney, NSW, Australia.

C3 Shenzhen University; Friedrich Schiller University of Jena; University

of Sydney

RP Cai, Y; Chen, XC (corresponding author), Shenzhen Univ, Sch Med, Dept Pathogen Biol, Guangdong Prov Key Lab Reg Immun & Dis, Shenzhen, Peoples R China.

EM caiyi0113@szu.edu.cn; chenxinchun@szu.edu.cn

RI 张, 克宏/GRJ-9476-2022; Lin, Dachuan/I-4731-2016; Cai, Yi/AAJ-2789-2020;

feng, carl/AFU-5845-2022

OI Lin, Dachuan/0000-0002-4404-7391; Cai, Yi/0000-0002-1363-2328; feng,

carl/0000-0003-4301-4640

FU Natural Science Foundation of China [81770013, 81525016, 81871255,

91942315]; National Science and Technology Major Project

[2017ZX10201301]; Science and Technology Project of Shenzhen

[JCYJ20170412101048337]; Guangdong Provincial Science and Technology

Program [2019B030301009]

FX This study was supported by the Natural Science Foundation of China

(81770013, 81525016, 81871255, and 91942315), the National Science and

Technology Major Project (2017ZX10201301), the Science and Technology

Project of Shenzhen (JCYJ20170412101048337), and the Guangdong

Provincial Science and Technology Program (2019B030301009). The funders

had no role in the study design, data collection and analysis, decision

to publish, or preparation of the manuscript.

CR An KC, 2016, ASIAN SPINE J, V10, P787, DOI 10.4184/asj.2016.10.4.787

[Anonymous], 2018, Global Tuberculosis Report

Azhar GS, 2012, LUNG INDIA, V29, P147, DOI 10.4103/0970-2113.95320

Bento CF, 2015, DNA CELL BIOL, V34, P228, DOI 10.1089/dna.2014.2745

Bolisetty S, 2013, INT J MOL SCI, V14, P6306, DOI 10.3390/ijms14036306

Brown RE, 2017, FRONT IMMUNOL, V8, DOI 10.3389/fimmu.2017.00078

Chen FC, 2014, PLOS ONE, V9, DOI 10.1371/journal.pone.0100829

de Chastellier C, 2009, IMMUNOBIOLOGY, V214, P526, DOI 10.1016/j.imbio.2008.12.005

Fabri M, 2011, CURR OPIN IMMUNOL, V23, P65, DOI 10.1016/j.coi.2010.10.010

Fanning SW, 2018, ELIFE, V7, DOI 10.7554/eLife.37161

Feng HR, 2018, CELL DEATH DIS, V9, DOI 10.1038/s41419-018-1054-3

Feng XX, 2015, P NATL ACAD SCI USA, V112, pE7073, DOI 10.1073/pnas.1521988112

Filomeni G, 2015, CELL DEATH DIFFER, V22, P377, DOI 10.1038/cdd.2014.150

Jang WS, 2015, J MICROBIOL BIOTECHN, V25, P946, DOI 10.4014/jmb.1412.12023

Kaufmann SHE, 2018, NAT REV DRUG DISCOV, V17, P35, DOI 10.1038/nrd.2017.162

Komm BS, 2005, ENDOCRINOLOGY, V146, P3999, DOI 10.1210/en.2005-0030

Köster S, 2017, P NATL ACAD SCI USA, V114, pE8711, DOI 10.1073/pnas.1707792114

Kung AWC, 2009, EXPERT OPIN PHARMACO, V10, P1377, DOI 10.1517/14656560902980228

Lam A, 2017, AM J PHYSIOL-LUNG C, V313, pL218, DOI 10.1152/ajplung.00162.2017

Leignadier J, 2017, BIOCHEM PHARMACOL, V144, P18, DOI 10.1016/j.bcp.2017.06.120

Levine B, 2008, CELL, V132, P27, DOI 10.1016/j.cell.2007.12.018

Levine B, 2007, NAT REV IMMUNOL, V7, P767, DOI 10.1038/nri2161

Lupien A, 2018, ANTIMICROB AGENTS CH, V62, DOI [10.1128/AAC.00840-18, 10.1128/aac.00840-18]

Mizushima N, 2008, NATURE, V451, P1069, DOI 10.1038/nature06639

Okerlund ND, 2017, NEURON, V93, P897, DOI 10.1016/j.neuron.2017.01.026

Palomino JC, 2014, ANTIBIOTICS-BASEL, V3, P317, DOI 10.3390/antibiotics3030317

Pinto Lancelot, 2011, Infect Drug Resist, V4, P129, DOI 10.2147/IDR.S10332

Rogov V, 2014, MOL CELL, V53, P167, DOI 10.1016/j.molcel.2013.12.014

Scherz-Shouval R, 2007, TRENDS CELL BIOL, V17, P422, DOI 10.1016/j.tcb.2007.07.009

Schmid D, 2007, IMMUNITY, V27, P11, DOI 10.1016/j.immuni.2007.07.004

Shimamoto K, 2011, J TOXICOL SCI, V36, P775, DOI 10.2131/jts.36.775

Shin DM, 2010, PLOS PATHOG, V6, DOI 10.1371/journal.ppat.1001230

Sun SY, 2010, CANCER BIOL THER, V9, P109, DOI 10.4161/cbt.9.2.10583

Thomas A, 2005, INT J TUBERC LUNG D, V9, P556

Vernon PJ, 2013, ANTIOXID REDOX SIGN, V18, P677, DOI 10.1089/ars.2012.4810

Wardell SE, 2013, CLIN CANCER RES, V19, P2420, DOI 10.1158/1078-0432.CCR-12-3771

Xue JF, 2017, BIOMED PHARMACOTHER, V89, P1252, DOI 10.1016/j.biopha.2017.01.130

Yamamoto A, 1998, CELL STRUCT FUNCT, V23, P33, DOI 10.1247/csf.23.33

NR 38

TC 34

Z9 40

U1 0

U2 9

PU AMER SOC MICROBIOLOGY

PI WASHINGTON

PA 1752 N ST NW, WASHINGTON, DC 20036-2904 USA

SN 2379-5042

J9 MSPHERE

JI mSphere

PD MAR-APR

PY 2020

VL 5

IS 2

AR e00124-20

DI 10.1128/mSphere.00124-20

PG 10

WC Microbiology

WE Science Citation Index Expanded (SCI-EXPANDED)

SC Microbiology

GA MK1JR

UT WOS:000548543100023

OA Green Submitted, gold

DA 2026-05-07

ER

PT J

AU Churchyard, GJ

Kaplan, G

Fallows, D

Wallis, RS

Onyebujoh, P

Rook, GA

AF Churchyard, Gavin J.

Kaplan, Gilla

Fallows, Dorothy

Wallis, Robert S.

Onyebujoh, Philip

Rook, Graham A.

TI Advances in Immunotherapy for Tuberculosis Treatment

SO CLINICS IN CHEST MEDICINE

LA English

DT Article

DE Tuberculosis; Immunotherapeutics; Antituberculous; Chemotherapy;

Pulmonary infection; Immunopathology; TB treatment

ID NECROSIS-FACTOR-ALPHA; KILLED MYCOBACTERIUM-VACCAE; MULTIDRUG-RESISTANT

TUBERCULOSIS; ADJUNCTIVE THALIDOMIDE THERAPY; PULMONARY TUBERCULOSIS;

IMMUNE-RESPONSE; IFN-GAMMA; T-CELLS; ANTITUBERCULOSIS TREATMENT;

ANTIINFLAMMATORY ACTIVITY

AB Immunotherapies have the potential to improve the outcome in all patients with tuberculosis (TB) including those with multidrug-resistant (MDR)-TB and extensively drug-resistant (XDR)-TB. Immunotherapy for TB may shorten duration of treatment and reduce pathology in individuals cured by chemotherapy, potentially preventing recurrence. Currently none of the available candidate agents have proof of efficacy for use in MDR-TB or XDR-TB. Further development and evaluation of existing immunotherapeutic agents is required to identify an effective agent that can be used adjunctively with chemotherapy to improve treatment outcomes for drug-susceptible TB, MDR-TB, and XDR-TB. With a range of potential immunotherapeutics, some of which have been produced to good manufacturing practice (GMP) standards and are registered for other indications in humans, the immunotherapy option should no longer be ignored.

C1 [Churchyard, Gavin J.] Aurum Inst Hlth Res, ZA-2041 Johannesburg, South Africa.

[Churchyard, Gavin J.] Univ KwaZulu Natal, Nelson R Mandela Sch Med, ZA-4041 Durban, South Africa.

[Churchyard, Gavin J.] Univ Cape Town, Fac Hlth Sci, ZA-7935 Cape Town, South Africa.

[Kaplan, Gilla; Fallows, Dorothy] Univ Med & Dent New Jersey, Publ Hlth Res Inst, Newark, NJ 07103 USA.

[Wallis, Robert S.] Pfizer Global Res & Dev, New London, CT 06320 USA.

[Onyebujoh, Philip] WHO, Trop Dis Res, CH-1211 Geneva 27, Switzerland.

[Rook, Graham A.] Royal Free & Univ Coll Med Sch, Ctr Infect Dis & Int Hlth, Windeyer Inst Med Sci, London W1P 7PP, England.

C3 University of Kwazulu Natal; University of Cape Town; Rutgers University

System; Rutgers University New Brunswick; Rutgers University Biomedical

& Health Sciences; Pfizer; Pfizer USA; World Health Organization;

University of London; University College London

RP Churchyard, GJ (corresponding author), Aurum Inst Hlth Res, Suite 300,Private Bag X 30500, ZA-2041 Johannesburg, South Africa.

EM gchurchyard@auruminstitute.org

RI ; Wallis, Robert S/A-8018-2009

OI Rook, Graham/0000-0002-8041-8110; Churchyard, Gavin/0000-0002-4269-3699;

Wallis, Robert S/0000-0001-6152-5183

FU NIAID NIH HHS [R01 AI066046, R01 AI054338] Funding Source: Medline

CR Algood HMS, 2004, J IMMUNOL, V172, P6846, DOI 10.4049/jimmunol.172.11.6846

[Anonymous], 2009, WHOHTMTB2009411

Anthony RM, 2008, SCIENCE, V320, P373, DOI 10.1126/science.1154315

Anthony RM, 2008, P NATL ACAD SCI USA, V105, P19571, DOI 10.1073/pnas.0810163105

Bilaçeroglu S, 1999, INT J TUBERC LUNG D, V3, P47

Bonilla Cesar A, 2008, PLoS One, V3, pe2957, DOI 10.1371/journal.pone.0002957

Cardona PJ, 2006, TUBERCULOSIS, V86, P273, DOI 10.1016/j.tube.2006.01.024

Cardona PJ, 2005, VACCINE, V23, P1393, DOI 10.1016/j.vaccine.2004.09.008

Chen ML, 2005, P NATL ACAD SCI USA, V102, P419, DOI 10.1073/pnas.0408197102

CHURCHYARD GJ, 2007, EXPERT CONSULTATION

Condos R, 1997, LANCET, V349, P1513, DOI 10.1016/S0140-6736(96)12273-X

Corral LG, 1999, ANN RHEUM DIS, V58, P107, DOI 10.1136/ard.58.2008.i107

Corral LG, 1996, MOL MED, V2, P506, DOI 10.1007/BF03401909

de Vallière S, 2004, INT J TUBERC LUNG D, V8, P767

Deretic V, 2006, CELL MICROBIOL, V8, P719, DOI 10.1111/j.1462-5822.2006.00705.x

Diacon AH, 2009, NEW ENGL J MED, V360, P2397, DOI 10.1056/NEJMoa0808427

Dlugovitzky D, 2006, RESP MED, V100, P1079, DOI 10.1016/j.rmed.2005.09.026

Elías-López AL, 2008, CLIN EXP IMMUNOL, V154, P123, DOI 10.1111/j.1365-2249.2008.03723.x

[范妙仪 FAN Miaoyi], 2007, [中国循证医学杂志, Chinese Journal of Evidence-Based Medicine], V7, P449

Fenton MJ, 1997, INFECT IMMUN, V65, P5149, DOI 10.1128/IAI.65.12.5149-5156.1997

FLYNN JL, 1995, IMMUNITY, V2, P561, DOI 10.1016/1074-7613(95)90001-2

Fortune SM, 2004, J IMMUNOL, V172, P6272, DOI 10.4049/jimmunol.172.10.6272

Gandhi NR, 2006, LANCET, V368, P1575, DOI 10.1016/S0140-6736(06)69573-1

Giosuè S, 2000, EUR CYTOKINE NETW, V11, P99

Gottlieb AB, 2008, CURR MED RES OPIN, V24, P1529, DOI [10.1185/030079908X301866, 10.1185/030079908X301866 ]

Gutzwiller MER, 2007, VET DERMATOL, V18, P87, DOI 10.1111/j.1365-3164.2007.00579.x

Harris J, 2007, IMMUNITY, V27, P505, DOI 10.1016/j.immuni.2007.07.022

Hernández-Pando R, 2006, CLIN EXP IMMUNOL, V144, P264, DOI 10.1111/j.1365-2249.2006.03049.x

Hernández-Pando R, 2005, J INFECT DIS, V191, P299, DOI 10.1086/426453

Hernandez-Pando R, 2004, EUR J IMMUNOL, V34, P174, DOI 10.1002/eji.200324253

Hernandez-Pando R, 2000, IMMUNOLOGY, V100, P391, DOI 10.1046/j.1365-2567.2000.00054.x

Hesseling AC, 2008, INT J TUBERC LUNG D, V12, P1376

Hewitson JP, 2009, MOL BIOCHEM PARASIT, V167, P1, DOI 10.1016/j.molbiopara.2009.04.008

HORNE NW, 1960, BRIT MED J, V2, P1751, DOI 10.1136/bmj.2.5215.1751

Johnson B, 1998, NOVART FDN SYMP, V217, P99, DOI 10.1002/0470846526.ch7

Johnson BJ, 1997, TUBERCLE LUNG DIS, V78, P195, DOI 10.1016/S0962-8479(97)90026-5

Johnson BJ, 1998, INFECT IMMUN, V66, P2426, DOI 10.1128/IAI.66.6.2426-2433.1998

Johnson JL, 2003, AM J RESP CRIT CARE, V168, P185, DOI 10.1164/rccm.200211-1359OC

Johnson JL, 2000, J INFECT DIS, V181, P1304, DOI 10.1086/315393

Kalamidas SA, 2006, J CELL SCI, V119, P3686, DOI 10.1242/jcs.03091

Kaplan G, 2003, INFECT IMMUN, V71, P7099, DOI 10.1128/IAI.71.12.7099-7108.2003

Klausner JD, 1996, J ACQ IMMUN DEF SYND, V11, P247, DOI 10.1097/00042560-199603010-00005

Koh WJ, 2004, J KOREAN MED SCI, V19, P167, DOI 10.3346/jkms.2004.19.2.167

Krutzik SR, 2008, J IMMUNOL, V181, P7115, DOI 10.4049/jimmunol.181.10.7115

Lewinsohn DA, 2007, PLOS PATHOG, V3, P1240, DOI 10.1371/journal.ppat.0030127

Lowrie DB, 1999, NATURE, V400, P269, DOI 10.1038/22326

Luo SJ, 2001, IEEE T COMPON PACK T, V24, P43, DOI 10.1109/6144.910801

Luo Y, 2000, Zhonghua Jie He He Hu Xi Za Zhi, V23, P85

Malik ZA, 2000, J EXP MED, V191, P287, DOI 10.1084/jem.191.2.287

Mariotti S, 2002, EUR J IMMUNOL, V32, P3050, DOI 10.1002/1521-4141(200211)32:11<3050::AID-IMMU3050>3.0.CO;2-K

Master SS, 2008, CELL HOST MICROBE, V3, P224, DOI 10.1016/j.chom.2008.03.003

Mayanja-Kizza H, 2005, J INFECT DIS, V191, P856, DOI 10.1086/427995

Mohan VP, 2001, INFECT IMMUN, V69, P1847, DOI 10.1128/IAI.69.3.1847-1855.2001

Mwinga A, 2002, LANCET, V360, P1050, DOI 10.1016/S0140-6736(02)11141-X

Nikolaeva LG, 2008, INT IMMUNOPHARMACOL, V8, P845, DOI 10.1016/j.intimp.2008.01.029

Nikolaeva LG, 2008, CYTOKINE, V44, P392, DOI 10.1016/j.cyto.2008.10.009

North RJ, 1998, CLIN EXP IMMUNOL, V113, P55, DOI 10.1046/j.1365-2249.1998.00636.x

Nuermberger E, 2005, AM J RESP CRIT CARE, V172, P1452, DOI 10.1164/rccm.200507-1047OC

Okada M, 2009, VACCINE, V27, P3267, DOI 10.1016/j.vaccine.2009.01.064

Olver S, 2006, CANCER RES, V66, P571, DOI 10.1158/0008-5472.CAN-05-1362

Orellana C, 2002, LANCET INFECT DIS, V2, P711, DOI 10.1016/S1473-3099(02)00465-6

Pasipanodya JG, 2007, CHEST, V131, P1817, DOI 10.1378/chest.06-2949

Pedral-Sampaio Diana Brasil, 2003, Braz J Infect Dis, V7, P245

PRIHODA N D., 2008, INT J BIOMED PHARM S, V2, P59

RADEMACHER TW, 1994, P NATL ACAD SCI USA, V91, P6123, DOI 10.1073/pnas.91.13.6123

Raju B, 2004, INFECT IMMUN, V72, P1275, DOI 10.1128/IAI.72.3.1275-1283.2004

Reading C, 2006, CLIN MICROBIOL INFEC, V12, P1082, DOI 10.1111/j.1469-0691.2006.01520.x

Reljic R, 2007, J INTERF CYTOK RES, V27, P353, DOI 10.1089/jir.2006.0103

ROOK GAW, 1994, IMMUNOLOGY, V81, P149

Rook GAW, 2007, J INFECT DIS, V196, P191, DOI 10.1086/518937

Rook GAW, 2009, J INFECT DIS, V199, P613, DOI 10.1086/596654

Roy E, 2005, INFECT IMMUN, V73, P6101, DOI 10.1128/IAI.73.9.6101-6109.2005

Roy E, 2008, TUBERCULOSIS, V88, P197, DOI [10.1016/j.tube.2007.11.005, 10.1016/S1472-9792(08)70011-8]

Roy E, 2007, CURR MOL MED, V7, P373, DOI 10.2174/156652407780831557

Ryan AA, 2007, J IMMUNOL, V179, P8418, DOI 10.4049/jimmunol.179.12.8418

Schoeman JF, 2004, J CHILD NEUROL, V19, P250, DOI 10.1177/088307380401900402

Schoeman JF, 2000, J CHILD NEUROL, V15, P497, DOI 10.1177/088307380001500801

Silva CL, 2005, GENE THER, V12, P281, DOI 10.1038/sj.gt.3302418

Simbirtsev Andrey, 2003, Russ J Immunol, V8, P11

Skinner MA, 1997, INFECT IMMUN, V65, P4525, DOI 10.1128/IAI.65.11.4525-4530.1997

Sonnenberg P, 2001, LANCET, V358, P1687, DOI 10.1016/S0140-6736(01)06712-5

Stewart GR, 1999, CLIN EXP IMMUNOL, V117, P517

Stickney DR, 2007, ANTIMICROB AGENTS CH, V51, P2639, DOI 10.1128/AAC.01446-06

Suárez-Méndez R, 2004, BMC INFECT DIS, V4, DOI 10.1186/1471-2334-4-44

Szeliga J, 2008, TUBERCULOSIS, V88, P7, DOI 10.1016/j.tube.2007.08.009

TRAMONTANA JM, 1995, MOL MED, V1, P384, DOI 10.1007/BF03401576

Tsenova L, 1998, J INFECT DIS, V177, P1563, DOI 10.1086/515327

Tsenova L, 2002, ANTIMICROB AGENTS CH, V46, P1887, DOI 10.1128/AAC.46.6.1887-1895.2002

Turner OC, 2000, INFECT IMMUN, V68, P3674, DOI 10.1128/IAI.68.6.3674-3679.2000

van Crevel R, 2000, J INFECT DIS, V181, P1194, DOI 10.1086/315325

Verver S, 2005, AM J RESP CRIT CARE, V171, P1430, DOI 10.1164/rccm.200409-1200OC

Victora GD, 2009, CANCER GENE THER, V16, P598, DOI 10.1038/cgt.2009.9

Wallis R.S., 2009, Tuberculosis, P718, DOI DOI 10.1016/B978-1-4160-3988-4.00070-6

Wallis RS, 2004, AIDS, V18, P257, DOI 10.1097/00002030-200401230-00015

Wallis RS, 2005, CLIN INFECT DIS, V41, P201, DOI 10.1086/430914

Yu DH, 2008, GENE THER, V15, P652, DOI 10.1038/gt.2008.13

Zárate-Bladés CR, 2009, J GENE MED, V11, P66, DOI 10.1002/jgm.1269

ZHANG M, 1994, J CLIN INVEST, V94, P2435, DOI 10.1172/JCI117611

Zhang XZ, 2007, VACCINE, V25, P1342, DOI 10.1016/j.vaccine.2006.09.089

Zuany-Amorim C, 2002, NAT MED, V8, P625, DOI 10.1038/nm0602-625

NR 100

TC 45

Z9 49

U1 0

U2 6

PU W B SAUNDERS CO-ELSEVIER INC

PI PHILADELPHIA

PA 1600 JOHN F KENNEDY BOULEVARD, STE 1800, PHILADELPHIA, PA 19103-2899 USA

SN 0272-5231

EI 1557-8216

J9 CLIN CHEST MED

JI Clin. Chest Med.

PD DEC

PY 2009

VL 30

IS 4

BP 769

EP +

DI 10.1016/j.ccm.2009.08.009

PG 15

WC Respiratory System

WE Science Citation Index Expanded (SCI-EXPANDED)

SC Respiratory System

GA 617BQ

UT WOS:000279256200010

DA 2026-05-07

ER

PT J

AU Mo, SW

Guo, JB

Ye, TS

Zhang, XM

Zeng, J

Xu, YZ

Bin P

Dai, YC

Xiao, W

Zhang, PZ

Deng, GF

Xu, DC

Long, XR

Cai, Y

Chen, XC

AF Mo, Siwei

Guo, Jiubiao

Ye, Taosheng

Zhang, Ximeng

Zeng, Jiang

Xu, Yuzhong

Bin Peng

Dai, Youchao

Xiao, Wei

Zhang, Peize

Deng, Guofang

Xu, Dechang

Long, Xiaoru

Cai, Yi

Chen, Xinchun

TI Mycobacterium tuberculosis Utilizes Host Histamine Receptor H1 to

Modulate Reactive Oxygen Species Production and Phagosome Maturation via

the p38MAPK-NOX2 Axis

SO MBIO

LA English

DT Article

DE Mycobacterium tuberculosis; HRH1; NOX2; p38MAPK; ROS

ID ACTIVATION; ROS

AB Once engulfed in macrophage phagosomes, M. tuberculosis adopts various strategies to take advantage of the host environment for its intracellular survival. Histamine is an organic nitrogen-containing compound that mediates a plethora of cellular processes via different receptors, but the crosstalk mechanism between M. tuberculosis and HRH1 in macrophages is not clear.

Tuberculosis (TB), which is caused by the single pathogenic bacterium, Mycobacterium tuberculosis, is among the top 10 lethal diseases worldwide. This situation has been exacerbated by the increasing number of cases of multidrug-resistant TB (MDR-TB) and extensively drug-resistant TB (XDR-TB). Histamine is an organic nitrogenous compound that mediates a plethora of cell processes via different receptors. The expression of histamine receptor H1 (HRH1), one of the four histamine receptors identified to date was previously reported to be augmented by M. tuberculosis infection, although the underlying mechanism is unclear. In the present study, we applied confocal microscopy, flow cytometry, and Western blotting to show that HRH1 expression was enhanced in macrophages following mycobacterial infection. Furthermore, by combining techniques of gene knockdown, immunoprecipitation, intracellular bacterial burden analysis, fluorescence labeling, and imaging, we found that M. tuberculosis targeted the host HRH1 to suppress NOX2-mediated cROS production and inhibit phagosome maturation and acidification via the GRK2-p38MAPK signaling pathway. Our findings clarified the underlying mechanism of the M. tuberculosis and host HRH1 interaction and may provide useful information for the development of novel antituberculosis treatments. IMPORTANCE Once engulfed in macrophage phagosomes, M. tuberculosis adopts various strategies to take advantage of the host environment for its intracellular survival. Histamine is an organic nitrogen-containing compound that mediates a plethora of cellular processes via different receptors, but the crosstalk mechanism between M. tuberculosis and HRH1 in macrophages is not clear. Our results revealed that M. tuberculosis infection enhanced HRH1 expression, which in turn restrained macrophage bactericidal activity by modulating the GRK2-p38MAPK signaling pathway, inhibiting NOX2-mediated cROS production and phagosome maturation. Clarification of the underlying mechanism by which M. tuberculosis utilizes host HRH1 to favor its intracellular survival may provide useful information for the development of novel antituberculosis treatments.

C1 [Mo, Siwei; Zhang, Ximeng; Bin Peng; Dai, Youchao; Xiao, Wei; Cai, Yi; Chen, Xinchun] Shenzhen Univ, Dept Pathogen Biol, Guangdong Prov Key Lab Reg Immun & Dis, Sch Med, Shenzhen, Peoples R China.

[Guo, Jiubiao] Shenzhen Technol Univ, Coll Pharm, Shenzhen, Peoples R China.

[Ye, Taosheng; Zhang, Peize; Deng, Guofang] Southern Univ Sci & Technol, Shenzhen Peoples Hosp 3, Natl Clin Res Ctr Infect Dis Shenzhen, Pulm Dis Dept 2,Guangdong Prov Clin Res Ctr Inf, Shenzhen, Peoples R China.

[Zeng, Jiang; Xu, Dechang] Fifth Peoples Hosp Ganzhou, Ganzhou, Jiangxi, Peoples R China.

[Xu, Yuzhong] Shenzhen Univ, Dept Clin Lab, Shenzhen Baoan Hosp, Shenzhen, Peoples R China.

[Mo, Siwei; Long, Xiaoru] Chongqing Med Univ, Dept Resp Med, Chongqing Key Lab Pediat, Childrens Hosp, Chongqing, Peoples R China.

[Mo, Siwei; Long, Xiaoru] Chongqing Med Univ, Childrens Hosp, Natl Clin Res Ctr Child Hlth & Disorders,Chongqin, Minist Educ,Key Lab Child Dev & Disorders,China I, Chongqing, Peoples R China.

C3 Shenzhen University; Shenzhen Technology University; Southern University

of Science & Technology; Shenzhen University; Chongqing Medical

University; Chongqing Medical University

RP Cai, Y; Chen, XC (corresponding author), Shenzhen Univ, Dept Pathogen Biol, Guangdong Prov Key Lab Reg Immun & Dis, Sch Med, Shenzhen, Peoples R China.

EM caiyi0113@szu.edu.cn; chenxinchun@szu.edu.cn

RI Cai, Yi/AAJ-2789-2020; Ye, TaoSheng/AAF-1503-2019

OI Ye, TaoSheng/0000-0001-7217-6214; MO, siwei/0000-0002-1276-8362; Zhang,

Peize/0000-0002-4023-0228; chen, xinchun/0000-0002-2101-9013

FU National Natural Science Foundation of China [82130066, 82072252,

31800064, 82070016]; Science and Technology Project of Shenzhen

[JCYJ20200109105012589, JCYJ20180507182049853]; Guangdong Provincial Key

Laboratory of Regional Immunity and Diseases [2019B030301009]; Natural

Science Foundation of Top Talent of SZTU [GDRC202120]; Natural Science

Foundation of Chongqing City [cstc2020jcyj-msxmX0580]; general project

of clinical medicine research of National Center for clinical medicine

of child health and disease [NCRCCHD-2020-GP-06]

FX We thank Jessica Tamanini (Shenzhen University) for editing and

polishing the manuscript before submission and gratefully acknowledge

the laboratory personnel who contributed to this study.This study was

supported by the National Natural Science Foundation of China (grant no.

82130066, 82072252, 31800064, and 82070016), the Science and Technology

Project of Shenzhen (grant no. JCYJ20200109105012589 and

JCYJ20180507182049853), Guangdong Provincial Key Laboratory of Regional

Immunity and Diseases (2019B030301009), Natural Science Foundation of

Top Talent of SZTU (grant no. GDRC202120), the Natural Science

Foundation of Chongqing City (cstc2020jcyj-msxmX0580) and the general

project of clinical medicine research of National Center for clinical

medicine of child health and disease (NCRCCHD-2020-GP-06).S. M., J. G.,

X. C., and Y. C. contributed to the acquisition, analysis, and

interpretation of the data, and writing of the manuscript; S. M., J. G.,

X. Z., B. P., Y. D, and W. X. contributed to the study design and data

interpretation and participated in performing the experiments; T. Y., J.

Z., Y. X., P. Z., G. D., X. L., and D. X. contributed to the data

analysis and interpretation.The funders had no role in the study design,

data collection, and analysis, decision to publish, or preparation of

the manuscript.We declare no conflict of interest.

CR Bedard K, 2007, PHYSIOL REV, V87, P245, DOI 10.1152/physrev.00044.2005

Bellot GL, 2013, MITOCHONDRION, V13, P155, DOI 10.1016/j.mito.2012.06.007

Brites D, 2015, IMMUNOL REV, V264, P6, DOI 10.1111/imr.12264

Canovas B, 2021, NAT REV MOL CELL BIO, V22, P346, DOI 10.1038/s41580-020-00322-w

Carlos D, 2009, INFECT IMMUN, V77, P5359, DOI 10.1128/IAI.01497-08

Carthy E, 2021, FRONT NEUROSCI-SWITZ, V15, DOI 10.3389/fnins.2021.680214

Chakaya J, 2022, INT J INFECT DIS, V124, pS26, DOI 10.1016/j.ijid.2022.03.011

Cheng HJ, 2022, CELL SIGNAL, V95, DOI 10.1016/j.cellsig.2022.110337

de Martino M, 2019, FRONT PEDIATR, V7, DOI 10.3389/fped.2019.00350

Ding SL, 2018, CELL DEATH DIS, V9, DOI 10.1038/s41419-018-0723-6

Ernst JD, 2012, NAT REV IMMUNOL, V12, P581, DOI 10.1038/nri3259

Fernández-Nogueira P, 2018, CANCER LETT, V424, P70, DOI 10.1016/j.canlet.2018.03.014

Glickman MS, 2001, CELL, V104, P477, DOI 10.1016/S0092-8674(01)00236-7

Huynh KK, 2007, EMBO J, V26, P313, DOI 10.1038/sj.emboj.7601511

Ji LT, 2020, MOL MED REP, V21, P161, DOI 10.3892/mmr.2019.10815

Jutel M, 2002, CURR OPIN IMMUNOL, V14, P735, DOI 10.1016/S0952-7915(02)00395-3

Jutel M, 2009, CLIN EXP ALLERGY, V39, P1786, DOI 10.1111/j.1365-2222.2009.03374.x

Lange C, 2019, LANCET, V394, P953, DOI 10.1016/S0140-6736(19)31882-3

Liu D, 2017, FRONT MICROBIOL, V8, DOI 10.3389/fmicb.2017.02231

Ma RLZ, 2002, SCIENCE, V297, P620, DOI 10.1126/science.1072810

Makni-Maalej K, 2015, J LEUKOCYTE BIOL, V97, P1081, DOI 10.1189/jlb.2A1214-623R

Megyeri K, 2006, MICROBES INFECT, V8, P1035, DOI 10.1016/j.micinf.2005.10.022

MEINDL W, 1990, ARCH PHARM, V323, P267, DOI 10.1002/ardp.19903230504

Mo SW, 2022, J LEUKOCYTE BIOL, V111, P1021, DOI 10.1002/JLB.4A0221-119RR

Muñoz S, 2003, J IMMUNOL, V170, P5590, DOI 10.4049/jimmunol.170.11.5590

O'Garra A, 2013, ANNU REV IMMUNOL, V31, P475, DOI 10.1146/annurev-immunol-032712-095939

Panday Arvind, 2015, Cell Mol Immunol, V12, P5, DOI 10.1038/cmi.2014.89

Panula P, 2015, PHARMACOL REV, V67, P601, DOI 10.1124/pr.114.010249

Penela P, 2010, BRIT J PHARMACOL, V160, P821, DOI 10.1111/j.1476-5381.2010.00727.x

Rocha SM, 2016, J NEUROINFLAMM, V13, DOI 10.1186/s12974-016-0600-0

Savina A, 2006, CELL, V126, P205, DOI 10.1016/j.cell.2006.05.035

Schneider E, 2002, TRENDS IMMUNOL, V23, P255, DOI 10.1016/S1471-4906(02)02215-9

Shah M, 2021, NEW ENGL J MED, V385, P2271, DOI 10.1056/NEJMcp2108501

Shi ZC, 2019, AM J PHYSIOL-GASTR L, V316, pG205, DOI 10.1152/ajpgi.00212.2018

Vasicek O, 2014, LIFE SCI, V100, P67, DOI 10.1016/j.lfs.2014.01.082

NR 35

TC 8

Z9 12

U1 0

U2 21

PU AMER SOC MICROBIOLOGY

PI WASHINGTON

PA 1752 N ST NW, WASHINGTON, DC 20036-2904 USA

SN 2150-7511

J9 MBIO

JI mBio

PD OCT 26

PY 2022

VL 13

IS 5

DI 10.1128/mbio.02004-22

EA AUG 2022

PG 14

WC Microbiology

WE Science Citation Index Expanded (SCI-EXPANDED)

SC Microbiology

GA 5X2SZ

UT WOS:000844488700001

OA Green Submitted, gold

DA 2026-05-07

ER

PT J

AU Mourenza, A

Gil, JA

Mateos, LM

Letek, M

AF Mourenza, Alvaro

Gil, Jose A.

Mateos, Luis M.

Letek, Michal

TI Novel Treatments againstMycobacterium tuberculosisBased on Drug

Repurposing

SO ANTIBIOTICS-BASEL

LA English

DT Review

DE Mycobacterium tuberculosis; multidrug-resistant strains; host-directed

therapies; drug repurposing

ID HOST-DIRECTED THERAPIES; MYCOBACTERIUM-TUBERCULOSIS; TYROSINE KINASE;

MACROPHAGES; AUTOPHAGY; INHIBITION; RESISTANCE; APOPTOSIS; SURVIVAL;

GROWTH

AB Tuberculosis is the leading cause of death, worldwide, due to a bacterial pathogen. This respiratory disease is caused by the intracellular pathogenMycobacterium tuberculosisand produces 1.5 million deaths every year. The incidence of tuberculosis has decreased during the last decade, but the emergence of MultiDrug-Resistant (MDR-TB) and Extensively Drug-Resistant (XDR-TB) strains ofM. tuberculosisis generating a new health alarm. Therefore, the development of novel therapies based on repurposed drugs against MDR-TB and XDR-TB have recently gathered significant interest. Recent evidence, focused on the role of host molecular factors onM. tuberculosisintracellular survival, allowed the identification of new host-directed therapies. Interestingly, the mechanism of action of many of these therapies is linked to the activation of autophagy (e.g., nitazoxanide or imatinib) and other well-known molecular pathways such as apoptosis (e.g., cisplatin and calycopterin). Here, we review the latest developments on the identification of novel antimicrobials against tuberculosis (including avermectins, eltrombopag, or fluvastatin), new host-targeting therapies (e.g., corticoids, fosfamatinib or carfilzomib) and the host molecular factors required for a mycobacterial infection that could be promising targets for future drug development.

C1 [Mourenza, Alvaro; Gil, Jose A.; Mateos, Luis M.; Letek, Michal] Univ Leon, Area Microbiol, Dept Biol Mol, Leon 24071, Spain.

[Gil, Jose A.; Mateos, Luis M.] Univ Leon, Inst Biol Mol Genom & Proteom INBIOMIC, Leon 24071, Spain.

[Letek, Michal] Univ Leon, Inst Desarrollo Ganadero & Sanidad Anim INDEGSAL, Leon 24071, Spain.

C3 Universidad de Leon; Universidad de Leon; Universidad de Leon

RP Mateos, LM; Letek, M (corresponding author), Univ Leon, Area Microbiol, Dept Biol Mol, Leon 24071, Spain.; Mateos, LM (corresponding author), Univ Leon, Inst Biol Mol Genom & Proteom INBIOMIC, Leon 24071, Spain.; Letek, M (corresponding author), Univ Leon, Inst Desarrollo Ganadero & Sanidad Anim INDEGSAL, Leon 24071, Spain.

EM amouf@unileon.es; jagils@unileon.es; luis.mateos@unileon.es;

michal.letek@unileon.es

RI Mourenza, Alvaro/AAA-5385-2020; Letek, Michal/K-3903-2019; Gil, José

A/L-1800-2019; Mateos, Luis/ABB-8848-2021

OI Mourenza Flórez, Álvaro/0000-0003-4926-3891; Letek,

Michal/0000-0002-9509-5174; Gil, José A/0000-0002-5069-2637; Mateos,

Luis/0000-0001-7763-582X

FU University of Leon; University of Roehampton

FX This research was funded by intramural funding from the University of

Leon to L.M.M. and from the University of Roehampton to M.L. A.M. was

granted an "Ayuda Puente" from the University of Leon.

CR An Q, 2020, EUR J MED CHEM, V192, DOI 10.1016/j.ejmech.2020.112175

Bah A, 2020, CELLS-BASEL, V9, DOI 10.3390/cells9030666

Battah B., 2018, MOLECULES, V24, P1

Bhaskar A, 2014, PLOS PATHOG, V10, DOI 10.1371/journal.ppat.1003902

Bilaçeroglu S, 1999, INT J TUBERC LUNG D, V3, P47

Bravo-Santano N, 2019, ANTIBIOTICS-BASEL, V8, DOI 10.3390/antibiotics8040241

Bravo-Santano N, 2019, SCI REP-UK, V9, DOI 10.1038/s41598-019-41260-8

Bussi C, 2019, FEMS MICROBIOL REV, V43, P341, DOI 10.1093/femsre/fuz006

Campbell GR, 2012, PLOS PATHOG, V8, DOI 10.1371/journal.ppat.1002689

Chiang CY, 2018, MBIO, V9, DOI [10.1128/mBio.01932-17, 10.1128/mbio.01932-17]

Coelho TS, 2020, LETT APPL MICROBIOL, V71, P146, DOI 10.1111/lam.13298

Cumming BM, 2015, P NATL ACAD SCI USA, V112, P13135, DOI 10.1073/pnas.1518171112

Dallmann-Sauer M, 2018, MAMM GENOME, V29, P523, DOI 10.1007/s00335-018-9765-4

Guerra-De-Blas PD, 2019, FRONT MICROBIOL, V10, DOI 10.3389/fmicb.2019.02097

Dheda K, 2016, LANCET, V387, P1211, DOI 10.1016/S0140-6736(15)00151-8

Divangahi M, 2009, NAT IMMUNOL, V10, P899, DOI 10.1038/ni.1758

Fang F, 2020, MOL IMMUNOL, V124, P18, DOI 10.1016/j.molimm.2020.05.001

Fischbach MA, 2009, SCIENCE, V325, P1089, DOI 10.1126/science.1176667

Gengenbacher M, 2020, ANTIMICROB AGENTS CH, V64, DOI 10.1128/AAC.02479-19

Hare NJ, 2017, J PROTEOME RES, V16, P247, DOI 10.1021/acs.jproteome.6b00685

Härtlova A, 2018, EMBO J, V37, DOI 10.15252/embj.201798694

Tong HV, 2017, TROP MED INT HEALTH, V22, P1063, DOI 10.1111/tmi.12923

Hu YL, 2020, J INFECTION, V80, pE19, DOI 10.1016/j.jinf.2020.03.003

Jasenosky LD, 2019, ISCIENCE, V19, P1265, DOI 10.1016/j.isci.2019.07.003

Kang MF, 2017, ONCOTARGETS THER, V10, P5667, DOI 10.2147/OTT.S143084

Kaur D, 2017, J TRANSL MED, V15, DOI 10.1186/s12967-017-1363-9

Kawatkar SP, 2020, BIOORG MED CHEM LETT, V30, DOI 10.1016/j.bmcl.2020.127393

Khan MT, 2020, SAUDI J BIOL SCI, V27, P3150, DOI 10.1016/j.sjbs.2020.07.015

Kim JH, 2013, P NATL ACAD SCI USA, V110, P19095, DOI 10.1073/pnas.1315860110

Konreddy AK, 2019, CURR MED CHEM, V26, P5363, DOI 10.2174/0929867325666180706101404

Korecka JA, 2020, MOL THER-NUCL ACIDS, V21, P623, DOI 10.1016/j.omtn.2020.06.027

Lam A, 2017, AM J PHYSIOL-LUNG C, V313, pL218, DOI 10.1152/ajplung.00162.2017

Lee H, 2020, STEM CELL REP, V14, P940, DOI 10.1016/j.stemcr.2020.04.001

Liao ZY, 2019, INTERDISCIP SCI, V11, P77, DOI 10.1007/s12539-019-00318-x

Lu J, 2013, FASEB J, V27, P1394, DOI 10.1096/fj.12-223305

Lu N, 2020, EUR J MED CHEM, V202, DOI 10.1016/j.ejmech.2020.112471

Maertzdorf J, 2011, GENES IMMUN, V12, P15, DOI 10.1038/gene.2010.51

Maier L, 2018, NATURE, V555, P623, DOI 10.1038/nature25979

McNab F, 2015, NAT REV IMMUNOL, V15, P87, DOI 10.1038/nri3787

Mehmood A, 2019, FRONT BIOENG BIOTECH, V7, DOI 10.3389/fbioe.2019.00404

Miro-Canturri Andrea, 2019, Front Microbiol, V10, P41, DOI 10.3389/fmicb.2019.00041

Mishra A, 2018, EMBO MOL MED, V10, DOI 10.15252/emmm.201708038

Mishra R, 2020, TUBERCULOSIS, V120, DOI 10.1016/j.tube.2020.101902

Moradi M, 2020, N-S ARCH PHARMACOL, V393, P2145, DOI 10.1007/s00210-020-01917-y

Mourenza A, 2020, ANTIOXIDANTS-BASEL, V9, DOI 10.3390/antiox9050361

Mourenza A, 2020, ANTIOXIDANTS-BASEL, V9, DOI 10.3390/antiox9020114

Naicker N, 2020, FRONT MICROBIOL, V11, DOI 10.3389/fmicb.2020.00435

Nair RR, 2019, MICROBIOL-SGM, V165, P668, DOI 10.1099/mic.0.000797

Nijjar JS, 2013, RHEUMATOLOGY, V52, P1556, DOI 10.1093/rheumatology/ket225

North TE, 2007, NATURE, V447, P1007, DOI 10.1038/nature05883

Odingo J, 2017, ACS OMEGA, V2, P5873, DOI 10.1021/acsomega.7b00892

Ordonez AA, 2020, MOL IMAGING BIOL, V22, P1489, DOI 10.1007/s11307-020-01494-9

Ouyang Q, 2020, MSPHERE, V5, DOI 10.1128/mSphere.00124-20

Pacios O, 2020, ANTIBIOTICS-BASEL, V9, DOI 10.3390/antibiotics9020065

Paik S, 2019, VIRULENCE, V10, P448, DOI 10.1080/21505594.2018.1536598

Parihar SP, 2014, J INFECT DIS, V209, P754, DOI 10.1093/infdis/jit550

Pedruzzi G, 2016, J THEOR BIOL, V389, P159, DOI 10.1016/j.jtbi.2015.10.025

Philips JA, 2012, ANNU REV PATHOL-MECH, V7, P353, DOI 10.1146/annurev-pathol-011811-132458

Pule CM, 2016, J ANTIMICROB CHEMOTH, V71, P17, DOI 10.1093/jac/dkv316

Rahman MA, 2020, P NATL ACAD SCI USA, V117, P6663, DOI 10.1073/pnas.1919211117

Ranjbar S, 2019, ISCIENCE, V22, P299, DOI 10.1016/j.isci.2019.11.001

Russell DG, 2011, IMMUNOL REV, V240, P252, DOI 10.1111/j.1600-065X.2010.00984.x

Salie S, 2019, REGUL TOXICOL PHARM, V109, DOI 10.1016/j.yrtph.2019.104508

Shi QH, 2020, ANN TRANSL MED, V8, DOI 10.21037/atm.2020-20-3048

Sly LM, 2003, J IMMUNOL, V170, P430, DOI 10.4049/jimmunol.170.1.430

Sogi KM, 2017, ACS INFECT DIS, V3, P564, DOI 10.1021/acsinfecdis.7b00046

Tiberi S, 2018, LANCET INFECT DIS, V18, pE183, DOI 10.1016/S1473-3099(18)30110-5

Torfs E, 2019, INT J MOL SCI, V20, DOI 10.3390/ijms20122868

Varma DM, 2020, TRANSL RES, V220, P98, DOI 10.1016/j.trsl.2020.03.009

Wallings RL, 2019, BIOCHEM SOC T, V47, P1581, DOI 10.1042/BST20180463

Wallis RS, 2016, OPEN FORUM INFECT DI, V3, DOI 10.1093/ofid/ofw151

Wallis RS, 2016, LANCET INFECT DIS, V16, pE34, DOI 10.1016/S1473-3099(16)00070-0

Wang Z, 2018, BMC SYST BIOL, V12, DOI 10.1186/s12918-017-0524-z

Yogalingam G, 2008, J BIOL CHEM, V283, P35941, DOI 10.1074/jbc.M804543200

Yuan QL, 2020, J CELL MOL MED, V24, P2004, DOI 10.1111/jcmm.14899

Zhan WH, 2019, J MED CHEM, V62, P9246, DOI 10.1021/acs.jmedchem.9b01187

Zhou XY, 2020, J INFECTION, V81, P231, DOI 10.1016/j.jinf.2020.05.030

Zumla Alimuddin, 2016, Int J Mycobacteriol, V5 Suppl 1, pS21, DOI [10.1016/j.ijmyco.2016.09.044, 10.1016/j.ijmyco.2016.09.044]

Zumla A, 2015, CLIN INFECT DIS, V61, P1432, DOI 10.1093/cid/civ631

NR 79

TC 28

Z9 32

U1 0

U2 9

PU MDPI

PI BASEL

PA ST ALBAN-ANLAGE 66, CH-4052 BASEL, SWITZERLAND

SN 2079-6382

J9 ANTIBIOTICS-BASEL

JI Antibiotics-Basel

PD SEP

PY 2020

VL 9

IS 9

AR 550

DI 10.3390/antibiotics9090550

PG 12

WC Infectious Diseases; Pharmacology & Pharmacy

WE Science Citation Index Expanded (SCI-EXPANDED)

SC Infectious Diseases; Pharmacology & Pharmacy

GA OE0TU

UT WOS:000580255000001

OA Green Submitted, Green Published, gold

DA 2026-05-07

ER

PT J

AU Hu, Y

Mathema, B

Zhao, Q

Zheng, XB

Li, DG

Jiang, WL

Wang, WB

Xu, B

AF Hu, Yi

Mathema, Barun

Zhao, Qi

Zheng, Xubin

Li, Dange

Jiang, Weili

Wang, Weibing

Xu, Biao

TI Comparison of the socio-demographic and clinical features of pulmonary

TB patients infected with sub-lineages within the W-Beijing and

non-Beijing Mycobacterium tuberculosis

SO TUBERCULOSIS

LA English

DT Article

DE Tuberculosis; Sublineage; Beijing family; Multidrug resistance

ID DRUG-RESISTANT TUBERCULOSIS; VARIABLE-NUMBER; GENETIC DIVERSITY;

NATIONAL-SURVEY; FAMILY; STRAINS; EPIDEMIOLOGY; CHINA; SUBLINEAGE;

GENOTYPE

AB Background: Highly lethal outbreaks of multidrug-resistant (MDR) and extensively drug-resistant (XDR) tuberculosis are increasing. Mycobacterium tuberculosis variant Beijing family and its members is regarded as a successful clone of M. tuberculosis that is associated with drug resistance in China. Understanding the genetic characteristics and molecular mechanism of drug resistant tuberculosis within Beijing family may help to clarify its origin and evolutionary history and the driving forces behind its emergence and current dissemination.

Methods: Totally of 1222 Mycobacterium tuberculosis isolates were recovered from patients in six counties of two provinces in eastern China within 2010/2012. Strain lineage and its major subgroups were studied respectively by using Spoligotyping and MIRU-VNTR The 1st-line drug susceptibility was analyzed by proportional method and 2nd-line drug susceptibility was determined by the HAINs MTBDRsI test. The genetic characterization of drug resistance was analyzed by sequencing the previously reported genes and loci associated with drug resistance together with the multiple genotyping including MIRU-VNTR, Spoligotyping and LSP genotyping.

Results: Of the 1222 Mtb isolates, 298 (24.4%) were resistant to 1st-line drug and 73 (5.9%) were simultaneously resistant to INH and RIF namely MDR-TB. Respectively 23.8% of 1st-line drug resistant TB and 12.0% of the drug susceptible TB contained the mutation associated with 2nd-line drugs by HAINs test. The Spoligotyping of 1222 Mtb isolates revealed the 967 (79.1%) of the isolates belonged to the W-Beijing family. Within W-Beijing family, 78.8% MDR-TB were observed in the isolates with simultaneous deletion of RD105 and RD207, with sub-lineage 181 accounting for 75% of MDR-TB. Analysis of 24 MIRU-VNTR loci revealed that 88.2% (15/17) of MDR and extensively drug resistant (XDR) clustered isolates were sub-lineage 181.

Conclusions: Sublineage 181 might have the capacity to spread throughout the general community in rural China. This is the first report on the extensive association of sub-lineage 181 with MDR TB and possibly pre-XDR TB and XDR TB. It is important to monitor sublineage 181 to verify its heightened transmission and understand its importance in the global MDR TB and XDR TB epidemics. (c) 2015 Elsevier Ltd. All rights reserved.

C1 [Hu, Yi; Zhao, Qi; Zheng, Xubin; Li, Dange; Jiang, Weili; Wang, Weibing; Xu, Biao] Fudan Univ, Dept Epidemiol, Sch Publ Hlth, Shanghai 200032, Peoples R China.

[Hu, Yi; Zhao, Qi; Zheng, Xubin; Li, Dange; Jiang, Weili; Wang, Weibing; Xu, Biao] Fudan Univ, Key Lab Publ Hlth Safety, Minist Educ, Shanghai 200032, Peoples R China.

[Mathema, Barun] Columbia Univ, Dept Epidemiol, Mailman Sch Publ Hlth, New York, NY USA.

C3 Fudan University; Fudan University; Columbia University

RP Xu, B (corresponding author), Fudan Univ, Sch Publ Hlth, 138 Yi Xue Yuan Rd, Shanghai 200032, Peoples R China.

EM yhu@fudan.edu.cn; barun.mathema@gmail.com; 14211020080@fudan.edu.cn;

14211020004@fudan.edu.cn; wljiang@fudan.edu.cn; bxu@shmu.edu.cn

RI Zheng, Xubin/LTE-6136-2024; zhao, qi/KGK-3760-2024

OI Mathema, Barun/0000-0003-2772-479X

FU National Institutes of Health (NIH)/NIAID [R01AI075463]; National

Natural Science Foundation of China (NSFC) [81361138019, 81373063]; TDR

small grant [PO201377850]

FX This work was supported by grants from the National Institutes of Health

(NIH)/NIAID (R01) (PI, Biao Xu, No. R01AI075463), National Natural

Science Foundation of China (NSFC) (PI, Biao Xu, No.81361138019; PI, Yi

Hu, No. 81373063) and TDR small grant (PI. Yi Hu, No. PO201377850).

CR Almeida D, 2005, CLIN INFECT DIS, V40, P881, DOI 10.1086/427940

Anh DD, 2000, EMERG INFECT DIS, V6, P302

[Anonymous], 2007, EURO SURVEILLANCE

Aziz MA, 2005, CLIN INFECT DIS, V41, pS258, DOI 10.1086/430786

Brosch R, 2002, P NATL ACAD SCI USA, V99, P3684, DOI 10.1073/pnas.052548299

Brudey K, 2006, BMC MICROBIOL, V6, DOI 10.1186/1471-2180-6-23

Caws M, 2008, PLOS PATHOG, V4, DOI 10.1371/journal.ppat.1000034

Chai Li-Quan, 2007, Zhonghua Liu Xing Bing Xue Za Zhi, V28, P785

Demay C, 2012, INFECT GENET EVOL, V12, P755, DOI 10.1016/j.meegid.2012.02.004

Faksri K, 2014, ASIAN PAC J ALLERGY, V32, P124, DOI 10.12932/AP0361.32.2.2013

Falzon D, 2011, EUR RESPIR J, V38, P516, DOI 10.1183/09031936.00073611

Filliol I, 2003, J CLIN MICROBIOL, V41, P1963, DOI 10.1128/JCM.41.5.1963-1970.2003

Gagneux S, 2006, P NATL ACAD SCI USA, V103, P2869, DOI 10.1073/pnas.0511240103

Gao GJ, 2014, INT J ANTIMICROB AGE

Hanekom M, 2007, J CLIN MICROBIOL, V45, P1483, DOI 10.1128/JCM.02191-06

Hirsh AE, 2004, P NATL ACAD SCI USA, V101, P4871, DOI 10.1073/pnas.0305627101

Iwamoto T, 2007, FEMS MICROBIOL LETT, V270, P67, DOI 10.1111/j.1574-6968.2007.00658.x

Jiao WW, 2007, CHINESE MED J-PEKING, V120, P814, DOI 10.1097/00029330-200705010-00014

Kamerbeek J, 1997, J CLIN MICROBIOL, V35, P907, DOI 10.1128/JCM.35.4.907-914.1997

Kang HY, 2010, J MED MICROBIOL, V59, P1191, DOI 10.1099/jmm.0.022103-0

Li D, 2014, INFECT GENET EVOL, V27, P294, DOI 10.1016/j.meegid.2014.08.004

Li Wei-min, 2003, Zhonghua Liu Xing Bing Xue Za Zhi, V24, P381

Maeda S, 2010, INT J TUBERC LUNG D, V14, P1201

PLIKAYTIS BB, 1994, J CLIN MICROBIOL, V32, P1542, DOI 10.1128/JCM.32.6.1542-1546.1994

Rad ME, 2003, EMERG INFECT DIS, V9, P838, DOI 10.3201/eid0907.020803

Shen Guo-miao, 2005, Zhonghua Jiehe He Huxi Zazhi, V28, P292

Supply P, 2001, J CLIN MICROBIOL, V39, P3563, DOI 10.1128/JCM.39.10.3563-3571.2001

Supply P, 2006, J CLIN MICROBIOL, V44, P4498, DOI 10.1128/JCM.01392-06

Tanveer M, 2008, BMC INFECT DIS, V8, DOI 10.1186/1471-2334-8-171

Thwaites G, 2008, J CLIN MICROBIOL, V46, P1363, DOI 10.1128/JCM.02180-07

VANSOOLINGEN D, 1991, J CLIN MICROBIOL, V29, P2578, DOI 10.1128/JCM.29.11.2578-2586.1991

Von Groll A, 2010, FEMS IMMUNOL MED MIC, V58, P119, DOI 10.1111/j.1574-695X.2009.00611.x

Wada T, 2009, INFECT GENET EVOL, V9, P921, DOI 10.1016/j.meegid.2009.06.012

Wang LX, 2014, LANCET, V383, P2057, DOI 10.1016/S0140-6736(13)62639-2

Weisenberg SA, 2012, INFECT GENET EVOL, V12, P664, DOI 10.1016/j.meegid.2011.07.018

Zhao YL, 2012, NEW ENGL J MED, V366, P2161, DOI 10.1056/NEJMoa1108789

NR 36

TC 15

Z9 17

U1 2

U2 13

PU CHURCHILL LIVINGSTONE

PI EDINBURGH

PA JOURNAL PRODUCTION DEPT, ROBERT STEVENSON HOUSE, 1-3 BAXTERS PLACE,

LEITH WALK, EDINBURGH EH1 3AF, MIDLOTHIAN, SCOTLAND

SN 1472-9792

J9 TUBERCULOSIS

JI Tuberculosis

PD MAR

PY 2016

VL 97

BP 18

EP 25

DI 10.1016/j.tube.2015.11.007

PG 8

WC Immunology; Microbiology; Respiratory System

WE Science Citation Index Expanded (SCI-EXPANDED)

SC Immunology; Microbiology; Respiratory System

GA DI5KS

UT WOS:000373538400003

DA 2026-05-07

ER

PT J

AU Riccardi, N

Giacomelli, A

Canetti, D

Comelli, A

Intini, E

Gaiera, G

Diaw, MM

Udwadia, Z

Besozzi, G

Codecasa, L

Di Biagio, A

AF Riccardi, Niccolo

Giacomelli, Andrea

Canetti, Diana

Comelli, Agnese

Intini, Enrica

Gaiera, Giovanni

Diaw, Mama M.

Udwadia, Zarir

Besozzi, Giorgio

Codecasa, Luigi

Di Biagio, Antonio

TI Clofazimine: an old drug for never-ending diseases

SO FUTURE MICROBIOLOGY

LA English

DT Review

DE clofazimine; leprosy; MDR-TB; NTM; XDR-TB

ID MULTIDRUG-RESISTANT TUBERCULOSIS; MYCOBACTERIUM-ABSCESSUS;

ANTITUBERCULOSIS DRUGS; LEPROSY; PHARMACOKINETICS; THERAPY; BEDAQUILINE;

COMBINATION; PREVALENCE; AMIKACIN

AB Clofazimine (CFZ), an old hydrophobic riminophenazine, has a wide range of antimycobacterial activity ranging from leprosy to nontuberculous mycobacterial diseases. CFZ has several advantages such as a favorable pharmacokinetic profile, dose-dependent side effects as well as low price. In this narrative review, we have assessed the clinical development of CFZ, starting from the potential in vitro mechanism of actions, to the spectrum of side effects and potential drug-drug interactions, highlighting its current place in therapy and future possible use in leprosy, nontuberculous mycobacterial diseases and drug-resistant tuberculosis.

C1 [Riccardi, Niccolo] IRCCS Sacro Cuore Don Calabria Hosp, Dept Infect Trop Dis & Microbiol, Verona, Italy.

[Riccardi, Niccolo; Giacomelli, Andrea; Canetti, Diana; Intini, Enrica; Diaw, Mama M.; Besozzi, Giorgio; Codecasa, Luigi; Di Biagio, Antonio] StopTB Italia Onlus, Milan, Italy.

[Giacomelli, Andrea] ASST Fatebenefratelli Sacco, Infect Dis Unit 3, Milan, Italy.

[Giacomelli, Andrea] Univ Milan, Dept Biomed & Clin Sci Luigi Sacco, Milan, Italy.

[Canetti, Diana] Univ Vita Salute San Raffaele, Milan, Italy.

[Canetti, Diana; Gaiera, Giovanni] IRCCS San Raffaele Sci Inst, Clin Infect Dis, Milan, Italy.

[Comelli, Agnese] Spedali Civil Brescia, Dept Infect & Trop Dis, Brescia, Italy.

[Intini, Enrica] Univ Cattolica Sacro Cuore, A Gemelli Univ Hosp, Div Resp Med, Rome, Italy.

[Diaw, Mama M.] Reg Med Thies, Thies, Senegal.

[Udwadia, Zarir] PD Hinduja Natl Hosp & Med Res Ctr, Dept Pulm Med, Mumbai, Maharashtra, India.

[Codecasa, Luigi] ASST Niguarda Ca Granda, Reg TB Reference Ctr & Lab, Villa Marelli Inst, Milan, Italy.

[Di Biagio, Antonio] IRCCS AOU San Martino IST, Clin Infect Dis, Genoa, Italy.

C3 IRCCS Sacro Cuore Don Calabria; University of Milan; Luigi Sacco

Hospital; Vita-Salute San Raffaele University; Vita-Salute San Raffaele

University; IRCCS Ospedale San Raffaele; Hospital Spedali Civili

Brescia; Catholic University of the Sacred Heart; IRCCS Policlinico

Gemelli; University of Genoa; IRCCS AOU San Martino IST

RP Riccardi, N (corresponding author), IRCCS Sacro Cuore Don Calabria Hosp, Dept Infect Trop Dis & Microbiol, Verona, Italy.; Riccardi, N (corresponding author), StopTB Italia Onlus, Milan, Italy.

EM niccolo.riccardi@yahoo.it

RI codecasa, luigi/K-2693-2018; Comelli, Agnese/AAZ-9857-2021; Riccardi,

Niccolo/ABG-7609-2020; Di Biagio, Antonio/J-4901-2019; Giacomelli,

Andrea/AAA-4642-2019

OI codecasa, luigi/0000-0001-6825-2050; Comelli,

Agnese/0000-0003-4817-8337; Riccardi, Niccolo/0000-0002-2947-4707;

intini, enrica/0000-0003-2981-270X; Giacomelli,

Andrea/0000-0003-3685-4289; Udwadia, Zarir/0000-0002-1830-3259

CR Access Campaign, 2018, WHO ISS NEW TB TREAT

Adjemian J, 2012, AM J RESP CRIT CARE, V185, P881, DOI 10.1164/rccm.201111-2016OC

Adler-Shohet FC, 2019, J PEDIAT INFECT DIS, DOI 10.1111/dth.12414

Ahmad N, 2018, LANCET, V392, P821, DOI 10.1016/S0140-6736(18)31644-1

Alcaide F, 2017, EUR J CLIN MICROBIOL, V36, P1425, DOI 10.1007/s10096-017-2949-0

[Anonymous], 2019, WHO Consolidated Guidelines on Drug-Resistant Tuberculosis Treatment

[Anonymous], 2009, ENH GLOB STRAT FURTH

[Anonymous], 2017, World Health Statistics 2017: Monitoring Health for The SDGs

[Anonymous], 1998, LAB PRESCR INF LAMPR

[Anonymous], 2018, Rapid communication: key changes to treatment of multidrug-and rifampicin-resistant tuberculosis (MDR/RR-TB)

AUSINA V, 1986, ANTIMICROB AGENTS CH, V29, P951, DOI 10.1128/AAC.29.5.951

Baik J, 2013, ANTIMICROB AGENTS CH, V57, P1218, DOI 10.1128/AAC.01731-12

Balestrino A, 2019, NEUROL SCI, V40, P1371, DOI 10.1007/s10072-019-03835-1

BARRY VC, 1957, NATURE, V179, P1013, DOI 10.1038/1791013a0

BROWNE S. G., 1962, LEPROSY REV, V33, P6

BROWNE S G, 1981, International Journal of Leprosy and Other Mycobacterial Diseases, V49, P167

Chaisson RE, 1997, AIDS, V11, P311, DOI 10.1097/00002030-199703110-00008

Diacon AH, 2015, AM J RESP CRIT CARE, V191, P943, DOI 10.1164/rccm.201410-1801OC

Duan H, 2019, CLIN MICROBIOL INFEC, V25, P190, DOI 10.1016/j.cmi.2018.07.012

Dutta R K, 1980, Lepr India, V52, P252

Ebenezer G J, 2002, Indian J Lepr, V74, P301

Faget G H, 1966, Int J Lepr Other Mycobact Dis, V34, P298

Ferro BE, 2016, ANTIMICROB AGENTS CH, V60, P1097, DOI 10.1128/AAC.02615-15

Griffith DE, 2007, AM J RESP CRIT CARE, V175, P367, DOI 10.1164/rccm.200604-571ST

Hartkoorn RC, 2014, ANTIMICROB AGENTS CH, V58, P2979, DOI 10.1128/AAC.00037-14

Haworth CS, 2017, THORAX, V72, DOI 10.1136/thoraxjnl-2017-210927

Horita Y, 2014, ANTIMICROB AGENTS CH, V58, P3168, DOI 10.1128/AAC.02278-13

Hwang TJ, 2014, BMJ OPEN, V4, DOI 10.1136/bmjopen-2013-004143

IMKAMP F M J H, 1973, Leprosy Review, V44, P127

IMKAMP FMJH, 1981, LEPROSY REV, V52, P135

Intini E, 2020, Indian J Tuberc, V67, P340, DOI [10.1016/j.ijtb.2019.06.005, 10.1016/j.ijtb.2019.06.005]

JACOBSON RR, 1976, LANCET, V2, P1304

Jarand J, 2016, CHEST, V149, P1285, DOI 10.1378/chest.15-0543

Jarand J, 2011, CLIN INFECT DIS, V52, P565, DOI 10.1093/cid/ciq237

Knight GM, 2019, LANCET INFECT DIS, V19, P903, DOI 10.1016/S1473-3099(19)30307-X

Lange C, 2019, CLIN MICROBIOL INFEC, V25, P128, DOI 10.1016/j.cmi.2018.11.010

Lechartier B, 2015, ANTIMICROB AGENTS CH, V59, P4457, DOI 10.1128/AAC.00395-15

Luo JJ, 2018, ANTIMICROB AGENTS CH, V62, DOI 10.1128/AAC.00072-18

Maartens G, 2018, INT J TUBERC LUNG D, V22, P26, DOI 10.5588/ijtld.17.0615

Maia MV, 2013, AN BRAS DERMATOL, V88, P205, DOI 10.1590/S0365-05962013000200003

Martiniano SL, 2017, CHEST, V152, P800, DOI 10.1016/j.chest.2017.04.175

Nahid P, 2019, AM J RESP CRIT CARE, V200, pE93, DOI 10.1164/rccm.201909-1874ST

Narang T, 2019, AM J TROP MED HYG, V100, P24, DOI 10.4269/ajtmh.18-0256

Nix DE, 2004, TUBERCULOSIS, V84, P365, DOI 10.1016/j.tube.2004.04.001

Ozturk Z, 2017, DERMATOL THER, V30, DOI 10.1111/dth.12414

Padayatchi N, 2014, J ANTIMICROB CHEMOTH, V69, P3103, DOI 10.1093/jac/dku235

Penna GO, 2017, PLOS NEGLECT TROP D, V11, DOI 10.1371/journal.pntd.0005725

Penna GO, 2011, LEPROSY REV, V82, P210

Prevots DR, 2017, EUR RESPIR J, V49, DOI 10.1183/13993003.00374-2017

Reddy VM, 1996, ANTIMICROB AGENTS CH, V40, P633, DOI 10.1128/AAC.40.3.633

Riccardi N, 2020, J INFECT CHEMOTHER, V26, P199, DOI 10.1016/j.jiac.2019.11.004

Riccardi N, 2019, BMC INFECT DIS, V19, DOI 10.1186/s12879-019-4211-0

Riccardi Niccolo, 2018, Recent Pat Antiinfect Drug Discov, V13, P3, DOI [10.2174/1574891x12666170619101904, 10.2174/1574891X12666170619101904]

Sangana R, 2018, DRUG METAB DISPOS, V46, P26, DOI 10.1124/dmd.117.077834

SCHAADLANYI Z, 1987, INT J LEPROSY, V55, P9

Shen GH, 2010, INT J ANTIMICROB AG, V35, P400, DOI 10.1016/j.ijantimicag.2009.12.008

Shimokawa Y, 2015, BIOL PHARM BULL, V38, P1425, DOI 10.1248/bpb.b15-00313

Singh S, 2014, CLIN MICROBIOL INFEC, V20, pO1124, DOI 10.1111/1469-0691.12780

Smith CS, 2017, LANCET INFECT DIS, V17, pE293, DOI 10.1016/S1473-3099(17)30418-8

Swanson RV, 2015, ANTIMICROB AGENTS CH, V59, P3042, DOI 10.1128/AAC.00260-15

Tang SJ, 2015, CLIN INFECT DIS, V60, P1361, DOI 10.1093/cid/civ027

van Ingen J, 2017, EUR RESPIR J, V49, DOI 10.1183/13993003.01855-2016

van Ingen J, 2012, ANTIMICROB AGENTS CH, V56, P6324, DOI 10.1128/AAC.01505-12

van Ingen J, 2010, INT J ANTIMICROB AG, V35, P169, DOI 10.1016/j.ijantimicag.2009.09.023

Venkatesan K, 1997, LEPROSY REV, V68, P242

Villellas C, 2017, J ANTIMICROB CHEMOTH, V72, P684, DOI 10.1093/jac/dkw502

WHO, 1977, TECHNICAL REPORT SER, V607

WHO, 2003, WHO LEPR EL PROJ STA

WHO Study Group on Chemotherapy, 1994, WHO TECHN REP SER, V847

Williams DL, 2012, LEPROSY REV, V83, P269

World Health Organisation, 1982, TECHNICAL REPORT SER, V675

Xu HB, 2012, CLIN MICROBIOL INFEC, V18, P1104, DOI 10.1111/j.1469-0691.2011.03716.x

Xu J, 2017, ANTIMICROB AGENTS CH, V61, DOI [10.1128/AAC.00239-17, 10.1128/aac.00239-17]

Yang B., 2017, SUSTAINABILITY, V9, P1

Yang B, 2017, ANTIMICROB AGENTS CH, V61, DOI [10.1128/AAC.02052-16, 10.1128/aac.02052-16]

Yano T, 2011, J BIOL CHEM, V286, P10276, DOI 10.1074/jbc.M110.200501

Yee D, 2003, AM J RESP CRIT CARE, V167, P1472, DOI 10.1164/rccm.200206-626OC

Zweijpfenning SMH, 2018, EUR RESPIR J, V52, DOI 10.1183/13993003.01386-2018

NR 78

TC 26

Z9 31

U1 1

U2 9

PU FUTURE MEDICINE LTD

PI LONDON

PA UNITEC HOUSE, 3RD FLOOR, 2 ALBERT PLACE, FINCHLEY CENTRAL, LONDON, N3

1QB, ENGLAND

SN 1746-0913

EI 1746-0921

J9 FUTURE MICROBIOL

JI Future Microbiol.

PD MAY

PY 2020

VL 15

IS 7

BP 557

EP 566

DI 10.2217/fmb-2019-0231

PG 10

WC Microbiology

WE Science Citation Index Expanded (SCI-EXPANDED)

SC Microbiology

GA QA2LB

UT WOS:000613278800010

DA 2026-05-07

ER

PT J

AU Nagdev, PK

Agnivesh, PK

Roy, A

Sau, S

Kalia, NP

AF Nagdev, Pavan Kumar

Agnivesh, Puja Kumari

Roy, Arnab

Sau, Shashikanta

Kalia, Nitin Pal

TI Exploring and exploiting the host cell autophagy during Mycobacterium

tuberculosis infection

SO EUROPEAN JOURNAL OF CLINICAL MICROBIOLOGY & INFECTIOUS DISEASES

LA English

DT Review

DE Tuberculosis; Autophagy; Host defense; Host cell inhibitors

ID INHALABLE PARTICLES; MTOR INHIBITORS; IFN-GAMMA; PROTEIN; MACROPHAGES;

MATURATION; MECHANISM; LIPOARABINOMANNAN; RAPAMYCIN; YEAST

AB Tuberculosis, caused by Mycobacterium tuberculosis, is a fatal infectious disease that prevails to be the second leading cause of death from a single infectious agent despite the availability of multiple drugs for treatment. The current treatment regimen involves the combination of several drugs for 6 months that remain ineffective in completely eradicating the infection because of several drawbacks, such as the long duration of treatment and the side effects of drugs causing non-adherence of patients to the treatment regimen. Autophagy is an intracellular degradative process that eliminates pathogens at the early stages of infection. Mycobacterium tuberculosis's unique autophagy-blocking capability makes it challenging to eliminate compared to usual pathogens. The present review discusses recent advances in autophagy-inhibiting factors and mechanisms that could be exploited to identify autophagy-inducing chemotherapeutics that could be used as adjunctive therapy with the existing first-line anti-TB agent to shorten the duration of therapy and enhance cure rates from multidrug-resistant tuberculosis (MDR-TB) and extreme drug-resistant tuberculosis (XDR-TB).

C1 [Nagdev, Pavan Kumar; Agnivesh, Puja Kumari; Roy, Arnab; Sau, Shashikanta; Kalia, Nitin Pal] Natl Inst Pharmaceut Educ & Res NIPER, Dept Biol Sci Pharmacol & Toxicol, Hyderabad 500037, Telangana, India.

C3 National Institute of Pharmaceutical Education & Research, S.A.S. Nagar

(Mohali); National Institute of Pharmaceutical Education & Research,

Hyderabad

RP Kalia, NP (corresponding author), Natl Inst Pharmaceut Educ & Res NIPER, Dept Biol Sci Pharmacol & Toxicol, Hyderabad 500037, Telangana, India.

EM nitin.kalia@niperhyd.ac.in

RI Kalia, Nitin Pal/AAE-5959-2019

OI Kalia, Nitin Pal/0000-0003-4346-6567; Roy, Arnab/0000-0003-3006-7997

FU We are thankful to the Department of Biotechnology, Ministry of Science

and Technology, New Delhi, India, for supporting tuberculosis research

by awarding Ramalingaswami Fellowship to NPK (D.O.NO.BT/HRD/35/02/2006;

No. BT/RLF/Re-entry 66/2017; GAP-39).

FX We are thankful to the Department of Biotechnology, Ministry of Science

and Technology, New Delhi, India, for supporting tuberculosis research

by awarding Ramalingaswami Fellowship to NPK (D.O.NO.BT/HRD/35/02/2006;

No. BT/RLF/Re-entry 66/2017; GAP-39).

CR Amano A, 2006, J BIOCHEM, V140, P161, DOI 10.1093/jb/mvj162

[Anonymous], 2014, First B, Paper E, P617, DOI [10.1182/blood-2013-11-535047, DOI 10.1182/BLOOD-2013-11-535047]

[Anonymous], 2021, Global tuberculosis report 2021

ARMSTRONG JA, 1971, J EXP MED, V134, P713, DOI 10.1084/jem.134.3.713

Ashley D, 2020, J CLIN MED, V9, DOI 10.3390/jcm9072043

Bach H, 2008, CELL HOST MICROBE, V3, P316, DOI 10.1016/j.chom.2008.03.008

Bafica A, 2005, J EXP MED, V202, P1715, DOI 10.1084/jem.20051782

Banaiee N, 2006, J IMMUNOL, V176, P3019, DOI 10.4049/jimmunol.176.5.3019

Barata P, 2018, INVEST NEW DRUG, V36, P451, DOI 10.1007/s10637-018-0574-0

Barnett TC, 2013, CELL HOST MICROBE, V14, P675, DOI 10.1016/j.chom.2013.11.003

Barrientos OM, 2021, LETT APPL MICROBIOL, V72, P351, DOI 10.1111/lam.13432

Bendell JC, 2015, INVEST NEW DRUG, V33, P187, DOI 10.1007/s10637-014-0177-3

Bento CF, 2015, DNA CELL BIOL, V34, P228, DOI 10.1089/dna.2014.2745

Bhatt K, 2021, DIS MODEL MECH, V14, DOI 10.1242/dmm.049018

Blay JY, 2011, SEMIN ONCOL, V38, pS3, DOI 10.1053/j.seminoncol.2011.01.016

Bruiners N, 2020, J LIPID RES, V61, P1617, DOI 10.1194/jlr.RA120000895

Cao Ruoqiong, 2021, BioMolecular Concepts, V12, P46, DOI [10.1515/bmc-2021-0006, 10.1515/bmc-2021-0006]

Cao WY, 2021, B CANCER, V108, P304, DOI 10.1016/j.bulcan.2020.11.004

Caron A, 2015, ANNU REV NUTR, V35, P321, DOI 10.1146/annurev-nutr-071714-034355

CHAN J, 1991, INFECT IMMUN, V59, P1755, DOI 10.1128/IAI.59.5.1755-1761.1991

Chauhan P, 2013, PLOS ONE, V8, DOI 10.1371/journal.pone.0077930

Chen W, 2011, Unusual regioversatility of acetyltransferase Eis, a cause of drug resistance in XDR-TB, V108, P1, DOI [10.1073/pnas.1105379108/-/DCSupplemental.www.pnas.org/cgi/doi/10.1073/pnas.1105379108, DOI 10.1073/PNAS.1105379108/-/DCSUPPLEMENTAL.WWW.PNAS.ORG/CGI/DOI/10.1073/PNAS.1105379108]

Chen WJ, 2012, BIOCHEMISTRY-US, V51, P4959, DOI 10.1021/bi3004473

Chen Z, 2015, JPN J INFECT DIS, V68, P420, DOI 10.7883/yoken.JJID.2014.466

Choi HH, 2010, FEBS LETT, V584, P2445, DOI 10.1016/j.febslet.2010.04.050

Choi SW, 2018, ANTIMICROB AGENTS CH, V62, DOI [10.1128/aac.01019-18, 10.1128/AAC.01019-18]

Cole ST, 1998, NATURE, V396, P190, DOI 10.1038/24206

Correia -Neves M, Biomarkers for tuberculosis: the case for lipoarabinomannan, DOI [10.1183/23120541.00115-2018, DOI 10.1183/23120541.00115-2018]

Cross GB, 2023, LANCET INFECT DIS, V23, P847, DOI 10.1016/S1473-3099(23)00067-1

Cui YY, 2016, TUBERCULOSIS, V97, P52, DOI 10.1016/j.tube.2015.12.009

CuiJ LiZ, 2021, IntImmu, Vnopharmacol91, DOI [10.1016/j, DOI 10.1016/J]

De P, 2021, J BIOL CHEM, V297, DOI 10.1016/j.jbc.2021.101265

Guerra-De-Blas PD, 2019, FRONT MICROBIOL, V10, DOI 10.3389/fmicb.2019.02097

Dikic I, 2018, NAT REV MOL CELL BIO, V19, P349, DOI 10.1038/s41580-018-0003-4

Ding SQ, 2019, J MICROBIOL BIOTECHN, V29, P989, DOI 10.4014/jmb.1811.11062

Ehlers S, 2013, FRONT IMMUNOL, V3, DOI 10.3389/fimmu.2012.00411

Essafi M., 2018, i v o r l a n o v l, DOI [10.3389/fcimb.2018.00327, DOI 10.3389/FCIMB.2018.00327]

Fassnacht M, 2015, LANCET ONCOL, V16, P426, DOI 10.1016/S1470-2045(15)70081-1

Fernandez-Soto P, 2019, SCI REP-UK, V9, DOI 10.1038/s41598-019-46731-6

Festjens N, 2011, EMBO MOL MED, V3, P222, DOI 10.1002/emmm.201000125

Floto RA, 2007, AUTOPHAGY, V3, P620, DOI 10.4161/auto.4898

Flynn JL, 2001, INFECT IMMUN, V69, P4195, DOI 10.1128/IAI.69.7.4195-4201.2001

Fratti RA, 2001, J CELL BIOL, V154, P631, DOI 10.1083/jcb.200106049

Fratti RA, 2003, P NATL ACAD SCI USA, V100, P5437, DOI 10.1073/pnas.0737613100

Furuta N, 2010, Combinational soluble N -ethylmaleimide-sensitive factor attachment protein receptor proteins VAMP8 and Vti1b mediate fusion of antimicrobial and canonical autophagosomes with lysosomes, V21, P1001, DOI [10.1091/mbc.E09, DOI 10.1091/MBC.E09]

Galanis E, 2015, Phase II trial of temsirolimus (CCI-779) in recurrent glioblastoma multiforme: a North Central Cancer Treatment Group Study, V23, DOI [10.1200/JCO.2005.23.622, DOI 10.1200/JCO.2005.23.622]

Gallagher EJ, 2012, ONCOGENE, V31, P3213, DOI 10.1038/onc.2011.495

Gannagé M, 2009, TRAFFIC, V10, P615, DOI 10.1111/j.1600-0854.2009.00883.x

Garg R, 2020, FRONT CELL INFECT MI, V10, DOI 10.3389/fcimb.2020.00461

Ge PP, 2022, AUTOPHAGY, V18, P576, DOI 10.1080/15548627.2021.1938912

Ghidini M, 2017, EXPERT OPIN INV DRUG, V26, P1229, DOI 10.1080/13543784.2017.1384813

Grange JM, 1996, J ETHNOPHARMACOL, V50, P49, DOI 10.1016/0378-8741(95)01331-8

Guo L, 2016, FRONT CELL INFECT MI, V6, DOI 10.3389/fcimb.2016.00134

Gupta A, 2012, IMMUNOBIOLOGY, V217, P363, DOI 10.1016/j.imbio.2011.07.008

Gupta A, 2016, PHARM RES-DORDR, V33, P1899, DOI 10.1007/s11095-016-1926-0

Gupta A, 2014, MOL PHARMACEUT, V11, P1201, DOI 10.1021/mp4006563

Ha J, 2015, Research Center for Tooth and. Mol. Aspects Med., DOI [10.1016/j.mam.2015.08.002, DOI 10.1016/J.MAM.2015.08.002]

Hong JY, 2014, INT J TUBERC LUNG D, V18, P73, DOI 10.5588/ijtld.13.0536

Hosokawa N., 2009, NUTRIENT DEPENDENT M, V20, P1981, DOI [10.1091/mbc.E08, DOI 10.1091/MBC.E08]

Houde VP, 2010, Deshaies Y, Chronic rapamycin treatment causes glucose intolerance and hyperlipidemia by upregulating hepatic gluconeogenesis and impairing lipid deposition in adipose tissue, V59, DOI [10.2337/db09-1324.V.P.H, DOI 10.2337/DB09-1324.V.P.H]

Hussain T, 2019, CELLS-BASEL, V8, DOI 10.3390/cells8050506

Hyttinen JMT, 2013, BBA-MOL CELL RES, V1833, P503, DOI 10.1016/j.bbamcr.2012.11.018

Itakura E, 2012, CELL, V151, P1256, DOI 10.1016/j.cell.2012.11.001

Jaimni V, 2021, PULM MED, V2021, DOI 10.1155/2021/5285841

Jayachandran R, 2007, CELL, V130, P37, DOI 10.1016/j.cell.2007.04.043

Juárez E, 2018, INT IMMUNOPHARMACOL, V65, P29, DOI 10.1016/j.intimp.2018.09.013

Kametaka S, 1998, J BIOL CHEM, V273, P22284, DOI 10.1074/jbc.273.35.22284

Keflie TS, 2015, NUTRITION, V31, P1204, DOI 10.1016/j.nut.2015.05.003

Khan MZ, 2021, ANTIMICROB AGENTS CH, V65, DOI 10.1128/AAC.02095-20

Khan MZ, 2017, J BIOL CHEM, V292, P16093, DOI 10.1074/jbc.M117.797563

Kirisako T, 1999, J CELL BIOL, V147, P435, DOI 10.1083/jcb.147.2.435

Klionsky DJ, 2003, DEV CELL, V5, P539, DOI 10.1016/S1534-5807(03)00296-X

Knight ZA, 2014, ELIFE, V3, DOI 10.7554/eLife.01913

Kolloli A, 2017, FRONT MED-LAUSANNE, V4, DOI 10.3389/fmed.2017.00171

Kumar R., 2017, EBP (3 regulate innate immune signaling, the polarization of macrophages and the trafficking of Mycobacterium tuberculosis to lysosomes during infection, P1, DOI DOI 10.1371/JOURNAL.PPAT.1006410

Labuzek K, 2010, N-S ARCH PHARMACOL, V381, P171, DOI 10.1007/s00210-009-0477-x

Lampson BL, 2016, BLOOD, V128, P195, DOI 10.1182/blood-2016-03-707133

Laopanupong T, 2021, SCI REP-UK, V11, DOI 10.1038/s41598-021-83835-4

Lee HJ, 2019, PLOS ONE, V14, DOI 10.1371/journal.pone.0199799

Liang C, 2006, NAT CELL BIOL, V8, P688, DOI 10.1038/ncb1426

Lima A, 2021, J PROTEOMICS, V244, DOI 10.1016/j.jprot.2021.104276

Liu F, 2018, NAT COMMUN, V9, DOI 10.1038/s41467-018-06836-4

Liu HR, 2022, CELLS-BASEL, V11, DOI 10.3390/cells11030361

Liu X, 2017, NAT CELL BIOL, V19, P626, DOI 10.1038/ncb3527

Liu YH, 2011, MOL IMMUNOL, V48, P1084, DOI 10.1016/j.molimm.2011.02.001

Lystad AH, 2019, AUTOPHAGY, V15, P1485, DOI 10.1080/15548627.2019.1618100

Mahadik K, 2018, FRONT IMMUNOL, V9, DOI 10.3389/fimmu.2018.00085

Mahairas GG, 1996, J BACTERIOL, V178, P1274, DOI 10.1128/jb.178.5.1274-1282.1996

Martineau AR, 2007, J IMMUNOL, V178, P7190, DOI 10.4049/jimmunol.178.11.7190

Martins F, 2013, ORAL ONCOL, V49, P293, DOI 10.1016/j.oraloncology.2012.11.008

Mawatwal S, 2017, BBA-GEN SUBJECTS, V1861, P3190, DOI 10.1016/j.bbagen.2017.09.010

Mily A, 2015, PLOS ONE, V10, DOI 10.1371/journal.pone.0138340

Mizushima N, 1998, NATURE, V395, P395, DOI 10.1038/26506

Mizushima N, 2011, COLD SH Q B, V76, P397, DOI 10.1101/sqb.2011.76.011023

Mizushima N, 1999, EMBO J, V18, P3888, DOI 10.1093/emboj/18.14.3888

Mizushima N, 2018, NAT CELL BIOL, V20, P521, DOI 10.1038/s41556-018-0092-5

Morelli E, 2014, AUTOPHAGY, V10, P2251, DOI 10.4161/15548627.2014.981913

Mori M, 2018, FRONT CELL INFECT MI, V8, DOI 10.3389/fcimb.2018.00077

Nascimbeni AC, 2017, EMBO J, V36, P2018, DOI 10.15252/embj.201797006

Neuhaus P, 2001, LIVER TRANSPLANT, V7, P473, DOI 10.1053/jlts.2001.24645

Neumann Y, 2016, AUTOPHAGY, V12, P2069, DOI 10.1080/15548627.2016.1226732

O'Brien J, 2018, FRONT ENDOCRINOL, V9, DOI 10.3389/fendo.2018.00402

Orme IM, 2014, SEMIN IMMUNOL, V26, P601, DOI 10.1016/j.smim.2014.09.009

Ouimet M, 2016, NAT IMMUNOL, V17, P677, DOI 10.1038/ni.3434

Ouyang Q, 2020, MSPHERE, V5, DOI 10.1128/mSphere.00124-20

Padhi A, 2019, J IMMUNOL, V203, P2665, DOI 10.4049/jimmunol.1801301

Padmapriydarsini C, 2022, Randomized trial of metfor min with anti-tuberculosis drugs for early sputum conversion in adults with pulmonary tuberculosis, V75, P425, DOI [10.1093/cid/ciab964, DOI 10.1093/CID/CIAB964]

Pan Q, 2018, SCI REP-UK, V8, DOI 10.1038/s41598-018-20802-6

Parzych KR, An overview of autophagy: morphology, mechanism, and regulation

Peng XL, 2016, TOXICON, V116, P29, DOI 10.1016/j.toxicon.2015.10.003

Pickar James H, 2015, Post Reprod Health, V21, P112, DOI [10.1177/2053369115599090, 10.1177/2053369115599090]

Polson HEJ, 2010, AUTOPHAGY, V6, P506, DOI 10.4161/auto.6.4.11863

Proikas-Cezanne T, 2004, ONCOGENE, V23, P9314, DOI 10.1038/sj.onc.1208331

Puri RV, 2013, PLOS ONE, V8, DOI 10.1371/journal.pone.0070514

Qu YL, 2019, CELL IMMUNOL, V337, P22, DOI 10.1016/j.cellimm.2019.01.004

Rabahi MF, 2017, J BRAS PNEUMOL, V43, P472, DOI [10.1590/S1806-37562016000000388, 10.1590/s1806-37562016000000388]

Rekha RS, 2018, BMC INFECT DIS, V18, DOI 10.1186/s12879-018-3203-9

Rekha RS, 2015, AUTOPHAGY, V11, P1688, DOI 10.1080/15548627.2015.1075110

Rugo HS, 2016, ANN ONCOL, V27, P519, DOI 10.1093/annonc/mdv595

Samuel LP, 2007, MICROBIOL-SGM, V153, P529, DOI 10.1099/mic.0.2006/002642-0

Schmid D, 2006, J MOL MED-JMM, V84, P194, DOI 10.1007/s00109-005-0014-4

Sengupta S, 2021, FRONT CELL INFECT MI, V11, DOI 10.3389/fcimb.2021.676456

Seto S, 2012, CELL MICROBIOL, V14, P710, DOI 10.1111/j.1462-5822.2012.01754.x

Shariq M, 2021, FRONT IMMUNOL, V12, DOI 10.3389/fimmu.2021.636644

Sharma A, 2020, ACS BIOMATER SCI ENG, V6, P4126, DOI 10.1021/acsbiomaterials.0c00823

Shin DM, 2010, PLOS PATHOG, V6, DOI 10.1371/journal.ppat.1001230

SIBLEY LD, 1988, INFECT IMMUN, V56, P1232, DOI 10.1128/IAI.56.5.1232-1236.1988

Singh P, 2018, FRONT MICROBIOL, V9, DOI 10.3389/fmicb.2018.00070

Singhal A, 2014, SCI TRANSL MED, V6, DOI 10.1126/scitranslmed.3009885

Sinha S, 2022, Coronin-1 levels in patients with tuberculosis, P866, DOI [10.4103/ijmr.IJMR, DOI 10.4103/IJMR.IJMR]

Sleat DE, 2004, P NATL ACAD SCI USA, V101, P5886, DOI 10.1073/pnas.0308456101

Strong EJ, 2022, MBIO, V13, DOI 10.1128/mbio.02974-21

Strong LM, 2021, ELIFE, V10, DOI [10.7554/eLife.70372, 10.7554/eLife.70372.sa1, 10.7554/eLife.70372.sa2]

Talat N, 2010, EMERG INFECT DIS, V16, P853, DOI 10.3201/eid1605.091693

Taylor P, 2011, andesio scienceo not distribute, DOI [10.4161/auto.7.8.15860, DOI 10.4161/AUTO.7.8.15860]

Taylor P, Syntaxin, V17, P4, DOI [10.4161/auto.24109, DOI 10.4161/AUTO.24109]

Tong YJ, 2015, AUTOPHAGY, V11, P1192, DOI 10.1080/15548627.2015.1054594

Turner J, 2018, Mannose-capped lipoarabinomannan in Mycobacterium tuberculosis pathogenesis, DOI [10.1093/femspd/fty026/4953419, DOI 10.1093/FEMSPD/FTY026/4953419]

Wallis RS, 2021, LANCET RESP MED, V9, P897, DOI 10.1016/S2213-2600(20)30448-3

Wallis RS, 2015, NAT REV IMMUNOL, V15, P255, DOI 10.1038/nri3813

Wang H, 2022, Human kinase IGF1R/IR inhibitor linsitinib controls the in vitro and intracellular growth of, DOI [10.1021/acsinfecdis.2c00278, DOI 10.1021/ACSINFECDIS.2C00278]

Wang J, 2022, MICROBIOL SPECTR, V10, DOI 10.1128/spectrum.02509-22

Wang J, 2017, NAT COMMUN, V8, DOI 10.1038/s41467-017-00279-z

Weidberg H, 2010, EMBO J, V29, P1792, DOI 10.1038/emboj.2010.74

Welin A, 2008, INFECT IMMUN, V76, P2882, DOI 10.1128/IAI.01549-07

Wen YL, 2022, BMC INFECT DIS, V22, DOI 10.1186/s12879-022-07427-x

Wong D, 2011, P NATL ACAD SCI USA, V108, P19371, DOI 10.1073/pnas.1109201108

Xu J, 2010, P NATL ACAD SCI USA, V107, P4764, DOI 10.1073/pnas.0910872107

Xu SJ, 2022, ACTA PHARM SIN B, V12, P4519, DOI 10.1016/j.apsb.2022.10.001

Yang AM, 2017, ELIFE, V6, DOI 10.7554/eLife.23905

Yang SJ, 2015, CELL PHYSIOL BIOCHEM, V35, P1276, DOI 10.1159/000373950

Zaunbrecher MA, 2009, P NATL ACAD SCI USA, V106, P20004, DOI 10.1073/pnas.0907925106

Zumla A, 2015, LANCET RESP MED, V3, P220, DOI 10.1016/S2213-2600(15)00063-6

NR 153

TC 12

Z9 12

U1 3

U2 9

PU SPRINGER

PI NEW YORK

PA ONE NEW YORK PLAZA, SUITE 4600, NEW YORK, NY, UNITED STATES

SN 0934-9723

EI 1435-4373

J9 EUR J CLIN MICROBIOL

JI Eur. J. Clin. Microbiol. Infect. Dis.

PD NOV

PY 2023

VL 42

IS 11

BP 1297

EP 1315

DI 10.1007/s10096-023-04663-0

EA SEP 2023

PG 19

WC Infectious Diseases; Microbiology

WE Science Citation Index Expanded (SCI-EXPANDED)

SC Infectious Diseases; Microbiology

GA U2LQ8

UT WOS:001093114600001

DA 2026-05-07

ER

PT J

AU Rao, M

Ippolito, G

Mfinanga, S

Ntoumi, F

Yeboah-Manu, D

Vilaplana, C

Zumla, A

Maeurer, M

AF Rao, Martin

Ippolito, Giuseppe

Mfinanga, Sayoki

Ntoumi, Francine

Yeboah-Manu, Dorothy

Vilaplana, Cris

Zumla, Alimuddin

Maeurer, Markus

TI Improving treatment outcomes for MDR-TB - Novel host-directed therapies

and personalised medicine of the future

SO INTERNATIONAL JOURNAL OF INFECTIOUS DISEASES

LA English

DT Article

DE Multidrug-resistant tuberculosis; Clinical studies; Host-directed

therapies; Personalised medicine; Immunotherapy; Biomarkers

ID DRUG-RESISTANT TUBERCULOSIS; CELLS; MULTIDRUG; METFORMIN; IMMUNE

AB Multidrug-resistant TB (MDR-TB) is a major threat to global health security. In 2017, only 50% of patients with MDR-TB who received WHO-recommended treatment were cured. Most MDR-TB patients who recover continue to suffer from functional disability due to long-term lung damage. Whilst new MDR-TB treatment regimens are becoming available, conventional drug therapies need to be complemented with host-directed therapies (HDTs) to reduce tissue damage and improve functional treatment outcomes. This viewpoint highlights recent data on biomarkers, immune cells, circulating effector molecules and genetics which could be utilised for developing personalised HDTs. Novel technologies currently used for cancer therapy which could facilitate in-depth understanding of host genetics and the microbiome in patients with MDR-TB are discussed. Against this background, personalised cell-based HDTs for adjunct MDR-TB treatment to improve clinical outcomes are proposed as a possibility for complementing standard therapy and other HDT agents. Insights into the molecular biology of the mechanisms of action of cellular HDTs may also aid to devise non-cell-based therapies targeting defined inflammatory pathway (s) in Mtb-driven immunopathology. (c) 2019 Published by Elsevier Ltd on behalf of International Society for Infectious Diseases. This is an open access article under the CC BY-NC-ND license (http://creativecommons.org/licenses/by-nc-nd/4.0/).

C1 [Rao, Martin; Maeurer, Markus] Champalimaud Ctr Unknown, ImmunoSurg Unit, Lisbon, Portugal.

[Ippolito, Giuseppe] Lazzaro Spallanzani, Natl Inst Infect Dis, Rome, Italy.

[Mfinanga, Sayoki] Natl Inst Med Res Muhimbili, Dar Es Salaam, Tanzania.

[Ntoumi, Francine] Univ Marten NGouabi, Brazzaville, Rep Congo.

[Ntoumi, Francine] FCRM, Brazzaville, Rep Congo.

[Ntoumi, Francine] Univ Tubingen, Inst Trop Med, Tubingen, Germany.

[Yeboah-Manu, Dorothy] Noguchi Mem Inst Med Res, Dept Bacteriol, Accra, Ghana.

[Vilaplana, Cris] UAB, Fdn Inst Germans Trias & Pujol IGTP, Expt TB Unit UTE, Badalona, Catalonia, Spain.

[Zumla, Alimuddin] UCL, Div Infect & Immun, London, England.

[Zumla, Alimuddin] UCL Hosp NHS Fdn Trust, NIHR Biomed Res Ctr, London, England.

[Maeurer, Markus] Krankenhaus NW Frankfurt, Dept Oncol & Haematol, Frankfurt, Germany.

C3 Fundacao Champalimaud; National Institute of Medical Research; Eberhard

Karls University of Tubingen; University of Ghana; Autonomous University

of Barcelona; University of London; University College London;

University of London; University College London; Krankenhaus Nordwest

RP Maeurer, M (corresponding author), Champalimaud Ctr Unknown, Ave Brasilia, P-1400038 Lisbon, Portugal.

EM martin.rao@research.fchampalimaud.org; giuseppe.ippolito@inmi.it;

gsmfinanga@yahoo.com; ffntoumi@hotmail.com;

dyeboah-manu@noguchi.ug.edu.gh; cvilaplana@gmail.com; a.zumla@ucl.ac.uk;

markus.maeurer@fundacaochampalimaud.pt

RI Vilaplana, Cristina/K-7216-2012; Rao, Martin/B-8996-2015; Ippolito,

Giuseppe/J-7207-2017; Zumla, Sir Alimuddin/ABA-8208-2020

OI Vilaplana, Cristina/0000-0002-2808-7270; Ippolito,

Giuseppe/0000-0002-1076-2979; Rao, Martin/0000-0002-0579-1097; Maeurer,

Markus/0000-0002-8436-8077; Zumla, Sir Alimuddin/0000-0002-5111-5735

FU European and Developing Countries Clinical Trials Partnership (EDCTP2)

programme [RIA2016E-1609]; Italian Ministry of Health [4]; Champalimaud

Foundation; National Institute for Health Research [NF-SI-0616-10016]

Funding Source: researchfish

FX Giuseppe Ippolito, Sayoki Mfinanga, Francine Ntoumi, Dorothy Yeboah-Manu

and Alimuddin Zumla are members of the PANDORA-ID-NET consortium

supported by the European and Developing Countries Clinical Trials

Partnership (EDCTP2) programme (Grant Agreement RIA2016E-1609). GI

received support from the Italian Ministry of Health (Grant Ricerca

Corrente, Research programme n.4: Tuberculosis). The views and opinions

of authors expressed herein do not necessarily state or reflect those of

EDCTP. Markus Maeurer reports receiving funding from the Champalimaud

Foundation.

CR [Anonymous], 2018, Global TB report, P243

Baraliakos X, 2018, LANCET, V392, P2415, DOI 10.1016/S0140-6736(18)32340-7

Castro-Garza J, 2018, MEM I OSWALDO CRUZ, V113, P161, DOI 10.1590/0074-02760170281

Cheng CF, 2018, SCI REP-UK, V8, DOI 10.1038/s41598-018-22061-x

Chowdhury RR, 2018, NATURE, V560, P644, DOI 10.1038/s41586-018-0439-x

Critchley JA, 2016, COCHRANE DB SYST REV

Degner NR, 2018, CLIN INFECT DIS, V66, P198, DOI 10.1093/cid/cix819

Galipeau J, 2018, CELL STEM CELL, V22, P824, DOI 10.1016/j.stem.2018.05.004

Garand M, 2018, FRONT IMMUNOL, V9, DOI 10.3389/fimmu.2018.00257

Gröschel MI, 2018, PLOS PATHOG, V14, DOI 10.1371/journal.ppat.1007297

Harishankar M, 2018, FRONT MED-LAUSANNE, V5, DOI 10.3389/fmed.2018.00213

Hong BY, 2018, TUBERCULOSIS, V109, P102, DOI 10.1016/j.tube.2018.02.006

Hong BY, 2016, CLIN MICROBIOL REV, V29, P915, DOI 10.1128/CMR.00096-15

Jarry U, 2016, ONCOIMMUNOLOGY, V5, DOI 10.1080/2162402X.2016.1168554

Kroesen VM, 2017, FRONT IMMUNOL, V8, DOI 10.3389/fimmu.2017.00772

Landskron G, 2014, J IMMUNOL RES, V2014, DOI 10.1155/2014/149185

Lange C, 2018, RESPIROLOGY, V23, P656, DOI 10.1111/resp.13304

Lee MC, 2018, PLOS ONE, V13, DOI 10.1371/journal.pone.0205807

Lee YJ, 2018, KOREAN J INTERN MED, V33, P933, DOI 10.3904/kjim.2017.249

Li D, 2017, PLOS ONE, V12

Maji A, 2018, ENVIRON MICROBIOL, V20, P402, DOI 10.1111/1462-2920.14015

Marupuru S, 2017, BRAZ J INFECT DIS, V21, P312, DOI 10.1016/j.bjid.2017.01.001

Mizukami A, 2018, STEM CELLS INT, V2018, DOI 10.1155/2018/4083921

Mpande CAM, 2018, FRONT IMMUNOL, V9, DOI 10.3389/fimmu.2018.00324

Nagu T, 2017, IMPROVING TREATMENT, P64

Rao M, 2018, ONCOIMMUNOLOGY PRACT, P41, DOI [10.1007/978-3-319-62431-0_4, DOI 10.1007/978-3-319-62431-0_4]

Robien K, 2013, NUTR CLIN PRACT, V28, P194, DOI 10.1177/0884533612467824

Rothschilds AM, 2019, TRENDS IMMUNOL, V40, P12, DOI 10.1016/j.it.2018.11.003

Singhal A, 2014, SCI TRANSL MED, V6, DOI 10.1126/scitranslmed.3009885

Skolimowska KH, 2012, PLOS ONE, V7, DOI 10.1371/journal.pone.0046481

Skrahin A, 2014, LANCET RESP MED, V2, P108, DOI 10.1016/S2213-2600(13)70234-0

Skrahin AE, 2016, EUR RESPIR J, V48, DOI 10.1183/13993003.congress-2016.PA1919

Stylianou E, 2018, INFECT IMMUN, V86, DOI 10.1128/IAI.00014-18

Suchman L., 2018, HEALTH POLICY PLANN, V33, P777, DOI [10.1093/heapol/czy053, DOI 10.1093/heapol/czy053]

Sun ET, 2017, MED SCI MONITOR, V23, P5330, DOI 10.12659/MSM.904309

Tiberi S, 2018, LANCET INFECT DIS, V18, pE183, DOI 10.1016/S1473-3099(18)30110-5

Veluchamy JP, 2017, FRONT IMMUNOL, V8, DOI 10.3389/fimmu.2017.00631

Wallis RS, 2016, OPEN FORUM INFECT DI, V3, DOI 10.1093/ofid/ofw151

Wallis RS, 2009, CLIN INFECT DIS, V48, P1429, DOI 10.1086/598504

Wang LT, 2016, J BIOMED SCI, V23, DOI 10.1186/s12929-016-0289-5

Wáng YXJ, 2018, QUANT IMAG MED SURG, V8, P161, DOI 10.21037/qims.2018.03.06

Wipperman MF, 2017, SCI REP-UK, V7, DOI 10.1038/s41598-017-10346-6

NR 42

TC 30

Z9 34

U1 0

U2 8

PU ELSEVIER SCI LTD

PI OXFORD

PA THE BOULEVARD, LANGFORD LANE, KIDLINGTON, OXFORD OX5 1GB, OXON, ENGLAND

SN 1201-9712

EI 1878-3511

J9 INT J INFECT DIS

JI Int. J. Infect. Dis.

PD MAR

PY 2019

VL 80

SU S

BP S62

EP S67

DI 10.1016/j.ijid.2019.01.039

PG 6

WC Infectious Diseases

WE Science Citation Index Expanded (SCI-EXPANDED)

SC Infectious Diseases

GA HO1JF

UT WOS:000460660800014

OA Green Submitted, gold

DA 2026-05-07

ER

PT J

AU Zhao, JW

Gao, SH

Chen, CG

Li, H

Wang, SH

Yu, YM

Ming, L

AF Zhao, Junwei

Gao, ShuHui

Chen, Chunguang

Li, Hui

Wang, Shaohua

Yu, Yongmin

Ming, Liang

TI Screening and identification of differentially expressed long non-coding

RNAs in multidrug-resistant tuberculosis

SO PEERJ

LA English

DT Article

DE Long non-coding RNAs; Multidrug-resistant tuberculosis; Biomarkers;

n335659; Serum

ID BIOMARKERS

AB Background. Efforts to eradicate tuberculosis are largely threatened by drug-resistant tuberculosis, particularly, multidrug-resistant tuberculosis (MDR-TB). Screening and identification potential biomarkers for MDR-TB is crucial to diagnose early and reduce the incidence of MDR-TB.

Methods. To screen the differentially expressed long non-coding RNAs in MDR-TB, the lncRNA and mRNA expression profiles in serum derived from healthy controls (HCs), individuals with MDR-TB and drug-sensitive tuberculosis (DS-TB) were analyzed by microarray assay and 10 lncRNAs were randomly selected for further validation by reverse transcription-quantitative real-time PCR(RT-qPCR). The biological functions of differentially expressed mRNAs as well as relationships between genes and signaling pathways were investigated using Gene Ontology (GO) and the Kyoto Encyclopedia of Genes and Genomes (KEGG), respectively.

Results. A total of 353 differentially expressed lncRNAs (312 upregulated) and 202 mRNAs (99 upregulated) were found in the MDR-TB group compared to HCs. And compared with the DS-TB group, 442 differentially expressed lncRNAs (115 upregulated) and 190 mRNAs (87 upregulated) were found in the MDR-TB group. The expression levels of lncRNA n335659 were found to differ significantly between each group by RT-qPCR. Compared with DS-TB group, the GO analysis showed that the differential mRNAs were mainly enriched in the processes associated with the detection of the chemical stimulus, the regulation of mRNA metabolic process and neutrophil activation in the MDR-TB group; the KEGG analysis indicated that the differential mRNAs between DS-TB and MDR-TB were mainly enriched in proteasome and Notch signaling pathway, which might reveal a fraction of the mechanism of MDR-TB. The discovery of the serum lncRNA n335659 might serve as a potential biomarker for MDR-TB and Notch signaling pathway provided a new clue for the investigation of the pathological mechanism of MDR-TB.

C1 [Zhao, Junwei; Gao, ShuHui; Ming, Liang] Zhengzhou Univ, Dept Clin Lab, Key Clin Lab Henan Prov, Affiliated Hosp 1, Zhengzhou, Peoples R China.

[Chen, Chunguang; Yu, Yongmin] Henan Prov Infect Dis Hosp, Dept Clin Lab, Zhengzhou, Peoples R China.

[Li, Hui; Wang, Shaohua] Ctr Dis Control & Prevent Henan Prov, TB Reference Lab, Zhengzhou, Peoples R China.

C3 Zhengzhou University

RP Ming, L (corresponding author), Zhengzhou Univ, Dept Clin Lab, Key Clin Lab Henan Prov, Affiliated Hosp 1, Zhengzhou, Peoples R China.

EM mingliang3072@163.com

RI Wang, Shaohua/G-8194-2018

FU National Natural Science Foundation of China [81501715]; Natural Science

Foundation for Young Teachers of Zhengzhou University, China

[JC21854032]

FX This work was supported by National Natural Science Foundation of China

(81501715) and Natural Science Foundation for Young Teachers of

Zhengzhou University, China (JC21854032). The funders had no role in

study design, data collection and analysis, decision to publish, or

preparation of the manuscript.

CR BeLow M, 2020, CELLS-BASEL, V9, DOI 10.3390/cells9102204

Chen LL, 2016, TRENDS BIOCHEM SCI, V41, P761, DOI 10.1016/j.tibs.2016.07.003

Chen ZL, 2017, SCI REP-UK, V7, DOI 10.1038/s41598-017-17146-y

Coll-Martínez B, 2019, BIOMOLECULES, V9, DOI 10.3390/biom9090395

Dheda K, 2019, LANCET RESP MED, V7, P820, DOI 10.1016/S2213-2600(19)30263-2

Dheda K, 2017, LANCET RESP MED, V5, P291, DOI [10.1016/s2213-2600(17)30079-6, 10.1016/S2213-2600(17)30079-6]

Farah E, 2019, J BIOL CHEM, V294, P8543, DOI 10.1074/jbc.RA118.006983

Geffner L, 2009, INFECT IMMUN, V77, P5025, DOI 10.1128/IAI.00224-09

Geisler S, 2013, NAT REV MOL CELL BIO, V14, P699, DOI 10.1038/nrm3679

Guenin-Macé L, 2009, TRANSBOUND EMERG DIS, V56, P255, DOI 10.1111/j.1865-1682.2009.01072.x

He AB, 2019, AGING-US, V11, P6237, DOI 10.18632/aging.102185

Hu XJ, 2020, J CLIN MICROBIOL, V58, DOI 10.1128/JCM.01973-19

Li G, 2019, J CELL BIOCHEM, V120, P18014, DOI 10.1002/jcb.29104

Li QF, 2018, INT IMMUNOPHARMACOL, V54, P24, DOI 10.1016/j.intimp.2017.10.026

Li ZB, 2020, INT J INFECT DIS, V92, P141, DOI 10.1016/j.ijid.2020.01.005

Lichter DI, 2012, BLOOD, V120, P4513, DOI 10.1182/blood-2012-05-426924

Liu SZ, 2020, INT J MOL SCI, V21, DOI 10.3390/ijms21249359

Martineau AR, 2007, J CLIN INVEST, V117, P1988, DOI 10.1172/JCI31097

McAuliffe SM, 2012, P NATL ACAD SCI USA, V109, pE2939, DOI 10.1073/pnas.1206400109

Millard J, 2015, BMJ-BRIT MED J, V350, DOI 10.1136/bmj.h882

Quinn JJ, 2016, NAT REV GENET, V17, P47, DOI 10.1038/nrg.2015.10

Singh R, 2020, J APPL MICROBIOL, V128, P1547, DOI 10.1111/jam.14478

Statello L, 2021, NAT REV MOL CELL BIO, V22, P96, DOI 10.1038/s41580-020-00315-9

Tang XR, 2018, LANCET ONCOL, V19, P382, DOI 10.1016/S1470-2045(18)30080-9

Walzl G, 2018, LANCET INFECT DIS, V18, pE199, DOI 10.1016/S1473-3099(18)30111-7

Wang C, 2016, INT J BIOL SCI, V12, P246, DOI 10.7150/ijbs.13805

Wang P, 2014, SCIENCE, V344, P310, DOI 10.1126/science.1251456

Wang Y, 2015, P NATL ACAD SCI USA, V112, pE3883, DOI 10.1073/pnas.1501662112

Yan H, 2018, INFECT DRUG RESIST, V11, P945, DOI 10.2147/IDR.S154255

Zhang SP, 2019, CELL IMMUNOL, V335, P85, DOI 10.1016/j.cellimm.2018.11.007

Zhang X, 2009, IMMUNITY, V31, P761, DOI 10.1016/j.immuni.2009.09.016

NR 31

TC 10

Z9 12

U1 0

U2 16

PU PEERJ INC

PI LONDON

PA 341-345 OLD ST, THIRD FLR, LONDON, EC1V 9LL, ENGLAND

SN 2167-8359

J9 PEERJ

JI PeerJ

PD JAN 17

PY 2022

VL 9

AR e12776

DI 10.7717/peerj.12776

PG 15

WC Multidisciplinary Sciences

WE Science Citation Index Expanded (SCI-EXPANDED)

SC Science & Technology - Other Topics

GA YK2MA

UT WOS:000745052600001

OA Green Submitted, gold

DA 2026-05-07

ER

PT J

AU Rodrigues, L

Cravo, P

Viveiros, M

AF Rodrigues, Liliana

Cravo, Pedro

Viveiros, Miguel

TI Efflux pump inhibitors as a promising adjunct therapy against drug

resistant tuberculosis: a new strategy to revisit mycobacterial targets

and repurpose old drugs

SO EXPERT REVIEW OF ANTI-INFECTIVE THERAPY

LA English

DT Review

DE Drug resistance; efflux pumps; efflux inhibitors; drug discovery; drug

repositioning; tuberculosis; oxidative phosphorylation

ID ETHIDIUM-BROMIDE EFFLUX; ANTITUBERCULOSIS DRUGS; ANTIBIOTIC-RESISTANCE;

MULTIDRUG-RESISTANCE; LEVEL RESISTANCE; P-GLYCOPROTEIN; VALPROIC ACID;

VERAPAMIL; BEDAQUILINE; TRANSPORTER

AB Introduction: In 2018, an estimated 377,000 people developed multidrug-resistant tuberculosis (MDR-TB), urging for new effective treatments. In the last years, it has been accepted that efflux pumps play an important role in the evolution of drug resistance. Strategies are required to mitigate the consequences of the activity of efflux pumps. Areas covered: Based upon the literature available in PubMed, up to February 2020, on the diversity of efflux pumps in Mycobacterium tuberculosis and their association with drug resistance, studies that identified efflux inhibitors and their effect on restoring the activity of antimicrobials subjected to efflux are reviewed. These support a new strategy for the development of anti-TB drugs, including efflux inhibitors, using in silico drug repurposing. Expert opinion: The current literature highlights the contribution of efflux pumps in drug resistance in M. tuberculosis and that efflux inhibitors may help to ensure the effectiveness of anti-TB drugs. However, despite the usefulness of efflux inhibitors in in vitro studies, in most cases their application in vivo is restricted due to toxicity. In a time when new drugs are needed to fight MDR-TB and extensively drug-resistant TB, cost-effective strategies to identify safer efflux inhibitors should be implemented in drug discovery programs.

C1 [Rodrigues, Liliana; Cravo, Pedro; Viveiros, Miguel] Univ Nova Lisboa, Inst Higiene & Med Trop, Global Hlth & Trop Med, Rua Junqueira 100, P-1349008 Lisbon, Portugal.

C3 Universidade Nova de Lisboa; Institute of Hygiene & Tropical Medicine -

UNL

RP Viveiros, M (corresponding author), Univ Nova Lisboa, Inst Higiene & Med Trop, Global Hlth & Trop Med, Rua Junqueira 100, P-1349008 Lisbon, Portugal.

EM mviveiros@ihmt.unl.pt

RI Rodrigues, Liliana/N-2463-2014; Cravo, Pedro/AET-0639-2022; Viveiros,

Miguel/A-7326-2008

OI Cravo, Pedro/0000-0003-1675-4504; Viveiros, Miguel/0000-0001-9676-6251

FU European Union [795924]; Marie Curie Actions (MSCA) [795924] Funding

Source: Marie Curie Actions (MSCA)

FX L Rodrigues has received funding from European Union's Horizon 2020

research and innovation programme under the Marie Sklodowska-Curie grant

agreement No 795924.

CR A-Rahman A, 2002, PEDIATR PULM, V34, P226, DOI 10.1002/ppul.10145

Abbate E, 2012, J ANTIMICROB CHEMOTH, V67, P473, DOI 10.1093/jac/dkr500

Adams KN, 2014, J INFECT DIS, V210, P456, DOI 10.1093/infdis/jiu095

Adams KN, 2011, CELL, V145, P39, DOI 10.1016/j.cell.2011.02.022

Aínsa JA, 1998, J BACTERIOL, V180, P5836

Al-Saeedi M, 2017, INFECT DRUG RESIST, V10, P332, DOI 10.2147/IDR.S144446

Da Silva PEA, 2011, J ANTIMICROB CHEMOTH, V66, P1417, DOI 10.1093/jac/dkr173

Almeida D, 2016, ANTIMICROB AGENTS CH, V60, P4590, DOI 10.1128/AAC.00753-16

Amaral L, 2007, J ANTIMICROB CHEMOTH, V59, P1237, DOI 10.1093/jac/dkl500

Amaral L, 2017, ANTIBIOTICS-BASEL, V6, DOI 10.3390/antibiotics6010003

Andrade CH, 2019, CURR MED CHEM, V26, P4355, DOI 10.2174/0929867325666180309114824

Andries K, 2005, SCIENCE, V307, P223, DOI 10.1126/science.1106753

Andries K, 2014, PLOS ONE, V9, DOI 10.1371/journal.pone.0102135

Pugazhendhi A, 2018, LIFE SCI, V200, P26, DOI 10.1016/j.lfs.2018.03.023

Ashburn TT, 2004, NAT REV DRUG DISCOV, V3, P673, DOI 10.1038/nrd1468

Ashkenazy H, 2016, NUCLEIC ACIDS RES, V44, pW344, DOI 10.1093/nar/gkw408

Bald D, 2017, MBIO, V8, DOI 10.1128/mBio.00272-17

Balganesh M, 2012, ANTIMICROB AGENTS CH, V56, P2643, DOI 10.1128/AAC.06003-11

Balganesh M, 2010, ANTIMICROB AGENTS CH, V54, P5167, DOI 10.1128/AAC.00610-10

Baluom M, 2013, BRIT J CLIN PHARMACO, V76, P78, DOI 10.1111/bcp.12048

Bazinet RP, 2006, PSYCHOPHARMACOLOGY, V184, P122, DOI 10.1007/s00213-005-0272-4

Bispo NA, 2013, PLOS ONE, V8, DOI 10.1371/journal.pone.0059288

Black PA, 2014, ANTIMICROB AGENTS CH, V58, P2491, DOI 10.1128/AAC.02293-13

Blaustein MP, 1998, CLIN EXP HYPERTENS, V20, P691, DOI 10.3109/10641969809053247

Bovill J. G., 2008, V182, P121, DOI 10.1007/978-3-540-74806-9_6

Boxberger KH, 2014, DRUG METAB DISPOS, V42, P990, DOI 10.1124/dmd.113.055095

Braselmann S, 2006, J PHARMACOL EXP THER, V319, P998, DOI 10.1124/jpet.106.109058

Briffotaux J, 2017, TUBERCULOSIS, V107, P13, DOI 10.1016/j.tube.2017.08.001

Cannalire R, 2017, EUR J MED CHEM, V140, P321, DOI 10.1016/j.ejmech.2017.09.014

Chen C, 2018, ANTIMICROB AGENTS CH, V62, DOI 10.1128/AAC.02107-17

Cheng KC, 2017, FRONT PHARMACOL, V8, DOI 10.3389/fphar.2017.00481

Choudhuri BS, 2002, BIOCHEM J, V367, P279, DOI 10.1042/BJ20020615

Chuang VTG, 2012, ANN PHARMACOTHER, V46, P1349, DOI 10.1345/aph.1Q677

Chung SY, 2005, ARCH PHARM RES, V28, P823, DOI 10.1007/BF02977349

Cohen K, 2019, EXPERT OPIN DRUG SAF, V18, P875, DOI 10.1080/14740338.2019.1648429

Colangeli R, 2005, MOL MICROBIOL, V55, P1829, DOI 10.1111/j.1365-2958.2005.04510.x

Costa SS, 2016, ASSAY DRUG DEV TECHN, V14, P524, DOI 10.1089/adt.2016.752

Danilchanka O, 2008, ANTIMICROB AGENTS CH, V52, P2503, DOI 10.1128/AAC.00298-08

de Knegt GJ, 2017, INT J ANTIMICROB AG, V49, P302, DOI 10.1016/j.ijantimicag.2016.11.027

De Rossi E, 2006, FEMS MICROBIOL REV, V30, P36, DOI 10.1111/j.1574-6976.2005.00000.x

De Rossi E, 1998, J BACTERIOL, V180, P6068, DOI 10.1128/JB.180.22.6068-6071.1998

De Rossi Edda, 2002, Molecular Medicine (Baltimore), V8, P714

Desai A, 1998, BIOESSAYS, V20, P523, DOI 10.1002/(SICI)1521-1878(199807)20:7<523::AID-BIES1>3.0.CO;2-L

Domenech P, 2005, INFECT IMMUN, V73, P3492, DOI 10.1128/IAI.73.6.3492-3501.2005

Doran JL, 1997, CLIN DIAGN LAB IMMUN, V4, P23, DOI 10.1128/CDLI.4.1.23-32.1997

Downing KH, 2000, ANNU REV CELL DEV BI, V16, P89, DOI 10.1146/annurev.cellbio.16.1.89

Du DJ, 2018, NAT REV MICROBIOL, V16, P523, DOI 10.1038/s41579-018-0048-6

Duan W, 2019, FUTURE MICROBIOL, V14, P47, DOI 10.2217/fmb-2018-0207

El-Habr EA, 2017, ACTA NEUROPATHOL, V133, P645, DOI 10.1007/s00401-016-1659-5

Gajadeera C, 2015, J ANTIBIOT, V68, P153, DOI 10.1038/ja.2014.131

Ghajavand H, 2019, ANTIMICROB AGENTS CH, V63, DOI [10.1128/AAC.02530-18, 10.1128/aac.02530-18]

Grossman TH, 2015, ANTIMICROB AGENTS CH, V59, P1539, DOI 10.1128/AAC.04271-14

Grzegorzewicz AE, 2012, NAT CHEM BIOL, V8, P334, DOI [10.1038/NCHEMBIO.794, 10.1038/nchembio.794]

Gupta AK, 2010, INDIAN J MED RES, V132, P176

Gupta AK, 2010, MICROB DRUG RESIST, V16, P21, DOI 10.1089/mdr.2009.0054

Gupta S, 2015, ANTIMICROB AGENTS CH, V59, P673, DOI 10.1128/AAC.04019-14

Gupta S, 2014, ANTIMICROB AGENTS CH, V58, P574, DOI 10.1128/AAC.01462-13

Gupta S, 2013, AM J RESP CRIT CARE, V188, P600, DOI 10.1164/rccm.201304-0650OC

Hartkoorn RC, 2014, ANTIMICROB AGENTS CH, V58, P2979, DOI 10.1128/AAC.00037-14

Hassan KA, 2015, MBIO, V6, DOI 10.1128/mBio.01982-14

Hegeto LA, 2018, TUBERCULOSIS, V111, P35, DOI 10.1016/j.tube.2018.05.006

Hsu WH, 2014, BMC GASTROENTEROL, V14, DOI 10.1186/1471-230X-14-91

Ismail N, 2019, ANTIMICROB AGENTS CH, V63, DOI 10.1128/AAC.00292-19

Jin J, 2010, MOLECULES, V15, P7750, DOI 10.3390/molecules15117750

Kanehiro Y, 2018, FRONT MICROBIOL, V9, DOI 10.3389/fmicb.2018.01517

Kapopoulou A, 2011, TUBERCULOSIS, V91, P8, DOI 10.1016/j.tube.2010.09.006

Kaur G, 2019, J BIOMOL STRUCT DYN, V37, P1254, DOI 10.1080/07391102.2018.1454852

Khanna A, 2010, ANTIMICROB AGENTS CH, V54, P4789, DOI 10.1128/AAC.00661-10

Khosravi AD, 2019, INFECT DRUG RESIST, V12, P3437, DOI 10.2147/IDR.S221823

Knudson SE, 2014, PLOS ONE, V9, DOI 10.1371/journal.pone.0093953

KOHLER P, 1978, MOL PHARMACOL, V14, P155

Kotwal P, 2019, J CHROMATOGR B, V1112, P48, DOI 10.1016/j.jchromb.2019.02.022

Kovoor PA, 2009, CLIN COLORECTAL CANC, V8, P200, DOI 10.3816/CCC.2009.n.034

Kristiansen JE, 2015, INT MICROBIOL, V18, P1, DOI 10.2436/20.1501.01.229

Lachmandas E, 2019, J INFECT DIS, V220, P139, DOI 10.1093/infdis/jiz064

Landaburu LU, 2020, NUCLEIC ACIDS RES, V48, pD992, DOI 10.1093/nar/gkz999

Lechner D, 2008, J ANTIMICROB CHEMOTH, V62, P345, DOI 10.1093/jac/dkn178

Lee MC, 2018, PLOS ONE, V13, DOI 10.1371/journal.pone.0205807

Li B, 2018, ANTIMICROB AGENTS CH, V62, DOI [10.1128/AAC.00175-18, 10.1128/aac.00175-18]

Li GL, 2015, J ANTIBIOT, V68, P431, DOI 10.1038/ja.2015.9

Li X, 2019, ARCH BIOCHEM BIOPHYS, V667, P59, DOI 10.1016/j.abb.2019.04.010

Li XZ, 2004, ANTIMICROB AGENTS CH, V48, P2415, DOI 10.1128/AAC.48.7.2415-2423.2004

Lienhardt C, 2016, PLOS MED, V13, DOI 10.1371/journal.pmed.1001965

Liu JY, 2019, FRONT MICROBIOL, V10, DOI 10.3389/fmicb.2019.00216

Lomovskaya O, 2006, BIOCHEM PHARMACOL, V71, P910, DOI 10.1016/j.bcp.2005.12.008

López-Muñoz F, 2012, ACTAS ESP PSIQUIATRI, V40, P34

Louw GE, 2009, ANTIMICROB AGENTS CH, V53, P3181, DOI 10.1128/AAC.01577-08

Louw GE, 2011, AM J RESP CRIT CARE, V184, P269, DOI 10.1164/rccm.201011-1924OC

Lund SS, 2007, DIABETES OBES METAB, V9, P394, DOI 10.1111/j.1463-1326.2007.00713.x

Ma S, 2020, BIOL CHEM, V401, P331, DOI 10.1515/hsz-2019-0326

MACHADO D, 2018, FRONT MICROBIOL, V9

Machado D, 2018, FUTURE MICROBIOL, V13, P1383, DOI 10.2217/fmb-2018-0110

Machado D, 2016, PLOS ONE, V11, DOI 10.1371/journal.pone.0149326

Machado D, 2012, PLOS ONE, V7, DOI 10.1371/journal.pone.0034538

Madiraju AK, 2018, NAT MED, V24, P1384, DOI 10.1038/s41591-018-0125-4

Martínez-Serrano M, 2017, REV ESP QUIM, V30, P84

Martins M, 2008, IN VIVO, V22, P69

Medeiros BC, 2017, LEUKEMIA, V31, P272, DOI 10.1038/leu.2016.275

Milano A, 2009, TUBERCULOSIS, V89, P84, DOI 10.1016/j.tube.2008.08.003

Mondesir J, 2016, J BLOOD MED, V7, P171, DOI 10.2147/JBM.S70716

Mordente A, 2009, CURR MED CHEM, V16, P1656, DOI 10.2174/092986709788186228

Narang A, 2019, PLOS ONE, V14, DOI 10.1371/journal.pone.0223163

Neves BJ, 2015, PLOS NEGLECT TROP D, V9, DOI 10.1371/journal.pntd.0003435

Nguyen ANT, 2007, J AM SOC NEPHROL, V18, P46, DOI 10.1681/ASN.2006010086

Opperman TJ, 2015, FRONT MICROBIOL, V6, DOI 10.3389/fmicb.2015.00421

Owens CP, 2013, J BIOL CHEM, V288, P21714, DOI 10.1074/jbc.M113.453076

Padmapriyadarsini C, 2019, BMJ OPEN, V9, DOI 10.1136/bmjopen-2018-024363

Pagès JM, 2009, BBA-PROTEINS PROTEOM, V1794, P826, DOI 10.1016/j.bbapap.2008.12.011

Pang Y, 2013, ANTIMICROB AGENTS CH, V57, P893, DOI 10.1128/AAC.01024-12

Pasca MR, 2005, ANTIMICROB AGENTS CH, V49, P4775, DOI 10.1128/AAC.49.11.4775-4777.2005

Pasca MR, 2004, ANTIMICROB AGENTS CH, V48, P3175, DOI 10.1128/AAC.48.8.3175-3178.2004

Pasipanodya JG, 2011, CURR OPIN PHARMACOL, V11, P457, DOI 10.1016/j.coph.2011.07.001

PEREIRA E, 1995, BIOCHEM PHARMACOL, V50, P451, DOI 10.1016/0006-2952(95)00174-X

Piddock LJV, 2006, CLIN MICROBIOL REV, V19, P382, DOI 10.1128/CMR.19.2.382-402.2006

Pieroni M, 2015, J MED CHEM, V58, P5842, DOI 10.1021/acs.jmedchem.5b00428

Pule CM, 2016, J ANTIMICROB CHEMOTH, V71, P17, DOI 10.1093/jac/dkv316

Puthiamadathil JM, 2017, CANCER MANAG RES, V9, P461, DOI 10.2147/CMAR.S113320

Ramón-García S, 2006, J ANTIMICROB CHEMOTH, V57, P252, DOI 10.1093/jac/dki436

Ramón-García S, 2007, J ANTIMICROB CHEMOTH, V59, P544, DOI 10.1093/jac/dkl510

Ramón-García S, 2012, ANTIMICROB AGENTS CH, V56, P2074, DOI 10.1128/AAC.05946-11

Ramón-García S, 2009, ANTIMICROB AGENTS CH, V53, P3675, DOI 10.1128/AAC.00550-09

Rao M, 2018, INT J INFECT DIS, V69, P78, DOI 10.1016/j.ijid.2018.02.021

Rao PS, 2012, INT J CANCER, V130, P2703, DOI 10.1002/ijc.26308

Rena G, 2017, DIABETOLOGIA, V60, P1577, DOI 10.1007/s00125-017-4342-z

Scaini JLR, 2019, J MOL GRAPH MODEL, V87, P98, DOI 10.1016/j.jmgm.2018.11.016

Rodrigues L, 2008, J ANTIMICROB CHEMOTH, V61, P1076, DOI 10.1093/jac/dkn070

Rodrigues L, 2017, DRUG DISCOV TODAY, V22, P592, DOI 10.1016/j.drudis.2017.01.002

Rodrigues L, 2013, ANTIMICROB AGENTS CH, V57, P751, DOI 10.1128/AAC.01482-12

Rodrigues L, 2012, INFECT GENET EVOL, V12, P695, DOI 10.1016/j.meegid.2011.08.009

Rodrigues Liliana, 2011, Recent Pat Antiinfect Drug Discov, V6, P118

Rodrigues L, 2009, INT J ANTIMICROB AG, V34, P529, DOI 10.1016/j.ijantimicag.2009.07.010

Rodriguez-Carlos A, 2020, MICROBES INFECT, V22, P111, DOI 10.1016/j.micinf.2019.10.002

ROLF MG, 2015, PHARMACOL RES PERSPE, V3, P5

Rosenberg G, 2007, CELL MOL LIFE SCI, V64, P2090, DOI 10.1007/s00018-007-7079-x

Rybniker J, 2015, NAT COMMUN, V6, DOI 10.1038/ncomms8659

Schiebler M, 2015, EMBO MOL MED, V7, P127, DOI 10.15252/emmm.201404137

Schmalstieg AM, 2012, ANTIMICROB AGENTS CH, V56, P4806, DOI 10.1128/AAC.05546-11

Schuster S, 2019, MOLECULES, V24, DOI 10.3390/molecules24030470

Seral C, 2003, J ANTIMICROB CHEMOTH, V51, P1167, DOI 10.1093/jac/dkg223

Sharma S, 2010, J ANTIMICROB CHEMOTH, V65, P1694, DOI 10.1093/jac/dkq186

Shugarts S, 2009, PHARM RES-DORDR, V26, P2039, DOI 10.1007/s11095-009-9924-0

Silva PEA, 2001, ANTIMICROB AGENTS CH, V45, P800, DOI 10.1128/AAC.45.3.800-804.2001

Singh M, 2011, INDIAN J MED RES, V133, P535

Singhal A, 2014, SCI TRANSL MED, V6, DOI 10.1126/scitranslmed.3009885

SLADE JM, 1993, ANAESTHESIA, V48, P1053

Slayden RA, 2006, MICROBIOL-SGM, V152, P1789, DOI 10.1099/mic.0.28762-0

Solnier J, 2020, MOLECULES, V25, DOI 10.3390/molecules25030734

SPEELMANS G, 1995, BBA-BIOMEMBRANES, V1238, P137, DOI 10.1016/0005-2736(95)00119-N

Srikrishna G, 2015, FUTURE MICROBIOL, V10, P1257, DOI 10.2217/FMB.15.56

Srivastava S, 2010, J INFECT DIS, V201, P1225, DOI 10.1086/651377

Stein EM, 2017, BLOOD, V130, P722, DOI 10.1182/blood-2017-04-779405

Sun ZG, 2014, MICROB DRUG RESIST, V20, P525, DOI 10.1089/mdr.2013.0171

Suzuki K, 2000, FEMS MICROBIOL LETT, V182, P69, DOI 10.1016/S0378-1097(99)00571-6

Sweeny DJ, 2010, DRUG METAB DISPOS, V38, P1166, DOI 10.1124/dmd.110.032151

Szklarczyk D, 2016, NUCLEIC ACIDS RES, V44, pD380, DOI 10.1093/nar/gkv1277

Tullius MV, 2011, P NATL ACAD SCI USA, V108, P5051, DOI 10.1073/pnas.1009516108

Udwadia Zarir F, 2011, Recent Pat Antiinfect Drug Discov, V6, P88

Ughachukwu PO, 2012, ANN MED HEALTH SCI R, V2, P191, DOI 10.4103/2141-9248.105671

Varela C, 2012, CHEM BIOL, V19, P498, DOI 10.1016/j.chembiol.2012.03.006

Vibe CB, 2016, NANOTOXICOLOGY, V10, P680, DOI 10.3109/17435390.2015.1107146

Viollet B, 2012, CLIN SCI, V122, P253, DOI 10.1042/CS20110386

Viveiros M, 2002, ANTIMICROB AGENTS CH, V46, P2804, DOI 10.1128/AAC.46.9.2804-2810.2002

Viveiros M, 2012, EXPERT REV ANTI-INFE, V10, P983, DOI [10.1586/ERI.12.89, 10.1586/eri.12.89]

Viveiros M, 2010, EXPERT REV ANTI-INFE, V8, P465, DOI [10.1586/eri.10.20, 10.1586/ERI.10.20]

Wasserstrom JA, 2005, AM J PHYSIOL-HEART C, V289, pH1781, DOI 10.1152/ajpheart.00707.2004

Weinstein EA, 2005, P NATL ACAD SCI USA, V102, P4548, DOI 10.1073/pnas.0500469102

WEISS RB, 1992, SEMIN ONCOL, V19, P670

Wescott HH, 2018, FRONT MICROBIOL, V9, DOI 10.3389/fmicb.2018.02542

*WHO, 2015, GLOB STRAT TARG TUB

*WHO, 2019, GLOBAL TUBERCULOSIS

*WHO, 2013, US BED TREATM MULT R

Wishart DS, 2018, NUCLEIC ACIDS RES, V46, pD1074, DOI 10.1093/nar/gkx1037

Xu J, 2017, ANTIMICROB AGENTS CH, V61, DOI [10.1128/AAC.00239-17, 10.1128/aac.00239-17]

Zhang Y, 1999, J BACTERIOL, V181, P2044, DOI 10.1128/JB.181.7.2044-2049.1999

Zhang YM, 2017, ANTIMICROB AGENTS CH, V61, DOI [10.1128/AAC.00940-17, 10.1128/aac.00940-17]

2020, MYCOBROWSER

2020, TDR TARGETS

NR 177

TC 46

Z9 51

U1 0

U2 48

PU TAYLOR & FRANCIS LTD

PI ABINGDON

PA 2-4 PARK SQUARE, MILTON PARK, ABINGDON OR14 4RN, OXON, ENGLAND

SN 1478-7210

EI 1744-8336

J9 EXPERT REV ANTI-INFE

JI Expert Rev. Anti-Infect. Ther.

PD AUG 2

PY 2020

VL 18

IS 8

BP 741

EP 757

DI 10.1080/14787210.2020.1760845

EA MAY 2020

PG 17

WC Infectious Diseases; Microbiology; Pharmacology & Pharmacy

WE Science Citation Index Expanded (SCI-EXPANDED)

SC Infectious Diseases; Microbiology; Pharmacology & Pharmacy

GA NI5IQ

UT WOS:000537903000001

OA Green Submitted, Green Published, Bronze

DA 2026-05-07

ER

PT J

AU Velayati, AA

Farnia, P

Hoffner, S

AF Velayati, Ali Akbar

Farnia, Parissa

Hoffner, Sven

TI Drug-resistant Mycobacterium tuberculosis: Epidemiology and role

of morphological alterations

SO JOURNAL OF GLOBAL ANTIMICROBIAL RESISTANCE

LA English

DT Review

DE Drug-resistant tuberculosis; Epidemiology; Morphology

ID TRANSMISSION ELECTRON-MICROSCOPY; MULTIDRUG-RESISTANT; CELL-WALL;

TDR-TB; EMERGENCE; STRAINS; PILI; IRAN; BEDAQUILINE; DELAMANID

AB In this brief review, various forms of drug-resistant tuberculosis (DR-TB) are discussed. The focus of the study is to highlight morphological alterations of DR-TB bacilli at the cellular level. (c) 2017 International Society for Chemotherapy of Infection and Cancer. Published by Elsevier Ltd. All rights reserved.

C1 [Velayati, Ali Akbar; Farnia, Parissa] Shahid Beheshti Univ Med Sci, MRC, NRITLD, Tehran, Iran.

[Hoffner, Sven] Karolinska Inst, Dept Microbiol Tumor & Cell Biol, Stockholm, Sweden.

C3 Shahid Beheshti University Medical Sciences; Karolinska Institutet

RP Farnia, P (corresponding author), Shahid Beheshti Univ Med Sci, MRC, NRITLD, Tehran, Iran.

EM velayati@theassm.org; farina@theaasm.org; sven.hoffner@ki.se

RI Farnia, parissa/G-7412-2017; Velayati, Ali Akbar/GQH-0049-2022

OI Farnia, parissa/0000-0003-3676-2018;

CR Acosta CD, 2014, PUBLIC HEALTH ACTION, V4, pS3, DOI 10.5588/pha.14.0087

Da Silva PEA, 2011, J ANTIMICROB CHEMOTH, V66, P1417, DOI 10.1093/jac/dkr173

Alsteens D, 2008, PFLUG ARCH EUR J PHY, V456, P117, DOI 10.1007/s00424-007-0386-0

Alteri CJ, 2007, P NATL ACAD SCI USA, V104, P5145, DOI 10.1073/pnas.0602304104

[Anonymous], 2016, Treatment Guidelines for Drug-Resistant Tuberculosis

[Anonymous], 2007, EURO SURVEILL

[Anonymous], 2012, GLOB TUB REP 2013

[Anonymous], 2012, TOT DRUG RES TUB WHO

[Anonymous], 2016, US DEL TREATM MULT R

[Anonymous], 2010, WHO/HTM/TB/2010.3

Boakye-Appiah JK, 2016, INT J MYCOBACT, V5, P226, DOI 10.1016/j.ijmyco.2016.02.004

Bose M, 1999, INT J IMMUNOPATH PH, V12, P69

Caminero LJA, 2010, INT J TUBERC LUNG D, V14, P131

Cardona PJ, 2004, EUR RESPIR J, V24, P1044, DOI 10.1183/09031936.04.00072604

Cegielski Peter, 2012, Emerg Infect Dis, V18, pe2, DOI 10.3201/eid1811.120526

Centers for Disease Control and Prevention (CDC), 2006, MMWR Morb Mortal Wkly Rep, V55, P301

Champion JA, 2006, P NATL ACAD SCI USA, V103, P4930, DOI 10.1073/pnas.0600997103

Cirillo Daniela M, 2016, Int J Mycobacteriol, V5 Suppl 1, pS252, DOI [10.1016/j.ijmyco.2016.09.053, 10.1016/j.ijmyco.2016.09.053]

Colijn C, 2011, PLOS ONE, V6, DOI 10.1371/journal.pone.0018327

DAVIES J, 1994, SCIENCE, V264, P375, DOI 10.1126/science.8153624

Doustdar F, 2008, MICROB DRUG RESIST, V14, P273, DOI 10.1089/mdr.2008.0842

Esposito S, 2015, EXPERT OPIN PHARMACO, V16, P2319, DOI 10.1517/14656566.2015.1080240

Falzon D, 2011, EUR RESPIR J, V38, P516, DOI 10.1183/09031936.00073611

Farnia P, 2004, J INFECTION, V49, P94, DOI 10.1016/j.jinf.2003.11.015

Farnia P, 2008, J CLIN MICROBIOL, V46, P796, DOI 10.1128/JCM.01435-07

Farnia P, 2010, INT J CLIN EXP MED, V3, P308

Grange J M, 1990, Bull Int Union Tuberc Lung Dis, V65, P57

Hoffmann C, 2008, P NATL ACAD SCI USA, V105, P3963, DOI 10.1073/pnas.0709530105

Hoffmann H, 2016, AM J RESP CRIT CARE, V193, P337, DOI 10.1164/rccm.201502-0372LE

Hosseini H, 2014, ASIAN PAC J TROP MED, V7, pS199, DOI 10.1016/S1995-7645(14)60232-7

Javed H, 2016, INT J MYCOBACT, V5, P128, DOI 10.1016/j.ijmyco.2015.12.004

Klopper M, 2013, EMERG INFECT DIS, V19, P449, DOI [10.3201/EID1903.120246, 10.3201//EID1903.120246]

Lacerda SN, 2014, INT ARCH MED, V7, P7

Lönnroth K, 2010, LANCET, V375, P1814, DOI 10.1016/S0140-6736(10)60483-7

Masjedi MR, 2006, CLIN INFECT DIS, V43, P841, DOI 10.1086/507542

Matteelli A, 2007, EXPERT REV ANTI-INFE, V5, P857, DOI 10.1586/14787210.5.5.857

Matteelli A, 2014, CLIN EPIDEMIOL, V6, P111, DOI 10.2147/CLEP.S35839

Mendoza M., 2009, 1 US CASE EXTREMELY

Migliori GB, 2017, INT J MOL SCI, V18, DOI 10.3390/ijms18020341

Migliori GB, 2013, EUR RESPIR J, V42, P169, DOI 10.1183/09031936.00136312

Moodley R, 2016, EUR RESPIR REV, V25, DOI 10.1183/16000617.0080-2015

Sarathy JP, 2012, PHARMACEUTICALS-BASE, V5, P1210, DOI 10.3390/ph5111210

Shah NS, 2007, EMERG INFECT DIS, V13, P380, DOI 10.3201/eid1303.061400

Shleeva MO, 2011, TUBERCULOSIS, V91, P146, DOI 10.1016/j.tube.2010.12.006

Skrahina A, 2012, EUR RESPIR J, V39, P1425, DOI 10.1183/09031936.00145411

Sotgiu G, 2013, INT J MYCOBACT, V2, P123, DOI 10.1016/j.ijmyco.2013.07.001

Sulis G, 2014, MEDITERR J HEMATOL I, V6, DOI 10.4084/MJHID.2014.070

Telenti A, 1998, THORAX, V53, P793, DOI 10.1136/thx.53.9.793

Udwadia ZF, 2012, CLIN INFECT DIS, V54, P579, DOI 10.1093/cid/cir889

Velayati AA, 2006, SCAND J INFECT DIS, V38, P463, DOI 10.1080/00365540500504117

Velayati AA, 2013, INT J MYCOBACT, V2, P71, DOI 10.1016/j.ijmyco.2013.04.005

Velayati AA, 2012, INT J MYCOBACT, V1, P57, DOI 10.1016/j.ijmyco.2012.04.002

Velayati AA, 2013, INT J CLIN EXP MED, V6, P307

Velayati AA, 2009, CHEMOTHERAPY, V55, P303, DOI 10.1159/000226425

Velayati AA, 2009, CHEST, V136, P420, DOI 10.1378/chest.08-2427

Velayati AA, 2007, ARCH IRAN MED, V10, P486

Vynnycky E, 2015, AM J EPIDEMIOL, V181, P619, DOI 10.1093/aje/kwu320

WHO, 2015, GLOB TUB REP 2015

Zignol M, 2006, J INFECT DIS, V194, P479, DOI 10.1086/505877

NR 59

TC 29

Z9 33

U1 0

U2 11

PU ELSEVIER SCI LTD

PI OXFORD

PA THE BOULEVARD, LANGFORD LANE, KIDLINGTON, OXFORD OX5 1GB, OXON, ENGLAND

SN 2213-7165

EI 2213-7173

J9 J GLOB ANTIMICROB RE

JI J. Glob. Antimicrob. Resist.

PD MAR

PY 2018

VL 12

BP 192

EP 196

DI 10.1016/j.jgar.2017.10.006

PG 5

WC Infectious Diseases; Pharmacology & Pharmacy

WE Science Citation Index Expanded (SCI-EXPANDED)

SC Infectious Diseases; Pharmacology & Pharmacy

GA GA4BB

UT WOS:000428273500051

DA 2026-05-07

ER

PT J

AU Saravanan, M

Niguse, S

Abdulkader, M

Tsegay, E

Hailekiros, H

Gebrekidan, A

Araya, T

Pugazhendhi, A

AF Saravanan, Muthupandian

Niguse, Selam

Abdulkader, Mahmud

Tsegay, Ephrem

Hailekiros, Haftamu

Gebrekidan, Atsbeha

Araya, Tadele

Pugazhendhi, Arivalagan

TI Review on emergence of drug -resistant tuberculosis (MDR & XDR-TB) and

its molecular diagnosis in Ethiopia

SO MICROBIAL PATHOGENESIS

LA English

DT Article

DE M. tuberculosis; Emergence; Drug resistance; Molecular diagnostics;

miRNA; MDR & XDR-TB; Anti-tuberculosis drugs

ID MYCOBACTERIUM-TUBERCULOSIS; RIFAMPIN RESISTANCE; IDENTIFICATION;

PREVALENCE

AB Tuberculosis (TB) remains a major global health problem and ranks as the second leading cause of death among deaths caused by infectious diseases worldwide. Although the availability of short-course regimens as first-line anti-tuberculosis drugs, the emergence of drug-resistant Mycobacterium tuberculosis strains pose a major challenge to the prevention and control efforts of national tuberculosis programs (NTPs). M. tuberculosis changes its cellular environment with the mechanisms that have been evolved since prehistoric times. The interactions between the bacteria and the host environment have been studied well. However, the studies at molecular level began to emerge recently including expression profiling of micro RNA (miRNA) and literature survey revealed that researchers find more information about their regulatory role in biological processes including immune response to infectious agents like mycobacteria. In developing countries, including Ethiopia, the burden of tuberculosis and or drug resistance profile of M. tuberculosis remains largely unexplored, mainly due to lack of quality controlled second-line laboratory tests and also lack of knowledge on molecular diagnostics. This review describes the disease etiology, pathogenesis, epidemiology, molecular mechanism and advanced molecular diagnostics for precision MDR-TB diagnosis.

C1 [Saravanan, Muthupandian; Niguse, Selam; Abdulkader, Mahmud; Tsegay, Ephrem; Hailekiros, Haftamu; Gebrekidan, Atsbeha; Araya, Tadele] Mekelle Univ, Sch Med, Dept Med Microbiol & Immunol, Coll Hlth Sci, Mekelle 1871, Ethiopia.

[Pugazhendhi, Arivalagan] Ton Duc Thang Univ, Fac Environm & Labour Safety, Innovat Green Prod Synth & Renewable Environm Dev, Ho Chi Minh City, Vietnam.

C3 Mekelle University; Ton Duc Thang University

RP Saravanan, M (corresponding author), Mekelle Univ, Sch Med, Dept Med Microbiol & Immunol, Coll Hlth Sci, Mekelle 1871, Ethiopia.; Pugazhendhi, A (corresponding author), Ton Duc Thang Univ, Fac Environm & Labour Safety, Innovat Green Prod Synth & Renewable Environm Dev, Ho Chi Minh City, Vietnam.

EM saravanan.muthupandian@mu.edu.ct; arivalagan.pugazhendhi@tdt.edu.vn

RI ; Muthupandian, Saravanan/F-3835-2012; Pugazhendhi,

Arivalagan/F-5411-2018

OI HAILEKIROS, HAFTAMU/0000-0001-5102-5222; Muthupandian,

Saravanan/0000-0002-1480-3555; Araya, Tadele/0000-0003-3729-6821;

Pugazhendhi, Arivalagan/0000-0002-9529-3306

CR Abebe G, 2011, J MICROBIOL METH, V84, P155, DOI 10.1016/j.mimet.2010.11.014

Ahmed Esmael Ahmed Esmael, 2014, Journal of Microbial and Biochemical Technology, V6, P075

Ani AE, 2008, ANN AFR MED, V7, P57, DOI 10.4103/1596-3519.55680

[Anonymous], GLOB TUB CONTR SURV

[Anonymous], 1994, TB GLOB EM

Aziz MA, 2006, LANCET, V368, P2142, DOI 10.1016/S0140-6736(06)69863-2

Bártfai Z, 2001, J CLIN MICROBIOL, V39, P3736, DOI 10.1128/JCM.39.10.3736-3739.2001

Berhan A, 2013, ETHIOP J HEALTH SCI, V23, P271, DOI 10.4314/ejhs.v23i3.10

Biadglegne F, 2014, ANTIMICROB RESIST IN, V3, DOI 10.1186/2047-2994-3-31

Biadglegne F, 2013, PLOS ONE, V8, DOI 10.1371/journal.pone.0081918

Coll P, 2005, MICROB DRUG RESIST, V11, P107, DOI 10.1089/mdr.2005.11.107

Drobniewski F, 2007, CLIN MICROBIOL INFEC, V13, P1144, DOI 10.1111/j.1469-0691.2007.01813.x

Dye C, 2005, JAMA-J AM MED ASSOC, V293, P2767, DOI 10.1001/jama.293.22.2767

Fu YR, 2011, J CLIN MICROBIOL, V49, P4246, DOI 10.1128/JCM.05459-11

Gegia M, 2008, ANTIMICROB AGENTS CH, V52, P725, DOI 10.1128/AAC.01124-07

Golub JE, 2006, INT J TUBERC LUNG D, V10, P24

Gutacker MM, 2006, J INFECT DIS, V193, P121, DOI 10.1086/498574

Hu Y, 2011, EPIDEMIOL INFECT, V139, P130, DOI 10.1017/S0950268810000890

ISEMAN MD, 1993, NEW ENGL J MED, V329, P784, DOI 10.1056/NEJM199309093291108

Kayser F., 2005, BASIC PRINCIPLES MIC, V22, P1

Ling Daphne I, 2008, Expert Rev Respir Med, V2, P583, DOI 10.1586/17476348.2.5.583

Luciani F, 2008, INFECT GENET EVOL, V8, P182, DOI 10.1016/j.meegid.2007.12.004

Ma F, 2011, NAT IMMUNOL, V12, P861, DOI 10.1038/ni.2073

Marais BJ, 2010, LANCET, V375, P2179, DOI 10.1016/S0140-6736(10)60554-5

Murray P.R.K., 2005, LANCET, V375, P1814

Negga B., 2004, PULMONARY TUBERCULOS, V15, P203

Park MY, 2009, J CLIN MICROBIOL, V47, P481, DOI 10.1128/JCM.01253-08

Pym A. S., 2002, BACTERIAL RESISTANCE, V33, P355

Shah NS, 2007, EMERG INFECT DIS, V13, P380, DOI 10.3201/eid1303.061400

Sharbati J, 2011, PLOS ONE, V6, DOI 10.1371/journal.pone.0020258

Somoskovi A, 2001, RESP RES, V2, P164, DOI 10.1186/rr54

SUDRE P, 1992, B WORLD HEALTH ORGAN, V70, P149

Tessema B, 2012, INT J TUBERC LUNG D, V16, P805, DOI 10.5588/ijtld.11.0522

Torres MJ, 2000, J CLIN MICROBIOL, V38, P3194, DOI 10.1128/JCM.38.9.3194-3199.2000

Troesch A, 1999, J CLIN MICROBIOL, V37, P49, DOI 10.1128/JCM.37.1.49-55.1999

Udoh M., 2009, BENIN J POSTGRAD MED, P55

van der Werf MJ, 2012, EUR RESPIR J, V39, P1511, DOI 10.1183/09031936.00125711

Van S. D., 1997, INT J SYST BACTERIOL, V47, P1200

Vander S., 2009, TUBERCULOSIS EDINB, V89, P120

Vernet G, 2004, J APPL MICROBIOL, V96, P59, DOI 10.1046/j.1365-2672.2003.02116.x

Wang C, 2011, PLOS ONE, V6, DOI 10.1371/journal.pone.0025832

WHO, 2003, TUB MAN MED STUD, P9

2015, MICROBE, V10, P354, DOI 10.1128/microbe.10.354.1

World Health Organization, 2010, ROADM ROLL OUT XPERT

Xing Z., 2013, PLOS ONE, V8

Zhang Y, 2009, INT J TUBERC LUNG D, V13, P1320

Zhang Y, 2000, MOLECULAR GENETICS OF MYCOBACTERIA, P235

Zignol M, 2006, J INFECT DIS, V194, P479, DOI 10.1086/505877

NR 48

TC 49

Z9 53

U1 0

U2 8

PU ACADEMIC PRESS LTD- ELSEVIER SCIENCE LTD

PI LONDON

PA 24-28 OVAL RD, LONDON NW1 7DX, ENGLAND

SN 0882-4010

EI 1096-1208

J9 MICROB PATHOGENESIS

JI Microb. Pathog.

PD APR

PY 2018

VL 117

BP 237

EP 242

DI 10.1016/j.micpath.2018.02.047

PG 6

WC Immunology; Microbiology

WE Science Citation Index Expanded (SCI-EXPANDED)

SC Immunology; Microbiology

GA GB5EG

UT WOS:000429085300034

DA 2026-05-07

ER

PT S

AU Pieroni, M

AF Pieroni, Marco

BE Chibale, K

TI Antituberculosis agents: Beyond medicinal chemistry rules

SO MEDICINAL CHEMISTRY APPROACHES TO TUBERCULOSIS AND TRYPANOSOMIASIS

SE Annual Reports in Medicinal Chemistry

LA English

DT Review; Book Chapter

ID EFFLUX PUMP INHIBITORS; DRUG-RESISTANT TUBERCULOSIS; HOST-DIRECTED

THERAPY; SMALL-MOLECULE EFFLUX; MYCOBACTERIUM-TUBERCULOSIS; ATP

SYNTHASE; IN-VITRO; ADAMANTYL GROUP; CELL-WALL; RIMINOPHENAZINE

DERIVATIVES

C1 [Pieroni, Marco] Univ Parma, P4T Grp, Parco Area Sci, Parma, Italy.

C3 University of Parma

RP Pieroni, M (corresponding author), Univ Parma, P4T Grp, Parco Area Sci, Parma, Italy.

EM marco.pieroni@unipr.it

RI Pieroni, Marco/K-4283-2017

OI Pieroni, Marco/0000-0001-9190-3712

CR Abbate E, 2012, J ANTIMICROB CHEMOTH, V67, P473, DOI 10.1093/jac/dkr500

Adams KN, 2014, J INFECT DIS, V210, P456, DOI 10.1093/infdis/jiu095

Adams KN, 2011, CELL, V145, P39, DOI 10.1016/j.cell.2011.02.022

AGRAWAL KC, 1979, J MED CHEM, V22, P583, DOI 10.1021/jm00191a025

Amaral L, 2014, FRONT PHARMACOL, V4, DOI 10.3389/fphar.2013.00168

Andries K, 2005, SCIENCE, V307, P223, DOI 10.1126/science.1106753

Andries K, 2014, PLOS ONE, V9, DOI 10.1371/journal.pone.0102135

[Anonymous], 2018, WHO | Global tuberculosis report

ASHTEKAR DR, 1993, ANTIMICROB AGENTS CH, V37, P183, DOI 10.1128/AAC.37.2.183

Azzali E, 2017, J MED CHEM, V60, P7108, DOI 10.1021/acs.jmedchem.7b00793

Bacon J, 2014, PLOS ONE, V9, DOI 10.1371/journal.pone.0087329

Bald D, 2017, MBIO, V8, DOI 10.1128/mBio.00272-17

Barry C.E., 2003, WO Patent, Patent No. [200,309,698,9, 2003096989]

Barry CE III, 2004, CURR PHARM DESIGN, V10, P3239, DOI 10.2174/1381612043383214

Bhardwaj Ashima K, 2012, Recent Pat Antiinfect Drug Discov, V7, P73

Bloemberg GV, 2015, NEW ENGL J MED, V373, P1986, DOI 10.1056/NEJMc1505196

Boechat N, 2015, MEM I OSWALDO CRUZ, V110, P492, DOI 10.1590/0074-02760140248

Boelsterli UA, 2006, CURR DRUG METAB, V7, P715, DOI 10.2174/138920006778520606

Bogatcheva E, 2006, J MED CHEM, V49, P3045, DOI 10.1021/jm050948+

Bogatcheva E, 2011, BIOORG MED CHEM LETT, V21, P5353, DOI 10.1016/j.bmcl.2011.07.015

Bogatcheva E, 2010, BIOORG MED CHEM LETT, V20, P201, DOI 10.1016/j.bmcl.2009.10.135

Bruns H, 2012, J IMMUNOL, V189, P4069, DOI 10.4049/jimmunol.1201538

Byrne ST, 2007, J ANTIMICROB CHEMOTH, V59, P313, DOI 10.1093/jac/dkl486

Caño-Muñiz S, 2018, CLIN MICROBIOL REV, V31, DOI 10.1128/CMR.00060-17

Chen P, 2006, J ANTIMICROB CHEMOTH, V58, P332, DOI 10.1093/jac/dkl227

Chiarelli LR, 2016, CURR MED CHEM, V23, P3813, DOI 10.2174/1389557516666160831164925

Cole ST, 1998, NATURE, V393, P537, DOI 10.1038/31159

Daniel J, 2014, PLOS ONE, V9, DOI 10.1371/journal.pone.0114877

Daniel WA, 2003, PROG NEURO-PSYCHOPH, V27, P65, DOI 10.1016/S0278-5846(02)00317-2

Dartois V, 2014, NAT REV MICROBIOL, V12, P159, DOI 10.1038/nrmicro3200

de Jonge MR, 2007, PROTEINS, V67, P971, DOI 10.1002/prot.21376

Diacon AH, 2015, AM J RESP CRIT CARE, V191, P943, DOI 10.1164/rccm.201410-1801OC

Doak BC, 2014, CHEM BIOL, V21, P1115, DOI 10.1016/j.chembiol.2014.08.013

Domenech P, 2005, INFECT IMMUN, V73, P3492, DOI 10.1128/IAI.73.6.3492-3501.2005

Dooley DP, 1997, CLIN INFECT DIS, V25, P872, DOI 10.1086/515543

Ekins S, 2011, TRENDS MICROBIOL, V19, P65, DOI 10.1016/j.tim.2010.10.005

Erve JCL, 2006, EXPERT OPIN DRUG MET, V2, P923, DOI 10.1517/17425255.2.6.923

Falzon D, 2011, EUR RESPIR J, V38, P516, DOI 10.1183/09031936.00073611

Fan YL, 2018, EUR J MED CHEM, V146, P554, DOI 10.1016/j.ejmech.2018.01.080

Favrot L, 2012, EXPERT REV ANTI-INFE, V10, P1023, DOI [10.1586/ERI.12.91, 10.1586/eri.12.91]

Ferreiro GR, 2002, TOXICOL LETT, V132, P109, DOI 10.1016/S0378-4274(02)00039-5

Fischbach MA, 2009, SCIENCE, V325, P1089, DOI 10.1126/science.1176667

Fox GJ, 2013, INFECT DIS THER, V2, P123, DOI 10.1007/s40121-013-0009-3

GERZON K, 1967, J MED CHEM, V10, P189, DOI 10.1021/jm00314a014

GERZON K, 1963, J MED CHEM, V6, P760, DOI 10.1021/jm00342a029

Gleeson MP, 2008, J MED CHEM, V51, P817, DOI 10.1021/jm701122q

Gler MT, 2012, NEW ENGL J MED, V366, P2151, DOI 10.1056/NEJMoa1112433

Goldman RC, 2013, TUBERCULOSIS, V93, P569, DOI 10.1016/j.tube.2013.09.003

Grzegorzewicz AE, 2012, NAT CHEM BIOL, V8, P334, DOI [10.1038/NCHEMBIO.794, 10.1038/nchembio.794]

Guglielmetti L, 2017, EUR RESPIR J, V49, DOI 10.1183/13993003.01799-2016

Gupta S, 2014, ANTIMICROB AGENTS CH, V58, P574, DOI 10.1128/AAC.01462-13

Haagsma AC, 2009, ANTIMICROB AGENTS CH, V53, P1290, DOI 10.1128/AAC.01393-08

HANSCH C, 1987, J PHARM SCI, V76, P663, DOI 10.1002/jps.2600760902

Harausz E, 2015, INT J TUBERC LUNG D, V19, P385, DOI 10.5588/ijtld.14.0335

Harrison C, 2014, NAT REV DRUG DISCOV, V13, P104, DOI [10.1038/nrd4250, DOI 10.1038/NRD4250]

Hartkoorn RC, 2014, ANTIMICROB AGENTS CH, V58, P2979, DOI 10.1128/AAC.00037-14

Heinrich N, 2015, J ANTIMICROB CHEMOTH, V70, P1558, DOI 10.1093/jac/dku553

Hoagland DT, 2016, ADV DRUG DELIVER REV, V102, P55, DOI 10.1016/j.addr.2016.04.026

Huitric E, 2010, ANTIMICROB AGENTS CH, V54, P1022, DOI 10.1128/AAC.01611-09

Hurdle JG, 2011, NAT REV MICROBIOL, V9, P62, DOI 10.1038/nrmicro2474

Hwang TJ, 2014, BMJ OPEN, V4, DOI 10.1136/bmjopen-2013-004143

Jang J, 2017, ANTIMICROB AGENTS CH, V61, DOI [10.1128/aac.02637-16, 10.1128/AAC.02637-16]

JARLIER V, 1994, FEMS MICROBIOL LETT, V123, P11, DOI 10.1111/j.1574-6968.1994.tb07194.x

Kakkar AK, 2014, TUBERCULOSIS, V94, P357, DOI 10.1016/j.tube.2014.04.001

Kalgutkar AS, 2005, CURR DRUG METAB, V6, P161, DOI 10.2174/1389200054021799

Kapp E, 2018, MINI-REV MED CHEM, V18, P72, DOI 10.2174/1389557517666170510105506

Kigondu EM, 2014, BIOORGAN MED CHEM, V22, P4453, DOI 10.1016/j.bmc.2014.06.012

Kigondu EM, 2014, MEDCHEMCOMM, V5, P502, DOI 10.1039/c3md00387f

Kiran D, 2016, SEMIN IMMUNOPATHOL, V38, P167, DOI 10.1007/s00281-015-0537-x

Koo MS, 2011, PLOS ONE, V6, DOI 10.1371/journal.pone.0017091

Kornhuber J, 2010, J CELL PHYSIOL, V224, P152, DOI 10.1002/jcp.22112

Koul A, 2008, J BIOL CHEM, V283, P25273, DOI 10.1074/jbc.M803899200

Koul A, 2007, NAT CHEM BIOL, V3, P323, DOI 10.1038/nchembio884

Kovtun V.Y., 1987, Pharm. Chem. J., V21, P555, DOI DOI 10.1007/BF00759430

Kumar M, 2018, BIOORGAN MED CHEM, V26, P833, DOI 10.1016/j.bmc.2017.12.047

Kumar M, 2016, ACS INFECT DIS, V2, P714, DOI 10.1021/acsinfecdis.6b00111

La Rosa V, 2012, ANTIMICROB AGENTS CH, V56, P324, DOI 10.1128/AAC.05270-11

Lamprecht DA, 2016, NAT COMMUN, V7, DOI 10.1038/ncomms12393

Laughon BE, 2017, NAT REV DIS PRIMERS, V3, DOI 10.1038/nrdp.2017.15

Lechartier B, 2014, EMBO MOL MED, V6, P158, DOI 10.1002/emmm.201201772

Lee RE, 2003, J COMB CHEM, V5, P172, DOI 10.1021/cc020071p

Li K, 2014, J MED CHEM, V57, P3126, DOI 10.1021/jm500131s

Li W, 2017, ANTIMICROB AGENTS CH, V61, DOI [10.1128/AAC.02399-16, 10.1128/aac.02399-16]

Li W, 2014, ANTIMICROB AGENTS CH, V58, P6413, DOI 10.1128/AAC.03229-14

Lipinski CA, 1997, ADV DRUG DELIVER REV, V23, P3, DOI 10.1016/S0169-409X(96)00423-1

Lu P, 2014, J ANTIBIOT, V67, P835, DOI 10.1038/ja.2014.74

Lun SC, 2013, NAT COMMUN, V4, DOI 10.1038/ncomms3907

Machado D, 2018, FUTURE MICROBIOL, V13, P1383, DOI 10.2217/fmb-2018-0110

Machado D, 2018, FRONT MICROBIOL, V9, DOI 10.3389/fmicb.2018.01367

Machado D, 2018, ANTIBIOTICS-BASEL, V7, DOI 10.3390/antibiotics7010018

Machado D, 2017, FRONT MICROBIOL, V8, DOI 10.3389/fmicb.2017.00711

Machado D, 2016, PLOS ONE, V11, DOI 10.1371/journal.pone.0149326

Machado D, 2012, PLOS ONE, V7, DOI 10.1371/journal.pone.0034538

Mahajan Rajiv, 2013, Int J Appl Basic Med Res, V3, P1, DOI 10.4103/2229-516X.112228

Makarov V, 2015, ANTIMICROB AGENTS CH, V59, P4446, DOI 10.1128/AAC.00778-15

Makarov V, 2014, EMBO MOL MED, V6, P372, DOI 10.1002/emmm.201303575

Makarov V, 2009, SCIENCE, V324, P801, DOI 10.1126/science.1171583

Manina G, 2010, MOL MICROBIOL, V77, P1172, DOI 10.1111/j.1365-2958.2010.07277.x

Manjunatha Ujjini, 2009, Commun Integr Biol, V2, P215

Mao JL, 2009, J MED CHEM, V52, P6966, DOI 10.1021/jm900340a

Martineau AR, 2017, BMJ-BRIT MED J, V356, DOI 10.1136/bmj.i6583

Martins M, 2007, INT J ANTIMICROB AG, V29, P338, DOI 10.1016/j.ijantimicag.2006.10.013

Matsumoto M, 2007, CURR TOP MED CHEM, V7, P499, DOI 10.2174/156802607780059727

Matsumoto M, 2006, PLOS MED, V3, P2131, DOI 10.1371/journal.pmed.0030466

MATSUNOYAGI A, 1993, J BIOL CHEM, V268, P1539

MITCHISON DA, 1979, CHEST, V76, P771, DOI 10.1378/chest.76.6.771

MITCHISON DA, 1985, TUBERCLE, V66, P219, DOI 10.1016/0041-3879(85)90040-6

Molinspiration, 2019, CALCULATION MOL PROP

Nagarajan K, 1989, PROG DRUG RES, V24, P631, DOI DOI 10.1016/0223-5234(89)90034-2

Nikonenko BV, 2007, ANTIMICROB AGENTS CH, V51, P1563, DOI 10.1128/AAC.01326-06

Onajole OK, 2013, J MED CHEM, V56, P4093, DOI 10.1021/jm4003878

Onajole OK, 2011, MED CHEM RES, V20, P1394, DOI 10.1007/s00044-010-9490-3

Ordonez AA, 2014, CURR MOL MED, V14, P385, DOI 10.2174/1566524013666131118112431

Pal P, 1996, PHOTOCHEM PHOTOBIOL, V63, P161, DOI 10.1111/j.1751-1097.1996.tb03008.x

Patterson S, 2014, TRENDS PARASITOL, V30, P289, DOI 10.1016/j.pt.2014.04.003

Payne DJ, 2007, NAT REV DRUG DISCOV, V6, P29, DOI 10.1038/nrd2201

Pethe K, 2013, NAT MED, V19, P1157, DOI 10.1038/nm.3262

Petrella S, 2006, ANTIMICROB AGENTS CH, V50, P2853, DOI 10.1128/AAC.00244-06

Piccaro G, 2015, J ANTIBIOT, V68, P711, DOI 10.1038/ja.2015.52

Pieroni M, 2015, J MED CHEM, V58, P5842, DOI 10.1021/acs.jmedchem.5b00428

Pieroni M, 2015, EUR J MED CHEM, V100, P44, DOI 10.1016/j.ejmech.2015.05.048

Pieroni M, 2014, EUR J MED CHEM, V72, P26, DOI 10.1016/j.ejmech.2013.11.007

Pieroni M, 2010, CHEMMEDCHEM, V5, P1667, DOI 10.1002/cmdc.201000169

Pule CM, 2016, J ANTIMICROB CHEMOTH, V71, P17, DOI 10.1093/jac/dkv316

Raether W, 2003, PARASITOL RES, V90, pS19, DOI 10.1007/s00436-002-0754-9

Rao SPS, 2013, SCI TRANSL MED, V5, DOI 10.1126/scitranslmed.3007355

RAPALA RT, 1965, J MED CHEM, V8, P580, DOI 10.1021/jm00329a007

Ravikumar B, 2004, NAT GENET, V36, P585, DOI 10.1038/ng1362

Reddy VM, 2010, ANTIMICROB AGENTS CH, V54, P2840, DOI 10.1128/AAC.01601-09

Sacksteder KA, 2012, FUTURE MICROBIOL, V7, P823, DOI [10.2217/fmb.12.56, 10.2217/FMB.12.56]

Salie S, 2014, J ANTIMICROB CHEMOTH, V69, P1551, DOI 10.1093/jac/dku036

Sasaki H, 2006, J MED CHEM, V49, P7854, DOI 10.1021/jm060957y

Scalacci N, 2017, EUR J MED CHEM, V127, P147, DOI 10.1016/j.ejmech.2016.12.042

Seiler P, 2003, J INFECT DIS, V188, P1326, DOI 10.1086/378563

SENSI P, 1983, REV INFECT DIS, V5, pS402

Sharma S, 2010, J ANTIMICROB CHEMOTH, V65, P1694, DOI 10.1093/jac/dkq186

Shigyo K, 2013, ANTIMICROB AGENTS CH, V57, P2834, DOI 10.1128/AAC.02542-12

Singh K, 2014, BIOORG MED CHEM LETT, V24, P2985, DOI 10.1016/j.bmcl.2014.05.022

Singh R, 2008, SCIENCE, V322, P1392, DOI 10.1126/science.1164571

Singhal A, 2014, SCI TRANSL MED, V6, DOI 10.1126/scitranslmed.3009885

Skerry C, 2012, PLOS ONE, V7, DOI 10.1371/journal.pone.0039680

Skripconoka V, 2013, EUR RESPIR J, V41, P1393, DOI 10.1183/09031936.00125812

Sotgiu G, 2015, EXPERT REV ANTI-INFE, V13, P305, DOI 10.1586/14787210.2015.1011127

Stanley SA, 2014, PLOS PATHOG, V10, DOI 10.1371/journal.ppat.1003946

Stover CK, 2000, NATURE, V405, P962, DOI 10.1038/35016103

Subbian S, 2011, PLOS PATHOG, V7, DOI 10.1371/journal.ppat.1002262

Sutherland HS, 2018, BIOORGAN MED CHEM, V26, P1797, DOI 10.1016/j.bmc.2018.02.026

Tabarrini O, 2012, CHEM BIOL DRUG DES, V80, P781, DOI 10.1111/cbdd.12022

Tacconelli E, 2018, LANCET INFECT DIS, V18, P318, DOI [10.1016/S1473-3099(17)30753-3, 10.1016/s1473-3099(17)30753-3]

Tahlan K, 2012, ANTIMICROB AGENTS CH, V56, P1797, DOI 10.1128/AAC.05708-11

Tang SJ, 2015, CLIN INFECT DIS, V60, P1361, DOI 10.1093/cid/civ027

Tong AST, 2017, ACS MED CHEM LETT, V8, P1019, DOI 10.1021/acsmedchemlett.7b00196

Trefzer C, 2010, J AM CHEM SOC, V132, P13663, DOI 10.1021/ja106357w

Uplekar M, 2015, LANCET, V385, P1799, DOI 10.1016/S0140-6736(15)60570-0

Van Bambeke Francoise, 2006, Recent Pat Antiinfect Drug Discov, V1, P157, DOI 10.2174/157489106777452692

van der Westhuyzen R, 2015, J MED CHEM, V58, P9371, DOI 10.1021/acs.jmedchem.5b01542

van Soolingen D, 2010, PLOS ONE, V5, DOI 10.1371/journal.pone.0012640

Veber DF, 2002, J MED CHEM, V45, P2615, DOI 10.1021/jm020017n

Veziris N, 2017, EUR RESPIR J, V49, DOI 10.1183/13993003.01719-2016

Viveiros M, 2012, EXPERT REV ANTI-INFE, V10, P983, DOI [10.1586/ERI.12.89, 10.1586/eri.12.89]

Vjecha MJ, 2018, NAT REV DRUG DISCOV, V17, P607, DOI 10.1038/nrd.2018.28

Wallis RS, 2015, NAT REV IMMUNOL, V15, P255, DOI 10.1038/nri3813

Wallis RS, 2004, AIDS, V18, P257, DOI 10.1097/00002030-200401230-00015

WALSH JS, 1987, J MED CHEM, V30, P150, DOI 10.1021/jm00384a025

Wanka L, 2013, CHEM REV, V113, P3516, DOI 10.1021/cr100264t

Warman AJ, 2013, J ANTIMICROB CHEMOTH, V68, P869, DOI 10.1093/jac/dks483

Weinstein EA, 2005, P NATL ACAD SCI USA, V102, P4548, DOI 10.1073/pnas.0500469102

Wright SH, 2004, ADV PHYSIOL EDUC, V28, P139, DOI 10.1152/advan.00029.2004

Yang B, 2017, ANTIMICROB AGENTS CH, V61, DOI [10.1128/AAC.02052-16, 10.1128/aac.02052-16]

Zhang B, 2019, CELL, V176, P636, DOI 10.1016/j.cell.2019.01.003

Zhang DF, 2014, MOLECULES, V19, P4380, DOI 10.3390/molecules19044380

Zhang DF, 2012, J MED CHEM, V55, P8409, DOI 10.1021/jm300828h

NR 172

TC 8

Z9 10

U1 0

U2 9

PU ELSEVIER ACADEMIC PRESS INC

PI SAN DIEGO

PA 525 B STREET, SUITE 1900, SAN DIEGO, CA 92101-4495 USA

SN 0065-7743

BN 978-0-12-817556-9

J9 ANNU REP MED CHEM

JI Annu. Rep. Med. Chem.

PY 2019

VL 52

BP 27

EP 69

DI 10.1016/bs.armc.2019.06.001

PG 43

WC Chemistry, Medicinal; Pharmacology & Pharmacy

WE Book Citation Index – Science (BKCI-S); Science Citation Index Expanded (SCI-EXPANDED)

SC Pharmacology & Pharmacy

GA BO1NX

UT WOS:000501595100003

DA 2026-05-07

ER

PT J

AU Anand, RS

Somasundaram, S

Doble, M

Paramasivan, CN

AF Anand, R. S.

Somasundaram, Sulochana

Doble, Mukesh

Paramasivan, C. N.

TI Docking studies on novel analogues of 8 methoxy fluoroquinolones against

GyrA mutants of Mycobacterium tuberculosis

SO BMC STRUCTURAL BIOLOGY

LA English

DT Article

DE Docking; Mycobacterium tuberculosis; GyrA; Fluoroquinolones;

gatifloxacin; moxifloxacin

ID STATIONARY-PHASE CULTURES; BACTERICIDAL ACTIVITY; EFFLUX PUMP;

RESISTANCE; OPTIMIZATION; GATIFLOXACIN; MOXIFLOXACIN; DISCOVERY;

SMEGMATIS; DRUGS

AB Background: Fluoroquinolone resistance is a serious threat in the battle against the treatment of multi drug resistant tuberculosis (MDR-TB) and extensively drug resistant tuberculosis (XDR-TB). Fluoroquinolone resistant isolates from India had shown to have evolved several mutants in the quinolone resistance determining region (QRDR) of DNA gyrase A subunit (GyrA), the target of fluoroquinolone. In view of high prevalence of mutations in the 'hot spot' region, a study on combinatorial drug design was carried out to identify better analogues for the treatment of MDR-TB. The gyrA subunit 'hot spot' region of codons 90, 94 and 95 were modeled into their corresponding protein folds and used as receptors for the docking studies. Further, invitro tests were carried using the parent compounds, namely gatifloxacin and moxifloxacin and correlated with the obtained docking scores.

Results: Molecular docking and in vitro studies correlated well in demonstrating the enhanced activity of moxifloxacin, when compared to gatifloxacin, on ofloxacin sensitive and resistant strains comprising of clinical isolates of MDR-TB. The evolved lead structures targeting against mutant QRDR receptors were guanosine and cholesteryl esters of gatifloxacin and moxifloxacin. They showed consistently high binding affinity values of -10.3 and -10.1 kcal/ mol respectively with the target receptors. Of these, the guanosine ester showed highest binding affinity score and its log P value lied within the Lipinski's range indicating that it could have better absorptivity when it is orally administered thereby having an enhanced activity against MTB.

Conclusions: The docking results showed that the addition of the cholesteryl and guanosine esters to the 'DNA gyrase binding' region of gatifloxacin and moxifloxacin enhanced the binding affinity of these parent molecules with the mutant DNA gyrase receptors. Viewing the positive correlation for the docking and in vitro results with the parent compounds, these lead structures could be further evaluated for their in vitro and in vivo activity against MDR-TB.

C1 [Somasundaram, Sulochana] Sri Venkateswara Coll Engn, Dept Biotechnol, Sriperumbudur 602105, India.

[Doble, Mukesh] Indian Inst Technol, Dept Biotechnol, Madras 600036, Tamil Nadu, India.

[Paramasivan, C. N.] Fdn Innovat, CH-1202 Geneva, Switzerland.

[Anand, R. S.] Anna Univ, Ctr Biotechnol, Madras 600025, Tamil Nadu, India.

C3 Indian Institute of Technology System (IIT System); Indian Institute of

Technology (IIT) - Madras; Foundation For Innovative New Diagnostics;

Anna University; Anna University Chennai

RP Somasundaram, S (corresponding author), Sri Venkateswara Coll Engn, Dept Biotechnol, Sriperumbudur 602105, India.

EM sulochana@svce.ac.in

RI ; RS, Anand/GPK-0913-2022

OI Doble, Mukeshd/0000-0002-4335-0698; , Anand/0000-0003-3513-9224;

CR Altschul SF, 1997, NUCLEIC ACIDS RES, V25, P3389, DOI 10.1093/nar/25.17.3389

[Anonymous], ACD CHEMSKETCH FREEW

Arnold K, 2006, BIOINFORMATICS, V22, P195, DOI 10.1093/bioinformatics/bti770

Brzostek A, 2007, FEMS MICROBIOL LETT, V275, P106, DOI 10.1111/j.1574-6968.2007.00865.x

Chan ED, 2004, AM J RESP CRIT CARE, V169, P1103, DOI 10.1164/rccm.200308-1159OC

Corbett EL, 2003, ARCH INTERN MED, V163, P1009, DOI 10.1001/archinte.163.9.1009

Crotty S, 2002, J MOL MED, V80, P86, DOI 10.1007/s00109-001-0308-0

Cynamon MH, 2003, ANTIMICROB AGENTS CH, V47, P2442, DOI 10.1128/AAC.47.8.2442-2444.2003

Emami S., 2010, Iranian Journal of Pharmaceutical Research, P123

Ginsburg AS, 2003, LANCET INFECT DIS, V3, P432, DOI 10.1016/S1473-3099(03)00671-6

Guzman CP, 2005, CHEST, V127, P643

HOOPER CD, 2003, QUINOLONE ANTIMICROB

Huang YY, 2006, NUCLEIC ACIDS RES, V34, P5650, DOI 10.1093/nar/gkl695

Jacobson KR, 2010, CLIN INFECT DIS, V51, P6, DOI 10.1086/653115

Kantardjieff K, 2004, CURR PHARM DESIGN, V10, P3195, DOI 10.2174/1381612043383205

Khan HA, 2007, HUM EXP TOXICOL, V26, P677, DOI 10.1177/0960327107083974

Kitchen DB, 2004, NAT REV DRUG DISCOV, V3, P935, DOI 10.1038/nrd1549

Kubendiran G, 2007, J CHEMOTHER, V18, P65

Liu J, 1996, J BACTERIOL, V178, P3791, DOI 10.1128/jb.178.13.3791-3795.1996

Masand V. H., 2010, Der Pharma Chemica, V2, P22

Mohammed AR, 2004, INT J PHARMACEUT, V285, P23, DOI 10.1016/j.ijpharm.2004.07.010

Muñoz S, 2009, SCAND J IMMUNOL, V70, P256, DOI 10.1111/j.1365-3083.2009.02295.x

Paramasivan CN, 2005, ANTIMICROB AGENTS CH, V49, P627, DOI 10.1128/AAC.49.2.627-631.2005

Piton J, 2010, PLOS ONE, V5, DOI 10.1371/journal.pone.0012245

Pletz MWR, 2004, ANTIMICROB AGENTS CH, V48, P780, DOI 10.1128/AAC.48.3.780-782.2004

Rattan A, 1998, EMERG INFECT DIS, V4, P195, DOI 10.3201/eid0402.980207

Schentag J, 1985, RES CLIN FORUMS, V7, P9

Schmalfuss U, 1997, J CONTROL RELEASE, V46, P279, DOI 10.1016/S0168-3659(96)01609-4

Schüttelkopf AW, 2004, ACTA CRYSTALLOGR D, V60, P1355, DOI 10.1107/S0907444904011679

Simoes MF, 2009, EUR J PHARM SCI, V37, P257, DOI 10.1016/j.ejps.2009.02.012

Sulochana S, 2007, J CHEMOTHERAPY, V19, P166, DOI 10.1179/joc.2007.19.2.166

Sulochana S, 2009, J CHEMOTHERAPY, V21, P127, DOI 10.1179/joc.2009.21.2.127

Sulochana S, 2005, J CHEMOTHER, V1, P81

Takiff HE, 1996, P NATL ACAD SCI USA, V93, P362, DOI 10.1073/pnas.93.1.362

Trott O, 2010, J COMPUT CHEM, V31, P455, DOI 10.1002/jcc.21334

World Health Organization, WHOTB97229

NR 36

TC 10

Z9 16

U1 0

U2 9

PU BIOMED CENTRAL LTD

PI LONDON

PA 236 GRAYS INN RD, FLOOR 6, LONDON WC1X 8HL, ENGLAND

SN 1472-6807

J9 BMC STRUCT BIOL

JI BMC Struct. Biol.

PD DEC 12

PY 2011

VL 11

AR 47

DI 10.1186/1472-6807-11-47

PG 13

WC Biophysics

WE Science Citation Index Expanded (SCI-EXPANDED)

SC Biophysics

GA 908LX

UT WOS:000301493000001

OA Green Submitted, hybrid

DA 2026-05-07

ER

PT J

AU Pule, CM

Sampson, SL

Warren, RM

Black, PA

van Helden, PD

Victor, TC

Louw, GE

AF Pule, Caroline M.

Sampson, Samantha L.

Warren, Robin M.

Black, Philippa A.

van Helden, Paul D.

Victor, Tommie C.

Louw, Gail E.

TI Efflux pump inhibitors: targeting mycobacterial efflux systems to

enhance TB therapy

SO JOURNAL OF ANTIMICROBIAL CHEMOTHERAPY

LA English

DT Review

ID ETHIDIUM-BROMIDE EFFLUX; GRAM-NEGATIVE BACTERIA; DRUG-RESISTANCE;

IN-VITRO; ANTIBIOTIC-RESISTANCE; MULTIDRUG TRANSPORTER; IONOPHORE

VALINOMYCIN; BACILLUS-SUBTILIS; ESCHERICHIA-COLI; P-GLYCOPROTEIN

AB The emergence of drug resistance continues to plague TB control, with a global increase in the prevalence of MDR-TB. This acts as a gateway to XDR-TB and thus emphasizes the urgency for drug development and optimal treatment options. Bedaquiline is the first new anti-TB drug approved by the FDA in 40 years and has been shown to be an effective treatment option for MDR Mycobacterium tuberculosis infection. Bedaquiline has also recently been included in clinical trials for new regimens with the aim of improving and shortening treatment periods. Alarmingly, efflux-mediated bedaquiline resistance, as well as efflux-mediated cross-resistance to clofazimine, has been identified in treatment failures. This mechanism of resistance results in efflux of a variety of anti-TB drugs from the bacterial cell, thereby decreasing the intracellular drug concentration. In doing so, the bacillus is able to render the antibiotic treatment ineffective. Recent studies have explored strategies to reverse the resistance phenotype conferred by efflux pump activation. It was observed that the addition of efflux pump inhibitors partially restored drug susceptibility in vitro and in vivo. This has significant clinical implications, especially in MDR-TB management where treatment options are extremely limited. This review aims to highlight the current efflux pump inhibitors effective against M. tuberculosis, the effect of efflux pump inhibitors on mycobacterial growth and the clinical promise of treatment with efflux pump inhibitors and standard anti-TB therapy.

C1 [Pule, Caroline M.; Sampson, Samantha L.; Warren, Robin M.; Black, Philippa A.; van Helden, Paul D.; Victor, Tommie C.; Louw, Gail E.] Univ Stellenbosch, Fac Med & Hlth Sci, Div Mol Biol & Human Genet, DST NRF Ctr Excellence Biomed TB Res,SAMRC Ctr TB, ZA-7505 Tygerberg, South Africa.

C3 National Research Foundation - South Africa; Stellenbosch University

RP Sampson, SL (corresponding author), Univ Stellenbosch, Fac Med & Hlth Sci, Div Mol Biol & Human Genet, DST NRF Ctr Excellence Biomed TB Res,SAMRC Ctr TB, ZA-7505 Tygerberg, South Africa.

EM ssampson@sun.ac.za

OI sampson, samantha/0000-0002-9596-8019; Warren, Robin/0000-0001-5741-7358

FU Medical Research Council of South Africa Scholarship; Claude Leon

post-doctoral fellowship; National Research Foundation grant [CPR

81776]; South African Research Chairs Initiative of Department of

Science and Technology; National Research Foundation of South Africa

[UID 86539]

FX This work was sponsored by a Medical Research Council of South Africa

Scholarship (to C. M. P.), a Claude Leon post-doctoral fellowship (to G.

E. L.) and a National Research Foundation grant (CPR 81776) (to R. M.

W.). S. L. S. is funded by the South African Research Chairs Initiative

of the Department of Science and Technology and National Research

Foundation of South Africa, award number UID 86539.

CR Adams KN, 2014, J INFECT DIS, V210, P456, DOI 10.1093/infdis/jiu095

Adams KN, 2011, CELL, V145, P39, DOI 10.1016/j.cell.2011.02.022

Amaral L, 1996, J ANTIMICROB CHEMOTH, V38, P1049, DOI 10.1093/jac/38.6.1049

Amaral L, 2007, J ANTIMICROB CHEMOTH, V59, P1237, DOI 10.1093/jac/dkl500

Amaral L, 2014, FRONT PHARMACOL, V4, DOI 10.3389/fphar.2013.00168

Amaral Leonard, 2011, Adv Enzymol Relat Areas Mol Biol, V77, P61

Andersen CL, 2006, BBA-BIOMEMBRANES, V1758, P1587, DOI 10.1016/j.bbamem.2006.05.022

Andries K, 2005, SCIENCE, V307, P223, DOI 10.1126/science.1106753

Andries K, 2014, PLOS ONE, V9, DOI 10.1371/journal.pone.0102135

[Anonymous], PHEN

ATAL CK, 1985, J PHARMACOL EXP THER, V232, P258

Balganesh M, 2012, ANTIMICROB AGENTS CH, V56, P2643, DOI 10.1128/AAC.06003-11

Banerjee SK, 1996, BIOCHEM BIOPH RES CO, V226, P362, DOI 10.1006/bbrc.1996.1362

Beck E, 2005, AM FAM PHYSICIAN, V71, P717

Bhadra K, 2011, MED RES REV, V31, P821, DOI 10.1002/med.20202

Bhardwaj RK, 2002, J PHARMACOL EXP THER, V302, P645, DOI 10.1124/jpet.102.034728

Choudhuri BS, 1999, BIOCHEM BIOPH RES CO, V256, P682, DOI 10.1006/bbrc.1999.0357

Colangeli R, 2005, MOL MICROBIOL, V55, P1829, DOI 10.1111/j.1365-2958.2005.04510.x

CORTI S, 1995, ANTIMICROB AGENTS CH, V39, P2466, DOI 10.1128/AAC.39.11.2466

DANIELE RP, 1976, P NATL ACAD SCI USA, V73, P3599, DOI 10.1073/pnas.73.10.3599

De Rossi E, 2006, FEMS MICROBIOL REV, V30, P36, DOI 10.1111/j.1574-6976.2005.00000.x

Diacon AH, 2014, NEW ENGL J MED, V371, P723, DOI 10.1056/NEJMoa1313865

Diacon AH, 2009, NEW ENGL J MED, V360, P2397, DOI 10.1056/NEJMoa0808427

ENDICOTT JA, 1989, ANNU REV BIOCHEM, V58, P137, DOI 10.1146/annurev.bi.58.070189.001033

Gibbons S, 2000, PHYTOTHER RES, V14, P139, DOI 10.1002/(SICI)1099-1573(200003)14:2<139::AID-PTR608>3.0.CO;2-8

Grossman TH, 2015, ANTIMICROB AGENTS CH, V59, P1539, DOI 10.1128/AAC.04271-14

Gupta AK, 2010, INDIAN J MED RES, V132, P176

Gupta S, 2014, ANTIMICROB AGENTS CH, V58, P574, DOI 10.1128/AAC.01462-13

Gupta S, 2013, AM J RESP CRIT CARE, V188, P600, DOI 10.1164/rccm.201304-0650OC

IWU MM, 1977, PLANTA MED, V32, P88, DOI 10.1055/s-0028-1097565

Jin J, 2011, J MED MICROBIOL, V60, P223, DOI 10.1099/jmm.0.025734-0

Jin J, 2010, MOLECULES, V15, P7750, DOI 10.3390/molecules15117750

JOHNSTON A, 1981, BRIT J CLIN PHARMACO, V12, P397, DOI 10.1111/j.1365-2125.1981.tb01233.x

Khan IA, 2006, ANTIMICROB AGENTS CH, V50, P810, DOI 10.1128/AAC.50.2.810-812.2006

Kim JB, 2010, PHYTOMEDICINE, V17, P436, DOI 10.1016/j.phymed.2009.08.012

Klyachko KA, 1997, J BACTERIOL, V179, P2189, DOI 10.1128/jb.179.7.2189-2193.1997

Koul A, 2008, J BIOL CHEM, V283, P25273, DOI 10.1074/jbc.M803899200

Kuo CL, 2004, CANCER LETT, V203, P127, DOI 10.1016/j.canlet.2003.09.002

Li XZ, 2004, DRUGS, V64, P159, DOI 10.2165/00003495-200464020-00004

Louw GE, 2011, AM J RESP CRIT CARE, V184, P269, DOI 10.1164/rccm.201011-1924OC

Machado D, 2012, PLOS ONE, V7, DOI 10.1371/journal.pone.0034538

Mahamoud A, 2007, J ANTIMICROB CHEMOTH, V59, P1223, DOI 10.1093/jac/dkl493

Markham PN, 1999, ANTIMICROB AGENTS CH, V43, P2404, DOI 10.1128/AAC.43.10.2404

Martins A, 2009, PLOS ONE, V4, DOI 10.1371/journal.pone.0006656

Martins M, 2008, IN VIVO, V22, P69

Martins M, 2007, INT J ANTIMICROB AG, V29, P338, DOI 10.1016/j.ijantimicag.2006.10.013

MCTAVISH D, 1989, DRUGS, V38, P19, DOI 10.2165/00003495-198938010-00003

Milano A, 2009, TUBERCULOSIS, V89, P84, DOI 10.1016/j.tube.2008.08.003

Molnar J, 1997, ANTICANCER RES, V17, P481

Nagabushan H, 2014, J POSTGRAD MED, V60, P300, DOI 10.4103/0022-3859.138772

NEYFAKH AA, 1991, P NATL ACAD SCI USA, V88, P4781, DOI 10.1073/pnas.88.11.4781

Ordway D, 2003, ANTIMICROB AGENTS CH, V47, P917, DOI 10.1128/AAC.47.3.917-922.2003

Park JW, 1997, BBA-LIPID LIPID MET, V1344, P132, DOI 10.1016/S0005-2760(96)00146-4

Pasca MR, 2005, ANTIMICROB AGENTS CH, V49, P4775, DOI 10.1128/AAC.49.11.4775-4777.2005

Pasca MR, 2004, ANTIMICROB AGENTS CH, V48, P3175, DOI 10.1128/AAC.48.8.3175-3178.2004

Paulsen IT, 1996, MICROBIOL REV, V60, P575, DOI 10.1128/MMBR.60.4.575-608.1996

Piddock LJV, 2000, J ANTIMICROB CHEMOTH, V45, P681, DOI 10.1093/jac/45.5.681

Piddock LJV, 2000, J ANTIMICROB CHEMOTH, V45, P159, DOI 10.1093/jac/45.2.159

PLUMMER ALBERT J., 1954, ANN NEW YORK ACAD SCI, V59, P8, DOI 10.1111/j.1749-6632.1954.tb45914.x

Poelarends GJ, 2002, BBA-BIOENERGETICS, V1555, P1, DOI 10.1016/S0005-2728(02)00246-3

Pos KM, 2009, BBA-PROTEINS PROTEOM, V1794, P782, DOI 10.1016/j.bbapap.2008.12.015

Rabori Eshagh Dortaj, 2006, Journal of Communicable Diseases, V37, P249

Ramón-García S, 2006, J ANTIMICROB CHEMOTH, V57, P252, DOI 10.1093/jac/dki436

Ramón-García S, 2009, ANTIMICROB AGENTS CH, V53, P3675, DOI 10.1128/AAC.00550-09

Rodrigues L, 2008, J ANTIMICROB CHEMOTH, V61, P1076, DOI 10.1093/jac/dkn070

Rodrigues L, 2013, ANTIMICROB AGENTS CH, V57, P751, DOI 10.1128/AAC.01482-12

Rodrigues L, 2012, INFECT GENET EVOL, V12, P695, DOI 10.1016/j.meegid.2011.08.009

Rodrigues Liliana, 2011, Recent Pat Antiinfect Drug Discov, V6, P118

Rodrigues L, 2009, INT J ANTIMICROB AG, V34, P529, DOI 10.1016/j.ijantimicag.2009.07.010

Rose L, 2007, BIOELECTROCHEMISTRY, V70, P387, DOI 10.1016/j.bioelechem.2006.05.009

Schmalstieg AM, 2012, ANTIMICROB AGENTS CH, V56, P4806, DOI 10.1128/AAC.05546-11

Seeger MA, 2008, CURR DRUG TARGETS, V9, P729, DOI 10.2174/138945008785747789

Sharma S, 2014, TUBERCULOSIS, V94, P389, DOI 10.1016/j.tube.2014.04.007

Sharma S, 2010, J ANTIMICROB CHEMOTH, V65, P1694, DOI 10.1093/jac/dkq186

Silva PEA, 2001, ANTIMICROB AGENTS CH, V45, P800, DOI 10.1128/AAC.45.3.800-804.2001

SINGH J, 1986, J PHARMACOL EXP THER, V236, P488

Singh K, 2014, BIOORG MED CHEM LETT, V24, P2985, DOI 10.1016/j.bmcl.2014.05.022

Singh M, 2011, INDIAN J MED RES, V133, P535

Stavri M, 2007, J ANTIMICROB CHEMOTH, V59, P1247, DOI 10.1093/jac/dkl460

Stermitz FR, 2000, P NATL ACAD SCI USA, V97, P1433, DOI 10.1073/pnas.030540597

Sun ZG, 2014, MICROB DRUG RESIST, V20, P525, DOI 10.1089/mdr.2013.0171

Szumowski JD, 2014, CURR TOP MICROBIOL, V374, P81, DOI 10.1007/82_2012_300

Thota N, 2008, BIOORGAN MED CHEM, V16, P6535, DOI 10.1016/j.bmc.2008.05.030

Van Bambeke Francoise, 2006, Recent Pat Antiinfect Drug Discov, V1, P157, DOI 10.2174/157489106777452692

Viveiros M, 2002, ANTIMICROB AGENTS CH, V46, P2804, DOI 10.1128/AAC.46.9.2804-2810.2002

Viveiros M, 2001, INT J ANTIMICROB AG, V17, P225, DOI 10.1016/S0924-8579(00)00343-5

Viveiros M, 2012, EXPERT REV ANTI-INFE, V10, P983, DOI [10.1586/ERI.12.89, 10.1586/eri.12.89]

Williams K, 2012, ANTIMICROB AGENTS CH, V56, P3114, DOI 10.1128/AAC.00384-12

Williams KJ, 1998, ANTIMICROB AGENTS CH, V42, P795, DOI 10.1128/AAC.42.4.795

Yamasaki S, 2013, NAT COMMUN, V4, DOI 10.1038/ncomms3078

Zhang Y, 2002, J MED MICROBIOL, V51, P42, DOI 10.1099/0022-1317-51-1-42

Zhang Y, 1999, J BACTERIOL, V181, P2044, DOI 10.1128/JB.181.7.2044-2049.1999

NR 92

TC 119

Z9 143

U1 0

U2 44

PU OXFORD UNIV PRESS

PI OXFORD

PA GREAT CLARENDON ST, OXFORD OX2 6DP, ENGLAND

SN 0305-7453

EI 1460-2091

J9 J ANTIMICROB CHEMOTH

JI J. Antimicrob. Chemother.

PD JAN

PY 2016

VL 71

IS 1

BP 17

EP 26

DI 10.1093/jac/dkv316

PG 10

WC Infectious Diseases; Microbiology; Pharmacology & Pharmacy

WE Science Citation Index Expanded (SCI-EXPANDED)

SC Infectious Diseases; Microbiology; Pharmacology & Pharmacy

GA DF3IS

UT WOS:000371238100004

OA Bronze

DA 2026-05-07

ER

PT J

AU Li, QM

Zhou, ML

Fan, XY

Yan, JL

Li, WM

Xie, JP

AF Li, Qiming

Zhou, Mingliang

Fan, Xiangyu

Yan, Jianlong

Li, Weimin

Xie, Jianping

TI Mycobacteriophage SWU1 gp39 can potentiate multiple antibiotics against

Mycobacterium via altering the cell wall permeability

SO SCIENTIFIC REPORTS

LA English

DT Article

ID INTRINSIC MULTIDRUG-RESISTANCE; DRUG-RESISTANCE; SMEGMATIS; GENE;

HOMEOSTASIS; ENDOLYSINS; MECHANISM; TARGET; GROWTH; INHA

AB M. tuberculosis is intrinsically tolerant to many antibiotics largely due to the imperviousness of its unusual mycolic acid-containing cell wall to most antimicrobials. The emergence and increasingly widespread of multidrug-resistant tuberculosis (MDR-TB) and extensively drug-resistant tuberculosis (XDR-TB) revitalized keen interest in phage-inspired therapy. SWU1gp39 is a novel gene from mycobacteriophage SWU1 with unknown function. SWU1gp39 expressed in M. smegmatis conferred the host cell increased susceptibility to multiple antibiotics, including isoniazid, erythromycin, norfloxacin, ampicillin, ciprofloxacin, ofloxacin, rifampicin and vancomycin, and multiple environment stresses such as H2O2, heat shock, low pH and SDS. By using EtBr/Nile red uptake assays, WT-pAL-gp39 strain showed higher cell wall permeability than control strain WT-pAL. Moreover, the WT-pAL-gp39 strain produced more reactive oxygen species and reduced NAD(+)/NADH ratio. RNA-Seq transcriptomes of the WT-pAL-gp39 and WT-pAL revealed that the transcription of 867 genes was differentially regulated, including genes associated with lipid metabolism. Taken together, our results implicated that SWU1gp39, a novel gene from mycobacteriophage, disrupted the lipid metabolism of host and increased cell wall permeability, ultimately potentiated the efficacy of multiple antibiotics and stresses against mycobacteria.

C1 [Li, Qiming; Zhou, Mingliang; Fan, Xiangyu; Yan, Jianlong; Xie, Jianping] Southwest Univ, State Key Lab Breeding Base Ecoenvironm & Bioreso, Key Lab Ecoenvironm Gorges Reservoir Reg 3,Minist, Inst Modern Biopharmaceut,Gorges Area 3,Sch Life, Chongqing 400715, Peoples R China.

[Fan, Xiangyu] Univ Jinan, Sch Biol Sci & Technol, Jinan 250022, Shandong, Peoples R China.

[Li, Weimin] Capital Med Univ, Beijing Chest Hosp, Beijing TB & Thorac Tumor Res Inst, Beijing Key Lab Drug Resistant TB Res,Natl TB Cli, Beijing 101149, Peoples R China.

C3 Southwest University - China; University of Jinan; Capital Medical

University

RP Xie, JP (corresponding author), Southwest Univ, State Key Lab Breeding Base Ecoenvironm & Bioreso, Key Lab Ecoenvironm Gorges Reservoir Reg 3,Minist, Inst Modern Biopharmaceut,Gorges Area 3,Sch Life, Chongqing 400715, Peoples R China.; Li, WM (corresponding author), Capital Med Univ, Beijing Chest Hosp, Beijing TB & Thorac Tumor Res Inst, Beijing Key Lab Drug Resistant TB Res,Natl TB Cli, Beijing 101149, Peoples R China.

EM lwm_18@aliyun.com; georgex@swu.edu.cn

RI xu, li/AAO-4684-2021; Fan, Xiangyu/D-2664-2009

OI Fan, Xiangyu/0000-0002-1376-0039

FU National Natural Science Foundation [81371851, 81071316, 81271882,

81301394, 81172806, 81471563]; New Century Excellent Talents in

Universities [NCET-11-0703]; National Megaprojects for Key Infectious

Diseases [2008ZX10003-006, 2008ZX10003-001]; Southwest University

[kb2010017, ky2011003]; Fundamental Research Funds for the Central

Universities [XDJK2014D040, XDJK2016D025]; Graduate research and

innovation project of graduate in Chongqing [CYS14044]; Chongqing

Municipal Committee of Education for postgraduates excellence program

[YJG123104]; undergraduates teaching reform program [2013JY201]

FX This work was supported by National Natural Science Foundation [grant

numbers 81371851, 81071316, 81271882, 81301394, 81172806, 81471563], New

Century Excellent Talents in Universities [grant number NCET-11-0703],

National Megaprojects for Key Infectious Diseases [grant numbers

2008ZX10003-006, 2008ZX10003-001], Excellent Ph.D. thesis fellowship of

Southwest University [grant numbers kb2010017, ky2011003], the

Fundamental Research Funds for the Central Universities [grant numbers

XDJK2014D040, XDJK2016D025], Graduate research and innovation project of

graduate in Chongqing (CYS14044), The Chongqing Municipal Committee of

Education for postgraduates excellence program [grant numbers

YJG123104], and The undergraduates teaching reform program [grant

numbers 2013JY201].

CR Anders S, 2015, BIOINFORMATICS, V31, P166, DOI 10.1093/bioinformatics/btu638

[Anonymous], 2015, J BIOMOLECULAR STRUC

BANERJEE A, 1994, SCIENCE, V263, P227, DOI 10.1126/science.8284673

BASS DA, 1983, J IMMUNOL, V130, P1910

BAUER AW, 1966, AM J CLIN PATHOL, V45, P493

Berney M, 2014, MBIO, V5, DOI 10.1128/mBio.01275-14

Bisson GP, 2012, J BACTERIOL, V194, P6441, DOI 10.1128/JB.01013-12

Bowman J, 2014, MOL MICROBIOL, V91, P121, DOI 10.1111/mmi.12448

BRANDT R, 1965, ANAL BIOCHEM, V11, P6, DOI 10.1016/0003-2697(65)90035-7

Cangelosi GA, 2001, MICROBIOL-UK, V147, P527, DOI 10.1099/00221287-147-3-527

Catalao MJ, 2011, PLOS ONE, V6, DOI 10.1371/journal.pone.0020515

Chuang YM, 2015, MBIO, V6, DOI 10.1128/mBio.02428-14

Costerton JW, 1999, SCIENCE, V284, P1318, DOI 10.1126/science.284.5418.1318

Dharmaraja AT, 2012, CHEM COMMUN, V48, P10325, DOI 10.1039/c2cc35343a

Dwyer DJ, 2014, P NATL ACAD SCI USA, V111, pE2100, DOI 10.1073/pnas.1401876111

Fair RJ, 2014, PERSPECT MED CHEM, V6, P25, DOI 10.4137/PMC.S14459

Fan XY, 2015, GENE, V561, P45, DOI 10.1016/j.gene.2015.02.053

Fan XY, 2012, J VIROL, V86, P10230, DOI 10.1128/JVI.01568-12

Gil F, 2010, MICROBIOL-SGM, V156, P1497, DOI 10.1099/mic.0.032821-0

Grover N, 2014, ENZYME MICROB TECH, V63, P1, DOI 10.1016/j.enzmictec.2014.04.018

Huang GT, 2014, BMC INFECT DIS, V14, DOI 10.1186/s12879-014-0681-2

Jankute M, 2015, ANNU REV MICROBIOL, V69, P405, DOI 10.1146/annurev-micro-091014-104121

Kashyap DR, 2014, PLOS PATHOG, V10, DOI 10.1371/journal.ppat.1004280

Koch A, 2014, EMERG MICROBES INFEC, V3, DOI 10.1038/emi.2014.17

Kohanski MA, 2007, CELL, V130, P797, DOI 10.1016/j.cell.2007.06.049

Kohanski MA, 2010, NAT REV MICROBIOL, V8, P423, DOI 10.1038/nrmicro2333

Lai MJ, 2015, MOLECULES, V20, P19277, DOI 10.3390/molecules201019277

Lee RE, 1996, CURR TOP MICROBIOL, V215, P1

Lewis T, 2000, J MICROBIOL METH, V43, P107, DOI 10.1016/S0167-7012(00)00217-7

Li GQ, 2015, BIOSCIENCE REP, V35, DOI 10.1042/BSR20140167

Li LP, 2014, MBIO, V5, DOI 10.1128/mBio.01708-14

Li QM, 2015, SCI REP-UK, V5, DOI 10.1038/srep11910

Loessner MJ, 2005, CURR OPIN MICROBIOL, V8, P480, DOI 10.1016/j.mib.2005.06.002

Lu P, 2015, SCI REP-UK, V5, DOI 10.1038/srep10333

Lu TK, 2009, P NATL ACAD SCI USA, V106, P4629, DOI 10.1073/pnas.0800442106

Mortazavi A, 2008, NAT METHODS, V5, P621, DOI 10.1038/nmeth.1226

Nandakumar M, 2014, NAT COMMUN, V5, DOI 10.1038/ncomms5306

Nguyen HT, 2010, MOL MICROBIOL, V76, P348, DOI 10.1111/j.1365-2958.2010.07110.x

Pang JM, 2012, J BACTERIOL, V194, P715, DOI 10.1128/JB.06304-11

Pawelczyk J, 2014, MICROBIOL SPECTR, V2, DOI 10.1128/microbiolspec.MGM2-0003-2013

Piccaro G, 2014, ANTIMICROB AGENTS CH, V58, P7527, DOI 10.1128/AAC.03169-14

Pohane AA, 2014, J BIOL CHEM, V289, P12085, DOI 10.1074/jbc.M113.529594

Ren H, 2006, ANTIMICROB AGENTS CH, V50, P250, DOI 10.1128/AAC.50.1.250-255.2006

Robinson MD, 2010, GENOME BIOL, V11, DOI 10.1186/gb-2010-11-3-r25

Rodrigues L, 2011, BMC MICROBIOL, V11, DOI 10.1186/1471-2180-11-35

Ronayne EA, 2016, PLOS GENET, V12, DOI 10.1371/journal.pgen.1005797

Royet J, 2011, NAT REV IMMUNOL, V11, P837, DOI 10.1038/nri3089

Rozwarski DA, 1998, SCIENCE, V279, P98, DOI 10.1126/science.279.5347.98

Salzman V, 2010, MOL MICROBIOL, V78, P64, DOI 10.1111/j.1365-2958.2010.07313.x

TAKAYAMA K, 1972, ANTIMICROB AGENTS CH, V2, P29, DOI 10.1128/AAC.2.1.29

Torres-Barceló C, 2016, TRENDS MICROBIOL, V24, P249, DOI 10.1016/j.tim.2015.12.011

Vandal OH, 2009, J BACTERIOL, V191, P625, DOI 10.1128/JB.00932-08

Vilcheze C., 2014, MICROBIOL SPECTR, V2, pMGM2, DOI [10.1128/microbiolspec.MGM2-0014-2013, DOI 10.1128/MICROBIOLSPEC.MGM2-0014-2013]

Vilchéze C, 2007, ANNU REV MICROBIOL, V61, P35, DOI 10.1146/annurev.micro.61.111606.122346

Wade MM, 2004, FRONT BIOSCI-LANDMRK, V9, P975, DOI 10.2741/1289

Wolff KA, 2009, ANTIMICROB AGENTS CH, V53, P3515, DOI 10.1128/AAC.00012-09

Wu X., 2011, PLOS ONE, V6

Xu WX, 2014, BIOCHEM BIOPH RES CO, V448, P255, DOI 10.1016/j.bbrc.2014.04.116

Yu J, 2012, INFECT IMMUN, V80, P1381, DOI 10.1128/IAI.06370-11

ZHANG Y, 1992, NATURE, V358, P591, DOI 10.1038/358591a0

NR 60

TC 36

Z9 40

U1 0

U2 50

PU NATURE PORTFOLIO

PI BERLIN

PA HEIDELBERGER PLATZ 3, BERLIN, 14197, GERMANY

SN 2045-2322

J9 SCI REP-UK

JI Sci Rep

PD JUN 28

PY 2016

VL 6

AR 28701

DI 10.1038/srep28701

PG 14

WC Multidisciplinary Sciences

WE Science Citation Index Expanded (SCI-EXPANDED)

SC Science & Technology - Other Topics

GA DQ0CU

UT WOS:000378866700001

OA Green Submitted, gold

DA 2026-05-07

ER

PT J

AU Mukonzo, J

Aklillu, E

Marconi, V

Schinazi, RF

AF Mukonzo, Jackson

Aklillu, Eleni

Marconi, Vincent

Schinazi, Raymond F.

TI Potential drug-drug interactions between antiretroviral therapy and

treatment regimens for multi-drug resistant tuberculosis: Implications

for HIV care of MDR-TB co-infected individuals

SO INTERNATIONAL JOURNAL OF INFECTIOUS DISEASES

LA English

DT Article

DE MDR-TB; HIV; Treatment; Drug-drug interactions

AB HIV-positive TB co-infected patients are at increased risk of multidrug-resistant (MDR)-TB compared to HIV-negative patients. Co-treatment of MDR-TB and HIV is common particularly in Sub-Saharan Africa where the co-morbidity is endemic. We discuss potential cellular metabolic pathway-mediated drug-drug interactions and the possible effect on HIV treatment outcomes of commonly prescribed antiretroviral therapy. (C) 2019 The Author(s). Published by Elsevier Ltd on behalf of International Society for Infectious Diseases.

C1 [Mukonzo, Jackson] Makerere Univ, Dept Pharmacol & Therapeut, Coll Hlth Sci, POB 7072, Kampala, Uganda.

[Aklillu, Eleni] Karolinska Univ Hosp Huddinge, Karolinska Inst, Dept Lab Med, Div Clin Pharmacol, Stockholm, Sweden.

[Marconi, Vincent] Emory Univ, Sch Med, Div Infect Dis, Atlanta, GA 30322 USA.

[Schinazi, Raymond F.] Emory Univ, Sch Med, Dept Pediat, Lab Biochem Pharmacol,Ctr AIDS Res, Atlanta, GA 30322 USA.

C3 Makerere University; Karolinska Institutet; Karolinska University

Hospital; Emory University; Emory University

RP Mukonzo, J (corresponding author), Makerere Univ, Dept Pharmacol & Therapeut, Coll Hlth Sci, POB 7072, Kampala, Uganda.

EM mukojack@yahoo.co.uk

RI Marconi, Vincent/N-3210-2014; Aklillu, Eleni/ABG-3461-2021

OI Marconi, Vincent/0000-0001-8409-4689; Aklillu,

Eleni/0000-0002-9788-0790; Mukonzo, Jackson/0000-0002-0573-1908

FU IAS -CIPHER [kij-216]; Swedish Research Council (Vetenskapsradet)

[2015-03295]; NIH CFAR [P30-AI-050409]; Swedish Research Council

[2015-03295] Funding Source: Swedish Research Council

FX Supported in part by an IAS -CIPHER grant kij-216 to JKM, supported in

part by The Swedish Research Council (Vetenskapsradet) grant 2015-03295

to JKM and EA.Supported in part by NIH CFAR grant P30-AI-050409 (to RFS

and VM). We thank Drs. James Kohler and Selwyn Hurwitz for their

critical comments.

CR Aliyu G, 2018, PLOS ONE, V13, DOI 10.1371/journal.pone.0191387

[Anonymous], 2013, Consolidated guidelines on the use of antiretroviral drugs for treating and preventing HIV infection

[Anonymous], 2015, HIV TREATM CAR WHATS

Boeree MJ, 2017, LANCET INFECT DIS, V17, P39, DOI 10.1016/S1473-3099(16)30274-2

Desta Z, 2001, ANTIMICROB AGENTS CH, V45, P382, DOI 10.1128/AAC.45.2.382-392.2001

Dickinson L, 2010, EXPERT OPIN DRUG MET, V6, P1575, DOI 10.1517/17425255.2010.535811

Essomba NE, 2017, MED SANTE TROP, V27, P286, DOI 10.1684/mst.2017.0713

Gurumurthy P, 2004, CLIN INFECT DIS, V38, P280, DOI 10.1086/380795

Harausz EP, 2018, PLOS MED, V15, DOI 10.1371/journal.pmed.1002591

Horita Y, 2018, ANTIMICROB AGENTS CH, V62, DOI [10.1128/aac.00008-18, 10.1128/AAC.00008-18]

Huang WL, 2004, DRUG METAB DISPOS, V32, P1434, DOI 10.1124/dmd.104.001313

Huang XJ, 2015, SCI REP-UK, V5, DOI 10.1038/srep08528

Katiyar SK, 2008, INT J TUBERC LUNG D, V12, P139

Lange C, 2018, J INTERN MED, V284, P163, DOI 10.1111/joim.12780

Marquez B, 2011, CURR DRUG TARGETS, V12, P600, DOI 10.2174/138945011795378504

Mesfin YM, 2014, PLOS ONE, V9, DOI 10.1371/journal.pone.0082235

Moodley R, 2016, EUR RESPIR REV, V25, DOI 10.1183/16000617.0080-2015

Moss L, 2015, XENOBIOTICA, V45, P60, DOI 10.3109/00498254.2014.942409

Moyle GJ, 1998, DRUGS, V55, P383, DOI 10.2165/00003495-199855030-00005

OCHS HR, 1983, BRIT J CLIN PHARMACO, V16, P743, DOI 10.1111/j.1365-2125.1983.tb02256.x

Sangana R, 2018, DRUG METAB DISPOS, V46, P26, DOI 10.1124/dmd.117.077834

Shimokawa Y, 2015, BIOL PHARM BULL, V38, P1425, DOI 10.1248/bpb.b15-00313

te Brake LHM, 2016, TUBERCULOSIS, V96, P150, DOI 10.1016/j.tube.2015.08.004

Tiberi S, 2017, PRESSE MED, V46, pE23, DOI 10.1016/j.lpm.2017.01.017

Van Deun A, 2010, AM J RESP CRIT CARE, V182, P684, DOI 10.1164/rccm.201001-0077OC

Zhang S, 2017, EMERG MICROBES INFEC, V6, DOI 10.1038/emi.2017.24

NR 26

TC 27

Z9 29

U1 0

U2 7

PU ELSEVIER SCI LTD

PI OXFORD

PA THE BOULEVARD, LANGFORD LANE, KIDLINGTON, OXFORD OX5 1GB, OXON, ENGLAND

SN 1201-9712

EI 1878-3511

J9 INT J INFECT DIS

JI Int. J. Infect. Dis.

PD JUN

PY 2019

VL 83

BP 98

EP 101

DI 10.1016/j.ijid.2019.04.009

PG 4

WC Infectious Diseases

WE Science Citation Index Expanded (SCI-EXPANDED)

SC Infectious Diseases

GA IA1LJ

UT WOS:000469322300019

OA Green Submitted, gold

DA 2026-05-07

ER

PT J

AU Ibrahim, MAA

Mahmoud, DGM

Ebada, SS

Sidhom, PA

Mekhemer, GAH

Hegazy, MEF

Han, YS

Abalkhail, T

AF Ibrahim, Mahmoud A. A.

Mahmoud, Doaa G. M.

Ebada, Sherif S.

Sidhom, Peter A.

Mekhemer, Gamal A. H.

Hegazy, Mohamed-Elamir F.

Han, Yanshuo

Abalkhail, Tarad

TI Exploring the Natural Products Atlas (NPAtlas) Database for Hunting

Prospective Irreversible Covalent DprE1 Inhibitors With Antitubercular

Activity: An Integrated In-Silico Approach

SO JOURNAL OF TROPICAL MEDICINE

LA English

DT Article

DE binding energy calculation; covalent docking; DprE1; molecular dynamics

simulation; NPAtlas

ID KILL MYCOBACTERIUM-TUBERCULOSIS; DRUG DISCOVERY; SIDE-CHAIN;

BENZOTHIAZINONES; DOCKING; IDENTIFICATION; GENERATION; ALGORITHM;

ACCURACY; ACCESS

AB As the second most deadly infectious disease worldwide after COVID-19, tuberculosis (TB) remains a pressing global health issue, further aggravated by multidrug-resistant TB (MDR-TB) and extensively drug-resistant TB (XDR-TB) strains. There is an urgent need to identify new anti-TB treatments and novel therapeutics to confront drug resistance. The decaprenylphosphoryl-D-ribose oxidase (DprE1) is an essential protein for the biosynthesis of the mycobacterial cell wall, and its inhibition features a promising antitubercular strategy. NPAtlas was utilized as a reference database, comprising natural products with confirmed biological effects. The aim of the current study is to identify and prioritize promising nitro-containing natural products from the NPAtlas as potential covalent DprE1 inhibitors using advanced in silico approaches. Herein, the docking scores of 133 nitro-containing NPAtlas compounds were assessed using a covalent docking technique. Thereafter, NPAtlas compounds with docking scores lower than PBTZ169 (calc. -7.8 kcal<middle dot>mol-1) were subjected to molecular dynamics simulation (MDS), accompanied by binding energy estimations utilizing the MM-GBSA approach. Based on MM-GBSA//250 ns MDS, NPA011203, NPA013234, NPA016048, NPA012944, NPA001712, and NPA002823 demonstrated higher binding affinities against DprE1 with Delta Gbinding values of -75.6, -62.7, -61.6, -57.6, -54.8, and -50.7 kcal<middle dot>mol-1, respectively, than PBTZ169 (calc. -49.4 kcal<middle dot>mol-1). The identified NPAtlas compounds also demonstrated structural and energetic stability within the DprE1 active site throughout 250 ns MDS. Physicochemical and ADMET predictions of the identified NPAtlas compounds indicated a suitable molecular size, favorable absorption, and negligible toxicity, suggesting their potential oral bioavailability. These in silico outcomes provide preliminary insights into the identified NPAtlas compounds as potential DprE1 inhibitors and can guide subsequent in vitro/in vivo experiments.

C1 [Ibrahim, Mahmoud A. A.; Mahmoud, Doaa G. M.; Mekhemer, Gamal A. H.] Minia Univ, Fac Sci, Dept Chem, Al Minya 61519, Egypt.

[Ibrahim, Mahmoud A. A.] Univ KwaZulu Natal, Sch Hlth Sci, Westville Campus, ZA-4000 Durban, South Africa.

[Ibrahim, Mahmoud A. A.] Univ Technol & Appl Sci, Dept Support Requirements, Nizwa 611, Oman.

[Ebada, Sherif S.] Ain Shams Univ, Dept Pharmacognosy, Fac Pharm, Cairo 11566, Egypt.

[Ebada, Sherif S.] Helmholtz Ctr Infect Res GmbH, Dept Microbial Drugs, D-38124 Braunschweig, Germany.

[Sidhom, Peter A.] Tanta Univ, Fac Pharm, Dept Pharmaceut Chem, Tanta 31527, Egypt.

[Hegazy, Mohamed-Elamir F.] Natl Res Ctr, Chem Med Plants Dept, Giza 12622, Egypt.

[Han, Yanshuo] Dalian Univ Technol, Sch Life & Pharmaceut Sci, Panjin, Peoples R China.

[Abalkhail, Tarad] King Saud Univ, Dept Bot & Microbiol, Coll Sci, Riyadh 11451, Saudi Arabia.

C3 Egyptian Knowledge Bank (EKB); Minia University; University of Kwazulu

Natal; Egyptian Knowledge Bank (EKB); Ain Shams University; Helmholtz

Association; Helmholtz-Center for Infection Research; Egyptian Knowledge

Bank (EKB); Tanta University; Egyptian Knowledge Bank (EKB); National

Research Centre (NRC); Dalian University of Technology; King Saud

University

RP Ibrahim, MAA (corresponding author), Minia Univ, Fac Sci, Dept Chem, Al Minya 61519, Egypt.; Ibrahim, MAA (corresponding author), Univ KwaZulu Natal, Sch Hlth Sci, Westville Campus, ZA-4000 Durban, South Africa.; Ibrahim, MAA (corresponding author), Univ Technol & Appl Sci, Dept Support Requirements, Nizwa 611, Oman.; Abalkhail, T (corresponding author), King Saud Univ, Dept Bot & Microbiol, Coll Sci, Riyadh 11451, Saudi Arabia.

EM m.ibrahim@compchem.net; tabalkhail@ksu.edu.sa

RI Sidhom, Peter/ISA-5193-2023; Abalkhail, Tarad/LIH-5677-2024; Hegazy,

Mohamed Elamir/AAA-3454-2019; Ebada, Sherif/AAE-5155-2020; Ibrahim,

Mahmoud/J-2936-2014

OI Hegazy, Mohamed Elamir/0000-0002-0343-4969; Ibrahim,

Mahmoud/0000-0003-4819-2040

FU King Saud University [ORF-2026-1438]

FX The authors extend their appreciation to the Ongoing Research Funding

Program (ORF-2026-1438), King Saud University, Riyadh, Saudi Arabia. The

computational work was completed with resources provided by the CompChem

Lab (Minia University, Egypt, hpc.compchem.net), Center for

High-Performance Computing (Cape Town, South Africa, ), and Bibliotheca

Alexandrina ().

CR Alsayed SSR, 2023, INT J MOL SCI, V24, DOI 10.3390/ijms24065202

Anand U, 2020, DRUG RESIST UPDATE, V51, DOI 10.1016/j.drup.2020.100695

Balganesh TS, 2008, TRENDS PHARMACOL SCI, V29, P576, DOI 10.1016/j.tips.2008.08.001

Batt SM, 2015, ACS INFECT DIS, V1, P615, DOI 10.1021/acsinfecdis.5b00065

Baumann S, 2014, ANGEW CHEM INT EDIT, V53, P14605, DOI 10.1002/anie.201409964

BAYLY CI, 1993, J PHYS CHEM-US, V97, P10269, DOI 10.1021/j100142a004

Belofsky GN, 1998, TETRAHEDRON, V54, P1715, DOI 10.1016/S0040-4020(97)10396-9

Bendre AD, 2021, Current Research in Pharmacology and Drug Discovery, V2, P100037, DOI [10.1016/j.crphar.2021.100037, DOI 10.1016/J.CRPHAR.2021.100037, 10.1016/j.crphar.2021.100037]

Benet LZ, 2016, ADV DRUG DELIVER REV, V101, P89, DOI 10.1016/j.addr.2016.05.007

BERENDSEN HJC, 1984, J CHEM PHYS, V81, P3684, DOI 10.1063/1.448118

Bianco G, 2016, PROTEIN SCI, V25, P295, DOI 10.1002/pro.2733

Bloom BR, 2017, DIS CONTROL PRIORITI, V3rd, P233, DOI [DOI 10.1596/978-1-4648-0524-0_CH11, 10.1596/978-1-4648-0524-0CH11]

Brecik M, 2015, ACS CHEM BIOL, V10, P1631, DOI 10.1021/acschembio.5b00237

Brosch R, 2002, P NATL ACAD SCI USA, V99, P3684, DOI 10.1073/pnas.052548299

Brüstle M, 2002, J MED CHEM, V45, P3345, DOI 10.1021/jm011027b

Chakaya J, 2022, INT J INFECT DIS, V124, pS26, DOI 10.1016/j.ijid.2022.03.011

Chikhale RV, 2018, J MED CHEM, V61, P8563, DOI 10.1021/acs.jmedchem.8b00281

Chopperla R, 2020, INT J MOL SCI, V21, DOI 10.3390/ijms21217974

Clark DE, 2000, DRUG DISCOV TODAY, V5, P49, DOI 10.1016/S1359-6446(99)01451-8

Cole ST, 1998, NATURE, V393, P537, DOI 10.1038/31159

Cragg GM, 1997, J NAT PROD, V60, P52, DOI 10.1021/np9604893

DARDEN T, 1993, J CHEM PHYS, V98, P10089, DOI 10.1063/1.464397

Dassault Systemes, 2019, BIOVIA DSV VERSION 2

de Jong BC, 2010, PLOS NEGLECT TROP D, V4, DOI 10.1371/journal.pntd.0000744

de Sousa NF, 2025, MICROORGANISMS, V13, DOI 10.3390/microorganisms13020396

De Vivo M, 2016, J MED CHEM, V59, P4035, DOI 10.1021/acs.jmedchem.5b01684

Egan WJ, 2002, CURR OPIN DRUG DISC, V5, P540

Elgaher WAM, 2020, CHEM-EUR J, V26, P7219, DOI 10.1002/chem.202000117

Forli S, 2016, NAT PROTOC, V11, P905, DOI 10.1038/nprot.2016.051

Frisch Gwt J., 2009, GAUSSIAN 09

Gao Y, 2019, BIOORG CHEM, V85, P168, DOI 10.1016/j.bioorg.2018.12.018

GASTEIGER J, 1980, TETRAHEDRON, V36, P3219, DOI 10.1016/0040-4020(80)80168-2

Gordon JC, 2005, NUCLEIC ACIDS RES, V33, pW368, DOI 10.1093/nar/gki464

Groom CR, 2016, ACTA CRYSTALLOGR B, V72, P171, DOI 10.1107/S2052520616003954

Gutierrez MC, 2005, PLOS PATHOG, V1, P55, DOI 10.1371/journal.ppat.0010005

Halgren TA, 1999, J COMPUT CHEM, V20, P720, DOI 10.1002/(SICI)1096-987X(199905)20:7<720::AID-JCC7>3.0.CO;2-X

Hariguchi N, 2020, ANTIMICROB AGENTS CH, V64, DOI 10.1128/AAC.02020-19

Hawkins PCD, 2010, J CHEM INF MODEL, V50, P572, DOI 10.1021/ci100031x

Hou TJ, 2011, J COMPUT CHEM, V32, P866, DOI 10.1002/jcc.21666

Huang ZJ, 2008, PHYTOCHEMISTRY, V69, P1604, DOI 10.1016/j.phytochem.2008.02.002

Huard RC, 2006, J BACTERIOL, V188, P4271, DOI 10.1128/JB.01783-05

Ibrahim MAA, 2024, PLOS ONE, V19, DOI 10.1371/journal.pone.0314422

Ibrahim MAA, 2025, SCI REP-UK, V15, DOI 10.1038/s41598-025-24162-w

Imran M, 2022, J INFECT PUBLIC HEAL, V15, P1097, DOI 10.1016/j.jiph.2022.08.016

ISHIGAMI K, 1994, J ANTIBIOT, V47, P1219, DOI 10.7164/antibiotics.47.1219

Izaguirre JA, 2001, J CHEM PHYS, V114, P2090, DOI 10.1063/1.1332996

JORGENSEN WL, 1983, J CHEM PHYS, V79, P926, DOI 10.1063/1.445869

Kerrigan John E, 2013, Methods Mol Biol, V993, P95, DOI 10.1007/978-1-62703-342-8_7

Li JWH, 2009, SCIENCE, V325, P161, DOI 10.1126/science.1168243

London N, 2014, NAT CHEM BIOL, V10, P1066, DOI 10.1038/nchembio.1666

Ma XC, 2020, RSC ADV, V10, P14410, DOI 10.1039/d0ra00750a

Mahmoud DGM, 2025, CHEMISTRYOPEN, V14, DOI 10.1002/open.202500237

Maier JA, 2015, J CHEM THEORY COMPUT, V11, P3696, DOI 10.1021/acs.jctc.5b00255

Mak KK, 2022, DRUG DISCOV TODAY, V27, P705, DOI 10.1016/j.drudis.2021.11.005

Makarov V, 2014, EMBO MOL MED, V6, P372, DOI 10.1002/emmm.201303575

Makarov V, 2009, SCIENCE, V324, P801, DOI 10.1126/science.1171583

Massova I, 2000, PERSPECT DRUG DISCOV, V18, P113, DOI 10.1023/A:1008763014207

MIYAMOTO S, 1992, J COMPUT CHEM, V13, P952, DOI 10.1002/jcc.540130805

Morris GM, 2009, J COMPUT CHEM, V30, P2785, DOI 10.1002/jcc.21256

Mullard A, 2018, NAT REV DRUG DISCOV, V17, P777, DOI 10.1038/nrd.2018.197

Neres J, 2015, ACS CHEM BIOL, V10, P705, DOI 10.1021/cb5007163

Neres J, 2012, SCI TRANSL MED, V4, DOI 10.1126/scitranslmed.3004395

Onufriev A, 2004, PROTEINS, V55, P383, DOI 10.1002/prot.20033

OpenEye Scientific Software, 2021, OMEGA 4110

OpenEye Scientific Software, 2021, SZYBKI 2400

OpenEye Scientific Software, 2021, QUACPAC 2130

Panda M, 2014, J MED CHEM, V57, P4761, DOI 10.1021/jm5002937

Perveen S, 2022, BIOCHEM PHARMACOL, V197, DOI 10.1016/j.bcp.2021.114906

Pietersen E, 2014, LANCET, V383, P1230, DOI 10.1016/S0140-6736(13)62675-6

Pires DEV, 2015, J MED CHEM, V58, P4066, DOI 10.1021/acs.jmedchem.5b00104

Poynton EF, 2024, NUCLEIC ACIDS RES, V53, pD691, DOI 10.1093/nar/gkae1093

Riccardi G, 2013, APPL MICROBIOL BIOT, V97, P8841, DOI 10.1007/s00253-013-5218-x

Rogacki MK, 2018, J MED CHEM, V61, P11221, DOI 10.1021/acs.jmedchem.8b01356

Shen MY, 2012, J CHEMINFORMATICS, V4, DOI 10.1186/1758-2946-4-31

Shirude PS, 2014, J MED CHEM, V57, P5728, DOI 10.1021/jm500571f

Singh R, 2020, J APPL MICROBIOL, V128, P1547, DOI 10.1111/jam.14478

Sommer R, 2018, ACS CHEM BIOL, V13, P3184, DOI 10.1021/acschembio.8b00790

Sun HY, 2018, PHYS CHEM CHEM PHYS, V20, P14450, DOI 10.1039/c7cp07623a

Tiwari R, 2013, J AM CHEM SOC, V135, P3539, DOI 10.1021/ja311058q

Trefzer C, 2012, J AM CHEM SOC, V134, P912, DOI 10.1021/ja211042r

Trefzer C, 2010, J AM CHEM SOC, V132, P13663, DOI 10.1021/ja106357w

University of California, 2020, AMBER 2020

van Ingen J, 2012, EMERG INFECT DIS, V18, P653, DOI 10.3201/eid1804.110888

van Noort CW, 2021, CURR OPIN STRUC BIOL, V70, P70, DOI 10.1016/j.sbi.2021.05.003

van Santen JA, 2019, ACS CENTRAL SCI, V5, P1824, DOI 10.1021/acscentsci.9b00806

Wang EC, 2019, CHEM REV, V119, P9478, DOI 10.1021/acs.chemrev.9b00055

Wang JM, 2004, J COMPUT CHEM, V25, P1157, DOI 10.1002/jcc.20035

Webb Benjamin, 2014, Curr Protoc Bioinformatics, V47, DOI [10.1002/cpbi.3, 10.1002/0471250953.bi0506s15, 10.1002/cpps.20, 10.1002/0471140864.ps0209s50, 10.1002/0471250953.bi0506s47]

Wilson BAP, 2020, NAT PROD REP, V37, P893, DOI 10.1039/c9np00068b

Zhang G, 2019, ACS MED CHEM LETT, V10, P348, DOI 10.1021/acsmedchemlett.8b00634

Zhang MW, 2021, EUR J MED RES, V26, DOI 10.1186/s40001-021-00502-0

NR 91

TC 0

Z9 0

U1 2

U2 2

PU WILEY

PI HOBOKEN

PA 111 RIVER ST, HOBOKEN 07030-5774, NJ USA

SN 1687-9686

EI 1687-9694

J9 J TROP MED-US

JI J. Trop. Med.

PY 2026

VL 2026

IS 1

AR 8879019

DI 10.1155/jotm/8879019

PG 20

WC Public, Environmental & Occupational Health; Tropical Medicine

WE Science Citation Index Expanded (SCI-EXPANDED)

SC Public, Environmental & Occupational Health; Tropical Medicine

GA DQ6MD

UT WOS:001689754300001

OA Green Submitted, gold

DA 2026-05-07

ER

PT J

AU De Siena, B

Campolattano, N

D'Abrosca, G

Russo, L

Cantillon, D

Marasco, R

Muscariello, L

Waddell, SJ

Sacco, M

AF De Siena, Barbara

Campolattano, Nicoletta

D'Abrosca, Gianluca

Russo, Luigi

Cantillon, Daire

Marasco, Rosangela

Muscariello, Lidia

Waddell, Simon J.

Sacco, Margherita

TI Characterization of the Mycobacterial MSMEG-3762/63 Efflux Pump in

Mycobacterium smegmatis Drug Efflux

SO FRONTIERS IN MICROBIOLOGY

LA English

DT Article

DE mycobacteria; efflux pump; antimicrobial drugs; multi drug resistance;

biofilm; transmembrane protein; 3D structure modeling

ID TUBERCULOSIS; VALIDATION; RESISTANCE

AB Multi-drug resistant tuberculosis (MDR-TB) represents a major health problem worldwide. Drug efflux and the activity of efflux transporters likely play important roles in the development of drug-tolerant and drug-resistant mycobacterial phenotypes. This study is focused on the action of a mycobacterial efflux pump as a mechanism of drug resistance. Previous studies demonstrated up-regulation of the TetR-like transcriptional regulator MSMEG_3765 in Mycobacterium smegmatis and its ortholog Rv1685c in Mycobacterium tuberculosis (Mtb) in acid-nitrosative stress conditions. MSMEG-3765 regulates the expression of the MSMEG_3762/63/65 operon, and of the orthologous region in Mtb (Rv1687c/86c/85c). MSMEG-3762 and Rv1687c are annotated as ATP-binding proteins, while MSMEG-3763 and Rv1686c are annotated as trans-membrane polypeptides, defining an ABC efflux pump in both M. smegmatis and Mtb. The two putative efflux systems share a high percentage of identity. To examine the role of the putative efflux system MSMEG-3762/63, we constructed and characterized a MSMEG-3763 deletion mutant in M. smegmatis ( increment MSMEG_3763). By comparative analysis of wild type, knockout, and complemented strains, together with structural modeling and molecular docking bioinformatics analyses of the MSMEG-3763 trans-membrane protein, we define the protein complex MSMEG-3762/63 as an efflux pump. Moreover, we demonstrate involvement of this pump in biofilm development and in the extrusion of rifampicin and ciprofloxacin (CIP), antimicrobial drugs used in first- and second-line anti-TB therapies.

C1 [De Siena, Barbara; Campolattano, Nicoletta; D'Abrosca, Gianluca; Russo, Luigi; Marasco, Rosangela; Muscariello, Lidia; Sacco, Margherita] Univ Campania Luigi Vanvitelli, Dipartimento Sci & Tecnol Ambientali Biol & Farma, Caserta, Italy.

[Cantillon, Daire; Waddell, Simon J.] Univ Sussex, Brighton & Sussex Med Sch, Dept Global Hlth & Infect, Brighton, E Sussex, England.

C3 Universita della Campania Vanvitelli; University of Brighton; University

of Sussex

RP Sacco, M (corresponding author), Univ Campania Luigi Vanvitelli, Dipartimento Sci & Tecnol Ambientali Biol & Farma, Caserta, Italy.

EM margherita.sacco@unicampania.it

RI Cantillon, Daire/AAD-9058-2022; D'Abrosca, Gianluca/T-7399-2018;

Waddell, Simon/A-9170-2013; RUSSO, Luigi/ABB-8650-2022; MUSCARIELLO,

Lidia/HKF-2665-2023

OI Cantillon, Daire/0000-0002-2180-373X; MARASCO,

Rosangela/0000-0003-4567-4431; Waddell, Simon/0000-0002-3684-9116;

RUSSO, Luigi/0000-0002-1577-176X; MUSCARIELLO, Lidia/0000-0003-1343-9314

FU Programma Valere 2018, University of Campania "Luigi Vanvitelli";

Programma Valere 2019, University of Campania "Luigi Vanvitelli";

Wellcome Trust Institutional Strategic Support Fund [G2306, 192470]

FX This work was partially supported by "Programma Valere 2018 to LR" and

"Programma Valere 2019 to LM," University of Campania "Luigi

Vanvitelli". SW and DC were supported by the Wellcome Trust

Institutional Strategic Support Fund, project G2306, grant number

192470.

CR Agrawal P, 2015, PLOS ONE, V10, DOI 10.1371/journal.pone.0122076

Ahmed S, 2020, ANTIBIOTICS-BASEL, V9, DOI 10.3390/antibiotics9010021

Alav I, 2018, J ANTIMICROB CHEMOTH, V73, P2003, DOI 10.1093/jac/dky042

Alcalde-Rico M, 2016, FRONT MICROBIOL, V7, DOI 10.3389/fmicb.2016.01483

Ayabina D, 2016, J R SOC INTERFACE, V13, DOI 10.1098/rsif.2016.0745

Balhana RJC, 2015, BMC GENOMICS, V16, DOI 10.1186/s12864-015-1696-9

Bekele S, 2018, TROP DIS TRAVEL MED, V4, DOI 10.1186/s40794-018-0075-3

Bogomolnaya LM, 2013, MBIO, V4, DOI 10.1128/mBio.00630-13

Botelho J, 2019, DRUG RESIST UPDATE, V44, P26, DOI 10.1016/j.drup.2019.07.002

Cossu A, 2013, MICROB PATHOGENESIS, V65, P89, DOI 10.1016/j.micpath.2013.10.004

Davis IW, 2007, NUCLEIC ACIDS RES, V35, pW375, DOI 10.1093/nar/gkm216

Dheda K, 2017, LANCET RESP MED, V5, P291, DOI [10.1016/s2213-2600(17)30079-6, 10.1016/S2213-2600(17)30079-6]

Du DJ, 2018, NAT REV MICROBIOL, V16, P523, DOI 10.1038/s41579-018-0048-6

Ehrt S, 2009, CELL MICROBIOL, V11, P1170, DOI 10.1111/j.1462-5822.2009.01335.x

Fernández LG, 2020, J INT AIDS SOC, V23, DOI 10.1002/jia2.25438

Gordon SV, 2018, MICROBIOL-SGM, V164, P437, DOI 10.1099/mic.0.000601

Grosdidier A, 2011, NUCLEIC ACIDS RES, V39, pW270, DOI 10.1093/nar/gkr366

Guo YL, 2020, FRONT CELL INFECT MI, V10, DOI 10.3389/fcimb.2020.00107

Hameed HMA, 2018, FRONT CELL INFECT MI, V8, DOI 10.3389/fcimb.2018.00114

Hassanzadeh S, 2020, MICROB PATHOGENESIS, V139, DOI 10.1016/j.micpath.2019.103850

Hayashi S, 2018, TROP MED INT HEALTH, V23, P1058, DOI 10.1111/tmi.13133

Hess B, 2008, J CHEM THEORY COMPUT, V4, P435, DOI 10.1021/ct700301q

Honeyborne I, 2016, BMC MED, V14, DOI 10.1186/s12916-016-0609-3

Huang J, 2013, J COMPUT CHEM, V34, P2135, DOI 10.1002/jcc.23354

Johnson EO, 2020, ACS INFECT DIS, V6, P56, DOI 10.1021/acsinfecdis.9b00373

Johnson EO, 2019, NATURE, V571, P72, DOI 10.1038/s41586-019-1315-z

Joosten RP, 2011, NUCLEIC ACIDS RES, V39, pD411, DOI 10.1093/nar/gkq1105

Krogh A, 2001, J MOL BIOL, V305, P567, DOI 10.1006/jmbi.2000.4315

Kumar M, 2016, ACS INFECT DIS, V2, P714, DOI 10.1021/acsinfecdis.6b00111

Laskowski RA, 1996, J BIOMOL NMR, V8, P477, DOI 10.1007/BF00228148

Machado D, 2017, FRONT MICROBIOL, V8, DOI 10.3389/fmicb.2017.00711

Minagawa S, 2012, BMC MICROBIOL, V12, DOI 10.1186/1471-2180-12-70

Moubareck CA, 2020, ANTIBIOTICS-BASEL, V9, DOI 10.3390/antibiotics9030119

Narang A, 2019, PLOS ONE, V14, DOI 10.1371/journal.pone.0223163

O'Toole GA, 2011, JOVE-J VIS EXP, DOI 10.3791/2437

Parish T, 1999, MICROBIOL-SGM, V145, P3497, DOI 10.1099/00221287-145-12-3497

Pasqua M, 2019, SCI REP-UK, V9, DOI 10.1038/s41598-019-39749-3

Perrone F, 2017, FRONT MICROBIOL, V8, DOI 10.3389/fmicb.2017.02039

Peters JS, 2019, LANCET INFECT DIS, V19, pE65, DOI 10.1016/S1473-3099(18)30477-8

Pettersen EF, 2004, J COMPUT CHEM, V25, P1605, DOI 10.1002/jcc.20084

Piddock LJV, 2006, NAT REV MICROBIOL, V4, P629, DOI 10.1038/nrmicro1464

Reza A, 2019, ANTIBIOTICS-BASEL, V8, DOI 10.3390/antibiotics8040229

Rodrigues L, 2013, ANTIMICROB AGENTS CH, V57, P751, DOI 10.1128/AAC.01482-12

Roy A, 2010, NAT PROTOC, V5, P725, DOI 10.1038/nprot.2010.5

Singh R, 2020, J APPL MICROBIOL, V128, P1547, DOI 10.1111/jam.14478

te Brake LHM, 2018, ANNU REV PHARMACOL, V58, P271, DOI 10.1146/annurev-pharmtox-010617-052438

Thapa J, 2019, TUBERCULOSIS, V117, P1, DOI 10.1016/j.tube.2019.05.004

Trauner A, 2012, J BIOL CHEM, V287, P24053, DOI 10.1074/jbc.M112.364851

Tsenova L, 2020, J PATHOL, V250, P636, DOI 10.1002/path.5407

Van Camp PJ, 2020, INT J MOL SCI, V21, DOI 10.3390/ijms21041363

Wildner LM, 2018, METHODS MOL BIOL, V1736, P117, DOI 10.1007/978-1-4939-7638-6_11

Zárate SG, 2019, ANTIBIOTICS-BASEL, V8, DOI 10.3390/antibiotics8010025

NR 52

TC 12

Z9 15

U1 1

U2 7

PU FRONTIERS MEDIA SA

PI LAUSANNE

PA AVENUE DU TRIBUNAL FEDERAL 34, LAUSANNE, CH-1015, SWITZERLAND

EI 1664-302X

J9 FRONT MICROBIOL

JI Front. Microbiol.

PD DEC 3

PY 2020

VL 11

AR 575828

DI 10.3389/fmicb.2020.575828

PG 11

WC Microbiology

WE Science Citation Index Expanded (SCI-EXPANDED)

SC Microbiology

GA PF7WB

UT WOS:000599259000001

OA Green Submitted, gold

DA 2026-05-07

ER

PT J

AU Zhang, C

Ouyang, Q

Zhou, XY

Huang, YF

Zeng, Y

Deng, L

Lin, DC

Zheng, WD

AF Zhang, Chi

Ouyang, Qi

Zhou, Xianyuan

Huang, Yingfeng

Zeng, Yu

Deng, Li

Lin, Dachuan

Zheng, Weidong

TI In vitro activity of tetracycline analogs against multidrug-resistant

and extensive drug resistance clinical isolates of Mycobacterium

tuberculosis

SO TUBERCULOSIS

LA English

DT Article

DE Tetracycline; Multiple -drug resistance; Doxycycline; Mycobacterium

tuberculosis

ID ANTITUBERCULOSIS DRUGS; ANTIBACTERIAL AGENTS; DOXYCYCLINE; GENES;

PHARMACOKINETICS; ANTIBIOTICS; COMBINATION; EXPRESSION; MUTATIONS;

PORINS

AB Background: Multidrug-resistant tuberculosis (MDR-TB) has become a big threaten to global health. The current strategy for treatment of MDR-TB and extensive drug resistant tuberculosis (XDR-TB) is with low efficacy and high side effect. While new drug is fundamental for cure MDR-TB, repurposing the Food and Drug Administration (FDA)-approved drugs represents an alternative soluation with less cost. Methods: The activity of 8 tetracycline-class antibiotics against mycobacterium tuberculosis (M.tb) were determined by Minimum Inhibitory Concentration (MIC) in vitro. A transposon M.smeg libraries was generated by using the Harm phage and then used to isolate the conditional growth mutants in doxycycline containing plate. Eleven mutants were isolated and genomic DNAs were extracted using the cetyltrimethyl ammonium bromide (CTAB) method and analyzed by whole genome sequencing. Results: We found that three of eight drugs efficiently inhibited mycobacteria growth under the peak plasma concentration in the human body. Further tests showed these three tetracycline analogs (demeclocycline, doxycycline and methacycline) had antimicrobial activity against seven clinical isolates, including MDR and XDR strains. Among them, Doxycycline had the lowest MICs in all mycobacteria strains tested in this study. By using a transposon library, we identify the insertion of transposon in two genes, porin and MshA, associatewith the resistant to doxycycline. Conclusions: Our findings show that tetracycline analogs such as doxycycline, has bactericidal activity against not only drug sensitive M.tb, but also clinical MDR and XDR strains, provided proof of concept to repurpose doxycycline to fight MDR-TB and XDR-TB. Further investigations are warranted to clarify the underlying mechanism and optimize the strategy in combination with other anti-TB drugs.

C1 [Zhang, Chi; Zhou, Xianyuan; Huang, Yingfeng; Zeng, Yu; Deng, Li; Zheng, Weidong] Shenzhen Univ, Shenzhen Univ Gen Hosp, Dept Lab Med, Shenzhen, Peoples R China.

[Ouyang, Qi; Lin, Dachuan] Shenzhen Univ, Sch Med, Dept Pathogen Biol, Guangdong Key Lab Reg Immun & Dis, Shenzhen, Peoples R China.

C3 Shenzhen University; Shenzhen University

RP Zheng, WD (corresponding author), Shenzhen Univ, Shenzhen Univ Gen Hosp, Dept Lab Med, Shenzhen, Peoples R China.; Lin, DC (corresponding author), Shenzhen Univ, Sch Med, Dept Pathogen Biol, Guangdong Key Lab Reg Immun & Dis, Shenzhen, Peoples R China.

EM lindachuan@szu.edu.cn; zwdin@szu.edu.cn

RI Lin, Dachuan/I-4731-2016; Deng, Li/A-7233-2015

OI Lin, Dachuan/0000-0002-4404-7391;

FU Shenzhen Science and Technology Innovation Commission

[JCYJ20220531102401003, KCXFZ202002011006190]; Shenzhen Key Laboratory

Foundation [ZDSYS20200811143757022]; Guangdong province Universities for

Special Research Program [2020kzdx1175]

FX This research was supported by Shenzhen Science and Technology

Innovation Commission (JCYJ20220531102401003 and KCXFZ202002011006190) ,

Shenzhen Key Laboratory Foundation (ZDSYS20200811143757022) and

Guangdong province Universities for Special Research Program for

COVID-19 control (2020kzdx1175) . The funders had no role in study

design, data collection and analysis, deci- sion to publish, or

preparation of the manuscript.

CR Agwuh KN, 2006, J ANTIMICROB CHEMOTH, V58, P256, DOI 10.1093/jac/dkl224

[Anonymous], 2019, Global tuberculosis report 2019

Arbex MA, 2010, J BRAS PNEUMOL, V36, P626, DOI 10.1590/S1806-37132010000500016

Balabanova Y, 2005, EUR J CLIN MICROBIOL, V24, P136, DOI 10.1007/s10096-004-1268-4

Brodersen DE, 2000, CELL, V103, P1143, DOI 10.1016/S0092-8674(00)00216-6

Buchmeier NA, 2006, J BACTERIOL, V188, P6245, DOI 10.1128/JB.00393-06

Carroll P, 2005, APPL ENVIRON MICROB, V71, P3077, DOI 10.1128/AEM.71.6.3077-3084.2005

Coban AY, 2011, AFR J MICROBIOL RES, V5, P311

Danilchanka O, 2008, ANTIMICROB AGENTS CH, V52, P3127, DOI 10.1128/AAC.00239-08

DUGGAR BM, 1948, ANN NY ACAD SCI, V51, P177, DOI 10.1111/j.1749-6632.1948.tb27262.x

Faller M, 2004, SCIENCE, V303, P1189, DOI 10.1126/science.1094114

Falzon D, 2011, EUR RESPIR J, V38, P516, DOI 10.1183/09031936.00073611

FINLAY AC, 1950, SCIENCE, V111, P85, DOI 10.1126/science.111.2874.85

Frenzel E, 2011, APPL ENVIRON MICROB, V77, P3068, DOI 10.1128/AEM.02492-10

Gonzaloa X, 2015, INT J ANTIMICROB AG, V45, P406, DOI 10.1016/j.ijantimicag.2014.11.017

Griffin MO, 2010, AM J PHYSIOL-CELL PH, V299, pC539, DOI 10.1152/ajpcell.00047.2010

GUILHOT C, 1994, J BACTERIOL, V176, P535, DOI 10.1128/JB.176.2.535-539.1994

Hillmann D, 2007, J BACTERIOL, V189, P958, DOI 10.1128/JB.01474-06

HOBBY GL, 1955, AM REV TUBERC PULM, V72, P367

Janin YL, 2007, BIOORGAN MED CHEM, V15, P2479, DOI 10.1016/j.bmc.2007.01.030

Joshi N, 1997, ARCH INTERN MED, V157, P1421, DOI 10.1001/archinte.157.13.1421

KING EQ, 1950, JAMA-J AM MED ASSOC, V143, P1

KLEIN NC, 1995, MED CLIN N AM, V79, P789, DOI 10.1016/S0025-7125(16)30039-6

MORTIMER PGS, 1993, J ANTIMICROB CHEMOTH, V32, P195, DOI 10.1093/jac/32.2.195

Nelson ML, 2011, ANN NY ACAD SCI, V1241, P17, DOI 10.1111/j.1749-6632.2011.06354.x

Newton GL, 2006, J BIOL CHEM, V281, P33910, DOI 10.1074/jbc.M604724200

Newton PN, 2005, ANTIMICROB AGENTS CH, V49, P1622, DOI 10.1128/AAC.49.4.1622-1625.2005

Njire M, 2016, ADV MED SCI-POLAND, V61, P63, DOI 10.1016/j.advms.2015.09.007

Parish T., 2008, Mycobacteria protocols

Pavlenok M, 2016, FEMS MICROBIOL LETT, V363, DOI 10.1093/femsle/fnw046

PUGSLEY AP, 1978, J BACTERIOL, V133, P1181, DOI 10.1128/JB.133.3.1181-1189.1978

Roberts MC, 1996, FEMS MICROBIOL REV, V19, P1, DOI 10.1111/j.1574-6976.1996.tb00251.x

Rubinstein E, 2005, DRUGS, V65, P1317, DOI 10.2165/00003495-200565100-00002

SARKAR S, 1968, P NATL ACAD SCI USA, V60, P1479, DOI 10.1073/pnas.60.4.1479

Sassetti CM, 2001, P NATL ACAD SCI USA, V98, P12712, DOI 10.1073/pnas.231275498

Seung KJ, 2015, CSH PERSPECT MED, V5, DOI 10.1101/cshperspect.a017863

Stephan J, 2004, ANTIMICROB AGENTS CH, V48, P4163, DOI 10.1128/AAC.48.11.4163-4170.2004

SUM PE, 1994, J MED CHEM, V37, P184, DOI 10.1021/jm00027a023

THANASSI DG, 1995, J BACTERIOL, V177, P998, DOI 10.1128/jb.177.4.998-1007.1995

Trieber CA, 2002, J BACTERIOL, V184, P2131, DOI 10.1128/JB.184.8.2131-2140.2002

Vilchèze C, 2008, MOL MICROBIOL, V69, P1316, DOI 10.1111/j.1365-2958.2008.06365.x

Vilchèze C, 2011, ANTIMICROB AGENTS CH, V55, P4422, DOI 10.1128/AAC.00564-11

Walker NF, 2012, AM J RESP CRIT CARE, V185, P989, DOI 10.1164/rccm.201110-1769OC

Wallace RJ, 2002, ANTIMICROB AGENTS CH, V46, P3164, DOI 10.1128/AAC.46.10.3164-3167.2002

Wiegand I, 2008, NAT PROTOC, V3, P163, DOI 10.1038/nprot.2007.521

Wilson DN, 2009, CRIT REV BIOCHEM MOL, V44, P393, DOI 10.3109/10409230903307311

Xu X, 2011, ANTIMICROB AGENTS CH, V55, P3133, DOI 10.1128/AAC.00020-11

NR 47

TC 9

Z9 10

U1 1

U2 19

PU CHURCHILL LIVINGSTONE

PI EDINBURGH

PA JOURNAL PRODUCTION DEPT, ROBERT STEVENSON HOUSE, 1-3 BAXTERS PLACE,

LEITH WALK, EDINBURGH EH1 3AF, MIDLOTHIAN, SCOTLAND

SN 1472-9792

EI 1873-281X

J9 TUBERCULOSIS

JI Tuberculosis

PD MAY

PY 2023

VL 140

AR 102336

DI 10.1016/j.tube.2023.102336

EA MAR 2023

PG 6

WC Immunology; Microbiology; Respiratory System

WE Science Citation Index Expanded (SCI-EXPANDED)

SC Immunology; Microbiology; Respiratory System

GA M5BB1

UT WOS:001030354300001

OA hybrid

DA 2026-05-07

ER

PT J

AU Mancuso, G

Midiri, A

De Gaetano, S

Ponzo, E

Biondo, C

AF Mancuso, Giuseppe

Midiri, Angelina

De Gaetano, Silvia

Ponzo, Elena

Biondo, Carmelo

TI Tackling Drug-Resistant Tuberculosis: New Challenges from the Old

Pathogen Mycobacterium tuberculosis

SO MICROORGANISMS

LA English

DT Review

DE M.tb pathogenesis; MDR-TB; XDR-TB; drug tolerance; new approaches

ID LATENT TUBERCULOSIS; EPIDEMIOLOGY; RIFAMPICIN; REEMERGENCE; INFECTION;

DETERMINANTS; MECHANISM; PROGRESS; ANCIENT; DEFENSE

AB Antibiotics have played a crucial role in the reduction in the incidence of TB globally as evidenced by the fact that before the mid-20th century, the mortality rate within five years of the onset of the disease was 50%. The use of antibiotics has eliminated TB as a devastating disease, but the challenge of resistance to anti-TB drugs, which had already been described at the time of the introduction of streptomycin, has become a major global issue in disease management. Mismanagement of multidrug-resistant tuberculosis (MDR-TB) cases, resulting from intermittent drug use, prescription errors, and non-compliance of patients, has been identified as a critical risk factor for the development of extensively drug-resistant tuberculosis (XDR-TB). Antimicrobial resistance (AMR) in TB is a multi-factorial, complex problem of microbes evolving to escape antibiotics, the gradual decline in antibiotic development, and different economic and social conditions. In this review, we summarize recent advances in our understanding of how Mycobacterium tuberculosis evolves drug resistance. We also highlight the importance of developing shorter regimens that rapidly reach bacteria in diverse host environments, eradicating all mycobacterial populations and preventing the evolution of drug resistance. Lastly, we also emphasize that the current burden of this ancient disease is driven by a combination of complex interactions between mycobacterial and host factors, and that only a holistic approach that effectively addresses all the critical issues associated with drug resistance will limit the further spread of drug-resistant strains throughout the community.

C1 [Mancuso, Giuseppe; Midiri, Angelina; De Gaetano, Silvia; Ponzo, Elena; Biondo, Carmelo] Univ Messina, Dept Human Pathol, Mycobacteriol Unit, I-98125 Messina, Italy.

C3 University of Messina

RP Biondo, C (corresponding author), Univ Messina, Dept Human Pathol, Mycobacteriol Unit, I-98125 Messina, Italy.

EM mancusog@unime.it; amidiri@unime.it; degaetano6@gmail.com;

elena.ponzo@studenti.unime.it; cbiondo@unime.it

RI PONZO, ELENA/ODL-1742-2025; Biondo, Carmelo/AAA-3391-2019

OI PONZO, ELENA/0009-0006-8544-4007;

CR Abascal E, 2021, SCI REP-UK, V11, DOI 10.1038/s41598-021-90568-x

Ahmad F, 2022, FRONT IMMUNOL, V13, DOI 10.3389/fimmu.2022.747799

Al-Humadi HW, 2017, FRONT PHARMACOL, V8, DOI 10.3389/fphar.2017.00689

Al-Saeedi M, 2017, INFECT DRUG RESIST, V10, P332, DOI 10.2147/IDR.S144446

Alexander KA, 2010, EMERG INFECT DIS, V16, P1296, DOI 10.3201/eid1608.100314

Allué-Guardia A, 2021, FRONT MICROBIOL, V12, DOI 10.3389/fmicb.2021.612675

Alsayed SSR, 2023, INT J MOL SCI, V24, DOI 10.3390/ijms24065202

[Anonymous], 2020, World Health Organization: Global Tuberculosis Report 2019

Assefa DG, 2023, ARCH PUBLIC HEALTH, V81, DOI 10.1186/s13690-023-01098-z

Bagcchi S, 2023, LANCET MICROBE, V4, pE20, DOI 10.1016/S2666-5247(22)00359-7

Bakhtiyariniya P, 2022, MOL BIOL REP, V49, P6135, DOI 10.1007/s11033-022-07404-2

Barberis I, 2017, J Prev Med Hyg, V58, pE9

Barry CE, 2011, CURR TOP MED CHEM, V11, P1216

Batt Sarah M, 2020, Cell Surf, V6, P100044, DOI [10.1016/j.tcsw.2020.100044, 10.1016/j.tcsw.2020.100044]

Behar SM, 2011, MUCOSAL IMMUNOL, V4, P279, DOI 10.1038/mi.2011.3

Behr MA, 2018, BMJ-BRIT MED J, V362, DOI 10.1136/bmj.k2738

Biondo C, 2023, LIFE-BASEL, V13, DOI 10.3390/life13061408

Biondo C, 2023, PATHOGENS, V12, DOI 10.3390/pathogens12010116

Biondo C, 2022, LIFE-BASEL, V12, DOI 10.3390/life12122087

Bloom B.R., 2017, Major Infectious Diseases, V3rd ed., P11

Borgdorff MW, 2013, CLIN MICROBIOL INFEC, V19, P889, DOI 10.1111/1469-0691.12253

Buzic I, 2020, J Prev Med Hyg, V61, pE3, DOI 10.15167/2421-4248/jpmh2020.61.1s1.1379

Cai L, 2023, FRONT PUBLIC HEALTH, V10, DOI 10.3389/fpubh.2022.1126429

Cambau E, 2014, CLIN MICROBIOL INFEC, V20, P196, DOI 10.1111/1469-0691.12555

Campbell EA, 2001, CELL, V104, P901, DOI 10.1016/S0092-8674(01)00286-0

Casco N, 2022, EUR RESPIR J, V59, DOI 10.1183/13993003.02538-2021

Cegielski JP, 2010, CLIN INFECT DIS, V50, pS195, DOI 10.1086/651491

Chakaya J, 2022, INT J INFECT DIS, V124, pS26, DOI 10.1016/j.ijid.2022.03.011

Chakaya J, 2021, INT J INFECT DIS, V113, pS7, DOI 10.1016/j.ijid.2021.02.107

Chandra P, 2022, NAT REV MICROBIOL, V20, P750, DOI 10.1038/s41579-022-00763-4

Chowdhury K, 2023, CUREUS J MED SCIENCE, V15, DOI 10.7759/cureus.35154

Cohen SB, 2018, CELL HOST MICROBE, V24, P439, DOI 10.1016/j.chom.2018.08.001

Comas I, 2011, TRENDS MICROBIOL, V19, P492, DOI 10.1016/j.tim.2011.07.002

Conradie F, 2022, NEW ENGL J MED, V387, P810, DOI 10.1056/NEJMoa2119430

Cooper AM, 2009, ANNU REV IMMUNOL, V27, P393, DOI 10.1146/annurev.immunol.021908.132703

Coscolla M, 2021, MICROB GENOMICS, V7, DOI 10.1099/mgen.0.000477

Cummings KJ, 2007, PUBLIC HEALTH REP, V122, P683, DOI 10.1177/003335490712200517

Daniel VS, 1999, CLIN INFECT DIS, V29, P1557, DOI 10.1086/313562

Dartois VA, 2022, NAT REV MICROBIOL, V20, P685, DOI 10.1038/s41579-022-00731-y

Davies PDO, 2003, ANN MED, V35, P235, DOI 10.1080/07853890310005713

Dean AS, 2022, LANCET INFECT DIS, V22, P191, DOI 10.1016/S1473-3099(21)00808-2

Delogu G, 2013, MEDITERR J HEMATOL I, V5, DOI 10.4084/MJHID.2013.070

Dheda K, 2022, LANCET RESP MED, V10, P603, DOI 10.1016/S2213-2600(22)00092-3

Dutour O, 2021, INT J PALEOPATHOL, V34, P130, DOI 10.1016/j.ijpp.2021.04.005

Esmail H, 2012, DRUG DISCOV TODAY, V17, P514, DOI 10.1016/j.drudis.2011.12.013

Espinosa-Pereiro J, 2022, MEDICINA-LITHUANIA, V58, DOI 10.3390/medicina58020188

Forrellad MA, 2013, VIRULENCE, V4, P3, DOI 10.4161/viru.22329

Fukunaga R, 2021, MMWR-MORBID MORTAL W, V70, P427, DOI 10.15585/mmwr.mm7012a4

García-Bengoa M, 2023, FRONT MICROBIOL, V14, DOI 10.3389/fmicb.2023.983299

Gengenbacher M, 2012, FEMS MICROBIOL REV, V36, P514, DOI 10.1111/j.1574-6976.2012.00331.x

Ghodbane R, 2013, TUBERCULOSIS, V93, P589, DOI 10.1016/j.tube.2013.09.005

Glaziou P, 2018, SEMIN RESP CRIT CARE, V39, P271, DOI 10.1055/s-0038-1651492

Gopal P, 2019, TRENDS PHARMACOL SCI, V40, P930, DOI 10.1016/j.tips.2019.10.005

Grange JM, 1996, J APPL BACTERIOL, V81, pS1, DOI 10.1111/j.1365-2672.1996.tb04827.x

Gygli SM, 2017, FEMS MICROBIOL REV, V41, P354, DOI 10.1093/femsre/fux011

Hamada Y, 2021, INT J STD AIDS, V32, P780, DOI 10.1177/0956462421992257

Hargreaves JR, 2011, AM J PUBLIC HEALTH, V101, P654, DOI 10.2105/AJPH.2010.199505

Hassan STS, 2018, J CLIN MED, V7, DOI 10.3390/jcm7080226

Hershkovitz I, 2008, PLOS ONE, V3, DOI 10.1371/journal.pone.0003426

Hildebrand RE, 2022, MICROBIOL SPECTR, V10, DOI 10.1128/spectrum.03075-22

Holloway KL, 2013, PLOS ONE, V8, DOI 10.1371/journal.pone.0062798

Huemer M, 2020, EMBO REP, V21, DOI 10.15252/embr.202051034

Jang Jong Geol, 2020, Yeungnam Univ J Med, V37, P277, DOI [10.12701/yujm.2020.00626, 10.12701/yujm.2020.00626]

Jian ZH, 2021, J BASIC MICROB, V61, P1049, DOI 10.1002/jobm.202100201

Jones RM, 2022, FRONT CELL INFECT MI, V12, DOI 10.3389/fcimb.2022.1027394

Kanabalan RD, 2021, MICROBIOL RES, V246, DOI 10.1016/j.micres.2020.126674

Kestler B, 2022, AM J PHYSIOL-LUNG C, V322, pL412, DOI 10.1152/ajplung.00217.2021

Khabibullina NF, 2022, TROP MED INFECT DIS, V7, DOI 10.3390/tropicalmed7030048

Khan A, 2019, J LEUKOCYTE BIOL, V106, P275, DOI 10.1002/JLB.MR0318-095RR

Khan SR, 2019, BRIT J PHARMACOL, V176, P4599, DOI 10.1111/bph.14867

Kherabi Y, 2022, EMERG INFECT DIS, V28, P1796, DOI 10.3201/eid2809.220458

Kim MJ, 2010, EMBO MOL MED, V2, P258, DOI 10.1002/emmm.201000079

Kostyukova I, 2023, MICROORGANISMS, V11, DOI 10.3390/microorganisms11020425

Lanni A, 2023, MICROORGANISMS, V11, DOI 10.3390/microorganisms11061511

Lee OYC, 2012, PLOS ONE, V7, DOI 10.1371/journal.pone.0041923

Li HC, 2018, INT J CLIN EXP PATHO, V11, P3903

Li T, 2023, CHINA CDC WEEKLY, V5, P259, DOI 10.46234/ccdcw2023.047

Luke Erica, 2022, Front Biosci (Schol Ed), V14, P6, DOI [10.31083/j.fbs1401006, 10.31083/j.fbs1401006]

Ma JN, 2022, BMC INFECT DIS, V22, DOI 10.1186/s12879-022-07891-5

Martin CJ, 2012, CELL HOST MICROBE, V12, P289, DOI 10.1016/j.chom.2012.06.010

Mayans MV, 1997, AIDS, V11, P499, DOI 10.1097/00002030-199704000-00014

McDonald SK, 2020, PHILOS T R SOC B, V375, DOI 10.1098/rstb.2019.0583

Nardell EA, 2016, CSH PERSPECT MED, V6, DOI 10.1101/cshperspect.a018192

Occhineri Sara, 2022, Curr Res Pharmacol Drug Discov, V3, P100128, DOI [10.1016/j.crphar.2022.100128, 10.1016/j.crphar.2022.100128]

Oh CE, 2022, CLIN EXP PEDIATR, V65, P214, DOI 10.3345/cep.2021.01186

Ojo OO, 2023, SCI REP-UK, V13, DOI 10.1038/s41598-023-38707-4

Oren E., 2017, Nutrition and Health in a Developing World

Pathak L, 2021, AM J PATHOL, V191, P1255, DOI 10.1016/j.ajpath.2021.03.011

Patterson B, 2019, TUBERCULOSIS, V117, P31, DOI 10.1016/j.tube.2019.05.003

Pawlowski A, 2012, PLOS PATHOG, V8, DOI 10.1371/journal.ppat.1002464

Pesapane F, 2015, AN BRAS DERMATOL, V90, P684, DOI 10.1590/abd1806-4841.20154262

Petersen HJ, 2013, FRONT IMMUNOL, V4, DOI 10.3389/fimmu.2013.00120

Phillips CJC, 2003, RES VET SCI, V74, P1, DOI 10.1016/S0034-5288(02)00145-5

PORTER JDH, 1994, ANNU REV PUBL HEALTH, V15, P303

Ravesloot-Chávez MM, 2021, ANNU REV IMMUNOL, V39, P611, DOI 10.1146/annurev-immunol-093019-010426

Riva MA, 2014, J ANTIBIOT, V67, P661, DOI 10.1038/ja.2014.108

Rossi G, 1996, Recenti Prog Med, V87, P487

Ryndak MB, 2019, FRONT CELL INFECT MI, V9, DOI 10.3389/fcimb.2019.00299

Saelens JW, 2019, FRONT IMMUNOL, V10, DOI 10.3389/fimmu.2019.00528

Sandhu GK, 2011, J GLOB INFECT DIS, V3, P143, DOI 10.4103/0974-777X.81691

Shuaib YA, 2022, GENES-BASEL, V13, DOI 10.3390/genes13060990

Silva ML, 2022, PLOS PATHOG, V18, DOI 10.1371/journal.ppat.1010490

Smith I, 2003, CLIN MICROBIOL REV, V16, P463, DOI 10.1128/CMR.16.3.463-496.2003

Soko RN, 2021, EMERG INFECT DIS, V27, P1831, DOI 10.3201/eid2707.210557

Somoskovi A, 2009, J CLIN MICROBIOL, V47, P255, DOI 10.1128/JCM.01268-08

Sterling TR, 2020, MMWR RECOMM REP, V69, DOI 10.15585/mmwr.rr6901a1

Sudomová M, 2019, MAR DRUGS, V17, DOI 10.3390/md17110641

Swain SS, 2020, EMERG MICROBES INFEC, V9, P1651, DOI 10.1080/22221751.2020.1785334

Tripp L, 2017, PLOS ONE, V12, DOI 10.1371/journal.pone.0183296

Ulrichs T, 2004, J PATHOL, V204, P217, DOI 10.1002/path.1628

Urbaniec J, 2022, FEMS MICROBIOL REV, V46, DOI 10.1093/femsre/fuab042

van Ingen J, 2012, EMERG INFECT DIS, V18, P653, DOI 10.3201/eid1804.110888

Vanino E, 2023, INT J INFECT DIS, V130, pS12, DOI 10.1016/j.ijid.2023.03.013

WHO, 2023, WHO TUBERCULOSIS

World Health Organization, 2022, Consolidated guidelines on HIV, viral hepatitis and STI prevention, diagnosis, treatment and care for key populations

Yang HL, 2012, FEMS IMMUNOL MED MIC, V66, P273, DOI 10.1111/j.1574-695X.2012.01002.x

Yang QL, 2022, MEDICINE, V101, DOI 10.1097/MD.0000000000030405

Zwerling A, 2016, AM J EPIDEMIOL, V183, P407, DOI 10.1093/aje/kwv176

NR 118

TC 55

Z9 67

U1 0

U2 12

PU MDPI

PI BASEL

PA MDPI AG, Grosspeteranlage 5, CH-4052 BASEL, SWITZERLAND

EI 2076-2607

J9 MICROORGANISMS

JI Microorganisms

PD SEP

PY 2023

VL 11

IS 9

AR 2277

DI 10.3390/microorganisms11092277

PG 16

WC Microbiology

WE Science Citation Index Expanded (SCI-EXPANDED)

SC Microbiology

GA AZ4A4

UT WOS:001122242800001

OA Green Submitted, gold

DA 2026-05-07

ER

PT J

AU Sun, QF

Li, SS

Gao, MQ

Pang, Y

AF Sun, Qingfeng

Li, Shanshan

Gao, Mengqiu

Pang, Yu

TI Therapeutic Strategies for Tuberculosis: Progress and Lessons Learned

SO BIOMEDICAL AND ENVIRONMENTAL SCIENCES

LA English

DT Review

ID MYCOBACTERIUM-TUBERCULOSIS; RESISTANT TUBERCULOSIS; PROTECTS MICE;

IMMUNOTHERAPY; CYTOKINE; REGIMENS; BEDAQUILINE; DISCOVERY; METFORMIN;

PA-824

AB Tuberculosis (TB) remains a significant global health challenge, ranking second only to COVID-19 as the leading cause of death from a single infectious agent, with 1.3 million TB-related deaths reported in 2022. Treatment efficacy has been compromised by the emergence of drug-resistant strains, including rifampin-resistant TB (RR-TB), multidrug-resistant TB (MDR-TB), and extensively drug-resistant TB (XDR-TB). Although first-line drugs like isoniazid, rifampicin, pyrazinamide, and ethambutol form the cornerstone of TB therapy, the rise of resistant strains necessitates the use of second-line drugs, which often come with increased toxicity and limited accessibility. Recent advances have focused on repurposing existing compounds and developing new drugs with novel mechanisms of action. Promising agents such as second-generation bedaquiline analogs (TBAJ-587, TBAJ-876), sudapyridine (WX-081), delamanid, pretomanid, and TBI-166 (pyrifazimine) have shown efficacy against resistant Mtb strains. Innovative treatment regimens like the BPaLM protocol-combining bedaquiline, pretomanid, linezolid, and moxifloxacin-offer shorter, all-oral therapies with higher cure rates. Personalized treatment durations and dose optimizations are becoming feasible through risk stratification algorithms and pharmacokinetic/ pharmacodynamic studies. Immunotherapy is emerging as a complementary strategy to enhance the host's immune response against Mtb. Agents such as vitamin D, corticosteroids, non-steroidal anti-inflammatory drugs (NSAIDs), statins, metformin, and biological agents like interleukins and granulocyte-macrophage colony-stimulating factor are under exploration. Additionally, cell therapies involving mesenchymal stem cells and immune effector cells present new therapeutic avenues. Despite these advancements, significant challenges remain in achieving the World Health Organization's "End TB Strategy" goals, particularly as the COVID-19 pandemic has diverted resources and attention. Ongoing research and global collaboration are crucial to develop novel therapeutic strategies, optimize treatment regimens, and ultimately reduce the global burden of TB.

C1 [Sun, Qingfeng; Li, Shanshan; Pang, Yu] Capital Med Univ, Beijing Chest Hosp, Beijing TB & Thorac Tumor Res Inst, Dept Bacteriol & Immunol, Beijing 101149, Peoples R China.

[Sun, Qingfeng; Gao, Mengqiu] Capital Med Univ, Beijing Chest Hosp, Beijing TB & Thorac Tumor Res Inst, Dept TB 2, Beijing 101149, Peoples R China.

C3 Capital Medical University; Capital Medical University

RP Pang, Y (corresponding author), Capital Med Univ, Beijing Chest Hosp, Beijing TB & Thorac Tumor Res Inst, Dept Bacteriol & Immunol, Beijing 101149, Peoples R China.; Gao, MQ (corresponding author), Capital Med Univ, Beijing Chest Hosp, Beijing TB & Thorac Tumor Res Inst, Dept TB 2, Beijing 101149, Peoples R China.

EM gaomqwdm@aliyun.com; pangyupound@163.com

RI Sun, qingfeng/MFJ-0579-2025

FU National Key Research and Development Program of China [2022YFC2302903]

FX * This work was supported by the National Key Research and Development

Program of China (Grant No. 2022YFC2302903) .

CR Abate G, 2016, IMMUNOTARGETS THER, V5, P37, DOI 10.2147/ITT.S81892

Abrahams KA, 2017, SCI REP-UK, V7, DOI 10.1038/s41598-017-09642-y

Aggarwal A, 2017, CELL, V170, P249, DOI 10.1016/j.cell.2017.06.025

Aldea M, 2020, EUR J CANCER, V141, P239, DOI 10.1016/j.ejca.2020.09.032

Ali AM, 2023, ANTIMICROB AGENTS CH, V67, DOI 10.1128/aac.01448-22

[Anonymous], 2021, Global tuberculosis report 2021

Bai XY, 2015, P NATL ACAD SCI USA, V112, P5111, DOI 10.1073/pnas.1424302112

Beites T, 2019, NAT COMMUN, V10, DOI 10.1038/s41467-019-12956-2

Bloom BR, 2023, J EXP MED, V220, DOI 10.1084/jem.20230859

Blum CA, 2015, LANCET, V385, P1511, DOI 10.1016/S0140-6736(14)62447-8

Brown KL, 2023, ANTIMICROB AGENTS CH, V67, DOI 10.1128/aac.01294-22

Bruhn DF, 2015, SCI REP-UK, V5, DOI 10.1038/srep13985

Cai L, 2022, FRONT NUTR, V9, DOI 10.3389/fnut.2022.873890

Cain DW, 2017, NAT REV IMMUNOL, V17, P233, DOI 10.1038/nri.2017.1

Cambau E, 2014, CLIN MICROBIOL INFEC, V20, P196, DOI 10.1111/1469-0691.12555

Campbell GR, 2012, PLOS PATHOG, V8, DOI 10.1371/journal.ppat.1002689

Cardona P, 2019, FRONT IMMUNOL, V10, DOI 10.3389/fimmu.2019.02139

Cardona PJ, 2006, TUBERCULOSIS, V86, P273, DOI 10.1016/j.tube.2006.01.024

Chackerian A, 2002, INFECT IMMUN, V70, P6302, DOI 10.1128/IAI.70.11.6302-6309.2002

Chatterjee S, 2017, PLOS ONE, V12, DOI 10.1371/journal.pone.0179701

Choi Y, 2018, J ANTIMICROB CHEMOTH, V73, P183, DOI 10.1093/jac/dkx367

Cohen KA, 2019, GENOME MED, V11, DOI 10.1186/s13073-019-0660-8

Conradie F, 2022, NEW ENGL J MED, V387, P810, DOI 10.1056/NEJMoa2119430

Dartois V, 2024, NAT REV DRUG DISCOV, V23, P381, DOI 10.1038/s41573-024-00897-5

Dartois VA, 2022, NAT REV MICROBIOL, V20, P685, DOI 10.1038/s41579-022-00731-y

Davies GR, 2023, CURR OPIN INFECT DIS, V36, P132, DOI 10.1097/QCO.0000000000000899

Degner NR, 2018, CLIN INFECT DIS, V66, P198, DOI 10.1093/cid/cix819

Dheda K, 2024, NAT REV DIS PRIMERS, V10, DOI 10.1038/s41572-024-00504-2

Diaz JMA, 2023, RESPIRATION, V102, P83, DOI 10.1159/000528274

Ding YM, 2022, ANTIMICROB AGENTS CH, V66, DOI 10.1128/aac.00658-22

Done MM, 2020, INFECTION, V48, P641, DOI 10.1007/s15010-020-01430-7

Dookie N, 2022, CLIN MICROBIOL REV, V35, DOI 10.1128/cmr.00180-19

Dorman SE, 2021, NEW ENGL J MED, V384, P1705, DOI 10.1056/NEJMoa2033400

Efremenko YV, 2013, HUM VACC IMMUNOTHER, V9, P1852, DOI 10.4161/hv.25280

Ejalonibu MA, 2021, INT J MOL SCI, V22, DOI 10.3390/ijms222413259

Fang C, 2018, BIOORG MED CHEM LETT, V28, P3529, DOI 10.1016/j.bmcl.2018.09.037

Floyd K, 2018, LANCET RESP MED, V6, P299, DOI 10.1016/S2213-2600(18)30057-2

Fowler CJ, 2012, TRENDS PHARMACOL SCI, V33, P468, DOI 10.1016/j.tips.2012.05.003

Francisco-Cruz A, 2016, TUBERCULOSIS, V100, P5, DOI 10.1016/j.tube.2016.05.015

Garcia-Prats AJ, 2018, CLIN INFECT DIS, V67, P1777, DOI 10.1093/cid/ciy416

Gong WP, 2020, MILITARY MED RES, V7, DOI 10.1186/s40779-020-00258-4

Green SR, 2022, NAT COMMUN, V13, DOI 10.1038/s41467-022-33736-5

Heinrichs MT, 2019, INT J ANTIMICROB AG, V53, P275, DOI 10.1016/j.ijantimicag.2018.10.012

Hoi DM, 2023, CELL, V186, DOI 10.1016/j.cell.2023.04.009

Hsu YC, 2016, PLOS ONE, V11, DOI 10.1371/journal.pone.0166552

Imperial MZ, 2021, AM J RESP CRIT CARE, V204, P1086, DOI 10.1164/rccm.202101-0117OC

Imran M, 2022, J INFECT PUBLIC HEAL, V15, P1097, DOI 10.1016/j.jiph.2022.08.016

Introna M, 2017, J AUTOIMMUN, V85, P32, DOI 10.1016/j.jaut.2017.06.008

Jeffreys LN, 2023, ACS INFECT DIS, V9, P221, DOI 10.1021/acsinfecdis.2c00283

Ju ZR, 2022, ACTA PHARM SIN B, V12, P2790, DOI 10.1016/j.apsb.2022.01.002

Kadura S, 2020, J ANTIMICROB CHEMOTH, V75, P2031, DOI 10.1093/jac/dkaa136

Kalfeist L, 2022, CELLS-BASEL, V11, DOI 10.3390/cells11050770

Khoshnood S, 2021, FRONT MICROBIOL, V12, DOI 10.3389/fmicb.2021.717045

Kim SH, 2005, IMMUNITY, V22, P131, DOI 10.1016/j.immuni.2004.12.003

Kumar NP, 2018, BMC INFECT DIS, V18, DOI 10.1186/s12879-018-3246-y

Lange C, 2018, RESPIROLOGY, V23, P656, DOI 10.1111/resp.13304

Lee BS, 2021, EMBO MOL MED, V13, DOI 10.15252/emmm.202013207

Lee M, 2019, TRIALS, V20, DOI 10.1186/s13063-018-3053-1

Li XF, 2017, J MED CHEM, V60, P8011, DOI 10.1021/acs.jmedchem.7b00631

Liang Y, 2022, FRONT IMMUNOL, V13, DOI 10.3389/fimmu.2022.876579

Liang Y, 2017, BMC IMMUNOL, V18, DOI 10.1186/s12865-017-0196-x

Liu JY, 2017, ACS INFECT DIS, V3, P72, DOI 10.1021/acsinfecdis.6b00158

Ma WQ, 2019, MOL CELL ENDOCRINOL, V479, P39, DOI 10.1016/j.mce.2018.08.012

Ma YF, 2011, MICROBES INFECT, V13, P1099, DOI 10.1016/j.micinf.2011.06.012

McQuaid CF, 2020, EUR RESPIR J, V56, DOI 10.1183/13993003.01718-2020

Mi J, 2021, FRONT CELL INFECT MI, V11, DOI 10.3389/fcimb.2021.763591

Mondoni M, 2021, CURR OPIN PHARMACOL, V59, P103, DOI 10.1016/j.coph.2021.05.007

Montoya D, 2014, SCI TRANSL MED, V6, DOI 10.1126/scitranslmed.3009546

Morris T, 2009, J IMMUNOL, V183, P2089, DOI 10.4049/jimmunol.0900477

Nahid P, 2019, AM J RESP CRIT CARE, V200, pE93, DOI 10.1164/rccm.201909-1874ST

Nair A, 2023, J NANOBIOTECHNOL, V21, DOI 10.1186/s12951-023-02156-y

Nie WJ, 2022, INFECTION, V50, P413, DOI 10.1007/s15010-021-01698-3

Novita Bernadette Dian, 2019, Indian J Tuberc, V66, P64, DOI [10.1016/j.ijtb.2018.04.003, 10.1016/j.ijtb.2018.04.003]

Nyang'wa BT, 2022, NEW ENGL J MED, V387, P2331, DOI 10.1056/NEJMoa2117166

Occhineri Sara, 2022, Curr Res Pharmacol Drug Discov, V3, P100128, DOI [10.1016/j.crphar.2022.100128, 10.1016/j.crphar.2022.100128]

Okada M, 2010, PROCEDIA VACCINOL, V2, P34, DOI 10.1016/j.provac.2010.03.007

Parida SK, 2015, INT J INFECT DIS, V32, P32, DOI 10.1016/j.ijid.2015.01.016

Parihar SP, 2014, J INFECT DIS, V209, P754, DOI 10.1093/infdis/jit550

Park JH, 2021, FRONT CELL INFECT MI, V11, DOI 10.3389/fcimb.2021.635335

Pedral-Sampaio Diana Brasil, 2003, Braz J Infect Dis, V7, P245

Peloquin CA, 2021, CLIN PHARMACOL THER, V110, P1455, DOI 10.1002/cpt.2261

Pi J, 2022, J NANOBIOTECHNOL, V20, DOI 10.1186/s12951-021-01234-3

Pontali E, 2016, EUR RESPIR J, V47, P394, DOI 10.1183/13993003.01891-2015

Prasad K, 2016, COCHRANE DB SYST REV, DOI 10.1002/14651858.CD002244.pub4

Ramos-Espinosa O, 2018, EXPERT REV RESP MED, V12, P427, DOI 10.1080/17476348.2018.1457439

Remmelts HHF, 2012, CLIN VACCINE IMMUNOL, V19, P1532, DOI 10.1128/CVI.00423-12

Rocha DMGC, 2021, GENES-BASEL, V12, DOI 10.3390/genes12122003

Rothchild AC, 2014, PLOS PATHOG, V10, DOI 10.1371/journal.ppat.1003805

Rudraraju RS, 2022, FRONT CELL INFECT MI, V12, DOI 10.3389/fcimb.2022.1008213

Sable SB, 2020, CLIN MICROBIOL REV, V33, DOI 10.1128/CMR.00100-19

Sagawa ZK, 2023, NAT COMMUN, V14, DOI 10.1038/s41467-023-36789-2

Sakai S, 2021, MUCOSAL IMMUNOL, V14, P199, DOI 10.1038/s41385-020-0332-4

Santos-Mena A, 2024, PULM PHARMACOL THER, V85, DOI 10.1016/j.pupt.2024.102300

Sarathy JP, 2022, ANTIMICROB AGENTS CH, V66, DOI 10.1128/aac.01237-22

Sarathy JP, 2020, ANTIMICROB AGENTS CH, V64, DOI 10.1128/AAC.02404-19

Sethiya JP, 2020, INT J MOL SCI, V21, DOI 10.3390/ijms21176202

Sharma SK, 2017, SCI REP-UK, V7, DOI 10.1038/s41598-017-03514-1

Shen H, 2015, ARCH MED SCI, V11, P584, DOI 10.5114/aoms.2015.52362

Shi H, 2022, NUTRIENTS, V14, DOI 10.3390/nu14224935

Singh R, 2008, SCIENCE, V322, P1392, DOI 10.1126/science.1164571

Skerry C, 2014, J ANTIMICROB CHEMOTH, V69, P2453, DOI 10.1093/jac/dku166

Stadler JAM, 2023, FRONT PHARMACOL, V14, DOI 10.3389/fphar.2023.1100488

Sturkenboom MGG, 2021, CLIN PHARMACOKINET, V60, P685, DOI 10.1007/s40262-021-00997-0

Tabernero P, 2023, BMJ GLOB HEALTH, V8, DOI 10.1136/bmjgh-2023-012039

Tait D, 2024, J INFECT DIS, V230, P1262, DOI 10.1093/infdis/jiae170

Tasneen R, Contribution of

Tasneen R, 2015, ANTIMICROB AGENTS CH, V59, P129, DOI 10.1128/AAC.03822-14

Tiberi S, 2017, INT J INFECT DIS, V56, P181, DOI 10.1016/j.ijid.2016.10.026

Tiwari Divya, 2023, Semin Immunol, V65, P101672, DOI [10.1016/j.smim.2022.101672, 10.1016/j.smim.2022.101672]

Tobin DM, 2013, CURR OPIN PHARMACOL, V13, P641, DOI 10.1016/j.coph.2013.06.007

Tobin DM, 2012, CELL, V148, P434, DOI 10.1016/j.cell.2011.12.023

Tonby K, 2016, BMC INFECT DIS, V16, DOI 10.1186/s12879-016-1938-8

Viksna A, 2023, BMC INFECT DIS, V23, DOI 10.1186/s12879-023-08629-7

Vilaplana C, 2013, J INFECT DIS, V208, P199, DOI 10.1093/infdis/jit152

von Groote-Bidlingmaier F, 2019, LANCET RESP MED, V7, P249, DOI [10.1016/s2213-2600(18)30426-0, 10.1016/S2213-2600(18)30426-0]

von Reyn CF, 2017, PLOS ONE, V12, DOI 10.1371/journal.pone.0175215

Wallis RS, 2023, NAT REV IMMUNOL, V23, P121, DOI 10.1038/s41577-022-00734-z

Wallis RS, 2016, OPEN FORUM INFECT DI, V3, DOI 10.1093/ofid/ofw151

Wallis RS, 2014, PLOS ONE, V9, DOI 10.1371/journal.pone.0094462

Wang N, 2023, IMMUN INFLAMM DIS, V11, DOI 10.1002/iid3.854

Weston DJ, 2024, DRUG METAB DISPOS, V52, P858, DOI 10.1124/dmd.124.001562

Won HI, 2024, NAT COMMUN, V15, DOI 10.1038/s41467-024-48506-8

Working Group for New TB Drugs, Discovery

Working Group on New Drugs, 2024, TBI-223

Xia H, 2021, MICROBIOL SPECTR, V9, DOI 10.1128/Spectrum.00409-21

Xiao SF, 2023, INT IMMUNOPHARMACOL, V118, DOI 10.1016/j.intimp.2023.109956

Xu J, 2021, ANTIMICROB AGENTS CH, V65, DOI 10.1128/AAC.02418-20

Xu J, 2019, ANTIMICROB AGENTS CH, V63, DOI [10.1128/aac.02155-18, 10.1128/AAC.02155-18]

Xu YN, 2017, J AM HEART ASSOC, V6, DOI 10.1161/JAHA.117.007134

Yan SY, 2019, PEERJ, V7, DOI 10.7717/peerj.7404

Yang XY, 2024, ENGINEERING-PRC, V38, P52, DOI 10.1016/j.eng.2024.02.009

Yew WW, 2019, TUBERCULOSIS, V115, P76, DOI 10.1016/j.tube.2019.02.004

Yu T, 2013, BMC MED, V11, DOI 10.1186/1741-7015-11-7

Yu XY, 2019, BMC INFECT DIS, V19, DOI 10.1186/s12879-019-4548-4

Zainabadi K, 2024, J INFECT DIS, V230, pe447, DOI 10.1093/infdis/jiad564

Zhang XY, 2021, FRONT IMMUNOL, V12, DOI 10.3389/fimmu.2021.695278

Zhang YR, 2012, SCI CHINA LIFE SCI, V55, P800, DOI 10.1007/s11427-012-4368-x

Zheng LY, 2024, MSPHERE, V9, DOI 10.1128/msphere.00518-23

Zou S, 2022, FRONT CELL INFECT MI, V12, DOI 10.3389/fcimb.2022.1071880

NR 139

TC 11

Z9 12

U1 4

U2 14

PU CHINESE CENTER DISEASE CONTROL & PREVENTION

PI BEIJING

PA 155 CHANGBAI RD, CHANGPING DISTRICT, BEIJING, 102206, PEOPLES R CHINA

SN 0895-3988

EI 2214-0190

J9 BIOMED ENVIRON SCI

JI Biomed. Environ. Sci.

PD NOV

PY 2024

VL 37

IS 11

BP 1310

EP 1323

DI 10.3967/bes2024.168

EA DEC 2024

PG 14

WC Environmental Sciences; Public, Environmental & Occupational Health

WE Science Citation Index Expanded (SCI-EXPANDED)

SC Environmental Sciences & Ecology; Public, Environmental & Occupational

Health

GA R3H7Y

UT WOS:001390408400009

DA 2026-05-07

ER

PT J

AU Kelishomi, FZ

Khanjani, S

Fardsanei, F

Sarabi, HS

Nikkhahi, F

Dehghani, B

AF Kelishomi, Fatemeh Zeynali

Khanjani, Susan

Fardsanei, Fatemeh

Sarabi, Hediyeh Saghi

Nikkhahi, Farhad

Dehghani, Behzad

TI Bacteriophages of Mycobacterium tuberculosis, their diversity,

and potential therapeutic uses: a review

SO BMC INFECTIOUS DISEASES

LA English

DT Review

DE Mycobacterium tuberculosis; Drug resistance; Mycobacteriophages; Phage

therapy

ID DRUG-RESISTANT TUBERCULOSIS; RISK-FACTORS; PHAGE; HOST; GENES

AB Tuberculosis (TB) caused by Mycobacterium tuberculosis (M. tuberculosis) is a highly infectious disease and worldwide health problem. Based on the WHO TB report, 9 million active TB cases are emerging, leading to 2 million deaths each year. The recent emergence of multidrug-resistant tuberculosis (MDR-TB) and extensively drug-resistant tuberculosis (XDR-TB) strains emphasizes the necessity to improve novel therapeutic plans. Among the various developing antibacterial approaches, phage therapy is thought to be a precise hopeful resolution. Mycobacteriophages are viruses that infect bacteria such as Mycobacterium spp., containing the M. tuberculosis complex. Phages and phage-derived proteins can act as promising antimicrobial agents. Also, phage cocktails can broaden the spectrum of lysis activity against bacteria. Recent researches have also shown the effective combination of antibiotics and phages to defeat the infective bacteria. There are limitations and concerns about phage therapy. For example, human immune response to phage therapy, transferring antibiotic resistance genes, emerging resistance to phages, and safety issues. So, in the present study, we introduced mycobacteriophages, their use as therapeutic agents, and their advantages and limitations as therapeutic applications.

C1 [Kelishomi, Fatemeh Zeynali; Khanjani, Susan; Fardsanei, Fatemeh; Sarabi, Hediyeh Saghi; Nikkhahi, Farhad] Qazvin Univ Med Sci, Med Microbiol Res Ctr, Qazvin, Iran.

[Dehghani, Behzad] Shiraz Univ Med Sci, Dept Bacteriol Virol, Shiraz, Iran.

C3 Qazvin University of Medical Sciences (QUMS); Shiraz University of

Medical Science

RP Nikkhahi, F (corresponding author), Qazvin Univ Med Sci, Med Microbiol Res Ctr, Qazvin, Iran.

EM farhadnikkhahi@gmail.com

RI Zeynali Kelishomi, Fatemeh/AEK-5268-2022; fardsanei,

fatemeh/AAZ-7223-2021

OI Zeynali Kelishomi, Fatemeh/0000-0002-1744-8800;

CR Abdelrahman F, 2021, ANTIBIOTICS-BASEL, V10, DOI 10.3390/antibiotics10020124

Abedon ST, 2021, PHARMACEUTICALS-BASE, V14, DOI 10.3390/ph14111157

Abedon ST, 2021, PHARMACEUTICALS-BASE, V14, DOI 10.3390/ph14101019

Alam CM, 2019, FRONT GENET, V10, DOI 10.3389/fgene.2019.00207

Allué-Guardia A, 2021, INT J MOL SCI, V22, DOI 10.3390/ijms22020735

Angkwanish T, 2021, FRONT VET SCI, V8, DOI 10.3389/fvets.2021.713663

Aslam B, 2021, INFECT DIS THER, V10, P1171, DOI 10.1007/s40121-021-00446-2

Azimi T, 2019, INFECT DRUG RESIST, V12, P2943, DOI 10.2147/IDR.S218638

Bajpai U, 2020, CAN J MICROBIOL, V66, P455, DOI 10.1139/cjm-2020-0104

Bajpai U, 2018, CAN J MICROBIOL, V64, P483, DOI 10.1139/cjm-2017-0387

Bebeacua C, 2013, J VIROL, V87, P1061, DOI 10.1128/JVI.02836-12

Beinhauerova M, 2021, MICROORGANISMS, V9, DOI 10.3390/microorganisms9020237

Brabban AD, 2005, FOODBORNE PATHOG DIS, V2, P287, DOI 10.1089/fpd.2005.2.287

Cahill J, 2019, ADV VIRUS RES, V103, P33, DOI 10.1016/bs.aivir.2018.09.003

Caratenuto RA, 2019, MICROBIOL RESOUR ANN, V8, DOI 10.1128/MRA.00399-19

Catalao MJ, 2019, FRONT MICROBIOL, V10, DOI 10.3389/fmicb.2019.00190

Catalao MJ, 2018, VIRUSES-BASEL, V10, DOI 10.3390/v10080428

Clokie Martha Rj, 2011, Bacteriophage, V1, P31, DOI 10.4161/bact.1.1.14942

de Leeuw M, 2020, VIRUSES-BASEL, V12, DOI 10.3390/v12101132

de Lima BF, 2014, CLIN RESPIR J, V8, P11, DOI 10.1111/crj.12044

Dedrick RM, 2019, TUBERCULOSIS, V115, P14, DOI 10.1016/j.tube.2019.01.002

Doekes HM, 2021, ELIFE, V10, DOI 10.7554/eLife.58410

Eniyan K, 2020, WORLD J MICROB BIOT, V36, DOI 10.1007/s11274-020-02858-7

Foglizzo V, 2021, PHARMACEUTICALS-BASE, V14, DOI 10.3390/ph14020161

Fraga AG, 2019, PLOS NEGLECT TROP D, V13, DOI 10.1371/journal.pntd.0007113

Gan Y, 2016, INDIAN J MED MICROBI, V34, P186, DOI 10.4103/0255-0857.180282

Garcia-Vilanova A, 2019, FRONT IMMUNOL, V10, DOI 10.3389/fimmu.2019.02909

Guerrero-Bustamante CA, 2021, MBIO, V12, DOI 10.1128/mBio.00973-21

Guo SL, 2012, FRONT BIOSCI-LANDMRK, V17, P2691, DOI 10.2741/4080

Haaber J, 2016, NAT COMMUN, V7, DOI 10.1038/ncomms13333

Hatfull GF, 2014, MICROBIOL SPECTR, V2, DOI 10.1128/microbiolspec.MGM2-0032-2013

Herridge WP, 2020, J MED MICROBIOL, V69, P176, DOI 10.1099/jmm.0.001141

Hjort K, 2022, J INFECT DIS, V225, P1011, DOI 10.1093/infdis/jiaa625

Jamal M, 2019, J BASIC MICROB, V59, P123, DOI 10.1002/jobm.201800412

Jiang Z, 2020, ANTIBIOTICS-BASEL, V9, DOI 10.3390/antibiotics9100714

Kalapala YC, 2020, FRONT MICROBIOL, V11, DOI 10.3389/fmicb.2020.583661

Khawbung JL, 2021, COMP IMMUNOL MICROB, V74, DOI 10.1016/j.cimid.2020.101574

Krut O, 2018, J IMMUNOL, V200, P3037, DOI 10.4049/jimmunol.1701745

Lange C, 2018, RESPIROLOGY, V23, P656, DOI 10.1111/resp.13304

Lee K, 2009, GLOB INST, P1

Li XX, 2021, MICROB PATHOGENESIS, V159, DOI 10.1016/j.micpath.2021.105135

Loewenberg S, 2012, LANCET, V379, P205, DOI 10.1016/S0140-6736(12)60085-3

Luong T, 2020, NAT PROTOC, V15, P2867, DOI 10.1038/s41596-020-0346-0

Maitra A, 2019, FEMS MICROBIOL REV, V43, P548, DOI 10.1093/femsre/fuz016

Mayer O, 2016, J BACTERIOL, V198, P3220, DOI 10.1128/JB.00592-16

Mohidem NA, 2021, REV ENVIRON HEALTH, V36, P493, DOI 10.1515/reveh-2020-0096

Nieth A, 2015, EXPERT OPIN DRUG DEL, V12, P1411, DOI 10.1517/17425247.2015.1043125

Paleckyte A, 2021, ANTIMICROB RESIST IN, V10, DOI 10.1186/s13756-021-00975-y

Parida SK, 2015, J INTERN MED, V277, P388, DOI 10.1111/joim.12264

Pawde DM, 2020, SAUDI PHARM J, V28, P1616, DOI 10.1016/j.jsps.2020.10.008

Pelfrene E, 2016, J ANTIMICROB CHEMOTH, V71, P2071, DOI 10.1093/jac/dkw083

Pereira C, 2021, INT J FOOD MICROBIOL, V338, DOI 10.1016/j.ijfoodmicro.2020.108995

Pires DP, 2020, FEMS MICROBIOL REV, V44, P684, DOI 10.1093/femsre/fuaa017

Pohane AA, 2014, J BIOL CHEM, V289, P12085, DOI 10.1074/jbc.M113.529594

Pope WH, 2015, ELIFE, V4, DOI 10.7554/eLife.06416

Puiu M, 2019, BIOCHEM SOC T, V47, P847, DOI 10.1042/BST20180506

Roach DR, 2017, CELL HOST MICROBE, V22, P38, DOI 10.1016/j.chom.2017.06.018

Russell DA, 2017, BIOINFORMATICS, V33, P784, DOI 10.1093/bioinformatics/btw711

Shen Y, 2021, CURR OPIN BIOTECH, V68, P166, DOI 10.1016/j.copbio.2020.11.004

Shield CG, 2021, MICROORGANISMS, V9, DOI 10.3390/microorganisms9112366

[舒薇 Shu Wei], 2022, [中国防痨杂志, Chinese Journal of Antituberculosis], V44, P45

Singla S, 2016, PLOS ONE, V11, DOI 10.1371/journal.pone.0153777

Siringan P, 2014, OPEN BIOL, V4, DOI 10.1098/rsob.130200

Starshinova A, 2022, ANTIBIOTICS-BASEL, V11, DOI 10.3390/antibiotics11111622

Sula L, 1981, Czech Med, V4, P209

Sweeney KA, 2011, NAT MED, V17, P1261, DOI 10.1038/nm.2420

Teytsa HMN, 2021, MATH MED BIOL, V38, P28, DOI 10.1093/imammb/dqaa010

Varshney K, 2021, INFECT DRUG RESIST, V14, P5429, DOI 10.2147/IDR.S339972

Velayati AA, 2009, CHEST, V136, P420, DOI 10.1378/chest.08-2427

Voigt E, 2021, PEERJ, V9, DOI 10.7717/peerj.12194

Wang JL, 2021, EPIDEMIOL INFECT, V149, DOI 10.1017/S0950268820003106

Wetzel KS, 2021, SCI REP-UK, V11, DOI 10.1038/s41598-021-86112-6

Wu ZF, 2021, VIRUSES-BASEL, V13, DOI 10.3390/v13101949

Xu H, 2021, FRONT MICROBIOL, V12, DOI 10.3389/fmicb.2021.705310

Yang Y, 2019, J BIOL CHEM, V294, P7615, DOI 10.1074/jbc.RA118.006968

Zaman K, 2010, J HEALTH POPUL NUTR, V28, P111

Zhou B, 2021, J MEMBRANE BIOL, V254, P397, DOI 10.1007/s00232-021-00187-w

NR 77

TC 24

Z9 29

U1 6

U2 26

PU BMC

PI LONDON

PA CAMPUS, 4 CRINAN ST, LONDON N1 9XW, ENGLAND

EI 1471-2334

J9 BMC INFECT DIS

JI BMC Infect. Dis.

PD DEC 22

PY 2022

VL 22

IS 1

DI 10.1186/s12879-022-07944-9

PG 14

WC Infectious Diseases

WE Science Citation Index Expanded (SCI-EXPANDED)

SC Infectious Diseases

GA 7H3UA

UT WOS:000903129700002

OA Green Submitted, gold

DA 2026-05-07

ER

PT J

AU Parida, KK

Lahiri, M

Ghosh, M

Dalal, A

Kalia, NP

AF Parida, Kishan Kumar

Lahiri, Monali

Ghosh, Mainak

Dalal, Aman

Kalia, Nitin Pal

TI P-glycoprotein inhibitors as an adjunct therapy for TB

SO DRUG DISCOVERY TODAY

LA English

DT Review

DE TB; drug efflux; fl ux; multidrug resistance; P-glycoprotein;

angiogenesis; granuloma; macrophages

ID RESISTANT MYCOBACTERIUM-TUBERCULOSIS; MULTIDRUG-RESISTANCE;

DRUG-BINDING; EFFLUX PUMP; ORGANIC ANION; MEMBRANE TRANSPORTERS;

COVALENT MODIFICATION; ABC TRANSPORTERS; MACROPHAGES; PROTEINS

AB The primary challenge in TB treatment is the emergence of multidrug-resistant TB (MDR-TB). One of the major factors responsible for MDR is the upregulation of efflux pumps. Permeation-glycoprotein (P-gp), an efflux pump, hinders the bioavailability of the administered drugs inside the infected cells. Simultaneously, angiogenesis, the formation of new blood vessels, contributes to drug delivery complexities. TB infection triggers a cascade of events that upregulates the expression of angiogenic factors and P-gp. The combined action of P-gp and angiogenesis foster the emergence of MDR-TB. Understanding these mechanisms is pivotal for developing targeted interventions to overcome MDR in TB. P-gp inhibitors, such as verapamil, and anti-angiogenic drugs, including bevacizumab, have shown improvement in TB drug delivery to granuloma. In this review, we discuss the potential of P-gp inhibitors as an adjunct therapy to shorten TB treatment.

C1 [Parida, Kishan Kumar; Lahiri, Monali; Ghosh, Mainak; Dalal, Aman; Kalia, Nitin Pal] Natl Inst Pharmaceut Educ & Res, Dept Biol Sci Pharmacol & Toxicol, Hyderabad, Telangana, India.

C3 National Institute of Pharmaceutical Education & Research, Hyderabad;

National Institute of Pharmaceutical Education & Research, S.A.S. Nagar

(Mohali)

RP Kalia, NP (corresponding author), Natl Inst Pharmaceut Educ & Res, Dept Biol Sci Pharmacol & Toxicol, Hyderabad, Telangana, India.

EM kalianpk@gmail.com

RI Kalia, Nitin/AAE-5959-2019

OI Parida, Kishan Kumar/0009-0008-7286-0444

FU NIPER Hyderabad; Department of Pharmaceuticals, Ministry of Chemicals

and Fertilizers, Government of India, New Delhi

FX The authors thank NIPER Hyderabad and the Department of Pharmaceuticals,

Ministry of Chemicals and Fertilizers, Government of India, New Delhi,

for providing fellowships.

CR Ali Y, 2020, CHIN MED-UK, V15, DOI 10.1186/s13020-020-00351-9

Aller SG, 2009, SCIENCE, V323, P1718, DOI 10.1126/science.1168750

Ambudkar SV, 2003, ONCOGENE, V22, P7468, DOI 10.1038/sj.onc.1206948

Amin ML, 2013, DRUG TARGET INSIGHT, V7, P27, DOI 10.4137/DTI.S12519

Annese T, 2022, DIAGNOSTICS, V12, DOI 10.3390/diagnostics12123120

[Anonymous], 2018, The Global Tuberculosis Report

BECK WT, 1988, BIOCHEM BIOPH RES CO, V153, P959, DOI 10.1016/S0006-291X(88)81321-4

Bhardwaj RK, 2002, J PHARMACOL EXP THER, V302, P645, DOI 10.1124/jpet.102.034728

Bolhuis H, 1997, FEMS MICROBIOL REV, V21, P55, DOI 10.1016/S0168-6445(97)00051-X

Borst P, 2002, ANNU REV BIOCHEM, V71, P537, DOI 10.1146/annurev.biochem.71.102301.093055

Borst P, 2007, PFLUG ARCH EUR J PHY, V453, P661, DOI 10.1007/s00424-006-0054-9

Brillault J, 2009, ANTIMICROB AGENTS CH, V53, P1457, DOI 10.1128/AAC.01253-08

Burckhardt G, 2000, AM J PHYSIOL-RENAL, V278, pF853, DOI 10.1152/ajprenal.2000.278.6.F853

Chan LMS, 2004, EUR J PHARM SCI, V21, P25, DOI 10.1016/j.ejps.2003.07.003

Colangeli R, 2005, MOL MICROBIOL, V55, P1829, DOI 10.1111/j.1365-2958.2005.04510.x

Cory TJ, 2016, PHARM RES-DORDR, V33, P2713, DOI 10.1007/s11095-016-1998-x

Cronan MR, 2022, FRONT IMMUNOL, V13, DOI 10.3389/fimmu.2022.820134

Datta M, 2024, PLOS COMPUT BIOL, V20, DOI 10.1371/journal.pcbi.1011847

Datta M, 2015, P NATL ACAD SCI USA, V112, P1827, DOI 10.1073/pnas.1424563112

Drenberg CD, 2017, CANCER RES, V77, P2102, DOI 10.1158/0008-5472.CAN-16-2548

Elmeliegy M, 2020, CLIN PHARMACOKINET, V59, P699, DOI 10.1007/s40262-020-00867-1

Faber KN, 2003, ADV DRUG DELIVER REV, V55, P107, DOI 10.1016/S0169-409X(02)00173-4

Fardel O, 2005, BIOMED PHARMACOTHER, V59, P104, DOI 10.1016/j.biopha.2005.01.005

Frank GA, 2016, MOL PHARMACOL, V90, P35, DOI 10.1124/mol.116.104190

Fu D, 2013, FRONT ONCOL, V3, DOI 10.3389/fonc.2013.00321

Fujita T, 2006, J PHARM SCI-US, V95, P25, DOI 10.1002/jps.20536

Giacomini KM, 2010, NAT REV DRUG DISCOV, V9, P215, DOI 10.1038/nrd3028

GOLLAPUDI S, 1994, BIOCHEM BIOPH RES CO, V199, P1181, DOI 10.1006/bbrc.1994.1355

Grant SS, 2013, VIRULENCE, V4, P273, DOI 10.4161/viru.23987

Guirado E, 2013, SEMIN IMMUNOPATHOL, V35, P563, DOI 10.1007/s00281-013-0388-2

Gupta S, 2014, ANTIMICROB AGENTS CH, V58, P574, DOI 10.1128/AAC.01462-13

Gupta S, 2013, AM J RESP CRIT CARE, V188, P600, DOI 10.1164/rccm.201304-0650OC

Gutmann DAP, 2010, TRENDS BIOCHEM SCI, V35, P36, DOI 10.1016/j.tibs.2009.07.009

Hagenbuch B, 2003, BBA-BIOMEMBRANES, V1609, P1, DOI 10.1016/S0005-2736(02)00633-8

Hartkoorn RC, 2007, TUBERCULOSIS, V87, P248, DOI 10.1016/j.tube.2006.12.001

Hasanuzzaman M, 2019, J PHARM SCI-US, V108, P3106, DOI 10.1016/j.xphs.2019.04.009

He Lei, 2009, Human Genomics, V3, P195

Hegeto LA, 2019, MICROB DRUG RESIST, V25, P120, DOI 10.1089/mdr.2018.0107

HIGGINS CF, 1992, TRENDS BIOCHEM SCI, V17, P18, DOI 10.1016/0968-0004(92)90419-A

Hoffmann U, 2004, DRUG METAB REV, V36, P669, DOI 10.1081/DMR-200033473

Homolya L, 2003, BIOFACTORS, V17, P103, DOI 10.1002/biof.5520170111

Hoosain FG, 2015, BIOMED RES INT, V2015, DOI 10.1155/2015/484963

Horita Y, 2014, ANTIMICROB AGENTS CH, V58, P3168, DOI 10.1128/AAC.02278-13

Jaishankar N, 2020, E. coli infections, P126

JamisDow CA, 1997, XENOBIOTICA, V27, P1015, DOI 10.1080/004982597239994

Jelokhani-Niaraki M, 2023, INT J MOL SCI, V24, DOI 10.3390/ijms24010468

Juvale IIA, 2022, HELIYON, V8, DOI 10.1016/j.heliyon.2022.e09777

Karthika C, 2018, ASIAN J PHARM, V12, P261

Kim Y, 2018, SCIENCE, V359, P915, DOI 10.1126/science.aar7389

Klaassen CD, 2010, PHARMACOL REV, V62, P1, DOI 10.1124/pr.109.002014

Kodan A, 2014, P NATL ACAD SCI USA, V111, P4049, DOI 10.1073/pnas.1321562111

KRAMER W, 1992, J BIOL CHEM, V267, P18598

Kumar R, 2012, CELL MICROBIOL, V14, P1620, DOI 10.1111/j.1462-5822.2012.01827.x

Lake MA, 2023, P NATL ACAD SCI USA, V120, DOI 10.1073/pnas.2215512120

Leitner I, 2011, J ANTIMICROB CHEMOTH, V66, P834, DOI 10.1093/jac/dkq526

Leslie EM, 2005, TOXICOL APPL PHARM, V204, P216, DOI 10.1016/j.taap.2004.10.012

Liang TT, 2021, INT J NANOMED, V16, P2703, DOI 10.2147/IJN.S298159

Liu YH, 2010, CLIN EXP PHARMACOL P, V37, P115, DOI 10.1111/j.1440-1681.2009.05252.x

Llaguno-Munive M, 2020, FRONT ONCOL, V10, DOI 10.3389/fonc.2020.581814

Loo TW, 2006, BIOCHEM J, V399, P351, DOI 10.1042/BJ20060715

Loo TW, 2012, J BIOL CHEM, V287, P26806, DOI 10.1074/jbc.M112.376202

Loo TW, 2003, J BIOL CHEM, V278, P1575, DOI 10.1074/jbc.M211307200

Loo TW, 2004, J BIOL CHEM, V279, P18232, DOI 10.1074/jbc.M400229200

Loo TW, 2003, J BIOL CHEM, V278, P20449, DOI 10.1074/jbc.C300154200

Loo TW, 2001, J BIOL CHEM, V276, P36877, DOI 10.1074/jbc.C100467200

Loo TW, 2001, J BIOL CHEM, V276, P14972, DOI 10.1074/jbc.M100407200

LOO TW, 1995, J BIOL CHEM, V270, P22957, DOI 10.1074/jbc.270.39.22957

Louw GE, 2011, AM J RESP CRIT CARE, V184, P269, DOI 10.1164/rccm.201011-1924OC

Lozano E, 2018, J PERS MED, V8, DOI 10.3390/jpm8020014

Mack U, 2009, EUR RESPIR J, V33, P956, DOI 10.1183/09031936.00120908

Martinec O, 2021, FRONT PHARMACOL, V12, DOI 10.3389/fphar.2021.684156

McCaffrey G, 2012, J NEUROCHEM, V122, P962, DOI 10.1111/j.1471-4159.2012.07831.x

Mercer SL, 2011, CURR TOP MED CHEM, V11, P1157

Mo Wei, 2012, Int J Biochem Mol Biol, V3, P1

Moon YS, 2016, TOXINS, V8, DOI 10.3390/toxins8080240

MOR N, 1994, ANTIMICROB AGENTS CH, V38, P1161, DOI 10.1128/AAC.38.5.1161

Nath Alok, 2020, Indian J Tuberc, V67, P172, DOI [10.1016/j.ijtb.2019.11.015, 10.1016/j.ijtb.2019.11.015]

Neves Ivan, 2012, EUROPEAN RESPIRATORY JOURNAL, V40

Ni B, 2014, J IMMUNOL, V193, P4537, DOI 10.4049/jimmunol.1400124

Nigam SK, 2015, NAT REV DRUG DISCOV, V14, P29, DOI 10.1038/nrd4461

Ottavi S, 2022, J MED CHEM, V65, P1996, DOI 10.1021/acs.jmedchem.1c01565

Otu A. A., 2013, Asian Pacific Journal of Tropical Disease, V3, P227, DOI 10.1016/S2222-1808(13)60045-6

Parvez MM, 2018, ANTIMICROB AGENTS CH, V62, DOI [10.1128/AAC.00512-18, 10.1128/aac.00512-18]

PEARSON ML, 1992, ANN INTERN MED, V117, P191, DOI 10.7326/0003-4819-117-3-191

Petzinger E, 2006, N-S ARCH PHARMACOL, V372, P465, DOI 10.1007/s00210-006-0042-9

Polena H, 2016, SCI REP-UK, V6, DOI 10.1038/srep33162

Pule CM, 2016, J ANTIMICROB CHEMOTH, V71, P17, DOI 10.1093/jac/dkv316

Rani, 2013, Global Journal of Pharmacology, V7, P87, DOI [10.5829/idosi.gjp.2013.7.1.1104, DOI 10.5829/IDOSI.GJP.2013.7.1.1104, DOI 10.5829/ID0SI.GJP.2013.7.1.1104]

RAVIV Y, 1990, J BIOL CHEM, V265, P3975

Remm S, 2022, FEMS MICROBIOL REV, V46, DOI 10.1093/femsre/fuab050

RONINSON IB, 1986, P NATL ACAD SCI USA, V83, P4538, DOI 10.1073/pnas.83.12.4538

Roth M, 2012, BRIT J PHARMACOL, V165, P1260, DOI 10.1111/j.1476-5381.2011.01724.x

Roy A, 2023, DRUG DISCOV TODAY, V28, DOI 10.1016/j.drudis.2023.103693

Roy U, 2015, FRONT MICROBIOL, V6, DOI 10.3389/fmicb.2015.00948

Saha P, 2024, RSC MED CHEM, V15, P1664, DOI 10.1039/d4md00118d

Sau S., 2023, Drugs and a Methodological Compendium: From Bench to Bedside, P357

Schaub TP, 1997, J AM SOC NEPHROL, V8, P1213

Seelig A, 2020, FRONT ONCOL, V10, DOI 10.3389/fonc.2020.576559

Shapiro AB, 1997, EUR J BIOCHEM, V250, P115, DOI 10.1111/j.1432-1033.1997.00115.x

Sharma S, 2014, TUBERCULOSIS, V94, P389, DOI 10.1016/j.tube.2014.04.007

Sharma S, 2010, J ANTIMICROB CHEMOTH, V65, P1694, DOI 10.1093/jac/dkq186

Sharom FJ, 1997, J MEMBRANE BIOL, V160, P161, DOI 10.1007/s002329900305

Sharom FJ, 2014, FRONT ONCOL, V4, DOI 10.3389/fonc.2014.00041

Song QZ, 2015, INT J CLIN EXP MED, V8, P15458

Tamai I, 2000, BIOCHEM BIOPH RES CO, V273, P251, DOI 10.1006/bbrc.2000.2922

te Brake LHM, 2018, ANNU REV PHARMACOL, V58, P271, DOI 10.1146/annurev-pharmtox-010617-052438

THIEBAUT F, 1987, P NATL ACAD SCI USA, V84, P7735, DOI 10.1073/pnas.84.21.7735

Thonghin N, 2018, BMC STRUCT BIOL, V18, DOI 10.1186/s12900-018-0098-z

Tsujimura S, 2017, RMD OPEN, V3, DOI 10.1136/rmdopen-2016-000423

Tsujimura S, 2018, MOD RHEUMATOL, V28, P276, DOI 10.1080/14397595.2017.1341458

Tweya H, 2013, PLOS ONE, V8, DOI 10.1371/journal.pone.0056248

Vaalburg W, 2005, Toxicol Appl Pharmacol, V207, P257, DOI 10.1016/j.taap.2005.03.027

Vahakangas KH, 2011, REPRODUCTIVE AND DEVELOPMENTAL TOXICOLOGY, P1051, DOI 10.1016/B978-0-12-382032-7.10079-7

Vora Agam, 2016, J Assoc Physicians India, V64, P20

Walton EM, 2018, CELL HOST MICROBE, V24, P514, DOI 10.1016/j.chom.2018.09.004

Wang GX, 2016, INT J NEUROSCI, V126, P385, DOI 10.3109/00207454.2015.1038710

Wróblewska A, 2023, INT J NANOMED, V18, P4521, DOI 10.2147/IJN.S421173

Wu Q, 2019, J INFECT DIS, V220, P1989, DOI 10.1093/infdis/jiz405

Xu J, 2018, ANTIMICROB AGENTS CH, V62, DOI 10.1128/AAC.01692-17

Xu YT, 2018, PLOS PATHOG, V14, DOI 10.1371/journal.ppat.1006974

Zennaro C, 2014, INT J MOL SCI, V15, P21366, DOI 10.3390/ijms151121366

Zhai WJ, 2016, EVID-BASED COMPL ALT, V2016, DOI 10.1155/2016/8597208

Zheng HL, 2019, CANCER CHEMOTH PHARM, V83, P361, DOI 10.1007/s00280-018-3735-0

NR 123

TC 7

Z9 8

U1 0

U2 19

PU ELSEVIER SCI LTD

PI London

PA 125 London Wall, London, ENGLAND

SN 1359-6446

EI 1878-5832

J9 DRUG DISCOV TODAY

JI Drug Discov. Today

PD SEP

PY 2024

VL 29

IS 9

AR 104108

DI 10.1016/j.drudis.2024.104108

EA JUL 2024

PG 13

WC Pharmacology & Pharmacy

WE Science Citation Index Expanded (SCI-EXPANDED)

SC Pharmacology & Pharmacy

GA A9R6Z

UT WOS:001285843000001

DA 2026-05-07

ER

PT J

AU Londt, R

Semple, L

Esmail, A

Pooran, A

Meldau, R

Davids, M

Dheda, K

Tomasicchio, M

AF Londt, Rolanda

Semple, Lynn

Esmail, Aliasgar

Pooran, Anil

Meldau, Richard

Davids, Malika

Dheda, Keertan

Tomasicchio, Michele

TI Autologous Human Dendritic Cells from XDR-TB Patients Polarize a Th1

Response Which Is Bactericidal to Mycobacterium tuberculosis

SO MICROORGANISMS

LA English

DT Article

DE dendritic cell vaccine; pre-extensively drug-resistant tuberculosis;

extensively drug-resistant tuberculosis; drug-resistant tuberculosis;

cellular vaccine; monocytic-derived dendritic cells

ID DRUG-RESISTANT TUBERCULOSIS; SUBUNIT VACCINATION; MELANOMA PATIENTS;

IMMUNE-RESPONSE; PE/PPE PROTEINS; GENE-EXPRESSION; ESAT-6; ANTIGEN;

IMMUNOGENICITY; TB10.4

AB Extensively drug-resistant tuberculosis (XDR-TB) is a public health concern as drug resistance is outpacing the drug development pipeline. Alternative immunotherapeutic approaches are needed. Peripheral blood mononuclear cells (PBMCs) were isolated from pre-XDR/XDR-TB (n = 25) patients and LTBI (n = 18) participants. Thereafter, monocytic-derived dendritic cells (mo-DCs) were co-cultured with M. tb antigens, with/without a maturation cocktail (interferon-gamma, interferon-alpha, CD40L, IL-1 beta, and TLR3 and TLR7/8 agonists). Two peptide pools were evaluated: (i) an ECAT peptide pool (ESAT6, CFP10, Ag85B, and TB10.4 peptides) and (ii) a PE/PPE peptide pool. Sonicated lysate of the M. tb HN878 strain served as a control. Mo-DCs were assessed for DC maturation markers, Th1 cytokines, and the ability of the DC-primed PBMCs to restrict the growth of M. tb-infected monocyte-derived macrophages. In pre-XDR/XDR-TB, mo-DCs matured with M. tb antigens (ECAT or PE/PPE peptide pool, or HN878 lysate) + cocktail, compared to mo-DCs matured with M. tb antigens only, showed higher upregulation of co-stimulatory molecules and IL-12p70 (p < 0.001 for both comparisons). The matured mo-DCs had enhanced antigen-specific CD8(+) T-cell responses to ESAT-6 (p = 0.05) and Ag85B (p = 0.03). Containment was higher with mo-DCs matured with the PE/PPE peptide pool cocktail versus mo-DCs matured with the PE/PPE peptide pool (p = 0.0002). Mo-DCs matured with the PE/PPE peptide pool + cocktail achieved better containment than the ECAT peptide pool + cocktail [50%, (IQR:39-75) versus 46%, (IQR:15-62); p = 0.02]. In patients with pre-XDR/XDR-TB, an effector response primed by mo-DCs matured with an ECAT or PE/PPE peptide pool + cocktail was capable of restricting the growth of M. tb in vitro.

C1 [Londt, Rolanda; Semple, Lynn; Esmail, Aliasgar; Pooran, Anil; Meldau, Richard; Davids, Malika; Dheda, Keertan; Tomasicchio, Michele] Univ Cape Town, Ctr Lung Infect & Immun, Dept Med, Div Pulmonol, ZA-7925 Cape Town, South Africa.

[Londt, Rolanda; Semple, Lynn; Esmail, Aliasgar; Pooran, Anil; Meldau, Richard; Davids, Malika; Dheda, Keertan; Tomasicchio, Michele] UCT Lung Inst, ZA-7925 CAPE TOWN, South Africa.

[Londt, Rolanda; Semple, Lynn; Esmail, Aliasgar; Pooran, Anil; Meldau, Richard; Davids, Malika; Dheda, Keertan; Tomasicchio, Michele] Univ Cape Town, South Africa MRC Ctr Study Antimicrobial Resistanc, ZA-7925 Cape Town, South Africa.

[Dheda, Keertan] Univ Cape Town, Inst Infect Dis & Mol Med, ZA-7925 Cape Town, South Africa.

[Dheda, Keertan] London Sch Hyg & Trop Med, Fac Infect & Trop Dis, Dept Immunol & Infect, London WC1E 7HT, England.

C3 University of Cape Town; University of Cape Town; University of Cape

Town; University of Cape Town; University of London; London School of

Hygiene & Tropical Medicine

RP Dheda, K (corresponding author), Univ Cape Town, Ctr Lung Infect & Immun, Dept Med, Div Pulmonol, ZA-7925 Cape Town, South Africa.; Dheda, K (corresponding author), UCT Lung Inst, ZA-7925 CAPE TOWN, South Africa.; Dheda, K (corresponding author), Univ Cape Town, South Africa MRC Ctr Study Antimicrobial Resistanc, ZA-7925 Cape Town, South Africa.; Dheda, K (corresponding author), Univ Cape Town, Inst Infect Dis & Mol Med, ZA-7925 Cape Town, South Africa.; Dheda, K (corresponding author), London Sch Hyg & Trop Med, Fac Infect & Trop Dis, Dept Immunol & Infect, London WC1E 7HT, England.

EM keertan.dheda@uct.ac.za

RI Esmail, Aliasgar/LJR-9915-2024

OI Esmail, Aliasgar/0000-0002-1835-9252; Tomasicchio,

Michele/0000-0003-2421-7215; Dheda, Keertan/0000-0001-7709-5341

FU Technology and Human Resources for Industry Programme (THRIP) and the

National Research Foundation (NRF) [TP14082193419]; Technology and Human

Resources for Industry Programme (THRIP); National Research Foundation

(NRF)

FX Funding (TP14082193419) was received from the Technology and Human

Resources for Industry Programme (THRIP) and the National Research

Foundation (NRF).

CR Afkhami S, 2020, FRONT IMMUNOL, V11, DOI 10.3389/fimmu.2020.557809

Ahonen CL, 1999, CELL IMMUNOL, V197, P62, DOI 10.1006/cimm.1999.1555

Anguille S, 2015, PHARMACOL REV, V67, P731, DOI 10.1124/pr.114.009456

[Anonymous], 2014, WHO Global Tuberculosis Report 2014

[Anonymous], 2018, The Global Tuberculosis Report

Arlehamn CSL, 2016, PLOS PATHOG, V12, DOI 10.1371/journal.ppat.1005760

Arlehamn CSL, 2015, P NATL ACAD SCI USA, V112, pE147, DOI 10.1073/pnas.1416537112

Babaki MKZ, 2017, MICROB PATHOGENESIS, V112, P20, DOI 10.1016/j.micpath.2017.08.040

Balan S, 2019, INT REV CEL MOL BIO, V348, P1, DOI 10.1016/bs.ircmb.2019.07.004

Beveridge NER, 2007, EUR J IMMUNOL, V37, P3089, DOI 10.1002/eji.200737504

Billeskov R, 2007, J IMMUNOL, V179, P3973, DOI 10.4049/jimmunol.179.6.3973

Brandt L, 2000, INFECT IMMUN, V68, P791, DOI 10.1128/IAI.68.2.791-795.2000

Brodin P, 2004, TRENDS MICROBIOL, V12, P500, DOI 10.1016/j.tim.2004.09.007

Caccamo N, 2010, EUR J IMMUNOL, V40, P2211, DOI 10.1002/eji.201040455

Carreno BM, 2013, J CLIN INVEST, V123, P3383, DOI 10.1172/JCI68395

Chaitra MG, 2008, MICROBES INFECT, V10, P858, DOI 10.1016/j.micinf.2008.04.017

Chaitra MG, 2008, J MED MICROBIOL, V57, P1079, DOI 10.1099/jmm.0.47565-0

Chaitra MG, 2008, SCAND J IMMUNOL, V67, P411, DOI 10.1111/j.1365-3083.2008.02078.x

Chemnitz JM, 2004, J IMMUNOL, V173, P945, DOI 10.4049/jimmunol.173.2.945

Cohen I, 2014, FRONT IMMUNOL, V5, DOI 10.3389/fimmu.2014.00236

Collin M, 2018, IMMUNOLOGY, V154, P3, DOI 10.1111/imm.12888

Collin M, 2013, IMMUNOLOGY, V140, P22, DOI 10.1111/imm.12117

Constantino J, 2017, IMMUNOL RES, V65, P798, DOI 10.1007/s12026-017-8931-1

Counoupas C, 2019, IMMUNOL CELL BIOL, V97, P656, DOI 10.1111/imcb.12275

Curtsinger JM, 2003, J IMMUNOL, V171, P5165, DOI 10.4049/jimmunol.171.10.5165

Dai FY, 2017, HUM VACC IMMUNOTHER, V13, P1425, DOI 10.1080/21645515.2017.1279771

Davids M, 2018, AM J RESP CRIT CARE, V198, P104, DOI 10.1164/rccm.201707-1441OC

de Vries IJM, 2005, NAT BIOTECHNOL, V23, P1407, DOI 10.1038/nbt1154

de Vries IJM, 2003, CANCER RES, V63, P12

DeBenedette MA, 2011, J IMMUNOTHER, V34, P45, DOI 10.1097/CJI.0b013e3181fb651a

Delogu G, 2001, INFECT IMMUN, V69, P5606, DOI 10.1128/IAI.69.9.5606-5611.2001

Dheda K, 2017, CLIN MICROBIOL INFEC, V23, P131, DOI 10.1016/j.cmi.2016.10.008

Dheda K, 2017, LANCET RESP MED, V5, P269, DOI [10.1016/S2213-2600(16)30433-7, 10.1016/s2213-2600(16)30433-7]

Domingo-Gonzalez R, 2016, MICROBIOL SPECTR, V4, DOI 10.1128/microbiolspec.TBTB2-0018-2016

Gastelum-Aviña P, 2015, EXPERT REV VACCINES, V14, P699, DOI 10.1586/14760584.2015.1015995

Golby P, 2007, MICROBIOL-SGM, V153, P3323, DOI 10.1099/mic.0.2007/009894-0

Hervas-Stubbs S, 2006, INFECT IMMUN, V74, P3396, DOI 10.1128/IAI.02086-05

Heufler C, 1996, EUR J IMMUNOL, V26, P659, DOI 10.1002/eji.1830260323

Huang H, 2020, NAT BIOTECHNOL, V38, P1194, DOI 10.1038/s41587-020-0505-4

Kamath AB, 2004, J EXP MED, V200, P1479, DOI 10.1084/jem.20041690

Kaufmann SHE, 2014, LANCET RESP MED, V2, P301, DOI 10.1016/S2213-2600(14)70033-5

Kim YR, 2017, J MICROBIOL BIOTECHN, V27, P1549, DOI 10.4014/jmb.1705.05032

Koh KW, 2009, INFECT IMMUN, V77, P3337, DOI 10.1128/IAI.01175-08

Kolloli A, 2017, FRONT MED-LAUSANNE, V4, DOI 10.3389/fmed.2017.00171

Lindenstrom T, 2009, J IMMUNOL, V182, P8047, DOI 10.4049/jimmunol.0801592

Lotze MT, 1997, ANN SURG, V226, P1, DOI 10.1097/00000658-199707000-00001

MacLeod MKL, 2009, SEMIN IMMUNOL, V21, P53, DOI 10.1016/j.smim.2009.02.006

Målen H, 2010, BMC MICROBIOL, V10, DOI 10.1186/1471-2180-10-132

Moguche AO, 2017, CELL HOST MICROBE, V21, P695, DOI 10.1016/j.chom.2017.05.012

Moliva Juan I, 2017, Front Immunol, V8, P407, DOI [10.3389/fimmu.2017.00407, 10.3389/fimmu.2017.00407]

Musvosvi M., 2015, H1:IC31

Napolitani G, 2005, NAT IMMUNOL, V6, P769, DOI 10.1038/ni1223

Okada H, 2011, J CLIN ONCOL, V29, P330, DOI 10.1200/JCO.2010.30.7744

Peng Q, 2020, NAT COMMUN, V11, DOI 10.1038/s41467-020-18570-x

Pietersen E, 2014, LANCET, V383, P1230, DOI 10.1016/S0140-6736(13)62675-6

Ravn P, 1999, J INFECT DIS, V179, P637, DOI 10.1086/314640

Rehren G, 2007, TUBERCULOSIS, V87, P347, DOI 10.1016/j.tube.2007.02.004

Sayes F, 2012, CELL HOST MICROBE, V11, P352, DOI 10.1016/j.chom.2012.03.003

Sayes F, 2016, PLOS PATHOG, V12, DOI 10.1371/journal.ppat.1005770

Schildberg FA, 2016, IMMUNITY, V44, P955, DOI 10.1016/j.immuni.2016.05.002

Scriba TJ, 2020, SEMIN IMMUNOL, V50, DOI 10.1016/j.smim.2020.101431

Scriba TJ, 2010, EUR J IMMUNOL, V40, P279, DOI 10.1002/eji.200939754

Shah NS, 2017, NEW ENGL J MED, V376, P243, DOI 10.1056/NEJMoa1604544

Skjot RLV, 2002, INFECT IMMUN, V70, P5446, DOI 10.1128/IAI.70.10.5446-5453.2002

Skjot RLV, 2000, INFECT IMMUN, V68, P214, DOI 10.1128/IAI.68.1.214-220.2000

Sparrow E, 2020, IMMUNOL LETT, V217, P126, DOI 10.1016/j.imlet.2019.11.005

Tameris M, 2014, PLOS ONE, V9, DOI 10.1371/journal.pone.0087340

Tena-Coki NG, 2010, AM J RESP CRIT CARE, V182, P120, DOI 10.1164/rccm.200912-1862OC

Tiberi S, 2018, LANCET INFECT DIS, V18, pE183, DOI 10.1016/S1473-3099(18)30110-5

Tomasicchio M, 2019, J INFECT DIS, V219, P1329, DOI 10.1093/infdis/jiy657

Tomasicchio M, 2019, CANCER IMMUNOL IMMUN, V68, P71, DOI 10.1007/s00262-018-2238-5

Ulrichs T, 1998, EUR J IMMUNOL, V28, P3949, DOI 10.1002/(SICI)1521-4141(199812)28:12<3949::AID-IMMU3949>3.3.CO;2-W

Vordermeier HM, 2012, PLOS ONE, V7, DOI 10.1371/journal.pone.0040890

Walch M, 2014, CELL, V157, P1309, DOI 10.1016/j.cell.2014.03.062

Winslow GM, 2008, IMMUNOL REV, V225, P284, DOI 10.1111/j.1600-065X.2008.00693.x

NR 75

TC 0

Z9 0

U1 0

U2 1

PU MDPI

PI BASEL

PA MDPI AG, Grosspeteranlage 5, CH-4052 BASEL, SWITZERLAND

EI 2076-2607

J9 MICROORGANISMS

JI Microorganisms

PD FEB

PY 2025

VL 13

IS 2

AR 345

DI 10.3390/microorganisms13020345

PG 31

WC Microbiology

WE Science Citation Index Expanded (SCI-EXPANDED)

SC Microbiology

GA Y3B2G

UT WOS:001430913800001

OA Green Submitted, gold

DA 2026-05-07

ER

PT J

AU Margaryan, H

Evangelopoulos, DD

Wildner, LM

McHugh, TD

AF Margaryan, Hasmik

Evangelopoulos, Dimitrios D.

Wildner, Leticia Muraro

McHugh, Timothy D.

TI Pre-Clinical Tools for Predicting Drug Efficacy in Treatment of

Tuberculosis

SO MICROORGANISMS

LA English

DT Article

DE tuberculosis; drug activity; in vitro preclinical modelling; MDR-TB;

synergism; transcriptomics; high order combinations; drug combinations;

drug efficacy

ID PARA-AMINOSALICYLIC ACID; IN-VITRO ACTIVITY; MYCOBACTERIUM-TUBERCULOSIS;

CROSS-RESISTANCE; PYRAZINAMIDE RESISTANCE; ANTITUBERCULOUS DRUGS;

GENE-MUTATIONS; ATP SYNTHASE; XDR-TB; CLOFAZIMINE

AB Combination therapy has, to some extent, been successful in limiting the emergence of drug-resistant tuberculosis. Drug combinations achieve this advantage by simultaneously acting on different targets and metabolic pathways. Additionally, drug combination therapies are shown to shorten the duration of therapy for tuberculosis. As new drugs are being developed, to overcome the challenge of finding new and effective drug combinations, systems biology commonly uses approaches that analyse mycobacterial cellular processes. These approaches identify the regulatory networks, metabolic pathways, and signaling programs associated with M. tuberculosis infection and survival. Different preclinical models that assess anti-tuberculosis drug activity are available, but the combination of models that is most predictive of clinical treatment efficacy remains unclear. In this structured literature review, we appraise the options to accelerate the TB drug development pipeline through the evaluation of preclinical testing assays of drug combinations.

C1 [Margaryan, Hasmik; Wildner, Leticia Muraro; McHugh, Timothy D.] UCL, Div Infect & Immun, UCL Ctr Clin Microbiol, Royal Free Campus, London NW3 2PF, England.

[Evangelopoulos, Dimitrios D.] UCL, Dept Microbial Dis, Eastman Dent Inst, Royal Free Campus,Rowland Hill St, London NW3 2PF, England.

C3 University of London; University College London; University of London;

University College London

RP Margaryan, H (corresponding author), UCL, Div Infect & Immun, UCL Ctr Clin Microbiol, Royal Free Campus, London NW3 2PF, England.

EM h.margaryan@ucl.ac.uk; d.evangelopoulos@ucl.ac.uk; l.wildner@ucl.ac.uk;

t.mchugh@ucl.ac.uk

RI Evangelopoulos, Dimitrios/F-8251-2013; McHugh, Timothy D/O-3683-2019

OI Evangelopoulos, Dimitrios/0000-0003-0472-7576; McHugh, Timothy

D/0000-0003-4658-8594; Muraro Wildner, Leticia/0000-0003-2553-3244

CR Andries K, 2005, SCIENCE, V307, P223, DOI 10.1126/science.1106753

[Anonymous], 2018, Global tuberculosis report 2018

[Anonymous], 2014, March 2014 supplement to the 2013 consolidated guidelines on the use of antiretroviral drugs for treating and preventing HIV infections: Recommendations for a public health approach

Aubry A, 2004, ANTIMICROB AGENTS CH, V48, P1281, DOI 10.1128/AAC.48.4.1281-1288.2004

Balasubramanian V, 2014, ANTIMICROB AGENTS CH, V58, P495, DOI 10.1128/AAC.01903-13

Bax HI, 2017, TUBERCULOSIS, V105, P80, DOI 10.1016/j.tube.2017.04.010

Beckert P, 2012, ANTIMICROB AGENTS CH, V56, P2743, DOI 10.1128/AAC.06227-11

Bergmann JS, 1998, INT J TUBERC LUNG D, V2, P621

Berry C, 2016, EUR RESPIR J, V47, P1591, DOI 10.1183/13993003.01646-2015

Bonapace CR, 2002, DIAGN MICR INFEC DIS, V44, P363, DOI 10.1016/S0732-8893(02)00473-X

Borrell S, 2019, PLOS ONE, V14, DOI 10.1371/journal.pone.0214088

Caleffi-Ferracioli KR, 2013, TUBERCULOSIS, V93, P660, DOI 10.1016/j.tube.2013.09.001

Carette X, 2012, NUCLEIC ACIDS RES, V40, P3018, DOI 10.1093/nar/gkr1113

Chandrasekaran S, 2010, P NATL ACAD SCI USA, V107, P17845, DOI 10.1073/pnas.1005139107

Cholo MC, 2017, J ANTIMICROB CHEMOTH, V72, P338, DOI 10.1093/jac/dkw426

Cokol M, 2017, SCI ADV, V3, DOI 10.1126/sciadv.1701881

Cokol-Cakmak M, 2018, JOVE-J VIS EXP, DOI 10.3791/57713

Conradie F, 2020, NEW ENGL J MED, V382, P893, DOI 10.1056/NEJMoa1901814

D'Ambrosio L, 2015, INT J INFECT DIS, V32, P156, DOI 10.1016/j.ijid.2014.12.024

Santos NCD, 2018, TUBERCULOSIS, V111, P41, DOI 10.1016/j.tube.2018.05.005

Deb Uttiya, 2021, Indian J Tuberc, V68, P287, DOI [10.1016/j.ijtb.2020.09.003, 10.1016/j.ijtb.2020.09.003]

den Hollander JG, 1998, ANTIMICROB AGENTS CH, V42, P744, DOI 10.1128/AAC.42.4.744

Dhar N, 2007, CURR OPIN MICROBIOL, V10, P30, DOI 10.1016/j.mib.2006.12.007

Drusano GL, 2014, PLOS ONE, V9, DOI 10.1371/journal.pone.0101311

Ekins S, 2014, J CHEM INF MODEL, V54, P1070, DOI 10.1021/ci500077v

Gillespie SH, 2014, NEW ENGL J MED, V371, P1577, DOI 10.1056/NEJMoa1407426

Gomez JE, 2004, TUBERCULOSIS, V84, P29, DOI 10.1016/j.tube.2003.08.003

Grant SS, 2016, CELL CHEM BIOL, V23, P666, DOI 10.1016/j.chembiol.2016.05.011

Hartkoorn RC, 2014, ANTIMICROB AGENTS CH, V58, P2979, DOI 10.1128/AAC.00037-14

Haver HL, 2015, ANTIMICROB AGENTS CH, V59, P5316, DOI 10.1128/AAC.00308-15

Huitric E, 2010, ANTIMICROB AGENTS CH, V54, P1022, DOI 10.1128/AAC.01611-09

Juréen P, 2008, ANTIMICROB AGENTS CH, V52, P1852, DOI 10.1128/AAC.00110-08

Koul A, 2007, NAT CHEM BIOL, V3, P323, DOI 10.1038/nchembio884

Lee BY, 2017, NAT COMMUN, V8, DOI 10.1038/ncomms14183

Li GL, 2019, INT J ANTIMICROB AG, V54, P642, DOI 10.1016/j.ijantimicag.2019.06.002

Lienhardt C., 2013, USE BEDAQUILINE TREA

López-Gavin A, 2015, INT J ANTIMICROB AG, V46, P582, DOI 10.1016/j.ijantimicag.2015.08.004

Louie A, 2018, ANTIMICROB AGENTS CH, V62, DOI [10.1128/AAC.01470-18, 10.1128/aac.01470-18]

Ma S, 2019, MBIO, V10, DOI 10.1128/mBio.02627-19

Ma SY, 2015, PLOS COMPUT BIOL, V11, DOI 10.1371/journal.pcbi.1004543

Flaviane MG, 2017, TUBERCULOSIS, V104, P24, DOI 10.1016/j.tube.2017.02.004

Manjunatha Ujjini, 2009, Commun Integr Biol, V2, P215

Massengo-Tiassé RP, 2009, CELL MOL LIFE SCI, V66, P1507, DOI 10.1007/s00018-009-8704-7

Mathys V, 2009, ANTIMICROB AGENTS CH, V53, P2100, DOI 10.1128/AAC.01197-08

Njire M, 2016, ADV MED SCI-POLAND, V61, P63, DOI 10.1016/j.advms.2015.09.007

Nosova EY, 2013, J MED MICROBIOL, V62, P108, DOI 10.1099/jmm.0.046821-0

Nunn AJ, 2019, NEW ENGL J MED, V380, P1201, DOI 10.1056/NEJMoa1811867

Nyang'wa BT, 2021, J INT AIDS SOC, V24, P70

Page MJ, 2021, REV ESP CARDIOL, V74, P790, DOI 10.1016/j.recesp.2021.06.016

Pang Y, 2019, DIAGN MICR INFEC DIS, V94, P361, DOI 10.1016/j.diagmicrobio.2019.02.012

Peterson EJR, 2016, NAT MICROBIOL, V1, DOI [10.1038/NMICROBIOL.2016.78, 10.1038/nmicrobiol.2016.78]

Peterson EJR, 2014, NUCLEIC ACIDS RES, V42, P11291, DOI 10.1093/nar/gku777

Pullan ST, 2016, BMC INFECT DIS, V16, DOI 10.1186/s12879-016-1533-z

Ramaswamy SV, 2003, ANTIMICROB AGENTS CH, V47, P1241, DOI 10.1128/AAC.47.4.1241-1250.2003

Reeves AZ, 2013, ANTIMICROB AGENTS CH, V57, P1857, DOI 10.1128/AAC.02191-12

Rengarajan J, 2004, MOL MICROBIOL, V53, P275, DOI 10.1111/j.1365-2958.2004.04120.x

Rey-Jurado E, 2012, TUBERCULOSIS, V92, P260, DOI 10.1016/j.tube.2012.01.005

Rueda J, 2015, ANTIMICROB AGENTS CH, V59, P7805, DOI 10.1128/AAC.01028-15

Sadouki Z, 2021, J ANTIMICROB CHEMOTH, V76, P2252, DOI 10.1093/jac/dkab160

Safi H, 2013, NAT GENET, V45, P1190, DOI 10.1038/ng.2743

Sharma D, 2007, J MOL BIOL, V374, P1065, DOI 10.1016/j.jmb.2007.10.003

Shetye GS, 2020, TRANSL RES, V220, P68, DOI 10.1016/j.trsl.2020.03.007

Silva CD, 2018, ANTIMICROB AGENTS CH, V62, DOI [10.1128/aac.00856-18, 10.1128/aac.00856-18, 10.1128/AAC.00856-18;e00856-18]

Silva CD, 2019, ANTIMICROB AGENTS CH, V63, DOI 10.1128/AAC.01695-18

Silva DR, 2018, J BRAS PNEUMOL, V44, P153, DOI [10.1590/s1806-37562017000000436, 10.1590/S1806-37562017000000436]

Smith T, 2014, CURR TOP MICROBIOL, V374, P53, DOI 10.1007/82_2012_279

Sotgiu G, 2012, EUR RESPIR J, V40, P1430, DOI 10.1183/09031936.00022912

Sowajassatakul A, 2014, BMC MICROBIOL, V14, DOI 10.1186/1471-2180-14-165

Springer B, 2001, ANTIMICROB AGENTS CH, V45, P2877, DOI 10.1128/AAC.45.10.2877-2884.2001

Swaney SM, 1998, ANTIMICROB AGENTS CH, V42, P3251, DOI 10.1128/AAC.42.12.3251

Thirumurugan R, 2015, J INFECT PUBLIC HEAL, V8, P619, DOI 10.1016/j.jiph.2015.05.003

Vilchèze C, 2006, NAT MED, V12, P1027, DOI 10.1038/nm1466

WHO, 2012, GLOBAL TUBERCULOSIS REPORT 2012, P1

WHO, 2020, MOD 4 TREATM DRUG RE

World Health O, 2021, Global tuberculosis report 2021

Yilancioglu K, 2019, SCI REP-UK, V9, DOI 10.1038/s41598-019-48410-y

Zhang QQ, 2019, FRONT CHEM, V7, DOI 10.3389/fchem.2019.00819

Zhang ZJ, 2015, INT J ANTIMICROB AG, V45, P71, DOI 10.1016/j.ijantimicag.2014.09.012

Zhao F, 2014, ANTIMICROB AGENTS CH, V58, P1479, DOI 10.1128/AAC.01775-13

Zhao WJ, 2016, INT J INFECT DIS, V52, P23, DOI 10.1016/j.ijid.2016.08.027

Zhu C, 2018, J BIOL CHEM, V293, P16741, DOI 10.1074/jbc.RA118.002693

Zimmer A, 2016, P NATL ACAD SCI USA, V113, P10442, DOI 10.1073/pnas.1606301113

Zumla A, 2013, NAT REV DRUG DISCOV, V12, P388, DOI 10.1038/nrd4001

NR 83

TC 5

Z9 6

U1 0

U2 17

PU MDPI

PI BASEL

PA ST ALBAN-ANLAGE 66, CH-4052 BASEL, SWITZERLAND

EI 2076-2607

J9 MICROORGANISMS

JI Microorganisms

PD MAR

PY 2022

VL 10

IS 3

AR 514

DI 10.3390/microorganisms10030514

PG 17

WC Microbiology

WE Science Citation Index Expanded (SCI-EXPANDED)

SC Microbiology

GA 0A7WS

UT WOS:000774160400001

OA Green Submitted, gold

DA 2026-05-07

ER

PT J

AU Shaikh, M

Patel, H

AF Shaikh, Matin

Patel, Harun

TI Structural toxicity relationship (STR) of linezolid to mitigate

myelosuppression and serotonergic toxicity

SO BIOORGANIC & MEDICINAL CHEMISTRY

LA English

DT Review

DE Linezolid; Structural toxicity relationship; Serotonergic toxicity;

Myelosuppression

ID DRUG-RESISTANT TUBERCULOSIS; MITOCHONDRIAL PROTEIN-SYNTHESIS;

MULTIDRUG-RESISTANT; ANTIBACTERIAL AGENTS; BIOLOGICAL EVALUATION;

LACTIC-ACIDOSIS; OXAZOLIDINONES; INHIBITION; SAFETY; SERIES

AB Tuberculosis (TB) remains a significant global health challenge, with multidrug-resistant (MDR-TB) and extensively drug-resistant (XDR-TB) strains posing severe threats to treatment efficacy. Linezolid, a key component of the BPaL (Bedaquiline, Pretomanid and Linezolid) regimen, has demonstrated substantial efficacy against MDRTB and XDR-TB. However, its clinical utility is often limited by side effects such as myelosuppression and monoamine oxidase (MAO) inhibition, linked to its mechanism of action. This perspective centres on the structural toxicity relationship (STR) of Linezolid and its analogues, exploring modifications to the C-ring and C-5 position that aim to reduce these toxicities while maintaining or enhancing antibacterial activity. Several promising analogues have been identified that exhibit reduced myelosuppression and MAO inhibition, highlighting the potential for developing safer Linezolid derivatives. The findings underscore the importance of continued research into the structure toxicity relationships of oxazolidinones to improve the therapeutic profiles of these essential drugs in combating drug-resistant TB.

C1 [Shaikh, Matin; Patel, Harun] RC Patel Inst Pharmaceut Educ & Res, Dept Pharmaceut Chem, Div Comp Aided Drug Design, Dhule 425405, Maharashtra, India.

[Shaikh, Matin] HR Patel Inst Pharmaceut Educ & Res, Dept Pharmaceut Chem, Dhule 425405, Maharashtra, India.

RP Patel, H (corresponding author), RC Patel Inst Pharmaceut Educ & Res, Dept Pharmaceut Chem, Div Comp Aided Drug Design, Dhule 425405, Maharashtra, India.

EM skm00707@gmail.com; hpatel_38@yahoo.com

RI Patel, Harun/L-7942-2019

FU Kavayitri Bahinabai Chaudhari North Maharashtra University, Jalgaon

[KBCNMU/11A/VCRMS/2023-24/Revised-351/2023]

FX MSS. would like to thank Kavayitri Bahinabai Chaudhari North Maharashtra

University, Jalgaon for providing financial assistance for research

project under the Vice Chancellor Research Motivation Scheme (VCRMS)

(Grant No. KBCNMU/11A/VCRMS/2023-24/Revised-351/2023) .

CR Ampomah-Wireko M, 2024, EUR J MED CHEM, V269, DOI 10.1016/j.ejmech.2024.116326

[Anonymous], 2023, Global tuberculosis report 2023

[Anonymous], 2022, Global tuberculosis report (research rep.)

Bahuguna A, 2020, MED RES REV, V40, P263, DOI 10.1002/med.21602

Barry CE, 2010, CURR OPIN CHEM BIOL, V14, P456, DOI 10.1016/j.cbpa.2010.04.008

Bhattarai D, 2014, CHEM PHARM BULL, V62, P1214, DOI 10.1248/cpb.c14-00510

Brickner SJ, 2008, J MED CHEM, V51, P1981, DOI 10.1021/jm800038g

Chen RH, 2024, PHARMACEUTICS, V16, DOI 10.3390/pharmaceutics16060818

Conradie F, 2020, NEW ENGL J MED, V382, P893, DOI 10.1056/NEJMoa1901814

Cox H, 2012, INT J TUBERC LUNG D, V16, P447, DOI 10.5588/ijtld.11.0451

De Vriese AS, 2006, CLIN INFECT DIS, V42, P1111, DOI 10.1086/501356

Du J, 2021, Front Microbiol, V12, P1

Ferrazzano L, 2016, EUR J MED CHEM, V124, P906, DOI 10.1016/j.ejmech.2016.09.007

Foti C, 2021, MOLECULES, V26, DOI 10.3390/molecules26144280

Gil CM, 2022, BREATHE, V18, DOI 10.1183/20734735.0149-2021

Girase R, 2024, J BIOMOL STRUCT DYN, V42, P2111, DOI 10.1080/07391102.2023.2203254

Girase RT, 2024, ACS MED CHEM LETT, V15, P924, DOI 10.1021/acsmedchemlett.4c00114

Gordeev MF, 2014, J MED CHEM, V57, P4487, DOI 10.1021/jm401931e

Hauck SI, 2007, BIOORG MED CHEM LETT, V17, P337, DOI 10.1016/j.bmcl.2006.10.063

Hou YL, 2019, BIOORG MED CHEM LETT, V29, DOI 10.1016/j.bmcl.2019.126746

Hoy SM, 2021, DRUGS, V81, P1587, DOI 10.1007/s40265-021-01576-0

Kumar A, 2022, EUR J MED CHEM, V242, DOI 10.1016/j.ejmech.2022.114655

Lawrence KR, 2006, CLIN INFECT DIS, V42, P1578, DOI 10.1086/503839

Leach KL, 2007, MOL CELL, V26, P393, DOI 10.1016/j.molcel.2007.04.005

Leach KL, 2011, ANN NY ACAD SCI, V1222, P49, DOI 10.1111/j.1749-6632.2011.05962.x

Lifan Z, 2019, INT J TUBERC LUNG D, V23, P1293, DOI 10.5588/ijtld.18.0822

Liu JQ, 2022, ACS MED CHEM LETT, V13, P1030, DOI 10.1021/acsmedchemlett.2c00191

Mahdi AS, 2023, Pharmacology &amp Pharmacy, V14, P19, DOI [10.4236/pp.2023.141002, 10.4236/pp.2023.141002, DOI 10.4236/PP.2023.141002]

Malik MS, 2023, PHARMACEUTICALS-BASE, V16, DOI 10.3390/ph16040516

Masbough F, 2022, CLIN CASE REP, V10, DOI 10.1002/ccr3.6341

McKee EE, 2006, ANTIMICROB AGENTS CH, V50, P2042, DOI 10.1128/AAC.01411-05

Michalska K, 2013, BIOORGAN MED CHEM, V21, P577, DOI 10.1016/j.bmc.2012.11.036

Milosevic TV, 2018, ANTIMICROB AGENTS CH, V62, DOI 10.1128/AAC.01599-17

Mondoni M, 2021, CURR OPIN PHARMACOL, V59, P103, DOI 10.1016/j.coph.2021.05.007

Narita M, 2007, PHARMACOTHERAPY, V27, P1189, DOI 10.1592/phco.27.8.1189

Natarajan Arvind, 2020, Indian J Tuberc, V67, P295, DOI [10.1016/j.ijtb.2020.02.005, 10.1016/j.ijtb.2020.02.005]

Oehadian A, 2022, TUBERC RESP DIS, V85, P111, DOI 10.4046/trd.2021.0122

Patel H, 2020, Reviews and Advances in Chemistry, V10, P112, DOI 10.1134/s2079978020030048

Phillips OA, 2015, EUR J MED CHEM, V106, P120, DOI 10.1016/j.ejmech.2015.10.025

Phillips OA, 2015, EUR J PHARM SCI, V71, P56, DOI 10.1016/j.ejps.2015.02.006

Piccionello AP, 2012, EUR J MED CHEM, V50, P441, DOI 10.1016/j.ejmech.2012.02.002

Poel TJ, 2007, J MED CHEM, V50, P5886, DOI 10.1021/jm070708p

Quinn Davin K, 2009, Prim Care Companion J Clin Psychiatry, V11, P353, DOI 10.4088/PCC.09r00853

Rao GG, 2020, THER DRUG MONIT, V42, P83, DOI 10.1097/FTD.0000000000000710

Reck F, 2005, J MED CHEM, V48, P499, DOI 10.1021/jm0400810

Renslo AR, 2007, BIOORG MED CHEM LETT, V17, P5036, DOI 10.1016/j.bmcl.2007.07.022

Renslo AR, 2010, EXPERT REV ANTI-INFE, V8, P565, DOI 10.1586/ERI.10.26

Santini A, 2017, EXPERT OPIN DRUG SAF, V16, P833, DOI 10.1080/14740338.2017.1335305

Sciotti RJ, 2002, BIOORG MED CHEM LETT, V12, P2121, DOI 10.1016/S0960-894X(02)00352-9

Sharma A, 2021, J MED CHEM, V64, P4359, DOI 10.1021/acs.jmedchem.0c01833

Sharma SK, 2006, CHEST, V130, P261, DOI [10.1378/chest.130.1.261, 10.1016/S0012-3692(15)50981-1]

Shin HN, 2013, BIOORG MED CHEM LETT, V23, P1193, DOI 10.1016/j.bmcl.2013.01.033

Singh B, 2019, COCHRANE DB SYST REV, DOI 10.1002/14651858.CD012836.pub2

Soriano A, 2005, NEW ENGL J MED, V353, P2305, DOI 10.1056/NEJM200511243532123

Stevens Dennis L, 2004, Expert Rev Anti Infect Ther, V2, P51, DOI 10.1586/14787210.2.1.51

Stratton CW, 1997, Antimicrob Infect Dis Newsl, V16, P69

Sun AW, 2019, BIOORG MED CHEM LETT, V29, P2686, DOI 10.1016/j.bmcl.2019.07.024

Sutton Jacob, 2016, Proc (Bayl Univ Med Cent), V29, P214, DOI 10.1080/08998280.2016.11929423

Suzuki H, 2013, ACS MED CHEM LETT, V4, P1074, DOI 10.1021/ml400280z

Uplekar M, 2015, LANCET, V385, P1799, DOI 10.1016/S0140-6736(15)60570-0

Van Deun A, 2010, AM J RESP CRIT CARE, V182, P684, DOI 10.1164/rccm.201001-0077OC

Vinh DC, 2009, J INFECTION, V59, pS59, DOI 10.1016/S0163-4453(09)60009-8

Wang W, 2021, CHEM RES TOXICOL, V34, P1348, DOI 10.1021/acs.chemrestox.0c00524

WHO consolidated guidelines on tuberculosis, 2022, MODULE 4 TREATMENT D

Woytowish MR, 2013, ANN PHARMACOTHER, V47, P388, DOI 10.1345/aph.1R386

Xu J, 2019, ANTIMICROB AGENTS CH, V63, DOI [10.1128/AAC.00021-19, 10.1128/aac.00021-19]

Zhang Y, 2012, ANTIBIOTIC DISCOVERY AND DEVELOPMENT, VOLS 1 AND 2, P719, DOI 10.1007/978-1-4614-1400-1_22

Zhao QQ, 2021, J MED CHEM, V64, P10557, DOI 10.1021/acs.jmedchem.1c00480

NR 68

TC 4

Z9 4

U1 1

U2 6

PU PERGAMON-ELSEVIER SCIENCE LTD

PI OXFORD

PA THE BOULEVARD, LANGFORD LANE, KIDLINGTON, OXFORD OX5 1GB, ENGLAND

SN 0968-0896

EI 1464-3391

J9 BIOORGAN MED CHEM

JI Bioorg. Med. Chem.

PD FEB 1

PY 2025

VL 118

AR 118025

DI 10.1016/j.bmc.2024.118025

EA DEC 2024

PG 18

WC Biochemistry & Molecular Biology; Chemistry, Medicinal; Chemistry,

Organic

WE Science Citation Index Expanded (SCI-EXPANDED)

SC Biochemistry & Molecular Biology; Pharmacology & Pharmacy; Chemistry

GA O8J2G

UT WOS:001373516100001

DA 2026-05-07

ER

PT J

AU Lee, JH

Ammerman, NC

Nolan, S

Geiman, DE

Lun, S

Guo, HD

Bishai, WR

AF Lee, Jong-Hee

Ammerman, Nicole C.

Nolan, Scott

Geiman, Deborah E.

Lun, Shichun

Guo, Haidan

Bishai, William R.

TI Isoniazid resistance without a loss of fitness in Mycobacterium

tuberculosis

SO NATURE COMMUNICATIONS

LA English

DT Article

ID MULTIDRUG-RESISTANT; CATALASE-PEROXIDASE; DRUG-RESISTANCE; SIGMA-FACTOR;

EXPRESSION; VIRULENCE; TARGET; GENE; INFECTIOUSNESS; MACROPHAGES

AB The emergence of multi- and extensively drug-resistant tuberculosis (MDR-TB and XDR-TB, respectively) has intensified the critical public health implications of this global disease. The fitness of Mycobacterium tuberculosis (M.tb.) strains exhibiting MDR and XDR phenotypes is of fundamental importance in predicting whether the MDR-/XDR-TB epidemic will be sustained across the human population. Here we describe a potential mechanism of M.tb. resistance to the TB drug isoniazid (INH) conferred by loss of a sigma factor, SigI. We demonstrate that the gain of INH resistance in the M.tb. Delta sigI mutant might not diminish the organism's fitness for causing disease. These findings have significant implications when considering the ability of drug-resistant M.tb. strains to initiate untreatable TB epidemics, as it is possible that loss or alteration of SigI function could have a role in the generation of MDR and XDR M.tb. strains of suitable fitness to spread in a community setting.

C1 [Lee, Jong-Hee; Ammerman, Nicole C.; Nolan, Scott; Geiman, Deborah E.; Lun, Shichun; Guo, Haidan; Bishai, William R.] Johns Hopkins Univ, Sch Med, Dept Med, Ctr TB Res, Baltimore, MD 21231 USA.

[Bishai, William R.] KwaZulu Natal Res Inst TB & HIV K RITH, ZA-4001 Durban, South Africa.

[Bishai, William R.] Howard Hughes Med Inst, Chevy Chase, MD 20815 USA.

C3 Johns Hopkins University; Howard Hughes Medical Institute

RP Bishai, WR (corresponding author), Johns Hopkins Univ, Sch Med, Dept Med, Ctr TB Res, Baltimore, MD 21231 USA.

EM wbishai@jhmi.edu

RI Ammerman, Nicole C/ACU-5022-2022; Lee, Jong-Hee/AAH-4129-2019

OI Ammerman, Nicole C/0000-0001-9359-9144; Lee,

Jong-Hee/0000-0001-9938-1306

FU Howard Hughes Medical Institute; National Institutes of Health

[AI036973, AI037856, AI079590]

FX We thank Andre Kubler and Mariama Maiga for their assistance in

processing experimental samples. We also thank Peter McCaffrey for his

assistance in assembling the figures. Support from the Howard Hughes

Medical Institute and the National Institutes of Health (awards

AI036973, AI037856 and AI079590) is gratefully acknowledged.

CR Agarwal N, 2009, NATURE, V460, P98, DOI 10.1038/nature08123

Ando H, 2011, MOL MICROBIOL, V79, P1615, DOI 10.1111/j.1365-2958.2011.07547.x

BANERJEE A, 1994, SCIENCE, V263, P227, DOI 10.1126/science.8284673

Blower SM, 2004, NAT MED, V10, P1111, DOI 10.1038/nm1102

Borrell S, 2009, INT J TUBERC LUNG D, V13, P1456

Cohen T, 2004, NAT MED, V10, P1117, DOI 10.1038/nm1110

Collins DM, 1996, TRENDS MICROBIOL, V4, P426, DOI 10.1016/0966-842X(96)10066-4

Davis JM, 2009, CELL, V136, P37, DOI 10.1016/j.cell.2008.11.014

DESSEN A, 1995, SCIENCE, V267, P1638, DOI 10.1126/science.7886450

Edgar R, 2002, NUCLEIC ACIDS RES, V30, P207, DOI 10.1093/nar/30.1.207

Gandhi NR, 2006, LANCET, V368, P1575, DOI 10.1016/S0140-6736(06)69573-1

Kaushal D, 2002, P NATL ACAD SCI USA, V99, P8330, DOI 10.1073/pnas.102055799

Kourbeti, 2000, Curr Infect Dis Rep, V2, P193, DOI 10.1007/s11908-000-0035-7

Lee JH, 2008, J BACTERIOL, V190, P699, DOI 10.1128/JB.01273-07

Manca C, 1999, INFECT IMMUN, V67, P74, DOI 10.1128/IAI.67.1.74-79.1999

Master S, 2001, J BACTERIOL, V183, P4033, DOI 10.1128/JB.183.13.4033-4039.2001

Ng VH, 2004, MOL MICROBIOL, V52, P1291, DOI 10.1111/j.1365-2958.2004.04078.x

Palmero D, 2002, MEDICINA-BUENOS AIRE, V62, P221

Pym AS, 2002, INFECT IMMUN, V70, P4955, DOI 10.1128/IAI.70.9.4955-4960.2002

Rozwarski DA, 1998, SCIENCE, V279, P98, DOI 10.1126/science.279.5347.98

Sachdeva P, 2010, FEBS J, V277, P605, DOI 10.1111/j.1742-4658.2009.07479.x

Tyagi R, 2004, BMC BIOTECHNOL, V4, DOI 10.1186/1472-6750-4-2

Vilchéze C, 2007, ANNU REV MICROBIOL, V61, P35, DOI 10.1146/annurev.micro.61.111606.122346

Volkman HE, 2010, SCIENCE, V327, P466, DOI 10.1126/science.1179663

Wirth T, 2008, PLOS PATHOG, V4, DOI 10.1371/journal.ppat.1000160

Zhang Y, 2009, INT J TUBERC LUNG D, V13, P1320

ZHANG Y, 1992, NATURE, V358, P591, DOI 10.1038/358591a0

NR 27

TC 33

Z9 37

U1 0

U2 8

PU NATURE PUBLISHING GROUP

PI LONDON

PA MACMILLAN BUILDING, 4 CRINAN ST, LONDON N1 9XW, ENGLAND

SN 2041-1723

J9 NAT COMMUN

JI Nat. Commun.

PD MAR

PY 2012

VL 3

AR 753

DI 10.1038/ncomms1724

PG 8

WC Multidisciplinary Sciences

WE Science Citation Index Expanded (SCI-EXPANDED)

SC Science & Technology - Other Topics

GA 923PB

UT WOS:000302630100042

OA Bronze

DA 2026-05-07

ER

PT J

AU Gao, XH

Feng, JH

Wei, LN

Dong, PZ

Chen, J

Zhang, LL

Yang, YH

Xu, L

Wang, HY

Luo, JM

Qin, M

AF Gao, Xuehan

Feng, Jihong

Wei, Linna

Dong, Pinzhi

Chen, Jin

Zhang, Langlang

Yang, Yuhan

Xu, Lin

Wang, Haiyan

Luo, Junmin

Qin, Ming

TI Defensins: A novel weapon against Mycobacterium

tuberculosis?

SO INTERNATIONAL IMMUNOPHARMACOLOGY

LA English

DT Article

DE Defensins; Mycobacterium tuberculosis; Tuberculosis; Macrophages;

Dendritic cells; Neutrophils

ID HUMAN NEUTROPHIL PEPTIDE-1; BETA-DEFENSIN; ANTIMICROBIAL PEPTIDES;

ALPHA-DEFENSIN; HOST-DEFENSE; CELL-WALL; DENDRITIC CELLS;

THETA-DEFENSIN; T-CELLS; ANTIBIOTIC ACTIVITIES

AB Tuberculosis (TB) is a serious airborne communicable disease caused by organisms of the Mycobacterium tuberculosis (Mtb) complex. Although the standard treatment antimicrobials, including isoniazid, rifampicin, pyrazinamide, and ethambutol, have made great progress in the treatment of TB, problems including the rising incidence of multidrug-resistant tuberculosis (MDR-TB) and extensively drug-resistant tuberculosis (XDR-TB), the severe toxicity and side effects of antimicrobials, and the low immunity of TB patients have become the bottlenecks of the current TB treatments. Therefore, both safe and effective new strategies to prevent and treat TB have become a top priority. As a subfamily of cationic antimicrobial peptides, defensins are rich in cysteine and play a vital role in resisting the invasion of microorganisms and regulating the immune response. Inspired by studies on the roles of defensins in host defence, we describe their research history and then review their structural features and antimicrobial mechanisms, specifically for fighting Mtb in detail. Finally, we discuss the clinical relevance, therapeutic potential, and potential challenges of defensins in anti-TB therapy. We further debate the possible solutions of the current application of defensins to provide new insights for eliminating Mtb.

C1 [Gao, Xuehan; Wei, Linna; Dong, Pinzhi; Chen, Jin; Zhang, Langlang; Yang, Yuhan; Xu, Lin; Luo, Junmin; Qin, Ming] Zunyi Med Univ, Ctr Immunomol Engn Innovat & Practice Base Grad St, Dept Immunol, Special Key Lab Gene Detect & Therapy, Zunyi 563000, Guizhou, Peoples R China.

[Feng, Jihong] Wenzhou Med Univ, Lishui Peoples Hosp, Dept Oncol, Affiliated Hosp 6, Lishui 323000, Peoples R China.

[Wang, Haiyan] Zunyi Med Univ, Dept Epidemiol & Hlth Stat, Zunyi 563000, Guizhou, Peoples R China.

[Qin, Ming] Zunyi Med Univ, Key Lab Basic Pharmacol, Minist Educ, Zunyi 563000, Guizhou, Peoples R China.

[Qin, Ming] Zunyi Med Univ, Joint Int Res Lab Ethnomed, Minist Educ, Zunyi 563000, Guizhou, Peoples R China.

[Luo, Junmin; Qin, Ming] Zunyi Med Univ, Dept Immunol, Zunyi 563000, Guizhou, Peoples R China.

C3 Zunyi Medical University; Wenzhou Medical University; Zunyi Medical

University; Zunyi Medical University; Zunyi Medical University; Zunyi

Medical University

RP Luo, JM; Qin, M (corresponding author), Zunyi Med Univ, Dept Immunol, Zunyi 563000, Guizhou, Peoples R China.

EM luojm16128@163.com; 158277563@qq.com

OI Gao, Xuehan/0009-0001-2613-3827; qin, ming/0000-0001-5633-3988

FU National Natural Science Foundation of China [81860291]; Academic new

seedling Cultivation and Innovation Exploration Project of Zunyi Medical

University [5772-015]; Program of Natural Science Foundation of Zhejiang

Province [2020ZB292]; Chinese Medicine Study Foundation of Zhejiang

Province [SJJG2023185]; [LY20H160017]

FX This work was supported by the National Natural Science Foundation of

China (No. 81860291) , Academic new seedling Cultivation and Innovation

Exploration Project of Zunyi Medical University (Guizhou Science and

Technology Cooperation Platform for Talents [2018] 5772-015) , the

reform project of teaching content and curriculum system in Guizhou

Province (SJJG2023185) , the Program of Natural Science Foundation of

Zhejiang Province (LY20H160017) , and Chinese Medicine Study Foundation

of Zhejiang Province (2020ZB292) .

CR Abrahams K, 2021, CURR OPIN MICROBIOL, V60, P58, DOI 10.1016/j.mib.2021.01.012

Adyns L, 2023, INT J MOL SCI, V24, DOI 10.3390/ijms24065268

Ahmad F, 2022, FRONT IMMUNOL, V13, DOI 10.3389/fimmu.2022.747799

Akdogan D, 2021, J INFECT CHEMOTHER, V27, P1591, DOI 10.1016/j.jiac.2021.07.003

Albrethsen J, 2005, BMC CANCER, V5, DOI 10.1186/1471-2407-5-8

Alvarez AH, 2018, INT J BIOCHEM CELL B, V104, P133, DOI 10.1016/j.biocel.2018.09.007

Alvarez-Jiménez VD, 2018, FRONT IMMUNOL, V9, DOI 10.3389/fimmu.2018.00272

Angala SK, 2014, CRIT REV BIOCHEM MOL, V49, P361, DOI 10.3109/10409238.2014.925420

[Anonymous], 2014, Innovative therapies for acute bacterial skin and skin-structure infections (ABSSSI) caused by methicillin-resistant Staphylococcus aureus

[Anonymous], 2022, A study to evaluate the efficacy and safety of brilacidin in hospitalized participants with COVID-19

[Anonymous], 2020, World Health Organization: Global Tuberculosis Report 2019

[Anonymous], 2017, Study of the effects of brilacidin oral rinse on radiation-induced oral mucositis in patients with head and neck cancer

Arnett E, 2011, CELL MICROBIOL, V13, P635, DOI 10.1111/j.1462-5822.2010.01563.x

Awang T, 2021, J MOL MODEL, V27, DOI 10.1007/s00894-021-04915-w

Barabas N, 2013, IMMUNOBIOLOGY, V218, P1005, DOI 10.1016/j.imbio.2012.11.007

Barry CE III, 2001, TRENDS MICROBIOL, V9, P237, DOI 10.1016/S0966-842X(01)02018-2

Barthold L, 2022, INT J MOL SCI, V23, DOI 10.3390/ijms23094509

Basso V, 2018, ANTIMICROB AGENTS CH, V62, DOI 10.1128/AAC.00111-18

Behr MA, 2015, CURR OPIN MICROBIOL, V23, P126, DOI 10.1016/j.mib.2014.11.015

Bharucha JP, 2021, FRONT CELL INFECT MI, V11, DOI 10.3389/fcimb.2021.535352

Bhat ZS, 2017, BIOMED PHARMACOTHER, V95, P1520, DOI 10.1016/j.biopha.2017.09.036

Biragyn A, 2002, SCIENCE, V298, P1025, DOI 10.1126/science.1075565

Biragyn A, 2008, J LEUKOCYTE BIOL, V83, P998, DOI 10.1189/jlb.1007700

Bo HT, 2023, FRONT CELL INFECT MI, V13, DOI 10.3389/fcimb.2023.1062963

Bohrer AC, 2022, CELL REP, V40, DOI 10.1016/j.celrep.2022.111144

Brandt CR, 2007, INVEST OPHTH VIS SCI, V48, P5118, DOI 10.1167/iovs.07-0302

Brogden KA, 2005, NAT REV MICROBIOL, V3, P238, DOI 10.1038/nrmicro1098

Brook M, 2016, P NATL ACAD SCI USA, V113, P4350, DOI 10.1073/pnas.1601831113

BROWN AE, 1987, J INFECT DIS, V156, P985, DOI 10.1093/infdis/156.6.985

Burgoyne RA, 2021, CELLS-BASEL, V10, DOI 10.3390/cells10102763

Bussi C, 2019, FEMS MICROBIOL REV, V43, P341, DOI 10.1093/femsre/fuz006

Cardot-Martin E, 2015, LETT APPL MICROBIOL, V61, P158, DOI 10.1111/lam.12438

Castagnini M, 2012, PLOS ONE, V7, DOI 10.1371/journal.pone.0041417

Castañeda-Sánchez JI, 2013, PATHOGENS, V2, P13, DOI 10.3390/pathogens2010013

Chai QY, 2020, CELL MOL IMMUNOL, V17, P901, DOI 10.1038/s41423-020-0502-z

Chakaya J, 2022, INT J INFECT DIS, V124, pS26, DOI 10.1016/j.ijid.2022.03.011

Chang TL, 2005, J CLIN INVEST, V115, P765, DOI 10.1172/JCI200521948

Charoenkwan P, 2022, EXCLI J, V21, P757, DOI 10.17179/excli2022-4913

Cheng FJ, 2015, INT J RHEUM DIS, V18, P533, DOI 10.1111/1756-185X.12433

Choi S, 2021, BIOMOLECULES, V11, DOI 10.3390/biom11091306

Cieslik M, 2021, CELLS-BASEL, V10, DOI 10.3390/cells10112991

Claret M, 2023, GUT, V72, P610, DOI 10.1136/gutjnl-2022-328158

Cole Alexander M, 2002, Am J Respir Med, V1, P249

Corrales-Garcia L, 2013, PROTEIN EXPRES PURIF, V89, P33, DOI 10.1016/j.pep.2013.02.007

Correia-Neves M, 2022, FRONT IMMUNOL, V13, DOI 10.3389/fimmu.2022.1035122

de Breij A, 2018, SCI TRANSL MED, V10, DOI 10.1126/scitranslmed.aan4044

de Leeuw E, 2010, FEBS LETT, V584, P1543, DOI 10.1016/j.febslet.2010.03.004

de Leeuw EPH, 2018, AVIAN DIS, V62, P381, DOI 10.1637/11912-061118-Reg.1

de Waal AM, 2022, THORAX, V77, P408, DOI 10.1136/thoraxjnl-2021-217997

DIAMOND G, 1991, P NATL ACAD SCI USA, V88, P3952, DOI 10.1073/pnas.88.9.3952

Dong HD, 2016, J IMMUNOL RES, V2016, DOI 10.1155/2016/7515687

Dong XT, 2022, IMMUNITY, V55, P1645, DOI 10.1016/j.immuni.2022.06.021

Dookie N, 2022, CLIN MICROBIOL REV, V35, DOI 10.1128/cmr.00180-19

Dorin JR, 2007, SCIENCE, V318, P1395, DOI 10.1126/science.1151370

Driss V, 2009, BLOOD, V113, P3235, DOI 10.1182/blood-2008-07-166595

Droin N, 2009, J PROTEOMICS, V72, P918, DOI 10.1016/j.jprot.2009.01.002

García-Pérez BE, 2011, IMMUNOBIOLOGY, V216, P925, DOI 10.1016/j.imbio.2011.01.004

Fait A, 2023, CURR BIOL, V33, DOI 10.1016/j.cub.2023.06.082

Falanga A, 2017, MOLECULES, V22, DOI 10.3390/molecules22071217

Fang XM, 2003, EUR J CLIN INVEST, V33, P82, DOI 10.1046/j.1365-2362.2003.01076.x

Faust KB, 2022, FRONT PEDIATR, V10, DOI 10.3389/fped.2022.909176

Ferris LK, 2013, J INVEST DERMATOL, V133, P460, DOI 10.1038/jid.2012.319

Forman MR, 2010, AM J PREV MED, V38, P646, DOI 10.1016/j.amepre.2010.03.002

Froy O, 2005, CELL MICROBIOL, V7, P1387, DOI 10.1111/j.1462-5822.2005.00590.x

Fu J, 2023, SIGNAL TRANSDUCT TAR, V8, DOI 10.1038/s41392-023-01553-x

Fu LM, 2003, INT J TUBERC LUNG D, V7, P1027

Futosi K, 2013, INT IMMUNOPHARMACOL, V17, P1185, DOI 10.1016/j.intimp.2013.11.010

Gácser A, 2014, MICROBES INFECT, V16, P80, DOI 10.1016/j.micinf.2013.09.003

Gaffney E, 2022, FRONT IMMUNOL, V13, DOI 10.3389/fimmu.2022.984293

GANZ T, 1985, J CLIN INVEST, V76, P1427, DOI 10.1172/JCI112120

Ganz T, 2002, SCIENCE, V298, P977, DOI 10.1126/science.1078708

Gao XH, 2021, ADV DRUG DELIVER REV, V179, DOI 10.1016/j.addr.2021.114008

Gbala ID, 2022, MOLECULES, V27, DOI 10.3390/molecules27144325

Giesemann T, 2008, GASTROENTEROLOGY, V134, P2049, DOI 10.1053/j.gastro.2008.03.008

Gong T, 2022, INT J MOL SCI, V23, DOI 10.3390/ijms23052789

Grein F, 2019, J MOL BIOL, V431, P3520, DOI 10.1016/j.jmb.2019.05.014

Grönberg A, 2014, WOUND REPAIR REGEN, V22, P613, DOI 10.1111/wrr.12211

Guler R, 2021, FRONT IMMUNOL, V12, DOI 10.3389/fimmu.2021.733853

Hein MJA, 2022, BIOCHEM SOC T, V50, P423, DOI 10.1042/BST20200884

Hernández-Santos N, 2018, CELL HOST MICROBE, V23, P511, DOI 10.1016/j.chom.2018.02.011

Hilda JN, 2020, INNATE IMMUN-LONDON, V26, P240, DOI 10.1177/1753425919881176

Holly MK, 2017, ANNU REV VIROL, V4, P369, DOI 10.1146/annurev-virology-101416-041734

Holzheimer M, 2021, CHEM REV, V121, P9554, DOI 10.1021/acs.chemrev.1c00043

Hooven TA, 2012, J ANTIMICROB CHEMOTH, V67, P2870, DOI 10.1093/jac/dks305

Howard NC, 2018, NAT MICROBIOL, V3, P1099, DOI 10.1038/s41564-018-0245-0

Huang J, 2022, ANTIVIR RES, V201, DOI 10.1016/j.antiviral.2022.105292

Islam S, 2023, HELIYON, V9, DOI 10.1016/j.heliyon.2023.e14158

Jackson M, 2014, CSH PERSPECT MED, V4, DOI 10.1101/cshperspect.a021105

Jacobo-Delgado YM, 2023, FRONT IMMUNOL, V14, DOI 10.3389/fimmu.2023.1194923

Jayne JG, 2018, AM J RESP CELL MOL, V58, P310, DOI 10.1165/rcmb.2016-0428OC

Jin J, 2022, CELL BIOSCI, V12, DOI 10.1186/s13578-022-00924-1

Judge CJ, 2015, CELL IMMUNOL, V297, P61, DOI 10.1016/j.cellimm.2015.06.004

Kalita A, 2004, J INFECT DIS, V190, P1476, DOI 10.1086/424463

Kang J, 2014, EUR J CLIN MICROBIOL, V33, P1823, DOI 10.1007/s10096-014-2152-5

Kang JJ, 2015, INT J TUBERC LUNG D, V19, P711, DOI 10.5588/ijtld.13.0272

Khatun A, 2019, J VET MED SCI, V81, P138, DOI 10.1292/jvms.18-0619

Khusro A, 2016, ASIAN PAC J TROP MED, V9, P1001, DOI 10.1016/j.apjtm.2016.09.005

Kim C, 2005, P NATL ACAD SCI USA, V102, P4830, DOI 10.1073/pnas.0500508102

Kim C, 2006, BIOCHEM J, V399, P225, DOI 10.1042/BJ20060425

Kim H, 2022, FRONT CELL INFECT MI, V12, DOI 10.3389/fcimb.2022.891878

Kim J, 2019, IMMUNOBIOLOGY, V224, P502, DOI 10.1016/j.imbio.2019.05.004

Kim TH, 2015, IMMUNOBIOLOGY, V220, P26, DOI 10.1016/j.imbio.2014.09.019

Kim WS, 2018, FRONT CELL INFECT MI, V8, DOI 10.3389/fcimb.2018.00095

Kisich KO, 2002, INFECT IMMUN, V70, P4591, DOI 10.1128/IAI.70.8.4591-4599.2002

Kisich KO, 2001, INFECT IMMUN, V69, P2692, DOI 10.1128/IAI.69.4.2692-2699.2001

Knyght I, 2016, LANGMUIR, V32, P7403, DOI 10.1021/acs.langmuir.6b01688

Kudryashova E, 2022, J MOL BIOL, V434, DOI 10.1016/j.jmb.2021.167225

Kudryashova E, 2017, BIOL CHEM, V398, P1069, DOI 10.1515/hsz-2017-0106

Kumar N.P., 2017, PLoS One, V12

Layre E, 2020, FRONT IMMUNOL, V11, DOI 10.3389/fimmu.2020.01230

Lee J, 2016, LANGMUIR, V32, P1782, DOI 10.1021/acs.langmuir.5b04113

Lehrer RI, 2007, CURR OPIN HEMATOL, V14, P16, DOI 10.1097/00062752-200701000-00005

Lehrer RI, 2012, J BIOL CHEM, V287, P27014, DOI 10.1074/jbc.R112.346098

Lehrer RI, 2009, INFECT IMMUN, V77, P4028, DOI 10.1128/IAI.00232-09

Li CW, 2016, FRONT CELL INFECT MI, V6, DOI 10.3389/fcimb.2016.00124

Li FJ, 2021, EXP APPL ACAROL, V83, P271, DOI 10.1007/s10493-020-00584-1

Liang ZM, 2020, PHARMACEUTICS, V12, DOI 10.3390/pharmaceutics12121172

LICHTENSTEIN A, 1986, BLOOD, V68, P1407

Lipkin RB, 2015, J MEMBRANE BIOL, V248, P469, DOI 10.1007/s00232-014-9759-4

Liu CH, 2017, CELL MOL IMMUNOL, V14, P963, DOI 10.1038/cmi.2017.88

Liu PT, 2009, PLOS ONE, V4, DOI 10.1371/journal.pone.0005810

Liu YT, 2022, COMMUN BIOL, V5, DOI 10.1038/s42003-022-03525-3

Lozza L, 2014, FRONT IMMUNOL, V5, DOI 10.3389/fimmu.2014.00324

Luo G, 2022, P NATL ACAD SCI USA, V119, DOI 10.1073/pnas.2117283119

Luo XF, 2023, EUR J PHARM SCI, V181, DOI 10.1016/j.ejps.2022.106363

Mackenzie-Dyck S, 2011, VET IMMUNOL IMMUNOP, V143, P87, DOI 10.1016/j.vetimm.2011.06.028

Mathew B, 2017, BIOORG MED CHEM LETT, V27, P3264, DOI 10.1016/j.bmcl.2017.06.031

Mattar EH, 2016, CYTOKINE GROWTH F R, V28, P95, DOI 10.1016/j.cytogfr.2015.11.002

Menendez A, 2007, CURR OPIN IMMUNOL, V19, P385, DOI 10.1016/j.coi.2007.06.008

Milewski L, 2011, J REPROD IMMUNOL, V91, P64, DOI 10.1016/j.jri.2011.05.008

Miyakawa Y, 1996, INFECT IMMUN, V64, P926, DOI 10.1128/IAI.64.3.926-932.1996

Moguche AO, 2017, CELL HOST MICROBE, V21, P695, DOI 10.1016/j.chom.2017.05.012

Morgan J, 2021, IMMUNOL REV, V301, P10, DOI 10.1111/imr.12963

Muefong CN, 2020, FRONT IMMUNOL, V11, DOI 10.3389/fimmu.2020.00962

Mumcu G, 2012, ARCH ORAL BIOL, V57, P642, DOI 10.1016/j.archoralbio.2011.11.003

Navid F, 2012, J IMMUNOL, V188, P735, DOI 10.4049/jimmunol.1100452

Nickel D, 2012, J IMMUNOL, V188, P4001, DOI 10.4049/jimmunol.1100976

Nicole W., 2018, The plant defensin HXP124 has the potential to be a new safe and effective topical treatment for onychomycosis

Nicole W., 2021, A phase I/IIa, randomized, double-blind, vehicle-controlled study of pezadeftide (HXP124), a novel topical treatment, in patients with onychomycosis

Niemann S, 2018, SCI REP-UK, V8, DOI 10.1038/s41598-018-20582-z

Oren Z, 1998, BIOPOLYMERS, V47, P451

Orme IM, 2015, NAT IMMUNOL, V16, P57, DOI 10.1038/ni.3048

Othumpangat S, 2023, PATHOGENS, V12, DOI 10.3390/pathogens12010123

Pace BT, 2017, MEDIAT INFLAMM, V2017, DOI 10.1155/2017/5186904

Pajuelo D, 2021, NAT COMMUN, V12, DOI 10.1038/s41467-021-26925-1

Pajuelo D, 2018, CELL REP, V24, P429, DOI 10.1016/j.celrep.2018.06.042

Park HS, 2021, INT J MOL SCI, V22, DOI 10.3390/ijms22147535

PATTERSONDELAFIELD J, 1980, INFECT IMMUN, V30, P180, DOI 10.1128/IAI.30.1.180-192.1980

Pellegrini A, 2001, BBA-GEN SUBJECTS, V1526, P131, DOI 10.1016/S0304-4165(01)00116-7

Porter E, 2005, INFECT IMMUN, V73, P4823, DOI 10.1128/IAI.73.8.4823-4833.2005

Prado-Montes de Oca E, 2010, INT J BIOCHEM CELL B, V42, P800, DOI 10.1016/j.biocel.2010.01.021

Presicce P, 2009, J LEUKOCYTE BIOL, V86, P941, DOI 10.1189/jlb.0708412

Quigley J, 2017, MBIO, V8, DOI 10.1128/mBio.00148-17

Ravin KA, 2016, CLIN REV ALLERG IMMU, V50, P214, DOI 10.1007/s12016-015-8525-4

Rivas-Santiago B, 2005, INFECT IMMUN, V73, P4505, DOI 10.1128/IAI.73.8.4505-4511.2005

Rivas-Santiago B, 2006, J INFECT DIS, V194, P697, DOI 10.1086/506454

Rivas-Santiago B, 2012, ARCH MED RES, V43, P324, DOI 10.1016/j.arcmed.2012.06.005

Rivas-Santiago CE, 2011, CLIN EXP IMMUNOL, V164, P80, DOI 10.1111/j.1365-2249.2010.04313.x

Salvatore M, 2007, J INFECT DIS, V196, P835, DOI 10.1086/521027

Sarmiento ME, 2019, TUBERCULOSIS, V115, P26, DOI 10.1016/j.tube.2019.01.003

Sass V, 2010, INFECT IMMUN, V78, P2793, DOI 10.1128/IAI.00688-09

Schneider T, 2010, SCIENCE, V328, P1168, DOI 10.1126/science.1185723

Schroeder BO, 2011, NATURE, V469, P419, DOI 10.1038/nature09674

Sciolla F, 2021, COLLOID SURFACE B, V208, DOI 10.1016/j.colsurfb.2021.112054

Selsted ME, 2005, NAT IMMUNOL, V6, P551, DOI 10.1038/ni1206

Semple CA, 2006, MOL DIVERS, V10, P575, DOI 10.1007/s11030-006-9031-7

Semple F, 2011, EUR J IMMUNOL, V41, P3291, DOI 10.1002/eji.201141648

Sengupta D, 2008, BBA-BIOMEMBRANES, V1778, P2308, DOI 10.1016/j.bbamem.2008.06.007

Shafee TMA, 2017, CELL MOL LIFE SCI, V74, P663, DOI 10.1007/s00018-016-2344-5

Sharma R, 2019, SCI REP-UK, V9, DOI 10.1038/s41598-019-44256-6

Sharma S, 2000, EUR RESPIR J, V16, P112, DOI 10.1034/j.1399-3003.2000.16a20.x

Shi CS, 2010, SCI SIGNAL, V3, DOI 10.1126/scisignal.2000751

Shi CS, 2008, J BIOL CHEM, V283, P33175, DOI 10.1074/jbc.M804478200

Shiba H, 2003, BIOCHEM BIOPH RES CO, V306, P867, DOI 10.1016/S0006-291X(03)01075-1

SILVA MT, 1989, MICROB PATHOGENESIS, V6, P369, DOI 10.1016/0882-4010(89)90079-X

Sinha S, 2003, ANTIMICROB AGENTS CH, V47, P494, DOI 10.1128/AAC.47.2.494-500.2003

Soehnlein O, 2008, J CLIN INVEST, V118, P3491, DOI 10.1172/JCI35740

Tai KP, 2014, INFECT IMMUN, V82, P2195, DOI 10.1128/IAI.01414-13

Taylor K, 2009, EUR J IMMUNOL, V39, P1353, DOI 10.1002/eji.200838566

Tecle T, 2007, J IMMUNOL, V178, P8046, DOI 10.4049/jimmunol.178.12.8046

Tewary P, 2013, J IMMUNOL, V191, P865, DOI 10.4049/jimmunol.1201648

Trabi M, 2001, BIOCHEMISTRY-US, V40, P4211, DOI 10.1021/bi002028t

Tsutsumi-Ishii Y, 2003, J IMMUNOL, V170, P4226, DOI 10.4049/jimmunol.170.8.4226

van der Does AM, 2019, ADV EXP MED BIOL, V1117, P149, DOI 10.1007/978-981-13-3588-4_10

Wang AP, 2013, J VIROL, V87, P2835, DOI 10.1128/JVI.02209-12

Wang C, 2020, GASTROENTEROLOGY, V159, P1145, DOI 10.1053/j.gastro.2020.05.015

Wang GS, 2014, PHARMACEUTICALS-BASE, V7, P545, DOI 10.3390/ph7050545

Wang W, 2006, J BIOL CHEM, V281, P32755, DOI 10.1074/jbc.M603614200

Wei G, 2009, J BIOL CHEM, V284, P29180, DOI 10.1074/jbc.M109.018085

Wilmes M, 2014, INT J MED MICROBIOL, V304, P93, DOI 10.1016/j.ijmm.2013.08.007

Wilmes M, 2011, NAT PROD REP, V28, P1350, DOI 10.1039/c1np00022e

Wilson SS, 2013, J MOL BIOL, V425, P4965, DOI 10.1016/j.jmb.2013.09.038

Wohlford-Lenane CL, 2009, J VIROL, V83, P11385, DOI 10.1128/JVI.01363-09

Wu JJ, 2018, PEPTIDES, V99, P82, DOI 10.1016/j.peptides.2017.11.009

Xu WX, 2021, IMMUNITY, V54, P526, DOI 10.1016/j.immuni.2021.01.003

Yamamoto-Furusho JK, 2010, INFLAMM BOWEL DIS, V16, P736, DOI 10.1002/ibd.21144

Yang D, 2007, CURR PHARM DESIGN, V13, P3131, DOI 10.2174/138161207782110453

Yeung AWK, 2023, FRONT PUBLIC HEALTH, V11, DOI 10.3389/fpubh.2023.1196596

Yuan XW, 2018, INT IMMUNOPHARMACOL, V55, P133, DOI 10.1016/j.intimp.2017.12.017

ZEYA HI, 1966, SCIENCE, V154, P1049, DOI 10.1126/science.154.3752.1049

Zhang CB, 2018, FASEB J, V32, P1354, DOI 10.1096/fj.201700936R

Zhao BC, 2016, CURR TOP MED CHEM, V16, P206, DOI 10.2174/1568026615666150701115911

Zhao YY, 2013, ACS APPL MATER INTER, V5, P8267, DOI 10.1021/am4021523

Zheng JB, 2018, RESP RES, V19, DOI 10.1186/s12931-018-0869-x

Zhu JH, 2023, CURR OPIN MICROBIOL, V72, DOI 10.1016/j.mib.2023.102266

Zou GZ, 2018, BIOCHEM BIOPH RES CO, V501, P454, DOI 10.1016/j.bbrc.2018.05.011

Zou ZY, 2022, ECLINICALMEDICINE, V53, DOI 10.1016/j.eclinm.2022.101646

NR 207

TC 4

Z9 6

U1 1

U2 25

PU ELSEVIER

PI AMSTERDAM

PA RADARWEG 29, 1043 NX AMSTERDAM, NETHERLANDS

SN 1567-5769

EI 1878-1705

J9 INT IMMUNOPHARMACOL

JI Int. Immunopharmacol.

PD JAN 25

PY 2024

VL 127

AR 111383

DI 10.1016/j.intimp.2023.111383

EA DEC 2023

PG 13

WC Immunology; Pharmacology & Pharmacy

WE Science Citation Index Expanded (SCI-EXPANDED)

SC Immunology; Pharmacology & Pharmacy

GA FA0J0

UT WOS:001142901100001

OA hybrid

DA 2026-05-07

ER

PT J

AU Zhao, JN

Zhang, XX

He, XC

Yang, GR

Zhang, XQ

Li, HC

AF Zhao, Jiang-nan

Zhang, Xian-xin

He, Xiao-chun

Yang, Guo-ru

Zhang, Xiao-qi

Li, Huai-chen

TI The Relationship between Extensively Drug-Resistant Tuberculosis and

Multidrug-Resistant Gram-Negative Bacilli

SO PLOS ONE

LA English

DT Article

ID ANTIMICROBIAL RESISTANCE; INFECTIONS; MANAGEMENT; CIRRHOSIS; ALBUMIN

AB Objective

The relationship between extensively drug-resistant tuberculosis (XDR-TB) and multidrug-resistant Gram-negative bacilli (MDR-GNB) is unclear. Identification of the relationship between XDR-TB and MDR-GNB would have important implications for patient care.

Methods

We conducted a retrospective study reviewing the records of patients admitted with a confirmed pulmonary TB from 2011 to 2014. To identify the relationship between XDR-TB and MDR-GNB, univariable comparison and multivariable logistic regression were performed.

Results

Among 2962 pulmonary TB patients, 45(1.5%) patients had a diagnosis of XDR-TB. A total of 165 MDR-GNB strains were detected in 143 (4.8%) pulmonary TB patients. XDR-TB patients had a significantly higher occurrence of MDR-GNB than non-XDR-TB patients (24.4% vs. 4.5%; P<0.001). Age (OR 1.02, 95% CI 1.01-1.03), hypoalbuminemia (OR 1.48, 95% CI 1.18-1.85), chronic renal failure (OR 6.67, 95% CI 1.42-31.47), chronic hepatic insufficiency (OR 1.99, 95% CI 1.15-3.43), presence of XDR-TB (OR 6.56, 95% CI 1.61-26.69), and duration of TB diagnostic delay (OR 1.01, 95% CI 1.00-1.02) were the independent risk factors for MDR-GNB infection.

Conclusions

Patients with XDR-TB have a significantly higher risk of being affected by MDR-GNB pathogen. The underlying mechanism association warrant further studies.

C1 [Zhao, Jiang-nan; He, Xiao-chun; Li, Huai-chen] Shandong Univ, Prov Hosp, Dept Resp Med, Jinan 250100, Peoples R China.

[Zhang, Xian-xin] Shandong Prov Chest Hosp, Dept Resp Med, Jinan, Peoples R China.

[Yang, Guo-ru] Chest Specially Hosp Weifang, Dept Resp Med, Weifang, Peoples R China.

[Zhang, Xiao-qi] Chest Specially Hosp Weifang, Dept TB Med, Weifang, Peoples R China.

C3 Shandong University; Shandong University

RP Li, HC (corresponding author), Shandong Univ, Prov Hosp, Dept Resp Med, Jinan 250100, Peoples R China.

EM zjn911016@126.com

RI XIAOQI, ZHANG/KIK-7052-2024

FU Science and technology development plan of Shandong Province

[2009GG10002054]

FX This work was supported by Science and technology development plan of

Shandong Province (Grant Number: 2009GG10002054). The funders had no

role in study design, data collection and analysis, decision to publish,

or preparation of the manuscript.

CR [Anonymous], 2006, Wkly Epidemiol Rec, V81, P430

[Anonymous], 2015, Guidelines for surveillance of drug resistance in tuberculosis

Arruda EAG, 1999, INFECT CONT HOSP EP, V20, P620, DOI 10.1086/501683

Bastos ML, 2014, CLIN INFECT DIS, V59, P1364, DOI 10.1093/cid/ciu619

Caminero JA, 2010, LANCET INFECT DIS, V10, P621, DOI 10.1016/S1473-3099(10)70139-0

Centers for Disease Control and Prevention (CDC), 2006, MMWR Morb Mortal Wkly Rep, V55, P301

Chang KC, 2013, RESPIROLOGY, V18, P8, DOI 10.1111/j.1440-1843.2012.02257.x

Clinical and Laboratory Standards Institute, 2008, M100S18 CLSI

D'Agata EMC, 2004, INFECT CONT HOSP EP, V25, P842, DOI 10.1086/502306

Daley CL, 2014, CLIN INFECT DIS, V59, P1064, DOI 10.1093/cid/ciu578

de Sande-Bruinsma NV, 2008, EMERG INFECT DIS, V14, P1722, DOI 10.3201/eid1411.070467

Gandhi NR, 2010, LANCET, V375, P1830, DOI 10.1016/S0140-6736(10)60410-2

GARNER JS, 1988, AM J INFECT CONTROL, V16, P128, DOI 10.1016/0196-6553(88)90053-3

Hsueh PR, 2005, INT J ANTIMICROB AG, V26, P463, DOI 10.1016/j.ijantimicag.2005.08.016

Jalan R, 2013, J HEPATOL, V59, P918, DOI 10.1016/j.jhep.2013.08.001

Jalan R, 2009, HEPATOLOGY, V50, P555, DOI 10.1002/hep.22913

Karyadi E, 2000, J NUTR, V130, P2953, DOI 10.1093/jn/130.12.2953

Kim HR, 2011, J HOSP INFECT, V77, P134, DOI 10.1016/j.jhin.2010.07.004

Kim HR, 2007, CLIN INFECT DIS, V45, P1290, DOI 10.1086/522537

Lai CC, 2011, J ANTIMICROB CHEMOTH, V66, P1374, DOI 10.1093/jac/dkr103

Lam E, 2014, EMERG INFECT DIS, V20, P400, DOI 10.3201/eid2003.130951

Mitnick CD, 2008, NEW ENGL J MED, V359, P563, DOI 10.1056/NEJMoa0800106

ONWUBALILI JK, 1988, EUR J CLIN NUTR, V42, P363

Pop-Vicas AE, 2005, CLIN INFECT DIS, V40, P1792, DOI 10.1086/430314

Safdar N, 2002, ANN INTERN MED, V136, P834, DOI 10.7326/0003-4819-136-11-200206040-00013

Shen GH, 2012, INT J ANTIMICROB AG, V39, P201, DOI 10.1016/j.ijantimicag.2011.11.014

Siege JD, 2006, MANAGEMENT MULTIDRUG

Simonsen GS, 2004, B WORLD HEALTH ORGAN, V82, P928

Temple B, 2008, CLIN INFECT DIS, V47, P1126, DOI 10.1086/592252

Wong SSY, 2007, PERITON DIALYSIS INT, V27, pS272

World Health Organization, 2014, WHOHTMTB201408

Zumla A, 2012, J INFECT DIS, V205, pS228, DOI 10.1093/infdis/jir858

NR 32

TC 0

Z9 0

U1 0

U2 2

PU PUBLIC LIBRARY SCIENCE

PI SAN FRANCISCO

PA 1160 BATTERY STREET, STE 100, SAN FRANCISCO, CA 94111 USA

SN 1932-6203

J9 PLOS ONE

JI PLoS One

PD JUL 31

PY 2015

VL 10

IS 7

AR e0134998

DI 10.1371/journal.pone.0134998

PG 10

WC Multidisciplinary Sciences

WE Science Citation Index Expanded (SCI-EXPANDED)

SC Science & Technology - Other Topics

GA CO0JY

UT WOS:000358838400190

OA Green Submitted, gold

DA 2026-05-07

ER

PT J

AU Yang, EZ

Yang, R

Guo, M

Huang, D

Wang, WD

Zhang, ZR

Chen, C

Wang, FF

Ho, WZ

Shen, L

Xiao, HP

Chen, ZW

Shen, HB

AF Yang, Enzhuo

Yang, Rui

Guo, Ming

Huang, Dan

Wang, Wandang

Zhang, Zhuoran

Chen, Crystal

Wang, Feifei

Ho, Wenzhe

Shen, Ling

Xiao, Heping

Chen, Zheng W.

Shen, Hongbo

TI Multidrug-resistant tuberculosis (MDR-TB) strain infection in macaques

results in high bacilli burdens in airways, driving broad

innate/adaptive immune responses

SO EMERGING MICROBES & INFECTIONS

LA English

DT Article

ID MYCOBACTERIUM-TUBERCULOSIS; DRUG-RESISTANCE; CELL SUBSET; IFN-GAMMA;

TNF-ALPHA; TRANSMISSION; FITNESS; EVOLUTION; APOPTOSIS; NECROSIS

AB Tuberculosis (TB) has become the most deadly infectious diseases due to epidemics of HIV/AIDS and multidrug-resistant/extensively drug-resistant TB (MDR-/XDR-TB). Although person-to-person transmission contributes to MDRTB, it remains unknown whether infection with MDR strains resembles infection with drug-sensitive (DS) TB strains, manipulating limited or broad immune responses. To address these questions, macaques were infected with MDR strain V791 and a drug-sensitive Erdman strain of TB. MDR bacilli burdens in the airway were significantly higher than those of the Erdman control after pulmonary exposure. This productive MDR strain infection upregulated the expression of caspase 3 in macrophages/monocytes and induced appreciable innate-like effector responses of CD3-negative lymphocytes and Ag-specific gamma delta T-cell subsets. Concurrently, MDR strain infection induced broad immune responses of T-cell subpopulations producing Th1, Th17, Th22, and CTL cytokines. Furthermore, MDR bacilli, like the Erdman strain, were capable of inducing typical TB disease characterized by weight loss, lymphocytopenia, and severe TB lesions. For the first time, our results suggest that MDR-TB infection acts like DS to induce high bacterial burdens in the airway (transmission advantage), innate/adaptive immune responses, and disease processes. Because nonhuman primates are biologically closer to humans than other species, our data may provide useful information for predicting the effects of primary MDR strain infection after person-to-person transmission. The findings also support the hypothesis that a vaccine or host-directed adjunctive modality that is effective for drug-sensitive TB is likely to also impact MDR-TB.

C1 [Yang, Enzhuo; Yang, Rui; Xiao, Heping; Shen, Hongbo] Tongji Univ, Sch Med, Shanghai Pulm Hosp, Clin & Res Ctr TB,Shanghai Key Lab TB, Shanghai, Peoples R China.

[Yang, Enzhuo; Huang, Dan; Wang, Wandang; Zhang, Zhuoran; Chen, Crystal; Shen, Ling; Chen, Zheng W.] Univ Illinois, Coll Med, Dept Microbiol & Immunol, Chicago, IL USA.

[Yang, Enzhuo; Huang, Dan; Wang, Wandang; Zhang, Zhuoran; Chen, Crystal; Shen, Ling; Chen, Zheng W.] Univ Illinois, Coll Med, Ctr Primate Biomed Res, Chicago, IL USA.

[Yang, Rui; Shen, Hongbo] Chinese Acad Sci, Inst Pasteur Shanghai, Unit AntiTB Immun, Shanghai 200031, Peoples R China.

[Guo, Ming; Ho, Wenzhe] Wuhan Univ, Coll Med, Wuhan 430072, Hubei, Peoples R China.

[Wang, Feifei] Fudan Univ, Shanghai Med Coll, Dept Med Microbiol & Parasitol, Shanghai 200032, Peoples R China.

C3 Tongji University; University of Illinois System; University of Illinois

Chicago; University of Illinois Chicago Hospital; University of Illinois

System; University of Illinois Chicago; University of Illinois Chicago

Hospital; Pasteur Network; Chinese Academy of Sciences; Shanghai

Institute of Immunity and Infection, CAS; Wuhan University; Fudan

University

RP Xiao, HP; Shen, HB (corresponding author), Tongji Univ, Sch Med, Shanghai Pulm Hosp, Clin & Res Ctr TB,Shanghai Key Lab TB, Shanghai, Peoples R China.; Chen, ZW (corresponding author), Univ Illinois, Coll Med, Dept Microbiol & Immunol, Chicago, IL USA.; Chen, ZW (corresponding author), Univ Illinois, Coll Med, Ctr Primate Biomed Res, Chicago, IL USA.

EM xiaoheping_sars@163.com; zchen@uic.edu; hongboshen109@hotmail.com

RI Yang, Rui/AAC-6246-2022

OI Yang, Rui/0000-0001-7679-9646; zhang, zhuoran/0000-0003-2266-8346

FU NIH [R01 HL64560/OD015092/HL129887]

FX We thank the other staff in the Chen laboratory for their technical

support. This work was supported by the following grants: NIH R01

HL64560/OD015092/HL129887 (to Z.W.C.).

CR Abate G, 1998, INT J TUBERC LUNG D, V2, P580

Alffenaar JWC, 2017, EXPERT REV ANTI-INFE, V15, P11, DOI 10.1080/14787210.2017.1247692

[Anonymous], 2017, World Health Statistics 2017: Monitoring Health for The SDGs

Apt A, 2009, TUBERCULOSIS, V89, P195, DOI 10.1016/j.tube.2009.02.002

Arcila ML, 2007, CELL IMMUNOL, V249, P80, DOI 10.1016/j.cellimm.2007.11.006

Belay M, 2015, APMIS, V123, P851, DOI 10.1111/apm.12423

Capuano SV, 2003, INFECT IMMUN, V71, P5831, DOI 10.1128/IAI.71.10.5831-5844.2003

Casali N, 2014, NAT GENET, V46, P279, DOI 10.1038/ng.2878

Chen CY, 2013, PLOS PATHOG, V9, DOI 10.1371/journal.ppat.1003501

Chen CY, 2012, J IMMUNOL, V188, P4278, DOI 10.4049/jimmunol.1101291

Chen CY, 2009, PLOS PATHOG, V5, DOI 10.1371/journal.ppat.1000392

Chen ZW, 2016, CURR OPIN IMMUNOL, V42, P105, DOI 10.1016/j.coi.2016.06.005

FLYNN JL, 1995, IMMUNITY, V2, P561, DOI 10.1016/1074-7613(95)90001-2

Fortin A, 2007, ANNU REV GENOM HUM G, V8, P163, DOI 10.1146/annurev.genom.8.080706.092315

Gagneux S, 2006, SCIENCE, V312, P1944, DOI 10.1126/science.1124410

Kendall EA, 2015, LANCET RESP MED, V3, P963, DOI 10.1016/S2213-2600(15)00458-0

Kundu M, 2009, NAT IMMUNOL, V10, P918, DOI 10.1038/ni.1754

Lin PL, 2009, INFECT IMMUN, V77, P4631, DOI 10.1128/IAI.00592-09

MacMicking JD, 2003, SCIENCE, V302, P654, DOI 10.1126/science.1088063

Maggioli MF, 2016, FRONT IMMUNOL, V7, DOI 10.3389/fimmu.2016.00421

Maitre T., 2016, MED MALADIES INFECT

Mariam DH, 2004, ANTIMICROB AGENTS CH, V48, P1289, DOI 10.1128/AAC.48.4.1289-1294.2004

Marks SM, 2016, INT J TUBERC LUNG D, V20, P435, DOI 10.5588/ijtld.15.0575

Matsuyoshi H, 2004, J IMMUNOL, V172, P776, DOI 10.4049/jimmunol.172.2.776

Mattila JT, 2011, J IMMUNOL, V186, P3527, DOI 10.4049/jimmunol.1003773

Naidoo CC, 2014, CLIN MICROBIOL INFEC, V20, pO361, DOI 10.1111/1469-0691.12415

O'Garra A, 2013, ANNU REV IMMUNOL, V31, P475, DOI 10.1146/annurev-immunol-032712-095939

Qaqish A, 2017, J IMMUNOL, V198, P4753, DOI 10.4049/jimmunol.1602019

Qiu YQ, 2012, PLOS PATHOG, V8, DOI 10.1371/journal.ppat.1002984

Robinson RT, 2015, IMMUNOL REV, V264, P46, DOI 10.1111/imr.12259

Ryan-Payseur B, 2012, J IMMUNOL, V189, P1285, DOI 10.4049/jimmunol.1200641

Schaaf K, 2017, SCI REP-UK, V7, DOI 10.1038/srep42101

Shen Y, 2002, SCIENCE, V295, P2255, DOI 10.1126/science.1068819

Torrado E, 2010, CYTOKINE GROWTH F R, V21, P455, DOI 10.1016/j.cytogfr.2010.10.004

Toungoussova OS, 2004, FEMS IMMUNOL MED MIC, V42, P281, DOI 10.1016/j.femsim.2004.05.012

Wang FF, 2015, ANTIMICROB AGENTS CH, V59, P818, DOI 10.1128/AAC.03504-14

Yao SY, 2014, J IMMUNOL, V192, P2120, DOI 10.4049/jimmunol.1301373

Yao SY, 2010, PLOS PATHOG, V6, DOI 10.1371/journal.ppat.1000789

Zeng GC, 2011, J IMMUNOL, V187, P190, DOI 10.4049/jimmunol.1004129

Zhang GL, 2016, CELL CYCLE, V15, P2527, DOI 10.1080/15384101.2016.1215386

NR 40

TC 14

Z9 17

U1 0

U2 6

PU TAYLOR & FRANCIS LTD

PI ABINGDON

PA 2-4 PARK SQUARE, MILTON PARK, ABINGDON OR14 4RN, OXON, ENGLAND

EI 2222-1751

J9 EMERG MICROBES INFEC

JI Emerg. Microbes Infect.

PD DEC 12

PY 2018

VL 7

AR 207

DI 10.1038/s41426-018-0213-z

PG 12

WC Immunology; Infectious Diseases; Microbiology

WE Science Citation Index Expanded (SCI-EXPANDED)

SC Immunology; Infectious Diseases; Microbiology

GA HE9PZ

UT WOS:000453786200001

OA Green Submitted, gold

DA 2026-05-07

ER

PT J

AU Pieterman, ED

te Brake, LHM

de Knegt, GJ

van der Meijden, A

Alffenaar, JWC

Bax, HI

Aarnoutse, RE

de Steenwinkel, JEM

AF Pieterman, Elise D.

te Brake, Lindsey H. M.

de Knegt, Gerjo J.

van der Meijden, Aart

Alffenaar, Jan-Willem C.

Bax, Hannelore I.

Aarnoutse, Rob E.

de Steenwinkel, Jurriaan E. M.

TI Assessment of the Additional Value of Verapamil to a Moxifloxacin and

Linezolid Combination Regimen in a Murine Tuberculosis Model

SO ANTIMICROBIAL AGENTS AND CHEMOTHERAPY

LA English

DT Article

DE Mycobacterium tuberculosis; efflux pump inhibitor; linezolid;

moxifloxacin; verapamil

ID DRUG-RESISTANT TUBERCULOSIS; MASS-SPECTROMETRY METHOD;

MYCOBACTERIUM-TUBERCULOSIS; MULTIDRUG-RESISTANT; IN-VITRO; EFFLUX PUMP;

ANTIMICROBIAL AGENTS; BEIJING GENOTYPE; MOUSE MODEL; MDR-TB

AB The favorable treatment outcome rate for multidrug-resistant tuberculosis (MDR-TB) is only 54%, and therefore new drug regimens are urgently needed. In this study, we evaluated the activity of the combination of moxifloxacin and linezolid as a possible new MDR-TB regimen in a murine TB model and the value of the addition of the efflux pump inhibitor verapamil to this backbone. BALB/c mice were infected with drug-sensitive Mycobacterium tuberculosis and were treated with human-equivalent doses of moxifloxacin (200 mg/kg of body weight) and linezolid (100 mg/kg) with or without verapamil (12.5 mg/kg) for 12 weeks. Pharmacokinetic parameters were collected during treatment at the steady state. After 12 weeks of treatment, a statistically significant decline in mycobacterial load in the lungs was observed with the moxifloxacin-linezolid regimen with and without verapamil (5.9 and 5.0 log CFU, respectively), but sterilization was not achieved yet. The spleens of all mice were culture negative after 12 weeks of treatment with both treatment modalities, and the addition of verapamil caused a significant reduction in relapse (14/14 positive spleens without versus 9/15 with verapamil, P = 0.017). In conclusion, treatment with a combination regimen of moxifloxacin and linezolid showed a strong decline in mycobacterial load in the mice. The addition of verapamil to this backbone had a modest additional effect in terms of reducing mycobacterial load in the lung as well as reducing the spleen relapse rate. These results warrant further studies on the role of efflux pump inhibition in improving the efficacy of MDR-TB backbone regimens.

C1 [Pieterman, Elise D.; de Knegt, Gerjo J.; van der Meijden, Aart; Bax, Hannelore I.; de Steenwinkel, Jurriaan E. M.] Erasmus MC, Dept Med Microbiol & Infect Dis, Rotterdam, Netherlands.

[te Brake, Lindsey H. M.; Aarnoutse, Rob E.] Radboud Univ Nijmegen, Med Ctr, Radboud Inst Hlth Sci, Dept Pharm, Nijmegen, Netherlands.

[te Brake, Lindsey H. M.] Radboud Univ Nijmegen, Med Ctr, Radboud Inst Mol Life Sci, Dept Pharmacol & Toxicol, Nijmegen, Netherlands.

[Alffenaar, Jan-Willem C.] Univ Groningen, Univ Med Ctr Groningen, Dept Clin Pharm & Pharmacol, Groningen, Netherlands.

[Bax, Hannelore I.] Erasmus Univ, Med Ctr, Sect Infect Dis, Dept Internal Med, Rotterdam, Netherlands.

C3 Erasmus University Rotterdam; Erasmus MC; Radboud University Nijmegen;

Radboud University Nijmegen; University of Groningen; Erasmus University

Rotterdam; Erasmus MC

RP Pieterman, ED (corresponding author), Erasmus MC, Dept Med Microbiol & Infect Dis, Rotterdam, Netherlands.

EM e.pieterman@erasmusmc.nl

RI Aarnoutse, R.E./L-4168-2015; Alffenaar, Jan-Willem/AFV-0554-2022; Brake,

Lindsey/C-4245-2014

FU Dutch Organization for Health Research and Development (ZonMw), Priority

Medicines Antimicrobial Resistance [205200002]

FX This work was funded by the Dutch Organization for Health Research and

Development (ZonMw), Priority Medicines Antimicrobial Resistance,

project 205200002.

CR Adams KN, 2014, J INFECT DIS, V210, P456, DOI 10.1093/infdis/jiu095

Alffenaar JWC, 2010, CLIN PHARMACOKINET, V49, P559, DOI 10.2165/11532080-000000000-00000

[Anonymous], C RETR OPP INF SEATT

[Anonymous], 2016, Treatment Guidelines for Drug-Resistant Tuberculosis

Brillault J, 2009, ANTIMICROB AGENTS CH, V53, P1457, DOI 10.1128/AAC.01253-08

Caminero JA, 2010, LANCET INFECT DIS, V10, P621, DOI 10.1016/S1473-3099(10)70139-0

Chang KC, 2013, ANTIMICROB AGENTS CH, V57, P4097, DOI 10.1128/AAC.00120-13

CLSI, 2012, SUSC TEST MYC NOC OT, P31

Coelho T, 2015, FRONT MICROBIOL, V6, DOI [10.3389/fmicb.2015.00330, 10.3389/fmicb.2015.01528]

de Knegt GJ, 2017, INT J ANTIMICROB AG, V49, P302, DOI 10.1016/j.ijantimicag.2016.11.027

De Steenwinkel JEM, 2009, INT J IMMUNOPATH PH, V22, P723, DOI 10.1177/039463200902200318

de Steenwinkel JEM, 2012, ANTIMICROB AGENTS CH, V56, P4937, DOI 10.1128/AAC.00124-12

de Steenwinkel JEM, 2012, EMERG INFECT DIS, V18, P660, DOI 10.3201/eid1804.110912

Deshpande D, 2016, CLIN INFECT DIS, V63, pS88, DOI 10.1093/cid/ciw473

Escribano I, 2007, CHEMOTHERAPY, V53, P397, DOI 10.1159/000109769

Fattorini L, 2003, ANTIMICROB AGENTS CH, V47, P360, DOI 10.1128/AAC.47.1.360-362.2003

Fuenmayor N T, 1992, Drugs, V44 Suppl 1, P1

Gee T, 2001, ANTIMICROB AGENTS CH, V45, P1843, DOI 10.1128/AAC.45.6.1843-1846.2001

Gengenbacher M, 2017, SCI REP-UK, V7, DOI 10.1038/s41598-017-09177-2

Grossman TH, 2015, ANTIMICROB AGENTS CH, V59, P1539, DOI 10.1128/AAC.04271-14

Gupta S, 2015, ANTIMICROB AGENTS CH, V59, P673, DOI 10.1128/AAC.04019-14

Gupta S, 2013, AM J RESP CRIT CARE, V188, P600, DOI 10.1164/rccm.201304-0650OC

Harmelink I.M., 2008, EJHP SCI, V14, P3

Harrigan EP, 2004, J CLIN PSYCHOPHARM, V24, P62, DOI 10.1097/01.jcp.0000104913.75206.62

Hu YM, 2003, ANTIMICROB AGENTS CH, V47, P653, DOI 10.1128/AAC.47.2.653-657.2003

Johnston JC, 2009, PLOS ONE, V4, DOI 10.1371/journal.pone.0006914

Kang YA, 2016, ANN AM THORAC SOC, V13, P364, DOI 10.1513/AnnalsATS.201510-690BC

Lee M, 2012, NEW ENGL J MED, V367, P1508, DOI 10.1056/NEJMoa1201964

López-Muñoz F, 2004, ACTAS ESP PSIQUIATRI, V32, P387

Louw GE, 2011, AM J RESP CRIT CARE, V184, P269, DOI 10.1164/rccm.201011-1924OC

Machado D, 2012, PLOS ONE, V7, DOI 10.1371/journal.pone.0034538

Mourik BC, 2018, SCI REP-UK, V8, DOI 10.1038/s41598-018-24067-x

Pang Y, 2017, J INFECTION, V75, P433, DOI 10.1016/j.jinf.2017.08.005

Pranger AD, 2011, EUR RESPIR J, V38, P888, DOI 10.1183/09031936.00176610

Pranger AD, 2010, J ANAL TOXICOL, V34, P135, DOI 10.1093/jat/34.3.135

Rabbaa L, 1996, ANTIMICROB AGENTS CH, V40, P2126, DOI 10.1128/AAC.40.9.2126

Rodrigues L, 2013, ANTIMICROB AGENTS CH, V57, P751, DOI 10.1128/AAC.01482-12

Ruslami R, 2013, LANCET INFECT DIS, V13, P27, DOI 10.1016/S1473-3099(12)70264-5

Ruslami R, 2010, ANTIMICROB AGENTS CH, V54, P1068, DOI 10.1128/AAC.00447-09

Seral C, 2003, ANTIMICROB AGENTS CH, V47, P1047, DOI 10.1128/AAC.47.3.1047-1051.2003

SHAND DG, 1981, CLIN PHARMACOL THER, V30, P701, DOI 10.1038/clpt.1981.223

Shandil RK, 2007, ANTIMICROB AGENTS CH, V51, P576, DOI 10.1128/AAC.00414-06

Sotgiu G, 2012, EUR RESPIR J, V40, P1430, DOI 10.1183/09031936.00022912

Srivastava S, 2017, ANTIMICROB AGENTS CH, V61, DOI 10.1128/AAC.00751-17

Tasneen R, 2016, ANTIMICROB AGENTS CH, V60, P270, DOI 10.1128/AAC.01691-15

te Brake LHM, 2018, ANNU REV PHARMACOL, V58, P271, DOI 10.1146/annurev-pharmtox-010617-052438

TSURUO T, 1981, CANCER RES, V41, P1967

WHO, 2017, 20171 WHO

Xu J, 2018, ANTIMICROB AGENTS CH, V62, DOI 10.1128/AAC.01692-17

Yang CE, 2012, JPN J INFECT DIS, V65, P240, DOI 10.7883/yoken.65.240

Zhang X, 2015, J THORAC DIS, V7, P603, DOI 10.3978/j.issn.2072-1439.2015.03.10

Zou L, 2015, TUBERCULOSIS, V95, P839, DOI 10.1016/j.tube.2015.07.003

NR 52

TC 8

Z9 10

U1 0

U2 12

PU AMER SOC MICROBIOLOGY

PI WASHINGTON

PA 1752 N ST NW, WASHINGTON, DC 20036-2904 USA

SN 0066-4804

EI 1098-6596

J9 ANTIMICROB AGENTS CH

JI Antimicrob. Agents Chemother.

PD SEP

PY 2018

VL 62

IS 9

AR e01354-18

DI 10.1128/AAC.01354-18

PG 9

WC Microbiology; Pharmacology & Pharmacy

WE Science Citation Index Expanded (SCI-EXPANDED)

SC Microbiology; Pharmacology & Pharmacy

GA GR6NN

UT WOS:000442771200077

OA Green Submitted, Bronze

DA 2026-05-07

ER

PT J

AU Chaiyachat, P

Kaewseekhao, B

Chaiprasert, A

Kamolwat, P

Nonghanphithak, D

Phetcharaburanin, J

Sirichoat, A

Ong, RTH

Faksri, K

AF Chaiyachat, Pratchakan

Kaewseekhao, Benjawan

Chaiprasert, Angkana

Kamolwat, Phalin

Nonghanphithak, Ditthawat

Phetcharaburanin, Jutarop

Sirichoat, Auttawit

Ong, Rick Twee-Hee

Faksri, Kiatichai

TI Metabolomic analysis of Mycobacterium tuberculosis reveals

metabolic profiles for identification of drug-resistant tuberculosis

SO SCIENTIFIC REPORTS

LA English

DT Article

ID BIOMARKERS

AB The detection of pre-extensively (pre-XDR) and extensively drug-resistant tuberculosis (XDR-TB) is challenging. Drug-susceptibility tests for some anti-TB drugs, especially ethambutol (ETH) and ethionamide (ETO), are problematic due to overlapping thresholds to differentiate between susceptible and resistant phenotypes. We aimed to identify possible metabolomic markers to detect Mycobacterium tuberculosis (Mtb) strains causing pre-XDR and XDR-TB. The metabolic patterns of ETH- and ETO-resistant Mtb isolates were also investigated. Metabolomics of 150 Mtb isolates (54 pre-XDR, 63 XDR-TB and 33 pan-susceptible; pan-S) were investigated. Metabolomics of ETH and ETO phenotypically resistant subgroups were analyzed using UHPLC-ESI-QTOF-MS/MS. Orthogonal partial least-squares discriminant analysis revealed distinct separation in all pairwise comparisons among groups. Two metabolites (meso-hydroxyheme and itaconic anhydride) were able to differentiate the pre-XDR and XDR-TB groups from the pan-S group with 100% sensitivity and 100% specificity. In comparisons of the ETH and ETO phenotypically resistant subsets, sets of increased (ETH = 15, ETO = 7) and decreased (ETH = 1, ETO = 6) metabolites specific for the resistance phenotype of each drug were found. We demonstrated the potential for metabolomics of Mtb to differentiate among types of DR-TB as well as between isolates that were phenotypically resistant to ETO and ETH. Thus, metabolomics might be further applied for DR-TB diagnosis and patient management.

C1 [Chaiyachat, Pratchakan; Kaewseekhao, Benjawan; Nonghanphithak, Ditthawat; Sirichoat, Auttawit; Faksri, Kiatichai] Khon Kaen Univ, Fac Med, Dept Microbiol, Khon Kaen, Thailand.

[Chaiyachat, Pratchakan; Kaewseekhao, Benjawan; Nonghanphithak, Ditthawat; Sirichoat, Auttawit; Faksri, Kiatichai] Khon Kaen Univ, Res & Diagnost Ctr Emerging Infect Dis RCEID, Khon Kaen, Thailand.

[Chaiprasert, Angkana] Mahidol Univ, Siriraj Hosp, Fac Med, Off Res & Dev, Bangkok, Thailand.

[Kamolwat, Phalin] Minist Publ Hlth, Bur TB, Dept Dis Control, Nonthaburi, Thailand.

[Phetcharaburanin, Jutarop] Khon Kaen Univ, Fac Med, Dept Syst Biosci & Computat Med, Khon Kaen, Thailand.

[Ong, Rick Twee-Hee] Natl Univ Singapore, Saw Swee Hock Sch Publ Hlth, Singapore, Singapore.

[Ong, Rick Twee-Hee] Natl Univ Hlth Syst, Singapore, Singapore.

C3 Khon Kaen University; Khon Kaen University; Mahidol University; Ministry

of Public Health - Thailand; Khon Kaen University; National University

of Singapore; National University of Singapore

RP Faksri, K (corresponding author), Khon Kaen Univ, Fac Med, Dept Microbiol, Khon Kaen, Thailand.; Faksri, K (corresponding author), Khon Kaen Univ, Res & Diagnost Ctr Emerging Infect Dis RCEID, Khon Kaen, Thailand.

EM kiatichai@kku.ac.th

RI Sirichoat, Auttawit/GQA-9663-2022

OI Sirichoat, Auttawit/0000-0001-7681-4295; Ong, Rick

Twee-Hee/0000-0002-0197-7820

FU National Research council of Thailand; Research and Diagnostic Center

for Emerging Infectious Diseases (RCEID), Khon Kaen University, Khon

Kaen, Thailand; Royal Golden Jubilee (RGJ)-Ph.D. program of the Thailand

Research Fund (TRF) [PHD/0044/2560]

FX This work was supported by National Research council of Thailand and

Research and Diagnostic Center for Emerging Infectious Diseases (RCEID),

Khon Kaen University, Khon Kaen, Thailand. This work was also supported

by the Royal Golden Jubilee (RGJ)-Ph.D. program Grant (PHD/0044/2560) of

the Thailand Research Fund (TRF). These funding sources had no role in

design of the study and collection, analysis, interpretation of data and

in writing the manuscript.

CR Ady CNAE, 2017, J NEUROSCI RES, V95, P2005, DOI 10.1002/jnr.24048

Ängeby K, 2012, B WORLD HEALTH ORGAN, V90, P693, DOI 10.2471/BLT.11.096644

[Anonymous], 2021, Global tuberculosis report 2021

[Anonymous], 1997, WHOTB97229 IUATLD

[Anonymous], 2013, Definitions and reporting framework for tuberculosis-2013 revision

Atkinson Beth, 2023, CRAN

Aung WW, 2015, ANN LAB MED, V35, P494, DOI 10.3343/alm.2015.35.5.494

Brandao AP, 2020, TUBERCULOSIS, V125, DOI 10.1016/j.tube.2020.102004

Caspi R, 2016, NUCLEIC ACIDS RES, V44, pD471, DOI 10.1093/nar/gkv1164

Chao A, 2019, CHEM REV, V119, P1193, DOI 10.1021/acs.chemrev.8b00285

Das MK, 2016, ANTIMICROB AGENTS CH, V60, P2257, DOI 10.1128/AAC.02586-15

de Carvalho LPS, 2010, CHEM BIOL, V17, P1122, DOI 10.1016/j.chembiol.2010.08.009

du Preez I, 2017, BIOMARK MED, V11, P179, DOI 10.2217/bmm-2016-0287

faahKO: Saghatelian, 2022, FAAH KNOCKOUT LC MS, DOI [10.1021/bi0480335, DOI 10.1021/BI0480335]

Faksri K, 2019, INT J ANTIMICROB AG, V54, P109, DOI 10.1016/j.ijantimicag.2019.04.004

Faksri K, 2018, SCI REP-UK, V8, DOI 10.1038/s41598-018-23337-y

Faksri K, 2016, BMC GENOMICS, V17, DOI 10.1186/s12864-016-3213-1

Fernández-Albert F, 2014, BIOINFORMATICS, V30, P1937, DOI 10.1093/bioinformatics/btu136

Grabowicz M, 2017, TRENDS BIOCHEM SCI, V42, P232, DOI 10.1016/j.tibs.2016.10.002

Graves AB, 2014, INORG CHEM, V53, P5931, DOI 10.1021/ic500033b

HOLLIGER C, 1992, J BACTERIOL, V174, P4435, DOI 10.1128/JB.174.13.4435-4443.1992

Huang H, 2020, EXP BIOL MED, V245, P501, DOI 10.1177/1535370220903464

Idle JR, 2007, CELL METAB, V6, P348, DOI 10.1016/j.cmet.2007.10.005

Jajou R, 2019, J ANTIMICROB CHEMOTH, V74, P2605, DOI 10.1093/jac/dkz215

Johnson RM, 2018, PATHOG DIS, V76, DOI 10.1093/femspd/fty048

Kardan-Yamchi J, 2021, ARCH MICROBIOL, V203, P3989, DOI 10.1007/s00203-021-02387-3

Kent PT., 1985, PUBLIC HLTH MYCOBACT

Klotoe BJ, 2019, BMC INFECT DIS, V19, DOI 10.1186/s12879-019-4201-2

Knapp GS, 2014, MICROBIOL SPECTR, V2, DOI 10.1128/microbiolspec.MGM2-0011-2013

Layre E, 2011, CHEM BIOL, V18, P1537, DOI 10.1016/j.chembiol.2011.10.013

Lee JH, 2012, NAT COMMUN, V3, DOI 10.1038/ncomms1724

Mahé P, 2019, PEERJ, V7, DOI 10.7717/peerj.6857

Matsui T, 2016, P NATL ACAD SCI USA, V113, P3779, DOI 10.1073/pnas.1523333113

May EE, 2013, COMPUT BIOL CHEM, V47, P167, DOI 10.1016/j.compbiolchem.2013.07.002

Nonghanphithak D, 2021, EMERG INFECT DIS, V27, P813, DOI 10.3201/eid2703.204364

Nonghanphithak D, 2020, PLOS ONE, V15, DOI 10.1371/journal.pone.0244829

Organization W. H., 2021, FACTSH GLOB TB REP

Pal R, 2017, 3 BIOTECH, V7, DOI 10.1007/s13205-017-0972-6

Pang ZQ, 2021, NUCLEIC ACIDS RES, V49, pW388, DOI 10.1093/nar/gkab382

Pillay S, 2022, COCHRANE DB SYST REV, DOI 10.1002/14651858.CD014841.pub2

Rafael LL, 2021, INFECT GENET EVOL, V96, DOI 10.1016/j.meegid.2021.105142

Rêgo AM, 2021, TUBERCULOSIS, V126, DOI 10.1016/j.tube.2020.102043

Sharma D, 2017, FRONT CELL INFECT MI, V7, DOI 10.3389/fcimb.2017.00240

Singh Pranveer, 2019, Front Biosci (Elite Ed), V11, P38, DOI 10.2741/e845

Skaar EP, 2010, PLOS PATHOG, V6, DOI 10.1371/journal.ppat.1000949

Sun YN, 2017, PLOS ONE, V12, DOI 10.1371/journal.pone.0168865

Thakuri B, 2021, BIOCHEMISTRY-US, V60, P918, DOI 10.1021/acs.biochem.0c00892

Thawornwattana Y, 2021, MICROB GENOMICS, V7, DOI 10.1099/mgen.0.000697

Trisakul K, 2022, EMERG MICROBES INFEC, V11, P1857, DOI 10.1080/22221751.2022.2099304

Vorkas PA, 2015, ANAL CHEM, V87, P4184, DOI 10.1021/ac503775m

WHO, 1998, INT J TUBERC LUNG D, V2, P71

Xu YH, 2020, BIOSAF HEALTH, V2, P135, DOI 10.1016/j.bsheal.2020.04.001

Zhang HN, 2017, SCI REP-UK, V7, DOI 10.1038/s41598-017-06289-7

Zhang PX, 2018, CLIN CHIM ACTA, V483, P57, DOI 10.1016/j.cca.2018.04.022

NR 54

TC 14

Z9 21

U1 0

U2 6

PU NATURE PORTFOLIO

PI BERLIN

PA HEIDELBERGER PLATZ 3, BERLIN, 14197, GERMANY

SN 2045-2322

J9 SCI REP-UK

JI Sci Rep

PD MAY 27

PY 2023

VL 13

IS 1

AR 8655

DI 10.1038/s41598-023-35882-2

PG 13

WC Multidisciplinary Sciences

WE Science Citation Index Expanded (SCI-EXPANDED)

SC Science & Technology - Other Topics

GA H6SD3

UT WOS:000997228700040

OA Green Submitted, gold

DA 2026-05-07

ER

PT J

AU Imran, M

Arora, MK

Chaudhary, A

Khan, SA

Kamal, M

Alshammari, MM

Alharbi, RM

Althomali, NA

Alzimam, IM

Alshammari, AA

Alharbi, BH

Alshengeti, A

Alsaleh, AA

Alqahtani, SA

Rabaan, AA

AF Imran, Mohd.

Arora, Mandeep Kumar

Chaudhary, Anurag

Khan, Shah Alam

Kamal, Mehnaz

Alshammari, Manal Mutlaq

Alharbi, Raghad Mohammad

Althomali, Nuha Abdullah

Alzimam, Ibrahim Mohammed

Alshammari, Abdullah Ayed

Alharbi, Bashair Hamed

Alshengeti, Amer

Alsaleh, Abdulmonem A.

Alqahtani, Shayea A.

Rabaan, Ali A.

TI MmpL3 Inhibition as a Promising Approach to Develop Novel Therapies

against Tuberculosis: A Spotlight on SQ109, Clinical Studies, and

Patents Literature

SO BIOMEDICINES

LA English

DT Review

DE tuberculosis; drug-resistance; Mmpl3; SQ109; clinical studies; patent

ID ANTITUBERCULAR DRUG; DISCOVERY; TRANSPORTER; RIFAMPICIN; DIAGNOSIS

AB Tuberculosis (TB) is accountable for considerable global morbidity and mortality. Effective TB therapy with multiple drugs completes in about six months. The longer duration of TB therapy challenges patient compliance and contributes to treatment collapse and drug resistance (DR) progress. Therefore, new medications with an innovative mechanism of action are desperately required to shorten the TB therapy's duration and effective TB control. The mycobacterial membrane protein Large 3 (MmpL3) is a novel, mycobacteria-conserved and recognized promiscuous drug target used in the development of better treatments for multi-drug resistance TB (MDR-TB) and extensively drug-resistant TB (XDR-TB). This article spotlights MmpL3, the clinical studies of its inhibitor (SQ109), and the patent literature. The literature on MmpL3 inhibitors was searched on PubMed and freely available patent databases (Espacenet, USPTO, and PatentScope). SQ109, an analog of ethambutol (EMB), is an established MmpL3 inhibitor and has completed Phase 2b-3 clinical trials. Infectex and Sequella are developing orally active SQ109 in partnership to treat MDR pulmonary TB. SQ109 has demonstrated activity against drug-sensitive (DS) and drug-resistant (DR) Mycobacterium tuberculosis (Mtb) and a synergistic effect with isoniazid (INH), rifampicin (RIF), clofazimine (CFZ), and bedaquiline (BNQ). The combination of SQ109, clofazimine, bedaquiline, and pyrazinamide (PZA) has been patented due to its excellent anti-TB activity against MDR-TB, XDR-TB, and latentTB. The combinations of SQ109 with other anti-TB drugs (chloroquine, hydroxychloroquine, and sutezolid) have also been claimed in the patent literature. SQ109 is more potent than EMB and could substitute EMB in the intensive stage of TB treatment with the three- or four-drug combination. Developing MmpL3 inhibitors is a promising approach to fighting the challenges associated with DS-TB and DR-TB. The authors foresee MmpL3 inhibitors such as SQ109 as future drugs for TB treatment.

C1 [Imran, Mohd.] Northern Border Univ, Dept Pharmaceut Chem, Fac Pharm, Rafha 91911, Saudi Arabia.

[Arora, Mandeep Kumar] DIT Univ, Sch Pharmaceut & Populat Hlth Informat, Dehra Dun 248009, Uttarakhand, India.

[Chaudhary, Anurag] Meerut Inst Engn & Technol, Dept Pharmaceut Technol, Meerut 250005, Uttar Pradesh, India.

[Khan, Shah Alam] Natl Univ Sci & Technol, Coll Pharm, Dept Pharmaceut Chem, Muscat 130, Oman.

[Kamal, Mehnaz] Prince Sattam Bin Abdulaziz Univ, Coll Pharm, Dept Pharmaceut Chem, Al Kharj 11942, Saudi Arabia.

[Alshammari, Manal Mutlaq] Hotat Bani Tamim Gen Hosp, Pharm Dept, Hotat Bani Tamim 16631, Saudi Arabia.

[Alharbi, Raghad Mohammad] Umm Alqura Univ, Dept Pharm, Mecca 24225, Saudi Arabia.

[Althomali, Nuha Abdullah] Kol Alosra, Dept Community Pharm, Taif 26512, Saudi Arabia.

[Alzimam, Ibrahim Mohammed] Northern Border Univ, Fac Pharm, Rafha 91911, Saudi Arabia.

[Alshammari, Abdullah Ayed] Nahdi Med Co, Riyadh 12211, Saudi Arabia.

[Alharbi, Bashair Hamed] Umm Alqura Univ, Dept Clin Pharm, Jeddah 21921, Saudi Arabia.

[Alshengeti, Amer] Taibah Univ, Coll Med, Dept Pediat, Al Madinah 41491, Saudi Arabia.

[Alshengeti, Amer] Prince Mohammad Bin Abdulaziz Hosp, Dept Infect Prevent & Control, Natl Guard Hlth Affairs, Al Madinah 41491, Saudi Arabia.

[Alsaleh, Abdulmonem A.] Mohammed Al Mana Coll Med Sci, Clin Lab Sci Dept, Dammam 34222, Saudi Arabia.

[Alqahtani, Shayea A.] Erhadh Hosp, Med Lab Dept, Dammam 32434, Saudi Arabia.

[Rabaan, Ali A.] Johns Hopkins Aramco Healthcare, Mol Diagnost Lab, Dhahran 31311, Saudi Arabia.

[Rabaan, Ali A.] Alfaisal Univ, Coll Med, Riyadh 11533, Saudi Arabia.

[Rabaan, Ali A.] Univ Haripur, Dept Publ Hlth & Nutr, Haripur 22610, Pakistan.

C3 Northern Border University; DIT University; Meerut Institute of

Engineering & Technology; National University of Science & Technology,

Oman; Prince Sattam Bin Abdulaziz University; Umm Al-Qura University;

Northern Border University; Umm Al-Qura University; Taibah University;

King Saud Bin Abdulaziz University for Health Sciences; Ministry of

National Guard - Health Affairs; Mohammed Al-Mana College for Medical

Sciences; Johns Hopkins Aramco Healthcare; Johns Hopkins University;

Johns Hopkins Medicine; Alfaisal University

RP Imran, M (corresponding author), Northern Border Univ, Dept Pharmaceut Chem, Fac Pharm, Rafha 91911, Saudi Arabia.

EM imran.pchem@gmail.com

RI Rabaan, Ali/AAI-3928-2020; Chaudhary, Anurag/AGF-7327-2022; Alsaleh,

Abdulmonem/LRU-9679-2024; khan, shah/AAX-8826-2020; KAMAL,

MEHNAZ/AAE-7681-2019; Ansari, Mohd. Imran/AAV-4144-2021; Arora,

Mandeep/AAQ-6056-2020; Alshammari, Abdullah/KVA-8799-2024

OI Rabaan, Ali/0000-0002-6774-9847; Chaudhary, Anurag/0000-0002-1657-811X;

Alsaleh, Abdulmonem/0000-0002-1188-1451; khan, shah/0000-0002-0729-3403;

KAMAL, MEHNAZ/0000-0002-3585-0828; Ansari, Mohd.

Imran/0000-0002-6064-1040; Arora, Mandeep/0000-0001-6977-2939;

CR Addison W, 2022, RSC MED CHEM, V13, P392, DOI 10.1039/d1md00359c

[Anonymous], 2008, Tuberculosis (Edinb), V88, P159, DOI 10.1016/S1472-9792(08)70026-X

[Anonymous], 2013, CONSOLIDATED GUIDELI

[Anonymous], 2021, Global Tuberculosis Report

[Anonymous], About Us

[Anonymous], SQ109

Bahuguna A, 2020, MED RES REV, V40, P263, DOI 10.1002/med.21602

Bajad Nilesh Gajanan, 2022, Curr Res Pharmacol Drug Discov, V3, P100119, DOI [10.1016/j.crphar.2022.100119, 10.1016/j.crphar.2022.100119]

Barry C.E., 2003, WO Patent, Patent No. [200,309,698,9, 2003096989]

Battaglia G., 2017, WO Patent, Patent No. [201,719,144,4, 2017191444]

Belardinelli JM, 2016, ACS INFECT DIS, V2, P702, DOI 10.1021/acsinfecdis.6b00095

Bishai W.R., 2015, WO Patent, Patent No. [201,516,448,2, 2015164482]

Black TA, 2021, J CLIN TUBER OTH MYC, V25, DOI 10.1016/j.jctube.2021.100285

Blumberg HM, 2016, JAMA-J AM MED ASSOC, V316, P931, DOI 10.1001/jama.2016.11021

Boeree MJ, 2017, LANCET INFECT DIS, V17, P39, DOI 10.1016/S1473-3099(16)30274-2

Bolla JR, 2020, BIOCHEM SOC T, V48, P1463, DOI 10.1042/BST20190950

Brickner S.J., 2013, EP Patent, Patent No. [234,002,2, 2340022]

Campaniço A, 2018, EUR J MED CHEM, V150, P525, DOI 10.1016/j.ejmech.2018.03.020

Dey R, 2020, CURR TOP MED CHEM, V20, P2662, DOI 10.2174/1568026620666200903163921

Feske ML, 2011, TUBERCULOSIS, V91, pS16, DOI 10.1016/j.tube.2011.10.005

Freundlich J.S., 2021, U.S. Patent, Patent No. [111,865,49, 11186549]

Freundlich J.S., 2019, WO Patent, Patent No. [201,904,646,5, 2019046465]

Frykman P.K., 2017, U.S. Patent, Patent No. [201,701,894,74, 20170189474]

Goldman Robert C., 2020, Infectious Disorders - Drug Targets, V20, P352, DOI [10.2174/1871526519666181205163810, 10.2174/1871526519666181205163810]

Hawn TR, 2014, MICROBIOL MOL BIOL R, V78, P650, DOI 10.1128/MMBR.00021-14

Heinrich N, 2015, J ANTIMICROB CHEMOTH, V70, P1558, DOI 10.1093/jac/dku553

Ho C.M., 2020, U.S. Patent, Patent No. [105,760,79, 10576079]

Ho C.M., 2018, WO Patent, Patent No. [201,819,162,8, 2018191628]

Iliev I., 2021, U.S. Patent, Patent No. [202,106,915,6, 2021069156]

Imran M, 2022, J INFECT PUBLIC HEAL, V15, P1097, DOI 10.1016/j.jiph.2022.08.016

Imran M, 2022, ANTIOXIDANTS-BASEL, V11, DOI 10.3390/antiox11050876

Imran M, 2022, NUTRIENTS, V14, DOI 10.3390/nu14061227

Imran M, 2022, EXPERT OPIN THER PAT, V32, P591, DOI 10.1080/13543776.2022.2045948

Imran M, 2021, BIOMEDICINES, V9, DOI 10.3390/biomedicines9121938

Imran M, 2021, PHARMACEUTICALS-BASE, V14, DOI 10.3390/ph14080710

Imran M, 2021, EXPERT OPIN THER PAT, V31, P759, DOI 10.1080/13543776.2021.1902990

Iqbal IK, 2018, PATHOGENS, V7, DOI 10.3390/pathogens7010024

Jiricek J., 2016, U.S. Patent, Patent No. [944,703,9, 9447039]

Joshua D., 2021, CN Patent, Patent No. [112,423,746, 112423746]

Kaneko T., 2021, WO Patent, Patent No. [202,106,231,9, 2021062319]

Kayigire XA, 2015, TUBERCULOSIS, V95, P770, DOI 10.1016/j.tube.2015.08.001

Kim YK, 2022, Patent, Patent No. [KR10-2022-0081273, 1020220081273]

Lee RE, 2003, J COMB CHEM, V5, P172, DOI 10.1021/cc020071p

Leibert E, 2010, EXPERT REV ANTI-INFE, V8, P801, DOI 10.1586/ERI.10.60

Liu D., 2022, CN Patent, Patent No. [112,028,811, 112028811]

Liu D., 2022, CN Patent, Patent No. [112,028,812, 112028812]

Liu S., 2021, CN Patent, Patent No. [113,768,909, 113768909]

Locher C.P., 2017, U.S. Patent, Patent No. [957,280,9, 9572809]

Malwal SR, 2021, ACS INFECT DIS, V7, P2492, DOI 10.1021/acsinfecdis.1c00259

Marais BJ, 2005, ARCH DIS CHILD, V90, P1162, DOI 10.1136/adc.2004.070797

Miggiano R, 2020, PATHOGENS, V9, DOI 10.3390/pathogens9050385

North J., 2019, U.S. Patent, Patent No. [103,838,48, 10383848]

North J., 2020, U.S. Patent, Patent No. [202,033,189,7, 2020331897]

Oldfield E, 2015, Patent No. [201,503,523,4, 2015035234, WO 2015035234 A2]

Oommen S, 2017, INDIAN J MED MICROBI, V35, P323, DOI 10.4103/ijmm.IJMM_16_204

Pages L.B., 2020, U.S. Patent, Patent No. [106,248,93, 10624893]

Prasad Rajendra, 2010, Indian J Tuberc, V57, P180

Protopopova M, 2005, J ANTIMICROB CHEMOTH, V56, P968, DOI 10.1093/jac/dki319

Protopopova M.N., 2012, U.S. Patent, Patent No. [826,889,4, 8268894]

Protopopova M.N., 2008, U.S. Patent, Patent No. [745,622,2, 7456222]

Protopopova M. N., 2012, Patent Appl. US, Patent No. [820,291,0, 8202910]

Protopopova M.N., 2010, U.S. Patent, Patent No. [784,272,9, 7842729]

Rao Z., 2020, WO Patent, Patent No. [202,010,385,6, 2020103856]

Rayasam GV, 2014, EXPERT OPIN THER TAR, V18, P247, DOI 10.1517/14728222.2014.859677

Reddy D.S., 2019, WO Patent, Patent No. [201,922,046,1, 2019220461]

Rendon A, 2016, J THORAC DIS, V8, P2666, DOI 10.21037/jtd.2016.10.14

Sacksteder KA, 2012, FUTURE MICROBIOL, V7, P823, DOI [10.2217/fmb.12.56, 10.2217/FMB.12.56]

Saxena AK, 2019, CURR TOP MED CHEM, V19, P337, DOI 10.2174/1568026619666190219105722

Sethiya JP, 2020, INT J MOL SCI, V21, DOI 10.3390/ijms21176202

Shao M, 2020, EUR J MED CHEM, V200, DOI 10.1016/j.ejmech.2020.112390

Sharma U.K., 2017, U.S. Patent, Patent No. [201,713,610,2, 2017136102]

Singh A., 2021, WO Patent, Patent No. [202,109,028,3, 2021090283]

Su CC, 2021, PLOS BIOL, V19, DOI 10.1371/journal.pbio.3001370

Su CC, 2019, P NATL ACAD SCI USA, V116, P11241, DOI 10.1073/pnas.1901346116

Swarts B.M., 2020, U.S. Patent, Patent No. [107,598,21, 10759821]

Umare MD, 2021, CHEMMEDCHEM, V16, P3136, DOI 10.1002/cmdc.202100359

Xu ZJ, 2017, P NATL ACAD SCI USA, V114, P7993, DOI 10.1073/pnas.1700062114

NR 77

TC 26

Z9 30

U1 1

U2 11

PU MDPI

PI BASEL

PA MDPI AG, Grosspeteranlage 5, CH-4052 BASEL, SWITZERLAND

EI 2227-9059

J9 BIOMEDICINES

JI Biomedicines

PD NOV

PY 2022

VL 10

IS 11

AR 2793

DI 10.3390/biomedicines10112793

PG 15

WC Biochemistry & Molecular Biology; Medicine, Research & Experimental;

Pharmacology & Pharmacy

WE Science Citation Index Expanded (SCI-EXPANDED)

SC Biochemistry & Molecular Biology; Research & Experimental Medicine;

Pharmacology & Pharmacy

GA 6A9NY

UT WOS:000880974500001

OA Green Submitted, gold

DA 2026-05-07

ER

PT J

AU Nair, V

Okello, MO

Mangu, NK

Seo, BI

Gund, MG

AF Nair, Vasu

Okello, Maurice O.

Mangu, Naveen K.

Seo, Byung I.

Gund, Machhindra G.

TI A novel molecule with notable activity against multi-drug resistant

tuberculosis

SO BIOORGANIC & MEDICINAL CHEMISTRY LETTERS

LA English

DT Article

DE Drug discovery; Anti-MDR TB; Synergism; CYP and UGT profiles

ID HIV INTEGRASE INHIBITORS; MYCOBACTERIUM-TUBERCULOSIS; DRUG DISCOVERY;

POTENT; AIDS; RALTEGRAVIR; METABOLISM

AB Multi-drug resistant tuberculosis (MDR-TB) is emerging as a serious global health problem, which has been elevated through co-infection involving HIV and MDR-Mtb. The discovery of new compounds with anti-MDR TB efficacy and favorable metabolism profiles is an important scientific challenge. Using computational biology and ligand docking data, we have conceived a multifunctional molecule, 2, as a potential anti-MDR TB agent. This compound was produced through a multi-step synthesis. It exhibited significant in vitro activity against MDR-TB (MIC 1.56 mu g/mL) and its half-life (t(1/2)) in human liver microsomes was 14.4 h. The metabolic profiles of compound 2 with respect to human cytochrome P450 (CYP) and uridine 5'-diphospho-glucuronosyltransferase (UGT) isozymes were favorable. Compound 2 also had relatively low in vitro cytotoxicity in uninfected macrophages. It displayed synergistic behavior against MDR-TB in combination with PA-824. Interestingly, compound 2 also displayed in vitro anti-HIV activity. (C) 2015 Elsevier Ltd. All rights reserved.

C1 [Nair, Vasu] Univ Georgia, UGA Ctr Drug Discovery, RC Wilson Pharm, Athens, GA 30602 USA.

Univ Georgia, Coll Pharm, RC Wilson Pharm, Athens, GA 30602 USA.

C3 University System of Georgia; University of Georgia; University System

of Georgia; University of Georgia

RP Nair, V (corresponding author), Univ Georgia, UGA Ctr Drug Discovery, RC Wilson Pharm, Room 320A, Athens, GA 30602 USA.

EM vnair@uga.edu

RI Seo, Byung-In/L-2810-2019; Mangu, Naveen/J-1738-2014

FU National Institutes of Health, United States [R01 AI 43181]; Terry

Endowment [RR10211184]; Georgia Research Alliance Eminent Scholar Award,

United States [GN012726]

FX Support of this research by the National Institutes of Health, United

States (R01 AI 43181) is gratefully acknowledged. The contents of this

Letter are solely the responsibility of the authors and do not

necessarily represent the official views of the NIH. One of us (V.N.)

also acknowledges support from the Terry Endowment (RR10211184) and from

the Georgia Research Alliance Eminent Scholar Award (GN012726), United

States. The in vitro anti-MDR TB and anti-HIV data were determined at

the Southern Research Institute, Birmingham, AL and Frederick, MD.

CR Da Silva PEA, 2011, J ANTIMICROB CHEMOTH, V66, P1417, DOI 10.1093/jac/dkr173

Andries K, 2005, SCIENCE, V307, P223, DOI 10.1126/science.1106753

[Anonymous], Global update on HIV treatment 2013: Results, impact and opportunities (PDF)

[Anonymous], 2012, WHO DRUG RES TUB

[Anonymous], GLOB REP UNAIDS REP

Baranczewski P, 2006, PHARMACOL REP, V58, P453

Campbell EA, 2001, CELL, V104, P901, DOI 10.1016/S0092-8674(01)00286-0

Chang CC, 2013, IMMUNOL REV, V254, P114, DOI 10.1111/imr.12063

Chauret N, 1997, DRUG METAB DISPOS, V25, P1130

de Montellano PR Ortiz., 2005, Cytochrome P450 Structure, Mechanism, and Biochemistry, VThird

Diacon AH, 2012, ANTIMICROB AGENTS CH, V56, P3027, DOI 10.1128/AAC.06125-11

Dye C, 2010, SCIENCE, V328, P856, DOI 10.1126/science.1185449

Kassahun K, 2007, DRUG METAB DISPOS, V35, P1657, DOI 10.1124/dmd.107.016196

Koul A., 2014, NAT COMMUN, P5

Koul A, 2011, NATURE, V469, P483, DOI 10.1038/nature09657

Nair V, 2006, J MED CHEM, V49, P445, DOI 10.1021/jm0508890

Nair V., 2014, INT C ANT AG CHEM

Nair V, 2007, REV MED VIROL, V17, P277, DOI 10.1002/rmv.539

Nair V, 2014, ANTIVIR RES, V108, P25, DOI 10.1016/j.antiviral.2014.05.001

Okello M, 2013, ORG BIOMOL CHEM, V11, P7852, DOI 10.1039/c3ob41728j

Okello MO, 2013, ANTIVIR RES, V98, P365, DOI 10.1016/j.antiviral.2013.04.005

Orenstein EW, 2009, LANCET INFECT DIS, V9, P153, DOI 10.1016/S1473-3099(09)70041-6

Pawlowski A, 2012, PLOS PATHOG, P8

Pethe K, 2013, NAT MED, V19, P1157, DOI 10.1038/nm.3262

Phillips L, 2013, NATURE, V493, P14, DOI 10.1038/493014a

Pommier Y, 2005, NAT REV DRUG DISCOV, V4, P236, DOI 10.1038/nrd1660

Reddy VM, 2010, ANTIMICROB AGENTS CH, V54, P2840, DOI 10.1128/AAC.01601-09

Russell DG, 2010, SCIENCE, V328, P852, DOI 10.1126/science.1184784

Seo BI, 2011, ACS MED CHEM LETT, V2, P877, DOI 10.1021/ml2001246

Siika A. M., 2013, PLOS ONE, P8

SMITH SD, 1984, CANCER RES, V44, P5657

Strassburg CP, 2002, GUT, V50, P259, DOI 10.1136/gut.50.2.259

Summa V, 2008, J MED CHEM, V51, P5843, DOI 10.1021/jm800245z

Taktakishvili M, 2000, J AM CHEM SOC, V122, P5671, DOI 10.1021/ja992528d

*WHO, 2013, WHO MULT RES TUB MDR

World Health Organization, 2014, ANT RES GLOB REP SUR

Zenkin N, 2005, ANTIMICROB AGENTS CH, V49, P1587, DOI 10.1128/AAC.49.4.1587-1590.2005

NR 37

TC 8

Z9 10

U1 0

U2 21

PU PERGAMON-ELSEVIER SCIENCE LTD

PI OXFORD

PA THE BOULEVARD, LANGFORD LANE, KIDLINGTON, OXFORD OX5 1GB, ENGLAND

SN 0960-894X

EI 1464-3405

J9 BIOORG MED CHEM LETT

JI Bioorg. Med. Chem. Lett.

PD MAR 15

PY 2015

VL 25

IS 6

BP 1269

EP 1273

DI 10.1016/j.bmcl.2015.01.050

PG 5

WC Chemistry, Medicinal; Chemistry, Organic

WE Science Citation Index Expanded (SCI-EXPANDED); Index Chemicus (IC)

SC Pharmacology & Pharmacy; Chemistry

GA CC9MZ

UT WOS:000350696000020

OA Green Submitted

DA 2026-05-07

ER

PT J

AU Imran, M

Alshrari, AS

Thabet, HK

Abida

Bakht, MA

AF Imran, Mohd

Alshrari, A. S.

Thabet, Hamdy Kh.

Abida

Bakht, Md. Afroz

TI Synthetic molecules as DprE1 inhibitors: A patent review

SO EXPERT OPINION ON THERAPEUTIC PATENTS

LA English

DT Review

DE Azaindole; benzothiazinone; dpre1 inhibitors; patent review; synthetic

molecules; tuberculosis

AB Introduction

In recent years, the advent of multidrug-resistant tuberculosis (MDR-TB), the extensively-resistant TB (XDR-TB), and the total drug-resistant-TB (TDR-TB) have led the community to develop new antitubercular molecules. The decaprenylphosphoryl-beta-D-ribose-2MODIFIER LETTER PRIME-epimerase-1 (DprE1) is an established target to developed new anti-TB drugs. This enzyme is required to synthesize the cell wall of Mycobacterium tuberculosis (Mtb).

Area Covered

This patent review focuses on the granted patents and patent applications related to the chemical entities developed as DprE1 inhibitors for TB treatment from the publication year of the BTZ-043 compound patent application (2007) till 30 September 2020.

Expert Opinion

The DprE1 has many advantages in the development of new antitubercular molecules, for example, its location in the periplasm of the Mtb cell wall and its absence in the human body. This indicates that the DprE1 inhibitors are selective for Mtb, and their toxic and side effects on the human body may be negligible or small. Accordingly, the use of DprE1 inhibitors may be benefic for patients with drug-resistant bacteria that require long-term medication. Four molecules are in clinical trials, which could become the drugs of the future for TB-therapy.

C1 [Imran, Mohd; Abida] Northern Border Univ, Dept Pharmaceut Chem, Fac Pharm, Rafha, Saudi Arabia.

[Alshrari, A. S.] Northern Border Univ, Dept Biol Sci, Fac Sci, Ar Ar, Saudi Arabia.

[Thabet, Hamdy Kh.] Northern Border Univ, Fac Arts & Sci, Dept Chem, Rafha, Saudi Arabia.

[Bakht, Md. Afroz] Prince Sattam Bin Abdulaziz Univ, Coll Sci & Humanities, Dept Chem, Al Kharj, Saudi Arabia.

C3 Northern Border University; Northern Border University; Northern Border

University; Prince Sattam Bin Abdulaziz University

RP Imran, M (corresponding author), Northern Border Univ, Dept Pharmaceut Chem, Fac Pharm, Rafha, Saudi Arabia.

EM imran.pchem@gmail.com

RI Alshrari, Ahmed/JJF-4976-2023; Bakht, Afroz/CAF-8716-2022; Ansari, Mohd.

Imran/AAV-4144-2021; Thabet, Hamdy/AGS-5648-2022; Khan,

Abida/KBB-2904-2024

OI Alshrari, Ahmed/0000-0002-3666-3848; Bakht, Afroz/0000-0002-3382-0457;

Ansari, Mohd. Imran/0000-0002-6064-1040; Thabet,

Hamdy/0000-0001-8387-0404; Khan, Abida/0000-0001-6186-461X

CR [Anonymous], 2019, WHO GUIDELINE RECOMM

[Anonymous], 2014, March 2014 supplement to the 2013 consolidated guidelines on the use of antiretroviral drugs for treating and preventing HIV infections: Recommendations for a public health approach

Aouina, 2015, REV PNEUMOL CLIN, V71

Bandodkar B, 2020, APPL SCI-BASEL, V10, DOI 10.3390/app10165704

Buroni S, 2012, APPL MICROBIOL BIOT, V94, P907, DOI 10.1007/s00253-012-4013-4

Campaniço A, 2018, EUR J MED CHEM, V150, P525, DOI 10.1016/j.ejmech.2018.03.020

Carlos, 2020, United States Patent Application Publication, Patent No. [US 20200231555 A1, 20200231555]

Carlos, 2019, International Publication Number, Patent No. [WO2019034729A1, 2019034729]

Changlun, 2020, Chinese Patent Application Publication, Patent No. 111393435

Chao, 2018, Chinese Patent Application Publication, Patent No. 108456204

Chatterjee, 2016, United States Patent Application Publication, Patent No. 20160194299

Chunhua, 2020, Chinese Patent Application Publication, Patent No. [CN111269197A, 111269197]

Chunhua, 2020, Chinese Patent Application Publication, Patent No. [CN111303075A, 111303075]

David, 2020, United States Patent Application Publication, Patent No. [US20200289462A1, 20200289462]

Degiacomi G, 2020, APPL SCI-BASEL, V10, DOI 10.3390/app10020623

Desai, 2019, International Publication Number, Patent No. 2019239382

Devnikar, 2020, INDIAN J TUBERC, V67

Dso V.N. Khachatryan, 2017, Patent No. [RU 2629369 C1 20170829, 2629369]

Esther, 2020, United States Patent Application Publication, Patent No. [US20200253967A1, 20200253967]

Esther, 2020, United States Patent Application Publication, Patent No. [US20200170997A1, 20200170997]

Florian, 2018, International Publication Number, Patent No. [WO2018055048A1, 2018055048]

Florian, 2019, European Patent Application Publication, Patent No. 3515920

Gawad J, 2018, CHEM CENT J, V12

Haihong, 2020, Chinese Patent Application Publication, Patent No. [CN110759889A, 110759889]

Hariguchi N, 2020, ANTIMICROB AGENTS CH, V64, DOI 10.1128/AAC.02020-19

Hoagland DT, 2016, ADV DRUG DELIVER REV, V102, P55, DOI 10.1016/j.addr.2016.04.026

Huiyuan, 2019, Chinese Patent Application Publication, Patent No. 109503631

Jie, 2018, Chinese Patent Application Publication, Patent No. [CN108623527A, 108623527]

Keam SJ, 2019, DRUGS, V79, P1797, DOI 10.1007/s40265-019-01207-9

Koo, 2020, MICROORGANISMS, V8

Lamichhane G, 2011, TRENDS MOL MED, V17, P25, DOI 10.1016/j.molmed.2010.10.004

Lin, 2018, Chinese Patent Application Publication, Patent No. [CN108558858A, 108558858]

Lluis BP., 2020, United States Patent, Patent No. [US 10624893 B2, 10624893]

Makarov, 2011, United States Patent, Patent No. [US7863268B2, 7863268]

Makarov, 2014, United States Patent, Patent No. [US8796264B2, 8796264]

Makarov V, 2020, APPL SCI-BASEL, V10, DOI 10.3390/app10072269

Makarov V, 2009, SCIENCE, V324, P801, DOI 10.1126/science.1171583

Mikusová K, 2017, DRUG DISCOV TODAY, V22, P534, DOI 10.1016/j.drudis.2016.09.025

Mikusová K, 2014, CURR PHARM DESIGN, V20, P4379

Miller, 2016, United States Patent, Patent No. [US9481683B2, 9481683]

Miller, 2017, United States Patent, Patent No. [US9708339B2, 9708339]

Nikolaevich, 2018, Russian Patent, Patent No. [RU2663848C1, 2663848]

Parekh MJ, 2013, THER ADV RESPIR DIS, V7, P351, DOI 10.1177/1753465813503028

Porras de Francisco E., 2019, [No title captured], Patent No. [2019034700, WO2019034700Novelcompounds]

Rabahi MF, 2017, J BRAS PNEUMOL, V43, P472, DOI [10.1590/S1806-37562016000000388, 10.1590/s1806-37562016000000388]

Rathwa, 2017, INDIAN J TUBERC, V64

Riccardi G, 2013, APPL MICROBIOL BIOT, V97, P8841, DOI 10.1007/s00253-013-5218-x

Shetye GS., 2020, TRANSL RES, V220

Shimizu, 2018, United States Patent, Patent No. [US 10,053,446, 10053446, US10053446B2]

Shimizu H, 2019, [No title captured], Patent No. [US10464926, 10464926]

Shirude, 2015, United States Patent, Patent No. [US9163020B2, 9163020]

Shirude, 2020, International Publication Number, Patent No. [WO2020188405A1, 2020188405]

Singh R, 2020, J APPL MICROBIOL, V128, P1547, DOI 10.1111/jam.14478

Smerdin, 2020, TERAPEVT ARKH, V92

Suma KB, 2020, J MOL GRAPH MODEL, V101, DOI 10.1016/j.jmgm.2020.107718

Trefzer C, 2010, J AM CHEM SOC, V132, P13663, DOI 10.1021/ja106357w

Unissa AN, 2016, INFECT GENET EVOL, V45, P474, DOI 10.1016/j.meegid.2016.09.004

WHO, 2012, GLOBAL TUBERCULOSIS REPORT 2012, P1

Yoshikazu, 2017, Japanese Patent Application Publication, Patent No. [JP2017141224A, 2017141224]

Yulevich LA., 2018, Pyranoindoles with antituberculosis activity. Russian Patent, Patent No. [RU2675240C1, 2675240]

Yuquan, 2014, Chinese Patent, Patent No. [CN102276598B, 102276598]

NR 61

TC 42

Z9 46

U1 0

U2 17

PU TAYLOR & FRANCIS LTD

PI ABINGDON

PA 2-4 PARK SQUARE, MILTON PARK, ABINGDON OR14 4RN, OXON, ENGLAND

SN 1354-3776

EI 1744-7674

J9 EXPERT OPIN THER PAT

JI Expert Opin. Ther. Patents

PD AUG 3

PY 2021

VL 31

IS 8

BP 759

EP 772

DI 10.1080/13543776.2021.1902990

EA APR 2021

PG 14

WC Chemistry, Medicinal; Pharmacology & Pharmacy

WE Science Citation Index Expanded (SCI-EXPANDED)

SC Pharmacology & Pharmacy

GA TR4XY

UT WOS:000639737200001

DA 2026-05-07

ER

PT J

AU Lee, HJ

Ko, HJ

Kim, SH

Jung, YJ

AF Lee, Hyo-Ji

Ko, Hyun-Jeong

Kim, Seung Hyun

Jung, Yu-Jin

TI Pasakbumin A controls the growth of Mycobacterium tuberculosis by

enhancing the autophagy and production of antibacterial mediators in

mouse macrophages

SO PLOS ONE

LA English

DT Article

ID EURYCOMA-LONGIFOLIA JACK; HOST-DIRECTED THERAPIES; CELL-DEATH; IMMUNITY;

ANTIBIOTICS; METABOLISM; APOPTOSIS; BACTERIAL

AB Tuberculosis (TB) is a chronic infectious disease caused by Mycobacterium tuberculosis (Mtb) and remains a major health problem worldwide. Thus, identification of new and more effective drugs to treat emerging multidrug-resistant TB (MDR-TB) and to reduce the side effects of anti-TB drugs, such as liver toxicity and other detrimental changes, is urgently needed. In this study, to develop a novel candidate drug for effective TB treatment with few side effects in the host, we selected pasakbumin A isolated from Eurycoma longifolia (E. longifolia) Jack, which protected host cells against Mtb infection-induced death. Pasakbumin A significantly inhibited intracellular Mtb growth by inducing the autophagy via the ERK1/2-mediated signaling pathway in Mtb-infected macrophages. We further investigated whether pasakbumin A could be used as a potential adjuvant for TB treatment. Treatment with pasakbumin A and anti-TB drug rifampicin (RMP) potently suppressed intracellular Mtb killing by promoting autophagy as well as TNF-alpha production via the ERK1/2-and NF-KB-mediated signaling pathways in Mtb-infected cells. Our results suggest that pasakbumin A could be developed as a novel anti-TB drug or host-directed therapeutic (HDT) strategy to protect against host cell death and improve host defense mechanisms against Mtb infection in macrophages.

C1 [Lee, Hyo-Ji; Jung, Yu-Jin] Kangwon Natl Univ, Dept Biol Sci, Chunchon, South Korea.

[Lee, Hyo-Ji; Jung, Yu-Jin] Kangwon Natl Univ, Inst Life Sci, Chunchon, South Korea.

[Ko, Hyun-Jeong] Kangwon Natl Univ, Coll Pharm, Chunchon, South Korea.

[Kim, Seung Hyun] Yonsei Univ, Coll Pharm, Incheon, South Korea.

C3 Kangwon National University; Kangwon National University; Kangwon

National University; Yonsei University

RP Jung, YJ (corresponding author), Kangwon Natl Univ, Dept Biol Sci, Chunchon, South Korea.; Jung, YJ (corresponding author), Kangwon Natl Univ, Inst Life Sci, Chunchon, South Korea.

EM yjjung@kangwon.ac.kr

RI Jung, Yu-Jin/GNH-4544-2022

OI Jung, Yu-Jin/0000-0002-4942-677X; Ko, Hyun-Jeong/0000-0002-3844-928X

FU Korea Health Technology R&D Project through the Korea Health Industry

Development Institute (KHIDI) - Ministry of Health & Welfare of the

Republic of Korea [HI15C0450]; Basic Science Research Program through

the National Research Foundation of Korea (NRF) - Ministry of Education,

Science and Technology [2017R1A6A3A11032251, 2018R1D1A1B07049097]

FX This research was supported by a grant from the Korea Health Technology

R&D Project through the Korea Health Industry Development Institute

(KHIDI), which is funded by the Ministry of Health & Welfare of the

Republic of Korea (HI15C0450), and the Basic Science Research Program

through the National Research Foundation of Korea (NRF), which is funded

by the Ministry of Education, Science and Technology

(2017R1A6A3A11032251, 2018R1D1A1B07049097).

CR [Anonymous], 2016, Treatment Guidelines for Drug-Resistant Tuberculosis

Arbex MA, 2010, J BRAS PNEUMOL, V36, P626, DOI 10.1590/S1806-37132010000500016

Bhat R, 2010, FITOTERAPIA, V81, P669, DOI 10.1016/j.fitote.2010.04.006

Castillo EF, 2012, P NATL ACAD SCI USA, V109, pE3168, DOI 10.1073/pnas.1210500109

Cho JH, 2017, ONCOTARGET, V8, P24932, DOI 10.18632/oncotarget.15326

Domingo-Gonzalez R, 2016, MICROBIOL SPECTR, V4, DOI 10.1128/microbiolspec.TBTB2-0018-2016

Duan L, 2016, BIOCHEM BIOPH RES CO, V473, P1229, DOI 10.1016/j.bbrc.2016.04.045

Farouk AE, 2007, SAUDI MED J, V28, P1422

Forget EJ, 2006, EXPERT OPIN DRUG SAF, V5, P231, DOI 10.1517/14740338.5.2.231

Gupta A, 2012, IMMUNOBIOLOGY, V217, P363, DOI 10.1016/j.imbio.2011.07.008

Gutierrez MG, 2004, CELL, V119, P753, DOI 10.1016/j.cell.2004.11.038

Harper Ian, 2010, Anthropol Med, V17, P201, DOI 10.1080/13648470.2010.493606

HAUSER WE, 1982, AM J MED, V72, P711, DOI 10.1016/0002-9343(82)90534-4

Hayat M., 2017, AUTOPHAGY CANC OTHER, P234

Hmama Z, 2015, IMMUNOL REV, V264, P220, DOI 10.1111/imr.12268

Huang J, 2014, NAT REV MICROBIOL, V12, P101, DOI 10.1038/nrmicro3160

Jo EK, 2013, FRONT IMMUNOL, V4, DOI 10.3389/fimmu.2013.00097

Kaufmann SHE, 2018, NAT REV DRUG DISCOV, V17, P35, DOI 10.1038/nrd.2017.162

Kim JJ, 2012, CELL HOST MICROBE, V11, P457, DOI 10.1016/j.chom.2012.03.008

Lee H, 2018, FRONT IMMUNOL, V9, DOI 10.3389/fimmu.2018.00924

Lee HJ, 2016, FRONT MICROBIOL, V7, DOI 10.3389/fmicb.2016.00541

Lee HJ, 2015, MOL CELLS, V38, P26, DOI 10.14348/molcells.2015.2136

Lee YK, 2016, BMB REP, V49, P424, DOI 10.5483/BMBRep.2016.49.8.081

Nguyen L, 2016, ARCH TOXICOL, V90, P1585, DOI 10.1007/s00204-016-1727-6

MENZIES R, 1993, TUBERCLE LUNG DIS, V74, P32, DOI 10.1016/0962-8479(93)90066-7

Moraco AH, 2014, SEMIN IMMUNOL, V26, P497, DOI 10.1016/j.smim.2014.10.001

Mortaz E, 2015, J CLIN IMMUNOL, V35, P1, DOI 10.1007/s10875-014-0103-7

North RJ, 2004, ANNU REV IMMUNOL, V22, P599, DOI 10.1146/annurev.immunol.22.012703.104635

Park S, 2014, BIOORG MED CHEM LETT, V24, P3835, DOI 10.1016/j.bmcl.2014.06.058

Park WB, 2010, J INFECTION, V61, P323, DOI 10.1016/j.jinf.2010.07.009

Petrini B, 1999, INT J ANTIMICROB AG, V13, P93, DOI 10.1016/S0924-8579(99)00111-9

Pieters J, 2008, CELL HOST MICROBE, V3, P399, DOI 10.1016/j.chom.2008.05.006

Rehman SU, 2016, MOLECULES, V21, DOI 10.3390/molecules21030331

Romagnoli A, 2012, AUTOPHAGY, V8, P1357, DOI 10.4161/auto.20881

Ryter SW, 2013, MOL CELLS, V36, P7, DOI 10.1007/s10059-013-0140-8

Saraav I, 2014, IMMUNOL CELL BIOL, V92, P741, DOI 10.1038/icb.2014.52

Shi LB, 2005, P NATL ACAD SCI USA, V102, P15629, DOI 10.1073/pnas.0507850102

Shibutani ST, 2015, NAT IMMUNOL, V16, P1014, DOI 10.1038/ni.3273

Stephenson HN, 2016, CURR OPIN IMMUNOL, V38, P59, DOI 10.1016/j.coi.2015.11.004

Sulis G, 2016, NPJ PRIM CARE RESP M, V26, DOI 10.1038/npjpcrm.2016.78

Tee TT, 2007, ANTICANCER RES, V27, P3425

Tousif S, 2014, J BIOL CHEM, V289, P30190, DOI 10.1074/jbc.C114.598946

Wallis RS, 2015, NAT REV IMMUNOL, V15, P255, DOI 10.1038/nri3813

Yakar F, 2013, MULTIDISCIP RESP MED, V8, DOI 10.1186/2049-6958-8-13

Yuk JM, 2009, CELL HOST MICROBE, V6, P231, DOI 10.1016/j.chom.2009.08.004

Zumla A, 2016, LANCET INFECT DIS, V16, pE47, DOI 10.1016/S1473-3099(16)00078-5

Zumla A, 2015, LANCET GLOB HEALTH, V3, pE10, DOI 10.1016/S2214-109X(14)70361-4

NR 47

TC 24

Z9 24

U1 0

U2 7

PU PUBLIC LIBRARY SCIENCE

PI SAN FRANCISCO

PA 1160 BATTERY STREET, STE 100, SAN FRANCISCO, CA 94111 USA

SN 1932-6203

J9 PLOS ONE

JI PLoS One

PD MAR 13

PY 2019

VL 14

IS 3

AR e0199799

DI 10.1371/journal.pone.0199799

PG 19

WC Multidisciplinary Sciences

WE Science Citation Index Expanded (SCI-EXPANDED)

SC Science & Technology - Other Topics

GA HO6OH

UT WOS:000461048900001

OA Green Submitted, gold

DA 2026-05-07

ER

PT J

AU Velayati, AA

Farnia, P

Ibrahim, TA

Haroun, RZ

Kuan, HO

Ghanavi, J

Farnia, P

Kabarei, AN

Tabarsi, P

Omar, AR

Varahram, M

Masjedi, MR

AF Velayati, Ali Akbar

Farnia, Parissa

Ibrahim, Tengku Azmi

Haroun, Rafiuz Zaman

Kuan, Ho Oi

Ghanavi, Jalaledin

Farnia, Poopak

Kabarei, Ali Naghee

Tabarsi, Payam

Omar, Abdul Rahman

Varahram, Mohammad

Masjedi, Mohammad Reza

TI Differences in Cell Wall Thickness between Resistant and Nonresistant

Strains of Mycobacterium tuberculosis: Using Transmission

Electron Microscopy

SO CHEMOTHERAPY

LA English

DT Article

DE Tuberculosis; Cell wall; TEM

ID MYCOLIC ACIDS; ANTITUBERCULOSIS DRUGS

AB The ultrastructure of the cell wall of extensively drug-resistant (XDR), multidrug-resistant (MDR) and susceptible tuberculosis ( TB) bacilli was viewed under transmission electron microscopy (TEM). Under the TEM, marked differences were observed in the thickness of their cell wall: 20.2 +/- 1.5 and 17.1 +/- 1.03 nm for the XDR and MDR TB bacilli, respectively, and 15.6 +/- 1.3 nm for the susceptible isolates (p < 0.05). In MDR bacilli, thickening of the cell wall was observed in the intermediate electron-transparent layer (ETL) and outer electron-opaque layer, whereas in XDR TB cells the basal peptidoglycan layer was denser and almost fused with the ETL. Five to seven percent of XDR TB bacilli had the appearance of the stationary phase with cell wall thickness ranging from 21 to 26 nm (p < 0.001). Information provided in this study is of significant importance in terms of drug selection for effective treatment of resistant strains. Copyright (C) 2009 S. Karger AG, Basel

C1 [Farnia, Poopak] Shaheed Beheshti Univ, WHO, NRITLD, Expt & Anim Res Lab, Tehran 19556, Iran.

[Velayati, Ali Akbar; Farnia, Parissa; Kuan, Ho Oi; Kabarei, Ali Naghee; Tabarsi, Payam; Varahram, Mohammad; Masjedi, Mohammad Reza] Shaheed Beheshti Univ, WHO Collaborating Ctr, NRITLD, Mycobacteriol Res Ctr, Tehran 19556, Iran.

[Farnia, Parissa; Ibrahim, Tengku Azmi; Haroun, Rafiuz Zaman; Omar, Abdul Rahman] Univ Putra Malaysia, Inst Biosci, Microscopy Unit, Serdang, Selangor Darul, Malaysia.

C3 World Health Organization; Shahid Beheshti University; Shahid Beheshti

University; World Health Organization; Universiti Putra Malaysia

RP Farnia, P (corresponding author), Shaheed Beheshti Univ, WHO, NRITLD, Expt & Anim Res Lab, Med Campus,PO 19575-154, Tehran 19556, Iran.

EM pfarnia@hotmail.com

RI Masjedi, Mohammad Reza/J-9776-2017; tabarsi, payam/G-8735-2017; ghanavi,

jalaledin/G-7394-2017; farnia, poopak/G-7412-2017; Velayati, Ali

Akbar/H-1448-2017; farnia, poopak/G-5859-2017; Varahram,

Mohammad/A-1395-2018; Farnia, parissa/G-7412-2017; Masjedi,

Mohammaeza/J-9776-2017

OI Masjedi, Mohammad Reza/0000-0002-6871-382X; tabarsi,

payam/0000-0002-8932-5420; ghanavi, jalaledin/0000-0002-4415-4840;

farnia, poopak/0000-0003-3190-2827; Velayati, Ali

Akbar/0000-0001-9398-5838; farnia, poopak/0000-0003-3190-2827; Varahram,

Mohammad/0000-0002-8033-1867; Farnia, parissa/0000-0003-3676-2018;

Masjedi, Mohammaeza/0000-0003-4964-3851

FU MRC/NRITLD/WHO [0031/A12/2007]

FX The authors wish to thank staff at the Microscopy Unit at the Institute

of Bioscience, University Putra Malaysia. We also thank for the kind

help of the TB laboratory staff at MRC/NRITLD. The study was sponsored

by grant No. 0031/A12/2007 from MRC/NRITLD/WHO.

CR ALDERWICK LJ, 2007, TRANSACTIONS, V55, P1325

Alsteens D, 2008, PFLUG ARCH EUR J PHY, V456, P117, DOI 10.1007/s00424-007-0386-0

[Anonymous], 2008, Global Tuberculosis Control: Surveillance, Planning, and Financing; WHO Report 2008

[Anonymous], 1985, Public health mycobacteriology: a guide for the level III laboratory

BRENNAN PJ, 1989, REV INFECT DIS, V11, P420

Chopra P, 2003, INDIAN J MED RES, V117, P1

Cremades R, 2009, CHEMOTHERAPY, V55, P114, DOI 10.1159/000194973

DYE C, 2000, P ROY SOC LOND B BIO, V267, P1

Escribano I, 2007, CHEMOTHERAPY, V53, P397, DOI 10.1159/000109769

FARNIA P, 2008, BMC INFECT DIS, V8, P109

Frothingham R, 1998, MICROBIOL-UK, V144, P1189, DOI 10.1099/00221287-144-5-1189

Gillespie SH, 2002, ANTIMICROB AGENTS CH, V46, P267, DOI 10.1128/AAC.46.2.267-274.2002

Hoffmann C, 2008, P NATL ACAD SCI USA, V105, P3963, DOI 10.1073/pnas.0709530105

Huang Q, 2007, CHEMOTHERAPY, V53, P338, DOI 10.1159/000107723

Liu J., 1999, MYCOBACTERIA MOL BIO, P220

Marcel N, 2008, CHEMOTHERAPY, V54, P404, DOI 10.1159/000153314

Masjedi MR, 2006, CLIN INFECT DIS, V43, P841, DOI 10.1086/507542

MITCHISON DA, 1985, TUBERCLE, V66, P219, DOI 10.1016/0041-3879(85)90040-6

Ramaswamy S., 1998, Tubercle and Lung Disease, V79, P3, DOI 10.1054/tuld.1998.0002

Raviglione MC, 2007, NEW ENGL J MED, V356, P656, DOI 10.1056/NEJMp068273

TELENTI A, 1993, LANCET, V341, P647, DOI 10.1016/0140-6736(93)90417-F

Toda T, 1960, P JAPAN ACAD, V36, P372

VANEMBDEN JD, 1993, J CLIN MICROBIOL, V31, P2406

Wang L, 2000, J BIOL CHEM, V275, P7224, DOI 10.1074/jbc.275.10.7224

Watanabe M, 2002, MICROBIOL-SGM, V148, P1881, DOI 10.1099/00221287-148-6-1881

Watanabe M, 2001, MICROBIOL-SGM, V147, P1825, DOI 10.1099/00221287-147-7-1825

*WHO, 2001, WHOCDCTB2001288

Wright A., 2006, Morbidity and Mortality Weekly Report, V55, P301

Zignol M, 2006, J INFECT DIS, V194, P479, DOI 10.1086/505877

NR 29

TC 91

Z9 106

U1 1

U2 22

PU KARGER

PI BASEL

PA ALLSCHWILERSTRASSE 10, CH-4009 BASEL, SWITZERLAND

SN 0009-3157

J9 CHEMOTHERAPY

JI Chemotherapy

PY 2009

VL 55

IS 5

BP 303

EP 307

DI 10.1159/000226425

PG 5

WC Oncology; Pharmacology & Pharmacy

WE Science Citation Index Expanded (SCI-EXPANDED)

SC Oncology; Pharmacology & Pharmacy

GA 501EH

UT WOS:000270361800001

DA 2026-05-07

ER

PT J

AU Stefan, MA

Velazquez, GM

Garcia, GA

AF Stefan, Maxwell A.

Velazquez, Glory M.

Garcia, George A.

TI High-throughput screening to discover inhibitors of the CarD.RNA

polymerase protein-protein interaction in Mycobacterium

tuberculosis

SO SCIENTIFIC REPORTS

LA English

DT Article

ID RNA-POLYMERASE; TRANSCRIPTION; COMPLEXES; TARGET

AB Multidrug-resistant Mycobacterium tuberculosis (MDR-TB) accounts for 3.7% of new cases of TB annually worldwide and is a major threat to global public health. Due to the prevalence of the MDR-TB and extensively drug resistant tuberculosis (XDR-TB) cases, there is an urgent need for new drugs with novel mechanisms of action. CarD, a global transcription regulator in MTB, binds RNAP and activates transcription by stabilizing the transcription initiation open-promoter complex (RPo). CarD is required for MTB viability and it has highly conserved homologues in many eubacteria. A fluorescence polarization (FP) assay which monitors the association of MTB RNAP, native rRNA promoter DNA and CarD has been developed. Overall, our objective is to identify and characterize small molecule inhibitors which block the CarD/RNAP interaction and to understand the mechanisms by which CarD interacts with the molecules. We expect that the development of a new and improved anti-TB compound with a novel mechanism of action will relieve the burden of resistance. This CarD FP assay is amenable to HTS and is an enabling tool for future novel therapeutic discovery.

C1 [Stefan, Maxwell A.; Velazquez, Glory M.; Garcia, George A.] Univ Michigan, Dept Med Chem, Ann Arbor, MI 48109 USA.

C3 University of Michigan System; University of Michigan

RP Garcia, GA (corresponding author), Univ Michigan, Dept Med Chem, Ann Arbor, MI 48109 USA.

EM gagarcia@umich.edu

FU NIAID [R01-AI110780]; University of Michigan, College of Pharmacy;

Horace H Rackham School of Graduate Studies

FX We would like to thank the University of Michigan Center for Chemical

Genomics their advice and supervision before, during, and after

screening. We would also like to thank Dr. Amanda Garner for allowing us

to screen her 237-compound natural product library. We would like to

thank Dr. Katsuhiko Murakami and Catherine Sutherland from Penn State

University for the M. smegmatis RbpA expression vector. We would also

like to thank Dr. Jennifer Schmidt for her help with characterizing the

CarD-dye conjugates by LCMS. This work was supported by NIAID grant

R01-AI110780 (G.A.G.) and the University of Michigan, College of

Pharmacy and Horace H Rackham School of Graduate Studies.

CR Bae B, 2015, ELIFE, V4, DOI 10.7554/eLife.08505

Boyaci H, 2018, ELIFE, V7, DOI 10.7554/eLife.34823

Buurman ET, 2012, J BACTERIOL, V194, P5504, DOI 10.1128/JB.01103-12

Chopra I, 2007, CURR OPIN INVEST DR, V8, P600

Davis E, 2015, NUCLEIC ACIDS RES, V43, P433, DOI 10.1093/nar/gku1231

Desikan P, 2018, INDIAN J MED RES, V147, P233, DOI 10.4103/ijmr.IJMR_652_17

Garner AL, 2014, MOL MICROBIOL, V93, P682, DOI 10.1111/mmi.12681

Gulten G, 2013, STRUCTURE, V21, P1859, DOI 10.1016/j.str.2013.08.014

Hubin EA, 2017, ELIFE, V6, DOI 10.7554/eLife.22520

Jadhav A, 2010, J MED CHEM, V53, P37, DOI 10.1021/jm901070c

Lin W, 2018, MOL CELL, V70, P60, DOI 10.1016/j.molcel.2018.02.026

Ma C, 2016, MICROBIOL MOL BIOL R, V80, P139, DOI 10.1128/MMBR.00055-15

Rammohan J, 2015, NUCLEIC ACIDS RES, V43, P3272, DOI 10.1093/nar/gkv078

Scharf NT, 2017, SLAS DISCOV, V22, P287, DOI 10.1177/2472555216679994

Srivastava DB, 2013, P NATL ACAD SCI USA, V110, P12619, DOI 10.1073/pnas.1308270110

Stallings CL, 2011, TRANSCR-AUSTIN, V2, P15, DOI 10.4161/trns.2.1.13628

Stallings CL, 2009, CELL, V138, P146, DOI 10.1016/j.cell.2009.04.041

Stefan MA, 2018, ANTIMICROB AGENTS CH, V62, DOI [10.1128/AAC.00164-18, 10.1128/aac.00164-18]

Weiss LA, 2012, J BACTERIOL, V194, P5621, DOI 10.1128/JB.00879-12

WHO, 2012, GLOBAL TUBERCULOSIS REPORT 2012, P1

Zhu DX, 2019, P NATL ACAD SCI USA, V116, P13573, DOI 10.1073/pnas.1900176116

NR 21

TC 9

Z9 14

U1 0

U2 11

PU NATURE PORTFOLIO

PI BERLIN

PA HEIDELBERGER PLATZ 3, BERLIN, 14197, GERMANY

SN 2045-2322

J9 SCI REP-UK

JI Sci Rep

PD DEC 4

PY 2020

VL 10

IS 1

AR 21309

DI 10.1038/s41598-020-78269-3

PG 12

WC Multidisciplinary Sciences

WE Science Citation Index Expanded (SCI-EXPANDED)

SC Science & Technology - Other Topics

GA PT8XC

UT WOS:000608893600010

OA Green Submitted, gold

DA 2026-05-07

ER

PT J

AU Song, SL

Gan, DH

Wu, D

Li, T

Zhang, SQ

Lu, YB

Jin, GQ

AF Song, Shulin

Gan, Donghui

Wu, Di

Li, Ting

Zhang, Shiqian

Lu, Yibo

Jin, Guanqiao

TI Molecular Indicator for Distinguishing Multi-drug-Resistant Tuberculosis

from Drug Sensitivity Tuberculosis and Potential Medications for

Treatment

SO MOLECULAR BIOTECHNOLOGY

LA English

DT Article

DE Multi-drug-resistant tuberculosis; Machine learning; Biomarker; Immune

infiltrating; Drug

ID MYCOBACTERIUM-TUBERCULOSIS; CELL; INFLAMMATION; INFECTION; TOOL

AB The issue of multi-drug-resistant tuberculosis (MDR-TB) presents a substantial challenge to global public health. Regrettably, the diagnosis of drug-resistant tuberculosis (DR-TB) frequently necessitates an extended period or more extensive laboratory resources. The swift identification of MDR-TB poses a particularly challenging endeavor. To identify the biomarkers indicative of multi-drug resistance, we conducted a screening of the GSE147689 dataset for differentially expressed genes (DEGs) and subsequently conducted a gene enrichment analysis. Our analysis identified a total of 117 DEGs, concentrated in pathways related to the immune response. Three machine learning methods, namely random forest, decision tree, and support vector machine recursive feature elimination (SVM-RFE), were implemented to identify the top 10 genes according to their feature importance scores. A4GALT and S1PR1, which were identified as common genes among the three methods, were selected as potential molecular markers for distinguishing between MDR-TB and drug-susceptible tuberculosis (DS-TB). These markers were subsequently validated using the GSE147690 dataset. The findings suggested that A4GALT exhibited area under the curve (AUC) values of 0.8571 and 0.7121 in the training and test datasets, respectively, for distinguishing between MDR-TB and DS-TB. S1PR1 demonstrated AUC values of 0.8163 and 0.5404 in the training and test datasets, respectively. When A4GALT and S1PR1 were combined, the AUC values in the training and test datasets were 0.881 and 0.7551, respectively. The relationship between hub genes and 28 immune cells infiltrating MDR-TB was investigated using single sample gene enrichment analysis (ssGSEA). The findings indicated that MDR-TB samples exhibited a higher proportion of type 1 T helper cells and a lower proportion of activated dendritic cells in contrast to DS-TB samples. A negative correlation was observed between A4GALT and type 1 T helper cells, whereas a positive correlation was found with activated dendritic cells. S1PR1 exhibited a positive correlation with type 1 T helper cells and a negative correlation with activated dendritic cells. Furthermore, our study utilized connectivity map analysis to identify nine potential medications, including verapamil, for treating MDR-TB. In conclusion, our research identified two molecular indicators for the differentiation between MDR-TB and DS-TB and identified a total of nine potential medications for MDR-TB.

C1 [Song, Shulin; Li, Ting; Jin, Guanqiao] Guangxi Med Univ, Dept Radiol, Canc Hosp, Nanning 530021, Guangxi, Peoples R China.

[Song, Shulin; Gan, Donghui; Wu, Di; Zhang, Shiqian; Lu, Yibo] Fourth Peoples Hosp Nanning, Dept Radiol, Nanning 530023, Guangxi, Peoples R China.

C3 Guangxi Medical University

RP Jin, GQ (corresponding author), Guangxi Med Univ, Dept Radiol, Canc Hosp, Nanning 530021, Guangxi, Peoples R China.; Lu, YB (corresponding author), Fourth Peoples Hosp Nanning, Dept Radiol, Nanning 530023, Guangxi, Peoples R China.

EM songshulin@sr.gxmu.edu.cn; gdh202209@163.com; Mango168188@outlook.com;

liting@sr.gxmu.edu.cn; zsq277792050@163.com; bobosunny@163.com;

jinguanqiao77@gxmu.edu.cn

OI Song, Shulin/0000-0003-1183-6228

FU Key Research and Development Program of Nanning

FX We thank Home for Researchers editorial team

(www.homefor-researchers.com) for language editing service.

CR Alsayed SSR, 2023, INT J MOL SCI, V24, DOI 10.3390/ijms24065202

Atzori L, 2010, COMPUT NETW, V54, P2787, DOI 10.1016/j.comnet.2010.05.010

Auais A, 2005, PEDIATR PULM, V40, P228, DOI 10.1002/ppul.20223

Çavusoglu C, 2022, TURK J MED SCI, V52, P649, DOI 10.55730/1300-0144.5357

Chiang CY, 2010, RESPIROLOGY, V15, P413, DOI 10.1111/j.1440-1843.2010.01738.x

Chung W, 2015, INT J TUBERC LUNG D, V19, P1476, DOI 10.5588/ijtld.15.0325

Dartois V, 2024, NAT REV DRUG DISCOV, V23, P381, DOI 10.1038/s41573-024-00897-5

Diaz JMA, 2023, RESPIRATION, V102, P83, DOI 10.1159/000528274

Druszczynska M, 2022, INT J MOL SCI, V23, DOI 10.3390/ijms23031112

Finci I, 2022, LANCET MICROBE, V3, pE672, DOI 10.1016/S2666-5247(22)00116-1

Flynn JL, 2022, CELL, V185, P4682, DOI 10.1016/j.cell.2022.10.025

Gannoun-Zaki L, 2013, ANTIMICROB AGENTS CH, V57, P629, DOI 10.1128/AAC.01972-12

Ghajavand H, 2019, ANTIMICROB RESIST IN, V8, DOI 10.1186/s13756-019-0516-4

Gopal R, 2013, AM J RESP CRIT CARE, V188, P1137, DOI 10.1164/rccm.201304-0803OC

Heyckendorf J, 2021, EUR RESPIR J, V58, DOI 10.1183/13993003.03492-2020

Ladislau L, 2018, CELL DEATH DIS, V9, DOI [10.1038/S41419-018-0426-z, 10.1038/s41419-018-0426-z]

Lake MA, 2023, P NATL ACAD SCI USA, V120, DOI 10.1073/pnas.2215512120

Lamb J, 2007, NAT REV CANCER, V7, P54, DOI 10.1038/nrc2044

Liang T, 2022, TUBERCULOSIS, V132, DOI 10.1016/j.tube.2021.102140

Lifshitz V, 2017, MOL CANCER THER, V16, P2516, DOI 10.1158/1535-7163.MCT-17-0379

Ling YX, 2023, J DIABETES, V15, P920, DOI 10.1111/1753-0407.13444

Liu YC, 2016, J EXP MED, V213, P809, DOI 10.1084/jem.20151248

Liu ZQ, 2016, BIOMED RES INT, V2016, DOI 10.1155/2016/7486425

Madamarandawala P, 2023, MOL BIOL REP, V50, P3935, DOI 10.1007/s11033-023-08307-6

Meehan CJ, 2019, NAT REV MICROBIOL, V17, P533, DOI 10.1038/s41579-019-0214-5

Mok J, 2022, LANCET, V400, P1522, DOI 10.1016/S0140-6736(22)01883-9

Noble WS, 2006, NAT BIOTECHNOL, V24, P1565, DOI 10.1038/nbt1206-1565

Ocaña-Guzmán R, 2021, J IMMUNOL RES, V2021, DOI 10.1155/2021/6654220

Powers RK, 2018, BIOINFORMATICS, V34, P555, DOI 10.1093/bioinformatics/bty271

Sharma A, 2016, ASIAN SPINE J, V10, P850, DOI 10.4184/asj.2016.10.5.850

Shen J, 2023, FRONT IMMUNOL, V14, DOI 10.3389/fimmu.2023.1159713

Stllberg B., 2020, LKARTIDNINGEN, V21, P20051

Szklarczyk D, 2015, NUCLEIC ACIDS RES, V43, pD447, DOI 10.1093/nar/gku1003

Tai AMY, 2019, ARTIF INTELL MED, V99, DOI 10.1016/j.artmed.2019.101704

Velayati AA, 2009, CHEMOTHERAPY, V55, P303, DOI 10.1159/000226425

Wang Y, 2023, J INFECTION, V86, P421, DOI 10.1016/j.jinf.2023.03.020

WHO, 2023, RAPID COMMUNICATION

WHO, 2014, High Priority Target Product Profiles For New Tuberculosis Diagnostics: Report of a Consensus Meeting

WHO, 2019, CONS GUID DRUG RES T

Wong AI, 2023, ELIFE, V12, DOI 10.7554/eLife.81177

Wu LW, 2021, J CELL MOL MED, V25, P7270, DOI 10.1111/jcmm.16756

Wu XC, 2023, EMERG MICROBES INFEC, V12, DOI 10.1080/22221751.2023.2261561

Xiao Y, 2022, CANCERS, V14, DOI 10.3390/cancers14133284

Yew WW, 2000, CHEST, V118, P567, DOI 10.1378/chest.118.2.567

Yu Y, 2023, FRONT MOL BIOSCI, V10, DOI 10.3389/fmolb.2023.1099654

Zhang M, 2023, AM J CANCER RES, V13, P4478

Zhang N, 2021, BRIEF BIOINFORM, V22, DOI 10.1093/bib/bbab071

Zhou CX, 2023, INT IMMUNOPHARMACOL, V116, DOI 10.1016/j.intimp.2022.109588

Zhou JX, 2022, FRONT AGING NEUROSCI, V14, DOI 10.3389/fnagi.2022.850217

Zumla A, 2013, NAT REV DRUG DISCOV, V12, P388, DOI 10.1038/nrd4001

NR 50

TC 1

Z9 2

U1 1

U2 8

PU SPRINGERNATURE

PI LONDON

PA CAMPUS, 4 CRINAN ST, LONDON, N1 9XW, ENGLAND

SN 1073-6085

EI 1559-0305

J9 MOL BIOTECHNOL

JI Mol. Biotechnol.

PD OCT

PY 2025

VL 67

IS 10

BP 3946

EP 3959

DI 10.1007/s12033-024-01299-z

EA OCT 2024

PG 14

WC Biochemistry & Molecular Biology; Biotechnology & Applied Microbiology

WE Science Citation Index Expanded (SCI-EXPANDED)

SC Biochemistry & Molecular Biology; Biotechnology & Applied Microbiology

GA 8BY1L

UT WOS:001340247200001

OA hybrid

DA 2026-05-07

ER

PT J

AU Wang, JY

Xiong, K

Zhao, SL

Zhang, C

Zhang, JW

Xu, L

Ma, AG

AF Wang, Jinyu

Xiong, Ke

Zhao, Shanliang

Zhang, Chao

Zhang, Jianwen

Xu, Lei

Ma, Aiguo

TI Long-Term Effects of Multi-Drug-Resistant Tuberculosis Treatment on Gut

Microbiota and Its Health Consequences

SO FRONTIERS IN MICROBIOLOGY

LA English

DT Article

DE antibiotics; gut microbiota; metabolic markers; multi-drug-resistant

tuberculosis; long-term effects

ID INTESTINAL MICROBIOTA; ANTIBIOTIC EXPOSURE; METABOLISM; ASSOCIATION;

COMMUNITIES; INFANCY; IMPACT; RISK

AB Gut microbiota dysbiosis has adverse health effects on human body. Multi-drug-resistant tuberculosis (MDR-TB) treatment uses a variety of antibiotics typically for more than 20 months, which may induce gut microbiota dysbiosis. The aim of this study is to investigate the long-term effects of MDR-TB treatment on human gut microbiota and its related health consequences. A total of 76 participants were recruited at a hospital in Linyi, China. The study included one active MDR-TB treatment group, one recovered group from MDR-TB and two treatment-naive tuberculosis groups as control. The two treatment-naive tuberculosis groups were constructed to match the sex and the age of the active MDR-TB treatment and the recovered group, respectively. The fecal and blood samples were collected and analyzed for gut microbiota and metabolic parameters. An altered gut microbiota community and a loss of richness were observed during the MDR-TB treatment. Strikingly, 3-8 years after recovery and discontinuing the treatment, the gut microbiota still exhibited an altered taxonomic composition (p = 0.001) and a 16% decrease in richness (p = 0.018) compared to the gut microbiota before the treatment. The abundance of fifty-eight bacterial genera was significantly changed in the MDR-TB recovered group versus the untreated control group. Although there were persistent and pervasive gut microbiota alterations, no gastrointestinal symptom such as abdominal pain, diarrhea, nausea, flatulence, and constipation was observed in the recovered group. However, chronic disorders may be indicated by the elevated level of low-density lipoprotein cholesterol (LDLC) (p = 0.034) and total cholesterol (TC) (p = 0.017). These adverse lipid changes were associated with the altered gut bacterial taxa, including phylum Firmicutes and Verrucomicrobia and genera Adlercreutzia, Akkermansia, Butyricicoccus, Coprococcus, Clostridioides, Eubacterium, Erysipelatoclostridium, Fusicatenibacter, Klebsiella, Psychrobacter, and Streptococcus. Collectively, MDR-TB treatment induced a lasting gut microbiota dysbiosis, which was associated with unfavorable changes in lipid profile.

C1 [Wang, Jinyu; Xiong, Ke; Zhang, Chao; Zhang, Jianwen; Xu, Lei; Ma, Aiguo] Qingdao Univ, Sch Publ Hlth, Inst Nutr & Hlth, Qingdao, Peoples R China.

[Zhao, Shanliang] Linyi Peoples Hosp, Linyi, Shandong, Peoples R China.

C3 Qingdao University

RP Ma, AG (corresponding author), Qingdao Univ, Sch Publ Hlth, Inst Nutr & Hlth, Qingdao, Peoples R China.

EM magfood@qdu.edu.cn

RI ; Wang, Jinyu/PKQ-8414-2026

OI Xu, Lei/0000-0002-9368-0652; Xiong, Ke/0000-0001-5290-3306; Ma,

Aiguo/0000-0001-9507-8844;

FU National Natural Science Foundation of China [81673160]; Qingdao

University [DC1900009730, DC1900009731]

FX This study was funded by the National Natural Science Foundation of

China (No. 81673160) and by Qingdao University (Nos. DC1900009730 and

DC1900009731). The funders had no role in the study design, data

collection, result interpretation, and the decision to submit work for

publication.

CR Allayee H, 2015, CIRC RES, V117, P750, DOI 10.1161/CIRCRESAHA.115.307409

[Anonymous], 2014, COMPANION HDB WHO GU

[Anonymous], 2017, GLOB TUB REP

Bailey LC, 2014, JAMA PEDIATR, V168, P1063, DOI 10.1001/jamapediatrics.2014.1539

Boursi B, 2015, EUR J ENDOCRINOL, V172, P639, DOI 10.1530/EJE-14-1163

BRAY JR, 1957, ECOL MONOGR, V27, P326, DOI 10.2307/1942268

Castrillo G, 2017, NATURE, V543, P513, DOI 10.1038/nature21417

Chang JY, 2008, J INFECT DIS, V197, P435, DOI 10.1086/525047

CHAO A, 1992, J AM STAT ASSOC, V87, P210, DOI 10.2307/2290471

Cho I, 2012, NATURE, V488, P621, DOI 10.1038/nature11400

Cox LM, 2014, CELL, V158, P705, DOI 10.1016/j.cell.2014.05.052

Croswell A, 2009, INFECT IMMUN, V77, P2741, DOI 10.1128/IAI.00006-09

Deshmukh HS, 2014, NAT MED, V20, P528, DOI 10.1038/nm.3542

Dethlefsen L, 2011, P NATL ACAD SCI USA, V108, P4554, DOI 10.1073/pnas.1000087107

Dethlefsen L, 2008, PLOS BIOL, V6, P2383, DOI 10.1371/journal.pbio.0060280

Dheda K, 2016, LANCET, V387, P1211, DOI 10.1016/S0140-6736(15)00151-8

Droste JHJ, 2000, CLIN EXP ALLERGY, V30, P1547

Edgar RC, 2011, BIOINFORMATICS, V27, P2194, DOI 10.1093/bioinformatics/btr381

Francino MP, 2016, FRONT MICROBIOL, V6, DOI 10.3389/fmicb.2015.01543

Friedman J, 2012, PLOS COMPUT BIOL, V8, DOI 10.1371/journal.pcbi.1002687

Fu JY, 2015, CIRC RES, V117, P817, DOI 10.1161/CIRCRESAHA.115.306807

Hviid A, 2011, GUT, V60, P49, DOI 10.1136/gut.2010.219683

Johnson CC, 2005, J ALLERGY CLIN IMMUN, V115, P1218, DOI 10.1016/j.jaci.2005.04.020

Jones BV, 2008, P NATL ACAD SCI USA, V105, P13580, DOI 10.1073/pnas.0804437105

Kimura I, 2013, NAT COMMUN, V4, DOI 10.1038/ncomms2852

Koeth RA, 2013, NAT MED, V19, P576, DOI 10.1038/nm.3145

Langille MGI, 2013, NAT BIOTECHNOL, V31, P814, DOI 10.1038/nbt.2676

Lawley TD, 2008, INFECT IMMUN, V76, P403, DOI 10.1128/IAI.01189-07

Lozupone C, 2006, BMC BIOINFORMATICS, V7, DOI 10.1186/1471-2105-7-371

Magoc T, 2011, BIOINFORMATICS, V27, P2957, DOI 10.1093/bioinformatics/btr507

MANN HB, 1947, ANN MATH STAT, V18, P50, DOI 10.1214/aoms/1177730491

Maynard CL, 2012, NATURE, V489, P231, DOI 10.1038/nature11551

Namasivayam S, 2017, MICROBIOME, V5, DOI 10.1186/s40168-017-0286-2

Ríos-Covián D, 2016, FRONT MICROBIOL, V7, DOI 10.3389/fmicb.2016.00185

Ryan KK, 2014, NATURE, V509, P183, DOI 10.1038/nature13135

Schloss PD, 2009, APPL ENVIRON MICROB, V75, P7537, DOI 10.1128/AEM.01541-09

Segata N, 2011, GENOME BIOL, V12, DOI 10.1186/gb-2011-12-6-r60

Shannon C., 1949, MATH THEORY INFORM

Sommer F, 2017, NAT REV MICROBIOL, V15, P630, DOI 10.1038/nrmicro.2017.58

Sommer F, 2013, NAT REV MICROBIOL, V11, P227, DOI 10.1038/nrmicro2974

Spearman C, 1904, AM J PSYCHOL, V15, P72, DOI 10.2307/1412159

Thomas C, 2009, CELL METAB, V10, P167, DOI 10.1016/j.cmet.2009.08.001

Vangay P, 2015, CELL HOST MICROBE, V17, P553, DOI 10.1016/j.chom.2015.04.006

Vrieze A, 2014, J HEPATOL, V60, P824, DOI 10.1016/j.jhep.2013.11.034

Wang Q, 2007, APPL ENVIRON MICROB, V73, P5261, DOI 10.1128/AEM.00062-07

Wang ZN, 2011, NATURE, V472, P57, DOI 10.1038/nature09922

Wipperman MF, 2017, SCI REP-UK, V7, DOI 10.1038/s41598-017-10346-6

World Health Organization, 2013, SYST SCREEN ACT TUB

NR 48

TC 39

Z9 49

U1 0

U2 18

PU FRONTIERS MEDIA SA

PI LAUSANNE

PA AVENUE DU TRIBUNAL FEDERAL 34, LAUSANNE, CH-1015, SWITZERLAND

SN 1664-302X

J9 FRONT MICROBIOL

JI Front. Microbiol.

PD JAN 30

PY 2020

VL 11

AR 53

DI 10.3389/fmicb.2020.00053

PG 10

WC Microbiology

WE Science Citation Index Expanded (SCI-EXPANDED)

SC Microbiology

GA KN5ZQ

UT WOS:000514915900001

OA Green Submitted, gold

DA 2026-05-07

ER

PT J

AU Abreu, MT

Carvalheiro, H

Rodrigues-Sousa, T

Domingos, A

Segorbe-Luis, A

Rodrigues-Santos, P

Souto-Carneiro, MM

AF Abreu, Monica T.

Carvalheiro, Helena

Rodrigues-Sousa, Tiago

Domingos, Antonio

Segorbe-Luis, Antonio

Rodrigues-Santos, Paulo

Margarida Souto-Carneiro, M.

TI Alterations in the peripheral blood B cell subpopulations of

multidrug-resistant tuberculosis patients

SO CLINICAL AND EXPERIMENTAL MEDICINE

LA English

DT Article

DE Memory B lymphocytes; Plasma cells; Multidrug-resistant tuberculosis;

CD21; TLR9; CD69

ID TUMOR-NECROSIS-FACTOR; PULMONARY TUBERCULOSIS; PLASMA-CELLS;

SJOGRENS-SYNDROME; DIFFERENTIATION; EXPRESSION; INFECTION;

RESPONSIVENESS; ORGANIZATION; LYMPHOCYTES

AB The function of B cells in the immune response against Mycobacterium tuberculosis (Mtb) is still regarded as secondary, although major findings in mouse models of tuberculosis (TB) support their participation as regulators and antibody producers. However, studies in cohorts of TB or multidrug-resistant TB (MDR-TB) patients have failed to clearly identify changes in the circulating B cell pool. Therefore, in the present study we aimed at identifying alterations in the different B cell subpopulations in peripheral blood samples of HIV-negative pulmonary MDR-TB patients when compared to healthy donors. The data show, for the first time, that MDR-TB patients, similarly to what has been observed in other chronic inflammatory diseases, have a much lower frequency of peripheral blood unswitched IgD(+) CD27(+) memory B cells. Equally novel are the findings that in MDR-TB patients there is a reduction in the circulating plasma cell pool and that in MDR-TB there is an increased frequency of circulating type 1 transitional IgD(+) CD38(++), CD69(+) and TLR9(+) B cells. These results document disease-related shifts in peripheral blood B cell subsets in MDR-TB and suggest that such changes should be taken into account when designing new strategies to boost the cellular and humoral immune response against Mtb.

C1 [Abreu, Monica T.; Carvalheiro, Helena; Rodrigues-Sousa, Tiago; Rodrigues-Santos, Paulo; Margarida Souto-Carneiro, M.] Univ Coimbra, Ctr Neurosci & Cell Biol, P-3004517 Coimbra, Portugal.

[Domingos, Antonio] Ctr Hosp Ctr Tejo, Hosp Torres Vedras, Dept Pneumol, Torres Vedras, Portugal.

[Segorbe-Luis, Antonio] Univ Coimbra, Fac Med, P-3004517 Coimbra, Portugal.

[Segorbe-Luis, Antonio] Hosp Univ Coimbra, Dept Immunoallergol, Coimbra, Portugal.

[Rodrigues-Santos, Paulo] Univ Coimbra, Fac Med, Inst Immunol, P-3004517 Coimbra, Portugal.

C3 Universidade de Coimbra; Universidade de Coimbra; Universidade de

Coimbra; Centro Hospitalar e Universitario de Coimbra (CHUC);

Universidade de Coimbra

RP Souto-Carneiro, MM (corresponding author), Univ Coimbra, Ctr Neurosci & Cell Biol, P-3004517 Coimbra, Portugal.

EM margarida.carneiro@cnc.uc.pt

RI Rodrigues Santos, Paulo/M-3175-2013; Carvalheiro, Helena/N-2179-2014;

Souto-Carneiro, Margarida/C-4386-2016; Abreu, Mónica/F-1098-2017

OI Rodrigues Santos, Paulo/0000-0001-7519-1620; Carvalheiro,

Helena/0000-0003-0698-818X; Souto-Carneiro,

Margarida/0000-0001-6923-0590; Abreu, Mónica/0000-0003-0626-7999;

Domingos, António/0000-0003-1417-5912

FU Fundacao para a Ciencia e Tecnologia [SFRH/BD/60467/2009]; Marie Curie

Reintegration Grant [FP7-PEOPLE-ERG-2008-LIF239422]; Fundação para a

Ciência e a Tecnologia [SFRH/BD/60467/2009] Funding Source: FCT

FX H Carvalheiro was supported by Fundacao para a Ciencia e Tecnologia

Grant SFRH/BD/60467/2009. This work was supported by the Marie Curie

Reintegration Grant FP7-PEOPLE-ERG-2008-LIF239422 to MM Souto-Carneiro.

CR Agematsu K, 1997, EUR J IMMUNOL, V27, P2073, DOI 10.1002/eji.1830270835

Allen CDC, 2004, NAT IMMUNOL, V5, P943, DOI 10.1038/ni1100

Almeida LP, 2011, GENET VACCIN THER, V9, P5

Bleesing JJ, 2006, J IMMUNOL, V176, P7096, DOI 10.4049/jimmunol.176.11.7096

Bockstal V, 2011, PLOS PATHOG, V7, DOI 10.1371/journal.ppat.1002089

Bohnhorst JO, 2001, J IMMUNOL, V167, P3610, DOI 10.4049/jimmunol.167.7.3610

Çagatay T, 2005, RESPIROLOGY, V10, P290, DOI 10.1111/j.1440-1843.2005.00711.x

Campbell DJ, 2003, IMMUNOL REV, V195, P58, DOI 10.1034/j.1600-065X.2003.00067.x

Haas KM, 2009, J IMMUNOL, V183, P3661, DOI 10.4049/jimmunol.0901218

Hansen A, 2007, ARTHRITIS RES THER, V9, DOI 10.1186/ar2210

Hargreaves DC, 2001, J EXP MED, V194, P45, DOI 10.1084/jem.194.1.45

Hernandez J, 2010, IMMUNOL INVEST, V39, P197, DOI 10.3109/08820130903586346

Hiepe F, 2011, NAT REV RHEUMATOL, V7, P170, DOI 10.1038/nrrheum.2011.1

Iseman MD, 1998, INT J TUBERC LUNG D, V2, P867

Jiang W, 2008, J VIROL, V82, P7837, DOI 10.1128/JVI.00660-08

Kondratieva TK, 2010, J IMMUNOL, V184, P1227, DOI 10.4049/jimmunol.0902011

Llinàs L, 2011, IMMUNOL LETT, V134, P113, DOI 10.1016/j.imlet.2010.10.009

Luger EO, 2009, J ALLERGY CLIN IMMUN, V124, P819, DOI 10.1016/j.jaci.2009.06.047

Maglione PJ, 2007, J IMMUNOL, V178, P7222, DOI 10.4049/jimmunol.178.11.7222

Muehlinghaus G, 2005, BLOOD, V105, P3965, DOI 10.1182/blood-2004-08-2992

Ozcan E, 2011, J ALLERGY CLIN IMMUN, V128, P601, DOI 10.1016/j.jaci.2011.04.052

Rodríguez-Bayona B, 2010, ARTHRITIS RES THER, V12, DOI 10.1186/ar3042

Sánchez-Rodríguez C, 2002, INT J TUBERC LUNG D, V6, P706

Sims GP, 2005, BLOOD, V105, P4390, DOI 10.1182/blood-2004-11-4284

Souto-Carneiro MM, 2009, ARTHRITIS RES THER, V11, DOI 10.1186/ar2718

TEDDER TF, 1985, J IMMUNOL, V135, P1786

Tuaillon E, 2009, J IMMUNOL, V182, P7155, DOI 10.4049/jimmunol.0803107

Vordermeier HM, 1996, CLIN EXP IMMUNOL, V106, P312, DOI 10.1046/j.1365-2249.1996.d01-845.x

Wehr C, 2004, CLIN IMMUNOL, V113, P161, DOI 10.1016/j.clim.2004.05.010

Weller S, 2004, BLOOD, V104, P3647, DOI 10.1182/blood-2004-01-0346

WILLCOX PA, 1989, RESP MED, V83, P195, DOI 10.1016/S0954-6111(89)80031-9

Wu YE, 2009, J INT MED RES, V37, P1742

Zarocostas J, 2011, BRIT MED J, V343, DOI 10.1136/bmj.d4485

Zhang MX, 2011, CELL IMMUNOL, V270, P217, DOI 10.1016/j.cellimm.2011.05.009

NR 34

TC 17

Z9 19

U1 0

U2 6

PU SPRINGER-VERLAG ITALIA SRL

PI MILAN

PA VIA DECEMBRIO, 28, MILAN, 20137, ITALY

SN 1591-8890

EI 1591-9528

J9 CLIN EXP MED

JI Clin. Exper. Med.

PD NOV

PY 2014

VL 14

IS 4

BP 423

EP 429

DI 10.1007/s10238-013-0258-1

PG 7

WC Medicine, Research & Experimental

WE Science Citation Index Expanded (SCI-EXPANDED)

SC Research & Experimental Medicine

GA AS8ZH

UT WOS:000344532900009

DA 2026-05-07

ER

PT J

AU Rani, C

Khan, IA

AF Rani, Chitra

Khan, Inshad Ali

TI UDP-GlcNAc pathway: Potential target for inhibitor discovery against

M-tuberculosis

SO EUROPEAN JOURNAL OF PHARMACEUTICAL SCIENCES

LA English

DT Article

DE Drug resistance; GlmU; M. tuberculosis; Peptidoglycan; Target based

screening; UDP-GlcNAc

ID N-ACETYLGLUCOSAMINE-1-PHOSPHATE URIDYLTRANSFERASE GLMU;

ESCHERICHIA-COLI; N-ACETYLGLUCOSAMINE; ACETYLTRANSFERASE ACTIVITY;

GLUCOSAMINE-1-PHOSPHATE ACETYLTRANSFERASE; CRYSTAL-STRUCTURES;

CANDIDA-ALBICANS; ACTIVE-SITE; 1-PHOSPHATE URIDYLTRANSFERASE;

PHOSPHOGLUCOSAMINE MUTASE

AB In the past five years, an alarming increase in the number of patients with multidrug resistant tuberculosis (MDR TB) and extensively drug-resistant tuberculosis (XDR TB) has been reported, particularly in Eastern Europe, Asia and Southern Africa. Current situation has challenged the control and treatment of tuberculosis (TB) which sparked an emergent need to find new anti-tubercular agents with different chemical scaffolds andmechanisms of action. A very fruitful way to identify novel anti-tubercular agents is the development of compounds that target the enzymes essentially required for the biosynthesis and assembly of the mycobacterial cell wall. Biosynthesis of uridine diphospho-N-acetylglucosamine (UDP-GlcNAc) represents one such pathway. Enzymes involved in UDP-GlcNAc biosynthesis have been predicted to be essential for Mycobacterium tuberculosis growth in vitro. It is a key precursor molecule of M. tuberculosis cell wall, being situated at the branched point of two essential biosynthetic pathways, namely peptidoglycan and a disaccharide linker, D-N-GlcNAc-1-rhamnose. This article provides a comprehensive overview of the present knowledge on the enzymes catalyzing the particular steps of the pathway in M. tuberculosis, with special emphasis put on N-acetylglucosamine-1-phosphate uridyltransferase (GlmU), a bifunctional enzyme, which catalyzes the last two steps of this pathway. It also gives an insight into the present knowledge about the inhibitors reported against the enzymes, which could be further used as chemical scaffold for the discovery of more potent anti-TB compounds. (C) 2015 Elsevier B.V. All rights reserved.

C1 [Khan, Inshad Ali] CSIR Indian Inst Integrat Med, Div Clin Microbiol, Canal Rd, Jammu 180001, India.

CSIR Indian Inst Integrat Med, Acad Sci & Innovat Res, Canal Rd, Jammu 180001, India.

C3 Council of Scientific & Industrial Research (CSIR) - India; CSIR -

Indian Institute of Integrative Medicine (IIIM); Academy of Scientific &

Innovative Research (AcSIR); Council of Scientific & Industrial Research

(CSIR) - India; CSIR - Indian Institute of Integrative Medicine (IIIM)

RP Khan, IA (corresponding author), CSIR Indian Inst Integrat Med, Div Clin Microbiol, Canal Rd, Jammu 180001, India.

EM iakhan@iiim.res.in

RI Rani, Chitra/LQK-6204-2024; Ali Khan, Inshad/AAV-2564-2021

OI Ali Khan, Inshad/0000-0003-4445-199X

CR Andries K, 2005, SCIENCE, V307, P223, DOI 10.1126/science.1106753

Tran AT, 2013, ORG BIOMOL CHEM, V11, P8113, DOI 10.1039/c3ob41896k

[Anonymous], 1994, WHO/TB/94

Barreteau H, 2008, FEMS MICROBIOL REV, V32, P168, DOI 10.1111/j.1574-6976.2008.00104.x

Barry Clifton E., 2007, Infectious Disorders - Drug Targets, V7, P182

BATES CJ, 1966, J BIOL CHEM, V241, P1705

Brown K, 1999, EMBO J, V18, P4096, DOI 10.1093/emboj/18.15.4096

Burton E, 2006, ANTIMICROB AGENTS CH, V50, P1835, DOI 10.1128/AAC.50.5.1835-1840.2006

Butkinaree C, 2010, BBA-GEN SUBJECTS, V1800, P96, DOI 10.1016/j.bbagen.2009.07.018

Buurman ET, 2011, J BIOL CHEM, V286, P40734, DOI 10.1074/jbc.M111.274068

Chan ED, 2002, BMJ-BRIT MED J, V325, P1282, DOI 10.1136/bmj.325.7375.1282

Cole ST, 1998, NATURE, V393, P537, DOI 10.1038/31159

Durand P, 2008, ARCH BIOCHEM BIOPHYS, V474, P302, DOI 10.1016/j.abb.2008.01.026

Elzinga G, 2004, LANCET, V363, P814, DOI 10.1016/S0140-6736(04)15698-5

Engohang-Ndong J, 2012, EXPERT OPIN INV DRUG, V21, P1789, DOI 10.1517/13543784.2012.724397

EUZEBY J., 2008, LIST PROKARYOTIC NAM

Favrot L, 2012, EXPERT REV ANTI-INFE, V10, P1023, DOI [10.1586/ERI.12.91, 10.1586/eri.12.91]

Floquet N, 2007, BIOORG MED CHEM LETT, V17, P1966, DOI 10.1016/j.bmcl.2007.01.052

Frothingham R, 2005, INT J INFECT DIS, V9, P297, DOI 10.1016/j.ijid.2005.04.001

Green OM, 2012, BIOORG MED CHEM LETT, V22, P1510, DOI 10.1016/j.bmcl.2012.01.016

Jagtap PKA, 2012, J BIOL CHEM, V287, DOI 10.1074/jbc.M112.390765

KENIG M, 1976, J GEN MICROBIOL, V94, P46, DOI 10.1099/00221287-94-1-46

Kostrewa D, 2001, J MOL BIOL, V305, P279, DOI 10.1006/jmbi.2000.4296

Li SF, 2012, PLOS ONE, V7, DOI 10.1371/journal.pone.0036513

Li YM, 2011, CARBOHYD RES, V346, P1714, DOI 10.1016/j.carres.2011.05.024

Lienhardt C, 2010, CURR OPIN PULM MED, V16, P186, DOI 10.1097/MCP.0b013e328337580c

Makarov V, 2009, SCIENCE, V324, P801, DOI 10.1126/science.1171583

March F, 1997, CLIN INFECT DIS, V25, P1044, DOI 10.1086/516065

Maruyama D, 2006, ACTA CRYSTALLOGR F, V62, P1206, DOI 10.1107/S1744309106044186

Matteelli A, 2010, FUTURE MICROBIOL, V5, P849, DOI [10.2217/fmb.10.50, 10.2217/FMB.10.50]

Mehra-Chaudhary R, 2011, J BACTERIOL, V193, P4081, DOI 10.1128/JB.00418-11

MENGINLECREULX D, 1994, J BACTERIOL, V176, P5788, DOI 10.1128/JB.176.18.5788-5795.1994

MenginLecreulx D, 1996, J BIOL CHEM, V271, P32, DOI 10.1074/jbc.271.1.32

MENGINLECREULX D, 1993, J BACTERIOL, V175, P6150, DOI 10.1128/JB.175.19.6150-6157.1993

Milewski S, 2006, YEAST, V23, P1, DOI 10.1002/yea.1337

MILEWSKI S, 1983, ARCH MICROBIOL, V135, P130, DOI 10.1007/BF00408022

Mio T, 1998, J BIOL CHEM, V273, P14392, DOI 10.1074/jbc.273.23.14392

Mio T, 2000, MICROBIOL-SGM, V146, P1753, DOI 10.1099/00221287-146-7-1753

Mochalkin I, 2008, PROTEIN SCI, V17, P577, DOI 10.1110/ps.073271408

Mochalkin I, 2007, PROTEIN SCI, V16, P2657, DOI 10.1110/ps.073135107

Narita M, 1998, AM J RESP CRIT CARE, V158, P157, DOI 10.1164/ajrccm.158.1.9712001

National Center for Biotechnology Information, PUBCHEM BIOASSAY DAT

Olsen LR, 2007, PROTEIN SCI, V16, P1230, DOI 10.1110/ps.072779707

Parikh A, 2009, J MOL BIOL, V386, P451, DOI 10.1016/j.jmb.2008.12.031

Peneff C, 2001, EMBO J, V20, P6191, DOI 10.1093/emboj/20.22.6191

Pereira MP, 2009, ANTIMICROB AGENTS CH, V53, P2306, DOI 10.1128/AAC.01572-08

PERRIENS JH, 1991, AM REV RESPIR DIS, V144, P750, DOI 10.1164/ajrccm/144.4.750

Pethe K, 2013, NAT MED, V19, P1157, DOI 10.1038/nm.3262

Pompeo F, 1998, J BACTERIOL, V180, P4799, DOI 10.1128/JB.180.18.4799-4803.1998

Rani C, 2015, TUBERCULOSIS, V95, P664, DOI 10.1016/j.tube.2015.06.003

Sassetti CM, 2003, MOL MICROBIOL, V48, P77, DOI 10.1046/j.1365-2958.2003.03425.x

SEPKOWITZ KA, 1995, CLIN MICROBIOL REV, V8, P180, DOI 10.1128/CMR.8.2.180

Singla Deepak, 2011, BMC Pharmacology, V11, P5, DOI 10.1186/1471-2210-11-5

Soni V, 2015, J MOL MODEL, V21, DOI 10.1007/s00894-015-2704-3

Stokes SS, 2012, BIOORG MED CHEM LETT, V22, P7019, DOI 10.1016/j.bmcl.2012.10.003

Sulzenbacher G, 2001, J BIOL CHEM, V276, P11844, DOI 10.1074/jbc.M011225200

Teplyakov A, 2002, NAT PROD REP, V19, P60, DOI 10.1039/b103713g

van Heijenoort J, 2001, NAT PROD REP, V18, P503, DOI 10.1039/a804532a

Vernon A, 1999, LANCET, V353, P1843, DOI 10.1016/S0140-6736(98)11467-8

Vertesy L, 2006, U.S. Patent, Patent No. [7,148,254, 7148254, 7,148, 254]

Volmink J., 2007, Directly observed therapy for treating tuberculosis, Cochrane Database Syst Rev

Wang-Gillam A, 2000, J BIOL CHEM, V275, P1433, DOI 10.1074/jbc.275.2.1433

WHO, 2010, TREATMENT OF TUBERCULOSIS: GUIDELINES, FOURTH EDITION, P1

WINTERBURN PJ, 1971, BIOCHEM J, V121, P721, DOI 10.1042/bj1210721

World Health Organization, 2014, GLOB TUB CONTR SURV

Yin J, 2008, ACTA CRYSTALLOGR F, V64, P805, DOI 10.1107/S1744309108024500

Zhang WL, 2008, INT J BIOCHEM CELL B, V40, P2560, DOI 10.1016/j.biocel.2008.05.003

Zhang Y, 2012, ANTIMICROB AGENTS CH, V56, P2223, DOI 10.1128/AAC.06288-11

Zhang Z, 2009, ACTA CRYSTALLOGR D, V65, P275, DOI 10.1107/S0907444909001036

Zhou Y, 2012, GLYCOCONJUGATE J, V29, P297, DOI 10.1007/s10719-012-9402-5

Zumla A, 2011, TROP MED INT HEALTH, V16, P79, DOI 10.1111/j.1365-3156.2010.02665.x

NR 71

TC 23

Z9 27

U1 0

U2 44

PU ELSEVIER SCIENCE BV

PI AMSTERDAM

PA PO BOX 211, 1000 AE AMSTERDAM, NETHERLANDS

SN 0928-0987

EI 1879-0720

J9 EUR J PHARM SCI

JI Eur. J. Pharm. Sci.

PD FEB 15

PY 2016

VL 83

BP 62

EP 70

DI 10.1016/j.ejps.2015.12.013

PG 9

WC Pharmacology & Pharmacy

WE Science Citation Index Expanded (SCI-EXPANDED)

SC Pharmacology & Pharmacy

GA DD8OU

UT WOS:000370187300009

DA 2026-05-07

ER

PT J

AU Chakraborti, S

Chakraborty, M

Bose, A

Srinivasan, N

Visweswariah, SS

AF Chakraborti, Sohini

Chakraborty, Moubani

Bose, Avipsa

Srinivasan, Narayanaswamy

Visweswariah, Sandhya S.

TI Identification of Potential Binders of Mtb Universal Stress

Protein (Rv1636) Through an in silico Approach and Insights Into

Compound Selection for Experimental Validation

SO FRONTIERS IN MOLECULAR BIOSCIENCES

LA English

DT Article

DE virtual screening; molecular docking; universal stress protein; Rv1636;

anti-tubercular compounds; computational drug discovery; MM-GBSA;

experimental insights

ID DRUG DISCOVERY; MYCOBACTERIUM-TUBERCULOSIS; CYCLIC-AMP; ACCURATE

PREDICTION; MOLECULAR DOCKING; GLIDE; CONFORMATIONS; BEDAQUILINE;

SOLUBILITY; PARAMETERS

AB Millions of deaths caused by Mycobacterium tuberculosis (Mtb) are reported worldwide every year. Treatment of tuberculosis (TB) involves the use of multiple antibiotics over a prolonged period. However, the emergence of resistance leading to multidrug-resistant TB (MDR-TB) and extensively drug-resistant TB (XDR-TB) is the most challenging aspect of TB treatment. Therefore, there is a constant need to search for novel therapeutic strategies that could tackle the growing problem of drug resistance. One such strategy could be perturbing the functions of novel targets in Mtb, such as universal stress protein (USP, Rv1636), which binds to cAMP with a higher affinity than ATP. Orthologs of these proteins are conserved in all mycobacteria and act as "sink" for cAMP, facilitating the availability of this second messenger for signaling when required. Here, we have used the cAMP-bound crystal structure of USP from Mycobacterium smegmatis, a closely related homolog of Mtb, to conduct a structure-guided hunt for potential binders of Rv1636, primarily employing molecular docking approach. A library of 1.9 million compounds was subjected to virtual screening to obtain an initial set of similar to 2,000 hits. An integrative strategy that uses the available experimental data and consensus indications from other computational analyses has been employed to prioritize 22 potential binders of Rv1636 for experimental validations. Binding affinities of a few compounds among the 22 prioritized compounds were tested through microscale thermophoresis assays, and two compounds of natural origin showed promising binding affinities with Rv1636. We believe that this study provides an important initial guidance to medicinal chemists and biochemists to synthesize and test an enriched set of compounds that have the potential to inhibit Mtb USP (Rv1636), thereby aiding the development of novel antitubercular lead candidates.

C1 [Chakraborti, Sohini; Srinivasan, Narayanaswamy] Indian Inst Sci, Mol Biophys Unit, Bengaluru, India.

[Chakraborty, Moubani; Bose, Avipsa; Visweswariah, Sandhya S.] Indian Inst Sci, Dept Mol Reprod Dev & Genet, Bengaluru, India.

C3 Indian Institute of Science (IISC) - Bangalore; Indian Institute of

Science (IISC) - Bangalore

RP Srinivasan, N (corresponding author), Indian Inst Sci, Mol Biophys Unit, Bengaluru, India.; Visweswariah, SS (corresponding author), Indian Inst Sci, Dept Mol Reprod Dev & Genet, Bengaluru, India.

EM ns@iisc.ac.in; sandhya@iisc.ac.in

RI ; Visweswariah, Sandhya/Q-7500-2019

OI Chakraborty, Moubani/0000-0001-8746-3937; Bose,

Avipsa/0000-0002-0155-5257; Visweswariah, Sandhya/0000-0003-2786-7344

FU Mathematical Biology program; FIST programs - Department of Science and

Technology (DST); DBT-IISc Partnership Program Phase-II

[BT/PR27952/INF/22/212/2018/21.01.2019]; DBT

[BT/PR15216/COE/34/02/2017]; UGC, India-Centre for Advanced Studies;

Ministry of Human Resource Development, India; DBT-Bioinformatics and

Computational Biology Centre; DST-INSPIRE; Council for Scientific and

Industrial Research

FX This research was supported by Mathematical Biology program and FIST

programs sponsored by the Department of Science and Technology (DST),

the DBT-IISc Partnership Program Phase-II

(BT/PR27952/INF/22/212/2018/21.01.2019) and a DBT grant to

SV(BT/PR15216/COE/34/02/2017). Support fromUGC, India-Centre for

Advanced Studies and Ministry of Human Resource Development, India is

gratefully acknowledged. The authors acknowledge the support from

DBT-Bioinformatics and Computational Biology Centre. SC acknowledges

DSTINSPIRE for her research fellowship and MC was supported by a Senior

Fellowship from the Council for Scientific and Industrial Research.

CR Aanandhi MV, 2014, J ADV PHARM TECHNOL, V5, P171, DOI 10.4103/2231-4040.143036

[Anonymous], **DATA OBJECT**, DOI DOI 10.2210/PDB1TQ8/PDB

Atanasov AG, 2021, NAT REV DRUG DISCOV, V20, P200, DOI 10.1038/s41573-020-00114-z

Baell J, 2014, NATURE, V513, P481, DOI 10.1038/513481a

Baell JB, 2010, FUTURE MED CHEM, V2, P1529, DOI 10.4155/FMC.10.237

Baell JB, 2010, J MED CHEM, V53, P2719, DOI 10.1021/jm901137j

Bahuguna A, 2020, MED RES REV, V40, P263, DOI 10.1002/med.21602

Ballell L, 2013, CHEMMEDCHEM, V8, P313, DOI 10.1002/cmdc.201200428

Banerjee A, 2015, J BIOL CHEM, V290, P12731, DOI 10.1074/jbc.M115.644856

Berman HM, 2000, NUCLEIC ACIDS RES, V28, P235, DOI 10.1093/nar/28.1.235

Bloemberg GV, 2015, NEW ENGL J MED, V373, P1986, DOI 10.1056/NEJMc1505196

Clark JJ, 2019, PLOS COMPUT BIOL, V15, DOI 10.1371/journal.pcbi.1006705

Cole ST, 1998, NATURE, V393, P537, DOI 10.1038/31159

Cozzini P, 2008, J MED CHEM, V51, P6237, DOI 10.1021/jm800562d

Dass BKM, 2008, J BACTERIOL, V190, P3824, DOI 10.1128/JB.00138-08

Deller MC, 2015, J COMPUT AID MOL DES, V29, P817, DOI 10.1007/s10822-015-9833-8

Duan JX, 2010, J MOL GRAPH MODEL, V29, P157, DOI 10.1016/j.jmgm.2010.05.008

Fradera X, 2002, BIOINFORMATICS, V18, P939, DOI 10.1093/bioinformatics/18.7.939

Friesner RA, 2004, J MED CHEM, V47, P1739, DOI 10.1021/jm0306430

Friesner RA, 2006, J MED CHEM, V49, P6177, DOI 10.1021/jm051256o

Ghodousi A, 2019, ANTIMICROB AGENTS CH, V63, DOI 10.1128/AAC.00915-19

Greenwood JR, 2010, J COMPUT AID MOL DES, V24, P591, DOI 10.1007/s10822-010-9349-1

Halgren TA, 2004, J MED CHEM, V47, P1750, DOI 10.1021/jm030644s

Holm L, 2020, PROTEIN SCI, V29, P128, DOI 10.1002/pro.3749

Huang SY, 2010, PHYS CHEM CHEM PHYS, V12, P12899, DOI 10.1039/c0cp00151a

James S. F, 2009, U.S. Patent, Patent No. [2009/0233955 A1, 20090233955]

Kalamidas SA, 2006, J CELL SCI, V119, P3686, DOI 10.1242/jcs.03091

Kim S, 2019, NUCLEIC ACIDS RES, V47, pD1102, DOI 10.1093/nar/gky1033

Koul A, 2007, NAT CHEM BIOL, V3, P323, DOI 10.1038/nchembio884

Lakshmanan M, 2013, J YOUNG PHARM, V5, P112, DOI 10.1016/j.jyp.2013.12.002

Lee K, 2009, GLOB INST, P1

Lipinski CA, 1997, ADV DRUG DELIVER REV, V23, P3, DOI 10.1016/S0169-409X(96)00423-1

Lipinski CA, 2000, J PHARMACOL TOX MET, V44, P235, DOI 10.1016/S1056-8719(00)00107-6

LOWRIE DB, 1979, J GEN MICROBIOL, V110, P431, DOI 10.1099/00221287-110-2-431

LOWRIE DB, 1975, NATURE, V254, P600, DOI 10.1038/254600a0

Lyne PD, 2006, J MED CHEM, V49, P4805, DOI 10.1021/jm060522a

Madeira F, 2019, NUCLEIC ACIDS RES, V47, pW636, DOI 10.1093/nar/gkz268

Manjunatha UH, 2015, BIOORGAN MED CHEM, V23, P5087, DOI 10.1016/j.bmc.2014.12.031

McGovern SL, 2003, J MED CHEM, V46, P2895, DOI 10.1021/jm0300330

Mendez D, 2019, NUCLEIC ACIDS RES, V47, pD930, DOI 10.1093/nar/gky1075

Meyder A, 2017, J CHEM INF MODEL, V57, P2437, DOI 10.1021/acs.jcim.7b00391

Mohs Richard C, 2017, Alzheimers Dement (N Y), V3, P651, DOI [10.1016/j.trci.2017.10.005, 10.1016/j.trci.2017.10.005]

Mugumbate G, 2015, PLOS ONE, V10, DOI 10.1371/journal.pone.0121492

Mushtaq S, 2018, EXCLI J, V17, P420, DOI 10.17179/excli2018-1174

PADH H, 1976, MICROBIOS, V16, P183

Ramirez LMN, 2020, ANTIBIOTICS-BASEL, V9, DOI 10.3390/antibiotics9030133

Robert X, 2014, NUCLEIC ACIDS RES, V42, pW320, DOI 10.1093/nar/gku316

Roos K, 2019, J CHEM THEORY COMPUT, V15, P1863, DOI 10.1021/acs.jctc.8b01026

Rueda M, 2010, J CHEM INF MODEL, V50, P186, DOI 10.1021/ci9003943

Sastry GM, 2013, J COMPUT AID MOL DES, V27, P221, DOI 10.1007/s10822-013-9644-8

Sastry M, 2010, J CHEM INF MODEL, V50, P771, DOI 10.1021/ci100062n

Sharma D, 2016, FRONT MICROBIOL, V7, DOI 10.3389/fmicb.2016.01816

Shelley JC, 2007, J COMPUT AID MOL DES, V21, P681, DOI 10.1007/s10822-007-9133-z

Shenoy AR, 2006, TRENDS MICROBIOL, V14, P543, DOI 10.1016/j.tim.2006.10.005

Shetye GS, 2020, TRANSL RES, V220, P68, DOI 10.1016/j.trsl.2020.03.007

Singh V, 2012, CELL HOST MICROBE, V12, P669, DOI 10.1016/j.chom.2012.09.012

Stefanachi A, 2018, MOLECULES, V23, DOI 10.3390/molecules23020250

Stinear TP, 2008, GENOME RES, V18, P729, DOI 10.1101/gr.075069.107

Syuib M, 2013, 2013 FIRST INTERNATIONAL CONFERENCE ON ARTIFICIAL INTELLIGENCE, MODELLING AND SIMULATION (AIMS 2013), P129, DOI 10.1109/AIMS.2013.28

Taylor SS, 2012, PHILOS T R SOC B, V367, P2517, DOI 10.1098/rstb.2012.0054

Walters WP, 1998, DRUG DISCOV TODAY, V3, P160, DOI 10.1016/S1359-6446(97)01163-X

WHO, 2012, GLOBAL TUBERCULOSIS REPORT 2012, P1

Wishart DS, 2006, NUCLEIC ACIDS RES, V34, pD668, DOI 10.1093/nar/gkj067

Xavier AS, 2014, J PHARMACOL PHARMACO, V5, P222, DOI 10.4103/0976-500X.136121

Yadav M, 2004, J IMMUNOL, V172, P5588, DOI 10.4049/jimmunol.172.9.5588

NR 65

TC 11

Z9 12

U1 1

U2 6

PU FRONTIERS MEDIA SA

PI LAUSANNE

PA AVENUE DU TRIBUNAL FEDERAL 34, LAUSANNE, CH-1015, SWITZERLAND

EI 2296-889X

J9 FRONT MOL BIOSCI

JI Front. Mol. Biosci.

PD MAY 3

PY 2021

VL 8

AR 599221

DI 10.3389/fmolb.2021.599221

PG 20

WC Biochemistry & Molecular Biology

WE Science Citation Index Expanded (SCI-EXPANDED)

SC Biochemistry & Molecular Biology

GA SC9NG

UT WOS:000650989800001

OA Green Accepted, Green Submitted, gold

DA 2026-05-07

ER

PT J

AU de Knegt, GJ

van der Meijden, A

de Vogel, CP

Aarnoutse, RE

de Steenwinkel, JEM

AF de Knegt, Gerjo J.

van der Meijden, Aart

de Vogel, Corne P.

Aarnoutse, Rob E.

de Steenwinkel, Jurriaan E. M.

TI Activity of moxifloxacin and linezolid against Mycobacterium

tuberculosis in combination with potentiator drugs verapamil,

timcodar, colistin and SQ109

SO INTERNATIONAL JOURNAL OF ANTIMICROBIAL AGENTS

LA English

DT Article

DE Mycobacterium tuberculosis; Moxifloxacin; Linezolid; Verapamil;

Colistin; SQ109

ID MDR-TB; ANTITUBERCULOSIS DRUGS; RESISTANT TUBERCULOSIS; BEIJING

GENOTYPE; MOUSE MODEL; IN-VITRO; EFFLUX; EFFICACY; BEDAQUILINE;

RIFAMPICIN

AB Current treatment for tuberculosis (TB) is complicated by the emergence of multidrug resistant TB (MDRTB). As a result, there is an urgent need for new powerful anti-TB regimens and novel strategies. In this study, we aimed to potentiate amoxifloxacin + linezolid backbone as treatment for MDR-TB with the efflux pump inhibitors verapamil and timcodar as well as with drugs that act on mycobacterial cell wall stability such as colistin and SQ109. Using a time-kill kinetics assay, the activities of moxifloxacin, linezolid, verapamil, timcodar, colistin and SQ109 as single drugs against Mycobacterium tuberculosis were evaluated. In addition, the activity of the moxifloxacin + linezolid backbone in combination with one of the potentiator drugs was assessed. As little as 0.125 mg/L moxifloxacin achieved 99% killing of M. tuberculosis after 6 days of exposure. Linezolid showedmoderate killing but 99% killingwas not achieved. Verapamil, timcodar and colistin only resulted in killing with the highest concentrations tested but 99% killing was not achieved. SQ109 resulted in complete elimination after 1 day of exposure to 256 mg/L and in 99% elimination after 6 days of exposure to 1mg/L. Furthermore, colistin added to the moxifloxacin + linezolid backbone resulted in increased elimination, whereas verapamil, timcodar and SQ109 showed no added value to the backbone. This finding that colistin potentiates the activity of the moxifloxacin + linezolid backbone against M. tuberculosis suggests its potential role in further studies on the applicability of a moxifloxacin + linezolid treatment of MDR-TB. (C) 2017 Elsevier B.V. and International Society of Chemotherapy. All rights reserved.

C1 [de Knegt, Gerjo J.; van der Meijden, Aart; de Vogel, Corne P.; de Steenwinkel, Jurriaan E. M.] Erasmus MC, Univ Med Ctr Rotterdam, Dept Med Microbiol & Infect Dis, Wytemaweg 80, NL-3015 CN Rotterdam, Netherlands.

[Aarnoutse, Rob E.] Radboud Univ Nijmegen, Med Ctr, Dept Pharm, Nijmegen, Netherlands.

C3 Erasmus University Rotterdam; Erasmus MC; Radboud University Nijmegen

RP de Knegt, GJ (corresponding author), Erasmus MC, Univ Med Ctr Rotterdam, Dept Med Microbiol & Infect Dis, Wytemaweg 80, NL-3015 CN Rotterdam, Netherlands.

EM g.deknegt@erasmusmc.nl

RI Aarnoutse, R.E./L-4168-2015

FU Dutch Organization for Health Research and Development (ZonMw), Priority

Medicines Antimicrobial Resistance [205200002]

FX This work was funded by the Dutch Organization for Health Research and

Development (ZonMw), Priority Medicines Antimicrobial Resistance

[project 205200002].

CR Abbate E, 2012, J ANTIMICROB CHEMOTH, V67, P473, DOI 10.1093/jac/dkr500

Adams KN, 2014, J INFECT DIS, V210, P456, DOI 10.1093/infdis/jiu095

Bax HI, 2015, J ANTIMICROB CHEMOTH, V70, P2828, DOI 10.1093/jac/dkv194

Boeree MJ, 2017, LANCET INFECT DIS, V17, P39, DOI 10.1016/S1473-3099(16)30274-2

Brown AN, 2015, MBIO, V6, DOI 10.1128/mBio.01741-15

Clinical and Laboratory Standards Institute, 2012, M24A2 CLSI

CRUMPLIN GC, 1984, J ANTIMICROB CHEMOTH, V13, P9, DOI 10.1093/jac/13.suppl_B.9

Dawson R, 2015, LANCET, V385, P1738, DOI 10.1016/S0140-6736(14)62002-X

de Knegt GJ, 2015, INT J ANTIMICROB AG, V46, P66, DOI 10.1016/j.ijantimicag.2015.02.025

de Knegt GJ, 2014, TUBERCULOSIS, V94, P701, DOI 10.1016/j.tube.2014.09.002

de Steenwinkel JEM, 2012, ANTIMICROB AGENTS CH, V56, P4937, DOI 10.1128/AAC.00124-12

de Steenwinkel JEM, 2012, EMERG INFECT DIS, V18, P660, DOI 10.3201/eid1804.110912

de Steenwinkel JEM, 2010, J ANTIMICROB CHEMOTH, V65, P2582, DOI 10.1093/jac/dkq374

Doern CD, 2014, J CLIN MICROBIOL, V52, P4124, DOI 10.1128/JCM.01121-14

Drlica K, 1996, ANTIMICROB AGENTS CH, V40, P1594, DOI 10.1128/AAC.40.7.1594

EAGLE H, 1948, J EXP MED, V88, P99, DOI 10.1084/jem.88.1.99

Escribano I, 2007, CHEMOTHERAPY, V53, P397, DOI 10.1159/000109769

Gillespie SH, 2014, NEW ENGL J MED, V371, P1577, DOI 10.1056/NEJMoa1407426

Gontijo AVL, 2014, ANTIMICROB AGENTS CH, V58, P3950, DOI 10.1128/AAC.02819-14

Grossman TH, 2015, ANTIMICROB AGENTS CH, V59, P1539, DOI 10.1128/AAC.04271-14

Günther G, 2015, EUR RESPIR J, V45, P1081, DOI 10.1183/09031936.00124614

Gupta S, 2015, ANTIMICROB AGENTS CH, V59, P673, DOI 10.1128/AAC.04019-14

Gupta S, 2014, ANTIMICROB AGENTS CH, V58, P574, DOI 10.1128/AAC.01462-13

Gupta S, 2013, AM J RESP CRIT CARE, V188, P600, DOI 10.1164/rccm.201304-0650OC

Hancock REW, 1999, ANTIMICROB AGENTS CH, V43, P1317, DOI 10.1128/AAC.43.6.1317

Heinrich N, 2015, J ANTIMICROB CHEMOTH, V70, P1558, DOI 10.1093/jac/dku553

HOOPER DC, 1987, AM J MED, V82, P12

Jeong BH, 2015, J ANTIMICROB CHEMOTH, V70, P3127, DOI 10.1093/jac/dkv215

Jia L, 2005, J PHARMACEUT BIOMED, V37, P793, DOI 10.1016/j.jpba.2004.11.036

Lee M, 2012, NEW ENGL J MED, V367, P1508, DOI 10.1056/NEJMoa1201964

LEWIN CS, 1991, EUR J CLIN MICROBIOL, V10, P240, DOI 10.1007/BF01966996

Mullin S, 2004, ANTIMICROB AGENTS CH, V48, P4171, DOI 10.1128/AAC.48.11.4171-4176.2004

Paulson T, 2013, NATURE, V502, pS2, DOI 10.1038/502S2a

Pule CM, 2016, J ANTIMICROB CHEMOTH, V71, P17, DOI 10.1093/jac/dkv316

Sacksteder KA, 2012, FUTURE MICROBIOL, V7, P823, DOI [10.2217/fmb.12.56, 10.2217/FMB.12.56]

Schuster A, 2013, THORAX, V68, P344, DOI 10.1136/thoraxjnl-2012-202059

Sotgiu G, 2012, EUR RESPIR J, V40, P1430, DOI 10.1183/09031936.00022912

Tasneen R, 2016, ANTIMICROB AGENTS CH, V60, P270, DOI 10.1128/AAC.01691-15

te Brake LHM, 2016, TUBERCULOSIS, V96, P150, DOI 10.1016/j.tube.2015.08.004

Wallis RS, 2014, PLOS ONE, V9, DOI 10.1371/journal.pone.0094462

WHO, 2015, GLOB TUB REP 2015

Williams KN, 2009, ANTIMICROB AGENTS CH, V53, P1314, DOI 10.1128/AAC.01182-08

NR 42

TC 18

Z9 20

U1 0

U2 11

PU ELSEVIER SCIENCE BV

PI AMSTERDAM

PA PO BOX 211, 1000 AE AMSTERDAM, NETHERLANDS

SN 0924-8579

EI 1872-7913

J9 INT J ANTIMICROB AG

JI Int. J. Antimicrob. Agents

PD MAR

PY 2017

VL 49

IS 3

BP 302

EP 307

DI 10.1016/j.ijantimicag.2016.11.027

PG 6

WC Infectious Diseases; Microbiology; Pharmacology & Pharmacy

WE Science Citation Index Expanded (SCI-EXPANDED)

SC Infectious Diseases; Microbiology; Pharmacology & Pharmacy

GA EP1MD

UT WOS:000397148100006

DA 2026-05-07

ER

PT J

AU Pitso, L

Potgieter, S

van Dijk, AV

AF Pitso, L.

Potgieter, S.

van Dijk, A. van der Spoel

TI Prevalence of isoniazid resistance-conferring mutations associated with

multidrug-resistant tuberculosis in Free State Province, South Africa

SO SAMJ SOUTH AFRICAN MEDICAL JOURNAL

LA English

DT Article

ID MTBDRPLUS VERSION 2; MYCOBACTERIUM-TUBERCULOSIS; DRUG-RESISTANCE; INHA;

ETHIONAMIDE; REGIMEN; ASSAY; KATG

AB Background. Multidrug- and extensively drug-resistant tuberculosis (MDR-TB and XDR-TB) threaten local and global control of the disease. The molecular line-probe assay (LPA) provides rapid diagnosis and early management of MDR-TB. The LPA detects mutations of katG and inhA genes associated with isoniazid (INH) resistance in Mycobacterium tuberculosis isolates. The katG and inhA genes are associated with high- and low-level INH resistance, respectively, as well as cross-resistance to ethionamide in the case of inhA gene mutations. Patients with MDR-TB due to an inhA mutation could benefit from the use of high- dose INH - instead of ethionamide - in their MDR-TB regimen.

Objectives. To determine the frequencies of katG and inhA mutations that conferred INH resistance among MDR-TB isolates during 2014 - 2016 in Free State (FS) Province of South Africa.

Methods. We retrospectively reviewed MDR-TB isolates assayed with GenoType MTBDRplus (Hain Lifescience, Germany) (LPA) at the central TB laboratory of Universitas Academic Hospital, Bloemfontein, FS, and calculated the frequencies of katG and inhA mutations.

Results. Among 918 MDR-TB isolates, the prevalence of katG, inhA and katG plus inhA mutations was 63.9%, 13.4% and 22.7%, respectively. Approximately 60% (n=536; 58.4%) of the isolates were obtained from male patients. The patients' ages ranged from 1 to 89 (median 37) years. The Xhariep district had the highest incidence of INH resistance-conferring mutations in the province.

Conclusions. katG-associated mutations are the predominant INH resistance-conferring mechanism among MDR-TB isolates in the FS. Patients infected with isolates that harbour the katG mutation are unlikely to benefit from high- dose INH therapy in the bedaquiline (BDQ)-containing modified short MDR-TB regimen. They may, however, benefit from the inclusion of ethionamide in the regimen. Dual katG and inhA gene mutations make these patients unlikely to respond to either high- dose INH or ethionamide and should now be considered for either the BDQ-containing long MDR-TB regimen or an individualised treatment regimen, depending on fluoroquinolone susceptibility. Clinicians should familiarise themselves with interpreting various INH resistance-conferring mutation results and their implications for management of MDR-TB treatment.

C1 [Pitso, L.] Univ Free State, Fac Hlth Sci, Dept Internal Med, Bloemfontein, South Africa.

[Potgieter, S.] Univ Free State, Fac Hlth Sci, Dept Internal Med, Div Infect Dis, Bloemfontein, South Africa.

[van Dijk, A. van der Spoel] Univ Free State, Fac Hlth Sci, Dept Med Microbiol, Bloemfontein, South Africa.

[van Dijk, A. van der Spoel] Natl Hlth Lab Serv, Bloemfontein, South Africa.

C3 University of the Free State; University of the Free State; University

of the Free State

RP Pitso, L (corresponding author), Univ Free State, Fac Hlth Sci, Dept Internal Med, Bloemfontein, South Africa.

EM pitsol@ufs.ac.za

RI ; Pitso, Lebohang/KMY-0071-2024

OI Potgieter, Samantha/0000-0002-7408-9787;

CR Abanda NN, 2017, BMC INFECT DIS, V17, DOI 10.1186/s12879-017-2489-3

[Anonymous], 2016, Treatment Guidelines for Drug-Resistant Tuberculosis

[Anonymous], 2014, COMPANION HDB WHO GU

[Anonymous], 2017, Global tuberculosis report 2017

[Anonymous], SHORT MDR TB REG

[Anonymous], 2016, Tuberculosis Diagnostics: Molecular Line-Probe Assay for the detection of resistance to Second-Line Anti-TB drugs (SL-LPA)

[Anonymous], 2018, Rapid communication: key changes to treatment of multidrug-and rifampicin-resistant tuberculosis (MDR/RR-TB)

Aung KJM, 2014, INT J TUBERC LUNG D, V18, P1180, DOI 10.5588/ijtld.14.0100

Aung WW, 2015, ANN LAB MED, V35, P494, DOI 10.3343/alm.2015.35.5.494

BANERJEE A, 1994, SCIENCE, V263, P227, DOI 10.1126/science.8284673

Barnard M, 2008, AM J RESP CRIT CARE, V177, P787, DOI 10.1164/rccm.200709-1436OC

Bollela VR, 2016, INT J TUBERC LUNG D, V20, P1099, DOI 10.5588/ijtld.15.0864

Falzon D, 2017, EUR RESPIR J, V49, DOI 10.1183/13993003.02308-2016

Hain Lifescience, RAP DIAGN TUB ITS RE

Jagielski T, 2014, J ANTIMICROB CHEMOTH, V69, P2369, DOI 10.1093/jac/dku161

Katiyar SK, 2008, INT J TUBERC LUNG D, V12, P139

Kiepiela P, 2000, TUBERCLE LUNG DIS, V80, P47, DOI 10.1054/tuld.1999.0231

Kuaban C, 2015, INT J TUBERC LUNG D, V19, P517, DOI 10.5588/ijtld.14.0535

Luo T, 2010, ANTIMICROB AGENTS CH, V54, P1075, DOI 10.1128/AAC.00964-09

Main O, 2018, LOCAL GOVT HDB S AFR

Massyn N, 2016, DISTRICT HLTH BAROME

Massyn N., 2017, District Health Barometer 2018/19

Maurya AK, 2013, INDIAN J MED MICROBI, V31, P230, DOI 10.4103/0255-0857.115625

Nathavitharana RR, 2016, J CLIN MICROBIOL, V54, P1624, DOI 10.1128/JCM.00251-16

National Department of Health, INT CLIN GUID IMPL I

National Department of Health, MAN DRUG RES TUB POL

National Department of Health, INTR NEW DRUGS DRUG

National Department of Health, NAT TUB MAN GUID 201

Nazir Ismail NN, 2017, NICD B, V15, P96

Niehaus AJ, 2015, PLOS ONE, V10, DOI 10.1371/journal.pone.0135003

Nunn AJ, 2014, TRIALS, V15, DOI 10.1186/1745-6215-15-353

Omer ZB, 2016, INT J MYCOBACT, V5, P475, DOI 10.1016/j.ijmyco.2016.06.005

Partners in Health, 2013, PIH GUIDE MED MANAGE

Piubello A, 2014, INT J TUBERC LUNG D, V18, P1188, DOI 10.5588/ijtld.13.0075

Resch SC, 2006, PLOS MED, V3, P1048, DOI 10.1371/journal.pmed.0030241

Schaaf HS, 2007, EUR J CLIN MICROBIOL, V26, P203, DOI 10.1007/s10096-007-0257-9

Seifert M, 2016, INT J TUBERC LUNG D, V20, P631, DOI 10.5588/ijtld.15.0788

Statistics South Africa, 2012, 030152 STATSSA

TBfacts.org, INF TUB TB STAT S AF

Tomasicchio M, 2016, SCI REP-UK, V6, DOI 10.1038/srep17850

Van Deun A, 2010, AM J RESP CRIT CARE, V182, P684, DOI 10.1164/rccm.201001-0077OC

Victor TC, 1997, J MED MICROBIOL, V46, P847, DOI 10.1099/00222615-46-10-847

Yadav RN, 2013, PLOS ONE, V8, DOI 10.1371/journal.pone.0072036

Zhang Y, 2015, INT J TUBERC LUNG D, V19, P1276, DOI 10.5588/ijtld.15.0389

NR 44

TC 17

Z9 22

U1 0

U2 4

PU SA MEDICAL ASSOC

PI PRETORIA

PA BLOCK F CASTLE WALK CORPORATE PARK, NOSSOB STREET, ERASMUSKLOOF EXT3,

PRETORIA, 0002, SOUTH AFRICA

SN 0256-9574

EI 2078-5135

J9 SAMJ S AFR MED J

JI SAMJ S. Afr. Med. J.

PD SEP

PY 2019

VL 109

IS 9

BP 659

EP 664

DI 10.7196/SAMJ.2019.v109i9.13730

PG 6

WC Medicine, General & Internal

WE Science Citation Index Expanded (SCI-EXPANDED)

SC General & Internal Medicine

GA IU2PO

UT WOS:000483422500012

OA gold

DA 2026-05-07

ER

PT J

AU Khusro, A

Aarti, C

Agastian, P

AF Khusro, Ameer

Aarti, Chirom

Agastian, Paul

TI Anti-tubercular peptides: A quest of future therapeutic weapon to combat

tuberculosis

SO ASIAN PACIFIC JOURNAL OF TROPICAL MEDICINE

LA English

DT Review

DE Anti-tubercular peptides; Mycobacterium tuberculosis; Therapeutic drugs;

Tuberculosis

ID MULTIDRUG-RESISTANT TUBERCULOSIS; UBIQUITIN-DERIVED PEPTIDES;

MYCOBACTERIUM-TUBERCULOSIS; HUMAN MACROPHAGES; HOST-DEFENSE;

ANTIMYCOBACTERIAL ACTIVITY; PHAGOSOME MATURATION; STREPTOMYCES SP;

CATHELICIDIN; AUTOPHAGY

AB Tuberculosis (TB) is a symbolic menace to mankind, infecting almost one third of the world's populace and causing over a million mortalities annually. Mycobacterium tuberculosis (M. tuberculosis) is the key pathogen of TB that invades and replicates inside the host's macrophage. With the emerging dilemma of multi-drug resistant tuberculosis (MDR-TB) and extensively drug resistant tuberculosis (XDR-TB), the exigency for developing new TB drugs is an obligation now for worldwide researchers. Among the propitious antimycobacterial agents examined in last few decades, anti-tubercular peptides have been substantiated to be persuasive with multiple advantages such as low immunogenicity, selective affinity to bacterial negatively charged cell envelopes and most importantly divergent mechanisms of action. In this review, we epitomized the current advances in the anti-tubercular peptides, focusing the sources and highlighting the mycobactericidal mechanisms of promising peptides. The review investigates the current anti-tubercular peptides exploited not only from human immune cells, human non-immune cells, bacteria and fungi but also from venoms, cyanobacteria, bacteriophages and several other unplumbed sources. The anti-tubercular peptides of those origins are also known to have unique second non-membrane targets within M. tuberculosis. The present context also describes the several cases that manifested the severe side effects of extant anti TB drugs. The downfall, failure to reach clinical trial phases, inept to MDR- or XDR-TB and severe complications of the currently available anti-tubercular drugs accentuate the imperative necessity to develop efficacious drugs from adequate anti-tubercular peptides. Keeping in view of the emerging trends of drug resistant M. tuberculosis globally and unexampled mycobactericidal characteristics of peptides, the anti-tubercular peptides of varied origins can be used as a potential weapon to eradicate TB in future by developing new therapeutic drugs.

C1 [Khusro, Ameer; Aarti, Chirom; Agastian, Paul] Loyola Coll, Res Dept Plant Biol & Biotechnol, Madras 600034, Tamil Nadu, India.

C3 Loyola College - Chennai

RP Agastian, P (corresponding author), Loyola Coll, Res Dept Plant Biol & Biotechnol, Madras 600034, Tamil Nadu, India.

EM armankhan0301@gmail.com; agastian@loyolacollege.edu

RI Khusro, Ameer/D-9546-2015

OI Agastian, Paul/0000-0001-6498-4940

FU Maulana Azad National Fellowship, University Grants Commission, Delhi,

India [F1-17.1/2015-16/MANF-2015-17-BIH-60730]

FX This study was supported by Maulana Azad National Fellowship

(F1-17.1/2015-16/MANF-2015-17-BIH-60730), University Grants Commission,

Delhi, India.

CR Ahsan Mohamed Jawed, 2015, Infectious Disorders - Drug Targets, V15, P32

Al-Amiry MH., 2013, J Pharmacovigil, V1, P103

Alonso S, 2007, P NATL ACAD SCI USA, V104, P6031, DOI 10.1073/pnas.0700036104

Amer K, 2013, INDIAN J PHARM PRACT, V6, P65

[Anonymous], 2013, Int. J. Pure App. Biosci

Atmaca HU, 2013, ISTANB MED J, V14, P215, DOI 10.5152/imj.2013.58

Bagley MC, 2005, CHEM REV, V105, P685, DOI 10.1021/cr0300441

Beebe Alexandria, 2015, J Clin Tuberc Other Mycobact Dis, V1, P13, DOI [10.1016/j.jctube.2015.09.001, 10.1016/j.jctube.2015.09.001]

Bento Joao, 2010, BMJ Case Rep, V2010, DOI 10.1136/bcr.12.2009.2554

Bhattacharyya S, 2012, J EVOL MED DENT SCI-, V1, P913, DOI 10.14260/jemds/146

Bhunia SK., 2015, Int J Trop Dis, V4, P1, DOI [10.1016/S2222-1808(15)60973-2, DOI 10.1016/S2222-1808(15)60973-2]

Bidarimath BC, 2016, International Journal of Pharmacological Research, V6, P84, DOI 10.7439/ijpr.v6i2.2908

Camacho LR, 2001, J BIOL CHEM, V276, P19845, DOI 10.1074/jbc.M100662200

Carroll James, 2010, Bioeng Bugs, V1, P408, DOI 10.4161/bbug.1.6.13642

Carroll J, 2010, INT J ANTIMICROB AG, V36, P132, DOI 10.1016/j.ijantimicag.2010.03.029

Chung HJ, 2000, LETT APPL MICROBIOL, V31, P416, DOI 10.1046/j.1472-765X.2000.00840.x

Dua Ruchi, 2010, Indian J Tuberc, V57, P53

Flynn JL, 2001, ANNU REV IMMUNOL, V19, P93, DOI 10.1146/annurev.immunol.19.1.93

Foss MH, 2012, BIOCHEMISTRY-US, V51, P9922, DOI 10.1021/bi301426j

Gamboa-Angulo M, 2013, PARASITOL RES, V112, P559, DOI 10.1007/s00436-012-3167-4

Ganz T, 2002, J CLIN INVEST, V109, P693, DOI 10.1172/JCI200215218

Garg R, 2009, Indian J Tuberc, V56, P51

Gavrish E, 2014, CHEM BIOL, V21, P509, DOI 10.1016/j.chembiol.2014.01.014

Guendouzi S.E., 2011, AFR J MICROBIOL RES, V5, P3468

Gutierrez MG, 2004, CELL, V119, P753, DOI 10.1016/j.cell.2004.11.038

Haritakun R, 2012, HELV CHIM ACTA, V95, P308, DOI 10.1002/hlca.201100335

Haritakun R, 2010, J NAT PROD, V73, P75, DOI 10.1021/np900520b

Hassi M, 2012, BRAZ J MICROBIOL, V43, P1516, DOI [10.1590/S1517-83822012000400036, 10.1590/S1517-838220120004000036]

Heunis TDJ, 2010, PROBIOTICS ANTIMICRO, V2, P46, DOI 10.1007/s12602-009-9024-9

Indrigo J, 2003, MICROBIOL-SGM, V149, P2049, DOI 10.1099/mic.0.26226-0

Iwatsuki M, 2006, J AM CHEM SOC, V128, P7486, DOI 10.1021/ja056780z

Jacobsen M, 2007, J MOL MED, V85, P613, DOI 10.1007/s00109-007-0157-6

Jamieson AG, 2013, CHEM BIOL DRUG DES, V81, P148, DOI 10.1111/cbdd.12042

Kaswala Dharmesh H, 2013, J Family Med Prim Care, V2, P83, DOI 10.4103/2249-4863.109958

Khan FY, 2010, INDIAN J CRIT CARE M, V14, P97, DOI 10.4103/0972-5229.68226

Khusro A, 2015, V8, P86

Koyama N, 2010, J ANTIBIOT, V63, P183, DOI 10.1038/ja.2010.14

Kusner DJ, 2001, J IMMUNOL, V167, P3308, DOI 10.4049/jimmunol.167.6.3308

Kwan CK, 2011, CLIN MICROBIOL REV, V24, P351, DOI 10.1128/CMR.00042-10

Lamkanfi M, 2010, CELL HOST MICROBE, V8, P44, DOI 10.1016/j.chom.2010.06.007

Li W, 2011, J CELL BIOCHEM, V112, P2688, DOI 10.1002/jcb.23218

Li YB, 2011, BIOORG MED CHEM LETT, V21, P6804, DOI 10.1016/j.bmcl.2011.09.031

Lin ZJ, 2014, J ANTIBIOT, V67, P121, DOI 10.1038/ja.2013.115

Liu PT, 2008, CURR OPIN IMMUNOL, V20, P371, DOI 10.1016/j.coi.2008.05.014

Magazine R, 2010, INDIAN J PHARMACOL, V42, P247, DOI 10.4103/0253-7613.68436

Malik ZA, 2003, J IMMUNOL, V170, P2811, DOI 10.4049/jimmunol.170.6.2811

Martineau AR, 2007, J IMMUNOL, V178, P7190, DOI 10.4049/jimmunol.178.11.7190

Martins MAP, 2013, BMC PHARMACOL TOXICO, V14, DOI 10.1186/2050-6511-14-27

Mattioni S, 2012, J PANCREAS, V13, P314

Mishra AK, 2011, FEMS MICROBIOL REV, V35, P1126, DOI 10.1111/j.1574-6976.2011.00276.x

Nemeth E, 2009, ACTA HAEMATOL-BASEL, V122, P78, DOI 10.1159/000243791

Okada M, 2011, HUM VACCINES, V7, P60, DOI 10.4161/hv.7.0.14563

Oninla SO, 2016, INT J MED PHARM, V6, P1

Otu AA, 2014, TROP J PHARM RES, V13, P303, DOI 10.4314/tjpr.v13i2.21

Palomino JC, 2013, CURR MED CHEM, V20, P3785, DOI 10.2174/09298673113209990166

Parul Kodan Parul Kodan, 2014, Journal of Medicine in the Tropics, V16, P32

Prabhu RD, 2013, Int J Pharmacol Clin Sci, V2, P126

Pruksakorn P, 2010, BIOORG MED CHEM LETT, V20, P3658, DOI 10.1016/j.bmcl.2010.04.100

Purdy GE, 2007, CELL MICROBIOL, V9, P2768, DOI 10.1111/j.1462-5822.2007.01039.x

Ramos DF, 2015, J VENOM ANIM TOXINS, V21

Rateb ME, 2011, NAT PROD REP, V28, P290, DOI 10.1039/c0np00061b

Rivas-Santiago CE, 2011, CLIN EXP IMMUNOL, V164, P80, DOI 10.1111/j.1365-2249.2010.04313.x

Rodríguez A, 2014, PLOS ONE, V9, DOI 10.1371/journal.pone.0101742

Saraf G, 2015, EGYPT J CHEST DIS TU, V64, P449, DOI 10.1016/j.ejcdt.2014.11.032

Sonawane A, 2011, CELL MICROBIOL, V13, P1601, DOI 10.1111/j.1462-5822.2011.01644.x

Sosunov V, 2007, J ANTIMICROB CHEMOTH, V59, P919, DOI 10.1093/jac/dkm053

Sow FB, 2007, J LEUKOCYTE BIOL, V82, P934, DOI 10.1189/jlb.0407216

Suda S, 2012, J BACTERIOL, V194, P708, DOI 10.1128/JB.06185-11

Verma S, 2007, INTERNET J PULM MED, V9, P1

Wang QM, 2009, MICROBIOL IMMUNOL, V53, P384, DOI 10.1111/j.1348-0421.2009.00138.x

Wei L, 2013, FASEB J, V27, P3067, DOI 10.1096/fj.13-227454

Welin A, 2011, J INNATE IMMUN, V3, P508, DOI 10.1159/000325297

WHO, 2012, GLOBAL TUBERCULOSIS REPORT 2012, P1

WHO, 2015, GLOB TUB REP 2015

Xie JP, 2003, INT J ANTIMICROB AG, V22, P172, DOI 10.1016/S0924-8579(03)00110-9

Xie YY, 2010, J ANTIBIOT, V63, P143, DOI 10.1038/ja.2010.6

Yang CS, 2009, J IMMUNOL, V182, P3696, DOI 10.4049/jimmunol.0802217

Yang H, 2013, PLOS ONE, V8, DOI 10.1371/journal.pone.0052848

Yasaswini B, 2015, WORLD J PHARM PHARM, V4, P1317

Yuk JM, 2009, CELL HOST MICROBE, V6, P231, DOI 10.1016/j.chom.2009.08.004

Zahir I, 2011, AFR J BIOTECHNOL, V10, P19473, DOI 10.5897/AJB11.2473

Zanetti M, 2004, J LEUKOCYTE BIOL, V75, P39, DOI 10.1189/jlb.0403147

Zhu LM, 2011, INT J TUBERC LUNG D, V15, P369

NR 83

TC 33

Z9 43

U1 0

U2 21

PU ELSEVIER SCI LTD

PI OXFORD

PA THE BOULEVARD, LANGFORD LANE, KIDLINGTON, OXFORD OX5 1GB, OXON, ENGLAND

SN 1995-7645

J9 ASIAN PAC J TROP MED

JI Asian Pac. J. Trop. Med.

PD NOV

PY 2016

VL 9

IS 11

BP 1001

EP 1012

DI 10.1016/j.apjtm.2016.09.005

PG 12

WC Public, Environmental & Occupational Health; Tropical Medicine

WE Science Citation Index Expanded (SCI-EXPANDED)

SC Public, Environmental & Occupational Health; Tropical Medicine

GA EB8IV

UT WOS:000387635600001

OA gold

DA 2026-05-07

ER

PT J

AU Zhang, L

Pang, Y

Yu, X

Wang, Y

Gao, M

Huang, H

Zhao, Y

AF Zhang, L.

Pang, Y.

Yu, X.

Wang, Y.

Gao, M.

Huang, H.

Zhao, Y.

TI Linezolid in the treatment of extensively drug-resistant tuberculosis

SO INFECTION

LA English

DT Article

DE Linezolid; Extensively drug-resistant; Tuberculosis

ID MYCOBACTERIUM-TUBERCULOSIS; MULTIDRUG-RESISTANT; MDR-TB; OXAZOLIDINONES;

MECHANISM

AB To investigate the linezolid (LZD) treatment outcome and correlation between in vitro susceptibility to LZD and clinical outcome.

We retrospectively reviewed records of tuberculosis (TB) patients who received treatment with linezolid between March 2012 and February 2013.

A total of 43 extensively drug-resistant (XDR) TB patients identified by drug susceptibility testing were enrolled in this study, including 15 (34.9 %) received LZD as part of individualized treatment regimens. Among the 43 XDR TB patients, 15 patients (34.9 %) obtained favorable clinical outcome, including 9 (60.0 %) from LZD group and 6 (21.4 %) from control group without LZD. Statistical analysis revealed that the percentage of favorable outcomes of LZD group was significantly higher than that of control group (P = 0.011). Furthermore, we analyzed the LZD minimum inhibitory concentrations of Mycobacterium tuberculosis (MTB) isolates from patients in LZD group and identified 4 (26.7 %) resistant to LZD. All of the patients with LZD resistance harbored adverse clinical outcome, while most of the patients infected with LZD sensitive MTB harbored favorable clinical outcome (81.8 %, 9/11). Statistical analysis revealed that the percentage of favorable outcome among the patients with LZD resistance was statistically lower than that among the LZD susceptible group (P = 0.011).

This study demonstrates that linezolid has efficacy against XDR pulmonary TB patients, even in shorter duration of administration. The XDR TB patients infected with LZD-resistant isolates were more likely to obtain the adverse clinical outcome under the treatment of regimen containing LZD.

C1 [Zhang, L.; Yu, X.; Wang, Y.; Gao, M.; Huang, H.] Capital Med Univ, Beijing Chest Hosp, Beijing TB & Thorac Tumor Res Inst, TB Dept, Beijing 101149, Peoples R China.

[Pang, Y.; Zhao, Y.] Chinese Ctr Dis Control & Prevent, Natl Ctr TB Control & Prevent, Beijing 102206, Peoples R China.

C3 Capital Medical University; Chinese Center for Disease Control &

Prevention

RP Gao, M (corresponding author), Capital Med Univ, Beijing Chest Hosp, Beijing TB & Thorac Tumor Res Inst, TB Dept, 97 Machang, Beijing 101149, Peoples R China.

EM gaomqwdm@aliyun.com; hairong.huangcn@gmail.com; zhaoyanlin@chinatb.org

RI Huang, Hairong/AGM-6480-2022

FU National Key Project [2013003ZX003]

FX The study was supported by National Key Project (2013003ZX003).

CR Anger HA, 2010, J ANTIMICROB CHEMOTH, V65, P775, DOI 10.1093/jac/dkq017

[Anonymous], 2009, WHOHTMTB2009422

[Anonymous], 2008, WHO/HTM/TB/2008.394

Bozdogan B, 2004, INT J ANTIMICROB AG, V23, P113, DOI 10.1016/j.ijantimicag.2003.11.003

Condos Rany, 2008, Chest, V134, P187, DOI 10.1378/chest.07-1988

Cynamon MH, 1999, ANTIMICROB AGENTS CH, V43, P1189, DOI 10.1128/AAC.43.5.1189

Dietze R, 2008, AM J RESP CRIT CARE, V178, P1180, DOI 10.1164/rccm.200806-892OC

Gandhi NR, 2010, LANCET, V375, P1830, DOI 10.1016/S0140-6736(10)60410-2

Hillemann D, 2008, ANTIMICROB AGENTS CH, V52, P800, DOI 10.1128/AAC.01189-07

Ippolito JA, 2008, J MED CHEM, V51, P3353, DOI 10.1021/jm800379d

Lee M, 2012, NEW ENGL J MED, V367, P1508, DOI 10.1056/NEJMoa1201964

Migliori GB, 2008, EUR RESPIR J, V31, P1155, DOI 10.1183/09031936.00028708

Ntziora F, 2007, INT J TUBERC LUNG D, V11, P606

Pang Y, 2013, ANTIMICROB AGENTS CH, V57, P893, DOI 10.1128/AAC.01024-12

Pang Y, 2012, PLOS ONE, V7, DOI 10.1371/journal.pone.0032976

Richter E, 2007, ANTIMICROB AGENTS CH, V51, P1534, DOI 10.1128/AAC.01113-06

Rodríguez JC, 2002, INT J ANTIMICROB AG, V20, P464, DOI 10.1016/S0924-8579(02)00239-X

Schecter GF, 2010, CLIN INFECT DIS, V50, P49, DOI 10.1086/648675

Sood R., 2006, Infectious Disorders - Drug Targets, V6, P343, DOI 10.2174/187152606779025860

Sotgiu G, 2012, EUR RESPIR J, V40, P1430, DOI 10.1183/09031936.00022912

Xu HB, 2012, INT J TUBERC LUNG D, V16, P358, DOI 10.5588/ijtld.11.0493

Yew WW, 2008, INT J TUBERC LUNG D, V12, P345

Yew WW, 2011, EUR RESPIR J, V37, P441, DOI 10.1183/09031936.00033010

Zhao YL, 2012, NEW ENGL J MED, V366, P2161, DOI 10.1056/NEJMoa1108789

Zurenko GE, 1996, ANTIMICROB AGENTS CH, V40, P839, DOI 10.1128/AAC.40.4.839

NR 25

TC 40

Z9 46

U1 0

U2 21

PU SPRINGER HEIDELBERG

PI HEIDELBERG

PA TIERGARTENSTRASSE 17, D-69121 HEIDELBERG, GERMANY

SN 0300-8126

EI 1439-0973

J9 INFECTION

JI Infection

PD AUG

PY 2014

VL 42

IS 4

BP 705

EP 711

DI 10.1007/s15010-014-0632-2

PG 7

WC Infectious Diseases

WE Science Citation Index Expanded (SCI-EXPANDED)

SC Infectious Diseases

GA AM3AC

UT WOS:000339722200013

DA 2026-05-07

ER

PT J

AU Cao, RQ

Islamoglu, H

Teskey, G

Gyurjian, K

Abrahem, R

Onajole, OK

Lun, SC

Bishai, W

Kozikowski, A

Fraix, MP

Sathananthan, A

Zhong, L

Stec, J

Venketaraman, V

AF Cao, Ruoqiong

Islamoglu, Hicret

Teskey, Garrett

Gyurjian, Karo

Abrahem, Rachel

Onajole, Oluseye K.

Lun, Shichun

Bishai, William

Kozikowski, Alan

Fraix, Marcel P.

Sathananthan, Airani

Zhong, Li

Stec, Jozef

Venketaraman, Vishwanath

TI The preclinical candidate indole-2-carboxamide improves immune responses

to Mycobacterium tuberculosis infection in healthy subjects and

individuals with type 2 diabetes

SO INTERNATIONAL MICROBIOLOGY

LA English

DT Article

DE Cytokines; Intracellular parasitology; Infection immunity; Innate

immunity; Mycobacterium tuberculosis; Drug discovery

ID GRANULOMA-FORMATION; T-CELLS; GROWTH; MODEL; INHIBITION; BLOOD; MDR

AB A novel group of agents known as the indole-2-carboxamides (often referred to as indoleamides) have been shown to demonstrate high antimycobacterial activity. Studies have demonstrated that the best indoleamides possess desirable ADME/Tox properties, with less adverse effects and increased efficacy against both MDR-TB (multi-drug resistant TB) and XDR-TB (extensively drug-resistant TB). The primary mechanism of killing Mycobacterium tuberculosis (Mtb) by indoleamides is by disrupting the function of the essential mycolic acid transporter MmpL3 protein (Mycobacterial membrane protein Large 3). Therefore, targeting this essential mycobacterial transporter by small molecules opens new possibility for the development of novel and effective anti-TB agents. In the present study, we characterized the effects of indoleamides in altering the viability of Mtb in an in vitro granuloma model using immune cells derived from healthy subjects and those with type 2 diabetes mellitus (T2DM). Our results indicate that treatment with the best indoleamide 3 resulted in a significant reduction in the viability of Mtb in both THP-1 macrophages as well as in granulomas derived from healthy individuals and subjects with T2DM.

Graphical

C1 [Cao, Ruoqiong; Zhong, Li] Hebei Univ, Coll Life Sci, Baoding 071000, Hebei, Peoples R China.

[Cao, Ruoqiong; Gyurjian, Karo; Zhong, Li; Venketaraman, Vishwanath] Western Univ Hlth Sci, Coll Osteopath Med Pacific, Dept Basic Med Sci, Pomona, CA 91766 USA.

[Islamoglu, Hicret] Calif State Polytech Univ Pomona, Dept Biol Sci, Pomona, CA 91768 USA.

[Teskey, Garrett; Abrahem, Rachel; Venketaraman, Vishwanath] Western Univ Hlth Sci, Grad Coll Biomed Sci, Pomona, CA 91766 USA.

[Onajole, Oluseye K.] Roosevelt Univ, Dept Biol Phys & Hlth Sci, 425 S Wabash Ave, Chicago, IL 60605 USA.

[Lun, Shichun; Bishai, William] JHU Ctr TB Res, Johns Hopkins Sch Med, 1550 Orleans St, Baltimore, MD USA.

[Kozikowski, Alan] StarWise Therapeut LLC, Univ Res Pk, Madison, WI USA.

[Fraix, Marcel P.] Western Univ Hlth Sci, Coll Osteopath Med Pacific, Dept Phys Med & Rehabil & Neuromusculoskeletal Me, Pomona, CA 91766 USA.

[Sathananthan, Airani] Western Univ Hlth Sci, Coll Osteopath Med Pacific, Dept Internal Med, Pomona, CA 91766 USA.

[Stec, Jozef] Marshall B Ketchum Univ, Coll Pharm, Dept Pharmaceut Sci, 2575 Yorba Linda Blvd, Fullerton, CA 92831 USA.

C3 Hebei University; Western University of Health Sciences; California

State University System; California State Polytechnic University Pomona;

Western University of Health Sciences; Roosevelt University; Johns

Hopkins University; Johns Hopkins Medicine; Western University of Health

Sciences; Western University of Health Sciences

RP Venketaraman, V (corresponding author), Western Univ Hlth Sci, Coll Osteopath Med Pacific, Dept Basic Med Sci, Pomona, CA 91766 USA.; Venketaraman, V (corresponding author), Western Univ Hlth Sci, Grad Coll Biomed Sci, Pomona, CA 91766 USA.

EM vvenketaraman@westernu.edu

RI venketaraman, Vishwanath/AAW-6945-2020; Liu, Zhiming/J-5328-2019

OI venketaraman, Vishwanath/0000-0002-2586-1160;

FU National Natural Science Foundation of China [No. 81272444] Funding

Source: Medline; National Natural Science Foundation of China (CN)

[81472744] Funding Source: Medline; Foundation for the National

Institutes of Health [HL 133190] Funding Source: Medline; Foundation for

the National Institutes of Health (US) [AI 37856] Funding Source:

Medline

CR American Diabetes Association, 2013, DAT STAT DIAB

[Anonymous], 2011, TUBERCULOSIS DIABETE

Birkness KA, 2007, IMMUNOL CELL BIOL, V85, P160, DOI 10.1038/sj.icb.7100019

Center for Disease Control, 2014, NAT DIAB STAT REP

Cheon SH, 2002, CLIN DIAGN LAB IMMUN, V9, P901, DOI 10.1128/CDLI.9.4.901-907.2002

Daoud AK, 2009, J IMMUNOTOXICOL, V6, P36, DOI 10.1080/15476910802604564

Davis JM, 2009, CELL, V136, P37, DOI 10.1016/j.cell.2008.11.014

Domenech P, 2005, INFECT IMMUN, V73, P3492, DOI 10.1128/IAI.73.6.3492-3501.2005

Dooley KE, 2009, LANCET INFECT DIS, V9, P737, DOI 10.1016/S1473-3099(09)70282-8

Dorman SE, 2007, NAT MED, V13, P295, DOI 10.1038/nm0307-295

Dreher D, 2002, AM J RESP CRIT CARE, V165, P1577, DOI 10.1164/rccm.2204010

Flynn JL, 2011, MUCOSAL IMMUNOL, V4, P271, DOI 10.1038/mi.2011.14

FRANKLIN GF, 1995, J IMMUNOL METHODS, V186, P285, DOI 10.1016/0022-1759(95)00153-2

Guirado E, 2015, MBIO, V6, DOI 10.1128/mBio.02537-14

Guirado E, 2013, FRONT IMMUNOL, V4, DOI 10.3389/fimmu.2013.00098

Henning LN, 2008, J IMMUNOL, V180, P7847, DOI 10.4049/jimmunol.180.12.7847

Hoft DF, 2002, J INFECT DIS, V186, P1448, DOI 10.1086/344359

Kapoor N, 2013, PLOS ONE, V8, DOI 10.1371/journal.pone.0053657

Koul A, 2011, NATURE, V469, P483, DOI 10.1038/nature09657

Lagman M, 2015, PLOS ONE, V10, DOI 10.1371/journal.pone.0118436

Lun SC, 2013, NAT COMMUN, V4, DOI 10.1038/ncomms3907

Ly J, 2015, J INTERF CYTOK RES, V35, P875, DOI 10.1089/jir.2014.0210

OKADA S, 1992, ENDOCRINOLOGY, V130, P2284, DOI 10.1210/en.130.4.2284

Onajole OK, 2013, J MED CHEM, V56, P4093, DOI 10.1021/jm4003878

Ormerod LP, 2005, BRIT MED BULL, V73-74, P17, DOI 10.1093/bmb/ldh047

Saunders BM, 2000, IMMUNOL CELL BIOL, V78, P334, DOI 10.1046/j.1440-1711.2000.00933.x

Saunders BM, 1999, IMMUNOLOGY, V98, P324

Seitzer U, 2003, CELLS TISSUES ORGANS, V174, P110, DOI 10.1159/000071151

Silver RF, 1998, J IMMUNOL, V160, P2408

Stec J, 2016, J MED CHEM, V59, P6232, DOI 10.1021/acs.jmedchem.6b00415

Stout RD, 2009, J LEUKOCYTE BIOL, V86, P1105, DOI 10.1189/jlb.0209073

Teskey G, 2018, FRONT IMMUNOL, V9, DOI 10.3389/fimmu.2018.02069

Udwadia ZF, 2012, THORAX, V67, P286, DOI 10.1136/thoraxjnl-2012-201663

WOLFF SP, 1987, BIOCHEM J, V245, P243, DOI 10.1042/bj2450243

Worku S, 2003, INFECT IMMUN, V71, P1763, DOI 10.1128/IAI.71.4.1763-1773.2003

World Health Organization, 2017, TUB REP

NR 36

TC 7

Z9 7

U1 0

U2 17

PU SPRINGER

PI NEW YORK

PA ONE NEW YORK PLAZA, SUITE 4600, NEW YORK, NY, UNITED STATES

SN 1139-6709

EI 1618-1905

J9 INT MICROBIOL

JI Int. Microbiol.

PD MAY

PY 2020

VL 23

IS 2

BP 161

EP 170

DI 10.1007/s10123-019-00086-0

PG 10

WC Biotechnology & Applied Microbiology; Microbiology

WE Science Citation Index Expanded (SCI-EXPANDED)

SC Biotechnology & Applied Microbiology; Microbiology

GA LO5SZ

UT WOS:000533688700004

DA 2026-05-07

ER

PT J

AU Kabir, S

Ejaz, H

Hussain, SZ

Rasheed, MA

Junaid, K

Rehman, A

AF Kabir, Saba

Ejaz, Hasan

Hussain, Syed Zajif

Rasheed, Muhammad Asif

Junaid, Kashaf

Rehman, Abdul

TI Effect of tellurite on growth of extensively drug resistant (XDR)

Mycobacterium tuberculosis and action on mycobacterial drug efflux pump

SO JOURNAL OF KING SAUD UNIVERSITY SCIENCE

LA English

DT Article

DE Tellurite; XDR-TB; mmpL7 gene; Drug efflux pump

ID OXIDATIVE STRESS; POTASSIUM TELLURITE; ESCHERICHIA-COLI; BACTERIA; GENE;

EXPRESSION; REDUCTION; MECHANISM

AB In the present study genotypic resistance of bacterial strain to first- and second-line anti-tuberculosis drugs was determined by Line probe assay. Toxic effect of tellurite on the growth of Mycobacterium tuberculosis (MTB) was determined by growing cells in different concentrations of tellurite (1 to 5 mM). Morphological effects of tellurite and uptake of metal in bacterial cells were confirmed by Scanning electron microscope (SEM) and energy dispersive X-ray (EDX) analysis. Change in fold expression of the efflux pump gene was measured by RT-PCR. Mycobacterial strain was characterized as XDR-TB based on the genotypic resistance to rifampicin and isoniazid, along with resistance to fluoroquinolones and second line injectable. XDR-TB showed black colonies in tellurite presence and growth was delayed (2-3 weeks) when compared with the control. The reduced cell size, metal accumulation and the characteristic tellurite peaks appeared in metal-treated cells. MTB showed MIC value of 1 mM and had high susceptibility for higher concentrations (2-5 mM). However, no significant metal inhibitory effect on the mmpL7 efflux system was determined. Tellurite shows significant growth reduction potential against XDR-TB strain. However, the exact mechanism of action needs to be elucidated with further research. & COPY; 2023 The Author(s). Published by Elsevier B.V. on behalf of King Saud University. This is an open access article under the CC BY-NC-ND license (http://creativecommons.org/licenses/by-nc-nd/4.0/).

C1 [Kabir, Saba] Univ Cent Punjab, Fac Life Sci, Dept Microbiol, Lahore, Pakistan.

[Ejaz, Hasan; Junaid, Kashaf] Jouf Univ, Coll Appl Med Sci, Dept Clin Lab Sci, Al Jouf 72388, Saudi Arabia.

[Hussain, Syed Zajif] Univ Management Sci, SBA Sch Sci & Engn, Dept Chem, Lahore, Pakistan.

[Rasheed, Muhammad Asif] COMSATS Univ Islamabad, Sahiwal Campus, Sahiwal, Pakistan.

[Rehman, Abdul] Univ Punjab, Inst Microbiol & Mol Genet, Quaid Azam Campus, Lahore 54590, Pakistan.

[Rehman, Abdul] Univ Punjab, Inst Microbiol & Mol Genet, New Campus, Lahore 54590, Pakistan.

C3 University of Central Punjab; Al Jouf University; COMSATS University

Islamabad (CUI); University of Punjab; University of Punjab

RP Rehman, A (corresponding author), Univ Punjab, Inst Microbiol & Mol Genet, New Campus, Lahore 54590, Pakistan.

EM rehman.mmg@pu.edu.pk

RI Ejaz, Hasan/V-8762-2018; Hussain, Syed Zajif/AAZ-3250-2021; Junaid,

Kashaf/AAU-3693-2020; Rehman, Abdul/KCZ-1929-2024

OI Ejaz, Hasan/0000-0002-6185-2042;

CR Acuña LG, 2009, ARCH MICROBIOL, V191, P473, DOI 10.1007/s00203-009-0473-z

Akhtar Asma, 2017, Punjab University Journal of Zoology, V32, P129

Allué-Guardia A, 2021, FRONT MICROBIOL, V12, DOI 10.3389/fmicb.2021.612675

da Silva PEA, 2011, FEMS IMMUNOL MED MIC, V63, P1, DOI 10.1111/j.1574-695X.2011.00831.x

[Anonymous], 2019, MOL BIOL+, DOI [DOI 10.1134/S0026893319040113, 10.1134/S0026893319040113]

Arenas FA, 2014, POLAR SCI, V8, P40, DOI 10.1016/j.polar.2014.01.001

Ba LA, 2010, ORG BIOMOL CHEM, V8, P4203, DOI 10.1039/c0Ob00086h

Chasteen TG, 2009, FEMS MICROBIOL REV, V33, P820, DOI 10.1111/j.1574-6976.2009.00177.x

Coral G, 2006, POL J ENVIRON STUD, V15, P517

de Souza JVP, 2020, INT J TUBERC LUNG D, V24, P591, DOI 10.5588/ijtld.19.0458

Fleming A, 1932, J PATHOL BACTERIOL, V35, P831, DOI 10.1002/path.1700350603

Kabir S, 2021, INT J INFECT DIS, V103, P56, DOI 10.1016/j.ijid.2020.11.007

Kabir S, 2020, BMC PULM MED, V20, DOI 10.1186/s12890-020-1172-4

Khan Z, 2015, APPL MICROBIOL BIOT, V99, P10745, DOI 10.1007/s00253-015-6901-x

Missen OP, 2020, EARTH-SCI REV, V204, DOI 10.1016/j.earscirev.2020.103150

Molina-Quiroz RC, 2012, PLOS ONE, V7, DOI 10.1371/journal.pone.0035452

Pasca MR, 2005, ANTIMICROB AGENTS CH, V49, P4775, DOI 10.1128/AAC.49.11.4775-4777.2005

Passet V, 2015, J CLIN MICROBIOL, V53, P1380, DOI 10.1128/JCM.03053-14

Pérez JM, 2007, PLOS ONE, V2, DOI 10.1371/journal.pone.0000211

Presentato A, 2019, ENVIRON CHEM, V16, P266, DOI 10.1071/EN18238

Pule CM, 2016, J ANTIMICROB CHEMOTH, V71, P17, DOI 10.1093/jac/dkv316

Rio DC., 2010, COLD SPRING HARBOR P, V6, DOI DOI 10.1101/PDB.PROT5439

Rodrigues L, 2012, INFECT GENET EVOL, V12, P695, DOI 10.1016/j.meegid.2011.08.009

Sandoval JM, 2012, MOL CELL TOXICOL, V8, P327, DOI 10.1007/s13273-012-0040-6

Sandoval JM, 2010, BIOMETALS, V23, P623, DOI 10.1007/s10534-010-9316-2

Swain SS, 2020, EMERG MICROBES INFEC, V9, P1651, DOI 10.1080/22221751.2020.1785334

Taylor DE, 1999, TRENDS MICROBIOL, V7, P111, DOI 10.1016/S0966-842X(99)01454-7

Tremaroli V, 2007, ARCH MICROBIOL, V187, P127, DOI 10.1007/s00203-006-0179-4

Nguyen VK, 2019, J IND ENG CHEM, V78, P246, DOI 10.1016/j.jiec.2019.06.006

NR 29

TC 3

Z9 3

U1 0

U2 5

PU ELSEVIER

PI AMSTERDAM

PA RADARWEG 29, 1043 NX AMSTERDAM, NETHERLANDS

SN 1018-3647

EI 2213-686X

J9 J KING SAUD UNIV SCI

JI J. King Saud Univ. Sci.

PD MAY

PY 2023

VL 35

IS 4

AR 102629

DI 10.1016/j.jksus.2023.102629

EA MAR 2023

PG 5

WC Multidisciplinary Sciences

WE Science Citation Index Expanded (SCI-EXPANDED)

SC Science & Technology - Other Topics

GA M4OU6

UT WOS:001030027300001

OA Green Submitted, gold

DA 2026-05-07

ER

PT J

AU Singh, R

Dwivedi, SP

Gaharwar, US

Meena, R

Rajamani, P

Prasad, T

AF Singh, R.

Dwivedi, S. P.

Gaharwar, U. S.

Meena, R.

Rajamani, P.

Prasad, T.

TI Recent updates on drug resistance in Mycobacterium tuberculosis

SO JOURNAL OF APPLIED MICROBIOLOGY

LA English

DT Review

DE anti-tubercular drugs; drug resistance; multidrug drug-resistant

tuberculosis; Mycobacterium tuberculosis; tuberculosis

ID FLUOROQUINOLONE RESISTANCE; MOLECULAR-MECHANISMS; MULTIDRUG-RESISTANCE;

EFFLUX PUMP; EVOLUTION; DELIVERY; TARGETS; TB; NANOPARTICLES; CHALLENGES

AB Tuberculosis (TB) along with acquired immune deficiency syndrome and malaria rank among the top three fatal infectious diseases which pose threat to global public health, especially in middle and low income countries. TB caused by Mycobacterium tuberculosis (Mtb) is an airborne infectious disease and one-third of the world's population gets infected with TB leading to nearly 1 center dot 6 million deaths annually. TB drugs are administered in different combinations of four first-line drugs (rifampicin, isoniazid, pyrazinamide and ethambutol) which form the core of treatment regimens in the initial treatment phase of 6-9 months. Several reasons account for the failure of TB therapy such as (i) late diagnosis, (ii) lack of timely and proper administration of effective drugs, (iii) lower availability of less toxic, inexpensive and effective drugs, (iv) long treatment duration, (v) nonadherence to drug regimen and (vi) evolution of drug-resistant TB strains. Drug-resistant TB poses a significant challenge to TB therapy and control programs. In the background of worldwide emergence of 558 000 new TB cases with resistance to rifampicin in the year 2017 and of them, 82% becoming multidrug-resistant TB (MDR-TB), it is essential to continuously update the knowledge on the mechanisms and molecular basis for evolution of Mtb drug resistance. This narrative and traditional review summarizes the progress on the anti-tubercular agents, their mode of action and drug resistance mechanisms in Mtb. The aim of this review is to provide recent updates on drug resistance mechanisms, newly developed/repurposed anti-TB agents in pipeline and international recommendations to manage MDR-TB. It is based on recent literature and WHO guidelines and aims to facilitate better understanding of drug resistance for effective TB therapy and clinical management.

C1 [Singh, R.; Prasad, T.] Jawaharlal Nehru Univ, AIRF, Room 14, New Delhi 110067, India.

[Singh, R.; Prasad, T.] Jawaharlal Nehru Univ, Special Ctr Nano Sci, New Delhi, India.

[Dwivedi, S. P.] IFTM Univ, Moradabad, Uttar Pradesh, India.

[Gaharwar, U. S.; Meena, R.; Rajamani, P.] Jawaharlal Nehru Univ, Sch Environm Sci, New Delhi, India.

C3 Jawaharlal Nehru University, New Delhi; Jawaharlal Nehru University, New

Delhi; Jawaharlal Nehru University, New Delhi

RP Prasad, T (corresponding author), Jawaharlal Nehru Univ, AIRF, Room 14, New Delhi 110067, India.

EM prasadtulika@hotmail.com

RI ; Dwivedi, Surya/GLS-4549-2022

OI Dwivedi, Surya Prakash/0000-0002-0294-5898; PRASAD,

TULIKA/0000-0003-1617-8375; MEENA, RAMOVATAR/0000-0002-3703-3274

FU Indian Funding Agency-Indian Council of Medical Research

[5/8/5/15/2012-ECD-I (2012-26130)]; Indian Council of Medical Research

(ICMR)

FX T.P. acknowledges Indian Funding Agency-Indian Council of Medical

Research [5/8/5/15/2012-ECD-I (2012-26130)] and R.S. gratefully thanks

Indian Council of Medical Research (ICMR) for the award of Junior

Research Fellowship.

CR Al-Saeedi M, 2017, INFECT DRUG RESIST, V10, P332, DOI 10.2147/IDR.S144446

Da Silva PEA, 2011, J ANTIMICROB CHEMOTH, V66, P1417, DOI 10.1093/jac/dkr173

[Anonymous], EURO SURVEILL

Ariza L, 2019, PLOS GENET, V15, DOI 10.1371/journal.pgen.1007971

Blair JMA, 2015, NAT REV MICROBIOL, V13, P42, DOI 10.1038/nrmicro3380

Byrne AL, 2015, INT J INFECT DIS, V32, P138, DOI 10.1016/j.ijid.2014.12.016

Caws M, 2006, J CLIN MICROBIOL, V44, P2333, DOI 10.1128/JCM.00330-06

Chen WJ, 2012, BIOCHEMISTRY-US, V51, P4959, DOI 10.1021/bi3004473

Conde MB, 2011, DRUG DEVELOP RES, V72, P501, DOI 10.1002/ddr.20456

Culyba MJ, 2015, BIOCHEMISTRY-US, V54, P3573, DOI 10.1021/acs.biochem.5b00109

DEMIRANDA SC, 2018, ANTIMICROB AGENTS CH, V63

Din FU, 2017, INT J NANOMED, V12, P7291, DOI 10.2147/IJN.S146315

Dookie N, 2018, J ANTIMICROB CHEMOTH, V73, P1138, DOI 10.1093/jac/dkx506

Drlica K, 2009, CURR TOP MED CHEM, V9, P981, DOI 10.2174/156802609789630947

Eldholm V, 2014, GENOME BIOL, V15, DOI 10.1186/s13059-014-0490-3

Falzon D, 2017, EUR RESPIR J, V49, DOI 10.1183/13993003.02308-2016

Farhat MR, 2016, J CLIN MICROBIOL, V54, P727, DOI 10.1128/JCM.02775-15

Ferlazzo G, 2018, LANCET INFECT DIS, V18, P536, DOI 10.1016/S1473-3099(18)30100-2

Gengenbacher M, 2012, FEMS MICROBIOL REV, V36, P514, DOI 10.1111/j.1574-6976.2012.00331.x

Ghajavand H, 2019, ANTIMICROB RESIST IN, V8, DOI 10.1186/s13756-019-0516-4

Gold B, 2017, MICROBIOL SPECTR, V5, DOI 10.1128/microbiolspec.TBTB2-0031-2016

Gomez JE, 2004, TUBERCULOSIS, V84, P29, DOI 10.1016/j.tube.2003.08.003

Gregory AE, 2013, FRONT CELL INFECT MI, V3, DOI 10.3389/fcimb.2013.00013

Gualano G, 2016, INFECT DIS REP, V8, P43, DOI 10.4081/idr.2016.6569

Gygli SM, 2017, FEMS MICROBIOL REV, V41, P354, DOI 10.1093/femsre/fux011

Hameed HMA, 2018, FRONT CELL INFECT MI, V8, DOI 10.3389/fcimb.2018.00114

Hari BNV, 2010, INDIAN J PHARMACOL, V42, P338, DOI 10.4103/0253-7613.71887

Heuvelings CC, 2017, INT J INFECT DIS, V56, P77, DOI 10.1016/j.ijid.2016.12.025

Hoagland DT, 2016, ADV DRUG DELIVER REV, V102, P55, DOI 10.1016/j.addr.2016.04.026

Honeyborne I, 2019, INT J INFECT DIS, V80, pS23, DOI 10.1016/j.ijid.2019.02.006

Houghton JL, 2013, CHEMBIOCHEM, V14, P2127, DOI 10.1002/cbic.201300359

Kardan-Yamchi J, 2019, J GLOB ANTIMICROB RE, V17, P201, DOI 10.1016/j.jgar.2019.01.003

Kaufmann SHE, 2017, INT J INFECT DIS, V56, P263, DOI 10.1016/j.ijid.2016.10.018

KHAZAEI S, 2017, SOUTH AFR J INFECT D, V32, P23

Kim H, 2015, SCI REP-UK, V5, DOI 10.1038/srep12737

Koch A, 2018, CURR OPIN PHARMACOL, V42, P7, DOI 10.1016/j.coph.2018.05.013

Koch A, 2014, EMERG MICROBES INFEC, V3, DOI 10.1038/emi.2014.17

Kurz SG, 2016, INFECT DIS CLIN N AM, V30, P509, DOI 10.1016/j.idc.2016.02.010

Labby KJ, 2013, FUTURE MED CHEM, V5, P1285, DOI 10.4155/fmc.13.80

Lambert N, 2017, J DRUG TARGET, V25, P296, DOI 10.1080/1061186X.2016.1258705

Lee ASG, 1999, ANTIMICROB AGENTS CH, V43, P2087, DOI 10.1128/AAC.43.8.2087

Lee JH, 2015, CHEM ENG SCI, V125, P75, DOI 10.1016/j.ces.2014.08.046

Lee SH, 2016, TUBERC RESPIR DIS, V79, P201, DOI 10.4046/trd.2016.79.4.201

Leibert E, 2010, EXPERT REV ANTI-INFE, V8, P801, DOI 10.1586/ERI.10.60

Lewis K, 2008, CURR TOP MICROBIOL, V322, P107

Li GL, 2015, PLOS ONE, V10, DOI 10.1371/journal.pone.0119013

Nguyen L, 2016, ARCH TOXICOL, V90, P1585, DOI 10.1007/s00204-016-1727-6

Lienhardt C, 2010, CURR OPIN PULM MED, V16, P186, DOI 10.1097/MCP.0b013e328337580c

Lin CH, 2017, J FOOD DRUG ANAL, V25, P219, DOI 10.1016/j.jfda.2017.02.001

Lin J, 2015, FRONT MICROBIOL, V6, DOI 10.3389/fmicb.2015.00034

Lipworth S, 2016, TUBERCULOSIS, V99, P131, DOI 10.1016/j.tube.2016.05.006

Luthra S, 2018, FRONT MICROBIOL, V9, DOI 10.3389/fmicb.2018.02179

Maisonneuve E, 2014, CELL, V157, P539, DOI 10.1016/j.cell.2014.02.050

Maningi NE, 2018, J CLIN MICROBIOL, V56, DOI [10.1128/JCM.01214-17, 10.1128/jcm.01214-17]

Marais BJ, 2013, LANCET INFECT DIS, V13, P436, DOI 10.1016/S1473-3099(13)70015-X

Matteelli A, 2018, EUR RESPIR REV, V27, DOI 10.1183/16000617.0035-2018

Melly G, 2019, MICROORGANISMS, V7, DOI 10.3390/microorganisms7030070

Migliori GB, 2007, EUR RESPIR J, V30, P623, DOI 10.1183/09031936.00077307

Migliori GB, 2007, EMERG INFECT DIS, V13, P780, DOI 10.3201/eid1305.070200

Mpagama SG, 2013, BMC INFECT DIS, V13, DOI 10.1186/1471-2334-13-432

Müller B, 2013, TRENDS GENET, V29, P160, DOI 10.1016/j.tig.2012.11.005

Narang A, 2017, INT J MYCOBACT, V6, P177, DOI 10.4103/ijmy.ijmy_26_17

Nasiri MJ, 2017, FRONT MICROBIOL, V8, DOI 10.3389/fmicb.2017.00681

Nasiruddin Mohammad, 2017, Tuberc Res Treat, V2017, P4920209, DOI [10.1155/2017/4920209, 10.1155/2017/4920209]

Pai M, 2016, NAT REV DIS PRIMERS, V2, DOI 10.1038/nrdp.2016.76

Pai M, 2015, INDIAN J MED RES, V141, P259, DOI 10.4103/0971-5916.156536

Palomino JC, 2014, ANTIBIOTICS-BASEL, V3, P317, DOI 10.3390/antibiotics3030317

Piddock LJV, 2006, CLIN MICROBIOL REV, V19, P382, DOI 10.1128/CMR.19.2.382-402.2006

Pinto Lancelot, 2011, Infect Drug Resist, V4, P129, DOI 10.2147/IDR.S10332

Prasad R, 2018, LUNG INDIA, V35, P78, DOI 10.4103/lungindia.lungindia_98_17

Ramirez MS, 2010, DRUG RESIST UPDATE, V13, P151, DOI 10.1016/j.drup.2010.08.003

Raviglione M, 2016, INFECT DIS REP, V8, P33, DOI 10.4081/idr.2016.6570

Ravimohan S, 2018, EUR RESPIR REV, V27, DOI 10.1183/16000617.0077-2017

Seifert M, 2015, PLOS ONE, V10, DOI 10.1371/journal.pone.0119628

Seung KJ, 2015, CSH PERSPECT MED, V5, DOI 10.1101/cshperspect.a017863

Shim TS, 2013, INFECT CHEMOTHER, V45, P367, DOI 10.3947/ic.2013.45.4.367

SINGLA R, 2017, ASTROCYTE, V4, P53

Sloan DJ, 2016, T ROY SOC TROP MED H, V110, P163, DOI 10.1093/trstmh/trv107

Smith T, 2014, CURR TOP MICROBIOL, V374, P53, DOI 10.1007/82_2012_279

Somoskovi A, 2001, RESP RES, V2, P164, DOI 10.1186/rr54

Sun G, 2012, J INFECT DIS, V206, P1724, DOI 10.1093/infdis/jis601

Tabarsi P, 2012, ARCH CLIN INFECT DIS, V7, P81, DOI 10.5812/archcid.14773

Tao J, 2013, NUCLEIC ACIDS RES, V41, P2370, DOI 10.1093/nar/gks1351

Tiberi S, 2018, PULMONOLOGY, V24, P86, DOI 10.1016/j.rppnen.2017.10.009

Trastoy R, 2018, CLIN MICROBIOL REV, V31, DOI 10.1128/CMR.00023-18

Udwadia ZF, 2012, CLIN INFECT DIS, V54, P579, DOI 10.1093/cid/cir889

Velayati AA, 2009, CHEST, V136, P420, DOI 10.1378/chest.08-2427

von Wintersdorff CJH, 2016, FRONT MICROBIOL, V7, DOI 10.3389/fmicb.2016.00173

Vyas A, 2014, SCI WORLD J, V2014, P1

Wang JY, 2015, PLOS ONE, V10, DOI 10.1371/journal.pone.0144136

Wang K, 2013, CURR MICROBIOL, V66, P222, DOI 10.1007/s00284-012-0215-3

Wang M, 2019, FRONT MICROBIOL, V10, DOI 10.3389/fmicb.2019.01001

*WHO, 2008, POL GUID DRUG SUSC T

*WHO, 2008, GUID PROGR MAN DRUG

*WHO, 2015, GLOB TUB REP

*WHO, 2018, GLOB TUB REP

*WHO, 2016, GLOB TUB REP

Xu J, 2019, ANTIMICROB AGENTS CH, V63, DOI [10.1128/aac.02155-18, 10.1128/AAC.02155-18]

Zammarchi L, 2014, MEDITERR J HEMATOL I, V6, DOI 10.4084/MJHID.2014.043

Zhang Y, 2009, INT J TUBERC LUNG D, V13, P1320

Zhang Y, 2012, ANTIMICROB AGENTS CH, V56, P2223, DOI 10.1128/AAC.06288-11

NR 101

TC 242

Z9 315

U1 1

U2 44

PU OXFORD UNIV PRESS

PI OXFORD

PA GREAT CLARENDON ST, OXFORD OX2 6DP, ENGLAND

SN 1364-5072

EI 1365-2672

J9 J APPL MICROBIOL

JI J. Appl. Microbiol.

PD JUN

PY 2020

VL 128

IS 6

BP 1547

EP 1567

DI 10.1111/jam.14478

EA OCT 2019

PG 21

WC Biotechnology & Applied Microbiology; Microbiology

WE Science Citation Index Expanded (SCI-EXPANDED)

SC Biotechnology & Applied Microbiology; Microbiology

GA LQ4EO

UT WOS:000492977900001

DA 2026-05-07

ER

PT J

AU Scalacci, N

Brown, AK

Pavan, FR

Ribeiro, CM

Manetti, F

Bhakta, S

Maitra, A

Smith, DL

Petricci, E

Castagnolo, D

AF Scalacci, Nicolo

Brown, Alistair K.

Pavan, Fernando R.

Ribeiro, Camila M.

Manetti, Fabrizio

Bhakta, Sanjib

Maitra, Arundhati

Smith, Darren L.

Petricci, Elena

Castagnolo, Daniele

TI Synthesis and SAR evaluation of novel thioridazine derivatives active

against drug-resistant tuberculosis

SO EUROPEAN JOURNAL OF MEDICINAL CHEMISTRY

LA English

DT Article

DE Thioridazine; Tuberculosis; MDR-TB; Efflux pump; Indole

ID MYCOBACTERIUM-TUBERCULOSIS; MULTIDRUG-RESISTANT; THERAPY;

PHENOTHIAZINES; IDENTIFICATION; DESIGN; SQ109; TB

AB The neuroleptic drug thioridazine has been recently repositioned as possible anti-tubercular drug. Thioridazine showed anti-tubercular activity against drug resistant mycobacteria but it is endowed with adverse side effects. A small library of thioridazine derivatives has been designed through the replacement of the piperidine and phenothiazine moieties, with the aim to improve the anti-tubercular activity and to reduce the cytotoxic effects. Among the resulting compounds, the indole derivative 12e showed an antimycobacterial activity significantly better than thioridazine and a cytotoxicity 15-fold lower. (C) 2016 Elsevier Masson SAS. All rights reserved.

C1 [Scalacci, Nicolo; Castagnolo, Daniele] Kings Coll London, Inst Pharmaceut Sci, 150 Stamford St, London SE1 9NH, England.

[Scalacci, Nicolo; Smith, Darren L.] Northumbria Univ Newcastle, Dept Appl Sci, Ellison Bldg,Ellison Pl, Newcastle Upon Tyne NE1 8ST, Tyne & Wear, England.

[Brown, Alistair K.] Univ Durham, Wolfson Res Inst, Sch Med Pharm & Hlth, Queens Campus, Stockton On Tees TS17 6BH, England.

[Pavan, Fernando R.; Ribeiro, Camila M.] Sao Paulo State Univ UNESP, Sch Pharmaceut Sci, Araraquara, Brazil.

[Manetti, Fabrizio; Petricci, Elena] Dipartimento Biotecnol Chim & Farm, Via A Moro 2, I-53100 Siena, Italy.

[Bhakta, Sanjib; Maitra, Arundhati] Birkbeck Univ London, Inst Struct & Mol Biol, Dept Biol Sci, Mycobacteria Res Lab, Malet St, London WC1E 7HX, England.

C3 University of London; King's College London; Northumbria University;

Durham University; Universidade Estadual Paulista; University of London;

Birkbeck University London

RP Castagnolo, D (corresponding author), Kings Coll London, Inst Pharmaceut Sci, 150 Stamford St, London SE1 9NH, England.

EM daniele.castagnolo@kcl.ac.uk

RI ; Pavan, Fernando Rogério/MZS-0713-2025; PETRICCI, ELENA/AAB-9275-2022;

Castagnolo, Daniele/C-2796-2014; Manetti, Fabrizio/Q-9412-2019; Maitra,

Arundhati/ABF-3375-2020

OI Bhakta, Sanjib/0000-0002-1165-9295; Pavan, Fernando

Rogério/0000-0002-6969-3963; PETRICCI, ELENA/0000-0002-9685-6342; Smith,

Darren/0000-0003-4925-467X; Brown, Alistair/0000-0003-0479-8294;

Castagnolo, Daniele/0000-0002-7517-5732; Manetti,

Fabrizio/0000-0002-9598-2339; Maitra, Arundhati/0000-0003-0367-6084

FU Northumbria University; Royal Society of Chemistry (Research Fund);

Birkbeck Anniversary/Wellcome Trust; MRC [G0801956, G0802079] Funding

Source: UKRI; Medical Research Council [G0802079, G0801956] Funding

Source: researchfish

FX Northumbria University is gratefully acknowledged for financial support.

DC thanks Royal Society of Chemistry (Research Fund 2015). AM thanks

Birkbeck Anniversary/Wellcome Trust PhD studentship.

CR Amaral L, 2001, J ANTIMICROB CHEMOTH, V47, P505, DOI 10.1093/jac/47.5.505

Amaral L, 2006, CURR DRUG TARGETS, V7, P887, DOI 10.2174/138945006777709539

Amaral L, 2012, PHARMACEUTICALS-BASE, V5, P1021, DOI 10.3390/ph5091021

Amaral L, 2012, IN VIVO, V26, P231

[Anonymous], 2007, INT J ANTIMICROB AG, DOI DOI 10.1016/j.ijantimicag.2006.10.013

Bhakta S, 2016, J MED CHEM, V59, P2780, DOI 10.1021/acs.jmedchem.6b00031

Caminero JA, 2006, INT J TUBERC LUNG D, V10, P829

Chan B, 2013, AM J HEALTH-SYST PH, V70, P1984, DOI 10.2146/ajhp130199

Chan ED, 2004, AM J RESP CRIT CARE, V169, P1103, DOI 10.1164/rccm.200308-1159OC

de Keijzer J, 2016, J PROTEOME RES, V15, P1776, DOI 10.1021/acs.jproteome.5b01037

Deshmukh RD, 2015, PLOS ONE, V10, DOI 10.1371/journal.pone.0135802

Eker B, 2008, EMERG INFECT DIS, V14, P1700, DOI 10.3201/eid1411.080729

Geldmacher C, 2012, CURR OPIN HIV AIDS, V7, P268, DOI 10.1097/COH.0b013e3283524e32

Hards K, 2015, J ANTIMICROB CHEMOTH, V70, P2028, DOI 10.1093/jac/dkv054

Johnston JC, 2009, PLOS ONE, V4, DOI 10.1371/journal.pone.0006914

Lee M, 2012, NEW ENGL J MED, V367, P1508, DOI 10.1056/NEJMoa1201964

Limanskaya OY, 2010, MOL BIOL+, V44, P559, DOI 10.1134/S0026893310040096

Machado D, 2012, PLOS ONE, V7, DOI 10.1371/journal.pone.0034538

Maitra A., 2016, VIRULENCE, P1

Mitnick CD, 2008, NEW ENGL J MED, V359, P563, DOI 10.1056/NEJMoa0800106

Miyata M, 2011, BIOMED PHARMACOTHER, V65, P456, DOI 10.1016/j.biopha.2011.04.021

O'Brien J, 2000, EUR J BIOCHEM, V267, P5421, DOI 10.1046/j.1432-1327.2000.01606.x

Palomino JC, 2002, ANTIMICROB AGENTS CH, V46, P2720, DOI 10.1128/AAC.46.8.2720-2722.2002

Pavan FR, 2010, EUR J MED CHEM, V45, P1898, DOI 10.1016/j.ejmech.2010.01.028

PELIZZETTI E, 1979, INORG CHEM, V18, P583, DOI 10.1021/ic50193a012

Pieroni M, 2015, J MED CHEM, V58, P5842, DOI 10.1021/acs.jmedchem.5b00428

Protopopova M, 2005, J ANTIMICROB CHEMOTH, V56, P968, DOI 10.1093/jac/dki319

Rodrigues L, 2011, BMC MICROBIOL, V11, DOI 10.1186/1471-2180-11-35

Ruan QL, 2016, EMERG MICROBES INFEC, V5, DOI 10.1038/emi.2016.12

Schlauderer F, 2013, ANGEW CHEM INT EDIT, V52, P10384, DOI 10.1002/anie.201304290

Tiemersma EW, 2011, PLOS ONE, V6, DOI 10.1371/journal.pone.0017601

Velayati AA, 2013, INT J CLIN EXP MED, V6, P307

NR 32

TC 27

Z9 32

U1 2

U2 25

PU ELSEVIER FRANCE-EDITIONS SCIENTIFIQUES MEDICALES ELSEVIER

PI ISSY-LES-MOULINEAUX

PA 65 RUE CAMILLE DESMOULINS, CS50083, 92442 ISSY-LES-MOULINEAUX, FRANCE

SN 0223-5234

EI 1768-3254

J9 EUR J MED CHEM

JI Eur. J. Med. Chem.

PD FEB 15

PY 2017

VL 127

BP 147

EP 158

DI 10.1016/j.ejmech.2016.12.042

PG 12

WC Chemistry, Medicinal

WE Science Citation Index Expanded (SCI-EXPANDED); Index Chemicus (IC)

SC Pharmacology & Pharmacy

GA EP1VQ

UT WOS:000397172800012

OA Green Submitted, Green Published, Green Accepted, Bronze

DA 2026-05-07

ER

PT J

AU Uddin, R

Siddiqui, QN

Azam, SS

Saima, B

Wadood, A

AF Uddin, Reaz

Siddiqui, Quratulain Nehal

Azam, Syed Sikander

Saima, Bibi

Wadood, Abdul

TI Identification and characterization of potential druggable targets among

hypothetical proteins of extensively drug resistant Mycobacterium

tuberculosis (XDR KZN 605) through subtractive genomics approach

SO EUROPEAN JOURNAL OF PHARMACEUTICAL SCIENCES

LA English

DT Article

DE Extensively resistant mycobacterium tuberculosis; Essential gene; Drug

targets; Subtractive genomics; Metabolic pathway analysis; Homology

modeling

ID IN-SILICO IDENTIFICATION; ANNOTATION; DATABASE; GENE; TB

AB Among the resistant isolates of tuberculosis (TB), the multidrug resistance tuberculosis (MDR-TB) and extensively drug resistant tuberculosis (XDR-TB) are the areas of growing concern for which the front-line antibiotics are no more effective. As a result, the search of new therapeutic targets against TB is an imperative need of time. On the other hand, the target identification is an a priori step in drug discovery based research. Furthermore, the availability of the complete proteomic data of extensively drug resistant Mycobacterium tuberculosis (XDR-MTB) made it possible to carry out in silico analysis for the discovery of new drug targets. In the current study, we aimed to prioritize the potential drug targets among the hypothetical proteins of XDR-TB via subtractive genomics approach. In the subtractive genomics, we stepwise reduced the complete proteome of XDR-MTB to only two hypothetical proteins and evidently proposed them as new therapeutic targets. The 3D structure of one of the two target proteins was predicted via homology modeling and later on, validated by various analysis tools. Our study suggested that the domains identified and the motif hits found in the sequences of the shortlisted drug targets are crucial for the survival of the XDR-MTB. To the best of our knowledge, the current study is the first attempt in which the complete proteomic data of XDR-MTB was subjected to the computational subtractive genomics approach and therefore, would provide an opportunity to identify the unique therapeutic targets against deadly XDR-MTB.

C1 [Uddin, Reaz; Siddiqui, Quratulain Nehal] Univ Karachi, Int Ctr Chem & Biol Sci, Dr Panjwani Ctr Mol Med & Drug Res, Lab 103 PCMD Ext, Karachi 75270, Pakistan.

[Azam, Syed Sikander] Quaid I Azam Univ, Natl Ctr Bioinformat, Islamabad, Pakistan.

[Saima, Bibi] Hazara Univ, Dept Biochem, Mansehra, Pakistan.

[Wadood, Abdul] Abdul Wali Khan Univ, Dept Biochem, Mardan, Pakistan.

C3 University of Karachi; Quaid I Azam University; Hazara University; Abdul

Wali Khan University

RP Uddin, R (corresponding author), Univ Karachi, Int Ctr Chem & Biol Sci, Dr Panjwani Ctr Mol Med & Drug Res, Lab 103 PCMD Ext, Karachi 75270, Pakistan.

EM mriazuddin@iccs.edu

RI Uddin, Reaz/I-5673-2017; Azam, Syed Sikander/AAD-4351-2021; Wadood,

Abdul/ABG-2789-2021

OI Uddin, Reaz/0000-0002-9928-4482; Siddiqui,

Quratulain/0000-0001-9383-4140; Azam, Syed Sikander/0000-0001-5624-6120;

FU Committee on Scientific and Technological Cooperation (COMSTECH)

[F/5378-1]; Higher Education Commission of Pakistan; International

Foundation for Science (IFS) [F/5378-1]

FX The authors would like to gratefully acknowledge the International

Foundation for Science (IFS) and Committee on Scientific and

Technological Cooperation (COMSTECH) to provide grant#F/5378-1 for the

research. The authors would like to acknowledge the Higher Education

Commission of Pakistan for providing the financial support to Ms.

QuratulAin Siddiqui during the course of this study.

CR Acland A, 2013, NUCLEIC ACIDS RES, V41, pD8, DOI [10.1093/nar/gkx1095, 10.1093/nar/gks1189, 10.1093/nar/gkq1172]

ALTSCHUL SF, 1990, J MOL BIOL, V215, P403, DOI 10.1016/S0022-2836(05)80360-2

Bakheet TM, 2010, BMC BIOINFORMATICS, V11, DOI 10.1186/1471-2105-11-195

Barh D, 2011, DRUG DEVELOP RES, V72, P162, DOI 10.1002/ddr.20413

Berman H. M., 2002, BIOL CRYSTALLOGR, P899

Broad Institute Mit H, 2009, MYC TUB COMP SEQ PRO

Cai CZ, 2003, NUCLEIC ACIDS RES, V31, P3692, DOI 10.1093/nar/gkg600

Camon E, 2004, NUCLEIC ACIDS RES, V32, pD262, DOI 10.1093/nar/gkh021

Chhabra G, 2010, BIOINFORMATION, V4, P278, DOI 10.6026/97320630004278

COLOVOS C, 1993, PROTEIN SCI, V2, P1511, DOI 10.1002/pro.5560020916

de Castro E, 2006, NUCLEIC ACIDS RES, V34, pW362, DOI 10.1093/nar/gkl124

Desler C, 2009, BMC BIOINFORMATICS, V10, DOI 10.1186/1471-2105-10-289

Dutta Anirban, 2006, In Silico Biology, V6, P43

Eisenberg D, 1997, METHOD ENZYMOL, V277, P396, DOI 10.1016/S0076-6879(97)77022-8

Garmory HS, 2004, INFECT IMMUN, V72, P6757, DOI 10.1128/IAI.72.12.6757-6763.2004

Goodacre NF, 2014, MBIO, V5, DOI 10.1128/mBio.00744-13

Hasan R, 2010, EMERG INFECT DIS, V16, P1473, DOI 10.3201/eid1609.100280

Izadi N, 2015, RESPIR MED CASE REP, V15, P12, DOI 10.1016/j.rmcr.2014.11.006

Jaiswal AK, 2017, INT J MOL SCI, V18, DOI 10.3390/ijms18020402

Kanabus A, 2016, INFORM TUBERCULOSIS

Kanehisa M, 2002, NUCLEIC ACIDS RES, V30, P42, DOI 10.1093/nar/30.1.42

Kanehisa M, 2004, NUCLEIC ACIDS RES, V32, pD277, DOI 10.1093/nar/gkh063

Kaur D, 2017, SCI REP-UK, V7, DOI 10.1038/srep46595

Kaur M, 2016, ARTIF CELL NANOMED B, V44, P478, DOI 10.3109/21691401.2014.962745

Kules J, 2016, MOL BIOSYST, V12, P2680, DOI 10.1039/c6mb00268d

Kwan CK, 2011, CLIN MICROBIOL REV, V24, P351, DOI 10.1128/CMR.00042-10

LASKOWSKI RA, 1993, J APPL CRYSTALLOGR, V26, P283, DOI 10.1107/S0021889892009944

Li WZ, 2006, BIOINFORMATICS, V22, P1658, DOI 10.1093/bioinformatics/btl158

Li WZ, 2001, BIOINFORMATICS, V17, P282, DOI 10.1093/bioinformatics/17.3.282

Magrane M, 2011, DATABASE-OXFORD, DOI 10.1093/database/bar009

McCann JR, 2007, MICROBIOL-SGM, V153, P3350, DOI 10.1099/mic.0.2007/008516-0

Mikusová K, 2017, DRUG DISCOV TODAY, V22, P534, DOI 10.1016/j.drudis.2016.09.025

Mirza IA, 2015, JCPSP-J COLL PHYSICI, V25, P337, DOI 05.2015/JCPSP.337341

Mizyed S, 2005, BIOCHEMISTRY-US, V44, P4011, DOI 10.1021/bi047704w

Moodley P., 2016, PLOS ONE

Moriya Y, 2007, NUCLEIC ACIDS RES, V35, pW182, DOI 10.1093/nar/gkm321

Raymond JB, 2005, J BIOL CHEM, V280, P326, DOI 10.1074/jbc.M411006200

Rizwan M, 2017, BMC BIOINFORMATICS, V18, DOI 10.1186/s12859-017-1540-0

Sanober G, 2017, GENE REP, V7, P147, DOI 10.1016/j.genrep.2017.04.008

Sarangi A.N., 2009, J. Comput. Sci. Syst. Biol, V2, P255, DOI DOI 10.4172/JCSB.1000038

Sathyanarayanan N, 2014, BIOINFORMATION, V10, P68, DOI 10.6026/97320630010068

Schultz J, 1998, P NATL ACAD SCI USA, V95, P5857, DOI 10.1073/pnas.95.11.5857

Sharma Vibhuti, 2008, In Silico Biology, V8, P331

Shawan MMAK, 2016, J PURE APPL MICROBIO, V10, P229

Uddin R, 2017, MED CHEM RES, V26, P1029, DOI 10.1007/s00044-017-1822-0

Uddin R, 2016, MED CHEM RES, V25, P473, DOI 10.1007/s00044-016-1501-6

Uddin R, 2015, GENE, V556, P213, DOI 10.1016/j.gene.2014.11.056

Uddin R, 2014, COMPUT BIOL CHEM, V48, P55, DOI 10.1016/j.compbiolchem.2013.11.005

Webb B, 2014, METHODS MOL BIOL, V1137, P1, DOI 10.1007/978-1-4939-0366-5_1

WHO, 2006, M WHO GLOB TASK FORC

WHO, 2013, GLOB TUB REP

WHO, 2015, GLOB TUB REP

Wishart DS, 2008, NUCLEIC ACIDS RES, V36, pD901, DOI 10.1093/nar/gkm958

Ying Z, 2005, ANNU REV PHARMACOL, V45, P529, DOI 10.1146/annurev.pharmtox.45.120403.100120

Yu NY, 2010, BIOINFORMATICS, V26, P1608, DOI 10.1093/bioinformatics/btq249

Zhang R, 2004, NUCLEIC ACIDS RES, V32, pD271, DOI 10.1093/nar/gkh024

NR 56

TC 26

Z9 32

U1 0

U2 15

PU ELSEVIER

PI AMSTERDAM

PA RADARWEG 29, 1043 NX AMSTERDAM, NETHERLANDS

SN 0928-0987

EI 1879-0720

J9 EUR J PHARM SCI

JI Eur. J. Pharm. Sci.

PD MAR 1

PY 2018

VL 114

BP 13

EP 23

DI 10.1016/j.ejps.2017.11.014

PG 11

WC Pharmacology & Pharmacy

WE Science Citation Index Expanded (SCI-EXPANDED)

SC Pharmacology & Pharmacy

GA FW0IN

UT WOS:000424977500002

DA 2026-05-07

ER

PT J

AU Shivangi

Meena, LS

AF Shivangi

Meena, Laxman S.

TI Calcium ATPase signaling: A must include mechanism in the radar of

therapeutics development against tuberculosis

SO BIOTECHNOLOGY AND APPLIED BIOCHEMISTRY

LA English

DT Review

DE ATP; calcium; cAMP; Mycobacterium tuberculosis

ID PHAGOSOME-LYSOSOME FUSION; MYCOBACTERIUM-TUBERCULOSIS; CA2+; SURVIVAL;

INHIBITION; CALMODULIN

AB An article presented by Peterson and Verkhratsky in 2016 explores the linkage of calcium and adenosine triphosphate (ATP) for a themed issue ofEvolution Brings Ca2+ and ATP Together to Control Life and Death. The article impacted due to several findings of their cooperative functioning such as the formulation of ATP necessitates a small concentration of cytosolic Ca2+, and therefore leads these two molecules together. The low cytosolic Ca(2+)concentration is a primary advantage of its function like a universal secondary messenger. The present article also explores the essential facts about Ca2+-ATP signaling in response to tuberculosis (TB). TB is still a major causal mortality of an important fraction of worldwide population and therefore we require an urgent regimen for its treatment. The new strategies that cover all forms of the disease multidrug-resistant TB, extensively drug-resistant TB, and TDR are necessity to overcome the disease pattern and its outcomes. Ionophores and channels-mediated transportation of Ca(2+)that leads to the elevation of intracellular Ca(2+)concentration provides an important clue to divert the focus toward this signaling mechanism. P2X7 pathway is an enormous Ca(2+)signaling pathway that incorporated Ca(2+)influx and potassium efflux upon activation by ATP molecule. After this activation, immune components like 1L-12 and IL-6 release and generate a protective response against pathogen. This review highlights importance of togetherness of Ca(2+)and ATP signaling with new and more emphasized view of Ca(2+)regulation.

C1 [Shivangi; Meena, Laxman S.] CSIR Inst Genom & Integrat Biol, Mall Rd, Delhi 110007, India.

[Shivangi; Meena, Laxman S.] Acad Sci & Innovat Res AcSIR, CSIR HRDC, Ghaziabad, Uttar Pradesh, India.

C3 Council of Scientific & Industrial Research (CSIR) - India; CSIR -

Institute of Genomics & Integrative Biology (IGIB); Council of

Scientific & Industrial Research (CSIR) - India; CSIR - Human Resource

Development Centre; Academy of Scientific & Innovative Research (AcSIR)

RP Meena, LS (corresponding author), CSIR Inst Genom & Integrat Biol, Mall Rd, Delhi 110007, India.

EM meena@igib.res.in

RI Shivangi, FNU/KTI-3890-2024

OI Shivangi, FNU/0000-0002-7344-541X; Meena, Laxman/0000-0002-1356-496X

FU Department of Science and Technology-SERB, Council of Scientific and

Industrial Research-Institute of Genomics and Integrative Biology

[GAP0145, EEQ/2016/000514]

FX The authors acknowledge financial support from the Department of Science

and Technology-SERB, Council of Scientific and Industrial

Research-Institute of Genomics and Integrative Biology under the

research project GAP0145 (SERB-DST Grant no: EEQ/2016/000514).

CR Antonio LS, 2011, BRIT J PHARMACOL, V163, P1069, DOI 10.1111/j.1476-5381.2011.01303.x

Beg Md Amjad, 2018, Advances in Bioinformatics, V2018, P6152014, DOI [10.1155/2018/6152014, 10.1155/2018/6152014]

Chin D, 2000, TRENDS CELL BIOL, V10, P322, DOI 10.1016/S0962-8924(00)01800-6

Chourasia M, 2012, CHEM BIOL DRUG DES, V79, P617, DOI 10.1111/j.1747-0285.2012.01334.x

Dey B, 2014, SEMIN IMMUNOL, V26, P486, DOI 10.1016/j.smim.2014.09.002

Dominguez DC, 2004, MOL MICROBIOL, V54, P291, DOI 10.1111/j.1365-2958.2004.04276.x

Domínguez DC, 2015, CELL CALCIUM, V57, P151, DOI 10.1016/j.ceca.2014.12.006

GANGOLA P, 1987, J BIOL CHEM, V262, P12570

Kumari P., 2016, APPL BIOCHEM BIOTECH, V174, P2663

Barrán-Berdón AL, 2011, ANAL BIOANAL CHEM, V400, P1015, DOI 10.1007/s00216-010-4209-3

LUTSENKO S, 1995, BIOCHEMISTRY-US, V34, P15607, DOI 10.1021/bi00048a001

Machado D, 2017, FRONT MICROBIOL, V8, DOI 10.3389/fmicb.2017.00711

Malik ZA, 2000, J EXP MED, V191, P287, DOI 10.1084/jem.191.2.287

Malik ZA, 2001, J IMMUNOL, V166, P3392, DOI 10.4049/jimmunol.166.5.3392

Meena LS, 2010, FEBS J, V277, P2416, DOI 10.1111/j.1742-4658.2010.07666.x

Mekahli D, 2011, CSH PERSPECT BIOL, V3, DOI 10.1101/cshperspect.a004317

Naseem R, 2009, J MOL BIOL, V391, P42, DOI 10.1016/j.jmb.2009.05.064

Norris V, 1996, J BACTERIOL, V178, P3677, DOI 10.1128/jb.178.13.3677-3682.1996

North RA, 2002, PHYSIOL REV, V82, P1013, DOI 10.1152/physrev.00015.2002

Novoa-Aponte L, 2014, BIOMED RES INT-UK, V2014, DOI 10.1155/2014/296986

Novoa-Aponte L, 2012, BMC STRUCT BIOL, V12, DOI 10.1186/1472-6807-12-25

ORDAL GW, 1977, NATURE, V270, P66, DOI 10.1038/270066a0

Padilla-Benavides T, 2013, J BIOL CHEM, V288, P11334, DOI 10.1074/jbc.M112.448175

Parham & Peter, 2009, IMMUNE SYSTEM

Paulsen IT, 2000, J MOL BIOL, V301, P75, DOI 10.1006/jmbi.2000.3961

Pearson G, 2001, ENDOCR REV, V22, P153, DOI 10.1210/er.22.2.153

Raeymaekers L, 2002, CELL CALCIUM, V32, P93, DOI 10.1016/S0143-4160(02)00125-2

Raman R, 2010, PLOS ONE, V5, DOI 10.1371/journal.pone.0014377

Ren DJ, 2001, SCIENCE, V294, P2372, DOI 10.1126/science.1065635

Salifu EY, 2020, COMPUT BIOL CHEM, V85, DOI 10.1016/j.compbiolchem.2020.107204

Santos P, 2020, BIOORGAN MED CHEM, V28, DOI 10.1016/j.bmc.2019.115256

Sharanbasappa K., 2018, BBA-PROTEINS PROTEOM, V1867, P317

Sharma S, 2017, APPL BIOCHEM BIOTECH, V181, P762, DOI 10.1007/s12010-016-2247-9

Shivangi, 2019, GENE REP, V15, DOI 10.1016/j.genrep.2019.100395

Shivangi, 2018, APPL BIOCHEM BIOTECH, V185, P815, DOI 10.1007/s12010-018-2695-5

Shivangi, 2019, RES REV J MICROBIOL, V8, P3

Shivangi, 2018, BIOMED J SCI TECHN R, V12, DOI [10.26717/BJSTR.2018.12.002231, DOI 10.26717/BJSTR.2018.12.002231]

Smith R J, 1995, Adv Microb Physiol, V37, P83, DOI 10.1016/S0065-2911(08)60144-7

Song LL, 2015, J MICROBIOL IMMUNOL, V48, P471, DOI 10.1016/j.jmii.2014.08.026

Trimple W. S., 2007, CELL, V130

Waditee R, 2004, J BIOL CHEM, V279, P4330, DOI 10.1074/jbc.M310282200

Ward SK, 2012, FRONT CELL INFECT MI, V2, DOI 10.3389/fcimb.2012.00063

Ward SK, 2010, MOL MICROBIOL, V77, P1096, DOI 10.1111/j.1365-2958.2010.07273.x

Welch RA, 2001, CURR TOP MICROBIOL, V257, P85

William G. T., 2010, TRENDS MICROBIOL, V158, P463

Zampese E, 2012, CELL MOL LIFE SCI, V69, P1077, DOI 10.1007/s00018-011-0845-9

NR 46

TC 0

Z9 1

U1 0

U2 6

PU WILEY

PI HOBOKEN

PA 111 RIVER ST, HOBOKEN 07030-5774, NJ USA

SN 0885-4513

EI 1470-8744

J9 BIOTECHNOL APPL BIOC

JI Biotechnol. Appl. Biochem.

PD DEC

PY 2021

VL 68

IS 6

BP 1167

EP 1173

DI 10.1002/bab.2038

EA OCT 2020

PG 7

WC Biochemistry & Molecular Biology; Biotechnology & Applied Microbiology

WE Science Citation Index Expanded (SCI-EXPANDED)

SC Biochemistry & Molecular Biology; Biotechnology & Applied Microbiology

GA XO5SU

UT WOS:000578565300001

DA 2026-05-07

ER

PT J

AU Singh, G

Shreya, S

Yadav, S

Akhter, Y

AF Singh, Garima

Shreya, Shriti

Yadav, Saurabh

Akhter, Yusuf

TI S292L mutation in Rv1258c efflux pump drives pyrazinamide efflux and a

novel inhibitor designed for co-therapy to improve MDR-TB treatment

outcomes

SO WORLD JOURNAL OF MICROBIOLOGY & BIOTECHNOLOGY

LA English

DT Article

DE Mycobacterium tuberculosis; Rv1258c efflux pump; Multidrug resistance;

Pyrazinamide; Molecular dynamics simulation; ADMET filtering; MM/GBSA

AB Tuberculosis (TB) remains a major global health challenge, exacerbated by the rise of drug-resistant Mycobacterium tuberculosis (Mtb) strains that undermine first-line therapies. Resistance to pyrazinamide (PZA), an essential anti-TB drug, is often linked to mutations in the pncA gene and efflux pump genes such as Rv1258c. The Rv1258c efflux pump not only expels antibiotics, reducing intracellular drug concentrations, but also enhances bacterial survival under stress, making it a promising therapeutic target. This study investigated the effects of two critical Rv1258c mutations, V219A and S292L, on drug efflux function and inhibitor binding. Molecular dynamics simulations revealed that both mutations significantly altered the thermodynamic stability and binding energetics of the efflux pump. Comparative MM/GBSA analysis showed that the binding free energy (Delta G) of PZA with wild-type Rv1258c (-17.08 +/- 1.66 kcal/mol) was moder-ately weakened in mutants S292L (-16.01 +/- 0.82 kcal/mol) and V219A (-11.68 +/- 3.31 kcal/mol), suggesting reduced drug affinity. Virtual screening further identified three potent inhibitors that met all ADMET criteria. MM/GBSA calculations indicated these inhibitors had substantially stronger binding affinities than PZA, with Delta G values of -46.49 +/- 3.97 kcal/mol, -19.61 +/- 4.58 kcal/mol, and -28.14 +/- 3.61 kcal/mol for ZINC000000035064, ZINC000000040452, and ZINC000000040453, respectively. These findings suggest that targeting Rv1258c could effectively restore intracellular drug concentrations even in resistant strains. Overall, this study provides a strong foundation for the development and experimental validation of efflux pump inhibitors as a novel therapeutic strategy against multidrug-resistant TB.

C1 [Singh, Garima; Shreya, Shriti; Yadav, Saurabh; Akhter, Yusuf] Babasaheb Bhimrao Ambedkar Univ, Dept Biotechnol, Lucknow 226025, Uttar Pradesh, India.

C3 Babasaheb Bhimrao Ambedkar University

RP Akhter, Y (corresponding author), Babasaheb Bhimrao Ambedkar Univ, Dept Biotechnol, Lucknow 226025, Uttar Pradesh, India.

EM yusuf@daad-alumni.de

RI yadav, saurabh/JZE-5132-2024; Singh, Garima/IVH-8654-2023; Akhter,

Yusuf/G-1779-2016

OI Akhter, Yusuf/0000-0001-9564-9790

FU Council of Scientific & Industrial Research (Ministry of Science &

Technology, Government of India)

FX GS is funded by the Council of Scientific & Industrial Research

(Ministry of Science & Technology, Government of India) for a Senior

Research Fellowship.

CR Al-Saeedi M, 2017, INFECT DRUG RESIST, V10, P332, DOI 10.2147/IDR.S144446

Baek M, 2021, SCIENCE, V373, P871, DOI 10.1126/science.abj8754

Balganesh M, 2012, ANTIMICROB AGENTS CH, V56, P2643, DOI 10.1128/AAC.06003-11

Bhattacharya D, 2016, NUCLEIC ACIDS RES, V44, pW406, DOI 10.1093/nar/gkw336

Biswas SS, 2024, AMB EXPRESS, V14, DOI 10.1186/s13568-024-01673-9

Cloete R, 2018, PLOS ONE, V13, DOI 10.1371/journal.pone.0207605

Daina A, 2017, SCI REP-UK, V7, DOI 10.1038/srep42717

Eberhardt J, 2021, J CHEM INF MODEL, V61, P3891, DOI 10.1021/acs.jcim.1c00203

Humphrey W, 1996, J MOL GRAPH MODEL, V14, P33, DOI 10.1016/0263-7855(96)00018-5

Irwin JJ, 2020, J CHEM INF MODEL, V60, P6065, DOI 10.1021/acs.jcim.0c00675

Jia HB, 2022, TUBERCULOSIS, V133, DOI 10.1016/j.tube.2022.102172

Jo S, 2008, J COMPUT CHEM, V29, P1859, DOI 10.1002/jcc.20945

L D.W., 2002, The PyMOL Molecular Graphics System

LASKOWSKI RA, 1993, J APPL CRYSTALLOGR, V26, P283, DOI 10.1107/S0021889892009944

Lemkul JustinA., 2018, Living J. Comput. Mol. Sci., V1, P5068, DOI [10.33011/livecoms.1.1.5068, 10.33011/livecoms.1.1.5068, DOI 10.33011/LIVECOMS.1.1.5068]

Li P, 2017, J MEMBRANE BIOL, V250, P573, DOI 10.1007/s00232-017-9982-x

Li XZ, 2009, DRUGS, V69, P1555, DOI 10.2165/11317030-000000000-00000

Liu JY, 2019, FRONT MICROBIOL, V10, DOI 10.3389/fmicb.2019.00216

Lomize AL, 2022, PROTEIN SCI, V31, P209, DOI 10.1002/pro.4219

Morris GM, 2009, J COMPUT CHEM, V30, P2785, DOI 10.1002/jcc.21256

O'Boyle NM, 2011, J CHEMINFORMATICS, V3, DOI 10.1186/1758-2946-3-33

Omasits U, 2014, BIOINFORMATICS, V30, P884, DOI 10.1093/bioinformatics/btt607

Pellegrini-Calace M, 2009, PLOS COMPUT BIOL, V5, DOI 10.1371/journal.pcbi.1000440

Radestock S, 2011, J MOL BIOL, V407, P698, DOI 10.1016/j.jmb.2011.02.008

Sauve S, 2023, MEMBRANES-BASEL, V13, DOI 10.3390/membranes13050462

Shahi F, 2021, HELIYON, V7, DOI 10.1016/j.heliyon.2021.e07566

Sharma A, 2019, INDIAN J MED RES, V149, P129, DOI 10.4103/ijmr.IJMR_2079_17

Sharma S, 2010, J ANTIMICROB CHEMOTH, V65, P1694, DOI 10.1093/jac/dkq186

Sun H, 2024, MICROBES INFECT, V26, DOI 10.1016/j.micinf.2023.105239

Tian W, 2018, NUCLEIC ACIDS RES, V46, pW363, DOI 10.1093/nar/gky473

Varadi M, 2024, NUCLEIC ACIDS RES, V52, pD368, DOI 10.1093/nar/gkad1011

WALLACE AC, 1995, PROTEIN ENG, V8, P127, DOI 10.1093/protein/8.2.127

Wellington S, 2018, ACS INFECT DIS, V4, P696, DOI 10.1021/acsinfecdis.7b00255

Zhang N, 2021, EUR RESPIR J, V58, DOI 10.1183/13993003.02013-2020

Zhang Y, 2003, J ANTIMICROB CHEMOTH, V52, P790, DOI 10.1093/jac/dkg446

Zhang Y, 2014, MICROBIOL SPECTR, V2, DOI 10.1128/microbiolspec.MGM2-0023-2013

Zomot E, 2018, J MOL BIOL, V430, P1368, DOI 10.1016/j.jmb.2018.02.026

NR 37

TC 2

Z9 2

U1 0

U2 0

PU SPRINGER

PI DORDRECHT

PA VAN GODEWIJCKSTRAAT 30, 3311 GZ DORDRECHT, NETHERLANDS

SN 0959-3993

EI 1573-0972

J9 WORLD J MICROB BIOT

JI World J. Microbiol. Biotechnol.

PD AUG 7

PY 2025

VL 41

IS 8

AR 300

DI 10.1007/s11274-025-04510-8

PG 16

WC Biotechnology & Applied Microbiology

WE Science Citation Index Expanded (SCI-EXPANDED)

SC Biotechnology & Applied Microbiology

GA 5WO6I

UT WOS:001546051100002

DA 2026-05-07

ER

PT J

AU Hegeto, LA

Caleffi-Ferracioli, KR

Nakamura-Vasconcelos, SS

de Almeida, AL

Baldin, VP

Nakamura, CV

Siqueira, VLD

Scodro, RBL

Cardoso, RF

AF Hegeto, Laise Adriane

Caleffi-Ferracioli, Katiany R.

Nakamura-Vasconcelos, Sandra S.

de Almeida, Aryadne Larissa

Baldin, Vanessa Pietrowski

Nakamura, Celso Vataru

Siqueira, Vera Lucia D.

Scodro, Regiane B. L.

Cardoso, Rosilene Fressatti

TI In vitro combinatory activity of piperine and anti-tuberculosis

drugs in Mycobacterium tuberculosis

SO TUBERCULOSIS

LA English

DT Article

DE Tuberculosis; Efflux pump; Inhibitor efflux pump; Piperine; Rifampicin;

Streptomycin; Synergism

ID NORA EFFLUX PUMP; RESISTANCE; ANALOGS; ASSAY

AB Tuberculosis (TB) is an important public health problem worldwide and the emergence of multidrug-resistant (MDR) TB and extensively drug-resistant (XDR) TB worsened the global context. The resistance in Mycobacterium tuberculosis, the causative agent of TB, can partially derive from efflux pumps (EPs) activity in plasma membrane. Due to the recent discovery of piperine (PIP), an organic alkaloid compound, increasing the bioavailability of various drugs, the current assay evaluated the combined activity of PIP and anti-TB drugs in susceptible and resistant M. tuberculosis clinical isolates. The minimum inhibitory concentrations for isoniazid, rifampicin, ethambutol, streptomycin and PIP were determined by resazurin microtiter assay and the combined effects of anti-TB drugs with PIP determined by resazurin drug combination microtiter assay and time-kill curve. The efflux pump inhibitor activity of PIP was determined by bromide accumulation assay and cytotoxicity carried out in VERO cells and J774. Al macrophages. PIP showed to have EPI activity and RIF + PIP and SM + PIP combinations showed synergistic effect, but low effect in enhancing the killing in M. tuberculosis H-37 Rv and in the clinical isolates studied, which had different resistance profiles. Future studies are needed to further clarify the importance of PIP as an adjunctive drug in the therapy against TB.

C1 [Hegeto, Laise Adriane; Scodro, Regiane B. L.; Cardoso, Rosilene Fressatti] Univ Estadual Maringa, Postgrad Program Hlth Sci, Maringa, Parana, Brazil.

[Hegeto, Laise Adriane; Caleffi-Ferracioli, Katiany R.; Nakamura-Vasconcelos, Sandra S.; de Almeida, Aryadne Larissa; Baldin, Vanessa Pietrowski; Siqueira, Vera Lucia D.; Scodro, Regiane B. L.; Cardoso, Rosilene Fressatti] Univ Estadual Maringa, Lab Med Bacteriol, Dept Clin Anal & Biomed, Maringa, Parana, Brazil.

[Nakamura, Celso Vataru] Univ Estadual Maringa, Dept Basic Hlth Sci, Maringa, Parana, Brazil.

C3 Universidade Estadual de Maringa; Universidade Estadual de Maringa;

Universidade Estadual de Maringa

RP Caleffi-Ferracioli, KR (corresponding author), Univ Estadual Maringa, Lab Med Bacteriol, Dept Clin Anal & Biomed, Maringa, Parana, Brazil.

EM katianyrcf@gmail.com

RI Bertin de Lima Scoo, Regiane/Y-9234-2019; Caleffi Ferracioli,

Katiany/AAX-9056-2021; Cardoso, Rosilene Fressatti/J-4796-2015;

Nakamura, Celso Vataru/AAA-5620-2019

OI Bertin de Lima Scoo, Regiane/0000-0002-9129-2445; Cardoso, Rosilene

Fressatti/0000-0001-6228-4780; Nakamura, Celso

Vataru/0000-0002-9911-7369

FU Capacitacao de Aperfeicoamento de Pessoal de Nivel Superior (CAPES)

FX This study was supported by grants from Capacitacao de Aperfeicoamento

de Pessoal de Nivel Superior (CAPES). The funder had no role in study

design, data collection and analysis, decision to publish or preparation

of the manuscript.

CR da Silva PEA, 2011, FEMS IMMUNOL MED MIC, V63, P1, DOI 10.1111/j.1574-695X.2011.00831.x

[Anonymous], 2017, Global tuberculosis report 2017

Bhusal Y, 2005, INT J ANTIMICROB AG, V26, P292, DOI 10.1016/j.ijantimicag.2005.05.005

Caleffi-Ferracioli KR, 2013, TUBERCULOSIS, V93, P660, DOI 10.1016/j.tube.2013.09.001

Caleffi-Ferracioli KR, 2016, MORPHOLOGICAL CHANGE, P1, DOI [10.1016/j.tube.2015.12.010, DOI 10.1016/J.TUBE.2015.12.010]

Chen C, 2018, ANTIMICROB AGENTS CH, V62, DOI 10.1128/AAC.02107-17

Coelho T, 2015, FRONT MICROBIOL, V6, DOI [10.3389/fmicb.2015.00330, 10.3389/fmicb.2015.01528]

de Steenwinkel JEM, 2010, J ANTIMICROB CHEMOTH, V65, P2582, DOI 10.1093/jac/dkq374

Demitto FD, 2015, PLOS ONE, V10, DOI 10.1371/journal.pone.0116545

Dubey RK, 2017, PHYTOTHER RES, V31, P1868, DOI 10.1002/ptr.5929

Escribano I, 2007, CHEMOTHERAPY, V53, P397, DOI 10.1159/000109769

Gupta S, 2013, AM J RESP CRIT CARE, V188, P600, DOI 10.1164/rccm.201304-0650OC

Islam MM, 2017, J GENET GENOMICS, V44, P21, DOI 10.1016/j.jgg.2016.10.002

Jiang X, 2008, MICROB DRUG RESIST, V14, P7, DOI 10.1089/mdr.2008.0772

Khajuria A, 1998, MOL CELL BIOCHEM, V189, P113, DOI 10.1023/A:1006877614411

Kumar A, 2008, J ANTIMICROB CHEMOTH, V61, P1270, DOI 10.1093/jac/dkn088

Laurenzo D, 2011, ACTA TROP, V119, P5, DOI 10.1016/j.actatropica.2011.04.008

MOON YS, 2016, TOXINS, V8, DOI DOI 10.3390/T0XINS8080240

MOSMANN T, 1983, J IMMUNOL METHODS, V65, P55, DOI 10.1016/0022-1759(83)90303-4

Murunikkara V, 2012, INFLAMMATION, V35, P1348, DOI 10.1007/s10753-012-9448-3

Palaksha M. N., 2013, World Journal of Pharmacy and Pharmaceutical Sciences (WJPPS), V2, P3840

Palomino JC, 2002, ANTIMICROB AGENTS CH, V46, P2720, DOI 10.1128/AAC.46.8.2720-2722.2002

Pillai S.K., 2005, Antimicrobial Combinations, V5

Protopopova M, 2017, IDENTIFICATION NEW A, DOI [10.1093/jac/dki319.968-74, DOI 10.1093/JAC/DKI319.968-74]

Rani, 2013, Global Journal of Pharmacology, V7, P87, DOI [10.5829/idosi.gjp.2013.7.1.1104, DOI 10.5829/IDOSI.GJP.2013.7.1.1104, DOI 10.5829/ID0SI.GJP.2013.7.1.1104]

Rodrigues L, 2013, ANTIMICROB AGENTS CH, V57, P751, DOI 10.1128/AAC.01482-12

SAMPAIO J, 2016, PLOS ONE, V11, DOI DOI 10.1371/JOURNAL.PONE.0149326

Sangwan PL, 2008, BIOORGAN MED CHEM, V16, P9847, DOI 10.1016/j.bmc.2008.09.042

Selvendiran K, 2006, PULM PHARMACOL THER, V19, P107, DOI 10.1016/j.pupt.2005.04.002

Sharma S, 2010, J ANTIMICROB CHEMOTH, V65, P1694, DOI 10.1093/jac/dkq186

Srinivasan K, 2007, CRIT REV FOOD SCI, V47, P735, DOI 10.1080/10408390601062054

te Brake LHM, 2018, ANNU REV PHARMACOL, V58, P271, DOI 10.1146/annurev-pharmtox-010617-052438

Vora Agam, 2016, J Assoc Physicians India, V64, P20

Zechini Barbara, 2009, Recent Pat Antiinfect Drug Discov, V4, P37

Zhai WJ, 2016, EVID-BASED COMPL ALT, V2016, DOI 10.1155/2016/8597208

NR 35

TC 29

Z9 40

U1 0

U2 7

PU CHURCHILL LIVINGSTONE

PI EDINBURGH

PA JOURNAL PRODUCTION DEPT, ROBERT STEVENSON HOUSE, 1-3 BAXTERS PLACE,

LEITH WALK, EDINBURGH EH1 3AF, MIDLOTHIAN, SCOTLAND

SN 1472-9792

J9 TUBERCULOSIS

JI Tuberculosis

PD JUL

PY 2018

VL 111

BP 35

EP 40

DI 10.1016/j.tube.2018.05.006

PG 6

WC Immunology; Microbiology; Respiratory System

WE Science Citation Index Expanded (SCI-EXPANDED)

SC Immunology; Microbiology; Respiratory System

GA GN5AN

UT WOS:000439054400006

DA 2026-05-07

ER

PT J

AU Dong, WQ

Wang, R

Li, P

Wang, GY

Ren, XX

Feng, JJ

Lu, H

Lu, WJ

Wang, XR

Chen, HC

Tan, C

AF Dong, Wenqi

Wang, Rui

Li, Pei

Wang, Gaoyan

Ren, Xuanxiu

Feng, Jiajia

Lu, Hao

Lu, Wenjia

Wang, Xiangru

Chen, Huanchun

Tan, Chen

TI Orphan response regulator Rv3143 increases antibiotic sensitivity by

regulating cell wall permeability in Mycobacterium smegmatis

SO ARCHIVES OF BIOCHEMISTRY AND BIOPHYSICS

LA English

DT Article

DE Rv3143; Two-component signal transduction systems; Cell wall

permeability; Antibiotic sensitivity

ID ESCHERICHIA-COLI; GENE-EXPRESSION; TUBERCULOSIS; RESISTANCE;

IDENTIFICATION; DRUG; BIOSYNTHESIS; CYCLOSERINE; VIRULENCE; ENVELOPE

AB About one quarter of people worldwide are infected with tuberculosis, and multi-drug resistant tuberculosis (MDR-TB) remains a health threat. It is known that two-Component Signal Transduction Systems (TCSs) of Mycobacterium tuberculosis are closely related to tuberculosis resistance, but the mechanism by which orphan response protein Rv3143 regulates strain sensitivity to drug is still unclear. This study found that Rv3143 overexpression resulted in approximately two-fold increase in Mycobacterium smegmatis antibiotic sensitivity. Transcriptome sequencing indicated that 198 potential genes were regulated by Rv3143, affecting the sensitivity of the strain to rifampicin (RIF). MSMEG_4740 promoter binding with Rv3143, was screened out by surface plasmon resonance (SPR). Ry1524, the homologous gene of MSMEG_4740, belonging to the glycosyltransferase (Gtf) family, was related to cell wall modification. By measuring ethidium bromide (EB) accumulation, we found when Rv3143 or MSMEG_4740, or Rv1524 was overexpressed, the cell wall permeability of Mycobacterium smegmatis was increased. In addition, a combination of Rv3143 and RIF was observed. Our findings provide a new strategy for treating drug-resistant tuberculosis by increasing the expression of Rv3143 to enhance the strain sensitivity to antibiotics.

C1 [Dong, Wenqi; Wang, Rui; Li, Pei; Wang, Gaoyan; Ren, Xuanxiu; Feng, Jiajia; Lu, Hao; Lu, Wenjia; Wang, Xiangru; Chen, Huanchun; Tan, Chen] Huazhong Agr Univ, Coll Vet Med, State Key Lab Agr Microbiol, Wuhan 430070, Hubei, Peoples R China.

[Wang, Xiangru; Chen, Huanchun; Tan, Chen] Key Lab Prevent Vet Med Hubei Prov, Wuhan 430070, Hubei, Peoples R China.

[Wang, Xiangru; Chen, Huanchun; Tan, Chen] Minist Agr Peoples Republ China, Key Lab Dev Vet Diagnost Prod, Wuhan 430070, Hubei, Peoples R China.

[Li, Pei] Jinan Univ, Dept Gastrointestinal Surg, Clin Med Coll 2, Shenzhen 518020, Guangdong, Peoples R China.

C3 Huazhong Agricultural University; Jinan University

RP Tan, C (corresponding author), Huazhong Agr Univ, Coll Vet Med, State Key Lab Agr Microbiol, Wuhan 430070, Hubei, Peoples R China.

EM tanchen@mail.hzau.edu.cn

RI lu, hao/HJA-4831-2022; Wang, Rui/C-7824-2012; Lu, wenjia/IYJ-4118-2023;

董, 文琪/HGF-1630-2022; Li, Pei/NIS-5216-2025; Wang, Gaoyan/N-8222-2013

OI lu, hao/0000-0003-0526-8172; Wang, Rui/0000-0003-3825-3620;

FU National Key R&D Program of China [2017YFD0500303]; outstanding youth

project of Natural Science Foundation in Hubei Province [2019CFA095]

FX This work was supported by grants from the National Key R&D Program of

China [2017YFD0500303], the outstanding youth project of Natural Science

Foundation in Hubei Province [2019CFA095].

CR Audrain B, 2013, APPL ENVIRON MICROB, V79, P7770, DOI 10.1128/AEM.02593-13

Bartek IL, 2009, TUBERCULOSIS, V89, P310, DOI 10.1016/j.tube.2009.06.001

Brennan PJ, 2003, TUBERCULOSIS, V83, P91, DOI 10.1016/S1472-9792(02)00089-6

Calabretta PJ, 2019, J AM CHEM SOC, V141, P9262, DOI 10.1021/jacs.9b02290

Cangelosi GA, 2006, ANTIMICROB AGENTS CH, V50, P461, DOI 10.1128/AAC.50.2.461-468.2006

Chatterjee A, 2013, PLOS ONE, V8, DOI 10.1371/journal.pone.0054717

Chen JZ, 2017, J ANTIMICROB CHEMOTH, V72, P3272, DOI 10.1093/jac/dkx316

COHEN ML, 1987, J CLIN MICROBIOL, V25, P1176, DOI 10.1128/JCM.25.7.1176-1180.1987

Dadura K, 2017, FRONT MICROBIOL, V8, DOI 10.3389/fmicb.2017.02145

Danilchanka O, 2008, ANTIMICROB AGENTS CH, V52, P2503, DOI 10.1128/AAC.00298-08

Deshayes C, 2005, J BACTERIOL, V187, P7283, DOI 10.1128/JB.187.21.7283-7291.2005

Edgar R, 2002, NUCLEIC ACIDS RES, V30, P207, DOI 10.1093/nar/30.1.207

Favrot L, 2012, EXPERT REV ANTI-INFE, V10, P1023, DOI [10.1586/ERI.12.91, 10.1586/eri.12.91]

Forrellad MA, 2013, VIRULENCE, V4, P3, DOI 10.4161/viru.22329

Foynes S, 2000, INFECT IMMUN, V68, P2016, DOI 10.1128/IAI.68.4.2016-2023.2000

Hirakawa H, 2003, J ANTIMICROB CHEMOTH, V52, P576, DOI 10.1093/jac/dkg406

Huang DW, 2009, NAT PROTOC, V4, P44, DOI 10.1038/nprot.2008.211

Jankute M, 2015, ANNU REV MICROBIOL, V69, P405, DOI 10.1146/annurev-micro-091014-104121

JARLIER V, 1994, FEMS MICROBIOL LETT, V123, P11, DOI 10.1111/j.1574-6968.1994.tb07194.x

Lairson LL, 2008, ANNU REV BIOCHEM, V77, P521, DOI 10.1146/annurev.biochem.76.061005.092322

Li QM, 2016, SCI REP-UK, V6, DOI 10.1038/srep28701

Li YQ, 2010, BMC MICROBIOL, V10, DOI 10.1186/1471-2180-10-242

Liu CF, 2013, P NATL ACAD SCI USA, V110, P6560, DOI 10.1073/pnas.1219704110

Livak KJ, 2001, METHODS, V25, P402, DOI 10.1006/meth.2001.1262

Matson JS, 2017, PLOS ONE, V12, DOI 10.1371/journal.pone.0186199

Muller C, 2018, MOL MICROBIOL, V107, P416, DOI 10.1111/mmi.13891

Palomino JC, 2002, ANTIMICROB AGENTS CH, V46, P2720, DOI 10.1128/AAC.46.8.2720-2722.2002

Parish T, 2014, MICROBIOL SPECTR, V2, DOI 10.1128/microbiolspec.MGM2-0010-2013

Piliarik Marek, 2009, V503, P65, DOI 10.1007/978-1-60327-567-5_5

Portevin D, 2005, J BIOL CHEM, V280, P8862, DOI 10.1074/jbc.M408578200

Portevin D, 2004, P NATL ACAD SCI USA, V101, P314, DOI 10.1073/pnas.0305439101

Prüss BM, 2017, J BACTERIOL, V199, DOI 10.1128/JB.00259-17

Sacksteder KA, 2012, FUTURE MICROBIOL, V7, P823, DOI [10.2217/fmb.12.56, 10.2217/FMB.12.56]

Singh A, 2005, J BACTERIOL, V187, P4173, DOI 10.1128/JB.187.12.4173-4186.2005

Singh P, 2016, SCI REP-UK, V6, DOI 10.1038/srep21624

Wang LK, 2010, BIOINFORMATICS, V26, P136, DOI 10.1093/bioinformatics/btp612

Williams KJ, 2015, MOL MICROBIOL, V97, P1142, DOI 10.1111/mmi.13091

World Health Organization, GLOB TUB REP 2019

Yu QY, 2018, J SCI FOOD AGR, V98, P3722, DOI 10.1002/jsfa.8880

Zhou L, 2015, INT J ANTIMICROB AG, V46, P73, DOI 10.1016/j.ijantimicag.2015.02.018

Zhou PF, 2012, CRIT REV EUKAR GENE, V22, P37, DOI 10.1615/CritRevEukarGeneExpr.v22.i1.30

NR 41

TC 10

Z9 10

U1 1

U2 23

PU ELSEVIER SCIENCE INC

PI NEW YORK

PA STE 800, 230 PARK AVE, NEW YORK, NY 10169 USA

SN 0003-9861

EI 1096-0384

J9 ARCH BIOCHEM BIOPHYS

JI Arch. Biochem. Biophys.

PD OCT 15

PY 2020

VL 692

AR 108522

DI 10.1016/j.abb.2020.108522

PG 9

WC Biochemistry & Molecular Biology; Biophysics

WE Science Citation Index Expanded (SCI-EXPANDED)

SC Biochemistry & Molecular Biology; Biophysics

GA NT4OJ

UT WOS:000572921900003

DA 2026-05-07

ER

PT J

AU Davids, M

Pooran, AS

Pietersen, E

Wainwright, HC

Binder, A

Warren, R

Dheda, K

AF Davids, Malika

Pooran, Anil S.

Pietersen, Elize

Wainwright, Helen C.

Binder, Anke

Warren, Robin

Dheda, Keertan

TI Regulatory T Cells Subvert Mycobacterial Containment in Patients Failing

Extensively Drug-Resistant Tuberculosis Treatment

SO AMERICAN JOURNAL OF RESPIRATORY AND CRITICAL CARE MEDICINE

LA English

DT Article

DE tuberculosis; TB host immunity; regulatory T cells; Tregs; XDR-TB

ID PULMONARY TUBERCULOSIS; ACTIVE TUBERCULOSIS; MULTIDRUG-RESISTANT;

IMMUNE-RESPONSES; BEDAQUILINE; DELAMANID; STRAINS; IMMUNOTHERAPY;

TRANSMISSION; INDUCTION

AB Rationale: The advent of extensively drug-resistant (XDR) tuberculosis (TB) and totally drug-resistant TB, with limited or no treatment options, has facilitated renewed interest in host-directed immunotherapy, particularly for therapeutically destitute patients. However, the selection and utility of such approaches depend on understanding the host immune response in XDR-TB, which hitherto remains unexplored.

Objectives: To determine the host immunological profile in patients with XDR-TB, compared with drug-sensitive TB (DS-TB), using peripheral blood and explanted lung tissue.

Methods: Blood and explanted lung tissue were obtained from patients with XDR-TB (n = 31), DS-TB (n = 20), and presumed latent TB infection (n = 20). T-cell phenotype (T-helper cell type 1 [Th1]/Th2/Th17/regulatory T cells [Tregs]) was evaluated in all patient groups, and Treg function assessed in XDR-TB nonresponders by coculturing PPD-preprimed effector T cells with H37Rv-infected monocyte-derived macrophages, with or without autologous Tregs. Mycobacterial

Measurements and Main Results: Patients failing XDR-TB treatment had an altered immunophenotype characterized by a substantial increase in the frequency (median; interquartile range) of CD4(+)CD25(+)FoxP3(+) Tregs (11.5%; 5.9-15.2%) compared with DS-TB (3.4%; 1.6-5.73%; P, 0.001) and presumed latent TB infection (1.8%; 1.2-2.3%; P = 0.001), which was unrelated to disease duration. Tregs isolated from patients with XDR-TB suppressed T-cell proliferation (up to 90%) and subverted containment of H37Rv-infected monocyte-derived macrophages (by 30%; P = 0.03) by impairing effector T-cell function through a mechanism independent of direct cell-to-cell contact, IL-10, TGF (transforming growth factor)-beta, and CTLA-4 (cytotoxic T-lymphocyte-associated protein 4).

Conclusions: Collectively, these data suggest that Tregs may be contributing to immune dysfunction, and bacterial persistence, in patients with XDR-TB. The relevant cellular pathways may serve as potential targets for immunotherapeutic intervention.

C1 [Davids, Malika; Pooran, Anil S.; Pietersen, Elize; Binder, Anke; Dheda, Keertan] Univ Cape Town, Dept Med, Cape Town, South Africa.

[Wainwright, Helen C.] Groote Schuur Hosp, Dept Pathol, Cape Town, South Africa.

[Warren, Robin] Stellenbosch Univ, South African Med Res Council Ctr TB Res, Dept Sci & Technol, Natl Res Fdn Ctr Excellence Biomed TB Res,Div Mol, Stellenbosch, South Africa.

C3 University of Cape Town; University of Cape Town; National Research

Foundation - South Africa; Stellenbosch University

RP Dheda, K (corresponding author), Div Pulmonol, Old Main Bldg, ZA-7439 Cape Town, South Africa.; Dheda, K (corresponding author), UCT Lung Inst, Dept Med, Old Main Bldg, ZA-7439 Cape Town, South Africa.

EM keertan.dheda@uct.ac.za

OI Dheda, Keertan/0000-0001-7709-5341; Warren, Robin/0000-0001-5741-7358

CR Abate G, 2016, IMMUNOTARGETS THER, V5, P37, DOI 10.2147/ITT.S81892

Basile JI, 2011, J INFECT DIS, V204, P1054, DOI 10.1093/infdis/jir460

Belkaid Y, 2002, NATURE, V420, P502, DOI 10.1038/nature01152

Bloemberg GV, 2015, NEW ENGL J MED, V373, P1986, DOI 10.1056/NEJMc1505196

Boussiotis VA, 2000, J CLIN INVEST, V105, P1317, DOI 10.1172/JCI9918

Bozzano F, 2014, MEDITERR J HEMATOL I, V6, DOI 10.4084/MJHID.2014.027

Chen XC, 2007, CLIN IMMUNOL, V123, P50, DOI 10.1016/j.clim.2006.11.009

Churina E G, 2012, Tuberc Res Treat, V2012, P931291, DOI 10.1155/2012/931291

Collison LW, 2010, NAT IMMUNOL, V11, P1093, DOI 10.1038/ni.1952

Comas I, 2012, NAT GENET, V44, P106, DOI 10.1038/ng.1038

de Cassan SC, 2010, CLIN VACCINE IMMUNOL, V17, P1066, DOI 10.1128/CVI.00047-10

Delgado JC, 2002, P NATL ACAD SCI USA, V99, P7576, DOI 10.1073/pnas.062056099

Dheda K, 2017, LANCET RESP MED, V5, P291, DOI [10.1016/s2213-2600(17)30079-6, 10.1016/S2213-2600(17)30079-6]

Dheda K, 2016, LANCET, V387, P1211, DOI 10.1016/S0140-6736(15)00151-8

Geffner L, 2014, PLOS ONE, V9, DOI 10.1371/journal.pone.0097837

Geffner L, 2009, INFECT IMMUN, V77, P5025, DOI 10.1128/IAI.00224-09

Guyot-Revol V, 2006, AM J RESP CRIT CARE, V173, P803, DOI 10.1164/rccm.200508-1294OC

Hoffmann H, 2016, AM J RESP CRIT CARE, V193, P337, DOI 10.1164/rccm.201502-0372LE

Hougardy JM, 2007, AM J RESP CRIT CARE, V176, P409, DOI 10.1164/rccm.200701-084OC

Huang ZK, 2015, PLOS ONE, V10, DOI 10.1371/journal.pone.0129744

HUNNINGHAKE GW, 1979, AM REV RESPIR DIS, V120, P49

Johnson JL, 2000, J INFECT DIS, V181, P1304, DOI 10.1086/315393

Kursar M, 2007, J IMMUNOL, V178, P2661, DOI 10.4049/jimmunol.178.5.2661

Li QJ, 2016, ANTIMICROB AGENTS CH, V60, P2807, DOI 10.1128/AAC.02358-15

Luo Y, 2012, SCAND J IMMUNOL, V75, P293, DOI 10.1111/j.1365-3083.2011.02666.x

Nell AS, 2014, PLOS ONE, V9, DOI 10.1371/journal.pone.0089612

Ordway D, 2007, J IMMUNOL, V179, P522, DOI 10.4049/jimmunol.179.1.522

Pietersen E, 2014, LANCET, V383, P1230, DOI 10.1016/S0140-6736(13)62675-6

Qin XJ, 2008, CHINESE MED J-PEKING, V121, P581, DOI 10.1097/00029330-200804010-00002

Riedhammer C, 2016, METHODS MOL BIOL, V1304, P53, DOI 10.1007/7651_2014_99

Roberts T, 2007, J INFECT DIS, V195, P870, DOI 10.1086/511277

Schmidt A, 2012, FRONT IMMUNOL, V3, DOI 10.3389/fimmu.2012.00051

Semple PL, 2013, AM J RESP CRIT CARE, V187, P1249, DOI 10.1164/rccm.201210-1934OC

Sharma MD, 2010, IMMUNITY, V33, P942, DOI 10.1016/j.immuni.2010.11.022

Sharma PK, 2009, AM J RESP CRIT CARE, V179, P1061, DOI 10.1164/rccm.200804-529OC

Singh A, 2015, INDIAN J MED RES, V141, P27, DOI 10.4103/0971-5916.154492

Smith T, 2014, CURR TOP MICROBIOL, V374, P53, DOI 10.1007/82_2012_279

Tadolini M, 2016, EUR RESPIR J, V48, P935, DOI 10.1183/13993003.00637-2016

Tan Q, 2012, EUR J CLIN MICROBIOL, V31, P1233, DOI 10.1007/s10096-011-1434-4

Tiemessen MM, 2007, P NATL ACAD SCI USA, V104, P19446, DOI 10.1073/pnas.0706832104

Velayati AA, 2009, CHEMOTHERAPY, V55, P303, DOI 10.1159/000226425

Wallis RS, 2015, NAT REV IMMUNOL, V15, P255, DOI 10.1038/nri3813

WHO, 2015, GLOB TUB REP 2015

NR 43

TC 27

Z9 29

U1 0

U2 11

PU AMER THORACIC SOC

PI NEW YORK

PA 25 BROADWAY, 18 FL, NEW YORK, NY 10004 USA

SN 1073-449X

EI 1535-4970

J9 AM J RESP CRIT CARE

JI Am. J. Respir. Crit. Care Med.

PD JUL 1

PY 2018

VL 198

IS 1

BP 104

EP 116

DI 10.1164/rccm.201707-1441OC

PG 13

WC Critical Care Medicine; Respiratory System

WE Science Citation Index Expanded (SCI-EXPANDED)

SC General & Internal Medicine; Respiratory System

GA GL3LQ

UT WOS:000437037000017

DA 2026-05-07

ER

PT J

AU Sharma, D

Late, M

Singh, R

Deo, N

Venkatesan, K

Bisht, D

AF Sharma, Divakar

Late, Manju

Singh, Rananjay

Deo, Nirmala

Venkatesan, Krishnamurthy

Bisht, Deepa

TI Cytosolic Proteome Profiling of Aminoglycosides Resistant

Mycobacterium tuberculosis Clinical Isolates Using MALDI-TOF/MS

SO FRONTIERS IN MICROBIOLOGY

LA English

DT Article

DE amikacin; bioinformatic tools; kanamycin; cytosolic proteome;

Mycobacterium tuberculosis; resistance

ID 2-DIMENSIONAL GEL-ELECTROPHORESIS; FAST INTERACTION REFINEMENT;

MOLECULAR DOCKING; PROTEINS; EXPRESSION; KANAMYCIN; AMIKACIN; BINDING;

GENE; OVEREXPRESSION

AB Emergence of extensively drug resistant tuberculosis (XDR-TB) is the consequence of the failure of second line TB treatment. Aminoglycosides are the important second line anti-TB drugs used to treat the multi drug resistant tuberculosis (MDR-TB). Main known mechanism of action of aminoglycosides is to inhibit the protein synthesis by inhibiting the normal functioning of ribosome. Primary target of aminoglycosides are the ribosomal RNA and its associated proteins. Various mechanisms have been proposed for aminoglycosides resistance but still some are unsolved. As proteins are involved in most of the biological processes, these act as a potential diagnostic markers and drug targets. In the present study we analyzed the purely cytosolic proteome of amikacin (AK) and kanamycin (KM) resistant Mycobacterium tuberculosis isolates by proteomic and bioinformatic approaches. Twenty protein spots were found to have over expressed in resistant isolates and were identified. Among these Ry3208A, Rv2623, Rv1360, Rv2140c, Rv1636, and Ry2185c are six proteins with unknown functions or undefined role. Docking results showed that AK and KM binds to the conserved domain (DUF, USP-A, Luciferase, PEBP and Polyketidecyclase/dehydrase domain) of these hypothetical proteins and over expression of these proteins might neutralize/modulate the effect of drug molecules. TBPred and GPS-PUP predicted cytoplasmic nature and potential pupylation sites within these identified proteins, respectively. String analysis also suggested that over expressed proteins along with their interactive partners might be involved in aminoglycosides resistance. Cumulative effect of these over expressed proteins could be involved in AK and KM resistance by mitigating the toxicity, repression of drug target and neutralizing affect. These findings need further exploitation for the expansion of newer therapeutics or diagnostic markers against AK and KM resistance so that an extreme condition like XDR-TB can be prevented.

C1 [Sharma, Divakar; Late, Manju; Singh, Rananjay; Deo, Nirmala; Venkatesan, Krishnamurthy; Bisht, Deepa] Natl JALMA Inst Leprosy & Other Mycobacterial Dis, Dept Biochem, Agra, Uttar Pradesh, India.

C3 Indian Council of Medical Research (ICMR); ICMR - National JALMA

Institute for Leprosy & Other Mycobacterial Diseases, Agra

RP Bisht, D (corresponding author), Natl JALMA Inst Leprosy & Other Mycobacterial Dis, Dept Biochem, Agra, Uttar Pradesh, India.

EM abd1109@rediffmail.com

RI ; Sharma, Divakar/AFX-9466-2022

OI Bisht, Deepa/0000-0001-5599-0775; Sharma, Divakar/0000-0002-8735-5562

FU ICMR, New Delhi [5/8/5/5/3/20111-ECD-1]

FX The research was financial supported by grant (No.

5/8/5/5/3/20111-ECD-1) from ICMR, New Delhi. DS is ICMR-P (ICMR, New

Delhi).

CR Andrusier N, 2007, PROTEINS, V69, P139, DOI 10.1002/prot.21495

[Anonymous], 2015, World Health Organization report

BEAUCLERK AAD, 1987, J MOL BIOL, V193, P661, DOI 10.1016/0022-2836(87)90349-4

Beste DJV, 2009, PLOS ONE, V4, DOI 10.1371/journal.pone.0005349

Betts JC, 2002, MOL MICROBIOL, V43, P717, DOI 10.1046/j.1365-2958.2002.02779.x

Bisht D, 2007, BIOCHEMISTRY-MOSCOW+, V72, P672, DOI 10.1134/S0006297907060119

BRADFORD MM, 1976, ANAL BIOCHEM, V72, P248, DOI 10.1016/0003-2697(76)90527-3

Brodie A F, 1979, Methods Enzymol, V55, P175

Burns KE, 2010, MOL CELL, V39, P821, DOI 10.1016/j.molcel.2010.07.019

CANETTI G, 1969, B WORLD HEALTH ORGAN, V41, P21

Darwin KH, 2003, SCIENCE, V302, P1963, DOI 10.1126/science.1091176

Santangelo MD, 2011, J BIOL CHEM, V286, P40219, DOI 10.1074/jbc.M111.259440

de Souza GA, 2011, MOL CELL PROTEOMICS, V10, DOI 10.1074/mcp.M110.002527

Eulenburg G, 2013, FEBS LETT, V587, P2936, DOI 10.1016/j.febslet.2013.07.038

Görg A, 2000, ELECTROPHORESIS, V21, P1037, DOI 10.1002/(SICI)1522-2683(20000401)21:6<1037::AID-ELPS1037>3.0.CO;2-V

Griffin JE, 2011, PLOS PATHOG, V7, DOI 10.1371/journal.ppat.1002251

Henriksson LM, 2004, EUR J BIOCHEM, V271, P4107, DOI 10.1111/j.1432-1033.2004.04348.x

Heym B, 1997, INFECT IMMUN, V65, P1395, DOI 10.1128/IAI.65.4.1395-1401.1997

Hu YM, 1999, J BACTERIOL, V181, P3486, DOI 10.1128/JB.181.11.3486-3493.1999

Hu YM, 2001, FEMS MICROBIOL LETT, V202, P59, DOI 10.1016/S0378-1097(01)00280-4

Kruh NA, 2010, PLOS ONE, V5, DOI 10.1371/journal.pone.0013938

Kumar B, 2013, J PROTEOMICS, V94, P68, DOI 10.1016/j.jprot.2013.08.025

Lata M, 2015, J PROTEOMICS, V127, P114, DOI 10.1016/j.jprot.2015.07.031

Lata M, 2015, PROTEIN PEPTIDE LETT, V22, P362, DOI 10.2174/0929866522666150209113708

Lee HN, 2014, PLOS ONE, V9, DOI 10.1371/journal.pone.0111680

Liu ZX, 2011, MOL BIOSYST, V7, P2737, DOI 10.1039/c1mb05217a

Magnet S, 2001, ANTIMICROB AGENTS CH, V45, P3375, DOI 10.1128/AAC.45.12.3375-3380.2001

Magnet S, 2003, ANTIMICROB AGENTS CH, V47, P1577, DOI 10.1128/AAC.47.5.1577-1583.2003

Mashiach E, 2008, NUCLEIC ACIDS RES, V36, pW229, DOI 10.1093/nar/gkn186

Maurya VK, 2014, INDIAN J EXP BIOL, V52, P773

Mawuenyega KG, 2005, MOL BIOL CELL, V16, P396, DOI 10.1091/mbc.e04-04-0329

Meena LS, 2003, BIOTECHNOL APPL BIOC, V38, P169, DOI 10.1042/BA20020122

Nienaber L, 2015, FEBS J, V282, P2708, DOI 10.1111/febs.13313

Nikaido H, 2003, MICROBIOL MOL BIOL R, V67, P593, DOI 10.1128/MMBR.67.4.593-656.2003

Palomino JC, 2002, ANTIMICROB AGENTS CH, V46, P2720, DOI 10.1128/AAC.46.8.2720-2722.2002

Puckett S, 2014, PLOS PATHOG, V10, DOI 10.1371/journal.ppat.1004144

Quevillon E, 2005, NUCLEIC ACIDS RES, V33, pW116, DOI 10.1093/nar/gki442

Raman Karthik, 2008, BMC Microbiol, V8, P234, DOI 10.1186/1471-2180-8-234

Rashid M, 2007, BMC BIOINFORMATICS, V8, DOI 10.1186/1471-2105-8-337

Rifat D, 2009, J INFECT DIS, V200, P1126, DOI 10.1086/605700

Schneidman-Duhovny D, 2005, NUCLEIC ACIDS RES, V33, pW363, DOI 10.1093/nar/gki481

Sharma D., 2014, BMC INFECT DIS S, V14, P60, DOI [10.1186/1471-2334-14-S3-P60, DOI 10.1186/1471-2334-14-S3-P60]

Sharma D, 2016, BIOCHEM BIOPH RES CO, V478, P908, DOI 10.1016/j.bbrc.2016.08.049

Sharma D, 2016, ELECTROPHORESIS, V37, P1187, DOI 10.1002/elps.201600025

Sharma D, 2015, PLOS ONE, V10, DOI 10.1371/journal.pone.0139414

Sharma D, 2015, CURR PROTEOMICS, V12, P96, DOI 10.2174/157016461202150903113053

Sharma P, 2010, INDIAN J MED RES, V132, P400

Sherman DR, 2001, P NATL ACAD SCI USA, V98, P7534, DOI 10.1073/pnas.121172498

Shevchenko A, 1996, ANAL CHEM, V68, P850, DOI 10.1021/ac950914h

Shires K, 2001, MOL MICROBIOL, V39, P994, DOI 10.1046/j.1365-2958.2001.02291.x

Singh V, 2011, CURR MED CHEM, V18, P1318, DOI 10.2174/092986711795029636

Starck J, 2004, MICROBIOL-SGM, V150, P3821, DOI 10.1099/mic.0.27284-0

Stewart GR, 2001, NAT MED, V7, P732, DOI 10.1038/89113

Vargas-Romero F, 2016, PATHOG DIS, V74, DOI 10.1093/femspd/ftv127

Welch KT, 2005, BIOORGAN MED CHEM, V13, P6252, DOI 10.1016/j.bmc.2005.06.059

Zhang H, 2015, MOL IMMUNOL, V66, P147, DOI 10.1016/j.molimm.2015.02.020

NR 56

TC 35

Z9 39

U1 0

U2 6

PU FRONTIERS MEDIA SA

PI LAUSANNE

PA PO BOX 110, EPFL INNOVATION PARK, BUILDING I, LAUSANNE, 1015,

SWITZERLAND

SN 1664-302X

J9 FRONT MICROBIOL

JI Front. Microbiol.

PD NOV 15

PY 2016

VL 7

AR 1816

DI 10.3389/fmicb.2016.01816

PG 13

WC Microbiology

WE Science Citation Index Expanded (SCI-EXPANDED)

SC Microbiology

GA ED2WW

UT WOS:000388711900003

OA Green Submitted, gold

DA 2026-05-07

ER

PT J

AU Kuan, CS

Chan, CL

Yew, SM

Toh, YF

Khoo, JS

Chong, J

Lee, KW

Tan, YC

Yee, WY

Ngeow, YF

Ng, KP

AF Kuan, Chee Sian

Chan, Chai Ling

Yew, Su Mei

Toh, Yue Fen

Khoo, Jia-Shiun

Chong, Jennifer

Lee, Kok Wei

Tan, Yung-Chie

Yee, Wai-Yan

Ngeow, Yun Fong

Ng, Kee Peng

TI Genome Analysis of the First Extensively Drug-Resistant (XDR)

Mycobacterium tuberculosis in Malaysia Provides Insights into the

Genetic Basis of Its Biology and Drug Resistance

SO PLOS ONE

LA English

DT Article

ID MULTIDRUG-RESISTANT; STREPTOMYCIN RESISTANCE; EFFLUX PUMP; COMPENSATORY

MUTATIONS; LOW-LEVEL; STRAINS; IDENTIFICATION; BIOSYNTHESIS; CHINA; KATG

AB The outbreak of extensively drug-resistant tuberculosis (XDR-TB) has become an increasing problem in many TB-burdened countries. The underlying drug resistance mechanisms, including the genetic variation favored by selective pressure in the resistant population, are partially understood. Recently, the first case of XDR-TB was reported in Malaysia. However, the detailed genotype family and mechanisms of the formation of multiple drugs resistance are unknown. We sequenced the whole genome of the UM 1072388579 strain with a 2-kb insert-size library and combined with that from previously sequenced 500-bp-insert paired-end reads to produce an improved sequence with maximal sequencing coverage across the genome. In silico spoligotyping and phylogenetic analyses demonstrated that UM 1072388579 strain belongs to an ancestral-like, non-Beijing clade of East Asia lineage. This is supported by the presence of a number of lineage-specific markers, including fadD28, embA, nuoD and pks7. Polymorphism analysis showed that the drug-susceptibility profile is correlated with the pattern of resistance mutations. Mutations in drug-efflux pumps and the cell wall biogenesis pathway such as mmpL, pks and fadD genes may play an important role in survival and adaptation of this strain to its surrounding environment. In this work, fifty-seven putative promoter SNPs were identified. Among them, we identified a novel SNP located at -4 T allele of TetR/acrR promoter as an informative marker to recognize strains of East Asian lineage. Our work indicates that the UM 1072388579 harbors both classical and uncommon SNPs that allow it to escape from inhibition by many antibiotics. This study provides a strong foundation to dissect the biology and underlying resistance mechanisms of the first reported XDR M. tuberculosis in Malaysia.

C1 [Kuan, Chee Sian; Chan, Chai Ling; Yew, Su Mei; Toh, Yue Fen; Chong, Jennifer; Ngeow, Yun Fong; Ng, Kee Peng] Univ Malaya, Fac Med, Dept Med Microbiol, Kuala Lumpur, Malaysia.

[Khoo, Jia-Shiun; Lee, Kok Wei; Tan, Yung-Chie; Yee, Wai-Yan] Codon Genom SB, Seri Kembangan, Selangor Darul, Malaysia.

C3 Universiti Malaya

RP Ng, KP (corresponding author), Univ Malaya, Fac Med, Dept Med Microbiol, Kuala Lumpur, Malaysia.

EM kpng@ummc.edu.my

RI NG, KEE/B-9225-2010; Kuan, Chee/J-8930-2015; Yee, Wai-Yan/J-8520-2015;

Ngeow, Yun Fong/B-9237-2010

OI Ngeow, Yun Fong/0000-0002-1011-762X; Tan, Yung-Chie/0000-0001-6221-6630

FU High Impact Research MoE Grant from the Ministry of Education Malaysia

[UM.C/625/1/HIR/MOHE/MED/31]; Codon Genomics SB

FX This study was supported by High Impact Research MoE Grant

UM.C/625/1/HIR/MOHE/MED/31 (Account no. H-20001-00-E000070) from the

Ministry of Education Malaysia. Codon Genomics SB provided support in

the form of salaries for authors JSK, KWL, YCT and WYY, but did not have

any additional role in the study design, data collection and analysis,

decision to publish, or preparation of the manuscript. The specific

roles of these authors are articulated in the 'author contributions'

section.

CR Da Silva PEA, 2011, J ANTIMICROB CHEMOTH, V66, P1417, DOI 10.1093/jac/dkr173

Ayres DL, 2012, SYST BIOL, V61, P170, DOI 10.1093/sysbio/syr100

Balganesh M, 2010, ANTIMICROB AGENTS CH, V54, P5167, DOI 10.1128/AAC.00610-10

Barnard A, 2004, CURR OPIN MICROBIOL, V7, P102, DOI 10.1016/j.mib.2004.02.011

Bifani PJ, 1996, JAMA-J AM MED ASSOC, V275, P452, DOI 10.1001/jama.275.6.452

Billman-Jacobe H, 1999, ANTIMICROB AGENTS CH, V43, P3011, DOI 10.1128/AAC.43.12.3011

Birch HL, 2008, MOL MICROBIOL, V69, P1191, DOI 10.1111/j.1365-2958.2008.06354.x

Boetzer M, 2012, GENOME BIOL, V13, DOI 10.1186/gb-2012-13-6-r56

Boetzer M, 2011, BIOINFORMATICS, V27, P578, DOI 10.1093/bioinformatics/btq683

Brandis G, 2013, J ANTIMICROB CHEMOTH, V68, P2493, DOI 10.1093/jac/dkt224

Brandis G, 2012, MOL MICROBIOL, V85, P142, DOI 10.1111/j.1365-2958.2012.08099.x

Brossier F, 2006, J CLIN MICROBIOL, V44, P3659, DOI 10.1128/JCM.01054-06

Brudey K, 2006, BMC MICROBIOL, V6, DOI 10.1186/1471-2180-6-23

Cade CE, 2010, PROTEIN SCI, V19, P458, DOI 10.1002/pro.324

Caminero JA, 2010, LANCET INFECT DIS, V10, P621, DOI 10.1016/S1473-3099(10)70139-0

Casali N, 2012, GENOME RES, V22, P735, DOI 10.1101/gr.128678.111

Chuang PC, 2010, J CLIN MICROBIOL, V48, P4245, DOI 10.1128/JCM.00970-10

Cole ST, 1998, NATURE, V393, P537, DOI 10.1038/31159

Coll F, 2014, NAT COMMUN, V5, DOI 10.1038/ncomms5812

Coll F, 2012, BIOINFORMATICS, V28, P2991, DOI 10.1093/bioinformatics/bts544

Comas I, 2009, PLOS ONE, V4, DOI 10.1371/journal.pone.0007815

Cox HS, 2008, NEW ENGL J MED, V359, P2398, DOI 10.1056/NEJMc0805644

Danilchanka O, 2008, ANTIMICROB AGENTS CH, V52, P2503, DOI 10.1128/AAC.00298-08

de Vos M, 2013, ANTIMICROB AGENTS CH, V57, P827, DOI 10.1128/AAC.01541-12

Demay C, 2012, INFECT GENET EVOL, V12, P755, DOI 10.1016/j.meegid.2012.02.004

Domenech P, 2005, INFECT IMMUN, V73, P3492, DOI 10.1128/IAI.73.6.3492-3501.2005

Farhat MR, 2013, NAT GENET, V45, P1183, DOI 10.1038/ng.2747

Feuerriegel Silke, 2012, BMC Microbiol, V12, P90, DOI 10.1186/1471-2180-12-90

Flores L, 2007, J CLIN MICROBIOL, V45, P3393, DOI 10.1128/JCM.00828-07

Gandhi N. R., 2006, Lancet (British edition), V368, P1575, DOI 10.1016/S0140-6736(06)69573-1

García P, 2009, APPL ENVIRON MICROB, V75, P7663, DOI 10.1128/AEM.01864-09

Gavalda S, 2009, J BIOL CHEM, V284, P19255, DOI 10.1074/jbc.M109.006940

Guio H, 2014, GENOME ANNOUNCEMENTS, V2, DOI 10.1128/genomeA.00759-14

Guo HL, 2006, J MED MICROBIOL, V55, P1527, DOI 10.1099/jmm.0.46718-0

Hasan R, 2010, EMERG INFECT DIS, V16, P1473, DOI 10.3201/eid1609.100280

HEYM B, 1993, J BACTERIOL, V175, P4255, DOI 10.1128/JB.175.13.4255-4259.1993

Hong WL, 2014, EXPERT OPIN THER TAR, V18, P691, DOI 10.1517/14728222.2014.902937

Ilina EN, 2013, PLOS ONE, V8, DOI 10.1371/journal.pone.0056577

Ioerger TR, 2013, PLOS ONE, V8, DOI 10.1371/journal.pone.0075245

Ioerger TR, 2010, BMC GENOMICS, V11, DOI 10.1186/1471-2164-11-670

Ioerger TR, 2009, PLOS ONE, V4, DOI 10.1371/journal.pone.0007778

Jassal M, 2009, LANCET INFECT DIS, V9, P19, DOI 10.1016/S1473-3099(08)70260-3

Johansen SK, 2006, MOL CELL, V23, P173, DOI 10.1016/j.molcel.2006.05.044

Karuthedath Vellarikkal S, 2013, GENOME ANNOUNC, V1

Kozinska Monika, 2011, Pneumonol Alergol Pol, V79, P278

Kurtz S, 2004, GENOME BIOL, V5, DOI 10.1186/gb-2004-5-2-r12

Lagesen K, 2007, NUCLEIC ACIDS RES, V35, P3100, DOI 10.1093/nar/gkm160

Lanzas F, 2013, J CLIN MICROBIOL, V51, P3277, DOI 10.1128/JCM.01122-13

Lin N, 2013, FEMS MICROBIOL LETT

Lomsadze A, 2005, NUCLEIC ACIDS RES, V33, P6494, DOI 10.1093/nar/gki937

Lowe TM, 1997, NUCLEIC ACIDS RES, V25, P955, DOI 10.1093/nar/25.5.955

Michael JS, 2012, INDIAN J MED RES, V136, P599

Migliori GB, 2007, EMERG INFECT DIS, V13, P780, DOI 10.3201/eid1305.070200

Murase Y, 2010, EMERG INFECT DIS, V16, P948, DOI 10.3201/eid1606.091844

Nadalin F, 2012, BMC BIOINFORMATICS, V13, DOI 10.1186/1471-2105-13-S14-S8

Ng KP, 2014, INT J MYCOBACT, V3, P82, DOI 10.1016/j.ijmyco.2014.03.005

Ng KP, 2013, GENOME ANNOUNC, V1

NIKAIDO H, 1994, SCIENCE, V264, P382, DOI 10.1126/science.8153625

O'Toole RF, 2014, GENOME ANNOUNC, V2

Okamoto S, 2007, MOL MICROBIOL, V63, P1096, DOI 10.1111/j.1365-2958.2006.05585.x

Pasca MR, 2004, ANTIMICROB AGENTS CH, V48, P3175, DOI 10.1128/AAC.48.8.3175-3178.2004

Pei H, 2011, FOLIA MICROBIOL, V56, P549, DOI 10.1007/s12223-011-0080-7

Reese MG, 2001, COMPUT CHEM, V26, P51, DOI 10.1016/S0097-8485(01)00099-7

Rutherford K, 2000, BIOINFORMATICS, V16, P944, DOI 10.1093/bioinformatics/16.10.944

Sampson SL, 2011, CLIN DEV IMMUNOL, DOI 10.1155/2011/497203

Schroeder E. K., 2002, Current Pharmaceutical Biotechnology, V3, P197, DOI 10.2174/1389201023378328

Sekiguchi J, 2007, J CLIN MICROBIOL, V45, P179, DOI 10.1128/JCM.00750-06

Shi R, 2007, ANTIMICROB AGENTS CH, V51, P4515, DOI 10.1128/AAC.00416-07

Somerville W, 2005, J CLIN MICROBIOL, V43, P2996, DOI 10.1128/JCM.43.6.2996-2997.2005

Sowajassatakul A, 2014, BMC MICROBIOL, V14, DOI 10.1186/1471-2180-14-165

Spies FS, 2011, J CLIN MICROBIOL, V49, P2625, DOI 10.1128/JCM.00168-11

Sreevatsan S, 1997, ANTIMICROB AGENTS CH, V41, P1677, DOI 10.1128/AAC.41.8.1677

Sreevatsan S, 1997, P NATL ACAD SCI USA, V94, P9869, DOI 10.1073/pnas.94.18.9869

Sreevatsan S, 1996, ANTIMICROB AGENTS CH, V40, P1024, DOI 10.1128/AAC.40.4.1024

Su CC, 2007, BIOCHEM BIOPH RES CO, V361, P85, DOI 10.1016/j.bbrc.2007.06.175

Tang SJ, 2011, SCAND J INFECT DIS, V43, P280, DOI 10.3109/00365548.2010.548080

van Doorn HR, 2001, J CLIN MICROBIOL, V39, P1591, DOI 10.1128/JCM.39.4.1591-1594.2001

Verma JS, 2014, J ANTIMICROB CHEMOTH, V69, P2935, DOI 10.1093/jac/dku273

Wan KL, 2011, PLOS ONE, V6, DOI 10.1371/journal.pone.0029190

Wang H, 2001, ANTIMICROB AGENTS CH, V45, P1515, DOI 10.1128/AAC.45.5.1515-1521.2001

Wang K, 2010, NUCLEIC ACIDS RES, V38, DOI 10.1093/nar/gkq603

Wang K, 2013, CURR MICROBIOL, V66, P222, DOI 10.1007/s00284-012-0215-3

Williams MJ, 2011, J BACTERIOL, V193, P98, DOI 10.1128/JB.00774-10

Xu C, 1996, J INFECT DIS, V174, P1127, DOI 10.1093/infdis/174.5.1127

Zaunbrecher MA, 2009, P NATL ACAD SCI USA, V106, P20004, DOI 10.1073/pnas.0907925106

Zerbino DR, 2008, GENOME RES, V18, P821, DOI 10.1101/gr.074492.107

Zhang HT, 2013, NAT GENET, V45, P1255, DOI 10.1038/ng.2735

zur Wiesch PS, 2010, ANTIMICROB AGENTS CH, V54, P2085, DOI 10.1128/AAC.01460-09

NR 88

TC 23

Z9 27

U1 0

U2 26

PU PUBLIC LIBRARY SCIENCE

PI SAN FRANCISCO

PA 1160 BATTERY STREET, STE 100, SAN FRANCISCO, CA 94111 USA

EI 1932-6203

J9 PLOS ONE

JI PLoS One

PD JUN 25

PY 2015

VL 10

IS 6

AR e0131694

DI 10.1371/journal.pone.0131694

PG 20

WC Multidisciplinary Sciences

WE Science Citation Index Expanded (SCI-EXPANDED)

SC Science & Technology - Other Topics

GA CL4OY

UT WOS:000356933800169

OA Green Submitted, gold

DA 2026-05-07

ER

PT J

AU Teng, TS

Liu, JF

Wei, HP

AF Teng, Tieshan

Liu, Jiafa

Wei, Hongping

TI Anti-Mycobacterial Peptides: From Human to Phage

SO CELLULAR PHYSIOLOGY AND BIOCHEMISTRY

LA English

DT Review

DE Anti-mycobacterial peptides; Mycobacterial; Tuberculosis

ID HUMAN NEUTROPHIL PEPTIDE-1; MYCOBACTERIUM-TUBERCULOSIS H37RV;

UBIQUITIN-DERIVED PEPTIDES; ANTIMICROBIAL-PEPTIDE; HOST-DEFENSE; HUMAN

CATHELICIDIN; DNA VACCINE; D-ALANINE; IN-VITRO; LL-37

AB Mycobacterium tuberculosis is the major pathogen of tuberculosis (TB). With the growing problem of M. tuberculosis resistant to conventional antibiotics, especially multi-drug resistant tuberculosis (MDR-TB) and extensively-drug resistant tuberculosis (XDR-TB), the need for new TB drugs is now more prominent than ever. Among the promising candidates for anti-TB drugs, anti-mycobacterial peptides have a few advantages, such as low immunogenicity, selective affinity to prokaryotic negatively charged cell envelopes, and diverse modes of action. In this review, we summarize the recent progress in the anti-mycobacterial peptides, highlighting the sources, effectiveness and bactericidal mechanisms of these antimicrobial peptides. Most of the current anti-mycobacterial peptides are derived either from host immune cells, bacterial extraction, or mycobacteriophages. Besides trans-membrane pore formation, which is considered to be the common bactericidal mechanism, many of the anti-mycobacterial peptides have the second non-membrane targets within mycobacteria. Additionally, some antimicrobial peptides play critical roles in innate immunity. However, a few obstacles, such as short half-life in vivo and resistance to antimicrobial peptides, need overcoming before clinical applications. Nevertheless, the multiple functions of anti-mycobacterial peptides, especially direct killing of pathogens and immune-modulators in infectious and inflammatory conditions, indicate that they are promising candidates for future drug development. Copyright (C) 2015 S. Karger AG, Basel

C1 [Teng, Tieshan; Wei, Hongping] Chinese Acad Sci, Wuhan Inst Virol, Key Lab Special Pathogens & Biosafety, Wuhan, Peoples R China.

[Teng, Tieshan] Univ Chinese Acad Sci, Beijing, Peoples R China.

[Liu, Jiafa] Hubei Ctr Dis Control & Prevent, Wuhan, Peoples R China.

C3 Chinese Academy of Sciences; Wuhan Institute of Virology, CAS; Chinese

Academy of Sciences; University of Chinese Academy of Sciences, CAS;

Hubei Provincial Center for Disease Prevention & Control

RP Wei, HP (corresponding author), Xiao Hong Shan 44, Wuhan, Peoples R China.

EM hpwei@wh.iov.cn

FU Chinese Academy of Sciences [KJZD-EW-L02]; Hubei Province's outstanding

medical academic leader program; Ministry of Health, China

[2009ZXl0003-019]

FX We would like to thank the financial supports from the Chinese Academy

of Sciences (grant No.: KJZD-EW-L02), Hubei Province's outstanding

medical academic leader program (to Jiafa Liu), and the infectious

diseases Mega-project of Ministry of Health, China (2009ZXl0003-019).

CR Akopian T, 2012, EMBO J, V31, P1529, DOI 10.1038/emboj.2012.5

Alonso S, 2007, P NATL ACAD SCI USA, V104, P6031, DOI 10.1073/pnas.0700036104

Andreu D, 1999, BIOCHEM J, V344, P845, DOI 10.1042/0264-6021:3440845

Arnusch CJ, 2012, PLOS ONE, V7, DOI 10.1371/journal.pone.0039768

Asaduzzaman SM, 2009, J BIOSCI BIOENG, V107, P475, DOI 10.1016/j.jbiosc.2009.01.003

Barthe C, 2011, PROTEIN PEPTIDE LETT, V18, P36, DOI 10.2174/092986611794328627

Büchau AS, 2010, J IMMUNOL, V184, P369, DOI 10.4049/jimmunol.0902110

Carmona G, 2013, PROTEIN J, V32, P456, DOI 10.1007/s10930-013-9505-2

Carroll James, 2010, Bioeng Bugs, V1, P408, DOI 10.4161/bbug.1.6.13642

Carroll J, 2010, INT J ANTIMICROB AG, V36, P132, DOI 10.1016/j.ijantimicag.2010.03.029

Chatterjee S, 2011, PLOS PATHOG, V7, DOI 10.1371/journal.ppat.1002378

Cho J, 2012, J MICROBIOL BIOTECHN, V22, P1457

Cotter PD, 2005, CURR PROTEIN PEPT SC, V6, P61, DOI 10.2174/1389203053027584

Cotter PD, 2005, P NATL ACAD SCI USA, V102, P18584, DOI 10.1073/pnas.0509371102

Dangel A, 2013, FASEB J, V27, P4476, DOI 10.1096/fj.13-234575

Daugherty A, 2011, J BACTERIOL, V193, P6824, DOI 10.1128/JB.05492-11

Devocelle M, 2012, FRONT IMMUNOL, V3, DOI 10.3389/fimmu.2012.00309

Draper LA, 2013, BMC MICROBIOL, V13, DOI 10.1186/1471-2180-13-212

Draper LA, 2009, MOL MICROBIOL, V71, P1043, DOI 10.1111/j.1365-2958.2008.06590.x

DUCLOHIER H, 1989, BIOPHYS J, V56, P1017, DOI 10.1016/S0006-3495(89)82746-8

Ernst CM, 2011, MOL MICROBIOL, V80, P290, DOI 10.1111/j.1365-2958.2011.07576.x

Falciani C, 2012, PLOS ONE, V7, DOI 10.1371/journal.pone.0046259

Fenton Mark, 2013, Int J Microbiol, V2013, P625341, DOI 10.1155/2013/625341

Field D, 2012, PLOS ONE, V7, DOI 10.1371/journal.pone.0046884

Filatova LY, 2013, BIOCHIMIE, V95, P1689, DOI 10.1016/j.biochi.2013.04.013

Foss MH, 2012, BIOCHEMISTRY-US, V51, P9922, DOI 10.1021/bi301426j

Gavrish E, 2014, CHEM BIOL, V21, P509, DOI 10.1016/j.chembiol.2014.01.014

Gonzalez-Curiel I, 2011, HUM IMMUNOL, V72, P656, DOI 10.1016/j.humimm.2011.03.027

Gorr SU, 2011, J CLIN PERIODONTOL, V38, P126, DOI 10.1111/j.1600-051X.2010.01664.x

Guralp SA, 2013, PLOS ONE, V8, DOI 10.1371/journal.pone.0059305

Hassan M, 2012, J APPL MICROBIOL, V113, P723, DOI 10.1111/j.1365-2672.2012.05338.x

Hilpert K, 2005, NAT BIOTECHNOL, V23, P1008, DOI 10.1038/nbt1113

Horgan M, 2009, APPL ENVIRON MICROB, V75, P872, DOI 10.1128/AEM.01831-08

Islam MR, 2012, BIOCHEM SOC T, V40, P1528, DOI 10.1042/BST20120190

Jena P, 2011, PEPTIDES, V32, P881, DOI 10.1016/j.peptides.2011.03.002

Jiang ZQ, 2011, PROTEIN PEPTIDE LETT, V18, P241, DOI 10.2174/092986611794578288

Joshi S, 2012, FEBS J, V279, P3776, DOI 10.1111/j.1742-4658.2012.08738.x

Kalita A, 2004, J INFECT DIS, V190, P1476, DOI 10.1086/424463

Kapoor R, 2011, ANTIMICROB AGENTS CH, V55, P3058, DOI 10.1128/AAC.01667-10

Kieffer AE, 2003, FASEB J, V17, P776, DOI 10.1096/fj.02-0699fje

Kisich KO, 2001, INFECT IMMUN, V69, P2692, DOI 10.1128/IAI.69.4.2692-2699.2001

Kolodkin-Gal I, 2010, SCIENCE, V328, P627, DOI 10.1126/science.1188628

Kurosaka K, 2005, J IMMUNOL, V174, P6257, DOI 10.4049/jimmunol.174.10.6257

Layoun A, 2012, INFLAMMATION, V35, P1500, DOI 10.1007/s10753-012-9463-4

Li YM, 2012, PEPTIDES, V37, P207, DOI 10.1016/j.peptides.2012.07.001

Liu PT, 2008, CURR OPIN IMMUNOL, V20, P371, DOI 10.1016/j.coi.2008.05.014

Liu PT, 2007, J IMMUNOL, V179, P2060, DOI 10.4049/jimmunol.179.4.2060

Loessner MJ, 2005, CURR OPIN MICROBIOL, V8, P480, DOI 10.1016/j.mib.2005.06.002

Lofton H, 2013, PLOS ONE, V8, DOI 10.1371/journal.pone.0068875

Lukacik P, 2012, P NATL ACAD SCI USA, V109, P9857, DOI 10.1073/pnas.1203472109

Martin NI, 2007, FUTURE MICROBIOL, V2, P513, DOI 10.2217/17460913.2.5.513

Martineau AR, 2007, J IMMUNOL, V178, P7190, DOI 10.4049/jimmunol.178.11.7190

McGrath DM, 2013, P NATL ACAD SCI USA, V110, P3477, DOI 10.1073/pnas.1221924110

Meher AK, 2006, FEBS J, V273, P1445, DOI 10.1111/j.1742-4658.2006.05166.x

Mendez-Samperio Patricia, 2013, Adv Biomed Res, V2, P50, DOI 10.4103/2277-9175.114192

Méndez-Samperio P, 2010, PEPTIDES, V31, P1791, DOI 10.1016/j.peptides.2010.06.016

Mohanty S, 2013, ANTIMICROB AGENTS CH, V57, P3688, DOI 10.1128/AAC.02475-12

Mooney C, 2012, PLOS ONE, V7, DOI 10.1371/journal.pone.0045012

Morgan SM, 2005, ANTIMICROB AGENTS CH, V49, P2606, DOI 10.1128/AAC.49.7.2606-2611.2005

Mueller H, 2011, PLOS ONE, V6, DOI 10.1371/journal.pone.0029367

Munk JK, 2014, ANTIMICROB AGENTS CH, V58, P1063, DOI 10.1128/AAC.02369-13

Nagaoka I, 2002, CLIN DIAGN LAB IMMUN, V9, P972, DOI 10.1128/CDLI.9.5.972-982.2002

Nemeth E, 2004, SCIENCE, V306, P2090, DOI 10.1126/science.1104742

Nicolas G, 2002, J CLIN INVEST, V110, P1037, DOI 10.1172/JCI200215686

Okada M, 2011, HUM VACCINES, V7, P60, DOI 10.4161/hv.7.0.14563

Osborne R, 2013, NAT BIOTECHNOL, V31, P89, DOI 10.1038/nbt0213-89

Patzer SI, 2012, J BIOL CHEM, V287, P23381, DOI 10.1074/jbc.M112.362913

Pazgier M, 2006, CELL MOL LIFE SCI, V63, P1294, DOI 10.1007/s00018-005-5540-2

Pitabut N, 2013, INT J MED SCI, V10, P1003, DOI 10.7150/ijms.6437

Pulido D, 2013, ANTIMICROB AGENTS CH, V57, P3797, DOI 10.1128/AAC.00428-13

Purdy GE, 2007, CELL MICROBIOL, V9, P2768, DOI 10.1111/j.1462-5822.2007.01039.x

Purdy GE, 2009, MOL MICROBIOL, V73, P844, DOI 10.1111/j.1365-2958.2009.06801.x

Rivas-Santiago B, 2008, INFECT IMMUN, V76, P935, DOI 10.1128/IAI.01218-07

Rivas-Santiago B, 2013, INT J ANTIMICROB AG, V41, P143, DOI 10.1016/j.ijantimicag.2012.09.015

Rivas-Santiago B, 2012, ARCH MED RES, V43, P324, DOI 10.1016/j.arcmed.2012.06.005

Rivas-Santiago CE, 2011, CLIN EXP IMMUNOL, V164, P80, DOI 10.1111/j.1365-2249.2010.04313.x

Ryan L, 2013, J BIOL CHEM, V288, P20162, DOI 10.1074/jbc.M113.459560

Ryan MP, 1999, J BIOL CHEM, V274, P37544, DOI 10.1074/jbc.274.53.37544

Samuchiwal SK, 2014, BMC INFECT DIS, V14, DOI 10.1186/1471-2334-14-355

Santos P, 2012, PEPTIDES, V36, P121, DOI 10.1016/j.peptides.2012.04.018

Saravanan R, 2014, BIOTECHNOL BIOENG, V111, P37, DOI 10.1002/bit.25003

Sassi M, 2013, J VIROL, V87, P8099, DOI 10.1128/JVI.01209-13

Schneider JJ, 2005, J MOL MED, V83, P587, DOI 10.1007/s00109-005-0657-1

Sharma S, 2001, CURR MICROBIOL, V43, P74, DOI 10.1007/s002840010263

Sharma S, 2001, ANTIMICROB AGENTS CH, V45, P639, DOI 10.1128/AAC.45.2.639-640.2001

Sharma S, 1999, ARCH MICROBIOL, V171, P338, DOI 10.1007/s002030050719

Sharma S, 2000, EUR RESPIR J, V16, P112, DOI 10.1034/j.1399-3003.2000.16a20.x

Silva T, 2014, ANTIMICROB AGENTS CH, V58, P3461, DOI 10.1128/AAC.02728-13

Singh D, 2013, J BIOL CHEM, V288, P8258, DOI 10.1074/jbc.M112.440883

Sonawane A, 2011, CELL MICROBIOL, V13, P1601, DOI 10.1111/j.1462-5822.2011.01644.x

Sorensen O, 1997, J IMMUNOL METHODS, V206, P53, DOI 10.1016/S0022-1759(97)00084-7

Sorensen OE, 2001, BLOOD, V97, P3951, DOI 10.1182/blood.V97.12.3951

Sosunov V, 2007, J ANTIMICROB CHEMOTH, V59, P919, DOI 10.1093/jac/dkm053

Sow FB, 2007, J LEUKOCYTE BIOL, V82, P934, DOI 10.1189/jlb.0407216

Sow FB, 2011, TUBERCULOSIS, V91, P314, DOI 10.1016/j.tube.2011.03.003

Sow FB, 2009, J LEUKOCYTE BIOL, V86, P1247, DOI 10.1189/jlb.1208719

Spencer JD, 2011, KIDNEY INT, V80, P175, DOI 10.1038/ki.2011.109

Stenger S, 1998, SCIENCE, V282, P121, DOI 10.1126/science.282.5386.121

Suda S, 2012, J BACTERIOL, V194, P708, DOI 10.1128/JB.06185-11

Toro JC, 2006, MICROBES INFECT, V8, P1985, DOI 10.1016/j.micinf.2006.02.030

Vandamme D, 2012, CELL IMMUNOL, V280, P22, DOI 10.1016/j.cellimm.2012.11.009

Waghu FH, 2014, NUCLEIC ACIDS RES, V42, pD1154, DOI 10.1093/nar/gkt1157

Wan Y, 2013, BIOMATERIALS, V34, P3020, DOI 10.1016/j.biomaterials.2012.12.051

Wang QM, 2012, SCAND J IMMUNOL, V76, P123, DOI 10.1111/j.1365-3083.2012.02719.x

Wang QM, 2009, MICROBIOL IMMUNOL, V53, P384, DOI 10.1111/j.1348-0421.2009.00138.x

Wang QM, 2012, DNA CELL BIOL, V31, P489, DOI 10.1089/dna.2011.1309

Wang Y, 2014, BIOMATERIALS, V35, P3102, DOI 10.1016/j.biomaterials.2013.12.049

Wang Z, 2000, J IMMUNOL, V165, P1486, DOI 10.4049/jimmunol.165.3.1486

Wei L, 2013, FASEB J, V27, P3067, DOI 10.1096/fj.13-227454

Yacoby Iftach, 2007, Infectious Disorders - Drug Targets, V7, P221, DOI 10.2174/187152607782109998

Llamas-González YY, 2013, MICROB DRUG RESIST, V19, P124, DOI 10.1089/mdr.2012.0149

Yamshchikov AV, 2010, AM J CLIN NUTR, V92, P603, DOI 10.3945/ajcn.2010.29411

Yan JX, 2013, ANTIMICROB AGENTS CH, V57, P220, DOI 10.1128/AAC.01619-12

Yang H, 2012, ANTIMICROB AGENTS CH, V56, P5031, DOI 10.1128/AAC.00891-12

Yang H, 2013, PLOS ONE, V8, DOI 10.1371/journal.pone.0052848

Zhao XW, 2013, PLOS ONE, V8, DOI 10.1371/journal.pone.0066557

Zhao YY, 2010, J AM CHEM SOC, V132, P12349, DOI 10.1021/ja1028843

Zhu LM, 2011, INT J TUBERC LUNG D, V15, P369

NR 118

TC 26

Z9 30

U1 0

U2 29

PU KARGER

PI BASEL

PA ALLSCHWILERSTRASSE 10, CH-4009 BASEL, SWITZERLAND

SN 1015-8987

EI 1421-9778

J9 CELL PHYSIOL BIOCHEM

JI Cell. Physiol. Biochem.

PY 2015

VL 35

IS 2

BP 452

EP 466

DI 10.1159/000369711

PG 15

WC Cell Biology; Physiology

WE Science Citation Index Expanded (SCI-EXPANDED)

SC Cell Biology; Physiology

GA CA6NY

UT WOS:000349032700004

OA Green Submitted, gold

DA 2026-05-07

ER

PT J

AU Cui, YX

Lanne, A

Peng, XD

Browne, E

Bhatt, A

Coltman, NJ

Craven, P

Cox, LR

Cundy, NJ

Dale, K

Feula, A

Frampton, J

Fung, M

Morton, M

Goff, A

Salih, M

Lang, XF

Li, XJ

Moon, C

Pascoe, J

Portman, V

Press, C

Schulz-Utermoehl, T

Lee, S

Tortorella, MD

Tu, ZC

Underwood, ZE

Wang, CW

Yoshizawa, A

Zhang, TY

Waddell, SJ

Bacon, J

Alderwick, L

Fossey, JS

Neagoie, C

AF Cui, Yixin

Lanne, Alice

Peng, Xudan

Browne, Edward

Bhatt, Apoorva

Coltman, Nicholas J.

Craven, Philip

Cox, Liam R.

Cundy, Nicholas J.

Dale, Katie

Feula, Antonio

Frampton, Jon

Fung, Martin

Morton, Michael

Goff, Aaron

Salih, Mariwan

Lang, Xingfen

Li, Xingjian

Moon, Chris

Pascoe, Jordan

Portman, Vanessa

Press, Cara

Schulz-Utermoehl, Timothy

Lee, Suki

Tortorella, Micky D.

Tu, Zhengchao

Underwood, Zoe E.

Wang, Changwei

Yoshizawa, Akina

Zhang, Tianyu

Waddell, Simon J.

Bacon, Joanna

Alderwick, Luke

Fossey, John S.

Neagoie, Cleopatra

TI Azetidines Kill Multidrug-Resistant Mycobacterium tuberculosis

without Detectable Resistance by Blocking Mycolate Assembly

SO JOURNAL OF MEDICINAL CHEMISTRY

LA English

DT Article

ID INTERFERENCE COMPOUNDS PAINS; TREHALOSE MONOMYCOLATE; GENE-EXPRESSION;

ASSAY; MECHANISM; INHIBITION; SYNTHASE; MMPL3; CHEMOTHERAPY; TRANSPORTER

AB Tuberculosis (TB) is the leading cause of global morbidity and mortality resulting from infectious disease, with over 10.6 million new cases and 1.4 million deaths in 2021. This global emergency is exacerbated by the emergence of multidrug-resistant MDR-TB and extensively drug-resistant XDR-TB; therefore, new drugs and new drug targets are urgently required. From a whole cell phenotypic screen, a series of azetidines derivatives termed BGAz, which elicit potent bactericidal activity with MIC99 values <10 mu M against drug-sensitive Mycobacterium tuberculosis and MDR-TB, were identified. These compounds demonstrate no detectable drug resistance. The mode of action and target deconvolution studies suggest that these compounds inhibit mycobacterial growth by interfering with cell envelope biogenesis, specifically late-stage mycolic acid biosynthesis. Transcriptomic analysis demonstrates that the BGAz compounds tested display a mode of action distinct from the existing mycobacterial cell wall inhibitors. In addition, the compounds tested exhibit toxicological and PK/PD profiles that pave the way for their development as antitubercular chemotherapies.

C1 [Cui, Yixin; Craven, Philip; Cox, Liam R.; Cundy, Nicholas J.; Feula, Antonio; Salih, Mariwan; Li, Xingjian; Yoshizawa, Akina; Fossey, John S.] Univ Birmingham, Sch Chem, Birmingham B15 2TT, W Midlands, England.

[Lanne, Alice; Bhatt, Apoorva; Dale, Katie; Press, Cara; Alderwick, Luke] Univ Birmingham, Inst Microbiol & Infect, Sch Biosci, Birmingham B15 2TT, W Midlands, England.

[Peng, Xudan; Lang, Xingfen; Li, Xingjian; Tortorella, Micky D.; Tu, Zhengchao; Wang, Changwei; Zhang, Tianyu; Neagoie, Cleopatra] Chinese Acad Sci, Guangzhou Inst Biomed & Hlth, State Key Lab Resp Dis, China New Zealand Joint Lab Biomed & Hlth, Guangzhou 510530, Peoples R China.

[Browne, Edward; Portman, Vanessa; Schulz-Utermoehl, Timothy] Sygnature Discovery, Nottingham NG1 1GR, England.

[Coltman, Nicholas J.] Univ Birmingham, Sch Biosci, Birmingham B15 2TT, W Midlands, England.

[Frampton, Jon] Univ Birmingham, Coll Med & Dent Sci, Birmingham B15 2TT, W Midlands, England.

[Fung, Martin; Lee, Suki; Tortorella, Micky D.; Neagoie, Cleopatra] Chinese Acad Sci, Hong Kong Inst Sci & Innovat, Ctr Regenerat Med & Hlth, Hong Kong, Peoples R China.

[Morton, Michael] ApconiX Ltd, Nether Alderly SK10 4TG, Cheshire, England.

[Goff, Aaron; Waddell, Simon J.] Univ Sussex, Brighton & Sussex Med Sch, Dept Global Hlth & Infect, Brighton BN1 9PX, England.

[Moon, Chris; Pascoe, Jordan; Underwood, Zoe E.; Bacon, Joanna] Natl Infect Serv, TB Res Grp, Publ Hlth England UKHSA, Salisbury SP4 0JG, England.

[Alderwick, Luke] Charles River Labs, Discovery Sci, Saffron Walden CB10 1XL, England.

[Neagoie, Cleopatra] Univ Birmingham, Sch Chem, Birmingham B15 2TT, W Midlands, England.

C3 University of Birmingham; University of Birmingham; Chinese Academy of

Sciences; Guangzhou Institute of Biomedicine & Health, CAS; State Key

Laboratory of Respiratory Disease; University of Birmingham; University

of Birmingham; Chinese Academy of Sciences; University of Brighton;

University of Sussex; University of Birmingham

RP Fossey, JS (corresponding author), Univ Birmingham, Sch Chem, Birmingham B15 2TT, W Midlands, England.; Alderwick, L (corresponding author), Univ Birmingham, Inst Microbiol & Infect, Sch Biosci, Birmingham B15 2TT, W Midlands, England.; Neagoie, C (corresponding author), Chinese Acad Sci, Guangzhou Inst Biomed & Hlth, State Key Lab Resp Dis, China New Zealand Joint Lab Biomed & Hlth, Guangzhou 510530, Peoples R China.; Neagoie, C (corresponding author), Chinese Acad Sci, Hong Kong Inst Sci & Innovat, Ctr Regenerat Med & Hlth, Hong Kong, Peoples R China.; Alderwick, L (corresponding author), Charles River Labs, Discovery Sci, Saffron Walden CB10 1XL, England.; Neagoie, C (corresponding author), Univ Birmingham, Sch Chem, Birmingham B15 2TT, W Midlands, England.

EM luke.alderwick@crl.com; j.s.fossey@bham.ac.uk;

cleopatra.neagoie@crmh-cas.org.hk

RI ; Yoshizawa, Akina/OOJ-9323-2025; Lee, Suki Man Yan/C-4596-2009; Cox,

Liam/AAG-9351-2020; Cui, Yixin/MDS-8323-2025; Zhang, Tianyu/K-4801-2015;

Craven, Philip/G-2578-2016; Press, Cara/MSY-5403-2025; Waddell,

Simon/A-9170-2013; Lee, Suki Man Yan/C-4596-2009; Fossey,

John/C-3172-2009

OI Hama Salih, Mariwan/0000-0002-1489-1671; Yoshizawa,

Akina/0000-0001-8625-0999; Lanne, Alice/0000-0003-2969-4336; Lee, Suki

Man Yan/0000-0002-1912-5511; Cox, Liam/0000-0001-7018-3904; Coltman,

Nicholas James/0000-0002-8210-9178; Alderwick, Luke/0000-0002-1257-6053;

Craven, Philip/0000-0002-7617-132X; Press, Cara/0000-0003-3706-4764;

Waddell, Simon/0000-0002-3684-9116; Cundy, Nicholas/0000-0001-6783-5422;

Lee, Suki Man Yan/0000-0002-0268-0789;

FU , National Key Research and Development Program of China; MRC

[EP/J003220/1]; EPSRC; University of Birmingham; Royal Society; Public

Health England [2021 YFA1300900]; National Key R&D Program of China

[YJKYYQ20210026, 154144KYSB20190005]; Chinese Academy of Sciences Grant;

National Centre for the Replacement [NC/R001669/1]; Reduction of Animals

in Research (NC3Rs)

FX We dedicate this study to the memory of Professor John Fossey, an

exceptional synthetic chemist who passed away on April 15, 2022.

Professor Fossey's passion for chirality and stereoselective synthesis

marked him as a distinguished scientific leader in the field of

chemistry. His legacy endures through his scientific integrity,

innovative thinking, and his role as a remarkable scientific mentor and

collaborator. Professor John Fossey will be profoundly missed by

colleagues, family, and friends, and we extend our deepest sympathies

and sincere condolences to his loved ones. We are grateful for the

support underpinning much of this study from MRC Confidence in Concepts

and EPSRC follow-on fund schemes. The University of Birmingham is

acknowledged for support, including travel funds permitting A.Y., X.L.,

and Y.C. to undertake training placements at GIBH. J.S.F. is grateful to

the Royal Society for the training provided because of a previous

Industrial Fellowship and the EPSRC for previous funding (EP/J003220/1).

Funding for part of this study was received from Public Health England.

This work was supported by the National Key R&D Program of China (2021

YFA1300900) and by the Chinese Academy of Sciences Grant

(YJKYYQ20210026, 154144KYSB20190005). S.J.W. and A.G. thank the National

Centre for the Replacement, Refinement. and Reduction of Animals in

Research (NC3Rs) for grant support (NC/R001669/1). Qiong Pan (GIBH),

Jingfang Xiong (GIBH). and Miaoqin She (GIBH) are acknowledged for

conducting aspects of the PK/PD studies of this report. Dr. Chi Tsang

(UoB), Dr. Peter Ashton (UoB), and Jiajia Wei (GIBH) are acknowledged

for their helpful discussions and practical support with aspects of mass

spectrometry. Dr. Cecile S. Le Duff (UoB) and Dr. Neil Spencer (UoB)

gave advice on aspects of NMR spectroscopy underpinning the preliminary

or previously reported, findings. Yingxue Liu (GIBH) is acknowledged for

help with the purification of final products by HPLC where required.

CR Abrahams KA, 2017, SCI REP-UK, V7, DOI 10.1038/s41598-017-09642-y

Abrahams KA, 2016, NAT COMMUN, V7, DOI 10.1038/ncomms12581

Abrahams KA, 2012, PLOS ONE, V7, DOI 10.1371/journal.pone.0052951

Amaral L, 2017, ANTIBIOTICS-BASEL, V6, DOI 10.3390/antibiotics6010003

Anderson CM, 2013, ORGANOMETALLICS, V32, P4199, DOI 10.1021/om400398g

Andries K, 2005, SCIENCE, V307, P223, DOI 10.1126/science.1106753

[Anonymous], BIRM DRUG DISC FAC

[Anonymous], Global Health Observatory

Bacon J, 2014, PLOS ONE, V9, DOI 10.1371/journal.pone.0087329

Baell JB, 2018, ACS CHEM BIOL, V13, P36, DOI 10.1021/acschembio.7b00903

Baell JB, 2010, J MED CHEM, V53, P2719, DOI 10.1021/jm901137j

Balázsi G, 2008, MOL SYST BIOL, V4, DOI 10.1038/msb.2008.63

BANERJEE A, 1994, SCIENCE, V263, P227, DOI 10.1126/science.8284673

Barry CS, 2011, J AM CHEM SOC, V133, P13232, DOI 10.1021/ja204249p

Batt SM, 2015, ACS INFECT DIS, V1, P615, DOI 10.1021/acsinfecdis.5b00065

Belisle JT, 1997, SCIENCE, V276, P1420, DOI 10.1126/science.276.5317.1420

Borgers K, 2019, BMC GENOMICS, V20, DOI 10.1186/s12864-019-5909-5

Boshoff HIM, 2004, J BIOL CHEM, V279, P40174, DOI 10.1074/jbc.M406796200

Briffotaux J, 2019, FRONT MICROBIOL, V10, DOI 10.3389/fmicb.2019.00249

Briffotaux J, 2017, TUBERCULOSIS, V107, P13, DOI 10.1016/j.tube.2017.08.001

Caspi R, 2016, NUCLEIC ACIDS RES, V44, pD471, DOI 10.1093/nar/gkv1164

Chang Y, 2006, BIOCHEMISTRY-US, V45, P13476, DOI 10.1021/bi0615285

CLARK PW, 1987, J ORGANOMET CHEM, V330, P447, DOI 10.1016/S0022-328X(00)99057-0

Colomer I, 2016, CHEM COMMUN, V52, P7209, DOI 10.1039/c6cc03244c

COMBS DL, 1990, ANN INTERN MED, V112, P397, DOI 10.7326/0003-4819-76-3-112-6-397

Cox JAG, 2016, NAT MICROBIOL, V1, DOI [10.1038/NMICROBIOL.2015.6, 10.1038/nmicrobiol.2015.6]

Crespo M, 1997, J CHEM SOC DALTON, P1231, DOI 10.1039/a606872c

Favrot L, 2013, NAT COMMUN, V4, DOI 10.1038/ncomms3748

Feula A, 2013, ORG BIOMOL CHEM, V11, P5083, DOI 10.1039/c3ob41007b

Feula A, 2010, ORG LETT, V12, P5044, DOI 10.1021/ol102215e

Gavalda S, 2014, CHEM BIOL, V21, P1660, DOI 10.1016/j.chembiol.2014.10.011

Gupta RS, 2018, FRONT MICROBIOL, V9, DOI 10.3389/fmicb.2018.00067

Hards K, 2015, J ANTIMICROB CHEMOTH, V70, P2028, DOI 10.1093/jac/dkv054

Hendon-Dunn CL, 2019, ANTIMICROB AGENTS CH, V63, DOI 10.1128/AAC.00570-19

Hendon-Dunn CL, 2016, ANTIMICROB AGENTS CH, V60, P3869, DOI 10.1128/AAC.02712-15

Hoagland DT, 2016, ADV DRUG DELIVER REV, V102, P55, DOI 10.1016/j.addr.2016.04.026

James BW, 2000, J APPL MICROBIOL, V88, P669, DOI 10.1046/j.1365-2672.2000.01020.x

JOHNSSON K, 1995, J AM CHEM SOC, V117, P5009, DOI 10.1021/ja00122a038

Kapopoulou A, 2011, TUBERCULOSIS, V91, P8, DOI 10.1016/j.tube.2010.09.006

Karp PD, 2019, BRIEF BIOINFORM, V20, P1085, DOI 10.1093/bib/bbx085

Lambert RJW, 2000, J APPL MICROBIOL, V88, P784, DOI 10.1046/j.1365-2672.2000.01017.x

Lambert RJW, 2000, J APPL MICROBIOL, V89, P275, DOI 10.1046/j.1365-2672.2000.01105.x

Lee RE, 2003, J COMB CHEM, V5, P172, DOI 10.1021/cc020071p

Ling LL, 2015, NATURE, V517, P455, DOI 10.1038/nature14098

Liu Y, 2019, NAT COMMUN, V10, DOI [10.1038/s41467-019-08464-y, 10.1038/s41467-019-11196-8]

Lovering F, 2013, MEDCHEMCOMM, V4, P515, DOI 10.1039/c2md20347b

Lovering F, 2009, J MED CHEM, V52, P6752, DOI 10.1021/jm901241e

Luo SH, 2010, MOL PHARMACEUT, V7, P412, DOI 10.1021/mp900193e

Ma ZK, 2010, LANCET, V375, P2100, DOI 10.1016/S0140-6736(10)60359-9

Makarov V, 2009, SCIENCE, V324, P801, DOI 10.1126/science.1171583

Manganelli R, 2001, MOL MICROBIOL, V41, P423, DOI 10.1046/j.1365-2958.2001.02525.x

McGillivray A, 2014, PLOS ONE, V9, DOI 10.1371/journal.pone.0093604

Mdluli K, 2015, CSH PERSPECT MED, V5, DOI 10.1101/cshperspect.a021154

MILANES CL, 1989, KIDNEY INT, V36, P802, DOI 10.1038/ki.1989.265

Miles AA, 1938, J HYG-CAMBRIDGE, V38, P732, DOI 10.1017/S002217240001158X

Mishra S, 2017, ELIFE, V6, DOI 10.7554/eLife.25624

Mosaei H, 2018, MOL CELL, V72, P263, DOI 10.1016/j.molcel.2018.08.028

Nateche F, 2006, J MED MICROBIOL, V55, P857, DOI 10.1099/jmm.0.46513-0

Neagoie C. D., 2020, Patent No. 2020206594

Palomino JC, 2002, ANTIMICROB AGENTS CH, V46, P2720, DOI 10.1128/AAC.46.8.2720-2722.2002

Portevin D, 2004, P NATL ACAD SCI USA, V101, P314, DOI 10.1073/pnas.0305439101

Prosser GA, 2013, FEBS J, V280, P1150, DOI 10.1111/febs.12108

Qin LH, 2019, BMC BIOL, V17, DOI 10.1186/s12915-019-0628-6

Raymer B, 2018, J MED CHEM, V61, P10375, DOI 10.1021/acs.jmedchem.8b00407

Rose JD, 2002, CARBOHYD RES, V337, P105, DOI 10.1016/S0008-6215(01)00288-9

Shah NS, 2007, EMERG INFECT DIS, V13, P380, DOI 10.3201/eid1303.061400

Sharma A, 2021, J MED CHEM, V64, P4359, DOI 10.1021/acs.jmedchem.0c01833

Sharma SK, 2006, CHEST, V130, P261, DOI [10.1378/chest.130.1.261, 10.1016/S0012-3692(15)50981-1]

Su CC, 2019, P NATL ACAD SCI USA, V116, P11241, DOI 10.1073/pnas.1901346116

Tahlan K, 2012, ANTIMICROB AGENTS CH, V56, P1797, DOI 10.1128/AAC.05708-11

TAKAYAMA K, 1989, ANTIMICROB AGENTS CH, V33, P1493, DOI 10.1128/AAC.33.9.1493

Viljoen A, 2018, J BIOL CHEM, V293, P2755, DOI 10.1074/jbc.RA117.000760

Waddell SJ, 2004, TUBERCULOSIS, V84, P263, DOI 10.1016/j.tube.2003.12.005

WHO, 2022, GLOBAL TUBERCULOSIS

Xu ZJ, 2017, P NATL ACAD SCI USA, V114, P7993, DOI 10.1073/pnas.1700062114

Yang F, 2015, PLOS ONE, V10, DOI 10.1371/journal.pone.0119341

Yang YD, 2010, J MED CHEM, V53, P7709, DOI 10.1021/jm1008456

Yoshizawa A, 2018, FRONT CHEM, V6, DOI 10.3389/fchem.2018.00211

Yoshizawa A, 2018, SCI REP-UK, V8, DOI 10.1038/s41598-018-24784-3

Zhang TW, 2012, PLOS ONE, V7, DOI 10.1371/journal.pone.0030531

Zumla A, 2013, NAT REV DRUG DISCOV, V12, P388, DOI 10.1038/nrd4001

NR 81

TC 5

Z9 6

U1 0

U2 15

PU AMER CHEMICAL SOC

PI WASHINGTON

PA 1155 16TH ST, NW, WASHINGTON, DC 20036 USA

SN 0022-2623

EI 1520-4804

J9 J MED CHEM

JI J. Med. Chem.

PD FEB 8

PY 2024

VL 67

IS 4

BP 2529

EP 2548

DI 10.1021/acs.jmedchem.3c01643

EA FEB 2024

PG 20

WC Chemistry, Medicinal

WE Science Citation Index Expanded (SCI-EXPANDED)

SC Pharmacology & Pharmacy

GA JP7X0

UT WOS:001167257200001

OA Green Submitted, hybrid

DA 2026-05-07

ER

PT J

AU AlMatar, M

Var, I

Kayar, B

Köksal, F

AF AlMatar, Manaf

Var, Isil

Kayar, Begum

Koksal, Fatih

TI Differential Expression of Resistant and Efflux Pump Genes in MDR-TB

Isolates

SO ENDOCRINE METABOLIC & IMMUNE DISORDERS-DRUG TARGETS

LA English

DT Article

DE Mycobacterium tuberculosis; antituberculosis agents; efflux pumps; gene

expression; RT-qPCR; drug resistance

ID CATALASE-PEROXIDASE GENE; LEVEL ISONIAZID RESISTANCE; OXIDATIVE STRESS

RESPONSES; INTRINSIC DRUG-RESISTANCE; MYCOBACTERIUM-TUBERCULOSIS;

MOLECULAR CHARACTERIZATION; RIFAMPIN RESISTANCE; ENZYMATIC

CHARACTERIZATION; COMPENSATORY MUTATIONS; ANTIBIOTIC-RESISTANCE

AB Background: Numerous investigations demonstrate efflux as a worldwide bacterial mode of action which contributes to the resistance of drugs. The activity of antibiotics, which subjects to efflux, can be improved by the combined usage of efflux inhibitors. However, the efflux role to the overall levels of antibiotic resistance of clinical M. tuberculosis isolates is inadequately comprehended and is still disregarded by many.

Methods: Here, we assessed the contribution of resistant genes associated with isoniazid (INH) and rifampin (R) resistance to the levels of drug resistance in the (27) clinical isolates of MDR-TB. Additionally, the role of the resistance for six putative drug efflux pump genes to the antibiotics was investigated. The level of katG expression was down-regulated in 24/27 (88.88%) of MDR-TB isolates. Of the 27 MDR-TB isolates, inhA, oxyR-ahpC, and rpoB showed either overexpression or up-regulation in 8 (29.62%), 4 (14.81 %), and 24 (88.88%), respectively. Moreover, the efflux pump genes drrA, drrB, efpA, Rv2459, Rv1634, and Rv1250 were overexpressed under INH/RIF plus fresh pomegranate juice (FPJ) stress signifying the efflux pumps contribution to the overall levels of the resistance of MDR-TB isolates.

Conclusion: These results displayed that the levels of drug resistance of MDR-TB clinical isolates are due to combination among drug efflux pump and the presence of mutations in target genes, a truth which is often ignored by the specialists of tuberculosis in favour of the almost undoubted significance of drug target-gene mutations for the resistance in M. tuberculosis.

C1 [AlMatar, Manaf] Cukurova Univ, Inst Nat & Appl Sci, Dept Biotechnol, Fen Bilimleri Enstitusu, Adana, Turkey.

[Var, Isil] Cukurova Univ, Agr Fac, Dept Food Engn, Adana, Turkey.

[Kayar, Begum; Koksal, Fatih] Cukurova Univ, Fac Med, Dept Med Microbiol, Adana, Turkey.

C3 Cukurova University; Cukurova University; Cukurova University

RP AlMatar, M (corresponding author), Cukurova Univ, Inst Nat & Appl Sci, Dept Biotechnol, Fen Bilimleri Enstitusu, Adana, Turkey.

EM manafmatar19@gmail.com

RI AlMatar, Manaf/AGT-5623-2022; Kayar, Begum/L-5173-2016; KÖKSAL,

fatih/AAG-6034-2019; Var, Işıl/B-6352-2018

CR Abate D, 2014, INT J TUBERC LUNG D, V18, P946, DOI 10.5588/ijtld.13.0926

Adami AG, 2017, DIAGN MICR INFEC DIS, V87, P108, DOI 10.1016/j.diagmicrobio.2016.10.023

Ahmad S, 2004, INT J ANTIMICROB AG, V23, P473, DOI 10.1016/j.ijantimicag.2003.10.004

AlMatar M., 2017, PHARM REP

AlMatar M, 2019, CURR PHARM BIOTECHNO, V20, P317, DOI 10.2174/1389201020666190308130343

AlMatar M, 2018, PHARMACOL RES, V128, P288, DOI 10.1016/j.phrs.2017.10.011

AlMatar M, 2017, BIOMED PHARMACOTHER, V91, P546, DOI 10.1016/j.biopha.2017.04.105

Ando H, 2011, MOL MICROBIOL, V79, P1615, DOI 10.1111/j.1365-2958.2011.07547.x

Ando H, 2010, ANTIMICROB AGENTS CH, V54, P1793, DOI 10.1128/AAC.01691-09

Arnvig KB, 2011, PLOS PATHOG, V7, DOI 10.1371/journal.ppat.1002342

Bakonyte D, 2003, ANTIMICROB AGENTS CH, V47, P2009, DOI 10.1128/AAC.47.6.2009-2011.2003

BANERJEE A, 1994, SCIENCE, V263, P227, DOI 10.1126/science.8284673

Bardou F, 1998, MICROBIOL-UK, V144, P2539, DOI 10.1099/00221287-144-9-2539

Basso LA, 1998, J INFECT DIS, V178, P769, DOI 10.1086/515362

Betts JC, 2003, ANTIMICROB AGENTS CH, V47, P2903, DOI 10.1128/AAC.47.9.2903-2913.2003

Bisson G. P., 2012, J BACTERIOL

Black P. A., 2014, ANTIMICROBIAL AGENTS, DOI [10.1128/AAC.02293-13, DOI 10.1128/AAC.02293-13]]

Brandis G, 2013, J ANTIMICROB CHEMOTH, V68, P2493, DOI 10.1093/jac/dkt224

Brandis G, 2012, MOL MICROBIOL, V85, P142, DOI 10.1111/j.1365-2958.2012.08099.x

Brossier F, 2006, J CLIN MICROBIOL, V44, P3659, DOI 10.1128/JCM.01054-06

Burian J, 2013, NUCLEIC ACIDS RES, V41, P10062, DOI 10.1093/nar/gkt751

Bustin SA, 2010, MIQE PRECIS PRACTICA

Cade CE, 2010, PROTEIN SCI, V19, P458, DOI 10.1002/pro.324

Campbell EA, 2001, CELL, V104, P901, DOI 10.1016/S0092-8674(01)00286-0

Cardosa RF, 2007, MEM I OSWALDO CRUZ, V102, P59, DOI 10.1590/S0074-02762007000100009

Cardoso RF, 2004, ANTIMICROB AGENTS CH, V48, P3373, DOI 10.1128/AAC.48.9.3373-3381.2004

Chatterjee A, 2013, PLOS ONE, V8, DOI 10.1371/journal.pone.0054717

Chihota V. N., 2011, J CLIN MICROBIOL JCM

Chikaonda T, 2017, AFR J LAB MED, V6, DOI 10.4102/ajlm.v6i2.463

Choudhuri BS, 2002, BIOCHEM J, V367, P279, DOI 10.1042/BJ20020615

Clark TG, 2013, PLOS ONE, V8, DOI 10.1371/journal.pone.0083012

Cole ST, 1998, NATURE, V393, P537, DOI 10.1038/31159

da Silva P. E. A., 2016, EFFLUX MEDIATED ANTI, P527, DOI [DOI 10.1007/978-3-319-39658-3_21, 10.1007/978-3-319-39658-3_21]

De Rossi E, 2006, FEMS MICROBIOL REV, V30, P36, DOI 10.1111/j.1574-6976.2005.00000.x

De Rossi Edda, 2002, Molecular Medicine (Baltimore), V8, P714

de Welzen L, 2017, ANTIMICROB AGENTS CH, V61, DOI 10.1128/AAC.01461-17

DeMarco CE, 2007, ANTIMICROB AGENTS CH, V51, P3235, DOI 10.1128/AAC.00430-07

DESSEN A, 1995, SCIENCE, V267, P1638, DOI 10.1126/science.7886450

Dhandayuthapani S, 1996, J BACTERIOL, V178, P3641, DOI 10.1128/jb.178.12.3641-3649.1996

Du Plessis J., 2014, DECIPHERING IMPACT R

du Preez I, 2012, OMICS, V16, P596, DOI 10.1089/omi.2012.0028

FARR SB, 1991, MICROBIOL REV, V55, P561, DOI 10.1128/MMBR.55.4.561-585.1991

Feng LS, 2010, EUR J MED CHEM, V45, P3407, DOI 10.1016/j.ejmech.2010.04.027

Fu LM, 2006, TUBERCULOSIS, V86, P134, DOI 10.1016/j.tube.2005.07.004

Gagneux S, 2006, PLOS PATHOG, V2, P603, DOI 10.1371/journal.ppat.0020061

Garcia de Viedma D, 2002, J CLIN MICROBIOL, V40, P988, DOI 10.1128/JCM.40.3.988-995.2002

Garima K, 2015, TUBERCULOSIS, V95, P155, DOI 10.1016/j.tube.2015.01.005

Georghiou SB, 2016, ANTIMICROB AGENTS CH, V60, P3994, DOI 10.1128/AAC.00222-16

Gupta AK, 2010, INDIAN J MED RES, V132, P176

Gupta AK, 2010, MICROB DRUG RESIST, V16, P21, DOI 10.1089/mdr.2009.0054

Hazbón MH, 2006, ANTIMICROB AGENTS CH, V50, P2640, DOI 10.1128/AAC.00112-06

Heep M, 2001, J CLIN MICROBIOL, V39, P107, DOI 10.1128/JCM.39.1.107-110.2001

Herrera L, 2004, INT J ANTIMICROB AG, V23, P572, DOI 10.1016/j.ijantimicag.2003.09.032

HEYM B, 1995, MOL MICROBIOL, V15, P235, DOI 10.1111/j.1365-2958.1995.tb02238.x

Heym B., 1999, Tubercle and Lung Disease, V79, P267, DOI 10.1054/tuld.1998.0208

Heym B, 1997, INFECT IMMUN, V65, P1395, DOI 10.1128/IAI.65.4.1395-1401.1997

HEYM B, 1992, RES MICROBIOL, V143, P721, DOI 10.1016/0923-2508(92)90067-X

Jagielski T, 2014, J ANTIMICROB CHEMOTH, V69, P2369, DOI 10.1093/jac/dku161

Kelley CL, 1997, ANTIMICROB AGENTS CH, V41, P2057, DOI 10.1128/AAC.41.9.2057

Khan SN, 2013, PAK J ZOOL, V45, P93

Khanna A, 2010, ANTIMICROB AGENTS CH, V54, P4789, DOI 10.1128/AAC.00661-10

Kolyva AS, 2012, UNDERSTANDING TUBERCULOSIS - NEW APPROACHES TO FIGHTING AGAINST DRUG RESISTANCE, P209

Lahiri N, 2016, J BIOL CHEM, V291, P14248, DOI 10.1074/jbc.M116.716704

Larsen MH, 2002, MOL MICROBIOL, V46, P453, DOI 10.1046/j.1365-2958.2002.03162.x

Lavender C, 2005, ANTIMICROB AGENTS CH, V49, P4068, DOI 10.1128/AAC.49.10.4068-4074.2005

Lee JH, 2012, NAT COMMUN, V3, DOI 10.1038/ncomms1724

Li G., 2015, PLOS ONE, V10, DOI [10.1371/journal.pone.0119013, DOI 10.1371/J0URNAL.P0NE.0119013]]

Li GL, 2015, J ANTIBIOT, V68, P431, DOI 10.1038/ja.2015.9

Li XZ, 2004, ANTIMICROB AGENTS CH, V48, P2415, DOI 10.1128/AAC.48.7.2415-2423.2004

Livak K. J., 2001, ANAL RELATIVE GENE E

Luo T, 2010, ANTIMICROB AGENTS CH, V54, P1075, DOI 10.1128/AAC.00964-09

Machado D, 2017, FRONT MICROBIOL, V8, DOI 10.3389/fmicb.2017.00711

Machado D, 2016, PLOS ONE, V11, DOI 10.1371/journal.pone.0149326

Machado D, 2013, J ANTIMICROB CHEMOTH, V68, P1728, DOI 10.1093/jac/dkt090

Machado D, 2012, PLOS ONE, V7, DOI 10.1371/journal.pone.0034538

Manganelli R, 1999, MOL MICROBIOL, V31, P715, DOI 10.1046/j.1365-2958.1999.01212.x

Mariam DH, 2004, ANTIMICROB AGENTS CH, V48, P1289, DOI 10.1128/AAC.48.4.1289-1294.2004

Marrakchi H, 2000, MICROBIOL-UK, V146, P289, DOI 10.1099/00221287-146-2-289

Master S, 2001, J BACTERIOL, V183, P4033, DOI 10.1128/JB.183.13.4033-4039.2001

McMurry LM, 1999, ANTIMICROB AGENTS CH, V43, P711, DOI 10.1128/AAC.43.3.711

Mdluli K, 1998, SCIENCE, V280, P1607, DOI 10.1126/science.280.5369.1607

Miesel L, 1998, J BACTERIOL, V180, P2459, DOI 10.1128/JB.180.9.2459-2467.1998

Mokrousov I, 2002, ANTIMICROB AGENTS CH, V46, P1417, DOI 10.1128/AAC.46.5.1417-1424.2002

Morlock GP, 2003, ANTIMICROB AGENTS CH, V47, P3799, DOI 10.1128/AAC.47.12.3799-3805.2003

Morris RP, 2005, P NATL ACAD SCI USA, V102, P12200, DOI 10.1073/pnas.0505446102

Müller B, 2011, INT J TUBERC LUNG D, V15, P344

MUSSER JM, 1995, CLIN MICROBIOL REV, V8, P496, DOI 10.1128/CMR.8.4.496

Narang A, 2017, INT J MYCOBACT, V6, P177, DOI 10.4103/ijmy.ijmy_26_17

Nathanson E, 2010, NEW ENGL J MED, V363, P1050, DOI 10.1056/NEJMra0908076

Ng VH, 2004, MOL MICROBIOL, V52, P1291, DOI 10.1111/j.1365-2958.2004.04078.x

Niehaus AJ, 2015, PLOS ONE, V10, DOI 10.1371/journal.pone.0135003

Organization W. H., 2016, GLOB TUB REP 2016

Organization WH, 2010, MULT EXT DRUG RES TB

Pagán-Ramos E, 2006, J BACTERIOL, V188, P2674, DOI 10.1128/JB.188.7.2674-2680.2006

Pang Y., 2012, ANTIMICROBIAL AGENTS

Peng JF, 2016, FRONT MICROBIOL, V7, DOI 10.3389/fmicb.2016.00618

Poon A, 2005, GENETICS, V170, P1323, DOI 10.1534/genetics.104.037259

Pym AS, 2002, INFECT IMMUN, V70, P4955, DOI 10.1128/IAI.70.9.4955-4960.2002

Pym AS, 2001, MOL MICROBIOL, V40, P879, DOI 10.1046/j.1365-2958.2001.02427.x

Qian LS, 2002, J CLIN MICROBIOL, V40, P1091, DOI 10.1128/JCM.40.3.1091-1094.2002

QUEMARD A, 1995, BIOCHEMISTRY-US, V34, P8235, DOI 10.1021/bi00026a004

Ramaswamy S., 1998, Tubercle and Lung Disease, V79, P3, DOI 10.1054/tuld.1998.0002

Ramaswamy SV, 2003, ANTIMICROB AGENTS CH, V47, P1241, DOI 10.1128/AAC.47.4.1241-1250.2003

Ramón-García S, 2012, ANTIMICROB AGENTS CH, V56, P2074, DOI 10.1128/AAC.05946-11

Ramón-García S, 2009, ANTIMICROB AGENTS CH, V53, P3675, DOI 10.1128/AAC.00550-09

Rifat D, 2017, FUTURE MICROBIOL, V12, P753, DOI 10.2217/fmb-2017-0022

Rindi L, 2005, INT J TUBERC LUNG D, V9, P94

Rodrigues L, 2012, INFECT GENET EVOL, V12, P695, DOI 10.1016/j.meegid.2011.08.009

Sala C, 2008, BMC MOL BIOL, V9, DOI 10.1186/1471-2199-9-33

Schmalstieg A. M., 2012, ANTIBIOTIC RESISTANC, DOI [10.1128/AAC.05546-11, DOI 10.1128/AAC.05546-11]]

Siddiqi N, 2004, INFECTION, V32, P109, DOI 10.1007/s15010-004-3097-x

Silva MSN, 2003, J CLIN MICROBIOL, V41, P4471, DOI 10.1128/JCM.41.9.4471-4474.2003

Song W, 2009, INT J ANTIMICROB AG, V33, P237, DOI 10.1016/j.ijantimicag.2008.05.022

Springer B, 2001, INFECT IMMUN, V69, P5967, DOI 10.1128/IAI.69.10.5967-5973.2001

Sreevatsan S, 1997, ANTIMICROB AGENTS CH, V41, P600, DOI 10.1128/AAC.41.3.600

TELENTI A, 1993, LANCET, V341, P647, DOI 10.1016/0140-6736(93)90417-F

Tracevska T, 2002, J CLIN MICROBIOL, V40, P3789, DOI 10.1128/JCM.40.10.3789-3792.2002

Trotochaud AE, 2005, NAT STRUCT MOL BIOL, V12, P313, DOI 10.1038/nsmb917

Tudó G, 2010, FUTURE MED CHEM, V2, P1371, DOI 10.4155/FMC.10.219

van Soolingen D, 2000, J INFECT DIS, V182, P1788, DOI 10.1086/317598

Vassylyev DG, 2007, NATURE, V448, P163, DOI 10.1038/nature05931

Vilchèze C, 2014, MICROBIOL SPECTR, V2, DOI 10.1128/microbiolspec.MGM2-0014-2013

Viuda-Martos M, 2010, COMPR REV FOOD SCI F, V9, P635, DOI 10.1111/j.1541-4337.2010.00131.x

Viveiros M, 2012, EXPERT REV ANTI-INFE, V10, P983, DOI [10.1586/ERI.12.89, 10.1586/eri.12.89]

Wade M. M, 2004, FRONT BIOSCI, P9975, DOI DOI 10.2741/1289]

Wassarman KM, 2000, CELL, V101, P613, DOI 10.1016/S0092-8674(00)80873-9

Wilson T, 1998, MICROBIOL-UK, V144, P2687, DOI 10.1099/00221287-144-10-2687

Wilson TM, 1996, MOL MICROBIOL, V19, P1025, DOI 10.1046/j.1365-2958.1996.449980.x

Yue J, 2003, J CLIN MICROBIOL, V41, P2209, DOI 10.1128/JCM.41.5.2209-2212.2003

Zahrt TC, 2001, MOL MICROBIOL, V39, P1174, DOI 10.1111/j.1365-2958.2001.02321.x

Zhang Jing-rui, 2013, Zhonghua Liu Xing Bing Xue Za Zhi, V34, P379

Zhang M, 2005, J CLIN MICROBIOL, V43, P5477, DOI 10.1128/JCM.43.11.5477-5482.2005

Zhang Y, 2009, INT J TUBERC LUNG D, V13, P1320

ZHANG Y, 1992, NATURE, V358, P591, DOI 10.1038/358591a0

NR 134

TC 19

Z9 19

U1 1

U2 16

PU BENTHAM SCIENCE PUBL LTD

PI SHARJAH

PA EXECUTIVE STE Y-2, PO BOX 7917, SAIF ZONE, 1200 BR SHARJAH, U ARAB

EMIRATES

SN 1871-5303

EI 2212-3873

J9 ENDOCR METAB IMMUNE

JI Endocr. Metab. Immune Disord.-Drug Targets

PY 2020

VL 20

IS 2

BP 271

EP 287

DI 10.2174/1871530319666191009153834

PG 17

WC Endocrinology & Metabolism; Immunology; Pharmacology & Pharmacy

WE Science Citation Index Expanded (SCI-EXPANDED)

SC Endocrinology & Metabolism; Immunology; Pharmacology & Pharmacy

GA KN2GW

UT WOS:000514660900013

OA hybrid

DA 2026-05-07

ER

PT J

AU Thakare, R

Dasgupta, A

Chopra, S

AF Thakare, R.

Dasgupta, A.

Chopra, S.

TI Pretomanid for the treatment of pulmonary tuberculosis

SO DRUGS OF TODAY

LA English

DT Article

DE Pretomanid; Tuberculosis; Bacterial infections; Nitric oxide (NO)

donors; Antibacterial drugs; Nitroimidazoles

ID NONREPLICATING MYCOBACTERIUM-TUBERCULOSIS; IN-VIVO ACTIVITIES;

BACTERICIDAL ACTIVITY; NITROIMIDAZOPYRAN PA-824; MOXIFLOXACIN; VITRO;

PHARMACOKINETICS; PYRAZINAMIDE; SAFETY; COMBINATIONS

AB Discovering novel drugs active against Mycobacterium tuberculosis (Mtb), the causative agent of tuberculosis (TB), is currently one of the most unmet medical needs. In this context, pretomanid (PA-824), a novel nitroimidazole prodrug that targets both replicating and nonreplicating cells, is being developed by TB Alliance under license from Novartis. In replicating Mtb, pretomanid inhibits mycolic acid biosynthesis, which is an important building block of Mtb cell wall. Under nonreplicating conditions, pretomanid is reduced by deazaflavin-dependent nitroreductase, leading to generation of reactive nitrogen species exhibiting potent antimycobacterial activity. The U.S. Food and Drug Administration (FDA) has approved pretomanid under the Limited Population Pathway for Antibacterial and Antifungal Drugs (LPAD pathway) for treatment of adult patients with treatmentintolerant or nonresponsive multidrug-resistant TB and extensively drug-resistant TB in combination with bedaquiline and linezolid as part of the oral bedaquiline + pretomanid + linezolid regimen. Pretomanid has also received U.S. FDA qualified infectious disease product (QIDP) designation under the Generating Antibiotic Incentives Now (GAIN) Act. It is the second drug to be approved under the LPAD pathway, and the third new anti-TB drug approved by the FDA in more than 40 years. In addition, the European Medicines Agency (EMA) has also recently approved pretomanid for the same indications.

C1 [Thakare, R.; Dasgupta, A.; Chopra, S.] CSIR Cent Drug Res Inst, Div Microbiol, Sect 10,Sitapur Rd, Lucknow 226031, Uttar Pradesh, India.

C3 Council of Scientific & Industrial Research (CSIR) - India; CSIR -

Central Drug Research Institute (CDRI)

RP Chopra, S (corresponding author), CSIR Cent Drug Res Inst, Div Microbiol, Sect 10,Sitapur Rd, Lucknow 226031, Uttar Pradesh, India.

EM skchopra007@gmail.com

RI Thakare, Ritesh/AFR-2456-2022; Chopra, Sidharth/G-1385-2012

OI Thakare, Ritesh/0000-0002-7426-2519; Chopra,

Sidharth/0000-0001-8823-6074; Dasgupta, Arunava/0000-0001-9014-1904

CR [Anonymous], 2020, PRET

[Anonymous], 2020, PRET SUMM OP

ASHTEKAR DR, 1993, ANTIMICROB AGENTS CH, V37, P183, DOI 10.1128/AAC.37.2.183

Dawson R, 2015, LANCET, V385, P1738, DOI 10.1016/S0140-6736(14)62002-X

Diacon AH, 2015, AM J RESP CRIT CARE, V191, P943, DOI 10.1164/rccm.201410-1801OC

Diacon AH, 2012, LANCET, V380, P986, DOI 10.1016/S0140-6736(12)61080-0

Diacon AH, 2012, ANTIMICROB AGENTS CH, V56, P3027, DOI 10.1128/AAC.06125-11

Dooley KE, 2014, ANTIMICROB AGENTS CH, V58, P5245, DOI 10.1128/AAC.03332-14

Food and Drug Administration (FDA), 2019, FDA BRIEF DOC PRET T

Ginsberg AM, 2009, ANTIMICROB AGENTS CH, V53, P3720, DOI 10.1128/AAC.00106-09

Gothi Dipti, 2011, Recent Pat Antiinfect Drug Discov, V6, P27, DOI 10.2174/157489111794407859

Iacobino A, 2017, INT J MYCOBACT, V6, P213, DOI 10.4103/ijmy.ijmy_85_17

Jamaati H, 2017, FRONT MICROBIOL, V8, DOI 10.3389/fmicb.2017.02008

Ji BH, 2006, ANTIMICROB AGENTS CH, V50, P1921, DOI 10.1128/AAC.00052-06

Lenaerts AJ, 2005, ANTIMICROB AGENTS CH, V49, P2294, DOI 10.1128/AAC.49.6.2294-2301.2005

López-Gavin A, 2015, INT J ANTIMICROB AG, V46, P582, DOI 10.1016/j.ijantimicag.2015.08.004

Manjunatha UH, 2006, P NATL ACAD SCI USA, V103, P431, DOI 10.1073/pnas.0508392103

Manjunatha Ujjini, 2009, Commun Integr Biol, V2, P215

Pretomanid, 2019, PRET HIGHL PRESCR IN

Salinger DH., 2019, ANTIMICROB AGENTS CH, V63

Silva CD, 2018, ANTIMICROB AGENTS CH, V62, DOI [10.1128/aac.00856-18, 10.1128/aac.00856-18, 10.1128/AAC.00856-18;e00856-18]

Singh R, 2008, SCIENCE, V322, P1392, DOI 10.1126/science.1164571

Stover CK, 2000, NATURE, V405, P962, DOI 10.1038/35016103

Tasneen R, 2015, ANTIMICROB AGENTS CH, V59, P129, DOI 10.1128/AAC.03822-14

Tweed CD, 2019, LANCET RESP MED, V7, P1048, DOI 10.1016/S2213-2600(19)30366-2

Tyagi S, 2005, ANTIMICROB AGENTS CH, V49, P2289, DOI 10.1128/AAC.49.6.2289-2293.2005

WHO, 2012, GLOBAL TUBERCULOSIS REPORT 2012, P1

Winter H, 2013, ANTIMICROB AGENTS CH, V57, P5516, DOI 10.1128/AAC.00798-13

World Health Organization, 2020, Tuberculosis: Key facts

Xu J, 2019, NUTR RES, V63, P21, DOI 10.1016/j.nutres.2018.12.006

NR 30

TC 10

Z9 15

U1 0

U2 17

PU PROUS SCIENCE, SAU-THOMSON REUTERS

PI BARCELONA

PA 398 PROVENCA, 08025 BARCELONA, SPAIN

SN 1699-3993

EI 1699-4019

J9 DRUG TODAY

JI Drugs Today

PD OCT

PY 2020

VL 56

IS 10

BP 655

EP 668

DI 10.1358/dot.2020.56.10.3161237

PG 14

WC Pharmacology & Pharmacy

WE Science Citation Index Expanded (SCI-EXPANDED)

SC Pharmacology & Pharmacy

GA ON3CP

UT WOS:000586583800003

DA 2026-05-07

ER

PT J

AU Kanji, A

Hasan, R

Ali, A

Zaver, A

Zhang, Y

Imtiaz, K

Shi, WL

Clark, TG

McNerney, R

Phelan, J

Rao, S

Shafiq, S

Hasan, Z

AF Kanji, Akbar

Hasan, Rumina

Ali, Asho

Zaver, Ambreen

Zhang, Ying

Imtiaz, Kehkashan

Shi, Wanliang

Clark, Taane G.

McNerney, Ruth

Phelan, Jody

Rao, Shoaib

Shafiq, Samreen

Hasan, Zahra

TI Single nucleotide polymorphisms in efflux pumps genes in extensively

drug resistant Mycobacterium tuberculosis isolates from Pakistan

SO TUBERCULOSIS

LA English

DT Article

DE Extensively drug resistant; Mycobacterium tuberculosis; Single

nucleotide polymorphism; Efflux pump

ID SMEGMATIS; EXPRESSION; STRAINS; GENOME; PCR; SUSCEPTIBILITY;

IDENTIFICATION; TRANSPORTERS; INHIBITORS; RIFAMPICIN

AB It is challenging to understand mechanisms of drug resistance in Mycobacterium tuberculosis (MTB) due to the large variability in resistance associated genes. Efflux pump genes contribute to drug resistance and thus add to this complexity. Efflux pump gene protein superfamilies have been characterized by genome analysis of drug resistant strains and through in vitro transcriptional studies. However, there is limited information regarding efflux pump genes in extensively drug resistant (XDR) tuberculosis (TB) isolates.

Whole genome sequencing (WGS) based analysis of 37 extensively drug resistant (XDR) and five drug sensitive (DS) MTB clinical isolates was performed. Single nucleotide polymorphisms (SNPs) in efflux pump genes Rv0194, Rv1217, Rv1218, drrA, drrB, Rv1258, Rv1634, Rv2688, Rv1273, Rv1819, Rv1458, Rv1877 and Rv1250 were determined in the clinical isolates as compared with the H37Rv reference strain. Allele frequencies of SNPs identified in XDR strains were compared with DS strains. Gene expression of Rv0194, Rv2688, Rv1634, drrA and drrB was determined in XDR - TB isolates (n = 9), DS-TB strains (n = 4) and H37Rv.

We identified SNPs in XDR-TB isolates which were either unique or present at very low frequencies in DS strains; Rv0194 G170V; Rv1217 L151R; Rv1258 P369T and G391R; Rv1273 S118G and I175T; Rv1877 I534T; Rv1250 V318X/A and S333A, and Rv2688 P156T. The expression of Rv2688 and drrB was found to be raised in XDR-TB as compared with DS-TB strains.

We identified unique SNPs in efflux pump genes which may be associated with increased drug resistance in the isolates. Increased levels of Rv2688 and drrB efflux pump gene expression observed in XDR strains even in the absence of antibiotics suggests that these clinical isolates may be more refractory to treatment. Further studies are required to directly associate these mutations with increased resistance in MTB. (C) 2017 Elsevier Ltd. All rights reserved.

C1 [Kanji, Akbar; Hasan, Rumina; Zaver, Ambreen; Imtiaz, Kehkashan; Rao, Shoaib; Shafiq, Samreen; Hasan, Zahra] Aga Khan Univ Hosp, Dept Pathol & Lab Med, POB 3500, Karachi 74800, Pakistan.

[Clark, Taane G.; Phelan, Jody] London Sch Hyg & Trop Med, Fac Infect & Trop Dis, London, England.

[Clark, Taane G.] London Sch Hyg & Trop Med, Fac Epidemiol & Populat Hlth, London, England.

[McNerney, Ruth] Univ Cape Town, Dept Med, Lung Infect & Immun Unit, Div Pulmonol, Cape Town, South Africa.

[McNerney, Ruth] UCT Lung Inst, Cape Town, South Africa.

[Zhang, Ying; Shi, Wanliang] Johns Hopkins Univ, Bloomberg Sch Publ Hlth, Dept Mol Microbiol & Immunol, Baltimore, MD 21205 USA.

[Ali, Asho] King Abdulaziz Univ, Fac Microbiol, Jeddah, Saudi Arabia.

C3 Aga Khan University; University of London; London School of Hygiene &

Tropical Medicine; University of London; London School of Hygiene &

Tropical Medicine; University of Cape Town; University of Cape Town;

Johns Hopkins University; Johns Hopkins Bloomberg School of Public

Health; King Abdulaziz University

RP Hasan, Z (corresponding author), Aga Khan Univ Hosp, Dept Pathol & Lab Med, POB 3500, Karachi 74800, Pakistan.

EM zahra.hasan@aku.edu

RI Phelan, Jody/GVR-7908-2022; Hasan, Zahra/AAF-2191-2020; Clark,

Taane/AAM-2570-2020; Clark, Taane/GVR-7908-2022

OI Phelan, Jody/0000-0001-8323-7019; Hasan, Zahra/0000-0001-7580-372X;

Clark, Taane/0000-0001-8985-9265; Clark, Taane/0000-0001-8985-9265

FU MRC [MR/N010469/1] Funding Source: UKRI; Biotechnology and Biological

Sciences Research Council [1351999] Funding Source: researchfish;

Medical Research Council [MR/N010469/1] Funding Source: researchfish;

Medical Research Council [MR/N010469/1] Funding Source: Medline

CR Aínsa JA, 1998, J BACTERIOL, V180, P5836

Ali A, 2015, PLOS ONE, V10, DOI 10.1371/journal.pone.0117771

Ayaz A, 2012, INT J INFECT DIS, V16, pE303, DOI 10.1016/j.ijid.2011.12.015

Balganesh M, 2010, ANTIMICROB AGENTS CH, V54, P5167, DOI 10.1128/AAC.00610-10

Bina XWR, 2009, J ANTIMICROB CHEMOTH, V63, P103, DOI 10.1093/jac/dkn466

Braibant M, 2000, FEMS MICROBIOL REV, V24, P449, DOI 10.1016/S0168-6445(00)00034-6

Camacho LR, 2001, J BIOL CHEM, V276, P19845, DOI 10.1074/jbc.M100662200

Carver TJ, 2005, BIOINFORMATICS, V21, P3422, DOI 10.1093/bioinformatics/bti553

Cattoir V, 2004, PATHOL BIOL, V52, P607, DOI 10.1016/j.patbio.2004.09.001

Cole ST, 1998, NATURE, V393, P537, DOI 10.1038/31159

Coll F, 2014, NAT COMMUN, V5, DOI 10.1038/ncomms5812

Danilchanka O, 2008, ANTIMICROB AGENTS CH, V52, P2503, DOI 10.1128/AAC.00298-08

De Rossi E, 2006, FEMS MICROBIOL REV, V30, P36, DOI 10.1111/j.1574-6976.2005.00000.x

De Rossi E, 1998, J BACTERIOL, V180, P6068, DOI 10.1128/JB.180.22.6068-6071.1998

De Rossi Edda, 2002, Molecular Medicine (Baltimore), V8, P714

Dheda K, 2004, BIOTECHNIQUES, V37, P112, DOI 10.2144/04371RR03

Garima K, 2015, TUBERCULOSIS, V95, P155, DOI 10.1016/j.tube.2015.01.005

Guerra-Assunçao JA, 2015, ELIFE, V4, DOI 10.7554/eLife.05166

Hasan R, 2010, EMERG INFECT DIS, V16, P1473, DOI 10.3201/eid1609.100280

Honoré-Bouakline S, 2003, J CLIN MICROBIOL, V41, P2323, DOI 10.1128/JCM.41.6.2323-2329.2003

Jiang X, 2008, MICROB DRUG RESIST, V14, P7, DOI 10.1089/mdr.2008.0772

Kim SY, 2008, FEMS MICROBIOL LETT, V282, P282, DOI 10.1111/j.1574-6968.2008.01143.x

Li XZ, 2004, ANTIMICROB AGENTS CH, V48, P2415, DOI 10.1128/AAC.48.7.2415-2423.2004

Liu J, 1996, J BACTERIOL, V178, P3791, DOI 10.1128/jb.178.13.3791-3795.1996

Livak KJ, 2001, METHODS, V25, P402, DOI 10.1006/meth.2001.1262

Nomura K, 2004, AM J INFECT CONTROL, V32, P185, DOI 10.1016/j.ajic.2003.07.007

Okamoto S, 2007, MOL MICROBIOL, V63, P1096, DOI 10.1111/j.1365-2958.2006.05585.x

Pal R, 2014, INT J CURR MICROBIOL, V3, DOI DOI 10.1007/s15010-004-3097-x

Pasca MR, 2004, ANTIMICROB AGENTS CH, V48, P3175, DOI 10.1128/AAC.48.8.3175-3178.2004

Paulsen IT, 2001, J MOL MICROB BIOTECH, V3, P145

Phelan J, 2016, BMC MED, V14, DOI 10.1186/s12916-016-0575-9

Piddock LJV, 2000, J ANTIMICROB CHEMOTH, V45, P159, DOI 10.1093/jac/45.2.159

Poelarends GJ, 2000, DRUG RESIST UPDATE, V3, P330, DOI 10.1054/drup.2000.0173

Ramón-García S, 2009, ANTIMICROB AGENTS CH, V53, P3675, DOI 10.1128/AAC.00550-09

Sarathy J.P., PLOS ONE, V8, DOI DOI 10.1155/2015/951706

Siddiqi N, 2004, INFECTION, V32, P109, DOI 10.1007/s15010-004-3097-x

Takiff HE, 1996, P NATL ACAD SCI USA, V93, P362, DOI 10.1073/pnas.93.1.362

TAKIFF HE, 1994, ANTIMICROB AGENTS CH, V38, P773, DOI 10.1128/AAC.38.4.773

Tamang DG, 2009, J MEMBRANE BIOL, V229, P53, DOI 10.1007/s00232-009-9168-2

Taniguchi H, 1997, J BACTERIOL, V179, P4795, DOI 10.1128/jb.179.15.4795-4801.1997

Taniguchi H, 1996, FEMS MICROBIOL LETT, V144, P103, DOI 10.1111/j.1574-6968.1996.tb08515.x

Tanveer M, 2008, BMC INFECT DIS, V8, DOI 10.1186/1471-2334-8-171

Tseng Tsai-Tien, 1999, Journal of Molecular Microbiology and Biotechnology, V1, P107

Wang K, 2013, CURR MICROBIOL, V66, P222, DOI 10.1007/s00284-012-0215-3

Wood G.D., 2003, Simulating Mechanical Systems in Simulink with SimMechanics, P1

World Health Organization, 2012, UPD CRIT CONC 1 LIN

Xu Y., 2015, BioMed research international, P951706

Yamchi JK, 2015, INFECT GENET EVOL, V36, P23, DOI 10.1016/j.meegid.2015.08.036

NR 48

TC 31

Z9 35

U1 0

U2 11

PU CHURCHILL LIVINGSTONE

PI EDINBURGH

PA JOURNAL PRODUCTION DEPT, ROBERT STEVENSON HOUSE, 1-3 BAXTERS PLACE,

LEITH WALK, EDINBURGH EH1 3AF, MIDLOTHIAN, SCOTLAND

SN 1472-9792

J9 TUBERCULOSIS

JI Tuberculosis

PD DEC

PY 2017

VL 107

BP 20

EP 30

DI 10.1016/j.tube.2017.07.012

PG 11

WC Immunology; Microbiology; Respiratory System

WE Science Citation Index Expanded (SCI-EXPANDED)

SC Immunology; Microbiology; Respiratory System

GA FS4WC

UT WOS:000419793500004

OA Green Submitted

DA 2026-05-07

ER

PT J

AU Wang, ZM

Tang, ZH

Heidari, H

Molaeipour, L

Ghanavati, R

Kazemian, H

Koohsar, F

Kouhsari, E

AF Wang, Zheming

Tang, Zhihua

Heidari, Hamid

Molaeipour, Leila

Ghanavati, Roya

Kazemian, Hossein

Koohsar, Faramarz

Kouhsari, Ebrahim

TI Global status of phenotypic pyrazinamide resistance in Mycobacterium

tuberculosis clinical isolates: an updated systematic review and

meta-analysis

SO JOURNAL OF CHEMOTHERAPY

LA English

DT Review

DE Tuberculosis; MDR; pyrazinamide; resistance; systematic review and

meta-analysis

ID ADDITIONAL DRUG-RESISTANCE; LINE-PROBE ASSAY; TREATMENT OUTCOMES;

MDR-TB; PNCA MUTATIONS; KHYBER PAKHTUNKHWA; RISK-FACTORS; MULTIDRUG;

PREVALENCE; PATTERNS

AB Pyrazinamide (PZA) is an essential first-line tuberculosis drug for its unique mechanism of action active against multidrug-resistant-TB (MDR-TB). Thus, the aim of updated meta-analysis was to estimate the PZA weighted pooled resistance (WPR) rate in M. tuberculosis isolates based on publication date and WHO regions. We systematically searched the related reports in PubMed, Scopus, and Embase (from January 2015 to July 2022). Statistical analyses were performed using STATA software. The 115 final reports in the analysis investigated phenotypic PZA resistance data. The WPR of PZA was 57% (95% CI 48-65%) in MDR-TB cases. According to the WHO regions, the higher WPRs of PZA were reported in the Western Pacific (32%; 95% CI 18-46%), South East Asian region (37%; 95% CI 31-43%), and the Eastern Mediterranean (78%; 95% CI 54-95%) among any-TB patients, high risk of MDR-TB patients, and MDR-TB patients, respectively. A negligible increase in the rate of PZA resistance were showed in MDR-TB cases (55% to 58%). The rate of PZA resistance has been rising in recent years among MDR-TB cases, underlines the essential for both standard and novel drug regimens development.

C1 [Wang, Zheming; Tang, Zhihua] Shaoxing Peoples Hosp, Dept Pharm, Shaoxing, Peoples R China.

[Heidari, Hamid] Shahid Sadoughi Univ Med Sci, Fac Med, Dept Microbiol, Yazd, Iran.

[Molaeipour, Leila] Univ Med Sci, Sch Publ Hlth, Dept Epidemiol, Tehran, Iran.

[Ghanavati, Roya] Behbahan Fac Med Sci, Behbahan, Iran.

[Kazemian, Hossein] Ilam Univ Med Sci, Clin Microbiol Res Ctr, Ilam, Iran.

[Koohsar, Faramarz; Kouhsari, Ebrahim] Golestan Univ Med Sci, Lab Sci Res Ctr, Gorgan, Iran.

[Kouhsari, Ebrahim] Golestan Univ Med Sci, Fac Paramed, Dept Lab Sci, Gorgan, Iran.

[Kouhsari, Ebrahim] Golestan Univ Med Sci, Lab Sci Res Ctr, Gorgan Sari Rd,POB 4918936316, Gorgan, Golestan Provin, Iran.

[Tang, Zhihua] Shaoxing Peoples Hosp, Dept Pharm, Shaoxing 312000, Peoples R China.

C3 Shahid Sadoughi University of Medical Sciences; Tehran University of

Medical Sciences; Golestan University of Medical Sciences; Golestan

University of Medical Sciences; Golestan University of Medical Sciences

RP Kouhsari, E (corresponding author), Golestan Univ Med Sci, Lab Sci Res Ctr, Gorgan Sari Rd,POB 4918936316, Gorgan, Golestan Provin, Iran.; Tang, ZH (corresponding author), Shaoxing Peoples Hosp, Dept Pharm, Shaoxing 312000, Peoples R China.

EM sxtzh@163.com; ekouhsari1987@gmail.com

RI Molaeipour, Leila/M-1102-2018; Ghanavati, Roya/AAX-3223-2020; Kazemian,

Hossein/AAE-7329-2020; KOOHSAR, faramarz/T-2475-2019; Heidari,

Hamid/M-2302-2019; Kouhsari, Ebrahim/GRJ-1090-2022

OI Heidari, Hamid/0000-0002-6869-2301; Kouhsari,

Ebrahim/0000-0001-5893-6483

FU Golestan University of Medical Sciences [14-112317]

FX This study was supported in Golestan University of Medical Sciences (SN:

14-112317) for which we are very grateful.

CR Ahmad B, 2017, J PAK MED ASSOC, V67, P1684

Ahmad N, 2015, INT J TUBERC LUNG D, V19, P1109, DOI 10.5588/ijtld.15.0167

Ahmad Nafees, 2016, Braz J Infect Dis, V20, P41, DOI 10.1016/j.bjid.2015.09.011

Aia P, 2016, PLOS ONE, V11, DOI 10.1371/journal.pone.0149806

Akbal AU, 2020, INT J MYCOBACT, V9, P274, DOI 10.4103/ijmy.ijmy_42_20

Al-Hayani AM, 2021, CUREUS J MED SCIENCE, V13, DOI 10.7759/cureus.17069

Al-Mutairi NM, 2019, EUR J MED RES, V24, DOI 10.1186/s40001-019-0397-2

Alene KA, 2020, TROP MED INFECT DIS, V5, DOI 10.3390/tropicalmed5030123

Ali S, 2020, POL J MICROBIOL, V69, P133, DOI 10.33073/pjm-2020-005

Anastasakos V, 2017, INT J TUBERC LUNG D, V21, P309, DOI 10.5588/ijtld.16.0155

Andres S, 2019, ANTIMICROB AGENTS CH, V63, DOI 10.1128/AAC.01798-18

Atif M, 2017, BMC INFECT DIS, V17, DOI 10.1186/s12879-017-2746-5

Aung WW, 2018, ANTIMICROB AGENTS CH, V62, DOI 10.1128/AAC.01984-17

Bai RL, 2019, BIOSCIENCE REP, V39, DOI 10.1042/BSR20181746

Gutiérrez-Aroca JB, 2018, MICROB DRUG RESIST, V24, P839, DOI 10.1089/mdr.2017.0353

Bitar I, 2020, J GLOB ANTIMICROB RE, V22, P275, DOI 10.1016/j.jgar.2020.03.013

Bouzouita I, 2019, EMERG INFECT DIS, V25, P547, DOI 10.3201/eid2503.181370

Bwalya P, 2018, TUBERCULOSIS, V109, P117, DOI 10.1016/j.tube.2017.12.007

Calderón RI, 2017, INT J TUBERC LUNG D, V21, P894, DOI 10.5588/ijtld.16.0850

Cambau E, 2015, J ANTIMICROB CHEMOTH, V70, P686, DOI 10.1093/jac/dku438

Cegielski JP, 2016, CLIN INFECT DIS, V62, P418, DOI 10.1093/cid/civ910

Chaidir L, 2019, J GLOB ANTIMICROB RE, V16, P170, DOI 10.1016/j.jgar.2018.08.018

Che Y, 2021, BMC INFECT DIS, V21, DOI 10.1186/s12879-021-06306-1

Chen XC, 2019, FRONT MICROBIOL, V10, DOI 10.3389/fmicb.2019.01741

Chernyaeva E, 2021, ANTIBIOTICS-BASEL, V10, DOI 10.3390/antibiotics10010027

Chuang PH, 2016, PLOS ONE, V11, DOI 10.1371/journal.pone.0165222

Daum LT, 2019, DIAGN MICR INFEC DIS, V93, P334, DOI 10.1016/j.diagmicrobio.2018.10.018

den Hertog AL, 2015, PATHOG DIS, V73, DOI 10.1093/femspd/ftv037

DERSIMONIAN R, 1986, CONTROL CLIN TRIALS, V7, P177, DOI 10.1016/0197-2456(86)90046-2

Diriba G, 2020, PLOS ONE, V15, DOI 10.1371/journal.pone.0243493

du Cros P, 2021, ERJ OPEN RES, V7, DOI 10.1183/23120541.00537-2020

Dudley MZ, 2016, AM J TROP MED HYG, V95, P1239, DOI 10.4269/ajtmh.15-0711

Ei PW, 2020, TUBERCULOSIS, V125, DOI 10.1016/j.tube.2020.102013

Elhassan Mogahid M., 2017, Infectious Disorders - Drug Targets, V17, P14, DOI [10.2174/1871526517666161116104242, 10.2174/1871526517666161116104242]

Feliciano CS, 2016, BRAZ J INFECT DIS, V20, P290, DOI 10.1016/j.bjid.2016.01.004

Finci I, 2022, LANCET MICROBE, V3, pE672, DOI 10.1016/S2666-5247(22)00116-1

Madrazo-Moya CF, 2019, PLOS ONE, V14, DOI 10.1371/journal.pone.0213046

Fregona G, 2017, REV SAUDE PUBL, V51, DOI 10.1590/S1518-8787.2017051006688

Furuya-Kanamori L, 2018, INT J EVID-BASED HEA, V16, P195, DOI 10.1097/XEB.0000000000000141

Gallo JF, 2018, CLIN MICROBIOL INFEC, V24, P889, DOI 10.1016/j.cmi.2017.11.015

Gautam SS, 2018, PLOS ONE, V13, DOI 10.1371/journal.pone.0192351

Genestet C, 2020, INT J ANTIMICROB AG, V55, DOI 10.1016/j.ijantimicag.2020.105912

Gu YT, 2016, DIAGN MICR INFEC DIS, V84, P207, DOI 10.1016/j.diagmicrobio.2015.10.017

Habous M, 2020, INT J MYCOBACT, V9, P391, DOI 10.4103/ijmy.ijmy_170_20

Hai HT, 2019, TUBERCULOSIS, V119, DOI 10.1016/j.tube.2019.101864

Hameed HMA, 2020, INFECT DRUG RESIST, V13, P217, DOI 10.2147/IDR.S230774

He GQ, 2020, INT J INFECT DIS, V96, P68, DOI 10.1016/j.ijid.2020.04.043

He WC, 2022, MICROBIOL SPECTR, V10, DOI 10.1128/spectrum.01543-21

Hu Y, 2017, CLIN MICROBIOL INFEC, V23, P835, DOI 10.1016/j.cmi.2017.03.028

Hu Yi, 2016, Int J Mycobacteriol, V5 Suppl 1, pS34, DOI [10.1016/j.ijmyco.2016.11.007, 10.1016/j.ijmyco.2016.11.007]

Ignatyeva O, 2015, TUBERCULOSIS, V95, P581, DOI 10.1016/j.tube.2015.05.018

Iwamoto T, 2019, PLOS ONE, V14, DOI 10.1371/journal.pone.0212798

Jou RW, 2019, INFECT GENET EVOL, V72, P141, DOI 10.1016/j.meegid.2018.12.031

Juma SP, 2019, BMC INFECT DIS, V19, DOI 10.1186/s12879-019-3757-1

Kazemian H, 2021, ADV RESPIR MED, V89, P110, DOI 10.5603/ARM.a2021.0033

Khan MT, 2018, MICROB DRUG RESIST, V24, P1417, DOI 10.1089/mdr.2017.0234

Khan MT, 2019, BMC INFECT DIS, V19, DOI 10.1186/s12879-019-3764-2

Khan MT, 2018, COMPUT STRUCT BIOTEC, V16, P379, DOI 10.1016/j.csbj.2018.09.004

Khoshnood S, 2021, FRONT MICROBIOL, V12, DOI 10.3389/fmicb.2021.717045

Khoshnood S, 2021, J GLOB ANTIMICROB RE, V25, P48, DOI 10.1016/j.jgar.2021.02.017

Ko DH, 2019, ANN CLIN MICROB ANTI, V18, DOI 10.1186/s12941-018-0300-y

Koch A, 2018, CURR OPIN PHARMACOL, V42, P7, DOI 10.1016/j.coph.2018.05.013

Kuhlin J, 2021, CLIN INFECT DIS, V73, pE3511, DOI 10.1093/cid/ciaa1509

Kuo CY, 2018, J MICROBIOL IMMUNOL, V51, P88, DOI 10.1016/j.jmii.2017.05.008

Kurbatova EV, 2013, CLIN INFECT DIS, V57, P1081, DOI 10.1093/cid/cit452

Lange C, 2018, LANCET RESP MED, V6, P246, DOI 10.1016/S2213-2600(18)30104-8

Li DG, 2016, ANTIMICROB AGENTS CH, V60, P5159, DOI 10.1128/AAC.02687-15

Li K, 2021, FRONT MICROBIOL, V11, DOI 10.3389/fmicb.2020.594171

Lin S, 2021, INFECT DRUG RESIST, V14, P3979, DOI [10.2147/idr.s331516, 10.2147/IDR.S331516]

Liu DX, 2022, CLIN MICROBIOL INFEC, V28, DOI 10.1016/j.cmi.2021.09.014

Liu W, 2018, CLIN MICROBIOL INFEC, V24, DOI 10.1016/j.cmi.2017.12.012

MacNeil A, 2020, MMWR-MORBID MORTAL W, V69, P281, DOI 10.15585/mmwr.mm6911a2

Maningi NE, 2018, J CLIN MICROBIOL, V56, DOI [10.1128/JCM.01214-17, 10.1128/jcm.01214-17]

Maningi NE, 2015, J CLIN MICROBIOL, V53, P3779, DOI 10.1128/JCM.01179-15

Mansjö M, 2017, INT J MYCOBACT, V6, P156, DOI 10.4103/ijmy.ijmy_23_17

Maslov DA, 2015, TUBERCULOSIS, V95, P608, DOI 10.1016/j.tube.2015.05.013

Mehmood A, 2019, FRONT BIOENG BIOTECH, V7, DOI 10.3389/fbioe.2019.00404

Meinzen C, 2016, TUBERCULOSIS, V99, P41, DOI 10.1016/j.tube.2016.03.011

Mekonnen B, 2019, PLOS ONE, V14, DOI 10.1371/journal.pone.0215679

Modesti PA, 2016, PLOS ONE, V11, DOI 10.1371/journal.pone.0147601

Modlin SJ, 2021, ANTIMICROB AGENTS CH, V65, DOI 10.1128/AAC.01916-20

Moher D, 2009, BMJ-BRIT MED J, V339, DOI 10.1136/bmj.b2535

Mok S, 2021, ANTIMICROB AGENTS CH, V65, DOI 10.1128/AAC.02617-20

Moradi J, 2017, J CLIN DIAGN RES, V11, pDC9, DOI 10.7860/JCDR/2017/29314.10915

Naluyange R, 2020, PLOS ONE, V15, DOI 10.1371/journal.pone.0232543

Ng KKM, 2021, HONG KONG MED J, V27, P421, DOI 10.12809/hkmj208553

Njire M, 2016, ADV MED SCI-POLAND, V61, P63, DOI 10.1016/j.advms.2015.09.007

Pandey S, 2018, TUBERCULOSIS, V111, P109, DOI 10.1016/j.tube.2018.06.006

Pang Y, 2017, BMC INFECT DIS, V17, DOI 10.1186/s12879-017-2761-6

Pang Y, 2017, ANTIMICROB AGENTS CH, V61, DOI 10.1128/AAC.02170-16

Pang Y, 2015, J MICROBIOL METH, V119, P142, DOI 10.1016/j.mimet.2015.10.014

Park J, 2018, ANN LAB MED, V38, P316, DOI 10.3343/alm.2018.38.4.316

Park M, 2022, BMC INFECT DIS, V22, DOI 10.1186/s12879-022-07329-y

Park S, 2020, INT J TUBERC LUNG D, V24, P233, DOI 10.5588/ijtld.19.0314

Pecerska J, 2021, EPIDEMICS-NETH, V36, DOI 10.1016/j.epidem.2021.100471

Piersimoni C, 2016, INT J TUBERC LUNG D, V20, P1677, DOI 10.5588/ijtld.16.0360

Rahman A, 2017, ANTIMICROB AGENTS CH, V61, DOI 10.1128/AAC.00511-17

Ramirez-Busby SM, 2017, SCI REP-UK, V7, DOI 10.1038/s41598-017-03452-y

Ramirez-Busby SM, 2015, ANTIMICROB AGENTS CH, V59, P5267, DOI 10.1128/AAC.00204-15

Reena K, 2016, RES J PHARM BIOL CHE, V7, P1884

Reena K, 2016, J PURE APPL MICROBIO, V10, P2153

Rienthong S, 2015, INT J TUBERC LUNG D, V19, P817, DOI 10.5588/ijtld.14.0311

Sekiguchi JI, 2007, J CLIN MICROBIOL, V45, P2802, DOI 10.1128/JCM.00352-07

Sengstake S, 2017, BMC INFECT DIS, V17, DOI 10.1186/s12879-017-2594-3

Shea J, 2017, J CLIN MICROBIOL, V55, P1871, DOI 10.1128/JCM.00298-17

Sheen P, 2017, BMC GENOMICS, V18, DOI 10.1186/s12864-017-4146-z

Shi J, 2020, INFECT DRUG RESIST, V13, P2929, DOI 10.2147/IDR.S260161

Shi WL, 2021, ANTIBIOTICS-BASEL, V10, DOI 10.3390/antibiotics10080909

Sodja E, 2022, J GLOB ANTIMICROB RE, V29, P507, DOI 10.1016/j.jgar.2021.09.019

Soetaert K, 2022, ACTA CLIN BELG, V77, P853, DOI 10.1080/17843286.2021.1999588

Spinato J, 2016, BMC MICROBIOL, V16, DOI 10.1186/s12866-016-0786-4

Suthum K, 2020, J INFECT DEV COUNTR, V14, P268, DOI 10.3855/jidc.11974

Tagliani E, 2017, SCI REP-UK, V7, DOI 10.1038/s41598-017-17705-3

Tam KKG, 2019, J CLIN MICROBIOL, V57, DOI [10.1128/JCM.00145-19, 10.1128/jcm.00145-19]

Velásquez GE, 2016, ANTIMICROB AGENTS CH, V60, P6766, DOI 10.1128/AAC.00632-16

Verdugo D, 2015, ANTIMICROB AGENTS CH, V59, P6140, DOI 10.1128/AAC.00764-15

Wang GR, 2021, J INFECTION, V82, P371, DOI 10.1016/j.jinf.2021.02.004

Wang ZR, 2022, INFECT DRUG RESIST, V15, P1793, DOI 10.2147/IDR.S361635

Werngren J, 2017, J CLIN MICROBIOL, V55, P1920, DOI 10.1128/JCM.02532-16

Whitfield MG, 2022, J CLIN MICROBIOL, V60, DOI 10.1128/JCM.01907-21

Whitfield MG, 2020, SCI REP-UK, V10, DOI 10.1038/s41598-020-61286-7

Whitfield MG, 2016, TUBERCULOSIS, V99, P128, DOI 10.1016/j.tube.2016.05.003

Whitfield MG, 2015, PLOS ONE, V10, DOI 10.1371/journal.pone.0133869

WHO, US

Wollenberg KR, 2017, J CLIN MICROBIOL, V55, P457, DOI 10.1128/JCM.02116-16

World Health Organization, 2021, CAT MUT MYC TUB COMP

World Health Organization, 2021, WHO CONS GUID TUB MO

World Health Organization, 2021, TREATM DRUG SUSC TUB

Wu XY, 2019, INFECT GENET EVOL, V72, P147, DOI 10.1016/j.meegid.2018.11.012

Wu XY, 2015, J ANTIMICROB CHEMOTH, V70, P2295, DOI 10.1093/jac/dkv112

Xia H, 2020, BMC INFECT DIS, V20, DOI 10.1186/s12879-020-4758-9

Xia Q, 2015, ANTIMICROB AGENTS CH, V59, P1695, DOI 10.1128/AAC.04541-14

Ye M, 2021, ANN CLIN MICROB ANTI, V20, DOI 10.1186/s12941-021-00478-z

Zenteno-Cuevas R, 2020, MICROB DRUG RESIST, V26, P732, DOI 10.1089/mdr.2019.0054

Zheng XB, 2017, INT J ANTIMICROB AG, V49, P183, DOI 10.1016/j.ijantimicag.2016.10.012

Zhu JH, 2021, INT J INFECT DIS, V108, P81, DOI 10.1016/j.ijid.2021.04.027

Zignol M, 2013, DRUG RESIST UPDATE, V16, P108, DOI 10.1016/j.drup.2014.02.003

Zignol M, 2012, B WORLD HEALTH ORGAN, V90, P111, DOI 10.2471/BLT.11.092585

Zürcher K, 2019, LANCET INFECT DIS, V19, P298, DOI 10.1016/S1473-3099(18)30673-X

NR 139

TC 7

Z9 7

U1 1

U2 16

PU TAYLOR & FRANCIS LTD

PI ABINGDON

PA 2-4 PARK SQUARE, MILTON PARK, ABINGDON OR14 4RN, OXON, ENGLAND

SN 1120-009X

EI 1973-9478

J9 J CHEMOTHERAPY

JI J. Chemother.

PD OCT 3

PY 2023

VL 35

IS 7

BP 583

EP 595

DI 10.1080/1120009X.2023.2214473

EA MAY 2023

PG 13

WC Oncology; Infectious Diseases; Pathology; Pharmacology & Pharmacy

WE Science Citation Index Expanded (SCI-EXPANDED)

SC Oncology; Infectious Diseases; Pathology; Pharmacology & Pharmacy

GA X6QN2

UT WOS:000993418800001

DA 2026-05-07

ER

PT J

AU Manina, G

Pasca, MR

Buroni, S

De Rossi, E

Riccardi, G

AF Manina, G.

Pasca, M. R.

Buroni, S.

De Rossi, E.

Riccardi, G.

TI Decaprenylphosphoryl-β-D-Ribose 2′-Epimerase from Mycobacterium

tuberculosis is a Magic Drug Target

SO CURRENT MEDICINAL CHEMISTRY

LA English

DT Review

DE Mycobacterium tuberculosis; Rv3790 (DprE1); cellular target; cell wall;

new drugs

ID CELL-WALL; RESISTANT TUBERCULOSIS; CURRENT CHALLENGES; ATP SYNTHASE;

D-ARABINOSE; BIOSYNTHESIS; STRATEGIES; GENES; BENZOTHIAZINONES;

ANTIBIOTICS

AB Tuberculosis is still a leading cause of death in developing countries and a resurgent disease in developed countries. The selection and soaring spread of Mycobacterium tuberculosis multidrug-resistant (MDR-TB) and extensively drug-resistant strains (XDR-TB) is a severe public health problem. Currently, there is an urgent need of new drugs for tuberculosis treatment, with novel mechanisms of action and, moreover, the necessity to identify new drug targets.

Several enzymes involved in various metabolic processes have been described as potential targets for the development of new drugs.

Recently, two different classes of most promising drugs, the benzothiazinones (BTZ) and the dinitrobenzamide derivatives (DNB), have been found to be highly active against M. tuberculosis, including XDR-TB strains. Interestingly, both drugs have the same target: the heteromeric decaprenylphosphoryl-beta-D-ribose 2'-epimerase encoded by dprE1 (Rv3790) and dprE2 (Rv3791) genes, respectively. DprE1 and DprE2 are involved in the biosynthesis of D-arabinose and, in particular, they are essential to perform the transformation of decaprenylphosphoryl-D-ribose to decaprenylphosphoryl-D-arabinose, which is a substrate for arabinosyltransferases in the synthesis of the cell-envelope arabinogalactan and liporabinomannan polysaccharides of mycobacteria. Arabinogalactan is a fundamental component of the mycobacterial cell wall, which co-valently binds the outer layer of mycolic acids to peptidoglycan.

The heteromeric decaprenylphosphoryl-beta-D-ribose 2'-epimerase thus represents a valid vulnerable antimycobacterial drug target which could result "magic" for tuberculosis treatment.

C1 [Manina, G.; Pasca, M. R.; Buroni, S.; De Rossi, E.; Riccardi, G.] Univ Pavia, Dipartimento Genet & Microbiol, I-27100 Pavia, Italy.

[Manina, G.] Ecole Polytech Fed Lausanne, Global Hlth Inst, Stn 19, CH-1015 Lausanne, Switzerland.

C3 University of Pavia; Swiss Federal Institutes of Technology Domain;

Ecole Polytechnique Federale de Lausanne

RP Riccardi, G (corresponding author), Univ Pavia, Dipartimento Genet & Microbiol, Via Ferrata 1, I-27100 Pavia, Italy.

EM giovanna.riccardi@unipv.it

RI MANINA, GIULIA/AAI-5496-2020; Buroni, Silvia/AAX-5454-2020; PASCA, Maria

Rosalia/J-8752-2016; RICCARDI, Giovanna/A-9269-2012

OI MANINA, GIULIA/0000-0001-9905-6095; Buroni, Silvia/0000-0002-6979-2275;

PASCA, Maria Rosalia/0000-0002-8906-4937; RICCARDI,

Giovanna/0000-0003-3295-0070

FU EC [LSHP-CT-2005-018923]

FX Our studies were funded by EC-VI Framework Contract no.

LSHP-CT-2005-018923.

CR Alderwick LJ, 2007, BIOCHEM SOC T, V35, P1325, DOI 10.1042/BST0351325

Andries K, 2005, SCIENCE, V307, P223, DOI 10.1126/science.1106753

Aubry A, 2006, ANTIMICROB AGENTS CH, V50, P104, DOI 10.1128/AAC.50.1.104-112.2006

Ayers B, 2009, CARBOHYD RES, V344, P739, DOI 10.1016/j.carres.2009.02.006

Baker Edward N., 2007, Journal of Structural and Functional Genomics, V8, P57, DOI 10.1007/s10969-007-9020-9

Barry Clifton E., 2007, Infectious Disorders - Drug Targets, V7, P182

Blasi P, 2009, EXPERT OPIN DRUG DEL, V6, P977, DOI 10.1517/17425240903130577

Böttger EC, 2008, EUR J PEDIATR, V167, P141, DOI 10.1007/s00431-007-0606-9

Centrone CA, 2004, BIOORGAN MED CHEM, V12, P5495, DOI 10.1016/j.bmc.2004.08.014

Chan ED, 2008, CURR OPIN INFECT DIS, V21, P587, DOI 10.1097/QCO.0b013e328319bce6

Christophe T, 2009, PLOS PATHOG, V5, DOI 10.1371/journal.ppat.1000645

Cole ST, 1998, NATURE, V393, P537, DOI 10.1038/31159

Comas I, 2009, PLOS PATHOG, V5, DOI 10.1371/journal.ppat.1000600

Darwin KH, 2003, SCIENCE, V302, P1963, DOI 10.1126/science.1091176

Dhar N, 2007, CURR OPIN MICROBIOL, V10, P30, DOI 10.1016/j.mib.2006.12.007

Fischbach MA, 2009, SCIENCE, V325, P1089, DOI 10.1126/science.1176667

Ginsberg AM, 2008, SEMIN RESP CRIT CARE, V29, P552, DOI 10.1055/s-0028-1085706

Ginsburg AS, 2003, LANCET INFECT DIS, V3, P432, DOI 10.1016/S1473-3099(03)00671-6

Glickman MS, 2000, MOL CELL, V5, P717, DOI 10.1016/S1097-2765(00)80250-6

Haagsma AC, 2009, ANTIMICROB AGENTS CH, V53, P1290, DOI 10.1128/AAC.01393-08

Jain A, 2008, FEMS IMMUNOL MED MIC, V53, P145, DOI 10.1111/j.1574-695X.2008.00400.x

Kaur Devinder, 2009, Adv Appl Microbiol, V69, P23, DOI 10.1016/S0065-2164(09)69002-X

Lamichhane G, 2005, INFECT IMMUN, V73, P2533, DOI 10.1128/IAI.73.4.2533-2540.2005

Lenaerts AJ, 2008, TRENDS MICROBIOL, V16, P48, DOI 10.1016/j.tim.2007.12.002

Lin G, 2009, NATURE, V461, P621, DOI 10.1038/nature08357

Lin May Young, 2008, Endocrine Metabolic & Immune Disorders-Drug Targets, V8, P15, DOI 10.2174/187153008783928398

LoBue P, 2009, CURR OPIN INFECT DIS, V22, P167, DOI 10.1097/QCO.0b013e3283229fab

Lönnroth K, 2008, SEMIN RESP CRIT CARE, V29, P481, DOI 10.1055/s-0028-1085700

Loerger TR, 2009, CURR OPIN MICROBIOL, V12, P318, DOI 10.1016/j.mib.2009.04.006

Maartens G, 2007, LANCET, V370, P2030, DOI 10.1016/S0140-6736(07)61262-8

Makarov V, 2009, SCIENCE, V324, P801, DOI 10.1126/science.1171583

McKinney JD, 2000, NATURE, V406, P735, DOI 10.1038/35021074

Meniche X, 2008, MICROBIOL-SGM, V154, P2315, DOI 10.1099/mic.0.2008/016378-0

Mikusová K, 2005, J BACTERIOL, V187, P8020, DOI 10.1128/JB.187.23.8020-8025.2005

Moir DT, 1999, ANTIMICROB AGENTS CH, V43, P439, DOI 10.1128/AAC.43.3.439

Molle V, 2010, MOL MICROBIOL, V75, P1064, DOI 10.1111/j.1365-2958.2009.07041.x

Palomino JC, 2009, CURR MED CHEM, V16, P1898, DOI 10.2174/092986709788186066

Park HD, 2003, MOL MICROBIOL, V48, P833, DOI 10.1046/j.1365-2958.2003.03474.x

Pasca MR, 2010, ANTIMICROB AGENTS CH, V54, P1616, DOI 10.1128/AAC.01676-09

Pathak AK, 2009, BIOORGAN MED CHEM, V17, P872, DOI 10.1016/j.bmc.2008.11.027

Riccardi G, 2009, FUTURE MICROBIOL, V4, P597, DOI [10.2217/fmb.09.20, 10.2217/FMB.09.20]

Rosamond L, 2000, SCIENCE, V287, P1973, DOI 10.1126/science.287.5460.1973

Rustad TR, 2009, CELL MICROBIOL, V11, P1151, DOI 10.1111/j.1462-5822.2009.01325.x

Sacchettini JC, 2008, NAT REV MICROBIOL, V6, P41, DOI 10.1038/nrmicro1816

Sassetti CM, 2003, P NATL ACAD SCI USA, V100, P12989, DOI 10.1073/pnas.2134250100

Sassetti CM, 2003, MOL MICROBIOL, V48, P77, DOI 10.1046/j.1365-2958.2003.03425.x

Schreiber M, 2009, CURR OPIN CELL BIOL, V21, P325, DOI 10.1016/j.ceb.2009.01.026

Selzer PM, 2000, INT J MED MICROBIOL, V290, P191

Umesiri FE, 2010, MED RES REV, V30, P290, DOI 10.1002/med.20190

Vicente M, 2006, FEMS MICROBIOL REV, V30, P841, DOI 10.1111/j.1574-6976.2006.00038.x

Wehenkel A, 2008, BBA-PROTEINS PROTEOM, V1784, P193, DOI 10.1016/j.bbapap.2007.08.006

Wei JR, 2008, TUBERCULOSIS, V88, pS19, DOI 10.1016/S1472-9792(08)70033-7

Williams KJ, 2007, CURR MOL MED, V7, P297, DOI 10.2174/156652407780598575

Wolucka BA, 2008, FEBS J, V275, P2691, DOI 10.1111/j.1742-4658.2008.06395.x

Ying Z, 2005, ANNU REV PHARMACOL, V45, P529, DOI 10.1146/annurev.pharmtox.45.120403.100120

Zhang Y, 2006, DRUG DISCOV TODAY, V11, P21, DOI 10.1016/S1359-6446(05)03626-3

Zhang Y, 2009, INT J TUBERC LUNG D, V13, P1320

Zignol M, 2006, J INFECT DIS, V194, P479, DOI 10.1086/505877

NR 58

TC 75

Z9 81

U1 0

U2 27

PU BENTHAM SCIENCE PUBL LTD

PI SHARJAH

PA EXECUTIVE STE Y-2, PO BOX 7917, SAIF ZONE, 1200 BR SHARJAH, U ARAB

EMIRATES

SN 0929-8673

EI 1875-533X

J9 CURR MED CHEM

JI Curr. Med. Chem.

PD SEP

PY 2010

VL 17

IS 27

BP 3099

EP 3108

DI 10.2174/092986710791959693

PG 10

WC Biochemistry & Molecular Biology; Chemistry, Medicinal; Pharmacology &

Pharmacy

WE Science Citation Index Expanded (SCI-EXPANDED)

SC Biochemistry & Molecular Biology; Pharmacology & Pharmacy

GA 633SE

UT WOS:000280524500010

OA Green Submitted

DA 2026-05-07

ER

PT J

AU Patel, RV

Keum, YS

Park, SW

AF Patel, Rahul V.

Keum, Young-Soo

Park, Se Won

TI Nitroimidazoles, Quinolones and Oxazolidinones as Fluorine Bearing

Antitubercular Clinical Candidates

SO MINI-REVIEWS IN MEDICINAL CHEMISTRY

LA English

DT Review

DE Antitubercular drugs; delamanid; fluorine-containing drugs;

gatifloxacin; linezolid; moxifloxacin; pretomanid; sutezolid

ID DRUG-RESISTANT TUBERCULOSIS; EARLY BACTERICIDAL ACTIVITY;

MYCOBACTERIUM-TUBERCULOSIS; IN-VITRO; PULMONARY TUBERCULOSIS;

MULTIDRUG-RESISTANT; HEALTHY-SUBJECTS; MURINE MODEL; PHASE-II;

MOXIFLOXACIN

AB Tuberculosis is a leading killer of lives worldwide and the global curse of multi-drug resistant tuberculosis is attaining really dangerous levels. Synergistic interaction of HIV and TB is the twin epidemics in resource-limited countries as each potentiate progression of the other. The increasing emergence of MDR-TB and XDR-TB place an immense burden for the treatment of TB with currently available drugs. The situation urgently demands for the discovery of new drugs with novel mode of action and differs in structural features in order to overcome resistance appears in conventional TB therapeutics. The present report covers the discovery of three classes of antituberculosis drugs, Nitroimidazoles, Quinolones and Oxazolidinones, undergoing clinical development with fluorine atom in their structures. Highly electronegative fluorine atom plays a signature role in advancing medicinal innovations as it existence in the drug compounds critically influences metabolic stability and lipophilicity thereby delaying its elimination by the body which results into a long term in vivo efficiency of the drug. Presence of fluorine atom(s) in the drug structures described in this report, has been associated with the several fold increase in the overall potency of the compound as demonstrated since the early discoveries. 6 Fluorinated derivatives from these three classes as pretomanid, delamanid, moxifloxacin, gatifloxacin, linezolid and sutezolid have been discussed with their antituberculosis effects, mode of action, chemical synthetic routes and results of clinical studies.

C1 [Patel, Rahul V.] Inst Expt Bot ASCR, Ctr Reg Hana Biotechnol & Agr Res, Lab Growth Regulators, Olomouc 78371, Czech Republic.

[Patel, Rahul V.] Palacky Univ, Olomouc 78371, Czech Republic.

[Keum, Young-Soo; Park, Se Won] Konkuk Univ, Coll Life & Environm Sci, Dept Bioresources & Food Sci, Organ Res Lab, Seoul 143701, South Korea.

C3 Czech Academy of Sciences; Institute of Experimental Botany of the Czech

Academy of Sciences; Palacky University Olomouc; Konkuk University

RP Patel, RV (corresponding author), Inst Expt Bot ASCR, Ctr Reg Hana Biotechnol & Agr Res, Lab Growth Regulators, Slechtitelu 27, Olomouc 78371, Czech Republic.

EM rahul.svnit11@gmail.com

RI Nile, Shivraj/HCH-4220-2022

OI Nile, Shivraj/0000-0003-3141-5754

FU Czech Ministry of Education grant from the National Program for

Sustainability I [LO1204]

FX This work was financed by Czech Ministry of Education grant from the

National Program for Sustainability I (LO1204).

CR Abubakar I, 2013, LANCET INFECT DIS, V6, P529

Andersson MI, 2003, J ANTIMICROB CHEMOTH, V51, P1, DOI 10.1093/jac/dkg212

[Anonymous], 2011, GUID PROGR MAN DRUG

[Anonymous], LIN TREAT EXT DRUG R

[Anonymous], GLOB TUB REP 2013

ASHTEKAR DR, 1993, ANTIMICROB AGENTS CH, V37, P183, DOI 10.1128/AAC.37.2.183

Baker W. R. S. C., 2000, U.S. Patent, Patent No. 6087358

Barbachyn MR, 2003, ANGEW CHEM INT EDIT, V42, P2010, DOI 10.1002/anie.200200528

Barbachyn MR, 1996, J MED CHEM, V39, P680, DOI 10.1021/jm950956y

Clercq D.E., 2012, ACTA PHARM SINICA B, V12, P535

ClinicalTrials.gov, TBTC STUD 28 MOX VER

ClinicalTrials.gov, SAF PHARM PK MULT RE

ClinicalTrials.gov, PLAC CONTR PHAS 2 TR

ClinicalTrials.gov, PHARM SAF MOX MFX468

ClinicalTrials.gov, SAF EFF PHARM DEL PA

Conde MB, 2009, LANCET, V373, P1183, DOI 10.1016/S0140-6736(09)60333-0

Conte JE Jr, 2002, ANTIMICROB AGENTS CH, V46, P1475, DOI 10.1128/AAC.46.5.1475-1480.2002

Cynamon MH, 1999, ANTIMICROB AGENTS CH, V43, P1189, DOI 10.1128/AAC.43.5.1189

Diacon AH, 2012, LANCET, V380, P986, DOI 10.1016/S0140-6736(12)61080-0

Diacon AH, 2010, ANTIMICROB AGENTS CH, V54, P3402, DOI 10.1128/AAC.01354-09

Dietze R, 2008, AM J RESP CRIT CARE, V178, P1180, DOI 10.1164/rccm.200806-892OC

Donnelly JP, 2004, CLIN MICROBIOL INFEC, V10, P107, DOI 10.1111/j.1470-9465.2004.00838.x

DUSCHINSKY R, 1957, J AM CHEM SOC, V79, P4559, DOI 10.1021/ja01573a087

Falzon D, 2011, EUR RESPIR J, V38, P516, DOI 10.1183/09031936.00073611

Filler R, 2009, FUTURE MED CHEM, V1, P777, DOI [10.4155/fmc.09.65, 10.4155/FMC.09.65]

Fortún J, 2005, J ANTIMICROB CHEMOTH, V56, P180, DOI 10.1093/jac/dki148

Gillespie SH, 1999, J ANTIMICROB CHEMOTH, V44, P393, DOI 10.1093/jac/44.3.393

Ginsberg AM, 2009, ANTIMICROB AGENTS CH, V53, P3720, DOI 10.1128/AAC.00106-09

Ginsberg AM, 2009, ANTIMICROB AGENTS CH, V53, P3726, DOI 10.1128/AAC.00112-09

HEIDELBERGER C, 1957, NATURE, V179, P663, DOI 10.1038/179663a0

Hooper DC, 1999, DRUGS, V58, P6, DOI 10.2165/00003495-199958002-00002

Howard M.R., 1995, 35 INT C ANT AG CHEM, P153

Jawahar MS, 2013, PLOS ONE, V8, DOI 10.1371/journal.pone.0067030

Koh WJ, 2012, J ANTIMICROB CHEMOTH, V67, P1503, DOI 10.1093/jac/dks078

Lee M, 2012, NEW ENGL J MED, V367, P1508, DOI 10.1056/NEJMoa1201964

Mandrioli R, 2008, CURR DRUG METAB, V9, P827, DOI 10.2174/138920008786049258

Marsini MA, 2010, J ORG CHEM, V75, P7479, DOI 10.1021/jo1015807

Masuzawa K., 1990, 8-Alkoxyquinolonecarboxylic acid and saltsthereof, Patent No. [US 04980470, 04980470, US 4,980,470 A, 4980470]

Matsumoto M, 2006, PLOS MED, V3, P2131, DOI 10.1371/journal.pmed.0030466

Merle CSC, 2012, TRIALS, V13, DOI 10.1186/1745-6215-13-61

Mukherjee T, 2011, FUTURE MED CHEM, V3, P1427, DOI [10.4155/FMC.11.90, 10.4155/fmc.11.90]

National Institutes of Health (U.S.), STUD PNU 100480 NEWL

Otsuka, 2004, Patent, Patent No. [WO 2004/035547, 2004035547]

Paramasivan CN, 2005, ANTIMICROB AGENTS CH, V49, P627, DOI 10.1128/AAC.49.2.627-631.2005

Park IN, 2006, J ANTIMICROB CHEMOTH, V58, P701, DOI 10.1093/jac/dkl298

Pawlowski A, 2012, PLOS PATHOG, V8, DOI 10.1371/journal.ppat.1002464

Raman J. V., 2012, European Patent, Patent No. [EP 2516408 A1, 2516408, 2516408 A1]

Rao DR, 2012, United States patent US, Patent No. 8198451

Richardson K., 1990, REV INFECT DIS, P267

Robert W, 2013, COMMUNICATION	0405

Rodríguez JC, 2002, INT J ANTIMICROB AG, V20, P464, DOI 10.1016/S0924-8579(02)00239-X

Rustomjee R, 2008, INT J TUBERC LUNG D, V12, P128

Samuelson J, 1999, ANTIMICROB AGENTS CH, V43, P1533, DOI 10.1128/AAC.43.7.1533

Sasaki H, 2006, J MED CHEM, V49, P7854, DOI 10.1021/jm060957y

Savarino A, 2003, LANCET INFECT DIS, V3, P722, DOI 10.1016/S1473-3099(03)00806-5

Schlagenhauf P, 2010, MALARIA J, V9, DOI 10.1186/1475-2875-9-357

Shandil RK, 2007, ANTIMICROB AGENTS CH, V51, P576, DOI 10.1128/AAC.00414-06

Shaw KJ, 2011, ANN NY ACAD SCI, V1241, P48, DOI 10.1111/j.1749-6632.2011.06330.x

Smythe W, 2013, ANTIMICROB AGENTS CH, V57, P4164, DOI 10.1128/AAC.00479-13

Stover CK, 2000, NATURE, V405, P962, DOI 10.1038/35016103

Takiff H, 2011, ANTIMICROB AGENTS CH, V55, P5421, DOI 10.1128/AAC.00695-11

TOMIOKA H, 1993, ANTIMICROB AGENTS CH, V37, P1259, DOI 10.1128/AAC.37.6.1259

Wallis R.S., 2012, THLBB02 INT AIDS C

Wallis RS, 2010, J INFECT DIS, V202, P745, DOI 10.1086/655471

Wang J, 2014, CHEM REV, V114, P2432, DOI 10.1021/cr4002879

Wang MG, 2004, ANTIMICROB AGENTS CH, V48, P1400, DOI 10.1128/AAC.48.4.1400-1401.2004

Williams KN, 2009, ANTIMICROB AGENTS CH, V53, P1314, DOI 10.1128/AAC.01182-08

Yasui Mitsuru, 2005, Nihon Shinkei Seishin Yakurigaku Zasshi, V25, P143

Zumla A, 2013, NAT REV DRUG DISCOV, V12, P388, DOI 10.1038/nrd4001

Zumla A, 2013, NEW ENGL J MED, V368, P745, DOI 10.1056/NEJMra1200894

Zumla AI, 2014, LANCET INFECT DIS, V14, P327, DOI 10.1016/S1473-3099(13)70328-1

NR 71

TC 11

Z9 12

U1 1

U2 29

PU BENTHAM SCIENCE PUBL LTD

PI SHARJAH

PA EXECUTIVE STE Y-2, PO BOX 7917, SAIF ZONE, 1200 BR SHARJAH, U ARAB

EMIRATES

SN 1389-5575

EI 1875-5607

J9 MINI-REV MED CHEM

JI Mini-Rev. Med. Chem.

PY 2015

VL 15

IS 14

BP 1174

EP 1186

DI 10.2174/1389557515666150709121153

PG 13

WC Chemistry, Medicinal

WE Science Citation Index Expanded (SCI-EXPANDED)

SC Pharmacology & Pharmacy

GA CT0WX

UT WOS:000362519700006

OA Green Submitted

DA 2026-05-07

ER

PT J

AU Yu, JJ

Ren, WC

Yuan, JF

Liu, RM

Ma, LP

Tang, SJ

Pang, Y

AF Yu, Jiajia

Ren, Weicong

Yuan, Jinfeng

Liu, Rongmei

Ma, Liping

Tang, Shenjie

Pang, Yu

TI Identification of urine biomarkers predictive of prolonged QTc interval

in multidrug-resistant tuberculosis patients treated with bedaquiline

SO FRONTIERS IN PHARMACOLOGY

LA English

DT Article

DE metabolomics; tuberculosis; bedaquiline; urine; biomarkers

ID PHOSPHOLIPASE A(2)

AB The most frequent adverse event associated with bedaquiline (BDQ) is the QTc interval prolongation; however, there was no biomarkers that could be used to predict the occurrence of QTc prolongation in BDQ-treated patients. In this study, we employed the ultra-high performance liquid chromatography-MS/MS (UHPLC-MS/MS) to generate metabolic profiling for the discovery of potential predictive urine biomarkers of QTc prolongation in these patients. Untargeted metabolomic technique was used to concentrate the differential metabolic pathway, and targeted metabolomic technique was subsequently performed to identify predictive biomarkers for QTc prolongation. A total of 45 rifampicin-resistant TB (RR-TB) and multidrug-resistant TB (MDR-TB) patients were enrolled in our study, including 15 RR/MDR-TB patients with QTc interval prolongation (QIP) and 30 RR/MDR-TB patients with QTc interval un-prolongations (QIU). Untargeted technique revealed that the lipid metabolism was the most differential metabolic pathway between two groups. Further targeted technique identified four differential metabolites, including betaine, LPE (18:2), LPE (20:3), and LPE (20:4). The combined analysis of metabolisms revealed that the combined use of LPE (20:3) and LPE (20:4) had the best performance for predicting the occurrence of QTc prolongation in TB patients, yielding a sensitivity of 87.4% and a specificity of 78.5%. In addition, with the progression of BDQ treatment, the LPEs exhibited persistent difference in the BDQ-treated TB patients experiencing QTc interval prolongation. In conclusion, our data demonstrate that the combined use of LPE (20:3) and LPE (20:4) yields promising performance for predicting the occurrence of QTc interval prolongation in BDQ-treated patients.

C1 [Yu, Jiajia; Ren, Weicong; Yuan, Jinfeng; Pang, Yu] Capital Med Univ, Beijing Chest Hosp, Beijing TB & Thorac Tumor Res Inst, Dept Bacteriol & Immunol, Beijing, Peoples R China.

[Liu, Rongmei; Ma, Liping] Capital Med Univ, Beijing Chest Hosp, Beijing TB & Thorac Tumor Res Inst, Dept TB, Beijing, Peoples R China.

[Tang, Shenjie] Capital Med Univ, Clin Ctr TB, Beijing Chest Hosp, Beijing TB & Thorac Tumor Res Inst, Beijing, Peoples R China.

C3 Capital Medical University; Capital Medical University; Capital Medical

University

RP Pang, Y (corresponding author), Capital Med Univ, Beijing Chest Hosp, Beijing TB & Thorac Tumor Res Inst, Dept Bacteriol & Immunol, Beijing, Peoples R China.; Tang, SJ (corresponding author), Capital Med Univ, Clin Ctr TB, Beijing Chest Hosp, Beijing TB & Thorac Tumor Res Inst, Beijing, Peoples R China.

EM pangyupound@163.com; tangsj1106@hotmail.com

RI Mark, Vinder/JHS-7837-2023; 袁, 金锋/LZE-8341-2025

FU 'Beijing Key Clinical Specialty Project [20201214]; Beijing Hospitals

Authority Ascent Plan [DFL20191601]; Beijing Hospitals Authority

Clinical Medicine Development of Special Funding [ZYLX202122]; Beijing

Nova Program [20220484169, 20230484295]

FX The author(s) declare financial support was received for the research,

authorship, and/or publication of this article. This work was supported

by 'Beijing Key Clinical Specialty Project (20201214)', and Beijing

Hospitals Authority Ascent Plan (DFL20191601); Beijing Hospitals

Authority Clinical Medicine Development of Special Funding (ZYLX202122)

and by Sponsored by Beijing Nova Program (20220484169&20230484295)r The

author(s) declare financial support was received for the research,

authorship, and/or publication of this article. This work was supported

by 'Beijing Key Clinical Specialty Project (20201214)', and Beijing

Hospitals Authority Ascent Plan (DFL20191601); Beijing Hospitals

Authority Clinical Medicine Development of Special Funding (ZYLX202122)

and by Sponsored by Beijing Nova Program (20220484169&20230484295).

CR [Anonymous], 2019, WHO Consolidated Guidelines on Drug-Resistant Tuberculosis Treatment

[Anonymous], 2013, The use of Bedaquiline in the treatment of m ultidrug-resistant tuberculosis. Interim policy guidance

[Anonymous], 2018, The Global Tuberculosis Report

Aragón IM, 2018, EUR UROL FOCUS, V4, P128, DOI 10.1016/j.euf.2016.11.001

Arking DE, 2014, NAT GENET, V46, P826, DOI 10.1038/ng.3014

Bi XJ, 2022, CELL REP, V38, DOI 10.1016/j.celrep.2021.110271

Chorell E, 2021, METABOLITES, V11, DOI 10.3390/metabo11010025

Diacon AH, 2014, NEW ENGL J MED, V371, P723, DOI 10.1056/NEJMoa1313865

Dookie N, 2022, CLIN MICROBIOL REV, V35, DOI 10.1128/cmr.00180-19

Dooley KE, 2021, LANCET INFECT DIS, V21, P975, DOI 10.1016/S1473-3099(20)30770-2

Duan L, 2001, J IMMUNOL, V166, P7469, DOI 10.4049/jimmunol.166.12.7469

Duflot T, 2021, METABOLITES, V11, DOI 10.3390/metabo11110784

Food and Drug Administration, 2012, Application number 204384Orig1-000

Gao M, 2021, CLIN MICROBIOL INFEC, V27, P597, DOI 10.1016/j.cmi.2020.06.004

Harpole M, 2016, EXPERT REV PROTEOMIC, V13, P609, DOI 10.1080/14789450.2016.1190651

Hurley BP, 2008, INFECT IMMUN, V76, P2259, DOI 10.1128/IAI.00059-08

Janssen Therapeutics, 2012, SIRTURO (bedaquiline) label

Kowalewski NN, 2021, JOVE-J VIS EXP, DOI 10.3791/62453

Lee JM, 2017, BIOMOL THER, V25, P194, DOI 10.4062/biomolther.2016.046

Lyu XL, 2022, CAN J INFECT DIS MED, V2022, DOI 10.1155/2022/2703635

Meikle PJ, 2017, NAT REV ENDOCRINOL, V13, P79, DOI 10.1038/nrendo.2016.169

Nyang'wa BT, 2024, LANCET RESP MED, V12, P117, DOI 10.1016/S2213-2600(23)00389-2

Piotrovsky V, 2005, AAPS J, V7, pE609, DOI 10.1208/aapsj070363

Pontali E, 2017, EUR RESPIR J, V50, DOI 10.1183/13993003.01462-2017

Tan ST, 2020, PROG LIPID RES, V80, DOI 10.1016/j.plipres.2020.101068

NR 25

TC 0

Z9 0

U1 1

U2 2

PU FRONTIERS MEDIA SA

PI LAUSANNE

PA AVENUE DU TRIBUNAL FEDERAL 34, LAUSANNE, CH-1015, SWITZERLAND

EI 1663-9812

J9 FRONT PHARMACOL

JI Front. Pharmacol.

PD MAY 30

PY 2024

VL 15

AR 1362544

DI 10.3389/fphar.2024.1362544

PG 10

WC Pharmacology & Pharmacy

WE Science Citation Index Expanded (SCI-EXPANDED)

SC Pharmacology & Pharmacy

GA TY5C6

UT WOS:001244822500001

OA Green Submitted, gold

DA 2026-05-07

ER

PT J

AU Jha, AK

Faheem, I

Nagaraja, V

AF Jha, Abodh Kumar

Faheem, Iqball

Nagaraja, Valakunja

TI Succinylation of GyrB Is a Mechanism Conferring Fluoroquinolone

Resistance

SO ACS INFECTIOUS DISEASES

LA English

DT Article

DE <italic>Mycobacterium tuberculosis</italic>; DNA gyrase;

post-translational modification; fluoroquinoloneresistance; gyrase

succinylation

ID MYCOBACTERIUM-TUBERCULOSIS STRAINS; IN-VIVO ACTIVITIES; DNA GYRASE;

ESCHERICHIA-COLI; MULTIDRUG-RESISTANT; ACETYLATION; SMEGMATIS;

MOXIFLOXACIN; OVEREXPRESSION; INHIBITORS

AB Being a persistent and deadly infection, tuberculosis (TB) caused by Mycobacterium tuberculosis remains a global health challenge. Despite having a well-established 4-drug combination therapy for drug-sensitive TB, the emergence of drug-resistant TB has rendered the treatment less effective. Although fluoroquinolones (FQs) are among the prominent drugs in the second-line treatment for multidrug-resistant tuberculosis (MDR-TB), FQ resistance has readily emerged in cases of extensively drug-resistant tuberculosis (XDR-TB). Other than the mutations in DNA gyrase, a universally conserved bacterial enzyme targeted by FQs, several mechanisms contribute to the emergence of FQ resistance. Recently, post-translational modification of DNA gyrase is implicated as one of the mechanisms for FQ resistance. Here, we describe succinylation of GyrB by a promiscuous acetyltransferase, Eis of M. tuberculosis, as a new mechanism contributing to FQ resistance in mycobacteria. Lysine succinylation of GyrB results in a reduced interaction of FQs with the enzyme, thereby decreasing the DNA cleavage by DNA gyrase in the presence of FQs. Accordingly, Eis overexpressing mycobacterial strains exhibit increased minimum inhibitory concentration (MIC) to FQs. Thus, succinylation of DNA gyrase is yet another resistance mechanism against the FQs.

C1 [Jha, Abodh Kumar; Faheem, Iqball; Nagaraja, Valakunja] Indian Inst Sci IISc, Dept Microbiol & Cell Biol, Bangalore 560012, India.

[Nagaraja, Valakunja] Indian Inst Sci Educ & Res IISER, Bhopal 462066, India.

C3 Indian Institute of Science (IISC) - Bangalore; Indian Institute of

Science Education & Research (IISER) - Bhopal

RP Nagaraja, V (corresponding author), Indian Inst Sci IISc, Dept Microbiol & Cell Biol, Bangalore 560012, India.; Nagaraja, V (corresponding author), Indian Inst Sci Educ & Res IISER, Bhopal 462066, India.

EM vraj@iisc.ac.in

RI Faheem, Iqball/OMN-2497-2025

OI Jha, Abodh Kumar/0000-0003-4829-4901

FU Indian Institute of Science [NA]; Department of Biotechnology, Ministry

of Science and Technology, India [BT/ PR41348/MED/29/1536/2020]

FX V.N. laboratory members are acknowledged for their valuable suggestions

and critical inputs. A.K.J. acknowledges the Indian Institute of

Science, Bangalore, India, for financial assistance through Senior

Research Fellowship (SRF).

CR Aldred KJ, 2016, P NATL ACAD SCI USA, V113, pE839, DOI 10.1073/pnas.1525055113

Alvirez-Freites EJ, 2002, ANTIMICROB AGENTS CH, V46, P1022, DOI 10.1128/AAC.46.4.1022-1025.2002

An DD, 2009, ANTIMICROB AGENTS CH, V53, P4835, DOI 10.1128/AAC.00541-09

Anand C, 2021, MICROBIOL-SGM, V167, DOI 10.1099/mic.0.001058

Arora G, 2021, FEBS J, V288, P3375, DOI 10.1111/febs.15582

Aubry A, 2004, ANTIMICROB AGENTS CH, V48, P1281, DOI 10.1128/AAC.48.4.1281-1288.2004

Birhanu AG, 2019, SCI REP-UK, V9, DOI 10.1038/s41598-019-39654-9

Birhanu AG, 2017, J PROTEOME RES, V16, P4045, DOI 10.1021/acs.jproteome.7b00429

Biswas P, 2023, RES MICROBIOL, V174, DOI 10.1016/j.resmic.2023.104093

Bloom B. R., 2017, MAJOR INFECT DIS, V6, P233

Blower TR, 2016, P NATL ACAD SCI USA, V113, P1706, DOI 10.1073/pnas.1525047113

Caminero JA, 2010, LANCET INFECT DIS, V10, P621, DOI 10.1016/S1473-3099(10)70139-0

Chatterji M, 2001, J ANTIMICROB CHEMOTH, V48, P479, DOI 10.1093/jac/48.4.479

Chatterji M, 2003, ARCH MICROBIOL, V180, P339, DOI 10.1007/s00203-003-0598-4

Chatterji M, 2002, EMBO REP, V3, P261, DOI 10.1093/embo-reports/kvf038

Cole ST, 1998, NATURE, V393, P537, DOI 10.1038/31159

Cui ZL, 2011, BMC INFECT DIS, V11, DOI 10.1186/1471-2334-11-78

Datta D, 2024, FEBS J, V291, P4433, DOI 10.1111/febs.17142

Dong YZ, 1998, ANTIMICROB AGENTS CH, V42, P2978, DOI 10.1128/AAC.42.11.2978

Drlica K, 2003, J ANTIMICROB CHEMOTH, V52, P11, DOI 10.1093/jac/dkg269

Drlica K, 2003, CURR TOP MED CHEM, V3, P249, DOI 10.2174/1568026033452537

Drlica K, 2020, BACTERIAL RESISTANCE TO ANTIBIOTICS - FROM MOLECULES TO MAN, P125

Faheem I, 2025, ACS INFECT DIS, V11, P366, DOI 10.1021/acsinfecdis.4c00880

Faheem I, 2023, MOL MICROBIOL, V119, P728, DOI 10.1111/mmi.15068

Feng LP, 2021, P NATL ACAD SCI USA, V118, DOI 10.1073/pnas.2016705118

Feuerriegel S, 2009, ANTIMICROB AGENTS CH, V53, P3353, DOI 10.1128/AAC.00050-09

GELLERT M, 1976, P NATL ACAD SCI USA, V73, P3872, DOI 10.1073/pnas.73.11.3872

GELLERT M, 1977, P NATL ACAD SCI USA, V74, P4772, DOI 10.1073/pnas.74.11.4772

Ghosh S, 2016, MOL MICROBIOL, V100, P577, DOI 10.1111/mmi.13339

Ginsburg AS, 2003, LANCET INFECT DIS, V3, P432, DOI 10.1016/S1473-3099(03)00671-6

Gupta R, 2006, BIOCHEM BIOPH RES CO, V343, P1141, DOI 10.1016/j.bbrc.2006.02.195

Houghton JL, 2013, J ANTIMICROB CHEMOTH, V68, P800, DOI 10.1093/jac/dks497

ITO T, 1992, ANTIMICROB AGENTS CH, V36, P1708, DOI 10.1128/AAC.36.8.1708

Jassal M, 2009, LANCET INFECT DIS, V9, P19, DOI 10.1016/S1473-3099(08)70260-3

Ji BH, 1998, ANTIMICROB AGENTS CH, V42, P2066, DOI 10.1128/AAC.42.8.2066

Kang YA, 2016, ANN AM THORAC SOC, V13, P364, DOI 10.1513/AnnalsATS.201510-690BC

Kouzarides T, 2000, EMBO J, V19, P1176, DOI 10.1093/emboj/19.6.1176

Kumar R, 2014, ANTIMICROB AGENTS CH, V58, P2013, DOI 10.1128/AAC.01958-13

Lelovic N, 2020, J ANTIBIOT, V73, P780, DOI 10.1038/s41429-020-0320-7

Nguyen L, 2016, ARCH TOXICOL, V90, P1585, DOI 10.1007/s00204-016-1727-6

Long Y, 2024, MICROBIOL SPECTR, V12, DOI 10.1128/spectrum.02510-23

Madhusudan K, 1995, MICROBIOL-SGM, V141, P3029, DOI 10.1099/13500872-141-12-3029

Malik M, 2005, ANTIMICROB AGENTS CH, V49, P2008, DOI 10.1128/AAC.49.5.2008-2014.2005

Malik M, 2006, ANTIMICROB AGENTS CH, V50, P2842, DOI 10.1128/AAC.00250-06

Manjunatha U. H., 2006, Journal of the Indian Institute of Science, V86, P751

Manjunatha UH, 2002, NUCLEIC ACIDS RES, V30, P2144, DOI 10.1093/nar/30.10.2144

Manjunatha UH, 2001, FEMS MICROBIOL LETT, V194, P87

Maruri F, 2012, J ANTIMICROB CHEMOTH, V67, P819, DOI 10.1093/jac/dkr566

Mayer C, 2014, MICROBIOL SPECTR, V2, DOI 10.1128/microbiolspec.MGM2-0009-2013

Mazzariol A, 2000, ANTIMICROB AGENTS CH, V44, P3441, DOI 10.1128/AAC.44.12.3441-3443.2000

Millán-Zambrano G, 2022, NAT REV GENET, V23, P563, DOI 10.1038/s41576-022-00468-7

Mohammed H, 2016, NAT PROTOC, V11, P316, DOI 10.1038/nprot.2016.020

Mokrousov I, 2008, ANTIMICROB AGENTS CH, V52, P2937, DOI 10.1128/AAC.00036-08

Mustaev A, 2014, J BIOL CHEM, V289, P12300, DOI 10.1074/jbc.M113.529164

Nagaraja V, 2022, EPIGENOMICS-UK, V14, P69, DOI 10.2217/epi-2021-0357

Palomino JC, 2002, ANTIMICROB AGENTS CH, V46, P2720, DOI 10.1128/AAC.46.8.2720-2722.2002

Pantel A, 2012, ANTIMICROB AGENTS CH, V56, P1990, DOI 10.1128/AAC.06272-11

Patel DJ, 2013, ANNU REV BIOCHEM, V82, P81, DOI 10.1146/annurev-biochem-072711-165700

Prisic S, 2010, P NATL ACAD SCI USA, V107, P7521, DOI 10.1073/pnas.0913482107

RevelViravau V, 1996, ANTIMICROB AGENTS CH, V40, P2054, DOI 10.1128/AAC.40.9.2054

Robicsek A, 2006, NAT MED, V12, P83, DOI 10.1038/nm1347

Sakatos A, 2018, SCI ADV, V4, DOI 10.1126/sciadv.aao1478

Sengupta S, 2008, NUCLEIC ACIDS RES, V36, P4310, DOI 10.1093/nar/gkn355

Sengupta S, 2008, FEMS MICROBIOL LETT, V279, P40, DOI 10.1111/j.1574-6968.2007.01005.x

Sengupta S, 2006, NUCLEIC ACIDS RES, V34, P5567, DOI 10.1093/nar/gkl704

Shen LL, 2000, METH MOL B, V95, P171

Singh PR, 2025, TRENDS MICROBIOL, V33, P354, DOI 10.1016/j.tim.2024.10.007

Takiff HE, 1996, P NATL ACAD SCI USA, V93, P362, DOI 10.1073/pnas.93.1.362

Tran JH, 2005, ANTIMICROB AGENTS CH, V49, P118, DOI 10.1128/AAC.49.1.118-125.2005

Vetting MW, 2008, BIOCHEMISTRY-US, V47, P9825, DOI 10.1021/bi800664x

Vetting MW, 2006, BIOCHEMISTRY-US, V45, P1, DOI 10.1021/bi052130w

Vetting MW, 2005, ARCH BIOCHEM BIOPHYS, V433, P212, DOI 10.1016/j.abb.2004.09.003

Walsh CT, 2005, ANGEW CHEM INT EDIT, V44, P7342, DOI 10.1002/anie.200501023

Wang JC, 1998, Q REV BIOPHYS, V31, P107, DOI 10.1017/S0033583598003424

Wei J, 2000, J BACTERIOL, V182, P377, DOI 10.1128/JB.182.2.377-384.2000

Xie LX, 2019, BIOSCIENCE REP, V39, DOI 10.1042/BSR20191661

Xie LX, 2015, J PROTEOME RES, V14, P107, DOI 10.1021/pr500859a

Zaunbrecher MA, 2009, P NATL ACAD SCI USA, V106, P20004, DOI 10.1073/pnas.0907925106

Zhou ML, 2017, J BIOMOL STRUCT DYN, V35, P1030, DOI 10.1080/07391102.2016.1169219

NR 79

TC 0

Z9 0

U1 1

U2 1

PU AMER CHEMICAL SOC

PI WASHINGTON

PA 1155 16TH ST, NW, WASHINGTON, DC 20036 USA

SN 2373-8227

J9 ACS INFECT DIS

JI ACS Infect. Dis.

PD DEC 12

PY 2025

VL 11

IS 12

BP 3607

EP 3619

DI 10.1021/acsinfecdis.5c00781

EA NOV 2025

PG 13

WC Chemistry, Medicinal; Infectious Diseases

WE Science Citation Index Expanded (SCI-EXPANDED)

SC Pharmacology & Pharmacy; Infectious Diseases

GA T9564

UT WOS:001616542500001

DA 2026-05-07

ER

PT J

AU Kovalishyn, V

Grouleff, J

Semenyuta, I

Sinenko, VO

Slivchuk, SR

Hodyna, D

Brovarets, V

Blagodatny, V

Poda, G

Tetko, IV

Metelytsia, L

AF Kovalishyn, Vasyl

Grouleff, Julie

Semenyuta, Ivan

Sinenko, Vitaliy O.

Slivchuk, Sergiy R.

Hodyna, Diana

Brovarets, Volodymyr

Blagodatny, Volodymyr

Poda, Gennady

Tetko, Igor V.

Metelytsia, Larysa

TI Rational design of isonicotinic acid hydrazide derivatives with

antitubercular activity: Machine learning, molecular docking, synthesis

and biological testing

SO CHEMICAL BIOLOGY & DRUG DESIGN

LA English

DT Article

DE antitubercular activity; isonicotinic acid hydrazide derivatives;

machine learning; molecular docking; Mycobacterium tuberculosis (Mtb);

OCHEM

ID APPLICABILITY DOMAIN; IN-VITRO; DISCOVERY; MODELS

AB The problem of designing new antitubercular drugs against multiple drug-resistant tuberculosis (MDR-TB) was addressed using advanced machine learning methods. As there are only few published measurements against MDR-TB, we collected a large literature data set and developed models against the non-resistant H37Rv strain. The predictive accuracy of these models had a coefficient of determination q(2)=.7-.8 (regression models) and balanced accuracies of about 80% (classification models) with cross-validation and independent test sets. The models were applied to screen a virtual chemical library, which was designed to have MDR-TB activity. The seven most promising compounds were identified, synthesized and tested. All of them showed activity against the H37Rv strain, and three molecules demonstrated activity against the MDR-TB strain. The docking analysis indicated that the discovered molecules could bind enoyl reductase, InhA, which is required in mycobacterial cell wall development. The models are freely available online () and can be used to predict potential anti-TB activity of new chemicals.

C1 [Kovalishyn, Vasyl; Semenyuta, Ivan; Sinenko, Vitaliy O.; Slivchuk, Sergiy R.; Hodyna, Diana; Brovarets, Volodymyr; Metelytsia, Larysa] Natl Acad Sci Ukraine, Inst Bioorgan Chem & Petrochem, Kiev, Ukraine.

[Grouleff, Julie; Poda, Gennady] Ontario Inst Canc Res, Drug Discovery Program, MaRS Ctr, Toronto, ON, Canada.

[Blagodatny, Volodymyr] PL Shupyk Natl Med Acad Postgrad Educ, Kiev, Ukraine.

[Poda, Gennady] Univ Toronto, Leslie Dan Fac Pharm, Toronto, ON, Canada.

[Tetko, Igor V.] German Res Ctr Environm Hlth GmbH, Helmholtz Zentrum Munchen, Neuherberg, Germany.

[Tetko, Igor V.] BIGCHEM GmbH, Neuherberg, Germany.

C3 National Academy of Sciences Ukraine; V. P. Kukhar Institute of

Bioorganic Chemistry & Petrochemistry of the National Academy of

Sciences of Ukraine; University of Toronto; Ontario Institute for Cancer

Research; Shupyk National Healthcare University of Ukraine; University

of Toronto; Helmholtz Association; Helmholtz-Center Munich - German

Research Center for Environmental Health

RP Tetko, IV (corresponding author), German Res Ctr Environm Hlth GmbH, Helmholtz Zentrum Munchen, Neuherberg, Germany.

EM itetko@vcclab.org

RI ; Kovalishyn, Vasyl/I-6823-2018; Tetko, Igor/B-1540-2010; Brovarets,

Volodymyr/AAG-7344-2020; Hodyna, Diana/AAZ-6131-2021; Grouleff,

Julie/D-5054-2011

OI , Serhii Slyvchuk/0009-0004-6936-4870; Semenyuta,

Ivan/0000-0001-8464-3692; Kovalishyn, Vasyl/0000-0002-9352-7332; Tetko,

Igor/0000-0002-6855-0012; Hodyna, Diana/0000-0001-6161-9833; Grouleff,

Julie Jungberg/0009-0000-4815-5368

FU Ontario Institute for Cancer Research; Government of Ontario

FX GP and JG would like to acknowledge the support of the Ontario Institute

for Cancer Research and its funding from the Government of Ontario. We

also thank ChemAxon (http://www.chemaxon.com) for academic license of

their software used in this study, and David Smil and Michael Withnall

for critical reading of the manuscript.

CR Ban FQ, 2017, J CHEM INF MODEL, V57, P1018, DOI 10.1021/acs.jcim.7b00137

Breiman L, 2001, MACH LEARN, V45, P5, DOI 10.1023/A:1010933404324

CANETTI G, 1963, B WORLD HEALTH ORGAN, V29, P565

CHEN T, 2016, ARXIV PREPRINT ARXIV, DOI DOI 10.1145/2939672.2939785

Fernandes GFD, 2017, PHARMACEUTICALS-BASE, V10, DOI 10.3390/ph10020051

Frank E, 2004, BIOINFORMATICS, V20, P2479, DOI 10.1093/bioinformatics/bth261

Gasteiger J, 2006, J MED CHEM, V49, P6429, DOI 10.1021/jm0608964

Islam MM, 2017, J GENET GENOMICS, V44, P21, DOI 10.1016/j.jgg.2016.10.002

KIER LB, 1990, PHARM RES-DORDR, V7, P801, DOI 10.1023/A:1015952613760

Kovalishyn Vasyl, 2017, Curr Drug Discov Technol, V14, P25, DOI [10.2174/1570163813666161108125227, 10.2174/1570163813666161108125227]

Kumar D, 2014, EUR J MED CHEM, V81, P301, DOI 10.1016/j.ejmech.2014.05.005

Mahajan Rajiv, 2013, Int J Appl Basic Med Res, V3, P1, DOI 10.4103/2229-516X.112228

Makam P, 2013, EUR J MED CHEM, V69, P564, DOI 10.1016/j.ejmech.2013.08.054

Martínez-Hoyos M, 2016, EBIOMEDICINE, V8, P291, DOI 10.1016/j.ebiom.2016.05.006

Martins F, 2014, EUR J MED CHEM, V81, P119, DOI 10.1016/j.ejmech.2014.04.077

Panda M, 2014, J MED CHEM, V57, P4761, DOI 10.1021/jm5002937

Shaikh MS, 2014, RSC ADV, V4, P62308, DOI 10.1039/c4ra11752b

Sinenko VO, 2016, RUSS J GEN CHEM+, V86, P1597, DOI 10.1134/S1070363216070100

Spagnuolo LA, 2017, J AM CHEM SOC, V139, P3417, DOI 10.1021/jacs.6b11148

Sushko I, 2012, J CHEM INF MODEL, V52, P2310, DOI 10.1021/ci300245q

Sushko I, 2011, J COMPUT AID MOL DES, V25, P533, DOI 10.1007/s10822-011-9440-2

Sushko I, 2010, J CHEMOMETR, V24, P202, DOI 10.1002/cem.1296

Tetko IV, 2008, J CHEM INF MODEL, V48, P1733, DOI 10.1021/ci800151m

Tetko Igor V., 2008, V458, P185

Tetko IV, 2002, J CHEM INF COMP SCI, V42, P1136, DOI 10.1021/ci025515j

Thomas KD, 2011, EUR J MED CHEM, V46, P5283, DOI 10.1016/j.ejmech.2011.07.033

Tiberi S, 2017, PRESSE MED, V46, pE41, DOI 10.1016/j.lpm.2017.01.016

Verbitskiy EV, 2016, BIOORGAN MED CHEM, V24, P3771, DOI 10.1016/j.bmc.2016.06.020

Villa AEP, 1999, J NEUROSCI METH, V86, P161, DOI 10.1016/S0165-0270(98)00164-2

Whitley DC, 2000, J CHEM INF COMP SCI, V40, P1160, DOI 10.1021/ci000384c

NR 30

TC 11

Z9 14

U1 4

U2 24

PU WILEY

PI HOBOKEN

PA 111 RIVER ST, HOBOKEN 07030-5774, NJ USA

SN 1747-0277

EI 1747-0285

J9 CHEM BIOL DRUG DES

JI Chem. Biol. Drug Des.

PD JUL

PY 2018

VL 92

IS 1

BP 1272

EP 1278

DI 10.1111/cbdd.13188

PG 7

WC Biochemistry & Molecular Biology; Chemistry, Medicinal

WE Science Citation Index Expanded (SCI-EXPANDED); Index Chemicus (IC)

SC Biochemistry & Molecular Biology; Pharmacology & Pharmacy

GA GK7QN

UT WOS:000436403500010

OA Green Submitted

DA 2026-05-07

ER

PT J

AU Wei, WJ

Yan, HM

Zhao, J

Li, HC

Li, ZY

Guo, HX

Wang, XZ

Zhou, Y

Zhang, XL

Zeng, JC

Chen, T

Zhou, L

AF Wei, Wenjing

Yan, Huimin

Zhao, Jiao

Li, Haicheng

Li, Zhenyan

Guo, Huixin

Wang, Xuezhi

Zhou, Ying

Zhang, Xiaoli

Zeng, Jincheng

Chen, Tao

Zhou, Lin

TI Multi-omics comparisons of p-aminosalicylic acid (PAS) resistance

in folC mutated and un-mutated Mycobacterium tuberculosis

strains

SO EMERGING MICROBES & INFECTIONS

LA English

DT Article

DE P-Aminosalicylic acid; multi-omics; Mycobacterium tuberculosis; folC

ID FOLATE METABOLISM; EFFLUX PUMP; SUSCEPTIBILITY; TRANSPORTERS; SUBSTRATE;

SYNTHASE; PATHWAY; GENES; CHINA; LIGHT

AB p-Aminosalicylic acid (PAS) is an important second-line antibiotic for treating multidrug-resistant tuberculosis (MDR-TB). Due to gastrointestinal disturbance and intolerance, its potent and efficacy in the treatment of extensively drug-resistant (XDR)-TB commonly are poor. Thus, it is important to reveal the mechanism of susceptibility and resistance of Mycobacterium tuberculosis (Mtb) to this drug. Herein, we screened and established PAS-resistant (PAS(r)) folC mutated and un-mutated Mtb strains, then utilized a multi-omics (genome, proteome, and metabolome) analysis to better characterize the mechanisms of PAS resistance in Mtb. Interestingly, we found that promotion of SAM-dependent methyltransferases and suppression of PAS uptake via inhibiting some drug transport associated membrane proteins were two key pathways for the folC mutated strain evolving into the PAS(r) Mtb strain. However, the folC un-mutated strain was resistant to PAS via uptake of exogenous methionine, mitigating the role of inhibitors, and promoting DfrA, ThyA and FolC expression. Beyond these findings, we also found PAS resistance in Mtb might be associated with the increasing phenylalanine metabolism pathway. Collectively, our findings uncovered the differences of resistant mechanism between folC mutated and un-mutated Mtb strains resistant to PAS using multi-omics analysis and targeting modulators to these pathways may be effective for treatment of PAS(r) Mtb strains.

C1 [Wei, Wenjing; Li, Haicheng; Li, Zhenyan; Guo, Huixin; Wang, Xuezhi; Chen, Tao; Zhou, Lin] Ctr TB Control Guangdong Prov, Guangzhou 510630, Guangdong, Peoples R China.

[Wei, Wenjing; Li, Haicheng; Li, Zhenyan; Guo, Huixin; Wang, Xuezhi; Chen, Tao; Zhou, Lin] Key Lab Translat Med Guangdong, Guangzhou, Guangdong, Peoples R China.

[Yan, Huimin; Zeng, Jincheng] Guangdong Med Univ, Dongguan Key Lab Med Bioact Mol Dev & Translat Re, Guangdong Prov Key Lab Med Mol Diagnost, Dongguan 523808, Guangdong, Peoples R China.

[Zhao, Jiao] Jinan Univ, Guangzhou, Guangdong, Peoples R China.

[Zhou, Ying; Zhang, Xiaoli] Foshan Univ, Sch Stomatol & Med, Foshan, Peoples R China.

[Chen, Tao] South China Inst Biomed, Guangzhou, Guangdong, Peoples R China.

C3 Guangdong Medical University; Jinan University; Foshan University

RP Chen, T; Zhou, L (corresponding author), Ctr TB Control Guangdong Prov, Guangzhou 510630, Guangdong, Peoples R China.; Zeng, JC (corresponding author), Guangdong Med Univ, Dongguan Key Lab Med Bioact Mol Dev & Translat Re, Guangdong Prov Key Lab Med Mol Diagnost, Dongguan 523808, Guangdong, Peoples R China.

EM zengjc@gdmu.edu.cn; chentaoibp@163.com; zhoulingjk@163.com

RI ; Zeng, Jincheng/N-3833-2018

OI wang, xuezhi/0000-0003-4746-5561;

FU National Science and Technology Key Projects [2017ZX10201302,

2018ZX10715004-002]; National Natural Science Foundation of China

[31800129, 81500007]; Science and Technology Planning Project of

Guangzhou City [201604020006]

FX This work was supported by National Science and Technology Key Projects

[Grant Number 2017ZX10201302, 2018ZX10715004-002]; the National Natural

Science Foundation of China: [Grant Number 31800129, 81500007]; Science

and Technology Planning Project of Guangzhou City [Grant Number

201604020006].

CR Agarwal N, 2006, NUCLEIC ACIDS RES, V34, P4245, DOI 10.1093/nar/gkl521

Akhtar P, 2009, J MED MICROBIOL, V58, P798, DOI 10.1099/jmm.0.002550-0

[Anonymous], 2017, Global tuberculosis report 2017

Chakraborty S, 2013, SCIENCE, V339, P88, DOI 10.1126/science.1228980

Dawadi S, 2017, ORG LETT, V19, P5220, DOI 10.1021/acs.orglett.7b02487

Desai U, 2018, LUNG INDIA, V35, P488, DOI 10.4103/lungindia.lungindia_141_18

Deshayes C, 2005, J BACTERIOL, V187, P7283, DOI 10.1128/JB.187.21.7283-7291.2005

FEREBEE SH, 1966, ANN NY ACAD SCI, V135, P910, DOI 10.1111/j.1749-6632.1966.tb45533.x

Giacomini KM, 2010, NAT REV DRUG DISCOV, V9, P215, DOI 10.1038/nrd3028

Griffin JE, 2011, PLOS PATHOG, V7, DOI 10.1371/journal.ppat.1002251

Hilbig M, 2015, J CHEM INF MODEL, V55, P2071, DOI 10.1021/acs.jcim.5b00292

Ho RH, 2005, CLIN PHARMACOL THER, V78, P260, DOI 10.1016/j.clpt.2005.05.011

Hoagland DT, 2016, ADV DRUG DELIVER REV, V102, P55, DOI 10.1016/j.addr.2016.04.026

Horai H, 2010, J MASS SPECTROM, V45, P703, DOI 10.1002/jms.1777

Howe MD, 2018, FRONT CELL INFECT MI, V8, DOI 10.3389/fcimb.2018.00399

KIM SJ, 1992, TUBERCLE LUNG DIS, V73, P219, DOI 10.1016/0962-8479(92)90090-7

Larsen Michelle H, 2007, Curr Protoc Microbiol, VChapter 10, DOI 10.1002/9780471729259.mc10a02s6

Lau SKP, 2015, EMERG MICROBES INFEC, V4, DOI 10.1038/emi.2015.6

LEHMANN J, 1946, LANCET, V250, P15

Li RQ, 2009, BIOINFORMATICS, V25, P1966, DOI 10.1093/bioinformatics/btp336

Loots DT, 2016, J ANTIMICROB CHEMOTH, V71, P655, DOI 10.1093/jac/dkv406

Mengatto L, 2006, MEM I OSWALDO CRUZ, V101, P535, DOI 10.1590/S0074-02762006000500009

Minato Y, 2015, ANTIMICROB AGENTS CH, V59, P5097, DOI 10.1128/AAC.00647-15

Nixon MR, 2014, CHEM BIOL, V21, P819, DOI 10.1016/j.chembiol.2014.04.009

Parish T., 2001, Mycobacterium tuberculosis protocols. Methods in Molecular Medicine

Patel BA, 2013, BIOORG MED CHEM LETT, V23, P5523, DOI 10.1016/j.bmcl.2013.08.059

Ramón-García S, 2012, ANTIMICROB AGENTS CH, V56, P2074, DOI 10.1128/AAC.05946-11

Rengarajan J, 2004, MOL MICROBIOL, V53, P275, DOI 10.1111/j.1365-2958.2004.04120.x

Sassetti CM, 2003, MOL MICROBIOL, V48, P77, DOI 10.1046/j.1365-2958.2003.03425.x

Somerville W, 2005, J CLIN MICROBIOL, V43, P2996, DOI 10.1128/JCM.43.6.2996-2997.2005

Sondén B, 2005, MOL MICROBIOL, V58, P426, DOI 10.1111/j.1365-2958.2005.04847.x

Srivastava R, 2017, DATA BRIEF, V13, P320, DOI 10.1016/j.dib.2017.05.049

Tanaka KJ, 2018, BBA-BIOMEMBRANES, V1860, P868, DOI 10.1016/j.bbamem.2017.08.011

Taylor SL, 2017, BIOINFORMATICS, V33, P17, DOI 10.1093/bioinformatics/btw578

Tsugawa H, 2015, NAT METHODS, V12, P523, DOI 10.1038/nmeth.3393

Veiga T, 2012, EUKARYOT CELL, V11, P238, DOI 10.1128/EC.05285-11

Wang K, 2013, CURR MICROBIOL, V66, P222, DOI 10.1007/s00284-012-0215-3

Wang XD, 2011, MOL MICROBIOL, V82, P1375, DOI 10.1111/j.1365-2958.2011.07892.x

Yang SS, 2017, ANTIMICROB AGENTS CH, V61, DOI [10.1128/AAC.00551-17, 10.1128/aac.00551-17]

YE QZ, 1990, P NATL ACAD SCI USA, V87, P9391, DOI 10.1073/pnas.87.23.9391

Zhang HT, 2013, NAT GENET, V45, P1255, DOI 10.1038/ng.2735

Zhang L, 2013, ANTIMICROB AGENTS CH, V57, P3659, DOI 10.1128/AAC.00670-13

Zhang XB, 2015, ANTIMICROB AGENTS CH, V59, P1320, DOI 10.1128/AAC.03695-14

Zhao F, 2014, ANTIMICROB AGENTS CH, V58, P1479, DOI 10.1128/AAC.01775-13

Zheng J, 2013, J BIOL CHEM, V288, P23447, DOI 10.1074/jbc.M113.475798

NR 45

TC 14

Z9 19

U1 0

U2 24

PU TAYLOR & FRANCIS LTD

PI ABINGDON

PA 2-4 PARK SQUARE, MILTON PARK, ABINGDON OR14 4RN, OXON, ENGLAND

SN 2222-1751

J9 EMERG MICROBES INFEC

JI Emerg. Microbes Infect.

PD FEB 11

PY 2019

VL 8

IS 1

BP 248

EP 261

DI 10.1080/22221751.2019.1568179

PG 14

WC Immunology; Infectious Diseases; Microbiology

WE Science Citation Index Expanded (SCI-EXPANDED)

SC Immunology; Infectious Diseases; Microbiology

GA HK8QQ

UT WOS:000458255100002

OA Green Submitted, gold

DA 2026-05-07

ER

PT J

AU Lee, JJ

Kang, HY

Lee, W

Cho, SY

Kim, YJ

Lee, HJ

AF Lee, J. J.

Kang, H. Y.

Lee, W-, I

Cho, S. Y.

Kim, Y. J.

Lee, H. J.

TI Efflux pump gene expression study using RNA-seq in multidrug-resistant

TB

SO INTERNATIONAL JOURNAL OF TUBERCULOSIS AND LUNG DISEASE

LA English

DT Article

DE RNA seq; efflux pump; kanamycin-resistant

ID MYCOBACTERIUM-TUBERCULOSIS; DRUG-RESISTANCE; ANTIBIOTIC-RESISTANCE;

RIFAMPICIN

AB BACKGROUND: The mechanism underlying kanamycin (KM) resistance in Mycobacterium tuberculosis is not well understood, although efflux pump proteins are thought to play a role. This study used RNA-seq data to investigate changes in the expression levels of efflux pump genes following exposure to KM. M ETH O D S : RNA expression of efflux pump and regu-latory genes following exposure to different concentra-tions of KM (minimum inhibitory concentration [MIC] 25 and MIC50) in rrs wild-type strain and rrs A1401G mutated strain were compared with the control group. RES U LT S : The selected strains had differential RNA expression patterns. Among the 71 putative efflux pump and regulatory genes, 46 had significant fold changes, and 12 genes (Rv0842, Rv1146, Rv1258c, Rv1473, Rv1686c, Rv1687c, Rv1877, Rv2038c, Rv3065, Rv3197a, Rv3728 and Rv3789) that were overex-pressed following exposure to KM were thought to contribute to drug resistance. Rv3197A (whiB7) showed a distinct fold change based on the concentration of KM. CON C L U S I O N : The significant changes in the expres-sion of the efflux pump and regulatory genes following exposure to KM may provide insights into the identifi-cation of a new resistance mechanism.

C1 [Lee, J. J.] Kyung Hee Univ, Grad Sch, Dept Lab Med, Seoul, South Korea.

[Lee, J. J.; Kang, H. Y.; Cho, S. Y.; Kim, Y. J.] Kyung Hee Univ, Kyung Hee Univ Hosp, Dept Lab Med, Sch Med, Seoul, South Korea.

[Lee, W-, I] Kyung Hee Univ, Kyung Hee Univ Hosp Gangdong, Dept Lab Med, Sch Med, Seoul, South Korea.

[Lee, H. J.] Korean Natl TB Assoc, Baumoe Ro 6 Gil 57, Seoul 06763, South Korea.

C3 Kyung Hee University; Kyung Hee University; Kyung Hee University

Hospital; Kyung Hee University; Kyung Hee University Hospital

RP Lee, HJ (corresponding author), Korean Natl TB Assoc, Baumoe Ro 6 Gil 57, Seoul 06763, South Korea.; Kim, YJ (corresponding author), Kyung Hee Univ, Sch Med, Dept Lab Med, 23 Kyungheedae Ro, Seoul 02447, South Korea.

EM khmclab@gmail.com; leehejo@gmail.com

RI Cho, Seung-Yeon/AAK-7954-2020

FU National Research Foundation (NRF) - Korean Government (Ministry of

Science and Information and Communication Technology)

[NRF-2014R1A1A2004931, 2019R1A2B5B01001909]

FX This work was supported by a National Research Foundation (NRF; Daejeon,

South Korea) of Korea grant funded by the Korean Government (Ministry of

Science and Information and Communication Technology)

(NRF-2014R1A1A2004931, 2019R1A2B5B01001909).

CR Aínsa JA, 1998, J BACTERIOL, V180, P5836

Alcalde-Rico M, 2016, FRONT MICROBIOL, V7, DOI 10.3389/fmicb.2016.01483

da Silva PEA, 2011, FEMS IMMUNOL MED MIC, V63, P1, DOI 10.1111/j.1574-695X.2011.00831.x

[Anonymous], 2019, WHO Consolidated Guidelines on Drug-Resistant Tuberculosis Treatment

Bai YY, 2016, PLOS ONE, V11, DOI 10.1371/journal.pone.0150321

Brossier F, 2016, J CLIN MICROBIOL, V54, P1573, DOI 10.1128/JCM.00051-16

De Rossi Edda, 2002, Molecular Medicine (Baltimore), V8, P714

Ghajavand H, 2019, ANTIMICROB RESIST IN, V8, DOI 10.1186/s13756-019-0516-4

Gupta AK, 2010, MICROB DRUG RESIST, V16, P21, DOI 10.1089/mdr.2009.0054

Halloum I, 2017, ANTIMICROB AGENTS CH, V61, DOI [10.1128/AAC.02509-16, 10.1128/aac.02509-16]

Hrdlickova R, 2017, WIRES RNA, V8, DOI 10.1002/wrna.1364

James HJ, 2015, SUSCEPTIBILITY TEST, V11th, P1356

Li GL, 2015, J ANTIBIOT, V68, P431, DOI 10.1038/ja.2015.9

Li XZ, 2004, ANTIMICROB AGENTS CH, V48, P2415, DOI 10.1128/AAC.48.7.2415-2423.2004

Liang YP, 2017, TUBERCULOSIS, V107, P1, DOI 10.1016/j.tube.2017.07.010

Lin G., 2011, Susceptibility Testing of Mycobacteria, Nocardiae, and Other Aerobic Actinomycetes

Lyu J, 2013, INT J TUBERC LUNG D, V17, P120, DOI 10.5588/ijtld.12.0197

Martin JA, 2011, NAT REV GENET, V12, P671, DOI 10.1038/nrg3068

Morris RP, 2005, P NATL ACAD SCI USA, V102, P12200, DOI 10.1073/pnas.0505446102

Nikaido H, 2001, SEMIN CELL DEV BIOL, V12, P215, DOI 10.1006/scdb.2000.0247

Oh TS, 2017, INFECT GENET EVOL, V49, P111, DOI 10.1016/j.meegid.2017.01.002

Pang Y, 2016, SCI REP-UK, V6, DOI 10.1038/srep25330

Pasca MR, 2005, ANTIMICROB AGENTS CH, V49, P4775, DOI 10.1128/AAC.49.11.4775-4777.2005

Rodrigues L, 2013, ANTIMICROB AGENTS CH, V57, P751, DOI 10.1128/AAC.01482-12

Rodrigues L, 2012, INFECT GENET EVOL, V12, P695, DOI 10.1016/j.meegid.2011.08.009

Schmalstieg AM, 2012, ANTIMICROB AGENTS CH, V56, P4806, DOI 10.1128/AAC.05546-11

Sgaragli G, 2016, CURR MED CHEM, V23, P1186, DOI 10.2174/0929867323666160405112820

Singh K, 2014, BIOORG MED CHEM LETT, V24, P2985, DOI 10.1016/j.bmcl.2014.05.022

Song LL, 2016, INT J ANTIMICROB AG, V47, P421, DOI 10.1016/j.ijantimicag.2016.04.007

Sowajassatakul A, 2018, ANN CLIN MICROB ANTI, V17, DOI 10.1186/s12941-018-0285-6

Szumowski JD, 2014, CURR TOP MICROBIOL, V374, P81, DOI 10.1007/82_2012_300

Vadwai V, 2011, J CLIN MICROBIOL, V49, P2540, DOI 10.1128/JCM.02319-10

World Health Organization, 2012, WHOHTMTB2008392

World Health Organization (WHO), 2018, Technical manual for drug susceptibility testing of medicines used in the treatment of tuberculosis, DOI DOI 10.2471/BLT.11.096644

NR 34

TC 8

Z9 9

U1 0

U2 4

PU INT UNION AGAINST TUBERCULOSIS LUNG DISEASE (I U A T L D)

PI PARIS

PA 68 BOULEVARD SAINT-MICHEL,, 75006 PARIS, FRANCE

SN 1027-3719

EI 1815-7920

J9 INT J TUBERC LUNG D

JI Int. J. Tuberc. Lung Dis.

PD DEC 1

PY 2021

VL 25

IS 12

BP 974

EP +

DI 10.5588/ijtld.21.0117

PG 9

WC Infectious Diseases; Respiratory System

WE Science Citation Index Expanded (SCI-EXPANDED)

SC Infectious Diseases; Respiratory System

GA YJ0HR

UT WOS:000744220900003

DA 2026-05-07

ER

PT J

AU Pais, JP

Policarpo, M

Pires, D

Francisco, AP

Madureira, AM

Testa, B

Anes, E

Constantino, L

AF Pais, Joao Pedro

Policarpo, Margarida

Pires, David

Francisco, Ana Paula

Madureira, Ana Margarida

Testa, Bernard

Anes, Elsa

Constantino, Luis

TI Fluoroquinolone Derivatives in the Treatment of Mycobacterium

tuberculosis Infection

SO PHARMACEUTICALS

LA English

DT Article

DE fluoroquinolones; esters; prodrugs; tuberculosis; mycobacteria

ID ANTIBACTERIAL ACTIVITY; MOXIFLOXACIN; PRODRUG; DESIGN; ESTER; ACID

AB Tuberculosis (TB) is currently one of the leading causes of death due to infective agents, and the growing rate of multidrug-resistant tuberculosis (MDR TB) cases poses an emergent public health threat. Fluoroquinolones are commonly used in the treatment of both MDR TB and drug-sensitive tuberculosis patients who are intolerant to first-line antitubercular agents. Unfortunately, these drugs have mild side effects, relevant to the prolonged treatment regimens and diminished bioavailability due to binding of metal ions. Moreover, the resistance to fluoroquinolones is also on the rise, a characteristic of extensively drug-resistant TB (XDR TB). Here, we developed esters as prodrugs of the fluoroquinolones levofloxacin and ciprofloxacin, with long-chain fatty alcohols. Both the alcohols and the quinolone have previously shown antimycobacterial activity and the aim was to develop esters with improved lipophilicity and capable of delivering the free acid inside mycobacterial cells. The carboxylic acid group of fluoroquinolones is essential to the mode of action but is also responsible for many of its side effects and metal-chelating properties. The synthesis, stability in biological media, and antibacterial activity were evaluated, the latter not only against Mycobacterium tuberculosis but also against other clinically relevant bacterial species, since the parent compounds display a broad spectrum of activity. The biological results show a reduction in the antitubercular activity of the synthesized derivatives, probably due to deficient activation of the ester prodrug. Despite this, it was found that the derivatives exhibit bioactivity against other fluoroquinolone-resistant bacteria, indicating a different mode of action and suggesting that it may be worthwhile to research further modifications to the carboxylic acid group. This might lead to new compounds that are efficient against resistant strains. This idea that the compounds may act by a different mechanism of action was further supported by a brief computer investigation that demonstrated the potential lack of selectivity of the esters to the fluoroquinolone target.

C1 [Pais, Joao Pedro; Policarpo, Margarida; Pires, David; Francisco, Ana Paula; Madureira, Ana Margarida; Anes, Elsa; Constantino, Luis] Res Inst Med & Pharmaceut Sci iMed UL, Av Prof Gama Pinto, P-1649003 Lisbon, Portugal.

[Francisco, Ana Paula; Madureira, Ana Margarida; Anes, Elsa; Constantino, Luis] Univ Lisbon, Fac Pharm, Av Prof Gama Pinto, P-1649003 Lisbon, Portugal.

[Testa, Bernard] Univ Lausanne, CH-1015 Lausanne, Switzerland.

C3 Universidade de Lisboa; Universidade de Lisboa; University of Lausanne

RP Constantino, L (corresponding author), Res Inst Med & Pharmaceut Sci iMed UL, Av Prof Gama Pinto, P-1649003 Lisbon, Portugal.; Constantino, L (corresponding author), Univ Lisbon, Fac Pharm, Av Prof Gama Pinto, P-1649003 Lisbon, Portugal.

EM constant@ff.ul.pt

RI Anes, Elsa/K-3124-2013; Pais, João/AAP-1360-2021; Madureira, Ana

Margarida/HTR-2820-2023; Constantino, Luis/M-7169-2013; Francisco,

Ana/M-4178-2013; Pires, David/D-8245-2012

OI Madureira, Ana Margarida/0000-0001-5973-2183; Almeida Pais, João

Pedro/0000-0002-3865-5913; Constantino, Luis/0000-0002-6847-4830;

Francisco, Ana/0000-0001-6602-2075; Anes, Elsa/0000-0001-5934-0198;

Pires, David/0000-0001-9602-1516

FU Fundacao para a Ciencia e Tecnologia (FCT) [PTDC/SAUINF/28080/2017,

EXPL/SAU-INF/1097/2021]; FCT [UIDB/04138/2020, UIDP/04138/2020]

FX This research was funded by Fundacao para a Ciencia e Tecnologia (FCT),

grant PTDC/SAUINF/28080/2017 and grant EXPL/SAU-INF/1097/2021. It also

received financial support from FCT (via ImedULisboa) from projects

UIDB/04138/2020 and UIDP/04138/2020.

CR Akhtar M, 2019, PAK J PHARM SCI, V32, P1301

Al-Qawasmeh RA, 2009, ARKIVOC, P322, DOI 10.3998/ark.5550190.0010.c28

Assali M, 2016, CHEMISTRYSELECT, V1, P1132, DOI 10.1002/slct.201600091

Bartzatt R, 2010, MED CHEM, V6, P51, DOI 10.2174/157340610791321442

Castro RAD, 2020, MOL BIOL EVOL, V37, P195, DOI 10.1093/molbev/msz214

Cheng AV, 2019, ACS INFECT DIS, V5, P816, DOI 10.1021/acsinfecdis.9b00019

Dorman SE, 2021, NEW ENGL J MED, V384, P1705, DOI 10.1056/NEJMoa2033400

Hooper DC, 2016, CSH PERSPECT MED, V6, DOI 10.1101/cshperspect.a025320

Kanji Akbar, 2019, Indian J Tuberc, V66, P20, DOI [10.1016/j.ijtb.2018.07.008, 10.1016/j.ijtb.2018.07.008]

Larsen EM, 2019, DRUG DEVELOP RES, V80, P33, DOI 10.1002/ddr.21468

Liu B, 2022, ANGEW CHEM INT EDIT, V61, DOI 10.1002/anie.202200120

Liu R, 2019, TETRAHEDRON, V75, P1781, DOI 10.1016/j.tet.2018.12.021

Mede T, 2018, CHEM SOC REV, V47, P7577, DOI 10.1039/c8cs00096d

Morris GM, 2009, J COMPUT CHEM, V30, P2785, DOI 10.1002/jcc.21256

Mukherjee K, 2013, FEMS MICROBIOL LETT, V338, P177, DOI 10.1111/1574-6968.12043

Pires D, 2015, ANTIMICROB AGENTS CH, V59, P7693, DOI 10.1128/AAC.00936-15

Saleh LH, 2021, EGYPT J CHEM, V64, P517, DOI 10.21608/EJCHEM.2020.34131.2720

Shinmachi K, 2020, PHARMAZIE, V75, P554, DOI 10.1691/ph.2020.0601

Tetko IV, 2005, J COMPUT AID MOL DES, V19, P453, DOI 10.1007/s10822-005-8694-y

Valente E, 2021, EUR J PHARM SCI, V162, DOI 10.1016/j.ejps.2021.105831

Valente E, 2011, J MICROBIOL METH, V85, P98, DOI 10.1016/j.mimet.2011.02.003

World Health Organization, 2020, WHO CONSOLIDATED GUI

NR 22

TC 4

Z9 4

U1 0

U2 21

PU MDPI

PI BASEL

PA MDPI AG, Grosspeteranlage 5, CH-4052 BASEL, SWITZERLAND

EI 1424-8247

J9 PHARMACEUTICALS-BASE

JI Pharmaceuticals

PD OCT

PY 2022

VL 15

IS 10

AR 1213

DI 10.3390/ph15101213

PG 13

WC Chemistry, Medicinal; Pharmacology & Pharmacy

WE Science Citation Index Expanded (SCI-EXPANDED)

SC Pharmacology & Pharmacy

GA 5P9SN

UT WOS:000873482300001

OA Green Submitted, Green Published, gold

DA 2026-05-07

ER

PT J

AU Andrews, JR

Gandhi, NR

Moodley, P

Shah, NS

Bohlken, L

Moll, AP

Pillay, M

Friedland, G

Sturm, AW

AF Andrews, Jason R.

Gandhi, Neel R.

Moodley, Prashini

Shah, N. Sarita

Bohlken, Louise

Moll, Anthony P.

Pillay, Manormoney

Friedland, Gerald

Sturm, A. Willem

CA Tugela Ferry Care & Res Collaborat

TI Exogenous Reinfection as a Cause of Multidrug-Resistant and Extensively

Drug-Resistant Tuberculosis in Rural South Africa

SO JOURNAL OF INFECTIOUS DISEASES

LA English

DT Article; Proceedings Paper

CT 15th Conference on Retroviruses and Opportunistic Infections

CY FEB 03-06, 2008

CL Boston, MA

ID MYCOBACTERIUM-TUBERCULOSIS; RECURRENT TUBERCULOSIS; MIXED INFECTION;

STRAINS; TRANSMISSION

AB Background. Multidrug-resistant (MDR) and extensively drug-resistant (XDR) tuberculosis (TB) are now major threats in areas of South Africa with a high prevalence of TB and human immunodeficiency virus (HIV) infection. The role of exogenous reinfection as a cause of MDR and XDR TB in these settings has not been determined.

Methods. We reviewed data from patients with culture-positive TB who later developed MDR or XDR TB in Tugela Ferry, KwaZulu-Natal, South Africa during 2005-2006. We performed spoligotyping on initial isolates (obtained at the time of treatment initiation) and follow-up isolates obtained from these patients.

Results. We identified 23 patients who developed MDR or XDR TB after being treated for less resistant TB between June 2005 and June 2006. Both initial and follow-up isolates were available for spoligotyping for 17 of these patients. In all cases, the follow-up isolates' spoligotypes differed from those of the initial isolate, indicating exogenous reinfection. Two genotypes (shared type [ST] 34 and ST 60, associated with MDR and XDR TB, respectively) were responsible for 85% of reinfections. All 17 patients had been hospitalized; all 15 whose HIV infection status was known were HIV-infected.

Conclusions. Exogenous reinfection is an important mechanism for the development of MDR and XDR TB. In addition to strengthening TB treatment programs, effective infection control strategies are urgently needed to reduce the transmission of MDR and XDR TB.

C1 [Gandhi, Neel R.] Montefiore Med Ctr, Div Gen Internal Med, Bronx, NY 10467 USA.

[Andrews, Jason R.] Univ Calif San Francisco, San Francisco Gen Hosp, Div Gen Internal Med, San Francisco, CA USA.

[Gandhi, Neel R.; Shah, N. Sarita] Albert Einstein Coll Med, Dept Med, Bronx, NY 10467 USA.

[Gandhi, Neel R.; Shah, N. Sarita] Albert Einstein Coll Med, Dept Epidemiol & Publ Hlth, Bronx, NY 10467 USA.

[Friedland, Gerald] Yale Univ, Sch Med, AIDS Program, New Haven, CT USA.

[Friedland, Gerald] Yale Univ, Sch Med, Dept Med, New Haven, CT 06510 USA.

[Moodley, Prashini; Bohlken, Louise; Pillay, Manormoney; Sturm, A. Willem] Nelson R Mandela Sch Med, Dept Med Microbiol, Durban, South Africa.

[Moll, Anthony P.] Philanjalo & Church Scotland Hosp, Tugela Ferry, South Africa.

C3 Montefiore Medical Center; Albert Einstein College of Medicine;

University of California System; University of California San Francisco;

Montefiore Medical Center; Albert Einstein College of Medicine; Yeshiva

University; Montefiore Medical Center; Albert Einstein College of

Medicine; Yeshiva University; Yale University; Yale University;

University of Kwazulu Natal

RP Gandhi, NR (corresponding author), Montefiore Med Ctr, Div Gen Internal Med, 111 E 210 St, Bronx, NY 10467 USA.

EM neelgandhi@alumni.williams.edu

RI Andrews, Jason/AAR-8914-2020

OI Andrews, Jason/0000-0002-5967-251X; Gandhi, Neel/0000-0002-1665-299X

CR Andrews JR, 2007, J INFECT DIS, V196, pS482, DOI 10.1086/521121

ANDREWS JR, 2007, 38 UN WORLD C LUNG H

[Anonymous], 2006, Wkly Epidemiol Rec, V81, P408

[Anonymous], 2006, LANCET INFECT DIS, V6, P679, DOI 10.1016/S1473-3099(06)70604-1

[Anonymous], 2008, GLOBAL TUBERCULOSIS

[Anonymous], 2003, Treatment of Tuberculosis: Guidelines for National Programmes

[Anonymous], REP GLOB AIDS EP

Baldeviano-Vidalón GC, 2005, INT J TUBERC LUNG D, V9, P1155

Bandera A, 2001, J CLIN MICROBIOL, V39, P2213, DOI 10.1128/JCM.39.6.2213-2218.2001

Basu S, 2007, LANCET, V370, P1500, DOI 10.1016/S0140-6736(07)61636-5

Bock NN, 2007, J INFECT DIS, V196, pS108, DOI 10.1086/518661

Brudey K, 2006, BMC MICROBIOL, V6, DOI 10.1186/1471-2180-6-23

BUTHELEZI S, 2008, 2 GLOB XDR TB TASK F

Caminero JA, 2001, AM J RESP CRIT CARE, V163, P717, DOI 10.1164/ajrccm.163.3.2003070

DAS S, 1993, TUBERCLE LUNG DIS, V74, P47, DOI 10.1016/0962-8479(93)90068-9

Das S, 2004, INT J TUBERC LUNG D, V8, P267

DEVIEDMA DG, 2002, ARCH INTERN MED, V162, P1873

El Sahly HM, 2004, INT J TUBERC LUNG D, V8, P333

Escombe A Roderick, 2007, PLoS Med, V4, pe68, DOI 10.1371/journal.pmed.0040068

Gandhi NR, 2006, LANCET, V368, P1575, DOI 10.1016/S0140-6736(06)69573-1

Glynn JR, 2004, J INFECT DIS, V190, P1158, DOI 10.1086/423144

Krüüner A, 2002, CLIN INFECT DIS, V35, P146, DOI 10.1086/340980

Li X, 2007, J INFECT DIS, V195, P864, DOI 10.1086/511985

MOODLEY P, 2007, 8 UN WORLD C LUNG HL

*NAT TUB CONTR ASS, 2004, GUID APPL GEN TUB PR

Nelson LJ, 2005, LANCET, V366, P488, DOI 10.1016/S0140-6736(05)67062-6

Nunes EA, 2005, INT J TUBERC LUNG D, V9, P494

Pillay M, 2007, CLIN INFECT DIS, V45, P1409, DOI 10.1086/522987

Shamputa IC, 2006, RESP RES, V7, DOI 10.1186/1465-9921-7-99

SMALL PM, 1993, NEW ENGL J MED, V328, P1137, DOI 10.1056/NEJM199304223281601

Sonnenberg P, 2001, LANCET, V358, P1687, DOI 10.1016/S0140-6736(01)06712-5

Umubyeyi AN, 2007, INT J TUBERC LUNG D, V11, P189

van Rie A, 2005, AM J RESP CRIT CARE, V172, P636, DOI 10.1164/rccm.200503-449OC

Van Rie A, 1999, NEW ENGL J MED, V341, P1174, DOI 10.1056/NEJM199910143411602

Van Rie A, 2006, LANCET, V368, P1554, DOI 10.1016/S0140-6736(06)69575-5

Warren RM, 2004, AM J RESP CRIT CARE, V169, P610, DOI 10.1164/rccm.200305-714OC

Wells CD, 2007, J INFECT DIS, V196, pS86, DOI 10.1086/518665

*WHO, 2008, 4 WHO IUATLD

NR 38

TC 122

Z9 135

U1 0

U2 7

PU OXFORD UNIV PRESS INC

PI CARY

PA JOURNALS DEPT, 2001 EVANS RD, CARY, NC 27513 USA

SN 0022-1899

EI 1537-6613

J9 J INFECT DIS

JI J. Infect. Dis.

PD DEC 1

PY 2008

VL 198

IS 11

BP 1582

EP 1589

DI 10.1086/592991

PG 8

WC Immunology; Infectious Diseases; Microbiology

WE Science Citation Index Expanded (SCI-EXPANDED); Conference Proceedings Citation Index - Science (CPCI-S)

SC Immunology; Infectious Diseases; Microbiology

GA 374NY

UT WOS:000261052200003

OA Bronze

DA 2026-05-07

ER

PT J

AU Royce, S

Khann, S

Yadav, RP

Mao, ET

Cattamanchi, A

Sam, S

Handley, MA

AF Royce, S.

Khann, S.

Yadav, R. P.

Mao, E. T.

Cattamanchi, A.

Sam, S.

Handley, M. A.

TI Identifying multidrug resistance in previously treated tuberculosis

patients: a mixed-methods study in Cambodia

SO INTERNATIONAL JOURNAL OF TUBERCULOSIS AND LUNG DISEASE

LA English

DT Article

DE MDR-TB case finding; diagnostic pathway; testing coverage; qualitative

methods; mixed methods; stakeholder engagement; implementation science

AB SETTING: Previously treated tuberculosis (TB) patients are a priority for drug susceptibility testing (DST) to identify cases with multidrug-resistant TB (MDR-TB). A Cambodia study found that one third of smear-positive previously treated patients had DST results.

OBJECTIVE: To quantify the gaps in the detection of MDR-TB in previously treated TB patients in Cambodia, and describe health workers' perspectives on barriers, facilitators and potential interventions.

DESIGN: Analysis of Cambodia's 2004-2012 case notifications and semi-structured interviews with stake-holders.

RESULTS: The proportion of previously treated notifications varied significantly across provinces in 2010 2012. If there had been no attrition along the path to detecting MDR-TB among smear-positive notified cases in 2012, an estimated 75 additional MDR-TB cases could have been identified, which would double the number actually detected. Most were lost due to misclassification of previously treated patients as 'new'. Barriers include patients' reluctance to disclose and staff difficulty in eliciting treatment history, partly attributed to the availability of streptomycin (SM) only in hospitals. Facilitators include collection of sputum for DST even if previously treated patients are not receiving SM, streamlining sputum transportation and prompt reporting of results.

CONCLUSION: Improved monitoring, supportive staff supervision and training, patient education, and correct classification of previously treated cases are essential for improving the detection of MDR-TB.

C1 [Royce, S.; Handley, M. A.] Univ Calif San Francisco, Dept Epidemiol & Biostat, San Francisco, CA 94110 USA.

[Khann, S.; Yadav, R. P.] WHO, Cambodia Off, Phnom Penh, Cambodia.

[Mao, E. T.] Natl Ctr TB & Leprosy Control, Phnom Penh, Cambodia.

[Cattamanchi, A.] Univ Calif San Francisco, San Francisco Gen Hosp, Dept Med, Div Pulm & Crit Care Med, San Francisco, CA 94143 USA.

[Sam, S.] Cambodian Hlth Comm, Phnom Penh, Cambodia.

[Handley, M. A.] Univ Calif San Francisco, Dept Med, Div Gen Internal Med, San Francisco Gen Hosp, San Francisco, CA USA.

C3 University of California System; University of California San Francisco;

World Health Organization; University of California System; University

of California San Francisco; University of California System; University

of California San Francisco

RP Handley, MA (corresponding author), Univ Calif San Francisco, San Francisco Gen Hosp, Ctr Vulnerable Populat, Dept Epidemiol & Biostat, San Francisco, CA 94110 USA.

EM handleym@medsfgh.ucsf.edu

OI Yadav, Rajendra Prasad/0000-0002-0398-1313

FU National Institutes of Health (NIH, Bethesda, MD, USA) [P60MD006902,

P30DK092924, K23HL094141]; National Center for Advancing Translational

Sciences, NIH, through UCSF-CTSI [UL1 TR000004]; National Institute of

Diabetes and Digestive and Kidney Diseases [P30DK092924] Funding Source:

NIH RePORTER

FX This project was supported by the following National Institutes of

Health (NIH, Bethesda, MD, USA) funds: P60MD006902 (MN), P30DK092924

(MH), K23HL094141 (AC) and by the National Center for Advancing

Translational Sciences, NIH, through UCSF-CTSI Grant Number UL1

TR000004. Its contents are solely the responsibility of the authors and

do not necessarily represent the official views of the NIH or the World

Health Organization.

CR [Anonymous], PRIOR OP RES IMPR TU

[Anonymous], 2009, WHOHTMTB2009422

[Anonymous], 2015, GLOBAL TUBERCULOSIS

Chadha SS, 2011, PLOS ONE, V6, DOI 10.1371/journal.pone.0026659

Green L.W., 2005, Health program planning: An educational and ecological approach, V4th

Handley MA, 2013, GLOB PUBLIC HEALTH, V8, P245, DOI 10.1080/17441692.2012.729218

Khann S, 2013, PLOS ONE, V8, DOI 10.1371/journal.pone.0059903

Kilale AM, 2013, PUBLIC HEALTH ACTION, V3, P156, DOI 10.5588/pha.12.0103

Menzies D, 2009, PLOS MED, V6, DOI [10.1371/journal.pmed.1000150, 10.1371/journal.pmed.1000146]

National Center for TB and Leprosy Control (CENAT) Ministry of Health Cambodia, 2011, REP NAT TUB DRUG RES

National Center for TB and Leprosy Control Ministry of Health Cambodia, 2013, NAT CTR TB LEPR CONT

Noeske Jurgen, 2012, BMC Res Notes, V5, P160, DOI 10.1186/1756-0500-5-160

Pluye P, 2014, ANNU REV PUBL HEALTH, V35, P29, DOI 10.1146/annurev-publhealth-032013-182440

Sandelowski M, 2012, J MIX METHOD RES, V6, P317, DOI 10.1177/1558689811427913

Strauss E, 1998, CLIN ORTHOP RELAT R, P2

Voils CI, 2008, FIELD METHOD, V20, P3, DOI 10.1177/1525822X07307463

World Health Organization, 2014, GLOB TB DAT CAS NOT

World Health Organization, 2010, Document WHO/HTM/TB/2009.420

Yagui M, 2006, INT J TUBERC LUNG D, V10, P838

NR 19

TC 3

Z9 3

U1 0

U2 21

PU INT UNION AGAINST TUBERCULOSIS LUNG DISEASE (I U A T L D)

PI PARIS

PA 68 BOULEVARD SAINT-MICHEL,, 75006 PARIS, FRANCE

SN 1027-3719

EI 1815-7920

J9 INT J TUBERC LUNG D

JI Int. J. Tuberc. Lung Dis.

PD NOV

PY 2014

VL 18

IS 11

BP 1299

EP 1306

DI 10.5588/ijtld.14.0116

PG 8

WC Infectious Diseases; Respiratory System

WE Science Citation Index Expanded (SCI-EXPANDED)

SC Infectious Diseases; Respiratory System

GA AR4AK

UT WOS:000343530200008

OA Green Submitted

DA 2026-05-07

ER

PT J

AU de Araújo, JA

Vasconcelos, AC

de Sousa, EM

Kipnis, A

Ribeiro, E

Junqueira-Kipnis, AP

AF de Araujo-Filho, Joso Alves

Vasconcelos, Arioldo Carvalho, Jr.

de Sousa, Eduardo Martins

Kipnis, Andre

Ribeiro, Elisangela

Junqueira-Kipnis, Ana Paula

TI Cellular responses to MPT-51, GlcB and ESAT-6 among MDR-TB and active

tuberculosis patients in Brazil

SO TUBERCULOSIS

LA English

DT Article

DE multi-drug resistant; tuberculosis; interferon-gamma; interleukin-10;

MPT-51; GlcB

ID MULTIDRUG-RESISTANT TUBERCULOSIS; BLOOD MONONUCLEAR-CELLS;

NECROSIS-FACTOR-ALPHA; GROWTH-FACTOR-BETA; MYCOBACTERIUM-TUBERCULOSIS;

IMMUNE-RESPONSES; PULMONARY TUBERCULOSIS; INTERFERON-GAMMA; MALATE

SYNTHASE; DIAGNOSIS

AB Multi-drug resistant pulmonary tuberculosis (MDR-TB) may result from either insufficiency of the host cellular immune response or mycobacterial. mechanisms of resistance. Mycobacterium tuberculosis-specific CD8(+) and CD4(+) T lymphocytes from MDR-TB patients are poorly studied. The aim of this study was to evaluate CD4(+)-IFN-gamma(+), CD4(+)-IL-10(+), CD8(+)IFN-gamma(+) and CD8(+)IL-10(+) cell populations by flow cytometry in non-resistant TB and multi-drug resistant tuberculosis (MDR-TB) patients from mid-central Brazil after stimulation with MPT-51, GlcB and ESAT-6 recombinant antigens from M. tuberculosis in comparison to tuberculin skin test negative (TST) healthy individuals. Non-resistant TB patients present specific cellular responses (CD4 and CD8, both IFN-gamma and IL-10) to GLcB, MPT-51 and ESAT-6; while MDR-TB patients present only CD8(+)IFN-gamma(+) responses to ESAT-6 and CD8(+)IL-10(+) responses to GlcB and ESAT-6. The results show that MDR-TB patients present impaired specific CD4 IFN-gamma and IL-10 responses and increased/normal specific CD8 IFN-gamma and IL-10 responses. This suggests an important role for CD8 function in these patients. (c) 2008 El.sevier Ltd. All rights reserved.

C1 [de Araujo-Filho, Joso Alves; Vasconcelos, Arioldo Carvalho, Jr.; de Sousa, Eduardo Martins; Kipnis, Andre; Junqueira-Kipnis, Ana Paula] Univ Fed Goias, Inst Patol Trop & Saude Publ, BR-74605050 Goiania, Go, Brazil.

[de Araujo-Filho, Joso Alves] Secretaria Saude Estado Goias, Hosp Doencas Tropicais Dr Anuar Auad, BR-74853120 Goiania, Go, Brazil.

[Ribeiro, Elisangela] Assoc Combate Ao Canc Goias, Hosp Araujo Jorge, BR-74605070 Goiania, Go, Brazil.

C3 Universidade Federal de Goias

RP Junqueira-Kipnis, AP (corresponding author), Univ Fed Goias, Inst Patol Trop & Saude Publ, Rua Dalenda Rezende Melo S-N,Setor Univ, BR-74605050 Goiania, Go, Brazil.

EM anapaula@iptsp.ufg.br

RI Kipnis, Andre/M-7485-2017; Kipnis, Andre/M-7485-2017; Filho,

João/V-5279-2019; Junqueira-Kipnis, Ana/I-5534-2012; de Sousa,

Eduardo/AAT-9294-2020

OI Kipnis, Andre/0000-0001-5950-106X;

FU Conselho Nacional de Desenvolvimento Cientifico e Tecnologico - CNPq -

Brasil [471348/2004-0]; Coordenacao de Aperfeicoamento de Pessoal de

Nivel Superior - CAPES - Brazil; CNPq - Brazil

FX Supported by grant from Conselho Nacional de Desenvolvimento Cientifico

e Tecnologico - CNPq - Brasil No 471348/2004-0. ACV Jr received a

fellowship from Coordenacao de Aperfeicoamento de Pessoal de Nivel

Superior - CAPES - Brazil and EMS received a fellowship from CNPq -

Brazil. ACV Jr and JAAF contributed equally to this work. The English

language edition was done by www.journalexperts.com.

CR Achkar JM, 2006, CLIN VACCINE IMMUNOL, V13, P1291, DOI 10.1128/CVI.00158-06

[Anonymous], 2007, WHO REPORT 2007 GLOB, P277

Antia R, 1996, P ROY SOC B-BIOL SCI, V263, P257, DOI 10.1098/rspb.1996.0040

Blöndal K, 2007, B WORLD HEALTH ORGAN, V85, P387, DOI 10.2471/BLT.06.035345

Bonecini-Almeida MG, 2004, INFECT IMMUN, V72, P2628, DOI 10.1128/IAI.72.5.2628-2634.2004

Çagatay T, 2005, RESPIROLOGY, V10, P290, DOI 10.1111/j.1440-1843.2005.00711.x

Caminero JA, 2006, INT J TUBERC LUNG D, V10, P829

CANETTI G, 1963, Rev Tuberc Pneumol (Paris), V27, P217

Almeida CM, 2008, CLIN VACCINE IMMUNOL, V15, P579, DOI 10.1128/CVI.00359-07

Castro AZ, 2005, J INFECTION, V51, P318, DOI 10.1016/j.jinf.2004.08.035

Chan ED, 2004, AM J RESP CRIT CARE, V169, P1103, DOI 10.1164/rccm.200308-1159OC

Cohen T, 2003, LANCET INFECT DIS, V3, P13, DOI 10.1016/S1473-3099(03)00483-3

Cosmi L, 2007, INT ARCH ALLERGY IMM, V143, P1, DOI 10.1159/000098220

Dlugovitzky D, 2000, CLIN EXP IMMUNOL, V122, P343, DOI 10.1046/j.1365-2249.2000.01394.x

Drobniewski F, 2002, THORAX, V57, P810, DOI 10.1136/thorax.57.9.810

Ferraz JC, 2006, BRAZ J MED BIOL RES, V39, P1387, DOI 10.1590/S0100-879X2006001100002

Flynn JL, 2001, ANNU REV IMMUNOL, V19, P93, DOI 10.1146/annurev.immunol.19.1.93

Fortes A, 2005, CLIN EXP IMMUNOL, V141, P541, DOI 10.1111/j.1365-2249.2005.02872.x

Frieden TR, 2003, LANCET, V362, P887, DOI 10.1016/S0140-6736(03)14333-4

Goletti D, 2006, J INFECT DIS, V194, P984, DOI 10.1086/507427

Junqueira-Kipnis AP, 2006, IMMUNOLOGY, V119, P224, DOI 10.1111/j.1365-2567.2006.02427.x

Kang YA, 2006, RESPIROLOGY, V11, P793, DOI 10.1111/j.1440-1843.2006.00948.x

KRISKI AL, 1996, AM J RESP CRIT CARE, V153, P331

Lalloo UG, 2006, CURR OPIN PULM MED, V12, P179, DOI 10.1097/01.mcp.0000219266.27439.52

Lazarevic V, 2005, J IMMUNOL, V175, P1107, DOI 10.4049/jimmunol.175.2.1107

Lee JH, 2008, J BACTERIOL, V190, P1128, DOI 10.1128/JB.00511-07

Lee JS, 2002, CLIN EXP IMMUNOL, V128, P516, DOI 10.1046/j.1365-2249.2002.01858.x

Meya DB, 2007, J INTERN MED, V261, P309, DOI 10.1111/j.1365-2796.2007.01795.x

*MIN SAUD, 2006, MULT RES TUB EP SURV

Palmero D, 2002, MEDICINA-BUENOS AIRE, V62, P221

Rattan A, 1998, EMERG INFECT DIS, V4, P195, DOI 10.3201/eid0402.980207

Richeldi L, 2006, AM J RESP CRIT CARE, V174, P736, DOI 10.1164/rccm.200509-1516PP

Sahiratmadja E, 2007, INFECT IMMUN, V75, P820, DOI 10.1128/IAI.00602-06

Shahemabadi AS, 2007, CLIN EXP IMMUNOL, V149, P285, DOI 10.1111/j.1365-2249.2007.03406.x

Singh KK, 2005, CLIN DIAGN LAB IMMUN, V12, P354, DOI 10.1128/CDLI.12.2.354-358.2005

Singh KK, 2003, J INFECT DIS, V188, P371, DOI 10.1086/376532

SINGH M, 2007, TUBERCULOSIS 2007

Smith CV, 2003, J BIOL CHEM, V278, P1735, DOI 10.1074/jbc.M209248200

WAYNE LG, 1982, INFECT IMMUN, V37, P1042, DOI 10.1128/IAI.37.3.1042-1049.1982

*WHO, 2006, GUID PROGR MAN DRUG, P174

Wright A., 2006, Morbidity and Mortality Weekly Report, V55, P301

NR 41

TC 15

Z9 17

U1 0

U2 3

PU CHURCHILL LIVINGSTONE

PI EDINBURGH

PA JOURNAL PRODUCTION DEPT, ROBERT STEVENSON HOUSE, 1-3 BAXTERS PLACE,

LEITH WALK, EDINBURGH EH1 3AF, MIDLOTHIAN, SCOTLAND

SN 1472-9792

J9 TUBERCULOSIS

JI Tuberculosis

PD SEP

PY 2008

VL 88

IS 5

BP 474

EP 481

DI 10.1016/j.tube.2008.06.002

PG 8

WC Immunology; Microbiology; Respiratory System

WE Science Citation Index Expanded (SCI-EXPANDED)

SC Immunology; Microbiology; Respiratory System

GA 353UE

UT WOS:000259593100014

DA 2026-05-07

ER

PT J

AU Yan, H

Xu, RF

Zhang, XR

Wang, Q

Pang, J

Zhang, X

Chang, XA

Zhang, YQ

AF Yan, Hong

Xu, Rufeng

Zhang, Xiangrong

Wang, Qian

Pang, Jing

Zhang, Xia

Chang, Xiaoai

Zhang, Yaqin

TI Identifying differentially expressed long non-coding RNAs in PBMCs in

response to the infection of multidrug-resistant tuberculosis

SO INFECTION AND DRUG RESISTANCE

LA English

DT Article

DE mycobacterium tuberculosis; MDR; molecular characterization; lncRNA;

infection

ID MYCOBACTERIUM-TUBERCULOSIS; GASTRIC-CANCER; BETA

AB Purpose: The aim of this paper was to identify differentially expressed long non-coding RNAs (lncRNAs) in peripheral blood mononuclear cells (PBMCs) influenced by the infection of multidrug-resistant tuberculosis (MDR-TB).

Materials and methods: IncRNA and mRNA expression profiles in PBMCs derived from healthy controls (HCs) and individuals with MDR-TB and drug-sensitive tuberculosis (DS-TB) were analyzed and compared by microarray assay. Six lncRNAs were randomly selected for validation by using real-time quantitative polymerase chain reaction (RT-qPCR). The biological functions and signaling pathways affected by the differentially expressed mRNAs were investigated by using Gene Ontology (GO) and Kyoto Encyclopedia of Genes and Genomes (KEGG) pathway-based approaches.

Results: Compared with the HC group, 1,429 lncRNAs (983 mRNAs) and 2,040 lncRNAs (1,407 mRNAs) were identified to be deregulated in the MDR-TB group and in the DS-TB group, respectively, and 1,511 lncRNAs and 1,047 mRNAs were identified to be differentially expressed in both MDR-TB and DS-TB groups. Between the three groups, 22 lncRNAs and 38 mRNAs were found deregulated. Most deregulated lncRNAs were from intergenic regions (similar to 55% of the total), natural antisense to protein-coding loci (similar to 32% of the total), or intronic antisense to protein-coding loci (similar to 5% of the total). Significantly enriched signaling pathways regulated by the deregulated mRNAs were mainly associated with natural killer cell-mediated cytotoxicity, antigen processing and presentation, graft-vs-host disease, the transforming growth factor-beta signaling pathway, and the Hippo signaling pathway.

Conclusion: This study is the first to report differentially expressed lncRNAs in PBMCs in response to MDR-TB infection. It revealed that some lncRNAs might be associated with regulating host immune response to MDR-TB infection. Further elucidation of the potential of these deregulated lncRNAs in MDR-TB and its reactivation requires further study.

C1 [Yan, Hong; Wang, Qian] Nanjing Chest Hosp, Dept Lab Med, Nanjing 210029, Jiangsu, Peoples R China.

[Xu, Rufeng; Pang, Jing; Chang, Xiaoai; Zhang, Yaqin] Nanjing Med Univ, Key Lab Human Funct Genom Jiangsu Prov, Dept Biochem & Mol Biol, 140 Hanzhong Rd, Nanjing 210029, Jiangsu, Peoples R China.

[Zhang, Xiangrong; Zhang, Xia] Nanjing Publ Hlth Clin Ctr, Nanjing 211133, Jiangsu, Peoples R China.

C3 Nanjing Medical University

RP Zhang, YQ (corresponding author), Nanjing Med Univ, Key Lab Human Funct Genom Jiangsu Prov, Dept Biochem & Mol Biol, 140 Hanzhong Rd, Nanjing 210029, Jiangsu, Peoples R China.

EM yaqinzhang@njmu.edu.cn

RI Wang, Zhiqiang/P-8347-2019; Yan, Hong/KIL-6604-2024

FU Outstanding Young scholar Foundation of Jiangsu Province, China

[BK20170106]; Collaborative Research Project of Southeast University

[2242017K3DN27]; Nanjing Medical University [2242017K3DN27]; Medical

Science and technology development Foundation, Nanjing Department of

Health [ZKX13042]; Foundation of Nanjing Medical Science and Technique

Development [QRX11229]

FX We thank Professor Xiao Han for his critical reading of the manuscript

and helpful comments. This work was supported by research grants from

the Outstanding Young scholar Foundation of Jiangsu Province, China

(BK20170106), the grant from Collaborative Research Project of Southeast

University and Nanjing Medical University (2242017K3DN27), the Key

Project supported by Medical Science and technology development

Foundation, Nanjing Department of Health (ZKX13042), and the Foundation

of Nanjing Medical Science and Technique Development (QRX11229).

CR Alam-Faruque Y, 2011, PLOS ONE, V6, DOI 10.1371/journal.pone.0027541

Aung H, 2000, TUBERCLE LUNG DIS, V80, P61, DOI 10.1054/tuld.2000.0235

CANETTI G, 1963, B WORLD HEALTH ORGAN, V29, P565

Cao L, 2016, ONCOL REP, V35, P757, DOI 10.3892/or.2015.4415

Comas I, 2012, NAT GENET, V44, P106, DOI 10.1038/ng.1038

Denholm J, 2010, J TRAVEL MED, V17, P216, DOI 10.1111/j.1708-8305.2009.00396.x

Corral-Fernández NE, 2017, TUBERCULOSIS, V105, P1, DOI 10.1016/j.tube.2017.03.007

Huarte M, 2010, CELL, V142, P409, DOI 10.1016/j.cell.2010.06.040

Johnston J, 2012, BMC INFECT DIS, V12, DOI 10.1186/1471-2334-12-266

Lee JT, 2012, SCIENCE, V338, P1435, DOI 10.1126/science.1231776

Li J, 2016, NAT COMMUN, V7, DOI 10.1038/ncomms11730

Loveday M, 2017, SAMJ S AFR MED J, V107, P176, DOI [10.7196/SAMJ.2017.v107i3.12203, 10.7196/samj.2017.v107i3.12203]

Ma YL, 2016, GUT, V65, P1494, DOI 10.1136/gutjnl-2014-308392

Malardo T, 2016, TUBERCULOSIS, V101, P151, DOI 10.1016/j.tube.2016.09.003

Pang Y, 2013, ANTIMICROB AGENTS CH, V57, P893, DOI 10.1128/AAC.01024-12

Qin FN, 2013, CELL BIOSCI, V3, DOI 10.1186/2045-3701-3-31

Seung KJ, 2015, CSH PERSPECT MED, V5, DOI 10.1101/cshperspect.a017863

Sun MY, 2017, BIOTECHNOL LETT, V39, P1477, DOI 10.1007/s10529-017-2392-4

Toossi Z, 1998, CLIN IMMUNOL IMMUNOP, V87, P107, DOI 10.1006/clin.1998.4528

Wang C, 2016, INT J BIOL SCI, V12, P246, DOI 10.7150/ijbs.13805

Wu M, 2012, SCAND J IMMUNOL, V75, P301, DOI 10.1111/j.1365-3083.2011.02668.x

Yao YL, 2016, ONCOTARGET, V7, P20549, DOI 10.18632/oncotarget.7823

Yi ZJ, 2014, J INFECTION, V69, P558, DOI 10.1016/j.jinf.2014.06.016

Zhang XW, 2015, BIOCHEM BIOPH RES CO, V462, P227, DOI 10.1016/j.bbrc.2015.04.121

Zumla A, 2015, LANCET GLOB HEALTH, V3, pE10, DOI 10.1016/S2214-109X(14)70361-4

NR 25

TC 34

Z9 38

U1 0

U2 11

PU DOVE MEDICAL PRESS LTD

PI ALBANY

PA PO BOX 300-008, ALBANY, AUCKLAND 0752, NEW ZEALAND

SN 1178-6973

J9 INFECT DRUG RESIST

JI Infect. Drug Resistance

PY 2018

VL 11

BP 945

EP 959

DI 10.2147/IDR.S154255

PG 15

WC Infectious Diseases; Pharmacology & Pharmacy

WE Science Citation Index Expanded (SCI-EXPANDED)

SC Infectious Diseases; Pharmacology & Pharmacy

GA GM9XO

UT WOS:000438609500001

OA Green Submitted, gold

DA 2026-05-07

ER

PT J

AU Usha, V

Hobrath, JV

Gurcha, SS

Reynolds, RC

Besra, GS

AF Usha, Veeraraghavan

Hobrath, Judith V.

Gurcha, Sudagar S.

Reynolds, Robert C.

Besra, Gurdyal S.

TI Identification of Novel Mt-Guab2 Inhibitor Series Active against M.

tuberculosis

SO PLOS ONE

LA English

DT Article

ID INOSINE MONOPHOSPHATE DEHYDROGENASE; IMP DEHYDROGENASE; 5'-MONOPHOSPHATE

DEHYDROGENASE; CRYSTAL-STRUCTURE; MYCOBACTERIUM-TUBERCULOSIS; CELL-WALL;

MECHANISM; DISCOVERY; COMPLEX; PURIFICATION

AB Tuberculosis (TB) remains a leading cause of mortality worldwide. With the emergence of multidrug resistant TB, extensively drug resistant TB and HIV-associated TB it is imperative that new drug targets be identified. The potential of Mycobacterium tuberculosis inosine monophosphate dehydrogenase (IMPDH) as a novel drug target was explored in the present study. IMPDH exclusively catalyzes the conversion of inosine monophosphate (IMP) to xanthosine monophosphate (XMP) in the presence of the cofactor nicotinamide adenine dinucleotide (NAD(+)). Although the enzyme is a dehydrogenase, the enzyme does not catalyze the reverse reaction i.e. the conversion of XMP to IMP. Unlike other bacteria, M. tuberculosis harbors three IMPDH-like genes, designated as Mt-guaB1, Mt-guaB2 and Mt-guaB3 respectively. Of the three putative IMPDH's, we previously confirmed that Mt-GuaB2 was the only functional ortholog by characterizing the enzyme kinetically. Using an in silico approach based on designed scaffolds, a series of novel classes of inhibitors was identified. The inhibitors possess good activity against M. tuberculosis with MIC values in the range of 0.4 to 11.4 mu g mL(-1). Among the identified ligands, two inhibitors have nanomolar K(i)s against the Mt-GuaB2 enzyme.

C1 [Usha, Veeraraghavan; Gurcha, Sudagar S.; Besra, Gurdyal S.] Univ Birmingham, Sch Biosci, Birmingham, W Midlands, England.

[Hobrath, Judith V.; Reynolds, Robert C.] So Res Inst, Drug Discovery Div, Birmingham, AL 35255 USA.

C3 University of Birmingham

RP Usha, V (corresponding author), Univ Birmingham, Sch Biosci, Birmingham, W Midlands, England.

EM g.besra@bham.ac.uk

OI Besra, Gurdyal/0000-0002-5605-0395

FU American Reinvestment and Recovery Act [1RC1AI086677-01]; National

Institutes of Health (NIH); National Institute of Allergy and Infectious

Diseases (NIAID); Tuberculosis Antimicrobial Acquisition and

Coordinating Facility through research and development [NO1 AI-95385];

Colorado State University; DHHS; NIH; NIAID-United States of America

IDIQ [HHSN266200600011C]

FX GSB acknowledges support in the form of a Personal Research Chair from

Mr. James Bardrick, Royal Society Wolfson Research Merit Award, as a

former Lister Institute-Jenner Fellow. RCR acknowledges American

Reinvestment and Recovery Act Grant 1RC1AI086677-01 (National Institutes

of Health (NIH), National Institute of Allergy and Infectious Diseases

(NIAID)) "Targeting MDR-TB''; the Tuberculosis Antimicrobial Acquisition

and Coordinating Facility through research and development contracts NO1

AI-95385 by Dr. Anne Lenaerts at Colorado State University (Program

Officer: Dr. Robert Goldman); DHHS, NIH, NIAID-United States of America

IDIQ contract number HHSN266200600011C by Dr. Anne Lenaerts at Colorado

State University (Program Officer: Dr. Tina Parker). The funders had no

role in study design, data collection and analysis, decision to publish,

or preparation of the manuscript.

CR Ananthan S, 2009, TUBERCULOSIS, V89, P334, DOI 10.1016/j.tube.2009.05.008

Arai M, 2008, BIOORGAN MED CHEM, V16, P6732, DOI 10.1016/j.bmc.2008.05.061

BRENNAN PJ, 1995, ANNU REV BIOCHEM, V64, P29, DOI 10.1146/annurev.bi.64.070195.000333

CARR SF, 1993, J BIOL CHEM, V268, P27286

Chaturvedi V, 2007, J GEN APPL MICROBIOL, V53, P333, DOI 10.2323/jgam.53.333

Chen LQ, 2010, J MED CHEM, V53, P4768, DOI 10.1021/jm100424m

Chen P, 2003, BIOORG MED CHEM LETT, V13, P1345, DOI 10.1016/S0960-894X(03)00107-0

Chiang CY, 2010, RESPIROLOGY, V15, P413, DOI 10.1111/j.1440-1843.2010.01738.x

Colby TD, 1999, P NATL ACAD SCI USA, V96, P3531, DOI 10.1073/pnas.96.7.3531

Cole ST, 1998, NATURE, V393, P537, DOI 10.1038/31159

COONEY DA, 1982, BIOCHEM PHARMACOL, V31, P2133, DOI 10.1016/0006-2952(82)90436-1

COOPER AM, 1993, J EXP MED, V178, P2243, DOI 10.1084/jem.178.6.2243

Dhar TGM, 2002, J MED CHEM, V45, P2127, DOI 10.1021/jm0105777

Digits JA, 1999, BIOCHEMISTRY-US, V38, P15388, DOI 10.1021/bi991558q

Digits JA, 1999, BIOCHEMISTRY-US, V38, P2295, DOI 10.1021/bi982305k

Dobie F, 2007, MOL BIOCHEM PARASIT, V152, P11, DOI 10.1016/j.molbiopara.2006.11.007

Dye C, 2010, SCIENCE, V328, P856, DOI 10.1126/science.1185449

Engelhardt H, 2002, J BIOL CHEM, V277, P37567, DOI 10.1074/jbc.M206983200

FRANKLIN TJ, 1969, BIOCHEM J, V113, P515, DOI 10.1042/bj1130515

Gan L, 2002, BIOCHEMISTRY-US, V41, P13309, DOI 10.1021/bi0203785

GILBERT HJ, 1979, BIOCHEM J, V183, P481, DOI 10.1042/bj1830481

Goldstein BM, 1999, CURR MED CHEM, V6, P519

Harries AD, 2006, ANN TROP MED PARASIT, V100, P415, DOI 10.1179/136485906X91477

HEDSTROM L, 1990, BIOCHEMISTRY-US, V29, P849, DOI 10.1021/bi00456a001

Hedstrom L, 2010, PCT Int Appl, Patent No. [WO201010 8187 A2 20100923, 2010108187]

Hedstrom L. K., 2007, Patent No. [WO 2007143557, 2007143557]

Hedstrom L, 2009, CHEM REV, V109, P2903, DOI 10.1021/cr900021w

JARLIER V, 1994, FEMS MICROBIOL LETT, V123, P11, DOI 10.1111/j.1574-6968.1994.tb07194.x

Lenaerts AJM, 2003, ANTIMICROB AGENTS CH, V47, P783, DOI 10.1128/AAC.47.2.783-785.2003

Lenaerts AJ, 2008, ANTIMICROB AGENTS CH, V52, P1513, DOI 10.1128/AAC.00897-07

Li XZ, 2004, ANTIMICROB AGENTS CH, V48, P2415, DOI 10.1128/AAC.48.7.2415-2423.2004

MacPherson IS, 2010, J AM CHEM SOC, V132, P1230, DOI 10.1021/ja909947a

Maddry JA, 2009, TUBERCULOSIS, V89, P354, DOI 10.1016/j.tube.2009.07.006

Martinson Neil A, 2011, Proc Am Thorac Soc, V8, P288, DOI 10.1513/pats.201010-064WR

Maurya SK, 2009, J MED CHEM, V52, P4623, DOI 10.1021/jm900410u

McMillan FM, 2000, BIOCHEMISTRY-US, V39, P4533, DOI 10.1021/bi992645l

OGara MJ, 1997, ANTIMICROB AGENTS CH, V41, P40, DOI 10.1128/AAC.41.1.40

Orme I, 2001, ANTIMICROB AGENTS CH, V45, P1943, DOI 10.1128/AAC.45.7.1943-1946.2001

Park SJ, 2004, PROTEOMICS, V4, P3632, DOI 10.1002/pmic.200400921

Pimkin M, 2009, J BIOL CHEM, V284, P7960, DOI 10.1074/jbc.M808541200

Prosise GL, 2002, J BIOL CHEM, V277, P50654, DOI 10.1074/jbc.M208330200

Reynolds RC, 2012, TUBERCULOSIS, V92, P72, DOI 10.1016/j.tube.2011.05.005

Riera TV, 2008, BIOCHEMISTRY-US, V47, P8689, DOI 10.1021/bi800674a

Rullas J, 2010, ANTIMICROB AGENTS CH, V54, P2262, DOI 10.1128/AAC.01423-09

Sassetti CM, 2003, MOL MICROBIOL, V48, P77, DOI 10.1046/j.1365-2958.2003.03425.x

Sassetti CM, 2001, P NATL ACAD SCI USA, V98, P12712, DOI 10.1073/pnas.231275498

Sharling L, 2010, PLOS NEGLECT TROP D, V4, DOI 10.1371/journal.pntd.0000794

Shu QN, 2008, MED RES REV, V28, P219, DOI 10.1002/med.20104

Sintchak MD, 1996, CELL, V85, P921, DOI 10.1016/S0092-8674(00)81275-1

Umejiego NN, 2004, J BIOL CHEM, V279, P40320, DOI 10.1074/jbc.M407121200

Usha V, 2010, MICROBIOLOGY, V715, P290

Wang W, 1998, BIOCHEMISTRY-US, V37, P11949, DOI 10.1021/bi981132w

Watterson SH, 2007, J MED CHEM, V50, P3730, DOI 10.1021/jm070299x

Whitby FG, 1997, BIOCHEMISTRY-US, V36, P10666, DOI 10.1021/bi9708850

Yew WW, 2011, AM J RESP CRIT CARE, V184, P180, DOI 10.1164/rccm.201102-0325UP

Zhang RG, 1999, BIOCHEMISTRY-US, V38, P4691, DOI 10.1021/bi982858v

Zhang XH, 2009, MICROB PATHOGENESIS, V47, P267, DOI 10.1016/j.micpath.2009.09.001

Zhou X, 1997, J BIOL CHEM, V272, P21977, DOI 10.1074/jbc.272.35.21977

NR 58

TC 30

Z9 34

U1 0

U2 10

PU PUBLIC LIBRARY SCIENCE

PI SAN FRANCISCO

PA 1160 BATTERY STREET, STE 100, SAN FRANCISCO, CA 94111 USA

SN 1932-6203

J9 PLOS ONE

JI PLoS One

PD MAR 29

PY 2012

VL 7

IS 3

AR e33886

DI 10.1371/journal.pone.0033886

PG 12

WC Multidisciplinary Sciences

WE Science Citation Index Expanded (SCI-EXPANDED)

SC Science & Technology - Other Topics

GA 948SR

UT WOS:000304523400042

OA Green Submitted, gold

DA 2026-05-07

ER

PT J

AU Lee, SFK

Laughon, BE

McHugh, TD

Lipman, M

AF Lee, Simon F. K.

Laughon, Barbara E.

McHugh, Timothy D.

Lipman, Marc

TI New drugs to treat difficult tuberculous and nontuberculous

mycobacterial pulmonary disease

SO CURRENT OPINION IN PULMONARY MEDICINE

LA English

DT Review

DE multidrug-resistant tuberculosis; mycobacteria; nontuberculous;

nontuberculous mycobacteria; regimen; tuberculosis

ID MULTIDRUG-RESISTANT TUBERCULOSIS; INTENSIVE PHASE TREATMENT; HIGH-DOSE

RIFAMPICIN; IN-VITRO ACTIVITY; CELL-WALL CORE; BACTERICIDAL ACTIVITY;

ANTITUBERCULOSIS AGENTS; ANTIMICROBIAL SUSCEPTIBILITY; RIMINOPHENAZINE

DERIVATIVES; CAPURAMYCIN ANALOGS

AB Purpose of review

Treatment of drug-sensitive tuberculosis (TB) is effective, whereas that of multidrug-resistant and extensively drug-resistant TB as well as nontuberculous mycobacterial (NTM) disease are less so. Therapy in general requires good adherence to potentially toxic drug regimens over prolonged periods. Poor adherence is associated with resistance development and poor outcome. This review will present promising new treatments, both new drugs and regimens, for difficult mycobacterial pulmonary infections.

Recent findings

A number of new and repurposed drugs including bedaquiline, delamanid, pretomanid, linezolid and clofazimine, and drug regimens, such as the STREAM trial regimens, are currently progressing from basic research through clinical trials.

Summary

(1) The role of bedaquiline and delamanid in TB and NTM treatment is still not clearly defined.

(2) New and repurposed drugs such as pretomanid, linezolid and clofazimine have the potential to advance TB and NTM treatment. Inhaled liposomal amikacin shows promise in pulmonary NTM disease.

(3) Patients with multidrug-resistant TB, extensively drug-resistant TB and NTM disease should be offered the choice to participate in drug trials that may shorten or otherwise improve their experience of treatment.

(4) The use of an effective regimen based on appropriate NTM-specific drug susceptibility testing should be a cornerstone of treatment for NTM as much as it is for Mycobacterium tuberculosis treatment.

(5) All new drugs identified for Mycobacterium tuberculosis should also be tested for activity against NTM, though robust tools for NTM drug susceptibility testing are required.

C1 [Lee, Simon F. K.] Chelsea & Westminster Hosp, HIV Med, London, England.

[Laughon, Barbara E.] NIAID, Div Microbiol & Infect Dis, NIH, 9000 Rockville Pike, Bethesda, MD 20892 USA.

[McHugh, Timothy D.] UCL, Dept Infect, UCL Ctr Clin Microbiol, London, England.

[Lipman, Marc] UCL, Div Med, UCL Resp, London, England.

C3 Imperial College London; National Institutes of Health (NIH) - USA; NIH

National Institute of Allergy & Infectious Diseases (NIAID); University

of London; University College London; University of London; University

College London

RP Lipman, M (corresponding author), Royal Free London NHS Fdn Trust, Resp Med, Pond St, London NW3 2QG, England.

EM marclipman@nhs.net

RI Lee, Simon/LOR-8298-2024; McHugh, Timothy D/O-3683-2019

OI Lee, Simon/0000-0002-7885-4714; McHugh, Timothy D/0000-0003-4658-8594;

Laughon, Barbara/0000-0002-8683-3681; Lipman, Marc/0000-0001-7501-4448

FU Insmed

FX M.L. is chair of NTM Network, UK, which has received administrative

support funding from Insmed.

CR Aarnoutse RE, 2017, ANTIMICROB AGENTS CH, V61, DOI [10.1128/AAC.01054-17, 10.1128/aac.01054-17]

Achar J, 2017, EMERG INFECT DIS, V23, P1711, DOI 10.3201/eid2310.170303

Aleksandrov A, 2019, EXPERT OPIN DRUG DIS, V14, P35, DOI 10.1080/17460441.2019.1550482

Alsaad N, 2013, EUR RESPIR J, V42, P504, DOI 10.1183/09031936.00114812

Andries K, 2005, SCIENCE, V307, P223, DOI 10.1126/science.1106753

[Anonymous], 1981, Tubercle, V62, P95

[Anonymous], 2018, ANTIMICROB AGENTS CH

[Anonymous], J THORAC DIS

[Anonymous], ASM MICROBE 2018

[Anonymous], LANCET

[Anonymous], CURR OPIN PULM MED

[Anonymous], ANTIMICROB AGENTS CH

[Anonymous], 2018, ANTIMICROB AGENTS CH

[Anonymous], 2017, ASM MICROBE 2017 SES

[Anonymous], AM J RESP CRIT CARE

[Anonymous], ASM MICROBE 2018

[Anonymous], 2018, Rapid communication: key changes to treatment of multidrug-and rifampicin-resistant tuberculosis (MDR/RR-TB)

Aung KJM, 2014, INT J TUBERC LUNG D, V18, P1180, DOI 10.5588/ijtld.14.0100

Bald D, 2017, MBIO, V8, DOI 10.1128/mBio.00272-17

Boeree MJ, 2017, LANCET INFECT DIS, V17, P39, DOI 10.1016/S1473-3099(16)30274-2

Boeree MJ, 2015, AM J RESP CRIT CARE, V191, P1058, DOI 10.1164/rccm.201407-1264OC

Broda A, 2013, J CLIN MICROBIOL, V51, P217, DOI 10.1128/JCM.02260-12

Brown-Elliott BA, 2018, ANTIMICROB AGENTS CH, V62, DOI [10.1128/AAC.01503-18, 10.1128/aac.01503-18]

Cariello PF, 2015, TRANSPL INFECT DIS, V17, P111, DOI 10.1111/tid.12340

Chabala C, 2018, TRIALS, V19, DOI 10.1186/s13063-018-2608-5

Chatterji M, 2014, ANTIMICROB AGENTS CH, V58, P5325, DOI 10.1128/AAC.03233-14

Chen Ray Y, 2017, Gates Open Res, V1, P9, DOI [10.12688/gatesopenres.12750.1, 10.12688/gatesopenres.12750.1]

Choi PJ, 2017, BIOORG MED CHEM LETT, V27, P5190, DOI 10.1016/j.bmcl.2017.10.042

Conde MB, 2016, PLOS ONE, V11, DOI 10.1371/journal.pone.0154778

Cowman S, 2016, J INFECTION, V72, P324, DOI 10.1016/j.jinf.2015.12.007

Cynamon M, 2012, ANTIMICROB AGENTS CH, V56, P3986, DOI 10.1128/AAC.00743-12

Dawson R, 2015, INT J TUBERC LUNG D, V19, P780, DOI 10.5588/ijtld.14.0868

de Carvalho LPS, 2009, J MED CHEM, V52, P5789, DOI 10.1021/jm9010719

De Groote MA, 2014, J ANTIMICROB CHEMOTH, V69, P1057, DOI 10.1093/jac/dkt451

De Lorenzo S, 2013, EUR RESPIR J, V41, P1386, DOI 10.1183/09031936.00124312

Dey T, 2013, J ANTIMICROB CHEMOTH, V68, P284, DOI 10.1093/jac/dks389

Dheda K, 2017, LANCET RESP MED, V5, P291, DOI [10.1016/s2213-2600(17)30079-6, 10.1016/S2213-2600(17)30079-6]

Diacon AH, 2015, AM J RESP CRIT CARE, V191, P943, DOI 10.1164/rccm.201410-1801OC

Diacon AH, 2014, NEW ENGL J MED, V371, P723, DOI 10.1056/NEJMoa1313865

Diacon AH, 2012, LANCET, V380, P986, DOI 10.1016/S0140-6736(12)61080-0

Diacon AH, 2009, NEW ENGL J MED, V360, P2397, DOI 10.1056/NEJMoa0808427

Disratthakit A, 2010, ANTIMICROB AGENTS CH, V54, P2684, DOI 10.1128/AAC.01545-09

Dorman SE, 2015, AM J RESP CRIT CARE, V191, P333, DOI 10.1164/rccm.201410-1843OC

Dorman SE, 2012, J INFECT DIS, V206, P1030, DOI 10.1093/infdis/jis461

Dubuisson T, 2010, J ANTIMICROB CHEMOTH, V65, P2590, DOI 10.1093/jac/dkq372

Esposito S, 2014, EUR RESPIR J, V44, P811, DOI 10.1183/09031936.00060414

Ferlazzo G, 2018, LANCET INFECT DIS, V18, P536, DOI 10.1016/S1473-3099(18)30100-2

Ferro BE, 2016, ANTIMICROB AGENTS CH, V60, P1097, DOI 10.1128/AAC.02615-15

Fox W, 1999, INT J TUBERC LUNG D, V3, pS231

Furin JJ, 2016, ANTIMICROB AGENTS CH, V60, P6591, DOI 10.1128/AAC.01163-16

Gillespie SH, 2014, NEW ENGL J MED, V371, P1577, DOI 10.1056/NEJMoa1407426

Gler MT, 2012, NEW ENGL J MED, V366, P2151, DOI 10.1056/NEJMoa1112433

Griffith D.E, 2018, AM J RESP CRIT CARE, V198, P1559, DOI [10.1164/rccm.201807-1318OC, DOI 10.1164/rccm.201807-1243ED]

Griffith DE, 2007, AM J RESP CRIT CARE, V175, P367, DOI 10.1164/rccm.200604-571ST

Gudzera OI, 2016, J ENZYM INHIB MED CH, V31, P201, DOI 10.1080/14756366.2016.1190712

Gudzera OI, 2016, BIOORGAN MED CHEM, V24, P1023, DOI 10.1016/j.bmc.2016.01.028

Harbut MB, 2015, P NATL ACAD SCI USA, V112, P4453, DOI 10.1073/pnas.1504022112

Haworth CS, 2017, THORAX, V72, DOI 10.1136/thoraxjnl-2017-210927

Heinrich N, 2015, J ANTIMICROB CHEMOTH, V70, P1558, DOI 10.1093/jac/dku553

Hewison C, 2017, EMERG INFECT DIS, V23, P1746, DOI 10.3201/eid2310.170468

Hotoda H, 2003, BIOORG MED CHEM LETT, V13, P2829, DOI 10.1016/S0960-894X(03)00596-1

Iqbal IK, 2018, PATHOGENS, V7, DOI 10.3390/pathogens7010024

Ishizaki Y, 2013, J BIOL CHEM, V288, P30309, DOI 10.1074/jbc.M113.492173

Jawahar MS, 2013, PLOS ONE, V8, DOI 10.1371/journal.pone.0067030

Jindani A, 2016, INT J TUBERC LUNG D, V20, P832, DOI 10.5588/ijtld.15.0577

Jindani A, 2014, NEW ENGL J MED, V371, P1599, DOI 10.1056/NEJMoa1314210

Kalia NP, 2017, P NATL ACAD SCI USA, V114, P7426, DOI 10.1073/pnas.1706139114

Kang S, 2017, EUR J MED CHEM, V136, P420, DOI 10.1016/j.ejmech.2017.05.021

Kang S, 2017, EUR J MED CHEM, V125, P807, DOI 10.1016/j.ejmech.2016.09.082

Kim TS, 2017, ANTIMICROB AGENTS CH, V61, DOI [10.1128/AAC.02752-16, 10.1128/aac.02752-16]

Kloss F, 2017, ANGEW CHEM INT EDIT, V56, P2187, DOI [10.1002/ange.201609737, 10.1002/anie.201609737]

Koga T, 2004, J ANTIMICROB CHEMOTH, V54, P755, DOI 10.1093/jac/dkh417

Lamprecht DA, 2016, NAT COMMUN, V7, DOI 10.1038/ncomms12393

Laughon BE, 2017, NAT REV DIS PRIMERS, V3, DOI 10.1038/nrdp.2017.15

Lee M, 2012, NEW ENGL J MED, V367, P1508, DOI 10.1056/NEJMoa1201964

Lee RE, 2014, NAT MED, V20, P152, DOI 10.1038/nm.3458

Lerat I, 2014, J INFECT DIS, V209, P905, DOI 10.1093/infdis/jit614

Li GL, 2017, INT J ANTIMICROB AG, V49, P364, DOI 10.1016/j.ijantimicag.2016.10.024

Li XF, 2017, J MED CHEM, V60, P8011, DOI 10.1021/acs.jmedchem.7b00631

Libardo MDJ, 2018, CURR OPIN PHARMACOL, V42, P81, DOI 10.1016/j.coph.2018.08.001

Liu JY, 2017, ACS INFECT DIS, V3, P72, DOI 10.1021/acsinfecdis.6b00158

Lounis N, 2009, ANTIMICROB AGENTS CH, V53, P4927, DOI 10.1128/AAC.00689-09

Lu P, 2018, SCI REP-UK, V8, DOI 10.1038/s41598-018-20989-8

Lv K, 2017, ACS MED CHEM LETT, V8, P636, DOI 10.1021/acsmedchemlett.7b00106

Makarov V, 2014, EMBO MOL MED, V6, P372, DOI 10.1002/emmm.201303575

Makarov V, 2009, SCIENCE, V324, P801, DOI 10.1126/science.1171583

Manjunatha Ujjini, 2009, Commun Integr Biol, V2, P215

Merle CS, 2014, NEW ENGL J MED, V371, P1588, DOI 10.1056/NEJMoa1315817

Moodley R, 2016, EUR RESPIR REV, V25, DOI 10.1183/16000617.0080-2015

Murray S., 2017, C RETR OPP INF BOST

Nash KA, 2009, ANTIMICROB AGENTS CH, V53, P1367, DOI 10.1128/AAC.01275-08

Nunn AJ, 2014, TRIALS, V15, DOI 10.1186/1745-6215-15-353

Olivier KN, 2017, AM J RESP CRIT CARE, V195, P814, DOI 10.1164/rccm.201604-0700OC

Ordway D.J., 2011, CURR PROTOC IMMUNOL

Palencia A, 2016, ANTIMICROB AGENTS CH, V60, P6271, DOI 10.1128/AAC.01339-16

Peloquin CA, 2017, ANTIMICROB AGENTS CH, V61

Pethe K, 2013, NAT MED, V19, P1157, DOI 10.1038/nm.3262

Philley JV, 2015, CHEST, V148, P499, DOI 10.1378/chest.14-2764

Phillips PPJ, 2012, J INFECT DIS, V205, pS250, DOI 10.1093/infdis/jis041

Piton J, 2018, ANTIMICROB AGENTS CH, V62, DOI [10.1128/AAC.00681-18, 10.1128/aac.00681-18]

Piubello A, 2014, INT J TUBERC LUNG D, V18, P1188, DOI 10.5588/ijtld.13.0075

Reddy VM, 2012, J ANTIMICROB CHEMOTH, V67, P1163, DOI 10.1093/jac/dkr589

Reddy VM, 2010, ANTIMICROB AGENTS CH, V54, P2840, DOI 10.1128/AAC.01601-09

Robertson GT, 2017, J ANTIMICROB CHEMOTH, V72, P770, DOI 10.1093/jac/dkw467

Sacksteder KA, 2012, FUTURE MICROBIOL, V7, P823, DOI [10.2217/fmb.12.56, 10.2217/FMB.12.56]

Salomon JJ, 2013, THER DELIV, V4, P915, DOI [10.4155/tde.13.62, 10.4155/TDE.13.62]

Savic RM, 2017, CLIN PHARMACOL THER, V102, P321, DOI 10.1002/cpt.634

Schwartz M, 2018, MICROB DRUG RESIST, V24, P1191, DOI 10.1089/mdr.2017.0286

Shigyo K, 2013, ANTIMICROB AGENTS CH, V57, P2834, DOI 10.1128/AAC.02542-12

Shoen C, 2018, ANTIMICROB AGENTS CH, V62, DOI [10.1128/aac.00493-18, 10.1128/ACC00493-18;e00493-18, 10.1128/AAC.00493-18, 10.1128/aac.00493-18]

Singh R, 2008, SCIENCE, V322, P1392, DOI 10.1126/science.1164571

Skripconoka V, 2013, EUR RESPIR J, V41, P1393, DOI 10.1183/09031936.00125812

Soni I, 2016, J MED MICROBIOL, V65, P1, DOI 10.1099/jmm.0.000198

Sotgiu G, 2017, INT J INFECT DIS, V56, P190, DOI 10.1016/j.ijid.2016.10.021

Sotgiu G, 2016, INT J MOL SCI, V17, DOI 10.3390/ijms17030373

Sotgiu G, 2012, EUR RESPIR J, V40, P1430, DOI 10.1183/09031936.00022912

Stein GE, 2006, CLIN INFECT DIS, V43, P518, DOI 10.1086/505494

Stover CK, 2000, NATURE, V405, P962, DOI 10.1038/35016103

Sutherland HS, 2018, BIOORGAN MED CHEM, V26, P1797, DOI 10.1016/j.bmc.2018.02.026

Svensson RJ, 2018, J INFECT DIS, V218, P991, DOI 10.1093/infdis/jiy242

Tahlan K, 2012, ANTIMICROB AGENTS CH, V56, P1797, DOI 10.1128/AAC.05708-11

Tong AST, 2017, ACS MED CHEM LETT, V8, P1019, DOI 10.1021/acsmedchemlett.7b00196

Tweed CD, 2018, BMC INFECT DIS, V18, DOI 10.1186/s12879-018-3230-6

Tweed CD, 2018, BMC MED, V16, DOI 10.1186/s12916-018-1033-7

Van Deun A, 2010, AM J RESP CRIT CARE, V182, P684, DOI 10.1164/rccm.201001-0077OC

van Ingen J, 2012, ANTIMICROB AGENTS CH, V56, P6324, DOI 10.1128/AAC.01505-12

Wallace RJ, 2014, J ANTIMICROB CHEMOTH, V69, P1945, DOI 10.1093/jac/dku062

Wallace RJ, 2002, ANTIMICROB AGENTS CH, V46, P3164, DOI 10.1128/AAC.46.10.3164-3167.2002

Wallis RS, 2014, PLOS ONE, V9, DOI 10.1371/journal.pone.0094462

Wallis RS, 2011, ANTIMICROB AGENTS CH, V55, P567, DOI 10.1128/AAC.01179-10

Wallis RS, 2010, J INFECT DIS, V202, P745, DOI 10.1086/655471

Yamaguchi T, 2016, PLOS NEGLECT TROP D, V10, DOI 10.1371/journal.pntd.0005013

Yunivita V, 2016, INT J ANTIMICROB AG, V48, P415, DOI 10.1016/j.ijantimicag.2016.06.016

Zhang DF, 2014, MOLECULES, V19, P4380, DOI 10.3390/molecules19044380

Zhang DF, 2012, J MED CHEM, V55, P8409, DOI 10.1021/jm300828h

Zong ZJ, 2018, ANTIMICROB AGENTS CH, V62, DOI [10.1128/aac.00165-18, 10.1128/aac.00165-18, 10.1128/AAC.00165-18;e00165-18]

NR 136

TC 22

Z9 28

U1 0

U2 13

PU LIPPINCOTT WILLIAMS & WILKINS

PI PHILADELPHIA

PA TWO COMMERCE SQ, 2001 MARKET ST, PHILADELPHIA, PA 19103 USA

SN 1070-5287

EI 1531-6971

J9 CURR OPIN PULM MED

JI Curr. Opin. Pulm. Med.

PD MAY

PY 2019

VL 25

IS 3

BP 271

EP 280

DI 10.1097/MCP.0000000000000570

PG 10

WC Respiratory System

WE Science Citation Index Expanded (SCI-EXPANDED)

SC Respiratory System

GA HX9XT

UT WOS:000467763000010

OA Bronze

DA 2026-05-07

ER

PT J

AU Wang, K

Pei, H

Huang, BA

Zhu, X

Zhang, J

Zhou, B

Zhu, L

Zhang, Y

Zhou, FF

AF Wang, Ke

Pei, Hao

Huang, Biao

Zhu, Xue

Zhang, Jue

Zhou, Bin

Zhu, Lan

Zhang, Yi

Zhou, Fan-Fan

TI The Expression of ABC Efflux Pump, Rv1217c-Rv1218c, and Its Association

with Multidrug Resistance of Mycobacterium tuberculosis in China

SO CURRENT MICROBIOLOGY

LA English

DT Article

ID DRUG-RESISTANCE; TRANSPORTER; PREVALENCE; DISCOVERY

AB Currently the treatment of Mycobacterium tuberculosis (TB) infection is largely limited due to the prevalence of multidrug resistance strains. Over-expressing the efflux pumps such as the ATP-binding cassette (ABC) transporter has been reported to significantly contribute to its resistance to several antibiotics. This study investigated the expression profile of one important ABC efflux pump, Rv1217c-Rv1218c, by quantitative real-time PCR (RT-qPCR) in clinical isolates from China, which also revealed its association with the multidrug resistance of M. tuberculosis. Significantly increased expressions of Rv1217c and Rv1218c at transcriptional level have been observed in multidrug-resistant TB group (MDR-TB) compared to those of the drug-susceptible group (P < 0.05), when H37Rv strain was used as the control. Furthermore, correlation analysis revealed that the over-expression of both Rv1217c and Rv1218c resulted in the higher minimum inhibition concentrations (MICs) of rifampicin (RIF) (OR = 1.01, P < 0.05 of Rv1217c; OR = 1.23, P < 0.05 of Rv1218c), while the over-expression of Rv1218c only led to the higher MICs of isoniazid (INH) (OR = 1.17, P < 0.05). Our findings contributed to the better understanding of the molecular mechanisms of ABC efflux pumps, in particular Rv1217c-Rv1218c, in M. tuberculosis and will assist in developing new antibiotic treatments for multidrug-resistant M. tuberculosis in the future.

C1 [Wang, Ke; Huang, Biao; Zhu, Xue; Zhang, Jue; Zhou, Bin; Zhu, Lan; Zhang, Yi] Jiangsu Inst Nucl Med, Key Lab Nucl Med, Minist Hlth, Jiangsu Key Lab Mol Nucl Med, Wuxi 214063, Jiangsu, Peoples R China.

[Pei, Hao] Wuxi Hosp Infect Dis, Wuxi 214005, Jiangsu, Peoples R China.

[Zhou, Fan-Fan] Univ Sydney, Fac Pharm, Sydney, NSW 2006, Australia.

C3 University of Sydney

RP Pei, H (corresponding author), Wuxi Hosp Infect Dis, Wuxi 214005, Jiangsu, Peoples R China.

EM cluseee@hotmail.com; peihao1008@sohu.com

RI Huang, B/HGA-7110-2022; Zhou, Fanfan/M-4404-2013; Zhu, Xue/AAR-9416-2020

OI Zhou, Fanfan/0000-0002-1982-1541;

FU Foundation of Department of Public Health of Jiangsu Province [H200954];

Natural Science Foundation of Jiangsu Province [BK2011168]

FX This work was financially supported by Grant from the Foundation of

Department of Public Health of Jiangsu Province (Grant No. H200954) and

the Natural Science Foundation of Jiangsu Province (Grant No.

BK2011168).

CR da Silva PEA, 2011, FEMS IMMUNOL MED MIC, V63, P1, DOI 10.1111/j.1574-695X.2011.00831.x

Balganesh M, 2012, ANTIMICROB AGENTS CH, V56, P2643, DOI 10.1128/AAC.06003-11

Balganesh M, 2010, ANTIMICROB AGENTS CH, V54, P5167, DOI 10.1128/AAC.00610-10

Braibant M, 2000, FEMS MICROBIOL REV, V24, P449, DOI 10.1016/S0168-6445(00)00034-6

Choudhuri BS, 2002, BIOCHEM J, V367, P279, DOI 10.1042/BJ20020615

Dover LG, 2011, J MED CHEM, V54, P6157, DOI 10.1021/jm200305q

Dye C, 2002, J INFECT DIS, V185, P1197, DOI 10.1086/339818

He GX, 2010, PLOS ONE, V5, DOI 10.1371/journal.pone.0010799

He GX, 2008, BMC INFECT DIS, V8, DOI 10.1186/1471-2334-8-166

Hu Y, 2008, MICROB DRUG RESIST, V14, P227, DOI 10.1089/mdr.2008.0823

Jean SS, 2011, INT J ANTIMICROB AG, V37, P291, DOI 10.1016/j.ijantimicag.2011.01.009

Jiang X, 2008, MICROB DRUG RESIST, V14, P7, DOI 10.1089/mdr.2008.0772

Koul A, 2011, NATURE, V469, P483, DOI 10.1038/nature09657

Molle V, 2004, FEMS MICROBIOL LETT, V234, P215, DOI 10.1016/j.femsle.2004.03.033

Moody JE, 2005, J BIOENERG BIOMEMBR, V37, P475, DOI 10.1007/s10863-005-9494-8

Orme IM, 2011, FUTURE MICROBIOL, V6, P161, DOI 10.2217/FMB.10.168

Pasca MR, 2004, ANTIMICROB AGENTS CH, V48, P3175, DOI 10.1128/AAC.48.8.3175-3178.2004

Pei H, 2011, FOLIA MICROBIOL, V56, P549, DOI 10.1007/s12223-011-0080-7

Shah NS, 2007, EMERG INFECT DIS, V13, P380, DOI 10.3201/eid1303.061400

Zhang Y, 2009, INT J TUBERC LUNG D, V13, P1320

Zignol M, 2006, J INFECT DIS, V194, P479, DOI 10.1086/505877

NR 21

TC 60

Z9 69

U1 2

U2 44

PU SPRINGER

PI NEW YORK

PA 233 SPRING ST, NEW YORK, NY 10013 USA

SN 0343-8651

EI 1432-0991

J9 CURR MICROBIOL

JI Curr. Microbiol.

PD MAR

PY 2013

VL 66

IS 3

BP 222

EP 226

DI 10.1007/s00284-012-0215-3

PG 5

WC Microbiology

WE Science Citation Index Expanded (SCI-EXPANDED)

SC Microbiology

GA 079PF

UT WOS:000314182400003

DA 2026-05-07

ER

PT J

AU Rajkhowa, S

Deka, RC

AF Rajkhowa, Sanchaita

Deka, Ramesh C.

TI DFT Based QSAR/QSPR Models in the Development of Novel Anti-tuberculosis

Drugs Targeting Mycobacterium tuberculosis

SO CURRENT PHARMACEUTICAL DESIGN

LA English

DT Article

DE Tuberculosis; 2D QSAR; 3D QSAR; DFT

ID DENSITY-FUNCTIONAL THEORY; ISONIAZID TARGET; SOFT ACIDS; AB-INITIO;

CELL-WALL; ARABINAN BIOSYNTHESIS; STRUCTURAL MECHANISM;

CHEMICAL-REACTIVITY; CATALASE-PEROXIDASE; MOLECULAR-DYNAMICS

AB Tuberculosis caused by Mycobacterium tuberculosis is an infectious bacterial disease which is a leading cause of mortality affecting more than 9 million people worldwide. The current standard regimens that are available for the treatment of TB are severely hampered due to the occurrence of multidrug-resistant (MDR-TB) and extensively drug-resistant (XDR-TB) strains of Mycobacterium tuberculosis. In the past few years, a huge and constantly expanding effort has been developed to understand the chemical-biological interaction of many new anti-tubercular drugs and their targets in mathematical terms. Here, we have elected to review only those studies concerning 2D and 3D QSAR models that contain different DFT based descriptors as their parameters.

C1 [Rajkhowa, Sanchaita; Deka, Ramesh C.] Tezpur Univ, Dept Chem Sci, Tezpur 784028, Assam, India.

C3 Tezpur University

RP Deka, RC (corresponding author), Tezpur Univ, Dept Chem Sci, Tezpur 784028, Assam, India.

EM ramesh@tezu.ernet.in

RI Rajkhowa, Sanchaita/HTM-9360-2023; Deka, Ramesh/AAA-5932-2020; Rajkhowa,

Sanchaita/HTM-9360-2023

OI Rajkhowa, Sanchaita/0000-0002-9407-1760; Rajkhowa,

Sanchaita/0000-0002-4834-2654

FU Department of Science and Technology (DST), New Delhi; DST under

DST/INSPIRE Fellowship program; INSPIRE [IF110258]; Tezpur University

FX Funding provided by Department of Science and Technology (DST), New

Delhi. SR would like to acknowledge financial support from DST under

DST/INSPIRE Fellowship program; INSPIRE Fellow code - IF110258, and

Tezpur University for providing the necessary resources during the

preparation of the manuscript.

CR Alcaide F, 1997, ANTIMICROB AGENTS CH, V41, P2270, DOI 10.1128/AAC.41.10.2270

Da Silva PEA, 2011, J ANTIMICROB CHEMOTH, V66, P1417, DOI 10.1093/jac/dkr173

Asif M, 2012, MINI-REV MED CHEM, V12, P1404

Bagchi MC, 2004, J MOL STRUC-THEOCHEM, V679, P179, DOI 10.1016/j.theochem.2004.04.013

BAMZAI AS, 1981, REV MOD PHYS, V53, P593, DOI 10.1103/RevModPhys.53.593

Banerjee R, 2008, EXPERT REV ANTI-INFE, V6, P713, DOI 10.1586/14787210.6.5.713

Barua N, 2012, CHEM BIOL DRUG DES, V79, P553, DOI 10.1111/j.1747-0285.2011.01289.x

Basak S.C., 1997, From Chemical Topology to Three-dimensional Geometry, P73

BASAK SC, 1987, MED SCI RES-BIOCHEM, V15, P605

Belanger AE, 1996, P NATL ACAD SCI USA, V93, P11919, DOI 10.1073/pnas.93.21.11919

Bertrand T, 2004, J BIOL CHEM, V279, P38991, DOI 10.1074/jbc.M402382200

BESRA GS, 1995, BIOCHEMISTRY-US, V34, P4257, DOI 10.1021/bi00013a015

BLESSINGTON B, 1990, J CHROMATOGR, V522, P195, DOI 10.1016/0021-9673(90)85189-3

BLOOM BR, 1992, SCIENCE, V257, P1055, DOI 10.1126/science.257.5073.1055

BRENNAN PJ, 1995, ANNU REV BIOCHEM, V64, P29, DOI 10.1146/annurev.bi.64.070195.000333

Campbell EA, 2001, CELL, V104, P901, DOI 10.1016/S0092-8674(01)00286-0

Car R, 2022, QUANT STRUCT ACT REL, V21920, P97

Carloni P, 2001, THEORETICAL BIOCH PR

Cavalli A, 2001, PROTEINS, V45, P478, DOI 10.1002/prot.10013

Chattaraj P K., 2003, COMPUTATIONAL MED CH, P295

Chattaraj PK, 2003, J PHYS CHEM A, V107, P4973, DOI 10.1021/jp034707u

CHATTARAJ PK, 1991, J AM CHEM SOC, V113, P1855, DOI 10.1021/ja00005a073

CHATTARAJ PK, 1994, J AM CHEM SOC, V116, P1067, DOI 10.1021/ja00082a031

Chermette H, 1999, J COMPUT CHEM, V20, P129, DOI 10.1002/(SICI)1096-987X(19990115)20:1<129::AID-JCC13>3.0.CO;2-A

Cho DH, 2001, B KOR CHEM SOC, V22, P388

CORNISHBOWDEN A, 1978, BIOCHEM J, V175, P969, DOI 10.1042/bj1750969

Cunha E.F.F.D., 2007, Computational and Mathematical Methods in Medicine, V8, P113, DOI [10.1080/17486700701374292, DOI 10.1080/17486700701374292]

Delley B, 2000, J CHEM PHYS, V113, P7756, DOI 10.1063/1.1316015

DESSEN A, 1995, SCIENCE, V267, P1638, DOI 10.1126/science.7886450

Díaz N, 2000, J AM CHEM SOC, V122, P4197, DOI 10.1021/ja994462s

DIETRICH SW, 1980, J MED CHEM, V23, P1201, DOI 10.1021/jm00185a010

Dover LG, 2011, J MED CHEM, V54, P6157, DOI 10.1021/jm200305q

Elstner M, 2003, J MOL STRUC-THEOCHEM, V632, P29, DOI 10.1016/S0166-1280(03)00286-0

Estrada E., 1999, Topological Indices and Related Descriptors in QSAR and QSPR, P403

Frisch MJ., 2009, Gaussian

FUKUI K, 1982, SCIENCE, V218, P747, DOI 10.1126/science.218.4574.747

Geerlings P, 2003, CHEM REV, V103, P1793, DOI 10.1021/cr990029p

GHOSH SK, 1982, PHYS REP, V92, P1, DOI 10.1016/0370-1573(82)90134-X

Gillespie SH, 2002, ANTIMICROB AGENTS CH, V46, P267, DOI 10.1128/AAC.46.2.267-274.2002

Gonze X, 2009, COMPUT PHYS COMMUN, V180, P2582, DOI 10.1016/j.cpc.2009.07.007

Gozalbes R., 2002, Current Drug Targets - Infectious Disorders, V2, P93

Guillemont J., 2009, CURR BIOACT COMPD, V5, P137, DOI [DOI 10.2174/157340709788451975, 10.2174/157340709788451975]

GUPTA SP, 1989, CHEM REV, V89, P1765, DOI 10.1021/cr00098a007

HANSCH C, 1964, J AM CHEM SOC, V86, P1616, DOI 10.1021/ja01062a035

Hazarika KK, 2009, STRUCT CHEM, V20, P1079, DOI 10.1007/s11224-009-9512-2

HEYM B, 1994, LANCET, V344, P293, DOI 10.1016/S0140-6736(94)91338-2

HOHENBERG P, 1964, PHYS REV B, V136, pB864, DOI 10.1103/PhysRevB.7.1912

Johnsson K, 1997, J BIOL CHEM, V272, P2834, DOI 10.1074/jbc.272.5.2834

JOHNSSON K, 1995, J AM CHEM SOC, V117, P5009, DOI 10.1021/ja00122a038

JOHNSSON K, 1994, J AM CHEM SOC, V116, P7425, DOI 10.1021/ja00095a063

Karelson M, 1996, CHEM REV, V96, P1027, DOI 10.1021/cr950202r

Khoo KH, 1996, J BIOL CHEM, V271, P28682, DOI 10.1074/jbc.271.45.28682

Klein ML, 2001, SCIENCE, V291, P2106, DOI 10.1126/science.1060087

KOHN W, 1965, PHYS REV, V140, P1133, DOI 10.1103/PhysRev.140.A1133

KONNO K, 1967, AM REV RESPIR DIS, V95, P461

Koopmans T.A., 1933, Physica, V1, P104, DOI [https://doi.org/10.1016/S0031-8914(34)90011-2, DOI 10.1016/S0031-8914(34)90011-2]

Kubinyi H, 1997, 3DQSAR DRUG DESIGN

Kuninyi H, 1993, QSAR HANSCH ANAL REL

Lin YM, 2002, BIOORGAN MED CHEM, V10, P2795, DOI 10.1016/S0968-0896(02)00094-9

Lipkowitz KB, 2007, MOL CONNECTIVITY CHI

Maddry JA, 1996, RES MICROBIOL, V147, P106, DOI 10.1016/0923-2508(96)80211-7

MOAZED D, 1987, NATURE, V327, P389, DOI 10.1038/327389a0

Monti E, 2005, J MED CHEM, V48, P857, DOI 10.1021/jm049508t

NALEWAJSKI RF, 1984, J AM CHEM SOC, V106, P944, DOI 10.1021/ja00316a020

Orme IM, 2005, TUBERCULOSIS, V86, P68

Parr R.G., 1989, DENSITY FUNCTIONAL T, DOI DOI 10.1093/OSO/9780195092769.001.0001

Parr RG, 1999, J AM CHEM SOC, V121, P1922, DOI 10.1021/ja983494x

PARR RG, 1983, J AM CHEM SOC, V105, P7512, DOI 10.1021/ja00364a005

PARR RG, 1983, ANNU REV PHYS CHEM, V34, P631, DOI 10.1146/annurev.pc.34.100183.003215

PARR RG, 1983, J PHYS CHEM-US, V87, P2810, DOI 10.1021/j100238a023

Pearson R.G., 1973, Hard and Soft Acids and Bases

PEARSON RG, 1963, J AM CHEM SOC, V85, P3533, DOI 10.1021/ja00905a001

PEARSON RG, 1987, J CHEM EDUC, V64, P561, DOI 10.1021/ed064p561

Penrose Roger., 1955, MATH PROC CAMBRIDGE, V51, P406, DOI [10.1017/S0305004100030401, DOI 10.1017/S0305004100030401]

Perlman DC, 1997, AIDS, V11, P1473, DOI 10.1097/00002030-199712000-00011

Platts JA, 2006, J INORG BIOCHEM, V100, P1199, DOI 10.1016/j.jinorgbio.2006.01.035

Rajagopal A.K., 1980, Adv. Chem. Phys, V41, P59

Rakesh, 2009, EUR J MED CHEM, V44, P460, DOI 10.1016/j.ejmech.2008.04.007

Rattan A, 1998, EMERG INFECT DIS, V4, P195, DOI 10.3201/eid0402.980207

Roy RK, 1998, J PHYS CHEM A, V102, P3746, DOI 10.1021/jp973450v

Rozwarski DA, 1998, SCIENCE, V279, P98, DOI 10.1126/science.279.5347.98

Sacchettini JC, 1996, RES MICROBIOL, V147, P36, DOI 10.1016/0923-2508(96)80201-4

Sagnella DE, 1996, BIOPHYS J, V71, P1172, DOI 10.1016/S0006-3495(96)79321-9

Saikia N, 2013, J COMPUT AID MOL DES, V27, P257, DOI 10.1007/s10822-013-9638-6

Saikia N, 2013, J MOL MODEL, V19, P215, DOI 10.1007/s00894-012-1534-9

Sarmah P, 2010, J MOL MODEL, V16, P411, DOI 10.1007/s00894-009-0551-9

Sarmah P, 2009, J COMPUT AID MOL DES, V23, P343, DOI 10.1007/s10822-009-9265-4

Schindler T, 2000, SCIENCE, V289, P1938, DOI 10.1126/science.289.5486.1938

SCHMIDT MW, 1993, J COMPUT CHEM, V14, P1347, DOI 10.1002/jcc.540141112

Scorpio A, 1996, NAT MED, V2, P662, DOI 10.1038/nm0696-662

Screnci D, 2000, BRIT J CANCER, V82, P966, DOI 10.1054/bjoc.1999.1026

Segall MD, 2002, J PHYS-CONDENS MAT, V14, P2717, DOI 10.1088/0953-8984/14/11/301

Sheen P, 2009, TUBERCULOSIS, V89, P109, DOI 10.1016/j.tube.2009.01.004

SOUCHARD JP, 1991, J MED CHEM, V34, P863, DOI 10.1021/jm00106a056

Speck-Planche A, 2010, CURR PHARM DESIGN, V16, P2656, DOI 10.2174/138161210792389289

Srivastava HK, 2005, INT J QUANTUM CHEM, V103, P237, DOI 10.1002/qua.20506

Stigliani JL, 2008, J MOL GRAPH MODEL, V27, P536, DOI 10.1016/j.jmgm.2008.09.006

Sulpizi M, 2002, QUANT STRUCT-ACT REL, V21, P173, DOI 10.1002/1521-3838(200207)21:2<173::AID-QSAR173>3.0.CO;2-B

TAKAYAMA K, 1979, ANTIMICROB AGENTS CH, V16, P240, DOI 10.1128/AAC.16.2.240

TAKAYAMA K, 1989, ANTIMICROB AGENTS CH, V33, P1493, DOI 10.1128/AAC.33.9.1493

Tangallapally RP, 2007, BIOORG MED CHEM LETT, V17, P6638, DOI 10.1016/j.bmcl.2007.09.048

Tangallapally RP, 2006, BIOORG MED CHEM LETT, V16, P2584, DOI 10.1016/j.bmcl.2006.02.048

Tangallapally RP, 2005, J MED CHEM, V48, P8261, DOI 10.1021/jm050765n

Tangallapally RP, 2004, J MED CHEM, V47, P5276, DOI 10.1021/jm049972y

Tawari NR, 2010, J COMPUT CHEM, V31, P739, DOI 10.1002/jcc.21371

Timmins GS, 2006, MOL MICROBIOL, V62, P1220, DOI 10.1111/j.1365-2958.2006.05467.x

Todeschini R., 2000, HDB MOL DESCRIPTORS

UGI I, 1990, CONCEPTS AND APPLICATIONS OF MOLECULAR SIMILARITY, P239

Van Damme S, 2010, J MOL STRUC-THEOCHEM, V943, P83, DOI 10.1016/j.theochem.2009.10.031

Venkataraman L, 2007, NANO LETT, V7, P502, DOI 10.1021/nl062923j

Vilchèze C, 2006, NAT MED, V12, P1027, DOI 10.1038/nm1466

Waisser K, 2003, ARCH PHARM, V336, P322, DOI 10.1002/ardp.200300725

Wan J, 2004, J COMPUT CHEM, V25, P1827, DOI 10.1002/jcc.20122

WEHRLI W, 1983, REV INFECT DIS, V5, pS407

WILKINSON R, 1961, J AM CHEM SOC, V83, P2212, DOI 10.1021/ja01470a052

WOLD S, 1991, QUANT STRUCT-ACT REL, V10, P191, DOI 10.1002/qsar.19910100302

WOLUCKA BA, 1994, J BIOL CHEM, V269, P23328

World Health organisation, 2011, PROGR PROJ GLOB TUB

World Health Organisation, 2013, WHO ISS INT GUID NEW

Yao SW, 2003, BIOORGAN MED CHEM, V11, P4999, DOI 10.1016/j.bmc.2003.09.005

Yuen LKW, 1999, J CLIN MICROBIOL, V37, P3844, DOI 10.1128/JCM.37.12.3844-3850.1999

Zhang Y, 2003, J ANTIMICROB CHEMOTH, V52, P790, DOI 10.1093/jac/dkg446

NR 122

TC 15

Z9 15

U1 0

U2 27

PU BENTHAM SCIENCE PUBL LTD

PI SHARJAH

PA EXECUTIVE STE Y-2, PO BOX 7917, SAIF ZONE, 1200 BR SHARJAH, U ARAB

EMIRATES

SN 1381-6128

EI 1873-4286

J9 CURR PHARM DESIGN

JI Curr. Pharm. Design

PY 2014

VL 20

IS 27

BP 4455

EP 4473

PG 19

WC Pharmacology & Pharmacy

WE Science Citation Index Expanded (SCI-EXPANDED)

SC Pharmacology & Pharmacy

GA AK6AL

UT WOS:000338509300010

DA 2026-05-07

ER

PT J

AU Sun, L

Zhang, LQ

Wang, T

Jiao, WW

Li, QJ

Yin, QQ

Li, JQ

Qi, H

Xu, F

Shen, C

Xiao, J

Liu, SP

Mokrousov, I

Huang, HR

Shen, A

AF Sun, Lin

Zhang, Liqun

Wang, Ting

Jiao, Weiwei

Li, Qinjing

Yin, Qingqin

Li, Jieqiong

Qi, Hui

Xu, Fang

Shen, Chen

Xiao, Jing

Liu, Shuping

Mokrousov, Igor

Huang, Hairong

Shen, Adong

TI Mutations of Mycobacterium tuberculosis induced by

anti-tuberculosis treatment result in metabolism changes and elevation

of ethambutol resistance

SO INFECTION GENETICS AND EVOLUTION

LA English

DT Article

DE Mycobacterium tuberculosis; Drug resistance; Whole-genome sequencing;

Metabolomics

ID DRUG-RESISTANCE; IDENTIFICATION; GENE; PATHWAY; KINASE

AB Selective pressure from antibiotic use is one of the most important risk factors associated with the development of drug resistance in Mycobacterium tuberculosis (MTB). However, the mechanisms underlying drug resistance at the molecular level remain partly unclear. Therefore, the purpose of this study was to investigate the potential functional effect of novel mutations arising from anti-tuberculosis treatment. We analyzed two multidrug-resistant TB (MDR-TB) isolates from the same patient; one collected before and one almost a year after commencing MDR-TB treatment. The post-treatment isolate exhibited elevated ethambutol resistance. We sequenced the whole genomes of the two clinical isolates and detected six novel polymorphisms affecting the genes Rv1026, nc0021, Rv2155c, Rv2437, and Rv3696c, and the intergenic region between Rv2764c and Rv2765. Metabolomics approach was used to reveal the effect of the found variation on the metabolic pathways of MTB. Partial least squares-discriminant analysis showed a clear differentiation between the two isolates, involving a total of 175 metabolites. Pathway analysis showed that these metabolites are mainly involved in amino sugar and nucleotide sugar metabolism, beta-alanine metabolism, sulfur metabolism, and galactose metabolism. The increased ethambutol resistance exhibited by the post-treatment MDR-TB strain could speculatively be linked to the identified genetic variations, which affected the synthesis of a number of metabolites associated with sources of carbon and energy. This may have been the main factor underlying the increased ethambutol resistance of this isolate.

C1 [Sun, Lin; Wang, Ting; Jiao, Weiwei; Li, Qinjing; Yin, Qingqin; Li, Jieqiong; Qi, Hui; Xu, Fang; Shen, Chen; Xiao, Jing; Liu, Shuping; Shen, Adong] Capital Med Univ, Key Lab Major Dis Children,Natl Clin Res Ctr Resp, Minist Educ,Beijing Pediat Res Inst,Beijing Child, Natl Key Discipline Pediat,Beijing Key Lab Pediat, 56 Nanlishi Rd, Beijing 100045, Peoples R China.

[Zhang, Liqun; Huang, Hairong] Capital Med Univ, Natl Clin Lab TB, Beijing Key Lab Drug Resistant TB Res, Beijing Chest Hosp,Beijing TB & Thorac Tumor Inst, 97 Beimachang Rd, Beijing 101149, Peoples R China.

[Mokrousov, Igor] St Petersburg Pasteur Inst, Lab Mol Epidemiol & Evolutionary Genet, St Petersburg, Russia.

C3 Capital Medical University; Capital Medical University; Saint-Petersburg

Pasteur Institute

RP Shen, A (corresponding author), Capital Med Univ, Key Lab Major Dis Children,Natl Clin Res Ctr Resp, Minist Educ,Beijing Pediat Res Inst,Beijing Child, Natl Key Discipline Pediat,Beijing Key Lab Pediat, 56 Nanlishi Rd, Beijing 100045, Peoples R China.; Huang, HR (corresponding author), Capital Med Univ, Natl Clin Lab TB, Beijing Key Lab Drug Resistant TB Res, Beijing Chest Hosp,Beijing TB & Thorac Tumor Inst, 97 Beimachang Rd, Beijing 101149, Peoples R China.; Mokrousov, I (corresponding author), St Petersburg Pasteur Inst, St Petersburg 197101, Russia.

EM imokrousov@mail.ru; huanghairong@tb123.org; shenad16@hotmail.com

RI shen, chen/AAC-2458-2020; 孙, 琳/HGE-6966-2022; Huang,

Hairong/AGM-6480-2022; Mokrousov, Igor/J-3640-2014

FU National Natural Science Foundation of China [81271889, 81450057]

FX This work was supported by the National Natural Science Foundation of

China [nos. 81271889 and 81450057].

CR Ali A, 2015, PLOS ONE, V10, DOI 10.1371/journal.pone.0117771

Anishetty S, 2005, COMPUT BIOL CHEM, V29, P368, DOI 10.1016/j.compbiolchem.2005.07.001

Bisson GP, 2012, J BACTERIOL, V194, P6441, DOI 10.1128/JB.01013-12

Chuang YM, 2015, MBIO, V6, DOI 10.1128/mBio.02428-14

Coll F, 2014, NAT COMMUN, V5, DOI 10.1038/ncomms5812

Cuevas-Córdoba B, 2015, ANTIMICROB AGENTS CH, V59, P5455, DOI 10.1128/AAC.00117-15

Farhat MR, 2016, AM J RESP CRIT CARE, V194, P621, DOI 10.1164/rccm.201510-2091OC

Franzblau SG, 1998, J CLIN MICROBIOL, V36, P362, DOI 10.1128/JCM.36.2.362-366.1998

Hasan Md. Anayet, 2014, Genomics & Informatics, V12, P268, DOI 10.5808/GI.2014.12.4.268

Hazbón MH, 2005, ANTIMICROB AGENTS CH, V49, P3794, DOI 10.1128/AAC.49.9.3794-3802.2005

Kanehisa M, 2006, NUCLEIC ACIDS RES, V34, pD354, DOI 10.1093/nar/gkj102

Lau SKP, 2015, EMERG MICROBES INFEC, V4, DOI 10.1038/emi.2015.6

Leung KL, 2010, J APPL MICROBIOL, V109, P2087, DOI 10.1111/j.1365-2672.2010.04840.x

Loots DT, 2014, ANTIMICROB AGENTS CH, V58, P2144, DOI 10.1128/AAC.02344-13

Marrero J, 2013, PLOS PATHOG, V9, DOI 10.1371/journal.ppat.1003116

MIKUSOVA K, 1995, ANTIMICROB AGENTS CH, V39, P2484, DOI 10.1128/AAC.39.11.2484

Miotto P, 2017, EUR RESPIR J, V50, DOI 10.1183/13993003.01354-2017

Mokrousov I, 2002, J CLIN MICROBIOL, V40, P3810, DOI 10.1128/JCM.40.10.3810-3813.2002

Nguyen L, 2009, ANNU REV PHARMACOL, V49, P427, DOI 10.1146/annurev-pharmtox-061008-103123

Nimmo C, 2017, INT J INFECT DIS, V62, P44, DOI 10.1016/j.ijid.2017.07.007

Olivier I, 2011, J CELL TISSUE RES, V11, P2655

Perdigao J, 2009, INT J ANTIMICROB AG, V33, P334, DOI 10.1016/j.ijantimicag.2008.09.021

Pérez-Lago L, 2014, J INFECT DIS, V209, P98, DOI 10.1093/infdis/jit439

Sharma K, 2017, OMICS, V21, P413, DOI 10.1089/omi.2017.0070

Singh M, 2016, SCI REP-UK, V6, DOI 10.1038/srep26900

Smith T, 2014, CURR TOP MICROBIOL, V374, P53, DOI 10.1007/82_2012_279

Subramanian D, 2015, GENE, V574, P149, DOI 10.1016/j.gene.2015.08.006

Supply P, 2006, J CLIN MICROBIOL, V44, P4498, DOI 10.1128/JCM.01392-06

TELENTI A, 1993, LANCET, V341, P647, DOI 10.1016/0140-6736(93)90417-F

Telenti A, 1997, NAT MED, V3, P567, DOI 10.1038/nm0597-567

Thakur M, 2008, BIOCHEM J, V415, P27, DOI 10.1042/BJ20080234

Tufariello JM, 2004, INFECT IMMUN, V72, P515, DOI 10.1128/IAI.72.1.515-526.2004

VANEMBDEN JDA, 1993, J CLIN MICROBIOL, V31, P406, DOI 10.1128/JCM.31.2.406-409.1993

Vilcheze C, 2014, MICROBIOL SPECTR, V2

WHO, 2017, GLOB TUB CONTR

Zhang Zong-de, 2007, Zhonghua Jiehe He Huxi Zazhi, V30, P683

Zignol M, 2018, LANCET INFECT DIS, V18, P675, DOI 10.1016/S1473-3099(18)30073-2

NR 37

TC 10

Z9 12

U1 0

U2 16

PU ELSEVIER

PI AMSTERDAM

PA RADARWEG 29, 1043 NX AMSTERDAM, NETHERLANDS

SN 1567-1348

EI 1567-7257

J9 INFECT GENET EVOL

JI Infect. Genet. Evol.

PD AUG

PY 2019

VL 72

SI SI

BP 151

EP 158

DI 10.1016/j.meegid.2018.09.027

PG 8

WC Infectious Diseases

WE Science Citation Index Expanded (SCI-EXPANDED)

SC Infectious Diseases

GA IE8AW

UT WOS:000472597300017

DA 2026-05-07

ER

PT J

AU Hong, H

Dooley, KE

Starbird, LE

Francis, HW

Farley, JE

AF Hong, Hyejeong

Dooley, Kelly E.

Starbird, Laura E.

Francis, Howard W.

Farley, Jason E.

TI Adverse outcome pathway for aminoglycoside ototoxicity in drug-resistant

tuberculosis treatment

SO ARCHIVES OF TOXICOLOGY

LA English

DT Article

DE Aminoglycoside; Ototoxicity; Sensorineural hearing loss; Tuberculosis

ID MITOCHONDRIAL 1555A-GREATER-THAN-G MUTATION; INDUCED HEARING-LOSS;

N-TERMINAL KINASE; OUTER HAIR-CELLS; OXIDATIVE STRESS; GENTAMICIN

OTOTOXICITY; MULTIDRUG-RESISTANT; PROTECTIVE ROLE; RENAL TOXICITY; ROS

PRODUCTION

AB Individuals treated for multidrug-resistant tuberculosis (MDR-TB) with aminoglycosides (AGs) in resource-limited settings often experience permanent hearing loss. However, AG ototoxicity has never been conceptually integrated or causally linked to MDR-TB patients' pre-treatment health condition. We sought to develop a framework that examines the relationships between pre-treatment conditions and AG-induced hearing loss among MDR-TB-infected individuals in sub-Saharan Africa. The adverse outcome pathway (AOP) approach was used to develop a framework linking key events (KEs) within a biological pathway that results in adverse outcomes (AO), which are associated with chemical perturbation of a molecular initiating event (MIE). This AOP describes pathways initiating from AG accumulation in hair cells, sound transducers of the inner ear immediately after AG administration. After administration, the drug catalyzes cellular oxidative stress due to overproduction of reactive oxygen species. Since oxidative stress inhibits mitochondrial protein synthesis, hair cells undergo apoptotic cell death, resulting in irreversible hearing loss (AO). We identified the following pre-treatment conditions that worsen the causal linkage between MIE and AO: HIV, malnutrition, aging, noise, smoking, and alcohol use. The KEs are: (1) nephrotoxicity, pre-existing hearing loss, and hypoalbuminemia that catalyzes AG accumulation; (2) immunodeficiency and antioxidant deficiency that trigger oxidative stress pathways; and (3) co-administration of mitochondrial toxic drugs that hinder mitochondrial protein synthesis, causing apoptosis. This AOP clearly warrants the development of personalized interventions for patients undergoing MDR-TB treatment. Such interventions (i.e., choosing less ototoxic drugs, scheduling frequent monitoring, modifying nutritional status, avoiding poly-pharmacy) will be required to limit the burden of AG ototoxicity.

C1 [Hong, Hyejeong; Farley, Jason E.] Johns Hopkins Univ, Sch Nursing, 525 North Wolfe St, Baltimore, MD 21205 USA.

[Hong, Hyejeong; Farley, Jason E.] Johns Hopkins Univ, Sch Nursing, REACH Initiat, 855 N Wolfe St, Baltimore, MD 21205 USA.

[Dooley, Kelly E.] Johns Hopkins Univ, Div Clin Pharmacol, Sch Med, 600 North Wolfe St, Baltimore, MD 21205 USA.

[Dooley, Kelly E.] Johns Hopkins Univ, Div Infect Dis, Sch Med, 600 North Wolfe St, Baltimore, MD 21205 USA.

[Starbird, Laura E.] Columbia Univ, Ctr Hlth Policy, Sch Nursing, 560 W 168 St, New York, NY 10032 USA.

[Francis, Howard W.] Duke Univ, Div Head & Neck Surg & Commun Sci, Sch Med, 40 Duke Med Circle, Durham, NC 27710 USA.

C3 Johns Hopkins University; Johns Hopkins University; Johns Hopkins

University; Johns Hopkins University; Columbia University; Duke

University

RP Hong, H (corresponding author), Johns Hopkins Univ, Sch Nursing, 525 North Wolfe St, Baltimore, MD 21205 USA.; Hong, H (corresponding author), Johns Hopkins Univ, Sch Nursing, REACH Initiat, 855 N Wolfe St, Baltimore, MD 21205 USA.

EM hhong13@jhu.edu

RI Dooley, Kelly/LUZ-8112-2024

OI Dooley, Kelly/0000-0001-9013-5256; Farley, Jason/0000-0002-6896-0134;

Starbird, Laura/0000-0003-4056-2422

FU National Institute of Allergy and Infectious Disease [R01

AI104488-01A1]; National Institute of Nursing Research of the National

Institutes of Health [F31 NR016910-01A1]; Sigma Theta Tau International

Global Nursing Research Grant; Sigma Theta Tau International/Association

of Nurses in AIDS Care Grant; Global Korean Nursing Foundation

Scientific Award; Dr. Scholl Foundation Dissertation Scholarship; Johns

Hopkins Center for Global Health Established Field Placements Grant;

National Institute of Nursing Research [T32NR014205] Funding Source: NIH

RePORTER

FX Research reported in this manuscript was funded by the National

Institute of Allergy and Infectious Disease (R01 AI104488-01A1 to J.

Farley), the National Institute of Nursing Research (F31 NR016910-01A1

to H. Hong) of the National Institutes of Health, Sigma Theta Tau

International Global Nursing Research Grant, Sigma Theta Tau

International/Association of Nurses in AIDS Care Grant, Global Korean

Nursing Foundation Scientific Award, Dr. Scholl Foundation Dissertation

Scholarship, the Johns Hopkins Center for Global Health Established

Field Placements Grant. We would like to express our appreciation to

Martin Blair for his editorial support. The content is solely the

responsibility of the authors and does not necessarily represent the

official views of the aforementioned organizations/institutions. The

authors declare that they have no conflict of interest.

CR Abi-Hachem Ralph N, 2010, Recent Pat CNS Drug Discov, V5, P147

Aladag I, 2016, J LARYNGOL OTOL, V130, P440, DOI 10.1017/S0022215116000992

Albano E, 2006, P NUTR SOC, V65, P278, DOI 10.1079/PNS2006496

Alford RL, 2014, GENET MED, V16, P347, DOI 10.1038/gim.2014.2

Alomar MJ, 2014, SAUDI PHARM J, V22, P83, DOI 10.1016/j.jsps.2013.02.003

Anema Aranka, 2009, Curr HIV/AIDS Rep, V6, P224, DOI 10.1007/s11904-009-0030-z

Ankley GT, 2010, ENVIRON TOXICOL CHEM, V29, P730, DOI 10.1002/etc.34

[Anonymous], 2013, GUID DOC DEV ASS ADV

[Anonymous], 2012, PROP TEMPL GUID DEV

[Anonymous], NUTR HIV AIDS

[Anonymous], 2017, World Health Statistics 2017: Monitoring Health for The SDGs

[Anonymous], 2014, World Health Organization

[Anonymous], SOC DET HLTH WHAT AR

ARAN JM, 1975, ACTA OTO-LARYNGOL, V79, P24, DOI 10.3109/00016487509124650

Ariano RE, 2008, ANN PHARMACOTHER, V42, P1282, DOI 10.1345/aph.1L001

Aukrust P, 2005, BLOOD, V105, P4730, DOI 10.1182/blood-2004-11-4272

Avent ML, 2011, INTERN MED J, V41, P441, DOI 10.1111/j.1445-5994.2011.02452.x

Bächinger D, 2018, TOXICOL LETT, V291, P121, DOI 10.1016/j.toxlet.2018.04.007

Bankaitis A E, 1995, Ear Nose Throat J, V74, P353

BAREGGI R, 1990, PHARMACOL RES, V22, P635, DOI 10.1016/S1043-6618(05)80056-8

Bartlett JG GJ., 2012, Medical Management of HIV Infection

Basile AS, 1996, NAT MED, V2, P1338, DOI 10.1038/nm1296-1338

Benhar M, 2002, EMBO REP, V3, P420, DOI 10.1093/embo-reports/kvf094

Benhar M, 2001, MOL CELL BIOL, V21, P6913, DOI 10.1128/MCB.21.20.6913-6926.2001

Bisaso Kuteesa R., 2014, In Silico Pharmacology, V2, P3, DOI 10.1186/s40203-014-0003-9

Bitner-Glindzicz M, 2009, NEW ENGL J MED, V360, P640, DOI 10.1056/NEJMc0806396

Blot SI, 2014, ADV DRUG DELIVER REV, V77, P3, DOI 10.1016/j.addr.2014.07.006

Bodmer D, 2002, LARYNGO RHINO OTOL, V81, P853, DOI 10.1055/s-2002-36100

Bosch J, 2014, OMICS, V18, P481, DOI 10.1089/omi.2013.0166

Calza L, 2011, SCAND J INFECT DIS, V43, P656, DOI 10.3109/00365548.2011.572906

Campbell KCM, 2016, INT J AUDIOL, V55, P273, DOI 10.3109/14992027.2016.1143980

Cernada M., 2014, NeoReviews, V15, pe449

CHANDRASEKHAR SS, 1992, AM J OTOL, V13, P207

CHOI DW, 1992, J NEUROBIOL, V23, P1261, DOI 10.1002/neu.480230915

Clerici WJ, 1996, HEARING RES, V98, P116, DOI 10.1016/0378-5955(96)00075-5

Corns LF, 2017, FRONT MOL NEUROSCI, V10, DOI 10.3389/fnmol.2017.00326

COX EC, 1964, P NATL ACAD SCI USA, V51, P703, DOI 10.1073/pnas.51.4.703

Cui Hang, 2012, J Signal Transduct, V2012, P646354, DOI 10.1155/2012/646354

Dai CF, 2006, HEARING RES, V213, P64, DOI 10.1016/j.heares.2005.11.011

DAVIES J, 1965, SCIENCE, V149, P1096, DOI 10.1126/science.149.3688.1096

de Grey ADJ., 1999, MITOCHONDRIAL FREE R

de Jager P, 2002, INT J TUBERC LUNG D, V6, P622

de Pee S, 2010, FOOD NUTR BULL, V31, pS313, DOI 10.1177/15648265100314S403

Dogan M, 2017, INT J PEDIATR OTORHI, V96, P140, DOI 10.1016/j.ijporl.2017.03.023

Donohue JF, 2006, THORAX, V61, P461, DOI 10.1136/thx.2005.053058

Elias A., 2013, Am. J. Pharmacol. Toxicol, V8, P187, DOI DOI 10.3844/AJPTSP.2013.187.196

Esterberg R, 2016, J CLIN INVEST, V126, P3556, DOI 10.1172/JCI84939

Farris HE, 2004, J PHYSIOL-LONDON, V558, P769, DOI 10.1113/jphysiol.2004.061267

Fetoni AR, 2004, INT J AUDIOL, V43, P166, DOI 10.1080/14992020400050023

Fiume G, 2012, NUCLEIC ACIDS RES, V40, P3548, DOI 10.1093/nar/gkr1224

Fox DJ, 2016, J CYST FIBROS, V15, P518, DOI 10.1016/j.jcf.2015.06.005

Fraisse T, 2014, AGE AGEING, V43, P676, DOI 10.1093/ageing/afu023

Freed EO., 2007, HIVs and Their Replication

García-Alcántara F, 2018, HEARING RES, V358, P10, DOI 10.1016/j.heares.2017.12.004

GARETZ SL, 1994, HEARING RES, V77, P81, DOI 10.1016/0378-5955(94)90255-0

Gonzalez LS, 1998, AM FAM PHYSICIAN, V58, P1811

Hamasaki K, 1997, BIOCHEMISTRY-US, V36, P12323, DOI 10.1021/bi970962r

Hashino E, 1995, BRAIN RES, V704, P135, DOI 10.1016/0006-8993(95)01198-6

HAYASHIDA T, 1985, ARCH OTO-RHINO-LARYN, V242, P257, DOI 10.1007/BF00453548

Henderson D, 2006, EAR HEARING, V27, P1, DOI 10.1097/01.aud.0000191942.36672.f3

HILL AB, 1965, P ROY SOC MED, V58, P295, DOI 10.1177/003591576505800503

Hirose K, 1997, HEARING RES, V104, P1, DOI 10.1016/S0378-5955(96)00169-4

Hirose K, 2014, JARO-J ASSOC RES OTO, V15, P555, DOI 10.1007/s10162-014-0458-8

Hobbie SN, 2008, P NATL ACAD SCI USA, V105, P20888, DOI 10.1073/pnas.0811258106

Hong H, 2018, INT J TUBERC LUNG D, V22, P667, DOI 10.5588/ijtld.17.0830

Hood MLH, 2013, EUR J CLIN NUTR, V67, P1122, DOI 10.1038/ejcn.2013.143

Hu WM, 2018, AGING DIS, V9, P133, DOI 10.14336/AD.2017.0119

Huth M E, 2011, Int J Otolaryngol, V2011, P937861, DOI 10.1155/2011/937861

HYDE GE, 1995, OTOLARYNG HEAD NECK, V113, P530, DOI 10.1016/S0194-5998(95)70043-9

Imamura S, 2003, JARO-J ASSOC RES OTO, V4, P176, DOI 10.1007/s10162-002-2036-8

Isik B, 2007, INHAL TOXICOL, V19, P767, DOI 10.1080/08958370701401418

Ivanov AV, 2017, OXID MED CELL LONGEV, V2017, DOI 10.1155/2017/3496043

Ivers LC, 2009, CLIN INFECT DIS, V49, P1096, DOI 10.1086/605573

Iwai H, 2008, BRAIN RES, V1208, P8, DOI 10.1016/j.brainres.2008.02.069

Jiang MY, 2017, FRONT CELL NEUROSCI, V11, DOI 10.3389/fncel.2017.00308

Jin S, 2015, INFECT CHEMOTHER, V47, P239, DOI 10.3947/ic.2015.47.4.239

Kabahuma RI, 2011, INT J PEDIATR OTORHI, V75, P611, DOI 10.1016/j.ijporl.2011.01.029

Kalinec GM, 2017, FRONT CELL NEUROSCI, V11, DOI 10.3389/fncel.2017.00192

Karasawa T, 2010, CELL DEATH DIS, V1, DOI 10.1038/cddis.2010.80

KEENE M, 1982, ARCH OTOLARYNGOL, V108, P65

Kelly P, 1999, AIDS, V13, P495, DOI 10.1097/00002030-199903110-00008

Kenyon C, 2011, S AFR J HIV MED, P43

Khare M, 2014, INT J PEDIAT, V2014, DOI 10.1155/2014/254396

Kim CW, 2018, YONSEI MED J, V59, P141, DOI 10.3349/ymj.2018.59.1.141

Kim SH, 2011, PLOS ONE, V6, DOI 10.1371/journal.pone.0021656

Kim S, 2017, AM SURGEON, V83, P1220

Kirkwood NK, 2017, FRONT CELL NEUROSCI, V11, DOI 10.3389/fncel.2017.00262

Koethe JR, 2009, CLIN INFECT DIS, V49, P787, DOI 10.1086/605285

Kohler JJ, 2009, LAB INVEST, V89, P513, DOI 10.1038/labinvest.2009.14

Koo JW, 2015, SCI TRANSL MED, V7, DOI 10.1126/scitranslmed.aac5546

Krause KM, 2016, CSH PERSPECT MED, V6, DOI 10.1101/cshperspect.a027029

Krause KH, 1997, CELL, V88, P439, DOI 10.1016/S0092-8674(00)81884-X

Ladrech S, 2017, HISTOCHEM CELL BIOL, V148, P129, DOI 10.1007/s00418-017-1548-6

Lasisi AO, 2014, INT J PEDIATR OTORHI, V78, P1870, DOI 10.1016/j.ijporl.2014.08.014

Le Prell CG, 2014, JARO-J ASSOC RES OTO, V15, P375, DOI 10.1007/s10162-014-0448-x

Lecain E, 2007, APOPTOSIS, V12, P333, DOI 10.1007/s10495-006-0580-0

Lee Kyu-Yup, 2013, Korean J Audiol, V17, P45, DOI 10.7874/kja.2013.17.2.45

Lemasters JJ, 2005, REJUV RES, V8, P3, DOI 10.1089/rej.2005.8.3

LENOIR M, 1987, HEARING RES, V26, P199, DOI 10.1016/0378-5955(87)90112-2

Lesniak W, 2005, CHEM RES TOXICOL, V18, P357, DOI 10.1021/tx0496946

Marcotti W, 2005, J PHYSIOL-LONDON, V567, P505, DOI 10.1113/jphysiol.2005.085951

Marí M, 2009, ANTIOXID REDOX SIGN, V11, P2685, DOI [10.1089/ars.2009.2695, 10.1089/ARS.2009.2695]

Marks SM, 2014, EMERG INFECT DIS, V20, P812, DOI 10.3201/eid2005.131037

Marra CM, 1997, ARCH NEUROL-CHICAGO, V54, P407, DOI 10.1001/archneur.1997.00550160049015

McNaghten AD, 2001, ARCH OTOLARYNGOL, V127, P1516

Michaels L., 2012, Ototoxic Damage to the Inner Ear

Miller ME, 2010, OTOL NEUROTOL, V31, P681, DOI 10.1097/MAO.0b013e3181dbb7e4

Mingeot-Leclercq MP, 1999, ANTIMICROB AGENTS CH, V43, P727, DOI 10.1128/AAC.43.4.727

Mohamed-Hussein A., 2016, Egyptian Journal of Bronchology, V10, P58

Morike K, 1997, DRUG AGING, V10, P259

Mostafa BE, 2007, ACTA OTO-LARYNGOL, V127, P234, DOI 10.1080/00016480600794495

Murphy MP, 2013, CELL METAB, V18, P145, DOI 10.1016/j.cmet.2013.07.006

Nakamagoe M, 2010, HEARING RES, V261, P67, DOI 10.1016/j.heares.2010.01.004

Nishida I, 1996, ORL J OTO-RHINO-LARY, V58, P68, DOI 10.1159/000276801

NORDSTROM L, 1990, J ANTIMICROB CHEMOTH, V25, P159, DOI 10.1093/jac/25.1.159

Op de Beeck K, 2011, HEAR RES, V281, P18

Owens KN, 2007, J COMP NEUROL, V502, P522, DOI 10.1002/cne.21345

Pagkalis S, 2011, DRUGS, V71, P2277, DOI 10.2165/11597020-000000000-00000

Park YH., 2018, Clinical and experimental otorhinolaryngology

Patel R, 2018, CLIN GERIATR MED, V34, P163, DOI 10.1016/j.cger.2018.01.001

Prasad HKC, 2006, AM J OTOLARYNG, V27, P179, DOI 10.1016/j.amjoto.2005.09.011

PREZANT TR, 1993, NAT GENET, V4, P289, DOI 10.1038/ng0793-289

Priuska EM, 1995, BIOCHEM PHARMACOL, V50, P1749, DOI 10.1016/0006-2952(95)02160-4

PUEL JL, 1991, HEARING RES, V51, P255, DOI 10.1016/0378-5955(91)90042-8

Rajopadhye SH, 2017, J CLIN DIAGN RES, V11, pBC24, DOI 10.7860/JCDR/2017/28478.10473

Ricci AJ, 2005, J NEUROSCI, V25, P7831, DOI 10.1523/JNEUROSCI.1127-05.2005

Roche M, 2008, FEBS LETT, V582, P1783, DOI 10.1016/j.febslet.2008.04.057

Rzewnicki I, 2012, MED SCI MONITOR, V18, pRA17, DOI 10.12659/MSM.882505

SAITOH Y, 1994, HEARING RES, V75, P27, DOI 10.1016/0378-5955(94)90052-3

Scherzer R, 2012, AIDS, V26, P867, DOI 10.1097/QAD.0b013e328351f68f

Sha SH, 1999, LAB INVEST, V79, P807

Sha SH, 1999, HEARING RES, V128, P112, DOI 10.1016/S0378-5955(98)00200-7

Shokolenko I, 2009, NUCLEIC ACIDS RES, V37, P2539, DOI 10.1093/nar/gkp100

Simdon J, 2001, CLIN INFECT DIS, V32, P1623, DOI 10.1086/320522

Sitar ME, 2013, CLIN LAB, V59, P945, DOI 10.7754/Clin.Lab.2012.121115

Son Y, 2013, METHOD ENZYMOL, P2748

STAAL FJT, 1992, LANCET, V339, P909, DOI 10.1016/0140-6736(92)90939-Z

Stepanyan RS, 2011, JARO-J ASSOC RES OTO, V12, P729, DOI 10.1007/s10162-011-0288-x

Sturdy A, 2011, J ANTIMICROB CHEMOTH, V66, P1815, DOI 10.1093/jac/dkr221

Torre P, 2015, JAMA OTOLARYNGOL, V141, P202, DOI 10.1001/jamaoto.2014.3302

TUNSTALL MJ, 1995, J PHYSIOL-LONDON, V485, P739, DOI 10.1113/jphysiol.1995.sp020765

Tysome JR KR., 2016, Hearing: An Introduction Practical Guide

van Lettow M, 2003, NUTR REV, V61, P81, DOI 10.1301/nr.2003.marr.81-90

Vandebona H, 2009, NEW ENGL J MED, V360, P642, DOI 10.1056/NEJMc0806397

Vinken M, 2013, TOXICOLOGY, V312, P158, DOI 10.1016/j.tox.2013.08.011

Wanchu A, 2009, AIDS RES HUM RETROV, V25, P1307, DOI 10.1089/aid.2009.0062

Wang AM, 2012, NEURAL REGEN RES, V7, P2793, DOI 10.3969/j.issn.1673-5374.2012.35.007

Wang J, 2003, J NEUROSCI, V23, P8596

Wang Q, 2009, JARO-J ASSOC RES OTO, V10, P205, DOI 10.1007/s10162-009-0160-4

Weder S, 2013, CASE REP NEUROL, V5, P62, DOI 10.1159/000350574

Weinstein JR, 2010, ADV CHRONIC KIDNEY D, V17, P302, DOI 10.1053/j.ackd.2010.05.002

WERSALL J, 1962, Acta Otolaryngol, V54, P1, DOI 10.3109/00016486209126917

WHO, 2016, GLOB TUB REP 2016

Wonkam A, 2015, SAMJ S AFR MED J, V105, P23, DOI 10.7196/SAMJ.8814

Wu CY, 2015, J APPL TOXICOL, V35, P273, DOI 10.1002/jat.3043

Yu XY, 2018, TOXICOL LETT, V285, P9, DOI 10.1016/j.toxlet.2017.12.026

Zorov DB, 2014, PHYSIOL REV, V94, P909, DOI 10.1152/physrev.00026.2013

NR 157

TC 15

Z9 17

U1 0

U2 11

PU SPRINGER HEIDELBERG

PI HEIDELBERG

PA TIERGARTENSTRASSE 17, D-69121 HEIDELBERG, GERMANY

SN 0340-5761

EI 1432-0738

J9 ARCH TOXICOL

JI Arch. Toxicol.

PD MAY

PY 2019

VL 93

IS 5

BP 1385

EP 1399

DI 10.1007/s00204-019-02407-8

PG 15

WC Toxicology

WE Science Citation Index Expanded (SCI-EXPANDED)

SC Toxicology

GA IA7UI

UT WOS:000469762400016

OA Green Submitted

DA 2026-05-07

ER

PT J

AU Inturi, B

Pujar, GV

Purohit, MN

AF Inturi, Bharathkumar

Pujar, Gurubasavaraj V.

Purohit, Madhusudhan N.

TI Recent Advances and Structural Features of Enoyl-ACP Reductase

Inhibitors of Mycobacterium tuberculosis

SO ARCHIV DER PHARMAZIE

LA English

DT Review

DE Antituberculosis activity; Aryl amides; Diphenyl ethers;

4-Hydroxy-2-pyridones; Mtb InhA

ID CARRIER PROTEIN REDUCTASE; DRUG-RESISTANT STRAINS; INHA INHIBITORS;

DIPHENYL ETHERS; ANTIMYCOBACTERIAL ACTIVITY; TRICLOSAN; AFFINITY;

DERIVATIVES; DESIGN; COMPLEX

AB Mycobacterium tuberculosis enoyl-ACP reductase (InhA) has been validated as a promising target for antitubercular agents. Isoniazid (INH), the most prescribed drug to treat tuberculosis (TB), inhibits a NADH-dependent InhA that provides precursors of mycolic acids, which are components of the mycobacterial cell wall. It is a pro-drug that needs activation to form the inhibitory INH-NAD adduct by KatG coding for catalase-peroxidase. The INH resistance of M. tuberculosis is caused by mutations in KatG, which may lead to multidrug-resistant TB (MDR-TB). Hence, there is a need for new drugs that can combat MDR-TB. The rationale for the development of new drugs to combat MDR-TB strains is the design of InhA inhibitors that can bypass bioactivation by KatG. In the present review, special attention was paid to discuss the chemical nature and recent developments of direct InhA inhibitors. The InhA inhibitors reported here have significant inhibitory effects against Mtb InhA. The diphenyl ether derivatives have shown slow onset, a tight-binding mechanism, and high affinity at the InhA active site. However, some of the diphenyl ethers have significant in vitro efficacy, which fails to transform into in vivo efficacy. Among the InhA inhibitors, 4-hydroxy-2-pyridones have emerged as a new chemical class with significant InhA inhibitory activity and better pharmacokinetic parameters when compared to diphenyl ethers.

C1 [Inturi, Bharathkumar; Pujar, Gurubasavaraj V.; Purohit, Madhusudhan N.] Jagadguru Sri Shivarathreeshwara Univ, Dept Pharmaceut Chem, JSS Coll Pharm, Mysore 570015, Karnataka, India.

C3 JSS Academy of Higher Education & Research; JSS College of Pharmacy,

Mysuru

RP Pujar, GV (corresponding author), Jagadguru Sri Shivarathreeshwara Univ, Dept Pharmaceut Chem, JSS Coll Pharm, Mysore 570015, Karnataka, India.

EM gvpujar@jssuni.edu.in

RI Inturi, Bharathkumar/AAB-3437-2019; Pujar, Gurubasavaraj/AAS-4920-2020

OI Inturi, Bharathkumar/0000-0001-8496-8606; Pujar,

Gurubasavaraj/0000-0002-0658-3636

FU DBT [BT/PR5594/MED/29/540/2012]

FX Authors are thankful to the Principal, Jagadguru Sri Shivarathreeshwara

College of Pharmacy, Jagadguru Sri Shivarathreeshwara University,

Mysore, India for providing necessary facilities. Authors are beholden

to DBT for the financial assistance (Ref. No:

BT/PR5594/MED/29/540/2012).

CR Alderwick L. J., 2015, PERSPECT MED, V5

[Anonymous], 2015, GLOBAL TUBERCULOSIS

Betts JC, 2003, ANTIMICROB AGENTS CH, V47, P2903, DOI 10.1128/AAC.47.9.2903-2913.2003

Boyne ME, 2007, ANTIMICROB AGENTS CH, V51, P3562, DOI 10.1128/AAC.00383-07

Brennan PJ, 2003, TUBERCULOSIS, V83, P91, DOI 10.1016/S1472-9792(02)00089-6

Chhibber M, 2006, BIOORGAN MED CHEM, V14, P8086, DOI 10.1016/j.bmc.2006.07.034

Chollet A, 2015, EUR J MED CHEM, V101, P218, DOI 10.1016/j.ejmech.2015.06.035

Chung-Delgado K, 2015, PLOS ONE, V10, DOI 10.1371/journal.pone.0119332

Lima CHD, 2015, INT J MOL SCI, V16, P23695, DOI 10.3390/ijms161023695

Freundlich JS, 2009, CHEMMEDCHEM, V4, P241, DOI 10.1002/cmdc.200800261

He X, 2006, J MED CHEM, V49, P6308, DOI 10.1021/jm060715y

Hea X, 2007, BIOORGAN MED CHEM, V15, P6649, DOI 10.1016/j.bmc.2007.08.013

Jankute M, 2015, ANNU REV MICROBIOL, V69, P405, DOI 10.1146/annurev-micro-091014-104121

Jones RD, 2000, AM J INFECT CONTROL, V28, P184, DOI 10.1016/S0196-6553(00)90027-0

Kamsri P, 2014, SAR QSAR ENVIRON RES, V25, P473, DOI 10.1080/1062936X.2014.898690

Kanetaka H, 2015, EUR J MED CHEM, V94, P378, DOI 10.1016/j.ejmech.2015.02.062

Kumar V, 2014, FUTURE MED CHEM, V6, P605, DOI [10.4155/fmc.14.27, 10.4155/FMC.14.27]

Kuo MR, 2003, J BIOL CHEM, V278, P20851, DOI 10.1074/jbc.M211968200

Lafeuille E, 2016, MED MALADIES INFECT, V46, P52, DOI 10.1016/j.medmal.2015.12.008

Manjunatha UH, 2015, SCI TRANSL MED, V7, DOI 10.1126/scitranslmed.3010597

Massengo-Tiassé RP, 2009, CELL MOL LIFE SCI, V66, P1507, DOI 10.1007/s00018-009-8704-7

McMurry LM, 1998, NATURE, V394, P531, DOI 10.1038/28970

Ng PS, 2015, EUR J MED CHEM, V106, P144, DOI 10.1016/j.ejmech.2015.10.008

Pajk S, 2016, EUR J MED CHEM, V112, P252, DOI 10.1016/j.ejmech.2016.02.008

Pan P, 2014, CHEMMEDCHEM, V9, P776, DOI 10.1002/cmdc.201300429

Parikh S, 1999, BIOCHEMISTRY-US, V38, P13623, DOI 10.1021/bi990529c

Pidugu LS, 2004, J MOL BIOL, V343, P147, DOI 10.1016/j.jmb.2004.08.033

Punkvang A, 2010, EUR J MED CHEM, V45, P5585, DOI 10.1016/j.ejmech.2010.09.008

Rawat R, 2003, P NATL ACAD SCI USA, V100, P13881, DOI 10.1073/pnas.2235848100

Rozwarski DA, 1999, J BIOL CHEM, V274, P15582, DOI 10.1074/jbc.274.22.15582

Seifert M, 2015, PLOS ONE, V10, DOI 10.1371/journal.pone.0119628

Sivaraman S, 2004, J MED CHEM, V47, P509, DOI 10.1021/jm030182i

STEAD WW, 1982, AM REV RESPIR DIS, V125, P94

Sullivan TJ, 2006, ACS CHEM BIOL, V1, P43, DOI 10.1021/cb0500042

Tonge PJ, 2007, CURR TOP MED CHEM, V7, P489, DOI 10.2174/156802607780059781

Vale N, 2013, CURR DRUG METAB, V14, P151, DOI 10.2174/138920013804545151

Velayati AA, 2013, INT J CLIN EXP MED, V6, P307

Wall MD, 2007, BIOORG MED CHEM LETT, V17, P2740, DOI 10.1016/j.bmcl.2007.02.078

Wang L., 2004, PHARMACOLOGY, V32, P1162

Ward WHJ, 1999, BIOCHEMISTRY-US, V38, P12514, DOI 10.1021/bi9907779

NR 40

TC 20

Z9 24

U1 1

U2 26

PU WILEY-V C H VERLAG GMBH

PI WEINHEIM

PA POSTFACH 101161, 69451 WEINHEIM, GERMANY

SN 0365-6233

EI 1521-4184

J9 ARCH PHARM

JI Arch. Pharm.

PD NOV

PY 2016

VL 349

IS 11

BP 817

EP 826

DI 10.1002/ardp.201600186

PG 10

WC Chemistry, Medicinal; Chemistry, Multidisciplinary; Pharmacology &

Pharmacy

WE Science Citation Index Expanded (SCI-EXPANDED)

SC Pharmacology & Pharmacy; Chemistry

GA EC6VZ

UT WOS:000388276100001

DA 2026-05-07

ER

PT J

AU Nirmal, CR

Rajadas, SE

Balasubramanian, M

Magdaline, D

Chilamakuru, NB

Dinesh, R

Radhakrishnan, A

Paraman, R

Mondal, R

Dusthackeer, VNA

AF Nirmal, Christy Rosaline

Rajadas, Sam Ebenezer

Balasubramanian, Mahizhaveni

Magdaline, Divya

Chilamakuru, Naresh Babu

Dinesh, Rajkumar

Radhakrishnan, A.

Paraman, Ramalingam

Mondal, Rajesh

Dusthackeer, V. N. Azger

TI Dodecanoic acid & palmitic acid disarms rifampicin resistance by

putatively targeting mycobacterial efflux pump Rv1218c

SO INDIAN JOURNAL OF MEDICAL RESEARCH

LA English

DT Article

DE Adjunct therapy; efflux inhibitors; efflux pump; rifampicin-resistance;

Rv1218c; tuberculosis

ID TUBERCULOSIS; PROTEIN; TRANSPORTERS; EXPRESSION; INHIBITORS; MUTATIONS;

MECHANISM; MODEL; ASSAY; GENE

AB Background & objectives: Drug-resistant tuberculosis (TB) jeopardizes the treatment process with poor outcomes. Efflux pumps (EPs) belonging to the ABC transporter family in Mycobacterium tuberculosis confer resistance to rifampicin (RMP) besides genetic mutations thus serving as a target for a potential adjunct therapeutic inhibitory molecule. Rv1218c is one such pump that was previously reported to be active in multidrug-resistant TB clinical isolates.

Methods: In this study, the inhibition potential of Rv1218c-EP was tested on 8 molecules that were shortlisted by in silico methods. These molecules were subjected to the minimum inhibitory concentration (MIC) determination, checkerboard drug combination assay, ethidium bromide-DNA binding assay, and in vitro and ex vivo cytotoxicity assay.

Results: Based on the outcome of the study, two molecules dodecanoic acid (DA) and palmitic acid (PA) were found to be potential enough to decrease the MIC of RMP by 8 to 1000 folds against multidrug-resistant clinical isolates and Rv1218c expressing recombinant Mycobacterium smegmatis.

Interpretation & conclusions: These molecules were also found to reduce the time taken by RMP to kill these drug-resistant Mycobacteria to 48 h, unlike control isolates that survived more than 240 h of RMP exposure. The functional concentration of both molecules was non-toxic to the epithelial and blood mononuclear cells. With further comprehensive scientific validation, PA and DA could be recommended as adjunct therapeutic molecules with first-line anti-TB drugs to treat drug-resistant TB.

C1 [Nirmal, Christy Rosaline; Rajadas, Sam Ebenezer; Balasubramanian, Mahizhaveni; Magdaline, Divya; Dinesh, Rajkumar; Dusthackeer, V. N. Azger] ICMR Natl Inst Res TB, Dept Bacteriol, Chennai, Tamil Nadu, India.

[Rajadas, Sam Ebenezer] VSB Engn Coll, Dept Biotechnol, Karur, Tamil Nadu, India.

[Nirmal, Christy Rosaline] Achira Labs Pvt Ltd, Bengaluru, Karnataka, India.

[Chilamakuru, Naresh Babu] Raghavendra Inst Pharmaceut Educ & Res, Dept Pharmaceut Chem, Chiyyedu, Andhra Pradesh, India.

[Paraman, Ramalingam] Natl Inst Pharmaceut Educ & Res, Dept Pharmaceut Anal, Hajipur, Bihar, India.

[Mondal, Rajesh] ICMR Natl Inst Res Environm Hlth, Dept Biochem, Bhopal, Madhya Pradesh, India.

C3 Indian Council of Medical Research (ICMR); ICMR - National Institute for

Research in Tuberculosis (NIRT); National Institute of Pharmaceutical

Education & Research, S.A.S. Nagar (Mohali); Indian Council of Medical

Research (ICMR); ICMR - National Institute for Research in Environmental

Health (NIREH)

RP Dusthackeer, VNA (corresponding author), ICMR Nat Inst Res TB, Chennai 600031, Tamil Nadu, India.

EM azgerdusthackeer.vn@icmr.gov.in

RI Dusthackeer, Azger/KHU-7767-2024; Rajadas, Sam Ebenezer/KQV-2787-2024;

Chilamakuru, Naresh Babu/AAF-7747-2021

OI Rajadas, Sam Ebenezer/0000-0002-7283-3584;

FU ICMR, New Delhi [3/1/3/PDF (16)/-2017-HRD 3, 3/1/3/PDF (16)/2019-HRD 3]

FX The authors CRN and RSE received financial support for Post-Doctoral

Fellowship [3/1/3/PDF (16)/-2017-HRD 3(16th batch)], and [3/1/3/PDF

(16)/2019-HRD 3] from ICMR, New Delhi.

CR Anzaku AA, 2017, ANN CLIN LAB RES, V05, DOI [10.21767/2386-5180.1000170, 10.21767/2386-5180.1000170, DOI 10.21767/2386-5180.1000170]

Balganesh M, 2012, ANTIMICROB AGENTS CH, V56, P2643, DOI 10.1128/AAC.06003-11

Balganesh M, 2010, ANTIMICROB AGENTS CH, V54, P5167, DOI 10.1128/AAC.00610-10

BALL PR, 1980, BIOCHEM BIOPH RES CO, V93, P74, DOI 10.1016/S0006-291X(80)80247-6

Barnabas V, 2022, ACS INFECT DIS, V8, P2374, DOI 10.1021/acsinfecdis.2c00460

Betts JC, 2002, MOL MICROBIOL, V43, P717, DOI 10.1046/j.1365-2958.2002.02779.x

Campbell EA, 2001, CELL, V104, P901, DOI 10.1016/S0092-8674(01)00286-0

Carta G, 2017, FRONT PHYSIOL, V8, DOI 10.3389/fphys.2017.00902

Caviedes L, 2002, J CLIN MICROBIOL, V40, P1873, DOI 10.1128/JCM.40.5.1873-1874.2002

Daina A, 2017, SCI REP-UK, V7, DOI 10.1038/srep42717

Dayrit FM, 2015, J AM OIL CHEM SOC, V92, P1, DOI 10.1007/s11746-014-2562-7

de Alencar DB, 2015, AN ACAD BRAS CIENC, V87, P1113

Delmar JA, 2015, J BIOL CHEM, V290, P28559, DOI 10.1074/jbc.M115.683797

FELMLEE TA, 1995, J CLIN MICROBIOL, V33, P1617, DOI 10.1128/JCM.33.6.1617-1623.1995

Friesner RA, 2006, J MED CHEM, V49, P6177, DOI 10.1021/jm051256o

Garcia de Viedma D, 2002, J CLIN MICROBIOL, V40, P988, DOI 10.1128/JCM.40.3.988-995.2002

Garlid KD, 1996, J BIOL CHEM, V271, P2615, DOI 10.1074/jbc.271.5.2615

Ghajavand H, 2019, ANTIMICROB RESIST IN, V8, DOI 10.1186/s13756-019-0516-4

Ghajavand H, 2019, ANTIMICROB AGENTS CH, V63, DOI [10.1128/AAC.02530-18, 10.1128/aac.02530-18]

Gopinath K, 2013, FUTURE MICROBIOL, V8, P1405, DOI 10.2217/fmb.13.113

Gupta AK, 2010, MICROB DRUG RESIST, V16, P21, DOI 10.1089/mdr.2009.0054

HSIEH MH, 1993, DIAGN MICR INFEC DIS, V16, P343, DOI 10.1016/0732-8893(93)90087-N

Li GL, 2015, J ANTIBIOT, V68, P431, DOI 10.1038/ja.2015.9

Machado D, 2012, PLOS ONE, V7, DOI 10.1371/journal.pone.0034538

Mahajan SD, 2012, METHOD ENZYMOL, V509, P41, DOI 10.1016/B978-0-12-391858-1.00003-4

Mancini A, 2015, MOLECULES, V20, P17339, DOI 10.3390/molecules200917339

Mazando S., 2017, Journal of Applied Biosciences, V115, P11465

Muniyan R, 2016, PHARM BIOL, V54, P2814, DOI 10.1080/13880209.2016.1184691

Narang A, 2019, PLOS ONE, V14, DOI 10.1371/journal.pone.0223163

Pule CM, 2016, J ANTIMICROB CHEMOTH, V71, P17, DOI 10.1093/jac/dkv316

Ramón-García S, 2007, J ANTIMICROB CHEMOTH, V59, P544, DOI 10.1093/jac/dkl510

Rempel S, 2020, NATURE, V580, P409, DOI 10.1038/s41586-020-2072-8

Rienthong D, 2009, SRIRAJ MED J, V61, P49

Rodrigues L, 2013, ANTIMICROB AGENTS CH, V57, P751, DOI 10.1128/AAC.01482-12

Rodríguez-Corrales JA, 2017, METHODS MOL BIOL, V1647, P207, DOI 10.1007/978-1-4939-7201-2_14

Saravanan P, 2021, SCI REP-UK, V11, DOI 10.1038/s41598-020-80439-2

Seukep AJ, 2020, J PHARM ANAL, V10, P277, DOI 10.1016/j.jpha.2019.11.002

Seung KJ, 2015, CSH PERSPECT MED, V5, DOI 10.1101/cshperspect.a017863

Shrestha SC, 2021, INT J NANOMED, V16, P1977, DOI 10.2147/IJN.S276301

Stavri M, 2007, J ANTIMICROB CHEMOTH, V59, P1247, DOI 10.1093/jac/dkl460

Tarling EJ, 2013, TRENDS ENDOCRIN MET, V24, P342, DOI 10.1016/j.tem.2013.01.006

TELENTI A, 1993, LANCET, V341, P647, DOI 10.1016/0140-6736(93)90417-F

World Health Organization, 2020, Tuberculosis and COVID-19 interaction

NR 43

TC 7

Z9 8

U1 0

U2 4

PU SCIENTIFIC SCHOLAR LLC

PI PITTSFORD

PA 50, WOODGREEN DR, PITTSFORD, NY 14534 USA

SN 0971-5916

J9 INDIAN J MED RES

JI Indian J. Med. Res.

PD FEB-MAR

PY 2023

VL 157

IS 2

BP 192

EP 203

DI 10.4103/ijmr.ijmr_1610_22

PG 12

WC Immunology; Medicine, General & Internal; Medicine, Research &

Experimental

WE Science Citation Index Expanded (SCI-EXPANDED)

SC Immunology; General & Internal Medicine; Research & Experimental

Medicine

GA O9VS0

UT WOS:001047227400011

OA Green Submitted, gold

DA 2026-05-07

ER

PT J

AU Lawlor, C

O'Connor, G

O'Leary, S

Gallagher, PJ

Cryan, SA

Keane, J

O'Sullivan, MP

AF Lawlor, Ciaran

O'Connor, Gemma

O'Leary, Seonadh

Gallagher, Paul J.

Cryan, Sally-Ann

Keane, Joseph

O'Sullivan, Mary P.

TI Treatment of Mycobacterium tuberculosis-Infected Macrophages with

Poly(Lactic-Co-Glycolic Acid) Microparticles Drives NFκB and Autophagy

Dependent Bacillary Killing

SO PLOS ONE

LA English

DT Article

ID DENDRITIC CELLS; INHALABLE MICROPARTICLES; ALVEOLAR MACROPHAGES;

IN-VITRO; PLGA-MICROSPHERES; DEFENSE-MECHANISM; PHAGOCYTOSIS; APOPTOSIS;

IMMUNOTHERAPY; INHIBITION

AB The emergence of multiple-drug-resistant tuberculosis (MDR-TB) has pushed our available repertoire of anti-TB therapies to the limit of effectiveness. This has increased the urgency to develop novel treatment modalities, and inhalable microparticle (MP) formulations are a promising option to target the site of infection. We have engineered poly(lactic-co-glycolic acid) (PLGA) MPs which can carry a payload of anti-TB agents, and are successfully taken up by human alveolar macrophages. Even without a drug cargo, MPs can be potent immunogens; yet little is known about how they influence macrophage function in the setting of Mycobacterium tuberculosis (Mtb) infection. To address this issue we infected THP-1 macrophages with Mtb H37Ra or H37Rv and treated with MPs. In controlled experiments we saw a reproducible reduction in bacillary viability when THP-1 macrophages were treated with drug-free MPs. NF kappa B activity was increased in MP-treated macrophages, although cytokine secretion was unaltered. Confocal microscopy of immortalized murine bone marrow-derived macrophages expressing GFP-tagged LC3 demonstrated induction of autophagy. Inhibition of caspases did not influence the MP-induced restriction of bacillary growth, however, blockade of NF kappa B or autophagy with pharmacological inhibitors reversed this MP effect on macrophage function. These data support harnessing inhaled PLGA MP-drug delivery systems as an immunotherapeutic in addition to serving as a vehicle for targeted drug delivery. Such "added value" could be exploited in the generation of inhaled vaccines as well as inhaled MDR-TB therapeutics when used as an adjunct to existing treatments.

C1 [Lawlor, Ciaran; O'Connor, Gemma; Gallagher, Paul J.; Cryan, Sally-Ann] Royal Coll Surgeons Ireland, Sch Pharm, Dublin 2, Ireland.

[Cryan, Sally-Ann] Trinity Coll Dublin, Trinity Ctr Bioengn, Dublin 2, Ireland.

[Lawlor, Ciaran; O'Connor, Gemma; O'Leary, Seonadh; Keane, Joseph; O'Sullivan, Mary P.] Trinity Coll Dublin, Inst Mol Med, Dept Clin Med, Dublin 2, Ireland.

[Lawlor, Ciaran; O'Connor, Gemma; O'Leary, Seonadh; Keane, Joseph; O'Sullivan, Mary P.] St James Hosp, Dublin 8, Ireland.

[Lawlor, Ciaran] Moderna Therapeut, Cambridge, MA USA.

C3 Royal College of Surgeons in Ireland - RCSI; Trinity College Dublin;

Trinity College Dublin; Trinity College Dublin

RP O'Sullivan, MP (corresponding author), Trinity Coll Dublin, Inst Mol Med, Dept Clin Med, Dublin 2, Ireland.

EM mary.osullivan@tcd.ie

RI O'Leary, Seónadh/C-9413-2012; Cryan, Sally-Ann/D-3510-2012; O'Sullivan,

Mary/C-9413-2012; Keane, Joseph/AAX-6658-2021

OI O'Leary, Seónadh/0000-0002-7903-2325; Cryan,

Sally-Ann/0000-0002-3941-496X; O'Leary, Seónadh/0000-0002-7903-2325;

O'Sullivan, Mary/0000-0002-6104-8943; Keane, Joseph/0000-0001-5313-385X

FU Irish Health Research Board [RP/2006/152, HRB HRAPOR/2012/43, HRB

CSA/2012/16]; Royal City of Dublin Hospital Trust; Science Foundation

Ireland [08/RFP/BMT1689]; Science Foundation Ireland (SFI)

[08/RFP/BMT1689] Funding Source: Science Foundation Ireland (SFI)

FX This work was supported by the Irish Health Research Board

(http://www.hrb.ie/home/) under grant RP/2006/152 and HRB HRAPOR/2012/43

(SAC), HRB CSA/2012/16 and the Royal City of Dublin Hospital Trust, (JK)

and Science Foundation Ireland (http://www.sfi.ie/) 08/RFP/BMT1689

(MOS). The funders had no role in study design, data collection and

analysis, decision to publish, or preparation of the manuscript.

CR Arcila ML, CELLULAR IMMUNOLOGY, V249, P80

Bonilla DL, 2013, IMMUNITY, V39, P537, DOI 10.1016/j.immuni.2013.08.026

Briken V, 2008, FUTURE MICROBIOL, V3, P415, DOI 10.2217/17460913.3.4.415

Deretic V, 2006, CELL MICROBIOL, V8, P719, DOI 10.1111/j.1462-5822.2006.00705.x

Edwards DA, 2002, ANNU REV BIOMED ENG, V4, P93, DOI 10.1146/annurev.bioeng.4.100101.132311

Evora C, 1998, J CONTROL RELEASE, V51, P143, DOI 10.1016/S0168-3659(97)00149-1

Getts DR, 2014, SCI TRANSL MED, V6, DOI 10.1126/scitranslmed.3007563

Gutierrez MG, 2004, CELL, V119, P753, DOI 10.1016/j.cell.2004.11.038

Harris J, 2011, J BIOL CHEM, V286, P9587, DOI 10.1074/jbc.M110.202911

Hartman ML, 2011, PLOS ONE, V6, DOI 10.1371/journal.pone.0027972

Hirota K, 2010, J CONTROL RELEASE, V142, P339, DOI 10.1016/j.jconrel.2009.11.020

Keane J, 1997, INFECT IMMUN, V65, P298, DOI 10.1128/IAI.65.1.298-304.1997

Keane J, 2000, J IMMUNOL, V164, P2016, DOI 10.4049/jimmunol.164.4.2016

Lawlor C, 2012, J MATER SCI-MATER M, V23, P89, DOI 10.1007/s10856-011-4511-4

Lawlor C, 2011, TUBERCULOSIS, V91, P93, DOI 10.1016/j.tube.2010.12.001

Lawlor C, 2011, MOL PHARMACEUT, V8, P1100, DOI 10.1021/mp1004178

Lewis JS, 2012, BIOMATERIALS, V33, P7221, DOI 10.1016/j.biomaterials.2012.06.049

Lu DM, 2007, PHARM RES-DORDR, V24, P1834, DOI 10.1007/s11095-007-9302-8

Malyala P, 2008, J PHARM SCI-US, V97, P1155, DOI 10.1002/jps.21065

Marques AP, 2004, J BIOMED MATER RES A, V71A, P419, DOI 10.1002/jbm.a.30155

Mueller M, 2012, J CONTROL RELEASE, V162, P159, DOI 10.1016/j.jconrel.2012.06.015

Muttil P, 2007, EUR J PHARM SCI, V32, P140, DOI 10.1016/j.ejps.2007.06.006

Muttil P, 2009, PHARM RES-DORDR, V26, P2401, DOI 10.1007/s11095-009-9957-4

Nicolete R, 2011, INT IMMUNOPHARMACOL, V11, P1557, DOI 10.1016/j.intimp.2011.05.014

O'Hara P, 2000, PHARMACEUT RES, V17, P955, DOI 10.1023/A:1007527204887

O'Leary S, 2011, AM J RESP CELL MOL, V45, P172, DOI 10.1165/rcmb.2010-0319OC

O'Sullivan MP, 2007, INFECT IMMUN, V75, P1984, DOI 10.1128/IAI.01107-06

Pham NLL, 2010, P NATL ACAD SCI USA, V107, P12198, DOI 10.1073/pnas.1004661107

Ramakrishnan L, 2012, NAT REV IMMUNOL, V12, P352, DOI 10.1038/nri3211

Roberts RA, 2013, PLOS ONE, V8, DOI 10.1371/journal.pone.0062115

Ryan RCM, 2011, BMC MICROBIOL, V11, DOI 10.1186/1471-2180-11-237

Salminen Antero, 2012, Int J Cell Biol, V2012, P849541, DOI 10.1155/2012/849541

Sanjuan MA, 2007, NATURE, V450, P1253, DOI 10.1038/nature06421

Schlosser E, 2008, VACCINE, V26, P1626, DOI 10.1016/j.vaccine.2008.01.030

Sharma R, 2007, J ANTIMICROB CHEMOTH, V59, P499, DOI 10.1093/jac/dkl533

Sharma R, 2011, TUBERCULOSIS, V91, P107, DOI 10.1016/j.tube.2010.11.003

Sharp FA, 2009, P NATL ACAD SCI USA, V106, P870, DOI 10.1073/pnas.0804897106

Shui WQ, 2008, P NATL ACAD SCI USA, V105, P16952, DOI 10.1073/pnas.0809218105

Stern ST, 2012, PART FIBRE TOXICOL, V9, DOI 10.1186/1743-8977-9-20

Thi EP, 2012, PLOS PATHOG, V8, DOI 10.1371/journal.ppat.1002551

van der Vaart M, 2014, CELL HOST MICROBE, V15, P753, DOI 10.1016/j.chom.2014.05.005

Waeckerle-Men Y, 2004, J IMMUNOL METHODS, V287, P109, DOI 10.1016/j.jim.2004.01.010

Wang CC, 2009, AAPS J, V11, P139, DOI 10.1208/s12248-009-9089-0

Yadav AB, 2010, TUBERCULOSIS, V90, P188, DOI 10.1016/j.tube.2010.03.001

Yadav AB, 2009, INDIAN J EXP BIOL, V47, P469

NR 45

TC 49

Z9 56

U1 0

U2 10

PU PUBLIC LIBRARY SCIENCE

PI SAN FRANCISCO

PA 1160 BATTERY STREET, STE 100, SAN FRANCISCO, CA 94111 USA

SN 1932-6203

J9 PLOS ONE

JI PLoS One

PD FEB 19

PY 2016

VL 11

IS 2

AR e0149167

DI 10.1371/journal.pone.0149167

PG 18

WC Multidisciplinary Sciences

WE Science Citation Index Expanded (SCI-EXPANDED)

SC Science & Technology - Other Topics

GA DF3DG

UT WOS:000371223400033

OA Green Submitted, gold

DA 2026-05-07

ER

PT J

AU Cole, ST

Riccardi, G

AF Cole, Stewart T.

Riccardi, Giovanna

TI New tuberculosis drugs on the horizon

SO CURRENT OPINION IN MICROBIOLOGY

LA English

DT Review

ID D-RIBOSE 2'-EPIMERASE; MYCOBACTERIUM-TUBERCULOSIS; ATP SYNTHASE;

RESISTANT TUBERCULOSIS; MECHANISMS; PNU-100480; ARABINAN; TMC207; HIV

AB Tuberculosis (TB) remains a major global health concern whose control has been exacerbated by HIV and the emergence of multidrug-resistant (MDR-TB) and extensively drug-resistant (XDR-TB) strains of Mycobacterium tuberculosis. The demand for new and faster acting TB drugs is thus greater than ever. In the past decade intensive efforts have been made to discover new leads for TB drug development using both target-based and cell-based approaches. Here, we describe the most promising anti-tubercular drug candidates that are in clinical development and introduce some nitro-aromatic compounds that inhibit a new target, DprE1, an essential enzyme involved in a crucial step in mycobacterial cell wall biosynthesis.

C1 [Riccardi, Giovanna] Univ Pavia, Dipartimento Genet & Microbiol, I-27100 Pavia, Italy.

[Cole, Stewart T.] Ecole Polytech Fed Lausanne, Global Hlth Inst, CH-1015 Lausanne, Switzerland.

C3 University of Pavia; Swiss Federal Institutes of Technology Domain;

Ecole Polytechnique Federale de Lausanne

RP Riccardi, G (corresponding author), Univ Pavia, Dipartimento Genet & Microbiol, Via Palestro 3, I-27100 Pavia, Italy.

EM giovanna.riccardi@unipv.it

RI RICCARDI, Giovanna/A-9269-2012

OI RICCARDI, Giovanna/0000-0003-3295-0070

FU European Commission [LSHP-CT-2005-018923, 260872]

FX We thank the European Commission for support (VI Framework, contract N.

LSHP-CT-2005-018923 and VII Framework, contract N. 260872).

CR Ahmad Z, 2011, ANTIMICROB AGENTS CH, V55, P239, DOI 10.1128/AAC.00849-10

Andries K, 2005, SCIENCE, V307, P223, DOI 10.1126/science.1106753

Bald D, 2010, FEMS MICROBIOL LETT, V308, P1, DOI 10.1111/j.1574-6968.2010.01959.x

Barry CE, 2010, CURR OPIN CHEM BIOL, V14, P456, DOI 10.1016/j.cbpa.2010.04.008

Bobkova EV, 2003, J BIOL CHEM, V278, P9802, DOI 10.1074/jbc.M209249200

Boshoff HIM, 2004, J BIOL CHEM, V279, P40174, DOI 10.1074/jbc.M406796200

Bozdogan B, 2004, INT J ANTIMICROB AG, V23, P113, DOI 10.1016/j.ijantimicag.2003.11.003

Burman WJ, 2010, CLIN INFECT DIS, V50, pS165, DOI 10.1086/651487

Carvalho Anna C C, 2010, Expert Rev Respir Med, V4, P189, DOI 10.1586/ers.10.7

Cegielski JP, 2010, CLIN INFECT DIS, V50, pS195, DOI 10.1086/651491

Chao MC, 2010, ANNU REV MICROBIOL, V64, P293, DOI 10.1146/annurev.micro.112408.134043

Christophe T, 2009, PLOS PATHOG, V5, DOI 10.1371/journal.ppat.1000645

De Bus L, 2010, J MED TOXICOL, V6, P322, DOI 10.1007/s13181-010-0047-0

Fischbach MA, 2009, SCIENCE, V325, P1089, DOI 10.1126/science.1176667

Fortún J, 2005, J ANTIMICROB CHEMOTH, V56, P180, DOI 10.1093/jac/dki148

Fu LM, 2003, INT J TUBERC LUNG D, V7, P1027

García-Tapia A, 2004, CHEMOTHERAPY, V50, P211, DOI 10.1159/000081707

Glaziou P, 2009, CLIN CHEST MED, V30, P621, DOI 10.1016/j.ccm.2009.08.017

Haagsma AC, 2009, ANTIMICROB AGENTS CH, V53, P1290, DOI 10.1128/AAC.01393-08

Harries AD, 2010, LANCET, V375, P1906, DOI 10.1016/S0140-6736(10)60409-6

Harrington M, 2010, CLIN INFECT DIS, V50, pS260, DOI 10.1086/651500

Huitric E, 2010, ANTIMICROB AGENTS CH, V54, P1022, DOI 10.1128/AAC.01611-09

Lalloo Umesh G, 2010, Curr HIV/AIDS Rep, V7, P143, DOI 10.1007/s11904-010-0054-4

Leibert E, 2010, EXPERT REV ANTI-INFE, V8, P801, DOI 10.1586/ERI.10.60

Ma ZK, 2010, LANCET, V375, P2100, DOI 10.1016/S0140-6736(10)60359-9

Magnet S, 2010, TUBERCULOSIS, V90, P354, DOI 10.1016/j.tube.2010.09.001

Makarov V, 2009, SCIENCE, V324, P801, DOI 10.1126/science.1171583

Manina G, 2010, CURR MED CHEM, V17, P3099, DOI 10.2174/092986710791959693

Manjunatha Ujjini, 2009, Commun Integr Biol, V2, P215

Manjunatha UH, 2006, ANTIMICROB AGENTS CH, V50, P3350, DOI 10.1128/AAC.00488-06

Matsumoto M, 2006, PLOS MED, V3, P2131, DOI 10.1371/journal.pmed.0030466

Matteelli A, 2010, FUTURE MICROBIOL, V5, P849, DOI [10.2217/fmb.10.50, 10.2217/FMB.10.50]

Mikusová K, 2005, J BACTERIOL, V187, P8020, DOI 10.1128/JB.187.23.8020-8025.2005

Pasca MR, 2010, ANTIMICROB AGENTS CH, V54, P1616, DOI 10.1128/AAC.01676-09

Payne DJ, 2007, NAT REV DRUG DISCOV, V6, P29, DOI 10.1038/nrd2201

Rao SPS, 2008, P NATL ACAD SCI USA, V105, P11945, DOI 10.1073/pnas.0711697105

Reddy VM, 2010, ANTIMICROB AGENTS CH, V54, P2840, DOI 10.1128/AAC.01601-09

Riccardi G, 2009, FUTURE MICROBIOL, V4, P597, DOI [10.2217/fmb.09.20, 10.2217/FMB.09.20]

Rosenthal IM, 2007, PLOS MED, V4, P1931, DOI 10.1371/journal.pmed.0040344

Shi RR, 2010, TOHOKU J EXP MED, V221, P97, DOI 10.1620/tjem.221.97

Singh R, 2008, SCIENCE, V322, P1392, DOI 10.1126/science.1164571

Trefzer C, 2010, J AM CHEM SOC, V132, P13663, DOI 10.1021/ja106357w

Velayati AA, 2009, CHEST, V136, P420, DOI 10.1378/chest.08-2427

Wallis RS, 2011, ANTIMICROB AGENTS CH, V55, P567, DOI 10.1128/AAC.01179-10

Wallis RS, 2010, J INFECT DIS, V202, P745, DOI 10.1086/655471

Williams KN, 2009, AM J RESP CRIT CARE, V180, P371, DOI 10.1164/rccm.200904-0611OC

Wolucka BA, 2008, FEBS J, V275, P2691, DOI 10.1111/j.1742-4658.2008.06395.x

NR 47

TC 84

Z9 97

U1 0

U2 26

PU CURRENT BIOLOGY LTD

PI LONDON

PA 84 THEOBALDS RD, LONDON WC1X 8RR, ENGLAND

SN 1369-5274

EI 1879-0364

J9 CURR OPIN MICROBIOL

JI Curr. Opin. Microbiol.

PD OCT

PY 2011

VL 14

IS 5

BP 570

EP 576

DI 10.1016/j.mib.2011.07.022

PG 7

WC Microbiology

WE Science Citation Index Expanded (SCI-EXPANDED)

SC Microbiology

GA 847BB

UT WOS:000296945800010

OA Green Submitted, Bronze

DA 2026-05-07

ER

PT J

AU Armstrong, T

Lamont, M

Lanne, A

Alderwick, LJ

Thomas, NR

AF Armstrong, Tom

Lamont, Malcolm

Lanne, Alice

Alderwick, Luke J.

Thomas, Neil R.

TI Inhibition of Mycobacterium tuberculosis InhA: Design, synthesis

and evaluation of new di-triclosan derivatives

SO BIOORGANIC & MEDICINAL CHEMISTRY

LA English

DT Article

DE InhA; Triclosan; Triazole; Isoniazid; Mycobacterium tuberculosis; TB

ID CARRIER PROTEIN REDUCTASE; AZIDE-ALKYNE CYCLOADDITION; DRUG-RESISTANT

STRAINS; ENOYL REDUCTASE; MULTIDRUG-RESISTANT; EFFLUX PUMPS; TARGET;

MECHANISM; POTENT; SCOPE

AB Multi-drug resistant tuberculosis (MDR-TB) represents a growing problem for global healthcare systems. In addition to 1.3 million deaths in 2018, the World Health Organisation reported 484,000 new cases of MDR-TB. Isoniazid is a key anti-TB drug that inhibits InhA, a crucial enzyme in the cell wall biosynthesis pathway and identical in Mycobacterium tuberculosis and M. bovis. Isoniazid is a pro-drug which requires activation by the enzyme KatG, mutations in KatG prevent activation and confer INH-resistance. 'Direct inhibitors' of InhA are attractive as they would circumvent the main clinically observed resistance mechanisms. A library of new 1,5triazoles, designed to mimic the structures of both triclosan molecules uniquely bound to InhA have been synthesised. The inhibitory activity of these compounds was evaluated using isolated enzyme assays with 2 (5-chloro-2-(4-(5-(((4-(4-chloro-2-hydroxyphenoxy)benzyl)oxy)methyl)-1H-1,2,3-triazol-1-yl)phenoxy)phenol) exhibiting an IC50 of 5.6 mu M. Whole-cell evaluation was also performed, with 11 (5-chloro-2-(4-(5-(((4-(cyclopropylmethoxy)benzyl)oxy)methyl)-1H-1,2,3-triazol-1-yl)phenoxy)phenol) showing the greatest potency, with an MIC99 of 12.9 mu M against M. bovis.

C1 [Armstrong, Tom; Lamont, Malcolm; Thomas, Neil R.] Univ Nottingham, Sch Chem, Biodiscovery Inst, Univ Pk, Nottingham NG7 2RD, England.

[Lanne, Alice; Alderwick, Luke J.] Univ Birmingham, Sch Biosci, Inst Microbiol & Infect, Birmingham B15 2TT, W Midlands, England.

C3 University of Nottingham; University of Birmingham

RP Thomas, NR (corresponding author), Univ Nottingham, Sch Chem, Biodiscovery Inst, Univ Pk, Nottingham NG7 2RD, England.

EM neil.thomas@nottingham.ac.uk

OI Lanne, Alice/0000-0003-2969-4336; Alderwick, Luke/0000-0002-1257-6053

FU Wellcome Trust Antimicrobials and Antimicrobial Resistance (AAMR)

doctoral training programme Birmingham/Nottingham [203974/Z/17/A,

215154/Z/18/Z]; Biotechnology and Biological Sciences Research Council

doctoral training studentship [BB/M008770/1]; Wellcome Trust

[203974/Z/17/A, 215154/Z/18/Z] Funding Source: Wellcome Trust

FX T.A. is supported by the Wellcome Trust Antimicrobials and Antimicrobial

Resistance (AAMR) doctoral training programme Birmingham/Nottingham

[203974/Z/17/A].M.L. is supported by a Biotechnology and Biological

Sciences Research Council doctoral training studentship [Grant Number

BB/M008770/1].A.L. is supported by the Wellcome Trust Antimicrobials and

Antimicrobial Resistance (AAMR) doctoral training programme

Birmingham/Nottingham [215154/Z/18/Z].

CR Balganesh M, 2012, ANTIMICROB AGENTS CH, V56, P2643, DOI 10.1128/AAC.06003-11

Barral K, 2007, ORG LETT, V9, P1809, DOI 10.1021/ol070527h

Bastos ML, 2017, EUR RESPIR J, V49, DOI 10.1183/13993003.00803-2016

Boren BC, 2008, J AM CHEM SOC, V130, P8923, DOI 10.1021/ja0749993

Chetty S, 2017, BIOORG MED CHEM LETT, V27, P370, DOI 10.1016/j.bmcl.2016.11.084

Creary X, 2012, J ORG CHEM, V77, P8756, DOI 10.1021/jo301265t

Deoghare S, 2013, INDIAN J PHARMACOL, V45, P536, DOI 10.4103/0253-7613.117765

Encinas L, 2014, J MED CHEM, V57, P1276, DOI 10.1021/jm401326j

Günther G, 2014, CLIN MED, V14, P279, DOI 10.7861/clinmedicine.14-3-279

Han J, 2013, J MATER CHEM A, V1, P4941, DOI 10.1039/c3ta00090g

He X, 2006, J MED CHEM, V49, P6308, DOI 10.1021/jm060715y

Hea X, 2007, BIOORGAN MED CHEM, V15, P6649, DOI 10.1016/j.bmc.2007.08.013

Johansson JR, 2016, CHEM REV, V116, P14726, DOI 10.1021/acs.chemrev.6b00466

Jones G, 1997, J MOL BIOL, V267, P727, DOI 10.1006/jmbi.1996.0897

Kuo MR, 2003, J BIOL CHEM, V278, P20851, DOI 10.1074/jbc.M211968200

Manjunatha UH, 2015, SCI TRANSL MED, V7, DOI 10.1126/scitranslmed.3010597

Marrakchi H, 2000, MICROBIOL-UK, V146, P289, DOI 10.1099/00221287-146-2-289

Martínez-Hoyos M, 2016, EBIOMEDICINE, V8, P291, DOI 10.1016/j.ebiom.2016.05.006

Ng PS, 2015, EUR J MED CHEM, V106, P144, DOI 10.1016/j.ejmech.2015.10.008

Parikh S, 1999, BIOCHEMISTRY-US, V38, P13623, DOI 10.1021/bi990529c

Parikh SL, 2000, BIOCHEMISTRY-US, V39, P7645, DOI 10.1021/bi0008940

Pontali E, 2017, EUR RESPIR J, V49, DOI 10.1183/13993003.00146-2017

Rodriguez F, 2020, BIOORG CHEM, V95, DOI 10.1016/j.bioorg.2019.103498

Rozman K, 2017, DRUG DISCOV TODAY, V22, P492, DOI 10.1016/j.drudis.2016.09.009

Rozwarski DA, 1998, SCIENCE, V279, P98, DOI 10.1126/science.279.5347.98

Shirude PS, 2013, J MED CHEM, V56, P8533, DOI 10.1021/jm4012033

Sivaraman S, 2004, J MED CHEM, V47, P509, DOI 10.1021/jm030182i

Slepikas L, 2016, J MED CHEM, V59, P10917, DOI 10.1021/acs.jmedchem.5b01620

Stec J, 2014, CHEMMEDCHEM, V9, P2528, DOI 10.1002/cmdc.201402255

Sullivan TJ, 2006, ACS CHEM BIOL, V1, P43, DOI 10.1021/cb0500042

Szumowski JD, 2014, CURR TOP MICROBIOL, V374, P81, DOI 10.1007/82_2012_300

Timmins GS, 2006, MOL MICROBIOL, V62, P1220, DOI 10.1111/j.1365-2958.2006.05467.x

Vosátka R, 2018, EUR J PHARM SCI, V114, P318, DOI 10.1016/j.ejps.2017.12.013

WHO, 2012, GLOBAL TUBERCULOSIS REPORT 2012, P1

Xavier AS, 2014, J PHARMACOL PHARMACO, V5, P222, DOI 10.4103/0976-500X.136121

Zhai WJ, 2019, INT J MOL SCI, V20, DOI 10.3390/ijms20020340

ZHANG Y, 1992, NATURE, V358, P591, DOI 10.1038/358591a0

Zhao XB, 2006, BIOCHEMISTRY-US, V45, P4131, DOI 10.1021/bi051967o

NR 38

TC 22

Z9 27

U1 2

U2 18

PU PERGAMON-ELSEVIER SCIENCE LTD

PI OXFORD

PA THE BOULEVARD, LANGFORD LANE, KIDLINGTON, OXFORD OX5 1GB, ENGLAND

SN 0968-0896

EI 1464-3391

J9 BIOORGAN MED CHEM

JI Bioorg. Med. Chem.

PD NOV 15

PY 2020

VL 28

IS 22

AR 115744

DI 10.1016/j.bmc.2020.115744

PG 8

WC Biochemistry & Molecular Biology; Chemistry, Medicinal; Chemistry,

Organic

WE Science Citation Index Expanded (SCI-EXPANDED); Index Chemicus (IC)

SC Biochemistry & Molecular Biology; Pharmacology & Pharmacy; Chemistry

GA PB1HK

UT WOS:000596080300007

OA Green Submitted

DA 2026-05-07

ER

PT J

AU Hernández-Bazán, S

Mata-Espinosa, D

Ramos-Espinosa, O

Lozano-Ordaz, V

Barrios-Payán, J

López-Casillas, F

Hernández-Pando, R

AF Hernandez-Bazan, Sujhey

Mata-Espinosa, Dulce

Ramos-Espinosa, Octavio

Lozano-Ordaz, Vasti

Barrios-Payan, Jorge

Lopez-Casillas, Fernando

Hernandez-Pando, Rogelio

TI Adenoviral Vector Codifying for TNF as a Co-Adjuvant Therapy against

Multi-Drug-Resistant Tuberculosis

SO MICROORGANISMS

LA English

DT Article

DE Tuberculosis; gene therapy; adenoviral vectors; multi-drug resistance;

Tumor Necrosis Factor

ID NECROSIS-FACTOR-ALPHA; MYCOBACTERIUM-TUBERCULOSIS; INTERFERON-GAMMA;

IMMUNE-RESPONSE; NITRIC-OXIDE; SERIOUS INFECTIONS; MURINE MODEL;

ACTIVATION; MECHANISMS; GRANULOMA

AB Mycobacterium tuberculosis is the main causal agent of pulmonary tuberculosis (TB); the treatment of this disease is long and involves a mix of at least four different antibiotics that frequently lead to abandonment, favoring the surge of drug-resistant mycobacteria (MDR-TB), whose treatment becomes more aggressive, being longer and more toxic. Thus, the search for novel strategies for treatment that improves time or efficiency is of relevance. In this work, we used a murine model of pulmonary TB produced by the MDR-TB strain to test the efficiency of gene therapy with adenoviral vectors codifying TNF (AdTNF), a pro-inflammatory cytokine that has protective functions in TB by inducing apoptosis, granuloma formation and expression of other Th1-like cytokines. When compared to the control group that received an adenoviral vector that codifies for the green fluorescent protein (AdGFP), a single dose of AdTNF at the chronic active stage of the disease produced total survival, decreasing bacterial load and tissue damage (pneumonia), which correlated with an increase in cells expressing IFN-gamma, iNOS and TNF in pneumonic areas and larger granulomas that efficiently contain and eliminate mycobacteria. Second-line antibiotic treatment against MDR-TB plus AdTNF gene therapy reduced bacterial load faster within a week of treatment compared to empty vector plus antibiotics or antibiotics alone, suggesting that AdTNF is a new potential type of treatment against MDR-TB that can shorten second-line chemotherapy but which requires further experimentation in other animal models (non-human primates) that develop a more similar disease to human pulmonary TB.

C1 [Hernandez-Bazan, Sujhey; Mata-Espinosa, Dulce; Ramos-Espinosa, Octavio; Lozano-Ordaz, Vasti; Barrios-Payan, Jorge; Hernandez-Pando, Rogelio] Inst Nacl Ciencias Med & Nutr Salvador Zubiran, Dept Patol, Secc Patol Expt, Mexico City 14080, Mexico.

[Lopez-Casillas, Fernando] Univ Nacl Autonoma Mexico, Dept Biol Celular, Inst Fisiol Celular, Mexico City 14080, Mexico.

C3 Instituto Nacional de Ciencias Medicas y Nutricion Salvador Zubiran -

Mexico; Universidad Nacional Autonoma de Mexico

RP Hernández-Pando, R (corresponding author), Inst Nacl Ciencias Med & Nutr Salvador Zubiran, Dept Patol, Secc Patol Expt, Mexico City 14080, Mexico.

EM sujheyhb@yahoo.com.mx; dulmat@yahoo.com.mx; octavioramos13@gmail.com;

loov1288@hotmail.com; qcjbp77@yahoo.com.mx; fcasilla@ifc.unam.mx;

rhdezpando@hotmail.com

OI Hernández-Bazán, Sujhey/0000-0002-3251-2479; Lozano,

Vasti/0000-0002-7395-0360; Ramos-Espinosa, Octavio/0000-0002-9010-4625;

Barrios-Payán, Jorge Alberto/0000-0002-5134-5573

FU Consejo Nacional de Ciencia y Tecnologa (CONACyT)

FX No Statement Available

CR [Anonymous], 2020, World Health Organization: Global Tuberculosis Report 2019

Auricchio A, 2017, HUM GENE THER, V28, P982, DOI 10.1089/hum.2017.164

Balikó Z, 1998, FEMS IMMUNOL MED MIC, V22, P199, DOI 10.1016/S0928-8244(98)00089-3

Burney S, 1999, MUTAT RES-FUND MOL M, V424, P37, DOI 10.1016/S0027-5107(99)00006-8

Chanthaphavong RS, 2012, J BIOL CHEM, V287, P35887, DOI 10.1074/jbc.M112.365171

Cinelli MA, 2020, MED RES REV, V40, P158, DOI 10.1002/med.21599

Cooper AM, 2011, MUCOSAL IMMUNOL, V4, P252, DOI 10.1038/mi.2011.13

Douglas JT, 2007, MOL BIOTECHNOL, V36, P71, DOI 10.1007/s12033-007-0021-5

Ehlers S, 2000, J IMMUNOL, V165, P483, DOI 10.4049/jimmunol.165.1.483

FLYNN JL, 1995, IMMUNITY, V2, P561, DOI 10.1016/1074-7613(95)90001-2

Flynn JL, 2022, CELL, V185, P4682, DOI 10.1016/j.cell.2022.10.025

Fotin-Mleczek M, 2002, J CELL SCI, V115, P2757

Francisco-Cruz A, 2013, CLIN EXP IMMUNOL, V171, P283, DOI 10.1111/cei.12015

Harris J, 2010, CLIN EXP IMMUNOL, V161, P1, DOI 10.1111/j.1365-2249.2010.04146.x

Hernandez-Bazan S, 2022, HUM GENE THER, V33, P1037, DOI 10.1089/hum.2022.030

HernandezPando R, 1996, IMMUNOLOGY, V89, P26

HERNANDEZPANDO R, 1995, FEMS IMMUNOL MED MIC, V12, P63, DOI 10.1016/0928-8244(95)00051-8

Kak Gunjan, 2018, BioMolecular Concepts, V9, P64, DOI [10.1515/bmc-2018-0007, 10.1515/bmc-2018-0007]

Kalliolias GD, 2016, NAT REV RHEUMATOL, V12, P49, DOI 10.1038/nrrheum.2015.169

Keane J, 2001, NEW ENGL J MED, V345, P1098, DOI 10.1056/NEJMoa011110

Kim HW, 2023, CLIN EXP RHEUMATOL, V41, P1491, DOI [10.55563/clinexprheumatol/qkiorp, 10.55563/clinexprheumatol/qkiorp]

Liebenberg D, 2022, FRONT CELL INFECT MI, V12, DOI 10.3389/fcimb.2022.943545

Mata-Espinosa DA, 2008, MOL THER, V16, P1065, DOI 10.1038/mt.2008.69

O'Garra A, 2013, ANNU REV IMMUNOL, V31, P475, DOI 10.1146/annurev-immunol-032712-095939

Pai M, 2016, NAT REV DIS PRIMERS, V2, DOI 10.1038/nrdp.2016.76

Pattanaik KP, 2021, MOL IMMUNOL, V130, P133, DOI 10.1016/j.molimm.2020.11.020

Ramakrishnan L, 2012, NAT REV IMMUNOL, V12, P352, DOI 10.1038/nri3211

Ramos-Espinosa O, 2021, J LEUKOCYTE BIOL, V110, P951, DOI 10.1002/JLB.4MA0920-627R

Ramos-Espinosa O, 2016, PATHOG DIS, V74, DOI 10.1093/femspd/ftw075

Ricobaraza A, 2020, INT J MOL SCI, V21, DOI 10.3390/ijms21103643

Roberts T, 2007, J INFECT DIS, V195, P870, DOI 10.1086/511277

Roca FJ, 2022, SCIENCE, V376, P1431, DOI 10.1126/science.abh2841

Rojas M, 1999, J IMMUNOL, V162, P6122

Russell WC, 2000, J GEN VIROL, V81, P2573, DOI 10.1099/0022-1317-81-11-2573

Scanga CA, 2001, INFECT IMMUN, V69, P7711, DOI 10.1128/IAI.69.12.7711-7717.2001

Schroder K, 2004, J LEUKOCYTE BIOL, V75, P163, DOI 10.1189/jlb.0603252

Sia JK, 2019, MICROBIOL SPECTR, V7, DOI 10.1128/microbiolspec.GPP3-0022-2018

Siegmund D, 2016, CELL DEATH DIS, V7, DOI 10.1038/cddis.2016.285

Suárez I, 2019, DTSCH ARZTEBL INT, V116, P729, DOI 10.3238/arztebl.2019.0729

Tatsis N, 2004, MOL THER, V10, P616, DOI 10.1016/j.ymthe.2004.07.013

Tsao TCY, 2002, INT J TUBERC LUNG D, V6, P720

Tseng WY, 2018, CYTOKINE, V101, P19, DOI 10.1016/j.cyto.2016.08.027

Vankayalapati R, 2003, CLIN INFECT DIS, V36, P24, DOI 10.1086/344903

Wilby KJ, 2020, EUR J DRUG METAB PH, V45, P305, DOI 10.1007/s13318-019-00604-5

Yoo IK, 2014, YONSEI MED J, V55, P442, DOI 10.3349/ymj.2014.55.2.442

Zhang C, 2016, HUM VACC IMMUNOTHER, V12, P2064, DOI 10.1080/21645515.2016.1165908

NR 46

TC 1

Z9 1

U1 0

U2 1

PU MDPI

PI BASEL

PA ST ALBAN-ANLAGE 66, CH-4052 BASEL, SWITZERLAND

EI 2076-2607

J9 MICROORGANISMS

JI Microorganisms

PD DEC

PY 2023

VL 11

IS 12

AR 2934

DI 10.3390/microorganisms11122934

PG 15

WC Microbiology

WE Science Citation Index Expanded (SCI-EXPANDED)

SC Microbiology

GA DI4D9

UT WOS:001131380300001

OA Green Submitted, gold

DA 2026-05-07

ER

PT J

AU Kaur, P

Potluri, V

Ahuja, VK

Naveenkumar, CN

Krishnamurthy, RV

Gangadharaiah, ST

Shivarudraiah, P

Eswaran, S

Nirmal, CR

Mahizhaveni, B

Dusthackeer, A

Mondal, R

Batt, SM

Richardson, EJ

Loman, NJ

Besra, GS

Shandil, RK

Narayanan, S

AF Kaur, Parvinder

Potluri, Vijay

Ahuja, Vijay Kamal

Naveenkumar, C. N.

Krishnamurthy, Ramya Vadageri

Gangadharaiah, Shruthi Thimmalapura

Shivarudraiah, Prasad

Eswaran, Sumesh

Nirmal, Christy Rosaline

Mahizhaveni, Balasubramanian

Dusthackeer, Azger

Mondal, Rajesh

Batt, Sarah M.

Richardson, Emily J.

Loman, Nicholas J.

Besra, Gurdyal Singh

Shandil, Radha Krishan

Narayanan, Shridhar

TI A multi-targeting pre-clinical candidate against drug-resistant

tuberculosis

SO TUBERCULOSIS

LA English

DT Article

DE Drug resistance; Mycobacterium tuberculosis; First-in-class;

Multi-target; Pre-clinical candidate

ID AEROSOL INFECTION MODEL; MYCOBACTERIUM-TUBERCULOSIS; ANTIMYCOBACTERIAL

ACTIVITY; AGENTS; DERIVATIVES; DISCOVERY; DESIGN; SAR

AB FNDR-20081 [4-{4-[5-(4-Isopropyl-phenyl)- [1,2,4]oxadiazol-3-ylmethyl]-piperazin-1-yl}-7-pyridin-3-yl-quinoline] is a novel, first in class anti-tubercular pre-clinical candidate against sensitive and drug-resistant Mycobacterium tuberculosis (Mtb). In-vitro combination studies of FNDR-20081 with first- and second-line drugs exhibited no antagonism, suggesting its compatibility for developing new combination-regimens. FNDR-20081, which is non-toxic with no CYP3A4 liability, demonstrated exposure-dependent killing of replicating-Mtb, as well as the non-replicating-Mtb, and efficacy in a mouse model of infection. Whole genome sequencing (WGS) of FNDR-20081 resistant mutants revealed the identification of pleotropic targets: marR (Rv0678), a regulator of MmpL5, a transporter/efflux pump mechanism for drug resistance; and Rv3683, a putative metalloprotease potentially involved in peptidoglycan biosynthesis. In summary, FNDR-20081 is a promising first in class compound with the potential to form a new combination regimen for MDR-TB treatment.

C1 [Kaur, Parvinder; Potluri, Vijay; Ahuja, Vijay Kamal; Naveenkumar, C. N.; Krishnamurthy, Ramya Vadageri; Shandil, Radha Krishan; Narayanan, Shridhar] Fdn Neglected Dis Res, Bangalore, Karnataka, India.

[Gangadharaiah, Shruthi Thimmalapura; Shivarudraiah, Prasad; Eswaran, Sumesh] Anthem BioSci Pvt Ltd, 49 Canara Bank Rd,Hosur Rd,Elect City Phase 1, Bengaluru 560099, Karnataka, India.

[Nirmal, Christy Rosaline; Mahizhaveni, Balasubramanian; Dusthackeer, Azger; Mondal, Rajesh] Natl Inst Res TB, 1 Mayor Sathiyamoorthy St, Chennai 600031, Tamil Nadu, India.

[Batt, Sarah M.; Richardson, Emily J.; Loman, Nicholas J.; Besra, Gurdyal Singh] Univ Birmingham, Sch Biosci, Insitiute Microbiol & Infect, Birmingham B15 2TT, W Midlands, England.

C3 Indian Council of Medical Research (ICMR); ICMR - National Institute for

Research in Tuberculosis (NIRT); University of Birmingham

RP Kaur, P (corresponding author), Fdn Neglected Dis Res FNDR, Plot 20A, Bangalore 561203, Karnataka, India.

EM parvinder.kaur@fndr.in

RI ; Loman, Nicholas/AFM-0891-2022; MONDAL, RAJESH/AAZ-1612-2021;

Dusthackeer, Azger/GLU-7879-2022

OI Batt, Sarah/0000-0003-1955-2967; Narayanan,

Shridhar/0000-0002-7781-7399; Kaur, Parvinder/0000-0001-9154-3278;

Dusthackeer, Azger/0000-0002-3616-3675; Ahuja, Vijay

Kamal/0000-0002-9117-473X; Besra, Gurdyal/0000-0002-5605-0395

FU Foundation for Neglected Disease Research

FX This study was funded by Foundation for Neglected Disease Research.

CR Almansour AI, 2012, EUR J MED CHEM, V53, P416, DOI 10.1016/j.ejmech.2012.04.021

Martinez-Grau MA, 2018, BIOORG MED CHEM LETT, V28, P1758, DOI 10.1016/j.bmcl.2018.04.028

[Anonymous], Tuberculosis (TB)

Batt SM, 2015, ACS INFECT DIS, V1, P615, DOI 10.1021/acsinfecdis.5b00065

Bhakta S, 2016, J MED CHEM, V59, P2780, DOI 10.1021/acs.jmedchem.6b00031

Chiarelli LR, 2018, SCI REP-UK, V8, DOI 10.1038/s41598-018-21614-4

CLSIClinical and Laboratory Standards Institute (CLSI), SUSCEPTIBILITY TESTI

Conradie F, 2020, NEW ENGL J MED, V382, P893, DOI 10.1056/NEJMoa1901814

de Souza MVN, 2009, BIOORGAN MED CHEM, V17, P1474, DOI 10.1016/j.bmc.2009.01.013

Early JV, 2019, PLOS ONE, V14, DOI 10.1371/journal.pone.0222970

Eswaran S, 2010, EUR J MED CHEM, V45, P3374, DOI 10.1016/j.ejmech.2010.04.022

Franzblau SG, 1998, J CLIN MICROBIOL, V36, P362, DOI 10.1128/JCM.36.2.362-366.1998

Giacobbo BC, 2017, EUR J MED CHEM, V126, P491, DOI 10.1016/j.ejmech.2016.11.048

Hartkoorn RC, 2014, ANTIMICROB AGENTS CH, V58, P2979, DOI 10.1128/AAC.00037-14

Hernández-Pando R, 2000, LANCET, V356, P2133, DOI 10.1016/S0140-6736(00)03493-0

Jayaram R, 2004, ANTIMICROB AGENTS CH, V48, P2951, DOI 10.1128/AAC.48.8.2951-2957.2004

Jayaram R, 2003, ANTIMICROB AGENTS CH, V47, P2118, DOI 10.1128/AAC.47.7.2118-2124.2003

Jones CM, 2014, P NATL ACAD SCI USA, V111, P1945, DOI 10.1073/pnas.1311402111

Karabanovich G, 2017, EUR J MED CHEM, V126, P369, DOI 10.1016/j.ejmech.2016.11.041

Karabanovich G, 2016, J MED CHEM, V59, P2362, DOI 10.1021/acs.jmedchem.5b00608

Kaur P, 2019, AAR736 ASM

Kaur P, 2016, PLOS ONE, V11, DOI 10.1371/journal.pone.0154513

Kaur P, 2015, PLOS ONE, V10, DOI 10.1371/journal.pone.0117577

Kumar RR, 2011, BIOORGAN MED CHEM, V19, P3444, DOI 10.1016/j.bmc.2011.04.033

Lee A, 2020, BMJ-BRIT MED J, V368, DOI 10.1136/bmj.m216

Li K, 2014, J MED CHEM, V57, P3126, DOI 10.1021/jm500131s

Lilienkampf A, 2009, J MED CHEM, V52, P2109, DOI 10.1021/jm900003c

Mabhula A, 2019, MEDCHEMCOMM, V10, P1342, DOI 10.1039/c9md00057g

Makarov V, 2020, J MED CHEM, V63, P8917, DOI 10.1021/acs.jmedchem.9b02075

Marganakop SB, 2012, MED CHEM RES, V21, P185, DOI 10.1007/s00044-010-9522-z

Milano A, 2009, TUBERCULOSIS, V89, P84, DOI 10.1016/j.tube.2008.08.003

Mitchison DA, 2004, FRONT BIOSCI-LANDMRK, V9, P1059, DOI 10.2741/1293

Naidu KM, 2016, BIOORG MED CHEM LETT, V26, P2245, DOI 10.1016/j.bmcl.2016.03.059

Phummarin N, 2016, MEDCHEMCOMM, V7, P2122, DOI 10.1039/c6md00236f

Pissinate K, 2016, ACS MED CHEM LETT, V7, P235, DOI 10.1021/acsmedchemlett.5b00324

Poole K, 1996, ANTIMICROB AGENTS CH, V40, P2021, DOI 10.1128/AAC.40.9.2021

Rane RA, 2012, BIOORG MED CHEM LETT, V22, P6429, DOI 10.1016/j.bmcl.2012.08.061

SANCHEZ JP, 1988, J MED CHEM, V31, P983, DOI 10.1021/jm00400a016

Sassetti CM, 2003, MOL MICROBIOL, V48, P77, DOI 10.1046/j.1365-2958.2003.03425.x

Shandil RK, 2007, ANTIMICROB AGENTS CH, V51, P576, DOI 10.1128/AAC.00414-06

Shapira T, 2020, FRONT MICROBIOL, V11, DOI 10.3389/fmicb.2020.553962

Shirude PS, 2013, J MED CHEM, V56, P9701, DOI 10.1021/jm401382v

Shruthi TG, 2019, BIOORG MED CHEM LETT, V29, P97, DOI 10.1016/j.bmcl.2018.11.002

Tahlan K, 2012, ANTIMICROB AGENTS CH, V56, P1797, DOI 10.1128/AAC.05708-11

Tekaia F., 1999, Tubercle and Lung Disease, V79, P329, DOI 10.1054/tuld.1999.0220

Thomas KD, 2011, EUR J MED CHEM, V46, P2503, DOI 10.1016/j.ejmech.2011.03.039

Upadhayaya RS, 2009, BIOORGAN MED CHEM, V17, P2830, DOI 10.1016/j.bmc.2009.02.026

Viana JD, 2018, CURR TOP MED CHEM, V18, P750, DOI 10.2174/1568026618666180528124414

Wells RM, 2013, PLOS PATHOG, V9, DOI 10.1371/journal.ppat.1003120

World Health Organization, 2020, GLOBAL TUBERCULOSIS

NR 50

TC 16

Z9 18

U1 1

U2 11

PU CHURCHILL LIVINGSTONE

PI EDINBURGH

PA JOURNAL PRODUCTION DEPT, ROBERT STEVENSON HOUSE, 1-3 BAXTERS PLACE,

LEITH WALK, EDINBURGH EH1 3AF, MIDLOTHIAN, SCOTLAND

SN 1472-9792

EI 1873-281X

J9 TUBERCULOSIS

JI Tuberculosis

PD JUL

PY 2021

VL 129

AR 102104

DI 10.1016/j.tube.2021.102104

EA JUN 2021

PG 11

WC Immunology; Microbiology; Respiratory System

WE Science Citation Index Expanded (SCI-EXPANDED)

SC Immunology; Microbiology; Respiratory System

GA TP6IP

UT WOS:000677701500009

OA Green Submitted

DA 2026-05-07

ER

PT J

AU Bwalya, P

Solo, ES

Chizimu, JY

Shrestha, D

Mbulo, G

Thapa, J

Nakajima, C

Suzuki, Y

AF Bwalya, Precious

Solo, Eddie S.

Chizimu, Joseph Y.

Shrestha, Dipti

Mbulo, Grace

Thapa, Jeewan

Nakajima, Chie

Suzuki, Yasuhiko

TI Characterization of embB mutations involved in ethambutol resistance in

multi-drug resistant Mycobacterium tuberculosis isolates in Zambia

SO TUBERCULOSIS

LA English

DT Article

DE Mycobacterium tuberculosis; Ethambutol; Multi-drug resistance; embB

mutations; Zambia

ID CODON 306; SUSCEPTIBILITY; EVOLUTION

AB Background: Ethambutol (EMB) is an important anti-tuberculosis drug used in the management of multi-drug resistant tuberculosis (MDR-TB). Mutations in embB are the major mechanism of resistance. This study investigated embB mutations among MDR-TB isolates and analyzed their correlations with phenotypic drug susceptibility testing (DST) in Zambia. Method: A total of 132 MDR-TB isolates were collected from January 2014 to April 2017 and characterized using MGIT 960 systems, embB sequencing, and spoligotyping. Results: Out of 61 phenotypically EMB resistant isolates, 53 had mutations in embB. Among the 71 EMB susceptible isolates, 47 had embB mutations. Sensitivity of embB mutations was 86.9% while specificity was 33.8%. CAS1_Kili (SIT21) had high odds of having embB mutations, particularly, G918A (Met306eIl) (Odds ratio 16.7, p < 0.0001). Conclusion: Molecular EMB resistance testing by DNA sequencing can improve detection of EMB resistance among MDR-TB patients in Zambia. Additionally, CAS1_Kili was associated with embB amino acid substitution Met306Ile suggesting transmission. A detailed investigation to track and determine transmission hotspot area for MDR-TB could help optimize control strategies.

C1 [Bwalya, Precious; Chizimu, Joseph Y.; Shrestha, Dipti; Thapa, Jeewan; Nakajima, Chie; Suzuki, Yasuhiko] Hokkaido Univ, Div Bioresources, Int Inst Zoonosis Control, Sapporo, Hokkaido 0010020, Japan.

[Bwalya, Precious; Solo, Eddie S.; Mbulo, Grace] Univ Teaching Hosp, Dept Pathol & Microbiol, Minist Hlth, Lusaka 10101, Zambia.

[Chizimu, Joseph Y.] Zambia Natl Publ Hlth Inst, Minist Hlth, Lusaka 10101, Zambia.

[Shrestha, Dipti] Tribhuvan Univ, Kathmandu Coll Sci & Technol, Dept Microbiol, Kathmandu, Nepal.

[Nakajima, Chie; Suzuki, Yasuhiko] Hokkaido Univ, Int Collaborat Unit, Int Inst Zoonosis Control, Sapporo, Hokkaido 0010020, Japan.

C3 Hokkaido University; University of Zambia; Tribhuvan University;

Hokkaido University

RP Suzuki, Y (corresponding author), Kita 20,Nishi 10,Kita Ku, Sapporo, Hokkaido 0010020, Japan.

EM suzuki@czc.hokudai.ac.jp

RI Thapa, Jeewan/GQZ-1233-2022; Nakajima, Chie/A-5020-2012; Suzuki,

Yasuhiko/F-6890-2012

OI Nakajima, Chie/0000-0002-9194-1062; Suzuki, Yasuhiko/0000-0002-1313-1494

FU Ministry of Education, Culture, Sports, Science and Technology (MEXT) ,

Japan; Joint Research Program of the Research Center for Zoonosis

Control, Hokkaido University; Japan Agency for Medical Research and

Development (AMED) [JP21wm0125008, JP21jm0510001, JP21jk0210005];

Grants-in-Aid for Scientific Research [21K14969] Funding Source: KAKEN

FX This work was supported in part by a grant from the Ministry of

Education, Culture, Sports, Science and Technology (MEXT) , Japan, and

the Joint Research Program of the Research Center for Zoonosis Control,

Hokkaido University to YS, and in part by Japan Agency for Medical

Research and Development (AMED) under Grant Number JP21wm0125008,

JP21jm0510001, and JP21jk0210005 to YS.

CR Abrahams KA, 2018, PARASITOLOGY, V145, P116, DOI 10.1017/S0031182016002377

Ahmad S, 2007, TUBERCULOSIS, V87, P123, DOI 10.1016/j.tube.2006.05.004

Al-Mutairi NM, 2019, EUR J MED RES, V24, DOI 10.1186/s40001-019-0397-2

[Anonymous], 2021, Global tuberculosis report 2021

[Anonymous], 2020, World Health Organization: Global Tuberculosis Report 2019

Bakula Z, 2013, BIOMED RES INT, V2013, DOI 10.1155/2013/167954

Bates M, 2012, PLOS ONE, V7, DOI 10.1371/journal.pone.0040774

Brudey K, 2006, BMC MICROBIOL, V6, DOI 10.1186/1471-2180-6-23

Bwalya P, 2021, ANTIBIOTICS-BASEL, V10, DOI 10.3390/antibiotics10101169

Casali N, 2014, NAT GENET, V46, P279, DOI 10.1038/ng.2878

Chizimu JY, INT J INFECT DIS ND

Cuevas-Córdoba B, 2015, ANTIMICROB AGENTS CH, V59, P5455, DOI 10.1128/AAC.00117-15

Dookie N, 2016, BMC INFECT DIS, V16, DOI 10.1186/s12879-016-1906-3

Hall T.A., 1999, NUCL ACIDS S SER, V41, P95

Hazbón MH, 2005, ANTIMICROB AGENTS CH, V49, P3794, DOI 10.1128/AAC.49.9.3794-3802.2005

Kagujje M, 2020, PLOS ONE, V15, DOI 10.1371/journal.pone.0237931

Kamerbeek J, 1997, J CLIN MICROBIOL, V35, P907, DOI 10.1128/JCM.35.4.907-914.1997

Katale BZ, 2020, BMC GENOMICS, V21, DOI 10.1186/s12864-020-6577-1

Li MC, 2020, FRONT MICROBIOL, V11, DOI 10.3389/fmicb.2020.00781

Park YK, 2012, J MED MICROBIOL, V61, P529, DOI 10.1099/jmm.0.037614-0

Payne JL, 2019, PLOS BIOL, V17, DOI 10.1371/journal.pbio.3000265

Phelan JE, 2019, GENOME MED, V11, DOI 10.1186/s13073-019-0650-x

Safi H, 2008, ANTIMICROB AGENTS CH, V52, P2027, DOI 10.1128/AAC.01486-07

Safi H, 2013, NAT GENET, V45, P1190, DOI 10.1038/ng.2743

Safi H, 2010, ANTIMICROB AGENTS CH, V54, P103, DOI 10.1128/AAC.01288-09

Solo ES, 2021, INT J INFECT DIS, V102, P489, DOI 10.1016/j.ijid.2020.10.014

Sun Q, 2018, ANTIMICROB AGENTS CH, V62, DOI 10.1128/AAC.01279-17

The National Tuberculosis and Leprosy Control Program M, 2017, GUID PROGR MAN DRUG

Tulyaprawat O, 2019, J GLOB ANTIMICROB RE, V18, P115, DOI 10.1016/j.jgar.2019.05.033

WHO, 2016, GLOB TUB REP 2016

World Health Organization, 2021, Recent description of the burden of MDR-TB among Ukrainian refugees in Europe

World Health Organization, 2021, TUB PROF ZAMB

World Health Organization (WHO), 2018, Technical manual for drug susceptibility testing of medicines used in the treatment of tuberculosis, DOI DOI 10.2471/BLT.11.096644

Xu YH, 2015, BIOMED RES INT, V2015, DOI 10.1155/2015/951706

Zhang D, 2014, TROP MED INT HEALTH, V19, P1259, DOI 10.1111/tmi.12359

Zhang L, 2020, SCIENCE, V368, P1211, DOI 10.1126/science.aba9102

Zhang ZJ, 2014, J CLIN MICROBIOL, V52, P638, DOI 10.1128/JCM.02713-13

Zhao LL, 2015, ANTIMICROB AGENTS CH, V59, P2045, DOI 10.1128/AAC.04933-14

NR 38

TC 9

Z9 9

U1 0

U2 3

PU CHURCHILL LIVINGSTONE

PI EDINBURGH

PA JOURNAL PRODUCTION DEPT, ROBERT STEVENSON HOUSE, 1-3 BAXTERS PLACE,

LEITH WALK, EDINBURGH EH1 3AF, MIDLOTHIAN, SCOTLAND

SN 1472-9792

EI 1873-281X

J9 TUBERCULOSIS

JI Tuberculosis

PD MAR

PY 2022

VL 133

AR 102184

DI 10.1016/j.tube.2022.102184

EA FEB 2022

PG 6

WC Immunology; Microbiology; Respiratory System

WE Science Citation Index Expanded (SCI-EXPANDED)

SC Immunology; Microbiology; Respiratory System

GA 0W7LV

UT WOS:000789205300006

OA Green Submitted

DA 2026-05-07

ER

PT J

AU Hussey, G

Hawkridge, T

Hanekom, W

AF Hussey, Gregory

Hawkridge, Tony

Hanekom, Willem

TI Childhood tuberculosis: old and new vaccines

SO PAEDIATRIC RESPIRATORY REVIEWS

LA English

DT Article

DE BCG; tuberculosis; TB vaccines; HIV

ID CALMETTE-GUERIN VACCINATION; TH1-TYPE IMMUNE-RESPONSE; VIRUS STRAIN MVA;

MYCOBACTERIUM-TUBERCULOSIS; BCG VACCINATION; PROTECTIVE IMMUNITY;

ENVIRONMENTAL MYCOBACTERIA; THERAPEUTIC VACCINATION; NORTHERN MALAWI;

NEONATAL CALVES

AB The world is witnessing an escalation of the tuberculosis (TB) epidemic, particularly in sub-Saharan Africa and South-East Asia. The problem has been compounded by the evolution of the human immunodeficiency virus pandemic, the increase in multidrug-resistant TB and the emergence of extensively drug-resistant TB. This has led to renewed interest in vaccines aimed at preventing tuberculosis. The current Bacille Calmette-Guerin (BCG) vaccine prevents the invasive complications of childhood tuberculosis, such as meningitis and miliary disease, but provides variable protection against adult pulmonary disease. This review discusses the history of the BCG vaccine, the reasons for its variable efficacy, protective immunity and TB, and the evolution of and obstacles to development of new candidate vaccines. Several new TB vaccines have demonstrated promising results in animal models; a number have gone into phase I clinical trials in humans, and it is anticipated that phase III trials will commence by 2009. Licensing of an effective new TB vaccine by 2015 is thus a possibility. (c) 2007 Elsevier Ltd. All rights reserved.

C1 Univ Cape Town, S African TB Vaccine Initat, ZA-7925 Cape Town, South Africa.

C3 University of Cape Town

RP Hussey, G (corresponding author), Univ Cape Town, S African TB Vaccine Initat, Anzio Rd, ZA-7925 Cape Town, South Africa.

EM gregory.hussey@uct.ac.za

OI Hanekom, Willem/0000-0003-3070-6154

CR *AER GLOB TUB VACC, 2004, CLIN TRIAL NEW TB VA

Agger EM, 2006, VACCINE, V24, P5452, DOI 10.1016/j.vaccine.2006.03.072

[Anonymous], 1999, WHOVB9923

[Anonymous], 2006, The STOP TB strategy: Building on and enhancing DOTS to meet the TB-related Millennium Development Goals

BCG vaccine, 2004, WKLY EPIDEMIOL REC, P25

Behr MA, 1999, SCIENCE, V284, P1520, DOI 10.1126/science.284.5419.1520

Bennekov T, 2006, EUR J IMMUNOL, V36, P3346, DOI 10.1002/eji.200636128

Black GF, 2001, J INFECT DIS, V184, P322, DOI 10.1086/322042

Brandt L, 2002, INFECT IMMUN, V70, P672, DOI 10.1128/IAI.70.2.672-678.2002

Buddle BM, 2003, INFECT IMMUN, V71, P6411, DOI 10.1128/IAI.71.11.6411-6419.2003

COLDITZ GA, 1994, JAMA-J AM MED ASSOC, V271, P698, DOI 10.1001/jama.271.9.698

Cole ST, 1998, NATURE, V393, P537, DOI 10.1038/31159

COMSTOCK GW, 1994, CONTROL CLIN TRIALS, V15, P247, DOI 10.1016/0197-2456(94)90042-6

Cosma A, 2003, VACCINE, V22, P21, DOI 10.1016/S0264-410X(03)00538-3

Davids V, 2006, J INFECT DIS, V193, P531, DOI 10.1086/499825

Dye C, 2006, LANCET, V367, P938, DOI 10.1016/S0140-6736(06)68384-0

FINE PEM, 1995, LANCET, V346, P1339, DOI 10.1016/S0140-6736(95)92348-9

Flynn JL, 2004, TUBERCULOSIS, V84, P93, DOI 10.1016/j.tube.2003.08.010

Flynn JL, 2006, MICROBES INFECT, V8, P1179, DOI 10.1016/j.micinf.2005.10.033

Freidag BL, 2000, INFECT IMMUN, V68, P2948, DOI 10.1128/IAI.68.5.2948-2953.2000

Ginsberg AM, 2002, B WORLD HEALTH ORGAN, V80, P483

Hanekom WA, 2005, ANN NY ACAD SCI, V1062, P69, DOI 10.1196/annals.1358.010

HANEKOM WA, 2006, UNPUB

Harrer E, 2005, ANTIVIR THER, V10, P285

HAWKRIDGE AJ, VACC WORLD C VIENN A

Hesseling AC, 2007, VACCINE, V25, P14, DOI 10.1016/j.vaccine.2006.07.020

Hoft DF, 2002, J INFECT DIS, V186, P1448, DOI 10.1086/344359

HOFT DF, 2005, US JAP COOP MED SCI

HOFT DF, 2006, COMMUNICATION

Hope JC, 2005, CLIN EXP IMMUNOL, V139, P48, DOI 10.1111/j.1365-2249.2005.02668.x

Horwitz MA, 2000, P NATL ACAD SCI USA, V97, P13853, DOI 10.1073/pnas.250480397

Horwitz MA, 2003, INFECT IMMUN, V71, P1672, DOI 10.1128/IAI.71.4.1672-1679.2003

Hovav AH, 2003, INFECT IMMUN, V71, P3146, DOI 10.1128/IAI.71.6.3146-3154.2003

Hussey GD, 2002, IMMUNOLOGY, V105, P314, DOI 10.1046/j.1365-2567.2002.01366.x

Huygen K, 1996, NAT MED, V2, P893, DOI 10.1038/nm0896-893

Kaufmann SHE, 2000, NAT MED, V6, P955, DOI 10.1038/79631

Kaufmann SHE, 2005, NAT MED, V11, pS33, DOI 10.1038/nm1221

Kaufmann SHE, 2004, ANN RHEUM DIS, V63, P50, DOI 10.1136/ard.2004.028258

Kaufmann SHE, 2007, NAT MED, V13, P299, DOI 10.1038/nm0307-299

Koch R., 1891, Dtsch. Med. Wschr, V17, P101, DOI 10.1055/s-0029-1206198

Kostense S, 2004, AIDS, V18, P1213, DOI 10.1097/00002030-200405210-00019

Kristensen I., 2000, BMJ, V321, P1

MAHNEL H, 1994, BERL MUNCH TIERARZTL, V107, P253

Marchant A, 1999, J IMMUNOL, V163, P2249

McShane H, 2004, NAT MED, V10, P1240, DOI 10.1038/nm1128

MCSHANE H, 2007, COMMUNICATION

Murray RA, 2006, J IMMUNOL, V177, P5647, DOI 10.4049/jimmunol.177.8.5647

*NAT I HLTH, TUB VACC STAT SCI

OFORI O, 2007, COMMUNICATION

Orme IM, 2005, TUBERCULOSIS, V85, P13, DOI 10.1016/j.tube.2004.08.001

RODRIGUES LC, 1993, INT J EPIDEMIOL, V22, P1154, DOI 10.1093/ije/22.6.1154

Skeiky YAW, 2006, NAT REV MICROBIOL, V4, P469, DOI 10.1038/nrmicro1419

Skeiky YAW, 2004, J IMMUNOL, V172, P7618, DOI 10.4049/jimmunol.172.12.7618

Skjot RLV, 2002, INFECT IMMUN, V70, P5446, DOI 10.1128/IAI.70.10.5446-5453.2002

Skjot RLV, 2000, INFECT IMMUN, V68, P214, DOI 10.1128/IAI.68.1.214-220.2000

Smith SM, 2000, J IMMUNOL, V165, P7088, DOI 10.4049/jimmunol.165.12.7088

STICKL H, 1974, DEUT MED WOCHENSCHR, V99, P2386, DOI 10.1055/s-0028-1108143

Taylor JL, 2003, INFECT IMMUN, V71, P2192, DOI 10.1128/IAI.71.4.2192-2198.2003

Trouiller P, 2002, LANCET, V359, P2188, DOI 10.1016/S0140-6736(02)09096-7

Trunz BB, 2006, LANCET, V367, P1173, DOI 10.1016/S0140-6736(06)68507-3

Vogels R, 2003, J VIROL, V77, P8263, DOI 10.1128/JVI.77.15.8263-8271.2003

von Reyn CF, 2002, CLIN INFECT DIS, V35, P465, DOI 10.1086/341901

Weir RE, 2006, CLIN EXP IMMUNOL, V146, P390, DOI 10.1111/j.1365-2249.2006.03222.x

*WHO, BCG CURR VACC TUB

*WHO, 2007, WER, V82, P2

*WHO, WHO DECL TB EM AFR

NR 66

TC 39

Z9 47

U1 0

U2 9

PU ELSEVIER SCI LTD

PI OXFORD

PA THE BOULEVARD, LANGFORD LANE, KIDLINGTON, OXFORD OX5 1GB, OXON, ENGLAND

SN 1526-0542

J9 PAEDIATR RESPIR REV

JI Paediatr. Respir. Rev.

PD JUN

PY 2007

VL 8

IS 2

BP 148

EP 154

DI 10.1016/j.prrv.2007.04.009

PG 7

WC Pediatrics; Respiratory System

WE Science Citation Index Expanded (SCI-EXPANDED)

SC Pediatrics; Respiratory System

GA 188NO

UT WOS:000247926800008

DA 2026-05-07

ER

PT J

AU Lan, Y

Lam, JT

Siu, GKH

Yam, WC

Mason, AJ

Lam, JKW

AF Lan, Yun

Lam, Jason T.

Siu, Gilman K. H.

Yam, Wing Cheong

Mason, A. James

Lam, Jenny K. W.

TI Cationic amphipathic D-enantiomeric antimicrobial peptides with in

vitro and ex vivo activity against drug-resistant

Mycobacterium tuberculosis

SO TUBERCULOSIS

LA English

DT Article

DE Tuberculosis; Multi-drug resistant; Antimicrobial peptides; THP-1;

Isoniazid; Clinical isolates

ID AMINO-ACID SUBSTITUTION; HOST-DEFENSE; MULTIDRUG-RESISTANT;

ISONIAZID-RESISTANT; CELL-WALL; IDENTIFICATION; MODEL; ASSOCIATION;

SELECTIVITY; MACROPHAGE

AB Tuberculosis (TB) is the leading cause of bacterial death worldwide. Due to the emergence of multi-drug resistant TB (MDR-TB) and extensively drug-resistant TB (XDR-TB), and the persistence of latent infections, a safe and effective TB therapy is highly sought after. Antimicrobial peptides (AMPs) have therapeutic potential against infectious diseases and have the ability to target microbial pathogens within eukaryotic cells. In the present study, we investigated the activity of a family of six AMPs containing all-D amino acids (D-LAK peptides) against MDR and XDR clinical strains of Mycobacterium tuberculosis (Mtb) both in vitro and, using THP-1 cells as a macrophage model, cultured ex vivo. All the DLAK peptides successfully inhibited the growth of Mtb in vitro and were similarly effective against MDR and XDR strains. D-LAK peptides effectively broke down the heavy clumping of mycobacteria in broth culture, consistent with a 'detergent-like effect' that could reduce the hydrophobic interactions between the highly lipidic cell walls of the mycobacteria, preventing bacteria cell aggregation. Furthermore, though not able to eradicate the intracellular mycobacteria, D-LAK peptides substantially inhibited the intracellular growth of drug-resistant Mtb clinical isolates at concentrations that were well tolerated by THP-1 cells. Finally, combining D-LAK peptide with isoniazid could enhance the anti-TB efficacy. D-LAK peptide, particularly D-LAK120-A, was effective as an adjunct agent at non-toxic concentration to potentiate the efficacy of isoniazid against drug-resistant Mtb in vitro, possibly by facilitating the access of isoniazid into the mycobacteria by increasing the surface permeability of the pathogen. (C) 2014 Elsevier Ltd. All rights reserved.

C1 [Lan, Yun; Lam, Jenny K. W.] Univ Hong Kong, Li Ka Shing Fac Med, Dept Pharmacol & Pharm, Pokfulam, Hong Kong, Peoples R China.

[Lam, Jason T.; Yam, Wing Cheong] Univ Hong Kong, Li Ka Shing Fac Med, Dept Microbiol, Hong Kong, Hong Kong, Peoples R China.

[Siu, Gilman K. H.] Hong Kong Polytech Univ, Fac Hlth & Social Sci, Dept Hlth Technol & Informat, Kowloon, Hong Kong, Peoples R China.

[Mason, A. James] Kings Coll London, Inst Pharmaceut Sci, London SE1 9NH, England.

C3 University of Hong Kong; University of Hong Kong; Hong Kong Polytechnic

University; University of London; King's College London

RP Lam, JKW (corresponding author), Univ Hong Kong, Li Ka Shing Fac Med, Dept Pharmacol & Pharm, 21 Sassoon Rd, Pokfulam, Hong Kong, Peoples R China.

EM jkwlam@hku.hk

RI lan, yunchun/PTE-3571-2026; Yam, Cheong/C-4765-2009; Lam,

Jenny/A-4842-2010; SIU, Kit Hang/L-7796-2017; Mason, James/J-2273-2012

OI Lam, Jenny/0000-0003-2997-010X; SIU, Kit Hang/0000-0002-4354-3393;

Mason, James/0000-0003-0411-602X

FU Research Fund for the Control of Infectious Diseases (RFCID) [11000562];

Food and Health Bureau, Hong Kong SAR Government; Medical Research

Council (NIRG) [G0801072/87482]; Medical Research Council [G0801072]

Funding Source: researchfish; MRC [G0801072] Funding Source: UKRI

FX This study was funded by the Research Fund for the Control of Infectious

Diseases (RFCID 11000562), Food and Health Bureau, Hong Kong SAR

Government and the Medical Research Council (NIRG G0801072/87482 to

AJM).

CR Ando H, 2010, ANTIMICROB AGENTS CH, V54, P1793, DOI 10.1128/AAC.01691-09

[Anonymous], GLOB TUB REP 2013

Borelli V, 2003, INFECT IMMUN, V71, P605, DOI 10.1128/IAI.71.2.605-613.2003

Borrell S, 2009, INT J TUBERC LUNG D, V13, P1456

Brogden KA, 2005, NAT REV MICROBIOL, V3, P238, DOI 10.1038/nrmicro1098

Caminero JA, 2010, LANCET INFECT DIS, V10, P621, DOI 10.1016/S1473-3099(10)70139-0

Dorman SE, 2007, NAT MED, V13, P295, DOI 10.1038/nm0307-295

Ernst WA, 2000, J IMMUNOL, V165, P7102, DOI 10.4049/jimmunol.165.12.7102

Fattorini L, 2004, PEPTIDES, V25, P1075, DOI 10.1016/j.peptides.2004.04.003

Fenton MJ, 1996, INFECT IMMUN, V64, P683, DOI 10.1128/IAI.64.3.683-690.1996

Gomes MS, 1999, INFECT IMMUN, V67, P3199, DOI 10.1128/IAI.67.7.3199-3206.1999

Grieco P, 2013, BBA-BIOMEMBRANES, V1828, P652, DOI 10.1016/j.bbamem.2012.08.027

Health Protection Agency Public Health England, 2013, TUB UK 2013 REP

Hong SY, 1999, BIOCHEM PHARMACOL, V58, P1775, DOI 10.1016/S0006-2952(99)00259-2

JARLIER V, 1994, FEMS MICROBIOL LETT, V123, P11, DOI 10.1111/j.1574-6968.1994.tb07194.x

Jiang ZQ, 2011, PROTEIN PEPTIDE LETT, V18, P241, DOI 10.2174/092986611794578288

Kalita A, 2004, J INFECT DIS, V190, P1476, DOI 10.1086/424463

Katiyar SK, 2008, INT J TUBERC LUNG D, V12, P139

Kaur Devinder, 2009, Adv Appl Microbiol, V69, P23, DOI 10.1016/S0065-2164(09)69002-X

Liu PT, 2008, CURR OPIN IMMUNOL, V20, P371, DOI 10.1016/j.coi.2008.05.014

Louw GE, 2009, ANTIMICROB AGENTS CH, V53, P3181, DOI 10.1128/AAC.01577-08

Melo MN, 2009, NAT REV MICROBIOL, V7, P245, DOI 10.1038/nrmicro2095

Méndez-Samperio P, 2008, PEPTIDES, V29, P1836, DOI 10.1016/j.peptides.2008.05.024

Meyers PR, 1998, J CLIN MICROBIOL, V36, P2752, DOI 10.1128/JCM.36.9.2752-2754.1998

Musser JM, 1996, J INFECT DIS, V173, P196, DOI 10.1093/infdis/173.1.196

Nicolas P, 2009, FEBS J, V276, P6483, DOI 10.1111/j.1742-4658.2009.07359.x

OrtaloMagne A, 1996, J BACTERIOL, V178, P456, DOI 10.1128/jb.178.2.456-461.1996

Peters BM, 2010, PLOS PATHOG, V6, DOI 10.1371/journal.ppat.1001067

Pieters J, 2008, CELL HOST MICROBE, V3, P399, DOI 10.1016/j.chom.2008.05.006

Ramaswamy S., 1998, Tubercle and Lung Disease, V79, P3, DOI 10.1054/tuld.1998.0002

RASTOGI N, 1990, ANTIMICROB AGENTS CH, V34, P2061, DOI 10.1128/AAC.34.11.2061

Reyrat JM, 2001, TRENDS MICROBIOL, V9, P472, DOI 10.1016/S0966-842X(01)02168-0

Rivas-Santiago B, 2013, INT J ANTIMICROB AG, V41, P143, DOI 10.1016/j.ijantimicag.2012.09.015

Schaaf HS, 2007, EUR J CLIN MICROBIOL, V26, P203, DOI 10.1007/s10096-007-0257-9

Song YM, 2004, BIOCHEM BIOPH RES CO, V314, P615, DOI 10.1016/j.bbrc.2003.12.142

Stinear TP, 2008, GENOME RES, V18, P729, DOI 10.1101/gr.075069.107

Velayati AA, 2009, CHEMOTHERAPY, V55, P303, DOI 10.1159/000226425

Vermeer LS, 2012, J BIOL CHEM, V287, P34120, DOI 10.1074/jbc.M112.359067

Welin A, 2011, J INNATE IMMUN, V3, P508, DOI 10.1159/000325297

Wong KC, 2007, CLIN VACCINE IMMUNOL, V14, P1279, DOI 10.1128/CVI.00190-07

Yang ST, 2006, FEBS J, V273, P4040, DOI 10.1111/j.1742-4658.2006.05407.x

Zasloff M, 2002, NATURE, V415, P389, DOI 10.1038/415389a

NR 42

TC 53

Z9 59

U1 0

U2 32

PU CHURCHILL LIVINGSTONE

PI EDINBURGH

PA JOURNAL PRODUCTION DEPT, ROBERT STEVENSON HOUSE, 1-3 BAXTERS PLACE,

LEITH WALK, EDINBURGH EH1 3AF, MIDLOTHIAN, SCOTLAND

SN 1472-9792

EI 1873-281X

J9 TUBERCULOSIS

JI Tuberculosis

PD DEC

PY 2014

VL 94

IS 6

BP 678

EP 689

DI 10.1016/j.tube.2014.08.001

PG 12

WC Immunology; Microbiology; Respiratory System

WE Science Citation Index Expanded (SCI-EXPANDED)

SC Immunology; Microbiology; Respiratory System

GA AW0KB

UT WOS:000345979900019

DA 2026-05-07

ER

PT J

AU Bolla, JR

Do, SV

Long, F

Dai, L

Su, CC

Lei, HT

Chen, X

Gerkey, JE

Murphy, DC

Rajashankar, KR

Zhang, QJ

Yu, EW

AF Bolla, Jani Reddy

Do, Sylvia V.

Long, Feng

Dai, Lei

Su, Chih-Chia

Lei, Hsiang-Ting

Chen, Xiao

Gerkey, Jillian E.

Murphy, Daniel C.

Rajashankar, Kanagalaghatta R.

Zhang, Qijing

Yu, Edward W.

TI Structural and functional analysis of the transcriptional regulator

Rv3066 of Mycobacterium tuberculosis

SO NUCLEIC ACIDS RESEARCH

LA English

DT Article

ID DRUG-RESISTANT TUBERCULOSIS; MULTIDRUG EFFLUX PUMP; CRYSTAL-STRUCTURE;

TETR FAMILY; XDR-TB; REPRESSOR; SOFTWARE; EXPRESSION; EVOLUTION; COMPLEX

AB The Mmr multidrug efflux pump recognizes and actively extrudes a broad range of antimicrobial agents, and promotes the intrinsic resistance to these antimicrobials in Mycobacterium tuberculosis. The expression of Mmr is controlled by the TetR-like transcriptional regulator Rv3066, whose open reading frame is located downstream of the mmr operon. To understand the structural basis of Rv3066 regulation, we have determined the crystal structures of Rv3066, both in the absence and presence of bound ethidium, revealing an asymmetric homodimeric two-domain molecule with an entirely helical architecture. The structures underscore the flexibility and plasticity of the regulator essential for multidrug recognition. Comparison of the apo-Rv3066 and Rv3066-ethidium crystal structures suggests that the conformational changes leading to drug-mediated derepression is primarily due to a rigid body rotational motion within the dimer interface of the regulator. The Rv3066 regulator creates a multidrug-binding pocket, which contains five aromatic residues. The bound ethidium is found buried within the multidrug-binding site, where extensive aromatic stacking interactions seemingly govern the binding. In vitro studies reveal that the dimeric Rv3066 regulator binds to a 14-bp palindromic inverted repeat sequence in the nanomolar range. These findings provide new insight into the mechanisms of ligand binding and Rv3066 regulation.

C1 [Bolla, Jani Reddy; Long, Feng; Su, Chih-Chia; Lei, Hsiang-Ting; Chen, Xiao; Gerkey, Jillian E.; Murphy, Daniel C.; Yu, Edward W.] Iowa State Univ Sci & Technol, Coll Vet Med, Dept Chem, Ames, IA 50011 USA.

[Do, Sylvia V.; Yu, Edward W.] Iowa State Univ, Coll Vet Med, Bioinformat & Computat Biol Interdept Grad Progra, Ames, IA 50011 USA.

[Dai, Lei; Zhang, Qijing] Iowa State Univ, Dept Vet Microbiol, Coll Vet Med, Ames, IA 50011 USA.

[Rajashankar, Kanagalaghatta R.] Cornell Univ, Argonne Natl Lab, Dept Chem & Chem Biol, Argonne, IL 60439 USA.

[Rajashankar, Kanagalaghatta R.] Cornell Univ, Argonne Natl Lab, NE CAT, Argonne, IL 60439 USA.

[Yu, Edward W.] Iowa State Univ, Dept Phys & Astron, Ames, IA 50011 USA.

C3 Iowa State University; Iowa State University; Iowa State University;

United States Department of Energy (DOE); Argonne National Laboratory;

Cornell University; Cornell University; United States Department of

Energy (DOE); Argonne National Laboratory; Iowa State University

RP Yu, EW (corresponding author), Iowa State Univ Sci & Technol, Coll Vet Med, Dept Chem, Ames, IA 50011 USA.

EM ewyu@iastate.edu

RI Bolla, Jani R/AAT-1370-2020; Long, Feng/F-5475-2011

OI Bolla, Jani R/0000-0003-4346-182X;

FU National Center for Research Resources at the National Institutes of

Health [RR-15301]; National Institutes of Health (NIH) [DK063008,

GM086431]; U.S. Department of Energy, Office of Basic Energy Sciences

[DE-AC02-06CH11357]; Community College Institute of Science Internship

(CCI) and Science Undergraduate Laboratory Internship (SULI) from the

DOE

FX The authors are grateful to Susan T. Howard at the University of Texas

Health Science Center (Tyler) for providing the pMV261plasmid used in

this study. This work is based upon research conducted at the

Northeastern Collaborative Access Team beamlines of the Advanced Photon

Source, supported by award RR-15301 from the National Center for

Research Resources at the National Institutes of Health. National

Institutes of Health (NIH) [DK063008 to Q.Z. and GM086431 to E.W.Y.];

Use of the Advanced Photon Source is supported by the U.S. Department of

Energy, Office of Basic Energy Sciences [DE-AC02-06CH11357]; Community

College Institute of Science Internship (CCI) and Science Undergraduate

Laboratory Internship (SULI) from the DOE (to J.E.G. and D. C. M.).

Funding for open access charge: NIH [GM086431].

CR Adams PD, 2002, ACTA CRYSTALLOGR D, V58, P1948, DOI 10.1107/S0907444902016657

Alekshun MN, 2000, MOL MICROBIOL, V35, P1394, DOI 10.1046/j.1365-2958.2000.01802.x

Alguel Y, 2007, J MOL BIOL, V369, P829, DOI 10.1016/j.jmb.2007.03.062

Amaral L, 2010, INT J ANTIMICROB AG, V35, P524, DOI 10.1016/j.ijantimicag.2009.12.019

Bellinzoni M, 2009, J BACTERIOL, V191, P7531, DOI 10.1128/JB.00631-09

Brunger AT, 1998, ACTA CRYSTALLOGR D, V54, P905, DOI 10.1107/S0907444998003254

Cowtan K, 2010, ACTA CRYSTALLOGR D, V66, P470, DOI 10.1107/S090744490903947X

Danilchanka O, 2008, ANTIMICROB AGENTS CH, V52, P2503, DOI 10.1128/AAC.00298-08

De Rossi E, 2006, FEMS MICROBIOL REV, V30, P36, DOI 10.1111/j.1574-6976.2005.00000.x

De Rossi E, 1998, J BACTERIOL, V180, P6068, DOI 10.1128/JB.180.22.6068-6071.1998

Dover LG, 2004, J MOL BIOL, V340, P1095, DOI 10.1016/j.jmb.2004.06.003

Dutta NK, 2010, PLOS ONE, V5, DOI 10.1371/journal.pone.0010069

Emsley P, 2004, ACTA CRYSTALLOGR D, V60, P2126, DOI 10.1107/S0907444904019158

Frénois F, 2004, MOL CELL, V16, P301, DOI 10.1016/j.molcel.2004.09.020

FRIEDEN TR, 1993, NEW ENGL J MED, V328, P521, DOI 10.1056/NEJM199302253280801

Goldman Robert C., 2007, Infectious Disorders - Drug Targets, V7, P73

Grkovic S, 2002, MICROBIOL MOL BIOL R, V66, P671, DOI 10.1128/MMBR.66.4.671-701.2002

Gu RY, 2007, J MOL BIOL, V372, P583, DOI 10.1016/j.jmb.2007.06.072

Hernández A, 2009, J BIOL CHEM, V284, P14428, DOI 10.1074/jbc.M809221200

HINRICHS W, 1994, SCIENCE, V264, P418, DOI 10.1126/science.8153629

Hoffmann KM, 2005, J BACTERIOL, V187, P5008, DOI 10.1128/JB.187.14.5008-5012.2005

ISEMAN MD, 1993, NEW ENGL J MED, V329, P784, DOI 10.1056/NEJM199309093291108

JACOBS WR, 1991, METHOD ENZYMOL, V204, P537

Le TBK, 2011, NUCLEIC ACIDS RES, V39, P9433, DOI 10.1093/nar/gkr640

Li M, 2007, J MOL BIOL, V374, P591, DOI 10.1016/j.jmb.2007.09.064

Li XZ, 2004, DRUGS, V64, P159, DOI 10.2165/00003495-200464020-00004

Li XZ, 2004, ANTIMICROB AGENTS CH, V48, P2415, DOI 10.1128/AAC.48.7.2415-2423.2004

Livak KJ, 2001, METHODS, V25, P402, DOI 10.1006/meth.2001.1262

Mccoy AJ, 2007, J APPL CRYSTALLOGR, V40, P658, DOI 10.1107/S0021889807021206

Natsume R, 2004, J MOL BIOL, V336, P409, DOI 10.1016/j.jmb.2003.12.040

Nikaido H, 2001, SEMIN CELL DEV BIOL, V12, P215, DOI 10.1006/scdb.2000.0247

Orth P, 2000, NAT STRUCT BIOL, V7, P215

Otwinowski Z, 1997, METHOD ENZYMOL, V276, P307, DOI 10.1016/S0076-6879(97)76066-X

Pape T, 2004, J APPL CRYSTALLOGR, V37, P843, DOI 10.1107/S0021889804018047

Pillay M, 2007, CLIN INFECT DIS, V45, P1409, DOI 10.1086/522987

Ramos JL, 2005, MICROBIOL MOL BIOL R, V69, P326, DOI 10.1128/MMBR.69.2.326-356.2005

Schneider TR, 2002, ACTA CRYSTALLOGR D, V58, P1772, DOI 10.1107/S0907444902011678

Schumacher MA, 2002, EMBO J, V21, P1210, DOI 10.1093/emboj/21.5.1210

Schumacher MA, 2001, SCIENCE, V294, P2158, DOI 10.1126/science.1066020

Trott O, 2010, J COMPUT CHEM, V31, P455, DOI 10.1002/jcc.21334

Udwadia ZF, 2012, CLIN INFECT DIS, V54, P579, DOI 10.1093/cid/cir889

Yu Z, 2010, J MOL BIOL, V400, P847, DOI 10.1016/j.jmb.2010.05.062

Zianni Michael, 2006, J Biomol Tech, V17, P103

NR 43

TC 49

Z9 61

U1 0

U2 16

PU OXFORD UNIV PRESS

PI OXFORD

PA GREAT CLARENDON ST, OXFORD OX2 6DP, ENGLAND

SN 0305-1048

EI 1362-4962

J9 NUCLEIC ACIDS RES

JI Nucleic Acids Res.

PD OCT

PY 2012

VL 40

IS 18

BP 9340

EP 9355

DI 10.1093/nar/gks677

PG 16

WC Biochemistry & Molecular Biology

WE Science Citation Index Expanded (SCI-EXPANDED)

SC Biochemistry & Molecular Biology

GA 022AJ

UT WOS:000309927100055

OA gold

DA 2026-05-07

ER

PT J

AU Gun, MA

Bozdogan, B

Coban, AY

AF Gun, Mehmet Akif

Bozdogan, Bulent

Coban, Ahmet Yilmaz

TI Tuberculosis and beta-lactam antibiotics

SO FUTURE MICROBIOLOGY

LA English

DT Review

DE beta-lactams; MDR; tuberculosis; XDR

ID DRUG-RESISTANT TUBERCULOSIS; MEROPENEM-CLAVULANIC ACID;

MYCOBACTERIUM-TUBERCULOSIS; IN-VITRO; ANTITUBERCULOSIS DRUGS; TREAT

MULTIDRUG; COMBINATION; CARBAPENEMS; EFFICACY; SUSCEPTIBILITY

AB Tuberculosis (TB) is one of the oldest health problems in the world and it remains unresolved. Multidrug-resistant-TB and extensively resistant-TB are a serious problem for control programs. The evaluation of available antibiotics has gained importance in recent years for the treatment of resistant TB. Beta-lactam antibiotics inhibit cell wall biosynthesis in the bacteria; the presence of beta-lactamase enzyme in TB bacilli raises the question of whether this group of antibiotics can be used in treatment. As a result, it has been reported that the combination of beta-lactam antibiotics with beta-lactamase is effective againstMycobacterium tuberculosisbothin vitroandin vivo. The aim of this article is to review and discuss up-to-date knowledge and future perspective on beta-lactam antibiotics and TB.

C1 [Gun, Mehmet Akif] Ondokuz Mayis Univ, Sch Med, Dept Med Microbiol, TR-55139 Samsun, Turkey.

[Bozdogan, Bulent] Adnan Menderes Univ, Recombinant DNA & Recombinant Prot Res Ctr REDPRO, TR-09010 Aydin, Turkey.

[Coban, Ahmet Yilmaz] Akdeniz Univ, TB Res Ctr, TR-07070 Antalya, Turkey.

[Coban, Ahmet Yilmaz] Akdeniz Univ, Dept Nutr & Dietet, Fac Hlth Sci, TR-07070 Antalya, Turkey.

C3 Ondokuz Mayis University; Adnan Menderes University; Akdeniz University;

Akdeniz University

RP Coban, AY (corresponding author), Akdeniz Univ, TB Res Ctr, TR-07070 Antalya, Turkey.; Coban, AY (corresponding author), Akdeniz Univ, Dept Nutr & Dietet, Fac Hlth Sci, TR-07070 Antalya, Turkey.

EM cobanay2003@gmail.com

RI BOZDOGAN, BÜLENT/JAC-5096-2023; Gun, Mehmet Akif/IZE-2791-2023; Coban,

Ahmet Yilmaz/I-9067-2017

OI BOZDOGAN, BÜLENT/0000-0003-2469-9728; Gun, Mehmet

Akif/0000-0003-1286-7157; Coban, Ahmet Yilmaz/0000-0002-8815-6063

CR Abate G, 1998, J ANTIMICROB CHEMOTH, V42, P735, DOI 10.1093/jac/42.6.735

Caminero JA, 2015, EUR RESPIR J, V46, P887, DOI 10.1183/13993003.00432-2015

Catalao MJ, 2019, FRONT MICROBIOL, V10, DOI 10.3389/fmicb.2019.00190

Cavanaugh JS, 2017, J ANTIMICROB CHEMOTH, V72, P1678, DOI 10.1093/jac/dkx022

CHAMBERS HF, 1995, ANTIMICROB AGENTS CH, V39, P2620, DOI 10.1128/AAC.39.12.2620

Chambers HF, 2005, ANTIMICROB AGENTS CH, V49, P2816, DOI 10.1128/AAC.49.7.2816-2821.2005

Daffé M, 2019, MICROBIOL SPECTR, V7, DOI 10.1128/microbiolspec.GPP3-0027-2018

De Lorenzo S, 2013, EUR RESPIR J, V41, P1386, DOI 10.1183/09031936.00124312

Deshpande D, 2018, ANTIMICROB AGENTS CH, V62, DOI 10.1128/AAC.02232-17

Deshpande D, 2017, SCI ADV, V3, DOI 10.1126/sciadv.1701102

Dinçer I, 2004, INT J ANTIMICROB AG, V23, P408, DOI 10.1016/j.ijantimicag.2003.09.023

Donald PR, 2001, SCAND J INFECT DIS, V33, P466, DOI 10.1080/00365540152029954

Dooley KE, 2013, J INFECT DIS, V207, P1352, DOI 10.1093/infdis/jis460

England K, 2012, ANTIMICROB AGENTS CH, V56, P3384, DOI 10.1128/AAC.05690-11

Forsman LD, 2015, ANTIMICROB AGENTS CH, V59, P3630, DOI 10.1128/AAC.00171-15

Pagliotto ADF, 2016, J MICROBIOL IMMUNOL, V49, P980, DOI 10.1016/j.jmii.2015.08.025

Gonzalo X, 2013, J ANTIMICROB CHEMOTH, V68, P366, DOI 10.1093/jac/dks395

Guo ZY, 2019, BIOMED ENVIRON SCI, V32, P235, DOI 10.3967/bes2019.033

Gupta R, 2010, NAT MED, V16, P466, DOI 10.1038/nm.2120

Horita Y, 2014, ANTIMICROB AGENTS CH, V58, P7010, DOI 10.1128/AAC.03539-14

Hugonnet JE, 2009, SCIENCE, V323, P1215, DOI 10.1126/science.1167498

Jaganath D, 2016, INT J TUBERC LUNG D, V20, P1436, DOI 10.5588/ijtld.16.0498

Jain A, 2018, EXPERT REV ANTI-INFE, V16, P513, DOI 10.1080/14787210.2018.1496821

Jeon AB, 2017, PLOS ONE, V12, DOI 10.1371/journal.pone.0180925

Jeon D, 2017, TUBERC RESPIR DIS, V80, P336, DOI 10.4046/trd.2017.0049

Kaushik A, 2017, J ANTIMICROB CHEMOTH, V72, P2320, DOI 10.1093/jac/dkx152

Kaushik A, 2015, ANTIMICROB AGENTS CH, V59, P6561, DOI 10.1128/AAC.01158-15

Kumar P, 2017, NAT CHEM BIOL, V13, P54, DOI [10.1038/nchembio.2237, 10.1038/NCHEMBIO.2237]

Kurz SG, 2012, EXPERT REV ANTI-INFE, V10, P999, DOI [10.1586/ERI.12.96, 10.1586/eri.12.96]

Li F, 2018, BIOMED RES INT, V2018, DOI 10.1155/2018/3579832

Mainardi JL, 2011, CLIN MICROBIOL INFEC, V17, P1755, DOI 10.1111/j.1469-0691.2011.03699.x

McHugh TD, 2019, LANCET INFECT DIS, V19, P233, DOI 10.1016/S1473-3099(19)30060-X

Palmero Domingo, 2015, Arch Bronconeumol, V51, pe49, DOI [10.1016/j.arbr.2015.07.003, 10.1016/j.arbres.2015.03.012]

PAYEN MC, 2012, INT J TUBERC LUNG D, V16, P558, DOI DOI 10.5588/IJTLD.11.0414

Pontali E, 2018, CURR OPIN PULM MED, V24, P244, DOI [10.1097/MCP.0000000000000477, 10.1097/mcp.0000000000000477]

Ramón-García S, 2016, SCI REP-UK, V6, DOI 10.1038/srep34293

Rendon A, 2016, J THORAC DIS, V8, P2666, DOI 10.21037/jtd.2016.10.14

Rullas J, 2015, ANTIMICROB AGENTS CH, V59, P4997, DOI 10.1128/AAC.01063-15

Segura C, 1998, ANTIMICROB AGENTS CH, V42, P1524, DOI 10.1128/AAC.42.6.1524

Solapure S, 2013, ANTIMICROB AGENTS CH, V57, P2506, DOI 10.1128/AAC.00023-13

Sotgiu G, 2016, INT J MOL SCI, V17, DOI 10.3390/ijms17030373

Squeglia F, 2018, CHEM-EUR J, V24, P2533, DOI 10.1002/chem.201702973

Srivastava S, 2016, CLIN INFECT DIS, V63, pS102, DOI 10.1093/cid/ciw483

Story-Roller E, 2018, IUBMB LIFE, V70, P881, DOI 10.1002/iub.1875

Tiberi S, 2016, EUR RESPIR J, V47, P333, DOI 10.1183/13993003.01278-2015

van Rijn SP, 2017, ANTIMICROB AGENTS CH, V61, DOI [10.1128/AAC.02039-16, 10.1128/aac.02039-16]

Veziris N, 2011, ANTIMICROB AGENTS CH, V55, P2597, DOI 10.1128/AAC.01824-10

Vincent AT, 2018, FRONT MICROBIOL, V9, DOI 10.3389/fmicb.2018.02341

Voladri RKR, 1998, ANTIMICROB AGENTS CH, V42, P1375, DOI 10.1128/AAC.42.6.1375

Wang F, 2006, ANTIMICROB AGENTS CH, V50, P2762, DOI 10.1128/AAC.00320-06

WHO, 2012, GLOBAL TUBERCULOSIS REPORT 2012, P1

Wivagg CN, 2014, J ANTIBIOT, V67, P645, DOI 10.1038/ja.2014.94

WONG CS, 1988, J ANTIMICROB CHEMOTH, V22, P863, DOI 10.1093/jac/22.6.863

NR 53

TC 8

Z9 11

U1 0

U2 17

PU FUTURE MEDICINE LTD

PI LONDON

PA UNITEC HOUSE, 3RD FLOOR, 2 ALBERT PLACE, FINCHLEY CENTRAL, LONDON, N3

1QB, ENGLAND

SN 1746-0913

EI 1746-0921

J9 FUTURE MICROBIOL

JI Future Microbiol.

PD AUG

PY 2020

VL 15

IS 10

BP 937

EP 944

DI 10.2217/fmb-2019-0318

EA AUG 2020

PG 8

WC Microbiology

WE Science Citation Index Expanded (SCI-EXPANDED)

SC Microbiology

GA NG2PS

UT WOS:000556798400001

DA 2026-05-07

ER

PT J

AU Nguyen, TD

Choi, JA

Son, D

Yuk, HJ

Lee, J

Son, S

Kim, J

Jo, S

Kim, DS

Song, CH

AF Nguyen, Tam Doan

Choi, Ji-Ae

Son, Doyi

Yuk, Heung Joo

Lee, Junghwan

Son, Sanghun

Kim, Jaewhan

Jo, Seoyeon

Kim, Dong-Seon

Song, Chang-Hwa

TI Philadelphus tenuifolius Leaf Extract Exhibits Anti-Tuberculosis

Activity by Enhancing Host Autophagy and Immunity: A Promising

Host-Directed Therapeutic Candidate

SO JOURNAL OF MICROBIOLOGY AND BIOTECHNOLOGY

LA English

DT Article

DE Tuberculosis; Host-directed therapy; Philadelphus tenuifolius; MAPK

signaling; Autophagy; Natural products

AB The global emergence of drug-resistant Mycobacterium tuberculosis (Mtb) necessitates the urgent discovery of novel anti-tuberculosis agents. Philadelphus tenuifolius Rupr., a Korean aromatic herb, has been historically recognized for its traditional medicinal uses. This study aimed to scientifically investigate the anti-tuberculosis (TB) potential of the P. tenuifolius leaf extract (PT-LE) and elucidate its underlying mechanism of action. PT-LE was prepared by 50% ethanolic extraction. Its anti-TB activity was evaluated against intracellular Mtb in BMDMs. Mechanistic studies focused on the activation of the MAPK pathway and autophagy flux. Synergistic effects with conventional anti-TB drugs were also assessed. For the in vivo evaluation, Mtb-infected mice were orally treated daily with PTLE (100 mg/kg), followed by the determination of the lung bacterial burden. PT-LE exhibits negligible host cell cytotoxicity and effectively reduced the bacterial load of intracellular Mtb within macrophages. Mechanistically, PT-LE was shown to significantly activate the MAPK signaling pathway, which subsequently enhanced autophagy flux - a critical host defense mechanism against Mtb. Furthermore, PT-LE demonstrated potent synergistic activity with existing anti-TB drugs and modulated the host immune response by increasing the production of the chemokine MCP-1. Critically, the in vivo experiments showed that oral administration of PT-LE significantly reduced the Mtb bacterial burden in the lungs of infected mice. These findings reveal a novel anti-TB function of PT-LE, which operates by enhancing host autophagy and immunity. PT-LE represents a promising and effective host-directed therapeutic candidate for both drug-sensitive and drug-resistant tuberculosis.

C1 [Nguyen, Tam Doan; Choi, Ji-Ae; Son, Doyi; Lee, Junghwan; Son, Sanghun; Kim, Jaewhan; Jo, Seoyeon; Song, Chang-Hwa] Chungnam Natl Univ, Coll Med, Dept Microbiol, Daejeon 35015, South Korea.

[Nguyen, Tam Doan; Choi, Ji-Ae; Son, Doyi; Lee, Junghwan; Son, Sanghun; Kim, Jaewhan; Jo, Seoyeon; Song, Chang-Hwa] Chungnam Natl Univ, Coll Med, Dept Med Sci, Daejeon 35015, South Korea.

[Son, Doyi; Lee, Junghwan; Jo, Seoyeon; Song, Chang-Hwa] Chungnam Natl Univ, Translat Immunol Inst, Daejeon 34134, South Korea.

[Yuk, Heung Joo; Kim, Dong-Seon] Korea Inst Oriental Med, Herbal Med Res Div, Daejeon 34054, South Korea.

C3 Chungnam National University; Chungnam National University; Chungnam

National University; Korea Institute of Oriental Medicine (KIOM)

RP Song, CH (corresponding author), Chungnam Natl Univ, Coll Med, Dept Microbiol, Daejeon 35015, South Korea.; Song, CH (corresponding author), Chungnam Natl Univ, Coll Med, Dept Med Sci, Daejeon 35015, South Korea.; Song, CH (corresponding author), Chungnam Natl Univ, Translat Immunol Inst, Daejeon 34134, South Korea.

EM songch@cnu.ac.kr

RI Song, Chang-Hwa/AAD-7917-2022

FU National Research Foundation of Korea (NRF) - Korean government (MSIT)

[RS-2025-00523465, RS-2025-02263643]; Chungnam National University and

BK21 FOUR Program by Chungnam National University Research Grant

FX This work was supported by grants from National Research Foundation of

Korea (NRF) funded by the Korean government (MSIT) (RS-2025-00523465 and

RS-2025-02263643) , research fund of Chungnam National University and

BK21 FOUR Program by Chungnam National University Research Grant, 2024.

The funders had no role in study design, data collection and analysis

decision to publish, or preparation of the manuscript.

CR Adikesavalu H, 2021, MEDICINA-LITHUANIA, V57, DOI 10.3390/medicina57060522

Amingad SP, 2025, MICROB PATHOGENESIS, V206, DOI 10.1016/j.micpath.2025.107787

[Anonymous], 2022, WHO CONSOLIDATED GUI

Chang Chin-Sung, 2005, [Korean Journal of plant Taxonomy, 식물분류학회지], V35, P247

Cheng CY, 2017, SCI IMMUNOL, V2, DOI 10.1126/sciimmunol.aaj1789

Chi WY, 2024, BMC BIOL, V22, DOI 10.1186/s12915-024-01946-6

Dartois VA, 2022, NAT REV MICROBIOL, V20, P685, DOI 10.1038/s41579-022-00731-y

Domachowske JB, 2025, INTRO CLIN INFECT DI, P153, DOI [10.1007/978-3-031-84089-0_14, DOI 10.1007/978-3-031-84089-0_14]

Domingo-Gonzalez R, 2016, MICROBIOL SPECTR, V4, DOI 10.1128/microbiolspec.TBTB2-0018-2016

Dwivedi VP, 2017, FRONT CELL INFECT MI, V7, DOI 10.3389/fcimb.2017.00149

Fatima S, 2021, MEDCOMM, V2, P494, DOI 10.1002/mco2.82

Gomes RN, 2013, SHOCK, V39, P63, DOI 10.1097/SHK.0b013e31827802b5

Grancai Daniel, 2014, Ceska Slov Farm, V63, P120

Guirado E, 2013, SEMIN IMMUNOPATHOL, V35, P563, DOI 10.1007/s00281-013-0388-2

Gupta PK, 2023, FRONT IMMUNOL, V14, DOI 10.3389/fimmu.2023.1233630

Huang XJ, 2024, BIOMED PHARMACOTHER, V171, DOI 10.1016/j.biopha.2023.116087

Jantová S, 2000, PHYTOTHER RES, V14, P601, DOI 10.1002/1099-1573(200012)14:8<601::AID-PTR771>3.0.CO;2-B

Kim YM, 2022, J ASIA-PAC BIODIVERS, V15, P442

Kumar S, 2019, J BIOL CHEM, V294, P8555, DOI 10.1074/jbc.RA119.008005

Lee CK, 1998, ARCH PHARM RES, V21, P62, DOI 10.1007/BF03216754

Liu YL, 2024, J NANOBIOTECHNOL, V22, DOI 10.1186/s12951-024-02875-w

Mahajan P, 2025, MICROBIOL RES, V299, DOI 10.1016/j.micres.2025.128253

Nguyen TD, 2025, J ANTIBIOT, V78, P181, DOI 10.1038/s41429-024-00799-z

Ning BZ, 2023, MICROBIOL SPECTR, V11, DOI 10.1128/spectrum.04711-22

Omoteso OA, 2025, MICROORGANISMS, V13, DOI [10.3390/microorganisms13040722, 10.3390/microorganisms13040722]

Paik S, 2019, VIRULENCE, V10, P448, DOI 10.1080/21505594.2018.1536598

Park D, 2016, SCI REP-UK, V6, DOI 10.1038/srep21772

Peto A, 2022, MOLECULES, V27, DOI 10.3390/molecules27092652

Raqib R, 2024, BIOMOLECULES, V14, DOI 10.3390/biom14121497

Schorey JS, 2003, CELL MICROBIOL, V5, P133, DOI 10.1046/j.1462-5822.2003.00263.x

Sharma A, 2020, ACS BIOMATER SCI ENG, V6, P4126, DOI 10.1021/acsbiomaterials.0c00823

Son SH, 2023, MBIO, V14, DOI 10.1128/mbio.01535-23

Sung YY, 2025, J FUNCT FOODS, V130, DOI 10.1016/j.jff.2025.106904

Valko V, 2011, Acta Facultatis Pharmaceuticae Universitatis Comenianae, V58, P87, DOI [10.2478/v10219-011-0010-4, DOI 10.2478/V10219-011-0010-4]

Zhang QW, 2017, FRONT MICROBIOL, V8, DOI 10.3389/fmicb.2017.02142

Zumla A, 2016, LANCET INFECT DIS, V16, pE47, DOI 10.1016/S1473-3099(16)00078-5

NR 36

TC 0

Z9 0

U1 0

U2 0

PU KOREAN SOC MICROBIOLOGY & BIOTECHNOLOGY

PI SEOUL

PA KOREA SCI TECHNOL CENTER #402, 635-4 YEOGSAM-DONG, KANGNAM-GU, SEOUL,

SOUTH KOREA

SN 1017-7825

EI 1738-8872

J9 J MICROBIOL BIOTECHN

JI J. Microbiol. Biotechnol.

PY 2026

VL 36

AR e2601032

DI 10.4014/jmb.2601.01032

PG 11

WC Biotechnology & Applied Microbiology; Microbiology

WE Science Citation Index Expanded (SCI-EXPANDED)

SC Biotechnology & Applied Microbiology; Microbiology

GA GB0VX

UT WOS:001732158700006

OA Green Submitted

DA 2026-05-07

ER

PT J

AU Rathored, J

Budhbaware, T

Shende, S

Upadhyay, V

AF Rathored, Jaishriram

Budhbaware, Tanushree

Shende, Sandesh

Upadhyay, Vishwanath

TI Association of vitamin D receptor genetic variants with therapeutic

response in multidrug-resistant pulmonary tuberculosis: a systematic

review

SO FRONTIERS IN CELLULAR AND INFECTION MICROBIOLOGY

LA English

DT Review

DE genetic variants; multi-drug-resistant tuberculosis (MDR-TB);

polymorphism; treatment response; tuberculosis; vitamin D; vitamin D

receptors (VDRs)

ID 1,25-DIHYDROXYVITAMIN D-3; D SUPPLEMENTATION; DOUBLE-BLIND; D

DEFICIENCY; POLYMORPHISMS; SUSCEPTIBILITY; POPULATION; PREVALENCE; RISK;

VDR

AB Introduction: In high-burden nations like India, tuberculosis (TB) continues to be a significant global public health concern. HIV infection, diabetes mellitus, and low socioeconomic status are examples of comorbid illnesses that increase susceptibility to tuberculosis (TB), and the introduction of multidrug-resistant tuberculosis (MDR-TB) has made disease control even more challenging. The time-consuming nature of conventional drug susceptibility testing (DST) emphasizes the critical need for quick biomarkers to forecast treatment outcomes and resistance. Because of their possible impact on host immunity and MDR-TB risk, genetic variations particularly vitamin D receptor (VDR) polymorphisms have drawn attention. Methods: Studies published between 2000 and 2024 were the subject of an extensive examination of the literature. Relevance led to the selection of 213 articles. Keywords including vitamin D, VDR polymorphisms, MDR-TB, pulmonary tuberculosis, and immune response were used to search databases such as PubMed, Web of Science, and Google Scholar. To guarantee comprehensive coverage, both original research articles and reviews were included. Results: Low serum vitamin D levels were consistently linked to an elevated risk of MDR-TB and pulmonary tuberculosis (PTB), according to the investigation. Certain VDR polymorphisms have often been associated with altered immunological responses and an increased risk of disease, especially mutant forms like FokI and TaqI. Treatment response and disease progression have also been discovered to be influenced by immunological modulation and dietary variables. Discussion: These results imply that vitamin D levels and VDR polymorphisms could be useful biomarkers for the diagnosis and prognosis of MDR-TB. Knowing the genetic susceptibility of the host may help develop individualized treatment plans and enhance the management of MDR-TB. Systematic Review Registration: https://www.crd.york.ac.uk/PROSPERO/view/CRD420261295571, identifier CRD420261295571.

C1 [Rathored, Jaishriram; Budhbaware, Tanushree; Shende, Sandesh] Datta Meghe Inst Higher Educ & Res, Cent Res Lab & Mol Diagnost, Wardha, Maharashtra, India.

[Upadhyay, Vishwanath] Jamia Hamdard, Dept Mol Med, New Delhi 110062, India.

C3 Datta Meghe Institute of Higher Education & Research (Deemed to be

University); Jamia Hamdard University

RP Rathored, J (corresponding author), Datta Meghe Inst Higher Educ & Res, Cent Res Lab & Mol Diagnost, Wardha, Maharashtra, India.

EM jaishriz@gmail.com

CR Amos W, 2011, P ROY SOC B-BIOL SCI, V278, P1183, DOI 10.1098/rspb.2010.1920

Amrein K, 2020, EUR J CLIN NUTR, V74, P1498, DOI 10.1038/s41430-020-0558-y

[Anonymous], IMMIGRANTS TUBERCULO

Aparna P, 2018, J Family Med Prim Care, V7, P324, DOI [10.4103/jfmpc.jfmpc_78_18, 10.4103/jfmpc.jfmpc_78_18]

Aravindan PP, 2019, LUNG INDIA, V36, P244, DOI 10.4103/lungindia.lungindia_146_15

Babb C, 2007, HUM GENET, V121, P521, DOI 10.1007/s00439-007-0335-1

Bavi H, 2024, ADV BIOMED RES-INDIA, V13, DOI 10.4103/abr.abr_76_24

Bergwitz C, 2010, ANNU REV MED, V61, P91, DOI 10.1146/annurev.med.051308.111339

Bikle D. D., 2000, ENDOTEXT

Bikle DD, 2014, CHEM BIOL, V21, P319, DOI 10.1016/j.chembiol.2013.12.016

Bishop EL, 2021, JBMR PLUS, V5, DOI 10.1002/jbm4.10405

Cao Y, 2015, INT J CLIN EXP MED, V8, P10187

Chai QY, 2020, CELL MOL LIFE SCI, V77, P1859, DOI 10.1007/s00018-019-03353-5

Chen BW, 2017, FRONT GENET, V8, DOI 10.3389/fgene.2017.00167

Clarke GM, 2011, NAT PROTOC, V6, P121, DOI 10.1038/nprot.2010.182

Conradie F, 2020, NEW ENGL J MED, V382, P893, DOI 10.1056/NEJMoa1901814

Dashian MA, 2025, INT J MOL SCI, V26, DOI 10.3390/ijms26199544

Dominguez LJ, 2021, METABOLITES, V11, DOI 10.3390/metabo11040255

Duan TH, 2022, FRONT IMMUNOL, V13, DOI 10.3389/fimmu.2022.812774

Elhoseiny SM, 2016, INDIAN J HEMATOL BLO, V32, P228, DOI 10.1007/s12288-015-0552-z

Ghaseminejad-Raeini A, 2023, FRONT IMMUNOL, V14, DOI 10.3389/fimmu.2023.950465

Griffith DE, 2015, AM J RESP CRIT CARE, V192, P754, DOI 10.1164/rccm.201503-0444OC

Gulumbe BH, 2025, Decoding Infection and Transmission, V3, P100044, DOI [10.1016/j.dcit.2025.100044, 10.1016/j.dcit.2025.100044, DOI 10.1016/J.DCIT.2025.100044]

Harinarayan CV, 2013, DERM-ENDOCRINOL, V5, DOI 10.4161/derm.23873

Hayeck TJ, 2024, GENOME BIOL EVOL, V16, DOI 10.1093/gbe/evae009

Hibma JE, 2020, AM J RESP CRIT CARE, V202, P866, DOI 10.1164/rccm.201912-2489OC

Hornsby E, 2018, J ALLERGY CLIN IMMUN, V141, P269, DOI 10.1016/j.jaci.2017.02.039

Huang SJ, 2017, DRUG DES DEV THER, V11, P91, DOI 10.2147/DDDT.S79870

Imperial MZ, 2018, NAT MED, V24, P1708, DOI 10.1038/s41591-018-0224-2

Jafari M, 2016, INFECT GENET EVOL, V39, P92, DOI 10.1016/j.meegid.2016.01.013

Kafentzi T, 2025, INT J MOL SCI, V26, DOI 10.3390/ijms26051848

Kafle S, 2021, CUREUS J MED SCIENCE, V13, DOI 10.7759/cureus.17883

Kearns MD, 2014, J CLIN TRANSL ENDOCR, V1, P167, DOI 10.1016/j.jcte.2014.08.002

Legesse T, 2021, BMC INFECT DIS, V21, DOI 10.1186/s12879-021-05828-y

Lin HW, 2010, HUM HERED, V69, P160, DOI 10.1159/000267996

Liu TT, 2025, FRONT NUTR, V12, DOI 10.3389/fnut.2025.1658961

Lonnie M, 2018, NUTRIENTS, V10, DOI 10.3390/nu10030360

Lu JT, 2022, CUREUS J MED SCIENCE, V14, DOI 10.7759/cureus.27333

Martineau AR, 2011, LANCET, V377, P242, DOI 10.1016/S0140-6736(10)61889-2

Mase SR, 2019, CLIN CHEST MED, V40, P775, DOI 10.1016/j.ccm.2019.08.002

Meca AD, 2021, EXP THER MED, V21, DOI 10.3892/etm.2021.10040

Mehta K, 2022, INT J PEPT RES THER, V28, DOI 10.1007/s10989-022-10435-9

Menon PM, 2024, DISCOV MED, V36, P739, DOI 10.24976/Discov.Med.202436183.70

Mily A, 2015, PLOS ONE, V10, DOI 10.1371/journal.pone.0138340

Möller M, 2018, FRONT IMMUNOL, V9, DOI 10.3389/fimmu.2018.02219

Morales F, 2024, NUTRIENTS, V16, DOI 10.3390/nu16010001

Nahid P, 2011, CLIN INFECT DIS, V53, P291, DOI 10.1093/cid/cir378

Papagni R, 2022, INT J MOL SCI, V23, DOI 10.3390/ijms23073860

Patel DG, 2024, J CLIN TUBER OTH MYC, V35, DOI 10.1016/j.jctube.2024.100428

Rathored J, 2012, INT J TUBERC LUNG D, V16, P1522, DOI 10.5588/ijtld.12.0122

Rathored J, 2024, ANN MED, V56, DOI 10.1080/07853890.2024.2407066

Rathored J, 2025, BMC INFECT DIS, V25, DOI 10.1186/s12879-025-11707-7

Ritu G, 2014, NUTRIENTS, V6, P729, DOI 10.3390/nu6020729

Roque-Borda CA, 2025, PHARMACEUTICALS-BASE, V18, DOI 10.3390/ph18101440

Roth DE, 2004, J INFECT DIS, V190, P920, DOI 10.1086/423212

Miraj SS, 2022, EXPERT REV ANTI-INFE, V20, P1179, DOI 10.1080/14787210.2022.2081546

Shah S, 2024, INDIAN J CLIN BIOCHE, V39, P60, DOI 10.1007/s12291-022-01091-3

Singh Shruti, 2025, Perspect Clin Res, V16, P69, DOI [10.4103/picr.picr_325_23, 10.4103/picr.picr_325_23]

Supriya R, 2021, FRONT IMMUNOL, V12, DOI 10.3389/fimmu.2021.761382

Sutaria N, 2014, J CLIN TRANSL ENDOCR, V1, P151, DOI 10.1016/j.jcte.2014.08.001

Gler MT, 2013, AM J TROP MED HYG, V89, P943, DOI 10.4269/ajtmh.13-0011

Theodoropoulos C, 2003, AM J PHYSIOL-ENDOC M, V284, pE138, DOI 10.1152/ajpendo.00303.2002

Tiwari Simmi, 2012, Indian J Tuberc, V59, P135

Torres N. M. C., 2015, PDF FACTORS PREDICTI

Tourkochristou E, 2023, INT J MOL SCI, V24, DOI 10.3390/ijms241411404

Uçar N, 2025, NUTRIENTS, V17, DOI 10.3390/nu17030386

Usategui-Martín R, 2022, NUTRIENTS, V14, DOI 10.3390/nu14020360

Varughese M, 2023, BMC INFECT DIS, V23, DOI 10.1186/s12879-023-08098-y

Velayutham BRV, 2014, PLOS ONE, V9, DOI 10.1371/journal.pone.0088045

Voltan G, 2023, GENES-BASEL, V14, DOI 10.3390/genes14091691

WALDMAN I, 1974, NEW ENGL J MED, V290, P633

Weeres MA, 2014, J IMMUNOL, V193, P3456, DOI 10.4049/jimmunol.1400698

Wejse C, 2009, AM J RESP CRIT CARE, V179, P843, DOI 10.1164/rccm.200804-567OC

Wilkinson RJ, 2000, LANCET, V355, P618, DOI 10.1016/S0140-6736(99)02301-6

Wingfield T, 2014, J INFECT DIS, V210, P774, DOI 10.1093/infdis/jiu121

Wu Q, 2022, ANN MED, V54, P1657, DOI 10.1080/07853890.2022.2085318

Yadav U, 2021, INFECT GENET EVOL, V92, DOI 10.1016/j.meegid.2021.104871

Yao MH, 2024, FRONT PHARMACOL, V15, DOI 10.3389/fphar.2024.1298181

Zhang Jia-Wei, 2017, Nan Fang Yi Ke Da Xue Xue Bao, V37, P704

Zhang J, 2019, CHINESE MED J-PEKING, V132, P2950, DOI 10.1097/CM9.0000000000000554

NR 80

TC 1

Z9 1

U1 0

U2 0

PU FRONTIERS MEDIA SA

PI LAUSANNE

PA AVENUE DU TRIBUNAL FEDERAL 34, LAUSANNE, CH-1015, SWITZERLAND

SN 2235-2988

J9 FRONT CELL INFECT MI

JI Front. Cell. Infect. Microbiol.

PD FEB 9

PY 2026

VL 16

AR 1692281

DI 10.3389/fcimb.2026.1692281

PG 21

WC Immunology; Microbiology

WE Science Citation Index Expanded (SCI-EXPANDED)

SC Immunology; Microbiology

GA EB8OG

UT WOS:001697370800001

OA Green Submitted, gold

DA 2026-05-07

ER

PT J

AU Eldehna, WM

Mahmoud, ST

Elshnawey, ER

Elsayed, ZM

Majrashi, TA

El-Ashrey, MK

Rashed, M

Hemeda, LR

Shoun, AA

Elkaeed, EB

Hassab, MAE

Abdel-Aziz, MM

Shahin, MI

AF Eldehna, Wagdy M.

Mahmoud, Sally Tarek

Elshnawey, Esraa R.

Elsayed, Zainab M.

Majrashi, Taghreed A.

El-Ashrey, Mohamed K.

Rashed, Mahmoud

Hemeda, Loah R.

Shoun, Aly A.

Elkaeed, Eslam B.

Hassab, Mahmoud A. El

Abdel-Aziz, Marwa M.

Shahin, Mai I.

TI Novel indolinone-tethered benzothiophenes as anti-tubercular agents

against MDR/XDR M. tuberculosis: Design, synthesis,

biological evaluation and in vivo pharmacokinetic study

SO BIOORGANIC CHEMISTRY

LA English

DT Article

DE Synthesis; DprE1; Anti-mycobacterial activity; Molecular docking;

Pharmacokinetics

ID ANTITUBERCULOSIS ACTIVITY; ANTIMICROBIAL ACTIVITY; DERIVATIVES; ISATIN;

INHIBITORS; DOCKING; HYBRIDS

AB Joining the global effort to eradicate tuberculosis, one of the deadliest infectious killers in the world, we disclose in this paper the design and synthesis of new indolinone-tethered benzothiophene hybrids 6a-i and 7a-i as potential anti-tubercular agents. The MICs were determined in vitro for the synthesized compounds against the sensitive M. tuberculosis strain ATCC 25177. Potent compounds 6b, 6d, 6f, 6h, 7a, 7b, 7d, 7f, 7h and 7i were furtherly assessed versus resistant MDR-TB and XDR-TB. Structure activity relationship investigation of the synthesized compounds was illustrated, accordingly. Superlative potency was unveiled for compound 6h (MIC = 0.48, 1.95 and 7.81 mu g/mL for ATCC 25177 sensitive TB strain, resistant MDR-TB and XDR-TB, respectively). Moreover, validated in vivo pharmacokinetic study was performed for the most potent derivative 6h revealing superior pharmacokinetic profile over the reference drug. For further exploration of the anti-tubercular mechanism of action, molecular docking was carried out for the former compound in DprE1 active site as one of the important biological targets of TB. The binding mode and the docking score uncovered exceptional binding when compared to the co-crystallized ligand suggesting that it maybe the underlying target for its outstanding anti-tubercular potency.

C1 [Eldehna, Wagdy M.] Kafrelsheikh Univ, Fac Pharm, Dept Pharmaceut Chem, Kafrelsheikh 33516, Egypt.

[Eldehna, Wagdy M.; Elshnawey, Esraa R.; Elsayed, Zainab M.] Kafrelsheikh Univ, Fac Pharm, Sci Res & Innovat Support Unit, Kafrelsheikh, Egypt.

[Mahmoud, Sally Tarek; El-Ashrey, Mohamed K.] Cairo Univ, Fac Pharm, Pharmaceut Chem Dept, Kasr Elini St, Cairo 11562, Egypt.

[Majrashi, Taghreed A.] King Khalid Univ, Coll Pharm, Dept Pharmacognosy, Asir 61421, Saudi Arabia.

[El-Ashrey, Mohamed K.; Hassab, Mahmoud A. El] King Salman Int Univ, Fac Pharm, Med Chem Dept, Ras Sedr 46612, South Sinai, Egypt.

[Rashed, Mahmoud] Al Azhar Univ, Fac Pharm, Pharmaceut Med Chem & Drug Design Dept, Cairo, Egypt.

[Hemeda, Loah R.] Beni Suef Univ, Fac Pharm, Dept Med Chem, Bani Suwayf 62514, Egypt.

[Shoun, Aly A.] El Saleheya El Gadida Univ, Fac Pharm, Microbiol & Immunol Dept, El Saleheya El Gadida 44813, Egypt.

[Elkaeed, Eslam B.] AlMaarefa Univ, Coll Pharm, Dept Pharmaceut Sci, Riyadh 13713, Saudi Arabia.

[Abdel-Aziz, Marwa M.] Al Azhar Univ, Reg Ctr Mycol & Biotechnol, Cairo 11651, Egypt.

[Shahin, Mai I.] Ain Shams Univ, Fac Pharm, Dept Pharmaceut Chem, Cairo 11566, Egypt.

C3 Egyptian Knowledge Bank (EKB); Kafrelsheikh University; Egyptian

Knowledge Bank (EKB); Kafrelsheikh University; Egyptian Knowledge Bank

(EKB); Cairo University; King Khalid University; King Salman

International University; Egyptian Knowledge Bank (EKB); Al Azhar

University; Egyptian Knowledge Bank (EKB); Beni Suef University;

Almaarefa University; Egyptian Knowledge Bank (EKB); Al Azhar

University; Egyptian Knowledge Bank (EKB); Ain Shams University

RP Eldehna, WM (corresponding author), Kafrelsheikh Univ, Fac Pharm, Dept Pharmaceut Chem, Kafrelsheikh 33516, Egypt.; Eldehna, WM (corresponding author), Kafrelsheikh Univ, Fac Pharm, Sci Res & Innovat Support Unit, Kafrelsheikh, Egypt.

EM wagdy2000@gmail.com

RI Elkaeed, Eslam/R-4249-2019; Hemeda, Loah/PCT-2941-2025; Majrashi,

Taghreed/GRJ-0481-2022; Rashed, Mahmoud/AAB-6548-2022; Shoun,

Aly/ABB-1266-2021; tarek, sally/AAB-4533-2021; Shahin,

Mai/JCD-9864-2023; El-Ashrey, Mohamed/JGD-8544-2023; El Hassab,

Mahmoud/ABE-7774-2020; Eldehna, Wagdy/AAK-6871-2020

OI Rashed, Mahmoud/0000-0003-0706-1044; Hemeda, Loah/0000-0001-6151-6965;

El-Ashrey, Mohamed/0000-0002-3548-3888; El Hassab,

Mahmoud/0000-0002-6795-4816; Eldehna, Wagdy/0000-0001-6996-4017

FU Deanship of Scientific Research at King Khalid University [RGP.2/311/44]

FX The authors extend their appreciation to the Deanship of Scientific

Research at King Khalid University for funding this work through Large

Groups Project under grant number (RGP.2/311/44).

CR Abdel-Aziz H.A., 2014, J. Chem, V2014

Abdelrahman MA, 2022, MOLECULES, V27, DOI 10.3390/molecules27248807

Abo-Ashour MF, 2018, EUR J MED CHEM, V160, P49, DOI 10.1016/j.ejmech.2018.10.008

Aboul-Fadl T, 2003, ARCH PHARM RES, V26, P778, DOI 10.1007/BF02980020

Al-Warhi T, 2022, RSC ADV, V12, P31466, DOI 10.1039/d2ra04385h

Al-Warhi T, 2022, DRUG DES DEV THER, V16, P1457, DOI 10.2147/DDDT.S356988

Amado PSM, 2022, ACS OMEGA, DOI 10.1021/acsomega.2c05307

[Anonymous], 2020, World Health Organization: Global Tuberculosis Report 2019

Banerjee T, 2011, IUBMB LIFE, V63, P1111, DOI 10.1002/iub.543

Bhandari R, 2013, INT J PHARMACEUT, V441, P202, DOI 10.1016/j.ijpharm.2012.11.042

Brandao P, 2021, EUR J MED CHEM, V211, DOI 10.1016/j.ejmech.2020.113102

Caburet J, 2022, EUR J MED CHEM, V240, DOI 10.1016/j.ejmech.2022.114599

Chandrasekera NS, 2014, PEERJ, V2, DOI 10.7717/peerj.612

Cheke RS, 2022, PHARMACEUTICALS-BASE, V15, DOI 10.3390/ph15030272

Cheke Rameshwar S., 2018, Central Nervous System Agents in Medicinal Chemistry, V18, P76, DOI [10.2174/1871524917666171113124112, 10.2174/1871524917666171113124112]

Chowdhary S, 2022, PHARMACEUTICALS-BASE, V15, DOI 10.3390/ph15050536

Duc DX, 2020, CURR ORG CHEM, V24, P2256, DOI 10.2174/1385272824999200820151545

Dhanya TM, 2023, PHOSPHORUS SULFUR, V198, P283, DOI 10.1080/10426507.2022.2145476

Eldehna WM, 2022, SCI REP-UK, V12, DOI 10.1038/s41598-022-15050-8

Eldehna WM, 2022, INT J PHARMACEUT, V612, DOI 10.1016/j.ijpharm.2021.121369

Eldehna WM, 2021, J ENZYM INHIB MED CH, V36, P1424, DOI 10.1080/14756366.2021.1944127

Eldehna WM, 2018, J ENZYM INHIB MED CH, V33, P867, DOI 10.1080/14756366.2018.1462802

Elnaggar MH, 2023, J ENZYM INHIB MED CH, V38, DOI 10.1080/14756366.2023.2234665

Elsayed ZM, 2021, J ENZYM INHIB MED CH, V36, DOI 10.1080/14756366.2020.1868450

Fda Cder., 2018, Bioanalytical Method Validation Guidance for Industry Biopharmaceutics Bioanalytical Method Validation Guidance for Industry Biopharmaceutics Contains Nonbinding Recommendations [Internet]

Fernandes GFS, 2022, J MED CHEM, V65, P7489, DOI 10.1021/acs.jmedchem.2c00227

Franzblau SG, 1998, J CLIN MICROBIOL, V36, P362, DOI 10.1128/JCM.36.2.362-366.1998

Ghosh A, 2020, IUBMB LIFE, V72, P2637, DOI 10.1002/iub.2394

Güzel Ö, 2008, BIOORGAN MED CHEM, V16, P8976, DOI 10.1016/j.bmc.2008.08.050

Haribabu J, 2016, J MOL STRUCT, V1110, P185, DOI 10.1016/j.molstruc.2016.01.044

Isloor AM, 2010, EUR J MED CHEM, V45, P825, DOI 10.1016/j.ejmech.2009.11.015

Jiang D, 2018, J HETEROCYCLIC CHEM, V55, P1263, DOI 10.1002/jhet.3189

Karali N, 2007, BIOORGAN MED CHEM, V15, P5888, DOI 10.1016/j.bmc.2007.05.063

KARKI SS, 2011, TURK J PHARM SCI, V8, P169

Keri RS, 2017, EUR J MED CHEM, V138, P1002, DOI 10.1016/j.ejmech.2017.07.038

Konstantinovic J, 2017, MOLECULES, V22, DOI 10.3390/molecules22030343

Kumara THS, 2009, PHOSPHORUS SULFUR, V184, P1866, DOI 10.1080/10426500802388433

Leonczak P, 2014, CHEMMEDCHEM, V9, P2587, DOI 10.1002/cmdc.201402234

Mahajan PS, 2016, ACS MED CHEM LETT, V7, P751, DOI 10.1021/acsmedchemlett.6b00077

Martorana A, 2015, EUR J MED CHEM, V90, P537, DOI 10.1016/j.ejmech.2014.12.002

MOSMANN T, 1983, J IMMUNOL METHODS, V65, P55, DOI 10.1016/0022-1759(83)90303-4

Mourey RJ, 2010, J PHARMACOL EXP THER, V333, P797, DOI 10.1124/jpet.110.166173

Naganagowda G, 2012, J CHIL CHEM SOC, V57, P1043

Naganagowda G, 2011, J SULFUR CHEM, V32, P235, DOI 10.1080/17415993.2011.583394

Naganagowda G, 2011, J SULFUR CHEM, V32, P223, DOI 10.1080/17415993.2011.575943

Naganagowda G, 2010, PHOSPHORUS SULFUR, V185, P1691, DOI 10.1080/10426500903241713

Rao GK, 2015, Chemical Sciences Journal, V6, DOI [10.4172/2150-3494.100092, DOI 10.4172/2150-3494.100092, 10.4172/2150-3494.100092]

Romagnoli R, 2007, J MED CHEM, V50, P2273, DOI 10.1021/jm070050f

Shaldam M, 2021, EUR J MED CHEM, V216, DOI 10.1016/j.ejmech.2021.113283

Tiberi S, 2022, INT J INFECT DIS, V124, pS20, DOI 10.1016/j.ijid.2022.03.026

Trott O, 2010, J COMPUT CHEM, V31, P455, DOI 10.1002/jcc.21334

Wang F, 2013, P NATL ACAD SCI USA, V110, pE2510, DOI 10.1073/pnas.1309171110

Wang PC, 2016, ACTA PHARM SIN B, V6, P384, DOI 10.1016/j.apsb.2016.07.014

Wang PX, 2021, J MED CHEM, V64, P6241, DOI 10.1021/acs.jmedchem.1c00263

Xu Z, 2017, CHINESE CHEM LETT, V28, P159, DOI 10.1016/j.cclet.2016.07.032

Zhao LY, 2022, EUR J MED CHEM, V228, DOI 10.1016/j.ejmech.2021.113987

NR 56

TC 6

Z9 6

U1 0

U2 8

PU ACADEMIC PRESS INC ELSEVIER SCIENCE

PI SAN DIEGO

PA 525 B ST, STE 1900, SAN DIEGO, CA 92101-4495 USA

SN 0045-2068

EI 1090-2120

J9 BIOORG CHEM

JI Bioorganic Chem.

PD FEB

PY 2024

VL 143

AR 107009

DI 10.1016/j.bioorg.2023.107009

EA DEC 2023

PG 10

WC Biochemistry & Molecular Biology; Chemistry, Organic

WE Science Citation Index Expanded (SCI-EXPANDED); Index Chemicus (IC)

SC Biochemistry & Molecular Biology; Chemistry

GA EP4X0

UT WOS:001140126300001

DA 2026-05-07

ER

PT J

AU Jaganath, D

Lamichhane, G

Shahs, M

AF Jaganath, D.

Lamichhane, G.

Shahs, M.

TI Carbapenems against Mycobacterium tuberculosis: a review of the

evidence

SO INTERNATIONAL JOURNAL OF TUBERCULOSIS AND LUNG DISEASE

LA English

DT Article

DE multidrug-resistant tuberculosis; extensively drug-resistant

tuberculosis; Group D; anti-tuberculosis; antibiotics

ID DRUG-RESISTANT TUBERCULOSIS; MEROPENEM/CLAVULANATE-CONTAINING REGIMENS;

MEROPENEM-CLAVULANIC ACID; IN-VITRO ACTIVITY; BETA-LACTAMASE;

MULTIDRUG-RESISTANT; ERTAPENEM; L,D-TRANSPEPTIDASE; SAFETY;

PHARMACOKINETICS

AB Carbapenems, a more recent beta-lactam class, represent a unique anti-tuberculosis option, as emerging evidence demonstrates that they target the Mycobacterium tuberculosis cell wall and beta-lactamase. This provides a potentially new agent against M. tuberculosis, in particular for multidrug-resistant (MDR) and extensively drug-resistant (XDR) tuberculosis (TB), where options are limited. In this review, we examine the current evidence on the activity of carbapenems against M. tuberculosis. The predominance of work is in vitro, and suggests that carbapenems kill M. tuberculosis at least in the active phase, with possible greater potency with the addition of a P-lactamase inhibitor. The few in vivo and clinical studies suggest that there are benefits and that they are generally tolerated, although the variability in duration, dosing, and background regimen and lack of pharmacokinetic analyses limit interpretation of efficacy. We outline further areas of research to better understand the role of carbapenems to add a needed new agent to the treatment of MDR- and XDR-TB.

C1 [Jaganath, D.] Johns Hopkins Sch Med, Dept Pediat, Baltimore, MD USA.

[Lamichhane, G.; Shahs, M.] Johns Hopkins Sch Med, Ctr TB Res, Div Infect Dis, Room 224,725 N Wolfe St, Baltimore, MD 21205 USA.

C3 Johns Hopkins University; Johns Hopkins Medicine; Johns Hopkins

University; Johns Hopkins Medicine

RP Shahs, M (corresponding author), Johns Hopkins Sch Med, Ctr TB Res, Div Infect Dis, Room 224,725 N Wolfe St, Baltimore, MD 21205 USA.

EM mshah28@jhmi.edu

OI Lamichhane, Gyanu/0000-0002-2214-0114

FU National Institute of Allergy and Infectious Diseases, National

Institutes of Health (NIH), Bethesda, MD, USA [K23AI089259]; NIH

[DP2OD008459]

FX MS is funded by the National Institute of Allergy and Infectious

Diseases, National Institutes of Health (NIH), Bethesda, MD, USA, under

grant #K23AI089259. GL was supported by the NIH grant #DP2OD008459.

CR [Anonymous], 2016, WHO treatment guidelines for drug-resistant tuberculosis, 2016 update

[Anonymous], 2015, GLOBAL TUBERCULOSIS

Arbex MA, 2015, J BRAS PNEUMOL, V41, P554, DOI [10.1590/S1806-37562015000000299, 10.1590/s1806-37562015000000299]

Boselli E, 2006, INTENS CARE MED, V32, P2059, DOI 10.1007/s00134-006-0401-5

Chambers HF, 2005, ANTIMICROB AGENTS CH, V49, P2816, DOI 10.1128/AAC.49.7.2816-2821.2005

Conte JE Jr, 2005, INT J ANTIMICROB AG, V26, P449, DOI 10.1016/j.ijantimicag.2005.08.015

Cordillot M, 2013, ANTIMICROB AGENTS CH, V57, P5940, DOI 10.1128/AAC.01663-13

Cox H, 2012, INT J TUBERC LUNG D, V16, P447, DOI 10.5588/ijtld.11.0451

Dauby N, 2011, PEDIATR INFECT DIS J, V30, P812, DOI 10.1097/INF.0b013e3182154b05

De Lorenzo S, 2013, EUR RESPIR J, V41, P1386, DOI 10.1183/09031936.00124312

Dhar N, 2015, ANTIMICROB AGENTS CH, V59, P1308, DOI 10.1128/AAC.03461-14

Diacon AH, 2016, NEW ENGL J MED, V375, P393, DOI 10.1056/NEJMc1513236

Dinesh N, 2013, ANTIMICROB AGENTS CH, V57, P1941, DOI 10.1128/AAC.01957-12

England K, 2012, ANTIMICROB AGENTS CH, V56, P3384, DOI 10.1128/AAC.05690-11

Erdemli SB, 2012, STRUCTURE, V20, P2103, DOI 10.1016/j.str.2012.09.016

European Committee on Antimicrobial Susceptibility Testing, 2016, CLIN BREAKP V 6 0

Falzon D, 2011, EUR RESPIR J, V38, P516, DOI 10.1183/09031936.00073611

Forsman LD, 2015, ANTIMICROB AGENTS CH, V59, P3630, DOI 10.1128/AAC.00171-15

Gonzalo X, 2013, J ANTIMICROB CHEMOTH, V68, P366, DOI 10.1093/jac/dks395

Gupta R, 2010, NAT MED, V16, P466, DOI 10.1038/nm.2120

Hazra S, 2014, BIOCHEMISTRY-US, V53, P3671, DOI 10.1021/bi500339j

Horita Y, 2014, ANTIMICROB AGENTS CH, V58, P7010, DOI 10.1128/AAC.03539-14

Hugonnet JE, 2007, BIOCHEMISTRY-US, V46, P11998, DOI 10.1021/bi701506h

Hugonnet JE, 2009, SCIENCE, V323, P1215, DOI 10.1126/science.1167498

Iannazzo L, 2016, J MED CHEM, V59, P3427, DOI 10.1021/acs.jmedchem.6b00096

Kaushik A, 2015, ANTIMICROB AGENTS CH, V59, P6561, DOI 10.1128/AAC.01158-15

Kim HS, 2013, ACTA CRYSTALLOGR D, V69, P420, DOI 10.1107/S0907444912048998

Kumar P, 2012, MOL MICROBIOL, V86, P367, DOI 10.1111/j.1365-2958.2012.08199.x

Lee M, 2012, NEW ENGL J MED, V367, P1508, DOI 10.1056/NEJMoa1201964

Li WJ, 2013, CELL RES, V23, P728, DOI 10.1038/cr.2013.53

Nguyen L, 2016, ARCH TOXICOL, V90, P1585, DOI 10.1007/s00204-016-1727-6

Lun SC, 2014, MBIO, V5, DOI 10.1128/mBio.01767-14

MULLERSERIEYS C, 1987, J ANTIMICROB CHEMOTH, V20, P618, DOI 10.1093/jac/20.4.618

Palmero Domingo, 2015, Arch Bronconeumol, V51, pe49, DOI [10.1016/j.arbr.2015.07.003, 10.1016/j.arbres.2015.03.012]

Payen MC, 2012, INT J TUBERC LUNG D, V16, P558, DOI 10.5588/ijtld.11.0414

Rice LB, 2012, MAYO CLIN PROC, V87, P198, DOI 10.1016/j.mayocp.2011.12.003

Rullas J, 2015, ANTIMICROB AGENTS CH, V59, P4997, DOI 10.1128/AAC.01063-15

Solapure S, 2013, ANTIMICROB AGENTS CH, V57, P2506, DOI 10.1128/AAC.00023-13

Soroka D, 2015, ANTIMICROB AGENTS CH, V59, P5714, DOI 10.1128/AAC.00598-15

Sotgiu G, 2016, INT J MOL SCI, V17, DOI 10.3390/ijms17030373

Srivastava S, 2016, ANTIMICROB AGENTS CH, V60, P3193, DOI 10.1128/AAC.02924-15

Tiberi S, 2016, CLIN INFECT DIS, V62, P1188, DOI 10.1093/cid/ciw088

Tiberi S, 2016, EUR RESPIR J, V47, P1235, DOI 10.1183/13993003.02146-2015

Tiberi S, 2016, EUR RESPIR J, V47, P333, DOI 10.1183/13993003.01278-2015

Tremblay LW, 2010, BIOCHEMISTRY-US, V49, P3766, DOI 10.1021/bi100232q

van Rijn SP, 2016, EUR RESPIR J, V47, P1229, DOI 10.1183/13993003.01654-2015

Veziris N, 2011, ANTIMICROB AGENTS CH, V55, P2597, DOI 10.1128/AAC.01824-10

Zhanel GG, 2007, DRUGS, V67, P1027, DOI 10.2165/00003495-200767070-00006

Zhang D, 2016, ANTIMICROB AGENTS CH, V60, P393, DOI 10.1128/AAC.01035-15

NR 49

TC 28

Z9 38

U1 2

U2 10

PU INT UNION AGAINST TUBERCULOSIS LUNG DISEASE (I U A T L D)

PI PARIS

PA 68 BOULEVARD SAINT-MICHEL,, 75006 PARIS, FRANCE

SN 1027-3719

EI 1815-7920

J9 INT J TUBERC LUNG D

JI Int. J. Tuberc. Lung Dis.

PD NOV

PY 2016

VL 20

IS 11

BP 1436

EP 1447

DI 10.5588/ijtld.16.0498

PG 12

WC Infectious Diseases; Respiratory System

WE Science Citation Index Expanded (SCI-EXPANDED)

SC Infectious Diseases; Respiratory System

GA EA9TD

UT WOS:000386986700006

DA 2026-05-07

ER

PT J

AU Deshpande, D

Pasipanodya, JG

Srivastava, S

Martin, KR

Athale, S

van Zyl, J

Antiabong, J

Koeuth, T

Lee, PS

Dheda, K

Gumbo, T

AF Deshpande, Devyani

Pasipanodya, Jotam G.

Srivastava, Shashikant

Martin, Katherine R.

Athale, Shruti

van Zyl, Johanna

Antiabong, John

Koeuth, Thearith

Lee, Pooi S.

Dheda, Keertan

Gumbo, Tawanda

TI Minocycline Immunomodulates via Sonic Hedgehog Signaling and Apoptosis

and Has Direct Potency Against Drug-Resistant Tuberculosis

SO JOURNAL OF INFECTIOUS DISEASES

LA English

DT Article

DE Smac; DIABLO; caspase 3; PTCH1; GLI1; lung remodeling; host-directed

therapy

ID HOLLOW-FIBER MODEL; VERATRUM CALIFORNICUM; MULTIDRUG-RESISTANT; MDR-TB;

DOXYCYCLINE; LUNG; PHARMACOKINETICS; TETRACYCLINE; MOXIFLOXACIN;

EXPRESSION

AB We are running out of treatments for multidrug-resistant tuberculosis. Minocycline, an old and safe antibiotic, was highly potent against Mycobacterium tuberculosis in preclinical models. It potentiated bacterial killing by the immune system and promoted immune pathways associated with healing.Drug-resistant tuberculosis represents a global emergency, requiring new drugs. We found that minocycline was highly potent in laboratory strains of Mycobacterium tuberculosis and that 30 drug-susceptible and multidrug/extensively drug-resistant clinical strains were susceptible to clinically achievable concentrations. In the hollow fiber system model, lung concentration-time profiles of 7 mg/kg/day human-equivalent minocycline dose achieved bacterial kill rates equivalent to those of first-line antituberculosis agents. Minocycline killed extracellular bacilli directly. Minocycline also killed intracellular bacilli indirectly, via concentration-dependent granzyme Adriven apoptosis. Moreover, minocycline demonstrated dose-dependent antiinflammatory activity and downregulation of extracellular matrix-based remodeling pathways and, thus, could protect patients from tuberculosis immunopathology. In RNA sequencing of repetitive samples from the hollow fiber system and in independent protein abundance experiments, minocycline demonstrated dose-dependent inhibition of sonic hedgehogpatchedgli signaling. These findings have implications for improved lung remodeling and for dual immunomodulation and direct microbial kill-based treatment shortening regimens for drug-susceptible and drug-resistant latent and active M. tuberculosis infection.

C1 [Deshpande, Devyani; Pasipanodya, Jotam G.; Srivastava, Shashikant; Martin, Katherine R.; Athale, Shruti; van Zyl, Johanna; Antiabong, John; Koeuth, Thearith; Lee, Pooi S.; Gumbo, Tawanda] Baylor Res Inst, Ctr Infect Dis Res & Expt Therapeut, Dallas, TX 75204 USA.

[Dheda, Keertan; Gumbo, Tawanda] Univ Cape Town, Lung Inst, Ctr Lung Infect & Immun, Div Pulmonol, Rondebosch, South Africa.

C3 Baylor University Medical Center; Baylor Scott & White Health;

University of Cape Town

RP Gumbo, T (corresponding author), Baylor Res Inst, Ctr Infect Dis Res & Expt Therapeut, Dallas, TX 75204 USA.; Gumbo, T (corresponding author), Univ Cape Town, Lung Inst, Ctr Lung Infect & Immun, Div Pulmonol, Rondebosch, South Africa.

EM tawanda.gumbo@BSWHealth.org

RI Srivastava, Shashikant/I-4704-2019

OI Srivastava, Shashikant/0000-0002-8786-8851; Van Zyl, Johanna

S/0000-0003-4205-9214; Dheda, Keertan/0000-0001-7709-5341

FU Baylor Research Institute

FX This work was supported by the Baylor Research Institute.

CR Agwuh KN, 2006, J ANTIMICROB CHEMOTH, V58, P256, DOI 10.1093/jac/dkl224

Alffenaar JWC, 2015, CLIN INFECT DIS, V60, P969, DOI 10.1093/cid/ciu1146

Alsaad N, 2014, EUR RESPIR J, V43, P884, DOI 10.1183/09031936.00113713

BINNS W, 1964, ANN NY ACAD SCI, V111, P571, DOI 10.1111/j.1749-6632.1964.tb53124.x

Bollong MJ, 2017, P NATL ACAD SCI USA, V114, P4679, DOI 10.1073/pnas.1702750114

Brennan D, 2012, VITAM HORM, V88, P55, DOI 10.1016/B978-0-12-394622-5.00003-1

Chilukuri D, 2015, CLIN INFECT DIS, V61, pS32, DOI 10.1093/cid/civ460

Daley WP, 2008, J CELL SCI, V121, P255, DOI 10.1242/jcs.006064

Deshpande D, 2018, ANTIMICROB AGENTS CH, V62, DOI 10.1128/AAC.02232-17

Deshpande D, 2017, SCI ADV, V3, DOI 10.1126/sciadv.1701102

Deshpande D, 2016, CLIN INFECT DIS, V63, pS88, DOI 10.1093/cid/ciw473

Deshpande D, 2016, CLIN INFECT DIS, V63, pS80, DOI 10.1093/cid/ciw482

Dheda K, 2017, LANCET RESP MED, V5, P291, DOI [10.1016/s2213-2600(17)30079-6, 10.1016/S2213-2600(17)30079-6]

Dheda K, 2017, LANCET RESP MED, V5, P269, DOI [10.1016/S2213-2600(16)30433-7, 10.1016/s2213-2600(16)30433-7]

Dorus S, 2006, HUM MOL GENET, V15, P2031, DOI 10.1093/hmg/ddl123

DUGGAR BM, 1948, ANN NY ACAD SCI, V51, P177, DOI 10.1111/j.1749-6632.1948.tb27262.x

Fagan SC, 2010, STROKE, V41, P2283, DOI 10.1161/STROKEAHA.110.582601

Fitch PM, 2011, INT J EXP PATHOL, V92, P8, DOI 10.1111/j.1365-2613.2010.00743.x

Fouda AY, 2017, STROKE, V48, P2885, DOI 10.1161/STROKEAHA.117.018658

Frisk A R, 1968, Antimicrob Agents Chemother (Bethesda), V8, P335

Ghorpade DS, 2013, MOL CELL BIOL, V33, P543, DOI 10.1128/MCB.01108-12

Giménez A, 2017, INT J MOL SCI, V18, DOI 10.3390/ijms18112431

Gonzaloa X, 2015, INT J ANTIMICROB AG, V45, P406, DOI 10.1016/j.ijantimicag.2014.11.017

Gumbo T, 2004, J INFECT DIS, V190, P1642, DOI 10.1086/424849

Gumbo T, 2015, CLIN INFECT DIS, V61, pS25, DOI 10.1093/cid/civ427

Gumbo T, 2015, J INFECT DIS, V211, pS96, DOI 10.1093/infdis/jiu610

KEELER RF, 1968, TERATOLOGY, V1, P5, DOI 10.1002/tera.1420010103

Kugler MC, 2015, AM J RESP CELL MOL, V52, P1, DOI 10.1165/rcmb.2014-0132TR

Lieberman J, 2010, IMMUNOL REV, V235, P93, DOI 10.1111/j.0105-2896.2010.00902.x

Marquis JF, 2009, J IMMUNOL, V182, P3757, DOI 10.4049/jimmunol.0802094

Metz LM, 2017, NEW ENGL J MED, V376, P2122, DOI 10.1056/NEJMoa1608889

Modongo C, 2014, BMC INFECT DIS, V14, DOI 10.1186/1471-2334-14-542

NALINE E, 1991, BRIT J CLIN PHARMACO, V32, P402, DOI 10.1111/j.1365-2125.1991.tb03920.x

Piera-Velazquez S, 2016, J CLIN MED, V5, DOI 10.3390/jcm5040045

PINES A, 1964, BRIT MED J, V1, P311, DOI 10.1136/bmj.1.5378.311-c

Ruiz-Moreno C, 2018, TOXICOL IN VITRO, V50, P336, DOI 10.1016/j.tiv.2018.03.012

SAIVIN S, 1988, CLIN PHARMACOKINET, V15, P355, DOI 10.2165/00003088-198815060-00001

Smith K, 2005, CLIN THER, V27, P1329, DOI 10.1016/j.clinthera.2005.09.005

Sotgiu G, 2012, EUR RESPIR J, V40, P1430, DOI 10.1183/09031936.00022912

Srivastava S, 2017, ANTIMICROB AGENTS CH, V61, DOI 10.1128/AAC.00751-17

Srivastava S, 2016, EBIOMEDICINE, V6, P126, DOI 10.1016/j.ebiom.2016.02.040

STEENKEN W, 1950, ANN NY ACAD SCI, V53, P309, DOI 10.1111/j.1749-6632.1950.tb42164.x

STEENKEN W, 1949, AM REV TUBERC PULM, V59, P221

Stewart GA, 2003, J PATHOL, V199, P488, DOI 10.1002/path.1295

Szeto GL, 2011, J BIOL CHEM, V286, P11275, DOI 10.1074/jbc.M110.210518

Tikka T, 2001, J NEUROCHEM, V78, P1409, DOI 10.1046/j.1471-4159.2001.00543.x

Walker NF, 2012, AM J RESP CRIT CARE, V185, P989, DOI 10.1164/rccm.201110-1769OC

Wallace WAH, 2007, INT J EXP PATHOL, V88, P103, DOI 10.1111/j.1365-2613.2006.00515.x

Yamamoto T, 1999, Am J Ther, V6, P157, DOI 10.1097/00045391-199905000-00006

Zink MC, 2005, JAMA-J AM MED ASSOC, V293, P2003, DOI 10.1001/jama.293.16.2003

NR 50

TC 18

Z9 19

U1 0

U2 7

PU OXFORD UNIV PRESS INC

PI CARY

PA JOURNALS DEPT, 2001 EVANS RD, CARY, NC 27513 USA

SN 0022-1899

EI 1537-6613

J9 J INFECT DIS

JI J. Infect. Dis.

PD MAR 15

PY 2019

VL 219

IS 6

BP 975

EP 985

DI 10.1093/infdis/jiy587

PG 11

WC Immunology; Infectious Diseases; Microbiology

WE Science Citation Index Expanded (SCI-EXPANDED)

SC Immunology; Infectious Diseases; Microbiology

GA HQ7KS

UT WOS:000462599600015

OA Bronze

DA 2026-05-07

ER

PT J

AU Qiao, M

Li, SS

Yuan, JF

Ren, WC

Shang, YY

Wang, W

Liu, RM

Zhang, FZ

Li, Q

Wu, X

Lu, J

Gao, MQ

Pang, Y

AF Qiao, Min

Li, Shanshan

Yuan, Jinfeng

Ren, Weicong

Shang, Yuanyuan

Wang, Wei

Liu, Rongmei

Zhang, Fuzhen

Li, Qing

Wu, Xiao

Lu, Jie

Gao, Mengqiu

Pang, Yu

TI Delamanid suppresses CXCL10 expression via regulation of

JAK/STAT1 signaling and correlates with reduced inflammation in

tuberculosis patients

SO FRONTIERS IN IMMUNOLOGY

LA English

DT Article

DE delamanid; CXCL10; JAK; STAT1; tuberculosis; inflammation

ID GAMMA-INDUCIBLE PROTEIN-10; IFN-GAMMA; DRUG; RESPONSES; CELLS

AB BackgroundApart from bactericidal effects, anti-tuberculosis drugs can interfere with the host's immune system. In this study, we analyzed the role of delamanid (DLM), an inhibitor of mycolic acid synthesis of mycobacterial cell wall, on human macrophages. MethodsBased on a cohort of multidrug-resistant tuberculosis (MDR-TB) patients treated with DLM, the levels of C-reaction protein (CRP) and cytokines in the plasma were monitored using immunoturbidimetric assay and flow cytometry, respectively. We investigated the role of DLM on CXCL10 expression in U937 cell model using the following methods: cell viability assay, reverse transcription-quantitative polymerase chain reaction, enzyme linked immunosorbent assay, immunoblot, and transwell co-culture assay. ResultsA total of 23 MDR-TB patients were included, comprising of 13 patients treated with optimized background therapeutic regimen (OBR) plus DLM regimen (OBR+DLM) and 10 patients treated with OBR plus placebo. DLM administration was associated with a significant reduce in circulating CRP level. Correspondingly, after treatment, the level of CXCL10 in patients treated with OBR+DLM was significantly lower than that with control. Using cell model, DLM dramatically suppressed CXCL10 expression, which majorly depended on inhibiting the JAK/STAT pathway, and impaired the migration of PBMCs. ConclusionOur data firstly demonstrate that DLM suppresses CXCL10 expression via regulation of JAK2/STAT1 signaling and correlates with reduced inflammation in MDR-TB patients. DLM could be used as a potential drug for immunotherapy of patients with overactive immune response due to CXCL10.

C1 [Qiao, Min; Li, Shanshan; Yuan, Jinfeng; Ren, Weicong; Shang, Yuanyuan; Wang, Wei; Zhang, Fuzhen; Li, Qing; Wu, Xiao; Pang, Yu] Capital Med Univ, Beijing Chest Hosp, Beijing TB & Thorac Tumor Res Inst, Dept Bacteriol & Immunol, Beijing, Peoples R China.

[Qiao, Min] Shanxi Med Univ, Shanxi Prov Peoples Hosp, Dept Gastroenterol, Taiyuan, Shanxi, Peoples R China.

[Qiao, Min; Liu, Rongmei; Gao, Mengqiu] Capital Med Univ, Beijing Chest Hosp, Beijing TB & Thorac Tumor Res Inst, Dept TB, Beijing, Peoples R China.

[Lu, Jie] Capital Med Univ, Beijing Childrens Hosp, Beijing Pediat Res Inst, Natl Ctr Childrens Hlth,Beijing Key Lab Pediat Dis, Beijing, Peoples R China.

C3 Capital Medical University; Shanxi Medical University; Shanxi People's

Hospital; Capital Medical University; Capital Medical University

RP Pang, Y (corresponding author), Capital Med Univ, Beijing Chest Hosp, Beijing TB & Thorac Tumor Res Inst, Dept Bacteriol & Immunol, Beijing, Peoples R China.; Gao, MQ (corresponding author), Capital Med Univ, Beijing Chest Hosp, Beijing TB & Thorac Tumor Res Inst, Dept TB, Beijing, Peoples R China.; Lu, J (corresponding author), Capital Med Univ, Beijing Childrens Hosp, Beijing Pediat Res Inst, Natl Ctr Childrens Hlth,Beijing Key Lab Pediat Dis, Beijing, Peoples R China.

EM lujiebch@163.com; gaomqwdm@aliyun.com; pangyupound@163.com

RI 袁, 金锋/LZE-8341-2025; Zhang, Fuzhen/LZE-8185-2025; Wang, Li/HZM-0281-2023

FU Beijing Science and Technology Planning Project; Capital's Funds for

Health Improvement and Research; [Z191100006619077]; [2020-1-1041]

FX Funding This work was supported by the Beijing Science and Technology

Planning Project (Z191100006619077), and the Capital's Funds for Health

Improvement and Research (2020-1-1041).

CR Abedin SM, 2020, EXPERT OPIN INV DRUG, V29, P423, DOI 10.1080/13543784.2020.1757069

[Anonymous], 2019, WHO Consolidated Guidelines on Drug-Resistant Tuberculosis Treatment

[Anonymous], 2021, Global tuberculosis report 2021

Aota K, 2021, INFLAMMATION, V44, P206, DOI 10.1007/s10753-020-01322-w

Bi W, 2011, BRAIN RES, V1395, P12, DOI 10.1016/j.brainres.2011.04.019

Blot M, 2020, CRIT CARE, V24, DOI 10.1186/s13054-020-03328-0

Bone-Larson CL, 2001, J IMMUNOL, V167, P7077, DOI 10.4049/jimmunol.167.12.7077

Brigden G, 2015, INFECT DRUG RESIST, V8, P367, DOI 10.2147/IDR.S68351

Brightling CE, 2005, AM J RESP CRIT CARE, V171, P1103, DOI 10.1164/rccm.200409-1220OC

Chai QY, 2020, CELL MOL IMMUNOL, V17, P901, DOI 10.1038/s41423-020-0502-z

de Martino M, 2019, FRONT PEDIATR, V7, DOI 10.3389/fped.2019.00350

Feng JY, 2021, J FORMOS MED ASSOC, V120, P1080, DOI 10.1016/j.jfma.2020.09.012

Giraud-Gatineau A, 2020, ELIFE, V9, DOI [10.1101/758318, 10.7554/eLife.55692]

Gler MT, 2012, NEW ENGL J MED, V366, P2151, DOI 10.1056/NEJMoa1112433

House IG, 2020, CLIN CANCER RES, V26, P487, DOI 10.1158/1078-0432.CCR-19-1868

Kim SY, 2018, BMC INFECT DIS, V18, DOI 10.1186/s12879-018-3144-3

Kumar NP, 2019, SCI REP-UK, V9, DOI 10.1038/s41598-019-54803-w

Kumar NP, 2021, BMC INFECT DIS, V21, DOI 10.1186/s12879-021-06749-6

Lange C, 2018, RESPIROLOGY, V23, P656, DOI 10.1111/resp.13304

Liu ML, 2011, CYTOKINE GROWTH F R, V22, P121, DOI 10.1016/j.cytogfr.2011.06.001

Matsumoto M, 2006, PLOS MED, V3, P2131, DOI 10.1371/journal.pmed.0030466

Méndez-Samperio P, 2009, CELL IMMUNOL, V256, P12, DOI 10.1016/j.cellimm.2008.12.002

Mvungi HC, 2019, INT J MYCOBACT, V8, P53, DOI 10.4103/ijmy.ijmy_30_19

Nie Wenjuan, 2020, Cytokine X, V2, P100028, DOI [10.1016/j.cytox.2020.100028, 10.1016/j.cytox.2020.100028]

O'Garra A, 2013, ANNU REV IMMUNOL, V31, P475, DOI 10.1146/annurev-immunol-032712-095939

Pontali E, 2019, EUR RESPIR REV, V28, DOI 10.1183/16000617.0035-2019

Qi XF, 2009, J CELL PHYSIOL, V220, P690, DOI 10.1002/jcp.21815

Qiao M, 2021, INT J INFECT DIS, V106, P295, DOI 10.1016/j.ijid.2021.04.036

Ravimohan S, 2018, EUR RESPIR REV, V27, DOI 10.1183/16000617.0077-2017

Roe B, 2007, J INFECT DIS, V196, P1053, DOI 10.1086/520935

Saliu OY, 2007, J ANTIMICROB CHEMOTH, V60, P994, DOI 10.1093/jac/dkm291

Sasahara K, 2015, DRUG METAB DISPOS, V43, P1267, DOI 10.1124/dmd.115.064527

Tousif S, 2014, J BIOL CHEM, V289, P30190, DOI 10.1074/jbc.C114.598946

Uplekar M, 2015, LANCET, V385, P1799, DOI 10.1016/S0140-6736(15)60570-0

Wallis RS, 2005, CLIN INFECT DIS, V41, P201, DOI 10.1086/430914

Wang XH, 2013, FASEB J, V27, P2713, DOI 10.1096/fj.12-222992

Zaheer RS, 2009, J IMMUNOL, V182, P4854, DOI 10.4049/jimmunol.0802401

Zhang N, 2020, EUR REV MED PHARMACO, V24, P7497, DOI 10.26355/eurrev_202007_21922

NR 38

TC 12

Z9 13

U1 1

U2 9

PU FRONTIERS MEDIA SA

PI LAUSANNE

PA AVENUE DU TRIBUNAL FEDERAL 34, LAUSANNE, CH-1015, SWITZERLAND

SN 1664-3224

J9 FRONT IMMUNOL

JI Front. Immunol.

PD NOV 8

PY 2022

VL 13

AR 923492

DI 10.3389/fimmu.2022.923492

PG 13

WC Immunology

WE Science Citation Index Expanded (SCI-EXPANDED)

SC Immunology

GA 6N8KV

UT WOS:000889800600001

OA Green Submitted, gold

DA 2026-05-07

ER

PT J

AU Shi, CY

Zhang, PZ

Zhen, JF

Zhang, J

Wang, YX

Xiao, GY

Yu, X

Wang, T

Wang, WF

Cai, Y

Chen, XC

Deng, GF

AF Shi, Chenyan

Zhang, Peize

Zhen, Junfeng

Zhang, Jing

Wang, Yuxiang

Xiao, Guangyang

Yu, Xue

Wang, Tong

Wang, Wenfei

Cai, Yi

Chen, Xinchun

Deng, Guofang

TI Transcriptomic signatures predict severe linezolid-induced peripheral

neuropathy and reveal pre-treatment immune and antioxidant dysregulation

SO TUBERCULOSIS

LA English

DT Article

DE Drug-resistant tuberculosis; Linezolid; Peripheral neuropathy;

Transcriptome; Risk prediction

AB Linezolid is an essential drug for treating multidrug-resistant and extensively drug-resistant tuberculosis, yet its long-term use is frequently limited by peripheral neuropathy, which in severe cases may be irreversible. To investigate biological factors underlying susceptibility, we analyzed transcriptomic profiles of peripheral blood mononuclear cells from 51 MDR/XDR-TB patients before and after six months of linezolid treatment, stratifying individuals into asymptomatic, mild, and severe neuropathy groups. Patients who later developed severe neuropathy already exhibited distinct transcriptional signatures at baseline, including evidence of immune dysregulation and impaired antioxidant defense, with downregulation of genes involved in immune receptor activity and the pentose phosphate pathway. Following treatment, these patients showed limited transcriptional responses compared with other groups, most notably a marked downregulation of IL10, a central antiinflammatory cytokine, suggesting maladaptive systemic regulation. Using a machine learning-based feature selection approach, we derived an 11-gene predictive model that achieved strong performance with a cross-validated AUC of 0.93. Model interpretation using SHAP values highlighted HP and HSPA1B as the most influential predictors. Overall, our findings suggest that severe linezolid-induced peripheral neuropathy is predisposed by baseline immune and metabolic alterations and that patients with this complication fail to mount appropriate systemic responses during treatment.

C1 [Shi, Chenyan; Zhang, Jing; Xiao, Guangyang; Yu, Xue; Wang, Tong] Shenzhen Univ, Med Sch, Sch Publ Hlth, Shenzhen, Peoples R China.

[Shi, Chenyan; Cai, Yi; Chen, Xinchun] Shenzhen Univ, Dept Pathogen Biol, Guangdong Prov Key Lab Infect Immun & Inflammat, Med Sch, Shenzhen, Peoples R China.

[Zhang, Peize; Zhen, Junfeng; Wang, Yuxiang; Deng, Guofang] Southern Univ Sci & Technol, Shenzhen Peoples Hosp 3, Natl Clin Res Ctr Infect Dis, Dept Pulm Med & TB, Shenzhen, Peoples R China.

[Wang, Wenfei] Southern Univ Sci & Technol, Shenzhen Peoples Hosp 3, Natl Clin Res Ctr Infect Dis, Shenzhen, Peoples R China.

C3 Shenzhen University; Shenzhen University; Southern University of Science

& Technology; Southern University of Science & Technology

RP Chen, XC (corresponding author), Shenzhen Univ, Dept Pathogen Biol, Guangdong Prov Key Lab Infect Immun & Inflammat, Med Sch, Shenzhen, Peoples R China.; Deng, GF (corresponding author), Southern Univ Sci & Technol, Shenzhen Peoples Hosp 3, Natl Clin Res Ctr Infect Dis, Dept Pulm Med & TB, Shenzhen, Peoples R China.

EM chenxinchun@szu.edu.cn; jxxk1035@yeah.net

RI Cai, Yi/AAJ-2789-2020

FU Shenzhen Medical Research Project [C2401026]; Project of Guangzhou

National Laboratory [GZNL2024A01030]

FX <BOLD>Funding</BOLD> This work was supported by Shenzhen Medical

Research Project (C2401026) and Project of Guangzhou National Laboratory

(Grant No. GZNL2024A01030) .

CR Anders S, 2015, BIOINFORMATICS, V31, P166, DOI 10.1093/bioinformatics/btu638

Bolhuis MS, 2018, CLIN INFECT DIS, V67, pS327, DOI 10.1093/cid/ciy625

Chen L, 2019, NAT METAB, V1, P404, DOI 10.1038/s42255-019-0043-x

Conradie F, 2020, NEW ENGL J MED, V382, P893, DOI 10.1056/NEJMoa1901814

Copits BA, 2012, NAT REV NEUROSCI, V13, P675, DOI 10.1038/nrn3335

CRAN.R-project, TRAINING CCAR

Francis Karen, 2003, Expert Reviews in Molecular Medicine, V5, P1, DOI 10.1017/S1462399403006252

Friedman J, 2010, J STAT SOFTW, V33, P1, DOI 10.18637/jss.v033.i01

github, TRIMGALORE

github, STAR

Livermore DM, 2003, J ANTIMICROB CHEMOTH, V51, P9, DOI 10.1093/jac/dkg249

Long D, 2019, J AUTOIMMUN, V99, P1, DOI 10.1016/j.jaut.2019.01.013

Mo YP, 2022, FRONT NEUROL, V13, DOI 10.3389/fneur.2022.985499

Mohammad MT, 2019, J DIABETES RES, V2019, DOI 10.1155/2019/2673105

Old EA, 2014, J CLIN INVEST, V124, P2023, DOI 10.1172/JCI71389

Pollini L, 2020, INT J MOL SCI, V21, DOI 10.3390/ijms21165802

Poon IKH, 2010, BLOOD, V115, P2473, DOI 10.1182/blood-2009-07-234013

Rao ZG, 2023, P NATL ACAD SCI USA, V120, DOI 10.1073/pnas.2302070120

Rotterman TM, 2019, J NEUROSCI, V39, P3412, DOI 10.1523/JNEUROSCI.2945-17.2019

Singh B, 2019, COCHRANE DB SYST REV, DOI 10.1002/14651858.CD012836.pub2

Tan XP, 2025, NEUROPHARMACOLOGY, V277, DOI 10.1016/j.neuropharm.2025.110525

Tortola L, 2010, BLOOD, V116, P5200, DOI 10.1182/blood-2010-05-284547

Wickham H, 2016, GGPLOT2 ELEGANT GRAP

World Health Organization, RAPID COMMUNICATION

World Health Organization, 2021, GLOB TUB REP

Yang SJ, 2013, NEUROL RES, V35, P976, DOI 10.1179/1743132813Y.0000000238

Yu GC, 2012, OMICS, V16, P284, DOI 10.1089/omi.2011.0118

Zak DE, 2016, LANCET, V387, P2312, DOI 10.1016/S0140-6736(15)01316-1

Zhang L, 2023, STEM CELLS INT, V2023, DOI 10.1155/2023/7284986

NR 29

TC 0

Z9 0

U1 0

U2 0

PU CHURCHILL LIVINGSTONE

PI EDINBURGH

PA JOURNAL PRODUCTION DEPT, ROBERT STEVENSON HOUSE, 1-3 BAXTERS PLACE,

LEITH WALK, EDINBURGH EH1 3AF, MIDLOTHIAN, SCOTLAND

SN 1472-9792

EI 1873-281X

J9 TUBERCULOSIS

JI Tuberculosis

PD MAY

PY 2026

VL 158

AR 102751

DI 10.1016/j.tube.2026.102751

PG 9

WC Immunology; Microbiology; Respiratory System

WE Science Citation Index Expanded (SCI-EXPANDED)

SC Immunology; Microbiology; Respiratory System

GA EZ2TG

UT WOS:001713285600001

DA 2026-05-07

ER

PT J

AU Lu, XY

Hu, XL

Liu, ZY

Zhang, TY

Wang, RB

Wan, BJ

Franzblau, SG

You, QD

AF Lu, Xiaoyun

Hu, Xianglong

Liu, Zhiyong

Zhang, Tianyu

Wang, Ruibing

Wan, Baojie

Franzblau, Scott G.

You, Qidong

TI Benzylsulfanyl benzo-heterocycle amides and hydrazones as new agents

against drug-susceptible and resistant Mycobacterium tuberculosis

SO MEDCHEMCOMM

LA English

DT Article

ID IN-VITRO; DERIVATIVES

AB A series of benzylsulfanyl benzo-heterocycle amides and hydrazones were synthesized and evaluated for anti-tubercular activities. The isonicotinyl hydrazone derivatives 12d, 12e and 12f exhibited good anti-tubercular activity against Mycobacterium tuberculosis H(37)Rv (ATCC #27294) with MIC values of 0.23, 0.24 and 0.24 mu M, respectively, and were also active against SDR-TB, MDR-TB and XDR-TB. More importantly, compound 12e also showed low cytotoxicity and good metabolic stability, and could significantly reduce the mycobacterial burden in a mouse model infected with autoluminescent H37Ra strain, which may serve as a lead compound for further development.

C1 [Lu, Xiaoyun; Hu, Xianglong] Jinan Univ, Sch Pharm, 601 Huangpu Ave West, Guangzhou 510632, Guangdong, Peoples R China.

[Lu, Xiaoyun; You, Qidong] China Pharmaceut Univ, Jiangsu Key Lab Drug Design & Optimizat, Nanjing 210009, Peoples R China.

[Liu, Zhiyong; Zhang, Tianyu; Wang, Ruibing] Chinese Acad Sci, TB Res Lab, State Key Lab Resp Dis, Guangzhou Inst Biomed & Hlth, 190,Kaiyuan Ave,Sci Pk, Guangzhou 510530, Guangdong, Peoples R China.

[Wan, Baojie; Franzblau, Scott G.] Univ Illinois, Coll Pharm, Inst TB Res, 833 South Wood St, Chicago, IL 60612 USA.

C3 Jinan University; China Pharmaceutical University; State Key Laboratory

of Respiratory Disease; Chinese Academy of Sciences; Guangzhou Institute

of Biomedicine & Health, CAS; University of Illinois System; University

of Illinois Chicago; University of Illinois Chicago Hospital

RP Lu, XY (corresponding author), Jinan Univ, Sch Pharm, 601 Huangpu Ave West, Guangzhou 510632, Guangdong, Peoples R China.; Lu, XY (corresponding author), China Pharmaceut Univ, Jiangsu Key Lab Drug Design & Optimizat, Nanjing 210009, Peoples R China.

EM luxy2016@jnu.edu.cn; youqd@163.com

RI Lu, xiaoyun/V-6699-2019; Hu, Xianglong/AAD-1653-2020; Zhang,

Tianyu/K-4801-2015

OI Lu, xiaoyun/0000-0001-7931-6873; Zhang, Tianyu/0000-0001-5647-6014

FU Guangdong Natural Science Funds [2016A030313106]; National Natural

Science Foundation of China [81673285]; National High Technology

Research and Development (863) for Young Scientists program

[2015AA020906]; Chinese Academy of Sciences Grant [154144KYSB20150045,

KFZD-SW-207]; Jinan University; [SKLRD2016ZJ003]

FX The authors gratefully acknowledge financial support from Guangdong

Natural Science Funds (2016A030313106), the National Natural Science

Foundation of China (81673285), National High Technology Research and

Development (863) for Young Scientists program (2015AA020906), Jinan

University, the Chinese Academy of Sciences Grant (154144KYSB20150045,

KFZD-SW-207), and the Key Project Grant (SKLRD2016ZJ003).

CR Andries K, 2005, SCIENCE, V307, P223, DOI 10.1126/science.1106753

Carvalho SA, 2008, BIOORG MED CHEM LETT, V18, P538, DOI 10.1016/j.bmcl.2007.11.091

Cohen J, 2013, SCIENCE, V339, P130, DOI 10.1126/science.339.6116.130

Collins LA, 1997, ANTIMICROB AGENTS CH, V41, P1004, DOI 10.1128/AAC.41.5.1004

Diacon AH, 2014, NEW ENGL J MED, V371, P723, DOI 10.1056/NEJMoa1313865

Falzari K, 2005, ANTIMICROB AGENTS CH, V49, P1447, DOI 10.1128/AAC.49.4.1447-1454.2005

Keri RS, 2015, EUR J MED CHEM, V89, P207, DOI 10.1016/j.ejmech.2014.10.059

Klimesová V, 2009, EUR J MED CHEM, V44, P2286, DOI 10.1016/j.ejmech.2008.06.027

Koul A, 2011, NATURE, V469, P483, DOI 10.1038/nature09657

Martins F, 2014, EUR J MED CHEM, V81, P119, DOI 10.1016/j.ejmech.2014.04.077

Tang J, 2015, ACS MED CHEM LETT, V6, P814, DOI 10.1021/acsmedchemlett.5b00176

World Health Organization, 2016, Global guidelines for the prevention of surgical site infection

NR 12

TC 8

Z9 8

U1 1

U2 24

PU ROYAL SOC CHEMISTRY

PI CAMBRIDGE

PA THOMAS GRAHAM HOUSE, SCIENCE PARK, MILTON RD, CAMBRIDGE CB4 0WF, CAMBS,

ENGLAND

SN 2040-2503

EI 2040-2511

J9 MEDCHEMCOMM

JI MedChemComm

PD JUN 1

PY 2017

VL 8

IS 6

BP 1303

EP 1306

DI 10.1039/c7md00146k

PG 4

WC Biochemistry & Molecular Biology; Chemistry, Medicinal

WE Science Citation Index Expanded (SCI-EXPANDED); Index Chemicus (IC)

SC Biochemistry & Molecular Biology; Pharmacology & Pharmacy

GA EY6EV

UT WOS:000404076200018

OA Green Submitted, Bronze

DA 2026-05-07

ER

PT J

AU Amaral, L

Martins, A

Molnar, J

Kristiansen, JE

Martins, M

Viveiros, M

Rodrigues, L

Spengler, G

Couto, I

Ramos, J

Dastidar, S

Fanning, S

Mccusker, M

Pages, JM

AF Amaral, Leonard

Martins, Ana

Molnar, Joseph

Kristiansen, Jette E.

Martins, Marta

Viveiros, Miguel

Rodrigues, Liliana

Spengler, Gabriella

Couto, Isabel

Ramos, Jorge

Dastidar, Sujata

Fanning, Seamus

Mccusker, Matt

Pages, Jean-Marie

TI Phenothiazines, Bacterial Efflux Pumps and Targeting the Macrophage for

Enhanced Killing of Intracellular XDRTB

SO IN VIVO

LA English

DT Review

DE Phenothiazines; thioridazine; pulmonary tuberculosis; MDR TB; XDR TB;

human macrophage; enhanced killing activity by macrophage; efflux pumps;

therapy with thioridazine; review

ID MULTIDRUG-RESISTANT TUBERCULOSIS; ESCHERICHIA-COLI K-12;

PENICILLIN-BINDING PROTEINS; ANTIPSYCHOTIC-DRUGS INHIBIT; IN-VITRO

ACTIVITY; MYCOBACTERIUM-TUBERCULOSIS; STAPHYLOCOCCUS-AUREUS;

P-GLYCOPROTEIN; ANTIBIOTIC-RESISTANCE; POTENTIAL USE

AB Phenothiazines have their primary effects on the plasma membrane of prokaryotes and eukaryotes. Among the components of the prokaryotic plasma membrane affected are efflux pumps, their energy sources, energy providing enzymes such as ATPases, and genes that regulate and code for permeability aspects of the bacterium. The responses of multi-drug (MDR) and extensively drug resistant (XDR) Mycobacterium tuberculosis to the neuroleptic phenothiazine thioridazine are reviewed. The information collated suggests that this phenothiazine has the potential to cure XDR and MDR tuberculosis infections, a potential that has been recently demonstrated by its ability to cure 10 patients who presented with XDR TB infections. The mechanism by which this phenothiazine produces the desired effects within the infected macrophage is also discussed.

C1 [Amaral, Leonard; Martins, Ana; Martins, Marta; Viveiros, Miguel; Rodrigues, Liliana; Spengler, Gabriella; Couto, Isabel; Ramos, Jorge] Univ Nova Lisboa, Inst Hyg & Trop Med, Unit Mycobacteriol, P-1349008 Lisbon, Portugal.

[Couto, Isabel] Univ Nova Lisboa, Fac Ciencias & Tecnol, Ctr Recursos Microbiol, P-1349008 Lisbon, Portugal.

[Molnar, Joseph] Univ Szeged, Inst Med Microbiol & Immunol, Szeged, Hungary.

[Kristiansen, Jette E.] So Danish Univ, Dept Clin Microbiol, Sonderborg, Denmark.

[Dastidar, Sujata] Herbicure Healthcare Bioherbal Res Fdn, Dept Microbiol, Kolkata, India.

[Fanning, Seamus; Mccusker, Matt] Univ Coll Dublin, Ctr Food Safety, Sch Agr Food Sci & Vet Med, Dublin 2, Ireland.

[Pages, Jean-Marie] Univ Mediterranee, Fac Med & Pharm, UMR MD 1, Marseille, France.

[Amaral, Leonard; Martins, Ana; Rodrigues, Liliana; Spengler, Gabriella; Ramos, Jorge] Univ Nova Lisboa, Inst Hyg & Trop Med, UPMM, P-1349008 Lisbon, Portugal.

C3 Universidade Nova de Lisboa; Institute of Hygiene & Tropical Medicine -

UNL; Universidade Nova de Lisboa; Szeged University; University of

Southern Denmark; University College Dublin; Aix-Marseille Universite;

Universidade Nova de Lisboa; Institute of Hygiene & Tropical Medicine -

UNL

RP Amaral, L (corresponding author), Univ Nova Lisboa, Inst Hyg & Trop Med, Unit Mycobacteriol, Rua Junqueira 96, P-1349008 Lisbon, Portugal.

EM lamaral@ihmt.unl.pt

RI Amaral, Leonard/I-2112-2012; Martins, Marta/I-3015-2012; Martins,

Ana/I-6432-2012; Couto, Isabel/I-2640-2012; Roigues,

Liliana/N-2463-2014; Martins, Marta/I-6432-2012; molnar,

joseph/D-4375-2013; Spengler, Gabriella/I-6433-2012; Viveiros,

Miguel/A-7326-2008; PAGES, jean-marie/F-2555-2013

OI Amaral, Leonard/0000-0003-3127-1295; Martins, Marta/0000-0002-0323-2857;

Martins, Ana/0000-0002-8541-7440; Couto, Isabel/0000-0003-3536-2733;

Roigues, Liliana/0000-0002-2094-2666; Ramos, Jorge/0000-0001-6419-5351;

Fanning, Séamus/0000-0002-1922-8836; Martins, Marta/0000-0002-0323-2857;

Spengler, Gabriella/0000-0001-8085-0950; Viveiros,

Miguel/0000-0001-9676-6251;

CR ABBATE E, 2007, REV ARGENTINA MED RE, V1, P19

ABERNATHY RS, 1957, J LAB CLIN MED, V49, P708

ABERNATHY RS, 1957, P SOC EXP BIOL MED, V95, P580

Ahluwalia J, 2004, NATURE, V427, P853, DOI 10.1038/nature02356

ALFREDSSON G, 1976, PSYCHOPHARMACOLOGY, V48, P123, DOI 10.1007/BF00423250

Amaral L, 2008, CURR DRUG TARGETS, V9, P816, DOI 10.2174/138945008785747798

Amaral L., 2007, Infectious Disorders - Drug Targets, V7, P257, DOI 10.2174/187152607782110022

Amaral L, 2004, IN VIVO, V18, P725

AMARAL L, 1991, ANTIMICROB AGENTS CH, V35, P1923, DOI 10.1128/AAC.35.9.1923

Amaral L, 2000, INT J ANTIMICROB AG, V14, P225, DOI 10.1016/S0924-8579(00)00136-9

AMARAL L, 1992, J ANTIMICROB CHEMOTH, V30, P556, DOI 10.1093/jac/30.4.556

Amaral L, 1996, J ANTIMICROB CHEMOTH, V38, P1049, DOI 10.1093/jac/38.6.1049

Amaral L, 2000, INT J ANTIMICROB AG, V14, P173, DOI 10.1016/S0924-8579(99)00153-3

Amaral L, 2001, TROP MED INT HEALTH, V6, P1016, DOI 10.1046/j.1365-3156.2001.00804.x

AMARAL L, 1986, DRUG EXP CLIN RES, V12, P653

Amaral L, 2001, J ANTIMICROB CHEMOTH, V47, P505, DOI 10.1093/jac/47.5.505

AMARAL L, 1999, NEWSLETTER INT SOC C, V3, P6

Amaral L, 2007, IN VIVO, V21, P237

Amaral L, 2006, CURR DRUG TARGETS, V7, P887, DOI 10.2174/138945006777709539

Amaral L, 2007, J ANTIMICROB CHEMOTH, V59, P1237, DOI 10.1093/jac/dkl500

APOSHIAN HV, 1959, P SOC EXP BIOL MED, V100, P512

Bachhawat Nandita, 2007, Genomics Proteomics & Bioinformatics, V5, P236, DOI 10.1016/S1672-0229(08)60010-8

Baker-Austin C, 2007, TRENDS MICROBIOL, V15, P165, DOI 10.1016/j.tim.2007.02.005

BARABAS K, 1980, ACTA MICROBIOL HUNG, V27, P55

BARTSEVICH VV, 1995, MICROBIOL-SGM, V141, P2915, DOI 10.1099/13500872-141-11-2915

Basta-Kaim A, 2006, NEUROPSYCHOPHARMACOL, V31, P853, DOI 10.1038/sj.npp.1300911

Bebawy M, 2001, BRIT J CANCER, V85, P1998, DOI 10.1054/bjoc.2001.2184

BENNISON WH, 1948, LANCET, V254, P885

Berecz R, 2004, CURR DRUG TARGETS, V5, P573, DOI 10.2174/1389450043345263

Bettencourt MV, 2000, INT J ANTIMICROB AG, V16, P69, DOI 10.1016/S0924-8579(00)00199-0

Bhatnagar K, 2004, CURR MICROBIOL, V49, P28, DOI 10.1007/s00284-003-4261-8

Bhatnagar K, 2003, CURR MICROBIOL, V46, P265, DOI 10.1007/s00284-002-3876-5

BOROWIECKA A, 1956, Pol Tyg Lek (Wars), V11, P1790

BOURDON JL, 1961, ANN I PASTEUR PARIS, V101, P876

Broeck DV, 2007, ACTA BIOCHIM POL, V54, P757, DOI 10.18388/abp.2007_3154

BULLOUGH DA, 1985, ARCH BIOCHEM BIOPHYS, V236, P567, DOI 10.1016/0003-9861(85)90660-5

Carranza ML, 1998, J PHYSIOL-LONDON, V511, P235, DOI 10.1111/j.1469-7793.1998.235bi.x

CARRERA G, 1962, G Batteriol Virol Immunol, V55, P330

Ceccarelli M, 2009, FRONT BIOSCI-LANDMRK, V14, P3222, DOI 10.2741/3446

Chadfield MS, 2004, J APPL MICROBIOL, V96, P1002, DOI 10.1111/j.1365-2672.2004.02225.x

Chang TM, 2007, J CHEMOTHERAPY, V19, P488, DOI 10.1179/joc.2007.19.5.488

CHEDID L, 1954, CR SOC BIOL, V148, P1039

Chen Li-jun, 2003, Zhonghua Er Ke Za Zhi, V41, P525

Chevalier J, 2008, PLOS ONE, V3, DOI 10.1371/journal.pone.0003203

CHUMAKOVA R. I., 1964, MIKROBIOLOGIYA, V33, P639

Chwalla R, 1965, Z Urol Nephrol, V58, P375

Cima F, 2000, AQUAT TOXICOL, V48, P419, DOI 10.1016/S0166-445X(99)00064-8

Conter A, 2002, J BACTERIOL, V184, P2850, DOI 10.1128/JB.184.10.2850-2853.2002

COSTA G, 1950, Minerva Med, V41, P733

Coutinho HDM, 2008, CHEMOTHERAPY, V54, P328, DOI 10.1159/000151267

CROWLE AJ, 1992, CHEMOTHERAPY, V38, P410, DOI 10.1159/000239036

CSISZAR K, 1992, ANTICANCER RES, V12, P2267

Davidson AL, 2008, MICROBIOL MOL BIOL R, V72, P317, DOI 10.1128/MMBR.00031-07

Davin-Regli A, 2008, CURR DRUG TARGETS, V9, P750, DOI 10.2174/138945008785747824

Delcour AH, 2009, BBA-PROTEINS PROTEOM, V1794, P808, DOI 10.1016/j.bbapap.2008.11.005

Deng L, 2006, MOL BIOL CELL, V17, P4790, DOI 10.1091/mbc.E06-06-0526

Dhople AM, 1999, MICROBIOS, V98, P113

Dijkshoorn L, 2007, NAT REV MICROBIOL, V5, P939, DOI 10.1038/nrmicro1789

Doleans-Jordheim A, 2008, ANN BIOL CLIN-PARIS, V66, P499, DOI [10.1168/abc.2008.0266, 10.1684/abc.2008.0266]

Eicher T, 2009, BIOL CHEM, V390, P693, DOI 10.1515/BC.2009.090

FAGUET M, 1962, C R Hebd Seances Acad Sci, V255, P155

Falagas ME, 2006, J MED MICROBIOL, V55, P1619, DOI 10.1099/jmm.0.46747-0

FINLAND M, 1953, ARCH INTERN MED, V91, P143, DOI 10.1001/archinte.1953.00240140003001

FIORE M., 1963, RIV IST SIEROTERAP ITAL, V38, P220

GALDIERO F., 1963, RIV IST SIEROTERAP ITALIANO, V38, P80

Garfield Simon., 2001, Mauve: How One Man Invented a Color that Changed the World

GLEISSNER M, 1994, EUR J BIOCHEM, V224, P983, DOI 10.1111/j.1432-1033.1994.00983.x

GOLDENBAUM EG, 1966, J INFECT DIS, V116, P447, DOI 10.1093/infdis/116.4.447

Gonda K, 2000, CELL STRUCT FUNCT, V25, P243, DOI 10.1247/csf.25.243

Gould IM, 2008, INT J ANTIMICROB AG, V32, pS2, DOI 10.1016/j.ijantimicag.2008.06.016

Gronow S, 2001, J ENDOTOXIN RES, V7, P3, DOI 10.1179/096805101101532468

GROSZ HJ, 1964, BRIT J PSYCHIAT, V110, P603, DOI 10.1192/bjp.110.467.603-a

Gunics G, 2000, INT J ANTIMICROB AG, V14, P239, DOI 10.1016/S0924-8579(00)00131-X

Gunn JS, 2008, TRENDS MICROBIOL, V16, P284, DOI 10.1016/j.tim.2008.03.007

Guttmann P., 1891, Berliner Klinische Wochenschrift, V28, P953

HAIT WN, 1992, BIOCHEM PHARMACOL, V43, P103, DOI 10.1016/0006-2952(92)90667-8

Hartog E, 2008, MICROBIOL IMMUNOL, V52, P565, DOI 10.1111/j.1348-0421.2008.00075.x

Hughes PJ, 2006, J CELL BIOCHEM, V98, P590, DOI 10.1002/jcb.20787

Ishizaki J, 1996, PHARMACEUT RES, V13, P902, DOI 10.1023/A:1016061330387

JENSEN K, 1964, Nord Med, V71, P599

Jonas BM, 2001, ANTIMICROB AGENTS CH, V45, P3574, DOI 10.1128/AAC.45.12.3574-3579.2001

Jones AT, 2007, J CELL MOL MED, V11, P670, DOI 10.1111/j.1582-4934.2007.00062.x

Justice SS, 2008, NAT REV MICROBIOL, V6, P162, DOI 10.1038/nrmicro1820

Kaatz GW, 2002, J ANTIMICROB CHEMOTH, V50, P833, DOI 10.1093/jac/dkf224

KAWAMUKAI M, 1989, J BACTERIOL, V171, P4525, DOI 10.1128/JB.171.8.4525-4529.1989

Kecskemeti Valeria, 2004, Neuropsychopharmacol Hung, V6, P5

KENT JH, 1993, MED CLIN N AM, V77, P1391, DOI 10.1016/S0025-7125(16)30200-0

KLEIN M, 1953, J BACTERIOL, V65, P454, DOI 10.1128/JB.65.4.454-465.1953

Klitgaard JK, 2008, J ANTIMICROB CHEMOTH, V62, P1215, DOI 10.1093/jac/dkn417

KOLAKOWSKA T, 1976, PSYCHOPHARMACOLOGY, V49, P101

Konings WN, 2002, ANTON LEEUW INT J G, V81, P61, DOI 10.1023/A:1020573408652

KRISTIANSEN JE, 1986, ACTA PATH MICRO IM B, V94, P393

Kristiansen JE, 1997, J ANTIMICROB CHEMOTH, V40, P319, DOI 10.1093/jac/40.3.319

KRISTIANSEN JEH, 1982, BIOCHIM BIOPHYS ACTA, V685, P379, DOI 10.1016/0005-2736(82)90079-7

KRISTIANSEN JEH, 1981, UGESKRIFT LAEGER, V43, P1900

Kristiansen MM, 2003, INT J ANTIMICROB AG, V22, P250, DOI 10.1016/S0924-8579(03)00200-0

Kumar A, 2002, J ANTIMICROB CHEMOTH, V50, P593, DOI 10.1093/jac/dkf147

Kupittayanant P, 2006, CELL CALCIUM, V39, P417, DOI 10.1016/j.ceca.2006.01.011

Langton KP, 2005, NAT PROD REP, V22, P439, DOI 10.1039/b413734p

LEBEK G, 1964, DEUT MED WOCHENSCHR, V89, P2464, DOI 10.1055/s-0028-1113305

Lee EW, 2003, BIOL PHARM BULL, V26, P266, DOI 10.1248/bpb.26.266

Lee SC, 1998, MOL CELLS, V8, P698

Lee SY, 2004, J CARDIOVASC PHARM, V43, P706, DOI 10.1097/00005344-200405000-00014

Lee YR, 2004, INFECT IMMUN, V72, P6157, DOI 10.1128/IAI.72.10.6157-6159.2004

Li XZ, 2003, ANTIMICROB AGENTS CH, V47, P27, DOI 10.1128/AAC.47.1.27-33.2003

LIBENSON V S, 1967, Byulleten' Eksperimental'noi Biologii i Meditsiny, V63, P61

Liu Zhang-Xu, 2002, Clin Liver Dis, V6, P755, DOI 10.1016/S1089-3261(02)00025-9

LORIAN V, 1975, ANTIMICROB AGENTS CH, V7, P864, DOI 10.1128/AAC.7.6.864

LORIAN V, 1980, ANTIMICROB AGENTS CH, V18, P807, DOI 10.1128/AAC.18.5.807

LUDANY G, 1955, Orv Hetil, V96, P1100

LUM BL, 1993, PHARMACOTHERAPY, V13, P88

Martins M, 2009, INT J TUBERC LUNG D, V13, P569

MARTINS M, 2005, J CHEMOTHER S3, V17, P38

Martins M, 2008, INT J ANTIMICROB AG, V31, P198, DOI 10.1016/j.ijantimicag.2007.10.025

Martins M, 2008, IN VIVO, V22, P69

Martins M, 2007, IN VIVO, V21, P771

Masaoka Y, 2000, J BACTERIOL, V182, P2307, DOI 10.1128/JB.182.8.2307-2310.2000

MASEK K, 1963, Cas Lek Cesk, V102, P185

MAUEL J, 1983, B EUR PHYSIOPATH RES, V19, P115

Mayur YC, 2006, MINI-REV MED CHEM, V6, P1383, DOI 10.2174/138955706778993021

MIDDLEBROOK JL, 1977, INFECT IMMUN, V16, P232, DOI 10.1128/IAI.16.1.232-239.1977

Mizrahi I, 2007, APPL ENVIRON MICROB, V73, P5951, DOI 10.1128/AEM.01067-07

MOLNAR J, 1977, ZBL BAKT-INT J MED M, V239, P521

MOLNAR J, 1992, ANTON LEEUW INT J G, V62, P315, DOI 10.1007/BF00572600

MOLNAR J, 1973, ACTA MICROBIOL HUNG, V20, P171

Moon YJ, 2004, PHOTOCHEM PHOTOBIOL, V79, P114, DOI 10.1111/j.1751-1097.2004.tb09865.x

Moussatova A, 2008, BBA-BIOMEMBRANES, V1778, P1757, DOI 10.1016/j.bbamem.2008.06.009

Mulkidjanian AY, 2006, BBA-BIOENERGETICS, V1757, P415, DOI 10.1016/j.bbabio.2006.04.023

Mulkidjanian AY, 2006, PHOTOCH PHOTOBIO SCI, V5, P577, DOI 10.1039/b516443e

Mulkidjanian AY, 2006, BBA-BIOENERGETICS, V1757, P913, DOI 10.1016/j.bbabio.2006.02.015

Murray M, 2006, J PHARM PHARMACOL, V58, P871, DOI 10.1211/jpp.58.7.0001

NAPP J H, 1952, Med Klin, V47, P709

NASSAU E, 1955, Tubercle, V36, P281, DOI 10.1016/S0041-3879(55)80116-X

NATHAN HA, 1961, NATURE, V192, P471, DOI 10.1038/192471b0

Ni YN, 2005, TALANTA, V65, P1295, DOI 10.1016/j.talanta.2004.09.008

Nie HG, 2007, J PHYSIOL SCI, V57, P167, DOI 10.2170/physiolsci.RP000507

Nishino K, 2009, BBA-PROTEINS PROTEOM, V1794, P834, DOI 10.1016/j.bbapap.2009.02.002

NOYES HE, 1956, P SOC EXP BIOL MED, V92, P617, DOI 10.3181/00379727-92-22562

O'Neill A, 2008, EXPERT OPIN INV DRUG, V17, P297, DOI [10.1517/13543784.17.3.297, 10.1517/13543784.17.3.297 ]

O'Regan E, 2009, ANTIMICROB AGENTS CH, V53, P1080, DOI 10.1128/AAC.01005-08

Ordway D, 2003, ANTIMICROB AGENTS CH, V47, P917, DOI 10.1128/AAC.47.3.917-922.2003

Otani K, 2000, THER DRUG MONIT, V22, P118, DOI 10.1097/00007691-200002000-00025

Pacheco Gladys, 1993, Acta Cientifica Venezolana, V44, P111

Pagès JM, 2008, NAT REV MICROBIOL, V6, P893, DOI 10.1038/nrmicro1994

Pagès JM, 2009, BBA-PROTEINS PROTEOM, V1794, P826, DOI 10.1016/j.bbapap.2008.12.011

Pantosti A, 2007, FUTURE MICROBIOL, V2, P323, DOI 10.2217/17460913.2.3.323

PARANT M, 1964, P SOC EXP BIOL MED, V116, P906, DOI 10.3181/00379727-116-29404

Pavek P, 2003, J PHARMACOL EXP THER, V305, P1239, DOI 10.1124/jpet.102.048470

Pávek P, 2001, J PHARM SCI-US, V90, P1583, DOI 10.1002/jps.1108

Pavlik L L, 2005, Neurosci Behav Physiol, V35, P453, DOI 10.1007/s11055-005-0077-2

Peschel A, 2002, TRENDS MICROBIOL, V10, P179, DOI 10.1016/S0966-842X(02)02333-8

Piddock LJV, 2006, NAT REV MICROBIOL, V4, P629, DOI 10.1038/nrmicro1464

Popenenkova Z A, 1969, Zh Mikrobiol Epidemiol Immunobiol, V46, P66

POPPER M, 1959, PRESSE MED, V67, P212

Pos KM, 2009, BBA-PROTEINS PROTEOM, V1794, P782, DOI 10.1016/j.bbapap.2008.12.015

Quadri Luis E. N., 2007, Infectious Disorders - Drug Targets, V7, P230, DOI 10.2174/187152607782110040

Rábano M, 2004, MOL CELL ENDOCRINOL, V222, P9, DOI 10.1016/j.mce.2004.05.006

RAFFEL S, 1960, AM REV RESPIR DIS, V81, P555

Rajyaguru JM, 1997, J ANTIMICROB CHEMOTH, V40, P345, DOI 10.1093/jac/40.3.345

RAMOS J, 2009, 30 ANN C EUR SOC MYC

RATNAKAR P, 1995, INT CLIN PSYCHOPHARM, V10, P39, DOI 10.1097/00004850-199503000-00005

Reddy PT, 2003, J BACTERIOL, V185, P5263, DOI 10.1128/JB.185.17.5263-5268.2003

Reeves EP, 2002, NATURE, V416, P291, DOI 10.1038/416291a

RIVERACALIMLIM L, 1976, AM J PSYCHIAT, V133, P646

RODRIGUES L, ACTIVATION SOX UNPUB

Rojas M, 2000, J INFECT DIS, V182, P240, DOI 10.1086/315676

ROMANOVSKAYA M G, 1971, Farmakologiya i Toksikologiya (Moscow), V34, P698

Rosenfeld Y, 2006, BBA-BIOMEMBRANES, V1758, P1513, DOI 10.1016/j.bbamem.2006.05.017

ROSENOW E C, 1955, J Nerv Ment Dis, V122, P321, DOI 10.1097/00005053-195510000-00003

Rossolini GM, 2007, NEW MICROBIOL, V30, P332

Roy CR, 2009, SCI SIGNAL, V2, DOI 10.1126/scisignal.262pe14

Ryu SD, 2009, CELL CALCIUM, V45, P99, DOI 10.1016/j.ceca.2008.07.002

Salih ISM, 2007, CLIN PHARMACOL THER, V82, P548, DOI 10.1038/sj.clpt.6100194

Sander P, 2000, FEMS MICROBIOL LETT, V193, P19, DOI 10.1111/j.1574-6968.2000.tb09396.x

Sandström R, 1998, J PHARM PHARMACOL, V50, P729, DOI 10.1111/j.2042-7158.1998.tb07133.x

Sawicke Luciana, 2008, Vertex, V19, P387

Schauer-Vukasinovic V, 1999, BIOTECHNOL PROGR, V15, P513, DOI 10.1021/bp990058l

Schulemann W, 1932, Proc R Soc Med, V25, P897

Seeger MA, 2008, CURR DRUG TARGETS, V9, P729, DOI 10.2174/138945008785747789

Singh UP, 2001, MICROBIOL RES, V156, P65, DOI 10.1078/0944-5013-00071

Spengler G, 2009, IN VIVO, V23, P81

SPRATT BG, 1975, NATURE, V254, P516, DOI 10.1038/254516a0

Stöllberger C, 2005, INT CLIN PSYCHOPHARM, V20, P243

STOWE DJ, 1989, BIOCHIMIE, V71, P1101, DOI 10.1016/0300-9084(89)90117-X

SUBTIL A, 1994, J CELL SCI, V107, P3461

SUGIURA A, 1994, MOL MICROBIOL, V14, P929, DOI 10.1111/j.1365-2958.1994.tb01328.x

Sun HY, 2006, J BIOL CHEM, V281, P5877, DOI 10.1074/jbc.M600072200

Suzuki K, 2001, INT J HEMATOL, V74, P134, DOI 10.1007/BF02981995

Suzuki S, 2000, J CARDIOVASC PHARM, V36, P609, DOI 10.1097/00005344-200011000-00010

Swaminathan S, 2008, J BIOSCIENCES, V33, P527, DOI 10.1007/s12038-008-0071-2

TAJOLI E., 1966, ACTA ANAESTH [PADOVA], V17, P757

Thomas D, 2003, BRIT J PHARMACOL, V139, P567, DOI 10.1038/sj.bjp.0705283

THOMSON EF, 1952, J CLIN PATHOL, V5, P169, DOI 10.1136/jcp.5.2.169

Trimble WS, 2007, CELL, V130, P12, DOI 10.1016/j.cell.2007.06.039

Tumah HN, 2009, J CHEMOTHERAPY, V21, P5, DOI 10.1080/1120009X.2009.12030920

Tyteca D, 2005, EXP CELL RES, V310, P293, DOI 10.1016/j.yexcr.2005.07.027

UNANUE ER, 1976, AM J PATHOL, V85, P465

UTSUMI R, 1989, J BACTERIOL, V171, P2909, DOI 10.1128/jb.171.5.2909-2912.1989

Van Bambeke F, 2000, BIOCHEM PHARMACOL, V60, P457, DOI 10.1016/S0006-2952(00)00291-4

VANSOOLIGEN D, COMMUNICATION

VANSOOLIGEN D, 2010, INT J ANTIMICROB AGE

Vemuri Mohan C., 1993, Cytobios, V76, P117

Vieweg WVR, 2004, PSYCHOSOMATICS, V45, P371, DOI 10.1176/appi.psy.45.5.371

Vila J, 2008, CURR DRUG TARGETS, V9, P797, DOI 10.2174/138945008785747806

Vilariça AS, 2008, REV PORT PNEUMOL, V14, P829

Viveiros M, 2008, CURR DRUG TARGETS, V9, P760, DOI 10.2174/138945008785747734

Viveiros M, 2005, ANTIMICROB AGENTS CH, V49, P3578, DOI 10.1128/AAC.49.8.3578-3582.2005

Viveiros M, 2008, INT J ANTIMICROB AG, V31, P458, DOI 10.1016/j.ijantimicag.2007.12.015

Viveiros M, 2007, PLOS ONE, V2, DOI 10.1371/journal.pone.0000365

WAKSMAN SA, 1945, P NATL ACAD SCI USA, V31, P157, DOI 10.1073/pnas.31.6.157

WALSH CT, 1974, J PHARMACOL EXP THER, V188, P277

Wang JS, 2008, BASIC CLIN PHARMACOL, V103, P336, DOI 10.1111/j.1742-7843.2008.00298.x

Wang JS, 2006, PSYCHOPHARMACOLOGY, V187, P415, DOI 10.1007/s00213-006-0437-9

WARING MJ, 1970, HUMANGENETIK, V9, P234, DOI 10.1007/BF00279229

Weingart H, 2008, CURR DRUG TARGETS, V9, P789, DOI 10.2174/138945008785747752

Wikinski Silvia, 2005, Vertex, V16, P438

Winje Brita Askeland, 2008, Tidsskr Nor Laegeforen, V128, P2588

Yang D, 2009, CURR MICROBIOL, V58, P366, DOI 10.1007/s00284-009-9364-4

Yang HY, 2008, MICROB DRUG RESIST, V14, P245, DOI 10.1089/mdr.2008.0847

Zakharov SD, 1996, J BIOENERG BIOMEMBR, V28, P483, DOI 10.1007/BF02110438

ZEBROWSKI T, 1955, Beitr Klin Tuberk Spezif Tuberkuloseforsch, V113, P269, DOI 10.1007/BF02148112

Zechini Barbara, 2009, Recent Pat Antiinfect Drug Discov, V4, P37

ZENYOJI H, 1961, Nihon Saikingaku Zasshi, V16, P1015

NR 223

TC 44

Z9 54

U1 0

U2 18

PU INT INST ANTICANCER RESEARCH

PI ATHENS

PA EDITORIAL OFFICE 1ST KM KAPANDRITIOU-KALAMOU RD KAPANDRITI, PO BOX 22,

ATHENS 19014, GREECE

SN 0258-851X

EI 1791-7549

J9 IN VIVO

JI In Vivo

PD JUL-AUG

PY 2010

VL 24

IS 4

BP 409

EP 424

PG 16

WC Medicine, Research & Experimental

WE Science Citation Index Expanded (SCI-EXPANDED)

SC Research & Experimental Medicine

GA 638JH

UT WOS:000280890000005

DA 2026-05-07

ER

PT J

AU Kumar, G

Adhikrao, PA

AF Kumar, Gautam

Adhikrao, Patil Amruta

TI Targeting Mycobacterium tuberculosis iron-scavenging tools: a

recent update on siderophores inhibitors

SO RSC MEDICINAL CHEMISTRY

LA English

DT Review

ID SALICYLATE SYNTHASE MBTI; THERAPEUTIC TARGET; DRUG DISCOVERY;

ANTITUBERCULOSIS AGENTS; CHOLESTEROL-METABOLISM; NUCLEOSIDE ANTIBIOTICS;

BIOSYNTHESIS PATHWAY; NATURAL-PRODUCTS; DPRE1 INHIBITORS; ATP SYNTHESIS

AB Among the various bacterial infections, tuberculosis (TB) remains a life-threatening infectious disease responsible as the most significant cause of mortality and morbidity worldwide. The co-infection of human immunodeficiency virus (HIV) in association with TB burdens the healthcare system substantially. Notably, M.tb possesses defence against most antitubercular antibiotic drugs, and the efficacy of existing frontline anti-TB drugs is waning. Also, new and recurring cases of TB from resistant bacteria such as multidrug-resistant TB (MDR), extensively drug-resistant TB (XDR), and totally drug-resistant TB (TDR) strains are increasing. Hence, TB begs the scientific community to explore the new therapeutic class of compounds with their novel mechanism. M.tb requires iron from host cells to sustain, grow, and carry out several biological processes. M.tb has developed strategic methods of acquiring iron from the surrounding environment. In this communication, we discuss an overview of M.tb iron-scavenging tools. Also, we have summarized recently identified MbtA and MbtI inhibitors, which prevent M.tb from scavenging iron. These iron-scavenging tool inhibitors have the potential to be developed as anti-TB agents/drugs.

C1 [Kumar, Gautam; Adhikrao, Patil Amruta] Natl Inst Pharmaceut Educ & Res Hyderabad NIPER Hy, Dept Nat Prod, Chem Sci, Hyderabad 500037, India.

RP Kumar, G (corresponding author), Natl Inst Pharmaceut Educ & Res Hyderabad NIPER Hy, Dept Nat Prod, Chem Sci, Hyderabad 500037, India.

EM gautam.kumar2@niperhyd.ac.in

OI Kumar, Gautam/0000-0003-4047-1416

FU We thank the Director, NIPER-Hyderabad, for providing the necessary

research facilities. Amruta Patil is thankful to NIPER Hyderabad for

providing a fellowship. All figures were created in the BioRender

software.; NIPER-Hyderabad; NIPER Hyderabad

FX We thank the Director, NIPER-Hyderabad, for providing the necessary

research facilities. Amruta Patil is thankful to NIPER Hyderabad for

providing a fellowship. All figures were created in the BioRender

software.

CR Abreu R, 2018, Sci Rep, V8, P1, DOI DOI 10.1038/s41598-017-17765-5

Abreu R, 2020, PLOS ONE, V15, DOI 10.1371/journal.pone.0240949

Abuhammad A, 2017, BRIT J PHARMACOL, V174, P2194, DOI 10.1111/bph.13694

Activity A, 2020, Antimicrob. Agents Chemother., V64, P1, DOI 10.1128/AAC.02020-19

Actor J. K., 2015, MEDIAT INFLAMM, V2015, P15

Alfarisi O, 2017, EXPERT REV CLIN PHAR, V10, P1027, DOI 10.1080/17512433.2017.1366311

Alffenaar JWC, 2011, ANTIMICROB AGENTS CH, V55, P1287, DOI 10.1128/AAC.01297-10

Almeida DV, 2020, ANTIMICROB AGENTS CH, V64, DOI 10.1128/AAC.00259-20

Altharawi A, 2023, SCI REP-UK, V13, DOI 10.1038/s41598-023-34222-8

Andrews JR, 2012, CLIN INFECT DIS, V54, P784, DOI 10.1093/cid/cir951

Arnold FM, 2020, NATURE, V580, P413, DOI 10.1038/s41586-020-2136-9

Aziz DB, 2017, ANTIMICROB AGENTS CH, V61, DOI 10.1128/AAC.00155-17

Balasubramanian V, 2014, ANTIMICROB AGENTS CH, V58, P495, DOI 10.1128/AAC.01903-13

Bald D, 2010, FEMS MICROBIOL LETT, V308, P1, DOI 10.1111/j.1574-6968.2010.01959.x

Banaschewski B, 2019, J CYST FIBROS, V18, P714, DOI 10.1016/j.jcf.2019.05.013

Banerjee R, 2014, TUBERCULOSIS, V94, P397, DOI 10.1016/j.tube.2014.03.008

Banerjee S, 2011, INFECT GENET EVOL, V11, P825, DOI 10.1016/j.meegid.2011.02.016

Bento CM, 2020, ANTIBIOTICS-BASEL, V9, DOI 10.3390/antibiotics9010018

Blair HA, 2015, DRUGS, V75, P91, DOI 10.1007/s40265-014-0331-4

Blondiaux N, 2017, SCIENCE, V355, P1206, DOI 10.1126/science.aag1006

Bolla JR, 2020, BIOCHEM SOC T, V48, P1463, DOI 10.1042/BST20190950

Boradia VM, 2014, NAT COMMUN, V5, DOI 10.1038/ncomms5730

Boudaher E, 2019, MEDCHEMCOMM, V10, P682, DOI 10.1039/c9md00076c

Bretl DJ, 2011, MICROBIOL MOL BIOL R, V75, P566, DOI 10.1128/MMBR.05004-11

Brown-Elliott BA, 2018, ANTIMICROB AGENTS CH, V62, DOI [10.1128/AAC.01503-18, 10.1128/aac.01503-18]

Bythrow GV, 2019, BIOCHEMISTRY-US, V58, P833, DOI 10.1021/acs.biochem.8b01153

Carroll CS, 2018, CRIT REV BIOCHEM MOL, V53, P356, DOI 10.1080/10409238.2018.1476449

Caza M, 2013, FRONT CELL INFECT MI, V3, DOI 10.3389/fcimb.2013.00080

Chalut C, 2016, TUBERCULOSIS, V100, P32, DOI 10.1016/j.tube.2016.06.004

Chao A, 2019, CHEM REV, V119, P1193, DOI 10.1021/acs.chemrev.8b00285

Chauhan A. S., 2015, SCI REP-UK, V5, P1

Chen XH, 2017, ANTIMICROB AGENTS CH, V61, DOI [10.1128/aac.02402-16, 10.1128/AAC.02402-16]

Cheon SA, 2016, J MICROBIOL METH, V123, P51, DOI 10.1016/j.mimet.2016.02.007

Chetty S, 2017, BIOORG MED CHEM LETT, V27, P370, DOI 10.1016/j.bmcl.2016.11.084

Chhabra S, 2021, ACS OMEGA, V6, P14430, DOI 10.1021/acsomega.1c01314

Chi G, 2012, BIOCHEMISTRY-US, V51, P4868, DOI 10.1021/bi3002067

Chiarelli LR, 2019, J ENZYM INHIB MED CH, V34, P823, DOI 10.1080/14756366.2019.1589462

Chiarelli LR, 2018, EUR J MED CHEM, V155, P754, DOI 10.1016/j.ejmech.2018.06.033

Chikhale RV, 2018, J MED CHEM, V61, P8563, DOI 10.1021/acs.jmedchem.8b00281

Cholo MC, 2017, J ANTIMICROB CHEMOTH, V72, P338, DOI 10.1093/jac/dkw426

Choo SW, 2016, PLOS ONE, V11, DOI 10.1371/journal.pone.0150413

Choudhury M, 2021, BIOMETALS, V34, P511, DOI 10.1007/s10534-021-00292-2

Conradie F, 2020, NEW ENGL J MED, V382, P893, DOI 10.1056/NEJMoa1901814

Converse PJ, 2009, INFECT IMMUN, V77, P1230, DOI 10.1128/IAI.01117-08

Coudevylle N, 2010, J BIOL CHEM, V285, P41646, DOI 10.1074/jbc.M110.123331

D'Souza C, 2023, IMMUNOBIOLOGY, V228, DOI 10.1016/j.imbio.2022.152321

Dahl SL, 2018, FRONT IMMUNOL, V9, DOI 10.3389/fimmu.2018.02717

Daleke MH, 2011, J BIOL CHEM, V286, P19024, DOI 10.1074/jbc.M110.204966

Dawadi S, 2018, ACS MED CHEM LETT, V9, P386, DOI 10.1021/acsmedchemlett.8b00090

Dawadi S, 2016, BIOORGAN MED CHEM, V24, P1314, DOI 10.1016/j.bmc.2016.02.002

Dawadi S, 2015, J ORG CHEM, V80, P4835, DOI 10.1021/acs.joc.5b00550

Degiacomi G, 2017, SCI REP-UK, V7, DOI 10.1038/srep43495

Dow A, 2021, PLOS PATHOG, V17, DOI 10.1371/journal.ppat.1009570

Dragset MS, 2015, ANTIMICROB AGENTS CH, V59, P2256, DOI 10.1128/AAC.05114-14

Duckworth BP, 2012, ACS CHEM BIOL, V7, P1653, DOI 10.1021/cb300112x

Dunn EA, 2014, BIOORGAN MED CHEM, V22, P5320, DOI 10.1016/j.bmc.2014.07.050

Durcik M, 2019, EXPERT OPIN THER PAT, V29, P171, DOI 10.1080/13543776.2019.1575362

El-Gamal MI, 2017, EUR J MED CHEM, V131, P185, DOI 10.1016/j.ejmech.2017.03.022

Engelhart CA, 2013, J ORG CHEM, V78, P7470, DOI 10.1021/jo400976f

Ennassiri W, 2017, J GLOB ANTIMICROB RE, V11, P75, DOI 10.1016/j.jgar.2017.07.002

Fang Z, 2015, TUBERCULOSIS, V95, P123, DOI 10.1016/j.tube.2015.01.004

Faustino C., 2020, STUDIES NATURAL PROD, V65

Ferguson L, 2019, CHEMMEDCHEM, V14, P1735, DOI 10.1002/cmdc.201900217

Ferreira D, 2016, J PROTEOMICS, V145, P153, DOI 10.1016/j.jprot.2016.04.006

Ferreras JA, 2005, NAT CHEM BIOL, V1, P29, DOI 10.1038/nchembio706

Ferreras JA, 2011, BIOORG MED CHEM LETT, V21, P6533, DOI 10.1016/j.bmcl.2011.08.052

Fisher, 2016, MED, V6, P1

Foss MH, 2016, ACS INFECT DIS, V2, P500, DOI 10.1021/acsinfecdis.6b00052

Gan YL, 2015, MED HYPOTHESES, V84, P477, DOI 10.1016/j.mehy.2015.01.046

Gao W, 2015, ANTIMICROB AGENTS CH, V59, P880, DOI 10.1128/AAC.04054-14

Garrett CK, 2015, PLOS ONE, V10, DOI 10.1371/journal.pone.0135447

Garrison A. T., 2015, ANGEW CHEM INT EDIT, V127, P15032, DOI DOI 10.1002/ANGE.201508155

Garzón-Posse F, 2019, EUR J ORG CHEM, V2019, P7747, DOI 10.1002/ejoc.201901257

Gavalda S, 2014, CHEM BIOL, V21, P1660, DOI 10.1016/j.chembiol.2014.10.011

Gil CM, 2022, BREATHE, V18, DOI 10.1183/20734735.0149-2021

Gokarn K., 2017, BMC COMPLEM ALTERN M, V17, P1, DOI DOI 10.1186/s12906-016-1505-2

Gokarn K., 2016, MOL BIOL+, V05, P1

Grosset Jacques H, 2013, Am J Respir Crit Care Med, V188, P608, DOI 10.1164/rccm.201304-0753OC

Grzegorzewicz AE, 2015, ACS INFECT DIS, V1, P91, DOI 10.1021/id500032q

Haagsma AC, 2010, FEMS MICROBIOL LETT, V313, P68, DOI 10.1111/j.1574-6968.2010.02123.x

Hameed Saif, 2015, Open Microbiol J, V9, P91, DOI [10.2174/1874285801509010091, 10.2174/1874285801509010091]

He JL, 2011, ACTA PHARM SIN B, V1, P8, DOI 10.1016/j.apsb.2011.04.008

Hider RC, 2010, NAT PROD REP, V27, P637, DOI 10.1039/b906679a

Hoagland DT, 2016, ADV DRUG DELIVER REV, V102, P55, DOI 10.1016/j.addr.2016.04.026

Hu ZJ, 2020, ACS INFECT DIS, V6, P25, DOI 10.1021/acsinfecdis.9b00286

Huang ZG, 2022, BIOORG MED CHEM LETT, V71, DOI 10.1016/j.bmcl.2022.128824

Hunter RL, 2016, TUBERCULOSIS, V97, P8, DOI 10.1016/j.tube.2015.11.010

Imran M, 2022, J INFECT PUBLIC HEAL, V15, P1097, DOI 10.1016/j.jiph.2022.08.016

Iqbal IK, 2018, PATHOGENS, V7, DOI 10.3390/pathogens7010024

Irfan A., 2023, PHARMACEUTICALS-BASE, V16, P1

Jaganath D, 2016, INT J TUBERC LUNG D, V20, P1436, DOI 10.5588/ijtld.16.0498

Jayaprakash, 2021, ECSOC 25 MDPI BAS SW, V8, P62

Jenkins HE, 2014, LANCET, V383, P1572, DOI 10.1016/S0140-6736(14)60195-1

Jian Y., 2020, EUR J MED CHEM, V15, P1

Johansen MD, 2020, NAT REV MICROBIOL, V18, P392, DOI 10.1038/s41579-020-0331-1

Jones CM, 2014, P NATL ACAD SCI USA, V111, P1945, DOI 10.1073/pnas.1311402111

Jones CM, 2010, J BACTERIOL, V192, P6411, DOI 10.1128/JB.00986-10

Juarez-Hernandez, 2013, SIDEROPHORE MEDIATED, P65

Kashyap A, 2018, TUBERCULOSIS, V113, P43, DOI 10.1016/j.tube.2018.09.001

Kawai K, 2014, EPIDEMIOL INFECT, V142, P1505, DOI 10.1017/S0950268813002495

Kazmaier U, 2021, MAR DRUGS, V19, DOI 10.3390/md19080446

Keam SJ, 2019, DRUGS, V79, P1797, DOI 10.1007/s40265-019-01207-9

Kem MP, 2015, BIOMETALS, V28, P445, DOI 10.1007/s10534-015-9827-y

Kiazyk S, 2017, Can Commun Dis Rep, V43, P62, DOI 10.14745/ccdr.v43i34a01

Kim TS, 2017, ANTIMICROB AGENTS CH, V61, DOI [10.1128/AAC.02752-16, 10.1128/aac.02752-16]

Koul A, 2011, NATURE, V469, P483, DOI 10.1038/nature09657

Krajczyk A, 2016, BIOORGAN MED CHEM, V24, P3133, DOI 10.1016/j.bmc.2016.05.037

Kumar GS, 2023, DISTRIB PARALLEL DAT, V41, P233, DOI 10.1007/s10619-023-07423-3

Kumar G, 2023, BIOORGAN MED CHEM, V81, DOI 10.1016/j.bmc.2023.117212

Kumar G, 2018, EUR J MED CHEM, V156, P871, DOI 10.1016/j.ejmech.2018.07.047

Kumar N, 2020, J APPL MICROBIOL, V129, P1733, DOI 10.1111/jam.14728

Kumar N, 2021, MOL ASPECTS MED, V81, DOI 10.1016/j.mam.2021.101002

Kurth C, 2016, ORG BIOMOL CHEM, V14, P8212, DOI [10.1039/c6ob01400c, 10.1039/C6OB01400C]

Kurthkoti K, 2015, MOL MICROBIOL, V98, P864, DOI 10.1111/mmi.13166

Kwofie SK, 2019, MOLECULES, V24, DOI 10.3390/molecules24122299

Laborde J, 2016, CHEMISTRYSELECT, V1, P172, DOI 10.1002/slct.201600040

Lange C, 2018, J INTERN MED, V284, P163, DOI 10.1111/joim.12780

Lange C, 2019, LANCET, V394, P953, DOI 10.1016/S0140-6736(19)31882-3

Larsson Lars-Olof, 2016, Int J Mycobacteriol, V5 Suppl 1, pS29, DOI [10.1016/j.ijmyco.2016.09.032, 10.1016/j.ijmyco.2016.09.032]

Leach KL, 2011, ANN NY ACAD SCI, V1222, P49, DOI 10.1111/j.1749-6632.2011.05962.x

Lechartier B, 2015, ANTIMICROB AGENTS CH, V59, P4457, DOI 10.1128/AAC.00395-15

Lee H, 2016, J IND MICROBIOL BIOT, V43, P205, DOI 10.1007/s10295-015-1709-3

Lee RE, 2014, NAT MED, V20, P152, DOI 10.1038/nm.3458

Lee SFK, 2019, CURR OPIN PULM MED, V25, P271, DOI 10.1097/MCP.0000000000000570

Lefebvre C., 2018, SCI REP-UK, V8, P1

Lefebvre C, 2020, SCI REP-UK, V10, DOI 10.1038/s41598-020-58967-8

Leon-Sicairos N, 2015, BIOMED RES INT, V2015, DOI 10.1155/2015/476534

Li KH, 2016, BIOMETALS, V29, P377, DOI 10.1007/s10534-016-9935-3

Li W, 2016, ANTIMICROB AGENTS CH, V60, P5198, DOI 10.1128/AAC.00826-16

Li W, 2013, CELL PHYSIOL BIOCHEM, V31, P1, DOI 10.1159/000343343

Lienhardt C, 2012, J INFECT DIS, V205, pS241, DOI 10.1093/infdis/jis034

Liu C, 2015, NAT COMMUN, V6, DOI 10.1038/ncomms7205

Liu H., 2023, ANTIMICROB AGENTS CH, V67, P1

Liu JY, 2017, ACS INFECT DIS, V3, P72, DOI 10.1021/acsinfecdis.6b00158

Liu Z, 2015, J ORG CHEM, V80, P6545, DOI 10.1021/acs.joc.5b00455

Locher CP, 2015, ANTIMICROB AGENTS CH, V59, P1455, DOI 10.1128/AAC.04347-14

Lociuro S., 2020, PRESS RELEASE BIOVER, P1

Lovewell RR, 2016, CURR OPIN MICROBIOL, V29, P30, DOI 10.1016/j.mib.2015.10.002

Lu P, 2014, BBA-BIOENERGETICS, V1837, P1208, DOI 10.1016/j.bbabio.2014.01.022

Lu Y, 2011, ANTIMICROB AGENTS CH, V55, P5185, DOI 10.1128/AAC.00699-11

Lun S, 2013, ANTIMICROB AGENTS CH, V57, P5138, DOI 10.1128/AAC.00918-13

Luo JJ, 2018, ANTIMICROB AGENTS CH, V62, DOI 10.1128/AAC.00072-18

Luthra S, 2018, FRONT MICROBIOL, V9, DOI 10.3389/fmicb.2018.02179

Madak JT, 2015, CURR TOP MED CHEM, V15, P745, DOI 10.2174/1568026615666150302105622

Madhura DB, 2016, MEDCHEMCOMM, V7, P114, DOI 10.1039/c5md00349k

Malhotra H, 2017, FRONT CELL INFECT MI, V7, DOI 10.3389/fcimb.2017.00245

Mandal S, 2019, MICROBIOL-SGM, V165, P492, DOI 10.1099/mic.0.000760

Manjunatha UH, 2015, BIOORGAN MED CHEM, V23, P5087, DOI 10.1016/j.bmc.2014.12.031

Manos-Turvey A, 2012, ORG BIOMOL CHEM, V10, P9223, DOI 10.1039/c2ob26736e

Mayanja-Kizza H, 2005, J INFECT DIS, V191, P856, DOI 10.1086/427995

McCoy LS, 2011, WIRES RNA, V2, P209, DOI 10.1002/wrna.60

McLean KJ, 2017, DRUG DISCOV TODAY, V22, P566, DOI 10.1016/j.drudis.2016.11.004

McMahon MD, 2012, J BACTERIOL, V194, P2809, DOI 10.1128/JB.00088-12

Meena LS, 2010, FEBS J, V277, P2416, DOI 10.1111/j.1742-4658.2010.07666.x

Melly G, 2019, MICROORGANISMS, V7, DOI 10.3390/microorganisms7030070

Miller MJ, 2011, J AM CHEM SOC, V133, P2076, DOI 10.1021/ja109665t

Momin MAM, 2018, INT J PHARMACEUT, V550, P398, DOI 10.1016/j.ijpharm.2018.08.061

Monfeli Ryan R., 2007, Infectious Disorders - Drug Targets, V7, P213, DOI 10.2174/187152607782110031

Mori M, 2022, PHARMACEUTICALS-BASE, V15, DOI 10.3390/ph15080992

Mori M, 2021, PHARMACEUTICALS-BASE, V14, DOI 10.3390/ph14020155

Mori M, 2020, J MED CHEM, V63, P7066, DOI 10.1021/acs.jmedchem.0c00373

Nandi S., 2023, Important Targets and Inhibitors of Mycobacterium tuberculosis. Tuberculosis: Integrated Studies for a Complex Disease, P429, DOI [10.1007/978-3-031-15955-8_21, DOI 10.1007/978-3-031-15955-8_21]

Narang R, 2019, EUR J MED CHEM, V182, DOI 10.1016/j.ejmech.2019.111644

Nasiruddin Mohammad, 2017, Tuberc Res Treat, V2017, P4920209, DOI [10.1155/2017/4920209, 10.1155/2017/4920209]

Nelson KM, 2015, J MED CHEM, V58, P5459, DOI 10.1021/acs.jmedchem.5b00391

Neyrolles O, 2015, IMMUNOL REV, V264, P249, DOI 10.1111/imr.12265

Nguta JM, 2015, INT J MYCOBACT, V4, P165, DOI 10.1016/j.ijmyco.2015.05.004

Ouellet H, 2011, TRENDS MICROBIOL, V19, P530, DOI 10.1016/j.tim.2011.07.009

Palencia A, 2016, ANTIMICROB AGENTS CH, V60, P6271, DOI 10.1128/AAC.01339-16

Parish T, 2014, MICROBIOL SPECTR, V2, DOI 10.1128/microbiolspec.MGM2-0010-2013

Park Y, 2019, ANTIMICROB AGENTS CH, V63, DOI 10.1128/AAC.01006-19

Patel K, 2018, EUR J MED CHEM, V157, P783, DOI 10.1016/j.ejmech.2018.08.030

Patil K, 2018, BIOMED PHARMACOTHER, V99, P735, DOI 10.1016/j.biopha.2018.01.115

Pawlowski A, 2012, PLOS PATHOG, V8, DOI 10.1371/journal.ppat.1002464

Pethe K, 2013, NAT MED, V19, P1157, DOI 10.1038/nm.3262

Pfaeffle HOI, 2021, PULM PHARMACOL THER, V70, DOI 10.1016/j.pupt.2021.102058

Pini E, 2018, MOLECULES, V23, DOI 10.3390/molecules23071506

Piton J, 2017, DRUG DISCOV TODAY, V22, P526, DOI 10.1016/j.drudis.2016.09.014

Piton J, 2010, PLOS ONE, V5, DOI 10.1371/journal.pone.0012245

Pushkaran AC, 2019, CHALL ADV COMPUT CHE, V27, P307, DOI 10.1007/978-3-030-05282-9_10

Quémard A, 2016, TRENDS MICROBIOL, V24, P725, DOI 10.1016/j.tim.2016.04.009

Rayasam GV, 2014, EXPERT OPIN THER TAR, V18, P247, DOI 10.1517/14728222.2014.859677

Reddy PV, 2013, J INFECT DIS, V208, P1255, DOI 10.1093/infdis/jit250

Reuter A, 2019, LANCET, V394, P967, DOI 10.1016/S0140-6736(19)32045-8

Ribeiro M, 2019, ENVIRON CHEM LETT, V17, P1485, DOI 10.1007/s10311-019-00887-9

Riccardi G, 2014, J ANTIBIOT, V67, P655, DOI 10.1038/ja.2014.109

Riccardi G, 2013, APPL MICROBIOL BIOT, V97, P8841, DOI 10.1007/s00253-013-5218-x

Rodriguez G. M., 2015, MOL GENETICS MYCOBAC, P377

Rohilla A, 2017, SCI REP-UK, V7, DOI 10.1038/s41598-017-04748-9

Rozman K, 2017, DRUG DISCOV TODAY, V22, P492, DOI 10.1016/j.drudis.2016.09.009

Ryan NJ, 2014, DRUGS, V74, P1041, DOI 10.1007/s40265-014-0241-5

Sah S, 2015, POLNOHOSPODARSTVO, V61, P97, DOI [10.1515/agri-2015-0015, 10.1515/agri-2015-0015, DOI 10.1515/AGRI-2015-0015]

Sandhu P, 2017, J INORG BIOCHEM, V170, P75, DOI 10.1016/j.jinorgbio.2017.02.013

Sarathy JP, 2019, ANTIMICROB AGENTS CH, V63, DOI 10.1128/AAC.01191-19

Saxena AK, 2013, INT J ANTIMICROB AG, V42, P27, DOI 10.1016/j.ijantimicag.2013.03.007

Saxena AK, 2019, CURR TOP MED CHEM, V19, P337, DOI 10.2174/1568026619666190219105722

Scherr N, 2018, NAT COMMUN, V9, DOI 10.1038/s41467-018-07804-8

Seidi K, 2013, J MED HYPOTHESES IDE, V7, P69, DOI 10.1016/j.jmhi.2013.04.002

Shirude PS, 2013, J MED CHEM, V56, P9701, DOI 10.1021/jm401382v

Shyam M, 2022, J MED CHEM, V65, P234, DOI 10.1021/acs.jmedchem.1c01349

Shyam M, 2021, J MED CHEM, V64, P71, DOI 10.1021/acs.jmedchem.0c01176

Siegrist MS, 2014, MBIO, V5, DOI 10.1128/mBio.01073-14

Singh VK, 2014, TUBERCULOSIS, V94, P252, DOI 10.1016/j.tube.2014.02.001

Sirgel FA, 2013, PLOS ONE, V8, DOI 10.1371/journal.pone.0059414

Sritharan M, 2016, J BACTERIOL, V198, P2399, DOI 10.1128/JB.00359-16

Srivastava A, 2011, CURR OPIN MICROBIOL, V14, P532, DOI 10.1016/j.mib.2011.07.030

Stephanie F, 2022, LIFE-BASEL, V12, DOI 10.3390/life12111774

Stokes SS, 2020, ACS INFECT DIS, V6, P1323, DOI 10.1021/acsinfecdis.0c00025

Sun CL, 2020, J NAT PROD, V83, P1646, DOI 10.1021/acs.jnatprod.0c00151

Sutherland HS, 2019, BIOORGAN MED CHEM, V27, P1292, DOI 10.1016/j.bmc.2019.02.026

Swayambhu G, 2021, CURR OPIN BIOTECH, V69, P242, DOI 10.1016/j.copbio.2021.01.021

Tarapdar A, 2018, BEILSTEIN J ORG CHEM, V14, P2646, DOI 10.3762/bjoc.14.242

Thee S, 2016, TUBERCULOSIS, V97, P126, DOI 10.1016/j.tube.2015.09.007

Tiberi S., 2018, LANCET INFECT DIS, V18, P1

Tufariello JM, 2016, P NATL ACAD SCI USA, V113, pE348, DOI 10.1073/pnas.1523321113

Uhía I, 2012, ENV MICROBIOL REP, V4, P168, DOI 10.1111/j.1758-2229.2011.00314.x

Umare MD, 2021, CHEMMEDCHEM, V16, P3136, DOI 10.1002/cmdc.202100359

Unissa AN, 2016, INFECT GENET EVOL, V45, P474, DOI 10.1016/j.meegid.2016.09.004

Urban M, 2021, EUR J MED CHEM, V212, DOI 10.1016/j.ejmech.2020.113139

van Ingen J, 2012, ANTIMICROB AGENTS CH, V56, P6324, DOI 10.1128/AAC.01505-12

Varela C, 2012, CHEM BIOL, V19, P498, DOI 10.1016/j.chembiol.2012.03.006

Vasan M, 2010, CHEMMEDCHEM, V5, P2079, DOI 10.1002/cmdc.201000275

Vasudevan D, 2013, J BIOL CHEM, V288, P30883, DOI 10.1074/jbc.M113.493767

Vestergaard M, 2022, J GLOB ANTIMICROB RE, V29, P29, DOI 10.1016/j.jgar.2022.01.026

Vilchèze C, 2020, APPL SCI-BASEL, V10, DOI 10.3390/app10072278

Vjecha MJ, 2018, NAT REV DRUG DISCOV, V17, P607, DOI 10.1038/nrd.2018.28

W. H. Organization, 2020, CONSOLIDATED GUIDELI

Wagh S, 2021, ANTIMICROB AGENTS CH, V65, DOI 10.1128/AAC.01744-20

Wallis RS, 2009, CLIN INFECT DIS, V48, P1429, DOI 10.1086/598504

Wang QF, 2018, ANTIMICROB AGENTS CH, V62, DOI 10.1128/AAC.02149-17

Ward SK, 2010, MOL MICROBIOL, V77, P1096, DOI 10.1111/j.1365-2958.2010.07273.x

Wellington S, 2018, ACS INFECT DIS, V4, P696, DOI 10.1021/acsinfecdis.7b00255

Wells RM, 2013, PLOS PATHOG, V9, DOI 10.1371/journal.ppat.1003120

Weyer K, 2013, EUR RESPIR J, V42, P252, DOI 10.1183/09031936.00157212

Wilson BR, 2016, TRENDS MOL MED, V22, P1077, DOI 10.1016/j.molmed.2016.10.005

Wohlkönig A, 2017, BIOCHEM BIOPH RES CO, V487, P403, DOI 10.1016/j.bbrc.2017.04.074

Xu Y., 2019, ANTIMICROB AGENTS CH, V63, P1

Yang L, 2022, DRUG DISCOV TODAY, V27, P326, DOI 10.1016/j.drudis.2021.09.003

Yano T, 2011, J BIOL CHEM, V286, P10276, DOI 10.1074/jbc.M110.200501

YAO Rong, 2022, MICROBIOL SPECTR, V10, P1, DOI [10.1128/SPEC TRUM.02477-21/FORMAT/EPUB, DOI 10.1128/SPECTRUM.02477-21/FORMAT/EPUB]

Yip PCW, 2013, INT J ANTIMICROB AG, V42, P96, DOI 10.1016/j.ijantimicag.2013.03.002

Zhang L, 2022, NAT COMMUN, V13, DOI 10.1038/s41467-022-29873-6

Zheng HQ, 2020, FUTURE MED CHEM, V12, P457, DOI 10.4155/fmc-2019-0263

Zhou B, 2020, J NAT PROD, V83, P657, DOI 10.1021/acs.jnatprod.9b01095

Zhou S, 2021, ELIFE, V10, DOI [10.7554/eLife.69418, 10.7554/eLife.69418.sa0, 10.7554/eLife.69418.sa1, 10.7554/eLife.69418.sa2]

Zumla A, 2015, LANCET RESP MED, V3, P220, DOI 10.1016/S2213-2600(15)00063-6

NR 246

TC 11

Z9 14

U1 2

U2 13

PU ROYAL SOC CHEMISTRY

PI CAMBRIDGE

PA THOMAS GRAHAM HOUSE, SCIENCE PARK, MILTON RD, CAMBRIDGE CB4 0WF, CAMBS,

ENGLAND

EI 2632-8682

J9 RSC MED CHEM

JI RSC Med. Chem.

PD OCT 18

PY 2023

VL 14

IS 10

BP 1885

EP 1913

DI 10.1039/d3md00201b

EA SEP 2023

PG 29

WC Biochemistry & Molecular Biology; Chemistry, Medicinal

WE Science Citation Index Expanded (SCI-EXPANDED)

SC Biochemistry & Molecular Biology; Pharmacology & Pharmacy

GA U3LJ0

UT WOS:001062480400001

OA Green Submitted

DA 2026-05-07

ER

PT J

AU van Rijn, SP

Zuur, MA

Anthony, R

Wilffert, B

van Altena, R

Akkerman, OW

de Lange, WCM

van der Werf, TS

Kosterink, JGW

Alffenaar, JWC

AF van Rijn, Sander P.

Zuur, Marlanka A.

Anthony, Richard

Wilffert, Bob

van Altena, Richard

Akkerman, Onno W.

de Lange, Wiel C. M.

van der Werf, Tjip S.

Kosterink, Jos G. W.

Alffenaar, Jan-Willem C.

TI Evaluation of Carbapenems for Treatment of Multi- and Extensively

Drug-Resistant Mycobacterium tuberculosis

SO ANTIMICROBIAL AGENTS AND CHEMOTHERAPY

LA English

DT Article

DE carbapenems; clinical; ertapenem; imipenem; in vitro; in vivo;

meropenem; tuberculosis

ID MEROPENEM-CLAVULANIC ACID; BETA-LACTAM ANTIBIOTICS; IN-VITRO ACTIVITY;

NONCLASSICAL TRANSPEPTIDASES; CONTAINING REGIMENS; INVITRO ACTIVITY;

CELL-WALL; L,D-TRANSPEPTIDASE; INACTIVATION; COMBINATION

AB Multi-and extensively drug-resistant tuberculosis (M/XDR-TB) has become an increasing threat not only in countries where the TB burden is high but also in affluent regions, due to increased international travel and globalization. Carbapenems are earmarked as potentially active drugs for the treatment of Mycobacterium tuberculosis. To better understand the potential of carbapenems for the treatment of M/XDR-TB, the aim of this review was to evaluate the literature on currently available in vitro, in vivo, and clinical data on carbapenems in the treatment of M. tuberculosis and to detect knowledge gaps, in order to target future research. In February 2018, a systematic literature search of PubMed and Web of Science was performed. Overall, the results of the studies identified in this review, which used a variety of carbapenem susceptibility tests on clinical and laboratory strains of M. tuberculosis, are consistent. In vitro, the activity of carbapenems against M. tuberculosis is increased when used in combination with clavulanate, a BLaC inhibitor. However, clavulanate is not commercially available alone, and therefore, it is impossible in practice to prescribe carbapenems in combination with clavulanate at this time. Few in vivo studies have been performed, including one prospective, two observational, and seven retrospective clinical studies to assess the effectiveness, safety, and tolerability of three different carbapenems (imipenem, meropenem, and ertapenem). We found no clear evidence at the present time to select one particular carbapenem among the different candidate compounds to design an effective M/XDR-TB regimen. Therefore, more clinical evidence and dose optimization substantiated by hollow-fiber infection studies are needed to support repurposing carbapenems for the treatment of M/XDR-TB.

C1 [van Rijn, Sander P.; Zuur, Marlanka A.; Wilffert, Bob; Kosterink, Jos G. W.; Alffenaar, Jan-Willem C.] Univ Groningen, Univ Med Ctr Groningen, Dept Clin Pharm & Pharmacol, Groningen, Netherlands.

[Anthony, Richard] Natl Inst Publ Hlth & Environm, Natl Mycobacteria Reference Lab, Bilthoven, Netherlands.

[Wilffert, Bob; Kosterink, Jos G. W.] Univ Groningen, Groningen Res Inst Pharm, Unit Pharmacotherapy Epidemiol & Pharmacoecon, Groningen, Netherlands.

[van Altena, Richard; Akkerman, Onno W.; de Lange, Wiel C. M.] Univ Groningen, Univ Med Ctr Groningen, TB Ctr Beatrixoord, Haren, Netherlands.

[van Altena, Richard; Akkerman, Onno W.; de Lange, Wiel C. M.; van der Werf, Tjip S.] Univ Groningen, Univ Med Ctr Groningen, Dept Pulm Dis & TB, Groningen, Netherlands.

[van der Werf, Tjip S.] Univ Groningen, Univ Med Ctr Groningen, Dept Internal Med, Groningen, Netherlands.

C3 University of Groningen; Netherlands National Institute for Public

Health & the Environment; University of Groningen; University of

Groningen; University of Groningen; University of Groningen

RP Alffenaar, JWC (corresponding author), Univ Groningen, Univ Med Ctr Groningen, Dept Clin Pharm & Pharmacol, Groningen, Netherlands.

EM j.w.c.alffenaar@umcg.nl

RI ; Anthony, Richard/ACG-9717-2022; van der Werf, Tjip S/J-1053-2014;

Alffenaar, Jan-Willem/AFV-0554-2022; van Rijn, S/N-9775-2016

OI Akkerman, Onno/0000-0001-5638-9260; Kosterink, Jos/0000-0001-8358-765X;

Anthony, Richard/0000-0002-0550-5273; van der Werf, Tjip

S/0000-0002-4824-1642;

CR Alffenaar JW, 2017, ANTIMICROB AGENTS CH, V61

Alsaad N, 2014, EUR RESPIR J, V43, P884, DOI 10.1183/09031936.00113713

[Anonymous], 2016, Treatment Guidelines for Drug-Resistant Tuberculosis

[Anonymous], 2017, Global tuberculosis report 2017

Arbex MA, 2016, REV PORT PNEUMOL, V22, P337, DOI 10.1016/j.rppnen.2016.06.006

Basta LAB, 2015, J BIOL CHEM, V290, P25670, DOI 10.1074/jbc.M115.660753

Cavaleri M, 2015, CLIN INFECT DIS, V61, pS1, DOI 10.1093/cid/civ484

Cavanaugh JS, 2017, J ANTIMICROB CHEMOTH, V72, P1678, DOI 10.1093/jac/dkx022

CHAMBERS HF, 1995, ANTIMICROB AGENTS CH, V39, P2620, DOI 10.1128/AAC.39.12.2620

Chambers HF, 2005, ANTIMICROB AGENTS CH, V49, P2816, DOI 10.1128/AAC.49.7.2816-2821.2005

Cohen KA, 2016, EBIOMEDICINE, V9, P170, DOI 10.1016/j.ebiom.2016.05.041

Cordillot M, 2013, ANTIMICROB AGENTS CH, V57, P5940, DOI 10.1128/AAC.01663-13

Craig WA, 2003, INFECT DIS CLIN N AM, V17, P479, DOI 10.1016/S0891-5520(03)00065-5

CYNAMON MH, 1983, ANTIMICROB AGENTS CH, V24, P429, DOI 10.1128/AAC.24.3.429

De Lorenzo S, 2013, EUR RESPIR J, V41, P1386, DOI 10.1183/09031936.00124312

Deshpande D, 2018, ANTIMICROB AGENTS CH, V62, DOI 10.1128/AAC.02232-17

Deshpande D, 2017, SCI ADV, V3, DOI 10.1126/sciadv.1701102

Deshpande D, 2016, CLIN INFECT DIS, V63, pS95, DOI 10.1093/cid/ciw474

Dhar N, 2015, ANTIMICROB AGENTS CH, V59, P1308, DOI 10.1128/AAC.03461-14

Drusano GL, 2010, MBIO, V1, DOI 10.1128/mBio.00139-10

Dubée V, 2012, ANTIMICROB AGENTS CH, V56, P4189, DOI 10.1128/AAC.00665-12

Dubée V, 2012, ANTIMICROB AGENTS CH, V56, P3409, DOI 10.1128/AAC.06398-11

England K, 2012, ANTIMICROB AGENTS CH, V56, P3384, DOI 10.1128/AAC.05690-11

Erdemli SB, 2012, STRUCTURE, V20, P2103, DOI 10.1016/j.str.2012.09.016

European Medicines Agency, 2016, EMACHMPSAWP472902015

Falzon D, 2017, EUR RESPIR J, V49, DOI 10.1183/13993003.02308-2016

Forsman LD, 2015, ANTIMICROB AGENTS CH, V59, P3630, DOI 10.1128/AAC.00171-15

Gonzalo X, 2013, J ANTIMICROB CHEMOTH, V68, P366, DOI 10.1093/jac/dks395

Gumbo T, 2004, J INFECT DIS, V190, P1642, DOI 10.1086/424849

Gurumurthy M, 2017, J ANTIMICROB CHEMOTH, V72, P2012, DOI 10.1093/jac/dkx081

Horita Y, 2014, ANTIMICROB AGENTS CH, V58, P7010, DOI 10.1128/AAC.03539-14

Hugonnet JE, 2007, BIOCHEMISTRY-US, V46, P11998, DOI 10.1021/bi701506h

Hugonnet JE, 2009, SCIENCE, V323, P1215, DOI 10.1126/science.1167498

Kaushik A, 2017, J ANTIMICROB CHEMOTH, V72, P2320, DOI 10.1093/jac/dkx152

Kaushik A, 2015, ANTIMICROB AGENTS CH, V59, P6561, DOI 10.1128/AAC.01158-15

Khan FA, 2017, EUR RESPIR J, V50, DOI 10.1183/13993003.00061-2017

Kim HS, 2013, ACTA CRYSTALLOGR D, V69, P420, DOI 10.1107/S0907444912048998

Kumar P, 2017, NAT CHEM BIOL, V13, P54, DOI [10.1038/nchembio.2237, 10.1038/NCHEMBIO.2237]

Kumar P, 2012, MOL MICROBIOL, V86, P367, DOI 10.1111/j.1365-2958.2012.08199.x

Kurz SG, 2013, ANTIMICROB AGENTS CH, V57, P6085, DOI 10.1128/AAC.01253-13

Lavollay M, 2008, J BACTERIOL, V190, P4360, DOI 10.1128/JB.00239-08

Lecoq L, 2013, ACS CHEM BIOL, V8, P1140, DOI 10.1021/cb4001603

Magis-Escurra C, 2018, LANCET RESP MED, V6, pE11, DOI 10.1016/S2213-2600(18)30044-4

Moher D, 2009, J CLIN EPIDEMIOL, V62, P1006, DOI 10.1016/j.jclinepi.2009.06.005

Nicolau DP, 2008, CLIN INFECT DIS, V47, pS32, DOI 10.1086/590064

Palmero D, 2014, ARCH BRONCONEUMOL, V51, pe49, DOI [10.1016/j.arbr.2015.07.003, DOI 10.1016/J.ARBR.2015.07.003]

Payen MC, 2018, INT J TUBERC LUNG D, V22, P34, DOI 10.5588/ijtld.17.0352

Payen MC, 2012, INT J TUBERC LUNG D, V16, P558, DOI 10.5588/ijtld.11.0414

Rullas J, 2015, ANTIMICROB AGENTS CH, V59, P4997, DOI 10.1128/AAC.01063-15

Sala C, 2010, ANTIMICROB AGENTS CH, V54, P4150, DOI 10.1128/AAC.00821-10

Schoonmaker MK, 2014, J BACTERIOL, V196, P1394, DOI 10.1128/JB.01396-13

Solapure S, 2013, ANTIMICROB AGENTS CH, V57, P2506, DOI 10.1128/AAC.00023-13

Srivastava S, 2016, CLIN INFECT DIS, V63, pS102, DOI 10.1093/cid/ciw483

Srivastava S, 2016, ANTIMICROB AGENTS CH, V60, P3193, DOI 10.1128/AAC.02924-15

Nguyen TVA, 2018, CLIN INFECT DIS, V66, P1625, DOI 10.1093/cid/cix992

Tiberi S, 2016, CLIN INFECT DIS, V62, P1188, DOI 10.1093/cid/ciw088

Tiberi S, 2016, EUR RESPIR J, V47, P1235, DOI 10.1183/13993003.02146-2015

Tiberi S, 2016, EUR RESPIR J, V47, P333, DOI 10.1183/13993003.01278-2015

Triboulet S, 2013, PLOS ONE, V8, DOI 10.1371/journal.pone.0067831

Triboulet S, 2011, J BIOL CHEM, V286, P22777, DOI 10.1074/jbc.M111.239988

van der Paardt AF, 2015, EUR RESPIR J, V46, P444, DOI 10.1183/09031936.00147014

van Rijn SP, 2017, ANTIMICROB AGENTS CH, V61, DOI [10.1128/AAC.01783-16, 10.1128/aac.01783-16]

van Rijn SP, 2016, EUR RESPIR J, V47, P1229, DOI 10.1183/13993003.01654-2015

Veziris N, 2011, ANTIMICROB AGENTS CH, V55, P2597, DOI 10.1128/AAC.01824-10

WATT B, 1992, TUBERCLE LUNG DIS, V73, P134, DOI 10.1016/0962-8479(92)90145-A

Zhang D, 2016, ANTIMICROB AGENTS CH, V60, P393, DOI 10.1128/AAC.01035-15

Zuur MA, 2018, ANTIMICROB AGENTS CH, V62, DOI 10.1128/AAC.02250-17

NR 67

TC 28

Z9 35

U1 0

U2 11

PU AMER SOC MICROBIOLOGY

PI WASHINGTON

PA 1752 N ST NW, WASHINGTON, DC 20036-2904 USA

SN 0066-4804

EI 1098-6596

J9 ANTIMICROB AGENTS CH

JI Antimicrob. Agents Chemother.

PD FEB

PY 2019

VL 63

IS 2

AR e01489-18

DI 10.1128/AAC.01489-18

PG 13

WC Microbiology; Pharmacology & Pharmacy

WE Science Citation Index Expanded (SCI-EXPANDED)

SC Microbiology; Pharmacology & Pharmacy

GA HJ3YN

UT WOS:000457110200050

OA Green Submitted, Bronze

DA 2026-05-07

ER

PT J

AU Shaikh, MS

Kanhed, AM

Chandrasekaran, B

Palkar, MB

Agrawar, N

Lherbet, C

Hampannavar, GA

Karpoormath, R

AF Shaikh, Mahamadhanif S.

Kanhed, Ashish M.

Chandrasekaran, Balakumar

Palkar, Mahesh B.

Agrawar, Nikhil

Lherbet, Christian

Hampannavar, Girish A.

Karpoormath, Rajshekhar

TI Discovery of novel N-methyl carbazole tethered rhodanine

derivatives as direct inhibitors of Mycobacterium tuberculosis

InhA

SO BIOORGANIC & MEDICINAL CHEMISTRY LETTERS

LA English

DT Article

DE Tuberculosis; InhA inhibitor; Carbazole; Hybridization; GLIDE; Molecular

dynamics

ID DESIGN; SCAFFOLD; DRUGS

AB InhA (Enoyl-ACP reductase) plays a crucial role in the biosynthetic pathway of cell wall synthesis in Mycobacterium tuberculosis (Mtb). Isoniazid (INH) is an important first-line drug, which inhibits InhA. The rapid increase in resistance to INH and currently marketed drugs as well as emergence of MDR-TB and XDR-TB has complicated the diagnosis and treatment of Mtb with ever increasing threat to human kind. Herein, we report novel N-methyl carbazole derivatives as potential anti-TB compounds acting directly via InhA inhibition. All the synthesized final compounds were screened against Mtb virulent cell line H(37)Rv and investigated the InhA enzyme inhibition. Interestingly, compound 9e displayed promising inhibition (91%) at 50 mu M concentration and IC50 of 2.82 mu M against InhA. To understand the ligand receptor interaction between compound 9e and InhA, molecular docking and molecular dynamics experiments were performed. The computational results were in agreement with the observed experimental data. Further, the cytotoxicity studies on mammalian cells revealed that all the compounds were safe.

C1 [Shaikh, Mahamadhanif S.; Kanhed, Ashish M.; Chandrasekaran, Balakumar; Palkar, Mahesh B.; Agrawar, Nikhil; Hampannavar, Girish A.; Karpoormath, Rajshekhar] Univ KwaZulu Natal UKZN, Coll Hlth Sci, Discipline Pharmaceut Sci, ZA-4001 Durban, South Africa.

[Lherbet, Christian] Univ Paul Sabatier Toulouse III, UMR CNRS 5068, Lab SPCMIB, 118 Route Narbonne, F-31062 Toulouse 9, France.

[Lherbet, Christian] Univ Paul Sabatier Toulouse III, CNRS, ITAV USR3505, Toulouse, France.

C3 University of Kwazulu Natal; Universite de Toulouse; Universite Toulouse

III - Paul Sabatier; Centre National de la Recherche Scientifique

(CNRS); Universite de Toulouse; Universite Toulouse III - Paul Sabatier;

Centre National de la Recherche Scientifique (CNRS); CNRS - National

Institute for Biology (INSB)

RP Karpoormath, R (corresponding author), Univ KwaZulu Natal UKZN, Coll Hlth Sci, Discipline Pharmaceut Sci, ZA-4001 Durban, South Africa.

EM karpoormath@ukzn.ac.za

RI Kanhed, Ashish/AAA-8648-2021; PALKAR, MAHESH/G-9028-2016;

Chandrasekaran, Balakumar/HPB-6900-2023; Agrawal, Nikhil/AAF-1653-2019;

Hampannavar, Girish/AAS-2746-2020; Lherbet, Christian/O-4408-2017;

Karpoormath, Rajshekhar/N-5950-2013

OI Chandrasekaran, Balakumar/0000-0003-4198-8679; Agrawal,

Nikhil/0000-0002-5365-6332; Hampannavar, Girish/0000-0002-2257-2718;

Lherbet, Christian/0000-0001-5427-5040; Karpoormath,

Rajshekhar/0000-0002-1247-5754

FU National Research Foundation (NRF), South Africa (SA) [99546]; NRF-SA

[103728, 112079]

FX Authors are grateful to Discipline of Pharmaceutical Sciences, College

of Health Sciences, University of KwaZulu-Natal (UKZN), Durban, South

Africa for providing access to necessary facilities. Author BC

gratefully acknowledge National Research Foundation (NRF), South Africa

(SA) for the research funding in the form of NRF-Innovation

Post-Doctoral Research Fellowship (Grant No. 99546). RK is also thankful

to NRF-SA for funding this project (Grant No. 103728 and 112079).

Authors also sincerely thank Centre for High Performance Computing

(CHPC), Cape Town, South Africa for computational resources. Authors

express heartfelt thanks to Mr. Dilip Jagjivan and Dr. Caryl Janse Van

Rensburg (UKZN, South Africa) for their assistance in NMR and HR-MS

experiments.

CR Agrawal N, 2018, MOL PHARMACEUT, V15, P289, DOI 10.1021/acs.molpharmaceut.7b00966

[Anonymous], 2017, SCHROD REL 2017 2 GU

Babaoglu K, 2008, J MED CHEM, V51, P2502, DOI 10.1021/jm701500e

Baell JB, 2010, J MED CHEM, V53, P2719, DOI 10.1021/jm901137j

Balakumar C, 2018, J BIOMOL STRUCT DYN, V36, P3687, DOI 10.1080/07391102.2017.1396255

Bowers KJ, 2006, ACM IEEE SC 2006 C S

Chollet A, 2018, EUR J MED CHEM, V146, P318, DOI 10.1016/j.ejmech.2018.01.047

Chollet A, 2015, EUR J MED CHEM, V101, P218, DOI 10.1016/j.ejmech.2015.06.035

Dahlin J.L., 2004, Assay Guidance Manual

Dorkhan M, 2007, VASC HEALTH RISK MAN, V3, P721

Fernandes GFD, 2017, CRIT REV ANAL CHEM, V47, P298, DOI 10.1080/10408347.2017.1281098

Duan XK, 2014, FEMS MICROBIOL LETT, V360, P87, DOI 10.1111/1574-6968.12597

Falzari K, 2005, ANTIMICROB AGENTS CH, V49, P1447, DOI 10.1128/AAC.49.4.1447-1454.2005

Hampannavar GA, 2016, ACS MED CHEM LETT, V7, P686, DOI 10.1021/acsmedchemlett.6b00088

He XY, 2011, BIOORGAN MED CHEM, V19, P6726, DOI 10.1016/j.bmc.2011.09.047

He X, 2006, J MED CHEM, V49, P6308, DOI 10.1021/jm060715y

Jenkins PA, 2001, THORAX, V56, P167

Kaminskyy D, 2017, EXPERT OPIN DRUG DIS, V12, P1233, DOI 10.1080/17460441.2017.1388370

Knölker HJ, 2002, CHEM REV, V102, P4303, DOI 10.1021/cr020059j

Kuo MR, 2003, J BIOL CHEM, V278, P20851, DOI 10.1074/jbc.M211968200

Liu HY, 2019, EUR J MED CHEM, V172, P163, DOI 10.1016/j.ejmech.2019.03.059

Matviiuk T, 2016, EUR J MED CHEM, V123, P462, DOI 10.1016/j.ejmech.2016.07.028

McFarland J, 1907, J AMER MED ASSOC, V49, P1176

Mendgen T, 2012, J MED CHEM, V55, P743, DOI 10.1021/jm201243p

Mousavi SM, 2019, ARTIF CELL NANOMED B, V47, P1132, DOI 10.1080/21691401.2019.1573824

Muhammad SA, 2017, RES CHEM INTERMEDIAT, V43, P5871, DOI 10.1007/s11164-017-2968-6

Navarrete-Vázquez G, 2007, BIOORGAN MED CHEM, V15, P5502, DOI 10.1016/j.bmc.2007.05.053

Ollinger J, 2013, PLOS ONE, V8, DOI 10.1371/journal.pone.0060531

Patpi SR, 2012, J MED CHEM, V55, P3911, DOI 10.1021/jm300125e

Shaikh MS, 2014, RSC ADV, V4, P62308, DOI 10.1039/c4ra11752b

Sink R, 2010, CURR MED CHEM, V17, P4231, DOI 10.2174/092986710793348545

Song MX, 2017, MOLECULES, V22, DOI 10.3390/molecules22060970

Suarez J, 2009, J BIOL CHEM, V284, P7017, DOI 10.1074/jbc.M808106200

Subhedar DD, 2017, EUR J MED CHEM, V125, P385, DOI 10.1016/j.ejmech.2016.09.059

Sun LP, 2019, BIOORG MED CHEM LETT, V29, P1187, DOI 10.1016/j.bmcl.2019.03.023

Timmins GS, 2004, ANTIMICROB AGENTS CH, V48, P3006, DOI 10.1128/AAC.48.8.3006-3009.2004

Tomasic T, 2009, CURR MED CHEM, V16, P1596, DOI 10.2174/092986709788186200

Tomasic T, 2012, EXPERT OPIN DRUG DIS, V7, P549, DOI 10.1517/17460441.2012.688743

TUCKERMAN M, 1992, J CHEM PHYS, V97, P1990, DOI 10.1063/1.463137

Vannelli TA, 2002, J BIOL CHEM, V277, P12824, DOI 10.1074/jbc.M110751200

Wu YC, 2019, BIOORG MED CHEM LETT, V29, P496, DOI 10.1016/j.bmcl.2018.12.012

Zelmer A, 2012, J ANTIMICROB CHEMOTH, V67, P1948, DOI 10.1093/jac/dks161

Zhang D, 2018, BIOORGAN MED CHEM, V26, P2928, DOI 10.1016/j.bmc.2018.02.043

NR 43

TC 27

Z9 32

U1 1

U2 13

PU PERGAMON-ELSEVIER SCIENCE LTD

PI OXFORD

PA THE BOULEVARD, LANGFORD LANE, KIDLINGTON, OXFORD OX5 1GB, ENGLAND

SN 0960-894X

EI 1464-3405

J9 BIOORG MED CHEM LETT

JI Bioorg. Med. Chem. Lett.

PD AUG 15

PY 2019

VL 29

IS 16

BP 2338

EP 2344

DI 10.1016/j.bmcl.2019.06.015

PG 7

WC Chemistry, Medicinal; Chemistry, Organic

WE Science Citation Index Expanded (SCI-EXPANDED); Index Chemicus (IC)

SC Pharmacology & Pharmacy; Chemistry

GA IP7NQ

UT WOS:000480235700059

OA Green Submitted

DA 2026-05-07

ER

PT J

AU Loiseau, C

Windels, EM

Gygli, SM

Jugheli, L

Maghradze, N

Brites, D

Ross, A

Goig, G

Reinhard, M

Borrell, S

Trauner, A

Dötsch, A

Aspindzelashvili, R

Denes, R

Reither, K

Beisel, C

Tukvadze, N

Avaliani, Z

Stadler, T

Gagneux, S

AF Loiseau, Chloe

Windels, Etthel M.

Gygli, Sebastian M.

Jugheli, Levan

Maghradze, Nino

Brites, Daniela

Ross, Amanda

Goig, Galo

Reinhard, Miriam

Borrell, Sonia

Trauner, Andrej

Dotsch, Anna

Aspindzelashvili, Rusudan

Denes, Rebecca

Reither, Klaus

Beisel, Christian

Tukvadze, Nestani

Avaliani, Zaza

Stadler, Tanja

Gagneux, Sebastien

TI The relative transmission fitness of multidrug-resistant

Mycobacterium tuberculosis in a drug resistance hotspot

SO NATURE COMMUNICATIONS

LA English

DT Article

ID COMPENSATORY MUTATIONS; RIFAMPIN RESISTANCE; ANTIBIOTIC-RESISTANCE;

BEIJING GENOTYPE; BIOLOGICAL COST; EVOLUTION; FRAMEWORK; EPIDEMICS;

DISCOVERY; REGIMENS

AB Geographical hotspots with high frequency of multi-drug resistant tuberculosis (MDR-TB) have been observed in several locations, such as the country of Georgia. Here, the authors analyse genomic sequences from tuberculosis bacteria isolated from Georgia to show that the transmission fitness of MDR-TB strains is heterogeneous, and highly drug-resistant and transmissible isolates contribute to the emergence and maintenance of MDR-TB hotspots.

Multidrug-resistant tuberculosis (MDR-TB) is among the most frequent causes of death due to antimicrobial resistance. Although only 3% of global TB cases are MDR, geographical hotspots with up to 40% of MDR-TB have been observed in countries of the former Soviet Union. While the quality of TB control and patient-related factors are known contributors to such hotspots, the role of the pathogen remains unclear. Here we show that in the country of Georgia, a known hotspot of MDR-TB, MDR Mycobacterium tuberculosis strains of lineage 4 (L4) transmit less than their drug-susceptible counterparts, whereas most MDR strains of L2 suffer no such defect. Our findings further indicate that the high transmission fitness of these L2 strains results from epistatic interactions between the rifampicin resistance-conferring mutation RpoB S450L, compensatory mutations in the RNA polymerase, and other pre-existing genetic features of L2/Beijing clones that circulate in Georgia. We conclude that the transmission fitness of MDR M. tuberculosis strains is heterogeneous, but can be as high as drug-susceptible forms, and that such highly drug-resistant and transmissible strains contribute to the emergence and maintenance of hotspots of MDR-TB. As these strains successfully overcome the metabolic burden of drug resistance, and given the ongoing rollout of new treatment regimens against MDR-TB, proper surveillance should be implemented to prevent these strains from acquiring resistance to the additional drugs.

C1 [Loiseau, Chloe; Gygli, Sebastian M.; Jugheli, Levan; Maghradze, Nino; Brites, Daniela; Ross, Amanda; Goig, Galo; Reinhard, Miriam; Borrell, Sonia; Trauner, Andrej; Dotsch, Anna; Reither, Klaus; Tukvadze, Nestani; Gagneux, Sebastien] Swiss Trop & Publ Hlth Inst, Allschwil, Switzerland.

[Loiseau, Chloe; Gygli, Sebastian M.; Jugheli, Levan; Maghradze, Nino; Brites, Daniela; Ross, Amanda; Goig, Galo; Reinhard, Miriam; Borrell, Sonia; Trauner, Andrej; Dotsch, Anna; Reither, Klaus; Tukvadze, Nestani; Gagneux, Sebastien] Univ Basel, Basel, Switzerland.

[Windels, Etthel M.; Denes, Rebecca; Beisel, Christian; Stadler, Tanja] Swiss Fed Inst Technol, Dept BioSyst Sci & Engn, Basel, Switzerland.

[Windels, Etthel M.; Stadler, Tanja] Swiss Inst Bioinformat, Lausanne, Switzerland.

[Jugheli, Levan; Maghradze, Nino; Aspindzelashvili, Rusudan; Tukvadze, Nestani; Avaliani, Zaza] Natl Ctr TB & Lung Dis, Tbilisi, Tbilisi, Georgia.

C3 University of Basel; Swiss Tropical & Public Health Institute;

University of Basel; Swiss Federal Institutes of Technology Domain; ETH

Zurich; Swiss Institute of Bioinformatics

RP Gagneux, S (corresponding author), Swiss Trop & Publ Hlth Inst, Allschwil, Switzerland.; Gagneux, S (corresponding author), Univ Basel, Basel, Switzerland.; Windels, EM (corresponding author), Swiss Fed Inst Technol, Dept BioSyst Sci & Engn, Basel, Switzerland.; Windels, EM (corresponding author), Swiss Inst Bioinformat, Lausanne, Switzerland.

EM etthel.windels@bsse.ethz.ch; sebastien.gagneux@swisstph.ch

RI Brites, Daniela/AGL-8378-2022; Gagneux, Sebastien/ABE-7909-2020; Goig,

Galo/ABE-3887-2020; Windels, Etthel/ADT-1072-2022; Stadler,

Tanja/J-4742-2013

OI Brites, Daniela/0000-0002-8090-2287; Reither, Klaus/0000-0001-6195-8237;

Maghradze, Nino/0000-0003-1227-1900; Gagneux,

Sebastien/0000-0001-7783-9048; Jugheli, Levan/0009-0005-7471-0149; Goig,

Galo/0000-0002-4136-6610; Windels, Etthel/0000-0003-2106-6765; Stadler,

Tanja/0000-0001-6431-535X; Avaliani, Zaza/0000-0002-8790-3282; Tukvadze,

Nestani/0000-0002-0276-1204; Ross, Amanda/0000-0001-6027-5645; Beisel,

Christian/0000-0001-5360-2193

FU FIC NIH HHS [D43 TW007124] Funding Source: Medline; National Institute

of Allergy and Infectious Diseases; John E. Fogarty International Center

for Advanced Study in the Health Sciences [D43TW007124] Funding Source:

NIH RePORTER

CR Andersson DI, 2010, NAT REV MICROBIOL, V8, P260, DOI 10.1038/nrmicro2319

Andersson DI, 1999, CURR OPIN MICROBIOL, V2, P489, DOI 10.1016/S1369-5274(99)00005-3

[Anonymous], 2018, The Global Tuberculosis Report

Antimicrobial Resistance C, 2022, LANCET

Behr MA, 2018, BMJ-BRIT MED J, V362, DOI 10.1136/bmj.k2738

Belisle J., 1998, MYCOBACTERIA PROTOCO

Billington OJ, 1999, ANTIMICROB AGENTS CH, V43, P1866, DOI 10.1128/AAC.43.8.1866

Bolger AM, 2014, BIOINFORMATICS, V30, P2114, DOI 10.1093/bioinformatics/btu170

Borrell S, 2011, CLIN MICROBIOL INFEC, V17, P815, DOI 10.1111/j.1469-0691.2011.03556.x

Borrell S, 2009, INT J TUBERC LUNG D, V13, P1456

Bouckaert R, 2019, PLOS COMPUT BIOL, V15, DOI 10.1371/journal.pcbi.1006650

Bouckaert R, 2014, PLOS COMPUT BIOL, V10, DOI 10.1371/journal.pcbi.1003537

Brandis G, 2012, MOL MICROBIOL, V85, P142, DOI 10.1111/j.1365-2958.2012.08099.x

Brown H, 2004, LANCET, V363, P951, DOI 10.1016/S0140-6736(04)15823-6

Casali N, 2014, NAT GENET, V46, P279, DOI 10.1038/ng.2878

Castro RAD, 2020, MOL BIOL EVOL, V37, P195, DOI 10.1093/molbev/msz214

Cingolani P, 2012, FLY, V6, P80, DOI 10.4161/fly.19695

Cohen T, 2004, NAT MED, V10, P1117, DOI 10.1038/nm1110

Comas I, 2012, NAT GENET, V44, P106, DOI 10.1038/ng.1038

Conradie F, 2022, NEW ENGL J MED, V387, P810, DOI 10.1056/NEJMoa2119430

de Sousa JM, 2017, PLOS BIOL, V15, DOI 10.1371/journal.pbio.2001741

de Vos M, 2013, ANTIMICROB AGENTS CH, V57, P827, DOI 10.1128/AAC.01541-12

Didelot X, 2017, MOL BIOL EVOL, V34, P997, DOI 10.1093/molbev/msw275

Dye C, 2002, SCIENCE, V295, P2042, DOI 10.1126/science.1063814

Ford CB, 2013, NAT GENET, V45, P784, DOI 10.1038/ng.2656

Gagneux S, 2006, SCIENCE, V312, P1944, DOI 10.1126/science.1124410

Gagneux S, 2018, NAT REV MICROBIOL, V16, P202, DOI 10.1038/nrmicro.2018.8

Gavryushkina A, 2014, PLOS COMPUT BIOL, V10, DOI 10.1371/journal.pcbi.1003919

Gu ZG, 2014, BIOINFORMATICS, V30, P2811, DOI 10.1093/bioinformatics/btu393

Gygli SM, 2021, NAT MED, V27, P1171, DOI 10.1038/s41591-021-01358-x

Gygli SM, 2017, FEMS MICROBIOL REV, V41, P354, DOI 10.1093/femsre/fux011

Ives AR, 2010, SYST BIOL, V59, P9, DOI 10.1093/sysbio/syp074

Kendall EA, 2015, LANCET RESP MED, V3, P963, DOI 10.1016/S2213-2600(15)00458-0

Knight GM, 2015, CLIN INFECT DIS, V61, pS147, DOI 10.1093/cid/civ579

Koboldt DC, 2012, GENOME RES, V22, P568, DOI 10.1101/gr.129684.111

Kühnert D, 2016, MOL BIOL EVOL, V33, P2102, DOI 10.1093/molbev/msw064

Leaché AD, 2015, SYST BIOL, V64, P1032, DOI 10.1093/sysbio/syv053

Lewis PO, 2001, SYST BIOL, V50, P913, DOI 10.1080/106351501753462876

Li H, 2011, BIOINFORMATICS, V27, P2987, DOI 10.1093/bioinformatics/btr509

Li H, 2010, BIOINFORMATICS, V26, P589, DOI 10.1093/bioinformatics/btp698

Li QJ, 2016, ANTIMICROB AGENTS CH, V60, P2807, DOI 10.1128/AAC.02358-15

Liu QY, 2018, EMERG MICROBES INFEC, V7, DOI 10.1038/s41426-018-0101-6

Luo ND, 2005, P NATL ACAD SCI USA, V102, P541, DOI 10.1073/pnas.0408966102

Mariam DH, 2004, ANTIMICROB AGENTS CH, V48, P1289, DOI 10.1128/AAC.48.4.1289-1294.2004

McKenna A, 2010, GENOME RES, V20, P1297, DOI 10.1101/gr.107524.110

Merker M, 2022, NAT COMMUN, V13, DOI 10.1038/s41467-022-32455-1

Merker M, 2018, ELIFE, V7, DOI [10.1101/334599, 10.7554/eLife.38200]

Nagaev I, 2001, MOL MICROBIOL, V40, P433, DOI 10.1046/j.1365-2958.2001.02389.x

O'Neill J., 2014, Tackling drug resistant infections globally:A crisis for the health and wealth of nations

Ortiz AT, 2021, NAT COMMUN, V12, DOI 10.1038/s41467-021-27616-7

Pecerska J, 2021, EPIDEMICS-NETH, V36, DOI 10.1016/j.epidem.2021.100471

Pym AS, 2002, INFECT IMMUN, V70, P4955, DOI 10.1128/IAI.70.9.4955-4960.2002

Qi Q, 2016, P ROY SOC B-BIOL SCI, V283, DOI 10.1098/rspb.2015.2452

Rad ME, 2003, EMERG INFECT DIS, V9, P838, DOI 10.3201/eid0907.020803

Rambaut A, 2018, SYST BIOL, V67, P901, DOI 10.1093/sysbio/syy032

Reynolds MG, 2000, GENETICS, V156, P1471

Schubert B, 2019, NAT MICROBIOL, V4, P328, DOI 10.1038/s41564-018-0309-1

Shitikov E, 2017, SCI REP-UK, V7, DOI 10.1038/s41598-017-10018-5

Song T, 2014, MOL MICROBIOL, V91, P1106, DOI 10.1111/mmi.12520

Stamatakis A, 2014, BIOINFORMATICS, V30, P1312, DOI 10.1093/bioinformatics/btu033

Stefan MA, 2018, ANTIMICROB AGENTS CH, V62, DOI [10.1128/AAC.00164-18, 10.1128/aac.00164-18]

Stucki D, 2016, NAT GENET, V48, P1535, DOI 10.1038/ng.3704

Stuckler D, 2008, P NATL ACAD SCI USA, V105, P13280, DOI 10.1073/pnas.0801200105

Trauer JM, 2014, J THEOR BIOL, V358, P74, DOI 10.1016/j.jtbi.2014.05.023

Trauner A, 2021, ANTIMICROB AGENTS CH, V65, DOI [10.1128/aac.00504-21, 10.1128/AAC.00504-21]

Van Rie A, 2022, LANCET INFECT DIS, V22, P1411, DOI 10.1016/S1473-3099(22)00543-6

van Soolingen D, 2000, J INFECT DIS, V182, P1788, DOI 10.1086/317598

Vogwill T, 2016, P ROY SOC B-BIOL SCI, V283, DOI 10.1098/rspb.2016.0151

Walker TM, 2022, LANCET MICROBE, V3, pE265, DOI 10.1016/S2666-5247(21)00301-3

Werngren J, 2003, J CLIN MICROBIOL, V41, P1520, DOI 10.1128/JCM.41.4.1520-1524.2003

Yu Guangchuang, 2020, Curr Protoc Bioinformatics, V69, pe96, DOI [10.1002/cpbi.96, 10.1002/cpbi.96]

NR 71

TC 50

Z9 53

U1 0

U2 16

PU NATURE PORTFOLIO

PI BERLIN

PA HEIDELBERGER PLATZ 3, BERLIN, 14197, GERMANY

EI 2041-1723

J9 NAT COMMUN

JI Nat. Commun.

PD APR 8

PY 2023

VL 14

IS 1

AR 1988

DI 10.1038/s41467-023-37719-y

PG 11

WC Multidisciplinary Sciences

WE Science Citation Index Expanded (SCI-EXPANDED)

SC Science & Technology - Other Topics

GA F2OW7

UT WOS:000980800100016

OA Green Submitted, gold

DA 2026-05-07

ER

PT J

AU Singh, A

Zhao, XL

Drlica, K

AF Singh, Amit

Zhao, Xilin

Drlica, Karl

TI Fluoroquinolone heteroresistance, antimicrobial tolerance, and lethality

enhancement

SO FRONTIERS IN CELLULAR AND INFECTION MICROBIOLOGY

LA English

DT Review

DE antimycobacterial; oxidative stress; fluoroquinolone; respiration;

N-acetyl cysteine; redox biosensor; reductive stress; resistance

ID MUTANT SELECTION WINDOW; EARLY BACTERICIDAL ACTIVITY;

MYCOBACTERIUM-TUBERCULOSIS; PULMONARY TUBERCULOSIS; DRUG-RESISTANCE;

ANTIBIOTIC TOLERANCE; DNA-DAMAGE; PHENOTYPIC HETEROGENEITY; PREVENTION

CONCENTRATION; FUNCTIONAL-ANALYSIS

AB With tuberculosis, the emergence of fluoroquinolone resistance erodes the ability of treatment to interrupt the progression of MDR-TB to XDR-TB. One way to reduce the emergence of resistance is to identify heteroresistant infections in which subpopulations of resistant mutants are likely to expand and make the infections fully resistant: treatment modification can be instituted to suppress mutant enrichment. Rapid DNA-based detection methods exploit the finding that fluoroquinolone-resistant substitutions occur largely in a few codons of DNA gyrase. A second approach for restricting the emergence of resistance involves understanding fluoroquinolone lethality through studies of antimicrobial tolerance, a condition in which bacteria fail to be killed even though their growth is blocked by lethal agents. Studies with Escherichia coli guide work with Mycobacterium tuberculosis. Lethal action, which is mechanistically distinct from blocking growth, is associated with a surge in respiration and reactive oxygen species (ROS). Mutations in carbohydrate metabolism that attenuate ROS accumulation create pan-tolerance to antimicrobials, disinfectants, and environmental stressors. These observations indicate the existence of a general death pathway with respect to stressors. M. tuberculosis displays a variation on the death pathway idea, as stress-induced ROS is generated by NADH-mediated reductive stress rather than by respiration. A third approach, which emerges from lethality studies, uses a small molecule, N-acetyl cysteine, to artificially increase respiration and additional ROS accumulation. That enhances moxifloxacin lethality with M. tuberculosis in culture, during infection of cultured macrophages, and with infection of mice. Addition of ROS stimulators to fluoroquinolone treatment of tuberculosis constitutes a new direction for suppressing the transition of MDR-TB to XDR-TB.

C1 [Singh, Amit] Indian Inst Sci, Dept Microbiol & Cell Biol, Bangalore, India.

[Singh, Amit] Indian Inst Sci, Ctr Infect Dis Res, Bangalore, India.

[Zhao, Xilin; Drlica, Karl] Rutgers State Univ, Dept Microbiol Biochem & Mol Genet, New Jersey Med Sch, Rutgers Biomed & Hlth Sci, Newark, NJ 07103 USA.

[Zhao, Xilin; Drlica, Karl] Rutgers State Univ, Publ Hlth Res Inst, New Jersey Med Sch, Rutgers Biomed & Hlth Sci, Newark, NJ 07103 USA.

[Zhao, Xilin] Xiamen Univ, Sch Publ Hlth, State Key Lab Mol Vaccinol & Mol Diagnost, Xiamen, Peoples R China.

C3 Indian Institute of Science (IISC) - Bangalore; Indian Institute of

Science (IISC) - Bangalore; Rutgers University System; Rutgers

University New Brunswick; Rutgers University Biomedical & Health

Sciences; Rutgers University Newark; Rutgers University System; Rutgers

University Newark; Rutgers University New Brunswick; Rutgers University

Biomedical & Health Sciences; Xiamen University

RP Singh, A (corresponding author), Indian Inst Sci, Dept Microbiol & Cell Biol, Bangalore, India.; Singh, A (corresponding author), Indian Inst Sci, Ctr Infect Dis Res, Bangalore, India.; Drlica, K (corresponding author), Rutgers State Univ, Dept Microbiol Biochem & Mol Genet, New Jersey Med Sch, Rutgers Biomed & Hlth Sci, Newark, NJ 07103 USA.; Drlica, K (corresponding author), Rutgers State Univ, Publ Hlth Res Inst, New Jersey Med Sch, Rutgers Biomed & Hlth Sci, Newark, NJ 07103 USA.

EM asingh@iisc.ac.in; drlicaka@njms.rutgers.edu

RI Zhao, Xilin/HLG-3186-2023

OI Zhao, Xilin/0000-0001-8375-8658

FU Wellcome Trust/DBT India Alliance [IA/S/16/2/502700]; Department of

Biotechnology (DBT) [BT/PR13522/COE/34/27/2015,

BT/PR29098/Med/29/1324/2018, BT/HRD/NBA/39/07/2018-19,

BT/PR39308/DRUG/134/86/2021]; Revati and Satya Nadham Atluri Chair

Professorship; National Natural Science Foundation of China [82172316]

FX The authors' work has been supported by the Wellcome Trust/DBT India

Alliance Grants, IA/S/16/2/502700 (AS); Department of Biotechnology

(DBT) Grants (BT/PR13522/COE/34/27/2015, BT/PR29098/Med/29/1324/2018,

BT/HRD/NBA/39/07/2018-19, and BT/PR39308/DRUG/134/86/2021 (AS); the

Revati and Satya Nadham Atluri Chair Professorship (AS), and the

National Natural Science Foundation of China (82172316, XZ).

CR Ajbani K, 2012, PLOS ONE, V7, DOI 10.1371/journal.pone.0049433

Aldred KJ, 2014, BIOCHEMISTRY-US, V53, P1565, DOI 10.1021/bi5000564

Allen M, 2015, FRONT IMMUNOL, V6, DOI 10.3389/fimmu.2015.00508

Almeida D, 2007, ANTIMICROB AGENTS CH, V51, P4261, DOI 10.1128/AAC.01123-07

Amaral EP, 2016, BMC MICROBIOL, V16, DOI 10.1186/s12866-016-0872-7

Aubry A, 2006, ANTIMICROB AGENTS CH, V50, P104, DOI 10.1128/AAC.50.1.104-112.2006

Band VI, 2019, PLOS PATHOG, V15, DOI 10.1371/journal.ppat.1007726

Baniasadi S, 2010, EUR J GASTROEN HEPAT, V22, P1235, DOI 10.1097/MEG.0b013e32833aa11b

Bernard C, 2016, J ANTIMICROB CHEMOTH, V71, P3465, DOI 10.1093/jac/dkw344

Bernardo J, 2009, AM J RESP CRIT CARE, V180, P288, DOI 10.1164/rccm.200906-0863ED

Bhaskar A, 2014, PLOS PATHOG, V10, DOI 10.1371/journal.ppat.1003902

Bouakaze C, 2011, J CLIN MICROBIOL, V49, P3292, DOI 10.1128/JCM.00744-11

Brace LE, 2016, NPJ AGING MECH DIS, V2, DOI 10.1038/npjamd.2016.22

Brauner A, 2016, NAT REV MICROBIOL, V14, P320, DOI 10.1038/nrmicro.2016.34

Brindle R, 2001, BMC Pulm Med, V1, P2, DOI 10.1186/1471-2466-1-2

CANETTI G, 1965, AM REV RESPIR DIS, V92, P687

CANETTI G, 1963, B WORLD HEALTH ORGAN, V29, P565

Cao RQ, 2018, ANTIMICROB AGENTS CH, V62, DOI 10.1128/AAC.01132-18

Chakravorty S, 2011, J CLIN MICROBIOL, V49, P932, DOI 10.1128/JCM.02271-10

Cui JC, 2006, J INFECT DIS, V194, P1601, DOI 10.1086/508752

Dahan-Grobgeld E, 1998, J BIOL CHEM, V273, P30232, DOI 10.1074/jbc.273.46.30232

DELENCASTRE H, 1991, ANTIMICROB AGENTS CH, V35, P632, DOI 10.1128/AAC.35.4.632

Diacon AH, 2007, ANTIMICROB AGENTS CH, V51, P2994, DOI 10.1128/AAC.01474-06

Dong YZ, 1998, ANTIMICROB AGENTS CH, V42, P2978, DOI 10.1128/AAC.42.11.2978

Dong YZ, 2000, ANTIMICROB AGENTS CH, V44, P2581, DOI 10.1128/AAC.44.9.2581-2584.2000

Dorman SE, 2021, NEW ENGL J MED, V384, P1705, DOI 10.1056/NEJMoa2033400

Dorsey-Oresto A, 2013, CELL REP, V3, P528, DOI 10.1016/j.celrep.2013.01.026

Drlica K, 2007, CLIN INFECT DIS, V44, P681, DOI 10.1086/511642

Drlica K, 2021, EXPERT REV ANTI-INFE, V19, P601, DOI 10.1080/14787210.2021.1840353

Drlica K, 2020, BACTERIAL RESISTANCE TO ANTIBIOTICS - FROM MOLECULES TO MAN, P125

Dwyer DJ, 2014, P NATL ACAD SCI USA, V111, pE2100, DOI 10.1073/pnas.1401876111

Eilertson B, 2014, ANTIMICROB AGENTS CH, V58, P3270, DOI 10.1128/AAC.02066-13

Ezerina D, 2018, CELL CHEM BIOL, V25, P447, DOI 10.1016/j.chembiol.2018.01.011

Folkvardsen DB, 2013, J CLIN MICROBIOL, V51, P4220, DOI 10.1128/JCM.01602-13

Forsman LD, 2021, EUR RESPIR J, V57, DOI 10.1183/13993003.03463-2020

Fridman O, 2014, NATURE, V513, P418, DOI 10.1038/nature13469

Ginsburg AS, 2003, CLIN INFECT DIS, V37, P1448, DOI 10.1086/379328

Gootenberg JS, 2017, SCIENCE, V356, P438, DOI 10.1126/science.aam9321

Gruber CC, 2022, MBIO, V13, DOI 10.1128/mbio.03756-21

Gruber CC, 2018, DNA REPAIR, V71, P108, DOI 10.1016/j.dnarep.2018.08.014

Guerra C, 2011, PLOS ONE, V6, DOI 10.1371/journal.pone.0028378

Gutierrez A, 2017, MOL CELL, V68, P1147, DOI 10.1016/j.molcel.2017.11.012

Hillemann D, 2009, J CLIN MICROBIOL, V47, P1767, DOI 10.1128/JCM.00081-09

Hoeijmakers JHJ, 2009, NEW ENGL J MED, V361, P1475, DOI 10.1056/NEJMra0804615

Hofmann-Thiel S, 2009, EUR RESPIR J, V33, P368, DOI 10.1183/09031936.00089808

Hong YZ, 2019, P NATL ACAD SCI USA, V116, P10064, DOI 10.1073/pnas.1901730116

Hu SY, 2014, J CLIN MICROBIOL, V52, P1644, DOI 10.1128/JCM.03395-13

Huang QY, 2011, PLOS ONE, V6, DOI 10.1371/journal.pone.0019206

Imlay JA, 2015, CURR OPIN MICROBIOL, V24, P124, DOI 10.1016/j.mib.2015.01.004

JAESCHKE H, 1992, CHEM-BIOL INTERACT, V81, P57, DOI 10.1016/0009-2797(92)90026-H

Kamakoli MK, 2017, SCI REP-UK, V7, DOI 10.1038/srep41410

Kaplan G, 2003, INFECT IMMUN, V71, P7099, DOI 10.1128/IAI.71.12.7099-7108.2003

Keren I, 2013, SCIENCE, V339, P1213, DOI 10.1126/science.1232688

Kim H, 2011, ANTIMICROB AGENTS CH, V55, P3661, DOI 10.1128/AAC.00042-11

Kohanski MA, 2007, CELL, V130, P797, DOI 10.1016/j.cell.2007.06.049

Kunota TTR, 2021, ANTIOXIDANTS-BASEL, V10, DOI 10.3390/antiox10081285

Lamprecht DA, 2016, NAT COMMUN, V7, DOI 10.1038/ncomms12393

Lange C, 2018, RESPIROLOGY, V23, P656, DOI 10.1111/resp.13304

Lau RWT, 2011, ANTIMICROB AGENTS CH, V55, P608, DOI 10.1128/AAC.00920-10

Levin-Reisman I, 2017, SCIENCE, V355, P826, DOI 10.1126/science.aaj2191

Lieberman TD, 2016, NAT MED, V22, P1470, DOI 10.1038/nm.4205

Liu JF, 2020, SCIENCE, V367, P200, DOI 10.1126/science.aay3041

Liu YL, 2012, ANTIMICROB AGENTS CH, V56, P6048, DOI 10.1128/AAC.00754-12

Liu YY, 2013, SCIENCE, V339, P1210, DOI 10.1126/science.1232751

Lobritz MA, 2015, P NATL ACAD SCI USA, V112, P8173, DOI 10.1073/pnas.1509743112

Long QX, 2015, ANTIMICROB AGENTS CH, V59, P1787, DOI 10.1128/AAC.04434-14

Luan G, 2018, ANTIMICROB AGENTS CH, V62, DOI 10.1128/AAC.01622-17

Mahakalkar Sunil M, 2017, Perspect Clin Res, V8, P132, DOI [10.4103/2229-3485.210450, 10.4103/2229-3485.210450]

Malik M, 2007, ANTIMICROB AGENTS CH, V51, P28, DOI 10.1128/AAC.00739-06

Malik M, 2006, ANTIMICROB AGENTS CH, V50, P2842, DOI 10.1128/AAC.00250-06

Malik M, 2006, MOL MICROBIOL, V61, P810, DOI 10.1111/j.1365-2958.2006.05275.x

Malik M, 2012, ANTIMICROB AGENTS CH, V56, P3879, DOI 10.1128/AAC.00474-12

Malik M, 2010, ANTIMICROB AGENTS CH, V54, P149, DOI 10.1128/AAC.01035-09

Malik S, 2012, PLOS ONE, V7, DOI 10.1371/journal.pone.0039754

Mavi PS, 2020, ANTIOXID REDOX SIGN, V32, P1348, DOI 10.1089/ars.2019.7867

McGrath M, 2014, J ANTIMICROB CHEMOTH, V69, P292, DOI 10.1093/jac/dkt364

Metcalfe JZ, 2017, ANTIMICROB AGENTS CH, V61, DOI 10.1128/AAC.00888-17

Mishra R, 2021, TRENDS MICROBIOL, V29, P606, DOI 10.1016/j.tim.2020.10.013

Mishra R, 2019, SCI TRANSL MED, V11, DOI 10.1126/scitranslmed.aaw6635

MITCHISON DA, 1984, BRIT MED BULL, V40, P84, DOI 10.1093/oxfordjournals.bmb.a071952

Morris D, 2013, J INTERF CYTOK RES, V33, P270, DOI 10.1089/jir.2012.0103

Mustaev A, 2014, J BIOL CHEM, V289, P12300, DOI 10.1074/jbc.M113.529164

Nagrale D, 2013, EUR RESPIR J, V42

Nair RR, 2019, MICROBIOL-SGM, V165, P668, DOI 10.1099/mic.0.000797

Ni W, 2014, EUR J CLIN MICROBIOL, V33, P385, DOI 10.1007/s10096-013-1968-8

Nimmo C, 2020, EBIOMEDICINE, V55, DOI 10.1016/j.ebiom.2020.102747

Nuermberger EL, 2004, AM J RESP CRIT CARE, V169, P421, DOI 10.1164/rccm.200310-1380OC

Operario DJ, 2017, PLOS ONE, V12, DOI 10.1371/journal.pone.0176522

Palanisamy GS, 2011, PLOS ONE, V6, DOI 10.1371/journal.pone.0026254

Park S, 2003, J BACTERIOL, V185, P1942, DOI 10.1128/JB.185.6.1942-1950.2003

Pawlowski A, 2012, PLOS PATHOG, V8, DOI 10.1371/journal.ppat.1002464

Pholwat S, 2013, PLOS ONE, V8, DOI 10.1371/journal.pone.0057238

Piccaro G, 2014, ANTIMICROB AGENTS CH, V58, P7527, DOI 10.1128/AAC.03169-14

Pletz MWR, 2004, ANTIMICROB AGENTS CH, V48, P780, DOI 10.1128/AAC.48.3.780-782.2004

Possuelo LG, 2008, EUR J CLIN PHARMACOL, V64, P673, DOI 10.1007/s00228-008-0484-8

Post FA, 2004, J INFECT DIS, V190, P99, DOI 10.1086/421501

Pournaras S, 2005, J ANTIMICROB CHEMOTH, V55, P1055, DOI 10.1093/jac/dki115

Prideaux B, 2015, NAT MED, V21, P1223, DOI 10.1038/nm.3937

Rice JE, 2012, NUCLEIC ACIDS RES, V40, DOI 10.1093/nar/gks731

Rinder H, 2001, INT J TUBERC LUNG D, V5, P339

Rock JM, 2017, NAT MICROBIOL, V2, DOI 10.1038/nmicrobiol.2016.274

Rodríguez JC, 2004, J ANTIMICROB CHEMOTH, V53, P441, DOI 10.1093/jac/dkh119

Safe IP, 2020, PLOS ONE, V15, DOI 10.1371/journal.pone.0235381

Sarathy J, 2019, ANTIMICROB AGENTS CH, V63, DOI [10.1128/AAC.02516-18, 10.1128/aac.02516-18]

Shamputa IC, 2004, J CLIN MICROBIOL, V42, P5528, DOI 10.1128/JCM.42.12.5528-5536.2004

Shee S, 2022, ANTIMICROB AGENTS CH, V66, DOI 10.1128/aac.00592-22

Singh A, 2017, MICROBIOL-SGM, V163, P1740, DOI 10.1099/mic.0.000578

Takahashi N, 2017, P NATL ACAD SCI USA, V114, P9164, DOI 10.1073/pnas.1707466114

Teskey G, 2018, FRONT IMMUNOL, V9, DOI 10.3389/fimmu.2018.02069

TUOMANEN E, 1986, ANTIMICROB AGENTS CH, V30, P521, DOI 10.1128/AAC.30.4.521

Tyagi P, 2015, FREE RADICAL BIO MED, V84, P344, DOI 10.1016/j.freeradbiomed.2015.03.008

Vadwai V, 2011, PLOS ONE, V6, DOI 10.1371/journal.pone.0024770

van Rie A, 2005, AM J RESP CRIT CARE, V172, P636, DOI 10.1164/rccm.200503-449OC

Vargas DY, 2016, PLOS ONE, V11, DOI 10.1371/journal.pone.0156546

Vargas R, 2021, ELIFE, V10, DOI 10.7554/eLife.61805

Venketaraman Vishwanath, 2006, AIDS Res Ther, V3, P5, DOI 10.1186/1742-6405-3-5

Vilchèze C, 2021, ANTIMICROB AGENTS CH, V65, DOI 10.1128/AAC.01703-20

Vilchèze C, 2017, P NATL ACAD SCI USA, V114, P4495, DOI 10.1073/pnas.1704376114

Vilchèze C, 2013, NAT COMMUN, V4, DOI 10.1038/ncomms2898

Vogelstein B, 1999, P NATL ACAD SCI USA, V96, P9236, DOI 10.1073/pnas.96.16.9236

Wald-Dickler N, 2018, CLIN INFECT DIS, V66, P1470, DOI 10.1093/cid/cix1127

Wang XH, 2009, ANTIMICROB AGENTS CH, V53, P1395, DOI 10.1128/AAC.01087-08

Warren RM, 2004, AM J RESP CRIT CARE, V169, P610, DOI 10.1164/rccm.200305-714OC

WHO, 2019, Global tuberculosis report 2019, V2019

WHO, 2013, GLOBAL TUBERCULOSIS, V2013

Wu XL, 2011, PLOS ONE, V6, DOI 10.1371/journal.pone.0023909

Xiong MP, 2016, FRONT MICROBIOL, V7, DOI 10.3389/fmicb.2016.00874

YAJKO DM, 1995, J CLIN MICROBIOL, V33, P1944, DOI 10.1128/JCM.33.7.1944-1947.1995

Yano T, 2011, J BIOL CHEM, V286, P10276, DOI 10.1074/jbc.M110.200501

YOSHIDA H, 1990, ANTIMICROB AGENTS CH, V34, P1271, DOI 10.1128/AAC.34.6.1271

Zeng J, 2022, P NATL ACAD SCI USA, V119, DOI 10.1073/pnas.2118566119

Zetola NM, 2014, J INFECT DIS, V209, P1754, DOI 10.1093/infdis/jiu040

Zhang BX, 2014, BMC VET RES, V10, DOI 10.1186/s12917-014-0297-1

Zhang XB, 2013, J ANTIMICROB CHEMOTH, V68, P1537, DOI 10.1093/jac/dkt082

Zhang XB, 2012, J CLIN MICROBIOL, V50, P1471, DOI 10.1128/JCM.05793-11

Zhao BY, 1999, ANTIMICROB AGENTS CH, V43, P661, DOI 10.1128/AAC.43.3.661

Zhao XL, 2001, CLIN INFECT DIS, V33, pS147, DOI 10.1086/321841

Zhou JF, 2000, J INFECT DIS, V182, P517, DOI 10.1086/315708

Zhu YL, 2012, J ANTIMICROB CHEMOTH, V67, P2700, DOI 10.1093/jac/dks280

NR 139

TC 15

Z9 18

U1 2

U2 24

PU FRONTIERS MEDIA SA

PI LAUSANNE

PA AVENUE DU TRIBUNAL FEDERAL 34, LAUSANNE, CH-1015, SWITZERLAND

SN 2235-2988

J9 FRONT CELL INFECT MI

JI Front. Cell. Infect. Microbiol.

PD SEP 29

PY 2022

VL 12

AR 938032

DI 10.3389/fcimb.2022.938032

PG 17

WC Immunology; Microbiology

WE Science Citation Index Expanded (SCI-EXPANDED)

SC Immunology; Microbiology

GA 5I7YV

UT WOS:000868568600001

OA Green Submitted, gold

DA 2026-05-07

ER

PT J

AU Jain, A

Kumar, R

Mothsra, P

Sharma, AK

Singh, AK

Kumar, Y

AF Jain, Akanksha

Kumar, Rajesh

Mothsra, Poonam

Sharma, Atul Kumar

Singh, Anil Kumar

Kumar, Yogesh

TI Recent Biochemical Advances in Antitubercular Drugs: Challenges and

Future

SO CURRENT TOPICS IN MEDICINAL CHEMISTRY

LA English

DT Review

DE Anti-tuberculosis drugs; First line anti-tubercular drugs; Second line

anti-tubercular drugs; Multidrug resistant tuberculosis; mode of action

of anti-tubercular drugs; mechanism of drug resistance

ID IN-VITRO ACTIVITIES; RESISTANT MYCOBACTERIUM-TUBERCULOSIS; D-ALANINE

RACEMASE; MOXIFLOXACIN BAY 12-8039; PARA-AMINOSALICYLIC ACID;

SITAFLOXACIN DU-6859A; ANTIMYCOBACTERIAL ACTIVITIES; ANTIMICROBIAL

ACTIVITIES; TOPOISOMERASE-IV; CROSS-RESISTANCE

AB Tuberculosis (TB) is one of the leading causes of death world-wide after AIDS. It infects around one-third of global population and approximately two million people die annually from this disease because it is a very contagious disease spread by Mycobacterium tuberculosis. The increasing number of drug-resistant strains and the failure of conventional treatments against this strain are the challenges of the coming decades. New therapeutic techniques aim to confirm cure without deterioration, to reduce deaths, contagions and the formation of drug-resistant strains. A plethora of new diagnostic tests are available to diagnose the active tuberculosis, screen latent M. tuberculosis infection, and to identify drug-resistant strains of M. tuberculosis. When effective prevention strategies do not prevail, high rates of early case detection and successive cures to control TB emergence would not be possible. In this review, we discussed the structural features of M. tuberculosis, Multi drug resistance tuberculosis (MDR-TB), extremely drug-resistant tuberculosis (XDR-TB), the mechanism of M. tuberculosis infection, the mode of action of first and second-line antitubercular drugs, the mechanism of resistance to the existing drugs, compounds in preclinical and clinical trial and drugs presently available for the treatment of tuberculosis. Moreover, the new diagnostic techniques to detect M. Tuberculosis are also discussed in this review.

C1 [Jain, Akanksha] Univ Delhi, Bhagini Nivedita Coll, Dept Food & Nutr, Kair Near Najafgarh, Delhi 110043, India.

[Kumar, Rajesh] B R A Bihar Univ, RDS Coll, PG Dept Chem, Muzaffarpur 842002, India.

[Mothsra, Poonam; Kumar, Yogesh] Univ Delhi, Bhagini Nivedita Coll, Dept Chem, Kair Near Najafgarh, Delhi 110043, India.

[Sharma, Atul Kumar] Univ Delhi, Deshbandhu Coll, Dept Chem, Delhi 110019, India.

[Singh, Anil Kumar] Mahatma Gandhi Cent Univ, Sch Phys Sci, Dept Chem, Motihari 845401, Bihar, India.

C3 University of Delhi; University of Delhi; University of Delhi

RP Kumar, Y (corresponding author), Univ Delhi, Bhagini Nivedita Coll, Dept Chem, Kair Near Najafgarh, Delhi 110043, India.; Singh, AK (corresponding author), Mahatma Gandhi Cent Univ, Sch Phys Sci, Dept Chem, Motihari 845401, Bihar, India.

EM anilchemdu@gmail.com; yogesh.dixit84@gmail.com

RI Kumar, Yogesh/KYR-5232-2024

FU Department of Science and Technology (DST-SERB), Ministry of Science and

Technology, India [YSS/2015/001966]

FX YK is thankful to the Department of Science and Technology (DST-SERB),

Ministry of Science and Technology, India, for financial support

(YSS/2015/001966).

CR Adams LB, 1999, ANTIMICROB AGENTS CH, V43, P1638, DOI 10.1128/AAC.43.7.1638

Adams RA, 2021, J ANTIBIOT, V74, P786, DOI 10.1038/s41429-021-00462-x

Adilakshmi T, 2000, J BACTERIOL, V182, P264, DOI 10.1128/JB.182.2.264-271.2000

Alahari A, 2007, PLOS ONE, V2, DOI 10.1371/journal.pone.0001343

Allen A, 2000, ANTIMICROB AGENTS CH, V44, P1604, DOI 10.1128/AAC.44.6.1604-1608.2000

Andini N, 2006, ANTIMICROB AGENTS CH, V50, P2560, DOI 10.1128/AAC.00264-06

Andries K, 2005, SCIENCE, V307, P223, DOI 10.1126/science.1106753

[Anonymous], 2018, The Global Tuberculosis Report

Aoki H, 2002, ANTIMICROB AGENTS CH, V46, P1080, DOI 10.1128/AAC.46.4.1080-1085.2002

ASHTEKAR DR, 1993, ANTIMICROB AGENTS CH, V37, P183, DOI 10.1128/AAC.37.2.183

Asif M, 2012, MINI-REV MED CHEM, V12, P1404

Aubry A, 2004, ANTIMICROB AGENTS CH, V48, P1281, DOI 10.1128/AAC.48.4.1281-1288.2004

Baek SH, 2011, PLOS BIOL, V9, DOI 10.1371/journal.pbio.1001065

Baker JJ, 2018, SCI REP-UK, V8, DOI 10.1038/s41598-018-22343-4

Balfour JAB, 2000, DRUGS, V59, P115

BANERJEE A, 1994, SCIENCE, V263, P227, DOI 10.1126/science.8284673

Barbachyn MR, 1996, J MED CHEM, V39, P680, DOI 10.1021/jm950956y

Barry CE III, 2009, NAT REV MICROBIOL, V7, P845, DOI 10.1038/nrmicro2236

BARRY VC, 1957, NATURE, V179, P1013, DOI 10.1038/1791013a0

Baulard AR, 2000, J BIOL CHEM, V275, P28326

Belanger AE, 1996, P NATL ACAD SCI USA, V93, P11919, DOI 10.1073/pnas.93.21.11919

Berrada ZL, 2016, DIAGN MICR INFEC DIS, V85, P177, DOI 10.1016/j.diagmicrobio.2016.01.019

BIEDENBACH DJ, 1995, ANTIMICROB AGENTS CH, V39, P2325, DOI 10.1128/AAC.39.10.2325

Blanchard JS, 1996, ANNU REV BIOCHEM, V65, P215, DOI 10.1146/annurev.bi.65.070196.001243

Bos KI, 2014, NATURE, V514, P494, DOI 10.1038/nature13591

Bottger E C, 1994, Trends Microbiol, V2, P416, DOI 10.1016/0966-842X(94)90622-X

BRENNAN PJ, 1995, ANNU REV BIOCHEM, V64, P29, DOI 10.1146/annurev.bi.64.070195.000333

BROWN BA, 1992, ANTIMICROB AGENTS CH, V36, P180, DOI 10.1128/AAC.36.1.180

BROWN KA, 1975, BIOCHIM BIOPHYS ACTA, V385, P207, DOI 10.1016/0304-4165(75)90349-9

Bruzzese T, 2000, ARZNEIMITTEL-FORSCH, V50, P60

Caceres NE, 1997, J BACTERIOL, V179, P5046, DOI 10.1128/jb.179.16.5046-5055.1997

Cardeñosa O, 2000, CHEMOTHERAPY, V46, P379, DOI 10.1159/000007317

CASAL M, 1987, TUBERCLE, V68, P141, DOI 10.1016/0041-3879(87)90030-4

Caws M, 2006, J CLIN MICROBIOL, V44, P2333, DOI 10.1128/JCM.00330-06

Chacon O, 2002, ANTIMICROB AGENTS CH, V46, P47, DOI 10.1128/AAC.46.2.47-54.2002

Chan-Tack KM, 1998, NEW ENGL J MED, V339, P1079, DOI 10.1056/NEJM199810083391511

Chauhan L S, 2004, Indian Pediatr, V41, P901

Cheng AFB, 2004, ANTIMICROB AGENTS CH, V48, P596, DOI 10.1128/AAC.48.2.596-601.2004

Cohen KA, 2014, MICROBIOL SPECTR, V2, DOI 10.1128/microbiolspec.MGM2-0036-2013

Colca JR, 2003, J BIOL CHEM, V278, P21972, DOI 10.1074/jbc.M302109200

Cole ST, 2005, SCIENCE, V307, P214, DOI 10.1126/science.1108379

Comas I, 2013, NAT GENET, V45, P1176, DOI 10.1038/ng.2744

Conradie F, 2020, NEW ENGL J MED, V382, P893, DOI 10.1056/NEJMoa1901814

Corti G, 2000, INT J ANTIMICROB AG, V16, P527, DOI 10.1016/S0924-8579(00)00290-9

CRUTHERS LR, 1979, AM J VET RES, V40, P676, DOI 10.2460/ajvr.1979.40.05.676

Currens MJ, 1996, J PHARMACOL EXP THER, V279, P645

Cynamon MH, 1999, ANTIMICROB AGENTS CH, V43, P1189, DOI 10.1128/AAC.43.5.1189

Damtie D., 2014, MYCOBACT DIS, V4, P174, DOI DOI 10.4172/2161-1068.1000174

Daniel J, 2004, J BACTERIOL, V186, P5017, DOI 10.1128/jb.186.15.5017-5030.2004

Danilchanka O, 2015, ANTIMICROB AGENTS CH, V59, P2328, DOI 10.1128/AAC.04222-14

DAVID HL, 1969, AM REV RESPIR DIS, V100, P579

DeBarber AE, 2000, P NATL ACAD SCI USA, V97, P9677, DOI 10.1073/pnas.97.17.9677

Del' Alamo L, 1999, Braz J Infect Dis, V3, P215

Deretic V, 1999, MOL MICROBIOL, V31, P1603, DOI 10.1046/j.1365-2958.1999.01279.x

Dharmaratne HRW, 1998, PLANTA MED, V64, P460, DOI 10.1055/s-2006-957483

Dhople AM, 2001, ARZNEIMITTEL-FORSCH, V51, P501

Dhople AM, 2001, INT J ANTIMICROB AG, V17, P57, DOI 10.1016/S0924-8579(00)00306-X

DICKINSON JM, 1987, TUBERCLE, V68, P177, DOI 10.1016/0041-3879(87)90053-5

DICKINSON JM, 1987, TUBERCLE, V68, P183, DOI 10.1016/0041-3879(87)90054-7

Diekema DJ, 2000, DRUGS, V59, P7, DOI 10.2165/00003495-200059010-00002

Dlugovitzky D, 1999, RESP MED, V93, P557, DOI 10.1016/S0954-6111(99)90155-5

Donald PR, 2015, LANCET INFECT DIS, V15, P1091, DOI 10.1016/S1473-3099(15)00263-7

Duncan K, 2004, CURR OPIN MICROBIOL, V7, P460, DOI 10.1016/j.mib.2004.08.011

Ehlers S, 1996, ZBL BAKT-INT J MED M, V284, P218

Esmail H, 2014, PHILOS T R SOC B, V369, DOI 10.1098/rstb.2013.0437

Farmer P, 1998, INT J TUBERC LUNG D, V2, P869

Feng ZY, 2003, ANTIMICROB AGENTS CH, V47, P283, DOI 10.1128/AAC.47.1.283-291.2003

FERRERO L, 1994, MOL MICROBIOL, V13, P641, DOI 10.1111/j.1365-2958.1994.tb00458.x

Field SK, 2003, CHEST, V124, P1482, DOI 10.1378/chest.124.4.1482

FINKEN M, 1993, MOL MICROBIOL, V9, P1239, DOI 10.1111/j.1365-2958.1993.tb01253.x

Fitzgerald D.W., 2015, MANDELL DOUGLAS BENN, P2787, DOI [DOI 10.1016/B978-1-4557-4801-3.00251-4, 10.1016/C2012-1-00075-6, DOI 10.1016/C2012-1-00075-6]

Frénois F, 2006, TUBERCULOSIS, V86, P110, DOI 10.1016/j.tube.2005.07.005

Frénois F, 2004, MOL CELL, V16, P301, DOI 10.1016/j.molcel.2004.09.020

Gale E.F., 1972, MOL BASIS ANTIBIOTIC

Galinis DL, 1996, J MED CHEM, V39, P4507, DOI 10.1021/jm9602827

Gelmanova IY, 2007, B WORLD HEALTH ORGAN, V85, P703, DOI 10.2471/BLT.06.038331

Georghiou SB, 2012, PLOS ONE, V7, DOI 10.1371/journal.pone.0033275

Gillespie SH, 1999, J ANTIMICROB CHEMOTH, V44, P393, DOI 10.1093/jac/44.3.393

Gillespie SH, 2002, ANTIMICROB AGENTS CH, V46, P267, DOI 10.1128/AAC.46.2.267-274.2002

Gopal M, 2013, INT J TUBERC LUNG D, V17, P1001, DOI 10.5588/ijtld.12.0144

Groll A.H., 2000, ATLAS INFECT DIS FUN, P135

Hartkoorn RC, 2014, ANTIMICROB AGENTS CH, V58, P2979, DOI 10.1128/AAC.00037-14

HEIFETS L, 1992, AM REV RESPIR DIS, V145, P1223, DOI 10.1164/ajrccm/145.5.1223

Heifets L.B., 1991, DRUG SUSCEPTIBILITY, V13, P57

Heifets LB, 1996, TUBERCLE LUNG DIS, V77, P19, DOI 10.1016/S0962-8479(96)90070-2

HELD HR, 1980, J PHARM SCI-US, V69, P1284, DOI 10.1002/jps.2600691114

HEYM B, 1995, MOL MICROBIOL, V15, P235, DOI 10.1111/j.1365-2958.1995.tb02238.x

Hidaka T, 1999, Kekkaku, V74, P53

Hofmann SG, 2014, DEPRESS ANXIETY, V31, P175, DOI 10.1002/da.22257

HOUSTON AK, 1994, DIAGN MICR INFEC DIS, V18, P57, DOI 10.1016/0732-8893(94)90134-1

Hutchinson DK, 2003, CURR TOP MED CHEM, V3, P1021, DOI 10.2174/1568026033452195

INDERLIED CB, 1994, ANTIMICROB AGENTS CH, V38, P1838, DOI 10.1128/AAC.38.8.1838

Jagannath C, 1999, ANTIMICROB AGENTS CH, V43, P2898, DOI 10.1128/AAC.43.12.2898

Ji BH, 1998, ANTIMICROB AGENTS CH, V42, P2066, DOI 10.1128/AAC.42.8.2066

JOHNSTON JP, 1967, J PHARM PHARMACOL, V19, P1, DOI 10.1111/j.2042-7158.1967.tb07986.x

Kalscheuer R, 2019, BIOCHEM J, V476, P1995, DOI 10.1042/BCJ20190324

Kelkar MS, 1998, INT J CLIN PHARM RES, V18, P137

Kenaope L, 2020, J GLOB ANTIMICROB RE, V21, P434, DOI 10.1016/j.jgar.2019.11.008

Khadka P, 2023, DRUG DELIV TRANSL RE, V13, P1246, DOI 10.1007/s13346-022-01238-y

Knutson KL, 1998, J BIOL CHEM, V273, P645, DOI 10.1074/jbc.273.1.645

KONNO K, 1967, AM REV RESPIR DIS, V95, P461

Kraljevic S, 2004, EMBO REP, V5, P837, DOI 10.1038/sj.embor.7400236

Lalvani A, 2012, CELL, V148, P389, DOI 10.1016/j.cell.2012.01.026

Lange C, 2023, NEW ENGL J MED, V388, P190, DOI 10.1056/NEJMc2213970

LEVIN ME, 1993, MOL MICROBIOL, V8, P277, DOI 10.1111/j.1365-2958.1993.tb01572.x

Li SQ, 2022, NAT MICROBIOL, V7, P766, DOI 10.1038/s41564-022-01130-y

Loddenkemper R, 2002, EUR RESPIR J, V20, p66S, DOI 10.1183/09031936.02.00401302

Manjunatha UH, 2006, P NATL ACAD SCI USA, V103, P431, DOI 10.1073/pnas.0508392103

MCCUNE RM, 1966, J EXP MED, V123, P469, DOI 10.1084/jem.123.3.469

McKee TC, 1998, J NAT PROD, V61, P1252, DOI 10.1021/np980140a

Mdluli K, 1998, SCIENCE, V280, P1607, DOI 10.1126/science.280.5369.1607

Milatovic D, 2000, ANTIMICROB AGENTS CH, V44, P1102, DOI 10.1128/AAC.44.4.1102-1107.2000

Minias A, 2021, FRONT MICROBIOL, V11, DOI 10.3389/fmicb.2020.618168

MITCHISON DA, 1985, TUBERCLE, V66, P219, DOI 10.1016/0041-3879(85)90040-6

Mitchison DA, 1998, INT J TUBERC LUNG D, V2, P863

Miyazaki E, 1999, ANTIMICROB AGENTS CH, V43, P85, DOI 10.1128/AAC.43.1.85

Miyazaki E, 1999, ANTIMICROB AGENTS CH, V43, P2126, DOI 10.1128/AAC.43.9.2126

Mukherjee S, 2023, TUBERCULOSIS, V140, DOI 10.1016/j.tube.2023.102340

Nahid P, 2016, CLIN INFECT DIS, V63, pe147, DOI 10.1093/cid/ciw376

Nair A, 2023, J NANOBIOTECHNOL, V21, DOI 10.1186/s12951-023-02156-y

NAKASHIMA M, 1995, ANTIMICROB AGENTS CH, V39, P170, DOI 10.1128/AAC.39.1.170

Nathavitharana RR, 2015, EUR RESPIR J, V46, P293, DOI 10.1183/13993003.00436-2015

Ness T, 2023, BREATHE, V19, DOI 10.1183/20734735.0084-2023

Nilius Angela M, 2003, Curr Opin Investig Drugs, V4, P149

Oh P, 2002, EMERG INFECT DIS, V8, P1290

Oliva B, 2004, J ANTIMICROB CHEMOTH, V53, P435, DOI 10.1093/jac/dkh114

Onodera Y, 1999, J ANTIMICROB CHEMOTH, V44, P533, DOI 10.1093/jac/44.4.533

Orme IM, 2015, NAT IMMUNOL, V16, P57, DOI 10.1038/ni.3048

ORTALOMAGNE A, 1995, MICROBIOL-UK, V141, P1609, DOI 10.1099/13500872-141-7-1609

OrtaloMagne A, 1996, J BACTERIOL, V178, P456, DOI 10.1128/jb.178.2.456-461.1996

Pablos-Méndez A, 1998, NEW ENGL J MED, V338, P1641, DOI 10.1056/NEJM199806043382301

Pai M, 2016, NAT REV DIS PRIMERS, V2, DOI 10.1038/nrdp.2016.76

PELOQUIN CA, 1994, PHARMACOTHERAPY, V14, P415

Phillips I., 1997, ANTIBIOT CHEMOTHER, P164

Potkar C, 1999, CHEMOTHERAPY, V45, P147, DOI 10.1159/000007176

PREMA K, 1976, BIOCHEM PHARMACOL, V25, P1299, DOI 10.1016/0006-2952(76)90093-9

Qian L, 2006, CHEM RES TOXICOL, V19, P443, DOI 10.1021/tx050328b

RASTOGI N, 1991, ANTIMICROB AGENTS CH, V35, P462, DOI 10.1128/AAC.35.3.462

Ratledge C, 2004, TUBERCULOSIS, V84, P110, DOI 10.1016/j.tube.2003.08.012

REDDY MV, 1994, TUBERCLE LUNG DIS, V75, P208, DOI 10.1016/0962-8479(94)90010-8

Reddy VM, 1996, ANTIMICROB AGENTS CH, V40, P633, DOI 10.1128/AAC.40.3.633

Reed MB, 2004, NATURE, V431, P84, DOI 10.1038/nature02837

Rengarajan J, 2004, MOL MICROBIOL, V53, P275, DOI 10.1111/j.1365-2958.2004.04120.x

Roblin PM, 2003, ANTIMICROB AGENTS CH, V47, P1135, DOI 10.1128/AAC.47.3.1135-1136.2003

Roth A, 1997, EUR RESPIR J, V10, P1877, DOI 10.1183/09031936.97.10081877

Rozwarski DA, 1998, SCIENCE, V279, P98, DOI 10.1126/science.279.5347.98

Ruiz-Serrano MJ, 2000, ANTIMICROB AGENTS CH, V44, P2567, DOI 10.1128/AAC.44.9.2567-2568.2000

Russell DG, 2011, IMMUNOL REV, V240, P252, DOI 10.1111/j.1600-065X.2010.00984.x

Russell DG, 2003, J VET MED EDUC, V30, P140, DOI 10.3138/jvme.30.2.140

SAITO H, 1994, ANTIMICROB AGENTS CH, V38, P2877, DOI 10.1128/AAC.38.12.2877

SAITO H, 1991, ANTIMICROB AGENTS CH, V35, P542, DOI 10.1128/AAC.35.3.542

Samstein M, 2013, ELIFE, V2, DOI 10.7554/eLife.01086

Saxena N, 2019, Journal of Drug Delivery and Therapeutics, V9, P802, DOI 10.22270/jddt.v9i3-s.2777

Scorpio A, 1996, NAT MED, V2, P662, DOI 10.1038/nm0696-662

Scorpio A, 1997, ANTIMICROB AGENTS CH, V41, P540, DOI 10.1128/AAC.41.3.540

Seung KJ, 2004, CLIN INFECT DIS, V39, P1321, DOI 10.1086/425005

Seung KJ, 2015, CSH PERSPECT MED, V5, DOI 10.1101/cshperspect.a017863

Sharma AK, 2001, IMMUNOL CELL BIOL, V79, P537, DOI 10.1046/j.1440-1711.2001.01044.x

Shetye GS, 2020, TRANSL RES, V220, P68, DOI 10.1016/j.trsl.2020.03.007

Shinabarger D, 1999, Expert Opin Investig Drugs, V8, P1195, DOI 10.1517/13543784.8.8.1195

Somoskovi A, 2001, RESP RES, V2, P164, DOI 10.1186/rr54

Sotgiu G, 2015, CSH PERSPECT MED, V5, DOI 10.1101/cshperspect.a017822

Spino C, 1998, BIOORG MED CHEM LETT, V8, P3475, DOI 10.1016/S0960-894X(98)00628-3

Stover CK, 2000, NATURE, V405, P962, DOI 10.1038/35016103

STRIPPOLI V, 1988, FARMACO-ED SCI, V43, P619

Strovel Jeffrey., 2016, Assay Guidance Manual

Sullivan JT, 1999, ANTIMICROB AGENTS CH, V43, P2793, DOI 10.1128/AAC.43.11.2793

Sun Z., 1999, Tubercle and Lung Disease, V79, P319, DOI 10.1054/tuld.1999.0212

Swaney SM, 1998, ANTIMICROB AGENTS CH, V42, P3251, DOI 10.1128/AAC.42.12.3251

Takahata M, 1999, ANTIMICROB AGENTS CH, V43, P1077, DOI 10.1128/AAC.43.5.1077

Telenti A, 1997, NAT MED, V3, P567, DOI 10.1038/nm0597-567

Tobin DM, 2012, CELL, V148, P434, DOI 10.1016/j.cell.2011.12.023

Tomioka H, 2000, J Infect Chemother, V6, P8

Tomioka H, 1999, ANTIMICROB AGENTS CH, V43, P3001, DOI 10.1128/AAC.43.12.3001

Tomioka Haruaki, 2000, Archivum Immunologiae et Therapiae Experimentalis, V48, P183

Vale N, 2020, RSC MED CHEM, V11, P1423, DOI 10.1039/d0md00253d

van der Paardt AF, 2015, EUR RESPIR J, V46, P444, DOI 10.1183/09031936.00147014

Vannelli TA, 2002, J BIOL CHEM, V277, P12824, DOI 10.1074/jbc.M110751200

Via LE, 2010, J CLIN MICROBIOL, V48, P402, DOI 10.1128/JCM.01476-09

Vilchéze C, 2007, ANNU REV MICROBIOL, V61, P35, DOI 10.1146/annurev.micro.61.111606.122346

Warner DF, 2015, TRENDS MICROBIOL, V23, P14, DOI 10.1016/j.tim.2014.10.005

Watford WT, 2001, J IMMUNOL, V167, P6593, DOI 10.4049/jimmunol.167.11.6593

Watt B, 1997, J ANTIMICROB CHEMOTH, V39, P567, DOI 10.1093/jac/39.5.567

Whitehead TC, 1998, EUR J CLIN MICROBIOL, V17, P794, DOI 10.1007/s100960050189

WIMPENNY JW, 1967, J GEN MICROBIOL, V47, P389, DOI 10.1099/00221287-47-3-389

Wolf AJ, 2008, J EXP MED, V205, P105, DOI 10.1084/jem.20071367

Wolff M.E., 1996, Am. J. Ther, V3, P608, DOI DOI 10.1097/00045391-199608000-00012

Woodcock JM, 1997, ANTIMICROB AGENTS CH, V41, P101, DOI 10.1128/AAC.41.1.101

World Health Organization, 2023, WHATS NEW TB SECTION

Xu ZQ, 2004, BIOORGAN MED CHEM, V12, P1199, DOI 10.1016/j.bmc.2003.11.012

YAMANE T, 1993, CHEM PHARM BULL, V41, P148

YOUATT J, 1969, AM REV RESPIR DIS, V99, P729

Yu WY, 2020, TUBERCULOSIS CONTROL IN MIGRATING POPULATION, P1, DOI 10.1007/978-981-32-9763-0_1

Zhao BY, 1999, ANTIMICROB AGENTS CH, V43, P661, DOI 10.1128/AAC.43.3.661

Zhu HZ, 2023, FRONT CELL INFECT MI, V13, DOI 10.3389/fcimb.2023.1183597

Zingue D, 2018, SCI REP-UK, V8, DOI 10.1038/s41598-018-29660-8

Zurenko GE, 1996, ANTIMICROB AGENTS CH, V40, P839, DOI 10.1128/AAC.40.4.839

NR 197

TC 4

Z9 4

U1 2

U2 11

PU BENTHAM SCIENCE PUBL LTD

PI SHARJAH

PA EXECUTIVE STE Y-2, PO BOX 7917, SAIF ZONE, 1200 BR SHARJAH, U ARAB

EMIRATES

SN 1568-0266

EI 1873-4294

J9 CURR TOP MED CHEM

JI Curr. Top. Med. Chem.

PY 2024

VL 24

IS 21

BP 1829

EP 1855

DI 10.2174/0115680266286294240610102911

EA JUN 2024

PG 27

WC Chemistry, Medicinal

WE Science Citation Index Expanded (SCI-EXPANDED)

SC Pharmacology & Pharmacy

GA E3E2C

UT WOS:001261424200001

DA 2026-05-07

ER

PT J

AU Yuan, Y

Li, JM

Chen, YH

Cai, QS

Xu, YY

Lin, LT

Lang, YZ

Guo, SH

Zhang, RY

Cai, XJ

AF Yuan, Yuan

Li, Jinmeng

Chen, Yanhong

Cai, Qingshan

Xu, Yingying

Lin, Luting

Lang, Yazhen

Guo, Suhang

Zhang, Ruoying

Cai, Xinjun

TI Mechanism underlying linezolid-induced peripheral neuropathy in

multidrug-resistant tuberculosis

SO FRONTIERS IN PHARMACOLOGY

LA English

DT Article

DE linezolid; multidrug-resistant tuberculosis; peripheral neuropathy;

schwann cells; autophagy

ID SPINAL-CORD-INJURY; NERVE REGENERATION; SAFETY; TOLERABILITY; AUTOPHAGY;

REGIMENS; EFFICACY; CELLS; TB

AB Multidrug-resistant tuberculosis (MDR-TB) remains a main global health concern as there is no comprehensive therapeutic intervention yet and numerous adverse effects follow the therapeutic process. In recent years, linezolid has been frequently used for treating MDR-TB. However, peripheral neuropathy associated with linezolid has reduced patient compliance. The current study explored the mechanism underlying linezolid-induced peripheral neuropathy in MDR-TB. Autophagy plays a neuroprotective role against peripheral nerve injury. We hypothesized that autophagy might also play a neuroprotective role against linezolid-induced peripheral neuropathy. In this study, we collected 12 questionnaires from MDR-TB patients in our hospital, and 10 of them developed linezolid-induced pain. The pain is mainly concentrated in the feet and accompanied by numbness. Subsequently, we used Sprague-Dawley (SD) rats and Schwann cells (SCs) to explore the mechanism. We found that linezolid causes a sparse arrangement of sciatic nerve tissue with associated loss of neurons, myelin sheaths, and down-regulation of LC3B expression. These results were also confirmed by in vitro experiments, showing that linezolid inhibited the proliferation of SCs. And the expression of P-AKT and P62 was elevated, and the expression of LC3B declined compared with the control group. Moreover, chloroquine (CQ), an autophagy inhibitor, also exhibited experimental results similar to linezolid. In summary, we conclude that linezolid-induced peripheral neuropathy is associated with the inhibition of autophagy flux.

C1 [Yuan, Yuan; Li, Jinmeng; Cai, Qingshan; Xu, Yingying; Lang, Yazhen; Guo, Suhang; Zhang, Ruoying; Cai, Xinjun] Zhejiang Univ, Sch Med, Affiliated Hangzhou Chest Hosp, Hangzhou, Zhejiang, Peoples R China.

[Chen, Yanhong] Zhejiang Univ, Lab Anim Ctr, Hangzhou, Zhejiang, Peoples R China.

[Lin, Luting] Zhejiang Chinese Med Univ, Coll Pharm, Hangzhou, Zhejiang, Peoples R China.

C3 Zhejiang University; Zhejiang University; Zhejiang Chinese Medical

University

RP Zhang, RY; Cai, XJ (corresponding author), Zhejiang Univ, Sch Med, Affiliated Hangzhou Chest Hosp, Hangzhou, Zhejiang, Peoples R China.

EM hzhhzry@163.com; zjtcmcxj@zcmu.edu.cn

RI yuan, yin/PGF-6240-2026; chen, yanhong/JVE-0289-2024

FU Natural Science Foundation of China [82003669]; Zhejiang Medical and

Health Science and Technology Plan Project [2022RC229]; Hangzhou Medical

and Health Science and Technology Project [A20210231, ZD20220049,

Z20220017]; Hospital Pharmacy Special Project of Zhejiang Pharmaceutical

Society [2021ZYY33]

FX This work was supported by the Natural Science Foundation of China

(82003669) Zhejiang Medical and Health Science and Technology Plan

Project (2022RC229); Hangzhou Medical and Health Science and Technology

Project (A20210231, ZD20220049, Z20220017); Hospital Pharmacy Special

Project of Zhejiang Pharmaceutical Society (2021ZYY33).

CR Abad E, 2019, MOL CELL PROTEOMICS, V18, P231, DOI 10.1074/mcp.RA118.001102

Amado S, 2008, BIOMATERIALS, V29, P4409, DOI 10.1016/j.biomaterials.2008.07.043

Bankston AN, 2019, GLIA, V67, P1745, DOI 10.1002/glia.23646

Belgrad J, 2020, J NEUROSCI, V40, P256, DOI 10.1523/JNEUROSCI.1066-19.2019

Bender JK, 2018, DRUG RESIST UPDATE, V40, P25, DOI 10.1016/j.drup.2018.10.002

Berry C, 2016, EUR RESPIR J, V47, P1591, DOI 10.1183/13993003.01646-2015

Bigelow KM, 2020, ANTIMICROB AGENTS CH, V64, DOI 10.1128/AAC.01178-20

Bolhuis MS, 2018, CLIN INFECT DIS, V67, pS327, DOI 10.1093/cid/ciy625

Chen PW, 2015, ACTA NEUROPATHOL, V130, P605, DOI 10.1007/s00401-015-1482-4

Conradie F, 2020, NEW ENGL J MED, V382, P893, DOI 10.1056/NEJMoa1901814

De Vriese AS, 2006, CLIN INFECT DIS, V42, P1111, DOI 10.1086/501356

Diekema DJ, 2001, LANCET, V358, P1975, DOI 10.1016/S0140-6736(01)06964-1

Falagas ME, 2007, INT J ANTIMICROB AG, V29, P233, DOI 10.1016/j.ijantimicag.2006.08.030

Fang XX, 2022, FRONT PHARMACOL, V13, DOI 10.3389/fphar.2022.921612

Frippiat F, 2004, J ANTIMICROB CHEMOTH, V53, P1114, DOI 10.1093/jac/dkh199

Garrabou G, 2017, ANTIMICROB AGENTS CH, V61, DOI [10.1128/aac.00542-17, 10.1128/AAC.00542-17]

Hu BB, 2022, FRONT PHARMACOL, V13, DOI 10.3389/fphar.2022.778680

Jaspard M, 2020, EMERG INFECT DIS, V26, P1792, DOI 10.3201/eid2608.191499

Lan ZY, 2020, LANCET RESP MED, V8, P383, DOI 10.1016/S2213-2600(20)30047-3

Lee JK, 2019, LANCET INFECT DIS, V19, P46, DOI [10.1016/S1473-3099(18)30480-8, 10.1016/s1473-3099(18)30480-8]

Li R, 2020, THERANOSTICS, V10, P1649, DOI 10.7150/thno.40919

Li R, 2018, BIOMATERIALS, V168, P24, DOI 10.1016/j.biomaterials.2018.03.044

Liu JY, 2022, FRONT ENDOCRINOL, V13, DOI 10.3389/fendo.2022.824215

Liu Q, 2022, J NEUROINFLAMM, V19, DOI 10.1186/s12974-022-02518-7

Mirzayev F, 2021, EUR RESPIR J, V57, DOI 10.1183/13993003.03300-2020

Nunes GDF, 2021, NAT COMMUN, V12, DOI 10.1038/s41467-021-23552-8

O'Daniel PI, 2014, J AM CHEM SOC, V136, P3664, DOI 10.1021/ja500053x

Pertici V, 2014, BIOMATERIALS, V35, P6248, DOI 10.1016/j.biomaterials.2014.04.020

Reed CB, 2020, J NEUROSCI, V40, P6165, DOI 10.1523/JNEUROSCI.0951-20.2020

Rubinstein E, 2003, ANTIMICROB AGENTS CH, V47, P1824, DOI 10.1128/AAC.47.6.1824-1831.2003

Sardella-Silva G, 2021, BIOMOLECULES, V11, DOI 10.3390/biom11121887

Sarhane KA, 2019, NEUROTHERAPEUTICS, V16, P1283, DOI 10.1007/s13311-019-00745-0

Swire M, 2021, J NEUROSCI, V41, P7954, DOI 10.1523/JNEUROSCI.2463-20.2021

Tang S, 2013, BIOMATERIALS, V34, P7086, DOI 10.1016/j.biomaterials.2013.05.080

Tasneen R, 2021, ANTIMICROB AGENTS CH, V65, DOI 10.1128/AAC.00253-21

Wang Q, 2022, FRONT CELL NEUROSCI, V16, DOI 10.3389/fncel.2022.836931

Watzlawick R, 2014, JAMA NEUROL, V71, P91, DOI 10.1001/jamaneurol.2013.4684

WHO, 2019, GLOBAL TUBERCULOSIS, V2022

WHO, 2021, GLOBAL TUBERCULOSIS, V2022

Wu YQ, 2019, AGING DIS, V10, P1233, DOI 10.14336/AD.2018.1024

Yun HY, 2022, OPEN FORUM INFECT DI, V9, DOI 10.1093/ofid/ofab660

Zhang X, 2015, J THORAC DIS, V7, P603, DOI 10.3978/j.issn.2072-1439.2015.03.10

Zotter B, 2022, J NEUROSCI, V42, P183, DOI 10.1523/JNEUROSCI.3096-20.2021

NR 43

TC 10

Z9 14

U1 0

U2 16

PU FRONTIERS MEDIA SA

PI LAUSANNE

PA AVENUE DU TRIBUNAL FEDERAL 34, LAUSANNE, CH-1015, SWITZERLAND

EI 1663-9812

J9 FRONT PHARMACOL

JI Front. Pharmacol.

PD SEP 9

PY 2022

VL 13

AR 946058

DI 10.3389/fphar.2022.946058

PG 12

WC Pharmacology & Pharmacy

WE Science Citation Index Expanded (SCI-EXPANDED)

SC Pharmacology & Pharmacy

GA 4V6NW

UT WOS:000859592800001

OA Green Submitted, gold

DA 2026-05-07

ER

PT J

AU Ioerger, TR

Feng, YC

Chen, XH

Dobos, KM

Victor, TC

Streicher, EM

Warren, RM

Gey van Pittius, NC

Van Helden, PD

Sacchettini, JC

AF Ioerger, Thomas R.

Feng, Yicheng

Chen, Xiaohua

Dobos, Karen M.

Victor, Thomas C.

Streicher, Elizabeth M.

Warren, Robin M.

Gey van Pittius, Nicolaas C.

Van Helden, Paul D.

Sacchettini, James C.

TI The non-clonality of drug resistance in Beijing-genotype isolates of

Mycobacterium tuberculosis from the Western Cape of South Africa

SO BMC GENOMICS

LA English

DT Article

ID PYRAZINAMIDE RESISTANCE; ETHAMBUTOL RESISTANCE; GLOBAL DISSEMINATION;

IMMUNE-RESPONSE; FAMILY STRAINS; MUTATIONS; SPREAD; EPIDEMIOLOGY;

VIRULENCE; COMPLEX

AB Background: The Beijing genotype of M. tuberculosis is a virulent strain that is disseminating worldwide and has a strong association with drug resistance. In the Western Cape of South Africa, epidemiological studies have identified the R220 cluster of the Beijing genotype as a major contributor to a recent outbreak of drug-resistant tuberculosis. Although the outbreak is considered to be due to clonal transmission, the relationship among drug resistant isolates has not yet been established.

Results: To better understand the evolution of drug resistance among these strains, 14 drug-resistant clinical isolates of the Beijing genotype were sequenced by whole-genome sequencing, including eight from R220 and six from a more ancestral Beijing cluster, R86, for comparison. While each cluster shares a distinct resistance mutation for isoniazid, mapping of other drug-resistance mutations onto a phylogenetic tree constructed from single nucleotide polymorphisms shows that resistance mutations to many drugs have arisen multiple times independently within each cluster of isolates. Thus, drug resistance among these isolates appears to be acquired, not clonally derived. This observation suggests that, although the Beijing genotype as a whole might have selective advantages enabling its rapid dissemination, the XDR isolates are relatively less fit and do not propagate well. Although it has been hypothesized that the increased frequency of drug resistance in some Beijing lineages might be caused by a mutator phenotype, no significant shift in synonymous substitution patterns is observed in the genomes.

Conclusion: While MDR-TB is spreading by transmission in the Western Cape, our data suggests that further drug resistance (i.e. XDR-TB) at this stage is acquired.

C1 [Ioerger, Thomas R.] Texas A&M Univ, Dept Comp Sci & Engn, College Stn, TX 77843 USA.

[Feng, Yicheng; Chen, Xiaohua; Sacchettini, James C.] Texas A&M Univ, Dept Biochem & Biophys, College Stn, TX 77843 USA.

[Dobos, Karen M.] Colorado State Univ, Dept Microbiol Immunol & Pathol, Ft Collins, CO 80523 USA.

[Victor, Thomas C.; Streicher, Elizabeth M.; Warren, Robin M.; Gey van Pittius, Nicolaas C.; Van Helden, Paul D.] Univ Stellenbosch, DST NRF Ctr Excellence Biomed TB Res, US MRC Ctr Mol & Cellular Biol,Fac Hlth Sci, Div Mol Biol & Human Genet,Dept Biomed Sci, ZA-7505 Tygerberg, South Africa.

C3 Texas A&M University System; Texas A&M University College Station; Texas

A&M University System; Texas A&M University College Station; Colorado

State University System; Colorado State University Fort Collins;

National Research Foundation - South Africa; Stellenbosch University

RP Ioerger, TR (corresponding author), Texas A&M Univ, Dept Comp Sci & Engn, College Stn, TX 77843 USA.

EM ioerger@cs.tamu.edu

RI Streicher, Elizabeth/O-9597-2019; Dobos, Karen/D-1170-2017; Gey van

Pittius, Nicolaas/D-3434-2015

OI Streicher, Elizabeth/0000-0003-3901-1981; Dobos,

Karen/0000-0001-7115-8524; Gey van Pittius,

Nicolaas/0000-0002-8807-4105; Warren, Robin/0000-0001-5741-7358

FU Robert A. Welch Foundation [A-0015]

FX This work was support in part by funding from the Robert A. Welch

Foundation, grant number A-0015 (JCS).

CR Almeida D, 2005, CLIN INFECT DIS, V40, P881, DOI 10.1086/427940

Anh DD, 2000, EMERG INFECT DIS, V6, P302

Aubry A, 2006, ANTIMICROB AGENTS CH, V50, P104, DOI 10.1128/AAC.50.1.104-112.2006

Bifani PJ, 2002, TRENDS MICROBIOL, V10, P45, DOI 10.1016/S0966-842X(01)02277-6

Bifani PJ, 1996, JAMA-J AM MED ASSOC, V275, P452, DOI 10.1001/jama.275.6.452

Borydorff MW, 2004, TUBERCULOSIS, V84, P337, DOI 10.1016/j.tube.2003.10.002

Calver AD, 2010, EMERG INFECT DIS, V16, P264, DOI 10.3201/eid1602.090968

Cheng SJ, 2000, ANTIMICROB AGENTS CH, V44, P528, DOI 10.1128/AAC.44.3.528-532.2000

Constant P, 2002, J BIOL CHEM, V277, P38148, DOI 10.1074/jbc.M206538200

DeBarber AE, 2000, P NATL ACAD SCI USA, V97, P9677, DOI 10.1073/pnas.97.17.9677

Delcher AL, 2002, NUCLEIC ACIDS RES, V30, P2478, DOI 10.1093/nar/30.11.2478

DESSEN A, 1995, SCIENCE, V267, P1638, DOI 10.1126/science.7886450

Dos Vultos T, 2006, J BACTERIOL, V188, P3159, DOI 10.1128/JB.188.8.3159-3161.2006

Drobniewski F, 2005, JAMA-J AM MED ASSOC, V293, P2726, DOI 10.1001/jama.293.22.2726

Durbach SI, 2003, INFECT IMMUN, V71, P997, DOI 10.1128/IAI.71.2.997-1000.2003

Felsenstein J., 1993, PHYLIP (Phylogeny Inference Package) version 3.5c

Frieden TR, 1996, JAMA-J AM MED ASSOC, V276, P1229, DOI 10.1001/jama.276.15.1229

Gagneux S, 2006, SCIENCE, V312, P1944, DOI 10.1126/science.1124410

Glynn JR, 2002, EMERG INFECT DIS, V8, P843

Glynn JR, 2006, EMERG INFECT DIS, V12, P736, DOI 10.3201/eid1205.050400

Hanekom M, 2007, J CLIN MICROBIOL, V45, P1483, DOI 10.1128/JCM.02191-06

Hillemann D, 2005, ANTIMICROB AGENTS CH, V49, P1229, DOI 10.1128/AAC.49.3.1229-1231.2005

Ioerger TR, 2009, PLOS ONE, V4, DOI 10.1371/journal.pone.0007778

Iwamoto T, 2008, ANTIMICROB AGENTS CH, V52, P3805, DOI 10.1128/AAC.00579-08

Johnson R, 2006, INT J TUBERC LUNG D, V10, P1412

Johnson R, 2006, INT J TUBERC LUNG D, V10, P68

Johnson R, 2010, INT J TUBERC LUNG D, V14, P119

Kamerbeek J, 1997, J CLIN MICROBIOL, V35, P907, DOI 10.1128/JCM.35.4.907-914.1997

Kurtz S, 2004, GENOME BIOL, V5, DOI 10.1186/gb-2004-5-2-r12

Larsen MH, 2007, CURR PROTOC MICROBIO, V10, P12

Li Q, 2002, INFECT IMMUN, V70, P6489, DOI 10.1128/IAI.70.11.6489-6493.2002

López B, 2003, CLIN EXP IMMUNOL, V133, P30, DOI 10.1046/j.1365-2249.2003.02171.x

Manca C, 1999, J IMMUNOL, V162, P6740

Manca C, 2001, P NATL ACAD SCI USA, V98, P5752, DOI 10.1073/pnas.091096998

Marais BJ, 2006, J CLIN MICROBIOL, V44, P3539, DOI 10.1128/JCM.01291-06

MIDDLEBROOK G, 1958, AM J PUBLIC HEALTH N, V48, P844, DOI 10.2105/AJPH.48.7.844

Mlambo CK, 2008, INT J TUBERC LUNG D, V12, P99

Mokrousov I, 2006, ANTIMICROB AGENTS CH, V50, P2820, DOI 10.1128/AAC.00324-06

Morlock GP, 2003, ANTIMICROB AGENTS CH, V47, P3799, DOI 10.1128/AAC.47.12.3799-3805.2003

Mphahlele M, 2008, J CLIN MICROBIOL, V46, P3459, DOI 10.1128/JCM.00973-08

Park YK, 2005, J MICROBIOL METH, V63, P165, DOI 10.1016/j.mimet.2005.03.002

Parwati I, 2010, LANCET INFECT DIS, V10, P103, DOI 10.1016/S1473-3099(09)70330-5

Parwati I, 2010, J INFECT DIS, V201, P553, DOI 10.1086/650311

Pfyffer GE, 2001, EMERG INFECT DIS, V7, P855, DOI 10.3201/eid0705.017514

Pillay M, 2007, CLIN INFECT DIS, V45, P1409, DOI 10.1086/522987

Rad ME, 2003, EMERG INFECT DIS, V9, P838, DOI 10.3201/eid0907.020803

Ramaswamy S., 1998, Tubercle and Lung Disease, V79, P3, DOI 10.1054/tuld.1998.0002

Reed MB, 2004, NATURE, V431, P84, DOI 10.1038/nature02837

Richardson M, 2002, INT J TUBERC LUNG D, V6, P1001

Sreevatsan S, 1997, ANTIMICROB AGENTS CH, V41, P1677, DOI 10.1128/AAC.41.8.1677

Sreevatsan S, 1997, P NATL ACAD SCI USA, V94, P9869, DOI 10.1073/pnas.94.18.9869

Sreevatsan S, 1997, ANTIMICROB AGENTS CH, V41, P636, DOI 10.1128/AAC.41.3.636

Sreevatsan S, 1996, ANTIMICROB AGENTS CH, V40, P1024, DOI 10.1128/AAC.40.4.1024

Strauss OJ, 2008, J CLIN MICROBIOL, V46, P1514, DOI 10.1128/JCM.01938-07

Streicher EM, 2004, J CLIN MICROBIOL, V42, P891, DOI 10.1128/JCM.42.2.891-894.2004

Suzuki Y, 1998, J CLIN MICROBIOL, V36, P1220, DOI 10.1128/JCM.36.5.1220-1225.1998

Toungoussova OS, 2004, FEMS IMMUNOL MED MIC, V42, P281, DOI 10.1016/j.femsim.2004.05.012

Tsenova L, 2005, J INFECT DIS, V192, P98, DOI 10.1086/430614

Tsolaki AG, 2005, J CLIN MICROBIOL, V43, P3185, DOI 10.1128/JCM.43.7.3185-3191.2005

van der Spuy GD, 2009, TUBERCULOSIS, V89, P120, DOI 10.1016/j.tube.2008.09.003

VANSOOLINGEN D, 1995, J CLIN MICROBIOL, V33, P3234, DOI 10.1128/JCM.33.12.3234-3238.1995

Via LE, 2010, J CLIN MICROBIOL, V48, P402, DOI 10.1128/JCM.01476-09

Victor TC, 2007, INT J TUBERC LUNG D, V11, P195

Wang F, 2007, J EXP MED, V204, P73, DOI 10.1084/jem.20062100

Warren R, 1999, ELECTROPHORESIS, V20, P1807, DOI 10.1002/(SICI)1522-2683(19990101)20:8<1807::AID-ELPS1807>3.3.CO;2-0

Warren RM, 2004, J CLIN MICROBIOL, V42, P5774, DOI 10.1128/JCM.42.12.5774-5782.2004

Werngren J, 2003, J CLIN MICROBIOL, V41, P1520, DOI 10.1128/JCM.41.4.1520-1524.2003

Zhang M, 1999, J INFECT DIS, V179, P1213, DOI 10.1086/314738

NR 68

TC 65

Z9 79

U1 0

U2 10

PU BMC

PI LONDON

PA CAMPUS, 4 CRINAN ST, LONDON N1 9XW, ENGLAND

SN 1471-2164

J9 BMC GENOMICS

JI BMC Genomics

PD NOV 26

PY 2010

VL 11

AR 670

DI 10.1186/1471-2164-11-670

PG 14

WC Biotechnology & Applied Microbiology; Genetics & Heredity

WE Science Citation Index Expanded (SCI-EXPANDED)

SC Biotechnology & Applied Microbiology; Genetics & Heredity

GA 697KH

UT WOS:000285512000001

OA Green Submitted, gold

DA 2026-05-07

ER

PT J

AU Singh, DK

Bhaskar, A

Pahuja, I

Shaji, A

Moitra, B

Shi, YF

Dwivedi, VP

Das, G

AF Singh, Dhiraj Kumar

Bhaskar, Ashima

Pahuja, Isha

Shaji, Aishwarya

Moitra, Barnani

Shi, Yufang

Dwivedi, Ved Prakash

Das, Gobardhan

TI Cotreatment With Clofazimine and Rapamycin Eliminates Drug-Resistant

Tuberculosis by Inducing Polyfunctional Central Memory T-Cell Responses

SO JOURNAL OF INFECTIOUS DISEASES

LA English

DT Article

DE central memory T cells; clofazimine; extensively drug-resistant

tuberculosis; helper T cells; immunotherapy; multidrug-resistance;

rapamycin; tuberculosis

ID VACCINE EFFICACY; TDR-TB; AUTOPHAGY; IMMUNITY; THERAPY; DEFENSE; MTOR;

BCG

AB Mycobacterium tuberculosis, the causative agent of tuberculosis, is acquiring drug resistance at a faster rate than the discovery of new antibiotics. Therefore, alternate therapies that can limit the drug resistance and disease recurrence are urgently needed. Emerging evidence indicates that combined treatment with antibiotics and an immunomodulator provides superior treatment efficacy. Clofazimine (CFZ) enhances the generation of T central memory (T-CM) cells by blocking the Kv1.3(+) potassium channels. Rapamycin (RAPA) facilitates M. tuberculosis clearance by inducing autophagy. In this study, we observed that cotreatment with CFZ and RAPA potently eliminates both multiple and extensively drug-resistant (MDR and XDR) clinical isolates of M. tuberculosis in a mouse model by inducing robust T-cell memory and polyfunctional T-CM responses. Furthermore, cotreatment reduces the expression of latency-associated genes of M. tuberculosis in human macrophages. Therefore, CFZ and RAPA cotherapy holds promise for treating patients infected with MDR and XDR strains of M. tuberculosis.

Emergence of drug-resistant variants of Mycobacterium tuberculosis is one of the major concerns limiting the WHO end tuberculosis program. Here, we have demonstrated the antimycobacterial and immunomodulatory potential of 2 repurposed drugs, clofazimine and rapamycin, against MDR and XDR tuberculosis.

C1 [Singh, Dhiraj Kumar; Bhaskar, Ashima; Pahuja, Isha; Shaji, Aishwarya; Dwivedi, Ved Prakash] Int Ctr Genet Engn & Biotechnol, Immunobiol Grp, New Delhi, India.

[Singh, Dhiraj Kumar; Moitra, Barnani; Das, Gobardhan] Jawaharlal Nehru Univ, Special Ctr Mol Med, New Delhi, India.

[Shi, Yufang] Soochow Univ, Suzhou Med Coll, State Key Lab Radiat Med & Protect, Suzhou, Jiangsu, Peoples R China.

[Das, Gobardhan] Jawaharlal Nehru Univ, Special Ctr Mol Med, Aruna Asaf Ali Marg, New Delhi 110067, India.

C3 Department of Biotechnology (DBT) India; International Center for

Genetic Engineering & Biotechnology (ICGEB); International Center for

Genetic Engineering & Biotechnology (ICGEB), New Delhi; Jawaharlal Nehru

University, New Delhi; Soochow University - China; Jawaharlal Nehru

University, New Delhi

RP Das, G (corresponding author), Jawaharlal Nehru Univ, Special Ctr Mol Med, Aruna Asaf Ali Marg, New Delhi 110067, India.

EM gobardhan.das07@gmail.com

RI ; Shi, Yufang/AAE-4431-2020

OI Dwivedi, Ved Prakash/0000-0003-4321-2567; Shi,

Yufang/0000-0001-8964-319X

FU Science and Engineering Research Board (SERB), Department of

Biotechnology (DBT), Department of Science and Technology, Government of

India; International Centre for Genetic Engineering and Biotechnology,

New Delhi, India; DBT [HGK-IYBA]; SERB; Jiangsu Province International

Science and Technology Cooperation Program [BZ2019017]

FX This work was support by Science and Engineering Research Board (SERB),

Department of Biotechnology (DBT), Department of Science and Technology,

Government of India; and International Centre for Genetic Engineering

and Biotechnology, New Delhi, India. A. V. is the recipient of a Junior

Research Fellowship from DBT. A. B. is the recipient of a Fellowship

from DBT (grant number HGK-IYBA); and a Women Excellence Award from

SERB. V. P. D. is the recipient of an Early Career Research Award from

SERB. Y. S. is supported by the Jiangsu Province International Science

and Technology Cooperation Program (grant number BZ2019017).

CR Agarwal N, 2009, NATURE, V460, P98, DOI 10.1038/nature08123

Ahsan Mohamed Jawed, 2015, Infectious Disorders - Drug Targets, V15, P32

[Anonymous], Global Tuberculosis Report 2013

Araki K, 2010, IMMUNOL REV, V235, P234, DOI 10.1111/j.0105-2896.2010.00898.x

Araki K, 2009, NATURE, V460, P108, DOI 10.1038/nature08155

Babu GR, 2012, TUBERCULOSIS, V92, P301, DOI 10.1016/j.tube.2012.02.009

Bai J, 2017, ONCOTARGET, V8, P110693, DOI 10.18632/oncotarget.22690

Bhaskar A, 2020, ELIFE, V9, DOI 10.7554/eLife.55415

Bhaskar A, 2018, IUBMB LIFE, V70, P836, DOI 10.1002/iub.1888

Bhaskar A, 2014, PLOS PATHOG, V10, DOI 10.1371/journal.ppat.1003902

Bhattacharya D, 2014, J BIOL CHEM, V289, P16508, DOI 10.1074/jbc.M114.558098

Chatterjee S, 2011, PLOS PATHOG, V7, DOI 10.1371/journal.ppat.1002378

Counoupas C, 2019, IMMUNOL CELL BIOL, V97, P656, DOI 10.1111/imcb.12275

Das B, 2013, SCI TRANSL MED, V5, DOI 10.1126/scitranslmed.3004912

Domingo-Gonzalez R, 2016, MICROBIOL SPECTR, V4, DOI 10.1128/microbiolspec.TBTB2-0018-2016

Dooley DP, 1997, CLIN INFECT DIS, V25, P872, DOI 10.1086/515543

Dwivedi VP, 2017, FRONT CELL INFECT MI, V7, DOI 10.3389/fcimb.2017.00149

Dwivedi VP, 2012, J BIOL CHEM, V287, P33656, DOI 10.1074/jbc.M112.375154

Fatima S, 2020, J CLIN INVEST, V130, P655, DOI 10.1172/JCI128043

Floto RA, 2007, AUTOPHAGY, V3, P620, DOI 10.4161/auto.4898

FLYNN JL, 1993, J EXP MED, V178, P2249, DOI 10.1084/jem.178.6.2249

Gandhi NR, 2010, LANCET, V375, P1830, DOI 10.1016/S0140-6736(10)60410-2

Gutierrez MG, 2004, CELL, V119, P753, DOI 10.1016/j.cell.2004.11.038

Hester J, 2012, AM J TRANSPLANT, V12, P2008, DOI 10.1111/j.1600-6143.2012.04065.x

Uc-Cachón AH, 2014, PULM PHARMACOL THER, V27, P114, DOI 10.1016/j.pupt.2013.08.001

Jagannath C, 2012, METHODS MOL BIOL, V821, P295, DOI 10.1007/978-1-61779-430-8_18

Jang Jong Geol, 2020, Yeungnam Univ J Med, V37, P277, DOI [10.12701/yujm.2020.00626, 10.12701/yujm.2020.00626]

Jassal M, 2009, LANCET INFECT DIS, V9, P19, DOI 10.1016/S1473-3099(08)70260-3

Jayaraman P, 2016, PLOS PATHOG, V12, DOI 10.1371/journal.ppat.1005490

Khader SA, 2007, NAT IMMUNOL, V8, P369, DOI 10.1038/ni1449

Khan A, 2017, SCI REP-UK, V7, DOI 10.1038/s41598-017-15290-z

Kumari A, 2023, MICROBIOL SPECTR, V11, DOI 10.1128/spectrum.00583-23

Lewinsohn DA, 2017, FRONT IMMUNOL, V8, DOI 10.3389/fimmu.2017.01262

Maggioli MF, 2016, FRONT IMMUNOL, V7, DOI 10.3389/fimmu.2016.00421

Orme IM, 2018, FRONT IMMUNOL, V9, DOI 10.3389/fimmu.2018.00461

Pahuja I, 2023, PLOS PATHOG, V19, DOI 10.1371/journal.ppat.1011165

Prezzemolo T, 2014, FRONT IMMUNOL, V5, DOI 10.3389/fimmu.2014.00180

Prusa J, 2018, PATHOG DIS, V76, DOI 10.1093/femspd/fty054

Puleston DJ, 2014, ELIFE, V3, DOI 10.7554/eLife.03706

Raghuvanshi S, 2010, P NATL ACAD SCI USA, V107, P21653, DOI 10.1073/pnas.1007967107

Rosario FJ, 2016, SCI REP-UK, V6, DOI 10.1038/srep31705

Shen L, 2016, SCI REP-UK, V6, DOI 10.1038/srep38362

Singh AK, 2009, TRANSPL P, V41, P418, DOI 10.1016/j.transproceed.2008.10.079

Singh DK, 2016, J INFECT DIS, V214, P1456, DOI 10.1093/infdis/jiw395

Torrado E, 2010, CYTOKINE GROWTH F R, V21, P455, DOI 10.1016/j.cytogfr.2010.10.004

Tousif S, 2014, J BIOL CHEM, V289, P30190, DOI 10.1074/jbc.C114.598946

Tyagi S, 2015, P NATL ACAD SCI USA, V112, P869, DOI 10.1073/pnas.1416951112

Udwadia Z, 2013, J EPIDEMIOL COMMUN H, V67, P471, DOI 10.1136/jech-2012-201640

Velayati AA, 2013, INT J MYCOBACT, V2, P71, DOI 10.1016/j.ijmyco.2013.04.005

Vergne I, 2006, AUTOPHAGY, V2, P175, DOI 10.4161/auto.2830

NR 50

TC 9

Z9 13

U1 0

U2 13

PU OXFORD UNIV PRESS INC

PI CARY

PA JOURNALS DEPT, 2001 EVANS RD, CARY, NC 27513 USA

SN 0022-1899

EI 1537-6613

J9 J INFECT DIS

JI J. Infect. Dis.

PD NOV 2

PY 2023

VL 228

IS 9

BP 1166

EP 1178

DI 10.1093/infdis/jiad214

EA JUL 2023

PG 13

WC Immunology; Infectious Diseases; Microbiology

WE Science Citation Index Expanded (SCI-EXPANDED)

SC Immunology; Infectious Diseases; Microbiology

GA X4YF4

UT WOS:001033151300001

OA Bronze

DA 2026-05-07

ER

PT J

AU Shyam, M

Verma, H

Bhattacharje, G

Mukherjee, P

Singh, S

Kamilya, S

Jalani, P

Das, S

Dasgupta, A

Mondal, A

Das, AK

Singh, A

Brucoli, F

Bagnéris, C

Dickman, R

Basavanakatti, VN

Babu, PN

Sankaran, V

Dev, A

Sinha, BN

Bhakta, S

Jayaprakash, V

AF Shyam, Mousumi

Verma, Harshita

Bhattacharje, Gourab

Mukherjee, Piyali

Singh, Samsher

Kamilya, Sujit

Jalani, Pushpendu

Das, Swetarka

Dasgupta, Arunava

Mondal, Abhishake

Das, Amit Kumar

Singh, Amit

Brucoli, Federico

Bagneris, Claire

Dickman, Rachael

Basavanakatti, Vinay N.

Babu, Patibandla Naresh

Sankaran, Vadivelan

Dev, Abhimanyu

Sinha, Barij Nayan

Bhakta, Sanjib

Jayaprakash, Venkatesan

TI Mycobactin Analogues with Excellent Pharmacokinetic Profile Demonstrate

Potent Antitubercular Specific Activity and Exceptional Efflux Pump

Inhibition

SO JOURNAL OF MEDICINAL CHEMISTRY

LA English

DT Article

ID DRUG-RESISTANT TUBERCULOSIS; GLYCOPEPTIDOLIPID ANTIGENS; STRUCTURAL

SIMILARITIES; BIOSYNTHESIS PATHWAY; IRON; SIDEROPHORE; DISCOVERY; MODEL;

ANTIMICROBIALS; ACETYLATION

AB In this study, we have designed and synthesized pyrazoline analogues that partially mimic the structure of mycobactin, to address the requirement of novel therapeutics to tackle the emerging global challenge of antimicrobial resistance (AMR). Our investigation resulted in the identification of novel lead compounds 44 and 49 as potential mycobactin biosynthesis inhibitors against mycobacteria. Moreover, candidates efficiently eradicated intracellularly surviving mycobacteria. Thermofluorimetric analysis and molecular dynamics simulations suggested that compounds 44 and 49 bind to salicyl-AMP ligase (MbtA), a key enzyme in the mycobactin biosynthetic pathway. To the best of our knowledge, these are the first rationally designed mycobactin inhibitors to demonstrate an excellent in vivo pharmacokinetic profile. In addition, these compounds also exhibited more potent whole-cell efflux pump inhibition than known efflux pump inhibitors verapamil and chlorpromazine. Results from this study pave the way for the development of 3-(2-hydroxyphenyl)-5-(aryl)-pyrazolines as a new weapon against superbug-associated AMR challenges.

C1 [Shyam, Mousumi; Dev, Abhimanyu; Sinha, Barij Nayan; Jayaprakash, Venkatesan] Birla Inst Technol, Dept Pharmaceut Sci & Technol, Ranchi 835215, Jharkhand, India.

[Shyam, Mousumi; Verma, Harshita; Bagneris, Claire; Bhakta, Sanjib] Birkbeck Univ London, Inst Struct & Mol Biol, Dept Biol Sci, Mycobacteria Res Lab, London WC1E 7HX, England.

[Bhattacharje, Gourab; Das, Amit Kumar] Indian Inst Technol Kharagpur, Dept Biotechnol, Kharagpur 721302, W Bengal, India.

[Mukherjee, Piyali; Singh, Samsher; Singh, Amit] Indian Inst Sci, Dept Microbiol & Cell Biol, Bangalore 560012, Karnataka, India.

[Mukherjee, Piyali; Singh, Samsher; Singh, Amit] Indian Inst Sci, Ctr Infect Dis Res, Bangalore 560012, Karnataka, India.

[Kamilya, Sujit; Mondal, Abhishake] Indian Inst Sci, Solid State & Struct Chem Unit, Bangalore 560012, Karnataka, India.

[Jalani, Pushpendu; Das, Swetarka; Dasgupta, Arunava] CSIR Cent Drug Res Inst, Microbiol Div, Lucknow 226031, Uttar Pradesh, India.

[Brucoli, Federico] De Montfort Univ, Leicester Sch Pharm, Leicester LE1 9BH, Leics, England.

[Dickman, Rachael] Univ London, UCL Sch Pharm, Pharmaceut & Biol Chem, London WC1N 1AX, England.

[Basavanakatti, Vinay N.; Babu, Patibandla Naresh; Sankaran, Vadivelan] Eurofins Advinus Ltd, Bengaluru 560058, India.

C3 Birla Institute of Technology Mesra; University of London; Birkbeck

University London; Indian Institute of Technology System (IIT System);

Indian Institute of Technology (IIT) - Kharagpur; Indian Institute of

Science (IISC) - Bangalore; Indian Institute of Science (IISC) -

Bangalore; Indian Institute of Science (IISC) - Bangalore; Council of

Scientific & Industrial Research (CSIR) - India; CSIR - Central Drug

Research Institute (CDRI); De Montfort University; University of London;

University College London; Eurofins Scientific

RP Jayaprakash, V (corresponding author), Birla Inst Technol, Dept Pharmaceut Sci & Technol, Ranchi 835215, Jharkhand, India.; Bhakta, S (corresponding author), Birkbeck Univ London, Inst Struct & Mol Biol, Dept Biol Sci, Mycobacteria Res Lab, London WC1E 7HX, England.

EM s.bhakta@bbk.ac.uk; venkatessanj@bitmesra.ac.in

RI Jayaprakash, Venkatesan/B-4367-2011; Sinha, Barij/G-5948-2012;

Mukherjee, Piyali/G-8034-2019; Dickman, Rachael/ABC-3201-2020; Dev,

Abhimanyu/AAJ-6767-2020; Basavanakatti, Vinay/PQV-8817-2026; Verma,

Harshita/JXM-8419-2024; Das, Amit Kumar/KIL-2919-2024; Mondal,

Abhishake/Q-9804-2016; Bhattacharje, Gourab/KRQ-8639-2024

OI Bagneris, Claire/0000-0002-0561-7574; kamilya,

sujit/0000-0003-4881-0638; Mukherjee, Piyali/0000-0002-3681-2559; Singh,

Saher/0000-0001-7075-931X; Dev, Abhimanyu/0000-0001-7034-6021; Verma,

Harshita/0000-0002-4610-3676; Bhakta, Sanjib/0000-0002-1165-9295; Das,

Amit Kumar/0000-0001-9703-1898; Basavanakatti,

Vinay/0000-0003-1113-6184; Dasgupta, Arunava/0000-0001-9014-1904;

Mondal, Abhishake/0000-0002-5061-2326; Bhattacharje,

Gourab/0000-0001-7823-8033; SHYAM, MOUSUMI/0000-0002-0791-3772

FU Global Challenges Research Fund [GCRF 105123-11]; Medical Research

Council [G0802079] Funding Source: researchfish

FX V.J., A.D., and M.S. acknowledge the Department of Science and

Technology Science and Engineering Research Board (DSTSERB File. No:

EMR/2016/000675 dt. 05/08/2016). M.S. is a Newton-Bhabha International

Fellow (BT/IN/NBPP/MS/20/2019-20 dt. 04/03/2020). S.B. acknowledges the

Global Challenges Research Fund (GCRF 105123-11) for the financial

support in building a UK-India capacity for tackling antimicrobial drug

resistance in tuberculosis. P.M. gratefully acknowledges DBT-RA Program

in Biotechnology and Life Sciences.

CR Abraham Mark James, 2015, SoftwareX, V1-2, P19, DOI [10.1016/j.softx.2015.06.001, 10.1016/j.softx.2015.06.001]

Agre N, 2020, CHEM BIOL DRUG DES, V95, P192, DOI 10.1111/cbdd.13624

Andries K, 2014, PLOS ONE, V9, DOI 10.1371/journal.pone.0102135

Barry C, 2001, TUBERCULOSIS, V81, P1, DOI 10.1054/tube.2001.0288

Berman HM, 2000, NUCLEIC ACIDS RES, V28, P235, DOI 10.1093/nar/28.1.235

Bhakta S, 2016, J MED CHEM, V59, P2780, DOI 10.1021/acs.jmedchem.6b00031

BRENNAN PJ, 1981, EUR J BIOCHEM, V115, P7, DOI 10.1111/j.1432-1033.1981.tb06190.x

BRENNAN PJ, 1995, ANNU REV BIOCHEM, V64, P29, DOI 10.1146/annurev.bi.64.070195.000333

Bruker, 2012, APEX APEX2 SMART SAI

Caminero JA, 2006, INT J TUBERC LUNG D, V10, P829

Carvalho R, 2011, PLOS ONE, V6, DOI 10.1371/journal.pone.0016779

Chan ED, 2004, AM J RESP CRIT CARE, V169, P1103, DOI 10.1164/rccm.200308-1159OC

Danquah Cynthia A, 2016, Curr Protoc Microbiol, V40, DOI [10.1002/9780471729259.mc1708s40, 10.1002/9780471729259.mc1708s40]

De Voss JJ, 1999, J BACTERIOL, V181, P4443, DOI 10.1128/JB.181.15.4443-4451.1999

De Voss JJ, 2000, P NATL ACAD SCI USA, V97, P1252, DOI 10.1073/pnas.97.3.1252

DeLano WL., 2004, PyMOL user's guide

Dodda LS, 2017, NUCLEIC ACIDS RES, V45, pW331, DOI 10.1093/nar/gkx312

Eker B, 2008, EMERG INFECT DIS, V14, P1700, DOI 10.3201/eid1411.080729

Evangelopoulos D, 2010, METHODS MOL BIOL, V642, P193, DOI 10.1007/978-1-60327-279-7_15

Falkinham JO, 2018, FRONT MICROBIOL, V9, DOI 10.3389/fmicb.2018.01613

Farrugia LJ, 2012, J APPL CRYSTALLOGR, V45, P849, DOI 10.1107/S0021889812029111

Ferguson L, 2019, CHEMMEDCHEM, V14, P1735, DOI 10.1002/cmdc.201900217

Ferreras JA, 2005, NAT CHEM BIOL, V1, P29, DOI 10.1038/nchembio706

Ferreras JA, 2011, BIOORG MED CHEM LETT, V21, P6533, DOI 10.1016/j.bmcl.2011.08.052

Fogolari F, 2002, J MOL RECOGNIT, V15, P377, DOI 10.1002/jmr.577

Gordien AY, 2009, J ETHNOPHARMACOL, V126, P500, DOI 10.1016/j.jep.2009.09.007

Guex N, 1997, ELECTROPHORESIS, V18, P2714, DOI 10.1002/elps.1150181505

Gulick AM, 2009, ACS CHEM BIOL, V4, P811, DOI 10.1021/cb900156h

Gupta A, 2012, J ANTIMICROB CHEMOTH, V67, P1380, DOI 10.1093/jac/dks056

Gupta A, 2012, IMMUNOBIOLOGY, V217, P363, DOI 10.1016/j.imbio.2011.07.008

Harding E, 2020, LANCET RESP MED, V8, P19, DOI 10.1016/S2213-2600(19)30418-7

Helmke, 2018, UBUNTU UNLEASHED 201

Horn HW, 2004, J CHEM PHYS, V120, P9665, DOI 10.1063/1.1683075

Huynh Kathy, 2015, Curr Protoc Protein Sci, V79, DOI [10.1002/0471140864.ps2809s79, 10.1002/0471140864.ps2809s79]

Jiang F, 2014, J PHYS CHEM B, V118, P6983, DOI 10.1021/jp5017449

Johnson EO, 2020, ACS INFECT DIS, V6, P56, DOI 10.1021/acsinfecdis.9b00373

Jones CM, 2014, P NATL ACAD SCI USA, V111, P1945, DOI 10.1073/pnas.1311402111

Jones CM, 2010, J BACTERIOL, V192, P6411, DOI 10.1128/JB.00986-10

KARPLUS M, 1990, NATURE, V347, P631, DOI 10.1038/347631a0

Khan A, 2008, J MICROBIOL METH, V73, P62, DOI 10.1016/j.mimet.2008.01.015

Kumar M, 2016, ACS INFECT DIS, V2, P714, DOI 10.1021/acsinfecdis.6b00111

Kumari R, 2014, J CHEM INF MODEL, V54, P1951, DOI 10.1021/ci500020m

Lipinski C.A., 1986, ANNU REP MED CHEM, V21, P283, DOI [10.1016/S0065-7743(08)61137-9, DOI 10.1016/S0065-7743(08)61137-9]

LOPEZMARIN LM, 1994, MICROBIOL-UK, V140, P1109, DOI 10.1099/13500872-140-5-1109

Lun S, 2013, ANTIMICROB AGENTS CH, V57, P5138, DOI 10.1128/AAC.00918-13

Martínez L, 2009, J COMPUT CHEM, V30, P2157, DOI 10.1002/jcc.21224

Matsumoto C, 2023, COMMUN BIOL, V6, DOI [10.1038/s42003-023-05527-1, 10.1186/1758-2946-4-17]

McMahon MD, 2012, J BACTERIOL, V194, P2809, DOI 10.1128/JB.00088-12

Migliori GB, 2007, EUR RESPIR J, V29, P423, DOI 10.1183/09031936.00001307

Milano A, 2009, TUBERCULOSIS, V89, P84, DOI 10.1016/j.tube.2008.08.003

Mitnick CD, 2008, NEW ENGL J MED, V359, P563, DOI 10.1056/NEJMoa0800106

Molla A, 2018, CHEMISTRYSELECT, V3, P8669, DOI 10.1002/slct.201801867

Mori M, 2020, J MED CHEM, V63, P7066, DOI 10.1021/acs.jmedchem.0c00373

Morris GM, 2009, J COMPUT CHEM, V30, P2785, DOI 10.1002/jcc.21256

Naik SK, 2014, BMC COMPLEM ALTERN M, V14, DOI 10.1186/1472-6882-14-87

O'Donnell G, 2007, PHYTOTHER RES, V21, P653, DOI 10.1002/ptr.2136

Quadri LEN, 1998, CHEM BIOL, V5, P631, DOI 10.1016/S1074-5521(98)90291-5

Quadri Luis E. N., 2007, Infectious Disorders - Drug Targets, V7, P230, DOI 10.2174/187152607782110040

RATLDGE C, 1982, J GEN MICROBIOL, V128, P1559

Ratledge C, 2004, TUBERCULOSIS, V84, P110, DOI 10.1016/j.tube.2003.08.012

Raviglione MC, 2003, TUBERCULOSIS, V83, P4, DOI 10.1016/S1472-9792(02)00071-9

Recht J, 2001, J BACTERIOL, V183, P5718, DOI 10.1128/JB.183.19.5718-5724.2001

Reger AS, 2007, BIOCHEMISTRY-US, V46, P6536, DOI 10.1021/bi6026506

RICHMOND TJ, 1984, J MOL BIOL, V178, P63, DOI 10.1016/0022-2836(84)90231-6

Sacksteder KA, 2012, FUTURE MICROBIOL, V7, P823, DOI [10.2217/fmb.12.56, 10.2217/FMB.12.56]

Senisterra G, 2012, ASSAY DRUG DEV TECHN, V10, P128, DOI 10.1089/adt.2011.0390

SHARMAN GJ, 1995, CHEM BIOL, V2, P553, DOI 10.1016/1074-5521(95)90189-2

Sheldrick G.M., 1996, SADABS

Sheldrick GM, 2015, ACTA CRYSTALLOGR C, V71, P3, DOI [10.1107/S2053229614024218, 10.1107/S0108767307043930, 10.1107/S2053273314026370]

Shyam M, 2021, J MED CHEM, V64, P71, DOI 10.1021/acs.jmedchem.0c01176

SNOW GA, 1970, BACTERIOL REV, V34, P99, DOI 10.1128/MMBR.34.2.99-125.1970

Solnier J, 2020, MOLECULES, V25, DOI 10.3390/molecules25030734

Spackman MA, 2009, CRYSTENGCOMM, V11, P19, DOI 10.1039/b818330a

Stirrett KL, 2008, BIOORG MED CHEM LETT, V18, P2662, DOI 10.1016/j.bmcl.2008.03.025

Tan SL, 2017, CHEM ENG J, V316, P243, DOI 10.1016/j.cej.2017.01.079

Tekaia F., 1999, Tubercle and Lung Disease, V79, P329, DOI 10.1054/tuld.1999.0220

Velayati AA, 2013, INT J CLIN EXP MED, V6, P307

Vergne AF, 2000, NAT PROD REP, V17, P99, DOI 10.1039/a809397k

Vergnolle O, 2016, J BIOL CHEM, V291, P22315, DOI 10.1074/jbc.M116.744532

Viljoen A, 2017, MOL MICROBIOL, V104, P889, DOI 10.1111/mmi.13675

Wells RM, 2013, PLOS PATHOG, V9, DOI 10.1371/journal.ppat.1003120

NR 81

TC 15

Z9 18

U1 0

U2 19

PU AMER CHEMICAL SOC

PI WASHINGTON

PA 1155 16TH ST, NW, WASHINGTON, DC 20036 USA

SN 0022-2623

EI 1520-4804

J9 J MED CHEM

JI J. Med. Chem.

PD JAN 13

PY 2022

VL 65

IS 1

BP 234

EP 256

DI 10.1021/acs.jmedchem.1c01349

EA JAN 2022

PG 23

WC Chemistry, Medicinal

WE Science Citation Index Expanded (SCI-EXPANDED); Index Chemicus (IC)

SC Pharmacology & Pharmacy

GA ZP6QF

UT WOS:000741107300001

OA Green Submitted

DA 2026-05-07

ER

PT J

AU Islam, MM

Tan, YJ

Hameed, HMA

Liu, Y

Chhotaray, C

Cai, XY

Liu, ZY

Lu, ZL

Wang, S

Cai, XS

Su, BY

Li, XJ

Tan, SY

Liu, JX

Zhang, TY

AF Islam, Md Mahmudul

Tan, Yaoju

Hameed, H. M. Adnan

Liu, Yang

Chhotaray, Chiranjibi

Cai, Xiaoyin

Liu, Zhiyong

Lu, Zhili

Wang, Shuai

Cai, Xingshan

Su, Biyi

Li, Xinjie

Tan, Shouyong

Liu, Jianxiong

Zhang, Tianyu

TI Prevalence and molecular characterization of amikacin resistance among

Mycobacterium tuberculosis clinical isolates from southern China

SO JOURNAL OF GLOBAL ANTIMICROBIAL RESISTANCE

LA English

DT Article

DE Amikacin; Tuberculosis; Mutation; Resistance; Injectable drug;

Susceptibility testing

ID KANAMYCIN RESISTANCE; CROSS-RESISTANCE; MUTATIONS; STRAINS

AB Objectives: Amikacin is the only second-line injectable antituberculosis (anti-TB) drug still recommended for multidrug-resistant tuberculosis (MDR-TB) treatment when a short MDR-TB regimen is designed. Mutations in rrs and eis are reported to be associated with resistance to amikacin. In this study, we investigated the incidence of rrs, eis, tap and whiB7 mutations in amikacin-resistant Mycobacterium tuberculosis clinical isolates to find the proportion of different mutations related to amikacin resistance.

Methods: A total of 395 clinical isolates of M. tuberculosis were used for phenotypic drug susceptibility testing (DST) to 10 drugs with the Lowenstein-Jensen (L-J) method. We sequenced rrs, eis, tap and whiB7 genes in 178 M. tuberculosis clinical isolates (89 amikacin-resistant isolates and 89 of 306 amikacin-susceptible isolates).

Results: Our data showed that 22.53% (89/395) M. tuberculosis clinical isolates were resistant to amikacin. Of the 89 amikacin-resistant isolates, 89.89% (80/89) were MDR-TB, of which 12.36% (11/89) were preextensively drug-resistant TB (pre-XDR-TB) and 77.53% (69/89) were XDR-TB. The rrs mutations were found in 82% (73/89) in amikacin-resistant M. tuberculosis clinical isolates. The A1401G alteration in the rrs gene was the most dominant mutation (80.90%; 72/89). Five mutations were detected as new in rrs, tap and whiB7. Notably, 13.48% (12/89) amikacin-resistant isolates had no known mutation in these genes.

Conclusions: Our data reveal that the rrs mutation is a predominant molecular marker of amikacin resistance in southern China. Analysis of the rrs gene mutations will significantly reduce the time and cost to diagnose amikacin resistance in TB patients. Other unknown amikacin resistance mechanism(s) exist. (C) 2020 The Author(s). Published by Elsevier Ltd on behalf of International Society for Antimicrobial Chemotherapy.

C1 [Islam, Md Mahmudul; Hameed, H. M. Adnan; Liu, Yang; Chhotaray, Chiranjibi; Cai, Xiaoyin; Liu, Zhiyong; Lu, Zhili; Wang, Shuai; Zhang, Tianyu] Chinese Acad Sci, Guangzhou Inst Biomed & Hlth GIBH, State Key Lab Resp Dis, Guangzhou 510530, Guangdong, Peoples R China.

[Islam, Md Mahmudul; Hameed, H. M. Adnan; Chhotaray, Chiranjibi; Cai, Xiaoyin; Wang, Shuai; Zhang, Tianyu] Univ Chinese Acad Sci UCAS, Beijing 100049, Peoples R China.

[Tan, Yaoju; Cai, Xingshan; Su, Biyi; Li, Xinjie; Tan, Shouyong; Liu, Jianxiong] Guangzhou Chest Hosp, Dept Clin Lab, State Key Lab Resp Dis, Guangzhou 510095, Peoples R China.

[Islam, Md Mahmudul; Hameed, H. M. Adnan; Chhotaray, Chiranjibi; Cai, Xiaoyin; Liu, Zhiyong; Lu, Zhili; Wang, Shuai; Zhang, Tianyu] Chinese Acad Sci, Guangzhou Inst Biomed & Hlth GIBH, Guangdong Hong Kong Macao Joint Lab Resp Infect D, Guangzhou 510530, Guangdong, Peoples R China.

C3 Chinese Academy of Sciences; Guangzhou Institute of Biomedicine &

Health, CAS; State Key Laboratory of Respiratory Disease; Chinese

Academy of Sciences; University of Chinese Academy of Sciences, CAS;

State Key Laboratory of Respiratory Disease; Chinese Academy of

Sciences; Guangzhou Institute of Biomedicine & Health, CAS

RP Zhang, TY (corresponding author), Chinese Acad Sci, Guangzhou Inst Biomed & Hlth GIBH, TB Res Lab, State Key Lab Resp Dis, Room A132,190 Kaiyuan Ave,Sci Pk, Guangzhou 510530, Guangdong, Peoples R China.

EM zhang_tianyu@gibh.ac.cn

RI li, xinjie/LJK-4266-2024; Zhang, Tianyu/K-4801-2015

OI Ali, Sagor/0009-0000-8890-7675; Islam, Mahmudul/0000-0001-7264-5180;

Zhang, Tianyu/0000-0001-5647-6014; Chhotaray,

Chiranjibi/0000-0002-5310-8758

FU National Mega Project of China for Main Infectious Diseases

[2017ZX10302301-003-002, 2019ZX09721001-003-003]; National Natural

Science Foundation of China [81572037, 81973372, 21920102003]; Chinese

Academy of Sciences [154144KYSB20150045]; Public Research and Capacity

Building Project of Guangdong Provenience [2017A020212004]; Science and

Technology Innovation Leader of Guangdong Province [2016TX03R095];

CAS-TWAS President's PhD Fellowship Program; UCAS Fellowship Program;

Key-Area Research and Development Program of Guangdong Provenience

[2019B110233003]

FX This work was supported by the National Mega Project of China for Main

Infectious Diseases [grant number 2017ZX10302301-003-002] and Innovative

Drugs [grant number 2019ZX09721001-003-003], the National Natural

Science Foundation of China [grant numbers

81572037,81973372,21920102003], the Chinese Academy of Sciences Grants

[grant number 154144KYSB20150045], the Public Research and Capacity

Building Project [grant number 2017A020212004 and the Key-Area Research

and Development Program [grant number 2019B110233003] of Guangdong

Provenience. The funders had no role in study design, data collection

and analysis, decision to publish or preparation of the manuscript. The

study was sponsored by the Science and Technology Innovation Leader of

Guangdong Province [grant number 2016TX03R095], to TZ, CAS-TWAS

President's PhD Fellowship Program (to MMI and CC) and the UCAS

Fellowship Program(to HMAH) for international PhD students.

CR Al-Mutairi NM, 2019, BMC INFECT DIS, V19, DOI 10.1186/s12879-018-3638-z

Andries K, 2014, PLOS ONE, V9, DOI 10.1371/journal.pone.0102135

[Anonymous], 2019, CONSOLIDATED GUIDELI

Collins LA, 1997, ANTIMICROB AGENTS CH, V41, P1004, DOI 10.1128/AAC.41.5.1004

Gikalo MB, 2012, J ANTIMICROB CHEMOTH, V67, P2107, DOI 10.1093/jac/dks178

Hameed HMA, 2018, FRONT CELL INFECT MI, V8, DOI 10.3389/fcimb.2018.00114

Hartkoorn RC, 2014, ANTIMICROB AGENTS CH, V58, P2979, DOI 10.1128/AAC.00037-14

Islam MM, 2019, CLIN MICROBIOL INFEC, V25, DOI 10.1016/j.cmi.2018.12.008

Islam MM, 2017, J GENET GENOMICS, V44, P21, DOI 10.1016/j.jgg.2016.10.002

Miotto P, 2017, EUR RESPIR J, V50, DOI 10.1183/13993003.01354-2017

Morris RP, 2005, P NATL ACAD SCI USA, V102, P12200, DOI 10.1073/pnas.0505446102

Osei-Wusu S, 2018, INFECT DRUG RESIST, V11, P239, DOI 10.2147/IDR.S152720

Oudghiri A, 2018, BMC INFECT DIS, V18, DOI 10.1186/s12879-018-3009-9

Perdigao J, 2010, J ANTIMICROB CHEMOTH, V65, P224, DOI 10.1093/jac/dkp452

Reeves AZ, 2013, ANTIMICROB AGENTS CH, V57, P1857, DOI 10.1128/AAC.02191-12

Siddiqi S, 2006, WHOHTMTB2008392

Sowajassatakul A, 2014, BMC MICROBIOL, V14, DOI 10.1186/1471-2180-14-165

World Health Organization, 2018, GLOB TUB REP

Yuan XL, 2012, J CLIN MICROBIOL, V50, P2404, DOI 10.1128/JCM.06860-11

Zaunbrecher MA, 2009, P NATL ACAD SCI USA, V106, P20004, DOI 10.1073/pnas.0907925106

NR 20

TC 13

Z9 17

U1 0

U2 4

PU ELSEVIER SCI LTD

PI OXFORD

PA THE BOULEVARD, LANGFORD LANE, KIDLINGTON, OXFORD OX5 1GB, OXON, ENGLAND

SN 2213-7165

EI 2213-7173

J9 J GLOB ANTIMICROB RE

JI J. Glob. Antimicrob. Resist.

PD SEP

PY 2020

VL 22

BP 290

EP 295

DI 10.1016/j.jgar.2020.02.019

PG 6

WC Infectious Diseases; Pharmacology & Pharmacy

WE Science Citation Index Expanded (SCI-EXPANDED)

SC Infectious Diseases; Pharmacology & Pharmacy

GA NS4CB

UT WOS:000572210500016

OA Green Submitted, gold

DA 2026-05-07

ER

PT J

AU de Knegt, GJ

Bruning, O

ten Kate, MT

de Jong, M

van Belkum, A

Endtz, HP

Breit, TM

Bakker-Woudenberg, IAJM

de Steenwinkel, JEM

AF de Knegt, Gerjo J.

Bruning, Oskar

ten Kate, Marian T.

de Jong, Mark

van Belkum, Alex

Endtz, Hubert P.

Breit, Timo M.

Bakker-Woudenberg, Irma A. J. M.

de Steenwinkel, Jurriaan E. M.

TI Rifampicin-induced transcriptome response in rifampicin-resistant

Mycobacterium tuberculosis

SO TUBERCULOSIS

LA English

DT Article

DE Rifampicin-resistance; Mycobacterium tuberculosis; Microarray analysis

ID DRUG-RESISTANCE; GENE-EXPRESSION; MICROARRAY ANALYSIS; CELL-WALL;

EFFLUX; VIRULENCE; MUTATIONS; BIOSYNTHESIS; VALIDATION; SALICYLATE

AB Tuberculosis (TB) is still a major life-threatening infectious disease, within which especially the rise of multidrug resistant TB (MDR-TB) is currently worrying. This study focuses on mechanisms of development of rifampicin resistance, since rifampicin seems to play an important role in the development of MDR-TB.

To provide further insight in rifampicin resistance, we performed a genome-wide transcriptional profile analysis for Mycobacterium tuberculosis (M. tuberculosis) using microarray technology and qRT-PCR analysis. We exposed a rifampicin-susceptible H37Rv wild type (H37Rv-WT) and a rifampicin-resistant progeny H37Rv strain with a H526Y mutation in the rpoB gene (H37Rv-H526Y) to several concentrations of rifampicin, to define the effect of rifampicin on the transcription profile.

Our study showed that there are resistance-dependant differences in response between both M. tuberculosis strains. Gene clusters associated with efflux, transport and virulence were altered in the rifampicin-resistant H37Rv mutant compared to the rifampicin-susceptible H37Rv-WT strain after exposure to rifampicin.

We conclude that the small gene cluster Rv0559c-Rv0560c in the H37Rv-H526Y strain was remarkably up-regulated in the microarray analysis and qRT-PCR results and appeared to be dependent on rifampicin concentration and time of exposure. Therefore this study suggests that Rv0559c and Rv0560c play a pivotal role in rifampicin resistance of M. tuberculosis. Further investigation of Rv0559c and Rv0560c is needed to reveal function and mechanism of both genes that were triggered upon rifampicin exposure. (C) 2012 Elsevier Ltd. All rights reserved.

C1 [de Knegt, Gerjo J.; ten Kate, Marian T.; van Belkum, Alex; Endtz, Hubert P.; Bakker-Woudenberg, Irma A. J. M.; de Steenwinkel, Jurriaan E. M.] Univ Med Ctr Rotterdam, Erasmus MC, Dept Med Microbiol & Infect Dis, NL-3000 CA Rotterdam, Netherlands.

[Bruning, Oskar; de Jong, Mark; Breit, Timo M.] Univ Amsterdam, Fac Sci, Swammerdam Inst Life Sci, MicroArray Dept, Amsterdam, Netherlands.

[Bruning, Oskar; de Jong, Mark; Breit, Timo M.] Univ Amsterdam, Fac Sci, Swammerdam Inst Life Sci, Integrat Bioinformat Unit, Amsterdam, Netherlands.

[van Belkum, Alex] Biomerieux, R&D Microbiol, La Balme Les Grottes, France.

C3 Erasmus University Rotterdam; Erasmus MC; University of Amsterdam;

University of Amsterdam; bioMerieux

RP de Knegt, GJ (corresponding author), Univ Med Ctr Rotterdam, Erasmus MC, Dept Med Microbiol & Infect Dis, POB 2040, NL-3000 CA Rotterdam, Netherlands.

EM g.deknegt@erasmusmc.nl

CR da Silva PEA, 2011, FEMS IMMUNOL MED MIC, V63, P1, DOI 10.1111/j.1574-695X.2011.00831.x

[Anonymous], WHO report 2021

Balganesh M, 2012, ANTIMICROB AGENTS CH, V56, P2643, DOI 10.1128/AAC.06003-11

Balganesh M, 2010, ANTIMICROB AGENTS CH, V54, P5167, DOI 10.1128/AAC.00610-10

Camacho LR, 2001, J BIOL CHEM, V276, P19845, DOI 10.1074/jbc.M100662200

Cui XG, 2005, BIOSTATISTICS, V6, P59, DOI 10.1093/biostatistics/kxh018

Denkin S, 2005, ARCH MICROBIOL, V184, P152, DOI 10.1007/s00203-005-0037-9

Deshayes C, 2010, MOL MICROBIOL, V78, P989, DOI 10.1111/j.1365-2958.2010.07385.x

Ding Yongzeng, 2007, J Biomol Tech, V18, P321

Domenech P, 2005, INFECT IMMUN, V73, P3492, DOI 10.1128/IAI.73.6.3492-3501.2005

Drobniewski FA, 1998, J MED MICROBIOL, V47, P189, DOI 10.1099/00222615-47-3-189

Gupta AK, 2010, MICROB DRUG RESIST, V16, P21, DOI 10.1089/mdr.2009.0054

Hotter GS, 2011, VET MICROBIOL, V151, P91, DOI 10.1016/j.vetmic.2011.02.030

Hotter GS, 2005, J BACTERIOL, V187, P2267, DOI 10.1128/JB.187.7.2267-2277.2005

Huitric E, 2006, ANTIMICROB AGENTS CH, V50, P2860, DOI 10.1128/AAC.00303-06

JARLIER V, 1994, FEMS MICROBIOL LETT, V123, P11, DOI 10.1111/j.1574-6968.1994.tb07194.x

Louw GE, 2009, ANTIMICROB AGENTS CH, V53, P3181, DOI 10.1128/AAC.01577-08

Louw GE, 2011, AM J RESP CRIT CARE, V184, P269, DOI 10.1164/rccm.201011-1924OC

Milano A, 2009, TUBERCULOSIS, V89, P84, DOI 10.1016/j.tube.2008.08.003

Morey JS, 2006, BIOL PROCED ONLINE, V8, P175, DOI 10.1251/bpo126

Nikaido H, 1998, CURR OPIN MICROBIOL, V1, P516, DOI 10.1016/S1369-5274(98)80083-0

Parish T, 2003, MICROBIOL-SGM, V149, P1423, DOI 10.1099/mic.0.26245-0

Rajeevan MS, 2001, J MOL DIAGN, V3, P26, DOI 10.1016/S1525-1578(10)60646-0

Schuessler DL, 2012, PLOS ONE, V7, DOI 10.1371/journal.pone.0034471

Siu GKH, 2011, J ANTIMICROB CHEMOTH, V66, P730, DOI 10.1093/jac/dkq519

Smyth GK., 2004, STAT APPL GENET MOL, V3, DOI DOI 10.2202/1544-6115.1027

Stephan J, 2004, ANTIMICROB AGENTS CH, V48, P4163, DOI 10.1128/AAC.48.11.4163-4170.2004

Stewart GR, 2005, MOL MICROBIOL, V55, P1127, DOI 10.1111/j.1365-2958.2004.04450.x

Storey JD, 2003, P NATL ACAD SCI USA, V100, P9440, DOI 10.1073/pnas.1530509100

Sun ZH, 2001, FEMS MICROBIOL LETT, V203, P211, DOI 10.1111/j.1574-6968.2001.tb10843.x

TELENTI A, 1993, LANCET, V341, P647, DOI 10.1016/0140-6736(93)90417-F

Wang F, 2007, CHEM BIOL, V14, P543, DOI 10.1016/j.chembiol.2007.04.005

Wolfinger RD, 2001, J COMPUT BIOL, V8, P625, DOI 10.1089/106652701753307520

Wurmbach E, 2003, METHODS, V31, P306, DOI 10.1016/S1046-2023(03)00161-0

NR 34

TC 54

Z9 61

U1 0

U2 57

PU CHURCHILL LIVINGSTONE

PI EDINBURGH

PA JOURNAL PRODUCTION DEPT, ROBERT STEVENSON HOUSE, 1-3 BAXTERS PLACE,

LEITH WALK, EDINBURGH EH1 3AF, MIDLOTHIAN, SCOTLAND

SN 1472-9792

J9 TUBERCULOSIS

JI Tuberculosis

PD JAN

PY 2013

VL 93

IS 1

BP 96

EP 101

DI 10.1016/j.tube.2012.10.013

PG 6

WC Immunology; Microbiology; Respiratory System

WE Science Citation Index Expanded (SCI-EXPANDED)

SC Immunology; Microbiology; Respiratory System

GA 094UC

UT WOS:000315288300013

DA 2026-05-07

ER

PT J

AU Prasasty, VD

Cindana, S

Ivan, FX

Zahroh, H

Sinaga, E

AF Prasasty, Vivitri Dewi

Cindana, Sandra

Ivan, Fransiskus Xaverius

Zahroh, Hilyatuz

Sinaga, Ernawati

TI Structure-based discovery of novel inhibitors of Mycobacterium

tuberculosis CYP121 from Indonesian natural products

SO COMPUTATIONAL BIOLOGY AND CHEMISTRY

LA English

DT Article

DE CYP121; Molecular docking; Molecular dynamic; Tuberculosis

ID DIAGRAMS; DOCKING; DRUGS

AB Tuberculosis (TB) continues to be a serious global health threat with the emergence of multidrug-resistant tuberculosis (MDR-TB) and extremely drug-resistant tuberculosis (XDR-TB). There is an urgent need to discover new drugs to deal with the advent of drug-resistant TB variants. This study aims to find new M. tuberculosis CYP121 inhibitors by the screening of Indonesian natural products using the principle of structure-based drug design and discovery. In this work, eight natural compounds isolated from Rhoeo spathacea and Pluchea indica were selected based on their antimycobacterial activity. Derivatives compound were virtually designed from these natural molecules to improve the interaction of ligands with CYP121. Virtual screening of ligands was carried out using AutoDock Vina followed by 50 ns molecular dynamics simulation using YASARA to study the inhibition mechanism of the ligands. Two ligands, i.e., kaempferol (KAE) and its benzyl derivative (KAE3), are identified as the best CYP121 inhibitors based on their binding affinities and adherence to the Lipinski's rule. Results of molecular dynamics simulation indicate that KAE and KAE3 possess a unique inhibitory mechanism against CYP121 that is different from GGJ (control ligand). The control ligand alters the overall dynamics of the receptor, which is indicated by changes in residue flexibility away from CYP121 binding site. Meanwhile, the dynamic changes caused by the binding of KAE and KAE3 are isolated around the binding site of CYP121. These ligands can be developed for further potential biological activities.

C1 [Prasasty, Vivitri Dewi] Atma Jaya Catholic Univ, Fac Biotechnol, Jakarta 12930, Dki Jakarta, Indonesia.

[Cindana, Sandra; Ivan, Fransiskus Xaverius] Surya Univ, Fac Life Sci, Dept Biotechnol, Banten 15810, Indonesia.

[Zahroh, Hilyatuz] Univ Yarsi, Genet Res Ctr, Jakarta 10510, Dki Jakarta, Indonesia.

[Sinaga, Ernawati] Univ Nasl, Fac Biol, Jakarta 12520, Indonesia.

C3 University Katolik Indonesia Atma Jaya; Surya University; YARSI

University

RP Prasasty, VD (corresponding author), Atma Jaya Catholic Univ, Fac Biotechnol, Jakarta 12930, Dki Jakarta, Indonesia.

EM vivitri.dewi@atmajaya.ac.id

RI Zahroh, Hilyatuz/KCY-6660-2024; Prasasty, Vivitri/JYQ-0910-2024; Sinaga,

Ernawati/AAI-5200-2021

OI Sinaga, Ernawati/0000-0002-4739-6909

FU Scientific Committee of UAJ; Scientific Committee of SU; Scientific

Committee of UY; Scientific Committee of UNAS

FX The authors would like to thank Dr. Bimo Ario Tejo, UCSI University,

Malaysia, for a valuable suggestion regarding the manuscript content.

Ultimately, the authors express great gratitude to the Scientific

Committee of UAJ, SU, UY and UNAS for supporting the CADD project.

CR Afendi FM, 2012, PLANT CELL PHYSIOL, V53, DOI 10.1093/pcp/pcr165

Alexandrov AI, 2008, STRUCTURE, V16, P351, DOI 10.1016/j.str.2008.02.004

Alonso H, 2006, MED RES REV, V26, P531, DOI 10.1002/med.20067

[Anonymous], INF DIS REL TRAV

[Anonymous], J FARM UDAYANA

[Anonymous], 2013, EC BURDEN TUBERCULOS

[Anonymous], MED PLANT RES ASIA

[Anonymous], STRATEGI NASIONAL PE

[Anonymous], BR J MED PRACTITIONE

[Anonymous], DEV ANTITUBERCULOSIS

[Anonymous], ARXIV11117183

Chinsembu KC, 2016, ACTA TROP, V153, P46, DOI 10.1016/j.actatropica.2015.10.004

Hoagland DT, 2016, ADV DRUG DELIVER REV, V102, P55, DOI 10.1016/j.addr.2016.04.026

Jayaram B, 2012, BMC BIOINFORMATICS, V13, DOI 10.1186/1471-2105-13-S17-S7

Kanabus A., 2016, Information about tuberculosis

Kim S, 2016, NUCLEIC ACIDS RES, V44, pD1202, DOI 10.1093/nar/gkv951

Koli U, 2014, J BIOMED NANOTECHNOL, V10, P1953, DOI 10.1166/jbn.2014.1928

Krieger E, 2002, PROTEINS, V47, P393, DOI 10.1002/prot.10104

Laskowski RA, 2011, J CHEM INF MODEL, V51, P2778, DOI 10.1021/ci200227u

Lechartier B, 2014, EMBO MOL MED, V6, P158, DOI 10.1002/emmm.201201772

Leys D, 2003, J BIOL CHEM, V278, P5141, DOI 10.1074/jbc.M209928200

Lipinski CA, 1997, ADV DRUG DELIVER REV, V23, P3, DOI 10.1016/S0169-409X(96)00423-1

Matsumoto C, 2023, COMMUN BIOL, V6, DOI [10.1038/s42003-023-05527-1, 10.1186/1758-2946-4-17]

Matteelli A, 2018, EUR RESPIR REV, V27, DOI 10.1183/16000617.0035-2018

McLean KJ, 2008, J BIOL CHEM, V283, P33406, DOI 10.1074/jbc.M802115200

Miller RL, 2015, STRUCTURE, V23, P2291, DOI 10.1016/j.str.2015.07.024

Mohamad S, 2011, J ETHNOPHARMACOL, V133, P1021, DOI 10.1016/j.jep.2010.11.037

Nussinov R, 2014, J AM CHEM SOC, V136, P17692, DOI 10.1021/ja510028c

Nzila A, 2011, FUTURE MED CHEM, V3, P1413, DOI [10.4155/FMC.11.95, 10.4155/fmc.11.95]

O'Boyle NM, 2011, J CHEMINFORMATICS, V3, DOI 10.1186/1758-2946-3-33

Oelschlaeger P, 2003, BIOCHEMISTRY-US, V42, P8945, DOI 10.1021/bi0300332

Poce G, 2014, EUR J MED CHEM, V86, P335, DOI 10.1016/j.ejmech.2014.08.066

Radji M., 2015, Journal of Applied Pharmaceutical Science, V5, P19, DOI 10.7324/japs.2015.50104

Saludes JP, 2002, PHYTOTHER RES, V16, P683, DOI 10.1002/ptr.1003

Skjærven L, 2011, FUTURE MED CHEM, V3, P2079, DOI [10.4155/FMC.11.159, 10.4155/fmc.11.159]

Sliwoski G, 2014, PHARMACOL REV, V66, P334, DOI 10.1124/pr.112.007336

Suriyaphan O., 2014, Mahidol University Journal of Pharmaceutical Sciences, V41, P1

Tan JBL, 2015, J FOOD SCI TECH MYS, V52, P2394, DOI 10.1007/s13197-013-1236-z

Tiberi S, 2018, PULMONOLOGY, V24, P86, DOI 10.1016/j.rppnen.2017.10.009

Trott O, 2010, J COMPUT CHEM, V31, P455, DOI 10.1002/jcc.21334

Vettoretti G, 2016, SCI REP-UK, V6, DOI 10.1038/srep23830

WALLACE AC, 1995, PROTEIN ENG, V8, P127, DOI 10.1093/protein/8.2.127

WHO, 2015, GLOB TUB REP 2015

Zhang XJ, 2015, TRENDS BIOCHEM SCI, V40, P79, DOI 10.1016/j.tibs.2014.12.005

Zoete V, 2011, J COMPUT CHEM, V32, P2359, DOI 10.1002/jcc.21816

NR 45

TC 16

Z9 20

U1 0

U2 11

PU ELSEVIER SCI LTD

PI London

PA 125 London Wall, London, ENGLAND

SN 1476-9271

EI 1476-928X

J9 COMPUT BIOL CHEM

JI Comput. Biol. Chem.

PD APR

PY 2020

VL 85

AR 107205

DI 10.1016/j.compbiolchem.2020.107205

PG 9

WC Biology; Computer Science, Interdisciplinary Applications

WE Science Citation Index Expanded (SCI-EXPANDED)

SC Life Sciences & Biomedicine - Other Topics; Computer Science

GA LG8JK

UT WOS:000528339900009

DA 2026-05-07

ER

PT J

AU Bei, JL

Bigi, MM

Lima, A

Zhang, Q

Blanco, FC

Lopez, B

Yu, T

Wang, ZL

Dai, ZY

Chen, Z

Cataldi, AA

Sasiain, MD

Ritacco, V

De la Barrera, S

Soria, MA

Durán, R

Bigi, F

AF Bei, Jinlong

Bigi, Maria Mercedes

Lima, Analia

Zhang, Qi

Blanco, Federico Carlos

Lopez, Beatriz

Yu, Ting

Wang, Zhilin

Dai, Zhangyan

Chen, Zhuang

Cataldi, Angel Adrian

Sasiain, Maria del Carmen

Ritacco, Viviana

De la Barrera, Silvia

Soria, Marcelo Abel

Duran, Rosario

Bigi, Fabiana

TI A Phenotypic Characterization of Two Isolates of a Multidrug-Resistant

Outbreak Strain of Mycobacterium tuberculosis with Opposite

Epidemiological Fitness

SO BIOMED RESEARCH INTERNATIONAL

LA English

DT Article

ID MYCOLIC ACIDS; CELL; CYCLOPROPANATION; POLYMORPHISMS; VIRULENCE;

ANTIGEN; CLUSTER

AB Tuberculosis (TB) is an infectious disease, caused by Mycobacterium tuberculosis, primarily affecting the lungs. The M. tuberculosis strain of the Haarlem family named M was responsible for a large multidrug-resistant TB (MDR-TB) outbreak in Buenos Aires. This outbreak started in the early 1990s and in the mid 2000s still accounted for 29% of all MDR-TB cases in Argentina. By contrast, a clonal variant of strain M, named 410, has caused a single tuberculosis case since the onset of the outbreak. The molecular bases of the high epidemiological fitness of the M strain remain unclear. To assess its unique molecular properties, herein, we performed a comparative protein and lipid analysis of a representative clone of the M strain (Mp) and the nonprosperous M variant 410. We also evaluated their growth in low pH. The variant 410 had higher levels of latency proteins under standard conditions and delayed growth at low pH, suggesting that it is more sensitive to stress stimuli than Mp. Moreover, Mp showed higher levels of mycolic acids covalently attached to the cell wall and lower accumulation of free mycolic acids in the outer layer than the 410 strain. The low expression of latency proteins together with the reduced content of surface mycolic acids may facilitate Mp to evade the host immune responses.

C1 [Bei, Jinlong; Zhang, Qi; Yu, Ting; Wang, Zhilin; Dai, Zhangyan; Chen, Zhuang] GDAAS, AGRObiol Gene Res Ctr, Guangzhou, Peoples R China.

[Bigi, Maria Mercedes; Soria, Marcelo Abel] UBA, Sch Agron, Fac Agron, Buenos Aires, DF, Argentina.

[Lima, Analia; Duran, Rosario] Inst Pasteur Montevideo, Montevideo, Uruguay.

[Lima, Analia; Duran, Rosario] Inst Invest Biol Clemente Estable, Montevideo, Uruguay.

[Blanco, Federico Carlos; Cataldi, Angel Adrian; Bigi, Fabiana] Consejo Nacl Invest Cient & Tecn, INTA, IABIMO, Inst Biotecnol, Buenos Aires, DF, Argentina.

[Lopez, Beatriz; Ritacco, Viviana] Natl Inst Infect Dis ANLIS Carlos Malbran, Buenos Aires, DF, Argentina.

[Sasiain, Maria del Carmen; De la Barrera, Silvia] Natl Acad Med, CONICET, IMEX, Buenos Aires, DF, Argentina.

C3 University of Buenos Aires; Pasteur Network; Institut Pasteur de

Montevideo; Instituto de Investigaciones Biologicas Clemente Estable

Uruguay; Instituto Nacional de Tecnologia Agropecuaria (INTA); Consejo

Nacional de Investigaciones Cientificas y Tecnicas (CONICET); Buenos

Aires National Academy of Medicine; IMEX - Instituto de Medicina

Experimental; Consejo Nacional de Investigaciones Cientificas y Tecnicas

(CONICET)

RP Bigi, F (corresponding author), Consejo Nacl Invest Cient & Tecn, INTA, IABIMO, Inst Biotecnol, Buenos Aires, DF, Argentina.

EM beijinlong@gdaas.cn; mebigi@hotmail.com; alima@pasteur.edu.uy;

zhangqi@gdaas.cn; blanco.federico@inta.gob.ar; bealopez@anlis.gov.ar;

yuting@gdaas.cn; 55424414@qq.com; daizhangyan@gdaas.cn;

chenzhuang@gdaas.cn; cataldi.angeladrian@inta.gob.ar;

mariadelcarmen.sasiain@hotmail.com; ritaccoviviana@gmail.com;

sildelabar@gmail.com; soria@agro.uba.ar; duran@pasteur.edu.uy;

bigi.fabiana@inta.gob.ar

RI Qi, Zhang/GZK-9710-2022; Ritacco, Viviana/E-8135-2011; Durán,

Rosario/GQH-8502-2022; Lima, Analía/ABD-7806-2022; BIGI,

Mercedes/KJL-8523-2024

OI Durán, Rosario/0000-0002-8575-9651; Lima, Analía/0000-0002-1855-2327;

BIGI, Mercedes/0000-0001-8711-1413; Bei, Jinlong/0000-0001-8421-162X

FU INTA [I105]; CONICET [22520170100001CO]; International Cooperation

Project China [12190076]; International Cooperation Project Argentina

[12190076]; Presidential Foundation of Guangdong Academy of Agricultural

Sciences of China (China) [201727]; Science and Technology Program of

Guangzhou (China) [201904010432]; Science and Technology Program of

Guangdong Province (China) [2014B020201002]; Academy of Finland (AKA)

[201727] Funding Source: Academy of Finland (AKA); Austrian Science Fund

(FWF) [I105] Funding Source: Austrian Science Fund (FWF)

FX We are grateful to Julia Sabio Garcia and Mary Jackson for the critical

reading of this paper. We thank Valeria Rocha for her valuable technical

help. This work was supported by I105-INTA, CONICET 22520170100001CO,

the International Cooperation Project between China and Argentina

(12190076, China), the Presidential Foundation of Guangdong Academy of

Agricultural Sciences of China (201727, China), the Science and

Technology Program of Guangzhou (201904010432, China), and the Science

and Technology Program of Guangdong Province (2014B020201002, China).

CR Albrethsen J, 2013, MOL CELL PROTEOMICS, V12, P1180, DOI 10.1074/mcp.M112.018846

Alderwick LJ, 2015, CSH PERSPECT MED, V5, DOI 10.1101/cshperspect.a021113

Amigo N, 2016, PLOS ONE, V11, DOI 10.1371/journal.pone.0166883

[Anonymous], 2013, INFOSTAT SOFWARE EST

Barkan D, 2012, INFECT IMMUN, V80, P1958, DOI 10.1128/IAI.00021-12

Basile JI, 2011, J INFECT DIS, V204, P1054, DOI 10.1093/infdis/jir460

Belisle JT, 1997, SCIENCE, V276, P1420, DOI 10.1126/science.276.5317.1420

Bigi MM, 2016, MICROBIOL IMMUNOL, V60, P552, DOI 10.1111/1348-0421.12402

Boon C, 2002, J BACTERIOL, V184, P6760, DOI 10.1128/JB.184.24.6760-6767.2002

Bunker RD, 2015, P NATL ACAD SCI USA, V112, P4310, DOI 10.1073/pnas.1422387112

Carvalho P. C., 2012, CURR PROTOC BIOINFOR, V13, P13, DOI DOI 10.1002/0471250953.BI1319S40

Carvalho PC, 2016, NAT PROTOC, V11, P102, DOI 10.1038/nprot.2015.133

Carvalho PC, 2009, BIOINFORMATICS, V25, P2734, DOI 10.1093/bioinformatics/btp489

Cho S, 2000, P NATL ACAD SCI USA, V97, P12210, DOI 10.1073/pnas.210391497

Datta P, 2006, MOL MICROBIOL, V62, P1655, DOI 10.1111/j.1365-2958.2006.05491.x

Santangelo MD, 2009, MICROBIOL-SGM, V155, P2245, DOI 10.1099/mic.0.027086-0

Dubnau E, 2000, MOL MICROBIOL, V36, P630, DOI 10.1046/j.1365-2958.2000.01882.x

Dulberger CL, 2020, NAT REV MICROBIOL, V18, P47, DOI 10.1038/s41579-019-0273-7

Duncan C, 2017, PLOS ONE, V12, DOI 10.1371/journal.pone.0179996

Eng JK, 2015, J AM SOC MASS SPECTR, V26, P1865, DOI 10.1007/s13361-015-1179-x

Forrellad MA, 2013, VIRULENCE, V4, P3, DOI 10.4161/viru.22329

Galagan JE, 2013, NATURE, V499, P178, DOI 10.1038/nature12337

Geffner L, 2013, CLIN EXP IMMUNOL, V3, P1

Geffner L, 2014, PLOS ONE, V9, DOI 10.1371/journal.pone.0097837

Geffner L, 2009, INFECT IMMUN, V77, P5025, DOI 10.1128/IAI.00224-09

Mariotti S, 2013, J IMMUNOL, V191, P274, DOI 10.4049/jimmunol.1202900

Bigi MM, 2017, TUBERCULOSIS, V103, P28, DOI 10.1016/j.tube.2016.12.007

Mi HY, 2016, NUCLEIC ACIDS RES, V44, pD336, DOI 10.1093/nar/gkv1194

Monteserin J, 2019, AM J TROP MED HYG, V101, P1308, DOI 10.4269/ajtmh.19-0397

Ramakers C, 2003, NEUROSCI LETT, V339, P62, DOI 10.1016/S0304-3940(02)01423-4

Rao V, 2005, J EXP MED, V201, P535, DOI 10.1084/jem.20041668

Rao V, 2006, J CLIN INVEST, V116, P1660, DOI 10.1172/JCI27335

Reed MB, 2007, J BACTERIOL, V189, P2583, DOI 10.1128/JB.01670-06

Ritacco V, 1997, J INFECT DIS, V176, P637, DOI 10.1086/514084

Ritacco V, 2012, INFECT GENET EVOL, V12, P711, DOI 10.1016/j.meegid.2011.06.006

Sherman DR, 2001, P NATL ACAD SCI USA, V98, P7534, DOI 10.1073/pnas.121172498

Stadthagen G, 2005, J BIOL CHEM, V280, P40699, DOI 10.1074/jbc.M508332200

Telenti A, 1997, NAT MED, V3, P567, DOI 10.1038/nm0597-567

Yokobori N, 2013, INFECT GENET EVOL, V16, P151, DOI 10.1016/j.meegid.2013.01.007

Yokobori N, 2012, FEMS IMMUNOL MED MIC, V66, P363, DOI 10.1111/j.1574-695X.2012.01024.x

NR 40

TC 2

Z9 2

U1 0

U2 1

PU HINDAWI LTD

PI LONDON

PA ADAM HOUSE, 3RD FLR, 1 FITZROY SQ, LONDON, W1T 5HF, ENGLAND

SN 2314-6133

EI 2314-6141

J9 BIOMED RES INT

JI Biomed Res. Int.

PD APR 8

PY 2020

VL 2020

AR 4741237

DI 10.1155/2020/4741237

PG 9

WC Biotechnology & Applied Microbiology; Medicine, Research & Experimental

WE Science Citation Index Expanded (SCI-EXPANDED)

SC Biotechnology & Applied Microbiology; Research & Experimental Medicine

GA LI0LC

UT WOS:000529175700004

OA Green Submitted, hybrid

DA 2026-05-07

ER

PT J

AU Rahim, MA

Seo, H

Barman, I

Hossain, MS

Shuvo, MSH

Song, HY

AF Rahim, Md Abdur

Seo, Hoonhee

Barman, Indrajeet

Hossain, Mohammed Solayman

Shuvo, Md Sarower Hossen

Song, Ho-Yeon

TI Insights into Autophagy in Microbiome Therapeutic Approaches for

Drug-Resistant Tuberculosis

SO CELLS

LA English

DT Review

DE drug-resistant tuberculosis; microbiome therapeutics; autophagy

ID MYCOBACTERIUM-TUBERCULOSIS; PHAGOSOME MATURATION; PULMONARY

TUBERCULOSIS; CULTURED MACROPHAGES; IMMUNE-RESPONSES; RAT-LIVER;

IFN-GAMMA; PROTEIN; PROBIOTICS; FUSION

AB Tuberculosis, primarily caused by Mycobacterium tuberculosis, is an airborne lung disease and continues to pose a significant global health threat, resulting in millions of deaths annually. The current treatment for tuberculosis involves a prolonged regimen of antibiotics, which leads to complications such as recurrence, drug resistance, reinfection, and a range of side effects. This scenario underscores the urgent need for novel therapeutic strategies to combat this lethal pathogen. Over the last two decades, microbiome therapeutics have emerged as promising next-generation drug candidates, offering advantages over traditional medications. In 2022, the Food and Drug Administration approved the first microbiome therapeutic for recurrent Clostridium infections, and extensive research is underway on microbiome treatments for various challenging diseases, including metabolic disorders and cancer. Research on microbiomes concerning tuberculosis commenced roughly a decade ago, and the scope of this research has broadened considerably over the last five years, with microbiome therapeutics now viewed as viable options for managing drug-resistant tuberculosis. Nevertheless, the understanding of their mechanisms is still in its infancy. Although autophagy has been extensively studied in other diseases, research into its role in tuberculosis is just beginning, with preliminary developments in progress. Against this backdrop, this comprehensive review begins by succinctly outlining tuberculosis' characteristics and assessing existing treatments' strengths and weaknesses, followed by a detailed examination of microbiome-based therapeutic approaches for drug-resistant tuberculosis. Additionally, this review focuses on establishing a basic understanding of microbiome treatments for tuberculosis, mainly through the lens of autophagy as a mechanism of action. Ultimately, this review aims to contribute to the foundational comprehension of microbiome-based therapies for tuberculosis, thereby setting the stage for the further advancement of microbiome therapeutics for drug-resistant tuberculosis.

C1 [Rahim, Md Abdur; Barman, Indrajeet; Hossain, Mohammed Solayman; Shuvo, Md Sarower Hossen; Song, Ho-Yeon] Soonchunhyang Univ, Sch Med, Dept Microbiol & Immunol, 31 Suncheonhyang 6 Gil, Cheonan Si 31151, South Korea.

[Rahim, Md Abdur; Seo, Hoonhee; Barman, Indrajeet; Hossain, Mohammed Solayman; Shuvo, Md Sarower Hossen; Song, Ho-Yeon] Soonchunhyang Univ, Sch Med, Human Microbiome Med Res Ctr HM MRC, 22 Soonchunhyang Ro, Asan 31538, South Korea.

[Seo, Hoonhee; Song, Ho-Yeon] Soonchunhyang Univ, Probiot Microbiome Commercializat Res Ctr PMC, 22 Soonchunhyang Ro, Asan 31538, South Korea.

C3 Soonchunhyang University; Soonchunhyang University; Soonchunhyang

University

RP Song, HY (corresponding author), Soonchunhyang Univ, Sch Med, Dept Microbiol & Immunol, 31 Suncheonhyang 6 Gil, Cheonan Si 31151, South Korea.; Song, HY (corresponding author), Soonchunhyang Univ, Sch Med, Human Microbiome Med Res Ctr HM MRC, 22 Soonchunhyang Ro, Asan 31538, South Korea.; Song, HY (corresponding author), Soonchunhyang Univ, Probiot Microbiome Commercializat Res Ctr PMC, 22 Soonchunhyang Ro, Asan 31538, South Korea.

EM songmic@sch.ac.kr

RI Rahim, Abdur/E-9099-2015; Song, Ho-Yeon/G-7168-2016; Shuvo, Md Sazzad

Mahmud/OIR-9803-2025; Barman, Indrajeet/GRR-4795-2022

OI Barman, Indrajeet/0000-0003-1180-0380; Hossain, Mohammed

Solayman/0009-0004-8313-0886

FU National Research Foundation of Korea's Leading Research Center

[RS-2023-00219563, P248400003]; National Research Foundation of Korea's

Leading Research Center project; Korea Institute for Advancement of

Technology (KIAT) under the Ministry of Trade, Industry and Energy

(MOTIE); Soonchunhyang University Research Fund

FX This research received support from the National Research Foundation of

Korea's Leading Research Center project (RS-2023-00219563) under the

Ministry of Science and ICT. It also gained support from the

"Establishment of innovative infrastructure for manufacturing process

for next-generation microbiome drugs (P248400003)" program, overseen by

the Korea Institute for Advancement of Technology (KIAT) under the

Ministry of Trade, Industry and Energy (MOTIE). Further support came

from the Soonchunhyang University Research Fund.

CR Abdallah AM, 2007, NAT REV MICROBIOL, V5, P883, DOI 10.1038/nrmicro1773

ADAMS DO, 1976, AM J PATHOL, V84, P164

Alam MZ, 2023, FRONT IMMUNOL, V13, DOI 10.3389/fimmu.2022.1046472

Algood HMS, 2005, CLIN INFECT DIS, V41, pS189, DOI 10.1086/429994

Anes E, 2003, NAT CELL BIOL, V5, P793, DOI 10.1038/ncb1036

Arbex MA, 2010, J BRAS PNEUMOL, V36, P641, DOI 10.1590/S1806-37132010000500017

ARMSTRONG JA, 1975, J EXP MED, V142, P1, DOI 10.1084/jem.142.1.1

ARMSTRONG JA, 1971, J EXP MED, V134, P713, DOI 10.1084/jem.134.3.713

Asahara T, 2016, ANTIMICROB AGENTS CH, V60, P3041, DOI 10.1128/AAC.02928-15

ASHFORD TP, 1962, J CELL BIOL, V12, P198, DOI 10.1083/jcb.12.1.198

Axe EL, 2008, J CELL BIOL, V182, P685, DOI 10.1083/jcb.200803137

Bach H, 2008, CELL HOST MICROBE, V3, P316, DOI 10.1016/j.chom.2008.03.008

Bajic SS, 2020, SCI REP-UK, V10, DOI 10.1038/s41598-020-58177-2

Beharka AA, 2002, J IMMUNOL, V169, P3565, DOI 10.4049/jimmunol.169.7.3565

Benech N, 2024, CLIN MICROBIOL INFEC, V30, P462, DOI 10.1016/j.cmi.2023.12.007

Bento CF, 2015, DNA CELL BIOL, V34, P228, DOI 10.1089/dna.2014.2745

Blaser MJ, 2007, NATURE, V449, P843, DOI 10.1038/nature06198

Bonfili L, 2017, SCI REP-UK, V7, DOI 10.1038/s41598-017-02587-2

Breasted J.H., 1927, Ancient Records of Egypt: Indices, VVolume 5

Brodin P, 2004, TRENDS MICROBIOL, V12, P500, DOI 10.1016/j.tim.2004.09.007

Callura JM, 2012, P NATL ACAD SCI USA, V109, P5850, DOI 10.1073/pnas.1203808109

Canfora EE, 2015, NAT REV ENDOCRINOL, V11, P577, DOI 10.1038/nrendo.2015.128

Castillo EF, 2012, P NATL ACAD SCI USA, V109, pE3168, DOI 10.1073/pnas.1210500109

Chandra P, 2022, NAT REV MICROBIOL, V20, P750, DOI 10.1038/s41579-022-00763-4

Chen C, 2022, GUT, V71, P2233, DOI 10.1136/gutjnl-2021-326269

Chen JW, 2014, CELL PHYSIOL BIOCHEM, V34, P1175, DOI 10.1159/000366330

CHIANG HL, 1989, SCIENCE, V246, P382, DOI 10.1126/science.2799391

Clemens DL, 2000, INFECT IMMUN, V68, P2671, DOI 10.1128/IAI.68.5.2671-2684.2000

Cooper AM, 2011, MUCOSAL IMMUNOL, V4, P252, DOI 10.1038/mi.2011.13

Cooper AM, 2009, ANNU REV IMMUNOL, V27, P393, DOI 10.1146/annurev.immunol.021908.132703

Cuervo AM, 1998, MOL BIOL CELL, V9, P1995, DOI 10.1091/mbc.9.8.1995

Cuervo Ana Maria, 1995, American Journal of Physiology, V269, pC1200

Das T, 2021, SCI REP-UK, V11, DOI 10.1038/s41598-021-82538-0

David LA, 2014, GENOME BIOL, V15, DOI 10.1186/gb-2014-15-7-r89

Day TA, 2010, EUR J IMMUNOL, V40, P1663, DOI 10.1002/eji.201040299

Demissie A, 2006, INFECT IMMUN, V74, P2817, DOI 10.1128/IAI.74.5.2817-2822.2006

Deretic V, 2004, CELL MICROBIOL, V6, P999, DOI 10.1111/j.1462-5822.2004.00449.x

DETER RL, 1967, J CELL BIOL, V35, pC11, DOI 10.1083/jcb.35.2.C11

Diallo D, 2021, FRONT CELL INFECT MI, V11, DOI 10.3389/fcimb.2021.673100

Dice J.F., 2000, LYSOSOMAL PATHWAYS P

Dikic I, 2018, NAT REV MOL CELL BIO, V19, P349, DOI 10.1038/s41580-018-0003-4

Dorhoi A, 2014, EUR J IMMUNOL, V44, P2380, DOI 10.1002/eji.201344219

Duan MM, 2021, PLOS ONE, V16, DOI 10.1371/journal.pone.0255446

DUVE CD, 1955, BIOCHEM J, V60, P604, DOI 10.1042/bj0600604

Elkington P, 2011, J CLIN INVEST, V121, P1827, DOI 10.1172/JCI45666

Engevik MA, 2019, MBIO, V10, DOI 10.1128/mBio.01087-19

Ernst JD, 1998, INFECT IMMUN, V66, P1277, DOI 10.1128/IAI.66.4.1277-1281.1998

Feng SW, 2024, NAT MICROBIOL, V9, P684, DOI 10.1038/s41564-024-01608-x

Fijan S, 2019, BIOMED RES INT, V2019, DOI 10.1155/2019/7585486

Fitzgibbon G, 2020, EUR J IMMUNOL, V50, P326, DOI 10.1002/eji.201948322

Franch HA, 2001, J BIOL CHEM, V276, P19126, DOI 10.1074/jbc.M101777200

Fratti RA, 2001, J CELL BIOL, V154, P631, DOI 10.1083/jcb.200106049

Fratti RA, 2003, P NATL ACAD SCI USA, V100, P5437, DOI 10.1073/pnas.0737613100

Fuller R., 1992, Probiotics: the scientific basis., P1

Ganaie AA, 2011, PLOS ONE, V6, DOI 10.1371/journal.pone.0027590

Gasbarrini G, 2016, J CLIN GASTROENTEROL, V50, pS116, DOI 10.1097/MCG.0000000000000697

GAYNOR CD, 1995, J IMMUNOL, V155, P5343

Ghadimi D, 2010, INT IMMUNOPHARMACOL, V10, P694, DOI 10.1016/j.intimp.2010.03.014

Ghodousi A, 2022, EUR RESPIR J, V59, DOI 10.1183/13993003.02102-2021

Ginsberg AM, 2007, NAT MED, V13, P290, DOI 10.1038/nm0307-290

Gioacchini G, 2013, REPROD FERT DEVELOP, V25, P1115, DOI 10.1071/RD12187

Gopalakrishnan V, 2018, CANCER CELL, V33, P570, DOI 10.1016/j.ccell.2018.03.015

Gutierrez MC, 2005, PLOS PATHOG, V1, P55, DOI 10.1371/journal.ppat.0010005

Gutierrez MG, 2004, CELL, V119, P753, DOI 10.1016/j.cell.2004.11.038

Ha J, 2015, MOL ASPECTS MED, V46, P46, DOI 10.1016/j.mam.2015.08.002

Hamilton-Miller JMT, 2003, BRIT J NUTR, V90, P845, DOI 10.1079/BJN2003954

Han CQ, 2016, CELL PHYSIOL BIOCHEM, V38, P2464, DOI 10.1159/000445597

Harris J, 2007, IMMUNITY, V27, P505, DOI 10.1016/j.immuni.2007.07.022

Hartstra AV, 2015, DIABETES CARE, V38, P159, DOI 10.2337/dc14-0769

Heckmann BL, 2017, J MOL BIOL, V429, P3561, DOI 10.1016/j.jmb.2017.08.012

Hill C, 2014, NAT REV GASTRO HEPAT, V11, P506, DOI 10.1038/nrgastro.2014.66

Hossain MS, 2024, J BACTERIOL VIROL, V54, P325, DOI [10.4167/jbv.2024.54.4.325, 10.4167/jbv.2024.54.4.325, DOI 10.4167/JBV.2024.54.4.325]

Huttenhower C, 2012, NATURE, V486, P207, DOI 10.1038/nature11234

Huynh KK, 2007, EMBO J, V26, P313, DOI 10.1038/sj.emboj.7601511

Hyttinen JMT, 2013, BBA-MOL CELL RES, V1833, P503, DOI 10.1016/j.bbamcr.2012.11.018

Inaba Y, 2016, AM J PHYSIOL-GASTR L, V311, pG634, DOI 10.1152/ajpgi.00092.2016

Itakura E, 2012, CELL, V151, P1256, DOI 10.1016/j.cell.2012.11.001

Itoh T, 2008, MOL BIOL CELL, V19, P2916, DOI 10.1091/mbc.E07-12-1231

Jia GC, 2020, FOOD FUNCT, V11, P270, DOI [10.1039/c9fo02476j, 10.1039/C9FO02476J]

Jiang WY, 2021, CELLS-BASEL, V10, DOI 10.3390/cells10112814

Karska-Wysocki B, 2010, MICROBIOL RES, V165, P674, DOI 10.1016/j.micres.2009.11.008

Kashyap PC, 2017, MAYO CLIN PROC, V92, P1855, DOI 10.1016/j.mayocp.2017.10.004

Kelley VA, 2003, MOL BIOL CELL, V14, P3366, DOI 10.1091/mbc.e02-12-0780

Khader SA, 2006, J EXP MED, V203, P1805, DOI 10.1084/jem.20052545

Kim KH, 2012, P NATL ACAD SCI USA, V109, P7729, DOI 10.1073/pnas.1120251109

Kiple KennethF., 2000, CAMBRIDGE WORLD HIST, V2

Klionsky DJ, 2000, SCIENCE, V290, P1717, DOI 10.1126/science.290.5497.1717

Kolloli A, 2017, FRONT MED-LAUSANNE, V4, DOI 10.3389/fmed.2017.00171

Kopp MV, 2008, CLIN EXP ALLERGY, V38, P602, DOI 10.1111/j.1365-2222.2007.02911.x

Krug S, 2021, FRONT IMMUNOL, V12, DOI 10.3389/fimmu.2021.660916

Kusner DJ, 2005, CLIN IMMUNOL, V114, P239, DOI 10.1016/j.clim.2004.07.016

Lee Y, 2021, J MICROBIOL, V59, P1019, DOI 10.1007/s12275-021-1332-0

Levy M, 2017, NAT REV IMMUNOL, V17, P219, DOI 10.1038/nri.2017.7

Li WW, 2012, CELL MOL LIFE SCI, V69, P1125, DOI 10.1007/s00018-011-0865-5

Li ZH, 2024, J HEMATOL ONCOL, V17, DOI 10.1186/s13045-024-01541-w

Liu YY, 2018, J CLIN PHARMACOL, V58, pS164, DOI 10.1002/jcph.1121

Lu R, 2020, INFLAMM BOWEL DIS, V26, P1199, DOI 10.1093/ibd/izaa049

Luzio JP, 2007, NAT REV MOL CELL BIO, V8, P622, DOI 10.1038/nrm2217

Lystad AH, 2019, AUTOPHAGY, V15, P1485, DOI 10.1080/15548627.2019.1618100

Manley KJ, 2007, MED J AUSTRALIA, V186, P454, DOI 10.5694/j.1326-5377.2007.tb00995.x

Martínez-Colón GJ, 2018, PHARMACOL THERAPEUT, V185, P135, DOI 10.1016/j.pharmthera.2017.12.008

McEwan DG, 2015, MOL CELL, V57, P39, DOI 10.1016/j.molcel.2014.11.006

McGovern PE, 2009, UNCORKING THE PAST: THE QUEST FOR WINE, BEER, AND OTHER ALCOHOLIC BEVERAGES, P1

Methé BA, 2012, NATURE, V486, P215, DOI 10.1038/nature11209

Miccoli A, 2015, CELL PHYSIOL BIOCHEM, V35, P1706, DOI 10.1159/000373983

Mimee M, 2016, ADV DRUG DELIVER REV, V105, P44, DOI 10.1016/j.addr.2016.04.032

Mizushima N, 2001, J CELL BIOL, V152, P657, DOI 10.1083/jcb.152.4.657

Mizushima N, 2010, CURR OPIN CELL BIOL, V22, P132, DOI 10.1016/j.ceb.2009.12.004

Mller F.M., 1879, The Sacred Books of the East, VVolume 3

Mogues T, 2001, J EXP MED, V193, P271, DOI 10.1084/jem.193.3.271

Mori G, 2021, PLOS PATHOG, V17, DOI 10.1371/journal.ppat.1009377

Mubashra, 2024, Role of Autophagy and Reactive Oxygen Species in Cancer Treatment: Principles and Current Strategies, P209

Nagasaki A, 2010, DIGESTION, V81, P204, DOI 10.1159/000236042

Nakatogawa H, 2009, NAT REV MOL CELL BIO, V10, P458, DOI 10.1038/nrm2708

Narendran G, 2016, ORAL DIS, V22, P46, DOI 10.1111/odi.12389

Ndlovu H, 2016, FRONT IMMUNOL, V7, DOI 10.3389/fimmu.2016.00434

Negi S, 2019, FRONT IMMUNOL, V10, DOI 10.3389/fimmu.2019.01142

Nemati M, 2021, BIOMED RES INT, V2021, DOI 10.1155/2021/2931580

Ohsumi Y, 2014, CELL RES, V24, P9, DOI 10.1038/cr.2013.169

Ozen M, 2015, BENEF MICROBES, V6, P159, DOI 10.3920/BM2014.0103

Paglin S, 2001, CANCER RES, V61, P439

Pamer EG, 2016, SCIENCE, V352, P535, DOI 10.1126/science.aad9382

Perlman DC, 1997, CLIN INFECT DIS, V25, P242, DOI 10.1086/514546

Peterson J, 2009, GENOME RES, V19, P2317, DOI 10.1101/gr.096651.109

PFEIFER U, 1977, ACTA BIOL MED GER, V36, P1691

Phalane KG, 2013, MEDIAT INFLAMM, V2013, DOI 10.1155/2013/981984

Ponpuak M, 2010, IMMUNITY, V32, P329, DOI 10.1016/j.immuni.2010.02.009

Prasad R., 2021, ANN NATL ACAD MED SC, V57, P15, DOI [10.1055/s-0040-1722535, 10.1055/s-0040-1722535, DOI 10.1055/S-0040-1722535]

Qin JJ, 2010, NATURE, V464, P59, DOI 10.1038/nature08821

Rahim MA, 2024, MED MICROBIOL IMMUN, V213, DOI 10.1007/s00430-024-00794-z

Rahim MA, 2022, SCI REP-UK, V12, DOI 10.1038/s41598-022-12413-z

Rahlwes KC, 2023, VIRULENCE, V14, DOI 10.1080/21505594.2022.2150449

Randow F, 2012, TRENDS IMMUNOL, V33, P475, DOI 10.1016/j.it.2012.06.003

Rastogi N, 2001, REV SCI TECH OIE, V20, P21, DOI 10.20506/rst.20.1.1265

Raviglione M, 2012, LANCET, V379, P1902, DOI 10.1016/S0140-6736(12)60727-2

Ravikumar B, 2009, J CELL SCI, V122, P1707, DOI 10.1242/jcs.031773

Roca FJ, 2013, CELL, V153, P521, DOI 10.1016/j.cell.2013.03.022

Romao S, 2014, AUTOPHAGY, V10, P526, DOI 10.4161/auto.27606

Russell DG, 2001, NAT REV MOL CELL BIO, V2, P569, DOI 10.1038/35085034

Russo E, 2020, WORLD J GASTROENTERO, V26, DOI 10.3748/wjg.v26.i33.4919

Sandor M, 2003, TRENDS IMMUNOL, V24, P44, DOI 10.1016/S1471-4906(02)00006-6

SCHLESINGER LS, 1990, J IMMUNOL, V144, P2771

SEGLEN PO, 1982, P NATL ACAD SCI-BIOL, V79, P1889, DOI 10.1073/pnas.79.6.1889

Sekyere JO, 2020, ANN NY ACAD SCI, V1467, P21, DOI 10.1111/nyas.14300

Seo H, 2024, INT J ANTIMICROB AG, V64, DOI 10.1016/j.ijantimicag.2024.107274

Shah T, 2021, BIOMED PHARMACOTHER, V143, DOI 10.1016/j.biopha.2021.112108

Shanahan F, 2014, GASTROENTEROLOGY, V146, P1554, DOI 10.1053/j.gastro.2014.01.050

Shang LB, 2011, AUTOPHAGY, V7, P924, DOI 10.4161/auto.7.8.15860

Shenoy A, 2019, DERMATOL THER, V32, DOI 10.1111/dth.12970

Siawaya JFD, 2009, CYTOKINE, V47, P132, DOI 10.1016/j.cyto.2009.05.016

Singh P, 2018, FRONT MICROBIOL, V9, DOI 10.3389/fmicb.2018.00070

Smith J, 2008, INFECT IMMUN, V76, P5478, DOI 10.1128/IAI.00614-08

Sorbara MT, 2022, NAT REV MICROBIOL, V20, P365, DOI 10.1038/s41579-021-00667-9

Sorokulova I.B., 2006, J. Travel Med, V4, P167

Strong LM, 2021, ELIFE, V10, DOI [10.7554/eLife.70372, 10.7554/eLife.70372.sa1, 10.7554/eLife.70372.sa2]

Suprapti B, 2018, INDONES J PHARM, V29, P80, DOI 10.14499/indonesianjpharm29iss2pp80

Tang X., 2019, Lactic acid Bacteria, P297, DOI [DOI 10.1007/978-981-13-7832-4_10, 10.1007/978-981-13-7832-410, DOI 10.1007/978-981-13-7832-410]

Tanida I, 2011, ANTIOXID REDOX SIGN, V14, P2201, DOI 10.1089/ars.2010.3482

Tanida Isei, 2008, V445, P77, DOI 10.1007/978-1-59745-157-4_4

THUMM M, 1994, FEBS LETT, V349, P275, DOI 10.1016/0014-5793(94)00672-5

TSUKADA M, 1993, FEBS LETT, V333, P169, DOI 10.1016/0014-5793(93)80398-E

Verma N, 2022, J DRUG DELIV SCI TEC, V75, DOI 10.1016/j.jddst.2022.103690

Oberweis CV, 2020, TISSUE ENG PART B-RE, V26, P181, DOI [10.1089/ten.TEB.2019.0315, 10.1089/ten.teb.2019.0315]

Wang Y, 2017, AMB EXPRESS, V7, DOI 10.1186/s13568-017-0353-x

Wasserman S.A., 2022, Ph.D. Thesis

Wassie L, 2008, PLOS ONE, V3, DOI 10.1371/journal.pone.0001522

Webb JL, 2003, J BIOL CHEM, V278, P25009, DOI 10.1074/jbc.M300227200

WHO, 2024, GLOBAL TUBERCULOSIS

Wolf AJ, 2008, J EXP MED, V205, P105, DOI 10.1084/jem.20071367

Wong KW, 2017, MICROBIOL SPECTR, V5, DOI 10.1128/microbiolspec.TBTB2-0001-2015

Wong Sook-San, 2013, BMC Res Notes, V6, P135, DOI 10.1186/1756-0500-6-135

Wu YP, 2023, J CELL PHYSIOL, V238, P1336, DOI 10.1002/jcp.31016

Wu YP, 2019, FRONT MICROBIOL, V10, DOI 10.3389/fmicb.2019.02185

Wu YP, 2017, FRONT MICROBIOL, V8, DOI 10.3389/fmicb.2017.00469

Xiong WJ, 2018, J INFECT DIS, V217, P1267, DOI 10.1093/infdis/jiy033

Yadav M, 2022, GASTROENTEROL REP, V10, DOI 10.1093/gastro/goab046

Yang ZF, 2010, NAT CELL BIOL, V12, P814, DOI 10.1038/ncb0910-814

Yesilyurt N., 2021, Biologics, V1, P89, DOI [10.3390/biologics1020006, 10.3390/biologics1020006, DOI 10.3390/BIOLOGICS1020006]

Yokotsuka T., 1998, Microbiology of Fermented Foods, V2nd, P351, DOI DOI 10.1007/978-1-4613-0309-1_12

Yoon Y, 2021, J MICROBIOL BIOTECHN, V31, P1632, DOI 10.4014/jmb.2107.07044

Zaman K, 2010, J HEALTH POPUL NUTR, V28, P111

Zaylaa Mazen, 2019, Cell Physiol Biochem, V53, P774, DOI [10.33594/000000172, 10.33594/000000172]

Zimmermann P, 2018, VACCINE, V36, P4433, DOI 10.1016/j.vaccine.2018.04.066

Zumla A, 2015, J INTERN MED, V277, P373, DOI 10.1111/joim.12256

NR 184

TC 1

Z9 1

U1 0

U2 5

PU MDPI

PI BASEL

PA MDPI AG, Grosspeteranlage 5, CH-4052 BASEL, SWITZERLAND

EI 2073-4409

J9 CELLS-BASEL

JI Cells

PD APR 3

PY 2025

VL 14

IS 7

AR 540

DI 10.3390/cells14070540

PG 23

WC Cell Biology

WE Science Citation Index Expanded (SCI-EXPANDED)

SC Cell Biology

GA 1FF2T

UT WOS:001463601900001

OA Green Submitted, gold

DA 2026-05-07

ER

PT J

AU Rajni, LSM

AF Rajni, Laxmi S. Meena

TI Guanosine triphosphatases as novel therapeutic targets in tuberculosis

SO INTERNATIONAL JOURNAL OF INFECTIOUS DISEASES

LA English

DT Review

DE Mycobacterium; GTPase; Tuberculosis; Prokaryotes; Virulence factor;

Signaling transduction

ID GTP-BINDING PROTEINS; MYCOBACTERIUM-TUBERCULOSIS; PHAGOSOME MATURATION;

CELL-WALL; BACILLUS-SUBTILIS; VIRULENCE; GTPASES; BIOSYNTHESIS;

TRANSLATION; BIOGENESIS

AB Tuberculosis (TB) is an infectious disease caused by the aerobic microbe Mycobacterium tuberculosis H(37)Rv. Despite the availability of the Bacille Calmette-Guerin (BCG) vaccine and directly observed treatment, short-course (DOTS), TB is a leading cause of death and affects a third of the world's population. The most important factor associated with disease severity is the development of antibiotic-resistant strains, including multidrug-resistant (MDR)-TB and extensively drug-resistant (XDR)-TB. In order to understand disease pathogenesis, it is necessary to delineate the specific features of M. tuberculosis that enable it to evade the host defense system and contribute to its virulence. Here, we have reviewed the various characteristics, such as cell wall components, virulence genes, and the role of small guanosine triphosphatases (GTPases) in the pathogenesis of TB. GTPases are known to play a crucial role in the survival and pathogenesis of various pathogens. The key role of these proteins involves interference in phagosome maturation arrest, enabling pathogens to survive by escaping from lysozymes and toxic free radicals. This observation provides a new avenue for the development of anti-TB drugs. (C) 2010 International Society for Infectious Diseases. Published by Elsevier Ltd. All rights reserved.

C1 Inst Genom & Integrat Biol, Delhi 110007, India.

C3 Council of Scientific & Industrial Research (CSIR) - India; CSIR -

Institute of Genomics & Integrative Biology (IGIB)

RP Rajni, LSM (corresponding author), Inst Genom & Integrat Biol, Mall Rd, Delhi 110007, India.

EM meena@igib.res.in

FU DST (Department of Science and Technology, Government of India); CSIR

(Council of Scientific and Industrial Research)

FX We thank Dr Rajesh S. Gokhale for making this work possible. We also

thank Dr Hemant Khanna (University of Michigan, USA) for valuable

suggestions. The authors acknowledge financial support from GAP0050 of

the DST (Department of Science and Technology, Government of India) and

CSIR (Council of Scientific and Industrial Research).

CR Alderwick LJ, 2007, BIOCHEM SOC T, V35, P1325, DOI 10.1042/BST0351325

Belisle JT, 1997, SCIENCE, V276, P1420, DOI 10.1126/science.276.5317.1420

Berthet FX, 1998, SCIENCE, V282, P759, DOI 10.1126/science.282.5389.759

BOURNE HR, 1995, PHILOS T R SOC B, V349, P283, DOI 10.1098/rstb.1995.0114

BOURNE HR, 1991, NATURE, V349, P117, DOI 10.1038/349117a0

Brennan PJ, 2003, TUBERCULOSIS, V83, P91, DOI 10.1016/S1472-9792(02)00089-6

Brock S, 1998, MOL MICROBIOL, V29, P409, DOI 10.1046/j.1365-2958.1998.00893.x

Caldon CE, 2003, CURR OPIN MICROBIOL, V6, P135, DOI 10.1016/S1369-5274(03)00037-7

Caldon CE, 2001, MOL MICROBIOL, V41, P289, DOI 10.1046/j.1365-2958.2001.02536.x

Cardona PJ, 2004, EUR RESPIR J, V24, P1044, DOI 10.1183/09031936.04.00072604

Fratti RA, 2003, P NATL ACAD SCI USA, V100, P5437, DOI 10.1073/pnas.0737613100

Golden MP, 2005, AM FAM PHYSICIAN, V72, P1761

GUALERZI CO, 1990, BIOCHEMISTRY-US, V29, P5881, DOI 10.1021/bi00477a001

Hauryliuk VV, 2006, MOL BIOL+, V40, P688, DOI 10.1134/S0026893306050025

Holtz TH, 2007, EUR RESPIR J, V30, P396, DOI 10.1183/09031936.00042607

Jain A, 2008, J BIOSCIENCES, V33, P605, DOI 10.1007/s12038-008-0078-8

Kartmann B, 1999, J BACTERIOL, V181, P6543, DOI 10.1128/JB.181.20.6543-6546.1999

KOK J, 1994, J BACTERIOL, V176, P7155, DOI 10.1128/JB.176.23.7155-7160.1994

Kumar V., ROBBINS BASIC PATHOL, V8th, P516

Kyei GB, 2006, EMBO J, V25, P5250, DOI 10.1038/sj.emboj.7601407

Lam THJ, 2008, MICROB PATHOGENESIS, V45, P12, DOI 10.1016/j.micpath.2008.01.006

Lamb HK, 2007, PROTEIN SCI, V16, P2391, DOI 10.1110/ps.072900907

Leipe DD, 2002, J MOL BIOL, V317, P41, DOI 10.1006/jmbi.2001.5378

MAGALLANES VR, 2007, INFECT IMMUN, V75, P504

Margalit DN, 2004, P NATL ACAD SCI USA, V101, P11821, DOI 10.1073/pnas.0404439101

Meena LS, 2008, ENZYME MICROB TECH, V42, P138, DOI 10.1016/j.enzmictec.2007.08.008

Migliori GB, 2007, EUR RESPIR J, V29, P423, DOI 10.1183/09031936.00001307

Minkovsky N, 2002, J BACTERIOL, V184, P6389, DOI 10.1128/JB.184.22.6389-6394.2002

Mittenhuber G, 2001, J MOL MICROB BIOTECH, V3, P21

Morimoto T, 2002, MICROBIOL-SGM, V148, P3539, DOI 10.1099/00221287-148-11-3539

Movahedzadeh F, 2004, MOL MICROBIOL, V51, P1003, DOI 10.1046/j.1365-2958.2003.03900.x

Mutlu GM, 2006, AM J MED, V119, P639, DOI 10.1016/j.amjmed.2006.01.015

Ramaswamy S., 1998, Tubercle and Lung Disease, V79, P3, DOI 10.1054/tuld.1998.0002

Raynaud C, 2002, MOL MICROBIOL, V45, P203, DOI 10.1046/j.1365-2958.2002.03009.x

Rindi L, 1999, BIOCHEM BIOPH RES CO, V258, P94, DOI 10.1006/bbrc.1999.0591

Roberts EA, 2006, J CELL BIOL, V174, P923, DOI 10.1083/jcb.200603026

Rodnina MV, 1999, FEMS MICROBIOL REV, V23, P317, DOI 10.1016/S0168-6445(99)00010-8

Rodnina MV, 2000, BIOL CHEM, V381, P377, DOI 10.1515/BC.2000.050

Sharma SK, 2004, INDIAN J MED RES, V120, P316

Spigelman MK, 2007, J INFECT DIS, V196, pS28, DOI 10.1086/518663

Sprang SR, 1997, ANNU REV BIOCHEM, V66, P639, DOI 10.1146/annurev.biochem.66.1.639

Velmurugan K, 2007, PLOS PATHOG, V3, P972, DOI 10.1371/journal.ppat.0030110

Vergne I, 2005, P NATL ACAD SCI USA, V102, P4033, DOI 10.1073/pnas.0409716102

Vergne I, 2003, TRAFFIC, V4, P600, DOI 10.1034/j.1600-0854.2003.00120.x

Via LE, 1997, J BIOL CHEM, V272, P13326, DOI 10.1074/jbc.272.20.13326

Voskuil MI, 2003, J EXP MED, V198, P705, DOI 10.1084/jem.20030205

Wilson KS, 1998, CELL, V92, P337, DOI 10.1016/S0092-8674(00)80927-7

World Health Organization (WHO), 2006, GLOB REG INC

Zhang Y, 2001, BIOCHEM BIOPH RES CO, V284, P542, DOI 10.1006/bbrc.2001.4993

NR 49

TC 16

Z9 20

U1 0

U2 4

PU ELSEVIER SCI LTD

PI OXFORD

PA THE BOULEVARD, LANGFORD LANE, KIDLINGTON, OXFORD OX5 1GB, OXON, ENGLAND

SN 1201-9712

EI 1878-3511

J9 INT J INFECT DIS

JI Int. J. Infect. Dis.

PD AUG

PY 2010

VL 14

IS 8

BP E682

EP E687

DI 10.1016/j.ijid.2009.11.016

PG 6

WC Infectious Diseases

WE Science Citation Index Expanded (SCI-EXPANDED)

SC Infectious Diseases

GA 660RS

UT WOS:000282662100006

OA gold

DA 2026-05-07

ER

PT J

AU Silva, C

Perdigao, J

Alverca, E

de Matos, APA

Carvalho, PA

Portugal, I

Jordao, L

AF Silva, Carla

Perdigao, Joao

Alverca, Elsa

Alves de Matos, Antonio P.

Carvalho, Patricia A.

Portugal, Isabel

Jordao, Luisa

TI Exploring the Contribution of Mycobacteria Characteristics in Their

Interaction with Human Macrophages

SO MICROSCOPY AND MICROANALYSIS

LA English

DT Article

DE Mycobacterium; tuberculosis; host-pathogen interaction; drug resistance;

TEM; SEM; MIRUS; cell envelope

ID DRUG-RESISTANT TUBERCULOSIS; CELL-WALL; MULTIDRUG-RESISTANT;

GENETIC-ANALYSIS; MUTATIONS; LISBON; MECHANISMS; KANAMYCIN; SMEGMATIS;

STRAINS

AB Tuberculosis (TB) is a major health problem. The emergence of multidrug resistant (MDR) Mycobacterium tuberculosis (Mtb) isolates confounds treatment strategies. In Portugal, cases of MDR-TB are reported annually with an increased incidence noted in Lisbon. The majority of these MDR-TB cases are due to closely related mycobacteria known collectively as the Lisboa family and Q1 cluster. Genetic determinants linked to drug resistance have been exhaustively studied resulting in the identification of family and cluster specific mutations. Nevertheless, little is known about other factors involved in development of mycobacteria drug resistance. Here, we complement genetic analysis with the study of morphological and structural features of the Lisboa family and Q1 cluster isolates by using scanning and transmission electron microscopy. This analysis allowed the identification of structural differences, such as cell envelope thickness, between Mtb clinical isolates that are correlated with antibiotic resistance. The infection of human monocyte derived macrophages allowed us to document the relative selective advantage of the Lisboa family isolates over other circulating Mtb isolates.

C1 [Silva, Carla; Perdigao, Joao; Portugal, Isabel] Univ Lisbon, Fac Farm, URIA, Ctr Patogenese Mol, P-1649003 Lisbon, Portugal.

[Alverca, Elsa] Inst Nacl Saude Dr Ricardo Jorge INSA, Dept Saude Ambiental, P-1649016 Lisbon, Portugal.

[Alves de Matos, Antonio P.] Hosp Curry Cabral, Ctr Hosp Lisboa Cent, Serv Anat Patol, P-1069166 Lisbon, Portugal.

[Carvalho, Patricia A.] Univ Tecn Lisboa, Inst Super Tecn, Dept Bioengn, ICEMS, P-1049001 Lisbon, Portugal.

[Jordao, Luisa] INSA, Dept Doencas Infeciosas, P-1649016 Lisbon, Portugal.

C3 Universidade de Lisboa; Instituto Nacional de Saude Dr. Ricardo Jorge;

Centro Hospitalar de Lisboa Central, EPE; Universidade de Lisboa;

Universidade de Lisboa

RP Jordao, L (corresponding author), INSA, Dept Doencas Infeciosas, Av Padre Cruz, P-1649016 Lisbon, Portugal.

EM maria.jordao@insa.min-saude.pt

RI Portugal, Isabel/B-1491-2008; Jordao, Luisa/T-6768-2019; Perdigão,

João/L-6893-2013; Matos, Antonio/A-4539-2013; Silva, Carla/H-6245-2017;

carvalho, patricia/D-8078-2015

OI Portugal, Isabel/0000-0002-7843-0006; Jordao, Luisa/0000-0002-5608-021X;

Perdigão, João/0000-0002-0339-1305; Matos, Antonio/0000-0001-9386-0349;

Silva, Carla/0000-0003-3707-6263; carvalho, patricia/0000-0002-5447-0409

FU Fundacao para a Ciencia e Tecnologia [SFRH/BD/73579/2010,

SFRH/BD/45388/2008, C2008-C2008_P2]; Fundação para a Ciência e a

Tecnologia [SFRH/BD/73579/2010, SFRH/BD/45388/2008] Funding Source: FCT

FX We are grateful to the Technology and Innovation Unit for technical

support, to Cristina Furtado for providing access to mycobacterial

strains from the Epidemiological Surveillance Systems

(VigILab-Tuberculosis), and to Joao Paulo Gomes for the critical reading

of the manuscript. The authors acknowledge the funding by Fundacao para

a Ciencia e Tecnologia (SFRH/BD/73579/2010; SFRH/BD/45388/2008; and

C2008-C2008_P2).

CR Aderem A, 1999, ANNU REV IMMUNOL, V17, P593, DOI 10.1146/annurev.immunol.17.1.593

Alsteens D, 2008, PFLUG ARCH EUR J PHY, V456, P117, DOI 10.1007/s00424-007-0386-0

[Anonymous], 2011 2012 TUB GLOB F

Anuchin AM, 2009, MICROBIOL-SGM, V155, P1071, DOI 10.1099/mic.0.023028-0

Barkan D, 2009, CHEM BIOL, V16, P499, DOI 10.1016/j.chembiol.2009.04.001

BOZZOLA JJ, 1973, STAIN TECHNOL, V48, P317, DOI 10.3109/10520297309116648

Brennan PJ, 2003, TUBERCULOSIS, V83, P91, DOI 10.1016/S1472-9792(02)00089-6

Cade CE, 2010, PROTEIN SCI, V19, P458, DOI 10.1002/pro.324

Cohen J, 2013, SCIENCE, V339, P130, DOI 10.1126/science.339.6116.130

Cole ST, 2011, CURR OPIN MICROBIOL, V14, P570, DOI 10.1016/j.mib.2011.07.022

Cunningham AF, 1998, J BACTERIOL, V180, P801, DOI 10.1128/JB.180.4.801-808.1998

Davies J, 2010, MICROBIOL MOL BIOL R, V74, P417, DOI 10.1128/MMBR.00016-10

Demangel C, 2004, INFECT IMMUN, V72, P2170, DOI 10.1128/IAI.72.4.2170-2176.2004

DRAPPER P., 2005, TUBERCULOSIS TUBERCL, P405

Ehrt S, 2007, NAT MED, V13, P284, DOI 10.1038/nm0307-284

Farmer P, 1997, SOC SCI MED, V44, P347, DOI 10.1016/S0277-9536(96)00143-8

Fenton MJ, 2005, TUBERCULOSIS AND THE TUBERCLE BACILLUS, P405

Flores AR, 2005, MICROBIOL-SGM, V151, P521, DOI 10.1099/mic.0.27629-0

Gagliardi MC, 2007, CELL MICROBIOL, V9, P2081, DOI 10.1111/j.1462-5822.2007.00940.x

Gagneux S, 2006, SCIENCE, V312, P1944, DOI 10.1126/science.1124410

Grodzki ACG, 2013, PLOS ONE, V8, DOI 10.1371/journal.pone.0054926

Jain M, 2007, P NATL ACAD SCI USA, V104, P5133, DOI 10.1073/pnas.0610634104

JARLIER V, 1994, FEMS MICROBIOL LETT, V123, P11, DOI 10.1111/j.1574-6968.1994.tb07194.x

Jordao L, 2008, CELL MICROBIOL, V10, P529, DOI 10.1111/j.1462-5822.2007.01067.x

Jordao Luisa, 2011, Int J Cell Biol, V2011, P403623, DOI 10.1155/2011/403623

KAPUR V, 1994, J CLIN MICROBIOL, V32, P1095, DOI 10.1128/JCM.32.4.1095-1098.1994

KHOMENKO AG, 1987, TUBERCLE, V68, P243, DOI 10.1016/0041-3879(87)90064-X

Kocagoz T, 1996, ANTIMICROB AGENTS CH, V40, P1768, DOI 10.1128/AAC.40.8.1768

Krüüner A, 2003, ANTIMICROB AGENTS CH, V47, P2971, DOI 10.1128/AAC.47.9.2971-2973.2003

Kusner DJ, 2005, CLIN IMMUNOL, V114, P239, DOI 10.1016/j.clim.2004.07.016

Larsen MH, 2002, MOL MICROBIOL, V46, P453, DOI 10.1046/j.1365-2958.2002.03162.x

Maus CE, 2005, ANTIMICROB AGENTS CH, V49, P3192, DOI 10.1128/AAC.49.8.3192-3197.2005

Maus CE, 2005, ANTIMICROB AGENTS CH, V49, P571, DOI 10.1128/AAC.49.2.571-577.2005

Meier A, 1996, ANTIMICROB AGENTS CH, V40, P2452, DOI 10.1128/AAC.40.11.2452

Méndez-Samperio P, 2004, J INFECTION, V48, P66, DOI 10.1016/j.jinf.2003.08.009

Perdigao J, 2008, MICROB DRUG RESIST, V14, P133, DOI 10.1089/mdr.2008.0798

Perdigao J, 2013, J ANTIMICROB CHEMOTH, V68, P27, DOI 10.1093/jac/dks371

Perdigao J, 2010, J ANTIMICROB CHEMOTH, V65, P224, DOI 10.1093/jac/dkp452

Plinke C, 2006, ANTIMICROB AGENTS CH, V50, P1900, DOI 10.1128/AAC.50.5.1900-1902.2006

Portugal I, 1999, INT J TUBERC LUNG D, V3, P207

Ramaswamy S., 1998, Tubercle and Lung Disease, V79, P3, DOI 10.1054/tuld.1998.0002

Rossetti MLR, 2002, REV SAUDE PUBL, V36, P525, DOI 10.1590/S0034-89102002000400021

Russell DG, 2001, NAT REV MOL CELL BIO, V2, P569, DOI 10.1038/35085034

SCHLESINGER L.S., 1997, LUNG MACROPHAGES DEN, P437

Sreevatsan S, 1997, ANTIMICROB AGENTS CH, V41, P636, DOI 10.1128/AAC.41.3.636

Sreevatsan S, 1996, ANTIMICROB AGENTS CH, V40, P1024, DOI 10.1128/AAC.40.4.1024

Supply P, 2006, J CLIN MICROBIOL, V44, P4498, DOI 10.1128/JCM.01392-06

Swanson JA, 2008, NAT REV MOL CELL BIO, V9, P639, DOI 10.1038/nrm2447

Torrelles JB, 2012, GLYCOBIOLOGY, V22, P1118, DOI 10.1093/glycob/cws078

Velayati AA, 2010, EUR RESPIR J, V36, P1490, DOI 10.1183/09031936.00064510

Velayati AA, 2009, CHEMOTHERAPY, V55, P303, DOI 10.1159/000226425

Via LE, 2008, INFECT IMMUN, V76, P2333, DOI 10.1128/IAI.01515-07

Vizcaíno C, 2010, PLOS COMPUT BIOL, V6, DOI 10.1371/journal.pcbi.1000824

Wayne LG, 2001, ANNU REV MICROBIOL, V55, P139, DOI 10.1146/annurev.micro.55.1.139

Weniger T, 2010, NUCLEIC ACIDS RES, V38, pW326, DOI 10.1093/nar/gkq351

WHO, 2012, GLOBAL TUBERCULOSIS REPORT 2012, P1

Zaunbrecher MA, 2009, P NATL ACAD SCI USA, V106, P20004, DOI 10.1073/pnas.0907925106

NR 57

TC 5

Z9 6

U1 0

U2 16

PU CAMBRIDGE UNIV PRESS

PI NEW YORK

PA 32 AVENUE OF THE AMERICAS, NEW YORK, NY 10013-2473 USA

SN 1431-9276

EI 1435-8115

J9 MICROSC MICROANAL

JI Microsc. microanal.

PD OCT

PY 2013

VL 19

IS 5

BP 1159

EP 1169

DI 10.1017/S1431927613001906

PG 11

WC Materials Science, Multidisciplinary; Microscopy

WE Science Citation Index Expanded (SCI-EXPANDED)

SC Materials Science; Microscopy

GA 219ZQ

UT WOS:000324550900007

DA 2026-05-07

ER

PT J

AU Usman, MM

Ismail, S

Teoh, TC

AF Usman, Mohammed Maikudi

Ismail, Salmah

Teoh, Teow Chong

TI Vaccine research and development: tuberculosis as a global health threat

SO CENTRAL EUROPEAN JOURNAL OF IMMUNOLOGY

LA English

DT Article

DE tuberculosis; Mycobacterium tuberculosis; vaccine; clinical trials; BCG

vaccine

ID BACILLE CALMETTE-GUERIN; DRUG-RESISTANT TUBERCULOSIS; HIV-INFECTED

ADULTS; MYCOBACTERIUM-TUBERCULOSIS; PROTECTIVE EFFICACY;

IMMUNE-RESPONSE; FUSION PROTEIN; BCG VACCINE; TB VACCINE; IFN-GAMMA

AB One of the aims of the World Health Organisation (WHO) Millennium Development Goals (MDG) is to reduce the number of cases of tuberculosis (TB) infection by the year 2015. However, 9 million new cases were reported in 2013, with an estimated 480,000 new cases of multi-drug resistant tuberculosis (MDR-TB) globally. Bacille Calmette-Guerin (BCG) is the most available and currently used candidate vaccine against tuberculosis; it prevents childhood TB, but its effectiveness against pulmonary TB in adults and adolescents is disputed. To achieve the goal of the WHO MDG, the need for a new improved vaccine is of primary importance. This review highlights several articles that have reported vaccine development. There are about 16 TB vaccines in different phases of clinical trials at the time of writing, which include recombinant peptide/protein, live-attenuated and recombinant live-attenuated, protein/adjuvant, viral-vectored, and immunotherapeutic vaccine. Further studies in reverse vaccinology and massive campaigns on vaccination are needed in order to achieve the target for TB eradication by 2050.

C1 [Usman, Mohammed Maikudi; Ismail, Salmah; Teoh, Teow Chong] Univ Malaya, Inst Biol Sci, Fac Sci, Kuala Lumpur 50603, Malaysia.

[Usman, Mohammed Maikudi] Modibbo Adama Univ Technol, Sch Pure & Appl Sci, Dept Biotechnol, Yola, Nigeria.

C3 Universiti Malaya

RP Ismail, S (corresponding author), Univ Malaya, Inst Biol Sci, Fac Sci, Kuala Lumpur 50603, Malaysia.

EM salmah_r@um.edu.my

RI TEOH, TEOW/B-9950-2010; ISMAIL, SALMAH/B-9680-2010

FU University of Malaya through PPP [PG238-2014B, RG064-12B10, RG523-13HTM]

FX The authors wish to thank the University of Malaya for supporting this

research through PPP grants (Project no: PG238-2014B) RG064-12B10 and

RG523-13HTM.

CR Abubakar I, 2013, LANCET INFECT DIS, V13, P529, DOI 10.1016/S1473-3099(13)70030-6

Ahmad S, 2011, CLIN DEV IMMUNOL, DOI 10.1155/2011/814943

Ahmed A, 2016, ORAL DIS, V22, P53, DOI 10.1111/odi.12390

Ahsan Mohamed Jawed, 2015, Ther Adv Vaccines, V3, P66, DOI [10.1177/2051013615593891, 10.1177/2051013615593891]

Andersen P, 2014, TRENDS IMMUNOL, V35, P387, DOI 10.1016/j.it.2014.04.006

Andersen P, 2014, CSH PERSPECT MED, V4, DOI 10.1101/cshperspect.a018523

[Anonymous], PIPELINE REPORT HIV

[Anonymous], 2015, GLOBAL TUBERCULOSIS

[Anonymous], 2015, GLOBAL TUBERCULOSIS

Arbues A, 2013, VACCINE, V31, P4867, DOI 10.1016/j.vaccine.2013.07.051

Barreto Mauricio L., 2006, J. Pediatr. (Rio J.), V82, ps45

Behar SM, 2011, MUCOSAL IMMUNOL, V4, P279, DOI 10.1038/mi.2011.3

Bernatowska E., 2007, Kardiol Polska, V65, P1307

Billeskov R, 2012, PLOS ONE, V7, DOI 10.1371/journal.pone.0039909

Bottai D, 2015, VACCINE, V33, P2710, DOI 10.1016/j.vaccine.2015.03.083

Bruffaerts N, 2014, VACCINES-BASEL, V2, P181, DOI 10.3390/vaccines2010181

Cardona PJ, 2006, TUBERCULOSIS, V86, P273, DOI 10.1016/j.tube.2006.01.024

Churchyard GJ, 2015, VACCINE, V33, P1890, DOI 10.1016/j.vaccine.2015.02.004

Copin R, 2014, VACCINE, V32, P5998, DOI 10.1016/j.vaccine.2014.07.113

da Costa AC, 2014, FRONT IMMUNOL, V5, DOI 10.3389/fimmu.2014.00152

da Costa C, 2015, INT J INFECT DIS, V32, P5, DOI 10.1016/j.ijid.2014.11.026

Deng YH, 2011, MICROBIOL IMMUNOL, V55, P798, DOI 10.1111/j.1348-0421.2011.00376.x

Desvignes L, 2012, J IMMUNOL, V188, P6205, DOI 10.4049/jimmunol.1200255

Devarajan S., 2002, WORLD BANK POLICY RE

Duque-Correa MA, 2014, P NATL ACAD SCI USA, V111, pE4024, DOI 10.1073/pnas.1408839111

FINE PEM, 1995, LANCET, V346, P1339, DOI 10.1016/S0140-6736(95)92348-9

Finnie RKC, 2011, TROP MED INT HEALTH, V16, P394, DOI 10.1111/j.1365-3156.2010.02718.x

Forrellad MA, 2013, VIRULENCE, V4, P3, DOI 10.4161/viru.22329

Frick M., 2015, Pipeline report HIV, hepatitis C virus and tuberculosis drugs, diagnostics, vaccines, preventive technologies towards a cure and immune-based and gene therapies in development, P163

Fukuda T, 2013, MBIO, V4, DOI 10.1128/mBio.00472-12

Galagan JE, 2014, NAT REV GENET, V15, P307, DOI 10.1038/nrg3664

Girard MP, 2005, VACCINE, V23, P5725, DOI 10.1016/j.vaccine.2005.07.045

Gopal R, 2014, PLOS PATHOG, V10, DOI 10.1371/journal.ppat.1004099

Gopal R, 2013, EXPERT REV VACCINES, V12, P829, DOI 10.1586/14760584.2013.814836

Green AM, 2013, J IMMUNOL, V190, P270, DOI 10.4049/jimmunol.1200061

Harries AD, 2010, LANCET, V375, P1906, DOI 10.1016/S0140-6736(10)60409-6

Hartman-Adams H, 2014, AM FAM PHYSICIAN, V89, P889

Hawkridge T, 2011, PAEDIATR RESPIR REV, V12, P46, DOI 10.1016/j.prrv.2010.09.013

Hoft DF, 2008, J INFECT DIS, V198, P1491, DOI 10.1086/592450

Horwitz MA, 2000, P NATL ACAD SCI USA, V97, P13853, DOI 10.1073/pnas.250480397

Horwitz MA, 2003, INFECT IMMUN, V71, P1672, DOI 10.1128/IAI.71.4.1672-1679.2003

Jefferys R, 2012, HIV TREATMENT B HIV, V251

Jones-López EC, 2013, AM J RESP CRIT CARE, V187, P1007, DOI 10.1164/rccm.201208-1422OC

Kakchapati S, 2014, SE ASIAN J TROP MED, V45, P95

Kaufmann SHE, 2015, VACCINE, V33, P3047, DOI 10.1016/j.vaccine.2015.03.056

Kaufmann SHE, 2014, CURR OPIN PULM MED, V20, P294, DOI 10.1097/MCP.0000000000000041

Kaufmann SHE, 2013, SEMIN IMMUNOL, V25, P172, DOI 10.1016/j.smim.2013.04.006

Kaufmann SHE, 2010, LANCET, V375, P2110, DOI 10.1016/S0140-6736(10)60393-5

Kozakiewicz L, 2013, ADV EXP MED BIOL, V783, P225, DOI 10.1007/978-1-4614-6111-1_12

Lambrecht B., 2013, MUCOSAL IMMUNOLOGY A, P49, DOI [10.1007/978-1-4614-5326-0_2, DOI 10.1007/978-1-4614-5326-0_2]

Li W, 2014, MOL IMMUNOL, V62, P86, DOI 10.1016/j.molimm.2014.06.007

Lin PL, 2012, J CLIN INVEST, V122, P303, DOI 10.1172/JCI46252

Luca Simona, 2013, Maedica (Bucur), V8, P53

Madan-Lala R, 2014, J IMMUNOL, V192, P4263, DOI 10.4049/jimmunol.1303185

Mangtani P, 2014, CLIN INFECT DIS, V58, P470, DOI 10.1093/cid/cit790

Marchant A, 1999, J IMMUNOL, V163, P2249

Matucci A, 2014, J RHEUMATOL, V41, P17, DOI 10.3899/jrheum.140098

McShane H, 2011, PHILOS T R SOC B, V366, P2782, DOI 10.1098/rstb.2011.0097

Mirlekar B., 2012, Indian Journal of Leprosy, V85, P65

Modjarrad K, 2010, LANCET INFECT DIS, V10, P455, DOI 10.1016/S1473-3099(10)70093-1

Moliva JI, 2015, VACCINE, V33, P5035, DOI 10.1016/j.vaccine.2015.08.033

Monin L, 2014, SEMIN IMMUNOL, V26, P552, DOI 10.1016/j.smim.2014.09.004

Montagnani C, 2014, BMC INFECT DIS, V14, DOI 10.1186/1471-2334-14-S1-S2

Morris D, 2013, BIOMED RES INT, V2013, DOI 10.1155/2013/402827

Müller B, 2013, TRENDS GENET, V29, P160, DOI 10.1016/j.tig.2012.11.005

Mustafa AS, 2011, MICROBIOL IMMUNOL, V55, P267, DOI 10.1111/j.1348-0421.2011.00307.x

Nell AS, 2014, PLOS ONE, V9, DOI 10.1371/journal.pone.0089612

Nguipdop-Djomo P, 2016, LANCET INFECT DIS, V16, P219, DOI 10.1016/S1473-3099(15)00400-4

Nikolova M, 2013, DIAGN MICR INFEC DIS, V75, P277, DOI 10.1016/j.diagmicrobio.2012.11.023

Norouzi S, 2012, J INFECTION, V64, P543, DOI 10.1016/j.jinf.2012.03.012

Ocampo M., 2015, Tuberculosis- expanding knowledge, P103

Orme IM, 2013, DRUGS, V73, P1015, DOI 10.1007/s40265-013-0081-8

Ottenhoff TH, 2010, HUM VACCINES, V6, P1007, DOI 10.4161/hv.6.12.13143

Ottenhoff THM, 2012, PLOS PATHOG, V8, DOI 10.1371/journal.ppat.1002607

Pac M, 2012, CENT EUR J IMMUNOL, V37, P378, DOI 10.5114/ceji.2012.32667

Poyntz HC, 2014, TUBERCULOSIS, V94, P226, DOI 10.1016/j.tube.2013.12.006

Principi N, 2015, TUBERCULOSIS, V95, P6, DOI 10.1016/j.tube.2014.10.004

Randall PJ, 2015, IMMUNOL CELL BIOL, V93, P789, DOI 10.1038/icb.2015.43

Reed SG, 2009, TRENDS IMMUNOL, V30, P23, DOI 10.1016/j.it.2008.09.006

Reither K, 2014, PLOS ONE, V9, DOI 10.1371/journal.pone.0114602

Rivas-Santiago B, 2013, PLOS ONE, V8, DOI 10.1371/journal.pone.0059119

Rivas-Santiago B, 2009, INFECT IMMUN, V77, P4690, DOI 10.1128/IAI.01515-08

Rosilawati ML, 2012, SE ASIAN J TROP MED, V43, P89

Manzano JR, 2014, ARCH BRONCONEUMOL, V50, P373, DOI 10.1016/j.arbres.2014.03.001

Russell DG, 2010, SCIENCE, V328, P852, DOI 10.1126/science.1184784

Ryan K.J., 2010, Sherris Medical Microbiology

Samuchiwal SK, 2014, BMC INFECT DIS, V14, DOI 10.1186/1471-2334-14-355

Saraav I, 2014, IMMUNOL CELL BIOL, V92, P741, DOI 10.1038/icb.2014.52

Sarhan MA, 2010, SAUDI MED J, V31, P9

Schito M, 2015, CLIN INFECT DIS, V61, pS102, DOI 10.1093/cid/civ609

Sharma S, 2014, TUBERCULOSIS, V94, P389, DOI 10.1016/j.tube.2014.04.007

Stuckler D, 2011, AM J PUBLIC HEALTH, V101, P524, DOI 10.2105/AJPH.2009.175646

Svenson S, 2010, HUM VACCINES, V6, P309, DOI 10.4161/hv.6.4.10711

Thacher EG, 2014, AIDS, V28, P1769, DOI 10.1097/QAD.0000000000000343

Theron G, 2014, LANCET, V383, P424, DOI 10.1016/S0140-6736(13)62073-5

Udwadia ZF, 2012, CLIN INFECT DIS, V54, P579, DOI 10.1093/cid/cir889

Unnikrishnan M, 2012, CURR OPIN IMMUNOL, V24, P337, DOI 10.1016/j.coi.2012.03.013

Vandemootele Jan, 2004, TARGETING DEV CRITIC, P124

von Reyn CF, 2010, AIDS, V24, P675, DOI 10.1097/QAD.0b013e3283350f1b

Wang C, 2012, CLIN DEV IMMUNOL, DOI 10.1155/2012/563838

Wareham AS, 2014, PLOS ONE, V9, DOI 10.1371/journal.pone.0088149

World Health Organization, 2012, WHOHTMTB20126

World Health Organization, 2014, WHOHTMTB201408

Yu DH, 2008, GENE THER, V15, P652, DOI 10.1038/gt.2008.13

Yuan W, 2012, VACCINE, V30, P2490, DOI 10.1016/j.vaccine.2011.06.029

Yuen CM, 2014, PLOS ONE, V9, DOI 10.1371/journal.pone.0099880

NR 106

TC 14

Z9 16

U1 0

U2 13

PU TERMEDIA PUBLISHING HOUSE LTD

PI POZNAN

PA KLEEBERGA ST 2, POZNAN, 61-615, POLAND

SN 1426-3912

EI 1644-4124

J9 CENT EUR J IMMUNOL

JI Central Eur. J. Immunol.

PY 2017

VL 42

IS 2

BP 196

EP 204

DI 10.5114/ceji.2017.69362

PG 9

WC Immunology

WE Science Citation Index Expanded (SCI-EXPANDED)

SC Immunology

GA FE2WE

UT WOS:000408077100011

OA Green Submitted, gold

DA 2026-05-07

ER

PT J

AU Kardan-Yamchi, J

Kazemian, H

Haeili, M

Harati, AA

Amini, S

Feizabadi, MM

AF Kardan-Yamchi, Jalil

Kazemian, Hossein

Haeili, Mehri

Harati, Ahad Ali

Amini, Sirus

Feizabadi, Mohammad Mehdi

TI Expression analysis of 10 efflux pump genes in multidrug-resistant and

extensively drug-resistant Mycobacterium tuberculosis clinical

isolates

SO JOURNAL OF GLOBAL ANTIMICROBIAL RESISTANCE

LA English

DT Article

DE Mycobacterium tuberculosis; Efflux pump; RT-qPCR; Drug resistance;

Expression; CCCP

ID CATALASE-PEROXIDASE GENE; ISONIAZID RESISTANCE; MOLECULAR-CLONING;

INHIBITORS; MUTATIONS; STRAINS; P55

AB Objectives: Active extrusion of antituberculosis drugs via efflux pumps (EPs) has been suggested as contributing to drug resistance in Mycobacterium tuberculosis. This study was conducted to determine the role of 10 drug efflux transporters in the development of drug resistance in a series of clinical M. tuberculosis isolates.

Methods: A total of 31 clinical M. tuberculosis isolates without drug exposure [21 multi/extensively drug-resistant (M/XDR-TB) and 10 drug-susceptible isolates] were studied. The expression profile of 10 EP genes, including efpA, mmr, stp, drrA, drrB, mmpL7, Rv1250, Rv1634, Rv2994 and Rv1258c, was investigated against the H37Rv standard strain by quantitative reverse transcription PCR (RT-qPCR).

Results: Among the 21 M/XDR-TB isolates, 10 showed significantly increased levels of gene expression (>4-fold) for at least one of the studied EPs. Moreover, of the isolates with overexpressed genes, three and seven lacked genetic alterations in the surveyed regions of the rpoB + katG + inhA and katG + inhA genes, respectively. Whilst no elevation was observed in the expression of mmr, Rv1250, Rv1634 and Rv1258c genes in any of the isolates, drrA, stp and drrB were found to be the most commonly overexpressed, being overexpressed in seven, five and three isolates, respectively. Decreased minimum inhibitory concentrations (MICs) of rifampicin, but not isoniazid, were observed in the presence of the efflux pump inhibitor carbonyl cyanide 3-chlorophenylhydrazone (CCCP).

Conclusion: Overexpression of EP genes can contribute to the emergence of a MDR phenotype in M. tuberculosis. Inhibition of EPs may provide a promising strategy for improving tuberculosis treatment outcomes in patients infected with M/XDR-TB isolates. (C) 2019 International Society for Chemotherapy of Infection and Cancer. Published by Elsevier Ltd. All rights reserved.

C1 [Kardan-Yamchi, Jalil] Univ Tehran Med Sci, Sch Publ Hlth, Dept Pathobiol, Div Microbiol, Tehran, Iran.

[Kardan-Yamchi, Jalil; Kazemian, Hossein; Harati, Ahad Ali; Feizabadi, Mohammad Mehdi] Univ Tehran Med Sci, Sch Med, Dept Med Microbiol, Tehran, Iran.

[Kazemian, Hossein] Ilam Univ Med Sci, Clin Microbiol Res Ctr, Ilam, Iran.

[Haeili, Mehri] Univ Tabriz, Fac Nat Sci, Dept Biol, Tabriz, Iran.

[Amini, Sirus] Reg TB Reference Lab, Tehran, Iran.

[Feizabadi, Mohammad Mehdi] Univ Tehran Med Sci, Imam Khomeini Hosp, Thorac Res Ctr, Tehran, Iran.

[Feizabadi, Mohammad Mehdi] Univ Tehran Med Sci, Sch Med, Dept Microbiol, Poursina St,Engelab e Eslami Ave, Tehran, Iran.

C3 Tehran University of Medical Sciences; Tehran University of Medical

Sciences; University of Tabriz; Tehran University of Medical Sciences;

Tehran University of Medical Sciences

RP Feizabadi, MM (corresponding author), Univ Tehran Med Sci, Sch Med, Dept Med Microbiol, Tehran, Iran.; Feizabadi, MM (corresponding author), Univ Tehran Med Sci, Imam Khomeini Hosp, Thorac Res Ctr, Tehran, Iran.; Feizabadi, MM (corresponding author), Univ Tehran Med Sci, Sch Med, Dept Microbiol, Poursina St,Engelab e Eslami Ave, Tehran, Iran.

EM mfeizabadi@tums.ac.ir

RI Haeili, Mehri/KVY-6462-2024; Kazemian, Hossein/AAE-7329-2020; Feizabadi,

Mohammad Mahdi/ABB-9485-2021

OI Haeili, Mehri/0000-0002-7390-0742; Kardan-Yamchi,

Jalil/0000-0001-8546-512X; Kazemian, Hossein/0000-0003-4590-396X;

Feizabadi, Mohammad Mahdi/0000-0002-4825-1170

FU Tehran University of Medical Sciences (Tehran, Iran) [29139]

FX This study was supported by Tehran University of Medical Sciences

(Tehran, Iran) [project no. 29139].

CR Aínsa JA, 1998, J BACTERIOL, V180, P5836

[Anonymous], 2018, The Global Tuberculosis Report

Baghbanian M, 2017, CELL MOL BIOL, V63, P14, DOI [10.14715/10.14715/cmb/2017.63.12.5, 10.14715/cmb/2017.63.12.5]

Balganesh M, 2012, ANTIMICROB AGENTS CH, V56, P2643, DOI 10.1128/AAC.06003-11

Banerjee SK, 2000, MOL GEN GENET, V262, P949, DOI 10.1007/PL00008663

Calgin MK, 2013, DIAGN MICR INFEC DIS, V76, P291, DOI 10.1016/j.diagmicrobio.2013.02.033

Coelho T, 2015, FRONT MICROBIOL, V6, DOI [10.3389/fmicb.2015.00330, 10.3389/fmicb.2015.01528]

De Rossi E, 1998, ANTIMICROB AGENTS CH, V42, P1931, DOI 10.1128/AAC.42.8.1931

De Rossi E, 2006, FEMS MICROBIOL REV, V30, P36, DOI 10.1111/j.1574-6976.2005.00000.x

Dias Rodrigues LI, 2010, THESIS

Fonseca JD, 2015, INT J INFECT DIS, V32, P94, DOI 10.1016/j.ijid.2015.01.014

Guo HL, 2006, J MED MICROBIOL, V55, P1527, DOI 10.1099/jmm.0.46718-0

Gupta AK, 2010, MICROB DRUG RESIST, V16, P21, DOI 10.1089/mdr.2009.0054

HEYM B, 1995, MOL MICROBIOL, V15, P235, DOI 10.1111/j.1365-2958.1995.tb02238.x

Jaiswal I, 2017, LUNG INDIA, V34, P499, DOI 10.4103/0970-2113.217567

Kaatz Glenn W, 2002, Expert Opin Emerg Drugs, V7, P223, DOI 10.1517/14728214.7.2.223

Kanji A, 2017, TUBERCULOSIS, V107, P20, DOI 10.1016/j.tube.2017.07.012

Kazemian H, 2015, INT J ANTIMICROB AG, V45, P668, DOI 10.1016/j.ijantimicag.2015.02.004

Levy SB, 2002, J APPL MICROBIOL, V92, p65S, DOI 10.1046/j.1365-2672.92.5s1.4.x

Li GL, 2015, PLOS ONE, V10, DOI 10.1371/journal.pone.0119013

Li XZ, 2004, ANTIMICROB AGENTS CH, V48, P2415, DOI 10.1128/AAC.48.7.2415-2423.2004

Livak KJ, 2001, METHODS, V25, P402, DOI 10.1006/meth.2001.1262

Lomovskaya O, 2006, BIOCHEM PHARMACOL, V71, P910, DOI 10.1016/j.bcp.2005.12.008

Machado D, 2018, ANTIBIOTICS-BASEL, V7, DOI 10.3390/antibiotics7010018

Machado D, 2017, FRONT MICROBIOL, V8, DOI 10.3389/fmicb.2017.00711

Machado D, 2012, PLOS ONE, V7, DOI 10.1371/journal.pone.0034538

MCMURRY L, 1980, P NATL ACAD SCI-BIOL, V77, P3974, DOI 10.1073/pnas.77.7.3974

NIKAIDO H, 1994, SCIENCE, V264, P382, DOI 10.1126/science.8153625

Pasca MR, 2005, ANTIMICROB AGENTS CH, V49, P4775, DOI 10.1128/AAC.49.11.4775-4777.2005

Pasca MR, 2004, ANTIMICROB AGENTS CH, V48, P3175, DOI 10.1128/AAC.48.8.3175-3178.2004

Piddock LJV, 2006, CLIN MICROBIOL REV, V19, P382, DOI 10.1128/CMR.19.2.382-402.2006

Poole K, 2007, ANN MED, V39, P162, DOI 10.1080/07853890701195262

Ramón-García S, 2009, ANTIMICROB AGENTS CH, V53, P3675, DOI 10.1128/AAC.00550-09

Silva PEA, 2001, ANTIMICROB AGENTS CH, V45, P800, DOI 10.1128/AAC.45.3.800-804.2001

te Brake LHM, 2016, THESIS

TELENTI A, 1993, LANCET, V341, P647, DOI 10.1016/0140-6736(93)90417-F

VANSOOLINGEN D, 1994, METHOD ENZYMOL, V235, P196

Viveiros M, 2003, INT J ANTIMICROB AG, V22, P274, DOI 10.1016/S0924-8579(03)00208-5

Yamchi JK, 2015, INFECT GENET EVOL, V36, P23, DOI 10.1016/j.meegid.2015.08.036

ZHANG Y, 1992, NATURE, V358, P591, DOI 10.1038/358591a0

NR 40

TC 34

Z9 36

U1 2

U2 14

PU ELSEVIER SCI LTD

PI OXFORD

PA THE BOULEVARD, LANGFORD LANE, KIDLINGTON, OXFORD OX5 1GB, OXON, ENGLAND

SN 2213-7165

EI 2213-7173

J9 J GLOB ANTIMICROB RE

JI J. Glob. Antimicrob. Resist.

PD JUN

PY 2019

VL 17

BP 201

EP 208

DI 10.1016/j.jgar.2019.01.003

PG 8

WC Infectious Diseases; Pharmacology & Pharmacy

WE Science Citation Index Expanded (SCI-EXPANDED)

SC Infectious Diseases; Pharmacology & Pharmacy

GA ID8RJ

UT WOS:000471951900042

DA 2026-05-07

ER

PT J

AU Mata-Espinosa, D

Lara-Espinosa, JV

Barrios-Payán, J

Hernández-Pando, R

AF Mata-Espinosa, Dulce

Lara-Espinosa, Jacqueline V.

Barrios-Payan, Jorge

Hernandez-Pando, Rogelio

TI The Use of Viral Vectors for Gene Therapy and Vaccination in

Tuberculosis

SO PHARMACEUTICALS

LA English

DT Review

DE tuberculosis; gene therapy; adenovirus; vaccines

ID COLONY-STIMULATING FACTOR; T-CELL IMMUNITY; MYCOBACTERIUM-TUBERCULOSIS;

ANTIMICROBIAL PEPTIDES; INTERFERON-GAMMA; MURINE MODEL; ADENOVIRUS;

VIRUS; BCG; EXPRESSION

AB Tuberculosis (TB), an infection caused by Mycobacterium tuberculosis (Mtb), is one of the primary causes of death globally. The treatment of TB is long and based on several drugs, producing problems in compliance and toxicity, increasing Mtb resistance to first-line antibiotics that result in multidrug-resistant TB and extensively drug-resistant TB. Thus, the need for new anti-TB treatments has increased. Here, we review some model strategies to study gene therapy based on the administration of a recombinant adenovirus that encodes diverse cytokines, such as IFN gamma, IL12, GM/CSF, OPN, TNF alpha, and antimicrobial peptides to enhance the protective immune response against Mtb. These models include a model of progressive pulmonary TB, a model of chronic infection similar to latent TB, and a murine model of pulmonary Mtb transmission to close contacts. We also review new vaccines that deliver Mtb antigens via particle- or virus-based vectors and trigger protective immune responses. The results obtained in this type of research suggest that this is an alternative therapy that has the potential to treat active TB as an adjuvant to conventional antibiotics and a promising preventive treatment for latent TB reactivation and Mtb transmission. Moreover, Ad vector vaccines are adequate for preventing infectious diseases, including TB.

C1 [Mata-Espinosa, Dulce; Lara-Espinosa, Jacqueline V.; Barrios-Payan, Jorge; Hernandez-Pando, Rogelio] Inst Nacl Ciencias Med & Nutr Salvador Zubiran, Secc Patol Expt, Vasco de Quiroga 15,Belisario Dominguez Secc 16, Mexico City 14080, Mexico.

C3 Instituto Nacional de Ciencias Medicas y Nutricion Salvador Zubiran -

Mexico

RP Mata-Espinosa, D; Hernández-Pando, R (corresponding author), Inst Nacl Ciencias Med & Nutr Salvador Zubiran, Secc Patol Expt, Vasco de Quiroga 15,Belisario Dominguez Secc 16, Mexico City 14080, Mexico.

EM dulce.matae@incmnsz.mx

OI Barrios-Payán, Jorge Alberto/0000-0002-5134-5573; Hernández-Pando,

Rogelio/0000-0003-1292-3833; Lara Espinosa, Jacqueline

Viridiana/0000-0001-6635-2939

CR Mata-Espinosa DA, 2019, SCAND J IMMUNOL, V89, DOI 10.1111/sji.12743

Anderson LF, 2013, EUROSURVEILLANCE, V18, P7, DOI 10.2807/1560-7917.ES2013.18.40.20601

[Anonymous], 2021, Global tuberculosis report 2021

Appaiahgari MB, 2015, EXPERT OPIN BIOL TH, V15, P337, DOI 10.1517/14712598.2015.993374

Arrey F, 2019, FRONT IMMUNOL, V10, DOI 10.3389/fimmu.2019.00089

Arriaga AK, 2002, CLIN EXP IMMUNOL, V128, P229, DOI 10.1046/j.1365-2249.2002.01832.x

Belnoue E, 2022, INT J MOL SCI, V23, DOI 10.3390/ijms23052700

Bharti R, 2022, TUBERCULOSIS, V134, DOI 10.1016/j.tube.2022.102198

Bharti R, 2020, MOL THER-NUCL ACIDS, V22, P1121, DOI 10.1016/j.omtn.2020.10.023

Calderon VE, 2013, PLOS ONE, V8, DOI 10.1371/journal.pone.0063331

Carrigy NB, 2019, ANTIMICROB AGENTS CH, V63, DOI 10.1128/AAC.00871-19

Castañeda-Delgado J, 2010, CLIN EXP IMMUNOL, V161, P542, DOI 10.1111/j.1365-2249.2010.04199.x

Cheng EM, 2022, CANCER IMMUNOL IMMUN, V71, P2057, DOI 10.1007/s00262-022-03144-1

Cruz J, 2014, CURR MED CHEM, V21, P2299, DOI 10.2174/0929867321666140217110155

de Martino M, 2019, FRONT PEDIATR, V7, DOI 10.3389/fped.2019.00350

Ferreira RG, 2021, PROCESSES, V9, DOI 10.3390/pr9081430

FLYNN JL, 1993, J EXP MED, V178, P2249, DOI 10.1084/jem.178.6.2249

Francisco-Cruz A, 2013, CLIN EXP IMMUNOL, V171, P283, DOI 10.1111/cei.12015

Francisco-Cruz A, 2016, TUBERCULOSIS, V100, P5, DOI 10.1016/j.tube.2016.05.015

Garbuglia AR, 2022, VIRUSES-BASEL, V14, DOI 10.3390/v14040748

García-Romo GS, 2004, IMMUNOLOGY, V112, P661, DOI 10.1046/j.1365-2567.2004.01904.x

Hameed HMA, 2018, FRONT CELL INFECT MI, V8, DOI 10.3389/fcimb.2018.00114

Hansen SG, 2018, NAT MED, V24, P130, DOI 10.1038/nm.4473

Happel KI, 2005, INFECT IMMUN, V73, P5782, DOI 10.1128/IAI.73.9.5782-5788.2005

Harrach B, 2019, FEBS LETT, V593, P3660, DOI 10.1002/1873-3468.13687

He TC, 1998, P NATL ACAD SCI USA, V95, P2509, DOI 10.1073/pnas.95.5.2509

Hernandez-Bazan S, 2022, HUM GENE THER, V33, P1037, DOI 10.1089/hum.2022.030

Hernandez-Pando R, 2000, INT J EXP PATHOL, V81, P199, DOI 10.1046/j.1365-2613.2000.00152.x

Hernandez-Pando R, 2009, ARCH IMMUNOL THER EX, V57, P355, DOI 10.1007/s00005-009-0042-9

HernandezPando R, 1997, IMMUNOLOGY, V90, P607, DOI 10.1046/j.1365-2567.1997.00193.x

HernandezPando R, 1996, IMMUNOLOGY, V89, P26

Hidaka C, 1999, J CLIN INVEST, V103, P579, DOI 10.1172/JCI5309

Hu ZD, 2022, FRONT IMMUNOL, V13, DOI 10.3389/fimmu.2022.895020

Kagina BMN, 2014, VACCINE, V32, P5908, DOI 10.1016/j.vaccine.2014.09.001

Keane J, 2001, NEW ENGL J MED, V345, P1098, DOI 10.1056/NEJMoa011110

Kreppel F, 2008, MOL THER, V16, P16, DOI 10.1038/sj.mt.6300321

Kulanayake S, 2021, VIRUSES-BASEL, V13, DOI 10.3390/v13030388

Carabalí-Isajar ML, 2023, WORLD J MICROB BIOT, V39, DOI 10.1007/s11274-023-03636-x

Liu KY, 2016, J AEROSOL MED PULM D, V29, P393, DOI 10.1089/jamp.2015.1233

Loustalot F, 2016, INT REV CEL MOL BIO, V322, P331, DOI 10.1016/bs.ircmb.2015.10.006

Marquina-Castillo B, 2009, IMMUNOLOGY, V128, P123, DOI 10.1111/j.1365-2567.2008.03004.x

Mata-Espinosa DA, 2008, MOL THER, V16, P1065, DOI 10.1038/mt.2008.69

Montes-Galindo DA, 2019, ARCH VIROL, V164, P775, DOI 10.1007/s00705-018-04132-6

Nusbaum RJ, 2016, SCI REP-UK, V6, DOI 10.1038/srep21522

O'Garra A, 2013, ANNU REV IMMUNOL, V31, P475, DOI 10.1146/annurev-immunol-032712-095939

Ojo OO, 2023, SCI REP-UK, V13, DOI 10.1038/s41598-023-38707-4

Pandit R., 2015, J TUBERCULOSIS RES, V3, P171, DOI [10.4236/jtr.2015.34024, 10.4236/jtr.2015.34024, DOI 10.4236/JTR.2015.34024]

Perera PY, 2009, VACCINE, V27, P2121, DOI 10.1016/j.vaccine.2009.01.132

de Val BP, 2012, CLIN VACCINE IMMUNOL, V19, P1339, DOI 10.1128/CVI.00275-12

Poladian N, 2023, INT J MOL SCI, V24, DOI 10.3390/ijms24021772

Rahlwes KC, 2023, VIRULENCE, V14, DOI 10.1080/21505594.2022.2150449

Ramos-Espinosa O, 2021, J LEUKOCYTE BIOL, V110, P951, DOI 10.1002/JLB.4MA0920-627R

Ramos-Espinosa O, 2018, EXPERT REV RESP MED, V12, P427, DOI 10.1080/17476348.2018.1457439

Ramos-Espinosa O, 2016, PATHOG DIS, V74, DOI 10.1093/femspd/ftw075

Rivas-Santiago B, 2008, SCAND J IMMUNOL, V67, P448, DOI 10.1111/j.1365-3083.2008.02088.x

Rivas-Santiago B, 2006, J INFECT DIS, V194, P697, DOI 10.1086/506454

Rivas-Santiago CE, 2013, IMMUNOTHERAPY-UK, V5, P1117, DOI 10.2217/imt.13.111

Roediger EK, 2008, MOL THER, V16, P1161, DOI 10.1038/mt.2008.59

Rook GAW, 2007, BRAIN BEHAV IMMUN, V21, P169, DOI 10.1016/j.bbi.2006.08.003

Sakamoto K, 2012, VET PATHOL, V49, P423, DOI 10.1177/0300985811429313

Santosuosso M, 2005, J IMMUNOL, V174, P7986, DOI 10.4049/jimmunol.174.12.7986

Satti I, 2014, LANCET INFECT DIS, V14, P939, DOI 10.1016/S1473-3099(14)70845-X

Schrager LK, 2020, LANCET INFECT DIS, V20, pE28, DOI 10.1016/S1473-3099(19)30625-5

Sharma SK, 2021, EXPERT REV RESP MED, V15, P931, DOI 10.1080/17476348.2021.1927718

Shirley JL, 2020, MOL THER, V28, P709, DOI 10.1016/j.ymthe.2020.01.001

Shurygina AP, 2023, INT J MOL SCI, V24, DOI 10.3390/ijms24087439

Sivakumaran D, 2021, FRONT IMMUNOL, V12, DOI 10.3389/fimmu.2021.673532

Smaill F, 2013, SCI TRANSL MED, V5, DOI 10.1126/scitranslmed.3006843

Srivastava S, 2023, VACCINES-BASEL, V11, DOI 10.3390/vaccines11051013

Stukova M., 2018, Randomized Open Label Phase 1 Clinical Trial of TB/FLU-01L Tuberculosis Vaccine Administered Intranasally or Sublingual in BCG-Vaccinated Healthy Adults

Stylianou E, 2015, VACCINE, V33, P6800, DOI 10.1016/j.vaccine.2015.10.017

Suárez I, 2019, DTSCH ARZTEBL INT, V116, P729, DOI 10.3238/arztebl.2019.0729

Walsh NC, 2017, ANNU REV PATHOL-MECH, V12, P187, DOI 10.1146/annurev-pathol-052016-100332

Wang KX, 2008, CYTOKINE GROWTH F R, V19, P333, DOI 10.1016/j.cytogfr.2008.08.001

Wilkie M, 2020, VACCINE, V38, P779, DOI 10.1016/j.vaccine.2019.10.102

Wirth T, 2013, GENE, V525, P162, DOI 10.1016/j.gene.2013.03.137

Xing Z, 2009, PLOS ONE, V4, DOI 10.1371/journal.pone.0005856

Young LS, 2001, GUT, V48, P733, DOI 10.1136/gut.48.5.733

Rodriguez-Takeuchi SY, 2019, RADIOGRAPHICS, V39, P2023, DOI 10.1148/rg.2019190109

NR 79

TC 5

Z9 7

U1 0

U2 7

PU MDPI

PI BASEL

PA ST ALBAN-ANLAGE 66, CH-4052 BASEL, SWITZERLAND

EI 1424-8247

J9 PHARMACEUTICALS-BASE

JI Pharmaceuticals

PD OCT

PY 2023

VL 16

IS 10

AR 1475

DI 10.3390/ph16101475

PG 19

WC Chemistry, Medicinal; Pharmacology & Pharmacy

WE Science Citation Index Expanded (SCI-EXPANDED)

SC Pharmacology & Pharmacy

GA W2AU6

UT WOS:001089718600001

OA Green Submitted, gold

DA 2026-05-07

ER

PT J

AU Kapp, E

Malan, SF

Joubert, J

Sampson, SL

AF Kapp, Erika

Malan, Sarel F.

Joubert, Jacques

Sampson, Samantha L.

TI Small Molecule Efflux Pump Inhibitors in Mycobacterium

tuberculosis: A Rational Drug Design Perspective

SO MINI-REVIEWS IN MEDICINAL CHEMISTRY

LA English

DT Review

DE Active efflux; antimicrobial active transport systems; drug resistance;

efflux pump inhibitor; Mycobacterium tuberculosis; rational drug design

ID P-GLYCOPROTEIN; RESISTANT TUBERCULOSIS; ANTIBIOTIC EFFLUX; IN-VITRO;

XDR-TB; VERAPAMIL; THIORIDAZINE; PIPERINE; BERBERINE; ANALOGS

AB Drug resistance in Mycobacterium tuberculosis (M. tuberculosis) complicates management of tuberculosis. Efflux pumps contribute to low level resistance and acquisition of additional high level resistance mutations through sub-therapeutic concentrations of intracellular antimycobacterials. Various efflux pump inhibitors (EPIs) have been described for M. tuberculosis but little is known regarding the mechanism of efflux inhibition. As knowledge relating to the mechanism of action and drug target is central to the rational drug design of safe and sufficiently selective EPIs, this review aims to examine recent developments in the study of EPIs in M. tuberculosis from a rational drug development perspective and to provide an overview to facilitate systematic development of therapeutically effective EPIs. Review of literature points to a reduction in cellular energy or direct binding to the efflux pump as likely mechanisms for most EPIs described for M. tuberculosis. This review demonstrates that, where a direct interaction with efflux pumps is expected, both molecular structure and general physicochemical properties should be considered to accurately predict efflux pump substrates and inhibitors. Non-competitive EPIs do not necessarily demonstrate the same requirements as competitive inhibitors and it is therefore essential to differentiate between competitive and non-competitive inhibition to accurately determine structure activity relationships for efflux pump inhibition. It is also evident that there are various similarities between inhibitors of prokaryotic and eukaryotic efflux pumps but, depending on the specific chemical scaffolds under investigation, it may be possible to design EPIs that are less prone to inhibition of human P-glycoprotein, thereby reducing side effects and drug-drug interactions.

C1 [Kapp, Erika; Malan, Sarel F.; Joubert, Jacques] Univ Western Cape, Sch Pharm Bellville, Bellville, South Africa.

[Sampson, Samantha L.] Univ Stellenbosh, Div Mol Biol & Human Genet, Fac Med & Hlth Sci Bellville, Bellville, Western Cape, South Africa.

C3 University of the Western Cape

RP Malan, SF (corresponding author), Univ Western Cape, Sch Pharm, Fac Nat Sci, Private Bag X17, ZA-7535 Bellville, South Africa.

EM sfmalan@uwc.ac.za

RI ; Malan, Sarel/AAA-4189-2020; Joubert, Jacques/E-2441-2013

OI Kapp, Erika/0000-0001-5747-4766; Malan, Sarel/0000-0002-1640-6457;

Joubert, Jacques/0000-0003-0378-7091; sampson,

samantha/0000-0002-9596-8019

FU National Research Foundation of South Africa; South African Research

Chairs Initiative of the Department of Science and Technology; National

Research Foundation (NRF) of South Africa [UID 86539]

FX We are grateful to the National Research Foundation of South Africa for

financial support. SLS is funded by the South African Research Chairs

Initiative of the Department of Science and Technology and National

Research Foundation (NRF) of South Africa, award number UID 86539. The

content is solely the responsibility of the authors and does not

necessarily represent the official views of the NRF.

CR Abbate E, 2012, J ANTIMICROB CHEMOTH, V67, P473, DOI 10.1093/jac/dkr500

Abdallah HM, 2015, J ADV RES, V6, P45, DOI 10.1016/j.jare.2014.11.008

Adams KN, 2014, J INFECT DIS, V210, P456, DOI 10.1093/infdis/jiu095

Adams KN, 2011, CELL, V145, P39, DOI 10.1016/j.cell.2011.02.022

Ahmad N., 2012, ASIAN PACIFIC J TROP, V2, pS1945

da Silva PEA, 2011, FEMS IMMUNOL MED MIC, V63, P1, DOI 10.1111/j.1574-695X.2011.00831.x

Amaral L, 2007, J ANTIMICROB CHEMOTH, V59, P1237, DOI 10.1093/jac/dkl500

Amaral L, 2014, IN VIVO, V28, P267

Amaral L, 2014, FRONT PHARMACOL, V4, DOI 10.3389/fphar.2013.00168

Amaral L, 2012, INT J ANTIMICROB AG, V39, P376, DOI 10.1016/j.ijantimicag.2012.01.012

Amaral L, 2010, IN VIVO, V24, P409

Amaral L, 2010, INT J ANTIMICROB AG, V35, P524, DOI 10.1016/j.ijantimicag.2009.12.019

Andersen CL, 2006, BBA-BIOMEMBRANES, V1758, P1587, DOI 10.1016/j.bbamem.2006.05.022

Balganesh M, 2012, ANTIMICROB AGENTS CH, V56, P2643, DOI 10.1128/AAC.06003-11

Bettencourt MV, 2000, INT J ANTIMICROB AG, V16, P69, DOI 10.1016/S0924-8579(00)00199-0

Bhardwaj Ashima K, 2012, Recent Pat Antiinfect Drug Discov, V7, P73

Bhardwaj RK, 2002, J PHARMACOL EXP THER, V302, P645, DOI 10.1124/jpet.102.034728

Black PA, 2014, ANTIMICROB AGENTS CH, V58, P2491, DOI 10.1128/AAC.02293-13

Böttger EC, 2011, PROG RESPIR RES, V40, P128

Broccatelli F, 2011, J MED CHEM, V54, P1740, DOI 10.1021/jm101421d

Bruhn DF, 2015, SCI REP-UK, V5, DOI 10.1038/srep13985

Busse D, 2006, EUR J CLIN PHARMACOL, V62, P613, DOI 10.1007/s00228-006-0154-7

CROWLE AJ, 1992, CHEMOTHERAPY, V38, P410, DOI 10.1159/000239036

Daniel WA, 2003, PROG NEURO-PSYCHOPH, V27, P65, DOI 10.1016/S0278-5846(02)00317-2

de Keijzer J, 2016, J PROTEOME RES, V15, P1776, DOI 10.1021/acs.jproteome.5b01037

De Rossi E, 2006, FEMS MICROBIOL REV, V30, P36, DOI 10.1111/j.1574-6976.2005.00000.x

Dinesh N, 2013, ANTIMICROB AGENTS CH, V57, P1941, DOI 10.1128/AAC.01957-12

Dutta NK, 2014, TUBERCULOSIS, V94, P708, DOI 10.1016/j.tube.2014.09.001

Dutta NK, 2010, PLOS ONE, V5, DOI 10.1371/journal.pone.0010069

Farha MA, 2013, CHEM BIOL, V20, P1168, DOI 10.1016/j.chembiol.2013.07.006

Fu LW, 2004, CANCER CHEMOTH PHARM, V53, P349, DOI 10.1007/s00280-003-0742-5

Golan D.E., 2012, Principles of pharmacology : the pathophysiologic basis of drug therapy

Grossman TH, 2015, ANTIMICROB AGENTS CH, V59, P1539, DOI 10.1128/AAC.04271-14

Gupta S, 2015, ANTIMICROB AGENTS CH, V59, P673, DOI 10.1128/AAC.04019-14

Gupta S, 2014, ANTIMICROB AGENTS CH, V58, P574, DOI 10.1128/AAC.01462-13

Hait W. N, 1996, DRUG RESISTANCE

HENRY JP, 1989, BIOCHEM PHARMACOL, V38, P2395, DOI 10.1016/0006-2952(89)90082-8

Hille B., 2001, Ion channels of excitable membranes, V507

HOON TJ, 1986, AM HEART J, V112, P396, DOI 10.1016/0002-8703(86)90281-4

Jaszczyszyn A, 2012, PHARMACOL REP, V64, P16, DOI 10.1016/S1734-1140(12)70726-0

Khan IA, 2006, ANTIMICROB AGENTS CH, V50, P810, DOI 10.1128/AAC.50.2.810-812.2006

Klyachko KA, 1997, J BACTERIOL, V179, P2189, DOI 10.1128/jb.179.7.2189-2193.1997

Kornhuber J, 2010, J CELL PHYSIOL, V224, P152, DOI 10.1002/jcp.22112

Koul S, 2000, BIOORGAN MED CHEM, V8, P251, DOI 10.1016/S0968-0896(99)00273-4

Kourtesi Christina, 2013, Open Microbiol J, V7, P34, DOI 10.2174/1874285801307010034

Kumar A, 2008, J ANTIMICROB CHEMOTH, V61, P1270, DOI 10.1093/jac/dkn088

Lamers RP, 2013, PLOS ONE, V8, DOI 10.1371/journal.pone.0060666

Lee RE, 2014, NAT MED, V20, P152, DOI 10.1038/nm.3458

Liu CS, 2016, FITOTERAPIA, V109, P274, DOI 10.1016/j.fitote.2016.02.001

LOGIN IS, 1985, AM J PHYSIOL, V248, pE15, DOI 10.1152/ajpendo.1985.248.1.E15

Lomovskaya O, 2006, BIOCHEM PHARMACOL, V71, P910, DOI 10.1016/j.bcp.2005.12.008

Louw GE, 2009, ANTIMICROB AGENTS CH, V53, P3181, DOI 10.1128/AAC.01577-08

Louw GE, 2011, AM J RESP CRIT CARE, V184, P269, DOI 10.1164/rccm.201011-1924OC

Machado D, 2016, PLOS ONE, V11, DOI 10.1371/journal.pone.0149326

Marquez B, 2005, BIOCHIMIE, V87, P1137, DOI 10.1016/j.biochi.2005.04.012

Martins A, 2011, INT J ANTIMICROB AG, V37, P410, DOI 10.1016/j.ijantimicag.2011.01.010

Martins M, 2009, INT J TUBERC LUNG D, V13, P569

Martins M, 2008, IN VIVO, V22, P69

Meghwal M, 2013, PHYTOTHER RES, V27, P1121, DOI 10.1002/ptr.4972

Misra R, 2015, J BACTERIOL, V197, P2479, DOI 10.1128/JB.02456-14

Montanari F, 2015, ADV DRUG DELIVER REV, V86, P17, DOI 10.1016/j.addr.2015.03.001

Mullin S, 2004, ANTIMICROB AGENTS CH, V48, P4171, DOI 10.1128/AAC.48.11.4171-4176.2004

Okumura Y, 2010, BIOSCI BIOTECH BIOCH, V74, P1068, DOI 10.1271/bbb.90964

Opperman TJ, 2015, FRONT MICROBIOL, V6, DOI 10.3389/fmicb.2015.00421

Pagès JM, 2009, BBA-PROTEINS PROTEOM, V1794, P826, DOI 10.1016/j.bbapap.2008.12.011

Pasca MR, 2005, ANTIMICROB AGENTS CH, V49, P4775, DOI 10.1128/AAC.49.11.4775-4777.2005

Pieroni M, 2015, J MED CHEM, V58, P5842, DOI 10.1021/acs.jmedchem.5b00428

Pule CM, 2016, J ANTIMICROB CHEMOTH, V71, P17, DOI 10.1093/jac/dkv316

Putman M, 2000, MICROBIOL MOL BIOL R, V64, P672, DOI 10.1128/MMBR.64.4.672-693.2000

Rodrigues L, 2008, J ANTIMICROB CHEMOTH, V61, P1076, DOI 10.1093/jac/dkn070

Rodrigues L, 2012, INFECT GENET EVOL, V12, P695, DOI 10.1016/j.meegid.2011.08.009

Rodrigues Liliana, 2011, Recent Pat Antiinfect Drug Discov, V6, P118

Roy K., 2015, Quantitative Structure-Activity Relationships in Drug Design, Predictive Toxicology, and Risk Assessment

Sarathy JP, 2012, PHARMACEUTICALS-BASE, V5, P1210, DOI 10.3390/ph5111210

Sharma S, 2010, J ANTIMICROB CHEMOTH, V65, P1694, DOI 10.1093/jac/dkq186

Singh IP, 2015, CURR TOP MED CHEM, V15, P1722, DOI 10.2174/1568026615666150427123213

Singh K, 2014, BIOORG MED CHEM LETT, V24, P2985, DOI 10.1016/j.bmcl.2014.05.022

Smith DA, 2010, RSC DRUG DISCOV, V1, P486

Stermitz FR, 2000, P NATL ACAD SCI USA, V97, P1433, DOI 10.1073/pnas.030540597

Subramanian N, 2016, NEUROCHEM INT, V98, P146, DOI 10.1016/j.neuint.2016.05.005

te Brake LHM, 2016, TUBERCULOSIS, V96, P150, DOI 10.1016/j.tube.2015.08.004

Tegos G, 2002, ANTIMICROB AGENTS CH, V46, P3133, DOI 10.1128/AAC.46.10.3133-3141.2002

Tegos GP, 2011, CURR PHARM DESIGN, V17, P1291

THIEBAUT F, 1987, P NATL ACAD SCI USA, V84, P7735, DOI 10.1073/pnas.84.21.7735

Van Bambeke F, 2003, J ANTIMICROB CHEMOTH, V51, P1067, DOI 10.1093/jac/dkg225

Van Bambeke Francoise, 2006, Recent Pat Antiinfect Drug Discov, V1, P157, DOI 10.2174/157489106777452692

Viveiros M, 2002, ANTIMICROB AGENTS CH, V46, P2804, DOI 10.1128/AAC.46.9.2804-2810.2002

Viveiros M, 2014, TRENDS MICROBIOL, V22, P170, DOI 10.1016/j.tim.2014.02.008

Viveiros M, 2012, EXPERT REV ANTI-INFE, V10, P983, DOI [10.1586/ERI.12.89, 10.1586/eri.12.89]

Wang YX, 2012, ACTA PHARM SIN B, V2, P581, DOI 10.1016/j.apsb.2012.10.008

Weaver-Agostoni J, 2013, AM FAM PHYSICIAN, V88, P122

WEISS B, 1982, BIOCHEM PHARMACOL, V31, P2217, DOI 10.1016/0006-2952(82)90104-6

Wojtyczka RD, 2014, MOLECULES, V19, P6583, DOI 10.3390/molecules19056583

Wright SH, 2004, ADV PHYSIOL EDUC, V28, P139, DOI 10.1152/advan.00029.2004

Zechini Barbara, 2009, Recent Pat Antiinfect Drug Discov, V4, P37

Zhang Y, 2002, J MED MICROBIOL, V51, P42, DOI 10.1099/0022-1317-51-1-42

Zhou XY, 2016, J ANTIBIOT, V69, P741, DOI 10.1038/ja.2016.15

NR 97

TC 24

Z9 30

U1 0

U2 40

PU BENTHAM SCIENCE PUBL LTD

PI SHARJAH

PA EXECUTIVE STE Y-2, PO BOX 7917, SAIF ZONE, 1200 BR SHARJAH, U ARAB

EMIRATES

SN 1389-5575

EI 1875-5607

J9 MINI-REV MED CHEM

JI Mini-Rev. Med. Chem.

PY 2018

VL 18

IS 1

BP 72

EP 86

DI 10.2174/1389557517666170510105506

PG 15

WC Chemistry, Medicinal

WE Science Citation Index Expanded (SCI-EXPANDED)

SC Pharmacology & Pharmacy

GA FS4CG

UT WOS:000419734400007

DA 2026-05-07

ER

PT J

AU Chen, Q

Zheng, XD

Li, Y

Ma, BL

Nie, XY

Li, MY

Liu, YY

Xu, JR

Yang, Y

AF Chen, Qi

Zheng, Xuedi

Li, Yong

Ma, Boli

Nie, Xueyi

Li, Mengyuan

Liu, Yueyang

Xu, Jinrui

Yang, Yi

TI Wnt5a regulates autophagy in Bacille Calmette-Guerin (BCG)-Infected

pulmonary epithelial cells

SO MICROBIAL PATHOGENESIS

LA English

DT Article

DE Wnt5a; BCG; Pulmonary epithelial cells; Autophagy

ID DRUG-RESISTANT TUBERCULOSIS; SURVIVAL; ROLES

AB Autophagy functions as a critical process that can suppress the proliferation of Mycobacterium tuberculosis (Mtb) within infected host cells. Wnt5a is a secreted protein that plays a range of physiological functions, activating several signaling pathways and thereby controlling cellular responses to particular stimuli. The importance of Wnt5a as a regulator of protection against Mtb infection, however, has yet to be fully characterized. Here, changes in murine pulmonary epithelial-like TC-1 cell morphology, autophagy, the Wnt/Ca2+ signaling pathway, and the mTOR autophagy pathway were analyzed following infection with the Mtb model pathogen Bacille Calmette-Guerin (BCG) in order to understand the regulatory role of Wnt5a in this context. These experiments revealed that Wnt5a was upregulated and autophagy was enhanced in TC-1 cells infected with BCG, and Wnt5a overexpression was found to drive BCG-induced autophagy in these cells upon infection, whereas the inhibition or knockdown of Wnt5a yielded the opposite effect. At the mechanistic level, Wnt5a was found to mediate non -canonical Wnt/Ca2+ signaling and thereby inhibit mTOR-dependent pathway activation, promoting autophagic induction within BCG-infected TC-1 cells. These data offer new insight regarding how Wnt5a influences Mtb-induced autophagy within pulmonary epithelial cells, providing a foundation for further research exploring the immunological control of this infection through the modulation of autophagy.

C1 [Chen, Qi; Zheng, Xuedi; Li, Yong; Ma, Boli; Nie, Xueyi; Li, Mengyuan; Liu, Yueyang; Xu, Jinrui; Yang, Yi] Ningxia Univ, Minist Educ Conservat & Utilizat Special Biol Reso, Key Lab, Yinchuan 750021, Ningxia, Peoples R China.

[Chen, Qi; Zheng, Xuedi; Li, Yong; Ma, Boli; Nie, Xueyi; Li, Mengyuan; Liu, Yueyang; Xu, Jinrui; Yang, Yi] Ningxia Univ, Sch Life Sci, Yinchuan 750021, Ningxia, Peoples R China.

[Li, Mengyuan] Ningxia Med Univ, Key Lab Hui Ethn Med Modernizat, Minist Educ, Yinchuan 750004, Peoples R China.

[Xu, Jinrui; Yang, Yi] Ningxia Univ, Sch Life Sci, 539 W Helanshan Rd, Yinchuan 750021, Ningxia, Peoples R China.

C3 Ningxia University; Ningxia University; Ningxia Medical University;

Ningxia University

RP Xu, JR; Yang, Y (corresponding author), Ningxia Univ, Sch Life Sci, 539 W Helanshan Rd, Yinchuan 750021, Ningxia, Peoples R China.

EM xujr@nxu.edu.cn; yangyi@nxu.edu.cn

OI Yang, Yi/0000-0002-6317-5460

FU National Natural Science Foundation of China (NSFC) , China;

[31960712]; [31960700]

FX This work was supported by grants from the National Natural Science

Foundation of China (NSFC) , China (Nos. 31960712, 31960700) .

CR Ardain A, 2020, IMMUNOLOGY, V159, P245, DOI 10.1111/imm.13143

Asem MS, 2016, CANCERS, V8, DOI 10.3390/cancers8090079

Astudillo P, 2020, FRONT CELL DEV BIOL, V8, DOI 10.3389/fcell.2020.00110

Blumenthal A, 2006, BLOOD, V108, P965, DOI 10.1182/blood-2005-12-5046

Bussi C, 2019, FEMS MICROBIOL REV, V43, P341, DOI 10.1093/femsre/fuz006

Castillo EF, 2012, P NATL ACAD SCI USA, V109, pE3168, DOI 10.1073/pnas.1210500109

Chakaya J, 2021, INT J INFECT DIS, V113, pS7, DOI 10.1016/j.ijid.2021.02.107

Chen DM, 2017, DNA CELL BIOL, V36, P58, DOI 10.1089/dna.2016.3418

Chen Xu, 2020, Chinese Medical Sciences Journal, V35, P357, DOI [10.24920/003659, 10.24920/003659]

Deretic V, 2016, J LEUKOCYTE BIOL, V100, P969, DOI 10.1189/jlb.4MR0216-079R

Dong WQ, 2022, ISCIENCE, V25, DOI 10.1016/j.isci.2022.104279

Flores-Hernández E, 2020, CELL SIGNAL, V72, DOI 10.1016/j.cellsig.2020.109636

Galluzzi L, 2019, CELL, V177, P1682, DOI 10.1016/j.cell.2019.05.026

Gao YC, 2019, J IMMUNOL, V203, P922, DOI 10.4049/jimmunol.1900169

Gutierrez MG, 2004, CELL, V119, P753, DOI 10.1016/j.cell.2004.11.038

Invernizzi R, 2020, IMMUNOLOGY, V160, P171, DOI 10.1111/imm.13195

Jati S, 2021, FRONT IMMUNOL, V11, DOI 10.3389/fimmu.2020.628191

Jati S, 2018, FRONT IMMUNOL, V9, DOI 10.3389/fimmu.2018.00679

Kim Yi Sak, 2019, Exp Mol Med, V51, P1, DOI 10.1038/s12276-019-0290-7

Kumar P, 2021, FRONT IMMUNOL, V12, DOI 10.3389/fimmu.2021.778028

Li CG, 2020, CELLS-BASEL, V9, DOI 10.3390/cells9020384

Li JZ, 2022, EXP THER MED, V23, DOI 10.3892/etm.2021.10927

Liu F, 2018, NAT COMMUN, V9, DOI 10.1038/s41467-018-06836-4

Liu J, 2021, INT IMMUNOPHARMACOL, V91, DOI 10.1016/j.intimp.2020.107215

Mareninova OA, 2020, AUTOPHAGY, V16, P2084, DOI 10.1080/15548627.2020.1715047

Maurya SK, 2020, BMC INFECT DIS, V20, DOI 10.1186/s12879-020-05372-1

Migliori GB, 2012, CLIN INFECT DIS, V54, P1379, DOI 10.1093/cid/cis128

Ndoye A, 2017, CANCER RES, V77, P5873, DOI 10.1158/0008-5472.CAN-17-0907

Paik S, 2019, VIRULENCE, V10, P448, DOI 10.1080/21505594.2018.1536598

Persaud R, 2022, CELL CHEM BIOL, V29, P870, DOI 10.1016/j.chembiol.2021.07.017

Qu YL, 2019, CELL IMMUNOL, V337, P22, DOI 10.1016/j.cellimm.2019.01.004

Rose PC, 2012, INT J TUBERC LUNG D, V16, P1588, DOI 10.5588/ijtld.12.0322

Salazar G, 2020, AUTOPHAGY, V16, P1092, DOI 10.1080/15548627.2019.1659612

Sharma S, 2017, APPL BIOCHEM BIOTECH, V181, P762, DOI 10.1007/s12010-016-2247-9

Shi YN, 2021, INT J BIOL SCI, V17, P2561, DOI 10.7150/ijbs.58715

Shorning BY, 2020, INT J MOL SCI, V21, DOI 10.3390/ijms21124507

Sun GS, 2021, CANCER MANAG RES, V13, P2483, DOI 10.2147/CMAR.S289819

Valdez-Miramontes CE, 2020, CLIN EXP IMMUNOL, V199, P230, DOI 10.1111/cei.13388

Wong KW, 2017, MICROBIOL SPECTR, V5, DOI 10.1128/microbiolspec.TBTB2-0001-2015

Wu XL, 2019, MICROB PATHOGENESIS, V127, P277, DOI 10.1016/j.micpath.2018.12.016

Yamamoto K, 2014, AM J RESP CELL MOL, V50, P253, DOI 10.1165/rcmb.2013-0114OC

Yang Y, 2018, MEDIAT INFLAMM, V2018, DOI 10.1155/2018/3685948

Zhang H, 2018, ONCOL LETT, V15, P610, DOI 10.3892/ol.2017.7315

Zheng XD, 2022, MICROB PATHOGENESIS, V167, DOI 10.1016/j.micpath.2022.105564

Zhou CH, 2012, AUTOPHAGY, V8, P1215, DOI 10.4161/auto.20284

Zumla A, 2012, J INFECT DIS, V205, pS228, DOI 10.1093/infdis/jir858

NR 46

TC 7

Z9 7

U1 0

U2 4

PU ACADEMIC PRESS LTD- ELSEVIER SCIENCE LTD

PI LONDON

PA 24-28 OVAL RD, LONDON NW1 7DX, ENGLAND

SN 0882-4010

EI 1096-1208

J9 MICROB PATHOGENESIS

JI Microb. Pathog.

PD DEC

PY 2022

VL 173

AR 105826

DI 10.1016/j.micpath.2022.105826

EA OCT 2022

PN A

PG 14

WC Immunology; Microbiology

WE Science Citation Index Expanded (SCI-EXPANDED)

SC Immunology; Microbiology

GA 6C5BL

UT WOS:000882029500005

DA 2026-05-07

ER

PT J

AU Soares-Bezerra, RJ

Pinho, RT

Bisaggio, RDC

Benévolo-de-Andrade, TC

Alves, LA

AF Soares-Bezerra, Romulo J.

Pinho, Rosa T.

Bisaggio, Rodrigo da C.

Benevolo-de-Andrade, Thereza Christina

Alves, Luiz A.

TI The Search for New Agonists to P2X7R for Clinical Use: Tuberculosis as a

Possible Target

SO CELLULAR PHYSIOLOGY AND BIOCHEMISTRY

LA English

DT Review

DE Agonists; P2X7R; Purinergic Receptors; Multidrug Resistance;

Tuberculosis

ID MYCOBACTERIUM-TUBERCULOSIS; EXTRACELLULAR ATP; P2X(7) RECEPTOR;

PURINERGIC RECEPTORS; HUMAN MACROPHAGES; ADENOSINE 5'-TRIPHOSPHATE;

INTRAVENOUS ATP; P2 RECEPTOR; XDR-TB; POLYMORPHISM

AB Treatment for tuberculosis is effective with the use of proper antibiotics, but the number of drug-resistant cases is increasing. Drug resistance occurred in 650,000 cases of the 20 million patients in treatment worldwide in 2011, which demonstrates the necessity of finding new therapeutic approaches. In this context, the search for new medicines and immunomodulators could help reduce the prevalence and incidence of multi-drug-resistant tuberculosis cases. Thus several preclinical studies demonstrate the involvement of the P2X7 receptor (P2X7R) in the control of Mycobacterium tuberculosis (MTB) infection. Adenosine triphosphate (ATP), a natural agonist for P2X7R, promotes MTB death and the induction of apoptosis in monocytes and macrophages infected with MTB via activation of P2X7R by extracellular ATP. In addition, P2X7R activation in the presence of ATP increases the expression of major histocompatibility complex (MHC) class II by macrophages infected with Mycobacterium bovis (BCG) or MTB, which contributes to the generation of the antimicrobial immune response via T cells. Nevertheless, one idea that seems overlooked by the "purinergic community" is the use of the high-conductance channel associated with P2X7R to increase the passage of hydrophilic drugs to the cytoplasm of cells that express the P2X7 pore, a potential method for a drug delivery system. In this work, we propose the use of P2X7 agonists in conjunction with low molecular weight anti-tuberculosis medicines for the treatment of multi-drug-resistant tuberculosis. Copyright (C) 2015 S. Karger AG, Basel

C1 [Soares-Bezerra, Romulo J.; Alves, Luiz A.] Oswaldo Cruz Inst FIOCRUZ, Lab Cellular Commun, BR-21040360 Rio De Janeiro, Brazil.

[Pinho, Rosa T.; Benevolo-de-Andrade, Thereza Christina] Oswaldo Cruz Inst FIOCRUZ, Lab Clin Immunol, BR-21040360 Rio De Janeiro, Brazil.

[Bisaggio, Rodrigo da C.] Rio Janeiro IFRJ, Fed Inst Educ Technol & Sci, Rio De Janeiro, Brazil.

C3 Fundacao Oswaldo Cruz; Fundacao Oswaldo Cruz; Instituto Federal do Rio

de Janeiro (IFRJ)

RP Soares-Bezerra, RJ (corresponding author), Oswaldo Cruz Inst FIOCRUZ, Lab Cellular Commun, BR-21040360 Rio De Janeiro, Brazil.

EM cyentysta@gmail.com

RI Bezerra, Romulo/OAJ-8793-2025

OI da Cunha Bisaggio, Roigo/0000-0001-8628-4873; Bezerra,

Rômulo/0000-0002-8576-2869

CR Abate D, 2014, INT J TUBERC LUNG D, V18, P946, DOI 10.5588/ijtld.13.0926

Agteresch HJ, 2000, CLIN SCI, V98, P689, DOI [10.1042/CS20000001, 10.1042/cs0980689]

Agteresch HJ, 2000, J NATL CANCER I, V92, P321, DOI 10.1093/jnci/92.4.321

Agteresch HJ, 2002, J CLIN ONCOL, V20, P371, DOI 10.1200/JCO.2002.20.2.371

Agteresch HJ, 2000, EUR J CLIN PHARMACOL, V56, P49, DOI 10.1007/s002280050719

Amaral EP, 2014, PLOS PATHOG, V10, DOI 10.1371/journal.ppat.1004188

Amaral L, 2007, J ANTIMICROB CHEMOTH, V59, P1237, DOI 10.1093/jac/dkl500

Areeshi MY, 2013, GENET TEST MOL BIOMA, V17, P662, DOI 10.1089/gtmb.2013.0202

Arts ICW, 2012, J INT SOC SPORT NUTR, V9, DOI 10.1186/1550-2783-9-16

Bahari G, 2013, GENET MOL RES, V12, P160, DOI 10.4238/2013.January.24.8

Beijer S, 2007, INVEST NEW DRUG, V25, P571, DOI 10.1007/s10637-007-9076-1

Bhattacharya A, 2013, BRIT J PHARMACOL, V170, P624, DOI 10.1111/bph.12314

Bulanova E, 2005, J IMMUNOL, V174, P3880, DOI 10.4049/jimmunol.174.7.3880

CARLSTROM B, 1949, ANN RHEUM DIS, V8, P293, DOI 10.1136/ard.8.4.293

Chen K, 2013, ANNU REV IMMUNOL, V31, P605, DOI 10.1146/annurev-immunol-032712-100019

Chen S, 2013, NEUROBIOL DIS, V58, P296, DOI 10.1016/j.nbd.2013.06.011

Chiang CY, 2013, RESPIROLOGY, V18, P596, DOI 10.1111/resp.12067

Coutinho-Silva R, 2003, IMMUNITY, V19, P403, DOI 10.1016/S1074-7613(03)00235-8

Deng WY, 2014, CELL PHYSIOL BIOCHEM, V33, P273, DOI 10.1159/000356668

Di Virgilio F, 2012, CANCER RES, V72, P5441, DOI 10.1158/0008-5472.CAN-12-1600

Eurosurveillance editorial t.. WHO publishes global tubercu-losis report 2013, 2013, EURO SURVEILL, V18

Fernando SL, 2005, J INFECT DIS, V192, P149, DOI 10.1086/430622

Fernando SL, 2007, AM J RESP CRIT CARE, V175, P360, DOI 10.1164/rccm.200607-970OC

Ferrari D, 2006, J IMMUNOL, V176, P3877, DOI 10.4049/jimmunol.176.7.3877

Friedle Scott A, 2010, Recent Pat CNS Drug Discov, V5, P35

Guo Y, 2014, CELL DEATH DIS, V5, DOI 10.1038/cddis.2014.254

Haskell CM, 1996, MED PEDIATR ONCOL, V27, P165, DOI 10.1002/(SICI)1096-911X(199609)27:3<165::AID-MPO6>3.0.CO;2-C

Hu Y, 1998, J BIOL CHEM, V273, P27170, DOI 10.1074/jbc.273.42.27170

Humphreys BD, 2000, J BIOL CHEM, V275, P26792

Jacob F, 2013, PURINERG SIGNAL, V9, P285, DOI 10.1007/s11302-013-9357-4

Jamnik S., 2002, PNEUMOLOGIA, V28, P245, DOI DOI 10.1590/50102-35862002000500002

Kaczmarek-Hájek K, 2012, PURINERG SIGNAL, V8, P375, DOI 10.1007/s11302-012-9314-7

Khakh BS, 2006, NATURE, V442, P527, DOI 10.1038/nature04886

Khakh BS, 2012, NEURON, V76, P51, DOI 10.1016/j.neuron.2012.09.024

Kusner DJ, 2001, J IMMUNOL, V167, P3308, DOI 10.4049/jimmunol.167.6.3308

Lammas DA, 1997, IMMUNITY, V7, P433, DOI 10.1016/S1074-7613(00)80364-7

Li W, 2013, CELL PHYSIOL BIOCHEM, V31, P1, DOI 10.1159/000343343

Lister Martin F, 2007, J Inflamm (Lond), V4, P5, DOI 10.1186/1476-9255-4-5

Long G, 2014, J ARTHROPLASTY, V29, P2347, DOI 10.1016/j.arth.2014.03.025

Loomis WH, 2003, J BIOL CHEM, V278, P4590, DOI 10.1074/jbc.M207868200

Martins M, 2009, INT J TUBERC LUNG D, V13, P569

Mehta VB, 2001, J BIOL CHEM, V276, P3820, DOI 10.1074/jbc.M006814200

Mortensen SP, 2011, J PHYSIOL-LONDON, V589, P1847, DOI 10.1113/jphysiol.2010.203034

Nörenberg W, 2011, J BIOL CHEM, V286, P11067, DOI 10.1074/jbc.M110.198879

Nogueira JA, 2008, REV ELETR ENG, V4, P979

O'Garra A, 2013, ANNU REV IMMUNOL, V31, P475, DOI 10.1146/annurev-immunol-032712-095939

Pellegatti P, 2008, PLOS ONE, V3, DOI 10.1371/journal.pone.0002599

Placido R, 2006, CELL IMMUNOL, V244, P10, DOI 10.1016/j.cellimm.2007.02.001

Ralevic V, 1998, PHARMACOL REV, V50, P413

Ramachandra L, 2010, INFECT IMMUN, V78, P5116, DOI 10.1128/IAI.01089-09

Raviglione M, 2006, INT J TUBERC LUNG D, V10, P1185

Reichman LB, 2014, INT J TUBERC LUNG D, V18, P884, DOI 10.5588/ijtld.14.0347

Santos AA, 2013, IMMUNOBIOLOGY, V218, P1104, DOI 10.1016/j.imbio.2013.03.003

Saunders BM, 2003, J IMMUNOL, V171, P5442, DOI 10.4049/jimmunol.171.10.5442

Sikora A, 1999, J IMMUNOL, V163, P558

Sluyter R, 2001, CELL TISSUE RES, V304, P231, DOI 10.1007/s004410100372

Smith RA, 2001, VET IMMUNOL IMMUNOP, V78, P249, DOI 10.1016/S0165-2427(01)00245-8

Songane M, 2012, TUBERCULOSIS, V92, P388, DOI 10.1016/j.tube.2012.05.004

Sperlágh B, 1998, NEUROCHEM INT, V33, P209, DOI 10.1016/S0197-0186(98)00025-4

Volonté C, 2012, CNS NEUROL DISORD-DR, V11, P705, DOI 10.2174/187152712803581137

White N, 2006, TRENDS PHARMACOL SCI, V27, P211, DOI 10.1016/j.tips.2006.02.004

Wilhelm K, 2010, NAT MED, V16, P1434, DOI 10.1038/nm.2242

Wilson JM, 2013, NUTR METAB, V10, DOI 10.1186/1743-7075-10-57

Woods LT, 2012, AM J PHYSIOL-CELL PH, V303, pC790, DOI 10.1152/ajpcell.00072.2012

Wu GN, 2014, INFECT GENET EVOL, V24, P82, DOI 10.1016/j.meegid.2014.03.006

Xu XJ, 2012, J CELL SCI, V125, P3776, DOI 10.1242/jcs.099374

Zhou JZ, 2015, ONCOGENE, V34, P1831, DOI 10.1038/onc.2014.113

Zumla A, 2013, NEW ENGL J MED, V368, P745, DOI 10.1056/NEJMra1200894

NR 68

TC 11

Z9 16

U1 0

U2 7

PU KARGER

PI BASEL

PA ALLSCHWILERSTRASSE 10, CH-4009 BASEL, SWITZERLAND

SN 1015-8987

EI 1421-9778

J9 CELL PHYSIOL BIOCHEM

JI Cell. Physiol. Biochem.

PY 2015

VL 37

IS 2

BP 409

EP 418

DI 10.1159/000430364

PG 10

WC Cell Biology; Physiology

WE Science Citation Index Expanded (SCI-EXPANDED)

SC Cell Biology; Physiology

GA CR2JM

UT WOS:000361155400001

OA Green Submitted, gold

DA 2026-05-07

ER

PT J

AU Li, HR

Yuan, JF

Duan, SJ

Pang, Y

AF Li, Haoran

Yuan, Jinfeng

Duan, Shujuan

Pang, Yu

TI Resistance and tolerance of Mycobacterium tuberculosis to

antimicrobial agents-How M. tuberculosis can escape

antibiotics

SO WIRES MECHANISMS OF DISEASE

LA English

DT Review

DE drug; Mycobacterium tuberculosis; resistance; tolerance

ID PARA-AMINOSALICYLIC ACID; D-ALANINE RACEMASE; DRUG-RESISTANCE;

MULTIDRUG-RESISTANT; MOLECULAR CHARACTERIZATION; FLUOROQUINOLONE

RESISTANCE; IN-VITRO; STREPTOMYCIN RESISTANCE; PYRAZINAMIDE RESISTANCE;

CONFERS RESISTANCE

AB Tuberculosis (TB) poses a serious threat to public health worldwide since it was discovered. Until now, TB has been one of the top 10 causes of death from a single infectious disease globally. The treatment of active TB cases majorly relies on various anti-tuberculosis drugs. However, under the selection pressure by drugs, the continuous evolution of Mycobacterium tuberculosis (Mtb) facilitates the emergence of drug-resistant strains, further resulting in the accumulation of tubercle bacilli with multiple drug resistance, especially deadly multidrug-resistant TB and extensively drug-resistant TB. Researches on the mechanism of drug action and drug resistance of Mtb provide a new scheme for clinical management of TB patients, and prevention of drug resistance. In this review, we summarized the molecular mechanisms of drug resistance of existing anti-TB drugs to better understand the evolution of drug resistance of Mtb, which will provide more effective strategies against drug-resistant TB, and accelerate the achievement of the EndTB Strategy by 2035. This article is categorized under: Infectious Diseases > Molecular and Cellular Physiology

C1 [Li, Haoran; Yuan, Jinfeng; Pang, Yu] Capital Med Univ, Beijing Chest Hosp, Dept Bacteriol & Immunol, Beijing TB & Thorac Tumor Res Inst, Postal 9,Beiguan St, Beijing 101149, Peoples R China.

[Duan, Shujuan] Guangdong Med Univ, Sch Med Technol, Dongguan, Peoples R China.

C3 Capital Medical University; Guangdong Medical University

RP Pang, Y (corresponding author), Capital Med Univ, Beijing Chest Hosp, Dept Bacteriol & Immunol, Beijing TB & Thorac Tumor Res Inst, Postal 9,Beiguan St, Beijing 101149, Peoples R China.

EM pangyupound@163.com

RI 袁, 金锋/LZE-8341-2025

FU Beijing Hospitals Authority Ascent Plan [DFL20191601]; Beijing Hospitals

Authority Clinical Medicine Development of Special Funding [ZYLX202122]

FX Beijing Hospitals Authority Ascent Plan, Grant/Award Number:

DFL20191601; Beijing Hospitals Authority Clinical Medicine Development

of Special Funding, Grant/Award Number: ZYLX202122

CR Abu El Asrar R, 2017, BIOCHEM PHARMACOL, V135, P69, DOI 10.1016/j.bcp.2017.03.017

Adams KN, 2011, CELL, V145, P39, DOI 10.1016/j.cell.2011.02.022

Aguilar-Ayala DA, 2017, J MED MICROBIOL, V66, P1140, DOI 10.1099/jmm.0.000537

Alexander DC, 2012, J CLIN MICROBIOL, V50, P3726, DOI 10.1128/JCM.00620-12

Allix-Béguec C, 2018, NEW ENGL J MED, V379, P1403, DOI 10.1056/NEJMoa1800474

Allué-Guardia A, 2021, FRONT MICROBIOL, V12, DOI 10.3389/fmicb.2021.612675

[Anonymous], 2013, Global Health Expenditure Database

Baek SH, 2011, PLOS BIOL, V9, DOI 10.1371/journal.pbio.1001065

Baker JJ, 2018, SCI REP-UK, V8, DOI 10.1038/s41598-018-22343-4

Bandekar AC, 2020, CURR BIOL, V30, P3961, DOI 10.1016/j.cub.2020.07.070

BANERJEE A, 1994, SCIENCE, V263, P227, DOI 10.1126/science.8284673

Bashiri G, 2019, NAT COMMUN, V10, DOI 10.1038/s41467-019-09534-x

Battaglia S, 2020, J CLIN MICROBIOL, V58, DOI 10.1128/JCM.01304-20

Belanger AE, 1996, P NATL ACAD SCI USA, V93, P11919, DOI 10.1073/pnas.93.21.11919

Black PA, 2014, ANTIMICROB AGENTS CH, V58, P2491, DOI 10.1128/AAC.02293-13

Blondiaux N, 2017, SCIENCE, V355, P1206, DOI 10.1126/science.aag1006

Bodiguel J, 2001, J AM CHEM SOC, V123, P3832, DOI 10.1021/ja002674f

Boonaiam S, 2010, CLIN MICROBIOL INFEC, V16, P397, DOI 10.1111/j.1469-0691.2009.02838.x

Briffotaux J, 2019, FRONT MICROBIOL, V10, DOI 10.3389/fmicb.2019.00249

Brimacombe M, 2007, ANTIMICROB AGENTS CH, V51, P4157, DOI 10.1128/AAC.00619-07

Brossier F, 2015, ANTIMICROB AGENTS CH, V59, P4800, DOI 10.1128/AAC.00150-15

Bruning JB, 2011, ANTIMICROB AGENTS CH, V55, P291, DOI 10.1128/AAC.00558-10

Burian J, 2012, EXPERT REV ANTI-INFE, V10, P1037, DOI [10.1586/ERI.12.90, 10.1586/eri.12.90]

Caceres NE, 1997, J BACTERIOL, V179, P5046, DOI 10.1128/jb.179.16.5046-5055.1997

Campbell PJ, 2011, ANTIMICROB AGENTS CH, V55, P2032, DOI 10.1128/AAC.01550-10

Chen YL, 2015, PLOS ONE, V10, DOI 10.1371/journal.pone.0127788

Cholo MC, 2012, J ANTIMICROB CHEMOTH, V67, P290, DOI 10.1093/jac/dkr444

Choudhuri BS, 2002, BIOCHEM J, V367, P279, DOI 10.1042/BJ20020615

Cohen SB, 2018, CELL HOST MICROBE, V24, P439, DOI 10.1016/j.chom.2018.08.001

Cooksey RC, 1996, ANTIMICROB AGENTS CH, V40, P1186, DOI 10.1128/AAC.40.5.1186

Cui ZL, 2011, BMC INFECT DIS, V11, DOI 10.1186/1471-2334-11-78

de Chiara C, 2020, NAT CHEM BIOL, V16, P686, DOI 10.1038/s41589-020-0498-9

De Rossi Edda, 2002, Molecular Medicine (Baltimore), V8, P714

Dersch P, 2017, FRONT MICROBIOL, V8, DOI 10.3389/fmicb.2017.00803

Desjardins CA, 2016, NAT GENET, V48, P544, DOI 10.1038/ng.3548

Diacon AH, 2015, AM J RESP CRIT CARE, V191, P943, DOI 10.1164/rccm.201410-1801OC

Divita KM, 2021, CURR TOP MED CHEM, V21, P1623, DOI 10.2174/1568026621666210913122346

Du J, 2021, FRONT MICROBIOL, V12, DOI 10.3389/fmicb.2021.655653

Du QL, 2013, DIAGN MICR INFEC DIS, V77, P138, DOI 10.1016/j.diagmicrobio.2013.06.031

Dupont C, 2017, ANTIMICROB AGENTS CH, V61, DOI [10.1128/AAC.01225-17, 10.1128/aac.01225-17]

El-Mowafi SA, 2015, ANTIMICROB AGENTS CH, V59, P193, DOI 10.1128/AAC.03979-14

Emane AKA, 2021, TUBERCULOSIS, V129, DOI 10.1016/j.tube.2021.102091

Engström A, 2011, J ANTIMICROB CHEMOTH, V66, P1247, DOI 10.1093/jac/dkr109

Farhat MR, 2017, CLIN INFECT DIS, V65, P1364, DOI 10.1093/cid/cix556

Feng ZY, 2003, ANTIMICROB AGENTS CH, V47, P283, DOI 10.1128/AAC.47.1.283-291.2003

Fivian-Hughes AS, 2012, MICROBIOL-SGM, V158, P308, DOI 10.1099/mic.0.053983-0

Fogel N, 2015, TUBERCULOSIS, V95, P527, DOI 10.1016/j.tube.2015.05.017

Fujiwara M, 2018, TUBERCULOSIS, V108, P186, DOI 10.1016/j.tube.2017.12.006

Gikalo MB, 2012, J ANTIMICROB CHEMOTH, V67, P2107, DOI 10.1093/jac/dks178

Gillespie SH, 2002, ANTIMICROB AGENTS CH, V46, P267, DOI 10.1128/AAC.46.2.267-274.2002

Gomes LC, 2021, MICROB GENOMICS, V17, DOI 10.1099/mgen.0.000724

Gómez-González PJ, 2021, SCI REP-UK, V11, DOI 10.1038/s41598-021-98862-4

Goossens SN, 2021, CLIN MICROBIOL REV, V34, DOI 10.1128/CMR.00141-20

Gordon AK, 2021, INT J INFECT DIS, V113, pS40, DOI 10.1016/j.ijid.2021.02.114

Gouzy A, 2013, FRONT CELL INFECT MI, V3, DOI 10.3389/fcimb.2013.00068

Grzegorzewicz AE, 2012, NAT CHEM BIOL, V8, P334, DOI [10.1038/NCHEMBIO.794, 10.1038/nchembio.794]

Guenin-Macé L, 2009, TRANSBOUND EMERG DIS, V56, P255, DOI 10.1111/j.1865-1682.2009.01072.x

Haning K, 2014, FRONT CELL INFECT MI, V4, DOI 10.3389/fcimb.2014.00096

Hazbón MH, 2006, ANTIMICROB AGENTS CH, V50, P2640, DOI 10.1128/AAC.00112-06

Hiasa H, 1996, J BIOL CHEM, V271, P26424, DOI 10.1074/jbc.271.42.26424

Hillemann D, 2005, ANTIMICROB AGENTS CH, V49, P1229, DOI 10.1128/AAC.49.3.1229-1231.2005

Hillemann D, 2008, ANTIMICROB AGENTS CH, V52, P800, DOI 10.1128/AAC.01189-07

Islam MM, 2020, J GLOB ANTIMICROB RE, V22, P290, DOI 10.1016/j.jgar.2020.02.019

Ismail N, 2018, J MICROBIOL METH, V153, P1, DOI 10.1016/j.mimet.2018.08.011

Ismail N, 2019, ANTIMICROB AGENTS CH, V63, DOI 10.1128/AAC.00292-19

Jankute M, 2012, FUTURE MICROBIOL, V7, P129, DOI [10.2217/FMB.11.123, 10.2217/fmb.11.123]

Jeeves RE, 2015, PLOS ONE, V10, DOI 10.1371/journal.pone.0138253

Jnawali HN, 2013, DIAGN MICR INFEC DIS, V76, P187, DOI 10.1016/j.diagmicrobio.2013.02.035

Karakousis PC, 2008, J ANTIMICROB CHEMOTH, V61, P323, DOI 10.1093/jac/dkm485

Kazemian H, 2021, ADV RESPIR MED, V89, P110, DOI 10.5603/ARM.a2021.0033

Khan SR, 2016, DATA BRIEF, V6, P823, DOI 10.1016/j.dib.2016.01.035

Khan SR, 2016, CHEM-BIOL INTERACT, V244, P37, DOI 10.1016/j.cbi.2015.11.026

Kocagoz T, 1996, ANTIMICROB AGENTS CH, V40, P1768, DOI 10.1128/AAC.40.8.1768

Koch A, 2018, TRENDS MICROBIOL, V26, P555, DOI 10.1016/j.tim.2018.02.012

Kopinc R, 2012, J ANTIMICROB CHEMOTH, V67, P2903, DOI 10.1093/jac/dks313

Kumar P, 2007, P NATL ACAD SCI USA, V104, P11221, DOI 10.1073/pnas.0611649104

Lange C, 2018, RESPIROLOGY, V23, P656, DOI 10.1111/resp.13304

Lau RWT, 2011, ANTIMICROB AGENTS CH, V55, P608, DOI 10.1128/AAC.00920-10

Le Chevalier F, 2014, FUTURE MICROBIOL, V9, P969, DOI [10.2217/FMB.14.70, 10.2217/fmb.14.70]

Leung KL, 2010, J APPL MICROBIOL, V109, P2087, DOI 10.1111/j.1365-2672.2010.04840.x

Lin W, 2017, MOL CELL, V66, P169, DOI [10.1101/099606, 10.1016/j.molcel.2017.03.001]

Lipin MY, 2007, CLIN MICROBIOL INFEC, V13, P620, DOI 10.1111/j.1469-0691.2007.01711.x

Liu JY, 2019, FRONT MICROBIOL, V10, DOI 10.3389/fmicb.2019.00216

Liu YG, 2018, TUBERCULOSIS, V111, P20, DOI 10.1016/j.tube.2018.04.008

Long KS, 2012, ANTIMICROB AGENTS CH, V56, P603, DOI 10.1128/AAC.05702-11

Long QX, 2012, INT J ANTIMICROB AG, V39, P486, DOI 10.1016/j.ijantimicag.2012.02.015

Lu P, 2011, ANTIMICROB AGENTS CH, V55, P5354, DOI 10.1128/AAC.00507-11

Luo M, 2019, INFECT DRUG RESIST, V12, P2269, DOI 10.2147/IDR.S207259

Luo Q, 2021, CURR PROTEIN PEPT SC, V22, P290, DOI 10.2174/1389203722666210421105733

Lyu LN, 2019, FRONT MICROBIOL, V10, DOI 10.3389/fmicb.2019.01174

Machado D, 2018, FRONT MICROBIOL, V9, DOI 10.3389/fmicb.2018.01367

Makafe GG, 2016, ANTIMICROB AGENTS CH, V60, P3202, DOI 10.1128/AAC.00152-16

Malik S, 2012, PLOS ONE, V7, DOI 10.1371/journal.pone.0039754

Manganelli R, 2014, MICROBIOL SPECTR, V2, DOI 10.1128/microbiolspec.MGM2-0007-2013

Manjunatha UH, 2006, P NATL ACAD SCI USA, V103, P431, DOI 10.1073/pnas.0508392103

Maurya RK, 2019, FRONT CELL INFECT MI, V8, DOI 10.3389/fcimb.2018.00450

Mavi PS, 2020, ANTIOXID REDOX SIGN, V32, P1348, DOI 10.1089/ars.2019.7867

Mdluli Khisimuzi, 2007, Infectious Disorders - Drug Targets, V7, P159

Melly G, 2019, MICROORGANISMS, V7, DOI 10.3390/microorganisms7030070

Metcalfe C, 2008, J BIOL CHEM, V283, P6193, DOI 10.1074/jbc.M707412200

Minato Y, 2015, ANTIMICROB AGENTS CH, V59, P5097, DOI 10.1128/AAC.00647-15

Miotto P, 2018, RESPIROLOGY, V23, P1098, DOI 10.1111/resp.13393

Mishra R, 2019, SCI TRANSL MED, V11, DOI 10.1126/scitranslmed.aaw6635

MITCHISON DA, 1985, TUBERCLE, V66, P219, DOI 10.1016/0041-3879(85)90040-6

MOAZED D, 1987, NATURE, V327, P389, DOI 10.1038/327389a0

Morris RP, 2005, P NATL ACAD SCI USA, V102, P12200, DOI 10.1073/pnas.0505446102

Mulet X, 2011, ANTIMICROB AGENTS CH, V55, P4560, DOI 10.1128/AAC.00519-11

MURDOCH JM, 1969, LANCET, V1, P1094

Nakatani Y, 2017, ANTIMICROB AGENTS CH, V61, DOI 10.1128/AAC.01575-17

Nandakumar M, 2014, NAT COMMUN, V5, DOI 10.1038/ncomms5306

Pandey B, 2019, SCI REP-UK, V9, DOI 10.1038/s41598-019-48940-5

Pantel A, 2012, ANTIMICROB AGENTS CH, V56, P1990, DOI 10.1128/AAC.06272-11

Pierattelli R, 2004, J BIOL CHEM, V279, P39000, DOI 10.1074/jbc.M402384200

Pisu D, 2017, ANTIMICROB AGENTS CH, V61, DOI [10.1128/aac.01596-17, 10.1128/AAC.01596-17]

Prieri M, 2018, EUR J MED CHEM, V159, P35, DOI 10.1016/j.ejmech.2018.09.038

Nguyen QH, 2018, EVOL APPL, V11, P1498, DOI 10.1111/eva.12654

Ramaswamy S., 1998, Tubercle and Lung Disease, V79, P3, DOI 10.1054/tuld.1998.0002

Ramón-García S, 2013, J BIOL CHEM, V288, P34514, DOI 10.1074/jbc.M113.516385

Rawat R, 2003, P NATL ACAD SCI USA, V100, P13881, DOI 10.1073/pnas.2235848100

Renau TE, 1996, ANTIMICROB AGENTS CH, V40, P2363, DOI 10.1128/AAC.40.10.2363

Rengarajan J, 2004, MOL MICROBIOL, V53, P275, DOI 10.1111/j.1365-2958.2004.04120.x

Riccardi N, 2020, FUTURE MICROBIOL, V15, P557, DOI 10.2217/fmb-2019-0231

Rienksma RA, 2019, FRONT CELL INFECT MI, V9, DOI 10.3389/fcimb.2019.00144

Rifat D, 2021, ANTIMICROB AGENTS CH, V65, DOI 10.1128/AAC.01948-20

ROBITZEK EH, 1952, AM REV TUBERC PULM, V65, P402

Rueda J, 2015, ANTIMICROB AGENTS CH, V59, P7805, DOI 10.1128/AAC.01028-15

Safi H, 2013, NAT GENET, V45, P1190, DOI 10.1038/ng.2743

Sarathy JP, 2012, PHARMACEUTICALS-BASE, V5, P1210, DOI 10.3390/ph5111210

Sarin R, 2021, LUNG INDIA, V38, P520, DOI 10.4103/lungindia.lungindia_120_21

Schildkraut JA, 2022, ANTIMICROB AGENTS CH, V66, DOI 10.1128/AAC.01509-21

Seifert M, 2015, PLOS ONE, V10, DOI 10.1371/journal.pone.0119628

Sharma D, 2007, J MOL BIOL, V374, P1065, DOI 10.1016/j.jmb.2007.10.003

Sharma S, 2017, APPL BIOCHEM BIOTECH, V181, P762, DOI 10.1007/s12010-016-2247-9

Shi WL, 2011, SCIENCE, V333, P1630, DOI 10.1126/science.1208813

Shimokawa Y, 2014, BIOL PHARM BULL, V37, P1727, DOI 10.1248/bpb.b14-00311

SHOEB HA, 1985, ANTIMICROB AGENTS CH, V27, P404, DOI 10.1128/AAC.27.3.404

Siddiqi N, 2002, ANTIMICROB AGENTS CH, V46, P443, DOI 10.1128/AAC.46.2.443-450.2002

Singh A, 2005, J BACTERIOL, V187, P4173, DOI 10.1128/JB.187.12.4173-4186.2005

Singh KS, 2017, TUBERCULOSIS, V106, P73, DOI 10.1016/j.tube.2017.07.006

Singh P, 2017, INDIAN J MICROBIOL, V57, P371, DOI 10.1007/s12088-017-0674-0

Singh R, 2008, SCIENCE, V322, P1392, DOI 10.1126/science.1164571

Slayden RA, 2018, PATHOG DIS, V76, DOI 10.1093/femspd/fty039

Smittipat N, 2016, J GLOB ANTIMICROB RE, V4, P5, DOI 10.1016/j.jgar.2015.11.009

Song LL, 2016, INT J ANTIMICROB AG, V47, P421, DOI 10.1016/j.ijantimicag.2016.04.007

Spies FS, 2011, J CLIN MICROBIOL, V49, P2625, DOI 10.1128/JCM.00168-11

Srivastava S, 2009, INT J MED MICROBIOL, V299, P269, DOI 10.1016/j.ijmm.2008.07.001

Storz G, 2011, MOL CELL, V43, P880, DOI 10.1016/j.molcel.2011.08.022

Sun HH, 2016, TUBERCULOSIS, V96, P102, DOI 10.1016/j.tube.2015.09.004

Sy SKB, 2015, ANTIMICROB AGENTS CH, V59, P4129, DOI 10.1128/AAC.04049-14

Tahlan K, 2012, ANTIMICROB AGENTS CH, V56, P1797, DOI 10.1128/AAC.05708-11

TAKAYAMA K, 1989, ANTIMICROB AGENTS CH, V33, P1493, DOI 10.1128/AAC.33.9.1493

TAKIFF HE, 1994, ANTIMICROB AGENTS CH, V38, P773, DOI 10.1128/AAC.38.4.773

Tan YJ, 2017, FRONT MICROBIOL, V8, DOI 10.3389/fmicb.2017.02358

Tan YJ, 2014, J CLIN MICROBIOL, V52, P291, DOI 10.1128/JCM.01903-13

Tantivitayakul P, 2020, SCI REP-UK, V10, DOI 10.1038/s41598-020-64895-4

Thakare R, 2020, DRUG TODAY, V56, P655, DOI 10.1358/dot.2020.56.10.3161237

Thakur Z, 2018, GENE, V641, P161, DOI 10.1016/j.gene.2017.10.054

Theron G, 2016, COCHRANE DB SYST REV, DOI 10.1002/14651858.CD010705.pub3

Thiede J. M., 2022, MBIO, V13

Tudó G, 2010, FUTURE MED CHEM, V2, P1371, DOI 10.4155/FMC.10.219

Unissa AN, 2016, INFECT GENET EVOL, V45, P474, DOI 10.1016/j.meegid.2016.09.004

Vale N, 2013, CURR DRUG METAB, V14, P151, DOI 10.2174/138920013804545151

Van Deun A, 2010, AM J RESP CRIT CARE, V182, P684, DOI 10.1164/rccm.201001-0077OC

Velayati AA, 2009, CHEMOTHERAPY, V55, P303, DOI 10.1159/000226425

Vilchèze C, 2014, MICROBIOL SPECTR, V2, DOI 10.1128/microbiolspec.MGM2-0014-2013

Vilchèze C, 2017, P NATL ACAD SCI USA, V114, P4495, DOI 10.1073/pnas.1704376114

Wade MM, 2004, FRONT BIOSCI-LANDMRK, V9, P975, DOI 10.2741/1289

Wagenlehner F M E, 2005, Curr Infect Dis Rep, V7, P9, DOI 10.1007/s11908-005-0018-9

Wan T, 2021, MOL CELL, V81, P2887, DOI 10.1016/j.molcel.2021.06.002

Wang GR, 2021, J INFECTION, V82, P371, DOI 10.1016/j.jinf.2021.02.004

WEHRLI W, 1983, REV INFECT DIS, V5, pS407

Wei WJ, 2019, EMERG MICROBES INFEC, V8, P248, DOI 10.1080/22221751.2019.1568179

Wilson DN, 2008, P NATL ACAD SCI USA, V105, P13339, DOI 10.1073/pnas.0804276105

Wilson W, 1999, P NATL ACAD SCI USA, V96, P12833, DOI 10.1073/pnas.96.22.12833

Wohlkönig A, 2017, BIOCHEM BIOPH RES CO, V487, P403, DOI 10.1016/j.bbrc.2017.04.074

Wong SY, 2011, ANTIMICROB AGENTS CH, V55, P2515, DOI 10.1128/AAC.01814-10

Xu G, 2021, TUBERCULOSIS, V128, DOI 10.1016/j.tube.2021.102083

Yang J, 2020, INT J INFECT DIS, V99, P163, DOI 10.1016/j.ijid.2020.07.044

Yang SS, 2017, ANTIMICROB AGENTS CH, V61, DOI [10.1128/AAC.00551-17, 10.1128/aac.00551-17]

Yin XM, 2010, J INFECTION, V61, P150, DOI 10.1016/j.jinf.2010.05.001

Zhai WJ, 2019, INT J MOL SCI, V20, DOI 10.3390/ijms20020340

Zhang H, 2008, FEBS J, V275, P753, DOI 10.1111/j.1742-4658.2007.06241.x

Zhang S, 2015, J ANTIMICROB CHEMOTH, V70, P2507, DOI 10.1093/jac/dkv150

Zhang Y, 2015, INT J TUBERC LUNG D, V19, P1276, DOI 10.5588/ijtld.15.0389

Zhang Y, 2003, INT J TUBERC LUNG D, V7, P6

Zhang ZJ, 2014, INT J ANTIMICROB AG, V43, P231, DOI 10.1016/j.ijantimicag.2013.12.007

Zhao F, 2014, ANTIMICROB AGENTS CH, V58, P1479, DOI 10.1128/AAC.01775-13

Zhao WJ, 2016, INT J INFECT DIS, V52, P23, DOI 10.1016/j.ijid.2016.08.027

Zheng F, 2012, CELL BIOCHEM BIOPHYS, V63, P103, DOI 10.1007/s12013-012-9348-z

Zheng HW, 2021, BMC INFECT DIS, V21, DOI 10.1186/s12879-021-06024-8

Zheng J, 2013, J BIOL CHEM, V288, P23447, DOI 10.1074/jbc.M113.475798

Zong ZJ, 2018, ANTIMICROB AGENTS CH, V62, DOI [10.1128/aac.00165-18, 10.1128/aac.00165-18, 10.1128/AAC.00165-18;e00165-18]

NR 192

TC 17

Z9 17

U1 1

U2 49

PU WILEY

PI HOBOKEN

PA 111 RIVER ST, HOBOKEN 07030-5774, NJ USA

SN 2692-9368

J9 WIRES MECH DIS

JI WIREs Mech. Dis.

PD NOV

PY 2022

VL 14

IS 6

AR e1573

DI 10.1002/wsbm.1573

EA JUN 2022

PG 20

WC Medicine, Research & Experimental

WE Science Citation Index Expanded (SCI-EXPANDED)

SC Research & Experimental Medicine

GA 6A4KC

UT WOS:000815651400001

DA 2026-05-07

ER

PT J

AU Shinde, Y

Ahmad, I

Surana, S

Patel, H

AF Shinde, Yashodeep

Ahmad, Iqrar

Surana, Sanjay

Patel, Harun

TI The Mur Enzymes Chink in the Armour of Mycobacterium tuberculosis

cell wall

SO EUROPEAN JOURNAL OF MEDICINAL CHEMISTRY

LA English

DT Review

DE Tuberculosis; MDR-TB; XDR-TB; Mur ligase

ID D-GLUTAMATE LIGASE; PEPTIDOGLYCAN BIOSYNTHESIS; CRYSTAL-STRUCTURE;

ENOLPYRUVYL TRANSFERASE; CYTOPLASMIC STEPS; NATURAL-PRODUCTS;

ANTIBACTERIAL; BEDAQUILINE; DRUGS; INHIBITOR

AB Tuberculosis: (TB) transmitted by Mycobacterium tuberculosis (Mtb) is one of the top 10 causes of death globally. Currently, the widespread occurrence of resistance toward Mtb strains is becoming a significant concern to public health. This scenario exaggerated the need for the discovery of novel targets and their inhibitors. Targeting the "Mtb cell wall peptidoglycan synthesis" is an attractive strategy to overcome drug resistance. Mur enzymes (MurA-MurF) play essential roles in the peptidoglycan synthesis by catalyzing the ligation of key amino acid residues to the stem peptide. These enzymes are unique and confined to the eubacteria and are absent in humans, representing potential targets for anti-tubercular drug discovery. Mtb Mur ligases with the same catalytic mechanism share conserved amino acid regions and structural features that can conceivably exploit for the designing of the inhibitors, which can simultaneously target more than one isoforms (MurC-MurF) of the enzyme. In light of these findings in the current review, we have discussed the recent advances in medicinal chemistry of Mtb Mur enzymes (MurA-MurF) and their inhibitors, offering attractive multi-targeted strategies to combat the problem of drug-resistant in M. tuberculosis. (C) 2021 Elsevier Masson SAS. All rights reserved.

C1 [Shinde, Yashodeep; Ahmad, Iqrar; Surana, Sanjay; Patel, Harun] RC Patel Inst Pharmaceut Educ & Res, Dept Pharmaceut Chem, Shirpur 425405, Maharashtra, India.

RP Patel, H (corresponding author), RC Patel Inst Pharmaceut Educ & Res, Dept Pharmaceut Chem, Shirpur 425405, Maharashtra, India.

EM hpatel_38@yahoo.com

RI Surana, Sanjay/GLU-1846-2022; Patel, Harun/L-7942-2019; Ahmad,

Iqrar/ACC-4241-2022

OI Surana, Sanjay/0000-0002-0741-9483;

FU Indian Council of Medical Research (ICMR), New Delhi, Govt. of India

[ISRM/12(11)/2019]

FX The authors would like to thank `Indian Council of Medical Research

(ICMR), New Delhi, Govt. of India' (Grant No. ISRM/12(11)/2019) for

funding the Adhoc Research Grant.

CR Ahmad I., SILICO PHARM, V9, P23, DOI [10.1007/s40203-021-00086-x16.www.accessdata.fda, DOI 10.1007/S40203-021-00086-X16.WWW.ACCESSDATA.FDA]

Alderwick LJ, 2015, CSH PERSPECT MED, V5, DOI 10.1101/cshperspect.a021113

Anuradha CM, 2010, J MOL MODEL, V16, P77, DOI 10.1007/s00894-009-0521-2

Arvind A, 2012, INTERDISCIP SCI, V4, P223, DOI 10.1007/s12539-012-0133-x

Babajan B, 2011, INTERDISCIP SCI, V3, P204, DOI 10.1007/s12539-011-0100-y

Barreteau H, 2008, FEMS MICROBIOL REV, V32, P168, DOI 10.1111/j.1574-6976.2008.00104.x

Basavannacharya C, 2010, TUBERCULOSIS, V90, P16, DOI 10.1016/j.tube.2009.10.007

BENSON TE, 1993, BIOCHEMISTRY-US, V32, P2024, DOI 10.1021/bi00059a019

Bertrand JA, 1999, J MOL BIOL, V289, P579, DOI 10.1006/jmbi.1999.2800

Bertrand JA, 1997, EMBO J, V16, P3416, DOI 10.1093/emboj/16.12.3416

Bigelow KM, 2020, ANTIMICROB AGENTS CH, V64, DOI 10.1128/AAC.01178-20

Boutte CC, 2016, ELIFE, V5, DOI 10.7554/eLife.14590

BRENNAN PJ, 1995, ANNU REV BIOCHEM, V64, P29, DOI 10.1146/annurev.bi.64.070195.000333

Brindha S, 2016, BIOINFORMATION, V12, P368, DOI 10.6026/97320630012368

Brown S, 2013, ANNU REV MICROBIOL, V67, P313, DOI 10.1146/annurev-micro-092412-155620

BUGG TDH, 1992, NAT PROD REP, V9, P199, DOI 10.1039/np9920900199

Cano P Alejandra, 2017, Rev. colomb. Gastroenterol., V32, P337

Catalao MJ, 2019, FRONT MICROBIOL, V10, DOI 10.3389/fmicb.2019.00190

Chen MJ, 2013, HEPATOLOGY, V58, P388, DOI 10.1002/hep.26208

Choi PJ, 2017, BIOORG MED CHEM LETT, V27, P5190, DOI 10.1016/j.bmcl.2017.10.042

Conradie F, 2020, NEW ENGL J MED, V382, P893, DOI 10.1056/NEJMoa1901814

De Smet KAL, 1999, MICROBIOL-UK, V145, P3177, DOI 10.1099/00221287-145-11-3177

Dooley KE, 2013, CURR OPIN HIV AIDS, V8, P579, DOI 10.1097/COH.0000000000000009

Dramsi S, 2008, FEMS MICROBIOL REV, V32, P307, DOI 10.1111/j.1574-6976.2008.00102.x

Du WS, 2000, J BACTERIOL, V182, P4146, DOI 10.1128/JB.182.15.4146-4152.2000

Eniyan K, 2020, SLAS DISCOV, V25, P70, DOI 10.1177/2472555219881148

Eniyan K, 2018, BBA-PROTEINS PROTEOM, V1866, P397, DOI 10.1016/j.bbapap.2017.11.013

Eniyan K, 2016, SCI REP-UK, V6, DOI 10.1038/srep35134

Eniyan Kandasamy, 2017, DU J UNDERGRAD RES I, V3, P97

Gautam A, 2011, CRIT REV BIOTECHNOL, V31, P295, DOI 10.3109/07388551.2010.525498

Guzman JD, 2015, J ANTIMICROB CHEMOTH, V70, P1691, DOI 10.1093/jac/dkv010

Guzman JD, 2010, J ANTIMICROB CHEMOTH, V65, P2101, DOI 10.1093/jac/dkq313

Guzman JD, 2011, J ANTIMICROB CHEMOTH, V66, P1766, DOI 10.1093/jac/dkr203

Healy VL, 2000, CHEM BIOL, V7, pR109, DOI 10.1016/S1074-5521(00)00116-2

Hervin V, 2020, MOLECULES, V25, DOI 10.3390/molecules25214953

Horcajo P, 2012, MICROB DRUG RESIST, V18, P306, DOI 10.1089/mdr.2012.0009

Isa Mustafa Alhaji, 2018, Informatics in Medicine Unlocked, V12, P56, DOI [10.1016/j.imu.2018.06.007, 10.1016/j.imu.2018.06.007]

Isa MA, 2020, LIFE SCI, V262, DOI 10.1016/j.lfs.2020.118466

Isa MA, 2019, COMPUT BIOL CHEM, V78, P116, DOI 10.1016/j.compbiolchem.2018.11.002

Konduri S, 2020, BIOORG MED CHEM LETT, V30, DOI 10.1016/j.bmcl.2020.127512

Kouidmi I, 2014, MOL MICROBIOL, V94, P242, DOI 10.1111/mmi.12758

Kumar P, 2020, J BIOMOL STRUCT DYN, V38, P4997, DOI 10.1080/07391102.2019.1696231

Kumar V, 2011, J MOL MODEL, V17, P939, DOI 10.1007/s00894-010-0788-3

Lam H, 2009, SCIENCE, V325, P1552, DOI 10.1126/science.1178123

LEDERER E, 1975, MOL CELL BIOCHEM, V7, P87, DOI 10.1007/BF01792076

Mahapatra S, 2005, J BACTERIOL, V187, P2341, DOI 10.1128/JB.187.7.2341-2347.2005

Maitra A, 2019, FEMS MICROBIOL REV, V43, P548, DOI 10.1093/femsre/fuz016

MENGINLECREULX D, 1982, J BACTERIOL, V151, P1109, DOI 10.1128/JB.151.3.1109-1117.1982

Mizyed S, 2005, BIOCHEMISTRY-US, V44, P4011, DOI 10.1021/bi047704w

Mol CD, 2003, J BACTERIOL, V185, P4152, DOI 10.1128/JB.185.14.4152-4162.2003

Moraes GL, 2015, TUBERCULOSIS, V95, P95, DOI 10.1016/j.tube.2015.01.006

Munshi T, 2013, PLOS ONE, V8, DOI 10.1371/journal.pone.0060143

Nirwan S, 2021, J BIOMOL STRUCT DYN, V39, P656, DOI 10.1080/07391102.2020.1712258

Osman K, 2012, INT J ANTIMICROB AG, V39, P124, DOI 10.1016/j.ijantimicag.2011.09.018

Patel H, 2019, TUBERCULOSIS, V117, P79, DOI 10.1016/j.tube.2019.06.005

Patel H, 2019, EUR J MED CHEM, V167, P1, DOI 10.1016/j.ejmech.2019.01.073

Patel RV, 2014, CURR TOP MED CHEM, V14, P1866, DOI 10.2174/1568026614666140929114822

Rani J, 2020, J BIOMOL STRUCT DYN, V38, P2521, DOI 10.1080/07391102.2019.1637280

Rivers EC, 2008, DRUG DISCOV TODAY, V13, P1090, DOI 10.1016/j.drudis.2008.09.004

Seung KJ, 2015, CSH PERSPECT MED, V5, DOI 10.1101/cshperspect.a017863

Sieniawska E, 2015, CHEM BIOL, V22, P1288, DOI 10.1016/j.chembiol.2015.08.012

Silhavy TJ, 2010, CSH PERSPECT BIOL, V2, DOI 10.1101/cshperspect.a000414

Svensson EM, 2015, J ANTIMICROB CHEMOTH, V70, P1106, DOI 10.1093/jac/dku504

TB Alliance, 2019, ANTIMICROBIAL DRUGS

Thakur M, 2008, BIOCHEM J, V415, P27, DOI 10.1042/BJ20080234

Tiberi S, 2018, PULMONOLOGY, V24, P86, DOI 10.1016/j.rppnen.2017.10.009

Turk S, 2009, BIOORGAN MED CHEM, V17, P1884, DOI 10.1016/j.bmc.2009.01.052

Walsh AW, 1999, J BACTERIOL, V181, P5395, DOI 10.1128/JB.181.17.5395-5401.1999

World Health Organization Tuberculosis Programme, 2020, GLOB TUB REP

Wube A, 2012, MOLECULES, V17, P8217, DOI 10.3390/molecules17078217

Xu LM, 2014, RES MICROBIOL, V165, P91, DOI 10.1016/j.resmic.2014.01.004

Yan YW, 2000, J MOL BIOL, V304, P435, DOI 10.1006/jmbi.2000.4215

NR 72

TC 21

Z9 28

U1 0

U2 25

PU ELSEVIER FRANCE-EDITIONS SCIENTIFIQUES MEDICALES ELSEVIER

PI ISSY-LES-MOULINEAUX

PA 65 RUE CAMILLE DESMOULINS, CS50083, 92442 ISSY-LES-MOULINEAUX, FRANCE

SN 0223-5234

EI 1768-3254

J9 EUR J MED CHEM

JI Eur. J. Med. Chem.

PD OCT 15

PY 2021

VL 222

AR 113568

DI 10.1016/j.ejmech.2021.113568

EA JUN 2021

PG 20

WC Chemistry, Medicinal

WE Science Citation Index Expanded (SCI-EXPANDED)

SC Pharmacology & Pharmacy

GA UB1EG

UT WOS:000685593800013

DA 2026-05-07

ER

PT J

AU Takii, T

Hasegawa, T

Itoh, S

Maeda, S

Wada, T

Horita, Y

Nishiyama, A

Matsumoto, S

Ohara, N

Kimishima, A

Asami, Y

Hida, S

Onozaki, K

AF Takii, Takemasa

Hasegawa, Tomohiro

Itoh, Saotomo

Maeda, Shinji

Wada, Takayuki

Horita, Yasuhiro

Nishiyama, Akihito

Matsumoto, Sohkichi

Ohara, Naoya

Kimishima, Aoi

Asami, Yukihiro

Hida, Shigeaki

Onozaki, Kikuo

TI Analysis of the drug target of the anti-tuberculosis compound OCT313:

phosphotransacetylase is a potential drug target for two

anti-mycobacterial agents

SO MSPHERE

LA English

DT Article

DE phosphotransacetylase; acetyl coenzyme A; dithiocarbamate;

<italic>N</italic>-acetyl glucosamine; anti-mycobacterial agents; latent

tuberculosis infection

ID MYCOBACTERIUM-TUBERCULOSIS; ESCHERICHIA-COLI; ENZYME; DISULFIRAM;

GROWTH; VIVO

AB Tuberculosis (TB) is one of the most common infectious diseases caused by bacteria worldwide. The increasing prevalence of multidrug-resistant TB (MDR-TB) and latent TB infection (LTBI) has intensified the global TB burden. Therefore, the development of new drugs for MDR-TB and LTBI is urgently required. We have reported that the derivative of dithiocarbamate sugar derivative, 2-acetamido-2-deoxy-beta-D-glucopyranosyl N,N-dimethyldithiocarbamate (OCT313), exhibits anti-mycobacterial activity against MDR-MTB. Here, we identified the target of OCT313. In experimentally generated OCT313-resistant bacteria, adenine at position 1,092 in the metabolic enzyme phosphotransacetylase (PTA) gene was replaced with cytosine. This mutation is a nonsynonymous mutation that converts methionine to leucine at position 365 in the PTA protein. OCT313 inhibited the enzymatic activity of recombinant wild-type PTA, but not of the mutant PTA (M365L). PTA is an enzyme that produces acetyl-coenzyme A (acetyl-CoA) from acetyl phosphate and CoA and is involved in metabolic pathways; therefore, it was expected to also be active against dormant Mycobacterium tuberculosis bacilli. OCT313 exhibits antibacterial activity in the Wayne model of dormancy using Mycobacterium bovis BCG, and overexpression of PTA in OCT313-resistant bacilli restored sensitivity to OCT313. Collectively, the target of OCT313 is PTA, and OCT313 is a promising antimicrobial candidate for MDR-TB and LTBI.IMPORTANCEThrough this study, we propose a new target for the development of medicines to treat multidrug-resistant tuberculosis and latent tuberculosis infection. The target enzyme phosphotransacetylase (PTA) is a key enzyme that functions in major metabolic pathways, and the homologous structures of PTA enzymes vary greatly among bacterial species. Since the treatment of mycobacterial disease is long term, the development of antibiotics targeting PTA is useful for species-specific therapy.

C1 [Takii, Takemasa] Japan AntiTB Assoc, Res Inst TB, Dept Mycobacterium Reference & Res, Kiyose, Tokyo, Japan.

[Takii, Takemasa; Hasegawa, Tomohiro; Itoh, Saotomo; Hida, Shigeaki; Onozaki, Kikuo] Nagoya City Univ, Grad Sch Pharmaceut Sci, Dept Hyg Chem, Nagoya, Aichi, Japan.

[Takii, Takemasa] Kitasato Univ, Omura Satoshi Mem Inst, Minato Ku, Tokyo 1088641, Japan.

[Itoh, Saotomo] Aichi Gakuin Univ, Sch Pharm, Lab Hyg Chem, Nagoya, Aichi, Japan.

[Maeda, Shinji] Hokkaido Univ, Grad Sch Pharmaceut Sci, Sapporo, Hokkaido, Japan.

[Wada, Takayuki] Osaka Metropolitan Univ, Grad Sch Human Life & Ecol, Dept Nutr, Osaka, Japan.

[Wada, Takayuki] Osaka Metropolitan Univ, Osaka Int Res Ctr Infect Dis, Osaka, Japan.

[Horita, Yasuhiro] Nagoya City Univ, Grad Sch Med Sci, Dept Clin Pharmaceut, Nagoya, Aichi, Japan.

[Nishiyama, Akihito; Matsumoto, Sohkichi] Niigata Univ, Sch Med, Dept Bacteriol, Niigata, Japan.

[Matsumoto, Sohkichi] Osaka Metropolitan Univ, Grad Sch Med, Dept Bacteriol, Osaka, Japan.

[Matsumoto, Sohkichi] Hokkaido Univ, Inst Vaccine Res & Dev, Div Res Support, Sapporo, Japan.

[Matsumoto, Sohkichi] Univ Airlangga, Inst Trop Dis, Lab TB, Surabaya, Indonesia.

[Ohara, Naoya] Okayama Univ, Dept Oral Microbiol, Grad Sch Med Dent & Pharmaceut Sci, Okayama, Japan.

[Kimishima, Aoi; Asami, Yukihiro] Kitasato Univ, Omura Satoshi Mem Inst, Lab Appl Microbial Chem, Minato ku, Tokyo, Japan.

C3 Nagoya City University; Kitasato University; Aichi Gakuin University;

Hokkaido University; Osaka Metropolitan University; Osaka Metropolitan

University; Nagoya City University; Niigata University; Osaka

Metropolitan University; Hokkaido University; Airlangga University;

Okayama University; Kitasato University

RP Takii, T (corresponding author), Japan AntiTB Assoc, Res Inst TB, Dept Mycobacterium Reference & Res, Kiyose, Tokyo, Japan.; Takii, T (corresponding author), Nagoya City Univ, Grad Sch Pharmaceut Sci, Dept Hyg Chem, Nagoya, Aichi, Japan.; Takii, T (corresponding author), Kitasato Univ, Omura Satoshi Mem Inst, Minato Ku, Tokyo 1088641, Japan.

EM t-takii@jata.or.jp

RI OHARA, Naoya/B-2481-2011

OI Matsumoto, Sohkichi/0000-0002-6106-7538

FU Japan Agency for Medical Research and Development [JP21fk0108590,

JP23fk0108591, JP223fa627005]; Japan Society for the Promotion of

Science [20K07125, 24K09872]

FX We appreciated Ms. Miki Tokuda and Ms. Erika Miyata for their technical

assistance.

CR Campos-Bermudez VA, 2010, FEBS J, V277, P1957, DOI 10.1111/j.1742-4658.2010.07617.x

Andries K, 2005, SCIENCE, V307, P223, DOI 10.1126/science.1106753

Bloemberg GV, 2015, NEW ENGL J MED, V373, P1986, DOI 10.1056/NEJMc1505196

Brigden G, 2014, B WORLD HEALTH ORGAN, V92, P68, DOI 10.2471/BLT.13.122028

Brinsmade SR, 2007, J BIOL CHEM, V282, P12629, DOI 10.1074/jbc.M611439200

Brinsmade SR, 2004, J BACTERIOL, V186, P1890, DOI 10.1128/JB.186.6.1890-1892.2004

Cole ST, 1998, NATURE, V393, P537, DOI 10.1038/31159

Dalecki AG, 2015, ANTIMICROB AGENTS CH, V59, P4835, DOI 10.1128/AAC.00692-15

El-Mansi M, 2006, CURR OPIN MICROBIOL, V9, P173, DOI 10.1016/j.mib.2006.02.002

El-Mansi M, 2023, MICROBIOL RES, V266, DOI 10.1016/j.micres.2022.127224

Fujiwara M, 2018, TUBERCULOSIS, V110, P122, DOI 10.1016/j.tube.2018.03.004

Horita Y, 2012, ANTIMICROB AGENTS CH, V56, P4140, DOI 10.1128/AAC.06445-11

Horita Y, 2011, BIOORG MED CHEM LETT, V21, P899, DOI 10.1016/j.bmcl.2010.12.084

Horita Y, 2009, BIOORG MED CHEM LETT, V19, P6313, DOI 10.1016/j.bmcl.2009.09.095

Ingram-Smith C, 2006, TRENDS MICROBIOL, V14, P249, DOI 10.1016/j.tim.2006.04.001

Klotzsch H.R., 1969, Methods Enzymol, V13, P381, DOI DOI 10.1016/0076-6879(69)13065-7

Kozarewa I, 2009, NAT METHODS, V6, P291, DOI [10.1038/NMETH.1311, 10.1038/nmeth.1311]

Marchler-Bauer A, 2009, NUCLEIC ACIDS RES, V37, pD205, DOI 10.1093/nar/gkn845

MOSMANN T, 1983, J IMMUNOL METHODS, V65, P55, DOI 10.1016/0022-1759(83)90303-4

Organization WH, 2024, GLOBAL TUBERCULOSIS

Parish T., 2001, MYCOBACTERIUM TUBERC, P97

Parish T., 1998, MYCOBACTERIA PROTOCO, P31

Polsfuss S, 2019, CLIN INFECT DIS, V69, P1229, DOI 10.1093/cid/ciz074

Rasche ME, 1997, J BACTERIOL, V179, P7712, DOI 10.1128/jb.179.24.7712-7717.1997

Rücker N, 2015, J BACTERIOL, V197, P3182, DOI 10.1128/JB.00259-15

Seki M, 2009, VACCINE, V27, P1710, DOI 10.1016/j.vaccine.2009.01.034

Somashekar BS, 2011, J PROTEOME RES, V10, P4186, DOI 10.1021/pr2003352

Subramaniyan S, 2025, J MOL GRAPH MODEL, V138, DOI 10.1016/j.jmgm.2025.109025

Taylor T, 2015, EUKARYOT CELL, V14, P652, DOI 10.1128/EC.00007-15

WALLACE RJ, 1986, J CLIN MICROBIOL, V24, P976, DOI 10.1128/JCM.24.6.976-981.1986

Wang YL, 2023, MOL GENET METAB, V138, DOI 10.1016/j.ymgme.2022.106966

Wayne LG, 1996, INFECT IMMUN, V64, P2062, DOI 10.1128/IAI.64.6.2062-2069.1996

NR 32

TC 0

Z9 0

U1 1

U2 1

PU AMER SOC MICROBIOLOGY

PI WASHINGTON

PA 1752 N ST NW, WASHINGTON, DC 20036-2904 USA

EI 2379-5042

J9 MSPHERE

JI mSphere

PD DEC 23

PY 2025

VL 10

IS 12

DI 10.1128/msphere.00463-25

EA NOV 2025

PG 19

WC Microbiology

WE Science Citation Index Expanded (SCI-EXPANDED)

SC Microbiology

GA AD3TB

UT WOS:001626152800001

OA Green Submitted, gold

DA 2026-05-07

ER

PT J

AU Balgouthi, K

Eker, E

AlMatar, M

AF Balgouthi, Khaoula

Eker, Emel

AlMatar, Manaf

TI MmpS5-MmpL5 Transporters Deliver M. tuberculosis Resistance to

Bedaquiline (BDQ) and Delamanid (DLM)

SO CURRENT PROTEIN & PEPTIDE SCIENCE

LA English

DT Review

DE <italic>M. Tuberculosis</italic>; BDQ; DLM; MmpS5-MmpL5; efflux; drug

resistance

ID MYCOBACTERIUM-TUBERCULOSIS; EFFLUX

AB Introduction One of the earliest illnesses that has been identified is tuberculosis (TB). The largest challenge in managing tuberculosis today is the growing number of individuals infected with TB bacilli, particularly those that are Extensively drug resistant-tuberculosis (XDR-TB) and Multidrug-resistant tuberculosis (MDR-TB). However, by figuring out the resistance's molecular mechanism, Advanced molecular methods may be used to rapidly determine therapy plans. Combining Delamanid (DLM) with Bedaquiline (BDQ), one of the recently authorized medications, indicates that the therapy is effective.Methods We aim to investigate efflux-mediated resistance mechanisms in M. Tuberculosis by using quantitative real-time PCR to assess the expression level of mmpS5 and mmpL5.Results The median (M) and interquartile range (Iqr) of mmpL5 and mmpS5 expression varied from 5.65 to 9.01 and 7.95 to 10.74, respectively, when resistant strains were compared with sensitive ones. M and Iqr of mmpL5 and mmpS5 expression, however, ranged from 0.08-3.04 and 0.05-1.61 for sensitive strains, correspondingly.Discussion Our findings have implications for the development of fast genotypic drug susceptibility testing (DST). Quantitative real-time PCR to measure the expression level of mmpS5 and mmpL5 of baseline and post-baseline isolates is important to track the development of BDQ and DLM resistance.Conclusion Thus, when developing anti-tuberculosis drugs, mycobacterial MmpS5-MmpL5 transporters should be taken into consideration early on, as they are an MDR-efflux system.

C1 [Balgouthi, Khaoula] Cukurova Univ, Inst Nat & Appl Sci, Dept Biotechnol, TR-01330 Balcali, Adana, Turkiye.

[Eker, Emel] Harran Univ, Fac Pharm, Dept Pharmaceut Microbiol, TR-63300 Sanliurfa, Turkiye.

[AlMatar, Manaf] Sohar Univ, Fac Educ & Arts, Dept Biol, Sohar 311, Oman.

C3 Cukurova University; Harran University; Sohar University

RP AlMatar, M (corresponding author), Sohar Univ, Fac Educ & Arts, Dept Biol, Sohar 311, Oman.

EM manafmatar1985@gmail.com

RI AlMatar, Manaf/AAG-9123-2020; Eker, Emel/ABQ-0041-2022

CR AlMatar M, 2020, ENDOCR METAB IMMUNE, V20, P271, DOI 10.2174/1871530319666191009153834

AlMatar M, 2019, CURR MOL PHARMACOL, V12, P83, DOI 10.2174/1874467212666181126151948

da Silva PEA, 2011, FEMS IMMUNOL MED MIC, V63, P1, DOI 10.1111/j.1574-695X.2011.00831.x

Andries K, 2014, PLOS ONE, V9, DOI 10.1371/journal.pone.0102135

Balgouthia K, 2024, CURR MOL PHARMACOL, VXXXX, DOI 10.2174/0118761429314641240815080447

Bekker O., 2012, RU Patent, Patent No. 2462466

Bekker O., 2015, RU Patent, Patent No. 2566998

Caminero JA, 2010, LANCET INFECT DIS, V10, P621, DOI 10.1016/S1473-3099(10)70139-0

Danilenko VN, 2011, CURR TOP MED CHEM, V11, P1352

Domenech P, 2005, INFECT IMMUN, V73, P3492, DOI 10.1128/IAI.73.6.3492-3501.2005

Gandhi NR, 2010, LANCET, V375, P1830, DOI 10.1016/S0140-6736(10)60410-2

Ioerger TR, 2013, PLOS ONE, V8, DOI 10.1371/journal.pone.0075245

Islam MM, 2024, FRONT MED-LAUSANNE, V10, DOI 10.3389/fmed.2023.1304857

Klopper M, 2013, EMERG INFECT DIS, V19, P449, DOI [10.3201/EID1903.120246, 10.3201//EID1903.120246]

Livak KJ, 2001, METHODS, V25, P402, DOI 10.1006/meth.2001.1262

Mansj?? M., 2024, ANTIMICROB AGENTS CH, V69, pe00946

Maslov DA, 2018, B RUSS STATE MED U, P19, DOI 10.24075/brsmu.2018.039

Maslov DA, 2017, B RUSS STATE MED U, P38

Maslov DA, 2020, PATHOGENS, V9, DOI 10.3390/pathogens9030166

Maslov DA, 2019, EUR J MED CHEM, V178, P39, DOI 10.1016/j.ejmech.2019.05.081

Milano A, 2009, TUBERCULOSIS, V89, P84, DOI 10.1016/j.tube.2008.08.003

Organization W.H., 2024, GLOBAL TUBERCULOSIS

Parish T, 2000, MICROBIOL-SGM, V146, P1969, DOI 10.1099/00221287-146-8-1969

Richard M, 2018, FRONT MICROBIOL, V9, DOI 10.3389/fmicb.2018.00649

Sandhu P, 2017, J INORG BIOCHEM, V170, P75, DOI 10.1016/j.jinorgbio.2017.02.013

Schön T, 2021, CLIN MICROBIOL INFEC, V27, DOI 10.1016/j.cmi.2020.10.019

Tiberi S, 2018, PULMONOLOGY, V24, P86, DOI 10.1016/j.rppnen.2017.10.009

Vatlin AA, 2019, MICROBIOL RESOUR ANN, V8, DOI 10.1128/MRA.00263-19

Velayati Ali A, 2016, Int J Mycobacteriol, V5 Suppl 1, pS161, DOI [10.1016/j.ijmyco.2016.09.066, 10.1016/j.ijmyco.2016.09.066]

NR 29

TC 0

Z9 0

U1 1

U2 1

PU BENTHAM SCIENCE PUBL LTD

PI SHARJAH

PA EXECUTIVE STE Y-2, PO BOX 7917, SAIF ZONE, 1200 BR SHARJAH, U ARAB

EMIRATES

SN 1389-2037

EI 1875-5550

J9 CURR PROTEIN PEPT SC

JI Curr. Protein Pept. Sci.

PY 2026

VL 27

IS 2

BP 182

EP 188

DI 10.2174/0113892037383330250807101618

EA SEP 2025

PG 7

WC Biochemistry & Molecular Biology

WE Science Citation Index Expanded (SCI-EXPANDED)

SC Biochemistry & Molecular Biology

GA FQ3RC

UT WOS:001600988500001

DA 2026-05-07

ER

PT J

AU Egbelowo, O

Sarathy, JP

Gausi, K

Zimmerman, MD

Wang, H

Wijnant, GJ

Kaya, F

Gengenbacher, M

Van, N

Degefu, Y

Nacy, C

Aldridge, BB

Carter, CL

Denti, P

Dartois, V

AF Egbelowo, Oluwaseun

Sarathy, Jansy P.

Gausi, Kamunkhwala

Zimmerman, Matthew D.

Wang, Han

Wijnant, Gert-Jan

Kaya, Firat

Gengenbacher, Martin

Van, Nhi

Degefu, Yonatan

Nacy, Carol

Aldridge, Bree B.

Carter, Claire L.

Denti, Paolo

Dartois, Veronique

TI Pharmacokinetics and Target Attainment of SQ109 in Plasma and Human-Like

Tuberculosis Lesions in Rabbits

SO ANTIMICROBIAL AGENTS AND CHEMOTHERAPY

LA English

DT Article

DE Mycobacterium tuberculosis; MDR-TB; SQ109; lesion penetration;

pharmacokinetics; PK-PD

ID MYCOBACTERIUM-TUBERCULOSIS; ANTITUBERCULAR DRUG; IN-VITRO;

DIARYLQUINOLINE TMC207; BEDAQUILINE TMC207; PENETRATION; RIFAMPICIN;

MECHANISM; DIAMINE; TB

AB SQ109 is a novel well-tolerated drug candidate in clinical development for the treatment of drug-resistant tuberculosis (TB). It is the only inhibitor of the MmpL3 mycolic acid transporter in clinical development. No SQ109-resistant mutant has been directly isolated thus far in vitro, in mice, or in patients, which is tentatively attributed to its multiple targets. It is considered a potential replacement for poorly tolerated components of multidrug-resistant TB regimens. To prioritize SQ109-containing combinations with the best potential for cure and treatment shortening, one must understand its contribution against different bacterial populations in pulmonary lesions. Here, we have characterized the pharmacokinetics of SQ109 in the rabbit model of active TB and its penetration at the sites of disease-lung tissue, cellular and necrotic lesions, and caseum. A two-compartment model with first-order absorption and elimination described the plasma pharmacokinetics. At the human-equivalent dose, parameter estimates fell within the ranges published for preclinical species. Tissue concentrations were modeled using an "effect" compartment, showing high accumulation in lung and cellular lesion areas with penetration coefficients in excess of 1,000 and lower passive diffusion in caseum after 7 daily doses. These results, together with the hydrophobic nature and high nonspecific caseum binding of SQ109, suggest that multi-week dosing would be required to reach steady state in caseum and poorly vascularized compartments, similar to bedaquiline. Linking lesion pharmacokinetics to SQ109 potency in assays against replicating, nonreplicating, and intracellular M. tuberculosis showed SQ109 concentrations markedly above pharmacokinetic-pharmacodynamic targets in lung and cellular lesions throughout the dosing interval.

C1 [Egbelowo, Oluwaseun; Gausi, Kamunkhwala; Denti, Paolo] Univ Cape Town, Dept Med, Div Clin Pharmacol, Cape Town, South Africa.

[Sarathy, Jansy P.; Zimmerman, Matthew D.; Wang, Han; Wijnant, Gert-Jan; Kaya, Firat; Gengenbacher, Martin; Carter, Claire L.; Dartois, Veronique] Hackensack Meridian Hlth, Ctr Discovery & Innovat, Nutley, NJ 07110 USA.

[Van, Nhi; Degefu, Yonatan; Aldridge, Bree B.] Tufts Univ, Sch Med, Dept Mol Biol & Microbiol, Boston, MA 02111 USA.

[Nacy, Carol] Sequella Inc, Rockville, MD USA.

[Dartois, Veronique] Hackensack Meridian Hlth, Hackensack Sch Med, Nutley, NJ 07110 USA.

C3 University of Cape Town; Tufts University

RP Dartois, V (corresponding author), Hackensack Meridian Hlth, Ctr Discovery & Innovat, Nutley, NJ 07110 USA.; Dartois, V (corresponding author), Hackensack Meridian Hlth, Hackensack Sch Med, Nutley, NJ 07110 USA.

EM veronique.dartois@hmh-cti.org

RI Egbelowo, Oluwaseun Francis/T-8180-2019; Gausi,

Kamunkhwala/LRD-2193-2024; Denti, Paolo/H-6393-2015; Gengenbacher,

Martin/C-6876-2015; Kaya, Firat/OEO-4702-2025; Sarathy,

Jansy/ISS-8181-2023

OI Egbelowo, Oluwaseun Francis/0000-0002-5636-4642; Gausi,

Kamunkhwala/0000-0002-9002-8087; Carter, Claire/0000-0003-2996-7621;

Degefu, Yonatan/0000-0002-6540-3148; Wijnant,

Gert-Jan/0000-0003-2083-4523; Denti, Paolo/0000-0001-7494-079X;

Gengenbacher, Martin/0000-0002-0208-5920; Aldridge,

Bree/0000-0003-2236-1424;

FU NIH [S10OD023524, S10OD018072]

FX The study was funded in part by NIH grants S10OD023524 and S10OD018072

to V.D.

CR Ammerman NC, 2017, J ANTIMICROB CHEMOTH, V72, P455, DOI 10.1093/jac/dkw417

Anderson BJ, 2008, ANNU REV PHARMACOL, V48, P303, DOI 10.1146/annurev.pharmtox.48.113006.094708

Anderson N, 2006, FEBS LETT, V580, P5533, DOI 10.1016/j.febslet.2006.08.061

Beal SL, 2001, J PHARMACOKINET PHAR, V28, P481, DOI 10.1023/A:1012299115260

Blanc L, 2018, J EXP MED, V215, P1975, DOI 10.1084/jem.20180518

Boeree MJ, 2017, LANCET INFECT DIS, V17, P39, DOI 10.1016/S1473-3099(16)30274-2

CHAPMAN EH, 1981, J CLIN PATHOL, V34, P1083, DOI 10.1136/jcp.34.10.1083

Chen C, 2018, ANTIMICROB AGENTS CH, V62, DOI 10.1128/AAC.02107-17

Chen P, 2006, J ANTIMICROB CHEMOTH, V58, P332, DOI 10.1093/jac/dkl227

Cho SH, 2007, ANTIMICROB AGENTS CH, V51, P1380, DOI 10.1128/AAC.00055-06

Clemens DL, 2019, PLOS ONE, V14, DOI 10.1371/journal.pone.0215607

Cokol M, 2017, SCI ADV, V3, DOI 10.1126/sciadv.1701881

Conradie F, 2020, NEW ENGL J MED, V382, P893, DOI 10.1056/NEJMoa1901814

Daniel J, 2011, PLOS PATHOG, V7, DOI 10.1371/journal.ppat.1002093

Davies G, 2019, PLOS MED, V16, DOI 10.1371/journal.pmed.1002851

Dheda K, 2018, AM J RESP CRIT CARE, V198, P1208, DOI [10.1164/rccm.201711-2333oc, 10.1164/rccm.201711-2333OC]

Diacon AH, 2012, ANTIMICROB AGENTS CH, V56, P3271, DOI 10.1128/AAC.06126-11

Diacon AH, 2009, NEW ENGL J MED, V360, P2397, DOI 10.1056/NEJMoa0808427

Dosne AG, 2016, J PHARMACOKINET PHAR, V43, P583, DOI 10.1007/s10928-016-9487-8

Fearns A, 2020, CORRELATIVE LIGHT EL

Gil Z, 2020, PARASITOL RES, V119, P649, DOI 10.1007/s00436-019-06560-y

Glaziou Philippe., 2020, Predicted impact of the COVID-19 pandemic on global tuberculosis deaths in 2020, DOI [10.1101/2020.04.28.20079582, DOI 10.1101/2020.04.28.20079582]

Gler MT, 2012, NEW ENGL J MED, V366, P2151, DOI 10.1056/NEJMoa1112433

Gold B, 2017, MICROBIOL SPECTR, V5, DOI 10.1128/microbiolspec.TBTB2-0031-2016

Greenwood DJ, 2019, SCIENCE, V364, P1279, DOI 10.1126/science.aat9689

Grzegorzewicz AE, 2012, NAT CHEM BIOL, V8, P334, DOI [10.1038/NCHEMBIO.794, 10.1038/nchembio.794]

Heinrich N, 2015, J ANTIMICROB CHEMOTH, V70, P1558, DOI 10.1093/jac/dku553

Hogan AB, 2020, LANCET GLOB HEALTH, V8, pE1132, DOI 10.1016/S2214-109X(20)30288-6

Hooker AC, 2007, PHARM RES, V24, P2187, DOI 10.1007/s11095-007-9361-x

Huang L, 2018, FRONT CELL INFECT MI, V8, DOI 10.3389/fcimb.2018.00275

Irwin SM, 2016, ACS INFECT DIS, V2, P251, DOI 10.1021/acsinfecdis.5b00127

Jia J, 2005, BRIT J PHARMACOL, V144, P80, DOI 10.1038/sj.bjp.0705984

Jia L, 2006, BRIT J PHARMACOL, V147, P476, DOI 10.1038/sj.bjp.0706650

Kayigire XA, 2015, TUBERCULOSIS, V95, P770, DOI 10.1016/j.tube.2015.08.001

Keizer RJ, 2013, CPT-PHARMACOMET SYST, V2, DOI 10.1038/psp.2013.24

Kjellsson MC, 2012, ANTIMICROB AGENTS CH, V56, P446, DOI 10.1128/AAC.05208-11

Lakshminarayana SB, 2015, J ANTIMICROB CHEMOTH, V70, P857, DOI 10.1093/jac/dku457

Lee BY, 2018, PLOS ONE, V13, DOI 10.1371/journal.pone.0207469

Lee RE, 2003, J COMB CHEM, V5, P172, DOI 10.1021/cc020071p

Lessem E., 2014, ACTIVISTS GUIDE DELA

Li K, 2014, J MED CHEM, V57, P3126, DOI 10.1021/jm500131s

Li W, 2014, ANTIMICROB AGENTS CH, V58, P6413, DOI 10.1128/AAC.03229-14

McLeay SC, 2014, ANTIMICROB AGENTS CH, V58, P5315, DOI 10.1128/AAC.01418-13

Moscow Municipal Scientific Practical Center of Tuberculosis Control Health Department of Moscow Russian Federation, 2018, Tuberculosis and lung diseases, V96, P6, DOI [10.21292/2075-1230-2018-96-3-6-18, 10.21292/2075-1230-2018-96-3-6-18, DOI 10.21292/2075-1230-2018-96-3-6-18]

Murray S, 2016, INT J TUBERC LUNG D, V20, pS38, DOI 10.5588/ijtld.16.0069

Nikonenko BV, 2007, ANTIMICROB AGENTS CH, V51, P1563, DOI 10.1128/AAC.01326-06

Nikonenko BV, 2004, ANTIMICROB AGENTS CH, V48, P4550, DOI 10.1128/AAC.48.12.4550-4555.2004

Ollinger J, 2019, PLOS ONE, V14, DOI 10.1371/journal.pone.0205479

Pei YH, 2016, J CONTROL RELEASE, V240, P202, DOI 10.1016/j.jconrel.2015.12.014

Pisu D, 2020, CELL REP, V30, P335, DOI 10.1016/j.celrep.2019.12.033

Prideaux B, 2015, NAT MED, V21, P1223, DOI 10.1038/nm.3937

Prosser G, 2017, MICROBES INFECT, V19, P177, DOI 10.1016/j.micinf.2016.10.001

Protopopova M, 2005, J ANTIMICROB CHEMOTH, V56, P968, DOI 10.1093/jac/dki319

Reddy VM, 2010, ANTIMICROB AGENTS CH, V54, P2840, DOI 10.1128/AAC.01601-09

Rustomjee R, 2008, ANTIMICROB AGENTS CH, V52, P2831, DOI 10.1128/AAC.01204-07

Sacksteder KA, 2012, FUTURE MICROBIOL, V7, P823, DOI [10.2217/fmb.12.56, 10.2217/FMB.12.56]

Sarathy J, 2019, ANTIMICROB AGENTS CH, V63, DOI [10.1128/AAC.02516-18, 10.1128/aac.02516-18]

Sarathy JP, 2018, ANTIMICROB AGENTS CH, V62, DOI 10.1128/AAC.02266-17

Sarathy JP, 2016, ACS INFECT DIS, V2, P552, DOI 10.1021/acsinfecdis.6b00051

Sarma P.R., 1990, Clinical Methods: The History, Physical, and Laboratory Examinations

Silva DR, 2020, J BRAS PNEUMOL, V46, DOI 10.36416/1806-3756/e20200009

Skripconoka V, 2013, EUR RESPIR J, V41, P1393, DOI 10.1183/09031936.00125812

Smith DA, 2019, DRUG METAB DISPOS, V47, P665, DOI 10.1124/dmd.118.085951

Strydom N, 2019, PLOS MED, V16, DOI 10.1371/journal.pmed.1002773

Subbian S, 2011, OPEN BIOL, V1, DOI 10.1098/rsob.110016

Svensson EM, 2018, CLIN INFECT DIS, V67, P34, DOI 10.1093/cid/ciy026

Tahlan K, 2012, ANTIMICROB AGENTS CH, V56, P1797, DOI 10.1128/AAC.05708-11

Tweed CD, 2019, LANCET RESP MED, V7, P1048, DOI 10.1016/S2213-2600(19)30366-2

van Heeswijk RPG, 2014, J ANTIMICROB CHEMOTH, V69, P2310, DOI 10.1093/jac/dku171

Vocat A, 2015, ANTIMICROB AGENTS CH, V59, P4012, DOI 10.1128/AAC.00528-15

WHO, 2019, WHO consolidated guidelines on tuberculosis: module 4: treatment: drug-resistant tuberculosis treatment

World Health Organization, 2020, GLOBAL TUBERCULOSIS

Zimmerman M, 2018, JOVE-J VIS EXP, DOI 10.3791/57402

Zimmerman M, 2017, ANTIMICROB AGENTS CH, V61, DOI 10.1128/AAC.00924-17

NR 74

TC 20

Z9 22

U1 0

U2 12

PU AMER SOC MICROBIOLOGY

PI WASHINGTON

PA 1752 N ST NW, WASHINGTON, DC 20036-2904 USA

SN 0066-4804

EI 1098-6596

J9 ANTIMICROB AGENTS CH

JI Antimicrob. Agents Chemother.

PD SEP

PY 2021

VL 65

IS 9

AR e00024-21

DI 10.1128/AAC.00024-21

PG 19

WC Microbiology; Pharmacology & Pharmacy

WE Science Citation Index Expanded (SCI-EXPANDED)

SC Microbiology; Pharmacology & Pharmacy

GA WH8DM

UT WOS:000707901200001

OA Green Submitted, hybrid

DA 2026-05-07

ER

PT J

AU Uddin, R

Zahra, NUA

Azam, SS

AF Uddin, Reaz

Zahra, Noor-ul-Ain

Azam, Syed Sikander

TI Identification of glucosyl-3-phosphoglycerate phosphatase as a novel

drug target against resistant strain of Mycobacterium

tuberculosis (XDR1219) by using comparative metabolic pathway

approach

SO COMPUTATIONAL BIOLOGY AND CHEMISTRY

LA English

DT Article

DE Resistant mycobacterium tuberculosis; Essential gene; Drug targets;

Subtractive genomics; Metabolic pathway analysis; Virtual screening

ID PROTEIN; ANNOTATION; GENERATION; INHIBITORS; RESOURCE; DATABASE; GENES

AB Tuberculosis (TB) is a major global health challenge. It has been afflicting human for thousands of years and is still severely affecting a huge population. The etiological agent of the disease is Mycobacterium tuberculosis (MTB) that survives in the human host in latent, dormant, and non-replicative state by evading the immune system. It is one of the leading causes of infection related death worldwide. The situation is exacerbated by the massive increase in the resistant strains such as multi-drug resistant TB (MDR-TB) and extensive drug-resistant TB (XDR-TB). The resistance is as severe that it resulted in failure of the current chemotherapy regimens (i.e. anti-tubercular drugs). It is therefore imperative to discover the new anti-tuberculosis drug targets and their potential inhibitors. Current study has made the use of in silico approaches to perform the comparative metabolic pathway analysis of the MTBXDR1219 with the host i.e. H. sapiens. We identified several metabolic pathways which are unique to pathogen only. By performing subtractive genomic analysis 05 proteins as potential drug target are retrieved. This study suggested that the identified proteins are essential for the bacterial survival and non-homolog to the host proteins. Furthermore, we selected glucosyl-3-phosoglycerate phosphatase (GpgP, EC 5.4.2.1) out of the 05 proteins for molecular docking analysis and virtual screening. The protein is involved in the biosynthesis of methylglucose lipopolysaccharides (MGLPs) which regulate the biosynthesis of mycolic acid. Mycolic acid is the building block of the unique cell wall of the MTB which is responsible for the resistance and pathogenicity. A relatively larger library consisting of 10,431 compounds was screened using AutoDock Vina to predict the binding modes and to rank the potential inhibitors. No potent inhibitor against MTB GpgP has been reported yet, therefore ranking of compounds is performed by making a comparison with the substrate i.e. glucosyl-3-phosphoglycerate. The obtained results provide the understanding of underlying mechanism of interactions of ligands with protein. Follow up study will include the study of the Protein-Protein Interactions (PPIs), and to propose the potential inhibitors against them.

C1 [Uddin, Reaz; Zahra, Noor-ul-Ain] Univ Karachi, Dr Panjwani Ctr Mol Med & Drug Res, Int Ctr Chem & Biol Sci, Lab 103 PCMD Ext, Karachi 75270, Pakistan.

[Azam, Syed Sikander] Quaid I Azam Univ, Computat Biol Lab, Natl Ctr Bioinformat, Islamabad, Pakistan.

C3 University of Karachi; Quaid I Azam University

RP Uddin, R (corresponding author), Univ Karachi, Dr Panjwani Ctr Mol Med & Drug Res, Int Ctr Chem & Biol Sci, Lab 103 PCMD Ext, Karachi 75270, Pakistan.

EM mriazuddin@iccs.edu

RI Uddin, Reaz/I-5673-2017; Azam, Syed Sikander/AAD-4351-2021

OI Uddin, Reaz/0000-0002-9928-4482; Azam, Syed Sikander/0000-0001-5624-6120

FU Higher Education Commission of Pakistan under NRPU program

FX The authors would gratefully acknowledge the financial support provided

by the Higher Education Commission of Pakistan under NRPU program.

CR Altschul SF, 1997, NUCLEIC ACIDS RES, V25, P3389, DOI 10.1093/nar/25.17.3389

ALTSCHUL SF, 1990, J MOL BIOL, V215, P403, DOI 10.1016/S0022-2836(05)80360-2

Amir Asad, 2014, Int J Evol Biol, V2014, P284170, DOI 10.1155/2014/284170

Anishetty S, 2005, COMPUT BIOL CHEM, V29, P368, DOI 10.1016/j.compbiolchem.2005.07.001

[Anonymous], CONTROL MECH FATTY A

Bairoch A, 2008, NUCLEIC ACIDS RES, V36, pD190, DOI 10.1093/nar/gkm895

BERNSTEIN FC, 1977, EUR J BIOCHEM, V80, P319, DOI 10.1111/j.1432-1033.1977.tb11885.x

Burley SK, 2018, PROTEIN SCI, V27, P316, DOI 10.1002/pro.3331

Chang MW, 2010, PLOS ONE, V5, DOI 10.1371/journal.pone.0011955

Chhabra G, 2010, BIOINFORMATION, V4, P278, DOI 10.6026/97320630004278

Chiba Y, 2013, J BIOL CHEM, V288, P11448, DOI 10.1074/jbc.M112.449561

Choi KJ, 2005, FEBS LETT, V579, P4903, DOI 10.1016/j.febslet.2005.07.055

Cosconati S, 2010, EXPERT OPIN DRUG DIS, V5, P597, DOI 10.1517/17460441.2010.484460

Daina A, 2017, SCI REP-UK, V7, DOI 10.1038/srep42717

Desai PV, 2004, J MED CHEM, V47, P6609, DOI 10.1021/jm0493717

Fiser A, 2003, METHOD ENZYMOL, V374, P461, DOI 10.1016/S0076-6879(03)74020-8

Johnson M, 2008, NUCLEIC ACIDS RES, V36, pW5, DOI 10.1093/nar/gkn201

Kanehisa M, 2000, NUCLEIC ACIDS RES, V28, P27, DOI 10.1093/nar/28.1.27

Maranha A, 2015, SCI REP-UK, V5, DOI 10.1038/srep13610

Mendes V, 2011, SCI REP-UK, V1, DOI 10.1038/srep00177

Moriya Y, 2007, NUCLEIC ACIDS RES, V35, pW182, DOI 10.1093/nar/gkm321

Morris GM, 2009, J COMPUT CHEM, V30, P2785, DOI 10.1002/jcc.21256

Naveed M, 2018, ADVAN LIFE SCI, V5, P73

Nunes-Costa D, 2017, GLYCOBIOLOGY, V27, P213, DOI 10.1093/glycob/cww132

O'Leary NA, 2016, NUCLEIC ACIDS RES, V44, pD733, DOI 10.1093/nar/gkv1189

Overbeek R, 1999, P NATL ACAD SCI USA, V96, P2896, DOI 10.1073/pnas.96.6.2896

Patro R, 2012, BIOINFORMATICS, V28, P3105, DOI 10.1093/bioinformatics/bts592

Pettersen EF, 2004, J COMPUT CHEM, V25, P1605, DOI 10.1002/jcc.20084

Rigden DJ, 2001, PROTEIN SCI, V10, P1835, DOI 10.1110/ps.15701

Saxena S, 2014, J MOL GRAPH MODEL, V47, P37, DOI 10.1016/j.jmgm.2013.08.005

Shukla H, 2015, INT J BIOL MACROMOL, V78, P137, DOI 10.1016/j.ijbiomac.2015.04.005

Sievers F, 2011, MOL SYST BIOL, V7, DOI 10.1038/msb.2011.75

Trott O, 2010, J COMPUT CHEM, V31, P455, DOI 10.1002/jcc.21334

Uddin R, 2016, PLOS ONE, V11, DOI 10.1371/journal.pone.0146796

Uddin R, 2015, GENE, V556, P213, DOI 10.1016/j.gene.2014.11.056

WALLACE AC, 1995, PROTEIN ENG, V8, P127, DOI 10.1093/protein/8.2.127

Watkins HA, 2006, J BACTERIOL, V188, P3589, DOI 10.1128/JB.188.10.3589-3599.2006

Watkins HA, 2010, J BACTERIOL, V192, P2878, DOI 10.1128/JB.01615-09

WHO, 2016, GLOB TUB REP 2016

Wu W, 2013, MOL GENET GENOMICS, V288, P425, DOI 10.1007/s00438-013-0758-4

Yellaboina S, 2006, FEBS LETT, V580, P2567, DOI 10.1016/j.febslet.2006.03.090

Zhang R, 2004, NUCLEIC ACIDS RES, V32, pD271, DOI 10.1093/nar/gkh024

Zhang X, 2015, SCI REP-UK, V5, DOI 10.1038/srep12561

Zheng QQ, 2014, J BIOL CHEM, V289, P21242, DOI 10.1074/jbc.M114.569913

NR 44

TC 9

Z9 10

U1 0

U2 4

PU ELSEVIER SCI LTD

PI London

PA 125 London Wall, London, ENGLAND

SN 1476-9271

EI 1476-928X

J9 COMPUT BIOL CHEM

JI Comput. Biol. Chem.

PD APR

PY 2019

VL 79

BP 91

EP 102

DI 10.1016/j.compbiolchem.2019.01.011

PG 12

WC Biology; Computer Science, Interdisciplinary Applications

WE Science Citation Index Expanded (SCI-EXPANDED)

SC Life Sciences & Biomedicine - Other Topics; Computer Science

GA HR4NV

UT WOS:000463124100011

DA 2026-05-07

ER

PT J

AU Chahine, EB

Karaoui, LR

Mansour, H

AF Chahine, Elias B.

Karaoui, Lamis R.

Mansour, Hanine

TI Bedaquiline: A Novel Diarylquinoline for Multidrug-Resistant

Tuberculosis

SO ANNALS OF PHARMACOTHERAPY

LA English

DT Review

DE bedaquiline; diarylquinoline; antimycobacterial; multi-drug resistant

tuberculosis; MDR-TB

ID EARLY BACTERICIDAL ACTIVITY; PULMONARY TUBERCULOSIS; ATP SYNTHASE;

MURINE MODEL; TMC207; R207910; DRUG; TB; PHARMACOKINETICS; PYRAZINAMIDE

AB Objective: To review the chemistry, pharmacology, microbiology, pharmacokinetics, pharmacodynamics, clinical efficacy, safety, dosage, and administration of bedaquiline, a novel oral diarylquinoline antimycobacterial agent. approved by the Food and Drug Administration for the treatment of adults with pulmonary multidrug-resistant tuberculosis (MDR-TB). Data Sources: A search of PubMed (January 2004-May 2013) and International Pharmaceutical Abstracts (January 2004-May 2013) using the search terms bedaquiline, diarylquinoline, R207910, and TMC207 was performed. Supplementary sources included proceedings of the Union World Conference on Lung Health. Study Selection and Data Extraction: Preclinical data as well as Phase 1 and 2 studies published in English were evaluated. Data Synthesis: Bedaquiline possesses a unique mechanism of action that disrupts the activity of the mycobacterial adenosine triphosphate synthase. Clinical trials have been conducted evaluating the use of bedaquiline in combination with a background regimen for the treatment of adults with pulmonary MDR-TB. Bedaquiline has an excellent in vitro activity against Mycobacterium tuberculosis, including multidrug resistant M tuberculosis; however, its side effect profile limits its use against MDR-TB when no other effective regimen can be provided. Bedaquiline carries Black Box warnings for increased risk of unexplained mortality and QT prolongation. Bedaquiline is metabolized via the CYP3A4 isoenzyme and thus interacts with rifamycins and several antiretrovirals. Conclusions: In an era of emerging resistance and given the suboptimal efficacy and toxicity of currently available regimens for MDR-TB, bedaquiline represents a great addition to the existing armamentarium of anti-TB agents particularly in areas of the world where the disease is endemic.

C1 [Chahine, Elias B.] Palm Beach Atlantic Univ, W Palm Beach, FL USA.

[Karaoui, Lamis R.; Mansour, Hanine] Lebanese Amer Univ, Byblos, Lebanon.

C3 Palm Beach Atlantic University West Palm Beach; Lebanese American

University

RP Mansour, H (corresponding author), Lebanese Amer Univ, Sch Pharm, POB 36-S 23, Byblos, Lebanon.

EM hanine.mansour@lau.edu.lb

RI Mansour, Hanine/AAH-2649-2019; Karaoui, Lamis/AAH-4446-2019

OI Mansour, Hanine/0000-0001-6383-0288; Karaoui, Lamis/0000-0002-7857-7374

CR Andries K, 2005, SCIENCE, V307, P223, DOI 10.1126/science.1106753

[Anonymous], REP TUB US 2011

Cohen J, 2013, SCIENCE, V339, P130, DOI 10.1126/science.339.6116.130

Cole ST, 2005, SCIENCE, V307, P214, DOI 10.1126/science.1108379

Dhillon J, 2010, TUBERCULOSIS, V90, P301, DOI 10.1016/j.tube.2010.07.004

Diacon AH, 2012, ANTIMICROB AGENTS CH, V56, P3271, DOI 10.1128/AAC.06126-11

Diacon AH, 2013, ANTIMICROB AGENTS CH, V57, P2199, DOI 10.1128/AAC.02243-12

Diacon AH, 2012, LANCET, V380, P986, DOI 10.1016/S0140-6736(12)61080-0

Diacon AH, 2009, NEW ENGL J MED, V360, P2397, DOI 10.1056/NEJMoa0808427

Diel R, 2012, EUR RESPIR J, V40, P143, DOI 10.1183/09031936.00204611

Dooley KE, 2012, JAIDS-J ACQ IMM DEF, V59, P455, DOI 10.1097/QAI.0b013e3182410503

Gandhi NR, 2006, LANCET, V368, P1575, DOI 10.1016/S0140-6736(06)69573-1

Gumbo T, 2011, GOODMAN GILMANS PHAR, P1561

Haxaire M., 2011, 42 UN WORLD C LUNG H

Haxaire M, 2012, 43 UN WORLD C LUNG H

Huitric E, 2007, ANTIMICROB AGENTS CH, V51, P4202, DOI 10.1128/AAC.00181-07

Ibrahim M, 2007, ANTIMICROB AGENTS CH, V51, P1011, DOI 10.1128/AAC.00898-06

Ibrahim M, 2009, AM J RESP CRIT CARE, V180, P553, DOI 10.1164/rccm.200807-1152OC

Jacobson KR, 2010, CLIN INFECT DIS, V51, P6, DOI 10.1086/653115

Janssen Therapeutics, 2012, SIRT BED PROD INF

Ji BH, 2006, ANTIMICROB AGENTS CH, V50, P1921, DOI 10.1128/AAC.00052-06

Leibert E, 2010, EXPERT REV ANTI-INFE, V8, P801, DOI 10.1586/ERI.10.60

Loddenkemper R, 2012, EUR RESPIR J, V40, P9, DOI 10.1183/09031936.00027612

Lounis N, 2006, ANTIMICROB AGENTS CH, V50, P3543, DOI 10.1128/AAC.00766-06

Matteelli A, 2010, FUTURE MICROBIOL, V5, P849, DOI [10.2217/fmb.10.50, 10.2217/FMB.10.50]

McNeely DF, 2010, 41 UN WORLD C LUNG H

Mitchison DA, 2005, NAT BIOTECHNOL, V23, P187, DOI 10.1038/nbt0205-187

Petrella S, 2006, ANTIMICROB AGENTS CH, V50, P2853, DOI 10.1128/AAC.00244-06

Protopopova M, 2007, MED CHEM, V3, P301, DOI 10.2174/157340607780620626

Rouan MC, 2012, ANTIMICROB AGENTS CH, V56, P1444, DOI 10.1128/AAC.00720-11

Rustomjee R, 2008, ANTIMICROB AGENTS CH, V52, P2831, DOI 10.1128/AAC.01204-07

Singh H., 2013, International Journal of Basic Clinical Pharmacology, V2, P96, DOI [10.5455/2319-2003.ijbcp20130301, DOI 10.5455/2319-2003.IJBCP20130301]

Sotgiu G, 2009, EUR RESPIR J, V33, P871, DOI 10.1183/09031936.00168008

Svensson EM, 2013, ANTIMICROB AGENTS CH, V57, P2780, DOI 10.1128/AAC.00191-13

NR 34

TC 96

Z9 117

U1 0

U2 52

PU SAGE PUBLICATIONS INC

PI THOUSAND OAKS

PA 2455 TELLER RD, THOUSAND OAKS, CA 91320 USA

SN 1060-0280

EI 1542-6270

J9 ANN PHARMACOTHER

JI Ann. Pharmacother.

PD JAN

PY 2014

VL 48

IS 1

BP 107

EP 115

DI 10.1177/1060028013504087

PG 9

WC Pharmacology & Pharmacy

WE Science Citation Index Expanded (SCI-EXPANDED)

SC Pharmacology & Pharmacy

GA 300ZU

UT WOS:000330503300013

DA 2026-05-07

ER

PT J

AU Zein-Eddine, R

Ramuz, M

Refrégier, G

Lutzeyer, JF

Aleksandrov, A

Myllykallio, H

AF Zein-Eddine, Rima

Ramuz, Masoud

Refregier, Guislaine

Lutzeyer, Johannes F.

Aleksandrov, Alexey

Myllykallio, Hannu

TI Understanding the key challenges in tuberculosis drug discovery: what

does the future hold?

SO EXPERT OPINION ON DRUG DISCOVERY

LA English

DT Article

DE Anti-microbial resistance (AMR); <italic>mycobacterium

tuberculosis</italic> (<italic>mtb</italic>); anti-evolution drugs; drug

discovery; drug-resistance mechanism; AI-driven drug discovery

ID MYCOBACTERIUM-TUBERCULOSIS; RESISTANT TUBERCULOSIS;

ANTIBIOTIC-RESISTANCE; ISOCITRATE LYASE; CELL-WALL; EMERGENCE;

EVOLUTION; MECHANISM; TARGET; INHIBITORS

AB IntroductionTuberculosis (TB), caused by Mycobacterium tuberculosis (Mtb), remains a major global health concern. It spreads through airborne droplets and has a high mortality rate, particularly without treatment. Drug resistance is rising, with treatments against multidrug-resistant TB (MDR-TB) showing poor treatment success rates. The thick, lipid-rich wall of Mtb and its slow growth reduce antibiotic effectiveness, requiring long treatment courses of 4-6 months. Current therapies often fail against drug-resistant strains, highlighting the urgent need for new, short-course treatment, affordable, and combination-friendly drugs.Areas coveredWithin this perspective, the authors review and comment on the following topics regarding Mtb resistance emergence and treatment strategies: i) Existing treatment ii) Resistance evolution in Mtb; iii) Key challenges in drug discovery targeting Mtb; iv) emerging strategies and recent advances in Mtb drug discovery, and v) Next-generation approaches. Literature was identified through a search of PubMed, google scholar, and web of science, from January 2010 to March 2025.Expert opinionAI is accelerating the discovery of bioavailable and safe preclinical drug candidates for TB, though data limitations and biological complexity remain challenging. Future progress requires multi-modal models, open-access datasets, and interdisciplinary collaboration.

C1 [Zein-Eddine, Rima; Aleksandrov, Alexey; Myllykallio, Hannu] Inst Polytech Paris, Ecole Polytech, Lab Opt & Biosci LOB, Inserm,U1182,CNRS,UMR7645, F-11128 Palaiseau, France.

[Ramuz, Masoud; Lutzeyer, Johannes F.] Inst Polytech Paris, Ecole Polytech, Lab Informat LIX, CNRS,UMR7161, Palaiseau, France.

[Refregier, Guislaine] Univ Paris Saclay, CNRS, AgroParisTech, Lab Ecol Soc & Evolut ESE, Gif Sur Yvette, France.

C3 Centre National de la Recherche Scientifique (CNRS); CNRS - Institute of

Physics (INP); Institut Polytechnique de Paris; Ecole Polytechnique;

Institut National de la Sante et de la Recherche Medicale (Inserm);

Centre National de la Recherche Scientifique (CNRS); CNRS - Institute

for Information Sciences & Technologies (INS2I); Institut Polytechnique

de Paris; Ecole Polytechnique; Universite Paris Saclay; AgroParisTech;

Centre National de la Recherche Scientifique (CNRS)

RP Zein-Eddine, R; Myllykallio, H (corresponding author), Inst Polytech Paris, Ecole Polytech, Lab Opt & Biosci LOB, Inserm,U1182,CNRS,UMR7645, F-11128 Palaiseau, France.

EM rima.zein-eddine@polytechnique.edu; hannu.myllykallio@polytechnique.edu

RI ; Myllykallio, Hannu/Y-5462-2019; Александров, Алексей/T-3561-2017

OI Zein Eddine, Rima/0000-0001-9965-0027; Myllykallio,

Hannu/0000-0002-0541-1197;

FU European Union's Horizon 2020 research and innovation programme under

the Marie Sklodowska-Curie grant [899987]; ANRS Maladies Infectieuses

Emergentes (ANRS-MIE 2023-2 programme) [23534, 285656]; Carnot Telecom &

Societe Numerique; French National Research Agency (ANR) through the

"GraspGNNs" JCJC grant [ANR-24-CE23-3888]; ANR NORESIST [24HRY113]; ANR

[ANR-18-CE44-0002]; ANR (MMRDNABREAKS) [ANR-22-CE12-0042]; The

"Prematuration IP Paris" programme for applications in antimicrobial

drug discovery; Agence Nationale de la Recherche (ANR)

[ANR-18-CE44-0002] Funding Source: Agence Nationale de la Recherche

(ANR)

FX R Zein-Eddine initially received Funding from the European Union's

Horizon 2020 research and innovation programme under the Marie

Sklodowska-Curie grant agreement No. 899987, and is currently funded by

the ANRS Maladies Infectieuses Emergentes (ANRS-MIE 2023-2 programme,

grant agreement No. 23534). M Ramuz is supported by funding from the

Carnot Telecom & Societe Numerique. J Lutzeyer received support from the

French National Research Agency (ANR) through the "GraspGNNs" JCJC grant

(ANR-24-CE23-3888). G Refregier is supported by the ANR NORESIST, no

24HRY113. A Aleksandrov was supported by the ANR under grant

ANR-18-CE44-0002 (ATMCADD). H Myllykallio is currently supported by the

ANR (MMRDNABREAKS, ANR-22-CE12-0042) for research on DNA repair in

prokaryotic systems. H Myllykallio also acknowledges early support from

the "Prematuration IP Paris" programme for applications in antimicrobial

drug discovery. This review study was supported by a grant from the ANRS

Maladies Infectieuses Emergentes (ANRS-MIE 2023-2 programme, ANRS No

285656).

CR Abramson J, 2024, NATURE, V630, DOI [10.1038/s41586-024-08416-7, 10.1038/s41586-024-07487-w]

Acharya MP, 2021, CLIN INFECT DIS, V73, P793, DOI 10.1093/cid/ciab144

Rani KGA, 2021, EXPERT OPIN DRUG DIS, V16, P365, DOI 10.1080/17460441.2021.1854221

Agnivesh PK, 2025, MICROB PATHOGENESIS, V198, DOI 10.1016/j.micpath.2024.107074

Aleksandrov A, 2019, EXPERT OPIN DRUG DIS, V14, P35, DOI 10.1080/17460441.2019.1550482

Alexander PE, 2007, CAN J INFECT DIS MED, V18, P289, DOI 10.1155/2007/986794

Andersson DI, 2006, CURR OPIN MICROBIOL, V9, P461, DOI 10.1016/j.mib.2006.07.002

Arbués A, 2020, PLOS PATHOG, V16, DOI 10.1371/journal.ppat.1008312

Azaldegui CA, 2021, BIOPHYS J, V120, P1123, DOI 10.1016/j.bpj.2020.09.023

BANERJEE A, 1994, SCIENCE, V263, P227, DOI 10.1126/science.8284673

Basta T, 2012, OPEN BIOL, V2, DOI 10.1098/rsob.120120

Beites T, 2019, NAT COMMUN, V10, DOI 10.1038/s41467-019-12956-2

Benjak A, 2018, NAT COMMUN, V9, DOI 10.1038/s41467-017-02576-z

Berg K, 2023, EUR J MED CHEM, V249, DOI 10.1016/j.ejmech.2023.115125

Blair JMA, 2015, NAT REV MICROBIOL, V13, P42, DOI 10.1038/nrmicro3380

Bogdanovic N, 2018, J STRUCT BIOL, V204, P420, DOI 10.1016/j.jsb.2018.10.006

Boshoff Helena I M, 2003, Cell, V113, P183

Cambier CJ, 2014, CELL, V159, P1497, DOI 10.1016/j.cell.2014.11.024

Campbell EA, 2001, CELL, V104, P901, DOI 10.1016/S0092-8674(01)00286-0

Casadevall A, 2004, NAT REV MICROBIOL, V2, P695, DOI 10.1038/nrmicro974

Castañeda-García A, 2017, NAT COMMUN, V8, DOI 10.1038/ncomms14246

Chandra P, 2022, NAT REV MICROBIOL, V20, P750, DOI 10.1038/s41579-022-00763-4

Chen ZC, 2008, NATURE, V453, P489, DOI 10.1038/nature06971

Chiang CY, 2010, RESPIROLOGY, V15, P413, DOI 10.1111/j.1440-1843.2010.01738.x

Chopra I, 2003, DRUG RESIST UPDATE, V6, P137, DOI 10.1016/S1368-7646(03)00041-4

Cirz RT, 2007, CRIT REV BIOCHEM MOL, V42, P341, DOI 10.1080/10409230701597741

Codlin AJ, 2021, SCI REP-UK, V11, DOI 10.1038/s41598-021-03265-0

Coll F, 2018, NAT GENET, V50, P307, DOI 10.1038/s41588-017-0029-0

Comas I, 2012, NAT GENET, V44, P106, DOI 10.1038/ng.1038

Couce A, 2015, P ROY SOC B-BIOL SCI, V282, DOI 10.1098/rspb.2014.2698

Cretu M., ARXIV

Croitoru A, 2025, J CHEM THEORY COMPUT, V21, P3044, DOI 10.1021/acs.jctc.5c00046

Daffe M, 1998, ADV MICROB PHYSIOL, V39, P131

Dartois V, 2024, NAT REV DRUG DISCOV, V23, P381, DOI 10.1038/s41573-024-00897-5

Denamur E, 2006, MOL MICROBIOL, V60, P820, DOI 10.1111/j.1365-2958.2006.05150.x

Dhiman RK, 2019, PLOS ONE, V14, DOI 10.1371/journal.pone.0214958

Djaout K, 2016, SCI REP-UK, V6, DOI 10.1038/srep27792

Doyle CR, 2018, INFECT IMMUN, V86, DOI 10.1128/IAI.00300-18

Dulberger CL, 2020, NAT REV MICROBIOL, V18, P47, DOI 10.1038/s41579-019-0273-7

Eastman P, 2023, J PHYS CHEM B, V128, P109, DOI 10.1021/acs.jpcb.3c06662

Eberhardt N, 2022, PURINERG SIGNAL, V18, P93, DOI 10.1007/s11302-021-09838-y

Esaulova E, 2021, CELL HOST MICROBE, V29, P165, DOI 10.1016/j.chom.2020.11.013

Esteban J, 2018, FRONT MICROBIOL, V8, DOI 10.3389/fmicb.2017.02651

Fonseca JD, 2015, INT J INFECT DIS, V32, P94, DOI 10.1016/j.ijid.2015.01.014

FRIEDEN TR, 1993, NEW ENGL J MED, V328, P521, DOI 10.1056/NEJM199302253280801

Frimodt-Moller J, 2019, TRENDS MICROBIOL, V27, P291, DOI 10.1016/j.tim.2019.01.005

Gallucci S, 2001, CURR OPIN IMMUNOL, V13, P114, DOI 10.1016/S0952-7915(00)00191-6

Gao Y, 2019, BIOORG CHEM, V85, P168, DOI 10.1016/j.bioorg.2018.12.018

Gautam S, 2023, ANTIBIOTICS-BASEL, V12, DOI 10.3390/antibiotics12030541

Gessner S, 2023, ELIFE, V12, DOI 10.7554/eLife.75628

Gillespie SH, 2002, ANTIMICROB AGENTS CH, V46, P267, DOI 10.1128/AAC.46.2.267-274.2002

Gillespie SH, 2005, J ANTIMICROB CHEMOTH, V56, P344, DOI 10.1093/jac/dki191

Goude R, 2009, ANTIMICROB AGENTS CH, V53, P4138, DOI 10.1128/AAC.00162-09

Gregersen LH, 2018, TRENDS BIOCHEM SCI, V43, P327, DOI 10.1016/j.tibs.2018.02.010

Grzelak EM, 2019, J ANTIBIOT, V72, P719, DOI 10.1038/s41429-019-0205-9

Gygli SM, 2017, FEMS MICROBIOL REV, V41, P354, DOI 10.1093/femsre/fux011

Hamada Y, 2022, EBIOMEDICINE, V82, DOI 10.1016/j.ebiom.2022.104174

Harrison GA, 2019, MSPHERE, V4, DOI 10.1128/mSphere.00606-19

Heid E, 2023, J CHEM INF MODEL, V64, P9, DOI 10.1021/acs.jcim.3c01250

Heyckendorf J, 2021, EUR RESPIR J, V58, DOI 10.1183/13993003.03492-2020

Hu XP, 2022, ACTA PHARMACOL SIN, V43, P1605, DOI 10.1038/s41401-021-00779-1

Hughes D, 2017, ANNU REV MICROBIOL, V71, P579, DOI 10.1146/annurev-micro-090816-093813

Hugonnet JE, 2007, BIOCHEMISTRY-US, V46, P11998, DOI 10.1021/bi701506h

Hwang EJ, 2024, RADIOL-ARTIF INTELL, V6, DOI 10.1148/ryai.230327

Ishino S, 2018, NUCLEIC ACIDS RES, V46, P6206, DOI 10.1093/nar/gky460

Jankute M, 2015, ANNU REV MICROBIOL, V69, P405, DOI 10.1146/annurev-micro-091014-104121

JARLIER V, 1994, FEMS MICROBIOL LETT, V123, P11, DOI 10.1111/j.1574-6968.1994.tb07194.x

Jayatunga MKP, 2024, DRUG DISCOV TODAY, V29, DOI 10.1016/j.drudis.2024.104009

Jing B., 2022, ARXIV

Jumper J, 2021, NATURE, V596, P583, DOI 10.1038/s41586-021-03819-2

Kalia NP, 2019, SCI REP-UK, V9, DOI 10.1038/s41598-019-44887-9

Kana BD, 2014, TUBERCULOSIS, V94, P551, DOI 10.1016/j.tube.2014.10.003

Kashyap A, 2018, TUBERCULOSIS, V112, P83, DOI 10.1016/j.tube.2018.08.005

Kobayashi SD, 2018, MBIO, V9, DOI 10.1128/mBio.00297-18

Korbee CJ, 2018, NAT COMMUN, V9, DOI 10.1038/s41467-017-02777-6

Koul A, 2008, J BIOL CHEM, V283, P25273, DOI 10.1074/jbc.M803899200

Koul A, 2011, NATURE, V469, P483, DOI 10.1038/nature09657

Kumar N, 2021, MOL ASPECTS MED, V81, DOI 10.1016/j.mam.2021.101002

Kumar P, 2018, MBIO, V9, DOI 10.1128/mBio.02101-17

Lin J, 2015, FRONT MICROBIOL, V6, DOI 10.3389/fmicb.2015.00034

Liu QY, 2021, P NATL ACAD SCI USA, V118, DOI 10.1073/pnas.2017831118

Louw GE, 2011, AM J RESP CRIT CARE, V184, P269, DOI 10.1164/rccm.201011-1924OC

Luo Y, 2001, CELL, V106, P585, DOI 10.1016/S0092-8674(01)00479-2

Marais BJ, 2009, EUR RESPIR J, V33, P943, DOI 10.1183/09031936.00173608

Mcclean M, 2024, BREATHE, V20, DOI 10.1183/20734735.0056-2024

Merker M, 2018, ELIFE, V7, DOI [10.1101/334599, 10.7554/eLife.38200]

Migliori GB, 2020, INT J INFECT DIS, V92, pS15, DOI 10.1016/j.ijid.2020.01.042

Million-Weaver S, 2015, P NATL ACAD SCI USA, V112, pE1096, DOI 10.1073/pnas.1416651112

Mosaei Hamed, 2020, EcoSal Plus, V9, DOI [10.1128/ecosalplus.esp-0017-2019, 10.1128/ecosalplus.ESP-0017-2019]

Motley MP, 2019, CURR OPIN INFECT DIS, V32, P210, DOI 10.1097/QCO.0000000000000539

Murase LS, 2019, ANTIMICROB AGENTS CH, V63, DOI 10.1128/AAC.02520-18

Myllykallio H, 2002, SCIENCE, V297, P105, DOI 10.1126/science.1072113

Myllykallio H, 2020, BIOPHYS J, V119, P2508, DOI 10.1016/j.bpj.2020.10.042

Nahid P, 2019, AM J RESP CRIT CARE, V200, pE93, DOI 10.1164/rccm.201909-1874ST

Nathan C, 2015, IMMUNOL REV, V264, P308, DOI 10.1111/imr.12275

Naz S, 2023, ELIFE, V12, DOI 10.7554/eLife.75860

Nizi MG, 2020, EUR J MED CHEM, V201, DOI 10.1016/j.ejmech.2020.112420

Nsubuga M, 2024, BMC GENOMICS, V25, DOI 10.1186/s12864-024-10214-4

Oliver A, 2010, CLIN MICROBIOL INFEC, V16, P798, DOI 10.1111/j.1469-0691.2010.03250.x

Palencia A, 2016, ANTIMICROB AGENTS CH, V60, P6271, DOI 10.1128/AAC.01339-16

Palomino JC, 2014, ANTIBIOTICS-BASEL, V3, P317, DOI 10.3390/antibiotics3030317

Park JS, 2002, CELL, V109, P757, DOI 10.1016/S0092-8674(02)00769-9

Peetluk LS, 2022, CLIN INFECT DIS, V74, P973, DOI 10.1093/cid/ciab598

Petit-Jentreau L, 2017, FRONT CELL INFECT MI, V7, P1, DOI 10.3389/fcimb.2017.00347

Qabel A., 2022, BIORXIV, DOI [10.1101/2022.10.06.511103, DOI 10.1101/2022.10.06.511103]

Qiu YD, 2021, J CHEM THEORY COMPUT, V17, P6262, DOI 10.1021/acs.jctc.1c00571

Rad ME, 2003, EMERG INFECT DIS, V9, P838, DOI 10.3201/eid0907.020803

Radchenko EV, 2023, MOLECULES, V28, DOI 10.3390/molecules28020633

Ragheb MN, 2019, MOL CELL, V73, P157, DOI 10.1016/j.molcel.2018.10.015

Rahman K, 2022, GENOM PROTEOM BIOINF, V20, P1180, DOI 10.1016/j.gpb.2021.01.008

Rajasekar AA., ARXIV

Rao SPS, 2008, P NATL ACAD SCI USA, V105, P11945, DOI 10.1073/pnas.0711697105

Rawat R, 2003, P NATL ACAD SCI USA, V100, P13881, DOI 10.1073/pnas.2235848100

Recacha E, 2017, MBIO, V8, DOI 10.1128/mBio.00971-17

Restrepo BI, 2021, TUBERCULOSIS, V126, DOI 10.1016/j.tube.2020.102047

Rock J, 2019, PLOS PATHOG, V15, DOI 10.1371/journal.ppat.1007975

Roden DM, 2002, NAT REV DRUG DISCOV, V1, P37, DOI 10.1038/nrd705

Rood JE, 2022, NAT MED, V28, P2486, DOI 10.1038/s41591-022-02104-7

Sadybekov AV, 2023, NATURE, V616, P673, DOI 10.1038/s41586-023-05905-z

Santosh KC, 2022, J MED SYST, V46, DOI 10.1007/s10916-022-01870-8

Schneuing A, 2024, NAT COMPUT SCI, V4, DOI 10.1038/s43588-024-00737-x

Sengupta P, 2025, ACS OMEGA, V10, P13966, DOI 10.1021/acsomega.4c10518

Shukla H, 2015, INT J BIOL MACROMOL, V78, P137, DOI 10.1016/j.ijbiomac.2015.04.005

Shukla R., 2021, INNOVATIONS IMPLEMEN, P295, DOI DOI 10.1007/978-981-15-8936-2_12

Simeone R, 2012, PLOS PATHOG, V8, DOI 10.1371/journal.ppat.1002507

Singh A, 2017, MICROBIOL-SGM, V163, P1740, DOI 10.1099/mic.0.000578

Smith T, 2014, CURR TOP MICROBIOL, V374, P53, DOI 10.1007/82_2012_279

Smollett KL, 2012, J BIOL CHEM, V287, P22004, DOI 10.1074/jbc.M112.357715

Sogi KM, 2017, ACS INFECT DIS, V3, P564, DOI 10.1021/acsinfecdis.7b00046

Songsiriritthigul C, 2022, PROTEINS, V90, P898, DOI 10.1002/prot.26268

Sotgiu G, 2015, CSH PERSPECT MED, V5, DOI 10.1101/cshperspect.a017822

Sprouffske K, 2018, PLOS GENET, V14, DOI 10.1371/journal.pgen.1007324

Stagg HR, 2014, EUR RESPIR J, V43, P632, DOI 10.1183/09031936.00140513

Stokes JM, 2020, CELL, V180, P688, DOI 10.1016/j.cell.2020.01.021

Tahlan K, 2012, ANTIMICROB AGENTS CH, V56, P1797, DOI 10.1128/AAC.05708-11

Takemoto N, 2018, NUCLEIC ACIDS RES, V46, P6152, DOI 10.1093/nar/gky481

TAKIFF HE, 1994, ANTIMICROB AGENTS CH, V38, P773, DOI 10.1128/AAC.38.4.773

te Brake LHM, 2018, ANNU REV PHARMACOL, V58, P271, DOI 10.1146/annurev-pharmtox-010617-052438

TELENTI A, 1993, LANCET, V341, P647, DOI 10.1016/0140-6736(93)90417-F

Tiemersma EW, 2011, PLOS ONE, V6, DOI 10.1371/journal.pone.0017601

Timo GO, 2019, PHARMACEUTICALS-BASE, V12, DOI 10.3390/ph12030135

Tizzano B, 2021, SCI REP-UK, V11, DOI 10.1038/s41598-021-81223-6

Tran SL, 2005, J BACTERIOL, V187, P5023, DOI 10.1128/JB.187.14.5023-5028.2005

Tremblay LW, 2010, BIOCHEMISTRY-US, V49, P3766, DOI 10.1021/bi100232q

Pham TV, 2021, ACS CHEM BIOL, V16, P463, DOI 10.1021/acschembio.0c00740

Velayati AA, 2009, CHEST, V136, P420, DOI 10.1378/chest.08-2427

Wang XY, 2024, NAT COMMUN, V15, DOI 10.1038/s41467-024-54072-w

Wen Y, 2023, EUR J MED CHEM, V255, DOI 10.1016/j.ejmech.2023.115351

WHO, 2024, GLOB TUB REP

WHO, 2023, CATALOGUE MUTATIONS

Winchell CG, 2023, J EXP MED, V220, DOI 10.1084/jem.20230707

Wirth T, 2008, PLOS PATHOG, V4, DOI 10.1371/journal.ppat.1000160

Yan Y, 2020, BMC GENET, V21, DOI 10.1186/s12863-020-00939-y

Yang W, 2014, BIOCHEMISTRY-US, V53, P2793, DOI 10.1021/bi500019s

Yee D, 2003, AM J RESP CRIT CARE, V167, P1472, DOI 10.1164/rccm.200206-626OC

Zein-Eddine R., 2025, BIORXIV, DOI [10.1101/2025.01.17.633521, DOI 10.1101/2025.01.17.633521]

Zein-Eddine R, 2023, J BIOMED SCI, V30, DOI 10.1186/s12929-023-00932-4

Zhang G, 2018, MOLECULES, V23, DOI 10.3390/molecules23030524

Zhang Y, 2003, INT J TUBERC LUNG D, V7, P6

ZHANG Y, 1992, NATURE, V358, P591, DOI 10.1038/358591a0

Zhao HY, 2022, J MED CHEM, V65, P10534, DOI 10.1021/acs.jmedchem.2c00718

Zumla A, 2015, LANCET RESP MED, V3, P220, DOI 10.1016/S2213-2600(15)00063-6

Zumla A, 2013, NAT REV DRUG DISCOV, V12, P388, DOI 10.1038/nrd4001

zur Wiesch PA, 2011, LANCET INFECT DIS, V11, P236, DOI 10.1016/S1473-3099(10)70264-4

NR 164

TC 3

Z9 3

U1 0

U2 5

PU TAYLOR & FRANCIS LTD

PI ABINGDON

PA 2-4 PARK SQUARE, MILTON PARK, ABINGDON OR14 4RN, OXON, ENGLAND

SN 1746-0441

EI 1746-045X

J9 EXPERT OPIN DRUG DIS

JI Expert. Opin. Drug Discov.

PD SEP 2

PY 2025

VL 20

IS 9

BP 1115

EP 1130

DI 10.1080/17460441.2025.2531229

EA JUL 2025

PG 16

WC Pharmacology & Pharmacy

WE Science Citation Index Expanded (SCI-EXPANDED)

SC Pharmacology & Pharmacy

GA 6WM9Q

UT WOS:001526984300001

OA Green Submitted

DA 2026-05-07

ER

PT J

AU Valafar, SJ

AF Valafar, Siavash J.

TI Systematic Review of Mutations Associated with Isoniazid Resistance

Points to Continuing Evolution and Subsequent Evasion of Molecular

Detection, and Potential for Emergence of Multidrug Resistance in

Clinical Strains of Mycobacterium tuberculosis

SO ANTIMICROBIAL AGENTS AND CHEMOTHERAPY

LA English

DT Review

DE isoniazid; drug resistance evolution; drug resistance mechanisms;

genotypic identification; phenotypic identification; tuberculosis

ID GENOTYPE MTBDRPLUS ASSAY; EFFLUX PUMP GENES; REAL-TIME-PCR;

DRUG-RESISTANCE; PULMONARY TUBERCULOSIS; RAPID DETECTION; RIFAMPICIN;

KATG; TB; INHA

AB Molecular testing is rapidly becoming an integral component of global tuberculosis (TB) control. Uncommon mechanisms of resistance escape detection by these platforms and undermine our ability to contain outbreaks. This article is a systematic review of published articles that reported isoniazid (INH) resistance-conferring mutations between September 2013 and December 2019. The genes katG, inhA, and fabG1, and the intergenic region oxyR'-ahpC were considered in this review. Fifty-two articles were included that described 9,306 clinical isolates (5,804 INH resistant [INHr] and 3,502 INH susceptible [INHs]) from 31 countries. The three most frequently mutated loci continue to be locus 315 of katG (kal G315; n = 4,271), locus -15 of inhA (inhA-15; n = 787), and locus -8 of inhA (inhA-8; 106). However, the diagnostic value of inhA-8 is far lower than previously thought, as it only appears in 25 (0.4%) of the INHr isolates lacking the first two mutations. I catalogued 45 new loci (29 katG, nine inhA, and seven ahpC) associated with INH resistance and identified 59 loci (common to this and previous reviews) as a reliable basis for molecular diagnostics. Including all observed mutations provides a cumulative sensitivity of 85.6%. In 14.4% of resistant isolates, no mechanism of resistance was detected, making them likely to escape molecular detection, and in the case of INH monoresistance, likely to convert to multidrug-resistant TB (MDR-TB). Integrating the information cataloged in this study into current diagnostic tools is essential for combating the emergence of MDR-TB, and its exclusion can lead to an unintended selection against common mechanisms and to diversifying evolution. Observation of many low-frequency resistance-conferring mutations points to an advantage of whole-genome sequencing (WGS) for diagnostics. Finally, I provide five recommendations for future diagnostic platforms.

C1 [Valafar, Siavash J.] Univ Calif Irvine, Dept Biol, Irvine, CA 92697 USA.

C3 University of California System; University of California Irvine

RP Valafar, SJ (corresponding author), Univ Calif Irvine, Dept Biol, Irvine, CA 92697 USA.

EM svalafar@uci.edu

CR Abate D, 2014, INT J TUBERC LUNG D, V18, P946, DOI 10.5588/ijtld.13.0926

Alame-Emane AK, 2015, INT J TUBERC LUNG D, V19, P679, DOI 10.5588/ijtld.14.0768

Ali Imani Fooladi Abbas, 2014, Asian Pacific Journal of Tropical Biomedicine, V4, pS165, DOI 10.12980/APJTB.4.2014C1186

Ando H, 2014, MOL MICROBIOL, V91, P538, DOI 10.1111/mmi.12476

[Anonymous], 2018, The Global Tuberculosis Report

[Anonymous], 2017, Global tuberculosis report 2017

Aye KS, 2016, J INFECT CHEMOTHER, V22, P174, DOI 10.1016/j.jiac.2015.12.009

Bainomugisa A, 2018, MICROB GENOMICS, V4, DOI 10.1099/mgen.0.000188

Bollela VR, 2016, INT J TUBERC LUNG D, V20, P1099, DOI 10.5588/ijtld.15.0864

Brhane M, 2017, INFECT DRUG RESIST, V10, P75, DOI 10.2147/IDR.S127903

Campbell PJ, 2011, ANTIMICROB AGENTS CH, V55, P2032, DOI 10.1128/AAC.01550-10

Castro-Wallace SL, 2017, SCI REP-UK, V7, DOI 10.1038/s41598-017-18364-0

CDC, 2019, GLOB HLTH REP TUB

Chen J, 2017, BMC INFECT DIS, V17, DOI 10.1186/s12879-017-2409-6

Dookie N, 2016, BMC INFECT DIS, V16, DOI 10.1186/s12879-016-1906-3

Fauver JR, 2020, CELL, V181, P990, DOI 10.1016/j.cell.2020.04.021

Folkvardsen DB, 2013, J CLIN MICROBIOL, V51, P1596, DOI 10.1128/JCM.00472-13

Georghiou SB, 2016, ANTIMICROB AGENTS CH, V60, P3994, DOI 10.1128/AAC.00222-16

Gupta A, 2014, INDIAN J MED RES, V139, P892

Haeili M, 2014, INDIAN J MED MICROBI, V32, P398, DOI 10.4103/0255-0857.142245

Hamze M, 2015, INT J MYCOBACT, V4, P228, DOI 10.1016/j.ijmyco.2015.05.007

Hofmann-Thiel S, 2019, INT J TUBERC LUNG D, V23, P458, DOI 10.5588/ijtld.18.0615

Horne DJ, 2013, J CLIN MICROBIOL, V51, P393, DOI 10.1128/JCM.02724-12

Hu SY, 2014, J CLIN MICROBIOL, V52, P1644, DOI 10.1128/JCM.03395-13

Huang ZK, 2014, INT J TUBERC LUNG D, V18, P461, DOI 10.5588/ijtld.13.0736

Igarashi Y, 2017, DIAGN MICR INFEC DIS, V89, P276, DOI 10.1016/j.diagmicrobio.2017.08.016

Isakova J, 2018, BMC MICROBIOL, V18, DOI 10.1186/s12866-018-1168-x

Jo KW, 2017, RESP MED, V122, P12, DOI 10.1016/j.rmed.2016.11.016

Martínez-Lirola MJ, 2014, ENFERM INFEC MICR CL, V32, P511, DOI 10.1016/j.eimc.2014.02.017

Kambli P, 2015, DIAGN MICR INFEC DIS, V82, P49, DOI 10.1016/j.diagmicrobio.2015.01.009

Karimi H, 2018, J GLOB ANTIMICROB RE, V12, P63, DOI 10.1016/j.jgar.2017.09.002

Karunaratne GHRE, 2018, MICROB DRUG RESIST, V24, P844, DOI 10.1089/mdr.2017.0330

Kazemian H, 2019, INFECT DRUG RESIST, V12, P3585, DOI 10.2147/IDR.S222905

Kebede Abebaw, 2017, BMC Res Notes, V10, P661, DOI [10.1186/s13104-017-2989-7, 10.1186/s13104-017-2989-7]

Keicho, 2019, VIETNAM SCI REP, V9, P1, DOI [10.1038/s41598, DOI 10.1038/S41598-019-51812-7]

Kumar P, 2014, TUBERCULOSIS, V94, P73, DOI 10.1016/j.tube.2013.10.001

Kumari R, 2016, PLOS ONE, V11, DOI 10.1371/journal.pone.0159651

Li QL, 2019, J GLOB ANTIMICROB RE, V18, P168, DOI 10.1016/j.jgar.2019.03.012

Liu Q, 2017, CHINESE MED J-PEKING, V130, P1521, DOI 10.4103/0366-6999.208248

Lu J, 2014, MOL MED REP, V9, P2031, DOI 10.3892/mmr.2014.2045

Narang A, 2017, INT J MYCOBACT, V6, P177, DOI 10.4103/ijmy.ijmy_26_17

Negi SS, 2018, INT J MYCOBACT, V7, P228, DOI 10.4103/ijmy.ijmy_100_18

Nikolayevskyy V, 2016, PLOS ONE, V11, DOI 10.1371/journal.pone.0152926

Ogari CO, 2019, BMC INFECT DIS, V19, DOI 10.1186/s12879-019-3911-9

Omer ZB, 2016, INT J MYCOBACT, V5, P475, DOI 10.1016/j.ijmyco.2016.06.005

Pagán-Ramos E, 2006, J BACTERIOL, V188, P2674, DOI 10.1128/JB.188.7.2674-2680.2006

Park J, 2018, ANN LAB MED, V38, P316, DOI 10.3343/alm.2018.38.4.316

Purkan P, 2018, J Med Life, V11, P160

Ramasubban G, 2015, J GLOB ANTIMICROB RE, V3, P1, DOI 10.1016/j.jgar.2014.10.002

Ramaswamy SV, 2003, ANTIMICROB AGENTS CH, V47, P1241, DOI 10.1128/AAC.47.4.1241-1250.2003

Imperiale BR, 2019, INT J MYCOBACT, V8, P223, DOI 10.4103/ijmy.ijmy_94_19

Rueda J, 2015, ANTIMICROB AGENTS CH, V59, P7805, DOI 10.1128/AAC.01028-15

Seifert M, 2015, PLOS ONE, V10, DOI 10.1371/journal.pone.0119628

Sharma B., 2014, ADV PHARMACOL, V2014, P1, DOI DOI 10.1155/2014/648294

Singhal R, 2014, INDIAN J MED RES, V140, P501

Sirous M, 2018, INFECT DRUG RESIST, V11, P1819, DOI 10.2147/IDR.S178831

Siu GKH, 2014, ANTIMICROB AGENTS CH, V58, P6093, DOI 10.1128/AAC.03277-14

Stephen S, 2019, INT J MYCOBACT, V8, P83, DOI 10.4103/ijmy.ijmy_170_18

Tadesse M, 2016, INT J MYCOBACT, V5, P185, DOI 10.1016/j.ijmyco.2016.02.009

Tam KKG, 2017, DIAGN MICR INFEC DIS, V89, P118, DOI 10.1016/j.diagmicrobio.2017.06.018

Torres JN, 2015, EMERG MICROBES INFEC, V4, DOI 10.1038/emi.2015.42

Tseng ST, 2015, J MICROBIOL IMMUNOL, V48, P249, DOI 10.1016/j.jmii.2013.08.018

Unissa AN, 2017, TUBERCULOSIS, V107, P144, DOI 10.1016/j.tube.2017.07.014

Vilcheze C., 2014, Microbiology Spectrum, V2, DOI [10.1128/microbiolspec.MGM2-0014-2013, DOI 10.1128/MICROBIOLSPEC.MGM2-0014-2013]

Vilchèze C, 2019, J MOL BIOL, V431, P3450, DOI 10.1016/j.jmb.2019.02.016

Wang HY, 2018, ANN LAB MED, V38, P569, DOI 10.3343/alm.2018.38.6.569

WHO, 2012, GLOBAL TUBERCULOSIS REPORT 2012, P1

WHO, 2015, GLOB TUB REP 2015

WHO, 2016, GLOB TUB REP 2016

Yakrus MA, 2014, J CLIN MICROBIOL, V52, P1932, DOI 10.1128/JCM.00417-14

Yu XL, 2014, J ANTIBIOT, V67, P291, DOI 10.1038/ja.2013.133

Zhao LL, 2014, ANTIMICROB AGENTS CH, V58, P3475, DOI 10.1128/AAC.02426-14

Zurita J, 2019, INT J TUBERC LUNG D, V23, P166, DOI 10.5588/ijtld.18.0095

NR 73

TC 26

Z9 29

U1 0

U2 21

PU AMER SOC MICROBIOLOGY

PI WASHINGTON

PA 1752 N ST NW, WASHINGTON, DC 20036-2904 USA

SN 0066-4804

EI 1098-6596

J9 ANTIMICROB AGENTS CH

JI Antimicrob. Agents Chemother.

PD MAR

PY 2021

VL 65

IS 3

AR e02091-20

DI 10.1128/AAC.02091-20

PG 15

WC Microbiology; Pharmacology & Pharmacy

WE Science Citation Index Expanded (SCI-EXPANDED)

SC Microbiology; Pharmacology & Pharmacy

GA QJ7KB

UT WOS:000619864100056

OA Green Submitted

DA 2026-05-07

ER

PT J

AU Semenya, D

Touitou, M

Ribeiro, CM

Pavan, FR

Pisano, L

Singh, V

Chibale, K

Bano, G

Toscani, A

Manetti, F

Gianibbi, B

Castagnolo, D

AF Semenya, Dorothy

Touitou, Meir

Ribeiro, Camila Maringolo

Pavan, Fernando Rogerio

Pisano, Luca

Singh, Vinayak

Chibale, Kelly

Bano, Georg

Toscani, Anita

Manetti, Fabrizio

Gianibbi, Beatrice

Castagnolo, Daniele

TI Structural Rigidification of N-Aryl-pyrroles into Indoles Active

against Intracellular and Drug-Resistant Mycobacteria

SO ACS MEDICINAL CHEMISTRY LETTERS

LA English

DT Article

DE Tuberculosis; MDR-TB; XDR-TB; Indole; Pyrrole; Antimicrobial resistance

ID MULTIDRUG-RESISTANT; TUBERCULOSIS; DERIVATIVES; INSIGHT; ANALOGS

AB A series of indolyl-3-methyleneamines incorporating lipophilic side chains were designed through a structural rigidification approach and synthesized for investigation as new chemical entities against Mycobacterium tuberculosis (Mtb). The screening led to the identification of a 6-chloroindole analogue 7j bearing an N-octyl chain and a cycloheptyl moiety, which displayed potent in vitro activity against laboratory and clinical Mtb strains, including a pre-extensively drug-resistant (pre-XDR) isolate. 7j also demonstrated a marked ability to restrict the intracellular growth of Mtb in murine macrophages. Further assays geared toward mechanism of action elucidation have thus far ruled out the involvement of various known promiscuous targets, thereby suggesting that the new indole 7j may inhibit Mtb via a unique mechanism.

C1 [Semenya, Dorothy; Touitou, Meir; Pisano, Luca; Bano, Georg; Toscani, Anita; Castagnolo, Daniele] Kings Coll London, Sch Canc & Pharmaceut Sci, London SE1 9NH, England.

[Ribeiro, Camila Maringolo; Pavan, Fernando Rogerio] Sao Paulo State Univ UNESP, Sch Pharmaceut Sci, TB Res Lab, BR-14800903 Araraquara, SP, Brazil.

[Singh, Vinayak; Chibale, Kelly] Univ Cape Town, Drug Discovery & Dev Ctr H3D, ZA-7701 Rondebosch, South Africa.

[Singh, Vinayak; Chibale, Kelly] Univ Cape Town, Dept Chem, South African Med Res Council, Drug Discovery & Dev Res Unit, ZA-7701 Rondebosch, South Africa.

[Singh, Vinayak; Chibale, Kelly] Univ Cape Town, Inst Infect Dis & Mol Med, ZA-7701 Rondebosch, South Africa.

[Manetti, Fabrizio; Gianibbi, Beatrice] Univ Siena, Dipartimento Biotecnol Chim & Farm, I-53100 Siena, Italy.

C3 University of London; King's College London; Universidade Estadual

Paulista; University of Cape Town; University of Cape Town; South

African Medical Research Council; University of Cape Town; University of

Siena

RP Castagnolo, D (corresponding author), Kings Coll London, Sch Canc & Pharmaceut Sci, London SE1 9NH, England.

EM daniele.castagnolo@kcl.ac.uk

RI ; Pisano, Luca/GWB-9289-2022; Pavan, Fernando Rogério/MZS-0713-2025;

Singh, Vinayak/O-5099-2019; Castagnolo, Daniele/C-2796-2014; Manetti,

Fabrizio/Q-9412-2019

OI Chibale, Kelly/0000-0002-4541-646X; Semenya,

Dorothy/0000-0001-6818-6540; Pavan, Fernando

Rogério/0000-0002-6969-3963; Semenya, Dorothy/0009-0003-1618-2566;

Singh, Vinayak/0000-0001-9002-2489; Castagnolo,

Daniele/0000-0002-7517-5732; Manetti, Fabrizio/0000-0002-9598-2339

FU South African National Research Foundation-SARChI; University of London;

South African Medical Research Council (SAMRC); South African Department

of Science and Innovation; South African National Research Foundation;

Sao Paulo Research Foundation (FAPESP)

FX We gratefully acknowledge EPSRC (Global Challenges Competition King's

College London). D.S. acknowledges the South African National Research

Foundation-SARChI for financial support. M.T. acknowledges King's

College London for a period of leave. D.C. and A.T. acknowledge the

University of London for the Maplethorpe Fellowship to A.T.. The South

African Medical Research Council (SAMRC), the South African Department

of Science and Innovation, and the South African National Research

Foundation are gratefully acknowledged for research funding (K.C. and

V.S.). F.R.P. acknowledges Sa~o Paulo Research Foundation (FAPESP) for

financial support.

CR Abubakar M, 2021, FRONT PHARMACOL, V12, DOI 10.3389/fphar.2021.640555

Adams O, 2021, STRUCTURE, V29, P1182, DOI 10.1016/j.str.2021.06.013

Ahmad S, 2018, EUR J MED CHEM, V157, P527, DOI 10.1016/j.ejmech.2018.08.002

[Anonymous], 2020, World Health Organization Meeting report of the WHO expert consultation on the definition of extensively drug-resistant tuberculosis

Bhakta S, 2016, J MED CHEM, V59, P2780, DOI 10.1021/acs.jmedchem.6b00031

Chakaya J, 2021, INT J INFECT DIS, V113, pS7, DOI 10.1016/j.ijid.2021.02.107

Daniel TM, 2006, RESP MED, V100, P1862, DOI 10.1016/j.rmed.2006.08.006

Fernandes GFD, 2017, J MED CHEM, V60, P8647, DOI 10.1021/acs.jmedchem.7b01332

Evangelopoulos D, 2015, CHEM BIOL DRUG DES, V86, P951, DOI 10.1111/cbdd.12549

Gawad Jineetkumar, 2018, Indian J Tuberc, V65, P15, DOI [10.1016/j.ijtb.2017.08.011, 10.1016/j.ijtb.2017.08.011]

Huszár S, 2020, DRUG DISCOV TODAY, V25, P772, DOI 10.1016/j.drudis.2020.02.003

Jayaprakash V., 2019, MED CHEM NEGLECTED T

Kolyva AS, 2012, UNDERSTANDING TUBERCULOSIS - NEW APPROACHES TO FIGHTING AGAINST DRUG RESISTANCE, P209

Kumari A, 2019, BIOORG CHEM, V89, DOI 10.1016/j.bioorg.2019.103021

Li M, 2020, ACS INFECT DIS, V6, P1882, DOI 10.1021/acsinfecdis.0c00121

Li M, 2017, ACS MED CHEM LETT, V8, P1165, DOI 10.1021/acsmedchemlett.7b00287

Nguyen L, 2016, ARCH TOXICOL, V90, P1585, DOI 10.1007/s00204-016-1727-6

Mabhula A, 2019, MEDCHEMCOMM, V10, P1342, DOI 10.1039/c9md00057g

Maggiora G, 2014, J MED CHEM, V57, P3186, DOI 10.1021/jm401411z

Martins Marta, 2011, Recent Pat Antiinfect Drug Discov, V6, P110

Masci D, 2019, EUR J MED CHEM, V178, P500, DOI 10.1016/j.ejmech.2019.05.087

Nyantakyi SA, 2018, J MED CHEM, V61, P5733, DOI 10.1021/acs.jmedchem.8b00777

Onajole OK, 2013, J MED CHEM, V56, P4093, DOI 10.1021/jm4003878

Palomino JC, 2002, ANTIMICROB AGENTS CH, V46, P2720, DOI 10.1128/AAC.46.8.2720-2722.2002

Santos-Lazaro D., SCI REP-UK, V11

Sharma A, 2021, J MED CHEM, V64, P4359, DOI 10.1021/acs.jmedchem.0c01833

Sheikh BA, 2021, CURR PHARM BIOTECHNO, V22, P480, DOI [10.2174/15734129mta3snzetw, 10.2174/1389201021666200628021702]

Silva DR, 2020, J BRAS PNEUMOL, V46, DOI 10.36416/1806-3756/e20200009

Stec J, 2016, J MED CHEM, V59, P6232, DOI 10.1021/acs.jmedchem.6b00415

Tan YJ, 2021, ACS MED CHEM LETT, V12, P704, DOI 10.1021/acsmedchemlett.0c00588

Thanikachalam PV, 2019, EUR J MED CHEM, V180, P562, DOI 10.1016/j.ejmech.2019.07.019

Tiberi S, 2018, LANCET INFECT DIS, V18, pE183, DOI 10.1016/S1473-3099(18)30110-5

Tiberi S, 2017, INT J INFECT DIS, V56, P181, DOI 10.1016/j.ijid.2016.10.026

Touitou M, 2020, ACS MED CHEM LETT, V11, P638, DOI 10.1021/acsmedchemlett.9b00515

World Health Organization, 2020, GLOB TUB REP

NR 35

TC 6

Z9 6

U1 2

U2 6

PU AMER CHEMICAL SOC

PI WASHINGTON

PA 1155 16TH ST, NW, WASHINGTON, DC 20036 USA

SN 1948-5875

J9 ACS MED CHEM LETT

JI ACS Med. Chem. Lett.

PD JAN 13

PY 2022

VL 13

IS 1

BP 63

EP 69

DI 10.1021/acsmedchemlett.1c00431

EA DEC 2021

PG 7

WC Chemistry, Medicinal

WE Science Citation Index Expanded (SCI-EXPANDED)

SC Pharmacology & Pharmacy

GA ZS1BF

UT WOS:000730341500001

OA Green Submitted

DA 2026-05-07

ER

PT J

AU Wang, QQ

Fu, HX

Zhang, YN

Zhang, M

Xu, J

Fu, J

AF Wang, Qingqing

Fu, Huixiao

Zhang, Yining

Zhang, Man

Xu, Jian

Fu, Jian

TI Bibliometric and Visualization Analysis of DprE1 Inhibitors to Combat

Tuberculosis

SO DRUG DESIGN DEVELOPMENT AND THERAPY

LA English

DT Article

DE DprE1 inhibitors; tuberculosis; drug target; CiteSpace; VOSviewer;

R-bibliometrix

ID KILL MYCOBACTERIUM-TUBERCULOSIS; TRENDS; IDENTIFICATION; COMBINATION;

DISCOVERY

AB Background: Tuberculosis (TB) poses a serious threat to public health, particularly owing to the increase in multidrug-resistant tuberculosis (MDR-TB) and extremely drug-resistant tuberculosis (XDR-TB); thus, there is an imperative need for novel treatments to tackle this issue. Decaprenylphosphoryl-beta-D-ribose 2 '-epimerase (DprE1) is essential for mycobacterial cell wall integrity and viability. As no relevant bibliometric study has been reported, we performed bibliometric and visual analyses to depict the knowledge framework of research related to the involvement of DprE1 in TB. Methods: Relevant studies were sourced from the Web of Science Core Collection database. VOSviewer, CiteSpace, and bibliometrics (http://bibliometric.com/) were used to construct networks based on an analysis of journals, countries, funding, institutions, authors, references, and keywords. Results: A total of 184 publications were retrieved; the total citations were 3405 times and the mean citation was 17.28 per article. The annual number of publications on DprE1 in TB has shown a significantly increasing trend. The European Journal of Medicinal Chemistry is the most published journal, with 19 articles. Lu Yu and Bin Wang contributed the most prolific authors with 18 articles. Stratified by the number of publications, India was the most prolific country that cooperated closely with the USA, UK, Japan, and United Arab Emirates. Burstness analysis of references and keywords showed that the developing research trends in this field mainly woven around "Mtb", "DprE1" and "inhibitors" during the past years. Conclusion: A systematic bibliometric study indicates that DprE1 remains a focal point in the anti-TB domain. These results can serve as a data-driven reference for future research and offer precise insights into the development of anti-TB agents associated with DprE1. To the best of our knowledge, this study is the first to comprehensively investigate DprE1 in TB by means of bibliometric analysis.

C1 [Wang, Qingqing; Zhang, Man; Xu, Jian; Fu, Jian] Guizhou Univ Tradit Chinese Med, Coll Pharm, Natl Engn Technol Res Ctr Miao Med, Guizhou Engn Technol Res Ctr Proc & Preparat Tradi, Guiyang, Peoples R China.

[Fu, Huixiao] First Peoples Hosp Guiyang, Dept Sci & Educ, Guiyang, Peoples R China.

[Zhang, Yining] Tianjin Univ Tradit Chinese Med, Coll Tradit Chinese Med, Tianjin, Peoples R China.

[Fu, Jian] Guizhou Med Univ, State Key Lab Funct & Applicat Med Plants, Guiyang, Peoples R China.

C3 Guizhou University of Traditional Chinese Medicine; Tianjin University

of Traditional Chinese Medicine; Guizhou Medical University

RP Fu, J (corresponding author), Guizhou Univ Tradit Chinese Med, Coll Pharm, Natl Engn Technol Res Ctr Miao Med, Guizhou Engn Technol Res Ctr Proc & Preparat Tradi, Guiyang, Peoples R China.; Fu, HX (corresponding author), First Peoples Hosp Guiyang, Dept Sci & Educ, Guiyang, Peoples R China.; Fu, J (corresponding author), Guizhou Med Univ, State Key Lab Funct & Applicat Med Plants, Guiyang, Peoples R China.

EM fuhuixiao@126.com; fu_jian2008@163.com

OI Zhang, Yining/0009-0005-4236-8182; Fu, Jian/0000-0002-9836-8131

FU Guizhou Province High-level Innovative Talent Training Program [[2022]

014, [2023] 037, QKHZYD [2023] 006, [2022] 022]

FX This study was supported by the Guizhou Provincial Science and

Technology Projects (QKHZC [2021] 411, QKHPTRC [2017] 5101) , the

Project of State Key Laboratory of Functions and Application Plants (No.

FAMP201806K) , the Guizhou Province High-level Innovative Talent

Training Program [2020] for J. F, GCC [2022] 014 for H. F and GCC [2023]

037 for J. X., National Engineering Technology Research Center for Miao

Medicine Improved Ability (QKHZYD [2023] 006) , Engineering Research

Center of New Dosage Form and New Preparation of Traditional Chinese

Medicine (Miao Medicine) in Higher Education Institutions of Guizhou

Province (QJJ [2022] 022) .

CR [Anonymous], 2024, Global Tuberculosis Report 2024: The second national TB inventory study in Indonesia

Aria M, 2017, J INFORMETR, V11, P959, DOI 10.1016/j.joi.2017.08.007

Balabon O, 2020, J MED CHEM, V63, P5367, DOI 10.1021/acs.jmedchem.0c00107

Baptista R, 2021, MOLECULES, V26, DOI 10.3390/molecules26020475

Batt SM, 2012, P NATL ACAD SCI USA, V109, P11354, DOI 10.1073/pnas.1205735109

Bhutani I, 2015, PLOS ONE, V10, DOI 10.1371/journal.pone.0119771

Chen CM, 2019, PLOS ONE, V14, DOI 10.1371/journal.pone.0223994

Chen CM, 2006, J AM SOC INF SCI TEC, V57, P359, DOI 10.1002/asi.20317

Chen CM, 2004, P NATL ACAD SCI USA, V101, P5303, DOI 10.1073/pnas.0307513100

Chen JF, 2024, J MULTIDISCIP HEALTH, V17, P3557, DOI 10.2147/JMDH.S467877

Chen KP, 2024, DRUG DISCOV TODAY, V29, DOI 10.1016/j.drudis.2024.103987

Chen ZP, 2024, DRUG DES DEV THER, V18, P3043, DOI 10.2147/DDDT.S459431

Chhabra S, 2021, ACS OMEGA, V6, P14430, DOI 10.1021/acsomega.1c01314

Chikhale R, 2015, EUR J MED CHEM, V96, P30, DOI 10.1016/j.ejmech.2015.04.011

Chikhale RV, 2018, J MED CHEM, V61, P8563, DOI 10.1021/acs.jmedchem.8b00281

Christophe T, 2009, PLOS PATHOG, V5, DOI 10.1371/journal.ppat.1000645

Dartois VA, 2022, NAT REV MICROBIOL, V20, P685, DOI 10.1038/s41579-022-00731-y

Dash S, 2024, SCI REP-UK, V14, DOI 10.1038/s41598-024-61901-x

Dash S, 2024, J BIOMOL STRUCT DYN, V42, P6472, DOI 10.1080/07391102.2023.2230312

Dawson R, 2023, ANTIMICROB AGENTS CH, V67, DOI 10.1128/aac.01477-22

Dheda K, 2022, LANCET RESP MED, V10, P603, DOI 10.1016/S2213-2600(22)00092-3

Diedrich CR, 2011, INFECT IMMUN, V79, P1407, DOI 10.1128/IAI.01126-10

Eckhardt E, 2023, ANTIMICROB AGENTS CH, V67, DOI 10.1128/aac.01438-22

Fu J, 2022, BIOORGAN MED CHEM, V69, DOI 10.1016/j.bmc.2022.116896

Fu J, 2021, ORG BIOMOL CHEM, V19, P1818, DOI 10.1039/d1ob00138h

Glanville J, 2020, J MED LIBR ASSOC, V108, P556, DOI 10.5195/jmla.2020.912

Gupta VK, 2017, PHARM BIOL, V55, P1536, DOI 10.1080/13880209.2017.1309440

Hariguchi N, 2020, ANTIMICROB AGENTS CH, V64, DOI 10.1128/AAC.02020-19

Hoelscher M, 2024, CLIN MICROBIOL INFEC, V30, P1131, DOI 10.1016/j.cmi.2024.06.016

Imran M, 2022, J INFECT PUBLIC HEAL, V15, P1097, DOI 10.1016/j.jiph.2022.08.016

Imran M, 2021, EXPERT OPIN THER PAT, V31, P759, DOI 10.1080/13543776.2021.1902990

Joshi H, 2021, VIRULENCE, V12, P2721, DOI 10.1080/21505594.2021.1990660

Kirby A, 2023, PUBLICATIONS, V11, DOI 10.3390/publications11010010

Koul A, 2011, NATURE, V469, P483, DOI 10.1038/nature09657

Lane TR, 2022, MOL PHARMACEUT, V19, P674, DOI 10.1021/acs.molpharmaceut.1c00791

Lechartier B, 2012, ANTIMICROB AGENTS CH, V56, P5790, DOI 10.1128/AAC.01476-12

Makarov V, 2014, EMBO MOL MED, V6, P372, DOI 10.1002/emmm.201303575

Makarov V, 2009, SCIENCE, V324, P801, DOI 10.1126/science.1171583

McKenna L, 2023, NAT MED, V29, P16, DOI 10.1038/s41591-022-02136-z

Mikusová K, 2014, CURR PHARM DESIGN, V20, P4379

Newman DJ, 2022, NATL SCI REV, V9, DOI 10.1093/nsr/nwac206

Newman DJ, 2020, J NAT PROD, V83, P770, DOI 10.1021/acs.jnatprod.9b01285

Nguyen PY, 2024, RES SYNTH METHODS, V15, P627, DOI 10.1002/jrsm.1716

Ramey ME, 2023, ANTIMICROB AGENTS CH, V67, DOI 10.1128/aac.00597-23

Riccardi G, 2013, APPL MICROBIOL BIOT, V97, P8841, DOI 10.1007/s00253-013-5218-x

Robertson GT, 2021, ANTIMICROB AGENTS CH, V65, DOI 10.1128/AAC.00583-21

Rozwarski DA, 1998, SCIENCE, V279, P98, DOI 10.1126/science.279.5347.98

Samoon R, 2024, ACS INFECT DIS, V10, P3320, DOI 10.1021/acsinfecdis.4c00415

Shi T, 2018, ANTIMICROB AGENTS CH, V62, DOI 10.1128/AAC.01314-18

Shirude PS, 2013, J MED CHEM, V56, P9701, DOI 10.1021/jm401382v

Tan YQ, 2023, FRONT PHARMACOL, V14, DOI 10.3389/fphar.2023.1237456

Trefzer C, 2010, J AM CHEM SOC, V132, P13663, DOI 10.1021/ja106357w

van Eck NJ, 2010, SCIENTOMETRICS, V84, P523, DOI 10.1007/s11192-009-0146-3

Verma A, 2022, FRONT CELL INFECT MI, V12, DOI 10.3389/fcimb.2022.1079569

Wang C, 2024, DRUG DES DEV THER, V18, P4681, DOI 10.2147/DDDT.S475205

Wang F, 2013, P NATL ACAD SCI USA, V110, pE2510, DOI 10.1073/pnas.1309171110

[王青青 Wang Qingqing], 2023, [化学通报, Chemistry], V86, P216

Xiang W, 2023, MICROBIOL SPECTR, V11, DOI 10.1128/spectrum.04721-22

Yadav S, 2023, CHEMMEDCHEM, V18, DOI 10.1002/cmdc.202300099

Zhang L, 2020, SCIENCE, V368, P1211, DOI 10.1126/science.aba9102

NR 60

TC 2

Z9 2

U1 8

U2 11

PU DOVE MEDICAL PRESS LTD

PI ALBANY

PA PO BOX 300-008, ALBANY, AUCKLAND 0752, NEW ZEALAND

SN 1177-8881

J9 DRUG DES DEV THER

JI Drug Des. Dev. Ther.

PY 2025

VL 19

BP 2577

EP 2596

DI 10.2147/DDDT.S515049

PG 20

WC Chemistry, Medicinal; Pharmacology & Pharmacy

WE Science Citation Index Expanded (SCI-EXPANDED)

SC Pharmacology & Pharmacy

GA 0ZU2Y

UT WOS:001459914500001

OA Green Submitted, gold

DA 2026-05-07

ER

PT J

AU He, SM

Fan, HY

Sun, B

Yang, MP

Liu, HX

Yang, JW

Liu, JX

Luo, SZ

Chen, ZH

Zhou, J

Xia, L

Zhang, SL

Yan, B

AF He, Shumei

Fan, Hongyan

Sun, Bin

Yang, Meipan

Liu, Hongxu

Yang, Jianwei

Liu, Jianxin

Luo, Sizhu

Chen, Zihan

Zhou, Jing

Xia, Lu

Zhang, Shulin

Yan, Bo

TI Tibetan medicine salidroside improves host anti-mycobacterial response

by boosting inflammatory cytokine production in zebrafish

SO FRONTIERS IN PHARMACOLOGY

LA English

DT Article

DE mycobacterium; salidroside; zebrafish; innate immunity; neutrophil;

macrophage; Tnfa

ID MEDIATES RESISTANCE; DISEASE RESISTANCE; IMMUNE-RESPONSE; TUBERCULOSIS;

SUSCEPTIBILITY; MYCOBACTERIA; INFECTION; INSIGHTS; MODEL

AB The treatment for tuberculosis (TB), especially multidrug-resistant TB (MDR-TB), has a prolonged cycle which can last up to a year. This is partially due to the lack of effective therapies. The development of novel anti-TB drugs from the perspective of host immune regulation can provide an important supplement for conventional treatment strategies. Salidroside (SAL), a bioactive component from the Tibetan medicine Rhodiola rosea, has been used in the treatment of TB, although its mechanism remains unclear. Here, the bacteriostatic effect of SAL in vivo was first demonstrated using a zebrafish-M. marinum infection model. To further investigate the underlying mechanism, we then examined the impact of SAL on immune cell recruitment during wound and infection. Increased macrophage and neutrophil infiltrations were found both in the vicinity of the wound and infection sites after SAL treatment compared with control, which might be due to the elevated chemokine expression levels after SAL treatment. SAL treatment alone was also demonstrated to improve the survival of infected zebrafish larvae, an effect that was amplified when combining SAL treatment with isoniazid or rifampicin. Interestingly, the reduced bacterial burden and improved survival rate under SAL treatment were compromised in tnf alpha-deficient embryos which suggests a requirement of Tnf alpha signaling on the anti-mycobacterial effects of SAL. In summary, this study provides not only the cellular and molecular mechanisms for the host anti-mycobacterial effects of the Tibetan medicine SAL but also proof of concept that combined application of SAL with traditional first-line anti-TB drugs could be a novel strategy to improve treatment efficacy.

C1 [He, Shumei; Fan, Hongyan; Yang, Meipan; Liu, Hongxu; Yang, Jianwei; Liu, Jianxin; Luo, Sizhu] Xizang Minzu Univ, Sch Med, Key Lab Mol Mechanist & Intervent Res Plateau Dis, Key Lab High Altitude Hypoxia Environm & Life Hlth, Xianyang, Peoples R China.

[Fan, Hongyan; Liu, Hongxu; Yang, Jianwei; Liu, Jianxin; Luo, Sizhu; Chen, Zihan; Zhou, Jing; Xia, Lu; Zhang, Shulin; Yan, Bo] Fudan Univ, Shanghai Publ Hlth Clin Ctr, Shanghai, Peoples R China.

[Sun, Bin] Shihezi Univ, Dept Stomatol, Affiliated Hosp 1, Med Coll, Shihezi, Peoples R China.

[Chen, Zihan; Zhou, Jing] China Three Gorges Univ, Med Coll, Yichang, Peoples R China.

[Zhang, Shulin] Shanghai Jiao Tong Univ, Dept Immunol & Microbiol, Sch Med, Shanghai, Peoples R China.

C3 Xizang Minzu University; Fudan University; Shihezi University; China

Three Gorges University; Shanghai Jiao Tong University

RP He, SM (corresponding author), Xizang Minzu Univ, Sch Med, Key Lab Mol Mechanist & Intervent Res Plateau Dis, Key Lab High Altitude Hypoxia Environm & Life Hlth, Xianyang, Peoples R China.; Zhang, SL; Yan, B (corresponding author), Fudan Univ, Shanghai Publ Hlth Clin Ctr, Shanghai, Peoples R China.; Zhang, SL (corresponding author), Shanghai Jiao Tong Univ, Dept Immunol & Microbiol, Sch Med, Shanghai, Peoples R China.

EM shumeihe2021@sina.com; shulinzhang@sjtu.edu.cn; bo.yan@shphc.org.cn

RI Chen, Zi-Han/U-1530-2019; lu, xia/LIF-7200-2024

FU National Natural Science Foundation of China; Shanghai "Science and

Technology Innovation Action Plan" Medical Innovation Research Special

Project [81801977, 81871613]; Outstanding Youth Training Program of

Shanghai Municipal Health Commission [22Y11920500]; Shanghai Sailing

Program [2018YQ54]; Natural Science Foundation of Xizang (Tibet)

Autonomous Region [18YF1420400]; [XZ2019ZR G-37(z)]

FX This work was supported by the National Natural Science Foundation of

China (81801977 to BY; 81871613 to SZ), the Shanghai "Science and

Technology Innovation Action Plan" Medical Innovation Research Special

Project (22Y11920500 to BY), the Outstanding Youth Training Program of

Shanghai Municipal Health Commission (2018YQ54 to BY), the Shanghai

Sailing Program (18YF1420400 to BY), and the Natural Science Foundation

of Xizang (Tibet) Autonomous Region [No. XZ2019ZR G-37(z)].

CR Baba E, 2018, FISH SHELLFISH IMMUN, V79, P28, DOI 10.1016/j.fsi.2018.04.063

Benard EL, 2016, ADV GENET, V95, P217, DOI 10.1016/bs.adgen.2016.04.004

Boisson-Dupuis S, 2015, IMMUNOL REV, V264, P103, DOI 10.1111/imr.12272

Choi BB, 2015, AM J CHINESE MED, V43, P167, DOI 10.1142/S0192415X15500111

Clay H, 2008, IMMUNITY, V29, P283, DOI 10.1016/j.immuni.2008.06.011

Cooper AM, 2011, MUCOSAL IMMUNOL, V4, P252, DOI 10.1038/mi.2011.13

Cortesia C, 2017, BRAZ J MICROBIOL, V48, P151, DOI 10.1016/j.bjm.2016.09.009

Cronan MR, 2014, DIS MODEL MECH, V7, P777, DOI 10.1242/dmm.016089

Dal NJK, 2022, DIS MODEL MECH, V15, DOI 10.1242/dmm.049147

Dharra R, 2017, PLOS ONE, V12, DOI 10.1371/journal.pone.0183060

Ding Shou-Yong, 2020, Biochem Biophys Res Commun, V527, P1057, DOI [10.1016/j.bbrc.2020.05.066, 10.1016/j.bbrc.2020.05.066]

Dong WY, 2021, P NATL ACAD SCI USA, V118, DOI 10.1073/pnas.2019305118

Dorhoi A, 2014, SEMIN IMMUNOL, V26, P203, DOI 10.1016/j.smim.2014.04.003

Gang Y., 2009, QINGHAI MED J

Gao H, 2020, DRUG DES DEV THER, V14, P1445, DOI 10.2147/DDDT.S242862

Gomes MC, 2020, TRENDS MICROBIOL, V28, P10, DOI 10.1016/j.tim.2019.08.005

Guan S, 2011, INT IMMUNOPHARMACOL, V11, P2194, DOI 10.1016/j.intimp.2011.09.018

Hao XZ, 2019, MOL MED REP, V19, P678, DOI 10.3892/mmr.2018.9621

Hodges HL, 2018, P NATL ACAD SCI USA, V115, P5271, DOI 10.1073/pnas.1720996115

Lee SY, 2013, EVID-BASED COMPL ALT, V2013, DOI 10.1155/2013/284150

Lee SY, 2013, EVID-BASED COMPL ALT, V2013, DOI 10.1155/2013/718739

Lu LL, 2013, AGE, V35, P637, DOI 10.1007/s11357-012-9394-x

Ma CY, 2015, J PHARMACEUT BIOMED, V105, P84, DOI 10.1016/j.jpba.2014.11.036

Mayer-Barber KD, 2014, NATURE, V511, P99, DOI 10.1038/nature13489

Myllymäki H, 2016, FRONT IMMUNOL, V7, DOI 10.3389/fimmu.2016.00196

Niu LF, 2022, FRONT IMMUNOL, V13, DOI 10.3389/fimmu.2022.893611

Niu LF, 2019, JOVE-J VIS EXP, DOI 10.3791/60698

Nunn AJ, 2019, NEW ENGL J MED, V380, P1201, DOI 10.1056/NEJMoa1811867

Qiu XC, 2014, J CANCER RES THER, V10, P8, DOI 10.4103/0973-1482.139744

Ramakrishnan L, 2020, CURR OPIN MICROBIOL, V54, P103, DOI 10.1016/j.mib.2020.01.011

Reingewertz TH, 2020, ANTIMICROB AGENTS CH, V64, DOI 10.1128/AAC.01899-19

Roach DR, 2002, J IMMUNOL, V168, P4620, DOI 10.4049/jimmunol.168.9.4620

Roca FJ, 2013, CELL, V153, P521, DOI 10.1016/j.cell.2013.03.022

Sharma N, 2016, ARCH VIROL, V161, P3331, DOI 10.1007/s00705-016-3034-1

Sommer F, 2021, DEV COMP IMMUNOL, V116, DOI 10.1016/j.dci.2020.103932

Stinear TP, 2008, GENOME RES, V18, P729, DOI 10.1101/gr.075069.107

Takaki K, 2012, CELL REP, V2, P175, DOI 10.1016/j.celrep.2012.06.008

Tobin DM, 2012, CELL, V148, P434, DOI 10.1016/j.cell.2011.12.023

Tobin DM, 2010, CELL, V140, P717, DOI 10.1016/j.cell.2010.02.013

Trede NS, 2004, IMMUNITY, V20, P367, DOI 10.1016/S1074-7613(04)00084-6

Wang TN, 2019, J GENET GENOMICS, V46, P291, DOI 10.1016/j.jgg.2019.06.002

Wiegand I, 2008, NAT PROTOC, V3, P163, DOI 10.1038/nprot.2007.521

World Health O, 2021, Global tuberculosis report 2021

Xie Lingling W.F., 2017, J MICROBES INFECT, V12, P156

Xu J, 2016, DEV CELL, V38, P214, DOI 10.1016/j.devcel.2016.06.018

Yan B, 2014, J IMMUNOL, V192, P5998, DOI 10.4049/jimmunol.1301645

Yang DW, 2016, INT J MOL MED, V38, P1864, DOI 10.3892/ijmm.2016.2781

Yang KC, 2020, FISH SHELLFISH IMMUN, V106, P1, DOI 10.1016/j.fsi.2020.07.054

Yu T, 2017, BLOOD, V129, P509, DOI 10.1182/blood-2016-07-727651

Yundangongbu Y., 1982, 4 MED CODES TIBETAN

Zemheri-Navruz F, 2019, FISH SHELLFISH IMMUN, V89, P672, DOI 10.1016/j.fsi.2019.04.037

Zhao XJ, 2013, INT IMMUNOPHARMACOL, V17, P1134, DOI 10.1016/j.intimp.2013.10.016

Zheng T, 2015, BRIT J PHARMACOL, V172, P3284, DOI 10.1111/bph.13120

NR 53

TC 5

Z9 9

U1 1

U2 25

PU FRONTIERS MEDIA SA

PI LAUSANNE

PA AVENUE DU TRIBUNAL FEDERAL 34, LAUSANNE, CH-1015, SWITZERLAND

EI 1663-9812

J9 FRONT PHARMACOL

JI Front. Pharmacol.

PD AUG 31

PY 2022

VL 13

AR 936295

DI 10.3389/fphar.2022.936295

PG 11

WC Pharmacology & Pharmacy

WE Science Citation Index Expanded (SCI-EXPANDED)

SC Pharmacology & Pharmacy

GA 4O4SU

UT WOS:000854690600001

OA Green Submitted, gold

DA 2026-05-07

ER

PT J

AU Omoteso, OA

Fadaka, AO

Walker, RB

Khamanga, SM

AF Omoteso, Omobolanle A.

Fadaka, Adewale O.

Walker, Roderick B.

Khamanga, Sandile M.

TI Innovative Strategies for Combating Multidrug-Resistant Tuberculosis:

Advances in Drug Delivery Systems and Treatment

SO MICROORGANISMS

LA English

DT Review

DE multidrug-resistant tuberculosis; drug delivery systems; nanotechnology;

drug resistance mechanisms; host-directed therapies

ID MYCOBACTERIUM-TUBERCULOSIS; ANTIBACTERIAL ACTIVITY; RETROSPECTIVE

COHORT; SILVER NANOPARTICLES; THERAPY; PHARMACOKINETICS; BEDAQUILINE;

CYCLOSERINE; INFECTION; EFFICACY

AB Multidrug-resistant tuberculosis (MDR-TB) is a significant public health challenge globally, exacerbated by the limited efficacy of existing therapeutic approaches, prolonged treatment duration, and severe side effects. As drug resistance continues to emerge, innovative drug delivery systems and treatment strategies are critical to combating this crisis. This review highlights the molecular mechanisms underlying resistance to drugs in Mycobacterium tuberculosis, such as genetic mutation, efflux pump activity, and biofilm formation, contributing to the persistence and difficulty in eradicating MDR-TB. Current treatment options, including second-line drugs, offer limited effectiveness, prompting the need for innovation of advanced therapies and drug delivery systems. The progression in drug discovery has resulted in the approval of innovative therapeutics, including bedaquiline and delamanid, amongst other promising candidates under investigation. However, overcoming the limitations of traditional drug delivery remains a significant challenge. Nanotechnology has emerged as a promising solution, with nanoparticle-based drug delivery systems offering improved bioavailability and targeted and controlled release delivery, particularly for pulmonary targeting and intracellular delivery to macrophages. Furthermore, the development of inhalable formulations and the potential of nanomedicines to bypass drug resistance mechanisms presents a novel approach to enhancing drug efficacy. Moreover, adjunctive therapies, including immune modulation and host-directed therapies, are being explored to improve treatment outcomes. Immunotherapies, such as cytokine modulation and novel TB vaccines, offer complementary strategies to the use of antibiotics in combating MDR-TB. Personalized medicine approaches, leveraging genomic profiling of both the pathogen and the host, offer promise in optimizing treatment regimens and minimizing drug resistance. This review underscores the importance of multidisciplinary approaches, combining drug discovery, advanced delivery system development, and immune modulation to address the complexities of treating MDR-TB. Continued innovation, global collaboration, and improved diagnostics are essential to developing practical, accessible, and affordable treatments for MDR-TB.

C1 [Omoteso, Omobolanle A.; Walker, Roderick B.; Khamanga, Sandile M.] Rhodes Univ, Fac Pharm, Div Pharmaceut, ZA-6139 Makhanda, South Africa.

[Fadaka, Adewale O.] Cincinnati Childrens Hosp, Med Ctr, Dept Anesthesia, Div Pain Management, Cincinnati, OH 45229 USA.

C3 Rhodes University; Cincinnati Children's Hospital Medical Center

RP Omoteso, OA (corresponding author), Rhodes Univ, Fac Pharm, Div Pharmaceut, ZA-6139 Makhanda, South Africa.

EM omobolanle.omoteso@ru.ac.za; adewale.fadaka@cchmc.org;

r.b.walker@ru.ac.za; s.khamanga@ru.ac.za

RI ; OMOTESO, OMOBOLANLE AYOYINKA/KYP-5792-2024; Fadaka,

Adewale/O-8760-2017

OI Walker, Roderick/0000-0003-2781-4154; OMOTESO, OMOBOLANLE

AYOYINKA/0000-0003-3059-7381; Khamanga, Sandile/0000-0003-2374-8460

FU Rhodes University

FX The authors R.B.W. and S.M.K. acknowledge financial support from Rhodes

University, and the author O.A.O. acknowledges funding from Rhodes

University for a Postdoctoral Fellowship. Rhodes University funded the

APC.

CR Abdelghany S, 2019, J DRUG DELIV SCI TEC, V52, P642, DOI 10.1016/j.jddst.2019.05.025

Agyeman AA, 2016, ANN CLIN MICROB ANTI, V15, DOI 10.1186/s12941-016-0156-y

Ahmed S, 2020, ANTIBIOTICS-BASEL, V9, DOI 10.3390/antibiotics9010021

Akinnawo CA, 2025, ADV THER-GERMANY, V8, DOI 10.1002/adtp.202400189

Ali A, 2015, PLOS ONE, V10, DOI 10.1371/journal.pone.0117771

Alyami MH, 2022, PHARMACEUTICALS-BASE, V15, DOI 10.3390/ph15030321

Angula KT, 2021, PHARMACEUTICALS-BASE, V14, DOI 10.3390/ph14050461

[Anonymous], 2021, Global tuberculosis report 2021

Appaiahgari MB, 2015, EXPERT OPIN BIOL TH, V15, P337, DOI 10.1517/14712598.2015.993374

Awuh JA, 2017, CELL MOL LIFE SCI, V74, P1625, DOI 10.1007/s00018-016-2422-8

Ayodele S, 2023, BIOMED PHARMACOTHER, V162, DOI 10.1016/j.biopha.2023.114588

Babel A, 2021, FRONT DIGIT HEALTH, V3, DOI 10.3389/fdgth.2021.669869

Bahlool AZ, 2022, PHARMACEUTICS, V14, DOI 10.3390/pharmaceutics14081745

Bahlool Ahmad Z, 2022, Curr Res Immunol, V3, P54, DOI [10.1016/j.crimmu.2022.03.003, 10.1016/j.crimmu.2022.03.003]

Batt J, 2019, CAN MED ASSOC J, V191, pE678, DOI 10.1503/cmaj.190081

Bekraki A. I., 2020, NANOTECHNOLOGY BASED, P107, DOI [DOI 10.1016/B978-0-12-819811-7.00007-2, 10.1016/B978-0-12-819811-7.00007-2]

Bhanu MLS, 2023, Int. J. Infect. Dis. Res., V4, P1, DOI [10.47485/2693-2326.1028, DOI 10.47485/2693-2326.1028]

Bharti R, 2022, TUBERCULOSIS, V134, DOI 10.1016/j.tube.2022.102198

Borah P, 2021, CURR TOP MED CHEM, V21, P547, DOI 10.2174/1568026621999201211200447

Braunstein M, 2019, PHARM RES-DORDR, V36, DOI 10.1007/s11095-019-2704-6

BROWNE S. G., 1962, LEPROSY REV, V33, P6

Buya AB, 2021, PHARMACEUTICS, V13, DOI 10.3390/pharmaceutics13122041

Caminero JA, 2018, ERS MONOGR, V82, P205, DOI 10.1183/2312508X.10021617

Campbell RE, 2023, KIDNEY INT REP, V8, P2211, DOI 10.1016/j.ekir.2023.08.031

Cano A, 2020, J NANOBIOTECHNOL, V18, DOI 10.1186/s12951-020-00714-2

Carranza C, 2021, FRONT IMMUNOL, V12, DOI 10.3389/fimmu.2021.760468

CDC, 2024, Clinical Overview of Latent Tuberculosis Infection

Chai QY, 2020, CELL MOL IMMUNOL, V17, P901, DOI 10.1038/s41423-020-0502-z

Chan YH, 2020, MED HYPOTHESES, V144, DOI 10.1016/j.mehy.2020.110298

Chandila S., 2023, World J Pharm Res, V12, P355, DOI DOI 10.20959/WJPR202310-28585

Chen SM, 2020, CELL SIGNAL, V70, DOI 10.1016/j.cellsig.2020.109577

Chiang CY, 2016, INT J TUBERC LUNG D, V20, P1143, DOI 10.5588/ijtld.15.0884

Chun Rene F, 2011, Expert Rev Clin Pharmacol, V4, P583, DOI 10.1586/ecp.11.41

Comas I., 2023, Genomic Approaches for Tuberculosis Management and Control: The Challenge of Tuberculosis in the 21st Century, V3rd ed.

Conradie F, 2020, NEW ENGL J MED, V382, P893, DOI 10.1056/NEJMoa1901814

D'Souza S, 2022, J Pharm Sci, V111, P469, DOI [10.1016/j.xphs.2021.09.012, 10.1016/j.xphs.2021.09.012]

Dartois VA, 2022, NAT REV MICROBIOL, V20, P685, DOI 10.1038/s41579-022-00731-y

de Chastellier C, 2009, IMMUNOBIOLOGY, V214, P526, DOI 10.1016/j.imbio.2008.12.005

de Sousa AK, 2018, MICROB PATHOGENESIS, V123, P368, DOI 10.1016/j.micpath.2018.07.040

Deretic V, 2013, NAT REV IMMUNOL, V13, P722, DOI 10.1038/nri3532

Dharmadhikari AS, 2013, ANTIMICROB AGENTS CH, V57, P2613, DOI 10.1128/AAC.02346-12

Dheda K, 2017, LANCET RESP MED, V5, P291, DOI [10.1016/s2213-2600(17)30079-6, 10.1016/S2213-2600(17)30079-6]

Dua K, 2018, BIOMED PHARMACOTHER, V107, P1218, DOI 10.1016/j.biopha.2018.08.101

Eedara BB, 2023, PHARMACEUTICS, V15, DOI 10.3390/pharmaceutics15092354

El-Ridy MS, 2015, DRUG DELIV, V22, P21, DOI 10.3109/10717544.2013.868556

EPSTZIN ISRAEL G., 1955, ANTIBIOT MED, V1, P80

Espinosa-Pereiro J, 2022, MEDICINA-LITHUANIA, V58, DOI 10.3390/medicina58020188

Farid Thariqulhaq M., 2023, JURNAL EDUHEALTH, V14, P1382, DOI [10.54209/jurnaleduhealth.v14i3.2678, 10.54209/jurnaleduhealth.v14i3.2678, DOI 10.54209/JURNALEDUHEALTH.V14I3.2678]

Fatima S, 2021, FRONT IMMUNOL, V12, DOI 10.3389/fimmu.2021.645485

Feldmann-Jensen S., 2024, Current and Emerging Trends in the Management of International Disasters

Feng S., A CRISPR-Guided Mutagenic DNA Polymerase Strategy for the Detection of Antibiotic-Resistant Mutations in M. Tuberculosis: Molecular Therapy Nucleic Acids

Fox GJ, 2024, NEW ENGL J MED, V391, P2304, DOI 10.1056/NEJMoa2314325

Gairola A, 2022, ADV THER-GERMANY, V5, DOI 10.1002/adtp.202100193

Gao C, 2016, SCI REP-UK, V6, DOI 10.1038/srep29717

Gengenbacher M, 2012, FEMS MICROBIOL REV, V36, P514, DOI 10.1111/j.1574-6976.2012.00331.x

Ghajavand H, 2019, ANTIMICROB RESIST IN, V8, DOI 10.1186/s13756-019-0516-4

Ginsberg AM, 2009, ANTIMICROB AGENTS CH, V53, P3720, DOI 10.1128/AAC.00106-09

Giraud-Gatineau A, 2020, ELIFE, V9, DOI [10.1101/758318, 10.7554/eLife.55692]

Goletti D, 2018, RESPIROLOGY, V23, P455, DOI 10.1111/resp.13272

Goodridge HS, 2009, IMMUNOL REV, V230, P38, DOI 10.1111/j.1600-065X.2009.00793.x

Gordon SB, 2014, LANCET RESP MED, V2, P823, DOI 10.1016/S2213-2600(14)70168-7

Guo H, 2021, NATURE, V589, P143, DOI 10.1038/s41586-020-3004-3

Gupta S, 2018, BIOSENS BIOELECTRON, V115, P14, DOI 10.1016/j.bios.2018.05.017

Gygli SM, 2017, FEMS MICROBIOL REV, V41, P354, DOI 10.1093/femsre/fux011

Haagsma AC, 2011, PLOS ONE, V6, DOI 10.1371/journal.pone.0023575

Hatami, 2022, J BRAS PNEUMOL, V48, pe20210384

Haver HL, 2015, ANTIMICROB AGENTS CH, V59, P5316, DOI 10.1128/AAC.00308-15

Heidary M, 2019, INFECT DRUG RESIST, V12, P3425, DOI 10.2147/IDR.S221408

Heinrich N., 2023, BTZ-043 Shows Good Safety and Strong Bactericidal Activity in a Combined Phase1b/2a Study in Tuberculosis Patients

Hernandez-Bazan S, 2022, HUM GENE THER, V33, P1037, DOI 10.1089/hum.2022.030

Hoelscher M, 2024, CLIN MICROBIOL INFEC, V30, P1131, DOI 10.1016/j.cmi.2024.06.016

Hussain A, 2019, CURR DRUG DELIV, V16, P400, DOI 10.2174/1567201816666190201144815

Hussen BM, 2024, CELL COMMUN SIGNAL, V22, DOI 10.1186/s12964-024-01713-8

Imran M, 2023, J INFECT PUBLIC HEAL, V16, P928, DOI 10.1016/j.jiph.2023.04.011

Jaeger Timo, 2007, V44, P207

Jang Jong Geol, 2020, Yeungnam Univ J Med, V37, P277, DOI [10.12701/yujm.2020.00626, 10.12701/yujm.2020.00626]

Kadura S, 2020, J ANTIMICROB CHEMOTH, V75, P2031, DOI 10.1093/jac/dkaa136

Kamaruzzaman NF, 2019, INT J MOL SCI, V20, DOI 10.3390/ijms20112747

Kang Ju-Seop, 2009, Korean Journal of Internal Medicine, V24, P1, DOI 10.3904/kjim.2009.24.1.1

Kanji Akbar, 2019, Indian J Tuberc, V66, P20, DOI [10.1016/j.ijtb.2018.07.008, 10.1016/j.ijtb.2018.07.008]

Kanji A, 2017, TUBERCULOSIS, V107, P20, DOI 10.1016/j.tube.2017.07.012

Keam SJ, 2019, DRUGS, V79, P1797, DOI 10.1007/s40265-019-01207-9

Khare G., 2019, Mycobacterium tuberculosis: Molecular Infection Biology, Pathogenesis, Diagnostics and New Interventions, P463, DOI [10.1007/978-981-32-9413-425, DOI 10.1007/978-981-32-9413-425]

Khawbung JL, 2021, COMP IMMUNOL MICROB, V74, DOI 10.1016/j.cimid.2020.101574

Khoza LJ, 2022, INT J PHARMACEUT, V622, DOI 10.1016/j.ijpharm.2022.121893

Kia P, 2023, INT J NANOMED, V18, P1159, DOI 10.2147/IJN.S364634

Kilinç G, 2021, IMMUNOL REV, V301, P62, DOI 10.1111/imr.12951

Kimmey JM, 2016, TRENDS MOL MED, V22, P1060, DOI 10.1016/j.molmed.2016.10.008

Kirtane AR, 2021, NAT NANOTECHNOL, V16, P369, DOI 10.1038/s41565-021-00866-8

Kolloli A, 2017, FRONT MED-LAUSANNE, V4, DOI 10.3389/fmed.2017.00171

Koul A, 2011, NATURE, V469, P483, DOI 10.1038/nature09657

Krah A, 2022, CURR RES STRUCT BIOL, V4, P278, DOI 10.1016/j.crstbi.2022.09.001

Kreytsberg G. N., 2011, Journal of Physics: Conference Series, V291, DOI 10.1088/1742-6596/291/1/012030

Kulkarni P, 2019, COLLOID SURFACE A, V565, P131, DOI 10.1016/j.colsurfa.2019.01.004

Kumar M, 2023, PHARMACEUTICALS-BASE, V16, DOI 10.3390/ph16101360

Kumar PV, 2006, J DRUG TARGET, V14, P546, DOI 10.1080/10611860600825159

Kumar R, 2012, CELL MICROBIOL, V14, P1620, DOI 10.1111/j.1462-5822.2012.01827.x

Lamprecht DA, 2016, NAT COMMUN, V7, DOI 10.1038/ncomms12393

Lange C, 2019, CLIN MICROBIOL INFEC, V25, P128, DOI 10.1016/j.cmi.2018.11.010

Lange C, 2020, FRONT IMMUNOL, V11, DOI 10.3389/fimmu.2020.566608

Lawn SD, 2013, LANCET INFECT DIS, V13, P349, DOI 10.1016/S1473-3099(13)70008-2

Lee CY, 2021, ANTIBIOTICS-BASEL, V10, DOI 10.3390/antibiotics10010091

Lee M, 2012, NEW ENGL J MED, V367, P1508, DOI 10.1056/NEJMoa1201964

Lee N., 2024, StatPearls

Lee SFK, 2019, CURR OPIN PULM MED, V25, P271, DOI 10.1097/MCP.0000000000000570

Lee YJ, 2018, KOREAN J INTERN MED, V33, P933, DOI 10.3904/kjim.2017.249

Li GJ, 2021, INT J NANOMED, V16, P6553, DOI 10.2147/IJN.S321631

Li GL, 2023, FRONT MICROBIOL, V14, DOI 10.3389/fmicb.2023.1257267

Li ML, 2023, NANOSCALE ADV, V5, P6278, DOI 10.1039/d3na00530e

Li Y, 2019, INFECT DRUG RESIST, V12, P721, DOI 10.2147/IDR.S195555

Liu F, 2018, NAT COMMUN, V9, DOI 10.1038/s41467-018-06836-4

Louw GE, 2009, CLIN CHEM LAB MED, V47, P500, DOI 10.1515/CCLM.2009.111

Lu J, 2014, FREE RADICAL BIO MED, V66, P75, DOI 10.1016/j.freeradbiomed.2013.07.036

Lu XY, 2019, ACS INFECT DIS, V5, P239, DOI 10.1021/acsinfecdis.8b00225

Machado D, 2012, PLOS ONE, V7, DOI 10.1371/journal.pone.0034538

Mackieh R, 2023, ANTIBIOTICS-BASEL, V12, DOI 10.3390/antibiotics12040650

Madkhali OA, 2023, POLYMERS-BASEL, V15, DOI 10.3390/polym15214327

Maison DP, 2022, J CLIN TUBER OTH MYC, V27, DOI 10.1016/j.jctube.2022.100300

Makled S, 2021, DRUG DELIV TRANSL RE, V11, P1037, DOI 10.1007/s13346-020-00815-3

Malik M.A., 2020, Combination Therapy Against Multidrug Resistance, P1, DOI DOI 10.1016/B978-0-12-820576-1.00001-1

Maphasa RE, 2021, FRONT CELL INFECT MI, V10, DOI 10.3389/fcimb.2020.618414

Maranchick NF, 2024, J CLIN TUBER OTH MYC, V36, DOI 10.1016/j.jctube.2024.100444

Marwitz F, 2024, ACS INFECT DIS, V10, P3222, DOI 10.1021/acsinfecdis.4c00192

Matteelli A, 2010, FUTURE MICROBIOL, V5, P849, DOI [10.2217/fmb.10.50, 10.2217/FMB.10.50]

Mbuagbaw L, 2019, EMERG INFECT DIS, V25, P936, DOI 10.3201/eid2505.181823

McGee B, 2009, ANTIMICROB AGENTS CH, V53, P3981, DOI 10.1128/AAC.01378-08

Mehta P, 2018, ARTIF CELL NANOMED B, V46, pS791, DOI 10.1080/21691401.2018.1513938

Mehta PP, 2020, J DRUG DELIV SCI TEC, V56, DOI 10.1016/j.jddst.2020.101509

Miotto P, 2018, RESPIROLOGY, V23, P1098, DOI 10.1111/resp.13393

Miretti M, 2019, CHEMISTRYSELECT, V4, P9726, DOI 10.1002/slct.201902039

Mitchell SL, 2018, ACS INFECT DIS, V4, P1432, DOI 10.1021/acsinfecdis.8b00138

Mittal M, 2014, ANTIOXID REDOX SIGN, V20, P1126, DOI 10.1089/ars.2012.5149

Montelongo-Peralta LZ, 2019, SCI REP-UK, V9, DOI 10.1038/s41598-019-42049-5

Mudde SE, 2022, J ANTIMICROB CHEMOTH, V77, P880, DOI 10.1093/jac/dkab505

Munoz-Bellido JL, 2000, INT J ANTIMICROB AG, V14, P177, DOI 10.1016/S0924-8579(99)00154-5

Nahid P, 2019, AM J RESP CRIT CARE, V200, pE93, DOI 10.1164/rccm.201909-1874ST

Naicker N, 2020, FRONT MICROBIOL, V11, DOI 10.3389/fmicb.2020.00435

Nainwal N, 2022, TUBERCULOSIS, V135, DOI 10.1016/j.tube.2022.102228

Nair A, 2023, J NANOBIOTECHNOL, V21, DOI 10.1186/s12951-023-02156-y

Naranjo MF, 2024, J MATER CHEM B, V12, P1558, DOI 10.1039/d3tb01800h

Nguyen TVA, 2023, INT J ANTIMICROB AG, V62, DOI 10.1016/j.ijantimicag.2023.106953

Nurkanto A, 2024, HELIYON, V10, DOI 10.1016/j.heliyon.2024.e35648

Olawade DB., 2024, Health Sciences Review, V12, P100185, DOI [10.1016/j.hsr.2024.100185, 10.1016/j.hsr.2024.100185, DOI 10.1016/J.HSR.2024.100185]

Paik S, 2019, VIRULENCE, V10, P448, DOI 10.1080/21505594.2018.1536598

Paliwal H, 2024, INT J PHARMACEUT, V655, DOI 10.1016/j.ijpharm.2024.124031

Pang X, 2019, ACS NANO, V13, P2427, DOI 10.1021/acsnano.8b09336

Parida KK, 2024, DRUG DISCOV TODAY, V29, DOI 10.1016/j.drudis.2024.104108

Patel MN, 2025, N-S ARCH PHARMACOL, V398, P2127, DOI 10.1007/s00210-024-03466-0

Pawde DM, 2020, SAUDI PHARM J, V28, P1616, DOI 10.1016/j.jsps.2020.10.008

Peloquin CA, 2021, CLIN PHARMACOL THER, V110, P1455, DOI 10.1002/cpt.2261

Portevin D, 2014, MICROBIOLOGYOPEN, V3, P823, DOI 10.1002/mbo3.193

Praphakar RA, 2018, POLYM CHEM-UK, V9, P656, DOI 10.1039/c7py02000g

Priya KS, 2024, CUREUS J MED SCIENCE, V16, DOI 10.7759/cureus.60280

Punjabi K, 2018, FRONT MICROBIOL, V9, DOI 10.3389/fmicb.2018.02207

Quesniaux VFJ, 2010, CURR DIR AUTOIMMUN, V11, P157, DOI 10.1159/000289204

Rahman K, 2022, GENOM PROTEOM BIOINF, V20, P1180, DOI 10.1016/j.gpb.2021.01.008

Ramachandran S, 2023, PHARM DEV TECHNOL, V28, P978, DOI 10.1080/10837450.2023.2279691

Ramaswamy S., 1998, Tubercle and Lung Disease, V79, P3, DOI 10.1054/tuld.1998.0002

Ramírez-Lapausa M, 2016, ENFERM INFEC MICR CL, V34, P85, DOI 10.1016/j.eimc.2015.04.003

Ramón-García S, 2009, ANTIMICROB AGENTS CH, V53, P3675, DOI 10.1128/AAC.00550-09

Ranjan R, 2025, J AEROSOL MED PULM D, V38, P149, DOI 10.1089/jamp.2024.0051

Rao M, 2019, INT J INFECT DIS, V80, pS62, DOI 10.1016/j.ijid.2019.01.039

Raviglione M, 2016, INFECT DIS REP, V8, P33, DOI 10.4081/idr.2016.6570

Rehman OU, 2024, J CLIN TUBER OTH MYC, V34, DOI 10.1016/j.jctube.2023.100405

Rudolph D, 2023, ACS NANO, V17, P9478, DOI 10.1021/acsnano.3c01664

Sabur NF, 2021, BMC INFECT DIS, V21, DOI 10.1186/s12879-021-05947-6

Sadhu P.K., 2019, World J. Pharm. Res, V8, P921

Saini S, 2023, MICROBIOL RES, V275, DOI 10.1016/j.micres.2023.127466

Santucci P, 2021, NAT COMMUN, V12, DOI 10.1038/s41467-021-24127-3

Sarkar K, 2022, J DRUG DELIV SCI TEC, V67, DOI 10.1016/j.jddst.2021.102850

Satalkar P, 2016, NANOMED-NANOTECHNOL, V12, P893, DOI 10.1016/j.nano.2015.12.376

Selim A, 2018, REV SCI TECH OIE, V37, P823, DOI 10.20506/rst.37.3.2888

Shah NS, 2017, NEW ENGL J MED, V376, P243, DOI 10.1056/NEJMoa1604544

Shao LQ, 2022, FRONT BIOENG BIOTECH, V10, DOI 10.3389/fbioe.2022.941077

Sharma S, 2021, CURR PATHOBIOL REP, V9, P133, DOI 10.1007/s40139-021-00227-z

Sheard DE, 2019, PURE APPL CHEM, V91, P199, DOI 10.1515/pac-2018-0707

Sheikhpour M, 2022, SCI REP-UK, V12, DOI 10.1038/s41598-022-13682-4

Shetty Achal, 2024, Cureus, V16, pe71697, DOI [10.7759/cureus.71697, 10.7759/cureus.71697]

Shibeshi W, 2019, BMC PHARMACOL TOXICO, V20, DOI 10.1186/s40360-019-0313-y

Singh A., 2020, NanoBioMedicine, P285, DOI DOI 10.1007/978-981-32-9898-912

Singh B, 2019, COCHRANE DB SYST REV, DOI 10.1002/14651858.CD012836.pub2

Singh M, 2022, ELECTRONICS-SWITZ, V11, DOI 10.3390/electronics11172634

Singh P, 2018, FRONT MICROBIOL, V9, DOI 10.3389/fmicb.2018.00070

Singh V, 2021, ACCOUNTS CHEM RES, V54, P2361, DOI 10.1021/acs.accounts.0c00878

Singhal A, 2014, SCI TRANSL MED, V6, DOI 10.1126/scitranslmed.3009885

Sivaramakrishnan S, 2013, BIOSENSORS-BASEL, V3, P259, DOI 10.3390/bios3030259

Song HW, 2024, FRONT PUBLIC HEALTH, V12, DOI 10.3389/fpubh.2024.1408316

Sparrow A, 2024, TROP MED INFECT DIS, V9, DOI 10.3390/tropicalmed9110280

Spirescu VA, 2021, POLYMERS-BASEL, V13, DOI 10.3390/polym13050724

Stover CK, 2000, NATURE, V405, P962, DOI 10.1038/35016103

Sun D, 2020, ACS NANO, V14, P2063, DOI 10.1021/acsnano.9b08667

Sundar S, 2010, J GLOB INFECT DIS, V2, P159, DOI 10.4103/0974-777X.62886

Sunny P.S., 2024, Ph.D. Thesis

Suri Sarabjeet Singh, 2007, J Occup Med Toxicol, V2, P16, DOI 10.1186/1745-6673-2-16

Szumowski JD, 2015, DRUG DES DEV THER, V9, P677, DOI 10.2147/DDDT.S60923

Täubel J, 2020, J CLIN PHARMACOL, V60, P400, DOI 10.1002/jcph.1534

Tiwari Divya, 2023, Semin Immunol, V65, P101672, DOI [10.1016/j.smim.2022.101672, 10.1016/j.smim.2022.101672]

Torfs E, 2019, INT J MOL SCI, V20, DOI 10.3390/ijms20122868

Tram TTB, 2023, J CLIN MICROBIOL, V61, DOI 10.1128/jcm.01634-22

Trevisi L, 2023, AM J RESP CRIT CARE, V207, P1525, DOI 10.1164/rccm.202211-2125OC

van Heeswijk RPG, 2014, J ANTIMICROB CHEMOTH, V69, P2310, DOI 10.1093/jac/dku171

van Staden D., 2020, Ph.D. Thesis

Vanino E, 2023, INT J INFECT DIS, V130, pS12, DOI 10.1016/j.ijid.2023.03.013

Vemuri N, 2016, CURR NANOSCI, V12, P110, DOI 10.2174/1573413711666150901203515

Verma N, 2022, J DRUG DELIV SCI TEC, V75, DOI 10.1016/j.jddst.2022.103690

Verma S, 2023, AAPS PHARMSCITECH, V24, DOI 10.1208/s12249-023-02585-w

Vilchèze C, 2005, ANTIMICROB AGENTS CH, V49, P708, DOI 10.1128/AAC.49.2.708-720.2005

Wadhwa R., 2020, Role of Oxidative Stress in Pathophysiology of Diseases, P107

Wallis RS, 2014, PLOS ONE, V9, DOI 10.1371/journal.pone.0094462

Wang J, 2019, INFECT DRUG RESIST, V12, P763, DOI 10.2147/IDR.S194484

WHO 2023b WHO, Publishes Information Notes on the Use of Bedaquiline and Delamanid in Children and Adolescents with Drug-Resistant Tuberculosis

Winkle M, 2021, NAT REV DRUG DISCOV, V20, P629, DOI 10.1038/s41573-021-00219-z

Winter H, 2013, ANTIMICROB AGENTS CH, V57, P5516, DOI 10.1128/AAC.00798-13

World Health Organization, 2024, TUBERCULOSIS TB

Worley MV, 2014, PHARMACOTHERAPY, V34, P1187, DOI 10.1002/phar.1482

Wu LP, 2020, MAT SCI ENG C-MATER, V106, DOI 10.1016/j.msec.2019.110302

Yadav S, 2024, IP Indian Journal of Immunology and Respiratory Medicine, V9, P85, DOI [10.18231/j.ijirm.2024.017, 10.18231/j.ijirm.2024.017, DOI 10.18231/J.IJIRM.2024.017]

Yaghubi Kalurazi Tofigh, 2021, Indian J Tuberc, V68, P195, DOI [10.1016/j.ijtb.2020.07.032, 10.1016/j.ijtb.2020.07.032]

Yan H, 2018, INFECT DRUG RESIST, V11, P945, DOI 10.2147/IDR.S154255

Yan MY, 2022, SCI ADV, V8, DOI 10.1126/sciadv.add5907

Yan MY, 2020, MBIO, V11, DOI 10.1128/mBio.02364-19

Yang XP, 2024, ADV FUNCT MATER, V34, DOI 10.1002/adfm.202402630

Yew WW, 2019, TUBERCULOSIS, V115, P76, DOI 10.1016/j.tube.2019.02.004

Young C, 2020, MUCOSAL IMMUNOL, V13, P190, DOI 10.1038/s41385-019-0226-5

Yu DW, 2021, CHEMISTRYSELECT, V6, P10682, DOI 10.1002/slct.202101973

Zhang FZ, 2024, J INFECT PUBLIC HEAL, V17, P632, DOI 10.1016/j.jiph.2024.02.012

Zhang Y, 2015, INT J TUBERC LUNG D, V19, P1276, DOI 10.5588/ijtld.15.0389

NR 227

TC 15

Z9 17

U1 3

U2 11

PU MDPI

PI BASEL

PA MDPI AG, Grosspeteranlage 5, CH-4052 BASEL, SWITZERLAND

EI 2076-2607

J9 MICROORGANISMS

JI Microorganisms

PD MAR 24

PY 2025

VL 13

IS 4

AR 722

DI 10.3390/microorganisms13040722

PG 42

WC Microbiology

WE Science Citation Index Expanded (SCI-EXPANDED)

SC Microbiology

GA 2BG9Q

UT WOS:001478554000001

OA Green Submitted, gold

DA 2026-05-07

ER

PT J

AU Song, LJ

Merceron, R

Gracia, B

Quintana, AL

Risseeuw, MDP

Hulpia, F

Cos, P

Ainsa, JA

Munier-Lehmann, H

Savvides, SN

Van Calenbergh, S

AF Song, Lijun

Merceron, Romain

Gracia, Begona

Lucia Quintana, Ainhoa

Risseeuw, Martijn D. P.

Hulpia, Fabian

Cos, Paul

Ainsa, Jose A.

Munier-Lehmann, Helene

Savvides, Savvas N.

Van Calenbergh, Serge

TI Structure Guided Lead Generation toward Nonchiral &ITM&IT.

&ITtuberculosis&IT Thymidylate Kinase Inhibitors

SO JOURNAL OF MEDICINAL CHEMISTRY

LA English

DT Article

ID DRUG-RESISTANT TUBERCULOSIS; MYCOBACTERIUM-TUBERCULOSIS; LIGAND

EFFICIENCY; EFFLUX PUMP; PYRIMIDINE; DISCOVERY; DESIGN; ASSAY

AB In recent years, thymidylate kinase (TMPK), an enzyme indispensable for bacterial DNA biosynthesis, has been pursued for the development of new antibacterial agents including against Mycobacterium tuberculosis, the causative agent for the widespread infectious disease tuberculosis (TB). In response to a growing need for more effective anti-TB drugs, we have built upon our previous efforts toward the exploration of novel and potent Mycobacterium tuberculosis TMPK (MtTMPK) inhibitors, and reported here the design of a novel series of non-nucleoside inhibitors of MtTMPK. The inhibitors display hitherto unexplored interactions in the active site of MtTMPK, offering new insights into structure-activity relationships. To investigate the discrepancy between enzyme inhibitory activity and the whole-cell activity, experiments with efflux pump inhibitors and efflux pump knockout mutants were performed. The minimum inhibitory concentrations of particular inhibitors increased significantly when determined for the efflux pump mmr knockout mutant, which partly explains the observed dissonance.

C1 [Song, Lijun; Risseeuw, Martijn D. P.; Hulpia, Fabian; Van Calenbergh, Serge] Univ Ghent, Lab Med Chem, FFW, Ottergemsesteenweg 460, B-9000 Ghent, Belgium.

[Merceron, Romain; Savvides, Savvas N.] VIB, Ctr Inflammat Res, B-9052 Ghent, Belgium.

[Merceron, Romain; Savvides, Savvas N.] Univ Ghent, Dept Biochem & Microbiol, B-9000 Ghent, Belgium.

[Gracia, Begona; Lucia Quintana, Ainhoa; Ainsa, Jose A.] Univ Zaragoza, Fac Med, Dept Microbiol, Grp Genet Micobacterias, Zaragoza, Spain.

[Gracia, Begona; Lucia Quintana, Ainhoa; Ainsa, Jose A.] Univ Zaragoza, BIFI, Zaragoza, Spain.

[Gracia, Begona; Lucia Quintana, Ainhoa; Ainsa, Jose A.] Inst Salud Carlos III, CIBER Enfermedades Resp, CIBERES, Madrid 28029, Spain.

[Cos, Paul] Univ Antwerp, Dept Pharmaceut Sci, LMPH, Campus Drie Eiken,Univ Pl 1, B-2610 Antwerp, Belgium.

[Munier-Lehmann, Helene] Inst Pasteur, CNRS, Dept Struct Biol & Chem, Unit Chem & Biocatalysis,UMR3523, 28 Rue Dr Roux, F-75724 Paris 15, France.

C3 Ghent University; Flanders Institute for Biotechnology (VIB); Ghent

University; University of Zaragoza; University of Zaragoza; CIBER -

Centro de Investigacion Biomedica en Red; CIBERES; Instituto de Salud

Carlos III; University of Antwerp; Pasteur Network; Universite Paris

Cite; Institut Pasteur Paris; Centre National de la Recherche

Scientifique (CNRS)

RP Van Calenbergh, S (corresponding author), Univ Ghent, Lab Med Chem, FFW, Ottergemsesteenweg 460, B-9000 Ghent, Belgium.

EM Serge.VanCalenbergh@UGent.be

RI Hulpia, Fabian/M-6976-2019; Risseeuw, Martijn/HLG-7261-2023; Lucia

Quintana, Ainhoa/F-1306-2016; Aínsa Claver, José Antonio/G-5635-2010;

Savvides, Savvas/E-1938-2011; Ainsa, Jose/G-5635-2010; Van Calenbergh,

Serge/A-3167-2008; cos, paul/AAU-3896-2020; Munier-Lehmann,

Hélène/Q-1867-2019; song, lijun/ABB-7049-2021

OI Hulpia, Fabian/0000-0002-7470-3484; Lucia Quintana,

Ainhoa/0000-0002-0111-4697; Aínsa Claver, José

Antonio/0000-0003-2076-844X; cos, paul/0000-0003-4361-8911;

Munier-Lehmann, Hélène/0000-0001-7579-8561;

FU Chinese scholarship council (CSC); FWO Flanders; Hercules Foundation

[AUGE-11-029]; Flanders Institute for Biotechnology (VIB)

FX L.S. thanks the financial support of the Chinese scholarship council

(CSC) for a Ph.D. scholarship. F.H. is indebted to the FWO Flanders for

a Ph.D. scholarship. The authors would also like to thank Aaron Vasseur

for his help in the synthesis part of this project. RM. and S.N.S.

gratefully acknowledge infra structural access and support at

crystallographic beamlines PX1 at SOLEIL synchrotron (Gif-sur-Yvette,

France) and P14 at PETRA3 synchrotron (Hamburg, Germany), as well as

grant support from the Hercules Foundation (AUGE-11-029) and the

Flanders Institute for Biotechnology (VIB).

CR Aínsa JA, 1998, J BACTERIOL, V180, P5836

BERNET B, 1990, CARBOHYD RES, V204, P11, DOI 10.1016/0008-6215(90)84017-O

BLONDIN C, 1994, ANAL BIOCHEM, V220, P219, DOI 10.1006/abio.1994.1326

Boinski T, 2015, J ORG CHEM, V80, P3488, DOI 10.1021/acs.joc.5b00099

Brennan PJ, 2003, TUBERCULOSIS, V83, P91, DOI 10.1016/S1472-9792(02)00089-6

Bricogne G., 2016, BUSTER VERSION 2 11

Calver AD, 2010, EMERG INFECT DIS, V16, P264, DOI 10.3201/eid1602.090968

Cambier CJ, 2014, CELL, V159, P1497, DOI 10.1016/j.cell.2014.11.024

Chalker Justin M, 2012, Angew Chem Int Ed Engl, V51, P1835, DOI 10.1002/anie.201106432

Chen VB, 2010, ACTA CRYSTALLOGR D, V66, P12, DOI 10.1107/S0907444909042073

Cole ST, 1998, NATURE, V393, P537, DOI 10.1038/31159

Dannenberg Jr A. M., 2006, PATHOGENESIS HUMAN P

Dartois V, 2014, NAT REV MICROBIOL, V12, P159, DOI 10.1038/nrmicro3200

de la Sierra IL, 2001, J MOL BIOL, V311, P87, DOI 10.1006/jmbi.2001.4843

De Rossi E, 2006, FEMS MICROBIOL REV, V30, P36, DOI 10.1111/j.1574-6976.2005.00000.x

DEFREES SA, 1988, SYNTHETIC COMMUN, V18, P213, DOI 10.1080/00397918808077347

Emsley P, 2010, ACTA CRYSTALLOGR D, V66, P486, DOI 10.1107/S0907444910007493

Fattorini Lanfranco, 2007, Ann Ist Super Sanita, V43, P317

Frick M., 2016, 2016 PIPELINE REPORT

Frieden TR, 2003, LANCET, V362, P887, DOI 10.1016/S0140-6736(03)14333-4

Gopinath VS, 2013, EUR J MED CHEM, V69, P527, DOI 10.1016/j.ejmech.2013.08.028

Haas F., 1996, TUBERCULOSIS, P3

Hoffmann H, 2016, AM J RESP CRIT CARE, V193, P337, DOI 10.1164/rccm.201502-0372LE

Hopkins AL, 2004, DRUG DISCOV TODAY, V9, P430, DOI 10.1016/S1359-6446(04)03069-7

Hopkins AL, 2014, NAT REV DRUG DISCOV, V13, P105, DOI 10.1038/nrd4163

Kabsch W, 2010, ACTA CRYSTALLOGR D, V66, P125, DOI 10.1107/S0907444909047337

Knight DW, 1998, J CHEM SOC PERK T 1, P3673, DOI 10.1039/a807313i

Kumpaty HJ, 2005, SYNTHESIS-STUTTGART, P2205, DOI 10.1055/s2005-872081

Lenaerts A, 2015, IMMUNOL REV, V264, P288, DOI 10.1111/imr.12252

Liu J, 1996, J BIOL CHEM, V271, P29545, DOI 10.1074/jbc.271.47.29545

Liu PT, 2008, CURR OPIN IMMUNOL, V20, P371, DOI 10.1016/j.coi.2008.05.014

Ludek OR, 2006, EUR J ORG CHEM, V2006, P941, DOI 10.1002/ejoc.200500801

Ma DW, 2003, ORG LETT, V5, P3799, DOI 10.1021/ol0350947

Martínez-Botella G, 2012, J MED CHEM, V55, P10010, DOI 10.1021/jm3011806

MITCHELL MA, 1990, INT J PEPT PROT RES, V36, P350

Moriarty NW, 2009, ACTA CRYSTALLOGR D, V65, P1074, DOI [10.1107/S0907444909052925, 10.1107/S0907444909029436]

Munier-Lehmann H, 2001, PROTEIN SCI, V10, P1195, DOI 10.1110/ps.45701

Naik M, 2015, J MED CHEM, V58, P753, DOI 10.1021/jm5012947

Nikaido H, 2001, SEMIN CELL DEV BIOL, V12, P215, DOI 10.1006/scdb.2000.0247

Ogasawara D, 2011, BIOORGAN MED CHEM, V19, P3702, DOI 10.1016/j.bmc.2010.12.024

Palomino JC, 2002, ANTIMICROB AGENTS CH, V46, P2720, DOI 10.1128/AAC.46.8.2720-2722.2002

Pozharski E, 2013, ACTA CRYSTALLOGR D, V69, P150, DOI 10.1107/S0907444912044423

Rodrigues L, 2013, ANTIMICROB AGENTS CH, V57, P751, DOI 10.1128/AAC.01482-12

Sagong HY, 2014, J MED CHEM, V57, P8086, DOI 10.1021/jm500958x

Singh S, 2015, J ENZYM INHIB MED CH, V30, P492, DOI 10.3109/14756366.2014.930454

Smith I, 2003, CLIN MICROBIOL REV, V16, P463, DOI 10.1128/CMR.16.3.463-496.2003

Smith NH, 2009, NAT REV MICROBIOL, V7, P537, DOI 10.1038/nrmicro2165

Song LJ, 2017, BIOORGAN MED CHEM, V25, P5889, DOI 10.1016/j.bmc.2017.08.023

Song LJ, 2016, BIOORGAN MED CHEM, V24, P5172, DOI 10.1016/j.bmc.2016.08.041

Tanaka K, 2008, Patent No. [WO 2008136324 A1, 2008136324]

Tully D., 2007, Patent No. [WO2007140117 A1, 2007140117]

Van Calenbergh S, 2012, CURR TOP MED CHEM, V12, P694, DOI 10.2174/156802612799984580

Walsh CT, 2014, J ANTIBIOT, V67, P7, DOI 10.1038/ja.2013.49

WHO, 2017, GLOB TUB REP 2017

Winn MD, 2011, ACTA CRYSTALLOGR D, V67, P235, DOI 10.1107/S0907444910045749

NR 55

TC 34

Z9 37

U1 0

U2 17

PU AMER CHEMICAL SOC

PI WASHINGTON

PA 1155 16TH ST, NW, WASHINGTON, DC 20036 USA

SN 0022-2623

EI 1520-4804

J9 J MED CHEM

JI J. Med. Chem.

PD APR 12

PY 2018

VL 61

IS 7

BP 2753

EP 2775

DI 10.1021/acs.jmedchem.7b01570

PG 23

WC Chemistry, Medicinal

WE Science Citation Index Expanded (SCI-EXPANDED); Index Chemicus (IC)

SC Pharmacology & Pharmacy

GA GD1JQ

UT WOS:000430256600009

DA 2026-05-07

ER

PT J

AU Dwivedi, M

Mukhopadhyay, S

Yadav, S

Dubey, KD

AF Dwivedi, Manish

Mukhopadhyay, Sutanu

Yadav, Shalini

Dubey, Kshatresh Dutta

TI A multidrug efflux protein in Mycobacterium tuberculosis; tap as a

potential drug target for drug repurposing

SO COMPUTERS IN BIOLOGY AND MEDICINE

LA English

DT Article

DE Mycobacterium tuberculosis; Tap; Homology modelling; MD simulations;

Molecular docking; Drug repurposing; In silico; FDA-Approved; MD

simulation

ID RESISTANCE; PREDICTION; SERVER; ACID; GENE; PUMP

AB Tuberculosis (TB) is a serious communicative disease caused by Mycobacterium tuberculosis. Although there are vaccines and drugs available to treat the disease, they are not efficient, moreover, multidrug-resistant TB (MDR TB) become a major hurdle in its therapy. These MDR strains utilize the multidrug efflux pump as a decisive weapon to fight against antitubercular drugs. Tap membrane protein was observed as a crucial multidrug efflux pump in M. tuberculosis and its critical implication in MDR-MTB development makes it an effective drug target. In the present study, we have utilized various in silico approaches to predict the applicability of FDA-approved ion channel inhibitors and blockers as therapeutic leads against Tuberculosis by targeting multidrug efflux protein; Tap in MTB. Tap protein structure is predicted by Phyre2 server followed by model refinement, validation, physio-chemical catheterization and target prediction. Further, the interaction between Tap protein and ligands were analysed by molecular docking and MD simulation run of 100 ns. Based on implication and compatibility, 18 FDA-approved ion channel inhibitors and blockers are selected as a ligand against the Tap protein and eventually observed five ligands; Glimepiride, Flecainide, Flupiritine, Nimodipine and Amlodipine as promising compounds which have exhibited the significant stable interaction with Tap protein and are proposed to modulate or interfere with its activity. These compounds illustrated the substantial docking score and total binding enthalpy more than-7 kcal/mol and-42 kcal/mol respectively which implies that the selected FDA approved compounds can spontaneously interact with the Tap protein to modulate its function. This study proposed Tap protein as a prominent drug target in MTB and investigated compounds that show considerable interaction with the Tap protein as potential therapeutic molecules. These interactions may lead to modulating or inhibit the activity of drug efflux protein thereby making MTB susceptible to antitubercular drugs.

C1 [Dwivedi, Manish] Amity Univ Uttar Pradesh, Amity Inst Biotechnol, Lucknow 226028, India.

[Mukhopadhyay, Sutanu] Ramakrishna Mission Vivekananda Centenary Coll, Dept Chem, Khardaha, West Bengal, India.

[Yadav, Shalini; Dubey, Kshatresh Dutta] Shiv Nadar Univ, Dept Chem, Greater Noida 201314, India.

C3 Shiv Nadar University

RP Dwivedi, M (corresponding author), Amity Univ Uttar Pradesh, Amity Inst Biotechnol, Lucknow 226028, India.

EM mdwivedi@lko.amity.edu

RI ; Dubey, Kshatresh/F-3097-2012; DWIVEDI, MANISH/AAI-8940-2021

OI YADAV, SHALINI/0000-0002-6176-4747; DWIVEDI, MANISH/0000-0001-5217-9039;

Mukhopadhyay, Sutanu/0000-0001-8243-2138

FU DST-INSPIRE Faculty award (2017), Govt. of India

FX MD acknowledges the DST-INSPIRE Faculty award (2017) , Govt. of India

and SM acknowledges the DST-INSPIRE SHE award (2016) by Govt. of India.

CR Abu-Saleh AA, 2020, PHYS CHEM CHEM PHYS, V22, P23099, DOI 10.1039/d0cp04326e

Aínsa JA, 1998, J BACTERIOL, V180, P5836

Andrikopoulos GK, 2015, WORLD J CARDIOL, V7, P76, DOI 10.4330/wjc.v7.i2.76

[Anonymous], 2016, Sky Journal of Biochemistry Research

[Anonymous], 2020, Tuberculosis

[Anonymous], 2022, US NATL LIB MED NAT

[Anonymous], 2017, GLOB TUB REP

Armanini D, 2005, STEROIDS, V70, P538, DOI 10.1016/j.steroids.2005.01.007

Armstrong JF, 2020, NUCLEIC ACIDS RES, V48, pD1006, DOI 10.1093/nar/gkz951

Arshia AH, 2021, COMPUT BIOL MED, V139, DOI 10.1016/j.compbiomed.2021.104967

Ashburn TT, 2004, NAT REV DRUG DISCOV, V3, P673, DOI 10.1038/nrd1468

Bajaj S, 2020, NAT PROD REP, V37, P703, DOI 10.1039/c9np00056a

Bateman A, 2019, NUCLEIC ACIDS RES, V47, pD506, DOI 10.1093/nar/gky1049

BOWIE JU, 1991, SCIENCE, V253, P164, DOI 10.1126/science.1853201

Bussi C, 2019, FEMS MICROBIOL REV, V43, P341, DOI 10.1093/femsre/fuz006

Chang KY, 2013, PLOS ONE, V8, DOI 10.1371/journal.pone.0070166

COLOVOS C, 1993, PROTEIN SCI, V2, P1511, DOI 10.1002/pro.5560020916

Combet C, 2000, TRENDS BIOCHEM SCI, V25, P147, DOI 10.1016/S0968-0004(99)01540-6

Daina A, 2019, NUCLEIC ACIDS RES, V47, pW357, DOI 10.1093/nar/gkz382

Damm KL, 2006, BIOPHYS J, V90, P4558, DOI 10.1529/biophysj.105.066654

DeLano W L., 2002, Protein Crystallogr

Devi SS, 2023, J BIOMOL STRUCT DYN, V41, P2713, DOI 10.1080/07391102.2022.2037462

Dong YW, 2018, P NATL ACAD SCI USA, V115, P1274, DOI 10.1073/pnas.1718910115

Du X, 2016, INT J MOL SCI, V17, DOI 10.3390/ijms17020144

Dubyak GR, 2004, ADV PHYSIOL EDUC, V28, P143, DOI 10.1152/advan.00046.2004

Dwivedi M, 2020, CURR CHEM BIOL, V14, P01

Fogolari F, 2003, BIOPHYS J, V85, P159, DOI 10.1016/S0006-3495(03)74462-2

Fu TT, 2022, NUCLEIC ACIDS RES, V50, pD1417, DOI 10.1093/nar/gkab1013

Fu TT, 2018, ACS CHEM NEUROSCI, V9, P1492, DOI 10.1021/acschemneuro.8b00059

Gfeller D, 2014, NUCLEIC ACIDS RES, V42, pW32, DOI 10.1093/nar/gku293

Gohlke H, 2004, J COMPUT CHEM, V25, P238, DOI [10.1002/jcc.10379, 10.1022/jcc.10379]

Gupta S, 2013, AM J RESP CRIT CARE, V188, P600, DOI 10.1164/rccm.201304-0650OC

Hariono M, 2016, SCI REP-UK, V6, DOI 10.1038/srep38692

HEYM B, 1994, LANCET, V344, P293, DOI 10.1016/S0140-6736(94)91338-2

Jomhori M, 2021, COMPUT BIOL MED, V136, DOI 10.1016/j.compbiomed.2021.104692

Jose S, 2022, J MOL STRUCT, V1254, DOI 10.1016/j.molstruc.2022.132369

Kelley LA, 2015, NAT PROTOC, V10, P845, DOI 10.1038/nprot.2015.053

Kim S, 2021, NUCLEIC ACIDS RES, V49, pD1388, DOI 10.1093/nar/gkaa971

Klyachko KA, 1998, J BACTERIOL, V180, P2817, DOI 10.1128/JB.180.11.2817-2821.1998

Ko J, 2012, NUCLEIC ACIDS RES, V40, pW294, DOI 10.1093/nar/gks493

Krzan M, 2020, BIOMOLECULES, V10, DOI 10.3390/biom10020196

LASKOWSKI RA, 1993, J APPL CRYSTALLOGR, V26, P283, DOI 10.1107/S0021889892009944

Li YH, 2018, NUCLEIC ACIDS RES, V46, pD1121, DOI 10.1093/nar/gkx1076

Liu J, 1996, J BACTERIOL, V178, P3791, DOI 10.1128/jb.178.13.3791-3795.1996

Louw GE, 2011, AM J RESP CRIT CARE, V184, P269, DOI 10.1164/rccm.201011-1924OC

LUTHY R, 1992, NATURE, V356, P83, DOI 10.1038/356083a0

Machado D, 2016, PLOS ONE, V11, DOI 10.1371/journal.pone.0149326

Martínez L, 2015, PLOS ONE, V10, DOI 10.1371/journal.pone.0119264

Morris GM, 2009, J COMPUT CHEM, V30, P2785, DOI 10.1002/jcc.21256

Morris RP, 2005, P NATL ACAD SCI USA, V102, P12200, DOI 10.1073/pnas.0505446102

Mugumbate G, 2017, FRONT PHARMACOL, V8, DOI 10.3389/fphar.2017.00681

Mujwar S, 2021, COMPUT BIOL MED, V136, DOI 10.1016/j.compbiomed.2021.104748

Mujwar S, 2020, ASSAY DRUG DEV TECHN, V18, P318, DOI 10.1089/adt.2020.976

Murugesan S, 2021, COMPUT BIOL MED, V136, DOI 10.1016/j.compbiomed.2021.104683

MUSSER JM, 1995, CLIN MICROBIOL REV, V8, P496, DOI 10.1128/CMR.8.4.496

National Center for Biotechnology Information, 3356 CID NAT CTR BIO

National Center for Biotechnology Information, 2022, PUBCHEM COMP SUMM CI

National Center for Biotechnology Information, 2022, 4497 CID NAT CTR BIO

NAYLER WG, 1991, J HUM HYPERTENS, V5, P55

NEYFAKH AA, 1991, P NATL ACAD SCI USA, V88, P4781, DOI 10.1073/pnas.88.11.4781

Nikaido H, 1996, J BACTERIOL, V178, P5853, DOI 10.1128/jb.178.20.5853-5859.1996

Ordway D, 2003, ANTIMICROB AGENTS CH, V47, P917, DOI 10.1128/AAC.47.3.917-922.2003

Pace CN, 2011, J MOL BIOL, V408, P514, DOI 10.1016/j.jmb.2011.02.053

Pasca MR, 2005, ANTIMICROB AGENTS CH, V49, P4775, DOI 10.1128/AAC.49.11.4775-4777.2005

Paulsen IT, 1996, MICROBIOL REV, V60, P575, DOI 10.1128/MMBR.60.4.575-608.1996

RAMACHANDRAN GN, 1963, J MOL BIOL, V7, P95, DOI 10.1016/S0022-2836(63)80023-6

Schrodinger L., 2020, PYMOL

Sergeev Yuri V, 2014, Curr Protoc Protein Sci, V77, DOI 10.1002/0471140864.ps2013s77

Shah K, 2019, ASSAY DRUG DEV TECHN, V17, P285, DOI 10.1089/adt.2019.943

Shetye GS, 2020, TRANSL RES, V220, P68, DOI 10.1016/j.trsl.2020.03.007

Siddiqi N, 2004, INFECTION, V32, P109, DOI 10.1007/s15010-004-3097-x

Takiff HE, 1996, P NATL ACAD SCI USA, V93, P362, DOI 10.1073/pnas.93.1.362

Telenti A, 1997, NAT MED, V3, P567, DOI 10.1038/nm0597-567

Wade R C, 1989, Prog Clin Biol Res, V289, P433

Wang XN, 2022, NUCLEIC ACIDS RES, V50, pD560, DOI 10.1093/nar/gkab926

Wang YX, 2020, NUCLEIC ACIDS RES, V48, pD1031, DOI 10.1093/nar/gkz981

Williams CJ, 2018, PROTEIN SCI, V27, P293, DOI 10.1002/pro.3330

Wishart DS, 2018, NUCLEIC ACIDS RES, V46, pD1074, DOI 10.1093/nar/gkx1037

Xue WW, 2022, ACS CHEM NEUROSCI, V13, P340, DOI 10.1021/acschemneuro.1c00694

Xue WW, 2018, ACS CHEM NEUROSCI, V9, P1128, DOI 10.1021/acschemneuro.7b00490

Xue WW, 2018, PHYS CHEM CHEM PHYS, V20, P6606, DOI 10.1039/c7cp07869b

Yin JY, 2021, NUCLEIC ACIDS RES, V49, pD1233, DOI 10.1093/nar/gkaa755

Yin JY, 2020, NUCLEIC ACIDS RES, V48, pD1042, DOI 10.1093/nar/gkz779

Zhang Y, 2019, ACS CHEM NEUROSCI, V10, P2467, DOI 10.1021/acschemneuro.8b00729

Zhou Y, 2022, NUCLEIC ACIDS RES, V50, pD1398, DOI 10.1093/nar/gkab953

NR 85

TC 10

Z9 10

U1 0

U2 8

PU PERGAMON-ELSEVIER SCIENCE LTD

PI OXFORD

PA THE BOULEVARD, LANGFORD LANE, KIDLINGTON, OXFORD OX5 1GB, ENGLAND

SN 0010-4825

EI 1879-0534

J9 COMPUT BIOL MED

JI Comput. Biol. Med.

PD JUL

PY 2022

VL 146

AR 105607

DI 10.1016/j.compbiomed.2022.105607

EA MAY 2022

PG 19

WC Biology; Computer Science, Interdisciplinary Applications; Engineering,

Biomedical; Mathematical & Computational Biology

WE Science Citation Index Expanded (SCI-EXPANDED)

SC Life Sciences & Biomedicine - Other Topics; Computer Science;

Engineering; Mathematical & Computational Biology

GA 2B4HF

UT WOS:000810150000003

DA 2026-05-07

ER

PT J

AU Zheng, F

Xie, JP

AF Zheng, Fei

Xie, Jianping

TI The Interaction Topology of Mycobacterium tuberculosis Genes

Response to Capreomycin and Novel Clues for More Drug Targets

SO JOURNAL OF CELLULAR BIOCHEMISTRY

LA English

DT Article

DE CAPREOMYCIN; TOPOLOGY; DRUG TARGETS; MYCOBACTERIUM TUBERCULOSIS

ID PHTHIOCEROL DIMYCOCEROSATE; SIGMA-FACTOR; PROTEINS; RESISTANCE;

ATTENUATION; ADAPTATION; MYCOTHIOL; VIRULENCE; VIOMYCIN; MUTATION

AB The resurgence of tuberculosis (TB) and emergence of multidrug-resistant TB (MDR-TB) are significant obstacles to stop TB treatment. Capreomycin (CPM) is regarded as an ideal second-line treatment for TB as well as for MDR-TB. However, the inexorable emergence of capreomycin resistant TB cases accentuates the urgent need for more detailed characterization of CPM targets. Most of these are single gene mutation, such as those involved in the complex formation of ribosomal 30S initiation, inhibit protein synthesis, affect 50S ribosomal protein L10, control transcription and translation of operon rpIJL-rpoBC. A new paradigm integrating gene, small metabolites, protein and underlying signaling pathway to shed light on the physiology, pathogenesis, and network of pathogen response is emerging. This model holds great promise to unravel the intricacy of drug action. However, to our knowledge, no such work regarding Mycobacterium tuberculosis response to capreomycin exposure was ever reported. We employed the data mining to construct an interaction topology of M. tuberculosis genes response to capreomycin. Most valuable genes were summarized for further experimental validation based on this topology. Dampening the virulence factors and respiratory of M. tuberculosis might be the new targets of CPM beyond Rv1364c, pe_pgrs38, pe_pgrs51 which are the salient nodes of the network and represent most promising new capreomycin targets meriting further exploration. This work will facilitate further investigation of capreomycin targets against M. tuberculosis and be conducive to novel TB drug discovery. J. Cell. Biochem. 112: 2716-2720, 2011. (C) 2011 Wiley-Liss, Inc.

C1 [Zheng, Fei; Xie, Jianping] Southwest Univ, Inst Modern Biopharmaceut, Sch Life Sci,GorgesArea 3, State Key Lab Breeding Base Eco Environm & Biores, Chongqing 400715, Peoples R China.

[Zheng, Fei; Xie, Jianping] Southwest Univ, Bioresource Three GorgesArea, Chongqing 400715, Peoples R China.

C3 Southwest University - China; Southwest University - China

RP Xie, JP (corresponding author), Southwest Univ, Inst Modern Biopharmaceut, Sch Life Sci,GorgesArea 3, State Key Lab Breeding Base Eco Environm & Biores, Chongqing 400715, Peoples R China.

EM jianpingxiefudan@gmail.com

RI xu, li/AAO-4684-2021

FU National Key Infectious Disease Project [2008ZX10003-006,

2008ZX10003-001]; Fundamental Research Funds for the Central

Universities [XDJK2009A003]; Natural Science Foundation of CQ CSTC

[2010BB5002]; National Natural Science Foundation of China [81071316];

National Major Infectious Diseases Key Project [2008ZX10003-006,

2008ZX10003-001]; Southwest University [kh2009010, 2009009]

FX Grant sponsor: National Key Infectious Disease Project; Grant numbers:

2008ZX10003-006, 2008ZX10003-001; Grant sponsor: Fundamental Research

Funds for the Central Universities; Grant number: XDJK2009A003; Grant

sponsor: Natural Science Foundation Project of CQ CSTC; Grant number:

2010BB5002; Grant sponsor: National Natural Science Foundation of China;

Grant number: 81071316. This work is supported by National Major

Infectious Diseases Key Project (nos. 2008ZX10003-006 and

2008ZX10003-001), Excellence PhD Thesis Fellowship of Southwest

University (nos. kh2009010 and 2009009), Fundamental Research Funds for

Central Universities (no. XDJK2009A003), and National Natural Science

Foundation of China (grant no. 81071316).

CR Alam MS, 2009, FEBS J, V276, P76, DOI 10.1111/j.1742-4658.2008.06755.x

Bentrup KHZ, 2001, TRENDS MICROBIOL, V9, P597, DOI 10.1016/S0966-842X(01)02238-7

Buchmeier NA, 2003, MOL MICROBIOL, V47, P1723, DOI 10.1046/j.1365-2958.2003.03416.x

Camacho LR, 2001, J BIOL CHEM, V276, P19845, DOI 10.1074/jbc.M100662200

Chautard E, 2009, PATHOL BIOL, V57, P324, DOI 10.1016/j.patbio.2008.10.004

Fu Li M, 2009, Int J Microbiol, V2009, P879621, DOI 10.1155/2009/879621

Garcia-Contreras L, 2007, ANTIMICROB AGENTS CH, V51, P2830, DOI 10.1128/AAC.01164-06

Geiman DE, 2004, INFECT IMMUN, V72, P1733, DOI 10.1128/IAI.72.3.1733-1745.2004

Gomez JE, 2000, P NATL ACAD SCI USA, V97, P8554, DOI 10.1073/pnas.140225297

Gordillo S, 2006, SCAND J IMMUNOL, V64, P30, DOI 10.1111/j.1365-3083.2006.01765.x

GOREN MB, 1974, INFECT IMMUN, V9, P150, DOI 10.1128/IAI.9.1.150-158.1974

Goulding CW, 2004, J BIOL CHEM, V279, P3516, DOI 10.1074/jbc.M311833200

Gregory ST, 2005, J BACTERIOL, V187, P2200, DOI 10.1128/JB.187.6.2200-2202.2005

Hahn MY, 2005, J BACTERIOL, V187, P7062, DOI 10.1128/JB.187.20.7062-7071.2005

HOLMES AM, 1970, THORAX, V25, P608, DOI 10.1136/thx.25.5.608

Lee DS, 2008, P NATL ACAD SCI USA, V105, P9880, DOI 10.1073/pnas.0802208105

LI M, 2007, INFECTION, V54, P277

Manganelli R, 2004, J BACTERIOL, V186, P895, DOI 10.1128/JB.186.4.895-902.2004

Maus CE, 2005, ANTIMICROB AGENTS CH, V49, P571, DOI 10.1128/AAC.49.2.571-577.2005

MCCLATCHY JK, 1977, TUBERCLE, V58, P29, DOI 10.1016/S0041-3879(77)80007-X

MODOLELL J, 1977, EUR J BIOCHEM, V81, P491, DOI 10.1111/j.1432-1033.1977.tb11974.x

Newton GL, 2000, BIOCHEMISTRY-US, V39, P10739, DOI 10.1021/bi000356n

Parida BK, 2005, TUBERCULOSIS, V85, P347, DOI 10.1016/j.tube.2005.08.001

Pinto R, 2004, MICROBIOL-SGM, V150, P1681, DOI 10.1099/mic.0.26894-0

Scandurra GM, 2007, MICROBES INFECT, V9, P87, DOI 10.1016/j.micinf.2006.10.013

Shin SY, 2004, CHEST, V125, P974, DOI 10.1378/chest.125.3.974

Singh KK, 2001, INFECT IMMUN, V69, P4185, DOI 10.1128/IAI.69.6.4185-4191.2001

Singh PP, 2008, MICROBIOL-SGM, V154, P3469, DOI 10.1099/mic.0.2008/019968-0

Smeulders MJ, 1999, J BACTERIOL, V181, P270, DOI 10.1128/JB.181.1.270-283.1999

Sone N, 2003, BBA-BIOENERGETICS, V1557, P125, DOI 10.1016/S0005-2728(02)00394-8

Williams EP, 2007, J BACTERIOL, V189, P4234, DOI 10.1128/JB.00201-07

NR 31

TC 3

Z9 3

U1 0

U2 10

PU WILEY-BLACKWELL

PI MALDEN

PA COMMERCE PLACE, 350 MAIN ST, MALDEN 02148, MA USA

SN 0730-2312

J9 J CELL BIOCHEM

JI J. Cell. Biochem.

PD OCT

PY 2011

VL 112

IS 10

BP 2716

EP 2720

DI 10.1002/jcb.23232

PG 5

WC Biochemistry & Molecular Biology; Cell Biology

WE Science Citation Index Expanded (SCI-EXPANDED)

SC Biochemistry & Molecular Biology; Cell Biology

GA 829YP

UT WOS:000295623300009

DA 2026-05-07

ER

PT J

AU Resendiz-Galvan, JE

Arora, PR

Abdelwahab, MT

Udwadia, ZF

Rodrigues, C

Gupta, A

Denti, P

Ashavaid, TF

Tornheim, JA

AF Resendiz-Galvan, Juan Eduardo

Arora, Prerna R.

Abdelwahab, Mahmoud Tareq

Udwadia, Zarir F.

Rodrigues, Camilla

Gupta, Amita

Denti, Paolo

Ashavaid, Tester F.

Tornheim, Jeffrey A.

TI Pharmacokinetic analysis of linezolid for multidrug resistant

tuberculosis at a tertiary care centre in Mumbai, India

SO FRONTIERS IN PHARMACOLOGY

LA English

DT Article

DE MDR-TB (multidrug resistant-TB); linezolid (LZD); pharmacokinetics;

NONMEM modelling; pharmacometrics

ID POPULATION PHARMACOKINETICS; HEALTHY-VOLUNTEERS; MODEL; RIFAMPIN;

MECHANISM; REGIMENS

AB Linezolid is an oxazolidinone used to treat multidrug-resistant tuberculosis (MDR-TB), including in the recently-endorsed shorter 6-month treatment regimens. Due to its narrow therapeutic index, linezolid is often either dose-adjusted or discontinued due to intolerance or toxicity during treatment, and the optimal balance between linezolid efficacy and toxicity remains unclear. India carries a significant burden of MDR-TB cases in the world, but limited information on the pharmacokinetics of linezolid and minimum inhibitory concentration (MIC) distribution is available from Indian MDR-TB patients. We enrolled participants from a tertiary care centre in Mumbai, India, treated for MDR-TB and receiving linezolid daily doses of 600 or 300 mg. Pharmacokinetic visits were scheduled between 1 and 15 months after treatment initiation to undergo intensive or sparse blood sampling. Linezolid concentration versus time data were analysed using non-linear mixed-effects modelling, with simulations to evaluate doses for different scenarios. We enrolled 183 participants (121 females), with a median age of 26 years (interquartile range [IQR] 21-35), weight 55.0 kg (IQR 45.6-65.8), and fat-free mass 38.7 kg (IQR 32.7-46.0). Linezolid pharmacokinetics was best described by a one-compartment model with first-order elimination allometrically scaled by fat-free mass and transit compartment absorption. The typical clearance value was 3.81 L/h. Simulations predicted that treatment with 300 mg daily achieves a high probability of target attainment (PTA) when linezolid MIC was & LE;0.25 mg/L (61.5% of participant samples tested), while 600 mg daily would be required if MIC were 0.5 mg/L (29% of samples). While linezolid 300 mg daily is predicted to achieve effective targets for the majority of adults with MDR-TB, it failed to achieve the therapeutic target for 21% participants. A dose of 600 mg had a PTA > 90% for all susceptible samples, but with a higher likelihood of exceeding toxicity thresholds (31% vs 9.6%). These data suggest potential benefit to individualized dosing taking host and microbial characteristics into account to improve the likelihood of treatment efficacy while minimizing risk of toxicity from linezolid for the treatment of MDR-TB. Further prospective evaluation in different clinical settings is urgently needed to inform safety and efficacy of these lower doses.

C1 [Resendiz-Galvan, Juan Eduardo; Abdelwahab, Mahmoud Tareq; Denti, Paolo] Univ Cape Town, Dept Med, Div Clin Pharmacol, Cape Town, South Africa.

[Arora, Prerna R.; Rodrigues, Camilla; Ashavaid, Tester F.] PD Hinduja Natl Hosp & Med Res Ctr, Res Labs, Mumbai, India.

[Udwadia, Zarir F.] PD Hinduja Natl Hosp & Med Res Ctr, Div Resp Med, Mumbai, India.

[Gupta, Amita; Tornheim, Jeffrey A.] Johns Hopkins Univ, Ctr Infect Dis India, Div Infect Dis, Sch Med, Baltimore, MD USA.

[Gupta, Amita; Tornheim, Jeffrey A.] Johns Hopkins Univ, Ctr TB Res, Div Infect Dis, Sch Med, Baltimore, MD USA.

[Gupta, Amita; Tornheim, Jeffrey A.] Johns Hopkins Univ, Johns Hopkins Bloomberg Sch Publ Hlth, Baltimore, MD USA.

C3 University of Cape Town; Johns Hopkins University; Johns Hopkins

University; Johns Hopkins University; Johns Hopkins Bloomberg School of

Public Health

RP Ashavaid, TF (corresponding author), PD Hinduja Natl Hosp & Med Res Ctr, Res Labs, Mumbai, India.

EM dr_tashavaid@hindujahospital.com

RI ; Denti, Paolo/H-6393-2015; Ashavaid, Tester/AGS-6003-2022; Rodrigues,

Cecilia/M-3572-2013; Tornheim, Jeffrey/AFJ-9808-2022

OI Rodrigues, Camilla/0000-0002-6105-6660; Denti,

Paolo/0000-0001-7494-079X; Udwadia, Zarir/0000-0002-1830-3259; Ashavaid,

Tester Framroze/0000-0002-2740-0819; Resendiz-Galvan, Juan

Eduardo/0000-0001-9111-8052;

FU P. D. Hinduja Hospital and Medical Research Centre (PDHNH); NIH/DBT

RePORT India; Government of India's (GOI) Department of Biotechnology

(DBT), Department of Science and Technology (DST); United States

National Institutes of Health (NIH); National Institute of Allergy and

Infectious Diseases (NIAID),; Office of AIDS Research (OAR),; DBT India;

DST India [BT/PR24492/MED/29/1219/2017, DST/INT/SOUTH AFRICA/P-24/2017];

DBT-South African Medical Research Council (MRC) Indo-South Africa;

NIH/NIAID [K23AI135102, R21AI122922, R01AI134430]; NIH/Fogarty Global

Health Fellows Program Consortium [R25TW009340]; NIH/Fogarty

International Center [D43TW010559]

FX This publication was made possible by support from the P. D. Hinduja

Hospital and Medical Research Centre (PDHNH) (established and managed by

National Health and Education Society) and the NIH/DBT RePORT India

Consortium with funding in whole or in part from the Government of

India's (GOI) Department of Biotechnology (DBT), Department of Science

and Technology (DST), the United States National Institutes of Health

(NIH), National Institute of Allergy and Infectious Diseases (NIAID),

Office of AIDS Research (OAR), and distributed in part by CRDF Global.

Participant recruitment and retention for the primary cohort was

supported by the National Health and Education Society (P. D. Hinduja

Hospital and MRC, Mumbai, India). Sample processing and pharmacokinetic

analysis was supported by the DBT India and DST India

(BT/PR24492/MED/29/1219/2017 and DST/INT/SOUTH AFRICA/P-24/2017 to TA),

a joint DBT-South African Medical Research Council (MRC) Indo-South

Africa collaboration. JT and associated MIC work was supported by

NIH/NIAID (K23AI135102, R21AI122922, and R01AI134430), and the

NIH/Fogarty Global Health Fellows Program Consortium (R25TW009340). MA

was supported by the NIH/Fogarty International Center (D43TW010559).

CR Abdelwahab MT, 2021, ANTIMICROB AGENTS CH, V65, DOI 10.1128/AAC.01381-21

Alghamdi WA, 2020, ANTIMICROB AGENTS CH, V64, DOI 10.1128/AAC.01174-20

Anderson BJ, 2008, ANNU REV PHARMACOL, V48, P303, DOI 10.1146/annurev.pharmtox.48.113006.094708

Beal S. L., 2013, NONMEM USERS GUIDES

Beal SL, 2001, J PHARMACOKINET PHAR, V28, P481, DOI 10.1023/A:1012299115260

Bharadwaj R, 2021, INDIAN J MED RES, V154, P16, DOI 10.4103/ijmr.IJMR_3537_20

Bolhuis MS, 2018, CLIN INFECT DIS, V67, pS327, DOI 10.1093/cid/ciy625

Chigutsa E, 2011, ANTIMICROB AGENTS CH, V55, P4122, DOI 10.1128/AAC.01833-10

Chirehwa MT, 2019, J ANTIMICROB CHEMOTH, V74, P139, DOI 10.1093/jac/dky378

COCKCROFT DW, 1976, NEPHRON, V16, P31, DOI 10.1159/000180580

Conradie F, 2022, NEW ENGL J MED, V387, P810, DOI 10.1056/NEJMoa2119430

Conradie F, 2020, NEW ENGL J MED, V382, P893, DOI 10.1056/NEJMoa1901814

Diacon AH, 2007, ANTIMICROB AGENTS CH, V51, P2994, DOI 10.1128/AAC.01474-06

Dosne AG, 2017, J PHARMACOKINET PHAR, V44, P509, DOI 10.1007/s10928-017-9542-0

Du J, 2021, FRONT MICROBIOL, V12, DOI 10.3389/fmicb.2021.655653

Garcia-Prats AJ, 2019, PLOS MED, V16, DOI 10.1371/journal.pmed.1002789

Imperial MZ, 2022, CLIN INFECT DIS, V74, P1736, DOI 10.1093/cid/ciab699

Jonsson EN, 1999, COMPUT METH PROG BIO, V58, P51, DOI 10.1016/S0169-2607(98)00067-4

KARLSSON MO, 1993, J PHARMACOKINET BIOP, V21, P735, DOI 10.1007/BF01113502

Keizer RJ, 2013, CPT-PHARMACOMET SYST, V2, DOI 10.1038/psp.2013.24

Keizer RJ, 2015, PHARMACOL RES PERSPE, V3, DOI 10.1002/prp2.131

Livermore DM, 2003, J ANTIMICROB CHEMOTH, V51, P9, DOI 10.1093/jac/dkg249

Maartens G, 2015, EBIOMEDICINE, V2, P1568, DOI 10.1016/j.ebiom.2015.10.014

MacGowan AP, 2003, J ANTIMICROB CHEMOTH, V51, P17, DOI 10.1093/jac/dkg248

McIlleron H, 2012, ANTIMICROB AGENTS CH, V56, P3232, DOI 10.1128/AAC.05526-11

Millard J, 2018, J ANTIMICROB CHEMOTH, V73, P1755, DOI 10.1093/jac/dky096

Ministry of Health Family Welfare Government of India, 2021, GUID PROGR MAN TB PR

Mockeliunas L, 2022, PHARMACEUTICS, V14, DOI 10.3390/pharmaceutics14040753

Obach RS, 2022, DRUG METAB DISPOS, V50, P413, DOI 10.1124/dmd.121.000776

Padmapriyadarsini C, 2023, CLIN INFECT DIS, V76, pE938, DOI 10.1093/cid/ciac528

Pepper DJ, 2010, BMC INFECT DIS, V10, DOI 10.1186/1471-2334-10-83

Plock N, 2007, DRUG METAB DISPOS, V35, P1816, DOI 10.1124/dmd.106.013755

Qin Y, 2022, BRIT J CLIN PHARMACO, V88, P4043, DOI 10.1111/bcp.15368

Savic RM, 2007, J PHARMACOKINET PHAR, V34, P711, DOI 10.1007/s10928-007-9066-0

Schecter GF, 2010, CLIN INFECT DIS, V50, P49, DOI 10.1086/648675

Smythe W. A., 2016, THESIS U CAPE TOWN C

Song T, 2015, EBIOMEDICINE, V2, P1627, DOI 10.1016/j.ebiom.2015.09.051

Sprent P., 2011, International Encyclopedia of Statistical Science, DOI DOI 10.1007/978-3-642-04898-2_253

Stalker DJ, 2003, CLIN PHARMACOKINET, V42, P1129, DOI 10.2165/00003088-200342130-00004

Tietjen AK, 2022, BRIT J CLIN PHARMACO, V88, P1835, DOI 10.1111/bcp.15102

Tornheim JA, 2020, J CLIN TUBER OTH MYC, V20, DOI 10.1016/j.jctube.2020.100175

Tornheim J, 2017, EUR RESPIR J, V50, DOI 10.1183/1393003.congress-2017.PA3486

Tornheim JA, 2022, OPEN FORUM INFECT DI, V9, DOI 10.1093/ofid/ofab615

Udwadia ZF, 2019, BMC INFECT DIS, V19, DOI 10.1186/s12879-019-3726-8

Wasserman S, 2019, J ANTIMICROB CHEMOTH, V74, P2377, DOI 10.1093/jac/dkz206

Wasserman S, 2019, ANTIMICROB AGENTS CH, V63, DOI 10.1128/AAC.02164-18

Wasserman S, 2016, EXPERT REV ANTI-INFE, V14, P901, DOI 10.1080/14787210.2016.1225498

*WHO, 2020, GLOBAL TUBERCULOSIS

Wilkins JJ, 2008, ANTIMICROB AGENTS CH, V52, P2138, DOI 10.1128/AAC.00461-07

World Health Organization, 2018, TECHN REP CRIT CONC

World Health Organization, 2022, RAPID COMMUNICATION

World Health Organization, 2020, WHO CONS GUID TUB MO, P98

Zhou WQ, 2022, INT J ANTIMICROB AG, V59, DOI 10.1016/j.ijantimicag.2022.106589

NR 53

TC 6

Z9 7

U1 1

U2 8

PU FRONTIERS MEDIA SA

PI LAUSANNE

PA AVENUE DU TRIBUNAL FEDERAL 34, LAUSANNE, CH-1015, SWITZERLAND

EI 1663-9812

J9 FRONT PHARMACOL

JI Front. Pharmacol.

PD JAN 4

PY 2023

VL 13

AR 1081123

DI 10.3389/fphar.2022.1081123

PG 10

WC Pharmacology & Pharmacy

WE Science Citation Index Expanded (SCI-EXPANDED)

SC Pharmacology & Pharmacy

GA 8B8OP

UT WOS:000917180800001

OA Green Submitted, gold

DA 2026-05-07

ER

PT J

AU Zwerling, A

Dowdy, D

von Delft, A

Taylor, H

Merritt, MW

AF Zwerling, A.

Dowdy, D.

von Delft, A.

Taylor, H.

Merritt, M. W.

TI Incorporating social justice and stigma in cost-effectiveness analysis:

drug-resistant tuberculosis treatment

SO INTERNATIONAL JOURNAL OF TUBERCULOSIS AND LUNG DISEASE

LA English

DT Article

DE stigma; human rights; economic evaluation; ethics; MDR-TB

ID TB-RELATED STIGMA; HEALTH; DETERMINANTS; BANGLADESH

AB Novel therapies for multidrug-resistant tuberculosis (MDR-TB) are likely to be expensive. The cost of novel drugs (e.g., bedaquiline, delamanid) may be so prohibitively high that a traditional cost-effectiveness analysis (CEA) would rate regimens containing these drugs as not cost-effective. Traditional CEA may not appropriately account for considerations of social justice, and may put the most disadvantaged populations at greater risk. Using the example of novel drug regimens for MDR-TB, we propose a novel methodology, 'justice-enhanced CEA', and demonstrate how such an approach can simultaneously assess social justice impacts alongside traditional cost-effectiveness ratios. Justice-enhanced CEA, as we envision it, is performed in three steps: 1) systematic data collection about patients' lived experiences, 2) use of empirical findings to inform social justice assessments, and 3) incorporation of data-informed social justice assessments into a decision analytic framework that includes traditional CEA. These components are organized around a core framework of social justice developed by Bailey et al. to compare impacts on disadvantage not otherwise captured by CEA. Formal social justice assessments can produce three composite levels: 'expected not to worsen...', 'may worsen...', and 'expected to worsen clustering of disadvantage'. Levels of social justice impact would be assessed for each major type of outcome under each policy scenario compared. Social justice assessments are then overlaid side-by-side with cost-effectiveness assessments corresponding to each branch pathway on the decision tree. In conclusion, we present a 'justiceenhanced' framework that enables the incorporation of social justice concerns into traditional CEA for the evaluation of new regimens for MDR-TB.

C1 [Zwerling, A.; Dowdy, D.; Taylor, H.; Merritt, M. W.] Johns Hopkins Bloomberg Sch Publ Hlth, Baltimore, MD USA.

[Zwerling, A.] Univ Ottawa, Sch Epidemiol & Publ Hlth, Room 301E,600 Peter Morand Crescent, Ottawa, ON K1G 5Z3, Canada.

[von Delft, A.] TB Proof, Cape Town, South Africa.

[von Delft, A.] Univ Cape Town, Sch Publ Hlth & Family Med, Fac Hlth Sci, Cape Town, South Africa.

[Taylor, H.; Merritt, M. W.] Johns Hopkins Berman Inst Bioeth, Baltimore, MD USA.

C3 Johns Hopkins University; Johns Hopkins Bloomberg School of Public

Health; University of Ottawa; University of Cape Town; Johns Hopkins

University

RP Zwerling, A (corresponding author), Univ Ottawa, Sch Epidemiol & Publ Hlth, Room 301E,600 Peter Morand Crescent, Ottawa, ON K1G 5Z3, Canada.

EM azwerlin@uottawa.ca

OI von Delft, Arne/0000-0002-8785-9672

FU US National Institutes of Health, Bethesda, MD, USA [1R56AI114458-01]

FX Financial support for this study was provided entirely by a grant

awarded to MWM by the US National Institutes of Health, Bethesda, MD,

USA (1R56AI114458-01). The funding agreement ensured the authors'

independence in designing the study, interpreting the data, writing, and

publishing the report. The authors are grateful to two anonymous journal

reviewers for valuable comments that enabled us to improve the

manuscript.

CR [Anonymous], 2002, Valuing freedoms: Sen's capability approach and poverty reduction

[Anonymous], 2010, WHO/HTM/TB/2010.3

Aung KJM, 2014, INT J TUBERC LUNG D, V18, P1180, DOI 10.5588/ijtld.14.0100

Bailey TC, 2015, AM J PUBLIC HEALTH, V105, P629, DOI 10.2105/AJPH.2014.302454

Baral SC, 2007, BMC PUBLIC HEALTH, V7, DOI 10.1186/1471-2458-7-211

Beauchamp TL., 2013, PRINCIPLES BIOMEDICA, VSeventh

Berezin M, 2016, SOC SCI MED, V165, P201, DOI 10.1016/j.socscimed.2016.07.040

Brock DW., 2017, COST EFFECTIVENESS H

Brock DW., 2004, Public Health, Ethics, and Equity

Clair M, 2016, SOC SCI MED, V165, P223, DOI 10.1016/j.socscimed.2016.03.021

Coreil J, 2010, SOC SCI MED, V71, P1409, DOI 10.1016/j.socscimed.2010.07.017

Craig GM, 2017, INT J INFECT DIS, V56, P90, DOI 10.1016/j.ijid.2016.10.011

Crocker DA, 2008, ETHICS OF GLOBAL DEVELOPMENT: AGENCY, CAPABILITY, AND DELIBERATIVE DEMOCRACY, P1, DOI 10.1017/CBO9780511492594

Daniels Norman., 2008, JUST HLTH M HLTH NEE

Diacon AH, 2012, LANCET, V380, P986, DOI 10.1016/S0140-6736(12)61080-0

Diacon AH, 2009, NEW ENGL J MED, V360, P2397, DOI 10.1056/NEJMoa0808427

Drummond M.F., 2005, Methods for the Economic Evaluation of Health Care Programmes

Faden R., 2010, The Stanford Encyclopedia of Philosophy

Hargreaves JR, 2011, AM J PUBLIC HEALTH, V101, P654, DOI 10.2105/AJPH.2010.199505

Lessells RJ, 2013, THORAX, V68, P730, DOI 10.1136/thoraxjnl-2012-202520

Lessem E., 2014, ACTIVISTS GUIDE DELA

Lönnroth K, 2009, SOC SCI MED, V68, P2240, DOI 10.1016/j.socscimed.2009.03.041

Nussbaum M., 2011, Creating Capabilties: The human development approach

Powers M., 2006, Social justice: The moral foundations of public health and health policy

Rawls J., 2001, Justice as Fairness-A Restatement

Rawls John., 1999, A Theory of justice, DOI [10.2307/j.ctvjf9z6v, DOI 10.4159/9780674042605]

Ruger JenniferPrah., 2010, HLTH SOCIAL JUSTICE

Sen A., 1995, Inequality Reexamined

Sen A.K., 1999, Development as Freedom

Shin SS, 2007, INT J TUBERC LUNG D, V11, P1314

Somma D, 2008, INT J TUBERC LUNG D, V12, P856

TBAlliance, PORTF CLIN DEV

United Nations Committee on Economic Social and Cultural Rights, 1990, NAT STAT PART OBL

Vassall A., 2013, COST -EFFECTIVENESS OF INTRODUCING BEDAQUILINE IN MDR-TB REGIMENS - A EXPLORATORY ANALYSIS

VENKATAPURAM S., 2013, HLTH JUSTICE ARGUMEN

Weiss Mitchell G, 2006, Psychol Health Med, V11, P277

WHO, 2013, USE BEDAQUILINE TREA, P57

Wolff J., 2007, DISADVANTAGE

Zumla A, 2002, CURR OPIN PULM MED, V8, P166, DOI 10.1097/00063198-200205000-00003

NR 39

TC 17

Z9 20

U1 0

U2 10

PU INT UNION AGAINST TUBERCULOSIS LUNG DISEASE (I U A T L D)

PI PARIS

PA 68 BOULEVARD SAINT-MICHEL,, 75006 PARIS, FRANCE

SN 1027-3719

EI 1815-7920

J9 INT J TUBERC LUNG D

JI Int. J. Tuberc. Lung Dis.

PD NOV

PY 2017

VL 21

IS 11

SU 1

BP S69

EP +

DI 10.5588/ijtld.16.0839

PG 7

WC Infectious Diseases; Respiratory System

WE Science Citation Index Expanded (SCI-EXPANDED); Social Science Citation Index (SSCI)

SC Infectious Diseases; Respiratory System

GA FL2EG

UT WOS:000414027000011

OA Green Submitted

DA 2026-05-07

ER

PT J

AU Morandi, M

Sali, M

Manganelli, R

Delogu, G

AF Morandi, Matteo

Sali, Michela

Manganelli, Riccardo

Delogu, Giovanni

TI Exploiting the mycobacterial cell wall to design improved vaccines

against tuberculosis

SO JOURNAL OF INFECTION IN DEVELOPING COUNTRIES

LA English

DT Review

DE tuberculosis; vaccine; BCG; cell wall; HBHA

ID HEPARIN-BINDING-HEMAGGLUTININ; BACILLUS-CALMETTE-GUERIN; DRUG-RESISTANT

TUBERCULOSIS; PE-PGRS PROTEINS; PROTECTIVE IMMUNITY; 19-KDA LIPOPROTEIN;

T-CELL; OUTER-MEMBRANE; GAMMA RELEASE; VII SECRETION

AB The only vaccine available against tuberculosis (TB), the Bacille Calmette-Guerin (BCG), does not provide effective protection against the most common forms of adult TB and in recent years efforts have been made to develop a new and improved vaccine. Among the strategies implemented, the generation of a new live attenuated mycobacterial strain is seen as one of the most promising and feasible, for scientific, ethical and practical reasons. The new understanding of the biology of the tubercle bacilli and of host-pathogen interaction processes, coupled with the possibility to engineer BCG or M. tuberculosis, opened new avenues to design "intelligent" vaccines, capable of eliciting the immune response associated with protection while avoiding the induction of the host immune response associated with immunopathology. The complex and highly immunogenic mycobacterial cell wall can shape the general and antigen specific immune response elicited following immunization, and the possibility to exploit this knowledge may lead to the development of new vaccines that could help conquer this ancient human disease.

C1 [Morandi, Matteo; Sali, Michela; Delogu, Giovanni] Univ Cattolica Sacro Cuore, Ist Microbiol, I-00168 Rome, Italy.

[Manganelli, Riccardo] Univ Padua, Dept Mol Med, Padua, Italy.

C3 Catholic University of the Sacred Heart; IRCCS Policlinico Gemelli;

University of Padua

RP Delogu, G (corresponding author), Univ Cattolica Sacro Cuore, Ist Microbiol, Largo A Gemelli 8, I-00168 Rome, Italy.

EM gdelogu@rm.unicatt.it

RI ; Delogu, Giovanni/I-3701-2012; MANGANELLI, RICCARDO/A-7361-2009

OI SALI, Michela/0000-0003-3609-2990; Delogu, Giovanni/0000-0003-0182-8267;

MANGANELLI, RICCARDO/0000-0003-0940-7336

FU PRIN grant from the Ministry of Education and Research (MIUR) [70103566]

FX This work was supported by PRIN grant from the Ministry of Education and

Research (MIUR) Nr. 70103566 (MS).

CR Aagaard C, 2011, NAT MED, V17, P189, DOI 10.1038/nm.2285

Abdallah AM, 2008, J IMMUNOL, V181, P7166, DOI 10.4049/jimmunol.181.10.7166

Abdallah AM, 2007, NAT REV MICROBIOL, V5, P883, DOI 10.1038/nrmicro1773

Adindla S, 2003, J BIOSCIENCES, V28, P169, DOI 10.1007/BF02706216

Andersen P, 2005, NAT REV MICROBIOL, V3, P656, DOI 10.1038/nrmicro1211

Asensio JG, 2006, J BIOL CHEM, V281, P1313, DOI 10.1074/jbc.C500388200

BALASUBRAMANIAN V, 1994, TUBERCLE LUNG DIS, V75, P168, DOI 10.1016/0962-8479(94)90002-7

Banu S, 2002, MOL MICROBIOL, V44, P9, DOI 10.1046/j.1365-2958.2002.02813.x

Behar SM, 2011, MUCOSAL IMMUNOL, V4, P279, DOI 10.1038/mi.2011.3

BOOTH RJ, 1993, INFECT IMMUN, V61, P1509, DOI 10.1128/IAI.61.4.1509-1515.1993

Bottai D, 2012, MOL MICROBIOL, V83, P1195, DOI 10.1111/j.1365-2958.2012.08001.x

Brennan MJ, 2007, PLOS MED, V4, P1299, DOI 10.1371/journal.pmed.0040252

Brennan MJ, 2005, TUBERCULOSIS, V85, P7, DOI 10.1016/j.tube.2004.09.001

Brennan MJ, 2002, TRENDS MICROBIOL, V10, P246, DOI 10.1016/S0966-842X(02)02335-1

Brennan MJ, 2001, INFECT IMMUN, V69, P7326, DOI 10.1128/IAI.69.12.7326-7333.2001

Brightbill HD, 1999, SCIENCE, V285, P732, DOI 10.1126/science.285.5428.732

Cardona PJ, 2009, VACCINE, V27, P2499, DOI 10.1016/j.vaccine.2009.02.060

Cascioferro A, 2007, MOL MICROBIOL, V66, P1536, DOI 10.1111/j.1365-2958.2007.06023.x

Cegielski JP, 2010, CLIN INFECT DIS, V50, pS195, DOI 10.1086/651491

Ciaramella A, 2004, J INFECT DIS, V190, P1167, DOI 10.1086/423850

COLDITZ GA, 1995, PEDIATRICS, V96, P29

Cole ST, 1998, NATURE, V393, P537, DOI 10.1038/31159

Corbière V, 2012, PLOS ONE, V7, DOI 10.1371/journal.pone.0043285

Daleke MH, 2011, J BIOL CHEM, V286, P19024, DOI 10.1074/jbc.M110.204966

Delogu G, 2004, MOL MICROBIOL, V52, P725, DOI 10.1111/j.1365-2958.2004.04007.x

Delogu G, 1999, J BACTERIOL, V181, P7464, DOI 10.1128/JB.181.24.7464-7469.1999

Delogu G, 2012, PROTEIN PEPTIDE LETT, V19, P1035, DOI 10.2174/092986612802762697

Delogu G, 2011, PLOS ONE, V6, DOI 10.1371/journal.pone.0018315

Diel R, 2011, EUR RESPIR J, V37, P88, DOI 10.1183/09031936.00115110

Dupres V, 2005, NAT METHODS, V2, P515, DOI 10.1038/NMETH769

Esposito C, 2011, BIOCHEM BIOPH RES CO, V410, P339, DOI 10.1016/j.bbrc.2011.05.159

FINE PEM, 1995, LANCET, V346, P1339, DOI 10.1016/S0140-6736(95)92348-9

Flynn JL, 2005, TRENDS MICROBIOL, V13, P98, DOI 10.1016/j.tim.2005.01.005

Frigui W, 2008, PLOS PATHOG, V4, DOI 10.1371/journal.ppat.0040033

Gagneux S, 2012, PHILOS T R SOC B, V367, P850, DOI 10.1098/rstb.2011.0316

Gey van Pittius NC, 2006, BMC EVOL BIOL, V6, DOI 10.1186/1471-2148-6-95

Gioffré A, 2005, MICROBES INFECT, V7, P325, DOI 10.1016/j.micinf.2004.11.007

Gonzalo-Asensio J, 2008, PLOS ONE, V3, DOI 10.1371/journal.pone.0003496

Gordon SV, 2001, TUBERCULOSIS, V81, P157, DOI 10.1054/tube.2000.0269

Grode L, 2005, J CLIN INVEST, V115, P2472, DOI 10.1172/JCI24617

Grode L, 2002, J IMMUNOL, V168, P1869, DOI 10.4049/jimmunol.168.4.1869

Henao-Tamayo M, 2007, VACCINE, V25, P7153, DOI 10.1016/j.vaccine.2007.07.042

Hess J, 1998, P NATL ACAD SCI USA, V95, P5299, DOI 10.1073/pnas.95.9.5299

Hinchey J, 2007, J CLIN INVEST, V117, P2279, DOI 10.1172/JCI31947

Hoffmann C, 2008, P NATL ACAD SCI USA, V105, P3963, DOI 10.1073/pnas.0709530105

Hoft DF, 2008, J INFECT DIS, V198, P1491, DOI 10.1086/592450

Hondalus MK, 2000, INFECT IMMUN, V68, P2888, DOI 10.1128/IAI.68.5.2888-2898.2000

Horwitz MA, 2000, P NATL ACAD SCI USA, V97, P13853, DOI 10.1073/pnas.250480397

Horwitz MA, 2003, INFECT IMMUN, V71, P1672, DOI 10.1128/IAI.71.4.1672-1679.2003

Hougardy JM, 2007, PLOS ONE, V2, DOI 10.1371/journal.pone.0000926

Hsu T, 2003, P NATL ACAD SCI USA, V100, P12420, DOI 10.1073/pnas.1635213100

Kaufmann SHE, 2005, J EXP MED, V201, P1693, DOI 10.1084/jem.20050842

Kaufmann SHE, 2012, CURR OPIN BIOTECH, V23, P900, DOI 10.1016/j.copbio.2012.03.007

Kaufmann SHE, 2010, IMMUNITY, V33, P567, DOI 10.1016/j.immuni.2010.09.015

Kohama H, 2008, VACCINE, V26, P924, DOI 10.1016/j.vaccine.2007.12.005

Li ZM, 1999, INFECT IMMUN, V67, P4780, DOI 10.1128/IAI.67.9.4780-4786.1999

López M, 2003, J IMMUNOL, V170, P2409, DOI 10.4049/jimmunol.170.5.2409

Maciag A, 2007, J BACTERIOL, V189, P730, DOI 10.1128/JB.01190-06

Manganelli R, 2001, MOL MICROBIOL, V41, P423, DOI 10.1046/j.1365-2958.2001.02525.x

Manganelli R, 2004, J BACTERIOL, V186, P895, DOI 10.1128/JB.186.4.895-902.2004

Martin C, 2006, VACCINE, V24, P3408, DOI 10.1016/j.vaccine.2006.03.017

Masungi C, 2002, J INFECT DIS, V185, P513, DOI 10.1086/338833

Menozzi FD, 1996, J EXP MED, V184, P993, DOI 10.1084/jem.184.3.993

Menozzi FD, 1998, P NATL ACAD SCI USA, V95, P12625, DOI 10.1073/pnas.95.21.12625

Mishra KC, 2008, INFECT IMMUN, V76, P127, DOI 10.1128/IAI.00410-07

Molicotti P, 2011, DIAGN MICR INFEC DIS, V71, P449, DOI 10.1016/j.diagmicrobio.2011.08.010

Olsen AW, 2001, INFECT IMMUN, V69, P2773, DOI 10.1128/IAI.69.5.2773-2778.2001

Pai RK, 2003, J IMMUNOL, V171, P175, DOI 10.4049/jimmunol.171.1.175

Pando RH, 2010, INFECT IMMUN, V78, P3168, DOI 10.1128/IAI.00023-10

Parra M, 2004, INFECT IMMUN, V72, P6799, DOI 10.1128/IAI.72.12.6799-6805.2004

Pérez E, 2001, MOL MICROBIOL, V41, P179, DOI 10.1046/j.1365-2958.2001.02500.x

Pethe K, 2001, NATURE, V412, P190, DOI 10.1038/35084083

Place S, 2010, AM J RESP CRIT CARE, V182, P848, DOI 10.1164/rccm.201001-0083OC

Pym AS, 2002, MOL MICROBIOL, V46, P709, DOI 10.1046/j.1365-2958.2002.03237.x

Rodrigue S, 2006, FEMS MICROBIOL REV, V30, P926, DOI 10.1111/j.1574-6976.2006.00040.x

Russell DG, 2007, NAT REV MICROBIOL, V5, P39, DOI 10.1038/nrmicro1538

Sali M, 2008, MICROBES INFECT, V10, P605, DOI 10.1016/j.micinf.2008.02.012

Sali M, 2010, INFECT IMMUN, V78, P5202, DOI 10.1128/IAI.00267-10

Sambandamurthy VK, 2005, MICROBES INFECT, V7, P955, DOI 10.1016/j.micinf.2005.04.001

Santucci MB, 2000, J INFECT DIS, V181, P1506, DOI 10.1086/315371

Sayes F, 2012, CELL HOST MICROBE, V11, P352, DOI 10.1016/j.chom.2012.03.003

Serafini A, 2009, J BACTERIOL, V191, P6340, DOI 10.1128/JB.00756-09

Shin AR, 2006, CLIN VACCINE IMMUNOL, V13, P869, DOI 10.1128/CVI.00103-06

Siegrist MS, 2009, P NATL ACAD SCI USA, V106, P18792, DOI 10.1073/pnas.0900589106

Stoop EJM, 2012, TRENDS MICROBIOL, V20, P477, DOI 10.1016/j.tim.2012.07.001

Strong M, 2006, P NATL ACAD SCI USA, V103, P8060, DOI 10.1073/pnas.0602606103

Sun R, 2009, VACCINE, V27, P4412, DOI 10.1016/j.vaccine.2009.05.048

Sutcliffe IC, 2004, FEMS MICROBIOL REV, V28, P645, DOI 10.1016/j.femsre.2004.06.002

Sweeney KA, 2011, NAT MED, V17, P1261, DOI 10.1038/nm.2420

Temmerman S, 2004, NAT MED, V10, P935, DOI 10.1038/nm1090

Temmerman ST, 2005, J INFECT DIS, V192, P226, DOI 10.1086/430930

Udwadia ZF, 2012, CLIN INFECT DIS, V54, P579, DOI 10.1093/cid/cir889

Van Pittius NG., 2001, Genome Biol, V2, p44.41, DOI [10.1186/gb-2001-2-10-research0044, DOI 10.1186/GB-2001-2-10-RESEARCH0044]

WHO, 2011, GLOB TUB CONTR SURV

Wilkinson KA, 2009, BMC MICROBIOL, V9, DOI 10.1186/1471-2180-9-93

Yeremeev VV, 2000, CLIN EXP IMMUNOL, V120, P274, DOI 10.1046/j.1365-2249.2000.01212.x

Yeremeev VV, 2000, TUBERCLE LUNG DIS, V80, P243, DOI 10.1054/tuld.2000.0252

Zanetti S, 2005, CLIN DIAGN LAB IMMUN, V12, P1135, DOI 10.1128/CDLI.12.9.1135-1138.2005

Zhao SM, 2012, PLOS ONE, V7, DOI 10.1371/journal.pone.0031908

Zuber B, 2008, J BACTERIOL, V190, P5672, DOI 10.1128/JB.01919-07

NR 100

TC 15

Z9 21

U1 0

U2 19

PU J INFECTION DEVELOPING COUNTRIES

PI TRAMANIGLIO

PA JIDC CENT OFF PORTO CONTE RICERCHE RES CTR, S P 55, PORTO CONTE CAPO

CACCIA KM 8.400 LOC, TRAMANIGLIO, 07041, ITALY

SN 1972-2680

J9 J INFECT DEV COUNTR

JI J. Infect. Dev. Ctries.

PD MAR

PY 2013

VL 7

IS 3

BP 169

EP 181

DI 10.3855/jidc.3114

PG 13

WC Infectious Diseases

WE Science Citation Index Expanded (SCI-EXPANDED)

SC Infectious Diseases

GA 107AY

UT WOS:000316188300003

OA Green Submitted, gold

DA 2026-05-07

ER

PT J

AU Pathak, AK

Pathaka, V

Seitz, L

Gurcha, SS

Besra, GS

Riordan, JM

Reynolds, RC

AF Pathak, Ashish K.

Pathaka, Vibha

Seitz, Lainne

Gurcha, Sudagar S.

Besra, Gurdyal S.

Riordan, James M.

Reynolds, Robert C.

TI Disaccharide analogs as probes for glycosyltransferases in

Mycobacterium tuberculosis

SO BIOORGANIC & MEDICINAL CHEMISTRY

LA English

DT Article

DE arabinofuranose; galactofuranose; fluorescent probe; photoaffinity

probe; glycosyltransferases.; arabinosyltransferases;

galactosyltransferases; tuberculosis

ID DRUG-RESISTANT TUBERCULOSIS; CELL-WALL; ARABINOSYLTRANSFERASE ACTIVITY;

ARABINOFURANOSYL DISACCHARIDE; MOLECULAR-MECHANISMS;

TRANSFERASE-ACTIVITY; MASS-SPECTROMETRY; BIOSYNTHESIS; ARABINOGALACTAN;

INFECTION

AB Glycosyltransferases (GTs) play a crucial role in mycobacterial cell wall biosynthesis and are necessary for the survival of mycobacteria. Hence, these enzymes are potential new drug targets for the treatment of tuberculosis (TB), especially multiple drug-resistant TB (MDR-TB). Herein, we report the efficient syntheses of Araf(alpha 1 -> 5)Araf, Ga1f(beta 1 -> 5)Galf and Galf(beta 1 -> 6)Galf disaccharides possessing a 5-N,N-dimethylaminonaphthalene-l-sulfonamidoethyl (dansyl) unit that were prepared as fluorescent disaccharide acceptors for arabinosyl- and galactosyl-transferases, respectively. Such analogs may offer advantages relative to radiolabeled acceptors or donors for studying the enzymes and for assay development and compound screening. Additionally, analogs possessing a 5-azidonaphthalene-1-sulfonamidoethyl unit were prepared as photoaffinity probes for then-potential utility in studying active site labeling of the GTs (arabinosyl and galactosyl) in MYcobacterium tuberculosis (MTB). Beyond their preparation, initial biological testing and kinetic analysis of these disaccharides as acceptors toward glycosyltransferases are also presented. (C) 2007 Elsevier Ltd. All rights reserved.

C1 So Res Inst, Drug Discovery Div, Birmingham, AL 35255 USA.

Univ Birmingham, Sch Biosci, Birmingham B15 2TT, W Midlands, England.

Western Illinois Univ, Dept Chem, Macomb, IL 61455 USA.

C3 University of Birmingham; Western Illinois University

RP Reynolds, RC (corresponding author), So Res Inst, Drug Discovery Div, PO Box 55305, Birmingham, AL 35255 USA.

EM reynolds@ocsri.org

RI ; Pathak, Ashish/AGG-1059-2022

OI Besra, Gurdyal/0000-0002-5605-0395; Pathak, Ashish/0000-0003-0143-7979

FU Medical Research Council [G0500590] Funding Source: Medline; NCI NIH HHS

[P30 CA013148, CA-13148] Funding Source: Medline; NIAID NIH HHS [R01

AI045317, R01AI45317] Funding Source: Medline; Wellcome Trust Funding

Source: Medline; MRC [G0500590] Funding Source: UKRI; Medical Research

Council [G0500590] Funding Source: researchfish; National Cancer

Institute [P30CA013148] Funding Source: NIH RePORTER

CR ABOUZEID C, 1982, EUR J BIOCHEM, V128, P363, DOI 10.1111/j.1432-1033.1982.tb06973.x

BASS JB, 1994, AM J RESP CRIT CARE, V149, P1359, DOI 10.1164/ajrccm.149.5.8173779

Bayley H, 1977, Methods Enzymol, V46, P69

BESRA GS, 1995, BIOCHEMISTRY-US, V34, P4257, DOI 10.1021/bi00013a015

Blanchard JS, 1996, ANNU REV BIOCHEM, V65, P215, DOI 10.1146/annurev.bi.65.070196.001243

BRENNAN PJ, 1995, ANNU REV BIOCHEM, V64, P29, DOI 10.1146/annurev.bi.64.070195.000333

CHITTENDEN GJ, 1972, CARBOHYD RES, V25, P35, DOI 10.1016/S0008-6215(00)82743-3

CORY PP, 1968, J AM CHEM SOC, P1643

CYR N, 1979, CAN J CHEM, V57, P2504, DOI 10.1139/v79-399

D'Souza FW, 1998, J ORG CHEM, V63, P9037, DOI 10.1021/jo9815406

DAFFE M, 1990, J BIOL CHEM, V265, P6734

de Jong BC, 2004, ANNU REV MED, V55, P283, DOI 10.1146/annurev.med.55.091902.103753

EBERLE AN, 1985, METHOD ENZYMOL, V109, P129

GREENE TW, 1999, PROTECTIVE GROUPS OR, P86

GUTHRIE RD, 1968, CHEM IND-LONDON, P547

HAZUM E, 1983, METHOD ENZYMOL, V103, P58

KAM BL, 1979, CARBOHYD RES, V69, P135, DOI 10.1016/S0008-6215(00)85758-4

Lee RE, 1996, CURR TOP MICROBIOL, V215, P1

Lee RE, 1997, GLYCOBIOLOGY, V7, P1121, DOI 10.1093/glycob/7.8.1121

Long R, 2000, CAN MED ASSOC J, V163, P425

MAUCH H, 1993, ZBL HYG UMWELTMED, V194, P152

Mikusová K, 2000, J BIOL CHEM, V275, P33890, DOI 10.1074/jbc.M006875200

Mikusová K, 2006, J BACTERIOL, V188, P6592, DOI 10.1128/JB.00489-06

MIZUTANI K, 1989, CARBOHYD RES, V185, P27, DOI 10.1016/0008-6215(89)84018-2

MURAMOTO K, 1984, AGR BIOL CHEM TOKYO, V48, P2695, DOI 10.1080/00021369.1984.10866569

Pathak A K, 2001, Carbohydr Lett, V4, P117

Pathak AK, 1998, TETRAHEDRON LETT, V39, P1497, DOI 10.1016/S0040-4039(98)00051-3

Pathak AK, 2004, CARBOHYD RES, V339, P683, DOI 10.1016/j.carres.2003.10.031

Pathak AK, 2002, BIOORGAN MED CHEM, V10, P923, DOI 10.1016/S0968-0896(01)00343-1

Pathak AK, 2002, BIOORG MED CHEM LETT, V12, P2749, DOI 10.1016/S0960-894X(02)00536-X

Pathak AK, 1999, BIOORGAN MED CHEM, V7, P2407, DOI 10.1016/S0968-0896(99)00199-6

Pathak AK, 2001, TETRAHEDRON LETT, V42, P979, DOI 10.1016/S0040-4039(00)02161-4

Pathak AK, 2001, BIOORGAN MED CHEM, V9, P3129, DOI 10.1016/S0968-0896(01)00179-1

Pathak AK, 2001, BIOORGAN MED CHEM, V9, P3145, DOI 10.1016/S0968-0896(01)00180-8

Rattan A, 1998, EMERG INFECT DIS, V4, P195, DOI 10.3201/eid0402.980207

Raviglione MC, 2000, SCOT MED J, V45, P52, DOI 10.1177/00369330000450S124

Reynolds RC, 1999, CARBOHYD RES, V317, P164, DOI 10.1016/S0008-6215(99)00069-5

Rose NL, 2006, J AM CHEM SOC, V128, P6721, DOI 10.1021/ja058254d

SIBILLE Y, 1990, AM REV RESPIR DIS, V141, P47

Van Scoy RE, 1999, MAYO CLIN PROC, V74, P1038

Walsh C, 2000, NATURE, V406, P775, DOI 10.1038/35021219

NR 41

TC 17

Z9 19

U1 0

U2 13

PU PERGAMON-ELSEVIER SCIENCE LTD

PI OXFORD

PA THE BOULEVARD, LANGFORD LANE, KIDLINGTON, OXFORD OX5 1GB, ENGLAND

SN 0968-0896

J9 BIOORGAN MED CHEM

JI Bioorg. Med. Chem.

PD AUG 15

PY 2007

VL 15

IS 16

BP 5629

EP 5650

DI 10.1016/j.bmc.2007.04.012

PG 22

WC Biochemistry & Molecular Biology; Chemistry, Medicinal; Chemistry,

Organic

WE Science Citation Index Expanded (SCI-EXPANDED)

SC Biochemistry & Molecular Biology; Pharmacology & Pharmacy; Chemistry

GA 192AI

UT WOS:000248172700027

OA Green Submitted

DA 2026-05-07

ER

PT J

AU Deshpande, D

Pasipanodya, JG

Mpagama, SG

Srivastava, S

Bendet, P

Koeuth, T

Lee, PS

Heysell, SK

Gumbo, T

AF Deshpande, Devyani

Pasipanodya, Jotam G.

Mpagama, Stellah G.

Srivastava, Shashikant

Bendet, Paula

Koeuth, Thearith

Lee, Pooi S.

Heysell, Scott K.

Gumbo, Tawanda

TI Ethionamide Pharmacokinetics/Pharmacodynamics-derived Dose, the Role of

MICs in Clinical Outcome, and the Resistance Arrow of Time in

Multidrug-resistant Tuberculosis

SO CLINICAL INFECTIOUS DISEASES

LA English

DT Article

DE efflux pumps; hollow fiber system model; artificial intelligence; MIC vs

clinical outcome; tuberculous meningitis

ID FIBER SYSTEM MODEL; MYCOBACTERIUM-TUBERCULOSIS; EFFLUX-PUMP;

SUSCEPTIBILITY BREAKPOINTS; ARTIFICIAL-INTELLIGENCE;

CEREBROSPINAL-FLUID; DRUG-RESISTANCE; PHARMACOKINETICS;

PHARMACODYNAMICS; MUTATIONS

AB Background. Ethionamide is used to treat multidrug-resistant tuberculosis (MDR-TB). The antimicrobial pharmacokinetics/pharmacodynamics, the contribution of ethionamide to the multidrug regimen, and events that lead to acquired drug resistance (ADR) are unclear.

Methods. We performed a multidose hollow fiber system model of tuberculosis (HFS-TB) study to identify the 0-24 hour area under the concentration-time curve (AUC(0-24)) to minimum inhibitory concentration (MIC) ratios that achieved maximal kill and ADR suppression, defined as target exposures. Ethionamide-resistant isolates underwent whole-genome and targeted Sanger sequencing. We utilized Monte Carlo experiments (MCEs) to identify ethionamide doses that would achieve the target exposures in 10 000 patients with pulmonary tuberculosis. We also identified predictors of time-to-sputum conversion in Tanzanian patients on ethionamide-and levofloxacin-based regimens using multivariate adaptive regression splines (MARS).

Results. An AUC(0-24)/MIC > 56.2 was identified as the target exposure in the HFS-TB. Early efflux pump induction to ethionamide monotherapy led to simultaneous ethambutol and isoniazid ADR, which abrogated microbial kill of an isoniazid-ethambutol- ethionamide regimen. Genome sequencing of isolates that arose during ethionamide monotherapy revealed mutations in both ethA and embA. In MCEs, 20 mg/kg/day achieved the AUC(0-24)/MIC > 56.2 in > 95% of patients, provided the Sensititre assay MIC was < 2.5 mg/L. In the clinic, MARS revealed that ethionamide Sensititre MIC had linear negative relationships with time-to-sputum conversion until an MIC of 2.5 mg/L, above which patients with MDR-TB failed combination therapy.

Conclusions. Ethionamide is an important contributor to MDR-TB treatment regimens, at Sensititre MIC < 2.5 mg/L. Suboptimal ethionamide exposures led to efflux pump-mediated ADR.

C1 [Deshpande, Devyani; Pasipanodya, Jotam G.; Srivastava, Shashikant; Bendet, Paula; Koeuth, Thearith; Lee, Pooi S.; Gumbo, Tawanda] Baylor Univ, Med Ctr, Ctr Infect Dis Res & Expt Therapeut, Baylor Res Inst, Dallas, TX USA.

[Mpagama, Stellah G.] Kibongoto Infect Dis Hosp, Sanya Juu, Tanzania.

[Heysell, Scott K.] Univ Virginia, Div Infect Dis & Int Hlth, Charlottesville, VA USA.

C3 Baylor University Medical Center; Baylor University; Baylor Scott &

White Health; University of Virginia

RP Gumbo, T (corresponding author), Baylor Res Inst, Ctr Infect Dis Res & Expt Therapeut, 3434 Live Oak St, Dallas, TX 75204 USA.

EM tawanda.gumbo@BSWHealth.org

RI ; Srivastava, Shashikant/I-4704-2019

OI Mpagama, Stellah George/0000-0002-0660-6930; Gumbo,

Tawanda/0000-0001-7592-8012; Srivastava, Shashikant/0000-0002-8786-8851

FU Baylor Research Institute, Dallas, Texas; National Institutes of Health;

National Institute of Allergy and Infectious Diseases [U01AI119954]

FX This work was supported by the Baylor Research Institute, Dallas, Texas,

and portions from Tanzania were funded by the National Institutes of

Health and the National Institute of Allergy and Infectious Diseases

(U01AI119954; to S. K. H.). Supplement sponsored by Baylor Research

Institute, Dallas, Texas.

CR Auclair B, 2001, ANTIMICROB AGENTS CH, V45, P810, DOI 10.1128/AAC.45.3.810-814.2001

BANERJEE A, 1994, SCIENCE, V263, P227, DOI 10.1126/science.8284673

Brown JB, 2012, CHEM BIOL, V19, P23, DOI 10.1016/j.chembiol.2011.12.012

Cavaleri M, 2015, CLIN INFECT DIS, V61, pS1, DOI 10.1093/cid/civ484

Chigutsa E, 2015, ANTIMICROB AGENTS CH, V59, P38, DOI 10.1128/AAC.03931-14

Chilukuri D, 2015, CLIN INFECT DIS, V61, pS32, DOI 10.1093/cid/civ460

Colangeli R, 2005, MOL MICROBIOL, V55, P1829, DOI 10.1111/j.1365-2958.2005.04510.x

Conte JE, 2000, ANTIMICROB AGENTS CH, V44, P1337, DOI 10.1128/AAC.44.5.1337-1341.2000

Cui ZL, 2014, ANTIMICROB AGENTS CH, V58, P6837, DOI 10.1128/AAC.03285-14

Deshpande D, 2018, CLIN INFECT DIS, V67, pS293, DOI 10.1093/cid/ciy611

Deshpande D, 2018, CLIN INFECT DIS, V67, pS308, DOI 10.1093/cid/ciy624

Deshpande D, 2018, CLIN INFECT DIS, V67, pS274, DOI 10.1093/cid/ciy618

Deshpande D, 2018, ANTIMICROB AGENTS CH, V62, DOI 10.1128/AAC.02232-17

Deshpande D, 2017, SCI ADV, V3, DOI 10.1126/sciadv.1701102

Deshpande D, 2016, CLIN INFECT DIS, V63, pS95, DOI 10.1093/cid/ciw474

DONALD PR, 1989, J PEDIATR-US, V115, P483, DOI 10.1016/S0022-3476(89)80862-5

Drusano GL, 2001, ANTIMICROB AGENTS CH, V45, P13, DOI 10.1128/AAC.45.1.13-22.2001

DRUSANO GL, 1993, ANTIMICROB AGENTS CH, V37, P483, DOI 10.1128/AAC.37.3.483

Ebers A, 2017, PLOS ONE, V12, DOI 10.1371/journal.pone.0170663

EMA, 2015, EMEAHSAB0491QO2014SM

Falzon D, 2017, EUR RESPIR J, V49, DOI 10.1183/13993003.02308-2016

Friedman J H, 1995, Stat Methods Med Res, V4, P197, DOI 10.1177/096228029500400303

FRIEDMAN JH, 1991, ANN STAT, V19, P1, DOI 10.1214/aos/1176347963

Gumbo T, 2004, J INFECT DIS, V190, P1642, DOI 10.1086/424849

Gumbo T, 2007, ANTIMICROB AGENTS CH, V51, P2329, DOI 10.1128/AAC.00185-07

Gumbo T, 2018, CLIN INFECT DIS, V67, pS267, DOI 10.1093/cid/ciy608

Gumbo T, 2015, J INFECT DIS, V211, pS83, DOI 10.1093/infdis/jiv183

Gumbo T, 2015, J INFECT DIS, V211, pS96, DOI 10.1093/infdis/jiu610

Gumbo T, 2013, NAT GENET, V45, P720, DOI 10.1038/ng.2675

Gumbo T, 2010, ANTIMICROB AGENTS CH, V54, P1484, DOI 10.1128/AAC.01474-09

Heysell SK, 2015, ANTIMICROB AGENTS CH, V59, P7104, DOI 10.1128/AAC.01117-15

Hopkins AL, 2009, NATURE, V462, P167, DOI 10.1038/462167a

Huang TS, 2004, J ANTIMICROB CHEMOTH, V53, P600, DOI 10.1093/jac/dkh120

HUGHES IE, 1962, LANCET, V1, P616

Huyen MNT, 2013, ANTIMICROB AGENTS CH, V57, P3620, DOI 10.1128/AAC.00077-13

KENAKIN T, 1995, TRENDS PHARMACOL SCI, V16, P188, DOI 10.1016/S0165-6147(00)89020-3

Modongo C, 2016, ANTIMICROB AGENTS CH, V60, P5928, DOI 10.1128/AAC.00962-16

Müller B, 2011, INT J TUBERC LUNG D, V15, P344

Niehaus AJ, 2015, PLOS ONE, V10, DOI 10.1371/journal.pone.0135003

Pasipanodya JG, 2015, CLIN INFECT DIS, V61, pS10, DOI 10.1093/cid/civ425

Pasipanodya JG, 2011, CURR OPIN PHARMACOL, V11, P457, DOI 10.1016/j.coph.2011.07.001

Pietersen E, 2015, PLOS ONE, V10, DOI 10.1371/journal.pone.0123655

Rockwood N, 2017, CLIN INFECT DIS, V64, P1350, DOI 10.1093/cid/cix158

Rogers Z, 2016, EBIOMEDICINE, V11, P118, DOI 10.1016/j.ebiom.2016.07.031

Schmalstieg AM, 2012, ANTIMICROB AGENTS CH, V56, P4806, DOI 10.1128/AAC.05546-11

Shaw JET, 2010, MEDICINE, V89, P189, DOI 10.1097/MD.0b013e3181df9070

Srivastava S, 2006, CURR MICROBIOL, V53, P401, DOI 10.1007/s00284-006-0135-1

Srivastava S, 2017, ANTIMICROB AGENTS CH, V61

Srivastava S, 2016, ANTIMICROB AGENTS CH, V60, P5922, DOI 10.1128/AAC.00961-16

Srivastava S, 2011, J INFECT DIS, V204, P1951, DOI 10.1093/infdis/jir658

Srivastava S, 2010, J INFECT DIS, V201, P1225, DOI 10.1086/651377

Sturm N, 2012, J CHEM INF MODEL, V52, P2410, DOI 10.1021/ci300196g

Wong K, 2014, TRENDS BIOCHEM SCI, V39, P8, DOI 10.1016/j.tibs.2013.11.002

Zhu M, 2002, TUBERCULOSIS, V82, P91, DOI 10.1054/tube.2002.0330

NR 54

TC 35

Z9 37

U1 0

U2 7

PU OXFORD UNIV PRESS INC

PI CARY

PA JOURNALS DEPT, 2001 EVANS RD, CARY, NC 27513 USA

SN 1058-4838

EI 1537-6591

J9 CLIN INFECT DIS

JI Clin. Infect. Dis.

PD DEC 15

PY 2018

VL 67

SU 3

BP S317

EP S326

DI 10.1093/cid/ciy609

PG 10

WC Immunology; Infectious Diseases; Microbiology

WE Science Citation Index Expanded (SCI-EXPANDED)

SC Immunology; Infectious Diseases; Microbiology

GA HM7CN

UT WOS:000459635000007

OA Green Published, Bronze

DA 2026-05-07

ER

PT J

AU Ahmed, S

Raqib, R

Gudmundsson, GH

Bergman, P

Agerberth, B

Rekha, RS

AF Ahmed, Sultan

Raqib, Rubhana

Gudmundsson, Gudmundur Hrafn

Bergman, Peter

Agerberth, Birgitta

Rekha, Rokeya Sultana

TI Host-Directed Therapy as a Novel Treatment Strategy to Overcome

Tuberculosis: Targeting Immune Modulation

SO ANTIBIOTICS-BASEL

LA English

DT Review

DE Mycobacterium tuberculosis; host-directed therapy; drug resistance;

immune response; autophagy; innate immunity; antimicrobial peptides

ID TUMOR-NECROSIS-FACTOR; NATURAL-KILLER-CELLS; NITRIC-OXIDE SYNTHASE;

MYCOBACTERIUM-TUBERCULOSIS; VITAMIN-D; T-CELLS; ALVEOLAR MACROPHAGES;

ANTIMICROBIAL PEPTIDES; SELECTIVE AUTOPHAGY; GROWTH-INHIBITION

AB Tuberculosis (TB) is one of the leading causes of mortality and morbidity, particularly in developing countries, presenting a major threat to the public health. The currently recommended long term treatment regimen with multiple antibiotics is associated with poor patient compliance, which in turn, may contribute to the emergence of multi-drug resistant TB (MDR-TB). The low global treatment efficacy of MDR-TB has highlighted the necessity to develop novel treatment options. Host-directed therapy (HDT) together with current standard anti-TB treatments, has gained considerable interest, as HDT targets novel host immune mechanisms. These immune mechanisms would otherwise bypass the antibiotic bactericidal targets to kill Mycobacterium tuberculosis (Mtb), which may be mutated to cause antibiotic resistance. Additionally, host-directed therapies against TB have been shown to be associated with reduced lung pathology and improved disease outcome, most likely via the modulation of host immune responses. This review will provide an update of host-directed therapies and their mechanism(s) of action against Mycobacterium tuberculosis.

C1 [Ahmed, Sultan; Gudmundsson, Gudmundur Hrafn; Bergman, Peter; Agerberth, Birgitta; Rekha, Rokeya Sultana] Karolinska Inst, Div Clin Microbiol, Dept Lab Med, S-14152 Stockholm, Sweden.

[Raqib, Rubhana] Bangladesh Icddr B, Int Ctr Diarrhoeal Dis Res, Infect Dis Div, Dhaka 1213, Bangladesh.

[Gudmundsson, Gudmundur Hrafn] Univ Iceland, Biomed Ctr, IS-101 Reykjavik, Iceland.

[Bergman, Peter] Karolinska Univ Hosp, Immunodeficiency Unit, Infect Dis Clin, S-14152 Stockholm, Sweden.

C3 Karolinska Institutet; International Centre for Diarrhoeal Disease

Research (ICDDR); University of Iceland; Karolinska Institutet;

Karolinska University Hospital

RP Rekha, RS (corresponding author), Karolinska Inst, Div Clin Microbiol, Dept Lab Med, S-14152 Stockholm, Sweden.

EM sultan.ahmed@ki.se; rubhana@icddrb.org; ghrafn@hi.is;

Peter.Bergman@ki.se; Birgitta.Agerberth@ki.se;

rokeya.sultana.rekha@ki.se

RI ; Gudmundsson, Gudmundur H/K-9178-2015

OI Bergman, Peter/0000-0003-3306-3713; Gudmundsson, Gudmundur

H/0000-0001-5793-0565

FU Karolinska Institutet; Swedish Research Council [2013-09299, 2013-02709,

2016-01496]; Swedish Heart and Lung Foundation [20170358]; Stockholm

County Council [20190016]; Scandinavian Society for Antimicrobial

Chemotherapy (SSAC) [SLS-885491]; Foundation Against Antibiotic

Resistance [4-2018]; Vinnova [2013-02709] Funding Source: Vinnova;

Swedish Research Council [2013-09299, 2013-02709, 2016-01496] Funding

Source: Swedish Research Council

FX This study had financial and logistic support from Karolinska

Institutet, the Swedish Research Council (2013-09299, 2013-02709,

2016-01496), the Swedish Heart and Lung Foundation (20170358), the

Stockholm County Council (20190016), the Scandinavian Society for

Antimicrobial Chemotherapy (SSAC) (SLS-885491), and the Foundation

Against Antibiotic Resistance (4-2018).

CR Adams JS, 2008, NAT CLIN PRACT ENDOC, V4, P80, DOI 10.1038/ncpendmet0716

Aibana O, 2017, CLIN INFECT DIS, V65, P900, DOI 10.1093/cid/cix476

Algood HMS, 2005, CLIN INFECT DIS, V41, pS189, DOI 10.1086/429994

Ali Wahid, 2014, J Nat Sci Biol Med, V5, P130, DOI 10.4103/0976-9668.127310

Allen M, 2015, FRONT IMMUNOL, V6, DOI 10.3389/fimmu.2015.00508

Anding AL, 2017, DEV CELL, V41, P10, DOI 10.1016/j.devcel.2017.02.016

[Anonymous], 2019, WHO | Global tuberculosis report 2018

Awuh JA, 2017, CELL MOL LIFE SCI, V74, P1625, DOI 10.1007/s00018-016-2422-8

Bah A, 2017, FRONT IMMUNOL, V8, DOI 10.3389/fimmu.2017.01483

Bates M, 2015, CSH PERSPECT MED, V5, DOI 10.1101/cshperspect.a017889

Bekele A, 2018, J INTERN MED, V284, P292, DOI 10.1111/joim.12767

Berry MPR, 2010, NATURE, V466, P973, DOI 10.1038/nature09247

Borrego F, 2002, MOL IMMUNOL, V38, P637, DOI 10.1016/S0161-5890(01)00107-9

Bourigault ML, 2013, AM J CLIN EXP IMMUNO, V2, P124

Braian C, 2013, J INNATE IMMUN, V5, P591, DOI 10.1159/000348676

Brighenti S, 2018, J INTERN MED, V284, P145, DOI 10.1111/joim.12777

Brighenti S, 2010, BIOCHEM BIOPH RES CO, V396, P50, DOI 10.1016/j.bbrc.2010.02.141

Brogden KA, 2005, NAT REV MICROBIOL, V3, P238, DOI 10.1038/nrmicro1098

Brown DM, 2018, PLOS ONE, V13, DOI 10.1371/journal.pone.0201481

Buter J, 2019, NAT CHEM BIOL, V15, P889, DOI 10.1038/s41589-019-0336-0

Byrne ST, 2007, J ANTIMICROB CHEMOTH, V59, P313, DOI 10.1093/jac/dkl486

Carlberg C, 2019, FRONT IMMUNOL, V10, DOI 10.3389/fimmu.2019.02211

Chai QY, 2019, NAT COMMUN, V10, DOI 10.1038/s41467-019-09955-8

Chandra P, 2016, AUTOPHAGY, V12, P608, DOI 10.1080/15548627.2016.1139263

Chandra P, 2015, SCI REP-UK, V5, DOI 10.1038/srep16320

Chee CBE, 2018, RESPIROLOGY, V23, P893, DOI 10.1111/resp.13346

Choreno Parra Jose Alberto, 2017, Front Immunol, V8, P967, DOI 10.3389/fimmu.2017.00967

Chowdhury RR, 2018, NATURE, V560, P644, DOI 10.1038/s41586-018-0439-x

Chun Y, 2018, CELLS-BASEL, V7, DOI 10.3390/cells7120278

Coussens AK, 2015, PLOS PATHOG, V11, DOI 10.1371/journal.ppat.1005007

Dallenga T, 2016, PATHOG DIS, V74, DOI 10.1093/femspd/ftw012

Datta M, 2015, P NATL ACAD SCI USA, V112, P1827, DOI 10.1073/pnas.1424563112

de Chastellier C, 2009, IMMUNOBIOLOGY, V214, P526, DOI 10.1016/j.imbio.2008.12.005

Deretic V, 2006, CELL MICROBIOL, V8, P719, DOI 10.1111/j.1462-5822.2006.00705.x

Dheda K, 2010, RESPIROLOGY, V15, P433, DOI 10.1111/j.1440-1843.2010.01739.x

Dutta NK, 2014, MICROBIOL MOL BIOL R, V78, P343, DOI 10.1128/MMBR.00010-14

Dutta NK, 2007, INT J ANTIMICROB AG, V30, P336, DOI 10.1016/j.ijantimicag.2007.04.016

Ernst JD, 2012, NAT REV IMMUNOL, V12, P581, DOI 10.1038/nri3259

Ernst WA, 2000, J IMMUNOL, V165, P7102, DOI 10.4049/jimmunol.165.12.7102

Fabri M, 2011, CURR OPIN IMMUNOL, V23, P65, DOI 10.1016/j.coi.2010.10.010

Faurschou M, 2003, MICROBES INFECT, V5, P1317, DOI 10.1016/j.micinf.2003.09.008

Firmani MA, 2002, INFECT IMMUN, V70, P3965, DOI 10.1128/IAI.70.7.3965-3968.2002

FLESCH I, 1987, J IMMUNOL, V138, P4408

Flynn JL, 2001, INFECT IMMUN, V69, P4195, DOI 10.1128/IAI.69.7.4195-4201.2001

Gangull G, 2019, FRONT MICROBIOL, V10, DOI 10.3389/fmicb.2019.01121

Ganmaa D, 2017, AM J RESP CRIT CARE, V196, P628, DOI [10.1164/rccm.201705-0936OC, 10.1164/rccm.201705-0936oc]

Gough ME, 2017, INNATE IMMUN-LONDON, V23, P506, DOI 10.1177/1753425917719143

Gröschel MI, 2016, NAT REV MICROBIOL, V14, P677, DOI 10.1038/nrmicro.2016.131

Gudmundsson GH, 1999, J IMMUNOL METHODS, V232, P45, DOI 10.1016/S0022-1759(99)00152-0

Gutierrez MG, 2004, CELL, V119, P753, DOI 10.1016/j.cell.2004.11.038

Gutsmann T, 2016, BBA-BIOMEMBRANES, V1858, P1034, DOI 10.1016/j.bbamem.2016.01.031

Houben Rein M G J, 2016, PLoS Med, V13, pe1002152, DOI 10.1371/journal.pmed.1002152

Idh J, 2017, PLOS ONE, V12, DOI 10.1371/journal.pone.0181221

Jaeger Timo, 2007, V44, P207

Jagannath C, 1998, NITRIC OXIDE-BIOL CH, V2, P174, DOI 10.1006/niox.1998.9999

Jamaati H, 2017, FRONT MICROBIOL, V8, DOI 10.3389/fmicb.2017.02008

Jellbauer S, 2016, INFECT IMMUN, V84, P2639, DOI 10.1128/IAI.00132-16

Jena P, 2012, PLOS ONE, V7, DOI 10.1371/journal.pone.0050345

Jolliffe DA, 2019, EUR RESPIR J, V53, DOI 10.1183/13993003.02003-2018

Joosten SA, 2019, VACCINE, V37, P3022, DOI 10.1016/j.vaccine.2019.04.050

Junqueira-Kipnis AP, 2003, J IMMUNOL, V171, P6039, DOI 10.4049/jimmunol.171.11.6039

Kai-Larsen Y, 2008, FRONT BIOSCI, V13, P3760, DOI 10.2741/2964

Keflie Tibebeselassie Seyoum, 2018, J Clin Tuberc Other Mycobact Dis, V12, P27, DOI [10.1016/j.jctube.2018.05.002, 10.1016/j.jctube.2018.05.002]

Kim J, 2011, ANNU REV BIOCHEM, V80, P1001, DOI 10.1146/annurev-biochem-062209-094414

Kim K, 2014, TUBERCULOSIS, V94, P494, DOI 10.1016/j.tube.2014.07.002

Korbel DS, 2008, MICROBES INFECT, V10, P995, DOI 10.1016/j.micinf.2008.07.039

Kuo HP, 2000, AM J RESP CRIT CARE, V161, P192, DOI 10.1164/ajrccm.161.1.9902113

Lange C, 2018, J INTERN MED, V284, P163, DOI 10.1111/joim.12780

Levine B, 2011, NATURE, V469, P323, DOI 10.1038/nature09782

Lin PL, 2010, ARTHRITIS RHEUM-US, V62, P340, DOI 10.1002/art.27271

Liu PT, 2007, J IMMUNOL, V179, P2060, DOI 10.4049/jimmunol.179.4.2060

Liu PT, 2006, SCIENCE, V311, P1770, DOI 10.1126/science.1123933

Lu LL, 2019, NAT MED, V25, P977, DOI 10.1038/s41591-019-0441-3

Lu LL, 2016, CELL, V167, P433, DOI 10.1016/j.cell.2016.08.072

Madan-Lala R, 2014, J IMMUNOL, V192, P4263, DOI 10.4049/jimmunol.1303185

Maglione PJ, 2007, J IMMUNOL, V178, P7222, DOI 10.4049/jimmunol.178.11.7222

Majeed M, 1998, MICROB PATHOGENESIS, V24, P309, DOI 10.1006/mpat.1997.0200

Pareja MEM, 2013, FRONT CELL INFECT MI, V3, DOI 10.3389/fcimb.2013.00054

Martineau AR, 2007, J CLIN INVEST, V117, P1988, DOI 10.1172/JCI31097

Martineau AR, 2007, J IMMUNOL, V178, P7190, DOI 10.4049/jimmunol.178.11.7190

Martineau AR, 2019, HEALTH TECHNOL ASSES, V23, P1, DOI 10.3310/hta23020

Martineau AR, 2017, BMJ-BRIT MED J, V356, DOI 10.1136/bmj.i6583

Mily A, 2015, PLOS ONE, V10, DOI 10.1371/journal.pone.0138340

Mizushima N, 2008, NATURE, V451, P1069, DOI 10.1038/nature06639

Nicholson S, 1996, J EXP MED, V183, P2293, DOI 10.1084/jem.183.5.2293

Nozaki Y, 1997, INFECT IMMUN, V65, P3644, DOI 10.1128/IAI.65.9.3644-3647.1997

O'Connor GM, 2007, CLIN IMMUNOL, V124, P277, DOI 10.1016/j.clim.2007.05.016

Oehlers SH, 2015, NATURE, V517, P612, DOI 10.1038/nature13967

Oh J, 2017, J INFECTION, V74, P272, DOI 10.1016/j.jinf.2016.10.009

Orme IM, 2014, SEMIN IMMUNOL, V26, P601, DOI 10.1016/j.smim.2014.09.009

Pagán AJ, 2018, ANNU REV IMMUNOL, V36, P639, DOI 10.1146/annurev-immunol-032712-100022

Pagán AJ, 2015, CSH PERSPECT MED, V5, DOI 10.1101/cshperspect.a018499

Pai M, 2016, NAT REV DIS PRIMERS, V2, DOI 10.1038/nrdp.2016.76

Palucci I, 2018, CHEMOTHERAPY, V63, P172, DOI 10.1159/000490478

Parihar SP, 2014, J INFECT DIS, V209, P754, DOI 10.1093/infdis/jit550

Pende D, 1999, J EXP MED, V190, P1505, DOI 10.1084/jem.190.10.1505

Persson YAZ, 2008, MICROBES INFECT, V10, P233, DOI 10.1016/j.micinf.2007.11.007

Qrafli Mounia, 2017, BMC Res Notes, V10, P421, DOI [10.1186/s13104-017-2737-z, 10.1186/s13104-017-2737-z]

Rahman S, 2015, CLIN IMMUNOL, V156, P85, DOI 10.1016/j.clim.2014.12.003

Raja A, 2004, INDIAN J MED RES, V120, P213

Rayasaml GV, 2015, TRENDS PHARMACOL SCI, V36, P506, DOI 10.1016/j.tips.2015.05.005

Rekha RS, 2018, BMC INFECT DIS, V18, DOI 10.1186/s12879-018-3203-9

Rekha RS, 2015, AUTOPHAGY, V11, P1688, DOI 10.1080/15548627.2015.1075110

Reljic R, 2006, CLIN EXP IMMUNOL, V143, P467, DOI 10.1111/j.1365-2249.2006.03012.x

Ribeiro-Rodrigues R, 2006, CLIN EXP IMMUNOL, V144, P25, DOI 10.1111/j.1365-2249.2006.03027.x

Rich EA, 1997, TUBERCLE LUNG DIS, V78, P247, DOI 10.1016/S0962-8479(97)90005-8

Rizvi I, 2019, NEUROL INDIA, V67, P993, DOI 10.4103/0028-3886.266232

Romagnoli A, 2012, AUTOPHAGY, V8, P1357, DOI 10.4161/auto.20881

Sakowski ET, 2015, PLOS PATHOG, V11, DOI 10.1371/journal.ppat.1005076

Sarkar S, 2016, CRIT REV CL LAB SCI, V53, P132, DOI 10.3109/10408363.2015.1094443

Schiebler M, 2015, EMBO MOL MED, V7, P127, DOI 10.15252/emmm.201404137

Schierloh P, 2007, INFECT IMMUN, V75, P5325, DOI 10.1128/IAI.00381-07

Scriba TJ, 2017, PLOS PATHOG, V13, DOI 10.1371/journal.ppat.1006687

Selvaraj P, 2015, CAN J PHYSIOL PHARM, V93, P377, DOI 10.1139/cjpp-2014-0386

Shah NS, 2017, NEW ENGL J MED, V376, P243, DOI 10.1056/NEJMoa1604544

Sharma V, 2018, FRONT CELL DEV BIOL, V6, DOI 10.3389/fcell.2018.00147

Shastri MD, 2018, OXID MED CELL LONGEV, V2018, DOI 10.1155/2018/7695364

Sherman DR, 1996, SCIENCE, V272, P1641, DOI 10.1126/science.272.5268.1641

Shibutani ST, 2014, CELL RES, V24, P58, DOI 10.1038/cr.2013.159

Shin DM, 2010, PLOS PATHOG, V6, DOI 10.1371/journal.ppat.1001230

Siddiqui S, 2015, FRONT IMMUNOL, V6, DOI 10.3389/fimmu.2015.00337

Singh P, 2018, FRONT MICROBIOL, V9, DOI 10.3389/fmicb.2018.00070

Singhal A, 2014, SCI TRANSL MED, V6, DOI 10.1126/scitranslmed.3009885

Sivori S, 1997, J EXP MED, V186, P1129, DOI 10.1084/jem.186.7.1129

Skerry C, 2012, PLOS ONE, V7, DOI 10.1371/journal.pone.0039680

Songane M, 2012, TUBERCULOSIS, V92, P388, DOI 10.1016/j.tube.2012.05.004

Steinmann J, 2009, ANTIMICROB AGENTS CH, V53, P5127, DOI 10.1128/AAC.00818-09

Suliman S, 2018, AM J RESP CRIT CARE, V197, P1198, DOI 10.1164/rccm.201711-2340OC

Sumner T, 2019, SCI REP-UK, V9, DOI 10.1038/s41598-019-47645-z

Tan SM, 2017, METHODS MOL BIOL, V1519, P333, DOI 10.1007/978-1-4939-6581-6_23

Tenforde MW, 2017, JAIDS-J ACQ IMM DEF, V75, pE71, DOI 10.1097/QAI.0000000000001308

Timmins GS, 2006, MOL MICROBIOL, V62, P1220, DOI 10.1111/j.1365-2958.2006.05467.x

Tobin DM, 2015, CSH PERSPECT MED, V5, DOI 10.1101/cshperspect.a021196

Torfs E, 2019, INT J MOL SCI, V20, DOI 10.3390/ijms20122868

Vilaplana C, 2013, J INFECT DIS, V208, P199, DOI 10.1093/infdis/jit152

Vilchèze C, 2005, ANTIMICROB AGENTS CH, V49, P708, DOI 10.1128/AAC.49.2.708-720.2005

Vitale M, 1998, J EXP MED, V187, P2065, DOI 10.1084/jem.187.12.2065

Vordermeier HM, 1996, CLIN EXP IMMUNOL, V106, P312, DOI 10.1046/j.1365-2249.1996.d01-845.x

Voskuil MI, 2011, FRONT MICROBIOL, V2, DOI 10.3389/fmicb.2011.00105

Wallis RS, 2016, OPEN FORUM INFECT DI, V3, DOI 10.1093/ofid/ofw151

Wallis RS, 2015, NAT REV IMMUNOL, V15, P255, DOI 10.1038/nri3813

Wallis RS, 2004, AIDS, V18, P257, DOI 10.1097/00002030-200401230-00015

Wang CH, 2001, INT J TUBERC LUNG D, V5, P283

Wang QZ, 2019, J NUTR SCI VITAMINOL, V65, P390, DOI 10.3177/jnsv.65.390

Watson RO, 2012, CELL, V150, P803, DOI 10.1016/j.cell.2012.06.040

Weiner J III, 2018, NAT COMMUN, V9, DOI 10.1038/s41467-018-07635-7

Weiss G, 2015, IMMUNOL REV, V264, P182, DOI 10.1111/imr.12266

Williams A, 2004, IMMUNOLOGY, V111, P328, DOI 10.1111/j.1365-2567.2004.01809.x

Wolf AJ, 2007, J IMMUNOL, V179, P2509, DOI 10.4049/jimmunol.179.4.2509

Wong KW, 2017, MICROBIOL SPECTR, V5, DOI 10.1128/microbiolspec.TBTB2-0001-2015

Woodworth JSM, 2006, CRIT REV IMMUNOL, V26, P317, DOI 10.1615/CritRevImmunol.v26.i4.30

Wu YW, 2019, VIRULENCE, V10, P352, DOI 10.1080/21505594.2019.1602020

Yang ZF, 2009, CURR TOP MICROBIOL, V335, P1, DOI 10.1007/978-3-642-00302-8_1

Zak DE, 2016, LANCET, V387, P2312, DOI 10.1016/S0140-6736(15)01316-1

Zhai WJ, 2019, INT J MOL SCI, V20, DOI 10.3390/ijms20020340

Zhao YS, 2018, J IMMUNOL RES, V2018, DOI 10.1155/2018/5081634

NR 156

TC 45

Z9 53

U1 0

U2 7

PU MDPI

PI BASEL

PA MDPI AG, Grosspeteranlage 5, CH-4052 BASEL, SWITZERLAND

SN 2079-6382

J9 ANTIBIOTICS-BASEL

JI Antibiotics-Basel

PD JAN

PY 2020

VL 9

IS 1

AR 21

DI 10.3390/antibiotics9010021

PG 19

WC Infectious Diseases; Pharmacology & Pharmacy

WE Science Citation Index Expanded (SCI-EXPANDED)

SC Infectious Diseases; Pharmacology & Pharmacy

GA KL6IK

UT WOS:000513524700025

OA Green Submitted, gold

DA 2026-05-07

ER

PT J

AU Gokhale, KM

Telvekar, VN

AF Gokhale, Kunal M.

Telvekar, Vikas N.

TI Novel peptidomimetic peptide deformylase (PDF) inhibitors

ofMycobacterium tuberculosis

SO CHEMICAL BIOLOGY & DRUG DESIGN

LA English

DT Article

DE docking; MABA; Mtb; Mycobacterium tuberculosis; peptide deformylase

(PDF) inhibitor; peptidomimetic

ID ANTIMYCOBACTERIAL ACTIVITY; POLYPEPTIDE DEFORMYLASE; PHARMACOKINETICS;

GSK1322322; SAFETY; TARGET; ACIDS

AB Emergence of MDR-TB and XDR-TB led to the failure of available anti-tubercular drugs. In order to explore, identify and develop new anti-tubercular drugs, novel peptidomimetic series of Mtb-peptide deformylase (PDF) inhibitors was designed and synthesized. In vitro antimycobacterial potential of compounds was established by screening of compounds againstMycobacterium tuberculosisH37Rv strain using MABA. Among them, ester series of compounds4a,4b,4c,4d, and4ewere found most active, with compound4cbeing highly active and exhibiting minimum inhibitory concentration of 6.25 mu g/ml againstM. tbH37Rv strain. Additionally, the compounds were docked to determine the probable binding interactions and understand the mechanism of action of most active molecules on Mtb-peptide deformylase (PDF), which is involved in the mycobacterium protein synthesis.

C1 [Gokhale, Kunal M.; Telvekar, Vikas N.] Inst Chem Technol, Dept Pharmaceut Sci & Technol, Mumbai 400019, Maharashtra, India.

C3 Institute of Chemical Technology - Mumbai

RP Telvekar, VN (corresponding author), Inst Chem Technol, Dept Pharmaceut Sci & Technol, Mumbai 400019, Maharashtra, India.

EM vikastelvekar@rediffmail.com

RI Gokhale, Kunal/AEQ-3590-2022

OI Gokhale, Kunal/0000-0002-0629-9686

CR ADAMS JM, 1968, J MOL BIOL, V33, P571, DOI 10.1016/0022-2836(68)90307-0

Aubart K, 2006, PROGR MED CHEM, V44, P109, DOI 10.1016/S0079-6468(05)44403-3

Biava M, 2008, J MED CHEM, V51, P3644, DOI 10.1021/jm701560p

BROUGHTON BJ, 1975, J CHEM SOC PERK T 1, P857, DOI 10.1039/p19750000857

Clements JM, 2001, ANTIMICROB AGENTS CH, V45, P563, DOI 10.1128/AAC.45.2.563-570.2001

DOMAGALA JM, 1981, J ORG CHEM, V46, P134, DOI 10.1021/jo00314a029

Giglione C, 2000, MOL MICROBIOL, V36, P1197, DOI 10.1046/j.1365-2958.2000.01908.x

HATANAKA M, 1973, J MED CHEM, V16, P978, DOI 10.1021/jm00267a005

Hoover J, 2016, ANTIMICROB AGENTS CH, V60, P180, DOI 10.1128/AAC.01842-15

Hu YJ, 1999, BIOCHEMISTRY-US, V38, P643, DOI 10.1021/bi9820412

Jain R, 2005, CURR MED CHEM, V12, P1607, DOI 10.2174/0929867054367194

JERCHEL D, 1955, CHEM BER-RECL, V88, P156, DOI 10.1002/cber.19550880125

Johnson K. W., 2005, Current Drug Targets - Infectious Disorders, V5, P39, DOI 10.2174/1568005053174618

Karanfil A, 2012, TETRAHEDRON, V68, P10218, DOI 10.1016/j.tet.2012.09.081

Lee SK, 2010, BIOORG MED CHEM LETT, V20, P4317, DOI 10.1016/j.bmcl.2010.06.088

Mazel D, 1997, J MOL BIOL, V266, P939, DOI 10.1006/jmbi.1996.0835

Naderer OJ, 2013, ANTIMICROB AGENTS CH, V57, P2005, DOI 10.1128/AAC.01779-12

Navarrete-Vázquez G, 2007, BIOORGAN MED CHEM, V15, P5502, DOI 10.1016/j.bmc.2007.05.053

Pichota A, 2008, BIOORG MED CHEM LETT, V18, P6568, DOI 10.1016/j.bmcl.2008.10.040

Rolan P, 2011, CLIN PHARMACOL THER, V90, P256, DOI 10.1038/clpt.2011.101

Smith KJ, 2003, PROTEIN SCI, V12, P349, DOI 10.1110/ps.0229303

Teo JWP, 2006, ANTIMICROB AGENTS CH, V50, P3665, DOI 10.1128/AAC.00555-06

Vaidya I. S., 2011, IJAPR, V2, P405

Verma S. K., 2011, PHARMACOPHORE, V2, P114

Wadhwa K, 2008, SYNTHETIC COMMUN, V38, P4434, DOI 10.1080/00397910802369554

Yuan ZY, 2006, BIOCHEM PHARMACOL, V71, P1042, DOI 10.1016/j.bcp.2005.10.015

NR 26

TC 11

Z9 12

U1 0

U2 21

PU WILEY

PI HOBOKEN

PA 111 RIVER ST, HOBOKEN 07030-5774, NJ USA

SN 1747-0277

EI 1747-0285

J9 CHEM BIOL DRUG DES

JI Chem. Biol. Drug Des.

PD JAN

PY 2021

VL 97

IS 1

BP 148

EP 156

DI 10.1111/cbdd.13769

EA AUG 2020

PG 9

WC Biochemistry & Molecular Biology; Chemistry, Medicinal

WE Science Citation Index Expanded (SCI-EXPANDED); Index Chemicus (IC)

SC Biochemistry & Molecular Biology; Pharmacology & Pharmacy

GA PK7XJ

UT WOS:000562004000001

DA 2026-05-07

ER

PT J

AU Alsayed, SSR

Lun, S

Payne, A

Bishai, WR

Gunosewoyo, H

AF Alsayed, Shahinda S. R.

Lun, Shichun

Payne, Alan

Bishai, William R.

Gunosewoyo, Hendra

TI Design, synthesis and antimycobacterial evaluation of novel adamantane

and adamantanol analogues effective against drug-resistant tuberculosis

SO BIOORGANIC CHEMISTRY

LA English

DT Article

DE Tuberculosis; MmpL3; Indoleamides; Adamantane; Adamantanol; MDR-TB;

XDR-TB; Cytotoxicity; Water solubility

ID MYCOLIC ACID TRANSPORT; MYCOBACTERIUM-TUBERCULOSIS; MULTIDRUG-RESISTANT;

BIOLOGICAL EVALUATION; MEMBRANE TRANSPORTER; CELL-WALL; MMPL3;

DISCOVERY; TARGETS; DERIVATIVES

AB The treacherous nature of tuberculosis (TB) combined with the ubiquitous presence of the drug-resistant (DR) forms pose this disease as a growing public health menace. Therefore, it is imperative to develop new chemotherapeutic agents with a novel mechanism of action to circumvent the cross-resistance problems. The unique architecture of the Mycobacterium tuberculosis (M. tb) outer envelope plays a predominant role in its pathogenesis, contributing to its intrinsic resistance against available therapeutic agents. The mycobacterial membrane protein large 3 (MmpL3), which is a key player in forging the M. tb rigid cell wall, represents an emerging target for TB drug development. Several indole-2-carboxamides were previously identified in our group as potent anti-TB agents that act as inhibitor of MmpL3 transporter protein. Despite their highly potent in vitro activities, the lingering Achilles heel of these indoleamides can be ascribed to their high lipophilicity as well as low water solubility. In this study, we report our attempt to improve the aqueous solubility of these indole-2-carboxamides while maintaining an adequate lipophilicity to allow effective M. tb cell wall penetration. A more polar adamantanol moiety was incorporated into the framework of several indole-2-carboxamides, whereupon the corresponding analogues were tested for their anti-TB activity against drug-sensitive (DS) M. tb H37Rv strain. Three adamantanol derivatives 8i, 8j and 8l showed nearly 2- and 4-fold higher activity (MIC = 1.32 - 2.89 mu M) than ethambutol (MIC = 4.89 mu M). Remarkably, the most potent adamantanol analogue 8j demonstrated high selectivity towards DS and DR M. tb strains over mammalian cells [IC50 (Vero cells) >= 169 mu M], On evincing its lack of cytotoxicity. The top eight active compounds 8b, 8d, 8f, 8i, 8j, 8k, 8l and 10a retained their in vitro potency against DR M. tb strains and were docked into the MmpL3 active site. The most potent adamantanol/adamantane-based indoleamides 8j/8k displayed a two-fold surge in potency against extensively DR (XDR) M. tb strains with MIC values of 0.66 and 0.012 mu M, respectively. The adamantanol-containing indole-2-carboxamides exhibited improved water solubility both in silico and experimentally, relative to the adamantane counterparts. Overall, the observed antimycobacterial and physicochemical profiles support the notion that adamantanol moiety is a suitable replacement to the adamantane scaffold within the series of indole-2-carboxamide-based MmpL3 inhibitors.

C1 [Alsayed, Shahinda S. R.; Gunosewoyo, Hendra] Curtin Univ, Fac Hlth Sci, Sch Pharm & Biomed Sci, Perth, WA 6102, Australia.

[Lun, Shichun; Bishai, William R.] Johns Hopkins Sch Med, Dept Med, Div Infect Dis, Ctr TB Res, 1550 Orleans St, Baltimore, MD 21231 USA.

[Payne, Alan] Curtin Univ, Sch Mol & Life Sci, Perth, WA 6102, Australia.

[Bishai, William R.] Howard Hughes Med Inst, 4000 Jones Bridge Rd, Chevy Chase, MD 20815 USA.

C3 Curtin University; Johns Hopkins University; Johns Hopkins Medicine;

Curtin University; Howard Hughes Medical Institute

RP Gunosewoyo, H (corresponding author), Curtin Univ, Fac Hlth Sci, Sch Pharm & Biomed Sci, Perth, WA 6102, Australia.; Bishai, WR (corresponding author), Johns Hopkins Sch Med, Dept Med, Div Infect Dis, Ctr TB Res, 1550 Orleans St, Baltimore, MD 21231 USA.; Bishai, WR (corresponding author), Howard Hughes Med Inst, 4000 Jones Bridge Rd, Chevy Chase, MD 20815 USA.

EM wbishai1@jhmi.edu; Hendra.Gunosewoyo@curtin.edu.au

RI Payne, Alan/G-4995-2015; Gunosewoyo, Hendra/AAR-6193-2020

OI Gunosewoyo, Hendra/0000-0003-3897-1948

FU Curtin International Postgraduate Research Scholarship (CIPRS); NIH [AI

37856, HL 133190]; Australian Research Council - Australian Government

[DE160100482]; Australian Research Council [DE160100482] Funding Source:

Australian Research Council

FX SSRA is thankful for the support of Curtin International Postgraduate

Research Scholarship (CIPRS). WRB acknowledges the support of NIH grants

AI 37856 and HL 133190. HG is the recipient of an Australian Research

Council Discovery Early Career Researcher Award (DE160100482) funded by

the Australian Government.

CR Alsayed SSR, 2020, RSC ADV, V10, P7523, DOI 10.1039/c9ra10663d

Alsayed SSR, 2019, CURR MOL PHARMACOL, V12, P27, DOI 10.2174/1874467211666181025141114

Andries K, 2005, SCIENCE, V307, P223, DOI 10.1126/science.1106753

[Anonymous], 2003, AM J RESP CRIT CARE, V167, P603

[Anonymous], 2019, GLOB TUB REP

Barberis I, 2017, J Prev Med Hyg, V58, pE9

Blaser MJ, 2007, NATURE, V449, P843, DOI 10.1038/nature06198

Blomgran R, 2012, CELL HOST MICROBE, V11, P81, DOI 10.1016/j.chom.2011.11.012

Brennan PJ, 2003, TUBERCULOSIS, V83, P91, DOI 10.1016/S1472-9792(02)00089-6

BRENNAN PJ, 1995, ANNU REV BIOCHEM, V64, P29, DOI 10.1146/annurev.bi.64.070195.000333

Brown JR, 2011, BIOORGAN MED CHEM, V19, P5585, DOI 10.1016/j.bmc.2011.07.034

Cambier CJ, 2014, CELL, V159, P1497, DOI 10.1016/j.cell.2014.11.024

Caminero JA, 2006, INT J TUBERC LUNG D, V10, P829

Caminero JA, 2010, LANCET INFECT DIS, V10, P621, DOI 10.1016/S1473-3099(10)70139-0

Campaniço A, 2018, EUR J MED CHEM, V150, P525, DOI 10.1016/j.ejmech.2018.03.020

Chackerian AA, 2002, INFECT IMMUN, V70, P4501, DOI 10.1128/IAI.70.8.4501-4509.2002

Chan ED, 2004, AM J RESP CRIT CARE, V169, P1103, DOI 10.1164/rccm.200308-1159OC

Collins LA, 1997, ANTIMICROB AGENTS CH, V41, P1004, DOI 10.1128/AAC.41.5.1004

Degiacomi G, 2017, SCI REP-UK, V7, DOI 10.1038/srep43495

Dheda K, 2016, LANCET, V387, P1211, DOI 10.1016/S0140-6736(15)00151-8

Eker B, 2008, EMERG INFECT DIS, V14, P1700, DOI 10.3201/eid1411.080729

Franz ND, 2017, BIOORGAN MED CHEM, V25, P3746, DOI 10.1016/j.bmc.2017.05.015

Grzegorzewicz AE, 2012, NAT CHEM BIOL, V8, P334, DOI [10.1038/NCHEMBIO.794, 10.1038/nchembio.794]

Hoffmann C, 2008, P NATL ACAD SCI USA, V105, P3963, DOI 10.1073/pnas.0709530105

Kaufmann SHE, 2005, NAT MED, V11, pS33, DOI 10.1038/nm1221

Keshavjee S, 2012, NEW ENGL J MED, V367, P931, DOI 10.1056/NEJMra1205429

Khader SA, 2006, J EXP MED, V203, P1805, DOI 10.1084/jem.20052545

Kozikowski AP, 2017, J MED CHEM, V60, P5876, DOI 10.1021/acs.jmedchem.7b00582

Li W, 2019, ACS INFECT DIS, V5, P1001, DOI 10.1021/acsinfecdis.9b00048

Li W, 2016, ANTIMICROB AGENTS CH, V60, P5198, DOI 10.1128/AAC.00826-16

Li W, 2014, ANTIMICROB AGENTS CH, V58, P6413, DOI 10.1128/AAC.03229-14

Lipinski CA, 1997, ADV DRUG DELIVER REV, V23, P3, DOI 10.1016/S0169-409X(96)00423-1

Liu J, 1996, J BIOL CHEM, V271, P29545, DOI 10.1074/jbc.271.47.29545

Lun SC, 2013, NAT COMMUN, V4, DOI 10.1038/ncomms3907

Migliori GB, 2007, EUR RESPIR J, V29, P423, DOI 10.1183/09031936.00001307

Mitnick CD, 2008, NEW ENGL J MED, V359, P563, DOI 10.1056/NEJMoa0800106

Nataraj V, 2015, MOL MICROBIOL, V98, P7, DOI 10.1111/mmi.13101

Onajole OK, 2013, J MED CHEM, V56, P4093, DOI 10.1021/jm4003878

Owens CP, 2013, FUTURE MED CHEM, V5, P1391, DOI 10.4155/fmc.13.109

Owens CP, 2013, J BIOL CHEM, V288, P21714, DOI 10.1074/jbc.M113.453076

Padiya KJ, 2012, ORG LETT, V14, P2814, DOI 10.1021/ol301009d

Parida SK, 2015, J INTERN MED, V277, P388, DOI 10.1111/joim.12264

Pawlowski A, 2012, PLOS PATHOG, V8, DOI 10.1371/journal.ppat.1002464

Pieroni M, 2011, CHEMMEDCHEM, V6, P334, DOI 10.1002/cmdc.201000490

Rao SPS, 2013, SCI TRANSL MED, V5, DOI 10.1126/scitranslmed.3007355

Scherman MS, 2012, BIOORGAN MED CHEM, V20, P3255, DOI 10.1016/j.bmc.2012.03.058

Stanley SA, 2012, ACS CHEM BIOL, V7, P1377, DOI 10.1021/cb300151m

Stec J, 2016, J MED CHEM, V59, P6232, DOI 10.1021/acs.jmedchem.6b00415

Sutherland HS, 2018, BIOORGAN MED CHEM, V26, P1797, DOI 10.1016/j.bmc.2018.02.026

Tahlan K, 2012, ANTIMICROB AGENTS CH, V56, P1797, DOI 10.1128/AAC.05708-11

Takayama K, 2005, CLIN MICROBIOL REV, V18, P81, DOI 10.1128/CMR.18.1.81-101.2005

Tullius MV, 2011, P NATL ACAD SCI USA, V108, P5051, DOI 10.1073/pnas.1009516108

Varela C, 2012, CHEM BIOL, V19, P498, DOI 10.1016/j.chembiol.2012.03.006

Velayati AA, 2013, INT J CLIN EXP MED, V6, P307

Wanka L, 2013, CHEM REV, V113, P3516, DOI 10.1021/cr100264t

WAYNE LG, 1994, EUR J CLIN MICROBIOL, V13, P908, DOI 10.1007/BF02111491

Worley MV, 2014, PHARMACOTHERAPY, V34, P1187, DOI 10.1002/phar.1482

Xu ZJ, 2017, P NATL ACAD SCI USA, V114, P7993, DOI 10.1073/pnas.1700062114

Yamamoto K, 2016, J DRUG DELIV SCI TEC, V33, P13, DOI 10.1016/j.jddst.2016.03.002

Zhang B, 2019, CELL, V176, P636, DOI 10.1016/j.cell.2019.01.003

NR 60

TC 16

Z9 21

U1 0

U2 15

PU ACADEMIC PRESS INC ELSEVIER SCIENCE

PI SAN DIEGO

PA 525 B ST, STE 1900, SAN DIEGO, CA 92101-4495 USA

SN 0045-2068

EI 1090-2120

J9 BIOORG CHEM

JI Bioorganic Chem.

PD JAN

PY 2021

VL 106

AR 104486

DI 10.1016/j.bioorg.2020.104486

PG 12

WC Biochemistry & Molecular Biology; Chemistry, Organic

WE Science Citation Index Expanded (SCI-EXPANDED); Index Chemicus (IC)

SC Biochemistry & Molecular Biology; Chemistry

GA PO2OK

UT WOS:000605008800004

OA Green Submitted

DA 2026-05-07

ER

PT J

AU Desikan, P

Rangnekar, A

AF Desikan, Prabha

Rangnekar, Aseem

TI Host-targeted therapy for tuberculosis: Time to revisit the concept

SO INDIAN JOURNAL OF MEDICAL RESEARCH

LA English

DT Review

DE Host-targeted therapy; micro RNA; multidrug-resistant/extensively

drug-resistant TB; VEGF

ID ENDOTHELIAL GROWTH-FACTOR; MYCOBACTERIUM-TUBERCULOSIS; PULMONARY

TUBERCULOSIS; HUMAN MACROPHAGES; DIRECTED THERAPY; IMMUNE-RESPONSE;

IFN-GAMMA; AUTOPHAGY; ENHANCEMENT; VACCINES

AB Tuberculosis (TB) is an important cause of morbidity and mortality worldwide. Every year millions of people die due to TB. Drug resistance has been a major factor that has obstructed successful control and treatment of TB. As the rate of spread of drug-resistant TB outpaces the rate of discovery of new anti-tubercular drugs, targeted therapy may provide a new approach to TB cure. In a scenario where drug resistance is spreading rapidly, and existing drugs regimens seem to be dwindling away, this review summarizes the concept of host-targeted therapy which may be the ray of hope for the effective management and control of the rapidly spreading drug-resistant TB (multidrug resistant and extensively drug resistant).

C1 [Desikan, Prabha; Rangnekar, Aseem] Bhopal Mem Hosp & Res Ctr, Dept Microbiol, Natl Reference Lab TB, Bhopal, India.

C3 Indian Council of Medical Research (ICMR); ICMR - Bhopal Memorial

Hospital & Research Center (BMHRC)

RP Desikan, P (corresponding author), Bhopal Mem Hosp & Res Ctr, Dept Microbiol, Bhopal 462038, Madhya Pradesh, India.

EM prabhadesikan@yahoo.com

CR Alatas F, 2004, CHEST, V125, P2156, DOI 10.1378/chest.125.6.2156

[Anonymous], INT GUID US BED TREA

Balgi AD, 2009, PLOS ONE, V4, DOI 10.1371/journal.pone.0007124

Bento CF, 2015, DNA CELL BIOL, V34, P228, DOI 10.1089/dna.2014.2745

Bradfute SB, 2013, CURR OPIN MICROBIOL, V16, P355, DOI 10.1016/j.mib.2013.05.003

Bruns H, 2012, J IMMUNOL, V189, P4069, DOI 10.4049/jimmunol.1201538

Campbell GR, 2012, PLOS PATHOG, V8, DOI 10.1371/journal.ppat.1002689

Cheallaigh CN, 2011, CLIN EXP IMMUNOL, V164, P291, DOI 10.1111/j.1365-2249.2011.04381.x

Fabri M, 2011, SCI TRANSL MED, V3, DOI 10.1126/scitranslmed.3003045

Gupta A, 2016, ADV DRUG DELIVER REV, V102, P10, DOI 10.1016/j.addr.2016.01.016

Gutierrez MG, 2004, CELL, V119, P753, DOI 10.1016/j.cell.2004.11.038

Harris RC, 2016, BMC INFECT DIS, V16, DOI 10.1186/s12879-016-1585-0

Hoagland DT, 2016, ADV DRUG DELIVER REV, V102, P55, DOI 10.1016/j.addr.2016.04.026

Iannaccone M, 2014, EXPERT OPIN THER TAR, V18, P491, DOI 10.1517/14728222.2014.897696

Kiran D, 2016, SEMIN IMMUNOPATHOL, V38, P167, DOI 10.1007/s00281-015-0537-x

Lam KKY, 2012, PLOS PATHOG, V8, DOI 10.1371/journal.ppat.1002691

Luciani F, 2009, P NATL ACAD SCI USA, V106, P14711, DOI 10.1073/pnas.0902437106

MacMicking JD, 2003, SCIENCE, V302, P654, DOI 10.1126/science.1088063

Madansein R, 2015, INT J INFECT DIS, V32, P61, DOI 10.1016/j.ijid.2015.01.019

Manca C, 2013, PLOS ONE, V8, DOI 10.1371/journal.pone.0074082

Matsuyama W, 2000, AM J RESP CRIT CARE, V162, P1120, DOI 10.1164/ajrccm.162.3.9911010

Meerak J, 2013, VACCINE, V31, P784, DOI 10.1016/j.vaccine.2012.11.075

Nagahara Y, 2013, BIOCHEM BIOPH RES CO, V435, P657, DOI 10.1016/j.bbrc.2013.05.040

Ong CWM, 2015, PLOS PATHOG, V11, DOI 10.1371/journal.ppat.1004917

Parihar SP, 2014, J INFECT DIS, V209, P754, DOI 10.1093/infdis/jit550

Pernicova I, 2014, NAT REV ENDOCRINOL, V10, P143, DOI 10.1038/nrendo.2013.256

Ray KK, 2010, ARCH INTERN MED, V170, P1024, DOI 10.1001/archinternmed.2010.182

Rivas-Santiago B, 2014, SCAND J INFECT DIS, V46, P161, DOI 10.3109/00365548.2013.871645

Rubinsztein DC, 2007, NAT REV DRUG DISCOV, V6, P304, DOI 10.1038/nrd2272

Sachan M, 2016, CURR PHARM DESIGN, V22, P2599, DOI 10.2174/1381612822666160128150636

Sarkar S, 2005, J CELL BIOL, V170, P1101, DOI 10.1083/jcb.200504035

Singh SB, 2006, SCIENCE, V313, P1438, DOI 10.1126/science.1129577

Singhal A, 2014, SCI TRANSL MED, V6, DOI 10.1126/scitranslmed.3009885

Sloan B, 2008, CURR OPIN INVEST DR, V9, P1324

Stanley SA, 2014, PLOS PATHOG, V10, DOI 10.1371/journal.ppat.1003946

Suárez-Méndez R, 2004, BMC INFECT DIS, V4, DOI 10.1186/1471-2334-4-44

Sundaramurthy V, 2013, CELL HOST MICROBE, V13, P129, DOI 10.1016/j.chom.2013.01.008

Villarreal DO, 2014, HUM VACC IMMUNOTHER, V10, P2188, DOI 10.4161/hv.29574

Wei YM, 2013, CELL PHYSIOL BIOCHEM, V31, P925, DOI 10.1159/000350111

WHO, 2016, GLOB TUB REP 2016

Wienecke R, 2006, AM J KIDNEY DIS, V48, pE27, DOI 10.1053/j.ajkd.2006.05.018

World Health Organization, BEND CURV END TB ANN

World Health Organization, INT GUID US DEL TREA

Yu XW, 2013, CELL SIGNAL, V25, P1272, DOI 10.1016/j.cellsig.2013.02.011

Zárate-Bladés CR, 2013, HUM VACC IMMUNOTHER, V9, P1093, DOI 10.4161/hv.23417

NR 45

TC 4

Z9 6

U1 1

U2 2

PU WOLTERS KLUWER MEDKNOW PUBLICATIONS

PI MUMBAI

PA WOLTERS KLUWER INDIA PVT LTD , A-202, 2ND FLR, QUBE, C T S NO 1498A-2

VILLAGE MAROL, ANDHERI EAST, MUMBAI, 400059, INDIA

SN 0971-5916

J9 INDIAN J MED RES

JI Indian J. Med. Res.

PD MAR

PY 2018

VL 147

BP 233

EP 238

DI 10.4103/ijmr.IJMR_652_17

PG 6

WC Immunology; Medicine, General & Internal; Medicine, Research &

Experimental

WE Science Citation Index Expanded (SCI-EXPANDED)

SC Immunology; General & Internal Medicine; Research & Experimental

Medicine

GA GK8TL

UT WOS:000436501200005

OA gold

DA 2026-05-07

ER

PT J

AU Kumar, D

Negi, B

Rawat, DS

AF Kumar, Deepak

Negi, Beena

Rawat, Diwan S.

TI The anti-tuberculosis agents under development and the challenges ahead

SO FUTURE MEDICINAL CHEMISTRY

LA English

DT Review

ID DRUG-RESISTANT TUBERCULOSIS; POTENT ANTIMYCOBACTERIAL ACTIVITY; KILL

MYCOBACTERIUM-TUBERCULOSIS; D-RIBOSE 2'-EPIMERASE; CELL-WALL CORE;

IN-VITRO; MULTIDRUG-RESISTANT; BACTERICIDAL ACTIVITY; MURINE MODEL;

PULMONARY TUBERCULOSIS

AB Tuberculosis (TB) is a serious health problem causing 1.5 million deaths worldwide. After the discovery of first-line anti-TB drugs, the mortality rate declined sharply, however, the emergence of drug-resistant strains and HIV co-infection have led to increased incidence of this disease. A number of new potential antitubercular drug candidates with novel modes of action have entered clinical trials in recent years. Compounds such as gatifloxacin, moxifloxacin and linezolid, the already known antibiotics are currently being evaluated for their anti-TB activity. OPC-67683 and TMC207 have been approved for the treatment of MDR-TB patients recently, while PA-824, SQ109, PNU-100480, AZD5847, LL3858, SQ609, SQ641, BTZ043, DC-159a, CPZEN-45, Q-203, DNB1, TBA-354 are in various phases of clinical and preclinical developments. This review evaluates the current status of TB drug development and future aspects.

C1 [Kumar, Deepak; Negi, Beena; Rawat, Diwan S.] Univ Delhi, Dept Chem, Delhi 110007, India.

C3 University of Delhi

RP Rawat, DS (corresponding author), Univ Delhi, Dept Chem, Delhi 110007, India.

EM dsrawat@chemistry.du.ac.in

RI Rawat, Diwan/B-1150-2008; Negi, Beena/AHB-6171-2022

OI Rawat, Diwan/0000-0002-5473-7476; Negi, Beena/0000-0003-1198-9478

FU DU-DST-PURSE; University of Delhi

FX DS Rawat thanks DU-DST-PURSE grant and University of Delhi for financial

assistance. The authors have no other relevant affiliations or financial

involvement with any organization or entity with a financial interest in

or financial conflict with the subject matter or materials discussed in

the manuscript apart from those disclosed.

CR Abrahams KA, 2012, PLOS ONE, V7, DOI 10.1371/journal.pone.0052951

Ahmad Z, 2011, ANTIMICROB AGENTS CH, V55, P1781, DOI 10.1128/AAC.01514-10

Albanna AS, 2011, DRUGS, V71, P815, DOI 10.2165/11585440-000000000-00000

Andersen P, 2005, NAT REV MICROBIOL, V3, P656, DOI 10.1038/nrmicro1211

Andries K, 2005, SCIENCE, V307, P223, DOI 10.1126/science.1106753

Anger HA, 2010, J ANTIMICROB CHEMOTH, V65, P775, DOI 10.1093/jac/dkq017

[Anonymous], 2006, PCT Int. Appl., Patent No. [W02006123792 Al, 2006123792]

[Anonymous], 1948, BMJ-BRIT MED J, V2, P769

Arora S. K., 2004, [No title captured], Patent No. [WO2004/026828A1, 2004026828]

Balasubramanian V, 2014, ANTIMICROB AGENTS CH, V58, P495, DOI 10.1128/AAC.01903-13

Barbachyn MR, 2003, ANGEW CHEM INT EDIT, V42, P2010, DOI 10.1002/anie.200200528

Barbachyn MR, 1996, BIOORG MED CHEM LETT, V6, P1009, DOI 10.1016/0960-894X(96)00155-2

Barbachyn MR, 1996, J MED CHEM, V39, P680, DOI 10.1021/jm950956y

Barbachyn MR., 1997, SPEC PUBL R SOC CHEM, V198, P15

Barnes PF, 2009, TUBERCULOSIS, V89, pS5, DOI 10.1016/S1472-9792(09)70004-6

BARRY VC, 1957, NATURE, V179, P1013, DOI 10.1038/1791013a0

Biava M., 2006, Infectious Disorders - Drug Targets, V6, P159, DOI 10.2174/187152606784112173

Blaser A, 2012, J MED CHEM, V55, P312, DOI 10.1021/jm2012276

Bogatcheva E, 2011, BIOORG MED CHEM LETT, V21, P5353, DOI 10.1016/j.bmcl.2011.07.015

Bogatcheva E, 2011, J ANTIMICROB CHEMOTH, V66, P578, DOI 10.1093/jac/dkq495

Bogatcheva E, 2010, BIOORG MED CHEM LETT, V20, P201, DOI 10.1016/j.bmcl.2009.10.135

Brecik M, 2015, ACS CHEM BIOL, V10, P1631, DOI 10.1021/acschembio.5b00237

Brickner SJ, 1996, J MED CHEM, V39, P673, DOI 10.1021/jm9509556

Buroni S, 2012, UNDERSTANDING TUBERCULOSIS - NEW APPROACHES TO FIGHTING AGAINST DRUG RESISTANCE, P273

Christophe T, 2009, PLOS PATHOG, V5, DOI 10.1371/journal.ppat.1000645

Crellin PK, 2011, PLOS ONE, V6, DOI 10.1371/journal.pone.0016869

Dartois V, 2014, NAT REV MICROBIOL, V12, P159, DOI 10.1038/nrmicro3200

Dartois V, 2013, BIOORG MED CHEM LETT, V23, P4741, DOI 10.1016/j.bmcl.2013.07.006

Davies Peter D O, 2003, Am J Respir Med, V2, P203

Deidda D, 1998, ANTIMICROB AGENTS CH, V42, P3035, DOI 10.1128/AAC.42.11.3035

Di Paolo A, 2010, CLIN PHARMACOKINET, V49, P439, DOI 10.2165/11319960-000000000-00000

Diacon AH, 2012, LANCET, V380, P986, DOI 10.1016/S0140-6736(12)61080-0

Diacon AH, 2010, ANTIMICROB AGENTS CH, V54, P3402, DOI 10.1128/AAC.01354-09

Pham DD, 2015, INT J PHARMACEUT, V478, P517, DOI 10.1016/j.ijpharm.2014.12.009

Disratthakit A, 2010, ANTIMICROB AGENTS CH, V54, P2684, DOI 10.1128/AAC.01545-09

Dong YZ, 1998, ANTIMICROB AGENTS CH, V42, P2978, DOI 10.1128/AAC.42.11.2978

Dooley KE, 2013, J INFECT DIS, V207, P1352, DOI 10.1093/infdis/jis460

Dorman SE, 2012, J INFECT DIS, V206, P1030, DOI 10.1093/infdis/jis461

Engohang-Ndong J, 2012, EXPERT OPIN INV DRUG, V21, P1789, DOI 10.1517/13543784.2012.724397

Gillespie SH, 2014, NEW ENGL J MED, V371, P1577, DOI 10.1056/NEJMoa1407426

Ginsberg AM, 2009, ANTIMICROB AGENTS CH, V53, P3720, DOI 10.1128/AAC.00106-09

Ginsberg AM, 2009, ANTIMICROB AGENTS CH, V53, P3726, DOI 10.1128/AAC.00112-09

*GLOB ALL TB DRUG, 2000, EX SUMM SCI BLUEPR T

Goldman RC, 2013, TUBERCULOSIS, V93, P569, DOI 10.1016/j.tube.2013.09.003

Gootz TD, 1996, MED RES REV, V16, P433, DOI 10.1002/(SICI)1098-1128(199609)16:5<433::AID-MED3>3.0.CO;2-W

Gravestock MB, 2003, BIOORG MED CHEM LETT, V13, P4179, DOI 10.1016/j.bmcl.2003.07.033

GRUMBACH F, 1956, CR HEBD ACAD SCI, V242, P2187

Grzegorzewicz AE, 2012, NAT CHEM BIOL, V8, P334, DOI [10.1038/NCHEMBIO.794, 10.1038/nchembio.794]

Haagsma AC, 2011, PLOS ONE, V6, DOI 10.1371/journal.pone.0023575

Haagsma AC, 2009, ANTIMICROB AGENTS CH, V53, P1290, DOI 10.1128/AAC.01393-08

Heinrich N, 2015, J ANTIMICROB CHEMOTH, V70, P1558, DOI 10.1093/jac/dku553

Hirano S, 2008, BIOORGAN MED CHEM, V16, P5123, DOI 10.1016/j.bmc.2008.03.020

Hu YM, 2003, ANTIMICROB AGENTS CH, V47, P653, DOI 10.1128/AAC.47.2.653-657.2003

Huang TS, 2008, ANTIMICROB AGENTS CH, V52, P2226, DOI 10.1128/AAC.00414-07

HUDGINS PC, 1955, AM REV TUBERC PULM, V72, P685

Hutchinson DK, 1995, 35 INT C ANT AG CHEM

Igarahi M, 2003, J ANTIBIOT, V56, P580, DOI 10.7164/antibiotics.56.580

Ippolito JA, 2008, J MED CHEM, V51, P3353, DOI 10.1021/jm800379d

Irwin SM, 2014, ANTIMICROB AGENTS CH, V58, P4026, DOI 10.1128/AAC.02565-14

Ishizaki Y, 2013, J BIOL CHEM, V288, P30309, DOI 10.1074/jbc.M113.492173

Jia J, 2005, BRIT J PHARMACOL, V144, P80, DOI 10.1038/sj.bjp.0705984

Jindani A, 2014, NEW ENGL J MED, V371, P1599, DOI 10.1056/NEJMoa1314210

Kang S, 2014, J MED CHEM, V57, P5293, DOI 10.1021/jm5003606

Kasim NA, 2004, MOL PHARMACEUT, V1, P85, DOI 10.1021/mp034006h

Kim P, 2009, J MED CHEM, V52, P1329, DOI 10.1021/jm801374t

Kim P, 2009, J MED CHEM, V52, P1317, DOI 10.1021/jm801246z

KLEMENS SP, 1993, ANTIMICROB AGENTS CH, V37, P2344, DOI 10.1128/AAC.37.11.2344

Kmentova I, 2010, J MED CHEM, V53, P8421, DOI 10.1021/jm101288t

Koga T, 2004, J ANTIMICROB CHEMOTH, V54, P755, DOI 10.1093/jac/dkh417

Koh WJ, 2012, J ANTIMICROB CHEMOTH, V67, P1503, DOI 10.1093/jac/dks078

KONNO K, 1967, AM REV RESPIR DIS, V95, P461

Koul A, 2007, NAT CHEM BIOL, V3, P323, DOI 10.1038/nchembio884

KRAJEWSKA MM, 1993, J INFECT DIS, V167, P899, DOI 10.1093/infdis/167.4.899

L'homme RFA, 2009, AIDS, V23, P863, DOI 10.1097/QAD.0b013e328329148e

Leach KL, 2011, ANN NY ACAD SCI, V1222, P49, DOI 10.1111/j.1749-6632.2011.05962.x

Lechartier B, 2012, ANTIMICROB AGENTS CH, V56, P5790, DOI 10.1128/AAC.01476-12

Lee E, 2003, CLIN INFECT DIS, V37, P1389, DOI 10.1086/379012

Lee M, 2012, NEW ENGL J MED, V367, P1508, DOI 10.1056/NEJMoa1201964

Lee RE, 2003, J COMB CHEM, V5, P172, DOI 10.1021/cc020071p

LEHMANN J, 1946, LANCET, V250, P15

Lenaerts AJ, 2005, ANTIMICROB AGENTS CH, V49, P2294, DOI 10.1128/AAC.49.6.2294-2301.2005

Lu Y, 2011, ANTIMICROB AGENTS CH, V55, P5185, DOI 10.1128/AAC.00699-11

Luetkemeyer AF, 2011, AM J RESP CRIT CARE, V184, P1107, DOI 10.1164/rccm.201106-0995PP

Magnet S, 2010, TUBERCULOSIS, V90, P354, DOI 10.1016/j.tube.2010.09.001

Makarov V, 2014, EMBO MOL MED, V6, P372, DOI 10.1002/emmm.201303575

Makarov V, 2009, SCIENCE, V324, P801, DOI 10.1126/science.1171583

Manina G, 2010, CURR MED CHEM, V17, P3099, DOI 10.2174/092986710791959693

Manjunatha Ujjini, 2009, Commun Integr Biol, V2, P215

Masjedi MR, 2006, CLIN INFECT DIS, V43, P841, DOI 10.1086/507542

Matsumoto M, 2006, PLOS MED, V3, P2131, DOI 10.1371/journal.pmed.0030466

MCCLURE WR, 1978, J BIOL CHEM, V253, P8949

Mdluli K, 2015, CSH PERSPECT MED, V5, DOI 10.1101/cshperspect.a021154

Merle CS, 2014, NEW ENGL J MED, V371, P1588, DOI 10.1056/NEJMoa1315817

Migliori GB., 2007, Euro Surveill, V12, P3194, DOI [10.2807/esw.12.20.03194-en, DOI 10.2807/ESW.12.20.03194-EN]

Mikusová K, 2014, CURR PHARM DESIGN, V20, P4379

Mitscher LA, 2005, CHEM REV, V105, P559, DOI 10.1021/cr030101q

Nahid Payam, 2006, Proc Am Thorac Soc, V3, P103, DOI 10.1513/pats.200511-119JH

Naik M, 2014, J MED CHEM, V57, P5419, DOI 10.1021/jm5005978

Neres J, 2012, SCI TRANSL MED, V4, DOI 10.1126/scitranslmed.3004395

Niemi M, 2003, CLIN PHARMACOKINET, V42, P819, DOI 10.2165/00003088-200342090-00003

Nikonenko B, 2014, ANTIMICROB AGENTS CH, V58, P587, DOI 10.1128/AAC.01254-13

Nikonenko BV, 2007, ANTIMICROB AGENTS CH, V51, P1563, DOI 10.1128/AAC.01326-06

Nikonenko BV, 2009, ANTIMICROB AGENTS CH, V53, P3138, DOI 10.1128/AAC.00366-09

Nikonenko BV, 2004, ANTIMICROB AGENTS CH, V48, P4550, DOI 10.1128/AAC.48.12.4550-4555.2004

Nuermberger EL, 2004, AM J RESP CRIT CARE, V170, P1131, DOI 10.1164/rccm.200407-885OC

Nuermberger EL, 2004, AM J RESP CRIT CARE, V169, P421, DOI 10.1164/rccm.200310-1380OC

Nuermberger E, 2008, ANTIMICROB AGENTS CH, V52, P1522, DOI 10.1128/AAC.00074-08

Nuermberger E, 2006, ANTIMICROB AGENTS CH, V50, P2621, DOI 10.1128/AAC.00451-06

Okumura R, 2008, J ANTIMICROB CHEMOTH, V62, P98, DOI 10.1093/jac/dkn136

Onajole OK, 2010, EUR J MED CHEM, V45, P2075, DOI 10.1016/j.ejmech.2010.01.046

Palomino JC, 2013, FUTURE MICROBIOL, V8, P1071, DOI 10.2217/fmb.13.85

Panda M, 2014, J MED CHEM, V57, P4761, DOI 10.1021/jm5002937

Paramasivan CN, 2005, ANTIMICROB AGENTS CH, V49, P627, DOI 10.1128/AAC.49.2.627-631.2005

Pethe K, 2013, NAT MED, V19, P1157, DOI 10.1038/nm.3262

Protopopova M, 2005, J ANTIMICROB CHEMOTH, V56, P968, DOI 10.1093/jac/dki319

Reddy VM, 2008, ANTIMICROB AGENTS CH, V52, P719, DOI 10.1128/AAC.01469-07

Reddy VM, 2010, ANTIMICROB AGENTS CH, V54, P2840, DOI 10.1128/AAC.01601-09

Reddy VM, 1999, J ANTIMICROB CHEMOTH, V43, P615, DOI 10.1093/jac/43.5.615

Ribeiro ALDL, 2011, PLOS ONE, V6, DOI 10.1371/journal.pone.0026675

ROBITZEK EH, 1952, AM REV TUBERC PULM, V65, P402

Rustomjee R, 2008, INT J TUBERC LUNG D, V12, P128

Sacksteder KA, 2012, FUTURE MICROBIOL, V7, P823, DOI [10.2217/fmb.12.56, 10.2217/FMB.12.56]

Sasaki H, 2006, J MED CHEM, V49, P7854, DOI 10.1021/jm060957y

Sekiguchi J, 2011, ANTIMICROB AGENTS CH, V55, P3958, DOI 10.1128/AAC.00417-10

SENSI P, 1983, REV INFECT DIS, V5, pS402

Shirude PS, 2013, J MED CHEM, V56, P9701, DOI 10.1021/jm401382v

Singh R, 2008, SCIENCE, V322, P1392, DOI 10.1126/science.1164571

Skripconoka V, 2013, EUR RESPIR J, V41, P1393, DOI 10.1183/09031936.00125812

Stanley SA, 2012, ACS CHEM BIOL, V7, P1377, DOI 10.1021/cb300151m

Stark WM., 1963, Antimicrob Agents Chemother, V1962, P596

STEAD WW, 1967, AM REV RESPIR DIS, V95, P729

Steel HC, 1999, J ANTIMICROB CHEMOTH, V44, P209, DOI 10.1093/jac/44.2.209

Stover CK, 2000, NATURE, V405, P962, DOI 10.1038/35016103

Swaney SM, 1998, ANTIMICROB AGENTS CH, V42, P3251, DOI 10.1128/AAC.42.12.3251

Tahlan K, 2012, ANTIMICROB AGENTS CH, V56, P1797, DOI 10.1128/AAC.05708-11

Tasneen R, 2008, ANTIMICROB AGENTS CH, V52, P3664, DOI 10.1128/AAC.00686-08

THOMAS JP, 1961, AM REV RESPIR DIS, V83, P891

Tillotson GS, 1996, J MED MICROBIOL, V44, P320, DOI 10.1099/00222615-44-5-320

Timmins GS, 2006, MOL MICROBIOL, V62, P1220, DOI 10.1111/j.1365-2958.2006.05467.x

Trefzer C, 2010, J AM CHEM SOC, V132, P13663, DOI 10.1021/ja106357w

Tyagi JS, 2002, TRENDS MICROBIOL, V10, P68, DOI 10.1016/S0966-842X(01)02296-X

Upton AM, 2015, ANTIMICROB AGENTS CH, V59, P136, DOI 10.1128/AAC.03823-14

Wallis RS, 2011, ANTIMICROB AGENTS CH, V55, P567, DOI 10.1128/AAC.01179-10

Wallis RS, 2010, J INFECT DIS, V202, P745, DOI 10.1086/655471

Wang F, 2013, P NATL ACAD SCI USA, V110, pE2510, DOI 10.1073/pnas.1309171110

Warner DF, 2014, NEW ENGL J MED, V371, P1642, DOI 10.1056/NEJMe1410977

WHO, GLOB TUB REP 2014

Williams KN, 2009, ANTIMICROB AGENTS CH, V53, P1314, DOI 10.1128/AAC.01182-08

Williams KN, 2009, AM J RESP CRIT CARE, V180, P371, DOI 10.1164/rccm.200904-0611OC

World Health Organisation, NEW TECHN TUB CONTR

Xavier AS, 2014, J PHARMACOL PHARMACO, V5, P222, DOI 10.4103/0976-500X.136121

YAMAGUCHI H, 1986, J ANTIBIOT, V39, P1047, DOI 10.7164/antibiotics.39.1047

YANAGISAWA K, 1957, J ANTIBIOT, V10, P233

Yano T, 2011, J BIOL CHEM, V286, P10276, DOI 10.1074/jbc.M110.200501

YEAGER RL, 1952, AM REV TUBERC PULM, V65, P523

Ying Z, 2005, ANNU REV PHARMACOL, V45, P529, DOI 10.1146/annurev.pharmtox.45.120403.100120

Zhang DF, 2012, J MED CHEM, V55, P8409, DOI 10.1021/jm300828h

Zumla A, 2013, NAT REV DRUG DISCOV, V12, P388, DOI 10.1038/nrd4001

NR 158

TC 34

Z9 41

U1 0

U2 33

PU FUTURE SCI LTD

PI LONDON

PA UNITED HOUSE, 2 ALBERT PL, LONDON, N3 1QB, ENGLAND

SN 1756-8919

EI 1756-8927

J9 FUTURE MED CHEM

JI Future Med. Chem.

PY 2015

VL 7

IS 15

BP 1981

EP 2003

DI 10.4155/fmc.15.128

PG 23

WC Chemistry, Medicinal

WE Science Citation Index Expanded (SCI-EXPANDED)

SC Pharmacology & Pharmacy

GA CW7PE

UT WOS:000365190600005

DA 2026-05-07

ER

PT J

AU Long, Y

Wang, B

Xie, TC

Luo, RX

Tang, J

Deng, JP

Wang, C

AF Long, Ying

Wang, Bin

Xie, Tiancheng

Luo, Ruixin

Tang, Jing

Deng, Jianping

Wang, Chuan

TI Overexpression of efflux pump genes is one of the mechanisms causing

drug resistance in Mycobacterium tuberculosis

SO MICROBIOLOGY SPECTRUM

LA English

DT Article

DE Mycobacterium tuberculosis; MIRU-VNTR; mutation; efflux pump gene;

verapamil; drug resistance mechanism

ID EXPRESSION; MULTIDRUG; RIFAMPICIN

AB Currently, drug resistance is a formidable obstacle to tuberculosisprevention globally. A deep understanding of the drug-resistance mechanism of Mycobacterium tuberculosis is helpful to deal with this problem. In this study, 46 clinical isolates of Mycobacterium tuberculosis from Zigong, Sichuan, China, including 5 sensitive isolates, 5 rifampicin (RIF) mono-resistant isolates, 18 isoniazid mono-resistant isolates, and 18 multi-drug-resistant isolates, were selected. We evaluated the impact of drug induction on the relative expression levels of 10 putative efflux pump genes, namely, Rv3065, Rv2836c, efpA, Rv1410c, Rv1250, Rv0876c, Rv1819c, Rv0933, Rv1217c, and Rv1218c. Meanwhile, we assessed the effect of verapamil, an efflux pump inhibitor, on the drug resistance of the isolates. The results showed that 100% (5/5) of the RIF mono-resistant isolates, 44.4% (8 of 18) of the isoniazid mono-resistant isolates, and 88.9% (16 of 8) of the multi-drug-resistant isolates overexpressed anyone of the 10 efflux pump genes; however, none of the 10 efflux pump genes were overexpressed on the sensitive isolates. Among drug-resistant strains, Rv1250 (51.2%) and Rv0933 (53.7%) were the genes with the highest frequency of overexpression. Only the rifampicin mono-resistant isolates showed a significant increase in the number of overexpressed efflux pump genes after drug induction, while the change trends of the numbers of overexpressed efflux pump genes in isoniazid mono-resistant isolates and multi-drug-resistant isolates after drug induction were not definite. Verapamil did not have a significant effect on the minimum inhibitory concentrations (MICs) of isoniazid mono-resistant isolates but reduced the MICs of all sensitive isolates and some multi-drug-resistant isolates. We also investigated the correlation between polymorphic loci of mycobacterial interspersed repetitive unit-variable number tandem repeat (MIRU-VNTR) typing, mutations in drug resistance-associated genes, and overexpression of the efflux pump genes. The results indicated that there was no significant association between the two most polymorphic loci, MIRU26 and MIRU31, and the incidence of overexpression of the efflux pump genes. However, isolates with mutations in drug resistance-associated genes exhibited a higher rate of overexpression of the efflux pump genes compared to those without mutations. In conclusion, we found that drug-resistant isolates had a higher background expression level of the efflux pump genes compared to sensitive isolates. This suggests that the overexpression of the efflux pump genes could be one of the mechanisms causing drug resistance in Mycobacterium tuberculosis. We also found that verapamil was able to reduce MICs in some drug-resistant isolates, indicating that inhibition of the efflux pump could be used as an adjuvant treatment option for drug-resistant tuberculosis patients.

C1 [Long, Ying; Xie, Tiancheng; Tang, Jing; Wang, Chuan] Sichuan Univ, West China Sch Publ Hlth, Chengdu, Peoples R China.

[Long, Ying; Xie, Tiancheng; Tang, Jing; Wang, Chuan] Sichuan Univ, West China Hosp 4, Chengdu, Peoples R China.

[Wang, Bin; Luo, Ruixin; Deng, Jianping] Zigong Ctr Dis Control & Prevent, Zigong, Peoples R China.

C3 Sichuan University; Sichuan University

RP Wang, C (corresponding author), Sichuan Univ, West China Sch Publ Hlth, Chengdu, Peoples R China.; Wang, C (corresponding author), Sichuan Univ, West China Hosp 4, Chengdu, Peoples R China.; Deng, JP (corresponding author), Zigong Ctr Dis Control & Prevent, Zigong, Peoples R China.

EM 232680892@qq.com; wangchuan@scu.edu.cn

OI Xie, Tiancheng/0000-0002-5204-5468

FU Major Scientific and Technological Application Demonstration Project of

Chengdu Science and Technology Bureau; International Cooperation Project

of Sichuan Province Science and Technology Department [2017HH0080]; Key

Science and Technology Planning Project of Zigong [2020YLSF13];

[2022-YF09-00011-SN]

FX This study is supported by the Major Scientific and Technological

Application Demonstration Project of Chengdu Science and Technology

Bureau (no. 2022-YF09-00011-SN), the International Cooperation Project

of Sichuan Province Science and Technology Department (no. 2017HH0080),

and the Key Science and Technology Planning Project of Zigong (no.

2020YLSF13).

CR [Anonymous], 2019, Global tuberculosis report

Arora G, 2021, FEBS J, V288, P3375, DOI 10.1111/febs.15582

Braibant M, 2000, FEMS MICROBIOL REV, V24, P449, DOI 10.1016/S0168-6445(00)00034-6

Bustin SA, 2010, BMC MOL BIOL, V11, DOI 10.1186/1471-2199-11-74

Caleffi-Ferracioli KR, 2016, TUBERCULOSIS, V97, P65, DOI 10.1016/j.tube.2015.12.010

Canezin PH, 2018, J ANTIMICROB CHEMOTH, V73, P1770, DOI 10.1093/jac/dky091

Costa SS, 2011, BMC MICROBIOL, V11, DOI 10.1186/1471-2180-11-241

de Souza JVP, 2020, INT J TUBERC LUNG D, V24, P591, DOI 10.5588/ijtld.19.0458

De Spiegelaere W, 2015, PLOS ONE, V10, DOI 10.1371/journal.pone.0122515

Domenech P, 2009, J BACTERIOL, V191, P477, DOI 10.1128/JB.01132-08

Duang-Nkern J, 2021, MICROBIOL RES, V251, DOI 10.1016/j.micres.2021.126816

Guo Y, 2022, MICROBIOL RES, V264, DOI 10.1016/j.micres.2022.127174

Gupta AK, 2010, MICROB DRUG RESIST, V16, P21, DOI 10.1089/mdr.2009.0054

Gupta S, 2014, ANTIMICROB AGENTS CH, V58, P574, DOI 10.1128/AAC.01462-13

Isakova J, 2018, BMC MICROBIOL, V18, DOI 10.1186/s12866-018-1168-x

Jang J, 2017, ANTIMICROB AGENTS CH, V61, DOI [10.1128/aac.02637-16, 10.1128/AAC.02637-16]

Kanabalan RD, 2021, MICROBIOL RES, V246, DOI 10.1016/j.micres.2020.126674

Kwara A, 2003, J CLIN MICROBIOL, V41, P2683, DOI 10.1128/JCM.41.6.2683-2685.2003

Larsen Michelle H, 2007, Curr Protoc Microbiol, VChapter 10, DOI 10.1002/9780471729259.mc10a02s6

Lee K, 2009, GLOB INST, P1

Li GL, 2015, J ANTIBIOT, V68, P431, DOI 10.1038/ja.2015.9

Li GL, 2015, PLOS ONE, V10, DOI 10.1371/journal.pone.0119013

Machado D, 2018, FRONT MICROBIOL, V9, DOI 10.3389/fmicb.2018.03072

Machado D, 2018, FUTURE MICROBIOL, V13, P1383, DOI 10.2217/fmb-2018-0110

Machado D, 2017, FRONT MICROBIOL, V8, DOI 10.3389/fmicb.2017.00711

Machado D, 2012, PLOS ONE, V7, DOI 10.1371/journal.pone.0034538

Mazando S., 2017, Journal of Applied Biosciences, V115, P11465

Mishra MN, 2013, J BACTERIOL, V195, P1610, DOI 10.1128/JB.01724-12

Narang A, 2019, PLOS ONE, V14, DOI 10.1371/journal.pone.0223163

Narang A, 2017, INT J MYCOBACT, V6, P177, DOI 10.4103/ijmy.ijmy_26_17

Nontaleerak B, 2022, MICROBIOL RES, V263, DOI 10.1016/j.micres.2022.127112

Ogonowska P, 2020, SCI REP-UK, V10, DOI 10.1038/s41598-020-73409-1

Palomino JC, 2002, ANTIMICROB AGENTS CH, V46, P2720, DOI 10.1128/AAC.46.8.2720-2722.2002

Pang Y, 2013, ANTIMICROB AGENTS CH, V57, P893, DOI 10.1128/AAC.01024-12

Rabori Eshagh Dortaj, 2006, Journal of Communicable Diseases, V37, P249

Ramón-García S, 2012, ANTIMICROB AGENTS CH, V56, P2074, DOI 10.1128/AAC.05946-11

Remm S, 2022, FEMS MICROBIOL REV, V46, DOI 10.1093/femsre/fuab050

Rodrigues L, 2012, INFECT GENET EVOL, V12, P695, DOI 10.1016/j.meegid.2011.08.009

Shahi F, 2021, HELIYON, V7, DOI 10.1016/j.heliyon.2021.e07566

Viveiros M, 2002, ANTIMICROB AGENTS CH, V46, P2804, DOI 10.1128/AAC.46.9.2804-2810.2002

Wang K, 2013, CURR MICROBIOL, V66, P222, DOI 10.1007/s00284-012-0215-3

WHO, 2012, GLOBAL TUBERCULOSIS REPORT 2012, P1

Xie F. L., 2012, Plant Molecular Biology, V80, P75, DOI 10.1007/s11103-012-9885-2

NR 43

TC 11

Z9 12

U1 3

U2 22

PU AMER SOC MICROBIOLOGY

PI WASHINGTON

PA 1752 N ST NW, WASHINGTON, DC 20036-2904 USA

SN 2165-0497

J9 MICROBIOL SPECTR

JI Microbiol. Spectr.

PD JAN 11

PY 2024

VL 12

IS 1

DI 10.1128/spectrum.02510-23

EA DEC 2023

PG 13

WC Microbiology

WE Science Citation Index Expanded (SCI-EXPANDED)

SC Microbiology

GA ET8N7

UT WOS:001113389800007

OA Green Submitted, gold

DA 2026-05-07

ER

PT J

AU Ferrian, S

Ross, M

Conradie, F

Omar, SV

Small, N

Little, F

Kaplan, G

Fallows, D

Gray, CM

AF Ferrian, Selena

Ross, Melinda

Conradie, Francesca

Omar, Shaheed Vally

Small, Nazir

Little, Francesca

Kaplan, Gilla

Fallows, Dorothy

Gray, Clive M.

TI Frequency of Circulating CD4+ Ki67+

HLA-DR- T Regulatory Cells Prior to Treatment for Multidrug

Resistant Tuberculosis Can Differentiate the Severity of Disease and

Predict Time to Culture Conversion

SO FRONTIERS IN IMMUNOLOGY

LA English

DT Article

DE Treg cells; Mycobacteria tuberculosis; multi-drug resistant

tuberculosis; lung cavities; sputum culture; smear grades; culture

conversion

ID IMMUNE-RESPONSE; EXPRESSION; BLOOD; EXPANSION; LYMPHOCYTES; INDUCTION;

ANTIGENS; SUBSETS

AB Identifying a blood circulating cellular biomarker that can be used to assess severity of disease and predict the time to culture conversion (TCC) in patients with multidrug resistant tuberculosis (MDR-TB) would facilitate monitoring response to treatment and may be of value in the design of future drug trials. We report on the frequency of blood Ki67(+) HLA-DR- CD4(+) T regulatory (Treg) cells in predicting microbiological outcome before initiating second-line treatment for MDR-TB. Fifty-one patients with MDR-TB were enrolled and followed over 18 months; a subset of patients was sputum culture (SC) negative at baseline (n = 9). SC positive patients were divided into two groups, based on median TCC: rapid responders (<71 days TCC; n = 21) and slow responders (>71 days TCC; n = 21). Whole blood at baseline, months 2 and 6 was stimulated with M tuberculosis (Mtb) antigens and Treg cells were then identified as CD3(+)CD4(+)CD25(hi)FoxP3(+)ECD127(-)CD69(-) and further delineated as Ki67(+) HLA-DR- Treg. The frequency of these cells was significantly enlarged at baseline in SC positive relative to SC negative and smear positive relative to smear negative patients and in those with lung cavitation. This difference was further supported by unsupervised hierarchical clustering showing a significant grouping at baseline of total and early differentiated memory Treg cells in slow responders. Conversely, there was a clustering of a lower proportion of Treg cells and activated IFN gamma-expressing T cells at baseline in the rapid responders. Examining changes over time revealed a more gradual reduction of Treg cells in slow responders relative to rapid responders to treatment. Receiver operating curve analysis showed that baseline Mtb-stimulated Ki67-FHLA-DR- Treg cells could predict the TCC of MDR-TB treatment response with 81.2% sensitivity and 85% specificity (AUC of 0.87, p < 0.0001), but this was not the case after 2 months of treatment. In conclusion, our data show that the frequency of a highly defined Mtb-stimulated blood Treg cell population at baseline can discriminate MDR-TB disease severity and predict time to culture clearance.

C1 [Ferrian, Selena; Gray, Clive M.] Univ Cape Town, Inst Infect Dis & Mol Med, Dept Pathol, Div Immunol, Cape Town, South Africa.

[Ross, Melinda; Little, Francesca] Univ Cape Town, Dept Stat Sci, Cape Town, South Africa.

[Conradie, Francesca] Univ Witwatersrand, Fac Hlth Sci, Johannesburg, South Africa.

[Omar, Shaheed Vally; Small, Nazir] Natl Inst Communicable Dis, Ctr TB, Johannesburg, South Africa.

[Small, Nazir] Univ Pretoria, Dept Med Microbiol, Pretoria, South Africa.

[Kaplan, Gilla; Fallows, Dorothy] Rutgers State Univ, Publ Hlth Res Inst, Newark, NJ USA.

[Gray, Clive M.] Natl Hlth Lab Serv, Cape Town, South Africa.

[Ferrian, Selena] Stanford Univ, Dept Pathol, Palo Alto, CA 94304 USA.

C3 University of Cape Town; University of Cape Town; University of

Witwatersrand; National Institute for Communicable Diseases (NICD);

University of Pretoria; Rutgers University System; Rutgers University

New Brunswick; Rutgers University Newark; Stanford University

RP Gray, CM (corresponding author), Univ Cape Town, Inst Infect Dis & Mol Med, Dept Pathol, Div Immunol, Cape Town, South Africa.; Gray, CM (corresponding author), Natl Hlth Lab Serv, Cape Town, South Africa.

EM clive.gray@uct.ac.za

RI Gray, Clive/LIH-6901-2024; Omar, Shaheed Vally/AAB-5071-2020; Ferrian,

Selena/ABC-4857-2021

OI Gray, Clive/0000-0002-9293-901X; Little, Francesca/0000-0002-5917-9928;

Omar, Shaheed Vally/0000-0001-6983-1636;

FU National Institute of Allergy and Infectious Diseases [RO1 AI80737]

FX This work was supported by a National Institute of Allergy and

Infectious Diseases grant (RO1 AI80737). The funders had no role in

study design, data collection and analysis, decision to publish, or

preparation of the manuscript.

CR Almeida AS, 2009, J IMMUNOL, V183, P718, DOI 10.4049/jimmunol.0801212

[Anonymous], 2017, GLOB TUB REP

Apostolou I, 2002, NAT IMMUNOL, V3, P756, DOI 10.1038/ni816

Baecher-Allan C, 2006, J IMMUNOL, V176, P4622, DOI 10.4049/jimmunol.176.8.4622

Beiranvand Elham, 2014, Inflammation & Allergy Drug Targets, V13, P330

Brighenti S, 2016, MICROBIOL SPECTR, V4, DOI 10.1128/microbiolspec.TBTB2-0006-2016

Chatila TA, 2005, J ALLERGY CLIN IMMUN, V116, P949, DOI 10.1016/j.jaci.2005.08.047

Chaudhary B, 2016, VACCINES-BASEL, V4, DOI 10.3390/vaccines4030028

Chen WJ, 2003, J EXP MED, V198, P1875, DOI 10.1084/jem.20030152

Chen XC, 2007, CLIN IMMUNOL, V123, P50, DOI 10.1016/j.clim.2006.11.009

Davids M, 2018, AM J RESP CRIT CARE, V198, P104, DOI 10.1164/rccm.201707-1441OC

Desbien AL, 2017, METHODS MOL BIOL, V1494, P305, DOI 10.1007/978-1-4939-6445-1_23

Ferrian S, 2017, PLOS ONE, V12, DOI 10.1371/journal.pone.0176660

Feruglio SL, 2015, CLIN EXP IMMUNOL, V179, P454, DOI 10.1111/cei.12468

Garg A, 2008, EUR J IMMUNOL, V38, P459, DOI 10.1002/eji.200737268

Geffner L, 2014, CLIN EXP IMMUNOL, V175, P235, DOI 10.1111/cei.12227

Geffner L, 2009, INFECT IMMUN, V77, P5025, DOI 10.1128/IAI.00224-09

Green AM, 2010, J INFECT DIS, V202, P533, DOI 10.1086/654896

Guyot-Revol V, 2006, AM J RESP CRIT CARE, V173, P803, DOI 10.1164/rccm.200508-1294OC

Hanekom WA, 2004, J IMMUNOL METHODS, V291, P185, DOI 10.1016/j.jim.2004.06.010

Hougardy JM, 2007, MICROBES INFECT, V9, P1325, DOI 10.1016/j.micinf.2007.06.004

Hougardy JM, 2007, AM J RESP CRIT CARE, V176, P409, DOI 10.1164/rccm.200701-084OC

Itoh M, 1999, J IMMUNOL, V162, P5317

Larson RP, 2013, ADV EXP MED BIOL, V783, P165, DOI 10.1007/978-1-4614-6111-1_9

Li L, 2011, PLOS ONE, V6, DOI 10.1371/journal.pone.0023700

Li N, 2015, INT J TUBERC LUNG D, V19, P1230, DOI 10.5588/ijtld.15.0148

Parkash O, 2015, IMMUNOL RES, V62, P386, DOI 10.1007/s12026-015-8654-0

Pathakumari B, 2018, IMMUNOLOGY, V153, P325, DOI 10.1111/imm.12833

Raats M. M., 1991, Food Quality and Preference, V3, P89, DOI 10.1016/0950-3293(91)90028-D

Ribeiro-Rodrigues R, 2006, CLIN EXP IMMUNOL, V144, P25, DOI 10.1111/j.1365-2249.2006.03027.x

Santegoets SJAM, 2015, CANCER IMMUNOL IMMUN, V64, P1271, DOI 10.1007/s00262-015-1729-x

Schwander S, 2018, AM J RESP CRIT CARE, V198, P11, DOI 10.1164/rccm.201802-0233ED

Scott-Browne JP, 2007, J EXP MED, V204, P2159, DOI 10.1084/jem.20062105

Serrano CJ, 2015, CLIN IMMUNOL, V157, P145, DOI 10.1016/j.clim.2015.02.008

Singh A, 2012, PLOS ONE, V7, DOI 10.1371/journal.pone.0044931

Smigiel KS, 2014, IMMUNOL REV, V259, P40, DOI 10.1111/imr.12170

Walker MR, 2003, J CLIN INVEST, V112, P1437, DOI 10.1172/JCI200319441

Wang CH, 1998, EUR RESPIR J, V11, P809, DOI 10.1183/09031936.98.11040809

Wergeland I, 2011, SCAND J IMMUNOL, V73, P234, DOI 10.1111/j.1365-3083.2010.02496.x

Wu YE, 2010, INT J INFECT DIS, V14, pE815, DOI 10.1016/j.ijid.2010.04.005

Zumla A, 2015, LANCET GLOB HEALTH, V3, pE10, DOI 10.1016/S2214-109X(14)70361-4

NR 41

TC 7

Z9 8

U1 0

U2 4

PU FRONTIERS MEDIA SA

PI LAUSANNE

PA AVENUE DU TRIBUNAL FEDERAL 34, LAUSANNE, CH-1015, SWITZERLAND

SN 1664-3224

J9 FRONT IMMUNOL

JI Front. Immunol.

PD OCT 25

PY 2018

VL 9

AR 2438

DI 10.3389/fimmu.2018.02438

PG 12

WC Immunology

WE Science Citation Index Expanded (SCI-EXPANDED)

SC Immunology

GA GY0TO

UT WOS:000448234700001

OA Green Submitted, gold

DA 2026-05-07

ER

PT J

AU Li, N

Xie, WP

Kong, H

Min, R

Hu, CM

Zhou, XB

Lu, ZM

Ji, XH

Wang, H

AF Li, N.

Xie, W-P.

Kong, H.

Min, R.

Hu, C-M.

Zhou, X-B.

Lu, Z-M.

Ji, X-H.

Wang, H.

TI Enrichment of regulatory T-cells in blood of patients with

multidrug-resistant tuberculosis

SO INTERNATIONAL JOURNAL OF TUBERCULOSIS AND LUNG DISEASE

LA English

DT Article

DE Treg cells; MDR-TB; PD-1; ICOS; CTLA-4

ID MYCOBACTERIUM-TUBERCULOSIS; EXPRESSION; PATHWAY

AB OBJECTIVE: To measure the percentage of regulatory T-cells (Treg) and the expression of signalling molecules in these cells from the peripheral blood of patients with multidrug-resistant tuberculosis (MDR-TB).

DESIGN: Patients with drug-susceptible tuberculosis (STB), MDR-TB and healthy controls (HCs) were recruited into the study. Levels of CD4(+)CD25(+)Foxp3(+) Treg cells from peripheral blood, and programmed death-1 (PD-1), cytotoxic T lymphocyte-associated antigen-4 (CTLA-4) and inducible costimulatory (ICOS) molecule expression in the cells were measured using flow cytometry. Suppression mediated by Treg cells was assessed in carboxyfluorescein succinimidyl ester (CFSE) based suppression assays with autologous CD4(+)CD25-T-effector (Teff) cells.

RESULTS: Presence of Mycobacterium tuberculosis resulted in a higher proportion of Treg cells in S-TB patients than in HCs, and even higher levels in MDR-TB patients. Moreover, Treg cells in MDR-TB patients constitutively expressed high-level PD-1, CTLA-4 and ICOS. In addition, when cultured with activated CD4(+)CD25(-) Teff cells, Treg cells potently suppressed proliferation of Teff cells.

CONCLUSIONS: The high level of Treg cells found in the peripheral blood of tuberculosis patients may partly explain the poor immune response against M. tuberculosis, and could be a marker of MDR-TB.

C1 [Li, N.; Xie, W-P.; Kong, H.; Min, R.; Wang, H.] Nanjing Med Univ, Dept Resp Med, Affiliated Hosp 1, Nanjing, Jiangsu, Peoples R China.

[Hu, C-M.] Nanjing Chest Hosp, Dept Resp Med, Nanjing, Jiangsu, Peoples R China.

[Zhou, X-B.; Lu, Z-M.; Ji, X-H.] Nanjing Med Univ, Dept Microbiol & Immunol, Nanjing, Jiangsu, Peoples R China.

C3 Nanjing Medical University; Nanjing Medical University

RP Wang, H (corresponding author), Nanjing Med Univ, Dept Resp Med, Affiliated Hosp 1, Nanjing, Jiangsu, Peoples R China.

EM hongwangnjmu@163.com

FU major projects for science and technology development '12th Five-Year

Plan' on Key Infectious Diseases of the National Department of

Technology, Beijing, China [2013ZX10003008-002]

FX The study was supported by grants from one of the major projects for

science and technology development '12th Five-Year Plan' on Key

Infectious Diseases of the National Department of Technology, Beijing,

China (no. 2013ZX10003008-002).

CR Alvarez IB, 2010, J INFECT DIS, V202, P524, DOI 10.1086/654932

Amarnath S, 2010, PLOS BIOL, V8, DOI 10.1371/journal.pbio.1000302

Burl S, 2007, CLIN EXP IMMUNOL, V149, P117, DOI 10.1111/j.1365-2249.2007.03399.x

Busse M, 2012, J IMMUNOL, V189, P1975, DOI 10.4049/jimmunol.1103581

Chatila TA, 2005, J ALLERGY CLIN IMMUN, V116, P949, DOI 10.1016/j.jaci.2005.08.047

Conrad C, 2012, CANCER RES, V72, P5240, DOI 10.1158/0008-5472.CAN-12-2271

Ernst JD, 2012, NAT REV IMMUNOL, V12, P581, DOI 10.1038/nri3259

Francisco LM, 2010, IMMUNOL REV, V236, P219, DOI 10.1111/j.1600-065X.2010.00923.x

Gandhi NR, 2010, LANCET, V375, P1830, DOI 10.1016/S0140-6736(10)60410-2

Geffner L, 2009, INFECT IMMUN, V77, P5025, DOI 10.1128/IAI.00224-09

Green AM, 2010, J INFECT DIS, V202, P533, DOI 10.1086/654896

Guyot-Revol V, 2006, AM J RESP CRIT CARE, V173, P803, DOI 10.1164/rccm.200508-1294OC

Ha SJ, 2008, J EXP MED, V205, P543, DOI 10.1084/jem.20071949

Harding CV, 2010, NAT REV MICROBIOL, V8, P296, DOI 10.1038/nrmicro2321

Kaufmann SHE, 2011, LANCET INFECT DIS, V11, P633, DOI 10.1016/S1473-3099(11)70146-3

Kursar M, 2007, J IMMUNOL, V178, P2661, DOI 10.4049/jimmunol.178.5.2661

Lim HJ, 2013, TUBERCULOSIS, V93, P523, DOI 10.1016/j.tube.2013.06.001

Mills KHG, 2004, SEMIN IMMUNOL, V16, P107, DOI 10.1016/j.smim.2003.12.006

Nouailles G, 2011, EUR J IMMUNOL, V41, P981, DOI 10.1002/eji.201040608

Paust S, 2005, IMMUNOL REV, V204, P195, DOI 10.1111/j.0105-2896.2005.00247.x

Philips JA, 2012, ANNU REV PATHOL-MECH, V7, P353, DOI 10.1146/annurev-pathol-011811-132458

Sakthivel P, 2014, PLOS ONE, V9, DOI 10.1371/journal.pone.0100970

Scott-Browne JP, 2007, J EXP MED, V204, P2159, DOI 10.1084/jem.20062105

Shafiani S, 2010, J EXP MED, V207, P1409, DOI 10.1084/jem.20091885

Sharma PK, 2009, AM J RESP CRIT CARE, V179, P1061, DOI 10.1164/rccm.200804-529OC

Sharpe AH, 2007, NAT IMMUNOL, V8, P239, DOI 10.1038/ni1443

Singh A, 2012, PLOS ONE, V7, DOI 10.1371/journal.pone.0044728

Suzuki S, 2015, VET IMMUNOL IMMUNOP, V163, P115, DOI 10.1016/j.vetimm.2014.10.006

Takahashi N, 2009, J IMMUNOL, V182, P5515, DOI 10.4049/jimmunol.0803256

Tan Q, 2012, EUR J CLIN MICROBIOL, V31, P1233, DOI 10.1007/s10096-011-1434-4

NR 30

TC 26

Z9 29

U1 0

U2 3

PU INT UNION AGAINST TUBERCULOSIS LUNG DISEASE (I U A T L D)

PI PARIS

PA 68 BOULEVARD SAINT-MICHEL,, 75006 PARIS, FRANCE

SN 1027-3719

EI 1815-7920

J9 INT J TUBERC LUNG D

JI Int. J. Tuberc. Lung Dis.

PD OCT

PY 2015

VL 19

IS 10

BP 1230

EP 1238

DI 10.5588/ijtld.15.0148

PG 9

WC Infectious Diseases; Respiratory System

WE Science Citation Index Expanded (SCI-EXPANDED)

SC Infectious Diseases; Respiratory System

GA CS5YR

UT WOS:000362154700019

DA 2026-05-07

ER

PT J

AU Li, GL

Zhang, JR

Guo, Q

Jiang, Y

Wei, JH

Zhao, LL

Zhao, XQ

Lu, JX

Wan, KL

AF Li, Guilian

Zhang, Jingrui

Guo, Qian

Jiang, Yi

Wei, Jianhao

Zhao, Li-li

Zhao, Xiuqin

Lu, Jianxin

Wan, Kanglin

TI Efflux Pump Gene Expression in Multidrug-Resistant

Mycobacterium tuberculosis Clinical Isolates

SO PLOS ONE

LA English

DT Article

ID INTRINSIC DRUG-RESISTANCE; ISONIAZID RESISTANCE; RIFAMPIN RESISTANCE;

MICROARRAY ANALYSIS; GENOME SEQUENCE; MECHANISM; SMEGMATIS;

SUSCEPTIBILITY; TRANSPORTERS; ETHAMBUTOL

AB Isoniazid (INH) and rifampicin (RIF) are the two most effective drugs in tuberculosis therapy. Understanding the molecular mechanisms of resistance to these two drugs is essential to quickly diagnose multidrug-resistant (MDR) tuberculosis and extensive drug-resistant tuberculosis. Nine clinical Mycobacterium tuberculosis isolates resistant to only INH and RIF and 10 clinical pan-sensitive isolates were included to evaluate the expression of 20 putative drug efflux pump genes and sequence mutations in rpoB (RIF), katG (INH), the inhA promoter (INH), and oxyR-ahpC (INH). Nine and three MDR isolates were induced to overexpress efflux pump genes by INH and RIF, respectively. Eight and two efflux pump genes were induced to overexpress by INH and RIF in MDR isolates, respectively. drrA, drrB, efpA, jefA (Rv2459), mmr, Rv0849, Rv1634, and Rv1250 were overexpressed under INH or RIF stress. Most efflux pump genes were overexpressed under INH stress in a MDR isolates that carried the wild-type katG, inhA, and oxyR-ahpC associated with INH resistance than in those that carried mutations. The expression levels of 11 genes (efpA, Rv0849, Rv1250, P55 (Rv1410c), Rv1634, Rv2994, stp, Rv2459, pstB, drrA, and drrB) without drug inducement were significantly higher (P < 0.05) in nine MDR isolates than in 10 pansensitive isolates. In conclusion, efflux pumps may play an important role in INH acquired resistance in MDR M. tuberculosis, especially in those strains having no mutations in genes associated with INH resistance; basal expression levels of some efflux pump genes are higher in MDR isolates than in pan-sensitive isolates and the basal expressional differences may be helpful to diagnose and treat resistant tuberculosis.

C1 [Li, Guilian; Zhang, Jingrui; Guo, Qian; Jiang, Yi; Wei, Jianhao; Zhao, Li-li; Zhao, Xiuqin; Wan, Kanglin] Chinese Ctr Dis Control & Prevent, Natl Inst Communicable Dis Control & Prevent, TB Branch, State Key Lab Infect Dis Prevent & Control, Beijing 102206, Peoples R China.

[Li, Guilian; Zhang, Jingrui; Guo, Qian; Jiang, Yi; Wei, Jianhao; Zhao, Li-li; Zhao, Xiuqin; Wan, Kanglin] Collaborat Innovat Ctr Diag & Treatment Infect Di, Hangzhou 310003, Zhejiang, Peoples R China.

[Zhang, Jingrui; Wei, Jianhao; Lu, Jianxin] Wenzhou Med Coll, Key Lab Lab Med, Wenzhou 325035, Zhejiang, Peoples R China.

[Zhang, Jingrui] Shijiazhuang Gynecol & Obstetr Hosp, Shijiazhuang 050000, Hebei, Peoples R China.

[Guo, Qian] South China Univ, Pathogen Biol Inst, Hengyang 421001, Hunan, Peoples R China.

C3 Chinese Center for Disease Control & Prevention; National Institute for

Communicable Disease Control & Prevention, Chinese Center for Disease

Control & Prevention; Collaborative Innovation Center for Diagnosis &

Treatment of Infectious Diseases; Wenzhou Medical University

RP Wan, KL (corresponding author), Chinese Ctr Dis Control & Prevent, Natl Inst Communicable Dis Control & Prevent, TB Branch, State Key Lab Infect Dis Prevent & Control, Beijing 102206, Peoples R China.

EM wankanglin@icdc.cn

FU National Key Programme of Mega Infectious Diseases [2013ZX10003002-001];

State Key Laboratory for Infectious Disease Prevention and Control

[2014SKLID104]

FX This work was financially supported by projects 2013ZX10003002-001 of

the National Key Programme of Mega Infectious Diseases and the key

project 2014SKLID104 of the State Key Laboratory for Infectious Disease

Prevention and Control. The funders had no role in the study design,

data collection and analysis, decision to publish, or manuscript

preparation.

CR da Silva PEA, 2011, FEMS IMMUNOL MED MIC, V63, P1, DOI 10.1111/j.1574-695X.2011.00831.x

Aslan G, 2008, JPN J INFECT DIS, V61, P255

Balganesh M, 2012, ANTIMICROB AGENTS CH, V56, P2643, DOI 10.1128/AAC.06003-11

Balganesh M, 2010, ANTIMICROB AGENTS CH, V54, P5167, DOI 10.1128/AAC.00610-10

Banerjee A, 1998, MICROBIOL-SGM, V144, P2697, DOI 10.1099/00221287-144-10-2697

Betts JC, 2003, ANTIMICROB AGENTS CH, V47, P2903, DOI 10.1128/AAC.47.9.2903-2913.2003

Brennan PJ, 2003, TUBERCULOSIS, V83, P91, DOI 10.1016/S1472-9792(02)00089-6

Bustin SA, 2010, BMC MOL BIOL, V11, DOI 10.1186/1471-2199-11-74

Camus JC, 2002, MICROBIOL-SGM, V148, P2967, DOI 10.1099/00221287-148-10-2967

Choudhuri BS, 2002, BIOCHEM J, V367, P279, DOI 10.1042/BJ20020615

Cole ST, 1998, NATURE, V393, P537, DOI 10.1038/31159

De Rossi E, 2006, FEMS MICROBIOL REV, V30, P36, DOI 10.1111/j.1574-6976.2005.00000.x

De Rossi E, 1998, J BACTERIOL, V180, P6068, DOI 10.1128/JB.180.22.6068-6071.1998

De Rossi Edda, 2002, Molecular Medicine (Baltimore), V8, P714

DeMarco CE, 2007, ANTIMICROB AGENTS CH, V51, P3235, DOI 10.1128/AAC.00430-07

Doustdar F, 2008, MICROB DRUG RESIST, V14, P273, DOI 10.1089/mdr.2008.0842

Escribano I, 2007, CHEMOTHERAPY, V53, P397, DOI 10.1159/000109769

Fu LM, 2006, TUBERCULOSIS, V86, P134, DOI 10.1016/j.tube.2005.07.004

Gupta AK, 2010, INDIAN J MED RES, V132, P176

Gupta AK, 2010, MICROB DRUG RESIST, V16, P21, DOI 10.1089/mdr.2009.0054

Jiang X, 2008, MICROB DRUG RESIST, V14, P7, DOI 10.1089/mdr.2008.0772

Kamerbeek J, 1997, J CLIN MICROBIOL, V35, P907, DOI 10.1128/JCM.35.4.907-914.1997

Kiepiela P, 2000, TUBERCLE LUNG DIS, V80, P47, DOI 10.1054/tuld.1999.0231

Leonard B, 2008, J CLIN MICROBIOL, V46, P3526, DOI 10.1128/JCM.02083-07

Li XZ, 2004, ANTIMICROB AGENTS CH, V48, P2415, DOI 10.1128/AAC.48.7.2415-2423.2004

Livak KJ, 2001, METHODS, V25, P402, DOI 10.1006/meth.2001.1262

Louw GE, 2011, AM J RESP CRIT CARE, V184, P269, DOI 10.1164/rccm.201011-1924OC

McCammon MT, 2005, ANTIMICROB AGENTS CH, V49, P2200, DOI 10.1128/AAC.49.6.2200-2209.2005

MCCLURE WR, 1978, J BIOL CHEM, V253, P8949

Mokrousov I, 2002, ANTIMICROB AGENTS CH, V46, P1417, DOI 10.1128/AAC.46.5.1417-1424.2002

Mukaigawa Jun, 2005, Kansenshogaku Zasshi, V79, P388

Niederweis M, 2003, MOL MICROBIOL, V49, P1167, DOI 10.1046/j.1365-2958.2003.03662.x

Pang Y, 2013, ANTIMICROB AGENTS CH, V57, P893, DOI 10.1128/AAC.01024-12

Pasca MR, 2005, ANTIMICROB AGENTS CH, V49, P4775, DOI 10.1128/AAC.49.11.4775-4777.2005

Piddock LJV, 2000, J ANTIMICROB CHEMOTH, V45, P159, DOI 10.1093/jac/45.2.159

Ramón-García S, 2009, ANTIMICROB AGENTS CH, V53, P3675, DOI 10.1128/AAC.00550-09

Rodrigues L, 2012, INFECT GENET EVOL, V12, P695, DOI 10.1016/j.meegid.2011.08.009

Sacchettini JC, 1996, RES MICROBIOL, V147, P36, DOI 10.1016/0923-2508(96)80201-4

Sekiguchi J, 2007, J CLIN MICROBIOL, V45, P179, DOI 10.1128/JCM.00750-06

Siddiqi S, 2006, WHOHTMTB2008392

Somoskovi A, 2001, RESP RES, V2, P164, DOI 10.1186/rr54

Waddell SJ, 2004, TUBERCULOSIS, V84, P263, DOI 10.1016/j.tube.2003.12.005

World Health Organization, 2012, 2012 UPD CRIT CONC 1

Zhang Jing-rui, 2013, Zhonghua Liu Xing Bing Xue Za Zhi, V34, P379

ZHANG Y, 1992, NATURE, V358, P591, DOI 10.1038/358591a0

NR 45

TC 91

Z9 110

U1 1

U2 21

PU PUBLIC LIBRARY SCIENCE

PI SAN FRANCISCO

PA 1160 BATTERY STREET, STE 100, SAN FRANCISCO, CA 94111 USA

SN 1932-6203

J9 PLOS ONE

JI PLoS One

PD FEB 19

PY 2015

VL 10

IS 2

AR e0119013

DI 10.1371/journal.pone.0119013

PG 12

WC Multidisciplinary Sciences

WE Science Citation Index Expanded (SCI-EXPANDED)

SC Science & Technology - Other Topics

GA CC0XE

UT WOS:000350062300059

OA Green Submitted, gold

DA 2026-05-07

ER

PT J

AU Ordonez, AA

Maiga, M

Gupta, S

Weinstein, EA

Bishai, WR

Jain, SK

AF Ordonez, A. A.

Maiga, M.

Gupta, S.

Weinstein, E. A.

Bishai, W. R.

Jain, S. K.

TI Novel Adjunctive Therapies for the Treatment of Tuberculosis

SO CURRENT MOLECULAR MEDICINE

LA English

DT Article

DE Corticosteroids; efflux pump inhibitors; microenvironment; inflammation;

TNF-alpha

ID VITAMIN-D DEFICIENCY; RESISTANT MYCOBACTERIUM-TUBERCULOSIS;

PLACEBO-CONTROLLED TRIAL; NECROSIS-FACTOR-ALPHA; DOUBLE-BLIND; PULMONARY

TUBERCULOSIS; EFFLUX PUMP; INTRACRANIAL TUBERCULOMAS;

1,25-DIHYDROXYVITAMIN D-3; CORTICOSTEROID-THERAPY

AB Despite significant efforts to control tuberculosis (TB), the disease remains a major global threat, with an estimated 8.6 million new cases and 1.3 million deaths in 2012 alone. Significant treatment challenges include HIV co-infection, the dramatic rise of multidrug-resistant TB and the vast reservoir of latently infected individuals, who will develop active disease years after the initial infection. The long duration of chemotherapy also remains a major barrier to effective large scale treatment of TB. Significant advances are being made in the development of shorter and effective TB drug regimens and there is growing evidence that host-directed and "non-antimicrobial" pathogen-directed therapies, could serve as novel approaches to enhance TB treatments. This review highlights the rationale for using these therapies and summarizes some of the progress in this field.

C1 [Ordonez, A. A.; Weinstein, E. A.; Jain, S. K.] Johns Hopkins Univ, Sch Med, Ctr Infect & Inflammat Imaging Res, Baltimore, MD USA.

[Ordonez, A. A.; Maiga, M.; Gupta, S.; Weinstein, E. A.; Bishai, W. R.; Jain, S. K.] Johns Hopkins Univ, Sch Med, Ctr TB Res, Baltimore, MD USA.

[Ordonez, A. A.; Jain, S. K.] Johns Hopkins Univ, Sch Med, Dept Pediat, Baltimore, MD 21205 USA.

[Maiga, M.; Gupta, S.; Weinstein, E. A.; Bishai, W. R.] Johns Hopkins Univ, Sch Med, Dept Med, Baltimore, MD 21205 USA.

C3 Johns Hopkins University; Johns Hopkins University; Johns Hopkins

University; Johns Hopkins University

RP Jain, SK (corresponding author), Ctr Infect & Inflammat Imaging Res, 1550 Orleans St,Rm 1-09, Baltimore, MD 21287 USA.

EM sjain5@jhmi.edu

RI Gupta, Shashank/E-7382-2016; Ordonez, Alvaro/AAC-9996-2022

OI Ordonez, Alvaro/0000-0002-8571-0655; Jain, Sanjay/0000-0001-9620-7070

FU NIH Director's New Innovator Award [OD006492]

FX This study was funded by the NIH Director's New Innovator Award OD006492

(S.K.J.). The funders had no role in study design, data collection and

analysis, decision to publish, or preparation of the manuscript.

CR Abbate E, 2012, J ANTIMICROB CHEMOTH, V67, P473, DOI 10.1093/jac/dkr500

Adams KN, 2011, CELL, V145, P39, DOI 10.1016/j.cell.2011.02.022

AFGHANI B, 1994, CLIN INFECT DIS, V19, P1092, DOI 10.1093/clinids/19.6.1092

Agarwal N, 2009, NATURE, V460, P98, DOI 10.1038/nature08123

Agerberth B, 2000, BLOOD, V96, P3086, DOI 10.1182/blood.V96.9.3086.h8003086_3086_3093

Amaral L, 2007, IN VIVO, V21, P237

Amaral L, 2012, INT J ANTIMICROB AG, V39, P376, DOI 10.1016/j.ijantimicag.2012.01.012

Annane D, 2011, ANN INTENSIVE CARE, V1, DOI 10.1186/2110-5820-1-7

[Anonymous], 2003, AM J RESP CRIT CARE, V167, P603

[Anonymous], 1983, TUBERCLE, V64, P73

[Anonymous], GLOB TUB REP 2013

Azuma A, 2012, THER ADV RESPIR DIS, V6, P107, DOI 10.1177/1753465812436663

Azuma Arata, 2010, Expert Rev Respir Med, V4, P301, DOI 10.1586/ers.10.32

Baeke F, 2010, CURR OPIN PHARMACOL, V10, P482, DOI 10.1016/j.coph.2010.04.001

Balganesh M, 2012, ANTIMICROB AGENTS CH, V56, P2643, DOI 10.1128/AAC.06003-11

Be NA, 2009, CURR MOL MED, V9, P94, DOI 10.2174/156652409787581655

Bekker LG, 1998, J INFECT DIS, V178, P580, DOI 10.1086/517479

Bilaçeroglu S, 1999, INT J TUBERC LUNG D, V3, P47

Blackmore TK, 2008, CLIN INFECT DIS, V47, pE83, DOI 10.1086/592695

BORNSTEIN PK, 1960, AM REV RESPIR DIS, V81, P421

CAPON AW, 1952, CAN MED ASSOC J, V67, P46

CHATTERJEE P K, 1958, J Indian Med Assoc, V31, P233

Chen S, 2007, J IMMUNOL, V179, P1634, DOI 10.4049/jimmunol.179.3.1634

Chun Rene F, 2011, Expert Rev Clin Pharmacol, V4, P583, DOI 10.1586/ecp.11.41

Churchyard G, 2007, WHO SPECIAL PROGRAMM

Conesa-Botella A, 2012, CLIN INFECT DIS, V55, P1004, DOI 10.1093/cid/cis577

Costamagna P., 2004, Morbidity and Mortality Weekly Report, V53, P683

Coussens AK, 2012, P NATL ACAD SCI USA, V109, P15449, DOI 10.1073/pnas.1200072109

De Bosscher K, 2010, CURR OPIN PHARMACOL, V10, P497, DOI 10.1016/j.coph.2010.04.007

Di Nardo A, 2003, J IMMUNOL, V170, P2274, DOI 10.4049/jimmunol.170.5.2274

Dooley DP, 1997, CLIN INFECT DIS, V25, P872, DOI 10.1086/515543

Dye C, 1999, JAMA-J AM MED ASSOC, V282, P677, DOI 10.1001/jama.282.7.677

ELDEWI T, 1962, SCHWEIZ MED WSCHR, V92, P1398

Elkington PTG, 2006, THORAX, V61, P259, DOI 10.1136/thx.2005.051979

Elliott AM, 2004, J INFECT DIS, V190, P869, DOI 10.1086/422257

Engel ME, 2007, COCHRANE DB SYST REV, DOI 10.1002/14651858.CD001876.pub2

ESCOBAR JA, 1975, PEDIATRICS, V56, P1050

Escribano I, 2007, CHEMOTHERAPY, V53, P397, DOI 10.1159/000109769

Flynn JL, 2005, TRENDS MICROBIOL, V13, P98, DOI 10.1016/j.tim.2005.01.005

Gans J. de, 2002, New England Journal of Medicine, V347, P1549, DOI 10.1056/NEJMoa021334

Gibney KB, 2008, CLIN INFECT DIS, V46, P443, DOI 10.1086/525268

Gilroy SA, 2011, SEMIN RESP CRIT CARE, V32, P775, DOI 10.1055/s-0031-1295725

GIRGIS NI, 1991, PEDIATR INFECT DIS J, V10, P179, DOI 10.1097/00006454-199103000-00002

Gold B, 2012, P NATL ACAD SCI USA, V109, P16004, DOI 10.1073/pnas.1214188109

Gomez JE, 2004, TUBERCULOSIS, V84, P29, DOI 10.1016/j.tube.2003.08.003

Green JA, 2010, J IMMUNOL, V184, P6492, DOI 10.4049/jimmunol.0903811

Grosset JH, 2012, INT J TUBERC LUNG D, V16, P1005, DOI 10.5588/ijtld.12.0277

Guo SL, 2012, FRONT BIOSCI-LANDMRK, V17, P2684, DOI 10.2741/4079

HAAPANEN JH, 1959, AM REV RESPIR DIS, V80, P1

Hakim JG, 2000, HEART, V84, P183, DOI 10.1136/heart.84.2.183

HANDLEY A E, 1956, S Afr Med J, V30, P1250

Harper J, 2012, J INFECT DIS, V205, P595, DOI 10.1093/infdis/jir786

Hewison M, 2011, NAT REV ENDOCRINOL, V7, P336, DOI 10.1038/nrendo.2010.226

HEYMAN A, 1959, J Med Soc N J, V56, P62

HORNE NW, 1960, BRIT MED J, V2, P1751, DOI 10.1136/bmj.2.5215.1751

Huang T-S, 2012, J FORMOSAN IN PRESS

Izzo AA, 2004, TUBERCULOSIS, V84, P387, DOI 10.1016/j.tube.2004.07.001

Jain SK, 2005, CLIN PEDIATR, V44, P443, DOI 10.1177/000992280504400510

KAOJARERN S, 1991, CLIN PHARMACOL THER, V49, P6, DOI 10.1038/clpt.1991.2

Keane J, 1997, INFECT IMMUN, V65, P298, DOI 10.1128/IAI.65.1.298-304.1997

Keane J, 2001, NEW ENGL J MED, V345, P1098, DOI 10.1056/NEJMoa011110

Koo MS, 2011, PLOS ONE, V6, DOI 10.1371/journal.pone.0017091

Kremer JM, 2009, ARTHRITIS RHEUM-US, V60, P1895, DOI 10.1002/art.24567

LAVERS K W, 1959, Tubercle, V40, P173, DOI 10.1016/S0041-3879(59)80036-2

Lazar-Molnar E, 2011, J IMMUNOL S, V186

Lázár-Molnár E, 2010, P NATL ACAD SCI USA, V107, P13402, DOI 10.1073/pnas.1007394107

LEE CH, 1988, CHEST, V94, P1256, DOI 10.1378/chest.94.6.1256

Lillebaek T, 2003, J INFECT DIS, V188, P1032, DOI 10.1086/378240

Liu PT, 2007, J IMMUNOL, V179, P2060, DOI 10.4049/jimmunol.179.4.2060

Louw GE, 2011, AM J RESP CRIT CARE, V184, P269, DOI 10.1164/rccm.201011-1924OC

Maiga M, 2012, PLOS ONE, V7, DOI 10.1371/journal.pone.0030749

Malhotra HS, 2009, ANN TROP MED PARASIT, V103, P625, DOI 10.1179/000349809X12502035776315

Martineau AR, 2007, J IMMUNOL, V178, P7190, DOI 10.4049/jimmunol.178.11.7190

Martineau AR, 2011, LANCET, V377, P242, DOI 10.1016/S0140-6736(10)61889-2

Martins M, 2009, INT J TUBERC LUNG D, V13, P569

Mayanja-Kizza H, 2005, J INFECT DIS, V191, P856, DOI 10.1086/427995

Mayosi BM, 2000, COCHRANE DB SYST REV, V2

McIntyre PB, 1997, JAMA-J AM MED ASSOC, V278, P925, DOI 10.1001/jama.278.11.925

Meagher LC, 1996, J IMMUNOL, V156, P4422

Meintjes G, 2012, AM J RESP CRIT CARE, V186, P369, DOI 10.1164/rccm.201201-0094OC

Meintjes G, 2010, AIDS, V24, P2381, DOI 10.1097/QAD.0b013e32833dfc68

Moens MJU, 2007, FRONT BIOSCI-LANDMRK, V12, P1814

Mohan VP, 2001, INFECT IMMUN, V69, P1847, DOI 10.1128/IAI.69.3.1847-1855.2001

Moitra K, 2011, CLIN PHARMACOL THER, V89, P491, DOI 10.1038/clpt.2011.14

Mootoo Amanda, 2009, Inflammation & Allergy Drug Targets, V8, P53

MORRIS D, 1963, Tubercle, V44, P484

Nursyam Elly Wijaya, 2006, Acta Med Indones, V38, P3

Omori K, 2007, CIRC RES, V100, P309, DOI 10.1161/01.RES.0000256354.95791.f1

Ordway D, 2003, ANTIMICROB AGENTS CH, V47, P917, DOI 10.1128/AAC.47.3.917-922.2003

Pan H, 2005, NATURE, V434, P767, DOI 10.1038/nature03419

Podlekareva DN, 2013, EUR RESP J

Prasad K, 2008, COCHRANE DB SYST REV, DOI 10.1002/14651858.CD002244.pub3

Ramakrishnan L, 2012, NAT REV IMMUNOL, V12, P352, DOI 10.1038/nri3211

REALE M, 1956, Minerva Med, V47, P1764

Roca FJ, 2013, CELL, V153, P521, DOI 10.1016/j.cell.2013.03.022

Rockett KA, 1998, INFECT IMMUN, V66, P5314, DOI 10.1128/IAI.66.11.5314-5321.1998

Ruslami R, 2013, LANCET INFECT DIS, V13, P27, DOI 10.1016/S1473-3099(12)70264-5

Sasidharan P K, 2002, J Assoc Physicians India, V50, P554

Schmalstieg AM, 2012, ANTIMICROB AGENTS CH, V56, P4806, DOI 10.1128/AAC.05546-11

Schoeman JF, 2004, J CHILD NEUROL, V19, P250, DOI 10.1177/088307380401900402

Schoeman JF, 1997, PEDIATRICS, V99, P226, DOI 10.1542/peds.99.2.226

Sharma S, 2010, J ANTIMICROB CHEMOTH, V65, P1694, DOI 10.1093/jac/dkq186

Skerry C, 2012, PLOS ONE, V7, DOI 10.1371/journal.pone.0039680

Smego RA, 2003, INT J TUBERC LUNG D, V7, P208

Spagnolo P, 2013, EUR RESPIR J, V42, P239, DOI 10.1183/09031936.00136712

Stahn C, 2008, NAT CLIN PRACT RHEUM, V4, P525, DOI 10.1038/ncprheum0898

STEAD WW, 1967, AM REV RESPIR DIS, V95, P729

Sternberg EM, 2006, NAT REV IMMUNOL, V6, P318, DOI 10.1038/nri1810

Subbian S, 2011, PLOS PATHOG, V7, DOI 10.1371/journal.ppat.1002262

Subbian S, 2011, AM J PATHOL, V179, P289, DOI 10.1016/j.ajpath.2011.03.039

Sunila ES, 2004, J ETHNOPHARMACOL, V90, P339, DOI 10.1016/j.jep.2003.10.016

Sweeny DJ, 2010, DRUG METAB DISPOS, V38, P1166, DOI 10.1124/dmd.110.032151

Szumowski JD, 2012, CURR TOP MICROBIOL I

Taylor JL, 2006, INFECT IMMUN, V74, P6135, DOI 10.1128/IAI.02048-05

Tegos GP, 2011, CURR PHARM DESIGN, V17, P1291

Thwaites GE, 2004, NEW ENGL J MED, V351, P1741, DOI 10.1056/NEJMoa040573

Thwaites G, 2009, J INFECTION, V59, P167, DOI 10.1016/j.jinf.2009.06.011

Tobin DM, 2012, CELL, V148, P434, DOI 10.1016/j.cell.2011.12.023

Török ME, 2011, PLOS ONE, V6, DOI 10.1371/journal.pone.0027821

Topalian SL, 2012, NEW ENGL J MED, V366, P2443, DOI 10.1056/NEJMoa1200690

Tsenova L, 1998, J INFECT DIS, V177, P1563, DOI 10.1086/515327

Tsenova L, 2002, ANTIMICROB AGENTS CH, V46, P1887, DOI 10.1128/AAC.46.6.1887-1895.2002

Udwadia Zarir F, 2011, Recent Pat Antiinfect Drug Discov, V6, P88

Ustianowski A, 2005, J INFECTION, V50, P432, DOI 10.1016/j.jinf.2004.07.006

van de Beek D, 2004, LANCET INFECT DIS, V4, P139, DOI 10.1016/S1473-3099(04)00937-5

VANDIVIERE H M, 1956, Am J Med Sci, V232, P30, DOI 10.1097/00000441-195607000-00006

Walker NF, 2012, AM J RESP CRIT CARE, V185, P989, DOI 10.1164/rccm.201110-1769OC

Wallis RS, 2009, CLIN INFECT DIS, V48, P1429, DOI 10.1086/598504

Wallis RS, 2004, AIDS, V18, P257, DOI 10.1097/00002030-200401230-00015

Wallis RS, 2005, CLIN INFECT DIS, V41, P201, DOI 10.1086/430914

Wallis RS, 2004, CLIN INFECT DIS, V38, P1261, DOI 10.1086/383317

Weinstein EA, 2005, P NATL ACAD SCI USA, V102, P4548, DOI 10.1073/pnas.0500469102

Wejse C, 2007, AM J CLIN NUTR, V86, P1376, DOI 10.1093/ajcn/86.5.1376

Wejse C, 2009, AM J RESP CRIT CARE, V179, P843, DOI 10.1164/rccm.200804-567OC

Williams B, 2008, PEDIATR INFECT DIS J, V27, P941, DOI 10.1097/INF.0b013e31817525df

Zasloff M, 2006, NAT MED, V12, P388, DOI 10.1038/nm0406-388

NR 136

TC 22

Z9 24

U1 0

U2 15

PU BENTHAM SCIENCE PUBL LTD

PI SHARJAH

PA EXECUTIVE STE Y-2, PO BOX 7917, SAIF ZONE, 1200 BR SHARJAH, U ARAB

EMIRATES

SN 1566-5240

EI 1875-5666

J9 CURR MOL MED

JI Curr. Mol. Med.

PD MAR

PY 2014

VL 14

IS 3

BP 385

EP 395

DI 10.2174/1566524013666131118112431

PG 11

WC Medicine, Research & Experimental

WE Science Citation Index Expanded (SCI-EXPANDED)

SC Research & Experimental Medicine

GA AD6BU

UT WOS:000333339100009

OA Green Submitted, Bronze

DA 2026-05-07

ER

PT J

AU Liu, YG

Matsumoto, M

Ishida, H

Ohguro, K

Yoshitake, M

Gupta, R

Geiter, L

Hafkin, J

AF Liu, Yongge

Matsumoto, Makoto

Ishida, Hidekaza

Ohguro, Kinue

Yoshitake, Masuhiro

Gupta, Rajesh

Geiter, Lawrence

Hafkin, Jeffrey

TI Delamanid: From discovery to its use for pulmonary multidrug-resistant

tuberculosis (MDR-TB)

SO TUBERCULOSIS

LA English

DT Review

DE Delamanid; Mycobacterium tuberculosis; MDR-TB; Nitroimidazole

ID IN-VIVO ACTIVITIES; MYCOBACTERIUM-TUBERCULOSIS; ANTITUBERCULOSIS DRUG;

NONREPLICATING PERSISTENCE; VITRO MODEL; OPC-67683; BEDAQUILINE;

MORTALITY; MECHANISM

AB Tuberculosis (TB), caused by Mycobacterium tuberculosis (MTB), is the leading cause of death from an infectious disease globally. The widespread and ever-increasing resistance to TB drugs is reducing the effectiveness of treatment and jeopardizing TB control. New effective drugs with acceptable safety profiles are needed to turn the tide. Since the early 1990s, Otsuka Pharmaceutical Co., Ltd. has had a TB drug development program that resulted in the selection and development of delamanid (OPC-67683, Deltyba (R)), a first-in-class bicyclic nitroimidazole. Delamanid was initially approved by the European Medicines Agency (EMA) in 2014 for the treatment of adult pulmonary multi-drug resistant (MDR)-TB when an effective treatment regimen cannot otherwise be composed for reasons of resistance or tolerability. It has since been approved by several other countries/regions. In this review, we describe the history of delamanid's development, including the screening process, in vitro and in vivo characterization, as well as various clinical studies. Delamanid possesses potent activity against replicating, dormant, and intracellular MTB bacilli, and is bactericidal in mouse and guinea pig TB models. Delamanid resistance mechanisms have been attributed to genes in the F-420-dependent deazaflavin nitroreductase bio-activation pathway, found in mycobacterium species but not in common bacterial or mammalian cells. Published susceptibility testing results from 744 clinical isolates from delamanid-naive patients indicate that the natural resistance rate to delamanid is very low (1.3%). Delamanid is largely metabolized by albumin in serum, and to a much less extent by cytochrome P-450 enzymes. Furthermore, it neither inhibits nor induces P450 enzymes. In terms of efficacy, delamanid demonstrated activity in an early bactericidal activity trial in drug susceptible pulmonary TB patients and increased 2-month sputum culture conversion rates when added to an optimized background regimen in MDR-TB patients in a phase 2b global clinical trial. In addition, recent results outside clinical studies show favourable responses in highly resistant TB patients including extensively drug resistant (XDR)-TB when treated with delamanid-containing regimens in routine programmatic settings. The primary safety concern with delamanid is QTcF interval prolongation, although this observation has thus far not been associated with any clinical cardiac events. Overall, delamanid appears to be a well-tolerated and safe anti-TB drug when compared to other drugs used to treat MDR-TB.

C1 [Liu, Yongge; Yoshitake, Masuhiro; Gupta, Rajesh; Geiter, Lawrence; Hafkin, Jeffrey] Otsuka Pharmaceut Dev & Commercializat Inc, 2440 Res Blvd, Rockville, MD 20850 USA.

[Matsumoto, Makoto] Otsuka Pharmaceut Co Ltd, Pharmaceut Business Div, Tokyo, Japan.

[Ishida, Hidekaza; Ohguro, Kinue] Otsuka Pharmaceut Co Ltd, Dept Med Innovat, New Drug Res Div, Tokushima, Japan.

C3 Otsuka Pharmaceutical; Otsuka Pharmaceutical; Otsuka Pharmaceutical

RP Liu, YG (corresponding author), Otsuka Pharmaceut Dev & Commercializat Inc, 2440 Res Blvd, Rockville, MD 20850 USA.

EM yongge.liu@otsuka-us.com

OI Gupta, Rajesh/0000-0002-3307-6541

CR Ängeby K, 2012, B WORLD HEALTH ORGAN, V90, P693, DOI 10.2471/BLT.11.096644

[Anonymous], 2016, Treatment Guidelines for Drug-Resistant Tuberculosis

[Anonymous], 46 UN WORLD C LUNG H

[Anonymous], 2017, GLOB INV TUB RES DEV

[Anonymous], UNITED REPUBLIC TANZ, P1

[Anonymous], 2018, WHO POS STAT US DEL

[Anonymous], 2014, Use of Convalescent Whole Blood or Plasma Collected from Patients Recovered from Ebola Virus Disease for Transfusion, as an Empirical Treatment during Outbreaks, P1, DOI DOI 10.1016/B978-0-12-374984-0.01018-4

[Anonymous], 2017, GLOB TUB REP

[Anonymous], 2013, GUID IND PULM TUB DE

ASHTEKAR DR, 1993, ANTIMICROB AGENTS CH, V37, P183, DOI 10.1128/AAC.37.2.183

Bashiri G, 2010, PLOS ONE, V5, DOI 10.1371/journal.pone.0015803

Bloemberg GV, 2015, NEW ENGL J MED, V373, P1986, DOI 10.1056/NEJMc1505196

Chen XH, 2017, ANTIMICROB AGENTS CH, V61, DOI [10.1128/aac.02402-16, 10.1128/AAC.02402-16]

Dartois V, 2013, BIOORG MED CHEM LETT, V23, P4741, DOI 10.1016/j.bmcl.2013.07.006

Dheda K, 2017, LANCET RESP MED, V5, P291, DOI [10.1016/s2213-2600(17)30079-6, 10.1016/S2213-2600(17)30079-6]

Diacon AH, 2011, INT J TUBERC LUNG D, V15, P949, DOI 10.5588/ijtld.10.0616

Doi N, 2006, INTERSCI C ANTIMICRO, V14

Esposito S, 2014, EUR RESPIR J, V44, P811, DOI 10.1183/09031936.00060414

European Medicines Agency, 2014, SUMM PROD CHAR DEL 2

European Medicines Agency, 2016, DRAFT ADD GUID EV ME, V44, P1

Fujiwara M, 2018, TUBERCULOSIS, V108, P186, DOI 10.1016/j.tube.2017.12.006

Gengenbacher M, 2017, SCI REP-UK, V7, DOI 10.1038/s41598-017-09177-2

Gler MT, 2012, NEW ENGL J MED, V366, P2151, DOI 10.1056/NEJMoa1112433

Gupta R, 2015, INT J TUBERC LUNG D, V19, P1261, DOI 10.5588/ijtld.15.0541

Gupta R, 2015, NEW ENGL J MED, V373, P291, DOI 10.1056/NEJMc1415332

Hafkin J, 2015, INTERSCI C ANTIMICRO

Hafkin J, 2017, EUR RESPIR J, V50, DOI 10.1183/13993003.00311-2017

Hanaki E, 2017, REGUL TOXICOL PHARM, V84, P1, DOI 10.1016/j.yrtph.2016.12.002

Haver HL, 2015, ANTIMICROB AGENTS CH, V59, P5316, DOI 10.1128/AAC.00308-15

Hewison C, 2017, EMERG INFECT DIS, V23, P1746, DOI 10.3201/eid2310.170468

Hoffmann H, 2016, AM J RESP CRIT CARE, V193, P337, DOI 10.1164/rccm.201502-0372LE

Keshavjee S, 2012, NEW ENGL J MED, V367, P931, DOI 10.1056/NEJMra1205429

Krieger D, 2016, EUR RESPIR J, V48, P1803, DOI 10.1183/13993003.01420-2016

Kuksa L, 2017, EUR RESPIR J, V50, DOI 10.1183/13993003.01105-2017

Mallikaarjun S, 2016, ANTIMICROB AGENTS CH, V60, P5976, DOI 10.1128/AAC.00509-16

Manjunatha UH, 2006, P NATL ACAD SCI USA, V103, P431, DOI 10.1073/pnas.0508392103

Matsumoto, 2016, SUCCESS DRUG DISCOVE, P137, DOI DOI 10.1002/9783527800315.CH7

Matsumoto M, 2007, CURR TOP MED CHEM, V7, P499, DOI 10.2174/156802607780059727

Matsumoto M, 2006, PLOS MED, V3, P2131, DOI 10.1371/journal.pmed.0030466

Mok J, 2018, J ANTIMICROB CHEMOTH, V73, P503, DOI 10.1093/jac/dkx373

Mukherjee T, 2011, FUTURE MED CHEM, V3, P1427, DOI [10.4155/FMC.11.90, 10.4155/fmc.11.90]

Nagarajan K, 1989, PROG DRUG RES, V24, P631, DOI DOI 10.1016/0223-5234(89)90034-2

Operario DJ, 2017, PLOS ONE, V12, DOI 10.1371/journal.pone.0176522

Pang Y, 2017, ANTIMICROB AGENTS CH, V61, DOI [10.1128/AAC.00900-17, 10.1128/aac.00900-17]

Patterson S, 2016, ELIFE, V5, DOI 10.7554/eLife.09744

Sasabe H, 2016, ANTIMICROB AGENTS CH, V60, P4431, DOI 10.1128/AAC.01094-16

Sasahara K, 2015, DRUG METAB DISPOS, V43, P1267, DOI 10.1124/dmd.115.064527

Sasaki H, 2006, J MED CHEM, V49, P7854, DOI 10.1021/jm060957y

Schena E, 2016, J ANTIMICROB CHEMOTH, V71, P1532, DOI 10.1093/jac/dkw044

Shibata M, 2017, BIOPHARM DRUG DISPOS, V38, P301, DOI 10.1002/bdd.2064

Shimokawa Y, 2015, DRUG METAB DISPOS, V43, P1277, DOI 10.1124/dmd.115.064550

Shimokawa Y, 2014, BIOL PHARM BULL, V37, P1727, DOI 10.1248/bpb.b14-00311

Skripconoka V, 2013, EUR RESPIR J, V41, P1393, DOI 10.1183/09031936.00125812

Sotgiu G, 2015, EXPERT REV ANTI-INFE, V13, P305, DOI 10.1586/14787210.2015.1011127

Stinson K, 2016, ANTIMICROB AGENTS CH, V60, P3316, DOI 10.1128/AAC.03014-15

Upton AM, 2015, ANTIMICROB AGENTS CH, V59, P136, DOI 10.1128/AAC.03823-14

Wayne L G, 2001, Methods Mol Med, V54, P247, DOI 10.1385/1-59259-147-7:247

Wayne LG, 1996, INFECT IMMUN, V64, P2062, DOI 10.1128/IAI.64.6.2062-2069.1996

Wells CD, 2015, EUR RESPIR J, V45, P1498, DOI 10.1183/09031936.00176314

Wyllie S, 2016, PLOS PATHOG, V12, DOI 10.1371/journal.ppat.1005971

Yuan Y, 1998, MOL MICROBIOL, V29, P1449, DOI 10.1046/j.1365-2958.1998.01026.x

NR 61

TC 129

Z9 164

U1 0

U2 38

PU CHURCHILL LIVINGSTONE

PI EDINBURGH

PA JOURNAL PRODUCTION DEPT, ROBERT STEVENSON HOUSE, 1-3 BAXTERS PLACE,

LEITH WALK, EDINBURGH EH1 3AF, MIDLOTHIAN, SCOTLAND

SN 1472-9792

J9 TUBERCULOSIS

JI Tuberculosis

PD JUL

PY 2018

VL 111

BP 20

EP 30

DI 10.1016/j.tube.2018.04.008

PG 11

WC Immunology; Microbiology; Respiratory System

WE Science Citation Index Expanded (SCI-EXPANDED)

SC Immunology; Microbiology; Respiratory System

GA GN5AN

UT WOS:000439054400004

DA 2026-05-07

ER

PT J

AU Mishra, S

Kumar, G

Singh, P

AF Mishra, Sahil

Kumar, Gobind

Singh, Parvesh

TI Isoniazid Hybrids As Potential Antitubercular Agents

SO CHEMISTRYSELECT

LA English

DT Review

DE Anti-TB; Drug-resistance; InhA; Isoniazid; MDR-TB; Molecular hybrids;

Quinoline

ID MYCOBACTERIUM-TUBERCULOSIS; ANTIMYCOBACTERIAL; PYRAZINAMIDE; CHALLENGES;

MOLECULES; MORTALITY; MECHANISM; INHA

AB The rapid emergence of various forms of drug-resistant tuberculosis (TB) has massively impacted people's health globally. According to the WHO, TB care and facilities have been downgraded by 21% since the COVID-19 pandemic, which has further slowed down the progress toward the "End TB Strategy." Multiple studies have shown drug-resistant tuberculosis to be resilient to front-line antituberculosis drugs, including isoniazid (INH), thus constantly prompting researchers worldwide to probe new potent chemical entities to replace/supplement the current artillery of anti-TB therapeutics. The molecular hybridization (MH) technique has recently been adopted by many synthetic and medicinal chemists to combine different pharmacophoric units into a single molecular architecture which in most cases has been reported to exhibit a better pharmacological profile as compared to its precursors. The present review provides a concise account of the MH hybridization strategies directed at the structural manipulations of isoniazid to develop new anti-TB agents and their structure-activity relationships. Furthermore, their toxicity profiles and docking studies with relevant receptors are also briefly discussed.

Molecular hybridization is a rational drug design strategy that combines two or more covalently binding pharmacological agents into a single multifunctional molecule. The present review discusses the recent functionalizing of isoniazid to new hybrids tackling the menace of various forms of drug-resistant tuberculosis and generating potential leads for further modifications. image

C1 [Mishra, Sahil; Kumar, Gobind; Singh, Parvesh] Univ KwaZulu Natal, Sch Chem & Phys, P Bag X54001, ZA-4000 Durban, South Africa.

C3 University of Kwazulu Natal

RP Singh, P (corresponding author), Univ KwaZulu Natal, Sch Chem & Phys, P Bag X54001, ZA-4000 Durban, South Africa.

EM singhp4@ukzn.ac.za

RI mishra, sahil/OFN-3440-2025; Singh, Parvesh/L-6161-2016

OI mishra, sahil/0000-0002-4722-9174; Singh, Parvesh/0000-0001-6742-6389

FU National Research Foundation [SRUG2204092857]; National Research

Foundation (South Africa)

FX PS gratefully acknowledges the National Research Foundation (South

Africa) for a competitive grant for rated researchers (Grant Number:

SRUG2204092857), and the Centre for High-Performance Computing (CHPC),

Cape Town for computational resources.

CR Aatif M, 2022, ANTIBIOTICS-BASEL, V11, DOI 10.3390/antibiotics11121750

Abdelrahman MA, 2022, MOLECULES, V27, DOI 10.3390/molecules27248807

Abdu-Allah HHM, 2017, ARCH PHARM RES, V40, P168, DOI 10.1007/s12272-016-0882-x

Alcaraz M, 2022, EUR J MED CHEM, V239, DOI 10.1016/j.ejmech.2022.114531

Aslan EK, 2022, PHARMACEUTICALS-BASE, V15, DOI 10.3390/ph15101301

Aslan EK, 2022, J MOL LIQ, V354, DOI 10.1016/j.molliq.2022.118873

Badar AD, 2020, J HETEROCYCLIC CHEM, V57, P3544, DOI 10.1002/jhet.4072

Beteck RM, 2019, MEDCHEMCOMM, V10, P326, DOI 10.1039/c8md00480c

Bhavani GV, 2023, DRUG DEVELOP RES, V84, P470, DOI 10.1002/ddr.22039

Bhoi MN, 2019, EUR J MED CHEM, V177, P12, DOI 10.1016/j.ejmech.2019.05.028

Bonvicini F, 2022, MOLECULES, V27, DOI 10.3390/molecules27185781

Borad MA, 2020, J MOL STRUCT, V1222, DOI 10.1016/j.molstruc.2020.128881

Chowdhury T, 2022, BANGLADESH J MED, V33, P168, DOI [10.3329/bjm.v33i2.59290, 10.3329/bjm.v33i2.59290, DOI 10.3329/BJM.V33I2.59290]

Cilloni L., 2020, The Potential Impact of the COVID19 Pandemic on the Tuberculosis Epidemic: A Modelling Analysis (preprint)

Conradie F, 2022, NEW ENGL J MED, V387, P810, DOI 10.1056/NEJMoa2119430

CROWLE AJ, 1990, ANTIMICROB AGENTS CH, V34, P2217, DOI 10.1128/AAC.34.11.2217

Daniel TM, 2006, RESP MED, V100, P1862, DOI 10.1016/j.rmed.2006.08.006

Dartois VA, 2022, NAT REV MICROBIOL, V20, P685, DOI 10.1038/s41579-022-00731-y

Dogan H, 2020, EUR J MED CHEM, V188, DOI 10.1016/j.ejmech.2020.112035

Dooley KE, 2020, AM J RESP CRIT CARE, V201, P1416, DOI 10.1164/rccm.201910-1960OC

Fernandes GFD, 2016, EUR J MED CHEM, V123, P523, DOI 10.1016/j.ejmech.2016.07.039

El-Sedik M, 2019, EGYPT J CHEM, V62, P2253, DOI 10.21608/ejchem.2019.12976.1810

Elsayed ZM, 2021, J ENZYM INHIB MED CH, V36, DOI 10.1080/14756366.2020.1868450

Eldin RRE, 2023, PHARMACEUTICALS-BASE, V16, DOI 10.3390/ph16091247

Fernandes GFS, 2022, J MED CHEM, V65, P7489, DOI 10.1021/acs.jmedchem.2c00227

Frieden TR, 2005, CLIN CHEST MED, V26, P197, DOI 10.1016/j.ccm.2005.02.001

Gavadia R, 2023, J MOL STRUCT, V1283, DOI 10.1016/j.molstruc.2023.135325

Ginsberg AM, 2007, NAT MED, V13, P290, DOI 10.1038/nm0307-290

Gomez GB, 2021, BMJ OPEN, V11, DOI 10.1136/bmjopen-2021-051521

Gouzy A, 2021, P NATL ACAD SCI USA, V118, DOI 10.1073/pnas.2024571118

Grobbelaar M, 2019, INFECT GENET EVOL, V74, DOI 10.1016/j.meegid.2019.103937

Guo MH, 2023, FITOTERAPIA, V168, DOI 10.1016/j.fitote.2023.105536

Jena L, 2014, INT J MYCOBACT, V3, P276, DOI 10.1016/j.ijmyco.2014.08.003

Johansen MD, 2021, ANTIMICROB AGENTS CH, V65, DOI 10.1128/AAC.00011-21

Kasetti AB, 2021, MOLECULES, V26, DOI 10.3390/molecules26102847

Kaur H, 2020, MOL DIVERS, V24, P949, DOI 10.1007/s11030-019-10004-1

Kayongo A, 2023, FRONT CELL INFECT MI, V13, DOI 10.3389/fcimb.2023.1146571

Khaleel EF, 2024, BIOORG CHEM, V144, DOI 10.1016/j.bioorg.2024.107138

Khan SA, 2023, PHARMACEUTICALS-BASE, V16, DOI 10.3390/ph16020179

Koli BP, 2023, SYNTHETIC COMMUN, V53, P1506, DOI 10.1080/00397911.2023.2235624

Lamont EA, 2020, MICROBIOL MOL BIOL R, V84, DOI 10.1128/MMBR.00070-19

Lei BF, 2000, J BIOL CHEM, V275, P2520, DOI 10.1074/jbc.275.4.2520

Lempens P, 2018, SCI REP-UK, V8, DOI 10.1038/s41598-018-21378-x

Li ZF, 2024, BIOORG MED CHEM LETT, V99, DOI 10.1016/j.bmcl.2024.129613

Lücking U, 2019, ORG CHEM FRONT, V6, P1319, DOI 10.1039/c8qo01233d

Marais BJ, 2016, INT J INFECT DIS, V45, P100, DOI 10.1016/j.ijid.2016.03.015

Marrakchi H, 2014, CHEM BIOL, V21, P67, DOI 10.1016/j.chembiol.2013.11.011

Mermer A, 2021, BIOORG CHEM, V114, DOI 10.1016/j.bioorg.2021.105076

Meunier B, 2008, ACCOUNTS CHEM RES, V41, P69, DOI 10.1021/ar7000843

Mishra S, 2018, EUR J MED CHEM, V155, P658, DOI 10.1016/j.ejmech.2018.06.039

Nalini R, 2022, J INDIAN CHEM SOC, V99, DOI 10.1016/j.jics.2021.100273

Nqoro X, 2017, MOLECULES, V22, DOI 10.3390/molecules22122268

Obaid Rami J., 2022, Process Biochemistry, P250, DOI [10.1016/j.procbio.2022.06.009, 10.1016/j.procbio.2022.06.009]

Osman M, 2021, SCI REP-UK, V11, DOI 10.1038/s41598-021-95331-w

Osman M, 2019, INT J INFECT DIS, V85, P57, DOI 10.1016/j.ijid.2019.05.024

Owusu E, 2022, DISEASES-BASEL, V10, DOI 10.3390/diseases10010010

Pai M, 2016, T ROY SOC TROP MED H, V110, P158, DOI 10.1093/trstmh/trw003

Pajk S, 2016, EUR J MED CHEM, V112, P252, DOI 10.1016/j.ejmech.2016.02.008

Panda SS, 2019, RSC ADV, V9, P20450, DOI 10.1039/c9ra03380g

Panti RIR, 2021, REV COLOMB QUIM, V50, P16, DOI 10.15446/rev.colomb.quim.v50n3.96424

Patel KD, 2020, ARAB J CHEM, V13, P1986, DOI 10.1016/j.arabjc.2018.02.017

Patil PS, 2020, BIOORG MED CHEM LETT, V30, DOI 10.1016/j.bmcl.2020.127434

Pawelczyk J, 2014, MICROBIOL SPECTR, V2, DOI 10.1128/microbiolspec.MGM2-0003-2013

Pranger AD, 2019, DRUGS, V79, P161, DOI 10.1007/s40265-018-1043-y

Prasad MS, 2021, BIOORG CHEM, V115, DOI 10.1016/j.bioorg.2021.105242

Prieri M, 2018, EUR J MED CHEM, V159, P35, DOI 10.1016/j.ejmech.2018.09.038

Rani A, 2020, BIOORG MED CHEM LETT, V30, DOI 10.1016/j.bmcl.2020.127576

Rasgania J, 2023, J MOL STRUCT, V1294, DOI 10.1016/j.molstruc.2023.136464

Ravimohan S, 2018, EUR RESPIR REV, V27, DOI 10.1183/16000617.0077-2017

Reis WJ, 2019, BIOORGAN MED CHEM, V27, P4143, DOI 10.1016/j.bmc.2019.07.045

Rycka D., 2020, Pharmaceuticals, V13, P465

Sanduja M, 2020, J SAUDI CHEM SOC, V24, P251, DOI 10.1016/j.jscs.2019.12.001

Shafiei M, 2020, BIOORG CHEM, V104, DOI 10.1016/j.bioorg.2020.104240

Shah SR, 2020, J HETEROCYCLIC CHEM, V57, P1682, DOI 10.1002/jhet.3893

Shao ZT, 2023, PHARM RES-DORDR, V40, P1073, DOI 10.1007/s11095-023-03488-y

Shaveta, 2016, EUR J MED CHEM, V124, P500, DOI 10.1016/j.ejmech.2016.08.039

Shibabaw A, 2020, PLOS ONE, V15, DOI 10.1371/journal.pone.0229040

Shiri P, 2023, FRONT CHEM, V11, DOI 10.3389/fchem.2023.1229825

Shivekar SS, 2020, SCI REP-UK, V10, DOI 10.1038/s41598-020-74432-y

Siddiqui SH, 2021, OSONG PUBLIC HEALTH, V12, P64, DOI 10.24171/j.phrp.2021.12.2.03

Sotgiu G, 2017, PRESSE MED, V46, pE5, DOI 10.1016/j.lpm.2017.01.013

Thakur RK, 2019, EUR J MED CHEM, V162, P448, DOI 10.1016/j.ejmech.2018.11.008

Torres M, 2019, THORAX, V74, P675, DOI 10.1136/thoraxjnl-2018-212529

Velayati AA, 2013, INT J CLIN EXP MED, V6, P307

Woimo TT, 2017, BMC PUBLIC HEALTH, V17, DOI 10.1186/s12889-017-4188-9

Yang W, 2021, POLYM CHEM-UK, V12, P2317, DOI 10.1039/d0py01726d

Zeng S, 2019, ANTIMICROB AGENTS CH, V63, DOI 10.1128/AAC.01841-18

Zhang YQ, 2023, J GLOB HEALTH, V13, DOI 10.7189/jogh.13.06043

Zheng XA, 2020, MOLECULES, V25, DOI 10.3390/molecules25071594

Zimmerman M., 2017, Antimicrob Agents Chemother, V61

Zimmermann G., 1971, Biological Aspects of Electrochemistry. Experientia Supplementum, P693, DOI [10.1007/978-3-0348-5848-966, DOI 10.1007/978-3-0348-5848-966]

Zitko J, 2018, MEDCHEMCOMM, V9, P685, DOI 10.1039/c8md00056e

NR 92

TC 9

Z9 9

U1 1

U2 12

PU WILEY-V C H VERLAG GMBH

PI WEINHEIM

PA POSTFACH 101161, 69451 WEINHEIM, GERMANY

SN 2365-6549

J9 CHEMISTRYSELECT

JI ChemistrySelect

PD OCT 8

PY 2024

VL 9

IS 38

AR e202402933

DI 10.1002/slct.202402933

PG 19

WC Chemistry, Multidisciplinary

WE Science Citation Index Expanded (SCI-EXPANDED)

SC Chemistry

GA I1U0S

UT WOS:001328165900001

OA hybrid

DA 2026-05-07

ER

PT J

AU Singh, A

Singh, A

Sinha, V

Rana, AK

Saraswati, SSK

Singh, A

Kalra, K

Thakur, S

Kumar, D

Natarajan, K

AF Singh, Akshita

Singh, Aarti

Sinha, Vishal

Rana, Ankush K.

Saraswati, Shakuntala S. K.

Singh, Aayushi

Kalra, Kanika

Thakur, Sunil

Kumar, Dhiraj

Natarajan, Krishnamurthy

TI Dectin-1 (CLEC-7A) and Mincle (CLEC-4E) suppress protective responses of

macrophages during mycobacterial infection

SO JOURNAL OF LEUKOCYTE BIOLOGY

LA English

DT Article

DE apoptosis; autophagy; C-Type Lectin receptor; oxidative burst;

Tuberculosis

ID LECTIN RECEPTORS; TREHALOSE 6,6'-DIMYCOLATE; IMMUNE-RESPONSES; INNATE

IMMUNE; TUBERCULOSIS; MITOCHONDRIA; CELLS; RECOGNITION; EXPRESSION;

APOPTOSIS

AB Mycobacterium tuberculosis (M. tb), the causative agent of tuberculosis (TB), is responsible for extreme mortality and morbidity across the globe. The bacteria have evolved multiple strategies for their successful prevalence. The emergence of multidrug-resistant TB (MDR-TB) has established the importance of eliciting host-pathogen interactions at cellular and molecular levels. Various pattern recognition receptors play determinant roles when encountering M. tb infection. Here, we investigated the regulation of key defense responses from Dectin-1 and Mincle during mycobacterial infection in THP-1-derived macrophages, the long-term hosts for mycobacteria. Our data indicate that infection of THP-1 macrophages with either M. bovis Bacillus Calmette-Gu & eacute;rin (BCG) or M. tb H37Rv increases the surface expression of Dectin-1 and Mincle. This increase translated directly to increased intracellular bacterial survival within macrophages. Likewise, M. bovis BCG infection of human peripheral blood mononuclear cell-derived macrophages also led to an increased expression of Dectin-1 and Mincle. Stimulation of Dectin-1 or Mincle along with BCG infection induces suppressor responses such as an attenuated oxidative burst and mitochondrial membrane potential intactness. In addition, decreased apoptosis and autophagy induction was also observed following stimulation of Dectin-1 and Mincle. Conversely, RNA interference-mediated knockdown of Dectin-1 or Mincle reversed the previous responses, resulting in higher oxidative burst, mitochondrial membrane potential disruption, increased mitochondrial reactive oxygen species production, and increased apoptosis. This results in a significant decrease in intracellular mycobacterial survival. These results point toward a well-orchestrated strategy of fine-tuning the host's defense machinery of Dectin-1 and Mincle adopted by mycobacteria to suppress protective responses mounted against it and prepare the macrophages for prolonged persistent infection.

Pathogenic mycobacteria upregulate expression of Dectin-1 and Mincle on THP-1 macrophages to suppress host cell defense responses and promote intracellular bacterial survival.

C1 [Singh, Akshita; Singh, Aarti; Sinha, Vishal; Rana, Ankush K.; Singh, Aayushi; Kalra, Kanika; Natarajan, Krishnamurthy] Univ Delhi, Univ Enclave, Infect Dis Immunol Lab, Dr B R Ambedkar Ctr Biomed Res, North Campus, Delhi 110007, India.

[Saraswati, Shakuntala S. K.; Kumar, Dhiraj] Int Ctr Genet Engn & Biotechnol, Cellular Immunol Grp, Aruna Asaf Ali Marg, New Delhi 110067, India.

[Thakur, Sunil] Univ Delhi, Univ Enclave, Art Fac, WUS Hlth Ctr, Chhatra Marg, New Delhi 110007, India.

C3 University of Delhi; Department of Biotechnology (DBT) India;

International Center for Genetic Engineering & Biotechnology (ICGEB);

International Center for Genetic Engineering & Biotechnology (ICGEB),

New Delhi; University of Delhi

RP Singh, A; Natarajan, K (corresponding author), Univ Delhi, Univ Enclave, Infect Dis Immunol Lab, Dr B R Ambedkar Ctr Biomed Res, North Campus, Delhi 110007, India.

EM aksinghacbr@gmail.com; knatarajan@acbr.du.ac.in

RI ; rana, ankush kumar/PIH-4907-2026

OI Saraswati, Shakuntala/0000-0003-4450-1002; rana, ankush

kumar/0009-0004-4536-7006; Singh, Akshita/0009-0009-0239-1854

FU Department of Biotechnology, Ministry of Science and Technology,

Government of India [BT/PR41505, MED/29/1551/2020]; University of Delhi;

Department of Biotechnology

FX This work was supported by the core grants received from the University

of Delhi and grants provided to KN by the Department of Biotechnology,

Ministry of Science and Technology, India (BT/PR41505/MED/29/1551/2020).

Ak.S. is a recipient of the Senior Research Fellowship from Department

of Biotechnology, Ministry of Science and Technology, India.

CR Anand SK, 2013, ADV VIROL, V2013, DOI 10.1155/2013/738794

Anang V, 2023, PLOS ONE, V18, DOI 10.1371/journal.pone.0283448

Anaya EU, 2023, BIOPHYS J, V122, P3749, DOI 10.1016/j.bpj.2023.07.021

Antony C, 2015, PLOS ONE, V10, DOI 10.1371/journal.pone.0124263

Bandyopadhyay U, 2017, J LEUKOCYTE BIOL, V102, P1249, DOI 10.1189/jlb.4A0217-042R

Behera A, 2022, INT J MOL SCI, V23, DOI 10.3390/ijms23052584

Behler-Janbeck F, 2016, PLOS PATHOG, V12, DOI 10.1371/journal.ppat.1006038

Bermejo-Jambrina M, 2018, FRONT IMMUNOL, V9, DOI 10.3389/fimmu.2018.00590

Brown GD, 2006, NAT REV IMMUNOL, V6, P33, DOI 10.1038/nri1745

Carlyon JA, 2004, INFECT IMMUN, V72, P4772, DOI 10.1128/IAI.72.8.4772-4783.2004

Chadha A, 2015, TUBERCULOSIS, V95, P599, DOI 10.1016/j.tube.2015.05.014

Chavez-Santoscoy AV, 2012, BIOMATERIALS, V33, P4762, DOI 10.1016/j.biomaterials.2012.03.027

Cheng QJ, 2024, SCI REP-UK, V14, DOI 10.1038/s41598-024-51620-8

Coates JTT, 2020, CELL DEATH DISCOV, V6, DOI 10.1038/s41420-020-00343-6

Deo P, 2018, PLOS PATHOG, V14, DOI 10.1371/journal.ppat.1006945

Drouin M, 2020, FRONT IMMUNOL, V11, DOI 10.3389/fimmu.2020.00251

Dunn JD, 2015, REDOX BIOL, V6, P472, DOI 10.1016/j.redox.2015.09.005

Faherty CS, 2008, TRENDS MICROBIOL, V16, P173, DOI 10.1016/j.tim.2008.02.001

Furukawa A, 2023, STRUCTURE, V31, P1077, DOI 10.1016/j.str.2023.05.018

Gupta D, 2010, J IMMUNOL, V184, P5444, DOI 10.4049/jimmunol.0903283

Gutierrez MG, 2004, CELL, V119, P753, DOI 10.1016/j.cell.2004.11.038

Heitmann L, 2013, IMMUNOBIOLOGY, V218, P506, DOI 10.1016/j.imbio.2012.06.005

Herb M, 2021, ANTIOXIDANTS-BASEL, V10, DOI 10.3390/antiox10020313

Herring SE, 2022, PLOS PATHOG, V18, DOI 10.1371/journal.ppat.1010700

Heyl KA, 2014, MBIO, V5, DOI 10.1128/mBio.01492-14

Hong J, 2022, FRONT ONCOL, V12, DOI 10.3389/fonc.2022.903874

Hoving JC, 2014, CELL MICROBIOL, V16, P185, DOI 10.1111/cmi.12249

Huang J, 2014, NAT REV MICROBIOL, V12, P101, DOI 10.1038/nrmicro3160

Huang XL, 2023, INT IMMUNOPHARMACOL, V114, DOI 10.1016/j.intimp.2022.109467

Indrigo J, 2003, MICROBIOL-SGM, V149, P2049, DOI 10.1099/mic.0.26226-0

Jiang JH, 2012, IUBMB LIFE, V64, P397, DOI 10.1002/iub.1021

Julián E, 2001, J IMMUNOL METHODS, V251, P21, DOI 10.1016/S0022-1759(01)00313-1

Kak Gunjan, 2020, BioMolecular Concepts, V11, P76, DOI [10.1515/bmc-2020-0006, 10.1515/bmc-2020-0006]

Kalia N, 2021, IMMUNOBIOLOGY, V226, DOI 10.1016/j.imbio.2021.152071

Kan-Sutton C, 2009, MICROBES INFECT, V11, P40, DOI 10.1016/j.micinf.2008.10.006

Katti MK, 2008, CELL MICROBIOL, V10, P1286, DOI 10.1111/j.1462-5822.2008.01126.x

Kaufmann SHE, 2002, ANN RHEUM DIS, V61, P54

Kawata K, 2012, J AUTOIMMUN, V39, P315, DOI 10.1016/j.jaut.2012.04.004

Khandia R, 2019, CELLS-BASEL, V8, DOI 10.3390/cells8070674

Kim JK, 2020, IMMUNE NETW, V20, DOI 10.4110/in.2020.20.e37

Kingeter LM, 2012, CELL MOL IMMUNOL, V9, P105, DOI 10.1038/cmi.2011.58

Klute S, 2024, VIRUSES-BASEL, V16, DOI 10.3390/v16040500

Kontchou CW, 2022, CELL DEATH DIFFER, V29, P2046, DOI 10.1038/s41418-022-00995-0

Krokowski S, 2016, FRONT CELL INFECT MI, V6, DOI 10.3389/fcimb.2016.00017

Kudchodkar SB, 2009, REV MED VIROL, V19, P359, DOI 10.1002/rmv.630

Kwon DH, 2017, BIOCHEM BIOPH RES CO, V490, P1093, DOI 10.1016/j.bbrc.2017.06.173

Lang R, 2013, FRONT IMMUNOL, V4, DOI 10.3389/fimmu.2013.00005

Lefèvre L, 2013, IMMUNITY, V38, P1038, DOI 10.1016/j.immuni.2013.04.010

Li DY, 2021, SIGNAL TRANSDUCT TAR, V6, DOI 10.1038/s41392-021-00687-0

Li H, 2021, FRONT MICROBIOL, V11, DOI 10.3389/fmicb.2020.622534

Liu CH, 2017, CELL MOL IMMUNOL, V14, P963, DOI 10.1038/cmi.2017.88

Marakalala MJ, 2017, PLOS PATHOG, V13, DOI 10.1371/journal.ppat.1006333

Matsunaga I, 2009, J EXP MED, V206, P2865, DOI 10.1084/jem.20092533

Maurice NM, 2023, PATHOGENS, V12, DOI 10.3390/pathogens12081005

Mehto S, 2015, PLOS ONE, V10, DOI 10.1371/journal.pone.0131767

Montecucco C, 2001, NAT REV MOL CELL BIO, V2, P457, DOI 10.1038/35073084

Naqvi KF, 2020, FRONT CELL INFECT MI, V10, DOI 10.3389/fcimb.2020.00263

Natarajan K, 2011, TUBERCULOSIS, V91, P427, DOI 10.1016/j.tube.2011.04.003

Neumann S, 2015, VIRUS RES, V209, P45, DOI 10.1016/j.virusres.2015.02.026

Newton HJ, 2010, CLIN MICROBIOL REV, V23, P274, DOI 10.1128/CMR.00052-09

Niu H, 2010, PLOS PATHOG, V6, DOI 10.1371/journal.ppat.1000774

Ochoa AE, 2023, INT J MOL SCI, V24, DOI 10.3390/ijms241311062

Ouyang YY, 2024, ELIFE, V13, DOI 10.7554/eLife.84282

Patin EC, 2017, FRONT IMMUNOL, V8, DOI 10.3389/fimmu.2017.00861

Rana AK, 2024, MICROBES INFECT, V26, DOI 10.1016/j.micinf.2023.105271

Ren XX, 2021, INFECT IMMUN, V89, DOI 10.1128/IAI.00315-21

Ruan HH, 2016, BIOCHEM BIOPH RES CO, V478, P618, DOI 10.1016/j.bbrc.2016.07.116

Rubio RM, 2019, P NATL ACAD SCI USA, V116, P26941, DOI 10.1073/pnas.1915139116

Ruggeri J, 2020, PLOS ONE, V15, DOI 10.1371/journal.pone.0229065

Sanin DE, 2018, IMMUNITY, V49, P1021, DOI 10.1016/j.immuni.2018.10.011

Saraswati SSK, 2023, MICROBES INFECT, V25, DOI 10.1016/j.micinf.2023.105126

Sato Y, 2020, INFECT IMMUN, V88, DOI 10.1128/IAI.00400-20

Selvakumar A, 2014, PLOS ONE, V9, DOI 10.1371/journal.pone.0096427

Sharma A, 2014, J INFECT DIS, V209, P1837, DOI 10.1093/infdis/jit820

Sharma D, 2016, PLOS ONE, V11, DOI 10.1371/journal.pone.0163845

Silwal P, 2020, FRONT IMMUNOL, V11, DOI 10.3389/fimmu.2020.01649

Singh Aayushi, 2021, BioMolecular Concepts, V12, P94, DOI [10.1515/bmc-2021-0010, 10.1515/bmc-2021-0010]

Singh A, 2020, CELL IMMUNOL, V357, DOI 10.1016/j.cellimm.2020.104198

Singhal J, 2012, J BIOL CHEM, V287, P11108, DOI 10.1074/jbc.M111.300319

Sinha V, 2024, J LEUKOCYTE BIOL, V116, P644, DOI 10.1093/jleuko/qiae068

Spooner R, 2011, INT J MOL SCI, V12, P334, DOI 10.3390/ijms12010334

Srivastava V, 2009, J BIOL CHEM, V284, P25532, DOI 10.1074/jbc.M109.006221

Sukumaran SK, 2010, MOL CELL, V37, P768, DOI 10.1016/j.molcel.2010.02.015

Thomson BJ, 2001, INT J EXP PATHOL, V82, P65, DOI 10.1111/j.1365-2613.2001.iep195.x

Tiku V, 2020, TRENDS CELL BIOL, V30, P263, DOI [10.1016/j.tcb.2020.01.006, 10.1016/j.tcb.2020.07.001]

Tirichen H, 2021, FRONT PHYSIOL, V12, DOI 10.3389/fphys.2021.627837

Vashishta M, 2015, PLOS ONE, V10, DOI 10.1371/journal.pone.0133601

Vatansever F, 2013, FEMS MICROBIOL REV, V37, P955, DOI 10.1111/1574-6976.12026

Venkatachalam G, 2020, ACS OMEGA, V5, P15973, DOI 10.1021/acsomega.0c01243

Vu A, 2024, INT J MOL SCI, V25, DOI 10.3390/ijms25116255

Wood SJ, 2015, J BIOL CHEM, V290, P29063, DOI 10.1074/jbc.M115.689950

World Health Organization, TB incidence

Yabaji SM, 2020, BBA-PROTEINS PROTEOM, V1868, DOI 10.1016/j.bbapap.2020.140470

Yuk JM, 2012, EXP MOL MED, V44, P99, DOI 10.3858/emm.2012.44.2.032

Zhai WJ, 2019, INT J MOL SCI, V20, DOI 10.3390/ijms20020340

Zhao QH, 2021, FRONT IMMUNOL, V12, DOI 10.3389/fimmu.2021.765615

NR 96

TC 0

Z9 0

U1 1

U2 1

PU OXFORD UNIV PRESS

PI OXFORD

PA GREAT CLARENDON ST, OXFORD OX2 6DP, ENGLAND

SN 0741-5400

EI 1938-3673

J9 J LEUKOCYTE BIOL

JI J. Leukoc. Biol.

PD NOV

PY 2025

VL 117

IS 11

AR qiaf146

DI 10.1093/jleuko/qiaf146

PG 16

WC Cell Biology; Hematology; Immunology

WE Science Citation Index Expanded (SCI-EXPANDED)

SC Cell Biology; Hematology; Immunology

GA 9YE2U

UT WOS:001617834900001

DA 2026-05-07

ER

PT J

AU Merget, B

Zilian, D

Müller, T

Sotriffer, CA

AF Merget, Benjamin

Zilian, David

Mueller, Tobias

Sotriffer, Christoph A.

TI MycPermCheck: the Mycobacterium tuberculosis permeability

prediction tool for small molecules

SO BIOINFORMATICS

LA English

DT Article

ID CARRIER PROTEIN REDUCTASE; CELL-WALL; DRUG DISCOVERY; INHIBITORS;

PACKAGE; INHA

AB Motivation: With >8 million new cases in 2010, particularly documented in developing countries, tuberculosis (TB) is still a highly present pandemic and often terminal. This is also due to the emergence of antibiotic-resistant strains (MDR-TB and XDR-TB) of the primary causative TB agent Mycobacterium tuberculosis (MTB). Efforts to develop new effective drugs against MTB are restrained by the unique and largely impermeable composition of the mycobacterial cell wall.

Results: Based on a database of antimycobacterial substances (CDD TB), 3815 compounds were classified as active and thus permeable. A data mining approach was conducted to gather the physico-chemical similarities of these substances and delimit them from a generic dataset of drug-like molecules. On the basis of the differences in these datasets, a regression model was generated and implemented into the online tool MycPermCheck to predict the permeability probability of small organic compounds.

Discussion: Given the current lack of precise molecular criteria determining mycobacterial permeability, MycPermCheck represents an unprecedented prediction tool intended to support antimycobacterial drug discovery. It follows a novel knowledge-driven approach to estimate the permeability probability of small organic compounds. As such, MycPermCheck can be used intuitively as an additional selection criterion for potential new inhibitors against MTB. Based on the validation results, its performance is expected to be of high practical value for virtual screening purposes.

C1 [Merget, Benjamin; Zilian, David; Sotriffer, Christoph A.] Univ Wurzburg, Inst Pharm & Food Chem, D-97074 Wurzburg, Germany.

[Mueller, Tobias] Univ Wurzburg, Dept Bioinformat, Bioctr, D-97074 Wurzburg, Germany.

C3 University of Wurzburg; University of Wurzburg

RP Sotriffer, CA (corresponding author), Univ Wurzburg, Inst Pharm & Food Chem, D-97074 Wurzburg, Germany.

EM sotriffer@uni-wuerzburg.de

FU German Research Foundation DFG [SFB 630]

FX This work was supported by the German Research Foundation DFG (SFB 630).

CR Ananthan S, 2009, TUBERCULOSIS, V89, P334, DOI 10.1016/j.tube.2009.05.008

[Anonymous], NUCL ACIDS RES

[Anonymous], 2013, COMMUNITY ECOLOGY PA

Beisser D, 2010, BIOINFORMATICS, V26, P1129, DOI 10.1093/bioinformatics/btq089

Bickerton GR, 2012, NAT CHEM, V4, P90, DOI [10.1038/NCHEM.1243, 10.1038/nchem.1243]

Cao Y, 2008, BIOINFORMATICS, V24, P1733, DOI 10.1093/bioinformatics/btn307

CARHART RE, 1985, J CHEM INF COMP SCI, V25, P64, DOI 10.1021/ci00046a002

Draper P, 2005, TUBERCULOSIS AND THE TUBERCLE BACILLUS, P261

Ekins S, 2010, MOL BIOSYST, V6, P840, DOI 10.1039/b917766c

Freundlich JS, 2009, CHEMMEDCHEM, V4, P241, DOI 10.1002/cmdc.200800261

He X, 2006, J MED CHEM, V49, P6308, DOI 10.1021/jm060715y

Hea X, 2007, BIOORGAN MED CHEM, V15, P6649, DOI 10.1016/j.bmc.2007.08.013

Hohman M, 2009, DRUG DISCOV TODAY, V14, P261, DOI 10.1016/j.drudis.2008.11.015

Hong X, 2004, BIOMACROMOLECULES, V5, P1066, DOI 10.1021/bm0345155

Irwin JJ, 2005, J CHEM INF MODEL, V45, P177, DOI 10.1021/ci049714+

JARLIER V, 1990, J BACTERIOL, V172, P1418, DOI 10.1128/jb.172.3.1418-1423.1990

Koul A, 2011, NATURE, V469, P483, DOI 10.1038/nature09657

Laneelle Marie-Antoinette, 2008, V465, P143, DOI 10.1007/978-1-59745-207-6_9

Lipinski CA, 1997, ADV DRUG DELIVER REV, V23, P3, DOI 10.1016/S0169-409X(96)00423-1

Liu J, 1996, J BIOL CHEM, V271, P29545, DOI 10.1074/jbc.271.47.29545

Luckner SR, 2010, J BIOL CHEM, V285, P14330, DOI 10.1074/jbc.M109.090373

Muddassar M, 2010, BIOORGAN MED CHEM, V18, P6914, DOI 10.1016/j.bmc.2010.07.010

Pounds S, 2003, BIOINFORMATICS, V19, P1236, DOI 10.1093/bioinformatics/btg148

QUEMARD A, 1995, BIOCHEMISTRY-US, V34, P8235, DOI 10.1021/bi00026a004

R Development Core Team, 2011, R: A Language and Environment for Statistical Computing

RASTOGI N, 1986, CURR MICROBIOL, V13, P237, DOI 10.1007/BF01568645

SADOWSKI J, 1994, J CHEM INF COMP SCI, V34, P1000, DOI 10.1021/ci00020a039

Sing T, 2005, BIOINFORMATICS, V21, P3940, DOI 10.1093/bioinformatics/bti623

Stubben C, 2007, J STAT SOFTW, V22, P1, DOI 10.18637/jss.v022.i11

Sullivan TJ, 2006, ACS CHEM BIOL, V1, P43, DOI 10.1021/cb0500042

TRIAS J, 1994, MOL MICROBIOL, V14, P283, DOI 10.1111/j.1365-2958.1994.tb01289.x

Yap CW, 2011, J COMPUT CHEM, V32, P1466, DOI 10.1002/jcc.21707

NR 32

TC 32

Z9 33

U1 0

U2 18

PU OXFORD UNIV PRESS

PI OXFORD

PA GREAT CLARENDON ST, OXFORD OX2 6DP, ENGLAND

SN 1367-4803

EI 1367-4811

J9 BIOINFORMATICS

JI Bioinformatics

PD JAN

PY 2013

VL 29

IS 1

BP 62

EP 68

DI 10.1093/bioinformatics/bts641

PG 7

WC Biochemical Research Methods; Biotechnology & Applied Microbiology;

Computer Science, Interdisciplinary Applications; Mathematical &

Computational Biology; Statistics & Probability

WE Science Citation Index Expanded (SCI-EXPANDED)

SC Biochemistry & Molecular Biology; Biotechnology & Applied Microbiology;

Computer Science; Mathematical & Computational Biology; Mathematics

GA 058SP

UT WOS:000312654600009

OA Green Published, Bronze

DA 2026-05-07

ER

PT J

AU Wu, X

Shang, YY

Ren, WC

Wang, W

Wang, YF

Xue, ZT

Li, SS

Pang, Y

AF Wu, Xiao

Shang, Yuanyuan

Ren, Weicong

Wang, Wei

Wang, Yufeng

Xue, Zhongtan

Li, Shanshan

Pang, Yu

TI Minimum inhibitory concentration of cycloserine against Mycobacterium

tuberculosis using the MGIT 960 system and a proposed critical

concentration

SO INTERNATIONAL JOURNAL OF INFECTIOUS DISEASES

LA English

DT Article

DE Cycloserine; Drug susceptibility testing; Tuberculosis

ID SUSCEPTIBILITY

AB Objectives: We aimed to determine the breakpoint of cycloserine (CS) susceptibility in MGIT and to describe the molecular characteristics of CS-resistant Mycobacterium tuberculosis (MTB) isolates.

Methods: A total of 124 MTB isolates were recruited in our analysis. Minimum inhibitory concentration (MIC) was determined using the MGIT system. The mutations of MTB isolates within alr, ddl, ald, and cycA, potentially conferring CS resistance were analyzed by the whole-genome sequencing.

Results: In vitro drug susceptibility testing of isolates with doubling concentrations of CS revealed that the modal MIC values was 4 mg/L for MGIT, accounting for 35.5% (44/124) of isolates tested. Seven isolates harbored mutations conferring CS resistance, consisting of five with alr mutations and two with ald mutations. On the basis of the MIC distributions of wild-type and resistotype populations, we proposed a tentative epidemiologic cut-off value of 16 mg/l. The proportion of CS resistance in extensively drug-resistant TB was significantly higher than that of multidrug-resistant TB.

Conclusion: In conclusion, we propose critical concentration for MGIT 960 to properly diagnose CS-resistant MTB and demonstrate that mutations in alr and ald genes are the major mechanism conferring CS resistance in clinical isolates. (C) 2022 The Authors. Published by Elsevier Ltd on behalf of International Society for Infectious Diseases.

C1 [Wu, Xiao; Shang, Yuanyuan; Ren, Weicong; Wang, Wei; Xue, Zhongtan; Li, Shanshan; Pang, Yu] Capital Med Univ, Beijing Key Lab Drug Resistant TB Res, Beijing TB & Thorac Tumor Res Inst, Dept Bacteriol & Immunol,Beijing Chest Hosp, Beijing, Peoples R China.

[Wang, Yufeng] Innovat Alliance TB Diag & Treatment, Beijing, Peoples R China.

C3 Capital Medical University

RP Li, SS; Pang, Y (corresponding author), 9 Beiguan St, Beijing 101149, Peoples R China.

EM lss9011@126.com; pangyupound@163.com

RI Wang, Li/HZM-0281-2023

FU Beijing Key Clinical Specialty Project [20201214]; Beijing Hospitals

Authority Ascent Plan [DFL20191601]; Beijing Hospitals Authority

Clinical Medicine Development of Special Funding [ZYLX202122]

FX This study was supported by the Beijing Key Clinical Specialty Project

(20201214), the Beijing Hospitals Authority Ascent Plan (DFL20191601),

and the Beijing Hospitals Authority Clinical Medicine Development of

Special Funding (ZYLX202122).

CR Alghamdi WA, 2019, ANTIMICROB AGENTS CH, V63

[Anonymous], 2019, WHO Consolidated Guidelines on Drug-Resistant Tuberculosis Treatment

[Anonymous], 2018, Technical report on critical concentrations for drug susceptibility testing of medicines used in the treatment of drug-resistant tuberculosis

Chen JZ, 2017, J ANTIMICROB CHEMOTH, V72, P3272, DOI 10.1093/jac/dkx316

de Chiara C, 2020, NAT CHEM BIOL, V16, P686, DOI 10.1038/s41589-020-0498-9

Deshpande D, 2018, CLIN INFECT DIS, V67, pS308, DOI 10.1093/cid/ciy624

Desjardins CA, 2016, NAT GENET, V48, P544, DOI 10.1038/ng.3548

Du YD, 2020, CLIN INFECT DIS, V71, P1047, DOI 10.1093/cid/ciz915

EPSTZIN ISRAEL G., 1955, ANTIBIOT MED, V1, P80

European Committee on Antimicrobial Susceptibility Testing, 2021, MIC DISTR SETT EP CU

Evangelopoulos D, 2019, NAT COMMUN, V10, DOI 10.1038/s41467-019-12074-z

Lee K, 2009, GLOB INST, P1

Nakatani Y, 2017, ANTIMICROB AGENTS CH, V61, DOI 10.1128/AAC.01575-17

Portelli S, 2018, SCI REP-UK, V8, DOI 10.1038/s41598-018-33370-6

Schena E, 2016, J ANTIMICROB CHEMOTH, V71, P1532, DOI 10.1093/jac/dkw044

Somerville W, 2005, J CLIN MICROBIOL, V43, P2996, DOI 10.1128/JCM.43.6.2996-2997.2005

The European Committee on Antimicrobial Susceptibility Testing, 2019, AR TECHN UNC ATU ANT

Zhu M, 2001, PHARMACOTHERAPY, V21, P891, DOI 10.1592/phco.21.11.891.34524

NR 18

TC 10

Z9 12

U1 0

U2 8

PU ELSEVIER SCI LTD

PI London

PA 125 London Wall, London, ENGLAND

SN 1201-9712

EI 1878-3511

J9 INT J INFECT DIS

JI Int. J. Infect. Dis.

PD AUG

PY 2022

VL 121

BP 148

EP 151

DI 10.1016/j.ijid.2022.05.030

EA MAY 2022

PG 4

WC Infectious Diseases

WE Science Citation Index Expanded (SCI-EXPANDED)

SC Infectious Diseases

GA 3Q2BQ

UT WOS:000838038500028

OA Green Submitted, gold

DA 2026-05-07

ER

PT J

AU Simonson, AW

Umstead, TM

Lawanprasert, A

Klein, B

Almarzooqi, S

Halstead, ES

Medina, SH

AF Simonson, Andrew W.

Umstead, Todd M.

Lawanprasert, Atip

Klein, Bailey

Almarzooqi, Sarah

Halstead, E. Scott

Medina, Scott H.

TI Extracellular matrix-inspired inhalable aerogels for rapid clearance of

pulmonary tuberculosis

SO BIOMATERIALS

LA English

DT Article

DE Peptide; Tuberculosis; Aerosol; Hyaluronic acid; Macrophage; Drug

delivery

ID RESPIRABLE PLGA MICROSPHERES; OCULAR SUSTAINED DELIVERY;

MYCOBACTERIUM-TUBERCULOSIS; MOXIFLOXACIN; HYALURONAN; RIFAMPICIN;

PROTEIN; SIZE

AB Tuberculosis (TB) remains a leading cause of death from a single infectious agent, and limiting the spread of multidrug-resistant TB (MDR-TB) is now an urgent global health priority. Essential to the persistence of this disease is the ability of Mycobacterium tuberculosis (Mtb) to circumvent host defenses by infecting lung macrophages to create a cellular niche for its survival and proliferation. This has urged the development of new therapeutic strategies that act through mechanisms distinct from conventional antibiotics, and thus are effective against MDR bacteria, while being able to efficiently kill persister Mtb cells in infected host macrophages. Here, we report a new class of gel-like microparticle aerosols, or 'aerogels', designed to exploit metabolic vulnerabilities of Mtb pathogens and TB-infected macrophages to enable preferential delivery of synergistic peptideantibiotic combinations for potent and rapid antitubercular therapy. This is achieved by formulating aerogels through the supramolecular assembly of a de novo designed anti-TB peptide and the extracellular matrix (ECM)derived polysaccharide, hyaluronic acid (HA). Importantly, HA serves as a nutrient source for Mtb cells during tissue invasion and proliferation, and is recognized by CD44 receptors highly expressed on lung macrophages during TB infection. By exploiting this metabolic substrate for pathogen targeting, HA aerogels are shown to avidly bind and kill both drug-sensitive and drug-resistant mycobacteria, while being efficiently internalized into macrophage host cells in vitro and in vivo to clear Mtb persisters. This multifaceted bioactivity suggests aerogels may serve as a versatile inhalable platform upon which novel biomaterials-enabled therapeutics can be developed to rapidly clear pulmonary MDR-TB.

C1 [Simonson, Andrew W.; Lawanprasert, Atip; Klein, Bailey; Almarzooqi, Sarah; Medina, Scott H.] Penn State Univ, Dept Biomed Engn, University Pk, PA 16802 USA.

[Umstead, Todd M.; Halstead, E. Scott] Penn State Coll Med, Dept Pediat, Hershey, PA 17033 USA.

[Umstead, Todd M.; Halstead, E. Scott] Penn State Coll Med, Pulm Immunol & Physiol Lab, Hershey, PA 17033 USA.

[Medina, Scott H.] Penn State Univ, Huck Inst Life Sci, University Pk, PA 16802 USA.

C3 Pennsylvania Commonwealth System of Higher Education (PCSHE);

Pennsylvania State University; Pennsylvania State University -

University Park; Pennsylvania Commonwealth System of Higher Education

(PCSHE); Pennsylvania State University; Penn State Health; Pennsylvania

Commonwealth System of Higher Education (PCSHE); Pennsylvania State

University; Penn State Health; Pennsylvania Commonwealth System of

Higher Education (PCSHE); Pennsylvania State University; Pennsylvania

State University - University Park

RP Medina, SH (corresponding author), Dept Biomed Engn, University Pk, PA 16802 USA.

EM shm126@psu.edu

OI Simonson, Anew/0000-0003-4789-3873; Klein, Bailey/0000-0002-7565-5277;

Halstead, E. Scott/0000-0002-1167-0363; Lawanprasert,

Atip/0009-0001-3163-8803

FU Penn State University - Huck Institutes of the Life Sciences Human

Health and the Environment seed program

FX We acknowledge John N. Alumasa for useful discussions and analytical

insight. Confocal and electron microscopy was performed at the Penn

State Microscopy and Cytometry Facility, University Park, PA. We also

acknowledge the Penn State Materials Characterization Laboratory,

University Park, PA for use of the dynamic light scattering

instrumentation. Funding for this work was provided by Penn State

University -Huck Institutes of the Life Sciences Human Health and the

Environment seed program.

CR Afacan NJ, 2012, CURR PHARM DESIGN, V18, P807, DOI 10.2174/138161212799277617

[Anonymous], 2000, Anti-tuberculosis drug resistance in the world report no 2: prevalence and trends publication

Aoki K, 2004, J BIOL CHEM, V279, P39798, DOI 10.1074/jbc.M402677200

Aronson MR, 2020, CELL MOL BIOENG, V13, P447, DOI 10.1007/s12195-020-00626-z

Arranz-Trullén J, 2017, FRONT IMMUNOL, V8, DOI 10.3389/fimmu.2017.01499

Bansal-Mutalik R, 2014, P NATL ACAD SCI USA, V111, P4958, DOI 10.1073/pnas.1403078111

Blank F, 2013, AM J RESP CELL MOL, V49, P67, DOI 10.1165/rcmb.2012-0387OC

Braakhuis HM, 2014, PART FIBRE TOXICOL, V11, DOI 10.1186/s12989-014-0049-1

Bussi C, 2019, FEMS MICROBIOL REV, V43, P341, DOI 10.1093/femsre/fuz006

Cryan SA, 2005, AAPS J, V7, pE20, DOI 10.1208/aapsj070104

Cyphert Jaime M, 2015, Int J Cell Biol, V2015, P563818, DOI [10.1155/2015/563818, 10.1155/2015/563818]

Danilchanka O, 2008, ANTIMICROB AGENTS CH, V52, P3127, DOI 10.1128/AAC.00239-08

Fjell CD, 2012, NAT REV DRUG DISCOV, V11, P37, DOI 10.1038/nrd3591

Gandhi NR, 2010, LANCET, V375, P1830, DOI 10.1016/S0140-6736(10)60410-2

Garantziotis S, 2016, AM J PHYSIOL-LUNG C, V310, pL785, DOI 10.1152/ajplung.00168.2015

GOGGINS JF, 1968, J HISTOCHEM CYTOCHEM, V16, P688, DOI 10.1177/16.11.688

Griffiths G, 2010, NAT REV MICROBIOL, V8, P827, DOI 10.1038/nrmicro2437

Guirado E, 2013, FRONT IMMUNOL, V4, DOI 10.3389/fimmu.2013.00098

Haney EF, 2019, FRONT CHEM, V7, DOI 10.3389/fchem.2019.00043

Hirayama Y, 2009, PLOS PATHOG, V5, DOI 10.1371/journal.ppat.1000643

Hirota K, 2010, J CONTROL RELEASE, V142, P339, DOI 10.1016/j.jconrel.2009.11.020

Hu B, 2018, ACS NANO, V12, P3385, DOI 10.1021/acsnano.7b08969

Jabbal S, 2017, J ALLERGY CLIN IMMUN, V139, P2013, DOI 10.1016/j.jaci.2016.11.036

Jo SH, 2017, ONCOTARGET, V8, P58686, DOI 10.18632/oncotarget.17419

Khusro A, 2016, ASIAN PAC J TROP MED, V9, P1001, DOI 10.1016/j.apjtm.2016.09.005

Kleinstreuer C, 2008, ANNU REV BIOMED ENG, V10, P195, DOI 10.1146/annurev.bioeng.10.061807.160544

Kotze A.F., BIODRUGS, V19

Kunisch R, 2012, BMC MICROBIOL, V12, DOI 10.1186/1471-2180-12-165

Lai SK, 2009, ADV DRUG DELIVER REV, V61, P158, DOI 10.1016/j.addr.2008.11.002

Lawlor C, 2011, MOL PHARMACEUT, V8, P1100, DOI 10.1021/mp1004178

Lee HK, 2014, INVEST OPHTH VIS SCI, V55

Lee HK, 2016, INVEST OPHTH VIS SCI, V57

Leemans JC, 2003, J CLIN INVEST, V111, P681, DOI 10.1172/JCI200316936

Lerner TR, 2017, J CELL BIOL, V216, P583, DOI 10.1083/jcb.201603040

Levitov A., 2005, J ANTIMICROB CHEMOTH, V57, P230

Liu D, 2018, INT J BIOL MACROMOL, V116, P1026, DOI 10.1016/j.ijbiomac.2018.05.113

Möller W, 2006, RESP RES, V7, DOI 10.1186/1465-9921-7-10

O'Hara P, 2000, PHARMACEUT RES, V17, P955, DOI 10.1023/A:1007527204887

Queval CJ, 2017, FRONT MICROBIOL, V8, DOI 10.3389/fmicb.2017.02284

Ramón-García S, 2013, ANTIMICROB AGENTS CH, V57, P2295, DOI 10.1128/AAC.00175-13

Rich M.L., COLD SPRING HARBOR P, V5

Silva JP, 2016, J CONTROL RELEASE, V235, P112, DOI 10.1016/j.jconrel.2016.05.064

Simonson AW, 2021, NAT BIOMED ENG, V5, P467, DOI 10.1038/s41551-020-00665-x

Simonson AW, 2019, NANOMED-NANOTECHNOL, V17, P391, DOI 10.1016/j.nano.2018.10.008

Singh B, 2017, PLOS ONE, V12, DOI 10.1371/journal.pone.0189606

Sosnik A, 2010, ADV DRUG DELIVER REV, V62, P547, DOI 10.1016/j.addr.2009.11.023

Suarez S, 2001, PHARMACEUT RES, V18, P1315, DOI 10.1023/A:1013094112861

To K., 2020, FRONT IMMUNOL, V11

Torrent M, 2012, PLOS PATHOG, V8, DOI 10.1371/journal.ppat.1003005

Weiss T, 2004, ANTIMICROB AGENTS CH, V48, P1974, DOI 10.1128/AAC.48.6.1974-1982.2004

WHO, 2010, WORLD MALARIA REPORT 2010, P1

World Health Organization, 2010, STOP TB TREATM TUB

Yathursan S, 2019, J PEPT SCI, V25, DOI 10.1002/psc.3213

Zimmermann GS, 2009, RESP RES, V10, DOI 10.1186/1465-9921-10-89

NR 54

TC 18

Z9 19

U1 1

U2 35

PU ELSEVIER SCI LTD

PI London

PA 125 London Wall, London, ENGLAND

SN 0142-9612

EI 1878-5905

J9 BIOMATERIALS

JI Biomaterials

PD JUN

PY 2021

VL 273

AR 120848

DI 10.1016/j.biomaterials.2021.120848

EA APR 2021

PG 11

WC Engineering, Biomedical; Materials Science, Biomaterials

WE Science Citation Index Expanded (SCI-EXPANDED)

SC Engineering; Materials Science

GA RZ6OO

UT WOS:000648720600003

DA 2026-05-07

ER

PT J

AU Tan, Q

Xie, WP

Min, R

Dai, GQ

Xu, CC

Pan, HQ

Miao, CD

Yang, Z

Xu, WG

Wang, H

AF Tan, Q.

Xie, W. P.

Min, R.

Dai, G. Q.

Xu, C-C.

Pan, H. Q.

Miao, C. D.

Yang, Z.

Xu, W. G.

Wang, H.

TI Characterization of Th1-and Th2-type immune response in human

multidrug-resistant tuberculosis

SO EUROPEAN JOURNAL OF CLINICAL MICROBIOLOGY & INFECTIOUS DISEASES

LA English

DT Article

ID GAMMA-INTERFERON PRODUCTION; T-CELL RESPONSES; IFN-GAMMA; MESSENGER-RNA;

TNF-ALPHA; CD4(+); INTERLEUKIN-4; MACROPHAGES; INHIBITION; IMMUNOLOGY

AB Multidrug-resistant tuberculosis (MDR-TB) has become a lethal global threat. Insights into the immune regulation of MDR-TB are urgently needed for the development of new treatments; however, the T cell response to an MDR-TB infection in human remains unclear. In the present study, the proportion of Th1 and Th2 cell subsets and the level of related T cell subset cytokines in peripheral blood were investigated. We detected that an MDR-TB infection resulted in suppressed Th1 and Th2 cell activation, which was more remarkable in patients with MDR-TB than that in drug-sensitive tuberculosis (DS-TB) sufferers when compared to healthy controls (HCs). In addition, MDR-TB infection down-regulated the expression of IFN-gamma, IL-2, and IL-10, and up-regulated IL-4, IL-6, and TNF-alpha expression. Our data suggest that the disturbance between protective and pathogenic effects induced by the immunosuppression of Th1- and Th2-type responses is a substantial characteristic of MDR-TB infections.

C1 [Tan, Q.; Xie, W. P.; Min, R.; Dai, G. Q.; Xu, C-C.; Wang, H.] Nanjing Med Univ, Dept Resp Med, Affiliated Hosp 1, Nanjing 210029, Jiangsu, Peoples R China.

[Pan, H. Q.] Third Hosp Zhenjiang City, Zhenjiang, Peoples R China.

[Miao, C. D.] Ctr Dis Control & Prevent CDC Taizhou City, Taizhou, Peoples R China.

[Yang, Z.] Jiangbei Hosp, Nanjing, Jiangsu, Peoples R China.

[Xu, W. G.] Ctr Dis Control & Prevent Jiangsu Prov, Nanjing, Jiangsu, Peoples R China.

C3 Nanjing Medical University; Jiangsu Provincial Center for Disease

Control & Prevention

RP Wang, H (corresponding author), Nanjing Med Univ, Dept Resp Med, Affiliated Hosp 1, Nanjing 210029, Jiangsu, Peoples R China.

EM hongwangnjmu@hotmail.com

RI tan, qi/KYR-4604-2024

FU National Department of Technology [2008ZX10003-014]

FX Funding for this study was provided by grants from one of the major

projects for science and technology development "11th Five-Year Plan" on

Key Infectious Diseases of the National Department of Technology (no.

2008ZX10003-014).

CR Annunziato F, 2007, J EXP MED, V204, P1849, DOI 10.1084/jem.20070663

Bai XY, 2004, TUBERCULOSIS, V84, P375, DOI 10.1016/j.tube.2004.05.001

Bergeron A, 1997, J IMMUNOL, V159, P3034

Biselli R, 2010, CLIN MICROBIOL INFEC, V16, P1282, DOI 10.1111/j.1469-0691.2009.03104.x

Boussiotis VA, 2000, J CLIN INVEST, V105, P1317, DOI 10.1172/JCI9918

COOPER AM, 1993, J EXP MED, V178, P2243, DOI 10.1084/jem.178.6.2243

Demangel C, 2000, IMMUNOL CELL BIOL, V78, P318, DOI 10.1046/j.1440-1711.2000.00935.x

Dominguez J, 2009, DIAGN MICR INFEC DIS, V63, P43, DOI 10.1016/j.diagmicrobio.2008.09.010

Fenhalls G, 2002, IMMUNOLOGY, V105, P325, DOI 10.1046/j.1365-2567.2002.01378.x

Fenton MJ, 1997, INFECT IMMUN, V65, P5149, DOI 10.1128/IAI.65.12.5149-5156.1997

FLESCH IEA, 1994, INT IMMUNOL, V6, P693, DOI 10.1093/intimm/6.5.693

FLYNN JL, 1993, J EXP MED, V178, P2249, DOI 10.1084/jem.178.6.2249

Flynn JL, 2004, TUBERCULOSIS, V84, P93, DOI 10.1016/j.tube.2003.08.010

Flynn JL, 2001, ANNU REV IMMUNOL, V19, P93, DOI 10.1146/annurev.immunol.19.1.93

Gor DO, 2003, NAT IMMUNOL, V4, P503, DOI 10.1038/ni0603-503

Handzel ZT, 2007, ISR MED ASSOC J, V9, P479

HernandezPando R, 1996, IMMUNOLOGY, V89, P26

Infante-Duarte C, 1999, SPRINGER SEMIN IMMUN, V21, P317, DOI 10.1007/BF00812260

Kant S, 2010, BIOSCI TRENDS, V4, P48

Khader SA, 2008, CYTOKINE, V41, P79, DOI 10.1016/j.cyto.2007.11.022

Khader SA, 2007, NAT IMMUNOL, V8, P369, DOI 10.1038/ni1449

Lucey DR, 1996, CLIN MICROBIOL REV, V9, P532, DOI 10.1128/CMR.9.4.532

Marino S, 2010, J THEOR BIOL, V265, P586, DOI 10.1016/j.jtbi.2010.05.012

Mattos AMM, 2010, INT IMMUNOL, V22, P775, DOI 10.1093/intimm/dxq429

MOSMANN TR, 1986, J IMMUNOL, V136, P2348

Nagabhushanam V, 2003, J IMMUNOL, V171, P4750, DOI 10.4049/jimmunol.171.9.4750

Nemeth J, 2011, CLIN IMMUNOL, V138, P50, DOI 10.1016/j.clim.2010.09.005

Ordway DJ, 2004, J INFECT DIS, V190, P756, DOI 10.1086/422532

Quesniaux VFJ, 2010, CURR DIR AUTOIMMUN, V11, P157, DOI 10.1159/000289204

Rengarajan J, 2000, IMMUNOL TODAY, V21, P479, DOI 10.1016/S0167-5699(00)01712-6

Rook G A, 2001, Curr Opin Pulm Med, V7, P116, DOI 10.1097/00063198-200105000-00002

Ruhwald M, 2008, PLOS ONE, V3, DOI 10.1371/journal.pone.0002858

Saunders BM, 2000, INFECT IMMUN, V68, P3322, DOI 10.1128/IAI.68.6.3322-3326.2000

Scriba TJ, 2008, J IMMUNOL, V180, P1962, DOI 10.4049/jimmunol.180.3.1962

Seah GT, 2000, J INFECT DIS, V181, P385, DOI 10.1086/315200

Seah GT, 2001, SCAND J INFECT DIS, V33, P106, DOI 10.1080/003655401750065472

Sharma SK, 2002, J CLIN IMMUNOL, V22, P345, DOI 10.1023/A:1020604331886

Sodhi A, 1997, CLIN INFECT DIS, V25, P617, DOI 10.1086/513769

van Crevel R, 2000, J INFECT DIS, V181, P1194, DOI 10.1086/315325

Verbon A, 1999, CLIN EXP IMMUNOL, V115, P110

Wang D, 2010, JPN J INFECT DIS, V63, P368

ZHANG M, 1995, INFECT IMMUN, V63, P3231, DOI 10.1128/IAI.63.8.3231-3234.1995

ZHANG YH, 1995, P NATL ACAD SCI USA, V92, P3632, DOI 10.1073/pnas.92.8.3632d

NR 43

TC 30

Z9 41

U1 0

U2 8

PU SPRINGER

PI NEW YORK

PA 233 SPRING ST, NEW YORK, NY 10013 USA

SN 0934-9723

EI 1435-4373

J9 EUR J CLIN MICROBIOL

JI Eur. J. Clin. Microbiol. Infect. Dis.

PD JUN

PY 2012

VL 31

IS 6

BP 1233

EP 1242

DI 10.1007/s10096-011-1434-4

PG 10

WC Infectious Diseases; Microbiology

WE Science Citation Index Expanded (SCI-EXPANDED)

SC Infectious Diseases; Microbiology

GA 940GQ

UT WOS:000303878400046

DA 2026-05-07

ER

PT J

AU Sheikh, BA

Bhat, BA

Ahmad, Z

Mir, MA

AF Sheikh, Bashir A.

Bhat, Basharat A.

Ahmad, Zahoor

Mir, Manzoor A.

TI Strategies Employed to Evade the Host Immune Response and the Mechanism

of Drug Resistance in Mycobacterium tuberculosis: In Search of

Finding New Targets

SO CURRENT PHARMACEUTICAL BIOTECHNOLOGY

LA English

DT Review

DE Tuberculosis; anti-tuberculosis drugs; drug delivery; drug resistance;

immunotherapy; cytokines; V5 immunitor

ID INNATE IMMUNITY; DENDRITIC CELLS; CD40 LIGAND; MULTIDRUG-RESISTANCE;

ANTIGEN PRESENTATION; INTERFERON-GAMMA; T-CELLS; BEDAQUILINE; APOPTOSIS;

COSTIMULATION

AB The partial effectiveness of the host immune response to M. tuberculosis drives bacteria into a latent state, but it is difficult to eliminate the bacteria completely. Usually, this latent condition of M. tuberculosis is reversible, and reactivation of tuberculosis is the leading cause of the majority of transmission. A number of studies performed on animal models and humans have not yet provided a detailed understanding of the mechanisms or correlates of immunity of M. tuberculosis infection or why there is a significant immunity failure to remove the pathogen. Moreover, the mechanism of resistance involved in drug-resistant M. tuberculosis leads to the emergence of strains of bacteria that show significant resistance to the majority of anti-tuberculosis drugs. We have also provided the recent findings and trends regarding the development of new drug molecules to treat drug and multidrug-resistant tuberculosis and the advancements in immunotherapy in the treatment of drug-resistant tuberculosis. This article provides an in-depth and critical analysis of various strategies employed by the drug-resistant M. tuberculosis to escape the host immune response. This bacterium persists in the host for a longer period of time and leads to the development of tuberculosis infection. Furthermore, we also discussed the new targets for the effective treatment of drug-resistant tuberculosis.

C1 [Sheikh, Bashir A.; Bhat, Basharat A.; Mir, Manzoor A.] Univ Kashmir, Sch Biol Sci, Dept Bioresources, Srinagar 190006, Jammu & Kashmir, India.

[Ahmad, Zahoor] IIIM, Clin Microbiol PK PD Lab, Srinagar 190005, India.

C3 University of Kashmir; Council of Scientific & Industrial Research

(CSIR) - India; CSIR - Indian Institute of Integrative Medicine (IIIM)

RP Mir, MA (corresponding author), Univ Kashmir, Sch Biol Sci, Dept Bioresources, Srinagar 190006, Jammu & Kashmir, India.

EM drmanzoor@kashmiruniversity.ac.in

RI Mir, Manzoor/W-8905-2018

OI Mir, Manzoor/0000-0003-3297-1402; sheikh, Bashir

ahmad/0000-0002-1087-564X; Ahmad, Zahoor/0000-0002-9663-2509

FU Science and Engineering Research Board, Department of Science and

Technology (SERB-DST) Govt. [TAR/001213/2018]; SERB-DST

FX The study was financially supported by Science and Engineering Research

Board, Department of Science and Technology (SERB-DST) Govt. of India

New Delhi; vide Project Grant No: TAR/001213/2018. We highly appreciate

SERB-DST for financial assistance.

CR Abate G, 2016, IMMUNOTARGETS THER, V5, P37, DOI 10.2147/ITT.S81892

Abebe M, 2011, CLIN DEV IMMUNOL, DOI 10.1155/2011/678570

Achkar JM, 2007, EXPERT REV ANTI-INFE, V5, P461, DOI 10.1586/14787210.5.3.461

Adrian J, 2019, CURR BIOL, V29, P616, DOI 10.1016/j.cub.2019.01.058

Agerberth B, 2006, CURR TOP MICROBIOL, V306, P67

Agrewala JN, 1998, CLIN EXP IMMUNOL, V111, P56

Alliance T.,, 2019, FDA APPR NEW TREATM

Alvarez IB, 2010, J INFECT DIS, V202, P524, DOI 10.1086/654932

Andries K, 2014, PLOS ONE, V9, DOI 10.1371/journal.pone.0102135

Ankley L, 2020, INFECT IMMUN, V88, DOI 10.1128/IAI.00916-19

[Anonymous], 2013, SEMIN IMMUNOPATHOL

Arias MA, 2007, J IMMUNOL, V179, P8381, DOI 10.4049/jimmunol.179.12.8381

Arrowsmith CH, 2012, NAT REV DRUG DISCOV, V11, P384, DOI 10.1038/nrd3674

Bahuguna A, 2020, MED RES REV, V40, P263, DOI 10.1002/med.21602

Balu S, 2011, J IMMUNOL, V186, P3113, DOI 10.4049/jimmunol.1003189

Barber DL, 2011, J IMMUNOL, V186, P1598, DOI 10.4049/jimmunol.1003304

Batdelger D, 2008, VACCINE, V26, P2733, DOI 10.1016/j.vaccine.2008.03.021

Behar SM, 2011, MUCOSAL IMMUNOL, V4, P279, DOI 10.1038/mi.2011.3

Behar SM, 2010, NAT REV MICROBIOL, V8, P668, DOI 10.1038/nrmicro2387

Bhatt K, 2009, J IMMUNOL, V182, P3793, DOI 10.4049/jimmunol.0802996

Blanc L, 2017, P NATL ACAD SCI USA, V114, P11205, DOI 10.1073/pnas.1707840114

Blomgran R, 2012, CELL HOST MICROBE, V11, P81, DOI 10.1016/j.chom.2011.11.012

Bojang A., 2020, IDENTIFYING INTRACEL

Bonato VLD, 2001, SCAND J IMMUNOL, V54, P564, DOI 10.1046/j.1365-3083.2001.00996.x

Braunstein M, 2019, PHARM RES-DORDR, V36, DOI 10.1007/s11095-019-2704-6

Briken V, 2008, FUTURE MICROBIOL, V3, P415, DOI 10.2217/17460913.3.4.415

Bruns H, 2014, FUTURE MICROBIOL, V9, P327, DOI 10.2217/fmb.13.164

Butov Dmitry A, 2011, J Immune Based Ther Vaccines, V9, P3, DOI 10.1186/1476-8518-9-3

Chai QY, 2020, CELL MOL IMMUNOL, V17, P901, DOI 10.1038/s41423-020-0502-z

Chandran A, 2015, FRONT CELL INFECT MI, V5, DOI 10.3389/fcimb.2015.00090

Chen CY, 2013, PLOS PATHOG, V9, DOI 10.1371/journal.ppat.1003501

Chen ST, 2020, BMC MICROBIOL, V20, DOI 10.1186/s12866-020-01940-2

Cicchese JM, 2020, FRONT PHARMACOL, V11, DOI 10.3389/fphar.2020.00333

Cohen K, 2019, EXPERT OPIN DRUG SAF, V18, P875, DOI 10.1080/14740338.2019.1648429

Compos-Neto A, 1998, J IMMUNOL, V160, P2037

Cooper AM, 2009, ANNU REV IMMUNOL, V27, P393, DOI 10.1146/annurev.immunol.021908.132703

Corleis B, 2012, CELL MICROBIOL, V14, P1109, DOI 10.1111/j.1462-5822.2012.01783.x

de Knegt GJ, 2017, INT J ANTIMICROB AG, V49, P302, DOI 10.1016/j.ijantimicag.2016.11.027

Dey B, 2015, NAT MED, V21, P401, DOI 10.1038/nm.3813

Diacon AH, 2012, ANTIMICROB AGENTS CH, V56, P3271, DOI 10.1128/AAC.06126-11

Diacon AH, 2012, LANCET, V380, P986, DOI 10.1016/S0140-6736(12)61080-0

Dookie N, 2018, J ANTIMICROB CHEMOTH, V73, P1138, DOI 10.1093/jac/dkx506

Dooley S.W., 1992, MULTIDRUG RESISTANT

Dwivedi M, 2020, Current Chemical Biology, V14, P14, DOI [10.2174/2212796814666200310100746, 10.2174/2212796814666200310100746, DOI 10.2174/2212796814666200310100746]

Elayati K.M., 2020, Inflammatory dendritic cells in the host immune response to mycobacteria: the role of cellular metabolism

Ernst JD, 2018, CELL HOST MICROBE, V24, P34, DOI 10.1016/j.chom.2018.06.004

Esposito S, 2015, EXPERT OPIN PHARMACO, V16, P2319, DOI 10.1517/14656566.2015.1080240

Fallahi-Sichani M, 2012, J IMMUNOL, V188, P3169, DOI 10.4049/jimmunol.1103298

Falodun O.I., 2020, MICROMEDICINE, V9, P8

FLESCH I, 1987, J IMMUNOL, V138, P4408

Foo CSY, 2016, ANTIMICROB AGENTS CH, V60, P6451, DOI 10.1128/AAC.01523-16

Fujiwara M, 2018, TUBERCULOSIS, V108, P186, DOI 10.1016/j.tube.2017.12.006

Fuse S, 2008, J IMMUNOL, V180, P1148, DOI 10.4049/jimmunol.180.2.1148

Ginsberg AM, 2009, ANTIMICROB AGENTS CH, V53, P3726, DOI 10.1128/AAC.00112-09

Godebo A, 2015, INT J PHARM BIOL SCI, V2, P1

Goldberg MF, 2014, MICROBIOL SPECTR, V2, DOI 10.1128/microbiolspec.MGM2-0005-2013

Grace PS, 2016, J IMMUNOL, V196, P357, DOI 10.4049/jimmunol.1501494

Grewal IS, 1997, CURR OPIN IMMUNOL, V9, P491, DOI 10.1016/S0952-7915(97)80100-8

Grewal IS, 1996, IMMUNOL REV, V153, P85, DOI 10.1111/j.1600-065X.1996.tb00921.x

Haberland M, 2009, NAT REV GENET, V10, P32, DOI 10.1038/nrg2485

Hanekom WA, 2003, J INFECT DIS, V188, P257, DOI 10.1086/376451

Hartkoorn RC, 2014, ANTIMICROB AGENTS CH, V58, P2979, DOI 10.1128/AAC.00037-14

Herold KC, 1997, J IMMUNOL, V159, P4150

Herzog EL, 2003, BLOOD, V102, P3483, DOI 10.1182/blood-2003-05-1664

Hmama Z, 2004, J CELL SCI, V117, P2131, DOI 10.1242/jcs.01072

Hu Y, 2008, INT J TUBERC LUNG D, V12, P69

Huff WX, 2019, INT J MOL SCI, V20, DOI 10.3390/ijms20112810

Hussain A, 2019, CURR DRUG DELIV, V16, P400, DOI 10.2174/1567201816666190201144815

JACOBS WR, 1993, SCIENCE, V260, P819, DOI 10.1126/science.8484123

Jia J, 2005, BRIT J PHARMACOL, V144, P80, DOI 10.1038/sj.bjp.0705984

Jiao XN, 2002, J IMMUNOL, V168, P1294, DOI 10.4049/jimmunol.168.3.1294

Jordao Luisa, 2011, Int J Cell Biol, V2011, P403623, DOI 10.1155/2011/403623

Joshi L, 2015, ARCH IMMUNOL THER EX, V63, P427, DOI 10.1007/s00005-015-0347-9

Joyce A.M, 2020, KNOWLEDGE ATTITUDE P

Jurado JO, 2008, J IMMUNOL, V181, P116, DOI 10.4049/jimmunol.181.1.116

Kan-Sutton C, 2009, MICROBES INFECT, V11, P40, DOI 10.1016/j.micinf.2008.10.006

Khan A, 2019, TUBERCULOSIS HOST-PATHOGEN INTERACTIONS, P95, DOI 10.1007/978-3-030-25381-3_5

Khan N, 2012, PLOS PATHOG, V8, DOI 10.1371/journal.ppat.1002676

Koh WJ, 2004, J KOREAN MED SCI, V19, P167, DOI 10.3346/jkms.2004.19.2.167

Korb VC, 2016, INT J MOL SCI, V17, DOI 10.3390/ijms17030131

Koul A, 2008, J BIOL CHEM, V283, P25273, DOI 10.1074/jbc.M803899200

Kruh-Garcia NA, 2015, TUBERCULOSIS, V95, P26, DOI 10.1016/j.tube.2014.10.010

Krutzik SR, 2001, CURR OPIN IMMUNOL, V13, P104, DOI 10.1016/S0952-7915(00)00189-8

Lam A, 2017, AM J PHYSIOL-LUNG C, V313, pL218, DOI 10.1152/ajplung.00162.2017

Lata M, 2015, J PROTEOMICS, V127, P114, DOI 10.1016/j.jprot.2015.07.031

Lázár-Molnár E, 2010, P NATL ACAD SCI USA, V107, P13402, DOI 10.1073/pnas.1007394107

Lazarevic V, 2003, IMMUNITY, V19, P823, DOI 10.1016/S1074-7613(03)00324-8

Levings MK, 2001, J EXP MED, V193, P1295, DOI 10.1084/jem.193.11.1295

Lewies A, 2019, PROBIOTICS ANTIMICRO, V11, P370, DOI 10.1007/s12602-018-9465-0

Liu CH, 2017, CELL MOL IMMUNOL, V14, P963, DOI 10.1038/cmi.2017.88

Longmore M., 2014, Oxford handbook of clinical medicine, V9th

Lugo-Villarino G, 2014, CSH PERSPECT MED, V4, DOI 10.1101/cshperspect.a018549

Lupien A, 2018, ANTIMICROB AGENTS CH, V62, DOI [10.1128/AAC.00840-18, 10.1128/aac.00840-18]

Machado D, 2018, FRONT MICROBIOL, V9, DOI 10.3389/fmicb.2018.01367

Makarov V, 2014, EMBO MOL MED, V6, P372, DOI 10.1002/emmm.201303575

Makarov V, 2009, SCIENCE, V324, P801, DOI 10.1126/science.1171583

Mallikaarjun S, 2021, ANTIMICROB AGENTS CH, V65, DOI 10.1128/AAC.01207-20

Manson AL, 2017, NAT GENET, V49, P395, DOI 10.1038/ng.3767

Mantegazza AR, 2013, TRAFFIC, V14, P135, DOI 10.1111/tra.12026

Marais BJ, 2010, LANCET, V375, P2179, DOI 10.1016/S0140-6736(10)60554-5

Mase S, 2013, MMWR RECOMM REP, V62, P1

Matsumoto M, 2006, PLOS MED, V3, P2131, DOI 10.1371/journal.pmed.0030466

Mir M., 2013, INT J BIOL PHARM ALL, V2, P1024

Mir M.A., 2013, J HEALTH SCI, V1, P77, DOI DOI 10.12816/0004774

Mir Manzoor A., 2007, Current Immunology Reviews, V3, P160

Mir MA, 2008, EXPERT OPIN THER TAR, V12, P969, DOI [10.1517/14728222.12.8.969, 10.1517/14728222.12.8.969 ]

Mir: Manzoor Ahmad., 2015, Developing Costimulatory Molecules for Immunotherapy of Diseases, P1

Mishra A, 2017, MOL IMMUNOL, V87, P240, DOI 10.1016/j.molimm.2017.05.001

Mohareer K, 2018, TUBERCULOSIS, V113, P99, DOI 10.1016/j.tube.2018.09.007

Murray RA, 2007, J IMMUNOL, V178, P338, DOI 10.4049/jimmunol.178.1.338

Nasiri MJ, 2017, FRONT MICROBIOL, V8, DOI 10.3389/fmicb.2017.00681

Nguyen L, 2009, ANNU REV PHARMACOL, V49, P427, DOI 10.1146/annurev-pharmtox-061008-103123

Olaru ID, 2015, EUR RESPIR J, V45, P1119, DOI 10.1183/09031936.00162314

Organization W.H, 2017, WHO BEST PRACT STAT

Organization WH, 2016, US DEL TREATM MULT R

Organization WH, 2020, WHO consolidated guidelines on tuberculosis: tuberculosis preventive treatment: World Health Organization

Pahari S, 2018, FRONT IMMUNOL, V9, DOI 10.3389/fimmu.2018.00193

Portela A, 2010, NAT BIOTECHNOL, V28, P1057, DOI 10.1038/nbt.1685

Qadri H., 2020, CURR DRUG TARGETS

Queval CJ, 2017, FRONT MICROBIOL, V8, DOI 10.3389/fmicb.2017.02284

Reddy VM, 2012, J ANTIMICROB CHEMOTH, V67, P1163, DOI 10.1093/jac/dkr589

Ribeiro ALDL, 2011, PLOS ONE, V6, DOI 10.1371/journal.pone.0026675

Romagnoli A, 2018, CELL DEATH DIS, V9, DOI 10.1038/s41419-018-0640-8

Roos D, 2003, MICROBES INFECT, V5, P1307, DOI 10.1016/j.micinf.2003.09.009

Sá-Nunes A, 2009, J IMMUNOL, V182, P7422, DOI 10.4049/jimmunol.0900075

SAHA B, 1994, EUR J IMMUNOL, V24, P2618, DOI 10.1002/eji.1830241108

Sakai S, 2010, INT IMMUNOL, V22, P915, DOI 10.1093/intimm/dxq446

Samten B, 2000, INFECT IMMUN, V68, P3002, DOI 10.1128/IAI.68.5.3002-3006.2000

Sandhaus S, 2018, SCI REP-UK, V8, DOI 10.1038/s41598-018-19944-4

Saravanan M, 2018, MICROB PATHOGENESIS, V117, P237, DOI 10.1016/j.micpath.2018.02.047

Schrager Lewis K, 2018, F1000Res, V7, P1732, DOI 10.12688/f1000research.16521.2

Seaworth B.J., 2017, TUBERCULOSIS NONTUBE, V5, DOI 10.1128/microbiolspec.TNMI7-0042-2017

SELWYN PA, 1989, AM J PUBLIC HEALTH, V79, P1358, DOI 10.2105/AJPH.79.10.1358

Sharma D, 2018, PROTEOMES, V6, DOI 10.3390/proteomes6020026

Sharma D, 2017, FRONT CELL INFECT MI, V7, DOI 10.3389/fcimb.2017.00240

Sharma D, 2017, FRONT MICROBIOL, V8, DOI 10.3389/fmicb.2017.00465

Sharma D, 2015, PLOS ONE, V10, DOI 10.1371/journal.pone.0139414

Sharma D, 2015, CURR PROTEOMICS, V12, P96, DOI 10.2174/157016461202150903113053

Shaw KJ, 2011, ANN NY ACAD SCI, V1241, P48, DOI 10.1111/j.1749-6632.2011.06330.x

Sheikh BA, 2021, CURR PHARM BIOTECHNO, V22, P480, DOI [10.2174/15734129mta3snzetw, 10.2174/1389201021666200628021702]

Shen H, 2015, ARCH MED SCI, V11, P584, DOI 10.5114/aoms.2015.52362

Shortman K, 2001, NAT IMMUNOL, V2, P988, DOI 10.1038/ni1101-988

Sia JK, 2015, J IMMUNOL RES, V2015, DOI 10.1155/2015/747543

Singh B, 2003, CLIN EXP IMMUNOL, V134, P70, DOI 10.1046/j.1365-2249.2003.02258.x

Singh R, 2008, SCIENCE, V322, P1392, DOI 10.1126/science.1164571

Singh V, 2021, ACCOUNTS CHEM RES, V54, P2361, DOI 10.1021/acs.accounts.0c00878

Somasundaram S, 2013, BMC MICROBIOL, V13, DOI 10.1186/1471-2180-13-218

Sommer R.C, 2019, INNOVATIVE TOOLS IN

Srivastava S, 2016, CELL HOST MICROBE, V19, P44, DOI 10.1016/j.chom.2015.12.003

Stewart GR, 2001, NAT MED, V7, P732, DOI 10.1038/89113

Stover CK, 2000, NATURE, V405, P962, DOI 10.1038/35016103

Swain SS, 2020, EMERG MICROBES INFEC, V9, P1651, DOI 10.1080/22221751.2020.1785334

Swanson B.I., 2017, GOOGLE PATENTS

Tasneen R, 2011, ANTIMICROB AGENTS CH, V55, P5485, DOI 10.1128/AAC.05293-11

TELENTI A, 1993, LANCET, V341, P647, DOI 10.1016/0140-6736(93)90417-F

Nguyen TVA, 2020, CLIN INFECT DIS, V71, P3252, DOI 10.1093/cid/ciaa755

Thind M, 2020, J CLIN PHARMACOL, V60, P284, DOI 10.1002/jcph.1551

Tiberi S, 2017, PRESSE MED, V46, pE41, DOI 10.1016/j.lpm.2017.01.016

Udwadia Z, 2013, J EPIDEMIOL COMMUN H, V67, P471, DOI 10.1136/jech-2012-201640

Unissa AN, 2017, TUBERCULOSIS, V105, P96, DOI 10.1016/j.tube.2017.04.008

Upadhyay S, 2018, PATHOG DIS, V76, DOI 10.1093/femspd/fty037

Urban CF, 2006, CELL MICROBIOL, V8, P1687, DOI 10.1111/j.1462-5822.2006.00792.x

Urdahl KB, 2011, MUCOSAL IMMUNOL, V4, P288, DOI 10.1038/mi.2011.10

Van Crevel R, 2003, ADV EXP MED BIOL, V531, P241

van der Werf MJ, 2012, EUR RESPIR J, V39, P1511, DOI 10.1183/09031936.00125711

Vasava Mahesh S, 2017, Indian J Tuberc, V64, P252, DOI [10.1016/j.ijtb.2017.03.002, 10.1016/j.ijtb.2017.03.002]

Velayati AA, 2009, CHEST, V136, P420, DOI 10.1378/chest.08-2427

Velmurugan K, 2007, PLOS PATHOG, V3, P972, DOI 10.1371/journal.ppat.0030110

Vergne I, 2015, FRONT CELL INFECT MI, V4, DOI 10.3389/fcimb.2014.00187

Wallis RS, 2011, ANTIMICROB AGENTS CH, V55, P567, DOI 10.1128/AAC.01179-10

Wallis RS, 2004, AIDS, V18, P257, DOI 10.1097/00002030-200401230-00015

Wang HM, 2019, SCI IMMUNOL, V4, DOI 10.1126/sciimmunol.aaw0402

Wang XL, 2018, PATHOG DIS, V76, DOI 10.1093/femspd/fty064

Witzky A, 2019, OPEN BIOL, V9, DOI 10.1098/rsob.190051

Xiao X, 2011, PLOS ONE, V6, DOI 10.1371/journal.pone.0020592

Yamauchi PS, 2000, J IMMUNOL, V165, P1506, DOI 10.4049/jimmunol.165.3.1506

Yang SJ, 2015, CELL PHYSIOL BIOCHEM, V35, P1276, DOI 10.1159/000373950

Young C, 2020, MUCOSAL IMMUNOL, V13, P190, DOI 10.1038/s41385-019-0226-5

Yu XW, 2019, DNA CELL BIOL, V38, P1178, DOI 10.1089/dna.2019.4852

Zhu T, 2014, ANTIMICROB AGENTS CH, V58, P3306, DOI 10.1128/AAC.01920-13

Zwane H, 2012, INVESTIGATION ROLE T

NR 181

TC 11

Z9 13

U1 0

U2 16

PU BENTHAM SCIENCE PUBL LTD

PI SHARJAH

PA EXECUTIVE STE Y-2, PO BOX 7917, SAIF ZONE, 1200 BR SHARJAH, U ARAB

EMIRATES

SN 1389-2010

EI 1873-4316

J9 CURR PHARM BIOTECHNO

JI Curr. Pharm. Biotechnol.

PY 2022

VL 23

IS 14

BP 1704

EP 1720

DI 10.2174/1389201023666211222164938

PG 17

WC Biochemistry & Molecular Biology; Pharmacology & Pharmacy

WE Science Citation Index Expanded (SCI-EXPANDED)

SC Biochemistry & Molecular Biology; Pharmacology & Pharmacy

GA 6C4TB

UT WOS:000882007700009

DA 2026-05-07

ER

PT J

AU Reeves, AZ

Campbell, PJ

Sultana, R

Malik, S

Murray, M

Plikaytis, BB

Shinnick, TM

Posey, JE

AF Reeves, Analise Z.

Campbell, Patricia J.

Sultana, Razvan

Malik, Seidu

Murray, Megan

Plikaytis, Bonnie B.

Shinnick, Thomas M.

Posey, James E.

TI Aminoglycoside Cross-Resistance in Mycobacterium tuberculosis Due

to Mutations in the 5′ Untranslated Region of whiB7

SO ANTIMICROBIAL AGENTS AND CHEMOTHERAPY

LA English

DT Article

ID LEVEL STREPTOMYCIN RESISTANCE; GENE-EXPRESSION; MOLECULAR ANALYSIS;

KANAMYCIN; TRANSCRIPTION; CAPREOMYCIN; MACROPHAGES; REGULATOR; VIOMYCIN;

SURVIVAL

AB Since the discovery of streptomycin's bactericidal activity against Mycobacterium tuberculosis, aminoglycosides have been utilized to treat tuberculosis (TB). Today, the aminoglycosides kanamycin and amikacin are used to treat multidrug-resistant (MDR) TB, and resistance to any of the second-line injectable antibiotics, including kanamycin, amikacin, or capreomycin, is a defining characteristic of extensively drug-resistant (XDR) TB. Resistance to kanamycin and streptomycin is thought to be due to the acquisition of unlinked chromosomal mutations. However, we identified eight independent mutations in the 5' untranslated region of the transcriptional activator whiB7 that confer low-level resistance to both aminoglycosides. The mutations lead to 23- to 145-fold increases in whiB7 transcripts and subsequent increased expression of both eis (Rv2416c) and tap (Rv1258c). Increased expression of eis confers kanamycin resistance in these mutants, while increased expression of tap, which encodes an efflux pump, is a previously uncharacterized mechanism of low-level streptomycin resistance. Additionally, high-level resistance to streptomycin arose at a much higher frequency in whiB7 mutants than in a wild-type (WT) strain. Although whiB7 is typically associated with intrinsic antibiotic resistance in M. tuberculosis, these data suggest that mutations in an uncharacterized regulatory region of whiB7 contribute to cross-resistance against clinically used second-line antibiotics. As drug resistance continues to develop and spread, understanding the mechanisms and molecular basis of antibiotic resistance is critical for the development of rapid molecular tests to diagnose drug-resistant TB strains and ultimately for designing regimens to treat drug-resistant cases of TB.

C1 [Reeves, Analise Z.; Campbell, Patricia J.] Emory Univ, Dept Microbiol & Immunol, Microbiol & Mol Genet Grad Program, Atlanta, GA 30322 USA.

[Reeves, Analise Z.; Campbell, Patricia J.; Malik, Seidu; Plikaytis, Bonnie B.; Shinnick, Thomas M.; Posey, James E.] Ctr Dis Control & Prevent, Div TB Eliminat, Natl Ctr HIV AIDS Viral Hepatitis STD & TB Preven, Atlanta, GA 30333 USA.

[Sultana, Razvan] Boston Univ, Dept Bioinformat, Boston, MA 02215 USA.

[Murray, Megan] Harvard Univ, Sch Publ Hlth, Dept Epidemiol, Boston, MA 02115 USA.

C3 Emory University; Centers for Disease Control & Prevention - USA; Boston

University; Harvard University; Harvard T.H. Chan School of Public

Health

RP Posey, JE (corresponding author), Ctr Dis Control & Prevent, Div TB Eliminat, Natl Ctr HIV AIDS Viral Hepatitis STD & TB Preven, Atlanta, GA 30333 USA.

EM jposey@cdc.gov

OI Murray, Megan/0000-0003-0443-1986

CR Adams KN, 2011, CELL, V145, P39, DOI 10.1016/j.cell.2011.02.022

Aínsa JA, 1998, J BACTERIOL, V180, P5836

Alangaden GJ, 1998, ANTIMICROB AGENTS CH, V42, P1295, DOI 10.1128/AAC.42.5.1295

[Anonymous], 1948, Br Med J, V2, P769

[Anonymous], 2006, Morbidity and Mortality Weekly Report, V55, P1176

[Anonymous], 2003, Susceptibility Testing of Mycobacteria, Nocardiae, and Other Aerobic Actinomycetes; Approved Standard. NCCLS document M24-A. NCCLS, 940 West Valley Road

[Anonymous], 2009, GLOBAL TUBERCULOSIS

Bardarov S, 2002, MICROBIOL-SGM, V148, P3007, DOI 10.1099/00221287-148-10-3007

Burian J, 2012, J BIOL CHEM, V287, P299, DOI 10.1074/jbc.M111.302588

Campbell PJ, 2011, ANTIMICROB AGENTS CH, V55, P2032, DOI 10.1128/AAC.01550-10

Geiman DE, 2006, ANTIMICROB AGENTS CH, V50, P2836, DOI 10.1128/AAC.00295-06

Gold B, 2001, MOL MICROBIOL, V42, P851, DOI 10.1046/j.1365-2958.2001.02684.x

Guo MM, 2009, GENOME RES, V19, P1301, DOI 10.1101/gr.086595.108

Jiang X, 2008, MICROB DRUG RESIST, V14, P7, DOI 10.1089/mdr.2008.0772

Larsson C, 2012, PLOS ONE, V7, DOI 10.1371/journal.pone.0037516

Lella RK, 2007, J BIOL CHEM, V282, P18671, DOI 10.1074/jbc.C600280200

Li H, 2008, GENOME RES, V18, P1851, DOI 10.1101/gr.078212.108

Louw GE, 2009, ANTIMICROB AGENTS CH, V53, P3181, DOI 10.1128/AAC.01577-08

Magnet S, 2005, CHEM REV, V105, P477, DOI 10.1021/cr0301088

Maus CE, 2005, ANTIMICROB AGENTS CH, V49, P3192, DOI 10.1128/AAC.49.8.3192-3197.2005

Meier A, 1996, ANTIMICROB AGENTS CH, V40, P2452, DOI 10.1128/AAC.40.11.2452

Morris RP, 2005, P NATL ACAD SCI USA, V102, P12200, DOI 10.1073/pnas.0505446102

Nusbaum C, 2009, NAT METHODS, V6, P67, DOI [10.1038/NMETH.1286, 10.1038/nmeth.1286]

Okamoto S, 2007, MOL MICROBIOL, V63, P1096, DOI 10.1111/j.1365-2958.2006.05585.x

Rohde KH, 2007, CELL HOST MICROBE, V2, P352, DOI 10.1016/j.chom.2007.09.006

Samuel LP, 2007, MICROBIOL-SGM, V153, P529, DOI 10.1099/mic.0.2006/002642-0

Schwab U, 2009, MICROB PATHOGENESIS, V46, P185, DOI 10.1016/j.micpath.2008.12.006

Shin DM, 2010, PLOS PATHOG, V6, DOI 10.1371/journal.ppat.1001230

Siddiqi N, 2004, INFECTION, V32, P109, DOI 10.1007/s15010-004-3097-x

Singh A, 2009, PLOS PATHOG, V5, DOI 10.1371/journal.ppat.1000545

Spies FS, 2008, ANTIMICROB AGENTS CH, V52, P2947, DOI 10.1128/AAC.01570-07

Suzuki Y, 1998, J CLIN MICROBIOL, V36, P1220, DOI 10.1128/JCM.36.5.1220-1225.1998

Taniguchi H, 1997, J BACTERIOL, V179, P4795, DOI 10.1128/jb.179.15.4795-4801.1997

Via LE, 2010, J CLIN MICROBIOL, V48, P402, DOI 10.1128/JCM.01476-09

Wong SY, 2011, ANTIMICROB AGENTS CH, V55, P2515, DOI 10.1128/AAC.01814-10

Wu SP, 2009, MICROBIOL-SGM, V155, P1272, DOI 10.1099/mic.0.024638-0

Zaunbrecher MA, 2009, P NATL ACAD SCI USA, V106, P20004, DOI 10.1073/pnas.0907925106

NR 37

TC 113

Z9 135

U1 0

U2 26

PU AMER SOC MICROBIOLOGY

PI WASHINGTON

PA 1752 N ST NW, WASHINGTON, DC 20036-2904 USA

SN 0066-4804

EI 1098-6596

J9 ANTIMICROB AGENTS CH

JI Antimicrob. Agents Chemother.

PD APR

PY 2013

VL 57

IS 4

BP 1857

EP 1865

DI 10.1128/AAC.02191-12

PG 9

WC Microbiology; Pharmacology & Pharmacy

WE Science Citation Index Expanded (SCI-EXPANDED)

SC Microbiology; Pharmacology & Pharmacy

GA 106CN

UT WOS:000316119100041

OA Green Submitted, Bronze

DA 2026-05-07

ER

PT J

AU Bai, XY

Oberley-Deegan, RE

Bai, A

Ovrutsky, AR

Kinney, WH

Weaver, M

Zhang, G

Honda, JR

Chan, ED

AF Bai, Xiyuan

Oberley-Deegan, Rebecca E.

Bai, An

Ovrutsky, Alida R.

Kinney, William H.

Weaver, Michael

Zhang, Gong

Honda, Jennifer R.

Chan, Edward D.

TI Curcumin enhances human macrophage control of Mycobacterium

tuberculosis infection

SO RESPIROLOGY

LA English

DT Article

DE apoptosis; autophagy; curcumin; nuclear-factor kappa B; tuberculosis

ID NF-KAPPA-B; MULTIDRUG-RESISTANT TUBERCULOSIS; NITRIC-OXIDE; IN-VIVO;

APOPTOSIS; CANCER; ACTIVATION; PATHWAY; LIPOARABINOMANNAN; AUTOPHAGOSOME

AB Background and objectiveWith the worldwide emergence of highly drug-resistant tuberculosis (TB), novel agents that have direct antimycobacterial effects or that enhance host immunity are urgently needed. Curcumin is a polyphenol responsible for the bright yellow-orange colour of turmeric, a spice derived from the root of the perennial herb Curcuma longa. Curcumin is a potent inducer of apoptosisan effector mechanism used by macrophages to kill intracellular Mycobacterium tuberculosis (MTB).

MethodsAn in vitro human macrophage infection model was used to determine the effects of curcumin on MTB survival.

ResultsWe found that curcumin enhanced the clearance of MTB in differentiated THP-1 human monocytes and in primary human alveolar macrophages. We also found that curcumin was an inducer of caspase-3-dependent apoptosis and autophagy. Curcumin mediated these anti-MTB cellular functions, in part, via inhibition of nuclear factor-kappa B (NFB) activation.

ConclusionCurcumin protects against MTB infection in human macrophages. The host-protective role of curcumin against MTB in macrophages needs confirmation in an animal model; if validated, the immunomodulatory anti-TB effects of curcumin would be less prone to drug resistance development.

The effects of curcumin (a polyphenol of tumeric) on Mycobacterium tuberculosis (MTB) infection using human macrophages were investigated in this study. Curcumin reduced the intracellular burden of MTB in infected macrophages through inhibition of NFB activation resulting in the induction of caspase-3-dependent apoptosis and autophagy.

C1 [Bai, Xiyuan; Bai, An; Chan, Edward D.] Denver Vet Affairs Med Ctr, Dept Med, Denver, CO USA.

[Bai, Xiyuan; Oberley-Deegan, Rebecca E.; Bai, An; Ovrutsky, Alida R.; Kinney, William H.; Weaver, Michael; Honda, Jennifer R.; Chan, Edward D.] Natl Jewish Hlth, Dept Med & Acad Affairs, Denver, CO USA.

[Bai, Xiyuan; Ovrutsky, Alida R.; Kinney, William H.; Honda, Jennifer R.; Chan, Edward D.] Univ Colorado, Sch Med, Dept Med, Div Pulm Sci & Crit Care Med, Aurora, CO USA.

[Oberley-Deegan, Rebecca E.] Univ Nebraska Med Ctr, Dept Biochem & Mol Biol, Omaha, NE USA.

[Zhang, Gong] Shaanxi Univ Chinese Med, Coll Pharm, Xianyang, Shaanxi, Peoples R China.

C3 US Department of Veterans Affairs; Veterans Health Administration (VHA);

Veterans Affairs Medical Center - Denver; National Jewish Health;

University of Colorado System; University of Colorado Anschutz;

University of Nebraska System; University of Nebraska Medical Center;

Shaanxi University of Chinese Medicine

RP Bai, XY (corresponding author), Natl Jewish Hlth, D509 Neustadt Bldg,1400 Jackson St, Denver, CO 80206 USA.

EM baix@njhealth.org

RI Weaver, Michael/MTB-3433-2025; Oberley-Deegan, Rebecca/E-4176-2013

CR Aggarwal BB, 2003, ANTICANCER RES, V23, P363

Avorn J, 2013, JAMA-J AM MED ASSOC, V309, P1349, DOI 10.1001/jama.2013.623

Bai XY, 2013, PLOS ONE, V8, DOI 10.1371/journal.pone.0061925

Bai XY, 2010, J IMMUNOL, V184, P3830, DOI 10.4049/jimmunol.0901913

Baldwin PR, 2015, EUR J MED CHEM, V92, P693, DOI 10.1016/j.ejmech.2015.01.020

Briken V, 2008, FUTURE MICROBIOL, V3, P415, DOI 10.2217/17460913.3.4.415

Chan ED, 2004, AM J RESP CRIT CARE, V169, P1103, DOI 10.1164/rccm.200308-1159OC

Chan ED, 2001, INFECT IMMUN, V69, P2001, DOI 10.1128/IAI.69.4.2001-2010.2001

Chan ED, 2008, NEW ENGL J MED, V359, P657, DOI 10.1056/NEJMc0706556

Changtam C, 2010, EUR J MED CHEM, V45, P4446, DOI 10.1016/j.ejmech.2010.07.003

Cox E, 2014, NEW ENGL J MED, V371, P689, DOI 10.1056/NEJMp1314385

Deretic Vojo, 2008, V445, P1, DOI 10.1007/978-1-59745-157-4_1

Diacon AH, 2014, NEW ENGL J MED, V371, P723, DOI 10.1056/NEJMoa1313865

Diacon AH, 2009, NEW ENGL J MED, V360, P2397, DOI 10.1056/NEJMoa0808427

Fenton M J, 1998, Curr Opin Hematol, V5, P72, DOI 10.1097/00062752-199801000-00012

Gler MT, 2012, NEW ENGL J MED, V366, P2151, DOI 10.1056/NEJMoa1112433

Goel A, 2008, BIOCHEM PHARMACOL, V75, P787, DOI 10.1016/j.bcp.2007.08.016

Gray JM, 2013, SEMIN RESP CRIT CARE, V34, P32, DOI 10.1055/s-0032-1333469

Guo CX, 2013, J NUTR BIOCHEM, V24, P754, DOI 10.1016/j.jnutbio.2012.04.002

Gupta PK, 2013, OPEN ANTIMICROB AGEN, V4, P1, DOI DOI 10.2174/1876518101304010001

Gupta SC, 2013, AAPS J, V15, P195, DOI 10.1208/s12248-012-9432-8

Gutierrez MG, 2004, CELL, V119, P753, DOI 10.1016/j.cell.2004.11.038

Hotchkiss RS, 2014, NEW ENGL J MED, V371, P380, DOI 10.1056/NEJMcibr1404664

Jäger R, 2014, NUTR J, V13, DOI 10.1186/1475-2891-13-11

Jagetia GC, 2007, J CLIN IMMUNOL, V27, P19, DOI 10.1007/s10875-006-9066-7

Kabeya Y, 2000, EMBO J, V19, P5720, DOI 10.1093/emboj/19.21.5720

Killian PH, 2012, CARCINOGENESIS, V33, P2507, DOI 10.1093/carcin/bgs312

Li MY, 2014, BIOCHEM BIOPH RES CO, V446, P626, DOI 10.1016/j.bbrc.2014.03.023

Liu PT, 2006, SCIENCE, V311, P1770, DOI 10.1126/science.1123933

Migliori GB, 2013, EUR RESPIR J, V42, P169, DOI 10.1183/09031936.00136312

Mizushima N, 2010, CELL, V140, P313, DOI 10.1016/j.cell.2010.01.028

Morris KR, 2003, INFECT IMMUN, V71, P1442, DOI 10.1128/IAI.71.3.1442-1452.2003

Morris KR, 2009, TUBERCULOSIS, V89, P294, DOI 10.1016/j.tube.2009.03.004

Placido R, 1997, J PATHOL, V181, P31, DOI 10.1002/(SICI)1096-9896(199701)181:1<31::AID-PATH722>3.0.CO;2-G

Prasad S, 2014, CANCER RES TREAT, V46, P2, DOI 10.4143/crt.2014.46.1.2

Sa G, 2008, CELL DIV, V3, DOI 10.1186/1747-1028-3-14

Schaible UE, 2003, NAT MED, V9, P1039, DOI 10.1038/nm906

Shehzad A, 2013, CHEM-BIOL INTERACT, V206, P394, DOI 10.1016/j.cbi.2013.10.007

Shishodia S, 2005, BIOCHEM PHARMACOL, V70, P700, DOI 10.1016/j.bcp.2005.04.043

SINGH S, 1995, J BIOL CHEM, V270, P24995, DOI 10.1074/jbc.270.42.24995

Srinivasan L, 2014, CSH PERSPECT MED, V4, DOI 10.1101/cshperspect.a022459

Wilken R, 2011, MOL CANCER, V10, DOI 10.1186/1476-4598-10-12

Yadav VR, 2010, BIOCHEM PHARMACOL, V80, P1021, DOI 10.1016/j.bcp.2010.06.022

Yamada H, 2001, INFECT IMMUN, V69, P7100, DOI 10.1128/IAI.69.11.7100-7105.2001

Yang CW, 2012, BMC COMPLEM ALTERN M, V12, DOI 10.1186/1472-6882-12-22

NR 45

TC 74

Z9 86

U1 0

U2 35

PU WILEY-BLACKWELL

PI HOBOKEN

PA 111 RIVER ST, HOBOKEN 07030-5774, NJ USA

SN 1323-7799

EI 1440-1843

J9 RESPIROLOGY

JI Respirology

PD JUL

PY 2016

VL 21

IS 5

BP 951

EP 957

DI 10.1111/resp.12762

PG 7

WC Respiratory System

WE Science Citation Index Expanded (SCI-EXPANDED)

SC Respiratory System

GA DQ2VQ

UT WOS:000379061900024

DA 2026-05-07

ER

PT J

AU Bahlool, AZ

Fattah, S

O'Sullivan, A

Cavanagh, B

MacLoughlin, R

Keane, J

O'Sullivan, MP

Cryan, SA

AF Bahlool, Ahmad Z.

Fattah, Sarinj

O'Sullivan, Andrew

Cavanagh, Brenton

MacLoughlin, Ronan

Keane, Joseph

O'Sullivan, Mary P.

Cryan, Sally-Ann

TI Development of Inhalable ATRA-Loaded PLGA Nanoparticles as Host-Directed

Immunotherapy against Tuberculosis

SO PHARMACEUTICS

LA English

DT Article

DE all trans retinoic acid; ATRA; tuberculosis; host-directed therapy;

inhalation; nebulization; pulmonary drug delivery; nanoparticles; PLGA;

immunotherapy

ID TRANS-RETINOIC ACID; VITAMIN-A; MYCOBACTERIUM-TUBERCULOSIS; PULMONARY

TUBERCULOSIS; ALVEOLAR MACROPHAGES; DENDRITIC CELLS; LUNG DEPOSITION;

MESH NEBULIZERS; DRUG-DELIVERY; EXPRESSION

AB Developing new effective treatment strategies to overcome the rise in multi-drug resistant tuberculosis cases (MDR-TB) represents a global challenge. A host-directed therapy (HDT), acting on the host immune response rather than Mtb directly, could address these resistance issues. We developed an HDT for targeted TB treatment, using All Trans Retinoic Acid (ATRA)-loaded nanoparticles (NPs) that are suitable for nebulization. Efficacy studies conducted on THP-1 differentiated cells infected with the H37Ra avirulent Mycobacterium tuberculosis (Mtb) strain, have shown a dose-dependent reduction in H37Ra growth as determined by the BACT/ALERT((R)) system. Confocal microscopy images showed efficient and extensive cellular delivery of ATRA-PLGA NPs into THP-1-derived macrophages. A commercially available vibrating mesh nebulizer was used to generate nanoparticle-loaded droplets with a mass median aerodynamic diameter of 2.13 mu m as measured by cascade impaction, and a volumetric median diameter of 4.09 mu m as measured by laser diffraction. In an adult breathing simulation experiment, 65.1% of the ATRA PLGA-NP dose was inhaled. This targeted inhaled HDT could offer a new adjunctive TB treatment option that could enhance current dosage regimens leading to better patient prognosis and a decreasing incidence of MDR-TB.

C1 [Bahlool, Ahmad Z.; Fattah, Sarinj; O'Sullivan, Andrew; MacLoughlin, Ronan; Cryan, Sally-Ann] Royal Coll Surg Ireland RCSI, Sch Pharm & Biomol Sci, 123 St Stephens Green, Dublin D02 YN77, Ireland.

[Bahlool, Ahmad Z.; Fattah, Sarinj; Cryan, Sally-Ann] Royal Coll Surg Ireland RCSI, Tissue Engn Res Grp, 123 St Stephens Green, Dublin D02 YN77, Ireland.

[Bahlool, Ahmad Z.; Keane, Joseph; O'Sullivan, Mary P.] Univ Dublin, Trinity Coll Dublin, St Jamess Hosp, Dept Clin Med,Trinity Translat Med Inst, Dublin D08 9WRT, Ireland.

[O'Sullivan, Andrew; MacLoughlin, Ronan] Aerogen Ltd, Res & Dev Sci & Emerging Technol, Galway Business Pk, Galway H91 HE94, Ireland.

[Cavanagh, Brenton] Royal Coll Surg Ireland RCSI, Cellular & Mol Imaging Core, Dublin D02 YN77, Ireland.

[MacLoughlin, Ronan] Trinity Coll Dublin, Sch Pharm & Pharmaceut Sci, Dublin D02 PN40, Ireland.

[Cryan, Sally-Ann] RCSI, SFI Adv Mat & Bioengn Res AMBER Ctr, Dublin D02 PN40, Ireland.

[Cryan, Sally-Ann] Trinity Coll Dublin, Dublin D02 PN40, Ireland.

[Cryan, Sally-Ann] NUIG, SFI Ctr Res Med Devices CURAM, Galway H91 W2TY, Ireland.

[Cryan, Sally-Ann] RCSI, Galway H91 W2TY, Ireland.

C3 Royal College of Surgeons in Ireland - RCSI; Royal College of Surgeons

in Ireland - RCSI; Trinity College Dublin; Royal College of Surgeons in

Ireland - RCSI; Trinity College Dublin; Royal College of Surgeons in

Ireland - RCSI; Trinity College Dublin; Royal College of Surgeons in

Ireland - RCSI

RP Cryan, SA (corresponding author), Royal Coll Surg Ireland RCSI, Sch Pharm & Biomol Sci, 123 St Stephens Green, Dublin D02 YN77, Ireland.; Cryan, SA (corresponding author), Royal Coll Surg Ireland RCSI, Tissue Engn Res Grp, 123 St Stephens Green, Dublin D02 YN77, Ireland.; Cryan, SA (corresponding author), RCSI, SFI Adv Mat & Bioengn Res AMBER Ctr, Dublin D02 PN40, Ireland.; Cryan, SA (corresponding author), Trinity Coll Dublin, Dublin D02 PN40, Ireland.; Cryan, SA (corresponding author), NUIG, SFI Ctr Res Med Devices CURAM, Galway H91 W2TY, Ireland.; Cryan, SA (corresponding author), RCSI, Galway H91 W2TY, Ireland.

EM scryan@rcsi.com

RI Keane, Joseph/AAX-6658-2021; Cavanagh, Brenton/D-2382-2009; Fattah,

Sarinj/AAP-5796-2021; Cryan, Sally-Ann/D-3510-2012; MacLoughlin,

Ronan/G-3530-2013; O'Sullivan, Mary/C-9413-2012; Bahlool,

Ahmad/LZF-5448-2025

OI Keane, Joseph/0000-0001-5313-385X; Cavanagh,

Brenton/0000-0002-1582-5756; Cryan, Sally-Ann/0000-0002-3941-496X;

MacLoughlin, Ronan/0000-0002-3164-1607; O'Sullivan,

Mary/0000-0002-6104-8943; O'Sullivan, Anew/0000-0002-5434-1260; Bahlool,

Ahmad/0000-0002-9992-7476

FU Royal College of Surgeons in Ireland; Science Foundation Ireland (SFI)

Center for Research in Medical Devices (CURAM); European Regional

Development Fund [13/RC/2073_P2]; Wellcome Trust; Health Research Board;

Royal City of Dublin Hospital Trust

FX The authors would like to acknowledge the Royal College of Surgeons in

Ireland for funding this project and the Servier medical art images that

were used to create the graphical abstract. S.A.C. would like to

acknowledge research funding from Science Foundation Ireland (SFI)

Center for Research in Medical Devices (CURAM) and the European Regional

Development Fund, grant number 13/RC/2073_P2. MOS and JK acknowledge

support from The Wellcome Trust, The Health Research Board and The Royal

City of Dublin Hospital Trust.

CR Agranoff D, 2006, LANCET, V368, P1012, DOI 10.1016/S0140-6736(06)69342-2

Aibana O, 2017, CLIN INFECT DIS, V65, P900, DOI 10.1093/cid/cix476

Alizadeh F, 2014, INT IMMUNOPHARMACOL, V18, P43, DOI 10.1016/j.intimp.2013.10.027

ALVAREZ JO, 1995, AM J CLIN NUTR, V61, P1273, DOI 10.1093/ajcn/61.6.1273

[Anonymous], LANGBRAC1601 PRODUCT

[Anonymous], European Pharmacopoeia, 6th Edition (6.5)

[Anonymous], STEP COMMITTEE MED P

[Anonymous], HLTH UN SUSTAINABLE

[Anonymous], GLOBAL TUBERCULOSIS

Ari A, 2015, J AEROSOL MED PULM D, V28, P281, DOI 10.1089/jamp.2014.1149

Babunovic GH, 2022, MBIO, V13, DOI 10.1128/mbio.03683-21

Bahlool Ahmad Z, 2022, Curr Res Immunol, V3, P54, DOI [10.1016/j.crimmu.2022.03.003, 10.1016/j.crimmu.2022.03.003]

Batalha IL, 2019, J CONTROL RELEASE, V314, P116, DOI 10.1016/j.jconrel.2019.10.009

Chikaura H, 2016, BIOSURF BIOTRIBOL, V2, P18, DOI [10.1016/j.bsbt.2016.02.003, 10.1016/j.bsbt.2016.02.003, DOI 10.1016/J.BSBT.2016.02.003]

Cohen SB, 2018, CELL HOST MICROBE, V24, P439, DOI 10.1016/j.chom.2018.08.001

Cohn DL, 2000, CLIN INFECT DIS, V31, P120, DOI 10.1086/313891

Coleman MM, 2018, AM J RESP CELL MOL, V59, P548, DOI 10.1165/rcmb.2017-0382OC

Coleman MM, 2013, AM J RESP CELL MOL, V48, P773, DOI 10.1165/rcmb.2012-0263OC

Contreras LG, 2015, MOL PHARMACEUT, V12, P2642, DOI 10.1021/acs.molpharmaceut.5b00046

Costet P, 2003, MOL CELL BIOL, V23, P7756, DOI 10.1128/MCB.23.21.7756-7766.2003

CROWLE AJ, 1989, INFECT IMMUN, V57, P840, DOI 10.1128/IAI.57.3.840-844.1989

De Santi C, 2020, MOL THER, V28, P1190, DOI 10.1016/j.ymthe.2020.02.001

Demoly P, 2014, RESP MED, V108, P1195, DOI 10.1016/j.rmed.2014.05.009

Desai TR, 2002, INT J PHARM, V241, P311, DOI 10.1016/S0378-5173(02)00245-4

DiNardo AR, 2020, medRxiv, DOI [10.1101/2020.05.13.20100776, 10.1101/2020.05.13.20100776, DOI 10.1101/2020.05.13.20100776]

DiNardo AR, 2021, MED-CAMBRIDGE, V2, P217, DOI 10.1016/j.medj.2020.11.003

Ding DW, 2018, MAT SCI ENG C-MATER, V92, P1041, DOI 10.1016/j.msec.2017.12.036

Dua K, 2018, BIOMED PHARMACOTHER, V107, P1218, DOI 10.1016/j.biopha.2018.08.101

Dugernier J, 2017, PHARM RES-DORDR, V34, P290, DOI 10.1007/s11095-016-2061-7

Duskey JT, 2020, POLYMERS-BASEL, V12, DOI 10.3390/polym12040823

Escher G, 2003, J BIOL CHEM, V278, P11015, DOI 10.1074/jbc.M212780200

Esmail H, 2014, PHILOS T R SOC B, V369, DOI 10.1098/rstb.2013.0437

Fatima S, 2021, FRONT IMMUNOL, V12, DOI 10.3389/fimmu.2021.645485

Feng Q, 2017, J THROMB HAEMOST, V15, P1845, DOI 10.1111/jth.13767

Fernández EF, 2018, MATERIALS, V11, DOI 10.3390/ma11010122

Fernández EF, 2021, ANTIBIOTICS-BASEL, V10, DOI 10.3390/antibiotics10050472

Gonçalves A, 2019, EUR J PHARM BIOPHARM, V143, P80, DOI 10.1016/j.ejpb.2019.08.014

Guerra JM, 2014, J FELINE MED SURG, V16, P243, DOI 10.1177/1098612X13516121

Hibbitts A, 2014, J AEROSOL MED PULM D, V27, P466, DOI 10.1089/jamp.2013.1045

Hickey AJ, 2013, J PHARM SCI-US, V102, P3900, DOI 10.1002/jps.23705

Hurst RJM, 2013, PARASITE IMMUNOL, V35, P229, DOI 10.1111/pim.12032

Ipek Y, 2012, CASE REP MED, V2012, DOI 10.1155/2012/236174

ITURRALDE M, 1992, BIOCHIM BIOPHYS ACTA, V1133, P241, DOI 10.1016/0167-4889(92)90043-B

Jabbal S, 2017, J ALLERGY CLIN IMMUN, V139, P2013, DOI 10.1016/j.jaci.2016.11.036

Kalombo L, 2019, NANOMATERIALS-BASEL, V9, DOI 10.3390/nano9081167

Keflie Tibebeselassie Seyoum, 2018, J Clin Tuberc Other Mycobact Dis, V12, P27, DOI [10.1016/j.jctube.2018.05.002, 10.1016/j.jctube.2018.05.002]

Keicho N, 2012, PLOS ONE, V7, DOI 10.1371/journal.pone.0038703

Kiran D, 2016, SEMIN IMMUNOPATHOL, V38, P167, DOI 10.1007/s00281-015-0537-x

Labiris NR, 2003, BRIT J CLIN PHARMACO, V56, P588, DOI 10.1046/j.1365-2125.2003.01892.x

Lawlor C, 2016, PLOS ONE, V11, DOI 10.1371/journal.pone.0149167

Lawson L, 2010, TROP MED INT HEALTH, V15, P1481, DOI 10.1111/j.1365-3156.2010.02638.x

Liang QZ, 2020, INT J NANOMED, V15, P7491, DOI 10.2147/IJN.S257758

Luo XM, 2005, J BIOL CHEM, V280, P36228, DOI 10.1074/jbc.M505749200

McWhorter FY, 2013, P NATL ACAD SCI USA, V110, P17253, DOI 10.1073/pnas.1308887110

Mora JR, 2008, NAT REV IMMUNOL, V8, P685, DOI 10.1038/nri2378

Mugusi FM, 2003, INT J TUBERC LUNG D, V7, P804

MUINDI J, 1992, BLOOD, V79, P299

Nahid P, 2019, AM J RESP CRIT CARE, V200, pE93, DOI 10.1164/rccm.201909-1874ST

Nahid P, 2016, CLIN INFECT DIS, V63, P853, DOI [10.1093/cid/ciw376, 10.1093/cid/ciw566]

O'Connor G, 2019, EUR J PHARM BIOPHARM, V134, P153, DOI 10.1016/j.ejpb.2018.10.020

O'Connor G, 2016, ADV DRUG DELIVER REV, V102, P33, DOI 10.1016/j.addr.2016.04.024

O'Leary S, 2021, JOVE-J VIS EXP, DOI 10.3791/62838

O'Leary S, 2011, AM J RESP CELL MOL, V45, P172, DOI 10.1165/rcmb.2010-0319OC

Oh J, 2017, J INFECTION, V74, P272, DOI 10.1016/j.jinf.2016.10.009

Olaru ID, 2016, J ANTIMICROB CHEMOTH, V71, P852, DOI 10.1093/jac/dkv354

Oprea Tudor I, 2011, Drug Discov Today Ther Strateg, V8, P61

Pai M, 2016, NAT REV DIS PRIMERS, V2, DOI 10.1038/nrdp.2016.76

Parumasivam T, 2016, ADV DRUG DELIVER REV, V102, P83, DOI 10.1016/j.addr.2016.05.011

Plit ML, 1998, INT J TUBERC LUNG D, V2, P590

Pritchard JN, 2018, THER DELIV, V9, P121, DOI 10.4155/tde-2017-0102

Ramachandran G, 2004, INT J TUBERC LUNG D, V8, P1130

Ramakrishnan L, 2012, NAT REV IMMUNOL, V12, P352, DOI 10.1038/nri3211

Roca FJ, 2013, CELL, V153, P521, DOI 10.1016/j.cell.2013.03.022

Ruge CA, 2011, NANOMED-NANOTECHNOL, V7, P690, DOI 10.1016/j.nano.2011.07.009

Saunders BM, 1999, IMMUNOLOGY, V98, P324

Schindelin J, 2012, NAT METHODS, V9, P676, DOI [10.1038/NMETH.2019, 10.1038/nmeth.2019]

Shariati A, 2022, FRONT CELL INFECT MI, V12, DOI 10.3389/fcimb.2022.926363

Sharp FA, 2009, P NATL ACAD SCI USA, V106, P870, DOI 10.1073/pnas.0804897106

STEPHENSEN CB, 1994, AM J CLIN NUTR, V60, P388, DOI 10.1093/ajcn/60.3.388

SZUTS EZ, 1991, ARCH BIOCHEM BIOPHYS, V287, P297, DOI 10.1016/0003-9861(91)90482-X

Tobin DM, 2012, CELL, V148, P434, DOI 10.1016/j.cell.2011.12.023

Vellozo NS, 2017, FRONT IMMUNOL, V8, DOI 10.3389/fimmu.2017.01560

Waldrep J. C., 2008, Current Drug Delivery, V5, P114, DOI 10.2174/156720108783954815

Wauthoz N, 2011, PHARM RES-DORDR, V28, P762, DOI 10.1007/s11095-010-0329-x

Wheelwright M, 2014, J IMMUNOL, V192, P2280, DOI 10.4049/jimmunol.1301686

Yadav KS, 2010, AAPS PHARMSCITECH, V11, P1456, DOI 10.1208/s12249-010-9519-4

Yamada H, 2007, J NUTR, V137, P2696, DOI 10.1093/jn/137.12.2696

Zhang HX, 2016, CELL PHYSIOL BIOCHEM, V40, P1603, DOI 10.1159/000453210

Zhou Q, 2015, ADV DRUG DELIVER REV, V85, P83, DOI 10.1016/j.addr.2014.10.022

NR 89

TC 24

Z9 25

U1 2

U2 18

PU MDPI

PI BASEL

PA ST ALBAN-ANLAGE 66, CH-4052 BASEL, SWITZERLAND

EI 1999-4923

J9 PHARMACEUTICS

JI Pharmaceutics

PD AUG

PY 2022

VL 14

IS 8

AR 1745

DI 10.3390/pharmaceutics14081745

PG 19

WC Pharmacology & Pharmacy

WE Science Citation Index Expanded (SCI-EXPANDED)

SC Pharmacology & Pharmacy

GA 4B4YT

UT WOS:000845785600001

OA Green Submitted, gold

DA 2026-05-07

ER

PT J

AU Fernandes, GFS

Campos, DL

Da Silva, IC

Prates, JLB

Pavan, AR

Pavan, FR

Dos Santos, JL

AF Fernandes, Guilherme F. S.

Campos, Debora L.

Da Silva, Isabel C.

Prates, Joao L. B.

Pavan, Aline R.

Pavan, Fernando R.

Dos Santos, Jean L.

TI Benzofuroxan Derivatives as Potent Agents against Multidrug-Resistant

Mycobacterium tuberculosis

SO CHEMMEDCHEM

LA English

DT Article

DE antitubercular agents; benzofuroxan; multidrug-resistant TB; N-oxide;

tuberculosis

ID DRUG-RESISTANCE; REACTIVE OXYGEN; EMERGENCE; PYRAZINAMIDE; RIFAMPICIN;

DISCOVERY; TOXICITY; DESIGN; PLATE; OXIDE

AB Tuberculosis (TB) is currently the leading cause of death related to infectious diseases worldwide, as reported by the World Health Organization. Moreover, the increasing number of multidrug-resistant tuberculosis (MDR-TB) cases has alarmed health agencies, warranting extensive efforts to discover novel drugs that are effective and also safe. In this study, 23 new compounds were synthesized and evaluated in vitro against the drug-resistant strains of M. tuberculosis. The compound 6-((3-fluoro-4-thiomorpholinophenyl)carbamoyl)benzo[c][1,2,5]oxadiazole 1-N-oxide (5 b) was particularly remarkable in this regard as it demonstrated MIC90 values below 0.28 mu M against all the MDR strains evaluated, thus suggesting that this compound might have a different mechanism of action. Benzofuroxans are an attractive new class of anti-TB agents, exemplified by compound 5 b, with excellent potency against the replicating and drug-resistant strains of M. tuberculosis.

C1 [Fernandes, Guilherme F. S.; Campos, Debora L.; Da Silva, Isabel C.; Prates, Joao L. B.; Pavan, Aline R.; Pavan, Fernando R.; Dos Santos, Jean L.] Sao Paulo State Univ UNESP, Sch Pharmaceut Sci, Araraquara Jau Highway KM 01, BR-14800903 Araraquara, SP, Brazil.

[Fernandes, Guilherme F. S.; Prates, Joao L. B.; Pavan, Aline R.; Dos Santos, Jean L.] Sao Paulo State Univ UNESP, Inst Chem, Francisco Degni St 55, BR-14800060 Araraquara, SP, Brazil.

C3 Universidade Estadual Paulista; Universidade Estadual Paulista

RP Fernandes, GFS; Pavan, FR; Dos Santos, JL (corresponding author), Sao Paulo State Univ UNESP, Sch Pharmaceut Sci, Araraquara Jau Highway KM 01, BR-14800903 Araraquara, SP, Brazil.; Fernandes, GFS; Dos Santos, JL (corresponding author), Sao Paulo State Univ UNESP, Inst Chem, Francisco Degni St 55, BR-14800060 Araraquara, SP, Brazil.

EM guilhermefelipe@outlook.com; fernando.pavan@unesp.br;

jean.santos@unesp.br

RI Pavan, Aline Renata/T-3889-2019; Silva, Isabel/P-2129-2016; Fernandes,

Guilherme/A-3668-2014; Dos Santos, Jean/O-9111-2019; Pavan, Fernando

Rogério/MZS-0713-2025

OI Pavan, Aline Renata/0000-0002-4932-7720; Silva,

Isabel/0000-0001-9617-9858; Fernandes, Guilherme/0000-0002-7644-5466;

Pavan, Fernando Rogério/0000-0002-6969-3963

FU FundacAo de Amparo a Pesquisa do Estado de SAo Paulo (FAPESP)

[2016/09502-7, 2018/17739-2, 2018/11079-0, 2018/00163-0, 2018/21778-3,

2018/24783-8]; CoordenacAo de Aperfeicoamento de Pessoal de Nivel

Superior-Brasil (CAPES) [001]; Conselho Nacional de Desenvolvimento

Cientifico e Tecnologico [CNPq 404181/2019-8, 429139/2018-7]; CNPq

[304731/2017-0, 303603/2018-6]

FX This study was supported by FundacAo de Amparo a Pesquisa do Estado de

SAo Paulo (FAPESP grants 2016/09502-7; 2018/17739-2; 2018/11079-0;

2018/00163-0; 2018/21778-3; 2018/24783-8). This study was financed in

part by the CoordenacAo de Aperfeicoamento de Pessoal de Nivel

Superior-Brasil (CAPES)-Finance Code 001 and Conselho Nacional de

Desenvolvimento Cientifico e Tecnologico (CNPq 404181/2019-8 and

429139/2018-7). J.L.S. is CNPq productivity fellows' level 2 (CNPq Ref.

Process: 304731/2017-0) and F.R.P. is CNPq productivity fellows' level

1D (CNPq Ref. Process: 303603/2018-6). Programa de Apoio ao

Desenvolvimento Cientifico da Faculdade de Ciencias Farmaceuticas da

UNESP (PADC-FCF UNESP).

CR Barberis I, 2017, J Prev Med Hyg, V58, pE9

Behar SM, 2011, MUCOSAL IMMUNOL, V4, P279, DOI 10.1038/mi.2011.3

Boiani M, 2010, BIOCHEM PHARMACOL, V79, P1736, DOI 10.1016/j.bcp.2010.02.009

Campos DL, 2020, J ANTIBIOT, V73, P120, DOI 10.1038/s41429-019-0243-3

Castro D, 2009, EUR J MED CHEM, V44, P5055, DOI 10.1016/j.ejmech.2009.09.009

Cerecetto H, 2005, MINI-REV MED CHEM, V5, P57, DOI 10.2174/1389557053402864

Chetty S, 2017, BIOORG MED CHEM LETT, V27, P370, DOI 10.1016/j.bmcl.2016.11.084

Daniel TM, 2006, RESP MED, V100, P1862, DOI 10.1016/j.rmed.2006.08.006

Dharmaraja AT, 2012, CHEM COMMUN, V48, P10325, DOI 10.1039/c2cc35343a

Fernandes GFD, 2018, CURR PHARM DESIGN, V24, P1325, DOI 10.2174/1381612824666180417122625

Fernandes GFD, 2017, J MED CHEM, V60, P8647, DOI 10.1021/acs.jmedchem.7b01332

Fernandes GFD, 2017, PHARMACEUTICALS-BASE, V10, DOI 10.3390/ph10020051

Fernandes GFD, 2016, EUR J MED CHEM, V123, P523, DOI 10.1016/j.ejmech.2016.07.039

Fernandes GFD, 2015, CURR MED CHEM, V22, P3133, DOI 10.2174/0929867322666150818103836

Fang FC, 2004, NAT REV MICROBIOL, V2, P820, DOI 10.1038/nrmicro1004

de Melo TRF, 2018, EUR J MED CHEM, V154, P341, DOI 10.1016/j.ejmech.2018.05.008

Frydenberg AR, 2009, TROP MED INT HEALTH, V14, P1329, DOI 10.1111/j.1365-3156.2009.02375.x

Ramos-Martinez AG, 2019, MEM I OSWALDO CRUZ, V114, DOI 10.1590/0074-02760190102

Ganihigama DU, 2015, EUR J MED CHEM, V89, P1, DOI 10.1016/j.ejmech.2014.10.026

GHOSH PB, 1968, J MED CHEM, V11, P305, DOI 10.1021/jm00308a027

Hernández P, 2013, EUR J MED CHEM, V59, P64, DOI 10.1016/j.ejmech.2012.10.047

Heunis T, 2017, J PROTEOME RES, V16, P3841, DOI 10.1021/acs.jproteome.7b00483

Heysell SK, 2015, ANTIMICROB AGENTS CH, V59, P7104, DOI 10.1128/AAC.01117-15

Johnson TW, 2018, J MED CHEM, V61, P6401, DOI 10.1021/acs.jmedchem.8b00077

Jorge SD, 2013, EUR J MED CHEM, V64, P200, DOI 10.1016/j.ejmech.2013.03.053

Keurulainen L, 2015, MEDCHEMCOMM, V6, P1673, DOI 10.1039/c5md00119f

Klopper M, 2013, EMERG INFECT DIS, V19, P449, DOI [10.3201/EID1903.120246, 10.3201//EID1903.120246]

Kots AY, 2000, BRIT J PHARMACOL, V129, P1163, DOI 10.1038/sj.bjp.0703156

Koul A, 2007, NAT CHEM BIOL, V3, P323, DOI 10.1038/nchembio884

KOYAMA M, 1987, J MED CHEM, V30, P552, DOI 10.1021/jm00386a019

Libardo MDJ, 2018, CURR OPIN PHARMACOL, V42, P81, DOI 10.1016/j.coph.2018.08.001

Ma ZK, 2010, LANCET, V375, P2100, DOI 10.1016/S0140-6736(10)60359-9

Maeurer M, 2014, LANCET RESP MED, V2, P256, DOI 10.1016/S2213-2600(14)70020-7

Manjunatha Ujjini, 2009, Commun Integr Biol, V2, P215

Matsumoto M, 2006, PLOS MED, V3, P2131, DOI 10.1371/journal.pmed.0030466

Mestre O, 2013, PLOS ONE, V8, DOI 10.1371/journal.pone.0053486

Miyata M, 2011, BIOMED PHARMACOTHER, V65, P456, DOI 10.1016/j.biopha.2011.04.021

Nahid P, 2019, AM J RESP CRIT CARE, V200, pE93, DOI 10.1164/rccm.201909-1874ST

O'Toole RF, 2017, GENOMICS, V109, P471, DOI 10.1016/j.ygeno.2017.07.004

Olea-Azar C, 2005, LETT DRUG DES DISCOV, V2, P294, DOI 10.2174/1570180054038341

Palomino JC, 2002, ANTIMICROB AGENTS CH, V46, P2720, DOI 10.1128/AAC.46.8.2720-2722.2002

Parida SK, 2015, J INTERN MED, V277, P388, DOI 10.1111/joim.12264

Pavan FR, 2010, EUR J MED CHEM, V45, P1898, DOI 10.1016/j.ejmech.2010.01.028

Perskvist N, 2002, J IMMUNOL, V168, P6358, DOI 10.4049/jimmunol.168.12.6358

Sahasrabudhe V, 2015, J INFECT DIS, V211, pS107, DOI 10.1093/infdis/jiv009

Sarlauskas J, 2009, CHEMIJA, V20, P109

Severina IS, 2003, BIOCHEMISTRY-MOSCOW+, V68, P1048, DOI 10.1023/A:1026029000085

Singh M, 2011, MED CHEM RES, V20, P1611, DOI 10.1007/s00044-010-9405-3

Slomski A, 2013, JAMA-J AM MED ASSOC, V309, P1097, DOI 10.1001/jama.2013.1802

Straniero V, 2016, EUR J MED CHEM, V120, P227, DOI 10.1016/j.ejmech.2016.03.068

Tasduq SA, 2007, HEPATOL RES, V37, P845, DOI 10.1111/j.1872-034X.2007.00129.x

Udwadia ZF, 2012, CLIN INFECT DIS, V54, P579, DOI 10.1093/cid/cir889

Velayati AA, 2009, CHEST, V136, P420, DOI 10.1378/chest.08-2427

Voskuil MI, 2011, FRONT MICROBIOL, V2, DOI 10.3389/fmicb.2011.00105

Wube AA, 2011, EUR J MED CHEM, V46, P2091, DOI 10.1016/j.ejmech.2011.02.062

Xiang P, 2012, MOLECULES, V17, P873, DOI 10.3390/molecules17010873

Xu BX, 2018, CHEM RES CHINESE U, V34, P51, DOI 10.1007/s40242-018-7302-3

Yee D, 2003, AM J RESP CRIT CARE, V167, P1472, DOI 10.1164/rccm.200206-626OC

Zumla A, 2015, LANCET RESP MED, V3, P220, DOI 10.1016/S2213-2600(15)00063-6

Zumla A, 2013, NAT REV DRUG DISCOV, V12, P388, DOI 10.1038/nrd4001

NR 60

TC 20

Z9 21

U1 0

U2 4

PU WILEY-V C H VERLAG GMBH

PI WEINHEIM

PA POSTFACH 101161, 69451 WEINHEIM, GERMANY

SN 1860-7179

EI 1860-7187

J9 CHEMMEDCHEM

JI ChemMedChem

PD APR 20

PY 2021

VL 16

IS 8

BP 1268

EP 1282

DI 10.1002/cmdc.202000899

EA FEB 2021

PG 15

WC Chemistry, Medicinal; Pharmacology & Pharmacy

WE Science Citation Index Expanded (SCI-EXPANDED); Index Chemicus (IC)

SC Pharmacology & Pharmacy

GA RQ2TC

UT WOS:000616364100001

OA Green Submitted

DA 2026-05-07

ER

PT J

AU Bai, XY

Feldman, NE

Chmura, K

Ovrutsky, AR

Su, WL

Griffin, L

Pyeon, D

McGibney, MT

Strand, MJ

Numata, M

Murakami, S

Gaido, L

Honda, JR

Kinney, WH

Oberley-Deegan, RE

Voelker, DR

Ordway, DJ

Chan, ED

AF Bai, Xiyuan

Feldman, Nicole E.

Chmura, Kathryn

Ovrutsky, Alida R.

Su, Wen-Lin

Griffin, Laura

Pyeon, Dohun

McGibney, Mischa T.

Strand, Matthew J.

Numata, Mari

Murakami, Seiji

Gaido, Loretta

Honda, Jennifer R.

Kinney, William H.

Oberley-Deegan, Rebecca E.

Voelker, Dennis R.

Ordway, Diane J.

Chan, Edward D.

TI Inhibition of Nuclear Factor-Kappa B Activation Decreases Survival of

Mycobacterium tuberculosis in Human Macrophages

SO PLOS ONE

LA English

DT Article

ID TUMOR-NECROSIS-FACTOR; DRUG-RESISTANT TUBERCULOSIS; TOLL-LIKE RECEPTORS;

NITRIC-OXIDE; TRANSCRIPTION FACTOR; HOST-DEFENSE; TNF-ALPHA; ALVEOLAR

MACROPHAGES; MURINE MACROPHAGES; INTERLEUKIN-8 GENE

AB Nuclear factor-kappa B (NF kappa B) is a ubiquitous transcription factor that mediates pro-inflammatory responses required for host control of many microbial pathogens; on the other hand, NF kappa B has been implicated in the pathogenesis of other inflammatory and infectious diseases. Mice with genetic disruption of the p50 subunit of NF kappa B are more likely to succumb to Mycobacterium tuberculosis (MTB). However, the role of NF kappa B in host defense in humans is not fully understood. We sought to examine the role of NFkB activation in the immune response of human macrophages to MTB. Targeted pharmacologic inhibition of NF kappa B activation using BAY 11-7082 (BAY, an inhibitor of I kappa B alpha kinase) or an adenovirus construct with a dominant-negative IkBa significantly decreased the number of viable intracellular mycobacteria recovered from THP-1 macrophages four and eight days after infection. The results with BAY were confirmed in primary human monocyte-derived macrophages and alveolar macrophages. NF kappa B inhibition was associated with increased macrophage apoptosis and autophagy, which are well-established killing mechanisms of intracellular MTB. Inhibition of the executioner protease caspase-3 or of the autophagic pathway significantly abrogated the effects of BAY. We conclude that NF kappa B inhibition decreases viability of intracellular MTB in human macrophages via induction of apoptosis and autophagy.

C1 [Bai, Xiyuan; Chan, Edward D.] Denver Vet Affairs Med Ctr, Dept Med, Denver, CO USA.

[Bai, Xiyuan; Feldman, Nicole E.; Chmura, Kathryn; Ovrutsky, Alida R.; McGibney, Mischa T.; Strand, Matthew J.; Numata, Mari; Murakami, Seiji; Honda, Jennifer R.; Kinney, William H.; Oberley-Deegan, Rebecca E.; Voelker, Dennis R.; Chan, Edward D.] Natl Jewish Hlth, Dept Med & Acad Affairs, Denver, CO USA.

[Bai, Xiyuan; Chmura, Kathryn; Ovrutsky, Alida R.; Honda, Jennifer R.; Kinney, William H.; Chan, Edward D.] Univ Colorado, Sch Med, Dept Med, Aurora, CO USA.

[Gaido, Loretta] Denver Hlth Med Ctr, Denver, CO USA.

[Griffin, Laura; Pyeon, Dohun] Univ Colorado, Sch Med, Dept Microbiol, Aurora, CO USA.

[Su, Wen-Lin] Natl Def Med Ctr, Dept Med, Tri Serv Gen Hosp, Taipei, Taiwan.

[Ordway, Diane J.] Colorado State Univ, Dept Microbiol Immunol & Pathol, Ft Collins, CO 80523 USA.

C3 US Department of Veterans Affairs; Veterans Health Administration (VHA);

Veterans Affairs Medical Center - Denver; National Jewish Health;

University of Colorado System; University of Colorado Anschutz; Denver

Health Medical Center; University of Colorado System; University of

Colorado Anschutz; Tri-Service General Hospital; National Defense

Medical University; Colorado State University System; Colorado State

University Fort Collins

RP Bai, XY (corresponding author), Denver Vet Affairs Med Ctr, Dept Med, Denver, CO USA.

EM baix@njhealth.org; chane@njhealth.org

RI ; Oberley-Deegan, Rebecca/E-4176-2013; Ordway, Diane/H-9732-2017;

Strand, Matthew/AAU-3762-2021

OI Pyeon, Dohun/0000-0003-1970-844X; Ordway, Diane/0000-0003-0003-326X;

Strand, Matthew/0000-0002-9083-5989

FU Department of Veterans Affairs, Veterans Health Administration, Office

of Research and Development [1 I01 BX001028-01A2]; National Institutes

of Health (NIH) [PHL073907]; General Clinical Research Center Program

Grant [M01-RR00051]

FX This work was supported by the Department of Veterans Affairs, Veterans

Health Administration, Office of Research and Development 1 I01

BX001028-01A2 (to EDC), National Institutes of Health (NIH) PHL073907

(to DRV), and General Clinical Research Center Program Grant No.

M01-RR00051. The funders had no role in study design, data collection

and analysis, decision to publish, or preparation of the manuscript.

CR Almeida PE, 2009, J IMMUNOL, V183, P1337, DOI 10.4049/jimmunol.0900365

Bafica A, 2005, J EXP MED, V202, P1715, DOI 10.1084/jem.20051782

Bai XY, 2010, J IMMUNOL, V184, P3830, DOI 10.4049/jimmunol.0901913

Bai XY, 2004, TUBERCULOSIS, V84, P375, DOI 10.1016/j.tube.2004.05.001

Baldwin AS, 1996, ANNU REV IMMUNOL, V14, P649, DOI 10.1146/annurev.immunol.14.1.649

Barnes PJ, 1997, NEW ENGL J MED, V336, P1066, DOI 10.1056/NEJM199704103361506

Bernardo J, 1998, INFECT IMMUN, V66, P28, DOI 10.1128/IAI.66.1.28-35.1998

Brightbill HD, 1999, SCIENCE, V285, P732, DOI 10.1126/science.285.5428.732

Chan ED, 2001, INFECT IMMUN, V69, P2001, DOI 10.1128/IAI.69.4.2001-2010.2001

Chan ED, 2001, AM J RESP CELL MOL, V25, P606, DOI 10.1165/ajrcmb.25.5.4487

Chan ED, 2008, CURR OPIN INFECT DIS, V21, P587, DOI 10.1097/QCO.0b013e328319bce6

Changsen C, 2003, ANTIMICROB AGENTS CH, V47, P3682, DOI 10.1128/AAC.47.12.3682-3687.2003

CHEN C, 1987, MOL CELL BIOL, V7, P2745, DOI 10.1128/MCB.7.8.2745

Chen MJ, 2006, J IMMUNOL, V176, P3707, DOI 10.4049/jimmunol.176.6.3707

Chiang CY, 2009, INT J TUBERC LUNG D, V13, P304

Cota-Gomez A, 2002, J BIOL CHEM, V277, P14390, DOI 10.1074/jbc.M108591200

Deretic V, 2006, CELL MICROBIOL, V8, P719, DOI 10.1111/j.1462-5822.2006.00705.x

Dhiman R, 2007, BBA-GEN SUBJECTS, V1770, P649, DOI 10.1016/j.bbagen.2006.11.016

Djavaheri-Mergny M, 2006, J BIOL CHEM, V281, P30373, DOI 10.1074/jbc.M602097200

Faherty CS, 2008, TRENDS MICROBIOL, V16, P173, DOI 10.1016/j.tim.2008.02.001

Ferrari G, 1999, CELL, V97, P435, DOI 10.1016/S0092-8674(00)80754-0

Foryst-Ludwig A, 2000, J BIOL CHEM, V275, P39779, DOI 10.1074/jbc.M007617200

Gutierrez MG, 2008, J IMMUNOL, V181, P2651, DOI 10.4049/jimmunol.181.4.2651

Gutierrez MG, 2009, CELL MICROBIOL, V11, P406, DOI 10.1111/j.1462-5822.2008.01263.x

Gutierrez MG, 2004, CELL, V119, P753, DOI 10.1016/j.cell.2004.11.038

Jagannath C, 2009, NAT MED, V15, P267, DOI 10.1038/nm.1928

Jobin C, 1998, J IMMUNOL, V160, P410

Kabeya Y, 2000, EMBO J, V19, P5720, DOI 10.1093/emboj/19.21.5720

Karin M, 2002, NAT IMMUNOL, V3, P221, DOI 10.1038/ni0302-221

Keane J, 1997, INFECT IMMUN, V65, P298, DOI 10.1128/IAI.65.1.298-304.1997

Keane J, 2000, J IMMUNOL, V164, P2016, DOI 10.4049/jimmunol.164.4.2016

Keane J, 2002, TUBERCULOSIS, V82, P55, DOI 10.1054/tube.2002.0322

Keane J, 2001, NEW ENGL J MED, V345, P1098, DOI 10.1056/NEJMoa011110

Kim SH, 2005, IMMUNITY, V22, P131, DOI 10.1016/j.immuni.2004.12.003

Kwon J, 1996, J BIOL CHEM, V271, P22383, DOI 10.1074/jbc.271.37.22383

Lee J, 2009, YONSEI MED J, V50, P1, DOI 10.3349/ymj.2009.50.1.1

Levine B, 2005, J CLIN INVEST, V115, P2679, DOI 10.1172/JCI26390

Liu PT, 2006, SCIENCE, V311, P1770, DOI 10.1126/science.1123933

Loeuillet C, 2006, J IMMUNOL, V177, P6245, DOI 10.4049/jimmunol.177.9.6245

Lurie M., 1964, Resistance to Tuberculosis: Experimental Studies in Native and Acquired Defensive Mechanisms

Macdonald SHF, 2012, PLOS ONE, V7, DOI 10.1371/journal.pone.0038488

Madariaga MG, 2008, AM J MED, V121, P835, DOI 10.1016/j.amjmed.2008.04.015

Mahajan S, 2012, J IMMUNOL, V188, P5593, DOI 10.4049/jimmunol.1103038

Means TK, 1999, J IMMUNOL, V163, P3920

Mizushima N, 2010, CELL, V140, P313, DOI 10.1016/j.cell.2010.01.028

Morris KR, 2003, INFECT IMMUN, V71, P1442, DOI 10.1128/IAI.71.3.1442-1452.2003

Morris KR, 2009, TUBERCULOSIS, V89, P294, DOI 10.1016/j.tube.2009.03.004

MUKAIDA N, 1990, J BIOL CHEM, V265, P21128

NABEL G, 1987, NATURE, V326, P711, DOI 10.1038/326711a0

Patel NR, 2007, J IMMUNOL, V179, P6973, DOI 10.4049/jimmunol.179.10.6973

Patel NR, 2009, J LEUKOCYTE BIOL, V86, P53, DOI [10.1189/jlb.0908574, 10.1189/JLB.0908574]

Peng H, 2011, J BIOL CHEM, V286, P24508, DOI 10.1074/jbc.M111.234062

Philpott DJ, 2000, J IMMUNOL, V165, P903, DOI 10.4049/jimmunol.165.2.903

Pierce JW, 1997, J BIOL CHEM, V272, P21096, DOI 10.1074/jbc.272.34.21096

Pieters J, 2001, MICROBES INFECT, V3, P249, DOI 10.1016/S1286-4579(01)01376-4

Qing GL, 2006, CELL RES, V16, P895, DOI 10.1038/sj.cr.7310109

Reiling N, 2002, J IMMUNOL, V169, P3480, DOI 10.4049/jimmunol.169.7.3480

Roebuck KA, 1999, J INTERF CYTOK RES, V19, P429, DOI 10.1089/107999099313866

Rojas M, 1997, J IMMUNOL, V159, P1352

Rojas M, 1999, J IMMUNOL, V162, P6122

Rossouw M, 2003, LANCET, V361, P1871, DOI 10.1016/S0140-6736(03)13491-5

SCHLESINGER LS, 1994, J IMMUNOL, V152, P4070

Schorey JS, 2003, CELL MICROBIOL, V5, P133, DOI 10.1046/j.1462-5822.2003.00263.x

SCHRECK R, 1991, EMBO J, V10, P2247, DOI 10.1002/j.1460-2075.1991.tb07761.x

SEGLEN PO, 1982, P NATL ACAD SCI-BIOL, V79, P1889, DOI 10.1073/pnas.79.6.1889

Shishodia S, 2002, J BIOCHEM MOL BIOL, V35, P28

Singh SB, 2006, SCIENCE, V313, P1438, DOI 10.1126/science.1129577

Spira A, 2003, AM J RESP CELL MOL, V29, P545, DOI 10.1165/rcmb.2002-0310OC

Tato CM, 2002, INFECT IMMUN, V70, P3311, DOI 10.1128/IAI.70.7.3311-3317.2002

Thoma-Uszynski S, 2001, SCIENCE, V291, P1544, DOI 10.1126/science.291.5508.1544

Underhill DM, 1999, P NATL ACAD SCI USA, V96, P14459, DOI 10.1073/pnas.96.25.14459

Yamada H, 2001, INFECT IMMUN, V69, P7100, DOI 10.1128/IAI.69.11.7100-7105.2001

Yuk JM, 2012, EXP MOL MED, V44, P99, DOI 10.3858/emm.2012.44.2.032

Zhang GL, 2001, J CLIN INVEST, V107, P13, DOI 10.1172/JCI11837

Zingarelli B, 2003, CRIT CARE MED, V31, pS105, DOI 10.1097/00003246-200301001-00015

NR 75

TC 74

Z9 84

U1 0

U2 25

PU PUBLIC LIBRARY SCIENCE

PI SAN FRANCISCO

PA 1160 BATTERY STREET, STE 100, SAN FRANCISCO, CA 94111 USA

SN 1932-6203

J9 PLOS ONE

JI PLoS One

PD APR 25

PY 2013

VL 8

IS 4

AR e61925

DI 10.1371/journal.pone.0061925

PG 13

WC Multidisciplinary Sciences

WE Science Citation Index Expanded (SCI-EXPANDED)

SC Science & Technology - Other Topics

GA 136DM

UT WOS:000318341400027

OA Green Submitted, gold

DA 2026-05-07

ER

PT J

AU Chasák, J

Oorts, L

Dak, M

Slachtová, V

Bazgier, V

Berka, K

De Vooght, L

Smiejkowska, N

Van Calster, K

Van Moll, L

Cappoen, D

Cos, P

Brulíková, L

AF Chasak, Jan

Oorts, Lauren

Dak, Milan

Slachtova, Veronika

Bazgier, Vaclav

Berka, Karel

De Vooght, Linda

Smiejkowska, Natalia

Van Calster, Kevin

Van Moll, Laurence

Cappoen, Davie

Cos, Paul

Brulikova, Lucie

TI Expanding the squaramide library as mycobacterial ATP synthase

inhibitors: Innovative synthetic pathway and biological evaluation

SO BIOORGANIC & MEDICINAL CHEMISTRY

LA English

DT Article

DE Squaramides; Tuberculosis; Mycobacterium tuberculosis; ATP synthase;

Antimycobacterial agents; MDR-TB

ID TUBERCULOSIS; BEDAQUILINE

AB Mycobacterial ATP synthase is a validated therapeutic target for combating drug-resistant tuberculosis. Inhibition of this enzyme has been featured as an efficient strategy for the development of new antimycobacterial agents against drug-resistant pathogens. In this study, we synthesised and explored two distinct series of squaric acid analogues designed to inhibit mycobacterial ATP synthase. Among the extensive array of compounds investigated, members of the phenyl-substituted sub-library emerged as primary hits. To gain deeper insights into their mechanisms of action, we conducted advanced biological studies, focusing on the compounds displaying a direct binding of a nitrogen heteroatom to the phenyl ring, resulting in the highest potency. Our investigations into spontaneous mutants led to the validation of a single point mutation within the atpB gene (Rv1304), responsible for encoding the ATP synthase subunit a. This genetic alteration sheds light on the molecular basis of resistance to squaramides. Furthermore, we explored the possibility of synergy between squaramides and the reference drug clofazimine using a checkerboard assay, highlighting the promising avenue for enhancing the effectiveness of existing treatments through combined therapeutic approaches. This study contributes to the expansion of investigating squaramides as promising drug candidates in the ongoing battle against drug-resistant tuberculosis.

C1 [Chasak, Jan; Dak, Milan; Slachtova, Veronika; Brulikova, Lucie] Palacky Univ, Fac Sci, Dept Organ Chem, 17 listopadu 12, Olomouc 77146, Czech Republic.

[Oorts, Lauren; De Vooght, Linda; Smiejkowska, Natalia; Van Calster, Kevin; Van Moll, Laurence; Cappoen, Davie; Cos, Paul] Univ Antwerp, Fac Pharmaceut Biomed & Vet Sci, Lab Microbiol Parasitol & Hyg LMPH, S7, Antwerp, Belgium.

[Bazgier, Vaclav; Berka, Karel] Palacky Univ, Fac Sci, Dept Phys Chem, 17 listopadu 12, Olomouc 77146, Czech Republic.

[Cappoen, Davie] Sci Inst Publ Hlth, Dept Food Med & Consumer Safety, Juliette Wytsmanstr 14, Brussels, Belgium.

C3 Palacky University Olomouc; University of Antwerp; Palacky University

Olomouc

RP Brulíková, L (corresponding author), Palacky Univ, Fac Sci, Dept Organ Chem, 17 listopadu 12, Olomouc 77146, Czech Republic.

EM lucie.brulikova@upol.cz

RI ; Van Moll, Laurence/LVS-0893-2024; Chasák, Jan/MTE-3851-2025; Bazgier,

Vaclav/AAF-3135-2020; Brulíková, Lucie/C-4338-2018; Berka,

Karel/E-2839-2010; cos, paul/AAU-3896-2020

OI Oorts, Lauren/0000-0003-1029-5407; Van Moll,

Laurence/0000-0002-9563-9581; Smiejkowska, Natalia/0000-0001-7488-6009;

Chasák, Jan/0000-0002-3235-5098; Bazgier, Vaclav/0000-0003-3393-3010;

Brulíková, Lucie/0000-0001-8380-073X; Šlachtová,

Veronika/0000-0001-8560-3916; Berka, Karel/0000-0001-9472-2589; Dak,

Milan/0000-0003-3034-0375; De Vooght, Linda/0000-0002-8425-8601;

FU Palacky University in Olomouc [JG_2019_002]; FWO [G066619N]; Ministry of

Education, Youth and Sports of the Czech Republic [LM2022123]

FX This work was supported by grant no. JG_2019_002 from Palacky University

in Olomouc, FWO grant no. G066619N and via infrastructure project ELIXIR

CZ no. LM2022123 from the Ministry of Education, Youth and Sports of the

Czech Republic.

CR Agnew-Francis KA, 2020, CHEM REV, V120, P11616, DOI 10.1021/acs.chemrev.0c00416

Ahmed S, 2022, EXPERT OPIN THER PAT, V32, P243, DOI 10.1080/13543776.2022.2012151

Andries K, 2005, SCIENCE, V307, P223, DOI 10.1126/science.1106753

Bald D, 2017, MBIO, V8, DOI 10.1128/mBio.00272-17

Banfi E, 2003, J ANTIMICROB CHEMOTH, V52, P796, DOI 10.1093/jac/dkg439

Barbaro L, 2021, RSC MED CHEM, V12, P943, DOI 10.1039/d1md00063b

Bhunia S, 2017, CHEM-EUR J, V23, P11234, DOI 10.1002/chem.201702459

Blaser A, 2019, BIOORGAN MED CHEM, V27, P1283, DOI 10.1016/j.bmc.2019.02.025

Chasák J, 2021, EUR J MED CHEM, V209, DOI 10.1016/j.ejmech.2020.112872

Choi PJ, 2017, BIOORG MED CHEM LETT, V27, P5190, DOI 10.1016/j.bmcl.2017.10.042

Courbon GM, 2023, EMBO J, V42, DOI 10.15252/embj.2023113687

Dartois V.A., Anti-tuberculosis treatment strategies and drug development: challenges and priorities, DOI [10.1038/s41579-022-00731-y, DOI 10.1038/S41579-022-00731-Y]

Guo H, 2021, NATURE, V589, P143, DOI 10.1038/s41586-020-3004-3

Ioerger TR, 2013, PLOS ONE, V8, DOI 10.1371/journal.pone.0075245

Koul A, 2007, NAT CHEM BIOL, V3, P323, DOI 10.1038/nchembio884

Kumar S, 2018, TUBERCULOSIS, V108, P56, DOI 10.1016/j.tube.2017.10.008

Li P, 2020, EUR J MED CHEM, V206, DOI 10.1016/j.ejmech.2020.112538

Lu P, 2014, BBA-BIOENERGETICS, V1837, P1208, DOI 10.1016/j.bbabio.2014.01.022

Luo M, 2020, COMMUN BIOL, V3, DOI 10.1038/s42003-020-01173-z

Montgomery M.G., 2021, Proc. Natl. Acad. Sci. U. S. A., V118, DOI [10.1073/pnas.2111899118CO-PNASA6, DOI 10.1073/PNAS.2111899118CO-PNASA6]

Morris GM, 2009, J COMPUT CHEM, V30, P2785, DOI 10.1002/jcc.21256

Nazarova EV, 2017, METHODS MOL BIOL, V1519, P325, DOI 10.1007/978-1-4939-6581-6_22

Nugraha RV, 2021, SCI PHARM, V89, DOI 10.3390/scipharm89020019

Qiao CJ, 2015, MOLECULES, V20, P22272, DOI 10.3390/molecules201219846

Rombouts JA, 2016, RSC ADV, V6, P108708, DOI 10.1039/c6ra22693k

Schön T, 2020, CLIN MICROBIOL INFEC, V26, P1488, DOI 10.1016/j.cmi.2020.07.036

Slachtová V, 2019, ACS OMEGA, V4, P19314, DOI 10.1021/acsomega.9b02702

Surase YB, 2017, BIOORG MED CHEM LETT, V27, P3454, DOI 10.1016/j.bmcl.2017.05.081

Sutherland HS, 2020, BIOORGAN MED CHEM, V28, DOI 10.1016/j.bmc.2019.115213

Tantry SJ, 2017, J MED CHEM, V60, P1379, DOI 10.1021/acs.jmedchem.6b01358

Trott O, 2010, J COMPUT CHEM, V31, P455, DOI 10.1002/jcc.21334

Urban M, 2021, EUR J MED CHEM, V212, DOI 10.1016/j.ejmech.2020.113139

Waterhouse A, 2018, NUCLEIC ACIDS RES, V46, pW296, DOI [10.7490/f1000research.1117296.1, 10.1093/nar/gky427]

NR 33

TC 5

Z9 5

U1 4

U2 11

PU PERGAMON-ELSEVIER SCIENCE LTD

PI OXFORD

PA THE BOULEVARD, LANGFORD LANE, KIDLINGTON, OXFORD OX5 1GB, ENGLAND

SN 0968-0896

EI 1464-3391

J9 BIOORGAN MED CHEM

JI Bioorg. Med. Chem.

PD NOV 15

PY 2023

VL 95

AR 117504

DI 10.1016/j.bmc.2023.117504

EA OCT 2023

PG 18

WC Biochemistry & Molecular Biology; Chemistry, Medicinal; Chemistry,

Organic

WE Science Citation Index Expanded (SCI-EXPANDED); Index Chemicus (IC)

SC Biochemistry & Molecular Biology; Pharmacology & Pharmacy; Chemistry

GA W9FN5

UT WOS:001094614900001

DA 2026-05-07

ER

PT J

AU Alvarado-Peña, N

Torrico, MM

Narváez-Díaz, L

Mejía-Ponce, PM

Vargas, EB

Zúñiga, J

Muñiz-Salazar, R

Laniado-Laborín, R

Licona-Cassani, C

Soberón, X

Silva-Herzog, E

AF Alvarado-Pena, Nestor

Torrico, Marcela Munoz

Narvaez-Diaz, Luis

Mejia-Ponce, Paulina M.

Vargas, Eduardo Becerril

Zuniga, Joaquin

Muniz-Salazar, Raquel

Laniado-Laborin, Rafael

Licona-Cassani, Cuauhtemoc

Soberon, Xavier

Silva-Herzog, Eugenia

TI Mycobacterium tuberculosis genomic surveillance in Mexico.

Characterization of variants in drug resistance and efflux pump genes

SO FRONTIERS IN MICROBIOLOGY

LA English

DT Article

DE tuberculosis; <italic>Mycobacterium tuberculosis</italic> complex;

drug-resistant tuberculosis; resistance-associated variants; efflux

pumps; whole genome sequencing

ID SOUTHEAST; MUTATIONS

AB Tuberculosis (TB) remains a persistent global public health challenge, with the rise of drug-resistant tuberculosis (DR-TB) complicating all the disease control efforts. The World Health Organization (WHO) has advocated for molecular diagnostic techniques, including whole-genome sequencing (WGS), to enhance TB diagnosis and treatment strategies. In this study, we performed WGS analysis on 49 pulmonary tuberculosis isolates from Mexican patients to identify mutations conferring resistance to 11 key antimicrobial agents: four first-line drugs (isoniazid, rifampicin, ethambutol, and pyrazinamide) and 7 second line drugs (fluoroquinolones, ethionamide/prothionamide, amikacin, kanamycin, capreomycin, streptomycin, and bedaquiline). We identified 89 novel variants: 48 in genes previously associated with drug resistance and 41 in genes not previously linked to resistance mechanisms, including potential novel mutations associated with delamanid resistance. Additionally, we detected 31 mutations across three efflux pump superfamilies (ABC, RND, and MFS); all of these variants warrant further investigation regarding their contribution to antibiotic resistance. This analysis represents approximately 10% of Mexico's national variant registry, providing substantial insight into the molecular epidemiology of drug-resistant tuberculosis within the country. The identification of new resistance-associated variants (RAV) from clinical isolates underrepresented in global databases, contributes to develop improved diagnostic tools, optimize treatment regimens, and probably to elucidate antibiotic resistance mechanisms. Specifically, the identification of RAVs for new drugs like bedaquiline, pretomanid, delamanid, and linezolid, which are central to the most recent schemes of treatment (BPaLM, BPaL, BDLLfxC, BLMZ), is key to the improvement of patient outcomes and preventing the emergence of resistance to these critical therapeutic options.

C1 [Alvarado-Pena, Nestor; Torrico, Marcela Munoz] Inst Nacl Enfermedades Respi Ismael Cosio Villegas, Clin TB, Mexico City, Mexico.

[Alvarado-Pena, Nestor; Silva-Herzog, Eugenia] Univ Nacl Autonoma Mexico, Fac Med, Lab Vinculac Cient, Inst Nacl Med Genom, Mexico City, Mexico.

[Narvaez-Diaz, Luis; Vargas, Eduardo Becerril] Inst Nacl Enfermedades Resp INER, Lab Microbiol, Mexico City, Mexico.

[Mejia-Ponce, Paulina M.; Licona-Cassani, Cuauhtemoc] Tecnol Monterrey, Escuela Ingn & Ciencias, Monterrey, Mexico.

[Zuniga, Joaquin] Inst Nacl Enfermedades Resp Ismael Cosio Villegas, Lab Inmunobiol & Genet, Mexico City, Mexico.

[Zuniga, Joaquin] Tecnol Monterrey, Escuela Med & Ciencias Salud, Mexico City, Mexico.

[Muniz-Salazar, Raquel] Univ Autonoma Baja California, Escuela Ciencias Salud, Ensenada, Mexico.

[Laniado-Laborin, Rafael] Univ Autonoma Baja California, Fac Med, Tijuana, Mexico.

[Licona-Cassani, Cuauhtemoc] Tecnol Monterrey, Inst Obes Res, Monterrey, Mexico.

[Soberon, Xavier] Univ Nacl Autonoma Mexico, Dept Ingn Celular & Biocatalisis, Inst Biotecnol, Cuernavaca, Mexico.

C3 Instituto Nacional de Medicina Genomica; Universidad Nacional Autonoma

de Mexico; Tecnologico de Monterrey; Tecnologico de Monterrey;

Universidad Autonoma de Baja California; Universidad Autonoma de Baja

California; Tecnologico de Monterrey; Universidad Nacional Autonoma de

Mexico

RP Silva-Herzog, E (corresponding author), Univ Nacl Autonoma Mexico, Fac Med, Lab Vinculac Cient, Inst Nacl Med Genom, Mexico City, Mexico.

EM esilvaherzog@inmegen.gob.mx

RI MUNIZ-SALAZAR, RAQUEL/F-5309-2019; Laniado-Laborin,

Rafael/AAU-9822-2021; Soberon, Xavier/A-8826-2008

FU ESH from CONAHCyT, Mexico [CF191978]

FX The author(s) declare that financial support was received for the

research and/or publication of this article. Funding was obtained from a

grant for ESH from CONAHCyT CF191978, Mexico.

CR Abrahams KA, 2023, NAT COMMUN, V14, DOI 10.1038/s41467-023-39300-z

Adams O, 2021, STRUCTURE, V29, P1182, DOI 10.1016/j.str.2021.06.013

Alvarez-Maya I, 2025, J GLOB ANTIMICROB RE, V41, P224, DOI 10.1016/j.jgar.2025.01.013

Malinga LA, 2016, Mycobacterial Diseases, V6, DOI [10.4172/2161-1068.1000222, 10.4172/2161-1068.1000222, DOI 10.4172/2161-1068.1000222]

Barbosa-Amezcua M, 2022, MICROBIOL SPECTR, V10, DOI 10.1128/spectrum.01252-22

Billows N, 2024, SCI REP-UK, V14, DOI 10.1038/s41598-024-62946-8

Brankin A, 2022, PLOS BIOL, V20, DOI 10.1371/journal.pbio.3001721

Chaitra R, 2023, MOL DIVERS, V27, P357, DOI 10.1007/s11030-022-10436-2

Comas I, 2012, NAT GENET, V44, P106, DOI 10.1038/ng.1038

Conceiçao EC, 2024, MICROORGANISMS, V12, DOI 10.3390/microorganisms12091817

Conkle-Gutierrez D, 2024, FRONT MICROBIOL, V14, DOI 10.3389/fmicb.2023.1265390

Cuevas-Córdoba B, 2021, PLOS ONE, V16, DOI 10.1371/journal.pone.0258774

D'Souza C, 2023, IMMUNOBIOLOGY, V228, DOI 10.1016/j.imbio.2022.152321

Guedes GMD, 2024, FUTURE MICROBIOL, V19, P495, DOI 10.2217/fmb-2023-0208

Domenech P, 2005, INFECT IMMUN, V73, P3492, DOI 10.1128/IAI.73.6.3492-3501.2005

El Meouche I, 2018, SCIENCE, V362, P686, DOI 10.1126/science.aar7981

Flores-Treviño S, 2015, ENFERM INFEC MICR CL, V33, P181, DOI 10.1016/j.eimc.2014.04.005

Ford CB, 2013, NAT GENET, V45, P784, DOI 10.1038/ng.2656

Madrazo-Moya CF, 2019, PLOS ONE, V14, DOI 10.1371/journal.pone.0213046

Gaurav A, 2023, MICROBIOL-SGM, V169, DOI 10.1099/mic.0.001333

Ghajavand H, 2019, ANTIMICROB RESIST IN, V8, DOI 10.1186/s13756-019-0516-4

Grimsey EM, 2020, ANTIMICROB AGENTS CH, V64, DOI 10.1128/AAC.02460-19

Han YQ, 2024, MICROBIOL RES, V287, DOI 10.1016/j.micres.2024.127825

Hasan Z, 2024, J GLOB ANTIMICROB RE, V38, P128, DOI 10.1016/j.jgar.2024.05.006

Tran HT, 2020, ANTIBIOTICS-BASEL, V9, DOI 10.3390/antibiotics9070390

Jiménez-Ruano AC, 2021, SCI REP-UK, V11, DOI 10.1038/s41598-020-80919-5

Kohl TA, 2018, PEERJ, V6, DOI 10.7717/peerj.5895

Laws M, 2022, TRENDS MICROBIOL, V30, P57, DOI 10.1016/j.tim.2021.05.001

Leconte A, 2024, MICROBIOL RES, V286, DOI 10.1016/j.micres.2024.127816

Li WW, 2024, MICROBIOL RES, V287, DOI 10.1016/j.micres.2024.127842

Lopez-Alvarez R, 2010, BMC MICROBIOL, V10, DOI 10.1186/1471-2180-10-82

Juarez-Eusebio DM, 2017, INFECT GENET EVOL, V55, P384, DOI 10.1016/j.meegid.2016.09.012

McHugh ML, 2012, BIOCHEM MEDICA, V22, P276, DOI 10.11613/bm.2012.031

Mejía-Ponce PM, 2023, PLOS ONE, V18, DOI 10.1371/journal.pone.0292965

Melly G, 2019, MICROORGANISMS, V7, DOI 10.3390/microorganisms7030070

Molina-Torres CA, 2022, FRONT CELL INFECT MI, V12, DOI 10.3389/fcimb.2022.875909

Monica M.-C. P., 2021, Am. J. Infect. Dis, DOI [10.3844/ajidsp.2021.138.149, DOI 10.3844/AJIDSP.2021.138.149]

Morey-León G, 2023, PLOS ONE, V18, DOI 10.1371/journal.pone.0294670

Naccache SN, 2014, GENOME RES, V24, P1180, DOI 10.1101/gr.171934.113

Napier G, 2023, SCI REP-UK, V13, DOI 10.1038/s41598-023-27516-4

Narang A, 2017, INT J MYCOBACT, V6, P177, DOI 10.4103/ijmy.ijmy_26_17

National Center for Preventive Programs and Disease Control, 2023, Programanacional para el control y prevencion de la tuberculosis

National Center for Preventive Programs and Disease Control, 2024, Programa nacional para el control y prevencion de la tuberculosis

Nimmo C, 2022, FRONT CELL INFECT MI, V12, DOI 10.3389/fcimb.2022.954074

Pei H, 2011, FOLIA MICROBIOL, V56, P549, DOI 10.1007/s12223-011-0080-7

Perez D., 2023, ITuberculosis Integrated Studies for a Complex Disease, P331, DOI [10.1007/978-3-031-15955-8, DOI 10.1007/978-3-031-15955-8]

Phelan JE, 2019, GENOME MED, V11, DOI 10.1186/s13073-019-0650-x

Puyén ZM, 2024, PLOS ONE, V19, DOI 10.1371/journal.pone.0304130

Raheem TY, 2020, Journal of Biosciences and Medicines, V08, P86, DOI [10.4236/jbm.2020.811008, 10.4236/jbm.2020.811008, DOI 10.4236/JBM.2020.811008]

Rahul Pal Rahul Pal, 2014, International Journal of Current Microbiology and Applied Sciences, V3, P528

Rao NT, 2024, INT J HEALTH SCI-IJH, V8, P830, DOI [10.53730/ijhs.v8ns1.14955, 10.53730/ijhs.v8ns1.14955, DOI 10.53730/IJHS.V8NS1.14955]

Remm S, 2022, FEMS MICROBIOL REV, V46, DOI 10.1093/femsre/fuab050

Roberts LW, 2024, LANCET MICROBE, V5, DOI 10.1016/S2666-5247(24)00053-3

Rodrigues L, 2020, EXPERT REV ANTI-INFE, V18, P741, DOI 10.1080/14787210.2020.1760845

Santos-Lazaro D, 2021, SCI REP-UK, V11, DOI 10.1038/s41598-021-88603-y

Tuberculosis en las Americas, 2021, Informe regional 2021

WHO, 2023, Catalogue of Mutations in Mycobacterium Tuberculosis Complex and Their Association with Drug Resistance, V2nd

WHO, 2024, WHO BACTERIAL PRIORI

WHO, 2016, GLOB TUB REP 2016

WHO, 2024, GLOBAL TUBERCULOSIS

World Health Organization, 2024, Module 3: Diagnosis WHO consolidated guidelines on tuberculosis Rapid diagnostics for tuberculosis detection 2024

Ye LW, 2024, MICROBIOL RES, V283, DOI 10.1016/j.micres.2024.127666

Zenteno-Cuevas R, 2009, MEM I OSWALDO CRUZ, V104, P468, DOI 10.1590/S0074-02762009000300012

Zenteno-Cuevas R, 2020, MICROB DRUG RESIST, V26, P732, DOI 10.1089/mdr.2019.0054

Zenteno-Cuevas R, 2015, INT J ANTIMICROB AG, V45, P671, DOI 10.1016/j.ijantimicag.2015.02.006

NR 65

TC 0

Z9 0

U1 1

U2 1

PU FRONTIERS MEDIA SA

PI LAUSANNE

PA AVENUE DU TRIBUNAL FEDERAL 34, LAUSANNE, CH-1015, SWITZERLAND

EI 1664-302X

J9 FRONT MICROBIOL

JI Front. Microbiol.

PD OCT 15

PY 2025

VL 16

AR 1666838

DI 10.3389/fmicb.2025.1666838

PG 12

WC Microbiology

WE Science Citation Index Expanded (SCI-EXPANDED)

SC Microbiology

GA 9CK2N

UT WOS:001603006700001

OA Green Submitted, gold

DA 2026-05-07

ER

PT J

AU Chen, B

Li, Y

Peng, Y

Zhou, L

Chen, XY

Ren, YL

Luo, D

Ling, YX

Zhou, YQ

Jiang, JM

Xu, B

AF Chen, Bin

Li, Yang

Peng, Ying

Zhou, Lin

Chen, Xin-yi

Ren, Yan-li

Luo, Dan

Ling, Yu-xiao

Zhou, Yi-qing

Jiang, Jian-min

Xu, Biao

TI Care-seeking and treatment pathways of multidrug-resistant tuberculosis

patients: an analysis of real-world data from regional health

information system in Ningbo City in Eastern China

SO ANNALS OF MEDICINE

LA English

DT Article

DE Multidrug-resistant tuberculosis; patient pathway analysis; real-world;

care seeking; ningbo

ID SERVICES; DIAGNOSIS

AB ObjectivesThe patient pathway of multidrug-resistant tuberculosis (MDR-TB) in China is an essential but not well-studied area. This study aimed to understand the alignment between patient-initiated care-seeking demands for MDR-TB and the availability of diagnostic and treatment services in Ningbo, a city in eastern China, using patient pathway analysis (PPA).MethodsWe collected the diagnostic and treatment data of 240 patients with MDR-TB in Ningbo from 2015 to 2019. Using patient pathway analysis, we matched the medical data of patients from different medical institutions and mapped their care pathways to illustrate their access to medical services.ResultOur study indicated that the proportion of patients with MDR-TB who chose non-TB-designated medical institutions (55%) was higher than those who chose TB-designated medical institutions (45%) at their initial visit. An estimated 69% of patients with MDR-TB patients received initial TB screening services during their first visit. In this study, 47% of patients needed to visit 4-7 medical institutions to be diagnosed with MDR-TB. Overall, 80% (n = 192) of patients were diagnosed with MDR-TB within four visits, while 13% (n = 30), 4% (n = 10), and 3% (n = 8) of patients were not diagnosed at the fourth visit and remained at level 2, 1, and 0 medical institutions, respectively.ConclusionThe care-seeking pathway of patients with MDR-TB in Ningbo is complex. This indicates room for improvement in local diagnosis and referral services. There is a need to promote the deployment of MDR-TB screening, diagnosis, and treatment services at lower-level institutions.

C1 [Chen, Bin; Peng, Ying; Zhou, Lin; Chen, Xin-yi; Jiang, Jian-min] Zhejiang Prov Ctr Dis Control & Prevent, Dept TB Control & Prevent, Hangzhou 310051, Zhejiang, Peoples R China.

[Li, Yang] Hangzhou Normal Univ, Sch Publ Hlth, Hangzhou, Peoples R China.

[Ren, Yan-li] Songzi Ctr Dis Control & Prevent, Jingzhou, Peoples R China.

[Luo, Dan; Zhou, Yi-qing] Hangzhou Med Coll, Sch Publ Hlth, Hangzhou, Peoples R China.

[Ling, Yu-xiao] Ningbo Univ, Hlth Sci Ctr, Sch Publ Hlth, Ningbo, Peoples R China.

[Jiang, Jian-min] Zhejiang Prov Ctr Dis Control & Prevent, Key Lab Vaccine Prevent & Control Infect Dis Zheji, Hangzhou, Peoples R China.

[Xu, Biao] Fudan Univ, Sch Publ Hlth, Dept Epidemiol, Shanghai 200032, Peoples R China.

[Xu, Biao] Fudan Univ, Key Lab Hlth Technol Assessment, Natl Hlth Commiss Peoples Republ China, Shanghai, Peoples R China.

C3 Zhejiang Provincial Center for Disease Control & Prevention; Hangzhou

Normal University; Hangzhou Medical College; Ningbo University; Zhejiang

Provincial Center for Disease Control & Prevention; Fudan University;

Fudan University

RP Jiang, JM (corresponding author), Zhejiang Prov Ctr Dis Control & Prevent, Dept TB Control & Prevent, Hangzhou 310051, Zhejiang, Peoples R China.; Xu, B (corresponding author), Fudan Univ, Sch Publ Hlth, Dept Epidemiol, Shanghai 200032, Peoples R China.

EM jmjiang@cdc.zj.cn; bxu@shmu.edu.cn

RI ling, yuxiao/JEP-6872-2023

FU National-Zhejiang Health Commission Major ST Project [WKJ-ZJ-2118];

Zhejiang Provincial Medical and Health Project [2023KY645]; Health

Commission of Zhejiang Province [2023KY642]

FX This work was supported by the National-Zhejiang Health Commission Major

S&T Project (grant number WKJ-ZJ-2118), Zhejiang Provincial Medical and

Health Project(Grant Nos. 2023KY645) and the Health Commission of

Zhejiang Province [2023KY642].

CR Ahmad S, 2014, J INFECT PUBLIC HEAL, V7, P75, DOI 10.1016/j.jiph.2013.09.001

[Anonymous], 2018, The Global Tuberculosis Report

[Anonymous], 2021, Global tuberculosis report 2021

Che Y, 2024, FRONT CELL INFECT MI, V14, DOI 10.3389/fcimb.2024.1327477

Che Y, 2022, FRONT PUBLIC HEALTH, V10, DOI 10.3389/fpubh.2022.956171

Chin DP, 2017, J INFECT DIS, V216, pS675, DOI 10.1093/infdis/jix368

Fatima R, 2017, J INFECT DIS, V216, pS733, DOI 10.1093/infdis/jix380

Fekadu L, 2017, J INFECT DIS, V216, pS696, DOI 10.1093/infdis/jix378

Garfin C, 2017, J INFECT DIS, V216, pS740, DOI 10.1093/infdis/jix391

Ge EJ, 2016, INFECT DIS POVERTY, V5, DOI 10.1186/s40249-016-0104-2

Hanson C, 2017, J INFECT DIS, V216, pS686, DOI 10.1093/infdis/jix388

Hanson CL, 2017, J INFECT DIS, V216, pS679, DOI 10.1093/infdis/jix387

Ho J, 2017, B WORLD HEALTH ORGAN, V95, P584, DOI 10.2471/BLT.17.193375

Huddart S, 2016, LANCET GLOB HEALTH, V4, pE907, DOI 10.1016/S2214-109X(16)30248-0

Kendall EA, 2021, AM J RESP CRIT CARE, V203, P168, DOI 10.1164/rccm.202006-2394PP

KNCV Tuberculosis Foundation, 2018, Operational Guide Find and Treat All Missing Persons with TB

Ku CC, 2020, BMJ GLOB HEALTH, V5, DOI 10.1136/bmjgh-2019-002187

Lange C, 2019, LANCET, V394, P953, DOI 10.1016/S0140-6736(19)31882-3

Li Y, 2014, PLOS ONE, V9, DOI 10.1371/journal.pone.0088330

Liu K, 2023, FRONT PUBLIC HEALTH, V11, DOI 10.3389/fpubh.2023.1233637

[柳正卫 Liu Zhengwei], 2021, [中国防痨杂志, Chinese Journal of Antituberculosis], V43, P790

Loveday M, 2012, INT J TUBERC LUNG D, V16, P209, DOI 10.5588/ijtld.11.0401

Lyakurwa D, 2021, HUM RESOUR HEALTH, V19, DOI 10.1186/s12960-021-00600-4

Masini E, 2017, J INFECT DIS, V216, pS714, DOI 10.1093/infdis/jix381

Naidoo P, 2017, J INFECT DIS, V216, pS702, DOI 10.1093/infdis/jix335

Reid MJA, 2019, LANCET, V393, P1331, DOI 10.1016/S0140-6736(19)30024-8

Seung KJ, 2015, CSH PERSPECT MED, V5, DOI 10.1101/cshperspect.a017863

Subbaraman R, 2019, PLOS MED, V16, DOI 10.1371/journal.pmed.1002754

Surya A, 2017, J INFECT DIS, V216, pS724, DOI 10.1093/infdis/jix379

The Centre for Applied Spatial Epidemiology (CASE) of KIT Royal Tropical Institute, 2018, The MATCH Manual

Titahong CN, 2021, TROP MED INFECT DIS, V6, DOI 10.3390/tropicalmed6040171

Parums DV, 2021, MED SCI MONITOR, V27, DOI 10.12659/MSM.934292

Vesga JF, 2019, LANCET GLOB HEALTH, V7, pE585, DOI 10.1016/S2214-109X(19)30037-3

Wang Y, 2019, PATIENT PREFER ADHER, V13, P1641, DOI 10.2147/PPA.S219920

Wells WA, 2017, INT J TUBERC LUNG D, V21, P1101, DOI 10.5588/ijtld.17.0271

Zhang L, 2021, CLIN MICROBIOL INFEC, V27, P1000, DOI 10.1016/j.cmi.2020.12.031

Zhao YL, 2012, NEW ENGL J MED, V366, P2161, DOI 10.1056/NEJMoa1108789

NR 37

TC 1

Z9 1

U1 5

U2 16

PU TAYLOR & FRANCIS LTD

PI ABINGDON

PA 2-4 PARK SQUARE, MILTON PARK, ABINGDON OR14 4RN, OXON, ENGLAND

SN 0785-3890

EI 1365-2060

J9 ANN MED

JI Ann. Med.

PD DEC 31

PY 2025

VL 57

IS 1

AR 2496405

DI 10.1080/07853890.2025.2496405

PG 12

WC Medicine, General & Internal

WE Science Citation Index Expanded (SCI-EXPANDED)

SC General & Internal Medicine

GA 1SM3N

UT WOS:001472595400001

OA Green Submitted, Green Accepted, gold

DA 2026-05-07

ER

PT J

AU Makarov, V

Manina, G

Mikusova, K

Möllmann, U

Ryabova, O

Saint-Joanis, B

Dhar, N

Pasca, MR

Buroni, S

Lucarelli, AP

Milano, A

De Rossi, E

Belanova, M

Bobovska, A

Dianiskova, P

Kordulakova, J

Sala, C

Fullam, E

Schneider, P

McKinney, JD

Brodin, P

Christophe, T

Waddell, S

Butcher, P

Albrethsen, J

Rosenkrands, I

Brosch, R

Nandi, V

Bharath, S

Gaonkar, S

Shandil, RK

Balasubramanian, V

Balganesh, T

Tyagi, S

Grosset, J

Riccardi, G

Cole, ST

AF Makarov, Vadim

Manina, Giulia

Mikusova, Katarina

Moellmann, Ute

Ryabova, Olga

Saint-Joanis, Brigitte

Dhar, Neeraj

Pasca, Maria Rosalia

Buroni, Silvia

Lucarelli, Anna Paola

Milano, Anna

De Rossi, Edda

Belanova, Martina

Bobovska, Adela

Dianiskova, Petronela

Kordulakova, Jana

Sala, Claudia

Fullam, Elizabeth

Schneider, Patricia

McKinney, John D.

Brodin, Priscille

Christophe, Thierry

Waddell, Simon

Butcher, Philip

Albrethsen, Jakob

Rosenkrands, Ida

Brosch, Roland

Nandi, Vrinda

Bharath, Sowmya

Gaonkar, Sheshagiri

Shandil, Radha K.

Balasubramanian, Venkataraman

Balganesh, Tanjore

Tyagi, Sandeep

Grosset, Jacques

Riccardi, Giovanna

Cole, Stewart T.

TI Benzothiazinones Kill Mycobacterium tuberculosis by Blocking

Arabinan Synthesis

SO SCIENCE

LA English

DT Article

ID CELL-WALL; IDENTIFICATION

AB New drugs are required to counter the tuberculosis (TB) pandemic. Here, we describe the synthesis and characterization of 1,3-benzothiazin-4-ones (BTZs), a new class of antimycobacterial agents that kill Mycobacterium tuberculosis in vitro, ex vivo, and in mouse models of TB. Using genetics and biochemistry, we identified the enzyme decaprenylphosphoryl-beta-D-ribose 2'-epimerase as a major BTZ target. Inhibition of this enzymatic activity abolishes the formation of decaprenylphosphoryl arabinose, a key precursor that is required for the synthesis of the cell-wall arabinans, thus provoking cell lysis and bacterial death. The most advanced compound, BTZ043, is a candidate for inclusion in combination therapies for both drug-sensitive and extensively drug-resistant TB.

C1 [Makarov, Vadim; Ryabova, Olga] Russian Acad Sci, AN Bakh Biochem Inst, Moscow 119071, Russia.

[Manina, Giulia; Pasca, Maria Rosalia; Buroni, Silvia; Lucarelli, Anna Paola; Milano, Anna; De Rossi, Edda; Riccardi, Giovanna] Univ Pavia, Dipartimento Genet & Microbiol, I-27100 Pavia, Italy.

[Mikusova, Katarina; Belanova, Martina; Bobovska, Adela; Dianiskova, Petronela; Kordulakova, Jana] Comenius Univ, Fac Nat Sci, Dept Biochem, Bratislava 84215, Slovakia.

[Moellmann, Ute] Hans Knoell Inst, Leibniz Inst Nat Prod Res & Infect Biol, Dept Mol & Appl Microbiol, D-07745 Jena, Germany.

[Saint-Joanis, Brigitte; Brosch, Roland] Inst Pasteur, F-75724 Paris 15, France.

[Dhar, Neeraj; Sala, Claudia; Fullam, Elizabeth; Schneider, Patricia; McKinney, John D.; Cole, Stewart T.] Ecole Polytech Fed Lausanne, Global Hlth Inst, CH-1015 Lausanne, Switzerland.

[Brodin, Priscille; Christophe, Thierry] Inst Pasteur Korea, Inserm Avenir Grp, Seoul 136791, South Korea.

[Waddell, Simon; Butcher, Philip] Univ London St Georges Hosp, Div Cellular & Mol Med, London SE17 ORE, England.

[Albrethsen, Jakob; Rosenkrands, Ida] Statens Serum Inst, Dept Infect Dis Immunol, DK-2300 Copenhagen S, Denmark.

[Nandi, Vrinda; Bharath, Sowmya; Gaonkar, Sheshagiri; Shandil, Radha K.; Balasubramanian, Venkataraman; Balganesh, Tanjore] AstraZeneca India, Bangalore, Karnataka, India.

[Tyagi, Sandeep; Grosset, Jacques] Johns Hopkins Univ, Sch Med, Ctr TB Res, Baltimore, MD 21231 USA.

C3 Research Center of Biotechnology RAS; Russian Academy of Sciences;

University of Pavia; Comenius University Bratislava; Leibniz

Association; Hans Knoll Institute (HKI); Pasteur Network; Universite

Paris Cite; Institut Pasteur Paris; Swiss Federal Institutes of

Technology Domain; Ecole Polytechnique Federale de Lausanne; Institut

National de la Sante et de la Recherche Medicale (Inserm); Institut

Pasteur Korea; City St Georges, University of London; Statens Serum

Institut; AstraZeneca; Johns Hopkins University

EM stewart.cole@epfl.ch

RI Sala, Claudia/R-6037-2017; MANINA, GIULIA/AAI-5496-2020; Riabova,

Olga/K-6030-2015; Waddell, Simon/A-9170-2013; Buroni,

Silvia/AAX-5454-2020; Brodin, Priscille/E-5802-2014; PASCA, Maria

Rosalia/J-8752-2016; Mikusova, Katarina/I-3622-2014; Korduláková,

Jana/I-3887-2014; RICCARDI, Giovanna/A-9269-2012; Rosenkrands,

Ida/Q-4392-2017; Dhar, Neeraj/K-8171-2013; Makarov, Vadim/HGB-7756-2022;

Brosch, Roland/AAZ-3850-2021

OI Sala, Claudia/0000-0003-4031-4648; MANINA, GIULIA/0000-0001-9905-6095;

Riabova, Olga/0000-0002-3794-6495; Waddell, Simon/0000-0002-3684-9116;

Buroni, Silvia/0000-0002-6979-2275; Brodin,

Priscille/0000-0003-0991-7344; PASCA, Maria Rosalia/0000-0002-8906-4937;

Mikusova, Katarina/0000-0002-0100-4877; Albrethsen,

Jakob/0000-0002-2842-1128; Butcher, Philip/0000-0003-1223-9097;

Korduláková, Jana/0000-0003-2834-0165; RICCARDI,

Giovanna/0000-0003-3295-0070; McKinney, John/0000-0002-0557-3479;

Rosenkrands, Ida/0000-0003-0085-7883; Dhar, Neeraj/0000-0002-5887-8137;

Makarov, Vadim/0000-0001-8746-2694; Brosch, Roland/0000-0003-2587-3863

FU European Commission [LHSP-CT-2005-018923]; Wellcome Trust [062511]

FX We thank A. Deshpande, I. Heinemann, P. Hojrup, K. Johnsson, M. K. N.

Kumar, N. Kumar, L. Pagani, P. Marone, M. R. McNeil, I. Old, J. Reddy,

S. Schmitt, P. Vachaspati, and C. Weigel for their help and support.

Patents related to this work have been filed (WO/2007/134625,

WO/2009/010163, and PCT/EP2008/001088). The NM4TB Consortium is funded

by the European Commission (LHSP-CT-2005-018923), and microarray work at

St Georges Hospital, University of London, is supported by the Wellcome

Trust (grant 062511). Microarray data are Minimum Information About a

Microarray Experiment (MIAME) compliant and deposited under accession

number E-BUGS-80 at http://bugs.sgul.ac.uk/E-BUGS-80.

CR Abraham VC, 2004, TRENDS BIOTECHNOL, V22, P15, DOI 10.1016/j.tibtech.2003.10.012

Alderwick LJ, 2005, J BIOL CHEM, V280, P32362, DOI 10.1074/jbc.M506339200

Andries K, 2005, SCIENCE, V307, P223, DOI 10.1126/science.1106753

Balaban NQ, 2004, SCIENCE, V305, P1622, DOI 10.1126/science.1099390

Balganesh TS, 2008, TRENDS PHARMACOL SCI, V29, P576, DOI 10.1016/j.tips.2008.08.001

Barry C, 2001, TUBERCULOSIS, V81, P1, DOI 10.1054/tube.2001.0288

Barry Clifton E., 2007, Infectious Disorders - Drug Targets, V7, P182

Dye C, 2006, LANCET, V367, P938, DOI 10.1016/S0140-6736(06)68384-0

Fenistein D, 2008, CYTOM PART A, V73A, P958, DOI 10.1002/cyto.a.20627

Gandhi NR, 2006, LANCET, V368, P1575, DOI 10.1016/S0140-6736(06)69573-1

MAKAROV V, 2007, Patent No. 2029583

Manjunatha UH, 2006, P NATL ACAD SCI USA, V103, P431, DOI 10.1073/pnas.0508392103

MARKAROV V, 2006, J ANTIMICROB CHEMOTH, V57, P1134

Matsumoto M, 2006, PLOS MED, V3, P2131, DOI 10.1371/journal.pmed.0030466

Mikusová K, 2005, J BACTERIOL, V187, P8020, DOI 10.1128/JB.187.23.8020-8025.2005

MIKUSOVA K, 1995, ANTIMICROB AGENTS CH, V39, P2484, DOI 10.1128/AAC.39.11.2484

Sassetti CM, 2001, P NATL ACAD SCI USA, V98, P12712, DOI 10.1073/pnas.231275498

Singh R, 2008, SCIENCE, V322, P1392, DOI 10.1126/science.1164571

Stover CK, 2000, NATURE, V405, P962, DOI 10.1038/35016103

Waddell SJ, 2007, CURR MOL MED, V7, P287, DOI 10.2174/156652407780598548

Wolucka BA, 2008, FEBS J, V275, P2691, DOI 10.1111/j.1742-4658.2008.06395.x

Young DB, 2008, J CLIN INVEST, V118, P1255, DOI 10.1172/JCI34614

Zhang Y, 2005, TUBERCULOSIS AND THE TUBERCLE BACILLUS, P115

Zignol M, 2006, J INFECT DIS, V194, P479, DOI 10.1086/505877

NR 24

TC 660

Z9 760

U1 3

U2 123

PU AMER ASSOC ADVANCEMENT SCIENCE

PI WASHINGTON

PA 1200 NEW YORK AVE, NW, WASHINGTON, DC 20005 USA

SN 0036-8075

EI 1095-9203

J9 SCIENCE

JI Science

PD MAY 8

PY 2009

VL 324

IS 5928

BP 801

EP 804

DI 10.1126/science.1171583

PG 4

WC Multidisciplinary Sciences

WE Science Citation Index Expanded (SCI-EXPANDED)

SC Science & Technology - Other Topics

GA 442HN

UT WOS:000265832400051

OA Green Submitted

DA 2026-05-07

ER

PT J

AU Rustomjee, R

Zumla, A

AF Rustomjee, Roxana

Zumla, Alimuddin

TI Delamanid expanded access novel treatment of drug resistant tuberculosis

SO INFECTION AND DRUG RESISTANCE

LA English

DT Review

DE delamanid; tuberculosis; drug resistance; MDR-TB; expanded access

ID PULMONARY TUBERCULOSIS; OPC-67683; MANAGEMENT; TRIALS

AB Tuberculosis (TB) remains a global emergency and is one of the most common infectious disease causes of death in developing countries. Current treatment regimens for multi-drug resistant TB are associated with low treatment success rates, are toxic, and require long duration of treatment. The need for shorter and more effective treatment regimens is urgent. Delamanid (Deltyba, or formerly known as OPC-67683) is a new dihydro-imidazooxazole anti-TB drug active against resistant forms of pulmonary TB. Delamanid kills Mycobacterium tuberculosis by inhibiting the synthesis of mycolic acids required for cell wall synthesis. Whilst delamanid has been included in the WHO Model List of Essential Medicine by the World Health Organization Expert Committee on Selection and Use of Essential Medicines and in international guidance for the treatment of multi-drug resistant TB since April 2014, its access in countries with the greatest need, has proven challenging. This review provides an update on currently available clinical safety and efficacy data on delamanid and offers a discussion on research priorities and recommendations for expedited, expanded access.

C1 [Rustomjee, Roxana] South African Med Res Council, ZA-7505 Cape Town, South Africa.

[Zumla, Alimuddin] UCL, Div Infect & Immun, London, England.

[Zumla, Alimuddin] Univ Coll Hosp NHS Fdn Trust, NIHR Biomed Res Ctr, London, England.

C3 South African Medical Research Council; University of London; University

College London; University of London; University College London

RP Rustomjee, R (corresponding author), South African Med Res Council, Francie van Zijl Dr, ZA-7505 Cape Town, South Africa.

EM roxana.rustomjee@mrc.ac.za

RI Zumla, Sir Alimuddin/ABA-8208-2020

OI Zumla, Sir Alimuddin/0000-0002-5111-5735

CR Ahuja SD, 2012, PLOS MED, V9, DOI 10.1371/journal.pmed.1001300

[Anonymous], 2014, WHO Global Tuberculosis Report 2014

[Anonymous], US DEL TREATM MULT R

[Anonymous], 2014, PHARM J, V293, P7830

Diacon AH, 2011, INT J TUBERC LUNG D, V15, P949, DOI 10.5588/ijtld.10.0616

Esposito S, 2014, EUR RESPIR J, V44, P811, DOI 10.1183/09031936.00060414

European Medicines Agency, 2013, EMACHMP7139092013

European Medicines Agency, 2014, SUMMARY PRODUCT CHAR

European Medicines Agency, 2014, DELT DEL AUTH DET

European Medicines Agency, 2014, EUR PUBL ASS REP DEL

Gler MT, 2012, NEW ENGL J MED, V366, P2151, DOI 10.1056/NEJMoa1112433

Guilloux A, 2014, HIDDEN PRICE TAGS DI

Gupta R, 2015, NEW ENGL J MED, V373, P291, DOI 10.1056/NEJMc1415332

Lewis JM, 2015, THER CLIN RISK MANAG, V11, P779, DOI 10.2147/TCRM.S71076

Ma ZK, 2010, LANCET, V375, P2100, DOI 10.1016/S0140-6736(10)60359-9

Matsumoto M, 2006, PLOS MED, V3, P2131, DOI 10.1371/journal.pmed.0030466

Nachman S, 2015, LANCET INFECT DIS, V15, P711, DOI 10.1016/S1473-3099(15)00007-9

NHS England Specialised Services Clinical Reference Groups for Infectious Diseases and Respiratory Medicine, 2014, CLIN COMM POL BED DE

Otsuka Pharmaceutical Development and Commercialization Inc., PHARM SAF TRIAL DET

Otsuka Pharmaceutical Development and Commercialization Inc., SAF EFF TRIAL DEL 6

Otsuka Pharmaceutical Development and Commercialization Inc., 6 MONTH SAF EFF PHAR

Paccaly A., 2012, 19 INT AIDS C JUL 22

Petersen C., 2012, 52 INT C ANT AG CHEM

Resist-tb, DR TB CLIN TRIAL PRO

Skripconoka V, 2013, EUR RESPIR J, V41, P1393, DOI 10.1183/09031936.00125812

Treatment Action Group, 2013, TREATM ACT GROUP APP

UNITAID, 2015, UNITAID JOINS HLTH P

WHO, GUID MED DON REV 201

World Health Organization, 2015, IN EXT ACC NEW TB DR

World Health Organization, SEL US ESS MED REP 2

World Health Organization, 2013, INT POL GUID US BED

Zhang Q, 2013, CELL BIOCHEM BIOPHYS, V67, P957, DOI 10.1007/s12013-013-9589-5

Zumla A, 2015, LANCET RESP MED, V3, P220, DOI 10.1016/S2213-2600(15)00063-6

NR 33

TC 17

Z9 22

U1 0

U2 10

PU DOVE MEDICAL PRESS LTD

PI ALBANY

PA PO BOX 300-008, ALBANY, AUCKLAND 0752, NEW ZEALAND

SN 1178-6973

J9 INFECT DRUG RESIST

JI Infect. Drug Resistance

PY 2015

VL 8

BP 359

EP 366

DI 10.2147/IDR.S62119

PG 8

WC Infectious Diseases; Pharmacology & Pharmacy

WE Science Citation Index Expanded (SCI-EXPANDED)

SC Infectious Diseases; Pharmacology & Pharmacy

GA CV2YA

UT WOS:000364123700001

OA Green Submitted, gold

DA 2026-05-07

ER

PT J

AU Jumat, MI

Sarmiento, ME

Acosta, A

Chin, KL

AF Jumat, Mohd Iskandar

Sarmiento, Maria E.

Acosta, Armando

Chin, Kai Ling

TI Role of non-coding RNAs in tuberculosis and their potential for clinical

applications

SO JOURNAL OF APPLIED MICROBIOLOGY

LA English

DT Review

DE tuberculosis; non-coding RNA; diagnosis; prognostic; therapeutic;

biomarkers

ID MYCOBACTERIUM-BOVIS BCG; IMMUNE-RESPONSES; PULMONARY TUBERCULOSIS;

TRANSCRIPTION FACTORS; IDENTIFICATION; EXPRESSION; MACROPHAGES;

PREDICTION; INFECTION; BIOMARKERS

AB Tuberculosis (TB), caused by Mycobacterium tuberculosis (Mtb), remains the leading cause of mortality due to infectious diseases, only surpassed in 2020 by COVID-19. Despite the development in diagnostics, therapeutics, and evaluation of new vaccines for TB, this infectious disease remains uncontrollable due to the emergence of multidrug-resistant (MDR) and extremely drug-resistant (XDR) TB, among other factors. The development in transcriptomics (RNomics) has enabled the study of gene expression in TB. It is considered that non-coding RNAs (ncRNAs) from host [microRNAs (miRNAs)] and Mtb [small RNAs (sRNAs)] are important elements in TB pathogenesis, immune resistance, and susceptibility. Many studies have shown the importance of host miRNAs in regulating immune response against Mtb via in vitro and in vivo mice models. The bacterial sRNAs play a major role in survival, adaptation, and virulence. Here, we review the characterization and function of host and bacteria ncRNAs in TB and their potential use in clinical applications as diagnostic, prognostic, and therapeutic biomarkers.

C1 [Jumat, Mohd Iskandar; Chin, Kai Ling] Univ Malaysia Sabah, Fac Med & Hlth Sci, Kota Kinabalu 88400, Sabah, Malaysia.

[Sarmiento, Maria E.; Acosta, Armando] Univ Sains Malaysia, Sch Hlth Sci, Kubang Kerian 16150, Kelantan, Malaysia.

C3 Universiti Malaysia Sabah; Universiti Sains Malaysia

RP Chin, KL (corresponding author), Univ Malaysia Sabah, Fac Med & Hlth Sci, Kota Kinabalu 88400, Sabah, Malaysia.; Sarmiento, ME; Acosta, A (corresponding author), Univ Sains Malaysia, Sch Hlth Sci, Kubang Kerian 16150, Kelantan, Malaysia.

EM mariesarmientogarcia@gmail.com; ducmar13@gmail.com; chinkl@ums.edu.my

RI ; Jumat, Mohd Iskandar/JVO-2786-2024; Sarmiento, Maria E/AAE-3620-2020

OI Acosta, Armando/0000-0001-5882-1360; Jumat, Mohd

Iskandar/0000-0001-5552-300X; Sarmiento, Maria E/0000-0002-6737-5961

FU Malaysia Ministry of Education [FRGS/1/2022/SKK12/UMS/01/2]; Universiti

Malaysia Sabah

FX Acknowledgement This research was funded by the Malaysia Ministry of

Education (grant no. FRGS/1/2022/SKK12/UMS/01/2) and Universiti Malaysia

Sabah (grant no. GUG0502-2/2020).

CR Abbas Q, 2016, GENES-BASEL, V7, DOI 10.3390/genes7120113

Afonso-Grunz F, 2015, CELL MOL LIFE SCI, V72, P3127, DOI 10.1007/s00018-015-1922-2

Agarwal V, 2015, ELIFE, V4, DOI 10.7554/eLife.05005

Akhtar MM, 2016, NUCLEIC ACIDS RES, V44, P24, DOI 10.1093/nar/gkv1221

Ami VKG, 2020, BMC GENOMICS, V21, DOI 10.1186/s12864-020-6573-5

An JY, 2013, NUCLEIC ACIDS RES, V41, P727, DOI 10.1093/nar/gks1187

[Anonymous], 2018, The Global Tuberculosis Report

Arnvig KB, 2011, PLOS PATHOG, V7, DOI 10.1371/journal.ppat.1002342

Arnvig KB, 2009, MOL MICROBIOL, V73, P397, DOI 10.1111/j.1365-2958.2009.06777.x

Badar Farheen, 2011, Oman Med J, V26, P210, DOI 10.5001/omj.2011.53

Bar A, 2021, FRONT MICROBIOL, V12, DOI 10.3389/fmicb.2021.635070

Benesova S, 2021, DIAGNOSTICS, V11, DOI 10.3390/diagnostics11060964

Borga C, 2019, NON-CODING RNA RES, V4, P80, DOI 10.1016/j.ncrna.2019.10.001

Busch A, 2008, BIOINFORMATICS, V24, P2849, DOI 10.1093/bioinformatics/btn544

Carpenter SM, 2022, FRONT IMMUNOL, V13, DOI 10.3389/fimmu.2022.830482

Chakaya J, 2022, INT J INFECT DIS, V124, pS26, DOI 10.1016/j.ijid.2022.03.011

Chandan K, 2020, FRONT IMMUNOL, V10, DOI 10.3389/fimmu.2019.03081

Chandra P, 2022, NAT REV MICROBIOL, V20, P750, DOI 10.1038/s41579-022-00763-4

Chen L, 2019, BRIEF BIOINFORM, V20, P1836, DOI 10.1093/bib/bby054

Chen X, 2008, CELL RES, V18, P997, DOI 10.1038/cr.2008.282

Chen XC, 2010, AM J RESP CRIT CARE, V181, P734, DOI 10.1164/rccm.200909-1463OC

Condrat CE, 2020, CELLS-BASEL, V9, DOI 10.3390/cells9020276

Cooper AM, 2008, IMMUNOL REV, V226, P191, DOI 10.1111/j.1600-065X.2008.00702.x

Cooper AM, 2009, ANNU REV IMMUNOL, V27, P393, DOI 10.1146/annurev.immunol.021908.132703

Cui JY, 2017, PLOS ONE, V12, DOI 10.1371/journal.pone.0184113

de Martino M, 2019, FRONT PEDIATR, V7, DOI 10.3389/fped.2019.00350

Dersch P, 2017, FRONT MICROBIOL, V8, DOI 10.3389/fmicb.2017.00803

DiChiara JM, 2010, NUCLEIC ACIDS RES, V38, P4067, DOI 10.1093/nar/gkq101

Du CS, 2009, NAT IMMUNOL, V10, P1252, DOI 10.1038/ni.1798

Eggenhofer F, 2011, NUCLEIC ACIDS RES, V39, pW149, DOI 10.1093/nar/gkr467

Corral-Fernández NE, 2017, TUBERCULOSIS, V105, P1, DOI 10.1016/j.tube.2017.03.007

Fay Elizabeth J., 2018, Non-Coding RNA, V4, P25, DOI [10.3390/ncrna4040025, 10.3390/ncrna4040025]

Fu YM, 2018, BIOCHEM BIOPH RES CO, V503, P490, DOI 10.1016/j.bbrc.2018.04.165

Fu YR, 2011, J CLIN MICROBIOL, V49, P4246, DOI 10.1128/JCM.05459-11

Gerrick ER, 2018, P NATL ACAD SCI USA, V115, P6464, DOI 10.1073/pnas.1718003115

Ghorpade DS, 2012, MOL CELL BIOL, V32, P2239, DOI 10.1128/MCB.06597-11

Girardin RC, 2020, MOL MICROBIOL, V113, P504, DOI 10.1111/mmi.14436

Glynn JR, 2006, EMERG INFECT DIS, V12, P736, DOI 10.3201/eid1205.050400

Gu X, 2017, EXP CELL RES, V354, P71, DOI 10.1016/j.yexcr.2017.03.039

Guo WR, 2010, PROTEIN CELL, V1, P419, DOI 10.1007/s13238-010-0056-4

He JH, 2021, FRONT ONCOL, V11, DOI 10.3389/fonc.2021.632834

Hodge J, 2020, ONCOIMMUNOLOGY, V9, DOI 10.1080/2162402X.2020.1724761

Huang HY, 2020, NUCLEIC ACIDS RES, V48, pD148, DOI 10.1093/nar/gkz896

Huang JC, 2007, NAT METHODS, V4, P1045, DOI 10.1038/nmeth1130

Jeeves RE, 2015, PLOS ONE, V10, DOI 10.1371/journal.pone.0138253

Jiang S, 2011, BLOOD, V118, P5487, DOI 10.1182/blood-2011-05-355644

Kaikkonen MU, 2011, CARDIOVASC RES, V90, P430, DOI 10.1093/cvr/cvr097

Kakati T, 2022, BMC BIOINFORMATICS, V23, DOI 10.1186/s12859-021-04527-4

Kallio MA, 2011, BMC GENOMICS, V12, DOI 10.1186/1471-2164-12-507

Kanniappan P, 2017, J CELL MOL MED, V21, P2276, DOI 10.1111/jcmm.13148

Khatun MS, 2022, CURR MED CHEM, V29, P865, DOI 10.2174/0929867328666210804090224

Kidd Parris, 2003, Altern Med Rev, V8, P223

Kim JK, 2015, J IMMUNOL, V194, P5355, DOI 10.4049/jimmunol.1402557

Kleinsteuber K, 2013, PLOS ONE, V8, DOI 10.1371/journal.pone.0061609

Korn T, 2009, ANNU REV IMMUNOL, V27, P485, DOI 10.1146/annurev.immunol.021908.132710

Kozomara A, 2019, NUCLEIC ACIDS RES, V47, pD155, DOI 10.1093/nar/gky1141

Kumar K, 2021, RNA BIOL, V18, P1152, DOI 10.1080/15476286.2020.1836455

Kumar M, 2015, CELL HOST MICROBE, V17, P345, DOI 10.1016/j.chom.2015.01.007

Kumar R, 2016, CELL MICROBIOL, V18, P679, DOI 10.1111/cmi.12540

Kumar R, 2012, CELL MICROBIOL, V14, P1620, DOI 10.1111/j.1462-5822.2012.01827.x

Lamichhane G, 2013, TUBERCULOSIS, V93, P26, DOI 10.1016/j.tube.2012.11.010

Latorre I, 2015, EUR RESPIR J, V45, P1173, DOI 10.1183/09031936.00221514

Law COK, 2019, MOL THER-NUCL ACIDS, V16, P218, DOI 10.1016/j.omtn.2019.02.011

Leitao AL, 2022, NON-CODING RNA, V8, DOI 10.3390/ncrna8010010

Lewis BP, 2003, CELL, V115, P787, DOI 10.1016/S0092-8674(03)01018-3

Li H, 2018, NAT REV IMMUNOL, V18, P591, DOI 10.1038/s41577-018-0028-0

Li HR, 2022, WIRES MECH DIS, V14, DOI 10.1002/wsbm.1573

Li S, 2013, PLOS ONE, V8, DOI 10.1371/journal.pone.0081438

Li ZX, 2011, J IMMUNOL, V186, P6182, DOI 10.4049/jimmunol.1000917

Liang SX, 2018, J IMMUNOL, V200, P3506, DOI 10.4049/jimmunol.1701448

Liu HY, 2016, CELL MOL BIOL, V62, P87

Long D, 2007, NAT STRUCT MOL BIOL, V14, P287, DOI 10.1038/nsmb1226

Lou J, 2017, EXP CELL RES, V358, P120, DOI 10.1016/j.yexcr.2017.06.007

Lu TX, 2011, J IMMUNOL, V187, P3362, DOI 10.4049/jimmunol.1101235

Lu TX, 2009, J IMMUNOL, V182, P4994, DOI 10.4049/jimmunol.0803560

Lu TP, 2012, PLOS ONE, V7, DOI 10.1371/journal.pone.0042390

Lukasik A, 2016, BIOINFORMATICS, V32, P2722, DOI 10.1093/bioinformatics/btw189

Lv Y, 2016, INT J INFECT DIS, V43, P68, DOI 10.1016/j.ijid.2015.12.014

Lyu MY, 2021, J CELL MOL MED, V25, P184, DOI 10.1111/jcmm.15903

Ma CY, 2014, MOL IMMUNOL, V62, P150, DOI 10.1016/j.molimm.2014.06.014

Ma F, 2011, NAT IMMUNOL, V12, P861, DOI 10.1038/ni.2073

Maertzdorf J, 2012, P NATL ACAD SCI USA, V109, P7853, DOI 10.1073/pnas.1121072109

Mahen EM, 2010, PLOS BIOL, V8, DOI 10.1371/journal.pbio.1000307

Mai JT, 2019, NUCLEIC ACIDS RES, V47, P4292, DOI 10.1093/nar/gkz149

Makeyev AV, 2002, RNA, V8, P265, DOI 10.1017/S1355838202024627

Mandin P, 2007, NUCLEIC ACIDS RES, V35, P962, DOI 10.1093/nar/gkl1096

Mourenza A, 2022, ANTIBIOTICS-BASEL, V11, DOI 10.3390/antibiotics11030356

Murugaiyan G, 2011, J IMMUNOL, V187, P2213, DOI 10.4049/jimmunol.1003952

Mycko MP, 2012, P NATL ACAD SCI USA, V109, pE1248, DOI 10.1073/pnas.1114325109

Nisar S, 2021, FRONT CELL DEV BIOL, V9, DOI 10.3389/fcell.2021.617281

Niu WY, 2018, CELL CYCLE, V17, P1931, DOI 10.1080/15384101.2018.1509636

O'Connell RM, 2010, IMMUNITY, V33, P607, DOI 10.1016/j.immuni.2010.09.009

Okamoto M, 2018, J BIOL CHEM, V293, P18585, DOI 10.1074/jbc.RA118.003862

Pánek J, 2008, BMC GENOMICS, V9, DOI 10.1186/1471-2164-9-217

Pelly S, 2012, GENE, V500, P85, DOI 10.1016/j.gene.2012.03.044

Peterson SM, 2014, FRONT GENET, V5, DOI 10.3389/fgene.2014.00023

Plotnikova O, 2019, FRONT GENET, V10, DOI 10.3389/fgene.2019.00933

Ponath F, 2022, FEMS MICROBIOL REV, V46, DOI 10.1093/femsre/fuac017

Qi YH, 2012, BMC INFECT DIS, V12, DOI 10.1186/1471-2334-12-384

Rajaram MVS, 2011, P NATL ACAD SCI USA, V108, P17408, DOI 10.1073/pnas.1112660108

Ren N, 2015, MOL MED REP, V12, P6561, DOI 10.3892/mmr.2015.4262

Riolo G, 2021, METHOD PROTOCOL, V4, DOI 10.3390/mps4010001

Roemhild R, 2022, NAT REV MICROBIOL, V20, P478, DOI 10.1038/s41579-022-00700-5

Salina EG, 2019, FRONT CELL INFECT MI, V9, DOI 10.3389/fcimb.2019.00405

Schulte C, 2020, TRENDS MOL MED, V26, P583, DOI 10.1016/j.molmed.2020.02.001

Singh NK, 2017, CELL MOL LIFE SCI, V74, P2239, DOI 10.1007/s00018-017-2469-1

Singh Y, 2013, J BIOL CHEM, V288, P5056, DOI 10.1074/jbc.C112.439778

Sittka A, 2008, PLOS GENET, V4, DOI 10.1371/journal.pgen.1000163

Smith KM, 2012, J IMMUNOL, V189, P1567, DOI 10.4049/jimmunol.1103171

Solans L, 2014, PLOS PATHOG, V10, DOI 10.1371/journal.ppat.1004183

Song QZ, 2015, INT J CLIN EXP MED, V8, P15458

Spinelli SV, 2013, MOL IMMUNOL, V53, P265, DOI 10.1016/j.molimm.2012.08.008

Steiner DF, 2011, IMMUNITY, V35, P169, DOI 10.1016/j.immuni.2011.07.009

Sun XG, 2002, NUCLEIC ACIDS RES, V30, P3662, DOI 10.1093/nar/gkf508

Tjaden B, 2008, NUCLEIC ACIDS RES, V36, pW109, DOI 10.1093/nar/gkn264

Tjaden B, 2006, NUCLEIC ACIDS RES, V34, P2791, DOI 10.1093/nar/gkl356

Tokar T, 2018, NUCLEIC ACIDS RES, V46, pD360, DOI 10.1093/nar/gkx1144

Voss B, 2009, BMC GENOMICS, V10, DOI 10.1186/1471-2164-10-123

Wagh V, 2017, TUBERCULOSIS, V102, P24, DOI 10.1016/j.tube.2016.10.007

Wang C, 2018, TUBERCULOSIS, V108, P26, DOI 10.1016/j.tube.2017.08.010

Wang C, 2011, PLOS ONE, V6, DOI 10.1371/journal.pone.0025832

Wang H, 2023, CANCERS, V15, DOI 10.3390/cancers15041007

Wang JL, 2013, PLOS PATHOG, V9, DOI 10.1371/journal.ppat.1003697

Wei L, 2021, FRONT IMMUNOL, V12, DOI 10.3389/fimmu.2021.664787

Wu ZW, 2012, FEBS LETT, V586, P2459, DOI 10.1016/j.febslet.2012.06.004

Xi XE, 2015, GENET TEST MOL BIOMA, V19, P650, DOI 10.1089/gtmb.2015.0090

Xu ZH, 2015, TUBERCULOSIS, V95, P395, DOI 10.1016/j.tube.2015.02.043

Yang TS, 2018, CANCER LETT, V431, P22, DOI 10.1016/j.canlet.2018.05.028

Yee PTI, 2016, CURR PHARM DESIGN, V22, P6694, DOI 10.2174/1381612822666160720165613

Yue D, 2009, CURR GENOMICS, V10, P478, DOI 10.2174/138920209789208219

Zare M, 2022, PHARMACEUTICS, V14, DOI 10.3390/pharmaceutics14081620

Zeuschner P, 2020, EXPERT REV MOL DIAGN, V20, P151, DOI 10.1080/14737159.2019.1665998

Zhai WJ, 2019, INT J MOL SCI, V20, DOI 10.3390/ijms20020340

Zhang Q, 2023, MOL CANCER, V22, DOI 10.1186/s12943-023-01749-3

Zhang XP, 2019, INT J MOL SCI, V20, DOI 10.3390/ijms20225573

Zhang Y, 2006, BIOCHEM BIOPH RES CO, V343, P950, DOI 10.1016/j.bbrc.2006.02.196

Zhang Y, 2017, BIOMED RES INT, V2017, DOI 10.1155/2017/9139504

Zhang ZM, 2017, EXP CELL RES, V352, P313, DOI 10.1016/j.yexcr.2017.02.025

Zheng L, 2015, PLOS ONE, V10, DOI 10.1371/journal.pone.0126018

Zheng ML, 2016, MICROB PATHOGENESIS, V100, P78, DOI 10.1016/j.micpath.2016.09.005

Zhou YL, 2013, BIOMATERIALS, V34, P8269, DOI 10.1016/j.biomaterials.2013.07.072

NR 141

TC 10

Z9 12

U1 0

U2 7

PU OXFORD UNIV PRESS

PI OXFORD

PA GREAT CLARENDON ST, OXFORD OX2 6DP, ENGLAND

SN 1364-5072

EI 1365-2672

J9 J APPL MICROBIOL

JI J. Appl. Microbiol.

PD JUN 1

PY 2023

VL 134

IS 6

AR lxad104

DI 10.1093/jambio/lxad104

EA JUN 2023

PG 14

WC Biotechnology & Applied Microbiology; Microbiology

WE Science Citation Index Expanded (SCI-EXPANDED)

SC Biotechnology & Applied Microbiology; Microbiology

GA I0ML7

UT WOS:000999801300002

OA Bronze

DA 2026-05-07

ER

PT J

AU Minakshi, P

Ghosh, M

Brar, B

Kumar, R

Lambe, UP

Ranjan, K

Manoj, J

Prasad, G

AF Minakshi, Prasad

Ghosh, Mayukh

Brar, Basanti

Kumar, Rajesh

Lambe, Upendra P.

Ranjan, Koushlesh

Manoj, Jinu

Prasad, Gaya

TI Nano-Antimicrobials: A New Paradigm for Combating Mycobacterial

Resistance

SO CURRENT PHARMACEUTICAL DESIGN

LA English

DT Review

DE Tuberculosis; nanoparticles; nano-carriers; nanoantibiotics;

nano-vaccine; microbial resistance

ID SOLID LIPID NANOPARTICLES; DRUG-DELIVERY-SYSTEM; ENCAPSULATED

ANTITUBERCULAR DRUGS; IN-VITRO EVALUATION; GUINEA-PIG MODEL;

MULTIDRUG-RESISTANT; AVIUM-COMPLEX; SILVER NANOPARTICLES; ALGINATE

NANOPARTICLES; INTRACELLULARE COMPLEX

AB Background: Mycobacterium group contains several pathogenic bacteria including M. tuberculosis where the emergence of multidrug-resistant tuberculosis (MDR-TB) and extensively drug-resistant tuberculosis (XDR-TB) is alarming for human and animal health around the world. The condition has further aggravated due to the speed of discovery of the newer drugs has been outpaced by the rate of resistance developed in microorganisms, thus requiring alternative combat strategies. For this purpose, nano-antimicrobials have emerged as a potential option.

Objective: The current review is focused on providing a detailed account of nanocarriers like liposome, micelles, dendrimers, solid lipid NPs, niosomes, polymeric nanoparticles, nano-suspensions, nano-emulsion, mesoporous silica and alginate-based drug delivery systems along with the recent updates on developments regarding nanoparticle-based therapeutics, vaccines and diagnostic methods developed or under pipeline with their potential benefits and limitations to combat mycobacterial diseases for their successful eradication from the world in future.

Results: Distinct morphology and the underlying mechanism of pathogenesis and resistance development in this group of organisms urge improved and novel methods for the early and efficient diagnosis, treatment and vaccination to eradicate the disease. Recent developments in nanotechnology have the potential to meet both the aspects: mno-materials are proven components of several efficient targeted drug delivery systems and the typical physicochemical properties of several nano-formulations have shown to possess distinct bacteriocidal properties. Along with the therapeutic aspects, nano-vaccines and theranostic applications of nano-formulations have grown in popularity in recent times as an effective alternative means to combat different microbial superbugs.

Conclusion: Nanomedicine holds a bright prospect to perform a key role in global tuberculosis elimination program.

C1 [Minakshi, Prasad; Brar, Basanti; Lambe, Upendra P.] LLR Univ Vet & Anim Sci, Dept Anim Biotechnol, Hisar 125004, Haryana, India.

[Ghosh, Mayukh] Birsa Agr Univ, Ranchi Vet Coll, Dept Vet Biochem, Ranchi 834006, Jharkhand, India.

[Kumar, Rajesh] KVASU, COVAS, Dept Vet Physiol, Wayanad 673576, Kerala, India.

[Ranjan, Koushlesh; Prasad, Gaya] SVP Univ Agr & Technol, Meerut, Uttar Pradesh, India.

[Manoj, Jinu] LUVAS, RVDEC Mahendergarh, Hisar, Haryana, India.

C3 Lala Lajpat Rai University of Veterinary & Animal Sciences; Birsa

Agricultural University; Sardar Vallabhbhai Patel University of

Agriculture & Technology; Lala Lajpat Rai University of Veterinary &

Animal Sciences

RP Minakshi, P (corresponding author), LLR Univ Vet & Anim Sci, Dept Anim Biotechnol, Hisar 125004, Haryana, India.

EM minakshi.abt@gmail.com

RI kumar, Rajesh/ABN-6491-2022; Prasad, Minakshi/ABD-7135-2020; Brar,

Basanti/JFL-0154-2023; Ranjan, Koushlesh/V-6683-2019; Manoj,

Jinu/AEW-6630-2022

OI kumar, Rajesh/0000-0003-4586-1923; Ranjan,

Koushlesh/0000-0003-3238-8057; Manoj, Jinu/0000-0002-9487-5275

CR Abdelghany S, 2017, J DRUG DELIV SCI TEC, V39, P442, DOI 10.1016/j.jddst.2017.04.034

Aboutaleb E, 2012, INT NANO LETT, V2, DOI 10.1186/2228-5326-2-33

AGARWAL A, 1994, ANTIMICROB AGENTS CH, V38, P588, DOI 10.1128/AAC.38.3.588

Ahmad Z, 2006, NANOSCIENCE, V1, P81

Ahmad Zahoor, 2006, Indian J Chest Dis Allied Sci, V48, P171

Ahmed M, 2008, PHARMAZIE, V63, P806, DOI 10.1691/ph.2008.8108

Alangaden GJ, 1998, ANTIMICROB AGENTS CH, V42, P1295, DOI 10.1128/AAC.42.5.1295

Ali HR, 2016, BIOCONJUGATE CHEM, V27, P2486, DOI 10.1021/acs.bioconjchem.6b00430

Da Silva PEA, 2011, J ANTIMICROB CHEMOTH, V66, P1417, DOI 10.1093/jac/dkr173

Andries K, 2014, PLOS ONE, V9, DOI 10.1371/journal.pone.0102135

[Anonymous], 2016, GLOB TUB REP

[Anonymous], 2000, ENDING NEGLECT ELIMI

[Anonymous], FRONTIERS IN BIOSCIE

[Anonymous], 2018, Tuberculosis

[Anonymous], 2015, PLOS NEGL TROP DIS, DOI DOI 10.1371/JOURNAL.PNTD.0004124

Arasteh K N, 2000, Eur J Med Res, V5, P424

Arranz-Trullén J, 2017, FRONT IMMUNOL, V8, DOI 10.3389/fimmu.2017.01499

Arunkumar N, 2009, ASIAN J PHARM, V3, P168, DOI 10.4103/0973-8398.56293

Baker JJ, 2014, MOL MICROBIOL, V94, P56, DOI 10.1111/mmi.12688

BANERJEE A, 1994, SCIENCE, V263, P227, DOI 10.1126/science.8284673

BANGHAM AD, 1965, J MOL BIOL, V13, P238, DOI 10.1016/S0022-2836(65)80093-6

Banu A, 2013, J NANOMED BIOTHERAPE, V3, P110, DOI [10.4172/2155-983X.1000110, DOI 10.4172/2155-983X.1000110]

Barry PM, 2012, EMERG INFECT DIS, V18, P113, DOI 10.3201/eid1801.110671

Bellaire B, 2013, US Patent, Patent No. [8449916 B1, 8449916]

Bellini RG, 2015, J MOL GRAPH MODEL, V60, P34, DOI 10.1016/j.jmgm.2015.05.012

Bermúdez JM, 2017, ANTIMICROBIAL NANOARCHITECTONICS: FROM SYNTHESIS TO APPLICATIONS, P1, DOI 10.1016/B978-0-323-52733-0.00001-X

Bhandari R, 2013, INT J PHARMACEUT, V441, P202, DOI 10.1016/j.ijpharm.2012.11.042

Bhat ZS, 2017, BIOMED PHARMACOTHER, V95, P1520, DOI 10.1016/j.biopha.2017.09.036

Bhave T, 2013, ADV NAT SCI-NANOSCI, V4, DOI 10.1088/2043-6262/4/4/045015

Biet F, 2005, VET RES, V36, P411, DOI 10.1051/vetres:2005001

Blanchard JS, 1996, ANNU REV BIOCHEM, V65, P215, DOI 10.1146/annurev.bi.65.070196.001243

Bloemberg GV, 2015, NEW ENGL J MED, V373, P1986, DOI 10.1056/NEJMc1505196

Bourdeaux F, 2018, ACS INFECT DIS, V4, P1082, DOI 10.1021/acsinfecdis.7b00237

Bouwstra JA, 1997, COLLOID SURFACE A, V123, P71, DOI 10.1016/S0927-7757(96)03800-9

Britton WJ, 2004, LANCET, V363, P1209, DOI 10.1016/S0140-6736(04)15952-7

Brossier F, 2011, ANTIMICROB AGENTS CH, V55, P355, DOI 10.1128/AAC.01030-10

Brown-Elliott BA, 2012, CLIN MICROBIOL REV, V25, P545, DOI 10.1128/CMR.05030-11

Caetano LA, 2016, MAR DRUGS, V14, DOI 10.3390/md14050090

Cardoso MJ, 2016, MAR DRUGS, V14, DOI 10.3390/md14020034

Caws M, 2006, J CLIN MICROBIOL, V44, P2333, DOI 10.1128/JCM.00330-06

Centers for Disease Control and Prevention (CDC), 2006, MMWR Morb Mortal Wkly Rep, V55, P301

Chaudhuri S, 2018, ELIFE, V7, DOI 10.7554/eLife.36782

Chellat F, 2005, BIOMATERIALS, V26, P7260, DOI 10.1016/j.biomaterials.2005.05.044

Chen CZS, 2002, BIOMATERIALS, V23, P3359, DOI 10.1016/S0142-9612(02)00036-4

Chen L, 2007, J NANOPART RES, V9, P777, DOI 10.1007/s11051-006-9103-8

Chiappetta DA, 2007, EUR J PHARM BIOPHARM, V66, P303, DOI 10.1016/j.ejpb.2007.03.022

Choi SR, 2017, PLOS ONE, V12, DOI 10.1371/journal.pone.0177987

Choi SR, 2017, ANTIMICROB AGENTS CH, V61, DOI [10.1128/aac.02505-16, 10.1128/AAC.02505-16]

Clemens DL, 2012, ANTIMICROB AGENTS CH, V56, P2535, DOI 10.1128/AAC.06049-11

Collins LF, 2017, OPEN FORUM INFECT DI, V4, DOI 10.1093/ofid/ofx120

Cooper AM, 2009, ANNU REV IMMUNOL, V27, P393, DOI 10.1146/annurev.immunol.021908.132703

Cosma CL, 2003, ANNU REV MICROBIOL, V57, P641, DOI 10.1146/annurev.micro.57.030502.091033

Costa-Gouveia J, 2017, SCI REP-UK, V7, DOI 10.1038/s41598-017-05453-3

Cox H, 2012, INT J TUBERC LUNG D, V16, P447, DOI 10.5588/ijtld.11.0451

Crosa JH, 2002, MICROBIOL MOL BIOL R, V66, P223, DOI 10.1128/MMBR.66.2.223-249.2002

Crucho CIC, 2017, MAT SCI ENG C-MATER, V80, P771, DOI 10.1016/j.msec.2017.06.004

Dahle UR, 2003, J CLIN MICROBIOL, V41, P67, DOI 10.1128/JCM.41.1.67-72.2003

Dannenberg AM, 2006, PATHOGENESIS OF HUMAN PULMONARY TUBERCULOSIS: INSIGHTS FROM THE RABBIT MODEL, P1

Das I, 2017, NANOTECHNOLOGY, V28, DOI 10.1088/1361-6528/aa60fd

Davies GR, 2010, TUBERCULOSIS, V90, P171, DOI 10.1016/j.tube.2010.03.007

Davis ME, 2008, NAT REV DRUG DISCOV, V7, P771, DOI 10.1038/nrd2614

de Faria TJ, 2012, ANTIMICROB AGENTS CH, V56, P2259, DOI 10.1128/AAC.05993-11

Deb C, 2009, PLOS ONE, V4, DOI 10.1371/journal.pone.0006077

DeBarber AE, 2000, P NATL ACAD SCI USA, V97, P9677, DOI 10.1073/pnas.97.17.9677

Delahay RJ, 2002, VET J, V164, P90, DOI 10.1053/tvjl.2001.0667

Deol P, 1997, ANTIMICROB AGENTS CH, V41, P1211, DOI 10.1128/AAC.41.6.1211

Dhar K, 2016, Ann Trop Med Public, V9, P194, DOI 10.4103/1755-6783.179100

Dhillon J, 2001, J ANTIMICROB CHEMOTH, V48, P869, DOI 10.1093/jac/48.6.869

Diacon AH, 2012, LANCET, V380, P986, DOI 10.1016/S0140-6736(12)61080-0

Diekema DJ, 2001, LANCET, V358, P1975, DOI 10.1016/S0140-6736(01)06964-1

Doktorovova S, 2014, EUR J PHARM BIOPHARM, V87, P1, DOI 10.1016/j.ejpb.2014.02.005

Dolatabadi JEN, 2015, ADV PHARM BULL, V5, P151, DOI 10.15171/apb.2015.022

Dookie N, 2018, J ANTIMICROB CHEMOTH, V73, P1138, DOI 10.1093/jac/dkx506

DOUCETPOPULAIRE F, 1995, J ANTIMICROB CHEMOTH, V36, P129, DOI 10.1093/jac/36.1.129

Dutt M, 2001, INT J ANTIMICROB AG, V17, P115, DOI 10.1016/S0924-8579(00)00330-7

Ehlers S, 1996, ZBL BAKT-INT J MED M, V284, P218

El-Ridy MS, 2015, DRUG DELIV, V22, P21, DOI 10.3109/10717544.2013.868556

El-Ridy MS, 2011, DRUG DEV IND PHARM, V37, P1110, DOI 10.3109/03639045.2011.560605

Eldholm V, 2015, NAT COMMUN, V6, DOI 10.1038/ncomms8119

Ellis T, 2018, ACS NANO, V12, P5228, DOI 10.1021/acsnano.7b08264

Fàbrega A, 2009, MICROB BIOTECHNOL, V2, P40, DOI 10.1111/j.1751-7915.2008.00063.x

Feng GZ, 2013, PLOS ONE, V8, DOI 10.1371/journal.pone.0061135

Ferreiro MG, 2002, INT J PHARMACEUT, V239, P47, DOI 10.1016/S0378-5173(02)00030-3

Feuerriegel S, 2010, ANTIMICROB AGENTS CH, V54, P4794, DOI 10.1128/AAC.00738-10

Field SK, 2004, CHEST, V126, P566, DOI 10.1378/chest.126.2.566

Fonseca JD, 2015, INT J INFECT DIS, V32, P94, DOI 10.1016/j.ijid.2015.01.014

Gabor F, 2004, ADV DRUG DELIVER REV, V56, P459, DOI 10.1016/j.addr.2003.10.015

Gajendiran M, 2016, MATER RES EXPRESS, V3, DOI 10.1088/2053-1591/3/6/065401

Gaspar DP, 2017, MOL PHARMACEUT, V14, P2977, DOI 10.1021/acs.molpharmaceut.7b00169

Gaspar MM, 2008, CURR TOP MED CHEM, V8, P579, DOI 10.2174/156802608783955629

Gaspar MM, 2008, INT J ANTIMICROB AG, V31, P37, DOI 10.1016/j.ijantimicag.2007.08.008

Ghiasi Z., 2007, Iran. J. Basic Med. Sci, V10, P90

Giongo J.L., 2015, Int. J. Pharm. Pharm. Sci, V7, P414

Giri S, 2007, NANOMEDICINE-UK, V2, P99, DOI 10.2217/17435889.2.1.99

Gliddon HD, 2016, NANOSCALE, V8, P10087, DOI 10.1039/c6nr00484a

GREGORIADIS G, 1972, EUR J BIOCHEM, V24, P485, DOI 10.1111/j.1432-1033.1972.tb19710.x

Grewal TK, 2018, INDIAN J MED RES, V147, P594, DOI [10.4103/ijmr.ijmr_2004_15, 10.4103/ijmr.IJMR_2004_15]

Griffith DE, 2007, AM J RESP CRIT CARE, V175, P367, DOI 10.1164/rccm.200604-571ST

Griffith DE, 2018, SEMIN RESP CRIT CARE, V39, P351, DOI 10.1055/s-0038-1660472

Griffith DE, 2016, ANN AM THORAC SOC, V13, P1881, DOI 10.1513/AnnalsATS.201609-666ED

Grosset JH, 2012, INT J TUBERC LUNG D, V16, P1005, DOI 10.5588/ijtld.12.0277

Hadad David Jamil, 1995, Revista do Instituto de Medicina Tropical de Sao Paulo, V37, P375, DOI 10.1590/S0036-46651995000500001

Hakkimane SS, 2018, INT J NANOMED, V13, P4303, DOI 10.2147/IJN.S163925

HANDJANIVILA RM, 1979, INT J COSMETIC SCI, V1, P303, DOI 10.1111/j.1467-2494.1979.tb00224.x

Hayashi M, 2012, AM J RESP CRIT CARE, V185, P575, DOI 10.1164/rccm.201107-1203OC

He XC, 2017, BMC INFECT DIS, V17, DOI 10.1186/s12879-017-2652-x

Henson D, 2014, CHEMOTHERAPY, V67, P1163

Hirlekar R, 2011, CURR DRUG THER, V6, P240, DOI 10.2174/157488511798109637

Ho YM, 2009, ANTIMICROB AGENTS CH, V53, P4010, DOI 10.1128/AAC.00433-09

Holladay RJ, 2006, US patent, Patent No. [72006;135:195, 72006135195]

Holladay RJ, 2004, US patent, Patent No. [62004;743:348, 62004743348]

Huang XH, 2007, NANOMEDICINE-UK, V2, P681, DOI 10.2217/17435889.2.5.681

Huang XL, 2010, BIOMATERIALS, V31, P438, DOI 10.1016/j.biomaterials.2009.09.060

Huh AJ, 2011, J CONTROL RELEASE, V156, P128, DOI 10.1016/j.jconrel.2011.07.002

Huitric E, 2010, ANTIMICROB AGENTS CH, V54, P1022, DOI 10.1128/AAC.01611-09

Ibrahim M, 2007, ANTIMICROB AGENTS CH, V51, P1011, DOI 10.1128/AAC.00898-06

ILLUM L, 1994, PHARMACEUT RES, V11, P1186, DOI 10.1023/A:1018901302450

Guerrero MI, 2014, BIOMEDICA, V34, P137, DOI [10.7705/biomedica.v34i0.1686, 10.1590/S0120-41572014000500016]

Islan GA, 2017, ACTA TROP, V170, P16, DOI 10.1016/j.actatropica.2017.02.019

Islan GA, 2016, COLLOID SURFACE B, V143, P168, DOI 10.1016/j.colsurfb.2016.03.040

Jafari A, 2018, J BASIC MICROB, V58, P41, DOI 10.1002/jobm.201700289

Jagielski T, 2014, PLOS ONE, V9, DOI 10.1371/journal.pone.0100078

Jain C. P., 2006, Indian Journal of Pharmaceutical Sciences, V68, P575

Jansson M, 2014, J CLIN MICROBIOL, V52, P1246, DOI 10.1128/JCM.03119-13

JARLIER V, 1994, FEMS MICROBIOL LETT, V123, P11, DOI 10.1111/j.1574-6968.1994.tb07194.x

Jayasingam SD, 2017, INT J MYCOBACT, V6, P387, DOI 10.4103/ijmy.ijmy_152_17

Jiang Ge, 2006, Yaoxue Xuebao, V41, P439

Johnson CM, 2005, ANTIMICROB AGENTS CH, V49, P4335, DOI 10.1128/AAC.49.10.4335-4338.2005

Justo OR, 2003, DRUG DELIV, V10, P201, DOI 10.1080/713840401

Kai M, 2011, CLIN INFECT DIS, V52, pE127, DOI 10.1093/cid/ciq217

KAILASAM S, 1994, TUBERCLE LUNG DIS, V75, P361, DOI 10.1016/0962-8479(94)90082-5

Karakousis PC, 2004, LANCET INFECT DIS, V4, P557, DOI 10.1016/S1473-3099(04)01130-2

Karki R., 2008, Rasayan J Chem, V1, P224

Kawatsu L, 2018, BMC INFECT DIS, V18, DOI 10.1186/s12879-018-3353-9

Khademi F, 2018, IRAN J BASIC MED SCI, V21, P116, DOI 10.22038/IJBMS.2017.22059.5648

Kim EJ, 2017, BIOSENS BIOELECTRON, V87, P150, DOI 10.1016/j.bios.2016.08.034

Kim HR, 2007, CLIN INFECT DIS, V45, P1290, DOI 10.1086/522537

Kiran D, 2016, SEMIN IMMUNOPATHOL, V38, P167, DOI 10.1007/s00281-015-0537-x

Kisich KO, 2007, INT J PHARM, V345, P154, DOI 10.1016/j.ijpharm.2007.05.062

KLEMENS SP, 1990, ANTIMICROB AGENTS CH, V34, P967, DOI 10.1128/AAC.34.6.967

Knight GM, 2018, J R SOC INTERFACE, V15, DOI 10.1098/rsif.2018.0025

Kobashi Y, 2007, RESP MED, V101, P130, DOI 10.1016/j.rmed.2006.04.002

Koh WJ, 2012, CHEST, V142, P1482, DOI 10.1378/chest.12-0494

Kulshreshtha NM, 2017, ANTIMICROBIAL NANOARCHITECTONICS: FROM SYNTHESIS TO APPLICATIONS, P29, DOI 10.1016/B978-0-323-52733-0.00002-1

Kumar GP, 2011, ACTA PHARM SIN B, V1, P208, DOI 10.1016/j.apsb.2011.09.002

Kumar N, 2017, RE VIEW, V19, P1

Kumar N., 2018, Egyptian Journal of Basic and Applied Sciences, V5, P87, DOI [10.1016/j.ejbas.2017.11.002, 10.1016/j.ejbas.2017.11.002, DOI 10.1016/J.EJBAS.2017.11.002]

Kumar P. Vijayaraj, 2007, Current Drug Delivery, V4, P11

Kumar PV, 2006, J DRUG TARGET, V14, P546, DOI 10.1080/10611860600825159

Kwok PCL, 2015, INT J PHARMACEUT, V491, P367, DOI 10.1016/j.ijpharm.2015.07.001

LADIGINA GA, 1986, BIOMED PHARMACOTHER, V40, P416

Lam PK, 2006, AM J RESP CRIT CARE, V173, P1283, DOI 10.1164/rccm.200509-1531OC

Lambe U, 2016, J EXP BIOL AGRIC SCI, V4, P307, DOI 10.18006/2016.4(3S).307.320

Larimer C, 2014, BIOMETALS, V27, P695, DOI 10.1007/s10534-014-9761-4

Lee JW, 2017, APPL ENVIRON MICROB, V83, DOI 10.1128/AEM.02289-16

Lee M, 2012, NEW ENGL J MED, V367, P1508, DOI 10.1056/NEJMoa1201964

Leidinger P, 2015, ANGEW CHEM INT EDIT, V54, P12597, DOI 10.1002/anie.201505493

Lemmer Y, 2015, J CONTROL RELEASE, V211, P94, DOI 10.1016/j.jconrel.2015.06.005

Lenaerts AJ, 2007, ANTIMICROB AGENTS CH, V51, P3338, DOI 10.1128/AAC.00276-07

Li B, 2017, FRONT MICROBIOL, V8, DOI 10.3389/fmicb.2017.01739

Li XN, 2014, ACS NANO, V8, P10682, DOI 10.1021/nn5042625

Li XY, 2008, BMC BIOTECHNOL, V8, DOI 10.1186/1472-6750-8-89

Lienhardt C, 2010, CURR OPIN PULM MED, V16, P186, DOI 10.1097/MCP.0b013e328337580c

Lin YSE, 1998, WATER RES, V32, P1997, DOI 10.1016/S0043-1354(97)00460-0

Lin YH, 2009, BIOMATERIALS, V30, P3332, DOI 10.1016/j.biomaterials.2009.02.036

Litvinov V, 2018, PLOS ONE, V13, DOI 10.1371/journal.pone.0203108

Lockwood DNJ, 2005, B WORLD HEALTH ORGAN, V83, P230

Lopes M, 2017, EXPERT OPIN DRUG DEL, V14, P769, DOI 10.1080/17425247.2016.1214564

Lounis N, 2006, ANTIMICROB AGENTS CH, V50, P3543, DOI 10.1128/AAC.00766-06

Maeda S, 2001, ANTIMICROB AGENTS CH, V45, P3635, DOI 10.1128/AAC.45.12.3635-3639.2001

Mahla RS, 2018, LANCET INFECT DIS, V18, P836, DOI 10.1016/S1473-3099(18)30401-8

Majoral JP, 2018, COORDIN CHEM REV, V358, P80, DOI 10.1016/j.ccr.2017.12.004

Malik YS, 2013, DEV BIOTECHNOLOGY

Manjunath K, 2005, J CONTROL RELEASE, V107, P215, DOI 10.1016/j.jconrel.2005.06.006

Manjunatha UH, 2006, P NATL ACAD SCI USA, V103, P431, DOI 10.1073/pnas.0508392103

Marianelli C, 2015, INT J INFECT DIS, V33, P39, DOI 10.1016/j.ijid.2014.12.043

Marks SM, 2014, EMERG INFECT DIS, V20, P812, DOI 10.3201/eid2005.131037

Matteelli A, 2010, FUTURE MICROBIOL, V5, P849, DOI [10.2217/fmb.10.50, 10.2217/FMB.10.50]

Meghana GS, 2017, INT J CHEMTECH RES, V10, P425

Mehnert W, 2001, ADV DRUG DELIVER REV, V47, P165, DOI 10.1016/S0169-409X(01)00105-3

MEHTA RT, 1993, ANTIMICROB AGENTS CH, V37, P2584, DOI 10.1128/AAC.37.12.2584

Mehta SK, 2008, PHARM RES-DORDR, V25, P227, DOI 10.1007/s11095-007-9355-8

Mehta SK, 2013, COLLOID SURFACE B, V101, P434, DOI 10.1016/j.colsurfb.2012.07.006

Mehta SK, 2010, AAPS PHARMSCITECH, V11, P143, DOI 10.1208/s12249-009-9356-5

Méndez-Samperio P, 2010, PEPTIDES, V31, P1791, DOI 10.1016/j.peptides.2010.06.016

Michel AL, 2010, VET MICROBIOL, V140, P371, DOI 10.1016/j.vetmic.2009.08.029

Migliori GB, 2008, EUR RESPIR J, V31, P1155, DOI 10.1183/09031936.00028708

Mignani S, 2013, NEW J CHEM, V37, P3337, DOI 10.1039/c3nj00300k

Mirsaeidi M, 2013, INT J MYCOBACT, V2, P1, DOI 10.1016/j.ijmyco.2013.01.004

Mitchison DA, 2006, J ANTIMICROB CHEMOTH, V58, P494, DOI 10.1093/jac/dkl260

MOAZED D, 1987, NATURE, V327, P389, DOI 10.1038/327389a0

Moazeni E, 2010, J MICROENCAPSUL, V27, P618, DOI 10.3109/02652048.2010.506579

Mohanty S, 2013, ANTIMICROB AGENTS CH, V57, P3688, DOI 10.1128/AAC.02475-12

Montoya J, 2013, J CLIN IMMUNOL, V33, P1360, DOI 10.1007/s10875-013-9949-3

Moretton MA, 2009, 1 WORKSH ART ORG BIO

Moretton MA, 2010, COLLOID SURFACE B, V79, P467, DOI 10.1016/j.colsurfb.2010.05.016

Morimoto K, 2016, ANN AM THORAC SOC, V13, P1904, DOI 10.1513/AnnalsATS.201604-246OC

Moss AR, 1997, INT J TUBERC LUNG D, V1, P115

Mujoriya R., 2011, J Appl Pharm Sci, V1, P20

Mullaicharam AR, 2004, J DRUG DELIV SCI TEC, V14, P99, DOI 10.1016/S1773-2247(04)50020-5

Nagarwal RC, 2009, J CONTROL RELEASE, V136, P2, DOI 10.1016/j.jconrel.2008.12.018

Narayanasamy P, 2015, SCI REP-UK, V5, DOI 10.1038/srep08824

Nasiruddin Mohammad, 2017, Tuberc Res Treat, V2017, P4920209, DOI [10.1155/2017/4920209, 10.1155/2017/4920209]

Neyrolles O, 2011, TUBERCULOSIS, V91, P187, DOI 10.1016/j.tube.2011.01.002

Nimje N, 2009, J DRUG TARGET, V17, P777, DOI 10.3109/10611860903115308

Ohashi K, 2009, J CONTROL RELEASE, V135, P19, DOI 10.1016/j.jconrel.2008.11.027

Okamoto S, 2007, MOL MICROBIOL, V63, P1096, DOI 10.1111/j.1365-2958.2006.05585.x

Olakanmi O, 2000, INFECT IMMUN, V68, P5619, DOI 10.1128/IAI.68.10.5619-5627.2000

OREILLY LM, 1995, TUBERCLE LUNG DIS, V76, P1, DOI 10.1016/0962-8479(95)90591-X

OROZCO LC, 1986, TUBERCLE, V67, P91, DOI 10.1016/0041-3879(86)90002-4

Owusu E, 2016, INT J MICROBIOL, V2016, P1, DOI [10.1155/2016/8304524, DOI 10.1155/2016/8304524]

Palomino JC, 2014, ANTIBIOTICS-BASEL, V3, P317, DOI 10.3390/antibiotics3030317

Pandey R, 2005, TUBERCULOSIS, V85, P415, DOI 10.1016/j.tube.2005.08.009

Pandey R, 2005, TUBERCULOSIS, V85, P227, DOI 10.1016/j.tube.2004.11.003

Pandey R, 2005, J ANTIMICROB CHEMOTH, V55, P430, DOI 10.1093/jac/dki027

Pandey R, 2004, J ANTIMICROB CHEMOTH, V54, P266, DOI 10.1093/jac/dkh260

Pandey R, 2004, INT J ANTIMICROB AG, V23, P414, DOI 10.1016/j.ijantimicag.2004.01.002

Pandey R, 2003, TUBERCULOSIS, V83, P373, DOI 10.1016/j.tube.2003.07.001

Pandey R, 2005, HANDBOOK OF CARBOHYDRATE ENGINEERING, P799, DOI 10.1201/9781420027631.ch27

Pandey R, 2007, CHEMOTHERAPY, V53, P437, DOI 10.1159/000110009

Pandey R, 2006, J ANTIMICROB CHEMOTH, V57, P1146, DOI 10.1093/jac/dkl128

Panyam J, 2003, ADV DRUG DELIVER REV, V55, P329, DOI 10.1016/S0169-409X(02)00228-4

Pardakhty A, 2013, NANOMED J, V1, P1, DOI 10.22038/NMJ.2013.697

Parlane NA, 2014, VET IMMUNOL IMMUNOP, V158, P8, DOI 10.1016/j.vetimm.2013.04.002

PARTHASARATHI G, 1994, J DRUG TARGET, V2, P173, DOI 10.3109/10611869409015907

Patel VR, 2011, J ADV PHARM TECHNOL, V2, P81, DOI 10.4103/2231-4040.82950

Patri AK, 2005, ADV DRUG DELIVER REV, V57, P2203, DOI 10.1016/j.addr.2005.09.014

Pawar KR, 2010, CRIT REV THER DRUG, V27, P419, DOI 10.1615/CritRevTherDrugCarrierSyst.v27.i5.20

Peppas NA, 2004, INT J PHARMACEUT, V277, P11, DOI 10.1016/j.ipharm.2003.03.001

Peters K, 2000, J ANTIMICROB CHEMOTH, V45, P77, DOI 10.1093/jac/45.1.77

Dong PV, 2012, INT NANO LETT, V2, DOI 10.1186/2228-5326-2-9

Phillips CJC, 2003, RES VET SCI, V74, P1, DOI 10.1016/S0034-5288(02)00145-5

Prasad M, 2018, BIOMED PHARMACOTHER, V97, P1521, DOI 10.1016/j.biopha.2017.11.026

Rai MK, 2012, J APPL MICROBIOL, V112, P841, DOI 10.1111/j.1365-2672.2012.05253.x

Ramaswamy SV, 2003, ANTIMICROB AGENTS CH, V47, P1241, DOI 10.1128/AAC.47.4.1241-1250.2003

Rani N.P., 2010, INT J PHARM BIO SCI, V1, P379

Reddy VM, 2012, J ANTIMICROB CHEMOTH, V67, P1163, DOI 10.1093/jac/dkr589

Reed C, 2006, AM J EPIDEMIOL, V164, P32, DOI 10.1093/aje/kwj159

Reisner BS, 1999, J CLIN MICROBIOL, V37, P2024, DOI 10.1128/JCM.37.6.2024-2026.1999

Rengarajan J, 2004, MOL MICROBIOL, V53, P275, DOI 10.1111/j.1365-2958.2004.04120.x

Restis Eva Marie, 2013, THESIS

Ribeiro R., 2014, DEV CHARACTERIZATION

Richards SJ, 2018, BIOMACROMOLECULES, V19, P256, DOI 10.1021/acs.biomac.7b01561

Rocha AD, 2012, J CLIN MICROBIOL, V50, P1912, DOI 10.1128/JCM.06561-11

Rohde K, 2007, IMMUNOL REV, V219, P37, DOI 10.1111/j.1600-065X.2007.00547.x

Safi H, 2013, NAT GENET, V45, P1190, DOI 10.1038/ng.2743

Santos-Magalhaes NS, 2010, ADV DRUG DELIVER REV, V62, P560, DOI 10.1016/j.addr.2009.11.024

Saraogi GK, 2011, J DRUG TARGET, V19, P219, DOI 10.3109/1061186X.2010.492522

Sarei F, 2013, INDIAN J PHARM SCI, V75, P442, DOI 10.4103/0250-474X.119829

Sarmento B, 2007, BIOMACROMOLECULES, V8, P3054, DOI 10.1021/bm0703923

Scheuch G, 2006, ADV DRUG DELIVER REV, V58, P996, DOI 10.1016/j.addr.2006.07.009

Scorpio A, 1996, NAT MED, V2, P662, DOI 10.1038/nm0696-662

Selim A, 2018, REV SCI TECH OIE, V37, P823, DOI 10.20506/rst.37.3.2888

SESSA G, 1968, J LIPID RES, V9, P310

Seth D, 2011, CURR MICROBIOL, V62, P715, DOI 10.1007/s00284-010-9770-7

Shah NS, 2018, PUBLIC HEALTH ACTION, V8, P7, DOI 10.5588/pha.17.0091

Shah NS, 2008, JAMA-J AM MED ASSOC, V300, P2153, DOI 10.1001/jama.300.18.2153

Sharma A, 2004, INT J ANTIMICROB AG, V24, P599, DOI 10.1016/j.ijantimicag.2004.07.010

Sharma A, 2004, J ANTIMICROB CHEMOTH, V54, P761, DOI 10.1093/jac/dkh411

Sharma S, 2012, J PHARM SCI-US, V101, P233, DOI 10.1002/jps.22763

SHEK PN, 1994, J DRUG TARGET, V2, P431, DOI 10.3109/10611869408996819

Shi WL, 2011, SCIENCE, V333, P1630, DOI 10.1126/science.1208813

Silva JP, 2016, J CONTROL RELEASE, V235, P112, DOI 10.1016/j.jconrel.2016.05.064

Silva MT, 2009, LANCET INFECT DIS, V9, P699, DOI 10.1016/S1473-3099(09)70234-8

Silva T, 2014, ANTIMICROB AGENTS CH, V58, P3461, DOI 10.1128/AAC.02728-13

Singh N., 2016, INDIAN RES J PHARM S, V3, P519

Singh R, 2008, SCIENCE, V322, P1392, DOI 10.1126/science.1164571

Singh R, 2015, INT J ANTIMICROB AG, V46, P183, DOI 10.1016/j.ijantimicag.2015.03.014

Sosnik A, 2010, ADV DRUG DELIVER REV, V62, P547, DOI 10.1016/j.addr.2009.11.023

Sreevatsan S, 1997, ANTIMICROB AGENTS CH, V41, P1677, DOI 10.1128/AAC.41.8.1677

Stewart LD, 2012, J CLIN MICROBIOL, V50, P1598, DOI 10.1128/JCM.05747-11

TAKIFF HE, 1994, ANTIMICROB AGENTS CH, V38, P773, DOI 10.1128/AAC.38.4.773

Tang TQ, 2016, TZU CHI MED J, V28, P29, DOI 10.1016/j.tcmj.2014.09.001

Tekin E, 2018, NPJ SYST BIOL APPL, V4, DOI 10.1038/s41540-018-0069-9

Telenti A, 1997, NAT MED, V3, P567, DOI 10.1038/nm0597-567

THACHARODI D, 1995, BIOMATERIALS, V16, P145, DOI 10.1016/0142-9612(95)98278-M

Tibotec Pharmaceuticals Limited, ANT ACT SAF TOL TMC

Tiuman TS, 2011, INT J INFECT DIS, V15, pE525, DOI 10.1016/j.ijid.2011.03.021

TOMIOKA H, 1991, AM REV RESPIR DIS, V144, P575, DOI 10.1164/ajrccm/144.3_Pt_1.575

Tonnesen HH, 2002, DRUG DEV IND PHARM, V28, P621, DOI 10.1081/DDC-120003853

Torres-Sangiao E, 2016, MOLECULES, V21, DOI 10.3390/molecules21070867

Tousif S, 2017, FRONT IMMUNOL, V8, DOI 10.3389/fimmu.2017.00739

Tsai TT, 2017, ACS SENSORS, V2, P1345, DOI 10.1021/acssensors.7b00450

UCHEGBU IF, 1995, ADV COLLOID INTERFAC, V58, P1, DOI 10.1016/0001-8686(95)00242-I

Uchegbu IF, 1998, INT J PHARM, V172, P33, DOI 10.1016/S0378-5173(98)00169-0

Vadlamudi HC, 2012, Indo Am J Pharm Res, V2

Vergne AF, 2000, NAT PROD REP, V17, P99, DOI 10.1039/a809397k

Verma JS, 2014, J ANTIMICROB CHEMOTH, V69, P2935, DOI 10.1093/jac/dku273

Vieira ACC, 2018, ARTIF CELL NANOMED B, V46, pS653, DOI 10.1080/21691401.2018.1434186

Vila A, 2004, EUR J PHARM BIOPHARM, V57, P123, DOI 10.1016/j.ejpb.2003.09.006

Vilchèze C, 2008, MOL MICROBIOL, V69, P1316, DOI 10.1111/j.1365-2958.2008.06365.x

VLADIMIRSKY MA, 1983, ANTIBIOT MED BIOTEK, V28, P23

Walker TM, 2018, LANCET INFECT DIS, V18, P431, DOI 10.1016/S1473-3099(18)30004-5

Wang XB, 2014, EMERG INFECT DIS, V20, P1252, DOI 10.3201/eid2007.131801

WHO, MULT RIF RES TB MDR

WHO, 2006, GLOB TASK FORC XDR T

Wijesekara I, 2011, CARBOHYD POLYM, V84, P14, DOI 10.1016/j.carbpol.2010.10.062

Wikberg M, 1997, ALIMENT PHARM THER, V11, P109

Williams DL, 2014, CLIN INFECT DIS, V58, P72, DOI 10.1093/cid/cit628

Williams DL, 2012, LEPROSY REV, V83, P269

Woodworth JS, 2017, MUCOSAL IMMUNOL, V10, P555, DOI 10.1038/mi.2016.70

World Health Organization, 2012, Treatment of Mycobacterium Ulcerans Disease (Buruli Ulcer): Guidance for Health Workers

World Health Organization, 2018, GLOB TUB REP

Wu HJ, 2014, ANAL CHEM, V86, P1988, DOI 10.1021/ac4027669

Xu KJ, 2018, ADV HEALTHC MATER, V7, DOI 10.1002/adhm.201700509

Xu W, 2013, J DRUG DELIV, V2013, DOI 10.1155/2013/340315

Xu YH, 2018, BMC GENOMICS, V19, DOI 10.1186/s12864-018-5010-5

Yang H, 2006, J BIOMAT SCI-POLYM E, V17, P3, DOI 10.1163/156856206774879171

Yang M, 2015, SCI REP-UK, V5, DOI 10.1038/srep13969

Yeo PL, 2017, ASIAN BIOMED, V11, P301, DOI 10.1515/abm-2018-0002

Zahoor A, 2005, INT J ANTIMICROB AG, V26, P298, DOI 10.1016/j.ijantimicag.2005.07.012

Zazo H, 2016, J CONTROL RELEASE, V224, P86, DOI 10.1016/j.jconrel.2016.01.008

Zhang L, 2008, CLIN PHARMACOL THER, V83, P761, DOI 10.1038/sj.clpt.6100400

Zhang L, 2010, CURR MED CHEM, V17, P585, DOI 10.2174/092986710790416290

Zhang S, 2015, J ANTIMICROB CHEMOTH, V70, P2507, DOI 10.1093/jac/dkv150

Zhang Y, 2003, INT J TUBERC LUNG D, V7, P6

Zhou J, 2015, ACS BIOMATER SCI ENG, V1, P1250, DOI 10.1021/acsbiomaterials.5b00274

Zimenkov DV, 2017, J ANTIMICROB CHEMOTH, V72, P1901, DOI 10.1093/jac/dkx094

Zimhony O, 2000, NAT MED, V6, P1043, DOI 10.1038/79558

NR 319

TC 18

Z9 21

U1 3

U2 60

PU BENTHAM SCIENCE PUBL LTD

PI SHARJAH

PA EXECUTIVE STE Y-2, PO BOX 7917, SAIF ZONE, 1200 BR SHARJAH, U ARAB

EMIRATES

SN 1381-6128

EI 1873-4286

J9 CURR PHARM DESIGN

JI Curr. Pharm. Design

PY 2019

VL 25

IS 13

BP 1554

EP 1579

DI 10.2174/1381612825666190620094041

PG 26

WC Pharmacology & Pharmacy

WE Science Citation Index Expanded (SCI-EXPANDED)

SC Pharmacology & Pharmacy

GA IR7JS

UT WOS:000481617400009

DA 2026-05-07

ER

PT J

AU Lucarelli, AP

Buroni, S

Pasca, MR

Rizzi, M

Cavagnino, A

Valentini, G

Riccardi, G

Chiarelli, LR

AF Lucarelli, Anna P.

Buroni, Silvia

Pasca, Maria R.

Rizzi, Menico

Cavagnino, Andrea

Valentini, Giovanna

Riccardi, Giovanna

Chiarelli, Laurent R.

TI +Mycobacterium tuberculosis Phosphoribosylpyrophosphate

Synthetase: Biochemical Features of a Crucial Enzyme for Mycobacterial

Cell Wall Biosynthesis

SO PLOS ONE

LA English

DT Article

ID MULTIDRUG-RESISTANT TUBERCULOSIS; ESCHERICHIA-COLI; DIPHOSPHATE

SYNTHASE; BACILLUS-SUBTILIS; PURIFICATION; BINDING; MECHANISM;

CATALYSIS; ARABINAN; RIBOSE

AB The selection and soaring spread of Mycobacterium tuberculosis multidrug-resistant (MDR-TB) and extensively drug-resistant strains (XDR-TB) is a severe public health problem. Currently, there is an urgent need for new drugs for tuberculosis treatment, with novel mechanisms of action and, moreover, the necessity to identify new drug targets. Mycobacterial phosphoribosylpyrophosphate synthetase (MtbPRPPase) is a crucial enzyme involved in the biosynthesis of decaprenylphosphoryl-arabinose, an essential precursor for the mycobacterial cell wall biosynthesis. Moreover, phosphoribosylpyrophosphate, which is the product of the PRPPase catalyzed reaction, is the precursor for the biosynthesis of nucleotides and of some amino acids such as histidine and tryptophan. In this context, the elucidation of the molecular and functional features of MtbPRPPase is mandatory. MtbPRPPase was obtained as a recombinant form, purified to homogeneity and characterized. According to its hexameric form, substrate specificity and requirement of phosphate for activity, the enzyme proved to belong to the class I of PRPPases. Although the sulfate mimicked the phosphate, it was less effective and required higher concentrations for the enzyme activation. MtbPRPPase showed hyperbolic response to ribose 5-phosphate, but sigmoidal behaviour towards Mg-ATP. The enzyme resulted to be allosterically activated by Mg(2+) or Mn(2+) and inhibited by Ca(2+) and Cu(2+) but, differently from other characterized PRPPases, it showed a better affinity for the Mn(2+) and Cu(2+) ions, indicating a different cation binding site geometry. Moreover, the enzyme from M. tuberculosis was allosterically inhibited by ADP, but less sensitive to inhibition by GDP. The characterization of M. tuberculosis PRPPase provides the starting point for the development of inhibitors for antitubercular drug design.

C1 [Lucarelli, Anna P.; Buroni, Silvia; Pasca, Maria R.; Riccardi, Giovanna] Univ Pavia, Dipartimento Genet & Microbiol, I-27100 Pavia, Italy.

[Valentini, Giovanna; Chiarelli, Laurent R.] Univ Pavia, Dipartimento Biochim A Castellani, I-27100 Pavia, Italy.

[Rizzi, Menico; Cavagnino, Andrea] Univ Piemonte Orientale, DISCAFF, Novara, Italy.

C3 University of Pavia; University of Pavia; University of Eastern Piedmont

Amedeo Avogadro

RP Lucarelli, AP (corresponding author), Univ Pavia, Dipartimento Genet & Microbiol, Via Palestro 3, I-27100 Pavia, Italy.

EM laurent.chiarelli@unipv.it

RI Rizzi, Michael/OEN-5286-2025; PASCA, Maria Rosalia/J-8752-2016;

RICCARDI, Giovanna/A-9269-2012; Chiarelli, Laurent Robert/J-8368-2016;

Buroni, Silvia/AAX-5454-2020

OI PASCA, Maria Rosalia/0000-0002-8906-4937; RICCARDI,

Giovanna/0000-0003-3295-0070; Chiarelli, Laurent

Robert/0000-0003-0348-9764; Buroni, Silvia/0000-0002-6979-2275

FU EC [LSHP-CT-2005-018923]

FX This work was supported by EC-VI Framework, Contract no.

LSHP-CT-2005-018923. The funders had no role in study design, data

collection and analysis, decision to publish, or preparation of the

manuscript.

CR [Anonymous], 2008, Global Tuberculosis Control: Surveillance, Planning, and Financing; WHO Report 2008

ARNVIG K, 1990, EUR J BIOCHEM, V192, P195, DOI 10.1111/j.1432-1033.1990.tb19214.x

Barry Clifton E., 2007, Infectious Disorders - Drug Targets, V7, P182

Blasi P, 2009, EXPERT OPIN DRUG DEL, V6, P977, DOI 10.1517/17425240903130577

Cho Y, 2008, J MED CHEM, V51, P5984, DOI 10.1021/jm800328v

CORPET F, 1988, NUCLEIC ACIDS RES, V16, P10881, DOI 10.1093/nar/16.22.10881

DeLano W. L., PYMOL MOL GRAPHICS S

Eriksen TA, 2002, PROTEIN SCI, V11, P271, DOI 10.1110/ps.28502

Eriksen TA, 2000, NAT STRUCT BIOL, V7, P303

FOX IH, 1971, J BIOL CHEM, V246, P5739

GIBSON KJ, 1982, J BIOL CHEM, V257, P2391

HOVEJENSEN B, 1986, J BIOL CHEM, V261, P6765

HOVEJENSEN B, 1988, J BACTERIOL, V170, P1148, DOI 10.1128/jb.170.3.1148-1152.1988

ISHIJIMA S, 1991, J BIOL CHEM, V266, P15693

Jain A, 2008, FEMS IMMUNOL MED MIC, V53, P145, DOI 10.1111/j.1574-695X.2008.00400.x

Kadziola A, 2005, J MOL BIOL, V354, P815, DOI 10.1016/j.jmb.2005.10.001

Krath BN, 1999, BBA-PROTEIN STRUCT M, V1430, P403, DOI 10.1016/S0167-4838(99)00022-9

Krath BN, 2001, J BIOL CHEM, V276, P17851, DOI 10.1074/jbc.M010172200

Krath BN, 2001, PROTEIN SCI, V10, P2317, DOI 10.1110/ps.11801

LASKOWSKI RA, 1993, J APPL CRYSTALLOGR, V26, P283, DOI 10.1107/S0021889892009944

Li S, 2007, BIOCHEM J, V401, P39, DOI 10.1042/BJ20061066

Loughran ST, 2006, ANAL BIOCHEM, V355, P148, DOI 10.1016/j.ab.2006.05.011

LOWRY OH, 1951, J BIOL CHEM, V193, P265

Makarov V, 2009, SCIENCE, V324, P801, DOI 10.1126/science.1171583

Manina G, 2010, CURR MED CHEM, V17, P3099, DOI 10.2174/092986710791959693

Mikusová K, 2005, J BACTERIOL, V187, P8020, DOI 10.1128/JB.187.23.8020-8025.2005

MILLER GA, 1975, ARCH BIOCHEM BIOPHYS, V171, P732, DOI 10.1016/0003-9861(75)90086-7

MONOD J, 1965, J MOL BIOL, V12, P88, DOI 10.1016/S0022-2836(65)80285-6

Nathanson E, 2006, EMERG INFECT DIS, V12, P1389, DOI 10.3201/eid1209.051618

NOSAL JM, 1993, J BIOL CHEM, V268, P10168

Palomino JC, 2009, CURR MED CHEM, V16, P1898, DOI 10.2174/092986709788186066

Pan F, 2001, J BACTERIOL, V183, P3991, DOI 10.1128/JB.183.13.3991-3998.2001

ROTH DG, 1974, J BIOL CHEM, V249, P297

Russell DG, 2010, SCIENCE, V328, P852, DOI 10.1126/science.1184784

Sassetti CM, 2003, MOL MICROBIOL, V48, P77, DOI 10.1046/j.1365-2958.2003.03425.x

Sharma SK, 2006, CHEST, V130, P261, DOI [10.1378/chest.130.1.261, 10.1016/S0012-3692(15)50981-1]

Studier FW, 2005, PROTEIN EXPRES PURIF, V41, P207, DOI 10.1016/j.pep.2005.01.016

SWITZER RL, 1973, J BIOL CHEM, V248, P1063

Willemoes M, 1997, BIOCHEMISTRY-US, V36, P5078, DOI 10.1021/bi962610a

Wolucka BA, 2008, FEBS J, V275, P2691, DOI 10.1111/j.1742-4658.2008.06395.x

NR 40

TC 24

Z9 26

U1 0

U2 9

PU PUBLIC LIBRARY SCIENCE

PI SAN FRANCISCO

PA 185 BERRY ST, STE 1300, SAN FRANCISCO, CA 94107 USA

SN 1932-6203

J9 PLOS ONE

JI PLoS One

PD NOV 15

PY 2010

VL 5

IS 11

AR e15494

DI 10.1371/journal.pone.0015494

PG 12

WC Multidisciplinary Sciences

WE Science Citation Index Expanded (SCI-EXPANDED)

SC Science & Technology - Other Topics

GA 680KW

UT WOS:000284231800038

OA Green Submitted, gold

DA 2026-05-07

ER

PT J

AU Chaitra, R

Gandhi, R

Jayanna, N

Satyanath, S

Pavadai, P

Murahari, M

AF Chaitra, R.

Gandhi, Rohit

Jayanna, N.

Satyanath, Sachin

Pavadai, Parasuraman

Murahari, Manikanta

TI Computational design of MmpL3 inhibitors for tuberculosis therapy

SO MOLECULAR DIVERSITY

LA English

DT Article

DE MmpL3; Tuberculosis; Pharmacophore; Docking; Hit compounds

ID MEMBRANE TRANSPORTER; CELL-WALL; MDR-TB; XDR-TB; PROTEINS; TARGETS

AB Tuberculosis is a chronic communicable disease caused by Mycobacterium tuberculosis (Mtb) and spreads from lungs to lymphatic system. The cell wall of mycobacterium plays a prominent role in maintaining the virulence and pathogenicity and also acts as prime target for drug discovery. Hence, this study has put into emphasis with target MmpLs (Mycobacterial membrane proteins Large) which are significant for the growth and survival of Mycobacterium tuberculosis. MmpLs belongs to the resistance, nodulation and division (RND) protein superfamily. MmpL3 is the only MmpL deemed essential for the replication and viability of mycobacterial cells. For the study, we have selected SQ109 derivatives as Mmpl3 inhibitor, which holds non-covalent property. Structure-based pharmacophore model of MmpL3 target protein with SQ109 as co-crystallized ligand (PDB: 6AJG) was generated to screen the ligand database. Compounds with decent fitness score and pharmacophoric features were compared with standard drug and taken for molecular docking studies. Further prime molecular mechanics- Poisson-Boltzmann surface area (MM-GBSA) and induced fit calculations identified potential molecules for further drug-likeness screening. Overall computational calculations identified ZINC000000016638 and ZINC000000003594 as potential in silico MmpL3 inhibitors. Molecular dynamics simulations integrated with MM-PBSA free energy calculations identified that MmpL3-ZINC000000016638 complex was more stable. Study can be further extended for synthesis and biological evaluation, derivatization of active compound to identify potential and safe lead compounds for effective tuberculosis therapy.

[GRAPHICS]

.

C1 [Chaitra, R.; Gandhi, Rohit; Jayanna, N.; Satyanath, Sachin; Pavadai, Parasuraman] MS Ramaiah Univ Appl Sci, Fac Pharm, Dept Pharmaceut Chem, Bangalore, Karnataka, India.

[Murahari, Manikanta] Koneru Lakshmaiah Educ Fdn, KL Coll Pharm, Med Chem Res Div, Vaddeswaram, Andhra Pradesh, India.

C3 M. S. Ramaiah University of Applied Sciences; Koneru Lakshmaiah

Education Foundation (K L Deemed to be University)

RP Murahari, M (corresponding author), Koneru Lakshmaiah Educ Fdn, KL Coll Pharm, Med Chem Res Div, Vaddeswaram, Andhra Pradesh, India.

EM manikanta.murahari@gmail.com

RI Pavadai, Parasuraman/AAF-5979-2019; Murahari,, Manikanta/AAF-6158-2019

OI Murahari,, Manikanta/0000-0002-5404-4426

CR [Anonymous], 2018, SCHRDING REL 2018 3

Arakal NG, 2021, COMPUT METH PROG BIO, V209, DOI 10.1016/j.cmpb.2021.106347

Babalola MO, 2015, Journal of Tuberculosis Research, V03, P184, DOI [10.4236/jtr.2015.34025, 10.4236/jtr.2015.34025, DOI 10.4236/JTR.2015.34025]

Bhakhar KA, 2021, J MOL STRUCT, V1244, DOI 10.1016/j.molstruc.2021.130941

Bolla JR, 2020, BIOCHEM SOC T, V48, P1463, DOI 10.1042/BST20190950

Bruchfeld J, 2015, CSH PERSPECT MED, V5, DOI 10.1101/cshperspect.a017871

Caminero JA, 2008, INT J TUBERC LUNG D, V12, P869

Chim N, 2015, CHEM BIOL, V22, P1098, DOI 10.1016/j.chembiol.2015.07.013

Degiacomi G, 2017, SCI REP-UK, V7, DOI 10.1038/srep43495

Dhameliya TM, 2022, J MOL STRUCT, V1248, DOI 10.1016/j.molstruc.2021.131473

Dorn-In S, 2020, TRANSBOUND EMERG DIS, V67, P308, DOI 10.1111/tbed.13353

GRANGE J, 2009, TUBERCULOSIS, P4459

Grobler L, 2016, COCHRANE DB SYST REV, DOI 10.1002/14651858.CD006086.pub4

Huante MB, 2019, TUBERCULOSIS HOST-PATHOGEN INTERACTIONS, P123, DOI 10.1007/978-3-030-25381-3_7

Huddart S, 2020, BMJ GLOB HEALTH, V5, DOI 10.1136/bmjgh-2019-002080

Ioerger TR, 2013, PLOS ONE, V8, DOI 10.1371/journal.pone.0075245

Irwin John J, 2008, Curr Protoc Bioinformatics, VChapter 14, DOI 10.1002/0471250953.bi1406s22

Krishna S, 2021, COMPUT BIOL MED, V134, DOI 10.1016/j.compbiomed.2021.104455

Lee GO, 2020, CURR DEV NUTR, V4, DOI 10.1093/cdn/nzaa001

Li W, 2019, ACS INFECT DIS, V5, P1001, DOI 10.1021/acsinfecdis.9b00048

Li W, 2018, FRONT MICROBIOL, V9, DOI 10.3389/fmicb.2018.01547

Li W, 2016, ANTIMICROB AGENTS CH, V60, P5198, DOI 10.1128/AAC.00826-16

Luo Q, 2021, CURR PROTEIN PEPT SC, V22, P290, DOI 10.2174/1389203722666210421105733

Ma S, 2020, BIOL CHEM, V401, P331, DOI 10.1515/hsz-2019-0326

Macallan DC, 1999, DIAGN MICR INFEC DIS, V34, P153, DOI 10.1016/S0732-8893(99)00007-3

Mayer KH, 2010, CLIN INFECT DIS, V50, pS67, DOI 10.1086/651475

Melly G, 2019, MICROORGANISMS, V7, DOI 10.3390/microorganisms7030070

Migliori GB, 2009, EUR RESPIR J, V34, P778, DOI 10.1183/09031936.00059409

Mondal R, 2007, EMERG INFECT DIS, V13, P1429, DOI 10.3201/eid1309.070443

Munnaluri R, 2019, COMPUT BIOL CHEM, V78, P81, DOI 10.1016/j.compbiolchem.2018.11.007

Nikaido H, 2018, RES MICROBIOL, V169, P363, DOI 10.1016/j.resmic.2018.03.001

Rayasam GV, 2014, EXPERT OPIN THER TAR, V18, P247, DOI 10.1517/14728222.2014.859677

Raynaud C, 2020, ACS INFECT DIS, V6, P324, DOI 10.1021/acsinfecdis.9b00389

Rodriguez-Campos S, 2014, RES VET SCI, V97, pS5, DOI 10.1016/j.rvsc.2014.02.009

Sandhu GK, 2011, J GLOB INFECT DIS, V3, P143, DOI 10.4103/0974-777X.81691

Shaji Bhagya, 2019, Indian J Tuberc, V66, P26, DOI [10.1016/j.ijtb.2018.10.001, 10.1016/j.ijtb.2018.10.001]

Shao M, 2020, EUR J MED CHEM, V200, DOI 10.1016/j.ejmech.2020.112390

Silva-Pereira TT, 2019, BMC GENOMICS, V20, DOI 10.1186/s12864-019-6407-5

Singh S, 2019, INT J PREVENTIVE MED, V10, DOI [10.4018/ijsds.2019070107, 10.4103/ijpvm.IJPVM_492_17, 10.4018/IJSDS.2019070107]

Smith I, 2003, CLIN MICROBIOL REV, V16, P463, DOI 10.1128/CMR.16.3.463-496.2003

Sotgiu G, 2015, CSH PERSPECT MED, V5, DOI 10.1101/cshperspect.a017822

Su CC, 2021, PLOS BIOL, V19, DOI 10.1371/journal.pbio.3001370

Tahlan K, 2012, ANTIMICROB AGENTS CH, V56, P1797, DOI 10.1128/AAC.05708-11

U.S. EPA, 2016, TOX EST SOFTW TOOL P

Vilchèze C, 2020, APPL SCI-BASEL, V10, DOI 10.3390/app10072278

Xu ZJ, 2017, P NATL ACAD SCI USA, V114, P7993, DOI 10.1073/pnas.1700062114

Zhang B, 2019, CELL, V176, P636, DOI 10.1016/j.cell.2019.01.003

NR 47

TC 7

Z9 8

U1 0

U2 12

PU SPRINGER

PI DORDRECHT

PA VAN GODEWIJCKSTRAAT 30, 3311 GZ DORDRECHT, NETHERLANDS

SN 1381-1991

EI 1573-501X

J9 MOL DIVERS

JI Mol. Divers.

PD FEB

PY 2023

VL 27

IS 1

BP 357

EP 369

DI 10.1007/s11030-022-10436-2

EA APR 2022

PG 13

WC Biochemistry & Molecular Biology; Chemistry, Applied; Chemistry,

Medicinal; Chemistry, Multidisciplinary

WE Science Citation Index Expanded (SCI-EXPANDED)

SC Biochemistry & Molecular Biology; Chemistry; Pharmacology & Pharmacy

GA 8W9FF

UT WOS:000794902400001

DA 2026-05-07

ER

PT J

AU Zhang, Y

Zhang, LY

Shi, LY

Wei, LL

Gan, L

Hu, YT

Huang, H

Xie, KP

Jiang, TT

Li, JC

AF Zhang, Yang

Zhang, Linyu

Shi, Liying

Wei, Liliang

Gan, Lin

Hu, Yuting

Huang, Huai

Xie, Keping

Jiang, Tingting

Li, Ji-Cheng

TI Plasma exosomal miR-122-5p_R-1, miR-23b-3p_R+1, and miR-15a-5p_R-1 are

associated with multidrug-resistant tuberculosis

SO BMC INFECTIOUS DISEASES

LA English

DT Article

DE MDR-TB; MiRNA; Exosome; Plasma; Biomarker.

ID MYCOBACTERIUM-TUBERCULOSIS; EXPRESSION; GROWTH; CANCER; MACROPHAGES;

MICRORNAS; COMPLEX; MIRNAS; ASSAY

AB BackgroundPatients with multidrug-resistant tuberculosis (MDR-TB) who are resistant to at least both rifampicin and isoniazid, lack effective treatment options in clinic. The gold standard for the diagnosis of MDR-TB is drug sensitivity test, which is time-consuming and has a relatively low positive detection rate. Screening early diagnostic biomarker for MDR-TB is urgent need in clinical practice.MethodsA total of 33 patients with MDR-TB, healthy controls and drug-sensitive tuberculosis (DS-TB) were included in this study. Total plasma exosomal RNA was extracted from the subjects, and the MDR-TB plasma-specific exosomal miRNAs were obtained by Illumina sequencing.ResultsThere were 644 and 647 differentially expressed miRNAs in the plasma exosomes of MDR-TB patients obtained by sequencing and biogenic analysis compared with DS-TB patients and healthy controls, respectively. Differential miRNAs are mainly involved in the biological function of regulation of transcription and protein binding, and enriched in the pathways in cancer and MAPK signaling pathway. Moreover, seven plasma exosomal miRNAs in MDR-TB patients were significantly different from those in DS-TB patients and healthy controls. Among them, three of the miRNAs (hsa-miR-122-5p_R-1, hsa-miR-23b-3p_R + 1, and hsa-miR-15a-5p_R-1) were found to be in target relationship with MDR-TB related genes (NTRK2, KIDINS220, NCKAP1, MAPK9, NFAT5, ATF6 and SLC11A2) by target gene prediction analysis. Further the bioinformatic analysis showed that hsa-miR-122-5p_R-1 targets the protein PGLYRP2, a diagnostic biomarker identified in our previous study.ConclusionsWe suggest that hsa-miR-122-5p_R-1, hsa-miR-23b-3p_R + 1, and hsa-miR-15a-5p_R-1 are closely related to MDR-TB.

C1 [Zhang, Yang; Zhang, Linyu; Gan, Lin; Hu, Yuting; Huang, Huai; Xie, Keping; Jiang, Tingting] South China Univ Technol, Sch Med, Ctr Pancreat Canc Res, 382 Waihuan Rd, Guangzhou 510006, Peoples R China.

[Shi, Liying] Zhejiang Hosp, Dept Clin Lab, Hangzhou 310030, Peoples R China.

[Wei, Liliang] Shaoxing Univ, Dept Pneumol, Affiliated Hosp, Shaoxing 312000, Peoples R China.

[Li, Ji-Cheng] Zhejiang Univ, Inst Cell Biol, Hangzhou 310031, Peoples R China.

C3 South China University of Technology; Shaoxing University; Zhejiang

University

RP Xie, KP; Jiang, TT (corresponding author), South China Univ Technol, Sch Med, Ctr Pancreat Canc Res, 382 Waihuan Rd, Guangzhou 510006, Peoples R China.; Li, JC (corresponding author), Zhejiang Univ, Inst Cell Biol, Hangzhou 310031, Peoples R China.

EM mcxiekeping@scut.edu.cn; ttjiang@scut.edu.cn; Zjulijicheng@163.com

RI ; Jiang, Tingting/MCK-2463-2025

OI Xie, Keping/0000-0001-7887-9586;

FU The Zhejiang Province Traditional Chinese Medicine Science and

Technology Plan Project; MDR-TB

FX We would like to express our sincere gratitude to all the MDR-TB and

DS-TB patients, as well as the healthy volunteers, who participated in

this study. We also extend our heartfelt thanks to the doctors and

nurses for their invaluable assistance in diagnosis and clinical data

collection.

CR Acharya B, 2020, MOL BIOL REP, V47, P4065, DOI 10.1007/s11033-020-05413-7

Ahmad S, 2007, TUBERCULOSIS, V87, P123, DOI 10.1016/j.tube.2006.05.004

An Y, 2015, CELL DEATH DIS, V6, DOI 10.1038/cddis.2015.123

Bandi N, 2009, CANCER RES, V69, P5553, DOI 10.1158/0008-5472.CAN-08-4277

Bhatnagar S, 2007, J BIOL CHEM, V282, P25779, DOI 10.1074/jbc.M702277200

Bhattacharya R, 2009, CANCER RES, V69, P9090, DOI 10.1158/0008-5472.CAN-09-2552

Bollaert E, 2021, INT J MOL SCI, V22, DOI 10.3390/ijms22105153

Bonci D, 2008, NAT MED, V14, P1271, DOI 10.1038/nm.1880

Bottoni A, 2005, J CELL PHYSIOL, V204, P280, DOI 10.1002/jcp.20282

Burroughs AM, 2010, GENOME RES, V20, P1398, DOI 10.1101/gr.106054.110

Calin GA, 2004, P NATL ACAD SCI USA, V101, P11755, DOI 10.1073/pnas.0404432101

Calin GA, 2008, P NATL ACAD SCI USA, V105, P5166, DOI 10.1073/pnas.0800121105

Carranza C, 2021, FRONT IMMUNOL, V12, DOI 10.3389/fimmu.2021.760468

Chen J, 2020, J CELL MOL MED, V24, P12537, DOI 10.1111/jcmm.15796

Chen QY, 2016, TUMOR BIOL, V37, P521, DOI 10.1007/s13277-015-3675-9

Chen XB, 2019, FRONT GENET, V10, DOI 10.3389/fgene.2019.00367

Chevillet JR, 2014, P NATL ACAD SCI USA, V111, P14888, DOI 10.1073/pnas.1408301111

Cloonan N, 2011, GENOME BIOL, V12, DOI 10.1186/gb-2011-12-12-r126

Cui XG, 2025, MICROBIOL RES, V290, DOI 10.1016/j.micres.2024.127943

Desikan P, 2017, INDIAN J MED RES, V145, P70, DOI 10.4103/ijmr.IJMR_831_14

Diaz G, 2016, SCI REP-UK, V6, DOI 10.1038/srep37975

Espín-Pérez A, 2018, ENVIRON POLLUT, V242, P182, DOI 10.1016/j.envpol.2018.06.051

Garzon R, 2007, ONCOGENE, V26, P4148, DOI 10.1038/sj.onc.1210186

Guo L, 2014, GENE, V544, P1, DOI 10.1016/j.gene.2014.04.039

Hu N, 2021, MOL MED REP, V23, DOI 10.3892/mmr.2021.12007

Hu YR, 2017, MOL CANCER, V16, DOI 10.1186/s12943-017-0743-3

Huang GY, 2022, CELL ONCOL, V45, P1329, DOI 10.1007/s13402-022-00722-4

Jain P, 2012, J CLIN MICROBIOL, V50, P1362, DOI 10.1128/JCM.06192-11

Jin YJ, 2024, J CLIN TUBER OTH MYC, V36, DOI 10.1016/j.jctube.2024.100453

Jing Z, 2020, CANCER MED-US, V9, P4324, DOI 10.1002/cam4.3020

Kaur R, 2022, GENES-BASEL, V13, DOI 10.3390/genes13081375

Kent PT., 1985, PUBLIC HLTH MYCOBACT

Kim DH, 2020, CANCER LETT, V475, P2, DOI 10.1016/j.canlet.2020.01.023

Kim H, 2021, APPL MICROBIOL BIOT, V105, P6059, DOI 10.1007/s00253-021-11446-0

Kim WR, 2020, MOL CELLS, V43, P953, DOI 10.14348/molcells.2020.0177

Kushwaha S, 2024, INDIAN J MED RES, V159, P653, DOI [10.25259/IJMR_1281_23, 10.25259/ijmr_1281_23]

Li D, 2023, CELL CYCLE, V22, P255, DOI 10.1080/15384101.2022.2112132

Madhavan B, 2015, INT J CANCER, V136, P2616, DOI 10.1002/ijc.29324

Mei ZX, 2015, BIOMED RES INT, V2015, DOI 10.1155/2015/651980

National Health and Family Planning Commission of the people's Republic of China, 2018, Electron J Emerg Infect Dis, V3, P59, DOI [DOI 10.3877/J.ISSN.2096-2738.2018.01.017, DOI 10.19871/J.CNKI.XFCRBZZ.2018.01.017]

Neilsen CT, 2012, TRENDS GENET, V28, P544, DOI 10.1016/j.tig.2012.07.005

Organization WH, 2024, Global tuberculosis report 2024

Pulimood AB, 2008, AM J CLIN PATHOL, V129, P846, DOI 10.1309/DKKECWQWMG4J23E3

Qiu R, 2022, CAN J GASTROENTEROL, V2022, DOI 10.1155/2022/1206134

Raitoharju E, 2016, SCI REP-UK, V6, DOI 10.1038/srep38262

Ren P, 2023, ANTICANCER RES, V43, P4379, DOI 10.21873/anticanres.16634

Saha S, 2009, CELL HOST MICROBE, V5, P137, DOI 10.1016/j.chom.2008.12.010

Seagar AL, 2008, J MED MICROBIOL, V57, P605, DOI 10.1099/jmm.0.47484-0

Singh PP, 2012, J IMMUNOL, V189, P777, DOI 10.4049/jimmunol.1103638

Sun ZC, 2023, FRONT MICROBIOL, V14, DOI 10.3389/fmicb.2023.1236012

Tan M, 2016, CELL PHYSIOL BIOCHEM, V38, P2348, DOI 10.1159/000445588

Vandewalle V, 2021, J CELL MOL MED, V25, P575, DOI 10.1111/jcmm.16110

Vasiliu A, 2022, BREATHE, V18, DOI 10.1183/20734735.0226-2022

Venter R, 2017, SCI REP-UK, V7, DOI 10.1038/s41598-017-14385-x

Xiao T, 2025, CELL SIGNAL, V127, DOI 10.1016/j.cellsig.2024.111561

Xie YL, 2024, LANCET MICROBE, V5, pe520, DOI 10.1016/S2666-5247(24)00001-6

Xiong M, 2024, PLACENTA, V153, P1, DOI 10.1016/j.placenta.2024.05.131

Yang JS, 2024, NEOPLASMA, V71, DOI 10.4149/neo_2024_230915N489

Yao HB, 2023, INT J MOL SCI, V24, DOI 10.3390/ijms24087204

Zhang W, 2019, J CELL BIOCHEM, V120, P16283, DOI 10.1002/jcb.28910

Zhou XN, 2020, CELL J, V21, P459, DOI 10.22074/cellj.2020.6281

NR 61

TC 0

Z9 0

U1 0

U2 6

PU BMC

PI LONDON

PA CAMPUS, 4 CRINAN ST, LONDON N1 9XW, ENGLAND

EI 1471-2334

J9 BMC INFECT DIS

JI BMC Infect. Dis.

PD AUG 25

PY 2025

VL 25

IS 1

AR 1063

DI 10.1186/s12879-025-11487-0

PG 10

WC Infectious Diseases

WE Science Citation Index Expanded (SCI-EXPANDED)

SC Infectious Diseases

GA 6PO3A

UT WOS:001558963300003

OA Green Submitted, gold

DA 2026-05-07

ER

PT J

AU Olson, DP

Day, CL

Magula, NP

Sahid, F

Moosa, MYS

AF Olson, Douglas P.

Day, Cheryl L.

Magula, Nombulelo P.

Sahid, Faieza

Moosa, Mahomed-Yunus S.

TI Case report: Cutaneous extensively drug-resistant tuberculosis

SO AMERICAN JOURNAL OF TROPICAL MEDICINE AND HYGIENE

LA English

DT Article

AB A 48-year-old immunocompetent man without known exposure to tuberculosis had a > 10-year history of recurrent skin lesions. Cutaneous tuberculosis without any current or past history of pulmonary tuberculosis was diagnosed. Culture of biopsy specimens showed the organism to be resistant to multiple first-line and second-line agents. The patient had a broad, vigorous CD4-specific immune response against multiple tuberculosis antigens. This case is the first report of cutaneous extensively drug-resistant tuberculosis.

C1 Univ KwaZulu Natal, Dept Infect Dis, Div Med, Durban, South Africa.

Univ KwaZulu Natal, HIV Pathogenesis Programme, Durban, South Africa.

George Washington Univ, Sch Med, Washington, DC USA.

Massachusetts Gen Hosp, Partners AIDS Res Ctr, Boston, MA 02114 USA.

Harvard Univ, Sch Med, Boston, MA USA.

C3 University of Kwazulu Natal; University of Kwazulu Natal; George

Washington University; Harvard University; Harvard University Medical

Affiliates; Massachusetts General Hospital; Harvard University; Harvard

Medical School

RP Olson, DP (corresponding author), Yale Univ, Sch Med, 26131 Town Walk Dr, Hamden, CT 06518 USA.

EM douglas.olson@yale.edu

RI , Cheryl/J-9844-2012; Day, Cheryl/J-9844-2012

OI , Cheryl/0000-0002-6497-4846;

CR [Anonymous], 1975, Tubercle, V56, P269

[Anonymous], 2006, WHO GLOB TASK FORC O

Barbagallo Joseph, 2002, Am J Clin Dermatol, V3, P319, DOI 10.2165/00128071-200203050-00004

Dye C, 1999, JAMA-J AM MED ASSOC, V282, P677, DOI 10.1001/jama.282.7.677

Friedman PC, 2005, J AM ACAD DERMATOL, V53, pS154, DOI 10.1016/j.jaad.2005.01.110

Ginsburg AS, 2003, CLIN INFECT DIS, V37, P1448, DOI 10.1086/379328

Golden MP, 2005, AM FAM PHYSICIAN, V72, P1761

Gopinathan R, 2001, Indian J Med Microbiol, V19, P193

Ho C. K., 2006, Hong Kong Medical Journal, V12, P272

Pitcher CJ, 1999, NAT MED, V5, P518, DOI 10.1038/8400

Ramam M, 2005, INT J DERMATOL, V44, P121, DOI 10.1111/j.1365-4632.2005.02063.x

Ramesh V, 2001, PEDIATR DERMATOL, V18, P393, DOI 10.1046/j.1525-1470.2001.01962.x

*S AFR NAT TUB CON, 2004, S AFR NAT TUB CONT P

*US CDCP, 2006, MMWR-MORBID MORTAL W, V55, P301

NR 14

TC 6

Z9 8

U1 0

U2 1

PU AMER SOC TROP MED & HYGIENE

PI MCLEAN

PA 8000 WESTPARK DR, STE 130, MCLEAN, VA 22101 USA

SN 0002-9637

J9 AM J TROP MED HYG

JI Am. J. Trop. Med. Hyg.

PD SEP

PY 2007

VL 77

IS 3

BP 551

EP 554

DI 10.4269/ajtmh.2007.77.551

PG 4

WC Public, Environmental & Occupational Health; Tropical Medicine

WE Science Citation Index Expanded (SCI-EXPANDED)

SC Public, Environmental & Occupational Health; Tropical Medicine

GA 208IE

UT WOS:000249312200027

OA Bronze

DA 2026-05-07

ER

PT J

AU Kristiansen, JE

Thomsen, VF

Martins, A

Viveiros, M

Amaral, L

AF Kristiansen, J. E.

Thomsen, V. F.

Martins, Ana

Viveiros, M.

Amaral, L.

TI Non-antibiotics Reverse Resistance of Bacteria to Antibiotics

SO IN VIVO

LA English

DT Article

DE Non-antibiotics; bacteria; antibiotics; reversal of resistance

ID STAPHYLOCOCCUS-AUREUS; EFFLUX PUMP; XDR-TB; PHENOTHIAZINES;

THIORIDAZINE; TUBERCULOSIS; MECHANISMS

AB Background: Most clinical isolates that exhibit a multi-drug resistant phenotype owe that resistance to over-expressed efflux pumps. Compounds that are efflux pump inhibitors (EPIs) reduce or reverse resistance to antibiotics to which the bacterial strain is initially resistant. We have evaluated non-antibiotics to reduce resistance of commonly encountered bacterial pathogens to antibiotics. Materials and Methods: The effect of non-antibiotics on the susceptibility of bacteria to antibiotics was conducted by minimum inhibition concentration determinations of the antibiotic in the absence and presence of the non-antibiotic. Results: Non-antibiotics such as chlorpromazine, amitryptiline and transchlorprothixene are shown to reduce or reverse resistance of a variety of bacteria to antibiotics. Conclusion: The results suggest that non-antibiotics may serve as adjuncts to conventional antibiotics for the therapy of problematic antibiotic infections caused by bacteria that owe their resistance to over-expressed efflux pumps.

C1 [Martins, Ana; Viveiros, M.; Amaral, L.] Univ Nova Lisboa, Inst Hyg & Trop Med, Unit Mycobacteriol, P-1349008 Lisbon, Portugal.

[Kristiansen, J. E.] So Danish Univ, Res Unit Clin Microbiol, Odense, Denmark.

[Amaral, L.] Univ Nova Lisboa, Inst Hyg & Trop Med, Unit Med Parasitol & Microbiol, P-1349008 Lisbon, Portugal.

[Thomsen, V. F.] Int Soc Antimicrobial Activ Nonantibiot ISN, Copenhagen, Denmark.

C3 Universidade Nova de Lisboa; Institute of Hygiene & Tropical Medicine -

UNL; University of Southern Denmark; Universidade Nova de Lisboa;

Institute of Hygiene & Tropical Medicine - UNL

RP Amaral, L (corresponding author), Univ Nova Lisboa, Inst Hyg & Trop Med, Unit Mycobacteriol, Rua Junqueira 100, P-1349008 Lisbon, Portugal.

EM lamaral@ihmt.unl.pt

RI Martins, Ana/I-6432-2012; Viveiros, Miguel/A-7326-2008; Amaral,

Leonard/I-2112-2012

OI Martins, Ana/0000-0002-8541-7440; Viveiros, Miguel/0000-0001-9676-6251;

Amaral, Leonard/0000-0003-3127-1295

CR ABBATE E, 2007, REV ARGENTINA MED RE, V1, P19

Amaral L, 2010, INT J ANTIMICROB AG, V35, P524, DOI 10.1016/j.ijantimicag.2009.12.019

Blair JMA, 2009, CURR OPIN MICROBIOL, V12, P512, DOI 10.1016/j.mib.2009.07.003

Couto I, 2008, J ANTIMICROB CHEMOTH, V62, P504, DOI 10.1093/jac/dkn217

Dasgupta A, 2010, INT J ANTIMICROB AG, V35, P174, DOI 10.1016/j.ijantimicag.2009.09.027

Kaatz GW, 2003, ANTIMICROB AGENTS CH, V47, P719, DOI 10.1128/AAC.47.2.719-726.2003

Kristiansen J.E., 1993, CURR OPIN INVEST DR, V2, P587

KRISTIANSEN JE, 1990, DAN MED BULL, V37, P165

Kristiansen JE, 2007, J ANTIMICROB CHEMOTH, V59, P1271, DOI 10.1093/jac/dkm071

Li XZ, 2009, DRUGS, V69, P1555, DOI 10.2165/11317030-000000000-00000

Martins A, 2009, INT J ANTIMICROB AG, V34, P602, DOI 10.1016/j.ijantimicag.2009.06.029

Martins M, 2009, INT J TUBERC LUNG D, V13, P569

Martins M, 2008, INT J ANTIMICROB AG, V31, P198, DOI 10.1016/j.ijantimicag.2007.10.025

Martins M, 2007, IN VIVO, V21, P771

Ordway D, 2002, INT J ANTIMICROB AG, V20, P34, DOI 10.1016/S0924-8579(02)00110-3

Pagès JM, 2009, BBA-PROTEINS PROTEOM, V1794, P826, DOI 10.1016/j.bbapap.2008.12.011

Van Bambeke Francoise, 2006, Recent Pat Antiinfect Drug Discov, V1, P157, DOI 10.2174/157489106777452692

Viveiros M, 2005, ANTIMICROB AGENTS CH, V49, P3578, DOI 10.1128/AAC.49.8.3578-3582.2005

NR 18

TC 47

Z9 56

U1 5

U2 85

PU INT INST ANTICANCER RESEARCH

PI ATHENS

PA EDITORIAL OFFICE 1ST KM KAPANDRITIOU-KALAMOU RD KAPANDRITI, PO BOX 22,

ATHENS 19014, GREECE

SN 0258-851X

EI 1791-7549

J9 IN VIVO

JI In Vivo

PD SEP-OCT

PY 2010

VL 24

IS 5

BP 751

EP 754

PG 4

WC Medicine, Research & Experimental

WE Science Citation Index Expanded (SCI-EXPANDED)

SC Research & Experimental Medicine

GA 670DD

UT WOS:000283400500021

DA 2026-05-07

ER

PT J

AU Ribeiro, ALDL

Degiacomi, G

Ewann, F

Buroni, S

Incandela, ML

Chiarelli, LR

Mori, G

Kim, J

Contreras-Dominguez, M

Park, YS

Han, SJ

Brodin, P

Valentini, G

Rizzi, M

Riccardi, G

Pasca, MR

AF Ribeiro, Ana Luisa de Jesus Lopes

Degiacomi, Giulia

Ewann, Fanny

Buroni, Silvia

Incandela, Maria Loreto

Chiarelli, Laurent R.

Mori, Giorgia

Kim, Jaeseung

Contreras-Dominguez, Monica

Park, Young-Sam

Han, Sung-Jun

Brodin, Priscille

Valentini, Giovanna

Rizzi, Menico

Riccardi, Giovanna

Pasca, Maria Rosalia

TI Analogous Mechanisms of Resistance to Benzothiazinones and

Dinitrobenzamides in Mycobacterium smegmatis

SO PLOS ONE

LA English

DT Article

ID D-RIBOSE 2'-EPIMERASE; TUBERCULOSIS; ARABINAN

AB Tuberculosis is still a leading cause of death worldwide. The selection and spread of Mycobacterium tuberculosis multidrug-resistant (MDR-TB) and extensively drug-resistant strains (XDR-TB) is a severe public health problem. Recently, two different classes of chemical series, the benzothiazinones (BTZ) and the dinitrobenzamide (DNB) derivatives have been found to be highly active against M. tuberculosis, including XDR-TB strains. The target of BTZs is DprE1 protein which works in concert with DprE2 to form the heteromeric decaprenylphosphoryl-beta-D-ribose 2'-epimerase, involved in Decaprenyl-Phospho-Arabinose (DPA) biosynthesis. Interestingly, it has been shown that the DNBs block the same pathway thus suggesting that both drugs could share the same target. Moreover, in Mycobacterium smegmatis the overexpression of the NfnB nitroreductase led to the inactivation of the BTZs by reduction of a critical nitro-group to an amino-group. In this work several spontaneous M. smegmatis mutants resistant to DNBs were isolated. Sixteen mutants, showing high levels of DNB resistance, exhibited a mutation in the Cys394 of DprE1. Using fluorescence titration and mass spectrometry it has been possible to monitor the binding between DprE1 and DNBs, achieving direct evidence that MSMEG_6382 is the cellular target of DNBs in mycobacteria. Additionally, M. smegmatis mutants having low levels of resistance to DNBs harbor various mutations in MSMEG_6503 gene encoding the transcriptional repressor of the nitroreductase NfnB. By LC/MS2 analysis it has been demonstrated that NfnB is responsible for DNB inactivation. Taken together, our data demonstrate that both DNB and BTZ drugs share common resistance mechanisms in M. smegmatis.

C1 [Ribeiro, Ana Luisa de Jesus Lopes; Degiacomi, Giulia; Buroni, Silvia; Incandela, Maria Loreto; Mori, Giorgia; Riccardi, Giovanna; Pasca, Maria Rosalia] Univ Pavia, Dipartimento Genet & Microbiol, I-27100 Pavia, Italy.

[Ribeiro, Ana Luisa de Jesus Lopes; Rizzi, Menico] Univ Piemonte Orientale, Dipartimento Sci Chim Alimentari Farmaceut & Farm, Novara, Italy.

[Ewann, Fanny; Kim, Jaeseung; Contreras-Dominguez, Monica; Park, Young-Sam; Han, Sung-Jun; Brodin, Priscille] Inst Pasteur Korea, Songnam, Gyeonggi Do, South Korea.

[Chiarelli, Laurent R.; Valentini, Giovanna] Univ Pavia, Dipartimento Biochim A Castellani, I-27100 Pavia, Italy.

C3 University of Pavia; University of Eastern Piedmont Amedeo Avogadro;

Institut Pasteur Korea; University of Pavia

RP Ribeiro, ALDL (corresponding author), Univ Pavia, Dipartimento Genet & Microbiol, Via Palestro 3, I-27100 Pavia, Italy.

EM mariarosalia.pasca@unipv.it

RI Mori, Giorgia/X-8670-2019; PASCA, Maria Rosalia/J-8752-2016; RICCARDI,

Giovanna/A-9269-2012; Buroni, Silvia/AAX-5454-2020; Brodin,

Priscille/E-5802-2014; Chiarelli, Laurent Robert/J-8368-2016; Han,

Sung-Jun/B-9547-2012; Degiacomi, Giulia/ABC-7235-2020

OI Mori, Giorgia/0000-0003-3469-5632; PASCA, Maria

Rosalia/0000-0002-8906-4937; RICCARDI, Giovanna/0000-0003-3295-0070;

Buroni, Silvia/0000-0002-6979-2275; Brodin,

Priscille/0000-0003-0991-7344; Chiarelli, Laurent

Robert/0000-0003-0348-9764; de Jesus Lopes Ribeiro, Ana

Luísa/0000-0002-3686-9213; Degiacomi, Giulia/0000-0002-3626-8311

FU European Commission [260872]; Korea Research Foundation

[K204EA000001-08E0100-00100, K204EA000001-09E0100-00100,

K20802001409-09B1300-03700]; National Research foundation of Korea

(NRF); Korea government(MEST) [2011-00244]; KISTI; Forte [2011-00244]

Funding Source: Forte

FX The funders had no role in study design, data collection and analysis,

decision to publish, or preparation of the manuscript. This work was

funded by the European Commission (contract no 260872 - More Medicines

for Tuberculosis, MM4TB), the Korea Research Foundation (Grant

K204EA000001-08E0100-00100, K204EA000001-09E0100-00100,

K20802001409-09B1300-03700) and INSERM-Avenir. This work was also

supported by the National Research foundation of Korea (NRF) grant

funded by the Korea government(MEST)(No. 2011-00244), Gyeonggi-do and

KISTI.

CR Brodin P., 2010, Pat., Patent No. [2010003533, WO2010003533]

Crellin PK, 2011, PLOS ONE, V6, DOI 10.1371/journal.pone.0016869

Jain A, 2008, FEMS IMMUNOL MED MIC, V53, P145, DOI 10.1111/j.1574-695X.2008.00400.x

Lalloo Umesh G, 2010, Curr HIV/AIDS Rep, V7, P143, DOI 10.1007/s11904-010-0054-4

Lenaerts AJ, 2008, TRENDS MICROBIOL, V16, P48, DOI 10.1016/j.tim.2007.12.002

Makarov V, 2009, SCIENCE, V324, P801, DOI 10.1126/science.1171583

Manina G, 2010, CURR MED CHEM, V17, P3099, DOI 10.2174/092986710791959693

Manina G, 2010, MOL MICROBIOL, V77, P1172, DOI 10.1111/j.1365-2958.2010.07277.x

Mikusová K, 2005, J BACTERIOL, V187, P8020, DOI 10.1128/JB.187.23.8020-8025.2005

Riccardi G, 2009, FUTURE MICROBIOL, V4, P597, DOI [10.2217/fmb.09.20, 10.2217/FMB.09.20]

Sambrook Joseph, 2006, CSH Protoc, V2006, DOI 10.1101/pdb.prot4022

Trefzer C, 2010, J AM CHEM SOC, V132, P13663, DOI 10.1021/ja106357w

Velayati AA, 2009, CHEST, V136, P420, DOI 10.1378/chest.08-2427

Wolucka BA, 2008, FEBS J, V275, P2691, DOI 10.1111/j.1742-4658.2008.06395.x

Zignol M, 2006, J INFECT DIS, V194, P479, DOI 10.1086/505877

Zumla A, 2009, LANCET INFECT DIS, V9, P197, DOI 10.1016/S1473-3099(09)70045-3

NR 16

TC 46

Z9 51

U1 0

U2 28

PU PUBLIC LIBRARY SCIENCE

PI SAN FRANCISCO

PA 1160 BATTERY STREET, STE 100, SAN FRANCISCO, CA 94111 USA

SN 1932-6203

J9 PLOS ONE

JI PLoS One

PD NOV 1

PY 2011

VL 6

IS 11

AR e26675

DI 10.1371/journal.pone.0026675

PG 7

WC Multidisciplinary Sciences

WE Science Citation Index Expanded (SCI-EXPANDED)

SC Science & Technology - Other Topics

GA 849UN

UT WOS:000297150900024

OA Green Submitted, gold

DA 2026-05-07

ER

PT J

AU Jadhav, K

Singh, R

Ray, E

Singh, AK

Verma, RK

AF Jadhav, Krishna

Singh, Raghuraj

Ray, Eupa

Singh, Amit Kumar

Verma, Rahul Kumar

TI Taming the Devil: Antimicrobial Peptides for Safer TB Therapeutics

SO CURRENT PROTEIN & PEPTIDE SCIENCE

LA English

DT Review

DE Antimicrobial peptides; tuberculosis; pro-inflammatory; drug delivery;

strategies; nanoparticles; microparticles

ID MYCOBACTERIUM-TUBERCULOSIS INFECTION; HUMAN NEUTROPHIL PEPTIDE-1;

HYALURONIC-ACID NANOGEL; IN-VITRO ACTIVITY; HOST-DEFENSE;

PHAGOSOME-MATURATION; CATHELICIDIN LL-37; ANTIBACTERIAL; DELIVERY;

MECHANISM

AB Tuberculosis (TB) is a highly contagious infection with extensive mortality and morbidity. The rise of TB-superbugs (drug-resistant strains) with the increase of their resistance to conventional antibiotics has prompted a further search for new anti-mycobacterial agents. It is difficult to breach the barriers around TB bacteria, including mycolic cell wall, granuloma, biofilm and mucus, by conventional antibiotics in a short span of time. Hence, there is an essential need for molecules with an unconventional mode of action and structure that can efficiently break the barriers around mycobacterium. Antimicrobial peptides (AMP) are essential components of innate immunity having cationic and amphipathic characteristics. Lines of evidence show that AMPs have good myco-bactericidal and anti-biofilm activity against normal as well as antibiotic-resistant TB bacteria. These peptides have shown direct killing of bacteria by membrane lysis and indirect killing by activation of innate immune response in host cells by interacting with the component of the bacterial membrane and intracellular targets through diverse mechanisms. Despite a good anti-mycobacterial activity, some undesirable characteristics are also associated with AMP, including hemolysis, cytotoxicity, susceptibility to proteolysis and poor pharmacokinetic profile, and hence only a few clinical studies have been conducted with these biomolecules. The design of new combinatorial therapies, including AMPs and particulate drug delivery systems, could be new potential alternatives to conventional antibiotics to fight MDR- and XDR-TB. This review outlined the array of AMP roles in TB therapy, possible mechanisms of actions, activities, and current advances in pragmatic strategies to improve challenges accompanying the delivery of AMP for tuberculosis therapeutics.

C1 [Jadhav, Krishna; Singh, Raghuraj; Ray, Eupa; Verma, Rahul Kumar] Habitat Ctr, Inst Nano Sci & Technol, Phase 10 Sect 64, Mohali 160062, Punjab, India.

[Singh, Amit Kumar] Natl JALMA Inst Leprosy & Other Mycobacterial Dis, Agra 282001, India.

C3 Department of Science & Technology (India); Institute of Nano Science &

Technology (INST); Indian Council of Medical Research (ICMR); ICMR -

National JALMA Institute for Leprosy & Other Mycobacterial Diseases,

Agra

RP Verma, RK (corresponding author), Habitat Ctr, Inst Nano Sci & Technol, Phase 10 Sect 64, Mohali 160062, Punjab, India.

EM rahulverma@inst.ac.in

RI Singh, Amit/GMW-5097-2022; Singh, Raghuraj/MTA-8191-2025; Jadhav,

Krishna/ABB-4859-2021; Jadhav, Krishna Sudhakar/ABB-4859-2021; Verma,

Rahul Kumar/C-6027-2012

OI Jadhav, Krishna Sudhakar/0000-0002-5875-2315; Verma, Rahul

Kumar/0000-0003-0719-8227

FU Indian Council of Medical Research (ICMR), India

[5/8/5/68/ADHOC/2020/ECD-1] Funding Source: Medline; SERB, Science and

Engineering Research Board [CRG/2019/001483] Funding Source: Medline

CR Abraham P, 2020, PEPTIDES, V132, DOI 10.1016/j.peptides.2020.170373

Adlakha S, 2020, CURR PROTEIN PEPT SC, V21, P369, DOI 10.2174/1389203721666191231110453

Agerberth B, 2006, CURR TOP MICROBIOL, V306, P67

Alderwick LJ, 2015, CSH PERSPECT MED, V5, DOI 10.1101/cshperspect.a021113

AlMatar M, 2018, PHARMACOL RES, V128, P288, DOI 10.1016/j.phrs.2017.10.011

[Anonymous], 2006, Wkly Epidemiol Rec, V81, P430

[Anonymous], 2020, GLOBAL TUBERCULOSIS

Aoki W, 2013, PHARMACEUTICALS-BASE, V6, P1055, DOI 10.3390/ph6081055

Ashitani J, 2002, CHEST, V121, P519, DOI 10.1378/chest.121.2.519

Barlow PG, 2010, AM J RESP CELL MOL, V43, P692, DOI 10.1165/rcmb.2009-0250OC

Batoni G, 2016, BBA-BIOMEMBRANES, V1858, P1044, DOI 10.1016/j.bbamem.2015.10.013

Bi L, 2011, J CONTROL RELEASE, V150, P150, DOI 10.1016/j.jconrel.2010.11.024

Bocchinfuso G, 2009, J PEPT SCI, V15, P550, DOI 10.1002/psc.1144

Brogden KA, 2005, NAT REV MICROBIOL, V3, P238, DOI 10.1038/nrmicro1098

Carroll J, 2010, INT J ANTIMICROB AG, V36, P132, DOI 10.1016/j.ijantimicag.2010.03.029

Chen YX, 2007, ANTIMICROB AGENTS CH, V51, P1398, DOI 10.1128/AAC.00925-06

COUVREUR P, 1993, ADV DRUG DELIVER REV, V10, P141, DOI 10.1016/0169-409X(93)90046-7

Daffé M, 2015, TUBERCULOSIS, V95, pS155, DOI 10.1016/j.tube.2015.02.024

Dartois V, 2014, NAT REV MICROBIOL, V12, P159, DOI 10.1038/nrmicro3200

Dathe M, 1997, FEBS LETT, V403, P208, DOI 10.1016/S0014-5793(97)00055-0

Dutta P, 2016, CURR TOP MED CHEM, V16, P99, DOI 10.2174/1568026615666150703121819

Ebenhan T, 2014, BIOMED RES INT, V2014, DOI 10.1155/2014/867381

Erdem B.M., 2021, J APPL MICROBIOL, V12, P3631

Fattorini L, 2004, PEPTIDES, V25, P1075, DOI 10.1016/j.peptides.2004.04.003

Fehlbaum P, 2000, P NATL ACAD SCI USA, V97, P12723, DOI 10.1073/pnas.220424597

Fratti RA, 2003, P NATL ACAD SCI USA, V100, P5437, DOI 10.1073/pnas.0737613100

Gagnon MG, 2016, NUCLEIC ACIDS RES, V44, P2439, DOI 10.1093/nar/gkw018

Gavrish E, 2014, CHEM BIOL, V21, P509, DOI 10.1016/j.chembiol.2014.01.014

Gazit E, 1996, J MOL BIOL, V258, P860, DOI 10.1006/jmbi.1996.0293

Giuliani A, 2007, CENT EUR J BIOL, V2, P1, DOI 10.2478/s11535-007-0010-5

Gupta S, 2017, J PATHOL, V242, P52, DOI 10.1002/path.4878

Gutsmann T, 2016, BBA-BIOMEMBRANES, V1858, P1034, DOI 10.1016/j.bbamem.2016.01.031

Hancock REW, 2006, NAT BIOTECHNOL, V24, P1551, DOI 10.1038/nbt1267

Haney EF, 2013, BIOPOLYMERS, V100, P572, DOI 10.1002/bip.22250

Hollmann A, 2018, FRONT CHEM, V6, DOI 10.3389/fchem.2018.00204

Jacobsen M, 2007, J MOL MED, V85, P613, DOI 10.1007/s00109-007-0157-6

Kalita A, 2004, J INFECT DIS, V190, P1476, DOI 10.1086/424463

Kapoor R, 2011, ANTIMICROB AGENTS CH, V55, P3058, DOI 10.1128/AAC.01667-10

Khara JS, 2014, BIOMATERIALS, V35, P2032, DOI 10.1016/j.biomaterials.2013.11.035

Kim H, 2014, J ANTIMICROB CHEMOTH, V69, P121, DOI 10.1093/jac/dkt322

Kisich KO, 2001, INFECT IMMUN, V69, P2692, DOI 10.1128/IAI.69.4.2692-2699.2001

Kwok PCL, 2015, INT J PHARMACEUT, V491, P367, DOI 10.1016/j.ijpharm.2015.07.001

Lan Y, 2014, TUBERCULOSIS, V94, P678, DOI 10.1016/j.tube.2014.08.001

Lau YE, 2005, INFECT IMMUN, V73, P583, DOI 10.1128/IAI.73.1.583-591.2005

Lee TH, 2016, CURR TOP MED CHEM, V16, P25, DOI 10.2174/1568026615666150703121700

Lei J, 2019, AM J TRANSL RES, V11, P3919

Liang ZM, 2020, PHARMACEUTICS, V12, DOI 10.3390/pharmaceutics12121172

Lienhardt C, 2012, J INFECT DIS, V205, pS241, DOI 10.1093/infdis/jis034

Linde CMA, 2001, J ANTIMICROB CHEMOTH, V47, P575, DOI 10.1093/jac/47.5.575

LoBue Philip, 2009, Morbidity and Mortality Weekly Report, V58, P1

Mahlapuu M, 2016, FRONT CELL INFECT MI, V6, DOI 10.3389/fcimb.2016.00194

Mardirossian M, 2018, CELL CHEM BIOL, V25, P530, DOI 10.1016/j.chembiol.2018.02.004

Martins S, 2007, INT J NANOMED, V2, P595

Mily A, 2015, PLOS ONE, V10, DOI 10.1371/journal.pone.0138340

Mily A, 2013, BMC PULM MED, V13, DOI 10.1186/1471-2466-13-23

Mir M, 2017, COLLOID SURFACE B, V159, P217, DOI 10.1016/j.colsurfb.2017.07.038

Mohanty S, 2013, ANTIMICROB AGENTS CH, V57, P3688, DOI 10.1128/AAC.02475-12

Mookherjee N, 2006, J IMMUNOL, V176, P2455, DOI 10.4049/jimmunol.176.4.2455

Oliveira GS, 2021, PHARMACEUTICALS-BASE, V14, DOI 10.3390/ph14040323

Oren Z, 1998, BIOPOLYMERS, V47, P451

Pasupuleti M, 2012, CRIT REV BIOTECHNOL, V32, P143, DOI 10.3109/07388551.2011.594423

PATHAK N, 1995, PROTEINS, V22, P182, DOI 10.1002/prot.340220210

Pedrosa SS, 2014, MACROMOL BIOSCI, V14, P1556, DOI 10.1002/mabi.201400135

Coyotl EPA, 2020, PHARMACEUTICS, V12, DOI 10.3390/pharmaceutics12111071

Peters BM, 2010, PLOS PATHOG, V6, DOI 10.1371/journal.ppat.1001067

Piras AM, 2015, FRONT MICROBIOL, V6, DOI 10.3389/fmicb.2015.00372

Portell-Buj E, 2019, J MED MICROBIOL, V68, P211, DOI 10.1099/jmm.0.000912

Pruksakorn P, 2010, BIOORG MED CHEM LETT, V20, P3658, DOI 10.1016/j.bmcl.2010.04.100

Pulido D, 2013, ANTIMICROB AGENTS CH, V57, P3797, DOI 10.1128/AAC.00428-13

Ramírez-Carreto S, 2015, PEPTIDES, V73, P51, DOI 10.1016/j.peptides.2015.08.014

Ramón-García S, 2013, ANTIMICROB AGENTS CH, V57, P2295, DOI 10.1128/AAC.00175-13

Rekha RS, 2018, BMC INFECT DIS, V18, DOI 10.1186/s12879-018-3203-9

Rekha RS, 2015, AUTOPHAGY, V11, P1688, DOI 10.1080/15548627.2015.1075110

Rima M, 2021, ANTIBIOTICS-BASEL, V10, DOI 10.3390/antibiotics10091095

Rivas-Santiago B, 2005, INFECT IMMUN, V73, P4505, DOI 10.1128/IAI.73.8.4505-4511.2005

Rivas-Santiago B, 2008, INFECT IMMUN, V76, P935, DOI 10.1128/IAI.01218-07

Rivas-Santiago B, 2018, CURR PHARM DESIGN, V24, P1138, DOI 10.2174/1381612824666180327162357

Rivas-Santiago B, 2013, PLOS ONE, V8, DOI 10.1371/journal.pone.0059119

Rivas-Santiago CE, 2011, CLIN EXP IMMUNOL, V164, P80, DOI 10.1111/j.1365-2249.2010.04313.x

Rivas-Santiago CE, 2013, IMMUNOTHERAPY-UK, V5, P1117, DOI 10.2217/imt.13.111

Rodríguez A, 2014, PLOS ONE, V9, DOI 10.1371/journal.pone.0101742

Rozek A, 2000, BIOCHEMISTRY-US, V39, P15765, DOI 10.1021/bi000714m

Santos AG, 2019, SCI REP-UK, V9, DOI 10.1038/s41598-018-37186-2

Santos P, 2012, PEPTIDES, V36, P121, DOI 10.1016/j.peptides.2012.04.018

Sarkar T, 2021, FRONT CHEM, V9, DOI 10.3389/fchem.2021.691532

Sato E, 2013, J DERMATOL SCI, V70, P166, DOI 10.1016/j.jdermsci.2013.01.011

Seung KJ, 2015, CSH PERSPECT MED, V5, DOI 10.1101/cshperspect.a017863

Shai Y, 1999, BBA-BIOMEMBRANES, V1462, P55, DOI 10.1016/S0005-2736(99)00200-X

Sharma A., 2018, J AEROSOL MED PULMON, VA17

Sharma A, 2020, J CONTROL RELEASE, V324, P17, DOI 10.1016/j.jconrel.2020.05.013

Sharma A, 2019, INT J PHARMACEUT, V558, P231, DOI 10.1016/j.ijpharm.2018.12.076

Sharma A, 2018, INT J PHARMACEUT, V536, P50, DOI 10.1016/j.ijpharm.2017.11.046

Sharma S, 2000, EUR RESPIR J, V16, P112, DOI 10.1034/j.1399-3003.2000.16a20.x

Silva JP, 2016, J CONTROL RELEASE, V235, P112, DOI 10.1016/j.jconrel.2016.05.064

Sonawane A, 2011, CELL MICROBIOL, V13, P1601, DOI 10.1111/j.1462-5822.2011.01644.x

Sosunov V, 2007, J ANTIMICROB CHEMOTH, V59, P919, DOI 10.1093/jac/dkm053

Sow FB, 2007, J LEUKOCYTE BIOL, V82, P934, DOI 10.1189/jlb.0407216

Swaminathan J, 2012, EXPERT OPIN DRUG DEL, V9, P1489, DOI 10.1517/17425247.2012.735658

Tenland E, 2019, PLOS ONE, V14, DOI 10.1371/journal.pone.0212858

Tenland E, 2018, TUBERCULOSIS, V113, P231, DOI 10.1016/j.tube.2018.10.008

Thapa RK, 2021, J PHARM INVEST, V51, P377

Tiberi S, 2018, PULMONOLOGY, V24, P86, DOI 10.1016/j.rppnen.2017.10.009

Torres-Juarez F, 2015, INFECT IMMUN, V83, P4495, DOI 10.1128/IAI.00936-15

TOSSI A, 1994, FEBS LETT, V339, P108, DOI 10.1016/0014-5793(94)80395-1

Vilchèze C, 2020, APPL SCI-BASEL, V10, DOI 10.3390/app10072278

Wan M, 2014, J LEUKOCYTE BIOL, V95, P971, DOI 10.1189/jlb.0513304

Wang TT, 2004, J IMMUNOL, V173, P2909, DOI 10.4049/jimmunol.173.5.2909

Wiesner J, 2010, VIRULENCE, V1, P440, DOI 10.4161/viru.1.5.12983

Xu D, 2020, FRONT IMMUNOL, V11, DOI 10.3389/fimmu.2020.00764

Yeaman MR, 2003, PHARMACOL REV, V55, P27, DOI 10.1124/pr.55.1.2

Yu J, 2007, J IMMUNOL, V179, P7684, DOI 10.4049/jimmunol.179.11.7684

Yuk JM, 2009, CELL HOST MICROBE, V6, P231, DOI 10.1016/j.chom.2009.08.004

Zasloff M, 2002, NATURE, V415, P389, DOI 10.1038/415389a

NR 113

TC 24

Z9 25

U1 2

U2 7

PU BENTHAM SCIENCE PUBL LTD

PI SHARJAH

PA EXECUTIVE STE Y-2, PO BOX 7917, SAIF ZONE, 1200 BR SHARJAH, U ARAB

EMIRATES

SN 1389-2037

EI 1875-5550

J9 CURR PROTEIN PEPT SC

JI Curr. Protein Pept. Sci.

PY 2022

VL 23

IS 10

BP 643

EP 656

DI 10.2174/1389203723666220526161109

PG 14

WC Biochemistry & Molecular Biology

WE Science Citation Index Expanded (SCI-EXPANDED)

SC Biochemistry & Molecular Biology

GA 7H0GN

UT WOS:000902890700001

DA 2026-05-07

ER

PT J

AU Khoza, LJ

Kumar, P

Dube, A

Demana, PH

Choonara, YE

AF Khoza, Leon J.

Kumar, Pradeep

Dube, Admire

Demana, Patrick H.

Choonara, Yahya E.

TI Insights into innovative therapeutics for drug-resistant tuberculosis:

Host-directed therapy and autophagy inducing modified nanoparticles

SO INTERNATIONAL JOURNAL OF PHARMACEUTICS

LA English

DT Article

DE Mycobacterium tuberculosis; Innate immunity; Autophagy induction;

Host-directed therapy; Nanoparticles; Alveolar macrophages

ID NITRIC-OXIDE SYNTHASE; NANOSTRUCTURED LIPID CARRIERS;

MYCOBACTERIUM-TUBERCULOSIS; VITAMIN-D; HUMAN MACROPHAGES;

PHAGOSOME-MATURATION; PULMONARY DELIVERY; EIS PROTEIN; RIFAMPICIN;

FORMULATION

C1 [Khoza, Leon J.; Kumar, Pradeep; Choonara, Yahya E.] Univ Witwatersrand, Fac Hlth Sci, Sch Therap Sci, Dept Pharm Pharm,Wits Adv Drug Deliv Platform Res, ZA-2193 Johannesburg, South Africa.

[Dube, Admire] Univ Western Cape, Fac Nat Sci, Sch Pharm, Cape Town, South Africa.

[Demana, Patrick H.] Sefako Makgatho Hlth Sci Univ, Sch Pharm, Div Pharmaceut Sci, ZA-0208 Pretoria, South Africa.

C3 University of Witwatersrand; University of the Western Cape; Sefako

Makgatho Health Sciences University

RP Choonara, YE (corresponding author), Univ Witwatersrand, Fac Hlth Sci, Sch Therap Sci, Dept Pharm Pharm,Wits Adv Drug Deliv Platform Res, ZA-2193 Johannesburg, South Africa.

EM yahya.choonara@wits.ac.za

RI Choonara, Yahya/B-2955-2013; Kumar, Pradeep/C-9424-2013; Demana,

Patrick/PJS-5162-2026

OI Choonara, Yahya/0000-0002-3889-1529; Khoza, Leon

Joseph/0000-0002-2289-4426;

FU National Research Foundation (NRF) South Africa [UID 64814]

FX This work was supported by the National Research Foundation (NRF) South

Africa [grant numbers: UID 64814] . The content is solely the authors?

responsibility and does not necessarily represent the official views of

the National Research Foundation.

CR Abed N, 2014, INT J ANTIMICROB AG, V43, P485, DOI 10.1016/j.ijantimicag.2014.02.009

Adikesavalu H, 2021, MEDICINA-LITHUANIA, V57, DOI 10.3390/medicina57060522

Alavi-Naini Roya, 2012, Int J High Risk Behav Addict, V1, P71, DOI 10.5812/ijhrba.5215

Allué-Guardia A, 2021, FRONT MICROBIOL, V12, DOI 10.3389/fmicb.2021.612675

Amaral EP, 2016, BMC MICROBIOL, V16, DOI 10.1186/s12866-016-0872-7

Andrade F, 2011, DRUG DELIVERY LETT, V1, P171

Andrade F, 2013, ADV DRUG DELIVER REV, V65, P1816, DOI 10.1016/j.addr.2013.07.020

Aranow C, 2011, J INVEST MED, V59, P881, DOI 10.2310/JIM.0b013e31821b8755

Atri C, 2018, INT J MOL SCI, V19, DOI 10.3390/ijms19061801

Banerjee D, 2014, MED HYPOTHESES, V83, P88, DOI 10.1016/j.mehy.2014.04.002

Banerjee S, 2020, J DRUG TARGET, V28, P55, DOI 10.1080/1061186X.2019.1613409

Baranyai Z, 2021, ADV THER-GERMANY, V4, DOI 10.1002/adtp.202000113

Barberis I, 2017, J Prev Med Hyg, V58, pE9

Bedard K, 2007, PHYSIOL REV, V87, P245, DOI 10.1152/physrev.00044.2005

Behar SM, 2011, MUCOSAL IMMUNOL, V4, P279, DOI 10.1038/mi.2011.3

Bekale RB, 2019, PHARM RES-DORDR, V36, DOI 10.1007/s11095-018-2528-9

Bento CF, 2015, DNA CELL BIOL, V34, P228, DOI 10.1089/dna.2014.2745

Bhandari R, 2013, INT J PHARMACEUT, V441, P202, DOI 10.1016/j.ijpharm.2012.11.042

Bigelow KM, 2020, ANTIMICROB AGENTS CH, V64, DOI 10.1128/AAC.01178-20

Booysen LLIJ, 2013, INT J PHARMACEUT, V444, P10, DOI 10.1016/j.ijpharm.2013.01.038

Bourigault ML, 2013, AM J CLIN EXP IMMUNO, V2, P124

Cadena AM, 2016, MBIO, V7, DOI 10.1128/mBio.00342-16

Cambau E, 2014, CLIN MICROBIOL INFEC, V20, P196, DOI 10.1111/1469-0691.12555

Campbell GR, 2011, J BIOL CHEM, V286, P18890, DOI 10.1074/jbc.M110.206110

Carranza C, 2019, AM J MED SCI, V357, P184, DOI 10.1016/j.amjms.2018.12.003

Chai QY, 2020, CELL MOL IMMUNOL, V17, P901, DOI 10.1038/s41423-020-0502-z

Chai QY, 2018, FRONT CELL INFECT MI, V8, DOI 10.3389/fcimb.2018.00158

Chuan JL, 2013, J NANOPART RES, V15, DOI 10.1007/s11051-013-1634-1

Chun Rene F, 2011, Expert Rev Clin Pharmacol, V4, P583, DOI 10.1586/ecp.11.41

Conradie F., 2017, C RETROVIRUSES OPPOR, P13

Conradie F, 2020, NEW ENGL J MED, V382, P893, DOI 10.1056/NEJMoa1901814

Costa A, 2016, ADV DRUG DELIVER REV, V102, P102, DOI 10.1016/j.addr.2016.04.012

Coussens AK, 2015, PLOS PATHOG, V11, DOI 10.1371/journal.ppat.1005007

Coussens AK, 2012, P NATL ACAD SCI USA, V109, P15449, DOI 10.1073/pnas.1200072109

Crowther RR, 2021, FRONT IMMUNOL, V11, DOI 10.3389/fimmu.2020.628432

D'Souza S, 2022, J Pharm Sci, V111, P469, DOI [10.1016/j.xphs.2021.09.012, 10.1016/j.xphs.2021.09.012]

Dahanayake MH, 2021, J MICROBIOL METH, V181, DOI 10.1016/j.mimet.2020.106127

Dahl JL, 2001, INFECT IMMUN, V69, P4295, DOI 10.1128/IAI.69.7.4295-4302.2001

Dartois V, 2014, NAT REV MICROBIOL, V12, P159, DOI 10.1038/nrmicro3200

Deretic V, 2014, CSH PERSPECT MED, V4, DOI 10.1101/cshperspect.a018481

Dhiman N, 2021, FRONT CHEM, V9, DOI 10.3389/fchem.2021.580118

Dobrovolskaia MA, 2016, TOXICOL APPL PHARM, V299, P78, DOI 10.1016/j.taap.2015.12.022

Dong XP, 2010, NAT COMMUN, V1, DOI 10.1038/ncomms1037

Dube A, 2014, NANOMED-NANOTECHNOL, V10, P831, DOI 10.1016/j.nano.2013.11.012

Efremenko YV, 2012, IMMUNOTHERAPY-UK, V4, P273, DOI [10.2217/IMT.11.176, 10.2217/imt.11.176]

Fang F, 2020, MOL IMMUNOL, V124, P18, DOI 10.1016/j.molimm.2020.05.001

Farazi Aliasghar, 2015, Tuberc Res Treat, V2015, P205016, DOI [10.1155/2015/205016, 10.1155/2015/205016]

Floto RA, 2007, AUTOPHAGY, V3, P620, DOI 10.4161/auto.4898

Gagneux S, 2018, NAT REV MICROBIOL, V16, P202, DOI 10.1038/nrmicro.2018.8

Galetti M., 2019, EXPOSURE ENGINEERED

Garcia I, 2000, LAB INVEST, V80, P1385, DOI 10.1038/labinvest.3780146

Garg T, 2016, ARTIF CELL NANOMED B, V44, P997, DOI 10.3109/21691401.2015.1008508

Ghazaei C, 2018, J RES MED SCI, V23, DOI 10.4103/jrms.JRMS_904_17

Gina P, 2019, Afr J Thorac Crit Care Med, V25, DOI 10.7196/AJTCCM.2019.v25i2.014

Goletti D, 2013, CYTOKINE GROWTH F R, V24, P335, DOI 10.1016/j.cytogfr.2013.01.002

Green D.R., 2011, MEANS END APOPTOSIS

Grotz E, 2018, PHARM RES-DORDR, V35, DOI 10.1007/s11095-018-2497-z

Gu F, 2008, P NATL ACAD SCI USA, V105, P2586, DOI 10.1073/pnas.0711714105

Guirado E, 2013, SEMIN IMMUNOPATHOL, V35, P563, DOI 10.1007/s00281-013-0388-2

Gutierrez MG, 2004, CELL, V119, P753, DOI 10.1016/j.cell.2004.11.038

Haimhoffer A, 2019, SCI PHARM, V87, DOI 10.3390/scipharm87040033

Hewison M, 2011, NAT REV ENDOCRINOL, V7, P336, DOI 10.1038/nrendo.2010.226

Hinchey J, 2007, J CLIN INVEST, V117, P2279, DOI 10.1172/JCI31947

Hmama Z, 2015, IMMUNOL REV, V264, P220, DOI 10.1111/imr.12268

Horváti K, 2018, BIOCONJUGATE CHEM, V29, P1495, DOI 10.1021/acs.bioconjchem.8b00156

Hunter RL, 2006, AM J PATHOL, V168, P1249, DOI 10.2353/ajpath.2006.050848

Hussain A, 2019, CURR DRUG DELIV, V16, P400, DOI 10.2174/1567201816666190201144815

Italiani P, 2014, PLOS ONE, V9, DOI 10.1371/journal.pone.0087680

Jaguin M, 2013, CELL IMMUNOL, V281, P51, DOI 10.1016/j.cellimm.2013.01.010

Jain Sanjay K, 2010, PDA J Pharm Sci Technol, V64, P278

Jhaveri AM, 2014, FRONT PHARMACOL, V5, DOI 10.3389/fphar.2014.00077

Kalscheuer R, 2014, MICROBIOL SPECTR, V2, DOI 10.1128/microbiolspec.MGM2-0002-2013

Kaufmann SHE, 2018, NAT REV DRUG DISCOV, V17, P35, DOI 10.1038/nrd.2017.162

Kaur IP, 2014, J CONTROL RELEASE, V184, P36, DOI 10.1016/j.jconrel.2014.04.009

Khosa A, 2018, BIOMED PHARMACOTHER, V103, P598, DOI 10.1016/j.biopha.2018.04.055

Kilinç G, 2021, IMMUNOL REV, V301, P62, DOI 10.1111/imr.12951

Koch A, 2018, CURR OPIN PHARMACOL, V42, P7, DOI 10.1016/j.coph.2018.05.013

Kolloli A, 2017, FRONT MED-LAUSANNE, V4, DOI 10.3389/fmed.2017.00171

Kulkarni P, 2019, COLLOID SURFACE A, V565, P131, DOI 10.1016/j.colsurfa.2019.01.004

Kumari P, 2014, APPL BIOCHEM BIOTECH, V174, P2663, DOI 10.1007/s12010-014-1217-3

Kutscher HL, 2019, PHARM RES-DORDR, V36, DOI 10.1007/s11095-019-2576-9

Lammas DA, 1997, IMMUNITY, V7, P433, DOI 10.1016/S1074-7613(00)80364-7

Lee CY, 2021, ANTIBIOTICS-BASEL, V10, DOI 10.3390/antibiotics10010091

Lee J, 2009, YONSEI MED J, V50, P1, DOI 10.3349/ymj.2009.50.1.1

Lemmer Y, 2015, J CONTROL RELEASE, V211, P94, DOI 10.1016/j.jconrel.2015.06.005

Liu CH, 2017, CELL MOL IMMUNOL, V14, P963, DOI 10.1038/cmi.2017.88

Long R, 1999, ANTIMICROB AGENTS CH, V43, P403, DOI 10.1128/AAC.43.2.403

Look M, 2010, ADV DRUG DELIVER REV, V62, P378, DOI 10.1016/j.addr.2009.11.011

Lu Y, 2013, INT J PHARMACEUT, V453, P198, DOI 10.1016/j.ijpharm.2012.08.042

Ma C, 2021, DRUG DELIV TRANSL RE, V11, P1218, DOI 10.1007/s13346-020-00849-7

Ma JJ, 2014, FASEB J, V28, P3238, DOI 10.1096/fj.13-247056

Ma ZK, 2010, LANCET, V375, P2100, DOI 10.1016/S0140-6736(10)60359-9

MacMicking JD, 1997, P NATL ACAD SCI USA, V94, P5243, DOI 10.1073/pnas.94.10.5243

Maphasa RE, 2021, FRONT CELL INFECT MI, V10, DOI 10.3389/fcimb.2020.618414

Maretti E, 2017, INT J PHARMACEUT, V528, P440, DOI 10.1016/j.ijpharm.2017.06.045

Maretti E, 2014, INT J PHARMACEUT, V462, P74, DOI 10.1016/j.ijpharm.2013.12.034

Martin CJ, 2014, CURR OPIN MICROBIOL, V17, P17, DOI 10.1016/j.mib.2013.10.007

Martinez J, 2016, NATURE, V533, P115, DOI 10.1038/nature17950

Martinez J, 2015, NAT CELL BIOL, V17, P893, DOI 10.1038/ncb3192

Martinez J, 2011, P NATL ACAD SCI USA, V108, P17396, DOI 10.1073/pnas.1113421108

McKell MC, 2021, FRONT IMMUNOL, V12, DOI 10.3389/fimmu.2021.653571

Mehta P, 2014, CURR OPIN IMMUNOL, V26, P69, DOI 10.1016/j.coi.2013.10.012

Mehta SK, 2013, COLLOID SURFACE B, V101, P434, DOI 10.1016/j.colsurfb.2012.07.006

Mily A, 2015, PLOS ONE, V10, DOI 10.1371/journal.pone.0138340

Mily A, 2013, BMC PULM MED, V13, DOI 10.1186/1471-2466-13-23

Mishra R, 2020, TUBERCULOSIS, V120, DOI 10.1016/j.tube.2020.101902

Mohamed F, 2008, J PHARM SCI-US, V97, P71, DOI 10.1002/jps.21082

Momin MAM, 2019, PHARMACEUTICS, V11, DOI 10.3390/pharmaceutics11100502

Monopoli MP, 2012, NAT NANOTECHNOL, V7, P779, DOI [10.1038/nnano.2012.207, 10.1038/NNANO.2012.207]

Mortaz E, 2015, J CLIN IMMUNOL, V35, P1, DOI 10.1007/s10875-014-0103-7

Mozafari M.R., 2007, NANOMATERIALS NANOSY

Muankaew C, 2018, BASIC CLIN PHARMACOL, V122, P46, DOI 10.1111/bcpt.12917

Murray PJ, 2017, ANNU REV PHYSIOL, V79, P541, DOI 10.1146/annurev-physiol-022516-034339

Nabi B, 2020, DRUG DELIV TRANSL RE, V10, P1111, DOI 10.1007/s13346-020-00786-5

Narita M., 2017, TRAVEL TROPICAL MED

Nasiruddin Mohammad, 2017, Tuberc Res Treat, V2017, P4920209, DOI [10.1155/2017/4920209, 10.1155/2017/4920209]

Niemi M, 2003, CLIN PHARMACOKINET, V42, P819, DOI 10.2165/00003088-200342090-00003

Olugbosi M, 2020, J INT AIDS SOC, V23, P20

Organization W. H., 2021, Global tuberculosis report 2021

Organization W.H., 2020, GLOB TUB REP

Organization WH, 2019, Global tuberculosis report 2019

Ouyang Q, 2020, MSPHERE, V5, DOI 10.1128/mSphere.00124-20

Pai M, 2022, NEW ENGL J MED, V386, P1490, DOI 10.1056/NEJMp2118145

Paik S, 2019, VIRULENCE, V10, P448, DOI 10.1080/21505594.2018.1536598

Pandey R, 2005, J ANTIMICROB CHEMOTH, V55, P430, DOI 10.1093/jac/dki027

Pauwels AM, 2017, TRENDS IMMUNOL, V38, P407, DOI 10.1016/j.it.2017.03.006

Pereira L, 2015, J NANOPART RES, V17, DOI 10.1007/s11051-015-2892-x

Periyasamy KM, 2020, MOL IMMUNOL, V127, P238, DOI 10.1016/j.molimm.2020.08.007

Pieters J, 2008, CELL HOST MICROBE, V3, P399, DOI 10.1016/j.chom.2008.05.006

Plapied L, 2011, CURR OPIN COLLOID IN, V16, P228, DOI 10.1016/j.cocis.2010.12.005

Poerio N, 2022, J INFECT DIS, V225, P1675, DOI 10.1093/infdis/jiab602

Poirier V, 2014, J BIOL CHEM, V289, P29376, DOI 10.1074/jbc.M114.582502

Pourshahab PS, 2011, J MICROENCAPSUL, V28, P605, DOI 10.3109/02652048.2011.599437

Praphakar RA, 2017, INT J PHARMACEUT, V524, P168, DOI 10.1016/j.ijpharm.2017.03.089

Prashar A, 2017, FRONT CELL INFECT MI, V7, DOI 10.3389/fcimb.2017.00435

Puri V., 2022, CURR RES PHARM DRUG, V3

Queval CJ, 2017, FRONT MICROBIOL, V8, DOI 10.3389/fmicb.2017.02284

Ralph AP, 2013, PLOS ONE, V8, DOI 10.1371/journal.pone.0070032

Ralph AP, 2013, J INFECT DIS, V208, P616, DOI 10.1093/infdis/jit248

Rani N.P., 2010, INT J PHARM BIO SCI, V1, P379

Rao M, 2019, INT J INFECT DIS, V80, pS62, DOI 10.1016/j.ijid.2019.01.039

Rivas-Santiago B, 2013, INT J ANTIMICROB AG, V41, P143, DOI 10.1016/j.ijantimicag.2012.09.015

Rodrigues C, 2003, BBA-GEN SUBJECTS, V1620, P151, DOI 10.1016/S0304-4165(02)00528-7

Roszer T, 2015, MEDIAT INFLAMM, V2015, DOI 10.1155/2015/816460

Russell DG, 2009, NAT IMMUNOL, V10, P943, DOI 10.1038/ni.1781

Salahuddin N, 2013, BMC INFECT DIS, V13, DOI 10.1186/1471-2334-13-22

Salamon H, 2014, J IMMUNOL, V193, P30, DOI 10.4049/jimmunol.1400736

Samuel LP, 2007, MICROBIOL-SGM, V153, P529, DOI 10.1099/mic.0.2006/002642-0

Saraogi GK, 2011, J DRUG TARGET, V19, P219, DOI 10.3109/1061186X.2010.492522

Saraogi GK, 2010, INT J PHARMACEUT, V385, P143, DOI 10.1016/j.ijpharm.2009.10.004

Scanga CA, 2001, INFECT IMMUN, V69, P7711, DOI 10.1128/IAI.69.12.7711-7717.2001

Schaible UE, 2003, NAT MED, V9, P1039, DOI 10.1038/nm906

Schiebler M, 2015, EMBO MOL MED, V7, P127, DOI 10.15252/emmm.201404137

Schön T, 2003, EUR RESPIR J, V21, P483, DOI 10.1183/09031936.03.00090702

Schüller S, 2001, CELL MICROBIOL, V3, P785, DOI 10.1046/j.1462-5822.2001.00155.x

Scolari IR, 2019, AAPS PHARMSCITECH, V20, DOI 10.1208/s12249-018-1278-7

Scriba TJ, 2020, SEMIN IMMUNOL, V50, DOI 10.1016/j.smim.2020.101431

Sharma A, 2018, INT J PHARMACEUT, V536, P50, DOI 10.1016/j.ijpharm.2017.11.046

Sharma V, 2021, AUTOPHAGY, V17, P476, DOI 10.1080/15548627.2020.1725374

Shehzad A, 2013, BRAZ J INFECT DIS, V17, P74, DOI 10.1016/j.bjid.2012.10.009

Shelley H, 2018, J PHARM SCI-US, V107, P1741, DOI 10.1016/j.xphs.2018.03.021

Shin DM, 2010, PLOS PATHOG, V6, DOI 10.1371/journal.ppat.1001230

Sia JK, 2015, J IMMUNOL RES, V2015, DOI 10.1155/2015/747543

Singh R, 2020, J APPL MICROBIOL, V128, P1547, DOI 10.1111/jam.14478

Song X, 2015, PHARM RES-DORDR, V32, P1741, DOI 10.1007/s11095-014-1572-3

Stanley SA, 2014, PLOS PATHOG, V10, DOI 10.1371/journal.ppat.1003946

STURGILLKOSZYCKI S, 1994, SCIENCE, V263, P678, DOI 10.1126/science.8303277

Sun J, 2013, PLOS PATHOG, V9, DOI 10.1371/journal.ppat.1003499

Sun-Wada GH, 2009, J CELL SCI, V122, P2504, DOI 10.1242/jcs.050443

Sunnetcioglu A, 2015, ANN CLIN MICROB ANTI, V14, DOI 10.1186/s12941-015-0092-2

Swanson B, 2002, NUTRITION, V18, P688, DOI 10.1016/S0899-9007(02)00786-4

Tarique AA, 2015, AM J RESP CELL MOL, V53, P676, DOI 10.1165/rcmb.2015-0012OC

Tian MP, 2018, FRONT IMMUNOL, V9, DOI 10.3389/fimmu.2018.00310

Tinsley-Bown AM, 2000, J CONTROL RELEASE, V66, P229, DOI 10.1016/S0168-3659(99)00275-8

Tobin DM, 2013, CURR OPIN PHARMACOL, V13, P641, DOI 10.1016/j.coph.2013.06.007

Trousil J, 2017, NANOMED-NANOTECHNOL, V13, P307, DOI 10.1016/j.nano.2016.08.031

Upadhyay S, 2018, PATHOG DIS, V76, DOI 10.1093/femspd/fty037

Upadhyay TK, 2019, ARTIF CELL NANOMED B, V47, P427, DOI 10.1080/21691401.2018.1559180

Vanaporn M, 2020, VIRULENCE, V11, P1192, DOI 10.1080/21505594.2020.1809326

Velmurugan K, 2007, PLOS PATHOG, V3, P972, DOI 10.1371/journal.ppat.0030110

Vergne I, 2003, J EXP MED, V198, P653, DOI 10.1084/jem.20030527

Walburger A, 2004, SCIENCE, V304, P1800, DOI 10.1126/science.1099384

Wallis RS, 2015, NAT REV IMMUNOL, V15, P255, DOI 10.1038/nri3813

WHO, 2017, GUIDELINES TREATMENT

Woodrow KA, 2009, NAT MATER, V8, P526, DOI [10.1038/NMAT2444, 10.1038/nmat2444]

Yang CS, 2012, CELL HOST MICROBE, V11, P264, DOI 10.1016/j.chom.2012.01.018

Yao YL, 2019, FRONT IMMUNOL, V10, DOI 10.3389/fimmu.2019.00792

Young C, 2020, MUCOSAL IMMUNOL, V13, P190, DOI 10.1038/s41385-019-0226-5

Zazo H, 2016, J CONTROL RELEASE, V224, P86, DOI 10.1016/j.jconrel.2016.01.008

Zhai WJ, 2019, INT J MOL SCI, V20, DOI 10.3390/ijms20020340

Zumla A, 2015, J INTERN MED, V277, P373, DOI 10.1111/joim.12256

Zumla A, 2016, BMC MED, V14, DOI 10.1186/s12916-016-0635-1

Zumla A, 2015, NAT REV DRUG DISCOV, V14, P511, DOI 10.1038/nrd4696

NR 193

TC 14

Z9 16

U1 4

U2 13

PU ELSEVIER

PI AMSTERDAM

PA RADARWEG 29, 1043 NX AMSTERDAM, NETHERLANDS

SN 0378-5173

EI 1873-3476

J9 INT J PHARMACEUT

JI Int. J. Pharm.

PD JUN 25

PY 2022

VL 622

AR 121893

DI 10.1016/j.ijpharm.2022.121893

EA JUN 2022

PG 16

WC Pharmacology & Pharmacy

WE Science Citation Index Expanded (SCI-EXPANDED)

SC Pharmacology & Pharmacy

GA 2J5PG

UT WOS:000815708000004

OA Green Submitted

DA 2026-05-07

ER

PT J

AU Paliwal, H

Tongkanarak, K

Aekwattanaphol, N

Srichana, T

AF Paliwal, Himanshu

Tongkanarak, Krittawan

Aekwattanaphol, Nattanit

Srichana, Teerapol

TI Antimycobacterial and autophagic activity of nebulized delamanid

microemulsion targeting alveolar macrophage

SO DRUG DEVELOPMENT AND INDUSTRIAL PHARMACY

LA English

DT Article

DE Delamanid; microemulsion; pulmonary tuberculosis; nebulizer; autophagy

ID SURFACTANT; PERFORMANCE; DEPOSITION; SYSTEMS; SIZE; JET

AB ObjectiveThe aerosol delivery of the novel anti-tubercular drug delamanid for pulmonary targeting holds promise for the effective treatment of multi-drug-resistant tuberculosis. Here, we developed a microemulsion formulation of delamanid for nebulization, evaluating its potential bioactivity.MethodsHerein, we attempted to develop microemulsion formulations loaded delamanid for nebulization and their physicochemical characteristics were assessed. The formulation was checked for aerosol characteristics, cytotoxic potential, in vitro antimycobacterial activity, and autophagy.ResultsThe optimized formulations, nebulized into a next-generation impactor, exhibited a fine particle fraction of 63.12%, ensuring efficient in vitro deposition. In vitro evaluations revealed that formulation did not demonstrate any cytotoxic potential on respiratory cell lines up to 2.5 mu g/mL and lack of inflammatory cytokines production and nitric oxide from macrophage NR8383 cells, signified its safety. The flow cytometry analysis revealed that the elimination of Mycobacterium bovis by formulation after day 4, with considerably low minimum bactericidal concentration. The formulation demonstrated adequate macrophage autophagic activity when evaluated using confocal laser scanning microscopy and Western blot.ConclusionConsequently, the aerosolized microemulsion of delamanid for pulmonary delivery may serve as an effective therapy for MDR-TB, necessitating further in vivo studies.

C1 [Paliwal, Himanshu] Marwadi Univ, Marwadi Univ Res Ctr, Dept Pharm, Rajkot, India.

[Tongkanarak, Krittawan; Aekwattanaphol, Nattanit; Srichana, Teerapol] Prince Songkla Univ, Fac Pharmaceut Sci, Drug Delivery Syst Excellence Ctr, Dept Pharmaceut Technol, Hat Yai, Thailand.

C3 Marwadi University; Prince of Songkla University

RP Srichana, T (corresponding author), Prince Songkla Univ, Drug Delivery Syst Excellence Ctr, Dept Pharmaceut Technol, Dept Pharmaceut Sci, Hat Yai, Thailand.

EM teerapol.s@psu.ac.th

RI ; Paliwal, Himanshu/HNI-1729-2023; Srichana, Teerapol/C-8908-2009

OI Tongkanarak, Krittawan/0009-0003-1025-8005; Paliwal,

Himanshu/0000-0001-5438-9881;

FU National Science, Research and Innovation Fund (NSRF) and Prince of

Songkla University; Drug Delivery System Excellence Center; Faculty of

Pharmaceutical Sciences at Prince of Songkla University

FX The authors acknowledge the financial support from the Drug Delivery

System Excellence Center and the Faculty of Pharmaceutical Sciences at

Prince of Songkla University for furnishing the necessary resources to

undertake this research. The graphical abstract in this article was

developed using BioRender.com.

CR Alghamdi WA, 2021, J ANTIMICROB CHEMOTH, V76, P1019, DOI 10.1093/jac/dkaa550

Arora S, 2018, IMMUNOBIOLOGY, V223, P383, DOI 10.1016/j.imbio.2017.11.001

Asmawi AA, 2019, DRUG DELIV TRANSL RE, V9, P543, DOI 10.1007/s13346-018-0526-4

Atzori L, 2010, COMPUT NETW, V54, P2787, DOI 10.1016/j.comnet.2010.05.010

Beck-Broichsitter M, 2014, EUR J PHARM BIOPHARM, V87, P524, DOI 10.1016/j.ejpb.2014.03.006

Bender L, 2024, BIOMATER ADV, V159, DOI 10.1016/j.bioadv.2024.213823

Bergfreund J, 2021, LANGMUIR, V37, P6722, DOI 10.1021/acs.langmuir.1c00668

Boe J, 2001, EUR RESPIR J, V18, P228

Bourguignon T, 2023, PHARMACEUTICS, V15, DOI 10.3390/pharmaceutics15020393

Bussi C, 2019, FEMS MICROBIOL REV, V43, P341, DOI 10.1093/femsre/fuz006

Chen J, 2018, INT J FOOD PROP, V21, P1152, DOI 10.1080/10942912.2018.1485032

Chen XH, 2017, ANTIMICROB AGENTS CH, V61, DOI [10.1128/aac.02402-16, 10.1128/AAC.02402-16]

Cheng YS, 2014, AAPS PHARMSCITECH, V15, P630, DOI 10.1208/s12249-014-0092-0

Chunhachaichana C, 2019, PHARM DEV TECHNOL, V24, P1218, DOI 10.1080/10837450.2019.1650766

Davodabadi F, 2023, EUR J PHARMACOL, V958, DOI 10.1016/j.ejphar.2023.176013

DING AH, 1988, J IMMUNOL, V141, P2407

Eedara BB, 2018, EUR J PHARM SCI, V118, P216, DOI 10.1016/j.ejps.2018.04.003

Elbardisy B, 2022, PHARMACEUTICS, V14, DOI 10.3390/pharmaceutics14122717

European Medicines Agency, 2020, DELTYBA EUROPEAN PUB

Tena AF, 2012, ARCH BRONCONEUMOL, V48, P240, DOI 10.1016/j.arbres.2012.02.003

Gil CM, 2022, BREATHE, V18, DOI 10.1183/20734735.0149-2021

Guo Y, 2021, ACTA PHARM SIN B, V11, P2565, DOI 10.1016/j.apsb.2021.05.015

Haghighi E, 2024, J NANOBIOTECHNOL, V22, DOI 10.1186/s12951-024-02972-w

Hatae Arthur Cavalcante, 2023, OpenNano, DOI [10.1016/j.onano.2023.100171, 10.1016/j.onano.2023.100171]

Khan I, 2021, J DRUG DELIV SCI TEC, V66, DOI 10.1016/j.jddst.2021.102822

Khoshnood S, 2021, FRONT MICROBIOL, V12, DOI 10.3389/fmicb.2021.717045

Kumar M, 2023, JCIS OPEN, V12, DOI [10.1016/j.jciso.2023.100095, 10.1016/j.jciso.2023.100095]

Lange C, 2014, EUR RESPIR J, V44, P23, DOI 10.1183/09031936.00188313

Laube B.L., 2014, Middletons' Allergy: Principles and Practice, P1066, DOI DOI 10.1016/B978-0-323-08593-9.00067-X

Man N, 2019, J DRUG DELIV SCI TEC, V52, P597, DOI 10.1016/j.jddst.2019.05.003

Migliori GB, 2020, INT J INFECT DIS, V92, pS15, DOI 10.1016/j.ijid.2020.01.042

Nair A, 2023, J NANOBIOTECHNOL, V21, DOI 10.1186/s12951-023-02156-y

Najlah M, 2014, INT J PHARMACEUT, V461, P234, DOI 10.1016/j.ijpharm.2013.11.022

Nicod LP, 2005, EUR RESPIR REV, V14, P45, DOI 10.1183/09059180.05.00009501

Paliwal H, 2024, INT J PHARMACEUT, V655, DOI 10.1016/j.ijpharm.2024.124031

Paul PK, 2024, INT J PHARMACEUT, V660, DOI 10.1016/j.ijpharm.2024.124309

Pochapski DJ, 2021, LANGMUIR, V37, P13379, DOI 10.1021/acs.langmuir.1c02056

Rajput A, 2021, FRONT PHARMACOL, V12, DOI 10.3389/fphar.2021.749945

Rao L, 2024, NANO TODAY, V56, DOI 10.1016/j.nantod.2024.102298

Ryndak MB, 2019, FRONT CELL INFECT MI, V9, DOI 10.3389/fcimb.2019.00299

Sarheed O., 2020, STUDIES EFFECT OIL S, V12, P1223, DOI [10.3390/pharmaceutics12121223, DOI 10.3390/PHARMACEUTICS12121223]

Sharma H, 2021, SN APPL SCI, V3, DOI 10.1007/s42452-021-04235-x

Silva HD, 2015, J FOOD ENG, V167, P89, DOI 10.1016/j.jfoodeng.2015.07.037

Smola M, 2008, INT J NANOMED, V3, P1

Steckel H, 2003, EUR J PHARM SCI, V19, P443, DOI 10.1016/S0928-0987(03)00148-9

Suhail N, 2021, FRONT NANOTECHNOL, V3, DOI 10.3389/fnano.2021.754889

Szumala P, 2023, MOL PHARMACEUT, V20, P232, DOI 10.1021/acs.molpharmaceut.2c00599

Talapatra A, 2024, ENERG FUEL, V38, P7746, DOI 10.1021/acs.energyfuels.3c04902

Tanneau L, 2022, CLIN PHARMACOKINET, V61, P1177, DOI 10.1007/s40262-022-01133-2

Tapak M, 2023, BMJ OPEN RESPIR RES, V10, DOI 10.1136/bmjresp-2022-001589

Ho TM, 2022, ADV COLLOID INTERFAC, V299, DOI 10.1016/j.cis.2021.102541

USP, 2011, Patent No. [USP1125NF, 1125]

Van Duong T, 2022, EUR J PHARM BIOPHARM, V174, P131, DOI 10.1016/j.ejpb.2022.04.002

Verma N, 2022, J DRUG DELIV SCI TEC, V75, DOI 10.1016/j.jddst.2022.103690

Xavier AS, 2014, J PHARMACOL PHARMACO, V5, P222, DOI 10.4103/0976-500X.136121

Xi Y, 2022, PLOS ONE, V17, DOI 10.1371/journal.pone.0270003

Zhai WJ, 2019, INT J MOL SCI, V20, DOI 10.3390/ijms20020340

Zhang J, 2021, ACS OMEGA, V6, P5001, DOI 10.1021/acsomega.0c06142

Zheng YJ, 2022, PHARMACEUTICS, V14, DOI 10.3390/pharmaceutics14030617

NR 59

TC 0

Z9 0

U1 0

U2 0

PU TAYLOR & FRANCIS LTD

PI ABINGDON

PA 2-4 PARK SQUARE, MILTON PARK, ABINGDON OR14 4RN, OXON, ENGLAND

SN 0363-9045

EI 1520-5762

J9 DRUG DEV IND PHARM

JI Drug Dev. Ind. Pharm.

PD OCT 3

PY 2025

VL 51

IS 10

BP 1347

EP 1362

DI 10.1080/03639045.2025.2532762

EA JUL 2025

PG 16

WC Chemistry, Medicinal; Pharmacology & Pharmacy

WE Science Citation Index Expanded (SCI-EXPANDED)

SC Pharmacology & Pharmacy

GA 7SG5P

UT WOS:001534840500001

DA 2026-05-07

ER

PT J

AU Villellas, C

Coeck, N

Meehan, CJ

Lounis, N

de Jong, B

Rigouts, L

Andries, K

AF Villellas, Cristina

Coeck, Nele

Meehan, Conor J.

Lounis, Nacer

de Jong, Bouke

Rigouts, Leen

Andries, Koen

TI Unexpected high prevalence of resistance-associated Rv0678

variants in MDR-TB patients without documented prior use of clofazimine

or bedaquiline

SO JOURNAL OF ANTIMICROBIAL CHEMOTHERAPY

LA English

DT Article

ID MYCOBACTERIUM-TUBERCULOSIS; ATP SYNTHASE; CROSS-RESISTANCE;

SUSCEPTIBILITY; MUTATIONS; DELAMANID; SYSTEM

AB Objectives: Resistance-associated variants (RAVs) in Rv0678, a regulator of the MmpS5-MmpL5 efflux pump, have been shown to lead to increased MICs of bedaquiline ( 2-to8-fold) and clofazimine (2- to 4-fold). The prevalence of these Rv0678 RAVs in clinical isolates and their impact on treatment outcomes are important factors to take into account in bedaquiline treatment guidelines.

Methods: Baseline isolates from two bedaquiline MDR-TB clinical trials were sequenced for Rv0678 RAVs and corresponding bedaquiline MICs were determined on 7H11 agar. Rv0678 RAVs were also investigated in non-MDRTB sequences of a population-based cohort.

Results: Rv0678 RAVs were identified in 23/347 (6.3%) of MDR-TB baseline isolates. Surprisingly, bedaquiline MICs for these isolates were high (> 0.24 mg/ L, n = 8), normal (0.03-0.24 mg/ L, n =11) or low(< 0.03 mg/ L, n = 4). A variant at position - 11 in the intergenic regionmmpS5- Rv0678 was identified in 39 isolates (11.3%) and appeared to increase the susceptibility to bedaquiline. In non-MDR-TB isolates, the frequency of Rv0678 RAVs was lower (6/852 or 0.7%). Competition experiments suggested that rifampicin was not the drug selecting for Rv0678 RAVs.

Conclusions: RAVs in Rv0678 occurmore frequently in MDR-TB patients than previously anticipated, are not associated with prior use of bedaquiline or clofazimine, and in themajority of cases do not lead to bedaquiline MICs above the provisional breakpoint (0.24mg/L). Their origin remains unknown. Given the variety of RAVs in Rv0678 and their variable effects on the MIC, only phenotypic drug-susceptibilitymethods can currently be used to assess bedaquiline susceptibility.

C1 [Villellas, Cristina; Lounis, Nacer; Andries, Koen] Janssen Res & Dev, Beerse, Belgium.

[Coeck, Nele; Meehan, Conor J.; de Jong, Bouke; Rigouts, Leen] Inst Trop Med, Antwerp, Belgium.

C3 Johnson & Johnson; Janssen Pharmaceuticals; Institute of Tropical

Medicine (ITM)

RP Andries, K (corresponding author), Janssen Res & Dev, Beerse, Belgium.

EM kandries@its.jnj.com

RI ; de Jong, Bouke/A-3636-2013

OI Meehan, Conor/0000-0003-0724-8343; de Jong, Bouke/0000-0002-1017-4675

FU Janssen Pharmaceutica; Baekeland PhD scholarship from the Flemish

Institute for Scientific Technology, Belgium [IWT 130308]; European

Research Council Starting Grant INTERRUPTB [311725]

FX This work was supported by Janssen Pharmaceutica. N.C. was supported by

a Baekeland PhD scholarship from the Flemish Institute for Scientific

Technology (IWT 130308, Belgium). C.J.M.,L.R. and B.d.J. were supported

by a European Research Council Starting Grant INTERRUPTB (311725).

CR Almeida D, 2016, ANTIMICROB AGENTS CH, V60, P4590, DOI 10.1128/AAC.00753-16

Andries K, 2005, SCIENCE, V307, P223, DOI 10.1126/science.1106753

Andries K, 2014, PLOS ONE, V9, DOI 10.1371/journal.pone.0102135

Bloemberg GV, 2015, NEW ENGL J MED, V373, P1986, DOI 10.1056/NEJMc1505196

Cole ST, 1998, NATURE, V393, P537, DOI 10.1038/31159

de Knegt GJ, 2013, TUBERCULOSIS, V93, P96, DOI 10.1016/j.tube.2012.10.013

Diacon AH, 2014, NEW ENGL J MED, V371, P723, DOI 10.1056/NEJMoa1313865

EUCAST, BED BREAKP

Fang Z, 2015, TUBERCULOSIS, V95, P123, DOI 10.1016/j.tube.2015.01.004

Feuerriegel S, 2015, J CLIN MICROBIOL, V53, P1908, DOI 10.1128/JCM.00025-15

Gullberg E, 2011, PLOS PATHOG, V7, DOI 10.1371/journal.ppat.1002158

Hartkoorn RC, 2014, ANTIMICROB AGENTS CH, V58, P2979, DOI 10.1128/AAC.00037-14

Hoffmann H, 2016, AM J RESP CRIT CARE, V193, P337, DOI 10.1164/rccm.201502-0372LE

Huitric E, 2010, ANTIMICROB AGENTS CH, V54, P1022, DOI 10.1128/AAC.01611-09

Manina G, 2015, CELL HOST MICROBE, V17, P32, DOI 10.1016/j.chom.2014.11.016

Milano A, 2009, TUBERCULOSIS, V89, P84, DOI 10.1016/j.tube.2008.08.003

Palomino JC, 2002, ANTIMICROB AGENTS CH, V46, P2720, DOI 10.1128/AAC.46.8.2720-2722.2002

Pym AS, 2016, EUR RESPIR J, V47, P564, DOI 10.1183/13993003.00724-2015

Schwebach JR, 2001, J CLIN MICROBIOL, V39, P769, DOI 10.1128/JCM.39.2.769-771.2001

Seemann T., 2015, SNIPPY FAST BACTERIA

Segala E, 2012, ANTIMICROB AGENTS CH, V56, P2326, DOI 10.1128/AAC.06154-11

Somoskovi A, 2015, EUR RESPIR J, V45, P554, DOI 10.1183/09031936.00142914

Stucki D, 2012, PLOS ONE, V7, DOI 10.1371/journal.pone.0041253

Torrea G, 2015, J ANTIMICROB CHEMOTH, V70, P2300, DOI 10.1093/jac/dkv117

Wakamoto Y, 2013, SCIENCE, V339, P91, DOI 10.1126/science.1229858

Walker TM, 2015, LANCET INFECT DIS, V15, P1193, DOI 10.1016/S1473-3099(15)00062-6

Walker TM, 2013, LANCET INFECT DIS, V13, P137, DOI 10.1016/S1473-3099(12)70277-3

Wells RM, 2013, PLOS PATHOG, V9, DOI 10.1371/journal.ppat.1003120

NR 28

TC 154

Z9 178

U1 0

U2 2

PU OXFORD UNIV PRESS

PI OXFORD

PA GREAT CLARENDON ST, OXFORD OX2 6DP, ENGLAND

SN 0305-7453

EI 1460-2091

J9 J ANTIMICROB CHEMOTH

JI J. Antimicrob. Chemother.

PD MAR

PY 2017

VL 72

IS 3

BP 684

EP 690

DI 10.1093/jac/dkw502

PG 7

WC Infectious Diseases; Microbiology; Pharmacology & Pharmacy

WE Science Citation Index Expanded (SCI-EXPANDED)

SC Infectious Diseases; Microbiology; Pharmacology & Pharmacy

GA EQ4IE

UT WOS:000398038800004

OA Green Published, Bronze

DA 2026-05-07

ER

PT J

AU Xu, XY

Dong, BY

Peng, LJ

Gao, C

He, ZQ

Wang, C

Zeng, JM

AF Xu, Xinyue

Dong, Baoyu

Peng, Lijun

Gao, Chao

He, Zhiqun

Wang, Chuan

Zeng, Jumei

TI Anti-tuberculosis drug development via targeting the cell

envelope of Mycobacterium tuberculosis

SO FRONTIERS IN MICROBIOLOGY

LA English

DT Review

DE Mycobacterium tuberculosis; anti-tuberculosis drug; cell envelope; drug

target; lead compounds

ID CARRIER PROTEIN REDUCTASE; MYCOLIC ACID BIOSYNTHESIS; ACYL-COA

CARBOXYLASE; NITROIMIDAZOPYRAN PA-824; BACTERICIDAL ACTIVITY; MEMBRANE

TRANSPORTER; MULTIDRUG-RESISTANT; INHA INHIBITORS; WALL CORE; MECHANISM

AB Mycobacterium tuberculosis possesses a dynamic cell envelope, which consists of a peptidoglycan layer, a mycolic acid layer, and an arabinogalactan polysaccharide. This envelope possesses a highly complex and unique structure representing a barrier that protects and assists the growth of M. tuberculosis and allows its adaptation to the host. It regulates the immune response of the host cells, causing their damage. Therefore, the cell envelope of M. tuberculosis is an attractive target for vaccine and drug development. The emergence of multidrug-resistant as well as extensively drug resistant tuberculosis and co-infection with HIV prevented an effective control of this disease. Thus, the discovery and development of new drugs is a major keystone for TB treatment and control. This review mainly summarizes the development of drug enzymes involved in the biosynthesis of the cell wall in M. tuberculosis, and other potential drug targets in this pathway, to provide more effective strategies for the development of new drugs.

C1 [Xu, Xinyue; Dong, Baoyu; Peng, Lijun; He, Zhiqun; Wang, Chuan; Zeng, Jumei] Sichuan Univ, West China PUMC CC Chen Inst Hlth, West China Sch Publ Hlth, Chengdu, Peoples R China.

[Xu, Xinyue; Dong, Baoyu; Peng, Lijun; He, Zhiqun; Wang, Chuan; Zeng, Jumei] Sichuan Univ, West China Hosp 4, Chengdu, Peoples R China.

[Gao, Chao] Sichuan Univ, State Key Lab Biotherapy, Chengdu, Peoples R China.

[Gao, Chao] Sichuan Univ, West China Hosp, Lab Human Dis & Immunotherapies, Chengdu, Peoples R China.

C3 Sichuan University; Sichuan University; Sichuan University; Sichuan

University

RP Zeng, JM (corresponding author), Sichuan Univ, West China PUMC CC Chen Inst Hlth, West China Sch Publ Hlth, Chengdu, Peoples R China.; Zeng, JM (corresponding author), Sichuan Univ, West China Hosp 4, Chengdu, Peoples R China.

EM ZengJumei@scu.edu.cn

FU National Natural Science Foundation of China; Fundamental Research Funds

for the Central Universities; [31400040]; [YJ201985]

FX Funding This work was supported by the National Natural Science

Foundation of China (31400040), the Fundamental Research Funds for the

Central Universities (YJ201985).

CR Agarwal M, 2021, BIOCHEM J, V478, P2081, DOI 10.1042/BCJ20210170

Aggarwal A, 2017, CELL, V170, P249, DOI 10.1016/j.cell.2017.06.025

Alderwick LJ, 2007, BIOCHEM SOC T, V35, P1325, DOI 10.1042/BST0351325

Alderwick LJ, 2015, CSH PERSPECT MED, V5, DOI 10.1101/cshperspect.a021113

Alfhili MA, 2021, APOPTOSIS, V26, P96, DOI 10.1007/s10495-020-01650-0

ASSELINEAU J, 1950, NATURE, V166, P782, DOI 10.1038/166782a0

Bahuguna A, 2020, MED RES REV, V40, P263, DOI 10.1002/med.21602

Belisle JT, 1997, SCIENCE, V276, P1420, DOI 10.1126/science.276.5317.1420

Bell LCK, 2018, NAT REV MICROBIOL, V16, P80, DOI 10.1038/nrmicro.2017.128

Bhat ZS, 2017, BIOMED PHARMACOTHER, V95, P1520, DOI 10.1016/j.biopha.2017.09.036

Bhatt A, 2005, J BACTERIOL, V187, P7596, DOI 10.1128/JB.187.22.7596-7606.2005

Bhutani I, 2015, PLOS ONE, V10, DOI 10.1371/journal.pone.0119771

Bukhdruker S, 2020, INT J MOL SCI, V21, DOI [10.1101/2020.08.27.269936, 10.3390/ijms21207683]

Burki T, 2019, LANCET INFECT DIS, V19, P1063, DOI 10.1016/S1473-3099(19)30489-X

Caminero JA, 2010, LANCET INFECT DIS, V10, P621, DOI 10.1016/S1473-3099(10)70139-0

Chatterji M, 2014, ANTIMICROB AGENTS CH, V58, P5325, DOI 10.1128/AAC.03233-14

Choi KH, 2000, J BIOL CHEM, V275, P28201

Conradie F, 2020, NEW ENGL J MED, V382, P893, DOI 10.1056/NEJMoa1901814

Daffé M, 2015, TUBERCULOSIS, V95, pS155, DOI 10.1016/j.tube.2015.02.024

De Jager V, 2022, AM J RESP CRIT CARE, V205, P1228, DOI 10.1164/rccm.202108-1976OC

Delaine T, 2010, EUR J MED CHEM, V45, P4554, DOI 10.1016/j.ejmech.2010.07.016

Deshpande D, 2017, SCI ADV, V3, DOI 10.1126/sciadv.1701102

Dhaked DK, 2019, PROG BIOPHYS MOL BIO, V145, P52, DOI 10.1016/j.pbiomolbio.2018.12.004

Domenech P, 2005, INFECT IMMUN, V73, P3492, DOI 10.1128/IAI.73.6.3492-3501.2005

Dubée V, 2012, ANTIMICROB AGENTS CH, V56, P4189, DOI 10.1128/AAC.00665-12

Edoo Z, 2018, CHEM-EUR J, V24, P8081, DOI 10.1002/chem.201800923

Farjallah A, 2021, ACS INFECT DIS, V7, P552, DOI 10.1021/acsinfecdis.0c00325

Freundlich JS, 2009, CHEMMEDCHEM, V4, P241, DOI 10.1002/cmdc.200800261

Gavalda S, 2009, J BIOL CHEM, V284, P19255, DOI 10.1074/jbc.M109.006940

Giri A, 2019, TUBERCULOSIS, V115, P49, DOI 10.1016/j.tube.2019.01.004

Grzegorzewicz AE, 2012, NAT CHEM BIOL, V8, P334, DOI [10.1038/NCHEMBIO.794, 10.1038/nchembio.794]

Guardia A, 2016, CHEMMEDCHEM, V11, P687, DOI 10.1002/cmdc.201600020

Gupta R, 2010, NAT MED, V16, P466, DOI 10.1038/nm.2120

Han XY, 2019, FRONT MICROBIOL, V10, DOI 10.3389/fmicb.2019.01799

Hariguchi N, 2020, ANTIMICROB AGENTS CH, V64, DOI 10.1128/AAC.02020-19

Hea X, 2007, BIOORGAN MED CHEM, V15, P6649, DOI 10.1016/j.bmc.2007.08.013

Heinrich N, 2015, J ANTIMICROB CHEMOTH, V70, P1558, DOI 10.1093/jac/dku553

Hugonnet JE, 2007, BIOCHEMISTRY-US, V46, P11998, DOI 10.1021/bi701506h

Inoyama D, 2020, CELL CHEM BIOL, V27, P560, DOI 10.1016/j.chembiol.2020.02.007

Ioerger TR, 2013, PLOS ONE, V8, DOI 10.1371/journal.pone.0075245

Ishizaki Y, 2013, J BIOL CHEM, V288, P30309, DOI 10.1074/jbc.M113.492173

Jankute M, 2015, ANNU REV MICROBIOL, V69, P405, DOI 10.1146/annurev-micro-091014-104121

Jankute M, 2012, FUTURE MICROBIOL, V7, P129, DOI [10.2217/FMB.11.123, 10.2217/fmb.11.123]

Kalscheuer R, 2019, BIOCHEM J, V476, P1995, DOI 10.1042/BCJ20190324

Kamsri P, 2014, SAR QSAR ENVIRON RES, V25, P473, DOI 10.1080/1062936X.2014.898690

Kaur P, 2021, TUBERCULOSIS, V129, DOI 10.1016/j.tube.2021.102104

Kolly GS, 2015, J BACTERIOL, V197, P3686, DOI 10.1128/JB.00628-15

Kouassi AF, 2015, INT J MOL SCI, V16, P29744, DOI 10.3390/ijms161226196

Kremer L, 2000, J BIOL CHEM, V275, P16857, DOI 10.1074/jbc.M000569200

Kremer L, 2001, J BIOL CHEM, V276, P27967, DOI 10.1074/jbc.M103687200

Kumar P, 2020, J BIOMOL STRUCT DYN, V38, P4997, DOI 10.1080/07391102.2019.1696231

Lai CY, 2003, J BIOL CHEM, V278, P51494, DOI 10.1074/jbc.M308638200

Lavollay M, 2008, J BACTERIOL, V190, P4360, DOI 10.1128/JB.00239-08

Lee W, 2013, J PHYS CHEM B, V117, P8095, DOI 10.1021/jp403067m

Lenaerts AJ, 2005, ANTIMICROB AGENTS CH, V49, P2294, DOI 10.1128/AAC.49.6.2294-2301.2005

Li W, 2006, BIOCHEM BIOPH RES CO, V342, P170, DOI 10.1016/j.bbrc.2006.01.130

Li W, 2017, ANTIMICROB AGENTS CH, V61, DOI [10.1128/AAC.02399-16, 10.1128/aac.02399-16]

Li W, 2014, ANTIMICROB AGENTS CH, V58, P6413, DOI 10.1128/AAC.03229-14

Lu ZK, 2020, MOL PHARMACOL, V97, P287, DOI 10.1124/mol.119.118042

Makarov V, 2014, EMBO MOL MED, V6, P372, DOI 10.1002/emmm.201303575

Makarov V, 2009, SCIENCE, V324, P801, DOI 10.1126/science.1171583

Manjunatha U., 2009, Communicative and Integrative Biology, V2, P215, DOI [10.4161/cib.2.3.7926, DOI 10.4161/CIB.2.3.7926]

Manjunatha UH, 2015, SCI TRANSL MED, V7, DOI 10.1126/scitranslmed.3010597

Matsumoto M, 2006, PLOS MED, V3, P2131, DOI 10.1371/journal.pmed.0030466

McNeil MB, 2020, MSPHERE, V5, DOI 10.1128/mSphere.00985-20

Miotto P, 2018, RESPIROLOGY, V23, P1098, DOI 10.1111/resp.13393

Oh TJ, 2006, J BIOL CHEM, V281, P3899, DOI 10.1074/jbc.M511761200

Ouyang FX, 2018, SCI TOTAL ENVIRON, V626, P451, DOI 10.1016/j.scitotenv.2018.01.102

Parish T, 2007, J BACTERIOL, V189, P3721, DOI 10.1128/JB.01740-06

Portevin D, 2005, J BIOL CHEM, V280, P8862, DOI 10.1074/jbc.M408578200

Portevin D, 2004, P NATL ACAD SCI USA, V101, P314, DOI 10.1073/pnas.0305439101

Prasad MS, 2021, BIOORG CHEM, V115, DOI 10.1016/j.bioorg.2021.105242

Priyanka, 2020, ENVIRON POLLUT, V257, DOI 10.1016/j.envpol.2019.113617

Prosser GA, 2013, FEBS J, V280, P1150, DOI 10.1111/febs.12108

Qu DH, 2021, FRONT MICROBIOL, V12, DOI 10.3389/fmicb.2021.643951

Quezada LL, 2020, FRONT MICROBIOL, V11, DOI 10.3389/fmicb.2020.01248

Raghavendra T, 2018, GLYCOCONJUGATE J, V35, P421, DOI 10.1007/s10719-018-9842-7

Rani C, 2016, EUR J PHARM SCI, V83, P62, DOI 10.1016/j.ejps.2015.12.013

Rani J, 2020, J BIOMOL STRUCT DYN, V38, P2521, DOI 10.1080/07391102.2019.1637280

Rao SPS, 2013, SCI TRANSL MED, V5, DOI 10.1126/scitranslmed.3007355

Ravichandran R, 2022, J BIOMOL STRUCT DYN, V40, P5069, DOI 10.1080/07391102.2020.1867641

Robertson GT, 2021, ANTIMICROB AGENTS CH, V65, DOI 10.1128/AAC.00583-21

Sacco E, 2007, P NATL ACAD SCI USA, V104, P14628, DOI 10.1073/pnas.0704132104

Sacksteder KA, 2012, FUTURE MICROBIOL, V7, P823, DOI [10.2217/fmb.12.56, 10.2217/FMB.12.56]

Salomon JJ, 2013, THER DELIV, V4, P915, DOI [10.4155/tde.13.62, 10.4155/TDE.13.62]

Sangshetti JN, 2017, CURR PHARM DESIGN, V23, P3164, DOI 10.2174/1381612823666170214115048

Shi T, 2018, ANTIMICROB AGENTS CH, V62, DOI 10.1128/AAC.01314-18

Shirude PS, 2013, J MED CHEM, V56, P9701, DOI 10.1021/jm401382v

Singh A, 2019, GENE, V693, P114, DOI 10.1016/j.gene.2019.01.029

Singh R, 2008, SCIENCE, V322, P1392, DOI 10.1126/science.1164571

Siricilla S, 2015, J ANTIBIOT, V68, P271, DOI 10.1038/ja.2014.133

Siricilla S, 2014, ANAL BIOCHEM, V461, P36, DOI 10.1016/j.ab.2014.05.018

Slama N, 2016, MOL MICROBIOL, V99, P794, DOI 10.1111/mmi.13266

Soroka D, 2015, ANTIMICROB AGENTS CH, V59, P5714, DOI 10.1128/AAC.00598-15

Squeglia F, 2018, CHEM-EUR J, V24, P2533, DOI 10.1002/chem.201702973

Tadolini M, 2020, EUR RESPIR J, V56, DOI 10.1183/13993003.02328-2020

Tahlan K, 2012, ANTIMICROB AGENTS CH, V56, P1797, DOI 10.1128/AAC.05708-11

Tan YZ, 2020, NAT COMMUN, V11, DOI 10.1038/s41467-020-17202-8

Tan YZ, 2020, MOL CELL, V78, P683, DOI 10.1016/j.molcel.2020.04.014

Nguyen TVA, 2020, CLIN INFECT DIS, V71, P3252, DOI 10.1093/cid/ciaa755

Tiberi S, 2016, EUR RESPIR J, V47, P1758, DOI 10.1183/13993003.00214-2016

Torrelles JB, 2009, GLYCOBIOLOGY, V19, P743, DOI 10.1093/glycob/cwp042

Trefzer C, 2010, J AM CHEM SOC, V132, P13663, DOI 10.1021/ja106357w

Tyagi S, 2005, ANTIMICROB AGENTS CH, V49, P2289, DOI 10.1128/AAC.49.6.2289-2293.2005

van der Beek SL, 2019, MOL MICROBIOL, V111, P951, DOI 10.1111/mmi.14197

van Rijn SP, 2019, ANTIMICROB AGENTS CH, V63, DOI 10.1128/AAC.01489-18

Vilchèze C, 2019, J MOL BIOL, V431, P3450, DOI 10.1016/j.jmb.2019.02.016

Vilchèze C, 2014, MICROBIOL SPECTR, V2, DOI 10.1128/microbiolspec.MGM2-0014-2013

Vilchèze C, 2014, PLOS PATHOG, V10, DOI 10.1371/journal.ppat.1004115

Vosátka R, 2018, EUR J PHARM SCI, V114, P318, DOI 10.1016/j.ejps.2017.12.013

Wall MD, 2007, BIOORG MED CHEM LETT, V17, P2740, DOI 10.1016/j.bmcl.2007.02.078

Wilson R, 2013, NAT CHEM BIOL, V9, P499, DOI 10.1038/nchembio.1277

World Health, 2021, GLOBAL TUBERCULOSIS

Xavier AS, 2014, J PHARMACOL PHARMACO, V5, P222, DOI 10.4103/0976-500X.136121

Xu J, 2019, ANTIMICROB AGENTS CH, V63, DOI [10.1128/AAC.00021-19, 10.1128/aac.00021-19]

Yu MJ, 2018, PEERJ, V6, DOI 10.7717/peerj.4728

Yu X, 2018, ANTIMICROB AGENTS CH, V62, DOI [10.1128/AAC.01824-17, 10.1128/aac.01824-17]

Zhang B, 2019, CELL, V176, P636, DOI 10.1016/j.cell.2019.01.003

Zhang G, 2018, MOLECULES, V23, DOI 10.3390/molecules23030524

Zhang J, 2011, ACS CHEM BIOL, V6, P819, DOI 10.1021/cb200091m

Zhang L, 2021, MBIO, V12, DOI 10.1128/mBio.03381-20

Zhang L, 2020, SCIENCE, V368, P1211, DOI 10.1126/science.aba9102

Zhang W, 2021, EUR J MED CHEM, V213, DOI 10.1016/j.ejmech.2021.113202

NR 123

TC 24

Z9 26

U1 1

U2 29

PU FRONTIERS MEDIA SA

PI LAUSANNE

PA AVENUE DU TRIBUNAL FEDERAL 34, LAUSANNE, CH-1015, SWITZERLAND

EI 1664-302X

J9 FRONT MICROBIOL

JI Front. Microbiol.

PD DEC 21

PY 2022

VL 13

AR 1056608

DI 10.3389/fmicb.2022.1056608

PG 12

WC Microbiology

WE Science Citation Index Expanded (SCI-EXPANDED)

SC Microbiology

GA 7M5PD

UT WOS:000906708100001

OA Green Submitted, gold

DA 2026-05-07

ER

PT J

AU Nathavitharana, RR

Daru, P

Barrera, AE

Kamal, SMM

Islam, S

ul-Alam, M

Sultana, R

Rahman, M

Hossain, MS

Lederer, P

Hurwitz, S

Chakraborty, K

Kak, N

Tierney, DB

Nardell, E

AF Nathavitharana, R. R.

Daru, P.

Barrera, A. E.

Kamal, S. M. Mostofa

Islam, S.

ul-Alam, M.

Sultana, R.

Rahman, M.

Hossain, Md. S.

Lederer, P.

Hurwitz, S.

Chakraborty, K.

Kak, N.

Tierney, D. B.

Nardell, E.

TI FAST implementation in Bangladesh: high frequency of unsuspected

tuberculosis justifies challenges of scale-up

SO INTERNATIONAL JOURNAL OF TUBERCULOSIS AND LUNG DISEASE

LA English

DT Article

DE TB; nosocomial transmission; FAST; implementation

ID HEALTH-CARE WORKERS; XPERT MTB/RIF; OCCUPATIONAL TUBERCULOSIS; RESISTANT

TUBERCULOSIS; TRANSMISSION; INTERVENTIONS; PREVENTION; AFRICA; IMPACT

AB SETTING: National Institute of Diseases of the Chest and Hospital, Dhaka; Bangladesh Institute of Research and Rehabilitation in Diabetes, Endocrine and Metabolic Disorders, Dhaka; and Chittagong Chest Disease Hospital, Chittagong, Bangladesh.

OBJECTIVE: To present operational data and discuss the challenges of implementing FAST (Find cases Actively, Separate safely and Treat effectively) as a tuberculosis (TB) transmission control strategy.

DESIGN: FAST was implemented sequentially at three hospitals.

RESULTS: Using Xpert MTB/RIF, 733/6028 (12.2%, 95 %CI 11.4-13.0) patients were diagnosed with unsuspected TB. Patients with a history of TB who were admitted with other lung diseases had more than twice the odds of being diagnosed with unsuspected TB as those with no history of TB (OR 2.6, 95%CI 2.2-3.0, P < 0.001). Unsuspected multidrug-resistant TB (MDR TB) was diagnosed in 89/1415 patients (6.3%, 95%CI 5.1-7.7). Patients with unsuspected TB had nearly five times the odds of being diagnosed with MDR-TB than those admitted with a known TB diagnosis (OR 4.9, 95 %CI 3.1-7.6, P < 0.001). Implementation challenges include staff shortages, diagnostic failure, supply-chain issues and reliance on external funding.

CONCLUSION: FAST implementation revealed a high frequency of unsuspected TB in hospitalized patients in Bangladesh. Patients with a previous history of TB have an increased risk of being diagnosed with unsuspected TB. Ensuring financial resources, stakeholder engagement and laboratory capacity are important for sustain ability and scalability.

C1 [Nathavitharana, R. R.] Beth Israel Deaconess Med Ctr, Div Infect Dis, Boston, MA 02215 USA.

[Daru, P.; Chakraborty, K.; Kak, N.] Univ Res Co, Washington, DC USA.

[Barrera, A. E.] Univ Penn, Fac Nursing Sci, Philadelphia, PA 19104 USA.

[Kamal, S. M. Mostofa; Islam, S.; ul-Alam, M.; Sultana, R.; Rahman, M.; Hossain, Md. S.] Chest Hosp, Natl Inst Dis, Dhaka, Bangladesh.

[Lederer, P.] Massachusetts Gen Hosp, Div Infect Dis, Boston, MA 02114 USA.

[Hurwitz, S.] Brigham & Womens Hosp, Ctr Clin Invest, Div Biostat, 75 Francis St, Boston, MA 02115 USA.

[Tierney, D. B.; Nardell, E.] Brigham & Womens Hosp, Div Global Hlth Equ, 75 Francis St, Boston, MA 02115 USA.

C3 Harvard University; Harvard University Medical Affiliates; Beth Israel

Deaconess Medical Center; University of Pennsylvania; Harvard

University; Harvard University Medical Affiliates; Massachusetts General

Hospital; Harvard University; Harvard University Medical Affiliates;

Brigham & Women's Hospital; Harvard University; Harvard University

Medical Affiliates; Brigham & Women's Hospital

RP Nardell, E (corresponding author), Brigham & Womens Hosp, Div Global Hlth Equ, 75 Francis St, Boston, MA 02115 USA.

EM enardell@gmail.com

OI Rubel, Manzur-Ul-Alam/0000-0002-5602-4808

FU American people through the United States Agency for International

Development (USAID) through the TB CARE II project [AID-OAA-A-10-00021];

Harvard Center for AIDS Research, Cambridge, MA, USA (National Institute

of Allergy and Infectious Diseases) [2P30A1060354-11]; Imperial College

Institutional Strategic Support Fund (London, UK) Global Health

Fellowship; National Institute of Allergy and Infectious Diseases

[T32AI007061] Funding Source: NIH RePORTER; National Institute on Drug

Abuse; National Institute of Nursing Research; National Institute of

Diabetes and Digestive and Kidney Diseases; National Institute of

Allergy and Infectious Diseases; National Cancer Institute; Eunice

Kennedy Shriver National Institute of Child Health and Human

Development; National Institute on Minority Health and Health

Disparities; National Heart Lung and Blood Institute; National Institute

of Dental and Craniofacial Research; National Institute on Aging

[P30AI060354] Funding Source: NIH RePORTER

FX This work was made possible by the support of the American people

through the United States Agency for International Development (USAID)

through the TB CARE II project under Cooperative Agreement Number

AID-OAA-A-10-00021. The findings of this study are the sole

responsibility of the investigators and University Research Co, LLC and

do not necessarily reflect the views of USAID or the United States

Government. RRN was supported by a grant from the Harvard Center for

AIDS Research, Cambridge, MA, USA (National Institute of Allergy and

Infectious Diseases 2P30A1060354-11,

http://cfar.globalhealth.harvard.edu) and an Imperial College

Institutional Strategic Support Fund (London, UK) Global Health

Fellowship.

CR Albert H, 2016, EUR RESPIR J, V48, P516, DOI 10.1183/13993003.00543-2016

[Anonymous], 2016, WHO/HTM/TB/2016.13

Atun R, 2010, HEALTH POLICY PLANN, V25, P104, DOI 10.1093/heapol/czp055

Auld S C, 2016, C RETR OPP INF 22 25

Aurin TH, 2014, PLOS ONE, V9, DOI 10.1371/journal.pone.0099810

Banu S, 2012, PLOS ONE, V7, DOI 10.1371/journal.pone.0040545

Barrera E, 2015, INT J TUBERC LUNG D, V19, P381, DOI 10.5588/ijtld.14.0680

Bates M, 2012, PLOS ONE, V7, DOI 10.1371/journal.pone.0040774

Bhanu NV, 2004, AM J TROP MED HYG, V70, P527, DOI 10.4269/ajtmh.2004.70.527

Boehme CC, 2011, LANCET, V377, P1495, DOI 10.1016/S0140-6736(11)60438-8

Boehme CC, 2010, NEW ENGL J MED, V363, P1005, DOI 10.1056/NEJMoa0907847

Brugha R, 2000, HEALTH POLICY PLANN, V15, P239, DOI 10.1093/heapol/15.3.239

Creswell J, 2014, BMC INFECT DIS, V14, DOI 10.1186/1471-2334-14-2

Damschroder LJ, 2009, IMPLEMENT SCI, V4, DOI 10.1186/1748-5908-4-50

Dharmadhikari AS, 2014, INT J TUBERC LUNG D, V18, P1019, DOI 10.5588/ijtld.13.0834

Gandhi NR, 2013, J INFECT DIS, V207, P9, DOI 10.1093/infdis/jis631

Hossain S, 2014, BMJ OPEN, V4, DOI 10.1136/bmjopen-2013-004766

Kamal SMM, 2015, INT J TUBERC LUNG D, V19, P151, DOI 10.5588/ijtld.14.0200

Marx FM, 2016, EUR RESPIR J, V48, P1227, DOI 10.1183/13993003.00716-2016

Nardell E A., NCT02355223

Nathavitharana RR, 2017, PRESSE MED, V46, pE53, DOI 10.1016/j.lpm.2017.01.014

Nathavitharana RR, 2016, LANCET INFECT DIS, V16, P883, DOI 10.1016/S1473-3099(16)30199-2

Rifat M, 2011, INT J TUBERC LUNG D, V15, P647, DOI 10.5588/ijtld.10.0205

Rifat M, 2015, BMC INFECT DIS, V15, DOI 10.1186/s12879-015-1253-9

Rifat M, 2015, BMJ OPEN, V5, DOI 10.1136/bmjopen-2015-008273

ROUILLON A, 1976, TUBERCLE, V57, P275, DOI 10.1016/S0041-3879(76)80006-2

Verkuijl S, 2016, CLIN INFECT DIS, V62, pS231, DOI 10.1093/cid/civ1184

Verver S, 2005, AM J RESP CRIT CARE, V171, P1430, DOI 10.1164/rccm.200409-1200OC

Willingham FF, 2001, EMERG INFECT DIS, V7, P123, DOI 10.3201/eid0701.010117

World Health Organization, 2015, BANGL COUNTR PROF TU

Yuen CM, 2015, LANCET, V386, P2334, DOI 10.1016/S0140-6736(15)00322-0

NR 31

TC 32

Z9 35

U1 0

U2 8

PU INT UNION AGAINST TUBERCULOSIS LUNG DISEASE (I U A T L D)

PI PARIS

PA 68 BOULEVARD SAINT-MICHEL,, 75006 PARIS, FRANCE

SN 1027-3719

EI 1815-7920

J9 INT J TUBERC LUNG D

JI Int. J. Tuberc. Lung Dis.

PD SEP

PY 2017

VL 21

IS 9

BP 1020

EP 1025

DI 10.5588/ijtld.16.0794

PG 6

WC Infectious Diseases; Respiratory System

WE Science Citation Index Expanded (SCI-EXPANDED)

SC Infectious Diseases; Respiratory System

GA FF6XA

UT WOS:000409159100013

OA Green Submitted

DA 2026-05-07

ER

PT J

AU Moraski, GC

Markley, LD

Chang, M

Cho, S

Franzblau, SG

Hwang, CH

Boshoff, H

Miller, MJ

AF Moraski, Garrett C.

Markley, Lowell D.

Chang, Mayland

Cho, Sanghyun

Franzblau, Scott G.

Hwang, Chang Hwa

Boshoff, Helena

Miller, Marvin J.

TI Generation and exploration of new classes of antitubercular agents: The

optimization of oxazolines, oxazoles, thiazolines, thiazoles to

imidazo[1,2-a]pyridines and isomeric 5,6-fused scaffolds

SO BIOORGANIC & MEDICINAL CHEMISTRY

LA English

DT Article

DE Mycobacterium tuberculosis; MDR-TB; XDR-TB; 5,6-Fused heteroaromatics;

Imidazo[1,2-a]pyridine

ID MYCOBACTERIUM-TUBERCULOSIS; GROWTH; HYDROXAMATE; RESISTANCE; POTENT;

ASSAY

AB Tuberculosis (TB) is a devastating disease resulting in a death every 20 s. Thus, new drugs are urgently needed. Herein we report ten classes of compounds-oxazoline, oxazole, thiazoline, thiazole, pyrazole, pyridine, isoxazole,imidazo[1,2-a] pyridine, imidazo[1,2-a] pyrimidine and imidazo[1,2-c] pyrimidine-which have good (micromolar) to excellent (sub-micromolar) antitubercular potency. The 5,6-fused heteroaromatic compounds were the most potent with MIC's as low as < 0.195 mu M (9 and 11). Overall, the imidazo[1,2-a] pyridine class was determined to be most promising, with potency similar to isoniazid and PA-824 against replicating Mtb H(37)Rv, clinically relevant drug sensitive, multi-and extensively resistant Mtb strains as well as having good in vitro metabolic stability. (C) 2012 Elsevier Ltd. All rights reserved.

C1 [Moraski, Garrett C.; Markley, Lowell D.; Chang, Mayland; Miller, Marvin J.] Univ Notre Dame, Dept Chem & Biochem, Notre Dame, IN 46656 USA.

[Cho, Sanghyun; Franzblau, Scott G.; Hwang, Chang Hwa] Univ Illinois, Inst TB Res, Coll Pharm, Chicago, IL 60612 USA.

[Boshoff, Helena] NIAID, TB Res Sect, Lab Clin Infect Dis, NIH, Bethesda, MD 20892 USA.

C3 University of Notre Dame; University of Illinois System; University of

Illinois Chicago; University of Illinois Chicago Hospital; National

Institutes of Health (NIH) - USA; NIH National Institute of Allergy &

Infectious Diseases (NIAID)

RP Miller, MJ (corresponding author), Univ Notre Dame, Dept Chem & Biochem, Notre Dame, IN 46656 USA.

EM mmiller1@nd.edu

RI ; Moraski, Garrett/AAH-3616-2021; Boshoff, Helena/MHR-1530-2025

OI Franzblau, Scott/0000-0002-8698-0243; Moraski,

Garrett/0000-0002-6992-5584; Boshoff, Helena/0000-0002-4333-206X; CHO,

SANG HYUN/0000-0003-0926-6246

FU NIH, NIAID; National Institutes of Health (NIH) [2R01AI054193,

CHE-0741793]

FX This research was supported in part by the Intramural Research Program

of the NIH, NIAID and by Grant 2R01AI054193 from the National Institutes

of Health ( NIH) and in part by intermediates provided from DAS. We

would like to thank the University of Notre Dame, especially the Mass

Spectrometry and Proteomics Facility (Bill Boggess, Michelle Joyce,

Nonka Sevova), which is supported by the grant CHE-0741793 from the NIH.

We thank Prof. Jennifer DuBois for regular and lasting scientific

discussions. The excellent technical assistance of Baojie Wan and

Yuehong Wang with antitubercular assays at UIC is greatly appreciated.

CR Al-Tel TH, 2010, EUR J MED CHEM, V45, P5848, DOI 10.1016/j.ejmech.2010.09.049

[Anonymous], 1993, World Health Forum, V14, P438

[Anonymous], 2011, WHOHTMTB201116

[Anonymous], 1995, US PHARM, P2053

Bergeron R. J, 2003, US Patent, Patent No. [6,559,315 B1, 6559315]

Cho SH, 2007, ANTIMICROB AGENTS CH, V51, P1380, DOI 10.1128/AAC.00055-06

Collins LA, 1997, ANTIMICROB AGENTS CH, V41, P1004, DOI 10.1128/AAC.41.5.1004

De Voss JJ, 2000, P NATL ACAD SCI USA, V97, P1252, DOI 10.1073/pnas.97.3.1252

Harth G, 1999, J EXP MED, V189, P1425, DOI 10.1084/jem.189.9.1425

Lilienkampf A, 2010, J MED CHEM, V53, P678, DOI 10.1021/jm901273n

Manjunatha UH, 2006, P NATL ACAD SCI USA, V103, P431, DOI 10.1073/pnas.0508392103

Miller M.J., 2011, WO, Patent No. [2011/057145 A2, 2011057145]

Miller M.J., 2002, US Patent, Patent No. [6,403,623 B1, 6403623]

Miller MJ, 2006, ORG BIOMOL CHEM, V4, P4178, DOI 10.1039/b612475e

MINNICK AA, 1992, ANTIMICROB AGENTS CH, V36, P840, DOI 10.1128/AAC.36.4.840

Moraski GC, 2011, ACS MED CHEM LETT, V2, P466, DOI 10.1021/ml200036r

Moraski GC, 2010, HETEROCYCLES, V80, P977, DOI 10.3987/COM-09-S(S)69

Moraski GC, 2010, EUR J MED CHEM, V45, P1703, DOI 10.1016/j.ejmech.2009.12.074

Odell LR, 2009, BIOORG MED CHEM LETT, V19, P4790, DOI 10.1016/j.bmcl.2009.06.045

Pethe K, 2010, NAT COMMUN, V1, DOI 10.1038/ncomms1060

Phillips AJ, 2000, ORG LETT, V2, P1165, DOI 10.1021/ol005777b

Raviglione M., 1994, GLOBAL BURDEN TUBERC

Sacchettini JC, 2008, NAT REV MICROBIOL, V6, P41, DOI 10.1038/nrmicro1816

Stover CK, 2000, NATURE, V405, P962, DOI 10.1038/35016103

Williams DR, 1997, TETRAHEDRON LETT, V38, P331, DOI 10.1016/S0040-4039(96)02344-1

NR 25

TC 110

Z9 117

U1 0

U2 42

PU PERGAMON-ELSEVIER SCIENCE LTD

PI OXFORD

PA THE BOULEVARD, LANGFORD LANE, KIDLINGTON, OXFORD OX5 1GB, ENGLAND

SN 0968-0896

EI 1464-3391

J9 BIOORGAN MED CHEM

JI Bioorg. Med. Chem.

PD APR 1

PY 2012

VL 20

IS 7

BP 2214

EP 2220

DI 10.1016/j.bmc.2012.02.025

PG 7

WC Biochemistry & Molecular Biology; Chemistry, Medicinal; Chemistry,

Organic

WE Science Citation Index Expanded (SCI-EXPANDED)

SC Biochemistry & Molecular Biology; Pharmacology & Pharmacy; Chemistry

GA 906WH

UT WOS:000301379300003

DA 2026-05-07

ER

PT J

AU Zhad, LL

Liu, HC

Sun, Q

Xiao, TY

Zhao, XQ

Li, GL

Zeng, CY

Wan, KL

AF Zhad, Li-li

Liu, Hai-can

Sun, Qing

Xiao, Tong-yang

Zhao, Xiu-qin

Li, Gui-lian

Zeng, Chun-yan

Wan, Kang-lin

TI Identification of mutations conferring streptomycin resistance in

multidrug-resistant tuberculosis of China

SO DIAGNOSTIC MICROBIOLOGY AND INFECTIOUS DISEASE

LA English

DT Article

DE Multidrug-resistant tuberculosis; Streptomycin resistance; Mutation;

Genotype

ID MYCOBACTERIUM-TUBERCULOSIS; DRUG-RESISTANCE; MOLECULAR CHARACTERISTICS;

EFFLUX PUMP; STRAINS; MECHANISM; RPSL; GENE; DNA

AB We investigated the spectrum and frequency of mutations in rpsL, rrs, and gidB among 140 multidrug-resistant tuberculosis (MDR-TB) clinical isolates from China. The association between mutations and different genotypes was also analyzed. Our data revealed that 65.7% of MDR-TB were resistant to streptomycin (STR), and 90.2% of STR-resistant isolates were Beijing strains. STR resistance was correlated with Beijing family (P = 0.00). Compared with phenotypic data, detection of mutations for the combination of these 3 genes exhibited 94.6% sensitivity, 91.7% specificity, and 93.6% accuracy. The most common mutations in SIR-resistant isolates were rpsL128, 262, and rrs514, of which rpsL128 showed association with Beijing lineage (P = 0.00). A combination of these 3 mutations can serve as the reliable predictors for SIR resistance, showing the sensitivity, specificity, and accuracy of 85.9%, 97.9%, and 90.0%, respectively. Furthermore, gidBA276C, not A615G, was Beijing lineage specific. These findings are useful to develop rapid molecular diagnostic methods for SIR resistance in China. (C) 2015 Elsevier Inc. All rights reserved.

C1 [Zhad, Li-li; Liu, Hai-can; Sun, Qing; Xiao, Tong-yang; Zhao, Xiu-qin; Li, Gui-lian; Wan, Kang-lin] Chinese Ctr Dis Control & Prevent, State Key Lab Infect Dis Prevent & Control, Natl Inst Communicable Dis Control & Prevent, Beijing 102206, Peoples R China.

[Zhad, Li-li; Liu, Hai-can; Sun, Qing; Xiao, Tong-yang; Zhao, Xiu-qin; Li, Gui-lian; Wan, Kang-lin] Collaborat Innovat Ctr Diag & Treatment Infect Di, Hangzhou 310003, Zhejiang, Peoples R China.

[Sun, Qing] Univ South China, Pathogen Biol Inst, Hengyang 421001, Hunan, Peoples R China.

[Zeng, Chun-yan] Hulunbeier Peoples Hosp, Hulunbeier 021000, Peoples R China.

C3 Chinese Center for Disease Control & Prevention; National Institute for

Communicable Disease Control & Prevention, Chinese Center for Disease

Control & Prevention; Collaborative Innovation Center for Diagnosis &

Treatment of Infectious Diseases; University of South China

RP Wan, KL (corresponding author), Chinese Ctr Dis Control & Prevent, State Key Lab Infect Dis Prevent & Control, Natl Inst Communicable Dis Control & Prevent, POB 5, Beijing 102206, Peoples R China.

EM wankanglin@icdc.cn

RI Liu, Guijian/M-9597-2014

FU National Natural Science Foundation of China [81201348]; National Basic

Research Program of China (973 Program) [2015CB554202]

FX This study was supported by the projects of National Natural Science

Foundation of China (grant no. 81201348) and National Basic Research

Program of China (973 Program, grant no. 2015CB554202). The funder had

no role in the study design, data collection and analysis, decision to

publish, or preparation of the manuscript.

CR da Silva PEA, 2011, FEMS IMMUNOL MED MIC, V63, P1, DOI 10.1111/j.1574-695X.2011.00831.x

Chen QY, 2014, TUBERCULOSIS, V94, P159, DOI 10.1016/j.tube.2013.03.004

Chen YY, 2015, PLOS ONE, V10, DOI 10.1371/journal.pone.0117061

Cuevas-Córdoba B, 2013, J MICROBIOL IMMUNOL, V46, P30, DOI 10.1016/j.jmii.2012.08.020

Danilchanka O, 2008, ANTIMICROB AGENTS CH, V52, P2503, DOI 10.1128/AAC.00298-08

Feuerriegel S, 2012, BMC MICROBIOL, V12, DOI 10.1186/1471-2180-12-90

Georghiou SB, 2012, PLOS ONE, V7, DOI 10.1371/journal.pone.0033275

Glynn JR, 2006, EMERG INFECT DIS, V12, P736, DOI 10.3201/eid1205.050400

HONORE N, 1994, ANTIMICROB AGENTS CH, V38, P238, DOI 10.1128/AAC.38.2.238

Jagielski T, 2014, PLOS ONE, V9, DOI 10.1371/journal.pone.0100078

Jiang X, 2008, MICROB DRUG RESIST, V14, P7, DOI 10.1089/mdr.2008.0772

Jnawali HN, 2013, DIAGN MICR INFEC DIS, V76, P187, DOI 10.1016/j.diagmicrobio.2013.02.035

Kamerbeek J, 1997, J CLIN MICROBIOL, V35, P907, DOI 10.1128/JCM.35.4.907-914.1997

Meier A, 1996, ANTIMICROB AGENTS CH, V40, P2452, DOI 10.1128/AAC.40.11.2452

MEIER A, 1994, ANTIMICROB AGENTS CH, V38, P228, DOI 10.1128/AAC.38.2.228

Shi DW, 2011, J ANTIMICROB CHEMOTH, V66, P2240, DOI 10.1093/jac/dkr284

Shi RR, 2007, MICROBES INFECT, V9, P1538, DOI 10.1016/j.micinf.2007.08.009

Siddiqi S, 2006, WHOHTMTB2008392

Somerville W, 2005, J CLIN MICROBIOL, V43, P2996, DOI 10.1128/JCM.43.6.2996-2997.2005

Spies FS, 2008, ANTIMICROB AGENTS CH, V52, P2947, DOI 10.1128/AAC.01570-07

Spies FS, 2011, J CLIN MICROBIOL, V49, P2625, DOI 10.1128/JCM.00168-11

Sun YJ, 2010, CLIN MICROBIOL INFEC, V16, P287, DOI 10.1111/j.1469-0691.2009.02800.x

Tudó G, 2010, J ANTIMICROB CHEMOTH, V65, P2341, DOI 10.1093/jac/dkq322

Wong SY, 2011, ANTIMICROB AGENTS CH, V55, P2515, DOI 10.1128/AAC.01814-10

World Health Organization, 2009, WHOHTMTB2009420

Xia Q, 2015, ANTIMICROB AGENTS CH, V59, P1695, DOI 10.1128/AAC.04541-14

Yadav R, 2013, INT J TUBERC LUNG D, V17, P251, DOI 10.5588/ijtld.12.0319

Zhao LL, 2014, ANTIMICROB AGENTS CH, V58, P3475, DOI 10.1128/AAC.02426-14

Zhao YL, 2012, NEW ENGL J MED, V366, P2161, DOI 10.1056/NEJMoa1108789

NR 29

TC 17

Z9 18

U1 0

U2 17

PU ELSEVIER SCIENCE INC

PI NEW YORK

PA 360 PARK AVE SOUTH, NEW YORK, NY 10010-1710 USA

SN 0732-8893

EI 1879-0070

J9 DIAGN MICR INFEC DIS

JI Diagn. Microbiol. Infect. Dis.

PD OCT

PY 2015

VL 83

IS 2

BP 150

EP 153

DI 10.1016/j.diagmicrobio.2015.06.020

PG 4

WC Infectious Diseases; Microbiology

WE Science Citation Index Expanded (SCI-EXPANDED)

SC Infectious Diseases; Microbiology

GA CS8CA

UT WOS:000362312100013

DA 2026-05-07

ER

PT J

AU Imperiale, BR

Cataldi, AA

Morcillo, NS

AF Imperiale, Belen R.

Cataldi, Angel A.

Morcillo, Nora S.

TI In vitro anti-tuberculosis activity of azole drugs against

Mycobacterium tuberculosis clinical isolates

SO REVISTA ARGENTINA DE MICROBIOLOGIA

LA English

DT Article

DE Drug resistance; Imidazole; Latent tuberculosis; Nitroimidazole

ID METRONIDAZOLE; ANTIFUNGALS; DORMANT; TRICHOMONIASIS; INHIBITORS;

EFFICACY

AB Background: Latent tuberculosis has been associated with the persistence of dormant Mycobacterium tuberculosis in the organism of infected individuals, who are reservoirs of the bacilli and the source for spreading the disease in the community. New active anti-TB drugs exerting their metabolic action at different stages and on latent/dormant bacilli are urgently required to avoid endogenous reactivations and to be part of treatments of multi- and extensively-drug resistant tuberculosis (M/XDR-TB). It was previously reported that azole drugs are active against M. tuberculosis. For that reason, the aims of this study were to determine the in vitro activity of azole drugs, imidazole (clotrimazole, CLO and econazole, ECO) and nitroimidazole (metronidazole, MZ and ipronidazole, IPZ), against a collection of MDR M. tuberculosis clinical isolates; and to analyze their potential use in both the LTB and the active forms of M/XDR-TB treatments.

Methods: A total of 55 MDR M. tuberculosis isolates and H37Rv were included. MZ and IPZ activity against M. tuberculosis isolates were tested using anaerobic culture conditions. The activity of ECO and CLO was measured by the minimal inhibitory concentration (MIC) using a microdilution colorimetric method.

Results: MZ and IPZ showed bacteriostatic activity against M. tuberculosis strains. MIC50 and MIC90 to ECO was 4.0 mu g/ml, while MIC50 to CLO was 4.0 mu g/ml and MIC90 was 8.0 mu g/ml respectively.

Conclusion: All azole compounds tested in the study showed inhibitory activity against MDR M. tuberculosis clinical isolates. (C) 2017 Asociacion Argentina de Microbiologia. Published by Elsevier Espana, S.L.U.

C1 [Imperiale, Belen R.; Morcillo, Nora S.] Dr Cetrangolo Hosp, Reference Lab TB Control Program Buenos Aires Pro, Italia 1750, RA-1602 Florida, Buenos Aires, Argentina.

[Cataldi, Angel A.] Natl Inst Agr Technol INTA, Biotechnol Inst, Los Reseros & Nicolas Repetto S-N, RA-1686 Hurlingham, Buenos Aires, Argentina.

C3 Instituto Nacional de Tecnologia Agropecuaria (INTA)

RP Cataldi, AA (corresponding author), Natl Inst Agr Technol INTA, Biotechnol Inst, Los Reseros & Nicolas Repetto S-N, RA-1686 Hurlingham, Buenos Aires, Argentina.

EM cataldi.angeladrian@inta.gob.ar

CR Ahmad Z, 2005, FEMS MICROBIOL LETT, V251, P19, DOI 10.1016/j.femsle.2005.07.022

Ahmad Z, 2006, FEMS MICROBIOL LETT, V258, P200, DOI 10.1111/j.1574-6968.2006.00224.x

[Anonymous], 2014, GLOB TUB REP, P54

[Anonymous], GLOB TUB REP 2013

Carroll MW, 2013, ANTIMICROB AGENTS CH, V57, P3903, DOI 10.1128/AAC.00753-13

EBDEN P, 1989, ANTIMICROB AGENTS CH, V33, P963, DOI 10.1128/AAC.33.6.963

EDWARDS DI, 1980, BRIT J VENER DIS, V56, P285

Filippini P, 2010, ANTIMICROB AGENTS CH, V54, P2712, DOI 10.1128/AAC.01736-09

GERDING DN, 1986, ANTIBIOTICS LABORATO, P938

Giavini E, 2010, TOXICOL LETT, V198, P106, DOI 10.1016/j.toxlet.2010.07.005

Gomez JE, 2004, TUBERCULOSIS, V84, P29, DOI 10.1016/j.tube.2003.08.003

Ioerger TR, 2013, PLOS ONE, V8, DOI 10.1371/journal.pone.0075245

Iona E, 2007, ANTIMICROB AGENTS CH, V51, P1537, DOI 10.1128/AAC.01468-06

Lamp KC, 1999, CLIN PHARMACOKINET, V36, P353, DOI 10.2165/00003088-199936050-00004

Leitsch D, 2007, PLOS BIOL, V5, P1820, DOI 10.1371/journal.pbio.0050211

Löffler J, 2000, FEMS MICROBIOL LETT, V185, P59, DOI 10.1016/S0378-1097(00)00071-9

Lupetti A, 2002, TRENDS MOL MED, V8, P76, DOI 10.1016/S1471-4914(02)02280-3

McLean KJ, 2007, ARCH BIOCHEM BIOPHYS, V464, P228, DOI 10.1016/j.abb.2007.03.026

McLean KJ, 2006, TRENDS MICROBIOL, V14, P220, DOI 10.1016/j.tim.2006.03.002

McLean KJ, 2002, MICROBIOL-SGM, V148, P2937, DOI 10.1099/00221287-148-10-2937

Menendez C, 2011, EUR J MED CHEM, V46, P5524, DOI 10.1016/j.ejmech.2011.09.013

Morcillo N, 2010, INT J TUBERC LUNG D, V14, P1169

Mukherjee T, 2011, FUTURE MED CHEM, V3, P1427, DOI [10.4155/FMC.11.90, 10.4155/fmc.11.90]

Ouellet H, 2010, ARCH BIOCHEM BIOPHYS, V493, P82, DOI 10.1016/j.abb.2009.07.011

Parish T, 2015, PLOS ONE, V10

Piccaro G, 2013, ANTIMICROB AGENTS CH, V57, P1428, DOI 10.1128/AAC.02154-12

SKIRROW S, 1985, J AM VET MED ASSOC, V187, P405

Taneja NK, 2007, J ANTIMICROB CHEMOTH, V60, P288, DOI 10.1093/jac/dkm207

WAYNE LG, 1994, ANTIMICROB AGENTS CH, V38, P2054, DOI 10.1128/AAC.38.9.2054

Wayne LG, 1996, INFECT IMMUN, V64, P2062, DOI 10.1128/IAI.64.6.2062-2069.1996

WILLIAMS OJ, 1987, AUST VET J, V64, P159, DOI 10.1111/j.1751-0813.1987.tb09671.x

World Health Organization, GLOB PLAN STOP TB

NR 32

TC 13

Z9 14

U1 0

U2 5

PU ASOCIACION ARGENTINA MICROBIOLOGIA

PI BUENOS AIRES

PA BULNES 44 PB	B, BUENOS AIRES, 00000, ARGENTINA

SN 0325-7541

EI 1851-7617

J9 REV ARGENT MICROBIOL

JI Rev. Argent. Microbiol.

PD OCT-DEC

PY 2017

VL 49

IS 4

BP 332

EP 338

DI 10.1016/j.ram.2017.02.008

PG 7

WC Microbiology

WE Science Citation Index Expanded (SCI-EXPANDED)

SC Microbiology

GA FO3SY

UT WOS:000416755700008

OA Green Submitted, Green Published, gold

DA 2026-05-07

ER

PT J

AU Singh, V

Chibale, K

AF Singh, Vinayak

Chibale, Kelly

TI Strategies to Combat Multi-Drug Resistance in Tuberculosis

SO ACCOUNTS OF CHEMICAL RESEARCH

LA English

DT Review

ID MYCOBACTERIUM-TUBERCULOSIS; CLINICAL CANDIDATE; MOLECULAR DOCKING;

VERAPAMIL ANALOGS; BINDING-SITE; DRUG; CHALLENGES; Q203

AB "Drug resistance is an unavoidable consequence of the use of drugs; however, the emergence of multi-drug resistance can be managed by accurate diagnosis and tailor-made regimens."

Antimicrobial resistance (AMR), is one of the most paramount health perils that has emerged in the 21st century. The global increase in drug-resistant strains of various bacterial pathogens prompted the World Health Organization (WHO) to develop a priority list of AMR pathogens. Mycobacterium tuberculosis (Mtb), an acid-fast bacillus that causes tuberculosis (TB), merits being one of the highest priority pathogens on this list since drug-resistant TB (DR-TB) accounts for similar to 29% of deaths attributable to AMR. In recent years, funded collaborative efforts of researchers from academia, not-for-profit virtual R&D organizations and industry have resulted in the continuous growth of the TB drug discovery and development pipeline. This has so far led to the accelerated regulatory approval of bedaquiline and delamanid for the treatment of DR-TB. However, despite the availability of drug regimes, the current cure rate for multi-drug-resistant TB (MDR-TB) and extensively drug-resistant TB (XDR-TB) treatment regimens is 50% and 30%, respectively. It is to be noted that these regimens are administered over a long duration and have a serious side effect profile. Coupled with poor patient adherence, this has led to further acquisition of drug resistance and treatment failure. There is therefore an urgent need to develop new TB drugs with novel mechanism of actions (MoAs) and associated regimens.

This Account recapitulates drug resistance in TB, existing challenges in addressing DR-TB, new drugs and regimens in development, and potential ways to treat DR-TB. We highlight our research aimed at identifying novel small molecule leads and associated targets against TB toward contributing to the global TB drug discovery and development pipeline. Our work mainly involves screening of various small molecule chemical libraries in phenotypic whole-cell based assays to identify hits for medicinal chemistry optimization, with attendant deconvolution of the MoA. We discuss the identification of small molecule chemotypes active against Mtb and subsequent structure-activity relationships (SAR) and MoA deconvolution studies. This is followed by a discussion on a chemical series identified by whole-cell cross-screening against Mtb, for which MoA deconvolution studies revealed a pathway that explained the lack of in vivo efficacy in a mouse model of TB and reiterated the importance of selecting an appropriate growth medium during phenotypic screening. We also discuss our efforts on drug repositioning toward addressing DR-TB. In the concluding section, we preview some promising future directions and the challenges inherent in advancing the drug pipeline to address DR-TB.

C1 [Singh, Vinayak; Chibale, Kelly] Univ Cape Town, Drug Discovery & Dev Ctr H3D, ZA-7701 Rondebosch, South Africa.

[Singh, Vinayak; Chibale, Kelly] Univ Cape Town, South African Med Res Council, Drug Discovery & Dev Res Unit, Dept Chem, ZA-7701 Rondebosch, South Africa.

[Singh, Vinayak; Chibale, Kelly] Univ Cape Town, Inst Infect Dis & Mol Med, ZA-7701 Rondebosch, South Africa.

C3 University of Cape Town; University of Cape Town; South African Medical

Research Council; University of Cape Town

RP Chibale, K (corresponding author), Univ Cape Town, Drug Discovery & Dev Ctr H3D, ZA-7701 Rondebosch, South Africa.; Chibale, K (corresponding author), Univ Cape Town, South African Med Res Council, Drug Discovery & Dev Res Unit, Dept Chem, ZA-7701 Rondebosch, South Africa.; Chibale, K (corresponding author), Univ Cape Town, Inst Infect Dis & Mol Med, ZA-7701 Rondebosch, South Africa.

EM kelly.chibale@uct.ac.za

RI ; Singh, Vinayak/O-5099-2019

OI Chibale, Kelly/0000-0002-4541-646X; Singh, Vinayak/0000-0001-9002-2489

FU Bill and Melinda Gates Foundation (Global Health) [OPP1066878]; South

African Medical Research Council (SAMRC); Strategic Health Innovation

Partnerships (SHIP) unit of the SAMRC; Technology Innovation Agency

(TIA); South African Department of Science and Innovation/National

Research Foundation Research

FX The Bill and Melinda Gates Foundation (Global Health Grant Number

OPP1066878), South African Medical Research Council (SAMRC), Strategic

Health Innovation Partnerships (SHIP) unit of the SAMRC, Technology

Innovation Agency (TIA), and South African Department of Science and

Innovation/National Research Foundation Research are gratefully

acknowledged for support.

CR Abate G, 2016, ANTIMICROB AGENTS CH, V60, P1216, DOI 10.1128/AAC.01567-15

Akester JN, 2020, ACS INFECT DIS, V6, P1951, DOI 10.1021/acsinfecdis.0c00252

Alffenaar JWC, 2017, EXPERT REV ANTI-INFE, V15, P11, DOI 10.1080/14787210.2017.1247692

[Anonymous], 1948, Br Med J, V2, P769

Blondiaux N, 2017, SCIENCE, V355, P1206, DOI 10.1126/science.aag1006

Brauner A, 2016, NAT REV MICROBIOL, V14, P320, DOI 10.1038/nrmicro.2016.34

Candice SD, 2015, BIOORGAN MED CHEM, V23, P7240, DOI 10.1016/j.bmc.2015.10.021

Cicek-Saydam C, 2001, CLIN MICROBIOL INFEC, V7, P700, DOI 10.1046/j.1469-0691.2001.00341.x

de Jager VR, 2020, NEW ENGL J MED, V382, P1280, DOI 10.1056/NEJMc1913327

de Melo CS, 2021, J MED CHEM, V64, P719, DOI 10.1021/acs.jmedchem.0c01727

Dziwornu GA, 2019, MEDCHEMCOMM, V10, P961, DOI 10.1039/c9md00161a

Early JV, 2019, PLOS ONE, V14, DOI 10.1371/journal.pone.0222970

Harris CJ, 2011, COMB CHEM HIGH T SCR, V14, P521

Huszár S, 2020, DRUG DISCOV TODAY, V25, P772, DOI 10.1016/j.drudis.2020.02.003

Huszár S, 2017, ANTIMICROB AGENTS CH, V61, DOI 10.1128/AAC.01310-17

Kigondu EM, 2014, MEDCHEMCOMM, V5, P502, DOI 10.1039/c3md00387f

Kinsella RL, 2021, ANNU REV PATHOL-MECH, V16, P377, DOI 10.1146/annurev-pathol-042120-032916

Koch A, 2018, CURR OPIN PHARMACOL, V42, P7, DOI 10.1016/j.coph.2018.05.013

Lin H, 2011, EPIDEMIOL INFECT, V139, P1784, DOI 10.1017/S0950268810002797

Migliori GB, 2020, INT J INFECT DIS, V92, pS15, DOI 10.1016/j.ijid.2020.01.042

Njoroge M, 2019, ACS INFECT DIS, V5, P1634, DOI 10.1021/acsinfecdis.9b00208

Nzila A, 2011, FUTURE MED CHEM, V3, P1413, DOI [10.4155/FMC.11.95, 10.4155/fmc.11.95]

Pang Y, 2017, ANTIMICROB AGENTS CH, V61, DOI [10.1128/AAC.00900-17, 10.1128/aac.00900-17]

Pethe K, 2013, NAT MED, V19, P1157, DOI 10.1038/nm.3262

Portelli S, 2018, SCI REP-UK, V8, DOI 10.1038/s41598-018-33370-6

Schön T, 2017, CLIN MICROBIOL INFEC, V23, P154, DOI 10.1016/j.cmi.2016.10.022

Sen Tiyas, 2009, J Assoc Physicians India, V57, P660

Shah NS, 2017, NEW ENGL J MED, V376, P243, DOI 10.1056/NEJMoa1604544

Singh K, 2014, BIOORG MED CHEM LETT, V24, P2985, DOI 10.1016/j.bmcl.2014.05.022

Singh V, 2017, MOL MICROBIOL, V103, P13, DOI 10.1111/mmi.13535

Singh V, 2017, ACS INFECT DIS, V3, P5, DOI 10.1021/acsinfecdis.6b00102

Strydom N, 2020, ACS INFECT DIS, V6, P459, DOI 10.1021/acsinfecdis.9b00405

Strydom N, 2019, PLOS MED, V16, DOI 10.1371/journal.pmed.1002773

Tuberculosis Chemotherapy Centre, 1959, Bull World Health Organ, V21, P51

van der Westhuyzen R, 2015, J MED CHEM, V58, P9371, DOI 10.1021/acs.jmedchem.5b01542

WHO, 2020, GLOB TUB REP

WHO, 2019, WHO consolidated guidelines on tuberculosis: module 4: treatment: drug-resistant tuberculosis treatment

Wilson CR, 2017, J MED CHEM, V60, P10118, DOI 10.1021/acs.jmedchem.7b01347

Younis Y, 2012, J MED CHEM, V55, P3479, DOI 10.1021/jm3001373

NR 39

TC 162

Z9 189

U1 1

U2 38

PU AMER CHEMICAL SOC

PI WASHINGTON

PA 1155 16TH ST, NW, WASHINGTON, DC 20036 USA

SN 0001-4842

EI 1520-4898

J9 ACCOUNTS CHEM RES

JI Accounts Chem. Res.

PD MAY 18

PY 2021

VL 54

IS 10

BP 2361

EP 2376

DI 10.1021/acs.accounts.0c00878

EA APR 2021

PG 16

WC Chemistry, Multidisciplinary

WE Science Citation Index Expanded (SCI-EXPANDED)

SC Chemistry

GA SH6ZP

UT WOS:000654284300004

OA Green Submitted, hybrid

DA 2026-05-07

ER

PT J

AU Coyotl, EPA

Palacios, JB

Muciño, G

Moreno-Blas, D

Costas, M

Montes, TM

Diener, C

Uribe-Carvajal, S

Massieu, L

Castro-Obregón, S

Espinosa, OR

Espinosa, DM

Barrios-Payan, J

Contreras, JCL

Corzo, G

Hernández-Pando, R

Del Rio, G

AF Pelaez Coyotl, Erika A.

Barrios Palacios, Jacqueline

Mucino, Gabriel

Moreno-Blas, Daniel

Costas, Miguel

Montiel Montes, Teresa

Diener, Christian

Uribe-Carvajal, Salvador

Massieu, Lourdes

Castro-Obregon, Susana

Espinosa, Octavio Ramos

Mata Espinosa, Dulce

Barrios-Payan, Jorge

Leon Contreras, Juan Carlos

Corzo, Gerardo

Hernandez-Pando, Rogelio

Del Rio, Gabriel

TI Antimicrobial Peptide against Mycobacterium Tuberculosis That

Activates Autophagy Is an Effective Treatment for Tuberculosis

SO PHARMACEUTICS

LA English

DT Article

DE antimicrobial peptide; tuberculosis; autophagy; iztli peptide; multidrug

resistant

ID INFLAMMATORY RESPONSES; ANTICANCER ACTIVITY; EXTRACELLULAR ATP; INNATE;

MACROPHAGES; RECEPTORS; KINETICS; PROTEIN; CELLS; BETA

AB Mycobacterium tuberculosis (MTB) is the principal cause of human tuberculosis (TB), which is a serious health problem worldwide. The development of innovative therapeutic modalities to treat TB is mainly due to the emergence of multi drug resistant (MDR) TB. Autophagy is a cell-host defense process. Previous studies have reported that autophagy-activating agents eliminate intracellular MDR MTB. Thus, combining a direct antibiotic activity against circulating bacteria with autophagy activation to eliminate bacteria residing inside cells could treat MDR TB. We show that the synthetic peptide, IP-1 (KFLNRFWHWLQLKPGQPMY), induced autophagy in HEK293T cells and macrophages at a low dose (10 mu M), while increasing the dose (50 mu M) induced cell death; IP-1 induced the secretion of TNF alpha in macrophages and killed Mtb at a dose where macrophages are not killed by IP-1. Moreover, IP-1 showed significant therapeutic activity in a mice model of progressive pulmonary TB. In terms of the mechanism of action, IP-1 sequesters ATP in vitro and inside living cells. Thus, IP-1 is the first antimicrobial peptide that eliminates MDR MTB infection by combining four activities: reducing ATP levels, bactericidal activity, autophagy activation, and TNF alpha secretion.

C1 [Pelaez Coyotl, Erika A.; Diener, Christian; Del Rio, Gabriel] Natl Autonomous Univ Mexico UNAM, Inst Cellular Physiol, Dept Biochem & Struct Biol, Mexico City 04510, DF, Mexico.

[Barrios Palacios, Jacqueline; Espinosa, Octavio Ramos; Mata Espinosa, Dulce; Barrios-Payan, Jorge; Leon Contreras, Juan Carlos; Hernandez-Pando, Rogelio] Natl Inst Med Sci & Nutr Salvador Zubiran, Expt Pathol Sect, Mexico City 14080, DF, Mexico.

[Mucino, Gabriel; Moreno-Blas, Daniel; Castro-Obregon, Susana] Univ Nacl Autonoma Mexico, Inst Fisiol Celular, Dept Neurodev & Physiol, Mexico City 04510, DF, Mexico.

[Costas, Miguel] Univ Nacl Autonoma Mexico, Fac Quim, Dept Fisicoquim, Lab Biofisicoquim, Mexico City 04510, DF, Mexico.

[Montiel Montes, Teresa; Massieu, Lourdes] Natl Autonomous Univ Mexico UNAM, Inst Cellular Physiol, Dept Mol Neuropathol, Mexico City 04510, DF, Mexico.

[Uribe-Carvajal, Salvador] Univ Nacl Autonoma Mexico, Inst Cellular Physiol, Dept Mol Genet, Mexico City 04510, DF, Mexico.

[Corzo, Gerardo] Natl Autonomous Univ Mexico UNAM, Inst Biotechnol, Dept Mol Med & Bioproc, Cuernavaca 62210, Morelos, Mexico.

[Diener, Christian] Inst Syst Biol, Seattle, WA 98109 USA.

C3 Universidad Nacional Autonoma de Mexico; Universidad Nacional Autonoma

de Mexico; Universidad Nacional Autonoma de Mexico; Universidad Nacional

Autonoma de Mexico; Universidad Nacional Autonoma de Mexico; Universidad

Nacional Autonoma de Mexico; Institute for Systems Biology (ISB)

RP Del Rio, G (corresponding author), Natl Autonomous Univ Mexico UNAM, Inst Cellular Physiol, Dept Biochem & Struct Biol, Mexico City 04510, DF, Mexico.; Hernández-Pando, R (corresponding author), Natl Inst Med Sci & Nutr Salvador Zubiran, Expt Pathol Sect, Mexico City 14080, DF, Mexico.

EM erika16pc@hotmail.com; panterarosa860919@hotmail.com;

gabrielm@ifc.unam.mx; dmoreno@ifc.unam.mx; costasmi@unam.mx;

tmontiel@ifc.unam.mx; Christian.Diener@systemsbiology.org;

suribe@ifc.unam.mx; lmassieu@ifc.unam.mx; scastro@ifc.unam.mx;

octavioramos13@gmail.com; dulmat@yahoo.com.mx; qcjbp77@yahoo.com.mx;

jcleonc@hotmail.com; corzo@ibt.unam.mx; rhdezpando@hotmail.com;

gdelrio@ifc.unam.mx

RI Corzo, Gerardo/B-3778-2014; Massieu, Lourdes/GNW-3740-2022; Diener,

Christian/X-9574-2019; LEON CONTRERAS, JUAN CARLOS/JKI-3017-2023;

Moreno-Blas, Daniel/AAQ-6291-2021; Castro-Obregon, Susana/JAC-9372-2023

OI Corzo, Gerardo/0000-0002-9513-1622; Ramos-Espinosa,

Octavio/0000-0002-9010-4625; Diener, Christian/0000-0002-7476-0868; LEON

CONTRERAS, JUAN CARLOS/0000-0001-8827-1670; BARRIOS PALACIOS,

JACQUELINE/0009-0007-4831-8068; Moreno-Blas, Daniel/0000-0002-8394-8205;

Del Rio, Gabriel/0000-0002-8118-4985; Castro-Obregon,

Susana/0000-0003-0549-5690; Costas, Miguel/0000-0001-8604-6381

FU Programa de apoyo a proyectos de investigacion e innovacion tecnologica

(PAPIIT) from DGAPA-UNAM [IG100513, IG100416, IN208014, IN208817,

IN206518]; CONACyT-Mexico [FOIN-219, CB252316, 2015-2 FC-921,

FC2015-1/115, 442901, 375612, 588372, 417724]; International Center for

Theoretical Physics and Elettra Sincrotrone Trieste [20130244]

FX This work was supported in part by Programa de apoyo a proyectos de

investigacion e innovacion tecnologica (PAPIIT) from

DGAPA-UNAM(IG100513, IG100416, IN208014, IN208817, IN206518),

CONACyT-Mexico (FOIN-219, CB252316, 2015-2 FC-921 and FC2015-1/115),

International Center for Theoretical Physics and Elettra Sincrotrone
[truncated: 7,489,611 more chars]
